# Mutational Landscape of Esophageal Squamous Cell Carcinoma in an Indian Cohort

**DOI:** 10.3389/fonc.2020.01457

**Published:** 2020-08-20

**Authors:** Kiran K. Mangalaparthi, Krishna Patel, Aafaque A. Khan, Malini Manoharan, Coral Karunakaran, Sakthivel Murugan, Ravi Gupta, Rohit Gupta, Arati Khanna-Gupta, Amitabha Chaudhuri, Prashant Kumar, Bipin Nair, Rekha V. Kumar, T. S. Keshava Prasad, Aditi Chatterjee, Akhilesh Pandey, Harsha Gowda

**Affiliations:** ^1^Institute of Bioinformatics, International Technology Park, Bangalore, India; ^2^Amrita School of Biotechnology, Amrita Vishwa Vidyapeetham, Kollam, India; ^3^Medgenome Labs Pvt. Ltd., Bangalore, India; ^4^Manipal Academy of Higher Education, Manipal, India; ^5^Department of Pathology, Kidwai Memorial Institute of Oncology, Bangalore, India; ^6^Center for Systems Biology and Molecular Medicine, Yenepoya Research Centre, Yenepoya (Deemed to be University), Mangalore, India; ^7^Department of Laboratory Medicine and Pathology, Mayo Clinic, Rochester, MN, United States; ^8^Center for Individualized Medicine, Mayo Clinic, Rochester, MN, United States; ^9^Center for Molecular Medicine, National Institute of Mental Health and Neurosciences, Bangalore, India; ^10^Genetics and Computational Biology, QIMR Berghofer Medical Research Institute, Brisbane, QLD, Australia

**Keywords:** tobacco, mutation signatures, squamous cell carcinoma, esophageal cancer, whole exome sequencing

## Abstract

Esophageal squamous cell carcinoma (ESCC) is the most common histological subtype of esophageal cancer in India. Cigarette smoking and chewing tobacco are known risk factors associated with ESCC. However, genomic alterations associated with ESCC in India are not well-characterized. In this study, we carried out exome sequencing to characterize the mutational landscape of ESCC tumors from subjects with a varied history of tobacco usage. Whole exome sequence analysis of ESCC from an Indian cohort revealed several genes that were mutated or had copy number changes. ESCC from tobacco chewers had a higher frequency of C:G > A:T transversions and 2-fold enrichment for mutation signature 4 compared to smokers and non-users of tobacco. Genes, such as *TP53, CSMD3, SYNE1, PIK3CA*, and *NOTCH1* were found to be frequently mutated in Indian cohort. Mutually exclusive mutation patterns were observed in *PIK3CA*–*NOTCH1, DNAH5*–*ZFHX4, MUC16*–*FAT1*, and *ZFHX4*–*NOTCH1* gene pairs. Recurrent amplifications were observed in 3q22-3q29, 11q13.3-q13.4, 7q22.1-q31.1, and 8q24 regions. Approximately 53% of tumors had genomic alterations in *PIK3CA* making this pathway a promising candidate for targeted therapy. In conclusion, we observe enrichment of mutation signature 4 in ESCC tumors from patients with a history of tobacco chewing. This is likely due to direct exposure of esophagus to tobacco carcinogens when it is chewed and swallowed. Genomic alterations were frequently observed in PIK3CA-AKT pathway members independent of the history of tobacco usage. PIK3CA pathway can be potentially targeted in ESCC which currently has no effective targeted therapeutic options.

## Introduction

Esophageal cancer is the eighth most common cancer in the world and the sixth most common cause of cancer-related mortality (1). Histologically, there are two major types of esophageal cancer—Esophageal Squamous Cell Carcinoma (ESCC) and Esophageal Adenocarcinoma (EAC) with distinct geographical patterns of incidences across the globe (2). China has the highest burden of esophageal cancer in the world. The incidence and mortality rate is projected to increase in the future, especially in regions extending from Northeast China to Middle East China which is termed as esophageal cancer belt (3, 4). ESCC is generally diagnosed at advanced stages and hence its prognosis is poor. The overall 5-years survival rate of patients with advanced-stage cancer is lower than 15% (5). Surgery remains a major therapeutic option for ESCC and the standard chemotherapy regimen includes cisplatin and 5-fluorouracil (6). Thus, identification of novel therapeutic targets is necessary for better treatment strategies in ESCC.

In India, cancers associated with tobacco consumption accounts for 42% of total cancer-related deaths (7). Tobacco is consumed in different forms including cigarette smoking and smokeless tobacco products, such as khaini, gutka, betel quid, etc. As per the Global Adult Tobacco Survey (GATS), 34.6% of the adult Indian population consumes tobacco as cigarette and/or smokeless form by chewing and swallowing the extract. Cigarette smoking is a known risk factor for ESCC. Smoking is associated with poor prognosis as overall survival among ESCC patients is poor in smokers compared to non-smokers (8–11). However, molecular alterations associated with varied tobacco consumption in ESCC have not been well-studied.

Multiple studies from Chinese and Japanese cohort have characterized genomic anomalies in ESCC tumors including those from cigarette smokers to investigate mutational landscape and elucidation of potential driver genes (12–15). For instance, Song et al. studied ESCC using whole genome sequencing (WGS) of 17 and whole exome sequencing (WES) of 71 ESCC samples and reported *ADAM29* and *FAM1335B* as two significantly mutated genes along with frequently mutated genes, such as *TP53, PIK3CA, NOTCH1*, and *NFE2L2* (13). Lin et al. carried out WES analysis of 139 ESCC samples and identified *ZNF750* and *FAT1* as tumor suppressors which are frequently disrupted in ESCC (12). A study on ESCC from the Japanese cohort has reported an enrichment of drinking and smoking-related mutation signature which are not observed in the Chinese cohort (14). Despite the high incidence of ESCC in India and known association of tobacco consumption in both smoke and smokeless form, there are no reports on the mutation landscape of ESCC tumors from an Indian cohort. Hence, we employed whole exome sequencing analysis of 28 ESCC samples from an Indian cohort to characterize genomic alterations from patients with history of smoking or chewing tobacco and those with no history of tobacco consumption.

## Materials and Methods

### Study Cohort

ESCC tumor and paired adjacent normal tissue samples were collected from Kidwai Memorial Institute of Oncology, Bangalore, India. Informed consent from all the patients and ethical clearance were obtained from Kidwai Memorial Institute of Oncology, Bangalore. History of smoking and/or chewing tobacco was determined based on self-reporting by patients. Patients in the non-users group had no history of tobacco consumption. Details of 28 ESCC patients enrolled in this study and their history of tobacco consumption are provided in Supplementary Table 1. All the samples were stored at −80°C until further processing. The project was approved by institutional biosafety committee at the Institute of Bioinformatics.

### Whole Exome Sequence Analysis

DNA library for whole exome sequencing was prepared using Agilent SureSelectXT Human All Exon V5 kit using genomic DNA extracted from tissue samples. Genomic DNA was subjected to sonication using M220 Focused-ultrasonicator™ to produce sheared fragments of 150–200 bp length. Sheared DNA fragments were end-repaired and phosphorylated, adenylated at 3′ ends followed by ligation of standard paired-end adaptors. Hybridization reaction was carried out at 65°C for 16 h using a DNA library with the addition of biotin-labeled RNA probe sets. Resulting DNA-RNA duplexes were captured using Dynabeads® MyOne™ Streptavidin T1 beads. Amplification of the specific libraries was done using indexed primers and Herculase II Fusion DNA Polymerase (Agilent Technologies Inc.). Subsequently, cluster amplification was performed according to the manufacturer's protocol (Illumina Inc.). Paired-end sequencing was performed on Illumina HiSeq 2500 platform with a read length of 100 bp. Raw reads acquired in FASTQ format were assessed for Phred score using FastQC (16). To increase the accuracy and specificity of read mapping, bases with Phred score <20 were removed and trimmed reads with length ≥35 bps were retained for further analysis. Reads were aligned against reference genome hg19 (GRCh37) using BWA (Burrows-Wheeler Aligner)-MEM (Maximal Exact Matches) (17) with default parameters. Binary alignment map (BAM) files were further processed using GATK tool suite (Genome Analysis Toolkit, Broad Institute) which includes removal of duplicates using MarkDuplicates of Picard tools, indel realignment using IndelRealigner, and base recalibration using BaseRecalibrator. High confidence somatic single nucleotide variants (SNVs) were filtered using Strelka (18) and annotated using VariMAT. Identification of genes with mutually exclusive variants, oncoprints, and lollipop plots of somatic SNVs were generated using cBioPortal (19, 20).

Mutational signatures were deduced using R package SomaticSignatures (21). Somatic mutational signatures for each cohort were further deconstructed using Mutalisk to determine the presence of different COSMIC signatures in each cohort (22). Copy number alterations (CNAs) were inferred using OncoCNV and amplification with fold change >3 was considered CNA gain and <1 was considered CNA loss with *p*-value threshold 1 × 10^−5^ (23). Genes recurrently affected by CNAs in at least 5 samples were queried against DGIdb 3.0 database to identify potential druggable targets (24). Clinically actionable therapeutic targets were predicted using The Drug Gene Interaction Database (DGIdb). The database has curated list of drug-gene interactions compiled from various resources. A list of genes with coding mutations and/or amplified in at least five samples were queried against the database by choosing “Clinically actionable” gene category and keeping default source databases of DGIdb as background. “Druggable Genome” category was used to identify potentially druggable targets in ESCC. The Integrative Genomics Viewer (IGV) was used to visualize WES datasets (25) and boxplots were generated using R. Genes affected by copy number alteration were further analyzed for their gene expression pattern in TCGA dataset using “TCGA analysis” module of UALCAN (http://ualcan.path.uab.edu/analysis.html). Genes were queried against “Esophageal carcinoma” database and explored for expression and survival analysis with *p*-value threshold of 0.05 (26).

### Neoantigen Prediction

Single nucleotide somatic variants were used to predict potential neoantigens using tumor-specific neoantigen detector (TSNAD) (27). The tool is based on NetMHCpan (28) which is designed using an artificial neural network approach. It is specifically designed to handle somatic SNVs identified using WGS or WES analysis for prediction of potential neoantigens that can bind to major histocompatibility complex (MHC) class I molecule. Human leukocyte antigen (HLA) allele subtype is a mandatory input of TSNAD to predict compatible neoantigen peptide. HLA subtypes for each patient were inferred using HLAScan which is specifically designed for whole exome datasets (29). Clinically relevant four-digit HLA allele predicted using HLAScan was used to restrict neoantigen prediction compatible for each patient.

### Gene Ontology and Pathway Enrichment Analysis

Gene ontology overrepresentation analysis was carried out using FunRich with default Human database with *p*-value threshold of 0.05 (30). Pathway enrichment analysis was done using recurrently mutated genes to identify the most affected pathways. Genes affected by non-synonymous SNVs in at least two samples and/or genes with CNV in at least four samples were queried using DAVID (31). Pathway enrichment was carried out using Human backend database with a *p*-value threshold of 0.05 (Fisher's exact test).

### Statistical Analysis

Statistical significance of the differences in mutation load between cohorts in each study was determined using unpaired *t*-test with assumption of similar standard deviation with 95% confidence level. Statistical significance of transition and transversion frequencies between cohorts was determined using unpaired two-tailed Mann-Whitney test and within cohort was determined using paired two tailed Wilcoxon test. *P*-values for gene expression differences were obtained from UALCAN platform. Estimation of gene expression difference was performed using *t*-test module of Comprehensive Perl Archive Network (CPAN) “Statistics::TTest” in UALCAN (26). Differences with *p*-values ≤ 0.05 were considered statistically significant. Kaplan-Meier curve was generated using the PROGgeneV2 platform by grouping the samples in TCGA-Esophageal Carcinoma dataset based on median gene expression.

## Results

### Whole Exome Sequence Analysis

We performed whole exome sequence analysis of 28 ESCC tumor samples and germline DNA from adjacent normal tissue to characterize mutation landscape. Approximately 100× target depth with ~99% target coverage was achieved with an average of 90 million reads for all samples (Supplementary Table 2). WES analysis led to the identification of 9,524 SNVs in 6,774 genes. Among 9,524 SNVs, 1,500 were synonymous, 3,944 were non-synonymous, and others were non-exonic variants. Non-synonymous exonic variants include 3,630 missense, 217 nonsense, 84 splice site mutations, 7 stop loss, and 6 start loss SNVs. We observed a median of 296 variants per sample with a mutation burden of 5.81/Mb (2.28 ns-SNVs/Mb) (Supplementary Table 3).

### Somatic Mutation Spectrum of Esophageal Squamous Cell Carcinoma

**Figure 1:**
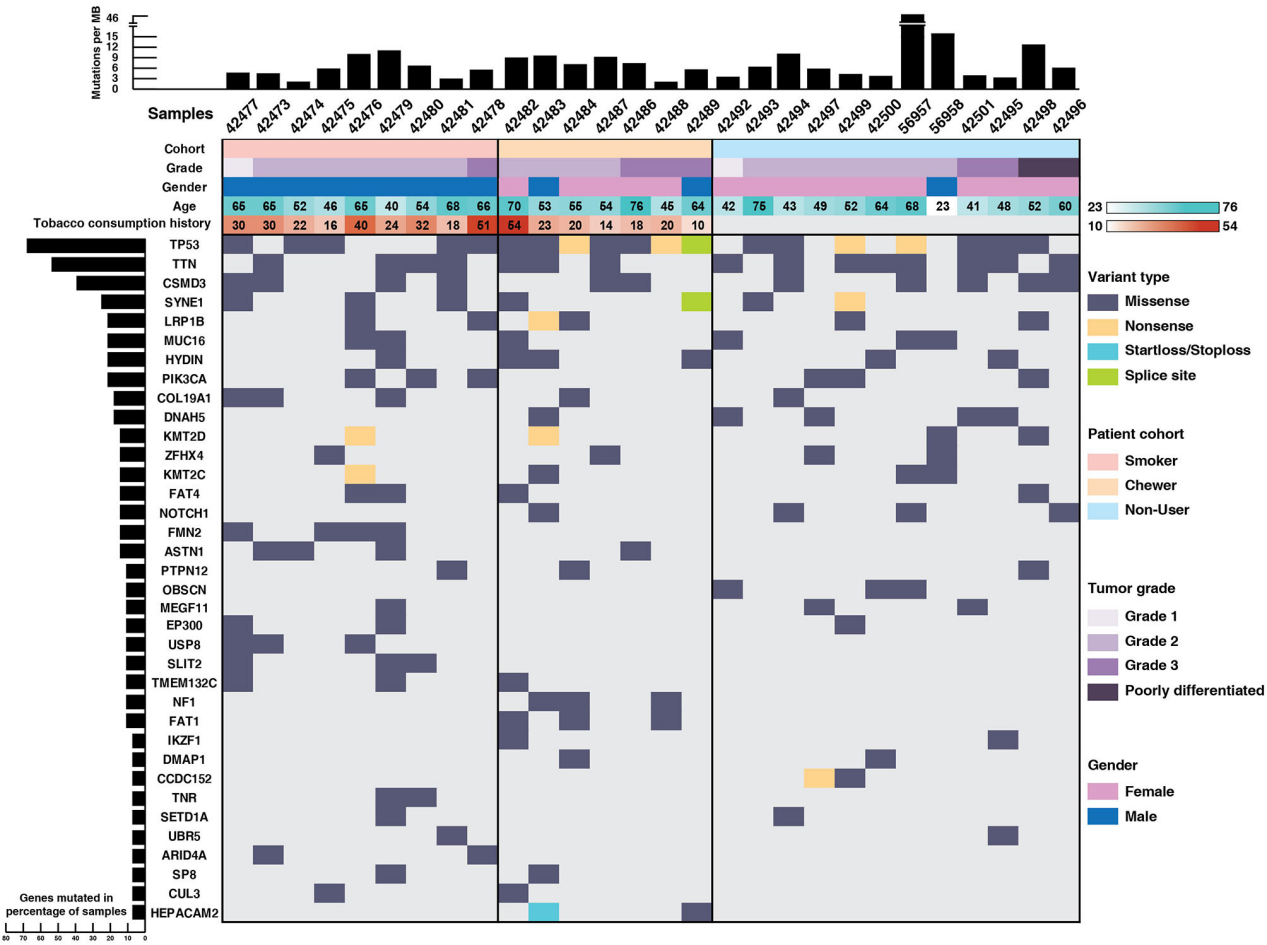
Mutational landscape of esophageal squamous cell carcinoma. Top panel depicts mutation load per Mb, middle panel depicts sample details including tumor grade, gender, age, and tobacco consumption history. Each column represents a sample and each row depicts a gene. Somatic mutations are colored based on mutation type and left panel depicts percentage of samples that harbor mutations.

*TP53* (71%), *TTN* (64%), *CSMD3* (43%), *SYNE1* (28%), *LRP1B* (25%), *PIK3CA* (25%), *DNAH5* (21%), and *NOTCH1* (14%) were recurrently mutated genes in our ESCC cohort (Figure 1). *TP53* mutations were identified in 71% of the samples which is in concordance with the reported mutation frequency of ~80% in previous studies (12, 13). *NF1* is a well-known tumor suppressor gene and it was mutated in 11% of the samples and *ZFHX4* in 21% of the samples. We identified SNVs in the cadherin repeat domains of FAT family genes, i.e., *FAT1* and *FAT4* in 21% samples, which are functionally characterized as tumor suppressor genes in multiple cancers including ESCC (13, 32–34). In concordance with ESCC genomic profiles from Chinese and Japanese cohorts, we identified several previously reported recurrently mutated genes (Supplementary Table 4). Gene ontology analysis revealed that most of the mutated genes are involved in cell communication, cell growth and maintenance (Supplementary Figure 1).

Notably, genes involved in epigenetic mechanisms, such as *SETD1A, CUL3, IKZF1, UBR5, EP300*, and *ARID4A* were recurrently mutated. *SETD1A*, also known as *KMT2F*, is a histone methyltransferase which methylates Lys4 of histone H3 and regulates transcriptional activation (35). Other genes from same family, i.e., *KMT2D* and *KMT2C* were also recurrently mutated. Gene expression analysis of *SETD1A* in EBI Expression atlas (36) revealed that *SETD1A* is downregulated in many cancers including triple-negative breast cancer, colorectal carcinoma and pancreatic adenocarcinoma. Also, its downregulation is significantly associated with poor overall survival in TCGA-Esophageal Carcinoma (ESCA) dataset (*p* = 0.019) as per the PROGeneV2 platform (Supplementary Figure 2). *EP300* and *CUL3* are well-known genes commonly mutated in ESCC (14, 37, 38). *IKZF1* is known to play a key role in enhancing the susceptibility of solid tumors to immunotherapy (39). Interestingly, we observed SNVs in epigenetic modulator *DMAP1* and mitotic kinetics regulator *HEPACAM2* that are not well-studied in ESCC. *DMAP1* (DNA methyltransferase 1 associated protein 1) forms a transcriptional repressive complex by directly interacting with *DNMT1*, a key enzyme responsible for epigenetic methylation patterns and transcriptional silencing process (40). Together with *DNMT1*, it plays an important role in DNA repair process (41–43). Mutations in various genes involved in epigenetic mechanisms suggest that epigenetic signatures could serve as a major hallmark of disease progression in ESCC. Apart from epigenetic modulators, mitotic kinetics regulator or HEPACAM family member 2 (*HEPACAM2*) was mutated in chewers cohort with a start loss mutation and a missense mutation. It is a cell adhesion molecule which belongs to Immunoglobulin superfamily and variants are observed in proximity to Immunoglobulin-like domain. *HEPACAM2* is involved in centrosome maturation during the mitotic cell division. Its expression is significantly downregulated in progressive manner from normal mucosa to adenoma to carcinoma in colorectal cancer and its higher expression is significantly associated with better survival (44).

### Mutually Exclusive Mutations in ESCC

**Figure 2:**
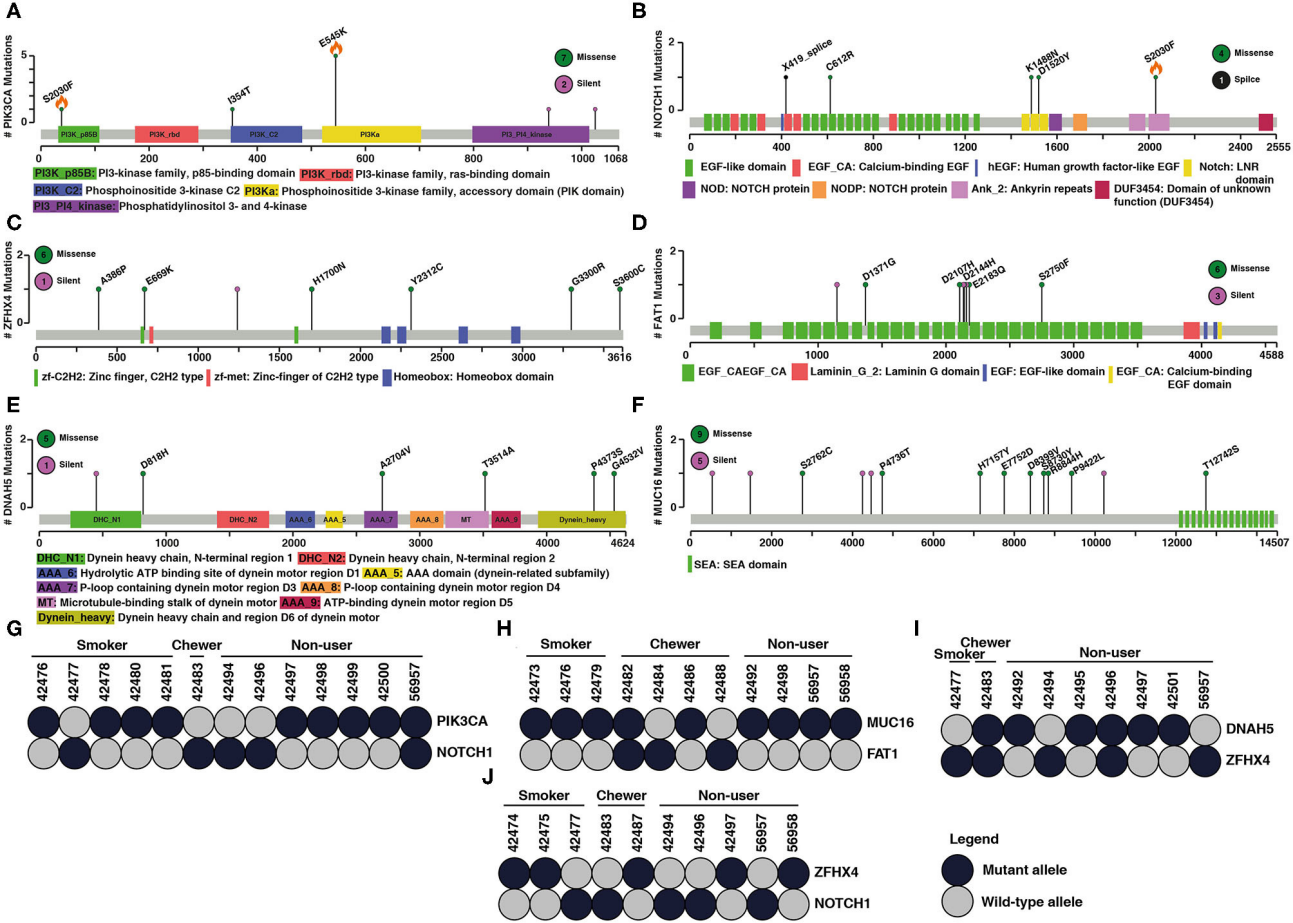
Examples of mutated genes that showed mutual exclusivity. Schematic representation of coding variant distribution on proteins **(A)** *PIK3CA*, **(B)** *NOTCH1*, **(C)** *ZFHX4*, **(D)** *FAT1*, **(E)** *DNAH5*, and **(F)** *MUC16*. Green indicates missense mutation, black indicates splice site and purple indicates silent mutation. Total number of different type of mutations for each gene is given within the circle on right side. X-axis represents length of the protein (amino acid) and y-axis represents number of samples. Orange flame indicates COSMIC hotspot mutations. Instances of gene pairs with mutually exclusive mutation pattern in ESCC **(G–J)** *PIK3CA* and *NOTCH1, MUC16* and *FAT1, DNAH5* and *ZFHX4, ZFHX4* and *NOTCH1*. Column represents samples and row represents genes. Dark shaded circles indicate the mutant allele and the gray circle indicates wild type allele.

Large-scale sequencing studies have shown several mutated genes exist in mutually exclusive manner across different tumors (45). Two major hypothesis associated with mutually exclusive mutated genes in cancers is functional redundancy in downstream pathways or synthetic lethality (46). We observed a mutually exclusive mutation pattern in *PIK3CA*–*NOTCH1, DNAH5*–*ZFHX4, MUC16*–*FAT1*, and *ZFHX4*–*NOTCH1* gene pairs in ESCC (Figure 2). A study by Song et al. has investigated mutually exclusive mutations of *PIK3CA* and *NOTCH1*. Based on clinicopathological parameters, they reported a better response to chemotherapy in patients with *PIK3CA* mutation compared to those with *NOTCH1* mutations. *PIK3CA* mutations are associated with longer overall survival and progression free survival compared to *NOTCH1* mutations (47). Mutations in *ZFHX4* and *DNAH5* were also associated with poor survival among ESCC patients. Knockdown of *ZFHX4* decreased migratory and invasive abilities of ESCC cell lines indicating a tumor-promoting ability in ESCC (48). *FAT1* is a significantly mutated gene in ESCC. Knockdown of *FAT1* leads to increased proliferation rate suggesting potential tumor suppressor function. (12). Few studies have investigated the role of *MUC16* in ESCC, and its overexpression is reported to have prognostic value in ESCC (49). A similar mutually exclusive pattern between these gene pairs is observed in cBioPortal, GDC (Supplementary Figure 3), and published Chinese and Japanese cohorts (Supplementary Figures 4–7). One of the genes in these gene pairs is often a known oncogene or tumor suppressor gene.

### Mutation Signatures Associated With Tobacco Usage

**Figure 3:**
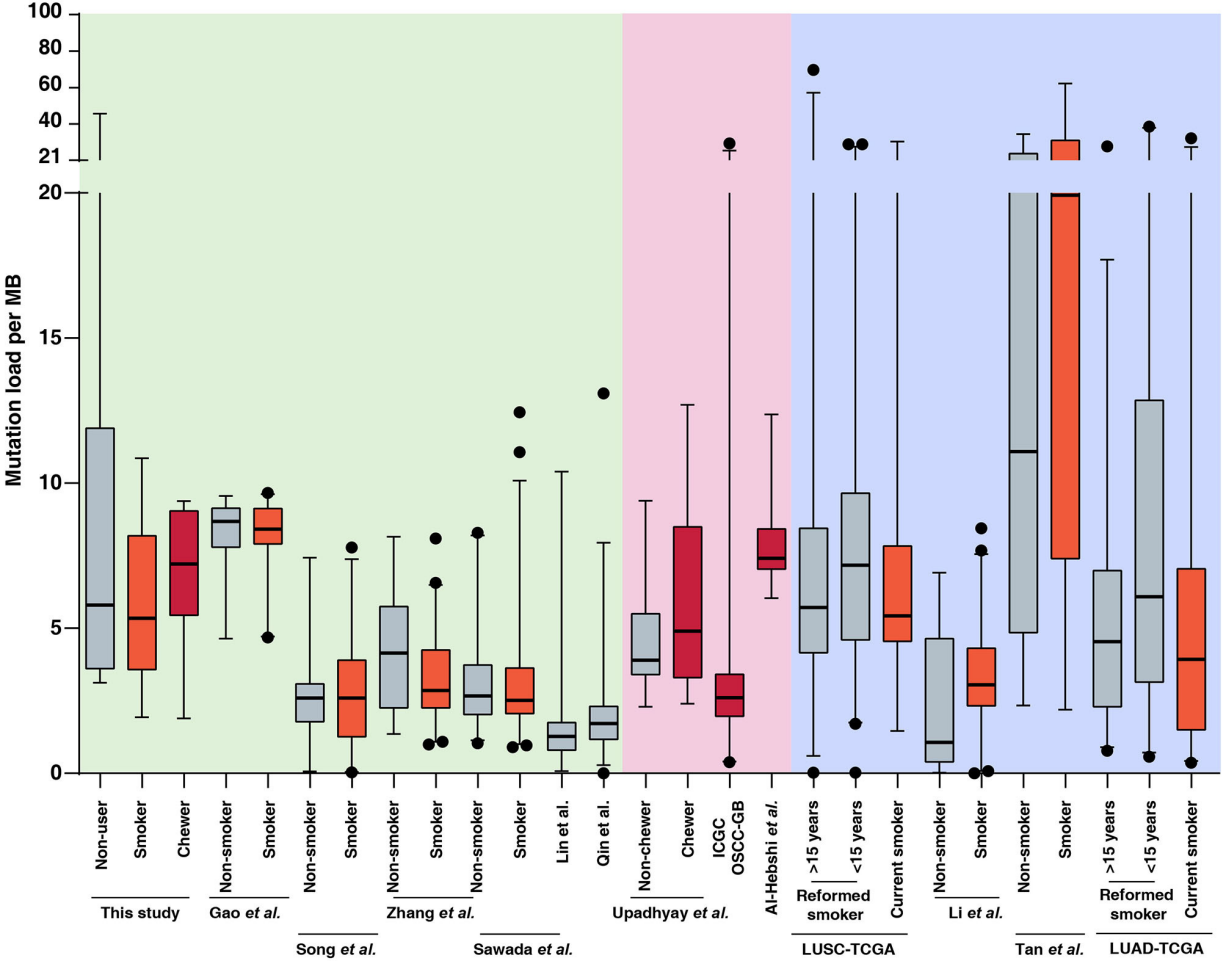
Mutation load observed in tumors from tobacco users and non-users in this study and publicly available datasets of esophageal squamous cell carcinoma (ESCC), oral squamous cell carcinoma (OSCC), lung squamous cell carcinoma (LUSC), and lung adenocarcinoma (LUAD). Black dots represents outliers identified using 97.5 percentile interval. Mutation burden for each sample in ESCC, OSCC, LUSC, and LUAD datasets is provided in Supplementary Table 5 and plotted as per tobacco consumption history.

In the present study, we sequenced 28 ESCC samples consisting of nine samples with tobacco smoking history, seven samples with tobacco chewing history and 12 samples from patients with no known history of tobacco consumption. The average mutation burden was 5.35 SNVs per Mb in smokers, 7.22 SNVs per Mb in chewers, and 5.8 SNVs per Mb in patients with no history of tobacco usage. We compared the mutation load in our cohort to published ESCC genomic profiling studies from Chinese and Japanese cohorts. We did not observe a significant difference in mutation load between smokers and non-smokers in Chinese as well as Japanese cohorts which was in line with observations from our cohort (12–14, 37, 50, 51). This is unlike lung cancers where lung squamous cell carcinoma and lung adenocarcinoma exhibit a high mutation load in smokers compared to non-smokers (5, 52–54) (Figure 3, Supplementary Table 5). Few studies have investigated the carcinogenic effects of chewing tobacco. Higher mutation load in tobacco chewers is reported in oral squamous cell carcinoma compared to non-chewers (55–57). We speculate that direct and prolonged exposure of chewing tobacco to esophagus and oral cavity may result in higher mutation burden compared to tobacco smoking.

**Figure 4:**
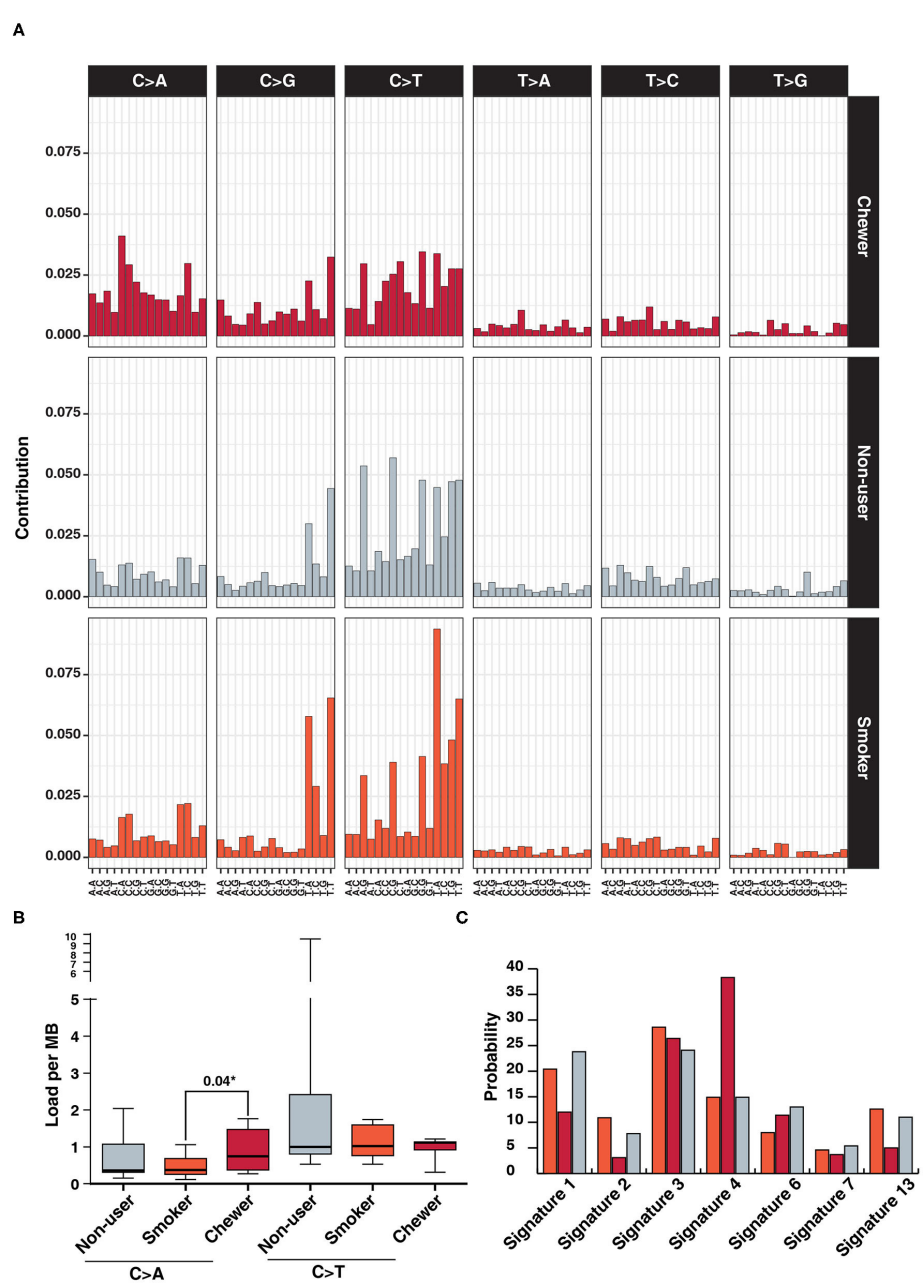
Analysis of mutation signatures enriched in ESCC from smokers, chewers and tobacco non-users. **(A)** Mutation signatures associated with ESCC from smokers, chewers and tobacco non-users. Height of the bar represents contribution of the base substitution across different trinucleotide contexts. **(B)** Mutation load of C > A transversions and C > T transitions in smokers, chewers and tobacco non-users. **(C)** Results of mutation signature decomposition in ESCC samples from chewers, smokers and tobacco non-users. (Red) Chewers, (Gray) Non-users, and (Orange) Smokers.

We further investigated mutational signatures in tumors from patients with and without history of tobacco usage. In concordance with previous ESCC studies, we observe a high frequency of C:G > T:A transitions regardless of tobacco usage history (13, 58) and higher frequency of C > A in chewer cohort (Figure 4A). C > A transversions were observed at a frequency of 0.75 SNVs/Mb in chewer cohort whereas 0.37 SNVs/Mb and 0.36 SNVs/Mb in smoker and non-user cohort, respectively (Figure 4B). Further, deconstruction of mutation signature for each cohort using Mutalisk revealed enrichment of COSMIC mutation signature 4 (38.3%) in chewer cohort whereas 14.9% in smoker and non-user cohort (Figure 4C). Signature 4 is enriched with C > A transversion and is associated with exposure to tobacco derived carcinogens, such as benzo[a]pyrene (58). Chronic treatment of mouse embryonic fibroblasts with tobacco carcinogen benzo[a]pyrene is reported to produce a mutation pattern with high C > A transversions (59). Higher frequency of C > A transversion due to direct exposure to tobacco mutagens is reported in multiple cigarette smoking associated cancers, such as lung and larynx cancer (58). Mutation signature of individual samples from three different cohorts is depicted in Supplementary Figure 8. In congruence with previous reports (58, 59), we propose that higher proportion of C > A transversions in chewers cohort could be attributed to direct exposure of esophageal tissue to tobacco mutagens whereas lack of enrichment of this signature in smoker's cohort may be due to passive exposure to cigarette smoke.

### Potential Neoantigens in ESCC

Cancer neoantigens derived from coding variants can be valuable candidates for developing cancer vaccines. T-cells can recognize neoantigens presented by cancer cells and eliminate them; which makes them valuable candidates for cancer immunotherapy. Efficacy of immune checkpoint inhibitors in treating melanoma and lung cancer is attributed to higher mutation load that potentially contributes to higher neoantigen load in these tumor types (60–62). Somatic non-synonymous single nucleotide variants identified using GATK pipeline were screened for such neoantigenic peptides using HLA-Scan and TSNAD. Elicitation of the immune response depends on the binding affinity of neoantigen with the MHC complex. A high confidence set of 91 neoantigens with wild type peptide binding affinity of ≥500 nM and corresponding mutant peptide binding affinity of ≤100 nM were predicted (63) (Supplementary Table 6). These 91 potential neoantigens were predicted based on 24 coding variants in chewers, 16 coding variants in smokers, and 51 coding variants in non-user cohort. Thirteen genes affected by this include *ATF6, DOCK5, IARS, KIAA0922, KIFAP3, MAN2B2, MBTPS1, MXRA8, NSD1, SKI, SLC12A9, TP53*, and *ZNF277*. Expression pattern of 6 out of 13 genes (*KIFAP3, MBTPS1, NSD1, SLC12A9, TP53*, and *ZNF277*) in the TCGA-Esophageal Carcinoma (ESCA) dataset indicates overexpression in ESCC samples (*n* = 95) (26). There are four predicted neoantigens harboring mutation p.Asp153Tyr in sample 42,482 that is compatible with predicted HLA-subtype of the patient, although with weak binding affinity. Similarly, two neoantigens with mutation p.Val320Phe were predicted in sample 42,483. As none of the variants in predicted neoantigens were positionally recurrent in our cohort, identifying neoantigens that can be targeted across multiple patients may prove challenging for immunotherapy. Validation of neoantigens in larger cohorts is warranted to determine their prevalence and potential use in immunotherapy.

### Copy Number Alterations in ESCC

**Figure 5:**
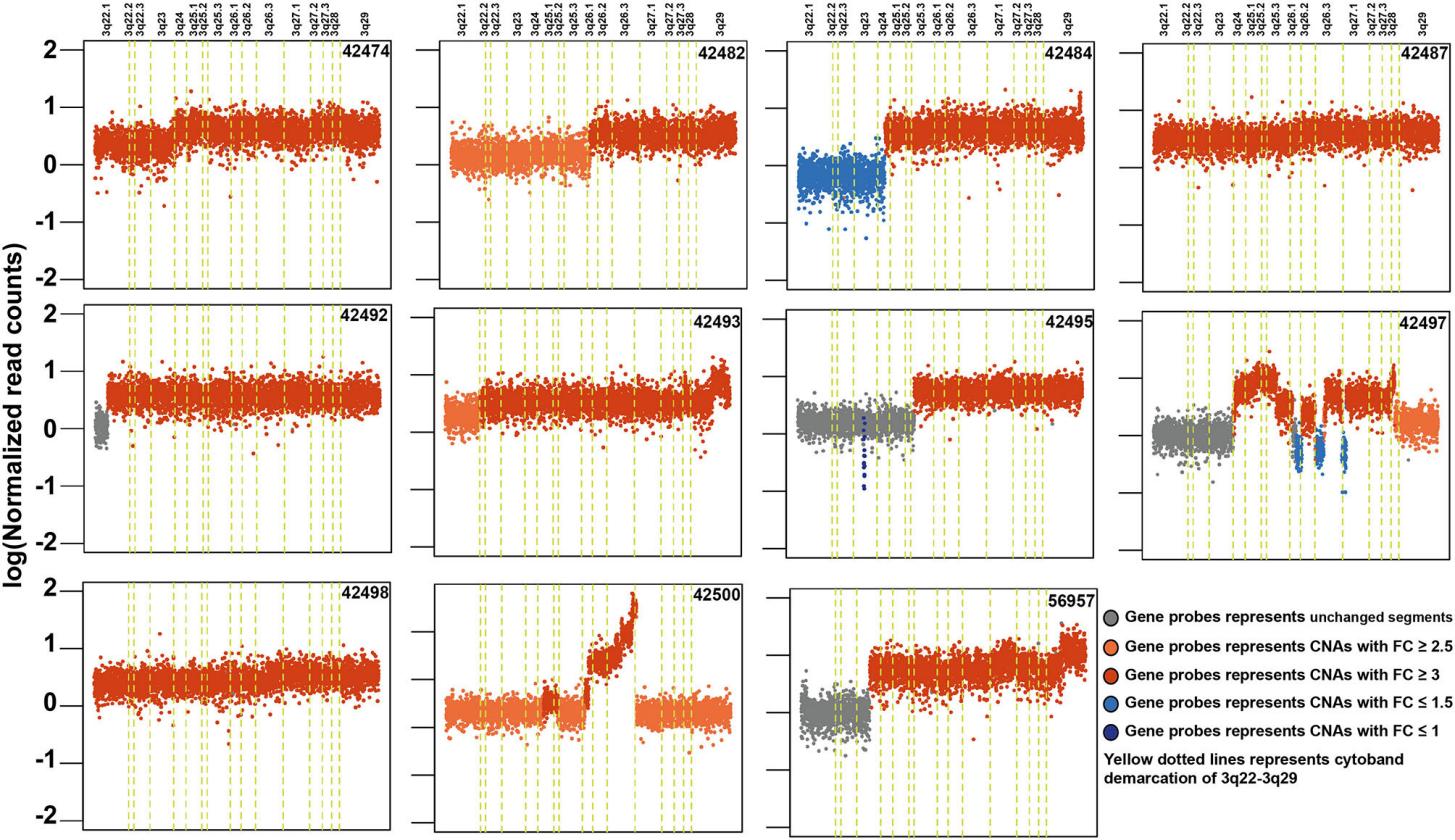
Schematic depicting recurrent genomic amplification in cytoband 3q22-3q29 in esophageal squamous cell carcinoma samples.

Copy number alteration analysis led to the identification of 2,368 affected genes in at least two samples (Supplementary Table 7). Surprisingly, we observed high copy number alterations in tumors from non-tobacco users compared to smokers or chewers. In non-users, 1,540 genes were affected compared to 358 in chewers and 102 in smokers. In concordance with previously published reports, we observed frequent amplifications in 3q24-26, 8q24, 11q13, 5p15, 9q34, 7q22, 16p13, and 20q11 regions in our cohort (Supplementary Figure 9) (51, 64). 11q13.3 region harbors key genes involved in cell growth, such as fibroblast growth factors (*FGF3, FGF4*, and *FGF19*) and recently reported oncogenes in ESCC including *ANO1* and *SHANK2* (65). CNV gains in 3q22-29 region are most frequently observed in ESCC and it contains several key oncogenes, such as *PIK3CA, TP63*, and *SOX2* (Figure 5). Out of 307 genes, 121 (40%) genes in 3q22-29 region are overexpressed in TCGA-Esophageal Carcinoma (ESCA) dataset as well as in ESCC *vs*. EAC based on the UALCAN database. This includes genes, such as *PFN2* and *CLSTN2* (26). Higher gene expression of *CLSTN2* is associated with poor overall survival in the TCGA-Esophageal Carcinoma dataset (*p*-value = 0.05) (Supplementary Figure 10). Genes in 3q26.33 (*n* = 15), 3q26.32 (*n* = 5) which is also referred to as OncoCassette (66) and 3q27.1 (*n* = 30) had copy number gain in ≥ 9 samples. Notably, *KCNMB2* and *KCNMB3* genes in 3q26.32 were amplified in 11 out of 28 samples. Overexpression of these genes is associated with overall poor survival in TCGA-Esophageal Carcinoma dataset (*p*-value ≤ 0.05). Genomic alterations, such as gain in 8q24 and 11q13 have been associated with poor prognosis in ESCC (67). Co-amplification of *SOX2* and *TP63* is often observed in squamous cell carcinomas which further validate our findings (68). Overall, our results indicate that amplification of 3q region could be a major driver in the development of ESCC.

### Identification of Potentially Druggable Genes

**Figure 6:**
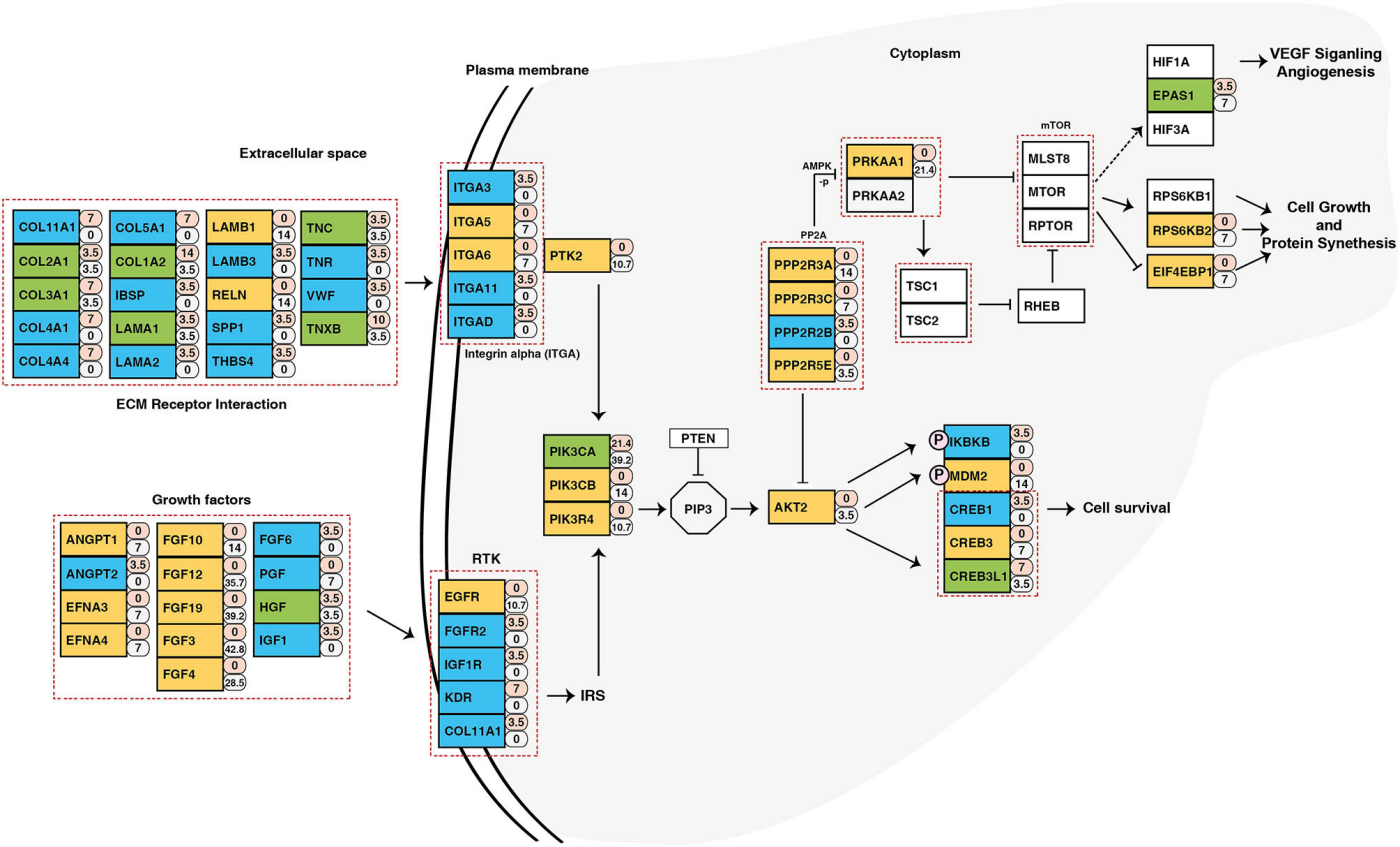
Schematic representation of PIK3CA/AKT pathway depicting genes altered in ESCC. The numbers beside rectangles indicate percentage of samples that carry mutations (pink) and/or copy number variations (white) in respective genes. Green color represents genes with both mutations and CNVs, yellow color represents genes with only CNVs, and blue color are genes with only mutations.

To identify potential druggable targets, we combined genes bearing somatic variants and genes amplified in at least 5 out of 28 samples and screened them against FDA approved anti-neoplastic drugs database DGIdb (24) (Supplementary Table 8). Analysis revealed *PIK3CA* as a potential therapeutic target. In our cohort, *PIK3CA* was recurrently mutated (Glu545Lys) in five samples and amplified in 11 samples. Pathway analysis of genes affected by genomic anomalies also revealed enrichment of PIK3CA-AKT pathway which suggests potential therapeutic benefit of targeting *PIK3CA* in ESCC (Figure 6). *PIK3CA* gene amplification is associated with advanced stage of tumor and overall poor disease-free survival (69). Other genes which have targeted drugs include *NOTCH1, NF1, FBXW7, PRKAA1, MYC, ATR* (Supplementary Table 9). Potential druggable targets include *USP13, LAMP3, BRD9, TNK2, PAK2*, and *PRKDC* which are overexpressed in ESCC based on RNA-Seq datasets from TCGA (Supplementary Figure 11) (26).

## Discussion

Esophageal squamous cell carcinoma is one of the deadliest cancers. Higher incidence of ESCC is observed in Asian countries including China, Japan and India. In Western countries, esophageal adenocarcinoma is more common. The reason for this difference is not well-understood, however, its complex etiology is frequently associated with environmental factors and food habits. Tobacco usage is a known risk factor for ESCC. It is predominantly consumed in the form of cigarette smoking in most countries. In India, it is consumed in different forms, such as gutka and betel nut quid which is a form of smokeless tobacco and cigarettes with/without filter. Epidemiological studies by Dar NA et al. in Kashmir, India and Nasrollahzadeh et al. in Iran have reported association of smokeless tobacco usage with increased risk of ESCC with an odds ratio of 2.88 and 2.91, respectively (70, 71). Multiple Chinese and Japanese studies have investigated genomic alterations in ESCC from patients with a history of cigarette smoking. However, a detailed investigation in an Indian cohort where tobacco is consumed in both smoking and chewing form has not been done.

In this study, we investigated mutational landscape of ESCC from an Indian cohort which included tobacco smokers, chewers and those with no history of tobacco usage. We identified several known and novel genomic alterations in ESCC. The observed non-synonymous mutation load (2.28 mutations per Mb) was comparable to reported mutation load of 1.9–3 mutations per Mb (12–14, 37, 64). We identified several frequently mutated genes, such as *TP53, PIK3CA, NOTCH1, FAT1, CUL3*, and *SLIT2* that have been previously reported in ESCC. Mutation and copy number alterations in *PIK3CA* are observed at a frequency of 4–45% in published studies. In our study, we observed *PIK3CA* alteration in 53% of the samples (64, 72). We observed mutually exclusive mutation patterns among samples with *PIK3CA* and *NOTCH1* variants as previously reported by Song et al. Knockdown of *NOTCH1* is known to enhance proliferation rate but not migration and invasive ability of KYSE150 and KYSE140 cells (47). We speculate that the concomitant presence of *PIK3CA* and *NOTCH1* mutation might result in synthetic lethality. However, functional validation of this hypothesis is warranted. We observed several genes that were recurrently mutated in patients with history of tobacco usage. This includes *USP8, FAT1, SLIT2, TMEM132C, NF1, ARID4A, CUL3*, and *HEPACAM2*. *FAT1* was mutated in 3 samples from patients with history of chewing tobacco. A previous study has shown that *FAT1* knockdown upregulates MAPK/ERK pathway and promotes epithelial-mesenchymal transition, a hallmark of cancer (73). *CUL3* was mutated in two samples with a history of tobacco consumption. It is known to have tumor suppressive property in ESCC and not in EAC (74). Knockdown of *CUL3*, a core member of BTB-CUL3-RBX1 E3 ubiquitin-protein ligase complex increased the proliferative capacity, migration and invasiveness in ESCC cell lines (38). *SLIT2* was mutated in 3 smoker samples and its lower expression is known to promote metastasis by activation of srGAP-Cdc42 pathway and is significantly correlated with poor overall survival and disease-free survival in ESCC patients (75). We did not identify mutations in genes *AJUBA* and *ZNF750* in our cohort which are reported to be commonly mutated in Chinese cohorts (50, 76).

We evaluated genomic signatures associated with ESCC based on the history of tobacco consumption. High frequency of C:G > T:A transition was observed in ESCC regardless of tobacco usage history. This transition is a result of oxidative damage to cytosine residues in DNA. Oxidatively modified cytosines are deaminated by cytidine deaminases leading to C > T change (77). We also observed 2-fold enrichment of signature 4 which is a known mutation signature associated with tobacco exposure in chewer cohort compared to smoker and non-user cohort. We did not observe enrichment of smoking associated signature in smokers cohort and non-user cohort as previously reported in the Chinese cohort (50). Overall, our findings suggest that exposure to chewing tobacco may damage DNA and increase the risk of ESCC.

Copy number alteration analysis showed genomic regions affected across different cohorts. Many amplified regions contain key driver genes, such as *PIK3CA, SOX2* in 3q26.32-33, *MYC* in 8q24.21, *FGF3/FGF4/FGF19* in 11q13.3, and *FGF12* in 3q28. These could be potentially associated with disease progression in ESCC. Combining mutations and copy number alterations, *PIK3CA* was affected in 53% of samples. *PIK3CA* hotspot mutations were not observed in chewer cohort (Supplementary Figure 12A). *PIK3CA* is overexpressed in ESCC compared to adenocarcinoma or normal esophagus (Supplementary Figure 12B) in TCGA-ESCA dataset (78). It is highly expressed in esophageal cancer in Asians compared to Caucasians and African-Americans (Supplementary Figure 12C). Comparing expression levels across TCGA datasets revealed that *PIK3CA* is relatively highly expressed in squamous cell carcinomas compared to adenocarcinomas across various cancer types (Supplementary Figure 12D). We also predicted potentially druggable targets among genes that were frequently mutated or amplified in ESCC using FDA approved anti-neoplastic drugs database DGIdb. For example, *USP13* (Ubiquitin specific peptidase 13) is a deubiquitinase enzyme which plays an important role in autophagy and endoplasmic reticulum-associated degradation. *USP13* is co-amplified with *PIK3CA* in high-grade serous ovarian cancer. Knockdown of *USP13* sensitized ovarian xenograft tumors to pan-AKT inhibitor MK-2206 (79). In a separate study, it was shown that inhibition of USP13 also sensitizes the tumor cells to BH3 mimetic inhibitor, ABT-263 (80). Thus, *USP13* is a potential therapeutic candidate in ESCC. *TNK2*, also known as *ACK1* is a non-receptor tyrosine kinase and amplification of *TNK2* is widely reported in multiple cancers (81–83). Knockdown of *TNK2* in xenograft model reduced the aggressiveness of triple negative breast cancer which further demonstrates the significance of developing an effective targeted therapy against TNK2 (84). Our study provides significant insights into molecular alterations in ESCC and reveals potential candidates for therapeutic targeting.

## Conclusions

ESCC is one of the prevalent cancers in India. Although genomic alterations associated with ESCC were previously reported in Chinese and Japanese cohorts, no data was available from Indian cohorts. Our study provides the first report of mutational landscape of ESCC in an Indian cohort. Tobacco usage is a well-known risk factor of ESCC. By carrying out exome sequencing of ESCC samples from cigarette smokers, tobacco chewers and non-users, we show that mutation signature 4 is enriched in patients with a history of tobacco chewing. We did not observe distinct signatures associated with ESCC from patients with a history of smoking. Unlike what is reported in lung cancers (85), high copy number alterations were observed in patients with no history of tobacco usage in our cohort. In addition to mutated genes known in ESCC, we identified several novel genes that have not been reported before. Alterations in the PIK3CA pathway were observed in half of the ESCC samples from Indian cohort. This was independent of the tobacco consumption history of the patients. Mutations in *PIK3CA* and *NOTCH1* were mutually exclusive potentially due to synthetic lethality. Inhibition of these pathways could be explored as a targeted therapeutic strategy to treat ESCC. We did not find specific gene targets based on the history of tobacco usage. Due to low sample numbers, we are not sufficiently powered to identify such gene targets. Further studies are warranted with larger sample size to validate these findings. We predicted potential neoantigens and novel drug targets that can be explored in ESCC.

## Data Availability Statement

The datasets generated for this study can be found in the Sequence Read Archive hosted by National Center for Biotechnology Information Search database (NCBI) with accession number PRJNA507919.

## Ethics Statement

The studies involving human participants were reviewed and approved by Kidwai Memorial Institute of Oncology, Bangalore. The patients/participants provided their written informed consent to participate in this study.

## Author Contributions

HG conceived and designed this study. KM and AK were involved in the sample collection. CK and SM were involved in DNA extraction, quality control, and next generation sequencing. KM, KP, MM, RaG, RoG, and AK-G were involved in the genomic data analyses and interpretation. KM and KP prepared the manuscript and manuscript figures. AChau, PK, BN, RK, AK-G, TP, AChat, AP, and HG edited, critically read, and revised the manuscript. All authors have read and approved the final manuscript.

## Conflict of Interest

MM, CK, SM, RaG, RoG, AK-G, and AChau were employed by the company Medgenome Labs Pvt. Ltd. The remaining authors declare that the research was conducted in the absence of any commercial or financial relationships that could be construed as a potential conflict of interest.

## Supplementary Material

Supplementary Figure 1 Gene ontology enrichment analysis of frequently mutated genes using FunRich keeping default human as background database.

Supplementary Figure 2 Kaplan–Meier curve for overall survival as a function of *SETD1A* expression using PROGgeneV2 platform [TCGA-Esophageal Carcinoma (ESCA) dataset].

Supplementary Figure 3 Schematic representation of gene pairs *PIK3CA* and *NOTCH1, MUC16* and *FAT1, DNAH5* and *ZFHX4, ZFHX4* and *NOTCH1* with mutually exclusive genomic alteration pattern derived using publicly available TCGA-Esophageal Carcinoma (ESCA) datasets hosted on cBioPortal and NCI GDC Data platform. Column represents examined samples and row represents genes.

Supplementary Figure 4 Schematic representation of mutual exclusivity of mutant *PIK3CA* and *NOTCH1* in published ESCC studies. **(A)** Zhang et al. (50), **(B)** Sawada et al. (14), **(C)** Gao et al. (37), and **(D)** Qin et al. (51). Blue circles indicate the presence of mutation in respective tumor sample and gray circle indicates presence of WT allele. Column represents examined samples and row represents genes.

Supplementary Figure 5 Schematic representation of mutual exclusivity of mutant *MUC16* and *FAT1* in published ESCC studies. **(A)** Zhang et al. (50), **(B)** Sawada et al. (14), **(C)** Gao et al. (37), and **(D)** Qin et al. (51). Blue circles indicate the presence of mutation in respective tumor sample and gray circle indicates presence of WT allele. Column represents examined samples and row represents genes.

Supplementary Figure 6 Schematic representation of mutual exclusivity of *DNAH5* and *ZFHX4* in published ESCC studies. **(A)** Zhang et al. (50), **(B)** Sawada et al. (14), **(C)** Gao et al. (37), and **(D)** Qin et al. (51). Blue circles indicate the presence of mutation in respective tumor sample and gray circle indicates presence of WT allele. Column represents examined samples and row represents genes.

Supplementary Figure 7 Schematic representation of mutual exclusivity of *NOTCH1* and *ZFHX4* in published ESCC studies. **(A)** Zhang et al. (50), **(B)** Sawada et al. (14), **(C)** Gao et al. (37), and **(D)** Qin et al. (51). Blue circles indicate the presence of mutation in respective tumor sample and gray circle indicates presence of WT allele. Column represents examined samples and row represents genes.

Supplementary Figure 8 Mutation signatures deduced by linear decomposition method using Mutalisk in each ESCC sample.

Supplementary Figure 9 Landscape of genomic amplifications and deletions in each esophageal squamous cell carcinoma sample investigated in the study. Green one-point-outlier, Dark gray surroundings: frequent one-point-outlier, Brown >1 level gain, Brown surroundings: 1-level gain, Blue: >1 level loss, Blue surroundings: 1-level loss.

Supplementary Figure 10 **(A)** Relative expression level of *CLSTN2* in normal esophagus, esophageal adenocarcinoma and esophageal squamous cell carcinoma. **(B)** Kaplan-Meier survival curve as a function of *CLSTN2* expression generated based on TCGA-Esophageal Carcinoma (ESCA) dataset using UALCAN platform.

Supplementary Figure 11 Relative expression level of **(A)** *USP13*, **(B)** *LAMP3*, **(C)** *BRD9*, **(D)** *TNK2*, **(E)** *PAK2*, **(F)** *PRKDC* in normal esophagus and ESCC (TCGA-Esophageal Carcinoma dataset) derived from UALCAN platform.

Supplementary Figure 12 *PIK3CA* is a recurrently altered and overexpressed gene in esophageal squamous cell carcinoma. **(A)** Schematic representation of ESCC samples with somatic variants or copy number alteration in *PIK3CA*. **(B)** *PIK3CA* expression levels in normal esophageal tissues, esophageal adenocarcinoma and esophageal squamous cell carcinoma based on UALCAN analysis (^***^*p*-value ≤ 0.001). **(C)** *PIK3CA* expression level in ESCC tumors from Asian, African-American and Caucasian cohorts based on UALCAN analysis (^***^*p*-value ≤ 0.001). **(D)** Pan-cancer expression levels of *PIK3CA* across different squamous cell carcinomas and adenocarcinomas compared to adjacent normal tissue samples. BLCA, Bladder Urothelial Carcinoma; BRCA, Breast Invasive Carcinoma; CESC, Cervical Squamous Cell Carcinoma; CHOL, Cholangiocarcinoma; ESCA, Esophageal Carcinoma; GBM, Glioblastoma Multiforme; HNSC, Head and Neck Squamous cell Carcinoma; KICH, Kidney Chromophobe; KIRC, Kidney Real Clear Cell Carcinoma; KIRP, Kidney Renal Papillary Cell Carcinoma; LIHC, Liver Hepatocellular Carcinoma; LUAD, Lung Adenocarcinoma; LUSC, Lung Squamous Cell Carcinoma; PAAD, Pancreatic Adenocarcinoma; PRAD, Prostate Adenocarcinoma; PCPG, Pheochromocytoma and Paraganglioma; READ, Rectum Adenocarcinoma; SARC, Sarcoma; SKCM, Skin Cutaneous Melanoma; THCA, Thyroid Carcinoma; THYM, Thymoma; STAD, Stomach Adenocarcinoma; UCEC, Uterine Corpus Endometrial Carcinoma.

Supplementary Table 1 Clinical characteristics of patients with ESCC that were subjected to whole exome sequencing.

Supplementary Table 2 Read statistics and QC details of whole exome sequencing for each sample.

Supplementary Table 3 List of different types of variants identified in ESCC samples.

Supplementary Table 4 List of somatic single nucleotide variants identified using whole exome sequencing of ESCC patients.

Supplementary Table 5 Mutation load in esophageal squamous cell carcinoma (ESCC), oral squamous cell carcinoma (OSCC), lung squamous cell carcinoma (LUSC), and Lung adenocarcinoma (LUAD).

Supplementary Table 6 List of neoantigens predicted using TSNAD.

Supplementary Table 7 **(A)** List of copy number alterations and affected genes in ESCC patients. **(B)** List of cytobands and number of genes affected by Copy number alterations (CNA).

Supplementary Table 8 List of genes screened for potential druggability using DGIdb resource.

Supplementary Table 9 List of identified genes with an available FDA-approved anti-neoplastic drug using DGIdb resource.

## Abbreviations

BAM: binary alignment map
BWA: Burrows-Wheeler Aligner
CAN: copy number alterations
EAC: esophageal adenocarcinoma
ESCA: esophageal carcinoma
ESCC: esophageal squamous cell carcinoma
FDR: false discovery rate
HLA: human leukocyte antigen
HNSCC: head and neck squamous cell carcinoma (HNSCC)
LUAD: lung adenocarcinoma
LUSC: lung squamous cell carcinoma
MEM: maximal exact matches
MHC: major histocompatibility complex
SNV: somatic single nucleotide variants
TSNAD: tumor-specific neoantigen detector
WES: whole exome sequence
WGS: whole genome sequence.
