# Supplementary material for: Mutational Landscape of Esophageal Squamous Cell Carcinoma in an Indian Cohort
Source: Front Oncol. 2020 Aug 20;10:1457. doi: 10.3389/fonc.2020.01457 (PMC7469928; doi:10.3389/fonc.2020.01457)

Supplementary Figure 1

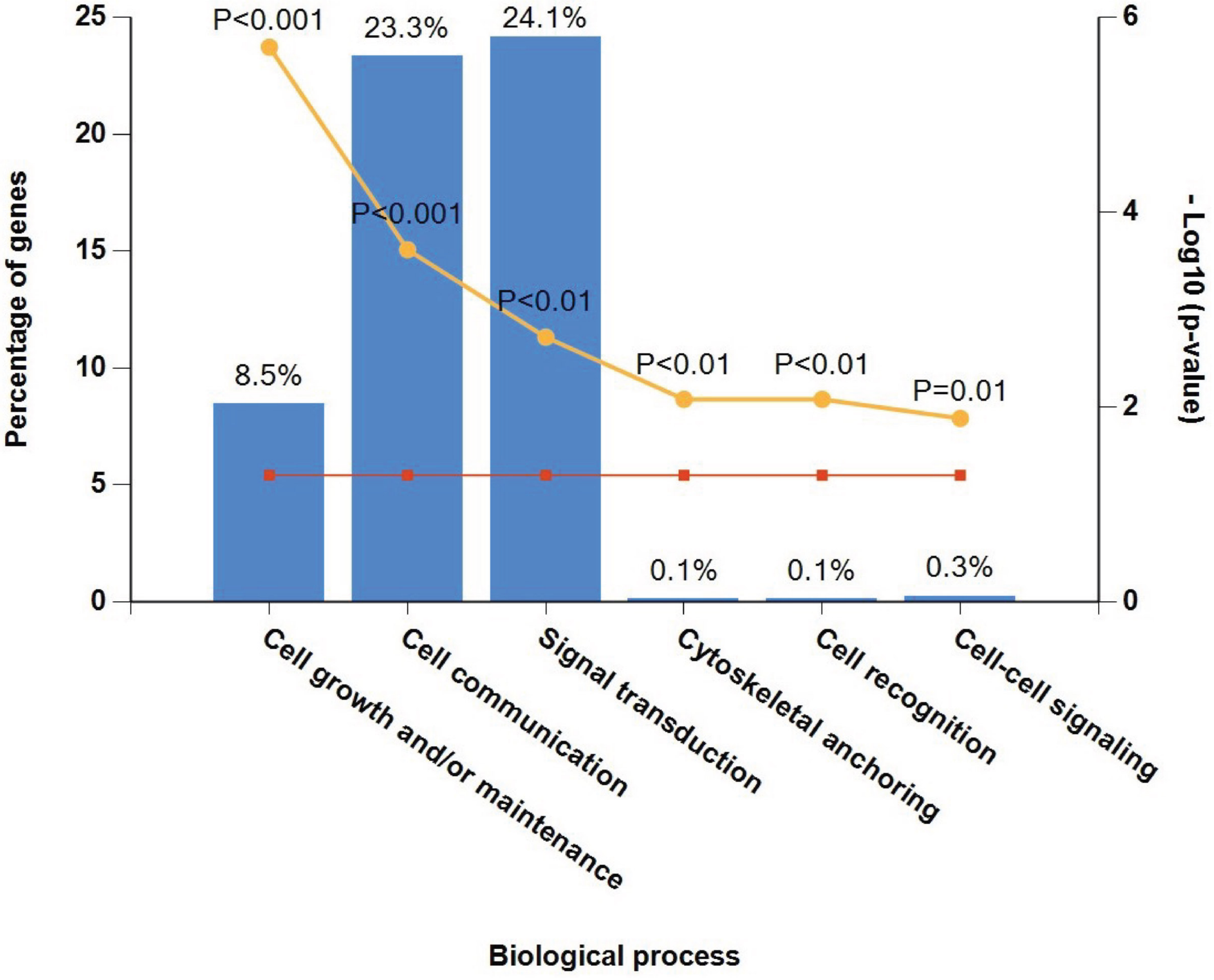

Supplementary figure 2

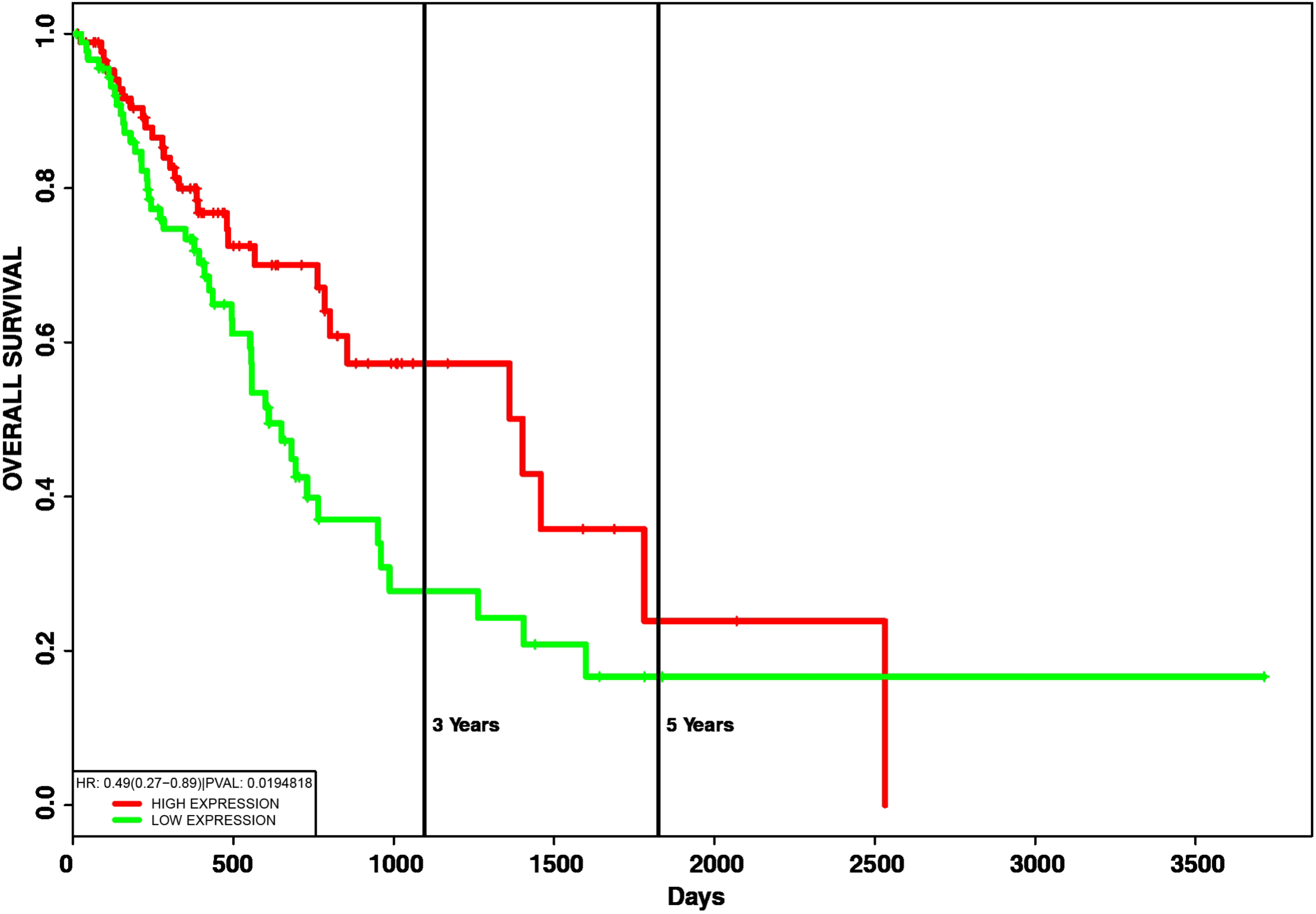

Supplementary figure 3

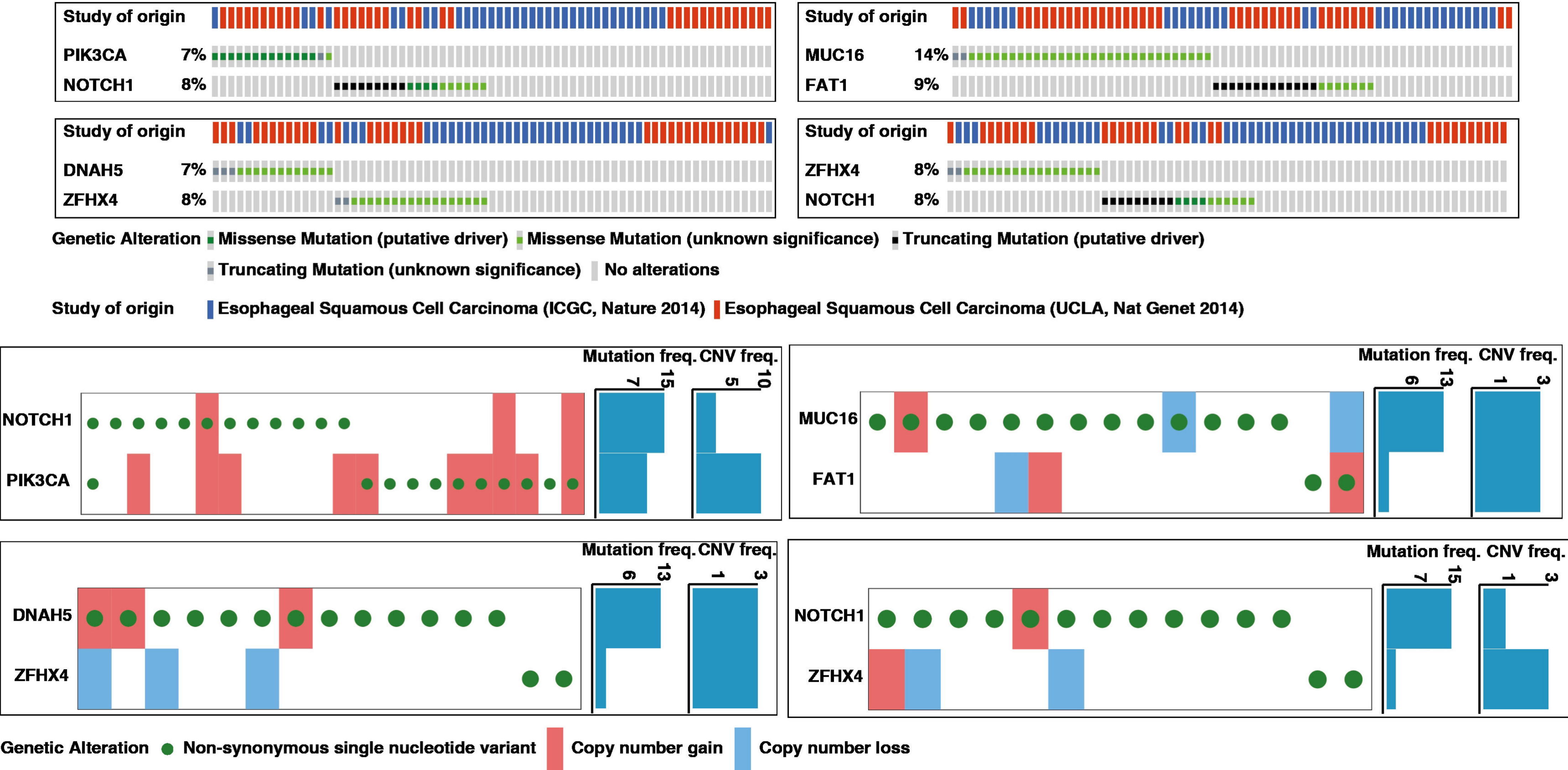

Supplementary figure 4

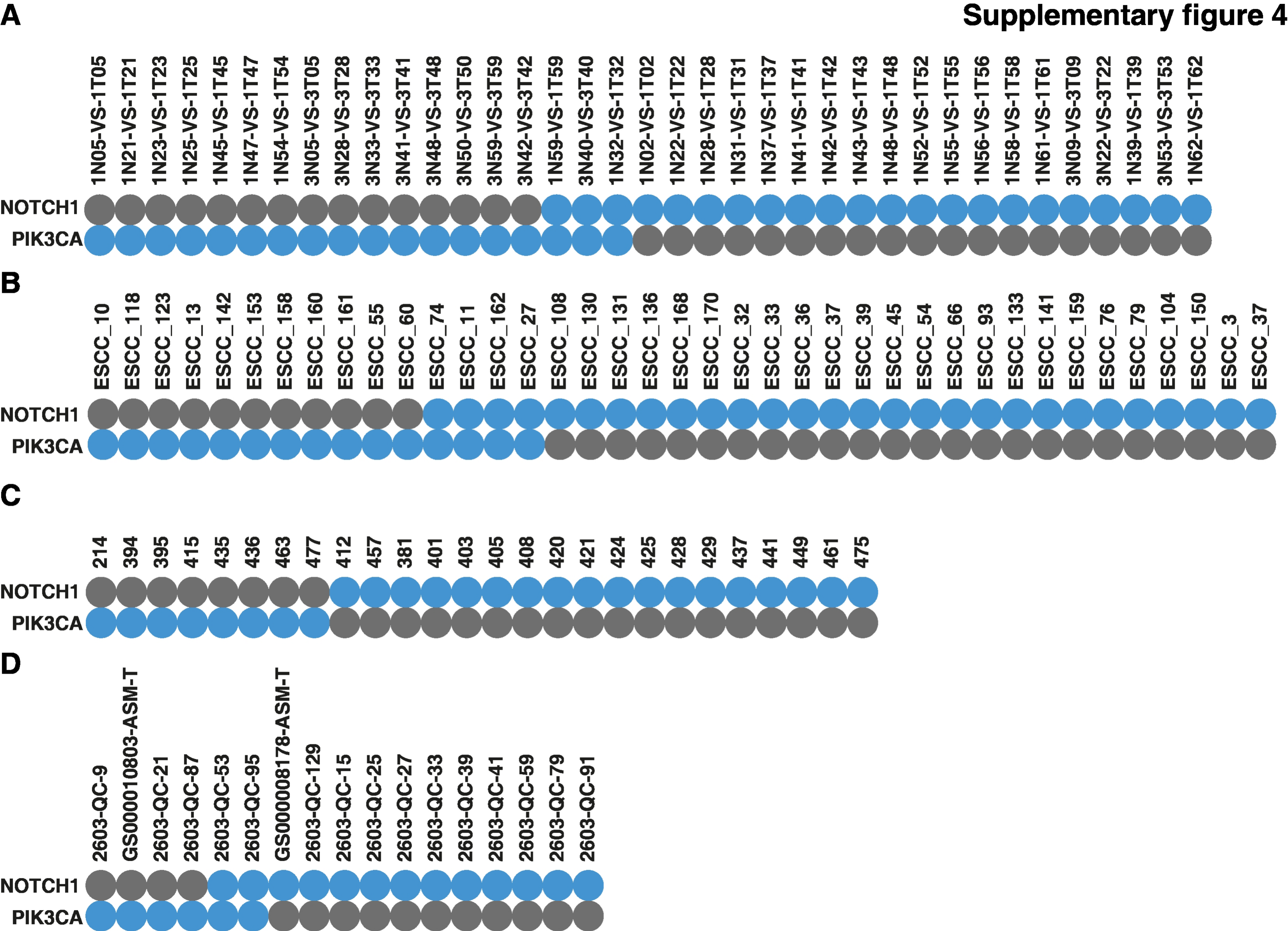

Supplementary figure 5

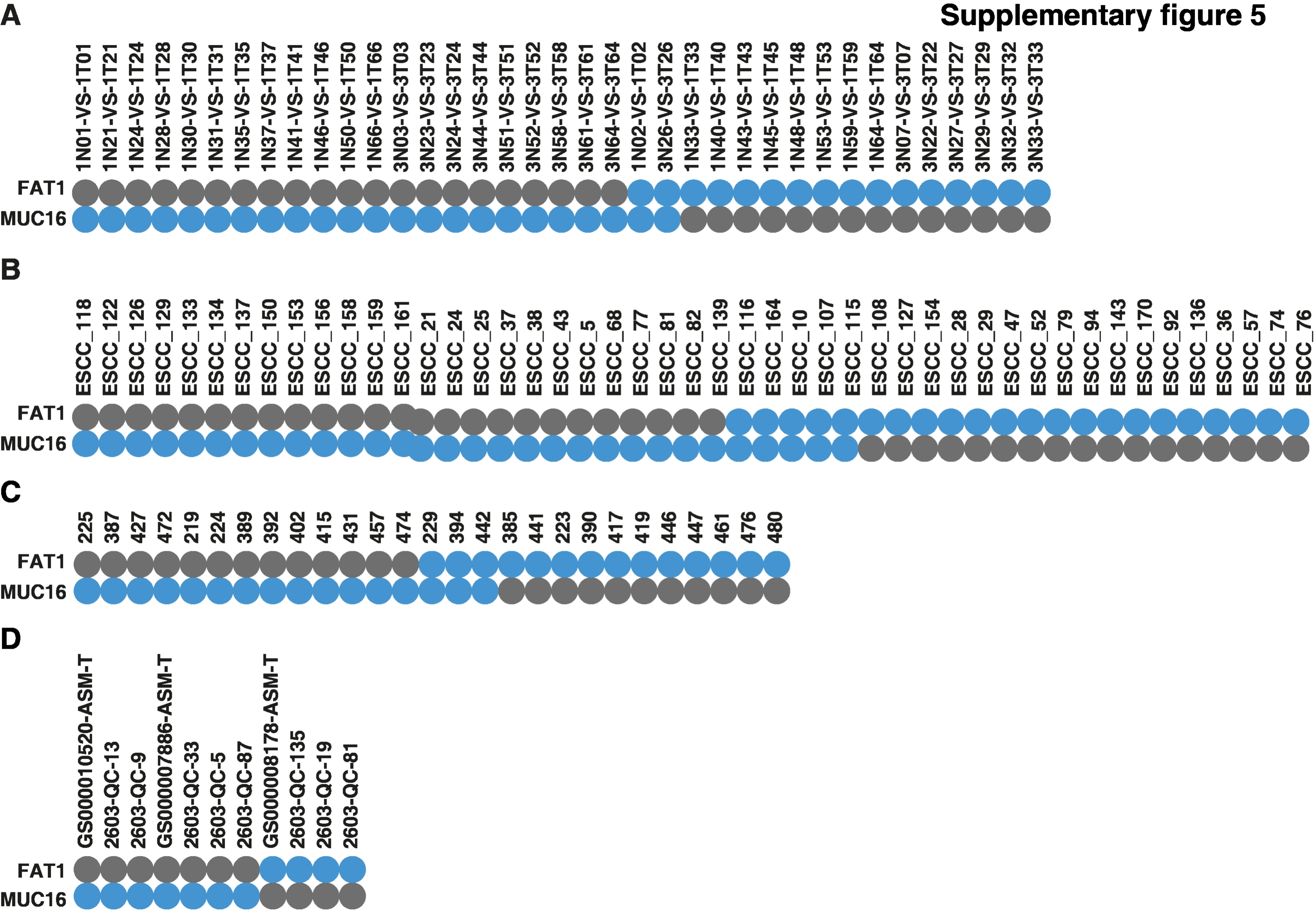

Supplementary figure 6

A

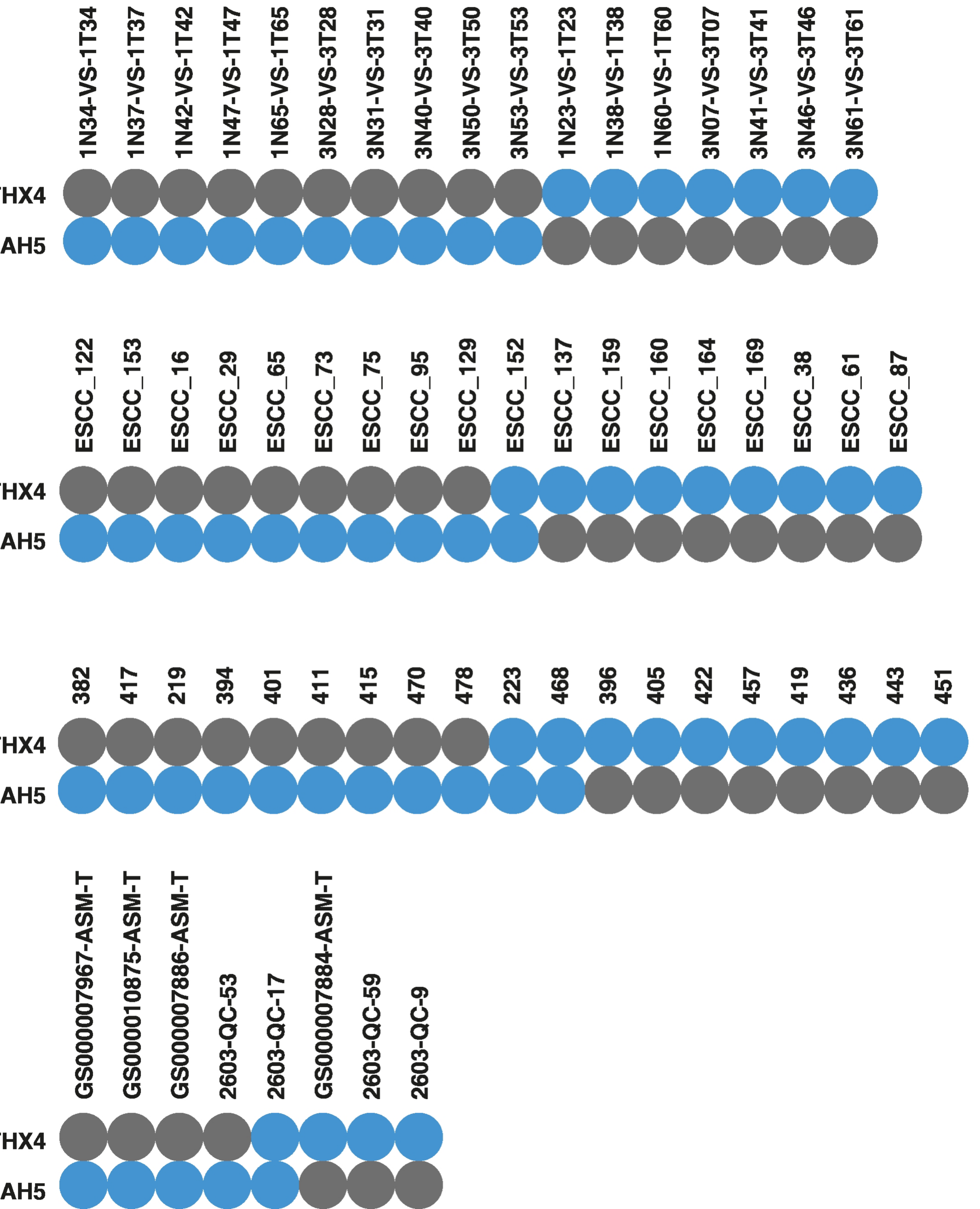

Supplementary figure 7

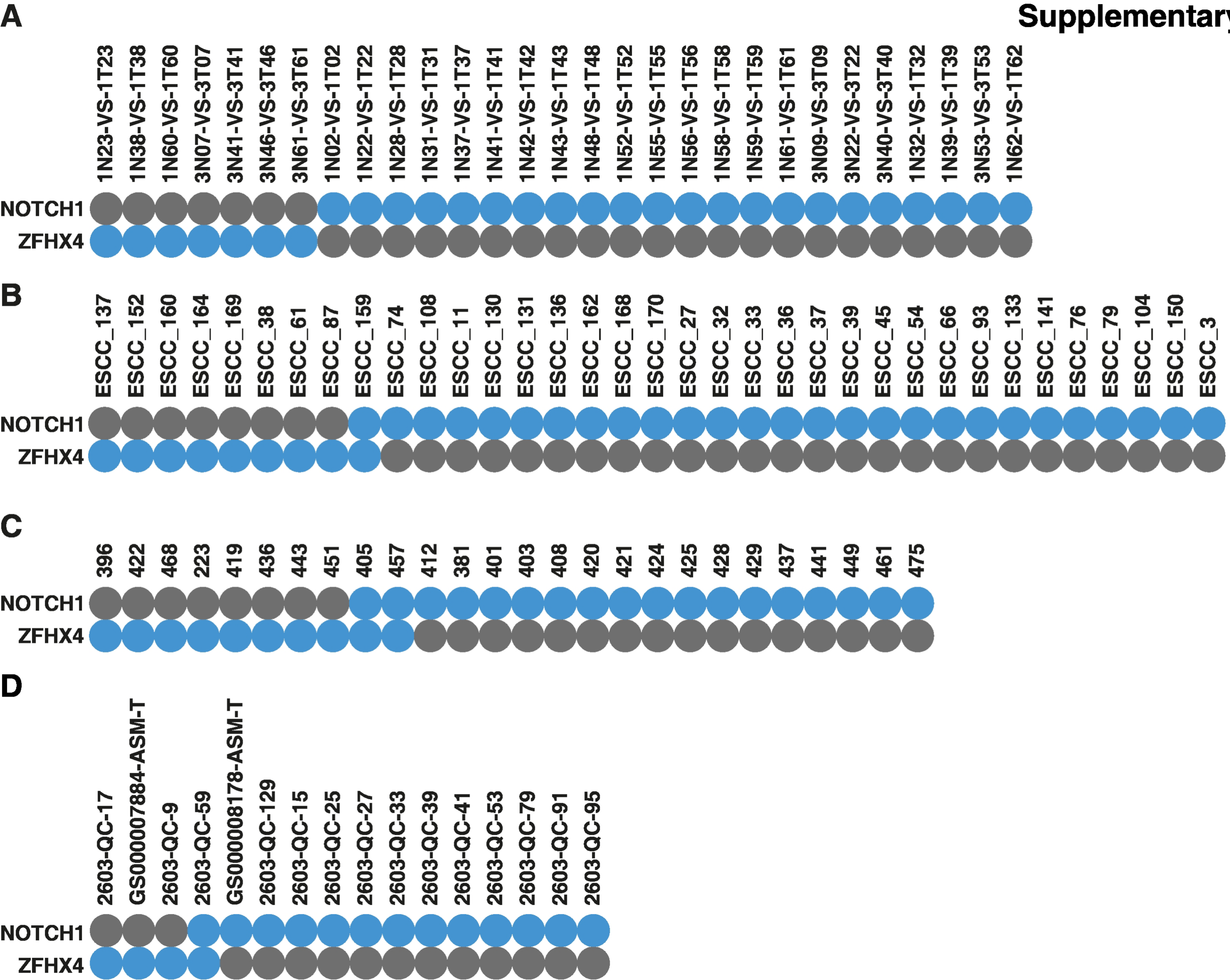

Supplementary figure 8

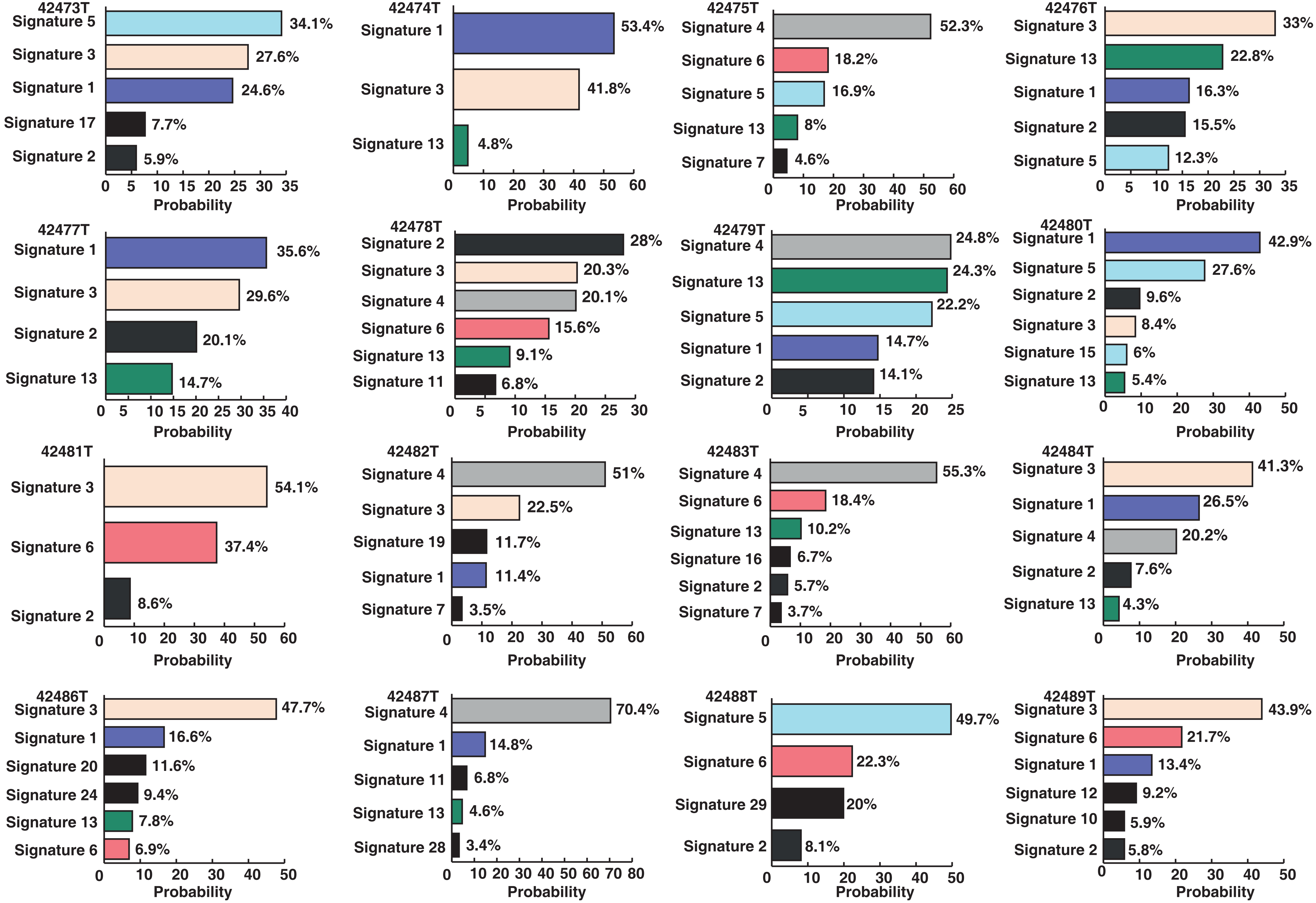

Supplementary figure 8

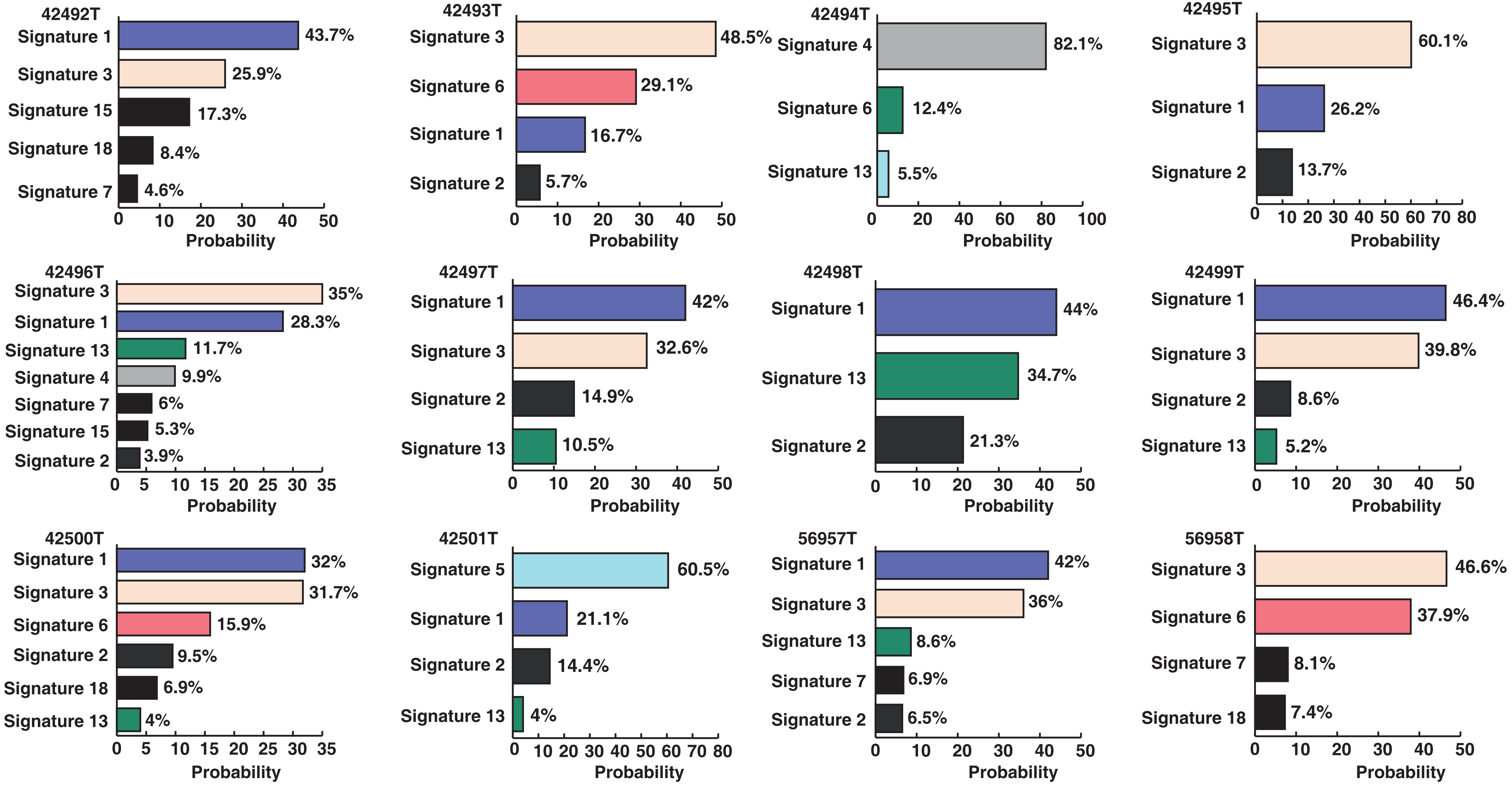

Supplementary figure 9

A

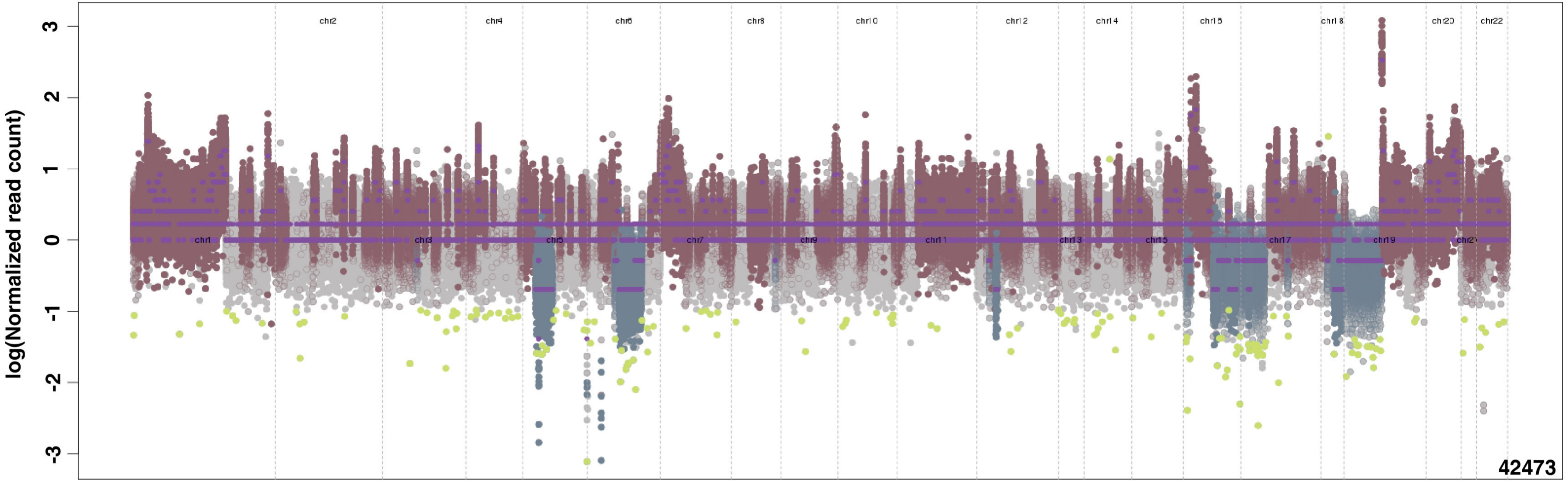

B

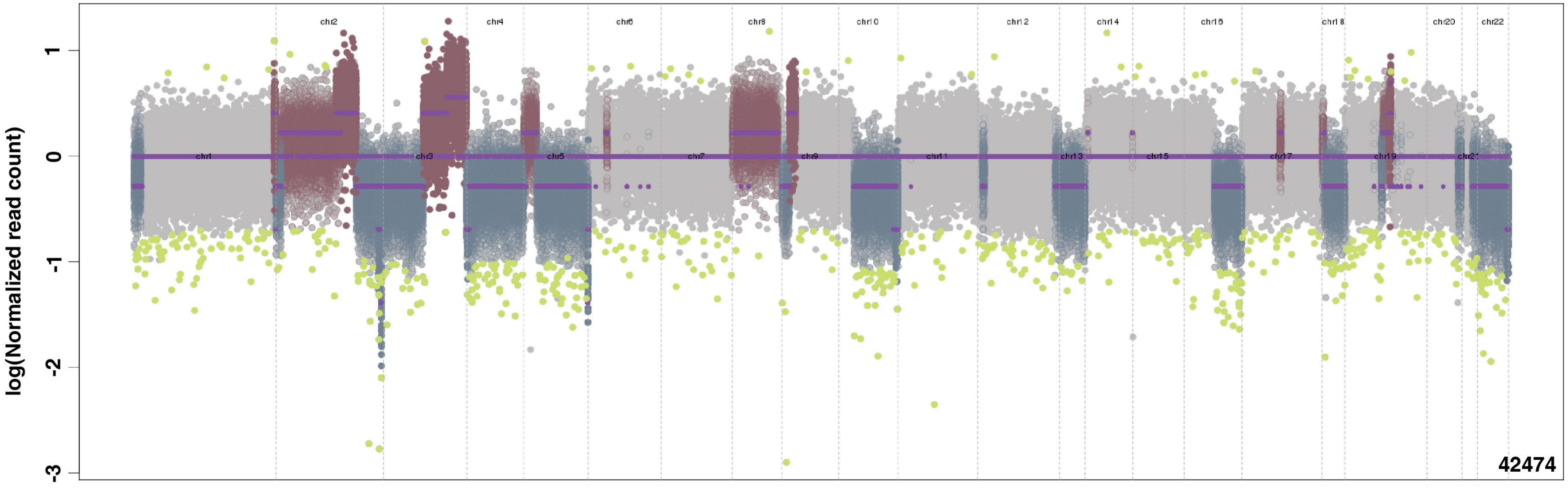

Supplementary figure 9

C

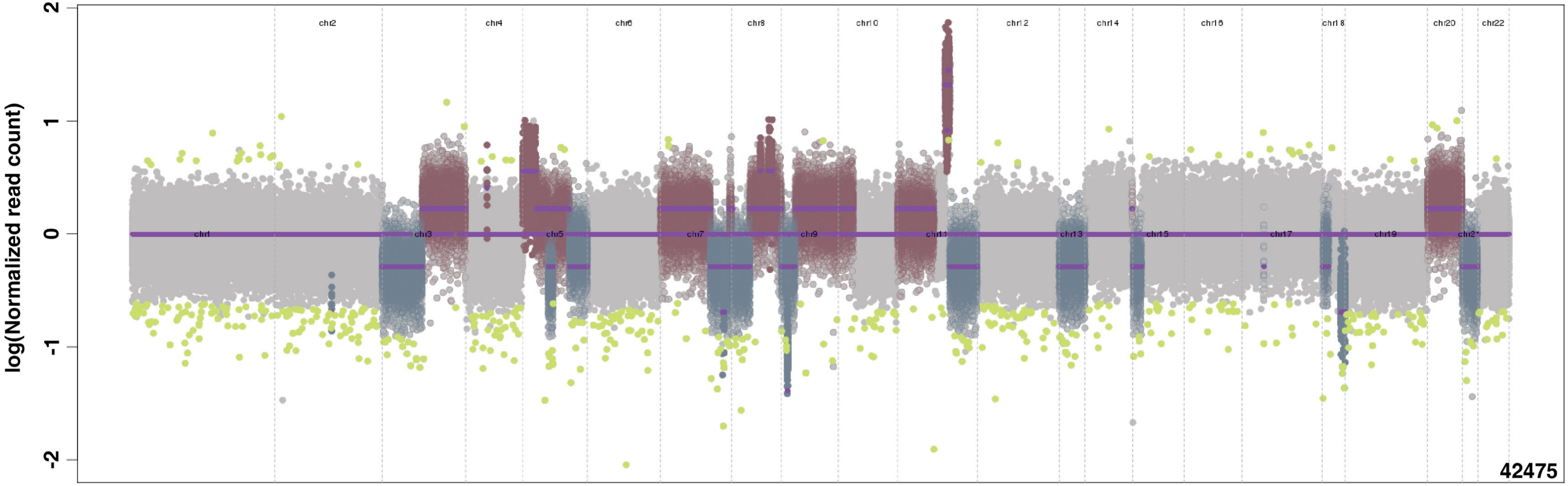

D

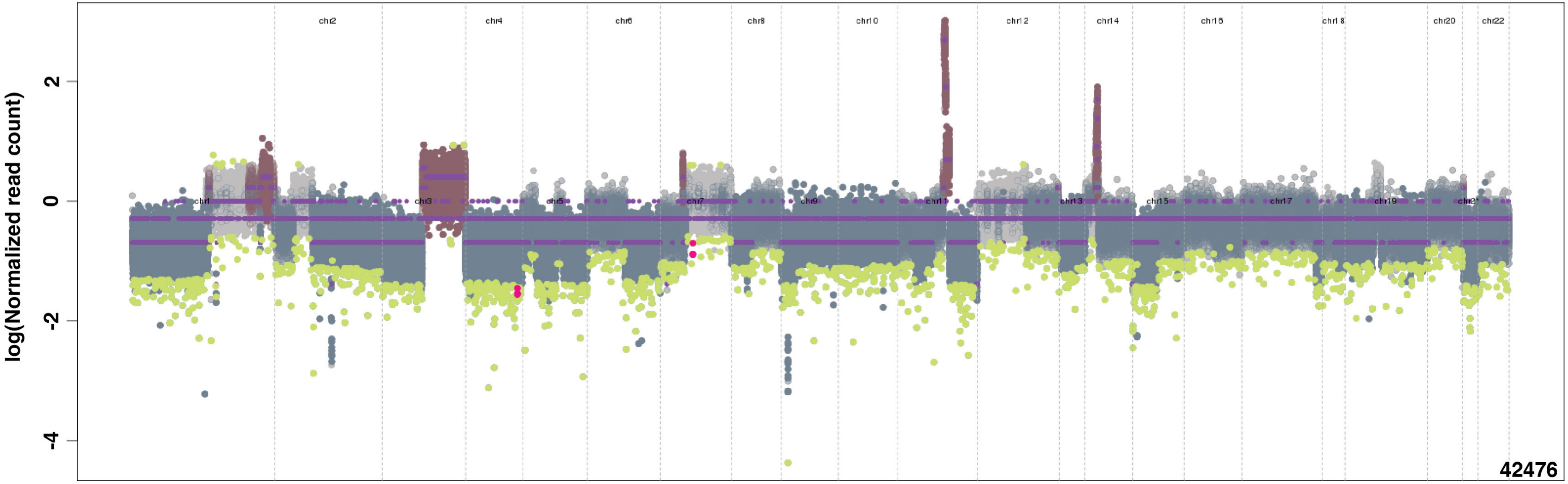

Supplementary figure 9

E

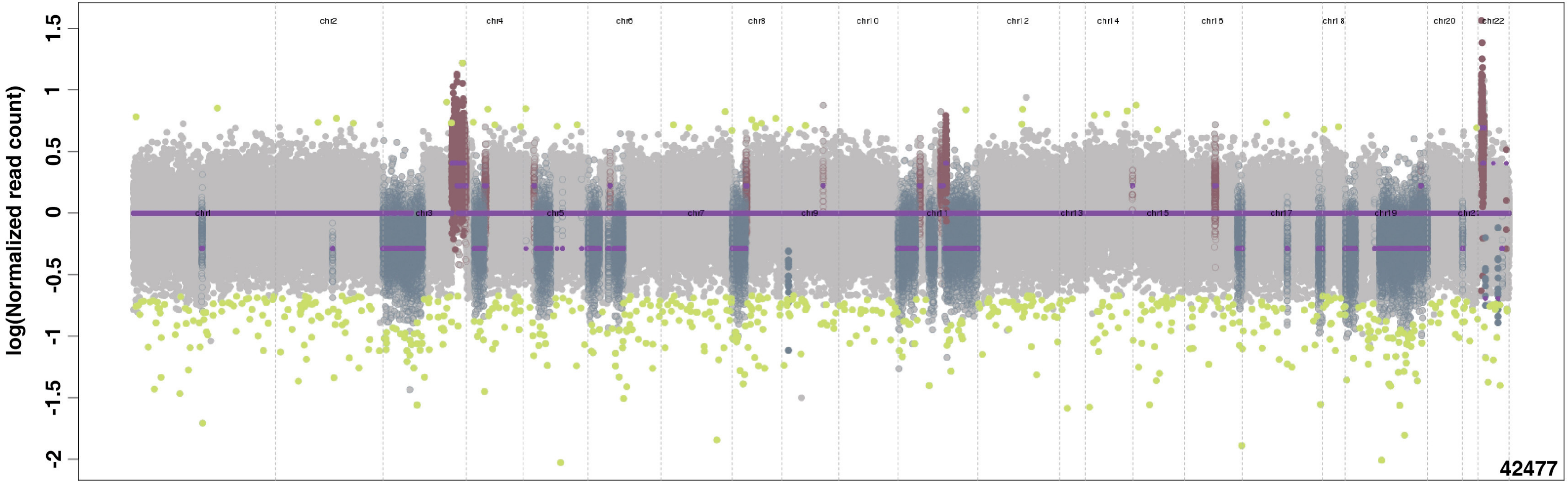

F

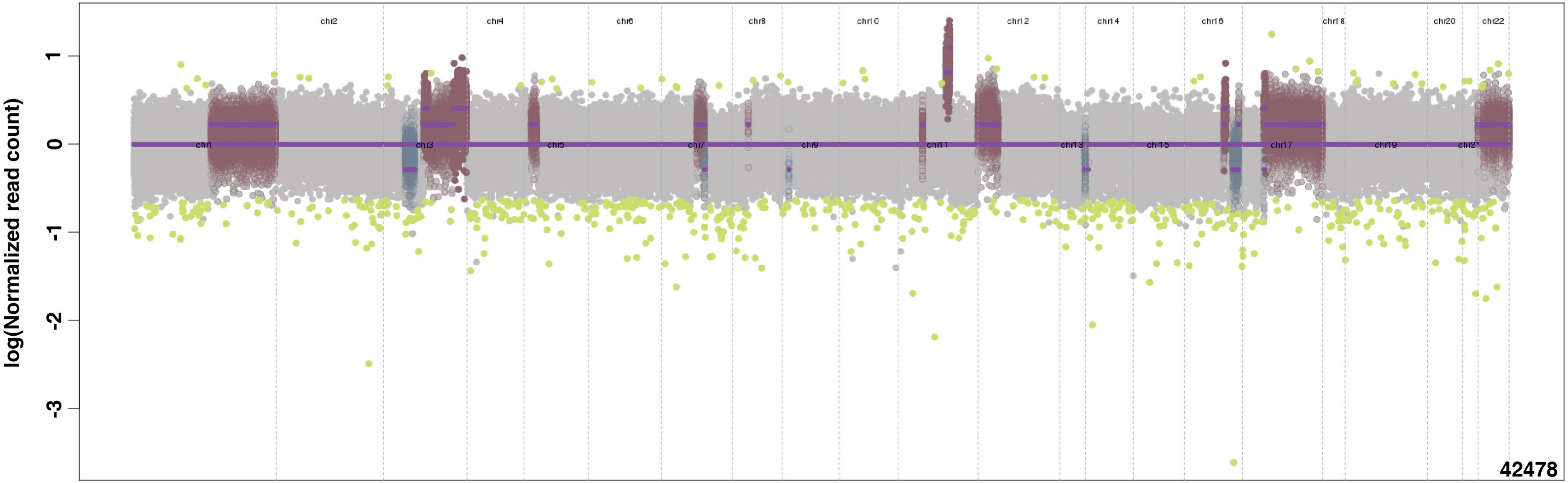

G

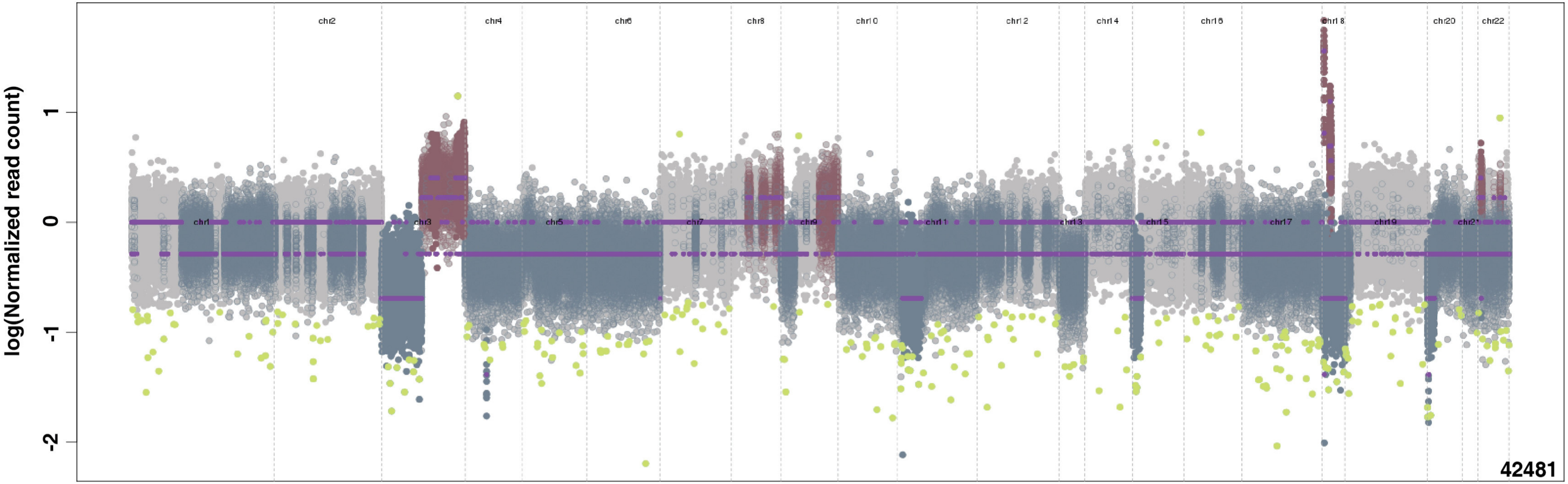
log(Normalized read count)
1
0
-1
-2
chr1
chr2
chr3
chr4
chr5
chr6
chr7
chr8
chr9
chr10
chr11
chr12
chr13
chr14
chr15
chr16
chr17
chr18
chr19
chr20
chr21
chr22
42481

H

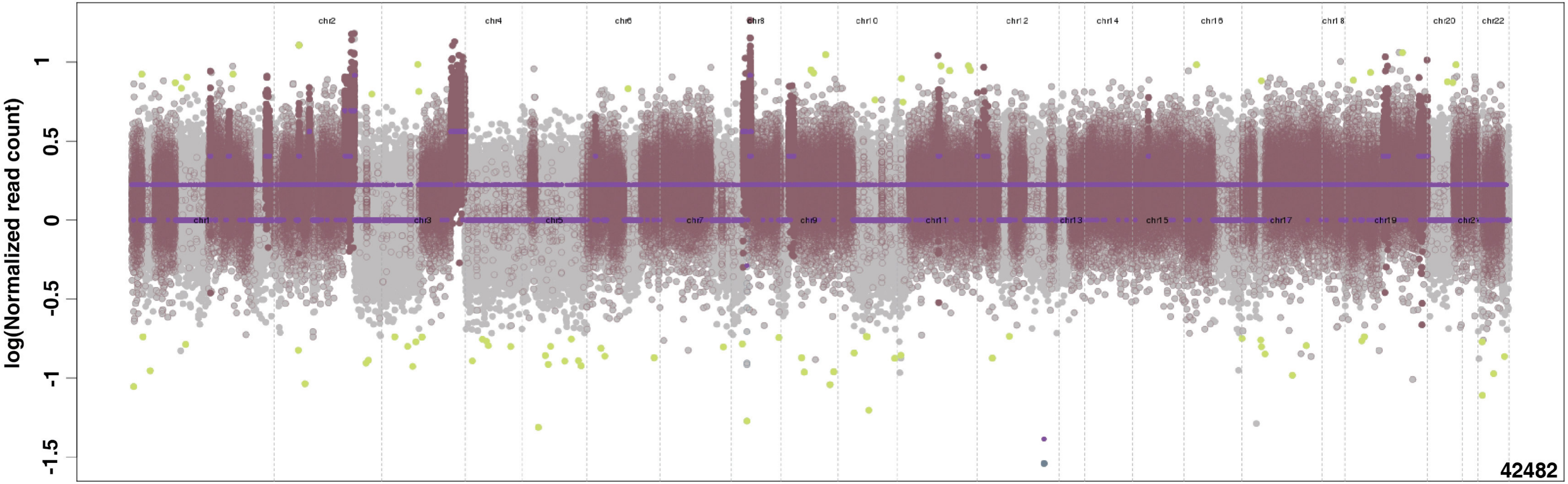
log(Normalized read count)
1
0.5
0
-0.5
-1
-1.5
chr1
chr2
chr3
chr4
chr5
chr6
chr7
chr8
chr9
chr10
chr11
chr12
chr13
chr14
chr15
chr16
chr17
chr18
chr19
chr20
chr21
chr22
42482

Supplementary figure 9

I

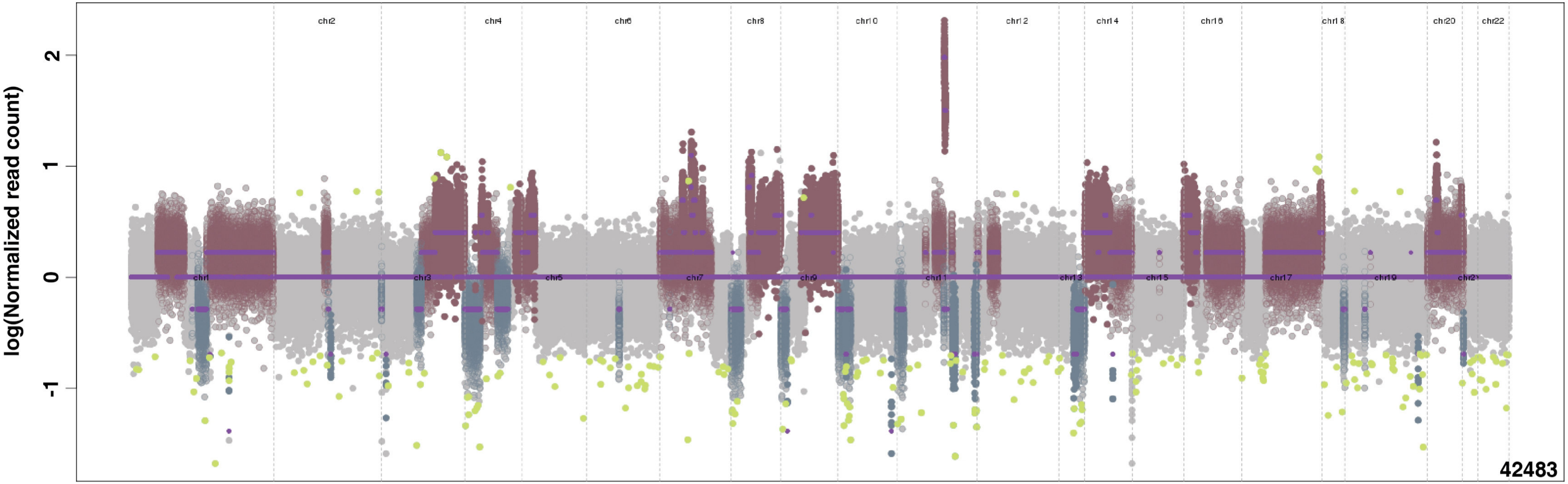

J

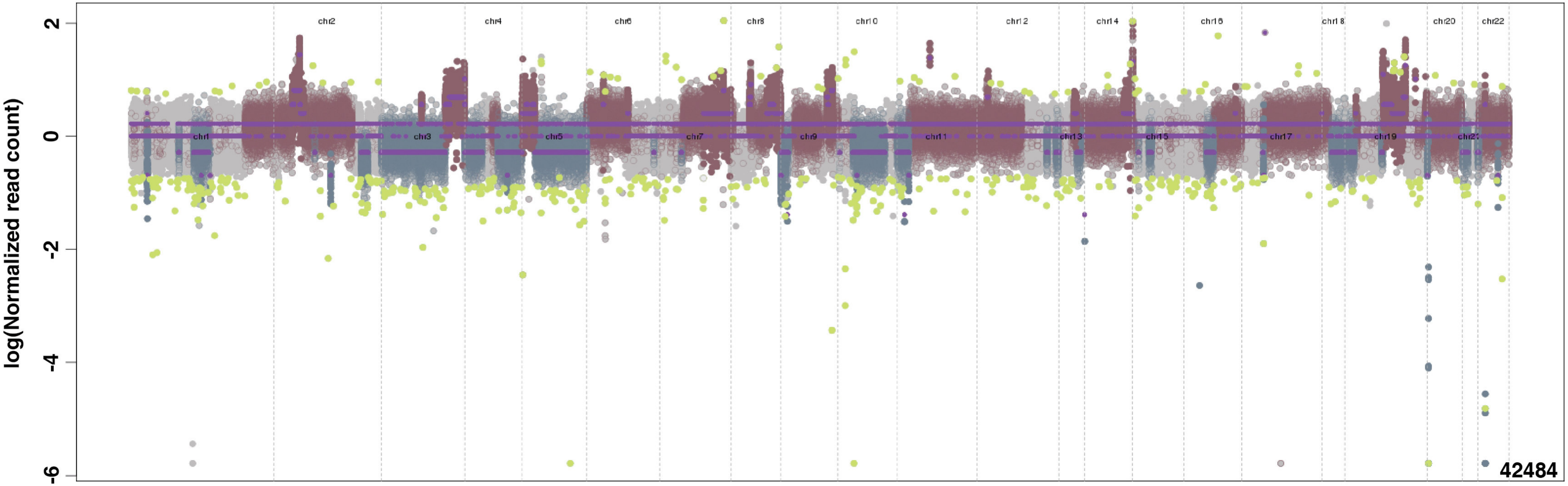

K

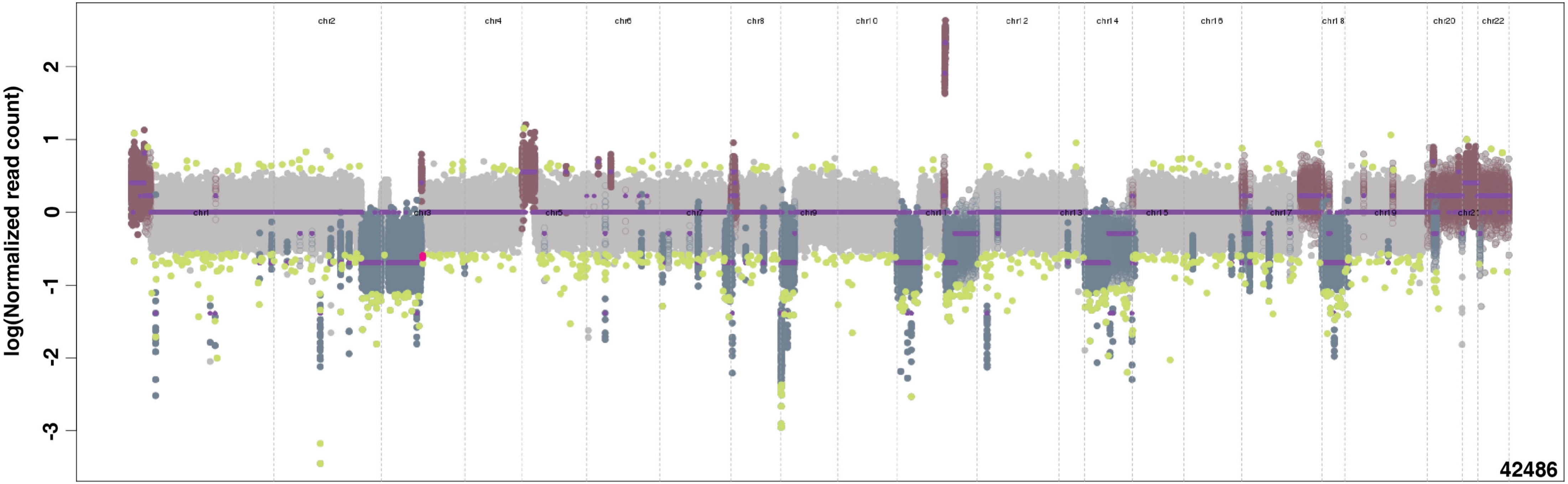
log(Normalized read count)
2
1
0
-1
-2
-3
chr1
chr2
chr3
chr4
chr5
chr6
chr7
chr8
chr9
chr10
chr11
chr12
chr13
chr14
chr15
chr16
chr17
chr18
chr19
chr20
chr21
chr22
42486

L

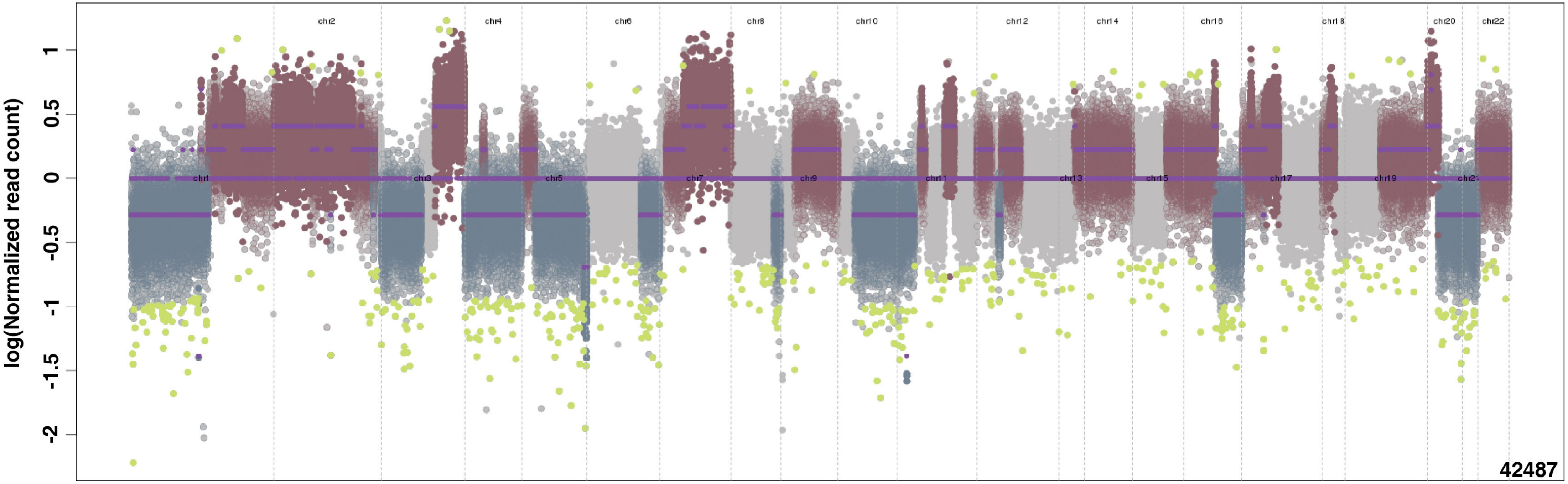
log(Normalized read count)
1
0.5
0
-0.5
-1
-1.5
-2
chr1
chr2
chr3
chr4
chr5
chr6
chr7
chr8
chr9
chr10
chr11
chr12
chr13
chr14
chr15
chr16
chr17
chr18
chr19
chr20
chr21
chr22
42487

Supplementary figure 9

M

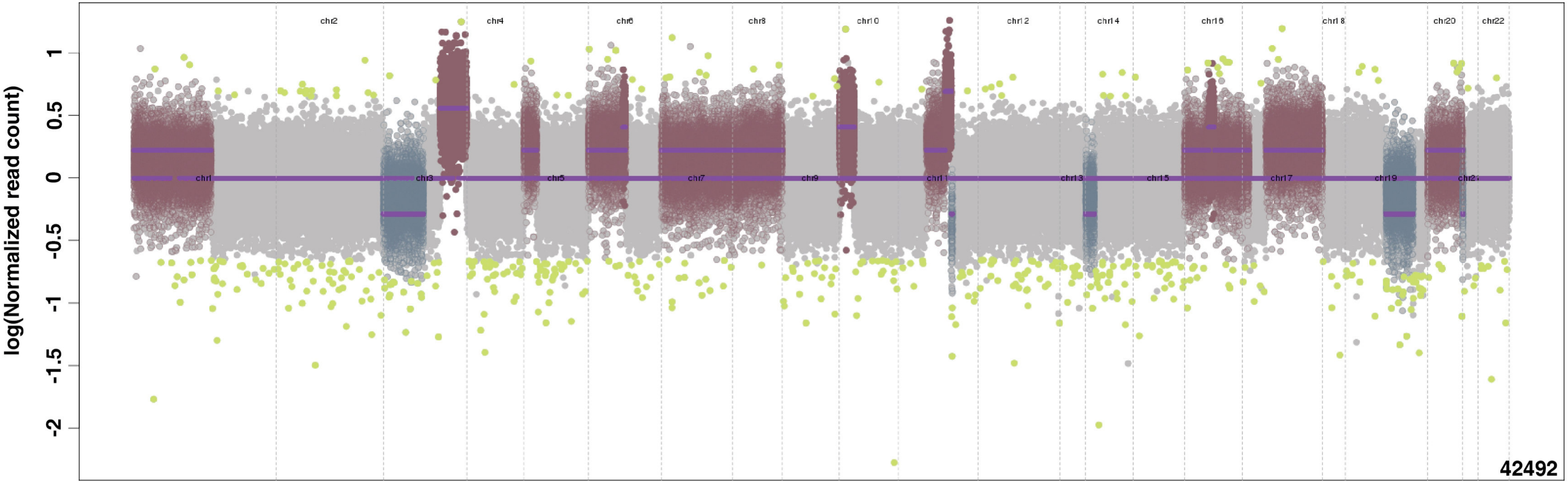

N

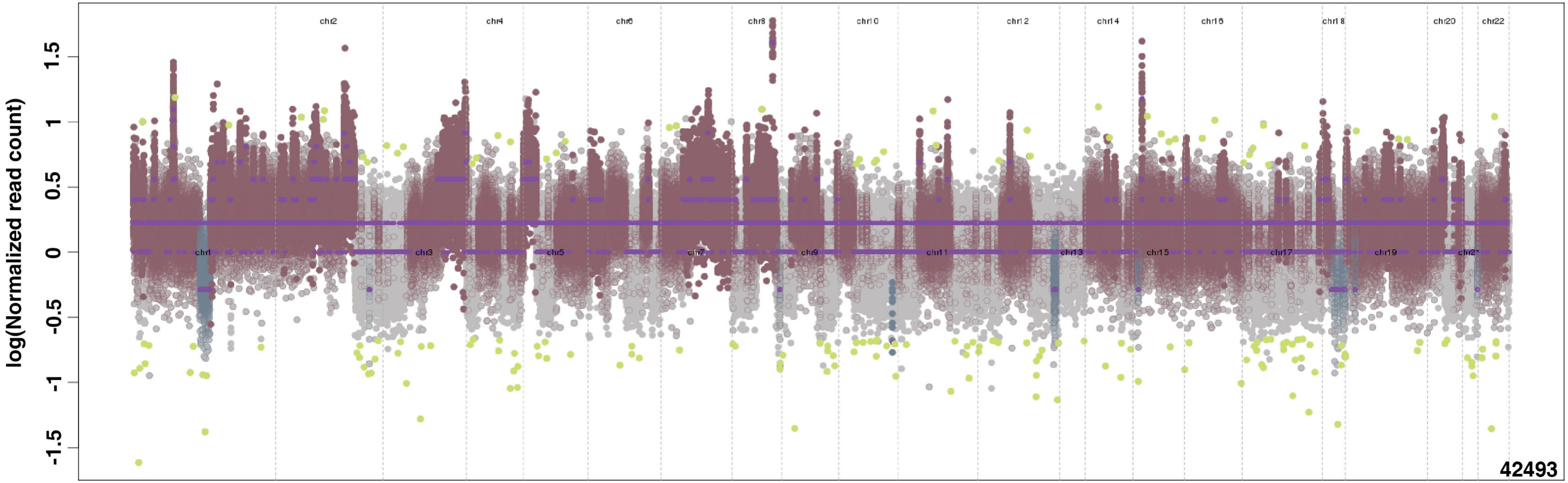

Supplementary figure 9

O

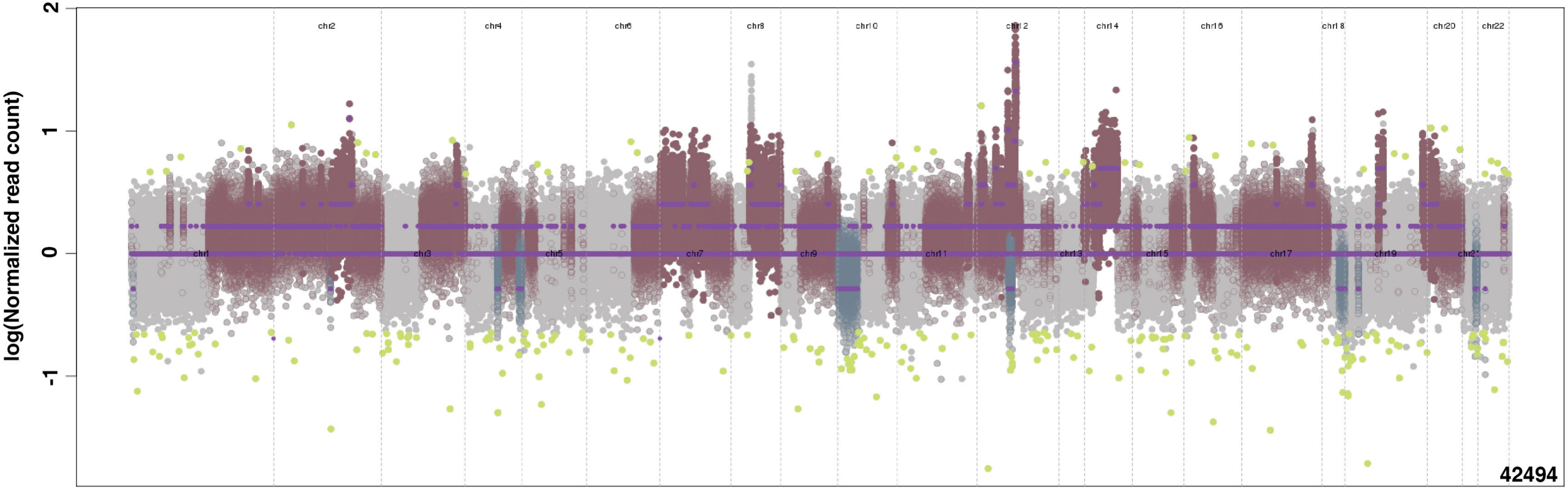

P

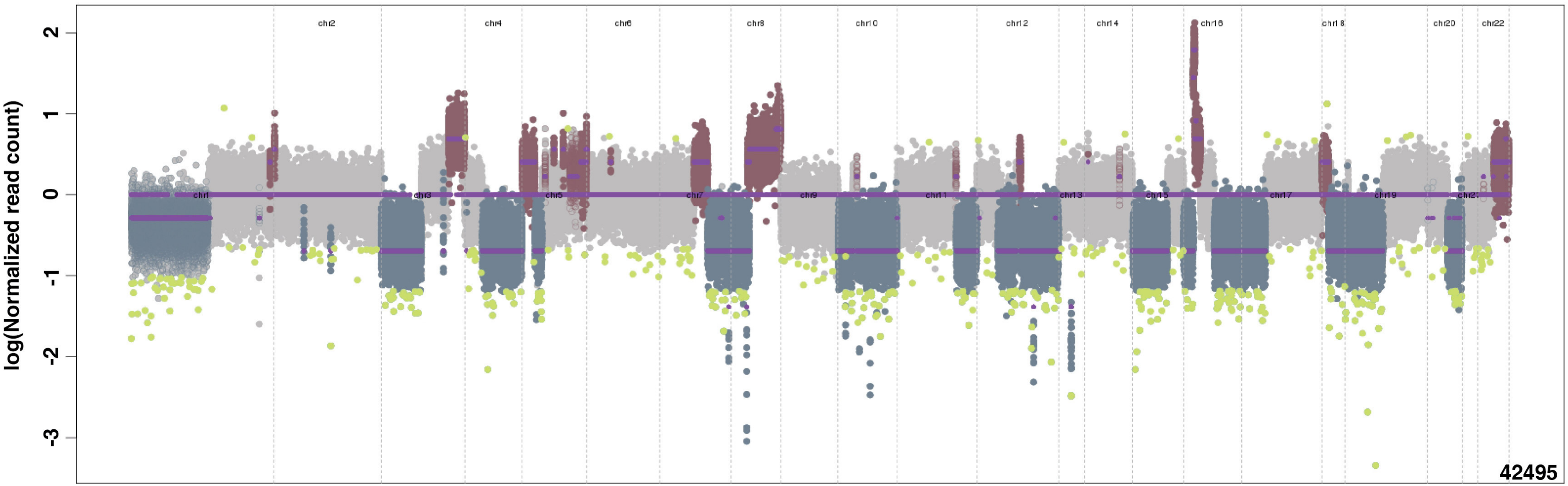

Q **Supplementary figure 9**

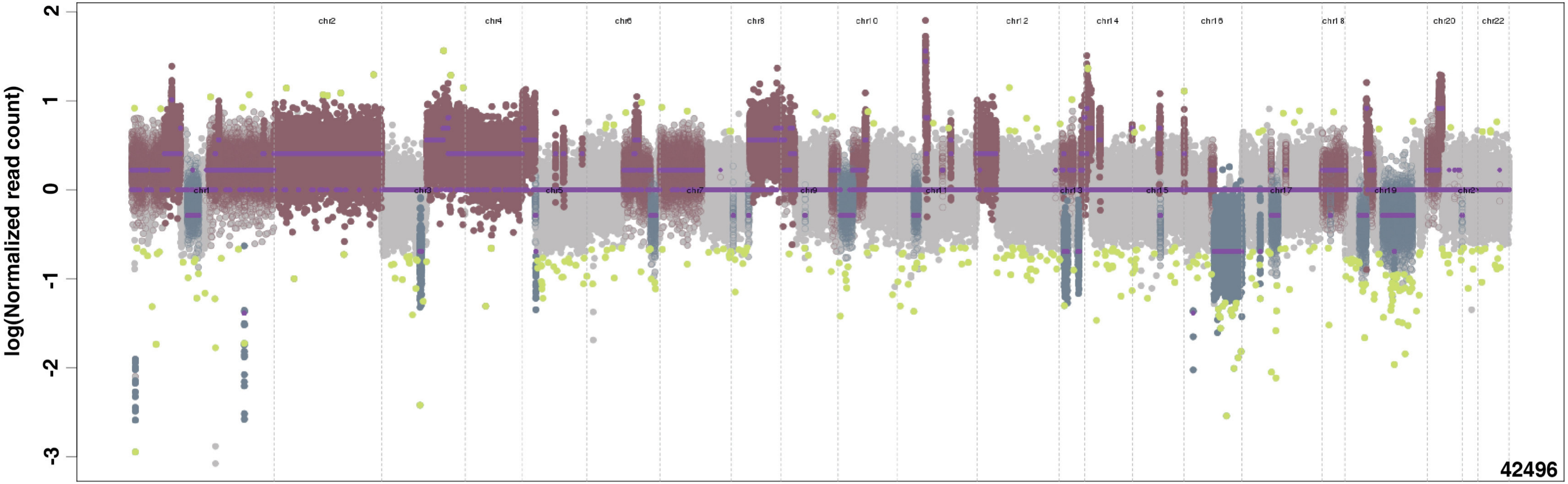

R

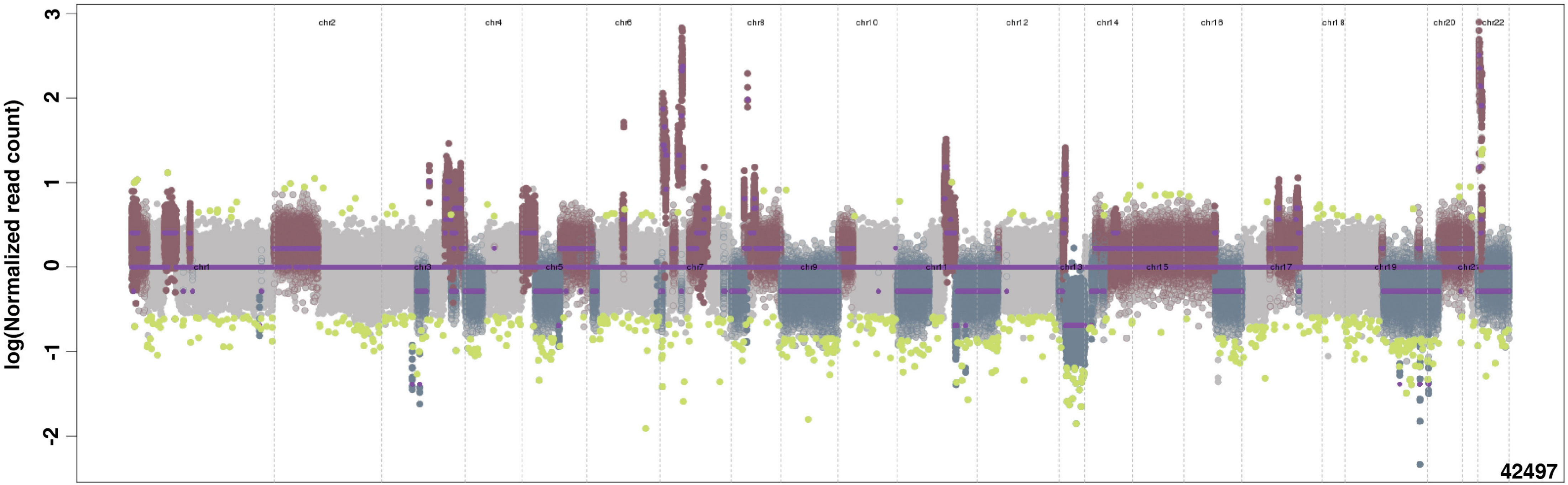

Supplementary figure 9

S

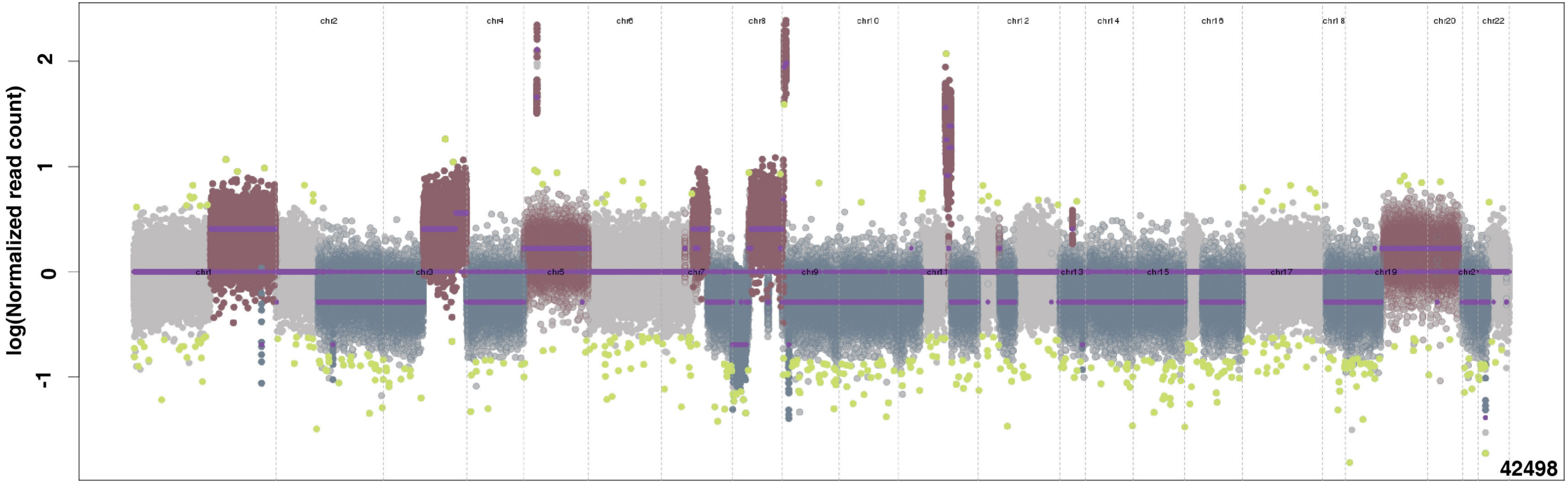

T

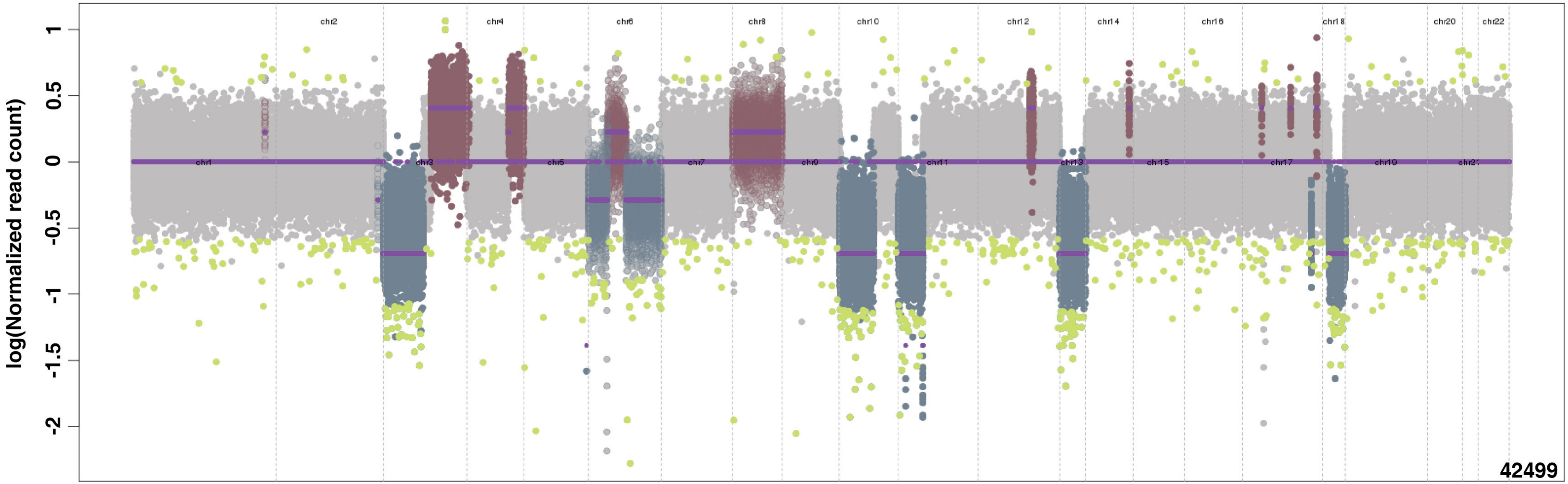

Supplementary figure 9

U

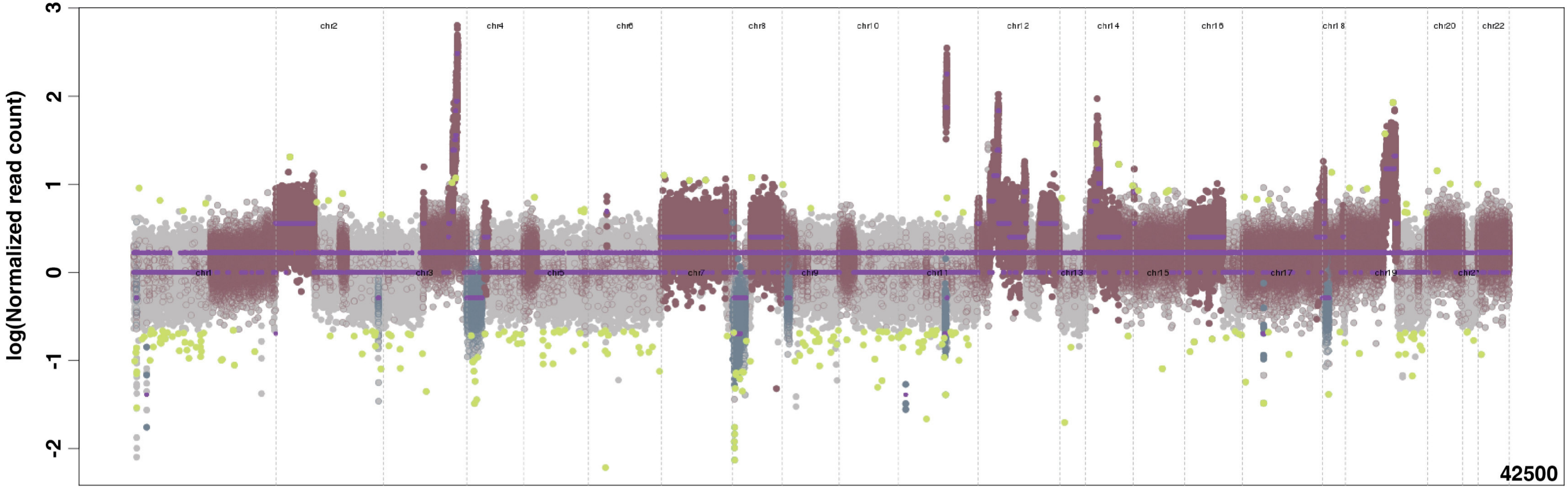

V

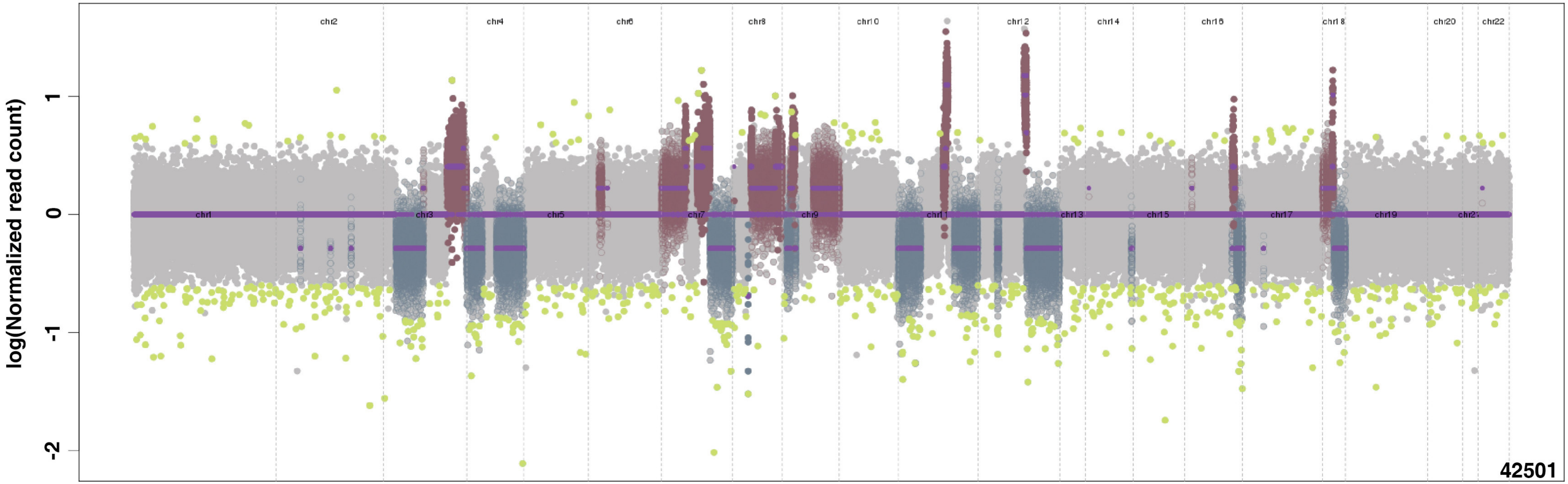

W **Supplementary figure 9**

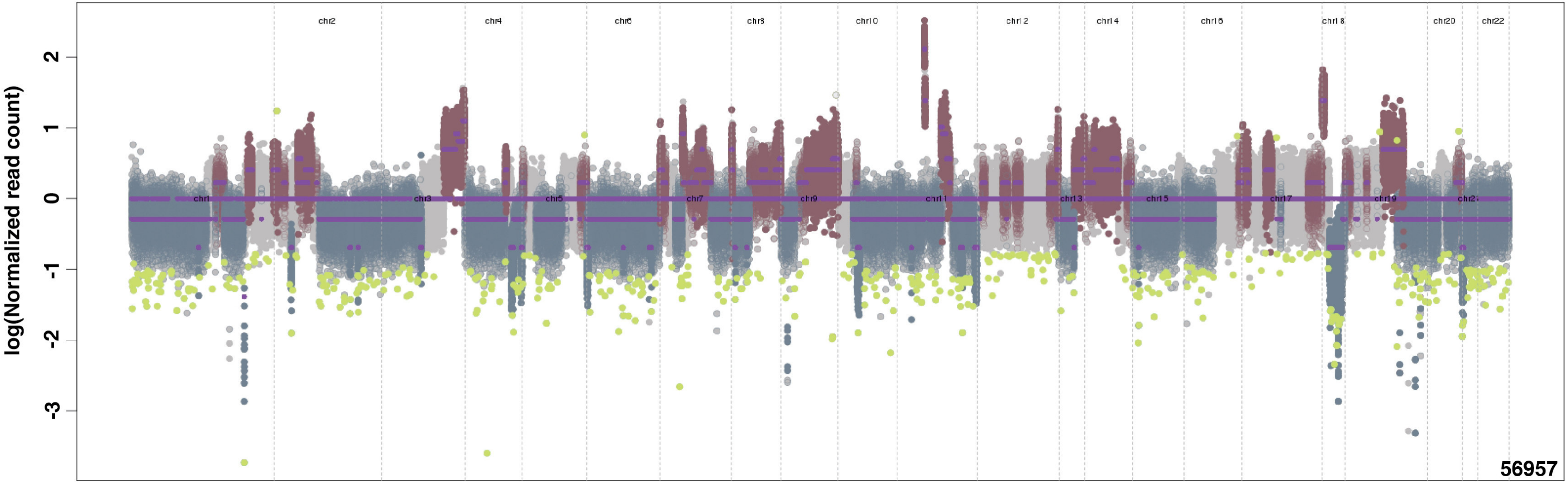

X

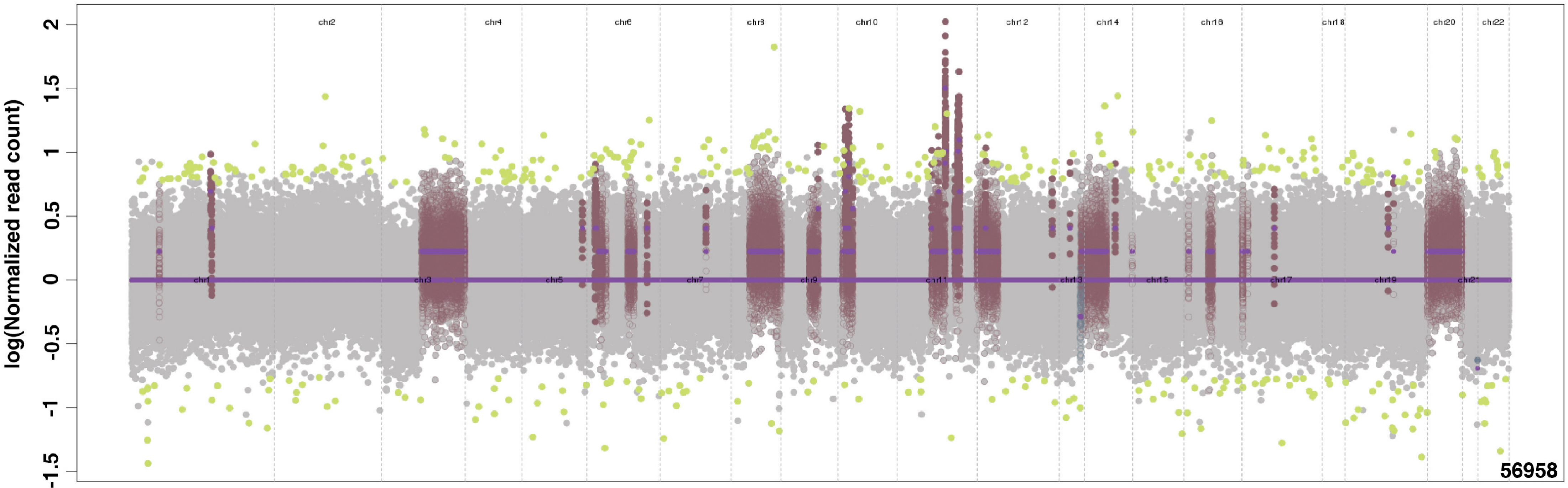

Supplementary figure 10

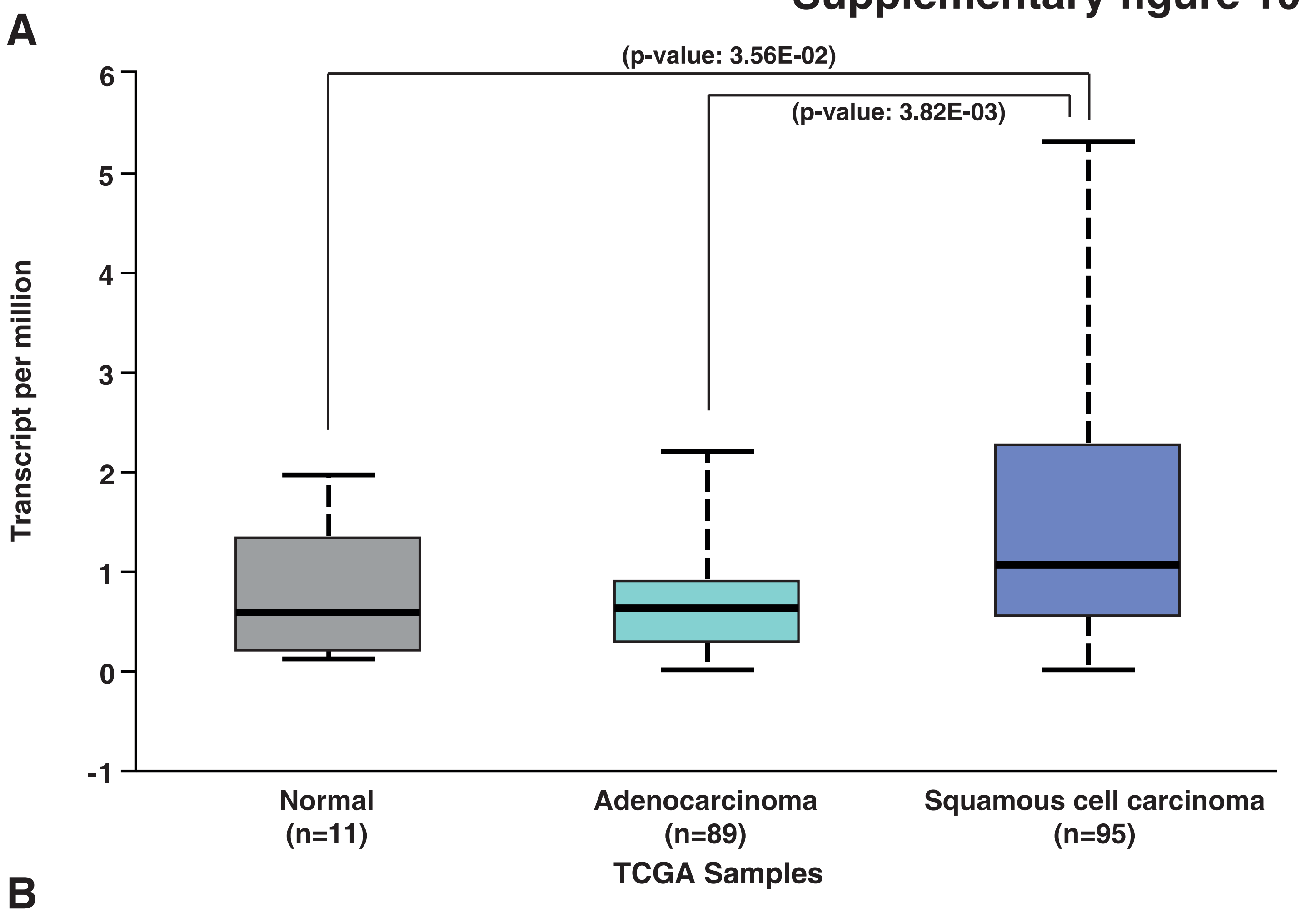

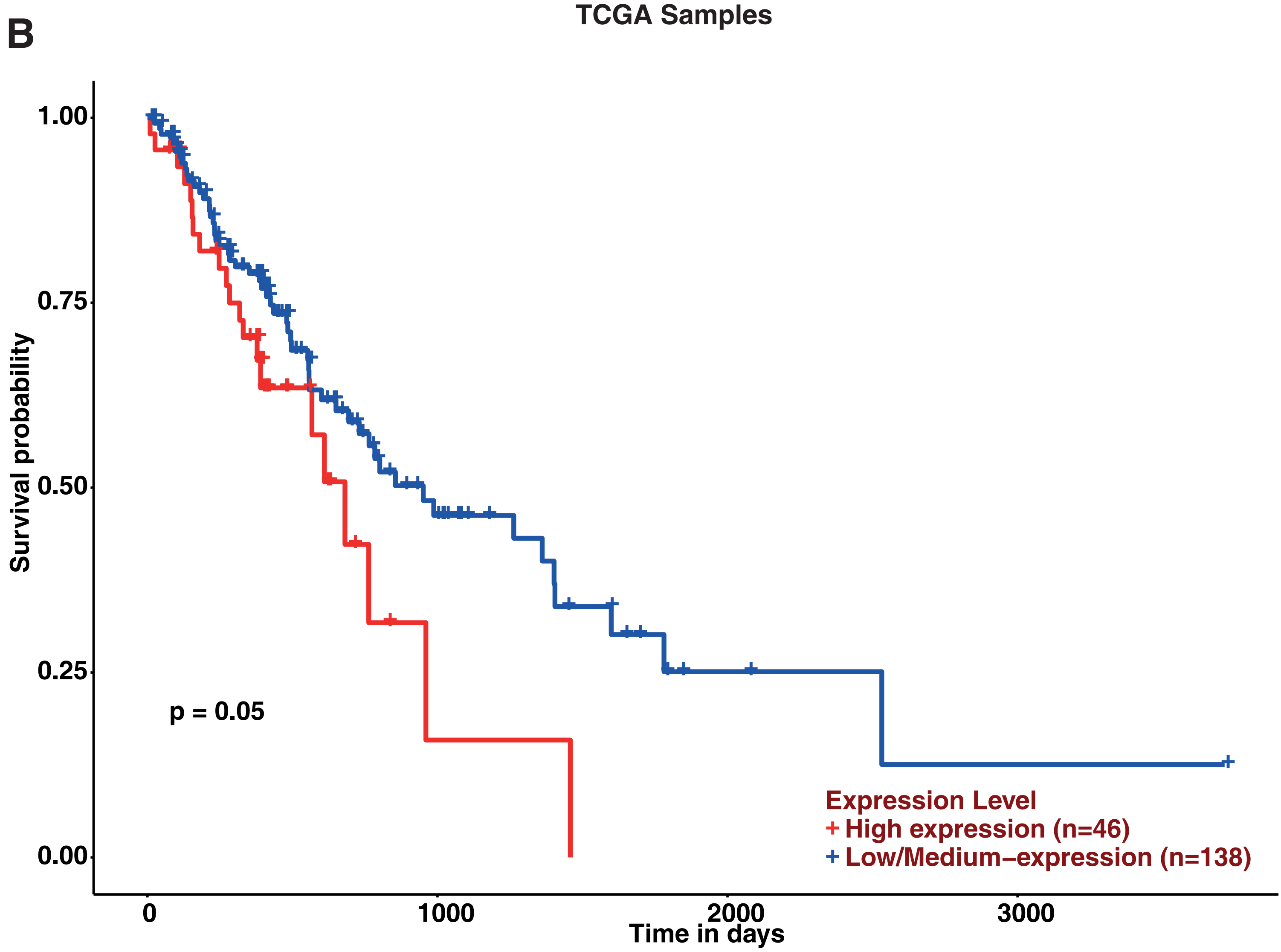

Supplementary figure 11

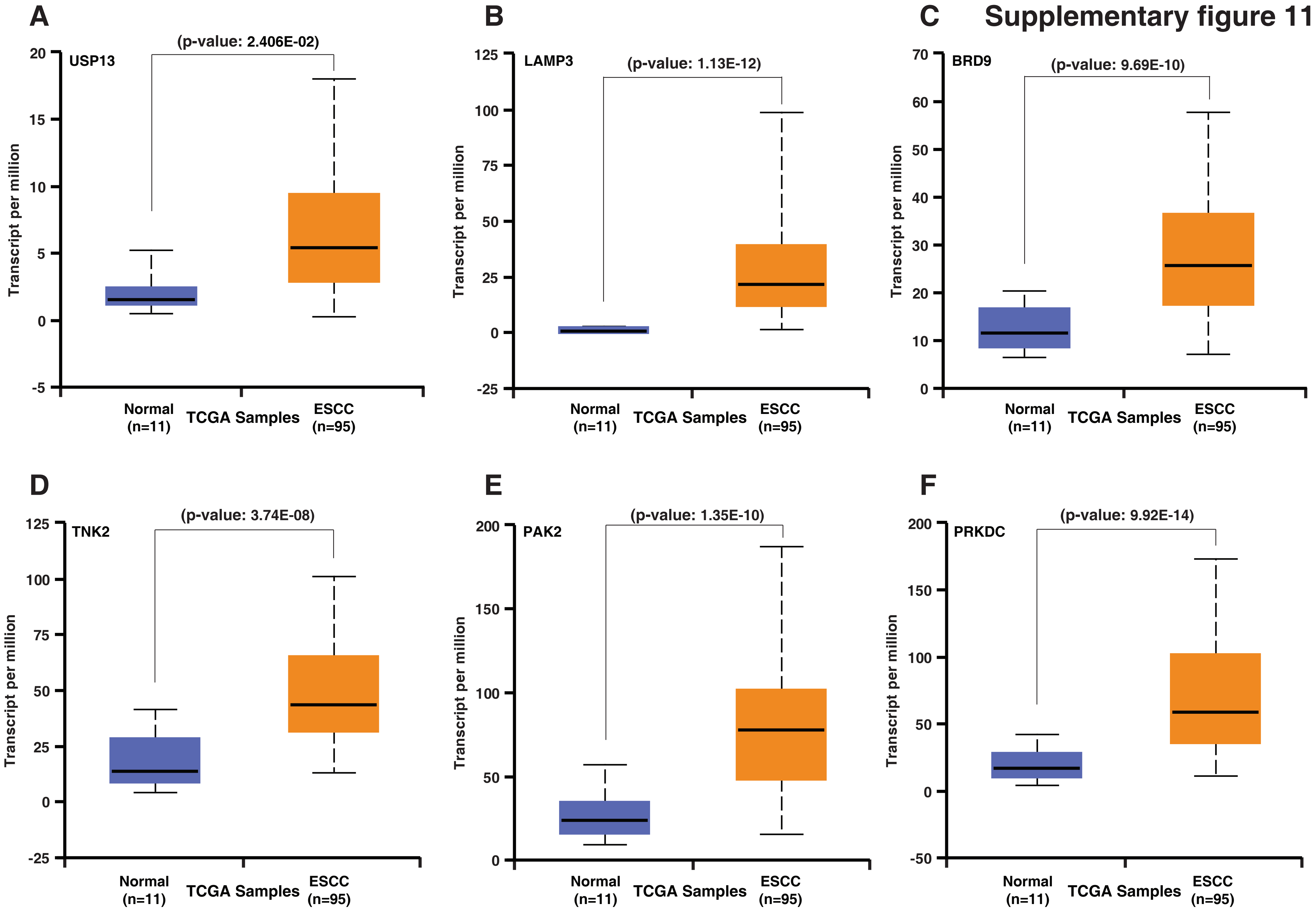

Supplementary Figure 12

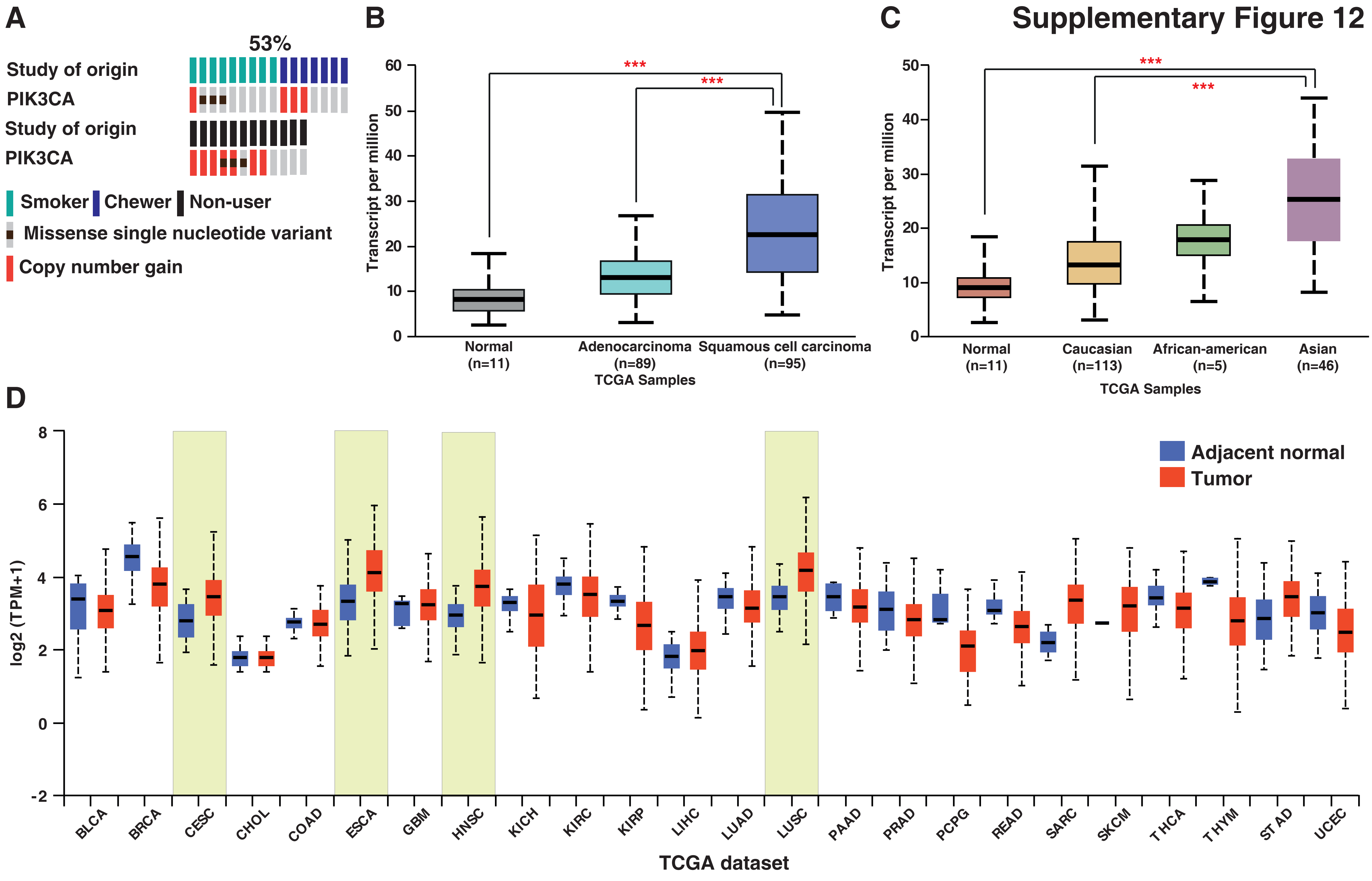

Supplement: Supplementary Figure 1 — Gene ontology enrichment analysis of frequently mutated genes using FunRich keeping default human as background database. [file Data_Sheet_1.pdf]
