# Supplementary material for: Mutational Landscape of Esophageal Squamous Cell Carcinoma in an Indian Cohort
Source: Front Oncol. 2020 Aug 20;10:1457. doi: 10.3389/fonc.2020.01457 (PMC7469928; doi:10.3389/fonc.2020.01457)
Supplement: Supplementary Table 1 — Clinical characteristics of patients with ESCC that were subjected to whole exome sequencing. [file Table_1.pdf]

Mangalaparthi *et al.* , 2020. Mutational landscape of esophageal squamous cell carcinoma in an Indian cohort
Supplementary Table 1. Clinical characteristics of patients with ESCC that were subjected to whole exome sequencing

Patient ID	Age	Gender	Grade	Tumor	Normal	Cohort	Alcohol
**IOB-N-15051/13**	65	M	Grade 1	42477T	42477N	Smoker	No
**IOB-N-12310/13**	65	M	Grade2	42476T	42476N	Smoker	Yes
**IOB-N-12754/13**	52	M	Grade2	42474T	42474N	Smoker	No
**IOB-N-13222/13**	46	M	Grade2	42475T	42475N	Smoker	No
**IOB-N-16271/14**	68	M	Grade2	42481T	42481N	Smoker	No
**IOB-N-16732/13**	40	M	Grade2	42479T	42479N	Smoker	Yes
**IOB-N-537/14**	54	M	Grade2	42480T	42480N	Smoker	Yes
**IOB-N-8419/13**	65	M	Grade2	42473T	42473N	Smoker	Yes
**IOB-N-16646/13**	66	M	Grade 3	42478T	42478N	Smoker	Yes
**IOB-N-1416/12**	70	F	Grade2	42482T	42482N	Chewer	No
**IOB-N-3866/13**	53	M	Grade2	42483T	42483N	Chewer	Yes
**IOB-N-6281/14**	55	F	Grade2	42484T	42484N	Chewer	No
**IOB-N-6953/14**	54	F	Grade2	42487T	42487N	Chewer	No
**IOB-N-6996/14**	76	F	Grade 3	42486T	42486N	Chewer	No
**IOB-N-12967/14**	45	F	Grade 3	42488T	42488N	Chewer	No
**IOB-N-12245/14**	64	M	Grade 3	42489T	42489N	Chewer	Yes
**IOB-N-756/13**	42	F	Grade 1	42492T	42492N	Non-user	No
**IOB-N-17458/12**	74	F	Grade 2	42493T	42493N	Non-user	No
**IOB-N-3665/13**	43	F	Grade2	42494T	42494N	Non-user	No
**IOB-N-6689/14**	49	F	Grade 2	42497T	42497N	Non-user	No
**IOB-N-17849/14**	52	F	Grade 2	42499T	42499N	Non-user	No
**IOB-N-17875/14**	64	F	Grade 2	42500T	42500N	Non-user	No
**IOB-N-8127/13**	23	M	Grade 2	56958T	56958N	Non-user	No
**IOB-N-15429/13**	68	F	Grade 2	56957T	56957N	Non-user	No
**IOB-N-13986/12**	48	F	Grade 3	42495T	42495N	Non-user	No
**IOB-N-13078/13**	60	F	Poorly differentiated	42496T	42496N	Non-user	No
**IOB-N-7896/14**	52	F	Poorly differentiated	42498T	42498N	Non-user	No
**IOB-N-3763/15**	41	F	Grade 3	42501T	42501N	Non-user	NA
