# Supplementary material for: Mutational Landscape of Esophageal Squamous Cell Carcinoma in an Indian Cohort
Source: Front Oncol. 2020 Aug 20;10:1457. doi: 10.3389/fonc.2020.01457 (PMC7469928; doi:10.3389/fonc.2020.01457)
Supplement: Supplementary Table 2 — Read statistics and QC details of whole exome sequencing for each sample. [file Table_2.pdf]

Mangalaparthi *et al.* , 2020. Mutational landscape of esophageal squamous cell carcinoma in an Indian cohort
Supplementary Table 2. Read statistics and QC details of whole exome sequencing for each sample

Cohort	Sample name	Sample type	Total read	Average Base Quality (Phred)	Alignment (%)	Crossmapped (%)	Unaligned (%)	OnTarget (%)	Panel length	Panel coverage	Ontarget region average depth	Panel depth (1x – 10x) (%)	Panel depth (11x – 30x) (%)	Panel depth (31x – 50x) (%)	Panel depth (51x – 100x) (%)	Panel depth (>100x) (%)
Smoker	42473T	Tumor	9,67,61,026	35.41	99.89	1.05	0.11	69.02	5,03,90,601	99.89	104.78	1.31	8.48	14.51	34.29	41.29
Smoker	42474T	Tumor	8,55,47,646	35.41	99.84	0.72	0.16	73.19	5,03,90,601	99.90	96.98	1.31	10.01	16.93	35.49	36.15
Smoker	42475T	Tumor	11,48,02,854	36.64	99.81	0.58	0.19	73.95	5,03,90,601	99.91	129.45	0.48	3.73	8.96	31.41	55.34
Smoker	42476T	Tumor	7,89,63,546	35.38	99.85	0.93	0.15	72.02	5,03,90,601	99.88	88.08	1.51	11.68	18.90	37.51	30.27
Smoker	42477T	Tumor	9,05,86,760	35.45	99.83	1.12	0.17	73.68	5,03,90,601	99.89	103.06	1.00	7.27	14.24	35.98	41.41
Smoker	42478T	Tumor	8,12,45,982	35.57	99.85	0.58	0.15	72.56	5,03,90,601	99.89	91.82	1.12	8.83	17.01	38.10	34.83
Smoker	42479T	Tumor	9,21,63,426	35.62	99.79	0.64	0.21	71.55	5,03,90,601	99.90	102.95	0.97	7.86	14.84	35.79	40.43
Smoker	42480T	Tumor	10,33,28,790	36.27	99.93	0.17	0.07	68.67	5,03,90,601	99.89	100.47	1.44	10.33	16.57	33.73	37.82
Smoker	42481T	Tumor	8,52,38,250	35.59	99.79	0.38	0.21	70.60	5,03,90,601	99.89	92.77	0.94	8.91	17.26	38.05	34.73
Smoker	42473N	Adjacent normal	7,58,41,354	35.30	99.97	0.19	0.03	70.48	5,03,90,601	99.87	79.96	2.58	16.56	20.34	32.56	27.82
Smoker	42474N	Adjacent normal	7,58,39,208	35.31	99.97	0.28	0.03	73.32	5,03,90,601	99.91	80.94	0.87	9.18	19.38	42.40	28.09
Smoker	42475N	Adjacent normal	7,61,99,182	35.23	99.97	0.33	0.03	71.82	5,03,90,601	99.91	82.10	0.87	8.87	18.89	42.19	29.09
Smoker	42476N	Adjacent normal	7,82,33,478	35.25	99.96	0.29	0.04	71.54	5,03,90,601	99.90	84.39	0.81	8.48	18.35	41.42	30.85
Smoker	42477N	Adjacent normal	7,71,62,994	35.37	99.97	0.31	0.03	73.03	5,03,90,601	99.90	85.78	0.79	8.27	17.74	41.15	31.95
Smoker	42478N	Adjacent normal	10,09,85,368	37.24	99.89	0.21	0.11	73.57	5,03,90,601	99.91	105.86	0.46	4.52	11.94	37.88	45.11
Smoker	42479N	Adjacent normal	8,65,90,274	35.41	99.97	0.30	0.03	73.01	5,03,90,601	99.91	95.64	0.71	6.64	15.04	39.50	38.02
Smoker	42480N	Adjacent normal	10,53,91,108	35.47	99.77	0.36	0.23	72.48	5,03,90,601	99.91	120.93	0.72	4.82	10.12	31.97	52.28
Smoker	42481N	Adjacent normal	11,19,09,366	35.32	99.97	0.23	0.03	70.71	5,03,90,601	99.89	119.01	0.81	5.76	11.67	33.01	48.64
Chewer	42482T	Tumor	11,56,19,110	35.58	99.45	0.48	0.55	72.85	5,03,90,601	99.83	124.53	0.37	3.34	9.09	32.71	54.32
Chewer	42483T	Tumor	8,95,97,734	35.47	99.97	0.31	0.03	73.38	5,03,90,601	99.92	94.22	0.74	7.54	15.94	39.25	36.46
Chewer	42484T	Tumor	9,30,93,268	36.58	99.94	0.97	0.06	71.20	5,03,90,601	99.81	90.05	0.74	8.28	16.85	39.99	33.94
Chewer	42486T	Tumor	9,81,11,280	35.54	99.82	0.85	0.18	71.93	5,03,90,601	99.80	108.45	0.72	6.03	12.78	35.34	44.94
Chewer	42487T	Tumor	7,93,35,078	35.57	99.81	0.83	0.19	70.54	5,03,90,601	99.79	86.28	1.36	11.36	18.79	37.46	30.82
Chewer	42488T	Tumor	10,63,82,232	36.84	99.82	0.48	0.18	72.05	5,03,90,601	99.81	116.71	0.56	4.18	10.35	34.57	50.15
Chewer	42489T	Tumor	10,03,15,163	36.97	99.84	0.64	0.16	72.01	5,03,90,601	99.90	107.87	1.03	8.47	14.92	34.26	41.22
Chewer	42482N	Adjacent normal	8,34,57,458	35.38	99.97	0.38	0.03	71.42	5,03,90,601	99.81	91.71	0.74	7.17	16.47	39.90	35.53
Chewer	42483N	Adjacent normal	9,06,65,038	35.41	99.79	0.57	0.21	71.48	5,03,90,601	99.91	99.83	0.71	6.32	14.35	38.29	40.24
Chewer	42484N	Adjacent normal	10,18,77,310	35.41	99.95	0.81	0.05	70.23	5,03,90,601	99.80	110.64	0.48	4.27	11.18	36.21	47.66
Chewer	42486N	Adjacent normal	9,04,79,158	35.47	99.84	0.42	0.16	68.93	5,03,90,601	99.80	98.59	0.75	6.20	14.19	38.18	40.48
Chewer	42487N	Adjacent normal	8,95,08,922	35.51	99.81	0.88	0.19	70.33	5,03,90,601	99.80	98.21	0.83	6.53	14.53	38.36	39.56
Chewer	42488N	Adjacent normal	8,43,88,080	35.45	99.96	0.79	0.04	70.12	5,03,90,601	99.81	91.29	0.75	7.06	16.05	40.15	35.79
Chewer	42489N	Adjacent normal	8,11,59,826	35.28	99.96	0.13	0.04	70.28	5,03,90,601	99.88	83.97	2.00	13.21	19.41	35.17	30.09
Non-user	42492T	Tumor	8,33,96,182	35.44	99.82	1.14	0.18	72.38	5,03,90,601	99.79	91.90	0.94	8.12	16.56	38.79	35.37
Non-user	42493T	Tumor	9,56,75,450	37.17	99.80	0.43	0.20	75.04	5,03,90,601	99.80	107.27	0.71	5.75	12.84	36.26	44.24
Non-user	42494T	Tumor	8,32,00,748	35.22	99.82	1.09	0.18	72.72	5,03,90,601	99.79	91.78	0.95	8.16	16.73	39.06	34.89
Non-user	42495T	Tumor	8,44,26,764	35.02	99.84	0.87	0.16	71.73	5,03,90,601	99.80	93.05	1.20	10.43	17.68	36.32	34.18
Non-user	42496T	Tumor	8,87,92,256	35.57	99.88	1.29	0.12	72.99	5,03,90,601	99.78	99.85	1.19	8.80	15.63	35.73	38.43
Non-user	42497T	Tumor	9,18,16,092	35.47	99.88	0.65	0.12	70.45	5,03,90,601	99.80	100.63	0.97	7.79	15.47	36.99	38.56
Non-user	42498T	Tumor	9,57,15,860	35.52	99.86	0.86	0.14	70.38	5,03,90,601	99.80	104.65	0.80	6.70	14.24	36.55	41.51
Non-user	42499T	Tumor	8,39,98,916	35.57	99.89	1.19	0.11	72.99	5,03,90,601	99.79	95.11	1.15	8.50	16.08	37.31	36.74
Non-user	42500T	Tumor	8,43,59,622	35.58	99.82	0.38	0.18	71.17	5,03,90,601	99.79	93.33	1.10	9.49	17.67	37.71	33.82
Non-user	42501T	Tumor	8,48,31,650	35.51	99.67	0.83	0.33	64.81	5,03,90,601	99.79	85.39	1.30	10.27	18.76	38.72	30.73
Non-user	56958T	Tumor	10,11,19,548	35.56	98.79	0.32	1.21	73.89	5,03,90,601	99.91	113.69	0.49	4.71	11.34	35.14	48.23
Non-user	56957T	Tumor	9,22,42,955	36.43	99.81	1.15	0.19	70.38	5,03,90,601	99.79	95.43	1.09	9.42	17.23	37.06	34.99
Non-user	42492N	Adjacent normal	7,21,75,094	35.37	99.77	0.70	0.23	73.16	5,03,90,601	99.79	79.51	1.27	11.25	20.59	39.46	27.21
Non-user	42493N	Adjacent normal	8,03,69,608	35.43	99.77	1.26	0.23	71.30	5,03,90,601	99.79	87.97	0.95	8.12	17.16	40.19	33.37
Non-user	42494N	Adjacent normal	8,59,45,018	35.37	99.78	0.85	0.22	70.19	5,03,90,601	99.80	92.27	0.75	6.95	15.98	39.81	36.30
Non-user	42495N	Adjacent normal	8,58,17,764	35.54	99.77	0.89	0.23	73.78	5,03,90,601	99.79	96.18	0.82	6.93	15.14	38.76	38.14
Non-user	42496N	Adjacent normal	8,27,29,738	35.51	99.76	0.68	0.24	72.58	5,03,90,601	99.78	92.68	0.86	7.44	16.11	39.30	36.07
Non-user	42497N	Adjacent normal	8,18,62,594	35.52	99.83	0.92	0.17	71.52	5,03,90,601	99.80	91.36	0.93	7.63	16.31	39.45	35.48
Non-user	42498N	Adjacent normal	7,88,60,972	35.36	99.84	0.67	0.16	71.08	5,03,90,601	99.79	87.32	1.05	8.68	17.36	39.72	32.97
Non-user	42499N	Adjacent normal	9,49,66,146	35.41	99.82	0.55	0.18	72.78	5,03,90,601	99.80	107.38	0.79	5.90	13.00	35.86	44.25
Non-user	42500N	Adjacent normal	9,86,70,014	35.42	99.86	0.78	0.14	73.69	5,03,90,601	99.80	112.81	0.74	4.90	11.24	34.70	48.21
Non-user	42501N	Adjacent normal	8,87,19,578	35.38	99.87	0.85	0.13	71.87	5,03,90,601	99.80	99.64	0.91	6.55	14.05	37.39	40.89
Non-user	56958N	Adjacent normal	7,03,35,214	35.39	99.83	0.55	0.17	72.12	5,03,90,601	99.88	79.27	1.38	11.33	20.08	39.80	27.30
Non-user	56957N	Adjacent normal	8,71,56,102	35.42	99.84	0.62	0.16	74.43	5,03,90,601	99.78	99.67	1.52	10.78	16.56	33.17	37.75
