# Supplementary material for: Mutational Landscape of Esophageal Squamous Cell Carcinoma in an Indian Cohort
Source: Front Oncol. 2020 Aug 20;10:1457. doi: 10.3389/fonc.2020.01457 (PMC7469928; doi:10.3389/fonc.2020.01457)
Supplement: Supplementary Table 3 — List of different types of variants identified in ESCC samples. [file Table_3.pdf]

Mangalaparthi *et al.* , 2020. Mutational landscape of esophageal squamous cell carcinoma in an Indian cohort
Supplementary Table 3. List of different types of variants identified in ESCC patients

S.No.	Cohort	Sample ID	Total mutations	Non-synonymous	Coding mutations						Non-coding mutations							Mutation rate	Cytoband with copy number alterations
					Missense	Nonsense	Nonstop	Startloss	Splice	Silent	5'UTR	3'UTR	Flanking	Intergenic	Intron	MicroRNA	Other non-coding genes		
1	Smoker	42473T	221	85	79	5	0	0	1	29	2	4	17	1	72	0	11	4.33	158
2	Smoker	42474T	99	41	37	1	0	0	3	16	2	2	3	2	22	0	11	1.94	27
3	Smoker	42475T	290	115	104	8	0	1	2	59	7	5	11	3	79	1	10	5.69	31
4	Smoker	42476T	503	205	178	19	2	0	6	57	5	9	21	4	183	1	18	9.86	18
5	Smoker	42477T	230	105	99	4	0	0	2	24	7	3	12	5	65	0	9	4.51	2
6	Smoker	42478T	273	109	102	7	0	0	0	46	2	7	6	2	92	1	8	5.35	2
7	Smoker	42479T	554	213	190	15	1	0	7	82	9	16	18	4	192	0	20	10.86	NA
8	Smoker	42480T	332	127	118	7	0	1	1	57	4	5	17	5	105	0	12	6.51	NA
9	Smoker	42481T	144	61	54	3	0	0	4	21	1	5	10	0	41	0	5	2.82	4
10	Chewer	42482T	451	203	196	6	0	0	1	63	4	12	17	4	125	0	23	8.84	31
11	Chewer	42483T	479	194	173	13	0	1	7	76	6	3	17	8	149	0	26	9.39	30
12	Chewer	42484T	353	160	151	5	1	0	3	50	1	6	13	3	103	0	17	6.92	66
13	Chewer	42486T	368	131	119	6	0	1	5	57	9	6	16	5	132	0	12	7.22	31
14	Chewer	42487T	461	194	177	13	0	0	4	68	5	5	25	3	144	1	16	9.04	63
15	Chewer	42488T	97	44	41	1	0	0	2	17	0	2	6	2	20	0	6	1.9	NA
16	Chewer	42489T	278	113	105	4	0	0	4	35	1	7	11	2	87	0	22	5.45	NA
17	Non-user	42492T	170	82	73	7	0	0	2	29	1	3	3	1	46	0	5	3.33	34
18	Non-user	42493T	316	124	118	5	0	0	1	53	4	3	7	8	102	0	15	6.2	107
19	Non-user	42494T	506	203	185	12	0	0	6	63	8	11	25	4	164	0	28	9.92	44
20	Non-user	42495T	159	68	64	3	0	0	1	14	1	7	4	0	57	0	8	3.12	71
21	Non-user	42496T	303	133	121	10	0	0	2	42	9	2	12	1	94	0	10	5.94	100
22	Non-user	42497T	289	118	112	5	0	0	1	39	4	3	9	1	107	0	8	5.67	55
23	Non-user	42498T	640	273	246	18	1	1	7	83	13	6	27	3	202	0	33	12.55	22
24	Non-user	42499T	210	96	85	10	0	0	1	28	4	3	8	3	59	0	9	4.12	1
25	Non-user	42500T	182	84	77	5	1	0	1	27	2	6	4	3	45	0	11	3.57	99
26	Non-user	42501T	190	72	65	5	0	1	1	30	2	3	4	3	67	0	9	3.73	22
27	Non-user	56957T	2324	483	461	16	1	0	5	278	26	51	74	273	903	3	233	45.57	65
28	Non-user	56958T	822	105	97	4	0	0	4	61	6	21	18	219	325	0	67	16.12	8
