# Supplementary material for: Mutational Landscape of Esophageal Squamous Cell Carcinoma in an Indian Cohort
Source: Front Oncol. 2020 Aug 20;10:1457. doi: 10.3389/fonc.2020.01457 (PMC7469928; doi:10.3389/fonc.2020.01457)
Supplement: Supplementary Table 5 — Mutation load in esophageal squamous cell carcinoma (ESCC), oral squamous cell carcinoma (OSCC), lung squamous cell carcinoma (LUSC), and Lung adenocarcinoma (LUAD). [file Table_5.pdf]

Mangalaparthi *et al.*, 2020. Mutational landscape of esophageal squamous cell carcinoma in an Indian cohort

Supplementary Table 5. Mutation load in esophageal squamous cell carcinoma (ESCC), oral squamous cell carcinoma (OSCC), lung squamous cell carcinoma (LUSC), and Lung adenocarcinoma (LUAD)

Esophageal squamous cell carcinoma (ESCC)													Oral squamous cell carcinoma (OSCC)				Lung squamous cell carcinoma (LUSC)							Lung adenocarcinoma		
ESCC_nonUser	ESCC_Smoker	ESCC_Chewer	25151357_2014_ nonSmoker	25151357_2014_ Smoker	24670651_2014_ nonSmoker	24670651_2014_ Smoker	25839328_2015_ nonSmoker	25839328_2015_ Smoker	26873401_2016_ nonSmoker	26873401_2016_ Smoker	24686850_2014_ noStratification	27058444_2016_ noStratification	28939077_2017_ nonChewer	28939077_2017_ Chewer	24292195_2013_ Chewer	26934577_2016_ Shamma	22960745_2012_ Current reformed smoker for > 15 years	22960745_2012_ Current reformed smoker for < or = 15 years	22960745_2012_ Current smoker	26503331_2015_ nonSmoker	26503331_2015_ Smoker	26943773_2016_ nonSmoker	26943773_2016_ Smoker	25079552_2014_ Current reformed smoker for > 15 years	25079552_2014_ Current reformed smoker for < or = 15 years	25079552_2014_ CurrentSmoker
3.33	4.33	8.84	6.16	4.72	2.92	0.744	4.65	1.54	5.757772965	2.473137381	10.4	13.09	3.9	4.9	1.39	8.5	6.77	8.07	10.56	6.92	8.45	5.82	7.27	8.47	37.78	19.72
6.2	1.94	9.39	4.64	4.69	2.06	2.919	8.16	4.66	6.975020269	2.820922325	6.26	6	4	2.4	9.5	7.4	69.60	2.97	7.72	5.55	7.68	11.09	2.2	3.5	14.41	4.16
9.92	5.69	6.92	4.76	4.9	1.991	1.066	5.95	4.78	2.801600939	4.501882888	0.7	5.86	3.9	3.3	2.21	7.18	10.45	11.37	4.56	5.18	6.87	34.2	31	6.43	10.19	6.92
3.12	9.86	7.22	4.77	4.82	0.276	2.473	2.84	5.02	2.608387081	1.680960563	1.82	5.18	3.7	3.6	2	10.7	7.42	13.99	5.35	4.47	6.5	5.11	11.64	6.34	28.42	7.52
5.94	4.51	9.04	4.71	4.83	3.023	2.687	3.65	5.37	2.106031051	2.666351239	1.08	4	5.5	3.3	2.37	8.89	5.00	13.50	9.94	3.42	6.18	5.2	62.14	13.46	5.11	17.17
5.67	5.35	1.9	4.81	4.81	4.167	1.894	2.06	3.28	2.453815995	2.589065695	1.32	3.53	2.3	4.7	2.41	7.43	16.02	3.90	2.84	3.21	5.95	34.45	4	3.53	7.41	1.06
12.55	10.86	5.45	7.63	4.82	2.35	4.985	5.14	4.3	7.689911543	5.931665437	1.54	3.38	3.5	7.6	11.78	12.37	6.76	3.71	2.52	1.11	5.82	21.39	7.8	0.98	1.35	3.1
4.12	6.51		7.68	4.89	3.546	3.019	1.36	2.86	1.97078135	11.07115406	0.8	3.1	7.7	6.1	2.19	7.03	7.02	12.41	5.43	1.03	5.76	4.43	6.41	1.41	19.84	2.99
3.57	2.82		7.76	4.94	2.783	2.122		3.66	1.951459964	3.130064497	1.24	3.03	9.4	8.5	3.31	8.08	7.38	1.71	7.59	1.03	5.63	27.52	17.68	1.09	7.72	7.15
3.73			7.88	7.67	2.916	6.928		2.77	2.028745508	2.144673822	0.62	3.02	3.1	11.4	4.67	6.96	5.00	4.48	8.89	0.82	5.53	2.86	36.7	3.9	2.64	3.7
45.57			8	7.67	7.43	0.072		1.75	4.018848244	2.550422924	0.12	2.96	3.4	12.7	3.52	7.09	6.35	9.79	4.72	0.42	5.34	5.32	25.32	9.3	0.95	1.84
16.12			8.1	7.7	2.769	1.213		3	2.724315396	0.908105132	1.32	2.89			6.47	6.04	7.79	10.72	6.20	0.32	5.32	4.45	18.64	4.74	18.69	1.29
			8.27	7.73	0.062	1.38		4.02	2.550422924	2.106031051	1.56	2.82			3.43	6.9	2.37	5.51	4.01	0.11	5.29	2.34	26.16	5.48	13.61	1.09
			8.37	7.74	3.246	3.867		2.98	2.260602137	2.144673822	1.16	2.76			5.21	8.18	15.03	4.36	5.22	0.03	4.97	22.61	21.2	11.02	28.94	1.49
			8.46	7.77	0.156	1.289		2.45	3.091421726	3.014136183	1.18	2.73			2.31	10.49	18.16	6.27	8.62		4.97	10.16	30.61	1.12	10.13	4.51
			8.54	7.78	0.124	2.441		6.07	2.028745508	4.154097944	0.82	2.45			3.76	6.87	5.25	9.54	8.77		4.74	24.8	37.84	1.09	10.77	2.87
			8.6	7.82	2.285	1.988		2.39	1.932138579	3.168707269	1.36	2.33			2.64	7.75	5.93	2.70	4.55		4.71	13.16		4.88	7.55	7.84
			8.64	7.88	1.861	3.282		2.25	3.400563899	2.434494609	0.08	2.24			4.35	7.16	4.82	4.46	30.33		4.71	31.18		5.54	1.95	3.73
			8.72	7.9	1.148	2.553		5.1	3.168707269	1.081997604	2.94	2.16			2.84	7.48	0.03	3.81	4.63		4.53	18.82		6.46	5.05	14.64
			8.78	7.91	2.798	0.156		1.19	1.932138579	3.033457569	1.84	2.15			2.84	7.11	9.29	4.00	3.81		4.5	4.59		7.78	9.67	26.99
			8.9	7.93	1.938	5.342		3.04	3.999526858	2.453815995		2.12			1.85		4.76	6.03	5.65		4.34	17.34		1.75	2.5	4.08
			8.88	7.95	3.298	1.798		2.86	1.777567492	4.424597345		2.08			5.12		6.70	5.56	5.57		4.29			4.97	10.02	2.04
			8.87	7.98	3.034	0.819		2.98	2.840243711	3.632420528		2.03			3.36		6.84	6.98	5.29		4.26			8.01	4.68	3.5
			8.87	8.01	2.47	4.117		1.33	5.255416934	2.56974431		1.92			2.6		19.06	12.62	13.39		4.16			7.23	5.34	1.29
			8.89	8.04	2.721	3.166		1.98	2.492458767	2.415173223		1.92			3.17		4.16	8.42	4.75		4.13			4.59	6.8	1.23
			9	8.06	0.218	2.796		1.43	2.357209066	2.762958168		1.91			1.03		3.46	10.45	3.65		3.97			2.21	3.47	11.77
			9.07	8.08	3.531	2.648		1.26	2.878886482	8.443445589		1.89			2.98		4.76	12.15	4.44		3.92			2.96	4.79	10.28
			9.16	8.08	1.928	2.699		1.95	2.492458767	4.057491015		1.84			1.14		3.58	3.53	5.83		3.89			4.13	3.13	1.67
			9.3	8.13	5.414	2.049		6.57	1.932138579	2.453815995		1.82			1.57		6.45	8.34	5.34		3.87			1.64	1.84	1.49
			9.31	8.12	1.528	0.899		5.59	3.651741914	2.415173223		1.78			2.82		4.14	6.93	16.54		3.76			6.06	7.64	6.49
			9.48	8.15		4.696		4.62	1.391139777	4.559847046		1.78			4.59		16.07	5.04	7.84		3.76			1.52	9.3	5.57
			9.41	8.25		2.245		1	1.449103934	2.840243711		1.74			2.42		4.74	7.89	5.89		3.68			3.36	4.16	4.77
			9.54	8.23		0.217		2.85	3.014136183	1.507068091		1.73			0.48		8.16	14.67	6.75		3.63			3.16	14.41	3.47
			9.55	8.29		2.259		2.7	6.260128995	2.395851838		1.73			3.16		4.93	3.66	2.85		3.5			27.67	0.57	6.95
			9.55	8.32		5.845		3.45	2.956172025	1.565032249		1.7			3.19		2.66	7.23	1.46		3.39			5.6	3.93	1.03
			9.56	8.26		1.182		3.43	1.043354832	5.583880492		1.68			2.27		2.95	7.38			3.37			14.38	4.74	0.43
				8.33		4.125		2.34	1.60367502	1.951459964		1.68			0.39		2.12	10.91			3.29			2.04	2.27	5.94
				8.41		4.19		2.72	2.415173223	3.284635584		1.64			29.29		4.21	7.92			3.26			2.27	2.99	0.57
				8.4		2.266		4.72	4.192740716	3.43920667		1.57			2.93		3.48	4.07			3.16			2.58	11.83	9.44
				8.44		4.252		4.97	1.951459964	3.400563899		1.5			1.88		7.28	0.03			3.16			2.61	11.66	6.4
				8.48		2.363		1.09	2.33788768	2.20263798		1.46			2.43		5.51	11.14			3.13			5.02	7.15	3.67
				8.49		2.718		2.64	2.511780152	2.086709665		1.44			2.26		4.67	21.93			3.13			0.95	2.7	3.96
				8.54		7.788		3.97	2.299244909	1.854853036		1.44			1.7		10.14	4.60			3.05			2.27	3.53	4.51
				8.52		2.106		1.44	4.192740716	2.473137381		1.36			1.54		5.46	8.88			3.05			7.95	11.86	14.7
				8.55		5.876		2.19	3.458528056	3.786991614		1.36			1.56		13.31	6.79			3			5.34	27.16	6.2
				8.69		0.125		2.15	7.941089558	2.086709665		1.35			1.96		3.33	4.59			2.95			5.94	27.13	12.72
				8.52		3.867		2.09	4.038169629	2.299244909		1.33			2.15		3.08	7.02			2.89			3.53	2.01	11.66
				8.67		1.187		1.85	3.477849442	2.183316594		1.25			2.62		9.48	8.13			2.87			4.54	15.56	2.24
				8.72		3.265		3.79	3.632420528	1.83553165		1.23					3.72	28.87			2.87			1.81	20.04	3.22
				8.73		3.992		2.92	8.288874503	3.632420528		1.21					24.26	4.73			2.84			0.98	1.78	1.29
				8.86		0.063		6.34		2.415173223		1.19						4.83			2.82			5.54	2.3	2.21
				8.81		1.172		4.24		1.391139777		1.16						2.80			2.82			7.26	3.16	1.46
				8.81		2.677		5.04		1.352497005		1.15						3.90			2.79			5.51	16.13	2.12
				8.9		4.031		2.57		4.385954574		1.12						10.16			2.74			3.79	4.77	1.35
				8.93		0.032		2.85		0.966069289		1.08						1.93			2.58			12	10.22	0.37
				9.09		3.129		2.34		2.724315396		1.05						7.54			2.55			3.62	5.25	1.35
				8.99		3.111		2.28		1.661639178		0.79						6.56			2.55			8.3	3.19	9.13
				9.1		4.833		3.33		1.83553165		0.73						7.81			2.53			2.38	5.77	0.66
				9.18				3.45		1.700281949		0.7						6.29			2.47			3.04	38.44	18.52
				9.15				2.54		2.163995208		0.7						7.17			2.47			2.01	6.09	5.97
				9.18				2.31		2.93685064		0.67						6.91			2.39			2.78	7.49	0.66
				9.27				2.65		4.250704873		0.65						8.27			2.39			3.33	1.03	1.21
				9.26				3.11		1.429782548		0.63						10.91			2.37			13.23	4.42	8.35
				9.23				1.1		1.429782548		0.58						9.09			2.34			6.75	0.75	6.06
				9.24				4.71		3.883598543		0.53						8.78			2.32			0.78	14.67	3.96
				9.37				3.48		2.473137381		0.46						12.06			2.26			11.51	2.81	2.3
				9.35				4.52		1.719603335		0.39						6.42			2.21			11.77	16.36	4.62
				9.32				4.14		2.318566294		0						7.72			1.79			9.73	5.91	4.19
				9.41				2.3		3.014136183								9.25			1.71			2.33	2.87	0.75
				9.48				3.53		3.226671426								2.51			1.71				28.31	8.99
				9.44				6.38		1.777567492								8.72			1.53				24.6	4.36
				9.45				1.89		4.753060904								8.18			1.5				4.33	1.52
				9.43				4.25		2.898207868								6.69			1.5				12.11	23.97
				9.5				2.15		1.990102736								4.08			1.47					3.93
				9.61				1.91		3.748348843								3.41			1.42					1.92
				9.66				8.09		1.487746706								8.05			1.21					32.12
				9.62				4.16		1.912817193								5.79			1.16					2.1
								4.35		2.724315396								28.87			1.08					
								2.58		2.260602137								5.68			0.76					
								1.58		4.463240117								15.44			0.61					
								2.58		12.44297245								5.17			0.37					
								2.53		1.526389477								9.65			0.34					
								5.29		2.956172025								4.71			0.13					
								2.02		3.613099142								7.62			0.08					
								4.37		4.038169629								11.95			0					
								2.11		2.086709665								5.27								
								2.58		6.124879294								10.57								
								3.08		3.709706071																
								3.63		4.753060904																
								6.08		2.376530452																
								2.66		4.965596147																
								2.66		2.395851838																
								3.92		1.912817193																
								1.93		2.724315396																
								2.55																		
								6.11																		
