# Supplementary material for: Mutational Landscape of Esophageal Squamous Cell Carcinoma in an Indian Cohort
Source: Front Oncol. 2020 Aug 20;10:1457. doi: 10.3389/fonc.2020.01457 (PMC7469928; doi:10.3389/fonc.2020.01457)
Supplement: Supplementary Table 6 — List of neoantigens predicted using TSNAD. [file Table_6.pdf]

Mangalaparthi *et al.* , 2020. Mutational landscape of esophageal squamous cell carcinoma in an Indian cohort

Supplementary Table 6. List of neoantigens predicted using TSNAD

Software	Variant accession	Position	HLA subtype	Wild type peptide	Wild binding	Wild rank	wild binding level	Mutant peptide	Mutant binding	Mutant rank	Mutant binding level	Gene symbol	Variant information	Habit	Sample ID	OncoMD	COSMIC	dbSNP	ExAC	ExAC_SAS	1000G	1000G_SAS	SIFT	Polyphen2	CONDEL	VEP	LRT	PhastCon7Way	Total read depth in Normal	Reference read support in Normal	Alternative read support in Normal	Total read depth in Tumor	Reference read support in Tumor	Alternate read support in Tumor	Alternate allele frequency in Tumor
HLA	ADPGK_K449T	10	HLA-A*33:03	NKPVVEWHR	4539.4	4.6391	-	NTPVVEWHR	48.2	0.181	SB	ADPGK	K449T	Smoker	42480	NA	NA	NA	NA	NA	NA	NA	T	BN	N	MODERATE	N	0.938	165	165	0	159	116	43	27.04
TSNAD	ADPGK_K449T	10	HLA-A*68:01	NKPVVEWHR	7168.3	9.1418	-	NTPVVEWHR	13.3	0.1499	SB	ADPGK	K449T	Smoker	42480	NA	NA	NA	NA	NA	NA	NA	T	BN	N	MODERATE	N	0.938	165	165	0	159	116	43	27.04
TSNAD	ADPGK_K449T	10	HLA-A*33:01	NKPVVEWHR	3803.2	3.2834	-	NTPVVEWHR	55.6	0.1578	SB	ADPGK	K449T	Smoker	42480	NA	NA	NA	NA	NA	NA	NA	T	BN	N	MODERATE	N	0.938	165	165	0	159	116	43	27.04
TSNAD	ADPGK_K449T	10	HLA-A*33:03	NKPVVEWHR	4539.4	4.6391	-	NTPVVEWHR	48.2	0.181	SB	ADPGK	K449T	Smoker	42480	NA	NA	NA	NA	NA	NA	NA	T	BN	N	MODERATE	N	0.938	165	165	0	159	116	43	27.04
HLA	AFF4_T973K	2	HLA-A*11:01	KSPFPMYSET	22056.7	19.4467	-	KSPFPMYSEK	29.2	0.1734	SB	AFF4	T973K	Chewer	42487	NA	NA	chr5*132223553	NA	NA	NA	NA	D	PrD_PrD	D	MODERATE	D	0.977	248	247	1	155	145	10	6.45
TSNAD	AFF4_T973K	2	HLA-A*30:01	KSPFPMYSET	1689.3	5.1696	-	KSPFPMYSEK	35.9	0.186	SB	AFF4	T973K	Chewer	42487	NA	NA	chr5*132223553	NA	NA	NA	NA	D	PrD_PrD	D	MODERATE	D	0.977	248	247	1	155	145	10	6.45
TSNAD	AFF4_T973K	2	HLA-A*03:01	KSPFPMYSET	23927.1	21.6078	-	KSPFPMYSEK	43.9	0.1623	SB	AFF4	T973K	Chewer	42487	NA	NA	chr5*132223553	NA	NA	NA	NA	D	PrD_PrD	D	MODERATE	D	0.977	248	247	1	155	145	10	6.45
TSNAD	AFF4_T973K	2	HLA-A*11:01	KSPFPMYSET	22056.7	19.4467	-	KSPFPMYSEK	29.2	0.1734	SB	AFF4	T973K	Chewer	42487	NA	NA	chr5*132223553	NA	NA	NA	NA	D	PrD_PrD	D	MODERATE	D	0.977	248	247	1	155	145	10	6.45
HLA	AKAP1_E784K	3	HLA-A*03:01	RAQVVASYE	16797.1	12.9087	-	RAQVVASYK	69.5	0.2629	SB	AKAP1	E784K	noHabit	56958	NA	NA	0.0001997	0.000066	0	0.000199681	0	T	BN_BN	N	MODERATE	D	0.986	172	171	0	243	224	18	7.41
TSNAD	AKAP1_E784K	3	HLA-A*03:01	RAQVVASYE	16797.1	12.9087	-	RAQVVASYK	69.5	0.2629	SB	AKAP1	E784K	noHabit	56958	NA	NA	0.0001997	0.000066	0	0.000199681	0	T	BN_BN	N	MODERATE	D	0.986	172	171	0	243	224	18	7.41
TSNAD	AKAP1_E784K	3	HLA-A*30:01	RAQVVASYE	826.1	3.0104	-	RAQVVASYK	11.4	0.0319	SB	AKAP1	E784K	noHabit	56958	NA	NA	0.0001997	0.000066	0	0.000199681	0	T	BN_BN	N	MODERATE	D	0.986	172	171	0	243	224	18	7.41
TSNAD	AKAP1_E784K	3	HLA-A*31:01	RAQVVASYE	7089.9	9.2623	-	RAQVVASYK	39.3	0.3223	SB	AKAP1	E784K	noHabit	56958	NA	NA	0.0001997	0.000066	0	0.000199681	0	T	BN_BN	N	MODERATE	D	0.986	172	171	0	243	224	18	7.41
TSNAD	AKAP1_E784K	3	HLA-A*11:01	RAQVVASYE	13681	11.5938	-	RAQVVASYK	33.4	0.2004	SB	AKAP1	E784K	noHabit	56958	NA	NA	0.0001997	0.000066	0	0.000199681	0	T	BN_BN	N	MODERATE	D	0.986	172	171	0	243	224	18	7.41
HLA	AMACR_N125I	10	HLA-A*02:01	INYLALSGV	4346.9	8.7462	-	IIYLALSGV	30.9	0.4129	SB	AMACR	N125I	noHabit	42494	NA	NA	NA	0.0000083	0	NA	NA	D_lc	PrD_PrD	D	MODERATE	NA	NA	76	76	0	61	50	11	18.03
TSNAD	AMACR_N125I	10	HLA-A*02:03	INYLALSGV	620	5.3245	-	IIYLALSGV	6.1	0.0871	SB	AMACR	N125I	noHabit	42494	NA	NA	NA	0.0000083	0	NA	NA	D_lc	PrD_PrD	D	MODERATE	NA	NA	76	76	0	61	50	11	18.03
TSNAD	AMACR_N125I	10	HLA-A*02:11	INYLALSGV	2100.7	9.8957	-	IIYLALSGV	7.7	0.4504	SB	AMACR	N125I	noHabit	42494	NA	NA	NA	0.0000083	0	NA	NA	D_lc	PrD_PrD	D	MODERATE	NA	NA	76	76	0	61	50	11	18.03
TSNAD	AMACR_N125I	10	HLA-A*02:09	INYLALSGV	4346.9	8.7462	-	IIYLALSGV	30.9	0.4129	SB	AMACR	N125I	noHabit	42494	NA	NA	NA	0.0000083	0	NA	NA	D_lc	PrD_PrD	D	MODERATE	NA	NA	76	76	0	61	50	11	18.03
TSNAD	AMACR_N125I	10	HLA-A*02:01	INYLALSGV	4346.9	8.7462	-	IIYLALSGV	30.9	0.4129	SB	AMACR	N125I	noHabit	42494	NA	NA	NA	0.0000083	0	NA	NA	D_lc	PrD_PrD	D	MODERATE	NA	NA	76	76	0	61	50	11	18.03
HLA	ARHGEF4_K34R	3	HLA-A*31:01	VVKSGTHVK	901.9	2.8897	-	VVKSGTHVR	52.1	0.4355	SB	ARHGEF4	K34R	Chewer	42483	NA	NA	NA	NA	NA	NA	NA	T	PoD_PoD	N	MODERATE	NA	NA	71	71	0	100	60	40	40
TSNAD	ARHGEF4_K34R	3	HLA-A*31:01	VVKSGTHVK	901.9	2.8897	-	VVKSGTHVR	52.1	0.4355	SB	ARHGEF4	K34R	Chewer	42483	NA	NA	NA	NA	NA	NA	NA	T	PoD_PoD	N	MODERATE	NA	NA	71	71	0	100	60	40	40
HLA	ASTN2_G1215R	3	HLA-A*68:01	NLYNGYTSG	32703.7	42.532	-	NLYNGYTSR	40.7	0.4672	SB	ASTN2	G1215R	Chewer	42488	NA	NA	NA	NA	NA	NA	NA	D	PrD_PrD	D	MODERATE	D	0.995	80	80	0	101	92	8	7.92
TSNAD	ASTN2_G1215R	3	HLA-A*33:01	NLYNGYTSG	32087.5	30.0106	-	NLYNGYTSR	32.9	0.0913	SB	ASTN2	G1215R	Chewer	42488	NA	NA	NA	NA	NA	NA	NA	D	PrD_PrD	D	MODERATE	D	0.995	80	80	0	101	92	8	7.92
TSNAD	ASTN2_G1215R	3	HLA-A*33:03	NLYNGYTSG	28792.6	28.7431	-	NLYNGYTSR	26.2	0.0941	SB	ASTN2	G1215R	Chewer	42488	NA	NA	NA	NA	NA	NA	NA	D	PrD_PrD	D	MODERATE	D	0.995	80	80	0	101	92	8	7.92
TSNAD	ASTN2_G1215R	3	HLA-A*31:01	NLYNGYTSG	26018.6	35.6749	-	NLYNGYTSR	51.8	0.4324	SB	ASTN2	G1215R	Chewer	42488	NA	NA	NA	NA	NA	NA	NA	D	PrD_PrD	D	MODERATE	D	0.995	80	80	0	101	92	8	7.92
TSNAD	ASTN2_G1215R	3	HLA-A*68:01	NLYNGYTSG	32703.7	42.532	-	NLYNGYTSR	40.7	0.4672	SB	ASTN2	G1215R	Chewer	42488	NA	NA	NA	NA	NA	NA	NA	D	PrD_PrD	D	MODERATE	D	0.995	80	80	0	101	92	8	7.92
TSNAD	ASTN2_G1215R	3	HLA-A*34:02	NLYNGYTSG	24280.7	28.5797	-	NLYNGYTSR	88	0.1459	SB	ASTN2	G1215R	Chewer	42488	NA	NA	NA	NA	NA	NA	NA	D	PrD_PrD	D	MODERATE	D	0.995	80	80	0	101	92	8	7.92
HLA	ATF6_G393V	2	HLA-A*02:11	VLAFIILNYG	1576.1	8.8133	-	VLAFIILNYV	5.7	0.3118	SB	ATF6	G393V	noHabit	56957	NA	NA	NA	NA	NA	NA	NA	D	PoD_PrD	D	MODERATE	D	0.992	101	37	64	56	3	53	94.64
HLA	ATF6_G393V	2	HLA-A*02:01	VLAFIILNYG	4457.5	8.8705	-	VLAFIILNYV	19.8	0.2692	SB	ATF6	G393V	noHabit	56957	NA	NA	NA	NA	NA	NA	NA	D	PoD_PrD	D	MODERATE	D	0.992	101	37	64	56	3	53	94.64
TSNAD	ATF6_G393V	2	HLA-A*02:09	VLAFIILNYG	4457.5	8.8705	-	VLAFIILNYV	19.8	0.2692	SB	ATF6	G393V	noHabit	56957	NA	NA	NA	NA	NA	NA	NA	D	PoD_PrD	D	MODERATE	D	0.992	101	37	64	56	3	53	94.64
TSNAD	ATF6_G393V	2	HLA-A*02:03	VLAFIILNYG	903.3	6.4007	-	VLAFIILNYV	7.3	0.1189	SB	ATF6	G393V	noHabit	56957	NA	NA	NA	NA	NA	NA	NA	D	PoD_PrD	D	MODERATE	D	0.992	101	37	64	56	3	53	94.64
TSNAD	ATF6_G393V	3	HLA-A*68:02	LAFIILNYG	6128.3	9.44	-	LAFIILNYV	37.7	0.3331	SB	ATF6	G393V	noHabit	56957	NA	NA	NA	NA	NA	NA	NA	D	PoD_PrD	D	MODERATE	D	0.992	101	37	64	56	3	53	94.64
TSNAD	ATF6_G393V	2	HLA-A*02:05	VLAFIILNYG	1437	5.3626	-	VLAFIILNYV	31	0.1978	SB	ATF6	G393V	noHabit	56957	NA	NA	NA	NA	NA	NA	NA	D	PoD_PrD	D	MODERATE	D	0.992	101	37	64	56	3	53	94.64
TSNAD	ATF6_G393V	2	HLA-A*02:11	VLAFIILNYG	1576.1	8.8133	-	VLAFIILNYV	5.7	0.3118	SB	ATF6	G393V	noHabit	56957	NA	NA	NA	NA	NA	NA	NA	D	PoD_PrD	D	MODERATE	D	0.992	101	37	64	56	3	53	94.64
TSNAD	ATF6_G393V	2	HLA-A*02:01	VLAFIILNYG	4457.5	8.8705	-	VLAFIILNYV	19.8	0.2692	SB	ATF6	G393V	noHabit	56957	NA	NA	NA	NA	NA	NA	NA	D	PoD_PrD	D	MODERATE	D	0.992	101	37	64	56	3	53	94.64
HLA	BMP2K_Q541K	1	HLA-A*11:01	SQYPTMMPQYQ	5798.9	6.3667	-	SQYPTMMPQY	21.1	0.1106	SB	BMP2K	Q541K	Chewer	42489	NA	NA	NA	NA	NA	NA	NA	D_lc	UN_UN	NA	MODERATE	N	1	65	65	0	64	48	16	25
TSNAD	BMP2K_Q541K	1	HLA-A*11:01	SQYPTMMPQYQ	5798.9	6.3667	-	SQYPTMMPQY	21.1	0.1106	SB	BMP2K	Q541K	Chewer	42489	NA	NA	NA	NA	NA	NA	NA	D_lc	UN_UN	NA	MODERATE	N	1	65	65	0	64	48	16	25
TSNAD	BMP2K_Q541K	1	HLA-A*03:01	SQYPTMMPQYQ	7614.4	6.2412	-	SQYPTMMPQY	34.1	0.1181	SB	BMP2K	Q541K	Chewer	42489	NA	NA	NA	NA	NA	NA	NA	D_lc	UN_UN	NA	MODERATE	N	1	65	65	0	64	48	16	25
HLA	BPIFB2_G61R	2	HLA-A*33:03	TVPHFLDWSG	24425.7	21.1855	-	TVPHFLDWSR	41.9	0.1564	SB	BPIFB2	G61R	Smoker	42480	NA	NA	NA	NA	NA	NA	NA	T	PoD	D	MODERATE	N	0.037	65	65	0	42	29	13	30.95
TSNAD	BPIFB2_G61R	2	HLA-A*33:03	TVPHFLDWSG	24425.7	21.1855	-	TVPHFLDWSR	41.9	0.1564	SB	BPIFB2	G61R	Smoker	42480	NA	NA	NA	NA	NA	NA	NA	T	PoD	D	MODERATE	N	0.037	65	65	0	42	29	13	30.95
TSNAD	BPIFB2_G61R	2	HLA-A*33:01	TVPHFLDWSG	26215	19.3645	-	TVPHFLDWSR	69.8	0.1936	SB	BPIFB2	G61R	Smoker	42480	NA	NA	NA	NA	NA	NA	NA	T	PoD	D	MODERATE	N	0.037	65	65	0	42	29	13	30.95
TSNAD	BPIFB2_G61R	2	HLA-A*68:01	TVPHFLDWSG	20686.8	21.6938	-	TVPHFLDWSR	10.3	0.1006	SB	BPIFB2	G61R	Smoker	42480	NA	NA	NA	NA	NA	NA	NA	T	PoD	D	MODERATE	N	0.037	65	65	0	42	29	13	30.95
HLA	C1orf220_R102W	5	HLA-A*02:01	LLSSRTRTL	2097.7	5.9657	-	LLSSRTWTL	14.5	0.1783	SB	C1orf220	R102W	noHabit	56957	NA	NA	0.004792	0.006629958	0.0144081	0.00479233	0.0245	NA	PoD_PrD	NA	MODERATE	NA	NA	125	61	64	96	15	81	84.38
TSNAD	C1orf220_R102W	5	HLA-A*02:01	LLSSRTRTL	2097.7	5.9657	-	LLSSRTWTL	14.5	0.1783	SB	C1orf220	R102W	noHabit	56957	NA	NA	0.004792	0.006629958	0.0144081	0.00479233	0.0245	NA	PoD_PrD	NA	MODERATE	NA	NA	125	61	64	96	15	81	84.38
TSNAD	C1orf220_R102W	5	HLA-A*02:05	LLSSRTRTL	1593.3	5.7612	-	LLSSRTWTL	39.6	0.2621	SB	C1orf220	R102W	noHabit	56957	NA	NA	0.004792	0.006629958	0.0144081	0.00479233	0.0245	NA	PoD_PrD	NA	MODERATE	NA	NA	125	61	64	96	15	81	84.38
TSNAD	C1orf220_R102W	5	HLA-A*02:09	LLSSRTRTL	2097.7	5.9657	-	LLSSRTWTL	14.5	0.1783	SB	C1orf220	R102W	noHabit	56957	NA	NA	0.004792	0.006629958	0.0144081	0.00479233	0.0245	NA	PoD_PrD	NA	MODERATE	NA	NA	125	61	64	96	15	81	84.38
HLA	C5orf24_N9I	8	HLA-A*11:01	SNPAFCGPGK	1062.5	2.604	-	SIPAFCGPGK	56.3	0.3466	SB	C5orf24	N9I	Chewer	42487	NA	NA	NA	NA	NA	NA	NA	D_lc	PrD_PrD	D	MODERATE	D	NA	84	84	0	61	24	37	60.66
TSNAD	C5orf24_N9I	8	HLA-A*11:01	SNPAFCGPGK	1062.5	2.604	-	SIPAFCGPGK	56.3	0.3466	SB	C5orf24	N9I	Chewer	42487	NA	NA	NA	NA	NA	NA	NA	D_lc	PrD_PrD	D	MODERATE	D	NA	84	84	0	61	24	37	60.66
HLA	CACNA1C_A520T	10	HLA-A*11:01	AAVKSNVFY	653.6	2.0209	-	ATVKSNVFY	62.1	0.3784	SB	CACNA1C	A520T	Chewer	42487	NA	NA	NA	0.0000196	0	NA	NA	D	BN_PoD	N	MODERATE	D	0.971	49	49	0	50	31	19	38
TSNAD	CACNA1C_A520T	10	HLA-A*11:01	AAVKSNVFY	653.6	2.0209	-	ATVKSNVFY	62.1	0.3784	SB	CACNA1C	A520T	Chewer	42487	NA	NA	NA	0.0000196	0	NA	NA	D	BN_PoD	N	MODERATE	D	0.971	49	49	0	50	31	19	38
HLA	CALCR_G106A	2	HLA-A*02:01	WLCWDDTPAG	3122.3	7.3277	-	WLCWDDTPA	31.1	0.4164	SB	CALCR	G106A	noHabit	42493	NA	NA	NA	NA	NA	NA	NA	D	PrD_PrD	D	MODERATE	NA	NA	167	167	0	273	225	48	17.58
TSNAD	CALCR_G106A	2	HLA-A*02:09	WLCWDDTPAG	3122.3	7.3277	-	WLCWDDTPA	31.1	0.4164	SB	CALCR	G106A	noHabit	42493	NA	NA	NA	NA	NA	NA	NA	D	PrD_PrD	D	MODERATE	NA	NA	167	167	0	273	225	48	17.58
TSNAD	CALCR_G106A	2	HLA-A*02:01	WLCWDDTPAG	3122.3	7.3277	-	WLCWDDTPA	31.1	0.4164	SB	CALCR	G106A	noHabit	42493	NA	NA	NA	NA	NA	NA	NA	D	PrD_PrD	D	MODERATE	NA	NA	167	167	0	273	225	48	17.58
HLA	CD19_R76S	9	HLA-A*02:01	HMRPLAIWL	802.3	3.6301	-	HMSPLAIWL	31.7	0.4235	SB	CD19	R76S	noHabit	42495	NA	NA	NA	NA	NA	NA	NA	D	BN_BN	N	MODERATE	N	0.01	45	45	0	42	21	21	50
TSNAD	CD19_R76S	10	HLA-B*57:03	MRPLAIWLF	14346.5	11.8764	-	MSPLAIWLF	55.5	0.118	SB	CD19	R76S	noHabit	42495	NA	NA	NA	NA	NA	NA	NA	D	BN_BN	N	MODERATE	N	0.01	45	45	0	42	21	21	50
TSNAD	CD19_R76S	9	HLA-A*02:09	HMRPLAIWL	802.3	3.6301	-	HMSPLAIWL	31.7	0.4235	SB	CD19	R76S	noHabit	42495	NA	NA	NA	NA	NA	NA	NA	D	BN_BN	N	MODERATE	N	0.01	45	45	0	42	21	21	50
TSNAD	CD19_R76S	9	HLA-A*02:05	HMRPLAIWL	710.9	3.2687	-	HMSPLAIWL	43.6	0.2882	SB	CD19	R76S	noHabit	42495	NA	NA	NA	NA	NA	NA	NA	D	BN_BN	N	MODERATE	N	0.01	45	45	0	42	21	21	50
TSNAD	CD19_R76S	10	HLA-B*58:01	MRPLAIWLF	12926	16.0163	-	MSPLAIWLF	98.5	0.4716	SB	CD19	R76S	noHabit	42495	NA	NA	NA	NA	NA	NA	NA	D	BN_BN	N	MODERATE	N	0.01	45	45	0	42	21	21	50
TSNAD	CD19_R76S	9	HLA-A*02:01	HMRPLAIWL	802.3	3.6301	-	HMSPLAIWL	31.7	0.4235	SB	CD19	R76S	noHabit	42495	NA	NA	NA	NA	NA	NA	NA	D	BN_BN	N	MODERATE	N	0.01	45	45	0	42	21	21	50
HLA	CHST1_E358K	2	HLA-A*11:01	TVRNSAATAE	23778.5	21.6743	-	TVRNSAATAK	81.1	0.4776	SB	CHST1	E358K	noHabit	42497	chr11*45671402	chr11*45671402	chr11*45671402	NA	NA	NA	NA	D	PoD_PrD	D	MODERATE	D	1	90	90	0	71	51	20	28.17
TSNAD	CHST1_E358K	2	HLA-A*11:01	TVRNSAATAE	23778.5	21.6743	-	TVRNSAATAK	81.1	0.4776	SB	CHST1	E358K	noHabit	42497	chr11*45671402	chr11*45671402	chr11*45671402	NA	NA	NA	NA	D	PoD_PrD	D	MODERATE	D	1	90	90	0	71	51	20	28.17
TSNAD	CHST1_E358K	2	HLA-A*30:01	TVRNSAATAE	4368.9	10.9713	-	TVRNSAATAK	98.9	0.494	SB	CHST1	E358K	noHabit	42497	chr11*45671402	chr11*45671402	chr11*45671402	NA	NA	NA	NA	D	PoD_PrD	D	MODERATE	D	1	90	90	0	71	51	20	28.17
TSNAD	CHST1_E358K	2	HLA-A*34:02	TVRNSAATAE	9491.7	9.6894	-	TVRNSAATAK	88.8	0.1472	SB	CHST1	E358K	noHabit	42497	chr11*45671402	chr11*45671402	chr11*45671402	NA	NA	NA	NA	D	PoD_PrD	D	MODERATE	D	1	90	90	0	71	51	20	28.17
TSNAD	CHST1_E358K	11	HLA-B*27:05	EKWRFRLSY	1794.4	2.8089	-	KKWRFRLSY	94	0.3123	SB	CHST1	E358K	noHabit	42497	chr11*45671402	chr11*45671402	chr11*45671402	NA	NA	NA	NA	D	PoD_PrD	D	MODERATE	D	1	90	90	0	71	51	20	28.17
HLA	CNTN6_D169Y	6	HLA-A*24:02	LYVQEDNRRF	701.4	0.8489	WB	LYVQEYNRRF	92.4	0.1989	SB	CNTN6	D169Y	noHabit	42494	NA	NA	NA	NA	NA	NA	NA	D	PoD_PoD	D	MODERATE	D	0.778	169	168	0	112	95	17	15.18
TSNAD	CNTN6_D169Y	6	HLA-A*24:02	LYVQEDNRRF	701.4	0.8489	WB	LYVQEYNRRF	92.4	0.1989	SB	CNTN6	D169Y	noHabit	42494	NA	NA	NA	NA	NA	NA	NA	D	PoD_PoD	D	MODERATE	D	0.778	169	168	0	112	95	17	15.18
HLA	CPA3_G370V	3	HLA-A*02:01	SLDWAYDLG	13958.5	18.3673	-	SLDWAYDLV	35.3	0.4665	SB	CPA3	G370V	Smoker	42478	NA	NA	NA	NA	NA	NA	NA	D	PrD	D	MODERATE	D	0.999	169	169	0	166	156	10	6.02
TSNAD	CPA3_G370V	10	HLA-A*29:01	LGIKHTFAF	1079.3	1.5813	WB	LVIKHTFAF	100	0.4094	SB	CPA3	G370V	Smoker	42478	NA	NA	NA	NA	NA	NA	NA	D	PrD	D	MODERATE	D	0.999	169	169	0	166	156	10	6.02
TSNAD	CPA3_G370V	10	HLA-A*29:02	LGIKHTFAF	1079.3	1.5813	WB	LVIKHTFAF	100	0.4094	SB	CPA3	G370V	Smoker	42478	NA	NA	NA	NA	NA	NA	NA	D	PrD	D	MODERATE	D	0.999	169	169	0	166	156	10	6.02
TSNAD	CPA3_G370V	3	HLA-A*02:11	SLDWAYDLG	6194.1	15.9923	-	SLDWAYDLV	6.3	0.3539	SB	CPA3	G370V	Smoker	42478	NA	NA	NA	NA	NA	NA	NA	D	PrD	D	MODERATE	D	0.999	169	169	0	166	156	10	6.02
TSNAD	CPA3_G370V	3	HLA-A*02:01	SLDWAYDLG	13958.5	18.3673	-	SLDWAYDLV	35.3	0.4665	SB	CPA3	G370V	Smoker	42478	NA	NA	NA	NA	NA	NA	NA	D	PrD	D	MODERATE	D	0.999	169	169	0	166	156	10	6.02
TSNAD	CPA3_G370V	10	HLA-A*32:01	LGIKHTFAF	1032.1	1.3313	WB	LVIKHTFAF	33.4	0.0599	SB	CPA3	G370V	Smoker	42478	NA	NA	NA	NA	NA	NA	NA	D	PrD	D	MODERATE	D	0.999	169	169	0	166	156	10	6.02
TSNAD	CPA3_G370V	3	HLA-A*02:09	SLDWAYDLG	13958.5	18.3673	-	SLDWAYDLV	35.3	0.4665	SB	CPA3	G370V	Smoker	42478	NA	NA	NA	NA	NA	NA	NA	D	PrD	D	MODERATE	D	0.999	169	169	0	166	156	10	6.02
HLA	CRYAA_R112L	10	HLA-A*33:03	SREFHRRYR	2484.1	3.2999	-	SLEFHRRYR	59.9	0.2296	SB	CRYAA	R112L	Smoker	42479	NA	NA	chr21*44592203	NA	NA	NA	NA	D	PrD	D	MODERATE	D	0.998	42	42	0	62	44	18	29.03
TSNAD	CRYAA_R112L	10	HLA-A*31:01	SREFHRRYR	1709	4.0752	-	SLEFHRRYR	25	0.1899	SB	CRYAA	R112L	Smoker	42479	NA	NA	chr21*44592203	NA	NA	NA	NA	D	PrD	D	MODERATE	D	0.998	42	42	0	62	44	18	29.03
TSNAD	CRYAA_R112L	10	HLA-A*33:03	SREFHRRYR	2484.1	3.2999	-	SLEFHRRYR	59.9	0.2296	SB	CRYAA	R112L	Smoker	42479	NA	NA	chr21*44592203	NA	NA	NA	NA	D	PrD	D	MODERATE	D	0.998	42	42	0	62	44	18	29.03
HLA	CSMD3_W47R	3	HLA-A*33:03	GIKSGFTFW	24928.1	21.938	-	GIKSGFTFR	37.9	0.1358	SB	CSMD3	W47R	Smoker	42479	chr8*114448945	chr8*114448945	NA	NA	NA	NA	NA	D_lc	BN	N	MODERATE	N	1	86	85	0	179	53	126	70.39
HLA	CSMD3_W47R	2	HLA-A*33:03	MGIKSGFTFW	13069.3	9.7915	-	MGIKSGFTFR	13.3	0.0253	SB	CSMD3	W47R	Smoker	42479	chr8*114448945	chr8*114448945	NA	NA	NA	NA	NA	D_lc	BN	N	MODERATE	N	1	86	85	0	179	53	126	70.39
TSNAD	CSMD3_W47R	2	HLA-A*33:03	MGIKSGFTFW	13069.3	9.7915	-	MGIKSGFTFR	13.3	0.0253	SB	CSMD3	W47R	Smoker	42479	chr8*114448945	chr8*114448945	NA	NA	NA	NA	NA	D_lc	BN	N	MODERATE	N	1	86	85	0	179	53	126	70.39
TSNAD	CSMD3_W47R	2	HLA-A*33:01	MGIKSGFTFW	17211.2	10.4501	-	MGIKSGFTFR	22.9	0.0565	SB	CSMD3	W47R	Smoker	42479	chr8*114448945	chr8*114448945	NA	NA	NA	NA	NA	D_lc	BN	N	MODERATE	N	1	86	85	0	179	53	126	70.39
TSNAD	CSMD3_W47R	3	HLA-A*31:01	GIKSGFTFW	11227.5	13.2877	-	GIKSGFTFR	8.6	0.0362	SB	CSMD3	W47R	Smoker	42479	chr8*114448945	chr8*114448945	NA	NA	NA	NA	NA	D_lc	BN	N	MODERATE	N	1	86	85	0	179	53	126	70.39
TSNAD	CSMD3_W47R	2	HLA-A*68:01	MGIKSGFTFW	11108	12.3743	-	MGIKSGFTFR	20.7	0.2598	SB	CSMD3	W47R	Smoker	42479	chr8*114448945	chr8*114448945	NA	NA	NA	NA	NA	D_lc	BN	N	MODERATE	N	1	86	85	0	179	53	126	70.39
TSNAD	CSMD3_W47R	1	HLA-A*31:01	KMGIKSGFTFW	7748.5	9.8764	-	KMGIKSGFTF	9.3	0.0423	SB	CSMD3	W47R	Smoker	42479	chr8*114448945	chr8*114448945	NA	NA	NA	NA	NA	D_lc	BN	N	MODERATE	N	1	86	85	0	179	53	126	70.39
TSNAD	CSMD3_W47R	3	HLA-A*33:03	GIKSGFTFW	24928.1	21.938	-	GIKSGFTFR	37.9	0.1358	SB	CSMD3	W47R	Smoker	42479	chr8*114448945	chr8*114448945	NA	NA	NA	NA	NA	D_lc	BN	N	MODERATE	N	1	86	85	0	179	53	126	70.39
TSNAD	CSMD3_W47R	2	HLA-A*31:01	MGIKSGFTFW	8836.7	10.8994	-	MGIKSGFTFR	9.2	0.0414	SB	CSMD3	W47R	Smoker	42479	chr8*114448945	chr8*114448945	NA	NA	NA	NA	NA	D_lc	BN	N	MODERATE	N	1	86	85	0	179	53	126	70.39
HLA	DOCK5_G639R	3	HLA-A*33:03	LTQNVDLLG	34274.1	42.7246	-	LTQNVDLLR	92.2	0.3549	SB	DOCK5	G639R	Smoker	42475	NA	NA	NA	NA	NA	NA	NA	D	PrD	D	MODERATE	D	0.994	179	178	0	176	83	93	52.84
TSNAD	DOCK5_G639R	3	HLA-A*33:03	LTQNVDLLG	34274.1	42.7246	-	LTQNVDLLR	92.2	0.3549	SB	DOCK5	G639R	Smoker	42475	NA	NA	NA	NA	NA	NA	NA	D	PrD	D	MODERATE	D	0.994	179	178	0	176	83	93	52.84
TSNAD	DOCK5_G639R	3	HLA-A*68:01	LTQNVDLLG	24634.5	26.865	-	LTQNVDLLR	21.7	0.2734	SB	DOCK5	G639R	Smoker	42475	NA	NA	NA	NA	NA	NA	NA	D	PrD	D	MODERATE	D	0.994	179	178	0	176	83	93	52.84
HLA	DPP4_P218S	10	HLA-A*01:01	SPNGTFLAY	6200.6	1.8638	WB	SSNGTFLAY	57	0.0556	SB	DPP4	P218S	Chewer	42483	NA	NA	NA	NA	NA	NA	NA	T	PrD_PrD	D	MODERATE	D	1	135	134	0	109	64	45	41.28
TSNAD	DPP4_P218S	10	HLA-A*01:02	SPNGTFLAY	1862	0.7888	WB	SSNGTFLAY	90.4	0.0206	SB	DPP4	P218S	Chewer	42483	NA	NA	NA	NA	NA	NA	NA	T	PrD_PrD	D	MODERATE	D	1	135	134	0	109	64	45	41.28
TSNAD	DPP4_P218S	10	HLA-A*30:02	SPNGTFLAY	995.1	1.4874	WB	SSNGTFLAY	18.7	0.0113	SB	DPP4	P218S	Chewer	42483	NA	NA	NA	NA	NA	NA	NA	T	PrD_PrD	D	MODERATE	D	1	135	134	0	109	64	45	41.28
TSNAD	DPP4_P218S	10	HLA-B*15:01	SPNGTFLAY	1650.6	3.6887	-	SSNGTFLAY	34.3	0.1651	SB	DPP4	P218S	Chewer	42483	NA	NA	NA	NA	NA	NA	NA	T	PrD_PrD	D	MODERATE	D	1	135	134	0	109	64	45	41.28
TSNAD	DPP4_P218S	10	HLA-A*36:01	SPNGTFLAY	2943.1	1.6068	WB	SSNGTFLAY	47.2	0.0288	SB	DPP4	P218S	Chewer	42483	NA	NA	NA	NA	NA	NA	NA	T	PrD_PrD	D	MODERATE	D	1	135	134	0	109	64	45	41.28
TSNAD	DPP4_P218S	10	HLA-A*30:04	SPNGTFLAY	2610	2.2536	-	SSNGTFLAY	95.4	0.0175	SB	DPP4	P218S	Chewer	42483	NA	NA	NA	NA	NA	NA	NA	T	PrD_PrD	D	MODERATE	D	1	135	134	0	109	64	45	41.28
TSNAD	DPP4_P218S	10	HLA-C*12:03	SPNGTFLAY	514	1.5759	WB	SSNGTFLAY	34.4	0.1437	SB	DPP4	P218S	Chewer	42483	NA	NA	NA	NA	NA	NA	NA	T	PrD_PrD	D	MODERATE	D	1	135	134	0	109	64	45	41.28
TSNAD	DPP4_P218S	10	HLA-A*11:01	SPNGTFLAY	6410.7	6.7597	-	SSNGTFLAY	34.6	0.2088	SB	DPP4	P218S	Chewer	42483	NA	NA	NA	NA	NA	NA	NA	T	PrD_PrD	D	MODERATE	D	1	135	134	0	109	64	45	41.28
TSNAD	DPP4_P218S	10	HLA-A*01:01	SPNGTFLAY	6200.6	1.8638	WB	SSNGTFLAY	57	0.0556	SB	DPP4	P218S	Chewer	42483	NA	NA	NA	NA	NA	NA	NA	T	PrD_PrD	D	MODERATE	D	1	135	134	0	109	64	45	41.28
TSNAD	DPP4_P218S	10	HLA-B*15:17	SPNGTFLAY	4140.4	9.0162	-	SSNGTFLAY	10.5	0.144	SB	DPP4	P218S	Chewer	42483	NA	NA	NA	NA	NA	NA	NA	T	PrD_PrD	D	MODERATE	D	1	135	134	0	109	64	45	41.28
TSNAD	DPP4_P218S	10	HLA-C*16:01	SPNGTFLAY	512.7	1.3963	WB	SSNGTFLAY	57.5	0.1925	SB	DPP4	P218S	Chewer	42483	NA	NA	NA	NA	NA	NA	NA	T	PrD_PrD	D	MODERATE	D	1	135	134	0	109	64	45	41.28
HLA	ELF3_E309K	11	HLA-A*11:01	EAVAQLWGQK	998.5	2.5156	-	KAVAQLWGQ	83.3	0.4877	SB	ELF3	E309K	noHabit	42500	NA	NA	NA	NA	NA	NA	NA	D	PrD_PrD	D	MODERATE	D	0.986	188	188	0	134	103	30	22.39
HLA	ELF3_E309K	3	HLA-A*11:01	GVFKFLRSE	9939.9	8.9665	-	GVFKFLRSK	20	0.1017	SB	ELF3	E309K	noHabit	42500	NA	NA	NA	NA	NA	NA	NA	D	PrD_PrD	D	MODERATE	D	0.986	188	188	0	134	103	30	22.39
TSNAD	ELF3_E309K	3	HLA-A*11:01	GVFKFLRSE	9939.9	8.9665	-	GVFKFLRSK	20	0.1017	SB	ELF3	E309K	noHabit	42500	NA	NA	NA	NA	NA	NA	NA	D	PrD_PrD	D	MODERATE	D	0.986	188	188	0	134	103	30	22.39
TSNAD	ELF3_E309K	3	HLA-A*03:01	GVFKFLRSE	12292.8	9.2686	-	GVFKFLRSK	26.5	0.0789	SB	ELF3	E309K	noHabit	42500	NA	NA	NA	NA	NA	NA	NA	D	PrD_PrD	D	MODERATE	D	0.986	188	188	0	134	103	30	22.39
TSNAD	ELF3_E309K	3	HLA-A*03:02	GVFKFLRSE	14110.1	11.2813	-	GVFKFLRSK	87.7	0.2556	SB	ELF3	E309K	noHabit	42500	NA	NA	NA	NA	NA	NA	NA	D	PrD_PrD	D	MODERATE	D	0.986	188	188	0	134	103	30	22.39
TSNAD	ELF3_E309K	11	HLA-A*11:01	EAVAQLWGQK	998.5	2.5156	-	KAVAQLWGQ	83.3	0.4877	SB	ELF3	E309K	noHabit	42500	NA	NA	NA	NA	NA	NA	NA	D	PrD_PrD	D	MODERATE	D	0.986	188	188	0	134	103	30	22.39
TSNAD	ELF3_E309K	7	HLA-B*08:01	FLRSEAVA	2452.6	2.8487	-	FLRSKAVA	78.4	0.1371	SB	ELF3	E309K	noHabit	42500	NA	NA	NA	NA	NA	NA	NA	D	PrD_PrD	D	MODERATE	D	0.986	188	188	0	134	103	30	22.39
TSNAD	ELF3_E309K	9	HLA-A*32:01	RSEAVAQLW	501	0.7609	WB	RSKAVAQLW	78.7	0.1458	SB	ELF3	E309K	noHabit	42500	NA	NA	NA	NA	NA	NA	NA	D	PrD_PrD	D	MODERATE	D	0.986	188	188	0	134	103	30	22.39
TSNAD	ELF3_E309K	7	HLA-B*57:01	FLRSEAVAQLW	560.6	0.8151	WB	FLRSKAVAQL	59.9	0.1585	SB	ELF3	E309K	noHabit	42500	NA	NA	NA	NA	NA	NA	NA	D	PrD_PrD	D	MODERATE	D	0.986	188	188	0	134	103	30	22.39
TSNAD	ELF3_E309K	2	HLA-A*34:02	EGVFKFLRSE	10471.7	10.59	-	EGVFKFLRSK	98.3	0.17	SB	ELF3	E309K	noHabit	42500	NA	NA	NA	NA	NA	NA	NA	D	PrD_PrD	D	MODERATE	D	0.986	188	188	0	134	103	30	22.39
TSNAD	ELF3_E309K	3	HLA-A*30:01	GVFKFLRSE	3390.3	8.9106	-	GVFKFLRSK	43.3	0.2273	SB	ELF3	E309K	noHabit	42500	NA	NA	NA	NA	NA	NA	NA	D	PrD_PrD	D	MODERATE	D	0.986	188	188	0	134	103	30	22.39
HLA	ELP3_E330K	2	HLA-A*11:01	LVIRGTGLYE	7297.4	7.3078	-	LVIRGTGLYK	14.3	0.0567	SB	ELP3	E330K	noHabit	42497	NA	NA	NA	NA	NA	NA	NA	T	BN_BN	N	MODERATE	D	1	60	60	0	46	33	13	28.26
TSNAD	ELP3_E330K	2	HLA-A*30:01	LVIRGTGLYE	2984.3	8.0195	-	LVIRGTGLYK	53	0.2755	SB	ELP3	E330K	noHabit	42497	NA	NA	NA	NA	NA	NA	NA	T	BN_BN	N	MODERATE	D	1	60	60	0	46	33	13	28.26
TSNAD	ELP3_E330K	11	HLA-A*30:01	ELWKSGRYK	2270.9	6.4782	-	KLWKSGRYK	34.8	0.1802	SB	ELP3	E330K	noHabit	42497	NA	NA	NA	NA	NA	NA	NA	T	BN_BN	N	MODERATE	D	1	60	60	0	46	33	13	28.26
TSNAD	ELP3_E330K	3	HLA-A*30:01	VIRGTGLYE	747.6	2.7995	-	VIRGTGLYK	14.7	0.0475	SB	ELP3	E330K	noHabit	42497	NA	NA	NA	NA	NA	NA	NA	T	BN_BN	N	MODERATE	D	1	60	60	0	46	33	13	28.26
TSNAD	ELP3_E330K	11	HLA-A*31:01	ELWKSGRYK	988	3.0297	-	KLWKSGRYK	37.1	0.3013	SB	ELP3	E330K	noHabit	42497	NA	NA	NA	NA	NA	NA	NA	T	BN_BN	N	MODERATE	D	1	60	60	0	46	33	13	28.26
TSNAD	ELP3_E330K	2	HLA-A*03:02	LVIRGTGLYE	11523.3	9.3876	-	LVIRGTGLYK	60.5	0.1736	SB	ELP3	E330K	noHabit	42497	NA	NA	NA	NA	NA	NA	NA	T	BN_BN	N	MODERATE	D	1	60	60	0	46	33	13	28.26
TSNAD	ELP3_E330K	2	HLA-A*11:01	LVIRGTGLYE	7297.4	7.3078	-	LVIRGTGLYK	14.3	0.0567	SB	ELP3	E330K	noHabit	42497	NA	NA	NA	NA	NA	NA	NA	T	BN_BN	N	MODERATE	D	1	60	60	0	46	33	13	28.26

Mangalaparthi *et al.* , 2020. Mutational landscape of esophageal squamous cell carcinoma in an Indian cohort

Supplementary Table 6. List of neoantigens predicted using TSNAD

Software	Variant accession	Position	HLA subtype	Wild type peptide	Wild binding	Wild rank	wild binding level	Mutant peptide	Mutant binding	Mutant rank	Mutant binding level	Gene symbol	Variant information	Habit	Sample ID	OncoMD	COSMIC	dbSNP	ExAC	ExAC_SAS	1000G	1000G_SAS	SIFT	Polyphen2	CONDEL	VEP	LRT	PhastCon7Way	Total read depth in Normal	Reference read support in Normal	Alternative read support in Normal	Total read depth in Tumor	Reference read support in Tumor	Alternate read support in Tumor	Alternate allele frequency in Tumor
TSNAD	ELP3_E330K	3	HLA-A*03:01	VIRGTGLYE	7361.1	6.0867	-	VIRGTGLYK	32.6	0.1106	SB	ELP3	E330K	noHabit	42497	NA	NA	NA	NA	NA	NA	NA	T	BN_BN	N	MODERATE	D	1	60	60	0	46	33	13	28.26
TSNAD	ELP3_E330K	2	HLA-A*03:01	LVIRGTGLYE	7265.8	6.0273	-	LVIRGTGLYK	20.4	0.0534	SB	ELP3	E330K	noHabit	42497	NA	NA	NA	NA	NA	NA	NA	T	BN_BN	N	MODERATE	D	1	60	60	0	46	33	13	28.26
TSNAD	ELP3_E330K	2	HLA-A*34:02	LVIRGTGLYE	2521.3	3.6021	-	LVIRGTGLYK	34.1	0.0281	SB	ELP3	E330K	noHabit	42497	NA	NA	NA	NA	NA	NA	NA	T	BN_BN	N	MODERATE	D	1	60	60	0	46	33	13	28.26
HLA	FAM133A_P37R	3	HLA-A*33:03	TIQDYLNRP	37322.5	53.7655	-	TIQDYLNRR	50.8	0.19	SB	FAM133A	P37R	Smoker	42480	NA	NA	NA	NA	NA	NA	NA	D_lc	PrD	D	MODERATE	D	0.995	60	58	1	70	43	27	38.57
TSNAD	FAM133A_P37R	3	HLA-A*68:01	TIQDYLNRP	36267.6	52.8253	-	TIQDYLNRR	42.2	0.4797	SB	FAM133A	P37R	Smoker	42480	NA	NA	NA	NA	NA	NA	NA	D_lc	PrD	D	MODERATE	D	0.995	60	58	1	70	43	27	38.57
TSNAD	FAM133A_P37R	3	HLA-A*33:03	TIQDYLNRP	37322.5	53.7655	-	TIQDYLNRR	50.8	0.19	SB	FAM133A	P37R	Smoker	42480	NA	NA	NA	NA	NA	NA	NA	D_lc	PrD	D	MODERATE	D	0.995	60	58	1	70	43	27	38.57
HLA	FAM46D_G334R	3	HLA-A*11:01	TMMALKVLG	19758.6	16.8658	-	TMMALKVLR	73.7	0.4403	SB	FAM46D	G334R	noHabit	42498	NA	NA	NA	NA	NA	NA	NA	D	PrD_PrD	D	MODERATE	U	1	234	233	0	306	262	44	14.38
TSNAD	FAM46D_G334R	3	HLA-A*03:02	TMMALKVLG	13578.4	10.8796	-	TMMALKVLR	30	0.0605	SB	FAM46D	G334R	noHabit	42498	NA	NA	NA	NA	NA	NA	NA	D	PrD_PrD	D	MODERATE	U	1	234	233	0	306	262	44	14.38
TSNAD	FAM46D_G334R	4	HLA-A*31:01	MMALKVLG	26569.9	36.914	-	MMALKVLR	59	0.497	SB	FAM46D	G334R	noHabit	42498	NA	NA	NA	NA	NA	NA	NA	D	PrD_PrD	D	MODERATE	U	1	234	233	0	306	262	44	14.38
TSNAD	FAM46D_G334R	3	HLA-A*33:01	TMMALKVLG	20556.3	13.0218	-	TMMALKVLR	11.5	0.0178	SB	FAM46D	G334R	noHabit	42498	NA	NA	NA	NA	NA	NA	NA	D	PrD_PrD	D	MODERATE	U	1	234	233	0	306	262	44	14.38
TSNAD	FAM46D_G334R	2	HLA-A*03:02	ITMMALKVLG	16758.2	13.5147	-	ITMMALKVLR	71.2	0.2084	SB	FAM46D	G334R	noHabit	42498	NA	NA	NA	NA	NA	NA	NA	D	PrD_PrD	D	MODERATE	U	1	234	233	0	306	262	44	14.38
TSNAD	FAM46D_G334R	3	HLA-A*33:03	TMMALKVLG	17214.6	12.9131	-	TMMALKVLR	9.2	0.01	SB	FAM46D	G334R	noHabit	42498	NA	NA	NA	NA	NA	NA	NA	D	PrD_PrD	D	MODERATE	U	1	234	233	0	306	262	44	14.38
TSNAD	FAM46D_G334R	4	HLA-A*33:01	MMALKVLG	33469.4	33.5357	-	MMALKVLR	98.5	0.269	SB	FAM46D	G334R	noHabit	42498	NA	NA	NA	NA	NA	NA	NA	D	PrD_PrD	D	MODERATE	U	1	234	233	0	306	262	44	14.38
TSNAD	FAM46D_G334R	3	HLA-A*11:01	TMMALKVLG	19758.6	16.8658	-	TMMALKVLR	73.7	0.4403	SB	FAM46D	G334R	noHabit	42498	NA	NA	NA	NA	NA	NA	NA	D	PrD_PrD	D	MODERATE	U	1	234	233	0	306	262	44	14.38
TSNAD	FAM46D_G334R	2	HLA-A*33:03	ITMMALKVLG	22911.4	19.0587	-	ITMMALKVLR	27.5	0.0992	SB	FAM46D	G334R	noHabit	42498	NA	NA	NA	NA	NA	NA	NA	D	PrD_PrD	D	MODERATE	U	1	234	233	0	306	262	44	14.38
TSNAD	FAM46D_G334R	1	HLA-A*31:01	LITMMALKVLG	23235.7	29.9275	-	LITMMALKVL	57.1	0.4815	SB	FAM46D	G334R	noHabit	42498	NA	NA	NA	NA	NA	NA	NA	D	PrD_PrD	D	MODERATE	U	1	234	233	0	306	262	44	14.38
TSNAD	FAM46D_G334R	2	HLA-A*74:01	ITMMALKVLG	21061.3	11.1817	-	ITMMALKVLR	98.1	0.0332	SB	FAM46D	G334R	noHabit	42498	NA	NA	NA	NA	NA	NA	NA	D	PrD_PrD	D	MODERATE	U	1	234	233	0	306	262	44	14.38
TSNAD	FAM46D_G334R	3	HLA-A*74:01	TMMALKVLG	19521.2	9.9362	-	TMMALKVLR	58.2	0.012	SB	FAM46D	G334R	noHabit	42498	NA	NA	NA	NA	NA	NA	NA	D	PrD_PrD	D	MODERATE	U	1	234	233	0	306	262	44	14.38
TSNAD	FAM46D_G334R	4	HLA-A*33:03	MMALKVLG	32932	38.7009	-	MMALKVLR	70.9	0.2774	SB	FAM46D	G334R	noHabit	42498	NA	NA	NA	NA	NA	NA	NA	D	PrD_PrD	D	MODERATE	U	1	234	233	0	306	262	44	14.38
TSNAD	FAM46D_G334R	2	HLA-A*31:01	ITMMALKVLG	11670	13.7397	-	ITMMALKVLR	11.8	0.0658	SB	FAM46D	G334R	noHabit	42498	NA	NA	NA	NA	NA	NA	NA	D	PrD_PrD	D	MODERATE	U	1	234	233	0	306	262	44	14.38
TSNAD	FAM46D_G334R	2	HLA-A*33:01	ITMMALKVLG	25971.6	19.0211	-	ITMMALKVLR	50.3	0.1453	SB	FAM46D	G334R	noHabit	42498	NA	NA	NA	NA	NA	NA	NA	D	PrD_PrD	D	MODERATE	U	1	234	233	0	306	262	44	14.38
TSNAD	FAM46D_G334R	2	HLA-A*68:01	ITMMALKVLG	21848.6	23.0797	-	ITMMALKVLR	19.7	0.2458	SB	FAM46D	G334R	noHabit	42498	NA	NA	NA	NA	NA	NA	NA	D	PrD_PrD	D	MODERATE	U	1	234	233	0	306	262	44	14.38
TSNAD	FAM46D_G334R	3	HLA-A*31:01	TMMALKVLG	9289.7	11.3404	-	TMMALKVLR	6.4	0.0201	SB	FAM46D	G334R	noHabit	42498	NA	NA	NA	NA	NA	NA	NA	D	PrD_PrD	D	MODERATE	U	1	234	233	0	306	262	44	14.38
TSNAD	FAM46D_G334R	3	HLA-A*68:01	TMMALKVLG	21266.5	22.3794	-	TMMALKVLR	16.2	0.1986	SB	FAM46D	G334R	noHabit	42498	NA	NA	NA	NA	NA	NA	NA	D	PrD_PrD	D	MODERATE	U	1	234	233	0	306	262	44	14.38
HLA	FOLH1_D567Y	11	HLA-A*02:03	DPMFKYHLTV	792.1	5.9954	-	YPMFKYHLTV	8.9	0.1709	SB	FOLH1	D567Y	Smoker	42479	NA	NA	NA	NA	NA	NA	NA	D	PrD	D	MODERATE	D	0.995	89	87	1	88	69	19	21.59
TSNAD	FOLH1_D567Y	2	HLA-A*29:02	TYELVEKFYD	10184.4	7.0299	-	TYELVEKFYY	43	0.2094	SB	FOLH1	D567Y	Smoker	42479	NA	NA	NA	NA	NA	NA	NA	D	PrD	D	MODERATE	D	0.995	89	87	1	88	69	19	21.59
TSNAD	FOLH1_D567Y	11	HLA-B*07:02	DPMFKYHLTV	2207.4	1.9741	WB	YPMFKYHLTV	40.6	0.1425	SB	FOLH1	D567Y	Smoker	42479	NA	NA	NA	NA	NA	NA	NA	D	PrD	D	MODERATE	D	0.995	89	87	1	88	69	19	21.59
TSNAD	FOLH1_D567Y	11	HLA-B*07:02	DPMFKYHL	8883.2	5.3632	-	YPMFKYHL	98.1	0.2925	SB	FOLH1	D567Y	Smoker	42479	NA	NA	NA	NA	NA	NA	NA	D	PrD	D	MODERATE	D	0.995	89	87	1	88	69	19	21.59
TSNAD	FOLH1_D567Y	10	HLA-A*24:02	YDPMFKYHL	13324.7	7.0526	-	YYPMFKYHL	35.6	0.0776	SB	FOLH1	D567Y	Smoker	42479	NA	NA	NA	NA	NA	NA	NA	D	PrD	D	MODERATE	D	0.995	89	87	1	88	69	19	21.59
TSNAD	FOLH1_D567Y	11	HLA-B*08:01	DPMFKYHLT	703.6	1.0021	WB	YPMFKYHLT	76.4	0.1328	SB	FOLH1	D567Y	Smoker	42479	NA	NA	NA	NA	NA	NA	NA	D	PrD	D	MODERATE	D	0.995	89	87	1	88	69	19	21.59
TSNAD	FOLH1_D567Y	2	HLA-A*29:01	TYELVEKFYD	10184.4	7.0299	-	TYELVEKFYY	43	0.2094	SB	FOLH1	D567Y	Smoker	42479	NA	NA	NA	NA	NA	NA	NA	D	PrD	D	MODERATE	D	0.995	89	87	1	88	69	19	21.59
TSNAD	FOLH1_D567Y	11	HLA-B*42:01	DPMFKYHL	818.4	1.6635	WB	YPMFKYHL	10	0.0321	SB	FOLH1	D567Y	Smoker	42479	NA	NA	NA	NA	NA	NA	NA	D	PrD	D	MODERATE	D	0.995	89	87	1	88	69	19	21.59
TSNAD	FOLH1_D567Y	10	HLA-A*23:01	YDPMFKYHL	10689.9	7.8541	-	YYPMFKYHL	33.6	0.1341	SB	FOLH1	D567Y	Smoker	42479	NA	NA	NA	NA	NA	NA	NA	D	PrD	D	MODERATE	D	0.995	89	87	1	88	69	19	21.59
TSNAD	FOLH1_D567Y	11	HLA-A*02:03	DPMFKYHLTV	792.1	5.9954	-	YPMFKYHLTV	8.9	0.1709	SB	FOLH1	D567Y	Smoker	42479	NA	NA	NA	NA	NA	NA	NA	D	PrD	D	MODERATE	D	0.995	89	87	1	88	69	19	21.59
TSNAD	FOLH1_D567Y	11	HLA-B*56:01	DPMFKYHLTVA	4476.2	2.1466	-	YPMFKYHLTV	59.3	0.0378	SB	FOLH1	D567Y	Smoker	42479	NA	NA	NA	NA	NA	NA	NA	D	PrD	D	MODERATE	D	0.995	89	87	1	88	69	19	21.59
TSNAD	FOLH1_D567Y	11	HLA-A*02:06	DPMFKYHLTV	3764.4	11.1379	-	YPMFKYHLTV	31.8	0.4027	SB	FOLH1	D567Y	Smoker	42479	NA	NA	NA	NA	NA	NA	NA	D	PrD	D	MODERATE	D	0.995	89	87	1	88	69	19	21.59
TSNAD	FOLH1_D567Y	11	HLA-B*56:01	DPMFKYHLTV	1636.4	0.9315	WB	YPMFKYHLTV	34.7	0.0214	SB	FOLH1	D567Y	Smoker	42479	NA	NA	NA	NA	NA	NA	NA	D	PrD	D	MODERATE	D	0.995	89	87	1	88	69	19	21.59
TSNAD	FOLH1_D567Y	11	HLA-A*02:09	DPMFKYHLTV	2419.3	6.4283	-	YPMFKYHLTV	10.7	0.1261	SB	FOLH1	D567Y	Smoker	42479	NA	NA	NA	NA	NA	NA	NA	D	PrD	D	MODERATE	D	0.995	89	87	1	88	69	19	21.59
TSNAD	FOLH1_D567Y	11	HLA-B*42:01	DPMFKYHLT	1884.3	2.8258	-	YPMFKYHLT	36.8	0.1845	SB	FOLH1	D567Y	Smoker	42479	NA	NA	NA	NA	NA	NA	NA	D	PrD	D	MODERATE	D	0.995	89	87	1	88	69	19	21.59
TSNAD	FOLH1_D567Y	11	HLA-B*07:05	DPMFKYHL	7155.5	5.1075	-	YPMFKYHL	69.2	0.2569	SB	FOLH1	D567Y	Smoker	42479	NA	NA	NA	NA	NA	NA	NA	D	PrD	D	MODERATE	D	0.995	89	87	1	88	69	19	21.59
TSNAD	FOLH1_D567Y	11	HLA-B*56:01	DPMFKYHLT	3795.6	1.8414	WB	YPMFKYHLT	49.4	0.0311	SB	FOLH1	D567Y	Smoker	42479	NA	NA	NA	NA	NA	NA	NA	D	PrD	D	MODERATE	D	0.995	89	87	1	88	69	19	21.59
TSNAD	FOLH1_D567Y	11	HLA-A*02:11	DPMFKYHLTV	743.3	6.5025	-	YPMFKYHLTV	3.7	0.1574	SB	FOLH1	D567Y	Smoker	42479	NA	NA	NA	NA	NA	NA	NA	D	PrD	D	MODERATE	D	0.995	89	87	1	88	69	19	21.59
TSNAD	FOLH1_D567Y	3	HLA-B*18:01	YELVEKFYD	8397.4	3.4376	-	YELVEKFYY	34.7	0.024	SB	FOLH1	D567Y	Smoker	42479	NA	NA	NA	NA	NA	NA	NA	D	PrD	D	MODERATE	D	0.995	89	87	1	88	69	19	21.59
TSNAD	FOLH1_D567Y	11	HLA-B*42:01	DPMFKYHLTVA	3324.9	4.0677	-	YPMFKYHLTV	75.5	0.3557	SB	FOLH1	D567Y	Smoker	42479	NA	NA	NA	NA	NA	NA	NA	D	PrD	D	MODERATE	D	0.995	89	87	1	88	69	19	21.59
TSNAD	FOLH1_D567Y	10	HLA-C*07:02	YDPMFKYHL	11936.7	8.1471	-	YYPMFKYHL	67.2	0.0334	SB	FOLH1	D567Y	Smoker	42479	NA	NA	NA	NA	NA	NA	NA	D	PrD	D	MODERATE	D	0.995	89	87	1	88	69	19	21.59
TSNAD	FOLH1_D567Y	11	HLA-A*02:01	DPMFKYHLTV	2419.3	6.4283	-	YPMFKYHLTV	10.7	0.1261	SB	FOLH1	D567Y	Smoker	42479	NA	NA	NA	NA	NA	NA	NA	D	PrD	D	MODERATE	D	0.995	89	87	1	88	69	19	21.59
TSNAD	FOLH1_D567Y	10	HLA-C*14:02	YDPMFKYHL	11584.1	16.4536	-	YYPMFKYHL	46.2	0.1561	SB	FOLH1	D567Y	Smoker	42479	NA	NA	NA	NA	NA	NA	NA	D	PrD	D	MODERATE	D	0.995	89	87	1	88	69	19	21.59
TSNAD	FOLH1_D567Y	11	HLA-B*55:01	DPMFKYHLTV	2742.4	0.9821	WB	YPMFKYHLTV	60.9	0.0151	SB	FOLH1	D567Y	Smoker	42479	NA	NA	NA	NA	NA	NA	NA	D	PrD	D	MODERATE	D	0.995	89	87	1	88	69	19	21.59
TSNAD	FOLH1_D567Y	11	HLA-B*07:05	DPMFKYHLTV	1615.7	1.8536	WB	YPMFKYHLTV	29.2	0.1189	SB	FOLH1	D567Y	Smoker	42479	NA	NA	NA	NA	NA	NA	NA	D	PrD	D	MODERATE	D	0.995	89	87	1	88	69	19	21.59
TSNAD	FOLH1_D567Y	11	HLA-A*02:02	DPMFKYHLTV	1736.5	8.8554	-	YPMFKYHLTV	12.2	0.2672	SB	FOLH1	D567Y	Smoker	42479	NA	NA	NA	NA	NA	NA	NA	D	PrD	D	MODERATE	D	0.995	89	87	1	88	69	19	21.59
HLA	FOXR2_P109S	10	HLA-A*11:01	SPFPQPPQK	7831.6	7.633	-	SSFPQPPQK	16.3	0.0708	SB	FOXR2	P109S	Smoker	42473	NA	NA	NA	0.0000115	0	NA	NA	T	BN	N	MODERATE	U	0.001	67	67	0	68	42	26	38.24
TSNAD	FOXR2_P109S	10	HLA-A*30:01	SPFPQPPQK	5404.8	13.0746	-	SSFPQPPQK	50.8	0.2654	SB	FOXR2	P109S	Smoker	42473	NA	NA	NA	0.0000115	0	NA	NA	T	BN	N	MODERATE	U	0.001	67	67	0	68	42	26	38.24
TSNAD	FOXR2_P109S	10	HLA-A*11:01	SPFPQPPQK	7831.6	7.633	-	SSFPQPPQK	16.3	0.0708	SB	FOXR2	P109S	Smoker	42473	NA	NA	NA	0.0000115	0	NA	NA	T	BN	N	MODERATE	U	0.001	67	67	0	68	42	26	38.24
TSNAD	FOXR2_P109S	10	HLA-A*34:02	SPFPQPPQK	2203.2	3.2655	-	SSFPQPPQK	88.4	0.1466	SB	FOXR2	P109S	Smoker	42473	NA	NA	NA	0.0000115	0	NA	NA	T	BN	N	MODERATE	U	0.001	67	67	0	68	42	26	38.24
TSNAD	FOXR2_P109S	10	HLA-A*03:01	SPFPQPPQK	10146	7.8093	-	SSFPQPPQK	89.2	0.3325	SB	FOXR2	P109S	Smoker	42473	NA	NA	NA	0.0000115	0	NA	NA	T	BN	N	MODERATE	U	0.001	67	67	0	68	42	26	38.24
HLA	FSIP2_D440Y	3	HLA-A*29:01	IMLVYPAGD	35668.7	47.0556	-	IMLVYPAGY	14.1	0.0578	SB	FSIP2	D440Y	Chewer	42482	NA	NA	NA	NA	NA	NA	NA	NA	BN_PoD	NA	MODERATE	N	0.102	75	75	0	130	89	41	31.54
TSNAD	FSIP2_D440Y	3	HLA-B*15:03	IMLVYPAGD	17566.4	36.5999	-	IMLVYPAGY	11	0.1753	SB	FSIP2	D440Y	Chewer	42482	NA	NA	NA	NA	NA	NA	NA	NA	BN_PoD	NA	MODERATE	N	0.102	75	75	0	130	89	41	31.54
TSNAD	FSIP2_D440Y	3	HLA-A*30:02	IMLVYPAGD	30196.1	57.0295	-	IMLVYPAGY	55	0.1136	SB	FSIP2	D440Y	Chewer	42482	NA	NA	NA	NA	NA	NA	NA	NA	BN_PoD	NA	MODERATE	N	0.102	75	75	0	130	89	41	31.54
TSNAD	FSIP2_D440Y	3	HLA-A*29:02	IMLVYPAGD	35668.7	47.0556	-	IMLVYPAGY	14.1	0.0578	SB	FSIP2	D440Y	Chewer	42482	NA	NA	NA	NA	NA	NA	NA	NA	BN_PoD	NA	MODERATE	N	0.102	75	75	0	130	89	41	31.54
TSNAD	FSIP2_D440Y	3	HLA-A*29:01	IMLVYPAGD	35668.7	47.0556	-	IMLVYPAGY	14.1	0.0578	SB	FSIP2	D440Y	Chewer	42482	NA	NA	NA	NA	NA	NA	NA	NA	BN_PoD	NA	MODERATE	N	0.102	75	75	0	130	89	41	31.54
TSNAD	FSIP2_D440Y	3	HLA-B*15:01	IMLVYPAGD	23964.9	36.1888	-	IMLVYPAGY	84	0.4498	SB	FSIP2	D440Y	Chewer	42482	NA	NA	NA	NA	NA	NA	NA	NA	BN_PoD	NA	MODERATE	N	0.102	75	75	0	130	89	41	31.54
HLA	HFM1_G1083V	3	HLA-A*02:11	NLISSEFVG	6530.7	16.4526	-	NLISSEFVV	6	0.3364	SB	HFM1	G1083V	Chewer	42482	NA	NA	NA	NA	NA	NA	NA	D	PrD_PrD	D	MODERATE	D	0.955	36	36	0	45	39	6	13.33
TSNAD	HFM1_G1083V	3	HLA-A*02:01	NLISSEFVG	9476	13.9482	-	NLISSEFVV	24.3	0.3335	SB	HFM1	G1083V	Chewer	42482	NA	NA	NA	NA	NA	NA	NA	D	PrD_PrD	D	MODERATE	D	0.955	36	36	0	45	39	6	13.33
TSNAD	HFM1_G1083V	3	HLA-A*02:09	NLISSEFVG	9476	13.9482	-	NLISSEFVV	24.3	0.3335	SB	HFM1	G1083V	Chewer	42482	NA	NA	NA	NA	NA	NA	NA	D	PrD_PrD	D	MODERATE	D	0.955	36	36	0	45	39	6	13.33
TSNAD	HFM1_G1083V	3	HLA-A*02:11	NLISSEFVG	6530.7	16.4526	-	NLISSEFVV	6	0.3364	SB	HFM1	G1083V	Chewer	42482	NA	NA	NA	NA	NA	NA	NA	D	PrD_PrD	D	MODERATE	D	0.955	36	36	0	45	39	6	13.33
HLA	IARS_E636V	1	HLA-A*02:11	YLINSPVVRAE	870.6	6.9358	-	YLINSPVVRA	6.7	0.3846	SB	IARS	E636V	noHabit	56957	NA	NA	NA	NA	NA	NA	NA	D	PrD_PrD	D	MODERATE	D	1	84	42	42	83	4	79	95.18
HLA	IARS_E636V	1	HLA-A*02:01	YLINSPVVRAE	2824.2	6.9575	-	YLINSPVVRA	31	0.4147	SB	IARS	E636V	noHabit	56957	NA	NA	NA	NA	NA	NA	NA	D	PrD_PrD	D	MODERATE	D	1	84	42	42	83	4	79	95.18
TSNAD	IARS_E636V	1	HLA-A*02:01	YLINSPVVRAE	2824.2	6.9575	-	YLINSPVVRA	31	0.4147	SB	IARS	E636V	noHabit	56957	NA	NA	NA	NA	NA	NA	NA	D	PrD_PrD	D	MODERATE	D	1	84	42	42	83	4	79	95.18
TSNAD	IARS_E636V	1	HLA-A*02:03	YLINSPVVRAE	1028.8	6.7989	-	YLINSPVVRA	11.2	0.2449	SB	IARS	E636V	noHabit	56957	NA	NA	NA	NA	NA	NA	NA	D	PrD_PrD	D	MODERATE	D	1	84	42	42	83	4	79	95.18
TSNAD	IARS_E636V	1	HLA-A*02:11	YLINSPVVRAE	870.6	6.9358	-	YLINSPVVRA	6.7	0.3846	SB	IARS	E636V	noHabit	56957	NA	NA	NA	NA	NA	NA	NA	D	PrD_PrD	D	MODERATE	D	1	84	42	42	83	4	79	95.18
TSNAD	IARS_E636V	1	HLA-A*02:09	YLINSPVVRAE	2824.2	6.9575	-	YLINSPVVRA	31	0.4147	SB	IARS	E636V	noHabit	56957	NA	NA	NA	NA	NA	NA	NA	D	PrD_PrD	D	MODERATE	D	1	84	42	42	83	4	79	95.18
HLA	IKBKB_S181L	10	HLA-A*02:11	TSFVGTLQYL	1955.4	9.6055	-	TLFVGTLQYL	7.2	0.4141	SB	IKBKB	S181L	noHabit	42497	NA	NA	NA	NA	NA	NA	NA	D	PrD_PrD	D	MODERATE	NA	NA	58	58	0	91	57	34	37.36
TSNAD	IKBKB_S181L	10	HLA-A*02:11	TSFVGTLQYL	1955.4	9.6055	-	TLFVGTLQYL	7.2	0.4141	SB	IKBKB	S181L	noHabit	42497	NA	NA	NA	NA	NA	NA	NA	D	PrD_PrD	D	MODERATE	NA	NA	58	58	0	91	57	34	37.36
TSNAD	IKBKB_S181L	10	HLA-A*02:01	TSFVGTLQYL	5097.9	9.577	-	TLFVGTLQYL	34.7	0.46	SB	IKBKB	S181L	noHabit	42497	NA	NA	NA	NA	NA	NA	NA	D	PrD_PrD	D	MODERATE	NA	NA	58	58	0	91	57	34	37.36
TSNAD	IKBKB_S181L	10	HLA-A*02:02	TSFVGTLQYL	1537	8.3166	-	TLFVGTLQYL	18.1	0.4329	SB	IKBKB	S181L	noHabit	42497	NA	NA	NA	NA	NA	NA	NA	D	PrD_PrD	D	MODERATE	NA	NA	58	58	0	91	57	34	37.36
TSNAD	IKBKB_S181L	1	HLA-B*40:02	KELDQGSLCTS	4367.5	4.2873	-	KELDQGSLCT	76.2	0.3099	SB	IKBKB	S181L	noHabit	42497	NA	NA	NA	NA	NA	NA	NA	D	PrD_PrD	D	MODERATE	NA	NA	58	58	0	91	57	34	37.36
TSNAD	IKBKB_S181L	10	HLA-A*02:09	TSFVGTLQYL	5097.9	9.577	-	TLFVGTLQYL	34.7	0.46	SB	IKBKB	S181L	noHabit	42497	NA	NA	NA	NA	NA	NA	NA	D	PrD_PrD	D	MODERATE	NA	NA	58	58	0	91	57	34	37.36
TSNAD	IKBKB_S181L	10	HLA-A*03:01	TSFVGTLQY	571.1	1.3252	WB	TLFVGTLQY	62.6	0.2367	SB	IKBKB	S181L	noHabit	42497	NA	NA	NA	NA	NA	NA	NA	D	PrD_PrD	D	MODERATE	NA	NA	58	58	0	91	57	34	37.36
HLA	ITGAD_D679Y	9	HLA-A*31:01	ALDPGRLTSR	530.5	2.1605	-	ALYPGRLTSR	35.8	0.289	SB	ITGAD	D679Y	Chewer	42483	NA	NA	NA	NA	NA	NA	NA	D	PrD_PrD	D	MODERATE	NA	NA	174	174	0	189	90	99	52.38
TSNAD	ITGAD_D679Y	11	HLA-B*07:05	DPGRLTSRAI	998.4	1.389	WB	YPGRLTSRAI	6.7	0.0136	SB	ITGAD	D679Y	Chewer	42483	NA	NA	NA	NA	NA	NA	NA	D	PrD_PrD	D	MODERATE	NA	NA	174	174	0	189	90	99	52.38
TSNAD	ITGAD_D679Y	11	HLA-B*07:02	DPGRLTSRAI	1080.4	1.2965	WB	YPGRLTSRAI	6.7	0.0119	SB	ITGAD	D679Y	Chewer	42483	NA	NA	NA	NA	NA	NA	NA	D	PrD_PrD	D	MODERATE	NA	NA	174	174	0	189	90	99	52.38
TSNAD	ITGAD_D679Y	2	HLA-A*29:02	SSVRFDLALD	36701.8	50.7989	-	SSVRFDLALY	90.7	0.3823	SB	ITGAD	D679Y	Chewer	42483	NA	NA	NA	NA	NA	NA	NA	D	PrD_PrD	D	MODERATE	NA	NA	174	174	0	189	90	99	52.38
TSNAD	ITGAD_D679Y	2	HLA-A*30:02	SSVRFDLALD	26214.1	44.1065	-	SSVRFDLALY	44.7	0.0834	SB	ITGAD	D679Y	Chewer	42483	NA	NA	NA	NA	NA	NA	NA	D	PrD_PrD	D	MODERATE	NA	NA	174	174	0	189	90	99	52.38
TSNAD	ITGAD_D679Y	9	HLA-A*31:01	ALDPGRLTSR	530.5	2.1605	-	ALYPGRLTSR	35.8	0.289	SB	ITGAD	D679Y	Chewer	42483	NA	NA	NA	NA	NA	NA	NA	D	PrD_PrD	D	MODERATE	NA	NA	174	174	0	189	90	99	52.38
TSNAD	ITGAD_D679Y	11	HLA-B*42:01	DPGRLTSRAI	741.5	1.5601	WB	YPGRLTSRAI	8.1	0.0198	SB	ITGAD	D679Y	Chewer	42483	NA	NA	NA	NA	NA	NA	NA	D	PrD_PrD	D	MODERATE	NA	NA	174	174	0	189	90	99	52.38
TSNAD	ITGAD_D679Y	9	HLA-A*03:01	ALDPGRLTSR	1708.1	2.5181	-	ALYPGRLTSR	49.2	0.1843	SB	ITGAD	D679Y	Chewer	42483	NA	NA	NA	NA	NA	NA	NA	D	PrD_PrD	D	MODERATE	NA	NA	174	174	0	189	90	99	52.38
TSNAD	ITGAD_D679Y	2	HLA-A*29:01	SSVRFDLALD	36701.8	50.7989	-	SSVRFDLALY	90.7	0.3823	SB	ITGAD	D679Y	Chewer	42483	NA	NA	NA	NA	NA	NA	NA	D	PrD_PrD	D	MODERATE	NA	NA	174	174	0	189	90	99	52.38
TSNAD	ITGAD_D679Y	3	HLA-B*15:01	SVRFDLALD	25830.7	40.313	-	SVRFDLALY	87.3	0.4664	SB	ITGAD	D679Y	Chewer	42483	NA	NA	NA	NA	NA	NA	NA	D	PrD_PrD	D	MODERATE	NA	NA	174	174	0	189	90	99	52.38
TSNAD	ITGAD_D679Y	2	HLA-B*15:17	SSVRFDLALD	4325.8	9.2444	-	SSVRFDLALY	15.7	0.2493	SB	ITGAD	D679Y	Chewer	42483	NA	NA	NA	NA	NA	NA	NA	D	PrD_PrD	D	MODERATE	NA	NA	174	174	0	189	90	99	52.38
TSNAD	ITGAD_D679Y	3	HLA-A*29:02	SVRFDLALD	38424.3	57.5897	-	SVRFDLALY	83.9	0.3623	SB	ITGAD	D679Y	Chewer	42483	NA	NA	NA	NA	NA	NA	NA	D	PrD_PrD	D	MODERATE	NA	NA	174	174	0	189	90	99	52.38
TSNAD	ITGAD_D679Y	3	HLA-A*30:02	SVRFDLALD	24673.8	39.4184	-	SVRFDLALY	35.3	0.0563	SB	ITGAD	D679Y	Chewer	42483	NA	NA	NA	NA	NA	NA	NA	D	PrD_PrD	D	MODERATE	NA	NA	174	174	0	189	90	99	52.38
TSNAD	ITGAD_D679Y	3	HLA-A*29:01	SVRFDLALD	38424.3	57.5897	-	SVRFDLALY	83.9	0.3623	SB	ITGAD	D679Y	Chewer	42483	NA	NA	NA	NA	NA	NA	NA	D	PrD_PrD	D	MODERATE	NA	NA	174	174	0	189	90	99	52.38
HLA	KCNMB4_E64K	3	HLA-A*03:01	VLSVQQIGE	18738.3	14.8076	-	VLSVQQIGK	63.7	0.2409	SB	KCNMB4	E64K	noHabit	42501	NA	NA	NA	NA	NA	NA	NA	D	BN_BN	N	MODERATE	D	0.999	135	135	0	291	249	42	14.43
TSNAD	KCNMB4_E64K	3	HLA-A*03:02	VLSVQQIGE	12990.4	10.4582	-	VLSVQQIGK	79.7	0.233	SB	KCNMB4	E64K	noHabit	42501	NA	NA	NA	NA	NA	NA	NA	D	BN_BN	N	MODERATE	D	0.999	135	135	0	291	249	42	14.43
TSNAD	KCNMB4_E64K	2	HLA-A*34:02	TVLSVQQIGE	10511.9	10.6266	-	TVLSVQQIGK	87.8	0.1454	SB	KCNMB4	E64K	noHabit	42501	NA	NA	NA	NA	NA	NA	NA	D	BN_BN	N	MODERATE	D	0.999	135	135	0	291	249	42	14.43
TSNAD	KCNMB4_E64K	3	HLA-A*03:01	VLSVQQIGE	18738.3	14.8076	-	VLSVQQIGK	63.7	0.2409	SB	KCNMB4	E64K	noHabit	42501	NA	NA	NA	NA	NA	NA	NA	D	BN_BN	N	MODERATE	D	0.999	135	135	0	291	249	42	14.43
TSNAD	KCNMB4_E64K	2	HLA-A*11:01	TVLSVQQIGE	16003.7	13.4184	-	TVLSVQQIGK	27.4	0.1614	SB	KCNMB4	E64K	noHabit	42501	NA	NA	NA	NA	NA	NA	NA	D	BN_BN	N	MODERATE	D	0.999	135	135	0	291	249	42	14.43
HLA	KIAA0922_H853Y	2	HLA-A*01:01	FTLNVTLPHH	7846.1	2.3757	-	FTLNVTLPHY	80.8	0.0763	SB	KIAA0922	H853Y	Smoker	42476	NA	NA	NA	NA	NA	NA	NA	D	PrD	D	MODERATE	D	0.998	53	53	0	47	29	17	36.17
TSNAD	KIAA0922_H853Y	3	HLA-B*15:01	TLNVTLPHH	6374.7	9.5937	-	TLNVTLPHY	89.5	0.4768	SB	KIAA0922	H853Y	Smoker	42476	NA	NA	NA	NA	NA	NA	NA	D	PrD	D	MODERATE	D	0.998	53	53	0	47	29	17	36.17
TSNAD	KIAA0922_H853Y	2	HLA-A*30:02	FTLNVTLPHH	4188.1	3.946	-	FTLNVTLPHY	78.5	0.193	SB	KIAA0922	H853Y	Smoker	42476	NA	NA	NA	NA	NA	NA	NA	D	PrD	D	MODERATE	D	0.998	53	53	0	47	29	17	36.17
TSNAD	KIAA0922_H853Y	2	HLA-A*29:01	FTLNVTLPHH	1268	1.7271	WB	FTLNVTLPHY	6.7	0.0128	SB	KIAA0922	H853Y	Smoker	42476	NA	NA	NA	NA	NA	NA	NA	D	PrD	D	MODERATE	D	0.998	53	53	0	47	29	17	36.17
TSNAD	KIAA0922_H853Y	2	HLA-B*15:01	FTLNVTLPHH	3726.1	6.3657	-	FTLNVTLPHY	52.3	0.2677	SB	KIAA0922	H853Y	Smoker	42476	NA	NA	NA	NA	NA	NA	NA	D	PrD	D	MODERATE	D	0.998	53	53	0	47	29	17	36.17
TSNAD	KIAA0922_H853Y	2	HLA-B*15:16	FTLNVTLPHH	4374.9	8.2	-	FTLNVTLPHY	98.3	0.3394	SB	KIAA0922	H853Y	Smoker	42476	NA	NA	NA	NA	NA	NA	NA	D	PrD	D	MODERATE	D	0.998	53	53	0	47	29	17	36.17
TSNAD	KIAA0922_H853Y	3	HLA-A*29:01	TLNVTLPHH	13027	9.0506	-	TLNVTLPHY	93.4	0.3898	SB	KIAA0922	H853Y	Smoker	42476	NA	NA	NA	NA	NA	NA	NA	D	PrD	D	MODERATE	D	0.998	53	53	0	47	29	17	36.17
TSNAD	KIAA0922_H853Y	2	HLA-A*36:01	FTLNVTLPHH	2372.9	1.348	WB	FTLNVTLPHY	60.6	0.0405	SB	KIAA0922	H853Y	Smoker	42476	NA	NA	NA	NA	NA	NA	NA	D	PrD	D	MODERATE	D	0.998	53	53	0	47	29	17	36.17
TSNAD	KIAA0922_H853Y	2	HLA-B*15:17	FTLNVTLPHH	2789.9	7.3182	-	FTLNVTLPHY	13.6	0.2109	SB	KIAA0922	H853Y	Smoker	42476	NA	NA	NA	NA	NA	NA	NA	D	PrD	D	MODERATE	D	0.998	53	53	0	47	29	17	36.17
TSNAD	KIAA0922_H853Y	2	HLA-A*29:02	FTLNVTLPHH	1268	1.7271	WB	FTLNVTLPHY	6.7	0.0128	SB	KIAA0922	H853Y	Smoker	42476	NA	NA	NA	NA	NA	NA	NA	D	PrD	D	MODERATE	D	0.998	53	53	0	47	29	17	36.17
TSNAD	KIAA0922_H853Y	2	HLA-A*01:01	FTLNVTLPHH	7846.1	2.3757	-	FTLNVTLPHY	80.8	0.0763	SB	KIAA0922	H853Y	Smoker	42476	NA	NA	NA	NA	NA	NA	NA	D	PrD	D	MODERATE	D	0.998	53	53	0	47	29	17	36.17
TSNAD	KIAA0922_H853Y	3	HLA-A*29:02	TLNVTLPHH	13027	9.0506	-	TLNVTLPHY	93.4	0.3898	SB	KIAA0922	H853Y	Smoker	42476	NA	NA	NA	NA	NA	NA	NA	D	PrD	D	MODERATE	D	0.998	53	53	0	47	29	17	36.17
TSNAD	KIAA0922_H853Y	2	HLA-C*03:02	FTLNVTLPHH	2487.8	4.3585	-	FTLNVTLPHY	54.9	0.2666	SB	KIAA0922	H853Y	Smoker	42476	NA	NA	NA	NA	NA	NA	NA	D	PrD	D	MODERATE	D	0.998	53	53	0	47	29	17	36.17
HLA	KIAA1462_E549K	3	HLA-A*11:01	VSSPYSQGE	22831	20.4265	-	VSSPYSQGK	58.7	0.3601	SB	KIAA1462	E549K	noHabit	42498	NA	NA	NA	0.00000828	0	NA	NA	D	BN_PoD	D	MODERATE	N	0.469	119	119	0	123	105	18	14.63
TSNAD	KIAA1462_E549K	3	HLA-A*11:01	VSSPYSQGE	22831	20.4265	-	VSSPYSQGK	58.7	0.3601	SB	KIAA1462	E549K	noHabit	42498	NA	NA	NA	0.00000828	0	NA	NA	D	BN_PoD	D	MODERATE	N	0.469	119	119	0	123	105	18	14.63
HLA	KIFAP3_E291K	2	HLA-A*11:01	ALYLLLNLAE	18868.8	15.9439	-	ALYLLLNLAK	75.6	0.4502	SB	KIFAP3	E291K	noHabit	42498	NA	NA	NA	NA	NA	NA	NA	D	PrD_PrD	D	MODERATE	D	NA	42	42	0	77	53	24	31.17
TSNAD	KIFAP3_E291K	2	HLA-A*03:02	ALYLLLNLAE	11440.6	9.3285	-	ALYLLLNLAK	69.2	0.2022	SB	KIFAP3	E291K	noHabit	42498	NA	NA	NA	NA	NA	NA	NA	D	PrD_PrD	D	MODERATE	D	NA	42	42	0	77	53	24	31.17
TSNAD	KIFAP3_E291K	2	HLA-A*11:01	ALYLLLNLAE	18868.8	15.9439	-	ALYLLLNLAK	75.6	0.4502	SB	KIFAP3	E291K	noHabit	42498	NA	NA	NA	NA	NA	NA	NA	D	PrD_PrD	D	MODERATE	D	NA	42	42	0	77	53	24	31.17
TSNAD	KIFAP3_E291K	2	HLA-A*03:01	ALYLLLNLAE	11721.6	8.8643	-	ALYLLLNLAK	30.4	0.0997	SB	KIFAP3	E291K	noHabit	42498	NA	NA	NA	NA	NA	NA	NA	D	PrD_PrD	D	MODERATE	D	NA	42	42	0	77	53	24	31.17

Mangalaparthi *et al.* , 2020. Mutational landscape of esophageal squamous cell carcinoma in an Indian cohort

Supplementary Table 6. List of neoantigens predicted using TSNAD

Software	Variant accession	Position	HLA subtype	Wild type peptide	Wild binding	Wild rank	wild binding level	Mutant peptide	Mutant binding	Mutant rank	Mutant binding level	Gene symbol	Variant information	Habit	Sample ID	OncoMD	COSMIC	dbSNP	ExAC	ExAC_SAS	1000G	1000G_SAS	SIFT	Polyphen2	CONDEL	VEP	LRT	PhastCon7Way	Total read depth in Normal	Reference read support in Normal	Alternative read support in Normal	Total read depth in Tumor	Reference read support in Tumor	Alternate read support in Tumor	Alternate allele frequency in Tumor
HLA	KIR3DL1_P184S	11	HLA-A*02:11	PMMLALAGT	980.1	7.2808	-	SMMLALAGT	8.5	0.4997	SB	KIR3DL1	P184S	noHabit	56957	NA	NA	chr19*55331362	0	0	NA	NA	T	BN_BN	N	MODERATE	N	0.032	185	99	86	166	152	14	8.43
TSNAD	KIR3DL1_P184S	11	HLA-A*02:06	PMMLALAGT	1711.1	6.9922	-	SMMLALAGT	29.9	0.3813	SB	KIR3DL1	P184S	noHabit	56957	NA	NA	chr19*55331362	0	0	NA	NA	T	BN_BN	N	MODERATE	N	0.032	185	99	86	166	152	14	8.43
TSNAD	KIR3DL1_P184S	11	HLA-A*02:11	PMMLALAGT	980.1	7.2808	-	SMMLALAGT	8.5	0.4997	SB	KIR3DL1	P184S	noHabit	56957	NA	NA	chr19*55331362	0	0	NA	NA	T	BN_BN	N	MODERATE	N	0.032	185	99	86	166	152	14	8.43
TSNAD	KIR3DL1_P184S	11	HLA-A*02:03	PMMLALAGT	728.4	5.7626	-	SMMLALAGT	11.2	0.2427	SB	KIR3DL1	P184S	noHabit	56957	NA	NA	chr19*55331362	0	0	NA	NA	T	BN_BN	N	MODERATE	N	0.032	185	99	86	166	152	14	8.43
HLA	LBR_R257I	3	HLA-A*02:11	ALYELWETR	754.8	6.5446	-	ALYELWETI	3	0.0968	SB	LBR	R257I	Smoker	42475	NA	NA	NA	0.00000826	0.0000606	NA	NA	T	PrD	D	MODERATE	N	0.681	80	80	0	113	69	44	38.94
TSNAD	LBR_R257I	3	HLA-A*02:03	ALYELWETR	7745.2	18.4725	-	ALYELWETI	17.2	0.4241	SB	LBR	R257I	Smoker	42475	NA	NA	NA	0.00000826	0.0000606	NA	NA	T	PrD	D	MODERATE	N	0.681	80	80	0	113	69	44	38.94
TSNAD	LBR_R257I	10	HLA-A*68:02	TRVFGVYLL	5647.1	8.9151	-	TIVFGVYLL	43.7	0.3769	SB	LBR	R257I	Smoker	42475	NA	NA	NA	0.00000826	0.0000606	NA	NA	T	PrD	D	MODERATE	N	0.681	80	80	0	113	69	44	38.94
TSNAD	LBR_R257I	3	HLA-A*02:05	ALYELWETR	5699.8	13.9803	-	ALYELWETI	63.2	0.4004	SB	LBR	R257I	Smoker	42475	NA	NA	NA	0.00000826	0.0000606	NA	NA	T	PrD	D	MODERATE	N	0.681	80	80	0	113	69	44	38.94
TSNAD	LBR_R257I	3	HLA-A*02:01	ALYELWETR	6208.5	10.7235	-	ALYELWETI	18.2	0.2433	SB	LBR	R257I	Smoker	42475	NA	NA	NA	0.00000826	0.0000606	NA	NA	T	PrD	D	MODERATE	N	0.681	80	80	0	113	69	44	38.94
TSNAD	LBR_R257I	3	HLA-A*02:11	ALYELWETR	754.8	6.5446	-	ALYELWETI	3	0.0968	SB	LBR	R257I	Smoker	42475	NA	NA	NA	0.00000826	0.0000606	NA	NA	T	PrD	D	MODERATE	N	0.681	80	80	0	113	69	44	38.94
TSNAD	LBR_R257I	3	HLA-A*02:06	ALYELWETR	4699.7	12.7642	-	ALYELWETI	26	0.3331	SB	LBR	R257I	Smoker	42475	NA	NA	NA	0.00000826	0.0000606	NA	NA	T	PrD	D	MODERATE	N	0.681	80	80	0	113	69	44	38.94
TSNAD	LBR_R257I	3	HLA-A*02:09	ALYELWETR	6208.5	10.7235	-	ALYELWETI	18.2	0.2433	SB	LBR	R257I	Smoker	42475	NA	NA	NA	0.00000826	0.0000606	NA	NA	T	PrD	D	MODERATE	N	0.681	80	80	0	113	69	44	38.94
TSNAD	LBR_R257I	3	HLA-A*32:01	ALYELWETR	7916.7	6.3578	-	ALYELWETI	30.8	0.0562	SB	LBR	R257I	Smoker	42475	NA	NA	NA	0.00000826	0.0000606	NA	NA	T	PrD	D	MODERATE	N	0.681	80	80	0	113	69	44	38.94
TSNAD	LBR_R257I	3	HLA-A*02:02	ALYELWETR	3426.2	12.3811	-	ALYELWETI	13.7	0.3138	SB	LBR	R257I	Smoker	42475	NA	NA	NA	0.00000826	0.0000606	NA	NA	T	PrD	D	MODERATE	N	0.681	80	80	0	113	69	44	38.94
HLA	LONP1_R459H	4	HLA-A*29:01	SSEFNVTRNY	651.7	1.2137	WB	SSEFNVTHNY	90.5	0.3819	SB	LONP1	R459H	noHabit	56958	NA	NA	NA	NA	NA	NA	NA	D	PrD_PrD	D	MODERATE	D	0.951	98	98	0	125	110	15	12
TSNAD	LONP1_R459H	4	HLA-A*29:01	SSEFNVTRNY	651.7	1.2137	WB	SSEFNVTHNY	90.5	0.3819	SB	LONP1	R459H	noHabit	56958	NA	NA	NA	NA	NA	NA	NA	D	PrD_PrD	D	MODERATE	D	0.951	98	98	0	125	110	15	12
TSNAD	LONP1_R459H	4	HLA-A*29:02	SSEFNVTRNY	651.7	1.2137	WB	SSEFNVTHNY	90.5	0.3819	SB	LONP1	R459H	noHabit	56958	NA	NA	NA	NA	NA	NA	NA	D	PrD_PrD	D	MODERATE	D	0.951	98	98	0	125	110	15	12
HLA	LPAR6_P83L	10	HLA-A*02:01	WPFGDLLCKI	15177.5	19.6479	-	WLFGDLLCKI	20	0.2716	SB	LPAR6	P83L	noHabit	56957	NA	NA	NA	NA	NA	NA	NA	T	BN_BN	N	MODERATE	D	0.996	158	158	0	85	53	32	37.65
HLA	LPAR6_P83L	10	HLA-A*02:11	WPFGDLLCKI	9916.8	20.6174	-	WLFGDLLCKI	8.3	0.4902	SB	LPAR6	P83L	noHabit	56957	NA	NA	NA	NA	NA	NA	NA	T	BN_BN	N	MODERATE	D	0.996	158	158	0	85	53	32	37.65
TSNAD	LPAR6_P83L	10	HLA-A*02:09	WPFGDLLCKI	15177.5	19.6479	-	WLFGDLLCKI	20	0.2716	SB	LPAR6	P83L	noHabit	56957	NA	NA	NA	NA	NA	NA	NA	T	BN_BN	N	MODERATE	D	0.996	158	158	0	85	53	32	37.65
TSNAD	LPAR6_P83L	10	HLA-A*02:03	WPFGDLLCKI	12968	25.4029	-	WLFGDLLCKI	15.8	0.3844	SB	LPAR6	P83L	noHabit	56957	NA	NA	NA	NA	NA	NA	NA	T	BN_BN	N	MODERATE	D	0.996	158	158	0	85	53	32	37.65
TSNAD	LPAR6_P83L	3	HLA-C*14:02	FYFTTRNWP	2357.3	4.4238	-	FYFTTRNWL	9.8	0.0178	SB	LPAR6	P83L	noHabit	56957	NA	NA	NA	NA	NA	NA	NA	T	BN_BN	N	MODERATE	D	0.996	158	158	0	85	53	32	37.65
TSNAD	LPAR6_P83L	10	HLA-A*03:02	WPFGDLLCK	13017.9	10.4783	-	WLFGDLLCK	98.6	0.2912	SB	LPAR6	P83L	noHabit	56957	NA	NA	NA	NA	NA	NA	NA	T	BN_BN	N	MODERATE	D	0.996	158	158	0	85	53	32	37.65
TSNAD	LPAR6_P83L	10	HLA-A*02:11	WPFGDLLCKI	9916.8	20.6174	-	WLFGDLLCKI	8.3	0.4902	SB	LPAR6	P83L	noHabit	56957	NA	NA	NA	NA	NA	NA	NA	T	BN_BN	N	MODERATE	D	0.996	158	158	0	85	53	32	37.65
TSNAD	LPAR6_P83L	3	HLA-C*07:02	FYFTTRNWP	7627.3	4.9064	-	FYFTTRNWL	27.8	0.0099	SB	LPAR6	P83L	noHabit	56957	NA	NA	NA	NA	NA	NA	NA	T	BN_BN	N	MODERATE	D	0.996	158	158	0	85	53	32	37.65
TSNAD	LPAR6_P83L	10	HLA-A*02:02	WPFGDLLCKI	7428.1	18.753	-	WLFGDLLCKI	15.2	0.3564	SB	LPAR6	P83L	noHabit	56957	NA	NA	NA	NA	NA	NA	NA	T	BN_BN	N	MODERATE	D	0.996	158	158	0	85	53	32	37.65
TSNAD	LPAR6_P83L	10	HLA-A*02:01	WPFGDLLCKI	15177.5	19.6479	-	WLFGDLLCKI	20	0.2716	SB	LPAR6	P83L	noHabit	56957	NA	NA	NA	NA	NA	NA	NA	T	BN_BN	N	MODERATE	D	0.996	158	158	0	85	53	32	37.65
TSNAD	LPAR6_P83L	10	HLA-A*02:06	WPFGDLLCKI	9979	20.8222	-	WLFGDLLCKI	38.3	0.4882	SB	LPAR6	P83L	noHabit	56957	NA	NA	NA	NA	NA	NA	NA	T	BN_BN	N	MODERATE	D	0.996	158	158	0	85	53	32	37.65
HLA	LRP2_F542L	10	HLA-A*11:01	AFMDGSNRK	640.9	1.9986	WB	ALMDGSNRK	44.5	0.274	SB	LRP2	F542L	noHabit	42498	NA	NA	NA	NA	NA	NA	NA	NA	PoD_PrD	NA	MODERATE	D	1	136	136	0	158	121	37	23.42
TSNAD	LRP2_F542L	9	HLA-A*03:02	RAFMDGSNRK	502.3	1.2202	WB	RALMDGSNRK	96.3	0.2843	SB	LRP2	F542L	noHabit	42498	NA	NA	NA	NA	NA	NA	NA	NA	PoD_PrD	NA	MODERATE	D	1	136	136	0	158	121	37	23.42
TSNAD	LRP2_F542L	10	HLA-A*03:02	AFMDGSNRK	1046.4	1.9748	WB	ALMDGSNRK	60.8	0.1744	SB	LRP2	F542L	noHabit	42498	NA	NA	NA	NA	NA	NA	NA	NA	PoD_PrD	NA	MODERATE	D	1	136	136	0	158	121	37	23.42
TSNAD	LRP2_F542L	10	HLA-A*11:01	AFMDGSNRK	640.9	1.9986	WB	ALMDGSNRK	44.5	0.274	SB	LRP2	F542L	noHabit	42498	NA	NA	NA	NA	NA	NA	NA	NA	PoD_PrD	NA	MODERATE	D	1	136	136	0	158	121	37	23.42
TSNAD	LRP2_F542L	10	HLA-A*03:01	AFMDGSNRK	2118.4	2.8361	-	ALMDGSNRK	62.1	0.2351	SB	LRP2	F542L	noHabit	42498	NA	NA	NA	NA	NA	NA	NA	NA	PoD_PrD	NA	MODERATE	D	1	136	136	0	158	121	37	23.42
HLA	MAB21L2_E281K	2	HLA-A*11:01	YHMKTLLLYE	16650.2	13.9564	-	YHMKTLLLYK	82.9	0.4859	SB	MAB21L2	E281K	noHabit	42497	NA	NA	NA	NA	NA	NA	NA	D	PrD_PrD	D	MODERATE	D	1	92	92	0	74	65	9	12.16
HLA	MAB21L2_E281K	3	HLA-A*11:01	HMKTLLLYE	10060.3	9.0443	-	HMKTLLLYK	29.7	0.1767	SB	MAB21L2	E281K	noHabit	42497	NA	NA	NA	NA	NA	NA	NA	D	PrD_PrD	D	MODERATE	D	1	92	92	0	74	65	9	12.16
TSNAD	MAB21L2_E281K	3	HLA-A*31:01	HMKTLLLYE	1945.2	4.3634	-	HMKTLLLYK	15.8	0.1031	SB	MAB21L2	E281K	noHabit	42497	NA	NA	NA	NA	NA	NA	NA	D	PrD_PrD	D	MODERATE	D	1	92	92	0	74	65	9	12.16
TSNAD	MAB21L2_E281K	3	HLA-A*11:01	HMKTLLLYE	10060.3	9.0443	-	HMKTLLLYK	29.7	0.1767	SB	MAB21L2	E281K	noHabit	42497	NA	NA	NA	NA	NA	NA	NA	D	PrD_PrD	D	MODERATE	D	1	92	92	0	74	65	9	12.16
TSNAD	MAB21L2_E281K	2	HLA-A*30:01	YHMKTLLLYE	5084.5	12.4364	-	YHMKTLLLYK	84.7	0.4254	SB	MAB21L2	E281K	noHabit	42497	NA	NA	NA	NA	NA	NA	NA	D	PrD_PrD	D	MODERATE	D	1	92	92	0	74	65	9	12.16
TSNAD	MAB21L2_E281K	3	HLA-A*34:02	HMKTLLLYE	6351.2	7.012	-	HMKTLLLYK	74	0.1157	SB	MAB21L2	E281K	noHabit	42497	NA	NA	NA	NA	NA	NA	NA	D	PrD_PrD	D	MODERATE	D	1	92	92	0	74	65	9	12.16
TSNAD	MAB21L2_E281K	2	HLA-A*03:01	YHMKTLLLYE	9610.6	7.4773	-	YHMKTLLLYK	23.9	0.0661	SB	MAB21L2	E281K	noHabit	42497	NA	NA	NA	NA	NA	NA	NA	D	PrD_PrD	D	MODERATE	D	1	92	92	0	74	65	9	12.16
TSNAD	MAB21L2_E281K	2	HLA-A*03:02	YHMKTLLLYE	10492	8.6621	-	YHMKTLLLYK	55.1	0.155	SB	MAB21L2	E281K	noHabit	42497	NA	NA	NA	NA	NA	NA	NA	D	PrD_PrD	D	MODERATE	D	1	92	92	0	74	65	9	12.16
TSNAD	MAB21L2_E281K	3	HLA-A*03:02	HMKTLLLYE	5273.3	5.2971	-	HMKTLLLYK	22.8	0.0388	SB	MAB21L2	E281K	noHabit	42497	NA	NA	NA	NA	NA	NA	NA	D	PrD_PrD	D	MODERATE	D	1	92	92	0	74	65	9	12.16
TSNAD	MAB21L2_E281K	3	HLA-A*03:01	HMKTLLLYE	4515.3	4.4026	-	HMKTLLLYK	11.6	0.0197	SB	MAB21L2	E281K	noHabit	42497	NA	NA	NA	NA	NA	NA	NA	D	PrD_PrD	D	MODERATE	D	1	92	92	0	74	65	9	12.16
TSNAD	MAB21L2_E281K	2	HLA-A*11:01	YHMKTLLLYE	16650.2	13.9564	-	YHMKTLLLYK	82.9	0.4859	SB	MAB21L2	E281K	noHabit	42497	NA	NA	NA	NA	NA	NA	NA	D	PrD_PrD	D	MODERATE	D	1	92	92	0	74	65	9	12.16
HLA	MAN2B2_Q737K	2	HLA-A*11:01	VIYSDNNGYQ	11040.2	9.6836	-	VIYSDNNGYK	41.7	0.2553	SB	MAN2B2	Q737K	Chewer	42487	NA	NA	NA	NA	NA	NA	NA	D	PoD_PoD	D	MODERATE	D	0.999	105	104	1	61	29	32	52.46
TSNAD	MAN2B2_Q737K	2	HLA-A*11:01	VIYSDNNGYQ	11040.2	9.6836	-	VIYSDNNGYK	41.7	0.2553	SB	MAN2B2	Q737K	Chewer	42487	NA	NA	NA	NA	NA	NA	NA	D	PoD_PoD	D	MODERATE	D	0.999	105	104	1	61	29	32	52.46
TSNAD	MAN2B2_Q737K	2	HLA-A*34:02	VIYSDNNGYQ	6073.6	6.7604	-	VIYSDNNGYK	94.2	0.16	SB	MAN2B2	Q737K	Chewer	42487	NA	NA	NA	NA	NA	NA	NA	D	PoD_PoD	D	MODERATE	D	0.999	105	104	1	61	29	32	52.46
TSNAD	MAN2B2_Q737K	11	HLA-A*30:01	QMQRRPYVSY	544.6	2.1967	-	KMQRRPYVSY	52	0.2711	SB	MAN2B2	Q737K	Chewer	42487	NA	NA	NA	NA	NA	NA	NA	D	PoD_PoD	D	MODERATE	D	0.999	105	104	1	61	29	32	52.46
TSNAD	MAN2B2_Q737K	2	HLA-A*03:02	VIYSDNNGYQ	12304.1	9.947	-	VIYSDNNGYK	86	0.2496	SB	MAN2B2	Q737K	Chewer	42487	NA	NA	NA	NA	NA	NA	NA	D	PoD_PoD	D	MODERATE	D	0.999	105	104	1	61	29	32	52.46
TSNAD	MAN2B2_Q737K	2	HLA-A*03:01	VIYSDNNGYQ	8457	6.7564	-	VIYSDNNGYK	30.8	0.1018	SB	MAN2B2	Q737K	Chewer	42487	NA	NA	NA	NA	NA	NA	NA	D	PoD_PoD	D	MODERATE	D	0.999	105	104	1	61	29	32	52.46
HLA	MAP10_L28F	10	HLA-A*33:03	FLRYIDLTPK	1015	1.9771	WB	FFRYIDLTPK	94.7	0.3629	SB	MAP10	L28F	Smoker	42480	NA	NA	NA	NA	NA	NA	NA	NA	PoD	NA	MODERATE	NA	NA	209	209	0	141	119	21	14.89
TSNAD	MAP10_L28F	10	HLA-A*33:03	FLRYIDLTPK	1015	1.9771	WB	FFRYIDLTPK	94.7	0.3629	SB	MAP10	L28F	Smoker	42480	NA	NA	NA	NA	NA	NA	NA	NA	PoD	NA	MODERATE	NA	NA	209	209	0	141	119	21	14.89
HLA	MATR3_E249A	9	HLA-A*33:03	DSEYERMGR	710	1.5725	WB	DSAYERMGR	78	0.305	SB	MATR3	E249A	Chewer	42489	NA	NA	NA	NA	NA	NA	NA	T	PrD_PoD	D	MODERATE	D	NA	129	129	0	116	90	26	22.41
TSNAD	MATR3_E249A	9	HLA-A*33:01	DSEYERMGR	510	0.9218	WB	DSAYERMGR	73.4	0.2048	SB	MATR3	E249A	Chewer	42489	NA	NA	NA	NA	NA	NA	NA	T	PrD_PoD	D	MODERATE	D	NA	129	129	0	116	90	26	22.41
TSNAD	MATR3_E249A	9	HLA-A*33:03	DSEYERMGR	710	1.5725	WB	DSAYERMGR	78	0.305	SB	MATR3	E249A	Chewer	42489	NA	NA	NA	NA	NA	NA	NA	T	PrD_PoD	D	MODERATE	D	NA	129	129	0	116	90	26	22.41
HLA	MBTPS1_G340R	3	HLA-A*33:03	MVSAIGNDG	31941.7	36.0036	-	MVSAIGNDR	63.3	0.245	SB	MBTPS1	G340R	Chewer	42489	chr16*84124493	chr16*84124493	chr16*84124493	NA	NA	NA	NA	D	PrD_PrD	D	MODERATE	D	0.997	178	177	1	150	17	133	88.67
TSNAD	MBTPS1_G340R	3	HLA-A*68:01	MVSAIGNDG	15909.8	16.604	-	MVSAIGNDR	8.7	0.0749	SB	MBTPS1	G340R	Chewer	42489	chr16*84124493	chr16*84124493	chr16*84124493	NA	NA	NA	NA	D	PrD_PrD	D	MODERATE	D	0.997	178	177	1	150	17	133	88.67
TSNAD	MBTPS1_G340R	3	HLA-A*33:03	MVSAIGNDG	31941.7	36.0036	-	MVSAIGNDR	63.3	0.245	SB	MBTPS1	G340R	Chewer	42489	chr16*84124493	chr16*84124493	chr16*84124493	NA	NA	NA	NA	D	PrD_PrD	D	MODERATE	D	0.997	178	177	1	150	17	133	88.67
HLA	MIEF2_E302K	3	HLA-A*11:01	MASEELLLE	22307	19.7654	-	MASEELLLK	58.9	0.361	SB	MIEF2	E302K	Chewer	42489	NA	NA	NA	0.0000168	0	NA	NA	D	BN_BN	N	MODERATE	N	0.782	36	36	0	42	27	15	35.71
HLA	MIEF2_E302K	2	HLA-A*11:01	SMASEELLLE	14800.8	12.4393	-	SMASEELLLK	21.9	0.1173	SB	MIEF2	E302K	Chewer	42489	NA	NA	NA	0.0000168	0	NA	NA	D	BN_BN	N	MODERATE	N	0.782	36	36	0	42	27	15	35.71
TSNAD	MIEF2_E302K	2	HLA-A*03:02	SMASEELLLE	12004.5	9.7345	-	SMASEELLLK	34.4	0.0729	SB	MIEF2	E302K	Chewer	42489	NA	NA	NA	0.0000168	0	NA	NA	D	BN_BN	N	MODERATE	N	0.782	36	36	0	42	27	15	35.71
TSNAD	MIEF2_E302K	3	HLA-A*11:01	MASEELLLE	22307	19.7654	-	MASEELLLK	58.9	0.361	SB	MIEF2	E302K	Chewer	42489	NA	NA	NA	0.0000168	0	NA	NA	D	BN_BN	N	MODERATE	N	0.782	36	36	0	42	27	15	35.71
TSNAD	MIEF2_E302K	2	HLA-A*03:01	SMASEELLLE	16653.8	12.7825	-	SMASEELLLK	36.9	0.1312	SB	MIEF2	E302K	Chewer	42489	NA	NA	NA	0.0000168	0	NA	NA	D	BN_BN	N	MODERATE	N	0.782	36	36	0	42	27	15	35.71
TSNAD	MIEF2_E302K	3	HLA-A*68:01	MASEELLLE	5442.2	7.7287	-	MASEELLLK	30.4	0.3691	SB	MIEF2	E302K	Chewer	42489	NA	NA	NA	0.0000168	0	NA	NA	D	BN_BN	N	MODERATE	N	0.782	36	36	0	42	27	15	35.71
TSNAD	MIEF2_E302K	2	HLA-A*11:01	SMASEELLLE	14800.8	12.4393	-	SMASEELLLK	21.9	0.1173	SB	MIEF2	E302K	Chewer	42489	NA	NA	NA	0.0000168	0	NA	NA	D	BN_BN	N	MODERATE	N	0.782	36	36	0	42	27	15	35.71
HLA	MRGPRX1_G233A	2	HLA-A*02:01	VFLLCGLPFG	2315.3	6.2848	-	VFLLCGLPFA	20.8	0.2844	SB	MRGPRX1	G233A	noHabit	42494	chr11*18955634	chr11*18955634	NA	NA	NA	NA	NA	D	PoD_PrD	D	MODERATE	N	0.057	113	113	0	96	78	17	17.71
TSNAD	MRGPRX1_G233A	2	HLA-A*02:06	VFLLCGLPFG	1465.8	6.3711	-	VFLLCGLPFA	24.1	0.3084	SB	MRGPRX1	G233A	noHabit	42494	chr11*18955634	chr11*18955634	NA	NA	NA	NA	NA	D	PoD_PrD	D	MODERATE	N	0.057	113	113	0	96	78	17	17.71
TSNAD	MRGPRX1_G233A	2	HLA-A*02:09	VFLLCGLPFG	2315.3	6.2848	-	VFLLCGLPFA	20.8	0.2844	SB	MRGPRX1	G233A	noHabit	42494	chr11*18955634	chr11*18955634	NA	NA	NA	NA	NA	D	PoD_PrD	D	MODERATE	N	0.057	113	113	0	96	78	17	17.71
TSNAD	MRGPRX1_G233A	2	HLA-A*02:01	VFLLCGLPFG	2315.3	6.2848	-	VFLLCGLPFA	20.8	0.2844	SB	MRGPRX1	G233A	noHabit	42494	chr11*18955634	chr11*18955634	NA	NA	NA	NA	NA	D	PoD_PrD	D	MODERATE	N	0.057	113	113	0	96	78	17	17.71
TSNAD	MRGPRX1_G233A	3	HLA-A*02:07	FLLCGLPFG	2921.3	0.5853	WB	FLLCGLPFA	51.9	0.009	SB	MRGPRX1	G233A	noHabit	42494	chr11*18955634	chr11*18955634	NA	NA	NA	NA	NA	D	PoD_PrD	D	MODERATE	N	0.057	113	113	0	96	78	17	17.71
HLA	MXRA8_S93L	10	HLA-A*02:03	YSAGEQRVYEA	3743.7	12.6526	-	YLAGEQRVYE	19.4	0.4845	SB	MXRA8	S93L	Smoker	42479	NA	NA	NA	NA	NA	NA	NA	D	PrD	D	MODERATE	D	0.944	25	25	0	39	27	12	30.77
TSNAD	MXRA8_S93L	10	HLA-A*02:09	YSAGEQRVYEA	10044.9	14.4942	-	YLAGEQRVYE	32.5	0.4335	SB	MXRA8	S93L	Smoker	42479	NA	NA	NA	NA	NA	NA	NA	D	PrD	D	MODERATE	D	0.944	25	25	0	39	27	12	30.77
TSNAD	MXRA8_S93L	10	HLA-A*02:03	YSAGEQRVYEA	3743.7	12.6526	-	YLAGEQRVYE	19.4	0.4845	SB	MXRA8	S93L	Smoker	42479	NA	NA	NA	NA	NA	NA	NA	D	PrD	D	MODERATE	D	0.944	25	25	0	39	27	12	30.77
TSNAD	MXRA8_S93L	10	HLA-A*02:11	YSAGEQRVYEA	2158.7	10.0065	-	YLAGEQRVYE	7	0.4002	SB	MXRA8	S93L	Smoker	42479	NA	NA	NA	NA	NA	NA	NA	D	PrD	D	MODERATE	D	0.944	25	25	0	39	27	12	30.77
TSNAD	MXRA8_S93L	10	HLA-A*02:02	YSAGEQRVYEA	3090.9	11.759	-	YLAGEQRVYE	17.1	0.4032	SB	MXRA8	S93L	Smoker	42479	NA	NA	NA	NA	NA	NA	NA	D	PrD	D	MODERATE	D	0.944	25	25	0	39	27	12	30.77
TSNAD	MXRA8_S93L	10	HLA-A*02:01	YSAGEQRVYEA	10044.9	14.4942	-	YLAGEQRVYE	32.5	0.4335	SB	MXRA8	S93L	Smoker	42479	NA	NA	NA	NA	NA	NA	NA	D	PrD	D	MODERATE	D	0.944	25	25	0	39	27	12	30.77
HLA	MYLK2_G393W	6	HLA-A*24:02	RQICDGILF	951.5	1.0439	WB	RQICDWILF	77.8	0.1698	SB	MYLK2	G393W	Chewer	42484	NA	NA	chr20*30414694	NA	NA	NA	NA	D	PrD_PrD	D	MODERATE	NA	NA	91	89	0	64	25	39	60.94
TSNAD	MYLK2_G393W	2	HLA-B*58:01	MVFVRQICDG	12935.5	16.0289	-	MVFVRQICDW	62.6	0.34	SB	MYLK2	G393W	Chewer	42484	NA	NA	chr20*30414694	NA	NA	NA	NA	D	PrD_PrD	D	MODERATE	NA	NA	91	89	0	64	25	39	60.94
TSNAD	MYLK2_G393W	6	HLA-A*24:02	RQICDGILF	951.5	1.0439	WB	RQICDWILF	77.8	0.1698	SB	MYLK2	G393W	Chewer	42484	NA	NA	chr20*30414694	NA	NA	NA	NA	D	PrD_PrD	D	MODERATE	NA	NA	91	89	0	64	25	39	60.94
HLA	MYO7A_P1244R	2	HLA-A*33:03	FVNGTRTQPP	34619.3	43.8523	-	FVNGTRTQPR	51.3	0.1919	SB	MYO7A	P1244R	Chewer	42484	NA	NA	NA	NA	NA	NA	NA	D	PrD_PrD	D	MODERATE	D	0.99	43	43	0	36	29	7	19.44
TSNAD	MYO7A_P1244R	9	HLA-B*07:05	QPPSWLEL	1295.7	1.6161	WB	QPRSWLEL	40.8	0.1571	SB	MYO7A	P1244R	Chewer	42484	NA	NA	NA	NA	NA	NA	NA	D	PrD_PrD	D	MODERATE	D	0.99	43	43	0	36	29	7	19.44
TSNAD	MYO7A_P1244R	9	HLA-B*07:02	QPPSWLEL	1902.7	1.7984	WB	QPRSWLEL	47.5	0.1665	SB	MYO7A	P1244R	Chewer	42484	NA	NA	NA	NA	NA	NA	NA	D	PrD_PrD	D	MODERATE	D	0.99	43	43	0	36	29	7	19.44
TSNAD	MYO7A_P1244R	11	HLA-A*03:01	PSWLELQATK	6454.4	5.5316	-	RSWLELQATK	94.9	0.3502	SB	MYO7A	P1244R	Chewer	42484	NA	NA	NA	NA	NA	NA	NA	D	PrD_PrD	D	MODERATE	D	0.99	43	43	0	36	29	7	19.44
TSNAD	MYO7A_P1244R	2	HLA-A*33:03	FVNGTRTQPP	34619.3	43.8523	-	FVNGTRTQPR	51.3	0.1919	SB	MYO7A	P1244R	Chewer	42484	NA	NA	NA	NA	NA	NA	NA	D	PrD_PrD	D	MODERATE	D	0.99	43	43	0	36	29	7	19.44
TSNAD	MYO7A_P1244R	9	HLA-B*42:01	QPPSWLEL	562.7	1.3138	WB	QPRSWLEL	55.3	0.2725	SB	MYO7A	P1244R	Chewer	42484	NA	NA	NA	NA	NA	NA	NA	D	PrD_PrD	D	MODERATE	D	0.99	43	43	0	36	29	7	19.44
TSNAD	MYO7A_P1244R	2	HLA-A*68:01	FVNGTRTQPP	28838.4	33.9622	-	FVNGTRTQPR	23.8	0.2982	SB	MYO7A	P1244R	Chewer	42484	NA	NA	NA	NA	NA	NA	NA	D	PrD_PrD	D	MODERATE	D	0.99	43	43	0	36	29	7	19.44
TSNAD	MYO7A_P1244R	11	HLA-A*11:01	PSWLELQATK	2197.2	3.7881	-	RSWLELQATK	67.3	0.4051	SB	MYO7A	P1244R	Chewer	42484	NA	NA	NA	NA	NA	NA	NA	D	PrD_PrD	D	MODERATE	D	0.99	43	43	0	36	29	7	19.44
HLA	N4BP1_E727K	3	HLA-A*11:01	IVTNDNFRE	22679.3	20.2358	-	IVTNDNFRK	64.3	0.3895	SB	N4BP1	E727K	Chewer	42487	NA	NA	NA	NA	NA	NA	NA	D	PrD_PrD	D	MODERATE	D	1	64	64	0	70	33	11	15.71
TSNAD	N4BP1_E727K	11	HLA-A*31:01	EFVNESVSWR	1260.4	3.4578	-	KFVNESVSWR	32.6	0.2583	SB	N4BP1	E727K	Chewer	42487	NA	NA	NA	NA	NA	NA	NA	D	PrD_PrD	D	MODERATE	D	1	64	64	0	70	33	11	15.71
TSNAD	N4BP1_E727K	3	HLA-A*11:01	IVTNDNFRE	22679.3	20.2358	-	IVTNDNFRK	64.3	0.3895	SB	N4BP1	E727K	Chewer	42487	NA	NA	NA	NA	NA	NA	NA	D	PrD_PrD	D	MODERATE	D	1	64	64	0	70	33	11	15.71
HLA	NDST4_K439M	4	HLA-A*32:01	QLYAAWKKVW	517.8	0.7808	WB	QLYAAWKMV	66.7	0.1224	SB	NDST4	K439M	noHabit	42496	NA	NA	NA	NA	NA	NA	NA	D	PrD_PrD	D	MODERATE	D	0.998	101	101	0	132	123	9	6.82
TSNAD	NDST4_K439M	6	HLA-B*58:01	YAAWKKVW	1001.1	2.2426	-	YAAWKMVW	90.7	0.4458	SB	NDST4	K439M	noHabit	42496	NA	NA	NA	NA	NA	NA	NA	D	PrD_PrD	D	MODERATE	D	0.998	101	101	0	132	123	9	6.82
TSNAD	NDST4_K439M	3	HLA-B*15:03	IQLYAAWKK	6166.2	16.5634	-	IQLYAAWKM	14.6	0.2547	SB	NDST4	K439M	noHabit	42496	NA	NA	NA	NA	NA	NA	NA	D	PrD_PrD	D	MODERATE	D	0.998	101	101	0	132	123	9	6.82
TSNAD	NDST4_K439M	4	HLA-A*32:01	QLYAAWKKVW	517.8	0.7808	WB	QLYAAWKMV	66.7	0.1224	SB	NDST4	K439M	noHabit	42496	NA	NA	NA	NA	NA	NA	NA	D	PrD_PrD	D	MODERATE	D	0.998	101	101	0	132	123	9	6.82
HLA	NFAT5_E754K	3	HLA-A*11:01	VVNLSQLTE	10175.6	9.12	-	VVNLSQLTK	26.5	0.1549	SB	NFAT5	E754K	noHabit	42500	NA	NA	NA	NA	NA	NA	NA	NA	BN_PoD	NA	MODERATE	N	0.947	212	212	0	127	79	48	37.8
HLA	NFAT5_E754K	2	HLA-A*11:01	TVVNLSQLTE	16798.2	14.0847	-	TVVNLSQLTK	23.3	0.1284	SB	NFAT5	E754K	noHabit	42500	NA	NA	NA	NA	NA	NA	NA	NA	BN_PoD	NA	MODERATE	N	0.947	212	212	0	127	79	48	37.8
TSNAD	NFAT5_E754K	3	HLA-A*11:01	VVNLSQLTE	10175.6	9.12	-	VVNLSQLTK	26.5	0.1549	SB	NFAT5	E754K	noHabit	42500	NA	NA	NA	NA	NA	NA	NA	NA	BN_PoD	NA	MODERATE	N	0.947	212	212	0	127	79	48	37.8
TSNAD	NFAT5_E754K	3	HLA-A*03:02	VVNLSQLTE	14453.6	11.544	-	VVNLSQLTK	93.3	0.2745	SB	NFAT5	E754K	noHabit	42500	NA	NA	NA	NA	NA	NA	NA	NA	BN_PoD	NA	MODERATE	N	0.947	212	212	0	127	79	48	37.8
TSNAD	NFAT5_E754K	3	HLA-A*03:01	VVNLSQLTE	15579.5	11.8219	-	VVNLSQLTK	58.4	0.2205	SB	NFAT5	E754K	noHabit	42500	NA	NA	NA	NA	NA	NA	NA	NA	BN_PoD	NA	MODERATE	N	0.947	212	212	0	127	79	48	37.8
TSNAD	NFAT5_E754K	2	HLA-A*34:02	TVVNLSQLTE	11611.7	11.6506	-	TVVNLSQLTK	83.1	0.1363	SB	NFAT5	E754K	noHabit	42500	NA	NA	NA	NA	NA	NA	NA	NA	BN_PoD	NA	MODERATE	N	0.947	212	212	0	127	79	48	37.8
TSNAD	NFAT5_E754K	2	HLA-A*11:01	TVVNLSQLTE	16798.2	14.0847	-	TVVNLSQLTK	23.3	0.1284	SB	NFAT5	E754K	noHabit	42500	NA	NA	NA	NA	NA	NA	NA	NA	BN_PoD	NA	MODERATE	N	0.947	212	212	0	127	79	48	37.8
HLA	NSD1_C1897Y	3	HLA-A*11:01	LSEIPRCNCK	877.9	2.3634	-	LSEIPRCNYK	81	0.4771	SB	NSD1	C1897Y	noHabit	42492	NA	NA	NA	NA	NA	NA	NA	D	PrD_PrD	D	MODERATE	D	1	122	122	0	146	69	77	52.74
TSNAD	NSD1_C1897Y	3	HLA-A*11:01	LSEIPRCNCK	877.9	2.3634	-	LSEIPRCNYK	81	0.4771	SB	NSD1	C1897Y	noHabit	42492	NA	NA	NA	NA	NA	NA	NA	D	PrD_PrD	D	MODERATE	D	1	122	122	0	146	69	77	52.74
HLA	OR11L1_F277L	10	HLA-A*02:01	VFYTVVTPL	5502.3	9.9944	-	VLYTVVTPL	27.2	0.3697	SB	OR11L1	F277L	noHabit	42494	NA	chr1*248004359	chr1*248004368	NA	NA	NA	NA	T	BN_PoD	N	MODERATE	U	1	70	70	0	99	94	5	5.05
TSNAD	OR11L1_F277L	10	HLA-A*02:11	VFYTVVTPL	1053.8	7.4949	-	VLYTVVTPL	3.7	0.1581	SB	OR11L1	F277L	noHabit	42494	NA	chr1*248004359	chr1*248004368	NA	NA	NA	NA	T	BN_PoD	N	MODERATE	U	1	70	70	0	99	94	5	5.05
TSNAD	OR11L1_F277L	10	HLA-A*02:03	VFYTVVTPL	2361.4	10.0907	-	VLYTVVTPL	11.2	0.245	SB	OR11L1	F277L	noHabit	42494	NA	chr1*248004359	chr1*248004368	NA	NA	NA	NA	T	BN_PoD	N	MODERATE	U	1	70	70	0	99	94	5	5.05
TSNAD	OR11L1_F277L	10	HLA-A*02:02	VFYTVVTPL	1541.9	8.3302	-	VLYTVVTPL	15	0.3503	SB	OR11L1	F277L	noHabit	42494	NA	chr1*248004359	chr1*248004368	NA	NA	NA	NA	T	BN_PoD	N	MODERATE	U	1	70	70	0	99	94	5	5.05
TSNAD	OR11L1_F277L	10	HLA-A*02:01	VFYTVVTPL	5502.3	9.9944	-	VLYTVVTPL	27.2	0.3697	SB	OR11L1	F277L	noHabit	42494	NA	chr1*248004359	chr1*248004368	NA	NA	NA	NA	T	BN_PoD	N	MODERATE	U	1	70	70	0	99	94	5	5.05
TSNAD	OR11L1_F277L	10	HLA-A*02:09	VFYTVVTPL	5502.3	9.9944	-	VLYTVVTPL	27.2	0.3697	SB	OR11L1	F277L	noHabit	42494	NA	chr1*248004359	chr1*248004368	NA	NA	NA	NA	T	BN_PoD	N	MODERATE	U	1	70	70	0	99	94	5	5.05
HLA	OR14I1_S149I	3	HLA-A*02:01	QMAVTTWLS	1875.9	5.6317	-	QMAVTTWLI	38.3	0.4991	SB	OR14I1	S149I	noHabit	42493	NA	NA	NA	NA	NA	NA	NA	T	BN_BN	N	MODERATE	N	0.028	128	128	0	213	199	14	6.57
TSNAD	OR14I1_S149I	1	HLA-A*02:06	CYQMAVTTWLS	807.8	4.4617	-	CYQMAVTTWI	34.3	0.4383	SB	OR14I1	S149I	noHabit	42493	NA	NA	NA	NA	NA	NA	NA	T	BN_BN	N	MODERATE	N	0.028	128	128	0	213	199	14	6.57
TSNAD	OR14I1_S149I	3	HLA-A*32:01	QMAVTTWLS	10533.7	8.1964	-	QMAVTTWLI	45.7	0.082	SB	OR14I1	S149I	noHabit	42493	NA	NA	NA	NA	NA	NA	NA	T	BN_BN	N	MODERATE	N	0.028	128	128	0	213	199	14	6.57
TSNAD	OR14I1_S149I	2	HLA-A*32:01	YQMAVTTWLS	8198.9	6.5518	-	YQMAVTTWLI	99.4	0.173	SB	OR14I1	S149I	noHabit	42493	NA	NA	NA	NA	NA	NA	NA	T	BN_BN	N	MODERATE	N	0.028	128	128	0	213	199	14	6.57
TSNAD	OR14I1_S149I	3	HLA-A*02:05	QMAVTTWLS	1297.9	4.9865	-	QMAVTTWLI	68.2	0.4377	SB	OR14I1	S149I	noHabit	42493	NA	NA	NA	NA	NA	NA	NA	T	BN_BN	N	MODERATE	N	0.028	128	128	0	213	199	14	6.57
TSNAD	OR14I1_S149I	3	HLA-A*02:09	QMAVTTWLS	1875.9	5.6317	-	QMAVTTWLI	38.3	0.4991	SB	OR14I1	S149I	noHabit	42493	NA	NA	NA	NA	NA	NA	NA	T	BN_BN	N	MODERATE	N	0.028	128	128	0	213	199	14	6.57
TSNAD	OR14I1_S149I	2	HLA-A*23:01	YQMAVTTWLS	8104.8	6.2406	-	YQMAVTTWLI	96.2	0.3288	SB	OR14I1	S149I	noHabit	42493	NA	NA	NA	NA	NA	NA	NA	T	BN_BN	N	MODERATE	N	0.028	128	128	0	213	199	14	6.57

Supplementary Table 6. List of neoantigens predicted using TSNAD

Software	Variant accession	Position	HLA subtype	Wild type peptide	Wild binding	Wild rank	wild binding level	Mutant peptide	Mutant binding	Mutant rank	Mutant binding level	Gene symbol	Variant information	Habit	Sample ID	OncoMD	COSMIC	dbSNP	ExAC	ExAC_SAS	1000G	1000G_SAS	SIFT	Polyphen2	CONDEL	VEP	LRT	PhastCon7Way	Total read depth in Normal	Reference read support in Normal	Alternative read support in Normal	Total read depth in Tumor	Reference read support in Tumor	Alternate read support in Tumor	Alternate allele frequency in Tumor
TSNAD	OR14I1_S149I	3	HLA-A*02:01	QMAVTTWLS	1875.9	5.6317	-	QMAVTTWLI	38.3	0.4991	SB	OR14I1	S149I	noHabit	42493	NA	NA	NA	NA	NA	NA	NA	T	BN_BN	N	MODERATE	N	0.028	128	128	0	213	199	14	6.57
TSNAD	OR14I1_S149I	2	HLA-B*13:02	YQMAVTTWLS	1688.5	0.538	WB	YQMAVTTWLI	63.2	0.0077	SB	OR14I1	S149I	noHabit	42493	NA	NA	NA	NA	NA	NA	NA	T	BN_BN	N	MODERATE	N	0.028	128	128	0	213	199	14	6.57
HLA	OR2T7_G290R	9	HLA-A*30:01	VTGALQKVV	5017.4	12.2995	-	VTRALQKVV	86.8	0.4361	SB	OR2T7	G290R	Smoker	42474	NA	NA	NA	NA	NA	NA	NA	T	BN	N	MODERATE	NA	NA	119	119	0	210	97	113	53.81
TSNAD	OR2T7_G290R	9	HLA-A*30:01	VTGALQKVV	5017.4	12.2995	-	VTRALQKVV	86.8	0.4361	SB	OR2T7	G290R	Smoker	42474	NA	NA	NA	NA	NA	NA	NA	T	BN	N	MODERATE	NA	NA	119	119	0	210	97	113	53.81
TSNAD	OR2T7_G290R	3	HLA-A*31:01	SLRNKDVTG	26771.4	37.3909	-	SLRNKDVTR	37.8	0.3078	SB	OR2T7	G290R	Smoker	42474	NA	NA	NA	NA	NA	NA	NA	T	BN	N	MODERATE	NA	NA	119	119	0	210	97	113	53.81
HLA	OR4A47_P127H	11	HLA-A*33:03	PLHYLVIMR	1302.9	2.307	-	HLHYLVIMR	59.7	0.2291	SB	OR4A47	P127H	Smoker	42480	NA	NA	NA	NA	NA	NA	NA	D_lc	PrD	D	MODERATE	D	0.943	247	245	1	245	209	36	14.69
TSNAD	OR4A47_P127H	11	HLA-A*33:03	PLHYLVIMR	1302.9	2.307	-	HLHYLVIMR	59.7	0.2291	SB	OR4A47	P127H	Smoker	42480	NA	NA	NA	NA	NA	NA	NA	D_lc	PrD	D	MODERATE	D	0.943	247	245	1	245	209	36	14.69
TSNAD	OR4A47_P127H	11	HLA-A*33:01	PLHYLVIMR	1874.6	2.1416	-	HLHYLVIMR	81.2	0.2263	SB	OR4A47	P127H	Smoker	42480	NA	NA	NA	NA	NA	NA	NA	D_lc	PrD	D	MODERATE	D	0.943	247	245	1	245	209	36	14.69
TSNAD	OR4A47_P127H	11	HLA-A*31:01	PLHYLVIMR	901.7	2.8893	-	HLHYLVIMR	53.7	0.4503	SB	OR4A47	P127H	Smoker	42480	NA	NA	NA	NA	NA	NA	NA	D_lc	PrD	D	MODERATE	D	0.943	247	245	1	245	209	36	14.69
TSNAD	OR4A47_P127H	11	HLA-A*68:01	PLHYLVIMR	1445	3.8742	-	HLHYLVIMR	36.8	0.4305	SB	OR4A47	P127H	Smoker	42480	NA	NA	NA	NA	NA	NA	NA	D_lc	PrD	D	MODERATE	D	0.943	247	245	1	245	209	36	14.69
HLA	OR4C3_P192L	10	HLA-A*02:11	LPFCGPNVI	12442.4	23.6552	-	LLFCGPNVI	7.9	0.4632	SB	OR4C3	P192L	noHabit	56957	NA	NA	NA	0.499950566	0.499939416	NA	NA	D	PrD_PrD	D	MODERATE	D	0.928	272	142	129	1539	1114	422	27.42
TSNAD	OR4C3_P192L	10	HLA-A*02:11	LPFCGPNVI	12442.4	23.6552	-	LLFCGPNVI	7.9	0.4632	SB	OR4C3	P192L	noHabit	56957	NA	NA	NA	0.499950566	0.499939416	NA	NA	D	PrD_PrD	D	MODERATE	D	0.928	272	142	129	1539	1114	422	27.42
TSNAD	OR4C3_P192L	10	HLA-A*02:03	LPFCGPNVI	15443.2	28.7204	-	LLFCGPNVI	14.1	0.3384	SB	OR4C3	P192L	noHabit	56957	NA	NA	NA	0.499950566	0.499939416	NA	NA	D	PrD_PrD	D	MODERATE	D	0.928	272	142	129	1539	1114	422	27.42
TSNAD	OR4C3_P192L	10	HLA-A*02:02	LPFCGPNVI	16281	31.2276	-	LLFCGPNVI	19.1	0.461	SB	OR4C3	P192L	noHabit	56957	NA	NA	NA	0.499950566	0.499939416	NA	NA	D	PrD_PrD	D	MODERATE	D	0.928	272	142	129	1539	1114	422	27.42
HLA	OR4C5_F198L	10	HLA-A*02:01	HFICDLFPL	781.6	3.5807	-	HLICDLFPL	3.4	0.0114	SB	OR4C5	F198L	noHabit	56957	NA	NA	NA	0.499403786	0.499889037	NA	NA	D_lc	PrD_PrD	D	MODERATE	D	0.994	276	154	122	1560	1151	405	25.96
HLA	OR4C5_F198L	8	HLA-A*02:01	IDHFICDLFPL	7936.4	12.4433	-	IDHLICDLFPL	29.6	0.3967	SB	OR4C5	F198L	noHabit	56957	NA	NA	NA	0.499403786	0.499889037	NA	NA	D_lc	PrD_PrD	D	MODERATE	D	0.994	276	154	122	1560	1151	405	25.96
HLA	OR4C5_F198L	10	HLA-A*02:01	HFICDLFPLL	521.2	2.8802	-	HLICDLFPLL	14.7	0.1813	SB	OR4C5	F198L	noHabit	56957	NA	NA	NA	0.499403786	0.499889037	NA	NA	D_lc	PrD_PrD	D	MODERATE	D	0.994	276	154	122	1560	1151	405	25.96
TSNAD	OR4C5_F198L	9	HLA-A*02:06	DHFICDLFPL	4067.9	11.6792	-	DHLICDLFPL	38.8	0.494	SB	OR4C5	F198L	noHabit	56957	NA	NA	NA	0.499403786	0.499889037	NA	NA	D_lc	PrD_PrD	D	MODERATE	D	0.994	276	154	122	1560	1151	405	25.96
TSNAD	OR4C5_F198L	10	HLA-A*03:02	HFICDLFPLLK	607.8	1.4018	WB	HLICDLFPLLK	82.2	0.2398	SB	OR4C5	F198L	noHabit	56957	NA	NA	NA	0.499403786	0.499889037	NA	NA	D_lc	PrD_PrD	D	MODERATE	D	0.994	276	154	122	1560	1151	405	25.96
TSNAD	OR4C5_F198L	10	HLA-A*02:03	HFICDLFPL	2378.7	10.1275	-	HLICDLFPL	6.6	0.1027	SB	OR4C5	F198L	noHabit	56957	NA	NA	NA	0.499403786	0.499889037	NA	NA	D_lc	PrD_PrD	D	MODERATE	D	0.994	276	154	122	1560	1151	405	25.96
TSNAD	OR4C5_F198L	10	HLA-A*02:09	HFICDLFPLL	521.2	2.8802	-	HLICDLFPLL	14.7	0.1813	SB	OR4C5	F198L	noHabit	56957	NA	NA	NA	0.499403786	0.499889037	NA	NA	D_lc	PrD_PrD	D	MODERATE	D	0.994	276	154	122	1560	1151	405	25.96
TSNAD	OR4C5_F198L	8	HLA-A*02:09	IDHFICDLFPL	7936.4	12.4433	-	IDHLICDLFPL	29.6	0.3967	SB	OR4C5	F198L	noHabit	56957	NA	NA	NA	0.499403786	0.499889037	NA	NA	D_lc	PrD_PrD	D	MODERATE	D	0.994	276	154	122	1560	1151	405	25.96
TSNAD	OR4C5_F198L	10	HLA-A*02:07	HFICDLFPL	5672.8	1.1934	WB	HLICDLFPL	86.1	0.0114	SB	OR4C5	F198L	noHabit	56957	NA	NA	NA	0.499403786	0.499889037	NA	NA	D_lc	PrD_PrD	D	MODERATE	D	0.994	276	154	122	1560	1151	405	25.96
TSNAD	OR4C5_F198L	10	HLA-A*02:09	HFICDLFPL	781.6	3.5807	-	HLICDLFPL	3.4	0.0114	SB	OR4C5	F198L	noHabit	56957	NA	NA	NA	0.499403786	0.499889037	NA	NA	D_lc	PrD_PrD	D	MODERATE	D	0.994	276	154	122	1560	1151	405	25.96
TSNAD	OR4C5_F198L	10	HLA-A*02:01	HFICDLFPLL	521.2	2.8802	-	HLICDLFPLL	14.7	0.1813	SB	OR4C5	F198L	noHabit	56957	NA	NA	NA	0.499403786	0.499889037	NA	NA	D_lc	PrD_PrD	D	MODERATE	D	0.994	276	154	122	1560	1151	405	25.96
TSNAD	OR4C5_F198L	10	HLA-A*02:01	HFICDLFPL	781.6	3.5807	-	HLICDLFPL	3.4	0.0114	SB	OR4C5	F198L	noHabit	56957	NA	NA	NA	0.499403786	0.499889037	NA	NA	D_lc	PrD_PrD	D	MODERATE	D	0.994	276	154	122	1560	1151	405	25.96
TSNAD	OR4C5_F198L	8	HLA-A*02:01	IDHFICDLFPL	7936.4	12.4433	-	IDHLICDLFPL	29.6	0.3967	SB	OR4C5	F198L	noHabit	56957	NA	NA	NA	0.499403786	0.499889037	NA	NA	D_lc	PrD_PrD	D	MODERATE	D	0.994	276	154	122	1560	1151	405	25.96
HLA	OR4F6_F177L	10	HLA-A*02:01	SFFCDLPRFI	2480.9	6.5111	-	SLFCDLPRFI	12.7	0.1532	SB	OR4F6	F177L	noHabit	42495	NA	NA	NA	NA	NA	NA	NA	D_lc	PrD_PrD	D	MODERATE	D	0.995	235	234	0	239	199	37	15.48
TSNAD	OR4F6_F177L	10	HLA-A*02:01	SFFCDLPRFI	2480.9	6.5111	-	SLFCDLPRFI	12.7	0.1532	SB	OR4F6	F177L	noHabit	42495	NA	NA	NA	NA	NA	NA	NA	D_lc	PrD_PrD	D	MODERATE	D	0.995	235	234	0	239	199	37	15.48
TSNAD	OR4F6_F177L	10	HLA-A*02:11	SFFCDLPRFI	685	6.2894	-	SLFCDLPRFI	4.9	0.2433	SB	OR4F6	F177L	noHabit	42495	NA	NA	NA	NA	NA	NA	NA	D_lc	PrD_PrD	D	MODERATE	D	0.995	235	234	0	239	199	37	15.48
TSNAD	OR4F6_F177L	10	HLA-A*02:09	SFFCDLPRFI	2480.9	6.5111	-	SLFCDLPRFI	12.7	0.1532	SB	OR4F6	F177L	noHabit	42495	NA	NA	NA	NA	NA	NA	NA	D_lc	PrD_PrD	D	MODERATE	D	0.995	235	234	0	239	199	37	15.48
TSNAD	OR4F6_F177L	10	HLA-A*02:03	SFFCDLPRFI	2291.3	9.9432	-	SLFCDLPRFI	14.3	0.343	SB	OR4F6	F177L	noHabit	42495	NA	NA	NA	NA	NA	NA	NA	D_lc	PrD_PrD	D	MODERATE	D	0.995	235	234	0	239	199	37	15.48
TSNAD	OR4F6_F177L	10	HLA-A*02:02	SFFCDLPRFI	1497.6	8.2054	-	SLFCDLPRFI	15.8	0.3716	SB	OR4F6	F177L	noHabit	42495	NA	NA	NA	NA	NA	NA	NA	D_lc	PrD_PrD	D	MODERATE	D	0.995	235	234	0	239	199	37	15.48
TSNAD	OR4F6_F177L	10	HLA-A*03:01	SFFCDLPRFIK	1192.6	2.0521	-	SLFCDLPRFIK	50	0.1873	SB	OR4F6	F177L	noHabit	42495	NA	NA	NA	NA	NA	NA	NA	D_lc	PrD_PrD	D	MODERATE	D	0.995	235	234	0	239	199	37	15.48
TSNAD	OR4F6_F177L	10	HLA-A*02:06	SFFCDLPRFI	1303.3	5.9383	-	SLFCDLPRFI	20.8	0.2636	SB	OR4F6	F177L	noHabit	42495	NA	NA	NA	NA	NA	NA	NA	D_lc	PrD_PrD	D	MODERATE	D	0.995	235	234	0	239	199	37	15.48
HLA	PCDHGA7_V662L	10	HLA-A*02:09	TVAVADSIPEV	1187.6	4.42	-	TLAVADSIPEV	34	0.4516	SB	PCDHGA7	V662L	Chewer	42487	chr5*140764450	chr5*140764450	chr5*140764450	NA	NA	NA	NA	D_lc	PoD_PoD	D	MODERATE	NA	NA	135	135	0	116	41	75	64.66
TSNAD	PCDHGA7_V662L	10	HLA-A*02:01	TVAVADSIPEV	1187.6	4.42	-	TLAVADSIPEV	34	0.4516	SB	PCDHGA7	V662L	Chewer	42487	chr5*140764450	chr5*140764450	chr5*140764450	NA	NA	NA	NA	D_lc	PoD_PoD	D	MODERATE	NA	NA	135	135	0	116	41	75	64.66
TSNAD	PCDHGA7_V662L	10	HLA-A*02:09	TVAVADSIPEV	1187.6	4.42	-	TLAVADSIPEV	34	0.4516	SB	PCDHGA7	V662L	Chewer	42487	chr5*140764450	chr5*140764450	chr5*140764450	NA	NA	NA	NA	D_lc	PoD_PoD	D	MODERATE	NA	NA	135	135	0	116	41	75	64.66
HLA	PPP2R2B_E145K	3	HLA-A*03:01	STFQSHEPE	20493.7	16.7893	-	STFQSHEPK	71.3	0.27	SB	PPP2R2B	E145K	noHabit	42501	NA	NA	NA	NA	NA	NA	NA	D	PoD_PrD	D	MODERATE	D	1	98	98	0	59	52	7	11.86
TSNAD	PPP2R2B_E145K	2	HLA-A*34:02	YSTFQSHEPE	11168.1	11.2314	-	YSTFQSHEPK	91.3	0.1528	SB	PPP2R2B	E145K	noHabit	42501	NA	NA	NA	NA	NA	NA	NA	D	PoD_PrD	D	MODERATE	D	1	98	98	0	59	52	7	11.86
TSNAD	PPP2R2B_E145K	2	HLA-A*68:01	YSTFQSHEPE	2446.1	5.0662	-	YSTFQSHEPK	12.6	0.1397	SB	PPP2R2B	E145K	noHabit	42501	NA	NA	NA	NA	NA	NA	NA	D	PoD_PrD	D	MODERATE	D	1	98	98	0	59	52	7	11.86
TSNAD	PPP2R2B_E145K	3	HLA-A*03:01	STFQSHEPE	20493.7	16.7893	-	STFQSHEPK	71.3	0.27	SB	PPP2R2B	E145K	noHabit	42501	NA	NA	NA	NA	NA	NA	NA	D	PoD_PrD	D	MODERATE	D	1	98	98	0	59	52	7	11.86
TSNAD	PPP2R2B_E145K	3	HLA-A*03:02	STFQSHEPE	13982.6	11.1822	-	STFQSHEPK	45.6	0.1189	SB	PPP2R2B	E145K	noHabit	42501	NA	NA	NA	NA	NA	NA	NA	D	PoD_PrD	D	MODERATE	D	1	98	98	0	59	52	7	11.86
TSNAD	PPP2R2B_E145K	2	HLA-A*11:01	YSTFQSHEPE	18808	15.8889	-	YSTFQSHEPK	34.5	0.2079	SB	PPP2R2B	E145K	noHabit	42501	NA	NA	NA	NA	NA	NA	NA	D	PoD_PrD	D	MODERATE	D	1	98	98	0	59	52	7	11.86
TSNAD	PPP2R2B_E145K	3	HLA-A*11:01	STFQSHEPE	5919.5	6.4418	-	STFQSHEPK	6.6	0.0115	SB	PPP2R2B	E145K	noHabit	42501	NA	NA	NA	NA	NA	NA	NA	D	PoD_PrD	D	MODERATE	D	1	98	98	0	59	52	7	11.86
TSNAD	PPP2R2B_E145K	3	HLA-A*68:01	STFQSHEPE	1082.8	3.3534	-	STFQSHEPK	9.1	0.0811	SB	PPP2R2B	E145K	noHabit	42501	NA	NA	NA	NA	NA	NA	NA	D	PoD_PrD	D	MODERATE	D	1	98	98	0	59	52	7	11.86
TSNAD	PPP2R2B_E145K	3	HLA-A*34:02	STFQSHEPE	5114.1	5.9264	-	STFQSHEPK	32.8	0.0261	SB	PPP2R2B	E145K	noHabit	42501	NA	NA	NA	NA	NA	NA	NA	D	PoD_PrD	D	MODERATE	D	1	98	98	0	59	52	7	11.86
TSNAD	PPP2R2B_E145K	3	HLA-A*30:01	STFQSHEPE	2997.1	8.0493	-	STFQSHEPK	31.8	0.161	SB	PPP2R2B	E145K	noHabit	42501	NA	NA	NA	NA	NA	NA	NA	D	PoD_PrD	D	MODERATE	D	1	98	98	0	59	52	7	11.86
HLA	PRDM13_G428W	2	HLA-A*24:02	RYAQLPPAPG	15274	8.1549	-	RYAQLPPAPW	97.8	0.2083	SB	PRDM13	G428W	noHabit	42494	NA	NA	chr6*100061793	NA	NA	NA	NA	D_lc	PrD_PrD	D	MODERATE	N	0.211	35	35	0	52	40	12	23.08
TSNAD	PRDM13_G428W	3	HLA-B*15:17	YAQLPPAPG	10380.9	15.4404	-	YAQLPPAPW	19.4	0.3193	SB	PRDM13	G428W	noHabit	42494	NA	NA	chr6*100061793	NA	NA	NA	NA	D_lc	PrD_PrD	D	MODERATE	N	0.211	35	35	0	52	40	12	23.08
TSNAD	PRDM13_G428W	3	HLA-B*58:01	YAQLPPAPG	10534.2	13.1195	-	YAQLPPAPW	19.2	0.1239	SB	PRDM13	G428W	noHabit	42494	NA	NA	chr6*100061793	NA	NA	NA	NA	D_lc	PrD_PrD	D	MODERATE	N	0.211	35	35	0	52	40	12	23.08
TSNAD	PRDM13_G428W	2	HLA-B*58:01	RYAQLPPAPG	11290.7	14.0119	-	RYAQLPPAPW	42.1	0.2534	SB	PRDM13	G428W	noHabit	42494	NA	NA	chr6*100061793	NA	NA	NA	NA	D_lc	PrD_PrD	D	MODERATE	N	0.211	35	35	0	52	40	12	23.08
TSNAD	PRDM13_G428W	3	HLA-C*03:02	YAQLPPAPG	1027.8	2.4076	-	YAQLPPAPW	43.7	0.2138	SB	PRDM13	G428W	noHabit	42494	NA	NA	chr6*100061793	NA	NA	NA	NA	D_lc	PrD_PrD	D	MODERATE	N	0.211	35	35	0	52	40	12	23.08
TSNAD	PRDM13_G428W	3	HLA-B*57:03	YAQLPPAPG	17348.3	14.8833	-	YAQLPPAPW	39.1	0.0797	SB	PRDM13	G428W	noHabit	42494	NA	NA	chr6*100061793	NA	NA	NA	NA	D_lc	PrD_PrD	D	MODERATE	N	0.211	35	35	0	52	40	12	23.08
TSNAD	PRDM13_G428W	3	HLA-B*53:01	YAQLPPAPG	10805.2	5.6557	-	YAQLPPAPW	27.8	0.0335	SB	PRDM13	G428W	noHabit	42494	NA	NA	chr6*100061793	NA	NA	NA	NA	D_lc	PrD_PrD	D	MODERATE	N	0.211	35	35	0	52	40	12	23.08
TSNAD	PRDM13_G428W	2	HLA-B*57:03	RYAQLPPAPG	19505.6	17.281	-	RYAQLPPAPW	90.4	0.1933	SB	PRDM13	G428W	noHabit	42494	NA	NA	chr6*100061793	NA	NA	NA	NA	D_lc	PrD_PrD	D	MODERATE	N	0.211	35	35	0	52	40	12	23.08
TSNAD	PRDM13_G428W	2	HLA-A*24:02	RYAQLPPAPG	15274	8.1549	-	RYAQLPPAPW	97.8	0.2083	SB	PRDM13	G428W	noHabit	42494	NA	NA	chr6*100061793	NA	NA	NA	NA	D_lc	PrD_PrD	D	MODERATE	N	0.211	35	35	0	52	40	12	23.08
TSNAD	PRDM13_G428W	2	HLA-A*23:01	RYAQLPPAPG	15063.6	10.8618	-	RYAQLPPAPW	90.4	0.3108	SB	PRDM13	G428W	noHabit	42494	NA	NA	chr6*100061793	NA	NA	NA	NA	D_lc	PrD_PrD	D	MODERATE	N	0.211	35	35	0	52	40	12	23.08
TSNAD	PRDM13_G428W	2	HLA-B*57:01	RYAQLPPAPG	11440.3	10.4532	-	RYAQLPPAPW	78.1	0.1956	SB	PRDM13	G428W	noHabit	42494	NA	NA	chr6*100061793	NA	NA	NA	NA	D_lc	PrD_PrD	D	MODERATE	N	0.211	35	35	0	52	40	12	23.08
HLA	PRSS8_E90K	3	HLA-A*11:01	SAAHCFPSE	9977.3	8.9899	-	SAAHCFPSK	16.7	0.0753	SB	PRSS8	E90K	noHabit	42498	NA	NA	NA	0.00000859	0	NA	NA	T	PoD_PoD	N	MODERATE	N	0.197	47	47	0	29	22	7	24.14
HLA	PRSS8_E90K	2	HLA-A*11:01	LSAAHCFPSE	12082.3	10.4317	-	LSAAHCFPSK	31.1	0.1861	SB	PRSS8	E90K	noHabit	42498	NA	NA	NA	0.00000859	0	NA	NA	T	PoD_PoD	N	MODERATE	N	0.197	47	47	0	29	22	7	24.14
TSNAD	PRSS8_E90K	3	HLA-A*34:02	SAAHCFPSE	6829.1	7.4194	-	SAAHCFPSK	71.1	0.1077	SB	PRSS8	E90K	noHabit	42498	NA	NA	NA	0.00000859	0	NA	NA	T	PoD_PoD	N	MODERATE	N	0.197	47	47	0	29	22	7	24.14
TSNAD	PRSS8_E90K	3	HLA-A*11:01	SAAHCFPSE	9977.3	8.9899	-	SAAHCFPSK	16.7	0.0753	SB	PRSS8	E90K	noHabit	42498	NA	NA	NA	0.00000859	0	NA	NA	T	PoD_PoD	N	MODERATE	N	0.197	47	47	0	29	22	7	24.14
TSNAD	PRSS8_E90K	2	HLA-A*11:01	LSAAHCFPSE	12082.3	10.4317	-	LSAAHCFPSK	31.1	0.1861	SB	PRSS8	E90K	noHabit	42498	NA	NA	NA	0.00000859	0	NA	NA	T	PoD_PoD	N	MODERATE	N	0.197	47	47	0	29	22	7	24.14
TSNAD	PRSS8_E90K	3	HLA-A*30:01	SAAHCFPSE	3953.5	10.0981	-	SAAHCFPSK	54.1	0.2807	SB	PRSS8	E90K	noHabit	42498	NA	NA	NA	0.00000859	0	NA	NA	T	PoD_PoD	N	MODERATE	N	0.197	47	47	0	29	22	7	24.14
TSNAD	PRSS8_E90K	2	HLA-A*30:01	LSAAHCFPSE	6682.1	15.7175	-	LSAAHCFPSK	95.9	0.4803	SB	PRSS8	E90K	noHabit	42498	NA	NA	NA	0.00000859	0	NA	NA	T	PoD_PoD	N	MODERATE	N	0.197	47	47	0	29	22	7	24.14
HLA	PSMD7_E60K	3	HLA-A*11:01	NSFAVPFDE	14253.5	11.9943	-	NSFAVPFDK	20	0.1019	SB	PSMD7	E60K	noHabit	42498	NA	NA	NA	NA	NA	NA	NA	D	PrD_PrD	D	MODERATE	D	1	54	54	0	50	39	11	22
HLA	PSMD7_E60K	1	HLA-A*11:01	VSNSFAVPFDE	22322.9	19.7855	-	VSNSFAVPFDK	58.9	0.3611	SB	PSMD7	E60K	noHabit	42498	NA	NA	NA	NA	NA	NA	NA	D	PrD_PrD	D	MODERATE	D	1	54	54	0	50	39	11	22
HLA	PSMD7_E60K	2	HLA-A*11:01	SNSFAVPFDE	12475.4	10.7183	-	SNSFAVPFDK	16.2	0.0694	SB	PSMD7	E60K	noHabit	42498	NA	NA	NA	NA	NA	NA	NA	D	PrD_PrD	D	MODERATE	D	1	54	54	0	50	39	11	22
TSNAD	PSMD7_E60K	3	HLA-A*34:02	NSFAVPFDE	10966.7	11.0355	-	NSFAVPFDK	82.9	0.1358	SB	PSMD7	E60K	noHabit	42498	NA	NA	NA	NA	NA	NA	NA	D	PrD_PrD	D	MODERATE	D	1	54	54	0	50	39	11	22
TSNAD	PSMD7_E60K	1	HLA-A*11:01	VSNSFAVPFDE	22322.9	19.7855	-	VSNSFAVPFDK	58.9	0.3611	SB	PSMD7	E60K	noHabit	42498	NA	NA	NA	NA	NA	NA	NA	D	PrD_PrD	D	MODERATE	D	1	54	54	0	50	39	11	22
TSNAD	PSMD7_E60K	2	HLA-A*11:01	SNSFAVPFDE	12475.4	10.7183	-	SNSFAVPFDK	16.2	0.0694	SB	PSMD7	E60K	noHabit	42498	NA	NA	NA	NA	NA	NA	NA	D	PrD_PrD	D	MODERATE	D	1	54	54	0	50	39	11	22
TSNAD	PSMD7_E60K	2	HLA-A*03:02	SNSFAVPFDE	17856.2	14.5674	-	SNSFAVPFDK	75.2	0.2203	SB	PSMD7	E60K	noHabit	42498	NA	NA	NA	NA	NA	NA	NA	D	PrD_PrD	D	MODERATE	D	1	54	54	0	50	39	11	22
TSNAD	PSMD7_E60K	3	HLA-A*68:01	NSFAVPFDE	1673.3	4.1765	-	NSFAVPFDK	9	0.08	SB	PSMD7	E60K	noHabit	42498	NA	NA	NA	NA	NA	NA	NA	D	PrD_PrD	D	MODERATE	D	1	54	54	0	50	39	11	22
TSNAD	PSMD7_E60K	3	HLA-A*11:01	NSFAVPFDE	14253.5	11.9943	-	NSFAVPFDK	20	0.1019	SB	PSMD7	E60K	noHabit	42498	NA	NA	NA	NA	NA	NA	NA	D	PrD_PrD	D	MODERATE	D	1	54	54	0	50	39	11	22
TSNAD	PSMD7_E60K	3	HLA-A*03:02	NSFAVPFDE	16370.1	13.1503	-	NSFAVPFDK	79.1	0.2314	SB	PSMD7	E60K	noHabit	42498	NA	NA	NA	NA	NA	NA	NA	D	PrD_PrD	D	MODERATE	D	1	54	54	0	50	39	11	22
HLA	RTL1_P558T	3	HLA-A*02:01	SLLPGLPHP	1131.4	4.3164	-	SLLPGLPHT	36.3	0.4773	SB	RTL1	P558T	noHabit	56957	NA	NA	0.007788	0.016757392	0.006889099	0.00778754	0.0031	T	BN_BN	N	MODERATE	N	0.878	102	44	58	144	137	7	4.86
TSNAD	RTL1_P558T	10	HLA-B*15:01	HPYSDLADVF	3732	6.3727	-	HTYSDLADVF	66.5	0.3572	SB	RTL1	P558T	noHabit	56957	NA	NA	0.007788	0.016757392	0.006889099	0.00778754	0.0031	T	BN_BN	N	MODERATE	N	0.878	102	44	58	144	137	7	4.86
TSNAD	RTL1_P558T	10	HLA-B*15:17	HPYSDLADVF	6914.3	12.0029	-	HTYSDLADVF	10.6	0.1455	SB	RTL1	P558T	noHabit	56957	NA	NA	0.007788	0.016757392	0.006889099	0.00778754	0.0031	T	BN_BN	N	MODERATE	N	0.878	102	44	58	144	137	7	4.86
TSNAD	RTL1_P558T	10	HLA-A*02:05	HPYSDLADV	17288.5	33.3175	-	HTYSDLADV	76.4	0.4933	SB	RTL1	P558T	noHabit	56957	NA	NA	0.007788	0.016757392	0.006889099	0.00778754	0.0031	T	BN_BN	N	MODERATE	N	0.878	102	44	58	144	137	7	4.86
TSNAD	RTL1_P558T	11	HLA-C*14:02	PYSDLADVF	2585.7	4.7388	-	TYSDLADVF	59.4	0.1973	SB	RTL1	P558T	noHabit	56957	NA	NA	0.007788	0.016757392	0.006889099	0.00778754	0.0031	T	BN_BN	N	MODERATE	N	0.878	102	44	58	144	137	7	4.86
TSNAD	RTL1_P558T	10	HLA-B*15:16	HPYSDLADVF	6204.9	10.7072	-	HTYSDLADVF	43.5	0.1157	SB	RTL1	P558T	noHabit	56957	NA	NA	0.007788	0.016757392	0.006889099	0.00778754	0.0031	T	BN_BN	N	MODERATE	N	0.878	102	44	58	144	137	7	4.86
TSNAD	RTL1_P558T	10	HLA-A*68:02	HPYSDLADV	2220.7	4.9039	-	HTYSDLADV	13.4	0.0969	SB	RTL1	P558T	noHabit	56957	NA	NA	0.007788	0.016757392	0.006889099	0.00778754	0.0031	T	BN_BN	N	MODERATE	N	0.878	102	44	58	144	137	7	4.86
TSNAD	RTL1_P558T	3	HLA-A*02:09	SLLPGLPHP	1131.4	4.3164	-	SLLPGLPHT	36.3	0.4773	SB	RTL1	P558T	noHabit	56957	NA	NA	0.007788	0.016757392	0.006889099	0.00778754	0.0031	T	BN_BN	N	MODERATE	N	0.878	102	44	58	144	137	7	4.86
TSNAD	RTL1_P558T	3	HLA-A*02:01	SLLPGLPHP	1131.4	4.3164	-	SLLPGLPHT	36.3	0.4773	SB	RTL1	P558T	noHabit	56957	NA	NA	0.007788	0.016757392	0.006889099	0.00778754	0.0031	T	BN_BN	N	MODERATE	N	0.878	102	44	58	144	137	7	4.86
TSNAD	RTL1_P558T	3	HLA-A*02:06	SLLPGLPHP	595.8	3.7401	-	SLLPGLPHT	23.8	0.3041	SB	RTL1	P558T	noHabit	56957	NA	NA	0.007788	0.016757392	0.006889099	0.00778754	0.0031	T	BN_BN	N	MODERATE	N	0.878	102	44	58	144	137	7	4.86
HLA	SARS2_S83L	10	HLA-A*02:11	RSADLPAII	2608.3	10.8227	-	RLADLPAII	7	0.3995	SB	SARS2	S83L	noHabit	56957	chr19*39421129	chr19*39421129	0.009185	0.009979883	0.027266028	0.0091853	0.0256	T	BN_BN	N	MODERATE	N	0.452	39	20	19	149	7	142	95.3
TSNAD	SARS2_S83L	9	HLA-A*02:02	LRSADLPAII	3036.8	11.6552	-	LRLADLPAII	19.9	0.4814	SB	SARS2	S83L	noHabit	56957	chr19*39421129	chr19*39421129	0.009185	0.009979883	0.027266028	0.0091853	0.0256	T	BN_BN	N	MODERATE	N	0.452	39	20	19	149	7	142	95.3
TSNAD	SARS2_S83L	10	HLA-A*02:02	RSADLPAII	1610.8	8.5181	-	RLADLPAII	10.4	0.1967	SB	SARS2	S83L	noHabit	56957	chr19*39421129	chr19*39421129	0.009185	0.009979883	0.027266028	0.0091853	0.0256	T	BN_BN	N	MODERATE	N	0.452	39	20	19	149	7	142	95.3
TSNAD	SARS2_S83L	10	HLA-A*02:03	RSADLPAII	1926	9.1384	-	RLADLPAII	10.9	0.2349	SB	SARS2	S83L	noHabit	56957	chr19*39421129	chr19*39421129	0.009185	0.009979883	0.027266028	0.0091853	0.0256	T	BN_BN	N	MODERATE	N	0.452	39	20	19	149	7	142	95.3
TSNAD	SARS2_S83L	10	HLA-A*02:11	RSADLPAII	2608.3	10.8227	-	RLADLPAII	7	0.3995	SB	SARS2	S83L	noHabit	56957	chr19*39421129	chr19*39421129	0.009185	0.009979883	0.027266028	0.0091853	0.0256	T	BN_BN	N	MODERATE	N	0.452	39	20	19	149	7	142	95.3
HLA	SCAF1_S856L	10	HLA-A*02:01	ASAGLGSIGV	3265.2	7.4944	-	ALAGLGSIGV	37.3	0.489	SB	SCAF1	S856L	noHabit	42494	NA	NA	NA	NA	NA	NA	NA	NA	UN_UN	NA	MODERATE	N	0.035	102	102	0	79	61	17	21.52
TSNAD	SCAF1_S856L	10	HLA-A*02:02	ASAGLGSIGV	800	6.0006	-	ALAGLGSIGV	13.4	0.3059	SB	SCAF1	S856L	noHabit	42494	NA	NA	NA	NA	NA	NA	NA	NA	UN_UN	NA	MODERATE	N	0.035	102	102	0	79	61	17	21.52
TSNAD	SCAF1_S856L	10	HLA-A*02:01	ASAGLGSIGV	3265.2	7.4944	-	ALAGLGSIGV	37.3	0.489	SB	SCAF1	S856L	noHabit	42494	NA	NA	NA	NA	NA	NA	NA	NA	UN_UN	NA	MODERATE	N	0.035	102	102	0	79	61	17	21.52
TSNAD	SCAF1_S856L	10	HLA-A*02:09	ASAGLGSIGV	3265.2	7.4944	-	ALAGLGSIGV	37.3	0.489	SB	SCAF1	S856L	noHabit	42494	NA	NA	NA	NA	NA	NA	NA	NA	UN_UN	NA	MODERATE	N	0.035	102	102	0	79	61	17	21.52
HLA	SCAMP4_P145L	10	HLA-A*02:11	SPGAAVVML	17145	29.5073	-	SLGAAVVML	7.4	0.4276	SB	SCAMP4	P145L	noHabit	56957	NA	NA	NA	0.000226799	0.000381777	NA	NA	T	BN_BN	N	MODERATE	NA	NA	37	24	13	55	6	49	89.09
TSNAD	SCAMP4_P145L	9	HLA-B*35:01	YSPGAAVVM	598.1	0.9763	WB	YSLGAAVVM	41.5	0.1609	SB	SCAMP4	P145L	noHabit	56957	NA	NA	NA	0.000226799	0.000381777	NA	NA	T	BN_BN	N	MODERATE	NA	NA	37	24	13	55	6	49	89.09
TSNAD	SCAMP4_P145L	10	HLA-A*02:03	SPGAAVVMLL	3760.2	12.68	-	SLGAAVVMLL	18.6	0.4645	SB	SCAMP4	P145L	noHabit	56957	NA	NA	NA	0.000226799	0.000381777	NA	NA	T	BN_BN	N	MODERATE	NA	NA	37	24	13	55	6	49	89.09
TSNAD	SCAMP4_P145L	10	HLA-A*02:11	SPGAAVVML	17145	29.5073	-	SLGAAVVML	7.4	0.4276	SB	SCAMP4	P145L	noHabit	56957	NA	NA	NA	0.000226799	0.000381777	NA	NA	T	BN_BN	N	MODERATE	NA	NA	37	24	13	55	6	49	89.09
TSNAD	SCAMP4_P145L	10	HLA-A*02:02	SPGAAVVMLL	1747.2	8.883	-	SLGAAVVMLL	10.3	0.1948	SB	SCAMP4	P145L	noHabit	56957	NA	NA	NA	0.000226799	0.000381777	NA	NA	T	BN_BN	N	MODERATE	NA	NA	37	24	13	55	6	49	89.09
TSNAD	SCAMP4_P145L	3	HLA-A*32:01	AIGFFQYSP	13001.3	10.0519	-	AIGFFQYSL	83.9	0.154	SB	SCAMP4	P145L	noHabit	56957	NA	NA	NA	0.000226799	0.000381777	NA	NA	T	BN_BN	N	MODERATE	NA	NA	37	24	13	55	6	49	89.09
TSNAD	SCAMP4_P145L	9	HLA-A*02:02	YSPGAAVVML	1764.4	8.927	-	YSLGAAVVML	19.7	0.4776	SB	SCAMP4	P145L	noHabit	56957	NA	NA	NA	0.000226799	0.000381777	NA	NA	T	BN_BN	N	MODERATE	NA	NA	37	24	13	55	6	49	89.09
TSNAD	SCAMP4_P145L	9	HLA-B*15:16	YSPGAAVVM	654.9	2.071	-	YSLGAAVVM	60	0.1904	SB	SCAMP4	P145L	noHabit	56957	NA	NA	NA	0.000226799	0.000381777	NA	NA	T	BN_BN	N	MODERATE	NA	NA	37	24	13	55	6	49	89.09
HLA	SERINC5_Q222R	3	HLA-A*03:02	VLMAVFYTQ	780.3	1.64	WB	VLMAVFYTR	29.4	0.059	SB	SERINC5	Q222R	noHabit	42495	NA	NA	NA	NA	NA	NA	NA	T	BN_BN	N	MODERATE	N	0.637	100	100	0	161	150	11	6.83
TSNAD	SERINC5_Q222R	3	HLA-A*74:01	VLMAVFYTQ	2982.5	1.3828	WB	VLMAVFYTR	37.7	0.0091	SB	SERINC5	Q222R	noHabit	42495	NA	NA	NA	NA	NA	NA	NA	T	BN_BN	N	MODERATE	N	0.637	100	100	0	161	150	11	6.83
TSNAD	SERINC5_Q222R	3	HLA-A*68:01	VLMAVFYTQ	3575.9	6.1569	-	VLMAVFYTR	31	0.3753	SB	SERINC5	Q222R	noHabit	42495	NA	NA	NA	NA	NA	NA	NA	T	BN_BN	N	MODERATE	N	0.637	100	100	0	161	150	11	6.83
TSNAD	SERINC5_Q222R	3	HLA-A*11:01	VLMAVFYTQ	1784	3.3948	-	VLMAVFYTR	67.4	0.4059	SB	SERINC5	Q222R	noHabit	42495	NA	NA	NA	NA	NA	NA	NA	T	BN_BN	N	MODERATE	N	0.637	100	100	0	161	150	11	6.83
TSNAD	SERINC5_Q222R	3	HLA-A*31:01	VLMAVFYTQ	2442.7	4.9154	-	VLMAVFYTR	8.6	0.0367	SB	SERINC5	Q222R	noHabit	42495	NA	NA	NA	NA	NA	NA	NA	T	BN_BN	N	MODERATE	N	0.637	100	100	0	161	150	11	6.83
TSNAD	SERINC5_Q222R	3	HLA-A*33:01	VLMAVFYTQ	9225.1	5.8892	-	VLMAVFYTR	25.8	0.0655	SB	SERINC5	Q222R	noHabit	42495	NA	NA	NA	NA	NA	NA	NA	T	BN_BN	N	MODERATE	N	0.637	100	100	0	161	150	11	6.83
TSNAD	SERINC5_Q222R	3	HLA-A*03:02	VLMAVFYTQ	780.3	1.64	WB	VLMAVFYTR	29.4	0.059	SB	SERINC5	Q222R	noHabit	42495	NA	NA	NA	NA	NA	NA	NA	T	BN_BN	N	MODERATE	N	0.637	100	100	0	161	150	11	6.83
TSNAD	SERINC5_Q222R	2	HLA-A*31:01	LVLMAVFYTQ	6337.3	8.5655	-	LVLMAVFYTR	18.2	0.126	SB	SERINC5	Q222R	noHabit	42495	NA	NA	NA	NA	NA	NA	NA	T	BN_BN	N	MODERATE	N	0.637	100	100	0	161	150	11	6.83
TSNAD	SERINC5_Q222R	2	HLA-A*33:01	LVLMAVFYTQ	11693.3	7.1031	-	LVLMAVFYTR	34.9	0.0961	SB	SERINC5	Q222R	noHabit	42495	NA	NA	NA	NA	NA	NA	NA	T	BN_BN	N	MODERATE	N	0.637	100	100	0	161	150	11	6.83
TSNAD	SERINC5_Q222R	2	HLA-A*68:01	LVLMAVFYTQ	1227.1	3.568	-	LVLMAVFYTR	14.7	0.1758	SB	SERINC5	Q222R	noHabit	42495	NA	NA	NA	NA	NA	NA	NA	T	BN_BN	N	MODERATE	N	0.637	100	100	0	161	150	11	6.83
TSNAD	SERINC5_Q222R	2	HLA-A*33:03	LVLMAVFYTQ	8071.9	6.6614	-	LVLMAVFYTR	28.1	0.1015	SB	SERINC5	Q222R	noHabit	42495	NA	NA	NA	NA	NA	NA	NA	T	BN_BN	N	MODERATE	N	0.637	100	100	0	161	150	11	6.83
TSNAD	SERINC5_Q222R	3	HLA-A*33:03	VLMAVFYTQ	6966.9	6.0258	-	VLMAVFYTR	22.1	0.0786	SB	SERINC5	Q222R	noHabit	42495	NA	NA	NA	NA	NA	NA	NA	T	BN_BN	N	MODERATE	N	0.637	100	100	0	161	150	11	6.83
HLA	SERPINA4_Q248K	3	HLA-A*11:01	TVRVPMMLQ	4179	5.3002	-	TVRVPMMLK	22.9	0.1246	SB	SERPINA4	Q248K	noHabit	42492	NA	NA	NA	NA	NA	NA	NA	D	PrD_PrD	D	MODERATE	N	0.035	65	65	0	56	51	5	8.93

Mangalaparthi *et al.* , 2020. Mutational landscape of esophageal squamous cell carcinoma in an Indian cohort

Supplementary Table 6. List of neoantigens predicted using TSNAD

Software	Variant accession	Position	HLA subtype	Wild type peptide	Wild binding	Wild rank	wild binding level	Mutant peptide	Mutant binding	Mutant rank	Mutant binding level	Gene symbol	Variant information	Habit	Sample ID	OncoMD	COSMIC	dbSNP	ExAC	ExAC_SAS	1000G	1000G_SAS	SIFT	Polyphen2	CONDEL	VEP	LRT	PhastCon7Way	Total read depth in Normal	Reference read support in Normal	Alternative read support in Normal	Total read depth in Tumor	Reference read support in Tumor	Alternate read support in Tumor	Alternate allele frequency in Tumor
HLA	SERPINA4_Q248K	2	HLA-A*11:01	TTVRVPMMLQ	3481.1	4.8162	-	TTVRVPMMLK	10.9	0.0314	SB	SERPINA4	Q248K	noHabit	42492	NA	NA	NA	NA	NA	NA	NA	D	PrD_PrD	D	MODERATE	N	0.035	65	65	0	56	51	5	8.93
TSNAD	SERPINA4_Q248K	3	HLA-A*11:01	TVRVPMMLQ	4179	5.3002	-	TVRVPMMLK	22.9	0.1246	SB	SERPINA4	Q248K	noHabit	42492	NA	NA	NA	NA	NA	NA	NA	D	PrD_PrD	D	MODERATE	N	0.035	65	65	0	56	51	5	8.93
TSNAD	SERPINA4_Q248K	2	HLA-A*30:01	TTVRVPMMLQ	1622.5	4.997	-	TTVRVPMMLK	37	0.1919	SB	SERPINA4	Q248K	noHabit	42492	NA	NA	NA	NA	NA	NA	NA	D	PrD_PrD	D	MODERATE	N	0.035	65	65	0	56	51	5	8.93
TSNAD	SERPINA4_Q248K	2	HLA-A*68:01	TTVRVPMMLQ	897.5	3.0437	-	TTVRVPMMLK	14.9	0.1785	SB	SERPINA4	Q248K	noHabit	42492	NA	NA	NA	NA	NA	NA	NA	D	PrD_PrD	D	MODERATE	N	0.035	65	65	0	56	51	5	8.93
TSNAD	SERPINA4_Q248K	2	HLA-A*03:02	TTVRVPMMLQ	11041.9	9.0376	-	TTVRVPMMLK	92.6	0.2721	SB	SERPINA4	Q248K	noHabit	42492	NA	NA	NA	NA	NA	NA	NA	D	PrD_PrD	D	MODERATE	N	0.035	65	65	0	56	51	5	8.93
TSNAD	SERPINA4_Q248K	3	HLA-A*03:01	TVRVPMMLQ	4452.4	4.365	-	TVRVPMMLK	23.3	0.0638	SB	SERPINA4	Q248K	noHabit	42492	NA	NA	NA	NA	NA	NA	NA	D	PrD_PrD	D	MODERATE	N	0.035	65	65	0	56	51	5	8.93
TSNAD	SERPINA4_Q248K	2	HLA-A*34:02	TTVRVPMMLQ	1761.6	2.7744	-	TTVRVPMMLK	25.7	0.0149	SB	SERPINA4	Q248K	noHabit	42492	NA	NA	NA	NA	NA	NA	NA	D	PrD_PrD	D	MODERATE	N	0.035	65	65	0	56	51	5	8.93
TSNAD	SERPINA4_Q248K	2	HLA-A*11:01	TTVRVPMMLQ	3481.1	4.8162	-	TTVRVPMMLK	10.9	0.0314	SB	SERPINA4	Q248K	noHabit	42492	NA	NA	NA	NA	NA	NA	NA	D	PrD_PrD	D	MODERATE	N	0.035	65	65	0	56	51	5	8.93
TSNAD	SERPINA4_Q248K	3	HLA-A*34:02	TVRVPMMLQ	1205.8	2.1215	-	TVRVPMMLK	23.7	0.0124	SB	SERPINA4	Q248K	noHabit	42492	NA	NA	NA	NA	NA	NA	NA	D	PrD_PrD	D	MODERATE	N	0.035	65	65	0	56	51	5	8.93
TSNAD	SERPINA4_Q248K	2	HLA-A*03:01	TTVRVPMMLQ	6927	5.826	-	TTVRVPMMLK	29	0.0928	SB	SERPINA4	Q248K	noHabit	42492	NA	NA	NA	NA	NA	NA	NA	D	PrD_PrD	D	MODERATE	N	0.035	65	65	0	56	51	5	8.93
HLA	SKI_S664L	10	HLA-A*02:01	YSAQIEDLQV	4915.7	9.3813	-	YLAQIEDLQV	21.7	0.2968	SB	SKI	S664L	noHabit	56957	NA	NA	NA	NA	NA	NA	NA	D	BN_PrD	D	MODERATE	D	1	21	21	0	37	22	15	40.54
HLA	SKI_S664L	10	HLA-A*02:11	YSAQIEDLQV	2629	10.8567	-	YLAQIEDLQV	7.7	0.4483	SB	SKI	S664L	noHabit	56957	NA	NA	NA	NA	NA	NA	NA	D	BN_PrD	D	MODERATE	D	1	21	21	0	37	22	15	40.54
TSNAD	SKI_S664L	10	HLA-A*02:03	YSAQIEDLQV	2159	9.665	-	YLAQIEDLQV	14.1	0.3382	SB	SKI	S664L	noHabit	56957	NA	NA	NA	NA	NA	NA	NA	D	BN_PrD	D	MODERATE	D	1	21	21	0	37	22	15	40.54
TSNAD	SKI_S664L	10	HLA-A*02:02	YSAQIEDLQV	573.6	5.0518	-	YLAQIEDLQV	6.4	0.0887	SB	SKI	S664L	noHabit	56957	NA	NA	NA	NA	NA	NA	NA	D	BN_PrD	D	MODERATE	D	1	21	21	0	37	22	15	40.54
TSNAD	SKI_S664L	10	HLA-A*02:11	YSAQIEDLQV	2629	10.8567	-	YLAQIEDLQV	7.7	0.4483	SB	SKI	S664L	noHabit	56957	NA	NA	NA	NA	NA	NA	NA	D	BN_PrD	D	MODERATE	D	1	21	21	0	37	22	15	40.54
TSNAD	SKI_S664L	10	HLA-A*02:01	YSAQIEDLQV	4915.7	9.3813	-	YLAQIEDLQV	21.7	0.2968	SB	SKI	S664L	noHabit	56957	NA	NA	NA	NA	NA	NA	NA	D	BN_PrD	D	MODERATE	D	1	21	21	0	37	22	15	40.54
TSNAD	SKI_S664L	10	HLA-A*02:09	YSAQIEDLQV	4915.7	9.3813	-	YLAQIEDLQV	21.7	0.2968	SB	SKI	S664L	noHabit	56957	NA	NA	NA	NA	NA	NA	NA	D	BN_PrD	D	MODERATE	D	1	21	21	0	37	22	15	40.54
HLA	SLC12A9_T261M	9	HLA-A*29:01	YTTGAVMNF	576.5	1.1322	WB	YTMGAVMNF	44.2	0.2149	SB	SLC12A9	T261M	Chewer	42482	chr7*100456481	chr7*100456481	NA	NA	NA	NA	NA	D	PrD_PrD	D	MODERATE	D	0.986	54	54	0	81	56	25	30.86
TSNAD	SLC12A9_T261M	8	HLA-A*24:02	DYTTGAVMNF	661.1	0.8132	WB	DYTMGAVMN	94.3	0.2022	SB	SLC12A9	T261M	Chewer	42482	chr7*100456481	chr7*100456481	NA	NA	NA	NA	NA	D	PrD_PrD	D	MODERATE	D	0.986	54	54	0	81	56	25	30.86
TSNAD	SLC12A9_T261M	9	HLA-A*29:02	YTTGAVMNF	576.5	1.1322	WB	YTMGAVMNF	44.2	0.2149	SB	SLC12A9	T261M	Chewer	42482	chr7*100456481	chr7*100456481	NA	NA	NA	NA	NA	D	PrD_PrD	D	MODERATE	D	0.986	54	54	0	81	56	25	30.86
TSNAD	SLC12A9_T261M	9	HLA-A*29:01	YTTGAVMNF	576.5	1.1322	WB	YTMGAVMNF	44.2	0.2149	SB	SLC12A9	T261M	Chewer	42482	chr7*100456481	chr7*100456481	NA	NA	NA	NA	NA	D	PrD_PrD	D	MODERATE	D	0.986	54	54	0	81	56	25	30.86
TSNAD	SLC12A9_T261M	9	HLA-A*23:01	YTTGAVMNF	750.6	1.3503	WB	YTMGAVMNF	46.4	0.1855	SB	SLC12A9	T261M	Chewer	42482	chr7*100456481	chr7*100456481	NA	NA	NA	NA	NA	D	PrD_PrD	D	MODERATE	D	0.986	54	54	0	81	56	25	30.86
TSNAD	SLC12A9_T261M	10	HLA-A*02:02	TTGAVMNFA	1051.6	6.8739	-	TMGAVMNFA	19.2	0.4642	SB	SLC12A9	T261M	Chewer	42482	chr7*100456481	chr7*100456481	NA	NA	NA	NA	NA	D	PrD_PrD	D	MODERATE	D	0.986	54	54	0	81	56	25	30.86
TSNAD	SLC12A9_T261M	10	HLA-A*02:03	TTGAVMNFA	971.3	6.6234	-	TMGAVMNFA	17.9	0.4455	SB	SLC12A9	T261M	Chewer	42482	chr7*100456481	chr7*100456481	NA	NA	NA	NA	NA	D	PrD_PrD	D	MODERATE	D	0.986	54	54	0	81	56	25	30.86
HLA	SLC22A1_W16L	10	HLA-A*02:11	GWFQKQAFL	3701	12.6184	-	GLFQKQAFL	6.2	0.3462	SB	SLC22A1	W16L	Chewer	42482	NA	NA	NA	NA	NA	NA	NA	T	BN_BN	N	MODERATE	NA	NA	90	90	0	104	89	14	13.46
TSNAD	SLC22A1_W16L	10	HLA-A*02:11	GWFQKQAFL	3701	12.6184	-	GLFQKQAFL	6.2	0.3462	SB	SLC22A1	W16L	Chewer	42482	NA	NA	NA	NA	NA	NA	NA	T	BN_BN	N	MODERATE	NA	NA	90	90	0	104	89	14	13.46
TSNAD	SLC22A1_W16L	10	HLA-A*02:02	GWFQKQAFL	3190.8	11.9457	-	GLFQKQAFL	13.2	0.2982	SB	SLC22A1	W16L	Chewer	42482	NA	NA	NA	NA	NA	NA	NA	T	BN_BN	N	MODERATE	NA	NA	90	90	0	104	89	14	13.46
HLA	SLC46A3_H326Y	9	HLA-A*24:02	DIHMAFIGIF	5170.3	3.1654	-	DIYMAFIGIF	98.9	0.2102	SB	SLC46A3	H326Y	Smoker	42477	NA	NA	chr13*29286900	NA	NA	NA	NA	T	BN	N	MODERATE	N	0.991	161	161	0	166	134	32	19.28
HLA	SLC46A3_H326Y	10	HLA-A*24:02	IHMAFIGIF	542.3	0.6982	WB	IYMAFIGIF	15.5	0.0237	SB	SLC46A3	H326Y	Smoker	42477	NA	NA	chr13*29286900	NA	NA	NA	NA	T	BN	N	MODERATE	N	0.991	161	161	0	166	134	32	19.28
TSNAD	SLC46A3_H326Y	3	HLA-C*03:02	FSYCMEDIH	1040	2.4256	-	FSYCMEDIY	23.5	0.1053	SB	SLC46A3	H326Y	Smoker	42477	NA	NA	chr13*29286900	NA	NA	NA	NA	T	BN	N	MODERATE	N	0.991	161	161	0	166	134	32	19.28
TSNAD	SLC46A3_H326Y	3	HLA-C*12:02	FSYCMEDIH	3937.5	4.1108	-	FSYCMEDIY	98.5	0.1193	SB	SLC46A3	H326Y	Smoker	42477	NA	NA	chr13*29286900	NA	NA	NA	NA	T	BN	N	MODERATE	N	0.991	161	161	0	166	134	32	19.28
TSNAD	SLC46A3_H326Y	2	HLA-A*29:01	LFSYCMEDIH	7204.3	5.2317	-	LFSYCMEDIY	62.7	0.289	SB	SLC46A3	H326Y	Smoker	42477	NA	NA	chr13*29286900	NA	NA	NA	NA	T	BN	N	MODERATE	N	0.991	161	161	0	166	134	32	19.28
TSNAD	SLC46A3_H326Y	3	HLA-A*29:02	FSYCMEDIH	6001.6	4.5238	-	FSYCMEDIY	43.8	0.2129	SB	SLC46A3	H326Y	Smoker	42477	NA	NA	chr13*29286900	NA	NA	NA	NA	T	BN	N	MODERATE	N	0.991	161	161	0	166	134	32	19.28
TSNAD	SLC46A3_H326Y	2	HLA-A*29:02	LFSYCMEDIH	7204.3	5.2317	-	LFSYCMEDIY	62.7	0.289	SB	SLC46A3	H326Y	Smoker	42477	NA	NA	chr13*29286900	NA	NA	NA	NA	T	BN	N	MODERATE	N	0.991	161	161	0	166	134	32	19.28
TSNAD	SLC46A3_H326Y	9	HLA-A*23:01	DIHMAFIGIF	1980.6	2.4399	-	DIYMAFIGIF	35.5	0.1419	SB	SLC46A3	H326Y	Smoker	42477	NA	NA	chr13*29286900	NA	NA	NA	NA	T	BN	N	MODERATE	N	0.991	161	161	0	166	134	32	19.28
TSNAD	SLC46A3_H326Y	3	HLA-B*15:16	FSYCMEDIH	2194.1	4.9711	-	FSYCMEDIY	42.3	0.1109	SB	SLC46A3	H326Y	Smoker	42477	NA	NA	chr13*29286900	NA	NA	NA	NA	T	BN	N	MODERATE	N	0.991	161	161	0	166	134	32	19.28
TSNAD	SLC46A3_H326Y	3	HLA-A*29:01	FSYCMEDIH	6001.6	4.5238	-	FSYCMEDIY	43.8	0.2129	SB	SLC46A3	H326Y	Smoker	42477	NA	NA	chr13*29286900	NA	NA	NA	NA	T	BN	N	MODERATE	N	0.991	161	161	0	166	134	32	19.28
TSNAD	SLC46A3_H326Y	9	HLA-A*24:02	DIHMAFIGIF	5170.3	3.1654	-	DIYMAFIGIF	98.9	0.2102	SB	SLC46A3	H326Y	Smoker	42477	NA	NA	chr13*29286900	NA	NA	NA	NA	T	BN	N	MODERATE	N	0.991	161	161	0	166	134	32	19.28
TSNAD	SLC46A3_H326Y	10	HLA-A*24:02	IHMAFIGIF	542.3	0.6982	WB	IYMAFIGIF	15.5	0.0237	SB	SLC46A3	H326Y	Smoker	42477	NA	NA	chr13*29286900	NA	NA	NA	NA	T	BN	N	MODERATE	N	0.991	161	161	0	166	134	32	19.28
TSNAD	SLC46A3_H326Y	10	HLA-A*23:01	IHMAFIGIFT	4847.9	4.3282	-	IYMAFIGIFT	75.2	0.2715	SB	SLC46A3	H326Y	Smoker	42477	NA	NA	chr13*29286900	NA	NA	NA	NA	T	BN	N	MODERATE	N	0.991	161	161	0	166	134	32	19.28
TSNAD	SLC46A3_H326Y	3	HLA-C*12:03	FSYCMEDIH	2834.4	5.4495	-	FSYCMEDIY	89.2	0.4042	SB	SLC46A3	H326Y	Smoker	42477	NA	NA	chr13*29286900	NA	NA	NA	NA	T	BN	N	MODERATE	N	0.991	161	161	0	166	134	32	19.28
TSNAD	SLC46A3_H326Y	3	HLA-B*35:01	FSYCMEDIH	2502.9	2.4251	-	FSYCMEDIY	54.6	0.209	SB	SLC46A3	H326Y	Smoker	42477	NA	NA	chr13*29286900	NA	NA	NA	NA	T	BN	N	MODERATE	N	0.991	161	161	0	166	134	32	19.28
TSNAD	SLC46A3_H326Y	3	HLA-B*15:17	FSYCMEDIH	3245	7.9389	-	FSYCMEDIY	19	0.3121	SB	SLC46A3	H326Y	Smoker	42477	NA	NA	chr13*29286900	NA	NA	NA	NA	T	BN	N	MODERATE	N	0.991	161	161	0	166	134	32	19.28
HLA	SOX3_P378R	3	HLA-A*33:03	MGSVVKSEP	37691.8	55.1969	-	MGSVVKSER	86.4	0.3355	SB	SOX3	P378R	Chewer	42484	NA	NA	NA	NA	NA	NA	NA	D	BN_BN	N	MODERATE	U	0.998	43	43	0	45	38	7	15.56
TSNAD	SOX3_P378R	3	HLA-A*31:01	MGSVVKSEP	33192.8	54.4977	-	MGSVVKSER	46.5	0.3844	SB	SOX3	P378R	Chewer	42484	NA	NA	NA	NA	NA	NA	NA	D	BN_BN	N	MODERATE	U	0.998	43	43	0	45	38	7	15.56
TSNAD	SOX3_P378R	3	HLA-A*33:03	MGSVVKSEP	37691.8	55.1969	-	MGSVVKSER	86.4	0.3355	SB	SOX3	P378R	Chewer	42484	NA	NA	NA	NA	NA	NA	NA	D	BN_BN	N	MODERATE	U	0.998	43	43	0	45	38	7	15.56
TSNAD	SOX3_P378R	9	HLA-B*45:01	SEPSSPPPA	657	0.5391	WB	SERSSPPPA	69.3	0.0765	SB	SOX3	P378R	Chewer	42484	NA	NA	NA	NA	NA	NA	NA	D	BN_BN	N	MODERATE	U	0.998	43	43	0	45	38	7	15.56
TSNAD	SOX3_P378R	8	HLA-A*30:01	KSEPSSPPPA	908.1	3.2418	-	KSERSSPPPA	11.5	0.0325	SB	SOX3	P378R	Chewer	42484	NA	NA	NA	NA	NA	NA	NA	D	BN_BN	N	MODERATE	U	0.998	43	43	0	45	38	7	15.56
HLA	SPPL3_D92Y	10	HLA-A*24:02	FDSVQVVFTI	17061.9	9.252	-	FYSVQVVFTI	72.3	0.1585	SB	SPPL3	D92Y	noHabit	42494	NA	NA	NA	NA	NA	NA	NA	D	PrD_PrD	D	MODERATE	D	1	84	83	0	86	69	15	17.44
TSNAD	SPPL3_D92Y	11	HLA-C*03:04	DSVQVVFTI	6595.1	5.0956	-	YSVQVVFTI	79.2	0.3097	SB	SPPL3	D92Y	noHabit	42494	NA	NA	NA	NA	NA	NA	NA	D	PrD_PrD	D	MODERATE	D	1	84	83	0	86	69	15	17.44
TSNAD	SPPL3_D92Y	11	HLA-B*15:16	DSVQVVFTI	1440.9	3.6629	-	YSVQVVFTI	41	0.1051	SB	SPPL3	D92Y	noHabit	42494	NA	NA	NA	NA	NA	NA	NA	D	PrD_PrD	D	MODERATE	D	1	84	83	0	86	69	15	17.44
TSNAD	SPPL3_D92Y	11	HLA-C*03:02	DSVQVVFTI	5048.7	7.2181	-	YSVQVVFTI	54.1	0.2618	SB	SPPL3	D92Y	noHabit	42494	NA	NA	NA	NA	NA	NA	NA	D	PrD_PrD	D	MODERATE	D	1	84	83	0	86	69	15	17.44
TSNAD	SPPL3_D92Y	11	HLA-C*12:03	DSVQVVFTI	2042.6	4.3063	-	YSVQVVFTI	44.3	0.1911	SB	SPPL3	D92Y	noHabit	42494	NA	NA	NA	NA	NA	NA	NA	D	PrD_PrD	D	MODERATE	D	1	84	83	0	86	69	15	17.44
TSNAD	SPPL3_D92Y	2	HLA-A*29:01	SLLVMFFFFD	10624.1	7.3512	-	SLLVMFFFFY	97.1	0.3999	SB	SPPL3	D92Y	noHabit	42494	NA	NA	NA	NA	NA	NA	NA	D	PrD_PrD	D	MODERATE	D	1	84	83	0	86	69	15	17.44
TSNAD	SPPL3_D92Y	11	HLA-C*03:03	DSVQVVFTI	6595.1	5.0956	-	YSVQVVFTI	79.2	0.3097	SB	SPPL3	D92Y	noHabit	42494	NA	NA	NA	NA	NA	NA	NA	D	PrD_PrD	D	MODERATE	D	1	84	83	0	86	69	15	17.44
TSNAD	SPPL3_D92Y	11	HLA-C*16:01	DSVQVVFTI	3784.7	6.0046	-	YSVQVVFTI	92.3	0.3227	SB	SPPL3	D92Y	noHabit	42494	NA	NA	NA	NA	NA	NA	NA	D	PrD_PrD	D	MODERATE	D	1	84	83	0	86	69	15	17.44
TSNAD	SPPL3_D92Y	3	HLA-A*29:01	LLVMFFFFD	30086.9	31.8173	-	LLVMFFFFY	62.1	0.2869	SB	SPPL3	D92Y	noHabit	42494	NA	NA	NA	NA	NA	NA	NA	D	PrD_PrD	D	MODERATE	D	1	84	83	0	86	69	15	17.44
TSNAD	SPPL3_D92Y	3	HLA-A*29:02	LLVMFFFFD	30086.9	31.8173	-	LLVMFFFFY	62.1	0.2869	SB	SPPL3	D92Y	noHabit	42494	NA	NA	NA	NA	NA	NA	NA	D	PrD_PrD	D	MODERATE	D	1	84	83	0	86	69	15	17.44
TSNAD	SPPL3_D92Y	11	HLA-B*58:01	DSVQVVFTI	2004.9	3.527	-	YSVQVVFTI	49.5	0.2838	SB	SPPL3	D92Y	noHabit	42494	NA	NA	NA	NA	NA	NA	NA	D	PrD_PrD	D	MODERATE	D	1	84	83	0	86	69	15	17.44
TSNAD	SPPL3_D92Y	10	HLA-A*24:02	FDSVQVVFTI	17061.9	9.252	-	FYSVQVVFTI	72.3	0.1585	SB	SPPL3	D92Y	noHabit	42494	NA	NA	NA	NA	NA	NA	NA	D	PrD_PrD	D	MODERATE	D	1	84	83	0	86	69	15	17.44
TSNAD	SPPL3_D92Y	10	HLA-A*23:01	FDSVQVVFTI	10494	7.7327	-	FYSVQVVFTI	39	0.1576	SB	SPPL3	D92Y	noHabit	42494	NA	NA	NA	NA	NA	NA	NA	D	PrD_PrD	D	MODERATE	D	1	84	83	0	86	69	15	17.44
TSNAD	SPPL3_D92Y	11	HLA-A*02:05	DSVQVVFTI	4900.5	12.5386	-	YSVQVVFTI	75.3	0.4862	SB	SPPL3	D92Y	noHabit	42494	NA	NA	NA	NA	NA	NA	NA	D	PrD_PrD	D	MODERATE	D	1	84	83	0	86	69	15	17.44
TSNAD	SPPL3_D92Y	11	HLA-A*02:06	DSVQVVFTI	4442.1	12.3266	-	YSVQVVFTI	30	0.3824	SB	SPPL3	D92Y	noHabit	42494	NA	NA	NA	NA	NA	NA	NA	D	PrD_PrD	D	MODERATE	D	1	84	83	0	86	69	15	17.44
TSNAD	SPPL3_D92Y	2	HLA-A*29:02	SLLVMFFFFD	10624.1	7.3512	-	SLLVMFFFFY	97.1	0.3999	SB	SPPL3	D92Y	noHabit	42494	NA	NA	NA	NA	NA	NA	NA	D	PrD_PrD	D	MODERATE	D	1	84	83	0	86	69	15	17.44
HLA	SYNE1_R2741K	3	HLA-A*11:01	SQWNDYVER	1091.1	2.6418	-	SQWNDYVEK	73.5	0.4396	SB	SYNE1	R2741K	Chewer	42489	NA	NA	0.0001997	0.0000659	0	0.000199681	0	NA	BN_BN	NA	MODERATE	N	0.92	193	193	0	238	186	51	21.43
TSNAD	SYNE1_R2741K	3	HLA-A*11:01	SQWNDYVER	1091.1	2.6418	-	SQWNDYVEK	73.5	0.4396	SB	SYNE1	R2741K	Chewer	42489	NA	NA	0.0001997	0.0000659	0	0.000199681	0	NA	BN_BN	NA	MODERATE	N	0.92	193	193	0	238	186	51	21.43
HLA	SYNE1_R689Q	10	HLA-A*02:01	GRWRELFMEV	5891.5	10.4068	-	GQWRELFMEV	16.8	0.2134	SB	SYNE1	R689Q	noHabit	42493	NA	NA	NA	0.00000824	0	NA	NA	NA	PrD_PrD	NA	MODERATE	D	0.899	161	161	0	141	118	22	15.6
TSNAD	SYNE1_R689Q	10	HLA-A*02:06	GRWRELFMEV	2610.1	8.9276	-	GQWRELFMEV	7.3	0.0636	SB	SYNE1	R689Q	noHabit	42493	NA	NA	NA	0.00000824	0	NA	NA	NA	PrD_PrD	NA	MODERATE	D	0.899	161	161	0	141	118	22	15.6
TSNAD	SYNE1_R689Q	10	HLA-A*02:09	GRWRELFMEV	5891.5	10.4068	-	GQWRELFMEV	16.8	0.2134	SB	SYNE1	R689Q	noHabit	42493	NA	NA	NA	0.00000824	0	NA	NA	NA	PrD_PrD	NA	MODERATE	D	0.899	161	161	0	141	118	22	15.6
TSNAD	SYNE1_R689Q	10	HLA-A*02:05	GRWRELFMEV	4921.2	12.5768	-	GQWRELFMEV	67	0.4286	SB	SYNE1	R689Q	noHabit	42493	NA	NA	NA	0.00000824	0	NA	NA	NA	PrD_PrD	NA	MODERATE	D	0.899	161	161	0	141	118	22	15.6
TSNAD	SYNE1_R689Q	10	HLA-A*02:01	GRWRELFMEV	5891.5	10.4068	-	GQWRELFMEV	16.8	0.2134	SB	SYNE1	R689Q	noHabit	42493	NA	NA	NA	0.00000824	0	NA	NA	NA	PrD_PrD	NA	MODERATE	D	0.899	161	161	0	141	118	22	15.6
HLA	TAF1A_R435Q	10	HLA-A*03:01	KRMKRSVKK	5620.1	5.0465	-	KQMKRSVKK	39.4	0.1423	SB	TAF1A	R435Q	noHabit	42501	chr1*222732051	chr1*222732051	0.003994	0.006256543	0.005673409	0.00399361	0.0031	T	BN_BN	N	MODERATE	N	0.83	47	47	0	29	24	5	17.24
TSNAD	TAF1A_R435Q	10	HLA-B*15:01	KRMKRSVKKY	685.5	2.0917	-	KQMKRSVKKY	43.3	0.2135	SB	TAF1A	R435Q	noHabit	42501	chr1*222732051	chr1*222732051	0.003994	0.006256543	0.005673409	0.00399361	0.0031	T	BN_BN	N	MODERATE	N	0.83	47	47	0	29	24	5	17.24
TSNAD	TAF1A_R435Q	10	HLA-A*03:01	KRMKRSVKK	5620.1	5.0465	-	KQMKRSVKK	39.4	0.1423	SB	TAF1A	R435Q	noHabit	42501	chr1*222732051	chr1*222732051	0.003994	0.006256543	0.005673409	0.00399361	0.0031	T	BN_BN	N	MODERATE	N	0.83	47	47	0	29	24	5	17.24
TSNAD	TAF1A_R435Q	10	HLA-A*11:01	KRMKRSVKK	8111	7.8079	-	KQMKRSVKK	52.5	0.3237	SB	TAF1A	R435Q	noHabit	42501	chr1*222732051	chr1*222732051	0.003994	0.006256543	0.005673409	0.00399361	0.0031	T	BN_BN	N	MODERATE	N	0.83	47	47	0	29	24	5	17.24
HLA	TMEM215_K79I	10	HLA-A*11:01	RKLPCFRKPK	664.3	2.0397	-	RILPCFRKPK	71.4	0.4279	SB	TMEM215	K79I	noHabit	42492	NA	NA	NA	NA	NA	NA	NA	T_lc	PrD_PrD	D	MODERATE	D	0.977	83	82	0	89	59	30	33.71
TSNAD	TMEM215_K79I	10	HLA-A*11:01	RKLPCFRKPK	664.3	2.0397	-	RILPCFRKPK	71.4	0.4279	SB	TMEM215	K79I	noHabit	42492	NA	NA	NA	NA	NA	NA	NA	T_lc	PrD_PrD	D	MODERATE	D	0.977	83	82	0	89	59	30	33.71
HLA	TP53_C141Y	2	HLA-A*30:02	KMFCQLAKTC	16700.5	19.8992	-	KMFCQLAKTY	47.8	0.0931	SB	TP53	C141Y	noHabit	42493	chr17*7578495*	chr17*7578491*	NA	NA	NA	NA	NA	D	PrD_PrD	D	MODERATE	D	0.991	49	49	0	62	40	22	35.48
TSNAD	TP53_C141Y	9	HLA-C*15:05	KTCPVQLWV	927.1	0.6074	WB	KTYPVQLWV	46.4	0.0117	SB	TP53	C141Y	noHabit	42493	chr17*7578495*	chr17*7578491*	NA	NA	NA	NA	NA	D	PrD_PrD	D	MODERATE	D	0.991	49	49	0	62	40	22	35.48
TSNAD	TP53_C141Y	2	HLA-B*15:03	KMFCQLAKTC	4241.9	13.0725	-	KMFCQLAKTY	10.6	0.1679	SB	TP53	C141Y	noHabit	42493	chr17*7578495*	chr17*7578491*	NA	NA	NA	NA	NA	D	PrD_PrD	D	MODERATE	D	0.991	49	49	0	62	40	22	35.48
TSNAD	TP53_C141Y	9	HLA-C*12:03	KTCPVQLWV	3143.2	5.8594	-	KTYPVQLWV	95.2	0.4298	SB	TP53	C141Y	noHabit	42493	chr17*7578495*	chr17*7578491*	NA	NA	NA	NA	NA	D	PrD_PrD	D	MODERATE	D	0.991	49	49	0	62	40	22	35.48
TSNAD	TP53_C141Y	2	HLA-B*15:01	KMFCQLAKTC	6070.6	9.2387	-	KMFCQLAKTY	20	0.0782	SB	TP53	C141Y	noHabit	42493	chr17*7578495*	chr17*7578491*	NA	NA	NA	NA	NA	D	PrD_PrD	D	MODERATE	D	0.991	49	49	0	62	40	22	35.48
TSNAD	TP53_C141Y	9	HLA-C*16:02	KTCPVQLWV	920.4	0.9795	WB	KTYPVQLWV	67.9	0.0348	SB	TP53	C141Y	noHabit	42493	chr17*7578495*	chr17*7578491*	NA	NA	NA	NA	NA	D	PrD_PrD	D	MODERATE	D	0.991	49	49	0	62	40	22	35.48
TSNAD	TP53_C141Y	2	HLA-A*30:02	KMFCQLAKTC	16700.5	19.8992	-	KMFCQLAKTY	47.8	0.0931	SB	TP53	C141Y	noHabit	42493	chr17*7578495*	chr17*7578491*	NA	NA	NA	NA	NA	D	PrD_PrD	D	MODERATE	D	0.991	49	49	0	62	40	22	35.48
HLA	TTC12_E679V	3	HLA-A*02:11	ALGKLCTAE	13292.3	24.7047	-	ALGKLCTAV	8.4	0.4967	SB	TTC12	E679V	noHabit	56957	NA	NA	NA	0.0000419	0.000306485	NA	NA	D	PrD_PrD	D	MODERATE	NA	NA	64	28	36	48	3	45	93.75
TSNAD	TTC12_E679V	3	HLA-A*02:11	ALGKLCTAE	13292.3	24.7047	-	ALGKLCTAV	8.4	0.4967	SB	TTC12	E679V	noHabit	56957	NA	NA	NA	0.0000419	0.000306485	NA	NA	D	PrD_PrD	D	MODERATE	NA	NA	64	28	36	48	3	45	93.75
TSNAD	TTC12_E679V	3	HLA-A*02:03	ALGKLCTAE	16740.3	30.5558	-	ALGKLCTAV	13.5	0.3178	SB	TTC12	E679V	noHabit	56957	NA	NA	NA	0.0000419	0.000306485	NA	NA	D	PrD_PrD	D	MODERATE	NA	NA	64	28	36	48	3	45	93.75
TSNAD	TTC12_E679V	11	HLA-B*07:02	EPRVPAESSHL	2099.4	1.9255	WB	VPRVPAESSHL	90.9	0.2761	SB	TTC12	E679V	noHabit	56957	NA	NA	NA	0.0000419	0.000306485	NA	NA	D	PrD_PrD	D	MODERATE	NA	NA	64	28	36	48	3	45	93.75
TSNAD	TTC12_E679V	11	HLA-B*07:05	EPRVPAESSHL	1691.5	1.9032	WB	VPRVPAESSHL	88.1	0.3162	SB	TTC12	E679V	noHabit	56957	NA	NA	NA	0.0000419	0.000306485	NA	NA	D	PrD_PrD	D	MODERATE	NA	NA	64	28	36	48	3	45	93.75
HLA	TTC18_R1029T	10	HLA-A*32:01	KRSPSCLTW	4002.9	3.7052	-	KTSPSCLTW	49.9	0.0883	SB	TTC18	R1029T	noHabit	42496	chr10*75032735	chr10*75032735	chr10*75032735	NA	NA	NA	NA	D	PoD_PrD	D	MODERATE	N	0.998	48	48	0	51	40	11	21.57
TSNAD	TTC18_R1029T	10	HLA-B*58:02	KRSPSCLTW	9349.9	0.8819	WB	KTSPSCLTW	51	0.0071	SB	TTC18	R1029T	noHabit	42496	chr10*75032735	chr10*75032735	chr10*75032735	NA	NA	NA	NA	D	PoD_PrD	D	MODERATE	N	0.998	48	48	0	51	40	11	21.57
TSNAD	TTC18_R1029T	10	HLA-A*32:01	KRSPSCLTW	4002.9	3.7052	-	KTSPSCLTW	49.9	0.0883	SB	TTC18	R1029T	noHabit	42496	chr10*75032735	chr10*75032735	chr10*75032735	NA	NA	NA	NA	D	PoD_PrD	D	MODERATE	N	0.998	48	48	0	51	40	11	21.57
TSNAD	TTC18_R1029T	10	HLA-B*57:01	KRSPSCLTW	2964.8	2.9126	-	KTSPSCLTW	6.1	0.0082	SB	TTC18	R1029T	noHabit	42496	chr10*75032735	chr10*75032735	chr10*75032735	NA	NA	NA	NA	D	PoD_PrD	D	MODERATE	N	0.998	48	48	0	51	40	11	21.57
TSNAD	TTC18_R1029T	10	HLA-B*15:16	KRSPSCLTW	5578.6	9.8778	-	KTSPSCLTW	17.4	0.0186	SB	TTC18	R1029T	noHabit	42496	chr10*75032735	chr10*75032735	chr10*75032735	NA	NA	NA	NA	D	PoD_PrD	D	MODERATE	N	0.998	48	48	0	51	40	11	21.57
TSNAD	TTC18_R1029T	10	HLA-B*15:17	KRSPSCLTW	3133	7.7855	-	KTSPSCLTW	4.2	0.0221	SB	TTC18	R1029T	noHabit	42496	chr10*75032735	chr10*75032735	chr10*75032735	NA	NA	NA	NA	D	PoD_PrD	D	MODERATE	N	0.998	48	48	0	51	40	11	21.57
TSNAD	TTC18_R1029T	9	HLA-B*15:16	CKRSPSCLTW	5857.6	10.2529	-	CKTSPSCLTW	85.4	0.2836	SB	TTC18	R1029T	noHabit	42496	chr10*75032735	chr10*75032735	chr10*75032735	NA	NA	NA	NA	D	PoD_PrD	D	MODERATE	N	0.998	48	48	0	51	40	11	21.57
TSNAD	TTC18_R1029T	9	HLA-B*57:01	CKRSPSCLTW	4143.6	3.8756	-	CKTSPSCLTW	24.1	0.058	SB	TTC18	R1029T	noHabit	42496	chr10*75032735	chr10*75032735	chr10*75032735	NA	NA	NA	NA	D	PoD_PrD	D	MODERATE	N	0.998	48	48	0	51	40	11	21.57
TSNAD	TTC18_R1029T	10	HLA-B*57:01	KRSPSCLTWL	5657.6	5.1347	-	KTSPSCLTWL	51.9	0.1407	SB	TTC18	R1029T	noHabit	42496	chr10*75032735	chr10*75032735	chr10*75032735	NA	NA	NA	NA	D	PoD_PrD	D	MODERATE	N	0.998	48	48	0	51	40	11	21.57
TSNAD	TTC18_R1029T	10	HLA-B*57:03	KRSPSCLTW	3710.3	3.5529	-	KTSPSCLTW	6.7	0.0076	SB	TTC18	R1029T	noHabit	42496	chr10*75032735	chr10*75032735	chr10*75032735	NA	NA	NA	NA	D	PoD_PrD	D	MODERATE	N	0.998	48	48	0	51	40	11	21.57
TSNAD	TTC18_R1029T	8	HLA-B*58:01	ACKRSPSCLTW	1647.9	3.1169	-	ACKTSPSCLTW	36.9	0.2301	SB	TTC18	R1029T	noHabit	42496	chr10*75032735	chr10*75032735	chr10*75032735	NA	NA	NA	NA	D	PoD_PrD	D	MODERATE	N	0.998	48	48	0	51	40	11	21.57
TSNAD	TTC18_R1029T	10	HLA-B*58:01	KRSPSCLTWL	4133.6	5.9589	-	KTSPSCLTWL	26.3	0.1763	SB	TTC18	R1029T	noHabit	42496	chr10*75032735	chr10*75032735	chr10*75032735	NA	NA	NA	NA	D	PoD_PrD	D	MODERATE	N	0.998	48	48	0	51	40	11	21.57
TSNAD	TTC18_R1029T	9	HLA-B*58:01	CKRSPSCLTW	1431.2	2.8463	-	CKTSPSCLTW	11.3	0.0708	SB	TTC18	R1029T	noHabit	42496	chr10*75032735	chr10*75032735	chr10*75032735	NA	NA	NA	NA	D	PoD_PrD	D	MODERATE	N	0.998	48	48	0	51	40	11	21.57
TSNAD	TTC18_R1029T	10	HLA-B*58:01	KRSPSCLTW	681.8	1.7312	WB	KTSPSCLTW	3.1	0.0077	SB	TTC18	R1029T	noHabit	42496	chr10*75032735	chr10*75032735	chr10*75032735	NA	NA	NA	NA	D	PoD_PrD	D	MODERATE	N	0.998	48	48	0	51	40	11	21.57
TSNAD	TTC18_R1029T	9	HLA-B*57:03	CKRSPSCLTW	8087.1	6.7303	-	CKTSPSCLTW	58.3	0.1246	SB	TTC18	R1029T	noHabit	42496	chr10*75032735	chr10*75032735	chr10*75032735	NA	NA	NA	NA	D	PoD_PrD	D	MODERATE	N	0.998	48	48	0	51	40	11	21.57
HLA	UBE2O_R1270L	9	HLA-A*11:01	SIRGVLTQFR	568.5	1.8964	WB	SILGVLTQFR	80.7	0.4757	SB	UBE2O	R1270L	Chewer	42487	NA	NA	NA	NA	NA	NA	NA	T_lc	BN_PoD	N	MODERATE	N	0.989	163	162	1	117	91	26	22.22
TSNAD	UBE2O_R1270L	9	HLA-A*11:01	SIRGVLTQFR	568.5	1.8964	WB	SILGVLTQFR	80.7	0.4757	SB	UBE2O	R1270L	Chewer	42487	NA	NA	NA	NA	NA	NA	NA	T_lc	BN_PoD	N	MODERATE	N	0.989	163	162	1	117	91	26	22.22
HLA	USP14_F186L	3	HLA-A*02:01	FLHMAFPQF	690.5	3.3564	-	FLHMAFPQL	12.2	0.146	SB	USP14	F186L	noHabit	42493	NA	NA	NA	NA	NA	NA	NA	D	PrD_PrD	D	MODERATE	N	1	118	118	0	242	212	29	11.98
TSNAD	USP14_F186L	3	HLA-A*02:03	FLHMAFPQF	618.9	5.3199	-	FLHMAFPQL	8.4	0.1575	SB	USP14	F186L	noHabit	42493	NA	NA	NA	NA	NA	NA	NA	D	PrD_PrD	D	MODERATE	N	1	118	118	0	242	212	29	11.98
TSNAD	USP14_F186L	3	HLA-A*02:01	FLHMAFPQF	690.5	3.3564	-	FLHMAFPQL	12.2	0.146	SB	USP14	F186L	noHabit	42493	NA	NA	NA	NA	NA	NA	NA	D	PrD_PrD	D	MODERATE	N	1	118	118	0	242	212	29	11.98
TSNAD	USP14_F186L	3	HLA-A*02:09	FLHMAFPQF	690.5	3.3564	-	FLHMAFPQL	12.2	0.146	SB	USP14	F186L	noHabit	42493	NA	NA	NA	NA	NA	NA	NA	D	PrD_PrD	D	MODERATE	N	1	118	118	0	242	212	29	11.98
HLA	VN1R2_R143I	10	HLA-A*32:01	KRIPETMATF	1635.8	1.871	WB	KIIPETMATF	57.3	0.1052	SB	VN1R2	R143I	noHabit	42496	chr19*53762056	chr19*53762056	chr19*53762056	NA	NA	NA	NA	D	BN_BN	N	MODERATE	NA	NA	164	164	0	152	103	49	32.24
TSNAD	VN1R2_R143I	10	HLA-A*32:01	KRIPETMATF	1635.8	1.871	WB	KIIPETMATF	57.3	0.1052	SB	VN1R2	R143I	noHabit	42496	chr19*53762056	chr19*53762056	chr19*53762056	NA	NA	NA	NA	D	BN_BN	N	MODERATE	NA	NA	164	164	0	152	103	49	32.24
TSNAD	VN1R2_R143I	10	HLA-B*15:01	KRIPETMATF	597.5	1.9075	WB	KIIPETMATF	28.6	0.1323	SB	VN1R2	R143I	noHabit	42496	chr19*53762056	chr19*53762056	chr19*53762056	NA	NA	NA	NA	D	BN_BN	N	MODERATE	NA	NA	164	164	0	152	103	49	32.24
HLA	YARS_P349L	10	HLA-A*03:01	KPMAKGPAK	7520.5	6.1845	-	KLMAKGPAK	11.4	0.019	SB	YARS	P349L	noHabit	42501	NA	NA	NA	NA	NA	NA	NA	T	BN_BN	N	MODERATE	N	0.817	76	76	0	44	31	13	29.55
HLA	YARS_P349L	8	HLA-A*03:01	KQKPMAKGPAK	784.3	1.581	WB	KQKLMAKGPA	87.7	0.3275	SB	YARS	P349L	noHabit	42501	NA	NA	NA	NA	NA	NA	NA	T	BN_BN	N	MODERATE	N	0.817	76	76	0	44	31	13	29.55
HLA	YARS_P349L	9	HLA-A*03:01	QKPMAKGPAK	16150.5	12.3307	-	QKLMAKGPAK	56.2	0.2117	SB	YARS	P349L	noHabit	42501	NA	NA	NA	NA	NA	NA	NA	T	BN_BN	N	MODERATE	N	0.817	76	76	0	44	31	13	29.55

Mangalaparthi *et al.* , 2020. Mutational landscape of esophageal squamous cell carcinoma in an Indian cohort

Supplementary Table 6. List of neoantigens predicted using TSNAD

Software	Variant accession	Position	HLA subtype	Wild type peptide	Wild binding	Wild rank	wild binding level	Mutant peptide	Mutant binding	Mutant rank	Mutant binding level	Gene symbol	Variant information	Habit	Sample ID	OncoMD	COSMIC	dbSNP	ExAC	ExAC_SAS	1000G	1000G_SAS	SIFT	Polyphen2	CONDEL	VEP	LRT	PhastCon7Way	Total read depth in Normal	Reference read support in Normal	Alternative read support in Normal	Total read depth in Tumor	Reference read support in Tumor	Alternate read support in Tumor	Alternate allele frequency in Tumor
TSNAD	YARS_P349L	10	HLA-A*03:02	KPMAKGPAK	8986.9	7.6447	-	KLMAKGPAK	30.2	0.061	SB	YARS	P349L	noHabit	42501	NA	NA	NA	NA	NA	NA	NA	T	BN_BN	N	MODERATE	N	0.817	76	76	0	44	31	13	29.55
TSNAD	YARS_P349L	3	HLA-B*07:05	YPDPSKQKP	13467.3	9.1418	-	YPDPSKQKL	76.8	0.2832	SB	YARS	P349L	noHabit	42501	NA	NA	NA	NA	NA	NA	NA	T	BN_BN	N	MODERATE	N	0.817	76	76	0	44	31	13	29.55
TSNAD	YARS_P349L	3	HLA-B*42:01	YPDPSKQKP	12292.4	11.8972	-	YPDPSKQKL	55.7	0.2742	SB	YARS	P349L	noHabit	42501	NA	NA	NA	NA	NA	NA	NA	T	BN_BN	N	MODERATE	N	0.817	76	76	0	44	31	13	29.55
TSNAD	YARS_P349L	10	HLA-A*11:01	KPMAKGPAK	8525	8.063	-	KLMAKGPAK	64.2	0.3893	SB	YARS	P349L	noHabit	42501	NA	NA	NA	NA	NA	NA	NA	T	BN_BN	N	MODERATE	N	0.817	76	76	0	44	31	13	29.55
TSNAD	YARS_P349L	9	HLA-A*03:01	QKPMAKGPAK	16150.5	12.3307	-	QKLMAKGPAK	56.2	0.2117	SB	YARS	P349L	noHabit	42501	NA	NA	NA	NA	NA	NA	NA	T	BN_BN	N	MODERATE	N	0.817	76	76	0	44	31	13	29.55
TSNAD	YARS_P349L	10	HLA-A*03:01	KPMAKGPAK	7520.5	6.1845	-	KLMAKGPAK	11.4	0.019	SB	YARS	P349L	noHabit	42501	NA	NA	NA	NA	NA	NA	NA	T	BN_BN	N	MODERATE	N	0.817	76	76	0	44	31	13	29.55
TSNAD	YARS_P349L	10	HLA-A*30:01	KPMAKGPAK	744	2.7896	-	KLMAKGPAK	25.9	0.114	SB	YARS	P349L	noHabit	42501	NA	NA	NA	NA	NA	NA	NA	T	BN_BN	N	MODERATE	N	0.817	76	76	0	44	31	13	29.55
TSNAD	YARS_P349L	8	HLA-A*03:01	KQKPMAKGPAK	784.3	1.581	WB	KQKLMAKGPA	87.7	0.3275	SB	YARS	P349L	noHabit	42501	NA	NA	NA	NA	NA	NA	NA	T	BN_BN	N	MODERATE	N	0.817	76	76	0	44	31	13	29.55
HLA	ZNF277_G184R	3	HLA-A*31:01	MFCNEEFLG	16446.4	19.3661	-	MFCNEEFLR	44.9	0.3718	SB	ZNF277	G184R	Chewer	42483	NA	NA	NA	0.0000165	0.000121462	NA	NA	D	PrD_PrD	D	MODERATE	D	0.744	50	50	0	45	17	27	60
HLA	ZNF277_G184R	2	HLA-A*31:01	CMFCNEEFLG	14140.5	16.484	-	CMFCNEEFLR	40.9	0.3373	SB	ZNF277	G184R	Chewer	42483	NA	NA	NA	0.0000165	0.000121462	NA	NA	D	PrD_PrD	D	MODERATE	D	0.744	50	50	0	45	17	27	60
TSNAD	ZNF277_G184R	2	HLA-A*33:01	CMFCNEEFLG	23391.1	15.8138	-	CMFCNEEFLR	75.3	0.2103	SB	ZNF277	G184R	Chewer	42483	NA	NA	NA	0.0000165	0.000121462	NA	NA	D	PrD_PrD	D	MODERATE	D	0.744	50	50	0	45	17	27	60
TSNAD	ZNF277_G184R	3	HLA-A*33:03	MFCNEEFLG	19334.2	14.9347	-	MFCNEEFLR	43.9	0.1646	SB	ZNF277	G184R	Chewer	42483	NA	NA	NA	0.0000165	0.000121462	NA	NA	D	PrD_PrD	D	MODERATE	D	0.744	50	50	0	45	17	27	60
TSNAD	ZNF277_G184R	3	HLA-A*33:01	MFCNEEFLG	19149.3	11.8791	-	MFCNEEFLR	44.8	0.1298	SB	ZNF277	G184R	Chewer	42483	NA	NA	NA	0.0000165	0.000121462	NA	NA	D	PrD_PrD	D	MODERATE	D	0.744	50	50	0	45	17	27	60
TSNAD	ZNF277_G184R	8	HLA-B*08:01	EFLGNRSVIL	907.8	1.2377	WB	EFLRNRSVIL	29.8	0.0499	SB	ZNF277	G184R	Chewer	42483	NA	NA	NA	0.0000165	0.000121462	NA	NA	D	PrD_PrD	D	MODERATE	D	0.744	50	50	0	45	17	27	60
TSNAD	ZNF277_G184R	2	HLA-A*31:01	CMFCNEEFLG	14140.5	16.484	-	CMFCNEEFLR	40.9	0.3373	SB	ZNF277	G184R	Chewer	42483	NA	NA	NA	0.0000165	0.000121462	NA	NA	D	PrD_PrD	D	MODERATE	D	0.744	50	50	0	45	17	27	60
TSNAD	ZNF277_G184R	2	HLA-A*33:03	CMFCNEEFLG	22380.7	18.3506	-	CMFCNEEFLR	63.4	0.2456	SB	ZNF277	G184R	Chewer	42483	NA	NA	NA	0.0000165	0.000121462	NA	NA	D	PrD_PrD	D	MODERATE	D	0.744	50	50	0	45	17	27	60
TSNAD	ZNF277_G184R	3	HLA-A*31:01	MFCNEEFLG	16446.4	19.3661	-	MFCNEEFLR	44.9	0.3718	SB	ZNF277	G184R	Chewer	42483	NA	NA	NA	0.0000165	0.000121462	NA	NA	D	PrD_PrD	D	MODERATE	D	0.744	50	50	0	45	17	27	60
TSNAD	ZNF277_G184R	9	HLA-B*08:01	FLGNRSVILN	2366.2	2.7635	-	FLRNRSVILN	61.8	0.1101	SB	ZNF277	G184R	Chewer	42483	NA	NA	NA	0.0000165	0.000121462	NA	NA	D	PrD_PrD	D	MODERATE	D	0.744	50	50	0	45	17	27	60
TSNAD	ZNF277_G184R	9	HLA-B*42:01	FLGNRSVIL	972.7	1.8426	WB	FLRNRSVIL	62.1	0.2973	SB	ZNF277	G184R	Chewer	42483	NA	NA	NA	0.0000165	0.000121462	NA	NA	D	PrD_PrD	D	MODERATE	D	0.744	50	50	0	45	17	27	60
