# Supplementary material for: Mutational Landscape of Esophageal Squamous Cell Carcinoma in an Indian Cohort
Source: Front Oncol. 2020 Aug 20;10:1457. doi: 10.3389/fonc.2020.01457 (PMC7469928; doi:10.3389/fonc.2020.01457)
Supplement: Supplementary Table 7 — (A) List of copy number alterations and affected genes in ESCC patients. (B) List of cytobands and number of genes affected by Copy number alterations (CNA). [file Table_7.pdf]

Mangalaparthi *et al.* , 2020. Mutational landscape of esophageal squamous cell carcinoma in an Indian cohort
Supplementary Table 7A. List of copy number alterations and affected genes in ESCC patients

Gene	Chromosome	Cytoband	Recurrence	Recurrence in smoker cohort	Recurrence in chewer cohort	Recurrence in No habit cohort	State	Samples with CNA gain	Samples with CNA loss	File info with CNA fold
A4GNT	chr3	3q22.3	4	0	1	3	Amplification	4	0	42496T: 3.5; 42492T: 3.5; 42487T: 3.5; 42493T: 3.5;
AACS	chr12	12q24.31	1	0	0	1	Amplification	1	0	42500T: 3.5;
AADAC	chr3	3q25.1	9	1	2	6	Amplification	9	0	42492T: 3.5; 42487T: 3.5; 42493T: 3.5; 42484T: 3.5; 42474T: 3.5; 56957T: 4; 42497T: 5.5; 42496T: 4; 42500T: 3.5;
AADACL2	chr3	3q25.1	9	1	2	6	Amplification	9	0	42487T: 3.5; 42493T: 3.5; 42492T: 3.5; 42474T: 3.5; 56957T: 4; 42484T: 3.5; 42500T: 3.5; 42497T: 5.5; 42496T: 4;
AAK1	chr2	2p13.3	1	0	0	1	Amplification	1	0	42500T: 3.5;
AAR2	chr20	20q11.23	1	1	0	0	Amplification	1	0	42473T: 3.5;
AARD	chr8	8q24.11	3	1	0	2	Amplification	3	0	42495T: 3.5; 42496T: 3.5; 42475T: 3.5;
AASS	chr7	7q31.32	1	0	1	0	Amplification	1	0	42487T: 3.5;
ABAT	chr16	16p13.2	2	1	0	1	Amplification	2	0	42473T: 5.5; 42495T: 4;
ABCA13	chr7	7p12.3	1	0	0	1	Amplification	2	0	42497T: 12,20.5;
ABCA2	chr9	9q34.3	2	1	0	1	Amplification	2	0	56957T: 3.5; 42473T: 5;
ABCA3	chr16	16p13.3	1	0	1	0	Amplification	1	0	42483T: 3.5;
ABCA7	chr19	19p13.3	1	0	0	1	Amplification	1	0	42493T: 3.5;
ABCB1	chr7	7q21.12	2	0	2	0	Amplification	2	0	42483T: 3.5; 42487T: 3.5;
ABCB4	chr7	7q21.12	2	0	2	0	Amplification	2	0	42487T: 3.5; 42483T: 3.5;
ABCB5	chr7	7p21.1	1	1	0	0	Amplification	1	0	42473T: 4;
ABCB9	chr12	12q24.31	1	0	0	1	Amplification	1	0	42500T: 3.5;
ABCC1	chr16	16p13.11	2	1	0	1	Amplification	2	0	42495T: 4; 42473T: 4;
ABCC3	chr17	17q21.33	1	1	0	0	Amplification	1	0	42473T: 4.5;
ABCC5	chr3	3q27.1	11	1	3	7	Amplification	11	0	42497T: 4; 42474T: 3.5; 42494T: 3.5; 42495T: 4; 56957T: 5; 42484T: 4; 42498T: 3.5; 42482T: 3.5; 42493T: 3.5; 42487T: 3.5; 42492T: 3.5;
ABCC6	chr16	16p13.11	2	1	0	1	Amplification	2	0	42495T: 4; 42473T: 4;
ABCC6P1	chr16	16p12.3	2	1	0	1	Amplification	2	0	42495T: 4; 42473T: 4;
ABCC9	chr12	12p12.1	1	0	0	1	Amplification	1	0	42500T: 6;
ABCD2	chr12	12q12	1	0	0	1	Amplification	1	0	42500T: 3.5;
ABCD4	chr14	14q24.3	2	0	0	2	Amplification	2	0	56957T: 3.5; 42494T: 4;
ABCF3	chr3	3q27.1	12	2	3	7	Amplification	12	0	42487T: 3.5; 42493T: 3.5; 42492T: 3.5; 42482T: 3.5; 42495T: 4; 42494T: 3.5; 42474T: 3.5; 56957T: 5; 42484T: 4; 42498T: 3.5; 42497T: 4; 42473T: 3.5;
ABCG1	chr21	21q22.3	1	1	0	0	Amplification	1	0	42473T: 3.5;
ABCG5	chr2	2p21	2	0	1	1	Amplification	2	0	42500T: 3.5; 42484T: 3.5;
ABCG8	chr2	2p21	2	0	1	1	Amplification	2	0	42484T: 3.5; 42500T: 3.5;
ABHD1	chr2	2p23.3	1	0	0	1	Amplification	1	0	42500T: 3.5;
ABHD10	chr3	3q13.2	1	0	0	1	Amplification	1	0	42496T: 3.5;
ABHD12B	chr14	14q22.1	1	0	0	1	Amplification	1	0	42494T: 4;
ABHD16A	chr6	6p21.33	1	1	0	0	Amplification	1	0	42473T: 3.5;

Mangalaparthi *et al.* , 2020. Mutational landscape of esophageal squamous cell carcinoma in an Indian cohort
Supplementary Table 7A. List of copy number alterations and affected genes in ESCC patients

Gene	Chromosome	Cytoband	Recurrence	Recurrence in smoker cohort	Recurrence in chewer cohort	Recurrence in No habit cohort	State	Samples with CNA gain	Samples with CNA loss	File info with CNA fold
ABHD4	chr14	14q11.2	1	0	0	1	Amplification	1	0	42496T: 4;
ABI1	chr10	10p12.1	1	0	0	1	Amplification	1	0	56958T: 4.5;
ABI3BP	chr3	3q12.2	1	1	0	0	Amplification	1	0	42476T: 3.5;
ABRA	chr8	8q23.1	2	0	0	2	Amplification	2	0	42496T: 3.5; 42495T: 3.5;
ABTB1	chr3	3q21.3	2	0	1	1	Amplification	2	0	42487T: 3.5; 42496T: 3.5;
ABTB2	chr11	11p13	1	1	0	0	Amplification	1	0	42473T: 4.5;
ACAA1	chr3	3p22.2	1	1	0	0	Amplification	1	0	42473T: 4.5;
ACACB	chr12	12q24.11	2	1	0	1	Amplification	2	0	42473T: 4.5; 42500T: 3.5;
ACAD10	chr12	12q24.12	1	0	0	1	Amplification	1	0	42500T: 3.5;
ACAD11	chr3	3q22.1	3	0	1	2	Amplification	3	0	42496T: 3.5; 42487T: 3.5; 42492T: 3.5;
ACAD9	chr3	3q21.3	3	0	1	2	Amplification	3	0	42496T: 3.5; 42493T: 3.5; 42487T: 3.5;
ACADS	chr12	12q24.31	1	0	0	1	Amplification	1	0	42500T: 3.5;
ACAP2	chr3	3q29	9	1	3	5	Amplification	9	0	42484T: 4; 42498T: 3.5; 42474T: 3.5; 42495T: 4; 56957T: 4.5; 42482T: 3.5; 42492T: 3.5; 42493T: 3.5; 42487T: 3.5;
ACBD6	chr1	1q25.2-q25.3	1	0	0	1	Amplification	1	0	42493T: 4;
ACER3	chr11	11q13.5	2	0	0	2	Amplification	2	0	42497T: 3.5; 42496T: 3.5;
ACHE	chr7	7q22.1	1	0	0	1	Amplification	1	0	42501T: 3.5;
ACIN1	chr14	14q11.2	1	0	0	1	Amplification	1	0	42496T: 4;
ACKR4	chr3	3q22.1	3	0	1	2	Amplification	3	0	42496T: 3.5; 42492T: 3.5; 42487T: 3.5;
ACN9	chr7	7q21.3	1	0	1	0	Amplification	1	0	42487T: 3.5;
ACOT1	chr14	14q24.3	2	0	0	2	Amplification	2	0	56957T: 3.5; 42494T: 4;
ACOT2	chr14	14q24.3	2	0	0	2	Amplification	2	0	42494T: 4; 56957T: 3.5;
ACOT4	chr14	14q24.3	2	0	0	2	Amplification	2	0	42494T: 4; 56957T: 3.5;
ACOT6	chr14	14q24.3	2	0	0	2	Amplification	2	0	56957T: 3.5; 42494T: 4;
ACOT8	chr20	20q13.12	1	1	0	0	Amplification	1	0	42473T: 5;
ACOX1	chr17	17q25.1	1	0	0	1	Amplification	1	0	42494T: 3.5;
ACP1	chr2	16p12.2	1	0	0	1	Amplification	1	0	42500T: 3.5;
ACP2	chr11	11p11.2|11p	1	1	0	0	Amplification	1	0	42473T: 3.5;
ACP6	chr1	1q21.2	1	1	0	0	Amplification	1	0	42473T: 4;
ACPL2	chr3	3q23	4	0	1	3	Amplification	4	0	42493T: 3.5; 42487T: 3.5; 42492T: 3.5; 42496T: 4;
ACPP	chr3	3q22.1	3	0	1	2	Amplification	3	0	42496T: 3.5; 42492T: 3.5; 42487T: 3.5;
ACSBG1	chr15	15q25.1	1	1	0	0	Amplification	1	0	42473T: 3.5;
ACSF2	chr17	17q21.33	1	1	0	0	Amplification	1	0	42473T: 4.5;
ACSM1	chr16	16p12.3	1	1	0	0	Amplification	1	0	42473T: 4;
ACSM2A	chr16	16p12.3	1	1	0	0	Amplification	1	0	42473T: 4;
ACSM2B	chr16	16p12.3	1	1	0	0	Amplification	1	0	42473T: 4;
ACSM3	chr16	16p12.3	1	1	0	0	Amplification	1	0	42473T: 4;
ACSM4	chr12	12p13.31	1	0	0	1	Amplification	1	0	42494T: 3.5;
ACSM5	chr16	16p12.3	1	1	0	0	Amplification	1	0	42473T: 4;
ACSS2	chr20	20q11.22	2	1	0	1	Amplification	2	0	42493T: 3.5; 42473T: 3.5;

Mangalaparthi *et al.* , 2020. Mutational landscape of esophageal squamous cell carcinoma in an Indian cohort
Supplementary Table 7A. List of copy number alterations and affected genes in ESCC patients

Gene	Chromosome	Cytoband	Recurrence	Recurrence in smoker cohort	Recurrence in chewer cohort	Recurrence in No habit cohort	State	Samples with CNA gain	Samples with CNA loss	File info with CNA fold
ACTB	chr7	2q21.1	2	1	0	1	Amplification	2	0	42497T: 8.5; 42473T: 4.5;
ACTL10	chr20	20q11.22	2	1	0	1	Amplification	2	0	42496T: 5; 42473T: 3.5;
ACTL6A	chr3	3q26.33	11	1	3	7	Amplification	11	0	42482T: 3.5; 42492T: 3.5; 42487T: 3.5; 42493T: 3.5; 42497T: 4; 42500T: 14; 42498T: 3.5; 42484T: 4; 56957T: 4; 42495T: 4; 42474T: 3.5;
ACTL8	chr1	1p36.13	1	1	0	0	Amplification	1	0	42473T: 4;
ACTN1	chr14	14q24.1|14q	1	0	0	1	Amplification	1	0	42494T: 4;
ACTN4	chr19	19q13.2	2	0	0	2	Amplification	2	0	42500T: 6.5; 56957T: 4;
ACTR10	chr14	14q23.1	1	0	0	1	Amplification	1	0	42494T: 4;
ACTR1B	chr2	2q11.2	1	0	0	1	Amplification	1	0	42493T: 3.5;
ACTR2	chr2	2p14	2	0	0	2	Amplification	2	0	56957T: 3.5; 42500T: 3.5;
ACTR5	chr20	20q11.23	1	1	0	0	Amplification	1	0	42473T: 3.5;
ACTRT3	chr3	3q26.2	9	1	3	5	Amplification	9	0	42482T: 3.5; 42487T: 3.5; 42493T: 3.5; 42492T: 3.5; 42500T: 8; 56957T: 4; 42495T: 4; 42474T: 3.5; 42484T: 4;
ACY3	chr11	11q13.2	2	1	0	1	Amplification	2	0	56957T: 5; 42473T: 3.5;
ACYP1	chr14	14q24.3	2	0	0	2	Amplification	2	0	42494T: 4; 56957T: 3.5;
ACYP2	chr2	2p16.2	2	0	1	1	Amplification	2	0	42484T: 4.5; 42500T: 3.5;
ADA	chr20	20q13.12	1	1	0	0	Amplification	1	0	42473T: 5;
ADAM15	chr1	1q21.3	2	1	0	1	Amplification	2	0	42473T: 6.5; 42496T: 3.5;
ADAM17	chr2	2p25.1	1	0	0	1	Amplification	1	0	42500T: 3.5;
ADAM1A	chr12	12q24.12-q2	1	0	0	1	Amplification	1	0	42500T: 3.5;
ADAM20	chr14	14q24.2	1	0	0	1	Amplification	1	0	42494T: 4;
ADAM21	chr14	14q24.2	1	0	0	1	Amplification	1	0	42494T: 4;
ADAM21P1	chr14	14q24.2	1	0	0	1	Amplification	1	0	42494T: 4;
ADAM22	chr7	7q21.12	2	0	2	0	Amplification	2	0	42487T: 3.5; 42483T: 3.5;
ADAM33	chr20	20p13	1	1	0	0	Amplification	1	0	42473T: 6;
ADAMTS12	chr5	5p13.3-p13.2	4	1	1	2	Amplification	4	0	42493T: 3.5; 42475T: 3.5; 42496T: 3.5; 42486T: 3.5;
ADAMTS16	chr5	5p15.32	4	1	1	2	Amplification	4	0	42475T: 3.5; 42496T: 3.5; 42486T: 3.5; 42493T: 4;
ADAMTS20	chr12	12q12	1	0	0	1	Amplification	1	0	42500T: 3.5;
ADAMTSL4	chr1	1q21.2	2	1	0	1	Amplification	2	0	42493T: 3.5; 42473T: 5;
ADAP1	chr7	7p22.3	1	1	0	0	Amplification	1	0	42473T: 5;
ADAP2	chr17	17q11.2	1	1	0	0	Amplification	1	0	42473T: 3.5;
ADAR	chr1	1q21.3	1	1	0	0	Amplification	1	0	42473T: 4.5;
ADCK1	chr14	14q24.3	1	0	0	1	Amplification	1	0	42494T: 4;
ADCK2	chr7	7q34	2	1	1	0	Amplification	2	0	42473T: 3.5; 42487T: 3.5;
ADCK4	chr19	19q13.2	2	0	0	2	Amplification	2	0	56957T: 4; 42500T: 3.5;
ADCK5	chr8	8q24.3	4	0	1	3	Amplification	4	0	42496T: 4; 42483T: 3.5; 56957T: 3.5; 42495T: 4.5;
ADCY1	chr7	7p12.3	1	1	0	0	Amplification	1	0	42473T: 4.5;
ADCY2	chr5	5p15.31	4	1	1	2	Amplification	4	0	42475T: 3.5; 42496T: 3.5; 42486T: 3.5; 42493T: 4;
ADCY3	chr2	8q24.22	1	0	0	1	Amplification	1	0	42500T: 3.5;

Mangalaparthi *et al.* , 2020. Mutational landscape of esophageal squamous cell carcinoma in an Indian cohort
Supplementary Table 7A. List of copy number alterations and affected genes in ESCC patients

Gene	Chromosome	Cytoband	Recurrence	Recurrence in smoker cohort	Recurrence in chewer cohort	Recurrence in No habit cohort	State	Samples with CNA gain	Samples with CNA loss	File info with CNA fold
ADCY5	chr3	3q21.1	2	1	0	1	Amplification	2	0	42496T: 3.5; 42473T: 3.5;
ADCY6	chr12	12q13.12	1	0	0	1	Amplification	1	0	42500T: 3.5;
ADCY8	chr8	8q24.22	3	0	1	2	Amplification	3	0	42496T: 3.5; 42484T: 3.5; 42495T: 3.5;
ADCY9	chr16	16p13.3	1	1	0	0	Amplification	1	0	42473T: 5.5;
ADCYAP1	chr18	18p11.32	3	0	0	3	Amplification	3	0	56957T: 8; 42493T: 3.5; 42500T: 4.5;
ADCYAP1R1	chr7	7p14.3	1	1	0	0	Amplification	1	0	42473T: 4;
ADD2	chr2	2p13.3	1	0	0	1	Amplification	1	0	42500T: 3.5;
ADHFE1	chr8	8q13.1	3	0	0	3	Amplification	3	0	42495T: 3.5; 42497T: 4; 42496T: 3.5;
ADI1	chr2	2p25.3	2	0	0	2	Amplification	2	0	42495T: 3.5; 42500T: 3.5;
ADIG	chr20	20q11.23	1	1	0	0	Amplification	1	0	42473T: 3.5;
ADIPOQ	chr3	3q27.3	10	1	3	6	Amplification	10	0	42492T: 3.5; 42487T: 3.5; 42493T: 3.5; 42482T: 3.5; 42484T: 4; 42498T: 3.5; 42495T: 4; 42474T: 3.5; 56957T: 4.5; 42497T: 4;
ADIPOR1	chr1	1q32.1	1	1	0	0	Amplification	1	0	42473T: 4;
ADNP	chr20	20q13.13	1	1	0	0	Amplification	1	0	42473T: 5;
ADORA1	chr1	1q32.1	1	1	0	0	Amplification	1	0	42473T: 4;
ADPRH	chr3	3q13.33	1	0	0	1	Amplification	1	0	42496T: 3.5;
ADPRHL1	chr13	13q34	1	0	0	1	Amplification	1	0	56957T: 3.5;
ADRA1D	chr20	20p13	1	1	0	0	Amplification	1	0	42473T: 4.5;
ADRB3	chr8	8p11.23	2	0	1	1	Amplification	2	0	42493T: 3.5; 42482T: 3.5;
ADRBK1	chr11	11q13.2	2	1	0	1	Amplification	2	0	42473T: 3.5; 56957T: 5;
ADRM1	chr20	20q13.33	2	1	1	0	Amplification	2	0	42473T: 6; 42486T: 3.5;
ADSL	chr22	22q13.1	1	1	0	0	Amplification	1	0	42473T: 3.5;
AEBP1	chr7	7p13	2	1	0	1	Amplification	2	0	42497T: 7.5; 42473T: 4.5;
AEBP2	chr12	12p12.3	1	0	0	1	Amplification	1	0	42500T: 4.5;
AFF3	chr2	2q11.2	1	0	0	1	Amplification	1	0	42493T: 3.5;
AFTPH	chr2	2p14	2	0	0	2	Amplification	2	0	42500T: 3.5; 56957T: 3.5;
AGBL2	chr11	11p11.2	1	1	0	0	Amplification	1	0	42473T: 3.5;
AGBL3	chr7	7q33	1	0	1	0	Amplification	1	0	42487T: 3.5;
AGBL5	chr2	2p23.3	1	0	0	1	Amplification	1	0	42500T: 3.5;
AGER	chr6	6p21.32	1	1	0	0	Amplification	1	0	42473T: 3.5;
AGK	chr7	7q34	1	0	1	0	Amplification	1	0	42487T: 3.5;
AGMO	chr7	7p21.2	1	1	0	0	Amplification	1	0	42473T: 3.5;
AGO2	chr8	8q24.3	3	0	1	2	Amplification	3	0	42496T: 3.5; 42483T: 3.5; 42495T: 3.5;
AGPAT1	chr6	6p21.32	1	1	0	0	Amplification	1	0	42473T: 3.5;
AGPAT2	chr9	9q34.3	1	0	0	1	Amplification	1	0	56957T: 3.5;
AGPS	chr2	2q31.2	1	0	0	1	Amplification	1	0	42493T: 4.5;
AGR2	chr7	7p21.1	1	1	0	0	Amplification	1	0	42473T: 7.5;
AGR3	chr7	7p21.1	1	1	0	0	Amplification	1	0	42473T: 7.5;
AGTR1	chr3	3q24	8	1	2	5	Amplification	8	0	42496T: 4; 42497T: 4.5; 42484T: 3.5; 42474T: 3.5; 56957T: 4; 42492T: 3.5; 42493T: 3.5; 42487T: 3.5;

Mangalaparthi *et al.* , 2020. Mutational landscape of esophageal squamous cell carcinoma in an Indian cohort
Supplementary Table 7A. List of copy number alterations and affected genes in ESCC patients

Gene	Chromosome	Cytoband	Recurrence	Recurrence in smoker cohort	Recurrence in chewer cohort	Recurrence in No habit cohort	State	Samples with CNA gain	Samples with CNA loss	File info with CNA fold
AGXT	chr2	2q37.3	1	1	0	0	Deletion	0	1	42474T: 0.5;
AGXT2	chr5	5p13.2	4	1	1	2	Amplification	4	0	42486T: 3.5; 42496T: 3.5; 42475T: 3.5; 42493T: 3.5;
AHCY	chr20	20q11.22	3	1	0	2	Amplification	3	0	42473T: 3.5; 42496T: 5; 42493T: 3.5;
AHCYL1	chr1	1p13.3	1	1	0	0	Amplification	1	0	42473T: 4.5;
AHCYL2	chr7	7q32.1	1	0	1	0	Amplification	1	0	42487T: 3.5;
AHDC1	chr1	1p36.11-p35	1	1	0	0	Amplification	1	0	42473T: 3.5;
AHR	chr7	7p21.1	1	1	0	0	Amplification	1	0	42473T: 7.5;
AHRR	chr5	5p15.33	5	2	1	2	Amplification	5	0	42496T: 4; 42473T: 3.5; 42486T: 3.5; 42475T: 3.5; 42493T: 3.5;
AHSA1	chr14	14q24.3	2	0	0	2	Amplification	2	0	42494T: 4; 56957T: 3.5;
AHSA2	chr2	2p15	3	0	1	2	Amplification	3	0	42500T: 3.5; 42484T: 4.5; 56957T: 3.5;
AHSG	chr3	3q27.3	10	1	3	6	Amplification	10	0	42482T: 3.5; 42492T: 3.5; 42487T: 3.5; 42493T: 3.5; 42497T: 4; 42498T: 3.5; 42484T: 4; 56957T: 4.5; 42495T: 4; 42474T: 3.5;
AIF1	chr6	6p21.33	1	1	0	0	Amplification	1	0	42473T: 3.5;
AIF1L	chr9	9q34.12-q34	1	0	1	0	Amplification	1	0	42484T: 4;
AIFM3	chr22	22q11.21	1	1	0	0	Amplification	1	0	42477T: 4;
AIM1	chr6	5p13.2	1	0	0	1	Amplification	1	0	42496T: 3.5;
AIMP2	chr7	7p22.1	1	1	0	0	Amplification	1	0	42473T: 4.5;
AIP	chr11	1p36.33	2	1	0	1	Amplification	2	0	42473T: 3.5; 56957T: 5;
AJUBA	chr14	14q11.2	1	0	0	1	Amplification	1	0	42496T: 4;
AK3	chr9	9p24.1	1	0	0	1	Amplification	1	0	42496T: 3.5;
AK9	chr6	6q21	1	0	0	1	Amplification	1	0	42496T: 3.5;
AKAP1	chr17	17q22	1	0	0	1	Amplification	1	0	42497T: 4;
AKAP2	chr9	9q31.3	1	0	1	0	Amplification	1	0	42483T: 3.5;
AKAP3	chr12	12p13.32	1	0	0	1	Amplification	1	0	42494T: 3.5;
AKAP5	chr14	14q23.3	1	0	0	1	Amplification	1	0	42494T: 4;
AKAP6	chr14	14q12	1	0	0	1	Amplification	1	0	42500T: 4.5;
AKAP9	chr7	7q21.2	3	0	2	1	Amplification	3	0	42487T: 3.5; 42494T: 3.5; 42483T: 4;
AKR1B1	chr7	7q33	1	0	1	0	Amplification	1	0	42487T: 3.5;
AKR1B10	chr7	7q33	1	0	1	0	Amplification	1	0	42487T: 3.5;
AKR1B15	chr7	7q33	1	0	1	0	Amplification	1	0	42487T: 3.5;
AKR1D1	chr7	7q33	1	0	1	0	Amplification	1	0	42487T: 3.5;
AKR7A2	chr1	1p36.13	1	1	0	0	Amplification	1	0	42473T: 4;
AKR7A3	chr1	1p36.13	1	1	0	0	Amplification	1	0	42473T: 4;
AKR7L	chr1	1p36.13|1p3	1	1	0	0	Amplification	1	0	42473T: 4;
AKT2	chr19	19q13.2	1	0	0	1	Amplification	1	0	42500T: 7.5;
ALDH1A1	chr9	9q21.13	1	1	0	0	Amplification	1	0	42473T: 4;
ALDH1L1	chr3	3q21.3	1	0	0	1	Amplification	1	0	42496T: 3.5;
ALDH2	chr12	12q24.12	1	0	0	1	Amplification	1	0	42500T: 3.5;
ALDH3B1	chr11	11q13.2	1	0	0	1	Amplification	1	0	56957T: 5;

Mangalaparthi *et al.* , 2020. Mutational landscape of esophageal squamous cell carcinoma in an Indian cohort
Supplementary Table 7A. List of copy number alterations and affected genes in ESCC patients

Gene	Chromosome	Cytoband	Recurrence	Recurrence in smoker cohort	Recurrence in chewer cohort	Recurrence in No habit cohort	State	Samples with CNA gain	Samples with CNA loss	File info with CNA fold
ALDH3B2	chr11	11q13.2	1	0	0	1	Amplification	1	0	56957T: 5;
ALDH4A1	chr1	1p36.13	1	1	0	0	Amplification	1	0	42473T: 4;
ALDH6A1	chr14	14q24.3	2	0	0	2	Amplification	2	0	42494T: 4; 56957T: 3.5;
ALG1	chr16	16p13.3	2	1	0	1	Amplification	2	0	42495T: 12; 42473T: 5.5;
ALG10	chr12	12q12	1	0	0	1	Amplification	1	0	42500T: 3.5;
ALG10B	chr12	12q12	1	0	0	1	Amplification	1	0	42500T: 3.5;
ALG1L	chr3	3q21.2	1	0	0	1	Amplification	1	0	42496T: 3.5;
ALG1L2	chr3	3q22.1	2	0	1	1	Amplification	2	0	42487T: 3.5; 42496T: 3.5;
ALG3	chr3	3q27.1	12	2	3	7	Amplification	12	0	42473T: 3.5; 42497T: 4; 56957T: 5; 42474T: 3.5; 42495T: 4; 42494T: 3.5; 42498T: 3.5; 42484T: 4; 42482T: 3.5; 42493T: 3.5; 42487T: 3.5; 42492T: 3.5;
ALK	chr2	2p23.2-p23.1	1	0	0	1	Amplification	1	0	42500T: 3.5;
ALKBH1	chr14	14q24.3	2	0	0	2	Amplification	2	0	42494T: 4; 56957T: 3.5;
ALKBH2	chr12	12q24.11	2	1	0	1	Amplification	2	0	42473T: 4.5; 42500T: 3.5;
ALKBH4	chr7	7q22.1	1	0	0	1	Amplification	1	0	42501T: 3.5;
ALKBH6	chr19	19q13.12	3	0	1	2	Amplification	3	0	42500T: 6.5; 56957T: 4; 42484T: 3.5;
ALLC	chr2	2p25.3	1	0	0	1	Amplification	1	0	42500T: 3.5;
ALMS1	chr2	2p13.1	1	0	0	1	Amplification	1	0	42500T: 3.5;
ALMS1P	chr2	2p13.1	1	0	0	1	Amplification	1	0	42500T: 3.5;
ALOX5AP	chr13	13q12.3	1	0	0	1	Amplification	1	0	42497T: 3.5;
ALPK3	chr15	15q25.3	1	1	0	0	Amplification	1	0	42473T: 4;
ALPL	chr1	1p36.12	1	1	0	0	Amplification	1	0	42473T: 4;
ALX3	chr1	1p13.3	1	1	0	0	Amplification	1	0	42473T: 4.5;
AMACR	chr5	5p13.2	4	1	1	2	Amplification	4	0	42496T: 3.5; 42486T: 3.5; 42475T: 3.5; 42493T: 3.5;
AMBRA1	chr11	11p11.2	2	1	0	1	Amplification/Deletio	1	1	42473T: 3.5; 42499T: 0.5;
AMD1	chr6	6q21	1	0	0	1	Amplification	1	0	42496T: 3.5;
AMN1	chr12	12p11.21	1	0	0	1	Amplification	1	0	42500T: 12.5;
AMOTL2	chr3	3q22.2	4	0	1	3	Amplification	4	0	42492T: 3.5; 42493T: 3.5; 42487T: 3.5; 42496T: 3.5;
AMPD2	chr1	1p13.3	1	1	0	0	Amplification	1	0	42473T: 4.5;
AMPH	chr7	7p14.1	1	1	0	0	Amplification	1	0	42473T: 3.5;
AMT	chr3	3p21.31	1	1	0	0	Amplification	1	0	42473T: 4;
AMZ1	chr7	7p22.3	1	1	0	0	Amplification	1	0	42473T: 5;
ANAPC13	chr3	3q22.2	4	0	1	3	Amplification	4	0	42496T: 3.5; 42493T: 3.5; 42487T: 3.5; 42492T: 3.5;
ANAPC15	chr11	11q13.4	8	3	0	5	Amplification	8	0	42475T: 5; 42501T: 6; 42498T: 5; 42478T: 4.5; 56957T: 3.5; 42476T: 4; 42492T: 4; 42493T: 3.5;
ANAPC2	chr9	9q34.3	2	1	0	1	Amplification	2	0	56957T: 3.5; 42473T: 5;
ANAPC5	chr12	12q24.31	1	0	0	1	Amplification	1	0	42500T: 3.5;
ANAPC7	chr12	12q24.11	2	1	0	1	Amplification	2	0	42473T: 4.5; 42500T: 3.5;
ANGEL1	chr14	14q24.3	2	0	0	2	Amplification	2	0	42494T: 4; 56957T: 3.5;
ANGPT1	chr8	8q23.1	2	0	0	2	Amplification	2	0	42496T: 3.5; 42495T: 3.5;
ANGPT4	chr20	20p13	1	1	0	0	Amplification	1	0	42473T: 3.5;

Mangalaparthi *et al.* , 2020. Mutational landscape of esophageal squamous cell carcinoma in an Indian cohort
Supplementary Table 7A. List of copy number alterations and affected genes in ESCC patients

Gene	Chromosome	Cytoband	Recurrence	Recurrence in smoker cohort	Recurrence in chewer cohort	Recurrence in No habit cohort	State	Samples with CNA gain	Samples with CNA loss	File info with CNA fold
ANGPTL5	chr11	11q22.1	1	0	0	1	Amplification	1	0	56958T: 5.5;
ANKAR	chr2	2q32.2	2	0	1	1	Amplification	2	0	42493T: 3.5; 42482T: 4;
ANKEF1	chr20	20p12.2	1	0	1	0	Amplification	1	0	42486T: 4;
ANKFN1	chr17	17q22	1	0	0	1	Amplification	1	0	42497T: 4;
ANKH	chr5	5p15.2	5	1	2	2	Amplification	5	0	42475T: 3.5; 42496T: 3.5; 42486T: 3.5; 42493T: 4; 42484T: 3.5;
ANKIB1	chr7	7q21.2	3	0	2	1	Amplification	3	0	42487T: 3.5; 42494T: 3.5; 42483T: 4;
ANKLE2	chr12	12q24.33	1	0	0	1	Amplification	1	0	56957T: 4;
ANKMY2	chr7	7p21.1	1	1	0	0	Amplification	1	0	42473T: 7.5;
ANKRD13A	chr12	12q24.11	2	1	0	1	Amplification	2	0	42500T: 3.5; 42473T: 4.5;
ANKRD13D	chr11	11q13.2	2	1	0	1	Amplification	2	0	56957T: 5; 42473T: 3.5;
ANKRD23	chr2	2q11.2	2	1	0	1	Amplification	2	0	42473T: 3.5; 42493T: 4.5;
ANKRD27	chr19	19q13.11	4	1	1	2	Amplification	4	0	56957T: 4; 42484T: 3.5; 42500T: 4.5; 42473T: 3.5;
ANKRD29	chr18	18q11.2	1	1	0	0	Amplification	1	0	42481T: 6;
ANKRD33B	chr5	5p15.2	4	1	1	2	Amplification	4	0	42475T: 3.5; 42486T: 3.5; 42496T: 3.5; 42493T: 4;
ANKRD34A	chr1	1q21.1	1	1	0	0	Amplification	1	0	42473T: 4;
ANKRD35	chr1	1q21.1	1	1	0	0	Amplification	1	0	42473T: 4;
ANKRD36	chr2	2q11.2	1	0	0	1	Amplification	1	0	42493T: 4.5;
ANKRD36B	chr2	2q11.2	1	0	0	1	Amplification	1	0	42493T: 3.5;
ANKRD36BP2	chr2	2p11.2	1	0	0	1	Amplification	1	0	42500T: 3.5;
ANKRD36C	chr2	2q11.1	2	1	0	1	Amplification	2	0	42473T: 3.5; 42493T: 4;
ANKRD39	chr2	2q11.2	2	1	0	1	Amplification	2	0	42473T: 3.5; 42493T: 4.5;
ANKRD40	chr17	17q21.33	1	1	0	0	Amplification	1	0	42473T: 4.5;
ANKRD44	chr2	2q33.1	1	0	1	0	Amplification	1	0	42482T: 4;
ANKRD46	chr8	8q22.3	2	0	0	2	Amplification	2	0	42495T: 3.5; 42496T: 3.5;
ANKRD52	chr12	12q13.3	1	0	0	1	Amplification	1	0	42494T: 7.5;
ANKRD53	chr2	2p13.3	1	0	0	1	Amplification	1	0	42500T: 3.5;
ANKRD54	chr22	22q13.1	1	1	0	0	Amplification	1	0	42473T: 3.5;
ANKRD60	chr20	20q13.32	1	1	0	0	Amplification	1	0	42473T: 4.5;
ANKRD61	chr7	7p22.1	1	1	0	0	Amplification	1	0	42473T: 4.5;
ANKRD62	chr18	18p11.21	1	0	0	1	Amplification	1	0	42493T: 3.5;
ANKRD63	chr15	15q15.1	1	1	0	0	Amplification	1	0	42473T: 3.5;
ANKRD7	chr7	7q31.31	1	0	1	0	Amplification	1	0	42487T: 3.5;
ANKS1B	chr12	12q23.1	1	0	0	1	Deletion	0	1	42495T: 0.5;
ANKS3	chr16	16p13.3	3	1	0	2	Amplification	3	0	42473T: 5.5; 42495T: 12; 42494T: 3.5;
ANKS4B	chr16	16p12.2	1	1	0	0	Amplification	1	0	42473T: 4;
ANKUB1	chr3	3q25.1	9	2	2	5	Amplification	9	0	42492T: 3.5; 42487T: 3.5; 42493T: 3.5; 42484T: 3.5; 42474T: 3.5; 56957T: 4; 42496T: 4; 42473T: 4; 42497T: 4.5;
ANLN	chr7	7p14.2	1	1	0	0	Amplification	1	0	42473T: 4.5;

Mangalaparthi *et al.* , 2020. Mutational landscape of esophageal squamous cell carcinoma in an Indian cohort
Supplementary Table 7A. List of copy number alterations and affected genes in ESCC patients

Gene	Chromosome	Cytoband	Recurrence	Recurrence in smoker cohort	Recurrence in chewer cohort	Recurrence in No habit cohort	State	Samples with CNA gain	Samples with CNA loss	File info with CNA fold
ANO1	chr11	11q13.3	12	3	2	7	Amplification	14	0	42498T: 7; 42478T: 4.5; 56957T: 5; 42497T: 4.5,6.5; 42483T: 9; 42486T: 13.5; 42500T: 13; 56958T: 9; 42501T: 6; 42475T: 7.5; 42492T: 4; 42476T: 29.5,13.5;
ANO6	chr12	12q12	1	0	0	1	Amplification	1	0	42500T: 3.5;
ANO7P1	chr1	1p36.13	1	1	0	0	Amplification	1	0	42473T: 8;
ANP32D	chr12	12q13.11	1	0	0	1	Amplification	1	0	42500T: 3.5;
ANP32E	chr1	1q21.2	1	1	0	0	Amplification	1	0	42473T: 5;
ANPEP	chr15	15q26.1	1	1	0	0	Amplification	1	0	42473T: 3.5;
ANTXR1	chr2	2p13.3	1	0	0	1	Amplification	1	0	42500T: 3.5;
ANXA1	chr9	9q21.13	1	1	0	0	Amplification	1	0	42473T: 4;
ANXA13	chr8	8q24.13	4	1	1	2	Amplification	4	0	42495T: 3.5; 42484T: 3.5; 42475T: 3.5; 42496T: 3.5;
ANXA2R	chr5	5p12	5	1	2	2	Amplification	5	0	42493T: 3.5; 42496T: 3.5; 42483T: 3.5; 42486T: 3.5; 42475T: 3.5;
ANXA4	chr2	2p13.3	1	0	0	1	Amplification	1	0	42500T: 3.5;
ANXA7	chr10	10q22.2	1	0	0	1	Amplification	1	0	42496T: 3.5;
ANXA9	chr1	1q21.3	1	1	0	0	Amplification	1	0	42473T: 4.5;
AOAH	chr7	7p14.2	1	1	0	0	Amplification	1	0	42473T: 4.5;
AP1G2	chr14	14q11.2	1	0	0	1	Amplification	1	0	42496T: 4;
AP1S1	chr7	7q22.1	2	0	0	2	Amplification	2	0	42493T: 3.5; 42501T: 3.5;
AP2M1	chr3	3q27.1	12	2	3	7	Amplification	12	0	42487T: 3.5; 42493T: 3.5; 42492T: 3.5; 42482T: 3.5; 56957T: 5; 42495T: 4; 42474T: 3.5; 42494T: 3.5; 42498T: 3.5; 42484T: 4; 42497T: 4; 42473T: 3.5;
AP3S2	chr15	15q26.1	1	1	0	0	Amplification	1	0	42473T: 3.5;
AP4S1	chr14	14q12	3	0	0	3	Amplification	3	0	42494T: 3.5; 56957T: 4; 42500T: 4.5;
AP5M1	chr14	14q22.3	1	0	0	1	Amplification	1	0	42494T: 4;
AP5S1	chr20	20p13	2	1	1	0	Amplification	2	0	42473T: 4.5; 42487T: 4.5;
AP5Z1	chr7	7p22.1	2	1	0	1	Amplification	2	0	42473T: 4.5; 42497T: 8.5;
APBB1IP	chr10	10p12.1	1	0	0	1	Amplification	1	0	56958T: 5;
APCDD1L	chr20	20q13.32	1	1	0	0	Amplification	1	0	42473T: 4.5;
APEH	chr3	3p21.31	1	1	0	0	Amplification	1	0	42473T: 4;
APH1A	chr1	1q21.2	1	1	0	0	Amplification	1	0	42473T: 5;
APIP	chr11	11p13	2	1	0	1	Amplification	2	0	42493T: 4; 42473T: 4.5;
APLF	chr2	2p13.3	1	0	0	1	Amplification	1	0	42500T: 3.5;
APLP1	chr19	19q13.12	3	0	1	2	Amplification	3	0	42484T: 3.5; 56957T: 4; 42500T: 6.5;
APOA1BP	chr1	1q22	1	1	0	0	Amplification	1	0	42473T: 7;
APOB	chr2	2p24.1	1	0	0	1	Amplification	1	0	42500T: 3.5;
APOBEC3A	chr22	22q13.1	1	1	0	0	Amplification	1	0	42473T: 3.5;
APOBEC3B	chr22	22q13.1	1	1	0	0	Amplification	1	0	42473T: 3.5;
APOBEC3C	chr22	22q13.1	1	1	0	0	Amplification	1	0	42473T: 3.5;
APOBEC3D	chr22	22q13.1	1	1	0	0	Amplification	1	0	42473T: 3.5;

Mangalaparthi *et al.* , 2020. Mutational landscape of esophageal squamous cell carcinoma in an Indian cohort
Supplementary Table 7A. List of copy number alterations and affected genes in ESCC patients

Gene	Chromosome	Cytoband	Recurrence	Recurrence in smoker cohort	Recurrence in chewer cohort	Recurrence in No habit cohort	State	Samples with CNA gain	Samples with CNA loss	File info with CNA fold
APOBEC3F	chr22	22q13.1	1	1	0	0	Amplification	1	0	42473T: 3.5;
APOBEC3G	chr22	22q13.1	1	1	0	0	Amplification	1	0	42473T: 3.5;
APOBEC3H	chr22	22q13.1	1	1	0	0	Amplification	1	0	42473T: 3.5;
APOC1	chr19	19q13.32	2	1	0	1	Amplification	2	0	56957T: 4; 42473T: 3.5;
APOC1P1	chr19	19q13.32	2	1	0	1	Amplification	2	0	42473T: 3.5; 56957T: 4;
APOC2	chr19	19q13.32	2	1	0	1	Amplification	2	0	56957T: 4; 42473T: 3.5;
APOC4	chr19	19q13.32	2	1	0	1	Amplification	2	0	42473T: 3.5; 56957T: 4;
APOD	chr3	3q29	9	1	3	5	Amplification	9	0	56957T: 6; 42474T: 3.5; 42495T: 4; 42498T: 3.5; 42484T: 4; 42482T: 3.5; 42487T: 3.5; 42493T: 3.5; 42492T: 3.5;
APOE	chr19	19q13.32	2	1	0	1	Amplification	2	0	56957T: 4; 42473T: 3.5;
APOL1	chr22	22q12.3	1	1	0	0	Amplification	1	0	42473T: 3.5;
APOL2	chr22	22q12.3	1	1	0	0	Amplification	1	0	42473T: 3.5;
APOL3	chr22	22q12.3	1	1	0	0	Amplification	1	0	42473T: 3.5;
APOL4	chr22	22q12.3	1	1	0	0	Amplification	1	0	42473T: 3.5;
APOL5	chr22	22q12.3	1	1	0	0	Amplification	1	0	42473T: 3.5;
APOL6	chr22	22q12.3	1	1	0	0	Amplification	1	0	42473T: 3.5;
APOLD1	chr12	12p13.1	1	0	0	1	Amplification	1	0	42500T: 4.5;
APOM	chr6	6p21.33	1	1	0	0	Amplification	1	0	42473T: 3.5;
AQP1	chr7	7p14.3	1	1	0	0	Amplification	1	0	42473T: 4;
AQP10	chr1	1q21.3	1	1	0	0	Amplification	1	0	42473T: 4.5;
AQP12A	chr2	2q37.3	1	1	0	0	Amplification	1	0	42473T: 4;
AQP12B	chr2	2q37.3	1	1	0	0	Amplification	1	0	42473T: 4;
AQP2	chr12	12q13.12	1	0	0	1	Amplification	1	0	42500T: 3.5;
AQP5	chr12	12q13.12	1	0	0	1	Amplification	1	0	42500T: 3.5;
AQP6	chr12	12q13.12	1	0	0	1	Amplification	1	0	42500T: 3.5;
AQP8	chr16	16p12.1	1	1	0	0	Amplification	1	0	42473T: 4;
ARAP1	chr11	11q13.4	6	3	0	3	Amplification	6	0	56957T: 3.5; 42492T: 4; 42498T: 5; 42478T: 4.5; 42475T: 8.5; 42476T: 4;
ARC	chr8	8q24.3	3	0	1	2	Amplification	3	0	42495T: 4.5; 42483T: 3.5; 42496T: 4;
AREL1	chr14	14q24.3	2	0	0	2	Amplification	2	0	56957T: 3.5; 42494T: 4;
ARF3	chr12	12q13.12	1	0	0	1	Amplification	1	0	42500T: 3.5;
ARF5	chr7	7q32.1	1	0	1	0	Amplification	1	0	42487T: 3.5;
ARF6	chr14	14q21.3	1	1	0	0	Amplification	1	0	42473T: 3.5;
ARFGAP1	chr20	20q13.33	1	1	0	0	Amplification	1	0	42473T: 6;
ARFGAP2	chr11	11p11.2	1	1	0	0	Amplification	1	0	42473T: 3.5;
ARFGEF1	chr8	8q13.2	3	0	0	3	Amplification	3	0	42497T: 4; 42496T: 3.5; 42495T: 3.5;
ARFGEF2	chr20	20q13.13	1	1	0	0	Amplification	1	0	42473T: 5;
ARFRP1	chr20	20q13.33	1	1	0	0	Amplification	1	0	42473T: 6;
ARG2	chr14	14q24.1	1	0	0	1	Amplification	1	0	42494T: 4;
ARGFX	chr3	3q13.33	1	0	0	1	Amplification	1	0	42496T: 3.5;

Mangalaparthi *et al.* , 2020. Mutational landscape of esophageal squamous cell carcinoma in an Indian cohort
Supplementary Table 7A. List of copy number alterations and affected genes in ESCC patients

Gene	Chromosome	Cytoband	Recurrence	Recurrence in smoker cohort	Recurrence in chewer cohort	Recurrence in No habit cohort	State	Samples with CNA gain	Samples with CNA loss	File info with CNA fold
ARHGAP1	chr11	11p11.2	1	1	0	0	Amplification	1	0	42473T: 3.5;
ARHGAP17	chr16	16p12.1	1	1	0	0	Amplification	1	0	42473T: 4;
ARHGAP23	chr17	17q12	1	1	0	0	Amplification	1	0	42473T: 3.5;
ARHGAP25	chr2	2p13.3	1	0	0	1	Amplification	1	0	42500T: 3.5;
ARHGAP28	chr18	18p11.31	1	0	0	1	Amplification	1	0	42493T: 3.5;
ARHGAP31	chr3	3q13.32-q13	1	0	0	1	Amplification	1	0	42496T: 3.5;
ARHGAP33	chr19	19q13.12	3	0	1	2	Amplification	3	0	42500T: 6.5; 56957T: 4; 42484T: 3.5;
ARHGAP39	chr8	8q24.3	5	0	1	4	Amplification	5	0	42496T: 4; 42483T: 3.5; 56957T: 3.5; 42495T: 4.5; 42494T: 3.5;
ARHGAP40	chr20	20q11.23	1	1	0	0	Amplification	1	0	42473T: 3.5;
ARHGAP5	chr14	14q12	1	0	0	1	Amplification	1	0	42500T: 4.5;
ARHGDIB	chr12	12p12.3	1	0	0	1	Amplification	1	0	42500T: 4.5;
ARHGDIG	chr16	16p13.3	1	0	1	0	Amplification	1	0	42483T: 3.5;
ARHGEF1	chr19	19q13.2	1	1	0	0	Amplification	1	0	42473T: 4.5;
ARHGEF10	chr8	8p23.3	1	0	0	1	Amplification	1	0	56957T: 4;
ARHGEF10L	chr1	1p36.13	1	1	0	0	Amplification	1	0	42473T: 4;
ARHGEF11	chr1	1q23.1	1	1	0	0	Amplification	1	0	42473T: 7;
ARHGEF17	chr11	11q13.4	6	3	0	3	Amplification	6	0	42478T: 4.5; 42492T: 4; 42498T: 6.5; 56957T: 3.5; 42476T: 4; 42475T: 8.5;
ARHGEF19	chr1	1p36.13	1	1	0	0	Amplification	1	0	42473T: 8;
ARHGEF2	chr1	1q22	2	1	0	1	Amplification	2	0	42493T: 4; 42473T: 4.5;
ARHGEF26	chr3	3q25.2	9	1	2	6	Amplification	9	0	42484T: 3.5; 56957T: 4; 42474T: 3.5; 42496T: 4.5; 42497T: 5.5; 42500T: 3.5; 42492T: 3.5; 42487T: 3.5; 42493T: 3.5;
ARHGEF33	chr2	2p22.1	1	0	0	1	Amplification	1	0	42500T: 3.5;
ARHGEF35	chr7	7q35	2	0	2	0	Amplification	2	0	42487T: 3.5; 42484T: 4.5;
ARHGEF39	chr9	9p13.3	3	1	0	2	Amplification	3	0	42501T: 3.5; 42473T: 3.5; 42496T: 4;
ARHGEF5	chr7	7q35	2	0	2	0	Amplification	2	0	42487T: 3.5; 42484T: 4.5;
ARID2	chr12	12q12	1	0	0	1	Amplification	1	0	42500T: 3.5;
ARID3A	chr19	19p13.3	1	0	0	1	Amplification	1	0	42493T: 3.5;
ARID3B	chr15	15q24.1	1	1	0	0	Amplification	1	0	42473T: 3.5;
ARID4A	chr14	14q23.1	1	0	0	1	Amplification	1	0	42494T: 4;
ARID4B	chr1	1q42.3	1	1	0	0	Amplification	1	0	42473T: 6.5;
ARID5A	chr2	2q11.2	2	1	0	1	Amplification	2	0	42473T: 3.5; 42493T: 3.5;
ARL13B	chr3	3q11.1-q11.2	2	0	1	1	Amplification	2	0	42500T: 3.5; 42484T: 3.5;
ARL14	chr3	3q25.33	8	1	2	5	Amplification	8	0	42492T: 3.5; 42487T: 3.5; 42493T: 3.5; 42484T: 4; 42495T: 4; 42474T: 3.5; 56957T: 4; 42497T: 3.5;
ARL4A	chr7	7p21.3	1	1	0	0	Amplification	1	0	42473T: 3.5;
ARL5C	chr17	17q12	1	1	0	0	Amplification	1	0	42473T: 4.5;
ARL6	chr3	3q11.2	2	1	0	1	Amplification	2	0	42476T: 3.5; 42500T: 3.5;
ARL6IP1	chr16	16p12.3	2	1	0	1	Amplification	2	0	42473T: 4; 42495T: 4;

Mangalaparthi *et al.* , 2020. Mutational landscape of esophageal squamous cell carcinoma in an Indian cohort
Supplementary Table 7A. List of copy number alterations and affected genes in ESCC patients

Gene	Chromosome	Cytoband	Recurrence	Recurrence in smoker cohort	Recurrence in chewer cohort	Recurrence in No habit cohort	State	Samples with CNA gain	Samples with CNA loss	File info with CNA fold
ARL6IP4	chr12	12q24.31	1	0	0	1	Amplification	1	0	42500T: 3.5;
ARMC1	chr8	8q13.1	2	0	0	2	Amplification	2	0	42495T: 3.5; 42496T: 3.5;
ARMC10	chr7	7q22.1	3	0	1	2	Amplification	3	0	42501T: 3.5; 42493T: 3.5; 42487T: 3.5;
ARMC10P1	chr3	3q11.2	2	0	1	1	Amplification	2	0	42484T: 3.5; 42500T: 3.5;
ARMC2	chr6	6q21	1	0	0	1	Amplification	1	0	42496T: 3.5;
ARMC8	chr3	3q22.3	4	0	1	3	Amplification	4	0	42496T: 3.5; 42492T: 3.5; 42493T: 3.5; 42487T: 3.5;
ARNT	chr1	1q41	1	1	0	0	Amplification	1	0	42473T: 4.5;
ARNTL2	chr12	12p11.23	1	0	0	1	Amplification	1	0	42500T: 6;
ARPC3	chr12	12q24.11	1	0	0	1	Amplification	1	0	42500T: 3.5;
ARRB1	chr11	11q13.4	2	0	0	2	Amplification	2	0	42498T: 8; 42492T: 4;
ARRDC1	chr9	9q34.3	2	1	0	1	Amplification	2	0	56957T: 3.5; 42473T: 5;
ART4	chr12	12p12.3	1	0	0	1	Amplification	1	0	42500T: 4.5;
ARVCF	chr22	22q11.21	1	0	0	1	Amplification	1	0	42497T: 17;
ASAP1	chr8	8q24.21-q24	3	0	1	2	Amplification	3	0	42484T: 3.5; 42495T: 3.5; 42496T: 3.5;
ASAP2	chr2	2p25.1|2p24	1	0	0	1	Amplification	1	0	42500T: 3.5;
ASAP3	chr1	1p36.12	1	1	0	0	Amplification	1	0	42473T: 3.5;
ASB15	chr7	7q31.32	2	0	1	1	Amplification	2	0	42493T: 3.5; 42487T: 3.5;
ASB3	chr2	2p16.2	2	0	1	1	Amplification	2	0	42500T: 3.5; 42484T: 4.5;
ASB4	chr7	7q21.3	1	0	1	0	Amplification	1	0	42487T: 3.5;
ASB8	chr12	12q13.11	1	0	0	1	Amplification	1	0	42500T: 3.5;
ASCC2	chr22	22q12.2	1	1	0	0	Amplification	1	0	42473T: 3.5;
ASH1L	chr1	1q22	1	1	0	0	Amplification	1	0	42473T: 6.5;
ASH2L	chr8	8p11.23	2	0	1	1	Amplification	2	0	42482T: 3.5; 42493T: 3.5;
ASIC1	chr12	12q13.12	1	0	0	1	Amplification	1	0	42500T: 3.5;
ASIP	chr20	10p11.22-p1	3	1	0	2	Amplification	3	0	42493T: 3.5; 42473T: 3.5; 42496T: 5;
ASL	chr7	7q11.21	1	0	0	1	Amplification	1	0	42501T: 3.5;
ASNSD1	chr2	2q32.2	2	0	1	1	Amplification	2	0	42493T: 3.5; 42482T: 4;
ASPH	chr8	8q12.3	2	0	0	2	Amplification	2	0	42496T: 3.5; 42495T: 3.5;
ASPRV1	chr2	2p13.3	1	0	0	1	Amplification	1	0	42500T: 3.5;
ASPSCR1	chr17	17q25.3	1	1	0	0	Amplification	1	0	42473T: 3.5;
ASTE1	chr3	3q22.1	3	0	1	2	Amplification	3	0	42496T: 3.5; 42487T: 3.5; 42492T: 3.5;
ASTL	chr2	2q11.2	2	1	0	1	Amplification	2	0	42493T: 3.5; 42473T: 3.5;
ASUN	chr12	12p11.23	1	0	0	1	Amplification	1	0	42500T: 6;
ASXL1	chr20	20q11.21	2	1	0	1	Amplification	2	0	42473T: 4; 42496T: 5;
ASXL2	chr2	2p23.3	1	0	0	1	Amplification	1	0	42500T: 3.5;
ASZ1	chr7	7q31.2	1	0	1	0	Amplification	1	0	42487T: 3.5;
ATAD2	chr8	8q24.13	4	1	1	2	Amplification	4	0	42495T: 3.5; 42484T: 3.5; 42475T: 3.5; 42496T: 3.5;
ATAD2B	chr2	2p24.1-p23.3	1	0	0	1	Amplification	1	0	42500T: 3.5;
ATAD5	chr17	17q11.2	1	1	0	0	Amplification	1	0	42473T: 3.5;
ATF1	chr12	5p13.2	2	0	0	2	Amplification	2	0	42500T: 3.5; 42494T: 3.5;
ATF2	chr2	5p13.2	1	0	0	1	Amplification	1	0	42493T: 3.5;

Mangalaparthi *et al.* , 2020. Mutational landscape of esophageal squamous cell carcinoma in an Indian cohort
Supplementary Table 7A. List of copy number alterations and affected genes in ESCC patients

Gene	Chromosome	Cytoband	Recurrence	Recurrence in smoker cohort	Recurrence in chewer cohort	Recurrence in No habit cohort	State	Samples with CNA gain	Samples with CNA loss	File info with CNA fold
ATF4	chr22	22q13.1	1	1	0	0	Amplification	1	0	42473T: 3.5;
ATF6B	chr6	6p21.32	1	1	0	0	Amplification	1	0	42473T: 3.5;
ATF7IP2	chr16	16p13.2-p13	2	1	0	1	Amplification	2	0	42473T: 5.5; 42495T: 4;
ATG13	chr11	11p11.2	1	1	0	0	Amplification	1	0	42473T: 3.5;
ATG14	chr14	14q22.3	1	0	0	1	Amplification	1	0	42494T: 4;
ATG16L1	chr2	2q37.1	1	1	0	0	Amplification	1	0	42473T: 4.5;
ATG16L2	chr11	11q13.4	5	3	0	2	Amplification	5	0	42476T: 4; 42475T: 8.5; 42492T: 4; 42478T: 4.5; 56957T: 3.5;
ATG3	chr3	3q13.2	1	0	0	1	Amplification	1	0	42496T: 3.5;
ATG5	chr6	6q21	1	0	0	1	Amplification	1	0	42496T: 3.5;
ATL1	chr14	14q32.2	2	0	0	2	Amplification	2	0	42494T: 4; 42496T: 3.5;
ATL2	chr2	2p22.2-p22.1	2	0	0	2	Amplification	2	0	42493T: 3.5; 42500T: 3.5;
ATN1	chr12	12p13.31	1	0	0	1	Amplification	1	0	42494T: 3.5;
ATOH8	chr2	2p11.2	1	0	0	1	Amplification	1	0	42500T: 3.5;
ATP11A	chr13	13q34	1	0	0	1	Amplification	1	0	56957T: 3.5;
ATP11B	chr3	3q26.33	10	1	3	6	Amplification	10	0	42500T: 24; 42498T: 3.5; 42484T: 4; 56957T: 4; 42495T: 4; 42474T: 3.5; 42482T: 3.5; 42492T: 3.5; 42487T: 3.5; 42493T: 3.5;
ATP13A3	chr3	3q29	9	1	3	5	Amplification	9	0	56957T: 4.5; 42495T: 4; 42474T: 3.5; 42498T: 3.5; 42484T: 4; 42493T: 3.5; 42487T: 3.5; 42492T: 3.5; 42482T: 3.5;
ATP13A4	chr3	3q29	10	1	3	6	Amplification	10	0	42482T: 3.5; 42492T: 3.5; 42487T: 3.5; 42493T: 3.5; 42501T: 3.5; 42484T: 4; 42498T: 3.5; 42474T: 3.5; 42495T: 4; 56957T: 4.5;
ATP13A5	chr3	3q29	10	1	3	6	Amplification	10	0	56957T: 4.5; 42495T: 4; 42474T: 3.5; 42498T: 3.5; 42484T: 4; 42501T: 3.5; 42487T: 3.5; 42493T: 3.5; 42492T: 3.5; 42482T: 3.5;
ATP1B3	chr3	3q23	4	0	1	3	Amplification	4	0	42496T: 4; 42492T: 3.5; 42487T: 3.5; 42493T: 3.5;
ATP2A2	chr12	12q24.11	2	1	0	1	Amplification	2	0	42500T: 3.5; 42473T: 4.5;
ATP2C1	chr3	3q22.1	3	0	1	2	Amplification	3	0	42496T: 3.5; 42492T: 3.5; 42487T: 3.5;
ATP4A	chr19	19q13.12	3	0	1	2	Amplification	3	0	42500T: 6.5; 42484T: 3.5; 56957T: 4;
ATP4B	chr13	13q34	1	0	0	1	Amplification	1	0	56957T: 3.5;
ATP5E	chr20	20q13.32	1	1	0	0	Amplification	1	0	42473T: 4.5;
ATP5G3	chr2	2q31.1	1	0	0	1	Amplification	1	0	42493T: 3.5;
ATP5S	chr14	14q21.3	1	0	0	1	Amplification	1	0	42496T: 3.5;
ATP5SL	chr19	19q13.2	1	1	0	0	Amplification	1	0	42473T: 4.5;
ATP6V0A2	chr12	12q24.31	1	0	0	1	Amplification	1	0	42500T: 3.5;
ATP6V0A4	chr7	7q34	1	0	1	0	Amplification	1	0	42487T: 3.5;
ATP6V0D2	chr8	8q21.3	2	0	0	2	Amplification	2	0	42496T: 3.5; 42495T: 3.5;
ATP6V1A	chr3	3q13.31	1	0	0	1	Amplification	1	0	42496T: 3.5;
ATP6V1B1	chr2	2p13.3	1	0	0	1	Amplification	1	0	42500T: 3.5;

Mangalaparthi *et al.* , 2020. Mutational landscape of esophageal squamous cell carcinoma in an Indian cohort
Supplementary Table 7A. List of copy number alterations and affected genes in ESCC patients

Gene	Chromosome	Cytoband	Recurrence	Recurrence in smoker cohort	Recurrence in chewer cohort	Recurrence in No habit cohort	State	Samples with CNA gain	Samples with CNA loss	File info with CNA fold
ATP6V1C1	chr8	8q22.3	2	0	0	2	Amplification	2	0	42496T: 3.5; 42495T: 3.5;
ATP6V1D	chr14	14q23.3	1	0	0	1	Amplification	1	0	42494T: 4;
ATP6V1E2	chr2	2p21|2p16-p	2	0	1	1	Amplification	2	0	42500T: 3.5; 42484T: 4.5;
ATP6V1F	chr7	7q32.1	1	0	1	0	Amplification	1	0	42487T: 3.5;
ATP6V1G1	chr9	9q32	1	0	0	1	Amplification	1	0	42493T: 3.5;
ATP6V1G2	chr6	6p21.33	1	1	0	0	Amplification	1	0	42473T: 3.5;
ATP6V1H	chr8	8q11.23	3	0	0	3	Amplification	3	0	42496T: 3.5; 42494T: 4; 42495T: 3.5;
ATP8B2	chr1	1q21.3	1	1	0	0	Amplification	1	0	42473T: 4.5;
ATP9A	chr20	20q13.2	1	1	0	0	Amplification	1	0	42473T: 5;
ATPIF1	chr1	1p35.3	1	1	0	0	Amplification	1	0	42473T: 3.5;
ATR	chr3	14q32.13	5	0	1	4	Amplification	5	0	56957T: 4; 42487T: 3.5; 42493T: 3.5; 42492T: 3.5; 42496T: 4;
ATRAID	chr2	2p23.3	1	0	0	1	Amplification	1	0	42500T: 3.5;
ATRN	chr20	20p13	1	1	0	0	Amplification	2	0	42473T: 3.5,6;
ATXN2	chr12	12q24.12	1	0	0	1	Amplification	1	0	42500T: 3.5;
ATXN7L1	chr7	7q22.3	4	0	1	3	Amplification	4	0	42497T: 4; 42501T: 3.5; 42493T: 3.5; 42487T: 3.5;
AUP1	chr2	2p13.1	1	0	0	1	Amplification	1	0	42500T: 3.5;
AURKA	chr20	20q13.2	1	1	0	0	Amplification	1	0	42473T: 5;
AVL9	chr7	7p14.3	1	1	0	0	Amplification	1	0	42473T: 4;
AVP	chr20	1q44	1	1	0	0	Amplification	1	0	42473T: 3.5;
AVPR1B	chr1	1q32.1	1	0	0	1	Amplification	1	0	42493T: 3.5;
AXIN1	chr16	16p13.3	1	0	1	0	Amplification	1	0	42483T: 3.5;
AXL	chr19	19q13.2	1	1	0	0	Amplification	1	0	42473T: 4.5;
AZIN1	chr8	8q22.3	2	0	0	2	Amplification	2	0	42496T: 3.5; 42495T: 3.5;
AZU1	chr19	19p13.3	1	0	0	1	Amplification	1	0	42493T: 3.5;
B3GALNT1	chr3	3q26.1	8	1	2	5	Amplification	8	0	42497T: 3.5; 56957T: 4; 42474T: 3.5; 42495T: 4; 42484T: 4; 42487T: 3.5; 42493T: 3.5; 42492T: 3.5;
B3GALT5	chr21	21q22.2	1	1	0	0	Amplification	1	0	42473T: 3.5;
B3GALTL	chr13	13q12.3	1	0	0	1	Amplification	1	0	42497T: 3.5;
B3GAT1	chr11	11q25	1	1	0	0	Deletion	0	1	42476T: 0.5;
B3GNT2	chr2	2p15	3	0	1	2	Amplification	3	0	42500T: 3.5; 56957T: 3.5; 42484T: 8.5;
B3GNT4	chr12	12q24.31	1	0	0	1	Amplification	1	0	42500T: 3.5;
B3GNT5	chr3	3q27.1	9	1	3	5	Amplification	9	0	56957T: 5; 42474T: 3.5; 42495T: 4; 42498T: 3.5; 42484T: 4; 42487T: 3.5; 42493T: 3.5; 42492T: 3.5; 42482T: 3.5;
B3GNT6	chr11	11q13.5	2	0	0	2	Amplification	2	0	42496T: 3.5; 42497T: 3.5;
B4GALNT3	chr12	12p13.33	1	0	0	1	Amplification	1	0	42500T: 3.5;
B4GALT4	chr3	3q13.32	1	0	0	1	Amplification	1	0	42496T: 3.5;
B4GALT5	chr20	20q13.13	1	1	0	0	Amplification	1	0	42473T: 5;
B9D2	chr19	19q13.2	1	1	0	0	Amplification	1	0	42473T: 4.5;
BAALC	chr8	8q22.3	2	0	0	2	Amplification	2	0	42495T: 3.5; 42496T: 3.5;

Mangalaparthi *et al.* , 2020. Mutational landscape of esophageal squamous cell carcinoma in an Indian cohort
Supplementary Table 7A. List of copy number alterations and affected genes in ESCC patients

Gene	Chromosome	Cytoband	Recurrence	Recurrence in smoker cohort	Recurrence in chewer cohort	Recurrence in No habit cohort	State	Samples with CNA gain	Samples with CNA loss	File info with CNA fold
BACE2	chr21	21q22.2-q22	1	1	0	0	Amplification	1	0	42473T: 3.5;
BAG2	chr6	6p12.1	1	0	0	1	Amplification	1	0	42497T: 3.5;
BAG4	chr8	8p11.23	2	0	1	1	Amplification	2	0	42482T: 3.5; 42493T: 3.5;
BAG6	chr6	6p21.33	1	1	0	0	Amplification	1	0	42473T: 3.5;
BAI1	chr8	8q24.3	3	0	1	2	Amplification	3	0	42496T: 4; 42483T: 3.5; 42495T: 4.5;
BAI2	chr1	1p35.2	1	1	0	0	Amplification	1	0	42473T: 3.5;
BAIAP2L2	chr22	22q13.1	1	1	0	0	Amplification	1	0	42473T: 3.5;
BAIAP3	chr16	16p13.3	1	0	1	0	Amplification	1	0	42483T: 3.5;
BASP1	chr5	5p15.1	4	1	1	2	Amplification	4	0	42493T: 4; 42475T: 3.5; 42486T: 3.5; 42496T: 3.5;
BATF	chr14	14q24.3	2	0	0	2	Amplification	2	0	56957T: 3.5; 42494T: 4;
BAZ1A	chr14	14q13.1-q13	2	1	0	1	Amplification	2	0	42500T: 4.5; 42476T: 5;
BBS1	chr11	11q13.2	1	0	0	1	Amplification	1	0	56957T: 5.5;
BBS9	chr7	7p14.3	1	1	0	0	Amplification	1	0	42473T: 4;
BCAM	chr19	19q13.32	2	1	0	1	Amplification	2	0	56957T: 4; 42473T: 3.5;
BCAN	chr1	1q23.1	1	1	0	0	Amplification	1	0	42473T: 7;
BCAP29	chr7	7q22.3	4	0	1	3	Amplification	4	0	42493T: 3.5; 42487T: 3.5; 42501T: 3.5; 42497T: 4;
BCAR4	chr16	16p13.13	2	1	0	1	Amplification	2	0	42473T: 12.5; 42495T: 4;
BCAS1	chr20	20q13.2	1	1	0	0	Amplification	1	0	42473T: 5;
BCAS4	chr20	20q13.13	1	1	0	0	Amplification	1	0	42473T: 5;
BCAT1	chr12	12p12.1	1	0	0	1	Amplification	1	0	42500T: 6;
BCDIN3D	chr12	12q13.12	1	0	0	1	Amplification	1	0	42500T: 3.5;
BCHE	chr3	3q26.1	9	1	3	5	Amplification	9	0	42500T: 4; 56957T: 4; 42474T: 3.5; 42495T: 4; 42484T: 4; 42482T: 3.5; 42493T: 3.5; 42487T: 3.5; 42492T: 3.5;
BCKDHA	chr19	19q13.2	1	1	0	0	Amplification	1	0	42473T: 4.5;
BCL11A	chr2	2p16.1	3	0	1	2	Amplification	3	0	42500T: 3.5; 42484T: 4.5; 56957T: 3.5;
BCL2L1	chr20	20q11.21	1	0	0	1	Amplification	1	0	42496T: 5;
BCL2L14	chr12	12p13.2	1	0	0	1	Amplification	1	0	42500T: 4.5;
BCL2L2	chr14	14q11.2	2	0	0	2	Amplification	2	0	42496T: 4; 42500T: 4;
BCL3	chr19	19q13.32	2	1	0	1	Amplification	2	0	42473T: 3.5; 56957T: 4;
BCL6	chr3	3q27.3	10	1	3	6	Amplification	10	0	42482T: 3.5; 42492T: 3.5; 42493T: 3.5; 42487T: 3.5; 42497T: 4; 42498T: 3.5; 42484T: 4; 56957T: 4.5; 42474T: 3.5; 42495T: 4;
BCL7A	chr12	12q24.31	1	0	0	1	Amplification	1	0	42500T: 3.5;
BCL9	chr1	1q21.2	1	1	0	0	Amplification	1	0	42473T: 4;
BCL9L	chr11	11q23.3	1	1	0	0	Amplification	1	0	42473T: 4;
BDH1	chr3	3q29	9	1	3	5	Amplification	9	0	42498T: 3.5; 42484T: 4; 56957T: 6; 42495T: 4; 42474T: 3.5; 42492T: 3.5; 42487T: 3.5; 42493T: 5; 42482T: 3.5;
BEND3	chr6	6q21	1	0	0	1	Amplification	1	0	42496T: 3.5;
BEND6	chr6	6p12.1	1	0	0	1	Amplification	1	0	42497T: 3.5;

Mangalaparthi *et al.* , 2020. Mutational landscape of esophageal squamous cell carcinoma in an Indian cohort
Supplementary Table 7A. List of copy number alterations and affected genes in ESCC patients

Gene	Chromosome	Cytoband	Recurrence	Recurrence in smoker cohort	Recurrence in chewer cohort	Recurrence in No habit cohort	State	Samples with CNA gain	Samples with CNA loss	File info with CNA fold
BEST3	chr12	12q15	2	0	0	2	Amplification	2	0	42500T: 5; 42501T: 5.5;
BET1	chr7	7q21.3	2	0	2	0	Amplification	2	0	42483T: 4; 42487T: 3.5;
BFAR	chr16	16p13.12	2	1	0	1	Amplification	2	0	42473T: 4; 42495T: 4;
BFSP2	chr3	3q22.1	3	0	1	2	Amplification	3	0	42487T: 3.5; 42492T: 3.5; 42496T: 3.5;
BGLAP	chr1	1q22	1	1	0	0	Amplification	1	0	42473T: 4.5;
BHLHE22	chr8	8q12.3	2	0	0	2	Amplification	2	0	42495T: 3.5; 42496T: 3.5;
BHLHE23	chr20	20q13.33	1	1	0	0	Amplification	1	0	42473T: 6;
BHLHE41	chr12	12p12.1	1	0	0	1	Amplification	1	0	42500T: 6;
BICD1	chr12	12p11.21	1	0	0	1	Amplification	1	0	42500T: 3.5;
BIN2	chr12	12q13.13	2	0	0	2	Amplification	2	0	42500T: 3.5; 42494T: 5.5;
BIRC2	chr11	11q22.2	1	0	0	1	Amplification	1	0	56958T: 5.5;
BIRC3	chr11	11q22.2	1	0	0	1	Amplification	1	0	56958T: 5.5;
BIRC6	chr2	2p22.3	1	0	0	1	Amplification	1	0	42500T: 3.5;
BIRC7	chr20	20q13.33	1	1	0	0	Amplification	1	0	42473T: 6;
BLCAP	chr20	20q11.23	1	1	0	0	Amplification	1	0	42473T: 3.5;
BLK	chr8	8p23.1	1	0	1	0	Amplification	1	0	42486T: 3.5;
BLOC1S1	chr12	12q13.2	1	0	0	1	Amplification	1	0	42494T: 3.5;
BLVRA	chr7	7p13	1	1	0	0	Amplification	1	0	42473T: 3.5;
BLVRB	chr19	19q13.2	2	0	0	2	Amplification	2	0	56957T: 4; 42500T: 3.5;
BMP10	chr2	2p13.3	1	0	0	1	Amplification	1	0	42500T: 3.5;
BMP3	chr4	4q21.21	1	1	0	0	Amplification	1	0	42473T: 4;
BMP4	chr14	14q22.2	1	0	0	1	Amplification	1	0	42494T: 4;
BMP7	chr20	20q13.31	1	1	0	0	Amplification	1	0	42473T: 7;
BMPER	chr7	7p14.3	1	1	0	0	Amplification	1	0	42473T: 4;
BNIP3P1	chr14	14q12	1	0	0	1	Amplification	1	0	42500T: 4.5;
BNIPL	chr1	1q21.3	1	1	0	0	Amplification	1	0	42473T: 4.5;
BOC	chr3	3q13.2	1	0	0	1	Amplification	1	0	42496T: 3.5;
BOLA1	chr1	1q21.2	1	1	0	0	Amplification	1	0	42473T: 5;
BOLA3	chr2	2p13.1	1	0	0	1	Amplification	1	0	42500T: 3.5;
BOLL	chr2	2q33.1	1	0	1	0	Amplification	1	0	42482T: 4;
BOP1	chr8	8q24.3	3	0	1	2	Amplification	3	0	42495T: 4.5; 42483T: 3.5; 42496T: 4;
BPGM	chr7	7q33	1	0	1	0	Amplification	1	0	42487T: 3.5;
BPI	chr20	20q11.23	1	1	0	0	Amplification	1	0	42473T: 3.5;
BPIFA1	chr20	20q11.21	2	1	0	1	Amplification	2	0	42496T: 5; 42473T: 3.5;
BPIFA2	chr20	20q11.21	2	1	0	1	Amplification	2	0	42496T: 5; 42473T: 3.5;
BPIFA3	chr20	20q11.21	2	1	0	1	Amplification	2	0	42473T: 3.5; 42496T: 5;
BPIFB1	chr20	20q11.21	2	1	0	1	Amplification	2	0	42473T: 3.5; 42496T: 5;
BPIFB2	chr20	20q11.21	2	1	0	1	Amplification	2	0	42473T: 3.5; 42496T: 5;
BPIFB3	chr20	20q11.21	2	1	0	1	Amplification	2	0	42496T: 5; 42473T: 3.5;
BPIFB4	chr20	20q11.21	2	1	0	1	Amplification	2	0	42473T: 3.5; 42496T: 5;
BPIFB6	chr20	20q11.21	2	1	0	1	Amplification	2	0	42496T: 5; 42473T: 3.5;

Mangalaparthi *et al.* , 2020. Mutational landscape of esophageal squamous cell carcinoma in an Indian cohort
Supplementary Table 7A. List of copy number alterations and affected genes in ESCC patients

Gene	Chromosome	Cytoband	Recurrence	Recurrence in smoker cohort	Recurrence in chewer cohort	Recurrence in No habit cohort	State	Samples with CNA gain	Samples with CNA loss	File info with CNA fold
BRAF	chr7	7q34	1	0	1	0	Amplification	1	0	42487T: 3.5;
BRAP	chr12	6p21.2	1	0	0	1	Amplification	1	0	42500T: 3.5;
BRAT1	chr7	7p22.3	1	1	0	0	Amplification	1	0	42473T: 5;
BRCA2	chr13	13q13.1	1	0	0	1	Amplification	1	0	42497T: 6;
BRD9	chr5	5p15.33	5	2	1	2	Amplification	5	0	42475T: 3.5; 42473T: 3.5; 42496T: 4; 42486T: 3.5; 42493T: 3.5;
BRE	chr2	2p23.2	1	0	0	1	Amplification	1	0	42500T: 3.5;
BRF2	chr8	8p11.23	1	0	1	0	Amplification	1	0	42482T: 3.5;
BRI3BP	chr12	12q24.31	1	0	0	1	Amplification	1	0	42500T: 3.5;
BRICD5	chr16	16p13.3	1	0	1	0	Amplification	1	0	42483T: 3.5;
BRIX1	chr5	5p13.2	4	1	1	2	Amplification	4	0	42493T: 3.5; 42486T: 3.5; 42496T: 3.5; 42475T: 3.5;
BRMS1L	chr14	14q13.2	2	1	0	1	Amplification	2	0	42500T: 8.5; 42476T: 8;
BRSK2	chr11	11p15.5	1	1	0	0	Amplification	1	0	42473T: 4;
BRWD1	chr21	21q22.2	1	1	0	0	Amplification	1	0	42473T: 3.5;
BSN	chr3	3p21.31	1	1	0	0	Amplification	1	0	42473T: 4;
BTBD18	chr11	11q12.1	1	0	0	1	Amplification	1	0	42496T: 4.5;
BTLA	chr3	3q13.2	1	0	0	1	Amplification	1	0	42496T: 3.5;
BTNL2	chr6	6p21.32	1	1	0	0	Amplification	1	0	42473T: 3.5;
BTNL3	chr5	5q35.3	2	1	0	1	Amplification/Deletio	1	1	42474T: 0.5; 42495T: 3.5;
BTNL8	chr5	5q35.3	2	1	0	1	Amplification/Deletio	1	1	42495T: 3.5; 42474T: 0.5;
BTNL9	chr5	5q35.3	2	1	0	1	Amplification/Deletio	1	1	42495T: 3.5; 42474T: 0.5;
BZRAP1	chr17	17q22	1	0	0	1	Amplification	1	0	42497T: 4;
BZW2	chr7	7p21.1	1	1	0	0	Amplification	1	0	42473T: 7.5;
C11orf24	chr11	11q13.2	1	0	0	1	Amplification	1	0	56957T: 5;
C11orf30	chr11	11q13.5	2	0	0	2	Amplification	2	0	42497T: 3.5; 42496T: 3.5;
C11orf31	chr11	11q12.1	1	0	0	1	Amplification	1	0	42496T: 4.5;
C11orf49	chr11	11p11.2	1	1	0	0	Amplification	1	0	42473T: 3.5;
C11orf70	chr11	11q22.1	1	0	0	1	Amplification	1	0	56958T: 5.5;
C11orf72	chr11	11q13.2	2	1	0	1	Amplification	2	0	42473T: 3.5; 56957T: 5;
C11orf80	chr11	11q13.2	2	1	0	1	Amplification	2	0	56957T: 5.5; 42473T: 3.5;
C11orf86	chr11	11q13.2	2	1	0	1	Amplification	2	0	56957T: 5.5; 42473T: 3.5;
C11orf94	chr11	11p11.2	1	1	0	0	Amplification	1	0	42473T: 3.5;
C12orf36	chr12	12p13.1	1	0	0	1	Amplification	1	0	42500T: 4.5;
C12orf39	chr12	12p12.1	1	0	0	1	Amplification	1	0	42500T: 6;
C12orf4	chr12	12p13.32	1	0	0	1	Amplification	1	0	42494T: 3.5;
C12orf40	chr12	12q12	1	0	0	1	Amplification	1	0	42500T: 3.5;
C12orf43	chr12	12q24.31	1	0	0	1	Amplification	1	0	42500T: 3.5;
C12orf49	chr12	12q24.22	1	0	0	1	Amplification	1	0	42500T: 3.5;
C12orf5	chr12	12p13.32	1	0	0	1	Amplification	1	0	42494T: 3.5;
C12orf52	chr12	12q24.13	1	0	0	1	Amplification	1	0	42500T: 3.5;
C12orf54	chr12	12q13.11	1	0	0	1	Amplification	1	0	42500T: 3.5;

Mangalaparthi *et al.* , 2020. Mutational landscape of esophageal squamous cell carcinoma in an Indian cohort
Supplementary Table 7A. List of copy number alterations and affected genes in ESCC patients

Gene	Chromosome	Cytoband	Recurrence	Recurrence in smoker cohort	Recurrence in chewer cohort	Recurrence in No habit cohort	State	Samples with CNA gain	Samples with CNA loss	File info with CNA fold
C12orf57	chr12	12p13.31	1	0	0	1	Amplification	1	0	42494T: 3.5;
C12orf60	chr12	12p13.1-p12	1	0	0	1	Amplification	1	0	42500T: 4.5;
C12orf65	chr12	12q24.31	1	0	0	1	Amplification	1	0	42500T: 3.5;
C12orf68	chr12	12q13.11	1	0	0	1	Amplification	1	0	42500T: 3.5;
C12orf71	chr12	12p11.23	1	0	0	1	Amplification	1	0	42500T: 6;
C12orf76	chr12	12q24.11	2	1	0	1	Amplification	2	0	42500T: 3.5; 42473T: 4.5;
C12orf77	chr12	12p12.1	1	0	0	1	Amplification	1	0	42500T: 6;
C14orf1	chr14	14q24.3	2	0	0	2	Amplification	2	0	42494T: 4; 56957T: 3.5;
C14orf105	chr14	14q22.3	1	0	0	1	Amplification	1	0	42494T: 4;
C14orf119	chr14	14q11.2	2	0	0	2	Amplification	2	0	42500T: 4; 42496T: 4;
C14orf164	chr14	14q11.2	2	0	0	2	Amplification	2	0	42500T: 4; 42496T: 4;
C14orf166	chr14	14q22.1	1	0	0	1	Amplification	1	0	42494T: 4;
C14orf166B	chr14	14q24.3	2	0	0	2	Amplification	2	0	42494T: 4; 56957T: 3.5;
C14orf169	chr14	14q24.3	2	0	0	2	Amplification	2	0	56957T: 3.5; 42494T: 4;
C14orf178	chr14	14q24.3	1	0	0	1	Amplification	1	0	42494T: 4;
C14orf183	chr14	14q21.3	1	0	0	1	Amplification	1	0	42496T: 3.5;
C14orf23	chr14	14q12	1	0	0	1	Amplification	1	0	42500T: 4.5;
C14orf28	chr14	14q21.2	1	0	0	1	Amplification	1	0	42500T: 5.5;
C14orf37	chr14	14q23.1	1	0	0	1	Amplification	1	0	42494T: 4;
C14orf39	chr14	14q23.1	2	0	1	1	Amplification	2	0	42483T: 3.5; 42494T: 4;
C14orf93	chr14	14q11.2	1	0	0	1	Amplification	1	0	42496T: 4;
C15orf38	chr15	15q26.1	1	1	0	0	Amplification	1	0	42473T: 3.5;
C15orf52	chr15	15q15.1	1	1	0	0	Amplification	1	0	42473T: 3.5;
C15orf59	chr15	15q24.1	1	1	0	0	Amplification	1	0	42473T: 3.5;
C15orf62	chr15	15q15.1	1	0	0	1	Amplification	1	0	42493T: 3.5;
C16orf11	chr16	16p13.3	2	0	1	1	Amplification	2	0	42493T: 3.5; 42483T: 3.5;
C16orf13	chr16	16p13.3	2	0	1	1	Amplification	2	0	42483T: 3.5; 42493T: 3.5;
C16orf45	chr16	16p13.11	2	1	0	1	Amplification	2	0	42495T: 4; 42473T: 4;
C16orf52	chr16	16p12.2	1	1	0	0	Amplification	1	0	42473T: 4;
C16orf59	chr16	16p13.3	1	0	1	0	Amplification	1	0	42483T: 3.5;
C16orf62	chr16	16p12.3	1	1	0	0	Amplification	1	0	42473T: 4;
C16orf71	chr16	16p13.3	3	1	0	2	Amplification	3	0	42473T: 5.5; 42494T: 3.5; 42495T: 12;
C16orf72	chr16	16p13.2	2	1	0	1	Amplification	2	0	42473T: 5.5; 42495T: 4;
C16orf89	chr16	16p13.3	2	1	0	1	Amplification	2	0	42495T: 12; 42473T: 5.5;
C16orf90	chr16	16p13.3	1	1	0	0	Amplification	1	0	42473T: 5.5;
C16orf91	chr16	16p13.3	1	0	1	0	Amplification	1	0	42483T: 3.5;
C16orf96	chr16	16p13.3	3	1	0	2	Amplification	3	0	42473T: 5.5; 42495T: 12; 42494T: 3.5;
C17orf47	chr17	17q22	1	0	0	1	Amplification	1	0	42497T: 4;
C17orf67	chr17	17q22	1	0	0	1	Amplification	1	0	42497T: 4;
C17orf96	chr17	17q12	1	1	0	0	Amplification	1	0	42473T: 3.5;
C17orf98	chr17	17q12	1	1	0	0	Amplification	1	0	42473T: 3.5;

Supplementary Table 7A. List of copy number alterations and affected genes in ESCC patients

Gene	Chromosome	Cytoband	Recurrence	Recurrence in smoker cohort	Recurrence in chewer cohort	Recurrence in No habit cohort	State	Samples with CNA gain	Samples with CNA loss	File info with CNA fold
C18orf42	chr18	18p11.31	1	0	0	1	Amplification	1	0	56957T: 8;
C18orf56	chr18	18p11.32	3	0	0	3	Amplification	3	0	42493T: 3.5; 56957T: 8; 42500T: 4.5;
C18orf8	chr18	18q11.2	1	1	0	0	Amplification	1	0	42481T: 4;
C19orf12	chr19	19q12	5	1	1	3	Amplification	5	0	42473T: 25; 42500T: 4.5; 42484T: 6; 42494T: 4; 56957T: 4;
C19orf33	chr19	19q13.2	4	1	1	2	Amplification	4	0	42500T: 6.5; 42474T: 4; 56957T: 4; 42484T: 3.5;
C19orf40	chr19	19q13.11	4	1	1	2	Amplification	4	0	56957T: 4; 42484T: 3.5; 42500T: 4.5; 42473T: 7;
C19orf47	chr19	19q13.2	2	0	0	2	Amplification	2	0	42500T: 7.5; 56957T: 4;
C19orf54	chr19	19q13.2	2	0	0	2	Amplification	2	0	42500T: 3.5; 56957T: 4;
C19orf55	chr19	19q13.12	3	0	1	2	Amplification	3	0	42500T: 6.5; 56957T: 4; 42484T: 3.5;
C19orf69	chr19	19q13.2	1	1	0	0	Amplification	1	0	42473T: 4.5;
C1D	chr2	2p14	1	0	0	1	Amplification	1	0	42500T: 3.5;
C1GALT1	chr7	7p22.1-p21.3	2	1	0	1	Amplification	2	0	42473T: 3.5; 42497T: 7.5;
C1orf134	chr1	1p36.13	1	1	0	0	Amplification	1	0	42473T: 8;
C1orf138	chr1	1q21.2	2	1	0	1	Amplification	2	0	42473T: 5; 42493T: 3.5;
C1orf186	chr1	1q32.1	1	0	0	1	Amplification	1	0	42493T: 3.5;
C1orf189	chr1	1q21.3	1	1	0	0	Amplification	1	0	42473T: 4.5;
C1orf210	chr1	1p34.2	1	0	0	1	Amplification	1	0	42493T: 3.5;
C1orf213	chr1	1p36.12	1	1	0	0	Amplification	1	0	42473T: 3.5;
C1orf234	chr1	1p36.12	1	1	0	0	Amplification	1	0	42473T: 3.5;
C1orf43	chr1	1q21.3	1	1	0	0	Amplification	1	0	42473T: 4.5;
C1orf50	chr1	1p34.2	2	0	0	2	Amplification	2	0	42496T: 5.5; 42493T: 3.5;
C1orf51	chr1	1q21.2	1	1	0	0	Amplification	1	0	42473T: 5;
C1orf54	chr1	1q21.2	1	1	0	0	Amplification	1	0	42473T: 5;
C1orf56	chr1	1q21.3	1	1	0	0	Amplification	1	0	42473T: 4.5;
C1orf61	chr1	1q22	1	1	0	0	Amplification	1	0	42473T: 4.5;
C1orf63	chr1	1p36.11	1	1	0	0	Amplification	1	0	42473T: 4.5;
C1orf64	chr1	1p36.13	1	1	0	0	Amplification	1	0	42473T: 8;
C1orf85	chr1	1q22	1	1	0	0	Amplification	1	0	42473T: 4.5;
C1orf86	chr1	1p36.33	1	0	0	1	Deletion	0	1	42496T: 0;
C1QA	chr1	1p36.12	1	1	0	0	Amplification	1	0	42473T: 4;
C1QB	chr1	1p36.12	1	1	0	0	Amplification	1	0	42473T: 4;
C1QC	chr1	1p36.12	1	1	0	0	Amplification	1	0	42473T: 4;
C1QL4	chr12	12q13.12	1	0	0	1	Amplification	1	0	42500T: 3.5;
C1QTNF3	chr5	5p13.2	4	1	1	2	Amplification	4	0	42493T: 3.5; 42486T: 3.5; 42496T: 3.5; 42475T: 3.5;
C1QTNF4	chr11	11p11.2	1	1	0	0	Amplification	1	0	42473T: 3.5;
C1QTNF6	chr22	22q12.3	1	1	0	0	Amplification	1	0	42473T: 3.5;
C1QTNF8	chr16	16p13.3	1	0	1	0	Amplification	1	0	42483T: 3.5;
C1R	chr12	12p13.31	1	0	0	1	Amplification	1	0	42494T: 3.5;
C1RL	chr12	12p13.31	1	0	0	1	Amplification	1	0	42494T: 3.5;
C1S	chr12	12p13.31	1	0	0	1	Amplification	1	0	42494T: 3.5;

Mangalaparthi *et al.* , 2020. Mutational landscape of esophageal squamous cell carcinoma in an Indian cohort
Supplementary Table 7A. List of copy number alterations and affected genes in ESCC patients

Gene	Chromosome	Cytoband	Recurrence	Recurrence in smoker cohort	Recurrence in chewer cohort	Recurrence in No habit cohort	State	Samples with CNA gain	Samples with CNA loss	File info with CNA fold
C2	chr6	14q11.2	1	1	0	0	Amplification	1	0	42473T: 3.5;
C20orf112	chr20	20q11.21	2	1	0	1	Amplification	2	0	42496T: 5; 42473T: 3.5;
C20orf141	chr20	20p13	1	1	0	0	Amplification	1	0	42473T: 3.5;
C20orf144	chr20	20q11.22	2	1	0	1	Amplification	2	0	42473T: 3.5; 42496T: 5;
C20orf166	chr20	20q13.33	1	1	0	0	Amplification	1	0	42473T: 6;
C20orf173	chr20	20q11.22	2	1	0	1	Amplification	2	0	42493T: 3.5; 42473T: 3.5;
C20orf194	chr20	20p13	1	1	0	0	Amplification	1	0	42473T: 3.5;
C20orf195	chr20	20q13.33	1	1	0	0	Amplification	1	0	42473T: 6;
C20orf197	chr20	20q13.33	1	1	0	0	Amplification	1	0	42473T: 4.5;
C20orf201	chr20	20q13.33	1	1	0	0	Amplification	1	0	42473T: 6;
C20orf202	chr20	20p13	1	1	0	0	Amplification	1	0	42473T: 3.5;
C20orf203	chr20	20q11.21	2	1	0	1	Amplification	2	0	42496T: 5; 42473T: 3.5;
C20orf24	chr20	20q11.23	1	1	0	0	Amplification	1	0	42473T: 3.5;
C20orf26	chr20	20p11.23	1	0	1	0	Amplification	1	0	42483T: 4;
C20orf27	chr20	20p13	1	1	0	0	Amplification	1	0	42473T: 6;
C20orf62	chr20	20q13.12	1	1	0	0	Amplification	1	0	42473T: 3.5;
C20orf96	chr20	20p13	1	1	0	0	Amplification	1	0	42473T: 3.5;
C21orf128	chr21	21q22.3	1	1	0	0	Amplification	1	0	42473T: 3.5;
C21orf88	chr21	21q22.2	1	1	0	0	Amplification	1	0	42473T: 3.5;
C22orf23	chr22	22q13.1	1	1	0	0	Amplification	1	0	42473T: 3.5;
C22orf29	chr22	22q11.21	1	0	0	1	Amplification	1	0	42497T: 17;
C22orf39	chr22	22q11.21	1	0	0	1	Amplification	1	0	42497T: 17;
C2CD2	chr21	21q22.3	1	1	0	0	Amplification	1	0	42473T: 3.5;
C2CD3	chr11	11q13.4	6	3	0	3	Amplification	6	0	42478T: 6; 42492T: 4; 42498T: 8; 56957T: 3.5; 42476T: 4; 42475T: 7.5;
C2CD5	chr12	12p12.1	1	0	0	1	Amplification	1	0	42500T: 6;
C2orf16	chr2	2p23.3	1	0	0	1	Amplification	1	0	42500T: 3.5;
C2orf42	chr2	2p13.3	1	0	0	1	Amplification	1	0	42500T: 3.5;
C2orf43	chr2	2p24.1	1	0	0	1	Amplification	1	0	42500T: 3.5;
C2orf44	chr2	2p23.3	1	0	0	1	Amplification	1	0	42500T: 3.5;
C2orf48	chr2	2p25.1	1	0	0	1	Amplification	1	0	42500T: 3.5;
C2orf49	chr2	2q12.2	1	0	0	1	Amplification	1	0	42493T: 3.5;
C2orf50	chr2	2p25.1	1	0	0	1	Amplification	1	0	42500T: 3.5;
C2orf54	chr2	2q37.3	1	1	0	0	Deletion	0	1	42474T: 0.5;
C2orf61	chr2	2p21	2	0	1	1	Amplification	2	0	42500T: 3.5; 42484T: 4.5;
C2orf66	chr2	2q33.1	1	0	1	0	Amplification	1	0	42482T: 4;
C2orf68	chr2	2p11.2	2	0	1	1	Amplification	2	0	42500T: 3.5; 42482T: 3.5;
C2orf70	chr2	2p23.3	1	0	0	1	Amplification	1	0	42500T: 3.5;
C2orf71	chr2	2p23.2	1	0	0	1	Amplification	1	0	42500T: 3.5;
C2orf73	chr2	2p16.2	2	0	1	1	Amplification	2	0	42484T: 4.5; 42500T: 3.5;
C2orf78	chr2	2p13.1	1	0	0	1	Amplification	1	0	42500T: 3.5;

Mangalaparthi *et al.* , 2020. Mutational landscape of esophageal squamous cell carcinoma in an Indian cohort
Supplementary Table 7A. List of copy number alterations and affected genes in ESCC patients

Gene	Chromosome	Cytoband	Recurrence	Recurrence in smoker cohort	Recurrence in chewer cohort	Recurrence in No habit cohort	State	Samples with CNA gain	Samples with CNA loss	File info with CNA fold
C2orf81	chr2	2p13.1	1	0	0	1	Amplification	1	0	42500T: 3.5;
C2orf88	chr2	2q32.2	3	0	1	2	Amplification	3	0	42493T: 3.5; 42494T: 3.5; 42482T: 4;
C2orf91	chr2	2p21	2	0	1	1	Amplification	2	0	42484T: 3.5; 42500T: 3.5;
C3orf17	chr3	3q13.2	1	0	0	1	Amplification	1	0	42496T: 3.5;
C3orf22	chr3	3q21.3	1	0	0	1	Amplification	1	0	42496T: 3.5;
C3orf27	chr3	3q21.3	2	0	1	1	Amplification	2	0	42496T: 3.5; 42487T: 3.5;
C3orf30	chr3	3q13.32	1	0	0	1	Amplification	1	0	42496T: 3.5;
C3orf33	chr3	3q25.31	8	1	2	5	Amplification	8	0	42497T: 5.5; 42496T: 4.5; 42484T: 3.5; 56957T: 4; 42474T: 3.5; 42492T: 3.5; 42487T: 3.5; 42493T: 3.5;
C3orf36	chr3	3q22.1	3	0	1	2	Amplification	3	0	42496T: 3.5; 42487T: 3.5; 42492T: 3.5;
C3orf37	chr3	3q21.3	2	0	1	1	Amplification	2	0	42487T: 3.5; 42496T: 3.5;
C3orf52	chr3	3q13.2	1	0	0	1	Amplification	1	0	42496T: 3.5;
C3orf55	chr3	3q25.32	8	1	2	5	Amplification	8	0	42492T: 3.5; 42487T: 3.5; 42493T: 3.5; 42484T: 3.5; 56957T: 4; 42495T: 4; 42474T: 3.5; 42497T: 5.5;
C3orf56	chr3	3q21.3	2	0	1	1	Amplification	2	0	42487T: 3.5; 42496T: 3.5;
C3orf58	chr3	3q24	7	1	1	5	Amplification	7	0	42496T: 4; 42497T: 3.5; 42474T: 3.5; 56957T: 4; 42493T: 3.5; 42487T: 3.5; 42492T: 3.5;
C3orf62	chr3	3p21.31	1	1	0	0	Amplification	1	0	42473T: 4;
C3orf65	chr3	3q27.2	10	1	3	6	Amplification	10	0	42497T: 4; 42484T: 4; 42498T: 3.5; 42495T: 4; 42474T: 3.5; 56957T: 4.5; 42482T: 3.5; 42492T: 3.5; 42487T: 3.5; 42493T: 3.5;
C3orf70	chr3	3q27.2	10	1	3	6	Amplification	10	0	42474T: 3.5; 42495T: 4; 56957T: 4.5; 42484T: 4; 42498T: 3.5; 42497T: 4; 42493T: 3.5; 42487T: 3.5; 42492T: 3.5; 42482T: 3.5;
C3orf72	chr3	3q22.3	4	0	1	3	Amplification	4	0	42492T: 3.5; 42487T: 3.5; 42493T: 3.5; 42496T: 3.5;
C3orf79	chr3	3q25.2	9	1	2	6	Amplification	9	0	42493T: 3.5; 42487T: 3.5; 42492T: 3.5; 42500T: 3.5; 42496T: 4; 42497T: 5.5; 42474T: 3.5; 56957T: 4; 42484T: 3.5;
C3orf80	chr3	3q25.33	8	1	2	5	Amplification	8	0	42497T: 3.5; 56957T: 4; 42495T: 4; 42474T: 3.5; 42484T: 4; 42493T: 3.5; 42487T: 3.5; 42492T: 3.5;
C4A	chr6	6p21.33	2	1	1	0	Amplification	2	0	42473T: 3.5; 42484T: 3.5;
C4B	chr6	6p21.33	2	1	1	0	Amplification	2	0	42484T: 3.5; 42473T: 3.5;
C4orf19	chr4	4p14	1	1	0	0	Amplification	1	0	42473T: 4.5;
C5orf17	chr5	5p14.2	4	1	1	2	Amplification	4	0	42493T: 4; 42475T: 3.5; 42486T: 3.5; 42496T: 3.5;
C5orf22	chr5	5p13.3	4	1	1	2	Amplification	4	0	42486T: 3.5; 42496T: 3.5; 42475T: 3.5; 42493T: 3.5;
C5orf24	chr5	5q31.1	1	1	0	0	Amplification	1	0	42473T: 3.5;
C5orf28	chr5	5p12	4	1	1	2	Amplification	4	0	42493T: 3.5; 42475T: 3.5; 42496T: 3.5; 42486T: 3.5;
C5orf34	chr5	5p12	4	1	1	2	Amplification	4	0	42493T: 3.5; 42486T: 3.5; 42496T: 3.5; 42475T: 3.5;
C5orf38	chr5	5p15.33	5	2	1	2	Amplification	5	0	42475T: 3.5; 42473T: 3.5; 42496T: 3.5; 42486T: 3.5; 42493T: 3.5;

Mangalaparthi *et al.* , 2020. Mutational landscape of esophageal squamous cell carcinoma in an Indian cohort
Supplementary Table 7A. List of copy number alterations and affected genes in ESCC patients

Gene	Chromosome	Cytoband	Recurrence	Recurrence in smoker cohort	Recurrence in chewer cohort	Recurrence in No habit cohort	State	Samples with CNA gain	Samples with CNA loss	File info with CNA fold
C5orf42	chr5	5p13.2	5	1	2	2	Amplification	5	0	42496T: 3.5; 42486T: 3.5; 42483T: 3.5; 42475T: 3.5; 42493T: 3.5;
C5orf49	chr5	5p15.31	4	1	1	2	Amplification	4	0	42493T: 4; 42486T: 3.5; 42496T: 3.5; 42475T: 3.5;
C5orf51	chr5	5p13.1	6	1	3	2	Amplification	6	0	42496T: 3.5; 42486T: 3.5; 42483T: 3.5; 42475T: 3.5; 42484T: 3.5; 42493T: 3.5;
C5orf55	chr5	5p15.33	5	2	1	2	Amplification	5	0	42493T: 3.5; 42475T: 3.5; 42486T: 3.5; 42473T: 3.5; 42496T: 4;
C6	chr5	20q13.33	6	1	3	2	Amplification	6	0	42484T: 3.5; 42493T: 3.5; 42496T: 3.5; 42483T: 3.5; 42486T: 3.5; 42475T: 3.5;
C6orf10	chr6	6p21.32	1	1	0	0	Amplification	1	0	42473T: 3.5;
C6orf183	chr6	6q21	1	0	0	1	Amplification	1	0	42496T: 3.5;
C6orf203	chr6	6q21	1	0	0	1	Amplification	1	0	42496T: 3.5;
C6orf25	chr6	6p21.33	1	1	0	0	Amplification	1	0	42473T: 3.5;
C6orf47	chr6	6p21.33	1	1	0	0	Amplification	1	0	42473T: 3.5;
C6orf48	chr6	6p21.33	1	1	0	0	Amplification	1	0	42473T: 3.5;
C6orf99	chr6	6q25.3	1	1	0	0	Amplification	1	0	42473T: 3.5;
C7	chr5	22q11.22	6	1	3	2	Amplification	6	0	42484T: 3.5; 42493T: 3.5; 42496T: 3.5; 42486T: 3.5; 42483T: 3.5; 42475T: 3.5;
C7orf10	chr7	7p14.1	1	1	0	0	Amplification	1	0	42473T: 3.5;
C7orf26	chr7	7p22.1	2	1	0	1	Amplification	2	0	42473T: 4.5; 42497T: 5;
C7orf31	chr7	7p15.3	1	1	0	0	Amplification	1	0	42473T: 4;
C7orf33	chr7	7q36.1	2	0	1	1	Amplification	2	0	42487T: 3.5; 42500T: 4;
C7orf34	chr7	7q34	1	0	1	0	Amplification	1	0	42487T: 3.5;
C7orf41	chr7	7p14.3	1	1	0	0	Amplification	1	0	42473T: 4;
C7orf49	chr7	7q33	1	0	1	0	Amplification	1	0	42487T: 3.5;
C7orf50	chr7	7p22.3	1	1	0	0	Amplification	1	0	42473T: 5;
C7orf55	chr7	7q34	1	0	1	0	Amplification	1	0	42487T: 3.5;
C7orf60	chr7	7q31.1	2	0	1	1	Amplification	2	0	42487T: 3.5; 42501T: 3.5;
C7orf62	chr7	7q21.13	2	0	2	0	Amplification	2	0	42483T: 3.5; 42487T: 3.5;
C7orf63	chr7	7q21.13	2	0	2	0	Amplification	2	0	42483T: 3.5; 42487T: 3.5;
C7orf66	chr7	7q31.1	3	0	1	2	Amplification	3	0	42501T: 3.5; 42497T: 4; 42487T: 3.5;
C7orf71	chr7	7p15.2	1	1	0	0	Amplification	1	0	42473T: 4;
C7orf72	chr7	7p12.2	1	0	0	1	Amplification	1	0	42497T: 20.5;
C7orf73	chr7	7q33	1	0	1	0	Amplification	1	0	42487T: 3.5;
C7orf76	chr7	7q21.3	1	0	1	0	Amplification	1	0	42487T: 3.5;
C8G	chr9	9q34.3	2	1	0	1	Amplification	2	0	56957T: 3.5; 42473T: 5;
C8orf12	chr8	8p23.1	1	0	1	0	Amplification	1	0	42486T: 3.5;
C8orf17	chr8	8q24.3	2	0	0	2	Amplification	2	0	42496T: 3.5; 42495T: 3.5;
C8orf22	chr8	8q11.21	5	0	2	3	Amplification	5	0	42484T: 3.5; 42494T: 3.5; 42495T: 3.5; 42482T: 3.5; 42496T: 3.5;
C8orf31	chr8	8q24.3	3	0	1	2	Amplification	3	0	42483T: 3.5; 42496T: 4; 42495T: 4.5;

Mangalaparthi *et al.* , 2020. Mutational landscape of esophageal squamous cell carcinoma in an Indian cohort
Supplementary Table 7A. List of copy number alterations and affected genes in ESCC patients

Gene	Chromosome	Cytoband	Recurrence	Recurrence in smoker cohort	Recurrence in chewer cohort	Recurrence in No habit cohort	State	Samples with CNA gain	Samples with CNA loss	File info with CNA fold
C8orf33	chr8	8q24.3	3	0	1	2	Amplification	3	0	42495T: 4.5; 42496T: 3.5; 42483T: 3.5;
C8orf34	chr8	8q13.2	2	0	0	2	Amplification	2	0	42495T: 3.5; 42496T: 3.5;
C8orf37	chr8	8q22.1	3	1	0	2	Amplification	3	0	42496T: 3.5; 42473T: 4.5; 42495T: 3.5;
C8orf44	chr8	8q13.1	3	0	0	3	Amplification	3	0	42495T: 3.5; 42496T: 3.5; 42497T: 4.5;
C8orf46	chr8	8q13.1	3	0	0	3	Amplification	3	0	42495T: 3.5; 42497T: 4.5; 42496T: 3.5;
C8orf47	chr8	8q22.2	2	0	0	2	Amplification	2	0	42495T: 3.5; 42496T: 3.5;
C8orf49	chr8	8p23.1	1	0	1	0	Amplification	1	0	42486T: 3.5;
C8orf56	chr8	8q22.3	2	0	0	2	Amplification	2	0	42495T: 3.5; 42496T: 3.5;
C8orf59	chr8	8q21.2	2	0	0	2	Amplification	2	0	42496T: 3.5; 42495T: 3.5;
C8orf74	chr8	8p23.1	1	0	1	0	Amplification	1	0	42486T: 3.5;
C8orf76	chr8	8q24.13	4	1	1	2	Amplification	4	0	42495T: 3.5; 42484T: 3.5; 42475T: 3.5; 42496T: 3.5;
C8orf82	chr8	8q24.3	5	0	1	4	Amplification	5	0	56957T: 3.5; 42494T: 3.5; 42495T: 4.5; 42496T: 4; 42483T: 3.5;
C8orf86	chr8	8p11.22	1	0	1	0	Amplification	1	0	42482T: 3.5;
C8orf87	chr8	8q22.1	2	0	0	2	Amplification	2	0	42496T: 3.5; 42495T: 3.5;
C9	chr5	10p15.1	6	1	3	2	Amplification	6	0	42496T: 3.5; 42486T: 3.5; 42483T: 3.5; 42475T: 3.5; 42484T: 3.5; 42493T: 3.5;
C9orf116	chr9	9q34.3	1	0	1	0	Amplification	1	0	42484T: 4.5;
C9orf123	chr9	9p24.1	1	0	0	1	Amplification	1	0	42496T: 3.5;
C9orf131	chr9	9p13.3	1	0	0	1	Amplification	1	0	42501T: 3.5;
C9orf139	chr9	9q34.3	2	1	0	1	Amplification	2	0	56957T: 3.5; 42473T: 5;
C9orf141	chr9	9q34.3	2	1	0	1	Amplification	2	0	42473T: 5; 56957T: 3.5;
C9orf142	chr9	9q34.3	2	1	0	1	Amplification	2	0	56957T: 3.5; 42473T: 5;
C9orf163	chr9	9q34.3	1	0	0	1	Amplification	1	0	56957T: 3.5;
C9orf169	chr9	9q34.3	2	1	0	1	Amplification	2	0	56957T: 3.5; 42473T: 5;
C9orf172	chr9	9q34.3	1	0	0	1	Amplification	1	0	56957T: 3.5;
C9orf173	chr9	9q34.3	2	1	0	1	Amplification	2	0	56957T: 3.5; 42473T: 5;
C9orf37	chr9	9q34.3	2	1	0	1	Amplification	2	0	42473T: 5; 56957T: 3.5;
C9orf53	chr9	9p21.3	2	1	1	0	Deletion	0	2	42486T: 0.5; 42475T: 0.5;
C9orf69	chr9	9q34.3	1	0	0	1	Amplification	1	0	56957T: 3.5;
C9orf91	chr9	9q32	1	0	0	1	Amplification	1	0	42493T: 3.5;
CA1	chr8	8q21.2	2	0	0	2	Amplification	2	0	42496T: 3.5; 42495T: 3.5;
CA13	chr8	8q21.2	2	0	0	2	Amplification	2	0	42496T: 3.5; 42495T: 3.5;
CA14	chr1	1q21.2	1	1	0	0	Amplification	1	0	42473T: 5;
CA2	chr8	8q21.2	2	0	0	2	Amplification	2	0	42496T: 3.5; 42495T: 3.5;
CA3	chr8	8q21.2	2	0	0	2	Amplification	2	0	42496T: 3.5; 42495T: 3.5;
CA8	chr8	8q12.1	2	0	0	2	Amplification	2	0	42496T: 3.5; 42495T: 3.5;
CA9	chr9	9p13.3	3	1	0	2	Amplification	3	0	42496T: 4; 42473T: 3.5; 42501T: 3.5;
CABLES1	chr18	18q11.2	1	1	0	0	Amplification	1	0	42481T: 4;
CABLES2	chr20	20q13.33	1	1	0	0	Amplification	1	0	42473T: 6;
CABP1	chr12	12q24.31	1	0	0	1	Amplification	1	0	42500T: 3.5;

Mangalaparthi *et al.* , 2020. Mutational landscape of esophageal squamous cell carcinoma in an Indian cohort
Supplementary Table 7A. List of copy number alterations and affected genes in ESCC patients

Gene	Chromosome	Cytoband	Recurrence	Recurrence in smoker cohort	Recurrence in chewer cohort	Recurrence in No habit cohort	State	Samples with CNA gain	Samples with CNA loss	File info with CNA fold
CABP2	chr11	11q13.2	2	1	0	1	Amplification	2	0	56957T: 5; 42473T: 3.5;
CABP4	chr11	11q13.2	2	1	0	1	Amplification	2	0	56957T: 5; 42473T: 3.5;
CABP7	chr22	22q12.2	1	1	0	0	Amplification	1	0	42473T: 3.5;
CABYR	chr18	18q11.2	1	1	0	0	Amplification	1	0	42481T: 4;
CACNA1G	chr17	17q21.33	1	1	0	0	Amplification	1	0	42473T: 4.5;
CACNA1H	chr16	16p13.3	1	0	1	0	Amplification	1	0	42483T: 3.5;
CACNA1I	chr22	22q13.1	1	1	0	0	Amplification	1	0	42473T: 3.5;
CACNA2D1	chr7	7q21.11	1	0	1	0	Amplification	1	0	42487T: 3.5;
CACNB1	chr17	17q12	1	1	0	0	Amplification	1	0	42473T: 4.5;
CACNB3	chr12	12q13.12	1	0	0	1	Amplification	1	0	42500T: 3.5;
CACNG2	chr22	22q12.3	1	1	0	0	Amplification	1	0	42473T: 3.5;
CACNG3	chr16	16p12.1	1	1	0	0	Amplification	1	0	42473T: 4;
CAD	chr2	13q22.3	1	0	0	1	Amplification	1	0	42500T: 3.5;
CADPS2	chr7	7q31.32	1	0	1	0	Amplification	1	0	42487T: 3.5;
CALB1	chr8	8q21.3	2	0	0	2	Amplification	2	0	42496T: 3.5; 42495T: 3.5;
CALCR	chr7	7q21.3	2	0	2	0	Amplification	2	0	42483T: 4; 42487T: 3.5;
CALCRL	chr2	2q32.1	1	0	0	1	Amplification	1	0	42493T: 3.5;
CALD1	chr7	7q33	1	0	1	0	Amplification	1	0	42487T: 3.5;
CALM1	chr14	14q32.11	1	1	0	0	Amplification	1	0	42473T: 3.5;
CALM2	chr2	2p21	2	0	1	1	Amplification	2	0	42484T: 4.5; 42500T: 3.5;
CALM3	chr19	19q13.32	1	0	1	0	Amplification	1	0	42484T: 7;
CALU	chr7	7q32.1	1	0	1	0	Amplification	1	0	42487T: 3.5;
CAMK2B	chr7	7p13	2	1	0	1	Amplification	2	0	42497T: 7.5; 42473T: 4.5;
CAMK2G	chr10	10q22.2	1	0	0	1	Amplification	1	0	42496T: 3.5;
CAMK2N1	chr1	1p36.12	1	1	0	0	Amplification	1	0	42473T: 4;
CAMK2N2	chr3	3q27.1	12	2	3	7	Amplification	12	0	42484T: 4; 42498T: 3.5; 42495T: 4; 42494T: 3.5; 42474T: 3.5; 56957T: 5; 42497T: 4; 42473T: 3.5; 42492T: 3.5; 42493T: 3.5; 42487T: 3.5; 42482T: 3.5;
CAMKK2	chr12	12q24.31	1	0	0	1	Amplification	1	0	42500T: 3.5;
CAMKMT	chr2	2p21	2	0	1	1	Amplification	2	0	42500T: 3.5; 42484T: 3.5;
CAMLG	chr5	5q31.1	1	1	0	0	Amplification	1	0	42473T: 3.5;
CAMSAP1	chr9	9q34.3	1	0	0	1	Amplification	1	0	56957T: 3.5;
CAND1	chr12	12q14.3-q15	2	0	0	2	Amplification	2	0	42501T: 6.5; 42500T: 4.5;
CAPG	chr2	4p15.31	1	0	0	1	Amplification	1	0	42500T: 3.5;
CAPN12	chr19	19q13.2	2	0	0	2	Amplification	2	0	42500T: 6.5; 56957T: 4;
CAPN13	chr2	2p23.1	1	0	0	1	Amplification	1	0	42500T: 3.5;
CAPN14	chr2	2p23.1	1	0	0	1	Amplification	1	0	42500T: 3.5;
CAPN15	chr16	16p13.3	1	0	1	0	Amplification	1	0	42483T: 3.5;
CAPNS1	chr19	19q13.12	3	0	1	2	Amplification	3	0	56957T: 4; 42484T: 3.5; 42500T: 6.5;
CAPRIN1	chr11	11p13	1	1	0	0	Amplification	1	0	42473T: 4.5;
CAPRIN2	chr12	12p11.21	1	0	0	1	Amplification	1	0	42500T: 8;

Mangalaparthi *et al.* , 2020. Mutational landscape of esophageal squamous cell carcinoma in an Indian cohort
Supplementary Table 7A. List of copy number alterations and affected genes in ESCC patients

Gene	Chromosome	Cytoband	Recurrence	Recurrence in smoker cohort	Recurrence in chewer cohort	Recurrence in No habit cohort	State	Samples with CNA gain	Samples with CNA loss	File info with CNA fold
CAPSL	chr5	5p13.2	4	1	1	2	Amplification	4	0	42493T: 3.5; 42486T: 3.5; 42496T: 3.5; 42475T: 3.5;
CAPZA2	chr7	7q31.2	1	0	1	0	Amplification	1	0	42487T: 3.5;
CAPZB	chr1	1p36.13	1	1	0	0	Amplification	1	0	42473T: 4;
CARD10	chr22	22q13.1	1	1	0	0	Amplification	1	0	42473T: 3.5;
CARD11	chr7	7p22.2	1	1	0	0	Amplification	1	0	42473T: 4.5;
CARD14	chr17	17q25.3	1	1	0	0	Amplification	1	0	42473T: 3.5;
CARD6	chr5	5p13.1	6	1	3	2	Amplification	6	0	42484T: 3.5; 42493T: 3.5; 42496T: 3.5; 42486T: 3.5; 42483T: 3.5; 42475T: 3.5;
CARD9	chr9	9q34.3	1	0	0	1	Amplification	1	0	56957T: 3.5;
CARHSP1	chr16	16p13.2	2	1	0	1	Amplification	2	0	42495T: 4; 42473T: 5.5;
CARNS1	chr11	11q13.2	2	1	0	1	Amplification	2	0	42473T: 3.5; 56957T: 5;
CASC1	chr12	12p12.1	1	0	0	1	Amplification	1	0	42500T: 6;
CASC3	chr17	17q21.1	1	0	0	1	Amplification	1	0	42497T: 4;
CASD1	chr7	7q21.3	1	0	1	0	Amplification	1	0	42487T: 3.5;
CASKIN1	chr16	16p13.3	1	0	1	0	Amplification	1	0	42483T: 3.5;
CASKIN2	chr17	17q25.1	1	1	0	0	Amplification	1	0	42473T: 4;
CASP16	chr16	16p13.3	1	1	0	0	Amplification	1	0	42473T: 5.5;
CASP2	chr7	7q34	1	0	1	0	Amplification	1	0	42487T: 3.5;
CASR	chr3	3q13.33-q21	1	0	0	1	Amplification	1	0	42496T: 3.5;
CASS4	chr20	20q13.31	1	1	0	0	Amplification	1	0	42473T: 5;
CAT	chr11	11q12.1	2	1	0	1	Amplification	2	0	42473T: 4.5; 42493T: 4;
CATSPER3	chr5	5q31.1	1	1	0	0	Amplification	1	0	42473T: 3.5;
CATSPERG	chr19	19q13.2	3	0	1	2	Amplification	3	0	56957T: 4; 42484T: 3.5; 42500T: 6.5;
CAV1	chr7	7q31.2	1	0	1	0	Amplification	1	0	42487T: 3.5;
CAV2	chr7	7q31.2	1	0	1	0	Amplification	1	0	42487T: 3.5;
CBFA2T2	chr20	20q11.21-q1	2	1	0	1	Amplification	2	0	42473T: 3.5; 42496T: 5;
CBLC	chr19	19q13.32	2	1	0	1	Amplification	2	0	56957T: 4; 42473T: 3.5;
CBLL1	chr7	7q22.3	4	0	1	3	Amplification	4	0	42501T: 3.5; 42497T: 4; 42487T: 3.5; 42493T: 3.5;
CBLN4	chr20	20q13.2	1	1	0	0	Amplification	1	0	42473T: 5;
CBWD1	chr9	9p24.3	2	0	1	1	Amplification/Deletio	1	1	42486T: 0.5; 42496T: 3.5;
CBX2	chr17	17q25.3	1	1	0	0	Amplification	1	0	42473T: 3.5;
CBX3	chr7	7p15.2	1	1	0	0	Amplification	1	0	42473T: 4;
CBX4	chr17	17q25.3	1	1	0	0	Amplification	1	0	42473T: 3.5;
CBX5	chr12	12q13.13	2	1	0	1	Amplification	2	0	42494T: 3.5; 42473T: 3.5;
CBX6	chr22	22q13.1	1	1	0	0	Amplification	1	0	42473T: 3.5;
CBX7	chr22	22q13.1	1	1	0	0	Amplification	1	0	42473T: 3.5;
CBX8	chr17	17q25.3	1	1	0	0	Amplification	1	0	42473T: 3.5;
CBY1	chr22	22q13.1	1	1	0	0	Amplification	1	0	42473T: 3.5;
CCDC104	chr2	2p16.1	3	0	1	2	Amplification	3	0	42484T: 4.5; 56957T: 3.5; 42500T: 3.5;
CCDC106	chr19	19q13.42	1	0	0	1	Amplification	1	0	42494T: 3.5;
CCDC107	chr9	9p13.3	2	1	0	1	Amplification	2	0	42473T: 3.5; 42501T: 3.5;

Mangalaparthi *et al.* , 2020. Mutational landscape of esophageal squamous cell carcinoma in an Indian cohort
Supplementary Table 7A. List of copy number alterations and affected genes in ESCC patients

Gene	Chromosome	Cytoband	Recurrence	Recurrence in smoker cohort	Recurrence in chewer cohort	Recurrence in No habit cohort	State	Samples with CNA gain	Samples with CNA loss	File info with CNA fold
CCDC108	chr2	2q35	1	1	0	0	Amplification	1	0	42473T: 3.5;
CCDC116	chr22	22q11.21	1	1	0	0	Amplification	1	0	42473T: 3.5;
CCDC121	chr2	2p23.3	1	0	0	1	Amplification	1	0	42500T: 3.5;
CCDC126	chr7	7p15.3	1	1	0	0	Amplification	1	0	42473T: 4;
CCDC127	chr5	5p15.33	5	2	1	2	Amplification	5	0	42486T: 3.5; 42496T: 4; 42473T: 3.5; 42475T: 3.5; 42493T: 3.5;
CCDC129	chr7	7p14.3	1	1	0	0	Amplification	1	0	42473T: 4;
CCDC132	chr7	7q21.2-q21.3	2	0	2	0	Amplification	2	0	42483T: 4; 42487T: 3.5;
CCDC136	chr7	7q32.1	1	0	1	0	Amplification	1	0	42487T: 3.5;
CCDC14	chr3	3q21.1	1	0	0	1	Amplification	1	0	42496T: 3.5;
CCDC141	chr2	2q31.2	1	0	0	1	Amplification	1	0	42493T: 3.5;
CCDC142	chr2	2p13.1	1	0	0	1	Amplification	1	0	42500T: 3.5;
CCDC146	chr7	7q11.23	1	0	1	0	Amplification	1	0	42487T: 3.5;
CCDC148	chr2	2q24.1	2	1	0	1	Amplification	2	0	42473T: 4; 42493T: 3.5;
CCDC150	chr2	2q33.1	1	0	1	0	Amplification	1	0	42482T: 4;
CCDC152	chr5	5p12	5	1	2	2	Amplification	5	0	42493T: 3.5; 42475T: 3.5; 42496T: 3.5; 42483T: 3.5; 42486T: 3.5;
CCDC154	chr16	16p13.3	1	0	1	0	Amplification	1	0	42483T: 3.5;
CCDC157	chr22	22q12.2	1	1	0	0	Amplification	1	0	42473T: 3.5;
CCDC162P	chr6	6q21	1	0	0	1	Amplification	1	0	42496T: 3.5;
CCDC166	chr8	8q24.3	3	0	1	2	Amplification	3	0	42483T: 3.5; 42496T: 4; 42495T: 4.5;
CCDC167	chr6	6p21.2	1	1	0	0	Amplification	1	0	42473T: 4.5;
CCDC175	chr14	14q23.1	2	0	1	1	Amplification	2	0	42494T: 4; 42483T: 3.5;
CCDC176	chr14	14q24.3	2	0	0	2	Amplification	2	0	42494T: 4; 56957T: 3.5;
CCDC177	chr14	14q24.1	1	0	0	1	Amplification	1	0	42494T: 4;
CCDC23	chr1	1p34.2	2	0	0	2	Amplification	2	0	42493T: 3.5; 42496T: 5.5;
CCDC26	chr8	8q24.21	3	0	1	2	Amplification	3	0	42484T: 3.5; 42495T: 3.5; 42496T: 3.5;
CCDC28B	chr1	1p35.2	1	1	0	0	Amplification	1	0	42473T: 4;
CCDC30	chr1	1p34.2	1	0	0	1	Amplification	1	0	42493T: 3.5;
CCDC33	chr15	15q24.1	1	1	0	0	Amplification	1	0	42473T: 3.5;
CCDC36	chr3	3p21.31	1	1	0	0	Amplification	1	0	42473T: 4;
CCDC37	chr3	3q21.3	1	0	0	1	Amplification	1	0	42496T: 3.5;
CCDC39	chr3	3q26.33	11	1	3	7	Amplification	11	0	42482T: 3.5; 42492T: 3.5; 42493T: 3.5; 42487T: 3.5; 42497T: 4; 42500T: 14; 42498T: 3.5; 42484T: 4; 56957T: 4; 42495T: 4; 42474T: 3.5;
CCDC40	chr17	17q25.3	1	1	0	0	Amplification	1	0	42473T: 3.5;
CCDC42B	chr12	12q24.13	1	0	0	1	Amplification	1	0	42500T: 3.5;
CCDC50	chr3	3q28	9	1	3	5	Amplification	9	0	42498T: 3.5; 42484T: 4; 56957T: 4.5; 42495T: 4; 42474T: 3.5; 42492T: 3.5; 42493T: 3.5; 42487T: 3.5; 42482T: 3.5;
CCDC58	chr3	3q21.1	1	0	0	1	Amplification	1	0	42496T: 3.5;

Mangalaparthi *et al.* , 2020. Mutational landscape of esophageal squamous cell carcinoma in an Indian cohort
Supplementary Table 7A. List of copy number alterations and affected genes in ESCC patients

Gene	Chromosome	Cytoband	Recurrence	Recurrence in smoker cohort	Recurrence in chewer cohort	Recurrence in No habit cohort	State	Samples with CNA gain	Samples with CNA loss	File info with CNA fold
CCDC60	chr12	12q24.23	1	0	0	1	Amplification	1	0	42500T: 3.5;
CCDC61	chr19	19q13.32	1	0	1	0	Amplification	1	0	42484T: 4;
CCDC62	chr12	12q24.31	1	0	0	1	Amplification	1	0	42500T: 3.5;
CCDC63	chr12	12q24.11	1	0	0	1	Amplification	1	0	42500T: 3.5;
CCDC64	chr12	12q24.23	1	0	0	1	Amplification	1	0	42500T: 3.5;
CCDC65	chr12	12q13.12	1	0	0	1	Amplification	1	0	42500T: 3.5;
CCDC71L	chr7	7q22.3	4	0	1	3	Amplification	4	0	42501T: 3.5; 42497T: 4; 42493T: 3.5; 42487T: 3.5;
CCDC77	chr12	12p13.33	1	0	0	1	Amplification	1	0	42500T: 3.5;
CCDC78	chr16	16p13.3	2	0	1	1	Amplification	2	0	42483T: 3.5; 42493T: 3.5;
CCDC8	chr19	19q13.32	1	0	1	0	Amplification	1	0	42484T: 4;
CCDC80	chr3	3q13.2	1	0	0	1	Amplification	1	0	42496T: 3.5;
CCDC84	chr11	11q23.3	1	1	0	0	Amplification	1	0	42473T: 4;
CCDC85A	chr2	2p16.1	3	0	1	2	Amplification	3	0	42500T: 3.5; 56957T: 3.5; 42484T: 4.5;
CCDC87	chr11	11q13.2	2	1	0	1	Amplification	2	0	42473T: 3.5; 56957T: 5.5;
CCDC88A	chr2	2p16.1	3	0	1	2	Amplification	3	0	42484T: 4.5; 56957T: 3.5; 42500T: 3.5;
CCDC91	chr12	12p11.22	2	0	0	2	Amplification	2	0	42500T: 6; 42494T: 4;
CCDC92	chr12	12q24.31	1	0	0	1	Amplification	1	0	42500T: 3.5;
CCDC97	chr19	19q13.2	1	1	0	0	Amplification	1	0	42473T: 4.5;
CCIN	chr9	9p13.3	1	0	0	1	Amplification	1	0	42501T: 3.5;
CCL19	chr9	9p13.3	1	0	0	1	Amplification	1	0	42501T: 3.5;
CCL21	chr9	9p13.3	1	0	0	1	Amplification	1	0	42501T: 3.5;
CCL28	chr5	5p12	4	1	1	2	Amplification	4	0	42486T: 3.5; 42496T: 3.5; 42475T: 3.5; 42493T: 3.5;
CCM2	chr7	7p13	1	1	0	0	Amplification	1	0	42473T: 4.5;
CCM2L	chr20	20q11.21	1	0	0	1	Amplification	1	0	42496T: 5;
CCND1	chr11	11q13.3	2	1	0	1	Amplification	2	0	42475T: 7.5; 42492T: 4;
CCND2	chr12	12p13.32	1	0	0	1	Amplification	1	0	42494T: 3.5;
CCNE1	chr19	19q12	5	1	1	3	Amplification	5	0	42494T: 4; 56957T: 4; 42484T: 6; 42500T: 4.5; 42473T: 25;
CCNE2	chr8	8q22.1	3	1	0	2	Amplification	3	0	42473T: 4.5; 42496T: 3.5; 42495T: 3.5;
CCNF	chr16	16p13.3	1	0	1	0	Amplification	1	0	42483T: 3.5;
CCNH	chr5	5q14.3	1	1	0	0	Deletion	0	1	42476T: 0.5;
CCNL1	chr3	3q25.31	8	1	2	5	Amplification	8	0	42497T: 5.5; 56957T: 4; 42474T: 3.5; 42495T: 4; 42484T: 3.5; 42487T: 3.5; 42493T: 3.5; 42492T: 3.5;
CCNT1	chr12	12q13.11-q1	1	0	0	1	Amplification	1	0	42500T: 3.5;
CCP110	chr16	16p12.3	1	1	0	0	Amplification	1	0	42473T: 4;
CCS	chr11	11q13.2	2	1	0	1	Amplification	2	0	56957T: 5.5; 42473T: 3.5;
CCT2	chr12	12q15	2	0	0	2	Amplification	2	0	42501T: 5.5; 42500T: 5;
CCT3	chr1	1q22	1	1	0	0	Amplification	1	0	42473T: 4.5;
CCT4	chr2	2p15	3	0	1	2	Amplification	3	0	56957T: 3.5; 42484T: 8.5; 42500T: 3.5;
CCT5	chr5	5p15.2	4	1	1	2	Amplification	4	0	42475T: 3.5; 42486T: 3.5; 42496T: 3.5; 42493T: 4;
CCT6A	chr7	7p11.2	2	0	1	1	Amplification	2	0	56957T: 5; 42483T: 4;

Mangalaparthi *et al.* , 2020. Mutational landscape of esophageal squamous cell carcinoma in an Indian cohort
Supplementary Table 7A. List of copy number alterations and affected genes in ESCC patients

Gene	Chromosome	Cytoband	Recurrence	Recurrence in smoker cohort	Recurrence in chewer cohort	Recurrence in No habit cohort	State	Samples with CNA gain	Samples with CNA loss	File info with CNA fold
CCT6P1	chr7	7q11.21	1	0	0	1	Amplification	1	0	42501T: 3.5;
CCT6P3	chr7	7q11.21	1	0	0	1	Amplification	1	0	42501T: 3.5;
CCT7	chr2	2p13.2	1	0	0	1	Amplification	1	0	42500T: 3.5;
CCZ1	chr7	7p22.1	2	1	0	1	Amplification	2	0	42497T: 8; 42473T: 4.5;
CCZ1B	chr7	7p22.1	2	1	0	1	Amplification	2	0	42497T: 7.5; 42473T: 6.5;
CD160	chr1	1q21.1	1	1	0	0	Amplification	1	0	42473T: 4;
CD163L1	chr12	12p13.31	1	0	0	1	Amplification	1	0	42494T: 3.5;
CD164	chr6	6q21	1	0	0	1	Amplification	1	0	42496T: 3.5;
CD164L2	chr1	1p36.11	1	1	0	0	Amplification	1	0	42473T: 5;
CD177	chr19	19q13.31	1	0	0	1	Amplification	1	0	56957T: 4;
CD200	chr3	3q13.2	1	0	0	1	Amplification	1	0	42496T: 3.5;
CD200R1	chr3	3q13.2	1	0	0	1	Amplification	1	0	42496T: 3.5;
CD200R1L	chr3	3q13.2	1	0	0	1	Amplification	1	0	42496T: 3.5;
CD207	chr2	2p13.3	1	0	0	1	Amplification	1	0	42500T: 3.5;
CD22	chr19	19q13.12	3	0	1	2	Amplification	3	0	42500T: 6.5; 56957T: 4; 42484T: 3.5;
CD274	chr9	9p24.1	2	0	0	2	Amplification	2	0	42496T: 3.5; 42498T: 14;
CD36	chr7	7q21.11	1	0	1	0	Amplification	1	0	42487T: 3.5;
CD3EAP	chr19	19q13.32	2	1	1	0	Amplification	2	0	42484T: 4; 42473T: 3.5;
CD4	chr12	12p13.31	2	1	0	1	Amplification	2	0	42473T: 3.5; 42494T: 3.5;
CD40	chr20	20q13.12	1	1	0	0	Amplification	1	0	42473T: 5;
CD44	chr11	11p13	1	1	0	0	Amplification	1	0	42473T: 4.5;
CD5L	chr1	1q23.1	1	1	0	0	Amplification	1	0	42473T: 5;
CD63	chr12	12q13.2	1	0	0	1	Amplification	1	0	42494T: 3.5;
CD72	chr9	9p13.3	2	1	0	1	Amplification	2	0	42501T: 3.5; 42473T: 3.5;
CD79A	chr19	19q13.2	1	1	0	0	Amplification	1	0	42473T: 4.5;
CD80	chr3	3q13.33	1	0	0	1	Amplification	1	0	42496T: 3.5;
CD82	chr11	11p11.2	1	1	0	0	Amplification	1	0	42473T: 3.5;
CD86	chr3	3q13.33	1	0	0	1	Amplification	1	0	42496T: 3.5;
CD8A	chr2	2p11.2	2	0	0	2	Amplification	2	0	42493T: 3.5; 42500T: 3.5;
CD8B	chr2	2p11.2	2	0	0	2	Amplification	2	0	42493T: 3.5; 42500T: 3.5;
CDA	chr1	1p36.12	1	1	0	0	Amplification	1	0	42473T: 4;
CDC16	chr13	13q34	2	0	0	2	Amplification	2	0	56957T: 3.5; 42496T: 4.5;
CDC20	chr1	1p34.2	1	0	0	1	Amplification	1	0	42493T: 3.5;
CDC25B	chr20	20p13	2	1	1	0	Amplification	2	0	42473T: 6; 42487T: 4.5;
CDC37L1	chr9	9p24.1	1	0	0	1	Amplification	1	0	42496T: 3.5;
CDC40	chr6	6q21	1	0	0	1	Amplification	1	0	42496T: 3.5;
CDC42	chr1	1p36.12	2	1	0	1	Amplification	2	0	42493T: 3.5; 42473T: 4;
CDC42EP1	chr22	22q13.1	1	1	0	0	Amplification	1	0	42473T: 3.5;
CDC42EP3	chr2	2p22.2	1	0	0	1	Amplification	1	0	42500T: 3.5;
CDC42SE1	chr1	1q21.3	1	1	0	0	Amplification	1	0	42473T: 4.5;
CDC45	chr22	22q11.21	1	0	0	1	Amplification	1	0	42497T: 17;

Mangalaparthi *et al.* , 2020. Mutational landscape of esophageal squamous cell carcinoma in an Indian cohort
Supplementary Table 7A. List of copy number alterations and affected genes in ESCC patients

Gene	Chromosome	Cytoband	Recurrence	Recurrence in smoker cohort	Recurrence in chewer cohort	Recurrence in No habit cohort	State	Samples with CNA gain	Samples with CNA loss	File info with CNA fold
CDC6	chr17	17q21.2	1	0	0	1	Amplification	1	0	42497T: 4;
CDCA3	chr12	12p13.31	2	1	0	1	Amplification	2	0	42494T: 3.5; 42473T: 3.5;
CDCA7	chr2	2q31.1	1	0	0	1	Amplification	1	0	42493T: 5;
CDCA7L	chr7	7p15.3	1	1	0	0	Amplification	1	0	42473T: 4;
CDH10	chr5	5p14.2-p14.1	4	1	1	2	Amplification	4	0	42493T: 4; 42496T: 3.5; 42486T: 3.5; 42475T: 3.5;
CDH12	chr5	5p14.3	4	1	1	2	Amplification	4	0	42493T: 4; 42486T: 3.5; 42496T: 3.5; 42475T: 3.5;
CDH17	chr8	8q22.1	2	0	0	2	Amplification	2	0	42496T: 3.5; 42495T: 3.5;
CDH18	chr5	5p14.3	4	1	1	2	Amplification	4	0	42493T: 4; 42475T: 3.5; 42496T: 3.5; 42486T: 3.5;
CDH22	chr20	20q13.12	1	1	0	0	Amplification	1	0	42473T: 5;
CDH24	chr14	14q11.2	1	0	0	1	Amplification	1	0	42496T: 4;
CDH26	chr20	20q13.33	1	1	0	0	Amplification	1	0	42473T: 4.5;
CDH4	chr20	20q13.33	1	1	0	0	Amplification	1	0	42473T: 6;
CDH6	chr5	5p13.3	4	1	1	2	Amplification	4	0	42493T: 3.5; 42475T: 3.5; 42486T: 3.5; 42496T: 3.5;
CDH9	chr5	5p14.1	4	1	1	2	Amplification	4	0	42493T: 3.5; 42486T: 3.5; 42496T: 3.5; 42475T: 3.5;
CDHR3	chr7	7q22.3	4	0	1	3	Amplification	4	0	42493T: 3.5; 42487T: 3.5; 42497T: 4; 42501T: 3.5;
CDIP1	chr16	16p13.3	3	1	0	2	Amplification	3	0	42473T: 5.5; 42495T: 8.5; 42494T: 3.5;
CDK12	chr17	17q12	2	1	0	1	Amplification	2	0	42473T: 6; 42497T: 3.5;
CDK13	chr7	7p14.1	1	1	0	0	Amplification	1	0	42473T: 3.5;
CDK14	chr7	7q21.13	3	0	2	1	Amplification	3	0	42483T: 4; 42487T: 3.5; 42494T: 3.5;
CDK19	chr6	6q21	1	0	0	1	Amplification	1	0	42496T: 3.5;
CDK2	chr12	12q13.2	1	0	0	1	Amplification	1	0	42494T: 5;
CDK2AP1	chr12	12q24.31	1	0	0	1	Amplification	1	0	42500T: 3.5;
CDK2AP2	chr11	11q13.2	2	1	0	1	Amplification	2	0	42473T: 3.5; 56957T: 5;
CDK3	chr17	17q25.1	1	0	0	1	Amplification	1	0	42494T: 3.5;
CDK5R2	chr2	2q35	1	1	0	0	Amplification	1	0	42473T: 3.5;
CDK5RAP1	chr20	20q11.21	2	1	0	1	Amplification	2	0	42473T: 3.5; 42496T: 5;
CDK6	chr7	7q21.2	2	0	2	0	Amplification	2	0	42483T: 4; 42487T: 3.5;
CDKL1	chr14	14q21.3	2	0	0	2	Amplification	2	0	42494T: 4; 42496T: 3.5;
CDKL3	chr5	5q31.1	1	1	0	0	Amplification	1	0	42473T: 3.5;
CDKL4	chr2	2p22.1	1	0	0	1	Amplification	1	0	42500T: 3.5;
CDKN1B	chr12	12p13.1	1	0	0	1	Amplification	1	0	42500T: 4.5;
CDKN2A	chr9	9p21.3	2	1	1	0	Deletion	0	2	42475T: 0.5; 42486T: 0.5;
CDKN2AIPNL	chr5	5q31.1	1	1	0	0	Amplification	1	0	42473T: 3.5;
CDKN2B	chr9	9p21.3	1	0	1	0	Deletion	0	1	42486T: 0.5;
CDKN3	chr14	14q22.2	1	0	0	1	Amplification	1	0	42494T: 4;
CDR2	chr16	16p12.2	1	1	0	0	Amplification	1	0	42473T: 4;
CDS2	chr20	20p12.3	1	1	0	0	Amplification	1	0	42473T: 4.5;
CDV3	chr3	3q22.1	3	0	1	2	Amplification	3	0	42492T: 3.5; 42487T: 3.5; 42496T: 3.5;
CEA	chr19	19q13.31	1	1	0	0	Amplification	1	0	42473T: 4.5;
CEACAM16	chr19	19q13.31-q1	2	1	0	1	Amplification	2	0	56957T: 4; 42473T: 3.5;
CEACAM19	chr19	19q13.31	2	1	0	1	Amplification	2	0	56957T: 4; 42473T: 3.5;

Mangalaparthi *et al.* , 2020. Mutational landscape of esophageal squamous cell carcinoma in an Indian cohort
Supplementary Table 7A. List of copy number alterations and affected genes in ESCC patients

Gene	Chromosome	Cytoband	Recurrence	Recurrence in smoker cohort	Recurrence in chewer cohort	Recurrence in No habit cohort	State	Samples with CNA gain	Samples with CNA loss	File info with CNA fold
CEACAM20	chr19	19q13.31	2	1	0	1	Amplification	2	0	42473T: 3.5; 56957T: 4;
CEACAM21	chr19	19q13.2	1	1	0	0	Amplification	1	0	42473T: 4.5;
CEACAM3	chr19	19q13.2	1	1	0	0	Amplification	1	0	42473T: 4.5;
CEACAM4	chr19	19q13.2	1	1	0	0	Amplification	1	0	42473T: 4.5;
CEACAM5	chr19	19q13.2	1	1	0	0	Amplification	1	0	42473T: 4.5;
CEACAM6	chr19	19q13.2	1	1	0	0	Amplification	1	0	42473T: 4.5;
CEACAM7	chr19	19q13.2	1	1	0	0	Amplification	1	0	42473T: 4.5;
CEBPB	chr20	20q13.13	1	1	0	0	Amplification	1	0	42473T: 6.5;
CEBPD	chr8	8q11.21	2	0	0	2	Amplification	2	0	42494T: 3.5; 42496T: 3.5;
CEBPE	chr14	14q11.2	2	0	0	2	Amplification	2	0	42500T: 4; 42496T: 4;
CEBPG	chr19	19q13.11	3	0	1	2	Amplification	3	0	42500T: 4.5; 56957T: 4; 42484T: 3.5;
CEBPZ	chr2	2p22.2	1	0	0	1	Amplification	1	0	42500T: 3.5;
CELA1	chr12	12q13.13	2	0	0	2	Amplification	2	0	42494T: 3.5; 42500T: 3.5;
CELA3A	chr1	1p36.12	2	1	0	1	Amplification	2	0	42473T: 4; 42493T: 3.5;
CELA3B	chr1	1p36.12	2	1	0	1	Amplification	2	0	42493T: 3.5; 42473T: 4;
CELF1	chr11	11p11.2	1	1	0	0	Amplification	1	0	42473T: 3.5;
CELF3	chr1	1q21.3	1	1	0	0	Amplification	1	0	42473T: 4.5;
CELSR1	chr22	22q13.31	1	0	0	1	Amplification	1	0	42495T: 4;
CENPA	chr2	2p23.3	1	0	0	1	Amplification	1	0	42500T: 3.5;
CENPB	chr20	20p13	2	1	1	0	Amplification	2	0	42473T: 6; 42487T: 4.5;
CENPO	chr2	2p23.3	1	0	0	1	Amplification	1	0	42500T: 3.5;
CEP19	chr3	3q29	9	1	3	5	Amplification	9	0	42487T: 3.5; 42493T: 5; 42492T: 3.5; 42482T: 3.5; 56957T: 6; 42495T: 4; 42474T: 3.5; 42498T: 3.5; 42484T: 4;
CEP250	chr20	20q11.22	2	1	0	1	Amplification	2	0	42473T: 3.5; 42493T: 3.5;
CEP41	chr7	7q32.2	1	0	1	0	Amplification	1	0	42487T: 3.5;
CEP57L1	chr6	6q21	1	0	0	1	Amplification	1	0	42496T: 3.5;
CEP63	chr3	3q22.2	4	0	1	3	Amplification	4	0	42493T: 3.5; 42487T: 3.5; 42492T: 3.5; 42496T: 3.5;
CEP68	chr2	2p14	2	0	0	2	Amplification	2	0	56957T: 3.5; 42500T: 3.5;
CEP70	chr3	3q22.3	4	0	1	3	Amplification	4	0	42496T: 3.5; 42487T: 3.5; 42493T: 3.5; 42492T: 3.5;
CEP72	chr5	5p15.33	5	2	1	2	Amplification	5	0	42493T: 3.5; 42475T: 3.5; 42486T: 3.5; 42473T: 3.5; 42496T: 4;
CEP89	chr19	19q13.11	4	1	1	2	Amplification	4	0	56957T: 4; 42484T: 3.5; 42500T: 4.5; 42473T: 7;
CEP97	chr3	3q12.3	1	1	0	0	Amplification	1	0	42476T: 3.5;
CER1	chr9	3p22-p21.2	1	0	0	1	Amplification	1	0	42498T: 14.5;
CERK	chr22	22q13.31	1	0	0	1	Amplification	1	0	42495T: 4;
CERKL	chr2	2q31.3	1	0	0	1	Amplification	1	0	42493T: 4;
CERS2	chr1	1q21.3	1	1	0	0	Amplification	1	0	42473T: 4.5;
CERS5	chr12	2q24.3	1	0	0	1	Amplification	1	0	42500T: 3.5;
CETN1	chr18	18p11.32	3	0	0	3	Amplification	3	0	42493T: 3.5; 56957T: 8; 42500T: 4.5;
CFB	chr6	6p21.33	1	1	0	0	Amplification	1	0	42473T: 3.5;

Mangalaparthi *et al.* , 2020. Mutational landscape of esophageal squamous cell carcinoma in an Indian cohort
Supplementary Table 7A. List of copy number alterations and affected genes in ESCC patients

Gene	Chromosome	Cytoband	Recurrence	Recurrence in smoker cohort	Recurrence in chewer cohort	Recurrence in No habit cohort	State	Samples with CNA gain	Samples with CNA loss	File info with CNA fold
CFD	chr19	2q34	1	0	0	1	Amplification	1	0	42493T: 3.5;
CFHR1	chr1	1q31.3	1	0	0	1	Amplification	1	0	42493T: 4.5;
CFHR3	chr1	1q31.3	1	0	0	1	Amplification	1	0	42493T: 4.5;
CFL2	chr14	14q13.1	1	0	0	1	Amplification	1	0	42500T: 4.5;
CFTR	chr7	7q31.2	1	0	1	0	Amplification	1	0	42487T: 3.5;
CGN	chr1	1q21.3	1	1	0	0	Amplification	1	0	42473T: 4.5;
CGREF1	chr2	2p23.3	1	0	0	1	Amplification	1	0	42500T: 3.5;
CGRRF1	chr14	14q22.2	1	0	0	1	Amplification	1	0	42494T: 4;
CHAC1	chr15	15q15.1	1	0	0	1	Amplification	1	0	42493T: 3.5;
CHAC2	chr2	2p16.2	2	0	1	1	Amplification	2	0	42500T: 3.5; 42484T: 4.5;
CHAD	chr17	17q21.33	1	1	0	0	Amplification	1	0	42473T: 4.5;
CHCHD1	chr10	10q22.2	2	1	0	1	Amplification	2	0	42496T: 3.5; 42473T: 4.5;
CHCHD2	chr7	7p11.2	2	0	1	1	Amplification	2	0	42483T: 4; 56957T: 5;
CHCHD3	chr7	7q32.3-q33	1	0	1	0	Amplification	1	0	42487T: 3.5;
CHCHD6	chr3	3q21.3	1	0	0	1	Amplification	1	0	42496T: 3.5;
CHCHD7	chr8	8q12.1	3	0	1	2	Amplification	3	0	42483T: 5; 42496T: 3.5; 42495T: 3.5;
CHD1L	chr1	1q21.1	1	1	0	0	Amplification	1	0	42473T: 4;
CHD6	chr20	20q12	1	1	0	0	Amplification	1	0	42473T: 3.5;
CHD8	chr14	14q11.2	1	0	0	1	Amplification	1	0	42496T: 5;
CHEK2P2	chr15	15q11.1	2	1	1	0	Amplification/Deletio	1	1	42484T: 7.5; 42476T: 0.5;
CHKA	chr11	11q13.2	1	0	0	1	Amplification	1	0	56957T: 5;
CHMP1B	chr18	18p11.21	1	0	0	1	Amplification	1	0	42493T: 3.5;
CHMP3	chr2	2p11.2	2	0	0	2	Amplification	2	0	42500T: 3.5; 42493T: 3.5;
CHMP4B	chr20	20q11.22	3	1	0	2	Amplification	3	0	42493T: 3.5; 42473T: 3.5; 42496T: 5;
CHMP4C	chr8	8q21.13	2	0	0	2	Amplification	2	0	42495T: 3.5; 42496T: 3.5;
CHN1	chr2	2q31.1	1	0	0	1	Amplification	1	0	42493T: 3.5;
CHN2	chr7	7p14.3	1	1	0	0	Amplification	1	0	42473T: 4;
CHP2	chr16	16p12.2	1	1	0	0	Amplification	1	0	42473T: 4;
CHRAC1	chr8	8q24.3	3	0	1	2	Amplification	3	0	42495T: 3.5; 42496T: 3.5; 42483T: 3.5;
CHRD	chr3	3q27.1	11	1	3	7	Amplification	11	0	42484T: 4; 42498T: 3.5; 42494T: 3.5; 42495T: 4; 42474T: 3.5; 56957T: 5; 42497T: 4; 42492T: 3.5; 42493T: 3.5; 42487T: 3.5; 42482T: 3.5;
CHRDL2	chr11	11q13.4	5	2	0	3	Amplification	5	0	56957T: 3.5; 42498T: 8; 42492T: 4; 42475T: 7.5; 42476T: 4;
CHRM2	chr7	7q33	1	0	1	0	Amplification	1	0	42487T: 3.5;
CHRM4	chr11	11p11.2	1	1	0	0	Amplification	1	0	42473T: 3.5;
CHRNA1	chr2	2q31.1	1	0	0	1	Amplification	1	0	42493T: 3.5;
CHRNA3	chr15	15q25.1	1	1	0	0	Amplification	1	0	42473T: 3.5;
CHRNA4	chr20	20q13.33	1	1	0	0	Amplification	1	0	42473T: 6;
CHRNA5	chr15	15q25.1	1	1	0	0	Amplification	1	0	42473T: 3.5;
CHRNA6	chr8	8p11.21	1	0	1	0	Amplification	1	0	42483T: 4.5;

Mangalaparthi *et al.* , 2020. Mutational landscape of esophageal squamous cell carcinoma in an Indian cohort
Supplementary Table 7A. List of copy number alterations and affected genes in ESCC patients

Gene	Chromosome	Cytoband	Recurrence	Recurrence in smoker cohort	Recurrence in chewer cohort	Recurrence in No habit cohort	State	Samples with CNA gain	Samples with CNA loss	File info with CNA fold
CHRNB2	chr1	1q21.3	1	1	0	0	Amplification	1	0	42473T: 4.5;
CHRNB3	chr8	8p11.21	1	0	1	0	Amplification	1	0	42483T: 4.5;
CHRNB4	chr15	15q25.1	1	1	0	0	Amplification	1	0	42473T: 3.5;
CHST10	chr2	2q11.2	1	0	0	1	Amplification	1	0	42493T: 3.5;
CHST12	chr7	7p22.3	1	1	0	0	Amplification	1	0	42473T: 5;
CHST13	chr3	3q21.3	1	0	0	1	Amplification	1	0	42496T: 3.5;
CHST8	chr19	19q13.11	3	0	1	2	Amplification	3	0	42500T: 4.5; 56957T: 4; 42484T: 3.5;
CHTF18	chr16	16p13.3	2	0	1	1	Amplification	2	0	42493T: 3.5; 42483T: 3.5;
CHTOP	chr1	1q21.3	1	1	0	0	Amplification	1	0	42473T: 4.5;
CHURC1	chr14	14q23.3	1	0	0	1	Amplification	1	0	42494T: 4;
CIAO1	chr2	2q11.2	2	1	0	1	Amplification	2	0	42473T: 3.5; 42493T: 3.5;
CIB1	chr15	15q26.1	1	1	0	0	Amplification	1	0	42473T: 3.5;
CIB2	chr15	15q25.1	1	1	0	0	Amplification	1	0	42473T: 3.5;
CIB4	chr2	2p23.3	1	0	0	1	Amplification	1	0	42500T: 3.5;
CIC	chr19	19q13.2	1	1	0	0	Amplification	1	0	42473T: 4.5;
CIDEA	chr18	18p11.21|18	1	0	0	1	Amplification	1	0	42493T: 3.5;
CIITA	chr16	16p13.13	2	1	0	1	Amplification	2	0	42473T: 5.5; 42495T: 5;
CIR1	chr2	2q31.1	1	0	0	1	Amplification	1	0	42493T: 3.5;
CISD3	chr17	17q12	1	1	0	0	Amplification	1	0	42473T: 3.5;
CIT	chr12	1p36.11	1	0	0	1	Amplification	1	0	42500T: 3.5;
CKAP5	chr11	11p11.2	1	1	0	0	Amplification	1	0	42473T: 3.5;
CKM	chr19	19q13.32	3	1	1	1	Amplification	3	0	56957T: 4; 42484T: 4; 42473T: 3.5;
CKS1B	chr1	1q21.3	2	1	0	1	Amplification	2	0	42496T: 3.5; 42473T: 6.5;
CLASRP	chr19	19q13.32	2	1	0	1	Amplification	2	0	42473T: 3.5; 56957T: 4;
CLC	chr19	11q13.2	2	0	0	2	Amplification	2	0	42500T: 7.5; 56957T: 4;
CLCA1	chr1	1p22.3	1	1	0	0	Amplification	1	0	42473T: 4;
CLCA2	chr1	1p22.3	1	1	0	0	Amplification	1	0	42473T: 4;
CLCA3P	chr1	1p22.3	1	1	0	0	Amplification	1	0	42473T: 4;
CLCA4	chr1	1p22.3	1	1	0	0	Amplification	1	0	42473T: 4;
CLCF1	chr11	11q13.2	2	1	0	1	Amplification	2	0	42473T: 3.5; 56957T: 5;
CLCN1	chr7	7q34	1	0	1	0	Amplification	1	0	42487T: 3.5;
CLCN2	chr3	3q27.1	11	1	3	7	Amplification	11	0	42493T: 3.5; 42487T: 3.5; 42492T: 3.5; 42482T: 3.5; 42474T: 3.5; 42495T: 4; 42494T: 3.5; 56957T: 5; 42484T: 4; 42498T: 3.5; 42497T: 4;
CLCN7	chr16	16p13.3	1	0	1	0	Amplification	1	0	42483T: 3.5;
CLCNKA	chr1	1p36.13	1	1	0	0	Amplification	1	0	42473T: 8;
CLCNKB	chr1	1p36.13	1	1	0	0	Amplification	1	0	42473T: 8;
CLDN1	chr3	3q28	9	1	3	5	Amplification	9	0	56957T: 4.5; 42474T: 3.5; 42495T: 4; 42498T: 3.5; 42484T: 4; 42482T: 3.5; 42493T: 3.5; 42487T: 3.5; 42492T: 3.5;

Mangalaparthi *et al.* , 2020. Mutational landscape of esophageal squamous cell carcinoma in an Indian cohort
Supplementary Table 7A. List of copy number alterations and affected genes in ESCC patients

Gene	Chromosome	Cytoband	Recurrence	Recurrence in smoker cohort	Recurrence in chewer cohort	Recurrence in No habit cohort	State	Samples with CNA gain	Samples with CNA loss	File info with CNA fold
CLDN11	chr3	3q26.2	9	1	3	5	Amplification	9	0	56957T: 4; 42495T: 4; 42474T: 3.5; 42484T: 4; 42500T: 8; 42487T: 3.5; 42493T: 3.5; 42492T: 3.5; 42482T: 3.5;
CLDN12	chr7	7q21.13	2	0	2	0	Amplification	2	0	42483T: 3.5; 42487T: 3.5;
CLDN15	chr7	7q22.1	2	0	0	2	Amplification	2	0	42493T: 3.5; 42501T: 3.5;
CLDN16	chr3	3q28	9	1	3	5	Amplification	9	0	42492T: 3.5; 42487T: 3.5; 42493T: 3.5; 42482T: 3.5; 42498T: 3.5; 42484T: 4; 56957T: 4.5; 42495T: 4; 42474T: 3.5;
CLDN18	chr3	3q22.3	4	0	1	3	Amplification	4	0	42496T: 3.5; 42493T: 3.5; 42487T: 3.5; 42492T: 3.5;
CLDN19	chr1	1p34.2	1	0	0	1	Amplification	1	0	42493T: 3.5;
CLDN5	chr22	22q11.21	1	0	0	1	Amplification	1	0	42497T: 17;
CLDND1	chr3	3q11.2	1	1	0	0	Amplification	1	0	42476T: 3.5;
CLEC14A	chr14	14q21.1	2	1	0	1	Amplification	2	0	42476T: 4; 42500T: 6.5;
CLEC16A	chr16	16p13.13	2	1	0	1	Amplification	2	0	42495T: 5; 42473T: 5.5;
CLEC19A	chr16	16p12.3	2	1	0	1	Amplification	2	0	42495T: 4; 42473T: 4;
CLEC2L	chr7	7q34	1	0	1	0	Amplification	1	0	42487T: 3.5;
CLEC4F	chr2	2p13.3	1	0	0	1	Amplification	1	0	42500T: 3.5;
CLEC5A	chr7	7q34	1	0	1	0	Amplification	1	0	42487T: 3.5;
CLHC1	chr2	2p16.1	3	0	1	2	Amplification	3	0	56957T: 3.5; 42484T: 4.5; 42500T: 3.5;
CLIC1	chr6	6p21.33	1	1	0	0	Amplification	1	0	42473T: 3.5;
CLIC3	chr9	9q34.3	2	1	0	1	Amplification	2	0	56957T: 3.5; 42473T: 5;
CLIC4	chr1	1p36.11	1	1	0	0	Amplification	1	0	42473T: 4.5;
CLIP1	chr12	12q24.31	1	0	0	1	Amplification	1	0	42500T: 3.5;
CLIP3	chr19	19q13.12	3	0	1	2	Amplification	3	0	56957T: 4; 42484T: 3.5; 42500T: 6.5;
CLIP4	chr2	2p23.2	1	0	0	1	Amplification	1	0	42500T: 3.5;
CLK2	chr1	16p13.3	1	1	0	0	Amplification	1	0	42473T: 6.5;
CLK3	chr15	15q24.1	1	1	0	0	Amplification	1	0	42473T: 3.5;
CLN8	chr8	8p23.3	1	0	0	1	Amplification	1	0	56957T: 4;
CLP1	chr11	18p11.32	1	0	0	1	Amplification	1	0	42496T: 4.5;
CLPB	chr11	11q13.4	7	3	0	4	Amplification	7	0	42475T: 5; 42476T: 4; 42493T: 3.5; 56957T: 3.5; 42498T: 5; 42492T: 4; 42478T: 4.5;
CLPTM1	chr19	19q13.32	2	1	0	1	Amplification	2	0	56957T: 4; 42473T: 3.5;
CLPTM1L	chr5	5p15.33	5	2	1	2	Amplification	5	0	42475T: 3.5; 42496T: 4; 42473T: 3.5; 42486T: 3.5; 42493T: 3.5;
CLRN1	chr3	3q25.1	9	1	2	6	Amplification	9	0	42487T: 3.5; 42493T: 3.5; 42492T: 3.5; 42474T: 3.5; 56957T: 4; 42484T: 3.5; 42500T: 3.5; 42497T: 4.5; 42496T: 4;
CLSTN2	chr3	3q23	4	0	1	3	Amplification	4	0	42496T: 4; 42487T: 3.5; 42493T: 3.5; 42492T: 3.5;
CLSTN3	chr12	12p13.31	1	0	0	1	Amplification	1	0	42494T: 3.5;
CLTA	chr9	9p13.3	1	0	0	1	Amplification	1	0	42501T: 3.5;
CLTCL1	chr22	22q11.21	1	0	0	1	Amplification	1	0	42497T: 21;

Mangalaparthi *et al.* , 2020. Mutational landscape of esophageal squamous cell carcinoma in an Indian cohort
Supplementary Table 7A. List of copy number alterations and affected genes in ESCC patients

Gene	Chromosome	Cytoband	Recurrence	Recurrence in smoker cohort	Recurrence in chewer cohort	Recurrence in No habit cohort	State	Samples with CNA gain	Samples with CNA loss	File info with CNA fold
CLUAP1	chr16	16p13.3	1	1	0	0	Amplification	1	0	42473T: 5.5;
CLUL1	chr18	18p11.32	3	0	0	3	Amplification	3	0	42493T: 3.5; 56957T: 8; 42500T: 4.5;
CLVS1	chr8	8q12.2-q12.3	2	0	0	2	Amplification	2	0	42496T: 3.5; 42495T: 3.5;
CMAS	chr12	12p12.1	1	0	0	1	Amplification	1	0	42500T: 6;
CMBL	chr5	5p15.2	4	1	1	2	Amplification	4	0	42496T: 3.5; 42486T: 3.5; 42475T: 3.5; 42493T: 4;
CMPK2	chr2	2p25.2	1	0	0	1	Amplification	1	0	42500T: 3.5;
CMSS1	chr3	3q12.1	1	1	0	0	Amplification	1	0	42476T: 3.5;
CMTM5	chr14	14q11.2	2	0	0	2	Amplification	2	0	42496T: 4; 42500T: 4;
CNBD1	chr8	8q21.3	2	0	0	2	Amplification	2	0	42496T: 3.5; 42495T: 3.5;
CNBD2	chr20	20q11.23	1	1	0	0	Amplification	1	0	42473T: 3.5;
CNBP	chr3	3q21.3	2	0	1	1	Amplification	2	0	42496T: 3.5; 42487T: 3.5;
CNFN	chr19	19q13.2	1	1	0	0	Amplification	1	0	42473T: 4.5;
CNGA3	chr2	2q11.2	1	0	0	1	Amplification	1	0	42493T: 3.5;
CNGB3	chr8	8q21.3	2	0	0	2	Amplification	2	0	42495T: 3.5; 42496T: 3.5;
CNIH	chr14	14q22.2	1	0	0	1	Amplification	1	0	42494T: 4;
CNN2	chr19	19p13.3	1	0	0	1	Amplification	1	0	42493T: 3.5;
CNNM3	chr2	2q11.2	2	1	0	1	Amplification	2	0	42473T: 3.5; 42493T: 4.5;
CNNM4	chr2	2q11.2	2	1	0	1	Amplification	2	0	42473T: 3.5; 42493T: 4.5;
CNOT11	chr2	2q11.2	1	0	0	1	Amplification	1	0	42493T: 3.5;
CNOT2	chr12	12q15	2	0	0	2	Amplification	2	0	42501T: 5.5; 42500T: 5;
CNOT4	chr7	7q33	1	0	1	0	Amplification	1	0	42487T: 3.5;
CNR2	chr1	5q31.3	1	1	0	0	Amplification	1	0	42473T: 3.5;
CNRIP1	chr2	2p14	1	0	0	1	Amplification	1	0	42500T: 3.5;
CNTD2	chr19	19q13.2	2	0	0	2	Amplification	2	0	42500T: 7.5; 56957T: 4;
CNTN1	chr12	12q12	1	0	0	1	Amplification	1	0	42500T: 3.5;
CNTNAP2	chr7	7q35-q36.1	2	0	1	1	Amplification	2	0	42487T: 3.5; 42500T: 4;
COA1	chr7	7p13	1	1	0	0	Amplification	1	0	42473T: 3.5;
COA4	chr11	11q13.4	6	3	0	3	Amplification	6	0	42478T: 6; 42492T: 4; 42498T: 8; 56957T: 3.5; 42476T: 4; 42475T: 7.5;
COA5	chr2	2q11.2	1	0	0	1	Amplification	1	0	42493T: 3.5;
COBL	chr7	7p12.1	1	0	0	1	Amplification	1	0	42497T: 20.5;
COCH	chr14	14q12	3	0	0	3	Amplification	3	0	42500T: 4.5; 56957T: 4; 42494T: 3.5;
COG5	chr7	7q22.3	4	0	1	3	Amplification	4	0	42501T: 3.5; 42497T: 4; 42493T: 3.5; 42487T: 3.5;
COG7	chr16	16p12.2	1	1	0	0	Amplification	1	0	42473T: 4;
COIL	chr17	17q22	1	0	0	1	Amplification	1	0	42497T: 4;
COL14A1	chr8	8q24.12	3	1	0	2	Amplification	3	0	42475T: 3.5; 42496T: 3.5; 42495T: 3.5;
COL16A1	chr1	1p35.2	1	1	0	0	Amplification	1	0	42473T: 3.5;
COL1A2	chr7	7q21.3	1	0	1	0	Amplification	1	0	42487T: 3.5;
COL20A1	chr20	20q13.33	1	1	0	0	Amplification	1	0	42473T: 6;
COL21A1	chr6	6p12.1|6p12	1	0	0	1	Amplification	1	0	42497T: 3.5;
COL22A1	chr8	8q24.23-q24	2	0	0	2	Amplification	2	0	42495T: 3.5; 42496T: 3.5;

Mangalaparthi *et al.* , 2020. Mutational landscape of esophageal squamous cell carcinoma in an Indian cohort
Supplementary Table 7A. List of copy number alterations and affected genes in ESCC patients

Gene	Chromosome	Cytoband	Recurrence	Recurrence in smoker cohort	Recurrence in chewer cohort	Recurrence in No habit cohort	State	Samples with CNA gain	Samples with CNA loss	File info with CNA fold
COL26A1	chr7	7q22.1	2	0	0	2	Amplification	2	0	42493T: 3.5; 42501T: 3.5;
COL28A1	chr7	7p21.3	2	1	0	1	Amplification	2	0	42473T: 3.5; 42497T: 7.5;
COL2A1	chr12	12q13.11	1	0	0	1	Amplification	1	0	42500T: 3.5;
COL3A1	chr2	2q32.2	1	0	0	1	Amplification	1	0	42493T: 3.5;
COL5A2	chr2	2q32.2	1	0	0	1	Amplification	1	0	42493T: 3.5;
COL6A5	chr3	3q22.1	2	0	1	1	Amplification	2	0	42496T: 3.5; 42487T: 3.5;
COL6A6	chr3	3q22.1	2	0	1	1	Amplification	2	0	42496T: 3.5; 42487T: 3.5;
COL8A1	chr3	3q12.1	1	1	0	0	Amplification	1	0	42476T: 3.5;
COL9A3	chr20	20q13.33	1	1	0	0	Amplification	1	0	42473T: 6;
COLEC10	chr8	8q24.12	3	1	0	2	Amplification	3	0	42475T: 3.5; 42496T: 3.5; 42495T: 3.5;
COLEC11	chr2	2p25.3	2	0	0	2	Amplification	2	0	42500T: 3.5; 42495T: 3.5;
COLEC12	chr18	18p11.32	3	0	0	3	Amplification	3	0	56957T: 8; 42493T: 3.5; 42500T: 4.5;
COMMD1	chr2	2p15	3	0	1	2	Amplification	3	0	42500T: 3.5; 42484T: 8.5; 56957T: 3.5;
COMMD2	chr3	3q25.1	8	1	2	5	Amplification	8	0	42492T: 3.5; 42487T: 3.5; 42493T: 3.5; 42496T: 4; 42497T: 4.5; 42484T: 3.5; 42474T: 3.5; 56957T: 4;
COMMD5	chr8	8q24.3	3	0	1	2	Amplification	3	0	42495T: 4.5; 42496T: 4; 42483T: 3.5;
COMMD7	chr20	20q11.21	2	1	0	1	Amplification	2	0	42496T: 5; 42473T: 3.5;
COMT	chr22	22q11.21	1	0	0	1	Amplification	1	0	42497T: 17;
COPB2	chr3	3q23	4	0	1	3	Amplification	4	0	42496T: 3.5; 42487T: 3.5; 42493T: 3.5; 42492T: 3.5;
COPG1	chr3	3q21.3	2	0	1	1	Amplification	2	0	42496T: 3.5; 42487T: 3.5;
COPG2	chr7	7q32.2	1	0	1	0	Amplification	1	0	42487T: 3.5;
COPRS	chr17	17q11.2	1	1	0	0	Amplification	1	0	42473T: 3.5;
COPS5	chr8	8q13.1	3	0	0	3	Amplification	3	0	42495T: 3.5; 42497T: 4; 42496T: 3.5;
COPZ1	chr12	12q13.13	2	1	0	1	Amplification	2	0	42494T: 3.5; 42473T: 3.5;
COQ10A	chr12	12q13.3	1	0	0	1	Amplification	1	0	42494T: 7.5;
COQ10B	chr2	2q33.1	1	0	1	0	Amplification	1	0	42482T: 4;
COQ5	chr12	12q24.31	1	0	0	1	Amplification	1	0	42500T: 3.5;
COQ6	chr14	14q24.3	2	0	0	2	Amplification	2	0	56957T: 3.5; 42494T: 4;
COQ7	chr16	16p12.3	2	1	0	1	Amplification	2	0	42473T: 4; 42495T: 4;
CORO1B	chr11	11q13.2	2	1	0	1	Amplification	2	0	56957T: 5; 42473T: 3.5;
CORO7	chr16	16p13.3	3	1	0	2	Amplification	3	0	42495T: 8.5; 42494T: 3.5; 42473T: 5.5;
COX11	chr17	6p22.1	1	0	0	1	Amplification	1	0	42497T: 3.5;
COX14	chr12	12q13.12	1	0	0	1	Amplification	1	0	42500T: 3.5;
COX16	chr14	14q24.2	1	0	0	1	Amplification	1	0	42494T: 4;
COX17	chr3	3q13.33	1	0	0	1	Amplification	1	0	42496T: 3.5;
COX19	chr7	7p22.3	1	1	0	0	Amplification	1	0	42473T: 5;
COX4I2	chr20	20q11.21	1	0	0	1	Amplification	1	0	42496T: 5;
COX5B	chr2	2q11.2	1	0	0	1	Amplification	1	0	42493T: 3.5;
COX6A1	chr12	12q24.31|12	1	0	0	1	Amplification	1	0	42500T: 3.5;
COX6B1	chr19	19q13.12	3	0	1	2	Amplification	3	0	56957T: 4; 42484T: 3.5; 42500T: 6.5;
COX6C	chr8	8q22.2	2	0	0	2	Amplification	2	0	42496T: 3.5; 42495T: 3.5;

Mangalaparthi *et al.* , 2020. Mutational landscape of esophageal squamous cell carcinoma in an Indian cohort
Supplementary Table 7A. List of copy number alterations and affected genes in ESCC patients

Gene	Chromosome	Cytoband	Recurrence	Recurrence in smoker cohort	Recurrence in chewer cohort	Recurrence in No habit cohort	State	Samples with CNA gain	Samples with CNA loss	File info with CNA fold
COX7A1	chr19	19q13.12	3	0	1	2	Amplification	3	0	42500T: 6.5; 56957T: 4; 42484T: 3.5;
COX7A2L	chr2	2p21	2	0	1	1	Amplification	2	0	42484T: 3.5; 42500T: 3.5;
COX7C	chr5	5q14.3	1	1	0	0	Deletion	0	1	42476T: 0.5;
CP	chr3	3q24-q25.1	8	1	2	5	Amplification	8	0	42492T: 3.5; 42487T: 3.5; 42493T: 3.5; 42484T: 3.5; 42474T: 3.5; 56957T: 4; 42497T: 4.5; 42496T: 4;
CPA1	chr7	7q32.2	1	0	1	0	Amplification	1	0	42487T: 3.5;
CPA2	chr7	7q32.2	1	0	1	0	Amplification	1	0	42487T: 3.5;
CPA3	chr3	7q32.2	7	1	2	4	Amplification	7	0	42492T: 3.5; 42484T: 3.5; 42493T: 3.5; 56957T: 4; 42487T: 3.5; 42474T: 3.5; 42497T: 4.5;
CPA4	chr7	7q32.2	1	0	1	0	Amplification	1	0	42487T: 3.5;
CPA5	chr7	7q32.2	1	0	1	0	Amplification	1	0	42487T: 3.5;
CPA6	chr8	8q13.2	3	0	0	3	Amplification	3	0	42497T: 4; 42496T: 3.5; 42495T: 3.5;
CPB1	chr3	3q24	8	1	2	5	Amplification	8	0	42492T: 3.5; 42493T: 3.5; 42487T: 3.5; 42484T: 3.5; 56957T: 4; 42474T: 3.5; 42497T: 4.5; 42496T: 4;
CPED1	chr7	7q31.31	1	0	1	0	Amplification	1	0	42487T: 3.5;
CPLX3	chr15	15q24.1	1	1	0	0	Amplification	1	0	42473T: 3.5;
CPM	chr12	12q15	2	0	0	2	Amplification	2	0	42501T: 6.5; 42500T: 5;
CPN2	chr3	16q13	9	1	3	5	Amplification	9	0	42487T: 3.5; 42493T: 3.5; 42492T: 3.5; 42482T: 3.5; 56957T: 4.5; 42495T: 4; 42474T: 3.5; 42498T: 3.5; 42484T: 4;
CPNE1	chr20	20q11.22	2	1	0	1	Amplification	2	0	42473T: 3.5; 42493T: 3.5;
CPNE3	chr8	8q21.3	2	0	0	2	Amplification	2	0	42496T: 3.5; 42495T: 3.5;
CPNE4	chr3	3q22.1	3	0	1	2	Amplification	3	0	42492T: 3.5; 42487T: 3.5; 42496T: 3.5;
CPNE8	chr12	12q12	1	0	0	1	Amplification	1	0	42500T: 3.5;
CPOX	chr3	3q11.2	1	1	0	0	Amplification	1	0	42476T: 3.5;
CPPED1	chr16	16p13.12	2	1	0	1	Amplification	2	0	42495T: 4; 42473T: 5.5;
CPQ	chr8	8q22.1	2	0	0	2	Amplification	2	0	42495T: 3.5; 42496T: 3.5;
CPSF1	chr8	8q24.3	4	0	1	3	Amplification	4	0	42483T: 3.5; 42496T: 4; 42495T: 4.5; 56957T: 3.5;
CPSF3	chr2	2p25.1	1	0	0	1	Amplification	1	0	42500T: 3.5;
CPSF6	chr12	12q15	2	0	0	2	Amplification	2	0	42500T: 5; 42501T: 5.5;
CPT1A	chr11	11q13.3	5	1	0	4	Amplification	5	0	42492T: 4; 42498T: 9.5; 56957T: 5; 42497T: 4.5; 42476T: 29.5;
CPVL	chr7	7p14.3	1	1	0	0	Amplification	1	0	42473T: 4;
CPXM1	chr20	20p13	1	1	0	0	Amplification	1	0	42473T: 3.5;
CRABP1	chr15	15q25.1	1	1	0	0	Amplification	1	0	42473T: 3.5;
CRABP2	chr1	1q23.1	1	1	0	0	Amplification	1	0	42473T: 7;
CRAMP1L	chr16	16p13.3	1	0	1	0	Amplification	1	0	42483T: 3.5;
CRCP	chr7	7q11.21	1	0	0	1	Amplification	1	0	42501T: 3.5;
CREB3	chr9	1q21.3	2	1	0	1	Amplification	2	0	42473T: 3.5; 42501T: 3.5;
CREB3L1	chr11	11p11.2	1	1	0	0	Amplification	1	0	42473T: 3.5;
CREB3L2	chr7	7q33	1	0	1	0	Amplification	1	0	42487T: 3.5;

Mangalaparthi *et al.* , 2020. Mutational landscape of esophageal squamous cell carcinoma in an Indian cohort
Supplementary Table 7A. List of copy number alterations and affected genes in ESCC patients

Gene	Chromosome	Cytoband	Recurrence	Recurrence in smoker cohort	Recurrence in chewer cohort	Recurrence in No habit cohort	State	Samples with CNA gain	Samples with CNA loss	File info with CNA fold
CREB3L4	chr1	1q21.3	1	1	0	0	Amplification	1	0	42473T: 4.5;
CREB5	chr7	7p15.1	1	1	0	0	Amplification	1	0	42473T: 4;
CREBBP	chr16	16p13.3	1	1	0	0	Amplification	1	0	42473T: 5.5;
CREBL2	chr12	12p13.1	1	0	0	1	Amplification	1	0	42500T: 4.5;
CREG2	chr2	2q11.2	1	0	0	1	Amplification	1	0	42493T: 3.5;
CRH	chr8	8q13.1	3	0	0	3	Amplification	3	0	42496T: 3.5; 42497T: 4; 42495T: 3.5;
CRHR2	chr7	7p14.3	1	1	0	0	Amplification	1	0	42473T: 4;
CRIM1	chr2	2p22.2	1	0	0	1	Amplification	1	0	42500T: 3.5;
CRIPT	chr2	2p21	2	0	1	1	Amplification	2	0	42500T: 3.5; 42484T: 4.5;
CRISPLD1	chr8	8q21.13	2	0	0	2	Amplification	2	0	42496T: 3.5; 42495T: 3.5;
CRKL	chr22	22q11.21	2	1	0	1	Amplification	2	0	42477T: 4; 42497T: 4;
CRLF3	chr17	17q11.2	1	1	0	0	Amplification	1	0	42473T: 3.5;
CRNKL1	chr20	20p11.23	1	0	1	0	Amplification	1	0	42483T: 4;
CRNN	chr1	1q21.3	1	1	0	0	Amplification	1	0	42473T: 4.5;
CROCC	chr1	1p36.13	1	1	0	0	Amplification	1	0	42473T: 4.5;
CROCCP2	chr1	1p36.13	1	1	0	0	Amplification	1	0	42473T: 4.5;
CROCCP3	chr1	1p36.13	1	1	0	0	Amplification	1	0	42473T: 4.5;
CROT	chr7	7q21.12	2	0	2	0	Amplification	2	0	42487T: 3.5; 42483T: 3.5;
CRTC2	chr1	1q21.3	1	1	0	0	Amplification	1	0	42473T: 4.5;
CRY2	chr11	11p11.2	1	1	0	0	Amplification	1	0	42473T: 3.5;
CRYBA2	chr2	2q35	1	1	0	0	Amplification	1	0	42473T: 3.5;
CRYBG3	chr3	3q11.2	2	1	0	1	Amplification	2	0	42476T: 3.5; 42500T: 3.5;
CRYGS	chr3	3q27.3	10	1	3	6	Amplification	10	0	42497T: 4; 42474T: 3.5; 42495T: 4; 56957T: 4.5; 42484T: 4; 42498T: 3.5; 42482T: 3.5; 42487T: 3.5; 42493T: 3.5; 42492T: 3.5;
CRYM	chr16	16p12.2	1	1	0	0	Amplification	1	0	42473T: 4;
CSE1L	chr20	20q13.13	1	1	0	0	Amplification	1	0	42473T: 5;
CSF1	chr1	1p13.3	1	1	0	0	Amplification	1	0	42473T: 4.5;
CSF2RB	chr22	22q12.3	1	1	0	0	Amplification	1	0	42473T: 3.5;
CSF3	chr17	17q21.1	1	0	0	1	Amplification	1	0	42497T: 3.5;
CSK	chr15	15q24.1	1	1	0	0	Amplification	1	0	42473T: 3.5;
CSMD1	chr8	8p23.2	1	0	1	0	Deletion	0	1	42486T: 0.5;
CSNK1E	chr22	22q13.1	1	1	0	0	Amplification	1	0	42473T: 3.5;
CSNK2A1	chr20	16q21	1	1	0	0	Amplification	1	0	42473T: 3.5;
CSNK2B	chr6	6p21.33	1	1	0	0	Amplification	1	0	42473T: 3.5;
CSPP1	chr8	8q13.1-q13.2	3	0	0	3	Amplification	3	0	42495T: 3.5; 42497T: 4; 42496T: 3.5;
CSRNP2	chr12	12q13.12	2	0	0	2	Amplification	2	0	42494T: 3.5; 42500T: 3.5;
CSTA	chr3	3q21.1	1	0	0	1	Amplification	1	0	42496T: 3.5;
CSTF1	chr20	20q13.2-q13	1	1	0	0	Amplification	1	0	42473T: 5;
CTAGE1	chr18	18q11.2	1	1	0	0	Amplification	1	0	42481T: 4;
CTAGE5	chr14	14q21.1	1	0	0	1	Amplification	1	0	42500T: 6.5;

Mangalaparthi *et al.* , 2020. Mutational landscape of esophageal squamous cell carcinoma in an Indian cohort
Supplementary Table 7A. List of copy number alterations and affected genes in ESCC patients

Gene	Chromosome	Cytoband	Recurrence	Recurrence in smoker cohort	Recurrence in chewer cohort	Recurrence in No habit cohort	State	Samples with CNA gain	Samples with CNA loss	File info with CNA fold
CTCFL	chr20	20q13.31	1	1	0	0	Amplification	1	0	42473T: 7;
CTDSPL	chr3	3p22.2	1	1	0	0	Amplification	1	0	42473T: 4.5;
CTHRC1	chr8	8q22.3	2	0	0	2	Amplification	2	0	42496T: 3.5; 42495T: 3.5;
CTNNA1	chr5	5q31.2	1	0	0	1	Amplification	1	0	42495T: 3.5;
CTNNA2	chr2	2p12	1	0	0	1	Amplification	1	0	42500T: 3.5;
CTNNBL1	chr20	20q11.23	1	1	0	0	Amplification	1	0	42473T: 3.5;
CTNND1	chr11	11q12.1	1	0	0	1	Amplification	1	0	42496T: 4.5;
CTNND2	chr5	5p15.2	4	1	1	2	Amplification	4	0	42496T: 3.5; 42486T: 3.5; 42475T: 3.5; 42493T: 4;
CTSA	chr20	20q13.12	1	1	0	0	Amplification	1	0	42473T: 5;
CTSB	chr8	8p23.1	1	0	1	0	Amplification	1	0	42486T: 3.5;
CTSE	chr1	1q32.1	1	0	0	1	Amplification	1	0	42493T: 3.5;
CTSF	chr11	11q13.2	1	0	0	1	Amplification	1	0	56957T: 5.5;
CTSK	chr1	1q21.3	1	1	0	0	Amplification	1	0	42473T: 4.5;
CTSS	chr1	1q21.3	1	1	0	0	Amplification	1	0	42473T: 4.5;
CTSZ	chr20	20q13.32	1	1	0	0	Amplification	1	0	42473T: 4.5;
CTTN	chr11	11q13.3	12	3	2	7	Amplification	12	0	42498T: 7; 42478T: 4.5; 56957T: 5; 42497T: 6.5; 42486T: 20.5; 42483T: 9; 56958T: 5.5; 42500T: 13; 42501T: 6; 42475T: 7.5; 42492T: 4; 42476T: 13.5;
CTTNBP2	chr7	7q31.31	1	0	1	0	Amplification	1	0	42487T: 3.5;
CTXN3	chr5	5q23.2	1	1	0	0	Amplification	1	0	42473T: 3.5;
CUBN	chr10	10p13	1	0	0	1	Amplification	1	0	56958T: 4;
CUEDC1	chr17	17q22	1	0	0	1	Amplification	1	0	42497T: 4;
CUL1	chr7	7q36.1	2	0	1	1	Amplification	2	0	42500T: 4; 42487T: 3.5;
CUL4A	chr13	13q34	1	0	0	1	Amplification	1	0	56957T: 3.5;
CUX1	chr7	7q22.1	2	0	0	2	Amplification	2	0	42501T: 3.5; 42493T: 3.5;
CUX2	chr12	12q24.11-q2	1	0	0	1	Amplification	1	0	42500T: 3.5;
CWC22	chr2	2q31.3	1	0	0	1	Amplification	1	0	42493T: 3.5;
CWC25	chr17	17q12	1	1	0	0	Amplification	1	0	42473T: 3.5;
CWH43	chr4	4p11	1	0	1	0	Amplification	1	0	42483T: 3.5;
CXCR5	chr11	11q23.3	1	1	0	0	Amplification	1	0	42473T: 4;
CYB5R1	chr1	1q32.1	1	1	0	0	Amplification	1	0	42473T: 4;
CYC1	chr8	4q21.1	3	0	1	2	Amplification	3	0	42495T: 4.5; 42483T: 3.5; 42496T: 4;
CYCS	chr7	7p15.3	1	1	0	0	Amplification	1	0	42473T: 4;
CYHR1	chr8	8q24.3	5	0	1	4	Amplification	5	0	42483T: 3.5; 42496T: 4; 42494T: 3.5; 42495T: 4.5; 56957T: 3.5;
CYP11A1	chr15	15q24.1	1	1	0	0	Amplification	1	0	42473T: 3.5;
CYP11B1	chr8	8q24.3	3	0	1	2	Amplification	3	0	42496T: 4; 42483T: 3.5; 42495T: 4.5;
CYP11B2	chr8	8q24.3	3	0	1	2	Amplification	3	0	42495T: 4.5; 42496T: 4; 42483T: 3.5;
CYP1A1	chr15	15q24.1	1	1	0	0	Amplification	1	0	42473T: 3.5;
CYP1A2	chr15	15q24.1	1	1	0	0	Amplification	1	0	42473T: 3.5;
CYP1B1	chr2	2p22.2	2	0	0	2	Amplification	2	0	42493T: 3.5; 42500T: 3.5;

Mangalaparthi *et al.* , 2020. Mutational landscape of esophageal squamous cell carcinoma in an Indian cohort
Supplementary Table 7A. List of copy number alterations and affected genes in ESCC patients

Gene	Chromosome	Cytoband	Recurrence	Recurrence in smoker cohort	Recurrence in chewer cohort	Recurrence in No habit cohort	State	Samples with CNA gain	Samples with CNA loss	File info with CNA fold
CYP21A2	chr6	6p21.33	1	1	0	0	Amplification	1	0	42473T: 3.5;
CYP24A1	chr20	20q13.2	1	1	0	0	Amplification	1	0	42473T: 5;
CYP26B1	chr2	2p13.2	1	0	0	1	Amplification	1	0	42500T: 3.5;
CYP2A13	chr19	19q13.2	1	0	0	1	Amplification	1	0	42500T: 3.5;
CYP2A6	chr19	19q13.2	2	0	0	2	Amplification	2	0	56957T: 4; 42500T: 3.5;
CYP2A7	chr19	19q13.2	2	0	0	2	Amplification	2	0	56957T: 4; 42500T: 3.5;
CYP2B6	chr19	19q13.2	1	0	0	1	Amplification	1	0	42500T: 3.5;
CYP2B7P1	chr19	19q13.2	2	0	0	2	Amplification	2	0	56957T: 4; 42500T: 3.5;
CYP2G1P	chr19	19q13.2	2	0	0	2	Amplification	2	0	56957T: 4; 42500T: 3.5;
CYP2W1	chr7	7p22.3	1	1	0	0	Amplification	1	0	42473T: 5;
CYP51A1	chr7	7q21.2	3	0	2	1	Amplification	3	0	42487T: 3.5; 42494T: 3.5; 42483T: 4;
CYP7A1	chr8	8q12.1	2	0	0	2	Amplification	2	0	42495T: 3.5; 42496T: 3.5;
CYP7B1	chr8	8q12.3	2	0	0	2	Amplification	2	0	42495T: 3.5; 42496T: 3.5;
CYS1	chr2	2p25.1	1	0	0	1	Amplification	1	0	42500T: 3.5;
CYTH3	chr7	7p22.1	1	1	0	0	Amplification	1	0	42473T: 4.5;
CYTH4	chr22	22q13.1	1	1	0	0	Amplification	1	0	42473T: 3.5;
DAAM1	chr14	14q23.1	1	0	0	1	Amplification	1	0	42494T: 4;
DAB2	chr5	5p13.1	6	1	3	2	Amplification	6	0	42483T: 3.5; 42486T: 3.5; 42496T: 3.5; 42475T: 3.5; 42484T: 3.5; 42493T: 3.5;
DACT1	chr14	14q23.1	1	0	0	1	Amplification	1	0	42494T: 4;
DACT3	chr19	19q13.32	1	0	1	0	Amplification	1	0	42484T: 7;
DAD1	chr14	14q11.2	1	0	0	1	Amplification	1	0	42496T: 4;
DAG1	chr3	3p21.31	1	1	0	0	Amplification	1	0	42473T: 4;
DAGLB	chr7	7p22.1	1	1	0	0	Amplification	1	0	42473T: 4.5;
DAO	chr12	12q24.11	2	1	0	1	Amplification	2	0	42473T: 4.5; 42500T: 3.5;
DAP	chr5	2q35	4	1	1	2	Amplification	4	0	42475T: 3.5; 42496T: 3.5; 42486T: 3.5; 42493T: 4;
DAP3	chr1	1p34.3	1	1	0	0	Amplification	1	0	42473T: 5.5;
DAZAP2	chr12	12q13.13	2	0	0	2	Amplification	2	0	42500T: 3.5; 42494T: 5.5;
DBF4	chr7	7q21.12	2	0	2	0	Amplification	2	0	42487T: 3.5; 42483T: 3.5;
DBNDD2	chr20	20q13.12	1	1	0	0	Amplification	1	0	42473T: 5;
DBNL	chr7	7p13	2	1	0	1	Amplification	2	0	42497T: 7.5; 42473T: 4.5;
DBR1	chr3	3q22.3	4	0	1	3	Amplification	4	0	42492T: 3.5; 42493T: 3.5; 42487T: 3.5; 42496T: 3.5;
DBX2	chr12	12q12	1	0	0	1	Amplification	1	0	42500T: 3.5;
DCAF13	chr8	8q22.3	2	0	0	2	Amplification	2	0	42496T: 3.5; 42495T: 3.5;
DCAF4	chr14	14q24.2	2	0	0	2	Amplification	2	0	56957T: 3.5; 42494T: 4;
DCAF4L2	chr8	8q21.3	2	0	0	2	Amplification	2	0	42495T: 3.5; 42496T: 3.5;
DCAF5	chr14	14q24.1	1	0	0	1	Amplification	1	0	42494T: 4;
DCBLD2	chr3	3q12.1|3	1	1	0	0	Amplification	1	0	42476T: 3.5;
DCD	chr12	12q13.2	1	0	0	1	Amplification	1	0	42494T: 3.5;
DCDC2B	chr1	1p35.2	1	1	0	0	Amplification	1	0	42473T: 4;
DCDC2C	chr2	2p25.3	1	0	0	1	Amplification	1	0	42500T: 3.5;

Mangalaparthi *et al.* , 2020. Mutational landscape of esophageal squamous cell carcinoma in an Indian cohort
Supplementary Table 7A. List of copy number alterations and affected genes in ESCC patients

Gene	Chromosome	Cytoband	Recurrence	Recurrence in smoker cohort	Recurrence in chewer cohort	Recurrence in No habit cohort	State	Samples with CNA gain	Samples with CNA loss	File info with CNA fold
DCST1	chr1	1q21.3	2	1	0	1	Amplification	2	0	42473T: 6.5; 42496T: 3.5;
DCST2	chr1	1q21.3	2	1	0	1	Amplification	2	0	42496T: 3.5; 42473T: 6.5;
DCSTAMP	chr8	8q22.3	2	0	0	2	Amplification	2	0	42495T: 3.5; 42496T: 3.5;
DCTN1	chr2	2p13.1	1	0	0	1	Amplification	1	0	42500T: 3.5;
DCTN5	chr16	16p12.2	1	1	0	0	Amplification	1	0	42473T: 4;
DCTN6	chr8	8p12	1	0	1	0	Amplification	1	0	42482T: 3.5;
DCUN1D1	chr3	3q26.33	10	1	3	6	Amplification	10	0	42482T: 3.5; 42487T: 3.5; 42493T: 3.5; 42492T: 3.5; 42500T: 24; 42495T: 4; 42474T: 3.5; 56957T: 4; 42484T: 4; 42498T: 3.5;
DCUN1D2	chr13	13q34	1	0	0	1	Amplification	1	0	56957T: 3.5;
DCUN1D3	chr16	16p12.3	1	1	0	0	Amplification	1	0	42473T: 4;
DCUN1D5	chr11	11q22.3	1	0	0	1	Amplification	1	0	56958T: 6;
DCXR	chr17	17q25.3	1	1	0	0	Amplification	1	0	42473T: 3.5;
DDAH2	chr6	6p21.33	1	1	0	0	Amplification	1	0	42473T: 3.5;
DDB2	chr11	11p11.2	1	1	0	0	Amplification	1	0	42473T: 3.5;
DDC	chr7	7p12.2-p12.1	1	0	0	1	Amplification	1	0	42497T: 20.5;
DDHD1	chr14	14q22.1	1	0	0	1	Amplification	1	0	42494T: 4;
DDHD2	chr8	8p11.23	2	0	1	1	Amplification	2	0	42493T: 3.5; 42482T: 3.5;
DDN	chr12	12q13.12	1	0	0	1	Amplification	1	0	42500T: 3.5;
DDO	chr6	6q21	1	0	0	1	Amplification	1	0	42496T: 3.5;
DDOST	chr1	1p36.12	1	1	0	0	Amplification	1	0	42473T: 4;
DDRGK1	chr20	20p13	1	1	0	0	Amplification	1	0	42473T: 3.5;
DDT	chr22	22q11.23	1	0	1	0	Amplification	1	0	42484T: 3.5;
DDTL	chr22	22q11.23	1	0	1	0	Amplification	1	0	42484T: 3.5;
DDX1	chr2	2p24.3	1	0	0	1	Amplification	1	0	42500T: 3.5;
DDX11	chr12	12p11.21	1	0	0	1	Amplification	1	0	42500T: 8;
DDX17	chr22	22q13.1	1	1	0	0	Amplification	1	0	42473T: 3.5;
DDX23	chr12	12q13.12	1	0	0	1	Amplification	1	0	42500T: 3.5;
DDX27	chr20	20q13.13	1	1	0	0	Amplification	1	0	42473T: 5;
DDX39B	chr6	6p21.33	1	1	0	0	Amplification	1	0	42473T: 3.5;
DDX46	chr5	5q31.1	1	1	0	0	Amplification	1	0	42473T: 3.5;
DDX47	chr12	12p13.1	1	0	0	1	Amplification	1	0	42500T: 4.5;
DDX52	chr17	17q12	1	1	0	0	Amplification	1	0	42473T: 3.5;
DDX54	chr12	12q24.13	1	0	0	1	Amplification	1	0	42500T: 3.5;
DDX55	chr12	12q24.31	1	0	0	1	Amplification	1	0	42500T: 3.5;
DDX56	chr7	7p13	2	1	0	1	Amplification	2	0	42473T: 4.5; 42497T: 7.5;
DDX6	chr11	11q23.3	1	1	0	0	Amplification	1	0	42473T: 4;
DEC1	chr9	9q33.1	1	0	0	1	Amplification	1	0	42493T: 3.5;
DECR1	chr8	8q21.3	3	1	0	2	Amplification	3	0	42495T: 3.5; 42496T: 3.5; 42475T: 3.5;
DECR2	chr16	16p13.3	1	0	1	0	Amplification	1	0	42483T: 3.5;
DEDD2	chr19	19q13.2	1	1	0	0	Amplification	1	0	42473T: 4.5;

Mangalaparthi *et al.* , 2020. Mutational landscape of esophageal squamous cell carcinoma in an Indian cohort
Supplementary Table 7A. List of copy number alterations and affected genes in ESCC patients

Gene	Chromosome	Cytoband	Recurrence	Recurrence in smoker cohort	Recurrence in chewer cohort	Recurrence in No habit cohort	State	Samples with CNA gain	Samples with CNA loss	File info with CNA fold
DEFA4	chr8	8p23.1	1	0	1	0	Amplification	1	0	42486T: 3.5;
DEFA5	chr8	8p23.1	1	0	1	0	Amplification	1	0	42486T: 3.5;
DEFA6	chr8	8p23.1	1	0	1	0	Amplification	1	0	42486T: 3.5;
DEFB1	chr8	8p23.1	1	0	1	0	Amplification	1	0	42486T: 3.5;
DEFB103A	chr8	8p23.1	1	0	1	0	Amplification	1	0	42486T: 3.5;
DEFB103B	chr8	8p23.1	1	0	1	0	Amplification	1	0	42486T: 3.5;
DEFB104A	chr8	8p23.1	1	0	1	0	Amplification	1	0	42486T: 3.5;
DEFB104B	chr8	8p23.1	1	0	1	0	Amplification	1	0	42486T: 3.5;
DEFB105A	chr8	8p23.1	1	0	1	0	Amplification	1	0	42486T: 3.5;
DEFB105B	chr8	8p23.1	1	0	1	0	Amplification	1	0	42486T: 3.5;
DEFB106A	chr8	8p23.1	1	0	1	0	Amplification	1	0	42486T: 3.5;
DEFB106B	chr8	8p23.1	1	0	1	0	Amplification	1	0	42486T: 3.5;
DEFB107A	chr8	8p23.1	1	0	1	0	Amplification	1	0	42486T: 3.5;
DEFB107B	chr8	8p23.1	1	0	1	0	Amplification	1	0	42486T: 3.5;
DEFB115	chr20	20q11.21	1	0	0	1	Amplification	1	0	42496T: 5;
DEFB116	chr20	20q11.21	1	0	0	1	Amplification	1	0	42496T: 5;
DEFB118	chr20	20q11.21	1	0	0	1	Amplification	1	0	42496T: 5;
DEFB119	chr20	20q11.21	1	0	0	1	Amplification	1	0	42496T: 5;
DEFB121	chr20	20q11.21	1	0	0	1	Amplification	1	0	42496T: 5;
DEFB123	chr20	20q11.21	1	0	0	1	Amplification	1	0	42496T: 5;
DEFB124	chr20	20q11.21	1	0	0	1	Amplification	1	0	42496T: 5;
DEFB125	chr20	20p13	1	1	0	0	Amplification	1	0	42473T: 3.5;
DEFB126	chr20	20p13	1	1	0	0	Amplification	1	0	42473T: 3.5;
DEFB127	chr20	20p13	1	1	0	0	Amplification	1	0	42473T: 3.5;
DEFB128	chr20	20p13	1	1	0	0	Amplification	1	0	42473T: 3.5;
DEFB129	chr20	20p13	1	1	0	0	Amplification	1	0	42473T: 3.5;
DEFB132	chr20	20p13	1	1	0	0	Amplification	1	0	42473T: 3.5;
DEFB134	chr8	8p23.1	1	0	1	0	Amplification	1	0	42486T: 3.5;
DEFB135	chr8	8p23.1	1	0	1	0	Amplification	1	0	42486T: 3.5;
DEFB136	chr8	8p23.1	1	0	1	0	Amplification	1	0	42486T: 3.5;
DEFB4A	chr8	8p23.1	1	0	1	0	Amplification	1	0	42486T: 3.5;
DEFB4B	chr8	8p23.1	1	0	1	0	Amplification	1	0	42486T: 3.5;
DENND2A	chr7	7q34	2	1	1	0	Amplification	2	0	42473T: 3.5; 42487T: 3.5;
DENND3	chr8	8q24.3	3	0	1	2	Amplification	3	0	42495T: 3.5; 42496T: 4; 42483T: 3.5;
DENND4B	chr1	1q21.3	1	1	0	0	Amplification	1	0	42473T: 4.5;
DENND5B	chr12	12p11.21	1	0	0	1	Amplification	1	0	42500T: 8;
DENR	chr12	12q24.31	1	0	0	1	Amplification	1	0	42500T: 3.5;
DEPTOR	chr8	8q24.12	3	1	0	2	Amplification	3	0	42495T: 3.5; 42496T: 3.5; 42475T: 3.5;
DERA	chr12	12p12.3	1	0	0	1	Amplification	1	0	42500T: 4.5;
DERL1	chr8	8q24.13	4	1	1	2	Amplification	4	0	42496T: 3.5; 42475T: 3.5; 42484T: 3.5; 42495T: 3.5;
DEXI	chr16	16p13.13	2	1	0	1	Amplification	2	0	42495T: 5; 42473T: 5.5;

Mangalaparthi *et al.* , 2020. Mutational landscape of esophageal squamous cell carcinoma in an Indian cohort
Supplementary Table 7A. List of copy number alterations and affected genes in ESCC patients

Gene	Chromosome	Cytoband	Recurrence	Recurrence in smoker cohort	Recurrence in chewer cohort	Recurrence in No habit cohort	State	Samples with CNA gain	Samples with CNA loss	File info with CNA fold
DFNA5	chr7	7p15.3	1	1	0	0	Amplification	1	0	42473T: 4;
DFNB59	chr2	2q31.2	1	0	0	1	Amplification	1	0	42493T: 3.5;
DGAT1	chr8	8q24.3	4	0	1	3	Amplification	4	0	42496T: 4; 42483T: 3.5; 56957T: 3.5; 42495T: 4.5;
DGAT2	chr11	11q13.5	2	0	0	2	Amplification	2	0	42492T: 4; 42498T: 6.5;
DGCR14	chr22	22q11.21|22	1	0	0	1	Amplification	1	0	42497T: 21;
DGCR2	chr22	22q11.21	1	0	0	1	Amplification	1	0	42497T: 21;
DGCR6	chr22	22q11.21	1	0	0	1	Amplification	1	0	42497T: 21;
DGCR6L	chr22	22q11.21	1	1	0	0	Amplification	1	0	42477T: 4;
DGKA	chr12	12q13.2	1	0	0	1	Amplification	1	0	42494T: 5;
DGKB	chr7	7p21.2	1	1	0	0	Amplification	1	0	42473T: 3.5;
DGKD	chr2	2q37.1	1	1	0	0	Amplification	1	0	42473T: 4.5;
DGKE	chr17	17q22	1	0	0	1	Amplification	1	0	42497T: 4;
DGKG	chr3	3q27.2-q27.3	10	1	3	6	Amplification	10	0	42492T: 3.5; 42493T: 3.5; 42487T: 3.5; 42482T: 3.5; 42484T: 4; 42498T: 3.5; 42474T: 3.5; 42495T: 4; 56957T: 4.5; 42497T: 4;
DGKI	chr7	7q33	1	0	1	0	Amplification	1	0	42487T: 3.5;
DGKZ	chr11	11p11.2	1	1	0	0	Amplification	1	0	42473T: 3.5;
DGUOK	chr2	2p13.1	1	0	0	1	Amplification	1	0	42500T: 3.5;
DHCR7	chr11	11q13.4	8	3	0	5	Amplification	8	0	56957T: 5; 42498T: 5; 42478T: 4.5; 42475T: 5; 42501T: 6; 42497T: 6.5; 42492T: 4; 42476T: 4;
DHFRL1	chr3	3q11.2	2	0	1	1	Amplification	2	0	42484T: 3.5; 42500T: 3.5;
DHH	chr12	12q13.12	1	0	0	1	Amplification	1	0	42500T: 3.5;
DHRS2	chr14	14q11.2	1	0	0	1	Amplification	1	0	42496T: 4;
DHRS7	chr14	14q23.1	2	0	1	1	Amplification	2	0	42494T: 4; 42483T: 3.5;
DHX35	chr20	20q11.23-q1	1	1	0	0	Amplification	1	0	42473T: 3.5;
DHX36	chr3	3q25.2	9	1	2	6	Amplification	9	0	42493T: 3.5; 42487T: 3.5; 42492T: 3.5; 42500T: 3.5; 42496T: 4.5; 42497T: 5.5; 42474T: 3.5; 56957T: 4; 42484T: 3.5;
DHX37	chr12	12q24.31	1	0	0	1	Amplification	1	0	42500T: 3.5;
DHX57	chr2	2p22.1	1	0	0	1	Amplification	1	0	42500T: 3.5;
DIABLO	chr12	12q24.31	1	0	0	1	Amplification	1	0	42500T: 3.5;
DIDO1	chr20	20q13.33	1	1	0	0	Amplification	1	0	42473T: 6;
DIO2	chr14	14q31.1	1	0	0	1	Amplification	1	0	42494T: 4;
DIP2B	chr12	12q13.12	2	0	0	2	Amplification	2	0	42494T: 3.5; 42500T: 3.5;
DIRC1	chr2	2q32.2	1	0	0	1	Amplification	1	0	42493T: 3.5;
DIRC2	chr3	3q21.1	1	0	0	1	Amplification	1	0	42496T: 3.5;
DISP2	chr15	15q15.1	1	1	0	0	Amplification	1	0	42473T: 3.5;
DLD	chr7	16q23.1	4	0	1	3	Amplification	4	0	42487T: 3.5; 42493T: 5; 42497T: 4; 42501T: 3.5;
DLEC1	chr3	3p22.2	1	1	0	0	Amplification	1	0	42473T: 4.5;

Mangalaparthi *et al.* , 2020. Mutational landscape of esophageal squamous cell carcinoma in an Indian cohort
Supplementary Table 7A. List of copy number alterations and affected genes in ESCC patients

Gene	Chromosome	Cytoband	Recurrence	Recurrence in smoker cohort	Recurrence in chewer cohort	Recurrence in No habit cohort	State	Samples with CNA gain	Samples with CNA loss	File info with CNA fold
DLG1	chr3	3q29	9	1	3	5	Amplification	9	0	42482T: 3.5; 42487T: 3.5; 42493T: 5; 42492T: 3.5; 56957T: 6; 42474T: 3.5; 42495T: 4; 42498T: 3.5; 42484T: 4;
DLGAP1	chr18	18p11.31	3	1	0	2	Amplification	3	0	42481T: 9.5; 56957T: 8; 42493T: 3.5;
DLGAP2	chr8	8p23.3	1	0	0	1	Amplification	1	0	56957T: 4;
DLGAP4	chr20	20q11.23	1	1	0	0	Amplification	1	0	42473T: 3.5;
DLGAP5	chr14	14q22.3	1	0	0	1	Amplification	1	0	42494T: 4;
DLL3	chr19	19q13.2	2	0	0	2	Amplification	2	0	56957T: 4; 42500T: 7.5;
DLL4	chr15	15q15.1	1	0	0	1	Amplification	1	0	42493T: 3.5;
DLST	chr14	14q24.3	2	0	0	2	Amplification	2	0	56957T: 3.5; 42494T: 4;
DLX1	chr2	2q31.1	2	1	0	1	Amplification	2	0	42473T: 3.5; 42493T: 5;
DLX2	chr2	2q31.1	2	1	0	1	Amplification	2	0	42473T: 3.5; 42493T: 5;
DLX5	chr7	7q21.3	1	0	1	0	Amplification	1	0	42487T: 3.5;
DLX6	chr7	7q21.3	1	0	1	0	Amplification	1	0	42487T: 3.5;
DMC1	chr22	17q25.1	1	1	0	0	Amplification	1	0	42473T: 3.5;
DMKN	chr19	19q13.12	3	0	1	2	Amplification	3	0	56957T: 4; 42484T: 3.5; 42500T: 6.5;
DMPK	chr19	19q13.32	1	0	1	0	Amplification	1	0	42484T: 4;
DMRT1	chr9	9p24.3	2	0	1	1	Amplification/Deletio	1	1	42496T: 3.5; 42486T: 0.5;
DMRT2	chr9	9p24.3	2	0	1	1	Amplification/Deletio	1	1	42486T: 0.5; 42496T: 3.5;
DMRT3	chr9	9p24.3	2	0	1	1	Amplification/Deletio	1	1	42486T: 0.5; 42496T: 3.5;
DMRTC2	chr19	19q13.2	1	1	0	0	Amplification	1	0	42473T: 4.5;
DMTF1	chr7	7q21.12	2	0	2	0	Amplification	2	0	42487T: 3.5; 42483T: 3.5;
DMWD	chr19	19q13.32	1	0	1	0	Amplification	1	0	42484T: 4;
DNAAF2	chr14	14q21.3	1	1	0	0	Amplification	1	0	42473T: 3.5;
DNAH10	chr12	12q24.31	1	0	0	1	Amplification	1	0	42500T: 3.5;
DNAH11	chr7	7p15.3	1	1	0	0	Amplification	1	0	42473T: 4;
DNAH3	chr16	16p12.3	1	1	0	0	Amplification	1	0	42473T: 4;
DNAH5	chr5	5p15.2	5	1	2	2	Amplification	5	0	42484T: 3.5; 42493T: 4; 42496T: 3.5; 42486T: 3.5; 42475T: 3.5;
DNAH6	chr2	2p11.2	1	0	0	1	Amplification	1	0	42500T: 3.5;
DNAH7	chr2	2q32.3	2	0	1	1	Amplification	2	0	42493T: 3.5; 42482T: 4;
DNAJA3	chr16	16p13.3	3	1	0	2	Amplification	3	0	42494T: 3.5; 42495T: 8.5; 42473T: 5.5;
DNAJA4	chr15	15q25.1	1	1	0	0	Amplification	1	0	42473T: 3.5;
DNAJB11	chr3	3q27.3	10	1	3	6	Amplification	10	0	42482T: 3.5; 42493T: 3.5; 42487T: 3.5; 42492T: 3.5; 42497T: 4; 56957T: 4.5; 42495T: 4; 42474T: 3.5; 42498T: 3.5; 42484T: 4;
DNAJB13	chr11	11q13.4	6	3	0	3	Amplification	6	0	42476T: 4; 42475T: 7.5; 42498T: 8; 42492T: 4; 42478T: 6; 56957T: 3.5;
DNAJB5	chr9	9p13.3	1	0	0	1	Amplification	1	0	42501T: 3.5;
DNAJB8	chr3	3q21.3	2	0	1	1	Amplification	2	0	42487T: 3.5; 42496T: 3.5;
DNAJB9	chr7	7q31.1|14q2	3	0	1	2	Amplification	3	0	42497T: 4; 42501T: 3.5; 42487T: 3.5;

Mangalaparthi *et al.* , 2020. Mutational landscape of esophageal squamous cell carcinoma in an Indian cohort
Supplementary Table 7A. List of copy number alterations and affected genes in ESCC patients

Gene	Chromosome	Cytoband	Recurrence	Recurrence in smoker cohort	Recurrence in chewer cohort	Recurrence in No habit cohort	State	Samples with CNA gain	Samples with CNA loss	File info with CNA fold
DNAJC10	chr2	2q32.1	1	0	0	1	Amplification	1	0	42493T: 4;
DNAJC13	chr3	3q22.1	3	0	1	2	Amplification	3	0	42487T: 3.5; 42492T: 3.5; 42496T: 3.5;
DNAJC17	chr15	15q15.1	1	0	0	1	Amplification	1	0	42493T: 3.5;
DNAJC19	chr3	3q26.33	11	1	3	7	Amplification	11	0	56957T: 4; 42495T: 4; 42474T: 3.5; 42498T: 3.5; 42484T: 4; 42500T: 24; 42497T: 4; 42487T: 3.5; 42493T: 3.5; 42492T: 3.5; 42482T: 3.5;
DNAJC2	chr7	7q22.1	4	0	1	3	Amplification	4	0	42487T: 3.5; 42493T: 3.5; 42501T: 3.5; 42497T: 4;
DNAJC21	chr5	5p13.2	4	1	1	2	Amplification	4	0	42493T: 3.5; 42486T: 3.5; 42496T: 3.5; 42475T: 3.5;
DNAJC22	chr12	12q13.12	1	0	0	1	Amplification	1	0	42500T: 3.5;
DNAJC27	chr2	2p23.3	1	0	0	1	Amplification	1	0	42500T: 3.5;
DNAJC5	chr20	20q13.33	2	1	1	0	Amplification	2	0	42473T: 6; 42483T: 3.5;
DNAJC5B	chr8	8q13.1	3	0	0	3	Amplification	3	0	42495T: 3.5; 42497T: 4; 42496T: 3.5;
DNAJC5G	chr2	2p23.3	1	0	0	1	Amplification	1	0	42500T: 3.5;
DNAJC8	chr1	1p35.3	1	1	0	0	Amplification	1	0	42473T: 3.5;
DNAL1	chr14	14q24.3	2	0	0	2	Amplification	2	0	56957T: 3.5; 42494T: 4;
DNAL4	chr22	22q13.1	1	1	0	0	Amplification	1	0	42473T: 3.5;
DNASE1	chr16	16p13.3	1	1	0	0	Amplification	1	0	42473T: 5.5;
DNASE1L2	chr16	16p13.3	1	0	1	0	Amplification	1	0	42483T: 3.5;
DNLZ	chr9	9q34.3	1	0	0	1	Amplification	1	0	56957T: 3.5;
DNM1L	chr12	12p11.21	1	0	0	1	Amplification	1	0	42500T: 3.5;
DNMT3A	chr2	2p23.3	1	0	0	1	Amplification	1	0	42500T: 3.5;
DNMT3B	chr20	20q11.21	2	1	0	1	Amplification	2	0	42473T: 3.5; 42496T: 5;
DNTTIP1	chr20	20q13.12	1	1	0	0	Amplification	1	0	42473T: 5;
DOCK4	chr7	7q31.1	2	0	1	1	Amplification	2	0	42487T: 3.5; 42501T: 3.5;
DOCK8	chr9	9p24.3	2	0	1	1	Amplification/Deletio	1	1	42486T: 0.5; 42496T: 3.5;
DOK1	chr2	2p13.1	1	0	0	1	Amplification	1	0	42500T: 3.5;
DOK5	chr20	20q13.2	1	1	0	0	Amplification	1	0	42473T: 5;
DOM3Z	chr6	6p21.33	1	1	0	0	Amplification	1	0	42473T: 3.5;
DPF1	chr19	19q13.2	3	0	1	2	Amplification	3	0	42484T: 3.5; 56957T: 4; 42500T: 6.5;
DPF3	chr14	14q24.2	2	0	0	2	Amplification	2	0	42494T: 4; 56957T: 3.5;
DPH7	chr9	9q34.3	2	1	0	1	Amplification	2	0	42473T: 5; 56957T: 3.5;
DPM1	chr20	20q13.13	1	1	0	0	Amplification	1	0	42473T: 5;
DPM3	chr1	1q22	1	1	0	0	Amplification	1	0	42473T: 6.5;
DPP3	chr11	11q13.2	1	0	0	1	Amplification	1	0	56957T: 5.5;
DPP7	chr9	9q34.3	2	1	0	1	Amplification	2	0	42473T: 5; 56957T: 3.5;
DPPA3P2	chr14	14q13.3	2	1	0	1	Amplification	2	0	42500T: 8.5; 42476T: 8;
DPY19L1	chr7	7p14.2	1	1	0	0	Amplification	1	0	42473T: 4;
DPY19L2P1	chr7	7p14.2	1	1	0	0	Amplification	1	0	42473T: 4;
DPY19L2P2	chr7	7q22.1	4	0	1	3	Amplification	4	0	42487T: 3.5; 42493T: 3.5; 42501T: 3.5; 42497T: 3.5;
DPY19L3	chr19	19q13.11	5	1	1	3	Amplification	5	0	42494T: 4; 56957T: 4; 42484T: 6; 42500T: 4.5; 42473T: 3.5;

Mangalaparthi *et al.* , 2020. Mutational landscape of esophageal squamous cell carcinoma in an Indian cohort
Supplementary Table 7A. List of copy number alterations and affected genes in ESCC patients

Gene	Chromosome	Cytoband	Recurrence	Recurrence in smoker cohort	Recurrence in chewer cohort	Recurrence in No habit cohort	State	Samples with CNA gain	Samples with CNA loss	File info with CNA fold
DPY19L4	chr8	8q22.1	2	0	0	2	Amplification	2	0	42496T: 3.5; 42495T: 3.5;
DPY30	chr2	2p22.3	1	0	0	1	Amplification	1	0	42500T: 3.5;
DPYS	chr8	8q22.3	2	0	0	2	Amplification	2	0	42496T: 3.5; 42495T: 3.5;
DPYSL5	chr2	2p23.3	1	0	0	1	Amplification	1	0	42500T: 3.5;
DQX1	chr2	2p13.1	1	0	0	1	Amplification	1	0	42500T: 3.5;
DRC1	chr2	2p23.3	1	0	0	1	Amplification	1	0	42500T: 3.5;
DRD3	chr3	3q13.31	1	0	0	1	Amplification	1	0	42496T: 3.5;
DROSHA	chr5	5p13.3	4	1	1	2	Amplification	4	0	42493T: 3.5; 42486T: 3.5; 42496T: 3.5; 42475T: 3.5;
DSCAM	chr21	21q22.2	1	1	0	0	Amplification	1	0	42473T: 3.5;
DSCC1	chr8	8q24.12	3	1	0	2	Amplification	3	0	42495T: 3.5; 42496T: 3.5; 42475T: 3.5;
DSN1	chr20	20q11.23	1	1	0	0	Amplification	1	0	42473T: 3.5;
DST	chr6	6p12.1	1	0	0	1	Amplification	1	0	42497T: 3.5;
DTD2	chr14	14q12	3	0	0	3	Amplification	3	0	56957T: 4; 42494T: 3.5; 42500T: 4.5;
DTNB	chr2	2p23.3	1	0	0	1	Amplification	1	0	42500T: 3.5;
DTX1	chr12	12q24.13	1	0	0	1	Amplification	1	0	42500T: 3.5;
DTX2	chr7	7q11.23	1	0	0	1	Amplification	1	0	42493T: 3.5;
DTX2P1	chr7	7q11.23	1	0	1	0	Amplification	1	0	42487T: 3.5;
DTX3L	chr3	3q21.1	1	0	0	1	Amplification	1	0	42496T: 3.5;
DUS1L	chr17	17q25.3	1	1	0	0	Amplification	1	0	42473T: 3.5;
DUS4L	chr7	7q22.3	4	0	1	3	Amplification	4	0	42487T: 3.5; 42493T: 3.5; 42497T: 4; 42501T: 3.5;
DUSP11	chr2	2p13.1	1	0	0	1	Amplification	1	0	42500T: 3.5;
DUSP14	chr17	17q12	1	1	0	0	Amplification	1	0	42473T: 3.5;
DUSP15	chr20	20q11.21	1	0	0	1	Amplification	1	0	42496T: 5;
DUSP19	chr2	2q32.1	1	0	0	1	Amplification	1	0	42493T: 3.5;
DUSP2	chr2	2q11.2	2	1	0	1	Amplification	2	0	42493T: 3.5; 42473T: 3.5;
DUSP26	chr8	2q37.3	2	0	1	1	Amplification	2	0	42482T: 3.5; 42497T: 4.5;
DUSP4	chr8	8p12	1	0	1	0	Amplification	1	0	42482T: 3.5;
DVL3	chr3	3q27.1	12	2	3	7	Amplification	12	0	42482T: 3.5; 42492T: 3.5; 42487T: 3.5; 42493T: 3.5; 42473T: 3.5; 42497T: 4; 42484T: 4; 42498T: 3.5; 42495T: 4; 42494T: 3.5; 42474T: 3.5; 56957T: 5;
DYNC1I1	chr7	7q21.3	1	0	1	0	Amplification	1	0	42487T: 3.5;
DYNC2H1	chr11	11q22.3	1	0	0	1	Amplification	2	0	56958T: 6,4;
DYNC2LI1	chr2	2p21	2	0	1	1	Amplification	2	0	42500T: 3.5; 42484T: 3.5;
DYNLL1	chr12	12q24.31	1	0	0	1	Amplification	1	0	42500T: 3.5;
DYNLL2	chr17	17q22	1	0	0	1	Amplification	1	0	42497T: 4;
DYNLRB1	chr20	20q11.22	3	1	0	2	Amplification	3	0	42493T: 3.5; 42473T: 3.5; 42496T: 5;
DYRK1B	chr19	19q13.2	2	0	0	2	Amplification	2	0	56957T: 4; 42500T: 7.5;
DYRK2	chr12	12q15	2	0	0	2	Amplification	2	0	42500T: 5; 42501T: 6.5;
DYSF	chr2	2p13.2	1	0	0	1	Amplification	1	0	42500T: 3.5;
DZIP1L	chr3	3q22.3	4	0	1	3	Amplification	4	0	42496T: 3.5; 42487T: 3.5; 42493T: 3.5; 42492T: 3.5;
E2F1	chr20	20q11.22	2	1	0	1	Amplification	2	0	42496T: 5; 42473T: 3.5;

Mangalaparthi *et al.* , 2020. Mutational landscape of esophageal squamous cell carcinoma in an Indian cohort
Supplementary Table 7A. List of copy number alterations and affected genes in ESCC patients

Gene	Chromosome	Cytoband	Recurrence	Recurrence in smoker cohort	Recurrence in chewer cohort	Recurrence in No habit cohort	State	Samples with CNA gain	Samples with CNA loss	File info with CNA fold
E2F2	chr1	1p36.12	1	1	0	0	Amplification	1	0	42473T: 3.5;
E2F5	chr8	8q21.2	2	0	0	2	Amplification	2	0	42496T: 3.5; 42495T: 3.5;
E2F6	chr2	2p25.1	1	0	0	1	Amplification	1	0	42500T: 3.5;
E4F1	chr16	16p13.3	1	0	1	0	Amplification	1	0	42483T: 3.5;
EAF2	chr3	1p34.1	1	0	0	1	Amplification	1	0	42496T: 3.5;
EAPP	chr14	14q13.1	1	0	0	1	Amplification	1	0	42500T: 4.5;
EARS2	chr16	16p12.2	1	1	0	0	Amplification	1	0	42473T: 4;
EBAG9	chr8	8q23.2	2	0	0	2	Amplification	2	0	42495T: 3.5; 42496T: 3.5;
EBF4	chr20	20p13	1	1	0	0	Amplification	1	0	42473T: 3.5;
EBNA1BP2	chr1	1p34.2	1	0	0	1	Amplification	1	0	42493T: 5.5;
ECE1	chr1	1p36.12	1	1	0	0	Amplification	1	0	42473T: 4;
ECE2	chr3	3q27.1	12	2	3	7	Amplification	12	0	42497T: 4; 42473T: 3.5; 42474T: 3.5; 42495T: 4; 42494T: 3.5; 56957T: 5; 42484T: 4; 42498T: 3.5; 42482T: 3.5; 42493T: 3.5; 42487T: 3.5; 42492T: 3.5;
ECH1	chr19	19q13.2	2	0	0	2	Amplification	2	0	56957T: 4; 42500T: 6.5;
ECI1	chr16	16p13.3	1	0	1	0	Amplification	1	0	42483T: 3.5;
ECM1	chr1	1q21.2	2	1	0	1	Amplification	2	0	42473T: 5; 42493T: 3.5;
ECT2	chr3	3q26.31	9	1	3	5	Amplification	9	0	42484T: 4; 56957T: 4; 42495T: 4; 42474T: 3.5; 42500T: 9; 42492T: 3.5; 42487T: 3.5; 42493T: 3.5; 42482T: 3.5;
EDC3	chr15	15q24.1	1	1	0	0	Amplification	1	0	42473T: 3.5;
EDEM2	chr20	20q11.22	2	1	0	1	Amplification	2	0	42493T: 3.5; 42473T: 3.5;
EDF1	chr9	9q34.3	2	1	0	1	Amplification	2	0	56957T: 3.5; 42473T: 5;
EDN3	chr20	20q13.32	1	1	0	0	Amplification	1	0	42473T: 4.5;
EEF1A2	chr20	20q13.33	1	1	0	0	Amplification	1	0	42473T: 6;
EEF1D	chr8	8q24.3	3	0	1	2	Amplification	3	0	42483T: 3.5; 42496T: 4; 42495T: 4.5;
EEF2K	chr16	16p12.2	1	1	0	0	Amplification	1	0	42473T: 4;
EEFSEC	chr3	3q21.3	2	0	1	1	Amplification	2	0	42496T: 3.5; 42487T: 3.5;
EEPD1	chr7	7p14.2	1	1	0	0	Amplification	1	0	42473T: 4.5;
EFCAB1	chr8	8q11.21	5	0	2	3	Amplification	5	0	42484T: 3.5; 42494T: 3.5; 42495T: 3.5; 42482T: 3.5; 42496T: 3.5;
EFCAB10	chr7	7q22.3	4	0	1	3	Amplification	4	0	42493T: 3.5; 42487T: 3.5; 42497T: 4; 42501T: 3.5;
EFCAB12	chr3	3q21.3	2	0	1	1	Amplification	2	0	42487T: 3.5; 42496T: 3.5;
EFCAB4B	chr12	12p13.32	1	0	0	1	Amplification	1	0	42494T: 3.5;
EFCAB8	chr20	20q11.21	2	1	0	1	Amplification	2	0	42496T: 5; 42473T: 3.5;
EFCC1	chr3	3q21.3	3	0	1	2	Amplification	3	0	42496T: 3.5; 42487T: 3.5; 42493T: 3.5;
EFEMP1	chr2	2p16.1	3	0	1	2	Amplification	3	0	56957T: 3.5; 42484T: 4.5; 42500T: 3.5;
EFNA1	chr1	1q22	1	1	0	0	Amplification	1	0	42473T: 6.5;
EFNA3	chr1	1q21.3	2	1	0	1	Amplification	2	0	42473T: 6.5; 42496T: 3.5;
EFNA4	chr1	1q21.3	2	1	0	1	Amplification	2	0	42496T: 3.5; 42473T: 6.5;
EFR3A	chr8	8q24.22	3	0	1	2	Amplification	3	0	42484T: 3.5; 42495T: 3.5; 42496T: 3.5;

Mangalaparthi *et al.* , 2020. Mutational landscape of esophageal squamous cell carcinoma in an Indian cohort
Supplementary Table 7A. List of copy number alterations and affected genes in ESCC patients

Gene	Chromosome	Cytoband	Recurrence	Recurrence in smoker cohort	Recurrence in chewer cohort	Recurrence in No habit cohort	State	Samples with CNA gain	Samples with CNA loss	File info with CNA fold
EFR3B	chr2	2p23.3	1	0	0	1	Amplification	1	0	42500T: 3.5;
EFS	chr14	14q11.2	2	0	0	2	Amplification	2	0	42496T: 4; 42500T: 4;
EGFEM1P	chr3	3q26.2	9	1	3	5	Amplification	9	0	42487T: 3.5; 42493T: 3.5; 42492T: 3.5; 42482T: 3.5; 42474T: 3.5; 42495T: 4; 56957T: 4; 42484T: 4; 42500T: 8;
EGFL7	chr9	9q34.3	1	0	0	1	Amplification	1	0	56957T: 3.5;
EGFL8	chr6	6p21.32	1	1	0	0	Amplification	1	0	42473T: 3.5;
EGFLAM	chr5	5p13.2-p13.1	6	1	3	2	Amplification	6	0	42493T: 3.5; 42484T: 3.5; 42475T: 3.5; 42486T: 3.5; 42483T: 3.5; 42496T: 3.5;
EGFR	chr7	7p11.2	3	0	1	2	Amplification	3	0	56957T: 5; 42483T: 4; 42497T: 21.5;
EGLN2	chr19	19q13.2	2	0	0	2	Amplification	2	0	56957T: 4; 42500T: 3.5;
EGLN3	chr14	14q13.1	1	0	0	1	Amplification	1	0	42500T: 4.5;
EGR1	chr5	5q31.2	1	0	0	1	Amplification	1	0	42495T: 3.5;
EGR4	chr2	2p13.2	1	0	0	1	Amplification	1	0	42500T: 3.5;
EHBP1	chr2	2p15	3	0	1	2	Amplification	3	0	42500T: 3.5; 42484T: 3.5; 56957T: 3.5;
EHD3	chr2	2p23.1	1	0	0	1	Amplification	1	0	42500T: 3.5;
EHF	chr11	11p13	2	1	0	1	Amplification	2	0	42493T: 4; 42473T: 4.5;
EHHADH	chr3	3q27.2	10	1	3	6	Amplification	10	0	42495T: 4; 42474T: 3.5; 56957T: 4.5; 42484T: 4; 42498T: 3.5; 42497T: 4; 42493T: 3.5; 42487T: 3.5; 42492T: 3.5; 42482T: 3.5;
EHMT1	chr9	9q34.3	1	0	0	1	Amplification	1	0	56957T: 3.5;
EHMT2	chr6	6p21.33	1	1	0	0	Amplification	1	0	42473T: 3.5;
EID2	chr19	19q13.2	2	0	0	2	Amplification	2	0	56957T: 4; 42500T: 7.5;
EID2B	chr19	19q13.2	2	0	0	2	Amplification	2	0	56957T: 4; 42500T: 7.5;
EIF2A	chr3	3q25.1	9	2	2	5	Amplification	9	0	56957T: 4; 42474T: 3.5; 42484T: 3.5; 42496T: 4; 42473T: 4; 42497T: 4.5; 42493T: 3.5; 42487T: 3.5; 42492T: 3.5;
EIF2AK1	chr7	7p22.1	1	1	0	0	Amplification	1	0	42473T: 4.5;
EIF2AK2	chr2	2p22.2	1	0	0	1	Amplification	1	0	42500T: 3.5;
EIF2AK3	chr2	2p11.2	1	0	0	1	Amplification	1	0	42500T: 3.5;
EIF2B1	chr12	12q24.31	1	0	0	1	Amplification	1	0	42500T: 3.5;
EIF2B2	chr14	14q24.3	2	0	0	2	Amplification	2	0	42494T: 4; 56957T: 3.5;
EIF2B4	chr2	2p23.3	1	0	0	1	Amplification	1	0	42500T: 3.5;
EIF2B5	chr3	3q27.1	11	1	3	7	Amplification	11	0	42482T: 3.5; 42492T: 3.5; 42487T: 3.5; 42493T: 3.5; 42497T: 4; 42484T: 4; 42498T: 3.5; 42474T: 3.5; 42494T: 3.5; 42495T: 4; 56957T: 5;
EIF2S1	chr14	14q23.3	1	0	0	1	Amplification	1	0	42494T: 4;
EIF2S2	chr20	20q11.22	3	1	0	2	Amplification	3	0	42496T: 5; 42473T: 3.5; 42493T: 3.5;
EIF3B	chr7	7p22.3	1	1	0	0	Amplification	1	0	42473T: 5;
EIF3D	chr22	22q12.3	1	1	0	0	Amplification	1	0	42473T: 3.5;
EIF3E	chr8	8q23.1	2	0	0	2	Amplification	2	0	42495T: 3.5; 42496T: 3.5;

Mangalaparthi *et al.* , 2020. Mutational landscape of esophageal squamous cell carcinoma in an Indian cohort
Supplementary Table 7A. List of copy number alterations and affected genes in ESCC patients

Gene	Chromosome	Cytoband	Recurrence	Recurrence in smoker cohort	Recurrence in chewer cohort	Recurrence in No habit cohort	State	Samples with CNA gain	Samples with CNA loss	File info with CNA fold
EIF3H	chr8	8q23.3-q24.1	3	1	0	2	Amplification	3	0	42496T: 3.5; 42475T: 3.5; 42495T: 3.5;
EIF3I	chr1	1p35.2	1	1	0	0	Amplification	1	0	42473T: 4;
EIF3K	chr19	19q13.2	2	0	0	2	Amplification	2	0	42500T: 6.5; 56957T: 4;
EIF3L	chr22	22q13.1	1	1	0	0	Amplification	1	0	42473T: 3.5;
EIF4A2	chr3	3q27.3	10	1	3	6	Amplification	10	0	42497T: 4; 42498T: 3.5; 42484T: 4; 56957T: 4.5; 42495T: 4; 42474T: 3.5; 42482T: 3.5; 42492T: 3.5; 42493T: 3.5; 42487T: 3.5;
EIF4A3	chr17	17q25.3	1	1	0	0	Amplification	1	0	42473T: 3.5;
EIF4B	chr12	12q13.13	1	1	0	0	Amplification	1	0	42473T: 4;
EIF4EBP1	chr8	8p11.23	2	0	1	1	Amplification	2	0	42493T: 3.5; 42482T: 3.5;
EIF4G1	chr3	3q27.1	12	2	3	7	Amplification	12	0	42487T: 3.5; 42493T: 3.5; 42492T: 3.5; 42482T: 3.5; 42495T: 4; 42494T: 3.5; 42474T: 3.5; 56957T: 5; 42484T: 4; 42498T: 3.5; 42473T: 3.5; 42497T: 4;
EIF4G3	chr1	1p36.12	1	1	0	0	Amplification	1	0	42473T: 4;
EIF5A2	chr3	3q26.2	9	1	3	5	Amplification	9	0	42482T: 3.5; 42492T: 3.5; 42487T: 3.5; 42493T: 3.5; 42500T: 8; 42484T: 4; 42474T: 3.5; 42495T: 4; 56957T: 4;
EIF5B	chr2	2q11.2	1	0	0	1	Amplification	1	0	42493T: 3.5;
EIF6	chr20	20q11.22	2	1	0	1	Amplification	2	0	42493T: 3.5; 42473T: 3.5;
ELAC1	chr18	18q21.2	1	0	0	1	Deletion	0	1	56957T: 0;
ELANE	chr19	19p13.3	1	0	0	1	Amplification	1	0	42493T: 3.5;
ELF5	chr11	11p13	2	1	0	1	Amplification	2	0	42493T: 4; 42473T: 4.5;
ELFN1	chr7	7p22.3	1	1	0	0	Amplification	1	0	42473T: 5;
ELFN2	chr22	22q13.1	1	1	0	0	Amplification	1	0	42473T: 3.5;
ELMO1	chr7	7p14.2-p14.1	1	1	0	0	Amplification	1	0	42473T: 4.5;
ELMO2	chr20	20q13.12	1	1	0	0	Amplification	1	0	42473T: 5;
ELMOD3	chr2	2p11.2	1	0	0	1	Amplification	1	0	42500T: 3.5;
ELMSAN1	chr14	14q24.3	2	0	0	2	Amplification	2	0	56957T: 3.5; 42494T: 4;
ELOVL1	chr1	1p34.2	1	0	0	1	Amplification	1	0	42493T: 3.5;
EMB	chr5	5q11.1	2	1	0	1	Amplification	2	0	42475T: 3.5; 42498T: 10.5;
EMC1	chr1	1p36.13	1	1	0	0	Amplification	1	0	42473T: 4;
EMC2	chr8	8q23.1	2	0	0	2	Amplification	2	0	42496T: 3.5; 42495T: 3.5;
EME1	chr17	17q21.33	1	1	0	0	Amplification	1	0	42473T: 3.5;
EME2	chr16	16p13.3	1	0	1	0	Amplification	1	0	42483T: 3.5;
EMILIN1	chr2	2p23.3	1	0	0	1	Amplification	1	0	42500T: 3.5;
EMILIN2	chr18	18p11.32-p1	2	0	0	2	Amplification	2	0	56957T: 8; 42500T: 4.5;
EMILIN3	chr20	20q12	1	1	0	0	Amplification	1	0	42473T: 3.5;
EML2	chr19	19q13.32	1	0	1	0	Amplification	1	0	42484T: 4;
EML4	chr2	2p21	2	0	1	1	Amplification	2	0	42500T: 3.5; 42484T: 3.5;
EMP1	chr12	12p13.1	1	0	0	1	Amplification	1	0	42500T: 4.5;
EMP2	chr16	16p13.13	2	1	0	1	Amplification	2	0	42495T: 5; 42473T: 5.5;

Mangalaparthi *et al.* , 2020. Mutational landscape of esophageal squamous cell carcinoma in an Indian cohort
Supplementary Table 7A. List of copy number alterations and affected genes in ESCC patients

Gene	Chromosome	Cytoband	Recurrence	Recurrence in smoker cohort	Recurrence in chewer cohort	Recurrence in No habit cohort	State	Samples with CNA gain	Samples with CNA loss	File info with CNA fold
EMX1	chr2	2p13.2	1	0	0	1	Amplification	1	0	42500T: 3.5;
ENAH	chr1	1q42.12	1	0	0	1	Amplification	1	0	42493T: 3.5;
ENO2	chr12	12p13.31	1	0	0	1	Amplification	1	0	42494T: 3.5;
ENOSF1	chr18	18p11.32	3	0	0	3	Amplification	3	0	42500T: 4.5; 56957T: 8; 42493T: 3.5;
ENPP2	chr8	8q24.12	3	1	0	2	Amplification	3	0	42496T: 3.5; 42475T: 3.5; 42495T: 3.5;
ENPP7	chr17	17q25.3	1	1	0	0	Amplification	1	0	42473T: 3.5;
ENSA	chr1	1q21.3	2	1	0	1	Amplification	2	0	42493T: 3.5; 42473T: 4.5;
ENTHD1	chr22	22q13.1	1	1	0	0	Amplification	1	0	42473T: 3.5;
ENTPD2	chr9	9q34.3	2	1	0	1	Amplification	2	0	56957T: 3.5; 42473T: 5;
ENTPD5	chr14	14q24.3	2	0	0	2	Amplification	2	0	56957T: 3.5; 42494T: 4;
ENTPD8	chr9	9q34.3	2	1	0	1	Amplification	2	0	42473T: 5; 56957T: 3.5;
ENY2	chr8	8q23.1	2	0	0	2	Amplification	2	0	42496T: 3.5; 42495T: 3.5;
EPAS1	chr2	2p21	2	0	1	1	Amplification	2	0	42500T: 3.5; 42484T: 4.5;
EPB41	chr1	1p35.3	1	1	0	0	Amplification	1	0	42473T: 3.5;
EPB41L1	chr20	20q11.23	1	1	0	0	Amplification	1	0	42473T: 3.5;
EPB41L3	chr18	18p11.31	2	1	0	1	Amplification/Deletio	1	1	56957T: 8; 42481T: 0.5;
EPB41L4B	chr9	9q31.3	1	0	1	0	Amplification	1	0	42483T: 3.5;
EPCAM	chr2	2p21	2	0	1	1	Amplification	2	0	42484T: 4.5; 42500T: 3.5;
EPDR1	chr7	7p14.1	1	1	0	0	Amplification	1	0	42473T: 4.5;
EPHA1	chr7	7q34-q35	1	0	1	0	Amplification	1	0	42487T: 3.5;
EPHA2	chr1	1p36.13	1	1	0	0	Amplification	1	0	42473T: 8;
EPHA6	chr3	3q11.2	2	1	0	1	Amplification	2	0	42476T: 3.5; 42500T: 3.5;
EPHA8	chr1	1p36.12	1	1	0	0	Amplification	1	0	42473T: 4;
EPHB1	chr3	3q22.2	4	0	1	3	Amplification	4	0	42496T: 3.5; 42492T: 3.5; 42487T: 3.5; 42493T: 3.5;
EPHB2	chr1	1p36.12	1	1	0	0	Amplification	1	0	42473T: 3.5;
EPHB3	chr3	3q27.1	12	2	3	7	Amplification	12	0	42482T: 3.5; 42493T: 3.5; 42487T: 3.5; 42492T: 3.5; 42473T: 4; 42497T: 4; 42474T: 3.5; 42495T: 4; 42494T: 3.5; 56957T: 5; 42484T: 4; 42498T: 3.5;
EPHB6	chr7	7q34	1	0	1	0	Amplification	1	0	42487T: 3.5;
EPHX1	chr1	1q42.12	1	0	0	1	Amplification	1	0	42493T: 3.5;
EPN1	chr19	19q13.42	1	0	0	1	Amplification	1	0	42494T: 3.5;
EPN3	chr17	17q21.33	1	1	0	0	Amplification	1	0	42473T: 4.5;
EPPIN	chr20	20q13.12	1	1	0	0	Amplification	1	0	42473T: 5;
EPPK1	chr8	8q24.3	3	0	1	2	Amplification	3	0	42496T: 4; 42483T: 3.5; 42495T: 4.5;
EPS8	chr12	12p12.3	1	0	0	1	Amplification	1	0	42500T: 4.5;
EPS8L3	chr1	1p13.3	1	1	0	0	Amplification	1	0	42473T: 4.5;
EPT1	chr2	2p23.3	1	0	0	1	Amplification	1	0	42500T: 3.5;
EPX	chr17	17q22	1	0	0	1	Amplification	1	0	42497T: 4;
ERBB2	chr17	17q12	2	1	0	1	Amplification	2	0	42473T: 6; 42497T: 3.5;
ERBB3	chr12	12q13.2	1	0	0	1	Amplification	1	0	42494T: 9.5;
ERCC1	chr19	19q13.32	2	1	1	0	Amplification	2	0	42484T: 4; 42473T: 3.5;

Mangalaparthi *et al.* , 2020. Mutational landscape of esophageal squamous cell carcinoma in an Indian cohort
Supplementary Table 7A. List of copy number alterations and affected genes in ESCC patients

Gene	Chromosome	Cytoband	Recurrence	Recurrence in smoker cohort	Recurrence in chewer cohort	Recurrence in No habit cohort	State	Samples with CNA gain	Samples with CNA loss	File info with CNA fold
ERCC2	chr19	19q13.32	3	1	1	1	Amplification	3	0	42484T: 4; 56957T: 4; 42473T: 3.5;
ERCC4	chr16	16p13.12	2	1	0	1	Amplification	2	0	42473T: 5.5; 42495T: 4;
ERF	chr19	5q31.2	1	1	0	0	Amplification	1	0	42473T: 4.5;
ERGIC2	chr12	12p11.22	1	0	0	1	Amplification	1	0	42500T: 6;
ERGIC3	chr20	20q11.22	2	1	0	1	Amplification	2	0	42473T: 3.5; 42493T: 3.5;
ERH	chr14	14q24.1	1	0	0	1	Amplification	1	0	42494T: 4;
ERI1	chr8	8p23.1	1	0	1	0	Amplification	1	0	42486T: 3.5;
ERI2	chr16	16p12.3	1	1	0	0	Amplification	1	0	42473T: 4;
ERLEC1	chr2	2p16.2	2	0	1	1	Amplification	2	0	42484T: 4.5; 42500T: 3.5;
ERLIN2	chr8	8p11.23	1	0	1	0	Amplification	1	0	42482T: 3.5;
ERMAP	chr1	1p34.2	2	0	0	2	Amplification	2	0	42496T: 5.5; 42493T: 6;
ERMP1	chr9	9p24.1	2	0	0	2	Amplification	2	0	42496T: 3.5; 42498T: 14;
ERN2	chr16	16p12.2	1	1	0	0	Amplification	1	0	42473T: 4;
ERO1L	chr14	14q22.1	1	0	0	1	Amplification	1	0	42494T: 4;
ERP27	chr12	12p12.3	1	0	0	1	Amplification	1	0	42500T: 4.5;
ERP29	chr12	12q24.13	1	0	0	1	Amplification	1	0	42500T: 3.5;
ERV3	chr7	7q11.21	1	0	0	1	Amplification	1	0	42501T: 3.5;
ESPNL	chr2	2q37.3	1	1	0	0	Amplification	1	0	42473T: 3.5;
ESPNP	chr1	1p36.13	1	1	0	0	Amplification	1	0	42473T: 4.5;
ESR2	chr14	14q23.2-q23	1	0	0	1	Amplification	1	0	42494T: 4;
ESRP1	chr8	8q22.1	2	0	0	2	Amplification	2	0	42496T: 3.5; 42495T: 3.5;
ESRRB	chr14	14q24.3	2	0	0	2	Amplification	2	0	42494T: 4; 56957T: 3.5;
ESYT1	chr12	12q13.2	1	0	0	1	Amplification	1	0	42494T: 7.5;
ESYT3	chr3	3q22.3	4	0	1	3	Amplification	4	0	42492T: 3.5; 42487T: 3.5; 42493T: 3.5; 42496T: 3.5;
ETF1	chr5	5q31.2	1	0	0	1	Amplification	1	0	42495T: 3.5;
ETHE1	chr19	19q13.31	1	0	0	1	Amplification	1	0	56957T: 4;
ETNK1	chr12	12p12.1	1	0	0	1	Amplification	1	0	42500T: 6;
ETS2	chr21	21q22.2	1	1	0	0	Amplification	1	0	42473T: 3.5;
ETV1	chr7	7p21.2	1	1	0	0	Amplification	1	0	42473T: 3.5;
ETV2	chr19	19q13.12	3	0	1	2	Amplification	3	0	56957T: 4; 42484T: 3.5; 42500T: 6.5;
ETV3	chr1	1q23.1	1	1	0	0	Amplification	1	0	42473T: 7;
ETV3L	chr1	1q23.1	1	1	0	0	Amplification	1	0	42473T: 7;
ETV5	chr3	3q27.2	10	1	3	6	Amplification	10	0	42497T: 4; 42498T: 3.5; 42484T: 4; 56957T: 4.5; 42495T: 4; 42474T: 3.5; 42482T: 3.5; 42492T: 3.5; 42487T: 3.5; 42493T: 3.5;
ETV6	chr12	12p13.2	1	0	0	1	Amplification	1	0	42500T: 4.5;
EVA1A	chr2	2p12	1	0	0	1	Amplification	1	0	42500T: 3.5;
EVI2A	chr17	17q11.2	1	1	0	0	Amplification	1	0	42473T: 3.5;
EVI2B	chr17	17q11.2	1	1	0	0	Amplification	1	0	42473T: 3.5;
EVPL	chr17	17q25.1	1	0	0	1	Amplification	1	0	42494T: 3.5;
EVX1	chr7	7p15.2	1	1	0	0	Amplification	1	0	42473T: 4;

Mangalaparthi *et al.* , 2020. Mutational landscape of esophageal squamous cell carcinoma in an Indian cohort
Supplementary Table 7A. List of copy number alterations and affected genes in ESCC patients

Gene	Chromosome	Cytoband	Recurrence	Recurrence in smoker cohort	Recurrence in chewer cohort	Recurrence in No habit cohort	State	Samples with CNA gain	Samples with CNA loss	File info with CNA fold
EVX2	chr2	2q31.1	1	0	0	1	Amplification	1	0	42493T: 3.5;
EXD2	chr14	14q24.1	1	0	0	1	Amplification	1	0	42494T: 4;
EXD3	chr9	9q34.3	2	1	0	1	Amplification	2	0	42473T: 5; 56957T: 3.5;
EXOC3	chr5	5p15.33	5	2	1	2	Amplification	5	0	42486T: 3.5; 42473T: 3.5; 42496T: 4; 42475T: 3.5; 42493T: 3.5;
EXOC3L2	chr19	19q13.32	2	1	0	1	Amplification	2	0	56957T: 4; 42473T: 3.5;
EXOC4	chr7	7q33	1	0	1	0	Amplification	1	0	42487T: 3.5;
EXOC5	chr14	14q22.3	1	0	0	1	Amplification	1	0	42494T: 4;
EXOC6B	chr2	2p13.2	1	0	0	1	Amplification	1	0	42500T: 3.5;
EXOSC4	chr8	8q24.3	1	0	1	0	Amplification	1	0	42483T: 3.5;
EXOSC5	chr19	19q13.2	1	1	0	0	Amplification	1	0	42473T: 4.5;
EXT1	chr8	8q24.11	3	1	0	2	Amplification	3	0	42495T: 3.5; 42496T: 3.5; 42475T: 3.5;
EYA1	chr8	8q13.3	2	0	0	2	Amplification	2	0	42495T: 3.5; 42496T: 3.5;
EYA2	chr20	20q13.12	1	1	0	0	Amplification	1	0	42473T: 5;
EYA3	chr1	1p35.3	1	1	0	0	Amplification	1	0	42473T: 3.5;
EZH2	chr7	7q36.1	2	0	1	1	Amplification	2	0	42500T: 4; 42487T: 3.5;
EZR	chr6	6q25.3	1	1	0	0	Amplification	1	0	42473T: 3.5;
F10	chr13	20p13	1	0	0	1	Amplification	1	0	56957T: 3.5;
F2	chr11	11p11.2	1	1	0	0	Amplification	1	0	42473T: 3.5;
F7	chr13	13q34	1	0	0	1	Amplification	1	0	56957T: 3.5;
FABP1	chr2	2p11.2	1	0	0	1	Amplification	1	0	42500T: 3.5;
FABP12	chr8	8q21.13	2	0	0	2	Amplification	2	0	42496T: 3.5; 42495T: 3.5;
FABP3	chr1	1p35.2	1	1	0	0	Amplification	1	0	42473T: 3.5;
FABP4	chr8	8q21.13	2	0	0	2	Amplification	2	0	42495T: 3.5; 42496T: 3.5;
FABP5	chr8	13q22.1	2	0	0	2	Amplification	2	0	42495T: 3.5; 42496T: 3.5;
FABP9	chr8	8q21.13	2	0	0	2	Amplification	2	0	42495T: 3.5; 42496T: 3.5;
FADD	chr11	11q13.3	12	3	2	7	Amplification	12	0	56957T: 5; 42478T: 4.5; 42498T: 7; 42475T: 7.5; 42501T: 6; 42500T: 13; 56958T: 9; 42483T: 9; 42486T: 13.5; 42497T: 6.5; 42492T: 4; 42476T: 13.5;
FAHD1	chr16	16p13.3	1	0	1	0	Amplification	1	0	42483T: 3.5;
FAHD2A	chr2	2q11.1	2	1	0	1	Amplification	2	0	42473T: 3.5; 42493T: 4;
FAHD2B	chr2	2q11.2	2	1	0	1	Amplification	2	0	42473T: 3.5; 42493T: 4.5;
FAHD2CP	chr2	2q11.2	2	1	0	1	Amplification	2	0	42473T: 3.5; 42493T: 4;
FAIM	chr3	3q22.3	4	0	1	3	Amplification	4	0	42487T: 3.5; 42493T: 3.5; 42492T: 3.5; 42496T: 3.5;
FAIM2	chr12	12q13.12	1	0	0	1	Amplification	1	0	42500T: 3.5;
FAM101A	chr12	12q24.31	1	0	0	1	Amplification	1	0	42500T: 3.5;
FAM105A	chr5	5p15.2	5	1	2	2	Amplification	5	0	42475T: 3.5; 42486T: 3.5; 42496T: 3.5; 42493T: 4; 42484T: 3.5;
FAM105B	chr5	5p15.2	5	1	2	2	Amplification	5	0	42484T: 3.5; 42493T: 4; 42486T: 3.5; 42496T: 3.5; 42475T: 3.5;
FAM109A	chr12	12q24.12	1	0	0	1	Amplification	1	0	42500T: 3.5;

Mangalaparthi *et al.* , 2020. Mutational landscape of esophageal squamous cell carcinoma in an Indian cohort
Supplementary Table 7A. List of copy number alterations and affected genes in ESCC patients

Gene	Chromosome	Cytoband	Recurrence	Recurrence in smoker cohort	Recurrence in chewer cohort	Recurrence in No habit cohort	State	Samples with CNA gain	Samples with CNA loss	File info with CNA fold
FAM110B	chr8	8q12.1	2	0	0	2	Amplification	2	0	42496T: 3.5; 42495T: 3.5;
FAM110C	chr2	2p25.3	1	0	0	1	Amplification	1	0	42500T: 3.5;
FAM114A1	chr4	4p14	1	1	0	0	Amplification	1	0	42473T: 3.5;
FAM115A	chr7	7q35	1	0	1	0	Amplification	1	0	42487T: 3.5;
FAM115C	chr7	7q35	1	0	1	0	Amplification	1	0	42487T: 3.5;
FAM126A	chr7	7p15.3	1	1	0	0	Amplification	1	0	42473T: 4;
FAM131A	chr3	3q27.1	11	1	3	7	Amplification	11	0	42498T: 3.5; 42484T: 4; 56957T: 5; 42474T: 3.5; 42495T: 4; 42494T: 3.5; 42497T: 4; 42492T: 3.5; 42493T: 3.5; 42487T: 3.5; 42482T: 3.5;
FAM131B	chr7	7q34	1	0	1	0	Amplification	1	0	42487T: 3.5;
FAM131C	chr1	1p36.13	1	1	0	0	Amplification	1	0	42473T: 8;
FAM132B	chr2	2q37.3	1	1	0	0	Amplification	1	0	42473T: 3.5;
FAM133B	chr7	7q21.2	2	0	2	0	Amplification	2	0	42483T: 4; 42487T: 3.5;
FAM134B	chr5	5p15.1	4	1	1	2	Amplification	4	0	42493T: 4; 42486T: 3.5; 42496T: 3.5; 42475T: 3.5;
FAM135B	chr8	8q24.23	2	0	0	2	Amplification	2	0	42496T: 3.5; 42495T: 3.5;
FAM136A	chr2	2p13.3	1	0	0	1	Amplification	1	0	42500T: 3.5;
FAM150A	chr8	8q11.23	4	0	1	3	Amplification	4	0	42496T: 3.5; 42495T: 3.5; 42494T: 3.5; 42484T: 5;
FAM150B	chr2	2p25.3	1	0	0	1	Amplification	1	0	42500T: 3.5;
FAM161A	chr2	2p15	3	0	1	2	Amplification	3	0	56957T: 3.5; 42484T: 8.5; 42500T: 3.5;
FAM161B	chr14	14q24.3	2	0	0	2	Amplification	2	0	42494T: 4; 56957T: 3.5;
FAM162A	chr3	3q21.1	1	0	0	1	Amplification	1	0	42496T: 3.5;
FAM166A	chr9	9q34.3	2	1	0	1	Amplification	2	0	42473T: 5; 56957T: 3.5;
FAM166B	chr9	9p13.3	2	1	0	1	Amplification	2	0	42501T: 3.5; 42473T: 3.5;
FAM167A	chr8	8p23.1	1	0	1	0	Amplification	1	0	42486T: 3.5;
FAM167B	chr1	1p35.2	1	1	0	0	Amplification	1	0	42473T: 4;
FAM168A	chr11	11q13.4	6	3	0	3	Amplification	6	0	42475T: 8.5; 42476T: 4; 56957T: 3.5; 42478T: 4.5; 42492T: 4; 42498T: 6.5;
FAM171B	chr2	2q32.1	1	0	0	1	Amplification	1	0	42493T: 3.5;
FAM173B	chr5	5p15.2	4	1	1	2	Amplification	4	0	42493T: 4; 42475T: 3.5; 42496T: 3.5; 42486T: 3.5;
FAM177A1	chr14	14q13.2	2	1	0	1	Amplification	2	0	42476T: 5; 42500T: 4.5;
FAM178B	chr2	2q11.2	2	1	0	1	Amplification	2	0	42473T: 3.5; 42493T: 4.5;
FAM179A	chr2	2p23.2	1	0	0	1	Amplification	1	0	42500T: 3.5;
FAM179B	chr14	14q21.2	1	0	0	1	Amplification	1	0	42500T: 5.5;
FAM180A	chr7	7q33	1	0	1	0	Amplification	1	0	42487T: 3.5;
FAM180B	chr11	11p11.2	1	1	0	0	Amplification	1	0	42473T: 3.5;
FAM183A	chr1	1p34.2	1	0	0	1	Amplification	1	0	42493T: 6;
FAM183B	chr7	7p14.1	1	1	0	0	Amplification	1	0	42473T: 3.5;
FAM185A	chr7	7q22.1	3	0	1	2	Amplification	3	0	42493T: 3.5; 42487T: 3.5; 42501T: 3.5;
FAM186A	chr12	12q13.12	1	0	0	1	Amplification	1	0	42500T: 3.5;
FAM186B	chr12	12q13.12	1	0	0	1	Amplification	1	0	42500T: 3.5;
FAM187B	chr19	19q13.12	3	0	1	2	Amplification	3	0	42500T: 6.5; 56957T: 4; 42484T: 3.5;

Mangalaparthi *et al.* , 2020. Mutational landscape of esophageal squamous cell carcinoma in an Indian cohort
Supplementary Table 7A. List of copy number alterations and affected genes in ESCC patients

Gene	Chromosome	Cytoband	Recurrence	Recurrence in smoker cohort	Recurrence in chewer cohort	Recurrence in No habit cohort	State	Samples with CNA gain	Samples with CNA loss	File info with CNA fold
FAM188B	chr7	7p14.3	1	1	0	0	Amplification	1	0	42473T: 4;
FAM188B2	chr3	3q25.1	10	2	2	6	Amplification	10	0	42474T: 3.5; 56957T: 4; 42484T: 3.5; 42500T: 3.5; 42497T: 4.5; 42473T: 4; 42496T: 4; 42487T: 3.5; 42493T: 3.5; 42492T: 3.5;
FAM189B	chr1	1q22	1	1	0	0	Amplification	1	0	42473T: 6.5;
FAM194A	chr3	3q25.1	10	2	2	6	Amplification	10	0	42500T: 3.5; 42473T: 4; 42497T: 4.5; 42496T: 4; 42474T: 3.5; 56957T: 4; 42484T: 3.5; 42493T: 3.5; 42487T: 3.5; 42492T: 3.5;
FAM195A	chr16	16p13.3	2	0	1	1	Amplification	2	0	42493T: 3.5; 42483T: 3.5;
FAM203A	chr8	8q24.3	3	0	1	2	Amplification	3	0	42483T: 3.5; 42496T: 4; 42495T: 4.5;
FAM203B	chr8	8q24.3	3	0	1	2	Amplification	3	0	42496T: 4; 42483T: 3.5; 42495T: 4.5;
FAM205A	chr9	9p13.3	1	0	0	1	Amplification	1	0	42501T: 3.5;
FAM205B	chr9	9p13.3	1	0	0	1	Amplification	1	0	42501T: 3.5;
FAM209A	chr20	20q13.31	1	1	0	0	Amplification	1	0	42473T: 5;
FAM209B	chr20	20q13.31	1	1	0	0	Amplification	1	0	42473T: 5;
FAM20C	chr7	7p22.3	1	1	0	0	Amplification	1	0	42473T: 5;
FAM210B	chr20	20q13.2	1	1	0	0	Amplification	1	0	42473T: 5;
FAM214B	chr9	9p13.3	1	0	0	1	Amplification	1	0	42501T: 3.5;
FAM216A	chr12	12q24.11	1	0	0	1	Amplification	1	0	42500T: 3.5;
FAM217B	chr20	20q13.33	1	1	0	0	Amplification	1	0	42473T: 4.5;
FAM220A	chr7	7p22.1	1	1	0	0	Amplification	1	0	42473T: 4.5;
FAM221A	chr7	7p15.3	1	1	0	0	Amplification	1	0	42473T: 4;
FAM221B	chr9	9p13.3	2	1	0	1	Amplification	2	0	42501T: 3.5; 42473T: 3.5;
FAM222A	chr12	12q24.11	2	1	0	1	Amplification	2	0	42473T: 4.5; 42500T: 3.5;
FAM227A	chr22	22q13.1	1	1	0	0	Amplification	1	0	42473T: 3.5;
FAM228A	chr2	2p23.3	1	0	0	1	Amplification	1	0	42500T: 3.5;
FAM228B	chr2	2p23.3	1	0	0	1	Amplification	1	0	42500T: 3.5;
FAM229A	chr1	1p35.1	1	1	0	0	Amplification	1	0	42473T: 4;
FAM229B	chr6	6q21	1	0	0	1	Amplification	1	0	42496T: 3.5;
FAM3B	chr21	21q22.3	1	1	0	0	Amplification	1	0	42473T: 3.5;
FAM3C	chr7	7q31.31	1	0	1	0	Amplification	1	0	42487T: 3.5;
FAM43B	chr1	1p36.12	1	1	0	0	Amplification	1	0	42473T: 4;
FAM49A	chr2	2p24.2	1	0	0	1	Amplification	1	0	42500T: 3.5;
FAM49B	chr8	8q24.21	3	0	1	2	Amplification	3	0	42495T: 3.5; 42484T: 3.5; 42496T: 3.5;
FAM60A	chr12	12p11.21	1	0	0	1	Amplification	1	0	42500T: 8;
FAM63A	chr1	1q21.3	1	1	0	0	Amplification	1	0	42473T: 4.5;
FAM65C	chr20	20q13.13	1	1	0	0	Amplification	1	0	42473T: 6.5;
FAM69B	chr9	9q34.3	1	0	0	1	Amplification	1	0	56957T: 3.5;
FAM71C	chr12	12q23.1	1	0	0	1	Deletion	0	1	42495T: 0.5;
FAM71D	chr14	14q23.3	1	0	0	1	Amplification	1	0	42494T: 4;
FAM71F1	chr7	7q32.1	1	0	1	0	Amplification	1	0	42487T: 3.5;

Gene	Chromosome	Cytoband	Recurrence	Recurrence in smoker cohort	Recurrence in chewer cohort	Recurrence in No habit cohort	State	Samples with CNA gain	Samples with CNA loss	File info with CNA fold
FAM71F2	chr7	7q32.1	1	0	1	0	Amplification	1	0	42487T: 3.5;
FAM76A	chr1	1p35.3	1	1	0	0	Amplification	1	0	42473T: 3.5;
FAM78A	chr9	9q34.13	1	0	1	0	Amplification	1	0	42484T: 4;
FAM83A	chr8	8q24.13	4	1	1	2	Amplification	4	0	42475T: 3.5; 42496T: 3.5; 42495T: 3.5; 42484T: 3.5;
FAM83C	chr20	20q11.22	2	1	0	1	Amplification	2	0	42493T: 3.5; 42473T: 3.5;
FAM83D	chr20	20q11.23	1	1	0	0	Amplification	1	0	42473T: 3.5;
FAM83F	chr22	22q13.1	1	1	0	0	Amplification	1	0	42473T: 3.5;
FAM83H	chr8	8q24.3	3	0	1	2	Amplification	3	0	42483T: 3.5; 42496T: 4; 42495T: 4.5;
FAM84B	chr8	8q24.21	4	1	1	2	Amplification	4	0	42484T: 3.5; 42495T: 3.5; 42496T: 3.5; 42475T: 3.5;
FAM86C2P	chr11	11q13.2	1	0	0	1	Amplification	1	0	56957T: 5;
FAM91A1	chr8	8q24.13	4	1	1	2	Amplification	4	0	42484T: 3.5; 42495T: 3.5; 42496T: 3.5; 42475T: 3.5;
FAM92A1	chr8	8q22.1	2	0	0	2	Amplification	2	0	42496T: 3.5; 42495T: 3.5;
FAM98A	chr2	2p22.3	1	0	0	1	Amplification	1	0	42500T: 3.5;
FAM98C	chr19	19q13.2	3	0	1	2	Amplification	3	0	42484T: 3.5; 56957T: 4; 42500T: 6.5;
FANCG	chr9	9p13.3	1	0	0	1	Amplification	1	0	42501T: 3.5;
FANCI	chr15	15q26.1	1	1	0	0	Amplification	1	0	42473T: 3.5;
FANCL	chr2	2p16.1	3	0	1	2	Amplification	3	0	42484T: 4.5; 56957T: 3.5; 42500T: 3.5;
FANCM	chr14	14q21.2	1	0	0	1	Amplification	1	0	42500T: 5.5;
FAR2	chr12	12p11.22	1	0	0	1	Amplification	1	0	42500T: 6;
FASN	chr17	17q25.3	1	1	0	0	Amplification	1	0	42473T: 3.5;
FASTKD3	chr5	5p15.31	4	1	1	2	Amplification	4	0	42486T: 3.5; 42496T: 3.5; 42475T: 3.5; 42493T: 4;
FASTKD5	chr20	20p13	1	1	0	0	Amplification	1	0	42473T: 3.5;
FBF1	chr17	17q25.1	1	0	0	1	Amplification	1	0	42494T: 3.5;
FBL	chr19	19q13.2	2	0	0	2	Amplification	2	0	42500T: 7.5; 56957T: 4;
FBRSL1	chr12	12q24.33	1	0	0	1	Amplification	1	0	56957T: 4;
FBXL13	chr7	7q22.1	3	0	1	2	Amplification	3	0	42487T: 3.5; 42493T: 3.5; 42501T: 3.5;
FBXL14	chr12	12p13.33	1	0	0	1	Amplification	1	0	42500T: 3.5;
FBXL16	chr16	16p13.3	2	0	1	1	Amplification	2	0	42483T: 3.5; 42493T: 3.5;
FBXL18	chr7	7p22.1	2	1	0	1	Amplification	2	0	42497T: 8.5; 42473T: 4.5;
FBXL20	chr17	17q12	2	1	0	1	Amplification	2	0	42497T: 3.5; 42473T: 4.5;
FBXL6	chr8	8q24.3	4	0	1	3	Amplification	4	0	42495T: 4.5; 56957T: 3.5; 42483T: 3.5; 42496T: 4;
FBXL7	chr5	5p15.1	5	1	2	2	Amplification	5	0	42475T: 3.5; 42496T: 3.5; 42486T: 3.5; 42493T: 4; 42484T: 3.5;
FBXO11	chr2	2p16.3	2	0	1	1	Amplification	2	0	42500T: 3.5; 42484T: 4.5;
FBXO17	chr19	19q13.2	2	0	0	2	Amplification	2	0	56957T: 4; 42500T: 6.5;
FBXO21	chr12	12q24.22	1	0	0	1	Amplification	1	0	42500T: 3.5;
FBXO27	chr19	19q13.2	2	0	0	2	Amplification	2	0	42500T: 6.5; 56957T: 4;
FBXO32	chr8	8q24.13	4	1	1	2	Amplification	4	0	42495T: 3.5; 42484T: 3.5; 42475T: 3.5; 42496T: 3.5;
FBXO33	chr14	14q21.1	1	0	0	1	Amplification	1	0	42500T: 6.5;
FBXO34	chr14	14q22.3	1	0	0	1	Amplification	1	0	42494T: 4;

Mangalaparthi *et al.* , 2020. Mutational landscape of esophageal squamous cell carcinoma in an Indian cohort
Supplementary Table 7A. List of copy number alterations and affected genes in ESCC patients

Gene	Chromosome	Cytoband	Recurrence	Recurrence in smoker cohort	Recurrence in chewer cohort	Recurrence in No habit cohort	State	Samples with CNA gain	Samples with CNA loss	File info with CNA fold
FBXO4	chr5	5p13.1	6	1	3	2	Amplification	6	0	42493T: 3.5; 42484T: 3.5; 42475T: 3.5; 42486T: 3.5; 42483T: 3.5; 42496T: 3.5;
FBXO40	chr3	3q13.33	1	0	0	1	Amplification	1	0	42496T: 3.5;
FBXO41	chr2	2p13.2	1	0	0	1	Amplification	1	0	42500T: 3.5;
FBXO42	chr1	1p36.13	1	1	0	0	Amplification	1	0	42473T: 8;
FBXO43	chr8	8q22.2	2	0	0	2	Amplification	2	0	42495T: 3.5; 42496T: 3.5;
FBXO45	chr3	3q29	9	1	3	5	Amplification	9	0	42482T: 3.5; 42487T: 3.5; 42493T: 5; 42492T: 3.5; 42474T: 3.5; 42495T: 4; 56957T: 6; 42484T: 4; 42498T: 3.5;
FBXO46	chr19	19q13.32	1	0	1	0	Amplification	1	0	42484T: 4;
FBXO47	chr17	17q12|17q12	1	1	0	0	Amplification	1	0	42473T: 3.5;
FBXO48	chr2	2p13.3	1	0	0	1	Amplification	1	0	42500T: 3.5;
FBXW5	chr9	9q34.3	2	1	0	1	Amplification	2	0	56957T: 3.5; 42473T: 5;
FBXW8	chr12	12q24.22	1	0	0	1	Amplification	1	0	42500T: 3.5;
FCF1	chr14	14q24.3	2	0	0	2	Amplification	2	0	42494T: 4; 56957T: 3.5;
FCGBP	chr19	19q13.2	2	0	0	2	Amplification	2	0	42500T: 7.5; 56957T: 4;
FCGR1A	chr1	1q21.2	1	1	0	0	Amplification	1	0	42473T: 4;
FCGR1B	chr1	1p11.2	1	1	0	0	Amplification	1	0	42473T: 4;
FCGR1C	chr1	1q21.1	1	1	0	0	Amplification	1	0	42473T: 4;
FCHSD2	chr11	11q13.4	6	3	0	3	Amplification	6	0	42476T: 4; 42475T: 8.5; 42478T: 4.5; 42492T: 4; 42498T: 6.5; 56957T: 3.5;
FCN3	chr1	1p36.11	1	1	0	0	Amplification	1	0	42473T: 5;
FCRL1	chr1	1q23.1	1	1	0	0	Amplification	1	0	42473T: 5;
FCRL2	chr1	1q23.3	1	1	0	0	Amplification	1	0	42473T: 5;
FCRL3	chr1	1q23.1	1	1	0	0	Amplification	1	0	42473T: 5;
FCRL4	chr1	1q23.1	1	1	0	0	Amplification	1	0	42473T: 5;
FCRL5	chr1	1q23.1	1	1	0	0	Amplification	1	0	42473T: 5;
FDFT1	chr8	8p23.1	1	0	1	0	Amplification	1	0	42486T: 3.5;
FDPS	chr1	1q22	1	1	0	0	Amplification	1	0	42473T: 6.5;
FER1L4	chr20	20q11.22	2	1	0	1	Amplification	2	0	42493T: 3.5; 42473T: 3.5;
FER1L6	chr8	8q24.13	4	1	1	2	Amplification	4	0	42475T: 3.5; 42496T: 3.5; 42495T: 3.5; 42484T: 3.5;
FERD3L	chr7	7p21.1	1	1	0	0	Amplification	1	0	42473T: 4;
FERMT2	chr14	14q22.1	1	0	0	1	Amplification	1	0	42494T: 4;
FETUB	chr3	3q27.3	10	1	3	6	Amplification	10	0	42482T: 3.5; 42487T: 3.5; 42493T: 3.5; 42492T: 3.5; 42497T: 4; 56957T: 4.5; 42474T: 3.5; 42495T: 4; 42498T: 3.5; 42484T: 4;
FEV	chr2	2q35	1	1	0	0	Amplification	1	0	42473T: 3.5;
FEZ2	chr2	2p22.2	1	0	0	1	Amplification	1	0	42500T: 3.5;
FEZF1	chr7	7q31.32	1	0	1	0	Amplification	1	0	42487T: 3.5;
FFAR1	chr19	19q13.12	3	0	1	2	Amplification	3	0	42500T: 6.5; 56957T: 4; 42484T: 3.5;
FFAR2	chr19	19q13.12	3	0	1	2	Amplification	3	0	42484T: 3.5; 56957T: 4; 42500T: 6.5;

Mangalaparthi *et al.* , 2020. Mutational landscape of esophageal squamous cell carcinoma in an Indian cohort
Supplementary Table 7A. List of copy number alterations and affected genes in ESCC patients

Gene	Chromosome	Cytoband	Recurrence	Recurrence in smoker cohort	Recurrence in chewer cohort	Recurrence in No habit cohort	State	Samples with CNA gain	Samples with CNA loss	File info with CNA fold
FFAR3	chr19	19q13.12	3	0	1	2	Amplification	3	0	42500T: 6.5; 42484T: 3.5; 56957T: 4;
FGD2	chr6	8q11.2-q13.2	1	0	1	0	Amplification	1	0	42486T: 3.5;
FGD4	chr12	12p11.21	1	0	0	1	Amplification	1	0	42500T: 3.5;
FGF10	chr5	5p12	4	1	1	2	Amplification	4	0	42493T: 3.5; 42475T: 3.5; 42496T: 3.5; 42486T: 3.5;
FGF12	chr3	3q28-q29	10	1	3	6	Amplification	10	0	42482T: 3.5; 42492T: 3.5; 42487T: 3.5; 42493T: 3.5; 42501T: 3.5; 42484T: 4; 42498T: 3.5; 42474T: 3.5; 42495T: 4; 56957T: 4.5;
FGF19	chr11	11q13.3	11	3	2	6	Amplification	11	0	42476T: 29.5; 42492T: 4; 42501T: 6; 42475T: 7.5; 42500T: 13; 56958T: 5; 42486T: 13.5; 42483T: 14.5; 42497T: 4.5; 42478T: 4.5; 42498T: 9.5;
FGF23	chr12	12p13.32	1	0	0	1	Amplification	1	0	42494T: 3.5;
FGF3	chr11	11q13.3	12	3	2	7	Amplification	12	0	42476T: 29.5; 42492T: 4; 42501T: 6; 42475T: 7.5; 56958T: 5; 42500T: 13; 42486T: 13.5; 42483T: 14.5; 42497T: 4.5; 56957T: 5; 42478T: 4.5; 42498T: 9.5;
FGF4	chr11	11q13.3	8	2	1	5	Amplification	8	0	56957T: 5; 42478T: 4.5; 42498T: 9.5; 42501T: 6; 42475T: 7.5; 56958T: 5; 42483T: 14.5; 42497T: 4.5;
FGFR1	chr8	8p11.23	1	0	1	0	Amplification	1	0	42482T: 3.5;
FGFR1OP2	chr12	12p11.23	1	0	0	1	Amplification	1	0	42500T: 6;
FGL2	chr7	7q11.23	1	0	1	0	Amplification	1	0	42487T: 3.5;
FGR	chr1	1p35.3	1	1	0	0	Amplification	1	0	42473T: 3.5;
FHL2	chr2	2q12.2	1	0	0	1	Amplification	1	0	42493T: 3.5;
FIG4	chr6	6q21	1	0	0	1	Amplification	1	0	42496T: 3.5;
FIGLA	chr2	2p13.3	1	0	0	1	Amplification	1	0	42500T: 3.5;
FILIP1L	chr3	3q12.1	1	1	0	0	Amplification	1	0	42476T: 3.5;
FIS1	chr7	7q22.1	2	0	0	2	Amplification	2	0	42501T: 3.5; 42493T: 3.5;
FITM2	chr20	20q13.12	1	1	0	0	Amplification	1	0	42473T: 3.5;
FIZ1	chr19	19q13.42	1	0	0	1	Amplification	1	0	42494T: 3.5;
FKBP11	chr12	12q13.12	1	0	0	1	Amplification	1	0	42500T: 3.5;
FKBP14	chr7	7p14.3	1	1	0	0	Amplification	1	0	42473T: 4;
FKBP1A	chr20	20p13	1	1	0	0	Amplification	1	0	42473T: 3.5;
FKBP1B	chr2	2p23.3	1	0	0	1	Amplification	1	0	42500T: 3.5;
FKBP3	chr14	14q21.2	1	0	0	1	Amplification	1	0	42500T: 5.5;
FKBP7	chr2	2q31.2	1	0	0	1	Amplification	1	0	42493T: 3.5;
FKBP8	chr19	19p13.11	1	0	0	1	Amplification	1	0	42494T: 4;
FKBP9	chr7	7p14.3	1	1	0	0	Amplification	1	0	42473T: 4;
FKBP9L	chr7	7p11.2	3	0	1	2	Amplification	3	0	42483T: 4; 42497T: 6.5; 56957T: 5;
FKBPL	chr6	6p21.32	1	1	0	0	Amplification	1	0	42473T: 3.5;
FLAD1	chr1	1q21.3	2	1	0	1	Amplification	2	0	42473T: 6.5; 42496T: 3.5;
FLG	chr1	1q21.3	1	1	0	0	Amplification	1	0	42473T: 4.5;
FLG2	chr1	1q21.3	1	1	0	0	Amplification	1	0	42473T: 4.5;
FLNC	chr7	7q32.1	1	0	1	0	Amplification	1	0	42487T: 3.5;

Mangalaparthi *et al.* , 2020. Mutational landscape of esophageal squamous cell carcinoma in an Indian cohort
Supplementary Table 7A. List of copy number alterations and affected genes in ESCC patients

Gene	Chromosome	Cytoband	Recurrence	Recurrence in smoker cohort	Recurrence in chewer cohort	Recurrence in No habit cohort	State	Samples with CNA gain	Samples with CNA loss	File info with CNA fold
FLVCR2	chr14	14q24.3	2	0	0	2	Amplification	2	0	42494T: 4; 56957T: 3.5;
FMNL3	chr12	12q13.12	1	0	0	1	Amplification	1	0	42500T: 3.5;
FMO5	chr1	1q21.1	1	1	0	0	Amplification	1	0	42473T: 4;
FNBP4	chr11	11p11.2	1	1	0	0	Amplification	1	0	42473T: 3.5;
FNDC3B	chr3	3q26.31	9	1	3	5	Amplification	9	0	42487T: 3.5; 42493T: 3.5; 42492T: 3.5; 42482T: 3.5; 56957T: 4; 42495T: 4; 42474T: 3.5; 42484T: 4; 42500T: 9;
FNDC4	chr2	2p23.3	1	0	0	1	Amplification	1	0	42500T: 3.5;
FNTA	chr8	8p11.21	1	0	1	0	Amplification	1	0	42483T: 4.5;
FNTB	chr14	14q23.3	1	0	0	1	Amplification	1	0	42494T: 4;
FOCAD	chr9	9p21.3	1	1	0	0	Deletion	0	1	42475T: 0.5;
FOLH1	chr11	11p11.12	1	0	0	1	Amplification	1	0	56957T: 8;
FOLR1	chr11	11q13.4	8	3	0	5	Amplification	8	0	42498T: 5; 42478T: 4.5; 56957T: 3.5; 42501T: 6; 42475T: 5; 42492T: 4; 42493T: 3.5; 42476T: 4;
FOLR2	chr11	11q13.4	8	3	0	5	Amplification	8	0	42476T: 4; 42492T: 4; 42493T: 3.5; 42475T: 5; 42501T: 6; 42498T: 5; 42478T: 4.5; 56957T: 3.5;
FOLR3	chr11	11q13.4	8	3	0	5	Amplification	8	0	42501T: 6; 42475T: 5; 42498T: 5; 42478T: 4.5; 56957T: 3.5; 42476T: 4; 42492T: 4; 42493T: 3.5;
FOPNL	chr16	16p13.11	2	1	0	1	Amplification	2	0	42473T: 4; 42495T: 4;
FOS	chr14	14q24.3	2	0	0	2	Amplification	2	0	56957T: 3.5; 42494T: 4;
FOSB	chr19	19q13.32	2	1	1	0	Amplification	2	0	42473T: 3.5; 42484T: 4;
FOSL2	chr2	2p23.2	1	0	0	1	Amplification	1	0	42500T: 3.5;
FOXA1	chr14	14q21.1	2	1	0	1	Amplification	2	0	42476T: 11; 42500T: 6.5;
FOXA3	chr19	19q13.32	1	0	1	0	Amplification	1	0	42484T: 4;
FOXG1	chr14	14q12	1	0	0	1	Amplification	1	0	42500T: 4.5;
FOXH1	chr8	8q24.3	5	0	1	4	Amplification	5	0	42483T: 3.5; 42496T: 4; 42495T: 4.5; 42494T: 3.5; 56957T: 3.5;
FOXI3	chr2	2p11.2	1	0	0	1	Amplification	1	0	42500T: 3.5;
FOXK1	chr7	7p22.1	2	1	0	1	Amplification	2	0	42497T: 8.5; 42473T: 4.5;
FOXL2	chr3	3q22.3	4	0	1	3	Amplification	4	0	42487T: 3.5; 42493T: 3.5; 42492T: 3.5; 42496T: 3.5;
FOXN2	chr2	2p16.3	2	0	1	1	Amplification	2	0	42484T: 4.5; 42500T: 3.5;
FOXN4	chr12	12q24.11	2	1	0	1	Amplification	2	0	42500T: 3.5; 42473T: 4.5;
FOXO3	chr6	6q21	1	0	0	1	Amplification	1	0	42496T: 3.5;
FOXP2	chr7	7q31.1	1	0	1	0	Amplification	1	0	42487T: 3.5;
FOXR1	chr11	11q23.3	1	1	0	0	Amplification	1	0	42473T: 4;
FOXRED2	chr22	22q12.3	1	1	0	0	Amplification	1	0	42473T: 3.5;
FOXS1	chr20	20q11.21	1	0	0	1	Amplification	1	0	42496T: 5;
FREM1	chr9	9p22.3	1	0	0	1	Amplification	1	0	42498T: 14.5;
FRMD6	chr14	14q22.1	1	0	0	1	Amplification	1	0	42494T: 4;
FRRS1L	chr9	9q31.3	1	0	1	0	Amplification	1	0	42483T: 3.5;
FRS2	chr12	12q15	2	0	0	2	Amplification	2	0	42501T: 5.5; 42500T: 5;

Mangalaparthi *et al.* , 2020. Mutational landscape of esophageal squamous cell carcinoma in an Indian cohort
Supplementary Table 7A. List of copy number alterations and affected genes in ESCC patients

Gene	Chromosome	Cytoband	Recurrence	Recurrence in smoker cohort	Recurrence in chewer cohort	Recurrence in No habit cohort	State	Samples with CNA gain	Samples with CNA loss	File info with CNA fold
FRY	chr13	13q13.1	1	0	0	1	Amplification	1	0	42497T: 3.5;
FRYL	chr4	4p11	1	0	1	0	Amplification	1	0	42483T: 3.5;
FRZB	chr2	2q32.1	1	0	0	1	Amplification	1	0	42493T: 3.5;
FSBP	chr8	8q22.1	2	0	0	2	Amplification	2	0	42496T: 3.5; 42495T: 3.5;
FSCB	chr14	14q21.2	1	0	0	1	Amplification	1	0	42500T: 5.5;
FSCN1	chr7	7p22.1	2	1	0	1	Amplification	2	0	42497T: 10.5; 42473T: 4.5;
FSCN3	chr7	7q32.1	1	0	1	0	Amplification	1	0	42487T: 3.5;
FSHR	chr2	2p16.3	2	0	1	1	Amplification	2	0	42484T: 4.5; 42500T: 3.5;
FSIP2	chr2	2q32.1	1	0	0	1	Amplification	1	0	42493T: 3.5;
FSTL1	chr3	3q13.33	1	0	0	1	Amplification	1	0	42496T: 3.5;
FTCDNL1	chr2	2q33.1	1	0	1	0	Amplification	1	0	42482T: 5;
FTSJ2	chr7	7p22.3	1	1	0	0	Amplification	1	0	42473T: 5;
FTSJD2	chr6	6p21.2	1	1	0	0	Amplification	1	0	42473T: 4.5;
FUCA1	chr1	1p36.11	1	1	0	0	Amplification	1	0	42473T: 3.5;
FUNDC2P2	chr2	2p11.2	1	0	0	1	Amplification	1	0	42500T: 3.5;
FUT10	chr8	8p12	2	0	1	1	Amplification	2	0	42497T: 4.5; 42482T: 3.5;
FUT11	chr10	10q22.2	2	1	0	1	Amplification	2	0	42473T: 4.5; 42496T: 3.5;
FUT7	chr9	9q34.3	2	1	0	1	Amplification	2	0	56957T: 3.5; 42473T: 5;
FUT8	chr14	14q23.3	1	0	0	1	Amplification	1	0	42494T: 4;
FXR1	chr3	3q26.33	11	1	3	7	Amplification	11	0	42492T: 3.5; 42493T: 3.5; 42487T: 3.5; 42482T: 3.5; 42498T: 3.5; 42484T: 4; 56957T: 4; 42495T: 4; 42474T: 3.5; 42497T: 4; 42500T: 14;
FXYD1	chr19	19q13.12	3	0	1	2	Amplification	3	0	56957T: 4; 42484T: 3.5; 42500T: 6.5;
FXYD3	chr19	19q13.12	3	0	1	2	Amplification	3	0	56957T: 4; 42484T: 3.5; 42500T: 6.5;
FXYD5	chr19	19q13.12	3	0	1	2	Amplification	3	0	42500T: 6.5; 56957T: 4; 42484T: 3.5;
FXYD7	chr19	19q13.12	3	0	1	2	Amplification	3	0	42500T: 6.5; 42484T: 3.5; 56957T: 4;
FYN	chr6	6q21	1	0	0	1	Amplification	1	0	42496T: 3.5;
FYTTD1	chr3	3q29	9	1	3	5	Amplification	9	0	42492T: 3.5; 42487T: 3.5; 42493T: 4; 42482T: 3.5; 42484T: 4; 42498T: 3.5; 42495T: 4; 42474T: 3.5; 56957T: 6;
FZD1	chr7	7q21.13	3	0	2	1	Amplification	3	0	42494T: 3.5; 42487T: 3.5; 42483T: 4;
FZD6	chr8	8q22.3	2	0	0	2	Amplification	2	0	42496T: 3.5; 42495T: 3.5;
G2E3	chr14	14q12	2	0	0	2	Amplification	2	0	56957T: 4; 42500T: 4.5;
GAA	chr17	17q25.3	1	1	0	0	Amplification	1	0	42473T: 3.5;
GABPB2	chr1	1q21.3	1	1	0	0	Amplification	1	0	42473T: 4.5;
GAL	chr11	11q13.2	2	1	0	1	Amplification	2	0	42476T: 29.5; 56957T: 5;
GALE	chr1	1p36.11	1	1	0	0	Amplification	1	0	42473T: 3.5;
GALK1	chr17	17q25.1	1	0	0	1	Amplification	1	0	42494T: 3.5;
GALM	chr2	2p22.1	2	0	0	2	Amplification	2	0	42500T: 3.5; 42493T: 3.5;
GALNT14	chr2	2p23.1	1	0	0	1	Amplification	1	0	42500T: 3.5;
GALNT16	chr14	7q11.22	1	0	0	1	Amplification	1	0	42494T: 4;

Mangalaparthi *et al.* , 2020. Mutational landscape of esophageal squamous cell carcinoma in an Indian cohort
Supplementary Table 7A. List of copy number alterations and affected genes in ESCC patients

Gene	Chromosome	Cytoband	Recurrence	Recurrence in smoker cohort	Recurrence in chewer cohort	Recurrence in No habit cohort	State	Samples with CNA gain	Samples with CNA loss	File info with CNA fold
GALNT6	chr12	12q13.13	2	0	0	2	Amplification	2	0	42500T: 3.5; 42494T: 3.5;
GALNT9	chr12	12q24.33	1	0	0	1	Amplification	1	0	56957T: 4;
GALR2	chr17	17q25.1	1	0	0	1	Amplification	1	0	42494T: 3.5;
GALR3	chr22	22q13.1	1	1	0	0	Amplification	1	0	42473T: 3.5;
GAP43	chr3	3q13.31	1	0	0	1	Amplification	1	0	42496T: 3.5;
GAPDHS	chr19	19q13.12	3	0	1	2	Amplification	3	0	42484T: 3.5; 56957T: 4; 42500T: 6.5;
GAREML	chr2	2p23.3	1	0	0	1	Amplification	1	0	42500T: 3.5;
GARS	chr7	21q22.11	1	1	0	0	Amplification	1	0	42473T: 4;
GAS6	chr13	13q34	1	0	0	1	Amplification	1	0	56957T: 3.5;
GATA2	chr3	3q21.3	2	0	1	1	Amplification	2	0	42496T: 3.5; 42487T: 3.5;
GATA4	chr8	8p23.1	1	0	1	0	Amplification	1	0	42486T: 3.5;
GATA5	chr20	20q13.33	1	1	0	0	Amplification	1	0	42473T: 6;
GATAD1	chr7	7q21.2	2	0	2	0	Amplification	2	0	42487T: 3.5; 42483T: 4;
GATAD2B	chr1	1q21.3	1	1	0	0	Amplification	1	0	42473T: 4.5;
GATC	chr12	12q24.31	1	0	0	1	Amplification	1	0	42500T: 3.5;
GATSL3	chr22	22q12.2	1	1	0	0	Amplification	1	0	42473T: 3.5;
GBA	chr1	1q22	1	1	0	0	Amplification	1	0	42473T: 6.5;
GBA2	chr9	9p13.3	2	1	0	1	Amplification	2	0	42501T: 3.5; 42473T: 3.5;
GBAP1	chr1	1q22	1	1	0	0	Amplification	1	0	42473T: 6.5;
GBAS	chr7	7p11.2	2	0	1	1	Amplification	2	0	42483T: 4; 56957T: 5;
GCAT	chr22	22q13.1	1	1	0	0	Amplification	1	0	42473T: 3.5;
GCC1	chr7	7q32.1	1	0	1	0	Amplification	1	0	42487T: 3.5;
GCFC2	chr2	2p12	1	0	0	1	Amplification	1	0	42500T: 3.5;
GCH1	chr14	14q22.2	1	0	0	1	Amplification	1	0	42494T: 4;
GCHFR	chr15	15q15.1	1	0	0	1	Amplification	1	0	42493T: 3.5;
GCK	chr7	11q13.1	2	1	0	1	Amplification	2	0	42473T: 4.5; 42497T: 7.5;
GCKR	chr2	14q22.1	1	0	0	1	Amplification	1	0	42500T: 3.5;
GCN1L1	chr12	12q24.23	1	0	0	1	Amplification	1	0	42500T: 3.5;
GCNT7	chr20	20q13.31	1	1	0	0	Amplification	1	0	42473T: 5;
GCSAM	chr3	3q13.2	1	0	0	1	Amplification	1	0	42496T: 3.5;
GDAP1L1	chr20	20q13.12	1	1	0	0	Amplification	1	0	42473T: 3.5;
GDE1	chr16	16p12.3	1	1	0	0	Amplification	1	0	42473T: 4;
GDF11	chr12	12q13.2	1	0	0	1	Amplification	1	0	42494T: 3.5;
GDF5	chr20	20q11.22	2	1	0	1	Amplification	2	0	42493T: 3.5; 42473T: 3.5;
GDF5OS	chr20	20q11.2	2	1	0	1	Amplification	2	0	42493T: 3.5; 42473T: 3.5;
GDF6	chr8	8q22.1	2	0	0	2	Amplification	2	0	42496T: 3.5; 42495T: 3.5;
GDF7	chr2	2p24.1	1	0	0	1	Amplification	1	0	42500T: 3.5;
GDNF	chr5	5p13.2	6	1	3	2	Amplification	6	0	42493T: 3.5; 42484T: 3.5; 42475T: 3.5; 42496T: 3.5; 42483T: 3.5; 42486T: 3.5;
GDPD5	chr11	11q13.4-q13	2	0	0	2	Amplification	2	0	42492T: 4; 42498T: 6.5;
GDPGP1	chr15	15q26.1	1	1	0	0	Amplification	1	0	42473T: 3.5;

Mangalaparthi *et al.* , 2020. Mutational landscape of esophageal squamous cell carcinoma in an Indian cohort
Supplementary Table 7A. List of copy number alterations and affected genes in ESCC patients

Gene	Chromosome	Cytoband	Recurrence	Recurrence in smoker cohort	Recurrence in chewer cohort	Recurrence in No habit cohort	State	Samples with CNA gain	Samples with CNA loss	File info with CNA fold
GEM	chr8	8q22.1	2	0	0	2	Amplification	2	0	42495T: 3.5; 42496T: 3.5;
GEMIN2	chr14	14q21.1	2	1	0	1	Amplification	2	0	42500T: 6.5; 42473T: 3.5;
GEMIN6	chr2	2p22.1	2	0	0	2	Amplification	2	0	42493T: 3.5; 42500T: 3.5;
GEMIN7	chr19	19q13.32	2	1	0	1	Amplification	2	0	42473T: 3.5; 56957T: 4;
GEN1	chr2	2p24.2	1	0	0	1	Amplification	1	0	42500T: 3.5;
GET4	chr7	7p22.3	1	1	0	0	Amplification	1	0	42473T: 5;
GFER	chr16	16p13.3	1	0	1	0	Amplification	1	0	42483T: 3.5;
GFM1	chr3	3q25.32	8	1	2	5	Amplification	8	0	42497T: 3.5; 42474T: 3.5; 42495T: 4; 56957T: 4; 42484T: 3.5; 42493T: 3.5; 42487T: 3.5; 42492T: 3.5;
GFPT1	chr2	2p13.3	1	0	0	1	Amplification	1	0	42500T: 3.5;
GFRA4	chr20	20p13	1	1	0	0	Amplification	1	0	42473T: 6;
GGA1	chr22	22q13.1	1	1	0	0	Amplification	1	0	42473T: 3.5;
GGA2	chr16	16p12.2	1	1	0	0	Amplification	1	0	42473T: 4;
GGA3	chr17	17q25.1	1	1	0	0	Amplification	1	0	42473T: 4;
GGCT	chr7	7p14.3	1	1	0	0	Amplification	1	0	42473T: 4;
GGCX	chr2	2p11.2	2	0	1	1	Amplification	2	0	42500T: 3.5; 42482T: 3.5;
GGH	chr8	8q12.3	2	0	0	2	Amplification	2	0	42495T: 3.5; 42496T: 3.5;
GGN	chr19	19q13.2	3	0	1	2	Amplification	3	0	56957T: 4; 42484T: 3.5; 42500T: 6.5;
GGT7	chr20	20q11.22	2	1	0	1	Amplification	2	0	42473T: 3.5; 42493T: 3.5;
GGTLC2	chr22	22q11.22	1	1	0	0	Amplification	1	0	42477T: 4;
GHR	chr5	5p13.1-p12	5	1	2	2	Amplification	5	0	42493T: 3.5; 42496T: 3.5; 42483T: 3.5; 42486T: 3.5; 42475T: 3.5;
GHRH	chr20	20q11.23	1	1	0	0	Amplification	1	0	42473T: 3.5;
GHRHR	chr7	7p14.3	1	1	0	0	Amplification	1	0	42473T: 4;
GHSR	chr3	3q26.31	9	1	3	5	Amplification	9	0	42482T: 3.5; 42492T: 3.5; 42493T: 3.5; 42487T: 3.5; 42500T: 9; 42484T: 4; 42495T: 4; 42474T: 3.5; 56957T: 4;
GIPR	chr19	19q13.32	1	0	1	0	Amplification	1	0	42484T: 4;
GIT2	chr12	12q24.11	2	1	0	1	Amplification	2	0	42473T: 4.5; 42500T: 3.5;
GJA5	chr1	1q21.2	1	1	0	0	Amplification	1	0	42473T: 4;
GJA8	chr1	1q21.2	1	1	0	0	Amplification	1	0	42473T: 4;
GK5	chr3	3q23	4	0	1	3	Amplification	4	0	42496T: 4; 42492T: 3.5; 42493T: 3.5; 42487T: 3.5;
GKN1	chr2	2p13.3	1	0	0	1	Amplification	1	0	42500T: 3.5;
GKN2	chr2	2p13.3	1	0	0	1	Amplification	1	0	42500T: 3.5;
GLB1L2	chr11	11q25	1	1	0	0	Deletion	0	1	42476T: 0.5;
GLB1L3	chr11	11q25	1	1	0	0	Deletion	0	1	42476T: 0.5;
GLCCI1	chr7	7p21.3	2	1	0	1	Amplification	2	0	42473T: 3.5; 42497T: 7.5;
GLDC	chr9	9p24.1	1	0	0	1	Amplification	1	0	42496T: 3.5;
GLI2	chr2	2q14.2	1	0	0	1	Amplification	1	0	42493T: 3.5;
GLI3	chr7	7p14.1	1	1	0	0	Amplification	1	0	42473T: 3.5;
GLI4	chr8	8q24.3	3	0	1	2	Amplification	3	0	42483T: 3.5; 42496T: 4; 42495T: 4.5;

Mangalaparthi *et al.* , 2020. Mutational landscape of esophageal squamous cell carcinoma in an Indian cohort
Supplementary Table 7A. List of copy number alterations and affected genes in ESCC patients

Gene	Chromosome	Cytoband	Recurrence	Recurrence in smoker cohort	Recurrence in chewer cohort	Recurrence in No habit cohort	State	Samples with CNA gain	Samples with CNA loss	File info with CNA fold
GLIPR2	chr9	9p13.3	1	0	0	1	Amplification	1	0	42501T: 3.5;
GLIS2	chr16	16p13.3	3	1	0	2	Amplification	3	0	42494T: 3.5; 42495T: 8.5; 42473T: 5.5;
GLIS3	chr9	9p24.2	2	0	0	2	Amplification	2	0	42498T: 4; 42496T: 3.5;
GLS	chr2	12q13.3	3	0	1	2	Amplification	3	0	42494T: 3.5; 42493T: 3.5; 42482T: 4;
GLT6D1	chr9	9q34.3	2	0	1	1	Amplification	2	0	56957T: 3.5; 42484T: 4.5;
GLTP	chr12	12q24.11	2	1	0	1	Amplification	2	0	42473T: 4.5; 42500T: 3.5;
GLUD1P3	chr10	10q22.2	2	1	0	1	Amplification	2	0	42496T: 3.5; 42473T: 4.5;
GLYCAM1	chr12	12q13.2	1	0	0	1	Amplification	1	0	42494T: 3.5;
GLYR1	chr16	16p13.3	2	1	0	1	Amplification	2	0	42473T: 5.5; 42495T: 12;
GMCL1	chr2	2p13.3	1	0	0	1	Amplification	1	0	42500T: 3.5;
GMEB1	chr1	1p35.3	1	1	0	0	Amplification	1	0	42473T: 3.5;
GMEB2	chr20	20q13.33	1	1	0	0	Amplification	1	0	42473T: 6;
GMFB	chr14	14q22.2	1	0	0	1	Amplification	1	0	42494T: 4;
GMFG	chr19	19q13.2	2	0	0	2	Amplification	2	0	56957T: 4; 42500T: 6.5;
GML	chr8	8q24.3	3	0	1	2	Amplification	3	0	42495T: 4.5; 42483T: 3.5; 42496T: 4;
GMNC	chr3	3q28	9	1	3	5	Amplification	9	0	42493T: 3.5; 42487T: 3.5; 42492T: 3.5; 42482T: 3.5; 42495T: 4; 42474T: 3.5; 56957T: 4.5; 42484T: 4; 42498T: 3.5;
GMPS	chr3	3q25.31	7	1	2	4	Amplification	7	0	42497T: 5.5; 42474T: 3.5; 42493T: 3.5; 56957T: 4; 42487T: 3.5; 42484T: 3.5; 42492T: 3.5;
GNA12	chr7	7p22.3-p22.2	1	1	0	0	Amplification	1	0	42473T: 4.5;
GNAI1	chr7	7q21.11	1	0	1	0	Amplification	1	0	42487T: 3.5;
GNAI3	chr1	1p13.3	1	1	0	0	Amplification	1	0	42473T: 4.5;
GNAL	chr18	18p11.21	1	0	0	1	Amplification	1	0	42493T: 3.5;
GNAS	chr20	20q13.32	1	1	0	0	Amplification	1	0	42473T: 4.5;
GNAT2	chr1	1p13.3	1	1	0	0	Amplification	1	0	42473T: 4.5;
GNAT3	chr7	7q21.11	1	0	1	0	Amplification	1	0	42487T: 3.5;
GNB1L	chr22	22q11.21	1	0	0	1	Amplification	1	0	42497T: 17;
GNB2L1	chr5	5q35.3	1	0	0	1	Amplification	1	0	42495T: 3.5;
GNB4	chr3	3q26.33	11	1	3	7	Amplification	11	0	42500T: 14; 42497T: 4; 42474T: 3.5; 42495T: 4; 56957T: 4; 42484T: 4; 42498T: 3.5; 42482T: 3.5; 42487T: 3.5; 42493T: 3.5; 42492T: 3.5;
GNE	chr9	9p13.3	1	0	0	1	Amplification	1	0	42501T: 3.5;
GNG11	chr7	7q21.3	2	0	2	0	Amplification	2	0	42483T: 4; 42487T: 3.5;
GNG13	chr16	16p13.3	2	0	1	1	Amplification	2	0	42483T: 3.5; 42493T: 3.5;
GNG2	chr14	14q22.1	1	0	0	1	Amplification	1	0	42494T: 4;
GNG8	chr19	19q13.32	1	0	1	0	Amplification	1	0	42484T: 7;
GNGT1	chr7	7q21.3	2	0	2	0	Amplification	2	0	42487T: 3.5; 42483T: 4;
GNLY	chr2	2p11.2	1	0	0	1	Amplification	1	0	42500T: 3.5;
GNPNAT1	chr14	14q22.1	1	0	0	1	Amplification	1	0	42494T: 4;
GNPTG	chr16	16p13.3	1	0	1	0	Amplification	1	0	42483T: 3.5;

Mangalaparthi *et al.* , 2020. Mutational landscape of esophageal squamous cell carcinoma in an Indian cohort
Supplementary Table 7A. List of copy number alterations and affected genes in ESCC patients

Gene	Chromosome	Cytoband	Recurrence	Recurrence in smoker cohort	Recurrence in chewer cohort	Recurrence in No habit cohort	State	Samples with CNA gain	Samples with CNA loss	File info with CNA fold
GNRH2	chr20	20p13	1	1	0	0	Amplification	1	0	42473T: 3.5;
GNRHR2	chr1	1q21.1	1	1	0	0	Amplification	1	0	42473T: 4;
GOLGB1	chr3	3q13.33	1	0	0	1	Amplification	1	0	42496T: 3.5;
GOLIM4	chr3	3q26.2	9	1	3	5	Amplification	9	0	42500T: 8; 56957T: 4; 42474T: 3.5; 42495T: 4; 42484T: 4; 42482T: 3.5; 42493T: 3.5; 42487T: 3.5; 42492T: 3.5;
GOLPH3	chr5	5p13.3	4	1	1	2	Amplification	4	0	42493T: 3.5; 42486T: 3.5; 42496T: 3.5; 42475T: 3.5;
GOLPH3L	chr1	1q21.3	2	1	0	1	Amplification	2	0	42473T: 4.5; 42493T: 3.5;
GOLT1B	chr12	12p12.1	1	0	0	1	Amplification	1	0	42500T: 6;
GON4L	chr1	1q22	1	1	0	0	Amplification	2	0	42473T: 5.5,4.5;
GOT1L1	chr8	8p11.23	2	0	1	1	Amplification	2	0	42493T: 3.5; 42482T: 3.5;
GP1BB	chr22	22q11.21	1	0	0	1	Amplification	1	0	42497T: 17;
GP2	chr16	16p12.3	1	1	0	0	Amplification	1	0	42473T: 4;
GP5	chr3	3q29	9	1	3	5	Amplification	9	0	42492T: 3.5; 42493T: 3.5; 42487T: 3.5; 42482T: 3.5; 42498T: 3.5; 42484T: 4; 56957T: 4.5; 42474T: 3.5; 42495T: 4;
GP9	chr3	3q21.3	3	0	1	2	Amplification	3	0	42493T: 3.5; 42487T: 3.5; 42496T: 3.5;
GPAA1	chr8	8q24.3	3	0	1	2	Amplification	3	0	42495T: 4.5; 42483T: 3.5; 42496T: 4;
GPANK1	chr6	6p21.33	1	1	0	0	Amplification	1	0	42473T: 3.5;
GPAT2	chr2	2q11.2	2	1	0	1	Amplification	2	0	42473T: 3.5; 42493T: 4;
GPATCH1	chr19	19q13.11	4	1	1	2	Amplification	4	0	56957T: 4; 42484T: 3.5; 42500T: 4.5; 42473T: 7;
GPATCH11	chr2	2p22.2	1	0	0	1	Amplification	1	0	42500T: 3.5;
GPATCH2L	chr14	14q24.3	2	0	0	2	Amplification	2	0	42494T: 4; 56957T: 3.5;
GPATCH4	chr1	1q22-q23.1	1	1	0	0	Amplification	1	0	42473T: 7;
GPD1	chr12	12q13.12	1	0	0	1	Amplification	1	0	42500T: 3.5;
GPER	chr7	7p22.3	1	1	0	0	Amplification	1	0	42473T: 5;
GPHB5	chr14	14q23.2	1	0	0	1	Amplification	1	0	42494T: 4;
GPHN	chr14	14q23.3-q24	1	0	0	1	Amplification	1	0	42494T: 4;
GPI	chr19	5q31.3	3	0	1	2	Amplification	3	0	56957T: 4; 42484T: 3.5; 42500T: 4.5;
GPIHBP1	chr8	8q24.3	3	0	1	2	Amplification	3	0	42495T: 4.5; 42483T: 3.5; 42496T: 4;
GPN1	chr2	2p23.3	1	0	0	1	Amplification	1	0	42500T: 3.5;
GPN3	chr12	12q24.11	1	0	0	1	Amplification	1	0	42500T: 3.5;
GPNMB	chr7	7p15.3	1	1	0	0	Amplification	1	0	42473T: 4;
GPR113	chr2	2p23.3	1	0	0	1	Amplification	1	0	42500T: 3.5;
GPR124	chr8	8p11.23	1	0	1	0	Amplification	1	0	42482T: 3.5;
GPR128	chr3	3q12.2	1	1	0	0	Amplification	1	0	42476T: 3.5;
GPR137C	chr14	14q22.1	1	0	0	1	Amplification	1	0	42494T: 4;
GPR139	chr16	16p12.3	1	1	0	0	Amplification	1	0	42473T: 4;
GPR141	chr7	7p14.1	1	1	0	0	Amplification	1	0	42473T: 4.5;
GPR146	chr7	7p22.3	1	1	0	0	Amplification	1	0	42473T: 5;

Mangalaparthi *et al.* , 2020. Mutational landscape of esophageal squamous cell carcinoma in an Indian cohort
Supplementary Table 7A. List of copy number alterations and affected genes in ESCC patients

Gene	Chromosome	Cytoband	Recurrence	Recurrence in smoker cohort	Recurrence in chewer cohort	Recurrence in No habit cohort	State	Samples with CNA gain	Samples with CNA loss	File info with CNA fold
GPR149	chr3	3q25.2	9	1	2	6	Amplification	9	0	42493T: 3.5; 42487T: 3.5; 42492T: 3.5; 42500T: 3.5; 42497T: 5.5; 42496T: 4.5; 56957T: 4; 42474T: 3.5; 42484T: 3.5;
GPR15	chr3	3q11.2	1	1	0	0	Amplification	1	0	42476T: 3.5;
GPR152	chr11	11q13.2	2	1	0	1	Amplification	2	0	56957T: 5; 42473T: 3.5;
GPR155	chr2	2q31.1	1	0	0	1	Amplification	1	0	42493T: 3.5;
GPR156	chr3	3q13.33	1	0	0	1	Amplification	1	0	42496T: 3.5;
GPR160	chr3	3q26.2	9	1	3	5	Amplification	9	0	42495T: 4; 42474T: 3.5; 56957T: 4; 42484T: 4; 42500T: 8; 42487T: 3.5; 42493T: 3.5; 42492T: 3.5; 42482T: 3.5;
GPR162	chr12	12p13.31	2	1	0	1	Amplification	2	0	42473T: 3.5; 42494T: 3.5;
GPR171	chr3	3q25.1	9	1	2	6	Amplification	9	0	42484T: 3.5; 42474T: 3.5; 56957T: 4; 42497T: 5.5; 42496T: 4; 42500T: 3.5; 42492T: 3.5; 42487T: 3.5; 42493T: 3.5;
GPR179	chr17	17q12	1	1	0	0	Amplification	1	0	42473T: 3.5;
GPR19	chr12	12p13.1	1	0	0	1	Amplification	1	0	42500T: 4.5;
GPR20	chr8	8q24.3	3	0	1	2	Amplification	3	0	42483T: 3.5; 42496T: 4; 42495T: 3.5;
GPR3	chr1	1p36.11	1	1	0	0	Amplification	1	0	42473T: 5;
GPR37	chr7	7q31.33	1	0	1	0	Amplification	1	0	42487T: 3.5;
GPR4	chr19	19q13.32	1	0	1	0	Amplification	1	0	42484T: 4;
GPR42	chr19	19q13.12	2	0	0	2	Amplification	2	0	56957T: 4; 42500T: 6.5;
GPR6	chr6	6q21	1	0	0	1	Amplification	1	0	42496T: 3.5;
GPR75	chr2	2p16.2	2	0	1	1	Amplification	2	0	42500T: 3.5; 42484T: 4.5;
GPR84	chr12	12q13.13	2	1	0	1	Amplification	2	0	42494T: 3.5; 42473T: 3.5;
GPR85	chr7	7q31.1	2	0	1	1	Amplification	2	0	42501T: 3.5; 42487T: 3.5;
GPR87	chr3	3q25.1	9	1	2	6	Amplification	9	0	42487T: 3.5; 42493T: 3.5; 42492T: 3.5; 42500T: 3.5; 42496T: 4; 42497T: 5.5; 56957T: 4; 42474T: 3.5; 42484T: 3.5;
GPR89A	chr1	1q21.1	1	1	0	0	Amplification	1	0	42473T: 4;
GPR89B	chr1	1q21.1	1	1	0	0	Amplification	1	0	42473T: 4;
GPR89C	chr1	1q21.2	1	1	0	0	Amplification	1	0	42473T: 4;
GPRC5A	chr12	12p13.1	1	0	0	1	Amplification	1	0	42500T: 4.5;
GPRC5B	chr16	16p12.3	1	1	0	0	Amplification	1	0	42473T: 4;
GPRC5D	chr12	12p13.1	1	0	0	1	Amplification	1	0	42500T: 4.5;
GPS1	chr17	17q25.3	1	1	0	0	Amplification	1	0	42473T: 3.5;
GPSM1	chr9	9q34.3	1	0	0	1	Amplification	1	0	56957T: 3.5;
GPSM3	chr6	6p21.32	1	1	0	0	Amplification	1	0	42473T: 3.5;
GPT	chr8	8q24.3	5	0	1	4	Amplification	5	0	42483T: 3.5; 42496T: 4; 42495T: 4.5; 42494T: 3.5; 56957T: 3.5;
GPX1	chr3	3p21.31	1	1	0	0	Amplification	1	0	42473T: 4;
GPX2	chr14	14q23.3	1	0	0	1	Amplification	1	0	42494T: 4;

Mangalaparthi *et al.* , 2020. Mutational landscape of esophageal squamous cell carcinoma in an Indian cohort
Supplementary Table 7A. List of copy number alterations and affected genes in ESCC patients

Gene	Chromosome	Cytoband	Recurrence	Recurrence in smoker cohort	Recurrence in chewer cohort	Recurrence in No habit cohort	State	Samples with CNA gain	Samples with CNA loss	File info with CNA fold
GRAMD1A	chr19	19q13.11	3	0	1	2	Amplification	3	0	42500T: 6.5; 42484T: 3.5; 56957T: 4;
GRAMD1C	chr3	3q13.31	1	0	0	1	Amplification	1	0	42496T: 3.5;
GRAMD4	chr22	22q13.31	1	0	0	1	Amplification	1	0	42495T: 4;
GRAP2	chr22	22q13.1	1	1	0	0	Amplification	1	0	42473T: 3.5;
GRB10	chr7	7p12.1	1	0	0	1	Amplification	1	0	42497T: 20.5;
GRB2	chr17	17q25.1	1	1	0	0	Amplification	1	0	42473T: 4;
GRB7	chr17	17q12	2	1	0	1	Amplification	2	0	42497T: 3.5; 42473T: 6;
GREB1	chr2	2p25.1	1	0	0	1	Amplification	1	0	42500T: 3.5;
GRHL1	chr2	2p25.1	1	0	0	1	Amplification	1	0	42500T: 3.5;
GRHL2	chr8	8q22.3	2	0	0	2	Amplification	2	0	42495T: 3.5; 42496T: 3.5;
GRHL3	chr1	1p36.11	1	1	0	0	Amplification	1	0	42473T: 3.5;
GRID2IP	chr7	7p22.1	2	1	0	1	Amplification	2	0	42473T: 4.5; 42497T: 5;
GRIFIN	chr7	7p22.3	1	1	0	0	Amplification	1	0	42473T: 5;
GRIK5	chr19	19q13.2	1	1	0	0	Amplification	1	0	42473T: 4.5;
GRIN1	chr9	5q35.2	2	1	0	1	Amplification	2	0	56957T: 3.5; 42473T: 5;
GRIN2A	chr16	16p13.2	2	1	0	1	Amplification	2	0	42473T: 5.5; 42495T: 4;
GRIN2B	chr12	12p13.1	1	0	0	1	Amplification	1	0	42500T: 4.5;
GRIN3B	chr19	19p13.3	1	0	0	1	Amplification	1	0	42493T: 3.5;
GRINA	chr8	8q24.3	3	0	1	2	Amplification	3	0	42495T: 4.5; 42483T: 3.5; 42496T: 4;
GRIP1	chr12	11q12.3	1	0	0	1	Amplification	1	0	42500T: 4.5;
GRK1	chr13	13q34	1	0	0	1	Amplification	1	0	56957T: 3.5;
GRK7	chr3	3q23	4	0	1	3	Amplification	4	0	42496T: 4; 42487T: 3.5; 42493T: 3.5; 42492T: 3.5;
GRM3	chr7	7q21.11-q21	2	0	2	0	Amplification	2	0	42483T: 6; 42487T: 3.5;
GRM8	chr7	7q31.33	1	0	1	0	Amplification	1	0	42487T: 3.5;
GRTP1	chr13	13q34	1	0	0	1	Amplification	1	0	56957T: 3.5;
GSAP	chr7	7q11.23	1	0	1	0	Amplification	1	0	42487T: 3.5;
GSC2	chr22	22q11.21	1	0	0	1	Amplification	1	0	42497T: 21;
GSDMA	chr17	17q21.1	1	0	0	1	Amplification	1	0	42497T: 3.5;
GSDMB	chr17	17q21.1	1	0	0	1	Amplification	1	0	42497T: 3.5;
GSDMC	chr8	8q24.21	3	0	1	2	Amplification	3	0	42496T: 3.5; 42484T: 3.5; 42495T: 3.5;
GSDMD	chr8	8q24.3	3	0	1	2	Amplification	3	0	42483T: 3.5; 42496T: 4; 42495T: 4.5;
GSG1	chr12	12p13.1	1	0	0	1	Amplification	1	0	42500T: 4.5;
GSK3A	chr19	19q13.2	1	1	0	0	Amplification	1	0	42473T: 4.5;
GSK3B	chr3	3q13.33	1	0	0	1	Amplification	1	0	42496T: 3.5;
GSPT1	chr16	16p13.13	2	1	0	1	Amplification	2	0	42473T: 5.5; 42495T: 4;
GSR	chr8	8p12	1	0	1	0	Amplification	1	0	42482T: 3.5;
GSS	chr20	20p13	2	1	0	1	Amplification	2	0	42473T: 3.5; 42493T: 3.5;
GSTK1	chr7	7q34	1	0	1	0	Amplification	1	0	42487T: 3.5;
GSTM1	chr1	1p13.3	2	1	1	0	Amplification	2	0	42487T: 4; 42473T: 4.5;
GSTM2	chr1	1p13.3	1	1	0	0	Amplification	1	0	42473T: 4.5;
GSTM3	chr1	1p13.3	1	1	0	0	Amplification	1	0	42473T: 4.5;

Mangalaparthi *et al.* , 2020. Mutational landscape of esophageal squamous cell carcinoma in an Indian cohort
Supplementary Table 7A. List of copy number alterations and affected genes in ESCC patients

Gene	Chromosome	Cytoband	Recurrence	Recurrence in smoker cohort	Recurrence in chewer cohort	Recurrence in No habit cohort	State	Samples with CNA gain	Samples with CNA loss	File info with CNA fold
GSTM4	chr1	1p13.3	1	1	0	0	Amplification	1	0	42473T: 4.5;
GSTM5	chr1	1p13.3	1	1	0	0	Amplification	1	0	42473T: 4.5;
GSTP1	chr11	11q13.2	2	1	0	1	Amplification	2	0	42473T: 3.5; 56957T: 5;
GSTT1	chr22	22q11.23	1	0	1	0	Deletion	0	1	42484T: 0;
GSTT2	chr22	22q11.23	1	0	1	0	Amplification	1	0	42484T: 3.5;
GSTT2B	chr22	22q11.23	1	0	1	0	Amplification	1	0	42484T: 3.5;
GSTZ1	chr14	14q24.3	2	0	0	2	Amplification	2	0	56957T: 3.5; 42494T: 4;
GTF2A1L	chr2	2p16.3	2	0	1	1	Amplification	2	0	42500T: 3.5; 42484T: 4.5;
GTF2E1	chr3	3q13.33	1	0	0	1	Amplification	1	0	42496T: 3.5;
GTF2E2	chr8	8p12	1	0	1	0	Amplification	1	0	42482T: 3.5;
GTF2H3	chr12	12q24.31	1	0	0	1	Amplification	1	0	42500T: 3.5;
GTF3C2	chr2	2p23.3	1	0	0	1	Amplification	1	0	42500T: 3.5;
GTF3C3	chr2	2q33.1	1	0	1	0	Amplification	1	0	42482T: 4;
GTF3C6	chr6	6q21	1	0	0	1	Amplification	1	0	42496T: 3.5;
GTPBP1	chr22	22q13.1	1	1	0	0	Amplification	1	0	42473T: 3.5;
GTPBP10	chr7	7q21.13	2	0	2	0	Amplification	2	0	42487T: 3.5; 42483T: 3.5;
GTPBP8	chr3	3q13.2	1	0	0	1	Amplification	1	0	42496T: 3.5;
GTSF1	chr12	12q13.13	2	1	0	1	Amplification	2	0	42473T: 3.5; 42494T: 3.5;
GTSF1L	chr20	20q13.12	1	1	0	0	Amplification	1	0	42473T: 3.5;
GUCY2C	chr12	12p12.3	1	0	0	1	Amplification	1	0	42500T: 4.5;
GUCY2EP	chr11	11q13.5	2	0	0	2	Amplification	2	0	42496T: 3.5; 42497T: 3.5;
GULP1	chr2	2q32.1-q32.2	1	0	0	1	Amplification	1	0	42493T: 3.5;
GUSB	chr7	7q11.21	1	0	0	1	Amplification	1	0	42501T: 3.5;
GUSBP1	chr5	5p14.3	4	1	1	2	Amplification	4	0	42486T: 3.5; 42496T: 3.5; 42475T: 3.5; 42493T: 4;
GXYLT1	chr12	12q12	1	0	0	1	Amplification	1	0	42500T: 3.5;
GYG1	chr3	3q24	8	1	2	5	Amplification	8	0	42497T: 4.5; 42496T: 4; 42484T: 3.5; 42474T: 3.5; 56957T: 4; 42492T: 3.5; 42493T: 3.5; 42487T: 3.5;
GYLTL1B	chr11	11p11.2	1	1	0	0	Amplification	1	0	42473T: 3.5;
GYS2	chr12	12p12.1	1	0	0	1	Amplification	1	0	42500T: 6;
H1F0	chr22	22q13.1	1	1	0	0	Amplification	1	0	42473T: 3.5;
H1FNT	chr12	12q13.11	1	0	0	1	Amplification	1	0	42500T: 3.5;
H1FOO	chr3	3q22.1	2	0	1	1	Amplification	2	0	42496T: 3.5; 42487T: 3.5;
H1FX	chr3	3q21.3	2	0	1	1	Amplification	2	0	42487T: 3.5; 42496T: 3.5;
H2AFJ	chr12	12p12.3	1	0	0	1	Amplification	1	0	42500T: 4.5;
H2AFV	chr7	7p13	1	1	0	0	Amplification	1	0	42473T: 4.5;
H3F3B	chr17	17q25.1	1	0	0	1	Amplification	1	0	42494T: 3.5;
H3F3C	chr12	12p11.21	1	0	0	1	Amplification	1	0	42500T: 12.5;
HAAO	chr2	2p21	2	0	1	1	Amplification	2	0	42500T: 3.5; 42484T: 3.5;
HADHA	chr2	2p23.3	1	0	0	1	Amplification	1	0	42500T: 3.5;
HADHB	chr2	2p23.3	1	0	0	1	Amplification	1	0	42500T: 3.5;
HAGH	chr16	16p13.3	1	0	1	0	Amplification	1	0	42483T: 3.5;

Mangalaparthi *et al.* , 2020. Mutational landscape of esophageal squamous cell carcinoma in an Indian cohort
Supplementary Table 7A. List of copy number alterations and affected genes in ESCC patients

Gene	Chromosome	Cytoband	Recurrence	Recurrence in smoker cohort	Recurrence in chewer cohort	Recurrence in No habit cohort	State	Samples with CNA gain	Samples with CNA loss	File info with CNA fold
HAGHL	chr16	16p13.3	2	0	1	1	Amplification	2	0	42493T: 3.5; 42483T: 3.5;
HAMP	chr19	19q13.12	3	0	1	2	Amplification	3	0	42500T: 6.5; 42484T: 3.5; 56957T: 4;
HAPLN2	chr1	1q23.1	1	1	0	0	Amplification	1	0	42473T: 7;
HARBI1	chr11	11p11.2	1	1	0	0	Amplification	1	0	42473T: 3.5;
HAS2	chr8	8q24.13	4	1	1	2	Amplification	4	0	42496T: 3.5; 42475T: 3.5; 42484T: 3.5; 42495T: 3.5;
HAT1	chr2	2q31.1	1	0	0	1	Amplification	1	0	42493T: 5;
HAUS4	chr14	14q11.2	1	0	0	1	Amplification	1	0	42496T: 4;
HAUS5	chr19	19q13.12	3	0	1	2	Amplification	3	0	42484T: 3.5; 56957T: 4; 42500T: 6.5;
HAX1	chr1	1q21.3	1	1	0	0	Amplification	1	0	42473T: 4.5;
HBA1	chr16	16p13.3	1	0	1	0	Amplification	1	0	42483T: 3.5;
HBA2	chr16	16p13.3	1	0	1	0	Amplification	1	0	42483T: 3.5;
HBM	chr16	11q13.2	1	0	1	0	Amplification	1	0	42483T: 3.5;
HBP1	chr7	7q22.3	4	0	1	3	Amplification	4	0	42487T: 3.5; 42493T: 3.5; 42497T: 4; 42501T: 3.5;
HBQ1	chr16	16p13.3	1	0	1	0	Amplification	1	0	42483T: 3.5;
HBZ	chr16	16p13.3	1	0	1	0	Amplification	1	0	42483T: 3.5;
HCAR1	chr12	12q24.31	1	0	0	1	Amplification	1	0	42500T: 3.5;
HCAR2	chr12	12q24.31	1	0	0	1	Amplification	1	0	42500T: 3.5;
HCAR3	chr12	12q24.31	1	0	0	1	Amplification	1	0	42500T: 3.5;
HCK	chr20	20q11.21	2	1	0	1	Amplification	2	0	42473T: 4.5; 42496T: 5;
HCLS1	chr3	3q13.33	1	0	0	1	Amplification	1	0	42496T: 3.5;
HCN1	chr5	5p12	4	1	1	2	Amplification	4	0	42475T: 3.5; 42486T: 3.5; 42496T: 3.5; 42493T: 3.5;
HCN3	chr1	1q22	1	1	0	0	Amplification	1	0	42473T: 6.5;
HCRTR1	chr1	1p35.2	1	1	0	0	Amplification	1	0	42473T: 3.5;
HCST	chr19	19q13.12	3	0	1	2	Amplification	3	0	42484T: 3.5; 56957T: 4; 42500T: 6.5;
HDAC1	chr1	1p35.2-p35.1	1	1	0	0	Amplification	1	0	42473T: 4;
HDAC7	chr12	12q13.11	1	0	0	1	Amplification	1	0	42500T: 3.5;
HDAC9	chr7	7p21.1	1	1	0	0	Amplification	1	0	42473T: 4;
HDGF	chr1	1q23.1	1	1	0	0	Amplification	1	0	42473T: 7;
HEATR2	chr7	7p22.3	1	1	0	0	Amplification	1	0	42473T: 5;
HEATR4	chr14	14q24.3	2	0	0	2	Amplification	2	0	56957T: 3.5; 42494T: 4;
HEATR5A	chr14	14q12	3	0	0	3	Amplification	3	0	56957T: 4; 42494T: 3.5; 42500T: 4.5;
HEATR5B	chr2	2p22.2	1	0	0	1	Amplification	1	0	42500T: 3.5;
HEBP1	chr12	12p13.1	1	0	0	1	Amplification	1	0	42500T: 4.5;
HEBP2	chr6	6q24.1	1	0	0	1	Amplification	1	0	42493T: 3.5;
HECTD1	chr14	14q12	3	0	0	3	Amplification	3	0	42494T: 3.5; 56957T: 4; 42500T: 4.5;
HECTD4	chr12	12q24.13	2	0	1	1	Amplification/Deletio	1	1	42482T: 0.5; 42500T: 3.5;
HECW1	chr7	7p14.1-p13	1	1	0	0	Amplification	1	0	42473T: 3.5;
HECW2	chr2	2q32.3	2	0	1	1	Amplification	2	0	42482T: 4; 42493T: 3.5;
HEG1	chr3	3q21.2	1	0	0	1	Amplification	1	0	42496T: 3.5;
HELB	chr12	12q14.3|12q	1	0	0	1	Amplification	1	0	42500T: 4.5;
HELZ2	chr20	20q13.33	1	1	0	0	Amplification	1	0	42473T: 6;

Mangalaparthi *et al.* , 2020. Mutational landscape of esophageal squamous cell carcinoma in an Indian cohort
Supplementary Table 7A. List of copy number alterations and affected genes in ESCC patients

Gene	Chromosome	Cytoband	Recurrence	Recurrence in smoker cohort	Recurrence in chewer cohort	Recurrence in No habit cohort	State	Samples with CNA gain	Samples with CNA loss	File info with CNA fold
HEPACAM2	chr7	7q21.2	2	0	2	0	Amplification	2	0	42487T: 3.5; 42483T: 4;
HERC2P3	chr15	15q11.1-q11	2	1	1	0	Amplification/Deletio	1	1	42476T: 0.5; 42484T: 7.5;
HERPUD2	chr7	7p14.2	1	1	0	0	Amplification	1	0	42473T: 4;
HES1	chr3	21q22.3	9	1	3	5	Amplification	9	0	42492T: 3.5; 42487T: 3.5; 42493T: 3.5; 42482T: 3.5; 42484T: 4; 42498T: 3.5; 42474T: 3.5; 42495T: 4; 56957T: 4.5;
HES6	chr2	2q37.3	1	1	0	0	Amplification	1	0	42473T: 3.5;
HEY1	chr8	8q21.13	2	0	0	2	Amplification	2	0	42495T: 3.5; 42496T: 3.5;
HFE2	chr1	1q21.1	1	1	0	0	Amplification	1	0	42473T: 4;
HGD	chr3	3q13.33	1	0	0	1	Amplification	1	0	42496T: 3.5;
HGF	chr7	2p22.1	1	0	1	0	Amplification	1	0	42487T: 3.5;
HGSNAT	chr8	8p11.21-p11	1	0	1	0	Amplification	1	0	42483T: 4.5;
HHLA1	chr8	8q24.22	3	0	1	2	Amplification	3	0	42495T: 3.5; 42484T: 3.5; 42496T: 3.5;
HIBADH	chr7	7p15.2	1	1	0	0	Amplification	1	0	42473T: 4;
HIBCH	chr2	2q32.2	3	0	1	2	Amplification	3	0	42493T: 3.5; 42494T: 3.5; 42482T: 4;
HIF1A	chr14	14q23.2	1	0	0	1	Amplification	1	0	42494T: 4;
HIF3A	chr19	19q13.32	1	0	1	0	Amplification	1	0	42484T: 4;
HIGD1C	chr12	12q13.12	2	0	0	2	Amplification	2	0	42494T: 3.5; 42500T: 3.5;
HINT2	chr9	9p13.3	2	1	0	1	Amplification	2	0	42473T: 3.5; 42501T: 3.5;
HIP1R	chr12	12q24.31	1	0	0	1	Amplification	1	0	42500T: 3.5;
HIPK2	chr7	7q34	1	0	1	0	Amplification	1	0	42487T: 3.5;
HIPK4	chr19	19q13.2	2	0	0	2	Amplification	2	0	42500T: 3.5; 56957T: 4;
HIRA	chr22	22q11.21	1	0	0	1	Amplification	2	0	42497T: 21,17;
HIST2H2AA3	chr1	1q21.2	2	1	0	1	Amplification	2	0	56958T: 4; 42473T: 4;
HIST2H2AA4	chr1	1q21.2	2	1	0	1	Amplification	2	0	56958T: 4; 42473T: 4;
HIST2H2AB	chr1	1q21.2	2	1	0	1	Amplification	2	0	42473T: 5; 56958T: 4;
HIST2H2AC	chr1	1q21.2	2	1	0	1	Amplification	2	0	56958T: 4; 42473T: 5;
HIST2H2BE	chr1	1q21.2	2	1	0	1	Amplification	2	0	56958T: 4; 42473T: 5;
HIST2H2BF	chr1	1q21.2	2	1	0	1	Amplification	2	0	42473T: 4; 56958T: 4;
HIST2H3A	chr1	1q21.2	2	1	0	1	Amplification	2	0	42473T: 4; 56958T: 4;
HIST2H3C	chr1	1q21.2	2	1	0	1	Amplification	2	0	42473T: 4; 56958T: 4;
HIST2H4A	chr1	1q21.2	2	1	0	1	Amplification	2	0	42473T: 4; 56958T: 4;
HIST2H4B	chr1	1q21.2	1	1	0	0	Amplification	1	0	42473T: 4;
HIST4H4	chr12	12p12.3	1	0	0	1	Amplification	1	0	42500T: 4.5;
HK2	chr2	19p13.13	1	0	0	1	Amplification	1	0	42500T: 3.5;
HKR1	chr19	19q13.12	3	0	1	2	Amplification	3	0	42484T: 3.5; 56957T: 4; 42500T: 6.5;
HLF	chr17	17q22	1	0	0	1	Amplification	1	0	42497T: 3.5;
HLTF	chr3	3q24	8	1	2	5	Amplification	8	0	42474T: 3.5; 56957T: 4; 42484T: 3.5; 42497T: 4.5; 42496T: 4; 42487T: 3.5; 42493T: 3.5; 42492T: 3.5;
HM13	chr20	20q11.21	1	0	0	1	Amplification	1	0	42496T: 5;
HMGB1	chr13	13q12.3	1	0	0	1	Amplification	1	0	42497T: 3.5;

Mangalaparthi *et al.* , 2020. Mutational landscape of esophageal squamous cell carcinoma in an Indian cohort
Supplementary Table 7A. List of copy number alterations and affected genes in ESCC patients

Gene	Chromosome	Cytoband	Recurrence	Recurrence in smoker cohort	Recurrence in chewer cohort	Recurrence in No habit cohort	State	Samples with CNA gain	Samples with CNA loss	File info with CNA fold
HMGCL	chr1	1p36.11	1	1	0	0	Amplification	1	0	42473T: 3.5;
HMGCS1	chr5	5p12	4	1	1	2	Amplification	4	0	42493T: 3.5; 42486T: 3.5; 42496T: 3.5; 42475T: 3.5;
HMGN1	chr21	21q22.2	1	1	0	0	Amplification	1	0	42473T: 3.5;
HMHA1	chr19	19p13.3	1	0	0	1	Amplification	1	0	42493T: 3.5;
HMOX2	chr16	16p13.3	3	1	0	2	Amplification	3	0	42473T: 5.5; 42494T: 3.5; 42495T: 8.5;
HN1L	chr16	16p13.3	1	0	1	0	Amplification	1	0	42483T: 3.5;
HNF1A	chr12	12q24.31	1	0	0	1	Amplification	1	0	42500T: 3.5;
HNF1B	chr17	17q12	1	1	0	0	Amplification	1	0	42473T: 3.5;
HNF4A	chr20	20q13.12	1	1	0	0	Amplification	1	0	42473T: 3.5;
HNF4G	chr8	8q21.13	2	0	0	2	Amplification	2	0	42496T: 3.5; 42495T: 3.5;
HNRNPA1	chr12	12q13.13	2	1	0	1	Amplification	2	0	42473T: 3.5; 42494T: 3.5;
HNRNPA2B1	chr7	7p15.2	1	1	0	0	Amplification	1	0	42473T: 4;
HNRNPA3	chr2	2q31.2	2	0	1	1	Amplification	2	0	42493T: 4.5; 42482T: 4;
HNRNPCL1	chr1	1p36.21	1	0	1	0	Amplification	1	0	42486T: 4.5;
HNRNPL	chr19	19q13.2	2	0	0	2	Amplification	2	0	56957T: 4; 42500T: 6.5;
HNRNPLL	chr2	2p22.1	2	0	0	2	Amplification	2	0	42500T: 3.5; 42493T: 3.5;
HNRNPR	chr1	1p36.12	1	1	0	0	Amplification	1	0	42473T: 3.5;
HNRNPUL1	chr19	19q13.2	1	1	0	0	Amplification	1	0	42473T: 4.5;
HOMEZ	chr14	14q11.2	2	0	0	2	Amplification	2	0	42496T: 4; 42500T: 4;
HOOK3	chr8	8p11.21	1	0	1	0	Amplification	1	0	42483T: 4.5;
HORMAD1	chr1	1q21.3	2	1	0	1	Amplification	2	0	42493T: 3.5; 42473T: 4.5;
HORMAD2	chr22	22q12.2	1	1	0	0	Amplification	1	0	42473T: 3.5;
HOXA1	chr7	7p15.2	1	1	0	0	Amplification	1	0	42473T: 4;
HOXA10	chr7	7p15.2	1	1	0	0	Amplification	1	0	42473T: 4;
HOXA11	chr7	7p15.2	1	1	0	0	Amplification	1	0	42473T: 4;
HOXA13	chr7	7p15.2	1	1	0	0	Amplification	1	0	42473T: 4;
HOXA2	chr7	7p15.2	1	1	0	0	Amplification	1	0	42473T: 4;
HOXA3	chr7	7p15.2	1	1	0	0	Amplification	1	0	42473T: 4;
HOXA4	chr7	7p15.2	1	1	0	0	Amplification	1	0	42473T: 4;
HOXA5	chr7	7p15.2	1	1	0	0	Amplification	1	0	42473T: 4;
HOXA6	chr7	7p15.2	1	1	0	0	Amplification	1	0	42473T: 4;
HOXA7	chr7	7p15.2	1	1	0	0	Amplification	1	0	42473T: 4;
HOXA9	chr7	7p15.2	1	1	0	0	Amplification	1	0	42473T: 4;
HOXC10	chr12	12q13.13	2	1	0	1	Amplification	2	0	42473T: 3.5; 42494T: 3.5;
HOXC11	chr12	12q13.13	1	1	0	0	Amplification	1	0	42473T: 3.5;
HOXC12	chr12	12q13.13	1	1	0	0	Amplification	1	0	42473T: 3.5;
HOXC4	chr12	12q13.13	2	1	0	1	Amplification	2	0	42473T: 3.5; 42494T: 3.5;
HOXC5	chr12	12q13.13	2	1	0	1	Amplification	2	0	42473T: 3.5; 42494T: 3.5;
HOXC6	chr12	12q13.13	2	1	0	1	Amplification	2	0	42494T: 3.5; 42473T: 3.5;
HOXC8	chr12	12q13.13	2	1	0	1	Amplification	2	0	42473T: 3.5; 42494T: 3.5;
HOXC9	chr12	12q13.13	2	1	0	1	Amplification	2	0	42473T: 3.5; 42494T: 3.5;

Mangalaparthi *et al.* , 2020. Mutational landscape of esophageal squamous cell carcinoma in an Indian cohort
Supplementary Table 7A. List of copy number alterations and affected genes in ESCC patients

Gene	Chromosome	Cytoband	Recurrence	Recurrence in smoker cohort	Recurrence in chewer cohort	Recurrence in No habit cohort	State	Samples with CNA gain	Samples with CNA loss	File info with CNA fold
HOXD10	chr2	2q31.1	1	0	0	1	Amplification	1	0	42493T: 3.5;
HOXD11	chr2	2q31.1	1	0	0	1	Amplification	1	0	42493T: 3.5;
HOXD12	chr2	2q31.1	1	0	0	1	Amplification	1	0	42493T: 3.5;
HOXD13	chr2	2q31.1	1	0	0	1	Amplification	1	0	42493T: 3.5;
HOXD3	chr2	2q31.1	1	0	0	1	Amplification	1	0	42493T: 3.5;
HOXD4	chr2	2q31.1	1	0	0	1	Amplification	1	0	42493T: 3.5;
HOXD8	chr2	2q31.1	1	0	0	1	Amplification	1	0	42493T: 3.5;
HOXD9	chr2	2q31.1	1	0	0	1	Amplification	1	0	42493T: 3.5;
HP1BP3	chr1	1p36.12	1	1	0	0	Amplification	1	0	42473T: 4;
HPCAL1	chr2	2p25.1	1	0	0	1	Amplification	1	0	42500T: 3.5;
HPD	chr12	12q24.31	1	0	0	1	Amplification	1	0	42500T: 3.5;
HPN	chr19	19q13.11	3	0	1	2	Amplification	3	0	42500T: 6.5; 56957T: 4; 42484T: 3.5;
HPS3	chr3	3q24	8	1	2	5	Amplification	8	0	42474T: 3.5; 56957T: 4; 42484T: 3.5; 42496T: 4; 42497T: 4.5; 42487T: 3.5; 42493T: 3.5; 42492T: 3.5;
HRASLS	chr3	3q29	10	1	3	6	Amplification	10	0	42501T: 3.5; 42498T: 3.5; 42484T: 4; 56957T: 4.5; 42474T: 3.5; 42495T: 4; 42482T: 3.5; 42492T: 3.5; 42487T: 3.5; 42493T: 3.5;
HRCT1	chr9	9p13.3	2	1	0	1	Amplification	2	0	42473T: 3.5; 42501T: 3.5;
HRG	chr3	3q27.3	10	1	3	6	Amplification	10	0	42482T: 3.5; 42493T: 3.5; 42487T: 3.5; 42492T: 3.5; 42497T: 4; 42495T: 4; 42474T: 3.5; 56957T: 4.5; 42484T: 4; 42498T: 3.5;
HRH3	chr20	20q13.33	1	1	0	0	Amplification	1	0	42473T: 6;
HRK	chr12	12q24.22	1	0	0	1	Amplification	1	0	42500T: 3.5;
HRNR	chr1	1q21.3	1	1	0	0	Amplification	1	0	42473T: 4.5;
HRSP12	chr8	8q22.2	2	0	0	2	Amplification	2	0	42495T: 3.5; 42496T: 3.5;
HS1BP3	chr2	2p24.1	1	0	0	1	Amplification	1	0	42500T: 3.5;
HS3ST2	chr16	16p12.2	1	1	0	0	Amplification	1	0	42473T: 4;
HS3ST4	chr16	16p12.1	1	1	0	0	Amplification	1	0	42473T: 4;
HS3ST6	chr16	16p13.3	1	0	1	0	Amplification	1	0	42483T: 3.5;
HSD17B7P2	chr10	10p11.1	1	0	0	1	Amplification	1	0	56958T: 3.5;
HSF1	chr8	8q24.3	4	0	1	3	Amplification	4	0	42495T: 4.5; 56957T: 3.5; 42483T: 3.5; 42496T: 4;
HSF5	chr17	17q22	1	0	0	1	Amplification	1	0	42497T: 4;
HSPA12B	chr20	20p13	1	1	0	0	Amplification	1	0	42473T: 6;
HSPA1A	chr6	6p21.33	1	1	0	0	Amplification	1	0	42473T: 3.5;
HSPA1B	chr6	6p21.33	1	1	0	0	Amplification	1	0	42473T: 3.5;
HSPA1L	chr6	6p21.33	1	1	0	0	Amplification	1	0	42473T: 3.5;
HSPA2	chr14	14q23.3	1	0	0	1	Amplification	1	0	42494T: 4;
HSPA9	chr5	5q31.2	1	0	0	1	Amplification	1	0	42495T: 3.5;
HSPB1	chr7	7q11.23	1	0	0	1	Amplification	1	0	42493T: 3.5;
HSPB6	chr19	19q13.12	3	0	1	2	Amplification	3	0	42500T: 6.5; 42484T: 3.5; 56957T: 4;
HSPB7	chr1	1p36.13	1	1	0	0	Amplification	1	0	42473T: 8;

Mangalaparthi *et al.* , 2020. Mutational landscape of esophageal squamous cell carcinoma in an Indian cohort
Supplementary Table 7A. List of copy number alterations and affected genes in ESCC patients

Gene	Chromosome	Cytoband	Recurrence	Recurrence in smoker cohort	Recurrence in chewer cohort	Recurrence in No habit cohort	State	Samples with CNA gain	Samples with CNA loss	File info with CNA fold
HSPB8	chr12	12q24.23	1	0	0	1	Amplification	1	0	42500T: 3.5;
HSPBAP1	chr3	3q21.1	1	0	0	1	Amplification	1	0	42496T: 3.5;
HSPD1	chr2	2q33.1	1	0	1	0	Amplification	1	0	42482T: 4;
HSPE1	chr2	2q33.1	1	0	1	0	Amplification	1	0	42482T: 4;
HSPG2	chr1	1p36.12	2	1	0	1	Amplification	2	0	42493T: 3.5; 42473T: 4;
HSPH1	chr13	13q12.3	1	0	0	1	Amplification	1	0	42497T: 3.5;
HTR1D	chr1	1p36.12	1	1	0	0	Amplification	1	0	42473T: 3.5;
HTR3C	chr3	3q27.1	11	1	3	7	Amplification	11	0	42482T: 3.5; 42492T: 3.5; 42493T: 3.5; 42487T: 3.5; 42497T: 4; 42484T: 4; 42498T: 3.5; 42474T: 3.5; 42494T: 3.5; 42495T: 4; 56957T: 5;
HTR3D	chr3	3q27.1	11	1	3	7	Amplification	11	0	42497T: 4; 42474T: 3.5; 42494T: 3.5; 42495T: 4; 56957T: 5; 42484T: 4; 42498T: 3.5; 42482T: 3.5; 42487T: 3.5; 42493T: 3.5; 42492T: 3.5;
HTR3E	chr3	3q27.1	12	2	3	7	Amplification	12	0	42497T: 4; 42473T: 3.5; 42494T: 3.5; 42495T: 4; 42474T: 3.5; 56957T: 5; 42484T: 4; 42498T: 3.5; 42482T: 3.5; 42487T: 3.5; 42493T: 3.5; 42492T: 3.5;
HTR6	chr1	1p36.13	1	1	0	0	Amplification	1	0	42473T: 4;
HTR7P1	chr12	12p13.1	1	0	0	1	Amplification	1	0	42500T: 4.5;
HTRA2	chr2	2p13.1	1	0	0	1	Amplification	1	0	42500T: 3.5;
HVCN1	chr12	12q24.11	1	0	0	1	Amplification	1	0	42500T: 3.5;
HYAL4	chr7	7q31.32	2	0	1	1	Amplification	2	0	42493T: 3.5; 42487T: 3.5;
HYDIN2	chr1	1q21.1	1	1	0	0	Amplification	1	0	42473T: 4;
HYKK	chr15	15q25.1	1	1	0	0	Amplification	1	0	42473T: 3.5;
HYOU1	chr11	11q23.3	1	1	0	0	Amplification	1	0	42473T: 4;
IAH1	chr2	2p25.1	1	0	0	1	Amplification	1	0	42500T: 3.5;
IAPP	chr12	12p12.1	1	0	0	1	Amplification	1	0	42500T: 6;
ICA1	chr7	7p21.3	2	1	0	1	Amplification	2	0	42473T: 3.5; 42497T: 7.5;
ID1	chr20	20q11.21	1	0	0	1	Amplification	1	0	42496T: 5;
ID2	chr2	2p25.1	1	0	0	1	Amplification	1	0	42500T: 3.5;
ID3	chr1	1p36.12	1	1	0	0	Amplification	1	0	42473T: 3.5;
IDH2	chr15	15q26.1	1	1	0	0	Amplification	1	0	42473T: 3.5;
IDH3A	chr15	15q25.1	1	1	0	0	Amplification	1	0	42473T: 3.5;
IDH3B	chr20	20p13	1	1	0	0	Amplification	1	0	42473T: 3.5;
IFFO2	chr1	1p36.13	1	1	0	0	Amplification	1	0	42473T: 4;
IFI6	chr1	1p35.3	1	1	0	0	Amplification	1	0	42473T: 3.5;
IFLTD1	chr12	12p12.1	1	0	0	1	Amplification	1	0	42500T: 6;
IFNA1	chr9	9p21.3	1	1	0	0	Deletion	0	1	42475T: 0.5;
IFNA13	chr9	9p21.3	1	1	0	0	Deletion	0	1	42475T: 0.5;
IFNA14	chr9	9p21.3	1	1	0	0	Deletion	0	1	42475T: 0.5;
IFNA16	chr9	9p21.3	1	1	0	0	Deletion	0	1	42475T: 0.5;
IFNA2	chr9	9p21.3	1	1	0	0	Deletion	0	1	42475T: 0.5;

Mangalaparthi *et al.* , 2020. Mutational landscape of esophageal squamous cell carcinoma in an Indian cohort
Supplementary Table 7A. List of copy number alterations and affected genes in ESCC patients

Gene	Chromosome	Cytoband	Recurrence	Recurrence in smoker cohort	Recurrence in chewer cohort	Recurrence in No habit cohort	State	Samples with CNA gain	Samples with CNA loss	File info with CNA fold
IFNA21	chr9	9p21.3	1	1	0	0	Deletion	0	1	42475T: 0.5;
IFNA5	chr9	9p21.3	1	1	0	0	Deletion	0	1	42475T: 0.5;
IFNA6	chr9	9p21.3	1	1	0	0	Deletion	0	1	42475T: 0.5;
IFNA7	chr9	9p21.3	1	1	0	0	Deletion	0	1	42475T: 0.5;
IFNA8	chr9	9p21.3	1	1	0	0	Deletion	0	1	42475T: 0.5;
IFNB1	chr9	9p21.3	1	1	0	0	Deletion	0	1	42475T: 0.5;
IFNE	chr9	9p21.3	1	1	0	0	Deletion	0	1	42475T: 0.5;
IFNG	chr12	12q15	2	0	0	2	Amplification	2	0	42501T: 6.5; 42500T: 5;
IFNL1	chr19	19q13.2	2	0	0	2	Amplification	2	0	56957T: 4; 42500T: 6.5;
IFNL2	chr19	19q13.2	2	0	0	2	Amplification	2	0	56957T: 4; 42500T: 6.5;
IFNL3	chr19	19q13.2	2	0	0	2	Amplification	2	0	42500T: 6.5; 56957T: 4;
IFNLR1	chr1	1p36.11	1	1	0	0	Amplification	1	0	42473T: 3.5;
IFNW1	chr9	9p21.3	1	1	0	0	Deletion	0	1	42475T: 0.5;
IFRD1	chr7	7q31.1	2	0	1	1	Amplification	2	0	42487T: 3.5; 42501T: 3.5;
IFT122	chr3	3q21.3-q22.1	2	0	1	1	Amplification	2	0	42487T: 3.5; 42496T: 3.5;
IFT140	chr16	16p13.3	1	0	1	0	Amplification	1	0	42483T: 3.5;
IFT172	chr2	2p23.3	1	0	0	1	Amplification	1	0	42500T: 3.5;
IFT27	chr22	22q12.3	1	1	0	0	Amplification	1	0	42473T: 3.5;
IFT43	chr14	14q24.3	2	0	0	2	Amplification	2	0	42494T: 4; 56957T: 3.5;
IFT52	chr20	20q13.12	1	1	0	0	Amplification	1	0	42473T: 3.5;
IFT80	chr3	3q25.33	8	1	2	5	Amplification	8	0	42484T: 4; 42495T: 4; 42474T: 3.5; 56957T: 4; 42497T: 3.5; 42492T: 3.5; 42493T: 3.5; 42487T: 3.5;
IFT81	chr12	12q24.11	2	1	0	1	Amplification	2	0	42473T: 4.5; 42500T: 3.5;
IGF2BP2	chr3	3q27.2	10	1	3	6	Amplification	10	0	42484T: 4; 42498T: 3.5; 42474T: 3.5; 42495T: 4; 56957T: 4.5; 42497T: 4; 42492T: 3.5; 42493T: 3.5; 42487T: 3.5; 42482T: 3.5;
IGF2BP3	chr7	7p15.3	1	1	0	0	Amplification	1	0	42473T: 4;
IGFALS	chr16	16p13.3	1	0	1	0	Amplification	1	0	42483T: 3.5;
IGFBP1	chr7	7p12.3	1	1	0	0	Amplification	1	0	42473T: 4.5;
IGFBP6	chr12	12q13.13	1	1	0	0	Amplification	1	0	42473T: 4;
IGFL1	chr19	19q13.32	1	0	1	0	Amplification	1	0	42484T: 4;
IGFL2	chr19	19q13.32	1	0	1	0	Amplification	1	0	42484T: 4;
IGFL3	chr19	19q13.32	1	0	1	0	Amplification	1	0	42484T: 4;
IGFL4	chr19	19q13.32	1	0	1	0	Amplification	1	0	42484T: 4;
IGFLR1	chr19	19q13.12	3	0	1	2	Amplification	3	0	42500T: 6.5; 42484T: 3.5; 56957T: 4;
IGHMBP2	chr11	11q13.3	7	1	1	5	Amplification	7	0	42492T: 4; 42498T: 9.5; 56957T: 5; 42497T: 4.5; 42476T: 29.5; 42483T: 14.5; 42501T: 3.5;
IGLL5	chr22	22q11.22	1	1	0	0	Amplification	1	0	42477T: 4;
IGSF10	chr3	3q25.1	9	1	2	6	Amplification	9	0	42484T: 3.5; 42474T: 3.5; 56957T: 4; 42497T: 5.5; 42496T: 4; 42500T: 3.5; 42492T: 3.5; 42493T: 3.5; 42487T: 3.5;

Mangalaparthi *et al.* , 2020. Mutational landscape of esophageal squamous cell carcinoma in an Indian cohort
Supplementary Table 7A. List of copy number alterations and affected genes in ESCC patients

Gene	Chromosome	Cytoband	Recurrence	Recurrence in smoker cohort	Recurrence in chewer cohort	Recurrence in No habit cohort	State	Samples with CNA gain	Samples with CNA loss	File info with CNA fold
IGSF11	chr3	3q13.32	1	0	0	1	Amplification	1	0	42496T: 3.5;
IGSF21	chr1	1p36.13	1	1	0	0	Amplification	1	0	42473T: 4;
IGSF23	chr19	19q13.31	2	1	0	1	Amplification	2	0	56957T: 4; 42473T: 3.5;
IGSF5	chr21	21q22.2	1	1	0	0	Amplification	1	0	42473T: 3.5;
IGSF6	chr16	16p12.2	1	1	0	0	Amplification	1	0	42473T: 4;
IHH	chr2	2q35	1	1	0	0	Amplification	1	0	42473T: 3.5;
IKZF1	chr7	7p12.2	1	0	0	1	Amplification	1	0	42497T: 20.5;
IKZF3	chr17	17q12-q21.1	1	0	0	1	Amplification	1	0	42497T: 3.5;
IKZF4	chr12	12q13.2	1	0	0	1	Amplification	1	0	42494T: 5;
IL11	chr19	19q13.42	1	0	0	1	Amplification	1	0	42494T: 3.5;
IL12A	chr3	3q25.33	8	1	2	5	Amplification	8	0	42493T: 3.5; 42487T: 3.5; 42492T: 3.5; 42497T: 3.5; 56957T: 4; 42474T: 3.5; 42495T: 4; 42484T: 4;
IL18BP	chr11	11q13.4	7	3	0	4	Amplification	7	0	42475T: 5; 42501T: 6; 42476T: 4; 56957T: 3.5; 42492T: 4; 42498T: 5; 42478T: 4.5;
IL18R1	chr2	2q12.1	1	0	0	1	Amplification	1	0	42493T: 3.5;
IL18RAP	chr2	2q12.1	1	0	0	1	Amplification	1	0	42493T: 3.5;
IL1R1	chr2	2q11.2-q12.1	1	0	0	1	Amplification	1	0	42493T: 3.5;
IL1R2	chr2	2q11.2	1	0	0	1	Amplification	1	0	42493T: 3.5;
IL1RAP	chr3	3q28	9	1	3	5	Amplification	9	0	42482T: 3.5; 42492T: 3.5; 42487T: 3.5; 42493T: 3.5; 42484T: 4; 42498T: 3.5; 42495T: 4; 42474T: 3.5; 56957T: 4.5;
IL1RL1	chr2	2q12.1	1	0	0	1	Amplification	1	0	42493T: 3.5;
IL1RL2	chr2	2q12.1	1	0	0	1	Amplification	1	0	42493T: 3.5;
IL20RB	chr3	3q22.3	4	0	1	3	Amplification	4	0	42496T: 3.5; 42487T: 3.5; 42493T: 3.5; 42492T: 3.5;
IL22	chr12	12q15	2	0	0	2	Amplification	2	0	42501T: 6.5; 42500T: 5;
IL22RA1	chr1	1p36.11	1	1	0	0	Amplification	1	0	42473T: 3.5;
IL25	chr14	14q11.2	2	0	0	2	Amplification	2	0	42496T: 4; 42500T: 4;
IL26	chr12	12q15	2	0	0	2	Amplification	2	0	42501T: 6.5; 42500T: 5;
IL2RB	chr22	22q12.3	1	1	0	0	Amplification	1	0	42473T: 3.5;
IL31	chr12	12q24.31	1	0	0	1	Amplification	1	0	42500T: 3.5;
IL33	chr9	9p24.1	1	0	0	1	Amplification	1	0	42496T: 3.5;
IL6	chr7	7p15.3	1	1	0	0	Amplification	1	0	42473T: 4;
IL6R	chr1	1q21.3	1	1	0	0	Amplification	1	0	42473T: 4.5;
IL7	chr8	8q21.13	2	0	0	2	Amplification	2	0	42495T: 3.5; 42496T: 3.5;
IL7R	chr5	5p13.2	4	1	1	2	Amplification	4	0	42496T: 3.5; 42486T: 3.5; 42475T: 3.5; 42493T: 3.5;
ILDR1	chr3	3q13.33	1	0	0	1	Amplification	1	0	42496T: 3.5;
ILF2	chr1	1q21.3	1	1	0	0	Amplification	1	0	42473T: 4.5;
ILKAP	chr2	2q37.3	1	1	0	0	Amplification	1	0	42473T: 3.5;
IMMP2L	chr7	7q31.1	2	0	1	1	Amplification	2	0	42487T: 3.5; 42501T: 3.5;
IMMT	chr2	2p11.2|2	1	0	0	1	Amplification	1	0	42500T: 3.5;
IMPA1	chr8	8q21.13	2	0	0	2	Amplification	2	0	42496T: 3.5; 42495T: 3.5;

Mangalaparthi *et al.* , 2020. Mutational landscape of esophageal squamous cell carcinoma in an Indian cohort
Supplementary Table 7A. List of copy number alterations and affected genes in ESCC patients

Gene	Chromosome	Cytoband	Recurrence	Recurrence in smoker cohort	Recurrence in chewer cohort	Recurrence in No habit cohort	State	Samples with CNA gain	Samples with CNA loss	File info with CNA fold
IMPA2	chr18	18p11.21	1	0	0	1	Amplification	1	0	42493T: 3.5;
IMPAD1	chr8	8q12.1	2	0	0	2	Amplification	2	0	42496T: 3.5; 42495T: 3.5;
IMPDH1	chr7	16p13.2	2	1	1	0	Amplification	2	0	42487T: 3.5; 42473T: 3.5;
IMPG2	chr3	3q12.3	1	1	0	0	Amplification	1	0	42476T: 3.5;
ING3	chr7	7q31.31	1	0	1	0	Amplification	1	0	42487T: 3.5;
INHBA	chr7	7p14.1	1	1	0	0	Amplification	1	0	42473T: 3.5;
INMT	chr7	7p14.3	1	1	0	0	Amplification	1	0	42473T: 4;
INO80	chr15	15q15.1	1	0	0	1	Amplification	1	0	42493T: 3.5;
INO80B	chr2	2p13.1	1	0	0	1	Amplification	1	0	42500T: 3.5;
INPP1	chr2	2q32.2	3	0	1	2	Amplification	3	0	42482T: 4; 42493T: 3.5; 42494T: 3.5;
INPP4A	chr2	2q11.2	1	0	0	1	Amplification	1	0	42493T: 3.5;
INPP5D	chr2	2q37.1	1	1	0	0	Amplification	1	0	42473T: 4.5;
INPP5E	chr9	9q34.3	1	0	0	1	Amplification	1	0	56957T: 3.5;
INPPL1	chr11	11q13.4	8	3	0	5	Amplification	8	0	42476T: 4; 42493T: 3.5; 42492T: 4; 42475T: 5; 42501T: 6; 56957T: 3.5; 42478T: 4.5; 42498T: 5;
INSL4	chr9	9p24.1	1	0	0	1	Amplification	1	0	42496T: 3.5;
INSL6	chr9	9p24.1	1	0	0	1	Amplification	1	0	42496T: 3.5;
INSM1	chr20	20p11.23	1	0	1	0	Amplification	1	0	42483T: 4;
INSM2	chr14	14q13.2	2	1	0	1	Amplification	2	0	42500T: 8.5; 42476T: 5;
INSRR	chr1	1q23.1	1	1	0	0	Amplification	1	0	42473T: 7;
INTS1	chr7	7p22.3	1	1	0	0	Amplification	1	0	42473T: 5;
INTS3	chr1	1q21.3	1	1	0	0	Amplification	1	0	42473T: 4.5;
INTS4L1	chr7	7q11.21	1	0	0	1	Amplification	1	0	42501T: 3.5;
INTS4L2	chr7	7q11.21	1	0	0	1	Amplification	1	0	42501T: 3.5;
INTS8	chr8	8q22.1	3	1	0	2	Amplification	3	0	42495T: 3.5; 42496T: 3.5; 42473T: 4.5;
IPO8	chr12	12p11.21	1	0	0	1	Amplification	1	0	42500T: 8;
IQCB1	chr3	3q13.33|3q2	1	0	0	1	Amplification	1	0	42496T: 3.5;
IQCC	chr1	1p35.2	1	1	0	0	Amplification	1	0	42473T: 4;
IQCD	chr12	12q24.13	1	0	0	1	Amplification	1	0	42500T: 3.5;
IQCE	chr7	7p22.3	1	1	0	0	Amplification	1	0	42473T: 5;
IQCG	chr3	3q29	9	1	3	5	Amplification	9	0	56957T: 6; 42495T: 4; 42474T: 3.5; 42498T: 3.5; 42484T: 3.5; 42482T: 3.5; 42493T: 4; 42487T: 3.5; 42492T: 3.5;
IQCJ	chr3	3q25.32	8	1	2	5	Amplification	8	0	42492T: 3.5; 42493T: 3.5; 42487T: 3.5; 42497T: 3.5; 42484T: 4; 42495T: 4; 42474T: 3.5; 56957T: 4;
IQCK	chr16	16p12.3	1	1	0	0	Amplification	1	0	42473T: 4;
IQGAP3	chr1	1q22	1	1	0	0	Amplification	1	0	42473T: 7;
IQSEC3	chr12	12p13.33	1	0	0	1	Amplification	1	0	42500T: 3.5;
IQUB	chr7	7q31.32	2	0	1	1	Amplification	2	0	42487T: 3.5; 42493T: 3.5;
IRAK3	chr12	12q14.3	1	0	0	1	Amplification	1	0	42500T: 4.5;
IRAK4	chr12	12q12	1	0	0	1	Amplification	1	0	42500T: 3.5;

Mangalaparthi *et al.* , 2020. Mutational landscape of esophageal squamous cell carcinoma in an Indian cohort
Supplementary Table 7A. List of copy number alterations and affected genes in ESCC patients

Gene	Chromosome	Cytoband	Recurrence	Recurrence in smoker cohort	Recurrence in chewer cohort	Recurrence in No habit cohort	State	Samples with CNA gain	Samples with CNA loss	File info with CNA fold
IREB2	chr15	15q25.1	1	1	0	0	Amplification	1	0	42473T: 3.5;
IRF2BP2	chr1	1q42.3	1	1	0	0	Amplification	1	0	42473T: 6.5;
IRF2BPL	chr14	14q24.3	2	0	0	2	Amplification	2	0	56957T: 3.5; 42494T: 4;
IRF5	chr7	7q32.1	1	0	1	0	Amplification	1	0	42487T: 3.5;
IRGQ	chr19	19q13.31	1	0	0	1	Amplification	1	0	56957T: 4;
IRX1	chr5	5p15.33	5	2	1	2	Amplification	5	0	42486T: 3.5; 42496T: 3.5; 42473T: 3.5; 42475T: 3.5; 42493T: 3.5;
IRX2	chr5	5p15.33	5	2	1	2	Amplification	5	0	42475T: 3.5; 42486T: 3.5; 42473T: 3.5; 42496T: 3.5; 42493T: 3.5;
IRX4	chr5	5p15.33	5	2	1	2	Amplification	5	0	42493T: 3.5; 42475T: 3.5; 42473T: 3.5; 42496T: 4; 42486T: 3.5;
ISCA2	chr14	14q24.3	2	0	0	2	Amplification	2	0	56957T: 3.5; 42494T: 4;
ISG20L2	chr1	1q23.1	1	1	0	0	Amplification	1	0	42473T: 7;
ISL1	chr5	5q11.1	2	1	0	1	Amplification	2	0	42475T: 3.5; 42498T: 16.5;
ISLR	chr15	15q24.1	1	1	0	0	Amplification	1	0	42473T: 3.5;
ISLR2	chr15	15q24.1	1	1	0	0	Amplification	1	0	42473T: 3.5;
ISM2	chr14	14q24.3	2	0	0	2	Amplification	2	0	56957T: 3.5; 42494T: 4;
ISOC2	chr19	19q13.42	1	0	0	1	Amplification	1	0	42494T: 3.5;
ISPD	chr7	7p21.2	1	1	0	0	Amplification	1	0	42473T: 3.5;
ISY1	chr3	3q21.3	3	0	1	2	Amplification	3	0	42493T: 3.5; 42487T: 3.5; 42496T: 3.5;
ITCH	chr20	20q11.22	3	1	0	2	Amplification	3	0	42473T: 3.5; 42496T: 5; 42493T: 3.5;
ITFG3	chr16	16p13.3	1	0	1	0	Amplification	1	0	42483T: 3.5;
ITGA10	chr1	1q21.1	1	1	0	0	Amplification	1	0	42473T: 4;
ITGA4	chr2	2q31.3	1	0	0	1	Amplification	1	0	42493T: 4;
ITGA5	chr12	12q13.13	2	1	0	1	Amplification	2	0	42494T: 3.5; 42473T: 3.5;
ITGA6	chr2	2q31.1	2	1	0	1	Amplification	2	0	42493T: 5; 42473T: 3.5;
ITGA7	chr12	12q13.2	1	0	0	1	Amplification	1	0	42494T: 3.5;
ITGAV	chr2	2q32.1	1	0	0	1	Amplification	1	0	42493T: 3.5;
ITGB1BP1	chr2	2p25.1	1	0	0	1	Amplification	1	0	42500T: 3.5;
ITGB4	chr17	17q25.1	1	0	0	1	Amplification	1	0	42494T: 3.5;
ITGB5	chr3	3q21.2	1	0	0	1	Amplification	1	0	42496T: 3.5;
ITGB8	chr7	7p21.1	1	1	0	0	Amplification	1	0	42473T: 4;
ITPA	chr20	20p13	1	1	0	0	Amplification	1	0	42473T: 3.5;
ITPKC	chr19	19q13.2	2	0	0	2	Amplification	2	0	56957T: 4; 42500T: 3.5;
ITPR2	chr12	12p11.23	1	0	0	1	Amplification	1	0	42500T: 6;
ITPRIPL1	chr2	2q11.2	2	1	0	1	Amplification	2	0	42493T: 3.5; 42473T: 3.5;
ITPRIPL2	chr16	16p12.3	2	1	0	1	Amplification	2	0	42473T: 4; 42495T: 4;
ITSN2	chr2	2p23.3	1	0	0	1	Amplification	1	0	42500T: 3.5;
JAK2	chr9	9p24.1	1	0	0	1	Amplification	1	0	42496T: 3.5;
JAM3	chr11	11q25	1	1	0	0	Deletion	0	1	42476T: 0.5;
JAZF1	chr7	7p15.2-p15.1	1	1	0	0	Amplification	1	0	42473T: 4;

Mangalaparthi *et al.* , 2020. Mutational landscape of esophageal squamous cell carcinoma in an Indian cohort
Supplementary Table 7A. List of copy number alterations and affected genes in ESCC patients

Gene	Chromosome	Cytoband	Recurrence	Recurrence in smoker cohort	Recurrence in chewer cohort	Recurrence in No habit cohort	State	Samples with CNA gain	Samples with CNA loss	File info with CNA fold
JDP2	chr14	14q24.3	2	0	0	2	Amplification	2	0	56957T: 3.5; 42494T: 4;
JHDM1D	chr7	7q34	1	0	1	0	Amplification	1	0	42487T: 3.5;
JKAMP	chr14	14q23.1	1	0	0	1	Amplification	1	0	42494T: 4;
JMJD8	chr16	16p13.3	2	0	1	1	Amplification	2	0	42483T: 3.5; 42493T: 3.5;
JOSD1	chr22	22q13.1	1	1	0	0	Amplification	1	0	42473T: 3.5;
JPH1	chr8	8q21.11	2	0	0	2	Amplification	2	0	42496T: 3.5; 42495T: 3.5;
JPH2	chr20	20q13.12	1	1	0	0	Amplification	1	0	42473T: 3.5;
JPH4	chr14	14q11.2	1	0	0	1	Amplification	1	0	42496T: 4;
JRK	chr8	8q24.3	3	0	1	2	Amplification	3	0	42496T: 4; 42483T: 3.5; 42495T: 4.5;
JTB	chr1	1q21.3	1	1	0	0	Amplification	1	0	42473T: 4.5;
KALRN	chr3	3q21.1-q21.2	1	0	0	1	Amplification	1	0	42496T: 3.5;
KANK1	chr9	9p24.3	2	0	1	1	Amplification/Deletio	1	1	42486T: 0.5; 42496T: 3.5;
KANSL2	chr12	12q13.11	1	0	0	1	Amplification	1	0	42500T: 3.5;
KANSL3	chr2	2q11.2	2	1	0	1	Amplification	2	0	42493T: 3.5; 42473T: 3.5;
KATNAL1	chr13	13q12.3	1	0	0	1	Amplification	1	0	42497T: 3.5;
KBTBD12	chr3	3q21.3	2	0	1	1	Amplification	2	0	42487T: 3.5; 42496T: 3.5;
KBTBD2	chr7	7p14.3	1	1	0	0	Amplification	1	0	42473T: 4;
KBTBD4	chr11	11p11.2	1	1	0	0	Amplification	1	0	42473T: 3.5;
KCMF1	chr2	2p11.2	1	0	0	1	Amplification	1	0	42500T: 3.5;
KCNAB1	chr3	3q25.31	8	1	2	5	Amplification	8	0	56957T: 4; 42474T: 3.5; 42495T: 4; 42484T: 3.5; 42497T: 5.5; 42487T: 3.5; 42493T: 3.5; 42492T: 3.5;
KCNB1	chr20	20q13.13	1	1	0	0	Amplification	1	0	42473T: 5;
KCNB2	chr8	8q21.11	2	0	0	2	Amplification	2	0	42496T: 3.5; 42495T: 3.5;
KCNE3	chr11	11q13.4	5	2	0	3	Amplification	5	0	42475T: 7.5; 42476T: 4; 56957T: 3.5; 42498T: 8; 42492T: 4;
KCNF1	chr2	2p25.1	1	0	0	1	Amplification	1	0	42500T: 3.5;
KCNG1	chr20	20q13.13	1	1	0	0	Amplification	1	0	42473T: 5;
KCNG3	chr2	2p21	2	0	1	1	Amplification	2	0	42484T: 3.5; 42500T: 3.5;
KCNH3	chr12	12q13.12	1	0	0	1	Amplification	1	0	42500T: 3.5;
KCNH5	chr14	14q23.2	1	0	0	1	Amplification	1	0	42494T: 4;
KCNIP3	chr2	2q11.1	2	1	0	1	Amplification	2	0	42493T: 4; 42473T: 3.5;
KCNJ4	chr22	22q13.1	1	1	0	0	Amplification	1	0	42473T: 3.5;
KCNJ8	chr12	12p12.1	1	0	0	1	Amplification	1	0	42500T: 6;
KCNK12	chr2	2p16.3	2	0	1	1	Amplification	2	0	42500T: 3.5; 42484T: 4.5;
KCNK13	chr14	14q32.11	1	1	0	0	Amplification	1	0	42473T: 3.5;
KCNK15	chr20	20q13.12	1	1	0	0	Amplification	1	0	42473T: 5;
KCNK3	chr2	2p23.3	1	0	0	1	Amplification	1	0	42500T: 3.5;
KCNK6	chr19	19q13.2	4	1	1	2	Amplification	4	0	42474T: 4; 56957T: 4; 42484T: 3.5; 42500T: 6.5;
KCNK9	chr8	8q24.3	2	0	0	2	Amplification	2	0	42496T: 3.5; 42495T: 3.5;

Mangalaparthi *et al.* , 2020. Mutational landscape of esophageal squamous cell carcinoma in an Indian cohort
Supplementary Table 7A. List of copy number alterations and affected genes in ESCC patients

Gene	Chromosome	Cytoband	Recurrence	Recurrence in smoker cohort	Recurrence in chewer cohort	Recurrence in No habit cohort	State	Samples with CNA gain	Samples with CNA loss	File info with CNA fold
KCNMB2	chr3	3q26.32	11	1	3	7	Amplification	11	0	42492T: 3.5; 42487T: 3.5; 42493T: 3.5; 42482T: 3.5; 42484T: 4; 42498T: 3.5; 42474T: 3.5; 42495T: 4; 56957T: 4; 42497T: 4; 42500T: 9.5;
KCNMB3	chr3	3q26.32	11	1	3	7	Amplification	11	0	42500T: 9.5; 42497T: 4; 42495T: 4; 42474T: 3.5; 56957T: 4; 42484T: 4; 42498T: 3.5; 42482T: 3.5; 42493T: 3.5; 42487T: 3.5; 42492T: 3.5;
KCNMB4	chr12	12q15	2	0	0	2	Amplification	2	0	42501T: 5.5; 42500T: 5;
KCNN3	chr1	1q21.3	1	1	0	0	Amplification	1	0	42473T: 4.5;
KCNN4	chr19	19q13.31	1	0	0	1	Amplification	1	0	56957T: 4;
KCNQ2	chr20	20q13.33	1	1	0	0	Amplification	1	0	42473T: 6;
KCNQ3	chr8	8q24.22	3	0	1	2	Amplification	3	0	42496T: 3.5; 42484T: 3.5; 42495T: 3.5;
KCNS1	chr20	20q13.12	1	1	0	0	Amplification	1	0	42473T: 5;
KCNS2	chr8	8q22.2	2	0	0	2	Amplification	2	0	42495T: 3.5; 42496T: 3.5;
KCNS3	chr2	2p24.2	1	0	0	1	Amplification	1	0	42500T: 3.5;
KCNT1	chr9	9q34.3	1	0	0	1	Amplification	1	0	56957T: 3.5;
KCNU1	chr8	8p11.23	1	0	1	0	Amplification	1	0	42482T: 3.5;
KCNV1	chr8	8q23.2	2	0	0	2	Amplification	2	0	42496T: 3.5; 42495T: 3.5;
KCNV2	chr9	9p24.2	2	0	1	1	Amplification/Deletio	1	1	42496T: 3.5; 42486T: 0.5;
KCP	chr7	7q32.1	1	0	1	0	Amplification	1	0	42487T: 3.5;
KCTD10	chr12	12q24.11	2	1	0	1	Amplification	2	0	42500T: 3.5; 42473T: 4.5;
KCTD15	chr19	19q13.11	3	0	1	2	Amplification	3	0	42484T: 3.5; 56957T: 4; 42500T: 4.5;
KCTD17	chr22	22q12.3	1	1	0	0	Amplification	1	0	42473T: 3.5;
KCTD7	chr7	7q11.21	1	0	0	1	Amplification	1	0	42501T: 3.5;
KDELR2	chr7	7p22.1	1	1	0	0	Amplification	1	0	42473T: 4.5;
KDELR3	chr22	22q13.1	1	1	0	0	Amplification	1	0	42473T: 3.5;
KDM1A	chr1	1p36.12	1	1	0	0	Amplification	1	0	42473T: 3.5;
KDM2A	chr11	11q13.2	1	1	0	0	Amplification	1	0	42473T: 3.5;
KDM2B	chr12	12q24.31	1	0	0	1	Amplification	1	0	42500T: 3.5;
KDM3A	chr2	2p11.2	2	0	0	2	Amplification	2	0	42493T: 3.5; 42500T: 3.5;
KDM4C	chr9	9p24.1	1	0	0	1	Amplification	1	0	42496T: 3.5;
KDM5A	chr12	12p13.33	1	0	0	1	Amplification	1	0	42500T: 3.5;
KDM5B	chr1	1q32.1	1	1	0	0	Amplification	1	0	42473T: 4;
KEL	chr7	7q34	1	0	1	0	Amplification	1	0	42487T: 3.5;
KHDRBS1	chr1	1p35.2	1	1	0	0	Amplification	1	0	42473T: 3.5;
KHDRBS3	chr8	8q24.23	2	0	0	2	Amplification	2	0	42496T: 3.5; 42495T: 3.5;
KHK	chr2	2p23.3	1	0	0	1	Amplification	1	0	42500T: 3.5;
KIAA0020	chr9	9p24.2	2	0	1	1	Amplification/Deletio	1	1	42496T: 3.5; 42486T: 0.5;
KIAA0087	chr7	7p15.2	1	1	0	0	Amplification	1	0	42473T: 4;
KIAA0195	chr17	17q25.1	1	1	0	0	Amplification	1	0	42473T: 4;
KIAA0196	chr8	8q24.13	4	1	1	2	Amplification	4	0	42484T: 3.5; 42495T: 3.5; 42496T: 3.5; 42475T: 3.5;

Mangalaparthi *et al.* , 2020. Mutational landscape of esophageal squamous cell carcinoma in an Indian cohort
Supplementary Table 7A. List of copy number alterations and affected genes in ESCC patients

Gene	Chromosome	Cytoband	Recurrence	Recurrence in smoker cohort	Recurrence in chewer cohort	Recurrence in No habit cohort	State	Samples with CNA gain	Samples with CNA loss	File info with CNA fold
KIAA0226	chr3	3q29	9	1	3	5	Amplification	9	0	56957T: 6; 42474T: 3.5; 42495T: 4; 42498T: 3.5; 42484T: 4; 42493T: 5; 42487T: 3.5; 42492T: 3.5; 42482T: 3.5;
KIAA0247	chr14	14q24.1	1	0	0	1	Amplification	1	0	42494T: 4;
KIAA0355	chr19	19q13.11	3	0	1	2	Amplification	3	0	42500T: 4.5; 42484T: 3.5; 56957T: 4;
KIAA0391	chr14	14q13.2	2	1	0	1	Amplification	2	0	42500T: 4.5; 42476T: 5;
KIAA0430	chr16	16p13.11	2	1	0	1	Amplification	2	0	42495T: 4; 42473T: 4;
KIAA0586	chr14	14q23.1	1	0	0	1	Amplification	1	0	42494T: 4;
KIAA0895	chr7	7p14.2	1	1	0	0	Amplification	1	0	42473T: 4.5;
KIAA0907	chr1	1q22	1	1	0	0	Amplification	1	0	42473T: 4.5;
KIAA0947	chr5	5p15.32	4	1	1	2	Amplification	4	0	42493T: 4; 42475T: 3.5; 42486T: 3.5; 42496T: 3.5;
KIAA1147	chr7	7q34	1	0	1	0	Amplification	1	0	42487T: 3.5;
KIAA1211L	chr2	2q11.2	1	0	0	1	Amplification	1	0	42493T: 3.5;
KIAA1239	chr4	4p14	1	1	0	0	Amplification	1	0	42473T: 4.5;
KIAA1244	chr6	6q23.3-q24.1	1	0	0	1	Amplification	1	0	42493T: 3.5;
KIAA1257	chr3	3q21.3	3	0	1	2	Amplification	3	0	42487T: 3.5; 42493T: 3.5; 42496T: 3.5;
KIAA1324L	chr7	7q21.12	2	0	2	0	Amplification	3	0	42483T: 6,3.5; 42487T: 3.5;
KIAA1377	chr11	11q22.1	1	0	0	1	Amplification	1	0	56958T: 5.5;
KIAA1407	chr3	3q13.31	1	0	0	1	Amplification	1	0	42496T: 3.5;
KIAA1429	chr8	8q22.1	2	0	0	2	Amplification	2	0	42496T: 3.5; 42495T: 3.5;
KIAA1432	chr9	9p24.1	2	0	0	2	Amplification	2	0	42496T: 3.5; 42498T: 14;
KIAA1467	chr12	12p13.1	1	0	0	1	Amplification	1	0	42500T: 4.5;
KIAA1549	chr7	7q34	1	0	1	0	Amplification	1	0	42487T: 3.5;
KIAA1551	chr12	12p11.21	1	0	0	1	Amplification	1	0	42500T: 12.5;
KIAA1586	chr6	6p12.1	1	0	0	1	Amplification	1	0	42497T: 3.5;
KIAA1614	chr1	1q25.3	1	0	0	1	Amplification	1	0	42493T: 4;
KIAA1715	chr2	2q31.1	1	0	0	1	Amplification	1	0	42493T: 3.5;
KIAA1737	chr14	14q24.3	2	0	0	2	Amplification	2	0	56957T: 3.5; 42494T: 4;
KIAA1755	chr20	20q11.23	1	1	0	0	Amplification	1	0	42473T: 3.5;
KIAA1841	chr2	2p15	3	0	1	2	Amplification	3	0	56957T: 3.5; 42484T: 4.5; 42500T: 3.5;
KIAA1875	chr8	8q24.3	3	0	1	2	Amplification	3	0	42495T: 4.5; 42483T: 3.5; 42496T: 4;
KIAA1919	chr6	6q21	1	0	0	1	Amplification	1	0	42496T: 3.5;
KIAA1984	chr9	9q34.3	1	0	0	1	Amplification	1	0	56957T: 3.5;
KIAA2018	chr3	3q13.2	1	0	0	1	Amplification	1	0	42496T: 3.5;
KIAA2026	chr9	9p24.1	2	0	0	2	Amplification	2	0	42498T: 14; 42496T: 3.5;
KIDINS220	chr2	2p25.1	1	0	0	1	Amplification	1	0	42500T: 3.5;
KIF13B	chr8	8p12	1	0	1	0	Amplification	1	0	42482T: 3.5;
KIF17	chr1	1p36.12	1	1	0	0	Amplification	1	0	42473T: 4;
KIF1A	chr2	2q37.3	2	2	0	0	Amplification/Deletio	1	1	42473T: 4; 42474T: 0.5;
KIF21A	chr12	12q12	1	0	0	1	Amplification	1	0	42500T: 3.5;
KIF2B	chr17	17q22	1	0	0	1	Amplification	1	0	42497T: 3.5;

Mangalaparthi *et al.* , 2020. Mutational landscape of esophageal squamous cell carcinoma in an Indian cohort
Supplementary Table 7A. List of copy number alterations and affected genes in ESCC patients

Gene	Chromosome	Cytoband	Recurrence	Recurrence in smoker cohort	Recurrence in chewer cohort	Recurrence in No habit cohort	State	Samples with CNA gain	Samples with CNA loss	File info with CNA fold
KIF3B	chr20	20q11.21	2	1	0	1	Amplification	2	0	42473T: 4.5; 42496T: 5;
KIF3C	chr2	2p23.3	1	0	0	1	Amplification	1	0	42500T: 3.5;
KIF7	chr15	15q26.1	1	1	0	0	Amplification	1	0	42473T: 3.5;
KIFC2	chr8	8q24.3	5	0	1	4	Amplification	5	0	42494T: 3.5; 42495T: 4.5; 56957T: 3.5; 42483T: 3.5; 42496T: 4;
KIRREL	chr1	1q23.1	1	1	0	0	Amplification	1	0	42473T: 5;
KIRREL2	chr19	19q13.12	3	0	1	2	Amplification	3	0	42500T: 6.5; 42484T: 3.5; 56957T: 4;
KISS1R	chr19	19p13.3	1	0	0	1	Amplification	1	0	42493T: 3.5;
KL	chr13	13q13.1	1	0	0	1	Amplification	1	0	42497T: 6;
KLC3	chr19	19q13.32	3	1	1	1	Amplification	3	0	56957T: 4; 42484T: 4; 42473T: 3.5;
KLF10	chr8	8q22.3	2	0	0	2	Amplification	2	0	42495T: 3.5; 42496T: 3.5;
KLF11	chr2	2p25.1	1	0	0	1	Amplification	1	0	42500T: 3.5;
KLF14	chr7	7q32.2	1	0	1	0	Amplification	1	0	42487T: 3.5;
KLF15	chr3	3q21.3	1	0	0	1	Amplification	1	0	42496T: 3.5;
KLF3	chr4	4p14	1	1	0	0	Amplification	1	0	42473T: 3.5;
KLHDC1	chr14	14q21.3	1	1	0	0	Amplification	1	0	42473T: 3.5;
KLHDC10	chr7	7q32.2	1	0	1	0	Amplification	1	0	42487T: 3.5;
KLHDC2	chr14	14q21.3	1	1	0	0	Amplification	1	0	42473T: 3.5;
KLHDC7A	chr1	1p36.13	1	1	0	0	Amplification	1	0	42473T: 4;
KLHL22	chr22	22q11.21	2	1	0	1	Amplification	2	0	42497T: 3.5; 42477T: 4;
KLHL24	chr3	3q27.1	10	1	3	6	Amplification	10	0	42497T: 4; 56957T: 5; 42474T: 3.5; 42495T: 4; 42498T: 3.5; 42484T: 4; 42482T: 3.5; 42493T: 3.5; 42487T: 3.5; 42492T: 3.5;
KLHL28	chr14	14q21.2	1	0	0	1	Amplification	1	0	42500T: 5.5;
KLHL29	chr2	2p24.1	1	0	0	1	Amplification	1	0	42500T: 3.5;
KLHL30	chr2	2q37.3	1	1	0	0	Amplification	1	0	42473T: 3.5;
KLHL35	chr11	11q13.4	2	0	0	2	Amplification	2	0	42492T: 4; 42498T: 6.5;
KLHL38	chr8	8q24.13	4	1	1	2	Amplification	4	0	42495T: 3.5; 42484T: 3.5; 42475T: 3.5; 42496T: 3.5;
KLHL42	chr12	12p11.22	2	0	0	2	Amplification	2	0	42494T: 4; 42500T: 6;
KLHL6	chr3	3q27.1	10	1	3	6	Amplification	10	0	42482T: 3.5; 42487T: 3.5; 42493T: 3.5; 42492T: 3.5; 42497T: 4; 56957T: 5; 42495T: 4; 42474T: 3.5; 42498T: 3.5; 42484T: 4;
KLHL7	chr7	7p15.3	1	1	0	0	Amplification	1	0	42473T: 4;
KLHL9	chr9	9p21.3	1	1	0	0	Deletion	0	1	42475T: 0.5;
KLRG2	chr7	7q34	1	0	1	0	Amplification	1	0	42487T: 3.5;
KMT2D	chr12	12q13.12	1	0	0	1	Amplification	1	0	42500T: 3.5;
KMT2E	chr7	7q22.3	4	0	1	3	Amplification	4	0	42497T: 4; 42501T: 3.5; 42493T: 3.5; 42487T: 3.5;
KNG1	chr3	3q27.3	10	1	3	6	Amplification	10	0	42482T: 3.5; 42493T: 3.5; 42487T: 3.5; 42492T: 3.5; 42497T: 4; 42495T: 4; 42474T: 3.5; 56957T: 4.5; 42484T: 4; 42498T: 3.5;
KNOP1	chr16	16p12.3	1	1	0	0	Amplification	1	0	42473T: 4;

Mangalaparthi *et al.* , 2020. Mutational landscape of esophageal squamous cell carcinoma in an Indian cohort
Supplementary Table 7A. List of copy number alterations and affected genes in ESCC patients

Gene	Chromosome	Cytoband	Recurrence	Recurrence in smoker cohort	Recurrence in chewer cohort	Recurrence in No habit cohort	State	Samples with CNA gain	Samples with CNA loss	File info with CNA fold
KNSTRN	chr15	15q15.1	1	1	0	0	Amplification	1	0	42473T: 3.5;
KNTC1	chr12	12q24.31	1	0	0	1	Amplification	1	0	42500T: 3.5;
KPNA1	chr3	3q21.1	1	0	0	1	Amplification	1	0	42496T: 3.5;
KPNA4	chr3	3q25.33	8	1	2	5	Amplification	8	0	42484T: 4; 42495T: 4; 42474T: 3.5; 56957T: 4; 42497T: 3.5; 42492T: 3.5; 42487T: 3.5; 42493T: 3.5;
KPNA6	chr1	1p35.2	1	1	0	0	Amplification	1	0	42473T: 3.5;
KRAS	chr12	12p12.1	1	0	0	1	Amplification	1	0	42500T: 6;
KRCC1	chr2	2p11.2	1	0	0	1	Amplification	1	0	42500T: 3.5;
KRIT1	chr7	7q21.2	3	0	2	1	Amplification	3	0	42487T: 3.5; 42494T: 3.5; 42483T: 4;
KRT121P	chr12	12q13.13	1	0	0	1	Amplification	1	0	42493T: 4;
KRT18	chr12	12q13.13	1	1	0	0	Amplification	1	0	42473T: 4;
KRT3	chr12	12q13.13	1	1	0	0	Amplification	1	0	42473T: 4;
KRT4	chr12	12q13.13	1	1	0	0	Amplification	1	0	42473T: 4;
KRT7	chr12	12q13.13	1	0	0	1	Amplification	1	0	42493T: 4;
KRT76	chr12	12q13.13	1	1	0	0	Amplification	1	0	42473T: 4;
KRT78	chr12	12q13.13	1	1	0	0	Amplification	1	0	42473T: 4;
KRT79	chr12	12q13.13	1	1	0	0	Amplification	1	0	42473T: 4;
KRT8	chr12	12q13.13	1	1	0	0	Amplification	1	0	42473T: 4;
KRT81	chr12	12q13.13	1	0	0	1	Amplification	1	0	42493T: 4;
KRT83	chr12	12q13.13	1	0	0	1	Amplification	1	0	42493T: 4;
KRT86	chr12	12q13.13	1	0	0	1	Amplification	1	0	42493T: 4;
KRTCAP2	chr1	1q22	1	1	0	0	Amplification	1	0	42473T: 6.5;
KRTCAP3	chr2	2p23.3	1	0	0	1	Amplification	1	0	42500T: 3.5;
KRTDAP	chr19	19q13.12	3	0	1	2	Amplification	3	0	42500T: 6.5; 56957T: 4; 42484T: 3.5;
KSR2	chr12	12q24.22-q2	1	0	0	1	Amplification	1	0	42500T: 3.5;
KTN1	chr14	14q22.3	1	0	0	1	Amplification	1	0	42494T: 4;
KY	chr3	3q22.2	4	0	1	3	Amplification	4	0	42492T: 3.5; 42487T: 3.5; 42493T: 3.5; 42496T: 3.5;
L2HGDH	chr14	14q21.3	1	0	0	1	Amplification	1	0	42496T: 3.5;
L3HYPDH	chr14	14q23.1	1	0	0	1	Amplification	1	0	42494T: 4;
L3MBTL1	chr20	20q13.12	1	1	0	0	Amplification	1	0	42473T: 3.5;
L3MBTL4	chr18	18p11.31	1	0	0	1	Amplification	1	0	42493T: 3.5;
LACE1	chr6	6q21	1	0	0	1	Amplification	1	0	42496T: 3.5;
LACRT	chr12	12q13.2	1	0	0	1	Amplification	1	0	42494T: 3.5;
LACTB2	chr8	8q13.3	2	0	0	2	Amplification	2	0	42495T: 3.5; 42496T: 3.5;
LACTBL1	chr1	1p36.12	1	1	0	0	Amplification	1	0	42473T: 3.5;
LAG3	chr12	12p13.31	2	1	0	1	Amplification	2	0	42473T: 3.5; 42494T: 3.5;
LALBA	chr12	12q13.11	1	0	0	1	Amplification	1	0	42500T: 3.5;
LAMA1	chr18	18p11.31	1	0	0	1	Amplification	1	0	42493T: 3.5;
LAMA3	chr18	6q21	1	1	0	0	Amplification	2	0	42481T: 6,4;
LAMA4	chr6	6q21	1	0	0	1	Amplification	1	0	42496T: 3.5;
LAMA5	chr20	20q13.33	1	1	0	0	Amplification	1	0	42473T: 6;

Mangalaparthi *et al.* , 2020. Mutational landscape of esophageal squamous cell carcinoma in an Indian cohort
Supplementary Table 7A. List of copy number alterations and affected genes in ESCC patients

Gene	Chromosome	Cytoband	Recurrence	Recurrence in smoker cohort	Recurrence in chewer cohort	Recurrence in No habit cohort	State	Samples with CNA gain	Samples with CNA loss	File info with CNA fold
LAMB1	chr7	7q31.1	4	0	1	3	Amplification	4	0	42493T: 5; 42487T: 3.5; 42501T: 3.5; 42497T: 4;
LAMB4	chr7	7q31.1	4	0	1	3	Amplification	4	0	42497T: 4; 42501T: 3.5; 42487T: 3.5; 42493T: 5;
LAMC3	chr9	9q34.12	1	0	1	0	Amplification	1	0	42484T: 4;
LAMP1	chr13	13q34	1	0	0	1	Amplification	1	0	56957T: 3.5;
LAMP3	chr3	3q27.1	9	1	3	5	Amplification	9	0	42484T: 4; 42498T: 3.5; 42495T: 4; 42474T: 3.5; 56957T: 5; 42492T: 3.5; 42493T: 3.5; 42487T: 3.5; 42482T: 3.5;
LAMTOR1	chr11	11q13.4	8	3	0	5	Amplification	8	0	56957T: 3.5; 42498T: 5; 42478T: 4.5; 42501T: 6; 42475T: 5; 42493T: 3.5; 42492T: 4; 42476T: 4;
LAMTOR2	chr1	1q22	2	1	0	1	Amplification	2	0	42493T: 4; 42473T: 4.5;
LANCL2	chr7	7p11.2	3	0	1	2	Amplification	3	0	56957T: 5; 42497T: 6.5; 42483T: 4;
LAPTM4A	chr2	2p24.1	2	1	0	1	Amplification	2	0	42473T: 4; 42500T: 3.5;
LAPTM4B	chr8	8q22.1	2	0	0	2	Amplification	2	0	42495T: 3.5; 42496T: 3.5;
LAPTM5	chr1	1p35.2	1	1	0	0	Amplification	1	0	42473T: 3.5;
LARP4	chr12	12q13.12	1	0	0	1	Amplification	1	0	42500T: 3.5;
LASP1	chr17	17q12	1	1	0	0	Amplification	1	0	42473T: 3.5;
LBH	chr2	6p21.31	1	0	0	1	Amplification	1	0	42500T: 3.5;
LBP	chr20	20q11.23	1	1	0	0	Amplification	1	0	42473T: 3.5;
LBX2	chr2	2p13.1	1	0	0	1	Amplification	1	0	42500T: 3.5;
LCA5L	chr21	21q22.2	1	1	0	0	Amplification	1	0	42473T: 3.5;
LCK	chr1	1p35.2	1	1	0	0	Amplification	1	0	42473T: 4;
LCLAT1	chr2	2p23.1	1	0	0	1	Amplification	1	0	42500T: 3.5;
LCMT1	chr16	16p12.1	1	1	0	0	Amplification	1	0	42473T: 4;
LCN1	chr9	9q34.3	1	0	1	0	Amplification	1	0	42484T: 4.5;
LCN10	chr9	9q34.3	1	0	0	1	Amplification	1	0	56957T: 3.5;
LCN12	chr9	9q34.3	2	1	0	1	Amplification	2	0	56957T: 3.5; 42473T: 5;
LCN15	chr9	9q34.3	1	0	0	1	Amplification	1	0	56957T: 3.5;
LCN6	chr9	9q34.3	1	0	0	1	Amplification	1	0	56957T: 3.5;
LCN8	chr9	9q34.3	1	0	0	1	Amplification	1	0	56957T: 3.5;
LCN9	chr9	9q34.3	2	0	1	1	Amplification	2	0	56957T: 3.5; 42484T: 4.5;
LCNL1	chr9	9q34.3	2	1	0	1	Amplification	2	0	56957T: 3.5; 42473T: 5;
LDHB	chr12	12p12.1	1	0	0	1	Amplification	1	0	42500T: 6;
LDLRAD2	chr1	1p36.12	1	1	0	0	Amplification	1	0	42473T: 4;
LEFTY1	chr1	1q42.12	1	0	0	1	Amplification	1	0	42493T: 3.5;
LEFTY2	chr1	1q42.12	1	0	0	1	Amplification	1	0	42493T: 3.5;
LEKR1	chr3	3q25.31	8	1	2	5	Amplification	8	0	42497T: 5.5; 42474T: 3.5; 42495T: 4; 56957T: 4; 42484T: 3.5; 42493T: 3.5; 42487T: 3.5; 42492T: 3.5;
LELP1	chr1	1q21.3	1	0	0	1	Amplification	1	0	42493T: 4;
LENEP	chr1	1q21.3	2	1	0	1	Amplification	2	0	42473T: 6.5; 42496T: 3.5;
LEP	chr7	7q32.1	1	0	1	0	Amplification	1	0	42487T: 3.5;
LEPRE1	chr1	1p34.2	2	0	0	2	Amplification	2	0	42496T: 5.5; 42493T: 3.5;

Mangalaparthi *et al.* , 2020. Mutational landscape of esophageal squamous cell carcinoma in an Indian cohort
Supplementary Table 7A. List of copy number alterations and affected genes in ESCC patients

Gene	Chromosome	Cytoband	Recurrence	Recurrence in smoker cohort	Recurrence in chewer cohort	Recurrence in No habit cohort	State	Samples with CNA gain	Samples with CNA loss	File info with CNA fold
LEPREL1	chr3	3q28	10	1	3	6	Amplification	10	0	42482T: 3.5; 42487T: 3.5; 42493T: 3.5; 42492T: 3.5; 42497T: 5; 42495T: 4; 42474T: 3.5; 56957T: 4.5; 42484T: 4; 42498T: 3.5;
LEPROTL1	chr8	8p12	1	0	1	0	Amplification	1	0	42482T: 3.5;
LETM2	chr8	8p11.23	1	0	1	0	Amplification	1	0	42482T: 3.5;
LETMD1	chr12	12q13.12	2	0	0	2	Amplification	2	0	42500T: 3.5; 42494T: 3.5;
LEUTX	chr19	19q13.2	2	0	0	2	Amplification	2	0	56957T: 4; 42500T: 7.5;
LFNG	chr7	7p22.3	1	1	0	0	Amplification	1	0	42473T: 5;
LGALS1	chr22	22q13.1	1	1	0	0	Amplification	1	0	42473T: 3.5;
LGALS13	chr19	19q13.2	2	0	0	2	Amplification	2	0	56957T: 4; 42500T: 7.5;
LGALS14	chr19	19q13.2	2	0	0	2	Amplification	2	0	56957T: 4; 42500T: 7.5;
LGALS16	chr19	19q13.2	2	0	0	2	Amplification	2	0	42500T: 7.5; 56957T: 4;
LGALS17A	chr19	19q13.2	2	0	0	2	Amplification	2	0	56957T: 4; 42500T: 7.5;
LGALS2	chr22	14q22.3	1	1	0	0	Amplification	1	0	42473T: 3.5;
LGALS3	chr14	14q22.3	1	0	0	1	Amplification	1	0	42494T: 4;
LGALS4	chr19	19q13.2	2	0	0	2	Amplification	2	0	42500T: 6.5; 56957T: 4;
LGALS7	chr19	19q13.2	2	0	0	2	Amplification	2	0	56957T: 4; 42500T: 6.5;
LGALS7B	chr19	19q13.2	2	0	0	2	Amplification	2	0	56957T: 4; 42500T: 6.5;
LGALSL	chr2	2p14	2	0	0	2	Amplification	2	0	42500T: 3.5; 56957T: 3.5;
LGI4	chr19	19q13.12|19(	3	0	1	2	Amplification	3	0	42500T: 6.5; 56957T: 4; 42484T: 3.5;
LGR5	chr12	12q21.1	1	0	0	1	Amplification	1	0	42501T: 5.5;
LHCGR	chr2	2p16.3	2	0	1	1	Amplification	2	0	42484T: 4.5; 42500T: 3.5;
LHFPL3	chr7	7q22.2-q22.3	4	0	1	3	Amplification	4	0	42501T: 3.5; 42497T: 4; 42493T: 3.5; 42487T: 3.5;
LHX3	chr9	9q34.3	1	0	0	1	Amplification	1	0	56957T: 3.5;
LHX4	chr1	1q25.2	1	0	0	1	Amplification	1	0	42493T: 4;
LHX5	chr12	12q24.13	1	0	0	1	Amplification	1	0	42500T: 3.5;
LIF	chr22	22q12.2	1	1	0	0	Amplification	1	0	42473T: 3.5;
LIFR	chr5	5p13.1	6	1	3	2	Amplification	6	0	42484T: 3.5; 42493T: 3.5; 42496T: 3.5; 42486T: 3.5; 42483T: 3.5; 42475T: 3.5;
LIMA1	chr12	12q13.12	1	0	0	1	Amplification	1	0	42500T: 3.5;
LIME1	chr20	20q13.33	1	1	0	0	Amplification	1	0	42473T: 6;
LIN37	chr19	19q13.12	3	0	1	2	Amplification	3	0	42500T: 6.5; 42484T: 3.5; 56957T: 4;
LIN52	chr14	14q24.3	2	0	0	2	Amplification	2	0	56957T: 3.5; 42494T: 4;
LINC00173	chr12	12q24.22	1	0	0	1	Amplification	1	0	42500T: 3.5;
LINC00174	chr7	7q11.21	1	0	0	1	Amplification	1	0	42501T: 3.5;
LINC00176	chr20	20q13.33	1	1	0	0	Amplification	1	0	42473T: 6;
LINC00293	chr8	8q11.1	3	0	1	2	Amplification	3	0	42496T: 3.5; 42494T: 3.5; 42484T: 3.5;
LINC00452	chr13	13q34	1	0	0	1	Amplification	1	0	56957T: 3.5;
LINC00461	chr5	5q14.3	1	1	0	0	Deletion	0	1	42476T: 0.5;
LINC00470	chr18	18p11.32	3	0	0	3	Amplification	3	0	56957T: 8; 42493T: 3.5; 42500T: 4.5;
LINC00477	chr12	12p12.1	1	0	0	1	Amplification	1	0	42500T: 6;

Mangalaparthi *et al.* , 2020. Mutational landscape of esophageal squamous cell carcinoma in an Indian cohort
Supplementary Table 7A. List of copy number alterations and affected genes in ESCC patients

Gene	Chromosome	Cytoband	Recurrence	Recurrence in smoker cohort	Recurrence in chewer cohort	Recurrence in No habit cohort	State	Samples with CNA gain	Samples with CNA loss	File info with CNA fold
LINC00479	chr21	21q22.3	1	1	0	0	Amplification	1	0	42473T: 3.5;
LINC00483	chr17	17q21.33	1	1	0	0	Amplification	1	0	42473T: 4.5;
LINC00529	chr8	8p23-p22	1	0	1	0	Amplification	1	0	42486T: 3.5;
LINC00552	chr13	13q34	1	0	0	1	Amplification	1	0	56957T: 3.5;
LINC00583	chr9	9p23	1	0	0	1	Amplification	1	0	42498T: 14.5;
LINC00599	chr8	8p23.1	1	0	1	0	Amplification	1	0	42486T: 3.5;
LINC00643	chr14	14q23.2	1	0	0	1	Amplification	1	0	42494T: 4;
LINC00657	chr20	20q11.23	1	1	0	0	Amplification	1	0	42473T: 3.5;
LINC00667	chr18	18p11.31	1	0	0	1	Amplification	1	0	56957T: 8;
LINC00668	chr18	18p11.31	1	0	0	1	Amplification	1	0	42493T: 3.5;
LINC00935	chr12	12q13.12	1	0	0	1	Amplification	1	0	42500T: 3.5;
LINC00941	chr12	12p11.21	1	0	0	1	Amplification	1	0	42500T: 8;
LINC00961	chr9	9p13.3	2	1	0	1	Amplification	2	0	42473T: 3.5; 42501T: 3.5;
LINC00964	chr8	8q24.13	4	1	1	2	Amplification	4	0	42496T: 3.5; 42475T: 3.5; 42484T: 3.5; 42495T: 3.5;
LINC00966	chr8	8q12.3	2	0	0	2	Amplification	2	0	42496T: 3.5; 42495T: 3.5;
LINC00967	chr8	8q13.1	3	0	0	3	Amplification	3	0	42496T: 3.5; 42497T: 4; 42495T: 3.5;
LINC00969	chr3	3q29	9	1	3	5	Amplification	9	0	42498T: 3.5; 42484T: 4; 56957T: 6; 42495T: 4; 42474T: 3.5; 42482T: 3.5; 42492T: 3.5; 42493T: 3.5; 42487T: 3.5;
LIPH	chr3	3q27.2	10	1	3	6	Amplification	10	0	42498T: 3.5; 42484T: 4; 56957T: 4.5; 42495T: 4; 42474T: 3.5; 42497T: 4; 42492T: 3.5; 42487T: 3.5; 42493T: 3.5; 42482T: 3.5;
LIPT1	chr2	2q11.2	1	0	0	1	Amplification	1	0	42493T: 3.5;
LIPT2	chr11	11q13.4	5	2	0	3	Amplification	5	0	42492T: 4; 42498T: 8; 56957T: 3.5; 42476T: 4; 42475T: 7.5;
LITAF	chr16	16p13.13	2	1	0	1	Amplification	2	0	42495T: 4; 42473T: 9.5;
LIX1L	chr1	1q21.1	1	1	0	0	Amplification	1	0	42473T: 4;
LMAN1L	chr15	15q24.1	1	1	0	0	Amplification	1	0	42473T: 3.5;
LMAN2L	chr2	2q11.2	2	1	0	1	Amplification	2	0	42493T: 4.5; 42473T: 3.5;
LMBR1L	chr12	12q13.12	1	0	0	1	Amplification	1	0	42500T: 3.5;
LMBRD2	chr5	5p13.2	4	1	1	2	Amplification	4	0	42475T: 3.5; 42496T: 3.5; 42486T: 3.5; 42493T: 3.5;
LMF1	chr16	16p13.3	1	0	1	0	Amplification	1	0	42483T: 3.5;
LMLN	chr3	3q29	9	1	3	5	Amplification	9	0	42482T: 3.5; 42493T: 4; 42487T: 3.5; 42492T: 3.5; 56957T: 6; 42474T: 3.5; 42495T: 4; 42498T: 3.5; 42484T: 3.5;
LMNA	chr1	1q22	2	1	0	1	Amplification	2	0	42473T: 4.5; 42493T: 4;
LMOD2	chr7	7q31.32	2	0	1	1	Amplification	2	0	42487T: 3.5; 42493T: 3.5;
LNP1	chr3	3q12.2	1	1	0	0	Amplification	1	0	42476T: 3.5;
LOH12CR1	chr12	12p13.2	1	0	0	1	Amplification	1	0	42500T: 4.5;
LOH12CR2	chr12	12p13.2	1	0	0	1	Amplification	1	0	42500T: 4.5;
LONRF2	chr2	2q11.2	1	0	0	1	Amplification	1	0	42493T: 3.5;

Mangalaparthi *et al.* , 2020. Mutational landscape of esophageal squamous cell carcinoma in an Indian cohort
Supplementary Table 7A. List of copy number alterations and affected genes in ESCC patients

Gene	Chromosome	Cytoband	Recurrence	Recurrence in smoker cohort	Recurrence in chewer cohort	Recurrence in No habit cohort	State	Samples with CNA gain	Samples with CNA loss	File info with CNA fold
LOR	chr1	8p21.3	1	0	0	1	Amplification	1	0	42493T: 4;
LOXL1	chr15	15q24.1	1	1	0	0	Amplification	1	0	42473T: 3.5;
LOXL3	chr2	2p13.1	1	0	0	1	Amplification	1	0	42500T: 3.5;
LPCAT1	chr5	5p15.33	5	2	1	2	Amplification	5	0	42475T: 3.5; 42486T: 3.5; 42496T: 4; 42473T: 3.5; 42493T: 3.5;
LPIN1	chr2	2p25.1	1	0	0	1	Amplification	1	0	42500T: 3.5;
LPIN2	chr18	18p11.31	2	0	0	2	Amplification	2	0	42500T: 4.5; 56957T: 8;
LPIN3	chr20	20q12	1	1	0	0	Amplification	1	0	42473T: 3.5;
LPO	chr17	17q22	1	0	0	1	Amplification	1	0	42497T: 4;
LPP	chr3	3q27.3-q28	10	1	3	6	Amplification	10	0	42484T: 4; 42498T: 3.5; 42474T: 3.5; 42495T: 4; 56957T: 4.5; 42497T: 5; 42492T: 3.5; 42487T: 3.5; 42493T: 3.5; 42482T: 3.5;
LPPR3	chr19	19p13.3	1	0	0	1	Amplification	1	0	42493T: 3.5;
LRCH3	chr3	3q29	9	1	3	5	Amplification	9	0	42495T: 4; 42474T: 3.5; 56957T: 6; 42484T: 5.5; 42498T: 3.5; 42493T: 4; 42487T: 3.5; 42492T: 3.5; 42482T: 3.5;
LRCOL1	chr12	12q24.33	1	0	0	1	Amplification	1	0	56957T: 4;
LRFN1	chr19	19q13.2	2	0	0	2	Amplification	2	0	56957T: 4; 42500T: 6.5;
LRFN3	chr19	19q13.12	3	0	1	2	Amplification	3	0	42500T: 6.5; 42484T: 3.5; 56957T: 4;
LRFN4	chr11	11q13.2	2	1	0	1	Amplification	2	0	56957T: 5.5; 42473T: 3.5;
LRFN5	chr14	14q21.1	1	0	0	1	Amplification	1	0	42500T: 5.5;
LRGUK	chr7	7q33	1	0	1	0	Amplification	1	0	42487T: 3.5;
LRMP	chr12	12p12.1	1	0	0	1	Amplification	1	0	42500T: 6;
LRP10	chr14	14q11.2	1	0	0	1	Amplification	1	0	42496T: 4;
LRP12	chr8	8q22.3	2	0	0	2	Amplification	2	0	42496T: 3.5; 42495T: 3.5;
LRP1B	chr2	2q22.1-q22.2	1	1	0	0	Deletion	0	1	42476T: 0;
LRP3	chr19	19q13.11	3	0	1	2	Amplification	3	0	56957T: 4; 42484T: 3.5; 42500T: 4.5;
LRP4	chr11	11p11.2	1	1	0	0	Amplification	1	0	42473T: 3.5;
LRP5	chr11	11q13.2	2	1	0	1	Amplification	2	0	42476T: 29.5; 56957T: 5;
LRP6	chr12	12p13.2	1	0	0	1	Amplification	1	0	42500T: 4.5;
LRPPRC	chr2	2p21	2	0	1	1	Amplification	2	0	42484T: 3.5; 42500T: 3.5;
LRR1	chr14	14q21.3	1	1	0	0	Amplification	1	0	42473T: 3.5;
LRRC10	chr12	12q15	2	0	0	2	Amplification	2	0	42501T: 5.5; 42500T: 5;
LRRC14	chr8	8q24.3	5	0	1	4	Amplification	5	0	42496T: 4; 42483T: 3.5; 56957T: 3.5; 42494T: 3.5; 42495T: 4.5;
LRRC14B	chr5	5p15.33	5	2	1	2	Amplification	5	0	42475T: 3.5; 42486T: 3.5; 42496T: 4; 42473T: 3.5; 42493T: 3.5;
LRRC15	chr3	3q29	9	1	3	5	Amplification	9	0	42492T: 3.5; 42493T: 3.5; 42487T: 3.5; 42482T: 3.5; 42498T: 3.5; 42484T: 4; 56957T: 4.5; 42474T: 3.5; 42495T: 4;
LRRC17	chr7	7q22.1	3	0	1	2	Amplification	3	0	42487T: 3.5; 42493T: 3.5; 42501T: 3.5;

Mangalaparthi *et al.* , 2020. Mutational landscape of esophageal squamous cell carcinoma in an Indian cohort
Supplementary Table 7A. List of copy number alterations and affected genes in ESCC patients

Gene	Chromosome	Cytoband	Recurrence	Recurrence in smoker cohort	Recurrence in chewer cohort	Recurrence in No habit cohort	State	Samples with CNA gain	Samples with CNA loss	File info with CNA fold
LRRC23	chr12	12p13.31	1	0	0	1	Amplification	1	0	42494T: 3.5;
LRRC30	chr18	18p11.23	1	0	0	1	Amplification	1	0	42493T: 3.5;
LRRC31	chr3	3q26.2	9	1	3	5	Amplification	9	0	42482T: 3.5; 42493T: 3.5; 42487T: 3.5; 42492T: 3.5; 42500T: 8; 42474T: 3.5; 42495T: 4; 56957T: 4; 42484T: 4;
LRRC32	chr11	11q13.5	2	0	0	2	Amplification	2	0	42497T: 3.5; 42496T: 3.5;
LRRC34	chr3	3q26.2	9	1	3	5	Amplification	9	0	42487T: 3.5; 42493T: 3.5; 42492T: 3.5; 42482T: 3.5; 56957T: 4; 42474T: 3.5; 42495T: 4; 42484T: 4; 42500T: 8;
LRRC37B	chr17	17q11.2	1	1	0	0	Amplification	1	0	42473T: 3.5;
LRRC3C	chr17	17q21.1	1	0	0	1	Amplification	1	0	42497T: 3.5;
LRRC4	chr7	7q32.1	1	0	1	0	Amplification	1	0	42487T: 3.5;
LRRC43	chr12	12q24.31	1	0	0	1	Amplification	1	0	42500T: 3.5;
LRRC45	chr17	17q25.3	1	1	0	0	Amplification	1	0	42473T: 3.5;
LRRC58	chr3	3q13.33	1	0	0	1	Amplification	1	0	42496T: 3.5;
LRRC59	chr17	17q21.33	1	1	0	0	Amplification	1	0	42473T: 3.5;
LRRC6	chr8	8q24.22	3	0	1	2	Amplification	3	0	42495T: 3.5; 42484T: 3.5; 42496T: 3.5;
LRRC69	chr8	8q21.3	2	0	0	2	Amplification	2	0	42495T: 3.5; 42496T: 3.5;
LRRC71	chr1	1q23.1	1	1	0	0	Amplification	1	0	42473T: 7;
LRRC72	chr7	7p21.1	1	1	0	0	Amplification	1	0	42473T: 5.5;
LRRC9	chr14	14q23.1	2	0	1	1	Amplification	2	0	42494T: 4; 42483T: 3.5;
LRRCC1	chr8	8q21.2	2	0	0	2	Amplification	2	0	42496T: 3.5; 42495T: 3.5;
LRRD1	chr7	7q21.2	3	0	2	1	Amplification	3	0	42487T: 3.5; 42494T: 3.5; 42483T: 4;
LRRIQ4	chr3	3q26.2	9	1	3	5	Amplification	9	0	42500T: 8; 56957T: 4; 42474T: 3.5; 42495T: 4; 42484T: 4; 42482T: 3.5; 42493T: 3.5; 42487T: 3.5; 42492T: 3.5;
LRRK2	chr12	12q12	1	0	0	1	Amplification	1	0	42500T: 3.5;
LRRN3	chr7	7q31.1	2	0	1	1	Amplification	2	0	42487T: 3.5; 42501T: 3.5;
LRRTM1	chr2	2p12	1	0	0	1	Amplification	1	0	42500T: 3.5;
LRRTM4	chr2	2p12	1	0	0	1	Amplification	1	0	42500T: 3.5;
LRTOMT	chr11	11q13.4	8	3	0	5	Amplification	8	0	42493T: 3.5; 42492T: 4; 42476T: 4; 56957T: 3.5; 42478T: 4.5; 42498T: 5; 42475T: 5; 42501T: 6;
LRWD1	chr7	7q22.1	2	0	0	2	Amplification	2	0	42493T: 3.5; 42501T: 3.5;
LSAMP	chr3	3q13.31	1	0	0	1	Amplification	1	0	42496T: 3.5;
LSG1	chr3	3q29	9	1	3	5	Amplification	9	0	56957T: 4.5; 42495T: 4; 42474T: 3.5; 42498T: 3.5; 42484T: 4; 42482T: 3.5; 42487T: 3.5; 42493T: 3.5; 42492T: 3.5;
LSM1	chr8	8p11.23	2	0	1	1	Amplification	2	0	42482T: 3.5; 42493T: 3.5;
LSM14A	chr19	19q13.11	3	0	1	2	Amplification	3	0	42500T: 4.5; 56957T: 4; 42484T: 3.5;
LSM14B	chr20	20q13.33	1	1	0	0	Amplification	1	0	42473T: 6;
LSM2	chr6	6p21.33	1	1	0	0	Amplification	1	0	42473T: 3.5;

Mangalaparthi *et al.* , 2020. Mutational landscape of esophageal squamous cell carcinoma in an Indian cohort
Supplementary Table 7A. List of copy number alterations and affected genes in ESCC patients

Gene	Chromosome	Cytoband	Recurrence	Recurrence in smoker cohort	Recurrence in chewer cohort	Recurrence in No habit cohort	State	Samples with CNA gain	Samples with CNA loss	File info with CNA fold
LSM5	chr7	7p14.3	1	1	0	0	Amplification	1	0	42473T: 4;
LSMEM1	chr7	7q31.1	2	0	1	1	Amplification	2	0	42501T: 3.5; 42487T: 3.5;
LSR	chr19	19q13.12	3	0	1	2	Amplification	3	0	42500T: 6.5; 42484T: 3.5; 56957T: 4;
LST1	chr6	12p12.1	1	1	0	0	Amplification	1	0	42473T: 3.5;
LTA	chr6	6p21.33	1	1	0	0	Amplification	1	0	42473T: 3.5;
LTB	chr6	6p21.33	1	1	0	0	Amplification	1	0	42473T: 3.5;
LTBP1	chr2	2p22.3	1	0	0	1	Amplification	1	0	42500T: 3.5;
LTBP2	chr14	11q13.1	2	0	0	2	Amplification	2	0	42494T: 4; 56957T: 3.5;
LTBP4	chr19	19q13.2	2	0	0	2	Amplification	2	0	56957T: 4; 42500T: 3.5;
LUC7L	chr16	16p13.3	1	0	1	0	Amplification	1	0	42483T: 3.5;
LUC7L2	chr7	7q34	1	0	1	0	Amplification	1	0	42487T: 3.5;
LUC7L3	chr17	17q21.33	1	1	0	0	Amplification	1	0	42473T: 4.5;
LUZP1	chr1	1p36.12	1	1	0	0	Amplification	1	0	42473T: 3.5;
LUZP6	chr7	7q33	1	0	1	0	Amplification	1	0	42487T: 3.5;
LXN	chr3	3q25.32	8	1	2	5	Amplification	8	0	42493T: 3.5; 42487T: 3.5; 42492T: 3.5; 42497T: 3.5; 42474T: 3.5; 42495T: 4; 56957T: 4; 42484T: 3.5;
LY6D	chr8	8q24.3	3	0	1	2	Amplification	3	0	42483T: 3.5; 42496T: 4; 42495T: 4.5;
LY6E	chr8	8q24.3	3	0	1	2	Amplification	3	0	42495T: 4.5; 42483T: 3.5; 42496T: 4;
LY6G5C	chr6	6p21.33	1	1	0	0	Amplification	1	0	42473T: 3.5;
LY6G6C	chr6	6p21.33	1	1	0	0	Amplification	1	0	42473T: 3.5;
LY6G6D	chr6	6p21.33	1	1	0	0	Amplification	1	0	42473T: 3.5;
LY6G6E	chr6	6p21.33	1	1	0	0	Amplification	1	0	42473T: 3.5;
LY6G6F	chr6	6p21.33	1	1	0	0	Amplification	1	0	42473T: 3.5;
LY6H	chr8	8q24.3	3	0	1	2	Amplification	3	0	42496T: 4; 42483T: 3.5; 42495T: 4.5;
LY6K	chr8	8q24.3	3	0	1	2	Amplification	3	0	42483T: 3.5; 42496T: 4; 42495T: 4.5;
LY96	chr8	8q21.11	2	0	0	2	Amplification	2	0	42496T: 3.5; 42495T: 3.5;
LYG1	chr2	2q11.2	1	0	0	1	Amplification	1	0	42493T: 3.5;
LYG2	chr2	2q11.2	1	0	0	1	Amplification	1	0	42493T: 3.5;
LYN	chr8	8q12.1	3	0	1	2	Amplification	3	0	42495T: 3.5; 42496T: 3.5; 42483T: 5;
LYNX1	chr8	8q24.3	3	0	1	2	Amplification	3	0	42495T: 4.5; 42483T: 3.5; 42496T: 4;
LYPD2	chr8	8q24.3	3	0	1	2	Amplification	3	0	42495T: 4.5; 42496T: 4; 42483T: 3.5;
LYPD3	chr19	19q13.31	1	0	0	1	Amplification	1	0	56957T: 4;
LYPD4	chr19	19q13.2	1	1	0	0	Amplification	1	0	42473T: 4.5;
LYPD5	chr19	19q13.31	1	0	0	1	Amplification	1	0	56957T: 4;
LYPLA1	chr8	8q11.23	3	0	0	3	Amplification	3	0	42495T: 3.5; 42494T: 4; 42496T: 3.5;
LYPLA2	chr1	1p36.11	1	1	0	0	Amplification	1	0	42473T: 3.5;
LYRM1	chr16	16p12.3	1	1	0	0	Amplification	1	0	42473T: 4;
LYRM5	chr12	12p12.1	1	0	0	1	Amplification	1	0	42500T: 6;
LYSMD1	chr1	1q21.3	1	1	0	0	Amplification	1	0	42473T: 4.5;
LYZ	chr12	12q15	2	0	0	2	Amplification	2	0	42500T: 5; 42501T: 5.5;
LZTR1	chr22	22q11.21|22	1	1	0	0	Amplification	1	0	42477T: 4;

Mangalaparthi *et al.* , 2020. Mutational landscape of esophageal squamous cell carcinoma in an Indian cohort
Supplementary Table 7A. List of copy number alterations and affected genes in ESCC patients

Gene	Chromosome	Cytoband	Recurrence	Recurrence in smoker cohort	Recurrence in chewer cohort	Recurrence in No habit cohort	State	Samples with CNA gain	Samples with CNA loss	File info with CNA fold
LZTS3	chr20	20p13	1	1	0	0	Amplification	1	0	42473T: 3.5;
M1AP	chr2	2p13.1	1	0	0	1	Amplification	1	0	42500T: 3.5;
MAATS1	chr3	3q13.33	1	0	0	1	Amplification	1	0	42496T: 3.5;
MACC1	chr7	7p21.1	1	1	0	0	Amplification	1	0	42473T: 4;
MAD1L1	chr7	7p22.3	1	1	0	0	Amplification	1	0	42473T: 5;
MADD	chr11	11p11.2	1	1	0	0	Amplification	1	0	42473T: 3.5;
MAF1	chr8	8q24.3	3	0	1	2	Amplification	3	0	42496T: 4; 42483T: 3.5; 42495T: 4.5;
MAFA	chr8	8q24.3	3	0	1	2	Amplification	3	0	42496T: 4; 42483T: 3.5; 42495T: 4.5;
MAFB	chr20	20q12	1	1	0	0	Amplification	1	0	42473T: 3.5;
MAFF	chr22	22q13.1	1	1	0	0	Amplification	1	0	42473T: 3.5;
MAFK	chr7	7p22.3	1	1	0	0	Amplification	1	0	42473T: 5;
MAG	chr19	19q13.12	3	0	1	2	Amplification	3	0	56957T: 4; 42484T: 3.5; 42500T: 6.5;
MAGEF1	chr3	3q27.1	12	2	3	7	Amplification	12	0	42497T: 4; 42473T: 4; 42498T: 3.5; 42484T: 4; 56957T: 5; 42495T: 4; 42474T: 3.5; 42494T: 3.5; 42482T: 3.5; 42492T: 3.5; 42487T: 3.5; 42493T: 3.5;
MAGI2	chr7	7q21.11	1	0	1	0	Amplification	1	0	42487T: 3.5;
MAK16	chr8	8p12	2	0	1	1	Amplification	2	0	42482T: 3.5; 42497T: 4.5;
MAL	chr2	22q13.1-q13	1	1	0	0	Amplification	1	0	42473T: 3.5;
MAL2	chr8	8q24.12	3	1	0	2	Amplification	3	0	42495T: 3.5; 42475T: 3.5; 42496T: 3.5;
MALSU1	chr7	7p15.3	1	1	0	0	Amplification	1	0	42473T: 4;
MAMDC4	chr9	9q34.3	2	1	0	1	Amplification	2	0	42473T: 5; 56957T: 3.5;
MAN1B1	chr9	9q34.3	2	1	0	1	Amplification	2	0	56957T: 3.5; 42473T: 5;
MAN1C1	chr1	1p36.11	1	1	0	0	Amplification	1	0	42473T: 4.5;
MANBAL	chr20	20q11.23	1	1	0	0	Amplification	1	0	42473T: 3.5;
MANSC1	chr12	12p13.2	1	0	0	1	Amplification	1	0	42500T: 4.5;
MANSC4	chr12	12p11.22	2	0	0	2	Amplification	2	0	42500T: 6; 42494T: 4;
MAP1LC3A	chr20	20q11.22	3	1	0	2	Amplification	3	0	42493T: 3.5; 42496T: 5; 42473T: 3.5;
MAP1LC3B2	chr12	12q24.22	1	0	0	1	Amplification	1	0	42500T: 3.5;
MAP3K10	chr19	19q13.2	2	0	0	2	Amplification	2	0	42500T: 7.5; 56957T: 4;
MAP3K13	chr3	3q27.2	10	1	3	6	Amplification	10	0	56957T: 4.5; 42474T: 3.5; 42495T: 4; 42498T: 3.5; 42484T: 4; 42497T: 4; 42493T: 3.5; 42487T: 3.5; 42492T: 3.5; 42482T: 3.5;
MAP3K6	chr1	1p36.11	1	1	0	0	Amplification	1	0	42473T: 5;
MAP3K9	chr14	14q24.2	1	0	0	1	Amplification	1	0	42494T: 4;
MAP4K1	chr19	19q13.2	2	0	0	2	Amplification	2	0	42500T: 6.5; 56957T: 4;
MAP4K3	chr2	2p22.1	1	0	0	1	Amplification	1	0	42500T: 3.5;
MAP4K4	chr2	2q11.2	1	0	0	1	Amplification	1	0	42493T: 3.5;
MAP4K5	chr14	14q22.1	2	0	0	2	Amplification	2	0	42496T: 3.5; 42494T: 4;
MAP6	chr11	11q13.5	2	0	0	2	Amplification	2	0	42498T: 6.5; 42492T: 4;

Mangalaparthi *et al.* , 2020. Mutational landscape of esophageal squamous cell carcinoma in an Indian cohort
Supplementary Table 7A. List of copy number alterations and affected genes in ESCC patients

Gene	Chromosome	Cytoband	Recurrence	Recurrence in smoker cohort	Recurrence in chewer cohort	Recurrence in No habit cohort	State	Samples with CNA gain	Samples with CNA loss	File info with CNA fold
MAP6D1	chr3	3q27.1	10	1	3	6	Amplification	10	0	42482T: 3.5; 42487T: 3.5; 42493T: 3.5; 42492T: 3.5; 42497T: 4; 42474T: 3.5; 42495T: 4; 56957T: 5; 42484T: 4; 42498T: 3.5;
MAPK1	chr22	22q11.22	1	1	0	0	Amplification	1	0	42473T: 3.5;
MAPK1IP1L	chr14	14q22.3	1	0	0	1	Amplification	1	0	42494T: 4;
MAPK8IP1	chr11	11p11.2	1	1	0	0	Amplification	1	0	42473T: 3.5;
MAPK8IP3	chr16	16p13.3	1	0	1	0	Amplification	1	0	42483T: 3.5;
MAPKAPK5	chr12	12q24.12-q2	1	0	0	1	Amplification	1	0	42500T: 3.5;
MAPRE1	chr20	20q11.21	2	1	0	1	Amplification	2	0	42496T: 5; 42473T: 3.5;
MAPRE3	chr2	2p23.3	1	0	0	1	Amplification	1	0	42500T: 3.5;
MARCH11	chr5	5p15.1	5	1	2	2	Amplification	5	0	42475T: 3.5; 42486T: 3.5; 42496T: 3.5; 42493T: 4; 42484T: 3.5;
MARCH6	chr5	5p15.2	4	1	1	2	Amplification	4	0	42486T: 3.5; 42496T: 3.5; 42475T: 3.5; 42493T: 4;
MARCKSL1	chr1	1p35.1	1	1	0	0	Amplification	1	0	42473T: 4;
MARK4	chr19	19q13.32	3	1	1	1	Amplification	3	0	42484T: 4; 56957T: 4; 42473T: 3.5;
MARS2	chr2	2q33.1	1	0	1	0	Amplification	1	0	42482T: 4;
MASP1	chr3	3q27.3	10	1	3	6	Amplification	10	0	42497T: 4; 42498T: 3.5; 42484T: 4; 56957T: 4.5; 42474T: 3.5; 42495T: 4; 42482T: 3.5; 42492T: 3.5; 42493T: 3.5; 42487T: 3.5;
MAT2A	chr2	2p11.2	2	0	1	1	Amplification	2	0	42482T: 3.5; 42500T: 3.5;
MATN1	chr1	1p35.2	1	1	0	0	Amplification	1	0	42473T: 3.5;
MATN2	chr8	8q22.1-q22.2	2	0	0	2	Amplification	2	0	42496T: 3.5; 42495T: 3.5;
MATN3	chr2	2p24.1	2	1	0	1	Amplification	2	0	42473T: 4; 42500T: 3.5;
MATN4	chr20	20q13.12	1	1	0	0	Amplification	1	0	42473T: 5;
MAVS	chr20	20p13	2	1	1	0	Amplification	2	0	42473T: 4.5; 42487T: 4;
MAX	chr14	14q23.3	1	0	0	1	Amplification	1	0	42494T: 4;
MB	chr22	22q12.3	1	1	0	0	Amplification	1	0	42473T: 3.5;
MB21D2	chr3	3q29	10	1	3	6	Amplification	10	0	42482T: 3.5; 42493T: 3.5; 42487T: 3.5; 42492T: 3.5; 42501T: 3.5; 42495T: 4; 42474T: 3.5; 56957T: 4.5; 42484T: 4; 42498T: 3.5;
MBD4	chr3	3q21.3	2	0	1	1	Amplification	2	0	42496T: 3.5; 42487T: 3.5;
MBIP	chr14	14q13.3	2	1	0	1	Amplification	2	0	42500T: 8.5; 42476T: 8;
MBNL1	chr3	3q25.1-q25.2	9	1	2	6	Amplification	9	0	42484T: 3.5; 56957T: 4; 42474T: 3.5; 42496T: 4; 42497T: 5.5; 42500T: 3.5; 42492T: 3.5; 42493T: 3.5; 42487T: 3.5;
MBOAT2	chr2	2p25.1	1	0	0	1	Amplification	1	0	42500T: 3.5;
MBOAT4	chr8	8p12	1	0	1	0	Amplification	1	0	42482T: 3.5;
MCCC1	chr3	3q27.1	10	1	3	6	Amplification	10	0	42495T: 4; 42474T: 3.5; 56957T: 5; 42484T: 4; 42498T: 3.5; 42500T: 24; 42487T: 3.5; 42493T: 3.5; 42492T: 3.5; 42482T: 3.5;
MCCD1	chr6	6p21.33	1	1	0	0	Amplification	1	0	42473T: 3.5;

Mangalaparthi *et al.* , 2020. Mutational landscape of esophageal squamous cell carcinoma in an Indian cohort
Supplementary Table 7A. List of copy number alterations and affected genes in ESCC patients

Gene	Chromosome	Cytoband	Recurrence	Recurrence in smoker cohort	Recurrence in chewer cohort	Recurrence in No habit cohort	State	Samples with CNA gain	Samples with CNA loss	File info with CNA fold
MCEE	chr2	2p13.3	1	0	0	1	Amplification	1	0	42500T: 3.5;
MCF2L	chr13	13q34	1	0	0	1	Amplification	1	0	56957T: 3.5;
MCF2L2	chr3	3q27.1	9	1	3	5	Amplification	9	0	42495T: 4; 42474T: 3.5; 56957T: 5; 42484T: 4; 42498T: 3.5; 42482T: 3.5; 42487T: 3.5; 42493T: 3.5; 42492T: 3.5;
MCFD2	chr2	2p21	2	0	1	1	Amplification	2	0	42500T: 3.5; 42484T: 4.5;
MCL1	chr1	1q21.2	2	1	0	1	Amplification	2	0	42493T: 3.5; 42473T: 5;
MCM2	chr3	7q22.1	2	0	1	1	Amplification	2	0	42496T: 3.5; 42487T: 3.5;
MCM4	chr8	8q11.21	5	0	2	3	Amplification	5	0	42496T: 3.5; 42482T: 5; 42494T: 3.5; 42495T: 3.5; 42484T: 3.5;
MCM5	chr22	22q12.3	1	1	0	0	Amplification	1	0	42473T: 3.5;
MCMDC2	chr8	8q13.1	3	0	0	3	Amplification	3	0	42495T: 3.5; 42496T: 3.5; 42497T: 4.5;
MCRS1	chr12	12q13.12	1	0	0	1	Amplification	1	0	42500T: 3.5;
MDFIC	chr7	7q31.1-q31.2	1	0	1	0	Amplification	1	0	42487T: 3.5;
MDGA1	chr6	6p21.2	1	1	0	0	Amplification	1	0	42473T: 4.5;
MDH1	chr2	2p15	3	0	1	2	Amplification	3	0	42484T: 3.5; 56957T: 3.5; 42500T: 3.5;
MDK	chr11	2q24-q32	1	1	0	0	Amplification	1	0	42473T: 3.5;
MDM1	chr12	12q15	2	0	0	2	Amplification	2	0	42500T: 5; 42501T: 6.5;
MDM2	chr12	12q15	2	0	0	2	Amplification	2	0	42501T: 6.5; 42500T: 5;
MECOM	chr3	3q26.2	9	1	3	5	Amplification	9	0	42482T: 3.5; 42492T: 3.5; 42487T: 3.5; 42493T: 3.5; 42500T: 8; 42484T: 4; 42495T: 4; 42474T: 3.5; 56957T: 4;
MECR	chr1	1p35.3	1	1	0	0	Amplification	1	0	42473T: 3.5;
MED1	chr17	3q21.3	2	1	0	1	Amplification	2	0	42473T: 6; 42497T: 3.5;
MED10	chr5	5p15.31	4	1	1	2	Amplification	4	0	42493T: 4; 42496T: 3.5; 42486T: 3.5; 42475T: 3.5;
MED12L	chr3	3q25.1	9	1	2	6	Amplification	9	0	42484T: 3.5; 42474T: 3.5; 56957T: 4; 42496T: 4; 42497T: 5.5; 42500T: 3.5; 42492T: 3.5; 42487T: 3.5; 42493T: 3.5;
MED13L	chr12	12q24.21	1	0	0	1	Amplification	1	0	42500T: 3.5;
MED15	chr22	22q11.21	2	1	0	1	Amplification	3	0	42477T: 4; 42497T: 3.5,13.5;
MED16	chr19	19p13.3	1	0	0	1	Amplification	1	0	42493T: 3.5;
MED18	chr1	1p35.3	1	1	0	0	Amplification	1	0	42473T: 3.5;
MED19	chr11	11q12.1	1	0	0	1	Amplification	1	0	42496T: 4.5;
MED21	chr12	12p11.23	1	0	0	1	Amplification	1	0	42500T: 6;
MED24	chr17	17q21.1	1	0	0	1	Amplification	1	0	42497T: 3.5;
MED29	chr19	19q13.2	2	0	0	2	Amplification	2	0	42500T: 6.5; 56957T: 4;
MED30	chr8	8q24.11	3	1	0	2	Amplification	3	0	42495T: 3.5; 42496T: 3.5; 42475T: 3.5;
MED6	chr14	14q24.2	1	0	0	1	Amplification	1	0	42494T: 4;
MED8	chr1	1p34.2	1	0	0	1	Amplification	1	0	42493T: 3.5;
MEDAG	chr13	13q12.3	1	0	0	1	Amplification	1	0	42497T: 3.5;
MEF2C	chr5	5q14.3	1	1	0	0	Deletion	0	1	42476T: 0.5;

Mangalaparthi *et al.* , 2020. Mutational landscape of esophageal squamous cell carcinoma in an Indian cohort
Supplementary Table 7A. List of copy number alterations and affected genes in ESCC patients

Gene	Chromosome	Cytoband	Recurrence	Recurrence in smoker cohort	Recurrence in chewer cohort	Recurrence in No habit cohort	State	Samples with CNA gain	Samples with CNA loss	File info with CNA fold
MEF2D	chr1	1q22	1	1	0	0	Amplification	1	0	42473T: 5;
MEFV	chr16	16p13.3	1	1	0	0	Amplification	1	0	42473T: 5.5;
MEGF8	chr19	19q13.2	1	1	0	0	Amplification	1	0	42473T: 4.5;
MEIOB	chr16	16p13.3	1	0	1	0	Amplification	1	0	42483T: 3.5;
MEIS1	chr2	2p14	1	0	0	1	Amplification	1	0	42500T: 3.5;
MEMO1	chr2	2p22.3	1	0	0	1	Amplification	1	0	42500T: 3.5;
MEOX2	chr7	7p21.2	1	1	0	0	Amplification	1	0	42473T: 3.5;
MESP1	chr15	15q26.1	1	1	0	0	Amplification	1	0	42473T: 3.5;
MESP2	chr15	15q26.1	1	1	0	0	Amplification	1	0	42473T: 3.5;
MEST	chr7	7q32.2	1	0	1	0	Amplification	1	0	42487T: 3.5;
MET	chr7	18p11.21	1	0	1	0	Amplification	1	0	42487T: 3.5;
METAP1D	chr2	2q31.1	2	1	0	1	Amplification	2	0	42493T: 5; 42473T: 3.5;
METRN	chr16	16p13.3	2	0	1	1	Amplification	2	0	42483T: 3.5; 42493T: 3.5;
METTL20	chr12	12p11.21	1	0	0	1	Amplification	1	0	42500T: 12.5;
METTL21D	chr14	14q21.3	1	0	0	1	Amplification	1	0	42496T: 3.5;
METTL22	chr16	16p13.2	2	1	0	1	Amplification	2	0	42495T: 4; 42473T: 5.5;
METTL24	chr6	6q21	1	0	0	1	Amplification	1	0	42496T: 3.5;
METTL2B	chr7	7q32.1	2	1	1	0	Amplification	2	0	42487T: 3.5; 42473T: 3.5;
METTL3	chr14	14q11.2	1	0	0	1	Amplification	1	0	42496T: 4;
METTL4	chr18	18p11.32	3	0	0	3	Amplification	3	0	42493T: 3.5; 56957T: 8; 42500T: 4.5;
METTL7A	chr12	12q13.12	2	0	0	2	Amplification	2	0	42500T: 3.5; 42494T: 3.5;
METTL7B	chr12	12q13.2	1	0	0	1	Amplification	1	0	42494T: 3.5;
METTL9	chr16	16p12.2	1	1	0	0	Amplification	1	0	42473T: 4;
MEX3A	chr1	1q22	2	1	0	1	Amplification	2	0	42473T: 4.5; 42493T: 4;
MFHAS1	chr8	8p23.1	1	0	1	0	Amplification	1	0	42486T: 3.5;
MFI2	chr3	3q29	9	1	3	5	Amplification	9	0	42484T: 4; 42498T: 3.5; 42474T: 3.5; 42495T: 4; 56957T: 6; 42482T: 3.5; 42492T: 3.5; 42493T: 5; 42487T: 3.5;
MFN1	chr3	3q26.33	11	1	3	7	Amplification	11	0	42493T: 3.5; 42487T: 3.5; 42492T: 3.5; 42482T: 3.5; 42474T: 3.5; 42495T: 4; 56957T: 4; 42484T: 4; 42498T: 3.5; 42500T: 14; 42497T: 4;
MFNG	chr22	22q13.1	1	1	0	0	Amplification	1	0	42473T: 3.5;
MFSD1	chr3	3q25.32	8	1	2	5	Amplification	8	0	42497T: 3.5; 42484T: 3.5; 56957T: 4; 42495T: 4; 42474T: 3.5; 42492T: 3.5; 42487T: 3.5; 42493T: 3.5;
MFSD2B	chr2	2p23.3	1	0	0	1	Amplification	1	0	42500T: 3.5;
MFSD3	chr8	8q24.3	5	0	1	4	Amplification	5	0	42483T: 3.5; 42496T: 4; 42494T: 3.5; 42495T: 4.5; 56957T: 3.5;
MFSD6	chr2	2q32.2	3	0	1	2	Amplification	3	0	42493T: 3.5; 42494T: 3.5; 42482T: 4;
MFSD9	chr2	2q12.1	1	0	0	1	Amplification	1	0	42493T: 3.5;
MGAM	chr7	7q34	1	0	1	0	Amplification	1	0	42487T: 3.5;
MGAT2	chr14	11q13.5	1	1	0	0	Amplification	1	0	42473T: 3.5;

Mangalaparthi *et al.* , 2020. Mutational landscape of esophageal squamous cell carcinoma in an Indian cohort
Supplementary Table 7A. List of copy number alterations and affected genes in ESCC patients

Gene	Chromosome	Cytoband	Recurrence	Recurrence in smoker cohort	Recurrence in chewer cohort	Recurrence in No habit cohort	State	Samples with CNA gain	Samples with CNA loss	File info with CNA fold
MGAT3	chr22	7q22.1	1	1	0	0	Amplification	1	0	42473T: 3.5;
MGAT4A	chr2	2q11.2	1	0	0	1	Amplification	1	0	42493T: 3.5;
MGLL	chr3	3q21.3	2	0	1	1	Amplification	2	0	42487T: 3.5; 42496T: 3.5;
MGP	chr12	12p12.3	1	0	0	1	Amplification	1	0	42500T: 4.5;
MGRN1	chr16	16p13.3	3	1	0	2	Amplification	3	0	42495T: 12; 42494T: 3.5; 42473T: 5.5;
MGST1	chr12	12p12.3	1	0	0	1	Amplification	1	0	42500T: 4.5;
MIA	chr19	19q13.2	2	0	0	2	Amplification	2	0	42500T: 3.5; 56957T: 4;
MICAL1	chr6	6q21	1	0	0	1	Amplification	1	0	42496T: 3.5;
MICALL1	chr22	22q13.1	1	1	0	0	Amplification	1	0	42473T: 3.5;
MICALL2	chr7	7p22.3	1	1	0	0	Amplification	1	0	42473T: 5;
MICB	chr6	6p21.33	1	1	0	0	Amplification	1	0	42473T: 3.5;
MIEN1	chr17	17q12	2	1	0	1	Amplification	2	0	42473T: 6; 42497T: 3.5;
MIF4GD	chr17	17q25.1	1	1	0	0	Amplification	1	0	42473T: 4;
MINA	chr3	3q11.2	1	1	0	0	Amplification	1	0	42476T: 3.5;
MINOS1	chr1	1p36.13	1	1	0	0	Amplification	1	0	42473T: 4;
MIOS	chr7	7p21.3	2	1	0	1	Amplification	2	0	42497T: 7.5; 42473T: 3.5;
MIPOL1	chr14	14q13.3-q21	2	1	0	1	Amplification	2	0	42500T: 6.5; 42476T: 11;
MIR296	chr20	20q13.32	1	1	0	0	Amplification	1	0	42473T: 4.5;
MIR4458	chr5	5p15.31	4	1	1	2	Amplification	4	0	42493T: 4; 42475T: 3.5; 42496T: 3.5; 42486T: 3.5;
MIR940	chr16	16p13.3	1	0	1	0	Amplification	1	0	42483T: 3.5;
MIS18BP1	chr14	14q21.2	1	0	0	1	Amplification	1	0	42500T: 5.5;
MISP	chr19	19p13.3	1	0	0	1	Amplification	1	0	42493T: 3.5;
MITD1	chr2	2q11.2	1	0	0	1	Amplification	1	0	42493T: 3.5;
MKKS	chr20	20p12.2	1	0	1	0	Amplification	1	0	42486T: 4;
MKL1	chr22	22q13.1-q13	1	1	0	0	Amplification	1	0	42473T: 3.5;
MKL2	chr16	16p13.12	2	1	0	1	Amplification	2	0	42473T: 4; 42495T: 4;
MKLN1	chr7	7q32.3	1	0	1	0	Amplification	1	0	42487T: 3.5;
MKRN1	chr7	7q34	2	1	1	0	Amplification	2	0	42487T: 3.5; 42473T: 3.5;
MKS1	chr17	17q22	1	0	0	1	Amplification	1	0	42497T: 4;
MLANA	chr9	9p24.1	2	0	0	2	Amplification	2	0	42496T: 3.5; 42498T: 14;
MLEC	chr12	12q24.31	1	0	0	1	Amplification	1	0	42500T: 3.5;
MLF1	chr3	3q25.32	8	1	2	5	Amplification	8	0	42492T: 3.5; 42493T: 3.5; 42487T: 3.5; 42497T: 3.5; 42484T: 3.5; 56957T: 4; 42495T: 4; 42474T: 3.5;
MLF2	chr12	12p13.31	1	1	0	0	Amplification	1	0	42473T: 3.5;
MLH3	chr14	14q24.3	2	0	0	2	Amplification	2	0	42494T: 4; 56957T: 3.5;
MLLT3	chr9	9p21.3	1	1	0	0	Deletion	0	1	42475T: 0.5;
MLLT6	chr17	17q12	1	1	0	0	Amplification	1	0	42473T: 3.5;
MLST8	chr16	16p13.3	1	0	1	0	Amplification	1	0	42483T: 3.5;
MLTK	chr2	2q31.1	2	1	0	1	Amplification	2	0	42473T: 6; 42493T: 5;
MLXIP	chr12	12q24.31	1	0	0	1	Amplification	1	0	42500T: 3.5;
MMAB	chr12	12q24.11	2	1	0	1	Amplification	2	0	42500T: 3.5; 42473T: 4.5;

Mangalaparthi *et al.* , 2020. Mutational landscape of esophageal squamous cell carcinoma in an Indian cohort
Supplementary Table 7A. List of copy number alterations and affected genes in ESCC patients

Gene	Chromosome	Cytoband	Recurrence	Recurrence in smoker cohort	Recurrence in chewer cohort	Recurrence in No habit cohort	State	Samples with CNA gain	Samples with CNA loss	File info with CNA fold
MMD	chr17	17q22	1	0	0	1	Amplification	1	0	42497T: 3.5;
MMD2	chr7	10p	2	1	0	1	Amplification	2	0	42473T: 4.5; 42497T: 8.5;
MME	chr3	11q22.2	7	1	2	4	Amplification	7	0	42497T: 5.5; 42492T: 3.5; 42484T: 3.5; 42493T: 3.5; 56957T: 4; 42487T: 3.5; 42474T: 3.5;
MMP12	chr11	11q22.2	1	0	0	1	Amplification	1	0	56958T: 6;
MMP13	chr11	11q22.2	1	0	0	1	Amplification	1	0	56958T: 6;
MMP14	chr14	14q11.2	1	0	0	1	Amplification	1	0	42496T: 4;
MMP16	chr8	8q21.3	3	1	0	2	Amplification	3	0	42496T: 3.5; 42475T: 3.5; 42495T: 3.5;
MMP19	chr12	12q13.2	1	0	0	1	Amplification	1	0	42494T: 5;
MMP24	chr20	20q11.22	2	1	0	1	Amplification	2	0	42493T: 3.5; 42473T: 3.5;
MMP7	chr11	11q22.2	1	0	0	1	Amplification	1	0	56958T: 5.5;
MMP9	chr20	20q13.12	1	1	0	0	Amplification	1	0	42473T: 5;
MNAT1	chr14	14q23.1	1	0	0	1	Amplification	1	0	42494T: 4;
MOB1A	chr2	2p13.1	1	0	0	1	Amplification	1	0	42500T: 3.5;
MOB2	chr11	11p15.5	1	1	0	0	Amplification	1	0	42473T: 4;
MOB4	chr2	2q33.1	1	0	1	0	Amplification	1	0	42482T: 4;
MOCS3	chr20	20q13.13	1	1	0	0	Amplification	1	0	42473T: 5;
MOGAT2	chr11	11q13.5	2	0	0	2	Amplification	2	0	42498T: 6.5; 42492T: 4;
MOGAT3	chr7	7q22.1	2	0	0	2	Amplification	2	0	42501T: 3.5; 42493T: 3.5;
MOGS	chr2	2p13.1	1	0	0	1	Amplification	1	0	42500T: 3.5;
MORN2	chr2	2p22.1	1	0	0	1	Amplification	1	0	42500T: 3.5;
MORN3	chr12	12q24.31	1	0	0	1	Amplification	1	0	42500T: 3.5;
MOS	chr8	18q12.2	3	0	1	2	Amplification	3	0	42496T: 3.5; 42483T: 5; 42495T: 3.5;
MPG	chr16	16p13.3	1	0	1	0	Amplification	1	0	42483T: 3.5;
MPHOSPH10	chr2	2p13.3	1	0	0	1	Amplification	1	0	42500T: 3.5;
MPHOSPH9	chr12	12q24.31	1	0	0	1	Amplification	1	0	42500T: 3.5;
MPI	chr15	20q13.12	1	1	0	0	Amplification	1	0	42473T: 3.5;
MPL	chr1	1p34.2	1	0	0	1	Amplification	1	0	42493T: 3.5;
MPLKIP	chr7	7p14.1	1	1	0	0	Amplification	1	0	42473T: 3.5;
MPO	chr17	17q22	1	0	0	1	Amplification	1	0	42497T: 4;
MPP5	chr14	14q23.3	1	0	0	1	Amplification	1	0	42494T: 4;
MPP6	chr7	7p15.3	1	1	0	0	Amplification	1	0	42473T: 4;
MPPE1	chr18	18p11.21	1	0	0	1	Amplification	1	0	42493T: 3.5;
MPST	chr22	22q12.3	1	1	0	0	Amplification	1	0	42473T: 3.5;
MPV17	chr2	2p23.3	1	0	0	1	Amplification	1	0	42500T: 3.5;
MPV17L	chr16	16p13.11	2	1	0	1	Amplification	2	0	42473T: 4; 42495T: 4;
MRAS	chr3	3q22.3	4	0	1	3	Amplification	4	0	42496T: 3.5; 42492T: 3.5; 42493T: 3.5; 42487T: 3.5;
MRGBP	chr20	20q13.33	1	1	0	0	Amplification	1	0	42473T: 6;
MRGPRD	chr11	11q13.3	7	1	1	5	Amplification	7	0	42498T: 9.5; 42492T: 4; 56957T: 5; 42497T: 4.5; 42476T: 29.5; 42483T: 14.5; 42501T: 3.5;

Mangalaparthi *et al.* , 2020. Mutational landscape of esophageal squamous cell carcinoma in an Indian cohort
Supplementary Table 7A. List of copy number alterations and affected genes in ESCC patients

Gene	Chromosome	Cytoband	Recurrence	Recurrence in smoker cohort	Recurrence in chewer cohort	Recurrence in No habit cohort	State	Samples with CNA gain	Samples with CNA loss	File info with CNA fold
MRGPRF	chr11	11q13.3	7	1	1	5	Amplification	7	0	42501T: 3.5; 42483T: 14.5; 42476T: 29.5; 42497T: 4.5; 56957T: 5; 42492T: 4; 42498T: 9.5;
MROH1	chr8	8q24.3	3	0	1	2	Amplification	3	0	42483T: 3.5; 42496T: 4; 42495T: 4.5;
MROH2B	chr5	5p13.1	6	1	3	2	Amplification	6	0	42493T: 3.5; 42484T: 3.5; 42475T: 3.5; 42483T: 3.5; 42486T: 3.5; 42496T: 3.5;
MROH5	chr8	8q24.3	3	0	1	2	Amplification	3	0	42495T: 4.5; 42483T: 3.5; 42496T: 4;
MROH6	chr8	8q24.3	3	0	1	2	Amplification	3	0	42495T: 4.5; 42496T: 4; 42483T: 3.5;
MROH8	chr20	20q11.23	1	1	0	0	Amplification	1	0	42473T: 3.5;
MRPL11	chr11	11q13.2	1	0	0	1	Amplification	1	0	56957T: 5.5;
MRPL13	chr8	8q24.12	3	1	0	2	Amplification	3	0	42496T: 3.5; 42475T: 3.5; 42495T: 3.5;
MRPL15	chr8	8q11.23	3	0	0	3	Amplification	3	0	42496T: 3.5; 42494T: 4; 42495T: 3.5;
MRPL19	chr2	2p12	1	0	0	1	Amplification	1	0	42500T: 3.5;
MRPL21	chr11	11q13.3	6	1	0	5	Amplification	6	0	42492T: 4; 42498T: 9.5; 56957T: 5; 42476T: 29.5; 42497T: 4.5; 42501T: 3.5;
MRPL24	chr1	1q23.1	1	1	0	0	Amplification	1	0	42473T: 7;
MRPL27	chr17	9q34.3	1	1	0	0	Amplification	1	0	42473T: 3.5;
MRPL28	chr16	2q11.2	1	0	1	0	Amplification	1	0	42483T: 3.5;
MRPL3	chr3	3q22.1	3	0	1	2	Amplification	3	0	42492T: 3.5; 42487T: 3.5; 42496T: 3.5;
MRPL30	chr2	2q11.2	1	0	0	1	Amplification	1	0	42493T: 3.5;
MRPL32	chr7	7p14.1	1	1	0	0	Amplification	1	0	42473T: 3.5;
MRPL33	chr2	2p23.2	1	0	0	1	Amplification	1	0	42500T: 3.5;
MRPL35	chr2	2p11.2	2	0	0	2	Amplification	2	0	42493T: 3.5; 42500T: 3.5;
MRPL36	chr5	5p15.33	5	2	1	2	Amplification	5	0	42475T: 3.5; 42473T: 3.5; 42496T: 4; 42486T: 3.5; 42493T: 3.5;
MRPL38	chr17	17q25.1	1	0	0	1	Amplification	1	0	42494T: 3.5;
MRPL40	chr22	22q11.21	1	0	0	1	Amplification	1	0	42497T: 17;
MRPL41	chr9	9q34.3	2	1	0	1	Amplification	2	0	56957T: 3.5; 42473T: 5;
MRPL45	chr17	17q12	1	1	0	0	Amplification	1	0	42473T: 3.5;
MRPL47	chr3	3q26.33	11	1	3	7	Amplification	11	0	42492T: 3.5; 42493T: 3.5; 42487T: 3.5; 42482T: 3.5; 42498T: 3.5; 42484T: 4; 56957T: 4; 42474T: 3.5; 42495T: 4; 42497T: 4; 42500T: 14;
MRPL48	chr11	11q13.4	6	3	0	3	Amplification	6	0	42476T: 4; 42475T: 8.5; 42478T: 6; 42492T: 4; 42498T: 8; 56957T: 3.5;
MRPL52	chr14	14q11.2	1	0	0	1	Amplification	1	0	42496T: 4;
MRPL53	chr2	2p13.1	1	0	0	1	Amplification	1	0	42500T: 3.5;
MRPL9	chr1	1q21.3	1	1	0	0	Amplification	1	0	42473T: 4.5;
MRPS12	chr19	16p13.3	2	0	0	2	Amplification	2	0	42500T: 6.5; 56957T: 4;
MRPS17	chr7	7p11.2	3	0	1	2	Amplification	3	0	56957T: 5; 42497T: 6.5; 42483T: 4;
MRPS2	chr9	9q34.3	1	0	1	0	Amplification	1	0	42484T: 4.5;
MRPS21	chr1	1q21.2	1	1	0	0	Amplification	1	0	42473T: 5;
MRPS22	chr3	3q23	4	0	1	3	Amplification	4	0	42487T: 3.5; 42493T: 3.5; 42492T: 3.5; 42496T: 3.5;

Mangalaparthi *et al.* , 2020. Mutational landscape of esophageal squamous cell carcinoma in an Indian cohort
Supplementary Table 7A. List of copy number alterations and affected genes in ESCC patients

Gene	Chromosome	Cytoband	Recurrence	Recurrence in smoker cohort	Recurrence in chewer cohort	Recurrence in No habit cohort	State	Samples with CNA gain	Samples with CNA loss	File info with CNA fold
MRPS23	chr17	17q22	1	0	0	1	Amplification	1	0	42497T: 4;
MRPS24	chr7	7p13	1	1	0	0	Amplification	1	0	42473T: 3.5;
MRPS26	chr20	20p13	1	1	0	0	Amplification	1	0	42473T: 3.5;
MRPS28	chr8	12p11.22	2	0	0	2	Amplification	2	0	42496T: 3.5; 42495T: 3.5;
MRPS30	chr5	5p12	4	1	1	2	Amplification	4	0	42486T: 3.5; 42496T: 3.5; 42475T: 3.5; 42493T: 3.5;
MRPS33	chr7	7q34	1	0	1	0	Amplification	1	0	42487T: 3.5;
MRPS34	chr16	16p13.3	1	0	1	0	Amplification	1	0	42483T: 3.5;
MRPS35	chr12	12p11.22	2	0	0	2	Amplification	2	0	42500T: 6; 42494T: 4;
MRPS5	chr2	2q11.1	1	1	0	0	Amplification	1	0	42473T: 3.5;
MRPS7	chr17	17q25.1	1	1	0	0	Amplification	1	0	42473T: 4;
MRPS9	chr2	2q12.1	1	0	0	1	Amplification	1	0	42493T: 3.5;
MSC	chr8	8p21.2	2	0	0	2	Amplification	2	0	42496T: 3.5; 42495T: 3.5;
MSGN1	chr2	2p24.2	1	0	0	1	Amplification	1	0	42500T: 3.5;
MSH2	chr2	2p21-p16.3	2	0	1	1	Amplification	2	0	42484T: 4.5; 42500T: 3.5;
MSH5	chr6	6p21.33	1	1	0	0	Amplification	1	0	42473T: 3.5;
MSH6	chr2	2p16.3	2	0	1	1	Amplification	2	0	42484T: 4.5; 42500T: 3.5;
MSI1	chr12	12q24.31	1	0	0	1	Amplification	1	0	42500T: 3.5;
MSI2	chr17	17q22	1	0	0	1	Amplification	1	0	42497T: 4;
MSL1	chr17	17q21.1	1	0	0	1	Amplification	1	0	42497T: 4;
MSL2	chr3	3q22.3	4	0	1	3	Amplification	4	0	42496T: 3.5; 42487T: 3.5; 42493T: 3.5; 42492T: 3.5;
MSLN	chr16	16p13.3	2	0	1	1	Amplification	2	0	42493T: 3.5; 42483T: 3.5;
MSLNL	chr16	16p13.3	2	0	1	1	Amplification	2	0	42483T: 3.5; 42493T: 3.5;
MSRA	chr8	8p23.1	1	0	1	0	Amplification	1	0	42486T: 3.5;
MSRB1	chr16	16p13.3	1	0	1	0	Amplification	1	0	42483T: 3.5;
MSS51	chr10	10q22.2	1	0	0	1	Amplification	1	0	42496T: 3.5;
MST1	chr3	20q13.12	1	1	0	0	Amplification	1	0	42473T: 4;
MST1L	chr1	1p36.13	1	1	0	0	Amplification	1	0	42473T: 4.5;
MSTN	chr2	2q32.2	3	0	1	2	Amplification	3	0	42494T: 3.5; 42493T: 3.5; 42482T: 4;
MSTO1	chr1	1q22	1	1	0	0	Amplification	1	0	42473T: 5.5;
MTA3	chr2	2p21	2	0	1	1	Amplification	2	0	42500T: 3.5; 42484T: 3.5;
MTAP	chr9	9p21.3	1	1	0	0	Deletion	0	1	42475T: 0.5;
MTBP	chr8	8q24.12	3	1	0	2	Amplification	3	0	42496T: 3.5; 42475T: 3.5; 42495T: 3.5;
MTCH1	chr6	6p21.2	1	0	1	0	Amplification	1	0	42486T: 3.5;
MTCH2	chr11	11p11.2	1	1	0	0	Amplification	1	0	42473T: 3.5;
MTDH	chr8	8q22.1	2	0	0	2	Amplification	2	0	42496T: 3.5; 42495T: 3.5;
MTERF	chr7	7q21.2	3	0	2	1	Amplification	3	0	42483T: 4; 42487T: 3.5; 42494T: 3.5;
MTERFD1	chr8	8q22.1	2	0	0	2	Amplification	2	0	42496T: 3.5; 42495T: 3.5;
MTFP1	chr22	22q12.2	1	1	0	0	Amplification	1	0	42473T: 3.5;
MTFR1	chr8	8q13.1	2	0	0	2	Amplification	2	0	42495T: 3.5; 42496T: 3.5;
MTFR1L	chr1	1p36.11	1	1	0	0	Amplification	1	0	42473T: 4.5;
MTG2	chr20	20q13.33	1	1	0	0	Amplification	1	0	42473T: 6;

Mangalaparthi *et al.* , 2020. Mutational landscape of esophageal squamous cell carcinoma in an Indian cohort
Supplementary Table 7A. List of copy number alterations and affected genes in ESCC patients

Gene	Chromosome	Cytoband	Recurrence	Recurrence in smoker cohort	Recurrence in chewer cohort	Recurrence in No habit cohort	State	Samples with CNA gain	Samples with CNA loss	File info with CNA fold
MTHFD1	chr14	14q23.3	1	0	0	1	Amplification	1	0	42494T: 4;
MTHFD2	chr2	2p13.1	1	0	0	1	Amplification	1	0	42500T: 3.5;
MTIF2	chr2	2p16.1	3	0	1	2	Amplification	3	0	42500T: 3.5; 42484T: 4.5; 56957T: 3.5;
MTL5	chr11	11q13.3	2	1	0	1	Amplification	2	0	56957T: 5; 42476T: 29.5;
MTMR11	chr1	1q21.2	1	1	0	0	Amplification	1	0	42473T: 5;
MTMR12	chr5	5p13.3	4	1	1	2	Amplification	4	0	42475T: 3.5; 42496T: 3.5; 42486T: 3.5; 42493T: 3.5;
MTMR3	chr22	22q12.2	1	1	0	0	Amplification	1	0	42473T: 3.5;
MTMR4	chr17	17q22	1	0	0	1	Amplification	1	0	42497T: 4;
MTMR9	chr8	8p23.1	1	0	1	0	Amplification	1	0	42486T: 3.5;
MTPN	chr7	7q33	1	0	1	0	Amplification	1	0	42487T: 3.5;
MTRR	chr5	5p15.31	4	1	1	2	Amplification	4	0	42475T: 3.5; 42496T: 3.5; 42486T: 3.5; 42493T: 4;
MTSS1	chr8	8q24.13	4	1	1	2	Amplification	4	0	42475T: 3.5; 42496T: 3.5; 42495T: 3.5; 42484T: 3.5;
MTX1	chr1	1q22	1	1	0	0	Amplification	1	0	42473T: 6.5;
MTX2	chr2	2q31.1	1	0	0	1	Amplification	1	0	42493T: 3.5;
MUC1	chr1	1q22	1	1	0	0	Amplification	1	0	42473T: 6.5;
MUC12	chr7	7q22.1	2	0	0	2	Amplification	2	0	42501T: 3.5; 42493T: 3.5;
MUC13	chr3	3q21.2	1	0	0	1	Amplification	1	0	42496T: 3.5;
MUC17	chr7	7q22.1	4	1	0	3	Amplification	4	0	42493T: 3.5; 56957T: 4; 42501T: 3.5; 42473T: 3.5;
MUC19	chr12	12q12	1	0	0	1	Amplification	1	0	42500T: 3.5;
MUC20	chr3	3q29	9	1	3	5	Amplification	9	0	42484T: 4; 42498T: 3.5; 42474T: 3.5; 42495T: 4; 56957T: 6; 42492T: 3.5; 42493T: 3.5; 42487T: 3.5; 42482T: 3.5;
MUC3A	chr7	7q22	2	0	0	2	Amplification	2	0	42501T: 3.5; 42493T: 3.5;
MUC4	chr3	3q29	9	1	3	5	Amplification	9	0	42484T: 4; 42498T: 3.5; 42495T: 4; 42474T: 3.5; 56957T: 6; 42492T: 3.5; 42487T: 3.5; 42493T: 3.5; 42482T: 3.5;
MUCL1	chr12	12q13.2	1	0	0	1	Amplification	1	0	42494T: 3.5;
MUL1	chr1	1p36.12	1	1	0	0	Amplification	1	0	42473T: 4;
MVK	chr12	12q24.11	2	1	0	1	Amplification	2	0	42473T: 4.5; 42500T: 3.5;
MX1	chr21	21q22.3	1	1	0	0	Amplification	1	0	42473T: 3.5;
MX2	chr21	21q22.3	1	1	0	0	Amplification	1	0	42473T: 3.5;
MXD1	chr2	2p13.3	1	0	0	1	Amplification	1	0	42500T: 3.5;
MYADML	chr2	2p22.3	1	0	0	1	Amplification	1	0	42500T: 3.5;
MYBL1	chr8	8q13.1	3	0	0	3	Amplification	3	0	42497T: 4.5; 42496T: 3.5; 42495T: 3.5;
MYBL2	chr20	20q13.12	1	1	0	0	Amplification	1	0	42473T: 3.5;
MYBPC3	chr11	11p11.2	1	1	0	0	Amplification	1	0	42473T: 3.5;
MYBPH	chr1	1q32.1	1	1	0	0	Amplification	1	0	42473T: 4;
MYC	chr8	8q24.21	5	1	1	3	Amplification	5	0	42496T: 3.5; 42475T: 3.5; 42484T: 3.5; 42495T: 3.5; 42493T: 10;
MYCBPAP	chr17	17q21.33	1	1	0	0	Amplification	1	0	42473T: 4.5;
MYCN	chr2	2p24.3	1	0	0	1	Amplification	1	0	42500T: 3.5;

Mangalaparthi *et al.* , 2020. Mutational landscape of esophageal squamous cell carcinoma in an Indian cohort
Supplementary Table 7A. List of copy number alterations and affected genes in ESCC patients

Gene	Chromosome	Cytoband	Recurrence	Recurrence in smoker cohort	Recurrence in chewer cohort	Recurrence in No habit cohort	State	Samples with CNA gain	Samples with CNA loss	File info with CNA fold
MYD88	chr3	3p22.2	1	1	0	0	Amplification	1	0	42473T: 4.5;
MYEOV	chr11	11q13.3	9	2	1	6	Amplification	9	0	42497T: 4.5; 42483T: 14.5; 56958T: 5; 42501T: 6; 42475T: 7.5; 42498T: 9.5; 56957T: 5; 42476T: 29.5; 42492T: 4;
MYH11	chr16	16p13.11	2	1	0	1	Amplification	2	0	42473T: 4; 42495T: 4;
MYH6	chr14	14q11.2	2	0	0	2	Amplification	2	0	42496T: 4; 42500T: 4;
MYH7	chr14	14q11.2	1	0	0	1	Amplification	1	0	42496T: 4;
MYH7B	chr20	20q11.22	2	1	0	1	Amplification	2	0	42493T: 3.5; 42473T: 3.5;
MYH9	chr22	22q12.3	1	1	0	0	Amplification	1	0	42473T: 3.5;
MYL10	chr7	7q22.1	2	0	0	2	Amplification	2	0	42501T: 3.5; 42493T: 3.5;
MYL12A	chr18	18p11.31	3	1	0	2	Amplification	3	0	56957T: 8; 42481T: 9.5; 42500T: 3.5;
MYL12B	chr18	18p11.31	3	1	0	2	Amplification	3	0	42500T: 3.5; 56957T: 8; 42481T: 9.5;
MYL2	chr12	12q24.11	1	0	0	1	Amplification	1	0	42500T: 3.5;
MYL6	chr12	12q13.2	1	0	0	1	Amplification	1	0	42494T: 7.5;
MYL6B	chr12	12q13.2	1	0	0	1	Amplification	1	0	42494T: 7.5;
MYL7	chr7	7p13	2	1	0	1	Amplification	2	0	42497T: 7.5; 42473T: 4.5;
MYL9	chr20	20q11.23	1	1	0	0	Amplification	1	0	42473T: 3.5;
MYLK	chr3	3q21.1	1	0	0	1	Amplification	1	0	42496T: 3.5;
MYLK2	chr20	20q11.21	1	0	0	1	Amplification	1	0	42496T: 5;
MYNN	chr3	3q26.2	9	1	3	5	Amplification	9	0	42482T: 3.5; 42492T: 3.5; 42487T: 3.5; 42493T: 3.5; 42500T: 8; 42484T: 4; 56957T: 4; 42474T: 3.5; 42495T: 4;
MYO10	chr5	5p15.1	4	1	1	2	Amplification	4	0	42475T: 3.5; 42496T: 3.5; 42486T: 3.5; 42493T: 4;
MYO1B	chr2	2q32.3	3	0	1	2	Amplification	3	0	42482T: 4; 42493T: 3.5; 42494T: 3.5;
MYO1G	chr7	7p13	1	1	0	0	Amplification	1	0	42473T: 4.5;
MYO1H	chr12	12q24.11	2	1	0	1	Amplification	2	0	42500T: 3.5; 42473T: 4.5;
MYOG	chr1	1q32.1	1	1	0	0	Amplification	1	0	42473T: 4;
MYOM1	chr18	18p11.31	2	0	0	2	Amplification	2	0	56957T: 8; 42500T: 3.5;
MYOM2	chr8	8p23.3	1	0	0	1	Amplification	1	0	56957T: 4;
MYOM3	chr1	1p36.11	1	1	0	0	Amplification	1	0	42473T: 3.5;
MYOZ1	chr10	10q22.2	2	1	0	1	Amplification	2	0	42473T: 4.5; 42496T: 3.5;
MYPOP	chr19	19q13.32	1	0	1	0	Amplification	1	0	42484T: 4;
MYRFL	chr12	12q15	2	0	0	2	Amplification	2	0	42500T: 5; 42501T: 5.5;
MYT1	chr20	16p13.3	1	1	0	0	Amplification	1	0	42473T: 6;
MYT1L	chr2	2p25.3	1	0	0	1	Amplification	1	0	42500T: 3.5;
N4BP2L1	chr13	13q13.1	1	0	0	1	Amplification	1	0	42497T: 6;
N4BP2L2	chr13	13q13.1	1	0	0	1	Amplification	1	0	42497T: 6;
NAA20	chr20	20p11.23	1	0	1	0	Amplification	1	0	42483T: 4;
NAA25	chr12	12q24.13	1	0	0	1	Amplification	1	0	42500T: 3.5;
NAA30	chr14	14q22.3	1	0	0	1	Amplification	1	0	42494T: 4;
NAA38	chr7	17p13.1	1	0	1	0	Amplification	1	0	42487T: 3.5;

Mangalaparthi *et al.* , 2020. Mutational landscape of esophageal squamous cell carcinoma in an Indian cohort
Supplementary Table 7A. List of copy number alterations and affected genes in ESCC patients

Gene	Chromosome	Cytoband	Recurrence	Recurrence in smoker cohort	Recurrence in chewer cohort	Recurrence in No habit cohort	State	Samples with CNA gain	Samples with CNA loss	File info with CNA fold
NAA50	chr3	3q13.31	1	0	0	1	Amplification	1	0	42496T: 3.5;
NAA60	chr16	16p13.3	1	1	0	0	Amplification	1	0	42473T: 5.5;
NAALADL2	chr3	3q26.31	9	1	3	5	Amplification	9	0	42484T: 4; 42474T: 3.5; 42495T: 4; 56957T: 4; 42500T: 12.5; 42492T: 3.5; 42493T: 3.5; 42487T: 3.5; 42482T: 3.5;
NAB1	chr2	2q32.2	3	0	1	2	Amplification	3	0	42494T: 3.5; 42493T: 3.5; 42482T: 4;
NABP1	chr2	2q32.3	3	0	1	2	Amplification	3	0	42482T: 4; 42494T: 3.5; 42493T: 3.5;
NABP2	chr12	12q13.3	1	0	0	1	Amplification	1	0	42494T: 7.5;
NACAD	chr7	7p13	1	1	0	0	Amplification	1	0	42473T: 4.5;
NACAP1	chr8	8q22.3	2	0	0	2	Amplification	2	0	42495T: 3.5; 42496T: 3.5;
NACC2	chr9	9q34.3	1	0	0	1	Amplification	1	0	56957T: 3.5;
NADK2	chr5	5p13.2	4	1	1	2	Amplification	4	0	42493T: 3.5; 42496T: 3.5; 42486T: 3.5; 42475T: 3.5;
NADSYN1	chr11	11q13.4	8	3	0	5	Amplification	8	0	42476T: 4; 42492T: 4; 42497T: 6.5; 42475T: 5; 42501T: 6; 42498T: 5; 42478T: 4.5; 56957T: 5;
NAGK	chr2	2p13.3	1	0	0	1	Amplification	1	0	42500T: 3.5;
NAGPA	chr16	16p13.3	2	1	0	1	Amplification	2	0	42473T: 5.5; 42495T: 12;
NAMPT	chr7	7q22.3	4	0	1	3	Amplification	4	0	42493T: 3.5; 42487T: 3.5; 42497T: 4; 42501T: 3.5;
NANOS2	chr19	19q13.32	1	0	1	0	Amplification	1	0	42484T: 4;
NAPEPLD	chr7	7q22.1	3	0	1	2	Amplification	3	0	42501T: 3.5; 42493T: 3.5; 42487T: 3.5;
NAPRT1	chr8	8q24.3	3	0	1	2	Amplification	3	0	42496T: 4; 42483T: 3.5; 42495T: 4.5;
NARFL	chr16	16p13.3	2	0	1	1	Amplification	2	0	42483T: 3.5; 42493T: 3.5;
NAT10	chr11	11p13	1	1	0	0	Amplification	1	0	42473T: 4.5;
NAT14	chr19	19q13.42	1	0	0	1	Amplification	1	0	42494T: 3.5;
NAT16	chr7	7q22.1	2	0	0	2	Amplification	2	0	42501T: 3.5; 42493T: 3.5;
NAT8	chr2	2p13.1	1	0	0	1	Amplification	1	0	42500T: 3.5;
NBAS	chr2	2p24.3	1	0	0	1	Amplification	1	0	42500T: 3.5;
NBL1	chr1	1p36.13	1	1	0	0	Amplification	1	0	42473T: 4;
NBN	chr8	1p34.1	3	1	0	2	Amplification	3	0	42475T: 3.5; 42496T: 3.5; 42495T: 3.5;
NBPF22P	chr5	5q14.3	1	1	0	0	Deletion	0	1	42476T: 0.5;
NCALD	chr8	8q22.3	2	0	0	2	Amplification	2	0	42495T: 3.5; 42496T: 3.5;
NCAPD3	chr11	11q25	1	1	0	0	Deletion	0	1	42476T: 0.5;
NCAPH	chr2	2q11.2	2	1	0	1	Amplification	2	0	42493T: 3.5; 42473T: 3.5;
NCBP2	chr3	3q29	9	1	3	5	Amplification	9	0	42482T: 3.5; 42493T: 5; 42487T: 3.5; 42492T: 3.5; 42474T: 3.5; 42495T: 4; 56957T: 6; 42484T: 4; 42498T: 3.5;
NCCRP1	chr19	19q13.2	2	0	0	2	Amplification	2	0	42500T: 6.5; 56957T: 4;
NCEH1	chr3	3q26.31	9	1	3	5	Amplification	9	0	42482T: 3.5; 42487T: 3.5; 42493T: 3.5; 42492T: 3.5; 42500T: 9; 56957T: 4; 42495T: 4; 42474T: 3.5; 42484T: 4;
NCF4	chr22	22q12.3	1	1	0	0	Amplification	1	0	42473T: 3.5;
NCK1	chr3	3q22.3	4	0	1	3	Amplification	4	0	42492T: 3.5; 42487T: 3.5; 42493T: 3.5; 42496T: 3.5;

Mangalaparthi *et al.* , 2020. Mutational landscape of esophageal squamous cell carcinoma in an Indian cohort
Supplementary Table 7A. List of copy number alterations and affected genes in ESCC patients

Gene	Chromosome	Cytoband	Recurrence	Recurrence in smoker cohort	Recurrence in chewer cohort	Recurrence in No habit cohort	State	Samples with CNA gain	Samples with CNA loss	File info with CNA fold
NCKAP1	chr2	2q32.1	1	0	0	1	Amplification	1	0	42493T: 3.5;
NCKAP1L	chr12	12q13.13-q1	2	1	0	1	Amplification	2	0	42473T: 3.5; 42494T: 3.5;
NCKAP5L	chr12	12q13.12	1	0	0	1	Amplification	1	0	42500T: 3.5;
NCMAP	chr1	1p36.11	1	1	0	0	Amplification	1	0	42473T: 3.5;
NCOA1	chr2	2p23.3	1	0	0	1	Amplification	1	0	42500T: 3.5;
NCOA2	chr8	8q13.3	2	0	0	2	Amplification	2	0	42496T: 3.5; 42495T: 3.5;
NCOA3	chr20	20q13.12	1	1	0	0	Amplification	1	0	42473T: 5;
NCOA5	chr20	20q13.12	1	1	0	0	Amplification	1	0	42473T: 5;
NCOA6	chr20	20q11.22	2	1	0	1	Amplification	2	0	42473T: 3.5; 42493T: 3.5;
NCOR2	chr12	12q24.31	1	0	0	1	Amplification	1	0	42500T: 3.5;
NCR3	chr6	6p21.33	1	1	0	0	Amplification	1	0	42473T: 3.5;
NDC80	chr18	18p11.32	2	0	0	2	Amplification	2	0	56957T: 8; 42500T: 4.5;
NDE1	chr16	16p13.11	2	1	0	1	Amplification	2	0	42473T: 4; 42495T: 4;
NDOR1	chr9	9q34.3	2	1	0	1	Amplification	2	0	56957T: 3.5; 42473T: 5;
NDRG1	chr8	8q24.22	3	0	1	2	Amplification	3	0	42496T: 3.5; 42484T: 3.5; 42495T: 3.5;
NDRG3	chr20	20q11.23	1	1	0	0	Amplification	1	0	42473T: 3.5;
NDST2	chr10	10q22.2	1	0	0	1	Amplification	1	0	42496T: 3.5;
NDUFA4	chr7	1p13.3	1	1	0	0	Amplification	1	0	42473T: 3.5;
NDUFA5	chr7	7q31.32	2	0	1	1	Amplification	2	0	42487T: 3.5; 42493T: 3.5;
NDUFAB1	chr16	16p12.2	1	1	0	0	Amplification	1	0	42473T: 4;
NDUFAF6	chr8	8q22.1	3	1	0	2	Amplification	3	0	42473T: 4.5; 42496T: 3.5; 42495T: 3.5;
NDUFAF7	chr2	2p22.2	1	0	0	1	Amplification	1	0	42500T: 3.5;
NDUFB10	chr16	16p13.3	1	0	1	0	Amplification	1	0	42483T: 3.5;
NDUFB2	chr7	7q34	1	0	1	0	Amplification	1	0	42487T: 3.5;
NDUFB4	chr3	3q13.33	1	0	0	1	Amplification	1	0	42496T: 3.5;
NDUFB5	chr3	3q26.33	11	1	3	7	Amplification	11	0	42492T: 3.5; 42487T: 3.5; 42493T: 3.5; 42482T: 3.5; 42484T: 4; 42498T: 3.5; 42474T: 3.5; 42495T: 4; 56957T: 4; 42497T: 4; 42500T: 14;
NDUFB9	chr8	8q24.13	4	1	1	2	Amplification	4	0	42484T: 3.5; 42495T: 3.5; 42496T: 3.5; 42475T: 3.5;
NDUFS3	chr11	11p11.2	1	1	0	0	Amplification	1	0	42473T: 3.5;
NDUFS6	chr5	5p15.33	5	2	1	2	Amplification	5	0	42493T: 3.5; 42486T: 3.5; 42473T: 3.5; 42496T: 4; 42475T: 3.5;
NDUFS8	chr11	11q13.2	1	0	0	1	Amplification	1	0	56957T: 5;
NDUFV1	chr11	11q13.2	2	1	0	1	Amplification	2	0	42473T: 3.5; 56957T: 5;
NECAB1	chr8	8q21.3	2	0	0	2	Amplification	2	0	42495T: 3.5; 42496T: 3.5;
NECAB3	chr20	20q11.22	2	1	0	1	Amplification	2	0	42496T: 5; 42473T: 3.5;
NECAP2	chr1	1p36.13	1	1	0	0	Amplification	1	0	42473T: 4.5;
NEIL2	chr8	8p23.1	1	0	1	0	Amplification	1	0	42486T: 3.5;
NEK11	chr3	3q22.1	3	0	1	2	Amplification	3	0	42496T: 3.5; 42487T: 3.5; 42492T: 3.5;
NEK9	chr14	14q24.3	2	0	0	2	Amplification	2	0	56957T: 3.5; 42494T: 4;
NELFB	chr9	9q34.3	2	1	0	1	Amplification	2	0	56957T: 3.5; 42473T: 5;

Mangalaparthi *et al.* , 2020. Mutational landscape of esophageal squamous cell carcinoma in an Indian cohort
Supplementary Table 7A. List of copy number alterations and affected genes in ESCC patients

Gene	Chromosome	Cytoband	Recurrence	Recurrence in smoker cohort	Recurrence in chewer cohort	Recurrence in No habit cohort	State	Samples with CNA gain	Samples with CNA loss	File info with CNA fold
NELFCD	chr20	20q13.32	1	1	0	0	Amplification	1	0	42473T: 4.5;
NELFE	chr6	6p21.33	1	1	0	0	Amplification	1	0	42473T: 3.5;
NELL2	chr12	12q12	1	0	0	1	Amplification	1	0	42500T: 3.5;
NEMF	chr14	14q21.3	1	1	0	0	Amplification	1	0	42473T: 3.5;
NES	chr1	1q23.1	1	1	0	0	Amplification	1	0	42473T: 7;
NEU1	chr6	9q34.3	1	1	0	0	Amplification	1	0	42473T: 3.5;
NEU2	chr2	2q37.1	1	1	0	0	Amplification	1	0	42473T: 4.5;
NEU3	chr11	11q13.4	2	0	0	2	Amplification	2	0	42492T: 4; 42498T: 8;
NEURL2	chr20	20q13.12	1	1	0	0	Amplification	1	0	42473T: 5;
NEURL3	chr2	2q11.2	2	1	0	1	Amplification	2	0	42473T: 3.5; 42493T: 3.5;
NEUROD1	chr2	2q31.3	1	0	0	1	Amplification	1	0	42493T: 4;
NEUROD2	chr17	17q12	2	1	0	1	Amplification	2	0	42473T: 6; 42497T: 3.5;
NEUROD4	chr12	12q13.2	1	0	0	1	Amplification	1	0	42494T: 3.5;
NEUROD6	chr7	7p14.3	1	1	0	0	Amplification	1	0	42473T: 4;
NF1	chr17	17q11.2	1	1	0	0	Amplification	1	0	42473T: 3.5;
NF2	chr22	22q12.2	1	1	0	0	Amplification	1	0	42473T: 3.5;
NFATC2	chr20	20q13.2	1	1	0	0	Amplification	1	0	42473T: 5;
NFE2	chr12	12q13.13	2	1	0	1	Amplification	2	0	42494T: 3.5; 42473T: 3.5;
NFE2L2	chr2	2q31.2	2	0	1	1	Amplification	2	0	42482T: 4; 42493T: 4.5;
NFE2L3	chr7	7p15.2	1	1	0	0	Amplification	1	0	42473T: 4;
NFIB	chr9	9p23-p22.3	1	0	0	1	Amplification	1	0	42498T: 14.5;
NFKBIA	chr14	14q13.2	2	1	0	1	Amplification	2	0	42500T: 8.5; 42476T: 5;
NFKBIB	chr19	19q13.2	2	0	0	2	Amplification	2	0	42500T: 6.5; 56957T: 4;
NFKBID	chr19	19q13.12	3	0	1	2	Amplification	3	0	42484T: 3.5; 56957T: 4; 42500T: 6.5;
NFKBIL1	chr6	6p21.33	1	1	0	0	Amplification	1	0	42473T: 3.5;
NFKBIZ	chr3	3q12.3	1	1	0	0	Amplification	1	0	42476T: 3.5;
NFS1	chr20	20q11.22	2	1	0	1	Amplification	2	0	42473T: 3.5; 42493T: 3.5;
NFU1	chr2	2p13.3	1	0	0	1	Amplification	1	0	42500T: 3.5;
NGB	chr14	14q24.3	2	0	0	2	Amplification	2	0	42494T: 4; 56957T: 3.5;
NGDN	chr14	14q11.2	1	0	0	1	Amplification	1	0	42496T: 4;
NHLRC4	chr16	16p13.3	2	0	1	1	Amplification	2	0	42493T: 3.5; 42483T: 3.5;
NICN1	chr3	3p21.31	1	1	0	0	Amplification	1	0	42473T: 4;
NID2	chr14	14q22.1	1	0	0	1	Amplification	1	0	42494T: 4;
NIM1	chr5	5p12	4	1	1	2	Amplification	4	0	42496T: 3.5; 42486T: 3.5; 42475T: 3.5; 42493T: 3.5;
NIN	chr14	14q22.1	1	0	0	1	Amplification	1	0	42494T: 4;
NINJ2	chr12	12p13.33	1	0	0	1	Amplification	1	0	42500T: 3.5;
NIPAL2	chr8	8q22.2	2	0	0	2	Amplification	2	0	42495T: 3.5; 42496T: 3.5;
NIPAL3	chr1	1p36.11	1	1	0	0	Amplification	1	0	42473T: 3.5;
NIPBL	chr5	5p13.2	5	1	2	2	Amplification	5	0	42486T: 3.5; 42483T: 3.5; 42496T: 3.5; 42475T: 3.5; 42493T: 3.5;
NIPSNAP1	chr22	22q12.2	1	1	0	0	Amplification	1	0	42473T: 3.5;

Mangalaparthi *et al.* , 2020. Mutational landscape of esophageal squamous cell carcinoma in an Indian cohort
Supplementary Table 7A. List of copy number alterations and affected genes in ESCC patients

Gene	Chromosome	Cytoband	Recurrence	Recurrence in smoker cohort	Recurrence in chewer cohort	Recurrence in No habit cohort	State	Samples with CNA gain	Samples with CNA loss	File info with CNA fold
NIT2	chr3	3q12.2	1	1	0	0	Amplification	1	0	42476T: 3.5;
NKAIN1	chr1	1p35.2	1	1	0	0	Amplification	1	0	42473T: 3.5;
NKAIN3	chr8	8q12.3	2	0	0	2	Amplification	2	0	42495T: 3.5; 42496T: 3.5;
NKAIN4	chr20	20q13.33	1	1	0	0	Amplification	1	0	42473T: 6;
NKD2	chr5	5p15.33	5	2	1	2	Amplification	5	0	42493T: 3.5; 42486T: 3.5; 42473T: 3.5; 42496T: 4; 42475T: 3.5;
NKPD1	chr19	19q13.32	2	1	0	1	Amplification	2	0	56957T: 4; 42473T: 3.5;
NLGN1	chr3	3q26.31	9	1	3	5	Amplification	9	0	42492T: 3.5; 42487T: 3.5; 42493T: 3.5; 42482T: 3.5; 42484T: 4; 56957T: 4; 42495T: 4; 42474T: 3.5; 42500T: 9;
NLRC3	chr16	16p13.3	1	1	0	0	Amplification	1	0	42473T: 5.5;
NLRC4	chr2	2p22.3	1	0	0	1	Amplification	1	0	42500T: 3.5;
NLRP11	chr19	19q13.42-q1	1	0	0	1	Amplification	1	0	42494T: 3.5;
NLRP13	chr19	19q13.43	1	0	0	1	Amplification	1	0	42494T: 3.5;
NLRP4	chr19	19q13.43	1	0	0	1	Amplification	1	0	42494T: 3.5;
NLRP9	chr19	19q13.42	1	0	0	1	Amplification	1	0	42494T: 3.5;
NMB	chr15	7p15.3	1	1	0	0	Amplification	1	0	42473T: 4;
NMD3	chr3	3q26.1	8	1	2	5	Amplification	8	0	42497T: 3.5; 42484T: 4; 56957T: 4; 42495T: 4; 42474T: 3.5; 42492T: 3.5; 42487T: 3.5; 42493T: 3.5;
NME1	chr17	9p13.3	1	1	0	0	Amplification	1	0	42473T: 4.5;
NME2	chr17	17q21.33	1	1	0	0	Amplification	1	0	42473T: 4.5;
NME3	chr16	16p13.3	1	0	1	0	Amplification	1	0	42483T: 3.5;
NME4	chr16	16p13.3	1	0	1	0	Amplification	1	0	42483T: 3.5;
NME8	chr7	7p14.1	1	1	0	0	Amplification	1	0	42473T: 4.5;
NME9	chr3	3q22.3	4	0	1	3	Amplification	4	0	42496T: 3.5; 42487T: 3.5; 42493T: 3.5; 42492T: 3.5;
NMNAT3	chr3	3q23	4	0	1	3	Amplification	4	0	42487T: 3.5; 42493T: 3.5; 42492T: 3.5; 42496T: 3.5;
NMRAL1	chr16	16p13.3	3	1	0	2	Amplification	3	0	42473T: 5.5; 42495T: 8.5; 42494T: 3.5;
NMS	chr2	2q11.2	1	0	0	1	Amplification	1	0	42493T: 3.5;
NNAT	chr20	20q11.23	1	1	0	0	Amplification	1	0	42473T: 3.5;
NNT	chr5	5p12	4	1	1	2	Amplification	4	0	42493T: 3.5; 42496T: 3.5; 42486T: 3.5; 42475T: 3.5;
NOBOX	chr7	7q35	1	0	1	0	Amplification	1	0	42487T: 3.5;
NOD1	chr7	7p14.3	1	1	0	0	Amplification	1	0	42473T: 4;
NOG	chr17	17q22	1	0	0	1	Amplification	1	0	42497T: 4;
NOL10	chr2	2p25.1	1	0	0	1	Amplification	1	0	42500T: 3.5;
NOMO1	chr16	16p13.11	2	1	0	1	Amplification	2	0	42473T: 4; 42495T: 4;
NOMO2	chr16	16p12.3	2	1	0	1	Amplification	2	0	42495T: 4; 42473T: 4;
NOMO3	chr16	16p13.11	2	1	0	1	Amplification	2	0	42495T: 4; 42473T: 4;
NOS1	chr12	10q26.11	1	0	0	1	Amplification	1	0	42500T: 3.5;
NOTCH1	chr9	9q34.3	1	0	0	1	Amplification	1	0	56957T: 3.5;
NOTCH2NL	chr1	1q21.1	1	1	0	0	Amplification	1	0	42473T: 4;
NOTCH4	chr6	6p21.32	1	1	0	0	Amplification	1	0	42473T: 3.5;

Mangalaparthi *et al.* , 2020. Mutational landscape of esophageal squamous cell carcinoma in an Indian cohort
Supplementary Table 7A. List of copy number alterations and affected genes in ESCC patients

Gene	Chromosome	Cytoband	Recurrence	Recurrence in smoker cohort	Recurrence in chewer cohort	Recurrence in No habit cohort	State	Samples with CNA gain	Samples with CNA loss	File info with CNA fold
NOTO	chr2	2p13.2	1	0	0	1	Amplification	1	0	42500T: 3.5;
NOV	chr8	Xq28	3	1	0	2	Amplification	3	0	42495T: 3.5; 42475T: 3.5; 42496T: 3.5;
NOVA2	chr19	19q13.32	1	0	1	0	Amplification	1	0	42484T: 4;
NOXA1	chr9	9q34.3	2	1	0	1	Amplification	2	0	56957T: 3.5; 42473T: 5;
NOXRED1	chr14	14q24.3	2	0	0	2	Amplification	2	0	42494T: 4; 56957T: 3.5;
NPAS2	chr2	2q11.2	1	0	0	1	Amplification	1	0	42493T: 3.5;
NPAS3	chr14	14q13.1	1	0	0	1	Amplification	1	0	42500T: 4.5;
NPAS4	chr11	11q13.2	1	0	0	1	Amplification	1	0	56957T: 5.5;
NPBWR1	chr8	8q11.23	4	0	1	3	Amplification	4	0	42496T: 3.5; 42482T: 3.5; 42494T: 3.5; 42495T: 3.5;
NPBWR2	chr20	20q13.33	1	1	0	0	Amplification	1	0	42473T: 6;
NPC1	chr18	4p15.1-q12	1	1	0	0	Amplification	1	0	42481T: 4;
NPC1L1	chr7	7p13	2	1	0	1	Amplification	2	0	42473T: 4.5; 42497T: 7.5;
NPC2	chr14	14q24.3	2	0	0	2	Amplification	2	0	56957T: 3.5; 42494T: 4;
NPDC1	chr9	9q34.3	2	1	0	1	Amplification	2	0	56957T: 3.5; 42473T: 5;
NPEPL1	chr20	20q13.32	1	1	0	0	Amplification	1	0	42473T: 4.5;
NPHP1	chr2	2q13	1	0	1	0	Deletion	0	1	42486T: 0.5;
NPHP3	chr3	3q22.1	3	0	1	2	Amplification	3	0	42496T: 3.5; 42487T: 3.5; 42492T: 3.5;
NPHS1	chr19	19q13.12	3	0	1	2	Amplification	3	0	42500T: 6.5; 42484T: 3.5; 56957T: 4;
NPR1	chr1	1q21.3	1	1	0	0	Amplification	1	0	42473T: 4.5;
NPR2	chr9	3p21.31	2	1	0	1	Amplification	2	0	42473T: 3.5; 42501T: 3.5;
NPR3	chr5	16p13.3	4	1	1	2	Amplification	4	0	42496T: 3.5; 42486T: 3.5; 42475T: 3.5; 42493T: 3.5;
NPRL3	chr16	16p13.3	1	0	1	0	Amplification	1	0	42483T: 3.5;
NPSR1	chr7	7p14.3	1	1	0	0	Amplification	1	0	42473T: 4;
NPTXR	chr22	22q13.1	1	1	0	0	Amplification	1	0	42473T: 3.5;
NPVF	chr7	7p15.3	1	1	0	0	Amplification	1	0	42473T: 4;
NPW	chr16	16p13.3	1	0	1	0	Amplification	1	0	42483T: 3.5;
NPY	chr7	7p15.3	1	1	0	0	Amplification	1	0	42473T: 4;
NR1D1	chr17	17q21.1	1	0	0	1	Amplification	1	0	42497T: 3.5;
NR1H3	chr11	11p11.2	1	1	0	0	Amplification	1	0	42473T: 3.5;
NR1I2	chr3	3q13.33	1	0	0	1	Amplification	1	0	42496T: 3.5;
NR2E1	chr6	6q21	1	0	0	1	Amplification	1	0	42496T: 3.5;
NRARP	chr9	9q34.3	2	1	0	1	Amplification	2	0	56957T: 3.5; 42473T: 5;
NRBP1	chr2	2p23.3	1	0	0	1	Amplification	1	0	42500T: 3.5;
NRBP2	chr8	8q24.3	3	0	1	2	Amplification	3	0	42495T: 4.5; 42496T: 4; 42483T: 3.5;
NRCAM	chr7	7q31.1	4	0	1	3	Amplification	4	0	42493T: 5; 42487T: 3.5; 42497T: 4; 42501T: 3.5;
NRDE2	chr14	14q32.11	1	1	0	0	Amplification	1	0	42473T: 3.5;
NRF1	chr7	7q32.2	1	0	1	0	Amplification	1	0	42487T: 3.5;
NRG1	chr8	8p12	2	0	1	1	Amplification	2	0	42482T: 3.5; 42497T: 4.5;
NRROS	chr3	3q29	9	1	3	5	Amplification	9	0	42482T: 3.5; 42492T: 3.5; 42493T: 5; 42487T: 3.5; 42498T: 3.5; 42484T: 4; 56957T: 6; 42474T: 3.5; 42495T: 4;

Mangalaparthi *et al.* , 2020. Mutational landscape of esophageal squamous cell carcinoma in an Indian cohort
Supplementary Table 7A. List of copy number alterations and affected genes in ESCC patients

Gene	Chromosome	Cytoband	Recurrence	Recurrence in smoker cohort	Recurrence in chewer cohort	Recurrence in No habit cohort	State	Samples with CNA gain	Samples with CNA loss	File info with CNA fold
NRSN2	chr20	20p13	1	1	0	0	Amplification	1	0	42473T: 3.5;
NRXN1	chr2	2p16.3	2	0	1	1	Amplification	2	0	42500T: 3.5; 42484T: 4.5;
NRXN3	chr14	14q24.3-q31	1	0	0	1	Amplification	1	0	42494T: 4;
NSFL1C	chr20	20p13	1	1	0	0	Amplification	1	0	42473T: 3.5;
NSMAF	chr8	8q12.1	2	0	0	2	Amplification	2	0	42496T: 3.5; 42495T: 3.5;
NSMCE2	chr8	8q24.13	4	1	1	2	Amplification	4	0	42496T: 3.5; 42475T: 3.5; 42484T: 3.5; 42495T: 3.5;
NSMF	chr9	9q34.3	2	1	0	1	Amplification	2	0	42473T: 5; 56957T: 3.5;
NSUN2	chr5	5p15.31	4	1	1	2	Amplification	4	0	42475T: 3.5; 42486T: 3.5; 42496T: 3.5; 42493T: 4;
NSUN3	chr3	3q11.2	2	0	1	1	Amplification	2	0	42500T: 3.5; 42484T: 3.5;
NT5C1B	chr2	2p24.2	1	0	0	1	Amplification	1	0	42500T: 3.5;
NT5C3A	chr7	7p14.3	1	1	0	0	Amplification	1	0	42473T: 4;
NTAN1	chr16	16p13.11	2	1	0	1	Amplification	2	0	42495T: 4; 42473T: 4;
NTHL1	chr16	16p13.3	1	0	1	0	Amplification	1	0	42483T: 3.5;
NTN3	chr16	16p13.3	1	0	1	0	Amplification	1	0	42483T: 3.5;
NTRK1	chr1	1q23.1	1	1	0	0	Amplification	1	0	42473T: 7;
NTSR1	chr20	20q13.33	1	1	0	0	Amplification	1	0	42473T: 6;
NTSR2	chr2	2p25.1	1	0	0	1	Amplification	1	0	42500T: 3.5;
NUBP1	chr16	16p13.3	2	1	0	1	Amplification	2	0	42473T: 5.5; 42495T: 5;
NUBP2	chr16	16p13.3	1	0	1	0	Amplification	1	0	42483T: 3.5;
NUBPL	chr14	14q12	1	0	0	1	Amplification	1	0	42500T: 4.5;
NUDCD1	chr8	8q23.1	2	0	0	2	Amplification	2	0	42495T: 3.5; 42496T: 3.5;
NUDCD3	chr7	7p13	2	1	0	1	Amplification	2	0	42497T: 7.5; 42473T: 4.5;
NUDT1	chr7	7p22.3	1	1	0	0	Amplification	1	0	42473T: 5;
NUDT16	chr3	3q22.1	3	0	1	2	Amplification	3	0	42487T: 3.5; 42492T: 3.5; 42496T: 3.5;
NUDT16L1	chr16	16p13.3	3	1	0	2	Amplification	3	0	42495T: 12; 42494T: 3.5; 42473T: 5.5;
NUDT17	chr1	1q21.1	1	1	0	0	Amplification	1	0	42473T: 4;
NUDT19	chr19	19q13.11	4	1	1	2	Amplification	4	0	42484T: 3.5; 56957T: 4; 42473T: 3.5; 42500T: 4.5;
NUDT4P1	chr1	1q21.2	1	1	0	0	Amplification	1	0	42473T: 4;
NUDT8	chr11	11q13.2	2	1	0	1	Amplification	2	0	42473T: 3.5; 56957T: 5;
NUMA1	chr11	11q13.4	8	3	0	5	Amplification	8	0	42498T: 5; 42478T: 4.5; 56957T: 3.5; 42501T: 6; 42475T: 5; 42492T: 4; 42493T: 3.5; 42476T: 4;
NUMB	chr14	14q24.2-q24	2	0	0	2	Amplification	2	0	42494T: 4; 56957T: 3.5;
NUMBL	chr19	19q13.2	2	0	0	2	Amplification	2	0	56957T: 4; 42500T: 3.5;
NUP107	chr12	12q15	2	0	0	2	Amplification	2	0	42500T: 5; 42501T: 6.5;
NUP155	chr5	5p13.2	5	1	2	2	Amplification	5	0	42493T: 3.5; 42475T: 3.5; 42483T: 3.5; 42486T: 3.5; 42496T: 3.5;
NUP160	chr11	11p11.2	1	1	0	0	Amplification	1	0	42473T: 3.5;
NUP205	chr7	7q33	1	0	1	0	Amplification	1	0	42487T: 3.5;
NUP210L	chr1	1q21.3	1	1	0	0	Amplification	1	0	42473T: 4.5;
NUP210P1	chr3	3q21.3	1	0	0	1	Amplification	1	0	42496T: 3.5;
NUP214	chr9	9q34.13	1	0	1	0	Amplification	1	0	42484T: 4;

Mangalaparthi *et al.* , 2020. Mutational landscape of esophageal squamous cell carcinoma in an Indian cohort
Supplementary Table 7A. List of copy number alterations and affected genes in ESCC patients

Gene	Chromosome	Cytoband	Recurrence	Recurrence in smoker cohort	Recurrence in chewer cohort	Recurrence in No habit cohort	State	Samples with CNA gain	Samples with CNA loss	File info with CNA fold
NUP35	chr2	2q32.1	1	0	0	1	Amplification	1	0	42493T: 3.5;
NUPL2	chr7	7p15.3	1	1	0	0	Amplification	1	0	42473T: 4;
NUPR1L	chr7	7p11.2	2	0	1	1	Amplification	2	0	56957T: 5; 42483T: 4;
NXPE3	chr3	3q12.3	1	1	0	0	Amplification	1	0	42476T: 3.5;
NXPH1	chr7	7p21.3	2	1	0	1	Amplification	2	0	42497T: 7.5; 42473T: 3.5;
OAS1	chr12	12q24.13	1	0	0	1	Amplification	1	0	42500T: 3.5;
OAS2	chr12	12q24.13	1	0	0	1	Amplification	1	0	42500T: 3.5;
OAS3	chr12	12q24.13	1	0	0	1	Amplification	1	0	42500T: 3.5;
OASL	chr12	12q24.31	1	0	0	1	Amplification	1	0	42500T: 3.5;
OAZ3	chr1	1q21.3	1	1	0	0	Amplification	1	0	42473T: 4.5;
OBP2A	chr9	9q34.3	1	0	1	0	Amplification	1	0	42484T: 4.5;
OC90	chr8	8q24.22	3	0	1	2	Amplification	3	0	42495T: 3.5; 42484T: 3.5; 42496T: 3.5;
OCIAD1	chr4	4p11	1	0	1	0	Amplification	1	0	42483T: 3.5;
OCIAD2	chr4	4p11	1	0	1	0	Amplification	1	0	42483T: 3.5;
OCM	chr7	7p22.1	2	1	0	1	Amplification	2	0	42473T: 4.5; 42497T: 10.5;
OCSTAMP	chr20	20q13.12	1	1	0	0	Amplification	1	0	42473T: 5;
ODF1	chr8	8q22.3	2	0	0	2	Amplification	2	0	42496T: 3.5; 42495T: 3.5;
ODF2L	chr1	1p22.3	1	1	0	0	Amplification	1	0	42473T: 4;
OGDH	chr7	7p13	2	1	0	1	Amplification	2	0	42497T: 7.5; 42473T: 4.5;
OGFOD2	chr12	12q24.31	1	0	0	1	Amplification	1	0	42500T: 3.5;
OGFR	chr20	20q13.33	1	1	0	0	Amplification	1	0	42473T: 6;
OLA1	chr2	2q31.1	1	0	0	1	Amplification	1	0	42493T: 3.5;
OMP	chr11	11q13.5	2	0	0	2	Amplification	2	0	42497T: 3.5; 42496T: 3.5;
OPA1	chr3	Xq13.1	10	1	3	6	Amplification	10	0	42501T: 3.5; 42495T: 4; 42474T: 3.5; 56957T: 4.5; 42484T: 4; 42498T: 3.5; 42482T: 3.5; 42487T: 3.5; 42493T: 3.5; 42492T: 3.5;
OPA3	chr19	19q13.32	1	0	1	0	Amplification	1	0	42484T: 4;
OPLAH	chr8	8q24.3	3	0	1	2	Amplification	3	0	42483T: 3.5; 42496T: 4; 42495T: 4.5;
OPN1SW	chr7	7q32.1	1	0	1	0	Amplification	1	0	42487T: 3.5;
OPRD1	chr1	1p35.3	1	1	0	0	Amplification	1	0	42473T: 3.5;
OPRK1	chr8	8q11.23	3	0	0	3	Amplification	3	0	42495T: 3.5; 42494T: 3.5; 42496T: 3.5;
OPRL1	chr20	20q13.33	1	1	0	0	Amplification	1	0	42473T: 6;
OR10AD1	chr12	12q13.11	1	0	0	1	Amplification	1	0	42500T: 3.5;
OR10G2	chr14	14q11.2	1	0	0	1	Amplification	1	0	42496T: 4;
OR10G3	chr14	14q11.2	1	0	0	1	Amplification	1	0	42496T: 4;
OR13J1	chr9	9p13.3	2	1	0	1	Amplification	2	0	42501T: 3.5; 42473T: 3.5;
OR1F1	chr16	16p13.3	1	1	0	0	Amplification	1	0	42473T: 5.5;
OR1F2P	chr16	16p13.3	1	1	0	0	Amplification	1	0	42473T: 5.5;
OR2A1	chr7	7q35	2	0	2	0	Amplification	2	0	42484T: 4.5; 42487T: 3.5;
OR2A12	chr7	7q35	1	0	1	0	Amplification	1	0	42487T: 3.5;
OR2A14	chr7	7q35	1	0	1	0	Amplification	1	0	42487T: 3.5;

Mangalaparthi *et al.* , 2020. Mutational landscape of esophageal squamous cell carcinoma in an Indian cohort
Supplementary Table 7A. List of copy number alterations and affected genes in ESCC patients

Gene	Chromosome	Cytoband	Recurrence	Recurrence in smoker cohort	Recurrence in chewer cohort	Recurrence in No habit cohort	State	Samples with CNA gain	Samples with CNA loss	File info with CNA fold
OR2A2	chr7	7q35	1	0	1	0	Amplification	1	0	42487T: 3.5;
OR2A20P	chr7	7q35	2	0	2	0	Amplification	2	0	42487T: 3.5; 42484T: 4.5;
OR2A25	chr7	7q35	1	0	1	0	Amplification	1	0	42487T: 3.5;
OR2A42	chr7	7q35	2	0	2	0	Amplification	2	0	42484T: 4.5; 42487T: 3.5;
OR2A5	chr7	7q35	1	0	1	0	Amplification	1	0	42487T: 3.5;
OR2A7	chr7	7q35	2	0	2	0	Amplification	2	0	42487T: 3.5; 42484T: 4.5;
OR2A9P	chr7	7q35	2	0	2	0	Amplification	2	0	42484T: 4.5; 42487T: 3.5;
OR2AP1	chr12	12q13.2	1	0	0	1	Amplification	1	0	42494T: 3.5;
OR2AT4	chr11	11q13.4	2	0	0	2	Amplification	2	0	42498T: 8; 42492T: 4;
OR2C1	chr16	16p13.3	1	1	0	0	Amplification	1	0	42473T: 5.5;
OR2F1	chr7	7q35	1	0	1	0	Amplification	1	0	42487T: 3.5;
OR2F2	chr7	7q35	1	0	1	0	Amplification	1	0	42487T: 3.5;
OR2S2	chr9	9p13.3	1	0	0	1	Amplification	1	0	42501T: 3.5;
OR2V1	chr5	5q35.3	1	0	0	1	Amplification	1	0	42495T: 3.5;
OR2V2	chr5	5q35.3	1	0	0	1	Amplification	1	0	42495T: 3.5;
OR2Y1	chr5	5q35.3	1	0	0	1	Amplification	1	0	42495T: 3.5;
OR4A15	chr11	11q11	1	0	0	1	Amplification	1	0	42496T: 8.5;
OR4A16	chr11	11q11	1	0	0	1	Amplification	1	0	42496T: 8.5;
OR4A47	chr11	11p11.2	1	0	0	1	Amplification	1	0	56957T: 8;
OR4A5	chr11	11q11	1	0	0	1	Amplification	1	0	42496T: 9.5;
OR4B1	chr11	11p11.2	1	0	0	1	Amplification	1	0	56957T: 16.5;
OR4C11	chr11	11q11	1	0	0	1	Amplification	1	0	42496T: 8.5;
OR4C12	chr11	11p11.12	1	0	0	1	Amplification	1	0	56957T: 8;
OR4C13	chr11	11p11.12	1	0	0	1	Amplification	1	0	56957T: 8;
OR4C15	chr11	11q11	1	0	0	1	Amplification	1	0	42496T: 8.5;
OR4C3	chr11	11p11.2	1	0	0	1	Amplification	1	0	56957T: 16.5;
OR4C46	chr11	11q11	1	0	0	1	Amplification	1	0	42496T: 9.5;
OR4C5	chr11	11p11.2	1	0	0	1	Amplification	1	0	56957T: 16.5;
OR4C6	chr11	11q11	1	0	0	1	Amplification	1	0	42496T: 8.5;
OR4D1	chr17	17q22	1	0	0	1	Amplification	1	0	42497T: 4;
OR4D2	chr17	17q22	1	0	0	1	Amplification	1	0	42497T: 4;
OR4M2	chr15	15q11.2	1	0	1	0	Amplification	1	0	42484T: 7.5;
OR4N4	chr15	15q11.2	1	0	1	0	Amplification	1	0	42484T: 7.5;
OR4P4	chr11	11q11	1	0	0	1	Amplification	1	0	42496T: 8.5;
OR4S1	chr11	11p11.2	1	0	0	1	Amplification	1	0	56957T: 16.5;
OR4S2	chr11	11q11	1	0	0	1	Amplification	1	0	42496T: 8.5;
OR4X1	chr11	11p11.2	1	0	0	1	Amplification	1	0	56957T: 16.5;
OR4X2	chr11	11p11.2	1	0	0	1	Amplification	1	0	56957T: 16.5;
OR5AC2	chr3	3q11.2	1	1	0	0	Amplification	1	0	42476T: 3.5;
OR5AK2	chr11	11q12.1	1	0	0	1	Amplification	1	0	42496T: 4.5;
OR5AP2	chr11	11q12.1	1	0	0	1	Amplification	1	0	42496T: 4.5;

Mangalaparthi *et al.* , 2020. Mutational landscape of esophageal squamous cell carcinoma in an Indian cohort
Supplementary Table 7A. List of copy number alterations and affected genes in ESCC patients

Gene	Chromosome	Cytoband	Recurrence	Recurrence in smoker cohort	Recurrence in chewer cohort	Recurrence in No habit cohort	State	Samples with CNA gain	Samples with CNA loss	File info with CNA fold
OR5AR1	chr11	11q12.1	1	0	0	1	Amplification	1	0	42496T: 4.5;
OR5AS1	chr11	11q12.1	1	0	0	1	Amplification	1	0	42496T: 4.5;
OR5D13	chr11	11q11	1	0	0	1	Amplification	1	0	42496T: 8.5;
OR5D14	chr11	11q11	1	0	0	1	Amplification	1	0	42496T: 8.5;
OR5D16	chr11	11q12.1	1	0	0	1	Amplification	1	0	42496T: 8.5;
OR5D18	chr11	11q12.1	1	0	0	1	Amplification	1	0	42496T: 8.5;
OR5F1	chr11	11q12.1	1	0	0	1	Amplification	1	0	42496T: 4.5;
OR5F2P	chr11	11q12.1	1	0	0	1	Amplification	1	0	42496T: 4.5;
OR5G5P	chr11	11q12.1	1	0	0	1	Amplification	1	0	42496T: 4.5;
OR5H1	chr3	3q11.2	1	1	0	0	Amplification	1	0	42476T: 3.5;
OR5H14	chr3	3q11.2	1	1	0	0	Amplification	1	0	42476T: 3.5;
OR5H15	chr3	3q11.2	1	1	0	0	Amplification	1	0	42476T: 3.5;
OR5H2	chr3	3q11.2	1	1	0	0	Amplification	1	0	42476T: 3.5;
OR5H6	chr3	3q11.2	1	1	0	0	Amplification	1	0	42476T: 3.5;
OR5J2	chr11	11q12.1	1	0	0	1	Amplification	1	0	42496T: 4.5;
OR5K1	chr3	3q11.2	1	1	0	0	Amplification	1	0	42476T: 3.5;
OR5K2	chr3	3q11.2	1	1	0	0	Amplification	1	0	42476T: 3.5;
OR5K3	chr3	3q11.2	1	1	0	0	Amplification	1	0	42476T: 3.5;
OR5K4	chr3	3q11.2	1	1	0	0	Amplification	1	0	42476T: 3.5;
OR5L1	chr11	11q12.1	1	0	0	1	Amplification	1	0	42496T: 8.5;
OR5L2	chr11	11q12.1	1	0	0	1	Amplification	1	0	42496T: 8.5;
OR5M1	chr11	11q12.1	1	0	0	1	Amplification	1	0	42496T: 4.5;
OR5T1	chr11	11q12.1	1	0	0	1	Amplification	1	0	42496T: 4.5;
OR5T2	chr11	11q12.1	1	0	0	1	Amplification	1	0	42496T: 4.5;
OR5T3	chr11	11q12.1	1	0	0	1	Amplification	1	0	42496T: 4.5;
OR6B1	chr7	7q35	1	0	1	0	Amplification	1	0	42487T: 3.5;
OR6C1	chr12	12q13.2	1	0	0	1	Amplification	1	0	42494T: 3.5;
OR6C2	chr12	12q13.2	1	0	0	1	Amplification	1	0	42494T: 3.5;
OR6C3	chr12	12q13.2	1	0	0	1	Amplification	1	0	42494T: 3.5;
OR6C4	chr12	12q13.2	1	0	0	1	Amplification	1	0	42494T: 3.5;
OR6C6	chr12	12q13.2	1	0	0	1	Amplification	1	0	42494T: 3.5;
OR6C65	chr12	12q13.2	1	0	0	1	Amplification	1	0	42494T: 3.5;
OR6C68	chr12	12q13.2	1	0	0	1	Amplification	1	0	42494T: 3.5;
OR6C70	chr12	12q13.2	1	0	0	1	Amplification	1	0	42494T: 3.5;
OR6C74	chr12	12q13.2	1	0	0	1	Amplification	1	0	42494T: 3.5;
OR6C75	chr12	12q13.2	1	0	0	1	Amplification	1	0	42494T: 3.5;
OR6C76	chr12	12q13.2	1	0	0	1	Amplification	1	0	42494T: 3.5;
OR6J1	chr14	14q11.2	1	0	0	1	Amplification	1	0	42496T: 4;
OR6V1	chr7	7q34	1	0	1	0	Amplification	1	0	42487T: 3.5;
OR6W1P	chr7	7q34	1	0	1	0	Amplification	1	0	42487T: 3.5;
OR8H2	chr11	11q12.1	1	0	0	1	Amplification	1	0	42496T: 4.5;

Mangalaparthi *et al.* , 2020. Mutational landscape of esophageal squamous cell carcinoma in an Indian cohort
Supplementary Table 7A. List of copy number alterations and affected genes in ESCC patients

Gene	Chromosome	Cytoband	Recurrence	Recurrence in smoker cohort	Recurrence in chewer cohort	Recurrence in No habit cohort	State	Samples with CNA gain	Samples with CNA loss	File info with CNA fold
OR8H3	chr11	11q12.1	1	0	0	1	Amplification	1	0	42496T: 4.5;
OR8I2	chr11	11q12.1	1	0	0	1	Amplification	1	0	42496T: 4.5;
OR8J3	chr11	11q12.1	1	0	0	1	Amplification	1	0	42496T: 4.5;
OR8K5	chr11	11q12.1	1	0	0	1	Amplification	1	0	42496T: 4.5;
OR8S1	chr12	12q13.11	1	0	0	1	Amplification	1	0	42500T: 3.5;
OR9A2	chr7	7q34	1	0	1	0	Amplification	1	0	42487T: 3.5;
OR9A4	chr7	7q34	1	0	1	0	Amplification	1	0	42487T: 3.5;
OR9G1	chr11	11q11	1	0	0	1	Amplification	1	0	42496T: 4.5;
OR9K2	chr12	12q13.2	1	0	0	1	Amplification	1	0	42494T: 3.5;
ORAI1	chr12	12q24.31	1	0	0	1	Amplification	1	0	42500T: 3.5;
ORAI2	chr7	7q22.1	2	0	0	2	Amplification	2	0	42493T: 3.5; 42501T: 3.5;
ORAOV1	chr11	11q13.3	12	3	2	7	Amplification	12	0	42476T: 29.5; 42492T: 4; 42497T: 4.5; 42486T: 13.5; 42483T: 14.5; 42500T: 13; 56958T: 5; 42475T: 7.5; 42501T: 6; 42498T: 9.5; 42478T: 4.5; 56957T: 5;
ORC5	chr7	7q22.1-q22.2	4	0	1	3	Amplification	4	0	42501T: 3.5; 42497T: 4; 42493T: 3.5; 42487T: 3.5;
ORMDL1	chr2	2q32.2	2	0	1	1	Amplification	2	0	42482T: 4; 42493T: 3.5;
ORMDL2	chr12	12q13.2	1	0	0	1	Amplification	1	0	42494T: 5;
ORMDL3	chr17	17q21.1	1	0	0	1	Amplification	1	0	42497T: 3.5;
OSBPL11	chr3	3q21.2	1	0	0	1	Amplification	1	0	42496T: 3.5;
OSBPL1A	chr18	18q11.2	1	1	0	0	Amplification	1	0	42481T: 3.5;
OSBPL2	chr20	20q13.33	2	1	1	0	Amplification	2	0	42486T: 3.5; 42473T: 6;
OSBPL3	chr7	7p15.3	1	1	0	0	Amplification	1	0	42473T: 4;
OSBPL6	chr2	2q31.2	1	0	0	1	Amplification	1	0	42493T: 3.5;
OSER1	chr20	20q13.12	1	1	0	0	Amplification	1	0	42473T: 3.5;
OSGEPL1	chr2	2q32.2	2	0	1	1	Amplification	2	0	42482T: 4; 42493T: 3.5;
OSGIN2	chr8	8q21.3	3	1	0	2	Amplification	3	0	42475T: 3.5; 42496T: 3.5; 42495T: 3.5;
OSM	chr22	7p13	1	1	0	0	Amplification	1	0	42473T: 3.5;
OSMR	chr5	5p13.1	6	1	3	2	Amplification	6	0	42484T: 3.5; 42493T: 3.5; 42496T: 3.5; 42483T: 3.5; 42486T: 3.5; 42475T: 3.5;
OSR1	chr2	2p24.1	1	0	0	1	Amplification	1	0	42500T: 3.5;
OSR2	chr8	8q22.2	2	0	0	2	Amplification	2	0	42496T: 3.5; 42495T: 3.5;
OST4	chr2	2p23.3	1	0	0	1	Amplification	1	0	42500T: 3.5;
OSTCP1	chr6	6q25.3	1	1	0	0	Amplification	1	0	42473T: 3.5;
OSTM1	chr6	6q21	1	0	0	1	Amplification	1	0	42496T: 3.5;
OSTN	chr3	3q28	9	1	3	5	Amplification	9	0	42482T: 3.5; 42487T: 3.5; 42493T: 3.5; 42492T: 3.5; 56957T: 4.5; 42495T: 4; 42474T: 3.5; 42498T: 3.5; 42484T: 4;
OTOA	chr16	16p12.2|16p	1	1	0	0	Amplification	1	0	42473T: 4;
OTOF	chr2	2p23.3	1	0	0	1	Amplification	1	0	42500T: 3.5;
OTOL1	chr3	3q26.1	8	1	2	5	Amplification	8	0	42497T: 3.5; 42484T: 4; 56957T: 4; 42495T: 4; 42474T: 3.5; 42492T: 3.5; 42487T: 3.5; 42493T: 3.5;

Mangalaparthi *et al.* , 2020. Mutational landscape of esophageal squamous cell carcinoma in an Indian cohort
Supplementary Table 7A. List of copy number alterations and affected genes in ESCC patients

Gene	Chromosome	Cytoband	Recurrence	Recurrence in smoker cohort	Recurrence in chewer cohort	Recurrence in No habit cohort	State	Samples with CNA gain	Samples with CNA loss	File info with CNA fold
OTUD3	chr1	1p36.13	1	1	0	0	Amplification	1	0	42473T: 4;
OTUD6B	chr8	8q21.3	2	0	0	2	Amplification	2	0	42496T: 3.5; 42495T: 3.5;
OTUD7B	chr1	1q21.2	1	1	0	0	Amplification	1	0	42473T: 5;
OTX1	chr2	2p15	3	0	1	2	Amplification	3	0	42500T: 3.5; 42484T: 3.5; 56957T: 3.5;
OTX2	chr14	14q22.3	1	0	0	1	Amplification	1	0	42494T: 4;
OVCH1	chr12	12p11.22	1	0	0	1	Amplification	1	0	42500T: 6;
OVOL3	chr19	19q13.12	3	0	1	2	Amplification	3	0	42484T: 3.5; 56957T: 4; 42500T: 6.5;
OXA1L	chr14	14q11.2	1	0	0	1	Amplification	1	0	42496T: 4;
OXCT1	chr5	5p13.1	6	1	3	2	Amplification	6	0	42484T: 3.5; 42493T: 3.5; 42496T: 3.5; 42483T: 3.5; 42486T: 3.5; 42475T: 3.5;
OXER1	chr2	2p21	2	0	1	1	Amplification	2	0	42484T: 3.5; 42500T: 3.5;
OXR1	chr8	8q23.1	2	0	0	2	Amplification	2	0	42495T: 3.5; 42496T: 3.5;
OXSR1	chr3	3p22.2	1	1	0	0	Amplification	1	0	42473T: 4.5;
OXT	chr20	20p13	1	1	0	0	Amplification	1	0	42473T: 3.5;
P2RX2	chr12	12q24.33	1	0	0	1	Amplification	1	0	56957T: 4;
P2RX4	chr12	12q24.31	1	0	0	1	Amplification	1	0	42500T: 3.5;
P2RX6	chr22	22q11.21	1	1	0	0	Amplification	1	0	42477T: 4;
P2RX7	chr12	12q24.31	1	0	0	1	Amplification	1	0	42500T: 3.5;
P2RY1	chr3	3q25.2	9	1	2	6	Amplification	9	0	42487T: 3.5; 42493T: 3.5; 42492T: 3.5; 42500T: 3.5; 42497T: 5.5; 42496T: 4; 42474T: 3.5; 56957T: 4; 42484T: 3.5;
P2RY12	chr3	3q25.1	9	1	2	6	Amplification	9	0	42484T: 3.5; 42474T: 3.5; 56957T: 4; 42497T: 5.5; 42496T: 4; 42500T: 3.5; 42492T: 3.5; 42493T: 3.5; 42487T: 3.5;
P2RY13	chr3	3q25.1	9	1	2	6	Amplification	9	0	42497T: 5.5; 42496T: 4; 42500T: 3.5; 42484T: 3.5; 42474T: 3.5; 56957T: 4; 42492T: 3.5; 42493T: 3.5; 42487T: 3.5;
P2RY2	chr11	11q13.4	5	2	0	3	Amplification	5	0	56957T: 3.5; 42492T: 4; 42498T: 6.5; 42475T: 8.5; 42476T: 4;
P2RY6	chr11	11q13.4	6	3	0	3	Amplification	6	0	56957T: 3.5; 42498T: 6.5; 42492T: 4; 42478T: 4.5; 42475T: 8.5; 42476T: 4;
P4HA3	chr11	11q13.4	5	2	0	3	Amplification	5	0	42476T: 4; 42475T: 7.5; 42492T: 4; 42498T: 8; 56957T: 3.5;
PA2G4	chr12	12q13.2	1	0	0	1	Amplification	1	0	42494T: 9.5;
PAAF1	chr11	11q13.4	6	3	0	3	Amplification	6	0	42476T: 4; 42475T: 7.5; 42498T: 8; 42492T: 4; 42478T: 6; 56957T: 3.5;
PABPC1	chr8	8q22.3	2	0	0	2	Amplification	2	0	42496T: 3.5; 42495T: 3.5;
PABPC1L	chr20	20q13.12	1	1	0	0	Amplification	1	0	42473T: 5;
PABPN1	chr14	14q11.2	2	0	0	2	Amplification	2	0	42500T: 4; 42496T: 4;
PACSIN3	chr11	11p11.2	1	1	0	0	Amplification	1	0	42473T: 3.5;
PAEP	chr9	9q34.3	2	0	1	1	Amplification	2	0	42484T: 4.5; 56957T: 3.5;

Mangalaparthi *et al.* , 2020. Mutational landscape of esophageal squamous cell carcinoma in an Indian cohort
Supplementary Table 7A. List of copy number alterations and affected genes in ESCC patients

Gene	Chromosome	Cytoband	Recurrence	Recurrence in smoker cohort	Recurrence in chewer cohort	Recurrence in No habit cohort	State	Samples with CNA gain	Samples with CNA loss	File info with CNA fold
PAF1	chr19	19q13.2	2	0	0	2	Amplification	2	0	42500T: 6.5; 56957T: 4;
PAFAH1B3	chr19	19q13.2	1	1	0	0	Amplification	1	0	42473T: 4.5;
PAG1	chr8	8q21.13	2	0	0	2	Amplification	2	0	42496T: 3.5; 42495T: 3.5;
PAIP1	chr5	5p12	4	1	1	2	Amplification	4	0	42475T: 3.5; 42496T: 3.5; 42486T: 3.5; 42493T: 3.5;
PAIP2B	chr2	2p13.3	1	0	0	1	Amplification	1	0	42500T: 3.5;
PAK2	chr3	1p22.2	9	1	3	5	Amplification	9	0	42498T: 3.5; 42484T: 4; 56957T: 6; 42474T: 3.5; 42495T: 4; 42482T: 3.5; 42492T: 3.5; 42487T: 3.5; 42493T: 5;
PAK4	chr19	19q13.2	2	0	0	2	Amplification	2	0	56957T: 4; 42500T: 6.5;
PAK6	chr15	15q15.1	1	1	0	0	Amplification	1	0	42473T: 3.5;
PALB2	chr16	16p12.2	1	1	0	0	Amplification	1	0	42473T: 4;
PALM	chr19	19p13.3	1	0	0	1	Amplification	1	0	42493T: 3.5;
PALM2	chr9	9q31.3	1	0	1	0	Amplification	1	0	42483T: 3.5;
PAM16	chr16	16p13.3	3	1	0	2	Amplification	3	0	42495T: 8.5; 42494T: 3.5; 42473T: 5.5;
PANK2	chr20	20p13	1	1	0	0	Amplification	1	0	42473T: 4.5;
PAPD7	chr5	5p15.31	4	1	1	2	Amplification	4	0	42493T: 4; 42475T: 3.5; 42486T: 3.5; 42496T: 3.5;
PAPL	chr19	19q13.2	2	0	0	2	Amplification	2	0	42500T: 6.5; 56957T: 4;
PAPLN	chr14	14q24.2	2	0	0	2	Amplification	2	0	56957T: 3.5; 42494T: 4;
PAPOLB	chr7	7p22.1	2	1	0	1	Amplification	2	0	42473T: 4.5; 42497T: 8.5;
PAPOLG	chr2	2p16.1	3	0	1	2	Amplification	3	0	42500T: 3.5; 56957T: 3.5; 42484T: 4.5;
PAQR6	chr1	1q22	1	1	0	0	Amplification	1	0	42473T: 4.5;
PAQR9	chr3	3q23	5	0	1	4	Amplification	5	0	42496T: 4; 42492T: 3.5; 56957T: 4; 42493T: 3.5; 42487T: 3.5;
PARD6B	chr20	20q13.13	1	1	0	0	Amplification	1	0	42473T: 5;
PARL	chr3	3q27.1	10	1	3	6	Amplification	10	0	42482T: 3.5; 42492T: 3.5; 42493T: 3.5; 42487T: 3.5; 42497T: 4; 42498T: 3.5; 42484T: 4; 56957T: 5; 42495T: 4; 42474T: 3.5;
PARN	chr16	16p13.12	2	1	0	1	Amplification	2	0	42495T: 4; 42473T: 4;
PARP10	chr8	8q24.3	3	0	1	2	Amplification	3	0	42495T: 4.5; 42483T: 3.5; 42496T: 4;
PARP11	chr12	12p13.32	1	0	0	1	Amplification	1	0	42494T: 3.5;
PARP12	chr7	7q34	1	0	1	0	Amplification	1	0	42487T: 3.5;
PARP14	chr3	3q21.1	1	0	0	1	Amplification	1	0	42496T: 3.5;
PARP15	chr3	3q21.1	1	0	0	1	Amplification	1	0	42496T: 3.5;
PARP8	chr5	5q11.1	2	1	0	1	Amplification	2	0	42475T: 3.5; 42498T: 10.5;
PARP9	chr3	3q21.1	1	0	0	1	Amplification	1	0	42496T: 3.5;
PAX1	chr20	20p11.22	1	0	1	0	Amplification	1	0	42483T: 4;
PAX4	chr7	7q32.1	1	0	1	0	Amplification	1	0	42487T: 3.5;
PAX7	chr1	1p36.13	1	1	0	0	Amplification	1	0	42473T: 4;
PAX9	chr14	14q13.3	2	1	0	1	Amplification	2	0	42500T: 8.5; 42476T: 8;
PBOV1	chr6	6q23.3	1	0	0	1	Amplification	1	0	42493T: 3.5;
PBRM1	chr3	3p21.1	1	0	0	1	Deletion	0	1	42497T: 0.5;

Mangalaparthi *et al.* , 2020. Mutational landscape of esophageal squamous cell carcinoma in an Indian cohort
Supplementary Table 7A. List of copy number alterations and affected genes in ESCC patients

Gene	Chromosome	Cytoband	Recurrence	Recurrence in smoker cohort	Recurrence in chewer cohort	Recurrence in No habit cohort	State	Samples with CNA gain	Samples with CNA loss	File info with CNA fold
PBX2	chr6	6p21.32	1	1	0	0	Amplification	1	0	42473T: 3.5;
PBX2P1	chr3	3q24	5	0	1	4	Amplification	5	0	42492T: 3.5; 56957T: 4; 42487T: 3.5; 42493T: 3.5; 42496T: 4;
PBXIP1	chr1	1q21.3	2	1	0	1	Amplification	2	0	42496T: 3.5; 42473T: 6.5;
PC	chr11	2q14.3	2	1	0	1	Amplification	2	0	42473T: 3.5; 56957T: 5.5;
PCBD2	chr5	5q31.1	1	1	0	0	Amplification	1	0	42473T: 3.5;
PCBP1	chr2	2p13.3	1	0	0	1	Amplification	1	0	42500T: 3.5;
PCCB	chr3	3q22.3	4	0	1	3	Amplification	4	0	42492T: 3.5; 42487T: 3.5; 42493T: 3.5; 42496T: 3.5;
PCED1A	chr20	20p13	1	1	0	0	Amplification	1	0	42473T: 3.5;
PCED1B	chr12	12q13.11	1	0	0	1	Amplification	1	0	42500T: 3.5;
PCGF1	chr2	2p13.1	1	0	0	1	Amplification	1	0	42500T: 3.5;
PCGF2	chr17	17q12	1	1	0	0	Amplification	1	0	42473T: 3.5;
PCID2	chr13	13q34	1	0	0	1	Amplification	1	0	56957T: 3.5;
PCIF1	chr20	20q13.12	1	1	0	0	Amplification	1	0	42473T: 5;
PCK1	chr20	20q13.31	1	1	0	0	Amplification	1	0	42473T: 7;
PCLO	chr7	7q21.11	1	0	1	0	Amplification	1	0	42487T: 3.5;
PCMTD1	chr8	8q11.23	5	0	2	3	Amplification	5	0	42482T: 3.5; 42496T: 3.5; 42484T: 5; 42495T: 3.5; 42494T: 3.5;
PCMTD2	chr20	20q13.33	1	1	0	0	Amplification	1	0	42473T: 6;
PCNA	chr20	20p12.3	1	1	0	0	Amplification	1	0	42473T: 4.5;
PCNP	chr3	3q12.3	1	1	0	0	Amplification	1	0	42476T: 3.5;
PCNX	chr14	14q24.2	1	0	0	1	Amplification	1	0	42494T: 4;
PCNXL4	chr14	14q23.1	2	0	1	1	Amplification	2	0	42494T: 4; 42483T: 3.5;
PCOLCE2	chr3	3q23	5	0	1	4	Amplification	5	0	42487T: 3.5; 56957T: 4; 42493T: 3.5; 42492T: 3.5; 42496T: 4;
PCP4	chr21	21q22.2	1	1	0	0	Amplification	1	0	42473T: 3.5;
PCTP	chr17	17q22	1	0	0	1	Amplification	1	0	42497T: 4;
PCYOX1	chr2	2p13.3	1	0	0	1	Amplification	1	0	42500T: 3.5;
PCYT1A	chr3	3q29	9	1	3	5	Amplification	9	0	42482T: 3.5; 42487T: 3.5; 42493T: 5; 42492T: 3.5; 56957T: 6; 42474T: 3.5; 42495T: 4; 42498T: 3.5; 42484T: 4;
PDCD10	chr3	3q26.1	9	1	3	5	Amplification	9	0	42482T: 3.5; 42493T: 3.5; 42487T: 3.5; 42492T: 3.5; 42500T: 8; 42474T: 3.5; 42495T: 4; 56957T: 4; 42484T: 4;
PDCD1LG2	chr9	9p24.1	2	0	0	2	Amplification	2	0	42498T: 14; 42496T: 3.5;
PDCD2L	chr19	19q13.11	3	0	1	2	Amplification	3	0	42500T: 4.5; 42484T: 3.5; 56957T: 4;
PDCD5	chr19	19q13.11	4	1	1	2	Amplification	4	0	42473T: 3.5; 42500T: 4.5; 42484T: 3.5; 56957T: 4;
PDCD6	chr5	5p15.33	5	2	1	2	Amplification	5	0	42475T: 3.5; 42473T: 3.5; 42496T: 4; 42486T: 3.5; 42493T: 3.5;
PDCL3	chr2	2q11.2	1	0	0	1	Amplification	1	0	42493T: 3.5;
PDE11A	chr2	2q31.2	1	0	0	1	Amplification	1	0	42493T: 4.5;

Mangalaparthi *et al.* , 2020. Mutational landscape of esophageal squamous cell carcinoma in an Indian cohort
Supplementary Table 7A. List of copy number alterations and affected genes in ESCC patients

Gene	Chromosome	Cytoband	Recurrence	Recurrence in smoker cohort	Recurrence in chewer cohort	Recurrence in No habit cohort	State	Samples with CNA gain	Samples with CNA loss	File info with CNA fold
PDE1A	chr2	2q32.1	1	0	0	1	Amplification	1	0	42493T: 4;
PDE1B	chr12	12q13.2	2	1	0	1	Amplification	2	0	42473T: 3.5; 42494T: 3.5;
PDE1C	chr7	7p14.3	1	1	0	0	Amplification	1	0	42473T: 4;
PDE2A	chr11	11q13.4	6	3	0	3	Amplification	6	0	56957T: 3.5; 42492T: 4; 42498T: 5; 42478T: 4.5; 42475T: 5; 42476T: 4;
PDE3A	chr12	12p12.2	1	0	0	1	Amplification	1	0	42500T: 4.5;
PDE4D	chr5	5q11.2-q12.	1	1	0	0	Deletion	0	1	42473T: 0.5;
PDE4DIP	chr1	1q21.2	1	1	0	0	Amplification	1	0	42473T: 4;
PDE6H	chr12	12p12.3	1	0	0	1	Amplification	1	0	42500T: 4.5;
PDE7A	chr8	8q13.1	2	0	0	2	Amplification	2	0	42496T: 3.5; 42495T: 3.5;
PDE8A	chr15	15q25.3	1	1	0	0	Amplification	1	0	42473T: 4;
PDE9A	chr21	21q22.3	1	1	0	0	Amplification	1	0	42473T: 3.5;
PDGFA	chr7	7p22.3	1	1	0	0	Amplification	1	0	42473T: 5;
PDGFB	chr22	22q13.1	1	1	0	0	Amplification	1	0	42473T: 3.5;
PDHX	chr11	11p13	1	1	0	0	Amplification	1	0	42473T: 4.5;
PDIA2	chr16	16p13.3	1	0	1	0	Amplification	1	0	42483T: 3.5;
PDIA3P	chr1	1q21.1	1	1	0	0	Amplification	1	0	42473T: 4;
PDIA4	chr7	7q36.1	2	0	1	1	Amplification	2	0	42500T: 4; 42487T: 3.5;
PDIA5	chr3	3q21.1	1	0	0	1	Amplification	1	0	42496T: 3.5;
PDIA6	chr2	2p25.1	1	0	0	1	Amplification	1	0	42500T: 3.5;
PDILT	chr16	16p12.3	1	1	0	0	Amplification	1	0	42473T: 4;
PDK1	chr2	16p13.3	2	1	0	1	Amplification	2	0	42473T: 4.5; 42493T: 5;
PDK4	chr7	7q21.3	1	0	1	0	Amplification	1	0	42487T: 3.5;
PDP1	chr8	9p24.1	2	0	0	2	Amplification	2	0	42496T: 3.5; 42495T: 3.5;
PDRG1	chr20	20q11.21	1	0	0	1	Amplification	1	0	42496T: 5;
PDS5B	chr13	13q13.1	1	0	0	1	Amplification	1	0	42497T: 6;
PDSS1	chr10	10p12.1	1	0	0	1	Amplification	1	0	56958T: 5;
PDSS2	chr6	6q21	1	0	0	1	Amplification	1	0	42496T: 3.5;
PDXDC1	chr16	16p13.11	2	1	0	1	Amplification	2	0	42495T: 4; 42473T: 4;
PDXP	chr22	22q13.1	1	1	0	0	Amplification	1	0	42473T: 3.5;
PDYN	chr20	20p13	1	1	0	0	Amplification	1	0	42473T: 3.5;
PDZD2	chr5	5p13.3	4	1	1	2	Amplification	4	0	42496T: 3.5; 42486T: 3.5; 42475T: 3.5; 42493T: 3.5;
PDZD9	chr16	16p12.2	1	1	0	0	Amplification	1	0	42473T: 4;
PDZK1	chr1	1q21.1	1	1	0	0	Amplification	1	0	42473T: 4;
PDZRN4	chr12	12q12	1	0	0	1	Amplification	1	0	42500T: 3.5;
PEAR1	chr1	1q23.1	1	1	0	0	Amplification	1	0	42473T: 7;
PEBP1	chr12	12q24.23	1	0	0	1	Amplification	1	0	42500T: 3.5;
PEF1	chr1	1p35.2	1	1	0	0	Amplification	1	0	42473T: 3.5;
PEG10	chr7	7q21.3	1	0	1	0	Amplification	1	0	42487T: 3.5;
PELI1	chr2	2p14	2	0	0	2	Amplification	2	0	56957T: 3.5; 42500T: 3.5;
PELI2	chr14	14q22.3	1	0	0	1	Amplification	1	0	42494T: 4;

Mangalaparthi *et al.* , 2020. Mutational landscape of esophageal squamous cell carcinoma in an Indian cohort
Supplementary Table 7A. List of copy number alterations and affected genes in ESCC patients

Gene	Chromosome	Cytoband	Recurrence	Recurrence in smoker cohort	Recurrence in chewer cohort	Recurrence in No habit cohort	State	Samples with CNA gain	Samples with CNA loss	File info with CNA fold
PELI3	chr11	11q13.2	1	0	0	1	Amplification	1	0	56957T: 5.5;
PELO	chr5	5q11.2	1	0	0	1	Amplification	1	0	42498T: 16.5;
PENK	chr8	8q12.1	2	0	0	2	Amplification	2	0	42496T: 3.5; 42495T: 3.5;
PEPD	chr19	19q13.11	3	0	1	2	Amplification	3	0	42484T: 3.5; 56957T: 4; 42500T: 4.5;
PER2	chr2	2q37.3	1	1	0	0	Amplification	1	0	42473T: 3.5;
PERP	chr6	6q23.3	1	0	0	1	Amplification	1	0	42493T: 3.5;
PEX1	chr7	19q13.12	2	0	2	0	Amplification	2	0	42483T: 4; 42487T: 3.5;
PEX11A	chr15	15q26.1	1	1	0	0	Amplification	1	0	42473T: 3.5;
PEX11B	chr1	1q21.1	1	1	0	0	Amplification	1	0	42473T: 4;
PEX13	chr2	2p15	3	0	1	2	Amplification	3	0	42484T: 4.5; 56957T: 3.5; 42500T: 3.5;
PEX16	chr11	11p11.2	1	1	0	0	Amplification	1	0	42473T: 3.5;
PEX2	chr8	8q21.13	2	0	0	2	Amplification	2	0	42496T: 3.5; 42495T: 3.5;
PEX26	chr22	22q11.21	1	0	0	1	Amplification	1	0	42497T: 24.5;
PEX5	chr12	12p13.31	1	0	0	1	Amplification	1	0	42494T: 3.5;
PEX5L	chr3	3q26.33	11	1	3	7	Amplification	11	0	42492T: 3.5; 42493T: 3.5; 42487T: 3.5; 42482T: 3.5; 42484T: 4; 42498T: 3.5; 42495T: 4; 42474T: 3.5; 56957T: 4; 42497T: 4; 42500T: 14;
PFDN4	chr20	20q13.2	1	1	0	0	Amplification	1	0	42473T: 5;
PFKM	chr12	12q13.11	1	0	0	1	Amplification	1	0	42500T: 3.5;
PFN2	chr3	3q25.1	9	2	2	5	Amplification	9	0	42474T: 3.5; 56957T: 4; 42484T: 3.5; 42496T: 4; 42497T: 4.5; 42473T: 4; 42487T: 3.5; 42493T: 3.5; 42492T: 3.5;
PFN4	chr2	2p23.3	1	0	0	1	Amplification	1	0	42500T: 3.5;
PGAM5	chr12	12q24.33	1	0	0	1	Amplification	1	0	56957T: 4;
PGAP1	chr2	2q33.1	1	0	1	0	Amplification	1	0	42482T: 4;
PGAP3	chr17	17q12	2	1	0	1	Amplification	2	0	42497T: 3.5; 42473T: 6;
PGF	chr14	14q24.3	2	0	0	2	Amplification	2	0	56957T: 3.5; 42494T: 4;
PGLYRP1	chr19	19q13.32	1	0	1	0	Amplification	1	0	42484T: 4;
PGLYRP3	chr1	1q21.3	1	0	0	1	Amplification	1	0	42493T: 4;
PGLYRP4	chr1	1q21.3	1	0	0	1	Amplification	1	0	42493T: 4;
PGM2	chr4	4p14	1	1	0	0	Amplification	1	0	42473T: 4.5;
PGM2L1	chr11	11q13.4	5	2	0	3	Amplification	5	0	56957T: 3.5; 42498T: 8; 42492T: 4; 42475T: 7.5; 42476T: 4;
PGP	chr16	16p13.3	1	0	1	0	Amplification	1	0	42483T: 3.5;
PHACTR3	chr20	20q13.32-q1	1	1	0	0	Amplification	1	0	42473T: 4.5;
PHACTR4	chr1	1p35.3	1	1	0	0	Amplification	1	0	42473T: 3.5;
PHB2	chr12	12p13.31	1	0	0	1	Amplification	1	0	42494T: 3.5;
PHC3	chr3	3q26.2	9	1	3	5	Amplification	9	0	42482T: 3.5; 42492T: 3.5; 42493T: 3.5; 42487T: 3.5; 42500T: 8; 42484T: 4; 42474T: 3.5; 42495T: 4; 56957T: 4;
PHF14	chr7	7p21.3	1	1	0	0	Amplification	1	0	42473T: 3.5;

Mangalaparthi *et al.* , 2020. Mutational landscape of esophageal squamous cell carcinoma in an Indian cohort
Supplementary Table 7A. List of copy number alterations and affected genes in ESCC patients

Gene	Chromosome	Cytoband	Recurrence	Recurrence in smoker cohort	Recurrence in chewer cohort	Recurrence in No habit cohort	State	Samples with CNA gain	Samples with CNA loss	File info with CNA fold
PHF15	chr5	5q31.1	1	1	0	0	Amplification	1	0	42473T: 3.5;
PHF20	chr20	20q11.22-q1	1	1	0	0	Amplification	1	0	42473T: 3.5;
PHF20L1	chr8	8q24.22	3	0	1	2	Amplification	3	0	42496T: 3.5; 42495T: 3.5; 42484T: 3.5;
PHF21A	chr11	11p11.2	1	1	0	0	Amplification	1	0	42473T: 3.5;
PHGR1	chr15	15q15.1	1	1	0	0	Amplification	1	0	42473T: 3.5;
PHKG1	chr7	7p11.2	2	0	1	1	Amplification	2	0	56957T: 5; 42483T: 4;
PHLDB2	chr3	3q13.2	1	0	0	1	Amplification	1	0	42496T: 3.5;
PHLDB3	chr19	19q13.31	1	0	0	1	Amplification	1	0	56957T: 4;
PHOX2A	chr11	11q13.4	7	3	0	4	Amplification	7	0	42475T: 5; 42476T: 4; 56957T: 3.5; 42493T: 3.5; 42478T: 4.5; 42498T: 5; 42492T: 4;
PHPT1	chr9	9q34.3	2	1	0	1	Amplification	2	0	42473T: 5; 56957T: 3.5;
PHTF2	chr7	7q11.23-q21	1	0	1	0	Amplification	1	0	42487T: 3.5;
PI15	chr8	8q21.13	2	0	0	2	Amplification	2	0	42496T: 3.5; 42495T: 3.5;
PI16	chr6	6p21.2	1	0	1	0	Amplification	1	0	42486T: 3.5;
PI3	chr20	20q13.12	1	1	0	0	Amplification	1	0	42473T: 5;
PI4KA	chr22	22q11.21	2	1	0	1	Amplification	3	0	42497T: 13.5,4; 42477T: 4;
PI4KAP1	chr22	22q11.21	1	1	0	0	Amplification	1	0	42477T: 4;
PI4KAP2	chr22	22q11.21	1	1	0	0	Amplification	1	0	42473T: 3.5;
PI4KB	chr1	1q21.3	1	1	0	0	Amplification	1	0	42473T: 4.5;
PIAS3	chr1	1q21.1	1	1	0	0	Amplification	1	0	42473T: 4;
PICK1	chr22	22q13.1	1	1	0	0	Amplification	1	0	42473T: 3.5;
PIGF	chr2	2p21	2	0	1	1	Amplification	2	0	42484T: 4.5; 42500T: 3.5;
PIGH	chr14	14q24.1	1	0	0	1	Amplification	1	0	42494T: 4;
PIGO	chr9	9p13.3	1	0	0	1	Amplification	1	0	42501T: 3.5;
PIGQ	chr16	16p13.3	2	0	1	1	Amplification	2	0	42493T: 3.5; 42483T: 3.5;
PIGT	chr20	20q13.12	1	1	0	0	Amplification	1	0	42473T: 5;
PIGU	chr20	20q11.22	2	1	0	1	Amplification	2	0	42473T: 3.5; 42493T: 3.5;
PIGX	chr3	3q29	9	1	3	5	Amplification	9	0	42482T: 3.5; 42493T: 5; 42487T: 3.5; 42492T: 3.5; 42495T: 4; 42474T: 3.5; 56957T: 6; 42484T: 4; 42498T: 3.5;
PIGZ	chr3	3q29	9	1	3	5	Amplification	9	0	56957T: 6; 42474T: 3.5; 42495T: 4; 42498T: 3.5; 42484T: 4; 42493T: 5; 42487T: 3.5; 42492T: 3.5; 42482T: 3.5;
PIK3C2G	chr12	12p12.3	1	0	0	1	Amplification	1	0	42500T: 4.5;
PIK3CA	chr3	3q26.32	11	1	3	7	Amplification	11	0	42500T: 9.5; 42497T: 4; 42495T: 4; 42474T: 3.5; 56957T: 4; 42484T: 4; 42498T: 3.5; 42482T: 3.5; 42493T: 3.5; 42487T: 3.5; 42492T: 3.5;
PIK3CB	chr3	3q22.3	4	0	1	3	Amplification	4	0	42496T: 3.5; 42487T: 3.5; 42493T: 3.5; 42492T: 3.5;
PIK3CG	chr7	7q22.3	4	0	1	3	Amplification	4	0	42501T: 3.5; 42497T: 4; 42487T: 3.5; 42493T: 3.5;
PIK3R4	chr3	3q22.1	3	0	1	2	Amplification	3	0	42487T: 3.5; 42492T: 3.5; 42496T: 3.5;
PINK1	chr1	1p36.12	1	1	0	0	Amplification	1	0	42473T: 4;

Mangalaparthi *et al.* , 2020. Mutational landscape of esophageal squamous cell carcinoma in an Indian cohort
Supplementary Table 7A. List of copy number alterations and affected genes in ESCC patients

Gene	Chromosome	Cytoband	Recurrence	Recurrence in smoker cohort	Recurrence in chewer cohort	Recurrence in No habit cohort	State	Samples with CNA gain	Samples with CNA loss	File info with CNA fold
PINLYP	chr19	19q13.31	1	0	0	1	Amplification	1	0	56957T: 4;
PINX1	chr8	8p23.1	1	0	1	0	Amplification	1	0	42486T: 3.5;
PIP	chr7	1q41	1	0	1	0	Amplification	1	0	42487T: 3.5;
PIP4K2B	chr17	17q12	1	1	0	0	Amplification	1	0	42473T: 3.5;
PIP5K1A	chr1	1q21.3	1	1	0	0	Amplification	1	0	42473T: 4.5;
PITHD1	chr1	1p36.11	1	1	0	0	Amplification	1	0	42473T: 3.5;
PITPNM1	chr11	11q13.2	2	1	0	1	Amplification	2	0	56957T: 5; 42473T: 3.5;
PITPNM2	chr12	12q24.31	1	0	0	1	Amplification	1	0	42500T: 3.5;
PITX1	chr5	5q31.1	1	1	0	0	Amplification	1	0	42473T: 3.5;
PKD1	chr16	16p13.3	1	0	1	0	Amplification	1	0	42483T: 3.5;
PKD1P5	chr16	16p12.3	1	0	1	0	Deletion	0	1	42484T: 0;
PKDCC	chr2	2p21	2	0	1	1	Amplification	2	0	42500T: 3.5; 42484T: 3.5;
PKHD1L1	chr8	8q23.1-q23.2	2	0	0	2	Amplification	2	0	42495T: 3.5; 42496T: 3.5;
PKIA	chr8	8q21.13	2	0	0	2	Amplification	2	0	42495T: 3.5; 42496T: 3.5;
PKIG	chr20	20q13.12	1	1	0	0	Amplification	1	0	42473T: 5;
PKLR	chr1	1q22	1	1	0	0	Amplification	1	0	42473T: 6.5;
PKP2	chr12	12p11.21	1	0	0	1	Amplification	1	0	42500T: 3.5;
PKP4	chr2	2q24.1	2	1	0	1	Amplification	2	0	42473T: 4; 42493T: 3.5;
PLA1A	chr3	3q13.33	1	0	0	1	Amplification	1	0	42496T: 3.5;
PLA2G10	chr16	16p13.12	2	1	0	1	Amplification	2	0	42495T: 4; 42473T: 4;
PLA2G1B	chr12	12q24.31	1	0	0	1	Amplification	1	0	42500T: 3.5;
PLA2G2A	chr1	1p36.13	1	1	0	0	Amplification	1	0	42473T: 4;
PLA2G2C	chr1	1p36.12	1	1	0	0	Amplification	1	0	42473T: 4;
PLA2G2D	chr1	1p36.12	1	1	0	0	Amplification	1	0	42473T: 4;
PLA2G2E	chr1	1p36.13	1	1	0	0	Amplification	1	0	42473T: 4;
PLA2G2F	chr1	1p36.12	1	1	0	0	Amplification	1	0	42473T: 4;
PLA2G5	chr1	1p36.13	1	1	0	0	Amplification	1	0	42473T: 4;
PLA2G6	chr22	22q13.1	1	1	0	0	Amplification	1	0	42473T: 3.5;
PLAG1	chr8	8q12.1	3	0	1	2	Amplification	3	0	42495T: 3.5; 42496T: 3.5; 42483T: 5;
PLAGL2	chr20	20q11.21	2	1	0	1	Amplification	2	0	42473T: 4.5; 42496T: 5;
PLAUR	chr19	19q13.31	1	0	0	1	Amplification	1	0	56957T: 4;
PLB1	chr2	2p23.2	1	0	0	1	Amplification	1	0	42500T: 3.5;
PLBD1	chr12	12p13.1	1	0	0	1	Amplification	1	0	42500T: 4.5;
PLBD2	chr12	12q24.13	1	0	0	1	Amplification	1	0	42500T: 3.5;
PLCB2	chr15	15q15.1	1	1	0	0	Amplification	1	0	42473T: 3.5;
PLCD1	chr3	3p22.2	1	1	0	0	Amplification	1	0	42473T: 4.5;
PLCG1	chr20	20q12	1	1	0	0	Amplification	1	0	42473T: 3.5;
PLCH1	chr3	3q25.31	8	1	2	5	Amplification	8	0	42496T: 4.5; 42497T: 5.5; 56957T: 4; 42474T: 3.5; 42484T: 3.5; 42493T: 3.5; 42487T: 3.5; 42492T: 3.5;
PLCL1	chr2	2q33.1	1	0	1	0	Amplification	1	0	42482T: 4;

Mangalaparthi *et al.* , 2020. Mutational landscape of esophageal squamous cell carcinoma in an Indian cohort
Supplementary Table 7A. List of copy number alterations and affected genes in ESCC patients

Gene	Chromosome	Cytoband	Recurrence	Recurrence in smoker cohort	Recurrence in chewer cohort	Recurrence in No habit cohort	State	Samples with CNA gain	Samples with CNA loss	File info with CNA fold
PLCXD3	chr5	5p13.1	6	1	3	2	Amplification	6	0	42484T: 3.5; 42493T: 3.5; 42486T: 3.5; 42483T: 3.5; 42496T: 3.5; 42475T: 3.5;
PLCZ1	chr12	12p12.3	1	0	0	1	Amplification	1	0	42500T: 4.5;
PLD1	chr3	19p13.2	9	1	3	5	Amplification	9	0	42493T: 3.5; 42487T: 3.5; 42492T: 3.5; 42482T: 3.5; 42495T: 4; 42474T: 3.5; 56957T: 4; 42484T: 4; 42500T: 9;
PLD3	chr19	19q13.2	2	0	0	2	Amplification	2	0	42500T: 3.5; 56957T: 4;
PLEC	chr8	8q24.3	3	0	1	2	Amplification	3	0	42483T: 3.5; 42496T: 4; 42495T: 4.5;
PLEK	chr2	2p14	1	0	0	1	Amplification	1	0	42500T: 3.5;
PLEK2	chr14	14q23.3-q24	1	0	0	1	Amplification	1	0	42494T: 4;
PLEKHA3	chr2	2q31.2	1	0	0	1	Amplification	1	0	42493T: 3.5;
PLEKHA5	chr12	12p12.3	2	1	0	1	Amplification	2	0	42473T: 3.5; 42500T: 4.5;
PLEKHA8	chr7	7p14.3	1	1	0	0	Amplification	1	0	42473T: 4;
PLEKHA8P1	chr12	12q12	1	0	0	1	Amplification	1	0	42500T: 3.5;
PLEKHB1	chr11	11q13.4	6	3	0	3	Amplification	6	0	42475T: 8.5; 42476T: 4; 56957T: 3.5; 42478T: 4.5; 42492T: 4; 42498T: 8;
PLEKHD1	chr14	14q24.1	1	0	0	1	Amplification	1	0	42494T: 4;
PLEKHF1	chr19	19q12	5	1	1	3	Amplification	5	0	42473T: 25; 42500T: 4.5; 42484T: 6; 42494T: 4; 56957T: 4;
PLEKHF2	chr8	8q22.1	3	1	0	2	Amplification	3	0	42495T: 3.5; 42473T: 4.5; 42496T: 3.5;
PLEKHG2	chr19	19q13.2	2	0	0	2	Amplification	2	0	56957T: 4; 42500T: 6.5;
PLEKHG3	chr14	14q23.3	1	0	0	1	Amplification	1	0	42494T: 4;
PLEKHG4B	chr5	5p15.33	4	1	1	2	Amplification	4	0	42493T: 3.5; 42486T: 3.5; 42496T: 4; 42475T: 3.5;
PLEKHH1	chr14	14q24.1	1	0	0	1	Amplification	1	0	42494T: 4;
PLEKHH2	chr2	2p21	2	0	1	1	Amplification	2	0	42500T: 3.5; 42484T: 3.5;
PLEKHO1	chr1	1q21.2	1	1	0	0	Amplification	1	0	42473T: 5;
PLGRKT	chr9	9p24.1	2	0	0	2	Amplification	2	0	42496T: 3.5; 42498T: 14;
PLIN1	chr15	15q26.1	1	1	0	0	Amplification	1	0	42473T: 3.5;
PLK1	chr16	16p12.2	1	1	0	0	Amplification	1	0	42473T: 4;
PLK1S1	chr20	20p11.23	1	0	1	0	Amplification	1	0	42483T: 4;
PLOD2	chr3	3q24	7	1	1	5	Amplification	7	0	42474T: 3.5; 42493T: 3.5; 56957T: 4; 42487T: 3.5; 42492T: 3.5; 42497T: 4.5; 42496T: 4;
PLOD3	chr7	7q22.1	2	0	0	2	Amplification	2	0	42501T: 3.5; 42493T: 3.5;
PLS1	chr3	3q23	5	0	1	4	Amplification	5	0	42487T: 3.5; 56957T: 4; 42493T: 3.5; 42492T: 3.5; 42496T: 4;
PLSCR1	chr3	3q24	7	1	1	5	Amplification	7	0	42497T: 4.5; 42496T: 4; 42487T: 3.5; 56957T: 4; 42493T: 3.5; 42474T: 3.5; 42492T: 3.5;
PLSCR2	chr3	3q24	7	1	1	5	Amplification	7	0	42492T: 3.5; 42474T: 3.5; 42487T: 3.5; 56957T: 4; 42493T: 3.5; 42496T: 4; 42497T: 4.5;
PLSCR4	chr3	3q24	7	1	1	5	Amplification	7	0	42496T: 4; 42497T: 4.5; 42474T: 3.5; 56957T: 4; 42493T: 3.5; 42487T: 3.5; 42492T: 3.5;

Mangalaparthi *et al.* , 2020. Mutational landscape of esophageal squamous cell carcinoma in an Indian cohort
Supplementary Table 7A. List of copy number alterations and affected genes in ESCC patients

Gene	Chromosome	Cytoband	Recurrence	Recurrence in smoker cohort	Recurrence in chewer cohort	Recurrence in No habit cohort	State	Samples with CNA gain	Samples with CNA loss	File info with CNA fold
PLSCR5	chr3	3q24	7	1	1	5	Amplification	7	0	42496T: 4; 42497T: 4.5; 42492T: 3.5; 56957T: 4; 42493T: 3.5; 42487T: 3.5; 42474T: 3.5;
PLTP	chr20	20q13.12	1	1	0	0	Amplification	1	0	42473T: 5;
PLXDC1	chr17	17q12	1	1	0	0	Amplification	1	0	42473T: 4.5;
PLXNA1	chr3	3q21.3	2	0	1	1	Amplification	2	0	42487T: 3.5; 42496T: 3.5;
PLXNA4	chr7	7q32.3	1	0	1	0	Amplification	1	0	42487T: 3.5;
PLXND1	chr3	3q22.1	2	0	1	1	Amplification	2	0	42487T: 3.5; 42496T: 3.5;
PM20D1	chr1	1q32.1	1	0	0	1	Amplification	1	0	42493T: 3.5;
PMEL	chr12	12q13.2	1	0	0	1	Amplification	1	0	42494T: 5;
PMEPA1	chr20	20q13.31	1	1	0	0	Amplification	1	0	42473T: 7;
PMF1	chr1	1q22	1	1	0	0	Amplification	1	0	42473T: 4.5;
PML	chr15	15q24.1	1	1	0	0	Amplification	1	0	42473T: 3.5;
PMM2	chr16	16p13.2	2	1	0	1	Amplification	2	0	42495T: 4; 42473T: 5.5;
PMPCA	chr9	9q34.3	1	0	0	1	Amplification	1	0	56957T: 3.5;
PMPCB	chr7	7q22.1	4	0	1	3	Amplification	4	0	42493T: 3.5; 42487T: 3.5; 42501T: 3.5; 42497T: 4;
PMS1	chr2	2q32.2	2	0	1	1	Amplification	2	0	42482T: 4; 42493T: 3.5;
PMS2	chr7	7p22.1	2	1	0	1	Amplification	2	0	42497T: 8; 42473T: 4.5;
PMS2CL	chr7	7p22.1	2	1	0	1	Amplification	2	0	42473T: 4.5; 42497T: 5;
PMS2P4	chr7	7q11.21	1	0	0	1	Amplification	1	0	42501T: 3.5;
PMVK	chr1	1q21.3	2	1	0	1	Amplification	2	0	42496T: 3.5; 42473T: 6.5;
PNMA1	chr14	14q24.3	2	0	0	2	Amplification	2	0	42494T: 4; 56957T: 3.5;
PNMAL1	chr19	19q13.32	1	0	1	0	Amplification	1	0	42484T: 4;
PNMAL2	chr19	19q13.32	1	0	1	0	Amplification	1	0	42484T: 4;
PNMT	chr17	17p11.2	2	1	0	1	Amplification	2	0	42497T: 3.5; 42473T: 6;
PNN	chr14	14q21.1	2	1	0	1	Amplification	2	0	42473T: 3.5; 42500T: 6.5;
PNO1	chr2	2p14	1	0	0	1	Amplification	1	0	42500T: 3.5;
PNPLA7	chr9	9q34.3	2	1	0	1	Amplification	2	0	42473T: 5; 56957T: 3.5;
PNPLA8	chr7	7q31.1	3	0	1	2	Amplification	3	0	42487T: 3.5; 42501T: 3.5; 42497T: 4;
PNPT1	chr2	2p16.1	3	0	1	2	Amplification	3	0	42500T: 3.5; 56957T: 3.5; 42484T: 4.5;
PNRC2	chr1	1p36.11	1	1	0	0	Amplification	1	0	42473T: 3.5;
PODXL	chr7	7q32.3	1	0	1	0	Amplification	1	0	42487T: 3.5;
PODXL2	chr3	3q21.3	2	0	1	1	Amplification	2	0	42496T: 3.5; 42487T: 3.5;
POFUT1	chr20	20q11.21	2	1	0	1	Amplification	2	0	42496T: 5; 42473T: 4.5;
POGLUT1	chr3	3q13.33	1	0	0	1	Amplification	1	0	42496T: 3.5;
POGZ	chr1	1q21.3	1	1	0	0	Amplification	1	0	42473T: 4.5;
POLD2	chr7	7p13	2	1	0	1	Amplification	2	0	42473T: 4.5; 42497T: 7.5;
POLD3	chr11	11q13.4	5	2	0	3	Amplification	5	0	42476T: 4; 42475T: 7.5; 42498T: 8; 42492T: 4; 56957T: 3.5;
POLD4	chr11	11q13.2	2	1	0	1	Amplification	2	0	42473T: 3.5; 56957T: 5;
POLE	chr12	12q24.33	1	0	0	1	Amplification	1	0	56957T: 4;
POLE2	chr14	14q21.3	1	1	0	0	Amplification	1	0	42473T: 3.5;

Mangalaparthi *et al.* , 2020. Mutational landscape of esophageal squamous cell carcinoma in an Indian cohort
Supplementary Table 7A. List of copy number alterations and affected genes in ESCC patients

Gene	Chromosome	Cytoband	Recurrence	Recurrence in smoker cohort	Recurrence in chewer cohort	Recurrence in No habit cohort	State	Samples with CNA gain	Samples with CNA loss	File info with CNA fold
POLE4	chr2	2p12	1	0	0	1	Amplification	1	0	42500T: 3.5;
POLG	chr15	15q26.1	1	1	0	0	Amplification	1	0	42473T: 3.5;
POLM	chr7	7p13	2	1	0	1	Amplification	2	0	42497T: 7.5; 42473T: 4.5;
POLQ	chr3	5q13.3	1	0	0	1	Amplification	1	0	42496T: 3.5;
POLR1A	chr2	2p11.2	1	0	0	1	Amplification	1	0	42500T: 3.5;
POLR2F	chr22	22q13.1	1	1	0	0	Amplification	1	0	42473T: 3.5;
POLR2H	chr3	3q27.1	11	1	3	7	Amplification	11	0	42482T: 3.5; 42487T: 3.5; 42493T: 3.5; 42492T: 3.5; 42497T: 4; 56957T: 5; 42474T: 3.5; 42495T: 4; 42494T: 3.5; 42498T: 3.5; 42484T: 4;
POLR2I	chr19	19q13.12	3	0	1	2	Amplification	3	0	56957T: 4; 42484T: 3.5; 42500T: 6.5;
POLR2J	chr7	7q22.1	2	0	0	2	Amplification	2	0	42493T: 3.5; 42501T: 3.5;
POLR2J2	chr7	7q22.1	3	0	1	2	Amplification	3	0	42501T: 3.5; 42487T: 3.5; 42493T: 3.5;
POLR2J3	chr7	7q22.1	3	0	1	2	Amplification	3	0	42501T: 3.5; 42487T: 3.5; 42493T: 3.5;
POLR2J4	chr7	7p13	2	1	0	1	Amplification	2	0	42497T: 7.5; 42473T: 3.5;
POLR2K	chr8	8q22.2	2	0	0	2	Amplification	2	0	42496T: 3.5; 42495T: 3.5;
POLR3C	chr1	1q21.1	1	1	0	0	Amplification	1	0	42473T: 4;
POLR3E	chr16	16p12.2	1	1	0	0	Amplification	1	0	42473T: 4;
POLR3GL	chr1	1q21.1	1	1	0	0	Amplification	1	0	42473T: 4;
POLR3K	chr16	16p13.3	1	0	1	0	Amplification	1	0	42483T: 3.5;
POM121L4P	chr22	22q11.21	2	1	0	1	Amplification	2	0	42477T: 4; 42497T: 13.5;
POMC	chr2	2p23.3	1	0	0	1	Amplification	1	0	42500T: 3.5;
POMT1	chr9	9q34.13	1	0	1	0	Amplification	1	0	42484T: 3.5;
POMT2	chr14	14q24.3	2	0	0	2	Amplification	2	0	42494T: 4; 56957T: 3.5;
PON1	chr7	7q21.3	1	0	1	0	Amplification	1	0	42487T: 3.5;
PON2	chr7	7q21.3	1	0	1	0	Amplification	1	0	42487T: 3.5;
PON3	chr7	7q21.3	1	0	1	0	Amplification	1	0	42487T: 3.5;
POP1	chr8	16p11.2	2	0	0	2	Amplification	2	0	42496T: 3.5; 42495T: 3.5;
POP4	chr19	Xp11.21	5	1	1	3	Amplification	5	0	42484T: 6; 42494T: 4; 56957T: 4; 42473T: 25; 42500T: 4.5;
POP5	chr12	12q24.31	1	0	0	1	Amplification	1	0	42500T: 3.5;
POPDC2	chr3	3q13.33	1	0	0	1	Amplification	1	0	42496T: 3.5;
POT1	chr7	7q31.33	1	0	1	0	Amplification	1	0	42487T: 3.5;
POTEA	chr8	8p11.1	1	0	1	0	Amplification	1	0	42483T: 4.5;
POTEB	chr15	15q11.2	1	0	1	0	Amplification	1	0	42484T: 7.5;
POTEB2	chr15	15q11.2	2	1	1	0	Amplification/Deletio	1	1	42476T: 0.5; 42484T: 7.5;
POU2F2	chr19	19q13.2	1	1	0	0	Amplification	1	0	42473T: 4.5;
POU3F3	chr2	2q12.1	1	0	0	1	Amplification	1	0	42493T: 3.5;
POU5F1B	chr8	8q24.21	5	1	1	3	Amplification	5	0	42495T: 3.5; 42493T: 10; 42484T: 3.5; 42475T: 3.5; 42496T: 3.5;
POU5F1P4	chr1	1q22	1	1	0	0	Amplification	1	0	42473T: 5.5;
POU6F1	chr12	12q13.13	2	0	0	2	Amplification	2	0	42494T: 3.5; 42500T: 3.5;

Mangalaparthi *et al.* , 2020. Mutational landscape of esophageal squamous cell carcinoma in an Indian cohort
Supplementary Table 7A. List of copy number alterations and affected genes in ESCC patients

Gene	Chromosome	Cytoband	Recurrence	Recurrence in smoker cohort	Recurrence in chewer cohort	Recurrence in No habit cohort	State	Samples with CNA gain	Samples with CNA loss	File info with CNA fold
POU6F2	chr7	7p14.1	1	1	0	0	Amplification	1	0	42473T: 3.5;
PPAPDC1B	chr8	8p11.23	2	0	1	1	Amplification	2	0	42482T: 3.5; 42493T: 3.5;
PPAPDC3	chr9	9q34.13	1	0	1	0	Amplification	1	0	42484T: 3.5;
PPDPF	chr20	20q13.33	1	1	0	0	Amplification	1	0	42473T: 6;
PPFIA1	chr11	11q13.3	12	3	2	7	Amplification	12	0	42501T: 6; 42475T: 7.5; 42500T: 13; 56958T: 5.5; 42483T: 9; 42486T: 20.5; 42497T: 6.5; 56957T: 5; 42478T: 4.5; 42498T: 7; 42476T: 13.5; 42492T: 4;
PPFIA4	chr1	1q32.1	1	1	0	0	Amplification	1	0	42473T: 4;
PPFIBP1	chr12	12p11.23-p1	1	0	0	1	Amplification	1	0	42500T: 6;
PPHLN1	chr12	12q12	1	0	0	1	Amplification	1	0	42500T: 3.5;
PPIA	chr7	7p13	1	1	0	0	Amplification	1	0	42473T: 4.5;
PPIH	chr1	1p34.2	1	0	0	1	Amplification	1	0	42493T: 3.5;
PPIL2	chr22	22q11.21	1	1	0	0	Amplification	1	0	42473T: 3.5;
PPIL6	chr6	6q21	1	0	0	1	Amplification	1	0	42496T: 3.5;
PPL	chr16	16p13.3	2	1	0	1	Amplification	2	0	42473T: 5.5; 42495T: 12;
PPM1A	chr14	14q23.1	2	0	1	1	Amplification	2	0	42483T: 3.5; 42494T: 4;
PPM1B	chr2	2p21	2	0	1	1	Amplification	2	0	42484T: 3.5; 42500T: 3.5;
PPM1G	chr2	2p23.3	1	0	0	1	Amplification	1	0	42500T: 3.5;
PPM1L	chr3	3q25.33-q26	8	1	2	5	Amplification	8	0	42492T: 3.5; 42487T: 3.5; 42493T: 3.5; 42497T: 3.5; 42484T: 4; 42474T: 3.5; 42495T: 4; 56957T: 4;
PPM1N	chr19	19q13.32	2	1	1	0	Amplification	2	0	42484T: 4; 42473T: 3.5;
PPME1	chr11	11q13.4	5	2	0	3	Amplification	5	0	42475T: 7.5; 42476T: 4; 56957T: 3.5; 42492T: 4; 42498T: 8;
PPP1CA	chr11	11q13.2	2	1	0	1	Amplification	2	0	42473T: 3.5; 56957T: 5;
PPP1CB	chr2	2p23.2	1	0	0	1	Amplification	1	0	42500T: 3.5;
PPP1CC	chr12	12q24.11	1	0	0	1	Amplification	1	0	42500T: 3.5;
PPP1R12B	chr1	1q32.1	1	1	0	0	Amplification	1	0	42473T: 4;
PPP1R13L	chr19	19q13.32	3	1	1	1	Amplification	3	0	42473T: 3.5; 42484T: 4; 56957T: 4;
PPP1R14A	chr19	19q13.2	4	1	1	2	Amplification	4	0	42484T: 3.5; 56957T: 4; 42474T: 4; 42500T: 6.5;
PPP1R14D	chr15	15q15.1	1	0	0	1	Amplification	1	0	42493T: 6.5;
PPP1R16A	chr8	8q24.3	5	0	1	4	Amplification	5	0	42483T: 3.5; 42496T: 4; 42494T: 3.5; 42495T: 4.5; 56957T: 3.5;
PPP1R16B	chr20	20q11.23	1	1	0	0	Amplification	1	0	42473T: 3.5;
PPP1R17	chr7	7p14.3	1	1	0	0	Amplification	1	0	42473T: 4;
PPP1R1A	chr12	12q13.2	2	1	0	1	Amplification	2	0	42473T: 3.5; 42494T: 3.5;
PPP1R1B	chr17	17q12	2	1	0	1	Amplification	2	0	42473T: 6; 42497T: 3.5;
PPP1R1C	chr2	2q31.3-q32.	1	0	0	1	Amplification	1	0	42493T: 4;
PPP1R2	chr3	3q29	9	1	3	5	Amplification	9	0	42482T: 3.5; 42492T: 3.5; 42487T: 3.5; 42493T: 3.5; 42498T: 3.5; 42484T: 4; 56957T: 4.5; 42474T: 3.5; 42495T: 4;
PPP1R21	chr2	2p16.3	2	0	1	1	Amplification	2	0	42484T: 4.5; 42500T: 3.5;

Mangalaparthi *et al.* , 2020. Mutational landscape of esophageal squamous cell carcinoma in an Indian cohort
Supplementary Table 7A. List of copy number alterations and affected genes in ESCC patients

Gene	Chromosome	Cytoband	Recurrence	Recurrence in smoker cohort	Recurrence in chewer cohort	Recurrence in No habit cohort	State	Samples with CNA gain	Samples with CNA loss	File info with CNA fold
PPP1R26	chr9	9q34.3	1	0	1	0	Amplification	1	0	42484T: 4.5;
PPP1R36	chr14	14q23.3	1	0	0	1	Amplification	1	0	42494T: 4;
PPP1R37	chr19	19q13.32	2	1	0	1	Amplification	2	0	42473T: 3.5; 56957T: 4;
PPP1R3A	chr7	7q31.1	1	0	1	0	Amplification	1	0	42487T: 3.5;
PPP1R3B	chr8	8p23.1	1	0	1	0	Amplification	1	0	42486T: 3.5;
PPP1R3D	chr20	20q13.33	1	1	0	0	Amplification	1	0	42473T: 4.5;
PPP1R3E	chr14	14q11.2	2	0	0	2	Amplification	2	0	42500T: 4; 42496T: 4;
PPP1R42	chr8	8q13.1	3	0	0	3	Amplification	3	0	42496T: 3.5; 42497T: 4.5; 42495T: 3.5;
PPP1R8	chr1	1p35.3	1	1	0	0	Amplification	1	0	42473T: 3.5;
PPP1R9A	chr7	7q21.3	1	0	1	0	Amplification	1	0	42487T: 3.5;
PPP2CA	chr5	5q31.1	1	1	0	0	Amplification	1	0	42473T: 3.5;
PPP2CB	chr8	8p12	1	0	1	0	Amplification	1	0	42482T: 3.5;
PPP2R3A	chr3	3q22.2-q22.3	4	0	1	3	Amplification	4	0	42496T: 3.5; 42487T: 3.5; 42493T: 3.5; 42492T: 3.5;
PPP2R3C	chr14	14q13.2	2	1	0	1	Amplification	2	0	42476T: 5; 42500T: 4.5;
PPP2R5E	chr14	14q23.2	1	0	0	1	Amplification	1	0	42494T: 4;
PPP3CB	chr10	10q22.2	1	0	0	1	Amplification	1	0	42496T: 3.5;
PPP3R1	chr2	2p14	1	0	0	1	Amplification	1	0	42500T: 3.5;
PPP4R1L	chr20	20q13.32	1	1	0	0	Amplification	1	0	42473T: 4.5;
PPP5C	chr19	19q13.32	1	0	1	0	Amplification	1	0	42484T: 4;
PPP5D1	chr19	19q13.32	1	0	1	0	Amplification	1	0	42484T: 4;
PPP6R3	chr11	11q13.2	2	1	0	1	Amplification	2	0	42476T: 29.5; 56957T: 5;
PPT2	chr6	6p21.32	1	1	0	0	Amplification	1	0	42473T: 3.5;
PPTC7	chr12	12q24.11	1	0	0	1	Amplification	1	0	42500T: 3.5;
PQLC2	chr1	1p36.13	1	1	0	0	Amplification	1	0	42473T: 4;
PQLC3	chr2	2p25.1	1	0	0	1	Amplification	1	0	42500T: 3.5;
PRADC1	chr2	2p13.2	1	0	0	1	Amplification	1	0	42500T: 3.5;
PRAME	chr22	22q11.22	1	1	0	0	Amplification	1	0	42477T: 4;
PRAMEF2	chr1	1p36.21	1	0	1	0	Amplification	1	0	42486T: 4.5;
PRAMEF22	chr1	1p36.21	1	0	1	0	Amplification	1	0	42486T: 4.5;
PRB2	chr12	12p13.2	1	0	0	1	Amplification	1	0	42500T: 4.5;
PRB4	chr12	12p13.2	1	0	0	1	Amplification	1	0	42500T: 4.5;
PRCC	chr1	1q23.1	1	1	0	0	Amplification	1	0	42473T: 7;
PRDM11	chr11	11p11.2	1	1	0	0	Amplification	1	0	42473T: 3.5;
PRDM14	chr8	8q13.3	2	0	0	2	Amplification	2	0	42495T: 3.5; 42496T: 3.5;
PRDM15	chr21	21q22.3	1	1	0	0	Amplification	1	0	42473T: 3.5;
PRDM9	chr5	5p14.2	4	1	1	2	Amplification	4	0	42475T: 3.5; 42496T: 3.5; 42486T: 3.5; 42493T: 4;
PREB	chr2	2p23.3	1	0	0	1	Amplification	1	0	42500T: 3.5;
PREPL	chr2	2p21	2	0	1	1	Amplification	2	0	42484T: 3.5; 42500T: 3.5;
PREX1	chr20	20q13.13	1	1	0	0	Amplification	1	0	42473T: 5;
PREX2	chr8	8q13.2	3	0	0	3	Amplification	3	0	42495T: 3.5; 42497T: 4; 42496T: 3.5;
PRICKLE1	chr12	12q12	1	0	0	1	Amplification	1	0	42500T: 3.5;

Mangalaparthi *et al.* , 2020. Mutational landscape of esophageal squamous cell carcinoma in an Indian cohort
Supplementary Table 7A. List of copy number alterations and affected genes in ESCC patients

Gene	Chromosome	Cytoband	Recurrence	Recurrence in smoker cohort	Recurrence in chewer cohort	Recurrence in No habit cohort	State	Samples with CNA gain	Samples with CNA loss	File info with CNA fold
PRIM2	chr6	6p11.2	1	0	0	1	Amplification	1	0	42497T: 3.5;
PRKAA1	chr5	5p13.1	6	1	3	2	Amplification	6	0	42486T: 3.5; 42483T: 3.5; 42496T: 3.5; 42475T: 3.5; 42484T: 3.5; 42493T: 3.5;
PRKAB1	chr12	12q24.23	1	0	0	1	Amplification	1	0	42500T: 3.5;
PRKAB2	chr1	1q21.1	1	1	0	0	Amplification	1	0	42473T: 4;
PRKAG1	chr12	12q13.12	1	0	0	1	Amplification	1	0	42500T: 3.5;
PRKAR1B	chr7	7p22.3	1	1	0	0	Amplification	1	0	42473T: 5;
PRKAR2B	chr7	7q22.3	4	0	1	3	Amplification	4	0	42487T: 3.5; 42493T: 3.5; 42501T: 3.5; 42497T: 4;
PRKCB	chr16	16p12.2-p12	1	1	0	0	Amplification	1	0	42473T: 4;
PRKCE	chr2	2p21	2	0	1	1	Amplification	2	0	42484T: 4.5; 42500T: 3.5;
PRKCH	chr14	14q23.1	1	0	0	1	Amplification	1	0	42494T: 4;
PRKCI	chr3	3q26.2	9	1	3	5	Amplification	9	0	42482T: 3.5; 42492T: 3.5; 42493T: 3.5; 42487T: 3.5; 42500T: 8; 42484T: 4; 42495T: 4; 42474T: 3.5; 56957T: 4;
PRKD1	chr14	14q12	2	0	0	2	Amplification	2	0	56957T: 4; 42500T: 4.5;
PRKD2	chr19	19q13.32	1	0	1	0	Amplification	1	0	42484T: 7;
PRKD3	chr2	2p22.2	1	0	0	1	Amplification	1	0	42500T: 3.5;
PRKDC	chr8	8q11.21	5	0	2	3	Amplification	5	0	42484T: 3.5; 42495T: 3.5; 42494T: 3.5; 42482T: 5; 42496T: 3.5;
PRKG2	chr4	4q21.21	1	1	0	0	Amplification	1	0	42473T: 4;
PRKRA	chr2	2q31.2	1	0	0	1	Amplification	1	0	42493T: 3.5;
PRKRIP1	chr7	7q22.1	2	0	0	2	Amplification	2	0	42493T: 3.5; 42501T: 3.5;
PRKRIR	chr11	11q13.5	1	0	0	1	Amplification	1	0	42497T: 3.5;
PRLR	chr5	5p13.2	4	1	1	2	Amplification	4	0	42493T: 3.5; 42486T: 3.5; 42496T: 3.5; 42475T: 3.5;
PRM1	chr16	8q11.22-q11	2	1	0	1	Amplification	2	0	42495T: 5; 42473T: 5.5;
PRM2	chr16	16p13.13	2	1	0	1	Amplification	2	0	42473T: 5.5; 42495T: 5;
PRM3	chr16	16p13.13	2	1	0	1	Amplification	2	0	42473T: 5.5; 42495T: 5;
PRMT5	chr14	14q11.2	1	0	0	1	Amplification	1	0	42496T: 4;
PRMT8	chr12	12p13.32	1	0	0	1	Amplification	1	0	42494T: 3.5;
PRND	chr20	20p13	1	1	0	0	Amplification	1	0	42473T: 4.5;
PRNP	chr20	20p13	1	1	0	0	Amplification	1	0	42473T: 4.5;
PRNT	chr20	20p13	1	1	0	0	Amplification	1	0	42473T: 4.5;
PROCR	chr20	20q11.22	2	1	0	1	Amplification	2	0	42473T: 3.5; 42493T: 3.5;
PRODH	chr22	22q11.21	1	0	0	1	Amplification	1	0	42497T: 21;
PRODH2	chr19	19q13.12	3	0	1	2	Amplification	3	0	42484T: 3.5; 56957T: 4; 42500T: 6.5;
PROKR1	chr2	2p13.3	1	0	0	1	Amplification	1	0	42500T: 3.5;
PROM2	chr2	2q11.1	2	1	0	1	Amplification	2	0	42473T: 3.5; 42493T: 4;
PROS1	chr3	3q11.1	2	0	1	1	Amplification	2	0	42484T: 3.5; 42500T: 3.5;
PROSC	chr8	8p11.23	1	0	1	0	Amplification	1	0	42482T: 3.5;
PROX2	chr14	14q24.3	2	0	0	2	Amplification	2	0	42494T: 4; 56957T: 3.5;
PROZ	chr13	13q34	1	0	0	1	Amplification	1	0	56957T: 3.5;

Mangalaparthi *et al.* , 2020. Mutational landscape of esophageal squamous cell carcinoma in an Indian cohort
Supplementary Table 7A. List of copy number alterations and affected genes in ESCC patients

Gene	Chromosome	Cytoband	Recurrence	Recurrence in smoker cohort	Recurrence in chewer cohort	Recurrence in No habit cohort	State	Samples with CNA gain	Samples with CNA loss	File info with CNA fold
PRPF3	chr1	1q21.2	1	1	0	0	Amplification	1	0	42473T: 5;
PRPF39	chr14	14q21.2	1	0	0	1	Amplification	1	0	42500T: 5.5;
PRPF40B	chr12	12q13.12	1	0	0	1	Amplification	1	0	42500T: 3.5;
PRPF6	chr20	20q13.33	1	1	0	0	Amplification	1	0	42473T: 6;
PRPH	chr12	6p21.1	1	0	0	1	Amplification	1	0	42500T: 3.5;
PRPS1L1	chr7	7p21.1	1	1	0	0	Amplification	1	0	42473T: 4;
PRR15	chr7	7p14.3	1	1	0	0	Amplification	1	0	42473T: 4;
PRR19	chr19	19q13.2	1	1	0	0	Amplification	1	0	42473T: 4.5;
PRR23A	chr3	3q23	4	0	1	3	Amplification	4	0	42493T: 3.5; 42487T: 3.5; 42492T: 3.5; 42496T: 3.5;
PRR23B	chr3	3q23	4	0	1	3	Amplification	4	0	42496T: 3.5; 42492T: 3.5; 42493T: 3.5; 42487T: 3.5;
PRR23C	chr3	3q23	4	0	1	3	Amplification	4	0	42496T: 3.5; 42492T: 3.5; 42493T: 3.5; 42487T: 3.5;
PRR25	chr16	16p13.3	2	0	1	1	Amplification	2	0	42483T: 3.5; 42493T: 3.5;
PRR9	chr1	1q21.3	1	0	0	1	Amplification	1	0	42493T: 4;
PRRC1	chr5	5q23.2	1	1	0	0	Amplification	1	0	42473T: 3.5;
PRRC2A	chr6	6p21.33	1	1	0	0	Amplification	1	0	42473T: 3.5;
PRRC2B	chr9	9q34.13	1	0	1	0	Amplification	1	0	42484T: 3.5;
PRRT1	chr6	6p21.32	1	1	0	0	Amplification	1	0	42473T: 3.5;
PRRT4	chr7	7q32.1	1	0	1	0	Amplification	1	0	42487T: 3.5;
PRSS1	chr7	7q34	1	0	1	0	Amplification	1	0	42487T: 3.5;
PRSS29P	chr16	16p13.3	1	0	1	0	Amplification	1	0	42483T: 3.5;
PRSS3	chr9	9p13.3	1	0	0	1	Amplification	1	0	42496T: 4;
PRSS37	chr7	7q34	1	0	1	0	Amplification	1	0	42487T: 3.5;
PRSS3P2	chr7	7q34	1	0	1	0	Amplification	1	0	42487T: 3.5;
PRSS55	chr8	8p23.1	1	0	1	0	Amplification	1	0	42486T: 3.5;
PRSS58	chr7	7q34	1	0	1	0	Amplification	1	0	42487T: 3.5;
PRTN3	chr19	19p13.3	1	0	0	1	Amplification	1	0	42493T: 3.5;
PRUNE	chr1	1q21.3	1	1	0	0	Amplification	1	0	42473T: 4.5;
PRX	chr19	19q13.2	2	0	0	2	Amplification	2	0	42500T: 3.5; 56957T: 4;
PSCA	chr8	8q24.3	3	0	1	2	Amplification	3	0	42495T: 4.5; 42483T: 3.5; 42496T: 4;
PSEN1	chr14	14q24.2	2	0	0	2	Amplification	2	0	42494T: 4; 56957T: 3.5;
PSENEN	chr19	19q13.12	3	0	1	2	Amplification	3	0	56957T: 4; 42484T: 3.5; 42500T: 6.5;
PSG1	chr19	19q13.2	1	0	0	1	Amplification	1	0	56957T: 4;
PSG10P	chr19	19q13.2	1	0	0	1	Amplification	1	0	56957T: 4;
PSG11	chr19	19q13.31	1	0	0	1	Amplification	1	0	56957T: 4;
PSG2	chr19	19q13.31	1	0	0	1	Amplification	1	0	56957T: 4;
PSG5	chr19	19q13.31	1	0	0	1	Amplification	1	0	56957T: 4;
PSG6	chr19	19q13.31	1	0	0	1	Amplification	1	0	56957T: 4;
PSG9	chr19	19q13.31	1	0	0	1	Amplification	1	0	56957T: 4;
PSKH2	chr8	8q21.3	2	0	0	2	Amplification	2	0	42495T: 3.5; 42496T: 3.5;
PSMA2	chr7	7p14.1	1	1	0	0	Amplification	1	0	42473T: 3.5;
PSMA3	chr14	14q23.1	1	0	0	1	Amplification	1	0	42494T: 4;

Mangalaparthi *et al.* , 2020. Mutational landscape of esophageal squamous cell carcinoma in an Indian cohort
Supplementary Table 7A. List of copy number alterations and affected genes in ESCC patients

Gene	Chromosome	Cytoband	Recurrence	Recurrence in smoker cohort	Recurrence in chewer cohort	Recurrence in No habit cohort	State	Samples with CNA gain	Samples with CNA loss	File info with CNA fold
PSMA4	chr15	15q25.1	1	1	0	0	Amplification	1	0	42473T: 3.5;
PSMA6	chr14	14q13.2	2	1	0	1	Amplification	2	0	42500T: 4.5; 42476T: 5;
PSMA7	chr20	20q13.33	1	1	0	0	Amplification	1	0	42473T: 6;
PSMB11	chr14	14q11.2	1	0	0	1	Amplification	1	0	42496T: 4;
PSMB3	chr17	17q12	1	1	0	0	Amplification	1	0	42473T: 3.5;
PSMB4	chr1	1q21.3	1	1	0	0	Amplification	1	0	42473T: 4.5;
PSMB5	chr14	14q11.2	1	0	0	1	Amplification	1	0	42496T: 4;
PSMC1	chr14	14q32.11	1	1	0	0	Amplification	1	0	42473T: 3.5;
PSMC2	chr7	7q22.1	4	0	1	3	Amplification	4	0	42493T: 3.5; 42487T: 3.5; 42497T: 4; 42501T: 3.5;
PSMC3	chr11	11p11.2	1	1	0	0	Amplification	1	0	42473T: 3.5;
PSMC4	chr19	19q13.2	2	0	0	2	Amplification	2	0	56957T: 4; 42500T: 7.5;
PSMC6	chr14	14q22.1	1	0	0	1	Amplification	1	0	42494T: 4;
PSMD2	chr3	3q27.1	12	2	3	7	Amplification	12	0	42492T: 3.5; 42487T: 3.5; 42493T: 3.5; 42482T: 3.5; 42484T: 4; 42498T: 3.5; 42495T: 4; 42494T: 3.5; 42474T: 3.5; 56957T: 5; 42473T: 3.5; 42497T: 4;
PSMD3	chr17	17q21.1	1	0	0	1	Amplification	1	0	42497T: 3.5;
PSMD4	chr1	1q21.3	1	1	0	0	Amplification	1	0	42473T: 4.5;
PSMD8	chr19	19q13.2	3	0	1	2	Amplification	3	0	56957T: 4; 42484T: 3.5; 42500T: 6.5;
PSMD9	chr12	12q24.31	1	0	0	1	Amplification	1	0	42500T: 3.5;
PSME4	chr2	2p16.2	2	0	1	1	Amplification	2	0	42500T: 3.5; 42484T: 4.5;
PSMF1	chr20	20p13	1	1	0	0	Amplification	1	0	42473T: 3.5;
PSMG1	chr21	21q22.2	1	1	0	0	Amplification	1	0	42473T: 3.5;
PSMG3	chr7	7p22.3	1	1	0	0	Amplification	1	0	42473T: 5;
PSPH	chr7	7p11.2	2	0	1	1	Amplification	2	0	56957T: 5; 42483T: 4;
PTAFR	chr1	1p35.3	1	1	0	0	Amplification	1	0	42473T: 3.5;
PTBP1	chr19	19p13.3	1	0	0	1	Amplification	1	0	42493T: 3.5;
PTCD3	chr2	2p11.2	1	0	0	1	Amplification	1	0	42500T: 3.5;
PTDSS1	chr8	8q22.1	2	0	0	2	Amplification	2	0	42495T: 3.5; 42496T: 3.5;
PTENP1	chr9	9p13.3	1	0	0	1	Amplification	1	0	42496T: 4;
PTGDR	chr14	14q22.1	1	0	0	1	Amplification	1	0	42494T: 4;
PTGDS	chr9	9q34.3	2	1	0	1	Amplification	2	0	42473T: 5; 56957T: 3.5;
PTGER2	chr14	14q22.1	1	0	0	1	Amplification	1	0	42494T: 4;
PTGER4	chr5	5p13.1	6	1	3	2	Amplification	6	0	42475T: 3.5; 42486T: 3.5; 42483T: 3.5; 42496T: 3.5; 42493T: 3.5; 42484T: 3.5;
PTGIR	chr19	19q13.32	1	0	1	0	Amplification	1	0	42484T: 7;
PTGIS	chr20	20q13.13	1	1	0	0	Amplification	1	0	42473T: 5;
PTGR2	chr14	14q24.3	2	0	0	2	Amplification	2	0	56957T: 3.5; 42494T: 4;
PTHLH	chr12	12p11.22	2	0	0	2	Amplification	2	0	42500T: 6; 42494T: 4;
PTK2	chr8	8q24.3	3	0	1	2	Amplification	3	0	42495T: 3.5; 42496T: 3.5; 42483T: 3.5;
PTK6	chr20	20q13.33	1	1	0	0	Amplification	1	0	42473T: 6;
PTMS	chr12	12p13.31	1	1	0	0	Amplification	1	0	42473T: 3.5;

Mangalaparthi *et al.* , 2020. Mutational landscape of esophageal squamous cell carcinoma in an Indian cohort
Supplementary Table 7A. List of copy number alterations and affected genes in ESCC patients

Gene	Chromosome	Cytoband	Recurrence	Recurrence in smoker cohort	Recurrence in chewer cohort	Recurrence in No habit cohort	State	Samples with CNA gain	Samples with CNA loss	File info with CNA fold
PTN	chr7	7q33	1	0	1	0	Amplification	1	0	42487T: 3.5;
PTP4A2	chr1	1p35.2	1	1	0	0	Amplification	1	0	42473T: 3.5;
PTP4A3	chr8	8q24.3	3	0	1	2	Amplification	3	0	42496T: 4; 42483T: 3.5; 42495T: 3.5;
PTPLAD2	chr9	9p21.3	1	1	0	0	Deletion	0	1	42475T: 0.5;
PTPLB	chr3	3q21.1	2	1	0	1	Amplification	2	0	42496T: 3.5; 42473T: 3.5;
PTPMT1	chr11	11p11.2	1	1	0	0	Amplification	1	0	42473T: 3.5;
PTPN1	chr20	20q13.13	1	1	0	0	Amplification	1	0	42473T: 6.5;
PTPN11	chr12	12q24.13	1	0	0	1	Amplification	1	0	42500T: 3.5;
PTPN12	chr7	7q11.23	1	0	1	0	Amplification	1	0	42487T: 3.5;
PTPN3	chr9	9q31.3	1	0	1	0	Amplification	1	0	42483T: 3.5;
PTPN6	chr12	12p13.31	1	0	0	1	Amplification	1	0	42494T: 3.5;
PTPRA	chr20	20p13	1	1	0	0	Amplification	1	0	42473T: 3.5;
PTPRB	chr12	12q15	2	0	0	2	Amplification	2	0	42500T: 5; 42501T: 5.5;
PTPRCAP	chr11	11q13.2	2	1	0	1	Amplification	2	0	56957T: 5; 42473T: 3.5;
PTPRD	chr9	9p24.1-p23	1	0	0	1	Amplification	1	0	42496T: 3.5;
PTPRJ	chr11	11p11.2	2	1	0	1	Amplification	2	0	56957T: 16.5; 42473T: 3.5;
PTPRO	chr12	1p35.3	1	0	0	1	Amplification	1	0	42500T: 4.5;
PTPRR	chr12	12q15	2	0	0	2	Amplification	2	0	42500T: 5; 42501T: 5.5;
PTPRT	chr20	20q12-q13.1	1	1	0	0	Amplification	1	0	42473T: 3.5;
PTPRU	chr1	1p35.3	1	1	0	0	Amplification	1	0	42473T: 3.5;
PTPRZ1	chr7	7q31.32	1	0	1	0	Amplification	1	0	42487T: 3.5;
PTRHD1	chr2	2p23.3	1	0	0	1	Amplification	1	0	42500T: 3.5;
PTTG2	chr4	4p14	1	1	0	0	Amplification	1	0	42473T: 7.5;
PTX3	chr3	3q25.32	8	1	2	5	Amplification	8	0	56957T: 4; 42474T: 3.5; 42495T: 4; 42484T: 3.5; 42497T: 5.5; 42493T: 3.5; 42487T: 3.5; 42492T: 3.5;
PTX4	chr16	16p13.3	1	0	1	0	Amplification	1	0	42483T: 3.5;
PUF60	chr8	8q24.3	3	0	1	2	Amplification	3	0	42495T: 4.5; 42496T: 4; 42483T: 3.5;
PUM1	chr1	1p35.2	1	1	0	0	Amplification	1	0	42473T: 3.5;
PUM2	chr2	2p24.1	1	0	0	1	Amplification	1	0	42500T: 3.5;
PURB	chr7	7p13	1	1	0	0	Amplification	1	0	42473T: 4.5;
PURG	chr8	8p12	1	0	1	0	Amplification	1	0	42482T: 3.5;
PUS10	chr2	2p16.1-p15	3	0	1	2	Amplification	3	0	42500T: 3.5; 56957T: 3.5; 42484T: 4.5;
PUS7	chr7	7q22.3	4	0	1	3	Amplification	4	0	42493T: 3.5; 42487T: 3.5; 42497T: 4; 42501T: 3.5;
PUS7L	chr12	12q12	1	0	0	1	Amplification	1	0	42500T: 3.5;
PVALB	chr22	22q12.3	1	1	0	0	Amplification	1	0	42473T: 3.5;
PVR	chr19	19q13.31	2	1	0	1	Amplification	2	0	42473T: 3.5; 56957T: 4;
PVRL2	chr19	19q13.32	2	1	0	1	Amplification	2	0	42473T: 3.5; 56957T: 4;
PVT1	chr8	8q24.21	5	1	1	3	Amplification	5	0	42475T: 3.5; 42496T: 3.5; 42495T: 3.5; 42493T: 10; 42484T: 3.5;
PXDN	chr2	2p25.3	1	0	0	1	Amplification	1	0	42500T: 3.5;

Mangalaparthi *et al.* , 2020. Mutational landscape of esophageal squamous cell carcinoma in an Indian cohort
Supplementary Table 7A. List of copy number alterations and affected genes in ESCC patients

Gene	Chromosome	Cytoband	Recurrence	Recurrence in smoker cohort	Recurrence in chewer cohort	Recurrence in No habit cohort	State	Samples with CNA gain	Samples with CNA loss	File info with CNA fold
PXDNL	chr8	8q11.22-q11	5	0	2	3	Amplification	5	0	42484T: 4; 42495T: 3.5; 42494T: 3.5; 42496T: 3.5; 42482T: 3.5;
PXMP2	chr12	12q24.33	1	0	0	1	Amplification	1	0	56957T: 4;
PXMP4	chr20	20q11.22	2	1	0	1	Amplification	2	0	42473T: 3.5; 42496T: 5;
PXN	chr12	2p25.3	1	0	0	1	Amplification	1	0	42500T: 3.5;
PYCR2	chr1	1q42.12	1	0	0	1	Amplification	1	0	42493T: 3.5;
PYCRL	chr8	8q24.3	3	0	1	2	Amplification	3	0	42495T: 4.5; 42483T: 3.5; 42496T: 4;
PYDC2	chr3	3q28	9	1	3	5	Amplification	9	0	42493T: 3.5; 42487T: 3.5; 42492T: 3.5; 42482T: 3.5; 42495T: 4; 42474T: 3.5; 56957T: 4.5; 42484T: 4; 42498T: 3.5;
PYGL	chr14	14q22.1	1	0	0	1	Amplification	1	0	42494T: 4;
PYGO2	chr1	1q21.3	2	1	0	1	Amplification	2	0	42473T: 6.5; 42496T: 3.5;
PYROXD1	chr12	12p12.1	1	0	0	1	Amplification	1	0	42500T: 6;
QPCT	chr2	2p22.2	1	0	0	1	Amplification	1	0	42500T: 3.5;
QPCTL	chr19	19q13.32	1	0	1	0	Amplification	1	0	42484T: 4;
QRSL1	chr6	6q21	1	0	0	1	Amplification	1	0	42496T: 3.5;
QSOX1	chr1	1q25.2	1	0	0	1	Amplification	1	0	42493T: 4;
QSOX2	chr9	9q34.3	1	0	0	1	Amplification	1	0	56957T: 3.5;
QTRTD1	chr3	3q13.31	1	0	0	1	Amplification	1	0	42496T: 3.5;
R3HDM4	chr19	19p13.3	1	0	0	1	Amplification	1	0	42493T: 3.5;
R3HDML	chr20	20q13.12	1	1	0	0	Amplification	1	0	42473T: 3.5;
RAB10	chr2	2p23.3	1	0	0	1	Amplification	1	0	42500T: 3.5;
RAB11FIP1	chr8	8p11.23	1	0	1	0	Amplification	1	0	42482T: 3.5;
RAB11FIP3	chr16	16p13.3	1	0	1	0	Amplification	1	0	42483T: 3.5;
RAB11FIP4	chr17	17q11.2	1	1	0	0	Amplification	1	0	42473T: 3.5;
RAB13	chr1	1q21.3	1	1	0	0	Amplification	1	0	42473T: 4.5;
RAB15	chr14	14q23.3	1	0	0	1	Amplification	1	0	42494T: 4;
RAB19	chr7	7q34	2	1	1	0	Amplification	2	0	42487T: 3.5; 42473T: 3.5;
RAB1A	chr2	2p14	2	0	0	2	Amplification	2	0	56957T: 3.5; 42500T: 3.5;
RAB21	chr12	12q21.1	1	0	0	1	Amplification	1	0	42501T: 6.5;
RAB22A	chr20	20q13.32	1	1	0	0	Amplification	1	0	42473T: 4.5;
RAB23	chr6	6p12.1-p11.2	1	0	0	1	Amplification	1	0	42497T: 3.5;
RAB25	chr1	1q22	2	1	0	1	Amplification	2	0	42493T: 4; 42473T: 4.5;
RAB26	chr16	16p13.3	1	0	1	0	Amplification	1	0	42483T: 3.5;
RAB2A	chr8	8q12.1-q12.2	2	0	0	2	Amplification	2	0	42495T: 3.5; 42496T: 3.5;
RAB2B	chr14	14q11.2	1	0	0	1	Amplification	1	0	42496T: 5;
RAB35	chr12	12q24.23	1	0	0	1	Amplification	1	0	42500T: 3.5;
RAB3IP	chr12	12q15	2	0	0	2	Amplification	2	0	42501T: 5.5; 42500T: 5;
RAB40C	chr16	16p13.3	2	0	1	1	Amplification	2	0	42483T: 3.5; 42493T: 3.5;
RAB42	chr1	14q32.11	1	1	0	0	Amplification	1	0	42473T: 3.5;
RAB43	chr3	3q21.3	3	0	1	2	Amplification	3	0	42496T: 3.5; 42487T: 3.5; 42493T: 3.5;

Mangalaparthi *et al.* , 2020. Mutational landscape of esophageal squamous cell carcinoma in an Indian cohort
Supplementary Table 7A. List of copy number alterations and affected genes in ESCC patients

Gene	Chromosome	Cytoband	Recurrence	Recurrence in smoker cohort	Recurrence in chewer cohort	Recurrence in No habit cohort	State	Samples with CNA gain	Samples with CNA loss	File info with CNA fold
RAB4B	chr19	19q13.2	2	0	0	2	Amplification	2	0	56957T: 4; 42500T: 3.5;
RAB5B	chr12	12q13.2	1	0	0	1	Amplification	1	0	42494T: 5;
RAB6A	chr11	11q13.4	6	3	0	3	Amplification	6	0	56957T: 3.5; 42478T: 6; 42492T: 4; 42498T: 8; 42475T: 8.5; 42476T: 4;
RAB6B	chr3	3q22.1	3	0	1	2	Amplification	3	0	42487T: 3.5; 42492T: 3.5; 42496T: 3.5;
RAB7A	chr3	3q21.3	3	0	1	2	Amplification	3	0	42493T: 3.5; 42487T: 3.5; 42496T: 3.5;
RAB7L1	chr1	1q32.1	1	0	0	1	Amplification	1	0	42493T: 3.5;
RABAC1	chr19	19q13.2	1	1	0	0	Amplification	1	0	42473T: 4.5;
RABIF	chr1	1q32.1	1	1	0	0	Amplification	1	0	42473T: 4;
RABL3	chr3	3q13.33	1	0	0	1	Amplification	1	0	42496T: 3.5;
RABL5	chr7	7q22.1	2	0	0	2	Amplification	2	0	42493T: 3.5; 42501T: 3.5;
RABL6	chr9	9q34.3	1	0	0	1	Amplification	1	0	56957T: 3.5;
RAC1	chr7	14q11.2	1	1	0	0	Amplification	1	0	42473T: 4.5;
RAC2	chr22	22q13.1	1	1	0	0	Amplification	1	0	42473T: 3.5;
RAC3	chr17	20q13.12	1	1	0	0	Amplification	1	0	42473T: 3.5;
RACGAP1	chr12	12q13.12	1	0	0	1	Amplification	1	0	42500T: 3.5;
RAD1	chr5	16q22.1	4	1	1	2	Amplification	4	0	42493T: 3.5; 42496T: 3.5; 42486T: 3.5; 42475T: 3.5;
RAD21	chr8	8q24.11	3	1	0	2	Amplification	3	0	42495T: 3.5; 42475T: 3.5; 42496T: 3.5;
RAD21L1	chr20	20p13	1	1	0	0	Amplification	1	0	42473T: 3.5;
RAD51AP1	chr12	12p13.32	1	0	0	1	Amplification	1	0	42494T: 3.5;
RAD51AP2	chr2	2p24.2	1	0	0	1	Amplification	1	0	42500T: 3.5;
RAD51B	chr14	14q24.1	1	0	0	1	Amplification	1	0	42494T: 4;
RAD51C	chr17	17q22	1	0	0	1	Amplification	1	0	42497T: 4;
RAD52	chr12	12p13.33	1	0	0	1	Amplification	1	0	42500T: 3.5;
RAD54B	chr8	8q22.1	2	0	0	2	Amplification	2	0	42495T: 3.5; 42496T: 3.5;
RAD9A	chr11	11q13.2	2	1	0	1	Amplification	2	0	56957T: 5; 42473T: 3.5;
RAD9B	chr12	12q24.11	1	0	0	1	Amplification	1	0	42500T: 3.5;
RADIL	chr7	7p22.1	2	1	0	1	Amplification	2	0	42497T: 8.5; 42473T: 4.5;
RAE1	chr20	14q11.2	1	1	0	0	Amplification	1	0	42473T: 7;
RAI14	chr5	5p13.2	4	1	1	2	Amplification	4	0	42493T: 3.5; 42475T: 3.5; 42496T: 3.5; 42486T: 3.5;
RALA	chr7	7p14.1	1	1	0	0	Amplification	1	0	42473T: 3.5;
RALGAPA1	chr14	14q13.2	2	1	0	1	Amplification	2	0	42500T: 8.5; 42476T: 5;
RALGAPA2	chr20	20p11.23	1	0	1	0	Amplification	1	0	42483T: 4;
RALGAPB	chr20	20q11.23	1	1	0	0	Amplification	1	0	42473T: 3.5;
RALY	chr20	20q11.22	3	1	0	2	Amplification	3	0	42493T: 3.5; 42473T: 3.5; 42496T: 5;
RALYL	chr8	8q21.2	2	0	0	2	Amplification	2	0	42496T: 3.5; 42495T: 3.5;
RAMP3	chr7	7p13	1	1	0	0	Amplification	1	0	42473T: 4.5;
RANBP3L	chr5	5p13.2	4	1	1	2	Amplification	4	0	42496T: 3.5; 42486T: 3.5; 42475T: 3.5; 42493T: 3.5;
RANBP6	chr9	9p24.1	2	0	0	2	Amplification	2	0	42496T: 3.5; 42498T: 14;
RAP1B	chr12	12q15	2	0	0	2	Amplification	2	0	42500T: 5; 42501T: 6.5;
RAP1GAP	chr1	1p36.12	1	1	0	0	Amplification	1	0	42473T: 4;

Mangalaparthi *et al.* , 2020. Mutational landscape of esophageal squamous cell carcinoma in an Indian cohort
Supplementary Table 7A. List of copy number alterations and affected genes in ESCC patients

Gene	Chromosome	Cytoband	Recurrence	Recurrence in smoker cohort	Recurrence in chewer cohort	Recurrence in No habit cohort	State	Samples with CNA gain	Samples with CNA loss	File info with CNA fold
RAP2B	chr3	3q25.2	9	1	2	6	Amplification	9	0	42492T: 3.5; 42493T: 3.5; 42487T: 3.5; 42497T: 5.5; 42496T: 4; 42500T: 3.5; 42484T: 3.5; 42474T: 3.5; 56957T: 4;
RAPGEF1	chr9	9q34.13	1	0	1	0	Amplification	1	0	42484T: 3.5;
RAPGEF3	chr12	12q13.11	1	0	0	1	Amplification	1	0	42500T: 3.5;
RAPGEF4	chr2	2q31.1	2	1	0	1	Amplification	2	0	42493T: 5; 42473T: 6;
RAPGEF5	chr7	7p15.3	1	1	0	0	Amplification	1	0	42473T: 4;
RAPGEFL1	chr17	17q21.1	1	0	0	1	Amplification	1	0	42497T: 4;
RAPSN	chr11	11p11.2	1	1	0	0	Amplification	1	0	42473T: 3.5;
RARRES1	chr3	3q25.32	8	1	2	5	Amplification	8	0	42497T: 3.5; 42484T: 3.5; 42495T: 4; 42474T: 3.5; 56957T: 4; 42492T: 3.5; 42487T: 3.5; 42493T: 3.5;
RASA1	chr5	6p21.32	1	1	0	0	Deletion	0	1	42476T: 0.5;
RASA2	chr3	3q23	4	0	1	3	Amplification	4	0	42496T: 4; 42492T: 3.5; 42493T: 3.5; 42487T: 3.5;
RASA3	chr13	13q34	1	0	0	1	Amplification	1	0	56957T: 3.5;
RASA4	chr7	7q22.1	3	0	1	2	Amplification	3	0	42493T: 3.5; 42487T: 3.5; 42501T: 3.5;
RASA4B	chr7	7q22.1	2	0	0	2	Amplification	2	0	42493T: 3.5; 42501T: 3.5;
RASA4CP	chr7	7p13	2	1	0	1	Amplification	2	0	42473T: 4.5; 42497T: 7.5;
RASAL1	chr12	12q24.13	1	0	0	1	Amplification	1	0	42500T: 3.5;
RASD2	chr22	22q12.3	1	1	0	0	Amplification	1	0	42473T: 3.5;
RASGEF1B	chr4	4q21.21	1	1	0	0	Amplification	1	0	42473T: 4;
RASGRP3	chr2	2p22.3	1	0	0	1	Amplification	1	0	42500T: 3.5;
RASSF2	chr20	20p13	1	1	0	0	Amplification	1	0	42473T: 4.5;
RASSF8	chr12	12p12.1	1	0	0	1	Amplification	1	0	42500T: 6;
RB1	chr13	13q14.2	1	0	0	1	Deletion	0	1	42495T: 0.5;
RBAK	chr7	7p22.1	2	1	0	1	Amplification	2	0	42497T: 8.5; 42473T: 4.5;
RBBP6	chr16	16p12.1	1	1	0	0	Amplification	1	0	42473T: 4;
RBBP8	chr18	18q11.2	1	1	0	0	Amplification	1	0	42481T: 4;
RBBP8NL	chr20	20q13.33	1	1	0	0	Amplification	1	0	42473T: 6;
RBCK1	chr20	20p13	1	1	0	0	Amplification	1	0	42473T: 3.5;
RBFOX1	chr16	16p13.3	2	1	0	1	Amplification	2	0	42473T: 5.5; 42495T: 4;
RBFOX2	chr22	22q12.3	1	1	0	0	Amplification	1	0	42473T: 3.5;
RBKS	chr2	2p23.2	1	0	0	1	Amplification	1	0	42500T: 3.5;
RBL1	chr20	20q11.23	1	1	0	0	Amplification	1	0	42473T: 3.5;
RBM12	chr20	20q11.22	2	1	0	1	Amplification	2	0	42493T: 3.5; 42473T: 3.5;
RBM12B	chr8	8q22.1	2	0	0	2	Amplification	2	0	42495T: 3.5; 42496T: 3.5;
RBM14	chr11	11q13.2	2	1	0	1	Amplification	2	0	56957T: 5.5; 42473T: 3.5;
RBM19	chr12	12q24.13-q2	1	0	0	1	Amplification	1	0	42500T: 3.5;
RBM23	chr14	14q11.2	1	0	0	1	Amplification	1	0	42496T: 4;
RBM25	chr14	14q24.2	2	0	0	2	Amplification	2	0	56957T: 3.5; 42494T: 4;
RBM28	chr7	7q32.1	1	0	1	0	Amplification	1	0	42487T: 3.5;
RBM34	chr1	1q42.3	1	1	0	0	Amplification	1	0	42473T: 6.5;

Mangalaparthi *et al.* , 2020. Mutational landscape of esophageal squamous cell carcinoma in an Indian cohort
Supplementary Table 7A. List of copy number alterations and affected genes in ESCC patients

Gene	Chromosome	Cytoband	Recurrence	Recurrence in smoker cohort	Recurrence in chewer cohort	Recurrence in No habit cohort	State	Samples with CNA gain	Samples with CNA loss	File info with CNA fold
RBM38	chr20	20q13.31	1	1	0	0	Amplification	1	0	42473T: 7;
RBM39	chr20	20q11.22	2	1	0	1	Amplification	2	0	42473T: 3.5; 42493T: 3.5;
RBM4	chr11	11q13.2	2	1	0	1	Amplification	2	0	56957T: 5.5; 42473T: 3.5;
RBM42	chr19	19q13.12	3	0	1	2	Amplification	3	0	42484T: 3.5; 56957T: 4; 42500T: 6.5;
RBM45	chr2	2q31.2	1	0	0	1	Amplification	1	0	42493T: 3.5;
RBM48	chr7	7q21.2	2	0	2	0	Amplification	2	0	42483T: 4; 42487T: 3.5;
RBM4B	chr11	11q13.2	2	1	0	1	Amplification	2	0	56957T: 5.5; 42473T: 3.5;
RBM8A	chr1	1q21.1	1	1	0	0	Amplification	1	0	42473T: 4;
RBP1	chr3	3q23	4	0	1	3	Amplification	4	0	42493T: 3.5; 42487T: 3.5; 42492T: 3.5; 42496T: 3.5;
RBP2	chr3	12q24.33	4	0	1	3	Amplification	4	0	42496T: 3.5; 42492T: 3.5; 42493T: 3.5; 42487T: 3.5;
RBP5	chr12	12p13.31	1	0	0	1	Amplification	1	0	42494T: 3.5;
RBPJL	chr20	20q13.12	1	1	0	0	Amplification	1	0	42473T: 5;
RBPMS	chr8	8p12	1	0	1	0	Amplification	1	0	42482T: 3.5;
RCAN3	chr1	1p36.11	1	1	0	0	Amplification	1	0	42473T: 3.5;
RCBTB2	chr13	13q14.2	1	0	0	1	Deletion	0	1	42495T: 0.5;
RCC1	chr1	1p35.3	1	1	0	0	Amplification	1	0	42473T: 3.5;
RCE1	chr11	11q13.2	2	1	0	1	Amplification	2	0	56957T: 5.5; 42473T: 3.5;
RCL1	chr9	9p24.1	1	0	0	1	Amplification	1	0	42496T: 3.5;
RDH10	chr8	8q21.11	2	0	0	2	Amplification	2	0	42496T: 3.5; 42495T: 3.5;
RDH11	chr14	14q24.1	1	0	0	1	Amplification	1	0	42494T: 4;
RDH12	chr14	14q24.1	1	0	0	1	Amplification	1	0	42494T: 4;
RDH14	chr2	2p24.2	1	0	0	1	Amplification	1	0	42500T: 3.5;
RDH5	chr12	12q13.2	1	0	0	1	Amplification	1	0	42494T: 3.5;
RECK	chr9	9p13.3	1	0	0	1	Amplification	1	0	42501T: 3.5;
RECQL	chr12	12p12.1	1	0	0	1	Amplification	1	0	42500T: 6;
REEP1	chr2	2p11.2	2	0	0	2	Amplification	2	0	42500T: 3.5; 42493T: 3.5;
REG1A	chr2	2p12	1	0	0	1	Amplification	1	0	42500T: 3.5;
REG1B	chr2	2p12	1	0	0	1	Amplification	1	0	42500T: 3.5;
REG3A	chr2	2p12	1	0	0	1	Amplification	1	0	42500T: 3.5;
REG3G	chr2	2p12	1	0	0	1	Amplification	1	0	42500T: 3.5;
REL	chr2	2p16.1	3	0	1	2	Amplification	3	0	42500T: 3.5; 42484T: 4.5; 56957T: 3.5;
RELB	chr19	19q13.32	2	1	0	1	Amplification	2	0	42473T: 3.5; 56957T: 4;
RELL1	chr4	4p14	1	1	0	0	Amplification	1	0	42473T: 4.5;
RELN	chr7	7q22.1	4	0	1	3	Amplification	4	0	42493T: 3.5; 42487T: 3.5; 42501T: 3.5; 42497T: 4;
RELT	chr11	11q13.4	6	3	0	3	Amplification	6	0	56957T: 3.5; 42498T: 6.5; 42492T: 4; 42478T: 4.5; 42475T: 8.5; 42476T: 4;
REM1	chr20	20q11.21	1	0	0	1	Amplification	1	0	42496T: 5;
REM2	chr14	14q11.2	1	0	0	1	Amplification	1	0	42496T: 4;
REP15	chr12	12p11.22	1	0	0	1	Amplification	1	0	42500T: 6;
RERG	chr12	12p12.3	1	0	0	1	Amplification	1	0	42500T: 4.5;
RETSAT	chr2	2p11.2	1	0	0	1	Amplification	1	0	42500T: 3.5;

Mangalaparthi *et al.* , 2020. Mutational landscape of esophageal squamous cell carcinoma in an Indian cohort
Supplementary Table 7A. List of copy number alterations and affected genes in ESCC patients

Gene	Chromosome	Cytoband	Recurrence	Recurrence in smoker cohort	Recurrence in chewer cohort	Recurrence in No habit cohort	State	Samples with CNA gain	Samples with CNA loss	File info with CNA fold
REV1	chr2	2q11.2	1	0	0	1	Amplification	1	0	42493T: 3.5;
REV3L	chr6	6q21	1	0	0	1	Amplification	1	0	42496T: 3.5;
RFC4	chr3	3q27.3	10	1	3	6	Amplification	10	0	42497T: 4; 42498T: 3.5; 42484T: 4; 56957T: 4.5; 42495T: 4; 42474T: 3.5; 42482T: 3.5; 42492T: 3.5; 42493T: 3.5; 42487T: 3.5;
RFC5	chr12	12q24.23	1	0	0	1	Amplification	1	0	42500T: 3.5;
RFNG	chr17	17q25.3	1	1	0	0	Amplification	1	0	42473T: 3.5;
RFPL4A	chr19	19q13.42	1	0	0	1	Amplification	1	0	42494T: 3.5;
RFPL4AL1	chr19	19q13.42	1	0	0	1	Amplification	1	0	42494T: 3.5;
RFPL4B	chr6	6q21	1	0	0	1	Amplification	1	0	42496T: 3.5;
RFTN2	chr2	2q33.1	1	0	1	0	Amplification	1	0	42482T: 4;
RFX3	chr9	9p24.2	3	0	1	2	Amplification/Deletio	2	1	42486T: 0.5; 42496T: 3.5; 42498T: 4;
RFX5	chr1	1q21.3	1	1	0	0	Amplification	1	0	42473T: 4.5;
RFX8	chr2	2q11.2	1	0	0	1	Amplification	1	0	42493T: 3.5;
RGP1	chr9	2p11.2	2	1	0	1	Amplification	2	0	42473T: 3.5; 42501T: 3.5;
RGPD2	chr2	2p11.2	1	0	0	1	Amplification	1	0	42500T: 3.5;
RGS11	chr16	16p13.3	1	0	1	0	Amplification	1	0	42483T: 3.5;
RGS19	chr20	20q13.33	1	1	0	0	Amplification	1	0	42473T: 6;
RGS20	chr8	8q11.23	3	0	0	3	Amplification	3	0	42494T: 4; 42495T: 3.5; 42496T: 3.5;
RGS22	chr8	8q22.2	2	0	0	2	Amplification	2	0	42495T: 3.5; 42496T: 3.5;
RGS6	chr14	14q24.2	2	0	0	2	Amplification	2	0	56957T: 3.5; 42494T: 4;
RGS9BP	chr19	19q13.11	4	1	1	2	Amplification	4	0	56957T: 4; 42484T: 3.5; 42500T: 4.5; 42473T: 3.5;
RHBDF1	chr16	16p13.3	1	0	1	0	Amplification	1	0	42483T: 3.5;
RHBDL1	chr16	16p13.3	2	0	1	1	Amplification	2	0	42483T: 3.5; 42493T: 3.5;
RHBG	chr1	1q22	1	1	0	0	Amplification	1	0	42473T: 4.5;
RHCE	chr1	1p36.11	1	1	0	0	Amplification	1	0	42473T: 4.5;
RHCG	chr15	15q26.1	1	1	0	0	Amplification	1	0	42473T: 3.5;
RHD	chr1	1p36.11	1	1	0	0	Amplification	1	0	42473T: 4.5;
RHEBL1	chr12	12q13.12	1	0	0	1	Amplification	1	0	42500T: 3.5;
RHO	chr3	3q22.1	2	0	1	1	Amplification	2	0	42487T: 3.5; 42496T: 3.5;
RHOA	chr3	3p21.31	1	1	0	0	Amplification	1	0	42473T: 4;
RHOB	chr2	2p24.1	1	0	0	1	Amplification	1	0	42500T: 3.5;
RHOD	chr11	11q13.2	1	0	0	1	Amplification	1	0	56957T: 5.5;
RHOF	chr12	12q24.31	1	0	0	1	Amplification	1	0	42500T: 3.5;
RHOJ	chr14	14q23.2	1	0	0	1	Amplification	1	0	42494T: 4;
RHOQ	chr2	2p21	2	0	1	1	Amplification	2	0	42484T: 4.5; 42500T: 3.5;
RHOT2	chr16	16p13.3	2	0	1	1	Amplification	2	0	42483T: 3.5; 42493T: 3.5;
RHOV	chr15	15q15.1	1	0	0	1	Amplification	1	0	42493T: 6.5;
RHPN1	chr8	8q24.3	3	0	1	2	Amplification	3	0	42495T: 4.5; 42483T: 3.5; 42496T: 4;
RHPN2	chr19	19q13.11	4	1	1	2	Amplification	4	0	42500T: 4.5; 42473T: 7; 56957T: 4; 42484T: 3.5;

Mangalaparthi *et al.* , 2020. Mutational landscape of esophageal squamous cell carcinoma in an Indian cohort
Supplementary Table 7A. List of copy number alterations and affected genes in ESCC patients

Gene	Chromosome	Cytoband	Recurrence	Recurrence in smoker cohort	Recurrence in chewer cohort	Recurrence in No habit cohort	State	Samples with CNA gain	Samples with CNA loss	File info with CNA fold
RICTOR	chr5	5p13.1	6	1	3	2	Amplification	6	0	42484T: 3.5; 42493T: 3.5; 42486T: 3.5; 42483T: 3.5; 42496T: 3.5; 42475T: 3.5;
RIIAD1	chr1	1q21.3	1	1	0	0	Amplification	1	0	42473T: 4.5;
RILPL1	chr12	12q24.31	1	0	0	1	Amplification	1	0	42500T: 3.5;
RILPL2	chr12	12q24.31	1	0	0	1	Amplification	1	0	42500T: 3.5;
RIMS2	chr8	8q22.3	2	0	0	2	Amplification	2	0	42496T: 3.5; 42495T: 3.5;
RIMS4	chr20	20q13.12	1	1	0	0	Amplification	1	0	42473T: 5;
RIN2	chr20	20p11.23	1	0	1	0	Amplification	1	0	42483T: 4;
RINL	chr19	19q13.2	2	0	0	2	Amplification	2	0	42500T: 6.5; 56957T: 4;
RINT1	chr7	7q22.3	4	0	1	3	Amplification	4	0	42501T: 3.5; 42497T: 4; 42487T: 3.5; 42493T: 3.5;
RIOK3	chr18	18q11.2	1	1	0	0	Amplification	1	0	42481T: 4;
RIPK2	chr8	8q21.3	3	1	0	2	Amplification	3	0	42495T: 3.5; 42475T: 3.5; 42496T: 3.5;
RIPK4	chr21	21q22.3	1	1	0	0	Amplification	1	0	42473T: 3.5;
RIT1	chr1	14q32.2	1	1	0	0	Amplification	1	0	42473T: 4.5;
RLN1	chr9	9p24.1	2	0	0	2	Amplification	2	0	42496T: 3.5; 42498T: 14;
RLN2	chr9	9p24.1	1	0	0	1	Amplification	1	0	42496T: 3.5;
RMDN1	chr8	8q21.3	2	0	0	2	Amplification	2	0	42496T: 3.5; 42495T: 3.5;
RMDN2	chr2	2p22.2	2	0	0	2	Amplification	2	0	42500T: 3.5; 42493T: 3.5;
RMI2	chr16	16p13.13	2	1	0	1	Amplification	2	0	42495T: 5; 42473T: 5.5;
RMND5A	chr2	2p11.2	2	0	0	2	Amplification	2	0	42493T: 3.5; 42500T: 3.5;
RNA5SP96	chr2	2p13.3	1	0	0	1	Amplification	1	0	42500T: 3.5;
RNASEH1	chr2	17p11.2	2	0	0	2	Amplification	2	0	42495T: 3.5; 42500T: 3.5;
RND1	chr12	12q13.12	1	0	0	1	Amplification	1	0	42500T: 3.5;
RNF10	chr12	12q24.31	1	0	0	1	Amplification	1	0	42500T: 3.5;
RNF103	chr2	2p11.2	2	0	0	2	Amplification	2	0	42500T: 3.5; 42493T: 3.5;
RNF114	chr20	20q13.13	1	1	0	0	Amplification	1	0	42473T: 5;
RNF115	chr1	1q21.1	1	1	0	0	Amplification	1	0	42473T: 4;
RNF121	chr11	11q13.4	7	3	0	4	Amplification	7	0	42501T: 6; 42475T: 5; 42476T: 4; 56957T: 3.5; 42498T: 5; 42492T: 4; 42478T: 4.5;
RNF122	chr8	8p12	2	0	1	1	Amplification	2	0	42497T: 4.5; 42482T: 3.5;
RNF123	chr3	3p21.31	1	1	0	0	Amplification	1	0	42473T: 3.5;
RNF126P1	chr17	17q22	1	0	0	1	Amplification	1	0	42497T: 4;
RNF13	chr3	3q25.1	9	2	2	5	Amplification	9	0	56957T: 4; 42474T: 3.5; 42484T: 3.5; 42496T: 4; 42497T: 4.5; 42473T: 4; 42493T: 3.5; 42487T: 3.5; 42492T: 3.5;
RNF135	chr17	17q11.2	1	1	0	0	Amplification	1	0	42473T: 3.5;
RNF139	chr8	8q24.13	4	1	1	2	Amplification	4	0	42484T: 3.5; 42495T: 3.5; 42496T: 3.5; 42475T: 3.5;
RNF144A	chr2	2p25.1	1	0	0	1	Amplification	1	0	42500T: 3.5;
RNF149	chr2	2q11.2	1	0	0	1	Amplification	1	0	42493T: 3.5;
RNF151	chr16	16p13.3	1	0	1	0	Amplification	1	0	42483T: 3.5;

Mangalaparthi *et al.* , 2020. Mutational landscape of esophageal squamous cell carcinoma in an Indian cohort
Supplementary Table 7A. List of copy number alterations and affected genes in ESCC patients

Gene	Chromosome	Cytoband	Recurrence	Recurrence in smoker cohort	Recurrence in chewer cohort	Recurrence in No habit cohort	State	Samples with CNA gain	Samples with CNA loss	File info with CNA fold
RNF168	chr3	3q29	9	1	3	5	Amplification	9	0	56957T: 6; 42495T: 4; 42474T: 3.5; 42498T: 3.5; 42484T: 4; 42482T: 3.5; 42487T: 3.5; 42493T: 5; 42492T: 3.5;
RNF169	chr11	11q13.4	4	1	0	3	Amplification	4	0	42492T: 4; 42498T: 8; 56957T: 3.5; 42475T: 7.5;
RNF170	chr8	8p11.21	1	0	1	0	Amplification	1	0	42483T: 4.5;
RNF181	chr2	2p11.2	2	0	1	1	Amplification	2	0	42500T: 3.5; 42482T: 3.5;
RNF186	chr1	1p36.13	1	1	0	0	Amplification	1	0	42473T: 4;
RNF19A	chr8	8q22.2	2	0	0	2	Amplification	2	0	42496T: 3.5; 42495T: 3.5;
RNF208	chr9	9q34.3	2	1	0	1	Amplification	2	0	56957T: 3.5; 42473T: 5;
RNF213	chr17	17q25.3	1	1	0	0	Amplification	1	0	42473T: 3.5;
RNF215	chr22	22q12.2	1	1	0	0	Amplification	1	0	42473T: 3.5;
RNF216	chr7	7p22.1	2	1	0	1	Amplification	2	0	42497T: 10.5; 42473T: 4.5;
RNF216P1	chr7	7p22.1	2	1	0	1	Amplification	2	0	42497T: 8.5; 42473T: 4.5;
RNF224	chr9	9q34.3	2	1	0	1	Amplification	2	0	56957T: 3.5; 42473T: 5;
RNF24	chr20	20p13	1	1	0	0	Amplification	1	0	42473T: 4.5;
RNF34	chr12	12q24.31	1	0	0	1	Amplification	1	0	42500T: 3.5;
RNF38	chr9	9p13.2	1	0	0	1	Amplification	1	0	42501T: 3.5;
RNF41	chr12	12q13.3	1	0	0	1	Amplification	1	0	42494T: 7.5;
RNF43	chr17	17q22	1	0	0	1	Amplification	1	0	42497T: 4;
RNF5	chr6	6p21.32	1	1	0	0	Amplification	1	0	42473T: 3.5;
RNF7	chr3	3q23	4	0	1	3	Amplification	4	0	42496T: 4; 42493T: 3.5; 42487T: 3.5; 42492T: 3.5;
RNF8	chr6	6p21.2	1	1	0	0	Amplification	1	0	42473T: 4.5;
RNFT2	chr12	12q24.22	1	0	0	1	Amplification	1	0	42500T: 3.5;
RNPS1	chr16	16p13.3	1	0	1	0	Amplification	1	0	42483T: 3.5;
ROCK1P1	chr18	18p11.32	3	0	0	3	Amplification	3	0	42500T: 4.5; 42493T: 3.5; 56957T: 8;
ROCK2	chr2	2p25.1	1	0	0	1	Amplification	1	0	42500T: 3.5;
ROGDI	chr16	16p13.3	2	1	0	1	Amplification	2	0	42495T: 12; 42473T: 5.5;
ROMO1	chr20	20q11.22	2	1	0	1	Amplification	2	0	42473T: 3.5; 42493T: 3.5;
ROPN1	chr3	3q21.1	1	0	0	1	Amplification	1	0	42496T: 3.5;
ROPN1B	chr3	3q21.2	1	0	0	1	Amplification	1	0	42496T: 3.5;
ROPN1L	chr5	5p15.2	4	1	1	2	Amplification	4	0	42486T: 3.5; 42496T: 3.5; 42475T: 3.5; 42493T: 4;
RORC	chr1	1q21.3	1	1	0	0	Amplification	1	0	42473T: 4.5;
RP11	chr1	19q13.42	4	1	2	1	Amplification/Deletio	13	1	42475T: 3.5,5,7.5; 42486T: 3.5,0.5; 42498T: 3.5,14.5,5,8; 42484T: 4.5,4,3.5,7.5,12.5;
RP1L1	chr8	8p23.1	1	0	1	0	Amplification	1	0	42486T: 3.5;
RP9	chr7	7p14.3	1	1	0	0	Amplification	1	0	42473T: 4;
RP9P	chr7	7p14.3	1	1	0	0	Amplification	1	0	42473T: 4;
RPA2	chr1	2q14.1	1	1	0	0	Amplification	1	0	42473T: 3.5;
RPAP3	chr12	12q13.11	1	0	0	1	Amplification	1	0	42500T: 3.5;
RPF2	chr6	6q21	1	0	0	1	Amplification	1	0	42496T: 3.5;
RPIA	chr2	2p11.2	1	0	0	1	Amplification	1	0	42500T: 3.5;

Mangalaparthi *et al.* , 2020. Mutational landscape of esophageal squamous cell carcinoma in an Indian cohort
Supplementary Table 7A. List of copy number alterations and affected genes in ESCC patients

Gene	Chromosome	Cytoband	Recurrence	Recurrence in smoker cohort	Recurrence in chewer cohort	Recurrence in No habit cohort	State	Samples with CNA gain	Samples with CNA loss	File info with CNA fold
RPL11	chr1	1p36.11	1	1	0	0	Amplification	1	0	42473T: 3.5;
RPL19	chr17	17q12	1	1	0	0	Amplification	1	0	42473T: 4.5;
RPL22L1	chr3	3q26.2	9	1	3	5	Amplification	9	0	42482T: 3.5; 42493T: 3.5; 42487T: 3.5; 42492T: 3.5; 42500T: 8; 42495T: 4; 42474T: 3.5; 56957T: 4; 42484T: 4;
RPL23	chr17	17q12	1	1	0	0	Amplification	1	0	42473T: 3.5;
RPL24	chr3	15q21.3	1	1	0	0	Amplification	1	0	42476T: 3.5;
RPL28	chr19	19q13.42	1	0	0	1	Amplification	1	0	42494T: 3.5;
RPL3	chr22	22q13.1	1	1	0	0	Amplification	1	0	42473T: 3.5;
RPL30	chr8	8q22.2	2	0	0	2	Amplification	2	0	42495T: 3.5; 42496T: 3.5;
RPL31	chr2	2q11.2	1	0	0	1	Amplification	1	0	42493T: 3.5;
RPL32P3	chr3	3q21.3	2	0	1	1	Amplification	2	0	42496T: 3.5; 42487T: 3.5;
RPL35A	chr3	3q29	9	1	3	5	Amplification	9	0	42493T: 4; 42487T: 3.5; 42492T: 3.5; 42482T: 3.5; 56957T: 6; 42474T: 3.5; 42495T: 4; 42498T: 3.5; 42484T: 3.5;
RPL36AL	chr14	14q21.3	1	1	0	0	Amplification	1	0	42473T: 3.5;
RPL37	chr5	5p13.1	6	1	3	2	Amplification	6	0	42493T: 3.5; 42484T: 3.5; 42475T: 3.5; 42483T: 3.5; 42486T: 3.5; 42496T: 3.5;
RPL39L	chr3	3q27.3	10	1	3	6	Amplification	10	0	42497T: 4; 56957T: 4.5; 42495T: 4; 42474T: 3.5; 42498T: 3.5; 42484T: 4; 42482T: 3.5; 42493T: 3.5; 42487T: 3.5; 42492T: 3.5;
RPL3L	chr16	16p13.3	1	0	1	0	Amplification	1	0	42483T: 3.5;
RPL41	chr12	12q13.2	1	0	0	1	Amplification	1	0	42494T: 7.5;
RPL6	chr12	12q24.13	1	0	0	1	Amplification	1	0	42500T: 3.5;
RPL7	chr8	8q21.11	2	0	0	2	Amplification	2	0	42495T: 3.5; 42496T: 3.5;
RPL8	chr8	8q24.3	3	0	1	2	Amplification	3	0	42495T: 4.5; 42496T: 4; 42483T: 3.5;
RPLP0	chr12	12q24.23	1	0	0	1	Amplification	1	0	42500T: 3.5;
RPLP0P6	chr2	2p22.1	2	0	0	2	Amplification	2	0	42500T: 3.5; 42493T: 3.5;
RPN1	chr3	3q21.3	2	0	1	1	Amplification	2	0	42487T: 3.5; 42496T: 3.5;
RPN2	chr20	20q11.23	1	1	0	0	Amplification	1	0	42473T: 3.5;
RPRD1B	chr20	20q11.23	1	1	0	0	Amplification	1	0	42473T: 3.5;
RPRD2	chr1	1q21.2	2	1	0	1	Amplification	2	0	42493T: 3.5; 42473T: 5;
RPS15A	chr16	16p12.3	2	1	0	1	Amplification	2	0	42495T: 4; 42473T: 4;
RPS16	chr19	19q13.2	2	0	0	2	Amplification	2	0	42500T: 6.5; 56957T: 4;
RPS19	chr19	19q13.2	1	1	0	0	Amplification	1	0	42473T: 4.5;
RPS19BP1	chr22	22q13.1	1	1	0	0	Amplification	1	0	42473T: 3.5;
RPS2	chr16	16p13.3	1	0	1	0	Amplification	1	0	42483T: 3.5;
RPS20	chr8	8q12.1	3	0	1	2	Amplification	3	0	42483T: 5; 42496T: 3.5; 42495T: 3.5;
RPS21	chr20	20q13.33	1	1	0	0	Amplification	1	0	42473T: 6;
RPS25	chr11	11q23.3	1	1	0	0	Amplification	1	0	42473T: 4;
RPS26	chr12	13q32.3	1	0	0	1	Amplification	1	0	42494T: 9.5;

Mangalaparthi *et al.* , 2020. Mutational landscape of esophageal squamous cell carcinoma in an Indian cohort
Supplementary Table 7A. List of copy number alterations and affected genes in ESCC patients

Gene	Chromosome	Cytoband	Recurrence	Recurrence in smoker cohort	Recurrence in chewer cohort	Recurrence in No habit cohort	State	Samples with CNA gain	Samples with CNA loss	File info with CNA fold
RPS27	chr1	1q21.3	1	1	0	0	Amplification	1	0	42473T: 4.5;
RPS27A	chr2	2p16.1	3	0	1	2	Amplification	3	0	42500T: 3.5; 42484T: 4.5; 56957T: 3.5;
RPS29	chr14	14q21.3	1	1	0	0	Amplification	1	0	42473T: 3.5;
RPS3	chr11	11q13.4	2	0	0	2	Amplification	2	0	42498T: 8; 42492T: 4;
RPS6KB2	chr11	11q13.2	2	1	0	1	Amplification	2	0	56957T: 5; 42473T: 3.5;
RPS6KL1	chr14	14q24.3	2	0	0	2	Amplification	2	0	42494T: 4; 56957T: 3.5;
RPS7	chr2	2p25.3	2	0	0	2	Amplification	2	0	42495T: 3.5; 42500T: 3.5;
RPTN	chr1	1q21.3	1	1	0	0	Amplification	1	0	42473T: 4.5;
RPUSD1	chr16	16p13.3	2	0	1	1	Amplification	2	0	42493T: 3.5; 42483T: 3.5;
RRM2	chr2	2p25.1	1	0	0	1	Amplification	1	0	42500T: 3.5;
RRM2B	chr8	8q22.3	2	0	0	2	Amplification	2	0	42496T: 3.5; 42495T: 3.5;
RRN3	chr16	16p13.11	2	1	0	1	Amplification	2	0	42473T: 4; 42495T: 4;
RRN3P1	chr16	16p12.2	1	1	0	0	Amplification	1	0	42473T: 4;
RRN3P3	chr16	16p12.2	1	1	0	0	Amplification	1	0	42473T: 4;
RRNAD1	chr1	1q23.1	1	1	0	0	Amplification	1	0	42473T: 7;
RRS1	chr8	8q13.1	3	0	0	3	Amplification	3	0	42495T: 3.5; 42496T: 3.5; 42497T: 4;
RSAD1	chr17	17q21.33	1	1	0	0	Amplification	1	0	42473T: 4.5;
RSAD2	chr2	2p25.2	1	0	0	1	Amplification	1	0	42500T: 3.5;
RSBN1L	chr7	7q11.23	1	0	1	0	Amplification	1	0	42487T: 3.5;
RSG1	chr1	1p36.13	1	1	0	0	Amplification	1	0	42473T: 8;
RSL1D1	chr16	16p13.13	2	1	0	1	Amplification	2	0	42473T: 5.5; 42495T: 4;
RSPH1	chr21	21q22.3	1	1	0	0	Amplification	1	0	42473T: 3.5;
RSPH10B	chr7	7p22.1	2	1	0	1	Amplification	2	0	42473T: 4.5; 42497T: 8;
RSPH10B2	chr7	7p22.1	2	1	0	1	Amplification	2	0	42473T: 5.5; 42497T: 7.5;
RSPH6A	chr19	19q13.32	1	0	1	0	Amplification	1	0	42484T: 4;
RSPO2	chr8	8q23.1	2	0	0	2	Amplification	2	0	42495T: 3.5; 42496T: 3.5;
RSPO4	chr20	20p13	1	1	0	0	Amplification	1	0	42473T: 3.5;
RSRC1	chr3	3q25.32	8	1	2	5	Amplification	8	0	42492T: 3.5; 42493T: 3.5; 42487T: 3.5; 42497T: 3.5; 42484T: 3.5; 56957T: 4; 42474T: 3.5; 42495T: 4;
RSRC2	chr12	12q24.31	1	0	0	1	Amplification	1	0	42500T: 3.5;
RTEL1	chr20	20q13.33	1	1	0	0	Amplification	1	0	42473T: 6;
RTFDC1	chr20	20q13.31	1	1	0	0	Amplification	1	0	42473T: 5;
RTKN	chr2	2p13.1	1	0	0	1	Amplification	1	0	42500T: 3.5;
RTN1	chr14	14q23.1	2	0	1	1	Amplification	2	0	42483T: 3.5; 42494T: 4;
RTN2	chr19	19q13.32	2	1	1	0	Amplification	2	0	42473T: 3.5; 42484T: 4;
RTN4	chr2	2p16.1	2	0	1	1	Amplification	2	0	42500T: 3.5; 42484T: 4.5;
RTN4IP1	chr6	6q21	1	0	0	1	Amplification	1	0	42496T: 3.5;
RTN4R	chr22	22q11.21	1	1	0	0	Amplification	1	0	42477T: 4;
RTP1	chr3	3q27.3	10	1	3	6	Amplification	10	0	42482T: 3.5; 42487T: 3.5; 42493T: 3.5; 42492T: 3.5; 42497T: 4; 42495T: 4; 42474T: 3.5; 56957T: 4.5; 42484T: 4; 42498T: 3.5;

Mangalaparthi *et al.* , 2020. Mutational landscape of esophageal squamous cell carcinoma in an Indian cohort
Supplementary Table 7A. List of copy number alterations and affected genes in ESCC patients

Gene	Chromosome	Cytoband	Recurrence	Recurrence in smoker cohort	Recurrence in chewer cohort	Recurrence in No habit cohort	State	Samples with CNA gain	Samples with CNA loss	File info with CNA fold
RTP2	chr3	3q27.3	10	1	3	6	Amplification	10	0	42484T: 4; 42498T: 3.5; 42495T: 4; 42474T: 3.5; 56957T: 4.5; 42497T: 4; 42492T: 3.5; 42493T: 3.5; 42487T: 3.5; 42482T: 3.5;
RTP4	chr3	3q27.3	10	1	3	6	Amplification	10	0	42492T: 3.5; 42493T: 3.5; 42487T: 3.5; 42482T: 3.5; 42498T: 3.5; 42484T: 4; 56957T: 4.5; 42495T: 4; 42474T: 3.5; 42497T: 4;
RUNDC3B	chr7	7q21.12	2	0	2	0	Amplification	2	0	42483T: 3.5; 42487T: 3.5;
RUNX1T1	chr8	8q21.3	2	0	0	2	Amplification	2	0	42495T: 3.5; 42496T: 3.5;
RUNX3	chr1	1p36.11	1	1	0	0	Amplification	1	0	42473T: 4.5;
RUSC1	chr1	1q22	1	1	0	0	Amplification	1	0	42473T: 6.5;
RUSC2	chr9	9p13.3	2	1	0	1	Amplification	2	0	42473T: 3.5; 42501T: 3.5;
RUVBL1	chr3	3q21.3	2	0	1	1	Amplification	2	0	42496T: 3.5; 42487T: 3.5;
RXFP2	chr13	13q13.1	1	0	0	1	Amplification	1	0	42497T: 3.5;
RXFP3	chr5	5p13.2	4	1	1	2	Amplification	4	0	42496T: 3.5; 42486T: 3.5; 42475T: 3.5; 42493T: 3.5;
RXFP4	chr1	1q22	1	1	0	0	Amplification	1	0	42473T: 4.5;
RYK	chr3	3q22.2	4	0	1	3	Amplification	4	0	42496T: 3.5; 42492T: 3.5; 42493T: 3.5; 42487T: 3.5;
RYR1	chr19	19q13.2	3	0	1	2	Amplification	3	0	56957T: 4; 42484T: 3.5; 42500T: 6.5;
S100A1	chr1	1q21.3	1	1	0	0	Amplification	1	0	42473T: 4.5;
S100A10	chr1	1q21.3	1	1	0	0	Amplification	1	0	42473T: 4.5;
S100A11	chr1	1q21.3	1	1	0	0	Amplification	1	0	42473T: 4.5;
S100A12	chr1	1q21.3	2	1	0	1	Amplification	2	0	42473T: 4.5; 42493T: 4;
S100A13	chr1	1q21.3	1	1	0	0	Amplification	1	0	42473T: 4.5;
S100A14	chr1	7q22.1	1	1	0	0	Amplification	1	0	42473T: 4.5;
S100A16	chr1	1q21.3	1	1	0	0	Amplification	1	0	42473T: 4.5;
S100A2	chr1	1q21.3	1	1	0	0	Amplification	1	0	42473T: 4.5;
S100A3	chr1	1q21.3	1	1	0	0	Amplification	1	0	42473T: 4.5;
S100A4	chr1	1q21.3	1	1	0	0	Amplification	1	0	42473T: 4.5;
S100A5	chr1	1q21.3	1	1	0	0	Amplification	1	0	42473T: 4.5;
S100A6	chr1	1q21.3	1	1	0	0	Amplification	1	0	42473T: 4.5;
S100A7	chr1	1q21.3	2	1	0	1	Amplification	2	0	42473T: 4.5; 42493T: 4;
S100A7A	chr1	1q21.3	2	1	0	1	Amplification	2	0	42473T: 4.5; 42493T: 4;
S100A7L2	chr1	1q21.3	2	1	0	1	Amplification	2	0	42493T: 4; 42473T: 4.5;
S100A8	chr1	1q21.3	2	1	0	1	Amplification	2	0	42473T: 4.5; 42493T: 4;
S100A9	chr1	1q21.3	2	1	0	1	Amplification	2	0	42473T: 4.5; 42493T: 4;
SAG	chr2	3q23	1	1	0	0	Amplification	1	0	42473T: 4.5;
SALL2	chr14	14q11.2	1	0	0	1	Amplification	1	0	42496T: 4;
SALL4	chr20	20q13.2	1	1	0	0	Amplification	1	0	42473T: 5;
SAMD10	chr20	20q13.33	1	1	0	0	Amplification	1	0	42473T: 6;
SAMD12	chr8	8q24.11-q24	3	1	0	2	Amplification	3	0	42475T: 3.5; 42496T: 3.5; 42495T: 3.5;
SAMD15	chr14	14q24.3	2	0	0	2	Amplification	2	0	42494T: 4; 56957T: 3.5;
SAMD4A	chr14	14q22.2	1	0	0	1	Amplification	1	0	42494T: 4;

Mangalaparthi *et al.* , 2020. Mutational landscape of esophageal squamous cell carcinoma in an Indian cohort
Supplementary Table 7A. List of copy number alterations and affected genes in ESCC patients

Gene	Chromosome	Cytoband	Recurrence	Recurrence in smoker cohort	Recurrence in chewer cohort	Recurrence in No habit cohort	State	Samples with CNA gain	Samples with CNA loss	File info with CNA fold
SAMD4B	chr19	19q13.2	2	0	0	2	Amplification	2	0	56957T: 4; 42500T: 6.5;
SAMD7	chr3	3q26.2	9	1	3	5	Amplification	9	0	42484T: 4; 42495T: 4; 42474T: 3.5; 56957T: 4; 42500T: 8; 42492T: 3.5; 42493T: 3.5; 42487T: 3.5; 42482T: 3.5;
SAMD9	chr7	7q21.2	2	0	2	0	Amplification	2	0	42483T: 4; 42487T: 3.5;
SAMD9L	chr7	7q21.2	2	0	2	0	Amplification	2	0	42483T: 4; 42487T: 3.5;
SAMHD1	chr20	20q11.23	1	1	0	0	Amplification	1	0	42473T: 3.5;
SAP30BP	chr17	17q25.1	1	0	0	1	Amplification	1	0	42494T: 3.5;
SAPCD1	chr6	6p21.33	1	1	0	0	Amplification	1	0	42473T: 3.5;
SAPCD2	chr9	9q34.3	2	1	0	1	Amplification	2	0	42473T: 5; 56957T: 3.5;
SAR1B	chr5	5q31.1	1	1	0	0	Amplification	1	0	42473T: 3.5;
SARNP	chr12	12q13.2	1	0	0	1	Amplification	1	0	42494T: 5;
SARS2	chr19	19q13.2	2	0	0	2	Amplification	2	0	42500T: 6.5; 56957T: 4;
SATB2	chr2	2q33.1	1	0	1	0	Amplification	1	0	42482T: 4;
SAV1	chr14	14q22.1	1	0	0	1	Amplification	1	0	42494T: 4;
SBDS	chr7	7q11.21	1	0	0	1	Amplification	1	0	42501T: 3.5;
SBK2	chr19	19q13.42	1	0	0	1	Amplification	1	0	42494T: 3.5;
SBNO1	chr12	12q24.31	1	0	0	1	Amplification	1	0	42500T: 3.5;
SBSN	chr19	19q13.12	3	0	1	2	Amplification	3	0	56957T: 4; 42484T: 3.5; 42500T: 6.5;
SBSPON	chr8	8q21.11	2	0	0	2	Amplification	2	0	42495T: 3.5; 42496T: 3.5;
SCAF11	chr12	12q12	1	0	0	1	Amplification	1	0	42500T: 3.5;
SCAMP2	chr15	15q24.1	1	1	0	0	Amplification	1	0	42473T: 3.5;
SCAMP3	chr1	1q22	1	1	0	0	Amplification	1	0	42473T: 6.5;
SCAND1	chr20	20q11.23	1	1	0	0	Amplification	1	0	42473T: 3.5;
SCAND2P	chr15	15q25.2	1	1	0	0	Amplification	1	0	42473T: 4;
SCARB1	chr12	12q24.31	1	0	0	1	Amplification	1	0	42500T: 3.5;
SCARF2	chr22	22q11.21	2	1	0	1	Amplification	2	0	42497T: 3.5; 42477T: 4;
SCFD1	chr14	14q12	3	0	0	3	Amplification	3	0	56957T: 4; 42494T: 3.5; 42500T: 4.5;
SCGB2B2	chr19	19q13.11	3	0	1	2	Amplification	3	0	42500T: 4.5; 42484T: 3.5; 56957T: 4;
SCHIP1	chr3	3q25.32-q25	8	1	2	5	Amplification	8	0	42495T: 4; 42474T: 3.5; 56957T: 4; 42484T: 4; 42497T: 3.5; 42487T: 3.5; 42493T: 3.5; 42492T: 3.5;
SCIN	chr7	7p21.3	1	1	0	0	Amplification	1	0	42473T: 3.5;
SCLY	chr2	2q37.3	1	1	0	0	Amplification	1	0	42473T: 3.5;
SCML4	chr6	6q21	1	0	0	1	Amplification	1	0	42496T: 3.5;
SCN1B	chr19	19q13.11	3	0	1	2	Amplification	3	0	42500T: 6.5; 56957T: 4; 42484T: 3.5;
SCN8A	chr12	12q13.13	1	0	0	1	Amplification	1	0	42494T: 5.5;
SCNM1	chr1	1q21.3	1	1	0	0	Amplification	1	0	42473T: 4.5;
SCNN1B	chr16	16p12.2	1	1	0	0	Amplification	1	0	42473T: 4;
SCNN1G	chr16	16p12.2	1	1	0	0	Amplification	1	0	42473T: 4;
SCPEP1	chr17	17q22	1	0	0	1	Amplification	1	0	42497T: 4;
SCRIB	chr8	8q24.3	3	0	1	2	Amplification	3	0	42483T: 3.5; 42496T: 4; 42495T: 4.5;

Mangalaparthi *et al.* , 2020. Mutational landscape of esophageal squamous cell carcinoma in an Indian cohort
Supplementary Table 7A. List of copy number alterations and affected genes in ESCC patients

Gene	Chromosome	Cytoband	Recurrence	Recurrence in smoker cohort	Recurrence in chewer cohort	Recurrence in No habit cohort	State	Samples with CNA gain	Samples with CNA loss	File info with CNA fold
SCRN1	chr7	7p14.3	1	1	0	0	Amplification	1	0	42473T: 4;
SCRN3	chr2	2q31.1	1	0	0	1	Amplification	1	0	42493T: 3.5;
SCRT1	chr8	8q24.3	4	0	1	3	Amplification	4	0	56957T: 3.5; 42495T: 4.5; 42496T: 4; 42483T: 3.5;
SCRT2	chr20	20p13	1	1	0	0	Amplification	1	0	42473T: 3.5;
SCXA	chr8	8q24.3	3	0	1	2	Amplification	3	0	42496T: 4; 42483T: 3.5; 42495T: 4.5;
SCXB	chr8	8q24.3	3	0	1	2	Amplification	3	0	42496T: 4; 42483T: 3.5; 42495T: 4.5;
SDC1	chr2	2p24.1	2	1	0	1	Amplification	2	0	42500T: 3.5; 42473T: 4;
SDC2	chr8	8q22.1	2	0	0	2	Amplification	2	0	42495T: 3.5; 42496T: 3.5;
SDC3	chr1	1p35.2	1	1	0	0	Amplification	1	0	42473T: 3.5;
SDC4	chr20	20q13.12	1	1	0	0	Amplification	1	0	42473T: 5;
SDCBP	chr8	8q12.1	2	0	0	2	Amplification	2	0	42495T: 3.5; 42496T: 3.5;
SDCBP2	chr20	20p13	1	1	0	0	Amplification	1	0	42473T: 3.5;
SDCCAG3	chr9	9q34.3	1	0	0	1	Amplification	1	0	56957T: 3.5;
SDE2	chr1	1q42.12	1	0	0	1	Amplification	1	0	42493T: 3.5;
SDF2L1	chr22	22q11.21	1	1	0	0	Amplification	1	0	42473T: 3.5;
SDHA	chr5	5p15.33	5	2	1	2	Amplification	5	0	42493T: 3.5; 42486T: 3.5; 42496T: 4; 42473T: 3.5; 42475T: 3.5;
SDHAF1	chr19	19q13.12	3	0	1	2	Amplification	3	0	42500T: 6.5; 56957T: 4; 42484T: 3.5;
SDHAP1	chr3	3q29	9	1	3	5	Amplification	9	0	56957T: 6; 42474T: 3.5; 42495T: 4; 42498T: 3.5; 42484T: 4; 42493T: 3.5; 42487T: 3.5; 42492T: 3.5; 42482T: 3.5;
SDHAP3	chr5	5p15.33	5	2	1	2	Amplification	5	0	42493T: 3.5; 42486T: 3.5; 42496T: 4; 42473T: 3.5; 42475T: 3.5;
SDK1	chr7	7p22.2	2	1	0	1	Amplification	3	0	42473T: 4.5; 42497T: 13,8.5;
SDPR	chr2	2q32.3	3	0	1	2	Amplification	3	0	42482T: 4; 42493T: 3.5; 42494T: 3.5;
SDR16C5	chr8	8q12.1	3	0	1	2	Amplification	3	0	42495T: 3.5; 42496T: 3.5; 42483T: 5;
SDR16C6P	chr8	8q12.1	2	0	0	2	Amplification	2	0	42496T: 3.5; 42495T: 3.5;
SDR42E2	chr16	16p12.2	1	1	0	0	Amplification	1	0	42473T: 4;
SDS	chr12	7q11.21	1	0	0	1	Amplification	1	0	42500T: 3.5;
SDSL	chr12	12q24.13	1	0	0	1	Amplification	1	0	42500T: 3.5;
SEC11A	chr15	15q25.2-q25	1	1	0	0	Amplification	1	0	42473T: 4;
SEC14L2	chr22	22q12.2	1	1	0	0	Amplification	1	0	42473T: 3.5;
SEC14L5	chr16	16p13.3	2	1	0	1	Amplification	2	0	42495T: 12; 42473T: 5.5;
SEC16A	chr9	9q34.3	1	0	0	1	Amplification	1	0	56957T: 3.5;
SEC22A	chr3	3q21.1	2	1	0	1	Amplification	2	0	42473T: 3.5; 42496T: 3.5;
SEC22B	chr1	1p12	1	1	0	0	Amplification	1	0	42473T: 4;
SEC23A	chr14	14q21.1	1	0	0	1	Amplification	1	0	42500T: 6.5;
SEC24A	chr5	5q31.1	1	1	0	0	Amplification	1	0	42473T: 3.5;
SEC24C	chr10	10q22.2	2	1	0	1	Amplification	2	0	42496T: 3.5; 42473T: 4.5;
SEC61A1	chr3	3q21.3	2	0	1	1	Amplification	2	0	42496T: 3.5; 42487T: 3.5;
SEC61G	chr7	7p11.2	3	0	1	2	Amplification	3	0	56957T: 5; 42497T: 21.5; 42483T: 4;

Mangalaparthi *et al.* , 2020. Mutational landscape of esophageal squamous cell carcinoma in an Indian cohort
Supplementary Table 7A. List of copy number alterations and affected genes in ESCC patients

Gene	Chromosome	Cytoband	Recurrence	Recurrence in smoker cohort	Recurrence in chewer cohort	Recurrence in No habit cohort	State	Samples with CNA gain	Samples with CNA loss	File info with CNA fold
SEC62	chr3	3q26.2	9	1	3	5	Amplification	9	0	42487T: 3.5; 42493T: 3.5; 42492T: 3.5; 42482T: 3.5; 42474T: 3.5; 42495T: 4; 56957T: 4; 42484T: 4; 42500T: 8;
SEC63	chr6	6q21	1	0	0	1	Amplification	1	0	42496T: 3.5;
SELENBP1	chr1	1q21.3	1	1	0	0	Amplification	1	0	42473T: 4.5;
SELT	chr3	3q25.1	10	2	2	6	Amplification	10	0	42474T: 3.5; 56957T: 4; 42484T: 3.5; 42500T: 3.5; 42473T: 4; 42496T: 4; 42497T: 4.5; 42487T: 3.5; 42493T: 3.5; 42492T: 3.5;
SELV	chr19	19q13.2	2	0	0	2	Amplification	2	0	42500T: 7.5; 56957T: 4;
SEMA3A	chr7	7q21.11	1	0	1	0	Amplification	1	0	42487T: 3.5;
SEMA3C	chr7	7q21.11	1	0	1	0	Amplification	1	0	42487T: 3.5;
SEMA3D	chr7	7q21.11	2	0	2	0	Amplification	2	0	42487T: 3.5; 42483T: 4.5;
SEMA3E	chr7	7q21.11	1	0	1	0	Amplification	1	0	42487T: 3.5;
SEMA4A	chr1	1q22	2	1	0	1	Amplification	2	0	42473T: 4.5; 42493T: 4;
SEMA4B	chr15	15q26.1	1	1	0	0	Amplification	1	0	42473T: 3.5;
SEMA4C	chr2	2q11.2	2	1	0	1	Amplification	2	0	42473T: 3.5; 42493T: 4.5;
SEMA4F	chr2	2p13.1	1	0	0	1	Amplification	1	0	42500T: 3.5;
SEMA5A	chr5	5p15.31	4	1	1	2	Amplification	4	0	42493T: 4; 42475T: 3.5; 42486T: 3.5; 42496T: 3.5;
SEMA5B	chr3	3q21.1	1	0	0	1	Amplification	1	0	42496T: 3.5;
SEMA6C	chr1	1q21.3	1	1	0	0	Amplification	1	0	42473T: 4.5;
SEMA7A	chr15	15q24.1	1	1	0	0	Amplification	1	0	42473T: 3.5;
SEMG1	chr20	20q13.12	1	1	0	0	Amplification	1	0	42473T: 5;
SEMG2	chr20	20q13.12	1	1	0	0	Amplification	1	0	42473T: 5;
SENP1	chr12	12q13.11	1	0	0	1	Amplification	1	0	42500T: 3.5;
SENP2	chr3	3q27.2	10	1	3	6	Amplification	10	0	42487T: 3.5; 42493T: 3.5; 42492T: 3.5; 42482T: 3.5; 42474T: 3.5; 42495T: 4; 56957T: 4.5; 42484T: 4; 42498T: 3.5; 42497T: 4;
SENP5	chr3	3q29	9	1	3	5	Amplification	9	0	42482T: 3.5; 42492T: 3.5; 42493T: 5; 42487T: 3.5; 42484T: 4; 42498T: 3.5; 42474T: 3.5; 42495T: 4; 56957T: 6;
SENP7	chr3	3q12.3	1	1	0	0	Amplification	1	0	42476T: 3.5;
SEPN1	chr1	1p36.11	1	1	0	0	Amplification	1	0	42473T: 4.5;
SEPP1	chr5	5p12	5	1	2	2	Amplification	5	0	42493T: 3.5; 42483T: 3.5; 42486T: 3.5; 42496T: 3.5; 42475T: 3.5;
SEPT12	chr16	16p13.3	2	1	0	1	Amplification	2	0	42495T: 12; 42473T: 5.5;
SEPT14	chr7	7p11.2	3	0	1	2	Amplification	3	0	56957T: 5; 42497T: 6.5; 42483T: 4;
SEPT4	chr17	17q22	1	0	0	1	Amplification	1	0	42497T: 4;
SEPT5	chr22	22q11.21	1	0	0	1	Amplification	1	0	42497T: 17;
SEPT7	chr7	7p14.2	1	1	0	0	Amplification	1	0	42473T: 4;
SEPT7P2	chr7	7p12.3	1	1	0	0	Amplification	1	0	42473T: 4.5;
SEPT7P9	chr10	10p11.1	1	0	0	1	Amplification	1	0	56958T: 3.5;

Mangalaparthi *et al.* , 2020. Mutational landscape of esophageal squamous cell carcinoma in an Indian cohort
Supplementary Table 7A. List of copy number alterations and affected genes in ESCC patients

Gene	Chromosome	Cytoband	Recurrence	Recurrence in smoker cohort	Recurrence in chewer cohort	Recurrence in No habit cohort	State	Samples with CNA gain	Samples with CNA loss	File info with CNA fold
SERINC2	chr1	1p35.2	1	1	0	0	Amplification	1	0	42473T: 3.5;
SERINC3	chr20	20q13.12	1	1	0	0	Amplification	1	0	42473T: 3.5;
SERP1	chr3	3q25.1	9	2	2	5	Amplification	9	0	42493T: 3.5; 42487T: 3.5; 42492T: 3.5; 56957T: 4; 42474T: 3.5; 42484T: 3.5; 42497T: 4.5; 42473T: 4; 42496T: 4;
SERPIND1	chr22	22q11.21	2	1	0	1	Amplification	2	0	42497T: 13.5; 42477T: 4;
SERPING1	chr11	11q12.1	1	0	0	1	Amplification	1	0	42496T: 4.5;
SERPINH1	chr11	11q13.5	2	0	0	2	Amplification	2	0	42492T: 4; 42498T: 6.5;
SERPINI1	chr3	3q26.1	9	1	3	5	Amplification	9	0	42482T: 3.5; 42492T: 3.5; 42487T: 3.5; 42493T: 3.5; 42500T: 8; 42484T: 4; 42495T: 4; 42474T: 3.5; 56957T: 4;
SERPINI2	chr3	3q26.1	9	1	3	5	Amplification	9	0	42482T: 3.5; 42487T: 3.5; 42493T: 3.5; 42492T: 3.5; 42500T: 8; 42474T: 3.5; 42495T: 4; 56957T: 4; 42484T: 4;
SERTAD1	chr19	19q13.2	2	0	0	2	Amplification	2	0	42500T: 3.5; 56957T: 4;
SERTAD2	chr2	2p14	2	0	0	2	Amplification	2	0	42500T: 3.5; 56957T: 3.5;
SERTAD3	chr19	19q13.2	2	0	0	2	Amplification	2	0	56957T: 4; 42500T: 3.5;
SESN1	chr6	6q21	1	0	0	1	Amplification	1	0	42496T: 3.5;
SESN2	chr1	1p35.3	1	1	0	0	Amplification	1	0	42473T: 3.5;
SESTD1	chr2	2q31.2	1	0	0	1	Amplification	1	0	42493T: 3.5;
SETD1B	chr12	12q24.31	1	0	0	1	Amplification	1	0	42500T: 3.5;
SETD8	chr12	12q24.31	1	0	0	1	Amplification	1	0	42500T: 3.5;
SETDB1	chr1	1q21.3	1	1	0	0	Amplification	1	0	42473T: 4.5;
SF3A1	chr22	22q12.2	1	1	0	0	Amplification	1	0	42473T: 3.5;
SF3B1	chr2	2q33.1	1	0	1	0	Amplification	1	0	42482T: 4;
SF3B14	chr2	2p23.3	1	0	0	1	Amplification	1	0	42500T: 3.5;
SF3B4	chr1	1q21.2	1	1	0	0	Amplification	1	0	42473T: 5;
SFRP4	chr7	7p14.1	1	1	0	0	Amplification	1	0	42473T: 4.5;
SFTA3	chr14	14q13.3	2	1	0	1	Amplification	2	0	42500T: 8.5; 42476T: 8;
SFTPB	chr2	2p11.2	1	0	0	1	Amplification	1	0	42500T: 3.5;
SFXN5	chr2	2p13.2	1	0	0	1	Amplification	1	0	42500T: 3.5;
SGCE	chr7	7q21.3	1	0	1	0	Amplification	1	0	42487T: 3.5;
SGK110	chr19	19q13.42	1	0	0	1	Amplification	1	0	42494T: 3.5;
SGK196	chr8	8p11.21	1	0	1	0	Amplification	1	0	42483T: 4.5;
SGK2	chr20	8q13.1	1	1	0	0	Amplification	1	0	42473T: 3.5;
SGK223	chr8	8p23.1	1	0	1	0	Amplification	1	0	42486T: 3.5;
SGK3	chr8	8q13.1	3	0	0	3	Amplification	3	0	42497T: 4.5; 42496T: 3.5; 42495T: 3.5;
SGPP1	chr14	14q23.2	1	0	0	1	Amplification	1	0	42494T: 4;
SGSH	chr17	17q25.3	1	1	0	0	Amplification	1	0	42473T: 3.5;
SGSM3	chr22	22q13.1	1	1	0	0	Amplification	1	0	42473T: 3.5;
SH2B2	chr7	7q22.1	2	0	0	2	Amplification	2	0	42493T: 3.5; 42501T: 3.5;

Mangalaparthi *et al.* , 2020. Mutational landscape of esophageal squamous cell carcinoma in an Indian cohort
Supplementary Table 7A. List of copy number alterations and affected genes in ESCC patients

Gene	Chromosome	Cytoband	Recurrence	Recurrence in smoker cohort	Recurrence in chewer cohort	Recurrence in No habit cohort	State	Samples with CNA gain	Samples with CNA loss	File info with CNA fold
SH2B3	chr12	12q24.12	1	0	0	1	Amplification	1	0	42500T: 3.5;
SH2D2A	chr1	1q23.1	1	1	0	0	Amplification	1	0	42473T: 7;
SH2D5	chr1	1p36.12	1	1	0	0	Amplification	1	0	42473T: 4;
SH2D6	chr2	2p11.2	2	0	1	1	Amplification	2	0	42482T: 3.5; 42500T: 3.5;
SH2D7	chr15	15q25.1	1	1	0	0	Amplification	1	0	42473T: 3.5;
SH3BGR	chr21	Xq21.1	1	1	0	0	Amplification	1	0	42473T: 3.5;
SH3BP1	chr22	22q13.1	1	1	0	0	Amplification	1	0	42473T: 3.5;
SH3GL1P1	chr17	17q11.2	1	1	0	0	Amplification	1	0	42473T: 3.5;
SH3GLB1	chr1	1p22.3	1	1	0	0	Amplification	1	0	42473T: 4;
SH3YL1	chr2	2p25.3	1	0	0	1	Amplification	1	0	42500T: 3.5;
SHANK2	chr11	11q13.3-q13	11	3	1	7	Amplification	14	0	42476T: 13.5,4; 42492T: 4; 42500T: 13,19; 56958T: 5.5; 42475T: 7.5; 42501T: 6; 42497T: 6.5; 42483T: 9; 56957T: 5; 42498T: 7,5; 42478T: 4.5;
SHARPIN	chr8	8q24.3	3	0	1	2	Amplification	3	0	42483T: 3.5; 42496T: 4; 42495T: 4.5;
SHC1	chr1	1q21.3	2	1	0	1	Amplification	2	0	42496T: 3.5; 42473T: 6.5;
SHE	chr1	1q21.3	1	1	0	0	Amplification	1	0	42473T: 4.5;
SHFM1	chr7	7q21.3	1	0	1	0	Amplification	1	0	42487T: 3.5;
SHISA9	chr16	16p13.12	2	1	0	1	Amplification	2	0	42473T: 5.5; 42495T: 4;
SHKBP1	chr19	19q13.2	2	0	0	2	Amplification	2	0	56957T: 4; 42500T: 3.5;
SHOX2	chr3	3q25.32	8	1	2	5	Amplification	8	0	42492T: 3.5; 42487T: 3.5; 42493T: 3.5; 42484T: 3.5; 56957T: 4; 42474T: 3.5; 42495T: 4; 42497T: 3.5;
SI	chr3	12q13.2	9	1	3	5	Amplification	9	0	42500T: 4; 42474T: 3.5; 42495T: 4; 56957T: 4; 42484T: 4; 42482T: 3.5; 42487T: 3.5; 42493T: 3.5; 42492T: 3.5;
SIAH2	chr3	3q25.1	10	2	2	6	Amplification	10	0	42474T: 3.5; 56957T: 4; 42484T: 3.5; 42500T: 3.5; 42496T: 4; 42473T: 4; 42497T: 4.5; 42487T: 3.5; 42493T: 3.5; 42492T: 3.5;
SIDT1	chr3	3q13.2	1	0	0	1	Amplification	1	0	42496T: 3.5;
SIGLEC1	chr20	20p13	1	1	0	0	Amplification	1	0	42473T: 6;
SIPA1L1	chr14	14q24.2	1	0	0	1	Amplification	1	0	42494T: 4;
SIPA1L3	chr19	19q13.13-q1	3	0	1	2	Amplification	3	0	56957T: 4; 42484T: 3.5; 42500T: 6.5;
SIRPA	chr20	20p13	1	1	0	0	Amplification	1	0	42473T: 3.5;
SIRPB1	chr20	20p13	1	1	0	0	Amplification	1	0	42473T: 3.5;
SIRPB2	chr20	20p13	1	1	0	0	Amplification	1	0	42473T: 3.5;
SIRPD	chr20	20p13	1	1	0	0	Amplification	1	0	42473T: 3.5;
SIRPG	chr20	20p13	1	1	0	0	Amplification	1	0	42473T: 3.5;
SIRT2	chr19	19q13.2	2	0	0	2	Amplification	2	0	56957T: 4; 42500T: 6.5;
SIRT4	chr12	12q24.23-q2	1	0	0	1	Amplification	1	0	42500T: 3.5;
SIT1	chr9	3p21.31	2	1	0	1	Amplification	2	0	42473T: 3.5; 42501T: 3.5;
SIX1	chr14	14q23.1	2	0	1	1	Amplification	2	0	42483T: 3.5; 42494T: 4;
SIX2	chr2	2p21	2	0	1	1	Amplification	2	0	42484T: 3.5; 42500T: 3.5;

Mangalaparthi *et al.* , 2020. Mutational landscape of esophageal squamous cell carcinoma in an Indian cohort
Supplementary Table 7A. List of copy number alterations and affected genes in ESCC patients

Gene	Chromosome	Cytoband	Recurrence	Recurrence in smoker cohort	Recurrence in chewer cohort	Recurrence in No habit cohort	State	Samples with CNA gain	Samples with CNA loss	File info with CNA fold
SIX3	chr2	2p21	2	0	1	1	Amplification	2	0	42500T: 3.5; 42484T: 3.5;
SIX4	chr14	14q23.1	2	0	1	1	Amplification	2	0	42483T: 3.5; 42494T: 4;
SIX5	chr19	19q13.32	1	0	1	0	Amplification	1	0	42484T: 4;
SIX6	chr14	14q23.1	2	0	1	1	Amplification	2	0	42494T: 4; 42483T: 3.5;
SKAP2	chr7	7p15.2	1	1	0	0	Amplification	1	0	42473T: 4;
SKIL	chr3	3q26.2	9	1	3	5	Amplification	9	0	42500T: 8; 42474T: 3.5; 42495T: 4; 56957T: 4; 42484T: 4; 42482T: 3.5; 42487T: 3.5; 42493T: 3.5; 42492T: 3.5;
SKIV2L	chr6	6p21.33	1	1	0	0	Amplification	1	0	42473T: 3.5;
SKP1	chr5	5q31.1	1	1	0	0	Amplification	1	0	42473T: 3.5;
SKP2	chr5	5p13.2	4	1	1	2	Amplification	4	0	42475T: 3.5; 42486T: 3.5; 42496T: 3.5; 42493T: 3.5;
SLA	chr8	4p15.2	3	0	1	2	Amplification	3	0	42484T: 3.5; 42495T: 3.5; 42496T: 3.5;
SLA2	chr20	20q11.23	1	1	0	0	Amplification	1	0	42473T: 3.5;
SLAIN2	chr4	4p11	1	0	1	0	Amplification	1	0	42483T: 3.5;
SLC10A1	chr14	14q24.1	1	0	0	1	Amplification	1	0	42494T: 4;
SLC10A4	chr4	4p11	1	0	1	0	Amplification	1	0	42483T: 3.5;
SLC10A5	chr8	8q21.13	2	0	0	2	Amplification	2	0	42496T: 3.5; 42495T: 3.5;
SLC11A2	chr12	12q13.12	2	0	0	2	Amplification	2	0	42494T: 3.5; 42500T: 3.5;
SLC12A2	chr5	5q23.3	1	1	0	0	Amplification	1	0	42473T: 3.5;
SLC12A5	chr20	20q13.12	1	1	0	0	Amplification	1	0	42473T: 5;
SLC12A7	chr5	5p15.33	5	2	1	2	Amplification	5	0	42493T: 3.5; 42475T: 3.5; 42473T: 3.5; 42496T: 4; 42486T: 3.5;
SLC12A8	chr3	3q21.2	1	0	0	1	Amplification	1	0	42496T: 3.5;
SLC12A9	chr7	7q22.1	1	0	0	1	Amplification	1	0	42501T: 3.5;
SLC13A1	chr7	7q31.32	2	0	1	1	Amplification	2	0	42487T: 3.5; 42493T: 3.5;
SLC13A3	chr20	20q13.12	1	1	0	0	Amplification	1	0	42473T: 5;
SLC13A4	chr7	7q33	1	0	1	0	Amplification	1	0	42487T: 3.5;
SLC15A2	chr3	3q13.33	1	0	0	1	Amplification	1	0	42496T: 3.5;
SLC15A5	chr12	12p12.3	1	0	0	1	Amplification	1	0	42500T: 4.5;
SLC16A10	chr6	6q21	1	0	0	1	Amplification	1	0	42496T: 3.5;
SLC16A8	chr22	22q13.1	1	1	0	0	Amplification	1	0	42473T: 3.5;
SLC17A9	chr20	20q13.33	1	1	0	0	Amplification	1	0	42473T: 6;
SLC1A1	chr9	9p24.2	1	0	0	1	Amplification	1	0	42496T: 3.5;
SLC1A3	chr5	5p13.2	4	1	1	2	Amplification	4	0	42493T: 3.5; 42475T: 3.5; 42486T: 3.5; 42496T: 3.5;
SLC1A4	chr2	2p14	2	0	0	2	Amplification	2	0	42500T: 3.5; 56957T: 3.5;
SLC1A7	chr1	1p32.3	1	0	0	1	Amplification	1	0	42496T: 4;
SLC22A13	chr3	3p22.2	1	1	0	0	Amplification	1	0	42473T: 4.5;
SLC22A16	chr6	6q21|6q21-q	1	0	0	1	Amplification	1	0	42496T: 3.5;
SLC22A17	chr14	14q11.2	2	0	0	2	Amplification	2	0	42496T: 4; 42500T: 4;
SLC23A2	chr20	5q31.2	1	1	0	0	Amplification	1	0	42473T: 4.5;
SLC24A3	chr20	20p11.23	1	0	1	0	Amplification	1	0	42483T: 4;

Mangalaparthi *et al.* , 2020. Mutational landscape of esophageal squamous cell carcinoma in an Indian cohort
Supplementary Table 7A. List of copy number alterations and affected genes in ESCC patients

Gene	Chromosome	Cytoband	Recurrence	Recurrence in smoker cohort	Recurrence in chewer cohort	Recurrence in No habit cohort	State	Samples with CNA gain	Samples with CNA loss	File info with CNA fold
SLC24A6	chr12	12q24.13	1	0	0	1	Amplification	1	0	42500T: 3.5;
SLC25A1	chr22	22q11.21	1	0	0	1	Amplification	1	0	42497T: 21;
SLC25A13	chr7	7q21.3	1	0	1	0	Amplification	1	0	42487T: 3.5;
SLC25A19	chr17	17q25.1	1	1	0	0	Amplification	1	0	42473T: 4;
SLC25A21	chr14	14q13.3	2	1	0	1	Amplification	2	0	42476T: 8; 42500T: 8.5;
SLC25A32	chr8	8q22.3	2	0	0	2	Amplification	2	0	42495T: 3.5; 42496T: 3.5;
SLC25A36	chr3	3q23	4	0	1	3	Amplification	4	0	42496T: 4; 42493T: 3.5; 42487T: 3.5; 42492T: 3.5;
SLC25A40	chr7	7q21.12	2	0	2	0	Amplification	2	0	42483T: 3.5; 42487T: 3.5;
SLC25A44	chr1	1q22	2	1	0	1	Amplification	2	0	42493T: 4; 42473T: 4.5;
SLC25A52	chr18	18q12.1	1	0	0	1	Amplification	1	0	42501T: 5.5;
SLC26A11	chr17	17q25.3	1	1	0	0	Amplification	1	0	42473T: 3.5;
SLC26A3	chr7	7q22.3-q31.1	4	0	1	3	Amplification	4	0	42487T: 3.5; 42493T: 3.5; 42501T: 3.5; 42497T: 4;
SLC26A4	chr7	7q22.3	4	0	1	3	Amplification	4	0	42497T: 4; 42501T: 3.5; 42487T: 3.5; 42493T: 3.5;
SLC26A5	chr7	7q22.1	4	0	1	3	Amplification	4	0	42497T: 4; 42501T: 3.5; 42487T: 3.5; 42493T: 3.5;
SLC26A7	chr8	8q21.3	2	0	0	2	Amplification	2	0	42495T: 3.5; 42496T: 3.5;
SLC26A9	chr1	1q32.1	1	0	0	1	Amplification	1	0	42493T: 3.5;
SLC27A3	chr1	1q21.3	1	1	0	0	Amplification	1	0	42473T: 4.5;
SLC28A1	chr15	15q25.3	1	1	0	0	Amplification	1	0	42473T: 4;
SLC29A2	chr11	11q13.2	1	0	0	1	Amplification	1	0	56957T: 5.5;
SLC29A4	chr7	7p22.1	2	1	0	1	Amplification	2	0	42473T: 4.5; 42497T: 8.5;
SLC2A1	chr1	1p34.2	2	0	0	2	Amplification	2	0	42496T: 5.5; 42493T: 6;
SLC2A10	chr20	20q13.12	1	1	0	0	Amplification	1	0	42473T: 5;
SLC2A13	chr12	12q12	1	0	0	1	Amplification	1	0	42500T: 3.5;
SLC2A2	chr3	3q26.2	9	1	3	5	Amplification	9	0	42482T: 3.5; 42493T: 3.5; 42487T: 3.5; 42492T: 3.5; 42500T: 8; 42495T: 4; 42474T: 3.5; 56957T: 4; 42484T: 4;
SLC2A4RG	chr20	20q13.33	1	1	0	0	Amplification	1	0	42473T: 6;
SLC30A3	chr2	2p23.3	1	0	0	1	Amplification	1	0	42500T: 3.5;
SLC30A6	chr2	2p22.3	1	0	0	1	Amplification	1	0	42500T: 3.5;
SLC30A8	chr8	8q24.11	3	1	0	2	Amplification	3	0	42495T: 3.5; 42475T: 3.5; 42496T: 3.5;
SLC32A1	chr20	20q11.23	1	1	0	0	Amplification	1	0	42473T: 3.5;
SLC33A1	chr3	3q25.31	8	1	2	5	Amplification	8	0	42492T: 3.5; 42487T: 3.5; 42493T: 3.5; 42484T: 3.5; 42474T: 3.5; 56957T: 4; 42497T: 5.5; 42496T: 4.5;
SLC34A3	chr9	9q34.3	2	1	0	1	Amplification	2	0	42473T: 5; 56957T: 3.5;
SLC35A5	chr3	3q13.2	1	0	0	1	Amplification	1	0	42496T: 3.5;
SLC35B4	chr7	7q33	1	0	1	0	Amplification	1	0	42487T: 3.5;
SLC35C1	chr11	11p11.2	1	1	0	0	Amplification	1	0	42473T: 3.5;
SLC35C2	chr20	20q13.12	1	1	0	0	Amplification	1	0	42473T: 5;
SLC35E3	chr12	12q15	2	0	0	2	Amplification	2	0	42500T: 5; 42501T: 6.5;
SLC35F4	chr14	14q22.3-q23	1	0	0	1	Amplification	1	0	42494T: 4;
SLC35F6	chr2	2p23.3	1	0	0	1	Amplification	1	0	42500T: 3.5;

Mangalaparthi *et al.* , 2020. Mutational landscape of esophageal squamous cell carcinoma in an Indian cohort
Supplementary Table 7A. List of copy number alterations and affected genes in ESCC patients

Gene	Chromosome	Cytoband	Recurrence	Recurrence in smoker cohort	Recurrence in chewer cohort	Recurrence in No habit cohort	State	Samples with CNA gain	Samples with CNA loss	File info with CNA fold
SLC35G5	chr8	8p23.1	1	0	1	0	Amplification	1	0	42486T: 3.5;
SLC37A1	chr21	21q22.3	1	1	0	0	Amplification	1	0	42473T: 3.5;
SLC37A3	chr7	7q34	2	1	1	0	Amplification	2	0	42473T: 3.5; 42487T: 3.5;
SLC37A4	chr11	11q23.3	1	1	0	0	Amplification	1	0	42473T: 4;
SLC38A1	chr12	12q13.11	1	0	0	1	Amplification	1	0	42500T: 3.5;
SLC38A2	chr12	12q13.11	1	0	0	1	Amplification	1	0	42500T: 3.5;
SLC38A4	chr12	12q13.11	1	0	0	1	Amplification	1	0	42500T: 3.5;
SLC38A6	chr14	14q23.1	1	0	0	1	Amplification	1	0	42494T: 4;
SLC39A1	chr1	1q21.3	1	1	0	0	Amplification	1	0	42473T: 4.5;
SLC39A10	chr2	2q32.3	2	0	1	1	Amplification	2	0	42482T: 4; 42493T: 3.5;
SLC39A13	chr11	11p11.2	1	1	0	0	Amplification	1	0	42473T: 3.5;
SLC39A4	chr8	8q24.3	4	0	1	3	Amplification	4	0	42496T: 4; 42483T: 3.5; 56957T: 3.5; 42495T: 4.5;
SLC39A5	chr12	12q13.3	1	0	0	1	Amplification	1	0	42494T: 7.5;
SLC39A9	chr14	14q24.1	1	0	0	1	Amplification	1	0	42494T: 4;
SLC3A1	chr2	2p21	2	0	1	1	Amplification	2	0	42500T: 3.5; 42484T: 3.5;
SLC40A1	chr2	2q32.2	2	0	1	1	Amplification	2	0	42493T: 3.5; 42482T: 4;
SLC41A1	chr1	1q32.1	1	0	0	1	Amplification	1	0	42493T: 3.5;
SLC41A3	chr3	3q21.2-q21.3	1	0	0	1	Amplification	1	0	42496T: 3.5;
SLC45A2	chr5	5p13.2	4	1	1	2	Amplification	4	0	42475T: 3.5; 42486T: 3.5; 42496T: 3.5; 42493T: 3.5;
SLC45A4	chr8	8q24.3	3	0	1	2	Amplification	3	0	42495T: 3.5; 42496T: 4; 42483T: 3.5;
SLC48A1	chr12	12q13.11	1	0	0	1	Amplification	1	0	42500T: 3.5;
SLC4A11	chr20	20p13	1	1	0	0	Amplification	1	0	42473T: 3.5;
SLC4A1AP	chr2	2p23.3	1	0	0	1	Amplification	1	0	42500T: 3.5;
SLC4A5	chr2	2p13.1	1	0	0	1	Amplification	1	0	42500T: 3.5;
SLC4A8	chr12	12q13.13	2	0	0	2	Amplification	2	0	42500T: 3.5; 42494T: 3.5;
SLC50A1	chr1	1q22	1	1	0	0	Amplification	1	0	42473T: 6.5;
SLC51A	chr3	3q29	9	1	3	5	Amplification	9	0	42498T: 3.5; 42484T: 4; 56957T: 6; 42495T: 4; 42474T: 3.5; 42482T: 3.5; 42492T: 3.5; 42487T: 3.5; 42493T: 5;
SLC52A2	chr8	8q24.3	4	0	1	3	Amplification	4	0	42496T: 4; 42483T: 3.5; 56957T: 3.5; 42495T: 4.5;
SLC52A3	chr20	20p13	1	1	0	0	Amplification	1	0	42473T: 3.5;
SLC5A11	chr16	16p12.1	1	1	0	0	Amplification	1	0	42473T: 4;
SLC5A6	chr2	2p23.3	1	0	0	1	Amplification	1	0	42500T: 3.5;
SLC6A12	chr12	12p13.33	1	0	0	1	Amplification	1	0	42500T: 3.5;
SLC6A13	chr12	12p13.33	1	0	0	1	Amplification	1	0	42500T: 3.5;
SLC6A18	chr5	5p15.33	5	2	1	2	Amplification	5	0	42475T: 3.5; 42486T: 3.5; 42496T: 4; 42473T: 3.5; 42493T: 3.5;
SLC6A19	chr5	5p15.33	5	2	1	2	Amplification	5	0	42486T: 3.5; 42473T: 3.5; 42496T: 4; 42475T: 3.5; 42493T: 3.5;
SLC6A3	chr5	5p15.33	5	2	1	2	Amplification	5	0	42473T: 3.5; 42496T: 4; 42486T: 3.5; 42475T: 3.5; 42493T: 3.5;

Supplementary Table 7A. List of copy number alterations and affected genes in ESCC patients

Gene	Chromosome	Cytoband	Recurrence	Recurrence in smoker cohort	Recurrence in chewer cohort	Recurrence in No habit cohort	State	Samples with CNA gain	Samples with CNA loss	File info with CNA fold
SLC7A10	chr19	19q13.11	3	0	1	2	Amplification	3	0	42500T: 4.5; 56957T: 4; 42484T: 3.5;
SLC7A14	chr3	3q26.2	9	1	3	5	Amplification	9	0	56957T: 4; 42474T: 3.5; 42495T: 4; 42484T: 4; 42500T: 8; 42493T: 3.5; 42487T: 3.5; 42492T: 3.5; 42482T: 3.5;
SLC7A4	chr22	22q11.21	1	1	0	0	Amplification	1	0	42477T: 4;
SLC7A7	chr14	14q11.2	1	0	0	1	Amplification	1	0	42496T: 4;
SLC7A8	chr14	14q11.2	2	0	0	2	Amplification	2	0	42496T: 4; 42500T: 4;
SLC7A9	chr19	19q13.11	4	1	1	2	Amplification	4	0	42500T: 4.5; 42473T: 7; 56957T: 4; 42484T: 3.5;
SLC8A1	chr2	2p22.1	1	0	0	1	Amplification	1	0	42500T: 3.5;
SLC8A3	chr14	14q24.2	1	0	0	1	Amplification	1	0	42494T: 4;
SLC9A2	chr2	2q12.1	1	0	0	1	Amplification	1	0	42493T: 3.5;
SLC9A3	chr5	5p15.33	5	2	1	2	Amplification	5	0	42493T: 3.5; 42486T: 3.5; 42473T: 3.5; 42496T: 4; 42475T: 3.5;
SLC9A3R2	chr16	16p13.3	1	0	1	0	Amplification	1	0	42483T: 3.5;
SLC9A4	chr2	2q12.1	1	0	0	1	Amplification	1	0	42493T: 3.5;
SLC9A8	chr20	20q13.13	1	1	0	0	Amplification	1	0	42473T: 5;
SLC9A9	chr3	3q24	6	1	1	4	Amplification	6	0	42474T: 3.5; 42487T: 3.5; 56957T: 4; 42493T: 3.5; 42492T: 3.5; 42496T: 4;
SLC9C1	chr3	3q13.2	1	0	0	1	Amplification	1	0	42496T: 3.5;
SLCO1A2	chr12	12p12.1	1	0	0	1	Amplification	1	0	42500T: 4.5;
SLCO1B1	chr12	12p12.1	1	0	0	1	Amplification	1	0	42500T: 4.5;
SLCO1B3	chr12	12p12.2	1	0	0	1	Amplification	1	0	42500T: 4.5;
SLCO1B7	chr12	12p12.2	1	0	0	1	Amplification	1	0	42500T: 4.5;
SLCO2A1	chr3	3q22.1-q22.2	3	0	1	2	Amplification	3	0	42496T: 3.5; 42487T: 3.5; 42492T: 3.5;
SLCO2B1	chr11	11q13.4	2	0	0	2	Amplification	2	0	42498T: 8; 42492T: 4;
SLCO4A1	chr20	20q13.33	1	1	0	0	Amplification	1	0	42473T: 6;
SLCO5A1	chr8	8q13.3	2	0	0	2	Amplification	2	0	42495T: 3.5; 42496T: 3.5;
SLIRP	chr14	14q24.3	2	0	0	2	Amplification	2	0	42494T: 4; 56957T: 3.5;
SLITRK3	chr3	3q26.1	9	1	3	5	Amplification	9	0	42500T: 4; 42495T: 4; 42474T: 3.5; 56957T: 4; 42484T: 4; 42482T: 3.5; 42487T: 3.5; 42493T: 3.5; 42492T: 3.5;
SLMO2	chr20	20q13.32	1	1	0	0	Amplification	1	0	42473T: 4.5;
SLPI	chr20	20q13.12	1	1	0	0	Amplification	1	0	42473T: 5;
SLURP1	chr8	8q24.3	3	0	1	2	Amplification	3	0	42483T: 3.5; 42496T: 4; 42495T: 4.5;
SLX4	chr16	16p13.3	1	1	0	0	Amplification	1	0	42473T: 5.5;
SMAD4	chr18	18q21.2	1	0	0	1	Deletion	0	1	56957T: 0;
SMAGP	chr12	12q13.13	2	0	0	2	Amplification	2	0	42494T: 5.5; 42500T: 3.5;
SMARCA2	chr9	9p24.3	2	0	1	1	Amplification/Deletio	1	1	42496T: 3.5; 42486T: 0.5;
SMARCC2	chr12	12q13.2	1	0	0	1	Amplification	1	0	42494T: 7.5;
SMARCD1	chr12	12q13.12	1	0	0	1	Amplification	1	0	42500T: 3.5;

Mangalaparthi *et al.* , 2020. Mutational landscape of esophageal squamous cell carcinoma in an Indian cohort
Supplementary Table 7A. List of copy number alterations and affected genes in ESCC patients

Gene	Chromosome	Cytoband	Recurrence	Recurrence in smoker cohort	Recurrence in chewer cohort	Recurrence in No habit cohort	State	Samples with CNA gain	Samples with CNA loss	File info with CNA fold
SMC4	chr3	3q25.33	8	1	2	5	Amplification	8	0	42493T: 3.5; 42487T: 3.5; 42492T: 3.5; 42495T: 4; 42474T: 3.5; 56957T: 4; 42484T: 4; 42497T: 3.5;
SMC6	chr2	2p24.2	1	0	0	1	Amplification	1	0	42500T: 3.5;
SMCHD1	chr18	18p11.32	2	0	0	2	Amplification	2	0	56957T: 8; 42500T: 4.5;
SMCO1	chr3	3q29	9	1	3	5	Amplification	9	0	56957T: 6; 42474T: 3.5; 42495T: 4; 42498T: 3.5; 42484T: 4; 42487T: 3.5; 42493T: 5; 42492T: 3.5; 42482T: 3.5;
SMCO2	chr12	12p11.23	1	0	0	1	Amplification	1	0	42500T: 6;
SMCO3	chr12	12p12.3	1	0	0	1	Amplification	1	0	42500T: 4.5;
SMCP	chr1	1q21.3	1	0	0	1	Amplification	1	0	42493T: 4;
SMCR7L	chr22	22q13.1	1	1	0	0	Amplification	1	0	42473T: 3.5;
SMEK2	chr2	2p16.1	3	0	1	2	Amplification	3	0	42500T: 3.5; 56957T: 3.5; 42484T: 4.5;
SMG1	chr16	16p12.3	2	1	0	1	Amplification	2	0	42495T: 4; 42473T: 4;
SMG5	chr1	1q22	1	1	0	0	Amplification	1	0	42473T: 4.5;
SMG9	chr19	19q13.31	1	0	0	1	Amplification	1	0	56957T: 4;
SMIM18	chr8	8p12	1	0	1	0	Amplification	1	0	42482T: 3.5;
SMIM19	chr8	8p11.21	1	0	1	0	Amplification	1	0	42483T: 4.5;
SMIM22	chr16	16p13.3	2	1	0	1	Amplification	2	0	42473T: 5.5; 42495T: 12;
SMKR1	chr7	7q32.1	1	0	1	0	Amplification	1	0	42487T: 3.5;
SMO	chr7	20p13	1	0	1	0	Amplification	1	0	42487T: 3.5;
SMOC1	chr14	14q24.2	1	0	0	1	Amplification	1	0	42494T: 4;
SMOX	chr20	20p13	1	1	0	0	Amplification	1	0	42473T: 4.5;
SMPD2	chr6	6q21	1	0	0	1	Amplification	1	0	42496T: 3.5;
SMPDL3B	chr1	1p35.3	1	1	0	0	Amplification	1	0	42473T: 3.5;
SMTNL1	chr11	11q12.1	1	0	0	1	Amplification	1	0	42496T: 4.5;
SMUG1	chr12	12q13.13	2	1	0	1	Amplification	2	0	42494T: 3.5; 42473T: 3.5;
SMYD1	chr2	2p11.2	1	0	0	1	Amplification	1	0	42500T: 3.5;
SMYD5	chr2	2p13.2	1	0	0	1	Amplification	1	0	42500T: 3.5;
SNAI1	chr20	20q13.13	1	1	0	0	Amplification	1	0	42473T: 6.5;
SNAI2	chr8	8q11.21	5	0	2	3	Amplification	5	0	42496T: 3.5; 42482T: 3.5; 42494T: 3.5; 42495T: 3.5; 42484T: 3.5;
SNAP25	chr20	20p12.2	1	0	1	0	Amplification	1	0	42486T: 4;
SNAP29	chr22	22q11.21	2	1	0	1	Amplification	2	0	42477T: 4; 42497T: 4;
SNAPC1	chr14	14q23.2	1	0	0	1	Amplification	1	0	42494T: 4;
SNAPC4	chr9	9q34.3	1	0	0	1	Amplification	1	0	56957T: 3.5;
SNAPIN	chr1	1q21.3	1	1	0	0	Amplification	1	0	42473T: 4.5;
SND1	chr7	7q32.1	1	0	1	0	Amplification	1	0	42487T: 3.5;
SNHG11	chr20	20q11.23	1	1	0	0	Amplification	1	0	42473T: 3.5;
SNHG15	chr7	7p13	1	1	0	0	Amplification	1	0	42473T: 4.5;
SNHG17	chr20	20q11.23	1	1	0	0	Amplification	1	0	42473T: 3.5;
SNHG6	chr8	8q13.1|8q13	3	0	0	3	Amplification	3	0	42495T: 3.5; 42497T: 4.5; 42496T: 3.5;

Mangalaparthi *et al.* , 2020. Mutational landscape of esophageal squamous cell carcinoma in an Indian cohort
Supplementary Table 7A. List of copy number alterations and affected genes in ESCC patients

Gene	Chromosome	Cytoband	Recurrence	Recurrence in smoker cohort	Recurrence in chewer cohort	Recurrence in No habit cohort	State	Samples with CNA gain	Samples with CNA loss	File info with CNA fold
SNHG9	chr16	16p13.3	1	0	1	0	Amplification	1	0	42483T: 3.5;
SNN	chr16	16p13.13	2	1	0	1	Amplification	2	0	42473T: 12.5; 42495T: 4;
SNORA71A	chr20	20q11.23	1	1	0	0	Amplification	1	0	42473T: 3.5;
SNORA71B	chr20	20q11.23	1	1	0	0	Amplification	1	0	42473T: 3.5;
SNORD56	chr20	20p13	1	1	0	0	Amplification	1	0	42473T: 3.5;
SNORD57	chr20	20p13	1	1	0	0	Amplification	1	0	42473T: 3.5;
SNPH	chr20	20p13	1	1	0	0	Amplification	1	0	42473T: 3.5;
SNRNP200	chr2	2q11.2	2	1	0	1	Amplification	2	0	42473T: 3.5; 42493T: 3.5;
SNRNP25	chr16	16p13.3	1	0	1	0	Amplification	1	0	42483T: 3.5;
SNRNP27	chr2	2p13.3	1	0	0	1	Amplification	1	0	42500T: 3.5;
SNRNP35	chr12	12q24.31	1	0	0	1	Amplification	1	0	42500T: 3.5;
SNRNP40	chr1	1p35.2	1	1	0	0	Amplification	1	0	42473T: 3.5;
SNRPA	chr19	19q13.2	2	0	0	2	Amplification	2	0	42500T: 3.5; 56957T: 4;
SNRPB	chr20	20p13	1	1	0	0	Amplification	1	0	42473T: 3.5;
SNRPD2	chr19	19q13.32	1	0	1	0	Amplification	1	0	42484T: 4;
SNRPG	chr2	2p13.3	1	0	0	1	Amplification	1	0	42500T: 3.5;
SNTA1	chr20	20q11.21	2	1	0	1	Amplification	2	0	42496T: 5; 42473T: 3.5;
SNTB1	chr8	8q24.12	4	1	1	2	Amplification	4	0	42475T: 3.5; 42496T: 3.5; 42495T: 3.5; 42484T: 3.5;
SNTG1	chr8	8q11.21	5	0	2	3	Amplification	5	0	42482T: 3.5; 42496T: 3.5; 42494T: 3.5; 42495T: 3.5; 42484T: 3.5;
SNTG2	chr2	2p25.3	1	0	0	1	Amplification	1	0	42500T: 3.5;
SNW1	chr14	14q24.3	1	0	0	1	Amplification	1	0	42494T: 4;
SNX10	chr7	7p15.2	1	1	0	0	Amplification	1	0	42473T: 4;
SNX13	chr7	7p21.1	1	1	0	0	Amplification	1	0	42473T: 4;
SNX16	chr8	8q21.13	2	0	0	2	Amplification	2	0	42496T: 3.5; 42495T: 3.5;
SNX17	chr2	2p23.3	1	0	0	1	Amplification	1	0	42500T: 3.5;
SNX21	chr20	20q13.12	1	1	0	0	Amplification	1	0	42473T: 5;
SNX27	chr1	1q21.3	1	1	0	0	Amplification	1	0	42473T: 4.5;
SNX29	chr16	16p13.13-p1	2	1	0	1	Amplification	2	0	42495T: 4; 42473T: 5.5;
SNX3	chr6	6q21	1	0	0	1	Amplification	1	0	42496T: 3.5;
SNX31	chr8	8q22.3	2	0	0	2	Amplification	2	0	42495T: 3.5; 42496T: 3.5;
SNX4	chr3	3q21.2	1	0	0	1	Amplification	1	0	42496T: 3.5;
SNX6	chr14	14q13.1	1	0	0	1	Amplification	1	0	42500T: 4.5;
SNX8	chr7	7p22.3	1	1	0	0	Amplification	1	0	42473T: 5;
SOBP	chr6	6q21	1	0	0	1	Amplification	1	0	42496T: 3.5;
SOCS1	chr16	16p13.13	2	1	0	1	Amplification	2	0	42473T: 5.5; 42495T: 5;
SOCS5	chr2	2p21	2	0	1	1	Amplification	2	0	42484T: 4.5; 42500T: 3.5;
SOCS7	chr17	14q22.3	1	1	0	0	Amplification	1	0	42473T: 3.5;
SOGA1	chr20	20q11.23	1	1	0	0	Amplification	1	0	42473T: 3.5;
SOHLH1	chr9	9q34.3	2	0	1	1	Amplification	2	0	56957T: 3.5; 42484T: 4.5;
SOS1	chr2	2p22.1	1	0	0	1	Amplification	1	0	42500T: 3.5;

Mangalaparthi *et al.* , 2020. Mutational landscape of esophageal squamous cell carcinoma in an Indian cohort
Supplementary Table 7A. List of copy number alterations and affected genes in ESCC patients

Gene	Chromosome	Cytoband	Recurrence	Recurrence in smoker cohort	Recurrence in chewer cohort	Recurrence in No habit cohort	State	Samples with CNA gain	Samples with CNA loss	File info with CNA fold
SOS2	chr14	14q21.3	1	0	0	1	Amplification	1	0	42496T: 3.5;
SOSTDC1	chr7	7p21.2	1	1	0	0	Amplification	1	0	42473T: 3.5;
SOX10	chr22	22q13.1	1	1	0	0	Amplification	1	0	42473T: 3.5;
SOX11	chr2	2p25.2	1	0	0	1	Amplification	1	0	42500T: 3.5;
SOX12	chr20	20p13	1	1	0	0	Amplification	1	0	42473T: 3.5;
SOX14	chr3	3q22.3	4	0	1	3	Amplification	4	0	42487T: 3.5; 42493T: 3.5; 42492T: 3.5; 42496T: 3.5;
SOX17	chr8	8q11.23	3	0	0	3	Amplification	3	0	42496T: 3.5; 42495T: 3.5; 42494T: 4;
SOX18	chr20	20q13.33	1	1	0	0	Amplification	1	0	42473T: 6;
SOX2	chr3	3q26.33	11	1	3	7	Amplification	11	0	42482T: 3.5; 42487T: 3.5; 42493T: 3.5; 42492T: 3.5; 42500T: 24; 42497T: 4; 42474T: 3.5; 42495T: 4; 56957T: 4; 42484T: 4; 42498T: 3.5;
SOX5	chr12	12p12.1	1	0	0	1	Amplification	1	0	42500T: 6;
SOX7	chr8	8p23.1	1	0	1	0	Amplification	1	0	42486T: 3.5;
SOX8	chr16	16p13.3	1	0	1	0	Amplification	1	0	42483T: 3.5;
SP3	chr2	2q31.1	1	0	0	1	Amplification	1	0	42493T: 5;
SP4	chr7	7p15.3	1	1	0	0	Amplification	1	0	42473T: 4;
SP8	chr7	7p21.1	1	1	0	0	Amplification	1	0	42473T: 4;
SP9	chr2	2q31.1	1	0	0	1	Amplification	1	0	42493T: 3.5;
SPAG1	chr8	8q22.2	2	0	0	2	Amplification	2	0	42496T: 3.5; 42495T: 3.5;
SPAG11A	chr8	8p23.1	1	0	1	0	Amplification	1	0	42486T: 3.5;
SPAG11B	chr8	8p23.1	1	0	1	0	Amplification	1	0	42486T: 3.5;
SPAG4	chr20	20q11.22	2	1	0	1	Amplification	2	0	42493T: 3.5; 42473T: 3.5;
SPAG8	chr9	9p13.3	2	1	0	1	Amplification	2	0	42501T: 3.5; 42473T: 3.5;
SPAG9	chr17	17q21.33	1	1	0	0	Amplification	1	0	42473T: 4.5;
SPAM1	chr7	7q31.32	1	0	1	0	Amplification	1	0	42487T: 3.5;
SPAST	chr2	2p22.3	1	0	0	1	Amplification	1	0	42500T: 3.5;
SPATA16	chr3	3q26.31	9	1	3	5	Amplification	9	0	42482T: 3.5; 42492T: 3.5; 42493T: 3.5; 42487T: 3.5; 42500T: 9; 42484T: 4; 42474T: 3.5; 42495T: 4; 56957T: 4;
SPATA2	chr20	20q13.13	1	1	0	0	Amplification	1	0	42473T: 5;
SPATA20	chr17	17q21.33	1	1	0	0	Amplification	1	0	42473T: 4.5;
SPATA21	chr1	1p36.13	1	1	0	0	Amplification	1	0	42473T: 4.5;
SPATA25	chr20	20q13.12	1	1	0	0	Amplification	1	0	42473T: 5;
SPATA6L	chr9	9p24.2-p24.1	1	0	0	1	Amplification	1	0	42496T: 3.5;
SPATC1	chr8	8q24.3	3	0	1	2	Amplification	3	0	42483T: 3.5; 42496T: 4; 42495T: 4.5;
SPATS2	chr12	12q13.12	1	0	0	1	Amplification	1	0	42500T: 3.5;
SPCS2	chr11	11q13.4	4	1	0	3	Amplification	4	0	56957T: 3.5; 42498T: 8; 42492T: 4; 42475T: 7.5;
SPDYA	chr2	2p23.2	1	0	0	1	Amplification	1	0	42500T: 3.5;
SPEF1	chr20	20p13	2	1	1	0	Amplification	2	0	42487T: 4.5; 42473T: 6;
SPEF2	chr5	5p13.2	4	1	1	2	Amplification	4	0	42475T: 3.5; 42486T: 3.5; 42496T: 3.5; 42493T: 3.5;
SPEN	chr1	1p13.3	1	1	0	0	Amplification	1	0	42473T: 8;

Mangalaparthi *et al.* , 2020. Mutational landscape of esophageal squamous cell carcinoma in an Indian cohort
Supplementary Table 7A. List of copy number alterations and affected genes in ESCC patients

Gene	Chromosome	Cytoband	Recurrence	Recurrence in smoker cohort	Recurrence in chewer cohort	Recurrence in No habit cohort	State	Samples with CNA gain	Samples with CNA loss	File info with CNA fold
SPI1	chr11	11p11.2	1	1	0	0	Amplification	1	0	42473T: 3.5;
SPICE1	chr3	3q13.2	1	0	0	1	Amplification	1	0	42496T: 3.5;
SPIDR	chr8	8q11.21	3	0	1	2	Amplification	3	0	42484T: 3.5; 42494T: 3.5; 42496T: 3.5;
SPINT1	chr15	15q15.1	1	0	0	1	Amplification	1	0	42493T: 6.5;
SPINT2	chr19	19q13.2	4	1	1	2	Amplification	4	0	42500T: 6.5; 42474T: 4; 56957T: 4; 42484T: 3.5;
SPINT3	chr20	20q13.12	1	1	0	0	Amplification	1	0	42473T: 5;
SPINT4	chr20	20q13.12	1	1	0	0	Amplification	1	0	42473T: 5;
SPO11	chr20	20q13.31	1	1	0	0	Amplification	1	0	42473T: 7;
SPOCD1	chr1	1p35.2	1	1	0	0	Amplification	1	0	42473T: 3.5;
SPPL3	chr12	12q24.31	1	0	0	1	Amplification	1	0	42500T: 3.5;
SPR	chr2	2p12	1	0	0	1	Amplification	1	0	42500T: 3.5;
SPRED2	chr2	2p14	2	0	0	2	Amplification	2	0	42500T: 3.5; 56957T: 3.5;
SPRED3	chr19	19q13.2	3	0	1	2	Amplification	3	0	42484T: 3.5; 56957T: 4; 42500T: 6.5;
SPRR1A	chr1	1q21.3	1	0	0	1	Amplification	1	0	42493T: 4;
SPRR1B	chr1	1q21.3	1	0	0	1	Amplification	1	0	42493T: 4;
SPRR2B	chr1	1q21.3	1	0	0	1	Amplification	1	0	42493T: 4;
SPRR2G	chr1	1q21.3	1	0	0	1	Amplification	1	0	42493T: 4;
SPRR3	chr1	1q21.3	1	0	0	1	Amplification	1	0	42493T: 4;
SPRR4	chr1	1q21.3	1	0	0	1	Amplification	1	0	42493T: 4;
SPRYD3	chr12	12q13.13	1	1	0	0	Amplification	1	0	42473T: 4;
SPSB2	chr12	12p13.31	1	0	0	1	Amplification	1	0	42494T: 3.5;
SPSB3	chr16	16p13.3	1	0	1	0	Amplification	1	0	42483T: 3.5;
SPSB4	chr3	3q23	4	0	1	3	Amplification	4	0	42496T: 4; 42492T: 3.5; 42487T: 3.5; 42493T: 3.5;
SPTB	chr14	14q23.3	1	0	0	1	Amplification	1	0	42494T: 4;
SPTBN1	chr2	2p16.2	2	0	1	1	Amplification	2	0	42500T: 3.5; 42484T: 4.5;
SPTBN2	chr11	11q13.2	2	1	0	1	Amplification	2	0	42473T: 3.5; 56957T: 5.5;
SPTBN4	chr19	19q13.2	2	0	0	2	Amplification	2	0	56957T: 4; 42500T: 3.5;
SPTLC2	chr14	14q24.3	2	0	0	2	Amplification	2	0	42494T: 4; 56957T: 3.5;
SPTSSA	chr14	14q13.1	1	0	0	1	Amplification	1	0	42500T: 4.5;
SPTSSB	chr3	3q26.1	8	1	2	5	Amplification	8	0	42493T: 3.5; 42487T: 3.5; 42492T: 3.5; 42497T: 3.5; 42495T: 4; 42474T: 3.5; 56957T: 4; 42484T: 4;
SQLE	chr8	8q24.13	4	1	1	2	Amplification	4	0	42484T: 3.5; 42495T: 3.5; 42496T: 3.5; 42475T: 3.5;
SRBD1	chr2	2p21	2	0	1	1	Amplification	2	0	42500T: 3.5; 42484T: 4.5;
SRC	chr20	20q11.23	1	1	0	0	Amplification	1	0	42473T: 3.5;
SRCIN1	chr17	17q12	1	1	0	0	Amplification	1	0	42473T: 3.5;
SRCRB4D	chr7	7q11.23	1	0	0	1	Amplification	1	0	42493T: 3.5;
SRD5A1	chr5	5p15.31	4	1	1	2	Amplification	4	0	42493T: 4; 42475T: 3.5; 42496T: 3.5; 42486T: 3.5;
SRD5A2	chr2	2p23.1	1	0	0	1	Amplification	1	0	42500T: 3.5;
SRGAP2B	chr1	1q21.1	1	1	0	0	Amplification	1	0	42473T: 4;
SRI	chr7	7q21.12	2	0	2	0	Amplification	2	0	42483T: 3.5; 42487T: 3.5;
SRL	chr16	16p13.3	3	1	0	2	Amplification	3	0	42473T: 5.5; 42494T: 3.5; 42495T: 8.5;

Mangalaparthi *et al.* , 2020. Mutational landscape of esophageal squamous cell carcinoma in an Indian cohort
Supplementary Table 7A. List of copy number alterations and affected genes in ESCC patients

Gene	Chromosome	Cytoband	Recurrence	Recurrence in smoker cohort	Recurrence in chewer cohort	Recurrence in No habit cohort	State	Samples with CNA gain	Samples with CNA loss	File info with CNA fold
SRMS	chr20	20q13.33	1	1	0	0	Amplification	1	0	42473T: 6;
SRP54	chr14	14q13.2	2	1	0	1	Amplification	2	0	42476T: 5; 42500T: 4.5;
SRP68	chr17	17q25.1	1	0	0	1	Amplification	1	0	42494T: 3.5;
SRP9	chr1	1q42.12	1	0	0	1	Amplification	1	0	42493T: 3.5;
SRPK2	chr7	7q22.3	4	0	1	3	Amplification	4	0	42497T: 4; 42501T: 3.5; 42487T: 3.5; 42493T: 3.5;
SRPRB	chr3	3q22.1	3	0	1	2	Amplification	3	0	42496T: 3.5; 42487T: 3.5; 42492T: 3.5;
SRRM1	chr1	1p36.11	1	1	0	0	Amplification	1	0	42473T: 3.5;
SRRM3	chr7	7q11.23	1	0	0	1	Amplification	1	0	42493T: 3.5;
SRRM4	chr12	12q24.23	1	0	0	1	Amplification	1	0	42500T: 3.5;
SRRT	chr7	7q22.1	1	0	0	1	Amplification	1	0	42501T: 3.5;
SRSF1	chr17	17q22	1	0	0	1	Amplification	1	0	42497T: 4;
SRSF10	chr1	1p36.11	1	1	0	0	Amplification	1	0	42473T: 3.5;
SRSF4	chr1	1p35.3	1	1	0	0	Amplification	1	0	42473T: 3.5;
SRSF5	chr14	14q24.1	1	0	0	1	Amplification	1	0	42494T: 4;
SRSF6	chr20	20q13.11	1	1	0	0	Amplification	1	0	42473T: 3.5;
SRSF7	chr2	2p22.1	2	0	0	2	Amplification	2	0	42500T: 3.5; 42493T: 3.5;
SRSF9	chr12	12q24.31	1	0	0	1	Amplification	1	0	42500T: 3.5;
SRXN1	chr20	20p13	1	1	0	0	Amplification	1	0	42473T: 3.5;
SS18L1	chr20	20q13.33	1	1	0	0	Amplification	1	0	42473T: 6;
SSBP1	chr7	7q34	1	0	1	0	Amplification	1	0	42487T: 3.5;
SSC5D	chr19	19q13.42	1	0	0	1	Amplification	1	0	42494T: 3.5;
SSFA2	chr2	2q31.3	1	0	0	1	Amplification	1	0	42493T: 4;
SSH1	chr12	12q24.11	2	1	0	1	Amplification	2	0	42473T: 4.5; 42500T: 3.5;
SSH3	chr11	11q13.2	2	1	0	1	Amplification	2	0	42473T: 3.5; 56957T: 5;
SSMEM1	chr7	7q32.2	1	0	1	0	Amplification	1	0	42487T: 3.5;
SSNA1	chr9	9q34.3	2	1	0	1	Amplification	2	0	56957T: 3.5; 42473T: 5;
SSPN	chr12	12p12.1	1	0	0	1	Amplification	1	0	42500T: 6;
SSR2	chr1	1q22	2	1	0	1	Amplification	2	0	42473T: 4.5; 42493T: 4;
SSR3	chr3	3q25.31	8	1	2	5	Amplification	8	0	42492T: 3.5; 42487T: 3.5; 42493T: 3.5; 42484T: 3.5; 56957T: 4; 42495T: 4; 42474T: 3.5; 42497T: 5.5;
SST	chr3	3q27.3	10	1	3	6	Amplification	10	0	42482T: 3.5; 42493T: 3.5; 42487T: 3.5; 42492T: 3.5; 42497T: 4; 56957T: 4.5; 42495T: 4; 42474T: 3.5; 42498T: 3.5; 42484T: 4;
SSTR1	chr14	14q21.1	2	1	0	1	Amplification	2	0	42500T: 6.5; 42476T: 4;
SSTR3	chr22	22q13.1	1	1	0	0	Amplification	1	0	42473T: 3.5;
SSTR5	chr16	16p13.3	1	0	1	0	Amplification	1	0	42483T: 3.5;
ST18	chr8	8q11.23	5	0	2	3	Amplification	5	0	42484T: 5; 42495T: 3.5; 42494T: 3.5; 42496T: 3.5; 42482T: 3.5;
ST3GAL1	chr8	8q24.22	2	0	0	2	Amplification	2	0	42496T: 3.5; 42495T: 3.5;
ST3GAL5	chr2	2p11.2	1	0	0	1	Amplification	1	0	42500T: 3.5;
ST3GAL6	chr3	3q12.1	1	1	0	0	Amplification	1	0	42476T: 3.5;

Mangalaparthi *et al.* , 2020. Mutational landscape of esophageal squamous cell carcinoma in an Indian cohort
Supplementary Table 7A. List of copy number alterations and affected genes in ESCC patients

Gene	Chromosome	Cytoband	Recurrence	Recurrence in smoker cohort	Recurrence in chewer cohort	Recurrence in No habit cohort	State	Samples with CNA gain	Samples with CNA loss	File info with CNA fold
ST6GAL1	chr3	3q27.3	10	1	3	6	Amplification	10	0	42487T: 3.5; 42493T: 3.5; 42492T: 3.5; 42482T: 3.5; 42495T: 4; 42474T: 3.5; 56957T: 4.5; 42484T: 4; 42498T: 3.5; 42497T: 4;
ST7	chr7	7q31.2	1	0	1	0	Amplification	1	0	42487T: 3.5;
ST8SIA1	chr12	12p12.1	1	0	0	1	Amplification	1	0	42500T: 6;
STAC2	chr17	17q12	1	1	0	0	Amplification	1	0	42473T: 4.5;
STAG1	chr3	20q13.31	4	0	1	3	Amplification	4	0	42496T: 3.5; 42492T: 3.5; 42493T: 3.5; 42487T: 3.5;
STAG3L4	chr7	7q11.21	1	0	0	1	Amplification	1	0	42501T: 3.5;
STAMBP	chr2	2p13.1	1	0	0	1	Amplification	1	0	42500T: 3.5;
STAR	chr8	Xq28	2	0	1	1	Amplification	2	0	42482T: 3.5; 42493T: 3.5;
STARD10	chr11	11q13.4	5	3	0	2	Amplification	5	0	42478T: 4.5; 42492T: 4; 56957T: 3.5; 42476T: 4; 42475T: 8.5;
STARD13	chr13	13q13.1-q13	1	0	0	1	Amplification	1	0	42497T: 6;
STARD3	chr17	17q12	2	1	0	1	Amplification	2	0	42473T: 6; 42497T: 3.5;
STARD3NL	chr7	7p14.1	1	1	0	0	Amplification	1	0	42473T: 4.5;
STARD7	chr2	2q11.2	2	1	0	1	Amplification	2	0	42473T: 3.5; 42493T: 3.5;
STAT1	chr2	2q32.2	3	0	1	2	Amplification	3	0	42493T: 3.5; 42494T: 3.5; 42482T: 4;
STAT4	chr2	2q32.2-q32.3	3	0	1	2	Amplification	3	0	42493T: 3.5; 42494T: 3.5; 42482T: 4;
STAU1	chr20	20q13.13	1	1	0	0	Amplification	1	0	42473T: 5;
STAU2	chr8	8q21.11	2	0	0	2	Amplification	2	0	42496T: 3.5; 42495T: 3.5;
STEAP1	chr7	7q21.13	2	0	2	0	Amplification	2	0	42487T: 3.5; 42483T: 3.5;
STEAP1B	chr7	7p15.3	1	1	0	0	Amplification	1	0	42473T: 4;
STEAP2	chr7	7q21.13	2	0	2	0	Amplification	2	0	42483T: 3.5; 42487T: 3.5;
STEAP4	chr7	7q21.12	2	0	2	0	Amplification	2	0	42487T: 3.5; 42483T: 3.5;
STK17A	chr7	7p13	1	1	0	0	Amplification	1	0	42473T: 3.5;
STK17B	chr2	2q32.3	2	0	1	1	Amplification	2	0	42482T: 4; 42493T: 3.5;
STK19	chr6	6p21.33	1	1	0	0	Amplification	1	0	42473T: 3.5;
STK3	chr8	13q32.2	2	0	0	2	Amplification	2	0	42495T: 3.5; 42496T: 3.5;
STK31	chr7	7p15.3	1	1	0	0	Amplification	1	0	42473T: 4;
STK35	chr20	20p13	1	1	0	0	Amplification	1	0	42473T: 3.5;
STK38L	chr12	12p11.23	1	0	0	1	Amplification	1	0	42500T: 6;
STK39	chr2	2q24.3	1	1	0	0	Amplification	1	0	42473T: 4;
STK4	chr20	20q13.12	1	1	0	0	Amplification	1	0	42473T: 5;
STMN2	chr8	8q21.13	2	0	0	2	Amplification	2	0	42495T: 3.5; 42496T: 3.5;
STMN3	chr20	20q13.33	1	1	0	0	Amplification	1	0	42473T: 6;
STOML1	chr15	15q24.1	1	1	0	0	Amplification	1	0	42473T: 3.5;
STOML2	chr9	9p13.3	1	0	0	1	Amplification	1	0	42501T: 3.5;
STON1	chr2	2p16.3	2	0	1	1	Amplification	2	0	42500T: 3.5; 42484T: 4.5;
STPG1	chr1	1p36.11	1	1	0	0	Amplification	1	0	42473T: 3.5;
STRA13	chr17	17q25.3	1	1	0	0	Amplification	1	0	42473T: 3.5;
STRA6	chr15	15q24.1	1	1	0	0	Amplification	1	0	42473T: 3.5;

Mangalaparthi *et al.* , 2020. Mutational landscape of esophageal squamous cell carcinoma in an Indian cohort
Supplementary Table 7A. List of copy number alterations and affected genes in ESCC patients

Gene	Chromosome	Cytoband	Recurrence	Recurrence in smoker cohort	Recurrence in chewer cohort	Recurrence in No habit cohort	State	Samples with CNA gain	Samples with CNA loss	File info with CNA fold
STRA8	chr7	7q33	1	0	1	0	Amplification	1	0	42487T: 3.5;
STRAP	chr12	5q23.1	1	0	0	1	Amplification	1	0	42500T: 4.5;
STRIP1	chr1	1p13.3	1	1	0	0	Amplification	1	0	42473T: 4.5;
STRIP2	chr7	7q32.1	1	0	1	0	Amplification	1	0	42487T: 3.5;
STRN	chr2	2p22.2	1	0	0	1	Amplification	1	0	42500T: 3.5;
STRN3	chr14	14q12	3	0	0	3	Amplification	3	0	42494T: 3.5; 56957T: 4; 42500T: 4.5;
STUB1	chr16	16p13.3	2	0	1	1	Amplification	2	0	42483T: 3.5; 42493T: 3.5;
STX12	chr1	1p35.3	1	1	0	0	Amplification	1	0	42473T: 3.5;
STX16	chr20	20q13.32	1	1	0	0	Amplification	1	0	42473T: 4.5;
STX6	chr1	1q25.3	1	0	0	1	Amplification	1	0	42493T: 4;
STXBP4	chr17	17q22	1	0	0	1	Amplification	1	0	42497T: 3.5;
STXBP5L	chr3	3q13.33	1	0	0	1	Amplification	1	0	42496T: 3.5;
STYX	chr14	14q22.1	1	0	0	1	Amplification	1	0	42494T: 4;
SUB1	chr5	5p13.3	4	1	1	2	Amplification	4	0	42493T: 3.5; 42486T: 3.5; 42496T: 3.5; 42475T: 3.5;
SUCLG1	chr2	2p11.2	1	0	0	1	Amplification	1	0	42500T: 3.5;
SUCNR1	chr3	3q25.1	9	1	2	6	Amplification	9	0	42484T: 3.5; 56957T: 4; 42474T: 3.5; 42496T: 4; 42497T: 5.5; 42500T: 3.5; 42492T: 3.5; 42493T: 3.5; 42487T: 3.5;
SUDS3	chr12	12q24.23	1	0	0	1	Amplification	1	0	42500T: 3.5;
SULF1	chr8	8q13.2-q13.3	2	0	0	2	Amplification	2	0	42495T: 3.5; 42496T: 3.5;
SULF2	chr20	20q13.12	1	1	0	0	Amplification	1	0	42473T: 5;
SULT6B1	chr2	2p22.2	1	0	0	1	Amplification	1	0	42500T: 3.5;
SUMF2	chr7	7p11.2	2	0	1	1	Amplification	2	0	42483T: 4; 56957T: 5;
SUN1	chr7	7p22.3	1	1	0	0	Amplification	1	0	42473T: 5;
SUN2	chr22	22q13.1	1	1	0	0	Amplification	1	0	42473T: 3.5;
SUN5	chr20	20q11.21	2	1	0	1	Amplification	2	0	42496T: 5; 42473T: 3.5;
SUOX	chr12	12q13.2	1	0	0	1	Amplification	1	0	42494T: 5;
SUPT4H1	chr17	17q22	1	0	0	1	Amplification	1	0	42497T: 4;
SUPT5H	chr19	19q13.2	2	0	0	2	Amplification	2	0	56957T: 4; 42500T: 6.5;
SUPT7L	chr2	2p23.3	1	0	0	1	Amplification	1	0	42500T: 3.5;
SUV420H1	chr11	11q13.2	1	0	0	1	Amplification	1	0	56957T: 5;
SUZ12	chr17	17q11.2	1	1	0	0	Amplification	1	0	42473T: 3.5;
SUZ12P	chr17	17q11.2	1	1	0	0	Amplification	1	0	42473T: 3.5;
SV2A	chr1	1q21.2	1	1	0	0	Amplification	1	0	42473T: 5;
SVOP	chr12	12q24.11	2	1	0	1	Amplification	2	0	42500T: 3.5; 42473T: 4.5;
SVOPL	chr7	7q34	1	0	1	0	Amplification	1	0	42487T: 3.5;
SYBU	chr8	8q23.2	2	0	0	2	Amplification	2	0	42496T: 3.5; 42495T: 3.5;
SYCN	chr19	19q13.2	2	0	0	2	Amplification	2	0	42500T: 6.5; 56957T: 4;
SYCP2	chr20	20q13.33	1	1	0	0	Amplification	1	0	42473T: 4.5;
SYF2	chr1	1p36.11	1	1	0	0	Amplification	1	0	42473T: 4.5;
SYMPK	chr19	19q13.32	1	0	1	0	Amplification	1	0	42484T: 4;

Mangalaparthi *et al.* , 2020. Mutational landscape of esophageal squamous cell carcinoma in an Indian cohort
Supplementary Table 7A. List of copy number alterations and affected genes in ESCC patients

Gene	Chromosome	Cytoband	Recurrence	Recurrence in smoker cohort	Recurrence in chewer cohort	Recurrence in No habit cohort	State	Samples with CNA gain	Samples with CNA loss	File info with CNA fold
SYNDIG1L	chr14	14q24.3	2	0	0	2	Amplification	2	0	56957T: 3.5; 42494T: 4;
SYNE2	chr14	14q23.2	1	0	0	1	Amplification	1	0	42494T: 4;
SYNE4	chr19	19q13.12	3	0	1	2	Amplification	3	0	56957T: 4; 42484T: 3.5; 42500T: 6.5;
SYNGR1	chr22	22q13.1	1	1	0	0	Amplification	1	0	42473T: 3.5;
SYNGR3	chr16	16p13.3	1	0	1	0	Amplification	1	0	42483T: 3.5;
SYNJ2BP	chr14	14q24.2	1	0	0	1	Amplification	1	0	42494T: 4;
SYNPO2L	chr10	10q22.2	2	1	0	1	Amplification	2	0	42473T: 4.5; 42496T: 3.5;
SYNRG	chr17	17q12	1	1	0	0	Amplification	1	0	42473T: 3.5;
SYPL1	chr7	7q22.3	4	0	1	3	Amplification	4	0	42493T: 3.5; 42487T: 3.5; 42497T: 4; 42501T: 3.5;
SYS1	chr20	20q13.12	1	1	0	0	Amplification	1	0	42473T: 5;
SYT10	chr12	12p11.1	1	0	0	1	Amplification	1	0	42500T: 3.5;
SYT11	chr1	11q13.2	1	1	0	0	Amplification	1	0	42473T: 4.5;
SYT12	chr11	11q13.2	2	1	0	1	Amplification	2	0	42473T: 3.5; 56957T: 5.5;
SYT13	chr11	11p11.2	1	1	0	0	Amplification	1	0	42473T: 3.5;
SYT16	chr14	14q23.2	1	0	0	1	Amplification	1	0	42494T: 4;
SYT17	chr16	16p12.3	2	1	0	1	Amplification	2	0	42495T: 4; 42473T: 4;
SYT2	chr1	1q32.1	1	1	0	0	Amplification	1	0	42473T: 4;
SYTL1	chr1	1p36.11	1	1	0	0	Amplification	1	0	42473T: 5;
SZRD1	chr1	1p36.13	1	1	0	0	Amplification	1	0	42473T: 4.5;
SZT2	chr1	1p34.2	1	0	0	1	Amplification	1	0	42493T: 3.5;
TAB1	chr22	22q13.1	1	1	0	0	Amplification	1	0	42473T: 3.5;
TAC1	chr7	7q21.3	1	0	1	0	Amplification	1	0	42487T: 3.5;
TACR1	chr2	2p12	1	0	0	1	Amplification	1	0	42500T: 3.5;
TADA2A	chr17	17q12	1	1	0	0	Amplification	1	0	42473T: 3.5;
TAF12	chr1	1p35.3	1	1	0	0	Amplification	1	0	42473T: 3.5;
TAF1B	chr2	2p25.1	1	0	0	1	Amplification	1	0	42500T: 3.5;
TAF2	chr8	8q24.12	3	1	0	2	Amplification	3	0	42495T: 3.5; 42496T: 3.5; 42475T: 3.5;
TAF4	chr20	20q13.33	1	1	0	0	Amplification	1	0	42473T: 6;
TAGLN3	chr3	3q13.2	1	0	0	1	Amplification	1	0	42496T: 3.5;
TAOK3	chr12	12q24.23	1	0	0	1	Amplification	1	0	42500T: 3.5;
TARS	chr5	5p13.3	4	1	1	2	Amplification	4	0	42493T: 3.5; 42475T: 3.5; 42496T: 3.5; 42486T: 3.5;
TARS2	chr1	1q21.2	2	1	0	1	Amplification	2	0	42473T: 5; 42493T: 3.5;
TAS1R2	chr1	1p36.13	1	1	0	0	Amplification	1	0	42473T: 4;
TAS2R1	chr5	5p15.31	4	1	1	2	Amplification	4	0	42493T: 4; 42496T: 3.5; 42486T: 3.5; 42475T: 3.5;
TAS2R16	chr7	7q31.32|7q3	2	0	1	1	Amplification	2	0	42487T: 3.5; 42493T: 3.5;
TAS2R3	chr7	7q34	1	0	1	0	Amplification	1	0	42487T: 3.5;
TAS2R38	chr7	7q34	1	0	1	0	Amplification	1	0	42487T: 3.5;
TAS2R39	chr7	7q34	1	0	1	0	Amplification	1	0	42487T: 3.5;
TAS2R4	chr7	7q34	1	0	1	0	Amplification	1	0	42487T: 3.5;
TAS2R40	chr7	7q34	1	0	1	0	Amplification	1	0	42487T: 3.5;
TAS2R41	chr7	7q35	1	0	1	0	Amplification	1	0	42487T: 3.5;

Mangalaparthi *et al.* , 2020. Mutational landscape of esophageal squamous cell carcinoma in an Indian cohort
Supplementary Table 7A. List of copy number alterations and affected genes in ESCC patients

Gene	Chromosome	Cytoband	Recurrence	Recurrence in smoker cohort	Recurrence in chewer cohort	Recurrence in No habit cohort	State	Samples with CNA gain	Samples with CNA loss	File info with CNA fold
TAS2R5	chr7	7q34	1	0	1	0	Amplification	1	0	42487T: 3.5;
TAS2R60	chr7	7q35	1	0	1	0	Amplification	1	0	42487T: 3.5;
TATDN1	chr8	8q24.13	4	1	1	2	Amplification	4	0	42484T: 3.5; 42495T: 3.5; 42496T: 3.5; 42475T: 3.5;
TAX1BP1	chr7	7p15.2	1	1	0	0	Amplification	1	0	42473T: 4;
TBC1D1	chr4	4p14	1	1	0	0	Amplification	1	0	42473T: 7;
TBC1D10A	chr22	22q12.2	1	1	0	0	Amplification	1	0	42473T: 3.5;
TBC1D10C	chr11	11q13.2	2	1	0	1	Amplification	2	0	42473T: 3.5; 56957T: 5;
TBC1D15	chr12	12q21.1	1	0	0	1	Amplification	1	0	42501T: 6.5;
TBC1D16	chr17	17q25.3	1	1	0	0	Amplification	1	0	42473T: 3.5;
TBC1D20	chr20	20p13	1	1	0	0	Amplification	1	0	42473T: 3.5;
TBC1D21	chr15	15q24.1	1	1	0	0	Amplification	1	0	42473T: 3.5;
TBC1D22B	chr6	6p21.2	1	1	0	0	Amplification	1	0	42473T: 4.5;
TBC1D23	chr3	3q12.1-q12.2	1	1	0	0	Amplification	1	0	42476T: 3.5;
TBC1D29	chr17	17q11.2	1	1	0	0	Amplification	1	0	42473T: 3.5;
TBC1D3	chr17	17q12	1	1	0	0	Amplification	1	0	42473T: 3.5;
TBC1D31	chr8	8q24.13	4	1	1	2	Amplification	4	0	42495T: 3.5; 42484T: 3.5; 42475T: 3.5; 42496T: 3.5;
TBC1D8	chr2	2q11.2	1	0	0	1	Amplification	1	0	42493T: 3.5;
TBCB	chr19	19q13.12	3	0	1	2	Amplification	3	0	42500T: 6.5; 42484T: 3.5; 56957T: 4;
TBCCD1	chr3	3q27.3	10	1	3	6	Amplification	10	0	42493T: 3.5; 42487T: 3.5; 42492T: 3.5; 42482T: 3.5; 42474T: 3.5; 42495T: 4; 56957T: 4.5; 42484T: 4; 42498T: 3.5; 42497T: 4;
TBL1XR1	chr3	3q26.32	9	1	3	5	Amplification	9	0	42493T: 3.5; 42487T: 3.5; 42492T: 3.5; 42482T: 3.5; 56957T: 4; 42474T: 3.5; 42495T: 4; 42484T: 4; 42500T: 9.5;
TBL3	chr16	16p13.3	1	0	1	0	Amplification	1	0	42483T: 3.5;
TBPL2	chr14	14q22.3	1	0	0	1	Amplification	1	0	42494T: 4;
TBRG4	chr7	7p13	1	1	0	0	Amplification	1	0	42473T: 4.5;
TBX1	chr22	22q11.21	1	0	0	1	Amplification	1	0	42497T: 17;
TBX10	chr11	11q13.2	2	1	0	1	Amplification	2	0	42473T: 3.5; 56957T: 5;
TBX20	chr7	7p14.2	1	1	0	0	Amplification	1	0	42473T: 4;
TBX3	chr12	12q24.21	1	0	0	1	Amplification	1	0	42500T: 3.5;
TBX5	chr12	12q24.21	1	0	0	1	Amplification	1	0	42500T: 3.5;
TBXAS1	chr7	7q34	1	0	1	0	Amplification	1	0	42487T: 3.5;
TCAP	chr17	17q12	2	1	0	1	Amplification	2	0	42497T: 3.5; 42473T: 6;
TCEA1	chr8	8q11.23	3	0	0	3	Amplification	3	0	42494T: 4; 42495T: 3.5; 42496T: 3.5;
TCEA2	chr20	20q13.33	1	1	0	0	Amplification	1	0	42473T: 6;
TCEA3	chr1	1p36.12	1	1	0	0	Amplification	1	0	42473T: 3.5;
TCEB1	chr8	8q21.11	2	0	0	2	Amplification	2	0	42495T: 3.5; 42496T: 3.5;
TCEB3	chr1	1p36.11	1	1	0	0	Amplification	1	0	42473T: 3.5;
TCF15	chr20	20p13	1	1	0	0	Amplification	1	0	42473T: 3.5;
TCF23	chr2	2p23.3	1	0	0	1	Amplification	1	0	42500T: 3.5;

Mangalaparthi *et al.* , 2020. Mutational landscape of esophageal squamous cell carcinoma in an Indian cohort
Supplementary Table 7A. List of copy number alterations and affected genes in ESCC patients

Gene	Chromosome	Cytoband	Recurrence	Recurrence in smoker cohort	Recurrence in chewer cohort	Recurrence in No habit cohort	State	Samples with CNA gain	Samples with CNA loss	File info with CNA fold
TCF24	chr8	8q13.1	3	0	0	3	Amplification	3	0	42496T: 3.5; 42497T: 4.5; 42495T: 3.5;
TCF7	chr5	5q31.1	1	1	0	0	Amplification	1	0	42473T: 3.5;
TCF7L1	chr2	2p11.2	1	0	0	1	Amplification	1	0	42500T: 3.5;
TCFL5	chr20	20q13.33	1	1	0	0	Amplification	1	0	42473T: 6;
TCHH	chr1	1q21.3	1	1	0	0	Amplification	1	0	42473T: 4.5;
TCHHL1	chr1	1q21.3	1	1	0	0	Amplification	1	0	42473T: 4.5;
TCHP	chr12	12q24.11	2	1	0	1	Amplification	2	0	42473T: 4.5; 42500T: 3.5;
TCIRG1	chr11	11q13.2	1	0	0	1	Amplification	1	0	56957T: 5;
TCTA	chr3	3p21.31	1	1	0	0	Amplification	1	0	42473T: 4;
TCTEX1D2	chr3	3q29	9	1	3	5	Amplification	9	0	42482T: 3.5; 42487T: 3.5; 42493T: 5; 42492T: 3.5; 42495T: 4; 42474T: 3.5; 56957T: 6; 42484T: 4; 42498T: 3.5;
TCTN1	chr12	12q24.11	1	0	0	1	Amplification	1	0	42500T: 3.5;
TCTN2	chr12	12q24.31	1	0	0	1	Amplification	1	0	42500T: 3.5;
TDH	chr8	8p23.1	1	0	1	0	Amplification	1	0	42486T: 3.5;
TDP1	chr14	14q32.11	1	1	0	0	Amplification	1	0	42473T: 3.5;
TDRD10	chr1	1q21.3	1	1	0	0	Amplification	1	0	42473T: 4.5;
TDRD12	chr19	19q13.11	4	1	1	2	Amplification	4	0	42500T: 4.5; 42473T: 3.5; 56957T: 4; 42484T: 3.5;
TDRD15	chr2	2p24.1	1	0	0	1	Amplification	1	0	42500T: 3.5;
TDRKH	chr1	1q21.3	1	1	0	0	Amplification	1	0	42473T: 4.5;
TEC	chr4	19q13.2	1	0	1	0	Amplification	1	0	42483T: 3.5;
TEFM	chr17	17q11.2	1	1	0	0	Amplification	1	0	42473T: 3.5;
TEKT4	chr2	2q11.1	1	1	0	0	Amplification	1	0	42473T: 3.5;
TEKT5	chr16	16p13.13	2	1	0	1	Amplification	2	0	42495T: 5; 42473T: 5.5;
TELO2	chr16	16p13.3	1	0	1	0	Amplification	1	0	42483T: 3.5;
TEN1	chr17	17q25.1	1	0	0	1	Amplification	1	0	42494T: 3.5;
TENC1	chr12	12q13.13	1	1	0	0	Amplification	1	0	42473T: 4;
TERC	chr3	3q26.2	9	1	3	5	Amplification	9	0	56957T: 4; 42474T: 3.5; 42495T: 4; 42484T: 4; 42500T: 8; 42487T: 3.5; 42493T: 3.5; 42492T: 3.5; 42482T: 3.5;
TERF1	chr8	8q21.11	2	0	0	2	Amplification	2	0	42496T: 3.5; 42495T: 3.5;
TERT	chr5	5p15.33	5	2	1	2	Amplification	5	0	42473T: 3.5; 42496T: 4; 42486T: 3.5; 42475T: 3.5; 42493T: 3.5;
TES	chr7	7q31.2	1	0	1	0	Amplification	1	0	42487T: 3.5;
TESC	chr12	12q24.22	1	0	0	1	Amplification	1	0	42500T: 3.5;
TESK1	chr9	9p13.3	2	1	0	1	Amplification	2	0	42501T: 3.5; 42473T: 3.5;
TESPA1	chr12	12q13.2	1	0	0	1	Amplification	1	0	42494T: 3.5;
TET3	chr2	2p13.1	1	0	0	1	Amplification	1	0	42500T: 3.5;
TEX101	chr19	19q13.31	1	0	0	1	Amplification	1	0	56957T: 4;
TEX14	chr17	17q22	1	0	0	1	Amplification	1	0	42497T: 4;
TEX15	chr8	8p12	1	0	1	0	Amplification	1	0	42482T: 3.5;

Mangalaparthi *et al.* , 2020. Mutational landscape of esophageal squamous cell carcinoma in an Indian cohort
Supplementary Table 7A. List of copy number alterations and affected genes in ESCC patients

Gene	Chromosome	Cytoband	Recurrence	Recurrence in smoker cohort	Recurrence in chewer cohort	Recurrence in No habit cohort	State	Samples with CNA gain	Samples with CNA loss	File info with CNA fold
TEX26	chr13	13q12.3	1	0	0	1	Amplification	1	0	42497T: 3.5;
TEX261	chr2	2p13.3	1	0	0	1	Amplification	1	0	42500T: 3.5;
TEX33	chr22	22q12.3	1	1	0	0	Amplification	1	0	42473T: 3.5;
TEX37	chr2	2p11.2	1	0	0	1	Amplification	1	0	42500T: 3.5;
TF	chr3	3q22.1	3	0	1	2	Amplification	3	0	42496T: 3.5; 42492T: 3.5; 42487T: 3.5;
TFAP2C	chr20	20q13.31	1	1	0	0	Amplification	1	0	42473T: 5;
TFAP4	chr16	16p13.3	3	1	0	2	Amplification	3	0	42473T: 5.5; 42495T: 8.5; 42494T: 3.5;
TFCP2	chr12	12q13.12-q1	2	0	0	2	Amplification	2	0	42500T: 3.5; 42494T: 3.5;
TFCP2L1	chr2	2q14.2	1	0	0	1	Amplification	1	0	42493T: 3.5;
TFDP1	chr13	13q34	1	0	0	1	Amplification	1	0	56957T: 3.5;
TFDP2	chr3	3q23	4	0	1	3	Amplification	4	0	42487T: 3.5; 42493T: 3.5; 42492T: 3.5; 42496T: 4;
TFEC	chr7	7q31.2	1	0	1	0	Amplification	1	0	42487T: 3.5;
TFF1	chr21	21q22.3	1	1	0	0	Amplification	1	0	42473T: 3.5;
TFF2	chr21	21q22.3	1	1	0	0	Amplification	1	0	42473T: 3.5;
TFF3	chr21	21q22.3	1	1	0	0	Amplification	1	0	42473T: 3.5;
TFG	chr3	3q12.2	1	1	0	0	Amplification	1	0	42476T: 3.5;
TFPI	chr2	2q32.1	1	0	0	1	Amplification	1	0	42493T: 3.5;
TFPI2	chr7	7q21.3	2	0	2	0	Amplification	2	0	42483T: 4; 42487T: 3.5;
TFRC	chr3	3q29	9	1	3	5	Amplification	9	0	42474T: 3.5; 42495T: 4; 56957T: 6; 42484T: 4; 42498T: 3.5; 42482T: 3.5; 42493T: 3.5; 42487T: 3.5; 42492T: 3.5;
TG	chr8	8q24.22	3	0	1	2	Amplification	3	0	42495T: 3.5; 42484T: 3.5; 42496T: 3.5;
TGFA	chr2	2p13.3	1	0	0	1	Amplification	1	0	42500T: 3.5;
TGFB1	chr19	19q13.2	1	1	0	0	Amplification	1	0	42473T: 4.5;
TGFB3	chr14	14q24.3	2	0	0	2	Amplification	2	0	56957T: 3.5; 42494T: 4;
TGFBRAP1	chr2	2q12.1-q12.2	1	0	0	1	Amplification	1	0	42493T: 3.5;
TGIF1	chr18	18p11.31	3	1	0	2	Amplification	3	0	56957T: 8; 42493T: 3.5; 42481T: 9.5;
TGIF2	chr20	20q11.23	1	1	0	0	Amplification	1	0	42473T: 3.5;
TGM2	chr20	20q11.23	1	1	0	0	Amplification	1	0	42473T: 3.5;
TGM3	chr20	20p13	1	1	0	0	Amplification	1	0	42473T: 3.5;
TGM6	chr20	20p13	1	1	0	0	Amplification	1	0	42473T: 3.5;
TGOLN2	chr2	2p11.2	1	0	0	1	Amplification	1	0	42500T: 3.5;
TGS1	chr8	1q42.12	3	0	1	2	Amplification	3	0	42496T: 3.5; 42483T: 5; 42495T: 3.5;
THADA	chr2	2p21	2	0	1	1	Amplification	2	0	42484T: 3.5; 42500T: 3.5;
THAP1	chr8	8p11.21	1	0	1	0	Amplification	1	0	42483T: 4.5;
THAP2	chr12	12q21.1	1	0	0	1	Amplification	1	0	42501T: 6.5;
THAP5	chr7	7q31.1	3	0	1	2	Amplification	3	0	42501T: 3.5; 42497T: 4; 42487T: 3.5;
THAP7	chr22	22q11.21	1	1	0	0	Amplification	1	0	42477T: 4;
THAP8	chr19	19q13.12	3	0	1	2	Amplification	3	0	42500T: 6.5; 56957T: 4; 42484T: 3.5;
THBS3	chr1	1q22	1	1	0	0	Amplification	1	0	42473T: 6.5;
THEM4	chr1	1q21.3	1	1	0	0	Amplification	1	0	42473T: 4.5;

Mangalaparthi *et al.* , 2020. Mutational landscape of esophageal squamous cell carcinoma in an Indian cohort
Supplementary Table 7A. List of copy number alterations and affected genes in ESCC patients

Gene	Chromosome	Cytoband	Recurrence	Recurrence in smoker cohort	Recurrence in chewer cohort	Recurrence in No habit cohort	State	Samples with CNA gain	Samples with CNA loss	File info with CNA fold
THEM5	chr1	1q21.3	1	1	0	0	Amplification	1	0	42473T: 4.5;
THEM6	chr8	8q24.3	3	0	1	2	Amplification	3	0	42495T: 4.5; 42496T: 4; 42483T: 3.5;
THEMIS2	chr1	1p35.3	1	1	0	0	Amplification	1	0	42473T: 3.5;
THNSL2	chr2	2p11.2	1	0	0	1	Amplification	1	0	42500T: 3.5;
THOC1	chr18	18p11.32	3	0	0	3	Amplification	3	0	42500T: 4.5; 56957T: 8; 42493T: 3.5;
THPO	chr3	3q27.1	11	1	3	7	Amplification	11	0	42497T: 4; 42498T: 3.5; 42484T: 4; 56957T: 5; 42474T: 3.5; 42494T: 3.5; 42495T: 4; 42482T: 3.5; 42492T: 3.5; 42487T: 3.5; 42493T: 3.5;
THRA	chr17	17q21.1	1	0	0	1	Amplification	1	0	42497T: 3.5;
THSD7A	chr7	7p21.3	1	1	0	0	Amplification	1	0	42473T: 3.5;
THTPA	chr14	14q11.2	1	0	0	1	Amplification	1	0	42496T: 4;
THUMPD1	chr16	16p12.3	1	1	0	0	Amplification	1	0	42473T: 4;
THUMPD2	chr2	2p22.1|2p22.	1	0	0	1	Amplification	1	0	42500T: 3.5;
THYN1	chr11	11q25	1	1	0	0	Deletion	0	1	42476T: 0.5;
TIA1	chr2	2p13.3	1	0	0	1	Amplification	1	0	42500T: 3.5;
TICRR	chr15	15q26.1	1	1	0	0	Amplification	1	0	42473T: 3.5;
TIE1	chr1	1p34.2	1	0	0	1	Amplification	1	0	42493T: 3.5;
TIGD5	chr8	8q24.3	3	0	1	2	Amplification	3	0	42495T: 4.5; 42483T: 3.5; 42496T: 4;
TIGD7	chr16	16p13.3	1	1	0	0	Amplification	1	0	42473T: 5.5;
TIGIT	chr3	3q13.31	1	0	0	1	Amplification	1	0	42496T: 3.5;
TIMM50	chr19	19q13.2	2	0	0	2	Amplification	2	0	56957T: 4; 42500T: 6.5;
TIMM9	chr14	14q23.1	1	0	0	1	Amplification	1	0	42494T: 4;
TIMMDC1	chr3	3q13.33	1	0	0	1	Amplification	1	0	42496T: 3.5;
TINAGL1	chr1	1p35.2	1	1	0	0	Amplification	1	0	42473T: 3.5;
TIPARP	chr3	3q25.31	8	1	2	5	Amplification	8	0	42492T: 3.5; 42487T: 3.5; 42493T: 3.5; 42497T: 5.5; 42484T: 3.5; 42474T: 3.5; 42495T: 4; 56957T: 4;
TLDC2	chr20	20q11.23	1	1	0	0	Amplification	1	0	42473T: 3.5;
TLN1	chr9	9p13.3	2	1	0	1	Amplification	2	0	42501T: 3.5; 42473T: 3.5;
TLN2	chr15	15q22.2	1	0	0	1	Amplification	1	0	42496T: 4;
TLR1	chr4	4p14	1	1	0	0	Amplification	1	0	42473T: 3.5;
TLR10	chr4	4p14	1	1	0	0	Amplification	1	0	42473T: 3.5;
TLR6	chr4	4p14	1	1	0	0	Amplification	1	0	42473T: 3.5;
TLX2	chr2	2p13.1	1	0	0	1	Amplification	1	0	42500T: 3.5;
TM4SF1	chr3	3q25.1	8	1	2	5	Amplification	8	0	42474T: 3.5; 56957T: 4; 42484T: 3.5; 42496T: 4; 42497T: 4.5; 42487T: 3.5; 42493T: 3.5; 42492T: 3.5;
TM4SF18	chr3	3q25.1	8	1	2	5	Amplification	8	0	42487T: 3.5; 42493T: 3.5; 42492T: 3.5; 42496T: 4; 42497T: 4.5; 42474T: 3.5; 56957T: 4; 42484T: 3.5;
TM4SF19	chr3	3q29	9	1	3	5	Amplification	9	0	42484T: 4; 42498T: 3.5; 42474T: 3.5; 42495T: 4; 56957T: 6; 42492T: 3.5; 42493T: 5; 42487T: 3.5; 42482T: 3.5;

Mangalaparthi *et al.* , 2020. Mutational landscape of esophageal squamous cell carcinoma in an Indian cohort
Supplementary Table 7A. List of copy number alterations and affected genes in ESCC patients

Gene	Chromosome	Cytoband	Recurrence	Recurrence in smoker cohort	Recurrence in chewer cohort	Recurrence in No habit cohort	State	Samples with CNA gain	Samples with CNA loss	File info with CNA fold
TM4SF4	chr3	3q25.1	8	1	2	5	Amplification	8	0	42484T: 3.5; 42474T: 3.5; 56957T: 4; 42497T: 4.5; 42496T: 4; 42492T: 3.5; 42493T: 3.5; 42487T: 3.5;
TM7SF3	chr12	12p11.23	1	0	0	1	Amplification	1	0	42500T: 6;
TM9SF4	chr20	20q11.21	2	1	0	1	Amplification	2	0	42496T: 5; 42473T: 4.5;
TMBIM4	chr12	12q14.3	1	0	0	1	Amplification	1	0	42500T: 4.5;
TMBIM6	chr12	12q13.12	1	0	0	1	Amplification	1	0	42500T: 3.5;
TMC1	chr9	9q21.13	1	1	0	0	Amplification	1	0	42473T: 4;
TMC2	chr20	20p13	1	1	0	0	Amplification	1	0	42473T: 3.5;
TMC5	chr16	16p12.3	1	1	0	0	Amplification	1	0	42473T: 4;
TMC7	chr16	16p12.3	2	1	0	1	Amplification	2	0	42473T: 4; 42495T: 4;
TMCC1	chr3	3q22.1	2	0	1	1	Amplification	2	0	42496T: 3.5; 42487T: 3.5;
TMCO3	chr13	13q34	1	0	0	1	Amplification	1	0	56957T: 3.5;
TMCO4	chr1	1p36.13	1	1	0	0	Amplification	1	0	42473T: 4;
TMED10	chr14	14q24.3	2	0	0	2	Amplification	2	0	56957T: 3.5; 42494T: 4;
TMED2	chr12	12q24.31	1	0	0	1	Amplification	1	0	42500T: 3.5;
TMED4	chr7	7p13	2	1	0	1	Amplification	2	0	42473T: 4.5; 42497T: 7.5;
TMED8	chr14	14q24.3	2	0	0	2	Amplification	2	0	42494T: 4; 56957T: 3.5;
TMEFF2	chr2	2q32.3	2	0	1	1	Amplification	2	0	42482T: 4; 42493T: 3.5;
TMEM100	chr17	17q22	1	0	0	1	Amplification	1	0	42497T: 4;
TMEM106B	chr7	7p21.3	1	1	0	0	Amplification	1	0	42473T: 3.5;
TMEM106C	chr12	12q13.11	1	0	0	1	Amplification	1	0	42500T: 3.5;
TMEM108	chr3	3q22.1	3	0	1	2	Amplification	3	0	42492T: 3.5; 42487T: 3.5; 42496T: 3.5;
TMEM114	chr16	16p13.2	2	1	0	1	Amplification	2	0	42495T: 4; 42473T: 5.5;
TMEM116	chr12	12q24.12-q2	1	0	0	1	Amplification	1	0	42500T: 3.5;
TMEM117	chr12	12q12	1	0	0	1	Amplification	1	0	42500T: 3.5;
TMEM120B	chr12	12q24.31	1	0	0	1	Amplification	1	0	42500T: 3.5;
TMEM123	chr11	11q22.2	1	0	0	1	Amplification	1	0	56958T: 5.5;
TMEM125	chr1	1p34.2	1	0	0	1	Amplification	1	0	42493T: 3.5;
TMEM127	chr2	2q11.2	2	1	0	1	Amplification	2	0	42473T: 3.5; 42493T: 3.5;
TMEM131	chr2	2q11.2	1	0	0	1	Amplification	1	0	42493T: 3.5;
TMEM132B	chr12	12q24.31-q2	1	0	0	1	Amplification	1	0	42500T: 3.5;
TMEM134	chr11	11q13.2	2	1	0	1	Amplification	2	0	42473T: 3.5; 56957T: 5;
TMEM139	chr7	7q34	1	0	1	0	Amplification	1	0	42487T: 3.5;
TMEM140	chr7	7q33	1	0	1	0	Amplification	1	0	42487T: 3.5;
TMEM141	chr9	9q34.3	1	0	0	1	Amplification	1	0	56957T: 3.5;
TMEM145	chr19	19q13.2	1	1	0	0	Amplification	1	0	42473T: 4.5;
TMEM147	chr19	19q13.12	3	0	1	2	Amplification	3	0	42484T: 3.5; 56957T: 4; 42500T: 6.5;
TMEM14E	chr3	3q25.2	9	1	2	6	Amplification	9	0	42492T: 3.5; 42487T: 3.5; 42493T: 3.5; 42496T: 4; 42497T: 5.5; 42500T: 3.5; 42484T: 3.5; 56957T: 4; 42474T: 3.5;
TMEM150A	chr2	2p11.2	2	0	1	1	Amplification	2	0	42500T: 3.5; 42482T: 3.5;

Mangalaparthi *et al.* , 2020. Mutational landscape of esophageal squamous cell carcinoma in an Indian cohort
Supplementary Table 7A. List of copy number alterations and affected genes in ESCC patients

Gene	Chromosome	Cytoband	Recurrence	Recurrence in smoker cohort	Recurrence in chewer cohort	Recurrence in No habit cohort	State	Samples with CNA gain	Samples with CNA loss	File info with CNA fold
TMEM156	chr4	4p14	1	1	0	0	Amplification	1	0	42473T: 3.5;
TMEM159	chr16	16p12.3	1	1	0	0	Amplification	1	0	42473T: 4;
TMEM161B	chr5	5q14.3	1	1	0	0	Deletion	0	1	42476T: 0.5;
TMEM168	chr7	7q31.1	2	0	1	1	Amplification	2	0	42487T: 3.5; 42501T: 3.5;
TMEM17	chr2	2p15	3	0	1	2	Amplification	3	0	42500T: 3.5; 56957T: 3.5; 42484T: 3.5;
TMEM178A	chr2	2p22.1	1	0	0	1	Amplification	1	0	42500T: 3.5;
TMEM178B	chr7	7q34	1	0	1	0	Amplification	1	0	42487T: 3.5;
TMEM18	chr2	2p25.3	1	0	0	1	Amplification	1	0	42500T: 3.5;
TMEM182	chr2	2q12.1	1	0	0	1	Amplification	1	0	42493T: 3.5;
TMEM183A	chr1	1q32.1	1	1	0	0	Amplification	1	0	42473T: 4;
TMEM184A	chr7	7p22.3	1	1	0	0	Amplification	1	0	42473T: 5;
TMEM184B	chr22	22q13.1	1	1	0	0	Amplification	1	0	42473T: 3.5;
TMEM186	chr16	16p13.2	2	1	0	1	Amplification	2	0	42473T: 5.5; 42495T: 4;
TMEM189	chr20	20q13.13	1	1	0	0	Amplification	1	0	42473T: 6.5;
TMEM19	chr12	12q21.1	1	0	0	1	Amplification	1	0	42501T: 6.5;
TMEM190	chr19	19q13.42	1	0	0	1	Amplification	1	0	42494T: 3.5;
TMEM191A	chr22	22q11.21	2	1	0	1	Amplification	2	0	42477T: 4; 42497T: 13.5;
TMEM194B	chr2	2q32.2	3	0	1	2	Amplification	3	0	42494T: 3.5; 42493T: 3.5; 42482T: 4;
TMEM196	chr7	7p21.1	1	1	0	0	Amplification	1	0	42473T: 4;
TMEM198B	chr12	12q13.2	1	0	0	1	Amplification	1	0	42494T: 5;
TMEM200B	chr1	1p35.3	1	1	0	0	Amplification	1	0	42473T: 3.5;
TMEM203	chr9	9q34.3	2	1	0	1	Amplification	2	0	56957T: 3.5; 42473T: 5;
TMEM204	chr16	16p13.3	1	0	1	0	Amplification	1	0	42483T: 3.5;
TMEM207	chr3	3q28	9	1	3	5	Amplification	9	0	56957T: 4.5; 42474T: 3.5; 42495T: 4; 42498T: 3.5; 42484T: 4; 42482T: 3.5; 42487T: 3.5; 42493T: 3.5; 42492T: 3.5;
TMEM209	chr7	7q32.2	1	0	1	0	Amplification	1	0	42487T: 3.5;
TMEM210	chr9	9q34.3	2	1	0	1	Amplification	2	0	56957T: 3.5; 42473T: 5;
TMEM212	chr3	3q26.31	9	1	3	5	Amplification	9	0	42482T: 3.5; 42487T: 3.5; 42493T: 3.5; 42492T: 3.5; 42500T: 9; 56957T: 4; 42495T: 4; 42474T: 3.5; 42484T: 4;
TMEM213	chr7	7q34	1	0	1	0	Amplification	1	0	42487T: 3.5;
TMEM214	chr2	2p23.3	1	0	0	1	Amplification	1	0	42500T: 3.5;
TMEM222	chr1	1p36.11	1	1	0	0	Amplification	1	0	42473T: 5;
TMEM229A	chr7	7q31.32	1	0	1	0	Amplification	1	0	42487T: 3.5;
TMEM229B	chr14	14q24.1	1	0	0	1	Amplification	1	0	42494T: 4;
TMEM230	chr20	20p13-p12.3	1	1	0	0	Amplification	1	0	42473T: 4.5;
TMEM233	chr12	12q24.23	1	0	0	1	Amplification	1	0	42500T: 3.5;
TMEM234	chr1	1p35.2	1	1	0	0	Amplification	1	0	42473T: 4;
TMEM239	chr20	20p13	1	1	0	0	Amplification	1	0	42473T: 3.5;
TMEM241	chr18	18q11.2	1	1	0	0	Amplification	1	0	42481T: 4;

Mangalaparthi *et al.* , 2020. Mutational landscape of esophageal squamous cell carcinoma in an Indian cohort
Supplementary Table 7A. List of copy number alterations and affected genes in ESCC patients

Gene	Chromosome	Cytoband	Recurrence	Recurrence in smoker cohort	Recurrence in chewer cohort	Recurrence in No habit cohort	State	Samples with CNA gain	Samples with CNA loss	File info with CNA fold
TMEM243	chr7	7q21.12	2	0	2	0	Amplification	2	0	42487T: 3.5; 42483T: 3.5;
TMEM245	chr9	9q31.3	1	0	1	0	Amplification	1	0	42483T: 3.5;
TMEM247	chr2	2p21	2	0	1	1	Amplification	2	0	42484T: 4.5; 42500T: 3.5;
TMEM248	chr7	7q11.21	1	0	0	1	Amplification	1	0	42501T: 3.5;
TMEM249	chr8	8q24.3	4	0	1	3	Amplification	4	0	42483T: 3.5; 42496T: 4; 42495T: 4.5; 56957T: 3.5;
TMEM255B	chr13	13q34	1	0	0	1	Amplification	1	0	56957T: 3.5;
TMEM259	chr19	19p13.3	1	0	0	1	Amplification	1	0	42493T: 3.5;
TMEM260	chr14	14q22.3	1	0	0	1	Amplification	1	0	42494T: 4;
TMEM30B	chr14	14q23.1	1	0	0	1	Amplification	1	0	42494T: 4;
TMEM30C	chr3	3q12.1	1	1	0	0	Amplification	1	0	42476T: 3.5;
TMEM39A	chr3	3q13.33	1	0	0	1	Amplification	1	0	42496T: 3.5;
TMEM39B	chr1	1p35.2	1	1	0	0	Amplification	1	0	42473T: 3.5;
TMEM41A	chr3	3q27.2	10	1	3	6	Amplification	10	0	42495T: 4; 42474T: 3.5; 56957T: 4.5; 42484T: 4; 42498T: 3.5; 42497T: 4; 42487T: 3.5; 42493T: 3.5; 42492T: 3.5; 42482T: 3.5;
TMEM44	chr3	3q29	9	1	3	5	Amplification	9	0	42484T: 4; 42498T: 3.5; 42495T: 4; 42474T: 3.5; 56957T: 4.5; 42482T: 3.5; 42492T: 3.5; 42487T: 3.5; 42493T: 3.5;
TMEM45A	chr3	3q12.2	1	1	0	0	Amplification	1	0	42476T: 3.5;
TMEM50A	chr1	1p36.11	1	1	0	0	Amplification	1	0	42473T: 4.5;
TMEM55A	chr8	8q21.3	2	0	0	2	Amplification	2	0	42495T: 3.5; 42496T: 3.5;
TMEM57	chr1	1p36.11|1p3	1	1	0	0	Amplification	1	0	42473T: 4.5;
TMEM60	chr7	7q11.23	1	0	1	0	Amplification	1	0	42487T: 3.5;
TMEM63A	chr1	1q42.12	1	0	0	1	Amplification	1	0	42493T: 3.5;
TMEM63C	chr14	14q24.3	2	0	0	2	Amplification	2	0	56957T: 3.5; 42494T: 4;
TMEM64	chr8	8q21.3	2	0	0	2	Amplification	2	0	42495T: 3.5; 42496T: 3.5;
TMEM65	chr8	8q24.13	4	1	1	2	Amplification	4	0	42475T: 3.5; 42496T: 3.5; 42495T: 3.5; 42484T: 3.5;
TMEM66	chr8	8p12	1	0	1	0	Amplification	1	0	42482T: 3.5;
TMEM67	chr8	8q22.1	2	0	0	2	Amplification	2	0	42495T: 3.5; 42496T: 3.5;
TMEM68	chr8	8q12.1	3	0	1	2	Amplification	3	0	42495T: 3.5; 42483T: 5; 42496T: 3.5;
TMEM70	chr8	8q21.11	2	0	0	2	Amplification	2	0	42496T: 3.5; 42495T: 3.5;
TMEM71	chr8	8q24.22	3	0	1	2	Amplification	3	0	42484T: 3.5; 42495T: 3.5; 42496T: 3.5;
TMEM74	chr8	8q23.1	2	0	0	2	Amplification	2	0	42496T: 3.5; 42495T: 3.5;
TMEM74B	chr20	20p13	1	1	0	0	Amplification	1	0	42473T: 3.5;
TMEM75	chr8	8q24.21	5	1	1	3	Amplification	5	0	42496T: 3.5; 42475T: 3.5; 42484T: 3.5; 42495T: 3.5; 42493T: 10;
TMEM79	chr1	1q22	1	1	0	0	Amplification	1	0	42473T: 4.5;
TMEM8A	chr16	16p13.3	1	0	1	0	Amplification	1	0	42483T: 3.5;
TMEM8B	chr9	9p13.3	2	1	0	1	Amplification	2	0	42501T: 3.5; 42473T: 3.5;
TMEM91	chr19	19q13.2	1	1	0	0	Amplification	1	0	42473T: 4.5;
TMOD4	chr1	1q21.3	1	1	0	0	Amplification	1	0	42473T: 4.5;

Mangalaparthi *et al.* , 2020. Mutational landscape of esophageal squamous cell carcinoma in an Indian cohort
Supplementary Table 7A. List of copy number alterations and affected genes in ESCC patients

Gene	Chromosome	Cytoband	Recurrence	Recurrence in smoker cohort	Recurrence in chewer cohort	Recurrence in No habit cohort	State	Samples with CNA gain	Samples with CNA loss	File info with CNA fold
TMPRSS12	chr12	12q13.12	2	0	0	2	Amplification	2	0	42494T: 3.5; 42500T: 3.5;
TMPRSS2	chr21	21q22.3	1	1	0	0	Amplification	1	0	42473T: 3.5;
TMPRSS3	chr21	21q22.3	1	1	0	0	Amplification	1	0	42473T: 3.5;
TMPRSS6	chr22	22q12.3	1	1	0	0	Amplification	1	0	42473T: 3.5;
TMPRSS7	chr3	3q13.2	1	0	0	1	Amplification	1	0	42496T: 3.5;
TMSB10	chr2	2p11.2	1	0	0	1	Amplification	1	0	42500T: 3.5;
TMTC1	chr12	12p11.22	1	0	0	1	Amplification	1	0	42500T: 6;
TMX1	chr14	14q22.1	1	0	0	1	Amplification	1	0	42494T: 4;
TMX2	chr11	11q12.1	1	0	0	1	Amplification	1	0	42496T: 4.5;
TNC	chr9	3p21.1	1	0	0	1	Amplification	1	0	42493T: 3.5;
TNF	chr6	6p21.33	1	1	0	0	Amplification	1	0	42473T: 3.5;
TNFAIP3	chr6	6q23.3	1	0	0	1	Amplification	1	0	42493T: 3.5;
TNFAIP8L2	chr1	1q21.3	1	1	0	0	Amplification	1	0	42473T: 4.5;
TNFRSF11B	chr8	8q24.12	3	1	0	2	Amplification	3	0	42496T: 3.5; 42475T: 3.5; 42495T: 3.5;
TNFRSF17	chr16	16p13.13	2	1	0	1	Amplification	2	0	42495T: 4; 42473T: 5.5;
TNFRSF6B	chr20	20q13.33	1	1	0	0	Amplification	1	0	42473T: 6;
TNFSF10	chr3	3q26.31	9	1	3	5	Amplification	9	0	42484T: 4; 56957T: 4; 42474T: 3.5; 42495T: 4; 42500T: 9; 42492T: 3.5; 42487T: 3.5; 42493T: 3.5; 42482T: 3.5;
TNFSF15	chr9	9q32	1	0	0	1	Amplification	1	0	42493T: 3.5;
TNFSF8	chr9	9q32-q33.1	1	0	0	1	Amplification	1	0	42493T: 3.5;
TNIK	chr3	3q26.2-q26.3	9	1	3	5	Amplification	9	0	42500T: 8; 42474T: 3.5; 42495T: 4; 56957T: 4; 42484T: 4; 42482T: 3.5; 42493T: 3.5; 42487T: 3.5; 42492T: 3.5;
TNK2	chr3	3q29	9	1	3	5	Amplification	9	0	42492T: 3.5; 42493T: 3.5; 42487T: 3.5; 42482T: 3.5; 42484T: 4; 42498T: 3.5; 42495T: 4; 42474T: 3.5; 56957T: 6;
TNKS	chr8	8p23.1	1	0	1	0	Amplification	1	0	42486T: 3.5;
TNNC2	chr20	20q13.12	1	1	0	0	Amplification	1	0	42473T: 5;
TNP2	chr16	16p13.13	2	1	0	1	Amplification	2	0	42495T: 5; 42473T: 5.5;
TNPO3	chr7	7q32.1	1	0	1	0	Amplification	1	0	42487T: 3.5;
TNRC18	chr7	7p22.1	2	1	0	1	Amplification	2	0	42497T: 8.5; 42473T: 4.5;
TNRC6A	chr16	16p12.1	1	1	0	0	Amplification	1	0	42473T: 4;
TNRC6B	chr22	22q13.1	1	1	0	0	Amplification	1	0	42473T: 3.5;
TNXB	chr6	6p21.33-p21	1	1	0	0	Amplification	1	0	42473T: 3.5;
TOB1	chr17	17q21.33	1	1	0	0	Amplification	1	0	42473T: 4.5;
TOLLIP	chr11	11p15.5	1	1	0	0	Amplification	1	0	42473T: 4;
TOM1	chr22	22q12.3	1	1	0	0	Amplification	1	0	42473T: 3.5;
TOM1L1	chr17	17q22	1	0	0	1	Amplification	1	0	42497T: 3.5;
TOMM20	chr1	1q42.3	1	1	0	0	Amplification	1	0	42473T: 6.5;
TOMM20L	chr14	14q23.1	1	0	0	1	Amplification	1	0	42494T: 4;

Mangalaparthi *et al.* , 2020. Mutational landscape of esophageal squamous cell carcinoma in an Indian cohort
Supplementary Table 7A. List of copy number alterations and affected genes in ESCC patients

Gene	Chromosome	Cytoband	Recurrence	Recurrence in smoker cohort	Recurrence in chewer cohort	Recurrence in No habit cohort	State	Samples with CNA gain	Samples with CNA loss	File info with CNA fold
TOMM22	chr22	22q13.1	1	1	0	0	Amplification	1	0	42473T: 3.5;
TOMM34	chr20	20q13.12	1	1	0	0	Amplification	1	0	42473T: 5;
TOMM40	chr19	19q13.32	2	1	0	1	Amplification	2	0	56957T: 4; 42473T: 3.5;
TOMM7	chr7	7p15.3	1	1	0	0	Amplification	1	0	42473T: 4;
TOMM70A	chr3	3q12.2	1	1	0	0	Amplification	1	0	42476T: 3.5;
TONSL	chr8	8q24.3	4	0	1	3	Amplification	4	0	42483T: 3.5; 42496T: 4; 42495T: 4.5; 56957T: 3.5;
TOP1	chr20	20q12	1	1	0	0	Amplification	1	0	42473T: 3.5;
TOP1MT	chr8	8q24.3	3	0	1	2	Amplification	3	0	42495T: 4.5; 42496T: 4; 42483T: 3.5;
TOPBP1	chr3	3q22.1	3	0	1	2	Amplification	3	0	42492T: 3.5; 42487T: 3.5; 42496T: 3.5;
TOR4A	chr9	9q34.3	2	1	0	1	Amplification	2	0	56957T: 3.5; 42473T: 5;
TOX	chr8	8q12.1	2	0	0	2	Amplification	2	0	42495T: 3.5; 42496T: 3.5;
TOX2	chr20	20q13.12	1	1	0	0	Amplification	1	0	42473T: 3.5;
TOX4	chr14	14q11.2	1	0	0	1	Amplification	1	0	42496T: 5;
TP53I11	chr11	11p11.2	1	1	0	0	Amplification	1	0	42473T: 3.5;
TP53I3	chr2	2p23.3	1	0	0	1	Amplification	1	0	42500T: 3.5;
TP53INP1	chr8	8q22.1	3	1	0	2	Amplification	3	0	42495T: 3.5; 42496T: 3.5; 42473T: 4.5;
TP53INP2	chr20	20q11.22	2	1	0	1	Amplification	2	0	42473T: 3.5; 42493T: 3.5;
TP53RK	chr20	20q13.12	1	1	0	0	Amplification	1	0	42473T: 5;
TP53TG5	chr20	20q13.12	1	1	0	0	Amplification	1	0	42473T: 5;
TP63	chr3	3q28	10	1	3	6	Amplification	10	0	42487T: 3.5; 42493T: 3.5; 42492T: 3.5; 42482T: 3.5; 56957T: 4.5; 42495T: 4; 42474T: 3.5; 42498T: 3.5; 42484T: 4; 42497T: 5;
TPCN1	chr12	12q24.13	1	0	0	1	Amplification	1	0	42500T: 3.5;
TPCN2	chr11	11q13.3	7	1	1	5	Amplification	7	0	42501T: 3.5; 42483T: 14.5; 42497T: 4.5; 42476T: 29.5; 56957T: 5; 42492T: 4; 42498T: 9.5;
TPD52	chr8	8q21.13	2	0	0	2	Amplification	2	0	42495T: 3.5; 42496T: 3.5;
TPD52L2	chr20	20q13.33	1	1	0	0	Amplification	1	0	42473T: 6;
TPD52L3	chr9	9p24.1	1	0	0	1	Amplification	1	0	42496T: 3.5;
TPH2	chr12	12q21.1	1	0	0	1	Amplification	1	0	42501T: 6.5;
TPI1	chr12	12p13.31	1	0	0	1	Amplification	1	0	42494T: 3.5;
TPK1	chr7	7q35	1	0	1	0	Amplification	1	0	42487T: 3.5;
TPM2	chr9	9p13.3	2	1	0	1	Amplification	2	0	42473T: 3.5; 42501T: 3.5;
TPM3	chr1	1q21.3	1	1	0	0	Amplification	1	0	42473T: 4.5;
TPO	chr2	2p25.3	1	0	0	1	Amplification	1	0	42500T: 3.5;
TPPP	chr5	5p15.33	5	2	1	2	Amplification	5	0	42493T: 3.5; 42475T: 3.5; 42473T: 3.5; 42496T: 4; 42486T: 3.5;
TPRA1	chr3	3q21.3	2	0	1	1	Amplification	2	0	42496T: 3.5; 42487T: 3.5;
TPRG1	chr3	3q28	10	1	3	6	Amplification	10	0	42484T: 4; 42498T: 3.5; 42474T: 3.5; 42495T: 4; 56957T: 4.5; 42497T: 5; 42492T: 3.5; 42487T: 3.5; 42493T: 3.5; 42482T: 3.5;
TPRKB	chr2	2p13.1	1	0	0	1	Amplification	1	0	42500T: 3.5;

Mangalaparthi *et al.* , 2020. Mutational landscape of esophageal squamous cell carcinoma in an Indian cohort
Supplementary Table 7A. List of copy number alterations and affected genes in ESCC patients

Gene	Chromosome	Cytoband	Recurrence	Recurrence in smoker cohort	Recurrence in chewer cohort	Recurrence in No habit cohort	State	Samples with CNA gain	Samples with CNA loss	File info with CNA fold
TPRN	chr9	9q34.3	2	1	0	1	Amplification	2	0	42473T: 5; 56957T: 3.5;
TPSAB1	chr16	16p13.3	1	0	1	0	Amplification	1	0	42483T: 3.5;
TPSB2	chr16	16p13.3	1	0	1	0	Amplification	1	0	42483T: 3.5;
TPSD1	chr16	16p13.3	1	0	1	0	Amplification	1	0	42483T: 3.5;
TPSG1	chr16	16p13.3	1	0	1	0	Amplification	1	0	42483T: 3.5;
TPST1	chr7	7q11.21	1	0	0	1	Amplification	1	0	42501T: 3.5;
TPX2	chr20	20q11.21	1	0	0	1	Amplification	1	0	42496T: 5;
TRA2A	chr7	7p15.3	1	1	0	0	Amplification	1	0	42473T: 4;
TRA2B	chr3	3q27.2	10	1	3	6	Amplification	10	0	42482T: 3.5; 42487T: 3.5; 42493T: 3.5; 42492T: 3.5; 42497T: 4; 56957T: 4.5; 42474T: 3.5; 42495T: 4; 42498T: 3.5; 42484T: 4;
TRABD2A	chr2	2p11.2	1	0	0	1	Amplification	1	0	42500T: 3.5;
TRAF2	chr9	2q24.2	2	1	0	1	Amplification	2	0	42473T: 5; 56957T: 3.5;
TRAF3IP1	chr2	2q37.3	1	1	0	0	Amplification	1	0	42473T: 3.5;
TRAF3IP2	chr6	6q21	1	0	0	1	Amplification	1	0	42496T: 3.5;
TRAF7	chr16	16p13.3	1	0	1	0	Amplification	1	0	42483T: 3.5;
TRAFD1	chr12	12q24.13	1	0	0	1	Amplification	1	0	42500T: 3.5;
TRAM1	chr8	8q13.3	2	0	0	2	Amplification	2	0	42496T: 3.5; 42495T: 3.5;
TRAP1	chr16	16p13.3	1	1	0	0	Amplification	1	0	42473T: 5.5;
TRAPPC12	chr2	2p25.3	2	0	0	2	Amplification	2	0	42495T: 3.5; 42500T: 3.5;
TRAPPC4	chr11	11q23.3	1	1	0	0	Amplification	1	0	42473T: 4;
TRAPPC6A	chr19	19q13.32	2	1	0	1	Amplification	2	0	56957T: 4; 42473T: 3.5;
TRAPPC6B	chr14	14q21.1	2	1	0	1	Amplification	2	0	42500T: 6.5; 42473T: 3.5;
TRAPPC8	chr18	18q12.1	1	0	0	1	Amplification	1	0	42501T: 5.5;
TRAPPC9	chr8	8q24.3	2	0	0	2	Amplification	2	0	42496T: 3.5; 42495T: 3.5;
TRDMT1	chr10	10p13	1	0	0	1	Amplification	1	0	56958T: 4;
TRH	chr3	3q22.1	2	0	1	1	Amplification	2	0	42496T: 3.5; 42487T: 3.5;
TRHDE	chr12	12q21.1	1	0	0	1	Amplification	1	0	42501T: 4;
TRHR	chr8	8q23.1	2	0	0	2	Amplification	2	0	42495T: 3.5; 42496T: 3.5;
TRIAP1	chr12	12q24.31	1	0	0	1	Amplification	1	0	42500T: 3.5;
TRIB1	chr8	8q24.13	4	1	1	2	Amplification	4	0	42475T: 3.5; 42496T: 3.5; 42495T: 3.5; 42484T: 3.5;
TRIB2	chr2	2p24.3	1	0	0	1	Amplification	1	0	42500T: 3.5;
TRIB3	chr20	20p13	1	1	0	0	Amplification	1	0	42473T: 3.5;
TRIL	chr7	7p14.3	1	1	0	0	Amplification	1	0	42473T: 4;
TRIM24	chr7	7q33-q34	1	0	1	0	Amplification	1	0	42487T: 3.5;
TRIM25	chr17	17q22	1	0	0	1	Amplification	1	0	42497T: 4;
TRIM41	chr5	5q35.3	1	0	0	1	Amplification	1	0	42495T: 3.5;
TRIM42	chr3	3q23	4	0	1	3	Amplification	4	0	42492T: 3.5; 42493T: 3.5; 42487T: 3.5; 42496T: 4;
TRIM43	chr2	2q11.1	2	1	0	1	Amplification	2	0	42473T: 3.5; 42493T: 4;
TRIM46	chr1	1q22	1	1	0	0	Amplification	1	0	42473T: 6.5;
TRIM47	chr17	17q25.1	1	0	0	1	Amplification	1	0	42494T: 3.5;

Mangalaparthi *et al.* , 2020. Mutational landscape of esophageal squamous cell carcinoma in an Indian cohort
Supplementary Table 7A. List of copy number alterations and affected genes in ESCC patients

Gene	Chromosome	Cytoband	Recurrence	Recurrence in smoker cohort	Recurrence in chewer cohort	Recurrence in No habit cohort	State	Samples with CNA gain	Samples with CNA loss	File info with CNA fold
TRIM48	chr11	11q11	1	0	0	1	Amplification	1	0	42496T: 8.5;
TRIM49B	chr11	11p11.12	1	0	0	1	Amplification	1	0	56957T: 8;
TRIM51	chr11	11q12.1	1	0	0	1	Amplification	1	0	42496T: 8.5;
TRIM52	chr5	5q35.3	1	0	0	1	Amplification	1	0	42495T: 3.5;
TRIM54	chr2	2p23.3	1	0	0	1	Amplification	1	0	42500T: 3.5;
TRIM55	chr8	8q13.1	3	0	0	3	Amplification	3	0	42497T: 4; 42496T: 3.5; 42495T: 3.5;
TRIM59	chr3	3q25.33	8	1	2	5	Amplification	8	0	42493T: 3.5; 42487T: 3.5; 42492T: 3.5; 56957T: 4; 42474T: 3.5; 42495T: 4; 42484T: 4; 42497T: 3.5;
TRIM64C	chr11	11p11.12	1	0	0	1	Amplification	1	0	56957T: 8;
TRIM65	chr17	17q25.1	1	0	0	1	Amplification	1	0	42494T: 3.5;
TRIM7	chr5	5q35.3	1	0	0	1	Amplification	1	0	42495T: 3.5;
TRIM9	chr14	14q22.1	1	0	0	1	Amplification	1	0	42494T: 4;
TRIO	chr5	5p15.2	5	1	2	2	Amplification	5	0	42493T: 4; 42484T: 3.5; 42475T: 3.5; 42496T: 3.5; 42486T: 3.5;
TRIOBP	chr22	22q13.1	1	1	0	0	Amplification	1	0	42473T: 3.5;
TRIP13	chr5	5p15.33	5	2	1	2	Amplification	5	0	42486T: 3.5; 42496T: 4; 42473T: 3.5; 42475T: 3.5; 42493T: 3.5;
TRIP6	chr7	7q22.1	1	0	0	1	Amplification	1	0	42501T: 3.5;
TRIQK	chr8	8q22.1	2	0	0	2	Amplification	2	0	42495T: 3.5; 42496T: 3.5;
TRMT10C	chr3	3q12.3	1	1	0	0	Amplification	1	0	42476T: 3.5;
TRMT5	chr14	14q23.1	1	0	0	1	Amplification	1	0	42494T: 4;
TRMT61B	chr2	2p23.2	1	0	0	1	Amplification	1	0	42500T: 3.5;
TRNAU1AP	chr1	1p35.3	1	1	0	0	Amplification	1	0	42473T: 3.5;
TROAP	chr12	12q13.12	1	0	0	1	Amplification	1	0	42500T: 3.5;
TRPA1	chr8	8q21.11	2	0	0	2	Amplification	2	0	42495T: 3.5; 42496T: 3.5;
TRPC1	chr3	3q23	5	0	1	4	Amplification	5	0	42496T: 4; 42492T: 3.5; 56957T: 4; 42493T: 3.5; 42487T: 3.5;
TRPC4AP	chr20	20q11.22	2	1	0	1	Amplification	2	0	42473T: 3.5; 42493T: 3.5;
TRPC6	chr11	11q22.1	1	0	0	1	Amplification	1	0	56958T: 5.5;
TRPS1	chr8	8q23.3	3	1	0	2	Amplification	3	0	42495T: 3.5; 42475T: 3.5; 42496T: 3.5;
TRPV4	chr12	12q24.11	2	1	0	1	Amplification	2	0	42473T: 4.5; 42500T: 3.5;
TRPV5	chr7	7q34	1	0	1	0	Amplification	1	0	42487T: 3.5;
TRPV6	chr7	7q34	1	0	1	0	Amplification	1	0	42487T: 3.5;
TSACC	chr1	1q22	1	1	0	0	Amplification	1	0	42473T: 4.5;
TSC2	chr16	16p13.3	1	0	1	0	Amplification	1	0	42483T: 3.5;
TSC22D2	chr3	3q25.1	9	2	2	5	Amplification	9	0	42492T: 3.5; 42487T: 3.5; 42493T: 3.5; 42484T: 3.5; 42474T: 3.5; 56957T: 4; 42473T: 4; 42496T: 4; 42497T: 4.5;
TSEN54	chr17	17q25.1	1	1	0	0	Amplification	1	0	42473T: 4;
TSGA10	chr2	2q11.2	1	0	0	1	Amplification	1	0	42493T: 3.5;
TSGA13	chr7	7q32.2	1	0	1	0	Amplification	1	0	42487T: 3.5;

Mangalaparthi *et al.* , 2020. Mutational landscape of esophageal squamous cell carcinoma in an Indian cohort
Supplementary Table 7A. List of copy number alterations and affected genes in ESCC patients

Gene	Chromosome	Cytoband	Recurrence	Recurrence in smoker cohort	Recurrence in chewer cohort	Recurrence in No habit cohort	State	Samples with CNA gain	Samples with CNA loss	File info with CNA fold
TSHZ2	chr20	20q13.2	1	1	0	0	Amplification	1	0	42473T: 5;
TSHZ3	chr19	19q12	5	1	1	3	Amplification	5	0	42500T: 4.5; 42473T: 25; 56957T: 4; 42494T: 4; 42484T: 6;
TSKU	chr11	11q13.5	2	0	0	2	Amplification	2	0	42496T: 3.5; 42497T: 3.5;
TSNARE1	chr8	8q24.3	3	0	1	2	Amplification	3	0	42483T: 3.5; 42496T: 4; 42495T: 4.5;
TSPAN11	chr12	12p11.21	1	0	0	1	Amplification	1	0	42500T: 8;
TSPAN12	chr7	7q31.31	1	0	1	0	Amplification	1	0	42487T: 3.5;
TSPAN13	chr7	7p21.1	1	1	0	0	Amplification	1	0	42473T: 7.5;
TSPAN18	chr11	11p11.2	1	1	0	0	Amplification	1	0	42473T: 3.5;
TSPAN33	chr7	7q32.1	1	0	1	0	Amplification	1	0	42487T: 3.5;
TSPAN8	chr12	12q21.1	1	0	0	1	Amplification	1	0	42501T: 5.5;
TSPY26P	chr20	20q11.21	2	1	0	1	Amplification	2	0	42473T: 4.5; 42496T: 5;
TSPYL6	chr2	2p16.2	2	0	1	1	Amplification	2	0	42500T: 3.5; 42484T: 4.5;
TSR3	chr16	16p13.3	1	0	1	0	Amplification	1	0	42483T: 3.5;
TSSC1	chr2	2p25.3	1	0	0	1	Amplification	1	0	42500T: 3.5;
TSSK2	chr22	22q11.21	1	0	0	1	Amplification	1	0	42497T: 21;
TSSK3	chr1	1p35.1	1	1	0	0	Amplification	1	0	42473T: 4;
TST	chr22	1q23.3	1	1	0	0	Amplification	1	0	42473T: 3.5;
TSTA3	chr8	8q24.3	3	0	1	2	Amplification	3	0	42495T: 4.5; 42496T: 4; 42483T: 3.5;
TTC14	chr3	3q26.33	11	1	3	7	Amplification	11	0	42484T: 4; 42498T: 3.5; 42474T: 3.5; 42495T: 4; 56957T: 4; 42497T: 4; 42500T: 14; 42492T: 3.5; 42493T: 3.5; 42487T: 3.5; 42482T: 3.5;
TTC18	chr10	10q22.2	1	0	0	1	Amplification	1	0	42496T: 3.5;
TTC23L	chr5	5p13.2	4	1	1	2	Amplification	4	0	42493T: 3.5; 42496T: 3.5; 42486T: 3.5; 42475T: 3.5;
TTC24	chr1	1q22	1	1	0	0	Amplification	1	0	42473T: 7;
TTC26	chr7	7q34	1	0	1	0	Amplification	1	0	42487T: 3.5;
TTC27	chr2	2p22.3	1	0	0	1	Amplification	1	0	42500T: 3.5;
TTC30A	chr2	2q31.2	1	0	0	1	Amplification	1	0	42493T: 4.5;
TTC30B	chr2	2q31.2	1	0	0	1	Amplification	1	0	42493T: 4.5;
TTC31	chr2	2p13.1	1	0	0	1	Amplification	1	0	42500T: 3.5;
TTC32	chr2	2p24.1	1	0	0	1	Amplification	1	0	42500T: 3.5;
TTC33	chr5	5p13.1	6	1	3	2	Amplification	6	0	42493T: 3.5; 42484T: 3.5; 42475T: 3.5; 42496T: 3.5; 42486T: 3.5; 42483T: 3.5;
TTC39C	chr18	18q11.2	1	1	0	0	Amplification	1	0	42481T: 4;
TTC6	chr14	14q21.1	2	1	0	1	Amplification	2	0	42500T: 6.5; 42476T: 4;
TTC7A	chr2	2p21	2	0	1	1	Amplification	2	0	42484T: 4.5; 42500T: 3.5;
TTC9	chr14	14q24.2	1	0	0	1	Amplification	1	0	42494T: 4;
TTC9B	chr19	19q13.2	2	0	0	2	Amplification	2	0	42500T: 7.5; 56957T: 4;
TTI1	chr20	20q11.23	1	1	0	0	Amplification	1	0	42473T: 3.5;
TTI2	chr8	8p12	2	0	1	1	Amplification	2	0	42497T: 4.5; 42482T: 3.5;
TTLL13	chr15	15q26.1	1	1	0	0	Amplification	1	0	42473T: 3.5;

Mangalaparthi *et al.* , 2020. Mutational landscape of esophageal squamous cell carcinoma in an Indian cohort
Supplementary Table 7A. List of copy number alterations and affected genes in ESCC patients

Gene	Chromosome	Cytoband	Recurrence	Recurrence in smoker cohort	Recurrence in chewer cohort	Recurrence in No habit cohort	State	Samples with CNA gain	Samples with CNA loss	File info with CNA fold
TTLL5	chr14	1p36.33	2	0	0	2	Amplification	2	0	42494T: 4; 56957T: 3.5;
TTLL9	chr20	20q11.21	1	0	0	1	Amplification	1	0	42496T: 5;
TTN	chr2	2q31.2	1	0	0	1	Amplification	1	0	42493T: 3.5;
TTPA	chr8	8q12.3	2	0	0	2	Amplification	2	0	42496T: 3.5; 42495T: 3.5;
TTPAL	chr20	20q13.12	1	1	0	0	Amplification	1	0	42473T: 3.5;
TTYH3	chr7	7p22.3	1	1	0	0	Amplification	1	0	42473T: 5;
TUBA1A	chr12	12q13.12	1	0	0	1	Amplification	1	0	42500T: 3.5;
TUBA1B	chr12	12q13.12	1	0	0	1	Amplification	1	0	42500T: 3.5;
TUBA1C	chr12	12q13.12	1	0	0	1	Amplification	1	0	42500T: 3.5;
TUBA8	chr22	22q11.21	1	0	0	1	Amplification	1	0	42497T: 24.5;
TUBB1	chr20	6p21.33	1	1	0	0	Amplification	1	0	42473T: 4.5;
TUBB4B	chr9	9q34.3	2	1	0	1	Amplification	2	0	56957T: 3.5; 42473T: 5;
TUBB6	chr18	18p11.21	1	0	0	1	Amplification	1	0	42493T: 3.5;
TUBBP1	chr8	8p12	1	0	1	0	Amplification	1	0	42482T: 3.5;
TUBE1	chr6	6q21	1	0	0	1	Amplification	1	0	42496T: 3.5;
TUFT1	chr1	1q21.3	1	1	0	0	Amplification	1	0	42473T: 4.5;
TVP23A	chr16	16p13.13	2	1	0	1	Amplification	2	0	42473T: 5.5; 42495T: 5;
TWF1	chr12	12q12	1	0	0	1	Amplification	1	0	42500T: 3.5;
TWIST1	chr7	7p21.1	1	1	0	0	Amplification	1	0	42473T: 4;
TWISTNB	chr7	7p21.1	1	1	0	0	Amplification	1	0	42473T: 4;
TXLNA	chr1	1p35.2	1	1	0	0	Amplification	1	0	42473T: 4;
TXN2	chr22	22q12.3	1	1	0	0	Amplification	1	0	42473T: 3.5;
TXNDC11	chr16	16p13.13	2	1	0	1	Amplification	2	0	42495T: 4; 42473T: 12.5;
TXNDC15	chr5	5q31.1	1	1	0	0	Amplification	1	0	42473T: 3.5;
TXNDC16	chr14	14q22.1	1	0	0	1	Amplification	1	0	42494T: 4;
TXNDC9	chr2	2q11.2	1	0	0	1	Amplification	1	0	42493T: 3.5;
TXNIP	chr1	1q21.1	1	1	0	0	Amplification	1	0	42473T: 4;
TXNRD2	chr22	22q11.21	1	0	0	1	Amplification	1	0	42497T: 17;
TXNRD3	chr3	3q21.3	1	0	0	1	Amplification	1	0	42496T: 3.5;
TXNRD3NB	chr3	3q21.3	1	0	0	1	Amplification	1	0	42496T: 3.5;
TYMS	chr18	18p11.32	3	0	0	3	Amplification	3	0	42493T: 3.5; 56957T: 8; 42500T: 4.5;
TYROBP	chr19	19q13.12	3	0	1	2	Amplification	3	0	42500T: 6.5; 42484T: 3.5; 56957T: 4;
TYW1	chr7	7q11.21	1	0	0	1	Amplification	1	0	42501T: 3.5;
U2AF1L4	chr19	19q13.12	3	0	1	2	Amplification	3	0	56957T: 4; 42484T: 3.5; 42500T: 6.5;
U2AF2	chr19	19q13.42	1	0	0	1	Amplification	1	0	42494T: 3.5;
U2SURP	chr3	3q23	5	0	1	4	Amplification	5	0	42496T: 4; 42492T: 3.5; 42493T: 3.5; 56957T: 4; 42487T: 3.5;
UAP1L1	chr9	9q34.3	2	1	0	1	Amplification	2	0	56957T: 3.5; 42473T: 5;
UBA2	chr19	19q13.11	3	0	1	2	Amplification	3	0	42500T: 4.5; 42484T: 3.5; 56957T: 4;
UBA5	chr3	3q22.1	3	0	1	2	Amplification	3	0	42492T: 3.5; 42487T: 3.5; 42496T: 3.5;
UBAC1	chr9	9q34.3	1	0	0	1	Amplification	1	0	56957T: 3.5;

Mangalaparthi *et al.* , 2020. Mutational landscape of esophageal squamous cell carcinoma in an Indian cohort
Supplementary Table 7A. List of copy number alterations and affected genes in ESCC patients

Gene	Chromosome	Cytoband	Recurrence	Recurrence in smoker cohort	Recurrence in chewer cohort	Recurrence in No habit cohort	State	Samples with CNA gain	Samples with CNA loss	File info with CNA fold
UBALD1	chr16	16p13.3	3	1	0	2	Amplification	3	0	42473T: 5.5; 42494T: 3.5; 42495T: 12;
UBAP2L	chr1	1q21.3	1	1	0	0	Amplification	1	0	42473T: 4.5;
UBASH3A	chr21	21q22.3	1	1	0	0	Amplification	1	0	42473T: 3.5;
UBC	chr12	12q24.31	1	0	0	1	Amplification	1	0	42500T: 3.5;
UBE2B	chr5	5q31.1	1	1	0	0	Amplification	1	0	42473T: 3.5;
UBE2C	chr20	20q13.12	1	1	0	0	Amplification	1	0	42473T: 5;
UBE2D4	chr7	7p13	2	1	0	1	Amplification	2	0	42473T: 3.5; 42497T: 7.5;
UBE2E3	chr2	2q31.3	1	0	0	1	Amplification	1	0	42493T: 3.5;
UBE2H	chr7	7q32.2	1	0	1	0	Amplification	1	0	42487T: 3.5;
UBE2I	chr16	16p13.3	1	0	1	0	Amplification	1	0	42483T: 3.5;
UBE2L3	chr22	22q11.21	1	1	0	0	Amplification	1	0	42473T: 3.5;
UBE2L6	chr11	11q12.1	1	0	0	1	Amplification	1	0	42496T: 4.5;
UBE2Q1	chr1	1q21.3	1	1	0	0	Amplification	1	0	42473T: 4.5;
UBE2QL1	chr5	5p15.31	4	1	1	2	Amplification	4	0	42493T: 4; 42475T: 3.5; 42486T: 3.5; 42496T: 3.5;
UBE2R2	chr9	9p13.3	1	0	0	1	Amplification	1	0	42496T: 4;
UBE2S	chr19	19q13.42	1	0	0	1	Amplification	1	0	42494T: 3.5;
UBE2V1	chr20	20q13.13	1	1	0	0	Amplification	1	0	42473T: 6.5;
UBE2V2	chr8	8q11.21	5	0	2	3	Amplification	5	0	42482T: 3.5; 42496T: 3.5; 42484T: 3.5; 42495T: 3.5; 42494T: 3.5;
UBE2W	chr8	8q21.11	2	0	0	2	Amplification	2	0	42495T: 3.5; 42496T: 3.5;
UBE3B	chr12	12q24.11	2	1	0	1	Amplification	2	0	42500T: 3.5; 42473T: 4.5;
UBFD1	chr16	16p12.2	1	1	0	0	Amplification	1	0	42473T: 4;
UBL7	chr15	15q24.1	1	1	0	0	Amplification	1	0	42473T: 3.5;
UBN1	chr16	16p13.3	2	1	0	1	Amplification	2	0	42473T: 5.5; 42495T: 12;
UBN2	chr7	7q34	1	0	1	0	Amplification	1	0	42487T: 3.5;
UBOX5	chr20	20p13	1	1	0	0	Amplification	1	0	42473T: 3.5;
UBQLN4	chr1	1q22	2	1	0	1	Amplification	2	0	42473T: 4.5; 42493T: 4;
UBR4	chr1	1p36.13	1	1	0	0	Amplification	1	0	42473T: 4;
UBR5	chr8	8q22.3	2	0	0	2	Amplification	2	0	42495T: 3.5; 42496T: 3.5;
UBXN10	chr1	1p36.12	1	1	0	0	Amplification	1	0	42473T: 4;
UBXN2A	chr2	2p23.3	1	0	0	1	Amplification	1	0	42500T: 3.5;
UBXN2B	chr8	8q12.1	2	0	0	2	Amplification	2	0	42495T: 3.5; 42496T: 3.5;
UBXN7	chr3	3q29	9	1	3	5	Amplification	9	0	42498T: 3.5; 42484T: 4; 56957T: 6; 42474T: 3.5; 42495T: 4; 42482T: 3.5; 42492T: 3.5; 42487T: 3.5; 42493T: 5;
UBXN8	chr8	8p12	1	0	1	0	Amplification	1	0	42482T: 3.5;
UCK1	chr9	9q34.13	1	0	1	0	Amplification	1	0	42484T: 3.5;
UCKL1	chr20	20q13.33	2	1	1	0	Amplification	2	0	42473T: 6; 42483T: 3.5;
UCN	chr2	2p23.3	1	0	0	1	Amplification	1	0	42500T: 3.5;
UCP2	chr11	11q13.4	6	3	0	3	Amplification	6	0	42478T: 6; 42492T: 4; 42498T: 8; 56957T: 3.5; 42476T: 4; 42475T: 7.5;

Mangalaparthi *et al.* , 2020. Mutational landscape of esophageal squamous cell carcinoma in an Indian cohort
Supplementary Table 7A. List of copy number alterations and affected genes in ESCC patients

Gene	Chromosome	Cytoband	Recurrence	Recurrence in smoker cohort	Recurrence in chewer cohort	Recurrence in No habit cohort	State	Samples with CNA gain	Samples with CNA loss	File info with CNA fold
UCP3	chr11	11q13.4	6	3	0	3	Amplification	6	0	42476T: 4; 42475T: 7.5; 42478T: 6; 42498T: 8; 42492T: 4; 56957T: 3.5;
UFD1L	chr22	22q11.21	1	0	0	1	Amplification	1	0	42497T: 17;
UFSP1	chr7	7q22.1	1	0	0	1	Amplification	1	0	42501T: 3.5;
UGP2	chr2	2p15	2	0	0	2	Amplification	2	0	56957T: 3.5; 42500T: 3.5;
UGT3A1	chr5	5p13.2	4	1	1	2	Amplification	4	0	42496T: 3.5; 42486T: 3.5; 42475T: 3.5; 42493T: 3.5;
UGT3A2	chr5	5p13.2	4	1	1	2	Amplification	4	0	42493T: 3.5; 42475T: 3.5; 42486T: 3.5; 42496T: 3.5;
UHRF2	chr9	9p24.1	1	0	0	1	Amplification	1	0	42496T: 3.5;
ULK3	chr15	15q24.1	1	1	0	0	Amplification	1	0	42473T: 3.5;
UMOD	chr16	16p12.3	1	1	0	0	Amplification	1	0	42473T: 4;
UMODL1	chr21	21q22.3	1	1	0	0	Amplification	1	0	42473T: 3.5;
UMPS	chr3	3q21.2	1	0	0	1	Amplification	1	0	42496T: 3.5;
UNC119B	chr12	12q24.31	1	0	0	1	Amplification	1	0	42500T: 3.5;
UNC13B	chr9	9p13.3	2	1	0	1	Amplification	2	0	42473T: 3.5; 42501T: 3.5;
UNC13D	chr17	17q25.1	1	0	0	1	Amplification	1	0	42494T: 3.5;
UNC50	chr2	2q11.2	1	0	0	1	Amplification	1	0	42493T: 3.5;
UNC5D	chr8	8p12	1	0	1	0	Amplification	1	0	42482T: 3.5;
UNC93B1	chr11	11q13.2	1	0	0	1	Amplification	1	0	56957T: 5;
UNCX	chr7	7p22.3	1	1	0	0	Amplification	1	0	42473T: 5;
UNG	chr12	12q24.11	2	1	0	1	Amplification	2	0	42473T: 4.5; 42500T: 3.5;
UNK	chr17	17q25.1	1	0	0	1	Amplification	1	0	42494T: 3.5;
UNKL	chr16	16p13.3	1	0	1	0	Amplification	1	0	42483T: 3.5;
UPF3A	chr13	13q34	2	0	0	2	Amplification	2	0	56957T: 3.5; 42496T: 4.5;
UPK1A	chr19	19q13.12	3	0	1	2	Amplification	3	0	42484T: 3.5; 56957T: 4; 42500T: 6.5;
UPK1B	chr3	3q13.32	1	0	0	1	Amplification	1	0	42496T: 3.5;
UPK2	chr11	11q23.3	1	1	0	0	Amplification	1	0	42473T: 4;
UPK3B	chr7	7q11.23	1	0	1	0	Amplification	1	0	42487T: 3.5;
UPK3BL	chr7	7q22.1	3	0	1	2	Amplification	3	0	42493T: 3.5; 42487T: 3.5; 42501T: 3.5;
UPP2	chr2	2q24.1	1	1	0	0	Amplification	1	0	42473T: 4;
UQCC	chr20	20q11.22	2	1	0	1	Amplification	2	0	42473T: 3.5; 42493T: 3.5;
UQCR10	chr22	22q12.2	1	1	0	0	Amplification	1	0	42473T: 3.5;
UQCRB	chr8	8q22.1	2	0	0	2	Amplification	2	0	42495T: 3.5; 42496T: 3.5;
UQCRC2	chr16	16p12.2	1	1	0	0	Amplification	1	0	42473T: 4;
UQCRFS1	chr19	19q12	5	1	1	3	Amplification	5	0	56957T: 4; 42494T: 4; 42484T: 6; 42500T: 4.5; 42473T: 25;
URGCP	chr7	7p13	1	1	0	0	Amplification	1	0	42473T: 3.5;
URI1	chr19	19q12	5	1	1	3	Amplification	5	0	42473T: 25; 42500T: 4.5; 42484T: 6; 42494T: 4; 56957T: 4;
UROC1	chr3	3q21.3	1	0	0	1	Amplification	1	0	42496T: 3.5;
USF2	chr19	19q13.12	3	0	1	2	Amplification	3	0	42484T: 3.5; 56957T: 4; 42500T: 6.5;

Mangalaparthi *et al.* , 2020. Mutational landscape of esophageal squamous cell carcinoma in an Indian cohort
Supplementary Table 7A. List of copy number alterations and affected genes in ESCC patients

Gene	Chromosome	Cytoband	Recurrence	Recurrence in smoker cohort	Recurrence in chewer cohort	Recurrence in No habit cohort	State	Samples with CNA gain	Samples with CNA loss	File info with CNA fold
USP13	chr3	3q26.33	11	1	3	7	Amplification	11	0	56957T: 4; 42474T: 3.5; 42495T: 4; 42498T: 3.5; 42484T: 4; 42500T: 14; 42497T: 4; 42493T: 3.5; 42487T: 3.5; 42492T: 3.5; 42482T: 3.5;
USP14	chr18	18p11.32	3	0	0	3	Amplification	3	0	42493T: 3.5; 56957T: 8; 42500T: 4.5;
USP18	chr22	22q11.21	1	0	0	1	Amplification	1	0	42497T: 24.5;
USP30	chr12	12q24.11	2	1	0	1	Amplification	2	0	42473T: 4.5; 42500T: 3.5;
USP31	chr16	1p36.12	1	1	0	0	Amplification	1	0	42473T: 4;
USP34	chr2	2p15	3	0	1	2	Amplification	3	0	56957T: 3.5; 42484T: 4.5; 42500T: 3.5;
USP39	chr2	2p11.2	2	0	1	1	Amplification	2	0	42482T: 3.5; 42500T: 3.5;
USP4	chr3	3p21.31	1	1	0	0	Amplification	1	0	42473T: 4;
USP40	chr2	2q37.1	1	1	0	0	Amplification	1	0	42473T: 4.5;
USP41	chr22	22q11.21	2	1	0	1	Amplification	2	0	42477T: 4; 42497T: 3.5;
USP42	chr7	7p22.1	1	1	0	0	Amplification	1	0	42473T: 4.5;
USP48	chr1	1p36.12	1	1	0	0	Amplification	1	0	42473T: 4;
USP54	chr10	10q22.2	1	0	0	1	Amplification	1	0	42496T: 3.5;
USP7	chr16	16p13.2	2	1	0	1	Amplification	2	0	42473T: 5.5; 42495T: 4;
USPL1	chr13	13q12.3	1	0	0	1	Amplification	1	0	42497T: 3.5;
UTP23	chr8	8q24.11	3	1	0	2	Amplification	3	0	42495T: 3.5; 42496T: 3.5; 42475T: 3.5;
UTP6	chr17	17q11.2	1	1	0	0	Amplification	1	0	42473T: 3.5;
UTS2B	chr3	3q28	9	1	3	5	Amplification	9	0	42484T: 4; 42498T: 3.5; 42474T: 3.5; 42495T: 4; 56957T: 4.5; 42492T: 3.5; 42487T: 3.5; 42493T: 3.5; 42482T: 3.5;
UVRAG	chr11	11q13.5	3	0	0	3	Amplification	3	0	42497T: 3.5; 42498T: 6.5; 42492T: 4;
VAMP5	chr2	2p11.2	2	0	1	1	Amplification	2	0	42482T: 3.5; 42500T: 3.5;
VAMP8	chr2	2p11.2	2	0	1	1	Amplification	2	0	42482T: 3.5; 42500T: 3.5;
VAPB	chr20	20q13.32	1	1	0	0	Amplification	1	0	42473T: 4.5;
VARS	chr6	6p21.33	1	1	0	0	Amplification	1	0	42473T: 3.5;
VASH1	chr14	14q24.3	2	0	0	2	Amplification	2	0	56957T: 3.5; 42494T: 4;
VASN	chr16	16p13.3	3	1	0	2	Amplification	3	0	42473T: 5.5; 42495T: 8.5; 42494T: 3.5;
VASP	chr19	19q13.32	2	1	1	0	Amplification	2	0	42473T: 3.5; 42484T: 4;
VAX2	chr2	2p13.3	1	0	0	1	Amplification	1	0	42500T: 3.5;
VCP	chr9	9p13.3	1	0	0	1	Amplification	1	0	42501T: 3.5;
VCPIP1	chr8	8q13.1	3	0	0	3	Amplification	3	0	42495T: 3.5; 42497T: 4.5; 42496T: 3.5;
VDAC1	chr5	5q31.1	1	1	0	0	Amplification	1	0	42473T: 3.5;
VDR	chr12	12q13.11	1	0	0	1	Amplification	1	0	42500T: 3.5;
VEPH1	chr3	3q25.31-q25	8	1	2	5	Amplification	8	0	42484T: 3.5; 42495T: 4; 42474T: 3.5; 56957T: 4; 42497T: 5.5; 42492T: 3.5; 42493T: 3.5; 42487T: 3.5;
VEZF1	chr17	17q22	1	0	0	1	Amplification	1	0	42497T: 4;
VGF	chr7	7q22.1	2	0	0	2	Amplification	2	0	42493T: 3.5; 42501T: 3.5;
VHLL	chr1	1q22	1	1	0	0	Amplification	1	0	42473T: 4.5;
VILL	chr3	3p22.2	1	1	0	0	Amplification	1	0	42473T: 4.5;

Mangalaparthi *et al.* , 2020. Mutational landscape of esophageal squamous cell carcinoma in an Indian cohort
Supplementary Table 7A. List of copy number alterations and affected genes in ESCC patients

Gene	Chromosome	Cytoband	Recurrence	Recurrence in smoker cohort	Recurrence in chewer cohort	Recurrence in No habit cohort	State	Samples with CNA gain	Samples with CNA loss	File info with CNA fold
VIM	chr10	10p13	1	0	0	1	Amplification	1	0	56958T: 4;
VIPAS39	chr14	14q24.3	2	0	0	2	Amplification	2	0	42494T: 4; 56957T: 3.5;
VIT	chr2	2p22.2	1	0	0	1	Amplification	1	0	42500T: 3.5;
VKORC1L1	chr7	7q11.21	1	0	0	1	Amplification	1	0	42501T: 3.5;
VLDLR	chr9	9p24.2	2	0	1	1	Amplification/Deletio	1	1	42486T: 0.5; 42496T: 3.5;
VOPP1	chr7	7p11.2	3	0	1	2	Amplification	3	0	56957T: 5; 42483T: 4; 42497T: 6.5;
VPS11	chr11	11q23.3	1	1	0	0	Amplification	1	0	42473T: 4;
VPS13B	chr8	8q22.2	2	0	0	2	Amplification	2	0	42495T: 3.5; 42496T: 3.5;
VPS16	chr20	20p13	1	1	0	0	Amplification	1	0	42473T: 3.5;
VPS18	chr15	15q15.1	1	0	0	1	Amplification	1	0	42493T: 6.5;
VPS26B	chr11	11q25	1	1	0	0	Deletion	0	1	42476T: 0.5;
VPS28	chr8	8q24.3	4	0	1	3	Amplification	4	0	42483T: 3.5; 42496T: 4; 42495T: 4.5; 56957T: 3.5;
VPS29	chr12	12q24.11	1	0	0	1	Amplification	1	0	42500T: 3.5;
VPS33A	chr12	12q24.31	1	0	0	1	Amplification	1	0	42500T: 3.5;
VPS37B	chr12	12q24.31	1	0	0	1	Amplification	1	0	42500T: 3.5;
VPS41	chr7	7p14.1	1	1	0	0	Amplification	1	0	42473T: 3.5;
VPS45	chr1	1q21.2	1	1	0	0	Amplification	1	0	42473T: 5;
VPS54	chr2	2p15-p14	2	0	0	2	Amplification	2	0	56957T: 3.5; 42500T: 3.5;
VPS72	chr1	1q21.3	1	1	0	0	Amplification	1	0	42473T: 4.5;
VPS8	chr3	3q27.2	10	1	3	6	Amplification	10	0	42484T: 4; 42498T: 3.5; 42474T: 3.5; 42495T: 4; 56957T: 4.5; 42497T: 4; 42492T: 3.5; 42493T: 3.5; 42487T: 3.5; 42482T: 3.5;
VRK2	chr2	2p16.1	3	0	1	2	Amplification	3	0	42500T: 3.5; 56957T: 3.5; 42484T: 4.5;
VRTN	chr14	14q24.3	2	0	0	2	Amplification	2	0	42494T: 4; 56957T: 3.5;
VSIG10	chr12	12q24.23	1	0	0	1	Amplification	1	0	42500T: 3.5;
VSNL1	chr2	2p24.2	1	0	0	1	Amplification	1	0	42500T: 3.5;
VSTM2A	chr7	7p11.2	2	0	0	2	Amplification	2	0	56957T: 5; 42497T: 21.5;
VSTM2B	chr19	19q12	5	1	1	3	Amplification	5	0	42484T: 6; 56957T: 4; 42494T: 4; 42473T: 25; 42500T: 4.5;
VSTM2L	chr20	20q11.23	1	1	0	0	Amplification	1	0	42473T: 3.5;
VSX2	chr14	14q24.3	2	0	0	2	Amplification	2	0	42494T: 4; 56957T: 3.5;
VTI1B	chr14	14q24.1	1	0	0	1	Amplification	1	0	42494T: 4;
VWA3A	chr16	16p12.2	1	1	0	0	Amplification	1	0	42473T: 4;
VWA3B	chr2	2q11.2	1	0	0	1	Amplification	1	0	42493T: 3.5;
VWA5B1	chr1	1p36.12	1	1	0	0	Amplification	1	0	42473T: 4;
VWA5B2	chr3	3q27.1	12	2	3	7	Amplification	12	0	42487T: 3.5; 42493T: 3.5; 42492T: 3.5; 42482T: 3.5; 42474T: 3.5; 42495T: 4; 42494T: 3.5; 56957T: 5; 42484T: 4; 42498T: 3.5; 42497T: 4; 42473T: 3.5;
VWA7	chr6	6p21.33	1	1	0	0	Amplification	1	0	42473T: 3.5;
VWC2	chr7	7p12.2	1	0	0	1	Amplification	1	0	42497T: 20.5;
VWDE	chr7	7p21.3	1	1	0	0	Amplification	1	0	42473T: 3.5;

Mangalaparthi *et al.* , 2020. Mutational landscape of esophageal squamous cell carcinoma in an Indian cohort
Supplementary Table 7A. List of copy number alterations and affected genes in ESCC patients

Gene	Chromosome	Cytoband	Recurrence	Recurrence in smoker cohort	Recurrence in chewer cohort	Recurrence in No habit cohort	State	Samples with CNA gain	Samples with CNA loss	File info with CNA fold
WASF1	chr6	6q21	1	0	0	1	Amplification	1	0	42496T: 3.5;
WASF2	chr1	1p36.11	1	1	0	0	Amplification	1	0	42473T: 5;
WASL	chr7	7q31.32	2	0	1	1	Amplification	2	0	42493T: 3.5; 42487T: 3.5;
WBP1	chr2	2p13.1	1	0	0	1	Amplification	1	0	42500T: 3.5;
WBP11	chr12	12p12.3	1	0	0	1	Amplification	1	0	42500T: 4.5;
WBP2	chr17	17q25.1	1	0	0	1	Amplification	1	0	42494T: 3.5;
WBP7	chr19	19q13.12	3	0	1	2	Amplification	3	0	42500T: 6.5; 42484T: 3.5; 56957T: 4;
WDHD1	chr14	14q22.2-q22	1	0	0	1	Amplification	1	0	42494T: 4;
WDPCP	chr2	2p15	3	0	1	2	Amplification	3	0	42500T: 3.5; 42484T: 3.5; 56957T: 3.5;
WDR18	chr19	19p13.3	1	0	0	1	Amplification	1	0	42493T: 3.5;
WDR24	chr16	16p13.3	2	0	1	1	Amplification	2	0	42493T: 3.5; 42483T: 3.5;
WDR35	chr2	2p24.1	1	0	0	1	Amplification	1	0	42500T: 3.5;
WDR4	chr21	21q22.3	1	1	0	0	Amplification	1	0	42473T: 3.5;
WDR43	chr2	2p23.2	1	0	0	1	Amplification	1	0	42500T: 3.5;
WDR49	chr3	3q26.1	9	1	3	5	Amplification	9	0	42492T: 3.5; 42493T: 3.5; 42487T: 3.5; 42482T: 3.5; 42484T: 4; 56957T: 4; 42495T: 4; 42474T: 3.5; 42500T: 8;
WDR52	chr3	3q13.2	1	0	0	1	Amplification	1	0	42496T: 3.5;
WDR53	chr3	3q29	9	1	3	5	Amplification	9	0	42493T: 5; 42487T: 3.5; 42492T: 3.5; 42482T: 3.5; 42495T: 4; 42474T: 3.5; 56957T: 6; 42484T: 4; 42498T: 3.5;
WDR54	chr2	2p13.1	1	0	0	1	Amplification	1	0	42500T: 3.5;
WDR5B	chr3	3q21.1	1	0	0	1	Amplification	1	0	42496T: 3.5;
WDR61	chr15	15q25.1	1	1	0	0	Amplification	1	0	42473T: 3.5;
WDR62	chr19	19q13.12	3	0	1	2	Amplification	3	0	42500T: 6.5; 56957T: 4; 42484T: 3.5;
WDR65	chr1	1p34.2	1	0	0	1	Amplification	1	0	42493T: 4.5;
WDR66	chr12	12q24.31	1	0	0	1	Amplification	1	0	42500T: 3.5;
WDR70	chr5	5p13.2	5	1	2	2	Amplification	5	0	42493T: 3.5; 42486T: 3.5; 42483T: 3.5; 42496T: 3.5; 42475T: 3.5;
WDR73	chr15	15q25.2	1	1	0	0	Amplification	1	0	42473T: 4;
WDR75	chr2	2q32.2	2	0	1	1	Amplification	2	0	42493T: 3.5; 42482T: 4;
WDR87	chr19	19q13.13	3	0	1	2	Amplification	3	0	56957T: 4; 42484T: 3.5; 42500T: 6.5;
WDR88	chr19	19q13.11	4	1	1	2	Amplification	4	0	42484T: 3.5; 56957T: 4; 42473T: 7; 42500T: 4.5;
WDR89	chr14	14q23.2	1	0	0	1	Amplification	1	0	42494T: 4;
WDR90	chr16	16p13.3	2	0	1	1	Amplification	2	0	42493T: 3.5; 42483T: 3.5;
WDR91	chr7	7q33	1	0	1	0	Amplification	1	0	42487T: 3.5;
WDR92	chr2	2p14	1	0	0	1	Amplification	1	0	42500T: 3.5;
WDR93	chr15	15q26.1	1	1	0	0	Amplification	1	0	42473T: 3.5;
WDYHV1	chr8	8q24.13	4	1	1	2	Amplification	4	0	42475T: 3.5; 42496T: 3.5; 42495T: 3.5; 42484T: 3.5;
WEE2	chr7	7q34	1	0	1	0	Amplification	1	0	42487T: 3.5;
WFDC10A	chr20	20q13.12	1	1	0	0	Amplification	1	0	42473T: 5;

Mangalaparthi *et al.* , 2020. Mutational landscape of esophageal squamous cell carcinoma in an Indian cohort
Supplementary Table 7A. List of copy number alterations and affected genes in ESCC patients

Gene	Chromosome	Cytoband	Recurrence	Recurrence in smoker cohort	Recurrence in chewer cohort	Recurrence in No habit cohort	State	Samples with CNA gain	Samples with CNA loss	File info with CNA fold
WFDC10B	chr20	20q13.12	1	1	0	0	Amplification	1	0	42473T: 5;
WFDC11	chr20	20q13.12	1	1	0	0	Amplification	1	0	42473T: 5;
WFDC12	chr20	20q13.12	1	1	0	0	Amplification	1	0	42473T: 5;
WFDC13	chr20	20q13.12	1	1	0	0	Amplification	1	0	42473T: 5;
WFDC2	chr20	20q13.12	1	1	0	0	Amplification	1	0	42473T: 5;
WFDC3	chr20	20q13.12	1	1	0	0	Amplification	1	0	42473T: 5;
WFDC5	chr20	20q13.12	1	1	0	0	Amplification	1	0	42473T: 5;
WFDC6	chr20	20q13.12	1	1	0	0	Amplification	1	0	42473T: 5;
WFDC8	chr20	20q13.12	1	1	0	0	Amplification	1	0	42473T: 5;
WFDC9	chr20	20q13.12	1	1	0	0	Amplification	1	0	42473T: 5;
WFIKKN1	chr16	16p13.3	2	0	1	1	Amplification	2	0	42483T: 3.5; 42493T: 3.5;
WFIKKN2	chr17	17q21.33	1	1	0	0	Amplification	1	0	42473T: 4.5;
WHSC1L1	chr8	8p11.23	2	0	1	1	Amplification	2	0	42482T: 3.5; 42493T: 3.5;
WIBG	chr12	12q13.2	1	0	0	1	Amplification	1	0	42494T: 5;
WIPF1	chr2	2q31.1	1	0	0	1	Amplification	1	0	42493T: 3.5;
WIPF2	chr17	17q21.2	1	0	0	1	Amplification	1	0	42497T: 4;
WIPF3	chr7	7p14.3	1	1	0	0	Amplification	1	0	42473T: 4;
WIPI2	chr7	7p22.1	2	1	0	1	Amplification	2	0	42473T: 4.5; 42497T: 8.5;
WISP1	chr8	8q24.22	3	0	1	2	Amplification	3	0	42484T: 3.5; 42495T: 3.5; 42496T: 3.5;
WISP2	chr20	20q13.12	1	1	0	0	Amplification	1	0	42473T: 5;
WISP3	chr6	6q21	1	0	0	1	Amplification	1	0	42496T: 3.5;
WNK1	chr12	12p13.33	1	0	0	1	Amplification	1	0	42500T: 3.5;
WNT1	chr12	12q13.12	1	0	0	1	Amplification	1	0	42500T: 3.5;
WNT10B	chr12	12q13.12	1	0	0	1	Amplification	1	0	42500T: 3.5;
WNT11	chr11	11q13.5	2	0	0	2	Amplification	2	0	42497T: 3.5; 42492T: 4;
WNT16	chr7	7q31.31	1	0	1	0	Amplification	1	0	42487T: 3.5;
WNT2	chr7	7q31.2	1	0	1	0	Amplification	1	0	42487T: 3.5;
WNT4	chr1	1p36.12	2	1	0	1	Amplification	2	0	42493T: 3.5; 42473T: 4;
WRB	chr21	21q22.2	1	1	0	0	Amplification	1	0	42473T: 3.5;
WRN	chr8	8p12	1	0	1	0	Amplification	1	0	42482T: 3.5;
WSB2	chr12	12q24.23	1	0	0	1	Amplification	1	0	42500T: 3.5;
WTIP	chr19	19q13.11	3	0	1	2	Amplification	3	0	42500T: 4.5; 56957T: 4; 42484T: 3.5;
WWP1	chr8	8q21.3	2	0	0	2	Amplification	2	0	42496T: 3.5; 42495T: 3.5;
WWTR1	chr3	3q25.1	8	1	2	5	Amplification	8	0	56957T: 4; 42474T: 3.5; 42484T: 3.5; 42497T: 4.5; 42496T: 4; 42487T: 3.5; 42493T: 3.5; 42492T: 3.5;
XDH	chr2	2p23.1	1	0	0	1	Amplification	1	0	42500T: 3.5;
XKR4	chr8	8q12.1	3	0	1	2	Amplification	3	0	42495T: 3.5; 42483T: 5; 42496T: 3.5;
XKR6	chr8	8p23.1	1	0	1	0	Amplification	1	0	42486T: 3.5;
XKR7	chr20	20q11.21	1	0	0	1	Amplification	1	0	42496T: 5;
XKR8	chr1	1p35.3	1	1	0	0	Amplification	1	0	42473T: 3.5;
XKR9	chr8	8q13.3	2	0	0	2	Amplification	2	0	42496T: 3.5; 42495T: 3.5;

Mangalaparthi *et al.* , 2020. Mutational landscape of esophageal squamous cell carcinoma in an Indian cohort
Supplementary Table 7A. List of copy number alterations and affected genes in ESCC patients

Gene	Chromosome	Cytoband	Recurrence	Recurrence in smoker cohort	Recurrence in chewer cohort	Recurrence in No habit cohort	State	Samples with CNA gain	Samples with CNA loss	File info with CNA fold
XPO1	chr2	2p15	3	0	1	2	Amplification	3	0	42500T: 3.5; 56957T: 3.5; 42484T: 4.5;
XPR1	chr1	1q25.3	1	0	0	1	Amplification	1	0	42493T: 4;
XRCC1	chr19	19q13.31	1	0	0	1	Amplification	1	0	56957T: 4;
XRN1	chr3	3q23	5	0	1	4	Amplification	5	0	42496T: 4; 42492T: 3.5; 42487T: 3.5; 56957T: 4; 42493T: 3.5;
XRN2	chr20	20p11.22	1	0	1	0	Amplification	1	0	42483T: 4;
XRRA1	chr11	11q13.4	4	1	0	3	Amplification	4	0	42475T: 7.5; 56957T: 3.5; 42498T: 8; 42492T: 4;
XXYLT1	chr3	3q29	9	1	3	5	Amplification	9	0	42493T: 3.5; 42487T: 3.5; 42492T: 3.5; 42482T: 3.5; 42474T: 3.5; 42495T: 4; 56957T: 4.5; 42484T: 4; 42498T: 3.5;
XYLT1	chr16	16p12.3	2	1	0	1	Amplification	2	0	42495T: 4; 42473T: 4;
XYLT2	chr17	17q21.33	1	1	0	0	Amplification	1	0	42473T: 3.5;
YAE1D1	chr7	7p14.1	1	1	0	0	Amplification	1	0	42473T: 3.5;
YAF2	chr12	12q12	1	0	0	1	Amplification	1	0	42500T: 3.5;
YAP1	chr11	11q22.1	1	0	0	1	Amplification	1	0	56958T: 5.5;
YARS2	chr12	12p11.21	1	0	0	1	Amplification	1	0	42500T: 3.5;
YBX1	chr1	1p34.2	1	0	0	1	Amplification	1	0	42493T: 3.5;
YDJC	chr22	22q11.21	1	1	0	0	Amplification	1	0	42473T: 3.5;
YEATS2	chr3	3q27.1	10	1	3	6	Amplification	10	0	42492T: 3.5; 42487T: 3.5; 42493T: 3.5; 42482T: 3.5; 42484T: 4; 42498T: 3.5; 42474T: 3.5; 42495T: 4; 56957T: 5; 42497T: 4;
YEATS4	chr12	12q15	2	0	0	2	Amplification	2	0	42500T: 5; 42501T: 5.5;
YES1	chr18	18p11.32	3	0	0	3	Amplification	3	0	56957T: 8; 42493T: 3.5; 42500T: 4.5;
YIF1B	chr19	19q13.2	4	1	1	2	Amplification	4	0	42500T: 6.5; 42474T: 4; 56957T: 4; 42484T: 3.5;
YIPF4	chr2	2p22.3	1	0	0	1	Amplification	1	0	42500T: 3.5;
YKT6	chr7	7p13	2	1	0	1	Amplification	2	0	42497T: 7.5; 42473T: 4.5;
YLPM1	chr14	14q24.3	2	0	0	2	Amplification	2	0	42494T: 4; 56957T: 3.5;
YPEL1	chr22	22q11.21-q1	1	1	0	0	Amplification	1	0	42473T: 3.5;
YPEL4	chr11	11q12.1	1	0	0	1	Amplification	1	0	42496T: 4.5;
YPEL5	chr2	2p23.1	1	0	0	1	Amplification	1	0	42500T: 3.5;
YTHDC2	chr5	5q22.2	1	0	0	1	Amplification	1	0	42495T: 3.5;
YTHDF1	chr20	20q13.33	1	1	0	0	Amplification	1	0	42473T: 6;
YTHDF2	chr1	1p35.3	1	1	0	0	Amplification	1	0	42473T: 3.5;
YTHDF3	chr8	8q12.3	2	0	0	2	Amplification	2	0	42496T: 3.5; 42495T: 3.5;
YWHAB	chr20	20q13.12	1	1	0	0	Amplification	1	0	42473T: 5;
YWHAG	chr7	7q11.23	1	0	0	1	Amplification	1	0	42493T: 3.5;
YWHAQ	chr2	2p25.1	1	0	0	1	Amplification	1	0	42500T: 3.5;
YWHAZ	chr8	8q22.3	2	0	0	2	Amplification	2	0	42496T: 3.5; 42495T: 3.5;
YY1AP1	chr1	1q22	1	1	0	0	Amplification	1	0	42473T: 5.5;
ZAP70	chr2	2q11.2	1	0	0	1	Amplification	1	0	42493T: 3.5;
ZAR1	chr4	4p11	1	0	1	0	Amplification	1	0	42483T: 3.5;

Mangalaparthi *et al.* , 2020. Mutational landscape of esophageal squamous cell carcinoma in an Indian cohort
Supplementary Table 7A. List of copy number alterations and affected genes in ESCC patients

Gene	Chromosome	Cytoband	Recurrence	Recurrence in smoker cohort	Recurrence in chewer cohort	Recurrence in No habit cohort	State	Samples with CNA gain	Samples with CNA loss	File info with CNA fold
ZAR1L	chr13	13q13.1	1	0	0	1	Amplification	1	0	42497T: 3.5;
ZBBX	chr3	3q26.1	9	1	3	5	Amplification	9	0	42482T: 3.5; 42487T: 3.5; 42493T: 3.5; 42492T: 3.5; 42500T: 8; 42495T: 4; 42474T: 3.5; 56957T: 4; 42484T: 4;
ZBP1	chr20	20q13.31	1	1	0	0	Amplification	1	0	42473T: 7;
ZBTB1	chr14	14q23.3	1	0	0	1	Amplification	1	0	42494T: 4;
ZBTB10	chr8	8q21.13	2	0	0	2	Amplification	2	0	42496T: 3.5; 42495T: 3.5;
ZBTB11	chr3	3q12.3	1	1	0	0	Amplification	1	0	42476T: 3.5;
ZBTB12	chr6	6p21.33	1	1	0	0	Amplification	1	0	42473T: 3.5;
ZBTB14	chr18	18p11.31	1	0	0	1	Amplification	1	0	56957T: 8;
ZBTB17	chr1	1p36.13	1	1	0	0	Amplification	1	0	42473T: 8;
ZBTB20	chr3	3q13.31	1	0	0	1	Amplification	1	0	42496T: 3.5;
ZBTB21	chr21	21q22.3	1	1	0	0	Amplification	1	0	42473T: 3.5;
ZBTB24	chr6	6q21	1	0	0	1	Amplification	1	0	42496T: 3.5;
ZBTB25	chr14	14q23.3	1	0	0	1	Amplification	1	0	42494T: 4;
ZBTB32	chr19	19q13.12	3	0	1	2	Amplification	3	0	42500T: 6.5; 42484T: 3.5; 56957T: 4;
ZBTB38	chr3	3q23	4	0	1	3	Amplification	4	0	42492T: 3.5; 42493T: 3.5; 42487T: 3.5; 42496T: 4;
ZBTB40	chr1	1p36.12	1	1	0	0	Amplification	1	0	42473T: 4;
ZBTB46	chr20	20q13.33	1	1	0	0	Amplification	1	0	42473T: 6;
ZBTB7B	chr1	1q21.3	2	1	0	1	Amplification	2	0	42496T: 3.5; 42473T: 6.5;
ZC2HC1A	chr8	8q21.13	2	0	0	2	Amplification	2	0	42496T: 3.5; 42495T: 3.5;
ZC2HC1C	chr14	14q24.3	2	0	0	2	Amplification	2	0	56957T: 3.5; 42494T: 4;
ZC3H15	chr2	2q32.1	1	0	0	1	Amplification	1	0	42493T: 3.5;
ZC3H3	chr8	8q24.3	3	0	1	2	Amplification	3	0	42483T: 3.5; 42496T: 4; 42495T: 4.5;
ZC3H7A	chr16	16p13.13	2	1	0	1	Amplification	2	0	42495T: 4; 42473T: 12.5;
ZC3HAV1	chr7	7q34	1	0	1	0	Amplification	1	0	42487T: 3.5;
ZC3HAV1L	chr7	7q34	1	0	1	0	Amplification	1	0	42487T: 3.5;
ZC3HC1	chr7	7q32.2	1	0	1	0	Amplification	1	0	42487T: 3.5;
ZCCHC17	chr1	1p35.2	1	1	0	0	Amplification	1	0	42473T: 3.5;
ZCCHC3	chr20	20p13	1	1	0	0	Amplification	1	0	42473T: 3.5;
ZCCHC8	chr12	12q24.31	1	0	0	1	Amplification	1	0	42500T: 3.5;
ZCRB1	chr12	12q12	1	0	0	1	Amplification	1	0	42500T: 3.5;
ZDHHC11	chr5	5p15.33	5	2	1	2	Amplification	5	0	42486T: 3.5; 42473T: 3.5; 42496T: 4; 42475T: 3.5; 42493T: 3.5;
ZDHHC11B	chr5	5p15.33	5	2	1	2	Amplification	5	0	42493T: 3.5; 42473T: 3.5; 42496T: 4; 42486T: 3.5; 42475T: 3.5;
ZDHHC19	chr3	3q29	9	1	3	5	Amplification	9	0	42495T: 4; 42474T: 3.5; 56957T: 6; 42484T: 4; 42498T: 3.5; 42493T: 5; 42487T: 3.5; 42492T: 3.5; 42482T: 3.5;
ZDHHC21	chr9	9p22.3	1	0	0	1	Amplification	1	0	42498T: 14.5;
ZDHHC22	chr14	14q24.3	2	0	0	2	Amplification	2	0	56957T: 3.5; 42494T: 4;

Mangalaparthi *et al.* , 2020. Mutational landscape of esophageal squamous cell carcinoma in an Indian cohort
Supplementary Table 7A. List of copy number alterations and affected genes in ESCC patients

Gene	Chromosome	Cytoband	Recurrence	Recurrence in smoker cohort	Recurrence in chewer cohort	Recurrence in No habit cohort	State	Samples with CNA gain	Samples with CNA loss	File info with CNA fold
ZDHHC23	chr3	3q13.31	1	0	0	1	Amplification	1	0	42496T: 3.5;
ZDHHC24	chr11	11q13.2	1	0	0	1	Amplification	1	0	56957T: 5.5;
ZDHHC4	chr7	7p22.1	2	1	0	1	Amplification	2	0	42497T: 5; 42473T: 4.5;
ZDHHC5	chr11	11q12.1	1	0	0	1	Amplification	1	0	42496T: 4.5;
ZFAND1	chr8	8q21.13	2	0	0	2	Amplification	2	0	42496T: 3.5; 42495T: 3.5;
ZFAND2A	chr7	7p22.3	1	1	0	0	Amplification	1	0	42473T: 5;
ZFAND3	chr6	6p21.2	1	1	0	0	Amplification	1	0	42473T: 4.5;
ZFAT	chr8	8q24.22	2	0	0	2	Amplification	2	0	42495T: 3.5; 42496T: 3.5;
ZFC3H1	chr12	12q21.1	1	0	0	1	Amplification	1	0	42501T: 6.5;
ZFHX2	chr14	14q11.2	1	0	0	1	Amplification	1	0	42496T: 4;
ZFHX4	chr8	8q21.13	2	0	0	2	Amplification	2	0	42495T: 3.5; 42496T: 3.5;
ZFP14	chr19	19q13.12	3	0	1	2	Amplification	3	0	42500T: 6.5; 56957T: 4; 42484T: 3.5;
ZFP30	chr19	19q13.12	3	0	1	2	Amplification	3	0	42484T: 3.5; 56957T: 4; 42500T: 6.5;
ZFP36	chr19	19q13.2	2	0	0	2	Amplification	2	0	42500T: 6.5; 56957T: 4;
ZFP36L1	chr14	14q24.1	1	0	0	1	Amplification	1	0	42494T: 4;
ZFP36L2	chr2	2p21	2	0	1	1	Amplification	2	0	42484T: 3.5; 42500T: 3.5;
ZFP41	chr8	8q24.3	3	0	1	2	Amplification	3	0	42495T: 4.5; 42483T: 3.5; 42496T: 4;
ZFP62	chr5	5q35.3	1	0	0	1	Amplification	1	0	42495T: 3.5;
ZFP64	chr20	20q13.2	1	1	0	0	Amplification	1	0	42473T: 5;
ZFP82	chr19	19q13.12	3	0	1	2	Amplification	3	0	42500T: 6.5; 42484T: 3.5; 56957T: 4;
ZFPM2	chr8	8q23.1	2	0	0	2	Amplification	2	0	42495T: 3.5; 42496T: 3.5;
ZFR	chr5	5p13.3	4	1	1	2	Amplification	4	0	42486T: 3.5; 42496T: 3.5; 42475T: 3.5; 42493T: 3.5;
ZFYVE1	chr14	14q24.2	2	0	0	2	Amplification	2	0	42494T: 4; 56957T: 3.5;
ZFYVE19	chr15	15q15.1	1	0	0	1	Amplification	1	0	42493T: 6.5;
ZFYVE26	chr14	14q24.1	1	0	0	1	Amplification	1	0	42494T: 4;
ZGPAT	chr20	20q13.33	1	1	0	0	Amplification	1	0	42473T: 6;
ZHX1	chr8	8q24.13	4	1	1	2	Amplification	4	0	42484T: 3.5; 42495T: 3.5; 42496T: 3.5; 42475T: 3.5;
ZHX2	chr8	8q24.13	4	1	1	2	Amplification	4	0	42496T: 3.5; 42475T: 3.5; 42484T: 3.5; 42495T: 3.5;
ZHX3	chr20	20q12	1	1	0	0	Amplification	1	0	42473T: 3.5;
ZIC1	chr3	3q24	7	1	1	5	Amplification	7	0	42492T: 3.5; 42474T: 3.5; 42493T: 3.5; 56957T: 4; 42487T: 3.5; 42497T: 4.5; 42496T: 4;
ZIC4	chr3	3q24	7	1	1	5	Amplification	7	0	42497T: 4.5; 42496T: 4; 42492T: 3.5; 56957T: 4; 42487T: 3.5; 42493T: 3.5; 42474T: 3.5;
ZKSCAN2	chr16	16p12.1	1	1	0	0	Amplification	1	0	42473T: 4;
ZMAT3	chr3	3q26.32	11	1	3	7	Amplification	11	0	42500T: 9.5; 42497T: 4; 56957T: 4; 42474T: 3.5; 42495T: 4; 42498T: 3.5; 42484T: 4; 42482T: 3.5; 42487T: 3.5; 42493T: 3.5; 42492T: 3.5;
ZMAT5	chr22	22q12.2	1	1	0	0	Amplification	1	0	42473T: 3.5;
ZMIZ2	chr7	7p13	1	1	0	0	Amplification	1	0	42473T: 4.5;
ZMYND19	chr9	9q34.3	2	1	0	1	Amplification	2	0	56957T: 3.5; 42473T: 5;
ZMYND8	chr20	20q13.12	1	1	0	0	Amplification	1	0	42473T: 5;

Mangalaparthi *et al.* , 2020. Mutational landscape of esophageal squamous cell carcinoma in an Indian cohort
Supplementary Table 7A. List of copy number alterations and affected genes in ESCC patients

Gene	Chromosome	Cytoband	Recurrence	Recurrence in smoker cohort	Recurrence in chewer cohort	Recurrence in No habit cohort	State	Samples with CNA gain	Samples with CNA loss	File info with CNA fold
ZNF107	chr7	7q11.21	1	0	0	1	Amplification	1	0	42501T: 3.5;
ZNF112	chr19	19q13.31	1	0	0	1	Amplification	1	0	56957T: 4;
ZNF117	chr7	7q11.21	1	0	0	1	Amplification	1	0	42501T: 3.5;
ZNF12	chr7	7p22.1	2	1	0	1	Amplification	2	0	42497T: 5; 42473T: 4.5;
ZNF131	chr5	5p12	5	1	2	2	Amplification	5	0	42493T: 3.5; 42475T: 3.5; 42483T: 3.5; 42486T: 3.5; 42496T: 3.5;
ZNF138	chr7	7q11.21	1	0	0	1	Amplification	1	0	42501T: 3.5;
ZNF146	chr19	19q13.12	3	0	1	2	Amplification	3	0	42484T: 3.5; 56957T: 4; 42500T: 6.5;
ZNF148	chr3	3q21.2	1	0	0	1	Amplification	1	0	42496T: 3.5;
ZNF155	chr19	19q13.31	1	0	0	1	Amplification	1	0	56957T: 4;
ZNF16	chr8	8q24.3	3	0	1	2	Amplification	3	0	42483T: 3.5; 42496T: 4; 42495T: 4.5;
ZNF174	chr16	16p13.3	1	1	0	0	Amplification	1	0	42473T: 5.5;
ZNF180	chr19	19q13.31	2	1	0	1	Amplification	2	0	56957T: 4; 42473T: 3.5;
ZNF181	chr19	19q13.11	3	0	1	2	Amplification	3	0	42500T: 4.5; 56957T: 4; 42484T: 3.5;
ZNF2	chr2	22p11.2	1	1	0	0	Amplification	1	0	42473T: 3.5;
ZNF200	chr16	16p13.3	1	1	0	0	Amplification	1	0	42473T: 5.5;
ZNF205	chr16	16p13.3	1	1	0	0	Amplification	1	0	42473T: 5.5;
ZNF212	chr7	7q36.1	1	0	1	0	Amplification	1	0	42487T: 3.5;
ZNF213	chr16	16p13.3	1	1	0	0	Amplification	1	0	42473T: 5.5;
ZNF217	chr20	20q13.2	1	1	0	0	Amplification	1	0	42473T: 5;
ZNF221	chr19	19q13.31	1	0	0	1	Amplification	1	0	56957T: 4;
ZNF222	chr19	19q13.31	1	0	0	1	Amplification	1	0	56957T: 4;
ZNF223	chr19	19q13.31	1	0	0	1	Amplification	1	0	56957T: 4;
ZNF224	chr19	19q13.31	1	0	0	1	Amplification	1	0	56957T: 4;
ZNF225	chr19	19q13.31	1	0	0	1	Amplification	1	0	56957T: 4;
ZNF226	chr19	19q13.31	1	0	0	1	Amplification	1	0	56957T: 4;
ZNF227	chr19	19q13.31	1	0	0	1	Amplification	1	0	56957T: 4;
ZNF229	chr19	19q13.31	1	0	0	1	Amplification	1	0	56957T: 4;
ZNF230	chr19	11p15.4|11p	1	0	0	1	Amplification	1	0	56957T: 4;
ZNF233	chr19	19q13.31	1	0	0	1	Amplification	1	0	56957T: 4;
ZNF234	chr19	19q13.31	1	0	0	1	Amplification	1	0	56957T: 4;
ZNF235	chr19	19q13.31	1	0	0	1	Amplification	1	0	56957T: 4;
ZNF250	chr8	8q24.3	3	0	1	2	Amplification	3	0	42496T: 4; 42483T: 3.5; 42495T: 4.5;
ZNF251	chr8	8q24.3	3	0	1	2	Amplification	3	0	42496T: 4; 42483T: 3.5; 42495T: 4.5;
ZNF252P	chr8	8q24.3	3	0	1	2	Amplification	3	0	42496T: 4; 42483T: 3.5; 42495T: 4.5;
ZNF260	chr19	19q13.12	3	0	1	2	Amplification	3	0	42500T: 6.5; 56957T: 4; 42484T: 3.5;
ZNF263	chr16	16p13.3	1	1	0	0	Amplification	1	0	42473T: 5.5;
ZNF273	chr7	7q11.21	1	0	0	1	Amplification	1	0	42501T: 3.5;
ZNF277	chr7	7q31.1	2	0	1	1	Amplification	2	0	42501T: 3.5; 42487T: 3.5;
ZNF280A	chr22	22q11.22	1	1	0	0	Amplification	1	0	42477T: 4;
ZNF280B	chr22	22q11.22	1	1	0	0	Amplification	1	0	42477T: 4;

Mangalaparthi *et al.* , 2020. Mutational landscape of esophageal squamous cell carcinoma in an Indian cohort
Supplementary Table 7A. List of copy number alterations and affected genes in ESCC patients

Gene	Chromosome	Cytoband	Recurrence	Recurrence in smoker cohort	Recurrence in chewer cohort	Recurrence in No habit cohort	State	Samples with CNA gain	Samples with CNA loss	File info with CNA fold
ZNF282	chr7	7q36.1	1	0	1	0	Amplification	1	0	42487T: 3.5;
ZNF283	chr19	19q13.31	1	0	0	1	Amplification	1	0	56957T: 4;
ZNF284	chr19	19q13.31	1	0	0	1	Amplification	1	0	56957T: 4;
ZNF285	chr19	19q13.31	1	0	0	1	Amplification	1	0	56957T: 4;
ZNF285B	chr19	19q13.31	2	1	0	1	Amplification	2	0	56957T: 4; 42473T: 3.5;
ZNF295	chr21	21q22.3	1	1	0	0	Amplification	1	0	42473T: 3.5;
ZNF296	chr19	19q13.32	2	1	0	1	Amplification	2	0	56957T: 4; 42473T: 3.5;
ZNF30	chr19	19q13.11	3	0	1	2	Amplification	3	0	42500T: 4.5; 56957T: 4; 42484T: 3.5;
ZNF302	chr19	19q13.11	3	0	1	2	Amplification	3	0	42500T: 4.5; 56957T: 4; 42484T: 3.5;
ZNF334	chr20	20q13.12	1	1	0	0	Amplification	1	0	42473T: 5;
ZNF335	chr20	20q13.12	1	1	0	0	Amplification	1	0	42473T: 5;
ZNF34	chr8	8q24.3	3	0	1	2	Amplification	3	0	42495T: 4.5; 42483T: 3.5; 42496T: 4;
ZNF341	chr20	20q11.22	2	1	0	1	Amplification	2	0	42473T: 3.5; 42496T: 5;
ZNF343	chr20	20p13	1	1	0	0	Amplification	1	0	42473T: 3.5;
ZNF345	chr19	19q13.12	3	0	1	2	Amplification	3	0	42500T: 6.5; 56957T: 4; 42484T: 3.5;
ZNF382	chr19	19q13.12	3	0	1	2	Amplification	3	0	42500T: 6.5; 42484T: 3.5; 56957T: 4;
ZNF383	chr19	19q13.12	3	0	1	2	Amplification	3	0	42500T: 6.5; 56957T: 4; 42484T: 3.5;
ZNF385A	chr12	12q13.13	2	1	0	1	Amplification	2	0	42473T: 3.5; 42494T: 3.5;
ZNF385B	chr2	2q31.2-q31.3	1	0	0	1	Amplification	1	0	42493T: 3.5;
ZNF398	chr7	7q36.1	2	0	1	1	Amplification	2	0	42487T: 3.5; 42500T: 4;
ZNF404	chr19	19q13.31	1	0	0	1	Amplification	1	0	56957T: 4;
ZNF408	chr11	16q24.2	1	1	0	0	Amplification	1	0	42473T: 3.5;
ZNF410	chr14	14q24.3	2	0	0	2	Amplification	2	0	56957T: 3.5; 42494T: 4;
ZNF420	chr19	19q13.12	3	0	1	2	Amplification	3	0	56957T: 4; 42484T: 3.5; 42500T: 6.5;
ZNF425	chr7	7q36.1	2	0	1	1	Amplification	2	0	42487T: 3.5; 42500T: 4;
ZNF428	chr19	19q13.31	1	0	0	1	Amplification	1	0	56957T: 4;
ZNF433	chr19	19p13.2	1	0	0	1	Amplification	1	0	42496T: 5;
ZNF436	chr1	1p36.12	1	1	0	0	Amplification	1	0	42473T: 3.5;
ZNF45	chr19	19q13.31	1	0	0	1	Amplification	1	0	56957T: 4;
ZNF461	chr19	19q13.12	3	0	1	2	Amplification	3	0	42500T: 6.5; 42484T: 3.5; 56957T: 4;
ZNF479	chr7	7p11.2	2	0	1	1	Amplification	2	0	56957T: 5; 42483T: 4;
ZNF500	chr16	16p13.3	3	1	0	2	Amplification	3	0	42495T: 12; 42494T: 3.5; 42473T: 5.5;
ZNF507	chr19	19q13.11	5	1	1	3	Amplification	5	0	42484T: 6; 56957T: 4; 42494T: 4; 42473T: 3.5; 42500T: 4.5;
ZNF512	chr2	2p23.3	1	0	0	1	Amplification	1	0	42500T: 3.5;
ZNF512B	chr20	20q13.33	2	1	1	0	Amplification	2	0	42483T: 3.5; 42473T: 6;
ZNF513	chr2	2p23.3	1	0	0	1	Amplification	1	0	42500T: 3.5;
ZNF514	chr2	2q11.1	1	1	0	0	Amplification	1	0	42473T: 3.5;
ZNF517	chr8	8q24.3	3	0	1	2	Amplification	3	0	42483T: 3.5; 42496T: 4; 42495T: 4.5;
ZNF524	chr19	19q13.42	1	0	0	1	Amplification	1	0	42494T: 3.5;
ZNF526	chr19	19q13.2	1	1	0	0	Amplification	1	0	42473T: 4.5;

Mangalaparthi *et al.* , 2020. Mutational landscape of esophageal squamous cell carcinoma in an Indian cohort
Supplementary Table 7A. List of copy number alterations and affected genes in ESCC patients

Gene	Chromosome	Cytoband	Recurrence	Recurrence in smoker cohort	Recurrence in chewer cohort	Recurrence in No habit cohort	State	Samples with CNA gain	Samples with CNA loss	File info with CNA fold
ZNF527	chr19	19q13.12	3	0	1	2	Amplification	3	0	42500T: 6.5; 42484T: 3.5; 56957T: 4;
ZNF529	chr19	19q13.12	3	0	1	2	Amplification	3	0	42484T: 3.5; 56957T: 4; 42500T: 6.5;
ZNF536	chr19	19q12	5	1	1	3	Amplification	5	0	42484T: 6; 56957T: 4; 42494T: 4; 42473T: 25; 42500T: 4.5;
ZNF540	chr19	19q13.12	3	0	1	2	Amplification	3	0	42484T: 3.5; 56957T: 4; 42500T: 6.5;
ZNF546	chr19	19q13.2	2	0	0	2	Amplification	2	0	56957T: 4; 42500T: 7.5;
ZNF566	chr19	19q13.12	3	0	1	2	Amplification	3	0	56957T: 4; 42484T: 3.5; 42500T: 6.5;
ZNF567	chr19	19q13.12	3	0	1	2	Amplification	3	0	42484T: 3.5; 56957T: 4; 42500T: 6.5;
ZNF568	chr19	19q13.12	3	0	1	2	Amplification	3	0	42484T: 3.5; 56957T: 4; 42500T: 6.5;
ZNF569	chr19	19q13.12	3	0	1	2	Amplification	3	0	42500T: 6.5; 56957T: 4; 42484T: 3.5;
ZNF570	chr19	19q13.12	3	0	1	2	Amplification	3	0	42500T: 6.5; 56957T: 4; 42484T: 3.5;
ZNF571	chr19	19q13.12	3	0	1	2	Amplification	3	0	56957T: 4; 42484T: 3.5; 42500T: 6.5;
ZNF572	chr8	8q24.13	4	1	1	2	Amplification	4	0	42484T: 3.5; 42495T: 3.5; 42496T: 3.5; 42475T: 3.5;
ZNF573	chr19	19q13.12	3	0	1	2	Amplification	3	0	56957T: 4; 42484T: 3.5; 42500T: 6.5;
ZNF574	chr19	19q13.2	1	1	0	0	Amplification	1	0	42473T: 4.5;
ZNF575	chr19	19q13.31	1	0	0	1	Amplification	1	0	56957T: 4;
ZNF576	chr19	19q13.31	1	0	0	1	Amplification	1	0	56957T: 4;
ZNF579	chr19	19q13.42	1	0	0	1	Amplification	1	0	42494T: 3.5;
ZNF580	chr19	19q13.42	1	0	0	1	Amplification	1	0	42494T: 3.5;
ZNF581	chr19	19q13.42	1	0	0	1	Amplification	1	0	42494T: 3.5;
ZNF585A	chr19	19q13.12	3	0	1	2	Amplification	3	0	42500T: 6.5; 42484T: 3.5; 56957T: 4;
ZNF585B	chr19	19q13.12	3	0	1	2	Amplification	3	0	42484T: 3.5; 56957T: 4; 42500T: 6.5;
ZNF592	chr15	15q25.3	1	1	0	0	Amplification	1	0	42473T: 4;
ZNF597	chr16	16p13.3	1	1	0	0	Amplification	1	0	42473T: 5.5;
ZNF598	chr16	16p13.3	1	0	1	0	Amplification	1	0	42483T: 3.5;
ZNF599	chr19	19q13.11	3	0	1	2	Amplification	3	0	56957T: 4; 42484T: 3.5; 42500T: 4.5;
ZNF607	chr19	19q13.12	3	0	1	2	Amplification	3	0	42484T: 3.5; 56957T: 4; 42500T: 6.5;
ZNF622	chr5	5p15.1	4	1	1	2	Amplification	4	0	42493T: 4; 42486T: 3.5; 42496T: 3.5; 42475T: 3.5;
ZNF623	chr8	8q24.3	3	0	1	2	Amplification	3	0	42483T: 3.5; 42496T: 4; 42495T: 4.5;
ZNF628	chr19	19q13.42	1	0	0	1	Amplification	1	0	42494T: 3.5;
ZNF638	chr2	2p13.3-p13.2	1	0	0	1	Amplification	1	0	42500T: 3.5;
ZNF639	chr3	3q26.33	11	1	3	7	Amplification	11	0	42500T: 9.5; 42497T: 4; 56957T: 4; 42495T: 4; 42474T: 3.5; 42498T: 3.5; 42484T: 4; 42482T: 3.5; 42493T: 3.5; 42487T: 3.5; 42492T: 3.5;
ZNF664	chr12	12q24.31	1	0	0	1	Amplification	1	0	42500T: 3.5;
ZNF680	chr7	7q11.21	1	0	0	1	Amplification	1	0	42501T: 3.5;
ZNF687	chr1	1q21.3	1	1	0	0	Amplification	1	0	42473T: 4.5;
ZNF691	chr1	1p34.2	2	0	0	2	Amplification	2	0	42496T: 5.5; 42493T: 6;
ZNF696	chr8	8q24.3	3	0	1	2	Amplification	3	0	42495T: 4.5; 42483T: 3.5; 42496T: 4;
ZNF7	chr8	8q24.3	3	0	1	2	Amplification	3	0	42496T: 4; 42483T: 3.5; 42495T: 4.5;
ZNF703	chr8	8p11.23	1	0	1	0	Amplification	1	0	42482T: 3.5;

Gene	Chromosome	Cytoband	Recurrence	Recurrence in smoker cohort	Recurrence in chewer cohort	Recurrence in No habit cohort	State	Samples with CNA gain	Samples with CNA loss	File info with CNA fold
ZNF704	chr8	8q21.13	2	0	0	2	Amplification	2	0	42495T: 3.5; 42496T: 3.5;
ZNF706	chr8	8q22.3	2	0	0	2	Amplification	2	0	42495T: 3.5; 42496T: 3.5;
ZNF707	chr8	8q24.3	3	0	1	2	Amplification	3	0	42495T: 4.5; 42496T: 4; 42483T: 3.5;
ZNF710	chr15	15q26.1	1	1	0	0	Amplification	1	0	42473T: 3.5;
ZNF713	chr7	7p11.2	2	0	1	1	Amplification	2	0	56957T: 5; 42483T: 4;
ZNF716	chr7	7p11.2	2	0	1	1	Amplification	2	0	56957T: 5; 42483T: 4;
ZNF733P	chr7	7q11.21	1	0	0	1	Amplification	1	0	56957T: 5;
ZNF74	chr22	22q11.21	2	1	0	1	Amplification	2	0	42497T: 3.5; 42477T: 4;
ZNF746	chr7	7q36.1	1	0	1	0	Amplification	1	0	42487T: 3.5;
ZNF75A	chr16	16p13.3	1	1	0	0	Amplification	1	0	42473T: 5.5;
ZNF763	chr19	19p13.2	1	0	0	1	Amplification	1	0	42496T: 5;
ZNF767	chr7	7q36.1	1	0	1	0	Amplification	1	0	42487T: 3.5;
ZNF777	chr7	7q36.1	1	0	1	0	Amplification	1	0	42487T: 3.5;
ZNF780A	chr19	19q13.2	2	0	0	2	Amplification	2	0	56957T: 4; 42500T: 7.5;
ZNF780B	chr19	19q13.2	2	0	0	2	Amplification	2	0	42500T: 7.5; 56957T: 4;
ZNF781	chr19	19q13.12	3	0	1	2	Amplification	3	0	42500T: 6.5; 56957T: 4; 42484T: 3.5;
ZNF783	chr7	7q36.1	1	0	1	0	Amplification	1	0	42487T: 3.5;
ZNF784	chr19	19q13.42	1	0	0	1	Amplification	1	0	42494T: 3.5;
ZNF786	chr7	7q36.1	2	0	1	1	Amplification	2	0	42500T: 4; 42487T: 3.5;
ZNF790	chr19	19q13.12	3	0	1	2	Amplification	3	0	42500T: 6.5; 56957T: 4; 42484T: 3.5;
ZNF792	chr19	19q13.11	3	0	1	2	Amplification	3	0	56957T: 4; 42484T: 3.5; 42500T: 4.5;
ZNF793	chr19	19q13.12	3	0	1	2	Amplification	3	0	42484T: 3.5; 56957T: 4; 42500T: 6.5;
ZNF80	chr3	3q13.31	1	0	0	1	Amplification	1	0	42496T: 3.5;
ZNF800	chr7	7q31.33	1	0	1	0	Amplification	1	0	42487T: 3.5;
ZNF804A	chr2	2q32.1	1	0	0	1	Amplification	1	0	42493T: 3.5;
ZNF804B	chr7	7q21.13	2	0	2	0	Amplification	2	0	42487T: 3.5; 42483T: 3.5;
ZNF815P	chr7	7p22.1	2	1	0	1	Amplification	2	0	42473T: 4.5; 42497T: 10.5;
ZNF829	chr19	19q13.12	3	0	1	2	Amplification	3	0	42484T: 3.5; 56957T: 4; 42500T: 6.5;
ZNF831	chr20	20q13.32	1	1	0	0	Amplification	1	0	42473T: 4.5;
ZNF850	chr19	19q13.12	3	0	1	2	Amplification	3	0	42500T: 6.5; 56957T: 4; 42484T: 3.5;
ZNF853	chr7	7p22.1	2	1	0	1	Amplification	2	0	42497T: 5; 42473T: 4.5;
ZNF865	chr19	19q13.42	1	0	0	1	Amplification	1	0	42494T: 3.5;
ZNF890P	chr7	7p22.1	2	1	0	1	Amplification	2	0	42473T: 4.5; 42497T: 8.5;
ZNF92	chr7	7q11.21	1	0	0	1	Amplification	1	0	42501T: 3.5;
ZNFX1	chr20	20q13.13	1	1	0	0	Amplification	1	0	42473T: 5;
ZNHIT1	chr7	7q22.1	2	0	0	2	Amplification	2	0	42501T: 3.5; 42493T: 3.5;
ZNRF2	chr7	7p14.3	1	1	0	0	Amplification	1	0	42473T: 4;
ZP2	chr16	16p12.3-p12	1	1	0	0	Amplification	1	0	42473T: 4;
ZP3	chr7	7q11.23	1	0	0	1	Amplification	1	0	42493T: 3.5;
ZPBP	chr7	7p12.2	1	0	0	1	Amplification	1	0	42497T: 20.5;
ZPBP2	chr17	17q21.1	1	0	0	1	Amplification	1	0	42497T: 3.5;

Mangalaparthi *et al.* , 2020. Mutational landscape of esophageal squamous cell carcinoma in an Indian cohort
Supplementary Table 7A. List of copy number alterations and affected genes in ESCC patients

Gene	Chromosome	Cytoband	Recurrence	Recurrence in smoker cohort	Recurrence in chewer cohort	Recurrence in No habit cohort	State	Samples with CNA gain	Samples with CNA loss	File info with CNA fold
ZPLD1	chr3	3q12.3	1	1	0	0	Amplification	1	0	42476T: 3.5;
ZSCAN10	chr16	16p13.3	1	1	0	0	Amplification	1	0	42473T: 5.5;
ZSCAN2	chr15	15q25.2	1	1	0	0	Amplification	1	0	42473T: 4;
ZSCAN32	chr16	16p13.3	1	1	0	0	Amplification	1	0	42473T: 5.5;
ZSWIM1	chr20	20q13.12	1	1	0	0	Amplification	1	0	42473T: 5;
ZSWIM2	chr2	2q32.1	1	0	0	1	Amplification	1	0	42493T: 3.5;
ZSWIM3	chr20	20q13.12	1	1	0	0	Amplification	1	0	42473T: 5;
ZSWIM8	chr10	10q22.2	2	1	0	1	Amplification	2	0	42473T: 3.5; 42496T: 3.5;
ZXDC	chr3	3q21.3	1	0	0	1	Amplification	1	0	42496T: 3.5;
ZYX	chr7	7q34	1	0	1	0	Amplification	1	0	42487T: 3.5;

Mangalaparthi *et al.* , 2020. Mutational landscape of esophageal squamous cell carcinoma in an Indian cohort

Supplementary Table 7B. List of cytobands and number of genes affected by Copy number alterations (CNA)

Cytoband	Number of genes affected by CNA	Number of samples
1p11.2	1	1
1p12	1	1
1p13.3	15	2
1p22.2	1	9
1p22.3	6	1
1p32.3	1	1
1p34.1	2	3
1p34.2	21	2
1p34.3	1	1
1p35.1	3	1
1p35.2	27	1
1p35.2-p35.1	1	1
1p35.3	27	2
1p36.11	36	2
1p36.11-p35.3	1	1
1p36.12	44	2
1p36.13	43	1
1p36.13|1p35-p36.1	1	1
1p36.21	3	1
1p36.33	3	4
1q21.1	26	1
1q21.2	38	3
1q21.3	116	4
1q22	47	2
1q22-q23.1	1	1
1q23.1	23	1
1q23.3	2	1
1q25.2	2	1
1q25.2-q25.3	1	1
1q25.3	3	1
1q31.3	2	1
1q32.1	18	2
1q41	2	2
1q42.12	9	4
1q42.3	4	1
1q44	1	1
2p11.2	44	5
2p12	13	1
2p13.1	33	1
2p13.2	11	1

Mangalaparthi *et al.* , 2020. Mutational landscape of esophageal squamous cell carcinoma in an Indian cohort

Supplementary Table 7B. List of cytobands and number of genes affected by Copy number alterations (CNA)

Cytoband	Number of genes affected by CNA	Number of samples
2p13.3	36	1
2p13.3-p13.2	1	1
2p14	16	2
2p15	15	3
2p15-p14	1	2
2p16.1	15	3
2p16.1-p15	1	3
2p16.2	9	2
2p16.3	10	2
2p21	34	2
2p21|2p16-p12	1	2
2p21-p16.3	1	2
2p22.1	14	3
2p22.1|2p22-p21	1	1
2p22.2	15	2
2p22.2-p22.1	1	2
2p22.3	12	1
2p23.1	8	1
2p23.2	12	1
2p23.2-p23.1	1	1
2p23.3	66	1
2p24.1	14	2
2p24.1-p23.3	1	1
2p24.2	9	1
2p24.3	4	1
2p25.1	26	1
2p25.1|2p24	1	1
2p25.2	3	1
2p25.3	16	2
2q11.1	8	2
2q11.2	58	3
2q11.2-q12.1	1	1
2q12.1	10	1
2q12.1-q12.2	1	1
2q12.2	2	1
2q13	1	1
2q14.1	1	1
2q14.2	2	1
2q14.3	1	2
2q21.1	1	2

Mangalaparthi *et al.* , 2020. Mutational landscape of esophageal squamous cell carcinoma in an Indian cohort

Supplementary Table 7B. List of cytobands and number of genes affected by Copy number alterations (CNA)

Cytoband	Number of genes affected by CNA	Number of samples
2q22.1-q22.2	1	1
2q24.1	3	2
2q24.2	1	2
2q24.3	2	2
2q24-q32	1	1
2q31.1	29	2
2q31.2	15	2
2q31.2-q31.3	1	1
2q31.3	6	1
2q31.3-q32.1	1	1
2q32.1	14	1
2q32.1-q32.2	1	1
2q32.2	18	3
2q32.2-q32.3	1	3
2q32.3	8	3
2q33.1	16	1
2q34	1	1
2q35	6	5
2q37.1	5	1
2q37.3	14	4
3p21.1	2	2
3p21.31	14	2
3p22.2	8	1
3p22-p21.2	1	1
3q11.1	1	2
3q11.1-q11.2	1	2
3q11.2	20	3
3q12.1	5	1
3q12.1|3	1	1
3q12.1-q12.2	1	1
3q12.2	7	1
3q12.3	9	1
3q13.2	21	1
3q13.31	12	1
3q13.32	4	1
3q13.32-q13.33	1	1
3q13.33	26	1
3q13.33-q21.1	1	1
3q21.1	19	2
3q21.1-q21.2	1	1

Mangalaparthi *et al.* , 2020. Mutational landscape of esophageal squamous cell carcinoma in an Indian cohort

Supplementary Table 7B. List of cytobands and number of genes affected by Copy number alterations (CNA)

Cytoband	Number of genes affected by CNA	Number of samples
3q21.2	10	1
3q21.2-q21.3	1	1
3q21.3	40	5
3q21.3-q22.1	1	2
3q22.1	29	3
3q22.1-q22.2	1	3
3q22.2	6	4
3q22.2-q22.3	1	4
3q22.3	18	4
3q23	26	6
3q24	15	8
3q24-q25.1	1	8
3q25.1	25	10
3q25.1-q25.2	1	9
3q25.2	7	9
3q25.31	9	9
3q25.31-q25.32	1	8
3q25.32	10	8
3q25.32-q25.33	1	8
3q25.33	7	8
3q25.33-q26.1	1	8
3q26.1	11	10
3q26.2	20	9
3q26.2-q26.31	1	9
3q26.31	9	9
3q26.32	5	11
3q26.33	15	11
3q27.1	30	13
3q27.2	11	10
3q27.2-q27.3	1	10
3q27.3	18	10
3q27.3-q28	1	10
3q28	12	10
3q28-q29	1	10
3q29	44	10
4p11	7	1
4p14	12	1
4p15.1-q12	1	1
4p15.2	1	3
4p15.31	1	1

Mangalaparthi *et al.* , 2020. Mutational landscape of esophageal squamous cell carcinoma in an Indian cohort

Supplementary Table 7B. List of cytobands and number of genes affected by Copy number alterations (CNA)

Cytoband	Number of genes affected by CNA	Number of samples
4q21.1	1	3
4q21.21	3	1
5p12	14	5
5p13.1	14	6
5p13.1-p12	1	5
5p13.2	28	8
5p13.2-p13.1	1	6
5p13.3	9	4
5p13.3-p13.2	1	4
5p14.1	1	4
5p14.2	2	4
5p14.2-p14.1	1	4
5p14.3	3	4
5p15.1	6	5
5p15.2	12	5
5p15.31	12	4
5p15.32	2	4
5p15.33	30	5
5q11.1	3	2
5q11.2	1	1
5q11.2-q12.1	1	1
5q13.3	1	1
5q14.3	6	1
5q22.2	1	1
5q23.1	1	1
5q23.2	2	1
5q23.3	1	1
5q31.1	17	1
5q31.2	6	2
5q31.3	2	4
5q35.2	1	2
5q35.3	11	2
6p11.2	1	1
6p12.1	4	1
6p12.1|6p12.3-p11.2	1	1
6p12.1-p11.2	1	1
6p21.1	1	1
6p21.2	9	3
6p21.31	1	1
6p21.32	14	2

Mangalaparthi *et al.* , 2020. Mutational landscape of esophageal squamous cell carcinoma in an Indian cohort

Supplementary Table 7B. List of cytobands and number of genes affected by Copy number alterations (CNA)

Cytoband	Number of genes affected by CNA	Number of samples
6p21.33	45	2
6p21.33-p21.32	1	1
6p22.1	1	1
6q21	46	2
6q21|6q21-q22.1	1	1
6q23.3	3	1
6q23.3-q24.1	1	1
6q24.1	1	1
6q25.3	3	1
7p11.2	18	3
7p12.1	2	1
7p12.2	4	1
7p12.2-p12.1	1	1
7p12.3	4	2
7p13	31	2
7p14.1	18	1
7p14.1-p13	1	1
7p14.2	9	1
7p14.2-p14.1	1	1
7p14.3	32	1
7p15.1	1	1
7p15.2	21	1
7p15.2-p15.1	1	1
7p15.3	25	1
7p21.1	18	1
7p21.2	6	1
7p21.3	11	2
7p22.1	34	2
7p22.1-p21.3	1	2
7p22.2	2	2
7p22.3	32	1
7p22.3-p22.2	1	1
7q11.21	25	3
7q11.22	1	1
7q11.23	14	2
7q11.23-q21.11	1	1
7q21.11	10	2
7q21.11-q21.12	1	2
7q21.12	12	2
7q21.13	9	3

Mangalaparthi *et al.* , 2020. Mutational landscape of esophageal squamous cell carcinoma in an Indian cohort

Supplementary Table 7B. List of cytobands and number of genes affected by Copy number alterations (CNA)

Cytoband	Number of genes affected by CNA	Number of samples
7q21.2	13	3
7q21.2-q21.3	1	2
7q21.3	23	2
7q22	1	2
7q22.1	44	7
7q22.1-q22.2	1	4
7q22.2-q22.3	1	4
7q22.3	18	4
7q22.3-q31.1	1	4
7q31.1	17	4
7q31.1|14q24.2-q24.3	1	3
7q31.1-q31.2	1	1
7q31.2	9	1
7q31.31	7	1
7q31.32	13	2
7q31.32|7q31	1	2
7q31.33	4	1
7q32.1	24	2
7q32.2	16	7
7q32.3	3	1
7q32.3-q33	1	1
7q33	25	1
7q33-q34	1	1
7q34	56	2
7q34-q35	1	1
7q35	21	2
7q35-q36.1	1	2
7q36.1	13	2
8p11.1	1	1
8p11.21	8	1
8p11.21-p11.1	1	1
8p11.22	1	1
8p11.23	18	2
8p12	22	2
8p21.2	1	2
8p21.3	1	1
8p23.1	45	1
8p23.2	1	1
8p23.3	4	1
8p23-p22	1	1

Mangalaparthi *et al.* , 2020. Mutational landscape of esophageal squamous cell carcinoma in an Indian cohort

Supplementary Table 7B. List of cytobands and number of genes affected by Copy number alterations (CNA)

Cytoband	Number of genes affected by CNA	Number of samples
8q11.1	1	3
8q11.21	9	5
8q11.22-q11.23	2	6
8q11.23	11	5
8q11.2-q13.2	1	1
8q12.1	17	3
8q12.1-q12.2	1	2
8q12.2-q12.3	1	2
8q12.3	8	2
8q13.1	19	4
8q13.1|8q13	1	3
8q13.1-q13.2	1	3
8q13.2	4	3
8q13.2-q13.3	1	2
8q13.3	7	2
8q21.11	12	2
8q21.13	22	2
8q21.2	8	2
8q21.3	20	3
8q22.1	26	3
8q22.1-q22.2	1	2
8q22.2	13	2
8q22.3	24	2
8q23.1	11	2
8q23.1-q23.2	1	2
8q23.2	3	2
8q23.3	1	3
8q23.3-q24.11	1	3
8q24.11	6	3
8q24.11-q24.12	1	3
8q24.12	11	4
8q24.13	25	4
8q24.21	8	5
8q24.21-q24.22	1	3
8q24.22	14	4
8q24.23	2	2
8q24.23-q24.3	1	2
8q24.3	97	5
9p13.2	1	1
9p13.3	39	3

Mangalaparthi *et al.* , 2020. Mutational landscape of esophageal squamous cell carcinoma in an Indian cohort

Supplementary Table 7B. List of cytobands and number of genes affected by Copy number alterations (CNA)

Cytoband	Number of genes affected by CNA	Number of samples
9p21.3	21	2
9p22.3	2	1
9p23	1	1
9p23-p22.3	1	1
9p24.1	23	3
9p24.1-p23	1	1
9p24.2	6	3
9p24.2-p24.1	1	1
9p24.3	7	2
9q21.13	3	1
9q31.3	6	1
9q32	3	1
9q32-q33.1	1	1
9q33.1	1	1
9q34.12	1	1
9q34.12-q34.13	1	1
9q34.13	7	1
9q34.3	86	3
10p	1	2
10p11.1	2	1
10p11.22-p11.21	1	3
10p12.1	3	1
10p13	3	1
10p15.1	1	6
10q22.2	14	2
10q26.11	1	1
11p11.12	5	1
11p11.2	51	3
11p11.2|11p12-p11	1	1
11p13	8	2
11p15.4|11p15	1	1
11p15.5	3	1
11q11	13	1
11q12.1	33	3
11q12.3	1	1
11q13.1	2	4
11q13.2	60	5
11q13.3	17	12
11q13.3-q13.4	1	11
11q13.4	49	9

Mangalaparthi *et al.* , 2020. Mutational landscape of esophageal squamous cell carcinoma in an Indian cohort

Supplementary Table 7B. List of cytobands and number of genes affected by Copy number alterations (CNA)

Cytoband	Number of genes affected by CNA	Number of samples
11q13.4-q13.5	1	2
11q13.5	15	5
11q22.1	5	1
11q22.2	7	8
11q22.3	2	1
11q23.3	11	1
11q25	7	1
12p11.1	1	1
12p11.21	16	1
12p11.22	11	4
12p11.23	9	1
12p11.23-p11.22	1	1
12p12.1	28	2
12p12.2	3	1
12p12.3	19	2
12p13.1	15	1
12p13.1-p12.3	1	1
12p13.2	8	1
12p13.31	22	2
12p13.32	9	1
12p13.33	10	1
12q12	27	1
12q13.11	22	1
12q13.11-q13.12	1	1
12q13.12	50	2
12q13.12-q13.13	1	2
12q13.13	41	4
12q13.13-q13.2	1	2
12q13.2	46	11
12q13.3	6	3
12q14.3	2	1
12q14.3-q15	1	2
12q15	23	2
12q21.1	9	1
12q23.1	2	1
12q24.11	34	2
12q24.11-q24.12	1	1
12q24.12	5	1
12q24.12-q24.13	3	1
12q24.13	20	2

Mangalaparthi *et al.* , 2020. Mutational landscape of esophageal squamous cell carcinoma in an Indian cohort

Supplementary Table 7B. List of cytobands and number of genes affected by Copy number alterations (CNA)

Cytoband	Number of genes affected by CNA	Number of samples
12q24.13-q24.21	1	1
12q24.21	3	1
12q24.22	8	1
12q24.22-q24.23	1	1
12q24.23	15	1
12q24.23-q24.31	1	1
12q24.31	77	1
12q24.31|12q24.2	1	1
12q24.31-q24.32	1	1
12q24.33	9	5
13q12.3	8	1
13q13.1	8	1
13q13.1-q13.2	1	1
13q14.2	2	1
13q22.1	1	2
13q22.3	1	1
13q32.2	1	2
13q32.3	1	1
13q34	21	2
14q11.2	48	3
14q12	15	3
14q13.1	6	1
14q13.1-q13.2	1	2
14q13.2	9	2
14q13.3	5	2
14q13.3-q21.1	1	2
14q21.1	11	3
14q21.2	8	1
14q21.3	15	3
14q22.1	22	3
14q22.2	7	1
14q22.2-q22.3	1	1
14q22.3	16	2
14q22.3-q23.1	1	1
14q23.1	26	2
14q23.2	11	1
14q23.2-q23.3	1	1
14q23.3	18	1
14q23.3-q24.1	2	1
14q24.1	19	1

Mangalaparthi *et al.* , 2020. Mutational landscape of esophageal squamous cell carcinoma in an Indian cohort

Supplementary Table 7B. List of cytobands and number of genes affected by Copy number alterations (CNA)

Cytoband	Number of genes affected by CNA	Number of samples
14q24.1|14q22-q24	1	1
14q24.2	19	2
14q24.2-q24.3	1	2
14q24.3	67	2
14q24.3-q31.1	1	1
14q31.1	1	1
14q32.11	6	1
14q32.13	1	5
14q32.2	2	3
15q11.1	1	2
15q11.1-q11.2	1	2
15q11.2	4	2
15q15.1	18	2
15q21.3	1	1
15q22.2	1	1
15q24.1	22	1
15q25.1	13	1
15q25.2	3	1
15q25.2-q25.3	1	1
15q25.3	4	1
15q26.1	19	1
16p11.2	1	2
16p12.1	9	1
16p12.2	28	2
16p12.2-p12.1	1	1
16p12.3	34	3
16p12.3-p12.2	1	1
16p13.11	13	2
16p13.12	7	2
16p13.13	19	2
16p13.13-p13.12	1	2
16p13.2	10	3
16p13.2-p13.13	1	2
16p13.3	167	10
16q13	1	9
16q21	1	1
16q22.1	1	4
16q23.1	1	4
16q24.2	1	1
17p11.2	2	4

Mangalaparthi *et al.* , 2020. Mutational landscape of esophageal squamous cell carcinoma in an Indian cohort

Supplementary Table 7B. List of cytobands and number of genes affected by Copy number alterations (CNA)

Cytoband	Number of genes affected by CNA	Number of samples
17p13.1	1	1
17q11.2	16	1
17q12	35	2
17q12-q21.1	1	1
17q21.1	13	1
17q21.2	2	1
17q21.33	18	1
17q22	37	1
17q25.1	26	2
17q25.3	20	1
18p11.21	7	2
18p11.21|18	1	1
18p11.23	1	1
18p11.31	14	4
18p11.32	16	4
18p11.32-p11.31	1	2
18q11.2	10	1
18q12.1	2	1
18q12.2	1	3
18q21.2	2	1
19p13.11	1	1
19p13.13	1	1
19p13.2	3	10
19p13.3	17	1
19q12	8	5
19q13.11	33	5
19q13.12	83	5
19q13.12|19q13.11	1	3
19q13.13	1	3
19q13.13-q13.2	1	3
19q13.2	125	6
19q13.31	45	2
19q13.31-q13.32	1	2
19q13.32	63	3
19q13.42	24	5
19q13.42-q13.43	1	1
19q13.43	2	1
20p11.22	2	1
20p11.23	8	1
20p12.2	3	1

Mangalaparthi *et al.* , 2020. Mutational landscape of esophageal squamous cell carcinoma in an Indian cohort

Supplementary Table 7B. List of cytobands and number of genes affected by Copy number alterations (CNA)

Cytoband	Number of genes affected by CNA	Number of samples
20p12.3	2	1
20p13	81	4
20p13-p12.3	1	1
20q11.2	1	2
20q11.21	44	2
20q11.21-q11.22	1	2
20q11.22	37	3
20q11.22-q11.23	1	1
20q11.23	42	1
20q11.23-q12	1	1
20q12	7	1
20q12-q13.11	1	1
20q13.11	1	1
20q13.12	81	1
20q13.13	24	1
20q13.2	13	1
20q13.2-q13.31	1	1
20q13.31	14	5
20q13.32	15	1
20q13.32-q13.33	1	1
20q13.33	66	7
21q22.11	1	1
21q22.2	11	1
21q22.2-q22.3	1	1
21q22.3	23	10
22p11.2	1	1
22q11.21	48	3
22q11.21|22q11.1-q11.2	1	1
22q11.21|22q11.2	1	1
22q11.21-q11.22	1	1
22q11.22	7	8
22q11.23	5	1
22q12.2	16	1
22q12.3	26	1
22q13.1	62	1
22q13.1-q13.2	2	1
22q13.31	3	1
