# Supplementary material for: Mutational Landscape of Esophageal Squamous Cell Carcinoma in an Indian Cohort
Source: Front Oncol. 2020 Aug 20;10:1457. doi: 10.3389/fonc.2020.01457 (PMC7469928; doi:10.3389/fonc.2020.01457)
Supplement: Supplementary Table 8 — List of genes screened for potential druggability using DGIdb resource. [file Table_8.pdf]

Mangalaparthi *et al.* , 2020. Mutational landscape of esophageal squamous cell carcinoma in an Indian cohort

Supplementary Table 8. List of genes screened for potential druggability using DGIdb resource

Somatic SNVs	Genes affected by copy number alteration in atleast 5 samples (434 genes)	All genes
TP53	ORAOV1	ORAOV1
CSMD3	FGF3	FGF3
TTN	CTTN	CTTN
ASTN1	FADD	FADD
DNAH5	PPFIA1	PPFIA1
LRP1B	ANO1	ANO1
PIK3CA	SHANK2	SHANK2
DNAH10	FGF19	FGF19
HYDIN	MYEOV	MYEOV
NF1	NADSYN1	NADSYN1
NOTCH1	KRTAP5	KRTAP5
RYR1	LRTOMT	LRTOMT
SLIT2	NUMA1	NUMA1
SLITRK2	INPPL1	INPPL1
SYNE1	FOLR3	FOLR3
TMEM132C	LAMTOR1	LAMTOR1
USH2A	FGF4	FGF4
VPS13B	ANAPC15	ANAPC15
ZFHX4	FOLR1	FOLR1
ADAMTSL1	DHCR7	DHCR7
ARID4A	FOLR2	FOLR2
CACNA1C	RNF121	RNF121
CDC42BPA	MRGPRF	MRGPRF
COL11A1	PHOX2A	PHOX2A
COL4A1	TPCN2	TPCN2
COL4A4	IL18BP	IL18BP
COL6A5	CLPB	CLPB
CPA3	IGHMBP2	IGHMBP2
CTNNA2	MRGPRD	MRGPRD
CTNND2	PDE2A	PDE2A
CTTNBP2	DNAJB13	DNAJB13
CUL3	P2RY6	P2RY6
DCHS1	FAM168A	FAM168A
DIAPH2	ARAP1	ARAP1
DMAP1	PAAF1	PAAF1

Mangalaparthi *et al.* , 2020. Mutational landscape of esophageal squamous cell carcinoma in an Indian cohort

Supplementary Table 8. List of genes screened for potential druggability using DGIdb resource

Somatic SNVs	Genes affected by copy number alteration in atleast 5 samples (434 genes)	All genes
DNM1	MRPL21	MRPL21
DPP4	C2CD3	C2CD3
DTNA	RELT	RELT
EIF4G3	RAB6A	RAB6A
EP300	COA4	COA4
ESYT2	ARHGEF17	ARHGEF17
FAT1	UCP2	UCP2
FAT4	MRPL48	MRPL48
FBN2	FCHSD2	FCHSD2
FBXW7	UCP3	UCP3
FRAS1	PLEKHB1	PLEKHB1
HAPLN4	LIPT2	LIPT2
HECTD2	PPME1	PPME1
HECW1	PGM2L1	PGM2L1
HEPACAM2	CHRDL2	CHRDL2
HMCN1	AP001372	AP001372
IKZF1	POLD3	POLD3
INPP5D	STARD10	STARD10
IQSEC3	ATG16L2	ATG16L2
KCNB1	KCNE3	KCNE3
KCNG2	P4HA3	P4HA3
KCNMB2	CPT1A	CPT1A
KIRREL3	P2RY2	P2RY2
KLHL1	ZNF507	ZNF507
LCT	UQCRFS1	UQCRFS1
LPAR6	CCNE1	CCNE1
LRRTM3	DPY19L3	DPY19L3
MMP16	URI1	URI1
MUC16	C19orf12	C19orf12
MYH4	PLEKHF1	PLEKHF1
MYO16	TSHZ3	TSHZ3
MYO7B	VSTM2B	VSTM2B
NEB	ZNF536	POP4
NFE2L2	POP4	AC011525
NTRK3	AC011525	MAGEF1

Mangalaparthi *et al.* , 2020. Mutational landscape of esophageal squamous cell carcinoma in an Indian cohort

Supplementary Table 8. List of genes screened for potential druggability using DGIdb resource

Somatic SNVs	Genes affected by copy number alteration in atleast 5 samples (434 genes)	All genes
NUP188	MAGEF1	HTR3E
NYAP1	HTR3E	PSMD2
PLB1	PSMD2	ECE2
PREX1	ECE2	EPHB3
PTPRB	EPHB3	AP2M1
RYR2	AP2M1	ABCF3
SETD1A	ABCF3	ALG3
SFRP1	ALG3	EIF4G1
SLC16A2	EIF4G1	DVL3
SLC7A14	DVL3	VWA5B2
SORCS1	VWA5B2	CAMK2N2
SP8	CAMK2N2	ZNF639
ST18	ZNF639	EIF2B5
ST6GALNAC5	EIF2B5	GNB4
STXBP5L	GNB4	KCNMB3
SYNE2	KCNMB3	CHRD
TRPM3	CHRD	ACTL6A
UBR5	ACTL6A	MFN1
UROC1	PIK3CA	HTR3D
WDR72	KCNMB2	FXR1
XKR7	MFN1	DNAJC19
ZNF536	HTR3D	ZMAT3
	FXR1	NDUFB5
	DNAJC19	USP13
	ZMAT3	FAM131A
	NDUFB5	TTC14
	USP13	POLR2H
	FAM131A	PEX5L
	TTC14	SOX2
	POLR2H	CCDC39
	PEX5L	MRPL47
	SOX2	ABCC5
	CCDC39	HTR3C
	MRPL47	CLCN2
	ABCC5	THPO

Mangalaparthi *et al.* , 2020. Mutational landscape of esophageal squamous cell carcinoma in an Indian cohort

Supplementary Table 8. List of genes screened for potential druggability using DGIdb resource

Somatic SNVs	Genes affected by copy number alteration in atleast 5 samples (434 genes)	All genes
	HTR3C	FGF12
	CLCN2	C3orf65
	THPO	SELT
	FGF12	MB21D2
	C3orf65	AHSG
	SELT	CRYGS
	MB21D2	FETUB
	AHSG	LPP
	CRYGS	MASP1
	FETUB	ETV5
	LPP	MAP6D1
	MASP1	ATP11B
	ETV5	FAM188B2
	MAP6D1	EHHADH
	ATP11B	ST6GAL1
	FAM188B2	YEATS2
	EHHADH	RTP4
	ST6GAL1	PARL
	YEATS2	ATP13A4
	RTP4	SST
	PARL	TRA2B
	ATP13A4	KNG1
	SST	KLHL24
	TRA2B	TP63
	KNG1	LEPREL1
	KLHL24	TPRG1
	TP63	FAM194A
	LEPREL1	HRASLS
	TPRG1	IGF2BP2
	FAM194A	SIAH2
	HRASLS	TBCCD1
	IGF2BP2	RPL39L
	SIAH2	RFC4
	TBCCD1	HRG
	RPL39L	DNAJB11

Mangalaparthi *et al.* , 2020. Mutational landscape of esophageal squamous cell carcinoma in an Indian cohort

Supplementary Table 8. List of genes screened for potential druggability using DGIdb resource

Somatic SNVs	Genes affected by copy number alteration in atleast 5 samples (434 genes)	All genes
	RFC4	RTP2
	HRG	MAP3K13
	DNAJB11	TMEM41A
	RTP2	BCL6
	MAP3K13	RTP1
	TMEM41A	DGKG
	BCL6	ADIPOQ
	RTP1	EIF4A2
	DGKG	DCUN1D1
	ADIPOQ	AC022498
	EIF4A2	OPA1
	DCUN1D1	SENP2
	AC022498	VPS8
	OPA1	C3orf70
	SENP2	ATP13A5
	VPS8	KLHL6
	C3orf70	MCCC1
	ATP13A5	LIPH
	KLHL6	MYNN
	MCCC1	PP13439
	LIPH	ACTRT3
	MYNN	SUCNR1
	PP13439	PRKCI
	ACTRT3	SI
	SUCNR1	ECT2
	PRKCI	TSC22D2
	SI	P2RY12
	ECT2	SLC51A
	TSC22D2	KIAA0226
	P2RY12	LRRIQ4
	SLC51A	SPATA16
	KIAA0226	SMCO1
	LRRIQ4	UBXN7
	SPATA16	GP5
	SMCO1	NAALADL2

Mangalaparthi *et al.* , 2020. Mutational landscape of esophageal squamous cell carcinoma in an Indian cohort

Supplementary Table 8. List of genes screened for potential druggability using DGIdb resource

Somatic SNVs	Genes affected by copy number alteration in atleast 5 samples (434 genes)	All genes
	UBXN7	LINC00969
	GP5	PHC3
	NAALADL2	GPR160
	LINC00969	C3orf79
	PHC3	ZDHHC19
	GPR160	ARHGEF26
	C3orf79	MED12L
	ZDHHC19	FNDC3B
	ARHGEF26	LSG1
	MED12L	GPR87
	FNDC3B	TERC
	LSG1	AADAC
	GPR87	NLGN1
	TERC	CLDN1
	AADAC	EIF2A
	NLGN1	IGSF10
	CLDN1	WDR49
	EIF2A	PDCD10
	IGSF10	EIF5A2
	WDR49	LMLN
	PDCD10	BDH1
	EIF5A2	MUC4
	LMLN	TNK2
	BDH1	CPN2
	MUC4	ACAP2
	TNK2	ATP13A3
	CPN2	NCEH1
	ACAP2	DHX36
	ATP13A3	B3GNT5
	NCEH1	CLDN16
	DHX36	RNF168
	B3GNT5	PYDC2
	CLDN16	TNIK
	RNF168	MBNL1
	PYDC2	TCTEX1D2

Mangalaparthi *et al.* , 2020. Mutational landscape of esophageal squamous cell carcinoma in an Indian cohort

Supplementary Table 8. List of genes screened for potential druggability using DGIdb resource

Somatic SNVs	Genes affected by copy number alteration in atleast 5 samples (434 genes)	All genes
	SLC7A14	HES1
	TNIK	UTS2B
	MBNL1	P2RY1
	TCTEX1D2	MFI2
	HES1	MCF2L2
	UTS2B	GMNC
	P2RY1	EGFEM1P
	MFI2	NCBP2
	MCF2L2	IL1RAP
	GMNC	TFRC
	EGFEM1P	LAMP3
	NCBP2	WDR53
	IL1RAP	CCDC50
	TFRC	DLG1
	LAMP3	FBXO45
	WDR53	CLDN11
	CCDC50	TM4SF19
	DLG1	TMEM212
	FBXO45	LRRC15
	CLDN11	PCYT1A
	TM4SF19	PPP1R2
	TMEM212	RPL35A
	LRRC15	SERPINI1
	PCYT1A	CLRN1
	PPP1R2	XXYLT1
	RPL35A	AADACL2
	SERPINI1	SAMD7
	CLRN1	GPR171
	XXYLT1	PAK2
	AADACL2	TMEM207
	SAMD7	SEC62
	GPR171	PIGZ
	PAK2	SERP1
	TMEM207	RPL22L1
	SEC62	RNF13

Mangalaparthi *et al.* , 2020. Mutational landscape of esophageal squamous cell carcinoma in an Indian cohort

Supplementary Table 8. List of genes screened for potential druggability using DGIdb resource

Somatic SNVs	Genes affected by copy number alteration in atleast 5 samples (434 genes)	All genes
	PIGZ	FYTTD1
	SERP1	PFN2
	RPL22L1	APOD
	RNF13	MUC20
	FYTTD1	CEP19
	PFN2	SENP5
	APOD	SKIL
	MUC20	IQCG
	CEP19	LRCH3
	SENP5	GPR149
	SKIL	BCHE
	IQCG	SERPINI2
	LRCH3	PIGX
	GPR149	LRRC34
	BCHE	PLD1
	SERPINI2	SLITRK3
	PIGX	RAP2B
	LRRC34	LRRC31
	PLD1	AC092964
	SLITRK3	ANKUB1
	RAP2B	TMEM14E
	LRRC31	GHSR
	AC092964	SDHAP1
	ANKUB1	NRROS
	TMEM14E	TBL1XR1
	GHSR	TNFSF10
	SDHAP1	ZBBX
	NRROS	MECOM
	TBL1XR1	SLC2A2
	TNFSF10	P2RY13
	ZBBX	GOLIM4
	MECOM	OSTN
	SLC2A2	TMEM44
	P2RY13	WWTR1
	GOLIM4	IL12A

Mangalaparthi *et al.* , 2020. Mutational landscape of esophageal squamous cell carcinoma in an Indian cohort

Supplementary Table 8. List of genes screened for potential druggability using DGIdb resource

Somatic SNVs	Genes affected by copy number alteration in atleast 5 samples (434 genes)	All genes
	OSTN	PTX3
	TMEM44	PLCH1
	WWTR1	VEPH1
	IL12A	AC104472
	PTX3	HLTF
	PLCH1	ARL14
	VEPH1	RARRES1
	AC104472	CP
	HLTF	C3orf33
	ARL14	AGTR1
	RARRES1	KCNAB1
	CP	PPM1L
	C3orf33	TRIM59
	AGTR1	SMC4
	KCNAB1	C3orf80
	PPM1L	MLF1
	TRIM59	CPB1
	SMC4	TM4SF4
	C3orf80	SCHIP1
	MLF1	CCNL1
	CPB1	MFSD1
	TM4SF4	SHOX2
	SCHIP1	RSRC1
	CCNL1	B3GALNT1
	MFSD1	TM4SF18
	SHOX2	HPS3
	RSRC1	NMD3
	B3GALNT1	OTOL1
	TM4SF18	C3orf55
	HPS3	KPNA4
	NMD3	LXN
	OTOL1	SLC33A1
	C3orf55	TM4SF1
	KPNA4	LEKR1
	LXN	GFM1

Mangalaparthi *et al.* , 2020. Mutational landscape of esophageal squamous cell carcinoma in an Indian cohort

Supplementary Table 8. List of genes screened for potential druggability using DGIdb resource

Somatic SNVs	Genes affected by copy number alteration in atleast 5 samples (434 genes)	All genes
	SLC33A1	SSR3
	TM4SF1	TIPARP
	LEKR1	COMMD2
	GFM1	IQCJ
	SSR3	IFT80
	TIPARP	GYG1
	COMMD2	SPTSSB
	IQCJ	PLSCR5
	IFT80	PLSCR1
	GYG1	PLOD2
	SPTSSB	PLSCR4
	PLSCR5	ZIC4
	CPA3	AC107021
	PLSCR1	ZIC1
	PLOD2	C3orf58
	PLSCR4	MME
	ZIC4	PLSCR2
	AC107021	GMPS
	ZIC1	SLC9A9
	C3orf58	PLS1
	MME	XRN1
	PLSCR2	TRPC1
	GMPS	PAQR9
	SLC9A9	ATR
	PLS1	U2SURP
	XRN1	PBX2P1
	TRPC1	PCOLCE2
	PAQR9	C5orf51
	ATR	RPL37
	U2SURP	OXCT1
	PBX2P1	EGFLAM
	PCOLCE2	FBXO4
	C5orf51	OSMR
	RPL37	C9
	OXCT1	PRKAA1

Mangalaparthi *et al.* , 2020. Mutational landscape of esophageal squamous cell carcinoma in an Indian cohort

Supplementary Table 8. List of genes screened for potential druggability using DGIdb resource

**Somatic SNVs**	**Genes affected by copy number alteration in atleast 5 samples (434 genes)**	**All genes**
	EGFLAM	MROH2B
	FBXO4	CARD6
	OSMR	LIFR
	C9	C6
	PRKAA1	PTGER4
	MROH2B	TTC33
	CARD6	GDNF
	LIFR	DAB2
	C6	C7
	PTGER4	RICTOR
	TTC33	PLCXD3
	GDNF	ZDHHC11B
	DAB2	FBXL7
	C7	SLC12A7
	RICTOR	SLC6A19
	PLCXD3	SLC9A3
	ZDHHC11B	CCDC152
	FBXL7	SLC6A3
	SLC12A7	LPCAT1
	SLC6A19	TERT
	SLC9A3	GHR
	CCDC152	EXOC3
	SLC6A3	SEPP1
	LPCAT1	TRIO
	TERT	IRX1
	GHR	NDUFS6
	EXOC3	WDR70
	SEPP1	SDHA
	TRIO	11-Mar
	IRX1	ANXA2R
	NDUFS6	MRPL36
	WDR70	PDCD6
	SDHA	CCDC127
	MARCH11	SLC6A18
	ANXA2R	C5orf38

Mangalaparthi *et al.* , 2020. Mutational landscape of esophageal squamous cell carcinoma in an Indian cohort

Supplementary Table 8. List of genes screened for potential druggability using DGIdb resource

Somatic SNVs	Genes affected by copy number alteration in atleast 5 samples (434 genes)	All genes
	MRPL36	AHRR
	PDCD6	TPPP
	CCDC127	ZDHHC11
	SLC6A18	ZNF131
	C5orf38	LRRC14B
	AHRR	CLPTM1L
	TPPP	FAM105A
	ZDHHC11	BRD9
	ZNF131	C5orf42
	LRRC14B	IRX4
	CLPTM1L	TRIP13
	FAM105A	ANKH
	BRD9	NIPBL
	C5orf42	CEP72
	IRX4	NKD2
	DNAH5	NUP155
	TRIP13	C5orf55
	ANKH	FAM105B
	NIPBL	SDHAP3
	CEP72	IRX2
	NKD2	C8orf22
	NUP155	POU5F1B
	C5orf55	CYHR1
	FAM105B	MYC
	SDHAP3	PPP1R16A
	IRX2	C8orf82
	C8orf22	PXDNL
	POU5F1B	PRKDC
	CYHR1	ARHGAP39
	MYC	LRRC14
	PPP1R16A	SNAI2
	C8orf82	EFCAB1
	PXDNL	MFSD3
	PRKDC	SNTG1
	ARHGAP39	MCM4

Mangalaparthi *et al.* , 2020. Mutational landscape of esophageal squamous cell carcinoma in an Indian cohort

Supplementary Table 8. List of genes screened for potential druggability using DGIdb resource

Somatic SNVs	Genes affected by copy number alteration in atleast 5 samples (434 genes)	All genes
	LRRC14	TMEM75
	SNAI2	KIFC2
	EFCAB1	GPT
	MFSD3	PCMTD1
	SNTG1	UBE2V2
	MCM4	FOXH1
	TMEM75	PVT1
	KIFC2	TP53
	GPT	CSMD3
	ST18	TTN
	PCMTD1	ASTN1
	UBE2V2	LRP1B
	FOXH1	DNAH10
	PVT1	HYDIN
		NF1
		NOTCH1
		RYR1
		SLIT2
		SLITRK2
		SYNE1
		TMEM132C
		USH2A
		VPS13B
		ZFHX4
		ADAMTSL1
		ARID4A
		CACNA1C
		CDC42BPA
		COL11A1
		COL4A1
		COL4A4
		COL6A5
		CTNNA2
		CTNND2
		CTTNBP2

Mangalaparthi *et al.* , 2020. Mutational landscape of esophageal squamous cell carcinoma in an Indian cohort

Supplementary Table 8. List of genes screened for potential druggability using DGIdb resource

Somatic SNVs	Genes affected by copy number alteration in atleast 5 samples (434 genes)	All genes
		CUL3
		DCHS1
		DIAPH2
		DMAP1
		DNM1
		DPP4
		DTNA
		EIF4G3
		EP300
		ESYT2
		FAT1
		FAT4
		FBN2
		FBXW7
		FRAS1
		HAPLN4
		HECTD2
		HECW1
		HEPACAM2
		HMCN1
		IKZF1
		INPP5D
		IQSEC3
		KCNB1
		KCNG2
		KIRREL3
		KLHL1
		LCT
		LPAR6
		LRRTM3
		MMP16
		MUC16
		MYH4
		MYO16
		MYO7B

Mangalaparthi *et al.* , 2020. Mutational landscape of esophageal squamous cell carcinoma in an Indian cohort

Supplementary Table 8. List of genes screened for potential druggability using DGIdb resource

Somatic SNVs	Genes affected by copy number alteration in atleast 5 samples (434 genes)	All genes
		NEB
		NFE2L2
		NTRK3
		NUP188
		NYAP1
		PLB1
		PREX1
		PTPRB
		RYR2
		SETD1A
		SFRP1
		SLC16A2
		SORCS1
		SP8
		ST6GALNAC5
		STXBP5L
		SYNE2
		TRPM3
		UBR5
		UROC1
		WDR72
		XKR7
		DNAH5
		PIK3CA
		CPA3
		KCNMB2
		SLC7A14
		ST18
		ZNF536
