# Supplementary material for: Mutational Landscape of Esophageal Squamous Cell Carcinoma in an Indian Cohort
Source: Front Oncol. 2020 Aug 20;10:1457. doi: 10.3389/fonc.2020.01457 (PMC7469928; doi:10.3389/fonc.2020.01457)
Supplement: Supplementary Table 9 — List of identified genes with an available FDA-approved anti-neoplastic drug using DGIdb resource. [file Table_9.pdf]

Mangalaparthi *et al.* , 2020. Mutational landscape of esophageal squamous cell carcinoma in an Indian cohort
Supplementary Table 9. List of identified genes with an available FDA-approved anti-neoplastic drug using DGIdb resource

**Gene**	**Drug**	**Interaction_types**	**Sources**	**PMIDs**
FGF3	SORAFENIB		CIViC	22890726
FOLR1	DEXAMETHASONE		NCI	15899836
P2RY2	TRETINOIN		NCI	18404486
CCNE1	PALBOCICLIB		CIViC	27020857
HTR3E	GRANISETRON		TdgClinicalTrial	
PSMD2	CARFILZOMIB	inhibitor	MyCancerGenome|ChemblInteractions	
PSMD2	BORTEZOMIB	inhibitor	MyCancerGenome|TdgClinicalTrial|ChemblInteractions|TEND	
EPHB3	VANDETANIB	inhibitor	ChemblInteractions	
HTR3D	GRANISETRON		TdgClinicalTrial	
ABCC5	FLUOROURACIL		PharmGKB	
HTR3C	GRANISETRON		TdgClinicalTrial	
ETV5	TRAMETINIB		CIViC	28178529
GPR87	CYCLOPHOSPHAMIDE	antagonist	GuideToPharmacologyInteractions	
TERC	IFOSFAMIDE		NCI	10769656
NLGN1	FLUOROURACIL		PharmGKB	
TNK2	BOSUTINIB	inhibitor	GuideToPharmacologyInteractions	
HES1	TRETINOIN		NCI	12080040
BCHE	IRINOTECAN		NCI	11345644
PLD1	TAMOXIFEN		NCI	12237338
SLC2A2	STREPTOZOTOCIN	ligand	DrugBank	9421374|7926307
RARRES1	TRETINOIN	agonist	TEND|DrugBank	15897880|15059893
AGTR1	DEXAMETHASONE		NCI	16482568
ATR	OLAPARIB		ClearityFoundationBiomarkers|CGI	
ATR	NIRAPARIB		ClearityFoundationBiomarkers	
ATR	RUCAPARIB		ClearityFoundationBiomarkers	
ATR	TEMOZOLOMIDE		CGI	
PRKAA1	ASPIRIN	activator	DrugBank	22517326|22406476
PRKAA1	PENTOSTATIN		NCI	11969266
PRKAA1	STREPTOZOTOCIN		NCI	15616011
PRKAA1	SIROLIMUS		NCI	16027121

Mangalaparthi *et al.* , 2020. Mutational landscape of esophageal squamous cell carcinoma in an Indian cohort
Supplementary Table 9. List of identified genes with an available FDA-approved anti-neoplastic drug using DGIdb resource

Gene	Drug	Interaction_types	Sources	PMIDs
PRKAA1	METFORMIN HYDROCHLORIDE		ClearityFoundationClinicalTrial	
PTGER4	ILOPROST	agonist	GuideToPharmacologyInteractions	
PTGER4	IBUPROFEN		NCI	9118476
PTGER4	STREPTOZOTOCIN		NCI	16954344
SLC6A3	PHENMETRAZINE	inhibitor	TdgClinicalTrial|TEND|DrugBank	12106802|17139284|17016423|17017961
SLC6A3	METHYLPHENIDATE	inhibitor|blocker	TdgClinicalTrial|GuideToPharmacologyInteractions|ChemblInteractions|NCI|TEND|TTD	15572278|12699766|15827573
TERT	BEVACIZUMAB		NCI	15687494
TERT	ARSENIC TRIOXIDE		NCI	16285558
TERT	OMACETAXINE MEPESUCCINATE		NCI	12744738
MYC	PREDNISONE		NCI	11911117
MYC	IMATINIB		NCI	15517875
MYC	VORINOSTAT		NCI	15583844
MYC	SULINDAC		NCI	12414619
MYC	TEMOZOLOMIDE		CGI	
MYC	THIOGUANINE		NCI	1988936
MYC	VERAPAMIL		NCI	1511424
MYC	AZACITIDINE		NCI	9006118
MYC	CALCITRIOL		NCI	8490200|15598784
GPT	TACROLIMUS		NCI	9825815
PVT1	IMATINIB MESYLATE		NCI	15251464
TP53	ASPIRIN	acetylation	DrugBank	21475861
TP53	BORTEZOMIB	inhibitor	TALC	
TP53	EPIRUBICIN		CKB	17388661|22903472
TP53	DECITABINE		CGI	
TP53	IRINOTECAN		CKB	25567130
TP53	TRAMETINIB		CKB	27659046
TP53	CYCLOPHOSPHAMIDE		CKB	17388661|16243804|26438783
TP53	ETOPOSIDE		CKB	25964101

Mangalaparthi *et al.* , 2020. Mutational landscape of esophageal squamous cell carcinoma in an Indian cohort
Supplementary Table 9. List of identified genes with an available FDA-approved anti-neoplastic drug using DGIdb resource

Gene	Drug	Interaction_types	Sources	PMIDs
TP53	VEMURAFENIB		CKB	26343583|28514312
TP53	PANITUMUMAB		CKB	28514312
TP53	INTERFERON ALFA-2B		NCI	16001656
TP53	GRANISETRON		ClearityFoundationClinicalTrial	
TP53	SIROLIMUS		CKB|NCI	26144316|16651424
TP53	PACLITAXEL		PharmGKB|CKB|NCI	16459017
TP53	OLAPARIB		CKB	22172724
TP53	PAZOPANIB		CKB	26646755|25669829
TP53	BEVACIZUMAB		CKB|NCI	27466356|21399868|23670029|11720743|17145525|15579019
TP53	TOPOTECAN		CKB	26438783
TP53	CARBOPLATIN		CKB|CIViC	25567130|25658463|11595686|26494859|27998224
TP53	DOCETAXEL		CKB|CIViC	21399868|22425996
TP53	RITUXIMAB		NCI	11895917
TP53	DASATINIB		CKB	26855149
TP53	TRIFLURIDINE		CKB	25700705
TP53	CYTARABINE		CKB	
TP53	CETUXIMAB		CIViC	24957073
TP53	PRAMLINTIDE		CGI	
TP53	DAUNORUBICIN		CKB	16243804
TP53	THIOTEPA		NCI	12058967
TP53	IFOSFAMIDE		CKB	23165797
TP53	PEMBROLIZUMAB		CKB	28039262
TP53	MYCOPHENOLIC ACID		NCI	16684279
TP53	CAPECITABINE		CIViC	24957073
TP53	TAMOXIFEN		CIViC	10786679
TP53	VORINOSTAT		CKB	26009011|25669829
TP53	OXALIPLATIN		CKB|CIViC	24957073|21468686

Mangalaparthi *et al.* , 2020. Mutational landscape of esophageal squamous cell carcinoma in an Indian cohort
Supplementary Table 9. List of identified genes with an available FDA-approved anti-neoplastic drug using DGIdb resource

**Gene**	**Drug**	**Interaction_types**	**Sources**	**PMIDs**
TP53	DABRAFENIB		CKB	27659046
TP53	ERLOTINIB		CKB	27659046
TP53	GEMCITABINE		CKB|CGI	27167172|23520471|21389100|27815358|26228206
TP53	FLUOROURACIL		PharmGKB	
TP53	DOXORUBICIN		CKB|CGI|CIViC	25658463|21399868|16243804|23165797|26826118|22698404|9569050
TP53	IBRUTINIB		CKB	26563132
TP53	TEMOZOLOMIDE		CKB	21730979
TP53	MITOMYCIN		CGI	
TP53	CRIZOTINIB		CKB	25971938|27149990|26438783
LRP1B	DOXORUBICIN		CIViC	22896685
NF1	TRAMETINIB		ClearityFoundationBiomarkers|OncoKB|CGI	24576830|21245089|25243813|26936308|24583796|2524381
NF1	NILOTINIB		CGI	
NF1	EVEROLIMUS		CGI	26859683|11010|2016|17557|2015
NF1	IMATINIB		CGI	
NF1	BEVACIZUMAB		CGI	24232489|2485933
NF1	VINBLASTINE		CGI	
NF1	COBIMETINIB		ClearityFoundationBiomarkers	
NF1	ERLOTINIB		CGI	
NF1	DASATINIB		CGI	
NF1	PAZOPANIB		CGI	
NF1	VEMURAFENIB		CGI|CIViC	23288408|231718
NF1	SORAFENIB		CGI	

Mangalaparthi *et al.* , 2020. Mutational landscape of esophageal squamous cell carcinoma in an Indian cohort
Supplementary Table 9. List of identified genes with an available FDA-approved anti-neoplastic drug using DGIdb resource

Gene	Drug	Interaction_types	Sources	PMIDs
NF1	TAMOXIFEN		CGI	
NF1	SIROLIMUS		CGI|CIViC	23171796
NF1	DABRAFENIB		CIViC	23171796
NOTCH1	CYTARABINE		CKB	25104330
NOTCH1	PREDNISOLONE		CKB	28151717
NOTCH1	BORTEZOMIB		CKB	28151717
NOTCH1	MERCAPTOPURINE		CKB	28151717
NOTCH1	EVEROLIMUS		CKB	28151717
NOTCH1	ASPARAGINASE		CKB	28151717
NOTCH1	METHOTREXATE		CKB	28151717
NOTCH1	TEMOZOLOMIDE		CKB	27154916
NOTCH1	DEXAMETHASONE		CKB	28151717
NOTCH1	Ribociclib		CKB	28151717
NOTCH1	DOCETAXEL		CKB	26202948
CACNA1C	VERAPAMIL	inhibitor|channel blocker	TdgClinicalTrial|GuideToPharmacologyInteractions|TEND|DrugBank	15880143|19125880|9846638|15286207
EP300	METHYLPHENIDATE	inhibitor	GuideToPharmacologyInteractions	
FBXW7	SIROLIMUS		CKB|CGI|CIViC	24586741|18787170|23558291
FBXW7	REGORAFENIB		CKB	27399335
FBXW7	DOCETAXEL		CKB	23274910
FBXW7	BELINOSTAT		CKB	23274910
FBXW7	VORINOSTAT		CKB	23274910
FBXW7	TEMSIROLIMUS		CKB	24360397
IKZF1	LENALIDOMIDE		CKB	24292625
MUC16	TOPOTECAN		NCI	17227902
MUC16	TAMOXIFEN		NCI	10629663
MUC16	DOCETAXEL		NCI	9288793|14756546
MUC16	ETOPOSIDE		NCI	1988893
NFE2L2	TAMOXIFEN		NCI	18539158
NFE2L2	SIROLIMUS		NCI	17652445

Mangalaparthi *et al.* , 2020. Mutational landscape of esophageal squamous cell carcinoma in an Indian cohort
Supplementary Table 9. List of identified genes with an available FDA-approved anti-neoplastic drug using DGIdb resource

Gene	Drug	Interaction_types	Sources	PMIDs
NFE2L2	FLUTAMIDE		NCI	16055512
NFE2L2	BLEOMYCIN (CHEMBL3039590)		NCI	15208274
NFE2L2	DEXAMETHASONE		NCI	15870285
NFE2L2	TRETINOIN		NCI	18048326
NFE2L2	DOXORUBICIN		NCI	18413364
PTPRB	SUNITINIB		CIViC	24633157
PIK3CA	CANDICIDIN	inhibitor	CKB|GuideToPharmacologyInteractions	26839307
PIK3CA	PHENMETRAZINE	inhibitor	OncoKB|CKB|GuideToPharmacologyInteractions	27672108|25877889|25172762|22653967|21169255|23721513|23258740|21362200|22355357|22188813|23136191|22065080|20881279|24076665|24900266|24561032|22294718|24608574|23394218|23850807|24310736|22049316|23726034|23258246|26137449|22340590|21668414|23662903|23903756|24244612
PIK3CA	IDELALISIB	inhibitor	CKB|GuideToPharmacologyInteractions|CIViC	26466009|26137449

Mangalaparthi *et al.* , 2020. Mutational landscape of esophageal squamous cell carcinoma in an Indian cohort
Supplementary Table 9. List of identified genes with an available FDA-approved anti-neoplastic drug using DGIdb resource

**Gene**	**Drug**	**Interaction_types**	**Sources**	**PMIDs**
PIK3CA	YOHIMBINE	inhibitor	OncoKB|GuideToPharmacologyInteractions	27672108|25877889|25172762|22915751|23275335|22653967|23085766|21169255|23721513|25425103|21362200|22355357|22188813|27126994|23136191|22065080|20881279|24076665|24900266|24561032|24608574|23850807|24310736|22049316|22915752|23726034|24198241|23258246|22340590|27402769|23662903|23903756|24244612
PIK3CA	PERTUZUMAB		CKB	23940356
PIK3CA	CAPECITABINE		CKB	26920887
PIK3CA	TAMOXIFEN		CKB	26116361
PIK3CA	CARBOPLATIN		CKB|NCI	16275998|21216929
PIK3CA	CABOZANTINIB		CIViC	25242168
PIK3CA	IRINOTECAN		CKB|NCI	24042735|16707468|15809721|25714871|21325073
PIK3CA	VENETOCLAX		CKB	27974663
PIK3CA	PONATINIB		CKB	26270481
PIK3CA	TEMSIROLIMUS		ClearityFoundationBiomarkers|CKB|CIViC	27893038|22271473|22245973|19706758|25527417|21216929|21289267|27016228
PIK3CA	FLOXURIDINE		CKB	25714871

Mangalaparthi *et al.* , 2020. Mutational landscape of esophageal squamous cell carcinoma in an Indian cohort
Supplementary Table 9. List of identified genes with an available FDA-approved anti-neoplastic drug using DGIdb resource

Gene	Drug	Interaction_types	Sources	PMIDs
PIK3CA	LAPATINIB		CKB|CIViC	27687302|26270481|26627007|23940356|26245675|22294718|26920887
PIK3CA	DOXORUBICIN		CKB|NCI	16905201|27893038|12761490
PIK3CA	Ribociclib		OncoKB|CIViC	25877889|22915751|23275335|23085766|25425103|22188813|27126994|24900266|22915752|23726034|24198241|27402769|25002028|23903756
PIK3CA	SIROLIMUS		CKB|NCI|CIViC	15647370|15878982|26882569|28514312|19029981|17376864
PIK3CA	COBIMETINIB		CKB	22084396
PIK3CA	BENZONATATE		CKB	27974663
PIK3CA	TOPOTECAN		CKB	21216929
PIK3CA	LETROZOLE		OncoKB|CKB	25877889|22915751|23275335|23085766|25425103|22188813|27126994|24900266|22915752|23726034|24198241|27402769|23903756
PIK3CA	BORTEZOMIB		CKB	21216929

Mangalaparthi *et al.* , 2020. Mutational landscape of esophageal squamous cell carcinoma in an Indian cohort
Supplementary Table 9. List of identified genes with an available FDA-approved anti-neoplastic drug using DGIdb resource

Gene	Drug	Interaction_types	Sources	PMIDs
PIK3CA	CETUXIMAB		CKB|CGI|CIViC	26715098|25714871|28446642|25724520|25838391|20619739|19223544|28424201|27002107
PIK3CA	PACLITAXEL		CKB|NCI	26469692|24170767|12761490|26245675|21216929
PIK3CA	OLAPARIB		OncoKB|CKB	25877889|26909613|22915751|23275335|23085766|25425103|22188813|27126994|24900266|22915752|23726034|24198241|27402769|23903756
PIK3CA	REGORAFENIB		CKB	23629727|25838391
PIK3CA	DASATINIB		CKB	26855149
PIK3CA	BEVACIZUMAB		CKB	27893038|23940356|21216929|25363205
PIK3CA	VINCRISTINE		NCI	12874004
PIK3CA	EVEROLIMUS		ClearityFoundationBiomarkers|OncoKB|CKB|CGI|CIViC	25877889|26951309|23629727|27797976|22915751|23275335|20664174|20664172|23085766|25425103|27893038|22188813|27126994|24900266|21358673|27091708|22915752|23726034|22662154|24198241|27402769|23903756

Mangalaparthi *et al.* , 2020. Mutational landscape of esophageal squamous cell carcinoma in an Indian cohort
Supplementary Table 9. List of identified genes with an available FDA-approved anti-neoplastic drug using DGIdb resource

**Gene**	**Drug**	**Interaction_types**	**Sources**	**PMIDs**
PIK3CA	SULINDAC		NCI	12654560
PIK3CA	TRASTUZUMAB		OncoKB|CKB|CGI|CIViC	25877889|17936563|22915751|23275335|21676217|24470511|27687302|23085766|25425103|26469692|22188813|27126994|28167203|24900266|23940356|20453058|26245675|22294718|22915752|23726034|21558396|24198241|27402769|23903756
PIK3CA	METFORMIN		CKB	23986086
PIK3CA	DABRAFENIB		CKB	27797976
PIK3CA	FLUOROURACIL		CKB	25714871|22336586|28424201
PIK3CA	SORAFENIB		CKB	23629727|25363205
PIK3CA	GEMCITABINE		CKB	28750271
PIK3CA	PANITUMUMAB		CIViC	19223544

Mangalaparthi *et al.* , 2020. Mutational landscape of esophageal squamous cell carcinoma in an Indian cohort
Supplementary Table 9. List of identified genes with an available FDA-approved anti-neoplastic drug using DGIdb resource

Gene	Drug	Interaction_types	Sources	PMIDs
PIK3CA	FULVESTRANT		OncoKB|CKB	27672108|25877889|25172762|22653967|27174596|21169255|23721513|21362200|22355357|22188813|23136191|22065080|20881279|24076665|24900266|24561032|24608574|23850807|24310736|22049316|23726034|23258246|22340590|23662903|23903756|24244612
PIK3CA	DOCETAXEL		PharmGKB	
PIK3CA	TRASTUZUMAB EMTANSINE		CKB	26920887
PIK3CA	VEMURAFENIB		CKB	24265153|26137449
PIK3CA	PALBOCICLIB		CKB|CIViC	27020857|25002028|26369631
PIK3CA	TRAMETINIB		CKB	27659046|27797976|26882569|26469692|26627007|26369631
PIK3CA	STREPTOZOTOCIN		NCI	16130182
