# Supplementary material for: Mutational Landscape of Esophageal Squamous Cell Carcinoma in an Indian Cohort
Source: Front Oncol. 2020 Aug 20;10:1457. doi: 10.3389/fonc.2020.01457 (PMC7469928; doi:10.3389/fonc.2020.01457)
Supplement: Supplementary Table 4 — List of somatic single nucleotide variants identified using whole exome sequencing of ESCC patients. [file Table_4.pdf]

Mangalaparthi *et al.* , 2020. Mutational landscape of esophageal squamous cell carcinoma in an Indian cohort
Supplementary Table 4. List of somatic single nucleotide variants identified using whole exome sequencing of ESCC patients

Cohort	Sample	Chromosome	Genomic position	Reference allele	Alternate allele	Gene symbol	Variant type	Variant annotation	OncoMD	COSMIC	dbSNP	ExAC	ExAC_SAS	1000G	1000G_SAS	SIFT	Polyphen2	CONDEL	VEP	LRT	PhastCon7 Way	Total read (Adjacent normal)	Reference allel read support (Adjacent normal)	Alternative read support (Adjacent normal)	Total read (Tumor)	Reference allel read support (Tumor)	Alternative read support (Tumor)	Alternate allele frequency
Smoker	42473	chr1	27023489	C	T	*ARID1A*	NONSENSE	ENSG00000117713:NONSENSE[ENST00000457599(c.595C>T,Cag>Tag):ENSP00000387636(p.Gln199Ter)];ENSG00000117713:NONSENSE[ENST00000324856(c.595C>T,Cag>Tag):ENSP00000320485(p.Gln199Ter)];	NA	NA	NA	NA	NA	NA	NA	NA	NA	NA	HIGH	NA	0.998	72	72	0	200	144	55	27.5
Smoker	42473	chr1	176853522	C	T	*ASTN1*	MISSENSE	ENSG00000152092:MISSENSE[ENST00000367654(c.3203G>A,cGt>cAt):ENSP00000356626(p.Arg1068His)];ENSG00000152092:MISSENSE[ENST00000424564(c.3179G>A,cGt>cAt):ENSP00000395041(p.Arg1060His)];ENSG00000152092:MISSENSE[ENST00000361833(c.3179G>A,cGt>cAt):ENSP00000354536(p.Arg1060His)];ENSG00000152092:MISSENSE[ENST00000367657(c.3179G>A,cGt>cAt):ENSP00000356629(p.Arg1060His)];	NA	NA	NA	5.77E-05	6.06E-05	NA	NA	T	PoD	N	MODERATE	D	0.989	71	70	1	92	51	41	44.57
Smoker	42473	chr1	181546968	T	A	*CACNA1E*	SILENT	ENSG00000198216:5UTR[ENST00000367567(c.-601T>A)];ENSG00000198216:SILENT[ENST00000360108(c.579T>A,cgT>cgA):ENSP00000353222(p.Arg193(=))];ENSG00000198216:SILENT[ENST00000524607(c.579T>A,cgT>cgA):ENSP00000432038(p.Arg193(=))];ENSG00000198216:SILENT[ENST00000358338(c.432T>A,cgT>cgA):ENSP00000351101(p.Arg144(=))];ENSG00000198216:SILENT[ENST00000357570(c.432T>A,cgT>cgA):ENSP00000350183(p.Arg144(=))];ENSG00000198216:SILENT[ENST00000526775(c.579T>A,cgT>cgA):ENSP00000434814(p.Arg193(=))];ENSG00000198216:SILENT[ENST00000367570(c.579T>A,cgT>cgA):ENSP00000356542(p.Arg193(=))];ENSG00000198216:SILENT[ENST00000367573(c.579T>A,cgT>cgA):ENSP00000356545(p.Arg193(=))];ENSG00000198216:EXONIC-NC[ENST00000533229(n.1013T>A)];	NA	NA	NA	NA	NA	NA	NA	NA	NA	NA	LOW	NA	NA	73	73	0	87	56	31	35.63
Smoker	42473	chr1	181719458	C	T	*CACNA1E*	INTRONIC	ENSG00000198216:[ENST00000358338(c.3528-1821C>T)];ENSG00000198216:[ENST00000367567(c.2541-1660C>T)];ENSG00000198216:[ENST00000357570(c.3585-1821C>T)];ENSG00000198216:[ENST00000360108(c.3663-154C>T)];ENSG00000198216:[ENST00000526775(c.3663-154C>T)];ENSG00000198216:[ENST00000367573(c.3720-154C>T)];ENSG00000198216:[ENST00000367570(c.3720-154C>T)];	NA	NA	NA	NA	NA	NA	NA	NA	NA	NA	MODIFIER	NA	NA	92	91	1	106	63	43	40.57
Smoker	42473	chr1	196648932	A	G	*CFH*	INTRONIC-SS-DNR	ENSG00000000971:[ENST00000466229(n.2806+9A>G)];ENSG00000000971:[ENST00000359637(c.598+9A>G)];ENSG00000000971:[ENST00000367429(c.790+9A>G)];ENSG00000000971:[ENST00000439155(c.790+9A>G)];	NA	NA	NA	8.58E-06	0	NA	NA	NA	NA	NA	MODIFIER	NA	NA	35	35	0	37	18	19	51.35
Smoker	42473	chr1	16948509	C	T	*CROCCP2*	INTRONIC	ENSG00000215908:[ENST00000540383(n.724-1976G>A)];ENSG00000215908:[ENST00000412962(n.1001-1990G>A)];	NA	NA	NA	NA	NA	NA	NA	NA	NA	NA	MODIFIER	NA	NA	36	35	1	63	40	23	36.51
Smoker	42473	chr1	36937750	G	C	*CSF3R*	INTRONIC-SS-ACR	ENSG00000119535:[ENST00000373106(c.998-10C>G)];ENSG00000119535:[ENST00000331941(c.998-10C>G)];ENSG00000119535:[ENST00000464365(n.673-10C>G)];ENSG00000119535:[ENST00000480825(n.2381-10C>G)];ENSG00000119535:[ENST00000361632(c.998-10C>G)];ENSG00000119535:[ENST00000373104(c.998-10C>G)];ENSG00000119535:[ENST00000487540(n.151-10C>G)];ENSG00000119535:[ENST00000418048(c.998-10C>G)];ENSG00000119535:[ENST00000338937(c.998-10C>G)];ENSG00000119535:[ENST00000373103(c.998-10C>G)];ENSG00000119535:[ENST00000440588(c.998-10C>G)];	NA	NA	NA	NA	NA	NA	NA	NA	NA	NA	MODIFIER	NA	NA	43	43	0	95	65	30	31.58
Smoker	42473	chr1	225521070	G	A	*DNAH14*	SILENT	ENSG00000185842:INTRONIC[ENST00000327794(c.2977+1791G>A)];ENSG00000185842:INTRONIC[ENST00000445597(c.7486-3898G>A)];ENSG00000185842:SILENT[ENST00000430092(c.9663G>A,gcG>gcA):ENSP00000414402(p.Ala3221(=))];ENSG00000185842:SILENT[ENST00000439375(c.9663G>A,gcG>gcA):ENSP00000392061(p.Ala3221(=))];	NA	NA	NA	NA	NA	NA	NA	NA	NA	NA	LOW	NA	NA	118	116	2	89	46	43	48.31
Smoker	42473	chr1	167097169	G	A	*DUSP27*	MISSENSE	ENSG00000198842:INTRONIC[ENST00000485151(n.419-809G>A)];ENSG00000198842:MISSENSE[ENST00000361200(c.2801G>A,aGt>aAt):ENSP00000354483(p.Ser934Asn)];ENSG00000198842:MISSENSE[ENST00000443333(c.2801G>A,aGt>aAt):ENSP00000404874(p.Ser934Asn)];ENSG00000198842:MISSENSE[ENST00000271385(c.2801G>A,aGt>aAt):ENSP00000271385(p.Ser934Asn)];	NA	NA	NA	NA	NA	NA	NA	T	BN	N	MODERATE	N	0.781	78	78	0	67	38	29	43.28
Smoker	42473	chr1	109260363	A	G	*FNDC7*	INTRONIC	ENSG00000143107:[ENST00000370017(c.83-36A>G)];ENSG00000143107:[ENST00000271311(c.86-36A>G)];	NA	NA	NA	NA	NA	NA	NA	NA	NA	NA	MODIFIER	NA	NA	119	118	0	196	136	59	30.1
Smoker	42473	chr1	29019413	A	T	*GMEB1*	INTRONIC	ENSG00000162419:[ENST00000294409(c.367-41A>T)];ENSG00000162419:[ENST00000373816(c.337-41A>T)];ENSG00000162419:[ENST00000480454(n.416-41A>T)];ENSG00000162419:[ENST00000361872(c.337-41A>T)];	NA	NA	NA	NA	NA	NA	NA	NA	NA	NA	MODIFIER	NA	NA	64	64	0	115	87	28	24.35
Smoker	42473	chr1	109732253	G	T	*KIAA1324*	MISSENSE	ENSG00000116299:MISSENSE[ENST00000529753(c.1127G>T,gGa>gTa):ENSP00000434595(p.Gly376Val)];ENSG00000116299:MISSENSE[ENST00000369939(c.1388G>T,gGa>gTa):ENSP00000358955(p.Gly463Val)];ENSG00000116299:MISSENSE[ENST00000457623(c.1238G>T,gGa>gTa):ENSP00000393964(p.Gly413Val)];ENSG00000116299:EXONIC-NC[ENST00000369936(n.545G>T)];ENSG00000116299:EXONIC-NC[ENST00000369938(n.1469G>T)];ENSG00000116299:EXONIC-NC[ENST00000490758(n.1189G>T)];ENSG00000116299:3UTR[ENST00000527996(c.*1078G>T)];	NA	NA	NA	NA	NA	NA	NA	NA	NA	NA	MODIFIER	NA	NA	28	28	0	56	35	20	35.71
Smoker	42473	chr1	72241981	C	G	*NEGR1*	INTRONIC-SS-ACR	ENSG00000172260:INTRONIC-SS-ACR[ENST00000467479(n.407-1G>C)];ENSG00000172260:INTRONIC-SS-ACR[ENST00000357731(c.410-1G>C)];ENSG00000172260:INTRONIC-SS-ACR[ENST00000306821(c.26-1G>C)];ENSG00000172260:INTRONIC-SS-ACR[ENST00000434200(c.404-1G>C)];	NA	NA	NA	NA	NA	NA	NA	NA	NA	NA	HIGH	NA	NA	61	61	0	71	53	18	25.35
Smoker	42473	chr1	156848916	C	T	*NTRK1*	MISSENSE-SS-PRX	ENSG00000198400:[ENST00000392302(c.1700C>T,tCc>tTc):ENSP00000376120(p.Ser567Phe)];ENSG00000198400:[ENST00000524377(c.1808C>T,tCc>tTc):ENSP00000431418(p.Ser603Phe)];ENSG00000198400:[ENST00000358660(c.1799C>T,tCc>tTc):ENSP00000351486(p.Ser600Phe)];ENSG00000198400:[ENST00000368196(c.1790C>T,tCc>tTc):ENSP00000357179(p.Ser597Phe)];ENSG00000198400:[ENST00000497019(c.*400C>T)];ENSG00000198400:[ENST00000530298(n.2261C>T)];	NA	NA	chr1*156848916*156848916*C*G*rs188270548*0*0.9998*0.0001997	NA	NA	NA	NA	NA	NA	NA	LOW	NA	NA	27	27	0	126	117	9	7.14
Smoker	42473	chr1	247886535	G	T	*OR14A2*	MISSENSE	ENSG00000241128:MISSENSE[ENST00000366485(c.811C>A,Ctg>Atg):ENSP00000355441(p.Leu271Met)];	NA	NA	NA	NA	NA	NA	NA	NA	NA	NA	MODIFIER	NA	NA	225	225	0	221	161	58	26.24
Smoker	42473	chr1	248756307	C	A	*OR2T10*	MISSENSE	ENSG00000184022:MISSENSE[ENST00000330500(c.763G>T,Gct>Tct):ENSP00000329210(p.Ala255Ser)];	chr1*24875	chr1*24875	NA	NA	NA	NA	NA	T	PoD	N	MODERATE	NA	NA	152	152	0	170	82	87	51.18

Mangalaparthi *et al.* , 2020. Mutational landscape of esophageal squamous cell carcinoma in an Indian cohort
Supplementary Table 4. List of somatic single nucleotide variants identified using whole exome sequencing of ESCC patients

Cohort	Sample	Chromosome	Genomic position	Reference allele	Alternate allele	Gene symbol	Variant type	Variant annotation	OncoMD	COSMIC	dbSNP	ExAC	ExAC_SAS	1000G	1000G_SAS	SIFT	Polyphen2	CONDEL	VEP	LRT	PhastCon7 Way	Total read (Adjacent normal)	Reference allel read support (Adjacent normal)	Alternative read support (Adjacent normal)	Total read (Tumor)	Reference allel read support (Tumor)	Alternative read support (Tumor)	Alternate allele frequency
Smoker	42473	chr1	151265386	C	T	*PI4KB*	MISSENSE	ENSG00000143393:MISSENSE[ENST00000368874(c.2348G>A,cGg>cAg):ENSP00000357868(p.Arg783Gln)];ENSG00000143393:MISSENSE[ENST00000529142(c.1397G>A,cGg>cAg):ENSP00000433149(p.Arg466Gln)];ENSG00000143393:MISSENSE[ENST00000368873(c.2393G>A,cGg>cAg):ENSP00000357867(p.Arg798Gln)];ENSG00000143393:MISSENSE[ENST00000368872(c.2348G>A,cGg>cAg):ENSP00000357866(p.Arg783Gln)];ENSG00000143393:MISSENSE[ENST00000271657(c.2429G>A,cGg>cAg):ENSP00000271657(p.Arg810Gln)];ENSG00000143393:MISSENSE[ENST00000368875(c.2429G>A,cGg>cAg):ENSP00000357869(p.Arg810Gln)];ENSG00000143393:MISSENSE[ENST00000455060(c.626G>A,cGg>cAg):ENSP00000410974(p.Arg209Gln)];ENSG00000143393:3UTR[ENST00000446339(c.*58G>A)];	NA	NA	NA	NA	NA	NA	NA	NA	NA	NA	MODIFIER	NA	NA	20	20	0	100	80	20	20
Smoker	42473	chr1	21991768	G	C	*RAP1GAP*	5UTR	ENSG00000076864:[ENST00000542643(c.-213C>G)];ENSG00000076864:[ENST00000317967(c.-213C>G)];ENSG00000076864:[ENST00000464457(n.156C>G)];ENSG00000076864:[ENST00000482984(n.127C>G)];ENSG00000076864:[ENST00000374765(c.-149+3979C>G)];ENSG00000076864:[ENST00000495204(c.-149+3979C>G)];	NA	NA	NA	NA	NA	NA	NA	NA	NA	NA	MODIFIER	NA	NA	65	65	0	293	201	92	31.4
Smoker	42473	chr1	182544644	G	C	*RNASEL*	SILENT	ENSG00000135828:SILENT[ENST00000444138(c.2109C>G,gtC>gtG):ENSP00000411147(p.Val703(=))];ENSG00000135828:SILENT[ENST00000367559(c.2109C>G,gtC>gtG):ENSP00000356530(p.Val703(=))];	NA	NA	chr1*182544644*182544644*G*A*rs535800688*0*0.9998*0.0001997	NA	NA	NA	NA	NA	NA	NA	LOW	NA	NA	71	71	0	62	27	35	56.45
Smoker	42473	chr1	247886535	G	T	*RP11-634B7.4*	INTRONIC	ENSG00000235749:[ENST00000449298(n.682-22970G>T)];	NA	NA	NA	NA	NA	NA	NA	NA	NA	NA	MODIFIER	NA	NA	225	225	0	221	161	58	26.24
Smoker	42473	chr1	247886535	G	T	*RP11-634B7.5*	EXONIC-NC-SS-PR	ENSG00000239395:[ENST00000426444(n.215C>A)];ENSG00000239395:[ENST00000419891(n.114+418C>A)];	NA	NA	NA	NA	NA	NA	NA	NA	NA	NA	MODIFIER	NA	NA	225	225	0	221	161	58	26.24
Smoker	42473	chr1	36552339	G	A	*TEKT2*	MISSENSE	ENSG00000092850:MISSENSE[ENST00000207457(c.523G>A,Gac>Aac):ENSP00000207457(p.Asp175Asn)];ENSG00000092850:3UTR[ENST00000469024(c.*327G>A)];	chr1*36552	chr1*36552	NA	8.24E-06	0	NA	NA	NA	NA	NA	MODIFIER	NA	NA	30	30	0	73	47	26	35.62
Smoker	42473	chr1	156020090	T	C	*UBQLN4*	MISSENSE	ENSG00000160803:MISSENSE[ENST00000368309(c.733A>G,Atg>Gtg):ENSP00000357292(p.Met245Val)];ENSG00000160803:EXONIC-NC[ENST00000472638(n.488A>G)];	NA	NA	NA	NA	NA	NA	NA	NA	NA	NA	MODIFIER	NA	NA	47	47	0	115	54	61	53.04
Smoker	42473	chr1	22838124	A	C	*ZBTB40*	INTRONIC	ENSG00000184677:[ENST00000404138(c.2003-45A>C)];ENSG00000184677:[ENST00000375647(c.2003-45A>C)];ENSG00000184677:[ENST00000374651(c.1667-45A>C)];	NA	NA	NA	NA	NA	NA	NA	NA	NA	NA	MODIFIER	NA	NA	50	50	0	102	72	30	29.41
Smoker	42473	chr1	200376534	T	C	*ZNF281*	MISSENSE	ENSG00000162702:MISSENSE[ENST00000367353(c.2300A>G,gAt>gGt):ENSP00000356322(p.Asp767Gly)];ENSG00000162702:MISSENSE[ENST00000367352(c.2192A>G,gAt>gGt):ENSP00000356321(p.Asp731Gly)];ENSG00000162702:MISSENSE[ENST00000294740(c.2300A>G,gAt>gGt):ENSP00000294740(p.Asp767Gly)];	NA	NA	NA	NA	NA	NA	NA	D	PrD	D	MODERATE	D	1	176	176	0	135	82	53	39.26
Smoker	42473	chr1	45501476	C	G	*ZSWIM5*	SILENT	ENSG00000162415:SILENT[ENST00000359600(c.2175G>C,ctG>ctC):ENSP00000352614(p.Leu725(=))];	NA	NA	NA	NA	NA	NA	NA	NA	NA	NA	LOW	NA	NA	80	80	0	157	111	46	29.3
Smoker	42473	chr10	1405306	C	T	*ADARB2*	MISSENSE	ENSG00000185736:MISSENSE[ENST00000381312(c.994G>A,Gcg>Acg):ENSP00000370713(p.Ala332Thr)];	chr10*1405	chr10*1405	NA	NA	NA	NA	NA	D	PrD	D	MODERATE	D	0.718	49	49	0	91	50	41	45.05
Smoker	42473	chr10	133787483	C	A	*BNIP3*	INTRONIC	ENSG00000176171:[ENST00000368636(c.47-36G>T)];ENSG00000176171:[ENST00000540159(c.47-36G>T)];	NA	NA	NA	2.58E-05	0	NA	NA	NA	NA	NA	MODIFIER	NA	NA	30	30	0	44	21	23	52.27
Smoker	42473	chr10	50534825	G	A	*C10orf71*	MISSENSE	ENSG00000177354:MISSENSE[ENST00000374144(c.4235G>A,gGt>gAt):ENSP00000363259(p.Gly1412Asp)];ENSG00000177354:INTRONIC[ENST00000323868(c.2075-151G>A)];	NA	NA	NA	NA	NA	NA	NA	NA	NA	NA	MODIFIER	NA	NA	21	21	0	37	23	14	37.84
Smoker	42473	chr10	68687042	A	G	*CTNNA3*	INTRONIC	ENSG00000183230:[ENST00000433211(c.1048-151760T>C)];ENSG00000183230:[ENST00000494580(c.128-151760T>C)];ENSG00000183230:[ENST00000373744(c.1048-151760T>C)];	NA	NA	NA	1.65E-05	0	NA	NA	T	PoD	N	MODERATE	D	1	258	258	0	195	116	79	40.51
Smoker	42473	chr10	44793326	G	A	*CXCL12*	3UTR	ENSG00000107562:[ENST00000395795(c.*13C>T)];	NA	NA	0.0001997	NA	NA	0.0001997	0.001	NA	NA	NA	MODIFIER	NA	NA	91	90	0	101	66	35	34.65
Smoker	42473	chr10	128780313	G	A	*DOCK1*	INTRONIC	ENSG00000150760:[ENST00000280333(c.227+76G>A)];	NA	NA	NA	NA	NA	NA	NA	NA	NA	NA	MODIFIER	NA	NA	92	92	0	104	56	48	46.15
Smoker	42473	chr10	7651468	A	T	*ITIH5*	INTRONIC	ENSG00000123243:[ENST00000397146(c.939+6477T>A)];ENSG00000123243:[ENST00000446830(c.285+6477T>A)];ENSG00000123243:[ENST00000434980(n.564+6477T>A)];ENSG00000123243:[ENST00000298441(c.297+6477T>A)];ENSG00000123243:[ENST00000256861(c.939+6477T>A)];ENSG00000123243:[ENST00000397145(c.939+6477T>A)];ENSG00000123243:[ENST00000461751(c.272-11T>A)];ENSG00000123243:[ENST00000476417(n.5482+6477T>A)];	NA	NA	NA	NA	NA	NA	NA	NA	NA	NA	MODIFIER	NA	NA	93	92	0	102	55	47	46.08
Smoker	42473	chr10	68687042	A	G	*LRRTM3*	MISSENSE	ENSG00000198739:MISSENSE[ENST00000361320(c.368A>G,tAt>tGt):ENSP00000355187(p.Tyr123Cys)];	NA	NA	NA	1.65E-05	0	NA	NA	T	PoD	N	MODERATE	D	1	258	258	0	195	116	79	40.51
Smoker	42473	chr10	69667905	G	A	*SIRT1*	INTRONIC	ENSG00000096717:[ENST00000432464(c.285+23G>A)];ENSG00000096717:[ENST00000403579(c.261+23G>A)];ENSG00000096717:[ENST00000212015(c.1170+23G>A)];ENSG00000096717:[ENST00000406900(c.261+23G>A)];	NA	NA	NA	NA	NA	NA	NA	NA	NA	NA	MODIFIER	NA	NA	42	42	0	52	35	17	32.69
Smoker	42473	chr10	108923780	C	T	*SORCS1*	MISSENSE	ENSG00000108018:MISSENSE[ENST00000263054(c.505G>A,Gga>Aga):ENSP00000263054(p.Gly169Arg)];ENSG00000108018:MISSENSE[ENST00000344440(c.505G>A,Gga>Aga):ENSP00000345964(p.Gly169Arg)];	NA	NA	NA	NA	NA	NA	NA	D	PoD	D	MODERATE	D	0.98	162	162	0	191	103	88	46.07
Smoker	42473	chr11	44297250	C	A	*ALX4*	INTRONIC	ENSG00000052850:[ENST00000329255(c.467-42G>T)];	NA	NA	chr11*44297250*44297251*CA*C*rs757563132*NA*NA*NA	NA	NA	NA	NA	NA	NA	NA	MODIFIER	NA	NA	49	49	0	57	38	19	33.33
Smoker	42473	chr11	94533436	C	T	*AMOTL1*	SILENT	ENSG00000166025:SILENT[ENST00000317837(c.1080C>T,gcC>gcT):ENSP00000323474(p.Ala360(=))];ENSG00000166025:SILENT[ENST00000317829(c.930C>T,gcC>gcT):ENSP00000320968(p.Ala310(=))];ENSG00000166025:SILENT[ENST00000433060(c.1080C>T,gcC>gcT):ENSP00000387739(p.Ala360(=))];	NA	NA	NA	5.05E-05	0	NA	NA	NA	NA	NA	LOW	NA	NA	98	98	0	150	65	85	56.67
Smoker	42473	chr11	926053	G	A	*AP2A2*	MISSENSE	ENSG00000183020:MISSENSE[ENST00000332231(c.32G>A,cGg>cAg):ENSP00000327694(p.Arg11Gln)];ENSG00000183020:MISSENSE[ENST00000528815(c.32G>A,cGg>cAg):ENSP00000431630(p.Arg11Gln)];ENSG00000183020:MISSENSE[ENST00000448903(c.32G>A,cGg>cAg):ENSP00000413234(p.Arg11Gln)];ENSG00000183020:MISSENSE[ENST00000534328(c.32G>A,cGg>cAg):ENSP00000436059(p.Arg11Gln)];ENSG00000183020:INTRONIC[ENST00000529818(n.44+1116G>A)];ENSG00000183020:INTRONIC[ENST00000525796(c.-46+53G>A)];ENSG00000183020:EXONIC-NC[ENST00000528195(n.109G>A)];ENSG00000183020:EXONIC-NC[ENST00000529858(n.184G>A)];	NA	NA	NA	NA	NA	NA	NA	NA	NA	NA	MODIFIER	NA	NA	52	52	0	77	42	35	45.45
Smoker	42473	chr11	111896421	G	A	*DLAT*	SILENT	ENSG00000150768:SILENT[ENST00000280346(c.225G>A,ctG>ctA):ENSP00000280346(p.Leu75(=))];ENSG00000150768:SILENT[ENST00000393051(c.225G>A,ctG>ctA):ENSP00000376771(p.Leu75(=))];ENSG00000150768:SILENT[ENST00000531306(c.102G>A,ctG>ctA):ENSP00000433432(p.Leu34(=))];ENSG00000150768:INTRONIC[ENST00000533297(c.152+73G>A)];ENSG00000150768:5UTR[ENST00000537636(c.-82G>A)];	NA	NA	NA	NA	NA	NA	NA	NA	NA	NA	LOW	NA	NA	79	78	1	153	69	83	54.25

Mangalaparthi *et al.* , 2020. Mutational landscape of esophageal squamous cell carcinoma in an Indian cohort
Supplementary Table 4. List of somatic single nucleotide variants identified using whole exome sequencing of ESCC patients

Cohort	Sample	Chromosome	Genomic position	Reference allele	Alternate allele	Gene symbol	Variant type	Variant annotation	OncoMD	COSMIC	dbSNP	ExAC	ExAC_SAS	1000G	1000G_SAS	SIFT	Polyphen2	CONDEL	VEP	LRT	PhastCon7 Way	Total read (Adjacent normal)	Reference allel read support (Adjacent normal)	Alternative read support (Adjacent normal)	Total read (Tumor)	Reference allel read support (Tumor)	Alternative read support (Tumor)	Alternate allele frequency
Smoker	42473	chr11	118922746	G	T	*HYOU1*	INTRONIC	ENSG00000149428:[ENST00000404233(c.1205+56C>A)];ENSG00000149428:[ENST00000543287(c.944+56C>A)];ENSG00000149428:[ENST00000529972(c.1205+56C>A)];ENSG00000149428:[ENST00000530473(c.1205+56C>A)];ENSG00000149428:[ENST00000525859(c.1205+56C>A)];ENSG00000149428:[ENST00000532519(c.*994+56C>A)];	NA	NA	NA	NA	NA	NA	NA	NA	NA	NA	MODIFIER	NA	NA	63	63	0	151	75	76	50.33
Smoker	42473	chr11	126326259	G	A	*KIRREL3*	MISSENSE	ENSG00000149571:MISSENSE[ENST00000529097(c.833C>T,gCt>gTt):ENSP00000434081(p.Ala278Val)];ENSG00000149571:MISSENSE[ENST00000525144(c.833C>T,gCt>gTt):ENSP00000435466(p.Ala278Val)];ENSG00000149571:MISSENSE[ENST00000525704(c.833C>T,gCt>gTt):ENSP00000435094(p.Ala278Val)];	NA	NA	NA	NA	NA	NA	NA	D	PoD	D	MODERATE	D	1	27	27	0	70	41	29	41.43
Smoker	42473	chr11	28352087	A	C	*METTL15*	MISSENSE	ENSG00000169519:MISSENSE[ENST00000407364(c.923A>C,aAg>aCg):ENSP00000384369(p.Lys308Thr)];ENSG00000169519:3UTR[ENST00000406787(c.*39A>C)];ENSG00000169519:3UTR[ENST00000451385(c.*537A>C)];ENSG00000169519:3UTR[ENST00000342303(c.*172A>C)];ENSG00000169519:3UTR[ENST00000303459(c.*172A>C)];ENSG00000169519:EXONIC-NC[ENST00000468295(n.730A>C)];	NA	NA	chr11*28352087*28352087*A*G*rs780739721*NA*NA*NA	NA	NA	NA	NA	NA	NA	NA	MODIFIER	NA	NA	170	169	1	157	92	65	41.4
Smoker	42473	chr11	48387284	G	A	*OR4C5*	MISSENSE	ENSG00000176540:MISSENSE[ENST00000319813(c.734C>T,tCa>tTa):ENSP00000321338(p.Ser245Leu)];	NA	NA	chr11*48387284*48387284*G*GAGTCTTTAGTA,GATGTGAGTCTTTAGTA*rs747433867*NA*NA*NA	NA	NA	NA	NA	D_lc	PrD	D	MODERATE	N	0.886	180	177	2	153	145	8	5.23
Smoker	42473	chr11	123810708	C	A	*OR4D5*	MISSENSE	ENSG00000171014:MISSENSE[ENST00000307033(c.385C>A,Ccc>Acc):ENSP00000305970(p.Pro129Thr)];	NA	NA	NA	NA	NA	NA	NA	D	PrD	D	MODERATE	D	0.967	97	97	0	126	56	70	55.56
Smoker	42473	chr11	5863138	A	T	*OR52E6*	INTRONIC-SS-ACR	ENSG00000205409:[ENST00000379946(c.8-6T>A)];	NA	NA	NA	NA	NA	NA	NA	NA	NA	NA	MODIFIER	NA	NA	245	244	0	212	125	86	40.57
Smoker	42473	chr11	67938615	C	A	*SUV420H1*	MISSENSE	ENSG00000110066:MISSENSE[ENST00000402789(c.844G>T,Gtg>Ttg):ENSP00000385005(p.Val282Leu)];ENSG00000110066:MISSENSE[ENST00000405515(c.844G>T,Gtg>Ttg):ENSP00000385640(p.Val282Leu)];ENSG00000110066:MISSENSE[ENST00000402185(c.775G>T,Gtg>Ttg):ENSP00000384724(p.Val259Leu)];ENSG00000110066:MISSENSE[ENST00000401547(c.844G>T,Gtg>Ttg):ENSP00000385965(p.Val282Leu)];ENSG00000110066:MISSENSE[ENST00000304363(c.844G>T,Gtg>Ttg):ENSP00000305899(p.Val282Leu)];ENSG00000110066:3UTR[ENST00000427752(c.*432G>T)];ENSG00000110066:3UTR[ENST00000441488(c.*52G>T)];ENSG00000110066:3UTR[ENST00000323599(c.*313G>T)];	NA	NA	NA	NA	NA	NA	NA	NA	NA	NA	MODIFIER	NA	NA	165	164	0	170	94	76	44.71
Smoker	42473	chr11	121016606	A	G	*TECTA*	MISSENSE	ENSG00000109927:MISSENSE[ENST00000264037(c.3886A>G,Aaa>Gaa):ENSP00000264037(p.Lys1296Glu)];ENSG00000109927:MISSENSE[ENST00000392793(c.3886A>G,Aaa>Gaa):ENSP00000376543(p.Lys1296Glu)];ENSG00000109927:EXONIC-NC[ENST00000478058(n.451A>G)];	NA	NA	NA	NA	NA	NA	NA	NA	NA	NA	MODIFIER	NA	NA	119	119	0	200	153	47	23.5
Smoker	42473	chr11	121023711	C	T	*TECTA*	SILENT	ENSG00000109927:SILENT[ENST00000264037(c.4227C>T,gaC>gaT):ENSP00000264037(p.Asp1409(=))];ENSG00000109927:SILENT[ENST00000392793(c.4227C>T,gaC>gaT):ENSP00000376543(p.Asp1409(=))];	chr11*1210	chr11*1210	NA	1.66E-05	0	NA	NA	NA	NA	NA	LOW	NA	0.983	51	51	0	85	23	62	72.94
Smoker	42473	chr11	5863138	A	T	*TRIM5*	INTRONIC	ENSG00000132256:[ENST00000412903(c.-62+95493T>A)];ENSG00000132256:[ENST00000380027(c.-441+13844T>A)];	NA	NA	NA	NA	NA	NA	NA	NA	NA	NA	MODIFIER	NA	NA	245	244	0	212	125	86	40.57
Smoker	42473	chr12	9243103	G	A	*A2M*	INTRONIC	ENSG00000175899:[ENST00000543436(c.213-25C>T)];ENSG00000175899:[ENST00000545828(n.348+11043C>T)];ENSG00000175899:[ENST00000318602(c.2470-25C>T)];ENSG00000175899:[ENST00000462568(n.24C>T)];	NA	NA	NA	NA	NA	NA	NA	NA	NA	NA	MODIFIER	NA	NA	105	105	0	78	47	31	39.74
Smoker	42473	chr12	9263989	T	G	*A2M*	INTRONIC	ENSG00000175899:[ENST00000404455(c.483+766A>C)];ENSG00000175899:[ENST00000539638(c.*50+766A>C)];ENSG00000175899:[ENST00000318602(c.483+766A>C)];	NA	NA	NA	NA	NA	NA	NA	NA	NA	NA	MODIFIER	NA	NA	102	101	1	102	61	41	40.2
Smoker	42473	chr12	91348301	C	T	*CCER1*	SILENT	ENSG00000197651:SILENT[ENST00000358859(c.219G>A,ccG>ccA):ENSP00000351727(p.Pro73(=))];ENSG00000197651:INTRONIC[ENST00000548187(n.52+599G>A)];	NA	NA	NA	NA	NA	NA	NA	NA	NA	NA	MODIFIER	NA	NA	74	74	0	89	44	44	49.44
Smoker	42473	chr12	132834337	C	A	*GALNT9*	MISSENSE	ENSG00000182870:MISSENSE[ENST00000328957(c.850G>T,Gtg>Ttg):ENSP00000329846(p.Val284Leu)];ENSG00000182870:EXONIC-NC[ENST00000424720(n.996G>T)];	NA	NA	NA	NA	NA	NA	NA	NA	NA	NA	MODIFIER	NA	NA	49	48	1	106	67	38	35.85
Smoker	42473	chr12	52865918	G	C	*KRT6C*	SILENT	ENSG00000170465:SILENT[ENST00000252250(c.687C>G,gtC>gtG):ENSP00000252250(p.Val229(=))];	NA	NA	chr12*52865918*52865918*G*A,C*rs425073*NA*NA*NA	0.2245138	0.147833312	NA	NA	NA	NA	NA	LOW	NA	NA	51	50	1	24	4	20	83.33
Smoker	42473	chr12	52865925	C	T	*KRT6C*	MISSENSE	ENSG00000170465:MISSENSE[ENST00000252250(c.680G>A,aGc>aAc):ENSP00000252250(p.Ser227Asn)];	chr12*5286	NA	NA	0.1938826	0.127581864	NA	NA	T	BN	N	MODERATE	N	0	45	45	0	15	4	11	73.33
Smoker	42473	chr12	40922015	A	C	*MUC19*	INTRONIC	ENSG00000205592:[ENST00000474954(n.2922-44A>C)];ENSG00000205592:[ENST00000424466(c.56+103A>C)];ENSG00000205592:[ENST00000454784(c.*5783-14349A>C)];	NA	NA	NA	NA	NA	NA	NA	NA	NA	NA	MODIFIER	NA	NA	228	228	0	211	141	70	33.18
Smoker	42473	chr12	20874913	T	C	*SLCO1C1*	SILENT	ENSG00000139155:SILENT[ENST00000381552(c.951T>C,ttT>ttC):ENSP00000370964(p.Phe317(=))];ENSG00000139155:SILENT[ENST00000545604(c.951T>C,ttT>ttC):ENSP00000444149(p.Phe317(=))];ENSG00000139155:SILENT[ENST00000545102(c.597T>C,ttT>ttC):ENSP00000444527(p.Phe199(=))];ENSG00000139155:SILENT[ENST00000266509(c.951T>C,ttT>ttC):ENSP00000266509(p.Phe317(=))];ENSG00000139155:SILENT[ENST00000540354(c.804T>C,ttT>ttC):ENSP00000438665(p.Phe268(=))];ENSG00000139155:3UTR[ENST00000539415(c.*535T>C)];	NA	NA	NA	NA	NA	NA	NA	NA	NA	NA	LOW	NA	0.949	60	59	0	79	53	26	32.91
Smoker	42473	chr13	35923280	G	A	*NBEA*	MISSENSE	ENSG00000172915:MISSENSE[ENST00000400445(c.5939G>A,cGt>cAt):ENSP00000383295(p.Arg1980His)];ENSG00000172915:MISSENSE[ENST00000540320(c.5939G>A,cGt>cAt):ENSP00000440951(p.Arg1980His)];ENSG00000172915:MISSENSE[ENST00000310336(c.5939G>A,cGt>cAt):ENSP00000308534(p.Arg1980His)];ENSG00000172915:MISSENSE[ENST00000379939(c.5930G>A,cGt>cAt):ENSP00000369271(p.Arg1977His)];	NA	NA	NA	1.69E-05	6.23E-05	NA	NA	D	PrD	D	MODERATE	D	0.996	149	145	4	167	109	58	34.73
Smoker	42473	chr13	61986080	C	T	*PCDH20*	MISSENSE	ENSG00000197991:MISSENSE[ENST00000409204(c.2152G>A,Ggt>Agt):ENSP00000387250(p.Gly718Ser)];ENSG00000197991:MISSENSE[ENST00000409186(c.2152G>A,Ggt>Agt):ENSP00000386653(p.Gly718Ser)];	NA	NA	NA	NA	NA	NA	NA	D	PrD	D	MODERATE	NA	0.998	198	198	0	150	98	52	34.67
Smoker	42473	chr13	32348775	C	A	*RXFP2*	NONSENSE	ENSG00000133105:NONSENSE[ENST00000380314(c.516C>A,tgC>tgA):ENSP00000369670(p.Cys172Ter)];ENSG00000133105:NONSENSE[ENST00000298386(c.516C>A,tgC>tgA):ENSP00000298386(p.Cys172Ter)];	NA	NA	NA	NA	NA	NA	NA	NA	NA	NA	HIGH	NA	0.989	78	75	1	64	34	30	46.88

Mangalaparthi *et al.* , 2020. Mutational landscape of esophageal squamous cell carcinoma in an Indian cohort
Supplementary Table 4. List of somatic single nucleotide variants identified using whole exome sequencing of ESCC patients

Cohort	Sample	Chromosome	Genomic position	Reference allele	Alternate allele	Gene symbol	Variant type	Variant annotation	OncoMD	COSMIC	dbSNP	ExAC	ExAC_SAS	1000G	1000G_SAS	SIFT	Polyphen2	CONDEL	VEP	LRT	PhastCon7 Way	Total read (Adjacent normal)	Reference allel read support (Adjacent normal)	Alternative read support (Adjacent normal)	Total read (Tumor)	Reference allel read support (Tumor)	Alternative read support (Tumor)	Alternate allele frequency
Smoker	42473	chr13	36903501	T	C	*SPG20*	MISSENSE-SS-PRX	ENSG00000133104:[ENST00000451493(c.1162A>G,Agg>Ggg):ENSP00000414147(p.Arg388Gly)];ENSG00000133104:[ENST00000494062(c.1162A>G,Agg>Ggg):ENSP00000473599(p.Arg388Gly)];ENSG00000133104:[ENST00000355182(c.1162A>G,Agg>Ggg):ENSP00000347314(p.Arg388Gly)];ENSG00000133104:[ENST00000438666(c.1162A>G,Agg>Ggg):ENSP00000406061(p.Arg388Gly)];ENSG00000133104:[ENST00000495510(n.1223A>G)];	NA	NA	NA	NA	NA	NA	NA	T	BN	N	MODERATE	N	0.967	111	109	1	112	58	54	48.21
Smoker	42473	chr14	58830980	G	A	*ARID4A*	MISSENSE	ENSG00000032219:MISSENSE[ENST00000355431(c.2173G>A,Gaa>Aaa):ENSP00000347602(p.Glu725Lys)];ENSG00000032219:MISSENSE[ENST00000417477(c.1207G>A,Gaa>Aaa):ENSP00000416053(p.Glu403Lys)];ENSG00000032219:MISSENSE[ENST00000395168(c.2173G>A,Gaa>Aaa):ENSP00000378597(p.Glu725Lys)];ENSG00000032219:MISSENSE[ENST00000431317(c.2173G>A,Gaa>Aaa):ENSP00000397368(p.Glu725Lys)];ENSG00000032219:MISSENSE[ENST00000348476(c.2173G>A,Gaa>Aaa):ENSP00000344556(p.Glu725Lys)];	NA	NA	NA	NA	NA	NA	NA	D_lc	BN	N	MODERATE	D	0.971	64	64	0	47	26	21	44.68
Smoker	42473	chr14	21215873	T	G	*EDDM3A*	MISSENSE	ENSG00000181562:MISSENSE[ENST00000326842(c.134T>G,tTc>tGc):ENSP00000315098(p.Phe45Cys)];	NA	NA	NA	NA	NA	NA	NA	D	PrD	D	MODERATE	D	0.023	138	136	1	105	60	45	42.86
Smoker	42473	chr14	67309305	C	A	*GPHN*	INTRONIC	ENSG00000171723:[ENST00000555668(c.*247-91C>A)];ENSG00000171723:[ENST00000459628(c.202-91C>A)];ENSG00000171723:[ENST00000556633(c.*83+18021C>A)];ENSG00000171723:[ENST00000543237(c.295-91C>A)];ENSG00000171723:[ENST00000305960(c.202-37352C>A)];ENSG00000171723:[ENST00000544752(n.342+18021C>A)];ENSG00000171723:[ENST00000557654(c.*246+18021C>A)];ENSG00000171723:[ENST00000555456(c.93+18021C>A)];ENSG00000171723:[ENST00000553936(c.*315+18021C>A)];ENSG00000171723:[ENST00000315266(c.294+18021C>A)];ENSG00000171723:[ENST00000478722(c.294+18021C>A)];	NA	NA	NA	NA	NA	NA	NA	NA	NA	NA	MODIFIER	NA	NA	101	101	0	81	76	5	6.17
Smoker	42473	chr14	39650926	T	A	*PNN*	MISSENSE	ENSG00000100941:MISSENSE[ENST00000216832(c.2013T>A,agT>agA):ENSP00000216832(p.Ser671Arg)];ENSG00000100941:INTRONIC[ENST00000557680(n.471-587T>A)];	NA	NA	NA	NA	NA	NA	NA	NA	NA	NA	MODIFIER	NA	NA	128	127	1	185	132	52	28.11
Smoker	42473	chr14	39650928	G	A	*PNN*	MISSENSE	ENSG00000100941:MISSENSE[ENST00000216832(c.2015G>A,aGt>aAt):ENSP00000216832(p.Ser672Asn)];ENSG00000100941:INTRONIC[ENST00000557680(n.471-585G>A)];	NA	NA	NA	NA	NA	NA	NA	NA	NA	NA	MODIFIER	NA	NA	131	131	0	192	135	56	29.17
Smoker	42473	chr14	25287331	G	A	*STXBP6*	INTRONIC	ENSG00000168952:[ENST00000358326(c.610-16C>T)];ENSG00000168952:[ENST00000548182(c.*574-16C>T)];ENSG00000168952:[ENST00000548369(c.303+912C>T)];ENSG00000168952:[ENST00000550887(c.609+912C>T)];ENSG00000168952:[ENST00000419632(c.609+912C>T)];ENSG00000168952:[ENST00000396700(c.609+912C>T)];ENSG00000168952:[ENST00000546511(c.609+912C>T)];ENSG00000168952:[ENST00000323944(c.609+912C>T)];ENSG00000168952:[ENST00000548724(c.609+912C>T)];	NA	NA	NA	NA	NA	NA	NA	NA	NA	NA	MODIFIER	NA	NA	120	119	1	137	76	61	44.53
Smoker	42473	chr15	80263321	T	A	*BCL2A1*	MISSENSE	ENSG00000140379:MISSENSE[ENST00000267953(c.141A>T,gaA>gaT):ENSP00000267953(p.Glu47Asp)];ENSG00000140379:MISSENSE[ENST00000335661(c.141A>T,gaA>gaT):ENSP00000335250(p.Glu47Asp)];	NA	NA	NA	NA	NA	NA	NA	T	BN	N	MODERATE	N	0.54	193	189	4	207	132	75	36.23
Smoker	42473	chr15	67663842	C	T	*IQCH*	INTRONIC	ENSG00000103599:[ENST00000535744(c.*198-607C>T)];ENSG00000103599:[ENST00000546225(c.-3-607C>T)];ENSG00000103599:[ENST00000561339(c.*69-607C>T)];ENSG00000103599:[ENST00000335894(c.754-607C>T)];ENSG00000103599:[ENST00000559568(c.*237-607C>T)];ENSG00000103599:[ENST00000358767(c.235-607C>T)];ENSG00000103599:[ENST00000514049(c.*313C>T)];	NA	NA	0.0001997	3.32E-05	0	0.0001997	0	NA	NA	NA	MODIFIER	NA	NA	104	103	1	63	38	25	39.68
Smoker	42473	chr15	81187412	C	T	*KIAA1199*	MISSENSE	ENSG00000103888:MISSENSE[ENST00000356249(c.1168C>T,Cgg>Tgg):ENSP00000348583(p.Arg390Trp)];ENSG00000103888:MISSENSE[ENST00000220244(c.1168C>T,Cgg>Tgg):ENSP00000220244(p.Arg390Trp)];ENSG00000103888:MISSENSE[ENST00000394685(c.1168C>T,Cgg>Tgg):ENSP00000378177(p.Arg390Trp)];	NA	NA	NA	0.0001565	0.001090116	NA	NA	D_lc	PoD	D	MODERATE	N	0.766	85	85	0	102	53	49	48.04
Smoker	42473	chr15	28230352	G	C	*OCA2*	INTRONIC	ENSG00000104044:[ENST00000354638(c.1240-18C>G)];ENSG00000104044:[ENST00000353809(c.1168-18C>G)];ENSG00000104044:[ENST00000382996(c.1240-18C>G)];	NA	NA	0.000599	0.0001285	0.00012458	0.000599	0	NA	NA	NA	MODIFIER	NA	NA	113	113	0	104	61	43	41.35
Smoker	42473	chr15	40993288	G	A	*RAD51*	SILENT	ENSG00000051180:SILENT[ENST00000525066(c.114G>A,gtG>gtA):ENSP00000431864(p.Val38(=))];ENSG00000051180:SILENT[ENST00000382643(c.114G>A,gtG>gtA):ENSP00000372088(p.Val38(=))];ENSG00000051180:SILENT[ENST00000531277(c.114G>A,gtG>gtA):ENSP00000436512(p.Val38(=))];ENSG00000051180:SILENT[ENST00000423169(c.114G>A,gtG>gtA):ENSP00000406602(p.Val38(=))];ENSG00000051180:SILENT[ENST00000526763(c.114G>A,gtG>gtA):ENSP00000431897(p.Val38(=))];ENSG00000051180:SILENT[ENST00000532743(c.114G>A,gtG>gtA):ENSP00000433924(p.Val38(=))];ENSG00000051180:SILENT[ENST00000527860(c.114G>A,gtG>gtA):ENSP00000432759(p.Val38(=))];ENSG00000051180:SILENT[ENST00000267868(c.114G>A,gtG>gtA):ENSP00000267868(p.Val38(=))];ENSG00000051180:SILENT[ENST00000557850(c.114G>A,gtG>gtA):ENSP00000454176(p.Val38(=))];	NA	NA	NA	NA	NA	NA	NA	NA	NA	NA	LOW	NA	NA	132	132	0	154	82	72	46.75
Smoker	42473	chr15	25429566	G	T	*SNHG14*	INTRONIC	ENSG00000224078:[ENST00000424208(n.450+344G>T)];	NA	NA	NA	NA	NA	NA	NA	NA	NA	NA	MODIFIER	NA	NA	73	73	0	192	133	59	30.73
Smoker	42473	chr15	25601120	C	T	*SNHG14*	INTRONIC	ENSG00000224078:[ENST00000554726(n.246-35623C>T)];	chr15*2560	chr15*2560	NA	NA	NA	NA	NA	NA	NA	NA	LOW	NA	1	83	83	0	52	29	23	44.23
Smoker	42473	chr15	42179537	C	A	*SPTBN5*	SILENT	ENSG00000137877:SILENT[ENST00000320955(c.762G>T,ctG>ctT):ENSP00000317790(p.Leu254(=))];	NA	NA	NA	NA	NA	NA	NA	NA	NA	NA	LOW	NA	NA	35	35	0	67	36	31	46.27
Smoker	42473	chr15	25601120	C	T	*UBE3A*	SILENT	ENSG00000114062:SILENT[ENST00000566215(c.1983G>A,caG>caA):ENSP00000457771(p.Gln661(=))];ENSG00000114062:SILENT[ENST00000397954(c.2052G>A,caG>caA):ENSP00000381045(p.Gln684(=))];ENSG00000114062:SILENT[ENST00000604860(c.224G>A,caG>caA):ENSP00000473822(p.Gln75(=))];ENSG00000114062:SILENT[ENST00000438097(c.1983G>A,caG>caA):ENSP00000411258(p.Gln661(=))];ENSG00000114062:SILENT[ENST00000428984(c.1983G>A,caG>caA):ENSP00000401265(p.Gln661(=))];ENSG00000114062:SILENT[ENST00000232165(c.2043G>A,caG>caA):ENSP00000232165(p.Gln681(=))];	chr15*2560	chr15*2560	NA	NA	NA	NA	NA	NA	NA	NA	LOW	NA	1	83	83	0	52	29	23	44.23
Smoker	42473	chr15	50784974	G	A	*USP8*	MISSENSE	ENSG00000138592:MISSENSE[ENST00000307179(c.2311G>A,Ggt>Agt):ENSP00000302239(p.Gly771Ser)];ENSG00000138592:MISSENSE[ENST00000396444(c.2311G>A,Ggt>Agt):ENSP00000379721(p.Gly771Ser)];ENSG00000138592:MISSENSE[ENST00000433963(c.2311G>A,Ggt>Agt):ENSP00000405537(p.Gly771Ser)];ENSG00000138592:MISSENSE[ENST00000425032(c.1993G>A,Ggt>Agt):ENSP00000412682(p.Gly665Ser)];	NA	NA	NA	0.001462	0.003408399	NA	NA	D	BN	N	MODERATE	N	1	164	161	2	192	182	8	4.17

Mangalaparthi *et al.* , 2020. Mutational landscape of esophageal squamous cell carcinoma in an Indian cohort
Supplementary Table 4. List of somatic single nucleotide variants identified using whole exome sequencing of ESCC patients

Cohort	Sample	Chromosome	Genomic position	Reference allele	Alternate allele	Gene symbol	Variant type	Variant annotation	OncoMD	COSMIC	dbSNP	ExAC	ExAC_SAS	1000G	1000G_SAS	SIFT	Polyphen2	CONDEL	VEP	LRT	PhastCon7 Way	Total read (Adjacent normal)	Reference allel read support (Adjacent normal)	Alternative read support (Adjacent normal)	Total read (Tumor)	Reference allel read support (Tumor)	Alternative read support (Tumor)	Alternate allele frequency
Smoker	42473	chr16	20494454	G	C	*ACSM2A*	MISSENSE	ENSG00000183747:MISSENSE[ENST00000575690(c.1584G>C,caG>caC):ENSP00000460349(p.Gln528His)];ENSG00000183747:MISSENSE[ENST00000573854(c.1584G>C,caG>caC):ENSP00000459451(p.Gln528His)];ENSG00000183747:MISSENSE[ENST00000536134(c.900G>C,caG>caC):ENSP00000445082(p.Gln300His)];ENSG00000183747:MISSENSE[ENST00000219054(c.1584G>C,caG>caC):ENSP00000219054(p.Gln528His)];ENSG00000183747:MISSENSE[ENST00000417235(c.1347G>C,caG>caC):ENSP00000392169(p.Gln449His)];ENSG00000183747:MISSENSE[ENST00000396104(c.1584G>C,caG>caC):ENSP00000379411(p.Gln528His)];ENSG00000183747:EXONIC-NC[ENST00000576119(n.863G>C)];ENSG00000183747:EXONIC-NC[ENST00000572843(n.1779G>C)];	NA	NA	NA	NA	NA	NA	NA	NA	NA	NA	MODIFIER	NA	NA	88	86	1	168	82	86	51.19
Smoker	42473	chr16	49411643	A	C	*C16orf78*	MISSENSE-SS-PRX	ENSG00000166152:[ENST00000299191(c.152A>C,aAg>aCg):ENSP00000299191(p.Lys51Thr)];	NA	NA	NA	NA	NA	NA	NA	T	PrD	D	MODERATE	NA	NA	32	32	0	27	8	19	70.37
Smoker	42473	chr16	1866938	C	A	*HAGH*	MISSENSE	ENSG00000063854:NONSENSE[ENST00000397356(c.703G>T,Gag>Tag):ENSP00000380514(p.Glu235Ter)];ENSG00000063854:NONSENSE[ENST00000397353(c.559G>T,Gag>Tag):ENSP00000380511(p.Glu187Ter)];ENSG00000063854:NONSENSE[ENST00000566709(c.559G>T,Gag>Tag):ENSP00000455422(p.Glu187Ter)];ENSG00000063854:NONSENSE[ENST00000564445(c.494G>T,Gag>Tag):ENSP00000455355(p.Glu166Ter)];ENSG00000063854:MISSENSE[ENST00000455446(c.594G>T,tgG>tgT):ENSP00000406552(p.Trp198Cys)];	NA	NA	chr16*1866938*1866938*C*T*rs759677550*NA*NA*NA	NA	NA	NA	NA	NA	NA	NA	HIGH	NA	NA	62	62	0	83	18	65	78.31
Smoker	42473	chr16	33962062	G	A	*LINC00273*	EXONIC-NC	ENSG00000256642:[ENST00000539813(n.442C>T)];	NA	NA	chr16*33962062*33962062*G*T*rs112992059*1*0.9565*0.04353	NA	NA	NA	NA	NA	NA	NA	MODIFIER	NA	NA	53	49	1	36	29	5	13.89
Smoker	42473	chr16	28990795	G	T	*RP11-264B17.3*	INTRONIC-SS-DNR	ENSG00000261067:[ENST00000569969(n.928+3G>T)];	NA	NA	chr16*28990766*28990809*GCTGGAGACTGGCACTGGGCTCTGAGGGTGAGTCTGGTCTTGGC*G*rs773522099*NA*NA*NA	NA	NA	NA	NA	NA	NA	NA	MODIFIER	NA	NA	36	36	0	90	52	36	40
Smoker	42473	chr16	29883607	G	A	*SEZ6L2*	INTRONIC-SS-ACR	ENSG00000174938:[ENST00000308713(c.2607-3C>T)];ENSG00000174938:[ENST00000350527(c.2436-3C>T)];ENSG00000174938:[ENST00000346932(c.2304-3C>T)];ENSG00000174938:[ENST00000537485(c.2514-3C>T)];	NA	NA	NA	NA	NA	NA	NA	NA	NA	NA	LOW	NA	NA	33	33	0	66	40	26	39.39
Smoker	42473	chr16	28990795	G	T	*SPNS1*	INTRONIC	ENSG00000169682:[ENST00000568829(c.107+1430G>T)];ENSG00000169682:[ENST00000323081(c.444+3G>T)];ENSG00000169682:[ENST00000564476(c.617+3G>T)];ENSG00000169682:[ENST00000311008(c.663+3G>T)];ENSG00000169682:[ENST00000561868(n.275+3G>T)];ENSG00000169682:[ENST00000352260(c.597+3G>T)];ENSG00000169682:[ENST00000565975(c.798+3G>T)];ENSG00000169682:[ENST00000334536(c.663+3G>T)];ENSG00000169682:[ENST00000566059(c.630+3G>T)];ENSG00000169682:[ENST00000568900(n.1040G>T)];	NA	NA	chr16*28990766*28990809*GCTGGAGACTGGCACTGGGCTCTGAGGGTGAGTCTGGTCTTGGC*G*rs773522099*NA*NA*NA	NA	NA	NA	NA	NA	NA	NA	MODIFIER	NA	NA	36	36	0	90	52	36	40
Smoker	42473	chr16	703398	G	A	*WDR90*	MISSENSE	ENSG00000161996:EXONIC-NC[ENST00000549648(n.1247G>A)];ENSG00000161996:EXONIC-NC[ENST00000550593(n.472G>A)];ENSG00000161996:EXONIC-NC[ENST00000420061(n.1244G>A)];ENSG00000161996:EXONIC-NC[ENST00000552943(n.1625G>A)];ENSG00000161996:MISSENSE[ENST00000549091(c.1180G>A,Gtc>Atc):ENSP00000448122(p.Val394Ile)];ENSG00000161996:MISSENSE[ENST00000293879(c.1180G>A,Gtc>Atc):ENSP00000293879(p.Val394Ile)];	NA	NA	NA	2.68E-05	6.32E-05	NA	NA	T	BN	N	MODERATE	U	0.016	30	30	0	70	11	59	84.29
Smoker	42473	chr17	14139787	C	T	*CDRT15*	INTRONIC	ENSG00000223510:[ENST00000420162(c.263-40G>A)];ENSG00000223510:[ENST00000431716(c.65-40G>A)];	NA	NA	NA	NA	NA	NA	NA	NA	NA	NA	MODIFIER	NA	NA	18	18	0	15	4	11	73.33
Smoker	42473	chr17	8133315	C	T	*CTC1*	INTRONIC	ENSG00000178971:[ENST00000578441(n.337-75G>A)];ENSG00000178971:[ENST00000578537(c.*232-75G>A)];ENSG00000178971:[ENST00000449476(c.2829-29G>A)];ENSG00000178971:[ENST00000315684(c.2934-29G>A)];ENSG00000178971:[ENST00000580299(c.105-29G>A)];ENSG00000178971:[ENST00000578240(n.1458G>A)];	NA	NA	NA	NA	NA	NA	NA	NA	NA	NA	MODIFIER	NA	NA	41	41	0	30	11	19	63.33
Smoker	42473	chr17	37872042	C	T	*ERBB2*	SILENT	ENSG00000141736:3UTR[ENST00000582648(c.*396C>T)];ENSG00000141736:3UTR[ENST00000578373(c.*1153C>T)];ENSG00000141736:EXONIC-NC[ENST00000583038(n.2055C>T)];ENSG00000141736:EXONIC-NC[ENST00000582788(n.852C>T)];ENSG00000141736:SILENT[ENST00000584450(c.1363C>T,Ctg>Ttg):ENSP00000463714(p.Leu455(=))];ENSG00000141736:SILENT[ENST00000540042(c.1273C>T,Ctg>Ttg):ENSP00000446382(p.Leu425(=))];ENSG00000141736:SILENT[ENST00000541774(c.1318C>T,Ctg>Ttg):ENSP00000446466(p.Leu440(=))];ENSG00000141736:SILENT[ENST00000445658(c.535C>T,Ctg>Ttg):ENSP00000404047(p.Leu179(=))];ENSG00000141736:SILENT[ENST00000578199(c.1273C>T,Ctg>Ttg):ENSP00000462808(p.Leu425(=))];ENSG00000141736:SILENT[ENST00000269571(c.1363C>T,Ctg>Ttg):ENSP00000269571(p.Leu455(=))];ENSG00000141736:SILENT[ENST00000406381(c.1273C>T,Ctg>Ttg):ENSP00000385185(p.Leu425(=))];ENSG00000141736:SILENT[ENST00000540147(c.1273C>T,Ctg>Ttg):ENSP00000443562(p.Leu425(=))];ENSG00000141736:SILENT[ENST00000584601(c.1273C>T,Ctg>Ttg):ENSP00000462438(p.Leu425(=))];	NA	NA	NA	NA	NA	NA	NA	NA	NA	NA	LOW	NA	NA	46	46	0	228	183	44	19.3
Smoker	42473	chr18	50432485	G	A	*DCC*	MISSENSE	ENSG00000187323:MISSENSE[ENST00000442544(c.484G>A,Gaa>Aaa):ENSP00000389140(p.Glu162Lys)];ENSG00000187323:MISSENSE[ENST00000412726(c.28G>A,Gaa>Aaa):ENSP00000397322(p.Glu10Lys)];ENSG00000187323:MISSENSE[ENST00000304775(c.285G>A,Gaa>Aaa):ENSP00000304146(p.Glu96Lys)];ENSG00000187323:3UTR[ENST00000579349(c.*182G>A)];ENSG00000187323:3UTR[ENST00000581559(c.*192G>A)];ENSG00000187323:EXONIC-NC[ENST00000580024(n.397G>A)];	NA	NA	NA	NA	NA	NA	NA	NA	NA	NA	MODIFIER	NA	NA	83	83	0	52	16	36	69.23
Smoker	42473	chr18	28934293	C	T	*DSG1*	MISSENSE	ENSG00000134760:MISSENSE[ENST00000462981(c.211C>T,Cgc>Tgc):ENSP00000462666(p.Arg71Cys)];ENSG00000134760:MISSENSE[ENST00000257192(c.2134C>T,Cgc>Tgc):ENSP00000257192(p.Arg712Cys)];	NA	NA	0.001198	0.0016969	0.00060562	0.0011981	0	NA	NA	NA	MODIFIER	NA	NA	164	164	0	260	202	58	22.31

Mangalaparthi *et al.* , 2020. Mutational landscape of esophageal squamous cell carcinoma in an Indian cohort
Supplementary Table 4. List of somatic single nucleotide variants identified using whole exome sequencing of ESCC patients

Cohort	Sample	Chromosome	Genomic position	Reference allele	Alternate allele	Gene symbol	Variant type	Variant annotation	OncoMD	COSMIC	dbSNP	ExAC	ExAC_SAS	1000G	1000G_SAS	SIFT	Polyphen2	CONDEL	VEP	LRT	PhastCon7 Way	Total read (Adjacent normal)	Reference allel read support (Adjacent normal)	Alternative read support (Adjacent normal)	Total read (Tumor)	Reference allel read support (Tumor)	Alternative read support (Tumor)	Alternate allele frequency
Smoker	42473	chr18	44113241	G	C	*LOXHD1*	MISSENSE	ENSG00000167210:MISSENSE[ENST00000582408(c.926C>G,tCt>tGt):ENSP00000461964(p.Ser309Cys)];ENSG00000167210:MISSENSE[ENST00000300591(c.926C>G,tCt>tGt):ENSP00000300591(p.Ser309Cys)];ENSG00000167210:MISSENSE[ENST00000441551(c.3641C>G,tCt>tGt):ENSP00000387621(p.Ser1214Cys)];ENSG00000167210:MISSENSE[ENST00000441893(c.1058C>G,tCt>tGt):ENSP00000409062(p.Ser353Cys)];ENSG00000167210:MISSENSE[ENST00000579038(c.638C>G,tCt>tGt):ENSP00000463285(p.Ser213Cys)];ENSG00000167210:MISSENSE[ENST00000536736(c.4259C>G,tCt>tGt):ENSP00000444586(p.Ser1420Cys)];ENSG00000167210:MISSENSE[ENST00000398722(c.3425C>G,tCt>tGt):ENSP00000381707(p.Ser1142Cys)];ENSG00000167210:3UTR[ENST00000536111(c.*98C>G)];ENSG00000167210:EXONIC-NC[ENST00000335730(n.3572C>G)];	NA	NA	NA	NA	NA	NA	NA	NA	NA	NA	MODIFIER	NA	NA	68	68	0	37	8	29	78.38
Smoker	42473	chr18	28934293	C	T	*RP11-534N16.1*	EXONIC-NC	ENSG00000266729:[ENST00000581856(n.219G>A)];ENSG00000266729:[ENST00000578477(n.417G>A)];ENSG00000266729:[ENST00000581452(n.350G>A)];	NA	NA	0.001198	0.0016969	0.00060562	0.0011981	0	NA	NA	NA	MODIFIER	NA	NA	164	164	0	260	202	58	22.31
Smoker	42473	chr19	14492366	T	G	*CD97*	INTRONIC-SS-DNR	ENSG00000123146:[ENST00000591080(c.22+9T>G)];ENSG00000123146:[ENST00000586517(c.22+9T>G)];ENSG00000123146:[ENST00000242786(c.22+9T>G)];ENSG00000123146:[ENST00000587606(c.22+9T>G)];ENSG00000123146:[ENST00000357355(c.22+9T>G)];ENSG00000123146:[ENST00000358600(c.22+9T>G)];ENSG00000123146:[ENST00000592261(c.22+9T>G)];ENSG00000123146:[ENST00000587535(n.181+9T>G)];ENSG00000123146:[ENST00000587728(n.116+938T>G)];	NA	NA	NA	NA	NA	NA	NA	NA	NA	NA	MODIFIER	NA	NA	70	70	0	84	15	69	82.14
Smoker	42473	chr19	57669872	C	T	*DUXA*	INTRONIC	ENSG00000258873:[ENST00000554048(c.293-31G>A)];	NA	NA	0.0003994	1.66E-05	0	0.0003994	0	NA	NA	NA	MODIFIER	NA	NA	40	40	0	69	55	14	20.29
Smoker	42473	chr19	9056795	G	A	*MUC16*	SILENT	ENSG00000181143:SILENT[ENST00000397910(c.30651C>T,gcC>gcT):ENSP00000381008(p.Ala10217(=))];	NA	NA	NA	NA	NA	NA	NA	NA	NA	NA	LOW	NA	NA	75	74	1	45	10	35	77.78
Smoker	42473	chr19	56422011	T	C	*NLRP13*	MISSENSE	ENSG00000173572:MISSENSE[ENST00000588751(c.2200A>G,Agt>Ggt):ENSP00000467899(p.Ser734Gly)];ENSG00000173572:MISSENSE[ENST00000342929(c.2200A>G,Agt>Ggt):ENSP00000343891(p.Ser734Gly)];	NA	NA	NA	NA	NA	NA	NA	T	PrD	D	MODERATE	NA	NA	100	100	0	106	62	44	41.51
Smoker	42473	chr19	36342567	C	T	*NPHS1*	SILENT	ENSG00000161270:EXONIC-NC[ENST00000591817(n.567G>A)];ENSG00000161270:SILENT[ENST00000378910(c.66G>A,gcG>gcA):ENSP00000368190(p.Ala22(=))];ENSG00000161270:SILENT[ENST00000353632(c.66G>A,gcG>gcA):ENSP00000343634(p.Ala22(=))];	NA	NA	chr19*36342567*36342567*C*G,T*rs750193555*NA*NA*NA	1.06E-05	0	NA	NA	NA	NA	NA	LOW	NA	NA	21	21	0	75	60	15	20
Smoker	42473	chr19	8467016	A	T	*RAB11B*	NONSENSE	ENSG00000185236:NONSENSE[ENST00000594216(c.283A>T,Aag>Tag):ENSP00000471148(p.Lys95Ter)];ENSG00000185236:NONSENSE[ENST00000328024(c.283A>T,Aag>Tag):ENSP00000333547(p.Lys95Ter)];ENSG00000185236:5UTR[ENST00000600719(c.-75A>T)];ENSG00000185236:5UTR[ENST00000601897(c.-75A>T)];	NA	NA	NA	NA	NA	NA	NA	NA	NA	NA	MODIFIER	NA	NA	63	63	0	87	20	67	77.01
Smoker	42473	chr19	31770676	G	T	*TSHZ3*	INTRONIC	ENSG00000121297:[ENST00000560707(c.*170-18C>A)];ENSG00000121297:[ENST00000240587(c.41-18C>A)];	NA	NA	NA	NA	NA	NA	NA	NA	NA	NA	MODIFIER	NA	NA	41	41	0	677	660	17	2.51
Smoker	42473	chr19	30935788	C	T	*ZNF536*	MISSENSE	ENSG00000198597:MISSENSE[ENST00000355537(c.1319C>T,aCc>aTc):ENSP00000347730(p.Thr440Ile)];ENSG00000198597:MISSENSE[ENST00000585628(c.1319C>T,aCc>aTc):ENSP00000465771(p.Thr440Ile)];	NA	NA	chr19*30935788*30935788*C*A,G*rs769608026*NA*NA*NA	NA	NA	NA	NA	T	BN	N	MODERATE	NA	NA	67	67	0	978	951	26	2.66
Smoker	42473	chr19	58564052	C	T	*ZSCAN1*	INTRONIC	ENSG00000152467:[ENST00000282326(c.604+56C>T)];	NA	NA	NA	NA	NA	NA	NA	NA	NA	NA	MODIFIER	NA	NA	34	34	0	61	40	21	34.43
Smoker	42473	chr2	169853223	G	A	*ABCB11*	SILENT	ENSG00000073734:SILENT[ENST00000263817(c.399C>T,aaC>aaT):ENSP00000263817(p.Asn133(=))];	NA	NA	0.001997	0.0008304	0.003937894	0.0019968	0.0102	NA	NA	NA	LOW	NA	0.942	172	169	0	126	73	52	41.27
Smoker	42473	chr2	241627951	C	A	*AC011298.2*	INTRONIC	ENSG00000219159:[ENST00000407635(n.1585+22C>A)];	NA	NA	NA	NA	NA	NA	NA	NA	NA	NA	MODIFIER	NA	NA	24	24	0	80	56	24	30
Smoker	42473	chr2	27558830	T	G	*AC109828.1*	INTRONIC	ENSG00000234945:[ENST00000416453(n.179+243T>G)];ENSG00000234945:[ENST00000608473(n.186+243T>G)];ENSG00000234945:[ENST00000585326(n.187T>G)];ENSG00000234945:[ENST00000592265(n.19T>G)];	NA	NA	NA	NA	NA	NA	NA	NA	NA	NA	MODIFIER	NA	NA	58	58	0	96	64	31	32.29
Smoker	42473	chr2	21228460	C	T	*APOB*	SILENT	ENSG00000084674:SILENT[ENST00000233242(c.11280G>A,tcG>tcA):ENSP00000233242(p.Ser3760(=))];	NA	NA	chr2*21228460*21228460*C*A,G,T*rs757222680*NA*NA*NA	1.65E-05	0	NA	NA	NA	NA	NA	LOW	NA	NA	223	222	1	222	123	97	43.69
Smoker	42473	chr2	131671515	G	A	*ARHGEF4*	INTRONIC	ENSG00000136002:[ENST00000526381(n.78-44G>A)];ENSG00000136002:[ENST00000409359(c.-951-44G>A)];	NA	NA	NA	NA	NA	NA	NA	NA	NA	NA	MODIFIER	NA	NA	43	43	0	55	36	17	30.91
Smoker	42473	chr2	130900912	A	G	*CCDC74B*	INTRONIC	ENSG00000152076:[ENST00000457413(c.375-26T>C)];ENSG00000152076:[ENST00000434929(c.*72-26T>C)];ENSG00000152076:[ENST00000409943(c.251-26T>C)];ENSG00000152076:[ENST00000409128(c.377-26T>C)];ENSG00000152076:[ENST00000409234(c.251-26T>C)];ENSG00000152076:[ENST00000392984(c.-331-26T>C)];ENSG00000152076:[ENST00000310463(c.251-26T>C)];ENSG00000152076:[ENST00000441670(c.377-26T>C)];ENSG00000152076:[ENST00000423263(c.251-26T>C)];	NA	NA	NA	NA	NA	NA	NA	NA	NA	NA	MODIFIER	NA	NA	31	31	0	52	21	31	59.62
Smoker	42473	chr2	227892791	T	A	*COL4A4*	INTRONIC	ENSG00000081052:[ENST00000329662(c.3974-66A>T)];ENSG00000081052:[ENST00000396625(c.3974-66A>T)];	NA	NA	NA	NA	NA	NA	NA	NA	NA	NA	MODIFIER	NA	NA	27	27	0	26	17	9	34.62
Smoker	42473	chr2	227924263	G	A	*COL4A4*	SILENT	ENSG00000081052:SILENT[ENST00000329662(c.2241C>T,ccC>ccT):ENSP00000328553(p.Pro747(=))];ENSG00000081052:SILENT[ENST00000396625(c.2241C>T,ccC>ccT):ENSP00000379866(p.Pro747(=))];	chr2*22792	chr2*22792	0.000599	0.0001164	0.000121168	0.000599	0.001	NA	NA	NA	LOW	NA	NA	94	94	0	93	51	42	45.16
Smoker	42473	chr2	80620504	A	G	*CTNNA2*	INTRONIC	ENSG00000066032:[ENST00000496558(c.1137+88A>G)];ENSG00000066032:[ENST00000465616(n.176+88A>G)];ENSG00000066032:[ENST00000361291(c.1239+88A>G)];ENSG00000066032:[ENST00000493024(n.287+88A>G)];ENSG00000066032:[ENST00000541047(c.1137+88A>G)];ENSG00000066032:[ENST00000343114(c.174+88A>G)];ENSG00000066032:[ENST00000540488(c.1137+88A>G)];ENSG00000066032:[ENST00000409550(c.132+88A>G)];ENSG00000066032:[ENST00000466387(c.1137+88A>G)];ENSG00000066032:[ENST00000402739(c.1137+88A>G)];	NA	NA	NA	NA	NA	NA	NA	NA	NA	NA	MODIFIER	NA	NA	40	40	0	43	23	20	46.51
Smoker	42473	chr2	39065884	C	T	*DHX57*	INTRONIC	ENSG00000163214:[ENST00000452978(c.396-795G>A)];ENSG00000163214:[ENST00000460193(n.3448-795G>A)];ENSG00000163214:[ENST00000457308(c.*724-795G>A)];ENSG00000163214:[ENST00000295373(c.2426-795G>A)];	NA	NA	NA	NA	NA	NA	NA	NA	NA	NA	MODIFIER	NA	NA	184	181	3	235	129	106	45.11
Smoker	42473	chr2	207527926	T	C	*DYTN*	MISSENSE	ENSG00000232125:MISSENSE[ENST00000452335(c.1334A>G,aAt>aGt):ENSP00000396593(p.Asn445Ser)];	NA	NA	NA	NA	NA	NA	NA	T	BN	N	MODERATE	NA	NA	220	219	1	224	126	98	43.75
Smoker	42473	chr2	212587213	G	T	*ERBB4*	MISSENSE	ENSG00000178568:MISSENSE[ENST00000402597(c.788C>A,cCc>cAc):ENSP00000385565(p.Pro263His)];ENSG00000178568:MISSENSE[ENST00000436443(c.788C>A,cCc>cAc):ENSP00000403204(p.Pro263His)];ENSG00000178568:MISSENSE[ENST00000342788(c.788C>A,cCc>cAc):ENSP00000342235(p.Pro263His)];ENSG00000178568:MISSENSE[ENST00000260943(c.787C>A,cCc>cAc):ENSP00000260943(p.Pro263His)];ENSG00000178568:EXONIC-NC[ENST00000484594(n.840C>A)];	NA	NA	NA	NA	NA	NA	NA	NA	NA	NA	MODIFIER	NA	NA	229	229	0	227	132	95	41.85

Mangalaparthi *et al.* , 2020. Mutational landscape of esophageal squamous cell carcinoma in an Indian cohort
Supplementary Table 4. List of somatic single nucleotide variants identified using whole exome sequencing of ESCC patients

Cohort	Sample	Chromosome	Genomic position	Reference allele	Alternate allele	Gene symbol	Variant type	Variant annotation	OncoMD	COSMIC	dbSNP	ExAC	ExAC_SAS	1000G	1000G_SAS	SIFT	Polyphen2	CONDEL	VEP	LRT	PhastCon7 Way	Total read (Adjacent normal)	Reference allel read support (Adjacent normal)	Alternative read support (Adjacent normal)	Total read (Tumor)	Reference allel read support (Tumor)	Alternative read support (Tumor)	Alternate allele frequency
Smoker	42473	chr2	24369723	G	T	*FAM228B*	INTRONIC	ENSG00000219626:[ENST00000407625(c.529+18G>T)];ENSG00000219626:[ENST00000468799(n.495+18G>T)];ENSG00000219626:[ENST00000420135(c.529+18G>T)];ENSG00000219626:[ENST00000417898(c.168+11666G>T)];	NA	NA	NA	NA	NA	NA	NA	NA	NA	NA	MODIFIER	NA	NA	105	103	1	115	76	39	33.91
Smoker	42473	chr2	11725303	C	T	*GREB1*	SILENT	ENSG00000196208:SILENT[ENST00000234142(c.918C>T,tcC>tcT):ENSP00000234142(p.Ser306(=))];ENSG00000196208:SILENT[ENST00000263834(c.918C>T,tcC>tcT):ENSP00000263834(p.Ser306(=))];ENSG00000196208:SILENT[ENST00000381483(c.918C>T,tcC>tcT):ENSP00000370892(p.Ser306(=))];ENSG00000196208:SILENT[ENST00000381486(c.918C>T,tcC>tcT):ENSP00000370896(p.Ser306(=))];	NA	NA	NA	2.86E-05	0	NA	NA	NA	NA	NA	LOW	NA	NA	60	60	0	93	57	36	38.71
Smoker	42473	chr2	27558830	T	G	*GTF3C2*	MISSENSE	ENSG00000115207:MISSENSE[ENST00000359541(c.1421A>C,aAg>aCg):ENSP00000352536(p.Lys474Thr)];ENSG00000115207:MISSENSE[ENST00000264720(c.1421A>C,aAg>aCg):ENSP00000264720(p.Lys474Thr)];ENSG00000115207:EXONIC-NC[ENST00000470115(n.528A>C)];ENSG00000115207:EXONIC-NC[ENST00000493511(n.636A>C)];	NA	NA	NA	NA	NA	NA	NA	NA	NA	NA	MODIFIER	NA	NA	58	58	0	96	64	31	32.29
Smoker	42473	chr2	89475967	G	T	*IGKV2-24*	MISSENSE	ENSG00000241294:MISSENSE[ENST00000484817(c.205C>A,Cca>Aca):ENSP00000419300(p.Pro69Thr)];	NA	NA	NA	NA	NA	NA	NA	D	PoD	D	MODERATE	NA	NA	208	208	0	147	87	60	40.82
Smoker	42473	chr2	18112994	G	A	*KCNS3*	MISSENSE	ENSG00000170745:MISSENSE[ENST00000304101(c.719G>A,cGg>cAg):ENSP00000305824(p.Arg240Gln)];ENSG00000170745:MISSENSE[ENST00000403915(c.719G>A,cGg>cAg):ENSP00000385968(p.Arg240Gln)];ENSG00000170745:INTRONIC[ENST00000465292(n.305+13856G>A)];	chr2*181129	NA	NA	NA	NA	NA	NA	NA	NA	NA	MODIFIER	NA	NA	161	158	2	198	105	93	46.97
Smoker	42473	chr2	228194404	G	A	*MFF*	INTRONIC	ENSG00000168958:[ENST00000409565(c.-40-938G>A)];ENSG00000168958:[ENST00000489696(n.306+899G>A)];ENSG00000168958:[ENST00000470090(n.158-2731G>A)];ENSG00000168958:[ENST00000354503(c.-41+899G>A)];ENSG00000168958:[ENST00000530359(c.-206-2731G>A)];ENSG00000168958:[ENST00000476924(n.208-2731G>A)];ENSG00000168958:[ENST00000349901(c.-41+899G>A)];ENSG00000168958:[ENST00000423098(c.-41+29G>A)];ENSG00000168958:[ENST00000531278(c.-206-2731G>A)];ENSG00000168958:[ENST00000304593(c.-41+899G>A)];ENSG00000168958:[ENST00000534203(c.-206-2731G>A)];ENSG00000168958:[ENST00000452930(c.-40-938G>A)];ENSG00000168958:[ENST00000337110(c.-41+899G>A)];ENSG00000168958:[ENST00000524634(c.-206-2731G>A)];ENSG00000168958:[ENST00000353339(c.-58G>A)];ENSG00000168958:[ENST00000392059(c.-58G>A)];ENSG00000168958:[ENST00000418961(c.-136G>A)];ENSG00000168958:[ENST00000443428(c.-58G>A)];ENSG00000168958:[ENST00000409616(c.-136G>A)];ENSG00000168958:[ENST00000525195(c.-50G>A)];ENSG00000168958:[ENST00000436237(c.-58G>A)];ENSG00000168958:[ENST00000460756(n.1174G>A)];	NA	NA	NA	NA	NA	NA	NA	NA	NA	NA	MODIFIER	NA	NA	62	62	0	70	43	27	38.57
Smoker	42473	chr2	234737446	C	A	*MROH2A*	INTRONIC	ENSG00000185038:[ENST00000389758(c.4263+21C>A)];ENSG00000185038:[ENST00000487979(n.225+21C>A)];	NA	NA	NA	NA	NA	NA	NA	NA	NA	NA	MODIFIER	NA	NA	32	32	0	54	29	25	46.3
Smoker	42473	chr2	133547662	G	T	*NCKAP5*	MISSENSE	ENSG00000176771:MISSENSE[ENST00000409261(c.1026C>A,agC>agA):ENSP00000387128(p.Ser342Arg)];ENSG00000176771:MISSENSE[ENST00000317721(c.1026C>A,agC>agA):ENSP00000380603(p.Ser342Arg)];ENSG00000176771:MISSENSE[ENST00000409213(c.1026C>A,agC>agA):ENSP00000386952(p.Ser342Arg)];ENSG00000176771:MISSENSE[ENST00000405974(c.1026C>A,agC>agA):ENSP00000385692(p.Ser342Arg)];	NA	NA	NA	NA	NA	NA	NA	D_lc	PrD	D	MODERATE	U	0.991	152	151	1	156	97	59	37.82
Smoker	42473	chr2	24369723	G	T	*RP11-507M3.1*	INTRONIC	ENSG00000266118:[ENST00000584973(c.244+18G>T)];	NA	NA	NA	NA	NA	NA	NA	NA	NA	NA	MODIFIER	NA	NA	105	103	1	115	76	39	33.91
Smoker	42473	chr2	114501227	C	A	*SLC35F5*	INTRONIC	ENSG00000115084:[ENST00000409106(c.544+63G>T)];ENSG00000115084:[ENST00000409342(c.544+63G>T)];ENSG00000115084:[ENST00000498768(n.740+63G>T)];ENSG00000115084:[ENST00000460863(n.186+63G>T)];ENSG00000115084:[ENST00000245680(c.562+63G>T)];	NA	NA	NA	NA	NA	NA	NA	NA	NA	NA	MODIFIER	NA	NA	63	63	0	56	24	32	57.14
Smoker	42473	chr2	37105199	A	G	*STRN*	INTRONIC	ENSG00000115808:[ENST00000263918(c.1187-29T>C)];ENSG00000115808:[ENST00000379213(c.1040-29T>C)];ENSG00000115808:[ENST00000495595(n.863-29T>C)];	NA	NA	NA	NA	NA	NA	NA	NA	NA	NA	MODIFIER	NA	NA	64	64	0	107	71	35	32.71
Smoker	42473	chr2	98377323	G	A	*TMEM131*	SILENT	ENSG00000075568:EXONIC-NC[ENST00000465767(n.338C>T)];ENSG00000075568:EXONIC-NC[ENST00000485245(n.4458C>T)];ENSG00000075568:SILENT[ENST00000186436(c.4944C>T,gcC>gcT):ENSP00000186436(p.Ala1648(=))];	NA	NA	NA	NA	NA	NA	NA	NA	NA	NA	LOW	NA	NA	82	82	0	128	76	52	40.62
Smoker	42473	chr2	152226638	C	A	*TNFAIP6*	MISSENSE	ENSG00000123610:MISSENSE[ENST00000243347(c.499C>A,Ctc>Atc):ENSP00000243347(p.Leu167Ile)];ENSG00000123610:EXONIC-NC[ENST00000460812(n.181C>A)];	NA	NA	NA	NA	NA	NA	NA	NA	NA	NA	MODIFIER	NA	NA	260	260	0	273	178	94	34.43
Smoker	42473	chr2	179574439	G	C	*TTN*	MISSENSE	ENSG00000155657:INTRONIC[ENST00000359218(c.13657+28370C>G)];ENSG00000155657:INTRONIC[ENST00000342175(c.13858+28370C>G)];ENSG00000155657:INTRONIC[ENST00000460472(c.13282+28370C>G)];ENSG00000155657:MISSENSE[ENST00000342992(c.24875C>G,cCa>cGa):ENSP00000343764(p.Pro8292Arg)];ENSG00000155657:MISSENSE[ENST00000589042(c.28607C>G,cCa>cGa):ENSP00000467141(p.Pro9536Arg)];ENSG00000155657:MISSENSE[ENST00000591111(c.27656C>G,cCa>cGa):ENSP00000465570(p.Pro9219Arg)];	NA	NA	NA	NA	NA	NA	NA	NA	NA	NA	MODIFIER	NA	NA	230	230	0	166	100	66	39.76
Smoker	42473	chr2	179614959	T	C	*TTN*	SILENT	ENSG00000155657:SILENT[ENST00000360870(c.12168A>G,gaA>gaG):ENSP00000354117(p.Glu4056(=))];ENSG00000155657:INTRONIC[ENST00000359218(c.10597+2892A>G)];ENSG00000155657:INTRONIC[ENST00000342992(c.10360+2892A>G)];ENSG00000155657:INTRONIC[ENST00000342175(c.10798+2892A>G)];ENSG00000155657:INTRONIC[ENST00000589042(c.11311+2892A>G)];ENSG00000155657:INTRONIC[ENST00000591111(c.10360+2892A>G)];ENSG00000155657:INTRONIC[ENST00000460472(c.10222+2892A>G)];	chr2*179614	NA	NA	NA	NA	NA	NA	NA	NA	NA	MODIFIER	NA	NA	182	181	0	143	73	68	47.55
Smoker	42473	chr2	179574439	G	C	*TTN-AS1*	INTRONIC	ENSG00000237298:[ENST00000592630(n.520-4042G>C)];ENSG00000237298:[ENST00000585451(n.199-51228G>C)];ENSG00000237298:[ENST00000431752(n.125-4042G>C)];ENSG00000237298:[ENST00000589830(n.212-4042G>C)];	NA	NA	NA	NA	NA	NA	NA	NA	NA	NA	MODIFIER	NA	NA	230	230	0	166	100	66	39.76
Smoker	42473	chr2	179614959	T	C	*TTN-AS1*	INTRONIC	ENSG00000237298:[ENST00000590773(n.244-6034T>C)];ENSG00000237298:[ENST00000585451(n.199-10708T>C)];ENSG00000237298:[ENST00000578746(n.64+5390T>C)];	chr2*179614	NA	NA	NA	NA	NA	NA	NA	NA	NA	MODIFIER	NA	NA	182	181	0	143	73	68	47.55
Smoker	42473	chr2	61558374	G	A	*USP34*	INTRONIC	ENSG00000115464:[ENST00000398571(c.2930+37C>T)];	NA	NA	0.000599	0.0007803	0.005490956	0.000599	0.0031	NA	NA	NA	MODIFIER	NA	NA	65	65	0	52	35	17	32.69

Supplementary Table 4. List of somatic single nucleotide variants identified using whole exome sequencing of ESCC patients

Cohort	Sample	Chromosome	Genomic position	Reference allele	Alternate allele	Gene symbol	Variant type	Variant annotation	OncoMD	COSMIC	dbSNP	ExAC	ExAC_SAS	1000G	1000G_SAS	SIFT	Polyphen2	CONDEL	VEP	LRT	PhastCon7 Way	Total read (Adjacent normal)	Reference allel read support (Adjacent normal)	Alternative read support (Adjacent normal)	Total read (Tumor)	Reference allel read support (Tumor)	Alternative read support (Tumor)	Alternate allele frequency
Smoker	42473	chr20	44439731	C	T	*DNTTIP1*	MISSENSE	ENSG00000101457:MISSENSE[ENST00000372622(c.887C>T,tCc>tTc):ENSP00000361705(p.Ser296Phe)];ENSG00000101457:MISSENSE[ENST00000456939(c.738C>T,tCc>tTc):ENSP00000401024(p.Ser247Phe)];ENSG00000101457:MISSENSE[ENST00000435014(c.595C>T,tCc>tTc):ENSP00000400573(p.Ser199Phe)];	NA	NA	NA	NA	NA	NA	NA	T	PoD	N	MODERATE	NA	NA	79	79	0	208	166	41	19.71
Smoker	42473	chr20	47989911	G	A	*KCNB1*	MISSENSE	ENSG00000158445:MISSENSE[ENST00000371741(c.2186C>T,gCa>gTa):ENSP00000360806(p.Ala729Val)];	NA	NA	NA	8.24E-06	0	NA	NA	D_lc	PoD	D	MODERATE	N	1	68	67	0	163	137	26	15.95
Smoker	42473	chr20	21143757	C	A	*PLK1S1*	EXONIC-NC	ENSG00000088970:[ENST00000428699(n.692C>A)];ENSG00000088970:[ENST00000458446(n.1442C>A)];ENSG00000088970:[ENST00000457464(n.973C>A)];ENSG00000088970:[ENST00000424111(n.1189C>A)];ENSG00000088970:[ENST00000427692(n.1442C>A)];ENSG00000088970:[ENST00000432487(n.1000C>A)];ENSG00000088970:[ENST00000447448(n.970C>A)];ENSG00000088970:[ENST00000246027(n.1371C>A)];	NA	NA	NA	NA	NA	NA	NA	NA	NA	NA	MODIFIER	NA	NA	162	162	0	231	152	78	33.77
Smoker	42473	chr20	21143757	C	A	*RP4-777D9.2*	INTRONIC	ENSG00000232712:[ENST00000591761(n.5052-230G>T)];	NA	NA	NA	NA	NA	NA	NA	NA	NA	NA	MODIFIER	NA	NA	162	162	0	231	152	78	33.77
Smoker	42473	chr20	44675057	G	A	*SLC12A5*	MISSENSE	ENSG00000124140:MISSENSE[ENST00000454036(c.1838G>A,cGc>cAc):ENSP00000387694(p.Arg613His)];ENSG00000124140:MISSENSE[ENST00000243964(c.1769G>A,cGc>cAc):ENSP00000243964(p.Arg590His)];ENSG00000124140:3UTR[ENST00000539566(c.*148G>A)];	chr20*4467	chr20*4467	NA	NA	NA	NA	NA	NA	NA	NA	MODIFIER	NA	NA	25	25	0	120	93	27	22.5
Smoker	42473	chr20	44443228	T	C	*UBE2C*	INTRONIC	ENSG00000175063:[ENST00000352551(c.130-952T>C)];ENSG00000175063:[ENST00000372568(c.99+119T>C)];ENSG00000175063:[ENST00000243893(c.129+1125T>C)];ENSG00000175063:[ENST00000356455(c.216+119T>C)];ENSG00000175063:[ENST00000405520(c.99+119T>C)];ENSG00000175063:[ENST00000335046(c.216+119T>C)];ENSG00000175063:[ENST00000496085(n.61+119T>C)];	NA	NA	0.01398	NA	NA	0.0139776	0	NA	NA	NA	MODIFIER	NA	NA	17	17	0	61	50	11	18.03
Smoker	42473	chr21	11098708	C	T	*BAGE2*	EXONIC-NC	ENSG00000187172:[ENST00000474011(n.273G>A)];ENSG00000187172:[ENST00000470054(n.218G>A)];	NA	NA	chr21*11098703*11098723*CACTCCAGCCGCCATCTTACT*C*rs761137294*NA*NA*NA|chr21*11098704*11098724*ACTCCAGCCGCCATCTTACTG*A*rs766882091*NA*NA*NA|chr21*11098707*11098716*CCAGCCGCCA*C*rs776756587*NA*NA*NA|chr21*11098708*11098708*C*A,T*rs75103008*NA*NA*NA	4.50E-05	0	NA	NA	NA	NA	NA	MODIFIER	NA	NA	34	33	0	67	60	6	8.96
Smoker	42473	chr21	37650530	G	A	*DOPEY2*	MISSENSE	ENSG00000142197:MISSENSE[ENST00000399151(c.5846G>A,cGg>cAg):ENSP00000382104(p.Arg1949Gln)];	NA	NA	NA	7.42E-05	0.000303398	NA	NA	D	PoD	D	MODERATE	D	0.993	121	121	0	179	170	9	5.03
Smoker	42473	chr22	37707572	G	A	*CYTH4*	INTRONIC-SS-DNR	ENSG00000100055:[ENST00000446506(c.214+4G>A)];ENSG00000100055:[ENST00000248901(c.957+4G>A)];ENSG00000100055:[ENST00000462927(n.1121G>A)];	NA	NA	NA	NA	NA	NA	NA	NA	NA	NA	LOW	NA	NA	45	45	0	138	85	53	38.41
Smoker	42473	chr22	42905119	G	A	*SERHL*	INTRONIC-SS-DNR	ENSG00000172250:[ENST00000359906(n.697+3G>A)];ENSG00000172250:[ENST00000421475(n.662+3G>A)];ENSG00000172250:[ENST00000455578(n.1064+3G>A)];	NA	NA	NA	0.0343879	0.031956352	NA	NA	NA	NA	NA	LOW	NA	NA	58	58	0	121	103	18	14.88
Smoker	42473	chr22	24579158	A	C	*SUSD2*	SILENT	ENSG00000099994:EXONIC-NC[ENST00000463101(n.614A>C)];ENSG00000099994:SILENT[ENST00000358321(c.210A>C,ggA>ggC):ENSP00000351075(p.Gly70(=))];	chr22*2457	chr22*2457	NA	0.47	0.457392389	NA	NA	NA	NA	NA	LOW	NA	NA	29	29	0	87	68	19	21.84
Smoker	42473	chr3	183905205	G	C	*ABCF3*	MISSENSE	ENSG00000161204:EXONIC-NC[ENST00000473311(n.495G>C)];ENSG00000161204:EXONIC-NC[ENST00000481116(n.372G>C)];ENSG00000161204:EXONIC-NC[ENST00000466416(n.313G>C)];ENSG00000161204:EXONIC-NC[ENST00000478288(n.369G>C)];ENSG00000161204:EXONIC-NC[ENST00000498136(n.334G>C)];ENSG00000161204:MISSENSE[ENST00000292808(c.304G>C,Gga>Cga):ENSP00000292808(p.Gly102Arg)];ENSG00000161204:MISSENSE[ENST00000429586(c.322G>C,Gga>Cga):ENSP00000411471(p.Gly108Arg)];ENSG00000161204:3UTR[ENST00000421340(c.*43G>C)];	NA	NA	NA	NA	NA	NA	NA	NA	NA	NA	MODIFIER	NA	NA	27	27	0	87	70	17	19.54
Smoker	42473	chr3	113508728	T	G	*ATP6V1A*	INTRONIC	ENSG00000114573:[ENST00000273398(c.988+41T>G)];ENSG00000114573:[ENST00000470455(c.*890+41T>G)];ENSG00000114573:[ENST00000538620(c.889+41T>G)];	NA	NA	NA	NA	NA	NA	NA	NA	NA	NA	MODIFIER	NA	NA	42	42	0	51	31	20	39.22
Smoker	42473	chr3	443282	T	C	*CHL1*	INTRONIC	ENSG00000134121:[ENST00000397491(c.3338-27T>C)];ENSG00000134121:[ENST00000445697(c.787-27T>C)];ENSG00000134121:[ENST00000256509(c.3386-27T>C)];	NA	NA	NA	NA	NA	NA	NA	NA	NA	NA	MODIFIER	NA	NA	81	79	1	62	31	31	50
Smoker	42473	chr3	1424782	G	T	*CNTN6*	NONSENSE	ENSG00000134115:NONSENSE[ENST00000539053(c.2107G>T,Gaa>Taa):ENSP00000442791(p.Glu703Ter)];ENSG00000134115:NONSENSE[ENST00000446702(c.2323G>T,Gaa>Taa):ENSP00000407822(p.Glu775Ter)];ENSG00000134115:NONSENSE[ENST00000350110(c.2323G>T,Gaa>Taa):ENSP00000341882(p.Glu775Ter)];ENSG00000134115:3UTR[ENST00000397479(c.*2461G>T)];	NA	NA	NA	NA	NA	NA	NA	NA	NA	NA	MODIFIER	NA	NA	165	165	0	136	76	59	43.38
Smoker	42473	chr3	39185254	A	T	*CSRNP1*	MISSENSE	ENSG00000144655:MISSENSE[ENST00000514182(c.1062T>A,gaT>gaA):ENSP00000422532(p.Asp354Glu)];ENSG00000144655:MISSENSE[ENST00000273153(c.1062T>A,gaT>gaA):ENSP00000273153(p.Asp354Glu)];	NA	NA	NA	NA	NA	NA	NA	T	BN	N	MODERATE	N	0.005	48	48	0	62	30	32	51.61
Smoker	42473	chr3	183905205	G	C	*EIF2B5*	INTRONIC	ENSG00000145191:[ENST00000444495(c.2106+42710G>C)];	NA	NA	NA	NA	NA	NA	NA	NA	NA	NA	MODIFIER	NA	NA	27	27	0	87	70	17	19.54
Smoker	42473	chr3	151163262	C	T	*IGSF10*	MISSENSE	ENSG00000152580:MISSENSE[ENST00000282466(c.4507G>A,Gtc>Atc):ENSP00000282466(p.Val1503Ile)];	NA	NA	NA	NA	NA	NA	NA	T	BN	N	MODERATE	N	0.003	190	190	0	199	107	92	46.23
Smoker	42473	chr3	169550942	A	C	*LRRIQ4*	MISSENSE	ENSG00000188306:MISSENSE[ENST00000340806(c.1501A>C,Aac>Cac):ENSP00000342188(p.Asn501His)];	NA	NA	NA	NA	NA	NA	NA	D	BN	D	MODERATE	N	0.444	101	101	0	130	73	56	43.08
Smoker	42473	chr3	194380870	A	C	*LSG1*	INTRONIC-SS-ACR	ENSG00000041802:[ENST00000427461(c.*130-8T>G)];ENSG00000041802:[ENST00000265245(c.522-8T>G)];	NA	NA	NA	NA	NA	NA	NA	NA	NA	NA	LOW	NA	NA	155	155	0	170	95	74	43.53

Mangalaparthi *et al.* , 2020. Mutational landscape of esophageal squamous cell carcinoma in an Indian cohort
Supplementary Table 4. List of somatic single nucleotide variants identified using whole exome sequencing of ESCC patients

Cohort	Sample	Chromosome	Genomic position	Reference allele	Alternate allele	Gene symbol	Variant type	Variant annotation	OncoMD	COSMIC	dbSNP	ExAC	ExAC_SAS	1000G	1000G_SAS	SIFT	Polyphen2	CONDEL	VEP	LRT	PhastCon7 Way	Total read (Adjacent normal)	Reference allel read support (Adjacent normal)	Alternative read support (Adjacent normal)	Total read (Tumor)	Reference allel read support (Tumor)	Alternative read support (Tumor)	Alternate allele frequency
Smoker	42473	chr3	154858001	C	T	*MME*	NONSENSE	ENSG00000196549:NONSENSE[ENST00000360490(c.877C>T,Cga>Tga):ENSP00000353679(p.Arg293Ter)];ENSG00000196549:NONSENSE[ENST00000460393(c.877C>T,Cga>Tga):ENSP00000418525(p.Arg293Ter)];ENSG00000196549:NONSENSE[ENST00000492661(c.877C>T,Cga>Tga):ENSP00000420389(p.Arg293Ter)];ENSG00000196549:NONSENSE[ENST00000493237(c.877C>T,Cga>Tga):ENSP00000417079(p.Arg293Ter)];ENSG00000196549:NONSENSE[ENST00000462745(c.877C>T,Cga>Tga):ENSP00000419653(p.Arg293Ter)];	NA	NA	NA	8.32E-06	0	NA	NA	NA	NA	NA	HIGH	NA	0.998	97	97	0	95	63	32	33.68
Smoker	42473	chr3	154858110	A	G	*MME*	INTRONIC	ENSG00000196549:[ENST00000360490(c.957+29A>G)];ENSG00000196549:[ENST00000460393(c.957+29A>G)];ENSG00000196549:[ENST00000492661(c.957+29A>G)];ENSG00000196549:[ENST00000493237(c.957+29A>G)];ENSG00000196549:[ENST00000462745(c.957+29A>G)];	NA	NA	NA	NA	NA	NA	NA	NA	NA	NA	MODIFIER	NA	NA	86	86	0	94	50	44	46.81
Smoker	42473	chr3	47039775	A	G	*NBEAL2*	INTRONIC	ENSG00000160796:[ENST00000450053(c.3118+59A>G)];ENSG00000160796:[ENST00000416683(c.1533+59A>G)];ENSG00000160796:[ENST00000383740(c.-2046+59A>G)];ENSG00000160796:[ENST00000292309(c.3118+59A>G)];	NA	NA	NA	NA	NA	NA	NA	NA	NA	NA	MODIFIER	NA	NA	29	28	0	60	9	51	85
Smoker	42473	chr3	128584100	C	T	*RP11-723O4.2*	INTRONIC	ENSG00000231305:[ENST00000498297(n.688+25G>A)];ENSG00000231305:[ENST00000480931(n.323+25G>A)];	NA	NA	NA	NA	NA	NA	NA	NA	NA	NA	MODIFIER	NA	NA	51	51	0	107	57	50	46.73
Smoker	42473	chr3	195690271	T	C	*SDHAP1*	EXONIC-NC	ENSG00000185485:[ENST00000440850(n.1935A>G)];ENSG00000185485:[ENST00000354559(n.319A>G)];ENSG00000185485:[ENST00000427149(n.143A>G)];ENSG00000185485:[ENST00000427841(n.2225A>G)];ENSG00000185485:[ENST00000354937(n.1970+2029A>G)];	NA	NA	NA	0.2494408	0.194671847	NA	NA	NA	NA	NA	MODIFIER	NA	NA	121	121	0	157	149	8	5.1
Smoker	42473	chr3	195690286	C	G	*SDHAP1*	EXONIC-NC	ENSG00000185485:[ENST00000440850(n.1920G>C)];ENSG00000185485:[ENST00000354559(n.304G>C)];ENSG00000185485:[ENST00000427149(n.128G>C)];ENSG00000185485:[ENST00000427841(n.2210G>C)];ENSG00000185485:[ENST00000354937(n.1970+2014G>C)];	NA	NA	NA	0.2500771	0.191166274	NA	NA	NA	NA	NA	MODIFIER	NA	NA	115	114	0	147	138	9	6.12
Smoker	42473	chr3	100091563	G	A	*TOMM70A*	MISSENSE	ENSG00000154174:MISSENSE[ENST00000284320(c.1339C>T,Cgc>Tgc):ENSP00000284320(p.Arg447Cys)];ENSG00000154174:EXONIC-NC[ENST00000483945(n.192C>T)];	chr3*10009	chr3*10009	NA	NA	NA	NA	NA	NA	NA	NA	MODIFIER	NA	NA	101	101	0	130	75	55	42.31
Smoker	42473	chr4	76956021	C	T	*ART3*	INTRONIC	ENSG00000156219:[ENST00000504914(c.-10+22625C>T)];ENSG00000156219:[ENST00000513353(c.-44+23548C>T)];ENSG00000156219:[ENST00000341029(c.-10+23548C>T)];ENSG00000156219:[ENST00000513122(c.-124-23122C>T)];ENSG00000156219:[ENST00000510669(n.121+23548C>T)];	NA	NA	chr4*76956021*76956021*C*G*rs764150862*NA*NA*NA	NA	NA	NA	NA	NA	NA	NA	MODIFIER	NA	NA	98	96	1	87	59	28	32.18
Smoker	42473	chr4	76956021	C	T	*CXCL11*	INTRONIC	ENSG00000169248:[ENST00000503860(c.262-52G>A)];ENSG00000169248:[ENST00000306621(c.262-52G>A)];	NA	NA	chr4*76956021*76956021*C*G*rs764150862*NA*NA*NA	NA	NA	NA	NA	NA	NA	NA	MODIFIER	NA	NA	98	96	1	87	59	28	32.18
Smoker	42473	chr4	123814224	T	A	*FGF2*	3UTR	ENSG00000138685:[ENST00000608478(c.*673T>A)];ENSG00000138685:[ENST00000264498(c.*673T>A)];	NA	NA	NA	NA	NA	NA	NA	NA	NA	NA	MODIFIER	NA	NA	196	196	0	203	107	95	46.8
Smoker	42473	chr4	162307178	G	A	*FSTL5*	SILENT	ENSG00000168843:SILENT[ENST00000379164(c.2262C>T,aaC>aaT):ENSP00000368462(p.Asn754(=))];ENSG00000168843:SILENT[ENST00000536695(c.2262C>T,aaC>aaT):ENSP00000440409(p.Asn754(=))];ENSG00000168843:SILENT[ENST00000427802(c.2235C>T,aaC>aaT):ENSP00000389270(p.Asn745(=))];ENSG00000168843:SILENT[ENST00000306100(c.2265C>T,aaC>aaT):ENSP00000305334(p.Asn755(=))];	NA	NA	NA	NA	NA	NA	NA	NA	NA	NA	MODIFIER	NA	NA	228	228	0	167	92	75	44.91
Smoker	42473	chr4	123207832	T	C	*KIAA1109*	SILENT	ENSG00000138688:SILENT[ENST00000455637(c.9174T>C,taT>taC):ENSP00000389925(p.Tyr3058(=))];ENSG00000138688:SILENT[ENST00000264501(c.9174T>C,taT>taC):ENSP00000264501(p.Tyr3058(=))];ENSG00000138688:SILENT[ENST00000388738(c.9174T>C,taT>taC):ENSP00000373390(p.Tyr3058(=))];ENSG00000138688:SILENT[ENST00000419325(c.3046T>C,taT>taC):ENSP00000393219(p.Tyr1016(=))];	NA	NA	NA	9.11E-05	6.06E-05	NA	NA	NA	NA	NA	LOW	NA	NA	275	275	0	242	233	9	3.72
Smoker	42473	chr4	123814224	T	A	*NUDT6*	MISSENSE	ENSG00000170917:MISSENSE[ENST00000304430(c.710A>T,aAt>aTt):ENSP00000306070(p.Asn237Ile)];ENSG00000170917:MISSENSE[ENST00000502270(c.203A>T,aAt>aTt):ENSP00000424117(p.Asn68Ile)];ENSG00000170917:MISSENSE[ENST00000339154(c.203A>T,aAt>aTt):ENSP00000344011(p.Asn68Ile)];ENSG00000170917:3UTR[ENST00000503370(c.*651A>T)];ENSG00000170917:3UTR[ENST00000512116(c.*801A>T)];ENSG00000170917:3UTR[ENST00000510735(c.*168A>T)];	NA	NA	NA	NA	NA	NA	NA	NA	NA	NA	MODIFIER	NA	NA	196	196	0	203	107	95	46.8
Smoker	42473	chr4	162307178	G	A	*RP11-234O6.2*	EXONIC-NC	ENSG00000249568:[ENST00000508189(n.449G>A)];	NA	NA	NA	NA	NA	NA	NA	NA	NA	NA	MODIFIER	NA	NA	228	228	0	167	92	75	44.91
Smoker	42473	chr4	25750028	G	C	*SEL1L3*	3UTR	ENSG00000091490:[ENST00000399878(c.*19C>G)];ENSG00000091490:[ENST00000502949(c.*19C>G)];ENSG00000091490:[ENST00000264868(c.*19C>G)];ENSG00000091490:[ENST00000513416(n.1372C>G)];ENSG00000091490:[ENST00000512286(n.397C>G)];	NA	NA	NA	NA	NA	NA	NA	NA	NA	NA	MODIFIER	NA	NA	41	41	0	40	21	19	47.5
Smoker	42473	chr4	98902361	C	A	*STPG2*	MISSENSE	ENSG00000163116:MISSENSE[ENST00000295268(c.721G>T,Ggt>Tgt):ENSP00000295268(p.Gly241Cys)];	NA	NA	NA	NA	NA	NA	NA	D	PrD	D	MODERATE	D	0.313	93	93	0	74	44	30	40.54
Smoker	42473	chr4	7006651	G	C	*TBC1D14*	MISSENSE-SS-PRX	ENSG00000132405:[ENST00000448507(c.1351G>C,Gat>Cat):ENSP00000404041(p.Asp451His)];ENSG00000132405:[ENST00000409757(c.1351G>C,Gat>Cat):ENSP00000386921(p.Asp451His)];ENSG00000132405:[ENST00000446947(c.190G>C,Gat>Cat):ENSP00000405875(p.Asp64His)];ENSG00000132405:[ENST00000439515(c.208G>C,Gat>Cat):ENSP00000389082(p.Asp70His)];ENSG00000132405:[ENST00000451522(c.511G>C,Gat>Cat):ENSP00000388886(p.Asp171His)];ENSG00000132405:[ENST00000410031(c.667G>C,Gat>Cat):ENSP00000386343(p.Asp223His)];	NA	NA	NA	NA	NA	NA	NA	T	BN	N	MODERATE	D	0.978	41	41	0	47	28	19	40.43
Smoker	42473	chr4	189060898	C	T	*TRIML1*	SILENT	ENSG00000184108:SILENT[ENST00000332517(c.186C>T,ggC>ggT):ENSP00000327738(p.Gly62(=))];	NA	NA	NA	NA	NA	NA	NA	NA	NA	NA	LOW	NA	NA	90	90	0	87	82	5	5.75
Smoker	42473	chr5	133642368	G	A	*CDKL3*	MISSENSE	ENSG00000006837:MISSENSE[ENST00000523054(c.823C>T,Cgc>Tgc):ENSP00000428500(p.Arg275Cys)];ENSG00000006837:MISSENSE[ENST00000609654(c.823C>T,Cgc>Tgc):ENSP00000477164(p.Arg275Cys)];ENSG00000006837:MISSENSE[ENST00000265334(c.1390C>T,Cgc>Tgc):ENSP00000265334(p.Arg464Cys)];ENSG00000006837:MISSENSE[ENST00000521118(c.1390C>T,Cgc>Tgc):ENSP00000428689(p.Arg464Cys)];ENSG00000006837:MISSENSE[ENST00000435240(c.505C>T,Cgc>Tgc):ENSP00000399807(p.Arg169Cys)];ENSG00000006837:MISSENSE[ENST00000536186(c.505C>T,Cgc>Tgc):ENSP00000441545(p.Arg169Cys)];ENSG00000006837:INTRONIC[ENST00000609383(c.479+1461C>T)];ENSG00000006837:3UTR[ENST00000520693(c.*485C>T)];ENSG00000006837:3UTR[ENST00000519312(c.*348C>T)];	chr5*13364	chr5*13364	0.0003994	0.0001117	0	0.0003994	0	NA	NA	NA	MODIFIER	NA	NA	122	121	0	212	19	193	91.04
Smoker	42473	chr5	167379571	T	A	*CTC-353G13.1*	INTRONIC	ENSG00000253660:[ENST00000523050(n.78+826A>T)];	NA	NA	NA	NA	NA	NA	NA	NA	NA	NA	MODIFIER	NA	NA	22	22	0	49	26	23	46.94
Smoker	42473	chr5	129083996	C	T	*CTC-575N7.1*	INTRONIC	ENSG00000251680:[ENST00000503616(n.285-13241G>A)];	NA	NA	NA	NA	NA	NA	NA	D_lc	BN	N	MODERATE	NA	NA	67	67	0	69	41	28	40.58
Smoker	42473	chr5	133642368	G	A	*CTD-2410N18.4*	INTRONIC	ENSG00000273345:[ENST00000518409(n.643+1461C>T)];ENSG00000273345:[ENST00000520515(n.897C>T)];	chr5*13364	chr5*13364	0.0003994	0.0001117	0	0.0003994	0	NA	NA	NA	MODIFIER	NA	NA	122	121	0	212	19	193	91.04

Mangalaparthi *et al.* , 2020. Mutational landscape of esophageal squamous cell carcinoma in an Indian cohort
Supplementary Table 4. List of somatic single nucleotide variants identified using whole exome sequencing of ESCC patients

Cohort	Sample	Chromosome	Genomic position	Reference allele	Alternate allele	Gene symbol	Variant type	Variant annotation	OncoMD	COSMIC	dbSNP	ExAC	ExAC_SAS	1000G	1000G_SAS	SIFT	Polyphen2	CONDEL	VEP	LRT	PhastCon7 Way	Total read (Adjacent normal)	Reference allel read support (Adjacent normal)	Alternative read support (Adjacent normal)	Total read (Tumor)	Reference allel read support (Tumor)	Alternative read support (Tumor)	Alternate allele frequency
Smoker	42473	chr5	150275002	G	C	*IRGM*	INTRONIC	ENSG00000237693:[ENST00000520549(c.*141-5149G>C)];	NA	NA	NA	NA	NA	NA	NA	NA	NA	NA	MODIFIER	NA	NA	74	72	2	52	15	37	71.15
Smoker	42473	chr5	129083996	C	T	*KIAA1024L*	MISSENSE	ENSG00000186367:MISSENSE[ENST00000564719(c.113C>T,cCg>cTg):ENSP00000454268(p.Pro38Leu)];	NA	NA	NA	NA	NA	NA	NA	D_lc	BN	N	MODERATE	NA	NA	67	67	0	69	41	28	40.58
Smoker	42473	chr5	177546668	G	T	*N4BP3*	MISSENSE	ENSG00000145911:MISSENSE[ENST00000274605(c.84G>T,gaG>gaT):ENSP00000274605(p.Glu28Asp)];	NA	NA	NA	NA	NA	NA	NA	T	PrD	D	MODERATE	D	0.991	38	38	0	86	48	38	44.19
Smoker	42473	chr5	177483076	C	T	*RP11-889L3.1*	EXONIC-NC	ENSG00000218227:[ENST00000477964(n.472C>T)];	NA	NA	NA	8.35E-06	0	NA	NA	NA	NA	NA	MODIFIER	NA	NA	55	55	0	76	70	6	7.89
Smoker	42473	chr5	177483087	C	T	*RP11-889L3.1*	EXONIC-NC	ENSG00000218227:[ENST00000477964(n.483C>T)];	NA	NA	NA	8.35E-06	0	NA	NA	NA	NA	NA	MODIFIER	NA	NA	48	48	0	68	62	6	8.82
Smoker	42473	chr5	167379571	T	A	*TENM2*	INTRONIC	ENSG00000145934:[ENST00000545108(c.713-22T>A)];ENSG00000145934:[ENST00000520394(c.140-22T>A)];ENSG00000145934:[ENST00000519204(c.350-22T>A)];ENSG00000145934:[ENST00000518693(n.60-22T>A)];ENSG00000145934:[ENST00000517586(n.430-22T>A)];ENSG00000145934:[ENST00000518659(c.713-22T>A)];ENSG00000145934:[ENST00000403607(c.212-22T>A)];ENSG00000145934:[ENST00000520393(n.264-22T>A)];ENSG00000145934:[ENST00000522488(n.192T>A)];	NA	NA	NA	NA	NA	NA	NA	NA	NA	NA	MODIFIER	NA	NA	22	22	0	49	26	23	46.94
Smoker	42473	chr5	150275002	G	C	*ZNF300*	MISSENSE	ENSG00000145908:MISSENSE[ENST00000418587(c.1691C>G,aCa>aGa):ENSP00000392593(p.Thr564Arg)];ENSG00000145908:MISSENSE[ENST00000274599(c.1799C>G,aCa>aGa):ENSP00000274599(p.Thr600Arg)];ENSG00000145908:MISSENSE[ENST00000394226(c.1799C>G,aCa>aGa):ENSP00000377773(p.Thr600Arg)];ENSG00000145908:MISSENSE[ENST00000446148(c.1847C>G,aCa>aGa):ENSP00000397178(p.Thr616Arg)];ENSG00000145908:3UTR[ENST00000427179(c.*2614C>G)];	NA	NA	NA	NA	NA	NA	NA	NA	NA	NA	MODIFIER	NA	NA	74	72	2	52	15	37	71.15
Smoker	42473	chr6	70639549	G	A	*COL19A1*	MISSENSE	ENSG00000082293:MISSENSE[ENST00000322773(c.623G>A,cGg>cAg):ENSP00000316030(p.Arg208Gln)];	NA	NA	NA	3.30E-05	0	NA	NA	D	UN	NA	MODERATE	D	0.979	258	258	0	84	24	60	71.43
Smoker	42473	chr6	38889135	T	G	*DNAH8*	INTRONIC-SS-ACR	ENSG00000124721:[ENST00000359357(c.9874-10T>G)];ENSG00000124721:[ENST00000327475(c.10489-10T>G)];ENSG00000124721:[ENST00000441566(c.9766-10T>G)];ENSG00000124721:[ENST00000449981(c.10525-10T>G)];	NA	NA	chr6*38889135*38889135*T*C*rs376660936*NA*NA*NA	NA	NA	NA	NA	NA	NA	NA	MODIFIER	NA	NA	118	117	1	81	9	72	88.89
Smoker	42473	chr6	65707458	A	G	*EYS*	INTRONIC	ENSG00000188107:[ENST00000370616(c.2259+17T>C)];ENSG00000188107:[ENST00000370621(c.2259+17T>C)];ENSG00000188107:[ENST00000503581(c.2259+17T>C)];	NA	NA	NA	NA	NA	NA	NA	NA	NA	NA	MODIFIER	NA	NA	105	105	0	71	18	53	74.65
Smoker	42473	chr6	6167650	G	A	*F13A1*	INTRONIC	ENSG00000124491:[ENST00000264870(c.1908+41C>T)];	NA	NA	NA	3.30E-05	6.06E-05	NA	NA	NA	NA	NA	MODIFIER	NA	NA	34	32	2	48	8	40	83.33
Smoker	42473	chr6	96651324	C	T	*FUT9*	MISSENSE	ENSG00000172461:MISSENSE[ENST00000302103(c.293C>T,tCa>tTa):ENSP00000302599(p.Ser98Leu)];	NA	NA	NA	NA	NA	NA	NA	T	BN	N	MODERATE	D	0.47	210	210	0	98	50	48	48.98
Smoker	42473	chr6	398899	C	A	*IRF4*	MISSENSE	ENSG00000137265:MISSENSE[ENST00000493114(c.709C>A,Cca>Aca):ENSP00000436094(p.Pro237Thr)];ENSG00000137265:MISSENSE[ENST00000380956(c.709C>A,Cca>Aca):ENSP00000370343(p.Pro237Thr)];	NA	NA	NA	NA	NA	NA	NA	T	BN	N	MODERATE	N	1	28	28	0	24	17	7	29.17
Smoker	42473	chr6	39880683	A	G	*MOCS1*	MISSENSE	ENSG00000124615:MISSENSE[ENST00000425303(c.823T>C,Tgg>Cgg):ENSP00000416478(p.Trp275Arg)];ENSG00000124615:MISSENSE[ENST00000373188(c.823T>C,Tgg>Cgg):ENSP00000362284(p.Trp275Arg)];ENSG00000124615:MISSENSE[ENST00000373195(c.562T>C,Tgg>Cgg):ENSP00000362291(p.Trp188Arg)];ENSG00000124615:MISSENSE[ENST00000373175(c.736T>C,Tgg>Cgg):ENSP00000362270(p.Trp246Arg)];ENSG00000124615:MISSENSE[ENST00000340692(c.823T>C,Tgg>Cgg):ENSP00000344794(p.Trp275Arg)];ENSG00000124615:MISSENSE[ENST00000308559(c.823T>C,Tgg>Cgg):ENSP00000309843(p.Trp275Arg)];ENSG00000124615:MISSENSE[ENST00000373186(c.823T>C,Tgg>Cgg):ENSP00000362282(p.Trp275Arg)];ENSG00000124615:MISSENSE[ENST00000432280(c.736T>C,Tgg>Cgg):ENSP00000410809(p.Trp246Arg)];ENSG00000124615:MISSENSE[ENST00000373181(c.562T>C,Tgg>Cgg):ENSP00000362277(p.Trp188Arg)];	NA	NA	NA	1.65E-05	0	NA	NA	D	PoD	D	MODERATE	NA	NA	28	28	0	53	7	46	86.79
Smoker	42473	chr6	57415236	T	G	*PRIM2*	INTRONIC	ENSG00000146143:[ENST00000389488(n.1107+16919T>G)];ENSG00000146143:[ENST00000607273(c.1020+16919T>G)];ENSG00000146143:[ENST00000550475(n.275+16919T>G)];	NA	NA	NA	NA	NA	NA	NA	NA	NA	NA	MODIFIER	NA	NA	350	349	1	221	185	36	16.29
Smoker	42473	chr6	152642979	T	C	*SYNE1*	SILENT	ENSG00000131018:EXONIC-NC[ENST00000490135(n.3306A>G)];ENSG00000131018:SILENT[ENST00000448038(c.15747A>G,caA>caG):ENSP00000390975(p.Gln5249(=))];ENSG00000131018:SILENT[ENST00000367255(c.15960A>G,caA>caG):ENSP00000356224(p.Gln5320(=))];ENSG00000131018:SILENT[ENST00000423061(c.15747A>G,caA>caG):ENSP00000396024(p.Gln5249(=))];ENSG00000131018:SILENT[ENST00000265368(c.15960A>G,caA>caG):ENSP00000265368(p.Gln5320(=))];ENSG00000131018:INTRONIC[ENST00000341594(c.14997-18A>G)];	NA	NA	chr6*152642979*152642979*T*A*rs144156489*0*0.9998*0.0001997	NA	NA	NA	NA	NA	NA	NA	LOW	NA	0.94	271	271	0	189	98	91	48.15
Smoker	42473	chr7	41739756	C	T	*AC005027.3*	INTRONIC	ENSG00000236310:[ENST00000416150(n.52+5126G>A)];	NA	NA	NA	8.24E-06	0	NA	NA	NA	NA	NA	MODIFIER	NA	NA	116	115	0	290	228	62	21.38
Smoker	42473	chr7	27135514	C	T	*HOTAIRM1*	INTRONIC	ENSG00000233429:[ENST00000495032(n.26+223C>T)];	NA	NA	NA	NA	NA	NA	NA	D	BN	N	MODERATE	N	1	33	33	0	128	104	23	17.97
Smoker	42473	chr7	27135514	C	T	*HOXA1*	MISSENSE	ENSG00000105991:MISSENSE[ENST00000343060(c.18G>A,atG>atA):ENSP00000343246(p.Met6Ile)];ENSG00000105991:MISSENSE[ENST00000355633(c.18G>A,atG>atA):ENSP00000347851(p.Met6Ile)];	NA	NA	NA	NA	NA	NA	NA	D	BN	N	MODERATE	N	1	33	33	0	128	104	23	17.97
Smoker	42473	chr7	41739756	C	T	*INHBA*	MISSENSE	ENSG00000122641:MISSENSE[ENST00000442711(c.217G>A,Gat>Aat):ENSP00000397197(p.Asp73Asn)];ENSG00000122641:MISSENSE[ENST00000242208(c.217G>A,Gat>Aat):ENSP00000242208(p.Asp73Asn)];	NA	NA	NA	8.24E-06	0	NA	NA	NA	NA	NA	MODIFIER	NA	NA	116	115	0	290	228	62	21.38
Smoker	42473	chr7	41739756	C	T	*INHBA-AS1*	INTRONIC	ENSG00000224116:[ENST00000420821(n.141+6070C>T)];ENSG00000224116:[ENST00000422822(n.152+6070C>T)];ENSG00000224116:[ENST00000415848(n.173+6070C>T)];	NA	NA	NA	8.24E-06	0	NA	NA	NA	NA	NA	MODIFIER	NA	NA	116	115	0	290	228	62	21.38
Smoker	42473	chr7	128544267	C	T	*KCP*	INTRONIC	ENSG00000135253:[ENST00000476647(n.616-76G>A)];ENSG00000135253:[ENST00000441244(n.617+79G>A)];ENSG00000135253:[ENST00000297801(n.618+79G>A)];ENSG00000135253:[ENST00000487494(n.616-76G>A)];	NA	NA	NA	NA	NA	NA	NA	NA	NA	NA	MODIFIER	NA	NA	33	33	0	55	31	24	43.64
Smoker	42473	chr7	151848517	A	T	*KMT2C*	INTRONIC-SS-DNR	ENSG00000055609:[ENST00000355193(c.12837+10T>A)];ENSG00000055609:[ENST00000424877(c.2517+10T>A)];ENSG00000055609:[ENST00000360104(c.5347+10T>A)];ENSG00000055609:[ENST00000558084(c.*10186+10T>A)];ENSG00000055609:[ENST00000262189(c.12666+10T>A)];ENSG00000055609:[ENST00000473186(n.10548+10T>A)];	NA	NA	NA	NA	NA	NA	NA	NA	NA	NA	MODIFIER	NA	NA	163	163	0	170	96	74	43.53

Supplementary Table 4. List of somatic single nucleotide variants identified using whole exome sequencing of ESCC patients

Cohort	Sample	Chromosome	Genomic position	Reference allele	Alternate allele	Gene symbol	Variant type	Variant annotation	OncoMD	COSMIC	dbSNP	ExAC	ExAC_SAS	1000G	1000G_SAS	SIFT	Polyphen2	CONDEL	VEP	LRT	PhastCon7 Way	Total read (Adjacent normal)	Reference allel read support (Adjacent normal)	Alternative read support (Adjacent normal)	Total read (Tumor)	Reference allel read support (Tumor)	Alternative read support (Tumor)	Alternate allele frequency
Smoker	42473	chr7	77885512	T	G	*MAGI2*	MISSENSE	ENSG00000187391:MISSENSE[ENST00000536571(c.1291A>C,Acc>Ccc):ENSP00000441584(p.Thr431Pro)];ENSG00000187391:MISSENSE[ENST00000419488(c.1795A>C,Acc>Ccc):ENSP00000405766(p.Thr599Pro)];ENSG00000187391:MISSENSE[ENST00000535697(c.1306A>C,Acc>Ccc):ENSP00000441603(p.Thr436Pro)];ENSG00000187391:MISSENSE[ENST00000354212(c.1795A>C,Acc>Ccc):ENSP00000346151(p.Thr599Pro)];ENSG00000187391:MISSENSE[ENST00000522391(c.1795A>C,Acc>Ccc):ENSP00000428389(p.Thr599Pro)];ENSG00000187391:EXONIC-NC[ENST00000519748(n.889A>C)];ENSG00000187391:EXONIC-NC[ENST00000520379(n.870A>C)];	NA	NA	NA	NA	NA	NA	NA	NA	NA	NA	MODIFIER	NA	NA	72	71	1	75	38	37	49.33
Smoker	42473	chr7	35414517	G	A	*NA*	INTERGENIC		NA	NA	NA	NA	NA	NA	NA	NA	NA	NA	MODIFIER	NA	NA	50	50	0	119	91	27	22.69
Smoker	42473	chr7	44715685	C	A	*OGDH*	MISSENSE	ENSG00000105953:MISSENSE[ENST00000443864(c.1143C>A,gaC>gaA):ENSP00000388084(p.Asp381Glu)];ENSG00000105953:MISSENSE[ENST00000543843(c.996C>A,gaC>gaA):ENSP00000443821(p.Asp332Glu)];ENSG00000105953:MISSENSE[ENST00000444676(c.1188C>A,gaC>gaA):ENSP00000414662(p.Asp396Glu)];ENSG00000105953:MISSENSE[ENST00000449767(c.1131C>A,gaC>gaA):ENSP00000392878(p.Asp377Glu)];ENSG00000105953:MISSENSE[ENST00000439616(c.693C>A,gaC>gaA):ENSP00000398576(p.Asp231Glu)];ENSG00000105953:MISSENSE[ENST00000222673(c.1143C>A,gaC>gaA):ENSP00000222673(p.Asp381Glu)];ENSG00000105953:MISSENSE[ENST00000447398(c.1176C>A,gaC>gaA):ENSP00000388183(p.Asp392Glu)];	NA	NA	NA	NA	NA	NA	NA	D	PoD	D	MODERATE	D	0.998	66	66	0	233	172	61	26.18
Smoker	42473	chr7	56717722	T	C	*RP11-10F11.2*	EXONIC-NC	ENSG00000228303:[ENST00000429248(n.171A>G)];	NA	NA	NA	NA	NA	NA	NA	NA	NA	NA	MODIFIER	NA	NA	92	92	0	129	94	35	27.13
Smoker	42473	chr7	150094002	A	G	*ZNF775*	MISSENSE	ENSG00000196456:MISSENSE[ENST00000329630(c.433A>G,Agc>Ggc):ENSP00000330838(p.Ser145Gly)];ENSG00000196456:MISSENSE[ENST00000490973(c.433A>G,Agc>Ggc):ENSP00000417483(p.Ser145Gly)];ENSG00000196456:STOPCODON-INCOMPLETE[ENST00000478789(c.433A>G,Ag>Gg):ENSP00000419336(UNDEFINED)];ENSG00000196456:INTRONIC[ENST00000483664(n.144+8413A>G)];ENSG00000196456:INTRONIC[ENST00000486297(n.187+8413A>G)];	NA	NA	chr7*150094002*150094002*A*C,G*rs759029217*NA*NA*NA	9.26E-06	0	NA	NA	NA	NA	NA	MODIFIER	NA	NA	66	66	0	111	67	44	39.64
Smoker	42473	chr8	124749589	G	C	*ANXA13*	5UTR	ENSG00000104537:[ENST00000262219(c.-15C>G)];ENSG00000104537:[ENST00000419625(c.-15C>G)];	NA	NA	NA	1.65E-05	0	NA	NA	NA	NA	NA	MODIFIER	NA	NA	187	187	0	197	112	84	42.64
Smoker	42473	chr8	113569125	A	T	*CSMD3*	MISSENSE	ENSG00000164796:MISSENSE[ENST00000352409(c.4101T>A,agT>agA):ENSP00000343124(p.Ser1367Arg)];ENSG00000164796:MISSENSE[ENST00000455883(c.3789T>A,agT>agA):ENSP00000412263(p.Ser1263Arg)];ENSG00000164796:MISSENSE[ENST00000297405(c.4101T>A,agT>agA):ENSP00000297405(p.Ser1367Arg)];ENSG00000164796:MISSENSE[ENST00000339701(c.2121T>A,agT>agA):ENSP00000341558(p.Ser707Arg)];ENSG00000164796:MISSENSE[ENST00000343508(c.3981T>A,agT>agA):ENSP00000345799(p.Ser1327Arg)];	NA	NA	NA	NA	NA	NA	NA	T	BN	N	MODERATE	D	1	79	77	1	72	52	20	27.78
Smoker	42473	chr8	15978549	G	A	*MSR1*	INTRONIC	ENSG00000038945:[ENST00000350896(c.1034-10822C>T)];ENSG00000038945:[ENST00000355282(c.1034-10822C>T)];ENSG00000038945:[ENST00000445506(c.1088-434C>T)];ENSG00000038945:[ENST00000262101(c.1034-434C>T)];ENSG00000038945:[ENST00000522672(c.*94C>T)];	NA	NA	NA	NA	NA	NA	NA	NA	NA	NA	MODIFIER	NA	NA	29	29	0	31	13	18	58.06
Smoker	42473	chr8	55538771	A	C	*RP1*	SILENT	ENSG00000104237:SILENT[ENST00000220676(c.2329A>C,Aga>Cga):ENSP00000220676(p.Arg777(=))];	NA	NA	NA	NA	NA	NA	NA	NA	NA	NA	LOW	NA	0.921	71	70	0	65	45	19	29.23
Smoker	42473	chr8	50449701	C	T	*RP11-738G5.2*	INTRONIC	ENSG00000253206:[ENST00000520800(c.163+15G>A)];	NA	NA	NA	NA	NA	NA	NA	NA	NA	NA	MODIFIER	NA	NA	63	63	0	84	34	50	59.52
Smoker	42473	chr8	15606014	C	A	*TUSC3*	INTRONIC	ENSG00000104723:[ENST00000382020(c.1028+40C>A)];ENSG00000104723:[ENST00000515859(c.*133+40C>A)];ENSG00000104723:[ENST00000511783(c.890+40C>A)];ENSG00000104723:[ENST00000508446(n.241+4893C>A)];ENSG00000104723:[ENST00000506802(c.937+4893C>A)];ENSG00000104723:[ENST00000507316(n.260+40C>A)];ENSG00000104723:[ENST00000510836(c.*42+4893C>A)];ENSG00000104723:[ENST00000503731(c.1028+40C>A)];ENSG00000104723:[ENST00000511342(n.214C>A)];	NA	NA	chr8*15606014*15606014*C*G,T*rs352781*1*0.9928*0.007188,.	NA	NA	NA	NA	NA	NA	NA	MODIFIER	NA	NA	109	109	0	75	47	28	37.33
Smoker	42473	chr8	146003723	C	T	*ZNF34*	INTRONIC	ENSG00000196378:[ENST00000534445(c.96+101G>A)];ENSG00000196378:[ENST00000429371(c.33+101G>A)];ENSG00000196378:[ENST00000343459(c.96+101G>A)];ENSG00000196378:[ENST00000534337(c.33+101G>A)];ENSG00000196378:[ENST00000527740(n.270G>A)];	NA	NA	NA	NA	NA	NA	NA	NA	NA	NA	MODIFIER	NA	NA	35	35	0	57	31	26	45.61
Smoker	42473	chr9	18684761	G	A	*ADAMTSL1*	MISSENSE	ENSG00000178031:MISSENSE[ENST00000380548(c.1537G>A,Gag>Aag):ENSP00000369921(p.Glu513Lys)];ENSG00000178031:MISSENSE[ENST00000276935(c.1537G>A,Gag>Aag):ENSP00000276935(p.Glu513Lys)];ENSG00000178031:MISSENSE[ENST00000327883(c.1537G>A,Gag>Aag):ENSP00000327887(p.Glu513Lys)];ENSG00000178031:EXONIC-NC[ENST00000546040(n.676G>A)];	NA	NA	NA	NA	NA	NA	NA	NA	NA	NA	MODIFIER	NA	NA	173	172	0	114	63	51	44.74
Smoker	42473	chr9	88836563	G	A	*C9orf153*	3UTR	ENSG00000187753:[ENST00000339137(c.*40C>T)];ENSG00000187753:[ENST00000469914(n.178C>T)];	NA	NA	NA	NA	NA	NA	NA	NA	NA	NA	MODIFIER	NA	NA	185	184	1	160	99	61	38.12
Smoker	42473	chr9	114520398	T	A	*C9orf84*	MISSENSE	ENSG00000165181:MISSENSE[ENST00000318737(c.482A>T,cAa>cTa):ENSP00000322108(p.Gln161Leu)];ENSG00000165181:MISSENSE[ENST00000374287(c.482A>T,cAa>cTa):ENSP00000363405(p.Gln161Leu)];ENSG00000165181:MISSENSE[ENST00000394779(c.365A>T,cAa>cTa):ENSP00000378259(p.Gln122Leu)];ENSG00000165181:MISSENSE[ENST00000374283(c.674A>T,cAa>cTa):ENSP00000363401(p.Gln225Leu)];ENSG00000165181:MISSENSE[ENST00000394777(c.365A>T,cAa>cTa):ENSP00000378257(p.Gln122Leu)];	NA	NA	NA	NA	NA	NA	NA	T	BN	N	MODERATE	NA	NA	60	60	0	24	16	8	33.33
Smoker	42473	chr9	86502092	G	A	*KIF27*	SILENT	ENSG00000165115:SILENT[ENST00000413982(c.2103C>T,ctC>ctT):ENSP00000401688(p.Leu701(=))];ENSG00000165115:SILENT[ENST00000376347(c.276C>T,ctC>ctT):ENSP00000365525(p.Leu92(=))];ENSG00000165115:SILENT[ENST00000334204(c.2103C>T,ctC>ctT):ENSP00000333928(p.Leu701(=))];ENSG00000165115:SILENT[ENST00000297814(c.2103C>T,ctC>ctT):ENSP00000297814(p.Leu701(=))];	NA	NA	NA	8.55E-06	0	NA	NA	NA	NA	NA	LOW	NA	NA	116	116	0	67	44	23	34.33
Smoker	42473	chr9	125391415	A	G	*OR1B1*	MISSENSE	ENSG00000171484:MISSENSE[ENST00000304833(c.400T>C,Tat>Cat):ENSP00000303151(p.Tyr134His)];	NA	NA	NA	8.24E-06	0	NA	NA	NA	NA	NA	MODIFIER	NA	NA	78	78	0	75	48	27	36
Smoker	42473	chr9	66457330	G	T	*RP11-262H14.1*	EXONIC-NC	ENSG00000238113:[ENST00000424345(n.42G>T)];ENSG00000238113:[ENST00000427509(n.46G>T)];	NA	NA	NA	NA	NA	NA	NA	NA	NA	NA	MODIFIER	NA	NA	112	81	1	156	104	8	5.13
Smoker	42473	chr9	125391415	A	G	*RP11-64P14.7*	INTRONIC	ENSG00000234156:[ENST00000419604(n.279-7970A>G)];ENSG00000234156:[ENST00000431442(n.137-7967A>G)];	NA	NA	NA	8.24E-06	0	NA	NA	NA	NA	NA	MODIFIER	NA	NA	78	78	0	75	48	27	36
Smoker	42473	chr9	114304978	T	G	*ZNF483*	MISSENSE	ENSG00000173258:MISSENSE[ENST00000309235(c.1763T>G,tTt>tGt):ENSP00000311679(p.Phe588Cys)];ENSG00000173258:INTRONIC[ENST00000358151(c.721+8345T>G)];	NA	NA	NA	NA	NA	NA	NA	NA	NA	NA	MODIFIER	NA	NA	84	83	1	69	41	28	40.58

Mangalaparthi *et al.* , 2020. Mutational landscape of esophageal squamous cell carcinoma in an Indian cohort
Supplementary Table 4. List of somatic single nucleotide variants identified using whole exome sequencing of ESCC patients

Cohort	Sample	Chromosome	Genomic position	Reference allele	Alternate allele	Gene symbol	Variant type	Variant annotation	OncoMD	COSMIC	dbSNP	ExAC	ExAC_SAS	1000G	1000G_SAS	SIFT	Polyphen2	CONDEL	VEP	LRT	PhastCon7 Way	Total read (Adjacent normal)	Reference allel read support (Adjacent normal)	Alternative read support (Adjacent normal)	Total read (Tumor)	Reference allel read support (Tumor)	Alternative read support (Tumor)	Alternate allele frequency
Smoker	42473	chrX	2835907	T	G	*ARSD*	MISSENSE	ENSG00000006756:MISSENSE[ENST00000381154(c.801A>C,caA>caC):ENSP00000370546(p.Gln267His)];ENSG00000006756:EXONIC-NC[ENST00000217890(n.801A>C)];ENSG00000006756:EXONIC-NC[ENST00000481340(n.13A>C)];ENSG00000006756:INTRONIC[ENST00000495294(n.119-9061A>C)];	NA	NA	NA	NA	NA	NA	NA	NA	NA	NA	MODIFIER	NA	NA	54	54	0	110	103	7	6.36
Smoker	42473	chrX	55650469	C	T	*FOXR2*	MISSENSE	ENSG00000189299:MISSENSE[ENST00000339140(c.325C>T,Cct>Tct):ENSP00000427329(p.Pro109Ser)];	NA	NA	NA	1.15E-05	0	NA	NA	T	BN	N	MODERATE	U	0.001	67	67	0	68	42	26	38.24
Smoker	42473	chrX	152960008	G	A	*SLC6A8*	MISSENSE	ENSG00000130821:MISSENSE[ENST00000430077(c.1171G>A,Gac>Aac):ENSP00000403041(p.Asp391Asn)];ENSG00000130821:MISSENSE[ENST00000253122(c.1516G>A,Gac>Aac):ENSP00000253122(p.Asp506Asn)];ENSG00000130821:MISSENSE[ENST00000413787(c.445G>A,Gac>Aac):ENSP00000400463(p.Asp149Asn)];ENSG00000130821:EXONIC-NC[ENST00000485324(n.1823G>A)];	NA	NA	NA	0.0004595	0	NA	NA	T_lc	PoD	N	MODERATE	NA	NA	11	11	0	31	2	29	93.55
Smoker	42473	chrY	6740668	T	C	*AMELY*	INTRONIC-SS-ACR	ENSG00000099721:[ENST00000215479(c.-12-7A>G)];	NA	NA	NA	NA	NA	NA	NA	NA	NA	NA	LOW	NA	NA	51	51	0	63	4	59	93.65
Smoker	42474	chr1	222860468	C	T	*AIDA*	INTRONIC	ENSG00000186063:[ENST00000355727(c.354-90G>A)];ENSG00000186063:[ENST00000470437(n.467-90G>A)];ENSG00000186063:[ENST00000541237(c.282-90G>A)];ENSG00000186063:[ENST00000474863(n.1603-90G>A)];ENSG00000186063:[ENST00000340020(c.354-90G>A)];ENSG00000186063:[ENST00000497112(n.426-90G>A)];	NA	NA	NA	NA	NA	NA	NA	NA	NA	NA	MODIFIER	NA	NA	79	78	0	90	53	36	40
Smoker	42474	chr1	110173803	G	A	*AMPD2*	3UTR	ENSG00000116337:[ENST00000528454(c.*29G>A)];ENSG00000116337:[ENST00000358729(c.*29G>A)];ENSG00000116337:[ENST00000256578(c.*29G>A)];ENSG00000116337:[ENST00000342115(c.*29G>A)];ENSG00000116337:[ENST00000369840(c.*29G>A)];ENSG00000116337:[ENST00000476688(c.*29G>A)];ENSG00000116337:[ENST00000393688(c.*29G>A)];ENSG00000116337:[ENST00000528958(n.395G>A)];	NA	NA	NA	0	0	NA	NA	NA	NA	NA	MODIFIER	NA	NA	41	41	0	58	48	10	17.24
Smoker	42474	chr1	156928784	C	A	*ARHGEF11*	INTRONIC	ENSG00000132694:[ENST00000361409(c.1259+54G>T)];ENSG00000132694:[ENST00000368194(c.1379+54G>T)];ENSG00000132694:[ENST00000486670(n.491+54G>T)];	NA	NA	0.0003994	NA	NA	0.0003994	0	NA	NA	NA	MODIFIER	NA	NA	82	82	0	106	80	26	24.53
Smoker	42474	chr1	176926897	G	A	*ASTN1*	MISSENSE	ENSG00000152092:MISSENSE[ENST00000367654(c.1828C>T,Cgc>Tgc):ENSP00000356626(p.Arg610Cys)];ENSG00000152092:MISSENSE[ENST00000424564(c.1804C>T,Cgc>Tgc):ENSP00000395041(p.Arg602Cys)];ENSG00000152092:MISSENSE[ENST00000361833(c.1804C>T,Cgc>Tgc):ENSP00000354536(p.Arg602Cys)];ENSG00000152092:MISSENSE[ENST00000367657(c.1804C>T,Cgc>Tgc):ENSP00000356629(p.Arg602Cys)];ENSG00000152092:EXONIC-NC[ENST00000281881(n.2101C>T)];	chr1*17692	chr1*17692	NA	NA	NA	NA	NA	NA	NA	NA	MODIFIER	NA	NA	43	43	0	65	54	11	16.92
Smoker	42474	chr1	1919511	T	C	*C1orf222*	INTRONIC	ENSG00000142609:[ENST00000270720(n.452+440A>G)];ENSG00000142609:[ENST00000378592(n.452+440A>G)];ENSG00000142609:[ENST00000468610(n.329+440A>G)];ENSG00000142609:[ENST00000378590(c.*52A>G)];	NA	NA	NA	NA	NA	NA	NA	NA	NA	NA	MODIFIER	NA	NA	48	48	0	43	24	19	44.19
Smoker	42474	chr1	115280186	T	C	*CSDE1*	INTRONIC-SS-ACR	ENSG00000009307:INTRONIC-SS-ACR[ENST00000358528(c.310-2A>G)];ENSG00000009307:INTRONIC-SS-ACR[ENST00000534699(c.310-2A>G)];ENSG00000009307:INTRONIC-SS-ACR[ENST00000438362(c.448-2A>G)];ENSG00000009307:INTRONIC[ENST00000369530(c.447+398A>G)];ENSG00000009307:INTRONIC[ENST00000261443(c.309+398A>G)];ENSG00000009307:INTRONIC[ENST00000339438(c.309+398A>G)];ENSG00000009307:INTRONIC[ENST00000529046(c.12+398A>G)];ENSG00000009307:INTRONIC[ENST00000530886(c.12+398A>G)];ENSG00000009307:INTRONIC[ENST00000525132(c.12+398A>G)];	NA	NA	NA	NA	NA	NA	NA	NA	NA	NA	MODIFIER	NA	NA	121	120	0	143	109	33	23.08
Smoker	42474	chr1	59248023	C	T	*JUN*	SILENT	ENSG00000177606:SILENT[ENST00000371222(c.720G>A,ccG>ccA):ENSP00000360266(p.Pro240(=))];	NA	NA	NA	NA	NA	NA	NA	NA	NA	NA	LOW	NA	NA	82	82	0	75	47	28	37.33
Smoker	42474	chr1	202702990	G	A	*KDM5B*	SILENT	ENSG00000117139:SILENT[ENST00000235790(c.2974C>T,Cta>Tta):ENSP00000235790(p.Leu992(=))];ENSG00000117139:SILENT[ENST00000367264(c.3556C>T,Cta>Tta):ENSP00000356233(p.Leu1186(=))];ENSG00000117139:SILENT[ENST00000367265(c.3448C>T,Cta>Tta):ENSP00000356234(p.Leu1150(=))];ENSG00000117139:EXONIC-NC[ENST00000472822(n.208C>T)];	NA	NA	NA	NA	NA	NA	NA	NA	NA	NA	LOW	NA	0.99	70	70	0	99	48	50	50.51
Smoker	42474	chr1	40431095	C	T	*MFSD2A*	INTRONIC	ENSG00000168389:[ENST00000480630(n.1125-48C>T)];ENSG00000168389:[ENST00000469745(n.390-48C>T)];ENSG00000168389:[ENST00000372811(c.478-48C>T)];ENSG00000168389:[ENST00000420632(c.10-48C>T)];ENSG00000168389:[ENST00000483824(n.613-48C>T)];ENSG00000168389:[ENST00000434861(c.472-48C>T)];ENSG00000168389:[ENST00000372809(c.517-48C>T)];	NA	NA	NA	NA	NA	NA	NA	NA	NA	NA	MODIFIER	NA	NA	150	150	0	137	102	35	25.55
Smoker	42474	chr1	248605375	G	A	*OR2T7*	MISSENSE	ENSG00000227152:MISSENSE[ENST00000460972(c.868G>A,Ggg>Agg):ENSP00000475521(p.Gly290Arg)];	NA	NA	NA	NA	NA	NA	NA	T	BN	N	MODERATE	NA	NA	119	119	0	210	97	113	53.81
Smoker	42474	chr1	111889571	G	A	*PIFO*	MISSENSE	ENSG00000173947:EXONIC-NC[ENST00000484512(n.112G>A)];ENSG00000173947:MISSENSE[ENST00000369737(c.59G>A,aGg>aAg):ENSP00000358752(p.Arg20Lys)];ENSG00000173947:MISSENSE[ENST00000369738(c.59G>A,aGg>aAg):ENSP00000358753(p.Arg20Lys)];	NA	NA	NA	NA	NA	NA	NA	T	BN	N	MODERATE	N	0.997	91	91	0	75	60	15	20
Smoker	42474	chr1	208390970	C	T	*PLXNA2*	MISSENSE	ENSG00000076356:MISSENSE[ENST00000367033(c.298G>A,Gtg>Atg):ENSP00000356000(p.Val100Met)];	NA	NA	NA	NA	NA	NA	NA	T	PrD	D	MODERATE	NA	NA	75	75	0	98	63	35	35.71
Smoker	42474	chr1	169599354	C	G	*SELP*	5UTR	ENSG00000174175:[ENST00000367793(c.-42G>C)];ENSG00000174175:[ENST00000367794(c.-42G>C)];ENSG00000174175:[ENST00000367792(c.-42G>C)];ENSG00000174175:[ENST00000367788(c.-42G>C)];ENSG00000174175:[ENST00000367791(c.-42G>C)];	NA	NA	NA	NA	NA	NA	NA	NA	NA	NA	MODIFIER	NA	NA	104	104	0	107	74	33	30.84
Smoker	42474	chr1	28146262	G	T	*STX12*	INTRONIC	ENSG00000117758:[ENST00000373943(c.732+19G>T)];	NA	NA	NA	NA	NA	NA	NA	NA	NA	NA	MODIFIER	NA	NA	169	169	0	213	133	80	37.56
Smoker	42474	chr1	51808702	G	C	*TTC39A*	SILENT	ENSG00000085831:SILENT[ENST00000527205(c.102C>G,acC>acG):ENSP00000432453(p.Thr34(=))];ENSG00000085831:INTRONIC[ENST00000413473(c.53+1961C>G)];ENSG00000085831:INTRONIC[ENST00000401051(c.53+1961C>G)];	NA	NA	NA	NA	NA	NA	NA	NA	NA	NA	MODIFIER	NA	NA	40	40	0	39	24	15	38.46
Smoker	42474	chr1	216498883	G	A	*USH2A*	MISSENSE	ENSG00000042781:MISSENSE[ENST00000366942(c.907C>T,Cgt>Tgt):ENSP00000355909(p.Arg303Cys)];ENSG00000042781:MISSENSE[ENST00000366943(c.907C>T,Cgt>Tgt):ENSP00000355910(p.Arg303Cys)];ENSG00000042781:MISSENSE[ENST00000307340(c.907C>T,Cgt>Tgt):ENSP00000305941(p.Arg303Cys)];	NA	NA	chr1*216498883*216498883*G*T*rs748465849*NA*NA*NA	NA	NA	NA	NA	NA	PrD	NA	MODERATE	NA	NA	71	71	0	99	74	25	25.25
Smoker	42474	chr10	93237987	G	C	*HECTD2*	MISSENSE	ENSG00000165338:MISSENSE[ENST00000298068(c.630G>C,aaG>aaC):ENSP00000298068(p.Lys210Asn)];ENSG00000165338:MISSENSE[ENST00000446394(c.630G>C,aaG>aaC):ENSP00000401023(p.Lys210Asn)];ENSG00000165338:5UTR[ENST00000536715(c.-507G>C)];	NA	NA	NA	NA	NA	NA	NA	NA	NA	NA	MODIFIER	NA	NA	72	72	0	85	47	38	44.71

Mangalaparthi *et al.* , 2020. Mutational landscape of esophageal squamous cell carcinoma in an Indian cohort
Supplementary Table 4. List of somatic single nucleotide variants identified using whole exome sequencing of ESCC patients

Cohort	Sample	Chromosome	Genomic position	Reference allele	Alternate allele	Gene symbol	Variant type	Variant annotation	OncoMD	COSMIC	dbSNP	ExAC	ExAC_SAS	1000G	1000G_SAS	SIFT	Polyphen2	CONDEL	VEP	LRT	PhastCon7 Way	Total read (Adjacent normal)	Reference allel read support (Adjacent normal)	Alternative read support (Adjacent normal)	Total read (Tumor)	Reference allel read support (Tumor)	Alternative read support (Tumor)	Alternate allele frequency
Smoker	42474	chr10	135273589	C	T	*RP11-108K14.4*	EXONIC-NC	ENSG00000214279:[ENST00000463137(n.2647C>T)];ENSG00000214279:[ENST00000488261(n.2795C>T)];ENSG00000214279:[ENST00000482993(n.3029C>T)];ENSG00000214279:[ENST00000356567(n.2795C>T)];	NA	NA	chr10*135273584*135273597*GGCCACGAGTCTGC*G*rs747547798*NA*NA*NA	NA	NA	NA	NA	NA	NA	NA	MODIFIER	NA	NA	40	40	0	26	9	17	65.38
Smoker	42474	chr10	135273589	C	T	*SPRN*	INTRONIC	ENSG00000203772:[ENST00000541506(c.-16-36388G>A)];	NA	NA	chr10*135273584*135273597*GGCCACGAGTCTGC*G*rs747547798*NA*NA*NA	NA	NA	NA	NA	NA	NA	NA	MODIFIER	NA	NA	40	40	0	26	9	17	65.38
Smoker	42474	chr11	67191327	G	A	*CARNS1*	MISSENSE	ENSG00000172508:INTRONIC[ENST00000531958(n.2122-1134G>A)];ENSG00000172508:MISSENSE[ENST00000307823(c.1739G>A,cGa>cAa):ENSP00000308268(p.Arg580Gln)];ENSG00000172508:MISSENSE[ENST00000531040(c.2030G>A,cGa>cAa):ENSP00000431670(p.Arg677Gln)];ENSG00000172508:MISSENSE[ENST00000423745(c.1739G>A,cGa>cAa):ENSP00000401519(p.Arg580Gln)];ENSG00000172508:MISSENSE[ENST00000445895(c.2108G>A,cGa>cAa):ENSP00000389009(p.Arg703Gln)];ENSG00000172508:EXONIC-NC[ENST00000531388(n.3316G>A)];	NA	NA	0.000599	0.0003764	0.001480933	0.000599	0.002	T	BN	N	MODERATE	N	0.558	114	113	0	105	67	38	36.19
Smoker	42474	chr11	93844104	C	G	*HEPHL1*	SILENT	ENSG00000181333:SILENT[ENST00000315765(c.3081C>G,ctC>ctG):ENSP00000313699(p.Leu1027(=))];	NA	NA	NA	NA	NA	NA	NA	NA	NA	NA	LOW	NA	NA	114	114	0	194	182	12	6.19
Smoker	42474	chr12	56868637	T	A	*GLS2*	MISSENSE	ENSG00000135423:MISSENSE[ENST00000311966(c.1040A>T,tAc>tTc):ENSP00000310447(p.Tyr347Phe)];ENSG00000135423:EXONIC-NC[ENST00000476991(n.277A>T)];ENSG00000135423:EXONIC-NC[ENST00000496006(n.244A>T)];ENSG00000135423:3UTR[ENST00000424141(c.*376A>T)];ENSG00000135423:3UTR[ENST00000479952(c.*33A>T)];ENSG00000135423:3UTR[ENST00000486896(c.*711A>T)];ENSG00000135423:3UTR[ENST00000486433(c.*129A>T)];	NA	NA	NA	NA	NA	NA	NA	NA	NA	NA	MODIFIER	NA	NA	42	42	0	26	16	10	38.46
Smoker	42474	chr12	69120323	A	C	*NUP107*	MISSENSE	ENSG00000111581:MISSENSE[ENST00000378905(c.1168A>C,Aat>Cat):ENSP00000368185(p.Asn390His)];ENSG00000111581:MISSENSE[ENST00000229179(c.1621A>C,Aat>Cat):ENSP00000229179(p.Asn541His)];ENSG00000111581:MISSENSE[ENST00000539906(c.1534A>C,Aat>Cat):ENSP00000441448(p.Asn512His)];ENSG00000111581:3UTR[ENST00000538993(c.*39A>C)];ENSG00000111581:3UTR[ENST00000535718(c.*1164A>C)];	NA	NA	chr12*69120323*69120323*A*G*rs767468734*NA*NA*NA	NA	NA	NA	NA	NA	NA	NA	MODIFIER	NA	NA	105	105	0	177	170	7	3.95
Smoker	42474	chr12	108931859	C	T	*SART3*	MISSENSE	ENSG00000075856:MISSENSE[ENST00000431469(c.1183G>A,Gat>Aat):ENSP00000414453(p.Asp395Asn)];ENSG00000075856:MISSENSE[ENST00000228284(c.1183G>A,Gat>Aat):ENSP00000228284(p.Asp395Asn)];ENSG00000075856:MISSENSE[ENST00000546815(c.1237G>A,Gat>Aat):ENSP00000449386(p.Asp413Asn)];ENSG00000075856:3UTR[ENST00000547528(c.*347G>A)];ENSG00000075856:3UTR[ENST00000546728(c.*77G>A)];ENSG00000075856:EXONIC-NC[ENST00000548119(n.50G>A)];	NA	NA	NA	NA	NA	NA	NA	T	BN	N	MODERATE	NA	NA	145	145	0	189	177	12	6.35
Smoker	42474	chr12	100784891	C	T	*SLC17A8*	MISSENSE	ENSG00000179520:MISSENSE[ENST00000392989(c.467C>T,gCt>gTt):ENSP00000376715(p.Ala156Val)];ENSG00000179520:MISSENSE[ENST00000323346(c.467C>T,gCt>gTt):ENSP00000316909(p.Ala156Val)];	chr12*1007[illegible]	chr12*1007[illegible]	NA	NA	NA	NA	NA	D	PoD	D	MODERATE	D	0.998	152	152	0	134	88	46	34.33
Smoker	42474	chr13	52515301	C	T	*ATP7B*	MISSENSE	ENSG00000123191:MISSENSE[ENST00000242839(c.3472G>A,Ggt>Agt):ENSP00000242839(p.Gly1158Ser)];ENSG00000123191:MISSENSE[ENST00000400366(c.3139G>A,Ggt>Agt):ENSP00000383217(p.Gly1047Ser)];ENSG00000123191:MISSENSE[ENST00000448424(c.3238G>A,Ggt>Agt):ENSP00000416738(p.Gly1080Ser)];ENSG00000123191:MISSENSE[ENST00000400370(c.2182G>A,Ggt>Agt):ENSP00000383221(p.Gly728Ser)];ENSG00000123191:MISSENSE[ENST00000418097(c.3277G>A,Ggt>Agt):ENSP00000393343(p.Gly1093Ser)];ENSG00000123191:MISSENSE[ENST00000344297(c.2851G>A,Ggt>Agt):ENSP00000342559(p.Gly951Ser)];ENSG00000123191:MISSENSE[ENST00000417240(c.1105G>A,Ggt>Agt):ENSP00000390360(p.Gly369Ser)];ENSG00000123191:EXONIC-NC[ENST00000482841(n.2407G>A)];	NA	NA	chr13*52515301*52515301*C*G,T*rs776086518*NA*NA*NA	8.28E-06	0	NA	NA	NA	NA	NA	MODIFIER	NA	NA	72	72	0	75	64	11	14.67
Smoker	42474	chr13	103394755	A	C	*NA*	INTERGENIC		NA	NA	NA	NA	NA	NA	NA	NA	NA	NA	MODIFIER	NA	NA	91	91	0	104	50	54	51.92
Smoker	42474	chr14	24642164	C	T	*REC8*	MISSENSE	ENSG00000100918:MISSENSE[ENST00000311457(c.182C>T,cCg>cTg):ENSP00000308699(p.Pro61Leu)];ENSG00000100918:MISSENSE[ENST00000559919(c.182C>T,cCg>cTg):ENSP00000453242(p.Pro61Leu)];ENSG00000100918:MISSENSE[ENST00000557806(c.182C>T,cCg>cTg):ENSP00000453763(p.Pro61Leu)];ENSG00000100918:EXONIC-NC[ENST00000558782(n.90C>T)];ENSG00000100918:EXONIC-NC[ENST00000557979(n.600C>T)];ENSG00000100918:EXONIC-NC[ENST00000560032(n.453C>T)];ENSG00000100918:5UTR[ENST00000560501(c.-146C>T)];	NA	NA	NA	NA	NA	NA	NA	NA	NA	NA	MODIFIER	NA	NA	91	91	0	77	43	34	44.16
Smoker	42474	chr15	30010328	T	A	*TJP1*	INTRONIC-SS-ACR	ENSG00000104067:[ENST00000356107(c.3877-6A>T)];ENSG00000104067:[ENST00000400011(c.3649-6A>T)];ENSG00000104067:[ENST00000346128(c.3877-6A>T)];ENSG00000104067:[ENST00000545208(c.3637-6A>T)];	NA	NA	chr15*30010328*30010328*T*TA,TAA*rs768185243*NA*NA*NA|chr15*30010328*30010328*T*TA,TAA*rs563006679*NA*NA*NA|chr15*30010328*30010329*TA*T*rs781451170*NA*NA*NA|chr15*30010328*30010330*TAA*T*rs796433006*NA*NA*NA|chr15*30010328*30010331*TAAA*T*rs776289841*NA*NA*NA|chr15*30010328*30010332*TAAAA*T*rs750918158*NA*NA*NA	NA	NA	NA	NA	NA	NA	NA	LOW	NA	NA	51	51	0	58	51	7	12.07

Mangalaparthi *et al.* , 2020. Mutational landscape of esophageal squamous cell carcinoma in an Indian cohort
Supplementary Table 4. List of somatic single nucleotide variants identified using whole exome sequencing of ESCC patients

Cohort	Sample	Chromosome	Genomic position	Reference allele	Alternate allele	Gene symbol	Variant type	Variant annotation	OncoMD	COSMIC	dbSNP	ExAC	ExAC_SAS	1000G	1000G_SAS	SIFT	Polyphen2	CONDEL	VEP	LRT	PhastCon7 Way	Total read (Adjacent normal)	Reference allel read support (Adjacent normal)	Alternative read support (Adjacent normal)	Total read (Tumor)	Reference allel read support (Tumor)	Alternative read support (Tumor)	Alternate allele frequency
Smoker	42474	chr15	38229075	G	T	*TMCO5A*	SILENT	ENSG00000166069:SILENT[ENST00000558158(c.168G>T,cgG>cgT):ENSP00000453047(p.Arg56(=))];ENSG00000166069:SILENT[ENST00000560653(c.168G>T,cgG>cgT):ENSP00000453561(p.Arg56(=))];ENSG00000166069:SILENT[ENST00000319669(c.168G>T,cgG>cgT):ENSP00000327234(p.Arg56(=))];ENSG00000166069:SILENT[ENST00000559502(c.168G>T,cgG>cgT):ENSP00000454112(p.Arg56(=))];ENSG00000166069:SILENT[ENST00000558625(c.168G>T,cgG>cgT):ENSP00000453271(p.Arg56(=))];ENSG00000166069:SILENT[ENST00000560841(c.168G>T,cgG>cgT):ENSP00000453162(p.Arg56(=))];ENSG00000166069:SILENT[ENST00000558148(c.168G>T,cgG>cgT):ENSP00000453369(p.Arg56(=))];ENSG00000166069:SILENT[ENST00000540944(c.168G>T,cgG>cgT):ENSP00000445914(p.Arg56(=))];ENSG00000166069:EXONIC-NC[ENST00000559813(n.270G>T)];	NA	NA	NA	NA	NA	NA	NA	NA	NA	NA	LOW	NA	NA	39	39	0	44	27	17	38.64
Smoker	42474	chr16	57760064	C	T	*CCDC135*	MISSENSE	ENSG00000159625:MISSENSE[ENST00000336825(c.1648C>T,Cgc>Tgc):ENSP00000338938(p.Arg550Cys)];ENSG00000159625:MISSENSE[ENST00000360716(c.1843C>T,Cgc>Tgc):ENSP00000353942(p.Arg615Cys)];ENSG00000159625:MISSENSE[ENST00000394337(c.1843C>T,Cgc>Tgc):ENSP00000377869(p.Arg615Cys)];ENSG00000159625:MISSENSE[ENST00000562250(c.612C>T,Cgc>Tgc):ENSP00000456323(p.Arg205Cys)];ENSG00000159625:EXONIC-NC[ENST00000565609(n.679C>T)];	NA	NA	NA	8.41E-06	0	NA	NA	NA	NA	NA	MODIFIER	NA	NA	50	49	1	41	16	24	58.54
Smoker	42474	chr16	335059	C	T	*PDIA2*	EXONIC-NC	ENSG00000185615:[ENST00000482665(n.758C>T)];ENSG00000185615:[ENST00000404312(c.670-25C>T)];ENSG00000185615:[ENST00000462950(n.142-25C>T)];ENSG00000185615:[ENST00000219406(c.679-25C>T)];ENSG00000185615:[ENST00000467212(n.750-25C>T)];ENSG00000185615:[ENST00000456379(c.624-25C>T)];	NA	NA	0.003195	0.0014072	0.000605767	0.0031949	0.002	NA	NA	NA	MODIFIER	NA	NA	50	50	0	55	29	25	45.45
Smoker	42474	chr16	626145	C	T	*PIGQ*	MISSENSE	ENSG00000007541:MISSENSE[ENST00000321878(c.833C>T,aCg>aTg):ENSP00000326674(p.Thr278Met)];ENSG00000007541:MISSENSE[ENST00000443147(c.875C>T,aCg>aTg):ENSP00000410434(p.Thr292Met)];ENSG00000007541:MISSENSE[ENST00000026218(c.833C>T,aCg>aTg):ENSP00000026218(p.Thr278Met)];ENSG00000007541:MISSENSE[ENST00000409527(c.833C>T,aCg>aTg):ENSP00000386760(p.Thr278Met)];ENSG00000007541:MISSENSE[ENST00000422307(c.833C>T,aCg>aTg):ENSP00000413753(p.Thr278Met)];ENSG00000007541:3UTR[ENST00000470411(c.*96C>T)];	NA	NA	0.0001997	0.0002053	0	0.0001997	0	T	BN	N	MODERATE	NA	NA	38	38	0	26	16	10	38.46
Smoker	42474	chr16	33965563	C	T	*RNA5-8SP2*	EXONIC-NC	ENSG00000200434:[ENST00000363564(n.138C>T)];	NA	NA	.	0.0158092	0.016436971	NA	NA	NA	NA	NA	MODIFIER	NA	NA	775	662	9	587	467	23	3.92
Smoker	42474	chr16	54274405	C	T	*RP11-324D17.2*	INTRONIC	ENSG00000259759:[ENST00000557953(n.311-21C>T)];	NA	NA	NA	NA	NA	NA	NA	NA	NA	NA	MODIFIER	NA	NA	84	84	0	77	71	6	7.79
Smoker	42474	chr17	78326558	G	A	*CTD-2047H16.4*	EXONIC-NC	ENSG00000263069:[ENST00000575034(n.2721C>T)];	NA	NA	NA	NA	NA	NA	NA	NA	NA	NA	MODIFIER	NA	NA	81	81	0	94	86	8	8.51
Smoker	42474	chr17	61895456	C	T	*DDX42*	MISSENSE	ENSG00000198231:MISSENSE[ENST00000389924(c.2515C>T,Cgc>Tgc):ENSP00000374574(p.Arg839Cys)];ENSG00000198231:MISSENSE[ENST00000578681(c.2515C>T,Cgc>Tgc):ENSP00000464050(p.Arg839Cys)];ENSG00000198231:MISSENSE[ENST00000583590(c.2515C>T,Cgc>Tgc):ENSP00000463561(p.Arg839Cys)];ENSG00000198231:MISSENSE[ENST00000359353(c.2158C>T,Cgc>Tgc):ENSP00000352308(p.Arg720Cys)];ENSG00000198231:MISSENSE[ENST00000457800(c.2515C>T,Cgc>Tgc):ENSP00000390121(p.Arg839Cys)];ENSG00000198231:EXONIC-NC[ENST00000578593(n.1768C>T)];ENSG00000198231:EXONIC-NC[ENST00000584010(n.2816C>T)];ENSG00000198231:EXONIC-NC[ENST00000581767(n.1024C>T)];ENSG00000198231:INTRONIC[ENST00000582985(n.378+751C>T)];	NA	NA	NA	NA	NA	NA	NA	NA	NA	NA	MODIFIER	NA	NA	181	181	0	219	138	81	36.99
Smoker	42474	chr17	17124920	G	A	*FLCN*	MISSENSE	ENSG00000154803:MISSENSE[ENST00000285071(c.802C>T,Cgg>Tgg):ENSP00000285071(p.Arg268Trp)];ENSG00000154803:MISSENSE[ENST00000389169(c.802C>T,Cgg>Tgg):ENSP00000373821(p.Arg268Trp)];ENSG00000154803:EXONIC-NC[ENST00000466317(n.645C>T)];ENSG00000154803:EXONIC-NC[ENST00000480316(n.768C>T)];	NA	NA	NA	6.87E-05	0.000244828	NA	NA	NA	NA	NA	MODIFIER	NA	NA	41	40	0	49	34	15	30.61
Smoker	42474	chr17	38975147	G	T	*KRT10*	MISSENSE	ENSG00000186395:MISSENSE[ENST00000269576(c.1640C>A,tCc>tAc):ENSP00000269576(p.Ser547Tyr)];	chr17*3897	chr17*3897	NA	0.0187951	0.023841414	NA	NA	D_lc	UN	NA	MODERATE	N	0	44	42	0	32	25	5	15.62
Smoker	42474	chr17	78326558	G	A	*RNF213*	INTRONIC	ENSG00000173821:[ENST00000582970(c.10304-182G>A)];ENSG00000173821:[ENST00000336301(c.4523-182G>A)];ENSG00000173821:[ENST00000508628(c.10451-182G>A)];	NA	NA	NA	NA	NA	NA	NA	NA	NA	NA	MODIFIER	NA	NA	81	81	0	94	86	8	8.51
Smoker	42474	chr17	17124920	G	A	*RP11-45M22.4*	INTRONIC	ENSG00000264187:[ENST00000427497(c.149-2552C>T)];	NA	NA	NA	6.87E-05	0.000244828	NA	NA	NA	NA	NA	MODIFIER	NA	NA	41	40	0	49	34	15	30.61
Smoker	42474	chr17	7577568	C	T	*TP53*	MISSENSE	ENSG00000141510:MISSENSE[ENST00000413465(c.713G>A,tGt>tAt):ENSP00000410739(p.Cys238Tyr)];ENSG00000141510:MISSENSE[ENST00000420246(c.713G>A,tGt>tAt):ENSP00000391127(p.Cys238Tyr)];ENSG00000141510:MISSENSE[ENST00000269305(c.713G>A,tGt>tAt):ENSP00000269305(p.Cys238Tyr)];ENSG00000141510:MISSENSE[ENST00000359597(c.713G>A,tGt>tAt):ENSP00000352610(p.Cys238Tyr)];ENSG00000141510:MISSENSE[ENST00000445888(c.713G>A,tGt>tAt):ENSP00000391478(p.Cys238Tyr)];ENSG00000141510:MISSENSE[ENST00000455263(c.713G>A,tGt>tAt):ENSP00000398846(p.Cys238Tyr)];ENSG00000141510:MISSENSE[ENST00000509690(c.317G>A,tGt>tAt):ENSP00000425104(p.Cys106Tyr)];ENSG00000141510:MISSENSE[ENST00000514944(c.434G>A,tGt>tAt):ENSP00000423862(p.Cys145Tyr)];ENSG00000141510:EXONIC-NC[ENST00000504937(n.595G>A)];ENSG00000141510:EXONIC-NC[ENST00000504290(n.595G>A)];ENSG00000141510:EXONIC-NC[ENST00000510385(n.595G>A)];	chr17*7577	chr17*7577	NA	1.65E-05	0	NA	NA	NA	NA	NA	MODIFIER	NA	NA	57	57	0	64	22	42	65.62
Smoker	42474	chr17	5984286	G	A	*WSCD1*	MISSENSE	ENSG00000179314:MISSENSE[ENST00000571494(c.223G>A,cGg>cAg):ENSP00000458339(p.Arg75Gln)];ENSG00000179314:MISSENSE[ENST00000576083(c.308G>A,cGg>cAg):ENSP00000460041(p.Arg103Gln)];ENSG00000179314:MISSENSE[ENST00000574232(c.308G>A,cGg>cAg):ENSP00000458374(p.Arg103Gln)];ENSG00000179314:MISSENSE[ENST00000574946(c.308G>A,cGg>cAg):ENSP00000460825(p.Arg103Gln)];ENSG00000179314:MISSENSE[ENST00000317744(c.308G>A,cGg>cAg):ENSP00000323087(p.Arg103Gln)];ENSG00000179314:MISSENSE[ENST00000539421(c.308G>A,cGg>cAg):ENSP00000446032(p.Arg103Gln)];ENSG00000179314:INTRONIC[ENST00000573634(c.80-7024G>A)];	NA	NA	NA	0.0001669	0.000125156	NA	NA	T	BN	N	MODERATE	N	0.122	65	65	0	58	36	22	37.93
Smoker	42474	chr19	18679345	G	C	*AC005253.4*	EXONIC-NC	ENSG00000268983:[ENST00000593791(n.403C>G)];	NA	NA	NA	NA	NA	NA	NA	NA	NA	NA	LOW	NA	NA	62	62	0	64	43	21	32.81
Smoker	42474	chr19	41316284	G	C	*CTC-490E21.12*	INTRONIC	ENSG00000268797:[ENST00000601627(c.119+8964G>C)];	NA	NA	NA	NA	NA	NA	NA	NA	NA	NA	MODIFIER	NA	NA	99	99	0	91	60	31	34.07
Smoker	42474	chr19	10395014	C	T	*CTD-2369P2.5*	INTRONIC	ENSG00000266978:[ENST00000592893(n.141+630G>A)];	NA	NA	0.0001997	0.0002458	0.001647145	0.0001997	0.001	NA	NA	NA	MODIFIER	NA	NA	94	94	0	75	42	33	44
Smoker	42474	chr19	41316284	G	C	*CYP2T2P*	EXONIC-NC	ENSG00000233622:[ENST00000432607(n.429C>G)];	NA	NA	NA	NA	NA	NA	NA	NA	NA	NA	MODIFIER	NA	NA	99	99	0	91	60	31	34.07
Smoker	42474	chr19	10395014	C	T	*ICAM1*	INTRONIC	ENSG00000090339:[ENST00000423829(c.259+18C>T)];ENSG00000090339:[ENST00000264832(c.925+18C>T)];	NA	NA	0.0001997	0.0002458	0.001647145	0.0001997	0.001	NA	NA	NA	MODIFIER	NA	NA	94	94	0	75	42	33	44

Mangalaparthi *et al.* , 2020. Mutational landscape of esophageal squamous cell carcinoma in an Indian cohort
Supplementary Table 4. List of somatic single nucleotide variants identified using whole exome sequencing of ESCC patients

Cohort	Sample	Chromosome	Genomic position	Reference allele	Alternate allele	Gene symbol	Variant type	Variant annotation	OncoMD	COSMIC	dbSNP	ExAC	ExAC_SAS	1000G	1000G_SAS	SIFT	Polyphen2	CONDEL	VEP	LRT	PhastCon7 Way	Total read (Adjacent normal)	Reference allel read support (Adjacent normal)	Alternative read support (Adjacent normal)	Total read (Tumor)	Reference allel read support (Tumor)	Alternative read support (Tumor)	Alternate allele frequency
Smoker	42474	chr19	18679345	G	C	*KXD1*	SILENT	ENSG00000105700:SILENT[ENST00000595073(c.435G>C,ctG>ctC):ENSP00000471554(p.Leu145(=))];ENSG00000105700:SILENT[ENST00000602094(c.435G>C,ctG>ctC):ENSP00000472836(p.Leu145(=))];ENSG00000105700:SILENT[ENST00000539106(c.435G>C,ctG>ctC):ENSP00000438903(p.Leu145(=))];ENSG00000105700:SILENT[ENST00000540691(c.435G>C,ctG>ctC):ENSP00000443549(p.Leu145(=))];ENSG00000105700:SILENT[ENST00000601630(c.492G>C,ctG>ctC):ENSP00000469648(p.Leu164(=))];ENSG00000105700:SILENT[ENST00000599319(c.435G>C,ctG>ctC):ENSP00000468990(p.Leu145(=))];ENSG00000105700:SILENT[ENST00000222307(c.435G>C,ctG>ctC):ENSP00000222307(p.Leu145(=))];ENSG00000105700:SILENT[ENST00000597438(c.435G>C,ctG>ctC):ENSP00000469294(p.Leu145(=))];ENSG00000105700:SILENT[ENST00000600654(c.435G>C,ctG>ctC):ENSP00000472678(p.Leu145(=))];ENSG00000105700:3UTR[ENST00000600099(c.*97G>C)];	NA	NA	NA	NA	NA	NA	NA	NA	NA	NA	LOW	NA	NA	62	62	0	64	43	21	32.81
Smoker	42474	chr19	54206028	G	A	*MIR518B*	EXONIC-NC	ENSG00000207862:[ENST00000385127(n.38G>A)];	NA	NA	NA	NA	NA	NA	NA	NA	NA	NA	MODIFIER	NA	NA	70	70	0	99	59	40	40.4
Smoker	42474	chr19	43780550	A	T	*NA*	INTERGENIC		NA	NA	NA	NA	NA	NA	NA	NA	NA	NA	MODIFIER	NA	NA	81	81	0	107	99	8	7.48
Smoker	42474	chr19	56895485	T	C	*ZNF582*	MISSENSE	ENSG00000018869:MISSENSE[ENST00000586929(c.1301A>G,cAt>cGt):ENSP00000465619(p.His434Arg)];ENSG00000018869:MISSENSE[ENST00000301310(c.1301A>G,cAt>cGt):ENSP00000301310(p.His434Arg)];ENSG00000018869:INTRONIC[ENST00000589143(c.232+5885A>G)];	NA	NA	NA	NA	NA	NA	NA	T	PoD	N	MODERATE	N	0.001	55	55	0	84	79	5	5.95
Smoker	42474	chr19	22585563	T	C	*ZNF98*	INTRONIC	ENSG00000197360:[ENST00000357774(c.253+28A>G)];ENSG00000197360:[ENST00000593657(c.130+28A>G)];ENSG00000197360:[ENST00000601553(c.253+28A>G)];	NA	NA	NA	NA	NA	NA	NA	NA	NA	NA	MODIFIER	NA	NA	101	101	0	119	78	40	33.61
Smoker	42474	chr19	56701310	C	A	*ZSCAN5B*	MISSENSE	ENSG00000197213:MISSENSE[ENST00000586855(c.1374G>T,caG>caT):ENSP00000466072(p.Gln458His)];ENSG00000197213:MISSENSE[ENST00000358992(c.1374G>T,caG>caT):ENSP00000351883(p.Gln458His)];	NA	NA	NA	NA	NA	NA	NA	T	PoD	D	MODERATE	NA	NA	61	61	0	104	98	6	5.77
Smoker	42474	chr2	133014591	C	T	*ANKRD30BL*	INTRONIC	ENSG00000163046:[ENST00000470729(n.441+511G>A)];	NA	NA	NA	0.0001142	0	NA	NA	NA	NA	NA	MODIFIER	NA	NA	179	156	1	154	128	6	3.9
Smoker	42474	chr2	36739512	C	T	*CRIM1*	SILENT	ENSG00000150938:SILENT[ENST00000280527(c.1755C>T,caC>caT):ENSP00000280527(p.His585(=))];	NA	NA	chr2*36739512*36739512*C*A,G,T*rs754851164*NA*NA*NA	4.12E-05	0.000121139	NA	NA	NA	NA	NA	LOW	NA	0.995	55	55	0	114	105	9	7.89
Smoker	42474	chr2	225365060	A	G	*CUL3*	INTRONIC	ENSG00000036257:[ENST00000344951(c.1412+20T>C)];ENSG00000036257:[ENST00000487558(n.35+20T>C)];ENSG00000036257:[ENST00000264414(c.1610+20T>C)];ENSG00000036257:[ENST00000409777(c.1538+20T>C)];ENSG00000036257:[ENST00000409096(c.1538+20T>C)];	NA	NA	NA	NA	NA	NA	NA	NA	NA	NA	MODIFIER	NA	NA	49	49	0	55	27	28	50.91
Smoker	42474	chr2	105858833	T	C	*GPR45*	MISSENSE	ENSG00000135973:MISSENSE[ENST00000258456(c.518T>C,gTg>gCg):ENSP00000258456(p.Val173Ala)];	chr2*10585	NA	NA	NA	NA	NA	NA	T	BN	N	MODERATE	N	0.941	70	70	0	78	65	13	16.67
Smoker	42474	chr2	133014591	C	T	*MIR663B*	EXONIC-NC	ENSG00000221288:[ENST00000408361(n.63G>A)];	NA	NA	NA	0.0001142	0	NA	NA	NA	NA	NA	MODIFIER	NA	NA	179	156	1	154	128	6	3.9
Smoker	42474	chr2	179494085	G	A	*TTN*	SILENT	ENSG00000155657:SILENT[ENST00000359218(c.17547C>T,taC>taT):ENSP00000352154(p.Tyr5849(=))];ENSG00000155657:SILENT[ENST00000342175(c.17748C>T,taC>taT):ENSP00000340554(p.Tyr5916(=))];ENSG00000155657:SILENT[ENST00000342992(c.36663C>T,taC>taT):ENSP00000343764(p.Tyr12221(=))];ENSG00000155657:SILENT[ENST00000591111(c.39444C>T,taC>taT):ENSP00000465570(p.Tyr13148(=))];ENSG00000155657:SILENT[ENST00000589042(c.44367C>T,taC>taT):ENSP00000467141(p.Tyr14789(=))];ENSG00000155657:SILENT[ENST00000460472(c.17172C>T,taC>taT):ENSP00000434586(p.Tyr5724(=))];	NA	NA	NA	2.50E-05	0	NA	NA	NA	NA	NA	MODIFIER	NA	NA	107	107	0	215	103	111	51.63
Smoker	42474	chr2	179500029	G	A	*TTN*	MISSENSE-SS-PRX	ENSG00000155657:[ENST00000359218(c.15067C>T,Cgt>Tgt):ENSP00000352154(p.Arg5023Cys)];ENSG00000155657:[ENST00000591111(c.36964C>T,Cgt>Tgt):ENSP00000465570(p.Arg12322Cys)];ENSG00000155657:[ENST00000589042(c.41887C>T,Cgt>Tgt):ENSP00000467141(p.Arg13963Cys)];ENSG00000155657:[ENST00000342992(c.34183C>T,Cgt>Tgt):ENSP00000343764(p.Arg11395Cys)];ENSG00000155657:[ENST00000342175(c.15268C>T,Cgt>Tgt):ENSP00000340554(p.Arg5090Cys)];ENSG00000155657:[ENST00000460472(c.14692C>T,Cgt>Tgt):ENSP00000434586(p.Arg4898Cys)];	NA	NA	NA	1.66E-05	0	NA	NA	NA	NA	NA	MODIFIER	NA	NA	158	158	0	438	323	115	26.26
Smoker	42474	chr2	179494085	G	A	*TTN-AS1*	INTRONIC	ENSG00000237298:[ENST00000592630(n.519+31677G>A)];ENSG00000237298:[ENST00000589487(n.111-14936G>A)];ENSG00000237298:[ENST00000590807(n.396-7216G>A)];ENSG00000237298:[ENST00000585451(n.198+105722G>A)];ENSG00000237298:[ENST00000589907(n.507-3017G>A)];ENSG00000237298:[ENST00000589830(n.87-7216G>A)];	NA	NA	NA	2.50E-05	0	NA	NA	NA	NA	NA	MODIFIER	NA	NA	107	107	0	215	103	111	51.63
Smoker	42474	chr2	179500029	G	A	*TTN-AS1*	INTRONIC	ENSG00000237298:[ENST00000592630(n.519+37621G>A)];ENSG00000237298:[ENST00000590807(n.396-1272G>A)];ENSG00000237298:[ENST00000585451(n.198+111666G>A)];ENSG00000237298:[ENST00000589830(n.87-1272G>A)];ENSG00000237298:[ENST00000589487(n.111-8992G>A)];	NA	NA	NA	1.66E-05	0	NA	NA	NA	NA	NA	MODIFIER	NA	NA	158	158	0	438	323	115	26.26
Smoker	42474	chr2	128464065	G	A	*WDR33*	SILENT	ENSG00000136709:SILENT[ENST00000322313(c.3843C>T,gaC>gaT):ENSP00000325377(p.Asp1281(=))];	chr2*12846	chr2*12846	NA	2.48E-05	6.08E-05	NA	NA	NA	NA	NA	LOW	NA	0.945	97	97	0	125	84	41	32.8
Smoker	42474	chr20	29628225	A	G	*FRG1B*	INTRONIC-SS-ACR	ENSG00000149531:INTRONIC-SS-ACR[ENST00000278882(c.229-2A>G)];ENSG00000149531:INTRONIC-SS-ACR[ENST00000439954(c.244-2A>G)];ENSG00000149531:INTRONIC-SS-ACR[ENST00000479318(c.113-2A>G)];ENSG00000149531:INTRONIC-SS-ACR[ENST00000358464(c.229-2A>G)];	NA	NA	NA	NA	NA	NA	NA	NA	NA	NA	HIGH	NA	NA	1527	1499	27	1824	1780	40	2.19
Smoker	42474	chr20	29633901	A	G	*FRG1B*	SILENT	ENSG00000149531:SILENT[ENST00000278882(c.540A>G,gaA>gaG):ENSP00000278882(p.Glu180(=))];ENSG00000149531:SILENT[ENST00000358464(c.540A>G,gaA>gaG):ENSP00000351249(p.Glu180(=))];	NA	NA	NA	0.0017097	0.00048585	NA	NA	NA	NA	NA	LOW	NA	NA	581	568	9	557	535	18	3.23
Smoker	42474	chr20	8709758	T	C	*PLCB1*	MISSENSE	ENSG00000182621:MISSENSE[ENST00000378641(c.1825T>C,Tat>Cat):ENSP00000367908(p.Tyr609His)];ENSG00000182621:MISSENSE[ENST00000338037(c.1825T>C,Tat>Cat):ENSP00000338185(p.Tyr609His)];ENSG00000182621:MISSENSE[ENST00000378637(c.1825T>C,Tat>Cat):ENSP00000367904(p.Tyr609His)];ENSG00000182621:MISSENSE[ENST00000487210(c.1047T>C,Tat>Cat):ENSP00000431704(p.Tyr350His)];ENSG00000182621:EXONIC-NC[ENST00000494924(n.977T>C)];	NA	NA	NA	NA	NA	NA	NA	NA	NA	NA	MODIFIER	NA	NA	223	223	0	317	202	115	36.28
Smoker	42474	chr20	47266570	G	A	*PREX1*	MISSENSE	ENSG00000124126:MISSENSE[ENST00000482556(c.957C>T,Cgc>Tgc):ENSP00000434632(p.Arg320Cys)];ENSG00000124126:MISSENSE[ENST00000371941(c.2992C>T,Cgc>Tgc):ENSP00000361009(p.Arg998Cys)];ENSG00000124126:MISSENSE[ENST00000396220(c.2992C>T,Cgc>Tgc):ENSP00000379522(p.Arg998Cys)];	NA	NA	NA	NA	NA	NA	NA	NA	PrD	NA	MODERATE	U	0.923	55	55	0	51	35	16	31.37
Smoker	42474	chr21	41143090	G	T	*IGSF5*	SILENT	ENSG00000183067:SILENT[ENST00000380588(c.666G>T,ctG>ctT):ENSP00000369962(p.Leu222(=))];ENSG00000183067:EXONIC-NC[ENST00000479378(n.772G>T)];	NA	NA	NA	NA	NA	NA	NA	NA	NA	NA	MODIFIER	NA	NA	74	74	0	76	53	23	30.26

Mangalaparthi *et al.* , 2020. Mutational landscape of esophageal squamous cell carcinoma in an Indian cohort
Supplementary Table 4. List of somatic single nucleotide variants identified using whole exome sequencing of ESCC patients

Cohort	Sample	Chromosome	Genomic position	Reference allele	Alternate allele	Gene symbol	Variant type	Variant annotation	OncoMD	COSMIC	dbSNP	ExAC	ExAC_SAS	1000G	1000G_SAS	SIFT	Polyphen2	CONDEL	VEP	LRT	PhastCon7 Way	Total read (Adjacent normal)	Reference allel read support (Adjacent normal)	Alternative read support (Adjacent normal)	Total read (Tumor)	Reference allel read support (Tumor)	Alternative read support (Tumor)	Alternate allele frequency
Smoker	42474	chr22	17072268	G	A	*CCT8L2*	SILENT	ENSG00000198445:SILENT[ENST00000359963(c.1173C>T,gcC>gcT):ENSP00000353048(p.Ala391(=))];	NA	NA	NA	NA	NA	NA	NA	NA	NA	NA	LOW	NA	NA	110	110	0	92	42	50	54.35
Smoker	42474	chr22	36156028	G	A	*RBFOX2*	MISSENSE	ENSG00000100320:MISSENSE[ENST00000262829(c.737C>T,gCc>gTc):ENSP00000262829(p.Ala246Val)];ENSG00000100320:MISSENSE[ENST00000414461(c.803C>T,gCc>gTc):ENSP00000407855(p.Ala268Val)];ENSG00000100320:MISSENSE[ENST00000359369(c.731C>T,gCc>gTc):ENSP00000352328(p.Ala244Val)];ENSG00000100320:MISSENSE[ENST00000397303(c.734C>T,gCc>gTc):ENSP00000380470(p.Ala245Val)];ENSG00000100320:MISSENSE[ENST00000416721(c.791C>T,gCc>gTc):ENSP00000405651(p.Ala264Val)];ENSG00000100320:MISSENSE[ENST00000438146(c.1016C>T,gCc>gTc):ENSP00000413035(p.Ala339Val)];ENSG00000100320:MISSENSE[ENST00000495377(c.349C>T,gCc>gTc):ENSP00000474464(p.Ala117Val)];ENSG00000100320:MISSENSE[ENST00000405409(c.794C>T,gCc>gTc):ENSP00000384944(p.Ala265Val)];ENSG00000100320:MISSENSE[ENST00000449924(c.803C>T,gCc>gTc):ENSP00000391670(p.Ala268Val)];	NA	NA	NA	NA	NA	NA	NA	T	PrD	N	MODERATE	D	1	22	22	0	19	3	16	84.21
Smoker	42474	chr3	52668640	G	A	*PBRM1*	MISSENSE	ENSG00000163939:MISSENSE[ENST00000409057(c.1279C>T,Ccc>Tcc):ENSP00000386593(p.Pro427Ser)];ENSG00000163939:MISSENSE[ENST00000410007(c.1279C>T,Ccc>Tcc):ENSP00000386529(p.Pro427Ser)];ENSG00000163939:MISSENSE[ENST00000356770(c.1183C>T,Ccc>Tcc):ENSP00000349213(p.Pro395Ser)];ENSG00000163939:MISSENSE[ENST00000296302(c.1279C>T,Ccc>Tcc):ENSP00000296302(p.Pro427Ser)];ENSG00000163939:MISSENSE[ENST00000409767(c.1279C>T,Ccc>Tcc):ENSP00000386601(p.Pro427Ser)];ENSG00000163939:MISSENSE[ENST00000337303(c.1279C>T,Ccc>Tcc):ENSP00000338302(p.Pro427Ser)];ENSG00000163939:MISSENSE[ENST00000409114(c.1279C>T,Ccc>Tcc):ENSP00000386643(p.Pro427Ser)];ENSG00000163939:MISSENSE[ENST00000394830(c.1279C>T,Ccc>Tcc):ENSP00000378307(p.Pro427Ser)];ENSG00000163939:MISSENSE[ENST00000412587(c.1279C>T,Ccc>Tcc):ENSP00000404579(p.Pro427Ser)];ENSG00000163939:MISSENSE[ENST00000423351(c.1279C>T,Ccc>Tcc):ENSP00000387775(p.Pro427Ser)];ENSG00000163939:MISSENSE[ENST00000446103(c.1111C>T,Ccc>Tcc):ENSP00000397662(p.Pro371Ser)];	chr3*52668	chr3*52668	NA	NA	NA	NA	NA	D	PrD	D	MODERATE	NA	NA	34	34	0	45	24	21	46.67
Smoker	42474	chr3	50308567	A	G	*SEMA3B*	EXONIC-NC	ENSG00000012171:[ENST00000454314(n.603A>G)];ENSG00000012171:[ENST00000418948(n.733A>G)];ENSG00000012171:[ENST00000419213(n.790A>G)];ENSG00000012171:[ENST00000414456(n.552A>G)];ENSG00000012171:[ENST00000454939(n.865A>G)];ENSG00000012171:[ENST00000426144(n.525A>G)];ENSG00000012171:[ENST00000316347(n.738A>G)];ENSG00000012171:[ENST00000441915(n.498A>G)];	NA	NA	NA	NA	NA	NA	NA	NA	NA	NA	MODIFIER	NA	NA	28	28	0	19	14	5	26.32
Smoker	42474	chr3	126208140	C	A	*UROC1*	MISSENSE	ENSG00000159650:MISSENSE[ENST00000383579(c.1867G>T,Gac>Tac):ENSP00000373073(p.Asp623Tyr)];ENSG00000159650:MISSENSE[ENST00000290868(c.1687G>T,Gac>Tac):ENSP00000290868(p.Asp563Tyr)];	chr3*12620	chr3*12620	chr3*126208140*126208140*C*T*rs372570937*NA*NA*NA	NA	NA	NA	NA	D	PrD	D	MODERATE	D	0.994	118	118	0	117	84	33	28.21
Smoker	42474	chr4	10502936	G	A	*CLNK*	MISSENSE	ENSG00000109684:MISSENSE[ENST00000226951(c.1084C>T,Cgc>Tgc):ENSP00000226951(p.Arg362Cys)];ENSG00000109684:MISSENSE[ENST00000515667(c.298C>T,Cgc>Tgc):ENSP00000427256(p.Arg100Cys)];	NA	NA	0.001398	0.0010437	0	0.0013978	0.00E+00	D	PrD	D	MODERATE	D	0.997	112	111	0	69	33	36	52.17
Smoker	42474	chr4	57877155	T	C	*POLR2B*	EXONIC-NC-SS-PR	ENSG00000047315:[ENST00000510355(n.313T>C)];ENSG00000047315:[ENST00000478188(n.1087T>C)];ENSG00000047315:[ENST00000441246(c.1668T>C,gaT>gaC):ENSP00000391452(p.Asp556(=))];ENSG00000047315:[ENST00000431623(c.1464T>C,gaT>gaC):ENSP00000391096(p.Asp488(=))];ENSG00000047315:[ENST00000381227(c.1689T>C,gaT>gaC):ENSP00000370625(p.Asp563(=))];ENSG00000047315:[ENST00000314595(c.1689T>C,gaT>gaC):ENSP00000312735(p.Asp563(=))];	NA	NA	NA	NA	NA	NA	NA	NA	NA	NA	LOW	NA	NA	63	63	0	54	45	9	16.67
Smoker	42474	chr5	170162738	C	T	*KCNIP1*	INTRONIC	ENSG00000182132:[ENST00000411494(c.637-25C>T)];ENSG00000182132:[ENST00000390656(c.604-25C>T)];ENSG00000182132:[ENST00000328939(c.604-25C>T)];ENSG00000182132:[ENST00000377360(c.631-25C>T)];ENSG00000182132:[ENST00000520740(c.520-25C>T)];ENSG00000182132:[ENST00000434108(c.679-25C>T)];	NA	NA	chr5*170162738*170162738*C*A*rs755540944*NA*NA*NA	NA	NA	NA	NA	NA	NA	NA	MODIFIER	NA	NA	118	118	0	103	49	53	51.46
Smoker	42474	chr6	109871432	T	C	*AK9*	MISSENSE	ENSG00000155085:MISSENSE[ENST00000424296(c.2825A>G,gAa>gGa):ENSP00000410186(p.Glu942Gly)];ENSG00000155085:MISSENSE[ENST00000355283(c.62A>G,gAa>gGa):ENSP00000347431(p.Glu21Gly)];ENSG00000155085:MISSENSE[ENST00000341338(c.62A>G,gAa>gGa):ENSP00000344637(p.Glu21Gly)];ENSG00000155085:EXONIC-NC[ENST00000466992(n.1536A>G)];	NA	NA	NA	NA	NA	NA	NA	NA	NA	NA	MODIFIER	NA	NA	233	233	0	314	207	107	34.08
Smoker	42474	chr6	46047970	C	T	*CLIC5*	MISSENSE	ENSG00000112782:MISSENSE[ENST00000185206(c.10G>A,Gaa>Aaa):ENSP00000185206(p.Glu4Lys)];	NA	NA	0.005192	0.0022428	0	0.0051917	0.00E+00	T_lc	BN	N	MODERATE	N	0.081	121	121	0	183	117	66	36.07
Smoker	42474	chr7	87150119	T	A	*ABCB1*	MISSENSE	ENSG00000085563:MISSENSE[ENST00000543898(c.2567A>T,tAt>tTt):ENSP00000444095(p.Tyr856Phe)];ENSG00000085563:MISSENSE[ENST00000265724(c.2759A>T,tAt>tTt):ENSP00000265724(p.Tyr920Phe)];ENSG00000085563:EXONIC-NC[ENST00000488737(n.401A>T)];ENSG00000085563:EXONIC-NC[ENST00000496821(n.387A>T)];ENSG00000085563:EXONIC-NC[ENST00000483831(n.317A>T)];	chr7*87150	chr7*87150	NA	NA	NA	NA	NA	NA	NA	NA	MODIFIER	NA	NA	185	184	0	290	280	10	3.45
Smoker	42474	chr7	134678321	C	T	*AGBL3*	MISSENSE	ENSG00000146856:MISSENSE[ENST00000458078(c.124C>T,Cgt>Tgt):ENSP00000395969(p.Arg42Cys)];ENSG00000146856:MISSENSE[ENST00000436302(c.202C>T,Cgt>Tgt):ENSP00000388275(p.Arg68Cys)];ENSG00000146856:MISSENSE[ENST00000435976(c.202C>T,Cgt>Tgt):ENSP00000401220(p.Arg68Cys)];ENSG00000146856:MISSENSE[ENST00000359383(c.202C>T,Cgt>Tgt):ENSP00000352343(p.Arg68Cys)];ENSG00000146856:MISSENSE[ENST00000275763(c.202C>T,Cgt>Tgt):ENSP00000275763(p.Arg68Cys)];ENSG00000146856:EXONIC-NC[ENST00000476700(n.401C>T)];ENSG00000146856:EXONIC-NC[ENST00000494702(n.408C>T)];ENSG00000146856:INTRONIC[ENST00000455283(c.124+4260C>T)];	NA	NA	NA	NA	NA	NA	NA	NA	NA	NA	MODIFIER	NA	NA	101	101	0	141	86	55	39.01
Smoker	42474	chr7	81334820	T	A	*HGF*	SILENT	ENSG00000019991:SILENT[ENST00000457544(c.1881A>T,gcA>gcT):ENSP00000391238(p.Ala627(=))];ENSG00000019991:SILENT[ENST00000222390(c.1896A>T,gcA>gcT):ENSP00000222390(p.Ala632(=))];	NA	NA	NA	NA	NA	NA	NA	NA	NA	NA	LOW	NA	NA	93	93	0	115	109	6	5.22
Smoker	42474	chr7	5925726	C	A	*OCM*	3UTR	ENSG00000122543:[ENST00000416608(c.*6C>A)];ENSG00000122543:[ENST00000242104(c.*6C>A)];	NA	NA	NA	NA	NA	NA	NA	NA	NA	NA	MODIFIER	NA	NA	107	107	0	116	89	26	22.41
Smoker	42474	chr7	39726244	T	C	*RALA*	5UTR	ENSG00000006451:[ENST00000436179(c.-23T>C)];ENSG00000006451:[ENST00000005257(c.-23T>C)];ENSG00000006451:[ENST00000468201(n.262-10040T>C)];	NA	NA	NA	NA	NA	NA	NA	NA	NA	NA	MODIFIER	NA	NA	78	78	0	120	78	42	35

Mangalaparthi *et al.* , 2020. Mutational landscape of esophageal squamous cell carcinoma in an Indian cohort
Supplementary Table 4. List of somatic single nucleotide variants identified using whole exome sequencing of ESCC patients

Cohort	Sample	Chromosome	Genomic position	Reference allele	Alternate allele	Gene symbol	Variant type	Variant annotation	OncoMD	COSMIC	dbSNP	ExAC	ExAC_SAS	1000G	1000G_SAS	SIFT	Polyphen2	CONDEL	VEP	LRT	PhastCon7 Way	Total read (Adjacent normal)	Reference allel read support (Adjacent normal)	Alternative read support (Adjacent normal)	Total read (Tumor)	Reference allel read support (Tumor)	Alternative read support (Tumor)	Alternate allele frequency
Smoker	42474	chr8	2823318	G	T	*CSMD1*	MISSENSE	ENSG00000183117:MISSENSE[ENST00000335551(c.7511C>A,Ccg>Acg):ENSP00000334828(p.Pro2505Thr)];ENSG00000183117:MISSENSE[ENST00000602557(c.9262C>A,Ccg>Acg):ENSP00000473359(p.Pro3088Thr)];ENSG00000183117:MISSENSE[ENST00000520002(c.9262C>A,Ccg>Acg):ENSP00000430733(p.Pro3088Thr)];ENSG00000183117:MISSENSE[ENST00000537824(c.9259C>A,Ccg>Acg):ENSP00000441462(p.Pro3087Thr)];ENSG00000183117:INTRONIC[ENST00000400186(c.8753-2401C>A)];ENSG00000183117:INTRONIC[ENST00000542608(c.8750-2401C>A)];ENSG00000183117:INTRONIC[ENST00000602723(c.8753-2401C>A)];	chr8*28233	chr8*28233	NA	NA	NA	NA	NA	NA	BN	NA	MODERATE	N	0.668	102	102	0	183	138	45	24.59
Smoker	42474	chr8	33451160	C	A	*DUSP26*	SILENT	ENSG00000133878:SILENT[ENST00000523956(c.327G>T,ctG>ctT):ENSP00000429176(p.Leu109(=))];ENSG00000133878:SILENT[ENST00000256261(c.327G>T,ctG>ctT):ENSP00000256261(p.Leu109(=))];ENSG00000133878:SILENT[ENST00000522982(c.327G>T,ctG>ctT):ENSP00000430922(p.Leu109(=))];	NA	NA	chr8*33451160*33451160*C*T*rs750309974*NA*NA*NA	NA	NA	NA	NA	NA	NA	NA	LOW	NA	NA	46	46	0	94	89	5	5.32
Smoker	42474	chr8	29062315	T	C	*KIF13B*	INTRONIC	ENSG00000197892:[ENST00000522355(c.150-18326A>G)];ENSG00000197892:[ENST00000521515(c.150-8599A>G)];ENSG00000197892:[ENST00000522846(n.209-13741A>G)];ENSG00000197892:[ENST00000524189(c.150-8599A>G)];ENSG00000197892:[ENST00000523968(c.*88-8599A>G)];	NA	NA	NA	NA	NA	NA	NA	NA	NA	NA	MODIFIER	NA	NA	88	88	0	116	103	13	11.21
Smoker	42474	chr8	133837418	G	A	*PHF20L1*	INTRONIC	ENSG00000129292:[ENST00000460236(c.*37+16G>A)];ENSG00000129292:[ENST00000395390(c.1562-91G>A)];ENSG00000129292:[ENST00000486199(c.*758+16G>A)];ENSG00000129292:[ENST00000220847(c.-203-91G>A)];ENSG00000129292:[ENST00000395386(c.1637-91G>A)];ENSG00000129292:[ENST00000469979(n.7715-91G>A)];ENSG00000129292:[ENST00000395383(n.3068G>A)];ENSG00000129292:[ENST00000315808(n.2058G>A)];	chr8*13383	NA	NA	NA	NA	NA	NA	NA	NA	NA	MODIFIER	NA	NA	187	186	0	304	237	67	22.04
Smoker	42474	chr8	110509424	G	T	*PKHD1L1*	NONSENSE	ENSG00000205038:NONSENSE[ENST00000526472(c.1306G>T,Gga>Tga):ENSP00000437376(p.Gly436Ter)];ENSG00000205038:NONSENSE[ENST00000378402(c.10522G>T,Gga>Tga):ENSP00000367655(p.Gly3508Ter)];	NA	NA	NA	NA	NA	NA	NA	NA	NA	NA	HIGH	NA	0.988	123	122	0	180	137	43	23.89
Smoker	42474	chr8	110520471	C	T	*PKHD1L1*	SILENT	ENSG00000205038:SILENT[ENST00000526472(c.2157C>T,aaC>aaT):ENSP00000437376(p.Asn719(=))];ENSG00000205038:SILENT[ENST00000378402(c.11373C>T,aaC>aaT):ENSP00000367655(p.Asn3791(=))];	chr8*11052	NA	NA	4.22E-05	0	NA	NA	NA	NA	NA	LOW	NA	0.939	111	111	0	202	150	52	25.74
Smoker	42474	chr8	21996915	G	C	*REEP4*	EXONIC-NC	ENSG00000168476:[ENST00000519074(n.717C>G)];ENSG00000168476:[ENST00000306306(c.417+14C>G)];ENSG00000168476:[ENST00000518664(c.417+14C>G)];ENSG00000168476:[ENST00000334530(c.417+14C>G)];ENSG00000168476:[ENST00000523293(c.417+14C>G)];	NA	NA	NA	NA	NA	NA	NA	NA	NA	NA	MODIFIER	NA	NA	32	32	0	30	19	11	36.67
Smoker	42474	chr8	110131656	C	A	*TRHR*	MISSENSE	ENSG00000174417:MISSENSE[ENST00000311762(c.1169C>A,gCt>gAt):ENSP00000309818(p.Ala390Asp)];ENSG00000174417:MISSENSE[ENST00000518632(c.1169C>A,gCt>gAt):ENSP00000430711(p.Ala390Asp)];	NA	NA	chr8*110131656*110131656*C*T*rs143795263*NA*NA*NA	NA	NA	NA	NA	T_lc	BN	N	MODERATE	N	0.998	94	94	0	198	136	62	31.31
Smoker	42474	chr8	30623823	G	A	*UBXN8*	EXONIC-NC	ENSG00000104691:[ENST00000520436(n.282G>A)];ENSG00000104691:[ENST00000341403(n.617G>A)];ENSG00000104691:[ENST00000517341(n.750G>A)];ENSG00000104691:[ENST00000518239(n.614G>A)];ENSG00000104691:[ENST00000523607(n.603G>A)];ENSG00000104691:[ENST00000265616(n.740G>A)];ENSG00000104691:[ENST00000380154(n.629G>A)];ENSG00000104691:[ENST00000519246(n.924G>A)];	NA	NA	NA	NA	NA	NA	NA	NA	NA	NA	MODIFIER	NA	NA	53	53	0	111	82	29	26.13
Smoker	42474	chr8	77761825	G	A	*ZFHX4*	SILENT	ENSG00000091656:SILENT[ENST00000455469(c.3588G>A,tcG>tcA):ENSP00000399605(p.Ser1196(=))];ENSG00000091656:SILENT[ENST00000518282(c.3645G>A,tcG>tcA):ENSP00000430848(p.Ser1215(=))];ENSG00000091656:SILENT[ENST00000050961(c.3588G>A,tcG>tcA):ENSP00000050961(p.Ser1196(=))];ENSG00000091656:SILENT[ENST00000521891(c.3723G>A,tcG>tcA):ENSP00000430497(p.Ser1241(=))];ENSG00000091656:3UTR[ENST00000523625(c.*623G>A)];	NA	NA	NA	8.28E-06	0	NA	NA	NA	NA	NA	LOW	NA	NA	54	54	0	94	40	54	57.45
Smoker	42474	chr9	34620458	C	T	*DCTN3*	MISSENSE	ENSG00000137100:MISSENSE[ENST00000378916(c.4G>A,Gcg>Acg):ENSP00000368196(p.Ala2Thr)];ENSG00000137100:MISSENSE[ENST00000341694(c.4G>A,Gcg>Acg):ENSP00000343986(p.Ala2Thr)];ENSG00000137100:MISSENSE[ENST00000378913(c.4G>A,Gcg>Acg):ENSP00000368193(p.Ala2Thr)];ENSG00000137100:MISSENSE[ENST00000259632(c.4G>A,Gcg>Acg):ENSP00000259632(p.Ala2Thr)];ENSG00000137100:MISSENSE[ENST00000477738(c.4G>A,Gcg>Acg):ENSP00000432915(p.Ala2Thr)];ENSG00000137100:MISSENSE[ENST00000447983(c.4G>A,Gcg>Acg):ENSP00000397046(p.Ala2Thr)];ENSG00000137100:MISSENSE[ENST00000378911(c.4G>A,Gcg>Acg):ENSP00000368191(p.Ala2Thr)];	NA	NA	chr9*34620457*34620477*GCCATCGCTACTACCGACCCA*G*rs776622875*NA*NA*NA	NA	NA	NA	NA	D_lc	PoD	D	MODERATE	NA	NA	61	61	0	85	63	22	25.88
Smoker	42474	chr9	77478962	G	A	*TRPM6*	INTRONIC	ENSG00000119121:[ENST00000449912(c.19-5298C>T)];ENSG00000119121:[ENST00000376871(c.34-5298C>T)];ENSG00000119121:[ENST00000359047(c.34-5298C>T)];ENSG00000119121:[ENST00000376872(c.34-5298C>T)];ENSG00000119121:[ENST00000451710(c.34-5298C>T)];ENSG00000119121:[ENST00000361255(c.19-5298C>T)];ENSG00000119121:[ENST00000360774(c.34-5298C>T)];ENSG00000119121:[ENST00000376864(c.34-5298C>T)];	NA	NA	NA	NA	NA	NA	NA	NA	NA	NA	MODIFIER	NA	NA	106	106	0	151	91	60	39.74
Smoker	42474	chr9	132631236	C	T	*USP20*	MISSENSE	ENSG00000136878:MISSENSE[ENST00000372429(c.1231C>T,Cgt>Tgt):ENSP00000361506(p.Arg411Cys)];ENSG00000136878:MISSENSE[ENST00000358355(c.1231C>T,Cgt>Tgt):ENSP00000351122(p.Arg411Cys)];ENSG00000136878:MISSENSE[ENST00000315480(c.1231C>T,Cgt>Tgt):ENSP00000313811(p.Arg411Cys)];ENSG00000136878:INTRONIC[ENST00000474895(n.140+84C>T)];ENSG00000136878:EXONIC-NC[ENST00000491731(n.179C>T)];	NA	NA	NA	3.40E-05	0	NA	NA	NA	NA	NA	MODIFIER	NA	NA	45	45	0	40	19	21	52.5
Smoker	42474	chrX	74293818	C	T	*ABCB7*	INTRONIC	ENSG00000131269:[ENST00000529949(c.778-27G>A)];ENSG00000131269:[ENST00000373394(c.856-27G>A)];ENSG00000131269:[ENST00000339447(c.736-27G>A)];ENSG00000131269:[ENST00000253577(c.859-27G>A)];	NA	NA	chrX*74293818*74293818*C*G,T*rs774953976*NA*NA*NA	3.97E-05	0	NA	NA	NA	NA	NA	MODIFIER	NA	NA	48	48	0	93	83	10	10.75
Smoker	42474	chrX	83359499	C	T	*RPS6KA6*	INTRONIC-SS-DNR	ENSG00000072133:INTRONIC-SS-DNR[ENST00000495332(n.353+1G>A)];ENSG00000072133:INTRONIC-SS-DNR-PRX[ENST00000262752(c.1614+8G>A)];ENSG00000072133:INTRONIC-SS-DNR-PRX[ENST00000543399(c.1614+8G>A)];	NA	NA	NA	NA	NA	NA	NA	NA	NA	NA	LOW	NA	NA	24	24	0	43	18	25	58.14
Smoker	42475	chr1	94510215	G	A	*ABCA4*	MISSENSE	ENSG00000198691:MISSENSE[ENST00000370225(c.3004C>T,Cgg>Tgg):ENSP00000359245(p.Arg1002Trp)];	NA	NA	NA	1.65E-05	0	NA	NA	D	PrD	D	MODERATE	D	0.976	143	143	0	191	156	35	18.32

Supplementary Table 4. List of somatic single nucleotide variants identified using whole exome sequencing of ESCC patients

Cohort	Sample	Chromosome	Genomic position	Reference allele	Alternate allele	Gene symbol	Variant type	Variant annotation	OncoMD	COSMIC	dbSNP	ExAC	ExAC_SAS	1000G	1000G_SAS	SIFT	Polyphen2	CONDEL	VEP	LRT	PhastCon7 Way	Total read (Adjacent normal)	Reference allel read support (Adjacent normal)	Alternative read support (Adjacent normal)	Total read (Tumor)	Reference allel read support (Tumor)	Alternative read support (Tumor)	Alternate allele frequency
Smoker	42475	chr1	26669350	C	T	*AIM1L*	SILENT	ENSG00000176092:3UTR[ENST00000374207(c.*73G>A)];ENSG00000176092:5UTR[ENST00000308182(c.-37G>A)];ENSG00000176092:SILENT[ENST00000527815(c.477G>A,gaG>gaA):ENSP00000433931(p.Glu159(=))];	NA	NA	NA	NA	NA	NA	NA	NA	NA	NA	LOW	NA	NA	61	61	0	107	84	23	21.5
Smoker	42475	chr1	75037361	C	T	*C1orf173*	MISSENSE	ENSG00000178965:MISSENSE[ENST00000326665(c.4033G>A,Gga>Aga):ENSP00000322609(p.Gly1345Arg)];ENSG00000178965:EXONIC-NC[ENST00000433746(n.2175G>A)];	NA	NA	NA	NA	NA	NA	NA	NA	NA	NA	MODIFIER	NA	NA	163	163	0	241	155	85	35.27
Smoker	42475	chr1	201042735	G	A	*CACNA1S*	MISSENSE	ENSG00000081248:MISSENSE[ENST00000367338(c.2099C>T,aCg>aTg):ENSP00000356307(p.Thr700Met)];ENSG00000081248:MISSENSE[ENST00000362061(c.2099C>T,aCg>aTg):ENSP00000355192(p.Thr700Met)];	NA	NA	0.0001997	0.0001812	6.06E-05	0.0001997	0	T	BN	N	MODERATE	U	0.131	114	114	0	150	99	51	34
Smoker	42475	chr1	15834434	T	A	*CASP9*	INTRONIC	ENSG00000132906:[ENST00000348549(c.418+10171A>T)];ENSG00000132906:[ENST00000546969(n.434-32A>T)];ENSG00000132906:[ENST00000400777(c.411-32A>T)];ENSG00000132906:[ENST00000333868(c.419-32A>T)];ENSG00000132906:[ENST00000447522(c.170-32A>T)];ENSG00000132906:[ENST00000440484(c.419-32A>T)];ENSG00000132906:[ENST00000546424(c.419-32A>T)];ENSG00000132906:[ENST00000375890(c.170-32A>T)];ENSG00000132906:[ENST00000474305(c.279-32A>T)];	NA	NA	NA	NA	NA	NA	NA	NA	NA	NA	MODIFIER	NA	NA	71	71	0	101	80	21	20.79
Smoker	42475	chr1	111860710	C	A	*CHIA*	SILENT	ENSG00000134216:SILENT[ENST00000369740(c.708C>A,ggC>ggA):ENSP00000358755(p.Gly236(=))];ENSG00000134216:SILENT[ENST00000343320(c.708C>A,ggC>ggA):ENSP00000341828(p.Gly236(=))];ENSG00000134216:SILENT[ENST00000489524(c.225C>A,ggC>ggA):ENSP00000433309(p.Gly75(=))];ENSG00000134216:SILENT[ENST00000430615(c.384C>A,ggC>ggA):ENSP00000391132(p.Gly128(=))];ENSG00000134216:SILENT[ENST00000483391(c.225C>A,ggC>ggA):ENSP00000436946(p.Gly75(=))];ENSG00000134216:SILENT[ENST00000353665(c.225C>A,ggC>ggA):ENSP00000338970(p.Gly75(=))];ENSG00000134216:SILENT[ENST00000451398(c.225C>A,ggC>ggA):ENSP00000390476(p.Gly75(=))];ENSG00000134216:SILENT[ENST00000422815(c.540C>A,ggC>ggA):ENSP00000387671(p.Gly180(=))];ENSG00000134216:3UTR[ENST00000352594(c.*217C>A)];ENSG00000134216:EXONIC-NC[ENST00000477918(n.3431C>A)];	NA	NA	NA	NA	NA	NA	NA	NA	NA	NA	MODIFIER	NA	NA	106	106	0	157	112	45	28.66
Smoker	42475	chr1	70897857	C	T	*CTH*	SILENT	ENSG00000116761:SILENT[ENST00000346806(c.684C>T,aaC>aaT):ENSP00000311554(p.Asn228(=))];ENSG00000116761:SILENT[ENST00000411986(c.720C>T,aaC>aaT):ENSP00000413407(p.Asn240(=))];ENSG00000116761:SILENT[ENST00000370938(c.816C>T,aaC>aaT):ENSP00000359976(p.Asn272(=))];	NA	NA	0.0001997	0.0006429	0.004058147	0.0001997	0.001	NA	NA	NA	LOW	NA	0.872	100	99	0	151	123	28	18.54
Smoker	42475	chr1	47398803	G	T	*CYP4A11*	INTRONIC	ENSG00000187048:[ENST00000465874(c.*21-84C>A)];ENSG00000187048:[ENST00000462347(c.929-84C>A)];ENSG00000187048:[ENST00000475477(c.*82-294C>A)];ENSG00000187048:[ENST00000468629(c.1127-294C>A)];ENSG00000187048:[ENST00000371904(c.1226-84C>A)];ENSG00000187048:[ENST00000371905(c.1223-84C>A)];ENSG00000187048:[ENST00000310638(c.1223-84C>A)];ENSG00000187048:[ENST00000474458(c.743-294C>A)];	NA	NA	NA	NA	NA	NA	NA	NA	NA	NA	MODIFIER	NA	NA	91	91	0	105	60	45	42.86
Smoker	42475	chr1	240256817	C	G	*FMN2*	MISSENSE	ENSG00000155816:MISSENSE[ENST00000319653(c.1408C>G,Cgg>Ggg):ENSP00000318884(p.Arg470Gly)];ENSG00000155816:INTRONIC[ENST00000447095(c.-87+25444C>G)];	chr1*24025	chr1*24025	NA	NA	NA	NA	NA	NA	NA	NA	MODIFIER	NA	NA	83	83	0	82	43	37	45.12
Smoker	42475	chr1	89732050	C	A	*GBP5*	MISSENSE	ENSG00000154451:MISSENSE[ENST00000443807(c.847G>T,Ggc>Tgc):ENSP00000403010(p.Gly283Cys)];ENSG00000154451:MISSENSE[ENST00000343435(c.847G>T,Ggc>Tgc):ENSP00000340396(p.Gly283Cys)];ENSG00000154451:MISSENSE[ENST00000370459(c.847G>T,Ggc>Tgc):ENSP00000359488(p.Gly283Cys)];ENSG00000154451:EXONIC-NC[ENST00000481145(n.213G>T)];ENSG00000154451:INTRONIC[ENST00000490568(n.202+1086G>T)];	NA	NA	NA	NA	NA	NA	NA	NA	NA	NA	MODIFIER	NA	NA	133	133	0	200	162	38	19
Smoker	42475	chr1	38047837	C	G	*GNL2*	MISSENSE	ENSG00000134697:MISSENSE[ENST00000373062(c.896G>C,cGg>cCg):ENSP00000362153(p.Arg299Pro)];ENSG00000134697:MISSENSE[ENST00000538069(c.247G>C,cGg>cCg):ENSP00000441288(p.Arg83Pro)];ENSG00000134697:EXONIC-NC[ENST00000469191(n.133G>C)];	NA	NA	chr1*38047837*38047837*C*T*rs147339918*0*0.9998*0.0001997	NA	NA	NA	NA	NA	NA	NA	MODIFIER	NA	NA	70	70	0	105	59	46	43.81
Smoker	42475	chr1	168054505	G	T	*GPR161*	3UTR	ENSG00000143147:[ENST00000271357(c.*264C>A)];ENSG00000143147:[ENST00000367838(c.*264C>A)];ENSG00000143147:[ENST00000478868(n.2511C>A)];	NA	NA	NA	NA	NA	NA	NA	NA	NA	NA	MODIFIER	NA	NA	20	20	0	43	24	19	44.19
Smoker	42475	chr1	44415530	G	A	*IPO13*	MISSENSE	ENSG00000117408:MISSENSE[ENST00000372343(c.526G>A,Gag>Aag):ENSP00000361418(p.Glu176Lys)];ENSG00000117408:INTRONIC[ENST00000489773(n.116-228G>A)];	NA	NA	NA	NA	NA	NA	NA	NA	NA	NA	MODIFIER	NA	NA	103	102	1	190	131	58	30.53
Smoker	42475	chr1	156509690	C	A	*IQGAP3*	SILENT	ENSG00000183856:SILENT[ENST00000491900(c.2703G>T,tcG>tcT):ENSP00000436603(p.Ser901(=))];ENSG00000183856:SILENT[ENST00000361170(c.2832G>T,tcG>tcT):ENSP00000354451(p.Ser944(=))];	NA	NA	chr1*156509690*156509690*C*T*rs778341679*NA*NA*NA	NA	NA	NA	NA	NA	NA	NA	LOW	NA	NA	93	92	0	140	90	50	35.71
Smoker	42475	chr1	211264083	G	T	*KCNH1*	INTRONIC	ENSG00000143473:[ENST00000367007(c.311-51C>A)];ENSG00000143473:[ENST00000271751(c.311-51C>A)];	NA	NA	NA	NA	NA	NA	NA	NA	NA	NA	MODIFIER	NA	NA	60	60	0	112	60	52	46.43
Smoker	42475	chr1	225605753	C	A	*LBR*	MISSENSE	ENSG00000143815:MISSENSE[ENST00000338179(c.770G>T,aGa>aTa):ENSP00000339883(p.Arg257Ile)];ENSG00000143815:MISSENSE[ENST00000272163(c.770G>T,aGa>aTa):ENSP00000272163(p.Arg257Ile)];ENSG00000143815:EXONIC-NC[ENST00000487054(n.230G>T)];	NA	NA	NA	8.26E-06	6.06E-05	NA	NA	NA	NA	NA	MODIFIER	NA	NA	80	80	0	113	69	44	38.94
Smoker	42475	chr1	65886408	A	T	*LEPR*	5UTR	ENSG00000116678:[ENST00000371059(c.-112A>T)];ENSG00000116678:[ENST00000349533(c.-112A>T)];ENSG00000116678:[ENST00000344610(c.-112A>T)];ENSG00000116678:[ENST00000371060(c.-112A>T)];ENSG00000116678:[ENST00000406510(c.-732A>T)];	NA	NA	NA	NA	NA	NA	NA	NA	NA	NA	MODIFIER	NA	NA	47	47	0	112	81	31	27.68
Smoker	42475	chr1	66102002	G	A	*LEPR*	MISSENSE	ENSG00000116678:STARTLOSS[ENST00000406510(c.3G>A,atG>atA):ENSP00000384025(p.Met1?)];ENSG00000116678:MISSENSE[ENST00000349533(c.2802G>A,atG>atA):ENSP00000330393(p.Met934Ile)];	NA	NA	NA	NA	NA	NA	NA	T	BN	N	MODERATE	N	0.986	158	158	0	219	175	44	20.09
Smoker	42475	chr1	65886408	A	T	*LEPROT*	STARTLOSS	ENSG00000213625:STARTLOSS[ENST00000371065(c.1A>T,Atg>Ttg):ENSP00000360104(p.Met1?)];ENSG00000213625:EXONIC-NC[ENST00000488747(n.18A>T)];ENSG00000213625:EXONIC-NC[ENST00000475108(n.139A>T)];ENSG00000213625:EXONIC-NC[ENST00000497874(n.58A>T)];ENSG00000213625:EXONIC-NC[ENST00000484243(n.16A>T)];	NA	NA	NA	NA	NA	NA	NA	NA	NA	NA	MODIFIER	NA	NA	47	47	0	112	81	31	27.68
Smoker	42475	chr1	75622644	C	A	*LHX8*	MISSENSE	ENSG00000162624:MISSENSE[ENST00000356261(c.847C>A,Cca>Aca):ENSP00000348597(p.Pro283Thr)];ENSG00000162624:MISSENSE[ENST00000294638(c.877C>A,Cca>Aca):ENSP00000294638(p.Pro293Thr)];	NA	NA	NA	NA	NA	NA	NA	T	BN	N	MODERATE	D	0.045	72	72	0	112	76	34	30.36

Mangalaparthi *et al.* , 2020. Mutational landscape of esophageal squamous cell carcinoma in an Indian cohort
Supplementary Table 4. List of somatic single nucleotide variants identified using whole exome sequencing of ESCC patients

Cohort	Sample	Chromosome	Genomic position	Reference allele	Alternate allele	Gene symbol	Variant type	Variant annotation	OncoMD	COSMIC	dbSNP	ExAC	ExAC_SAS	1000G	1000G_SAS	SIFT	Polyphen2	CONDEL	VEP	LRT	PhastCon7 Way	Total read (Adjacent normal)	Reference allel read support (Adjacent normal)	Alternative read support (Adjacent normal)	Total read (Tumor)	Reference allel read support (Tumor)	Alternative read support (Tumor)	Alternate allele frequency
Smoker	42475	chr1	144994708	C	G	*PDE4DIP*	SILENT	ENSG00000178104:EXONIC-NC[ENST00000530472(n.218G>C)];ENSG00000178104:EXONIC-NC[ENST00000533845(n.218G>C)];ENSG00000178104:INTRONIC[ENST00000533259(c.-82-39416G>C)];ENSG00000178104:INTRONIC[ENST00000532801(c.195+21185G>C)];ENSG00000178104:INTRONIC-SS-ACR-PRX[ENST00000369348(c.442-7G>C)];ENSG00000178104:INTRONIC-SS-ACR-PRX[ENST00000527063(n.591-7G>C)];ENSG00000178104:INTRONIC-SS-ACR-PRX[ENST00000531369(c.232-7G>C)];ENSG00000178104:INTRONIC-SS-ACR-PRX[ENST00000464103(n.128-7G>C)];ENSG00000178104:INTRONIC-SS-ACR-PRX[ENST00000530822(c.229-7G>C)];ENSG00000178104:INTRONIC-SS-ACR-PRX[ENST00000497529(n.685-7G>C)];ENSG00000178104:INTRONIC-SS-ACR-PRX[ENST00000496572(c.80-7G>C)];ENSG00000178104:INTRONIC-SS-ACR-PRX[ENST00000528552(n.569-7G>C)];ENSG00000178104:INTRONIC-SS-ACR-PRX[ENST00000533163(n.564-7G>C)];ENSG00000178104:INTRONIC-SS-ACR-PRX[ENST00000528129(c.40-7G>C)];ENSG00000178104:INTRONIC-SS-ACR-PRX[ENST00000369359(c.442-7G>C)];ENSG00000178104:INTRONIC-SS-ACR-PRX[ENST00000530740(c.442-7G>C)];ENSG00000178104:INTRONIC-SS-ACR-PRX[ENST00000530078(n.575-7G>C)];ENSG00000178104:INTRONIC-SS-ACR-PRX[ENST00000533396(n.471-7G>C)];ENSG00000178104:INTRONIC-SS-ACR-PRX[ENST00000532803(n.657-7G>C)];ENSG00000178104:INTRONIC-SS-ACR-PRX[ENST00000313382(c.229-7G>C)];ENSG00000178104:INTRONIC-SS-ACR-PRX[ENST00000534536(c.40-7G>C)];ENSG00000178104:SILENT[ENST00000369356(c.24G>C,ctG>ctC):ENSP00000358363(p.Leu8(=))];ENSG00000178104:SILENT[ENST00000369354(c.24G>C,ctG>ctC):ENSP00000358360(p.Leu8(=))];ENSG00000178104:SILENT[ENST00000369347(c.24G>C,ctG>ctC):ENSP00000358353(p.Leu8(=))];ENSG00000178104:SILENT[ENST00000369349(c.24G>C,ctG>ctC):ENSP00000358355(p.Leu8(=))];ENSG00000178104:SILENT[ENST00000369351(c.24G>C,ctG>ctC):ENSP00000358357(p	NA	NA	NA	NA	NA	NA	NA	NA	NA	NA	LOW	NA	NA	262	261	0	436	401	35	8.03
Smoker	42475	chr1	79002359	C	G	*PTGFR*	NONSENSE	ENSG00000122420:NONSENSE[ENST00000370757(c.1067C>G,tCa>tGa):ENSP00000359793(p.Ser356Ter)];ENSG00000122420:NONSENSE[ENST00000370758(c.1067C>G,tCa>tGa):ENSP00000359794(p.Ser356Ter)];ENSG00000122420:3UTR[ENST00000497923(c.*385C>G)];ENSG00000122420:3UTR[ENST00000370756(c.*244C>G)];	NA	NA	chr1*79002359*79002359*C*T*rs762675383*NA*NA*NA	NA	NA	NA	NA	NA	NA	NA	MODIFIER	NA	NA	94	94	0	142	110	32	22.54
Smoker	42475	chr1	241033455	C	A	*RGS7*	INTRONIC	ENSG00000182901:[ENST00000331110(c.308-36G>T)];ENSG00000182901:[ENST00000446183(c.134-36G>T)];ENSG00000182901:[ENST00000401882(c.227-36G>T)];ENSG00000182901:[ENST00000366564(c.386-36G>T)];ENSG00000182901:[ENST00000366565(c.386-36G>T)];ENSG00000182901:[ENST00000366562(c.386-36G>T)];ENSG00000182901:[ENST00000407727(c.386-36G>T)];ENSG00000182901:[ENST00000366563(c.386-36G>T)];ENSG00000182901:[ENST00000348120(c.227-36G>T)];	NA	NA	NA	NA	NA	NA	NA	NA	NA	NA	MODIFIER	NA	NA	78	78	0	113	91	22	19.47
Smoker	42475	chr1	45079757	G	C	*RNF220*	INTRONIC	ENSG00000187147:[ENST00000372247(c.626-80G>C)];ENSG00000187147:[ENST00000361799(c.626-80G>C)];ENSG00000187147:[ENST00000355387(c.626-80G>C)];ENSG00000187147:[ENST00000443020(c.-92-80G>C)];	NA	NA	NA	NA	NA	NA	NA	NA	NA	NA	MODIFIER	NA	NA	89	89	0	138	88	49	35.51
Smoker	42475	chr1	120925999	G	C	*RP11-439A17.10*	INTRONIC	ENSG00000234998:[ENST00000426275(n.76-9379G>C)];	NA	NA	NA	NA	NA	NA	NA	NA	NA	NA	MODIFIER	NA	NA	20	20	0	33	25	8	24.24
Smoker	42475	chr1	120925999	G	C	*RP11-439A17.9*	EXONIC-NC	ENSG00000233029:[ENST00000457996(n.366G>C)];	NA	NA	NA	NA	NA	NA	NA	NA	NA	NA	MODIFIER	NA	NA	20	20	0	33	25	8	24.24
Smoker	42475	chr1	89732050	C	A	*RP4-620F22.2*	INTRONIC	ENSG00000237568:[ENST00000437128(n.168-3231C>A)];	NA	NA	NA	NA	NA	NA	NA	NA	NA	NA	MODIFIER	NA	NA	133	133	0	200	162	38	19
Smoker	42475	chr1	111860710	C	A	*RP5-1125M8.2*	INTRONIC	ENSG00000229283:[ENST00000426321(n.149-161G>T)];	NA	NA	NA	NA	NA	NA	NA	NA	NA	NA	MODIFIER	NA	NA	106	106	0	157	112	45	28.66
Smoker	42475	chr1	169599373	C	T	*SELP*	5UTR	ENSG00000174175:[ENST00000367793(c.-61G>A)];ENSG00000174175:[ENST00000367794(c.-61G>A)];ENSG00000174175:[ENST00000367792(c.-61G>A)];ENSG00000174175:[ENST00000367788(c.-61G>A)];ENSG00000174175:[ENST00000367791(c.-61G>A)];	NA	NA	NA	NA	NA	NA	NA	NA	NA	NA	MODIFIER	NA	NA	96	96	0	141	103	38	26.95
Smoker	42475	chr1	160461062	C	T	*SLAMF6*	MISSENSE	ENSG00000162739:MISSENSE[ENST00000368055(c.166G>A,Gag>Aag):ENSP00000357034(p.Glu56Lys)];ENSG00000162739:MISSENSE[ENST00000368057(c.499G>A,Gag>Aag):ENSP00000357036(p.Glu167Lys)];ENSG00000162739:MISSENSE[ENST00000368059(c.499G>A,Gag>Aag):ENSP00000357038(p.Glu167Lys)];	NA	NA	NA	NA	NA	NA	NA	T	BN	N	MODERATE	N	0.001	71	71	0	138	112	26	18.84
Smoker	42475	chr1	75822849	G	C	*SLC44A5*	INTRONIC	ENSG00000137968:[ENST00000535611(c.-253-17534C>G)];ENSG00000137968:[ENST00000469525(n.246-17534C>G)];ENSG00000137968:[ENST00000370855(c.53-17534C>G)];ENSG00000137968:[ENST00000370859(c.53-17534C>G)];	NA	NA	NA	NA	NA	NA	NA	NA	NA	NA	MODIFIER	NA	NA	114	114	0	193	155	38	19.69
Smoker	42475	chr1	27440740	C	T	*SLC9A1*	SILENT	ENSG00000090020:SILENT[ENST00000263980(c.390G>A,ccG>ccA):ENSP00000263980(p.Pro130(=))];ENSG00000090020:SILENT[ENST00000374084(c.390G>A,ccG>ccA):ENSP00000363197(p.Pro130(=))];ENSG00000090020:SILENT[ENST00000374086(c.390G>A,ccG>ccA):ENSP00000363199(p.Pro130(=))];ENSG00000090020:INTRONIC[ENST00000545949(c.-204-4472G>A)];	NA	NA	0.0001997	5.00E-05	0.000246548	0.0001997	0.001	NA	NA	NA	LOW	NA	NA	66	66	0	88	51	36	40.91
Smoker	42475	chr1	165697340	C	A	*TMCO1*	MISSENSE	ENSG00000143183:MISSENSE[ENST00000392129(c.487G>T,Ggc>Tgc):ENSP00000375975(p.Gly163Cys)];ENSG00000143183:MISSENSE[ENST00000464650(c.235G>T,Ggc>Tgc):ENSP00000463951(p.Gly79Cys)];ENSG00000143183:MISSENSE[ENST00000580248(c.235G>T,Ggc>Tgc):ENSP00000462588(p.Gly79Cys)];ENSG00000143183:MISSENSE[ENST00000367881(c.640G>T,Ggc>Tgc):ENSP00000356856(p.Gly214Cys)];ENSG00000143183:3UTR[ENST00000476143(c.*277G>T)];	NA	NA	NA	NA	NA	NA	NA	NA	NA	NA	MODIFIER	NA	NA	62	61	0	95	70	25	26.32
Smoker	42475	chr1	19428053	C	A	*UBR4*	SILENT	ENSG00000127481:SILENT[ENST00000543981(c.57G>T,ccG>ccT):ENSP00000444070(p.Pro19(=))];ENSG00000127481:SILENT[ENST00000375267(c.12984G>T,ccG>ccT):ENSP00000364416(p.Pro4328(=))];ENSG00000127481:SILENT[ENST00000375224(c.105G>T,ccG>ccT):ENSP00000364372(p.Pro35(=))];ENSG00000127481:SILENT[ENST00000375254(c.12984G>T,ccG>ccT):ENSP00000364403(p.Pro4328(=))];ENSG00000127481:SILENT[ENST00000375217(c.12963G>T,ccG>ccT):ENSP00000364365(p.Pro4321(=))];ENSG00000127481:SILENT[ENST00000375226(c.12912G>T,ccG>ccT):ENSP00000364374(p.Pro4304(=))];	chr1*19428	chr1*19428	chr1*19428053*19428053*C*T*rs766838647*NA*NA*NA	NA	NA	NA	NA	NA	NA	NA	LOW	NA	NA	37	37	0	61	31	30	49.18

Mangalaparthi *et al.* , 2020. Mutational landscape of esophageal squamous cell carcinoma in an Indian cohort
Supplementary Table 4. List of somatic single nucleotide variants identified using whole exome sequencing of ESCC patients

Cohort	Sample	Chromosome	Genomic position	Reference allele	Alternate allele	Gene symbol	Variant type	Variant annotation	OncoMD	COSMIC	dbSNP	ExAC	ExAC_SAS	1000G	1000G_SAS	SIFT	Polyphen2	CONDEL	VEP	LRT	PhastCon7 Way	Total read (Adjacent normal)	Reference allel read support (Adjacent normal)	Alternative read support (Adjacent normal)	Total read (Tumor)	Reference allel read support (Tumor)	Alternative read support (Tumor)	Alternate allele frequency
Smoker	42475	chr10	52596066	C	A	*A1CF*	SILENT	ENSG00000148584:SILENT[ENST00000414883(c.372G>T,ggG>ggT):ENSP00000397953(p.Gly124(=))];ENSG00000148584:SILENT[ENST00000373993(c.372G>T,ggG>ggT):ENSP00000363105(p.Gly124(=))];ENSG00000148584:SILENT[ENST00000282641(c.372G>T,ggG>ggT):ENSP00000282641(p.Gly124(=))];ENSG00000148584:SILENT[ENST00000395495(c.372G>T,ggG>ggT):ENSP00000378873(p.Gly124(=))];ENSG00000148584:SILENT[ENST00000374001(c.372G>T,ggG>ggT):ENSP00000363113(p.Gly124(=))];ENSG00000148584:SILENT[ENST00000373995(c.396G>T,ggG>ggT):ENSP00000363107(p.Gly132(=))];ENSG00000148584:SILENT[ENST00000373997(c.372G>T,ggG>ggT):ENSP00000363109(p.Gly124(=))];ENSG00000148584:SILENT[ENST00000395489(c.351G>T,ggG>ggT):ENSP00000378868(p.Gly117(=))];ENSG00000148584:EXONIC-NC[ENST00000473480(n.416G>T)];	NA	NA	NA	NA	NA	NA	NA	NA	NA	NA	LOW	NA	NA	124	123	1	148	114	34	22.97
Smoker	42475	chr10	114170244	A	G	*ACSL5*	SILENT	ENSG00000197142:SILENT[ENST00000356116(c.903A>G,agA>agG):ENSP00000348429(p.Arg301(=))];ENSG00000197142:SILENT[ENST00000354273(c.735A>G,agA>agG):ENSP00000346223(p.Arg245(=))];ENSG00000197142:SILENT[ENST00000433418(c.735A>G,agA>agG):ENSP00000403647(p.Arg245(=))];ENSG00000197142:SILENT[ENST00000354655(c.735A>G,agA>agG):ENSP00000346680(p.Arg245(=))];ENSG00000197142:SILENT[ENST00000393081(c.735A>G,agA>agG):ENSP00000376796(p.Arg245(=))];ENSG00000197142:SILENT[ENST00000369410(c.81A>G,agA>agG):ENSP00000358418(p.Arg27(=))];ENSG00000197142:EXONIC-NC[ENST00000495539(n.177A>G)];	NA	NA	NA	NA	NA	NA	NA	NA	NA	NA	MODIFIER	NA	NA	89	89	0	110	76	34	30.91
Smoker	42475	chr10	99052403	T	C	*ARHGAP19*	5UTR	ENSG00000213390:[ENST00000358531(c.-19A>G)];	NA	NA	NA	NA	NA	NA	NA	NA	NA	NA	MODIFIER	NA	NA	166	165	1	164	90	74	45.12
Smoker	42475	chr10	99052403	T	C	*ARHGAP19-SLIT1*	5UTR	ENSG00000269891:[ENST00000358308(c.-19A>G)];ENSG00000269891:[ENST00000316676(c.-19A>G)];	NA	NA	NA	NA	NA	NA	NA	NA	NA	NA	MODIFIER	NA	NA	166	165	1	164	90	74	45.12
Smoker	42475	chr10	18803432	T	G	*CACNB2*	INTRONIC	ENSG00000165995:[ENST00000377328(c.456+16026T>G)];ENSG00000165995:[ENST00000396576(c.639+134T>G)];ENSG00000165995:[ENST00000377329(c.642+134T>G)];ENSG00000165995:[ENST00000324631(c.804+134T>G)];ENSG00000165995:[ENST00000377315(c.660+134T>G)];ENSG00000165995:[ENST00000282343(c.720+134T>G)];ENSG00000165995:[ENST00000377331(c.587-477T>G)];ENSG00000165995:[ENST00000352115(c.671-477T>G)];ENSG00000165995:[ENST00000377319(c.506-8T>G)];	NA	NA	chr10*18803431*18803432*CT*C*rs758678651*NA*NA*NA	NA	NA	NA	NA	NA	NA	NA	MODIFIER	NA	NA	107	107	0	276	193	82	29.71
Smoker	42475	chr10	68688128	C	A	*CTNNA3*	INTRONIC	ENSG00000183230:[ENST00000433211(c.1048-152846G>T)];ENSG00000183230:[ENST00000494580(c.128-152846G>T)];ENSG00000183230:[ENST00000373744(c.1048-152846G>T)];	NA	NA	NA	8.29E-06	0	NA	NA	D	PoD	D	MODERATE	D	0.998	87	87	0	142	93	49	34.51
Smoker	42475	chr10	394632	C	T	*DIP2C*	SILENT	ENSG00000151240:SILENT[ENST00000280886(c.3180G>A,ccG>ccA):ENSP00000280886(p.Pro1060(=))];	NA	NA	0.0009984	0.0005357	0	0.0009984	0	NA	NA	NA	LOW	NA	NA	98	98	0	194	144	49	25.26
Smoker	42475	chr10	129172033	G	T	*DOCK1*	INTRONIC	ENSG00000150760:[ENST00000280333(c.3370-12G>T)];	NA	NA	NA	NA	NA	NA	NA	NA	NA	NA	MODIFIER	NA	NA	76	76	0	130	100	30	23.08
Smoker	42475	chr10	26506507	C	A	*GAD2*	INTRONIC	ENSG00000136750:[ENST00000259271(c.77-32C>A)];ENSG00000136750:[ENST00000376261(c.77-32C>A)];ENSG00000136750:[ENST00000428517(c.77-32C>A)];	NA	NA	NA	NA	NA	NA	NA	NA	NA	NA	MODIFIER	NA	NA	72	72	0	143	102	41	28.67
Smoker	42475	chr10	68688128	C	A	*LRRTM3*	MISSENSE	ENSG00000198739:MISSENSE[ENST00000361320(c.1454C>A,cCc>cAc):ENSP00000355187(p.Pro485His)];	NA	NA	NA	8.29E-06	0	NA	NA	D	PoD	D	MODERATE	D	0.998	87	87	0	142	93	49	34.51
Smoker	42475	chr10	73587844	G	A	*PSAP*	MISSENSE	ENSG00000197746:MISSENSE[ENST00000394934(c.647C>T,tCc>tTc):ENSP00000378392(p.Ser216Phe)];ENSG00000197746:MISSENSE[ENST00000394936(c.647C>T,tCc>tTc):ENSP00000378394(p.Ser216Phe)];	NA	NA	NA	NA	NA	NA	NA	D	PrD	D	MODERATE	N	0.868	74	74	0	113	59	54	47.79
Smoker	42475	chr10	18803432	T	G	*RP11-499P20.2*	INTRONIC	ENSG00000240291:[ENST00000425669(n.483-843A>C)];	NA	NA	chr10*18803431*18803432*CT*C*rs758678651*NA*NA*NA	NA	NA	NA	NA	NA	NA	NA	MODIFIER	NA	NA	107	107	0	276	193	82	29.71
Smoker	42475	chr11	62379857	G	T	*EML3*	INTRONIC	ENSG00000149499:[ENST00000278845(c.22+51C>A)];ENSG00000149499:[ENST00000394773(c.22+51C>A)];ENSG00000149499:[ENST00000419857(c.-66+189C>A)];ENSG00000149499:[ENST00000529309(c.22+51C>A)];	NA	NA	NA	NA	NA	NA	NA	NA	NA	NA	MODIFIER	NA	NA	23	23	0	41	29	12	29.27
Smoker	42475	chr11	5528853	C	T	*HBG2*	INTRONIC	ENSG00000196565:[ENST00000380259(c.-565+83202G>A)];	NA	NA	chr11*5528853*5528853*C*G*rs754655830*NA*NA*NA	NA	NA	NA	NA	T	PrD	N	MODERATE	N	0.994	72	72	0	132	81	51	38.64
Smoker	42475	chr11	46898080	C	T	*LRP4*	MISSENSE	ENSG00000134569:MISSENSE[ENST00000378623(c.3473G>A,cGg>cAg):ENSP00000367888(p.Arg1158Gln)];	NA	NA	NA	2.47E-05	6.06E-05	NA	NA	D	BN	N	MODERATE	D	0.999	124	123	1	240	201	38	15.83
Smoker	42475	chr11	62455614	C	T	*LRRN4CL*	MISSENSE	ENSG00000177363:MISSENSE[ENST00000317449(c.367G>A,Gag>Aag):ENSP00000325808(p.Glu123Lys)];	NA	NA	NA	NA	NA	NA	NA	T	BN	N	MODERATE	U	0.36	107	107	0	171	114	57	33.33
Smoker	42475	chr11	60496873	G	T	*MS4A18*	MISSENSE	ENSG00000214782:MISSENSE[ENST00000398983(c.85G>T,Gtg>Ttg):ENSP00000381953(p.Val29Leu)];	NA	NA	NA	NA	NA	NA	NA	T	BN	N	MODERATE	NA	NA	69	69	0	137	96	41	29.93
Smoker	42475	chr11	1248993	C	T	*MUC5B*	SILENT	ENSG00000117983:SILENT[ENST00000529681(c.756C>T,gcC>gcT):ENSP00000436812(p.Ala252(=))];ENSG00000117983:SILENT[ENST00000447027(c.756C>T,gcC>gcT):ENSP00000415793(p.Ala252(=))];ENSG00000117983:EXONIC-NC[ENST00000525715(n.814C>T)];	NA	NA	chr11*1248993*1248993*C*A*rs781163769*NA*NA*NA	NA	NA	NA	NA	NA	NA	NA	MODIFIER	NA	NA	71	71	0	130	93	37	28.46
Smoker	42475	chr11	71184765	G	T	*NADSYN1*	INTRONIC	ENSG00000172890:[ENST00000525200(c.566+33G>T)];ENSG00000172890:[ENST00000530534(n.24+33G>T)];ENSG00000172890:[ENST00000528509(c.666+33G>T)];ENSG00000172890:[ENST00000319023(c.666+33G>T)];	NA	NA	NA	NA	NA	NA	NA	NA	NA	NA	MODIFIER	NA	NA	38	38	0	148	126	22	14.86
Smoker	42475	chr11	55563273	C	A	*OR5D14*	MISSENSE	ENSG00000186113:MISSENSE[ENST00000335605(c.242C>A,cCc>cAc):ENSP00000334456(p.Pro81His)];	chr11*5556	chr11*5556	NA	NA	NA	NA	NA	D	PrD	D	MODERATE	D	0.996	100	99	1	189	143	46	24.34
Smoker	42475	chr11	124742316	C	A	*ROBO3*	MISSENSE	ENSG00000154134:MISSENSE[ENST00000538940(c.1132C>A,Cgc>Agc):ENSP00000441797(p.Arg378Ser)];ENSG00000154134:MISSENSE[ENST00000397801(c.1198C>A,Cgc>Agc):ENSP00000380903(p.Arg400Ser)];	NA	NA	NA	NA	NA	NA	NA	D	PoD	D	MODERATE	D	0.681	44	44	0	47	19	28	59.57
Smoker	42475	chr11	62379857	G	T	*ROM1*	INTRONIC	ENSG00000149489:[ENST00000525801(c.-39+294G>T)];ENSG00000149489:[ENST00000534093(c.-39+465G>T)];	NA	NA	NA	NA	NA	NA	NA	NA	NA	NA	MODIFIER	NA	NA	23	23	0	41	29	12	29.27
Smoker	42475	chr11	18269563	C	G	*SAA2*	INTRONIC-SS-ACR	ENSG00000134339:INTRONIC-SS-ACR[ENST00000529528(c.-4-1G>C)];ENSG00000134339:INTRONIC-SS-ACR[ENST00000526900(c.-4-1G>C)];ENSG00000134339:INTRONIC-SS-ACR[ENST00000414546(c.-4-1G>C)];ENSG00000134339:INTRONIC-SS-ACR[ENST00000528349(c.-4-1G>C)];ENSG00000134339:INTRONIC-SS-ACR[ENST00000256733(c.-4-1G>C)];ENSG00000134339:5UTR[ENST00000530400(c.-5G>C)];	NA	NA	NA	NA	NA	NA	NA	NA	NA	NA	HIGH	NA	NA	87	87	0	129	89	40	31.01
Smoker	42475	chr11	18269563	C	G	*SAA2-SAA4*	INTRONIC-SS-ACR	ENSG00000255071:INTRONIC-SS-ACR[ENST00000524555(n.34-1G>C)];	NA	NA	NA	NA	NA	NA	NA	NA	NA	NA	HIGH	NA	NA	87	87	0	129	89	40	31.01
Smoker	42475	chr11	10064392	T	A	*SBF2*	MISSENSE-SS-PRX	ENSG00000133812:[ENST00000256190(c.278A>T,cAg>cTg):ENSP00000256190(p.Gln93Leu)];ENSG00000133812:[ENST00000533661(n.146A>T)];ENSG00000133812:[ENST00000527019(n.225A>T)];ENSG00000133812:[ENST00000533770(n.193A>T)];	NA	NA	NA	NA	NA	NA	NA	NA	NA	NA	LOW	NA	NA	61	61	0	135	127	8	5.93

Mangalaparthi *et al.* , 2020. Mutational landscape of esophageal squamous cell carcinoma in an Indian cohort
Supplementary Table 4. List of somatic single nucleotide variants identified using whole exome sequencing of ESCC patients

Cohort	Sample	Chromosome	Genomic position	Reference allele	Alternate allele	Gene symbol	Variant type	Variant annotation	OncoMD	COSMIC	dbSNP	ExAC	ExAC_SAS	1000G	1000G_SAS	SIFT	Polyphen2	CONDEL	VEP	LRT	PhastCon7 Way	Total read (Adjacent normal)	Reference allel read support (Adjacent normal)	Alternative read support (Adjacent normal)	Total read (Tumor)	Reference allel read support (Tumor)	Alternative read support (Tumor)	Alternate allele frequency
Smoker	42475	chr11	70331886	G	T	*SHANK2*	SILENT	ENSG00000162105:SILENT[ENST00000409161(c.2724C>A,ctC>ctA):ENSP00000386491(p.Leu908(=))];ENSG00000162105:SILENT[ENST00000424924(c.2349C>A,ctC>ctA):ENSP00000402944(p.Leu783(=))];ENSG00000162105:SILENT[ENST00000423696(c.3375C>A,ctC>ctA):ENSP00000394536(p.Leu1125(=))];ENSG00000162105:SILENT[ENST00000294018(c.3384C>A,ctC>ctA):ENSP00000294018(p.Leu1128(=))];ENSG00000162105:SILENT[ENST00000338508(c.4515C>A,ctC>ctA):ENSP00000345193(p.Leu1505(=))];ENSG00000162105:SILENT[ENST00000449833(c.2727C>A,ctC>ctA):ENSP00000399423(p.Leu909(=))];ENSG00000162105:INTRONIC[ENST00000412252(c.755+4522C>A)];	NA	NA	chr11*70331886*70331886*G*A*rs782438906*NA*NA*NA	NA	NA	NA	NA	NA	NA	NA	LOW	NA	NA	45	45	0	264	241	22	8.33
Smoker	42475	chr11	70777806	G	C	*SHANK2*	INTRONIC	ENSG00000162105:[ENST00000460048(n.66+19307C>G)];ENSG00000162105:[ENST00000338508(c.1072+1472C>G)];	NA	NA	NA	NA	NA	NA	NA	NA	NA	NA	MODIFIER	NA	NA	47	47	0	239	213	25	10.46
Smoker	42475	chr11	64359269	C	A	*SLC22A12*	MISSENSE	ENSG00000197891:MISSENSE[ENST00000377574(c.241C>A,Ccc>Acc):ENSP00000366797(p.Pro81Thr)];ENSG00000197891:MISSENSE[ENST00000336464(c.241C>A,Ccc>Acc):ENSP00000336836(p.Pro81Thr)];ENSG00000197891:MISSENSE[ENST00000377567(c.241C>A,Ccc>Acc):ENSP00000366790(p.Pro81Thr)];ENSG00000197891:MISSENSE[ENST00000377572(c.241C>A,Ccc>Acc):ENSP00000366795(p.Pro81Thr)];ENSG00000197891:5UTR[ENST00000473690(c.-268C>A)];	NA	NA	chr11*64359269*64359269*C*T*rs773911731*NA*NA*NA	NA	NA	NA	NA	NA	NA	NA	MODIFIER	NA	NA	49	49	0	64	48	16	25
Smoker	42475	chr11	5528853	C	T	*UBQLN3*	MISSENSE	ENSG00000175520:MISSENSE[ENST00000311659(c.1936G>A,Gat>Aat):ENSP00000347997(p.Asp646Asn)];	NA	NA	chr11*5528853*5528853*C*G*rs754655830*NA*NA*NA	NA	NA	NA	NA	T	PrD	N	MODERATE	N	0.994	72	72	0	132	81	51	38.64
Smoker	42475	chr11	18591854	G	A	*UEVLD*	SILENT	ENSG00000151116:SILENT[ENST00000379387(c.198C>T,ttC>ttT):ENSP00000368697(p.Phe66(=))];ENSG00000151116:SILENT[ENST00000535484(c.150C>T,ttC>ttT):ENSP00000441092(p.Phe50(=))];ENSG00000151116:SILENT[ENST00000300038(c.264C>T,ttC>ttT):ENSP00000300038(p.Phe88(=))];ENSG00000151116:SILENT[ENST00000396197(c.264C>T,ttC>ttT):ENSP00000379500(p.Phe88(=))];ENSG00000151116:SILENT[ENST00000320750(c.198C>T,ttC>ttT):ENSP00000323353(p.Phe66(=))];ENSG00000151116:SILENT[ENST00000543987(c.264C>T,ttC>ttT):ENSP00000442974(p.Phe88(=))];ENSG00000151116:INTRONIC[ENST00000396196(c.194-3825C>T)];ENSG00000151116:INTRONIC[ENST00000541984(c.193+5040C>T)];ENSG00000151116:INTRONIC[ENST00000540666(n.129-3825C>T)];ENSG00000151116:EXONIC-NC[ENST00000535340(n.315C>T)];ENSG00000151116:EXONIC-NC[ENST00000540917(n.206C>T)];	NA	NA	NA	NA	NA	NA	NA	NA	NA	NA	LOW	NA	1	106	106	0	230	153	77	33.48
Smoker	42475	chr12	97186274	T	C	*C12orf55*	SILENT	ENSG00000188596:SILENT[ENST00000524981(c.8847T>C,ccT>ccC):ENSP00000431759(p.Pro2949(=))];	NA	NA	NA	NA	NA	NA	NA	NA	NA	NA	LOW	NA	NA	95	95	0	141	98	43	30.5
Smoker	42475	chr12	41316302	A	T	*CNTN1*	INTRONIC	ENSG00000018236:[ENST00000347616(c.400+72A>T)];ENSG00000018236:[ENST00000348761(c.367+72A>T)];ENSG00000018236:[ENST00000551295(c.400+72A>T)];ENSG00000018236:[ENST00000360099(c.400+72A>T)];ENSG00000018236:[ENST00000547702(c.400+72A>T)];ENSG00000018236:[ENST00000547849(c.400+72A>T)];	NA	NA	NA	NA	NA	NA	NA	NA	NA	NA	MODIFIER	NA	NA	112	112	0	135	82	53	39.26
Smoker	42475	chr12	56221438	C	G	*DNAJC14*	SILENT	ENSG00000135392:SILENT[ENST00000317287(c.1005G>C,ctG>ctC):ENSP00000317500(p.Leu335(=))];ENSG00000135392:SILENT[ENST00000357606(c.1005G>C,ctG>ctC):ENSP00000350223(p.Leu335(=))];ENSG00000135392:SILENT[ENST00000317269(c.1005G>C,ctG>ctC):ENSP00000316240(p.Leu335(=))];	NA	NA	chr12*56221438*56221438*C*T*rs749808594*NA*NA*NA	NA	NA	NA	NA	NA	NA	NA	LOW	NA	NA	81	81	0	110	64	46	41.82
Smoker	42475	chr12	132464274	C	T	*EP400*	SILENT	ENSG00000183495:SILENT[ENST00000389562(c.1368C>T,gcC>gcT):ENSP00000374213(p.Ala456(=))];ENSG00000183495:SILENT[ENST00000332482(c.1368C>T,gcC>gcT):ENSP00000331737(p.Ala456(=))];ENSG00000183495:SILENT[ENST00000389561(c.1371C>T,gcC>gcT):ENSP00000374212(p.Ala457(=))];ENSG00000183495:SILENT[ENST00000333577(c.1479C>T,gcC>gcT):ENSP00000333602(p.Ala493(=))];ENSG00000183495:SILENT[ENST00000330386(c.1371C>T,gcC>gcT):ENSP00000330620(p.Ala457(=))];	NA	NA	chr12*132464274*132464274*C*G,T*rs140869465*NA*NA*NA	8.41E-05	6.27E-05	NA	NA	NA	NA	NA	LOW	NA	NA	116	116	0	135	88	46	34.07
Smoker	42475	chr12	100550844	G	A	*GOLGA2P5*	INTRONIC-SS-ACR	ENSG00000238105:[ENST00000397112(n.1986-8C>T)];ENSG00000238105:[ENST00000545820(n.4230-8C>T)];ENSG00000238105:[ENST00000456437(n.383-8C>T)];ENSG00000238105:[ENST00000304813(n.2404-8C>T)];ENSG00000238105:[ENST00000537988(n.4647-8C>T)];ENSG00000238105:[ENST00000266746(n.2041-8C>T)];	NA	NA	chr12*100550844*100550844*G*C*rs745954764*NA*NA*NA	NA	NA	NA	NA	NA	NA	NA	LOW	NA	NA	18	18	0	18	12	6	33.33
Smoker	42475	chr12	110643192	G	T	*IFT81*	INTRONIC	ENSG00000122970:[ENST00000550156(c.1553-56G>T)];ENSG00000122970:[ENST00000242591(c.1645-56G>T)];ENSG00000122970:[ENST00000552912(c.1645-56G>T)];ENSG00000122970:[ENST00000550748(n.2053G>T)];	NA	NA	chr12*110643192*110643192*G*A*rs566958016*0*0.9998*0.0001997	NA	NA	NA	NA	NA	NA	NA	MODIFIER	NA	NA	30	30	0	40	32	8	20
Smoker	42475	chr12	56418893	G	A	*IKZF4*	MISSENSE	ENSG00000123411:SILENT[ENST00000547791(c.102G>A,tcG>tcA):ENSP00000450020(p.Ser34(=))];ENSG00000123411:SILENT[ENST00000262032(c.237G>A,tcG>tcA):ENSP00000262032(p.Ser79(=))];ENSG00000123411:SILENT[ENST00000547167(c.237G>A,tcG>tcA):ENSP00000448419(p.Ser79(=))];ENSG00000123411:EXONIC-NC[ENST00000549519(n.396G>A)];ENSG00000123411:EXONIC-NC[ENST00000550860(n.470G>A)];ENSG00000123411:EXONIC-NC[ENST00000551701(n.209G>A)];ENSG00000123411:EXONIC-NC[ENST00000551124(n.370G>A)];ENSG00000123411:EXONIC-NC[ENST00000548601(n.174G>A)];ENSG00000123411:MISSENSE[ENST00000551103(c.143G>A,cGg>cAg):ENSP00000449857(p.Arg48Gln)];ENSG00000123411:5UTR[ENST00000431367(c.-70G>A)];	NA	NA	NA	0.0002644	0.000104254	NA	NA	NA	NA	NA	MODIFIER	NA	NA	123	123	0	175	110	65	37.14
Smoker	42475	chr12	85546805	C	G	*LRRIQ1*	MISSENSE	ENSG00000133640:MISSENSE[ENST00000393217(c.4423C>G,Cct>Gct):ENSP00000376910(p.Pro1475Ala)];	NA	NA	NA	NA	NA	NA	NA	T	BN	N	MODERATE	NA	NA	55	54	0	60	50	10	16.67
Smoker	42475	chr12	18435293	G	C	*PIK3C2G*	MISSENSE	ENSG00000139144:MISSENSE[ENST00000546003(c.278G>C,aGc>aCc):ENSP00000441618(p.Ser93Thr)];ENSG00000139144:MISSENSE[ENST00000535651(c.278G>C,aGc>aCc):ENSP00000443850(p.Ser93Thr)];ENSG00000139144:MISSENSE[ENST00000538779(c.278G>C,aGc>aCc):ENSP00000445381(p.Ser93Thr)];ENSG00000139144:MISSENSE[ENST00000266497(c.278G>C,aGc>aCc):ENSP00000266497(p.Ser93Thr)];ENSG00000139144:MISSENSE[ENST00000433979(c.278G>C,aGc>aCc):ENSP00000404845(p.Ser93Thr)];	NA	NA	NA	NA	NA	NA	NA	NA	NA	NA	MODIFIER	NA	NA	201	201	0	321	271	50	15.58

Mangalaparthi *et al.* , 2020. Mutational landscape of esophageal squamous cell carcinoma in an Indian cohort
Supplementary Table 4. List of somatic single nucleotide variants identified using whole exome sequencing of ESCC patients

Cohort	Sample	Chromosome	Genomic position	Reference allele	Alternate allele	Gene symbol	Variant type	Variant annotation	OncoMD	COSMIC	dbSNP	ExAC	ExAC_SAS	1000G	1000G_SAS	SIFT	Polyphen2	CONDEL	VEP	LRT	PhastCon7 Way	Total read (Adjacent normal)	Reference allel read support (Adjacent normal)	Alternative read support (Adjacent normal)	Total read (Tumor)	Reference allel read support (Tumor)	Alternative read support (Tumor)	Alternate allele frequency
Smoker	42475	chr12	11286239	A	G	*PRR4*	INTRONIC	ENSG00000111215:[ENST00000536668(n.177+37782T>C)];ENSG00000111215:[ENST00000546265(n.188-12460T>C)];ENSG00000111215:[ENST00000541175(n.40-12460T>C)];ENSG00000111215:[ENST00000541977(n.123+37782T>C)];ENSG00000111215:[ENST00000535024(n.192+37782T>C)];	NA	NA	NA	0.0002641	0	NA	NA	D	PoD	D	MODERATE	NA	NA	333	332	1	483	470	13	2.69
Smoker	42475	chr12	18435293	G	C	*RERGL*	INTRONIC	ENSG00000111404:[ENST00000541632(n.149+2946C>G)];	NA	NA	NA	NA	NA	NA	NA	NA	NA	NA	MODIFIER	NA	NA	201	201	0	321	271	50	15.58
Smoker	42475	chr12	129283880	C	T	*SLC15A4*	SILENT	ENSG00000139370:SILENT[ENST00000544112(c.486G>A,ggG>ggA):ENSP00000439946(p.Gly162(=))];ENSG00000139370:SILENT[ENST00000266771(c.1497G>A,ggG>ggA):ENSP00000266771(p.Gly499(=))];ENSG00000139370:SILENT[ENST00000545031(c.48G>A,ggG>ggA):ENSP00000444276(p.Gly16(=))];ENSG00000139370:3UTR[ENST00000376744(c.*449G>A)];	NA	NA	NA	NA	NA	NA	NA	NA	NA	NA	LOW	NA	NA	61	61	0	85	67	17	20
Smoker	42475	chr12	11286239	A	G	*TAS2R14*	INTRONIC	ENSG00000212127:[ENST00000381852(n.152+37782T>C)];	NA	NA	NA	0.0002641	0	NA	NA	D	PoD	D	MODERATE	NA	NA	333	332	1	483	470	13	2.69
Smoker	42475	chr12	11286239	A	G	*TAS2R30*	MISSENSE	ENSG00000256188:MISSENSE[ENST00000539585(c.605T>C,cTg>cCg):ENSP00000444736(p.Leu202Pro)];	NA	NA	NA	0.0002641	0	NA	NA	D	PoD	D	MODERATE	NA	NA	333	332	1	483	470	13	2.69
Smoker	42475	chr12	133583664	A	T	*ZNF26*	MISSENSE	ENSG00000198393:MISSENSE[ENST00000544181(c.169A>T,Acc>Tcc):ENSP00000445494(p.Thr57Ser)];ENSG00000198393:MISSENSE[ENST00000540238(c.70A>T,Acc>Tcc):ENSP00000443888(p.Thr24Ser)];ENSG00000198393:MISSENSE[ENST00000534834(c.10A>T,Acc>Tcc):ENSP00000437420(p.Thr4Ser)];ENSG00000198393:MISSENSE[ENST00000328654(c.70A>T,Acc>Tcc):ENSP00000333725(p.Thr24Ser)];	NA	NA	NA	NA	NA	NA	NA	T	BN	N	MODERATE	NA	NA	172	171	0	253	172	80	31.62
Smoker	42475	chr13	72204874	G	T	*DACH1*	INTRONIC	ENSG00000165659:[ENST00000305425(c.971-25C>A)];ENSG00000165659:[ENST00000313174(c.971-25C>A)];ENSG00000165659:[ENST00000354591(c.970+51053C>A)];ENSG00000165659:[ENST00000359684(c.971-25C>A)];	NA	NA	NA	NA	NA	NA	NA	NA	NA	NA	MODIFIER	NA	NA	51	51	0	48	25	22	45.83
Smoker	42475	chr13	25356075	G	C	*RNF17*	MISSENSE	ENSG00000132972:MISSENSE[ENST00000255324(c.604G>C,Gat>Cat):ENSP00000255324(p.Asp202His)];ENSG00000132972:MISSENSE[ENST00000381921(c.604G>C,Gat>Cat):ENSP00000371346(p.Asp202His)];ENSG00000132972:MISSENSE[ENST00000255325(c.604G>C,Gat>Cat):ENSP00000255325(p.Asp202His)];ENSG00000132972:EXONIC-NC[ENST00000255326(n.607G>C)];	NA	NA	NA	NA	NA	NA	NA	NA	NA	NA	MODIFIER	NA	NA	67	65	2	68	33	35	51.47
Smoker	42475	chr14	35245362	C	G	*BAZ1A*	MISSENSE	ENSG00000198604:MISSENSE[ENST00000358716(c.2500G>C,Gaa>Caa):ENSP00000351555(p.Glu834Gln)];ENSG00000198604:MISSENSE[ENST00000360310(c.2596G>C,Gaa>Caa):ENSP00000353458(p.Glu866Gln)];ENSG00000198604:MISSENSE[ENST00000382422(c.2596G>C,Gaa>Caa):ENSP00000371859(p.Glu866Gln)];	NA	NA	NA	NA	NA	NA	NA	T	PrD	D	MODERATE	N	0.995	126	125	0	177	117	60	33.9
Smoker	42475	chr14	42360826	C	A	*LRFN5*	MISSENSE	ENSG00000165379:MISSENSE[ENST00000298119(c.1759C>A,Cag>Aag):ENSP00000298119(p.Gln587Lys)];ENSG00000165379:INTRONIC[ENST00000554120(c.1385+3613C>A)];ENSG00000165379:INTRONIC[ENST00000554171(c.1385+3613C>A)];	NA	NA	NA	NA	NA	NA	NA	NA	NA	NA	MODIFIER	NA	NA	141	141	0	208	134	74	35.58
Smoker	42475	chr14	42368136	T	A	*LRFN5*	MISSENSE	ENSG00000165379:MISSENSE[ENST00000298119(c.2115T>A,aaT>aaA):ENSP00000298119(p.Asn705Lys)];ENSG00000165379:INTRONIC[ENST00000554120(c.1386-5225T>A)];ENSG00000165379:3UTR[ENST00000554171(c.*1T>A)];	NA	NA	NA	NA	NA	NA	NA	NA	NA	NA	MODIFIER	NA	NA	104	104	0	161	110	51	31.68
Smoker	42475	chr14	60485180	T	C	*LRRC9*	MISSENSE	ENSG00000131951:MISSENSE[ENST00000445360(c.3409T>C,Tat>Cat):ENSP00000454748(p.Tyr1137His)];ENSG00000131951:3UTR[ENST00000254271(c.*98T>C)];	NA	NA	NA	NA	NA	NA	NA	NA	NA	NA	MODIFIER	NA	NA	36	36	0	62	41	21	33.87
Smoker	42475	chr14	79117576	C	T	*NRXN3*	SILENT	ENSG00000021645:SILENT[ENST00000554719(c.9C>T,ggC>ggT):ENSP00000451648(p.Gly3(=))];ENSG00000021645:SILENT[ENST00000553363(c.445C>T,ggC>ggT):ENSP00000451754(p.Gly149(=))];ENSG00000021645:SILENT[ENST00000554738(c.1122C>T,ggC>ggT):ENSP00000450683(p.Gly374(=))];ENSG00000021645:SILENT[ENST00000335750(c.9C>T,ggC>ggT):ENSP00000338349(p.Gly3(=))];ENSG00000021645:EXONIC-NC[ENST00000556496(n.472C>T)];ENSG00000021645:INTRONIC[ENST00000553631(c.-67+48744C>T)];	NA	NA	chr14*79117576*79117576*C*A*rs145345072*1*0.9968*0.003195	NA	NA	NA	NA	NA	NA	NA	LOW	NA	NA	66	66	0	129	93	36	27.91
Smoker	42475	chr14	30063337	A	T	*PRKD1*	MISSENSE	ENSG00000184304:INTRONIC[ENST00000415220(c.2458+3360T>A)];ENSG00000184304:INTRONIC[ENST00000331968(c.2434+3360T>A)];ENSG00000184304:MISSENSE[ENST00000490795(c.217T>A,Tgc>Agc):ENSP00000447723(p.Cys73Ser)];	NA	NA	NA	NA	NA	NA	NA	D_lc	BN	N	MODERATE	NA	NA	114	114	0	186	123	63	33.87
Smoker	42475	chr14	60485180	T	C	*RP11-16B13.1*	INTRONIC	ENSG00000258553:[ENST00000555432(n.153+11834A>G)];ENSG00000258553:[ENST00000554123(n.164+11834A>G)];	NA	NA	NA	NA	NA	NA	NA	NA	NA	NA	MODIFIER	NA	NA	36	36	0	62	41	21	33.87
Smoker	42475	chr14	77242415	C	T	*RP11-488C13.6*	INTRONIC	ENSG00000259081:[ENST00000556368(n.120-1159G>A)];	NA	NA	NA	NA	NA	NA	NA	NA	NA	NA	MODIFIER	NA	NA	87	87	0	163	155	8	4.91
Smoker	42475	chr14	64547237	G	T	*SYNE2*	MISSENSE	ENSG00000054654:MISSENSE[ENST00000394768(c.313G>T,Gac>Tac):ENSP00000378249(p.Asp105Tyr)];ENSG00000054654:MISSENSE[ENST00000357395(c.313G>T,Gac>Tac):ENSP00000349969(p.Asp105Tyr)];ENSG00000054654:MISSENSE[ENST00000555002(c.1129G>T,Gac>Tac):ENSP00000450831(p.Asp377Tyr)];ENSG00000054654:MISSENSE[ENST00000554584(c.11326G>T,Gac>Tac):ENSP00000452570(p.Asp3776Tyr)];ENSG00000054654:MISSENSE[ENST00000344113(c.11227G>T,Gac>Tac):ENSP00000341781(p.Asp3743Tyr)];ENSG00000054654:MISSENSE[ENST00000358025(c.11227G>T,Gac>Tac):ENSP00000350719(p.Asp3743Tyr)];ENSG00000054654:EXONIC-NC[ENST00000557060(n.510G>T)];	NA	NA	NA	NA	NA	NA	NA	NA	NA	NA	MODIFIER	NA	NA	166	164	0	272	188	84	30.88
Smoker	42475	chr14	105059810	C	T	*TMEM179*	MISSENSE	ENSG00000258986:MISSENSE[ENST00000556320(c.89G>A,Gtg>Atg):ENSP00000452113(p.Val31Met)];ENSG00000258986:3UTR[ENST00000556573(c.*6G>A)];ENSG00000258986:INTRONIC[ENST00000415614(c.522+1692G>A)];	NA	NA	NA	NA	NA	NA	NA	NA	NA	NA	MODIFIER	NA	NA	61	61	0	117	71	46	39.32
Smoker	42475	chr14	103372165	G	T	*TRAF3*	3UTR	ENSG00000131323:[ENST00000392745(c.*44G>T)];ENSG00000131323:[ENST00000560371(c.*44G>T)];ENSG00000131323:[ENST00000351691(c.*44G>T)];ENSG00000131323:[ENST00000539721(c.*44G>T)];ENSG00000131323:[ENST00000347662(c.*44G>T)];	NA	NA	NA	NA	NA	NA	NA	NA	NA	NA	MODIFIER	NA	NA	37	37	0	40	27	13	32.5
Smoker	42475	chr14	77242415	C	T	*VASH1*	SILENT	ENSG00000071246:SILENT[ENST00000167106(c.711C>T,agC>agT):ENSP00000167106(p.Ser237(=))];ENSG00000071246:EXONIC-NC[ENST00000556038(n.484C>T)];	NA	NA	NA	NA	NA	NA	NA	NA	NA	NA	MODIFIER	NA	NA	87	87	0	163	155	8	4.91
Smoker	42475	chr15	65489531	G	C	*CILP*	SILENT	ENSG00000138615:SILENT[ENST00000261883(c.3093C>G,ggC>ggG):ENSP00000261883(p.Gly1031(=))];	NA	NA	NA	NA	NA	NA	NA	NA	NA	NA	LOW	NA	NA	140	140	0	196	130	66	33.67
Smoker	42475	chr15	48760715	C	A	*FBN1*	SILENT	ENSG00000166147:3UTR[ENST00000537463(c.*239G>T)];ENSG00000166147:SILENT[ENST00000316623(c.4476G>T,ctG>ctT):ENSP00000325527(p.Leu1492(=))];	NA	NA	NA	NA	NA	NA	NA	NA	NA	NA	LOW	NA	NA	50	50	0	63	50	13	20.63

Mangalaparthi *et al.* , 2020. Mutational landscape of esophageal squamous cell carcinoma in an Indian cohort
Supplementary Table 4. List of somatic single nucleotide variants identified using whole exome sequencing of ESCC patients

Cohort	Sample	Chromosome	Genomic position	Reference allele	Alternate allele	Gene symbol	Variant type	Variant annotation	OncoMD	COSMIC	dbSNP	ExAC	ExAC_SAS	1000G	1000G_SAS	SIFT	Polyphen2	CONDEL	VEP	LRT	PhastCon7 Way	Total read (Adjacent normal)	Reference allel read support (Adjacent normal)	Alternative read support (Adjacent normal)	Total read (Tumor)	Reference allel read support (Tumor)	Alternative read support (Tumor)	Alternate allele frequency
Smoker	42475	chr15	88678502	G	T	*NTRK3*	MISSENSE	ENSG00000140538:MISSENSE[ENST00000317501(c.1034C>A,tCc>tAc):ENSP00000318328(p.Ser345Tyr)];ENSG00000140538:MISSENSE[ENST00000557856(c.1034C>A,tCc>tAc):ENSP00000453959(p.Ser345Tyr)];ENSG00000140538:MISSENSE[ENST00000540489(c.1034C>A,tCc>tAc):ENSP00000444673(p.Ser345Tyr)];ENSG00000140538:MISSENSE[ENST00000357724(c.1034C>A,tCc>tAc):ENSP00000350356(p.Ser345Tyr)];ENSG00000140538:MISSENSE[ENST00000394480(c.1034C>A,tCc>tAc):ENSP00000377990(p.Ser345Tyr)];ENSG00000140538:MISSENSE[ENST00000542733(c.740C>A,tCc>tAc):ENSP00000437773(p.Ser247Tyr)];ENSG00000140538:MISSENSE[ENST00000355254(c.1034C>A,tCc>tAc):ENSP00000347397(p.Ser345Tyr)];ENSG00000140538:MISSENSE[ENST00000360948(c.1034C>A,tCc>tAc):ENSP00000354207(p.Ser345Tyr)];ENSG00000140538:MISSENSE[ENST00000558676(c.1034C>A,tCc>tAc):ENSP00000453511(p.Ser345Tyr)];ENSG00000140538:EXONIC-NC[ENST00000559764(n.281C>A)];ENSG00000140538:INTRONIC[ENST00000560017(c.40+628C>A)];	NA	NA	NA	NA	NA	NA	NA	NA	NA	NA	MODIFIER	NA	NA	104	104	0	167	119	48	28.74
Smoker	42475	chr15	60797689	A	G	*RORA*	INTRONIC	ENSG00000069667:[ENST00000335670(c.942+18T>C)];ENSG00000069667:[ENST00000261523(c.1041+18T>C)];ENSG00000069667:[ENST00000449337(c.777+18T>C)];ENSG00000069667:[ENST00000309157(c.1017+18T>C)];	NA	NA	NA	NA	NA	NA	NA	NA	NA	NA	MODIFIER	NA	NA	177	176	0	282	269	13	4.61
Smoker	42475	chr15	60797689	A	G	*RP11-219B17.1*	INTRONIC	ENSG00000245534:[ENST00000559824(n.290-4918A>G)];ENSG00000245534:[ENST00000558140(n.248-4918A>G)];ENSG00000245534:[ENST00000558235(n.193-4918A>G)];ENSG00000245534:[ENST00000501579(n.287-4918A>G)];	NA	NA	NA	NA	NA	NA	NA	NA	NA	NA	MODIFIER	NA	NA	177	176	0	282	269	13	4.61
Smoker	42475	chr15	20433934	G	A	*RP11-492D6.3*	EXONIC-NC	ENSG00000258628:[ENST00000557586(n.2451C>T)];	NA	NA	NA	NA	NA	NA	NA	NA	NA	NA	MODIFIER	NA	NA	316	313	2	325	310	15	4.62
Smoker	42475	chr15	53907702	T	C	*WDR72*	MISSENSE	ENSG00000166415:MISSENSE[ENST00000559418(c.2731A>G,Ata>Gta):ENSP00000452765(p.Ile911Val)];ENSG00000166415:MISSENSE[ENST00000557913(c.2692A>G,Ata>Gta):ENSP00000453378(p.Ile898Val)];ENSG00000166415:MISSENSE[ENST00000396328(c.2701A>G,Ata>Gta):ENSP00000379619(p.Ile901Val)];ENSG00000166415:MISSENSE[ENST00000360509(c.2701A>G,Ata>Gta):ENSP00000353699(p.Ile901Val)];	NA	NA	NA	8.49E-06	0	NA	NA	T	BN	N	MODERATE	N	0.798	44	44	0	65	51	14	21.54
Smoker	42475	chr15	85327310	G	T	*ZNF592*	SILENT	ENSG00000166716:SILENT[ENST00000559607(c.1404G>T,cgG>cgT):ENSP00000453491(p.Arg468(=))];ENSG00000166716:SILENT[ENST00000299927(c.1404G>T,cgG>cgT):ENSP00000299927(p.Arg468(=))];ENSG00000166716:SILENT[ENST00000560079(c.1404G>T,cgG>cgT):ENSP00000452877(p.Arg468(=))];	NA	NA	NA	NA	NA	NA	NA	NA	NA	NA	LOW	NA	NA	119	119	0	153	87	66	43.14
Smoker	42475	chr16	15711245	G	T	*C16orf45*	INTRONIC	ENSG00000166780:[ENST00000565857(c.*13-7502G>T)];	NA	NA	NA	NA	NA	NA	NA	NA	NA	NA	LOW	NA	NA	37	37	0	74	46	28	37.84
Smoker	42475	chr16	1262409	C	A	*CACNA1H*	INTRONIC	ENSG00000196557:[ENST00000562079(c.983+271C>A)];ENSG00000196557:[ENST00000348261(c.4760-102C>A)];ENSG00000196557:[ENST00000564231(c.984-102C>A)];ENSG00000196557:[ENST00000565831(c.4759+271C>A)];ENSG00000196557:[ENST00000358590(c.4759+271C>A)];ENSG00000196557:[ENST00000564927(c.408+271C>A)];ENSG00000196557:[ENST00000569107(c.1016+238C>A)];	NA	NA	NA	NA	NA	NA	NA	NA	NA	NA	MODIFIER	NA	NA	28	28	0	46	31	14	30.43
Smoker	42475	chr16	27692791	G	T	*CTD-2049O4.1*	INTRONIC	ENSG00000261329:[ENST00000564893(n.190-78C>A)];	NA	NA	chr16*27692791*27692791*G*A*rs747793821*NA*NA*NA	NA	NA	NA	NA	NA	NA	NA	MODIFIER	NA	NA	102	102	0	158	95	62	39.24
Smoker	42475	chr16	19184163	G	A	*CTD-2349B8.1*	INTRONIC	ENSG00000261427:[ENST00000568526(c.*99+64G>A)];ENSG00000261427:[ENST00000564808(c.*346+64G>A)];	NA	NA	NA	NA	NA	NA	NA	NA	NA	NA	MODIFIER	NA	NA	76	76	0	148	92	56	37.84
Smoker	42475	chr16	9858660	C	T	*GRIN2A*	MISSENSE	ENSG00000183454:MISSENSE[ENST00000535259(c.2270G>A,aGa>aAa):ENSP00000441572(p.Arg757Lys)];ENSG00000183454:MISSENSE[ENST00000330684(c.2741G>A,aGa>aAa):ENSP00000332549(p.Arg914Lys)];ENSG00000183454:MISSENSE[ENST00000396573(c.2741G>A,aGa>aAa):ENSP00000379818(p.Arg914Lys)];ENSG00000183454:MISSENSE[ENST00000404927(c.2741G>A,aGa>aAa):ENSP00000385872(p.Arg914Lys)];ENSG00000183454:MISSENSE[ENST00000396575(c.2741G>A,aGa>aAa):ENSP00000379820(p.Arg914Lys)];ENSG00000183454:MISSENSE[ENST00000562109(c.2741G>A,aGa>aAa):ENSP00000454998(p.Arg914Lys)];	NA	NA	NA	NA	NA	NA	NA	T_lc	BN	N	MODERATE	N	0.98	148	148	0	298	193	103	34.56
Smoker	42475	chr16	27895933	C	T	*GSG1L*	MISSENSE	ENSG00000169181:MISSENSE[ENST00000447459(c.424G>A,Gag>Aag):ENSP00000394954(p.Glu142Lys)];ENSG00000169181:MISSENSE[ENST00000562611(c.188G>A,Gag>Aag):ENSP00000454942(p.Glu64Lys)];ENSG00000169181:5UTR[ENST00000380897(c.-42G>A)];ENSG00000169181:5UTR[ENST00000380898(c.-42G>A)];ENSG00000169181:INTRONIC[ENST00000395724(c.398-39551G>A)];	NA	NA	NA	NA	NA	NA	NA	NA	NA	NA	MODIFIER	NA	NA	102	101	0	188	169	18	9.57
Smoker	42475	chr16	22826349	G	T	*HS3ST2*	MISSENSE	ENSG00000122254:MISSENSE[ENST00000261374(c.418G>T,Gtg>Ttg):ENSP00000261374(p.Val140Leu)];ENSG00000122254:MISSENSE[ENST00000473392(c.418G>T,Gtg>Ttg):ENSP00000454505(p.Val140Leu)];	NA	NA	chr16*22826349*22826349*G*A*rs371820994*NA*NA*NA	NA	NA	NA	NA	D	PoD	D	MODERATE	NA	NA	96	96	0	163	108	55	33.74
Smoker	42475	chr16	15711245	G	T	*KIAA0430*	SILENT	ENSG00000166783:SILENT[ENST00000551742(c.2868C>A,ggC>ggA):ENSP00000450309(p.Gly956(=))];ENSG00000166783:SILENT[ENST00000602337(c.2859C>A,ggC>ggA):ENSP00000473601(p.Gly953(=))];ENSG00000166783:SILENT[ENST00000552553(c.2868C>A,ggC>ggA):ENSP00000449329(p.Gly956(=))];ENSG00000166783:SILENT[ENST00000540441(c.2373C>A,ggC>ggA):ENSP00000439819(p.Gly791(=))];ENSG00000166783:SILENT[ENST00000344181(c.1794C>A,ggC>ggA):ENSP00000341939(p.Gly598(=))];ENSG00000166783:SILENT[ENST00000548025(c.2859C>A,ggC>ggA):ENSP00000449376(p.Gly953(=))];ENSG00000166783:SILENT[ENST00000396368(c.2868C>A,ggC>ggA):ENSP00000379654(p.Gly956(=))];ENSG00000166783:EXONIC-NC[ENST00000546864(n.625C>A)];	NA	NA	NA	NA	NA	NA	NA	NA	NA	NA	LOW	NA	NA	37	37	0	74	46	28	37.84
Smoker	42475	chr16	27692791	G	T	*KIAA0556*	NONSENSE	ENSG00000047578:NONSENSE[ENST00000261588(c.880G>T,Gag>Tag):ENSP00000261588(p.Glu294Ter)];ENSG00000047578:EXONIC-NC[ENST00000567894(n.741G>T)];	NA	NA	chr16*27692791*27692791*G*A*rs747793821*NA*NA*NA	NA	NA	NA	NA	NA	NA	NA	MODIFIER	NA	NA	102	102	0	158	95	62	39.24

Supplementary Table 4. List of somatic single nucleotide variants identified using whole exome sequencing of ESCC patients

Cohort	Sample	Chromosome	Genomic position	Reference allele	Alternate allele	Gene symbol	Variant type	Variant annotation	OncoMD	COSMIC	dbSNP	ExAC	ExAC_SAS	1000G	1000G_SAS	SIFT	Polyphen2	CONDEL	VEP	LRT	PhastCon7 Way	Total read (Adjacent normal)	Reference allel read support (Adjacent normal)	Alternative read support (Adjacent normal)	Total read (Tumor)	Reference allel read support (Tumor)	Alternative read support (Tumor)	Alternate allele frequency
Smoker	42475	chr16	812729	G	A	*MSLN*	MISSENSE	ENSG00000102854:MISSENSE[ENST00000563651(c.49G>A,Gcc>Acc):ENSP00000456132(p.Ala17Thr)];ENSG00000102854:MISSENSE[ENST00000382862(c.49G>A,Gcc>Acc):ENSP00000372313(p.Ala17Thr)];ENSG00000102854:MISSENSE[ENST00000566549(c.49G>A,Gcc>Acc):ENSP00000456702(p.Ala17Thr)];ENSG00000102854:MISSENSE[ENST00000563941(c.49G>A,Gcc>Acc):ENSP00000456008(p.Ala17Thr)];ENSG00000102854:MISSENSE[ENST00000569566(c.49G>A,Gcc>Acc):ENSP00000458003(p.Ala17Thr)];ENSG00000102854:MISSENSE[ENST00000545450(c.49G>A,Gcc>Acc):ENSP00000442965(p.Ala17Thr)];	NA	NA	NA	9.59E-05	0	NA	NA	T	BN	N	MODERATE	U	0.007	44	44	0	85	45	40	47.06
Smoker	42475	chr16	813265	G	T	*MSLN*	NONSENSE	ENSG00000102854:NONSENSE[ENST00000563651(c.118G>T,Gag>Tag):ENSP00000456132(p.Glu40Ter)];ENSG00000102854:NONSENSE[ENST00000382862(c.118G>T,Gag>Tag):ENSP00000372313(p.Glu40Ter)];ENSG00000102854:NONSENSE[ENST00000566549(c.118G>T,Gag>Tag):ENSP00000456702(p.Glu40Ter)];ENSG00000102854:NONSENSE[ENST00000563941(c.118G>T,Gag>Tag):ENSP00000456008(p.Glu40Ter)];ENSG00000102854:NONSENSE[ENST00000569566(c.118G>T,Gag>Tag):ENSP00000458003(p.Glu40Ter)];ENSG00000102854:NONSENSE[ENST00000545450(c.118G>T,Gag>Tag):ENSP00000442965(p.Glu40Ter)];	NA	NA	NA	NA	NA	NA	NA	NA	NA	NA	HIGH	NA	0.002	27	27	0	62	43	19	30.65
Smoker	42475	chr16	50744911	C	G	*NOD2*	MISSENSE	ENSG00000167207:MISSENSE[ENST00000300589(c.1089C>G,atC>atG):ENSP00000300589(p.Ile363Met)];	NA	NA	NA	NA	NA	NA	NA	D	PoD	D	MODERATE	N	0.836	158	157	0	249	159	90	36.14
Smoker	42475	chr16	4951052	C	G	*PPL*	SILENT	ENSG00000118898:INTRONIC[ENST00000592772(c.252+1388G>C)];ENSG00000118898:SILENT[ENST00000590782(c.477G>C,ccG>ccC):ENSP00000465640(p.Pro159(=))];ENSG00000118898:SILENT[ENST00000345988(c.477G>C,ccG>ccC):ENSP00000340510(p.Pro159(=))];	NA	NA	chr16*4951052*4951052*C*T*rs145770210*NA*NA*NA	NA	NA	NA	NA	NA	NA	NA	LOW	NA	NA	81	81	0	136	96	39	28.68
Smoker	42475	chr16	33965563	C	T	*RNA5-8SP2*	EXONIC-NC	ENSG00000200434:[ENST00000363564(n.138C>T)];	NA	NA	.	0.0158092	0.016436971	NA	NA	NA	NA	NA	MODIFIER	NA	NA	1025	884	12	1392	1168	31	2.23
Smoker	42475	chr16	2550471	G	T	*RP11-20I23.1*	INTRONIC	ENSG00000260272:[ENST00000564543(c.965+3357G>T)];	NA	NA	NA	NA	NA	NA	NA	D	BN	N	MODERATE	N	0.773	18	18	0	21	16	5	23.81
Smoker	42475	chr16	19184163	G	A	*SYT17*	5UTR	ENSG00000103528:[ENST00000562034(c.-739G>A)];ENSG00000103528:[ENST00000568115(c.-136-603G>A)];ENSG00000103528:[ENST00000355377(c.33+64G>A)];ENSG00000103528:[ENST00000566261(c.-150-589G>A)];ENSG00000103528:[ENST00000562711(c.21+64G>A)];ENSG00000103528:[ENST00000570264(c.-151+64G>A)];	NA	NA	NA	NA	NA	NA	NA	NA	NA	NA	MODIFIER	NA	NA	76	76	0	148	92	56	37.84
Smoker	42475	chr16	2550471	G	T	*TBC1D24*	MISSENSE	ENSG00000162065:MISSENSE[ENST00000564879(c.374G>T,gGc>gTc):ENSP00000457266(p.Gly125Val)];ENSG00000162065:MISSENSE[ENST00000293970(c.1505G>T,gGc>gTc):ENSP00000293970(p.Gly502Val)];ENSG00000162065:MISSENSE[ENST00000434757(c.1505G>T,gGc>gTc):ENSP00000390106(p.Gly502Val)];ENSG00000162065:MISSENSE[ENST00000567020(c.1487G>T,gGc>gTc):ENSP00000454408(p.Gly496Val)];	NA	NA	NA	NA	NA	NA	NA	D	BN	N	MODERATE	N	0.773	18	18	0	21	16	5	23.81
Smoker	42475	chr16	1291318	G	C	*TPSAB1*	MISSENSE	ENSG00000172236:MISSENSE[ENST00000338844(c.226G>C,Gtg>Ctg):ENSP00000343577(p.Val76Leu)];ENSG00000172236:MISSENSE[ENST00000461509(c.247G>C,Gtg>Ctg):ENSP00000418247(p.Val83Leu)];ENSG00000172236:MISSENSE[ENST00000561736(c.67G>C,Gtg>Ctg):ENSP00000456821(p.Val23Leu)];ENSG00000172236:EXONIC-NC[ENST00000562432(n.227G>C)];	chr16*1291	NA	chr16*1291318*1291318*G*A,C*rs151324823*NA*NA*NA	0.2017587	0.21261506	NA	NA	NA	NA	NA	MODIFIER	NA	NA	32	32	0	45	39	6	13.33
Smoker	42475	chr17	67148336	G	A	*ABCA10*	INTRONIC	ENSG00000154263:[ENST00000416101(c.*2680-12C>T)];ENSG00000154263:[ENST00000523419(c.*200-12C>T)];ENSG00000154263:[ENST00000519732(n.1480-12C>T)];ENSG00000154263:[ENST00000269081(c.4257-12C>T)];ENSG00000154263:[ENST00000522406(c.*3185-12C>T)];ENSG00000154263:[ENST00000518929(c.*3658-12C>T)];	NA	NA	chr17*67148336*67148337*GA*G*rs767131832*NA*NA*NA	NA	NA	NA	NA	NA	NA	NA	MODIFIER	NA	NA	58	58	0	110	75	35	31.82
Smoker	42475	chr17	8018856	A	C	*ALOXE3*	INTRONIC	ENSG00000179148:[ENST00000448843(c.434+69T>G)];ENSG00000179148:[ENST00000380149(c.902+69T>G)];ENSG00000179148:[ENST00000318227(c.830+69T>G)];	NA	NA	NA	NA	NA	NA	NA	NA	NA	NA	MODIFIER	NA	NA	53	53	0	71	48	23	32.39
Smoker	42475	chr17	8451560	C	A	*MYH10*	INTRONIC	ENSG00000133026:[ENST00000459986(n.674+71G>T)];ENSG00000133026:[ENST00000396239(c.1129+71G>T)];ENSG00000133026:[ENST00000269243(c.1129+71G>T)];ENSG00000133026:[ENST00000379980(c.1177+71G>T)];ENSG00000133026:[ENST00000360416(c.1159+71G>T)];	NA	NA	NA	NA	NA	NA	NA	NA	NA	NA	MODIFIER	NA	NA	92	92	0	136	91	45	33.09
Smoker	42475	chr17	40925388	C	A	*NA*	INTERGENIC		NA	NA	NA	NA	NA	NA	NA	NA	NA	NA	MODIFIER	NA	NA	40	40	0	54	37	16	29.63
Smoker	42475	chr17	6367652	G	C	*PITPNM3*	INTRONIC	ENSG00000091622:[ENST00000262483(c.2008-14C>G)];ENSG00000091622:[ENST00000421306(c.1900-14C>G)];ENSG00000091622:[ENST00000576664(n.757-14C>G)];ENSG00000091622:[ENST00000572795(n.4514-14C>G)];	NA	NA	NA	NA	NA	NA	NA	NA	NA	NA	MODIFIER	NA	NA	38	38	0	65	49	16	24.62
Smoker	42475	chr17	7416922	G	T	*POLR2A*	MISSENSE	ENSG00000181222:MISSENSE[ENST00000322644(c.5339G>T,aGc>aTc):ENSP00000314949(p.Ser1780Ile)];	NA	NA	chr17*7416922*7416922*G*A*rs755161570*NA*NA*NA	NA	NA	NA	NA	NA	UN	NA	MODERATE	U	0.972	285	285	0	316	216	99	31.33
Smoker	42475	chr17	57043192	C	T	*PPM1E*	MISSENSE	ENSG00000175175:MISSENSE[ENST00000308249(c.721C>T,Cgc>Tgc):ENSP00000312411(p.Arg241Cys)];	NA	NA	NA	NA	NA	NA	NA	D	PrD	D	MODERATE	D	1	59	58	1	120	86	34	28.33
Smoker	42475	chr17	7470318	A	G	*SENP3*	MISSENSE	ENSG00000161956:MISSENSE[ENST00000429205(c.1337A>G,aAa>aGa):ENSP00000403712(p.Lys446Arg)];ENSG00000161956:MISSENSE[ENST00000321337(c.1334A>G,aAa>aGa):ENSP00000314029(p.Lys445Arg)];ENSG00000161956:EXONIC-NC[ENST00000578868(n.1738A>G)];ENSG00000161956:EXONIC-NC[ENST00000580042(n.269A>G)];ENSG00000161956:EXONIC-NC[ENST00000578813(n.54A>G)];ENSG00000161956:INTRONIC[ENST00000580231(c.330+1237A>G)];	NA	NA	NA	NA	NA	NA	NA	NA	NA	NA	MODIFIER	NA	NA	80	80	0	121	90	31	25.62
Smoker	42475	chr17	7470318	A	G	*SENP3-EIF4A1*	EXONIC-NC	ENSG00000265500:[ENST00000579777(n.1128A>G)];	NA	NA	NA	NA	NA	NA	NA	NA	NA	NA	MODIFIER	NA	NA	80	80	0	121	90	31	25.62

Mangalaparthi *et al.* , 2020. Mutational landscape of esophageal squamous cell carcinoma in an Indian cohort
Supplementary Table 4. List of somatic single nucleotide variants identified using whole exome sequencing of ESCC patients

Cohort	Sample	Chromosome	Genomic position	Reference allele	Alternate allele	Gene symbol	Variant type	Variant annotation	OncoMD	COSMIC	dbSNP	ExAC	ExAC_SAS	1000G	1000G_SAS	SIFT	Polyphen2	CONDEL	VEP	LRT	PhastCon7 Way	Total read (Adjacent normal)	Reference allel read support (Adjacent normal)	Alternative read support (Adjacent normal)	Total read (Tumor)	Reference allel read support (Tumor)	Alternative read support (Tumor)	Alternate allele frequency
Smoker	42475	chr17	7577556	C	A	*TP53*	MISSENSE	ENSG00000141510:MISSENSE[ENST00000413465(c.725G>T,tGc>tTc):ENSP00000410739(p.Cys242Phe)];ENSG00000141510:MISSENSE[ENST00000420246(c.725G>T,tGc>tTc):ENSP00000391127(p.Cys242Phe)];ENSG00000141510:MISSENSE[ENST00000269305(c.725G>T,tGc>tTc):ENSP00000269305(p.Cys242Phe)];ENSG00000141510:MISSENSE[ENST00000359597(c.725G>T,tGc>tTc):ENSP00000352610(p.Cys242Phe)];ENSG00000141510:MISSENSE[ENST00000445888(c.725G>T,tGc>tTc):ENSP00000391478(p.Cys242Phe)];ENSG00000141510:MISSENSE[ENST00000455263(c.725G>T,tGc>tTc):ENSP00000398846(p.Cys242Phe)];ENSG00000141510:MISSENSE[ENST00000509690(c.329G>T,tGc>tTc):ENSP00000425104(p.Cys110Phe)];ENSG00000141510:MISSENSE[ENST00000514944(c.446G>T,tGc>tTc):ENSP00000423862(p.Cys149Phe)];ENSG00000141510:EXONIC-NC[ENST00000504937(n.607G>T)];ENSG00000141510:EXONIC-NC[ENST00000504290(n.607G>T)];ENSG00000141510:EXONIC-NC[ENST00000510385(n.607G>T)];	chr17*7577	chr17*7577	chr17*7577539*7577563*GGTTCATGCCGCCCATGCAGGAACT*G*rs397516437*NA*NA*NA|chr17*7577556*7577556*C*G,T*rs121912655*NA*NA*NA	NA	NA	NA	NA	NA	NA	NA	MODIFIER	NA	NA	57	57	0	78	32	46	58.97
Smoker	42475	chr17	7577558	G	A	*TP53*	SILENT	ENSG00000141510:EXONIC-NC[ENST00000504937(n.605C>T)];ENSG00000141510:EXONIC-NC[ENST00000504290(n.605C>T)];ENSG00000141510:EXONIC-NC[ENST00000510385(n.605C>T)];ENSG00000141510:SILENT[ENST00000413465(c.723C>T,tcC>tcT):ENSP00000410739(p.Ser241(=))];ENSG00000141510:SILENT[ENST00000420246(c.723C>T,tcC>tcT):ENSP00000391127(p.Ser241(=))];ENSG00000141510:SILENT[ENST00000269305(c.723C>T,tcC>tcT):ENSP00000269305(p.Ser241(=))];ENSG00000141510:SILENT[ENST00000509690(c.327C>T,tcC>tcT):ENSP00000425104(p.Ser109(=))];ENSG00000141510:SILENT[ENST00000359597(c.723C>T,tcC>tcT):ENSP00000352610(p.Ser241(=))];ENSG00000141510:SILENT[ENST00000514944(c.444C>T,tcC>tcT):ENSP00000423862(p.Ser148(=))];ENSG00000141510:SILENT[ENST00000445888(c.723C>T,tcC>tcT):ENSP00000391478(p.Ser241(=))];ENSG00000141510:SILENT[ENST00000455263(c.723C>T,tcC>tcT):ENSP00000398846(p.Ser241(=))];	chr17*7577	chr17*7577	chr17*7577539*7577563*GGTTCATGCCGCCCATGCAGGAACT*G*rs397516437*NA*NA*NA	NA	NA	NA	NA	NA	NA	NA	LOW	NA	NA	56	56	0	77	30	47	61.04
Smoker	42475	chr18	29099772	A	T	*DSG2*	MISSENSE	ENSG00000046604:MISSENSE[ENST00000585206(c.88A>T,Agc>Tgc):ENSP00000462503(p.Ser30Cys)];ENSG00000046604:MISSENSE[ENST00000261590(c.88A>T,Agc>Tgc):ENSP00000261590(p.Ser30Cys)];	NA	NA	NA	NA	NA	NA	NA	D	PoD	D	MODERATE	U	0.029	108	108	0	153	121	32	20.92
Smoker	42475	chr18	55919166	C	G	*NEDD4L*	INTRONIC	ENSG00000049759:[ENST00000456173(c.-120-67C>G)];ENSG00000049759:[ENST00000588494(c.-120-67C>G)];ENSG00000049759:[ENST00000256832(c.-120-67C>G)];ENSG00000049759:[ENST00000587634(n.384-67C>G)];ENSG00000049759:[ENST00000587190(c.-120-67C>G)];ENSG00000049759:[ENST00000356462(c.244-67C>G)];ENSG00000049759:[ENST00000592846(c.-120-67C>G)];ENSG00000049759:[ENST00000586263(c.220-67C>G)];ENSG00000049759:[ENST00000357895(c.220-67C>G)];ENSG00000049759:[ENST00000589054(c.49-138712C>G)];ENSG00000049759:[ENST00000588066(n.223-67C>G)];ENSG00000049759:[ENST00000400345(c.244-67C>G)];ENSG00000049759:[ENST00000431212(c.-120-67C>G)];ENSG00000049759:[ENST00000256830(c.244-67C>G)];ENSG00000049759:[ENST00000586268(c.-69-67C>G)];ENSG00000049759:[ENST00000382850(c.244-67C>G)];ENSG00000049759:[ENST00000435432(c.-120-67C>G)];ENSG00000049759:[ENST00000456986(c.-120-67C>G)];ENSG00000049759:[ENST00000585323(n.308-67C>G)];	NA	NA	NA	NA	NA	NA	NA	NA	NA	NA	MODIFIER	NA	NA	42	41	0	61	49	12	19.67
Smoker	42475	chr19	46806995	G	T	*HIF3A*	INTRONIC	ENSG00000124440:[ENST00000533789(c.27-160G>T)];ENSG00000124440:[ENST00000472815(c.-35-160G>T)];ENSG00000124440:[ENST00000475432(n.58-160G>T)];ENSG00000124440:[ENST00000244302(n.58-160G>T)];ENSG00000124440:[ENST00000377670(c.27-160G>T)];ENSG00000124440:[ENST00000300862(c.20+51G>T)];ENSG00000124440:[ENST00000524533(n.218-160G>T)];ENSG00000124440:[ENST00000244303(c.-35-160G>T)];ENSG00000124440:[ENST00000291300(n.58-160G>T)];ENSG00000124440:[ENST00000526506(c.-35-160G>T)];ENSG00000124440:[ENST00000528563(c.-35-160G>T)];ENSG00000124440:[ENST00000533145(c.-35-160G>T)];ENSG00000124440:[ENST00000339613(c.-142-160G>T)];	NA	NA	NA	NA	NA	NA	NA	NA	NA	NA	MODIFIER	NA	NA	47	47	0	82	60	22	26.83
Smoker	42475	chr19	55044022	C	A	*KIR3DX1*	MISSENSE	ENSG00000104970:MISSENSE[ENST00000446586(c.8C>A,cCc>cAc):ENSP00000396692(p.Pro3His)];ENSG00000104970:MISSENSE[ENST00000221567(c.8C>A,cCc>cAc):ENSP00000221567(p.Pro3His)];ENSG00000104970:MISSENSE[ENST00000335056(c.8C>A,cCc>cAc):ENSP00000335388(p.Pro3His)];ENSG00000104970:MISSENSE[ENST00000434659(c.8C>A,cCc>cAc):ENSP00000401224(p.Pro3His)];ENSG00000104970:MISSENSE[ENST00000447145(c.8C>A,cCc>cAc):ENSP00000395115(p.Pro3His)];ENSG00000104970:EXONIC-NC[ENST00000465702(n.114C>A)];ENSG00000104970:EXONIC-NC[ENST00000471931(n.114C>A)];	NA	NA	NA	NA	NA	NA	NA	NA	NA	NA	MODIFIER	NA	NA	68	68	0	82	55	27	32.93
Smoker	42475	chr19	55089096	C	T	*LILRA2*	INTRONIC	ENSG00000239998:[ENST00000251376(c.1255+1520C>T)];ENSG00000239998:[ENST00000391738(c.1255+1520C>T)];ENSG00000239998:[ENST00000391737(c.1219+1520C>T)];ENSG00000239998:[ENST00000251377(c.1255+1520C>T)];ENSG00000239998:[ENST00000472992(n.203C>T)];	NA	NA	NA	NA	NA	NA	NA	NA	NA	NA	MODIFIER	NA	NA	32	32	0	43	38	5	11.63
Smoker	42475	chr19	55089096	C	T	*LILRB1*	INTRONIC	ENSG00000104972:[ENST00000418536(c.70+3518C>T)];ENSG00000104972:[ENST00000448689(c.70+3518C>T)];ENSG00000104972:[ENST00000396321(c.70+3518C>T)];	NA	NA	NA	NA	NA	NA	NA	NA	NA	NA	MODIFIER	NA	NA	32	32	0	43	38	5	11.63
Smoker	42475	chr19	55174468	C	T	*LILRB4*	5UTR	ENSG00000186818:[ENST00000270452(c.-18C>T)];ENSG00000186818:[ENST00000391736(c.-18C>T)];ENSG00000186818:[ENST00000391734(c.-18C>T)];ENSG00000186818:[ENST00000430952(c.-18C>T)];ENSG00000186818:[ENST00000494796(n.268C>T)];	NA	NA	NA	NA	NA	NA	NA	NA	NA	NA	MODIFIER	NA	NA	71	71	0	94	55	39	41.49

Supplementary Table 4. List of somatic single nucleotide variants identified using whole exome sequencing of ESCC patients

Cohort	Sample	Chromosome	Genomic position	Reference allele	Alternate allele	Gene symbol	Variant type	Variant annotation	OncoMD	COSMIC	dbSNP	ExAC	ExAC_SAS	1000G	1000G_SAS	SIFT	Polyphen2	CONDEL	VEP	LRT	PhastCon7 Way	Total read (Adjacent normal)	Reference allel read support (Adjacent normal)	Alternative read support (Adjacent normal)	Total read (Tumor)	Reference allel read support (Tumor)	Alternative read support (Tumor)	Alternate allele frequency
Smoker	42475	chr19	19737565	G	A	*LPAR2*	MISSENSE	ENSG00000064547:MISSENSE[ENST00000586703(c.529C>T,Cgc>Tgc):ENSP00000465280(p.Arg177Cys)];ENSG00000064547:MISSENSE[ENST00000542587(c.529C>T,Cgc>Tgc):ENSP00000443256(p.Arg177Cys)];ENSG00000064547:MISSENSE[ENST00000407877(c.529C>T,Cgc>Tgc):ENSP00000384665(p.Arg177Cys)];	NA	NA	NA	NA	NA	NA	NA	D	BN	N	MODERATE	N	0.948	60	60	0	63	43	19	30.16
Smoker	42475	chr19	15302604	C	A	*NOTCH3*	MISSENSE	ENSG00000074181:MISSENSE[ENST00000263388(c.754G>T,Gtg>Ttg):ENSP00000263388(p.Val252Leu)];ENSG00000074181:MISSENSE[ENST00000601011(c.751G>T,Gtg>Ttg):ENSP00000473138(p.Val251Leu)];	NA	NA	chr19*15302599*15302644*ATCCACGCATGTCCCCCCATTGAGACATCGGTGTCCTGGACAGTCG*A*rs864621965*NA*NA*NA|chr19*15302604*15302604*C*T*rs115836330*NA*NA*NA	NA	NA	NA	NA	T	PrD	D	MODERATE	NA	NA	190	190	0	318	210	108	33.96
Smoker	42475	chr19	12825627	G	A	*TNPO2*	INTRONIC-SS-DNR	ENSG00000105576:[ENST00000356861(c.890+8C>T)];ENSG00000105576:[ENST00000592287(c.890+8C>T)];ENSG00000105576:[ENST00000585886(c.890+8C>T)];ENSG00000105576:[ENST00000588151(n.266+8C>T)];ENSG00000105576:[ENST00000441499(c.890+8C>T)];ENSG00000105576:[ENST00000425528(c.890+8C>T)];ENSG00000105576:[ENST00000450764(c.890+8C>T)];ENSG00000105576:[ENST00000588216(c.890+8C>T)];ENSG00000105576:[ENST00000589956(n.432+897C>T)];	NA	NA	NA	NA	NA	NA	NA	NA	NA	NA	LOW	NA	NA	49	49	0	58	34	24	41.38
Smoker	42475	chr19	38135512	C	A	*ZFP30*	SILENT-SS-PRX	ENSG00000120784:[ENST00000588979(c.135G>T,ctG>ctT):ENSP00000465496(p.Leu45(=))];ENSG00000120784:[ENST00000392144(c.135G>T,ctG>ctT):ENSP00000375988(p.Leu45(=))];ENSG00000120784:[ENST00000351218(c.135G>T,ctG>ctT):ENSP00000343581(p.Leu45(=))];ENSG00000120784:[ENST00000514101(c.135G>T,ctG>ctT):ENSP00000422930(p.Leu45(=))];ENSG00000120784:[ENST00000587809(c.135G>T,ctG>ctT):ENSP00000466563(p.Leu45(=))];ENSG00000120784:[ENST00000589018(c.135G>T,ctG>ctT):ENSP00000467387(p.Leu45(=))];ENSG00000120784:[ENST00000477900(n.334G>T)];	NA	NA	NA	NA	NA	NA	NA	NA	NA	NA	LOW	NA	NA	166	166	0	284	186	97	34.15
Smoker	42475	chr19	3810914	C	A	*ZFR2*	INTRONIC	ENSG00000105278:[ENST00000262961(c.2338-71G>T)];	NA	NA	chr19*3810914*3810914*C*T*rs77579744*0*0.9996*0.0003994	NA	NA	NA	NA	NA	NA	NA	MODIFIER	NA	NA	50	50	0	71	57	14	19.72
Smoker	42475	chr19	22193582	C	T	*ZNF208*	INTRONIC	ENSG00000160321:[ENST00000601773(c.3+12G>A)];ENSG00000160321:[ENST00000601993(c.-270+12G>A)];ENSG00000160321:[ENST00000599916(c.3+12G>A)];ENSG00000160321:[ENST00000597040(c.-188+12G>A)];ENSG00000160321:[ENST00000397126(c.3+12G>A)];	NA	NA	NA	NA	NA	NA	NA	NA	NA	NA	MODIFIER	NA	NA	104	104	0	159	126	33	20.75
Smoker	42475	chr19	37117873	G	T	*ZNF382*	SILENT	ENSG00000161298:SILENT[ENST00000435416(c.1071G>T,ggG>ggT):ENSP00000410113(p.Gly357(=))];ENSG00000161298:SILENT[ENST00000292928(c.1074G>T,ggG>ggT):ENSP00000292928(p.Gly358(=))];ENSG00000161298:SILENT[ENST00000439428(c.1071G>T,ggG>ggT):ENSP00000407593(p.Gly357(=))];ENSG00000161298:SILENT[ENST00000423582(c.927G>T,ggG>ggT):ENSP00000389722(p.Gly309(=))];	NA	NA	chr19*37117873*37117873*G*A*rs764921340*NA*NA*NA	NA	NA	NA	NA	NA	NA	NA	LOW	NA	NA	107	107	0	131	76	55	41.98
Smoker	42475	chr19	31025721	A	T	*ZNF536*	INTRONIC	ENSG00000198597:[ENST00000585628(c.2171-33A>T)];ENSG00000198597:[ENST00000355537(c.2171-33A>T)];	NA	NA	NA	NA	NA	NA	NA	NA	NA	NA	MODIFIER	NA	NA	46	46	0	92	79	13	14.13
Smoker	42475	chr19	37413860	A	C	*ZNF568*	INTRONIC	ENSG00000198453:[ENST00000415168(c.-58+5310A>C)];ENSG00000198453:[ENST00000455427(c.-116-2242A>C)];ENSG00000198453:[ENST00000333987(c.76+112A>C)];ENSG00000198453:[ENST00000587130(c.-209-2242A>C)];ENSG00000198453:[ENST00000587857(c.-116-2242A>C)];ENSG00000198453:[ENST00000444991(c.76+112A>C)];ENSG00000198453:[ENST00000427117(c.76+112A>C)];	NA	NA	NA	NA	NA	NA	NA	NA	NA	NA	MODIFIER	NA	NA	36	36	0	54	26	28	51.85
Smoker	42475	chr19	55994454	G	T	*ZNF628*	MISSENSE	ENSG00000197483:MISSENSE[ENST00000391718(c.1882G>T,Gtt>Ttt):ENSP00000375598(p.Val628Phe)];ENSG00000197483:MISSENSE[ENST00000598519(c.1894G>T,Gtt>Ttt):ENSP00000469591(p.Val632Phe)];	NA	NA	NA	NA	NA	NA	NA	D	PrD	D	MODERATE	U	0.894	100	100	0	99	62	36	36.36
Smoker	42475	chr2	239148424	C	A	*AC096574.4*	INTRONIC	ENSG00000225057:[ENST00000456601(n.1043+3786C>A)];	NA	NA	chr2*239148424*239148425*CG*C*rs777343242*NA*NA*NA	NA	NA	NA	NA	NA	NA	NA	MODIFIER	NA	NA	36	36	0	34	20	14	41.18
Smoker	42475	chr2	228162530	G	A	*AC097662.2*	INTRONIC	ENSG00000236432:[ENST00000433324(n.35-16025C>T)];ENSG00000236432:[ENST00000396588(n.397+3979C>T)];ENSG00000236432:[ENST00000439598(n.243+7646C>T)];	NA	NA	NA	NA	NA	NA	NA	NA	NA	NA	MODIFIER	NA	NA	49	49	0	71	54	17	23.94
Smoker	42475	chr2	228384791	G	A	*AGFG1*	INTRONIC	ENSG00000173744:[ENST00000409171(c.377+12G>A)];ENSG00000173744:[ENST00000456594(c.143+12G>A)];ENSG00000173744:[ENST00000409315(c.377+12G>A)];ENSG00000173744:[ENST00000409979(c.377+12G>A)];ENSG00000173744:[ENST00000373671(c.377+12G>A)];ENSG00000173744:[ENST00000486932(n.260+12G>A)];ENSG00000173744:[ENST00000310078(c.377+12G>A)];	NA	NA	NA	NA	NA	NA	NA	NA	NA	NA	MODIFIER	NA	NA	49	49	0	71	52	19	26.76
Smoker	42475	chr2	97818241	T	G	*ANKRD36*	MISSENSE-SS-PRX	ENSG00000135976:[ENST00000461153(c.1163T>G,gTg>gGg):ENSP00000419530(p.Val388Gly)];ENSG00000135976:[ENST00000420699(c.1163T>G,gTg>gGg):ENSP00000391950(p.Val388Gly)];	NA	NA	NA	6.06E-05	0	NA	NA	D_lc	BN	N	MODERATE	NA	NA	89	87	1	126	110	7	5.56
Smoker	42475	chr2	87085460	G	A	*CD8B*	SILENT	ENSG00000172116:SILENT[ENST00000349455(c.123C>T,tgC>tgT):ENSP00000340592(p.Cys41(=))];ENSG00000172116:SILENT[ENST00000390655(c.123C>T,tgC>tgT):ENSP00000375070(p.Cys41(=))];ENSG00000172116:SILENT[ENST00000393759(c.123C>T,tgC>tgT):ENSP00000377356(p.Cys41(=))];ENSG00000172116:SILENT[ENST00000393761(c.123C>T,tgC>tgT):ENSP00000377358(p.Cys41(=))];ENSG00000172116:SILENT[ENST00000331469(c.123C>T,tgC>tgT):ENSP00000331172(p.Cys41(=))];ENSG00000172116:INTRONIC[ENST00000431506(c.43+3486C>T)];	NA	NA	NA	7.43E-05	0	NA	NA	NA	NA	NA	LOW	NA	0.009	77	77	0	131	69	62	47.33
Smoker	42475	chr2	189866147	C	A	*COL3A1*	MISSENSE	ENSG00000168542:MISSENSE[ENST00000317840(c.2308C>A,Cct>Act):ENSP00000315243(p.Pro770Thr)];ENSG00000168542:MISSENSE[ENST00000304636(c.2308C>A,Cct>Act):ENSP00000304408(p.Pro770Thr)];	NA	NA	chr2*189866147*189866147*C*T*rs770241798*NA*NA*NA	NA	NA	NA	NA	NA	UN	NA	MODERATE	D	0.992	48	48	0	72	48	24	33.33

Cohort	Sample	Chromosome	Genomic position	Reference allele	Alternate allele	Gene symbol	Variant type	Variant annotation	OncoMD	COSMIC	dbSNP	ExAC	ExAC_SAS	1000G	1000G_SAS	SIFT	Polyphen2	CONDEL	VEP	LRT	PhastCon7 Way	Total read (Adjacent normal)	Reference allel read support (Adjacent normal)	Alternative read support (Adjacent normal)	Total read (Tumor)	Reference allel read support (Tumor)	Alternative read support (Tumor)	Alternate allele frequency
Smoker	42475	chr2	228162530	G	A	*COL4A3*	MISSENSE	ENSG00000169031:MISSENSE[ENST00000396578(c.3706G>A,Ggc>Agc):ENSP00000379823(p.Gly1236Ser)];ENSG00000169031:EXONIC-NC[ENST00000468753(n.369G>A)];ENSG00000169031:EXONIC-NC[ENST00000471862(n.124G>A)];	NA	NA	NA	NA	NA	NA	NA	NA	NA	NA	MODIFIER	NA	NA	49	49	0	71	54	17	23.94
Smoker	42475	chr2	225367714	C	T	*CUL3*	MISSENSE	ENSG00000036257:MISSENSE[ENST00000344951(c.1255G>A,Gat>Aat):ENSP00000343601(p.Asp419Asn)];ENSG00000036257:MISSENSE[ENST00000264414(c.1453G>A,Gat>Aat):ENSP00000264414(p.Asp485Asn)];ENSG00000036257:MISSENSE[ENST00000409777(c.1381G>A,Gat>Aat):ENSP00000386525(p.Asp461Asn)];ENSG00000036257:MISSENSE[ENST00000409096(c.1381G>A,Gat>Aat):ENSP00000387200(p.Asp461Asn)];ENSG00000036257:EXONIC-NC[ENST00000481135(n.749G>A)];	NA	NA	chr2*225367714*225367717*CCAT*C*rs762745891*NA*NA*NA	NA	NA	NA	NA	NA	NA	NA	MODIFIER	NA	NA	144	144	0	264	218	45	17.05
Smoker	42475	chr2	225669872	C	A	*DOCK10*	INTRONIC	ENSG00000135905:[ENST00000422684(c.697-31G>T)];ENSG00000135905:[ENST00000258390(c.4052-31G>T)];ENSG00000135905:[ENST00000409592(c.4034-31G>T)];	NA	NA	NA	NA	NA	NA	NA	NA	NA	NA	MODIFIER	NA	NA	75	75	0	88	67	20	22.73
Smoker	42475	chr2	176948136	G	T	*EVX2*	SILENT	ENSG00000174279:SILENT[ENST00000308618(c.369C>A,tcC>tcA):ENSP00000312385(p.Ser123(=))];	chr2*176948	NA	NA	NA	NA	NA	NA	NA	NA	NA	LOW	NA	NA	67	66	1	86	57	29	33.72
Smoker	42475	chr2	239148424	C	A	*HES6*	SILENT	ENSG00000144485:INTRONIC[ENST00000409574(c.82-36G>T)];ENSG00000144485:INTRONIC[ENST00000409002(c.82-36G>T)];ENSG00000144485:INTRONIC[ENST00000450098(c.81+42G>T)];ENSG00000144485:INTRONIC[ENST00000272937(c.82-36G>T)];ENSG00000144485:INTRONIC[ENST00000409160(c.82-36G>T)];ENSG00000144485:INTRONIC[ENST00000436051(c.272-36G>T)];ENSG00000144485:INTRONIC[ENST00000409182(c.81+42G>T)];ENSG00000144485:SILENT[ENST00000409356(c.123G>T,ccG>ccT):ENSP00000387107(p.Pro41(=))];	NA	NA	chr2*239148424*239148425*CG*C*rs777343242*NA*NA*NA	NA	NA	NA	NA	NA	NA	NA	MODIFIER	NA	NA	36	36	0	34	20	14	41.18
Smoker	42475	chr2	223574465	G	T	*MOGAT1*	SILENT	ENSG00000124003:SILENT[ENST00000446656(c.864G>T,ccG>ccT):ENSP00000406674(p.Pro288(=))];	NA	NA	chr2*223574465*223574465*G*A*rs769213168*NA*NA*NA	NA	NA	NA	NA	NA	NA	NA	LOW	NA	NA	87	87	0	102	62	40	39.22
Smoker	42475	chr2	89521147	G	T	*NA*	INTERGENIC		NA	NA	NA	NA	NA	NA	NA	NA	NA	NA	MODIFIER	NA	NA	74	74	0	102	58	44	43.14
Smoker	42475	chr2	15701272	C	A	*NBAS*	INTRONIC	ENSG00000151779:[ENST00000441750(c.117+40G>T)];ENSG00000151779:[ENST00000281513(c.117+40G>T)];	NA	NA	chr2*15701272*15701272*C*G*rs768927364*NA*NA*NA	NA	NA	NA	NA	NA	NA	NA	MODIFIER	NA	NA	61	61	0	68	43	23	33.82
Smoker	42475	chr2	51255220	G	T	*NRXN1*	SILENT	ENSG00000179915:SILENT[ENST00000405472(c.192C>A,cgC>cgA):ENSP00000434015(p.Arg64(=))];ENSG00000179915:SILENT[ENST00000404971(c.192C>A,cgC>cgA):ENSP00000385142(p.Arg64(=))];ENSG00000179915:SILENT[ENST00000406316(c.192C>A,cgC>cgA):ENSP00000384311(p.Arg64(=))];ENSG00000179915:SILENT[ENST00000401669(c.192C>A,cgC>cgA):ENSP00000385017(p.Arg64(=))];ENSG00000179915:SILENT[ENST00000406859(c.192C>A,cgC>cgA):ENSP00000385681(p.Arg64(=))];ENSG00000179915:SILENT[ENST00000405581(c.192C>A,cgC>cgA):ENSP00000385310(p.Arg64(=))];ENSG00000179915:SILENT[ENST00000402717(c.192C>A,cgC>cgA):ENSP00000385434(p.Arg64(=))];	chr2*51255	chr2*51255	chr2*51255220*51255220*G*A*rs748944452*NA*NA*NA	NA	NA	NA	NA	NA	NA	NA	LOW	NA	NA	73	72	1	101	77	24	23.76
Smoker	42475	chr2	25384019	C	A	*POMC*	MISSENSE	ENSG00000115138:MISSENSE[ENST00000264708(c.735G>T,aaG>aaT):ENSP00000264708(p.Lys245Asn)];ENSG00000115138:MISSENSE[ENST00000380794(c.735G>T,aaG>aaT):ENSP00000370171(p.Lys245Asn)];ENSG00000115138:MISSENSE[ENST00000449220(c.735G>T,aaG>aaT):ENSP00000387993(p.Lys245Asn)];ENSG00000115138:MISSENSE[ENST00000395826(c.735G>T,aaG>aaT):ENSP00000379170(p.Lys245Asn)];ENSG00000115138:MISSENSE[ENST00000405623(c.735G>T,aaG>aaT):ENSP00000384092(p.Lys245Asn)];	NA	NA	NA	NA	NA	NA	NA	D	PrD	D	MODERATE	N	1	120	120	0	202	124	78	38.61
Smoker	42475	chr2	89037508	A	C	*RPIA*	SILENT	ENSG00000153574:SILENT[ENST00000283646(c.753A>C,acA>acC):ENSP00000283646(p.Thr251(=))];	NA	NA	NA	NA	NA	NA	NA	NA	NA	NA	LOW	NA	NA	75	75	0	105	75	30	28.57
Smoker	42475	chr2	1094087	G	A	*SNTG2*	MISSENSE	ENSG00000172554:MISSENSE[ENST00000308624(c.316G>A,Gac>Aac):ENSP00000311837(p.Asp106Asn)];ENSG00000172554:MISSENSE[ENST00000452177(c.316G>A,Gac>Aac):ENSP00000412249(p.Asp106Asn)];ENSG00000172554:MISSENSE[ENST00000450962(c.316G>A,Gac>Aac):ENSP00000401997(p.Asp106Asn)];ENSG00000172554:INTRONIC[ENST00000407292(c.210+14746G>A)];	NA	NA	NA	NA	NA	NA	NA	T	BN	N	MODERATE	NA	NA	75	74	1	105	80	25	23.81
Smoker	42475	chr2	180309599	T	C	*ZNF385B*	INTRONIC-SS-DNR	ENSG00000144331:[ENST00000466398(n.1460+4A>G)];ENSG00000144331:[ENST00000409692(c.891+4A>G)];ENSG00000144331:[ENST00000409343(c.969+4A>G)];ENSG00000144331:[ENST00000410066(c.1197+4A>G)];ENSG00000144331:[ENST00000336917(c.891+4A>G)];	NA	NA	NA	NA	NA	NA	NA	NA	NA	NA	LOW	NA	NA	68	68	0	106	87	19	17.92
Smoker	42475	chr20	14306689	G	A	*FLRT3*	SILENT	ENSG00000125848:SILENT[ENST00000378053(c.1464C>T,taC>taT):ENSP00000367292(p.Tyr488(=))];ENSG00000125848:SILENT[ENST00000341420(c.1464C>T,taC>taT):ENSP00000339912(p.Tyr488(=))];	NA	NA	NA	NA	NA	NA	NA	NA	NA	NA	MODIFIER	NA	NA	156	156	0	354	246	107	30.23
Smoker	42475	chr20	29628205	T	A	*FRG1B*	INTRONIC	ENSG00000149531:[ENST00000278882(c.229-22T>A)];ENSG00000149531:[ENST00000439954(c.244-22T>A)];ENSG00000149531:[ENST00000479318(c.113-22T>A)];ENSG00000149531:[ENST00000358464(c.229-22T>A)];	NA	NA	NA	7.46E-05	0	NA	NA	NA	NA	NA	MODIFIER	NA	NA	1277	1253	22	1766	1699	62	3.51
Smoker	42475	chr20	29628233	A	G	*FRG1B*	MISSENSE	ENSG00000149531:MISSENSE[ENST00000278882(c.235A>G,Atg>Gtg):ENSP00000278882(p.Met79Val)];ENSG00000149531:MISSENSE[ENST00000439954(c.250A>G,Atg>Gtg):ENSP00000408863(p.Met84Val)];ENSG00000149531:MISSENSE[ENST00000358464(c.235A>G,Atg>Gtg):ENSP00000351249(p.Met79Val)];ENSG00000149531:MISSENSE[ENST00000479318(c.119A>G,Atg>Gtg):ENSP00000436872(p.Met41Val)];	NA	NA	NA	0.0004632	0.000121197	NA	NA	T	BN	N	MODERATE	NA	NA	1564	1541	18	2213	2162	46	2.08
Smoker	42475	chr20	29632634	A	G	*FRG1B*	MISSENSE	ENSG00000149531:MISSENSE[ENST00000278882(c.449A>G,gAc>gGc):ENSP00000278882(p.Asp150Gly)];ENSG00000149531:MISSENSE[ENST00000358464(c.449A>G,gAc>gGc):ENSP00000351249(p.Asp150Gly)];	NA	NA	NA	NA	NA	NA	NA	D	PoD	D	MODERATE	N	1	1361	1343	17	1570	1530	40	2.55
Smoker	42475	chr20	14306689	G	A	*MACROD2*	INTRONIC	ENSG00000172264:[ENST00000490428(n.263-167436G>A)];ENSG00000172264:[ENST00000217246(c.272-167436G>A)];ENSG00000172264:[ENST00000494602(n.299-167436G>A)];ENSG00000172264:[ENST00000477147(n.541-167436G>A)];ENSG00000172264:[ENST00000310348(c.272-167436G>A)];	NA	NA	NA	NA	NA	NA	NA	NA	NA	NA	MODIFIER	NA	NA	156	156	0	354	246	107	30.23
Smoker	42475	chr20	61443591	C	T	*OGFR*	SILENT	ENSG00000060491:SILENT[ENST00000370461(c.468C>T,caC>caT):ENSP00000359491(p.His156(=))];ENSG00000060491:SILENT[ENST00000290291(c.624C>T,caC>caT):ENSP00000290291(p.His208(=))];	NA	NA	NA	NA	NA	NA	NA	NA	NA	NA	LOW	NA	0.999	50	50	0	91	62	28	30.77

Mangalaparthi *et al.* , 2020. Mutational landscape of esophageal squamous cell carcinoma in an Indian cohort
Supplementary Table 4. List of somatic single nucleotide variants identified using whole exome sequencing of ESCC patients

Cohort	Sample	Chromosome	Genomic position	Reference allele	Alternate allele	Gene symbol	Variant type	Variant annotation	OncoMD	COSMIC	dbSNP	ExAC	ExAC_SAS	1000G	1000G_SAS	SIFT	Polyphen2	CONDEL	VEP	LRT	PhastCon7 Way	Total read (Adjacent normal)	Reference allel read support (Adjacent normal)	Alternative read support (Adjacent normal)	Total read (Tumor)	Reference allel read support (Tumor)	Alternative read support (Tumor)	Alternate allele frequency
Smoker	42475	chr20	9376220	C	T	*PLCB4*	MISSENSE	ENSG00000101333:MISSENSE[ENST00000334005(c.1465C>T,Cca>Tca):ENSP00000334105(p.Pro489Ser)];ENSG00000101333:MISSENSE[ENST00000414679(c.1465C>T,Cca>Tca):ENSP00000390616(p.Pro489Ser)];ENSG00000101333:MISSENSE[ENST00000278655(c.1465C>T,Cca>Tca):ENSP00000278655(p.Pro489Ser)];ENSG00000101333:MISSENSE[ENST00000378473(c.1465C>T,Cca>Tca):ENSP00000367734(p.Pro489Ser)];ENSG00000101333:MISSENSE[ENST00000378493(c.1465C>T,Cca>Tca):ENSP00000367754(p.Pro489Ser)];ENSG00000101333:MISSENSE[ENST00000378501(c.1465C>T,Cca>Tca):ENSP00000367762(p.Pro489Ser)];ENSG00000101333:EXONIC-NC[ENST00000492632(n.1480C>T)];ENSG00000101333:EXONIC-NC[ENST00000473151(n.1156C>T)];ENSG00000101333:EXONIC-NC[ENST00000482123(n.1156C>T)];ENSG00000101333:EXONIC-NC[ENST00000464199(n.1242C>T)];	NA	NA	NA	NA	NA	NA	NA	T	BN	N	MODERATE	N	0.642	125	125	0	232	163	69	29.74
Smoker	42475	chr21	31709740	C	A	*KRTAP27-1*	NONSENSE	ENSG00000206107:NONSENSE[ENST00000382835(c.247G>T,Gga>Tga):ENSP00000372286(p.Gly83Ter)];	chr21*3170[illegible]	chr21*3170[illegible]	chr21*31709740*31709740*C*T*rs147427257*NA*NA*NA	NA	NA	NA	NA	NA	NA	NA	HIGH	NA	0.076	100	99	0	130	70	60	46.15
Smoker	42475	chr21	22583029	C	G	*NCAM2*	INTRONIC	ENSG00000154654:[ENST00000400546(c.56-69869C>G)];ENSG00000154654:[ENST00000284894(c.56-81395C>G)];ENSG00000154654:[ENST00000535285(c.130+19C>G)];	NA	NA	NA	NA	NA	NA	NA	NA	NA	NA	MODIFIER	NA	NA	68	66	1	80	39	41	51.25
Smoker	42475	chr22	19504131	G	C	*CDC45*	MISSENSE	ENSG00000093009:MISSENSE[ENST00000407835(c.1522G>C,Ggc>Cgc):ENSP00000385240(p.Gly508Arg)];ENSG00000093009:MISSENSE[ENST00000263201(c.1522G>C,Ggc>Cgc):ENSP00000263201(p.Gly508Arg)];ENSG00000093009:MISSENSE[ENST00000404724(c.1384G>C,Ggc>Cgc):ENSP00000384978(p.Gly462Arg)];ENSG00000093009:MISSENSE[ENST00000437685(c.1618G>C,Ggc>Cgc):ENSP00000405726(p.Gly540Arg)];ENSG00000093009:EXONIC-NC[ENST00000493724(n.175G>C)];	NA	NA	NA	NA	NA	NA	NA	NA	NA	NA	MODIFIER	NA	NA	77	77	0	98	57	40	40.82
Smoker	42475	chr22	26291081	C	T	*CTA-125H2.2*	INTRONIC	ENSG00000231933:[ENST00000599080(n.473-2474G>A)];ENSG00000231933:[ENST00000594542(n.534-2474G>A)];ENSG00000231933:[ENST00000600211(n.396-2474G>A)];ENSG00000231933:[ENST00000609809(n.601+1882G>A)];ENSG00000231933:[ENST00000597614(n.575+1159G>A)];ENSG00000231933:[ENST00000608115(n.566-2474G>A)];ENSG00000231933:[ENST00000608257(n.432-2474G>A)];ENSG00000231933:[ENST00000609889(n.489-2474G>A)];ENSG00000231933:[ENST00000595102(n.454-2474G>A)];ENSG00000231933:[ENST00000609820(n.633+224G>A)];ENSG00000231933:[ENST00000609823(n.279-2474G>A)];ENSG00000231933:[ENST00000597284(n.437-2474G>A)];ENSG00000231933:[ENST00000597548(n.531-2474G>A)];ENSG00000231933:[ENST00000607895(n.174-2474G>A)];ENSG00000231933:[ENST00000609275(n.787G>A)];ENSG00000231933:[ENST00000453457(n.542G>A)];ENSG00000231933:[ENST00000609570(n.469G>A)];ENSG00000231933:[ENST00000608507(n.604G>A)];ENSG00000231933:[ENST00000594585(n.508G>A)];ENSG00000231933:[ENST00000600269(n.260G>A)];ENSG00000231933:[ENST00000593715(n.482G>A)];ENSG00000231933:[ENST00000599792(n.369G>A)];ENSG00000231933:[ENST00000609157(n.568G>A)];ENSG00000231933:[ENST00000608921(n.636G>A)];ENSG00000231933:[ENST00000596813(n.530G>A)];	NA	NA	NA	NA	NA	NA	NA	NA	NA	NA	MODIFIER	NA	NA	65	65	0	87	56	31	35.63
Smoker	42475	chr22	23077539	G	T	*IGLV2-18*	MISSENSE	ENSG00000211664:MISSENSE[ENST00000390310(c.316G>T,Gat>Tat):ENSP00000374845(p.Asp106Tyr)];	NA	NA	NA	NA	NA	NA	NA	D_lc	PoD	D	MODERATE	NA	NA	80	80	0	89	72	17	19.1
Smoker	42475	chr22	26291081	C	T	*MYO18B*	INTRONIC	ENSG00000133454:[ENST00000418374(n.3115-42C>T)];ENSG00000133454:[ENST00000335473(c.4544-42C>T)];ENSG00000133454:[ENST00000536101(c.4544-42C>T)];ENSG00000133454:[ENST00000407587(c.4547-42C>T)];ENSG00000133454:[ENST00000539302(c.*2002-42C>T)];	NA	NA	NA	NA	NA	NA	NA	NA	NA	NA	MODIFIER	NA	NA	65	65	0	87	56	31	35.63
Smoker	42475	chr22	32108503	C	A	*PRR14L*	SILENT	ENSG00000183530:SILENT[ENST00000434485(c.5322G>T,ggG>ggT):ENSP00000388314(p.Gly1774(=))];ENSG00000183530:SILENT[ENST00000397493(c.5322G>T,ggG>ggT):ENSP00000380630(p.Gly1774(=))];ENSG00000183530:SILENT[ENST00000330495(c.229G>T,ggG>ggT):ENSP00000332169(p.Gly77(=))];ENSG00000183530:SILENT[ENST00000327423(c.5322G>T,ggG>ggT):ENSP00000331845(p.Gly1774(=))];ENSG00000183530:SILENT[ENST00000431684(c.1329G>T,ggG>ggT):ENSP00000389527(p.Gly443(=))];	NA	NA	NA	NA	NA	NA	NA	NA	NA	NA	LOW	NA	NA	94	94	0	140	87	52	37.14
Smoker	42475	chr3	170204046	C	T	*CLDN11*	INTRONIC	ENSG00000013297:[ENST00000486975(c.391+62931C>T)];ENSG00000013297:[ENST00000480067(n.218+9385C>T)];ENSG00000013297:[ENST00000451576(c.391+62931C>T)];ENSG00000013297:[ENST00000471373(n.373-16554C>T)];	NA	NA	NA	NA	NA	NA	NA	T	BN	N	MODERATE	D	0.99	39	38	1	87	54	33	37.93
Smoker	42475	chr3	150416517	T	A	*FAM194A*	INTRONIC	ENSG00000163645:[ENST00000474463(c.475+61A>T)];ENSG00000163645:[ENST00000491361(c.115+61A>T)];ENSG00000163645:[ENST00000295910(c.553+61A>T)];	NA	NA	NA	NA	NA	NA	NA	NA	NA	NA	MODIFIER	NA	NA	119	119	0	236	177	59	25
Smoker	42475	chr3	42949655	C	A	*KRBOX1*	INTRONIC	ENSG00000273291:[ENST00000451200(n.169-30687C>A)];ENSG00000240747:[ENST00000426937(c.-163-630C>A)];	NA	NA	chr3*42949655*42949655*C*G*rs775648224*NA*NA*NA	NA	NA	NA	NA	NA	NA	NA	MODIFIER	NA	NA	86	86	0	92	78	14	15.22
Smoker	42475	chr3	129156525	C	T	*MBD4*	INTRONIC	ENSG00000129071:[ENST00000509587(n.442+38G>A)];ENSG00000129071:[ENST00000505883(n.351+126G>A)];ENSG00000129071:[ENST00000393278(c.247+126G>A)];ENSG00000129071:[ENST00000503197(c.335+38G>A)];ENSG00000129071:[ENST00000249910(c.335+38G>A)];ENSG00000129071:[ENST00000509828(c.104+2048G>A)];ENSG00000129071:[ENST00000429544(c.335+38G>A)];ENSG00000129071:[ENST00000507208(c.335+38G>A)];	NA	NA	NA	NA	NA	NA	NA	NA	NA	NA	MODIFIER	NA	NA	159	159	0	373	313	58	15.55

Mangalaparthi *et al.* , 2020. Mutational landscape of esophageal squamous cell carcinoma in an Indian cohort
Supplementary Table 4. List of somatic single nucleotide variants identified using whole exome sequencing of ESCC patients

Cohort	Sample	Chromosome	Genomic position	Reference allele	Alternate allele	Gene symbol	Variant type	Variant annotation	OncoMD	COSMIC	dbSNP	ExAC	ExAC_SAS	1000G	1000G_SAS	SIFT	Polyphen2	CONDEL	VEP	LRT	PhastCon7 Way	Total read (Adjacent normal)	Reference allel read support (Adjacent normal)	Alternative read support (Adjacent normal)	Total read (Tumor)	Reference allel read support (Tumor)	Alternative read support (Tumor)	Alternate allele frequency
Smoker	42475	chr3	127429459	C	T	*MGLL*	MISSENSE	ENSG00000074416:MISSENSE[ENST00000265052(c.560G>A,gGg>gAg):ENSP00000265052(p.Gly187Glu)];ENSG00000074416:MISSENSE[ENST00000398104(c.530G>A,gGg>gAg):ENSP00000381176(p.Gly177Glu)];ENSG00000074416:MISSENSE[ENST00000398101(c.452G>A,gGg>gAg):ENSP00000381173(p.Gly151Glu)];ENSG00000074416:MISSENSE[ENST00000434178(c.530G>A,gGg>gAg):ENSP00000402798(p.Gly177Glu)];ENSG00000074416:MISSENSE[ENST00000496306(c.247G>A,gGg>gAg):ENSP00000417060(p.Gly83Glu)];ENSG00000074416:MISSENSE[ENST00000487473(c.302G>A,gGg>gAg):ENSP00000420125(p.Gly101Glu)];ENSG00000074416:INTRONIC[ENST00000453507(c.510+10437G>A)];ENSG00000074416:INTRONIC[ENST00000484451(c.252+10437G>A)];ENSG00000074416:EXONIC-NC[ENST00000476682(n.3531G>A)];	NA	NA	NA	NA	NA	NA	NA	NA	NA	NA	MODIFIER	NA	NA	92	92	0	151	102	49	32.45
Smoker	42475	chr3	49721561	A	C	*MST1*	MISSENSE	ENSG00000173531:MISSENSE[ENST00000449682(c.2078T>G,aTa>aGa):ENSP00000414287(p.Ile693Arg)];ENSG00000173531:MISSENSE[ENST00000448220(c.486T>G,aTa>aGa):ENSP00000394756(p.Ile163Arg)];ENSG00000173531:EXONIC-NC[ENST00000479115(n.2133T>G)];ENSG00000173531:EXONIC-NC[ENST00000488350(n.4000T>G)];ENSG00000173531:EXONIC-NC[ENST00000493836(n.844T>G)];ENSG00000173531:EXONIC-NC[ENST00000492329(n.1854T>G)];	NA	NA	NA	NA	NA	NA	NA	NA	NA	NA	MODIFIER	NA	NA	55	55	0	76	70	6	7.89
Smoker	42475	chr3	193332716	A	G	*OPA1*	SILENT	ENSG00000198836:5UTR[ENST00000419435(c.-136A>G)];ENSG00000198836:SILENT[ENST00000392437(c.237A>G,aaA>aaG):ENSP00000376232(p.Lys79(=))];ENSG00000198836:SILENT[ENST00000361715(c.237A>G,aaA>aaG):ENSP00000355311(p.Lys79(=))];ENSG00000198836:SILENT[ENST00000361828(c.237A>G,aaA>aaG):ENSP00000354429(p.Lys79(=))];ENSG00000198836:SILENT[ENST00000392438(c.237A>G,aaA>aaG):ENSP00000376233(p.Lys79(=))];ENSG00000198836:SILENT[ENST00000361510(c.237A>G,aaA>aaG):ENSP00000355324(p.Lys79(=))];ENSG00000198836:SILENT[ENST00000392436(c.237A>G,aaA>aaG):ENSP00000376231(p.Lys79(=))];ENSG00000198836:SILENT[ENST00000361150(c.237A>G,aaA>aaG):ENSP00000354781(p.Lys79(=))];ENSG00000198836:SILENT[ENST00000361908(c.237A>G,aaA>aaG):ENSP00000354681(p.Lys79(=))];ENSG00000198836:EXONIC-NC[ENST00000487986(n.140A>G)];	NA	NA	chr3*193332716*193332716*A*T*rs752258799*NA*NA*NA	NA	NA	NA	NA	NA	NA	NA	LOW	NA	0.982	249	247	2	424	296	127	29.95
Smoker	42475	chr3	97983602	T	C	*OR5H6*	SILENT	ENSG00000230301:SILENT[ENST00000383696(c.474T>C,atT>atC):ENSP00000373196(p.Ile158(=))];	chr3*97983	chr3*97983	NA	0.0520571	0.068520227	NA	NA	NA	NA	NA	MODIFIER	NA	NA	227	224	3	489	461	27	5.52
Smoker	42475	chr3	155199435	C	A	*PLCH1*	MISSENSE	ENSG00000114805:MISSENSE[ENST00000334686(c.4290G>T,atG>atT):ENSP00000335469(p.Met1430Ile)];ENSG00000114805:MISSENSE[ENST00000414191(c.4290G>T,atG>atT):ENSP00000412977(p.Met1430Ile)];ENSG00000114805:MISSENSE[ENST00000340059(c.4404G>T,atG>atT):ENSP00000345988(p.Met1468Ile)];ENSG00000114805:MISSENSE[ENST00000460012(c.4290G>T,atG>atT):ENSP00000417502(p.Met1430Ile)];ENSG00000114805:3UTR[ENST00000447496(c.*1423G>T)];ENSG00000114805:INTRONIC[ENST00000494598(c.2938+3710G>T)];	chr3*15519	NA	NA	NA	NA	NA	NA	NA	NA	NA	MODIFIER	NA	NA	118	118	0	263	184	79	30.04
Smoker	42475	chr3	121168262	C	A	*POLQ*	SILENT	ENSG00000051341:EXONIC-NC[ENST00000474243(n.480G>T)];ENSG00000051341:SILENT[ENST00000264233(c.7164G>T,ggG>ggT):ENSP00000264233(p.Gly2388(=))];	NA	NA	NA	NA	NA	NA	NA	NA	NA	NA	LOW	NA	NA	107	107	0	209	167	41	19.62
Smoker	42475	chr3	75986741	G	T	*ROBO2*	NONSENSE	ENSG00000185008:NONSENSE[ENST00000602589(c.97G>T,Gga>Tga):ENSP00000473268(p.Gly33Ter)];ENSG00000185008:NONSENSE[ENST00000487694(c.97G>T,Gga>Tga):ENSP00000417335(p.Gly33Ter)];	NA	chr3*75986	NA	0.0695896	0.059116323	NA	NA	NA	NA	NA	HIGH	NA	NA	138	137	1	186	177	9	4.84
Smoker	42475	chr3	97983602	T	C	*RP11-325B23.2*	INTRONIC	ENSG00000251088:[ENST00000508616(n.26+31082T>C)];	chr3*97983	chr3*97983	NA	0.0520571	0.068520227	NA	NA	NA	NA	NA	MODIFIER	NA	NA	227	224	3	489	461	27	5.52
Smoker	42475	chr3	170204046	C	T	*SLC7A14*	MISSENSE	ENSG00000013293:MISSENSE[ENST00000231706(c.871G>A,Gcc>Acc):ENSP00000231706(p.Ala291Thr)];	NA	NA	NA	NA	NA	NA	NA	T	BN	N	MODERATE	D	0.99	39	38	1	87	54	33	37.93
Smoker	42475	chr3	87018242	A	G	*VGLL3*	SILENT	ENSG00000206538:SILENT[ENST00000494229(c.235T>C,agT>agC):ENSP00000419115(p.Ser79(=))];ENSG00000206538:SILENT[ENST00000398399(c.435T>C,agT>agC):ENSP00000381436(p.Ser145(=))];ENSG00000206538:SILENT[ENST00000383698(c.435T>C,agT>agC):ENSP00000373199(p.Ser145(=))];	NA	NA	NA	NA	NA	NA	NA	NA	NA	NA	LOW	NA	0.998	44	43	0	40	15	25	62.5
Smoker	42475	chr3	42949655	C	A	*ZNF662*	INTRONIC	ENSG00000182983:[ENST00000422021(c.34+15C>A)];ENSG00000182983:[ENST00000328199(c.217+15C>A)];ENSG00000182983:[ENST00000440367(c.34+15C>A)];ENSG00000182983:[ENST00000541208(c.34+15C>A)];ENSG00000182983:[ENST00000475386(n.410C>A)];	NA	NA	chr3*42949655*42949655*C*G*rs775648224*NA*NA*NA	NA	NA	NA	NA	NA	NA	NA	MODIFIER	NA	NA	86	86	0	92	78	14	15.22
Smoker	42475	chr4	92246294	G	T	*CCSER1*	INTRONIC	ENSG00000184305:[ENST00000509176(c.2217+239149G>T)];ENSG00000184305:[ENST00000333691(c.2217+239149G>T)];	NA	NA	NA	NA	NA	NA	NA	NA	PoD	NA	MODERATE	NA	NA	57	57	0	87	38	49	56.32
Smoker	42475	chr4	153897318	C	T	*FHDC1*	MISSENSE	ENSG00000137460:MISSENSE[ENST00000511601(c.2875C>T,Cgg>Tgg):ENSP00000427567(p.Arg959Trp)];ENSG00000137460:MISSENSE[ENST00000260008(c.2875C>T,Cgg>Tgg):ENSP00000260008(p.Arg959Trp)];	NA	NA	chr4*153897318*153897319*CG*C*rs768285338*NA*NA*NA	NA	NA	NA	NA	D	PrD	D	MODERATE	N	0.024	61	60	1	71	23	48	67.61
Smoker	42475	chr4	190878571	G	A	*FRG1*	MISSENSE	ENSG00000109536:MISSENSE[ENST00000226798(c.451G>A,Gcc>Acc):ENSP00000226798(p.Ala151Thr)];ENSG00000109536:MISSENSE[ENST00000531991(c.262G>A,Gcc>Acc):ENSP00000435943(p.Ala88Thr)];ENSG00000109536:MISSENSE[ENST00000524583(c.67G>A,Gcc>Acc):ENSP00000435067(p.Ala23Thr)];ENSG00000109536:EXONIC-NC[ENST00000514482(n.437G>A)];	NA	NA	NA	0.0007346	0.000371858	NA	NA	NA	NA	NA	MODIFIER	NA	NA	535	522	6	608	576	21	3.45
Smoker	42475	chr4	190394548	G	T	*HSP90AA4P*	EXONIC-NC	ENSG00000205100:[ENST00000378770(n.248G>T)];ENSG00000205100:[ENST00000506915(n.430G>T)];	NA	NA	NA	NA	NA	NA	NA	NA	NA	NA	MODIFIER	NA	NA	224	224	0	340	130	209	61.47
Smoker	42475	chr4	38690494	C	T	*KLF3*	MISSENSE	ENSG00000109787:MISSENSE[ENST00000514033(c.346C>T,Ccc>Tcc):ENSP00000421252(p.Pro116Ser)];ENSG00000109787:MISSENSE[ENST00000261438(c.346C>T,Ccc>Tcc):ENSP00000261438(p.Pro116Ser)];	NA	NA	NA	NA	NA	NA	NA	T	BN	N	MODERATE	D	0.928	214	214	0	240	80	159	66.25

Mangalaparthi *et al.* , 2020. Mutational landscape of esophageal squamous cell carcinoma in an Indian cohort
Supplementary Table 4. List of somatic single nucleotide variants identified using whole exome sequencing of ESCC patients

Cohort	Sample	Chromosome	Genomic position	Reference allele	Alternate allele	Gene symbol	Variant type	Variant annotation	OncoMD	COSMIC	dbSNP	ExAC	ExAC_SAS	1000G	1000G_SAS	SIFT	Polyphen2	CONDEL	VEP	LRT	PhastCon7 Way	Total read (Adjacent normal)	Reference allel read support (Adjacent normal)	Alternative read support (Adjacent normal)	Total read (Tumor)	Reference allel read support (Tumor)	Alternative read support (Tumor)	Alternate allele frequency
Smoker	42475	chr4	62542644	G	T	*LPHN3*	MISSENSE	ENSG00000150471:MISSENSE[ENST00000506700(c.370G>T,Gtc>Ttc):ENSP00000424120(p.Val124Phe)];ENSG00000150471:MISSENSE[ENST00000508693(c.574G>T,Gtc>Ttc):ENSP00000424030(p.Val192Phe)];ENSG00000150471:MISSENSE[ENST00000514157(c.370G>T,Gtc>Ttc):ENSP00000425201(p.Val124Phe)];ENSG00000150471:MISSENSE[ENST00000512091(c.370G>T,Gtc>Ttc):ENSP00000423388(p.Val124Phe)];ENSG00000150471:MISSENSE[ENST00000511324(c.574G>T,Gtc>Ttc):ENSP00000425033(p.Val192Phe)];ENSG00000150471:MISSENSE[ENST00000506720(c.574G>T,Gtc>Ttc):ENSP00000420931(p.Val192Phe)];ENSG00000150471:MISSENSE[ENST00000545650(c.370G>T,Gtc>Ttc):ENSP00000439831(p.Val124Phe)];ENSG00000150471:MISSENSE[ENST00000507625(c.574G>T,Gtc>Ttc):ENSP00000421372(p.Val192Phe)];ENSG00000150471:MISSENSE[ENST00000504896(c.370G>T,Gtc>Ttc):ENSP00000423434(p.Val124Phe)];ENSG00000150471:MISSENSE[ENST00000508946(c.370G>T,Gtc>Ttc):ENSP00000421627(p.Val124Phe)];ENSG00000150471:MISSENSE[ENST00000514591(c.370G>T,Gtc>Ttc):ENSP00000422533(p.Val124Phe)];ENSG00000150471:MISSENSE[ENST00000514996(c.370G>T,Gtc>Ttc):ENSP00000424258(p.Val124Phe)];ENSG00000150471:MISSENSE[ENST00000506746(c.574G>T,Gtc>Ttc):ENSP00000425884(p.Val192Phe)];ENSG00000150471:MISSENSE[ENST00000509896(c.574G>T,Gtc>Ttc):ENSP00000423787(p.Val192Phe)];ENSG00000150471:MISSENSE[ENST00000507164(c.574G>T,Gtc>Ttc):ENSP00000421476(p.Val192Phe)];ENSG00000150471:EXONIC-NC[ENST00000509089(n.174G>T)];	NA	NA	NA	NA	NA	NA	NA	NA	NA	NA	MODIFIER	NA	NA	49	49	0	74	31	43	58.11
Smoker	42475	chr4	146567369	G	A	*MMAA*	EXONIC-NC	ENSG00000151611:[ENST00000506919(n.1282G>A)];ENSG00000151611:[ENST00000281317(c.733+61G>A)];ENSG00000151611:[ENST00000541599(c.-25+61G>A)];ENSG00000151611:[ENST00000511969(c.733+61G>A)];	NA	NA	NA	NA	NA	NA	NA	NA	NA	NA	MODIFIER	NA	NA	56	56	0	66	22	44	66.67
Smoker	42475	chr4	82096007	G	T	*PRKG2*	MISSENSE	ENSG00000138669:MISSENSE[ENST00000418486(c.568C>A,Cag>Aag):ENSP00000389038(p.Gln190Lys)];ENSG00000138669:MISSENSE[ENST00000395578(c.568C>A,Cag>Aag):ENSP00000378945(p.Gln190Lys)];ENSG00000138669:MISSENSE[ENST00000264399(c.568C>A,Cag>Aag):ENSP00000264399(p.Gln190Lys)];	NA	NA	chr4*82096007*82096007*G*A*rs867102677*NA*NA*NA	NA	NA	NA	NA	NA	NA	NA	MODIFIER	NA	NA	229	227	2	353	151	201	56.94
Smoker	42475	chr4	82096007	G	T	*RP11-100N20.1*	INTRONIC	ENSG00000251059:[ENST00000512502(n.232-591G>T)];ENSG00000251059:[ENST00000505175(n.57-591G>T)];	NA	NA	chr4*82096007*82096007*G*A*rs867102677*NA*NA*NA	NA	NA	NA	NA	NA	NA	NA	MODIFIER	NA	NA	229	227	2	353	151	201	56.94
Smoker	42475	chr4	146567369	G	A	*RP11-557J10.4*	INTRONIC	ENSG00000248356:[ENST00000504555(n.254-3390C>T)];	NA	NA	NA	NA	NA	NA	NA	NA	NA	NA	MODIFIER	NA	NA	56	56	0	66	22	44	66.67
Smoker	42475	chr4	92246294	G	T	*RP11-763F8.1*	MISSENSE	ENSG00000249049:MISSENSE[ENST00000502368(c.109C>A,Cca>Aca):ENSP00000422924(p.Pro37Thr)];	NA	NA	NA	NA	NA	NA	NA	NA	PoD	NA	MODERATE	NA	NA	57	57	0	87	38	49	56.32
Smoker	42475	chr4	100571345	C	T	*RP11-766F14.2*	SILENT	ENSG00000248713:SILENT[ENST00000511828(c.4461G>A,agG>agA):ENSP00000427555(p.Arg1487(=))];	NA	NA	NA	NA	NA	NA	NA	NA	NA	NA	LOW	NA	NA	95	94	0	117	46	71	60.68
Smoker	42475	chr4	52926993	C	A	*SPATA18*	MISSENSE	ENSG00000163071:MISSENSE[ENST00000295213(c.239C>A,cCt>cAt):ENSP00000295213(p.Pro80His)];ENSG00000163071:MISSENSE[ENST00000419395(c.239C>A,cCt>cAt):ENSP00000415309(p.Pro80His)];ENSG00000163071:MISSENSE[ENST00000505038(c.239C>A,cCt>cAt):ENSP00000424656(p.Pro80His)];ENSG00000163071:MISSENSE[ENST00000505320(c.239C>A,cCt>cAt):ENSP00000421204(p.Pro80His)];ENSG00000163071:MISSENSE[ENST00000503055(c.133C>A,Ctt>Att):ENSP00000424695(p.Leu45Ile)];ENSG00000163071:EXONIC-NC[ENST00000511028(n.525C>A)];ENSG00000163071:INTRONIC[ENST00000514670(n.383+9036C>A)];ENSG00000163071:INTRONIC[ENST00000506829(n.380+9036C>A)];ENSG00000163071:INTRONIC[ENST00000508915(c.88-8994C>A)];	NA	NA	NA	NA	NA	NA	NA	NA	NA	NA	MODIFIER	NA	NA	103	103	0	122	52	70	57.38
Smoker	42475	chr4	1976959	C	T	*WHSC1*	INTRONIC	ENSG00000109685:[ENST00000508803(c.3515-62C>T)];ENSG00000109685:[ENST00000382888(c.1559-62C>T)];ENSG00000109685:[ENST00000382892(c.3515-62C>T)];ENSG00000109685:[ENST00000382895(c.3515-62C>T)];ENSG00000109685:[ENST00000515695(n.219-62C>T)];ENSG00000109685:[ENST00000353275(c.*1665-62C>T)];ENSG00000109685:[ENST00000382891(c.3515-62C>T)];ENSG00000109685:[ENST00000312087(c.*1798-62C>T)];ENSG00000109685:[ENST00000482415(n.2132-62C>T)];	NA	NA	NA	NA	NA	NA	NA	NA	NA	NA	MODIFIER	NA	NA	99	99	0	96	36	60	62.5
Smoker	42475	chr5	33988396	G	A	*AMACR*	3UTR	ENSG00000242110:[ENST00000506639(c.*1273C>T)];ENSG00000242110:[ENST00000382072(c.*1193C>T)];ENSG00000242110:[ENST00000335606(c.*802C>T)];ENSG00000242110:[ENST00000514195(n.1845C>T)];	NA	NA	NA	NA	NA	NA	NA	NA	NA	NA	MODIFIER	NA	NA	109	109	0	261	200	61	23.37
Smoker	42475	chr5	115394566	C	A	*ARL14EPL*	MISSENSE	ENSG00000268223:MISSENSE[ENST00000601302(c.381C>A,aaC>aaA):ENSP00000472409(p.Asn127Lys)];	NA	NA	NA	NA	NA	NA	NA	NA	NA	NA	MODERATE	NA	NA	51	51	0	93	77	16	17.2
Smoker	42475	chr5	878460	C	T	*BRD9*	INTRONIC-SS-DNR	ENSG00000028310:[ENST00000467963(c.1271+10G>A)];ENSG00000028310:[ENST00000493082(n.438+10G>A)];ENSG00000028310:[ENST00000323510(c.983+10G>A)];ENSG00000028310:[ENST00000388890(c.923+10G>A)];ENSG00000028310:[ENST00000483234(n.961+10G>A)];ENSG00000028310:[ENST00000489816(c.*963+10G>A)];ENSG00000028310:[ENST00000495265(c.*1023+10G>A)];ENSG00000028310:[ENST00000495794(n.3615+10G>A)];ENSG00000028310:[ENST00000519838(n.343+10G>A)];ENSG00000028310:[ENST00000519112(c.133+10G>A)];ENSG00000028310:[ENST00000466684(c.*887+10G>A)];ENSG00000028310:[ENST00000435709(c.*202+10G>A)];ENSG00000028310:[ENST00000483173(c.1112+10G>A)];ENSG00000028310:[ENST00000475706(n.305+10G>A)];ENSG00000028310:[ENST00000490814(c.*1236+10G>A)];ENSG00000028310:[ENST00000518251(c.86+10G>A)];	NA	NA	chr5*878460*878460*C*A,T*rs112921031*NA*NA*NA	0.0465886	0.034103899	NA	NA	NA	NA	NA	MODIFIER	NA	NA	97	97	0	260	244	16	6.15
Smoker	42475	chr5	40955588	G	C	*C7*	MISSENSE	ENSG00000112936:MISSENSE[ENST00000313164(c.1193G>C,gGa>gCa):ENSP00000322061(p.Gly398Ala)];	NA	NA	NA	NA	NA	NA	NA	T	BN	N	MODERATE	N	0.993	80	80	0	263	181	81	30.8
Smoker	42475	chr5	1324850	C	A	*CLPTM1L*	INTRONIC	ENSG00000049656:[ENST00000508765(n.548+28G>T)];ENSG00000049656:[ENST00000507195(n.293+28G>T)];ENSG00000049656:[ENST00000505605(n.95+28G>T)];ENSG00000049656:[ENST00000503534(n.128+28G>T)];ENSG00000049656:[ENST00000503042(n.2619+28G>T)];ENSG00000049656:[ENST00000320927(c.1089+28G>T)];ENSG00000049656:[ENST00000503151(n.466+28G>T)];ENSG00000049656:[ENST00000513250(n.530+28G>T)];ENSG00000049656:[ENST00000320895(c.1197+28G>T)];ENSG00000049656:[ENST00000507807(c.690+28G>T)];ENSG00000049656:[ENST00000506641(n.358+28G>T)];	NA	NA	NA	NA	NA	NA	NA	NA	NA	NA	MODIFIER	NA	NA	57	57	0	194	165	29	14.95
Smoker	42475	chr5	115394566	C	A	*CTD-2287O16.3*	MISSENSE	ENSG00000268714:MISSENSE[ENST00000600981(c.361C>A,aaC>aaA):ENSP00000470389(p.Asn121Lys)];	NA	NA	NA	NA	NA	NA	NA	NA	NA	NA	MODERATE	NA	NA	51	51	0	93	77	16	17.2

Mangalaparthi *et al.* , 2020. Mutational landscape of esophageal squamous cell carcinoma in an Indian cohort
Supplementary Table 4. List of somatic single nucleotide variants identified using whole exome sequencing of ESCC patients

Cohort	Sample	Chromosome	Genomic position	Reference allele	Alternate allele	Gene symbol	Variant type	Variant annotation	OncoMD	COSMIC	dbSNP	ExAC	ExAC_SAS	1000G	1000G_SAS	SIFT	Polyphen2	CONDEL	VEP	LRT	PhastCon7 Way	Total read (Adjacent normal)	Reference allel read support (Adjacent normal)	Alternative read support (Adjacent normal)	Total read (Tumor)	Reference allel read support (Tumor)	Alternative read support (Tumor)	Alternate allele frequency
Smoker	42475	chr5	158511755	C	A	*EBF1*	INTRONIC-SS-ACR	ENSG00000164330:[ENST00000380654(c.412-8G>T)];ENSG00000164330:[ENST00000518836(n.572-8G>T)];ENSG00000164330:[ENST00000313708(c.412-8G>T)];ENSG00000164330:[ENST00000522192(n.321-8G>T)];ENSG00000164330:[ENST00000519739(n.180-8G>T)];ENSG00000164330:[ENST00000523315(n.417-8G>T)];ENSG00000164330:[ENST00000517373(c.412-8G>T)];	NA	NA	NA	NA	NA	NA	NA	NA	NA	NA	LOW	NA	NA	132	128	4	159	142	9	5.66
Smoker	42475	chr5	127648423	G	T	*FBN2*	MISSENSE	ENSG00000138829:MISSENSE[ENST00000262464(c.4782C>A,aaC>aaA):ENSP00000262464(p.Asn1594Lys)];ENSG00000138829:MISSENSE[ENST00000508053(c.4782C>A,aaC>aaA):ENSP00000424571(p.Asn1594Lys)];	NA	NA	NA	NA	NA	NA	NA	T	BN	N	MODERATE	D	0.998	147	147	0	202	151	50	24.75
Smoker	42475	chr5	140572261	G	T	*PCDHB10*	MISSENSE	ENSG00000120324:MISSENSE[ENST00000239446(c.136G>T,Gtc>Ttc):ENSP00000239446(p.Val46Phe)];	NA	NA	chr5*140572258*140572265*GTGGTCAA*G*rs782214541*NA*NA*NA	NA	NA	NA	NA	D_lc	PoD	D	MODERATE	NA	NA	45	45	0	54	47	6	11.11
Smoker	42475	chr5	140572267	C	A	*PCDHB10*	MISSENSE	ENSG00000120324:MISSENSE[ENST00000239446(c.142C>A,Ctg>Atg):ENSP00000239446(p.Leu48Met)];	NA	NA	NA	NA	NA	NA	NA	D_lc	PrD	D	MODERATE	NA	NA	42	42	0	53	40	13	24.53
Smoker	42475	chr5	33988396	G	A	*RP11-1084J3.4*	3UTR	ENSG00000273294:[ENST00000382079(c.*1377C>T)];	NA	NA	NA	NA	NA	NA	NA	NA	NA	NA	MODIFIER	NA	NA	109	109	0	261	200	61	23.37
Smoker	42475	chr5	9318395	C	A	*SEMA5A*	INTRONIC	ENSG00000112902:[ENST00000513968(c.270+89G>T)];ENSG00000112902:[ENST00000382496(c.270+89G>T)];ENSG00000112902:[ENST00000509486(c.270+89G>T)];	NA	NA	NA	NA	NA	NA	NA	NA	NA	NA	MODIFIER	NA	NA	58	58	0	134	88	46	34.33
Smoker	42475	chr5	101735260	G	T	*SLCO6A1*	SILENT-SS-PRX	ENSG00000205359:[ENST00000379807(c.1813C>A,Cgg>Agg):ENSP00000369135(p.Arg605(=))];ENSG00000205359:[ENST00000506729(c.1813C>A,Cgg>Agg):ENSP00000421339(p.Arg605(=))];ENSG00000205359:[ENST00000513675(c.1054C>A,Cgg>Agg):ENSP00000421990(p.Arg352(=))];ENSG00000205359:[ENST00000389019(c.1627C>A,Cgg>Agg):ENSP00000373671(p.Arg543(=))];ENSG00000205359:[ENST00000379810(c.1054C>A,Cgg>Agg):ENSP00000369138(p.Arg352(=))];	NA	NA	chr5*101735260*101735260*G*A*rs777289666*NA*NA*NA	NA	NA	NA	NA	NA	NA	NA	LOW	NA	0.06	58	58	0	41	24	16	39.02
Smoker	42475	chr5	151052690	T	A	*SPARC*	MISSENSE-SS-PRX	ENSG00000113140:[ENST00000231061(c.208A>T,Aat>Tat):ENSP00000231061(p.Asn70Tyr)];ENSG00000113140:[ENST00000539687(c.208A>T,Aat>Tat):ENSP00000444998(p.Asn70Tyr)];ENSG00000113140:[ENST00000538026(c.-65-1435A>T)];ENSG00000113140:[ENST00000521327(n.332A>T)];ENSG00000113140:[ENST00000521569(c.-66A>T)];	NA	NA	NA	NA	NA	NA	NA	NA	NA	NA	LOW	NA	NA	83	82	0	102	49	53	51.96
Smoker	42475	chr6	131540792	C	T	*AKAP7*	INTRONIC-SS-ACR	ENSG00000118507:[ENST00000263050(c.-90-9C>T)];ENSG00000118507:[ENST00000537868(c.-90-9C>T)];ENSG00000118507:[ENST00000543815(n.323-9C>T)];ENSG00000118507:[ENST00000368123(c.637-9C>T)];ENSG00000118507:[ENST00000535150(n.448-9C>T)];ENSG00000118507:[ENST00000541650(c.700-9C>T)];ENSG00000118507:[ENST00000431975(c.703-9C>T)];	NA	NA	NA	NA	NA	NA	NA	NA	NA	NA	MODIFIER	NA	NA	20	20	0	47	23	23	48.94
Smoker	42475	chr6	55739272	C	G	*BMP5*	MISSENSE	ENSG00000112175:MISSENSE[ENST00000370830(c.392G>C,cGg>cCg):ENSP00000359866(p.Arg131Pro)];ENSG00000112175:MISSENSE[ENST00000446683(c.392G>C,cGg>cCg):ENSP00000391818(p.Arg131Pro)];	chr6*55739	chr6*55739	chr6*55739272*55739272*C*T*rs184688249*0*0.9998*0.0001997	NA	NA	NA	NA	T	BN	N	MODERATE	N	0.685	92	92	0	156	127	29	18.59
Smoker	42475	chr6	38893845	G	A	*DNAH8*	SILENT	ENSG00000124721:SILENT[ENST00000359357(c.10326G>A,ggG>ggA):ENSP00000352312(p.Gly3442(=))];ENSG00000124721:SILENT[ENST00000327475(c.10941G>A,ggG>ggA):ENSP00000333363(p.Gly3647(=))];ENSG00000124721:SILENT[ENST00000441566(c.10218G>A,ggG>ggA):ENSP00000402294(p.Gly3406(=))];ENSG00000124721:SILENT[ENST00000449981(c.10977G>A,ggG>ggA):ENSP00000415331(p.Gly3659(=))];	NA	NA	NA	NA	NA	NA	NA	NA	NA	NA	MODIFIER	NA	NA	88	88	0	118	95	22	18.64
Smoker	42475	chr6	56468408	A	G	*DST*	MISSENSE	ENSG00000151914:MISSENSE[ENST00000446842(c.9284T>C,cTg>cCg):ENSP00000393645(p.Leu3095Pro)];ENSG00000151914:MISSENSE[ENST00000370754(c.10796T>C,cTg>cCg):ENSP00000359790(p.Leu3599Pro)];ENSG00000151914:MISSENSE[ENST00000361203(c.10262T>C,cTg>cCg):ENSP00000354508(p.Leu3421Pro)];ENSG00000151914:MISSENSE[ENST00000370769(c.10262T>C,cTg>cCg):ENSP00000359805(p.Leu3421Pro)];ENSG00000151914:MISSENSE[ENST00000312431(c.10262T>C,cTg>cCg):ENSP00000307959(p.Leu3421Pro)];ENSG00000151914:INTRONIC[ENST00000370788(c.4651-3389T>C)];ENSG00000151914:INTRONIC[ENST00000244364(c.3673-3389T>C)];ENSG00000151914:INTRONIC[ENST00000421834(c.4651-3389T>C)];	NA	NA	NA	NA	NA	NA	NA	NA	NA	NA	MODIFIER	NA	NA	58	58	0	88	73	15	17.05
Smoker	42475	chr6	346308	T	C	*DUSP22*	INTRONIC	ENSG00000112679:[ENST00000419235(c.263+380T>C)];ENSG00000112679:[ENST00000605863(c.-47+380T>C)];ENSG00000112679:[ENST00000603726(c.*48+380T>C)];ENSG00000112679:[ENST00000605315(c.-47+380T>C)];ENSG00000112679:[ENST00000603005(n.185+380T>C)];ENSG00000112679:[ENST00000603453(c.-47+380T>C)];ENSG00000112679:[ENST00000604988(n.151+380T>C)];ENSG00000112679:[ENST00000344450(c.263+380T>C)];ENSG00000112679:[ENST00000603795(c.*48+380T>C)];ENSG00000112679:[ENST00000605391(n.488+380T>C)];ENSG00000112679:[ENST00000604971(c.-47+380T>C)];ENSG00000112679:[ENST00000604914(n.224+380T>C)];ENSG00000112679:[ENST00000603881(c.-75+380T>C)];ENSG00000112679:[ENST00000605035(c.-47+380T>C)];ENSG00000112679:[ENST00000603296(c.134+380T>C)];	NA	NA	NA	NA	NA	NA	NA	NA	NA	NA	MODIFIER	NA	NA	342	342	0	551	459	91	16.52
Smoker	42475	chr6	656803	C	T	*EXOC2*	INTRONIC	ENSG00000112685:[ENST00000230449(c.-43-18942G>A)];ENSG00000112685:[ENST00000448181(c.-24+36216G>A)];ENSG00000112685:[ENST00000443083(c.-44+360G>A)];	NA	NA	chr6*656803*656803*C*G*rs778160260*NA*NA*NA	NA	NA	NA	NA	T	BN	N	MODERATE	N	0.035	71	71	0	89	55	34	38.2
Smoker	42475	chr6	89977382	G	A	*GABRR2*	INTRONIC	ENSG00000111886:[ENST00000602399(c.811+16C>T)];ENSG00000111886:[ENST00000402938(c.736+16C>T)];ENSG00000111886:[ENST00000602432(n.567+16C>T)];	NA	NA	NA	NA	NA	NA	NA	NA	NA	NA	MODIFIER	NA	NA	83	83	0	99	82	17	17.17
Smoker	42475	chr6	656803	C	T	*HUS1B*	MISSENSE	ENSG00000188996:MISSENSE[ENST00000380907(c.142G>A,Ggc>Agc):ENSP00000370293(p.Gly48Ser)];	NA	NA	chr6*656803*656803*C*G*rs778160260*NA*NA*NA	NA	NA	NA	NA	T	BN	N	MODERATE	N	0.035	71	71	0	89	55	34	38.2
Smoker	42475	chr6	84871482	C	A	*KIAA1009*	NONSENSE	ENSG00000135315:NONSENSE[ENST00000403245(c.2659G>T,Gag>Tag):ENSP00000385215(p.Glu887Ter)];ENSG00000135315:NONSENSE[ENST00000257766(c.2431G>T,Gag>Tag):ENSP00000257766(p.Glu811Ter)];ENSG00000135315:EXONIC-NC[ENST00000461137(n.1783G>T)];	NA	NA	NA	NA	NA	NA	NA	NA	NA	NA	MODIFIER	NA	NA	37	37	0	65	44	21	32.31

Mangalaparthi *et al.* , 2020. Mutational landscape of esophageal squamous cell carcinoma in an Indian cohort
Supplementary Table 4. List of somatic single nucleotide variants identified using whole exome sequencing of ESCC patients

Cohort	Sample	Chromosome	Genomic position	Reference allele	Alternate allele	Gene symbol	Variant type	Variant annotation	OncoMD	COSMIC	dbSNP	ExAC	ExAC_SAS	1000G	1000G_SAS	SIFT	Polyphen2	CONDEL	VEP	LRT	PhastCon7 Way	Total read (Adjacent normal)	Reference allel read support (Adjacent normal)	Alternative read support (Adjacent normal)	Total read (Tumor)	Reference allel read support (Tumor)	Alternative read support (Tumor)	Alternate allele frequency
Smoker	42475	chr6	138528240	C	A	*KIAA1244*	MISSENSE	ENSG00000112379:MISSENSE[ENST00000251691(c.199C>A,Cat>Aat):ENSP00000251691(p.His67Asn)];	NA	NA	NA	NA	NA	NA	NA	T	BN	N	MODERATE	NA	NA	105	104	0	111	64	47	42.34
Smoker	42475	chr6	30995416	C	T	*MUC22*	SILENT	ENSG00000261272:SILENT[ENST00000561890(c.2208C>T,acC>acT):ENSP00000455906(p.Thr736(=))];	NA	NA	NA	NA	NA	NA	NA	NA	NA	NA	LOW	NA	NA	441	439	2	524	300	224	42.75
Smoker	42475	chr6	167907877	G	T	*NA*	INTERGENIC		NA	NA	NA	NA	NA	NA	NA	NA	NA	NA	MODIFIER	NA	NA	76	76	0	105	86	19	18.1
Smoker	42475	chr6	105726120	C	T	*PREP*	MISSENSE	ENSG00000085377:MISSENSE[ENST00000369110(c.2032G>A,Gcg>Acg):ENSP00000358106(p.Ala678Thr)];	NA	NA	chr6*105726120*105726120*C*A,T*rs773468354*NA*NA*NA	8.24E-06	0	NA	NA	D	PoD	D	MODERATE	D	0.999	60	60	0	104	84	20	19.23
Smoker	42475	chr6	38893845	G	A	*RP1-207H1.3*	INTRONIC	ENSG00000231150:[ENST00000418399(n.76-1018C>T)];ENSG00000231150:[ENST00000416948(n.153-1018C>T)];	NA	NA	NA	NA	NA	NA	NA	NA	NA	NA	MODIFIER	NA	NA	88	88	0	118	95	22	18.64
Smoker	42475	chr6	25773753	G	T	*SLC17A4*	MISSENSE	ENSG00000146039:MISSENSE[ENST00000397076(c.148G>T,Ggc>Tgc):ENSP00000380266(p.Gly50Cys)];ENSG00000146039:MISSENSE[ENST00000377905(c.838G>T,Ggc>Tgc):ENSP00000367137(p.Gly280Cys)];ENSG00000146039:INTRONIC[ENST00000439485(c.298-3070G>T)];	NA	NA	NA	NA	NA	NA	NA	NA	NA	NA	MODIFIER	NA	NA	179	179	0	244	195	48	19.67
Smoker	42475	chr6	134323187	G	A	*SLC2A12*	MISSENSE	ENSG00000146411:MISSENSE[ENST00000275230(c.1648C>T,Cct>Tct):ENSP00000275230(p.Pro550Ser)];	NA	NA	NA	NA	NA	NA	NA	D	PrD	D	MODERATE	D	0.997	71	71	0	105	50	55	52.38
Smoker	42475	chr6	158514160	C	A	*SYNJ2*	INTRONIC	ENSG00000078269:[ENST00000367121(c.3744+24C>A)];ENSG00000078269:[ENST00000355585(c.3744+24C>A)];ENSG00000078269:[ENST00000367112(c.999+24C>A)];ENSG00000078269:[ENST00000367122(c.3609+24C>A)];	NA	NA	NA	NA	NA	NA	NA	NA	NA	NA	MODIFIER	NA	NA	26	26	0	55	27	28	50.91
Smoker	42475	chr6	149699879	A	G	*TAB2*	SILENT	ENSG00000055208:SILENT[ENST00000538427(c.828A>G,caA>caG):ENSP00000445752(p.Gln276(=))];ENSG00000055208:SILENT[ENST00000286332(c.828A>G,caA>caG):ENSP00000286332(p.Gln276(=))];ENSG00000055208:SILENT[ENST00000392282(c.828A>G,caA>caG):ENSP00000376106(p.Gln276(=))];ENSG00000055208:SILENT[ENST00000536230(c.732A>G,caA>caG):ENSP00000443206(p.Gln244(=))];ENSG00000055208:SILENT[ENST00000470466(c.828A>G,caA>caG):ENSP00000432709(p.Gln276(=))];ENSG00000055208:SILENT[ENST00000367456(c.828A>G,caA>caG):ENSP00000356426(p.Gln276(=))];	NA	NA	NA	8.24E-06	0	NA	NA	NA	NA	NA	LOW	NA	0.999	158	157	0	191	124	66	34.55
Smoker	42475	chr6	80717696	G	A	*TTK*	MISSENSE	ENSG00000112742:MISSENSE[ENST00000369798(c.310G>A,Ggc>Agc):ENSP00000358813(p.Gly104Ser)];ENSG00000112742:MISSENSE[ENST00000509894(c.310G>A,Ggc>Agc):ENSP00000422936(p.Gly104Ser)];ENSG00000112742:MISSENSE[ENST00000230510(c.310G>A,Ggc>Agc):ENSP00000230510(p.Gly104Ser)];ENSG00000112742:MISSENSE[ENST00000511260(c.310G>A,Ggc>Agc):ENSP00000421636(p.Gly104Ser)];ENSG00000112742:MISSENSE[ENST00000504040(c.310G>A,Ggc>Agc):ENSP00000427483(p.Gly104Ser)];ENSG00000112742:MISSENSE[ENST00000502580(c.310G>A,Ggc>Agc):ENSP00000424851(p.Gly104Ser)];ENSG00000112742:3UTR[ENST00000509313(c.*141G>A)];ENSG00000112742:EXONIC-NC[ENST00000430061(n.213G>A)];	NA	NA	NA	NA	NA	NA	NA	T	BN	N	MODERATE	NA	NA	100	100	0	107	80	26	24.3
Smoker	42475	chr7	48545920	T	C	*ABCA13*	INTRONIC	ENSG00000179869:[ENST00000435803(c.13292-12T>C)];ENSG00000179869:[ENST00000544596(c.482-12T>C)];ENSG00000179869:[ENST00000411975(c.611-12T>C)];ENSG00000179869:[ENST00000453246(c.2088-12T>C)];	NA	NA	NA	8.29E-06	0	NA	NA	NA	NA	NA	MODIFIER	NA	NA	43	43	0	96	67	29	30.21
Smoker	42475	chr7	87913177	G	C	*AC003991.3*	EXONIC-NC	ENSG00000228113:[ENST00000595121(n.576G>C)];ENSG00000228113:[ENST00000434733(n.200G>C)];ENSG00000228113:[ENST00000600908(n.609G>C)];ENSG00000228113:[ENST00000447758(n.353-8326G>C)];	NA	NA	chr7*87913177*87913177*G*A*rs557496437*0*0.9998*0.0001997	NA	NA	NA	NA	NA	NA	NA	LOW	NA	NA	90	90	0	181	144	37	20.44
Smoker	42475	chr7	73771663	G	A	*CLIP2*	SILENT	ENSG00000106665:SILENT[ENST00000395060(c.1071G>A,ttG>ttA):ENSP00000378500(p.Leu357(=))];ENSG00000106665:SILENT[ENST00000361545(c.1071G>A,ttG>ttA):ENSP00000355151(p.Leu357(=))];ENSG00000106665:SILENT[ENST00000223398(c.1071G>A,ttG>ttA):ENSP00000223398(p.Leu357(=))];	NA	NA	NA	NA	NA	NA	NA	NA	NA	NA	LOW	NA	0.987	83	83	0	140	101	38	27.14
Smoker	42475	chr7	73771664	C	A	*CLIP2*	MISSENSE	ENSG00000106665:MISSENSE[ENST00000395060(c.1072C>A,Cag>Aag):ENSP00000378500(p.Gln358Lys)];ENSG00000106665:MISSENSE[ENST00000361545(c.1072C>A,Cag>Aag):ENSP00000355151(p.Gln358Lys)];ENSG00000106665:MISSENSE[ENST00000223398(c.1072C>A,Cag>Aag):ENSP00000223398(p.Gln358Lys)];	NA	NA	NA	NA	NA	NA	NA	D	BN	D	MODERATE	D	0.992	83	83	0	141	102	38	26.95
Smoker	42475	chr7	135098264	C	T	*CNOT4*	SILENT	ENSG00000080802:SILENT[ENST00000356162(c.660G>A,gcG>gcA):ENSP00000348485(p.Ala220(=))];ENSG00000080802:SILENT[ENST00000541284(c.660G>A,gcG>gcA):ENSP00000445508(p.Ala220(=))];ENSG00000080802:SILENT[ENST00000451834(c.660G>A,gcG>gcA):ENSP00000388491(p.Ala220(=))];ENSG00000080802:SILENT[ENST00000414802(c.660G>A,gcG>gcA):ENSP00000416532(p.Ala220(=))];ENSG00000080802:SILENT[ENST00000423368(c.660G>A,gcG>gcA):ENSP00000406777(p.Ala220(=))];ENSG00000080802:SILENT[ENST00000361528(c.660G>A,gcG>gcA):ENSP00000354673(p.Ala220(=))];ENSG00000080802:SILENT[ENST00000428680(c.660G>A,gcG>gcA):ENSP00000399108(p.Ala220(=))];ENSG00000080802:SILENT[ENST00000315544(c.660G>A,gcG>gcA):ENSP00000326731(p.Ala220(=))];	NA	NA	0.0001997	2.49E-05	6.06E-05	0.0001997	0	NA	NA	NA	LOW	NA	NA	143	143	0	200	93	107	53.5
Smoker	42475	chr7	51096174	G	T	*COBL*	SILENT	ENSG00000106078:SILENT[ENST00000431948(c.2274C>A,gcC>gcA):ENSP00000413498(p.Ala758(=))];ENSG00000106078:SILENT[ENST00000265136(c.2619C>A,gcC>gcA):ENSP00000265136(p.Ala873(=))];ENSG00000106078:SILENT[ENST00000395542(c.2865C>A,gcC>gcA):ENSP00000378912(p.Ala955(=))];ENSG00000106078:SILENT[ENST00000445054(c.2295C>A,gcC>gcA):ENSP00000401204(p.Ala765(=))];ENSG00000106078:EXONIC-NC[ENST00000462395(n.6259C>A)];	NA	NA	NA	NA	NA	NA	NA	NA	NA	NA	LOW	NA	NA	153	152	0	267	156	111	41.57
Smoker	42475	chr7	95439882	A	T	*DYNC1I1*	INTRONIC	ENSG00000158560:[ENST00000359388(c.223+64A>T)];ENSG00000158560:[ENST00000518089(c.223+64A>T)];ENSG00000158560:[ENST00000457059(c.223+64A>T)];ENSG00000158560:[ENST00000524053(c.223+64A>T)];ENSG00000158560:[ENST00000537881(c.223+64A>T)];ENSG00000158560:[ENST00000447467(c.223+64A>T)];ENSG00000158560:[ENST00000437599(c.223+64A>T)];ENSG00000158560:[ENST00000324972(c.223+64A>T)];ENSG00000158560:[ENST00000413338(c.223+64A>T)];	NA	NA	NA	NA	NA	NA	NA	NA	NA	NA	MODIFIER	NA	NA	81	81	0	109	70	39	35.78
Smoker	42475	chr7	30793464	G	T	*INMT*	MISSENSE	ENSG00000241644:MISSENSE[ENST00000409539(c.269G>T,cGg>cTg):ENSP00000386961(p.Arg90Leu)];ENSG00000241644:MISSENSE[ENST00000013222(c.272G>T,cGg>cTg):ENSP00000013222(p.Arg91Leu)];ENSG00000241644:EXONIC-NC[ENST00000484180(n.418G>T)];	chr7*30793	chr7*30793	chr7*30793464*30793464*G*A*rs200629016*NA*NA*NA	NA	NA	NA	NA	T	BN	N	MODERATE	NA	NA	84	84	0	123	82	41	33.33

Mangalaparthi *et al.* , 2020. Mutational landscape of esophageal squamous cell carcinoma in an Indian cohort
Supplementary Table 4. List of somatic single nucleotide variants identified using whole exome sequencing of ESCC patients

Cohort	Sample	Chromosome	Genomic position	Reference allele	Alternate allele	Gene symbol	Variant type	Variant annotation	OncoMD	COSMIC	dbSNP	ExAC	ExAC_SAS	1000G	1000G_SAS	SIFT	Polyphen2	CONDEL	VEP	LRT	PhastCon7 Way	Total read (Adjacent normal)	Reference allel read support (Adjacent normal)	Alternative read support (Adjacent normal)	Total read (Tumor)	Reference allel read support (Tumor)	Alternative read support (Tumor)	Alternate allele frequency
Smoker	42475	chr7	30793464	G	T	*INMT-FAM188B*	MISSENSE	ENSG00000254959:MISSENSE[ENST00000458257(c.269G>T,cGg>cTg):ENSP00000456039(p.Arg90Leu)];ENSG00000254959:MISSENSE[ENST00000451002(c.270G>T,cGg>cTg):ENSP00000456833(p.Arg91Leu)];	chr7*30793	chr7*30793	chr7*30793464*30793464*G*A*rs200629016*NA*NA*NA	NA	NA	NA	NA	T	BN	N	MODERATE	NA	NA	84	84	0	123	82	41	33.33
Smoker	42475	chr7	2598784	A	G	*IQCE*	EXONIC-NC	ENSG00000106012:[ENST00000476665(n.72A>G)];ENSG00000106012:[ENST00000470731(n.103A>G)];ENSG00000106012:[ENST00000325997(c.-32A>G)];ENSG00000106012:[ENST00000404984(c.-32A>G)];ENSG00000106012:[ENST00000402050(c.-32A>G)];ENSG00000106012:[ENST00000415271(c.-32A>G)];ENSG00000106012:[ENST00000438376(c.-32A>G)];	NA	NA	chr7*2598784*2598784*A*T*rs555742939*0*0.9996*0.0003994	NA	NA	NA	NA	NA	NA	NA	MODIFIER	NA	NA	26	26	0	45	31	14	31.11
Smoker	42475	chr7	44179147	G	T	*MYL7*	SILENT	ENSG00000106631:SILENT[ENST00000457314(c.465C>A,acC>acA):ENSP00000389202(p.Thr155(=))];ENSG00000106631:SILENT[ENST00000446581(c.180C>A,acC>acA):ENSP00000416010(p.Thr60(=))];ENSG00000106631:SILENT[ENST00000447951(c.486C>A,acC>acA):ENSP00000403988(p.Thr162(=))];ENSG00000106631:SILENT[ENST00000458240(c.318C>A,acC>acA):ENSP00000403360(p.Thr106(=))];ENSG00000106631:SILENT[ENST00000223364(c.399C>A,acC>acA):ENSP00000223364(p.Thr133(=))];ENSG00000106631:EXONIC-NC[ENST00000476118(n.345C>A)];ENSG00000106631:EXONIC-NC[ENST00000434895(n.453C>A)];	NA	NA	NA	NA	NA	NA	NA	NA	NA	NA	MODIFIER	NA	NA	81	80	0	113	81	32	28.32
Smoker	42475	chr7	82579098	A	T	*PCLO*	SILENT	ENSG00000186472:SILENT[ENST00000437081(c.966T>A,tcT>tcA):ENSP00000393760(p.Ser322(=))];ENSG00000186472:SILENT[ENST00000333891(c.10806T>A,tcT>tcA):ENSP00000334319(p.Ser3602(=))];ENSG00000186472:SILENT[ENST00000423517(c.10806T>A,tcT>tcA):ENSP00000388393(p.Ser3602(=))];	NA	NA	NA	NA	NA	NA	NA	NA	NA	NA	LOW	NA	NA	143	142	0	314	224	89	28.34
Smoker	42475	chr7	82582622	T	A	*PCLO*	SILENT	ENSG00000186472:SILENT[ENST00000333891(c.7647A>T,tcA>tcT):ENSP00000334319(p.Ser2549(=))];ENSG00000186472:SILENT[ENST00000423517(c.7647A>T,tcA>tcT):ENSP00000388393(p.Ser2549(=))];	NA	NA	chr7*82582622*82582622*T*C*rs755467556*NA*NA*NA	NA	NA	NA	NA	NA	NA	NA	LOW	NA	NA	137	135	0	277	199	77	27.8
Smoker	42475	chr7	82763797	T	G	*PCLO*	SILENT	ENSG00000186472:SILENT[ENST00000333891(c.3069A>C,acA>acC):ENSP00000334319(p.Thr1023(=))];ENSG00000186472:SILENT[ENST00000423517(c.3069A>C,acA>acC):ENSP00000388393(p.Thr1023(=))];ENSG00000186472:EXONIC-NC[ENST00000461143(n.130A>C)];	NA	NA	chr7*82763797*82763797*T*C*rs769949632*NA*NA*NA	NA	NA	NA	NA	NA	NA	NA	MODIFIER	NA	NA	157	155	0	228	145	83	36.4
Smoker	42475	chr7	103191645	G	T	*RELN*	SILENT	ENSG00000189056:SILENT[ENST00000424685(c.6171C>A,ctC>ctA):ENSP00000388446(p.Leu2057(=))];ENSG00000189056:SILENT[ENST00000428762(c.6171C>A,ctC>ctA):ENSP00000392423(p.Leu2057(=))];ENSG00000189056:SILENT[ENST00000343529(c.6171C>A,ctC>ctA):ENSP00000345694(p.Leu2057(=))];	NA	NA	NA	NA	NA	NA	NA	NA	NA	NA	LOW	NA	NA	97	97	0	132	89	43	32.58
Smoker	42475	chr7	122821181	A	G	*SLC13A1*	5UTR	ENSG00000081800:[ENST00000539873(c.-119T>C)];ENSG00000081800:[ENST00000439260(c.*133-26T>C)];ENSG00000081800:[ENST00000194130(c.100-26T>C)];ENSG00000081800:[ENST00000427975(c.*43-26T>C)];	NA	NA	NA	NA	NA	NA	NA	NA	NA	NA	MODIFIER	NA	NA	106	106	0	121	96	25	20.66
Smoker	42475	chr7	87913177	G	C	*STEAP4*	SILENT	ENSG00000127954:SILENT[ENST00000301959(c.408C>G,acC>acG):ENSP00000305545(p.Thr136(=))];ENSG00000127954:SILENT[ENST00000380079(c.408C>G,acC>acG):ENSP00000369419(p.Thr136(=))];ENSG00000127954:SILENT[ENST00000414498(c.408C>G,acC>acG):ENSP00000394399(p.Thr136(=))];	NA	NA	chr7*87913177*87913177*G*A*rs557496437*0*0.9998*0.0001997	NA	NA	NA	NA	NA	NA	NA	LOW	NA	NA	90	90	0	181	144	37	20.44
Smoker	42475	chr7	142119918	C	G	*TRBV7-7*	SILENT	ENSG00000253291:SILENT[ENST00000390377(c.249G>C,cgG>cgC):ENSP00000374900(p.Arg83(=))];	NA	NA	NA	NA	NA	NA	NA	NA	NA	NA	LOW	NA	NA	210	202	1	285	263	9	3.16
Smoker	42475	chr7	134889119	C	A	*WDR91*	SILENT	ENSG00000105875:EXONIC-NC[ENST00000485942(n.259G>T)];ENSG00000105875:EXONIC-NC[ENST00000466182(n.824G>T)];ENSG00000105875:SILENT[ENST00000354475(c.792G>T,tcG>tcT):ENSP00000346466(p.Ser264(=))];ENSG00000105875:SILENT[ENST00000344400(c.792G>T,tcG>tcT):ENSP00000340877(p.Ser264(=))];ENSG00000105875:SILENT[ENST00000423565(c.687G>T,tcG>tcT):ENSP00000392555(p.Ser229(=))];	NA	NA	chr7*134889119*134889119*C*G*rs369872179*0*0.9998*0.0001997	NA	NA	NA	NA	NA	NA	NA	LOW	NA	NA	52	52	0	67	33	34	50.75
Smoker	42475	chr8	145757746	T	A	*ARHGAP39*	NONSENSE	ENSG00000147799:NONSENSE[ENST00000276826(c.2830A>T,Aag>Tag):ENSP00000276826(p.Lys944Ter)];ENSG00000147799:NONSENSE[ENST00000540274(c.2830A>T,Aag>Tag):ENSP00000445075(p.Lys944Ter)];ENSG00000147799:NONSENSE[ENST00000377307(c.2923A>T,Aag>Tag):ENSP00000366522(p.Lys975Ter)];	NA	NA	NA	NA	NA	NA	NA	NA	NA	NA	HIGH	NA	0.999	57	57	0	133	111	22	16.54
Smoker	42475	chr8	139626131	G	T	*COL22A1*	SILENT	ENSG00000169436:SILENT[ENST00000303045(c.3957C>A,ccC>ccA):ENSP00000303153(p.Pro1319(=))];ENSG00000169436:SILENT[ENST00000435777(c.3897C>A,ccC>ccA):ENSP00000387655(p.Pro1299(=))];ENSG00000169436:EXONIC-NC[ENST00000341807(n.1642C>A)];ENSG00000169436:EXONIC-NC[ENST00000487854(n.624C>A)];	NA	NA	NA	NA	NA	NA	NA	NA	NA	NA	LOW	NA	NA	23	23	0	74	41	33	44.59
Smoker	42475	chr8	104432700	C	T	*DCAF13*	SILENT	ENSG00000164934:INTRONIC-SS-DNR-PRX[ENST00000521971(c.258+9C>T)];ENSG00000164934:INTRONIC-SS-DNR-PRX[ENST00000297579(c.726+9C>T)];ENSG00000164934:INTRONIC-SS-DNR-PRX[ENST00000519682(c.258+9C>T)];ENSG00000164934:INTRONIC-SS-DNR-PRX[ENST00000518554(n.305+9C>T)];ENSG00000164934:SILENT[ENST00000521716(c.267C>T,gtC>gtT):ENSP00000430645(p.Val89(=))];ENSG00000164934:INTRONIC[ENST00000521999(n.132+4956C>T)];	NA	NA	NA	NA	NA	NA	NA	NA	NA	NA	MODIFIER	NA	NA	61	61	0	132	101	31	23.48
Smoker	42475	chr8	25189778	G	C	*DOCK5*	MISSENSE	ENSG00000147459:MISSENSE[ENST00000276440(c.1915G>C,Ggc>Cgc):ENSP00000276440(p.Gly639Arg)];ENSG00000147459:MISSENSE[ENST00000444569(c.1229G>C,Ggc>Cgc):ENSP00000414125(p.Gly411Arg)];ENSG00000147459:MISSENSE[ENST00000467709(c.134G>C,Ggc>Cgc):ENSP00000428479(p.Gly46Arg)];	NA	NA	NA	NA	NA	NA	NA	D	PoD	D	MODERATE	NA	NA	179	178	0	176	83	93	52.84
Smoker	42475	chr8	82441705	C	A	*FABP12*	NONSENSE	ENSG00000197416:NONSENSE[ENST00000519696(c.167G>T,Gag>Tag):ENSP00000427973(p.Glu57Ter)];ENSG00000197416:NONSENSE[ENST00000360464(c.214G>T,Gag>Tag):ENSP00000353650(p.Glu72Ter)];	NA	NA	NA	NA	NA	NA	NA	NA	NA	NA	MODIFIER	NA	NA	129	128	0	311	183	127	40.84
Smoker	42475	chr8	89053752	G	A	*MMP16*	SILENT	ENSG00000156103:SILENT[ENST00000286614(c.1761C>T,ttC>ttT):ENSP00000286614(p.Phe587(=))];	chr8*89053	chr8*89053	NA	NA	NA	NA	NA	NA	NA	NA	LOW	NA	1	71	71	0	210	175	35	16.67

Mangalaparthi *et al.* , 2020. Mutational landscape of esophageal squamous cell carcinoma in an Indian cohort
Supplementary Table 4. List of somatic single nucleotide variants identified using whole exome sequencing of ESCC patients

Cohort	Sample	Chromosome	Genomic position	Reference allele	Alternate allele	Gene symbol	Variant type	Variant annotation	OncoMD	COSMIC	dbSNP	ExAC	ExAC_SAS	1000G	1000G_SAS	SIFT	Polyphen2	CONDEL	VEP	LRT	PhastCon7 Way	Total read (Adjacent normal)	Reference allel read support (Adjacent normal)	Alternative read support (Adjacent normal)	Total read (Tumor)	Reference allel read support (Tumor)	Alternative read support (Tumor)	Alternate allele frequency
Smoker	42475	chr8	67513943	C	G	*MYBL1*	MISSENSE	ENSG00000185697:MISSENSE[ENST00000522677(c.188G>C,aGt>aCt):ENSP00000429633(p.Ser63Thr)];ENSG00000185697:MISSENSE[ENST00000524176(c.188G>C,aGt>aCt):ENSP00000428011(p.Ser63Thr)];ENSG00000185697:MISSENSE[ENST00000517885(c.188G>C,aGt>aCt):ENSP00000428265(p.Ser63Thr)];ENSG00000185697:EXONIC-NC[ENST00000518079(n.276G>C)];	NA	NA	NA	NA	NA	NA	NA	NA	NA	NA	MODIFIER	NA	NA	83	83	0	186	153	33	17.74
Smoker	42475	chr8	145003276	C	A	*PLEC*	SILENT	ENSG00000178209:SILENT[ENST00000322810(c.3666G>T,ctG>ctT):ENSP00000323856(p.Leu1222(=))];ENSG00000178209:SILENT[ENST00000527096(c.3324G>T,ctG>ctT):ENSP00000434583(p.Leu1108(=))];ENSG00000178209:SILENT[ENST00000345136(c.3255G>T,ctG>ctT):ENSP00000344848(p.Leu1085(=))];ENSG00000178209:SILENT[ENST00000357649(c.3267G>T,ctG>ctT):ENSP00000350277(p.Leu1089(=))];ENSG00000178209:SILENT[ENST00000398774(c.3159G>T,ctG>ctT):ENSP00000381756(p.Leu1053(=))];ENSG00000178209:SILENT[ENST00000354589(c.3255G>T,ctG>ctT):ENSP00000346602(p.Leu1085(=))];ENSG00000178209:SILENT[ENST00000356346(c.3213G>T,ctG>ctT):ENSP00000348702(p.Leu1071(=))];ENSG00000178209:SILENT[ENST00000436759(c.3336G>T,ctG>ctT):ENSP00000388180(p.Leu1112(=))];ENSG00000178209:SILENT[ENST00000354958(c.3189G>T,ctG>ctT):ENSP00000347044(p.Leu1063(=))];	NA	NA	NA	NA	NA	NA	NA	NA	NA	NA	LOW	NA	NA	29	29	0	45	38	6	13.33
Smoker	42475	chr8	94746468	C	T	*RBM12B*	MISSENSE	ENSG00000183808:MISSENSE[ENST00000399300(c.2171G>A,cGg>cAg):ENSP00000382239(p.Arg724Gln)];ENSG00000183808:INTRONIC[ENST00000517700(c.1863-52G>A)];ENSG00000183808:INTRONIC[ENST00000520961(n.242-3342G>A)];	NA	NA	NA	1.77E-05	0	NA	NA	NA	NA	NA	MODIFIER	NA	NA	50	50	0	101	65	36	35.64
Smoker	42475	chr8	82441705	C	A	*RP11-257P3.3*	INTRONIC	ENSG00000253374:[ENST00000523380(n.505+3014C>A)];	NA	NA	NA	NA	NA	NA	NA	NA	NA	NA	MODIFIER	NA	NA	129	128	0	311	183	127	40.84
Smoker	42475	chr8	77618328	G	A	*ZFHX4*	MISSENSE	ENSG00000091656:MISSENSE[ENST00000518282(c.2005G>A,Gag>Aag):ENSP00000430848(p.Glu669Lys)];ENSG00000091656:MISSENSE[ENST00000455469(c.2005G>A,Gag>Aag):ENSP00000399605(p.Glu669Lys)];ENSG00000091656:MISSENSE[ENST00000050961(c.2005G>A,Gag>Aag):ENSP00000050961(p.Glu669Lys)];ENSG00000091656:MISSENSE[ENST00000521891(c.2005G>A,Gag>Aag):ENSP00000430497(p.Glu669Lys)];ENSG00000091656:INTRONIC[ENST00000517683(n.382+24473G>A)];ENSG00000091656:EXONIC-NC[ENST00000458716(n.2392G>A)];	NA	NA	NA	NA	NA	NA	NA	NA	PoD	NA	MODERATE	U	0.998	130	130	0	239	199	40	16.74
Smoker	42475	chr8	77764255	C	A	*ZFHX4*	MISSENSE	ENSG00000091656:MISSENSE[ENST00000455469(c.4963C>A,Cac>Aac):ENSP00000399605(p.His1655Asn)];ENSG00000091656:MISSENSE[ENST00000518282(c.5020C>A,Cac>Aac):ENSP00000430848(p.His1674Asn)];ENSG00000091656:MISSENSE[ENST00000050961(c.4963C>A,Cac>Aac):ENSP00000050961(p.His1655Asn)];ENSG00000091656:MISSENSE[ENST00000521891(c.5098C>A,Cac>Aac):ENSP00000430497(p.His1700Asn)];	NA	NA	NA	NA	NA	NA	NA	NA	BN	NA	MODERATE	U	0.988	268	268	0	459	341	117	25.49
Smoker	42475	chr9	68415359	T	C	*MIR4477A*	MATURE-MIRNA	ENSG00000266017:[ENST00000581659(n.52T>C)];	NA	NA	NA	0.0071633	0.010416667	NA	NA	NA	NA	NA	MODIFIER	NA	NA	168	160	1	235	221	9	3.83
Smoker	42475	chr9	35092605	C	T	*PIGO*	MISSENSE	ENSG00000165282:EXONIC-NC[ENST00000465745(n.2280G>A)];ENSG00000165282:EXONIC-NC[ENST00000474436(n.2737G>A)];ENSG00000165282:MISSENSE[ENST00000341666(c.1279G>A,Gag>Aag):ENSP00000339382(p.Glu427Lys)];ENSG00000165282:MISSENSE[ENST00000361778(c.1279G>A,Gag>Aag):ENSP00000354678(p.Glu427Lys)];ENSG00000165282:MISSENSE[ENST00000298004(c.1279G>A,Gag>Aag):ENSP00000298004(p.Glu427Lys)];ENSG00000165282:MISSENSE[ENST00000378617(c.1279G>A,Gag>Aag):ENSP00000367880(p.Glu427Lys)];	NA	NA	NA	NA	NA	NA	NA	T	BN	N	MODERATE	N	0.128	72	72	0	97	76	21	21.65
Smoker	42475	chr9	68427867	A	T	*RP11-764K9.4*	EXONIC-NC	ENSG00000215548:[ENST00000376334(n.925T>A)];	NA	NA	NA	NA	NA	NA	NA	NA	NA	NA	MODIFIER	NA	NA	498	478	4	589	551	29	4.92
Smoker	42475	chrX	3030308	G	T	*ARSF*	MISSENSE	ENSG00000062096:MISSENSE[ENST00000381127(c.1484G>T,gGa>gTa):ENSP00000370519(p.Gly495Val)];ENSG00000062096:MISSENSE[ENST00000359361(c.1484G>T,gGa>gTa):ENSP00000352319(p.Gly495Val)];ENSG00000062096:MISSENSE[ENST00000537104(c.1484G>T,gGa>gTa):ENSP00000445594(p.Gly495Val)];	NA	NA	NA	NA	NA	NA	NA	D	PrD	D	MODERATE	U	0.01	61	60	1	96	36	60	62.5
Smoker	42475	chrX	49079112	C	T	*CACNA1F*	INTRONIC	ENSG00000102001:[ENST00000480889(n.337-50G>A)];ENSG00000102001:[ENST00000376251(c.2045-50G>A)];ENSG00000102001:[ENST00000376265(c.2240-50G>A)];ENSG00000102001:[ENST00000323022(c.2207-50G>A)];	NA	NA	NA	NA	NA	NA	NA	NA	NA	NA	MODIFIER	NA	NA	32	32	0	42	24	18	42.86
Smoker	42475	chrX	49773643	G	T	*CLCN5*	INTRONIC	ENSG00000171365:[ENST00000376091(c.17-33282G>T)];ENSG00000171365:[ENST00000482218(c.17-33282G>T)];ENSG00000171365:[ENST00000376088(c.17-33282G>T)];	NA	NA	NA	NA	NA	NA	NA	NA	NA	NA	MODIFIER	NA	NA	42	42	0	84	31	52	61.9
Smoker	42475	chrX	125299639	C	G	*DCAF12L2*	MISSENSE	ENSG00000198354:MISSENSE[ENST00000538699(c.269G>C,cGc>cCc):ENSP00000441489(p.Arg90Pro)];ENSG00000198354:MISSENSE[ENST00000360028(c.269G>C,cGc>cCc):ENSP00000353128(p.Arg90Pro)];	NA	NA	NA	NA	NA	NA	NA	D	PoD	D	MODERATE	NA	NA	60	60	0	128	49	77	60.16
Smoker	42475	chrX	53279971	A	G	*IQSEC2*	MISSENSE	ENSG00000124313:MISSENSE[ENST00000375365(c.1172T>C,aTt>aCt):ENSP00000364514(p.Ile391Thr)];ENSG00000124313:MISSENSE[ENST00000396435(c.1787T>C,aTt>aCt):ENSP00000379712(p.Ile596Thr)];ENSG00000124313:MISSENSE[ENST00000375368(c.1757T>C,aTt>aCt):ENSP00000364517(p.Ile586Thr)];	NA	NA	NA	1.43E-05	0	NA	NA	NA	PrD	NA	MODERATE	U	1	46	46	0	45	12	32	71.11
Smoker	42475	chrX	140985173	G	T	*MAGEC3*	MISSENSE	ENSG00000165509:MISSENSE[ENST00000544766(c.735G>T,atG>atT):ENSP00000440444(p.Met245Ile)];ENSG00000165509:MISSENSE[ENST00000409007(c.735G>T,atG>atT):ENSP00000386566(p.Met245Ile)];ENSG00000165509:MISSENSE[ENST00000536088(c.735G>T,atG>atT):ENSP00000441107(p.Met245Ile)];ENSG00000165509:MISSENSE[ENST00000298296(c.1629G>T,atG>atT):ENSP00000298296(p.Met543Ile)];ENSG00000165509:MISSENSE[ENST00000443323(c.495G>T,atG>atT):ENSP00000438254(p.Met165Ile)];	NA	NA	NA	NA	NA	NA	NA	T	PoD	N	MODERATE	NA	NA	78	78	0	92	28	64	69.57
Smoker	42475	chrX	83319224	G	T	*RPS6KA6*	3UTR	ENSG00000072133:[ENST00000262752(c.*61C>A)];	NA	NA	NA	NA	NA	NA	NA	NA	NA	NA	MODIFIER	NA	NA	43	43	0	61	46	15	24.59
Smoker	42475	chrX	155004068	G	C	*SPRY3*	MISSENSE	ENSG00000168939:MISSENSE[ENST00000302805(c.535G>C,Gag>Cag):ENSP00000302978(p.Glu179Gln)];	NA	NA	NA	NA	NA	NA	NA	T	BN	N	MODERATE	D	0.984	113	112	0	246	201	45	18.29
Smoker	42475	chrX	37981384	G	A	*SYTL5*	MISSENSE	ENSG00000147041:MISSENSE[ENST00000357972(c.1757G>A,gGa>gAa):ENSP00000350657(p.Gly586Glu)];ENSG00000147041:MISSENSE[ENST00000297875(c.1757G>A,gGa>gAa):ENSP00000297875(p.Gly586Glu)];ENSG00000147041:MISSENSE[ENST00000456733(c.1823G>A,gGa>gAa):ENSP00000395220(p.Gly608Glu)];	NA	NA	NA	NA	NA	NA	NA	NA	NA	NA	MODIFIER	NA	NA	88	88	0	123	38	85	69.11

Supplementary Table 4. List of somatic single nucleotide variants identified using whole exome sequencing of ESCC patients

Cohort	Sample	Chromosome	Genomic position	Reference allele	Alternate allele	Gene symbol	Variant type	Variant annotation	OncoMD	COSMIC	dbSNP	ExAC	ExAC_SAS	1000G	1000G_SAS	SIFT	Polyphen2	CONDEL	VEP	LRT	PhastCon7 Way	Total read (Adjacent normal)	Reference allel read support (Adjacent normal)	Alternative read support (Adjacent normal)	Total read (Tumor)	Reference allel read support (Tumor)	Alternative read support (Tumor)	Alternate allele frequency
Smoker	42475	chrX	70627919	C	T	*TAF1*	SILENT	ENSG00000147133:SILENT[ENST00000373790(c.4299C>T,aaC>aaT):ENSP00000362895(p.Asn1433(=))];ENSG00000147133:SILENT[ENST00000437147(c.263C>T,aaC>aaT):ENSP00000406517(p.Asn88(=))];ENSG00000147133:SILENT[ENST00000276072(c.4362C>T,aaC>aaT):ENSP00000276072(p.Asn1454(=))];ENSG00000147133:SILENT[ENST00000373775(c.263C>T,aaC>aaT):ENSP00000362880(p.Asn88(=))];ENSG00000147133:SILENT[ENST00000423759(c.4362C>T,aaC>aaT):ENSP00000406549(p.Asn1454(=))];ENSG00000147133:SILENT[ENST00000449580(c.4299C>T,aaC>aaT):ENSP00000389000(p.Asn1433(=))];ENSG00000147133:SILENT[ENST00000463163(c.296C>T,aaC>aaT):ENSP00000421611(p.Asn99(=))];	NA	NA	NA	1.14E-05	0	NA	NA	NA	NA	NA	LOW	NA	NA	55	55	0	97	86	11	11.34
Smoker	42475	chrX	37981384	G	A	*TM4SF2*	INTRONIC	ENSG00000250349:[ENST00000465127(c.172-543991G>A)];	NA	NA	NA	NA	NA	NA	NA	NA	NA	NA	MODIFIER	NA	NA	88	88	0	123	38	85	69.11
Smoker	42475	chrX	54951114	G	T	*TRO*	MISSENSE	ENSG00000067445:MISSENSE[ENST00000375022(c.1353G>T,ttG>ttT):ENSP00000364162(p.Leu451Phe)];ENSG00000067445:MISSENSE[ENST00000431115(c.162G>T,ttG>ttT):ENSP00000407996(p.Leu54Phe)];ENSG00000067445:MISSENSE[ENST00000399736(c.162G>T,ttG>ttT):ENSP00000382641(p.Leu54Phe)];ENSG00000067445:MISSENSE[ENST00000319167(c.1353G>T,ttG>ttT):ENSP00000318278(p.Leu451Phe)];ENSG00000067445:MISSENSE[ENST00000445561(c.1353G>T,ttG>ttT):ENSP00000404794(p.Leu451Phe)];ENSG00000067445:MISSENSE[ENST00000375041(c.162G>T,ttG>ttT):ENSP00000364181(p.Leu54Phe)];ENSG00000067445:MISSENSE[ENST00000173898(c.1353G>T,ttG>ttT):ENSP00000173898(p.Leu451Phe)];ENSG00000067445:EXONIC-NC[ENST00000484031(n.450G>T)];ENSG00000067445:EXONIC-NC[ENST00000492706(n.80G>T)];ENSG00000067445:EXONIC-NC[ENST00000492142(n.190G>T)];ENSG00000067445:5UTR[ENST00000420798(c.-55G>T)];	NA	NA	NA	NA	NA	NA	NA	NA	NA	NA	MODIFIER	NA	NA	48	48	0	73	21	52	71.23
Smoker	42475	chrX	154467076	G	T	*VBP1*	MISSENSE	ENSG00000155959:MISSENSE[ENST00000535916(c.532G>T,Gat>Tat):ENSP00000438694(p.Asp178Tyr)];ENSG00000155959:MISSENSE[ENST00000286428(c.547G>T,Gat>Tat):ENSP00000286428(p.Asp183Tyr)];ENSG00000155959:EXONIC-NC[ENST00000459836(n.707G>T)];ENSG00000155959:EXONIC-NC[ENST00000460509(n.420G>T)];	NA	NA	NA	NA	NA	NA	NA	NA	NA	NA	MODIFIER	NA	NA	22	22	0	75	49	26	34.67
Smoker	42475	chrX	73064026	C	A	*XIST*	EXONIC-NC	ENSG00000229807:[ENST00000429829(n.8563G>T)];	NA	NA	NA	NA	NA	NA	NA	NA	NA	NA	MODIFIER	NA	NA	70	70	0	132	48	84	63.64
Smoker	42476	chr1	94473865	G	A	*ABCA4*	INTRONIC	ENSG00000198691:[ENST00000536513(c.646-12C>T)];ENSG00000198691:[ENST00000370225(c.5836-12C>T)];ENSG00000198691:[ENST00000465352(n.252-12C>T)];ENSG00000198691:[ENST00000535881(c.193-12C>T)];	NA	NA	NA	NA	NA	NA	NA	NA	NA	NA	MODIFIER	NA	NA	80	80	0	70	65	5	7.14
Smoker	42476	chr1	236897854	C	T	*ACTN2*	INTRONIC	ENSG00000077522:[ENST00000492634(n.930+36C>T)];ENSG00000077522:[ENST00000542672(c.783+36C>T)];ENSG00000077522:[ENST00000366578(c.698-1081C>T)];ENSG00000077522:[ENST00000546208(c.-448+36C>T)];	NA	NA	NA	NA	NA	NA	NA	NA	NA	NA	MODIFIER	NA	NA	95	94	0	183	168	15	8.2
Smoker	42476	chr1	150525401	C	A	*ADAMTSL4*	MISSENSE	ENSG00000143382:MISSENSE[ENST00000271643(c.106C>A,Cct>Act):ENSP00000271643(p.Pro36Thr)];ENSG00000143382:MISSENSE[ENST00000369038(c.106C>A,Cct>Act):ENSP00000358034(p.Pro36Thr)];ENSG00000143382:MISSENSE[ENST00000369039(c.106C>A,Cct>Act):ENSP00000358035(p.Pro36Thr)];ENSG00000143382:MISSENSE[ENST00000369041(c.106C>A,Cct>Act):ENSP00000358037(p.Pro36Thr)];	NA	NA	NA	NA	NA	NA	NA	NA	NA	NA	MODIFIER	NA	NA	55	55	0	99	94	5	5.05
Smoker	42476	chr1	203134557	G	A	*ADORA1*	SILENT	ENSG00000163485:SILENT[ENST00000367236(c.510G>A,gaG>gaA):ENSP00000356205(p.Glu170(=))];ENSG00000163485:SILENT[ENST00000309502(c.510G>A,gaG>gaA):ENSP00000308549(p.Glu170(=))];ENSG00000163485:SILENT[ENST00000337894(c.510G>A,gaG>gaA):ENSP00000338435(p.Glu170(=))];ENSG00000163485:EXONIC-NC[ENST00000467253(n.282G>A)];ENSG00000163485:EXONIC-NC[ENST00000472535(n.316G>A)];ENSG00000163485:EXONIC-NC[ENST00000464019(n.439G>A)];ENSG00000163485:3UTR[ENST00000367235(c.*337G>A)];	chr1*20313	chr1*20313	chr1*203134557*203134557*G*T*rs758690370*NA*NA*NA	NA	NA	NA	NA	NA	NA	NA	MODIFIER	NA	NA	142	142	0	238	200	38	15.97
Smoker	42476	chr1	116935553	G	T	*ATP1A1*	MISSENSE	ENSG00000163399:MISSENSE[ENST00000537345(c.1410G>T,atG>atT):ENSP00000445306(p.Met470Ile)];ENSG00000163399:MISSENSE[ENST00000295598(c.1410G>T,atG>atT):ENSP00000295598(p.Met470Ile)];ENSG00000163399:MISSENSE[ENST00000369496(c.1317G>T,atG>atT):ENSP00000358508(p.Met439Ile)];	chr1*11693	chr1*11693	NA	NA	NA	NA	NA	NA	NA	NA	MODIFIER	NA	NA	53	53	0	42	35	7	16.67
Smoker	42476	chr1	116935553	G	T	*ATP1A1OS*	EXONIC-NC	ENSG00000203865:[ENST00000608511(n.496C>A)];	chr1*11693	chr1*11693	NA	NA	NA	NA	NA	NA	NA	NA	MODIFIER	NA	NA	53	53	0	42	35	7	16.67
Smoker	42476	chr1	60456410	G	A	*C1orf87*	MISSENSE	ENSG00000162598:MISSENSE[ENST00000395552(c.478C>T,Cgc>Tgc):ENSP00000378921(p.Arg160Cys)];ENSG00000162598:MISSENSE[ENST00000371201(c.1576C>T,Cgc>Tgc):ENSP00000360244(p.Arg526Cys)];ENSG00000162598:MISSENSE[ENST00000450089(c.889C>T,Cgc>Tgc):ENSP00000389432(p.Arg297Cys)];ENSG00000162598:EXONIC-NC[ENST00000486478(n.589C>T)];ENSG00000162598:EXONIC-NC[ENST00000488027(n.517C>T)];	NA	NA	0.0001997	8.24E-06	0	0.0001997	0	NA	NA	NA	MODIFIER	NA	NA	53	53	0	31	14	17	54.84
Smoker	42476	chr1	200776560	C	T	*CAMSAP2*	SILENT	ENSG00000118200:SILENT[ENST00000413307(c.456C>T,gtC>gtT):ENSP00000416800(p.Val152(=))];ENSG00000118200:SILENT[ENST00000358823(c.456C>T,gtC>gtT):ENSP00000351684(p.Val152(=))];ENSG00000118200:SILENT[ENST00000236925(c.456C>T,gtC>gtT):ENSP00000236925(p.Val152(=))];	NA	NA	NA	NA	NA	NA	NA	NA	NA	NA	LOW	NA	NA	152	151	0	282	256	26	9.22
Smoker	42476	chr1	22405184	T	A	*CDC42*	INTRONIC	ENSG00000070831:[ENST00000400259(c.105+108T>A)];ENSG00000070831:[ENST00000344548(c.105+108T>A)];ENSG00000070831:[ENST00000315554(c.105+108T>A)];ENSG00000070831:[ENST00000498236(n.192-15T>A)];ENSG00000070831:[ENST00000421089(c.56-15T>A)];ENSG00000070831:[ENST00000411827(c.105+108T>A)];	NA	NA	NA	NA	NA	NA	NA	NA	NA	NA	MODIFIER	NA	NA	70	70	0	67	53	14	20.9
Smoker	42476	chr1	86283703	G	A	*COL24A1*	INTRONIC	ENSG00000171502:[ENST00000436319(c.3843+14C>T)];ENSG00000171502:[ENST00000426639(c.*1293+14C>T)];ENSG00000171502:[ENST00000370571(c.3843+14C>T)];	NA	NA	NA	NA	NA	NA	NA	NA	NA	NA	MODIFIER	NA	NA	147	147	0	109	69	39	35.78
Smoker	42476	chr1	57535035	G	C	*DAB1*	MISSENSE-SS-PRX	ENSG00000173406:[ENST00000371231(c.661C>G,Cag>Gag):ENSP00000360275(p.Gln221Glu)];ENSG00000173406:[ENST00000371236(c.661C>G,Cag>Gag):ENSP00000360280(p.Gln221Glu)];ENSG00000173406:[ENST00000371234(c.661C>G,Cag>Gag):ENSP00000360278(p.Gln221Glu)];ENSG00000173406:[ENST00000414851(c.558+2160C>G)];ENSG00000173406:[ENST00000371232(c.307-6419C>G)];ENSG00000173406:[ENST00000332102(c.558+2160C>G)];ENSG00000173406:[ENST00000420954(c.558+2160C>G)];ENSG00000173406:[ENST00000439789(c.307-6419C>G)];ENSG00000173406:[ENST00000485760(n.1422C>G)];	chr1*57535	NA	NA	NA	NA	NA	NA	NA	NA	NA	MODIFIER	NA	NA	67	67	0	70	43	27	38.57

Mangalaparthi *et al.* , 2020. Mutational landscape of esophageal squamous cell carcinoma in an Indian cohort
Supplementary Table 4. List of somatic single nucleotide variants identified using whole exome sequencing of ESCC patients

Cohort	Sample	Chromosome	Genomic position	Reference allele	Alternate allele	Gene symbol	Variant type	Variant annotation	OncoMD	COSMIC	dbSNP	ExAC	ExAC_SAS	1000G	1000G_SAS	SIFT	Polyphen2	CONDEL	VEP	LRT	PhastCon7 Way	Total read (Adjacent normal)	Reference allel read support (Adjacent normal)	Alternative read support (Adjacent normal)	Total read (Tumor)	Reference allel read support (Tumor)	Alternative read support (Tumor)	Alternate allele frequency
Smoker	42476	chr1	21205808	C	T	*EIF4G3*	INTRONIC-SS-DNR	ENSG00000075151:[ENST00000400422(c.2454+8G>A)];ENSG00000075151:[ENST00000537738(c.924+8G>A)];ENSG00000075151:[ENST00000536266(c.1266+8G>A)];ENSG00000075151:[ENST00000374937(c.2472+8G>A)];ENSG00000075151:[ENST00000374935(c.1614+8G>A)];ENSG00000075151:[ENST00000602326(c.2472+8G>A)];ENSG00000075151:[ENST00000264211(c.2454+8G>A)];	NA	NA	NA	NA	NA	NA	NA	NA	NA	NA	LOW	NA	NA	74	74	0	60	46	14	23.33
Smoker	42476	chr1	149676329	C	A	*FAM231D*	MISSENSE	ENSG00000203815:MISSENSE[ENST00000369173(c.260C>A,tCc>tAc):ENSP00000358171(p.Ser87Tyr)];	NA	NA	NA	NA	NA	NA	NA	NA	NA	NA	MODIFIER	NA	NA	202	202	0	325	313	12	3.69
Smoker	42476	chr1	157491031	G	A	*FCRL5*	MISSENSE	ENSG00000143297:MISSENSE[ENST00000361835(c.2291C>T,gCg>gTg):ENSP00000354691(p.Ala764Val)];ENSG00000143297:MISSENSE[ENST00000356953(c.2291C>T,gCg>gTg):ENSP00000349434(p.Ala764Val)];ENSG00000143297:EXONIC-NC[ENST00000461387(n.1554C>T)];ENSG00000143297:EXONIC-NC[ENST00000497286(n.1384C>T)];	NA	NA	NA	1.68E-05	0	NA	NA	NA	NA	NA	MODIFIER	NA	NA	64	64	0	89	66	23	25.84
Smoker	42476	chr1	213032466	C	G	*FLVCR1*	MISSENSE	ENSG00000162769:MISSENSE[ENST00000366971(c.672C>G,atC>atG):ENSP00000355938(p.Ile224Met)];ENSG00000162769:MISSENSE[ENST00000419102(c.209C>G,atC>atG):ENSP00000414680(p.Ile70Met)];	NA	NA	chr1*213032466*213032466*C*A*rs757289916*NA*NA*NA	NA	NA	NA	NA	D	PoD	D	MODERATE	NA	NA	67	67	0	54	48	6	11.11
Smoker	42476	chr1	240256006	C	G	*FMN2*	MISSENSE	ENSG00000155816:MISSENSE[ENST00000319653(c.597C>G,atC>atG):ENSP00000318884(p.Ile199Met)];ENSG00000155816:INTRONIC[ENST00000447095(c.-87+24633C>G)];	NA	NA	NA	NA	NA	NA	NA	NA	NA	NA	MODIFIER	NA	NA	50	50	0	93	84	9	9.68
Smoker	42476	chr1	240256018	C	T	*FMN2*	SILENT	ENSG00000155816:SILENT[ENST00000319653(c.609C>T,atC>atT):ENSP00000318884(p.Ile203(=))];ENSG00000155816:INTRONIC[ENST00000447095(c.-87+24645C>T)];	chr1*24025	chr1*24025	NA	NA	NA	NA	NA	NA	NA	NA	MODIFIER	NA	NA	41	41	0	78	71	7	8.97
Smoker	42476	chr1	155253762	C	G	*HCN3*	INTRONIC-SS-ACR	ENSG00000143630:[ENST00000368358(c.709-3C>G)];ENSG00000143630:[ENST00000496230(n.601-3C>G)];ENSG00000143630:[ENST00000467204(n.304-568C>G)];	chr1*15525	chr1*15525	NA	NA	NA	NA	NA	NA	NA	NA	MODIFIER	NA	NA	175	175	0	258	224	34	13.18
Smoker	42476	chr1	22191363	C	T	*HSPG2*	SILENT	ENSG00000142798:SILENT[ENST00000374695(c.4599G>A,ccG>ccA):ENSP00000363827(p.Pro1533(=))];	chr1*22191	NA	NA	3.41E-05	0.000153657	NA	NA	NA	NA	NA	LOW	NA	NA	75	75	0	63	37	26	41.27
Smoker	42476	chr1	201781021	C	G	*IPO9-AS1*	INTRONIC	ENSG00000231871:[ENST00000413035(n.684+5334G>C)];	NA	NA	NA	NA	NA	NA	NA	NA	NA	NA	MODIFIER	NA	NA	122	122	0	186	172	14	7.53
Smoker	42476	chr1	152884059	A	G	*IVL*	3UTR	ENSG00000163207:[ENST00000368764(c.*28A>G)];ENSG00000163207:[ENST00000392667(c.*28A>G)];	NA	NA	NA	3.71E-05	0.000405762	NA	NA	NA	NA	NA	MODIFIER	NA	NA	24	24	0	18	7	10	55.56
Smoker	42476	chr1	90179079	C	G	*LRRC8C*	NONSENSE	ENSG00000171488:NONSENSE[ENST00000370454(c.950C>G,tCa>tGa):ENSP00000359483(p.Ser317Ter)];ENSG00000171488:INTRONIC[ENST00000479252(n.393+26909C>G)];	NA	NA	NA	NA	NA	NA	NA	NA	NA	NA	MODIFIER	NA	NA	179	179	0	164	151	13	7.93
Smoker	42476	chr1	98510658	T	C	*MIR137HG*	INTRONIC	ENSG00000225206:[ENST00000602852(n.218-25A>G)];ENSG00000225206:[ENST00000424528(n.984+906A>G)];ENSG00000225206:[ENST00000538428(n.357A>G)];ENSG00000225206:[ENST00000602984(n.367A>G)];ENSG00000225206:[ENST00000413670(n.357A>G)];	NA	NA	NA	NA	NA	NA	NA	NA	NA	NA	MODIFIER	NA	NA	87	86	0	59	45	14	23.73
Smoker	42476	chr1	155178698	T	C	*MTX1*	MISSENSE	ENSG00000173171:MISSENSE[ENST00000368376(c.103T>C,Tgg>Cgg):ENSP00000357360(p.Trp35Arg)];ENSG00000173171:MISSENSE[ENST00000316721(c.103T>C,Tgg>Cgg):ENSP00000317106(p.Trp35Arg)];	NA	NA	NA	NA	NA	NA	NA	NA	NA	NA	LOW	NA	NA	65	65	0	89	74	15	16.85
Smoker	42476	chr1	201781021	C	G	*NAV1*	INTRONIC-SS-ACR	ENSG00000134369:[ENST00000367297(c.4929-10C>G)];ENSG00000134369:[ENST00000367300(c.4773-10C>G)];ENSG00000134369:[ENST00000295624(c.4944-10C>G)];ENSG00000134369:[ENST00000367302(c.4812-10C>G)];ENSG00000134369:[ENST00000367296(c.4953-10C>G)];ENSG00000134369:[ENST00000367295(c.3771-10C>G)];	NA	NA	NA	NA	NA	NA	NA	NA	NA	NA	MODIFIER	NA	NA	122	122	0	186	172	14	7.53
Smoker	42476	chr1	120502094	A	T	*NOTCH2*	NONSENSE	ENSG00000134250:NONSENSE[ENST00000256646(c.1947T>A,tgT>tgA):ENSP00000256646(p.Cys649Ter)];ENSG00000134250:EXONIC-NC[ENST00000479412(n.2085T>A)];	NA	NA	NA	NA	NA	NA	NA	NA	NA	NA	MODIFIER	NA	NA	166	166	0	302	256	45	14.9
Smoker	42476	chr1	52302008	G	A	*NRD1*	INTRONIC	ENSG00000078618:[ENST00000485608(n.299+33C>T)];ENSG00000078618:[ENST00000354831(c.834+33C>T)];ENSG00000078618:[ENST00000352171(c.631-124C>T)];ENSG00000078618:[ENST00000544028(c.235-124C>T)];ENSG00000078618:[ENST00000539524(c.438+33C>T)];ENSG00000078618:[ENST00000475715(n.187+33C>T)];	NA	NA	NA	NA	NA	NA	NA	NA	NA	NA	MODIFIER	NA	NA	62	62	0	52	39	13	25
Smoker	42476	chr1	247921079	G	T	*OR1C1*	SILENT	ENSG00000221888:SILENT[ENST00000408896(c.630C>A,ccC>ccA):ENSP00000386138(p.Pro210(=))];	NA	NA	NA	NA	NA	NA	NA	NA	NA	NA	LOW	NA	NA	93	93	0	129	13	115	89.15
Smoker	42476	chr1	248487466	G	A	*OR2M7*	SILENT	ENSG00000177186:SILENT[ENST00000317965(c.405C>T,ctC>ctT):ENSP00000324557(p.Leu135(=))];	NA	NA	NA	NA	NA	NA	NA	NA	NA	NA	LOW	NA	NA	321	319	1	548	522	26	4.74
Smoker	42476	chr1	186919850	C	G	*PLA2G4A*	MISSENSE	ENSG00000116711:MISSENSE[ENST00000442353(c.1146C>G,caC>caG):ENSP00000406892(p.His382Gln)];ENSG00000116711:MISSENSE[ENST00000367466(c.1326C>G,caC>caG):ENSP00000356436(p.His442Gln)];	chr1*18691	chr1*18691	chr1*186919850*186919850*C*T*rs370896190*NA*NA*NA	NA	NA	NA	NA	T	BN	N	MODERATE	N	0.857	241	240	0	328	320	8	2.44
Smoker	42476	chr1	220383810	G	C	*RAB3GAP2*	MISSENSE	ENSG00000118873:MISSENSE[ENST00000358951(c.532C>G,Cag>Gag):ENSP00000351832(p.Gln178Glu)];ENSG00000118873:MISSENSE[ENST00000484658(c.236C>G,Cag>Gag):ENSP00000418560(p.Gln80Glu)];ENSG00000118873:3UTR[ENST00000474178(c.*77C>G)];ENSG00000118873:EXONIC-NC[ENST00000237724(n.327C>G)];ENSG00000118873:INTRONIC[ENST00000478976(n.292-20043C>G)];	NA	NA	NA	NA	NA	NA	NA	T	BN	N	MODERATE	NA	NA	84	84	0	132	114	17	12.88
Smoker	42476	chr1	174188383	C	T	*RABGAP1L*	NONSENSE	ENSG00000152061:NONSENSE[ENST00000251507(c.88C>T,Cag>Tag):ENSP00000251507(p.Gln30Ter)];ENSG00000152061:NONSENSE[ENST00000457696(c.88C>T,Cag>Tag):ENSP00000403136(p.Gln30Ter)];ENSG00000152061:INTRONIC[ENST00000357444(c.27+61C>T)];ENSG00000152061:INTRONIC[ENST00000367689(c.-922+61C>T)];	NA	NA	NA	NA	NA	NA	NA	NA	NA	NA	MODIFIER	NA	NA	71	71	0	93	84	9	9.68
Smoker	42476	chr1	167673863	C	T	*RCSD1*	INTRONIC	ENSG00000198771:[ENST00000537350(c.1129-38C>T)];ENSG00000198771:[ENST00000367854(c.1219-38C>T)];	NA	NA	NA	NA	NA	NA	NA	NA	NA	NA	MODIFIER	NA	NA	96	95	1	132	126	6	4.55
Smoker	42476	chr1	90179079	C	G	*RP11-302M6.4*	INTRONIC	ENSG00000271949:[ENST00000370453(c.138+26909C>G)];	NA	NA	NA	NA	NA	NA	NA	NA	NA	NA	MODIFIER	NA	NA	179	179	0	164	151	13	7.93
Smoker	42476	chr1	149676329	C	A	*RP11-353N4.4*	INTRONIC	ENSG00000223759:[ENST00000443602(n.1447-417C>A)];	NA	NA	NA	NA	NA	NA	NA	NA	NA	NA	MODIFIER	NA	NA	202	202	0	325	313	12	3.69
Smoker	42476	chr1	149676329	C	A	*RP11-353N4.5*	INTRONIC	ENSG00000234232:[ENST00000608683(n.378+24587G>T)];	NA	NA	NA	NA	NA	NA	NA	NA	NA	NA	MODIFIER	NA	NA	202	202	0	325	313	12	3.69
Smoker	42476	chr1	150525401	C	A	*RP11-54A4.2*	INTRONIC	ENSG00000237781:[ENST00000442435(n.476+1610G>T)];	NA	NA	NA	NA	NA	NA	NA	NA	NA	NA	MODIFIER	NA	NA	55	55	0	99	94	5	5.05
Smoker	42476	chr1	156705635	C	T	*RRNAD1*	MISSENSE	ENSG00000143303:MISSENSE[ENST00000368218(c.755C>T,tCt>tTt):ENSP00000357201(p.Ser252Phe)];ENSG00000143303:MISSENSE[ENST00000476229(c.386C>T,tCt>tTt):ENSP00000430787(p.Ser129Phe)];ENSG00000143303:EXONIC-NC[ENST00000497515(n.222C>T)];ENSG00000143303:SILENT[ENST00000368216(c.1240C>T,Cta>Tta):ENSP00000357199(p.Leu414(=))];	NA	NA	NA	NA	NA	NA	NA	NA	NA	NA	LOW	NA	0.91	72	71	0	118	110	8	6.78
Smoker	42476	chr1	156244557	T	C	*SMG5*	INTRONIC	ENSG00000198952:[ENST00000368267(c.455-80A>G)];ENSG00000198952:[ENST00000361813(c.455-80A>G)];	NA	NA	NA	NA	NA	NA	NA	NA	NA	NA	MODIFIER	NA	NA	61	61	0	93	88	5	5.38

Mangalaparthi *et al.* , 2020. Mutational landscape of esophageal squamous cell carcinoma in an Indian cohort
Supplementary Table 4. List of somatic single nucleotide variants identified using whole exome sequencing of ESCC patients

Cohort	Sample	Chromosome	Genomic position	Reference allele	Alternate allele	Gene symbol	Variant type	Variant annotation	OncoMD	COSMIC	dbSNP	ExAC	ExAC_SAS	1000G	1000G_SAS	SIFT	Polyphen2	CONDEL	VEP	LRT	PhastCon7 Way	Total read (Adjacent normal)	Reference allel read support (Adjacent normal)	Alternative read support (Adjacent normal)	Total read (Tumor)	Reference allel read support (Tumor)	Alternative read support (Tumor)	Alternate allele frequency
Smoker	42476	chr1	32266109	G	C	*SPOCD1*	NONSENSE	ENSG00000134668:NONSENSE[ENST00000525930(c.14C>G,tCa>tGa):ENSP00000436239(p.Ser5Ter)];ENSG00000134668:NONSENSE[ENST00000257100(c.14C>G,tCa>tGa):ENSP00000257100(p.Ser5Ter)];ENSG00000134668:NONSENSE[ENST00000360482(c.1535C>G,tCa>tGa):ENSP00000353670(p.Ser512Ter)];ENSG00000134668:NONSENSE[ENST00000528791(c.14C>G,tCa>tGa):ENSP00000435385(p.Ser5Ter)];ENSG00000134668:NONSENSE[ENST00000533231(c.1535C>G,tCa>tGa):ENSP00000435851(p.Ser512Ter)];ENSG00000134668:INTRONIC[ENST00000485944(n.222-367C>G)];ENSG00000134668:INTRONIC[ENST00000373648(c.1426-367C>G)];ENSG00000134668:INTRONIC[ENST00000529396(c.-135-367C>G)];	NA	NA	NA	NA	NA	NA	NA	NA	NA	NA	MODIFIER	NA	NA	70	70	0	62	41	21	33.87
Smoker	42476	chr1	144013880	C	G	*SRGAP2B*	INTRONIC	ENSG00000196369:[ENST00000467933(n.916-21C>G)];ENSG00000196369:[ENST00000491897(n.68-21C>G)];	NA	NA	NA	NA	NA	NA	NA	NA	NA	NA	MODIFIER	NA	NA	178	178	0	259	246	13	5.02
Smoker	42476	chr1	155178698	T	C	*THBS3*	EXONIC-NC-SS-PR	ENSG00000169231:[ENST00000486260(n.145A>G)];	NA	NA	NA	NA	NA	NA	NA	NA	NA	NA	LOW	NA	NA	65	65	0	89	74	15	16.85
Smoker	42476	chr1	173155648	C	T	*TNFSF4*	3UTR	ENSG00000117586:[ENST00000367718(c.*7G>A)];ENSG00000117586:[ENST00000281834(c.*7G>A)];	NA	NA	NA	NA	NA	NA	NA	NA	NA	NA	MODIFIER	NA	NA	90	90	0	117	104	13	11.11
Smoker	42476	chr10	27506975	C	T	*ACBD5*	MISSENSE	ENSG00000107897:MISSENSE[ENST00000375901(c.436G>A,Gaa>Aaa):ENSP00000365066(p.Glu146Lys)];ENSG00000107897:MISSENSE[ENST00000375905(c.658G>A,Gaa>Aaa):ENSP00000365070(p.Glu220Lys)];ENSG00000107897:MISSENSE[ENST00000412279(c.691G>A,Gaa>Aaa):ENSP00000393398(p.Glu231Lys)];ENSG00000107897:MISSENSE[ENST00000426079(c.817G>A,Gaa>Aaa):ENSP00000401591(p.Glu273Lys)];ENSG00000107897:MISSENSE[ENST00000375888(c.790G>A,Gaa>Aaa):ENSP00000365049(p.Glu264Lys)];ENSG00000107897:MISSENSE[ENST00000396271(c.763G>A,Gaa>Aaa):ENSP00000379568(p.Glu255Lys)];ENSG00000107897:EXONIC-NC[ENST00000476758(n.850G>A)];ENSG00000107897:INTRONIC[ENST00000375897(c.298+1677G>A)];	NA	NA	NA	NA	NA	NA	NA	T	BN	N	MODERATE	N	0.094	134	134	0	110	76	34	30.91
Smoker	42476	chr10	37430914	T	G	*ANKRD30A*	MISSENSE	ENSG00000148513:MISSENSE[ENST00000374660(c.921T>G,agT>agG):ENSP00000363792(p.Ser307Arg)];ENSG00000148513:MISSENSE[ENST00000361713(c.921T>G,agT>agG):ENSP00000354432(p.Ser307Arg)];ENSG00000148513:MISSENSE[ENST00000602533(c.921T>G,agT>agG):ENSP00000473551(p.Ser307Arg)];	NA	NA	NA	NA	NA	NA	NA	T	BN	N	MODERATE	NA	NA	186	184	2	134	121	7	5.22
Smoker	42476	chr10	98778437	G	A	*ARHGAP19-SLIT1*	3UTR	ENSG00000269891:[ENST00000453547(c.*2598C>T)];	NA	NA	NA	NA	NA	NA	NA	NA	NA	NA	HIGH	NA	0.989	32	32	0	46	31	15	32.61
Smoker	42476	chr10	23270565	G	A	*ARMC3*	MISSENSE	ENSG00000165309:MISSENSE[ENST00000409983(c.1111G>A,Gag>Aag):ENSP00000386943(p.Glu371Lys)];ENSG00000165309:MISSENSE[ENST00000409049(c.1111G>A,Gag>Aag):ENSP00000387288(p.Glu371Lys)];ENSG00000165309:MISSENSE[ENST00000298032(c.1111G>A,Gag>Aag):ENSP00000298032(p.Glu371Lys)];ENSG00000165309:MISSENSE[ENST00000376528(c.322G>A,Gag>Aag):ENSP00000365711(p.Glu108Lys)];	NA	NA	NA	NA	NA	NA	NA	T	BN	N	MODERATE	N	0.738	125	125	0	149	107	42	28.19
Smoker	42476	chr10	116853712	C	G	*ATRNL1*	MISSENSE	ENSG00000107518:MISSENSE[ENST00000355044(c.203C>G,tCc>tGc):ENSP00000347152(p.Ser68Cys)];ENSG00000107518:MISSENSE[ENST00000527407(c.203C>G,tCc>tGc):ENSP00000473412(p.Ser68Cys)];ENSG00000107518:EXONIC-NC[ENST00000529665(n.589C>G)];ENSG00000107518:INTRONIC[ENST00000526946(c.92+317C>G)];ENSG00000107518:INTRONIC[ENST00000485327(n.272+317C>G)];	NA	NA	NA	NA	NA	NA	NA	NA	NA	NA	MODIFIER	NA	NA	33	33	0	25	16	9	36
Smoker	42476	chr10	116930894	A	G	*ATRNL1*	MISSENSE	ENSG00000107518:MISSENSE[ENST00000355044(c.1192A>G,Aaa>Gaa):ENSP00000347152(p.Lys398Glu)];ENSG00000107518:SILENT[ENST00000527407(c.1104A>G,caA>caG):ENSP00000473412(p.Gln368(=))];ENSG00000107518:EXONIC-NC[ENST00000529665(n.1578A>G)];ENSG00000107518:EXONIC-NC[ENST00000485327(n.1468A>G)];	NA	NA	NA	NA	NA	NA	NA	NA	NA	NA	MODIFIER	NA	NA	122	122	0	134	89	45	33.58
Smoker	42476	chr10	43297603	G	C	*BMS1*	MISSENSE	ENSG00000165733:MISSENSE[ENST00000374518(c.2266G>C,Gat>Cat):ENSP00000363642(p.Asp756His)];	NA	NA	NA	5.82E-05	0	NA	NA	D	PrD	D	MODERATE	D	0.999	189	189	0	195	175	20	10.26
Smoker	42476	chr10	33143501	C	G	*C10ORF68*	INTRONIC	ENSG00000150076:[ENST00000375028(c.1852+84C>G)];ENSG00000150076:[ENST00000375030(c.1717+84C>G)];ENSG00000150076:[ENST00000302316(c.1840+84C>G)];ENSG00000150076:[ENST00000375025(c.2032+84C>G)];	NA	NA	NA	NA	NA	NA	NA	NA	NA	NA	MODIFIER	NA	NA	46	46	0	47	37	10	21.28
Smoker	42476	chr10	104679287	C	T	*CNNM2*	SILENT	ENSG00000148842:SILENT[ENST00000369878(c.1050C>T,atC>atT):ENSP00000358894(p.Ile350(=))];ENSG00000148842:SILENT[ENST00000433628(c.1050C>T,atC>atT):ENSP00000392875(p.Ile350(=))];ENSG00000148842:SILENT[ENST00000369875(c.1050C>T,atC>atT):ENSP00000358891(p.Ile350(=))];	NA	NA	NA	NA	NA	NA	NA	NA	NA	NA	LOW	NA	0.996	134	134	0	172	126	46	26.74
Smoker	42476	chr10	94836816	G	C	*CYP26A1*	MISSENSE	ENSG00000095596:MISSENSE[ENST00000394139(c.1042G>C,Gac>Cac):ENSP00000377695(p.Asp348His)];ENSG00000095596:MISSENSE[ENST00000224356(c.1249G>C,Gac>Cac):ENSP00000224356(p.Asp417His)];ENSG00000095596:MISSENSE[ENST00000371531(c.1042G>C,Gac>Cac):ENSP00000360586(p.Asp348His)];	NA	NA	NA	NA	NA	NA	NA	D	PoD	D	MODERATE	D	0.971	90	90	0	72	58	14	19.44
Smoker	42476	chr10	82111711	C	G	*DYDC1*	SILENT	ENSG00000170788:SILENT[ENST00000453477(c.195G>C,ctG>ctC):ENSP00000388160(p.Leu65(=))];ENSG00000170788:SILENT[ENST00000372204(c.195G>C,ctG>ctC):ENSP00000361278(p.Leu65(=))];ENSG00000170788:SILENT[ENST00000421924(c.195G>C,ctG>ctC):ENSP00000402890(p.Leu65(=))];ENSG00000170788:SILENT[ENST00000372202(c.195G>C,ctG>ctC):ENSP00000361276(p.Leu65(=))];ENSG00000170788:SILENT[ENST00000454362(c.195G>C,ctG>ctC):ENSP00000414798(p.Leu65(=))];	NA	NA	NA	NA	NA	NA	NA	NA	NA	NA	MODIFIER	NA	NA	95	94	0	73	59	14	19.18
Smoker	42476	chr10	82111711	C	G	*DYDC2*	INTRONIC	ENSG00000133665:[ENST00000372199(c.-527-482C>G)];ENSG00000133665:[ENST00000372197(c.-309-4342C>G)];ENSG00000133665:[ENST00000372198(c.33+7140C>G)];	NA	NA	NA	NA	NA	NA	NA	NA	NA	NA	MODIFIER	NA	NA	95	94	0	73	59	14	19.18
Smoker	42476	chr10	15760747	A	G	*ITGA8*	INTRONIC	ENSG00000077943:[ENST00000378076(c.343+18T>C)];	NA	NA	NA	NA	NA	NA	NA	NA	NA	NA	MODIFIER	NA	NA	69	69	0	66	53	12	18.18
Smoker	42476	chr10	85994045	C	T	*LRIT1*	MISSENSE	ENSG00000148602:MISSENSE[ENST00000372105(c.679G>A,Gag>Aag):ENSP00000361177(p.Glu227Lys)];	chr10*8599	NA	NA	NA	NA	NA	NA	D	PoD	D	MODERATE	D	0.35	94	94	0	72	53	19	26.39
Smoker	42476	chr10	88702770	G	A	*MMRN2*	NONSENSE	ENSG00000173269:NONSENSE[ENST00000372027(c.1771C>T,Cag>Tag):ENSP00000361097(p.Gln591Ter)];	NA	NA	NA	NA	NA	NA	NA	NA	NA	NA	HIGH	NA	0.003	74	74	0	46	34	12	26.09
Smoker	42476	chr10	99221701	C	G	*MMS19*	INTRONIC	ENSG00000155229:[ENST00000478452(n.528-48G>C)];ENSG00000155229:[ENST00000415383(c.*1929-48G>C)];ENSG00000155229:[ENST00000370782(c.2116-48G>C)];ENSG00000155229:[ENST00000327238(c.1822-48G>C)];ENSG00000155229:[ENST00000438925(c.2116-48G>C)];ENSG00000155229:[ENST00000327277(c.1030-48G>C)];ENSG00000155229:[ENST00000355839(c.1987-48G>C)];ENSG00000155229:[ENST00000495415(n.329-48G>C)];ENSG00000155229:[ENST00000434538(c.840-48G>C)];	NA	NA	NA	NA	NA	NA	NA	NA	NA	NA	MODIFIER	NA	NA	58	58	0	51	40	11	21.57

Mangalaparthi *et al.* , 2020. Mutational landscape of esophageal squamous cell carcinoma in an Indian cohort
Supplementary Table 4. List of somatic single nucleotide variants identified using whole exome sequencing of ESCC patients

Cohort	Sample	Chromosome	Genomic position	Reference allele	Alternate allele	Gene symbol	Variant type	Variant annotation	OncoMD	COSMIC	dbSNP	ExAC	ExAC_SAS	1000G	1000G_SAS	SIFT	Polyphen2	CONDEL	VEP	LRT	PhastCon7 Way	Total read (Adjacent normal)	Reference allel read support (Adjacent normal)	Alternative read support (Adjacent normal)	Total read (Tumor)	Reference allel read support (Tumor)	Alternative read support (Tumor)	Alternate allele frequency
Smoker	42476	chr10	50955202	C	T	*OGDHL*	MISSENSE	ENSG00000197444:MISSENSE[ENST00000432695(c.413G>A,cGc>cAc):ENSP00000390240(p.Arg138His)];ENSG00000197444:MISSENSE[ENST00000419399(c.869G>A,cGc>cAc):ENSP00000401356(p.Arg290His)];ENSG00000197444:MISSENSE[ENST00000374103(c.1040G>A,cGc>cAc):ENSP00000363216(p.Arg347His)];	NA	NA	0.0001997	4.12E-05	0	0.0001997	0	T	BN	N	MODERATE	D	0.98	77	77	0	60	36	24	40
Smoker	42476	chr10	6264827	G	A	*PFKFB3*	MISSENSE	ENSG00000170525:MISSENSE[ENST00000540253(c.1135G>A,Gac>Aac):ENSP00000446384(p.Asp379Asn)];ENSG00000170525:MISSENSE[ENST00000317350(c.1093G>A,Gac>Aac):ENSP00000369105(p.Asp365Asn)];ENSG00000170525:MISSENSE[ENST00000379775(c.1093G>A,Gac>Aac):ENSP00000369100(p.Asp365Asn)];ENSG00000170525:MISSENSE[ENST00000379785(c.1093G>A,Gac>Aac):ENSP00000369111(p.Asp365Asn)];ENSG00000170525:MISSENSE[ENST00000379782(c.1093G>A,Gac>Aac):ENSP00000369108(p.Asp365Asn)];ENSG00000170525:MISSENSE[ENST00000379789(c.1033G>A,Gac>Aac):ENSP00000369115(p.Asp345Asn)];ENSG00000170525:MISSENSE[ENST00000360521(c.1093G>A,Gac>Aac):ENSP00000353712(p.Asp365Asn)];ENSG00000170525:MISSENSE[ENST00000467491(c.1093G>A,Gac>Aac):ENSP00000434628(p.Asp365Asn)];ENSG00000170525:MISSENSE[ENST00000477914(c.1093G>A,Gac>Aac):ENSP00000432356(p.Asp365Asn)];ENSG00000170525:MISSENSE[ENST00000461744(c.1093G>A,Gac>Aac):ENSP00000433771(p.Asp365Asn)];ENSG00000170525:MISSENSE[ENST00000450232(c.134G>A,Gac>Aac):ENSP00000390285(p.Asp46Asn)];ENSG00000170525:MISSENSE[ENST00000490474(c.1093G>A,Gac>Aac):ENSP00000436885(p.Asp365Asn)];ENSG00000170525:EXONIC-NC[ENST00000487989(n.157G>A)];ENSG00000170525:INTRONIC[ENST00000536985(c.*414-1094G>A)];	NA	NA	NA	NA	NA	NA	NA	NA	PrD	NA	MODERATE	NA	NA	44	44	0	44	38	6	13.64
Smoker	42476	chr10	98778437	G	A	*SLIT1*	NONSENSE	ENSG00000187122:NONSENSE[ENST00000371070(c.2875C>T,Cga>Tga):ENSP00000360109(p.Arg959Ter)];ENSG00000187122:NONSENSE[ENST00000266058(c.2875C>T,Cga>Tga):ENSP00000266058(p.Arg959Ter)];	NA	NA	NA	NA	NA	NA	NA	NA	NA	NA	HIGH	NA	0.989	32	32	0	46	31	15	32.61
Smoker	42476	chr10	106581790	C	G	*SORCS3*	INTRONIC	ENSG00000156395:[ENST00000369701(c.628-20760C>G)];	NA	NA	NA	NA	NA	NA	NA	NA	NA	NA	MODIFIER	NA	NA	133	133	0	118	94	24	20.34
Smoker	42476	chr10	17726870	C	T	*STAM*	MISSENSE	ENSG00000136738:MISSENSE[ENST00000377524(c.241C>T,Cat>Tat):ENSP00000366746(p.His81Tyr)];ENSG00000136738:EXONIC-NC[ENST00000486183(n.87C>T)];ENSG00000136738:INTRONIC[ENST00000540523(c.-36-3156C>T)];ENSG00000136738:INTRONIC[ENST00000377500(c.-36-3156C>T)];ENSG00000136738:3UTR[ENST00000445846(c.*216C>T)];	NA	NA	NA	NA	NA	NA	NA	NA	NA	NA	MODIFIER	NA	NA	104	104	0	91	74	17	18.68
Smoker	42476	chr10	30989097	C	T	*SVILP1*	EXONIC-NC	ENSG00000234814:[ENST00000429171(n.775C>T)];ENSG00000234814:[ENST00000422642(n.1413C>T)];	NA	NA	NA	NA	NA	NA	NA	NA	NA	NA	MODIFIER	NA	NA	190	190	0	212	200	12	5.66
Smoker	42476	chr10	75849829	C	T	*VCL*	NONSENSE	ENSG00000035403:NONSENSE[ENST00000436396(c.241C>T,Cga>Tga):ENSP00000415489(p.Arg81Ter)];ENSG00000035403:NONSENSE[ENST00000211998(c.1225C>T,Cga>Tga):ENSP00000211998(p.Arg409Ter)];ENSG00000035403:NONSENSE[ENST00000372755(c.1225C>T,Cga>Tga):ENSP00000361841(p.Arg409Ter)];ENSG00000035403:INTRONIC[ENST00000478896(n.332-10983C>T)];ENSG00000035403:INTRONIC[ENST00000417648(c.391-18948C>T)];	NA	NA	chr10*75849829*75849829*C*G,T*rs202005455*NA*NA*NA	1.65E-05	0	NA	NA	NA	NA	NA	MODIFIER	NA	NA	55	55	0	53	38	15	28.3
Smoker	42476	chr10	76977676	T	G	*VDAC2*	INTRONIC	ENSG00000165637:[ENST00000298468(c.151-1145T>G)];ENSG00000165637:[ENST00000332211(c.151-1145T>G)];ENSG00000165637:[ENST00000413289(c.196-1145T>G)];ENSG00000165637:[ENST00000344036(c.151-1145T>G)];ENSG00000165637:[ENST00000447677(c.151-1145T>G)];ENSG00000165637:[ENST00000543351(c.151-1145T>G)];ENSG00000165637:[ENST00000472137(n.340-15T>G)];ENSG00000165637:[ENST00000481876(n.301-1145T>G)];ENSG00000165637:[ENST00000313132(c.196-1145T>G)];ENSG00000165637:[ENST00000535553(c.-41T>G)];ENSG00000165637:[ENST00000468285(n.281T>G)];ENSG00000165637:[ENST00000498394(n.289T>G)];ENSG00000165637:[ENST00000470745(n.341T>G)];ENSG00000165637:[ENST00000475142(n.332T>G)];	NA	NA	NA	NA	NA	NA	NA	NA	NA	NA	MODIFIER	NA	NA	62	62	0	54	44	10	18.52
Smoker	42476	chr10	114202100	C	T	*ZDHHC6*	MISSENSE	ENSG00000023041:MISSENSE[ENST00000369404(c.357G>A,atG>atA):ENSP00000358412(p.Met119Ile)];ENSG00000023041:MISSENSE[ENST00000369405(c.369G>A,atG>atA):ENSP00000358413(p.Met123Ile)];	NA	NA	NA	NA	NA	NA	NA	T	BN	N	MODERATE	D	1	58	58	0	42	30	12	28.57
Smoker	42476	chr11	44072823	T	C	*ACCSL*	INTRONIC	ENSG00000205126:[ENST00000378832(c.636-62T>C)];ENSG00000205126:[ENST00000527145(c.*155-62T>C)];	NA	NA	NA	NA	NA	NA	NA	NA	NA	NA	MODIFIER	NA	NA	90	90	0	48	16	32	66.67
Smoker	42476	chr11	122775119	C	G	*C11orf63*	MISSENSE	ENSG00000109944:MISSENSE[ENST00000531316(c.831C>G,caC>caG):ENSP00000431669(p.His277Gln)];ENSG00000109944:MISSENSE[ENST00000227349(c.831C>G,caC>caG):ENSP00000227349(p.His277Gln)];ENSG00000109944:MISSENSE[ENST00000307257(c.831C>G,caC>caG):ENSP00000307695(p.His277Gln)];	NA	NA	NA	8.39E-06	0	NA	NA	D	PrD	D	MODERATE	NA	NA	89	89	0	35	26	9	25.71
Smoker	42476	chr11	32720738	T	C	*CCDC73*	INTRONIC	ENSG00000186714:[ENST00000531481(c.390+59A>G)];ENSG00000186714:[ENST00000534415(n.451+59A>G)];ENSG00000186714:[ENST00000335185(c.390+59A>G)];	NA	NA	NA	NA	NA	NA	NA	NA	NA	NA	MODIFIER	NA	NA	52	52	0	44	35	9	20.45
Smoker	42476	chr11	6291951	C	T	*CCKBR*	SILENT	ENSG00000110148:SILENT[ENST00000525462(c.729C>T,cgC>cgT):ENSP00000435534(p.Arg243(=))];ENSG00000110148:SILENT[ENST00000532715(c.477C>T,cgC>cgT):ENSP00000432079(p.Arg159(=))];ENSG00000110148:SILENT[ENST00000334619(c.729C>T,cgC>cgT):ENSP00000335544(p.Arg243(=))];	NA	NA	.	NA	NA	NA	NA	NA	NA	NA	LOW	NA	NA	154	154	0	122	94	28	22.95
Smoker	42476	chr11	70261691	C	T	*CTTN*	INTRONIC	ENSG00000085733:[ENST00000376561(c.403-78C>T)];ENSG00000085733:[ENST00000301843(c.403-78C>T)];ENSG00000085733:[ENST00000525276(n.43-78C>T)];ENSG00000085733:[ENST00000415461(c.348-78C>T)];ENSG00000085733:[ENST00000346329(c.403-78C>T)];	NA	NA	NA	NA	NA	NA	NA	NA	NA	NA	MODIFIER	NA	NA	65	65	0	494	480	14	2.83

Mangalaparthi *et al.* , 2020. Mutational landscape of esophageal squamous cell carcinoma in an Indian cohort

Supplementary Table 4. List of somatic single nucleotide variants identified using whole exome sequencing of ESCC patients

Cohort	Sample	Chromosome	Genomic position	Reference allele	Alternate allele	Gene symbol	Variant type	Variant annotation	OncoMD	COSMIC	dbSNP	ExAC	ExAC_SAS	1000G	1000G_SAS	SIFT	Polyphen2	CONDEL	VEP	LRT	PhastCon7 Way	Total read (Adjacent normal)	Reference allel read support (Adjacent normal)	Alternative read support (Adjacent normal)	Total read (Tumor)	Reference allel read support (Tumor)	Alternative read support (Tumor)	Alternate allele frequency
Smoker	42476	chr11	83983379	C	G	*DLG2*	INTRONIC	ENSG00000150672:[ENST00000524982(c.205-21045G>C)];ENSG00000150672:[ENST00000529111(n.483-21045G>C)];ENSG00000150672:[ENST00000280241(c.322-21045G>C)];ENSG00000150672:[ENST00000330014(c.21+815G>C)];ENSG00000150672:[ENST00000398309(c.205-21045G>C)];ENSG00000150672:[ENST00000376104(c.520-21045G>C)];ENSG00000150672:[ENST00000532653(c.205-21045G>C)];ENSG00000150672:[ENST00000537455(c.-535+815G>C)];ENSG00000150672:[ENST00000529401(c.106-36G>C)];ENSG00000150672:[ENST00000398301(c.322-21045G>C)];ENSG00000150672:[ENST00000531015(c.105+815G>C)];ENSG00000150672:[ENST00000376106(c.-1351+815G>C)];ENSG00000150672:[ENST00000527466(n.423-21045G>C)];ENSG00000150672:[ENST00000543673(c.520-21045G>C)];ENSG00000150672:[ENST00000418306(c.105+815G>C)];	NA	NA	NA	NA	NA	NA	NA	NA	NA	NA	MODIFIER	NA	NA	37	37	0	23	16	7	30.43
Smoker	42476	chr11	61593551	C	T	*FADS1*	INTRONIC	ENSG00000149485:[ENST00000448607(c.-49+2371G>A)];ENSG00000149485:[ENST00000421879(c.-49+3122G>A)];	NA	NA	NA	NA	NA	NA	NA	NA	NA	NA	MODIFIER	NA	NA	95	94	0	93	65	28	30.11
Smoker	42476	chr11	61593551	C	T	*FADS2*	5UTR	ENSG00000134824:[ENST00000574708(c.-7C>T)];ENSG00000134824:[ENST00000522639(c.-7C>T)];ENSG00000134824:[ENST00000517839(c.*44-204C>T)];ENSG00000134824:[ENST00000257261(c.141+9653C>T)];ENSG00000134824:[ENST00000522056(c.114+9383C>T)];	NA	NA	NA	NA	NA	NA	NA	NA	NA	NA	MODIFIER	NA	NA	95	94	0	93	65	28	30.11
Smoker	42476	chr11	106647202	G	A	*GUCY1A2*	MISSENSE	ENSG00000152402:MISSENSE[ENST00000282249(c.1799C>T,tCa>tTa):ENSP00000282249(p.Ser600Leu)];ENSG00000152402:MISSENSE[ENST00000526355(c.1799C>T,tCa>tTa):ENSP00000431245(p.Ser600Leu)];ENSG00000152402:MISSENSE[ENST00000347596(c.1862C>T,tCa>tTa):ENSP00000344874(p.Ser621Leu)];	NA	NA	NA	NA	NA	NA	NA	D	PrD	D	MODERATE	U	1	49	48	0	23	14	9	39.13
Smoker	42476	chr11	76912497	C	T	*MYO7A*	SILENT	ENSG00000137474:EXONIC-NC-SS-PRX[ENST00000481328(n.2393C>T)];ENSG00000137474:SILENT-SS-PRX[ENST00000458169(c.2283C>T,ggC>ggT):ENSP00000417017(p.Gly761(=))];ENSG00000137474:SILENT[ENST00000409619(c.4710C>T,ggC>ggT):ENSP00000386635(p.Gly1570(=))];ENSG00000137474:SILENT[ENST00000409709(c.4857C>T,ggC>ggT):ENSP00000386331(p.Gly1619(=))];ENSG00000137474:SILENT[ENST00000458637(c.4743C>T,ggC>ggT):ENSP00000392185(p.Gly1581(=))];	NA	NA	NA	1.90E-05	7.08E-05	NA	NA	NA	NA	NA	LOW	NA	NA	71	71	0	49	29	20	40.82
Smoker	42476	chr11	8159805	G	A	*RIC3*	INTRONIC	ENSG00000166405:[ENST00000529035(n.296+14C>T)];ENSG00000166405:[ENST00000526962(c.125-11454C>T)];ENSG00000166405:[ENST00000425599(c.427+14C>T)];ENSG00000166405:[ENST00000539720(c.280+14C>T)];ENSG00000166405:[ENST00000524799(n.288+14C>T)];ENSG00000166405:[ENST00000531450(c.427+14C>T)];ENSG00000166405:[ENST00000528463(c.*344+14C>T)];ENSG00000166405:[ENST00000309737(c.427+14C>T)];ENSG00000166405:[ENST00000335425(c.125-27121C>T)];ENSG00000166405:[ENST00000530060(n.507+14C>T)];ENSG00000166405:[ENST00000529271(n.154+14C>T)];ENSG00000166405:[ENST00000343202(c.427+14C>T)];	NA	NA	chr11*8159802*8159805*TGAG*T*rs375008019*NA*NA*NA	NA	NA	NA	NA	NA	NA	NA	MODIFIER	NA	NA	156	156	0	126	95	31	24.6
Smoker	42476	chr11	67074370	G	A	*SSH3*	MISSENSE	ENSG00000172830:EXONIC-NC[ENST00000532181(n.330G>A)];ENSG00000172830:3UTR[ENST00000532881(c.*391G>A)];ENSG00000172830:3UTR[ENST00000532600(c.*237G>A)];ENSG00000172830:MISSENSE[ENST00000308127(c.401G>A,cGa>cAa):ENSP00000312081(p.Arg134Gln)];ENSG00000172830:MISSENSE[ENST00000308298(c.401G>A,cGa>cAa):ENSP00000310055(p.Arg134Gln)];ENSG00000172830:MISSENSE[ENST00000376757(c.401G>A,cGa>cAa):ENSP00000365948(p.Arg134Gln)];	NA	NA	NA	3.35E-05	0	NA	NA	T	BN	N	MODERATE	NA	NA	41	41	0	49	41	8	16.33
Smoker	42476	chr11	35827934	G	T	*TRIM44*	STOPLOSS	ENSG00000166326:STOPLOSS[ENST00000299413(c.1034G>T,tGa>tTa):ENSP00000299413(p.Ter345LeuextTer17)];ENSG00000166326:EXONIC-NC[ENST00000532066(n.400G>T)];	NA	NA	chr11*35827934*35827934*G*A*rs200834184*0*0.9996*0.0003994	NA	NA	NA	NA	NA	NA	NA	MODIFIER	NA	NA	112	112	0	73	55	18	24.66
Smoker	42476	chr12	9241932	T	C	*A2M*	INTRONIC	ENSG00000175899:[ENST00000543436(c.451+576A>G)];ENSG00000175899:[ENST00000545828(n.348+12214A>G)];ENSG00000175899:[ENST00000462568(n.298-85A>G)];ENSG00000175899:[ENST00000318602(c.2719-85A>G)];	NA	NA	NA	NA	NA	NA	NA	NA	NA	NA	MODIFIER	NA	NA	76	76	0	95	74	21	22.11
Smoker	42476	chr12	110777378	C	A	*ATP2A2*	MISSENSE	ENSG00000174437:MISSENSE[ENST00000539276(c.1613C>A,tCt>tAt):ENSP00000440045(p.Ser538Tyr)];ENSG00000174437:MISSENSE[ENST00000548169(c.1284C>A,tCt>tAt):ENSP00000449454(p.Ser429Tyr)];ENSG00000174437:MISSENSE[ENST00000308664(c.1613C>A,tCt>tAt):ENSP00000311186(p.Ser538Tyr)];ENSG00000174437:MISSENSE[ENST00000395494(c.1532C>A,tCt>tAt):ENSP00000378872(p.Ser511Tyr)];ENSG00000174437:3UTR[ENST00000377685(c.*1453C>A)];	NA	NA	NA	NA	NA	NA	NA	NA	NA	NA	MODIFIER	NA	NA	87	86	0	84	64	20	23.81
Smoker	42476	chr12	2789529	G	C	*CACNA1C*	INTRONIC	ENSG00000151067:[ENST00000399644(c.5444+567G>C)];ENSG00000151067:[ENST00000399595(c.5468+567G>C)];ENSG00000151067:[ENST00000399617(c.5444+567G>C)];ENSG00000151067:[ENST00000347598(c.5588+567G>C)];ENSG00000151067:[ENST00000402845(c.5501+567G>C)];ENSG00000151067:[ENST00000327702(c.5444+567G>C)];ENSG00000151067:[ENST00000399641(c.5444+567G>C)];ENSG00000151067:[ENST00000399603(c.5444+567G>C)];ENSG00000151067:[ENST00000399629(c.5495+567G>C)];ENSG00000151067:[ENST00000399601(c.5444+567G>C)];ENSG00000151067:[ENST00000399597(c.5444+567G>C)];ENSG00000151067:[ENST00000399655(c.5444+567G>C)];ENSG00000151067:[ENST00000399606(c.5504+567G>C)];ENSG00000151067:[ENST00000399637(c.5501+567G>C)];ENSG00000151067:[ENST00000335762(c.5519+567G>C)];ENSG00000151067:[ENST00000399591(c.5468+567G>C)];ENSG00000151067:[ENST00000399621(c.5501+567G>C)];ENSG00000151067:[ENST00000399638(c.5528+567G>C)];ENSG00000151067:[ENST00000344100(c.5567+567G>C)];ENSG00000151067:[ENST00000399649(c.5462+567G>C)];ENSG00000151067:[ENST00000406454(c.5445G>C,agG>agC):ENSP00000385896(p.Arg1815Ser)];ENSG00000151067:[ENST00000399634(c.5445G>C,agG>agC):ENSP00000382542(p.Arg1815Ser)];	NA	NA	NA	NA	NA	NA	NA	NA	NA	NA	MODIFIER	NA	NA	65	65	0	87	73	14	16.09
Smoker	42476	chr12	2789529	G	C	*CACNA1C-AS1*	INTRONIC	ENSG00000246627:[ENST00000501371(n.295-2466C>G)];	NA	NA	NA	NA	NA	NA	NA	NA	NA	NA	MODIFIER	NA	NA	65	65	0	87	73	14	16.09
Smoker	42476	chr12	124416206	C	T	*CCDC92*	INTRONIC	ENSG00000119242:[ENST00000544798(n.288-7371G>A)];ENSG00000119242:[ENST00000542348(n.191+11085G>A)];	NA	NA	NA	8.31E-06	0	NA	NA	NA	NA	NA	MODIFIER	NA	NA	111	111	0	80	66	14	17.5

Mangalaparthi *et al.* , 2020. Mutational landscape of esophageal squamous cell carcinoma in an Indian cohort
Supplementary Table 4. List of somatic single nucleotide variants identified using whole exome sequencing of ESCC patients

Cohort	Sample	Chromosome	Genomic position	Reference allele	Alternate allele	Gene symbol	Variant type	Variant annotation	OncoMD	COSMIC	dbSNP	ExAC	ExAC_SAS	1000G	1000G_SAS	SIFT	Polyphen2	CONDEL	VEP	LRT	PhastCon7 Way	Total read (Adjacent normal)	Reference allel read support (Adjacent normal)	Alternative read support (Adjacent normal)	Total read (Tumor)	Reference allel read support (Tumor)	Alternative read support (Tumor)	Alternate allele frequency
Smoker	42476	chr12	10150921	G	A	*CLEC1B*	SILENT	ENSG00000165682:INTRONIC[ENST00000348658(c.64+715C>T)];ENSG00000165682:INTRONIC[ENST00000428126(c.64+715C>T)];ENSG00000165682:SILENT[ENST00000298527(c.123C>T,tgC>tgT):ENSP00000298527(p.Cys41(=))];	chr12*1015(	chr12*1015(	NA	0.000116	0.000242248	NA	NA	NA	NA	NA	MODIFIER	NA	NA	67	67	0	97	82	15	15.46
Smoker	42476	chr12	9840618	C	G	*CLEC2D*	NONSENSE	ENSG00000069493:NONSENSE[ENST00000536355(c.167C>G,tCa>tGa):ENSP00000438735(p.Ser56Ter)];ENSG00000069493:NONSENSE[ENST00000460309(c.122C>G,tCa>tGa):ENSP00000443177(p.Ser41Ter)];ENSG00000069493:NONSENSE[ENST00000543300(c.293C>G,tCa>tGa):ENSP00000443065(p.Ser98Ter)];ENSG00000069493:NONSENSE[ENST00000544322(c.215C>G,tCa>tGa):ENSP00000437861(p.Ser72Ter)];ENSG00000069493:NONSENSE[ENST00000444971(c.293C>G,tCa>tGa):ENSP00000399744(p.Ser98Ter)];ENSG00000069493:NONSENSE[ENST00000325960(c.293C>G,tCa>tGa):ENSP00000371409(p.Ser98Ter)];ENSG00000069493:NONSENSE[ENST00000290855(c.293C>G,tCa>tGa):ENSP00000290855(p.Ser98Ter)];ENSG00000069493:NONSENSE[ENST00000545918(c.182C>G,tCa>tGa):ENSP00000444818(p.Ser61Ter)];ENSG00000069493:NONSENSE[ENST00000430909(c.230C>G,tCa>tGa):ENSP00000413045(p.Ser77Ter)];ENSG00000069493:NONSENSE[ENST00000261339(c.182C>G,tCa>tGa):ENSP00000261339(p.Ser61Ter)];ENSG00000069493:NONSENSE[ENST00000466035(c.164C>G,tCa>tGa):ENSP00000446028(p.Ser55Ter)];ENSG00000069493:NONSENSE[ENST00000479877(c.293C>G,tCa>tGa):ENSP00000441653(p.Ser98Ter)];ENSG00000069493:NONSENSE[ENST00000261340(c.293C>G,tCa>tGa):ENSP00000261340(p.Ser98Ter)];ENSG00000069493:NONSENSE[ENST00000476198(c.167C>G,tCa>tGa):ENSP00000443627(p.Ser56Ter)];ENSG00000069493:3UTR[ENST00000492359(c.*190C>G)];	NA	NA	NA	NA	NA	NA	NA	NA	NA	NA	MODIFIER	NA	NA	140	139	0	133	108	25	18.8
Smoker	42476	chr12	31247692	C	T	*DDX11*	MISSENSE	ENSG00000013573:MISSENSE[ENST00000542838(c.1418C>T,aCg>aTg):ENSP00000443426(p.Thr473Met)];ENSG00000013573:MISSENSE[ENST00000545668(c.1418C>T,aCg>aTg):ENSP00000440402(p.Thr473Met)];ENSG00000013573:MISSENSE[ENST00000228264(c.1340C>T,aCg>aTg):ENSP00000228264(p.Thr447Met)];ENSG00000013573:MISSENSE[ENST00000407793(c.1418C>T,aCg>aTg):ENSP00000384703(p.Thr473Met)];ENSG00000013573:MISSENSE[ENST00000350437(c.1418C>T,aCg>aTg):ENSP00000309965(p.Thr473Met)];ENSG00000013573:3UTR[ENST00000435753(c.*734C>T)];ENSG00000013573:3UTR[ENST00000251758(c.*739C>T)];ENSG00000013573:3UTR[ENST00000539049(c.*519C>T)];ENSG00000013573:INTRONIC[ENST00000538345(c.50-7693C>T)];ENSG00000013573:EXONIC-NC[ENST00000543511(n.344C>T)];ENSG00000013573:EXONIC-NC[ENST00000536580(n.495C>T)];ENSG00000013573:EXONIC-NC[ENST00000539673(n.160C>T)];ENSG00000013573:EXONIC-NC[ENST00000536265(n.2018C>T)];	NA	NA	NA	3.03E-05	0	NA	NA	NA	NA	NA	MODIFIER	NA	NA	181	179	2	279	265	14	5.02
Smoker	42476	chr12	124362454	C	T	*DNAH10*	NONSENSE-SS-PRX	ENSG00000197653:[ENST00000409039(c.8017C>T,Cga>Tga):ENSP00000386770(p.Arg2673Ter)];ENSG00000197653:[ENST00000497783(c.*1067C>T)];	NA	NA	NA	NA	NA	NA	NA	NA	NA	NA	LOW	NA	NA	150	150	0	167	136	31	18.56
Smoker	42476	chr12	124416206	C	T	*DNAH10*	MISSENSE	ENSG00000197653:MISSENSE[ENST00000409039(c.12586C>T,Cgc>Tgc):ENSP00000386770(p.Arg4196Cys)];ENSG00000197653:EXONIC-NC[ENST00000492261(n.1731C>T)];	NA	NA	NA	8.31E-06	0	NA	NA	NA	NA	NA	MODIFIER	NA	NA	111	111	0	80	66	14	17.5
Smoker	42476	chr12	124416206	C	T	*DNAH10OS*	INTRONIC	ENSG00000250091:[ENST00000514254(c.*28-1116G>A)];	NA	NA	NA	8.31E-06	0	NA	NA	NA	NA	NA	MODIFIER	NA	NA	111	111	0	80	66	14	17.5
Smoker	42476	chr12	10370729	C	G	*GABARAPL1*	MISSENSE	ENSG00000139112:MISSENSE[ENST00000421801(c.158C>G,tCt>tGt):ENSP00000411256(p.Ser53Cys)];ENSG00000139112:MISSENSE[ENST00000543602(c.158C>G,tCt>tGt):ENSP00000445857(p.Ser53Cys)];ENSG00000139112:MISSENSE[ENST00000266458(c.158C>G,tCt>tGt):ENSP00000266458(p.Ser53Cys)];ENSG00000139112:MISSENSE[ENST00000545887(c.158C>G,tCt>tGt):ENSP00000444186(p.Ser53Cys)];ENSG00000139112:MISSENSE[ENST00000545859(c.158C>G,tCt>tGt):ENSP00000444108(p.Ser53Cys)];ENSG00000139112:5UTR[ENST00000539170(c.-113C>G)];ENSG00000139112:5UTR[ENST00000544284(c.-113C>G)];ENSG00000139112:5UTR[ENST00000535576(c.-113C>G)];ENSG00000139112:5UTR[ENST00000545290(c.-113C>G)];ENSG00000139112:5UTR[ENST00000546017(c.-113C>G)];ENSG00000139112:INTRONIC[ENST00000545047(c.91-2310C>G)];ENSG00000139112:INTRONIC[ENST00000541453(c.91-2310C>G)];ENSG00000139112:3UTR[ENST00000542722(c.*378C>G)];ENSG00000139112:3UTR[ENST00000541960(c.*1406C>G)];ENSG00000139112:3UTR[ENST00000540424(c.*489C>G)];ENSG00000139112:EXONIC-NC[ENST00000539289(n.468C>G)];	NA	NA	NA	NA	NA	NA	NA	NA	NA	NA	MODIFIER	NA	NA	59	59	0	75	51	24	32
Smoker	42476	chr12	89918035	C	G	*GALNT4*	MISSENSE	ENSG00000257594:MISSENSE[ENST00000529983(c.292G>C,Gag>Cag):ENSP00000436604(p.Glu98Gln)];ENSG00000257594:INTRONIC[ENST00000413530(c.361-585G>C)];	NA	NA	NA	NA	NA	NA	NA	T	BN	N	MODERATE	NA	NA	113	113	0	155	149	6	3.87
Smoker	42476	chr12	131471773	C	T	*GPR133*	SILENT	ENSG00000111452:SILENT[ENST00000535015(c.720C>T,ctC>ctT):ENSP00000444425(p.Leu240(=))];ENSG00000111452:SILENT[ENST00000542091(c.444C>T,ctC>ctT):ENSP00000442501(p.Leu148(=))];ENSG00000111452:SILENT[ENST00000261654(c.624C>T,ctC>ctT):ENSP00000261654(p.Leu208(=))];ENSG00000111452:5UTR[ENST00000537600(c.-100C>T)];ENSG00000111452:EXONIC-NC[ENST00000541143(n.6094C>T)];	chr12*1314	NA	NA	NA	NA	NA	NA	NA	NA	NA	MODIFIER	NA	NA	91	91	0	102	91	10	9.8
Smoker	42476	chr12	102811615	C	G	*IGF1*	MISSENSE	ENSG00000017427:MISSENSE[ENST00000307046(c.569G>C,aGa>aCa):ENSP00000302665(p.Arg190Thr)];ENSG00000017427:INTRONIC[ENST00000424202(c.354+1672G>C)];ENSG00000017427:INTRONIC[ENST00000392905(c.394+118G>C)];ENSG00000017427:INTRONIC[ENST00000456098(c.451+118G>C)];ENSG00000017427:INTRONIC[ENST00000337514(c.402+1672G>C)];ENSG00000017427:INTRONIC[ENST00000392904(c.451+118G>C)];	NA	NA	NA	NA	NA	NA	NA	NA	NA	NA	MODIFIER	NA	NA	129	129	0	135	108	26	19.26
Smoker	42476	chr12	10573049	G	A	*KLRC3*	MISSENSE	ENSG00000205810:MISSENSE[ENST00000381904(c.101C>T,aCc>aTc):ENSP00000371329(p.Thr34Ile)];ENSG00000205810:MISSENSE[ENST00000396439(c.101C>T,aCc>aTc):ENSP00000379716(p.Thr34Ile)];ENSG00000205810:MISSENSE[ENST00000381903(c.101C>T,aCc>aTc):ENSP00000371328(p.Thr34Ile)];	NA	NA	NA	NA	NA	NA	NA	NA	NA	NA	MODIFIER	NA	NA	156	156	0	220	211	9	4.09
Smoker	42476	chr12	49426373	C	A	*KMT2D*	NONSENSE	ENSG00000167548:NONSENSE[ENST00000301067(c.12115G>T,Gag>Tag):ENSP00000301067(p.Glu4039Ter)];	NA	NA	NA	NA	NA	NA	NA	NA	NA	NA	HIGH	NA	0.338	157	156	0	207	93	114	55.07

Mangalaparthi *et al.* , 2020. Mutational landscape of esophageal squamous cell carcinoma in an Indian cohort
Supplementary Table 4. List of somatic single nucleotide variants identified using whole exome sequencing of ESCC patients

Cohort	Sample	Chromosome	Genomic position	Reference allele	Alternate allele	Gene symbol	Variant type	Variant annotation	OncoMD	COSMIC	dbSNP	ExAC	ExAC_SAS	1000G	1000G_SAS	SIFT	Polyphen2	CONDEL	VEP	LRT	PhastCon7 Way	Total read (Adjacent normal)	Reference allel read support (Adjacent normal)	Alternative read support (Adjacent normal)	Total read (Tumor)	Reference allel read support (Tumor)	Alternative read support (Tumor)	Alternate allele frequency
Smoker	42476	chr12	49496278	C	G	*LMBR1L*	MISSENSE	ENSG00000139636:MISSENSE[ENST00000395141(c.727G>C,Gag>Cag):ENSP00000378573(p.Glu243Gln)];ENSG00000139636:MISSENSE[ENST00000267102(c.742G>C,Gag>Cag):ENSP00000267102(p.Glu248Gln)];ENSG00000139636:MISSENSE[ENST00000547382(c.742G>C,Gag>Cag):ENSP00000447329(p.Glu248Gln)];ENSG00000139636:MISSENSE[ENST00000547675(c.442G>C,Gag>Cag):ENSP00000447240(p.Glu148Gln)];ENSG00000139636:EXONIC-NC[ENST00000552879(n.141G>C)];ENSG00000139636:EXONIC-NC[ENST00000552577(n.873G>C)];ENSG00000139636:EXONIC-NC[ENST00000552141(n.272G>C)];ENSG00000139636:EXONIC-NC[ENST00000550867(n.854G>C)];ENSG00000139636:3UTR[ENST00000457164(c.*273G>C)];ENSG00000139636:3UTR[ENST00000417750(c.*719G>C)];ENSG00000139636:3UTR[ENST00000547670(c.*717G>C)];	NA	NA	chr12*49496278*49496278*C*T*rs748006998*NA*NA*NA	NA	NA	NA	NA	NA	NA	NA	MODIFIER	NA	NA	59	58	0	64	56	8	12.5
Smoker	42476	chr12	40643682	C	G	*LRRK2*	MISSENSE	ENSG00000188906:MISSENSE[ENST00000343742(c.893C>G,gCt>gGt):ENSP00000341930(p.Ala298Gly)];ENSG00000188906:MISSENSE[ENST00000298910(c.893C>G,gCt>gGt):ENSP00000298910(p.Ala298Gly)];ENSG00000188906:MISSENSE[ENST00000416796(c.545C>G,gCt>gGt):ENSP00000398726(p.Ala182Gly)];	NA	NA	chr12*40643682*40643682*C*T*rs281865059*NA*NA*NA	NA	NA	NA	NA	D	BN	N	MODERATE	NA	NA	150	150	0	214	181	33	15.42
Smoker	42476	chr12	40687398	G	A	*LRRK2*	MISSENSE	ENSG00000188906:MISSENSE[ENST00000343742(c.2741G>A,gGa>gAa):ENSP00000341930(p.Gly914Glu)];ENSG00000188906:MISSENSE[ENST00000298910(c.2741G>A,gGa>gAa):ENSP00000298910(p.Gly914Glu)];	NA	NA	NA	NA	NA	NA	NA	T	PoD	N	MODERATE	N	0.977	78	77	0	88	82	6	6.82
Smoker	42476	chr12	133031941	G	C	*NA*	INTERGENIC		NA	NA	NA	NA	NA	NA	NA	NA	NA	NA	MODIFIER	NA	NA	58	58	0	77	68	8	10.39
Smoker	42476	chr12	10573049	G	A	*NKG2-E*	INTRONIC	ENSG00000255641:[ENST00000539033(c.332-1952C>T)];	NA	NA	NA	NA	NA	NA	NA	NA	NA	NA	MODIFIER	NA	NA	156	156	0	220	211	9	4.09
Smoker	42476	chr12	52473634	C	T	*OR7E47P*	EXONIC-NC	ENSG00000257542:[ENST00000546390(n.155C>T)];	NA	NA	NA	NA	NA	NA	NA	NA	NA	NA	MODIFIER	NA	NA	16	16	0	24	19	5	20.83
Smoker	42476	chr12	45568092	T	G	*PLEKHA8P1*	EXONIC-NC	ENSG00000134297:[ENST00000256692(n.594A>C)];ENSG00000134297:[ENST00000336399(n.405A>C)];	NA	NA	NA	8.24E-06	0	NA	NA	NA	NA	NA	MODIFIER	NA	NA	145	144	0	217	127	89	41.01
Smoker	42476	chr12	89918035	C	G	*POC1B*	INTRONIC	ENSG00000139323:[ENST00000541909(c.-119+862G>C)];ENSG00000139323:[ENST00000539190(n.198+862G>C)];ENSG00000139323:[ENST00000546830(c.100+862G>C)];ENSG00000139323:[ENST00000552563(n.296+862G>C)];ENSG00000139323:[ENST00000393179(c.-119+862G>C)];ENSG00000139323:[ENST00000549504(c.-119+862G>C)];ENSG00000139323:[ENST00000549035(c.-27+862G>C)];ENSG00000139323:[ENST00000548715(c.100+862G>C)];ENSG00000139323:[ENST00000547274(c.100+862G>C)];ENSG00000139323:[ENST00000313546(c.100+862G>C)];ENSG00000139323:[ENST00000547496(c.100+862G>C)];	NA	NA	NA	NA	NA	NA	NA	T	BN	N	MODERATE	NA	NA	113	113	0	155	149	6	3.87
Smoker	42476	chr12	89918035	C	G	*POC1B-GALNT4*	MISSENSE	ENSG00000259075:INTRONIC[ENST00000547474(c.101-585G>C)];ENSG00000259075:MISSENSE[ENST00000548729(c.283G>C,Gag>Cag):ENSP00000447852(p.Glu95Gln)];	NA	NA	NA	NA	NA	NA	NA	T	BN	N	MODERATE	NA	NA	113	113	0	155	149	6	3.87
Smoker	42476	chr12	70928428	C	T	*PTPRB*	MISSENSE	ENSG00000127329:MISSENSE[ENST00000538708(c.5284G>A,Gaa>Aaa):ENSP00000438927(p.Glu1762Lys)];ENSG00000127329:MISSENSE[ENST00000550857(c.5284G>A,Gaa>Aaa):ENSP00000447302(p.Glu1762Lys)];ENSG00000127329:MISSENSE[ENST00000261266(c.5554G>A,Gaa>Aaa):ENSP00000261266(p.Glu1852Lys)];ENSG00000127329:MISSENSE[ENST00000334414(c.6208G>A,Gaa>Aaa):ENSP00000334928(p.Glu2070Lys)];ENSG00000127329:MISSENSE[ENST00000550358(c.5944G>A,Gaa>Aaa):ENSP00000448058(p.Glu1982Lys)];ENSG00000127329:MISSENSE[ENST00000451516(c.5284G>A,Gaa>Aaa):ENSP00000393028(p.Glu1762Lys)];	NA	NA	chr12*70928428*70928428*C*G*rs771119207*NA*NA*NA	NA	NA	NA	NA	NA	NA	NA	MODIFIER	NA	NA	42	42	0	90	78	12	13.33
Smoker	42476	chr12	52473634	C	T	*RP11-1100L3.7*	INTRONIC	ENSG00000257663:[ENST00000550301(n.33+2202G>A)];	NA	NA	NA	NA	NA	NA	NA	NA	NA	NA	MODIFIER	NA	NA	16	16	0	24	19	5	20.83
Smoker	42476	chr12	70928428	C	T	*RP11-588H23.3*	INTRONIC	ENSG00000258168:[ENST00000547656(n.78-3379C>T)];ENSG00000258168:[ENST00000548687(n.913-3402C>T)];ENSG00000258168:[ENST00000551438(n.441-3402C>T)];ENSG00000258168:[ENST00000546836(n.170-3402C>T)];ENSG00000258168:[ENST00000549460(n.585-3402C>T)];	NA	NA	chr12*70928428*70928428*C*G*rs771119207*NA*NA*NA	NA	NA	NA	NA	NA	NA	NA	MODIFIER	NA	NA	42	42	0	90	78	12	13.33
Smoker	42476	chr12	122359305	G	C	*RP11-87C12.2*	3UTR	ENSG00000256950:[ENST00000546333(c.*199G>C)];	NA	NA	NA	NA	NA	NA	NA	D_lc	BN	N	MODERATE	N	0.005	90	90	0	77	58	19	24.68
Smoker	42476	chr12	21358911	T	G	*SLCO1B1*	MISSENSE	ENSG00000134538:MISSENSE[ENST00000256958(c.1441T>G,Tac>Gac):ENSP00000256958(p.Tyr481Asp)];	NA	NA	chr12*21358911*21358911*T*C*rs745708956*NA*NA*NA	NA	NA	NA	NA	D	PrD	D	MODERATE	D	0.991	102	102	0	126	110	15	11.9
Smoker	42476	chr12	23728709	T	G	*SOX5*	MISSENSE	ENSG00000134532:MISSENSE[ENST00000309359(c.1189A>C,Aca>Cca):ENSP00000308927(p.Thr397Pro)];ENSG00000134532:MISSENSE[ENST00000546136(c.1189A>C,Aca>Cca):ENSP00000437487(p.Thr397Pro)];ENSG00000134532:MISSENSE[ENST00000537393(c.1123A>C,Aca>Cca):ENSP00000439832(p.Thr375Pro)];ENSG00000134532:MISSENSE[ENST00000451604(c.1228A>C,Aca>Cca):ENSP00000398273(p.Thr410Pro)];ENSG00000134532:MISSENSE[ENST00000396007(c.70A>C,Aca>Cca):ENSP00000379328(p.Thr24Pro)];ENSG00000134532:MISSENSE[ENST00000545921(c.1198A>C,Aca>Cca):ENSP00000443520(p.Thr400Pro)];ENSG00000134532:MISSENSE[ENST00000367206(c.1198A>C,Aca>Cca):ENSP00000356174(p.Thr400Pro)];ENSG00000134532:INTRONIC[ENST00000381381(c.1125+28612A>C)];ENSG00000134532:INTRONIC[ENST00000535530(n.489-12372A>C)];ENSG00000134532:INTRONIC[ENST00000541536(c.1125+28612A>C)];ENSG00000134532:EXONIC-NC[ENST00000542241(n.260A>C)];	NA	NA	NA	NA	NA	NA	NA	NA	NA	NA	MODIFIER	NA	NA	52	52	0	53	43	10	18.87
Smoker	42476	chr12	22354862	G	A	*ST8SIA1*	MISSENSE	ENSG00000111728:MISSENSE[ENST00000396037(c.695C>T,tCt>tTt):ENSP00000379353(p.Ser232Phe)];ENSG00000111728:MISSENSE[ENST00000539510(c.266C>T,tCt>tTt):ENSP00000446363(p.Ser89Phe)];ENSG00000111728:MISSENSE[ENST00000540824(c.548C>T,tCt>tTt):ENSP00000441707(p.Ser183Phe)];ENSG00000111728:INTRONIC[ENST00000545524(n.249+47078C>T)];ENSG00000111728:INTRONIC[ENST00000544732(n.151+47078C>T)];ENSG00000111728:EXONIC-NC[ENST00000508924(n.298C>T)];ENSG00000111728:EXONIC-NC[ENST00000545494(n.416C>T)];ENSG00000111728:3UTR[ENST00000261197(c.*177C>T)];	NA	NA	NA	NA	NA	NA	NA	NA	PrD	NA	MODERATE	NA	NA	91	91	0	133	113	20	15.04
Smoker	42476	chr12	112371885	C	A	*TMEM116*	INTRONIC	ENSG00000198270:[ENST00000552374(c.589-51G>T)];ENSG00000198270:[ENST00000549537(c.31-51G>T)];ENSG00000198270:[ENST00000547878(c.*563-51G>T)];ENSG00000198270:[ENST00000550831(c.313-51G>T)];ENSG00000198270:[ENST00000437003(c.313-51G>T)];ENSG00000198270:[ENST00000552801(n.659-51G>T)];ENSG00000198270:[ENST00000355445(c.484-51G>T)];ENSG00000198270:[ENST00000354825(c.313-51G>T)];ENSG00000198270:[ENST00000548283(n.3370G>T)];	NA	NA	NA	NA	NA	NA	NA	NA	NA	NA	MODIFIER	NA	NA	84	83	1	73	43	29	39.73

Supplementary Table 4. List of somatic single nucleotide variants identified using whole exome sequencing of ESCC patients

Cohort	Sample	Chromosome	Genomic position	Reference allele	Alternate allele	Gene symbol	Variant type	Variant annotation	OncoMD	COSMIC	dbSNP	ExAC	ExAC_SAS	1000G	1000G_SAS	SIFT	Polyphen2	CONDEL	VEP	LRT	PhastCon7 Way	Total read (Adjacent normal)	Reference allel read support (Adjacent normal)	Alternative read support (Adjacent normal)	Total read (Tumor)	Reference allel read support (Tumor)	Alternative read support (Tumor)	Alternate allele frequency
Smoker	42476	chr12	122359305	G	C	*WDR66*	MISSENSE	ENSG00000158023:MISSENSE[ENST00000397454(c.94G>C,Gaa>Caa):ENSP00000380595(p.Glu32Gln)];ENSG00000158023:MISSENSE[ENST00000288912(c.94G>C,Gaa>Caa):ENSP00000288912(p.Glu32Gln)];	NA	NA	NA	NA	NA	NA	NA	D_lc	BN	N	MODERATE	N	0.005	90	90	0	77	58	19	24.68
Smoker	42476	chr13	25458490	C	T	*CENPJ*	MISSENSE	ENSG00000151849:MISSENSE[ENST00000381884(c.3589G>A,Gtg>Atg):ENSP00000371308(p.Val1197Met)];ENSG00000151849:EXONIC-NC[ENST00000471870(n.479G>A)];ENSG00000151849:3UTR[ENST00000545981(c.*243G>A)];	NA	NA	NA	0.0001071	0	NA	NA	NA	NA	NA	MODIFIER	NA	NA	104	104	0	84	13	71	84.52
Smoker	42476	chr13	111135017	C	G	*COL4A2*	INTRONIC	ENSG00000134871:[ENST00000360467(c.2902+11C>G)];ENSG00000134871:[ENST00000483683(n.557+11C>G)];	NA	NA	NA	8.30E-06	6.09E-05	NA	NA	NA	NA	NA	MODIFIER	NA	NA	29	29	0	25	17	8	32
Smoker	42476	chr13	41525506	T	C	*ELF1*	MISSENSE	ENSG00000120690:MISSENSE[ENST00000239882(c.320A>G,aAt>aGt):ENSP00000239882(p.Asn107Ser)];ENSG00000120690:MISSENSE[ENST00000442101(c.320A>G,aAt>aGt):ENSP00000405580(p.Asn107Ser)];ENSG00000120690:EXONIC-NC[ENST00000498824(n.626A>G)];	NA	NA	NA	NA	NA	NA	NA	NA	NA	NA	MODIFIER	NA	NA	114	112	0	85	33	52	61.18
Smoker	42476	chr13	70456566	T	G	*KLHL1*	MISSENSE	ENSG00000150361:MISSENSE[ENST00000545028(c.497A>C,cAt>cCt):ENSP00000439602(p.His166Pro)];ENSG00000150361:MISSENSE[ENST00000377844(c.1076A>C,cAt>cCt):ENSP00000367075(p.His359Pro)];	NA	NA	chr13*70456566*70456566*T*C*rs372441278*NA*NA*NA	NA	NA	NA	NA	T	BN	N	MODERATE	D	1	136	136	0	81	21	60	74.07
Smoker	42476	chr13	97995235	A	G	*MBNL2*	INTRONIC	ENSG00000139793:[ENST00000343600(c.340-35A>G)];ENSG00000139793:[ENST00000445661(c.339+8541A>G)];ENSG00000139793:[ENST00000376673(c.340-35A>G)];ENSG00000139793:[ENST00000469707(c.340-35A>G)];ENSG00000139793:[ENST00000345429(c.340-35A>G)];ENSG00000139793:[ENST00000397601(c.340-35A>G)];	NA	NA	NA	NA	NA	NA	NA	NA	NA	NA	MODIFIER	NA	NA	61	61	0	82	16	66	80.49
Smoker	42476	chr13	35785266	A	T	*NBEA*	SILENT	ENSG00000172915:SILENT[ENST00000400445(c.5598A>T,ccA>ccT):ENSP00000383295(p.Pro1866(=))];ENSG00000172915:SILENT[ENST00000540320(c.5598A>T,ccA>ccT):ENSP00000440951(p.Pro1866(=))];ENSG00000172915:SILENT[ENST00000310336(c.5598A>T,ccA>ccT):ENSP00000308534(p.Pro1866(=))];ENSG00000172915:SILENT[ENST00000379939(c.5589A>T,ccA>ccT):ENSP00000369271(p.Pro1863(=))];	NA	NA	NA	NA	NA	NA	NA	NA	NA	NA	LOW	NA	NA	142	142	0	81	64	17	20.99
Smoker	42476	chr13	25671795	C	T	*PABPC3*	MISSENSE	ENSG00000151846:MISSENSE[ENST00000281589(c.1459C>T,Cgt>Tgt):ENSP00000281589(p.Arg487Cys)];	chr13*2567	chr13*2567	0.03494	0.001317	0.001093427	0.0349441	4.91E-02	D	BN	N	MODERATE	U	0.998	165	164	1	175	159	14	8
Smoker	42476	chr13	25671809	T	A	*PABPC3*	SILENT	ENSG00000151846:SILENT[ENST00000281589(c.1473T>A,gcT>gcA):ENSP00000281589(p.Ala491(=))];	NA	NA	chr13*25671803*25671812*AGCTGCTGCT*A*rs763555033*NA*NA*NA|chr13*25671806*25671821*TGCTGCTGCTGCTGCA*T*rs537105482*1*0.999*0.0009984|chr13*25671809*25671809*T*TGCTGCTGCTGCAGCTACC*rs767831154*NA*NA*NA	NA	NA	NA	NA	NA	NA	NA	LOW	NA	NA	159	159	0	159	144	8	5.03
Smoker	42476	chr13	39587605	G	A	*PROSER1*	MISSENSE	ENSG00000120685:INTRONIC[ENST00000484434(n.709-1279C>T)];ENSG00000120685:MISSENSE[ENST00000352251(c.1784C>T,tCa>tTa):ENSP00000332034(p.Ser595Leu)];ENSG00000120685:MISSENSE[ENST00000350125(c.1718C>T,tCa>tTa):ENSP00000339123(p.Ser573Leu)];	NA	NA	NA	8.24E-06	0	NA	NA	T_lc	BN	N	MODERATE	NA	NA	68	68	0	47	7	40	85.11
Smoker	42476	chr13	39608346	A	C	*PROSER1*	INTRONIC	ENSG00000120685:[ENST00000352251(c.46-13T>G)];ENSG00000120685:[ENST00000602534(n.98-13T>G)];ENSG00000120685:[ENST00000418503(c.46-2578T>G)];ENSG00000120685:[ENST00000350125(c.46-2578T>G)];	chr13*3960	NA	.	NA	NA	NA	NA	NA	NA	NA	MODIFIER	NA	NA	117	116	1	103	96	5	4.85
Smoker	42476	chr13	61057993	G	A	*TDRD3*	INTRONIC	ENSG00000083544:[ENST00000377882(c.288+13G>A)];ENSG00000083544:[ENST00000377894(c.288+13G>A)];ENSG00000083544:[ENST00000196169(c.288+13G>A)];ENSG00000083544:[ENST00000377881(c.288+13G>A)];ENSG00000083544:[ENST00000535286(c.567+13G>A)];ENSG00000083544:[ENST00000484389(c.288+13G>A)];	NA	NA	NA	NA	NA	NA	NA	NA	NA	NA	MODIFIER	NA	NA	89	89	0	79	73	6	7.59
Smoker	42476	chr14	95593018	C	T	*DICER1*	MISSENSE	ENSG00000100697:MISSENSE[ENST00000343455(c.802G>A,Gaa>Aaa):ENSP00000343745(p.Glu268Lys)];ENSG00000100697:MISSENSE[ENST00000526495(c.802G>A,Gaa>Aaa):ENSP00000437256(p.Glu268Lys)];ENSG00000100697:MISSENSE[ENST00000541352(c.802G>A,Gaa>Aaa):ENSP00000444719(p.Glu268Lys)];ENSG00000100697:MISSENSE[ENST00000393063(c.802G>A,Gaa>Aaa):ENSP00000376783(p.Glu268Lys)];ENSG00000100697:MISSENSE[ENST00000527414(c.802G>A,Gaa>Aaa):ENSP00000435681(p.Glu268Lys)];	NA	NA	NA	NA	NA	NA	NA	T	BN	N	MODERATE	N	0.993	71	70	0	78	38	40	51.28
Smoker	42476	chr14	59930943	G	T	*GPR135*	SILENT	ENSG00000181619:SILENT[ENST00000481661(c.1002C>A,ctC>ctA):ENSP00000432696(p.Leu334(=))];ENSG00000181619:SILENT[ENST00000395116(c.1002C>A,ctC>ctA):ENSP00000378548(p.Leu334(=))];	NA	NA	NA	NA	NA	NA	NA	NA	NA	NA	MODIFIER	NA	NA	58	58	0	64	54	10	15.62
Smoker	42476	chr14	35735965	G	C	*KIAA0391*	SILENT	ENSG00000100890:SILENT[ENST00000557404(c.192G>C,cgG>cgC):ENSP00000450898(p.Arg64(=))];ENSG00000258790:SILENT[ENST00000557565(c.1308G>C,cgG>cgC):ENSP00000454657(p.Arg436(=))];ENSG00000100890:SILENT[ENST00000605870(c.192G>C,cgG>cgC):ENSP00000474299(p.Arg64(=))];ENSG00000100890:SILENT[ENST00000604948(c.1023G>C,cgG>cgC):ENSP00000474620(p.Arg341(=))];ENSG00000100890:SILENT[ENST00000250377(c.1023G>C,cgG>cgC):ENSP00000250377(p.Arg341(=))];ENSG00000100890:SILENT[ENST00000603544(c.1260G>C,cgG>cgC):ENSP00000473856(p.Arg420(=))];ENSG00000100890:SILENT[ENST00000534898(c.1308G>C,cgG>cgC):ENSP00000440915(p.Arg436(=))];ENSG00000100890:SILENT[ENST00000321130(c.1260G>C,cgG>cgC):ENSP00000324697(p.Arg420(=))];	NA	NA	NA	NA	NA	NA	NA	NA	NA	NA	LOW	NA	NA	120	118	0	459	429	30	6.54
Smoker	42476	chr14	59930943	G	T	*L3HYPDH*	INTRONIC	ENSG00000126790:[ENST00000466522(n.31-3052C>A)];	NA	NA	NA	NA	NA	NA	NA	NA	NA	NA	MODIFIER	NA	NA	58	58	0	64	54	10	15.62
Smoker	42476	chr14	47311098	C	T	*MDGA2*	3UTR	ENSG00000139915:[ENST00000399222(c.*36G>A)];ENSG00000139915:[ENST00000357362(c.*36G>A)];ENSG00000139915:[ENST00000426342(c.*36G>A)];ENSG00000139915:[ENST00000399232(c.*36G>A)];	NA	NA	NA	NA	NA	NA	NA	NA	NA	NA	MODIFIER	NA	NA	133	132	0	104	99	5	4.81

Supplementary Table 4. List of somatic single nucleotide variants identified using whole exome sequencing of ESCC patients

Cohort	Sample	Chromosome	Genomic position	Reference allele	Alternate allele	Gene symbol	Variant type	Variant annotation	OncoMD	COSMIC	dbSNP	ExAC	ExAC_SAS	1000G	1000G_SAS	SIFT	Polyphen2	CONDEL	VEP	LRT	PhastCon7 Way	Total read (Adjacent normal)	Reference allel read support (Adjacent normal)	Alternative read support (Adjacent normal)	Total read (Tumor)	Reference allel read support (Tumor)	Alternative read support (Tumor)	Alternate allele frequency
Smoker	42476	chr14	101302559	G	C	*MEG3*	EXONIC-NC	ENSG00000214548:[ENST00000522618(n.503G>C)];ENSG00000214548:[ENST00000398474(n.1236G>C)];ENSG00000214548:[ENST00000554639(n.1222G>C)];ENSG00000214548:[ENST00000519709(n.1254G>C)];ENSG00000214548:[ENST00000521404(n.973G>C)];ENSG00000214548:[ENST0000398518(n.1216G>C)];ENSG00000214548:[ENST00000520714(n.1239G>C)];ENSG00000214548:[ENST00000524035(n.2368G>C)];ENSG00000214548:[ENST00000452514(n.322G>C)];ENSG00000214548:[ENST00000524131(n.441G>C)];ENSG00000214548:[ENST00000556736(n.1217G>C)];ENSG00000214548:[ENST00000423456(n.1337G>C)];ENSG00000214548:[ENST00000412736(n.1174G>C)];ENSG00000214548:[ENST00000424076(n.1087G>C)];ENSG00000214548:[ENST00000429159(n.1121G>C)];ENSG00000214548:[ENST00000555928(n.230G>C)];ENSG00000214548:[ENST00000522771(n.2906G>C)];ENSG00000214548:[ENST00000398461(n.2967G>C)];ENSG00000214548:[ENST00000451743(n.1087G>C)];ENSG00000214548:[ENST00000523671(n.2290G>C)];ENSG00000214548:[ENST00000455531(n.2945G>C)];ENSG00000214548:[ENST00000521812(n.634G>C)];	NA	NA	NA	NA	NA	NA	NA	NA	NA	NA	MODIFIER	NA	NA	97	97	0	72	61	10	13.89
Smoker	42476	chr14	45696111	G	C	*MIS18BP1*	INTRONIC	ENSG00000129534:[ENST00000310806(c.1747-72C>G)];ENSG00000129534:[ENST00000454990(c.*375-72C>G)];	NA	NA	NA	NA	NA	NA	NA	NA	NA	NA	MODIFIER	NA	NA	33	33	0	29	15	13	44.83
Smoker	42476	chr14	51197604	C	G	*NIN*	INTRONIC	ENSG00000100503:[ENST00000389868(c.3799+37G>C)];ENSG00000100503:[ENST00000245441(c.5878-1163G>C)];ENSG00000100503:[ENST00000476352(c.5938+37G>C)];ENSG00000100503:[ENST00000324330(c.5938+37G>C)];ENSG00000100503:[ENST00000382041(c.5878-1163G>C)];ENSG00000100503:[ENST00000389869(c.4349-1163G>C)];ENSG00000100503:[ENST00000530853(c.4349-1163G>C)];ENSG00000100503:[ENST00000382043(c.3739-1163G>C)];ENSG00000100503:[ENST00000453196(c.5878-1163G>C)];ENSG00000100503:[ENST00000530997(c.5878-1163G>C)];ENSG00000100503:[ENST00000485005(c.391+37G>C)];	NA	NA	NA	NA	NA	NA	NA	NA	NA	NA	MODIFIER	NA	NA	156	155	0	116	94	22	18.97
Smoker	42476	chr14	20585621	C	G	*OR4K17*	MISSENSE	ENSG00000176230:MISSENSE[ENST00000315543(c.56C>G,tCt>tGt):ENSP00000319197(p.Ser19Cys)];	NA	NA	NA	NA	NA	NA	NA	D_lc	BN	N	MODERATE	NA	NA	98	98	0	147	120	27	18.37
Smoker	42476	chr14	101302559	G	C	*RP11-123M6.2*	INTRONIC	ENSG00000258663:[ENST00000554041(n.144-1645C>G)];	NA	NA	NA	NA	NA	NA	NA	NA	NA	NA	MODIFIER	NA	NA	97	97	0	72	61	10	13.89
Smoker	42476	chr15	86205564	C	A	*AKAP13*	INTRONIC	ENSG00000170776:[ENST00000560676(n.1062-2230C>A)];ENSG00000170776:[ENST00000559486(c.462-55C>A)];ENSG00000170776:[ENST00000394518(c.4800-2230C>A)];ENSG00000170776:[ENST00000559362(c.4746-2230C>A)];ENSG00000170776:[ENST00000361243(c.4746-55C>A)];ENSG00000170776:[ENST00000560579(n.648-2230C>A)];	NA	NA	NA	NA	NA	NA	NA	NA	NA	NA	MODIFIER	NA	NA	206	205	0	190	169	20	10.53
Smoker	42476	chr15	45253763	A	C	*C15orf43*	MISSENSE	ENSG00000167014:MISSENSE[ENST00000340827(c.329A>C,cAa>cCa):ENSP00000340644(p.Gln110Pro)];ENSG00000167014:INTRONIC[ENST00000557864(c.*252+3053A>C)];ENSG00000167014:3UTR[ENST00000559137(c.*295A>C)];	NA	NA	NA	NA	NA	NA	NA	NA	NA	NA	MODIFIER	NA	NA	74	74	0	62	44	18	29.03
Smoker	42476	chr15	68570969	C	A	*FEM1B*	MISSENSE	ENSG00000169018:MISSENSE[ENST00000306917(c.214C>A,Cag>Aag):ENSP00000307298(p.Gln72Lys)];	NA	NA	NA	NA	NA	NA	NA	D	BN	N	MODERATE	D	1	55	55	0	74	63	11	14.86
Smoker	42476	chr15	28366615	G	T	*HERC2*	INTRONIC	ENSG00000128731:[ENST00000261609(c.13189-40C>A)];	NA	NA	NA	NA	NA	NA	NA	NA	NA	NA	MODIFIER	NA	NA	66	66	0	35	22	13	37.14
Smoker	42476	chr15	90176402	G	C	*KIF7*	MISSENSE	ENSG00000166813:MISSENSE[ENST00000394412(c.2688C>G,aaC>aaG):ENSP00000377934(p.Asn896Lys)];	NA	NA	chr15*90176402*90176402*G*A*rs779076505*NA*NA*NA	NA	NA	NA	NA	T	PoD	D	MODERATE	D	0.999	35	35	0	40	30	10	25
Smoker	42476	chr15	66853590	C	T	*LCTL*	MISSENSE	ENSG00000188501:MISSENSE[ENST00000341509(c.544G>A,Gcc>Acc):ENSP00000343490(p.Ala182Thr)];ENSG00000188501:MISSENSE[ENST00000537670(c.25G>A,Gcc>Acc):ENSP00000445419(p.Ala9Thr)];ENSG00000188501:MISSENSE[ENST00000565875(c.25G>A,Gcc>Acc):ENSP00000455793(p.Ala9Thr)];	NA	NA	NA	NA	NA	NA	NA	D_lc	PoD	D	MODERATE	NA	NA	85	85	0	78	63	15	19.23
Smoker	42476	chr15	40585188	C	A	*PLCB2*	SILENT	ENSG00000137841:SILENT[ENST00000558588(c.2265G>T,gtG>gtT):ENSP00000452642(p.Val755(=))];ENSG00000137841:SILENT[ENST00000260402(c.2265G>T,gtG>gtT):ENSP00000260402(p.Val755(=))];ENSG00000137841:SILENT[ENST00000557821(c.2253G>T,gtG>gtT):ENSP00000453975(p.Val751(=))];ENSG00000137841:SILENT[ENST00000456256(c.2265G>T,gtG>gtT):ENSP00000411991(p.Val755(=))];ENSG00000137841:EXONIC-NC[ENST00000561378(n.102G>T)];	NA	NA	NA	NA	NA	NA	NA	NA	NA	NA	MODIFIER	NA	NA	71	71	0	38	31	7	18.42
Smoker	42476	chr15	72700187	C	G	*RP11-1007O24.3*	INTRONIC-SS-ACR	ENSG00000261423:INTRONIC-SS-ACR[ENST00000562573(n.656-1G>C)];	NA	NA	chr15*72700184*72700187*TCTC*T*rs751625758*NA*NA*NA|chr15*72700187*72700187*C*CT*rs755185406*NA*NA*NA	NA	NA	NA	NA	NA	NA	NA	MODIFIER	NA	NA	38	38	0	24	14	10	41.67
Smoker	42476	chr15	20466479	G	A	*RP11-492D6.3*	INTRONIC	ENSG00000258628:[ENST00000557586(n.1047+646C>T)];	NA	NA	NA	NA	NA	NA	NA	NA	NA	NA	MODIFIER	NA	NA	121	121	0	55	48	7	12.73
Smoker	42476	chr15	90798984	C	T	*RP11-697E2.6*	INTRONIC	ENSG00000261147:[ENST00000561573(c.418-1386C>T)];	NA	NA	NA	NA	NA	NA	NA	NA	NA	NA	LOW	NA	NA	111	110	0	105	97	8	7.62
Smoker	42476	chr15	86205564	C	A	*RP11-815J21.4*	INTRONIC	ENSG00000259367:[ENST00000558980(n.264+8352G>T)];	NA	NA	NA	NA	NA	NA	NA	NA	NA	NA	MODIFIER	NA	NA	206	205	0	190	169	20	10.53
Smoker	42476	chr15	62995045	G	C	*TLN2*	MISSENSE	ENSG00000171914:MISSENSE[ENST00000561311(c.1986G>C,gaG>gaC):ENSP00000453508(p.Glu662Asp)];ENSG00000171914:MISSENSE[ENST00000306829(c.1986G>C,gaG>gaC):ENSP00000303476(p.Glu662Asp)];	NA	NA	NA	NA	NA	NA	NA	T	BN	N	MODERATE	D	0.995	63	63	0	59	29	30	50.85
Smoker	42476	chr15	72700187	C	G	*TMEM202*	MISSENSE	ENSG00000187806:MISSENSE[ENST00000341689(c.775C>G,Cta>Gta):ENSP00000340212(p.Leu259Val)];ENSG00000187806:3UTR[ENST00000567679(c.*201C>G)];ENSG00000187806:3UTR[ENST00000568167(c.*333C>G)];	NA	NA	chr15*72700184*72700187*TCTC*T*rs751625758*NA*NA*NA|chr15*72700187*72700187*C*CT*rs755185406*NA*NA*NA	NA	NA	NA	NA	NA	NA	NA	MODIFIER	NA	NA	38	38	0	24	14	10	41.67
Smoker	42476	chr15	90798984	C	T	*TTLL13*	INTRONIC-SS-ACR	ENSG00000213471:[ENST00000339615(c.418-3C>T)];ENSG00000213471:[ENST00000438251(c.418-3C>T)];	NA	NA	NA	NA	NA	NA	NA	NA	NA	NA	LOW	NA	NA	111	110	0	105	97	8	7.62
Smoker	42476	chr15	50784955	C	A	*USP8*	MISSENSE	ENSG00000138592:MISSENSE[ENST00000307179(c.2292C>A,aaC>aaA):ENSP00000302239(p.Asn764Lys)];ENSG00000138592:MISSENSE[ENST00000396444(c.2292C>A,aaC>aaA):ENSP00000379721(p.Asn764Lys)];ENSG00000138592:MISSENSE[ENST00000433963(c.2292C>A,aaC>aaA):ENSP00000405537(p.Asn764Lys)];ENSG00000138592:MISSENSE[ENST00000425032(c.1974C>A,aaC>aaA):ENSP00000412682(p.Asn658Lys)];	chr15*5078	chr15*5078	NA	0.3443056	0.382427148	NA	NA	D	BN	N	MODERATE	N	0.999	187	184	2	120	114	6	5

Supplementary Table 4. List of somatic single nucleotide variants identified using whole exome sequencing of ESCC patients

Cohort	Sample	Chromosome	Genomic position	Reference allele	Alternate allele	Gene symbol	Variant type	Variant annotation	OncoMD	COSMIC	dbSNP	ExAC	ExAC_SAS	1000G	1000G_SAS	SIFT	Polyphen2	CONDEL	VEP	LRT	PhastCon7 Way	Total read (Adjacent normal)	Reference allel read support (Adjacent normal)	Alternative read support (Adjacent normal)	Total read (Tumor)	Reference allel read support (Tumor)	Alternative read support (Tumor)	Alternate allele frequency
Smoker	42476	chr16	24979992	C	G	*ARHGAP17*	MISSENSE	ENSG00000140750:EXONIC-NC[ENST00000571575(n.418G>C)];ENSG00000140750:EXONIC-NC[ENST00000575447(n.450G>C)];ENSG00000140750:EXONIC-NC[ENST00000575975(n.374G>C)];ENSG00000140750:MISSENSE[ENST00000455311(c.371G>C,gGc>gCc):ENSP00000406701(p.Gly124Ala)];ENSG00000140750:MISSENSE[ENST00000441763(c.374G>C,gGc>gCc):ENSP00000406950(p.Gly125Ala)];ENSG00000140750:MISSENSE[ENST00000289968(c.374G>C,gGc>gCc):ENSP00000289968(p.Gly125Ala)];ENSG00000140750:MISSENSE[ENST00000303665(c.374G>C,gGc>gCc):ENSP00000303130(p.Gly125Ala)];ENSG00000140750:MISSENSE[ENST00000573449(c.143G>C,gGc>gCc):ENSP00000461426(p.Gly48Ala)];ENSG00000140750:MISSENSE[ENST00000571480(c.370G>C,Gca>Cca):ENSP00000459074(p.Ala124Pro)];ENSG00000140750:3UTR[ENST00000573625(c.*196G>C)];	NA	NA	NA	NA	NA	NA	NA	D_lc	UN	NA	MODERATE	NA	NA	42	41	0	63	55	7	11.11
Smoker	42476	chr16	80667008	G	A	*CDYL2*	MISSENSE	ENSG00000166446:MISSENSE[ENST00000563890(c.745C>T,Cgg>Tgg):ENSP00000455111(p.Arg249Trp)];ENSG00000166446:MISSENSE[ENST00000566173(c.745C>T,Cgg>Tgg):ENSP00000456934(p.Arg249Trp)];ENSG00000166446:MISSENSE[ENST00000562812(c.745C>T,Cgg>Tgg):ENSP00000454546(p.Arg249Trp)];ENSG00000166446:MISSENSE[ENST00000570137(c.742C>T,Cgg>Tgg):ENSP00000476295(p.Arg248Trp)];ENSG00000166446:MISSENSE[ENST00000561616(c.193C>T,Cgg>Tgg):ENSP00000456498(p.Arg65Trp)];ENSG00000166446:EXONIC-NC[ENST00000567924(n.70C>T)];	NA	NA	chr16*80667008*80667008*G*T*rs754339035*NA*NA*NA	NA	NA	NA	NA	NA	NA	NA	MODIFIER	NA	NA	75	75	0	90	78	12	13.33
Smoker	42476	chr16	24828294	A	C	*CTD-2515A14.1*	INTRONIC	ENSG00000261669:[ENST00000568895(n.134+2633T>G)];	NA	NA	NA	NA	NA	NA	NA	NA	NA	NA	MODIFIER	NA	NA	107	107	0	148	76	72	48.65
Smoker	42476	chr16	67912065	C	T	*EDC4*	INTRONIC-SS-ACR	ENSG00000038358:[ENST00000536072(n.900-4C>T)];ENSG00000038358:[ENST00000358933(c.895-4C>T)];ENSG00000038358:[ENST00000574770(n.659-4C>T)];ENSG00000038358:[ENST00000573992(n.274-4C>T)];ENSG00000038358:[ENST00000572221(n.1605C>T)];	NA	NA	NA	NA	NA	NA	NA	NA	NA	NA	MODIFIER	NA	NA	59	59	0	60	49	11	18.33
Smoker	42476	chr16	20335275	G	T	*GP2*	MISSENSE	ENSG00000169347:MISSENSE[ENST00000381362(c.398C>A,aCc>aAc):ENSP00000370767(p.Thr133Asn)];ENSG00000169347:MISSENSE[ENST00000302555(c.398C>A,aCc>aAc):ENSP00000304044(p.Thr133Asn)];ENSG00000169347:MISSENSE[ENST00000572478(c.203C>A,aCc>aAc):ENSP00000461061(p.Thr68Asn)];ENSG00000169347:MISSENSE[ENST00000572347(c.134C>A,aCc>aAc):ENSP00000461632(p.Thr45Asn)];ENSG00000169347:INTRONIC[ENST00000341642(c.95-974C>A)];ENSG00000169347:INTRONIC[ENST00000574982(c.*36-974C>A)];ENSG00000169347:INTRONIC[ENST00000575582(c.95-974C>A)];ENSG00000169347:INTRONIC[ENST00000381360(c.95-564C>A)];	NA	NA	chr16*20335275*20335275*G*C*rs775621858*NA*NA*NA	NA	NA	NA	NA	NA	NA	NA	MODIFIER	NA	NA	58	58	0	84	63	21	25
Smoker	42476	chr16	33965582	C	T	*NA*	INTERGENIC		NA	NA	NA	0.2372648	0.168472975	NA	NA	NA	NA	NA	MODIFIER	NA	NA	2037	1892	29	937	813	27	2.88
Smoker	42476	chr16	68255254	G	C	*NFATC3*	INTRONIC	ENSG00000072736:[ENST00000346183(c.3107-4999G>C)];ENSG00000072736:[ENST00000329524(c.*4-4999G>C)];ENSG00000072736:[ENST00000566301(n.3256-4999G>C)];ENSG00000072736:[ENST00000563288(n.3206-4999G>C)];ENSG00000072736:[ENST00000563796(n.3169-4999G>C)];ENSG00000072736:[ENST00000570212(c.3060-4999G>C)];ENSG00000072736:[ENST00000563319(n.3538-4999G>C)];ENSG00000072736:[ENST00000349223(c.3195+67G>C)];ENSG00000072736:[ENST00000569766(n.3434-4999G>C)];ENSG00000072736:[ENST00000567152(n.3065-4999G>C)];ENSG00000072736:[ENST00000562926(c.*4-4999G>C)];ENSG00000072736:[ENST00000535127(n.3610-4999G>C)];	NA	NA	NA	NA	NA	NA	NA	NA	NA	NA	MODIFIER	NA	NA	52	52	0	41	31	10	24.39
Smoker	42476	chr16	33965563	C	T	*RNA5-8SP2*	EXONIC-NC	ENSG00000200434:[ENST00000363564(n.138C>T)];	NA	NA	.	0.0158092	0.016436971	NA	NA	NA	NA	NA	MODIFIER	NA	NA	1513	1302	22	709	586	28	3.95
Smoker	42476	chr16	68255254	G	C	*RP11-96D1.5*	INTRONIC	ENSG00000260891:[ENST00000569088(n.223+98C>G)];	NA	NA	NA	NA	NA	NA	NA	NA	NA	NA	MODIFIER	NA	NA	52	52	0	41	31	10	24.39
Smoker	42476	chr16	3647978	G	A	*SLX4*	SILENT	ENSG00000188827:SILENT[ENST00000294008(c.1186C>T,Ctg>Ttg):ENSP00000294008(p.Leu396(=))];ENSG00000188827:EXONIC-NC[ENST00000466154(n.2407C>T)];	NA	NA	NA	NA	NA	NA	NA	NA	NA	NA	MODIFIER	NA	NA	83	83	0	91	79	11	12.09
Smoker	42476	chr16	24828294	A	C	*TNRC6A*	INTRONIC	ENSG00000090905:[ENST00000395799(c.4972+17A>C)];ENSG00000090905:[ENST00000569634(n.372+17A>C)];ENSG00000090905:[ENST00000315183(c.4825+17A>C)];ENSG00000090905:[ENST00000450465(c.1804+17A>C)];ENSG00000090905:[ENST00000569376(c.*170+17A>C)];ENSG00000090905:[ENST00000432286(c.406+17A>C)];ENSG00000090905:[ENST00000491718(c.*4497+17A>C)];	NA	NA	NA	NA	NA	NA	NA	NA	NA	NA	MODIFIER	NA	NA	107	107	0	148	76	72	48.65
Smoker	42476	chr16	683438	C	G	*WFIKKN1*	MISSENSE	ENSG00000127578:MISSENSE[ENST00000319070(c.1028C>G,aCc>aGc):ENSP00000324763(p.Thr343Ser)];ENSG00000127578:EXONIC-NC[ENST00000573440(n.4200C>G)];	NA	NA	NA	NA	NA	NA	NA	NA	NA	NA	MODIFIER	NA	NA	89	89	0	88	78	10	11.36
Smoker	42476	chr16	30106657	C	A	*YPEL3*	MISSENSE	ENSG00000090238:MISSENSE[ENST00000566134(c.33G>T,caG>caT):ENSP00000457598(p.Gln11His)];ENSG00000090238:MISSENSE[ENST00000398838(c.33G>T,caG>caT):ENSP00000381818(p.Gln11His)];ENSG00000090238:MISSENSE[ENST00000566595(c.33G>T,caG>caT):ENSP00000456427(p.Gln11His)];ENSG00000090238:MISSENSE[ENST00000398841(c.147G>T,caG>caT):ENSP00000381821(p.Gln49His)];ENSG00000090238:MISSENSE[ENST00000562641(c.147G>T,caG>caT):ENSP00000456825(p.Gln49His)];ENSG00000090238:MISSENSE[ENST00000563788(c.33G>T,caG>caT):ENSP00000457976(p.Gln11His)];ENSG00000090238:MISSENSE[ENST00000565110(c.33G>T,caG>caT):ENSP00000454350(p.Gln11His)];ENSG00000090238:MISSENSE[ENST00000566401(c.33G>T,caG>caT):ENSP00000456212(p.Gln11His)];ENSG00000090238:INTRONIC-SS-ACR[ENST00000568674(n.113-1G>T)];ENSG00000090238:INTRONIC-SS-ACR[ENST00000565479(n.110-1G>T)];ENSG00000090238:EXONIC-NC[ENST00000568681(n.253G>T)];	NA	NA	NA	NA	NA	NA	NA	NA	NA	NA	MODIFIER	NA	NA	76	76	0	80	37	43	53.75
Smoker	42476	chr16	72821147	C	T	*ZFHX3*	SILENT	ENSG00000140836:SILENT[ENST00000397992(c.8286G>A,gcG>gcA):ENSP00000438926(p.Ala2762(=))];ENSG00000140836:SILENT[ENST00000268489(c.11028G>A,gcG>gcA):ENSP00000268489(p.Ala3676(=))];	NA	NA	NA	NA	NA	NA	NA	NA	NA	NA	LOW	NA	NA	103	103	0	97	88	9	9.28
Smoker	42476	chr17	79095898	G	T	*AATK*	NONSENSE	ENSG00000181409:NONSENSE[ENST00000374792(c.1730C>A,tCg>tAg):ENSP00000363924(p.Ser577Ter)];ENSG00000181409:NONSENSE[ENST00000326724(c.1838C>A,tCg>tAg):ENSP00000324196(p.Ser613Ter)];ENSG00000181409:NONSENSE[ENST00000417379(c.1529C>A,tCg>tAg):ENSP00000398796(p.Ser510Ter)];ENSG00000181409:INTRONIC[ENST00000573469(n.432-3776C>A)];	chr17*7909[illegible]	chr17*7909[illegible]	chr17*79095898*79095898*G*C*rs753408863*NA*NA*NA	NA	NA	NA	NA	NA	NA	NA	MODIFIER	NA	NA	48	48	0	44	34	10	22.73
Smoker	42476	chr17	61271486	C	A	*AC037445.1*	INTRONIC	ENSG00000233635:[ENST00000581421(n.352-55G>T)];ENSG00000233635:[ENST00000435892(n.165-55G>T)];	NA	NA	NA	NA	NA	NA	NA	T	BN	N	MODERATE	NA	NA	75	75	0	67	53	14	20.9

Mangalaparthi *et al.* , 2020. Mutational landscape of esophageal squamous cell carcinoma in an Indian cohort
Supplementary Table 4. List of somatic single nucleotide variants identified using whole exome sequencing of ESCC patients

Cohort	Sample	Chromosome	Genomic position	Reference allele	Alternate allele	Gene symbol	Variant type	Variant annotation	OncoMD	COSMIC	dbSNP	ExAC	ExAC_SAS	1000G	1000G_SAS	SIFT	Polyphen2	CONDEL	VEP	LRT	PhastCon7 Way	Total read (Adjacent normal)	Reference allel read support (Adjacent normal)	Alternative read support (Adjacent normal)	Total read (Tumor)	Reference allel read support (Tumor)	Alternative read support (Tumor)	Alternate allele frequency
Smoker	42476	chr17	61271489	G	A	*AC037445.1*	INTRONIC	ENSG00000233635:[ENST00000581421(n.352-58C>T)];ENSG00000233635:[ENST00000435892(n.165-58C>T)];	NA	NA	NA	NA	NA	NA	NA	T	BN	N	MODERATE	NA	NA	74	74	0	66	52	14	21.21
Smoker	42476	chr17	27937758	C	T	*ANKRD13B*	MISSENSE	ENSG00000198720:MISSENSE[ENST00000394859(c.878C>T,tCa>tTa):ENSP00000378328(p.Ser293Leu)];ENSG00000198720:MISSENSE[ENST00000488766(c.878C>T,tCa>tTa):ENSP00000431958(p.Ser293Leu)];ENSG00000198720:EXONIC-NC[ENST00000493506(n.827C>T)];ENSG00000198720:EXONIC-NC[ENST00000578943(n.735C>T)];ENSG00000198720:EXONIC-NC[ENST00000487527(n.841C>T)];	NA	NA	NA	8.33E-06	0	NA	NA	NA	NA	NA	MODIFIER	NA	NA	72	72	0	64	53	11	17.19
Smoker	42476	chr17	12859102	G	C	*ARHGAP44*	INTRONIC	ENSG00000006740:[ENST00000544416(c.1137-82G>C)];ENSG00000006740:[ENST00000340825(c.1137-82G>C)];ENSG00000006740:[ENST00000538915(n.132-82G>C)];ENSG00000006740:[ENST00000580768(c.*482-82G>C)];ENSG00000006740:[ENST00000379672(c.1137-82G>C)];ENSG00000006740:[ENST00000262444(c.1137-82G>C)];ENSG00000006740:[ENST00000583608(c.-134G>C)];	NA	NA	NA	NA	NA	NA	NA	NA	NA	NA	MODIFIER	NA	NA	64	64	0	62	26	36	58.06
Smoker	42476	chr17	73499469	C	G	*CASKIN2*	INTRONIC-SS-DNR	ENSG00000177303:INTRONIC-SS-DNR[ENST00000321617(c.1819+1G>C)];ENSG00000177303:INTRONIC-SS-DNR[ENST00000580021(n.461+1G>C)];ENSG00000177303:INTRONIC-SS-DNR[ENST00000433559(c.1573+1G>C)];	NA	NA	NA	NA	NA	NA	NA	NA	NA	NA	HIGH	NA	NA	146	146	0	117	95	21	17.95
Smoker	42476	chr17	20266354	G	T	*CCDC144CP*	EXONIC-NC	ENSG00000154898:[ENST00000425519(n.1991G>T)];ENSG00000154898:[ENST00000433419(n.1755G>T)];	NA	NA	NA	NA	NA	NA	NA	NA	NA	NA	MODIFIER	NA	NA	49	49	0	51	42	8	15.69
Smoker	42476	chr17	62144127	G	C	*ERN1*	MISSENSE	ENSG00000178607:MISSENSE[ENST00000433197(c.746C>G,aCc>aGc):ENSP00000401445(p.Thr249Ser)];ENSG00000178607:EXONIC-NC[ENST00000583896(n.218C>G)];	NA	NA	NA	NA	NA	NA	NA	NA	NA	NA	MODIFIER	NA	NA	79	79	0	92	73	19	20.65
Smoker	42476	chr17	27903345	T	C	*GIT1*	MISSENSE	ENSG00000108262:MISSENSE[ENST00000394869(c.1531A>G,Agg>Ggg):ENSP00000378338(p.Arg511Gly)];ENSG00000108262:MISSENSE[ENST00000225394(c.1504A>G,Agg>Ggg):ENSP00000225394(p.Arg502Gly)];ENSG00000108262:MISSENSE[ENST00000581348(c.1531A>G,Agg>Ggg):ENSP00000462775(p.Arg511Gly)];ENSG00000108262:MISSENSE[ENST00000579937(c.1504A>G,Agg>Ggg):ENSP00000464388(p.Arg502Gly)];ENSG00000108262:MISSENSE[ENST00000578670(c.108A>G,Agg>Ggg):ENSP00000463746(p.Arg37Gly)];ENSG00000108262:EXONIC-NC[ENST00000473217(n.1294A>G)];ENSG00000108262:EXONIC-NC[ENST00000491377(n.457A>G)];	NA	NA	NA	NA	NA	NA	NA	NA	NA	NA	MODIFIER	NA	NA	84	84	0	93	70	23	24.73
Smoker	42476	chr17	42478762	C	G	*GPATCH8*	MISSENSE	ENSG00000186566:MISSENSE[ENST00000585614(c.608G>C,gGa>gCa):ENSP00000465781(p.Gly203Ala)];ENSG00000186566:MISSENSE[ENST00000591680(c.683G>C,gGa>gCa):ENSP00000467556(p.Gly228Ala)];ENSG00000186566:MISSENSE[ENST00000434000(c.449G>C,gGa>gCa):ENSP00000395016(p.Gly150Ala)];ENSG00000186566:EXONIC-NC[ENST00000335500(n.1999G>C)];ENSG00000186566:3UTR[ENST00000587228(c.*569G>C)];	NA	NA	chr17*42478762*42478762*C*A*rs746986203*NA*NA*NA	NA	NA	NA	NA	NA	NA	NA	MODIFIER	NA	NA	87	86	0	103	85	18	17.48
Smoker	42476	chr17	21199362	C	T	*MAP2K3*	INTRONIC	ENSG00000034152:[ENST00000526076(c.-38-2363C>T)];ENSG00000034152:[ENST00000583508(n.260-2363C>T)];ENSG00000034152:[ENST00000529517(c.246-36C>T)];ENSG00000034152:[ENST00000496046(c.50-2363C>T)];ENSG00000034152:[ENST00000316920(c.-163-36C>T)];ENSG00000034152:[ENST00000483928(c.*4-36C>T)];ENSG00000034152:[ENST00000361818(c.-38-2363C>T)];ENSG00000034152:[ENST00000395491(c.50-36C>T)];ENSG00000034152:[ENST00000479129(c.*3+281C>T)];ENSG00000034152:[ENST00000342679(c.50-2363C>T)];	NA	NA	NA	NA	NA	NA	NA	NA	NA	NA	MODIFIER	NA	NA	83	83	0	91	85	6	6.59
Smoker	42476	chr17	3571816	G	C	*P2RX5-TAX1BP3*	INTRONIC	ENSG00000257950:[ENST00000550383(c.*3396+536C>G)];	NA	NA	NA	NA	NA	NA	NA	D_lc	BN	N	MODERATE	U	0.729	44	44	0	46	34	11	23.91
Smoker	42476	chr17	33433162	G	A	*RAD51D*	INTRONIC	ENSG00000185379:[ENST00000590380(n.527+243C>T)];ENSG00000185379:[ENST00000587977(c.*316+243C>T)];ENSG00000185379:[ENST00000586210(c.*170+243C>T)];ENSG00000185379:[ENST00000360276(c.441+243C>T)];ENSG00000185379:[ENST00000586044(c.*307+243C>T)];ENSG00000185379:[ENST00000590016(c.636+243C>T)];ENSG00000185379:[ENST00000335858(c.240+243C>T)];ENSG00000185379:[ENST00000587405(c.219+243C>T)];ENSG00000185379:[ENST00000590631(c.45+243C>T)];ENSG00000185379:[ENST00000592577(c.582+243C>T)];ENSG00000185379:[ENST00000588594(c.*172+243C>T)];ENSG00000185379:[ENST00000460118(c.219+243C>T)];ENSG00000185379:[ENST00000394589(c.576+243C>T)];ENSG00000185379:[ENST00000345365(c.576+243C>T)];ENSG00000185379:[ENST00000587982(n.612C>T)];ENSG00000185379:[ENST00000415064(n.833C>T)];ENSG00000185379:[ENST00000592430(n.693C>T)];ENSG00000185379:[ENST00000588372(c.*44C>T)];	NA	NA	NA	NA	NA	NA	NA	NA	NA	NA	MODIFIER	NA	NA	61	61	0	63	55	8	12.7
Smoker	42476	chr17	33433162	G	A	*RAD51L3-RFFL*	INTRONIC	ENSG00000267618:[ENST00000591723(c.45+243C>T)];ENSG00000267618:[ENST00000592181(c.219+243C>T)];ENSG00000267618:[ENST00000593039(c.99+243C>T)];	NA	NA	NA	NA	NA	NA	NA	NA	NA	NA	MODIFIER	NA	NA	61	61	0	63	55	8	12.7
Smoker	42476	chr17	61782231	C	G	*RP11-51F16.8*	INTRONIC	ENSG00000125695:[ENST00000580553(c.*773-289G>C)];	NA	NA	NA	NA	NA	NA	NA	NA	NA	NA	MODIFIER	NA	NA	46	46	0	48	42	6	12.5
Smoker	42476	chr17	27896315	C	G	*RP11-68I3.2*	INTRONIC	ENSG00000264031:[ENST00000581474(n.153+8598C>G)];	NA	NA	NA	8.83E-06	0	NA	NA	NA	NA	NA	MODIFIER	NA	NA	64	64	0	64	57	7	10.94
Smoker	42476	chr17	27903345	T	C	*RP11-68I3.2*	INTRONIC	ENSG00000264031:[ENST00000581474(n.153+15628T>C)];	NA	NA	NA	NA	NA	NA	NA	NA	NA	NA	MODIFIER	NA	NA	84	84	0	93	70	23	24.73
Smoker	42476	chr17	27937758	C	T	*RP11-68I3.2*	INTRONIC	ENSG00000264031:[ENST00000581474(n.153+50041C>T)];	NA	NA	NA	8.33E-06	0	NA	NA	NA	NA	NA	MODIFIER	NA	NA	72	72	0	64	53	11	17.19
Smoker	42476	chr17	61782231	C	G	*STRADA*	INTRONIC	ENSG00000266173:[ENST00000582137(c.772-289G>C)];ENSG00000266173:[ENST00000336174(c.859-289G>C)];ENSG00000266173:[ENST00000579340(c.513-289G>C)];ENSG00000266173:[ENST00000392950(c.748-289G>C)];ENSG00000266173:[ENST00000375840(c.685-289G>C)];ENSG00000266173:[ENST00000580039(n.710-289G>C)];ENSG00000266173:[ENST00000447001(c.727-289G>C)];ENSG00000266173:[ENST00000245865(c.*8G>C)];ENSG00000266173:[ENST00000578801(c.*19G>C)];ENSG00000266173:[ENST00000578008(c.*8G>C)];ENSG00000266173:[ENST00000583085(n.1654G>C)];	NA	NA	NA	NA	NA	NA	NA	NA	NA	NA	MODIFIER	NA	NA	46	46	0	48	42	6	12.5
Smoker	42476	chr17	61271486	C	A	*TANC2*	MISSENSE	ENSG00000170921:MISSENSE[ENST00000389520(c.346C>A,Cag>Aag):ENSP00000374171(p.Gln116Lys)];ENSG00000170921:MISSENSE[ENST00000424789(c.346C>A,Cag>Aag):ENSP00000387593(p.Gln116Lys)];ENSG00000170921:MISSENSE[ENST00000583356(c.132C>A,Cag>Aag):ENSP00000462109(p.Gln45Lys)];	NA	NA	NA	NA	NA	NA	NA	T	BN	N	MODERATE	NA	NA	75	75	0	67	53	14	20.9

Mangalaparthi *et al.* , 2020. Mutational landscape of esophageal squamous cell carcinoma in an Indian cohort
Supplementary Table 4. List of somatic single nucleotide variants identified using whole exome sequencing of ESCC patients

Cohort	Sample	Chromosome	Genomic position	Reference allele	Alternate allele	Gene symbol	Variant type	Variant annotation	OncoMD	COSMIC	dbSNP	ExAC	ExAC_SAS	1000G	1000G_SAS	SIFT	Polyphen2	CONDEL	VEP	LRT	PhastCon7 Way	Total read (Adjacent normal)	Reference allel read support (Adjacent normal)	Alternative read support (Adjacent normal)	Total read (Tumor)	Reference allel read support (Tumor)	Alternative read support (Tumor)	Alternate allele frequency
Smoker	42476	chr17	61271489	G	A	*TANC2*	MISSENSE	ENSG00000170921:MISSENSE[ENST00000389520(c.349G>A,Gat>Aat):ENSP00000374171(p.Asp117Asn)];ENSG00000170921:MISSENSE[ENST00000424789(c.349G>A,Gat>Aat):ENSP00000387593(p.Asp117Asn)];ENSG00000170921:MISSENSE[ENST00000583356(c.135G>A,Gat>Aat):ENSP00000462109(p.Asp46Asn)];	NA	NA	NA	NA	NA	NA	NA	T	BN	N	MODERATE	NA	NA	74	74	0	66	52	14	21.21
Smoker	42476	chr17	3571816	G	C	*TAX1BP3*	MISSENSE	ENSG00000213977:MISSENSE[ENST00000225525(c.5C>G,tCc>tGc):ENSP00000225525(p.Ser2Cys)];	NA	NA	NA	NA	NA	NA	NA	D_lc	BN	N	MODERATE	U	0.729	44	44	0	46	34	11	23.91
Smoker	42476	chr17	53014585	T	A	*TOM1L1*	NONSENSE	ENSG00000141198:NONSENSE-SS-PRX[ENST00000540336(c.695T>A,tTa>tAa):ENSP00000441242(p.Leu232Ter)];ENSG00000141198:NONSENSE-SS-PRX[ENST00000445275(c.1031T>A,tTa>tAa):ENSP00000408958(p.Leu344Ter)];ENSG00000141198:NONSENSE-SS-PRX[ENST00000348161(c.800T>A,tTa>tAa):ENSP00000343901(p.Leu267Ter)];ENSG00000141198:NONSENSE-SS-PRX[ENST00000572158(c.1010T>A,tTa>tAa):ENSP00000461284(p.Leu337Ter)];ENSG00000141198:NONSENSE-SS-PRX[ENST00000536554(c.800T>A,tTa>tAa):ENSP00000443099(p.Leu267Ter)];ENSG00000141198:NONSENSE-SS-PRX[ENST00000575882(c.1031T>A,tTa>tAa):ENSP00000460823(p.Leu344Ter)];ENSG00000141198:NONSENSE-SS-PRX[ENST00000576932(c.800T>A,tTa>tAa):ENSP00000461876(p.Leu267Ter)];ENSG00000141198:NONSENSE[ENST00000570371(c.1031T>A,tTa>tAa):ENSP00000458553(p.Leu344Ter)];ENSG00000141198:NONSENSE[ENST00000575333(c.1031T>A,tTa>tAa):ENSP00000458918(p.Leu344Ter)];ENSG00000141198:EXONIC-NC[ENST00000570623(n.413T>A)];ENSG00000141198:3UTR-SS-PRX[ENST00000571319(c.*655T>A)];	NA	NA	NA	NA	NA	NA	NA	NA	NA	NA	LOW	NA	NA	49	49	0	48	43	5	10.42
Smoker	42476	chr17	27896315	C	G	*TP53I13*	INTRONIC	ENSG00000167543:[ENST00000583940(c.67-21C>G)];ENSG00000167543:[ENST00000577934(c.102-21C>G)];ENSG00000167543:[ENST00000580183(c.67-21C>G)];ENSG00000167543:[ENST00000581411(c.67-21C>G)];ENSG00000167543:[ENST00000301057(c.142-21C>G)];ENSG00000167543:[ENST00000578749(c.67-21C>G)];ENSG00000167543:[ENST00000582829(c.67-21C>G)];ENSG00000167543:[ENST00000580132(n.351C>G)];	NA	NA	NA	8.83E-06	0	NA	NA	NA	NA	NA	MODIFIER	NA	NA	64	64	0	64	57	7	10.94
Smoker	42476	chr17	33513392	C	G	*UNC45B*	SILENT	ENSG00000141161:SILENT[ENST00000378449(c.2367C>G,gtC>gtG):ENSP00000367710(p.Val789(=))];ENSG00000141161:SILENT[ENST00000433649(c.2604C>G,gtC>gtG):ENSP00000412840(p.Val868(=))];ENSG00000141161:SILENT[ENST00000268876(c.2610C>G,gtC>gtG):ENSP00000268876(p.Val870(=))];ENSG00000141161:SILENT[ENST00000591048(c.2367C>G,gtC>gtG):ENSP00000468335(p.Val789(=))];ENSG00000141161:SILENT[ENST00000394570(c.2604C>G,gtC>gtG):ENSP00000378071(p.Val868(=))];	NA	NA	NA	NA	NA	NA	NA	NA	NA	NA	LOW	NA	NA	28	28	0	35	24	10	28.57
Smoker	42476	chr17	6665376	C	T	*XAF1*	INTRONIC	ENSG00000132530:[ENST00000576724(c.*78+20C>T)];ENSG00000132530:[ENST00000574962(c.421+1456C>T)];ENSG00000132530:[ENST00000441631(c.422-98C>T)];ENSG00000132530:[ENST00000572546(c.*78+20C>T)];ENSG00000132530:[ENST00000575369(c.*441-98C>T)];ENSG00000132530:[ENST00000574907(c.520-98C>T)];ENSG00000132530:[ENST00000361842(c.422-98C>T)];ENSG00000132530:[ENST00000571217(c.169-98C>T)];ENSG00000132530:[ENST00000346752(c.365-98C>T)];ENSG00000132530:[ENST00000571673(c.364+1456C>T)];ENSG00000132530:[ENST00000573518(n.340C>T)];	NA	NA	0.0003994	0.0001313	0	0.0003994	0	NA	NA	NA	MODIFIER	NA	NA	78	78	0	84	73	11	13.1
Smoker	42476	chr17	18566494	G	A	*ZNF286B*	MISSENSE	ENSG00000249459:NONSENSE[ENST00000545289(c.325C>T,Caa>Taa):ENSP00000461413(p.Gln109Ter)];ENSG00000249459:MISSENSE[ENST00000285274(c.173C>T,cCa>cTa):ENSP00000460210(p.Pro58Leu)];	NA	NA	NA	NA	NA	NA	NA	NA	UN	NA	MODERATE	NA	NA	271	271	0	314	262	52	16.56
Smoker	42476	chr18	13042254	T	C	*CEP192*	MISSENSE	ENSG00000101639:MISSENSE[ENST00000510237(c.788T>C,cTt>cCt):ENSP00000423147(p.Leu263Pro)];ENSG00000101639:MISSENSE[ENST00000325971(c.200T>C,cTt>cCt):ENSP00000317156(p.Leu67Pro)];ENSG00000101639:MISSENSE[ENST00000589596(c.1211T>C,cTt>cCt):ENSP00000466258(p.Leu404Pro)];ENSG00000101639:MISSENSE[ENST00000430049(c.563T>C,cTt>cCt):ENSP00000389190(p.Leu188Pro)];ENSG00000101639:MISSENSE[ENST00000506447(c.1988T>C,cTt>cCt):ENSP00000427550(p.Leu663Pro)];ENSG00000101639:MISSENSE[ENST00000511820(c.604T>C,cTt>cCt):ENSP00000467038(p.Leu202Pro)];ENSG00000101639:3UTR[ENST00000513432(c.*156T>C)];	NA	NA	NA	NA	NA	NA	NA	NA	NA	NA	MODIFIER	NA	NA	156	156	0	132	90	42	31.82
Smoker	42476	chr18	65180004	A	C	*CTD-2541J13.2*	EXONIC-NC	ENSG00000263424:[ENST00000583493(n.2026A>C)];	NA	NA	NA	NA	NA	NA	NA	NA	NA	NA	HIGH	NA	0.986	116	116	0	86	41	45	52.33
Smoker	42476	chr18	65180004	A	C	*DSEL*	NONSENSE	ENSG00000171451:NONSENSE[ENST00000310045(c.1872T>G,taT>taG):ENSP00000310565(p.Tyr624Ter)];	NA	NA	NA	NA	NA	NA	NA	NA	NA	NA	HIGH	NA	0.986	116	116	0	86	41	45	52.33
Smoker	42476	chr18	31463371	A	T	*NOL4*	SILENT	ENSG00000101746:SILENT[ENST00000261592(c.1560T>A,gcT>gcA):ENSP00000261592(p.Ala520(=))];ENSG00000101746:SILENT[ENST00000589544(c.1254T>A,gcT>gcA):ENSP00000465450(p.Ala418(=))];ENSG00000101746:SILENT[ENST00000269185(c.912T>A,gcT>gcA):ENSP00000269185(p.Ala304(=))];ENSG00000101746:SILENT[ENST00000538587(c.1338T>A,gcT>gcA):ENSP00000443472(p.Ala446(=))];ENSG00000101746:SILENT[ENST00000535384(c.705T>A,gcT>gcA):ENSP00000445733(p.Ala235(=))];ENSG00000101746:SILENT[ENST00000535475(c.903T>A,gcT>gcA):ENSP00000438190(p.Ala301(=))];ENSG00000101746:SILENT[ENST00000586553(c.615T>A,gcT>gcA):ENSP00000467015(p.Ala205(=))];ENSG00000101746:SILENT[ENST00000590712(c.1320T>A,gcT>gcA):ENSP00000467629(p.Ala440(=))];ENSG00000101746:3UTR[ENST00000586314(c.*977T>A)];	NA	NA	chr18*31463371*31463371*A*C*rs78761172*1*0.999*0.0009984	NA	NA	NA	NA	NA	NA	NA	LOW	NA	NA	38	38	0	39	23	16	41.03

Mangalaparthi *et al.* , 2020. Mutational landscape of esophageal squamous cell carcinoma in an Indian cohort
Supplementary Table 4. List of somatic single nucleotide variants identified using whole exome sequencing of ESCC patients

Cohort	Sample	Chromosome	Genomic position	Reference allele	Alternate allele	Gene symbol	Variant type	Variant annotation	OncoMD	COSMIC	dbSNP	ExAC	ExAC_SAS	1000G	1000G_SAS	SIFT	Polyphen2	CONDEL	VEP	LRT	PhastCon7 Way	Total read (Adjacent normal)	Reference allel read support (Adjacent normal)	Alternative read support (Adjacent normal)	Total read (Tumor)	Reference allel read support (Tumor)	Alternative read support (Tumor)	Alternate allele frequency
Smoker	42476	chr18	12427072	G	C	*SLMO1*	MISSENSE	ENSG00000141391:MISSENSE[ENST00000587735(c.54G>C,gaG>gaC):ENSP00000464848(p.Glu18Asp)];ENSG00000141391:MISSENSE[ENST00000440960(c.324G>C,gaG>gaC):ENSP00000404700(p.Glu108Asp)];ENSG00000141391:MISSENSE[ENST00000592149(c.261G>C,gaG>gaC):ENSP00000466737(p.Glu87Asp)];ENSG00000141391:MISSENSE[ENST00000336990(c.324G>C,gaG>gaC):ENSP00000338988(p.Glu108Asp)];ENSG00000141391:MISSENSE[ENST00000590956(c.54G>C,gaG>gaC):ENSP00000467026(p.Glu18Asp)];ENSG00000141391:MISSENSE[ENST00000587862(c.387G>C,gaG>gaC):ENSP00000465996(p.Glu129Asp)];ENSG00000141391:MISSENSE[ENST00000588729(c.465G>C,gaG>gaC):ENSP00000467896(p.Glu155Asp)];ENSG00000141391:MISSENSE[ENST00000589952(c.261G>C,gaG>gaC):ENSP00000467457(p.Glu87Asp)];ENSG00000141391:3UTR[ENST00000589565(c.*4G>C)];	NA	NA	NA	NA	NA	NA	NA	NA	NA	NA	MODIFIER	NA	NA	65	65	0	59	44	15	25.42
Smoker	42476	chr18	45371793	G	A	*SMAD2*	NONSENSE	ENSG00000175387:NONSENSE[ENST00000402690(c.1198C>T,Cag>Tag):ENSP00000384449(p.Gln400Ter)];ENSG00000175387:NONSENSE[ENST00000591214(c.1108C>T,Cag>Tag):ENSP00000467075(p.Gln370Ter)];ENSG00000175387:NONSENSE[ENST00000356825(c.1108C>T,Cag>Tag):ENSP00000349282(p.Gln370Ter)];ENSG00000175387:NONSENSE[ENST00000262160(c.1198C>T,Cag>Tag):ENSP00000262160(p.Gln400Ter)];ENSG00000175387:NONSENSE[ENST00000586040(c.1108C>T,Cag>Tag):ENSP00000466193(p.Gln370Ter)];	NA	NA	chr18*45371793*45371793*G*C*rs754313157*NA*NA*NA	NA	NA	NA	NA	NA	NA	NA	HIGH	NA	1	144	143	0	95	87	7	7.37
Smoker	42476	chr18	71825478	G	A	*TIMM21*	SILENT	ENSG00000075336:SILENT[ENST00000579071(c.278G>A,ggG>ggA):ENSP00000464228(p.Gly93(=))];ENSG00000075336:SILENT[ENST00000169551(c.609G>A,ggG>ggA):ENSP00000169551(p.Gly203(=))];ENSG00000075336:EXONIC-NC[ENST00000584925(n.456G>A)];	NA	NA	chr18*71825478*71825478*G*C*rs778789995*NA*NA*NA	NA	NA	NA	NA	NA	NA	NA	MODIFIER	NA	NA	177	176	0	151	121	30	19.87
Smoker	42476	chr18	66350277	G	C	*TMX3*	INTRONIC	ENSG00000166479:[ENST00000564631(c.*533-59C>G)];ENSG00000166479:[ENST00000578765(n.424-59C>G)];ENSG00000166479:[ENST00000299608(c.849-59C>G)];	NA	NA	NA	NA	NA	NA	NA	NA	NA	NA	MODIFIER	NA	NA	73	73	0	59	43	16	27.12
Smoker	42476	chr18	72775258	G	C	*ZNF407*	MISSENSE	ENSG00000215421:EXONIC-NC[ENST00000579200(n.2021G>C)];ENSG00000215421:EXONIC-NC[ENST00000582214(n.299G>C)];ENSG00000215421:MISSENSE[ENST00000299687(c.5581G>C,Gag>Cag):ENSP00000299687(p.Glu1861Gln)];	NA	NA	NA	8.35E-06	0	NA	NA	T	BN	N	MODERATE	NA	NA	63	63	0	38	31	7	18.42
Smoker	42476	chr19	1042554	G	C	*ABCA7*	EXONIC-NC	ENSG00000064687:[ENST00000526885(n.867G>C)];ENSG00000064687:[ENST00000263094(c.498+158G>C)];ENSG00000064687:[ENST00000433129(c.498+158G>C)];	NA	NA	chr19*1042554*1042554*G*T*rs73920555*1*0.9716*0.02835	NA	NA	NA	NA	NA	NA	NA	MODIFIER	NA	NA	80	80	0	91	76	15	16.48
Smoker	42476	chr19	17405326	G	A	*ABHD8*	INTRONIC	ENSG00000127220:[ENST00000247706(c.933-13C>T)];	NA	NA	NA	NA	NA	NA	NA	NA	NA	NA	MODIFIER	NA	NA	68	67	0	75	69	6	8
Smoker	42476	chr19	1556653	C	G	*AC027307.1*	MATURE-MIRNA	ENSG00000222720:[ENST00000410788(n.50C>G)];	NA	NA	NA	NA	NA	NA	NA	D	PrD	D	MODERATE	NA	NA	93	93	0	110	94	16	14.55
Smoker	42476	chr19	51161329	C	T	*C19orf81*	SILENT	ENSG00000235034:SILENT[ENST00000458538(c.267C>T,agC>agT):ENSP00000391035(p.Ser89(=))];ENSG00000235034:SILENT[ENST00000425202(c.345C>T,agC>agT):ENSP00000417035(p.Ser115(=))];	NA	NA	NA	NA	NA	NA	NA	NA	NA	NA	MODIFIER	NA	NA	51	50	1	42	30	12	28.57
Smoker	42476	chr19	35827075	G	A	*CD22*	MISSENSE	ENSG00000012124:INTRONIC[ENST00000600655(n.37-4709G>A)];ENSG00000012124:INTRONIC[ENST00000597433(n.430+3248G>A)];ENSG00000012124:INTRONIC[ENST00000601329(n.44-1583G>A)];ENSG00000012124:INTRONIC[ENST00000594349(c.380-79G>A)];ENSG00000012124:INTRONIC[ENST00000600424(c.406+3248G>A)];ENSG00000012124:INTRONIC[ENST00000598815(n.45-4709G>A)];ENSG00000012124:INTRONIC[ENST00000599811(c.412+3248G>A)];ENSG00000012124:EXONIC-NC[ENST00000598138(n.579G>A)];ENSG00000012124:EXONIC-NC[ENST00000601732(n.340G>A)];ENSG00000012124:EXONIC-NC[ENST00000598028(n.181G>A)];ENSG00000012124:EXONIC-NC[ENST00000600905(n.512G>A)];ENSG00000012124:MISSENSE[ENST00000270311(c.189G>A,atG>atA):ENSP00000270311(p.Met63Ile)];ENSG00000012124:MISSENSE[ENST00000341773(c.549G>A,atG>atA):ENSP00000339349(p.Met183Ile)];ENSG00000012124:MISSENSE[ENST00000594250(c.549G>A,atG>atA):ENSP00000469984(p.Met183Ile)];ENSG00000012124:MISSENSE[ENST00000601769(c.430G>A,Gag>Aag):ENSP00000470193(p.Glu144Lys)];ENSG00000012124:MISSENSE[ENST00000593867(c.549G>A,atG>atA):ENSP00000471972(p.Met183Ile)];ENSG00000012124:MISSENSE[ENST00000419549(c.33G>A,atG>atA):ENSP00000403822(p.Met11Ile)];ENSG00000012124:MISSENSE[ENST00000544992(c.549G>A,atG>atA):ENSP00000441237(p.Met183Ile)];ENSG00000012124:MISSENSE[ENST00000085219(c.549G>A,atG>atA):ENSP00000085219(p.Met183Ile)];ENSG00000012124:MISSENSE[ENST00000536635(c.549G>A,atG>atA):ENSP00000442279(p.Met183Ile)];	NA	NA	NA	NA	NA	NA	NA	NA	NA	NA	MODIFIER	NA	NA	107	107	0	79	32	47	59.49
Smoker	42476	chr19	32968410	G	C	*DPY19L3*	EXONIC-NC	ENSG00000178904:[ENST00000608291(n.22G>C)];ENSG00000178904:[ENST00000342179(c.1698-18G>C)];ENSG00000178904:[ENST00000592142(n.1868-18G>C)];ENSG00000178904:[ENST00000392250(c.1698-18G>C)];ENSG00000178904:[ENST00000588648(c.*222-18G>C)];ENSG00000178904:[ENST00000586987(c.1698-18G>C)];ENSG00000178904:[ENST00000592832(c.399-18G>C)];	NA	NA	NA	NA	NA	NA	NA	NA	NA	NA	MODIFIER	NA	NA	146	146	0	104	85	19	18.27
Smoker	42476	chr19	40318137	C	T	*DYRK1B*	INTRONIC	ENSG00000105204:[ENST00000597639(c.954+13G>A)];ENSG00000105204:[ENST00000593685(c.954+13G>A)];ENSG00000105204:[ENST00000348817(c.954+13G>A)];ENSG00000105204:[ENST00000430012(c.954+13G>A)];ENSG00000105204:[ENST00000323039(c.954+13G>A)];	NA	NA	NA	NA	NA	NA	NA	NA	NA	NA	MODIFIER	NA	NA	77	77	0	79	68	11	13.92
Smoker	42476	chr19	3983202	G	A	*EEF2*	SILENT	ENSG00000167658:SILENT[ENST00000309311(c.306C>T,ctC>ctT):ENSP00000307940(p.Leu102(=))];	NA	NA	NA	2.50E-05	0	NA	NA	NA	NA	NA	LOW	NA	NA	129	128	0	119	113	6	5.04
Smoker	42476	chr19	8181534	C	G	*FBN3*	INTRONIC	ENSG00000142449:[ENST00000600128(c.3712+24G>C)];ENSG00000142449:[ENST00000601739(c.3712+24G>C)];ENSG00000142449:[ENST00000270509(c.3712+24G>C)];	NA	NA	NA	8.46E-06	0	NA	NA	NA	NA	NA	MODIFIER	NA	NA	46	46	0	43	29	14	32.56
Smoker	42476	chr19	35843162	C	T	*FFAR1*	SILENT	ENSG00000126266:SILENT[ENST00000246553(c.708C>T,tgC>tgT):ENSP00000246553(p.Cys236(=))];	NA	NA	NA	NA	NA	NA	NA	NA	NA	NA	LOW	NA	0.897	37	37	0	32	15	17	53.12
Smoker	42476	chr19	14162418	G	T	*IL27RA*	INTRONIC-SS-ACR	ENSG00000104998:INTRONIC-SS-ACR[ENST00000263379(c.1529-1G>T)];	NA	NA	chr19*14162418*14162418*G*C*rs746165316*NA*NA*NA	NA	NA	NA	NA	NA	NA	NA	HIGH	NA	NA	98	98	0	52	46	6	11.54

Supplementary Table 4. List of somatic single nucleotide variants identified using whole exome sequencing of ESCC patients

Cohort	Sample	Chromosome	Genomic position	Reference allele	Alternate allele	Gene symbol	Variant type	Variant annotation	OncoMD	COSMIC	dbSNP	ExAC	ExAC_SAS	1000G	1000G_SAS	SIFT	Polyphen2	CONDEL	VEP	LRT	PhastCon7 Way	Total read (Adjacent normal)	Reference allel read support (Adjacent normal)	Alternative read support (Adjacent normal)	Total read (Tumor)	Reference allel read support (Tumor)	Alternative read support (Tumor)	Alternate allele frequency
Smoker	42476	chr19	18368021	C	T	*KIAA1683*	MISSENSE	ENSG00000130518:MISSENSE[ENST00000600359(c.3374G>A,aGc>aAc):ENSP00000472912(p.Ser1125Asn)];ENSG00000130518:MISSENSE[ENST00000600328(c.3512G>A,aGc>aAc):ENSP00000470780(p.Ser1171Asn)];ENSG00000130518:MISSENSE[ENST00000392413(c.4073G>A,aGc>aAc):ENSP00000376213(p.Ser1358Asn)];ENSG00000130518:EXONIC-NC[ENST00000599638(n.5408G>A)];	NA	NA	NA	NA	NA	NA	NA	T	BN	N	MODERATE	N	0	89	89	0	147	139	8	5.44
Smoker	42476	chr19	1556653	C	G	*MEX3D*	MISSENSE	ENSG00000181588:MISSENSE[ENST00000402693(c.865G>C,Gtg>Ctg):ENSP00000384398(p.Val289Leu)];ENSG00000181588:MISSENSE[ENST00000388824(c.865G>C,Gtg>Ctg):ENSP00000373476(p.Val289Leu)];ENSG00000181588:MISSENSE[ENST00000605173(c.337G>C,Gtg>Ctg):ENSP00000475059(p.Val113Leu)];	NA	NA	NA	NA	NA	NA	NA	D	PrD	D	MODERATE	NA	NA	93	93	0	110	94	16	14.55
Smoker	42476	chr19	17405326	G	A	*MRPL34*	INTRONIC	ENSG00000130312:[ENST00000600434(c.-129+1663G>A)];ENSG00000130312:[ENST00000595444(c.214+1663G>A)];	NA	NA	NA	NA	NA	NA	NA	NA	NA	NA	MODIFIER	NA	NA	68	67	0	75	69	6	8
Smoker	42476	chr19	9064190	C	G	*MUC16*	MISSENSE	ENSG00000181143:MISSENSE[ENST00000397910(c.23256G>C,gaG>gaC):ENSP00000381008(p.Glu7752Asp)];	NA	NA	NA	NA	NA	NA	NA	NA	UN	NA	MODERATE	NA	NA	129	129	0	97	80	17	17.53
Smoker	42476	chr19	55997811	C	A	*NAT14*	MISSENSE	ENSG00000090971:MISSENSE[ENST00000205194(c.109C>A,Cat>Aat):ENSP00000205194(p.His37Asn)];ENSG00000090971:MISSENSE[ENST00000588985(c.23C>A,Cat>Aat):ENSP00000471807(p.His9Asn)];ENSG00000090971:3UTR[ENST00000591590(c.*577C>A)];ENSG00000090971:INTRONIC[ENST00000592719(n.106-685C>A)];ENSG00000090971:INTRONIC[ENST00000587400(c.10+726C>A)];	NA	NA	NA	NA	NA	NA	NA	NA	NA	NA	MODIFIER	NA	NA	61	61	0	51	44	7	13.73
Smoker	42476	chr19	49652029	A	T	*PPFIA3*	INTRONIC-SS-DNR	ENSG00000177380:[ENST00000334186(c.3109+9A>T)];ENSG00000177380:[ENST00000602655(c.*927+9A>T)];ENSG00000177380:[ENST00000602351(c.3082+9A>T)];ENSG00000177380:[ENST00000602905(n.191+9A>T)];ENSG00000177380:[ENST00000602897(c.256+9A>T)];ENSG00000177380:[ENST00000602848(c.358+9A>T)];	NA	NA	NA	NA	NA	NA	NA	NA	NA	NA	MODIFIER	NA	NA	21	21	0	29	19	10	34.48
Smoker	42476	chr19	50101049	C	T	*PRR12*	MISSENSE	ENSG00000126464:MISSENSE[ENST00000418929(c.3457C>T,Cgg>Tgg):ENSP00000394510(p.Arg1153Trp)];	NA	NA	NA	NA	NA	NA	NA	NA	PoD	NA	MODERATE	D	0.844	38	37	0	35	27	8	22.86
Smoker	42476	chr19	56041193	C	T	*SBK2*	SILENT	ENSG00000187550:SILENT[ENST00000413299(c.954G>A,gtG>gtA):ENSP00000389015(p.Val318(=))];ENSG00000187550:SILENT[ENST00000344158(c.954G>A,gtG>gtA):ENSP00000345044(p.Val318(=))];	NA	NA	NA	NA	NA	NA	NA	NA	NA	NA	LOW	NA	NA	27	27	0	32	24	8	25
Smoker	42476	chr19	17983443	C	T	*SLC5A5*	SILENT	ENSG00000105641:SILENT[ENST00000222248(c.315C>T,ttC>ttT):ENSP00000222248(p.Phe105(=))];	NA	NA	NA	NA	NA	NA	NA	NA	NA	NA	LOW	NA	0.95	80	80	0	108	55	53	49.07
Smoker	42476	chr19	47226210	G	A	*STRN4*	INTRONIC-SS-ACR	ENSG00000090372:[ENST00000601631(n.1035-3C>T)];ENSG00000090372:[ENST00000595357(n.1034-3C>T)];ENSG00000090372:[ENST00000600615(c.99-3C>T)];ENSG00000090372:[ENST00000539396(c.1409-3C>T)];ENSG00000090372:[ENST00000391910(c.1787-3C>T)];ENSG00000090372:[ENST00000263280(c.1766-3C>T)];ENSG00000090372:[ENST00000594581(n.1135-3C>T)];ENSG00000090372:[ENST00000594287(c.808-3C>T)];ENSG00000090372:[ENST00000594357(n.834-3C>T)];ENSG00000090372:[ENST00000598074(n.418C>T)];	NA	NA	NA	NA	NA	NA	NA	NA	NA	NA	LOW	NA	NA	51	51	0	45	34	11	24.44
Smoker	42476	chr19	15221436	C	T	*SYDE1*	MISSENSE	ENSG00000105137:MISSENSE[ENST00000600440(c.979C>T,Cgc>Tgc):ENSP00000470733(p.Arg327Cys)];ENSG00000105137:MISSENSE[ENST00000602203(c.45C>T,Cgc>Tgc):ENSP00000471553(p.Arg16Cys)];ENSG00000105137:MISSENSE[ENST00000600252(c.151C>T,Cgc>Tgc):ENSP00000469489(p.Arg51Cys)];ENSG00000105137:MISSENSE[ENST00000342784(c.1180C>T,Cgc>Tgc):ENSP00000341489(p.Arg394Cys)];	NA	NA	NA	NA	NA	NA	NA	T	PrD	D	MODERATE	D	0.627	60	60	0	29	23	6	20.69
Smoker	42476	chr19	51161329	C	T	*SYT3*	INTRONIC	ENSG00000213023:[ENST00000544769(c.-518+10281G>A)];	NA	NA	NA	NA	NA	NA	NA	NA	NA	NA	MODIFIER	NA	NA	51	50	1	42	30	12	28.57
Smoker	42476	chr19	12825775	G	A	*TNPO2*	INTRONIC	ENSG00000105576:[ENST00000586775(c.*413-22C>T)];ENSG00000105576:[ENST00000356861(c.772-22C>T)];ENSG00000105576:[ENST00000592287(c.772-22C>T)];ENSG00000105576:[ENST00000585886(c.772-22C>T)];ENSG00000105576:[ENST00000589956(n.432+749C>T)];ENSG00000105576:[ENST00000588151(n.148-22C>T)];ENSG00000105576:[ENST00000441499(c.772-22C>T)];ENSG00000105576:[ENST00000425528(c.772-22C>T)];ENSG00000105576:[ENST00000450764(c.772-22C>T)];ENSG00000105576:[ENST00000588216(c.772-22C>T)];	NA	NA	NA	NA	NA	NA	NA	NA	NA	NA	MODIFIER	NA	NA	98	98	0	102	78	23	22.55
Smoker	42476	chr19	36164235	G	A	*UPK1A*	INTRONIC	ENSG00000105668:[ENST00000222275(c.360+27G>A)];ENSG00000105668:[ENST00000379013(c.360+27G>A)];	NA	NA	NA	1.65E-05	0	NA	NA	NA	NA	NA	MODIFIER	NA	NA	127	125	0	100	63	37	37
Smoker	42476	chr19	38376384	G	C	*WDR87*	MISSENSE	ENSG00000171804:MISSENSE[ENST00000447313(c.7927C>G,Cct>Gct):ENSP00000405012(p.Pro2643Ala)];ENSG00000171804:MISSENSE[ENST00000303868(c.7810C>G,Cct>Gct):ENSP00000368025(p.Pro2604Ala)];	NA	NA	NA	NA	NA	NA	NA	NA	UN	NA	MODERATE	NA	NA	155	155	0	164	137	27	16.46
Smoker	42476	chr19	15536314	G	A	*WIZ*	SILENT	ENSG00000011451:SILENT[ENST00000545156(c.1860C>T,ccC>ccT):ENSP00000445824(p.Pro620(=))];ENSG00000011451:SILENT[ENST00000263381(c.1347C>T,ccC>ccT):ENSP00000263381(p.Pro449(=))];ENSG00000011451:SILENT[ENST00000599686(c.1470C>T,ccC>ccT):ENSP00000469534(p.Pro490(=))];ENSG00000011451:SILENT[ENST00000389282(c.3918C>T,ccC>ccT):ENSP00000373933(p.Pro1306(=))];ENSG00000011451:SILENT[ENST00000599910(c.1869C>T,ccC>ccT):ENSP00000469022(p.Pro623(=))];	NA	NA	NA	7.56E-05	0	NA	NA	NA	NA	NA	LOW	NA	NA	41	41	0	51	21	30	58.82
Smoker	42476	chr19	47572434	C	T	*ZC3H4*	SILENT	ENSG00000130749:SILENT[ENST00000253048(c.2313G>A,ctG>ctA):ENSP00000253048(p.Leu771(=))];ENSG00000130749:INTRONIC[ENST00000601973(c.1218-1308G>A)];ENSG00000130749:INTRONIC[ENST00000594019(n.797-1856G>A)];	chr19*4757	chr19*4757	NA	NA	NA	NA	NA	NA	NA	NA	MODIFIER	NA	NA	86	86	0	82	53	29	35.37
Smoker	42476	chr19	58946248	G	C	*ZNF132*	NONSENSE	ENSG00000131849:NONSENSE[ENST00000254166(c.563C>G,tCa>tGa):ENSP00000254166(p.Ser188Ter)];ENSG00000131849:EXONIC-NC[ENST00000599148(n.704C>G)];	NA	NA	NA	1.65E-05	0	NA	NA	NA	NA	NA	MODIFIER	NA	NA	68	68	0	79	56	23	29.11
Smoker	42476	chr19	44764203	G	T	*ZNF233*	INTRONIC	ENSG00000159915:[ENST00000592581(c.-48+112G>T)];ENSG00000159915:[ENST00000334152(c.262+48G>T)];ENSG00000159915:[ENST00000391958(c.-48+48G>T)];ENSG00000159915:[ENST00000590668(c.-34+112G>T)];ENSG00000159915:[ENST00000588489(c.-35G>T)];	NA	NA	NA	NA	NA	NA	NA	NA	NA	NA	MODIFIER	NA	NA	61	61	0	46	40	6	13.04
Smoker	42476	chr19	44764203	G	T	*ZNF235*	INTRONIC	ENSG00000159917:[ENST00000592844(c.239-25181C>A)];ENSG00000159917:[ENST00000589799(c.239-23321C>A)];	NA	NA	NA	NA	NA	NA	NA	NA	NA	NA	MODIFIER	NA	NA	61	61	0	46	40	6	13.04

Mangalaparthi *et al.* , 2020. Mutational landscape of esophageal squamous cell carcinoma in an Indian cohort
Supplementary Table 4. List of somatic single nucleotide variants identified using whole exome sequencing of ESCC patients

Cohort	Sample	Chromosome	Genomic position	Reference allele	Alternate allele	Gene symbol	Variant type	Variant annotation	OncoMD	COSMIC	dbSNP	ExAC	ExAC_SAS	1000G	1000G_SAS	SIFT	Polyphen2	CONDEL	VEP	LRT	PhastCon7 Way	Total read (Adjacent normal)	Reference allel read support (Adjacent normal)	Alternative read support (Adjacent normal)	Total read (Tumor)	Reference allel read support (Tumor)	Alternative read support (Tumor)	Alternate allele frequency
Smoker	42476	chr19	37203837	G	A	*ZNF567*	INTRONIC	ENSG00000189042:[ENST00000392163(c.130+68G>A)];ENSG00000189042:[ENST00000589264(c.160+68G>A)];ENSG00000189042:[ENST00000591308(c.118+68G>A)];ENSG00000189042:[ENST00000360729(c.130+68G>A)];ENSG00000189042:[ENST00000536254(c.223+68G>A)];ENSG00000189042:[ENST00000585696(c.130+68G>A)];ENSG00000189042:[ENST00000588311(c.130+68G>A)];	NA	NA	NA	NA	NA	NA	NA	NA	NA	NA	MODIFIER	NA	NA	82	82	0	80	65	14	17.5
Smoker	42476	chr2	215882786	G	A	*ABCA12*	SILENT	ENSG00000144452:SILENT[ENST00000389661(c.774C>T,tcC>tcT):ENSP00000374312(p.Ser258(=))];ENSG00000144452:SILENT[ENST00000272895(c.1728C>T,tcC>tcT):ENSP00000272895(p.Ser576(=))];	NA	NA	NA	NA	NA	NA	NA	NA	NA	NA	LOW	NA	NA	163	163	0	140	133	7	5
Smoker	42476	chr2	43902779	C	G	*AC011242.6*	EXONIC-NC	ENSG00000223658:[ENST00000475092(n.683G>C)];ENSG00000223658:[ENST00000542399(n.683G>C)];	chr2*43902	NA	chr2*43902779*43902779*C*T*rs747589003*NA*NA*NA	NA	NA	NA	NA	NA	NA	NA	MODIFIER	NA	NA	138	138	0	228	202	26	11.4
Smoker	42476	chr2	144704544	G	C	*AC016910.1*	INTRONIC	ENSG00000232377:[ENST00000422799(n.715+5813G>C)];	NA	NA	NA	NA	NA	NA	NA	NA	NA	NA	MODIFIER	NA	NA	39	39	0	49	32	17	34.69
Smoker	42476	chr2	96799688	C	T	*ASTL*	EXONIC-NC	ENSG00000188886:[ENST00000470582(n.375G>A)];ENSG00000188886:[ENST00000342380(c.337+16G>A)];	NA	NA	0.0001997	0.0001133	0.000126247	0.0001997	0	NA	NA	NA	MODIFIER	NA	NA	71	71	0	87	82	5	5.75
Smoker	42476	chr2	27460635	C	T	*CAD*	MISSENSE	ENSG00000084774:MISSENSE[ENST00000264705(c.4613C>T,tCg>tTg):ENSP00000264705(p.Ser1538Leu)];ENSG00000084774:MISSENSE[ENST00000458503(c.568C>T,tCg>tTg):ENSP00000414742(p.Ser190Leu)];ENSG00000084774:MISSENSE[ENST00000403525(c.4424C>T,tCg>tTg):ENSP00000384510(p.Ser1475Leu)];	NA	NA	NA	NA	NA	NA	NA	D	BN	N	MODERATE	D	0.995	88	88	0	83	71	12	14.46
Smoker	42476	chr2	202140306	G	T	*CASP8*	INTRONIC	ENSG00000064012:[ENST00000450491(c.306+630G>T)];ENSG00000064012:[ENST00000392266(c.551-1244G>T)];ENSG00000064012:[ENST00000323492(c.615+630G>T)];ENSG00000064012:[ENST00000264275(c.711+630G>T)];ENSG00000064012:[ENST00000358485(c.837+630G>T)];ENSG00000064012:[ENST00000447616(c.551-1244G>T)];ENSG00000064012:[ENST00000444430(c.139+2807G>T)];ENSG00000064012:[ENST00000392259(c.596-1244G>T)];ENSG00000064012:[ENST00000392258(c.596-1244G>T)];ENSG00000064012:[ENST00000339403(n.745+630G>T)];ENSG00000064012:[ENST00000392263(c.615+630G>T)];ENSG00000064012:[ENST00000432109(c.660+630G>T)];ENSG00000064012:[ENST00000264274(c.550+2807G>T)];ENSG00000064012:[ENST00000424461(c.140-1244G>T)];	NA	NA	NA	NA	NA	NA	NA	NA	NA	NA	MODIFIER	NA	NA	144	144	0	133	23	110	82.71
Smoker	42476	chr2	238253862	A	C	*COL6A3*	INTRONIC-SS-ACR	ENSG00000163359:[ENST00000472056(c.5272-6T>G)];ENSG00000163359:[ENST00000409809(c.6475-6T>G)];ENSG00000163359:[ENST00000295550(c.7093-6T>G)];ENSG00000163359:[ENST00000347401(c.6490-6T>G)];ENSG00000163359:[ENST00000346358(c.6493-6T>G)];ENSG00000163359:[ENST00000353578(c.6475-6T>G)];ENSG00000163359:[ENST00000491769(n.1347-6T>G)];	NA	NA	NA	NA	NA	NA	NA	NA	NA	NA	LOW	NA	NA	99	99	0	82	64	18	21.95
Smoker	42476	chr2	72362038	T	C	*CYP26B1*	MISSENSE	ENSG00000003137:MISSENSE[ENST00000412253(c.140A>G,cAg>cGg):ENSP00000401465(p.Gln47Arg)];ENSG00000003137:MISSENSE[ENST00000546307(c.488A>G,cAg>cGg):ENSP00000443304(p.Gln163Arg)];ENSG00000003137:MISSENSE[ENST00000001146(c.713A>G,cAg>cGg):ENSP00000001146(p.Gln238Arg)];ENSG00000003137:MISSENSE[ENST00000474509(c.488A>G,cAg>cGg):ENSP00000430888(p.Gln163Arg)];	NA	NA	NA	NA	NA	NA	NA	T	BN	N	MODERATE	NA	NA	64	64	0	91	85	6	6.59
Smoker	42476	chr2	169759086	C	T	*G6PC2*	INTRONIC	ENSG00000152254:[ENST00000421979(c.218+1027C>T)];ENSG00000152254:[ENST00000282075(c.218+1027C>T)];ENSG00000152254:[ENST00000375363(c.328+22C>T)];ENSG00000152254:[ENST00000429379(c.328+22C>T)];	NA	NA	NA	NA	NA	NA	NA	NA	NA	NA	MODIFIER	NA	NA	166	165	0	124	23	101	81.45
Smoker	42476	chr2	154800940	C	T	*GALNT13*	5UTR	ENSG00000144278:[ENST00000392825(c.-71C>T)];ENSG00000144278:[ENST00000434213(c.-71C>T)];	NA	NA	NA	NA	NA	NA	NA	NA	NA	NA	MODIFIER	NA	NA	88	88	0	71	58	13	18.31
Smoker	42476	chr2	144704544	G	C	*GTDC1*	3UTR	ENSG00000121964:[ENST00000463875(c.*75C>G)];ENSG00000121964:[ENST00000344850(c.*75C>G)];ENSG00000121964:[ENST00000409214(c.*75C>G)];ENSG00000121964:[ENST00000415569(c.*1483C>G)];ENSG00000121964:[ENST00000241391(c.*75C>G)];ENSG00000121964:[ENST00000392871(c.*176C>G)];ENSG00000121964:[ENST00000409298(c.*75C>G)];ENSG00000121964:[ENST00000392867(c.*75C>G)];ENSG00000121964:[ENST00000542155(c.*75C>G)];ENSG00000121964:[ENST00000392869(c.*75C>G)];	NA	NA	NA	NA	NA	NA	NA	NA	NA	NA	MODIFIER	NA	NA	39	39	0	49	32	17	34.69
Smoker	42476	chr2	242650832	G	A	*ING5*	MISSENSE	ENSG00000168395:MISSENSE[ENST00000406941(c.317G>A,cGc>cAc):ENSP00000385937(p.Arg106His)];ENSG00000168395:MISSENSE[ENST00000313552(c.317G>A,cGc>cAc):ENSP00000322142(p.Arg106His)];ENSG00000168395:MISSENSE[ENST00000445620(c.317G>A,cGc>cAc):ENSP00000416878(p.Arg106His)];ENSG00000168395:EXONIC-NC[ENST00000482774(n.341G>A)];ENSG00000168395:EXONIC-NC[ENST00000493261(n.337G>A)];ENSG00000168395:EXONIC-NC[ENST00000484145(n.338G>A)];ENSG00000168395:EXONIC-NC[ENST00000489509(n.318G>A)];ENSG00000168395:EXONIC-NC[ENST00000492488(n.348G>A)];	NA	NA	NA	NA	NA	NA	NA	NA	NA	NA	MODIFIER	NA	NA	132	131	0	86	11	75	87.21
Smoker	42476	chr2	234072485	A	G	*INPP5D*	MISSENSE	ENSG00000168918:MISSENSE[ENST00000538935(c.1334A>G,gAg>gGg):ENSP00000441010(p.Glu445Gly)];ENSG00000168918:MISSENSE[ENST00000455936(c.629A>G,gAg>gGg):ENSP00000404610(p.Glu210Gly)];ENSG00000168918:MISSENSE[ENST00000450745(c.629A>G,gAg>gGg):ENSP00000407916(p.Glu210Gly)];ENSG00000168918:MISSENSE[ENST00000359570(c.1337A>G,gAg>gGg):ENSP00000352575(p.Glu446Gly)];ENSG00000168918:MISSENSE[ENST00000445964(c.236A>G,gAg>gGg):ENSP00000405338(p.Glu79Gly)];ENSG00000168918:MISSENSE[ENST00000415617(c.236A>G,gAg>gGg):ENSP00000397421(p.Glu79Gly)];ENSG00000168918:MISSENSE[ENST00000435188(c.236A>G,gAg>gGg):ENSP00000400151(p.Glu79Gly)];ENSG00000168918:EXONIC-NC[ENST00000493078(n.171A>G)];	NA	NA	NA	NA	NA	NA	NA	NA	NA	NA	MODIFIER	NA	NA	70	69	0	50	4	46	92
Smoker	42476	chr2	160980474	G	C	*ITGB6*	INTRONIC	ENSG00000115221:[ENST00000428609(c.1758-75C>G)];ENSG00000115221:[ENST00000409583(c.*1498-75C>G)];ENSG00000115221:[ENST00000409872(c.1884-75C>G)];ENSG00000115221:[ENST00000409967(c.1661-11764C>G)];ENSG00000115221:[ENST00000283249(c.1884-75C>G)];	NA	NA	NA	NA	NA	NA	NA	NA	NA	NA	MODIFIER	NA	NA	77	77	0	63	47	16	25.4

Supplementary Table 4. List of somatic single nucleotide variants identified using whole exome sequencing of ESCC patients

Cohort	Sample	Chromosome	Genomic position	Reference allele	Alternate allele	Gene symbol	Variant type	Variant annotation	OncoMD	COSMIC	dbSNP	ExAC	ExAC_SAS	1000G	1000G_SAS	SIFT	Polyphen2	CONDEL	VEP	LRT	PhastCon7 Way	Total read (Adjacent normal)	Reference allel read support (Adjacent normal)	Alternative read support (Adjacent normal)	Total read (Tumor)	Reference allel read support (Tumor)	Alternative read support (Tumor)	Alternate allele frequency
Smoker	42476	chr2	176866891	C	T	*KIAA1715*	5UTR	ENSG00000144320:[ENST00000392540(c.-86G>A)];ENSG00000144320:[ENST00000544803(c.-66G>A)];ENSG00000144320:[ENST00000272748(c.-66G>A)];ENSG00000144320:[ENST00000409660(c.-205G>A)];ENSG00000144320:[ENST00000466445(n.275+349G>A)];ENSG00000144320:[ENST00000445472(c.-63+349G>A)];ENSG00000144320:[ENST00000535310(c.-435+349G>A)];ENSG00000144320:[ENST00000483230(n.117G>A)];	NA	NA	NA	NA	NA	NA	NA	NA	NA	NA	MODIFIER	NA	NA	30	30	0	8	3	5	62.5
Smoker	42476	chr2	74762643	C	A	*LOXL3*	INTRONIC	ENSG00000115318:[ENST00000470907(n.800-62G>T)];ENSG00000115318:[ENST00000264094(c.1417-62G>T)];ENSG00000115318:[ENST00000409986(c.982-62G>T)];ENSG00000115318:[ENST00000409249(c.1093+775G>T)];ENSG00000115318:[ENST00000481835(n.545-62G>T)];ENSG00000115318:[ENST00000409549(c.1249-62G>T)];ENSG00000115318:[ENST00000393937(c.982-62G>T)];	NA	NA	NA	NA	NA	NA	NA	NA	NA	NA	MODIFIER	NA	NA	102	102	0	144	102	42	29.17
Smoker	42476	chr2	141680600	C	T	*LRP1B*	MISSENSE	ENSG00000168702:MISSENSE[ENST00000434794(c.688G>A,Gaa>Aaa):ENSP00000413239(p.Glu230Lys)];ENSG00000168702:MISSENSE[ENST00000389484(c.3253G>A,Gaa>Aaa):ENSP00000374135(p.Glu1085Lys)];	NA	NA	NA	NA	NA	NA	NA	NA	PrD	NA	MODERATE	U	1	112	112	0	140	120	20	14.29
Smoker	42476	chr2	87426086	C	T	*NA*	INTERGENIC		NA	NA	NA	NA	NA	NA	NA	NA	NA	NA	MODIFIER	NA	NA	51	51	0	96	84	12	12.5
Smoker	42476	chr2	15448453	C	G	*NBAS*	MISSENSE	ENSG00000151779:MISSENSE[ENST00000281513(c.4684G>C,Gaa>Caa):ENSP00000281513(p.Glu1562Gln)];ENSG00000151779:MISSENSE[ENST00000441750(c.4324G>C,Gaa>Caa):ENSP00000413201(p.Glu1442Gln)];ENSG00000151779:MISSENSE[ENST00000442506(c.1827G>C,Gaa>Caa):ENSP00000398411(p.Glu610Gln)];	NA	NA	NA	NA	NA	NA	NA	D	PrD	D	MODERATE	NA	NA	81	80	0	71	54	17	23.94
Smoker	42476	chr2	178098815	T	C	*NFE2L2*	MISSENSE	ENSG00000116044:MISSENSE[ENST00000423513(c.182A>G,gAt>gGt):ENSP00000410015(p.Asp61Gly)];ENSG00000116044:MISSENSE[ENST00000397062(c.230A>G,gAt>gGt):ENSP00000380252(p.Asp77Gly)];ENSG00000116044:MISSENSE[ENST00000446151(c.182A>G,gAt>gGt):ENSP00000411575(p.Asp61Gly)];ENSG00000116044:MISSENSE[ENST00000464747(c.182A>G,gAt>gGt):ENSP00000467401(p.Asp61Gly)];ENSG00000116044:MISSENSE[ENST00000397063(c.182A>G,gAt>gGt):ENSP00000380253(p.Asp61Gly)];ENSG00000116044:MISSENSE[ENST00000449627(c.182A>G,gAt>gGt):ENSP00000391590(p.Asp61Gly)];ENSG00000116044:MISSENSE[ENST00000588123(c.182A>G,gAt>gGt):ENSP00000468089(p.Asp61Gly)];ENSG00000116044:MISSENSE[ENST00000586532(c.227A>G,gAt>gGt):ENSP00000464920(p.Asp76Gly)];ENSG00000116044:MISSENSE[ENST00000421929(c.182A>G,gAt>gGt):ENSP00000412191(p.Asp61Gly)];ENSG00000116044:MISSENSE[ENST00000448782(c.182A>G,gAt>gGt):ENSP00000400073(p.Asp61Gly)];ENSG00000116044:EXONIC-NC[ENST00000462023(n.313A>G)];ENSG00000116044:EXONIC-NC[ENST00000477534(n.320A>G)];ENSG00000116044:INTRONIC[ENST00000430047(c.63+167A>G)];	chr2*17809	chr2*17809	NA	NA	NA	NA	NA	NA	NA	NA	MODIFIER	NA	NA	112	112	0	106	15	91	85.85
Smoker	42476	chr2	242054689	G	C	*PASK*	INTRONIC	ENSG00000115687:[ENST00000493544(n.3631+14C>G)];ENSG00000115687:[ENST00000405260(c.3198+14C>G)];ENSG00000115687:[ENST00000358649(c.3198+14C>G)];ENSG00000115687:[ENST00000403638(c.3198+14C>G)];ENSG00000115687:[ENST00000544142(c.2640+14C>G)];ENSG00000115687:[ENST00000539818(c.2550+14C>G)];ENSG00000115687:[ENST00000234040(c.3198+14C>G)];	NA	NA	NA	NA	NA	NA	NA	NA	NA	NA	MODIFIER	NA	NA	153	152	0	142	130	12	8.45
Smoker	42476	chr2	197750647	G	C	*PGAP1*	INTRONIC	ENSG00000197121:[ENST00000374738(c.514-43C>G)];ENSG00000197121:[ENST00000482051(n.31-43C>G)];ENSG00000197121:[ENST00000409475(c.1174-43C>G)];ENSG00000197121:[ENST00000423035(c.*1105-43C>G)];ENSG00000197121:[ENST00000409188(c.*77-43C>G)];ENSG00000197121:[ENST00000470179(n.638-43C>G)];ENSG00000197121:[ENST00000354764(c.1174-43C>G)];	NA	NA	NA	NA	NA	NA	NA	NA	NA	NA	MODIFIER	NA	NA	36	36	0	37	31	6	16.22
Smoker	42476	chr2	28826896	C	G	*PLB1*	SILENT	ENSG00000163803:SILENT[ENST00000327757(c.2838C>G,gcC>gcG):ENSP00000330442(p.Ala946(=))];ENSG00000163803:SILENT[ENST00000422425(c.2805C>G,gcC>gcG):ENSP00000416440(p.Ala935(=))];ENSG00000163803:SILENT[ENST00000404858(c.2801C>G,gcC>gcG):ENSP00000384187(p.Ala934(=))];ENSG00000163803:INTRONIC[ENST00000411743(c.147+1108C>G)];ENSG00000163803:INTRONIC[ENST00000479065(n.179+1108C>G)];ENSG00000163803:INTRONIC[ENST00000541605(c.-49+1108C>G)];	NA	NA	NA	NA	NA	NA	NA	NA	NA	NA	LOW	NA	NA	68	68	0	45	22	23	51.11
Smoker	42476	chr2	43902779	C	G	*PLEKHH2*	INTRONIC	ENSG00000152527:[ENST00000405000(n.401-3223C>G)];ENSG00000152527:[ENST00000405223(n.207-3223C>G)];ENSG00000152527:[ENST00000282406(c.124-3223C>G)];ENSG00000152527:[ENST00000491692(c.124-3223C>G)];	chr2*43902	NA	chr2*43902779*43902779*C*T*rs747589003*NA*NA*NA	NA	NA	NA	NA	NA	NA	NA	MODIFIER	NA	NA	138	138	0	228	202	26	11.4
Smoker	42476	chr2	152330496	G	A	*RIF1*	MISSENSE	ENSG00000080345:MISSENSE[ENST00000444746(c.7114G>A,Gaa>Aaa):ENSP00000390181(p.Glu2372Lys)];ENSG00000080345:MISSENSE[ENST00000453091(c.7036G>A,Gaa>Aaa):ENSP00000414615(p.Glu2346Lys)];ENSG00000080345:MISSENSE[ENST00000428287(c.7036G>A,Gaa>Aaa):ENSP00000415691(p.Glu2346Lys)];ENSG00000080345:MISSENSE[ENST00000243326(c.7114G>A,Gaa>Aaa):ENSP00000243326(p.Glu2372Lys)];ENSG00000080345:MISSENSE[ENST00000430328(c.7036G>A,Gaa>Aaa):ENSP00000416123(p.Glu2346Lys)];ENSG00000080345:MISSENSE[ENST00000454583(c.1935G>A,Gaa>Aaa):ENSP00000404882(p.Glu646Lys)];	NA	NA	NA	NA	NA	NA	NA	D	PrD	D	MODERATE	NA	NA	37	36	0	52	40	12	23.08
Smoker	42476	chr2	200137389	G	A	*SATB2*	MISSENSE	ENSG00000119042:MISSENSE[ENST00000260926(c.1747C>T,Cat>Tat):ENSP00000260926(p.His583Tyr)];ENSG00000119042:MISSENSE[ENST00000428695(c.1393C>T,Cat>Tat):ENSP00000388581(p.His465Tyr)];ENSG00000119042:MISSENSE[ENST00000443023(c.1570C>T,Cat>Tat):ENSP00000388764(p.His524Tyr)];ENSG00000119042:MISSENSE[ENST00000417098(c.1747C>T,Cat>Tat):ENSP00000401112(p.His583Tyr)];ENSG00000119042:MISSENSE[ENST00000457245(c.1747C>T,Cat>Tat):ENSP00000405420(p.His583Tyr)];	NA	NA	NA	NA	NA	NA	NA	T	BN	N	MODERATE	D	0.999	49	49	0	31	18	13	41.94
Smoker	42476	chr2	169759086	C	T	*SPC25*	INTRONIC	ENSG00000152253:[ENST00000451987(c.-173+10624G>A)];ENSG00000152253:[ENST00000472216(n.176+10620G>A)];	NA	NA	NA	NA	NA	NA	NA	NA	NA	NA	MODIFIER	NA	NA	166	165	0	124	23	101	81.45
Smoker	42476	chr2	45808959	G	C	*SRBD1*	INTRONIC-SS-ACR	ENSG00000068784:[ENST00000263736(c.816-10C>G)];	NA	NA	NA	NA	NA	NA	NA	NA	NA	NA	MODIFIER	NA	NA	69	69	0	95	84	11	11.58
Smoker	42476	chr2	108872147	C	T	*SULT1C3*	SILENT	ENSG00000196228:SILENT[ENST00000376700(c.519C>T,tcC>tcT):ENSP00000365890(p.Ser173(=))];ENSG00000196228:SILENT[ENST00000329106(c.519C>T,tcC>tcT):ENSP00000333310(p.Ser173(=))];	NA	NA	NA	2.50E-05	0	NA	NA	NA	NA	NA	LOW	NA	NA	64	63	1	46	11	35	76.09

Mangalaparthi *et al.* , 2020. Mutational landscape of esophageal squamous cell carcinoma in an Indian cohort
Supplementary Table 4. List of somatic single nucleotide variants identified using whole exome sequencing of ESCC patients

Cohort	Sample	Chromosome	Genomic position	Reference allele	Alternate allele	Gene symbol	Variant type	Variant annotation	OncoMD	COSMIC	dbSNP	ExAC	ExAC_SAS	1000G	1000G_SAS	SIFT	Polyphen2	CONDEL	VEP	LRT	PhastCon7 Way	Total read (Adjacent normal)	Reference allel read support (Adjacent normal)	Alternative read support (Adjacent normal)	Total read (Tumor)	Reference allel read support (Tumor)	Alternative read support (Tumor)	Alternate allele frequency
Smoker	42476	chr2	10059155	C	G	*TAF1B*	INTRONIC	ENSG00000115750:[ENST00000263663(c.1272-15C>G)];ENSG00000115750:[ENST00000434858(c.*533-15C>G)];ENSG00000115750:[ENST00000396242(c.507-15C>G)];	NA	NA	NA	NA	NA	NA	NA	NA	NA	NA	MODIFIER	NA	NA	128	128	0	150	119	31	20.67
Smoker	42476	chr2	85051184	G	A	*TRABD2A*	SILENT	ENSG00000186854:SILENT[ENST00000335459(c.1080C>T,gcC>gcT):ENSP00000335004(p.Ala360(=))];ENSG00000186854:SILENT[ENST00000409520(c.1227C>T,gcC>gcT):ENSP00000387075(p.Ala409(=))];ENSG00000186854:EXONIC-NC[ENST00000496500(n.572C>T)];ENSG00000186854:EXONIC-NC[ENST00000479944(n.3353C>T)];	NA	NA	NA	NA	NA	NA	NA	NA	NA	NA	MODIFIER	NA	NA	57	57	0	94	73	21	22.34
Smoker	42476	chr2	179610969	A	G	*TTN*	SILENT	ENSG00000155657:SILENT[ENST00000360870(c.16158T>C,atT>atC):ENSP00000354117(p.Ile5386(=))];ENSG00000155657:INTRONIC[ENST00000359218(c.10598-4321T>C)];ENSG00000155657:INTRONIC[ENST00000342175(c.10799-4321T>C)];ENSG00000155657:INTRONIC[ENST00000342992(c.10360+6882T>C)];ENSG00000155657:INTRONIC[ENST00000589042(c.11312-4321T>C)];ENSG00000155657:INTRONIC[ENST00000591111(c.10361-4321T>C)];ENSG00000155657:INTRONIC[ENST00000460472(c.10223-4321T>C)];	NA	NA	NA	NA	NA	NA	NA	NA	NA	NA	MODIFIER	NA	NA	60	60	0	49	40	8	16.33
Smoker	42476	chr2	179610969	A	G	*TTN-AS1*	INTRONIC	ENSG00000237298:[ENST00000590773(n.243+3272A>G)];ENSG00000237298:[ENST00000585451(n.199-14698A>G)];ENSG00000237298:[ENST00000578746(n.64+1400A>G)];	NA	NA	NA	NA	NA	NA	NA	NA	NA	NA	MODIFIER	NA	NA	60	60	0	49	40	8	16.33
Smoker	42476	chr2	234394167	A	T	*USP40*	INTRONIC-SS-DNR	ENSG00000085982:[ENST00000251722(c.3501+10T>A)];ENSG00000085982:[ENST00000450966(c.3537+10T>A)];ENSG00000085982:[ENST00000483519(n.649+10T>A)];ENSG00000085982:[ENST00000427112(c.3501+10T>A)];	NA	NA	chr2*234394167*234394169*AAC*A*rs775957185*NA*NA*NA	NA	NA	NA	NA	NA	NA	NA	MODIFIER	NA	NA	78	77	0	56	43	13	23.21
Smoker	42476	chr20	30898360	G	C	*KIF3B*	MISSENSE	ENSG00000101350:MISSENSE[ENST00000375712(c.780G>C,gaG>gaC):ENSP00000364864(p.Glu260Asp)];ENSG00000101350:5UTR[ENST00000418717(c.-157G>C)];	NA	NA	NA	NA	NA	NA	NA	NA	NA	NA	MODIFIER	NA	NA	86	86	0	123	107	16	13.01
Smoker	42476	chr20	59053230	C	G	*MIR4533*	EXONIC-NC	ENSG00000266140:[ENST00000581943(n.62C>G)];	NA	NA	NA	NA	NA	NA	NA	NA	NA	NA	MODIFIER	NA	NA	122	122	0	138	106	32	23.19
Smoker	42476	chr20	41419810	A	C	*PTPRT*	INTRONIC	ENSG00000196090:[ENST00000373201(c.486+25T>G)];ENSG00000196090:[ENST00000356100(c.486+25T>G)];ENSG00000196090:[ENST00000373198(c.486+25T>G)];ENSG00000196090:[ENST00000373193(c.486+25T>G)];ENSG00000196090:[ENST00000373184(c.486+25T>G)];ENSG00000196090:[ENST00000373190(c.486+25T>G)];ENSG00000196090:[ENST00000373187(c.486+25T>G)];	NA	NA	NA	NA	NA	NA	NA	NA	NA	NA	MODIFIER	NA	NA	49	49	0	50	13	37	74
Smoker	42476	chr20	55953149	G	A	*RAE1*	SILENT	ENSG00000101146:EXONIC-NC[ENST00000462438(n.2881G>A)];ENSG00000101146:SILENT[ENST00000395840(c.1101G>A,aaG>aaA):ENSP00000379181(p.Lys367(=))];ENSG00000101146:SILENT[ENST00000395841(c.1101G>A,aaG>aaA):ENSP00000379182(p.Lys367(=))];ENSG00000101146:SILENT[ENST00000371242(c.1101G>A,aaG>aaA):ENSP00000360286(p.Lys367(=))];ENSG00000101146:3UTR[ENST00000492498(c.*340G>A)];	NA	NA	chr20*55953146*55953149*TAAG*T*rs774816755*NA*NA*NA	NA	NA	NA	NA	NA	NA	NA	MODIFIER	NA	NA	90	90	0	135	110	24	17.78
Smoker	42476	chr20	37203595	G	A	*RALGAPB*	SILENT	ENSG00000170471:EXONIC-NC[ENST00000461147(n.1937G>A)];ENSG00000170471:SILENT[ENST00000397038(c.3807G>A,aaG>aaA):ENSP00000380231(p.Lys1269(=))];ENSG00000170471:SILENT[ENST00000397040(c.4470G>A,aaG>aaA):ENSP00000380233(p.Lys1490(=))];ENSG00000170471:SILENT[ENST00000397042(c.4461G>A,aaG>aaA):ENSP00000380235(p.Lys1487(=))];ENSG00000170471:SILENT[ENST00000262879(c.4470G>A,aaG>aaA):ENSP00000262879(p.Lys1490(=))];ENSG00000170471:SILENT[ENST00000438490(c.3957G>A,aaG>aaA):ENSP00000416646(p.Lys1319(=))];ENSG00000170471:INTRONIC[ENST00000490114(n.807+12G>A)];	NA	NA	NA	NA	NA	NA	NA	NA	NA	NA	LOW	NA	NA	87	87	0	72	57	15	20.83
Smoker	42476	chr20	59053230	C	G	*RP5-1043L13.1*	INTRONIC	ENSG00000228340:[ENST00000437035(n.428-49215C>G)];	NA	NA	NA	NA	NA	NA	NA	NA	NA	NA	MODIFIER	NA	NA	122	122	0	138	106	32	23.19
Smoker	42476	chr20	50408601	C	T	*SALL4*	MISSENSE	ENSG00000101115:MISSENSE[ENST00000217086(c.421G>A,Gag>Aag):ENSP00000217086(p.Glu141Lys)];ENSG00000101115:MISSENSE[ENST00000395997(c.421G>A,Gag>Aag):ENSP00000379319(p.Glu141Lys)];ENSG00000101115:EXONIC-NC[ENST00000483130(n.703G>A)];ENSG00000101115:INTRONIC[ENST00000371539(c.131-2921G>A)];	NA	NA	NA	8.24E-06	0	NA	NA	NA	NA	NA	MODIFIER	NA	NA	82	82	0	79	63	16	20.25
Smoker	42476	chr20	44418624	G	C	*WFDC3*	INTRONIC	ENSG00000124116:[ENST00000465935(n.38-926C>G)];ENSG00000124116:[ENST00000493693(n.70-926C>G)];ENSG00000124116:[ENST00000481847(n.101+1847C>G)];ENSG00000124116:[ENST00000462017(n.72+1847C>G)];ENSG00000124116:[ENST00000471401(n.50+1847C>G)];ENSG00000124116:[ENST00000487343(n.73-926C>G)];ENSG00000124116:[ENST00000467679(n.85-926C>G)];ENSG00000124116:[ENST00000337205(c.12-40C>G)];ENSG00000124116:[ENST00000490877(n.45-926C>G)];ENSG00000124116:[ENST00000243938(c.-7-3C>G)];ENSG00000124116:[ENST00000372630(c.-10C>G)];ENSG00000124116:[ENST00000372632(c.-10C>G)];	NA	NA	chr20*44418624*44418624*G*GTT*rs777184518*NA*NA*NA	NA	NA	NA	NA	NA	NA	NA	MODIFIER	NA	NA	32	32	0	42	34	8	19.05
Smoker	42476	chr20	39833427	C	T	*ZHX3*	MISSENSE	ENSG00000174306:MISSENSE[ENST00000558993(c.130G>A,Gaa>Aaa):ENSP00000454208(p.Glu44Lys)];ENSG00000174306:MISSENSE[ENST00000544979(c.130G>A,Gaa>Aaa):ENSP00000443783(p.Glu44Lys)];ENSG00000174306:MISSENSE[ENST00000373261(c.130G>A,Gaa>Aaa):ENSP00000362358(p.Glu44Lys)];ENSG00000174306:MISSENSE[ENST00000436440(c.130G>A,Gaa>Aaa):ENSP00000403048(p.Glu44Lys)];ENSG00000174306:MISSENSE[ENST00000441102(c.130G>A,Gaa>Aaa):ENSP00000399433(p.Glu44Lys)];ENSG00000174306:MISSENSE[ENST00000557816(c.130G>A,Gaa>Aaa):ENSP00000453548(p.Glu44Lys)];ENSG00000174306:MISSENSE[ENST00000560364(c.130G>A,Gaa>Aaa):ENSP00000453647(p.Glu44Lys)];ENSG00000174306:MISSENSE[ENST00000540170(c.130G>A,Gaa>Aaa):ENSP00000442290(p.Glu44Lys)];ENSG00000174306:MISSENSE[ENST00000432768(c.130G>A,Gaa>Aaa):ENSP00000415498(p.Glu44Lys)];ENSG00000174306:MISSENSE[ENST00000559234(c.130G>A,Gaa>Aaa):ENSP00000452965(p.Glu44Lys)];ENSG00000174306:MISSENSE[ENST00000436099(c.130G>A,Gaa>Aaa):ENSP00000401852(p.Glu44Lys)];ENSG00000174306:MISSENSE[ENST00000419740(c.130G>A,Gaa>Aaa):ENSP00000400441(p.Glu44Lys)];ENSG00000174306:MISSENSE[ENST00000309060(c.130G>A,Gaa>Aaa):ENSP00000312222(p.Glu44Lys)];ENSG00000174306:MISSENSE[ENST00000560361(c.130G>A,Gaa>Aaa):ENSP00000454006(p.Glu44Lys)];	NA	NA	NA	NA	NA	NA	NA	T	BN	N	MODERATE	N	0.241	101	99	0	93	76	17	18.28

Mangalaparthi *et al.* , 2020. Mutational landscape of esophageal squamous cell carcinoma in an Indian cohort
Supplementary Table 4. List of somatic single nucleotide variants identified using whole exome sequencing of ESCC patients

Cohort	Sample	Chromosome	Genomic position	Reference allele	Alternate allele	Gene symbol	Variant type	Variant annotation	OncoMD	COSMIC	dbSNP	ExAC	ExAC_SAS	1000G	1000G_SAS	SIFT	Polyphen2	CONDEL	VEP	LRT	PhastCon7 Way	Total read (Adjacent normal)	Reference allel read support (Adjacent normal)	Alternative read support (Adjacent normal)	Total read (Tumor)	Reference allel read support (Tumor)	Alternative read support (Tumor)	Alternate allele frequency
Smoker	42476	chr21	31973494	G	C	*KRTAP22-1*	MISSENSE	ENSG00000186924:MISSENSE[ENST00000334680(c.55G>C,Ggc>Cgc):ENSP00000333887(p.Gly19Arg)];	NA	NA	NA	NA	NA	NA	NA	NA	UN	NA	MODERATE	N	0.763	70	70	0	36	29	7	19.44
Smoker	42476	chr21	16339261	G	C	*NRIP1*	NONSENSE	ENSG00000180530:NONSENSE[ENST00000400202(c.1253C>G,tCa>tGa):ENSP00000383063(p.Ser418Ter)];ENSG00000180530:NONSENSE[ENST00000318948(c.1253C>G,tCa>tGa):ENSP00000327213(p.Ser418Ter)];ENSG00000180530:NONSENSE[ENST00000400199(c.1253C>G,tCa>tGa):ENSP00000383060(p.Ser418Ter)];	NA	NA	NA	NA	NA	NA	NA	NA	NA	NA	HIGH	NA	0.13	176	176	0	196	157	39	19.9
Smoker	42476	chr21	45170329	G	A	*PDXK*	INTRONIC	ENSG00000160209:[ENST00000468392(n.510-52G>A)];ENSG00000160209:[ENST00000498040(n.659-52G>A)];ENSG00000160209:[ENST00000468090(c.381-52G>A)];ENSG00000160209:[ENST00000481512(n.414-52G>A)];ENSG00000160209:[ENST00000398078(n.514-52G>A)];ENSG00000160209:[ENST00000398085(n.639-52G>A)];ENSG00000160209:[ENST00000490666(n.348-52G>A)];ENSG00000160209:[ENST00000343528(n.435-52G>A)];ENSG00000160209:[ENST00000467908(c.345-52G>A)];ENSG00000160209:[ENST00000291565(c.465-52G>A)];	NA	NA	NA	NA	NA	NA	NA	NA	NA	NA	MODIFIER	NA	NA	77	77	0	44	28	16	36.36
Smoker	42476	chr22	24040137	C	G	*AP000347.2*	INTRONIC	ENSG00000272578:[ENST00000435868(n.711+16261G>C)];	NA	NA	NA	NA	NA	NA	NA	NA	NA	NA	MODIFIER	NA	NA	45	45	0	45	34	11	24.44
Smoker	42476	chr22	18081058	G	C	*ATP6V1E1*	INTRONIC-SS-ACR	ENSG00000131100:[ENST00000399798(c.370-4C>G)];ENSG00000131100:[ENST00000253413(c.436-4C>G)];ENSG00000131100:[ENST00000481365(n.405-4C>G)];ENSG00000131100:[ENST00000413576(c.439-4C>G)];ENSG00000131100:[ENST00000399796(c.346-4C>G)];	NA	NA	chr22*18081058*18081059*GA*G*rs771519246*NA*NA*NA	NA	NA	NA	NA	NA	NA	NA	LOW	NA	NA	97	97	0	114	90	21	18.42
Smoker	42476	chr22	41633690	C	G	*CHADL*	SILENT	ENSG00000100399:INTRONIC[ENST00000455425(c.79-202G>C)];ENSG00000100399:SILENT[ENST00000417999(c.1379G>C,gcG>gcC):ENSP00000392046(p.Ala460(=))];ENSG00000100399:SILENT[ENST00000216241(c.1386G>C,gcG>gcC):ENSP00000216241(p.Ala462(=))];	NA	NA	NA	NA	NA	NA	NA	NA	NA	NA	LOW	NA	NA	54	54	0	54	48	6	11.11
Smoker	42476	chr22	46719190	A	T	*GTSE1*	INTRONIC	ENSG00000075218:[ENST00000454366(c.1505+31A>T)];ENSG00000075218:[ENST00000479645(n.449+31A>T)];ENSG00000075218:[ENST00000466510(n.449+31A>T)];	NA	NA	chr22*46719190*46719190*A*G*rs748886308*NA*NA*NA	NA	NA	NA	NA	NA	NA	NA	MODIFIER	NA	NA	79	79	0	70	19	51	72.86
Smoker	42476	chr22	24040137	C	G	*GUSBP11*	INTRONIC	ENSG00000228315:[ENST00000422506(n.976-2433G>C)];ENSG00000228315:[ENST00000452737(n.927+2776G>C)];ENSG00000228315:[ENST00000445682(n.1096-2433G>C)];ENSG00000228315:[ENST00000451837(n.864-2433G>C)];	NA	NA	NA	NA	NA	NA	NA	NA	NA	NA	MODIFIER	NA	NA	45	45	0	45	34	11	24.44
Smoker	42476	chr22	22516716	G	C	*IGLV4-60*	INTRONIC	ENSG00000211639:[ENST00000390284(c.53-49G>C)];	NA	NA	chr22*22516716*22516716*G*T*rs749785922*NA*NA*NA	NA	NA	NA	NA	NA	NA	NA	MODIFIER	NA	NA	102	101	0	102	82	20	19.61
Smoker	42476	chr22	24040137	C	G	*KB-1572G7.2*	INTRONIC	ENSG00000273000:[ENST00000421064(n.890-2433G>C)];	NA	NA	NA	NA	NA	NA	NA	NA	NA	NA	MODIFIER	NA	NA	45	45	0	45	34	11	24.44
Smoker	42476	chr22	18354653	T	C	*MICAL3*	MISSENSE	ENSG00000243156:MISSENSE[ENST00000585038(c.2750A>G,tAc>tGc):ENSP00000462033(p.Tyr917Cys)];ENSG00000243156:MISSENSE[ENST00000429452(c.2750A>G,tAc>tGc):ENSP00000414846(p.Tyr917Cys)];ENSG00000243156:MISSENSE[ENST00000207726(c.2462A>G,tAc>tGc):ENSP00000207726(p.Tyr821Cys)];ENSG00000243156:MISSENSE[ENST00000444520(c.2378A>G,tAc>tGc):ENSP00000410315(p.Tyr793Cys)];ENSG00000243156:MISSENSE[ENST00000383094(c.2378A>G,tAc>tGc):ENSP00000372574(p.Tyr793Cys)];ENSG00000243156:MISSENSE[ENST00000414725(c.2462A>G,tAc>tGc):ENSP00000391827(p.Tyr821Cys)];ENSG00000243156:MISSENSE[ENST00000461307(c.1018A>G,tAc>tGc):ENSP00000462531(p.Tyr340Cys)];ENSG00000243156:MISSENSE[ENST00000400561(c.2378A>G,tAc>tGc):ENSP00000383406(p.Tyr793Cys)];ENSG00000243156:MISSENSE[ENST00000441493(c.2378A>G,tAc>tGc):ENSP00000416015(p.Tyr793Cys)];ENSG00000243156:3UTR[ENST00000495076(c.*1269A>G)];ENSG00000243156:EXONIC-NC[ENST00000462645(n.433A>G)];	NA	NA	NA	NA	NA	NA	NA	NA	NA	NA	MODIFIER	NA	NA	129	129	0	98	77	21	21.43
Smoker	42476	chr22	24040137	C	G	*RGL4*	INTRONIC	ENSG00000159496:[ENST00000460003(n.2110+89C>G)];ENSG00000159496:[ENST00000452208(c.304+89C>G)];ENSG00000159496:[ENST00000441897(c.*119+89C>G)];ENSG00000159496:[ENST00000290691(c.1260+89C>G)];ENSG00000159496:[ENST00000423392(c.1260+89C>G)];ENSG00000159496:[ENST00000401461(c.852+89C>G)];ENSG00000159496:[ENST00000467354(n.2110+89C>G)];	NA	NA	NA	NA	NA	NA	NA	NA	NA	NA	MODIFIER	NA	NA	45	45	0	45	34	11	24.44
Smoker	42476	chr22	30783262	G	A	*RNF215*	MISSENSE	ENSG00000099999:INTRONIC[ENST00000431544(c.1-514C>T)];ENSG00000099999:MISSENSE[ENST00000382363(c.41C>T,cCg>cTg):ENSP00000371800(p.Pro14Leu)];	NA	NA	NA	NA	NA	NA	NA	T_lc	UN	NA	MODERATE	U	0.032	12	12	0	8	0	8	100
Smoker	42476	chr22	43619159	G	T	*SCUBE1*	MISSENSE	ENSG00000159307:MISSENSE[ENST00000360835(c.1271C>A,gCa>gAa):ENSP00000354080(p.Ala424Glu)];	NA	NA	NA	NA	NA	NA	NA	T	BN	N	MODERATE	N	0.504	55	55	0	51	43	8	15.69
Smoker	42476	chr3	169487153	G	C	*ACTRT3*	MISSENSE	ENSG00000184378:MISSENSE[ENST00000330368(c.156C>G,tgC>tgG):ENSP00000333037(p.Cys52Trp)];	NA	NA	NA	NA	NA	NA	NA	D	PoD	D	MODERATE	N	0.19	64	64	0	125	120	5	4
Smoker	42476	chr3	193220431	G	C	*ATP13A4*	INTRONIC-SS-ACR	ENSG00000127249:[ENST00000490925(n.343-3C>G)];ENSG00000127249:[ENST00000489140(n.466-3C>G)];ENSG00000127249:[ENST00000392443(c.235-3C>G)];ENSG00000127249:[ENST00000342695(c.235-3C>G)];ENSG00000127249:[ENST00000295548(c.235-3C>G)];ENSG00000127249:[ENST00000450950(c.235-103C>G)];	NA	NA	NA	NA	NA	NA	NA	NA	NA	NA	LOW	NA	NA	89	89	0	184	171	13	7.07
Smoker	42476	chr3	33648832	T	A	*CLASP2*	INTRONIC	ENSG00000163539:[ENST00000333778(c.857+44A>T)];ENSG00000163539:[ENST00000307312(c.-8+44A>T)];ENSG00000163539:[ENST00000359576(c.1526+44A>T)];ENSG00000163539:[ENST00000461133(c.827+44A>T)];ENSG00000163539:[ENST00000468888(c.1529+44A>T)];ENSG00000163539:[ENST00000487200(c.845+44A>T)];ENSG00000163539:[ENST00000313350(c.845+44A>T)];ENSG00000163539:[ENST00000399362(c.1526+44A>T)];ENSG00000163539:[ENST00000539981(c.782+44A>T)];ENSG00000163539:[ENST00000480013(c.827+44A>T)];	NA	NA	0.01777	0.0156778	0.011745862	0.0177716	0.0082	NA	NA	NA	MODIFIER	NA	NA	36	36	0	22	13	9	40.91
Smoker	42476	chr3	139654315	C	G	*CLSTN2*	SILENT	ENSG00000158258:SILENT[ENST00000458420(c.99C>G,ctC>ctG):ENSP00000402460(p.Leu33(=))];ENSG00000158258:EXONIC-NC[ENST00000511524(n.287C>G)];	NA	NA	NA	NA	NA	NA	NA	NA	NA	NA	MODIFIER	NA	NA	43	43	0	69	61	8	11.59
Smoker	42476	chr3	99569144	G	C	*CMSS1*	INTRONIC	ENSG00000184220:[ENST00000421999(c.64+32257G>C)];ENSG00000184220:[ENST00000491299(c.64+32257G>C)];ENSG00000184220:[ENST00000463526(c.-114+32257G>C)];ENSG00000184220:[ENST00000496116(n.132+32257G>C)];	NA	NA	NA	2.49E-05	0	NA	NA	NA	NA	NA	MODIFIER	NA	NA	85	85	0	169	72	96	56.8

Mangalaparthi *et al.* , 2020. Mutational landscape of esophageal squamous cell carcinoma in an Indian cohort
Supplementary Table 4. List of somatic single nucleotide variants identified using whole exome sequencing of ESCC patients

Cohort	Sample	Chromosome	Genomic position	Reference allele	Alternate allele	Gene symbol	Variant type	Variant annotation	OncoMD	COSMIC	dbSNP	ExAC	ExAC_SAS	1000G	1000G_SAS	SIFT	Polyphen2	CONDEL	VEP	LRT	PhastCon7 Way	Total read (Adjacent normal)	Reference allel read support (Adjacent normal)	Alternative read support (Adjacent normal)	Total read (Tumor)	Reference allel read support (Tumor)	Alternative read support (Tumor)	Alternate allele frequency
Smoker	42476	chr3	148601503	G	A	*CPA3*	SILENT	ENSG00000163751:EXONIC-NC[ENST00000477926(n.359G>A)];ENSG00000163751:SILENT[ENST00000296046(c.882G>A,ctG>ctA):ENSP00000296046(p.Leu294(=))];	NA	NA	NA	NA	NA	NA	NA	NA	NA	NA	MODIFIER	NA	NA	119	119	0	301	257	44	14.62
Smoker	42476	chr3	99569144	G	C	*FILIP1L*	MISSENSE	ENSG00000168386:MISSENSE[ENST00000487087(c.104C>G,tCt>tGt):ENSP00000417774(p.Ser35Cys)];ENSG00000168386:MISSENSE[ENST00000354552(c.1376C>G,tCt>tGt):ENSP00000346560(p.Ser459Cys)];ENSG00000168386:MISSENSE[ENST00000331335(c.1376C>G,tCt>tGt):ENSP00000327880(p.Ser459Cys)];ENSG00000168386:MISSENSE[ENST00000383694(c.656C>G,tCt>tGt):ENSP00000373192(p.Ser219Cys)];ENSG00000168386:MISSENSE[ENST00000471562(c.656C>G,tCt>tGt):ENSP00000419642(p.Ser219Cys)];ENSG00000168386:MISSENSE[ENST00000495625(c.656C>G,tCt>tGt):ENSP00000419874(p.Ser219Cys)];ENSG00000168386:INTRONIC[ENST00000476723(n.103-19695C>G)];	NA	NA	NA	2.49E-05	0	NA	NA	NA	NA	NA	MODIFIER	NA	NA	85	85	0	169	72	96	56.8
Smoker	42476	chr3	121433816	G	C	*GOLGB1*	SILENT	ENSG00000173230:SILENT[ENST00000393667(c.1296C>G,ctC>ctG):ENSP00000377275(p.Leu432(=))];ENSG00000173230:SILENT[ENST00000340645(c.1281C>G,ctC>ctG):ENSP00000341848(p.Leu427(=))];ENSG00000173230:SILENT[ENST00000494517(c.1173C>G,ctC>ctG):ENSP00000418231(p.Leu391(=))];ENSG00000173230:SILENT[ENST00000489400(c.893C>G,ctC>ctG):ENSP00000417767(p.Leu298(=))];ENSG00000173230:3UTR[ENST00000482512(c.*1204C>G)];	chr3*12143	chr3*12143	NA	NA	NA	NA	NA	NA	NA	NA	LOW	NA	NA	43	43	0	86	72	14	16.28
Smoker	42476	chr3	185541076	G	A	*IGF2BP2*	INTRONIC	ENSG00000073792:[ENST00000466476(n.111-75C>T)];ENSG00000073792:[ENST00000457616(c.179-75C>T)];ENSG00000073792:[ENST00000382199(c.179-75C>T)];ENSG00000073792:[ENST00000346192(c.179-75C>T)];	NA	NA	NA	NA	NA	NA	NA	NA	NA	NA	MODIFIER	NA	NA	72	72	0	128	105	23	17.97
Smoker	42476	chr3	124048747	G	T	*KALRN*	NONSENSE	ENSG00000160145:NONSENSE[ENST00000240874(c.1318G>T,Gaa>Taa):ENSP00000240874(p.Glu440Ter)];ENSG00000160145:NONSENSE[ENST00000460856(c.1318G>T,Gaa>Taa):ENSP00000418611(p.Glu440Ter)];ENSG00000160145:NONSENSE[ENST00000354186(c.1251G>T,Gaa>Taa):ENSP00000346122(p.Glu418Ter)];ENSG00000160145:NONSENSE[ENST00000360013(c.1318G>T,Gaa>Taa):ENSP00000353109(p.Glu440Ter)];	NA	NA	chr3*124048747*124048747*G*A*rs774623456*NA*NA*NA	NA	NA	NA	NA	NA	NA	NA	HIGH	NA	0.984	141	141	0	203	188	15	7.39
Smoker	42476	chr3	9594280	G	C	*LHFPL4*	MISSENSE	ENSG00000156959:MISSENSE[ENST00000287585(c.84C>G,atC>atG):ENSP00000287585(p.Ile28Met)];ENSG00000156959:EXONIC-NC[ENST00000498277(n.278C>G)];	NA	NA	NA	NA	NA	NA	NA	NA	NA	NA	MODIFIER	NA	NA	71	71	0	51	39	12	23.53
Smoker	42476	chr3	46563182	C	G	*LRRC2*	INTRONIC	ENSG00000163827:[ENST00000296144(c.930-34G>C)];ENSG00000163827:[ENST00000395905(c.930-34G>C)];	NA	NA	NA	NA	NA	NA	NA	NA	NA	NA	MODIFIER	NA	NA	50	50	0	53	31	22	41.51
Smoker	42476	chr3	186971055	G	C	*MASP1*	MISSENSE	ENSG00000127241:MISSENSE[ENST00000392470(c.715C>G,Cca>Gca):ENSP00000376262(p.Pro239Ala)];ENSG00000127241:MISSENSE[ENST00000169293(c.793C>G,Cca>Gca):ENSP00000169293(p.Pro265Ala)];ENSG00000127241:MISSENSE[ENST00000296280(c.793C>G,Cca>Gca):ENSP00000296280(p.Pro265Ala)];ENSG00000127241:MISSENSE[ENST00000337774(c.793C>G,Cca>Gca):ENSP00000336792(p.Pro265Ala)];ENSG00000127241:MISSENSE[ENST00000392472(c.454C>G,Cca>Gca):ENSP00000376264(p.Pro152Ala)];ENSG00000127241:INTRONIC[ENST00000495249(n.67-16880C>G)];	NA	NA	NA	NA	NA	NA	NA	NA	NA	NA	MODIFIER	NA	NA	59	59	0	115	109	6	5.22
Smoker	42476	chr3	186974488	G	T	*MASP1*	MISSENSE	ENSG00000127241:MISSENSE[ENST00000337774(c.708C>A,gaC>gaA):ENSP00000336792(p.Asp236Glu)];ENSG00000127241:MISSENSE[ENST00000392470(c.630C>A,gaC>gaA):ENSP00000376262(p.Asp210Glu)];ENSG00000127241:MISSENSE[ENST00000296280(c.708C>A,gaC>gaA):ENSP00000296280(p.Asp236Glu)];ENSG00000127241:MISSENSE[ENST00000392472(c.369C>A,gaC>gaA):ENSP00000376264(p.Asp123Glu)];ENSG00000127241:MISSENSE[ENST00000169293(c.708C>A,gaC>gaA):ENSP00000169293(p.Asp236Glu)];ENSG00000127241:EXONIC-NC[ENST00000460839(n.655C>A)];ENSG00000127241:INTRONIC[ENST00000495249(n.67-20313C>A)];	NA	NA	NA	NA	NA	NA	NA	NA	NA	NA	MODIFIER	NA	NA	41	41	0	92	83	9	9.78
Smoker	42476	chr3	108703512	C	T	*MORC1*	INTRONIC	ENSG00000114487:[ENST00000483760(c.2261+51G>A)];ENSG00000114487:[ENST00000232603(c.2324+51G>A)];	NA	NA	NA	NA	NA	NA	NA	NA	NA	NA	MODIFIER	NA	NA	55	53	0	112	100	12	10.71
Smoker	42476	chr3	125295203	G	A	*OSBPL11*	MISSENSE	ENSG00000144909:MISSENSE[ENST00000296220(c.496C>T,Cct>Tct):ENSP00000296220(p.Pro166Ser)];	NA	NA	NA	NA	NA	NA	NA	T	PrD	N	MODERATE	N	1	56	55	1	135	120	15	11.11
Smoker	42476	chr3	142681286	C	T	*PAQR9*	MISSENSE	ENSG00000188582:MISSENSE[ENST00000340634(c.893G>A,cGc>cAc):ENSP00000341564(p.Arg298His)];ENSG00000188582:MISSENSE[ENST00000492509(c.114G>A,cGc>cAc):ENSP00000417945(p.Arg39His)];	chr3*14268	chr3*14268	NA	NA	NA	NA	NA	D	PrD	D	MODERATE	NA	NA	103	103	0	217	194	22	10.14
Smoker	42476	chr3	122278477	C	T	*PARP9*	5UTR	ENSG00000138496:[ENST00000471785(c.-10G>A)];ENSG00000138496:[ENST00000492382(c.-10G>A)];ENSG00000138496:[ENST00000477522(c.-10G>A)];ENSG00000138496:[ENST00000462315(c.-10G>A)];ENSG00000138496:[ENST00000360356(c.-10G>A)];ENSG00000138496:[ENST00000466126(c.-10G>A)];	NA	NA	NA	8.27E-06	6.18E-05	NA	NA	NA	NA	NA	MODIFIER	NA	NA	115	115	0	266	224	42	15.79
Smoker	42476	chr3	142607660	C	G	*PCOLCE2*	MISSENSE	ENSG00000163710:MISSENSE[ENST00000485766(c.79G>C,Gag>Cag):ENSP00000419842(p.Glu27Gln)];ENSG00000163710:MISSENSE[ENST00000295992(c.79G>C,Gag>Cag):ENSP00000295992(p.Glu27Gln)];ENSG00000163710:EXONIC-NC[ENST00000461818(n.205G>C)];ENSG00000163710:EXONIC-NC[ENST00000495732(n.244G>C)];ENSG00000163710:EXONIC-NC[ENST00000483454(n.110G>C)];	NA	NA	NA	NA	NA	NA	NA	NA	NA	NA	MODIFIER	NA	NA	75	75	0	140	133	7	5
Smoker	42476	chr3	178936091	G	A	*PIK3CA*	MISSENSE	ENSG00000121879:MISSENSE[ENST00000263967(c.1633G>A,Gag>Aag):ENSP00000263967(p.Glu545Lys)];	chr3*17893	chr3*17893	NA	8.34E-06	0	NA	NA	D	PrD	D	MODERATE	D	1	98	98	0	183	155	28	15.3
Smoker	42476	chr3	145917771	C	G	*PLSCR4*	MISSENSE	ENSG00000114698:MISSENSE[ENST00000354952(c.453G>C,caG>caC):ENSP00000347038(p.Gln151His)];ENSG00000114698:MISSENSE[ENST00000493382(c.453G>C,caG>caC):ENSP00000419040(p.Gln151His)];ENSG00000114698:MISSENSE[ENST00000446574(c.453G>C,caG>caC):ENSP00000399315(p.Gln151His)];ENSG00000114698:MISSENSE[ENST00000476202(c.453G>C,caG>caC):ENSP00000418173(p.Gln151His)];ENSG00000114698:MISSENSE[ENST00000460350(c.453G>C,caG>caC):ENSP00000417896(p.Gln151His)];ENSG00000114698:INTRONIC[ENST00000433593(c.310-3191G>C)];ENSG00000114698:INTRONIC[ENST00000475019(n.418+1051G>C)];ENSG00000114698:INTRONIC[ENST00000383083(c.355-3191G>C)];ENSG00000114698:INTRONIC[ENST00000460885(c.355-3191G>C)];	NA	NA	NA	NA	NA	NA	NA	NA	NA	NA	MODIFIER	NA	NA	68	68	0	101	86	15	14.85
Smoker	42476	chr3	121215820	C	G	*POLQ*	INTRONIC	ENSG00000051341:[ENST00000264233(c.2154-41G>C)];	NA	NA	NA	NA	NA	NA	NA	NA	NA	NA	MODIFIER	NA	NA	86	86	0	255	243	12	4.71
Smoker	42476	chr3	148601503	G	A	*RP11-680B3.2*	INTRONIC	ENSG00000240521:[ENST00000488190(n.163-13627C>T)];	NA	NA	NA	NA	NA	NA	NA	NA	NA	NA	MODIFIER	NA	NA	119	119	0	301	257	44	14.62

Mangalaparthi *et al.* , 2020. Mutational landscape of esophageal squamous cell carcinoma in an Indian cohort
Supplementary Table 4. List of somatic single nucleotide variants identified using whole exome sequencing of ESCC patients

Cohort	Sample	Chromosome	Genomic position	Reference allele	Alternate allele	Gene symbol	Variant type	Variant annotation	OncoMD	COSMIC	dbSNP	ExAC	ExAC_SAS	1000G	1000G_SAS	SIFT	Polyphen2	CONDEL	VEP	LRT	PhastCon7 Way	Total read (Adjacent normal)	Reference allel read support (Adjacent normal)	Alternative read support (Adjacent normal)	Total read (Tumor)	Reference allel read support (Tumor)	Alternative read support (Tumor)	Alternate allele frequency
Smoker	42476	chr3	14523152	C	G	*SLC6A6*	INTRONIC	ENSG00000131389:[ENST00000360861(c.1552-27C>G)];ENSG00000131389:[ENST00000458124(c.*347-27C>G)];ENSG00000131389:[ENST00000452151(c.77-27C>G)];ENSG00000131389:[ENST00000454876(c.1552-27C>G)];	NA	NA	NA	NA	NA	NA	NA	NA	NA	NA	MODIFIER	NA	NA	59	59	0	29	21	7	24.14
Smoker	42476	chr3	119154817	G	C	*TMEM39A*	INTRONIC	ENSG00000176142:[ENST00000438581(c.*780+821C>G)];ENSG00000176142:[ENST00000319172(c.1112+821C>G)];ENSG00000176142:[ENST00000473684(c.27-35C>G)];	NA	NA	NA	NA	NA	NA	NA	NA	NA	NA	MODIFIER	NA	NA	37	37	0	69	59	10	14.49
Smoker	42476	chr3	170825841	G	A	*TNIK*	MISSENSE	ENSG00000154310:INTRONIC[ENST00000470834(c.2295+13C>T)];ENSG00000154310:INTRONIC[ENST00000341852(c.2130+13C>T)];ENSG00000154310:INTRONIC[ENST00000436636(c.2382+13C>T)];ENSG00000154310:INTRONIC[ENST00000475336(c.2130+13C>T)];ENSG00000154310:INTRONIC[ENST00000488470(c.2217+13C>T)];ENSG00000154310:INTRONIC[ENST00000284483(c.2382+13C>T)];ENSG00000154310:INTRONIC[ENST00000460047(c.2217+13C>T)];ENSG00000154310:INTRONIC[ENST00000357327(c.2295+13C>T)];ENSG00000154310:MISSENSE[ENST00000538048(c.2230C>T,Ctc>Ttc):ENSP00000443278(p.Leu744Phe)];ENSG00000154310:MISSENSE[ENST00000369326(c.2308C>T,Ctc>Ttc):ENSP00000358332(p.Leu770Phe)];	NA	NA	NA	NA	NA	NA	NA	NA	NA	NA	MODIFIER	NA	NA	68	68	0	114	101	13	11.4
Smoker	42476	chr3	140401955	C	T	*TRIM42*	SILENT	ENSG00000155890:SILENT[ENST00000286349(c.993C>T,tcC>tcT):ENSP00000286349(p.Ser331(=))];	NA	NA	NA	1.65E-05	0	NA	NA	NA	NA	NA	LOW	NA	NA	189	189	0	357	277	79	22.13
Smoker	42476	chr3	126362846	C	A	*TXNRD3*	SILENT	ENSG00000197763:SILENT[ENST00000523403(c.681G>T,ccG>ccT):ENSP00000429584(p.Pro227(=))];ENSG00000197763:SILENT[ENST00000524230(c.735G>T,ccG>ccT):ENSP00000430031(p.Pro245(=))];	NA	NA	NA	NA	NA	NA	NA	NA	NA	NA	LOW	NA	NA	189	188	0	428	341	87	20.33
Smoker	42476	chr3	126224605	C	G	*UROC1*	MISSENSE	ENSG00000159650:MISSENSE[ENST00000383579(c.752G>C,gGa>gCa):ENSP00000373073(p.Gly251Ala)];ENSG00000159650:MISSENSE[ENST00000290868(c.752G>C,gGa>gCa):ENSP00000290868(p.Gly251Ala)];	chr3*12622-	NA	NA	NA	NA	NA	NA	D	PrD	D	MODERATE	D	0.978	139	139	0	306	275	31	10.13
Smoker	42476	chr4	122590808	G	C	*ANXA5*	MISSENSE	ENSG00000164111:MISSENSE[ENST00000515017(c.552C>G,atC>atG):ENSP00000424199(p.Ile184Met)];ENSG00000164111:MISSENSE[ENST00000296511(c.852C>G,atC>atG):ENSP00000296511(p.Ile284Met)];ENSG00000164111:MISSENSE[ENST00000501272(c.672C>G,atC>atG):ENSP00000424106(p.Ile224Met)];ENSG00000164111:3UTR[ENST00000506395(c.*423C>G)];ENSG00000164111:EXONIC-NC[ENST00000509648(n.750C>G)];ENSG00000164111:EXONIC-NC[ENST00000515717(n.253C>G)];	NA	NA	NA	NA	NA	NA	NA	NA	NA	NA	MODIFIER	NA	NA	98	97	0	50	34	16	32
Smoker	42476	chr4	5528112	C	A	*C4orf6*	INTRONIC	ENSG00000082929:[ENST00000515342(n.368+52C>A)];ENSG00000082929:[ENST00000195455(c.*2+52C>A)];ENSG00000082929:[ENST00000505296(c.*2+52C>A)];	NA	NA	NA	NA	NA	NA	NA	NA	NA	NA	MODIFIER	NA	NA	57	57	0	28	21	7	25
Smoker	42476	chr4	111398093	G	A	*ENPEP*	MISSENSE	ENSG00000138792:INTRONIC[ENST00000510961(n.73-11604G>A)];ENSG00000138792:MISSENSE[ENST00000265162(c.523G>A,Gaa>Aaa):ENSP00000265162(p.Glu175Lys)];	NA	NA	NA	NA	NA	NA	NA	T	BN	N	MODERATE	N	0.678	62	62	0	24	12	12	50
Smoker	42476	chr4	152498566	C	A	*FAM160A1*	INTRONIC	ENSG00000164142:[ENST00000435205(c.106-36C>A)];ENSG00000164142:[ENST00000505231(c.106-36C>A)];ENSG00000164142:[ENST00000503146(c.106-36C>A)];ENSG00000164142:[ENST00000512597(c.106-36C>A)];ENSG00000164142:[ENST00000513962(c.106-36C>A)];	NA	NA	NA	NA	NA	NA	NA	NA	NA	NA	MODIFIER	NA	NA	51	51	0	56	27	28	50
Smoker	42476	chr4	77138881	G	A	*FAM47E*	INTRONIC	ENSG00000189157:[ENST00000339906(c.81+40G>A)];ENSG00000189157:[ENST00000510197(c.81+40G>A)];ENSG00000189157:[ENST00000512895(c.150+3304G>A)];	NA	NA	NA	NA	NA	NA	NA	NA	NA	NA	MODIFIER	NA	NA	82	82	0	42	32	9	21.43
Smoker	42476	chr4	77177402	G	T	*FAM47E*	MISSENSE	ENSG00000189157:MISSENSE[ENST00000424749(c.146G>T,aGa>aTa):ENSP00000409423(p.Arg49Ile)];ENSG00000189157:INTRONIC[ENST00000502320(n.88+4429G>T)];ENSG00000189157:INTRONIC[ENST00000510328(n.121+4429G>T)];ENSG00000189157:INTRONIC[ENST00000510197(c.82-7455G>T)];ENSG00000189157:INTRONIC[ENST00000512895(c.151-7455G>T)];ENSG00000189157:INTRONIC[ENST00000339906(c.82-7455G>T)];ENSG00000189157:EXONIC-NC[ENST00000515589(n.456G>T)];	NA	NA	NA	NA	NA	NA	NA	NA	NA	NA	MODIFIER	NA	NA	76	76	0	53	41	12	22.64
Smoker	42476	chr4	77177402	G	T	*FAM47E-STBD1*	MISSENSE	ENSG00000272414:MISSENSE[ENST00000515604(c.146G>T,aGa>aTa):ENSP00000422067(p.Arg49Ile)];ENSG00000272414:INTRONIC[ENST00000514365(c.74+4429G>T)];ENSG00000118804:INTRONIC[ENST00000539752(c.-723+4429G>T)];ENSG00000272414:INTRONIC[ENST00000509377(c.74+4429G>T)];	NA	NA	NA	NA	NA	NA	NA	NA	NA	NA	MODIFIER	NA	NA	76	76	0	53	41	12	22.64
Smoker	42476	chr4	126400905	G	C	*FAT4*	MISSENSE	ENSG00000196159:MISSENSE[ENST00000394329(c.12483G>C,agG>agC):ENSP00000377862(p.Arg4161Ser)];ENSG00000196159:INTRONIC[ENST00000335110(c.7322-1771G>C)];	NA	NA	NA	NA	NA	NA	NA	NA	NA	NA	MODIFIER	NA	NA	79	79	0	71	62	9	12.68
Smoker	42476	chr4	144811284	G	A	*GYPE*	INTRONIC	ENSG00000197465:[ENST00000437468(c.38-9622C>T)];ENSG00000197465:[ENST00000358615(c.38-9622C>T)];ENSG00000197465:[ENST00000506264(c.38-9622C>T)];	NA	NA	NA	NA	NA	NA	NA	NA	NA	NA	MODIFIER	NA	NA	76	76	0	38	22	16	42.11
Smoker	42476	chr4	154523797	C	T	*KIAA0922*	MISSENSE	ENSG00000121210:MISSENSE[ENST00000409663(c.2554C>T,Cac>Tac):ENSP00000386574(p.His852Tyr)];ENSG00000121210:MISSENSE[ENST00000409959(c.2557C>T,Cac>Tac):ENSP00000386787(p.His853Tyr)];ENSG00000121210:MISSENSE[ENST00000440693(c.2305C>T,Cac>Tac):ENSP00000409663(p.His769Tyr)];ENSG00000121210:MISSENSE[ENST00000240487(c.1888C>T,Cac>Tac):ENSP00000240487(p.His630Tyr)];ENSG00000121210:EXONIC-NC[ENST00000497247(n.488C>T)];	NA	NA	NA	NA	NA	NA	NA	NA	NA	NA	MODIFIER	NA	NA	53	53	0	47	29	17	36.17
Smoker	42476	chr4	123165055	G	T	*KIAA1109*	MISSENSE	ENSG00000138688:MISSENSE[ENST00000446180(c.507G>T,Gat>Tat):ENSP00000394909(p.Asp170Tyr)];ENSG00000138688:MISSENSE[ENST00000455637(c.4789G>T,Gat>Tat):ENSP00000389925(p.Asp1597Tyr)];ENSG00000138688:MISSENSE[ENST00000264501(c.4789G>T,Gat>Tat):ENSP00000264501(p.Asp1597Tyr)];ENSG00000138688:MISSENSE[ENST00000388738(c.4789G>T,Gat>Tat):ENSP00000373390(p.Asp1597Tyr)];	NA	NA	chr4*123165013*123165059*CCGTATACTCCATTGGAAAAAAAACTCGCTGATAACACAGATGATGA*C*rs757839255*NA*NA*NA	NA	NA	NA	NA	NA	PrD	NA	MODERATE	U	1	116	116	0	71	46	25	35.21
Smoker	42476	chr4	151772075	G	T	*LRBA*	INTRONIC	ENSG00000198589:[ENST00000535741(c.3826-21C>A)];ENSG00000198589:[ENST00000507224(c.3826-21C>A)];ENSG00000198589:[ENST00000357115(c.3826-21C>A)];ENSG00000198589:[ENST00000510413(c.3826-21C>A)];	NA	NA	NA	NA	NA	NA	NA	NA	NA	NA	MODIFIER	NA	NA	88	88	0	47	29	18	38.3

Mangalaparthi *et al.* , 2020. Mutational landscape of esophageal squamous cell carcinoma in an Indian cohort
Supplementary Table 4. List of somatic single nucleotide variants identified using whole exome sequencing of ESCC patients

Cohort	Sample	Chromosome	Genomic position	Reference allele	Alternate allele	Gene symbol	Variant type	Variant annotation	OncoMD	COSMIC	dbSNP	ExAC	ExAC_SAS	1000G	1000G_SAS	SIFT	Polyphen2	CONDEL	VEP	LRT	PhastCon7 Way	Total read (Adjacent normal)	Reference allel read support (Adjacent normal)	Alternative read support (Adjacent normal)	Total read (Tumor)	Reference allel read support (Tumor)	Alternative read support (Tumor)	Alternate allele frequency
Smoker	42476	chr4	170327796	C	T	*NEK1*	MISSENSE	ENSG00000137601:MISSENSE[ENST00000510533(c.3109G>A,Gaa>Aaa):ENSP00000427653(p.Glu1037Lys)];ENSG00000137601:MISSENSE[ENST00000511633(c.3193G>A,Gaa>Aaa):ENSP00000423332(p.Glu1065Lys)];ENSG00000137601:MISSENSE[ENST00000512193(c.3034G>A,Gaa>Aaa):ENSP00000424938(p.Glu1012Lys)];ENSG00000137601:MISSENSE[ENST00000507142(c.3325G>A,Gaa>Aaa):ENSP00000424757(p.Glu1109Lys)];ENSG00000137601:MISSENSE[ENST00000439128(c.3241G>A,Gaa>Aaa):ENSP00000408020(p.Glu1081Lys)];	NA	NA	NA	NA	NA	NA	NA	D	BN	N	MODERATE	N	0.729	42	41	0	29	19	8	27.59
Smoker	42476	chr4	170327805	C	G	*NEK1*	MISSENSE	ENSG00000137601:MISSENSE[ENST00000510533(c.3100G>C,Gaa>Caa):ENSP00000427653(p.Glu1034Gln)];ENSG00000137601:MISSENSE[ENST00000511633(c.3184G>C,Gaa>Caa):ENSP00000423332(p.Glu1062Gln)];ENSG00000137601:MISSENSE[ENST00000439128(c.3232G>C,Gaa>Caa):ENSP00000408020(p.Glu1078Gln)];ENSG00000137601:MISSENSE[ENST00000512193(c.3025G>C,Gaa>Caa):ENSP00000424938(p.Glu1009Gln)];ENSG00000137601:MISSENSE[ENST00000507142(c.3316G>C,Gaa>Caa):ENSP00000424757(p.Glu1106Gln)];	NA	NA	NA	NA	NA	NA	NA	D	PrD	D	MODERATE	D	0.997	45	44	0	29	20	9	31.03
Smoker	42476	chr4	56212636	A	G	*SRD5A3*	MISSENSE	ENSG00000128039:MISSENSE[ENST00000505210(c.58A>G,Atc>Gtc):ENSP00000424714(p.Ile20Val)];ENSG00000128039:MISSENSE[ENST00000264228(c.133A>G,Atc>Gtc):ENSP00000264228(p.Ile45Val)];	NA	NA	NA	NA	NA	NA	NA	T	BN	N	MODERATE	N	0.696	144	144	0	88	61	27	30.68
Smoker	42476	chr5	150514092	G	C	*ANXA6*	INTRONIC	ENSG00000197043:[ENST00000354546(c.490-48C>G)];ENSG00000197043:[ENST00000521749(c.394-48C>G)];ENSG00000197043:[ENST00000356496(c.490-48C>G)];ENSG00000197043:[ENST00000519644(c.262+4203C>G)];ENSG00000197043:[ENST00000521512(c.224+4241C>G)];ENSG00000197043:[ENST00000523714(c.394-48C>G)];ENSG00000197043:[ENST00000517677(c.366-1344C>G)];ENSG00000197043:[ENST00000517486(c.318+4147C>G)];ENSG00000197043:[ENST00000377751(c.204+4822C>G)];	NA	NA	NA	NA	NA	NA	NA	NA	NA	NA	MODIFIER	NA	NA	31	31	0	24	14	10	41.67
Smoker	42476	chr5	37245647	C	G	*C5orf42*	MISSENSE	ENSG00000197603:MISSENSE[ENST00000508244(c.271G>C,Gat>Cat):ENSP00000421690(p.Asp91His)];ENSG00000197603:MISSENSE[ENST00000425232(c.271G>C,Gat>Cat):ENSP00000389014(p.Asp91His)];ENSG00000197603:5UTR[ENST00000274258(c.-3087G>C)];	NA	NA	NA	NA	NA	NA	NA	NA	NA	NA	MODIFIER	NA	NA	83	83	0	105	82	22	20.95
Smoker	42476	chr5	79351817	G	A	*CTD-2201I18.1*	INTRONIC	ENSG00000249825:[ENST00000503007(n.429-3093C>T)];	NA	NA	chr5*79351817*79351817*G*C*rs780799770*NA*NA*NA	NA	NA	NA	NA	T	BN	N	MODERATE	N	0.066	146	146	0	89	64	25	28.09
Smoker	42476	chr5	96478255	C	G	*CTD-2215E18.1*	INTRONIC	ENSG00000251606:[ENST00000509481(c.32+39457C>G)];	NA	NA	NA	NA	NA	NA	NA	T_lc	BN	N	MODERATE	N	0.998	124	124	0	62	44	18	29.03
Smoker	42476	chr5	61686818	A	G	*DIMT1*	INTRONIC-SS-ACR	ENSG00000086189:[ENST00000199320(c.793-9T>C)];ENSG00000086189:[ENST00000514605(n.377T>C)];	NA	NA	NA	NA	NA	NA	NA	NA	NA	NA	MODIFIER	NA	NA	49	48	0	31	9	22	70.97
Smoker	42476	chr5	61686818	A	G	*KIF2A*	INTRONIC	ENSG00000068796:[ENST00000509663(n.331+84455A>G)];	NA	NA	NA	NA	NA	NA	NA	NA	NA	NA	MODIFIER	NA	NA	49	48	0	31	9	22	70.97
Smoker	42476	chr5	96478255	C	G	*LIX1*	MISSENSE	ENSG00000145721:5UTR[ENST00000512378(c.-136G>C)];ENSG00000145721:MISSENSE[ENST00000274382(c.26G>C,aGa>aCa):ENSP00000274382(p.Arg9Thr)];	NA	NA	NA	NA	NA	NA	NA	T_lc	BN	N	MODERATE	N	0.998	124	124	0	62	44	18	29.03
Smoker	42476	chr5	16754900	G	C	*MYO10*	INTRONIC	ENSG00000145555:[ENST00000513882(c.1962+37C>G)];ENSG00000145555:[ENST00000510401(n.190+37C>G)];ENSG00000145555:[ENST00000513610(c.1929+37C>G)];	NA	NA	chr5*16754900*16754900*G*A*rs764712819*NA*NA*NA	NA	NA	NA	NA	NA	NA	NA	MODIFIER	NA	NA	87	87	0	102	85	17	16.67
Smoker	42476	chr5	6828103	G	T	*NA*	INTERGENIC		NA	NA	NA	NA	NA	NA	NA	NA	NA	NA	MODIFIER	NA	NA	54	54	0	42	26	16	38.1
Smoker	42476	chr5	43656109	T	A	*NNT*	MISSENSE	ENSG00000112992:MISSENSE[ENST00000512996(c.1834T>A,Tac>Aac):ENSP00000426343(p.Tyr612Asn)];ENSG00000112992:MISSENSE[ENST00000344920(c.2227T>A,Tac>Aac):ENSP00000343873(p.Tyr743Asn)];ENSG00000112992:MISSENSE[ENST00000264663(c.2227T>A,Tac>Aac):ENSP00000264663(p.Tyr743Asn)];	NA	NA	NA	NA	NA	NA	NA	D	PrD	D	MODERATE	D	0.995	84	84	0	104	99	5	4.81
Smoker	42476	chr5	140554702	C	T	*PCDHB7*	SILENT	ENSG00000113212:SILENT[ENST00000231137(c.2286C>T,ttC>ttT):ENSP00000231137(p.Phe762(=))];	NA	NA	NA	NA	NA	NA	NA	NA	NA	NA	LOW	NA	NA	109	109	0	72	67	5	6.94
Smoker	42476	chr5	140753731	G	A	*PCDHGA1*	INTRONIC	ENSG00000204956:[ENST00000517417(c.2421+41059G>A)];	NA	NA	chr5*140753731*140753731*G*C*rs753941386*NA*NA*NA	NA	NA	NA	NA	NA	NA	NA	MODIFIER	NA	NA	57	57	0	38	26	12	31.58
Smoker	42476	chr5	140871314	G	C	*PCDHGA1*	INTRONIC	ENSG00000204956:[ENST00000517417(c.2422-3060G>C)];	NA	NA	NA	NA	NA	NA	NA	NA	NA	NA	MODIFIER	NA	NA	95	95	0	53	34	19	35.85
Smoker	42476	chr5	140871314	G	C	*PCDHGA10*	INTRONIC	ENSG00000253846:[ENST00000398610(c.2437-3060G>C)];	NA	NA	NA	NA	NA	NA	NA	NA	NA	NA	MODIFIER	NA	NA	95	95	0	53	34	19	35.85
Smoker	42476	chr5	140871314	G	C	*PCDHGA11*	INTRONIC	ENSG00000253873:[ENST00000398587(c.2434-3060G>C)];ENSG00000253873:[ENST00000518882(c.1879-3060G>C)];	NA	NA	NA	NA	NA	NA	NA	NA	NA	NA	MODIFIER	NA	NA	95	95	0	53	34	19	35.85
Smoker	42476	chr5	140871314	G	C	*PCDHGA12*	INTRONIC	ENSG00000253159:[ENST00000252085(c.2425-3060G>C)];	NA	NA	NA	NA	NA	NA	NA	NA	NA	NA	MODIFIER	NA	NA	95	95	0	53	34	19	35.85
Smoker	42476	chr5	140753731	G	A	*PCDHGA2*	INTRONIC	ENSG00000081853:[ENST00000394576(c.2424+32769G>A)];	NA	NA	chr5*140753731*140753731*G*C*rs753941386*NA*NA*NA	NA	NA	NA	NA	NA	NA	NA	MODIFIER	NA	NA	57	57	0	38	26	12	31.58
Smoker	42476	chr5	140871314	G	C	*PCDHGA2*	INTRONIC	ENSG00000081853:[ENST00000394576(c.2425-3060G>C)];	NA	NA	NA	NA	NA	NA	NA	NA	NA	NA	MODIFIER	NA	NA	95	95	0	53	34	19	35.85
Smoker	42476	chr5	140753731	G	A	*PCDHGA3*	INTRONIC	ENSG00000254245:[ENST00000253812(c.2424+27707G>A)];	NA	NA	chr5*140753731*140753731*G*C*rs753941386*NA*NA*NA	NA	NA	NA	NA	NA	NA	NA	MODIFIER	NA	NA	57	57	0	38	26	12	31.58
Smoker	42476	chr5	140871314	G	C	*PCDHGA3*	INTRONIC	ENSG00000254245:[ENST00000253812(c.2425-3060G>C)];	NA	NA	NA	NA	NA	NA	NA	NA	NA	NA	MODIFIER	NA	NA	95	95	0	53	34	19	35.85
Smoker	42476	chr5	140753731	G	A	*PCDHGA4*	INTRONIC	ENSG00000262576:[ENST00000571252(c.2421+16543G>A)];	NA	NA	chr5*140753731*140753731*G*C*rs753941386*NA*NA*NA	NA	NA	NA	NA	NA	NA	NA	MODIFIER	NA	NA	57	57	0	38	26	12	31.58
Smoker	42476	chr5	140871314	G	C	*PCDHGA4*	INTRONIC	ENSG00000262576:[ENST00000571252(c.2422-3060G>C)];	NA	NA	NA	NA	NA	NA	NA	NA	NA	NA	MODIFIER	NA	NA	95	95	0	53	34	19	35.85
Smoker	42476	chr5	140753731	G	A	*PCDHGA5*	INTRONIC	ENSG00000253485:[ENST00000518069(c.2421+7413G>A)];	NA	NA	chr5*140753731*140753731*G*C*rs753941386*NA*NA*NA	NA	NA	NA	NA	NA	NA	NA	MODIFIER	NA	NA	57	57	0	38	26	12	31.58
Smoker	42476	chr5	140871314	G	C	*PCDHGA5*	INTRONIC	ENSG00000253485:[ENST00000518069(c.2422-3060G>C)];	NA	NA	NA	NA	NA	NA	NA	NA	NA	NA	MODIFIER	NA	NA	95	95	0	53	34	19	35.85
Smoker	42476	chr5	140753731	G	A	*PCDHGA6*	SILENT	ENSG00000253731:SILENT[ENST00000517434(c.81G>A,gcG>gcA):ENSP00000429601(p.Ala27(=))];	NA	NA	chr5*140753731*140753731*G*C*rs753941386*NA*NA*NA	NA	NA	NA	NA	NA	NA	NA	MODIFIER	NA	NA	57	57	0	38	26	12	31.58
Smoker	42476	chr5	140871314	G	C	*PCDHGA6*	INTRONIC	ENSG00000253731:[ENST00000517434(c.2425-3060G>C)];	NA	NA	NA	NA	NA	NA	NA	NA	NA	NA	MODIFIER	NA	NA	95	95	0	53	34	19	35.85
Smoker	42476	chr5	140871314	G	C	*PCDHGA7*	INTRONIC	ENSG00000253537:[ENST00000518325(c.2425-3060G>C)];	NA	NA	NA	NA	NA	NA	NA	NA	NA	NA	MODIFIER	NA	NA	95	95	0	53	34	19	35.85
Smoker	42476	chr5	140871314	G	C	*PCDHGA8*	INTRONIC	ENSG00000253767:[ENST00000398604(c.2425-3060G>C)];	NA	NA	NA	NA	NA	NA	NA	NA	NA	NA	MODIFIER	NA	NA	95	95	0	53	34	19	35.85
Smoker	42476	chr5	140871314	G	C	*PCDHGA9*	INTRONIC	ENSG00000261934:[ENST00000573521(c.2425-3060G>C)];	NA	NA	NA	NA	NA	NA	NA	NA	NA	NA	MODIFIER	NA	NA	95	95	0	53	34	19	35.85

Mangalaparthi *et al.* , 2020. Mutational landscape of esophageal squamous cell carcinoma in an Indian cohort
Supplementary Table 4. List of somatic single nucleotide variants identified using whole exome sequencing of ESCC patients

Cohort	Sample	Chromosome	Genomic position	Reference allele	Alternate allele	Gene symbol	Variant type	Variant annotation	OncoMD	COSMIC	dbSNP	ExAC	ExAC_SAS	1000G	1000G_SAS	SIFT	Polyphen2	CONDEL	VEP	LRT	PhastCon7 Way	Total read (Adjacent normal)	Reference allel read support (Adjacent normal)	Alternative read support (Adjacent normal)	Total read (Tumor)	Reference allel read support (Tumor)	Alternative read support (Tumor)	Alternate allele frequency
Smoker	42476	chr5	140753731	G	A	*PCDHGB1*	INTRONIC	ENSG00000254221:[ENST00000523390(c.2409+21495G>A)];	NA	NA	chr5*140753731*140753731*G*C*rs753941386*NA*NA*NA	NA	NA	NA	NA	NA	NA	NA	MODIFIER	NA	NA	57	57	0	38	26	12	31.58
Smoker	42476	chr5	140871314	G	C	*PCDHGB1*	INTRONIC	ENSG00000254221:[ENST00000523390(c.2410-3060G>C)];	NA	NA	NA	NA	NA	NA	NA	NA	NA	NA	MODIFIER	NA	NA	95	95	0	53	34	19	35.85
Smoker	42476	chr5	140753731	G	A	*PCDHGB2*	INTRONIC	ENSG00000253910:[ENST00000522605(c.2421+11608G>A)];	NA	NA	chr5*140753731*140753731*G*C*rs753941386*NA*NA*NA	NA	NA	NA	NA	NA	NA	NA	MODIFIER	NA	NA	57	57	0	38	26	12	31.58
Smoker	42476	chr5	140871314	G	C	*PCDHGB2*	INTRONIC	ENSG00000253910:[ENST00000522605(c.2422-3060G>C)];	NA	NA	NA	NA	NA	NA	NA	NA	NA	NA	MODIFIER	NA	NA	95	95	0	53	34	19	35.85
Smoker	42476	chr5	140753731	G	A	*PCDHGB3*	INTRONIC	ENSG00000262209:[ENST00000576222(c.2415+1355G>A)];	NA	NA	chr5*140753731*140753731*G*C*rs753941386*NA*NA*NA	NA	NA	NA	NA	NA	NA	NA	MODIFIER	NA	NA	57	57	0	38	26	12	31.58
Smoker	42476	chr5	140871314	G	C	*PCDHGB3*	INTRONIC	ENSG00000262209:[ENST00000576222(c.2416-3060G>C)];	NA	NA	NA	NA	NA	NA	NA	NA	NA	NA	MODIFIER	NA	NA	95	95	0	53	34	19	35.85
Smoker	42476	chr5	140871314	G	C	*PCDHGB4*	INTRONIC	ENSG00000253953:[ENST00000519479(c.2398-3060G>C)];	NA	NA	NA	NA	NA	NA	NA	NA	NA	NA	MODIFIER	NA	NA	95	95	0	53	34	19	35.85
Smoker	42476	chr5	140871314	G	C	*PCDHGB6*	INTRONIC	ENSG00000253305:[ENST00000520790(c.2419-3060G>C)];	NA	NA	NA	NA	NA	NA	NA	NA	NA	NA	MODIFIER	NA	NA	95	95	0	53	34	19	35.85
Smoker	42476	chr5	140871314	G	C	*PCDHGB7*	INTRONIC	ENSG00000254122:[ENST00000398594(c.2416-3060G>C)];	NA	NA	NA	NA	NA	NA	NA	NA	NA	NA	MODIFIER	NA	NA	95	95	0	53	34	19	35.85
Smoker	42476	chr5	140871314	G	C	*PCDHGC3*	INTRONIC	ENSG00000240184:[ENST00000308177(c.2431-3060G>C)];	NA	NA	NA	NA	NA	NA	NA	NA	NA	NA	MODIFIER	NA	NA	95	95	0	53	34	19	35.85
Smoker	42476	chr5	140871314	G	C	*PCDHGC4*	INTRONIC	ENSG00000242419:[ENST00000306593(c.2443-3060G>C)];	NA	NA	NA	NA	NA	NA	NA	NA	NA	NA	MODIFIER	NA	NA	95	95	0	53	34	19	35.85
Smoker	42476	chr5	140871314	G	C	*PCDHGC5*	INTRONIC	ENSG00000240764:[ENST00000252087(c.2460+47G>C)];	NA	NA	NA	NA	NA	NA	NA	NA	NA	NA	MODIFIER	NA	NA	95	95	0	53	34	19	35.85
Smoker	42476	chr5	170395644	C	G	*RANBP17*	INTRONIC	ENSG00000204764:[ENST00000518492(c.122+263C>G)];ENSG00000204764:[ENST00000519949(c.1710+263C>G)];ENSG00000204764:[ENST00000389118(c.1710+263C>G)];ENSG00000204764:[ENST00000519256(c.1710+263C>G)];ENSG00000204764:[ENST00000522533(c.1710+263C>G)];ENSG00000204764:[ENST00000522380(n.599+263C>G)];ENSG00000204764:[ENST00000522066(c.1710+263C>G)];ENSG00000204764:[ENST00000523189(c.1710+263C>G)];ENSG00000204764:[ENST00000520864(c.1643+263C>G)];	NA	NA	NA	NA	NA	NA	NA	NA	NA	NA	MODIFIER	NA	NA	30	30	0	36	28	8	22.22
Smoker	42476	chr5	175717610	A	T	*SIMC1*	MISSENSE	ENSG00000170085:INTRONIC[ENST00000467472(n.132-4423A>T)];ENSG00000170085:INTRONIC[ENST00000495423(n.257-4423A>T)];ENSG00000170085:INTRONIC[ENST00000430704(c.130-4423A>T)];ENSG00000170085:INTRONIC[ENST00000341199(c.130-4423A>T)];ENSG00000170085:MISSENSE[ENST00000443967(c.1026A>T,agA>agT):ENSP00000406571(p.Arg342Ser)];ENSG00000170085:MISSENSE[ENST00000429602(c.1083A>T,agA>agT):ENSP00000410552(p.Arg361Ser)];	NA	NA	NA	NA	NA	NA	NA	NA	NA	NA	MODIFIER	NA	NA	266	265	0	157	62	95	60.51
Smoker	42476	chr5	79351817	G	A	*THBS4*	MISSENSE	ENSG00000113296:MISSENSE[ENST00000350881(c.502G>A,Gag>Aag):ENSP00000339730(p.Glu168Lys)];ENSG00000113296:MISSENSE[ENST00000511733(c.229G>A,Gag>Aag):ENSP00000422298(p.Glu77Lys)];	NA	NA	chr5*79351817*79351817*G*C*rs780799770*NA*NA*NA	NA	NA	NA	NA	T	BN	N	MODERATE	N	0.066	146	146	0	89	64	25	28.09
Smoker	42476	chr6	11766540	A	G	*ADTRP*	SILENT	ENSG00000111863:SILENT[ENST00000414691(c.357T>C,gaT>gaC):ENSP00000404416(p.Asp119(=))];ENSG00000111863:SILENT[ENST00000229583(c.411T>C,gaT>gaC):ENSP00000229583(p.Asp137(=))];ENSG00000111863:SILENT[ENST00000379413(c.357T>C,gaT>gaC):ENSP00000368723(p.Asp119(=))];ENSG00000111863:SILENT[ENST00000505099(c.26T>C,gaT>gaC):ENSP00000424680(p.Asp9(=))];ENSG00000111863:SILENT[ENST00000506810(c.357T>C,gaT>gaC):ENSP00000422927(p.Asp119(=))];ENSG00000111863:SILENT[ENST00000379415(c.357T>C,gaT>gaC):ENSP00000368726(p.Asp119(=))];ENSG00000111863:3UTR[ENST00000485323(c.*155T>C)];ENSG00000111863:EXONIC-NC[ENST00000512139(n.220T>C)];	NA	NA	NA	NA	NA	NA	NA	NA	NA	NA	LOW	NA	NA	138	138	0	167	89	78	46.71
Smoker	42476	chr6	38472333	C	G	*BTBD9*	STOPLOSS	ENSG00000183826:STOPLOSS[ENST00000328403(c.956G>C,tGa>tCa):ENSP00000328328(p.Ter319SerextTer12)];ENSG00000183826:INTRONIC[ENST00000419706(c.977+73043G>C)];ENSG00000183826:INTRONIC[ENST00000314100(c.950+73043G>C)];ENSG00000183826:INTRONIC[ENST00000481247(c.1154+73043G>C)];ENSG00000183826:INTRONIC[ENST00000408958(c.950+73043G>C)];ENSG00000183826:INTRONIC[ENST00000403056(c.1154+73043G>C)];	NA	NA	NA	NA	NA	NA	NA	NA	NA	NA	MODIFIER	NA	NA	142	142	0	125	110	15	12
Smoker	42476	chr6	33133709	G	A	*COL11A2*	MISSENSE-SS-PRX	ENSG00000204248:[ENST00000395197(c.4301C>T,cCg>cTg):ENSP00000378623(p.Pro1434Leu)];ENSG00000204248:[ENST00000374713(c.4340C>T,cCg>cTg):ENSP00000363845(p.Pro1447Leu)];ENSG00000204248:[ENST00000361917(c.4160C>T,cCg>cTg):ENSP00000355123(p.Pro1387Leu)];ENSG00000204248:[ENST00000357486(c.4418C>T,cCg>cTg):ENSP00000350079(p.Pro1473Leu)];ENSG00000204248:[ENST00000374712(c.4238C>T,cCg>cTg):ENSP00000363844(p.Pro1413Leu)];ENSG00000204248:[ENST00000374708(c.4223C>T,cCg>cTg):ENSP00000363840(p.Pro1408Leu)];ENSG00000204248:[ENST00000341947(c.4481C>T,cCg>cTg):ENSP00000339915(p.Pro1494Leu)];ENSG00000204248:[ENST00000374714(c.4403C>T,cCg>cTg):ENSP00000363846(p.Pro1468Leu)];ENSG00000204248:[ENST00000477772(n.273-116C>T)];	NA	NA	NA	2.54E-05	6.07E-05	NA	NA	NA	NA	NA	MODIFIER	NA	NA	62	62	0	59	34	25	42.37
Smoker	42476	chr6	43016369	G	C	*CUL7*	INTRONIC	ENSG00000044090:[ENST00000535468(c.2078-62C>G)];ENSG00000044090:[ENST00000265348(c.1826-62C>G)];	NA	NA	NA	NA	NA	NA	NA	NA	NA	NA	MODIFIER	NA	NA	43	43	0	35	26	9	25.71
Smoker	42476	chr6	43171171	G	A	*CUL9*	MISSENSE	ENSG00000112659:MISSENSE[ENST00000354495(c.3536G>A,cGg>cAg):ENSP00000346490(p.Arg1179Gln)];ENSG00000112659:MISSENSE[ENST00000372647(c.3866G>A,cGg>cAg):ENSP00000361730(p.Arg1289Gln)];ENSG00000112659:MISSENSE[ENST00000252050(c.3866G>A,cGg>cAg):ENSP00000252050(p.Arg1289Gln)];ENSG00000112659:EXONIC-NC[ENST00000515344(n.161G>A)];ENSG00000112659:EXONIC-NC[ENST00000515773(n.4320G>A)];	chr6*43171	chr6*43171	NA	8.27E-06	0	NA	NA	NA	NA	NA	MODIFIER	NA	NA	47	47	0	62	48	14	22.58
Smoker	42476	chr6	116757018	A	T	*DSE*	MISSENSE	ENSG00000111817:MISSENSE[ENST00000452085(c.1387A>T,Aat>Tat):ENSP00000404049(p.Asn463Tyr)];ENSG00000111817:MISSENSE[ENST00000331677(c.1387A>T,Aat>Tat):ENSP00000332151(p.Asn463Tyr)];ENSG00000111817:MISSENSE[ENST00000537543(c.1444A>T,Aat>Tat):ENSP00000441152(p.Asn482Tyr)];ENSG00000111817:MISSENSE[ENST00000359564(c.1387A>T,Aat>Tat):ENSP00000352567(p.Asn463Tyr)];ENSG00000111817:EXONIC-NC[ENST00000606712(n.1291A>T)];	NA	NA	NA	NA	NA	NA	NA	NA	NA	NA	MODIFIER	NA	NA	164	164	0	144	136	8	5.56
Smoker	42476	chr6	170627323	A	G	*FAM120B*	MISSENSE	ENSG00000112584:MISSENSE[ENST00000476287(c.845A>G,aAa>aGa):ENSP00000417970(p.Lys282Arg)];ENSG00000112584:MISSENSE[ENST00000537664(c.914A>G,aAa>aGa):ENSP00000440125(p.Lys305Arg)];ENSG00000112584:MISSENSE[ENST00000540480(c.881A>G,aAa>aGa):ENSP00000444125(p.Lys294Arg)];ENSG00000112584:INTRONIC[ENST00000252510(c.-90+11393A>G)];	NA	NA	NA	NA	NA	NA	NA	T	BN	N	MODERATE	N	0.853	113	113	0	70	62	7	10

Mangalaparthi *et al.* , 2020. Mutational landscape of esophageal squamous cell carcinoma in an Indian cohort
Supplementary Table 4. List of somatic single nucleotide variants identified using whole exome sequencing of ESCC patients

Cohort	Sample	Chromosome	Genomic position	Reference allele	Alternate allele	Gene symbol	Variant type	Variant annotation	OncoMD	COSMIC	dbSNP	ExAC	ExAC_SAS	1000G	1000G_SAS	SIFT	Polyphen2	CONDEL	VEP	LRT	PhastCon7 Way	Total read (Adjacent normal)	Reference allel read support (Adjacent normal)	Alternative read support (Adjacent normal)	Total read (Tumor)	Reference allel read support (Tumor)	Alternative read support (Tumor)	Alternate allele frequency
Smoker	42476	chr6	71212429	C	T	*FAM135A*	SILENT	ENSG00000082269:SILENT[ENST00000457062(c.913C>T,Ctg>Ttg):ENSP00000409201(p.Leu305(=))];ENSG00000082269:SILENT[ENST00000505868(c.964C>T,Ctg>Ttg):ENSP00000423307(p.Leu322(=))];ENSG00000082269:SILENT[ENST00000361499(c.964C>T,Ctg>Ttg):ENSP00000354913(p.Leu322(=))];ENSG00000082269:SILENT[ENST00000505769(c.964C>T,Ctg>Ttg):ENSP00000423785(p.Leu322(=))];ENSG00000082269:SILENT[ENST00000418814(c.964C>T,Ctg>Ttg):ENSP00000410768(p.Leu322(=))];ENSG00000082269:SILENT[ENST00000370479(c.913C>T,Ctg>Ttg):ENSP00000359510(p.Leu305(=))];ENSG00000082269:SILENT[ENST00000515323(c.964C>T,Ctg>Ttg):ENSP00000422406(p.Leu322(=))];ENSG00000082269:EXONIC-NC[ENST00000425415(n.241C>T)];ENSG00000082269:EXONIC-NC[ENST00000393299(n.999C>T)];ENSG00000082269:3UTR[ENST00000194672(c.*464C>T)];	NA	NA	NA	NA	NA	NA	NA	NA	NA	NA	LOW	NA	NA	78	78	0	82	73	9	10.98
Smoker	42476	chr6	76063230	G	C	*FILIP1*	INTRONIC	ENSG00000118407:[ENST00000370020(c.332+25C>G)];ENSG00000118407:[ENST00000393004(c.629+25C>G)];ENSG00000118407:[ENST00000237172(c.629+25C>G)];	NA	NA	NA	NA	NA	NA	NA	NA	NA	NA	MODIFIER	NA	NA	47	46	0	24	15	9	37.5
Smoker	42476	chr6	52993561	C	T	*GCM1*	MISSENSE	ENSG00000137270:MISSENSE[ENST00000259803(c.754G>A,Gac>Aac):ENSP00000259803(p.Asp252Asn)];	NA	NA	chr6*52993548*52993579*GTGGAAGTCTGGTCAGTCAGATCTGTGATTCC*G*rs761544740*NA*NA*NA	NA	NA	NA	NA	T	BN	N	MODERATE	N	0.003	70	70	0	107	87	20	18.69
Smoker	42476	chr6	117127781	C	G	*GPRC6A*	MISSENSE	ENSG00000173612:MISSENSE[ENST00000310357(c.1087G>C,Gcc>Ccc):ENSP00000309493(p.Ala363Pro)];ENSG00000173612:MISSENSE[ENST00000368549(c.1087G>C,Gcc>Ccc):ENSP00000357537(p.Ala363Pro)];ENSG00000173612:INTRONIC[ENST00000530250(c.810+277G>C)];	chr6*11712[illegible]	NA	NA	NA	NA	NA	NA	NA	NA	NA	MODIFIER	NA	NA	182	182	0	163	157	6	3.68
Smoker	42476	chr6	146755467	C	A	*GRM1*	MISSENSE	ENSG00000152822:MISSENSE[ENST00000282753(c.3120C>A,aaC>aaA):ENSP00000282753(p.Asn1040Lys)];ENSG00000152822:MISSENSE[ENST00000361719(c.3120C>A,aaC>aaA):ENSP00000354896(p.Asn1040Lys)];ENSG00000152822:3UTR[ENST00000392299(c.*484C>A)];ENSG00000152822:3UTR[ENST00000507907(c.*449C>A)];ENSG00000152822:3UTR[ENST00000355289(c.*358C>A)];ENSG00000152822:3UTR[ENST00000492807(c.*484C>A)];	NA	NA	NA	NA	NA	NA	NA	NA	NA	NA	MODIFIER	NA	NA	63	63	0	26	15	11	42.31
Smoker	42476	chr6	26124467	G	A	*HIST1H2AC*	MISSENSE	ENSG00000180573:MISSENSE[ENST00000377791(c.7G>A,Gga>Aga):ENSP00000367022(p.Gly3Arg)];ENSG00000180573:MISSENSE[ENST00000602637(c.7G>A,Gga>Aga):ENSP00000473534(p.Gly3Arg)];ENSG00000180573:MISSENSE[ENST00000314088(c.7G>A,Gga>Aga):ENSP00000321389(p.Gly3Arg)];	NA	NA	NA	NA	NA	NA	NA	D_lc	UN	NA	MODERATE	NA	NA	78	78	0	90	77	13	14.44
Smoker	42476	chr6	32548113	G	A	*HLA-DRB1*	INTRONIC	ENSG00000196126:[ENST00000360004(c.764-66C>T)];	NA	NA	NA	NA	NA	NA	NA	NA	NA	NA	MODIFIER	NA	NA	44	44	0	59	50	8	13.56
Smoker	42476	chr6	114378879	G	C	*HS3ST5*	MISSENSE	ENSG00000249853:MISSENSE[ENST00000411826(c.583C>G,Cag>Gag):ENSP00000440332(p.Gln195Glu)];ENSG00000249853:MISSENSE[ENST00000312719(c.583C>G,Cag>Gag):ENSP00000427888(p.Gln195Glu)];	NA	NA	NA	NA	NA	NA	NA	NA	NA	NA	MODIFIER	NA	NA	290	287	0	238	209	29	12.18
Smoker	42476	chr6	76660491	C	T	*IMPG1*	MISSENSE	ENSG00000112706:MISSENSE[ENST00000369950(c.1612G>A,Gaa>Aaa):ENSP00000358966(p.Glu538Lys)];ENSG00000112706:3UTR[ENST00000369963(c.*3405G>A)];	NA	NA	NA	5.78E-05	0.000181862	NA	NA	NA	NA	NA	MODIFIER	NA	NA	91	91	0	49	43	6	12.24
Smoker	42476	chr6	76715238	C	G	*IMPG1*	MISSENSE	ENSG00000112706:MISSENSE[ENST00000369950(c.901G>C,Gag>Cag):ENSP00000358966(p.Glu301Gln)];ENSG00000112706:3UTR[ENST00000369963(c.*2694G>C)];	NA	NA	chr6*76715238*76715238*C*T*rs748397771*NA*NA*NA	NA	NA	NA	NA	NA	NA	NA	MODIFIER	NA	NA	64	64	0	24	14	10	41.67
Smoker	42476	chr6	33653845	C	G	*ITPR3*	MISSENSE	ENSG00000096433:MISSENSE[ENST00000374316(c.5683C>G,Cag>Gag):ENSP00000363435(p.Gln1895Glu)];ENSG00000096433:MISSENSE[ENST00000605930(c.5683C>G,Cag>Gag):ENSP00000475177(p.Gln1895Glu)];	NA	NA	NA	NA	NA	NA	NA	D	PrD	D	MODERATE	D	1	64	64	0	98	83	14	14.29
Smoker	42476	chr6	43016369	G	C	*KLC4*	INTRONIC	ENSG00000137171:[ENST00000467906(c.-553+5123G>C)];	NA	NA	NA	NA	NA	NA	NA	NA	NA	NA	MODIFIER	NA	NA	43	43	0	35	26	9	25.71
Smoker	42476	chr6	97561818	G	T	*KLHL32*	NONSENSE	ENSG00000186231:NONSENSE[ENST00000539200(c.580G>T,Gag>Tag):ENSP00000441527(p.Glu194Ter)];ENSG00000186231:NONSENSE[ENST00000536676(c.679G>T,Gag>Tag):ENSP00000440382(p.Glu227Ter)];ENSG00000186231:NONSENSE[ENST00000369261(c.787G>T,Gag>Tag):ENSP00000358265(p.Glu263Ter)];ENSG00000186231:3UTR[ENST00000447886(c.*23G>T)];ENSG00000186231:INTRONIC[ENST00000369254(c.412-13462G>T)];ENSG00000186231:INTRONIC[ENST00000544166(c.23-13462G>T)];	NA	NA	chr6*97561818*97561818*G*A*rs762493875*NA*NA*NA	NA	NA	NA	NA	NA	NA	NA	MODIFIER	NA	NA	100	100	0	57	34	23	40.35
Smoker	42476	chr6	70506704	C	A	*LMBRD1*	INTRONIC-SS-DNR	ENSG00000168216:INTRONIC-SS-DNR[ENST00000472827(c.69+1G>T)];ENSG00000168216:INTRONIC-SS-DNR[ENST00000370577(c.69+1G>T)];	NA	NA	NA	NA	NA	NA	NA	NA	NA	NA	HIGH	NA	NA	90	90	0	53	36	17	32.08
Smoker	42476	chr6	37616875	C	T	*MDGA1*	MISSENSE	ENSG00000112139:MISSENSE[ENST00000297153(c.1786G>A,Gag>Aag):ENSP00000297153(p.Glu596Lys)];ENSG00000112139:MISSENSE[ENST00000505425(c.1777G>A,Gag>Aag):ENSP00000422042(p.Glu593Lys)];ENSG00000112139:MISSENSE[ENST00000434837(c.1777G>A,Gag>Aag):ENSP00000402584(p.Glu593Lys)];	NA	NA	NA	0	0	NA	NA	T	BN	N	MODERATE	N	0.086	53	52	0	58	46	11	18.97
Smoker	42476	chr6	76558153	G	T	*MYO6*	MISSENSE	ENSG00000196586:MISSENSE[ENST00000369985(c.983G>T,gGt>gTt):ENSP00000359002(p.Gly328Val)];ENSG00000196586:MISSENSE[ENST00000369975(c.983G>T,gGt>gTt):ENSP00000358992(p.Gly328Val)];ENSG00000196586:MISSENSE[ENST00000369977(c.983G>T,gGt>gTt):ENSP00000358994(p.Gly328Val)];ENSG00000196586:MISSENSE[ENST00000369981(c.983G>T,gGt>gTt):ENSP00000358998(p.Gly328Val)];	NA	NA	NA	NA	NA	NA	NA	D	UN	NA	MODERATE	D	1	154	153	0	110	101	9	8.18
Smoker	42476	chr6	29231532	C	G	*OR2U1P*	EXONIC-NC	ENSG00000204697:[ENST00000377164(n.325G>C)];	NA	NA	NA	NA	NA	NA	NA	NA	NA	NA	MODIFIER	NA	NA	181	181	0	215	180	35	16.28
Smoker	42476	chr6	28269751	T	G	*PGBD1*	MISSENSE	ENSG00000137338:MISSENSE[ENST00000405948(c.2120T>G,gTc>gGc):ENSP00000385213(p.Val707Gly)];ENSG00000137338:MISSENSE[ENST00000259883(c.2120T>G,gTc>gGc):ENSP00000259883(p.Val707Gly)];	NA	NA	NA	NA	NA	NA	NA	D	BN	D	MODERATE	U	0.304	210	209	0	250	209	40	16
Smoker	42476	chr6	41708152	C	T	*PGC*	INTRONIC	ENSG00000096088:[ENST00000373025(c.767+77G>A)];	NA	NA	chr6*41708152*41708154*CTG*C*rs141886611*NA*NA*NA	NA	NA	NA	NA	NA	NA	NA	MODIFIER	NA	NA	64	64	0	59	38	21	35.59
Smoker	42476	chr6	31593589	G	A	*PRRC2A*	SILENT	ENSG00000204469:SILENT[ENST00000376033(c.780G>A,ccG>ccA):ENSP00000365201(p.Pro260(=))];ENSG00000204469:SILENT[ENST00000376007(c.780G>A,ccG>ccA):ENSP00000365175(p.Pro260(=))];ENSG00000204469:EXONIC-NC[ENST00000464890(n.59G>A)];ENSG00000204469:EXONIC-NC[ENST00000469577(n.625G>A)];	NA	NA	NA	NA	NA	NA	NA	NA	NA	NA	MODIFIER	NA	NA	111	110	0	115	64	51	44.35

Mangalaparthi *et al.* , 2020. Mutational landscape of esophageal squamous cell carcinoma in an Indian cohort
Supplementary Table 4. List of somatic single nucleotide variants identified using whole exome sequencing of ESCC patients

Cohort	Sample	Chromosome	Genomic position	Reference allele	Alternate allele	Gene symbol	Variant type	Variant annotation	OncoMD	COSMIC	dbSNP	ExAC	ExAC_SAS	1000G	1000G_SAS	SIFT	Polyphen2	CONDEL	VEP	LRT	PhastCon7 Way	Total read (Adjacent normal)	Reference allel read support (Adjacent normal)	Alternative read support (Adjacent normal)	Total read (Tumor)	Reference allel read support (Tumor)	Alternative read support (Tumor)	Alternate allele frequency
Smoker	42476	chr6	31599593	G	C	*PRRC2A*	MISSENSE	ENSG00000204469:MISSENSE[ENST00000376033(c.3143G>C,gGa>gCa):ENSP00000365201(p.Gly1048Ala)];ENSG00000204469:MISSENSE[ENST00000376007(c.3143G>C,gGa>gCa):ENSP00000365175(p.Gly1048Ala)];	NA	NA	NA	NA	NA	NA	NA	NA	UN	NA	MODERATE	D	0.994	99	99	0	88	76	12	13.64
Smoker	42476	chr6	111697098	T	A	*REV3L*	MISSENSE	ENSG00000009413:MISSENSE[ENST00000435970(c.2226A>T,aaA>aaT):ENSP00000402003(p.Lys742Asn)];ENSG00000009413:MISSENSE[ENST00000358835(c.2460A>T,aaA>aaT):ENSP00000351697(p.Lys820Asn)];ENSG00000009413:MISSENSE[ENST00000368802(c.2460A>T,aaA>aaT):ENSP00000357792(p.Lys820Asn)];ENSG00000009413:MISSENSE[ENST00000368805(c.2460A>T,aaA>aaT):ENSP00000357795(p.Lys820Asn)];ENSG00000009413:3UTR[ENST00000434009(c.*2551A>T)];ENSG00000009413:3UTR[ENST00000422377(c.*2444A>T)];	NA	NA	NA	NA	NA	NA	NA	NA	NA	NA	MODIFIER	NA	NA	106	106	0	67	50	17	25.37
Smoker	42476	chr6	114378879	G	C	*RP3-399L15.3*	INTRONIC	ENSG00000228624:[ENST00000519270(n.87-916G>C)];ENSG00000228624:[ENST00000523087(n.106+61655G>C)];ENSG00000228624:[ENST00000519104(n.1311-31222G>C)];	NA	NA	NA	NA	NA	NA	NA	NA	NA	NA	MODIFIER	NA	NA	290	287	0	238	209	29	12.18
Smoker	42476	chr6	152614823	G	A	*SYNE1*	MISSENSE	ENSG00000131018:MISSENSE[ENST00000540663(c.437C>T,tCc>tTc):ENSP00000437411(p.Ser146Phe)];ENSG00000131018:MISSENSE[ENST00000537033(c.578C>T,tCc>tTc):ENSP00000443879(p.Ser193Phe)];ENSG00000131018:MISSENSE[ENST00000356820(c.1484C>T,tCc>tTc):ENSP00000349276(p.Ser495Phe)];ENSG00000131018:MISSENSE[ENST00000423061(c.17699C>T,tCc>tTc):ENSP00000396024(p.Ser5900Phe)];ENSG00000131018:MISSENSE[ENST00000448038(c.17699C>T,tCc>tTc):ENSP00000390975(p.Ser5900Phe)];ENSG00000131018:MISSENSE[ENST00000367255(c.17912C>T,tCc>tTc):ENSP00000356224(p.Ser5971Phe)];ENSG00000131018:MISSENSE[ENST00000341594(c.16748C>T,tCc>tTc):ENSP00000341887(p.Ser5583Phe)];ENSG00000131018:MISSENSE[ENST00000265368(c.17912C>T,tCc>tTc):ENSP00000265368(p.Ser5971Phe)];ENSG00000131018:EXONIC-NC[ENST00000367256(n.1604C>T)];ENSG00000131018:EXONIC-NC[ENST00000409694(n.1496C>T)];ENSG00000131018:EXONIC-NC[ENST00000489156(n.631C>T)];	NA	NA	chr6*152614823*152614823*G*C*rs753835774*NA*NA*NA	NA	NA	NA	NA	NA	PoD	NA	MODERATE	N	0.996	40	40	0	27	20	6	22.22
Smoker	42476	chr6	152712453	C	A	*SYNE1*	MISSENSE	ENSG00000131018:MISSENSE[ENST00000448038(c.7984G>T,Ggg>Tgg):ENSP00000390975(p.Gly2662Trp)];ENSG00000131018:MISSENSE[ENST00000367255(c.7963G>T,Ggg>Tgg):ENSP00000356224(p.Gly2655Trp)];ENSG00000131018:MISSENSE[ENST00000423061(c.7984G>T,Ggg>Tgg):ENSP00000396024(p.Gly2662Trp)];ENSG00000131018:MISSENSE[ENST00000341594(c.8080G>T,Ggg>Tgg):ENSP00000341887(p.Gly2694Trp)];ENSG00000131018:MISSENSE[ENST00000265368(c.7963G>T,Ggg>Tgg):ENSP00000265368(p.Gly2655Trp)];ENSG00000131018:EXONIC-NC[ENST00000461872(n.8181G>T)];	NA	NA	NA	NA	NA	NA	NA	NA	NA	NA	MODIFIER	NA	NA	69	69	0	41	25	16	39.02
Smoker	42476	chr6	126333864	A	T	*TRMT11*	INTRONIC	ENSG00000066651:[ENST00000334379(c.926-53A>T)];ENSG00000066651:[ENST00000368332(c.926-53A>T)];ENSG00000066651:[ENST00000466316(c.-114-53A>T)];ENSG00000066651:[ENST00000453993(c.321-53A>T)];ENSG00000066651:[ENST00000479748(c.*341-53A>T)];	NA	NA	NA	NA	NA	NA	NA	NA	NA	NA	MODIFIER	NA	NA	85	85	0	66	58	8	12.12
Smoker	42476	chr6	29231532	C	G	*XXbac-BPG308J9.3*	INTRONIC	ENSG00000232505:[ENST00000606648(n.53-26371C>G)];ENSG00000232505:[ENST00000441381(n.80-77C>G)];	NA	NA	NA	NA	NA	NA	NA	NA	NA	NA	MODIFIER	NA	NA	181	181	0	215	180	35	16.28
Smoker	42476	chr6	28336528	G	C	*ZKSCAN3*	3UTR	ENSG00000189298:[ENST00000377255(c.*2466G>C)];	NA	NA	NA	NA	NA	NA	NA	NA	NA	NA	MODIFIER	NA	NA	95	95	0	91	75	15	16.48
Smoker	42476	chr7	142962193	T	C	*AC073342.12*	INTRONIC	ENSG00000231840:[ENST00000427392(n.435-9679A>G)];	NA	NA	NA	NA	NA	NA	NA	NA	NA	NA	LOW	NA	NA	78	78	0	98	87	11	11.22
Smoker	42476	chr7	74131336	C	G	*AC083884.8*	INTRONIC	ENSG00000232729:[ENST00000434256(n.266+10404G>C)];ENSG00000232729:[ENST00000601921(n.472+5719G>C)];ENSG00000232729:[ENST00000594967(n.284+10404G>C)];ENSG00000232729:[ENST00000450426(n.182+10404G>C)];	NA	NA	NA	NA	NA	NA	NA	NA	NA	NA	MODIFIER	NA	NA	26	26	0	34	28	5	14.71
Smoker	42476	chr7	100253117	G	C	*ACTL6B*	MISSENSE	ENSG00000077080:MISSENSE[ENST00000160382(c.195C>G,ttC>ttG):ENSP00000160382(p.Phe65Leu)];ENSG00000077080:EXONIC-NC[ENST00000487225(n.423C>G)];ENSG00000077080:EXONIC-NC[ENST00000485601(n.276C>G)];ENSG00000077080:5UTR[ENST00000461605(c.-49C>G)];	chr7*10025	NA	NA	NA	NA	NA	NA	NA	NA	NA	MODIFIER	NA	NA	100	99	0	114	102	11	9.65
Smoker	42476	chr7	99047838	G	A	*ATP5J2*	INTRONIC	ENSG00000241468:[ENST00000414062(c.*130-1022C>T)];ENSG00000241468:[ENST00000466753(n.56-1022C>T)];	NA	NA	NA	NA	NA	NA	NA	NA	NA	NA	MODIFIER	NA	NA	90	90	0	118	96	22	18.64
Smoker	42476	chr7	99047838	G	A	*ATP5J2-PTCD1*	INTRONIC	ENSG00000248919:[ENST00000451138(c.122-8728C>T)];ENSG00000248919:[ENST00000413834(c.121+9871C>T)];ENSG00000248919:[ENST00000437572(n.70-2027C>T)];	NA	NA	NA	NA	NA	NA	NA	NA	NA	NA	MODIFIER	NA	NA	90	90	0	118	96	22	18.64
Smoker	42476	chr7	99569656	G	A	*AZGP1*	INTRONIC	ENSG00000160862:[ENST00000411734(c.68-27C>T)];ENSG00000160862:[ENST00000495765(n.99-27C>T)];ENSG00000160862:[ENST00000292401(c.77-27C>T)];	NA	NA	NA	NA	NA	NA	NA	NA	NA	NA	MODIFIER	NA	NA	27	27	0	69	59	10	14.49
Smoker	42476	chr7	33392498	A	C	*BBS9*	INTRONIC	ENSG00000122507:[ENST00000396127(c.1447+13A>C)];ENSG00000122507:[ENST00000242067(c.1552+13A>C)];ENSG00000122507:[ENST00000355070(c.1537+1563A>C)];ENSG00000122507:[ENST00000434373(c.251+13A>C)];ENSG00000122507:[ENST00000433714(c.*313+13A>C)];ENSG00000122507:[ENST00000354265(c.1441+3621A>C)];ENSG00000122507:[ENST00000350941(c.1432+3716A>C)];	NA	NA	NA	NA	NA	NA	NA	NA	NA	NA	MODIFIER	NA	NA	90	90	0	79	59	18	22.78
Smoker	42476	chr7	80299255	C	T	*CD36*	INTRONIC	ENSG00000135218:[ENST00000433696(c.702-1038C>T)];ENSG00000135218:[ENST00000447544(c.749-14C>T)];ENSG00000135218:[ENST00000534394(c.521-14C>T)];ENSG00000135218:[ENST00000538969(c.569-14C>T)];ENSG00000135218:[ENST00000309881(c.749-14C>T)];ENSG00000135218:[ENST00000544133(c.749-14C>T)];ENSG00000135218:[ENST00000435819(c.749-14C>T)];ENSG00000135218:[ENST00000432207(c.749-14C>T)];ENSG00000135218:[ENST00000419819(c.749-14C>T)];ENSG00000135218:[ENST00000464213(n.577-14C>T)];ENSG00000135218:[ENST00000394788(c.749-14C>T)];	NA	NA	0.000599	0.0014754	0	0.000599	0	NA	NA	NA	MODIFIER	NA	NA	103	103	0	172	149	23	13.37
Smoker	42476	chr7	148080877	C	G	*CNTNAP2*	MISSENSE	ENSG00000174469:MISSENSE[ENST00000361727(c.3612C>G,caC>caG):ENSP00000354778(p.His1204Gln)];ENSG00000174469:MISSENSE[ENST00000538075(c.789C>G,caC>caG):ENSP00000440732(p.His263Gln)];ENSG00000174469:EXONIC-NC[ENST00000463592(n.299C>G)];ENSG00000174469:EXONIC-NC[ENST00000602734(n.297C>G)];	NA	NA	NA	NA	NA	NA	NA	NA	NA	NA	MODIFIER	NA	NA	61	61	0	90	79	11	12.22

Cohort	Sample	Chromosome	Genomic position	Reference allele	Alternate allele	Gene symbol	Variant type	Variant annotation	OncoMD	COSMIC	dbSNP	ExAC	ExAC_SAS	1000G	1000G_SAS	SIFT	Polyphen2	CONDEL	VEP	LRT	PhastCon7 Way	Total read (Adjacent normal)	Reference allel read support (Adjacent normal)	Alternative read support (Adjacent normal)	Total read (Tumor)	Reference allel read support (Tumor)	Alternative read support (Tumor)	Alternate allele frequency
Smoker	42476	chr7	99047838	G	A	*CPSF4*	INTRONIC	ENSG00000160917:[ENST00000471455(n.184-61G>A)];ENSG00000160917:[ENST00000484112(n.352-61G>A)];ENSG00000160917:[ENST00000436336(c.308-61G>A)];ENSG00000160917:[ENST00000451876(c.308-487G>A)];ENSG00000160917:[ENST00000430038(c.*345-61G>A)];ENSG00000160917:[ENST00000412686(c.149-61G>A)];ENSG00000160917:[ENST00000292476(c.308-61G>A)];ENSG00000160917:[ENST00000452047(c.209-61G>A)];ENSG00000160917:[ENST00000441580(c.149-61G>A)];	NA	NA	NA	NA	NA	NA	NA	NA	NA	NA	MODIFIER	NA	NA	90	90	0	118	96	22	18.64
Smoker	42476	chr7	117431452	C	T	*CTTNBP2*	MISSENSE	ENSG00000077063:MISSENSE[ENST00000160373(c.1798G>A,Gag>Aag):ENSP00000160373(p.Glu600Lys)];ENSG00000077063:MISSENSE[ENST00000446636(c.383G>A,Gag>Aag):ENSP00000389576(p.Glu129Lys)];ENSG00000077063:MISSENSE[ENST00000441556(c.1798G>A,Gag>Aag):ENSP00000397678(p.Glu600Lys)];	NA	NA	NA	NA	NA	NA	NA	D_lc	PrD	D	MODERATE	NA	NA	80	79	0	125	108	17	13.6
Smoker	42476	chr7	117432586	C	T	*CTTNBP2*	MISSENSE	ENSG00000077063:MISSENSE[ENST00000441556(c.664G>A,Gaa>Aaa):ENSP00000397678(p.Glu222Lys)];ENSG00000077063:MISSENSE[ENST00000160373(c.664G>A,Gaa>Aaa):ENSP00000160373(p.Glu222Lys)];	NA	NA	NA	NA	NA	NA	NA	T	BN	N	MODERATE	D	0.554	144	144	0	220	185	35	15.91
Smoker	42476	chr7	137076106	A	G	*DGKI*	INTRONIC	ENSG00000157680:[ENST00000494390(n.704-24T>C)];ENSG00000157680:[ENST00000477835(n.284-24T>C)];ENSG00000157680:[ENST00000453654(c.2089-24T>C)];ENSG00000157680:[ENST00000497321(n.451-24T>C)];ENSG00000157680:[ENST00000288490(c.3082-24T>C)];ENSG00000157680:[ENST00000424189(c.3121-24T>C)];ENSG00000157680:[ENST00000486153(n.377-24T>C)];ENSG00000157680:[ENST00000446122(c.3028-24T>C)];	NA	NA	NA	NA	NA	NA	NA	NA	NA	NA	MODIFIER	NA	NA	65	65	0	95	79	15	15.79
Smoker	42476	chr7	107569794	G	C	*DLD*	INTRONIC	ENSG00000091140:[ENST00000417551(c.*125-2082G>C)];	NA	NA	NA	NA	NA	NA	NA	NA	NA	NA	MODIFIER	NA	NA	195	195	0	334	297	36	10.78
Smoker	42476	chr7	102953441	C	T	*DNAJC2*	MISSENSE	ENSG00000105821:MISSENSE[ENST00000249270(c.1585G>A,Gaa>Aaa):ENSP00000249270(p.Glu529Lys)];ENSG00000105821:MISSENSE[ENST00000379263(c.1744G>A,Gaa>Aaa):ENSP00000368565(p.Glu582Lys)];ENSG00000105821:EXONIC-NC[ENST00000492497(n.1695G>A)];ENSG00000105821:EXONIC-NC[ENST00000464253(n.1687G>A)];ENSG00000105821:EXONIC-NC[ENST00000475065(n.1780G>A)];	NA	NA	NA	NA	NA	NA	NA	NA	NA	NA	MODIFIER	NA	NA	125	125	0	172	77	94	54.65
Smoker	42476	chr7	102963145	C	G	*DNAJC2*	INTRONIC-SS-DNR	ENSG00000105821:[ENST00000249270(c.811+5G>C)];ENSG00000105821:[ENST00000379263(c.811+5G>C)];ENSG00000105821:[ENST00000426036(c.696+5G>C)];ENSG00000105821:[ENST00000464253(n.689G>C)];ENSG00000105821:[ENST00000475065(n.782G>C)];	NA	NA	NA	NA	NA	NA	NA	NA	NA	NA	MODIFIER	NA	NA	50	50	0	82	73	9	10.98
Smoker	42476	chr7	76134891	G	A	*DTX2*	SILENT	ENSG00000091073:SILENT[ENST00000446600(c.1569G>A,gtG>gtA):ENSP00000397648(p.Val523(=))];ENSG00000091073:SILENT[ENST00000307569(c.1701G>A,gtG>gtA):ENSP00000305242(p.Val567(=))];ENSG00000091073:SILENT[ENST00000324432(c.1842G>A,gtG>gtA):ENSP00000322885(p.Val614(=))];ENSG00000091073:SILENT[ENST00000413936(c.1842G>A,gtG>gtA):ENSP00000390218(p.Val614(=))];ENSG00000091073:SILENT[ENST00000446820(c.1701G>A,gtG>gtA):ENSP00000392545(p.Val567(=))];ENSG00000091073:SILENT[ENST00000430490(c.1842G>A,gtG>gtA):ENSP00000411986(p.Val614(=))];ENSG00000091073:EXONIC-NC[ENST00000465488(n.512G>A)];ENSG00000091073:EXONIC-NC[ENST00000468546(n.1021G>A)];ENSG00000091073:EXONIC-NC[ENST00000479915(n.1331G>A)];	NA	NA	NA	NA	NA	NA	NA	NA	NA	NA	LOW	NA	NA	87	87	0	135	116	19	14.07
Smoker	42476	chr7	76135013	G	C	*DTX2*	3UTR	ENSG00000091073:[ENST00000446600(c.*95G>C)];ENSG00000091073:[ENST00000307569(c.*95G>C)];ENSG00000091073:[ENST00000324432(c.*95G>C)];ENSG00000091073:[ENST00000413936(c.*95G>C)];ENSG00000091073:[ENST00000430490(c.*95G>C)];ENSG00000091073:[ENST00000465488(n.634G>C)];ENSG00000091073:[ENST00000468546(n.1143G>C)];ENSG00000091073:[ENST00000479915(n.1453G>C)];	NA	NA	NA	NA	NA	NA	NA	NA	NA	NA	MODIFIER	NA	NA	57	57	0	64	53	11	17.19
Smoker	42476	chr7	142566367	G	A	*EPHB6*	MISSENSE	ENSG00000106123:MISSENSE[ENST00000411471(c.1325G>A,cGg>cAg):ENSP00000409061(p.Arg442Gln)];ENSG00000106123:MISSENSE[ENST00000442129(c.2156G>A,cGg>cAg):ENSP00000410789(p.Arg719Gln)];ENSG00000106123:MISSENSE[ENST00000392957(c.2156G>A,cGg>cAg):ENSP00000376684(p.Arg719Gln)];ENSG00000106123:EXONIC-NC[ENST00000486511(n.1200G>A)];ENSG00000106123:EXONIC-NC[ENST00000466783(n.3306G>A)];ENSG00000106123:3UTR[ENST00000425995(c.*1938G>A)];ENSG00000106123:3UTR[ENST00000422643(c.*1301G>A)];	chr7*14256	chr7*14256	NA	7.66E-05	0.00024266	NA	NA	NA	NA	NA	MODIFIER	NA	NA	57	57	0	73	64	9	12.33
Smoker	42476	chr7	158552790	C	G	*ESYT2*	MISSENSE	ENSG00000117868:MISSENSE[ENST00000251527(c.1426G>C,Gag>Cag):ENSP00000251527(p.Glu476Gln)];ENSG00000117868:MISSENSE[ENST00000275418(c.1336G>C,Gag>Cag):ENSP00000275418(p.Glu446Gln)];	NA	NA	NA	NA	NA	NA	NA	D	PrD	D	MODERATE	NA	NA	109	109	0	176	153	23	13.07
Smoker	42476	chr7	102614165	C	T	*FBXL13*	INTRONIC	ENSG00000161040:[ENST00000379306(c.496-5601G>A)];ENSG00000161040:[ENST00000393772(c.496-5601G>A)];ENSG00000161040:[ENST00000456695(c.496-5601G>A)];ENSG00000161040:[ENST00000313221(c.496-5601G>A)];ENSG00000161040:[ENST00000455112(c.496-5601G>A)];ENSG00000161040:[ENST00000448002(c.766-5601G>A)];ENSG00000161040:[ENST00000379305(c.496-5601G>A)];ENSG00000161040:[ENST00000471074(n.764-5601G>A)];ENSG00000161040:[ENST00000436908(c.496-5601G>A)];ENSG00000161040:[ENST00000379308(c.496-5601G>A)];	NA	NA	chr7*102614161*102614169*TCCTCAGCC*T*rs756444944*NA*NA*NA	NA	NA	NA	NA	NA	NA	NA	MODIFIER	NA	NA	63	63	0	86	76	9	10.47
Smoker	42476	chr7	126173692	G	T	*GRM8*	MISSENSE	ENSG00000179603:MISSENSE[ENST00000358373(c.1744C>A,Ccc>Acc):ENSP00000351142(p.Pro582Thr)];ENSG00000179603:MISSENSE[ENST00000444921(c.1744C>A,Ccc>Acc):ENSP00000409790(p.Pro582Thr)];ENSG00000179603:MISSENSE[ENST00000339582(c.1744C>A,Ccc>Acc):ENSP00000344173(p.Pro582Thr)];ENSG00000179603:MISSENSE[ENST00000472701(c.1744C>A,Ccc>Acc):ENSP00000419832(p.Pro582Thr)];ENSG00000179603:EXONIC-NC[ENST00000480995(n.1291C>A)];ENSG00000179603:3UTR[ENST00000341617(c.*309C>A)];	NA	NA	chr7*126173692*126173692*G*A*rs776841029*NA*NA*NA	NA	NA	NA	NA	NA	NA	NA	MODIFIER	NA	NA	122	122	0	140	123	17	12.14

Supplementary Table 4. List of somatic single nucleotide variants identified using whole exome sequencing of ESCC patients

Cohort	Sample	Chromosome	Genomic position	Reference allele	Alternate allele	Gene symbol	Variant type	Variant annotation	OncoMD	COSMIC	dbSNP	ExAC	ExAC_SAS	1000G	1000G_SAS	SIFT	Polyphen2	CONDEL	VEP	LRT	PhastCon7 Way	Total read (Adjacent normal)	Reference allel read support (Adjacent normal)	Alternative read support (Adjacent normal)	Total read (Tumor)	Reference allel read support (Tumor)	Alternative read support (Tumor)	Alternate allele frequency
Smoker	42476	chr7	142962193	T	C	*GSTK1*	MISSENSE	ENSG00000197448:INTRONIC-SS-DNR-PRX[ENST00000409500(c.384+8T>C)];ENSG00000197448:INTRONIC-SS-DNR-PRX[ENST00000443571(c.255+8T>C)];ENSG00000197448:INTRONIC-SS-DNR-PRX[ENST00000442394(c.384+8T>C)];ENSG00000197448:INTRONIC-SS-DNR-PRX[ENST00000358406(c.384+8T>C)];ENSG00000197448:INTRONIC-SS-DNR-PRX[ENST00000473649(n.726+8T>C)];ENSG00000197448:MISSENSE[ENST00000479303(c.392T>C,gTg>gCg):ENSP00000431049(p.Val131Ala)];	NA	NA	NA	NA	NA	NA	NA	NA	NA	NA	LOW	NA	NA	78	78	0	98	87	11	11.22
Smoker	42476	chr7	74131336	C	G	*GTF2I*	3UTR	ENSG00000077809:[ENST00000443166(c.*61C>G)];ENSG00000077809:[ENST00000324896(c.880+66C>G)];ENSG00000077809:[ENST00000353920(c.820+66C>G)];ENSG00000077809:[ENST00000346152(c.880+66C>G)];ENSG00000077809:[ENST00000484840(n.339+66C>G)];ENSG00000077809:[ENST00000416070(c.820+66C>G)];	NA	NA	NA	NA	NA	NA	NA	NA	NA	NA	MODIFIER	NA	NA	26	26	0	34	28	5	14.71
Smoker	42476	chr7	139288933	G	A	*HIPK2*	MISSENSE	ENSG00000064393:MISSENSE[ENST00000342645(c.2149C>T,Ccc>Tcc):ENSP00000343108(p.Pro717Ser)];ENSG00000064393:MISSENSE[ENST00000428878(c.2068C>T,Ccc>Tcc):ENSP00000413724(p.Pro690Ser)];ENSG00000064393:MISSENSE[ENST00000406875(c.2149C>T,Ccc>Tcc):ENSP00000385571(p.Pro717Ser)];	chr7*13928	NA	NA	NA	NA	NA	NA	D	PoD	D	MODERATE	NA	NA	77	77	0	99	92	7	7.07
Smoker	42476	chr7	138595980	G	A	*KIAA1549*	SILENT	ENSG00000122778:SILENT[ENST00000422774(c.3057C>T,aaC>aaT):ENSP00000416040(p.Asn1019(=))];ENSG00000122778:SILENT[ENST00000242365(c.2907C>T,aaC>aaT):ENSP00000242365(p.Asn969(=))];ENSG00000122778:SILENT[ENST00000440172(c.3057C>T,aaC>aaT):ENSP00000406661(p.Asn1019(=))];	NA	NA	NA	NA	NA	NA	NA	NA	NA	NA	LOW	NA	0.979	161	161	0	220	193	27	12.27
Smoker	42476	chr7	151970850	G	A	*KMT2C*	NONSENSE	ENSG00000055609:NONSENSE[ENST00000355193(c.952C>T,Cag>Tag):ENSP00000347325(p.Gln318Ter)];ENSG00000055609:NONSENSE[ENST00000262189(c.952C>T,Cag>Tag):ENSP00000262189(p.Gln318Ter)];ENSG00000055609:NONSENSE[ENST00000558084(c.952C>T,Cag>Tag):ENSP00000453752(p.Gln318Ter)];	NA	NA	NA	NA	NA	NA	NA	NA	NA	NA	HIGH	NA	NA	652	651	0	563	535	28	4.97
Smoker	42476	chr7	107569794	G	C	*LAMB1*	INTRONIC	ENSG00000091136:[ENST00000393561(c.4817+63C>G)];ENSG00000091136:[ENST00000222399(c.4745+63C>G)];ENSG00000091136:[ENST00000472714(n.629C>G)];	NA	NA	NA	NA	NA	NA	NA	NA	NA	NA	MODIFIER	NA	NA	195	195	0	334	297	36	10.78
Smoker	42476	chr7	112127113	A	G	*LSMEM1*	INTRONIC-SS-DNR	ENSG00000181016:[ENST00000486022(n.446+7A>G)];ENSG00000181016:[ENST00000312849(c.256+7A>G)];ENSG00000181016:[ENST00000439068(c.256+7A>G)];ENSG00000181016:[ENST00000471030(n.211+7A>G)];ENSG00000181016:[ENST00000455302(c.*164+7A>G)];	NA	NA	NA	NA	NA	NA	NA	NA	NA	NA	LOW	NA	NA	72	72	0	126	93	33	26.19
Smoker	42476	chr7	100550202	T	A	*MUC3A*	SILENT	ENSG00000228273:SILENT[ENST00000379458(c.783T>A,tcT>tcA):ENSP00000368771(p.Ser261(=))];ENSG00000169894:EXONIC-NC[ENST00000483366(n.853T>A)];	NA	NA	NA	NA	NA	NA	NA	NA	NA	NA	MODIFIER	NA	NA	288	250	3	344	291	12	3.49
Smoker	42476	chr7	100551048	C	G	*MUC3A*	EXONIC-NC	ENSG00000169894:[ENST00000483133(n.719C>G)];	NA	NA	NA	NA	NA	NA	NA	NA	NA	NA	MODIFIER	NA	NA	630	418	6	643	393	17	2.64
Smoker	42476	chr7	102614165	C	T	*NFE4*	EXONIC-NC	ENSG00000230257:[ENST00000420058(n.197C>T)];	NA	NA	chr7*102614161*102614169*TCCTCAGCC*T*rs756444944*NA*NA*NA	NA	NA	NA	NA	NA	NA	NA	MODIFIER	NA	NA	63	63	0	86	76	9	10.47
Smoker	42476	chr7	100086001	G	C	*NYAP1*	MISSENSE	ENSG00000166924:MISSENSE[ENST00000454988(c.486G>C,atG>atC):ENSP00000394424(p.Met162Ile)];ENSG00000166924:MISSENSE[ENST00000423930(c.657G>C,atG>atC):ENSP00000411861(p.Met219Ile)];ENSG00000166924:MISSENSE[ENST00000300179(c.657G>C,atG>atC):ENSP00000300179(p.Met219Ile)];	NA	NA	NA	NA	NA	NA	NA	D	PrD	D	MODERATE	D	1	111	111	0	143	124	19	13.29
Smoker	42476	chr7	82545025	C	T	*PCLO*	MISSENSE	ENSG00000186472:MISSENSE[ENST00000437081(c.2437G>A,Gat>Aat):ENSP00000393760(p.Asp813Asn)];ENSG00000186472:MISSENSE[ENST00000333891(c.12277G>A,Gat>Aat):ENSP00000334319(p.Asp4093Asn)];ENSG00000186472:MISSENSE[ENST00000423517(c.12277G>A,Gat>Aat):ENSP00000388393(p.Asp4093Asn)];	NA	NA	NA	NA	NA	NA	NA	NA	PrD	NA	MODERATE	NA	NA	114	114	0	196	126	70	35.71
Smoker	42476	chr7	100205291	G	A	*PCOLCE*	SILENT	ENSG00000106333:SILENT[ENST00000223061(c.1044G>A,cgG>cgA):ENSP00000223061(p.Arg348(=))];ENSG00000106333:EXONIC-NC[ENST00000472348(n.586G>A)];ENSG00000106333:EXONIC-NC[ENST00000486440(n.690G>A)];	NA	NA	NA	NA	NA	NA	NA	NA	NA	NA	MODIFIER	NA	NA	107	107	0	184	156	28	15.22
Smoker	42476	chr7	102953441	C	T	*PMPCB*	INTRONIC	ENSG00000105819:[ENST00000444457(c.*1431+863C>T)];ENSG00000105819:[ENST00000420236(c.1090+863C>T)];ENSG00000105819:[ENST00000249269(c.*723C>T)];	NA	NA	NA	NA	NA	NA	NA	NA	NA	NA	MODIFIER	NA	NA	125	125	0	172	77	94	54.65
Smoker	42476	chr7	102963145	C	G	*PMPCB*	INTRONIC	ENSG00000105819:[ENST00000444457(c.*1432-6233C>G)];ENSG00000105819:[ENST00000420236(c.1091-6233C>G)];	NA	NA	NA	NA	NA	NA	NA	NA	NA	NA	MODIFIER	NA	NA	50	50	0	82	73	9	10.98
Smoker	42476	chr7	106710724	A	G	*PRKAR2B*	INTRONIC-SS-ACR	ENSG00000005249:INTRONIC-SS-ACR[ENST00000393613(n.363-2A>G)];ENSG00000005249:INTRONIC-SS-ACR[ENST00000265717(c.308-2A>G)];	NA	NA	NA	NA	NA	NA	NA	NA	NA	NA	HIGH	NA	NA	62	62	0	86	79	7	8.14
Smoker	42476	chr7	99047838	G	A	*PTCD1*	INTRONIC	ENSG00000106246:[ENST00000555673(c.121+9871C>T)];ENSG00000106246:[ENST00000430982(c.-26-14947C>T)];	NA	NA	NA	NA	NA	NA	NA	NA	NA	NA	MODIFIER	NA	NA	90	90	0	118	96	22	18.64
Smoker	42476	chr7	5779296	C	G	*RNF216*	INTRONIC	ENSG00000011275:[ENST00000389900(c.874-44G>C)];ENSG00000011275:[ENST00000389902(c.1045-313G>C)];ENSG00000011275:[ENST00000425013(c.874-313G>C)];	NA	NA	NA	NA	NA	NA	NA	NA	NA	NA	MODIFIER	NA	NA	124	124	0	97	71	26	26.8
Smoker	42476	chr7	123672536	C	T	*RP5-921G16.1*	INTRONIC	ENSG00000242593:[ENST00000484322(n.75+203C>T)];	NA	NA	NA	NA	NA	NA	NA	NA	NA	NA	LOW	NA	NA	73	73	0	62	51	11	17.74
Smoker	42476	chr7	149498903	T	A	*SSPO*	EXONIC-NC	ENSG00000197558:[ENST00000378016(n.7355T>A)];ENSG00000197558:[ENST00000484709(n.21T>A)];	NA	NA	chr7*149498903*149498903*T*C*rs2074689*1*0.7855*0.2145|chr7*149498903*149498930*TGGCCCTGCCTGGGCTTCCAGCCTCAAG*T*rs762816124*NA*NA*NA	NA	NA	NA	NA	NA	NA	NA	MODIFIER	NA	NA	75	75	0	123	51	71	57.72
Smoker	42476	chr7	74131336	C	G	*STAG3L2*	INTRONIC	ENSG00000160828:[ENST00000380775(n.1218+6248G>C)];	NA	NA	NA	NA	NA	NA	NA	NA	NA	NA	MODIFIER	NA	NA	26	26	0	34	28	5	14.71
Smoker	42476	chr7	98449030	G	C	*TMEM130*	INTRONIC	ENSG00000166448:[ENST00000345589(c.700+14C>G)];ENSG00000166448:[ENST00000546258(c.949+14C>G)];ENSG00000166448:[ENST00000416379(c.1006+14C>G)];ENSG00000166448:[ENST00000450876(c.754+14C>G)];ENSG00000166448:[ENST00000339375(c.1006+14C>G)];	NA	NA	chr7*98449030*98449030*G*A*rs111432171*0*0.9998*0.0001997	NA	NA	NA	NA	NA	NA	NA	MODIFIER	NA	NA	75	75	0	121	105	16	13.22

Mangalaparthi *et al.* , 2020. Mutational landscape of esophageal squamous cell carcinoma in an Indian cohort
Supplementary Table 4. List of somatic single nucleotide variants identified using whole exome sequencing of ESCC patients

Cohort	Sample	Chromosome	Genomic position	Reference allele	Alternate allele	Gene symbol	Variant type	Variant annotation	OncoMD	COSMIC	dbSNP	ExAC	ExAC_SAS	1000G	1000G_SAS	SIFT	Polyphen2	CONDEL	VEP	LRT	PhastCon7 Way	Total read (Adjacent normal)	Reference allel read support (Adjacent normal)	Alternative read support (Adjacent normal)	Total read (Tumor)	Reference allel read support (Tumor)	Alternative read support (Tumor)	Alternate allele frequency
Smoker	42476	chr7	123672536	C	T	*TMEM229A*	SILENT	ENSG00000234224:SILENT[ENST00000455783(c.522G>A,ctG>ctA):ENSP00000395244(p.Leu174(=))];	NA	NA	NA	NA	NA	NA	NA	NA	NA	NA	LOW	NA	NA	73	73	0	62	51	11	17.74
Smoker	42476	chr7	142448553	G	T	*TRBV29-1*	MISSENSE	ENSG00000232869:MISSENSE[ENST00000422143(c.147G>T,tgG>tgT):ENSP00000395459(p.Trp49Cys)];	NA	NA	chr7*142448553*142448553*G*A*rs761280802*NA*NA*NA	NA	NA	NA	NA	D	PrD	D	MODERATE	NA	NA	116	116	0	176	102	74	42.05
Smoker	42476	chr7	148875982	G	C	*ZNF398*	MISSENSE	ENSG00000197024:MISSENSE[ENST00000335901(c.505G>C,Gag>Cag):ENSP00000338984(p.Glu169Gln)];ENSG00000197024:MISSENSE[ENST00000483892(c.505G>C,Gag>Cag):ENSP00000418564(p.Glu169Gln)];ENSG00000197024:MISSENSE[ENST00000426851(c.505G>C,Gag>Cag):ENSP00000389972(p.Glu169Gln)];ENSG00000197024:MISSENSE[ENST00000420008(c.505G>C,Gag>Cag):ENSP00000416751(p.Glu169Gln)];ENSG00000197024:MISSENSE[ENST00000491174(c.505G>C,Gag>Cag):ENSP00000419391(p.Glu169Gln)];ENSG00000197024:MISSENSE[ENST00000475153(c.1018G>C,Gag>Cag):ENSP00000420418(p.Glu340Gln)];ENSG00000197024:MISSENSE[ENST00000540950(c.1033G>C,Gag>Cag):ENSP00000439340(p.Glu345Gln)];	NA	NA	NA	NA	NA	NA	NA	T	PoD	D	MODERATE	N	0.969	89	89	0	96	74	22	22.92
Smoker	42476	chr7	148979304	A	T	*ZNF783*	MISSENSE	ENSG00000204946:MISSENSE[ENST00000434415(c.1511A>T,aAc>aTc):ENSP00000410890(p.Asn504Ile)];ENSG00000204946:INTRONIC[ENST00000476295(c.802+3686A>T)];ENSG00000204946:INTRONIC[ENST00000378052(c.802+3686A>T)];ENSG00000204946:INTRONIC[ENST00000489518(n.240+3686A>T)];ENSG00000204946:INTRONIC[ENST00000481519(c.-102+3686A>T)];	NA	NA	chr7*148979304*148979304*A*G*rs375019406*NA*NA*NA	NA	NA	NA	NA	NA	NA	NA	MODIFIER	NA	NA	103	103	0	112	55	56	50
Smoker	42476	chr8	39613297	C	T	*ADAM2*	MISSENSE	ENSG00000104755:INTRONIC[ENST00000521880(c.1608+5403G>A)];ENSG00000104755:MISSENSE[ENST00000265708(c.1747G>A,Gac>Aac):ENSP00000265708(p.Asp583Asn)];ENSG00000104755:MISSENSE[ENST00000347580(c.1690G>A,Gac>Aac):ENSP00000343854(p.Asp564Asn)];ENSG00000104755:MISSENSE[ENST00000379853(c.1279G>A,Gac>Aac):ENSP00000369182(p.Asp427Asn)];	NA	NA	NA	NA	NA	NA	NA	T	PoD	N	MODERATE	NA	NA	61	61	0	56	37	19	33.93
Smoker	42476	chr8	132052290	C	G	*ADCY8*	MISSENSE	ENSG00000155897:MISSENSE[ENST00000286355(c.290G>C,gGa>gCa):ENSP00000286355(p.Gly97Ala)];ENSG00000155897:MISSENSE[ENST00000377928(c.290G>C,gGa>gCa):ENSP00000367161(p.Gly97Ala)];	NA	NA	NA	NA	NA	NA	NA	T	BN	N	MODERATE	N	0.165	36	36	0	27	21	6	22.22
Smoker	42476	chr8	68179136	C	T	*ARFGEF1*	SILENT	ENSG00000066777:SILENT[ENST00000262215(c.1896G>A,gtG>gtA):ENSP00000262215(p.Val632(=))];ENSG00000066777:SILENT[ENST00000520381(c.258G>A,gtG>gtA):ENSP00000428429(p.Val86(=))];	NA	NA	NA	NA	NA	NA	NA	NA	NA	NA	LOW	NA	NA	67	67	0	57	51	6	10.53
Smoker	42476	chr8	139729139	T	G	*COL22A1*	INTRONIC	ENSG00000169436:[ENST00000303045(c.2356-27A>C)];ENSG00000169436:[ENST00000341807(n.101-27A>C)];ENSG00000169436:[ENST00000435777(c.2356-27A>C)];	NA	NA	NA	NA	NA	NA	NA	NA	NA	NA	MODIFIER	NA	NA	111	111	0	167	143	24	14.37
Smoker	42476	chr8	120650774	G	C	*ENPP2*	INTRONIC-SS-ACR	ENSG00000136960:[ENST00000518958(c.34-7C>G)];ENSG00000136960:[ENST00000259486(c.34-7C>G)];ENSG00000136960:[ENST00000520066(c.34-7C>G)];ENSG00000136960:[ENST00000075322(c.34-7C>G)];ENSG00000136960:[ENST00000427067(c.22-7C>G)];ENSG00000136960:[ENST00000522826(c.34-7C>G)];	NA	NA	NA	NA	NA	NA	NA	NA	NA	NA	LOW	NA	NA	143	143	0	188	107	80	42.55
Smoker	42476	chr8	125107136	G	T	*FER1L6*	INTRONIC-SS-ACR	ENSG00000214814:INTRONIC-SS-ACR[ENST00000522917(c.4553-1G>T)];ENSG00000214814:INTRONIC-SS-ACR[ENST00000399018(c.4553-1G>T)];	NA	NA	NA	NA	NA	NA	NA	NA	NA	NA	MODIFIER	NA	NA	44	44	0	51	40	11	21.57
Smoker	42476	chr8	125107136	G	T	*FER1L6-AS2*	INTRONIC	ENSG00000253868:[ENST00000520031(n.96-34103C>A)];	NA	NA	NA	NA	NA	NA	NA	NA	NA	NA	MODIFIER	NA	NA	44	44	0	51	40	11	21.57
Smoker	42476	chr8	104336793	C	G	*FZD6*	SILENT	ENSG00000164930:INTRONIC[ENST00000519011(c.178-489C>G)];ENSG00000164930:INTRONIC[ENST00000540287(c.33-489C>G)];ENSG00000164930:INTRONIC[ENST00000523933(c.279-470C>G)];ENSG00000164930:SILENT[ENST00000358755(c.459C>G,gtC>gtG):ENSP00000351605(p.Val153(=))];ENSG00000164930:SILENT[ENST00000522484(c.459C>G,gtC>gtG):ENSP00000428301(p.Val153(=))];ENSG00000164930:SILENT[ENST00000522566(c.459C>G,gtC>gtG):ENSP00000429055(p.Val153(=))];ENSG00000164930:SILENT[ENST00000523739(c.363C>G,gtC>gtG):ENSP00000429528(p.Val121(=))];ENSG00000164930:3UTR[ENST00000521195(c.*394C>G)];	NA	NA	NA	NA	NA	NA	NA	NA	NA	NA	LOW	NA	NA	134	134	0	127	73	54	42.52
Smoker	42476	chr8	122627101	C	G	*HAS2*	MISSENSE	ENSG00000170961:MISSENSE[ENST00000303924(c.907G>C,Gaa>Caa):ENSP00000306991(p.Glu303Gln)];	NA	NA	NA	NA	NA	NA	NA	T	BN	N	MODERATE	D	0.998	172	172	0	229	200	29	12.66
Smoker	42476	chr8	43016728	C	G	*HGSNAT*	INTRONIC	ENSG00000165102:[ENST00000517319(c.*132+78C>G)];ENSG00000165102:[ENST00000458501(c.647+78C>G)];ENSG00000165102:[ENST00000379644(c.563+78C>G)];ENSG00000165102:[ENST00000520704(c.413+78C>G)];	NA	NA	NA	NA	NA	NA	NA	NA	NA	NA	MODIFIER	NA	NA	92	90	0	71	58	13	18.31
Smoker	42476	chr8	144512462	C	G	*MAFA*	MISSENSE	ENSG00000182759:MISSENSE[ENST00000333480(c.115G>C,Gag>Cag):ENSP00000328364(p.Glu39Gln)];	NA	NA	chr8*144512462*144512462*C*A*rs868758709*NA*NA*NA	NA	NA	NA	NA	T	UN	NA	MODERATE	U	0.999	52	52	0	55	44	11	20
Smoker	42476	chr8	48874617	C	G	*MCM4*	MISSENSE	ENSG00000104738:EXONIC-NC[ENST00000520934(n.529C>G)];ENSG00000104738:EXONIC-NC[ENST00000519138(n.201C>G)];ENSG00000104738:MISSENSE[ENST00000518221(c.240C>G,atC>atG):ENSP00000430329(p.Ile80Met)];ENSG00000104738:MISSENSE[ENST00000262105(c.240C>G,atC>atG):ENSP00000262105(p.Ile80Met)];ENSG00000104738:MISSENSE[ENST00000519170(c.90C>G,atC>atG):ENSP00000428833(p.Ile30Met)];ENSG00000104738:MISSENSE[ENST00000523944(c.240C>G,atC>atG):ENSP00000430194(p.Ile80Met)];ENSG00000104738:INTRONIC[ENST00000524086(c.236-21C>G)];	NA	NA	chr8*48874617*48874617*C*T*rs148927296*NA*NA*NA	NA	NA	NA	NA	T	BN	N	MODERATE	N	0.994	62	62	0	43	31	12	27.91
Smoker	42476	chr8	27931956	C	G	*NUGGC*	5UTR	ENSG00000189233:[ENST00000413272(c.-29G>C)];ENSG00000189233:[ENST00000418860(c.-29G>C)];ENSG00000189233:[ENST00000341513(c.-29G>C)];	NA	NA	chr8*27931956*27931956*C*A*rs757131299*NA*NA*NA	NA	NA	NA	NA	NA	NA	NA	MODIFIER	NA	NA	93	93	0	90	84	6	6.67
Smoker	42476	chr8	105839748	G	C	*RP11-127H5.1*	INTRONIC	ENSG00000253350:[ENST00000518180(c.6+112545G>C)];ENSG00000253350:[ENST00000521923(c.128+42G>C)];	NA	NA	NA	NA	NA	NA	NA	NA	NA	NA	MODIFIER	NA	NA	59	59	0	53	42	11	20.75
Smoker	42476	chr8	145557168	G	C	*SCRT1*	SILENT	ENSG00000170616:SILENT[ENST00000332135(c.726C>G,ctC>ctG):ENSP00000331692(p.Leu242(=))];	NA	NA	chr8*145557168*145557168*G*A*rs781832749*NA*NA*NA	NA	NA	NA	NA	NA	NA	NA	LOW	NA	NA	62	62	0	94	86	8	8.51
Smoker	42476	chr8	70533314	C	G	*SULF1*	MISSENSE	ENSG00000137573:MISSENSE[ENST00000260128(c.1422C>G,caC>caG):ENSP00000260128(p.His474Gln)];ENSG00000137573:MISSENSE[ENST00000402687(c.1422C>G,caC>caG):ENSP00000385704(p.His474Gln)];ENSG00000137573:MISSENSE[ENST00000419716(c.1422C>G,caC>caG):ENSP00000390315(p.His474Gln)];ENSG00000137573:MISSENSE[ENST00000458141(c.1422C>G,caC>caG):ENSP00000403040(p.His474Gln)];ENSG00000137573:EXONIC-NC[ENST00000521946(n.762C>G)];	chr8*70533	NA	NA	NA	NA	NA	NA	NA	NA	NA	MODIFIER	NA	NA	153	153	0	196	172	24	12.24

Mangalaparthi *et al.* , 2020. Mutational landscape of esophageal squamous cell carcinoma in an Indian cohort
Supplementary Table 4. List of somatic single nucleotide variants identified using whole exome sequencing of ESCC patients

Cohort	Sample	Chromosome	Genomic position	Reference allele	Alternate allele	Gene symbol	Variant type	Variant annotation	OncoMD	COSMIC	dbSNP	ExAC	ExAC_SAS	1000G	1000G_SAS	SIFT	Polyphen2	CONDEL	VEP	LRT	PhastCon7 Way	Total read (Adjacent normal)	Reference allel read support (Adjacent normal)	Alternative read support (Adjacent normal)	Total read (Tumor)	Reference allel read support (Tumor)	Alternative read support (Tumor)	Alternate allele frequency
Smoker	42476	chr8	70533324	G	T	*SULF1*	NONSENSE	ENSG00000137573:NONSENSE[ENST00000260128(c.1432G>T,Gga>Tga):ENSP00000260128(p.Gly478Ter)];ENSG00000137573:NONSENSE[ENST00000402687(c.1432G>T,Gga>Tga):ENSP00000385704(p.Gly478Ter)];ENSG00000137573:NONSENSE[ENST00000419716(c.1432G>T,Gga>Tga):ENSP00000390315(p.Gly478Ter)];ENSG00000137573:NONSENSE[ENST00000458141(c.1432G>T,Gga>Tga):ENSP00000403040(p.Gly478Ter)];ENSG00000137573:EXONIC-NC[ENST00000521946(n.772G>T)];	NA	NA	chr8*70533324*70533324*G*A*rs773425918*NA*NA*NA	NA	NA	NA	NA	NA	NA	NA	MODIFIER	NA	NA	154	154	0	186	163	23	12.37
Smoker	42476	chr8	30703512	G	A	*TEX15*	MISSENSE	ENSG00000133863:MISSENSE[ENST00000256246(c.3022C>T,Cat>Tat):ENSP00000256246(p.His1008Tyr)];	NA	NA	NA	NA	NA	NA	NA	D	PrD	D	MODERATE	N	0.992	87	86	0	73	51	22	30.14
Smoker	42476	chr9	88248144	G	A	*AGTPBP1*	MISSENSE	ENSG00000135049:MISSENSE[ENST00000357081(c.1448C>T,tCt>tTt):ENSP00000349592(p.Ser483Phe)];ENSG00000135049:MISSENSE[ENST00000376083(c.1328C>T,tCt>tTt):ENSP00000365251(p.Ser443Phe)];ENSG00000135049:MISSENSE[ENST00000376109(c.1484C>T,tCt>tTt):ENSP00000365277(p.Ser495Phe)];ENSG00000135049:MISSENSE[ENST00000432218(c.962C>T,tCt>tTt):ENSP00000402804(p.Ser321Phe)];ENSG00000135049:3UTR[ENST00000337006(c.*549C>T)];	NA	NA	NA	NA	NA	NA	NA	T	BN	N	MODERATE	N	0.256	134	134	0	110	83	26	23.64
Smoker	42476	chr9	112899775	G	A	*AKAP2*	MISSENSE	ENSG00000241978:MISSENSE[ENST00000480388(c.1405G>A,Gag>Aag):ENSP00000419268(p.Glu469Lys)];ENSG00000241978:MISSENSE[ENST00000374525(c.1525G>A,Gag>Aag):ENSP00000363649(p.Glu509Lys)];ENSG00000241978:MISSENSE[ENST00000259318(c.1258G>A,Gag>Aag):ENSP00000259318(p.Glu420Lys)];ENSG00000241978:MISSENSE[ENST00000434623(c.1525G>A,Gag>Aag):ENSP00000404782(p.Glu509Lys)];ENSG00000241978:MISSENSE[ENST00000510514(c.1951G>A,Gag>Aag):ENSP00000421522(p.Glu651Lys)];ENSG00000241978:MISSENSE[ENST00000555236(c.1951G>A,Gag>Aag):ENSP00000451476(p.Glu651Lys)];ENSG00000241978:INTRONIC[ENST00000495980(c.157-18824G>A)];	NA	NA	NA	NA	NA	NA	NA	T	PrD	N	MODERATE	NA	0.95	65	65	0	67	6	61	91.04
Smoker	42476	chr9	72090884	A	G	*APBA1*	INTRONIC	ENSG00000107282:[ENST00000265381(c.1296+80T>C)];	NA	NA	NA	NA	NA	NA	NA	NA	NA	NA	MODIFIER	NA	NA	62	62	0	48	43	5	10.42
Smoker	42476	chr9	123253728	G	A	*CDK5RAP2*	MISSENSE	ENSG00000136861:MISSENSE[ENST00000349780(c.1339C>T,Cgc>Tgc):ENSP00000343818(p.Arg447Cys)];ENSG00000136861:MISSENSE[ENST00000360822(c.1339C>T,Cgc>Tgc):ENSP00000354065(p.Arg447Cys)];ENSG00000136861:MISSENSE[ENST00000480112(c.1336C>T,Cgc>Tgc):ENSP00000418418(p.Arg446Cys)];ENSG00000136861:MISSENSE[ENST00000360190(c.1339C>T,Cgc>Tgc):ENSP00000353317(p.Arg447Cys)];ENSG00000136861:MISSENSE[ENST00000359309(c.1339C>T,Cgc>Tgc):ENSP00000352258(p.Arg447Cys)];ENSG00000136861:EXONIC-NC[ENST00000483412(n.743C>T)];ENSG00000136861:3UTR[ENST00000473282(c.*83C>T)];	NA	NA	NA	9.26E-05	0.000122444	NA	NA	NA	NA	NA	MODIFIER	NA	NA	39	39	0	48	43	5	10.42
Smoker	42476	chr9	123924397	G	C	*CNTRL*	MISSENSE	ENSG00000119397:MISSENSE[ENST00000373850(c.3615G>C,caG>caC):ENSP00000362956(p.Gln1205His)];ENSG00000119397:MISSENSE[ENST00000373855(c.5271G>C,caG>caC):ENSP00000362962(p.Gln1757His)];ENSG00000119397:MISSENSE[ENST00000238341(c.5271G>C,caG>caC):ENSP00000238341(p.Gln1757His)];ENSG00000119397:EXONIC-NC[ENST00000373845(n.1750G>C)];	NA	NA	NA	NA	NA	NA	NA	NA	PoD	NA	MODERATE	NA	NA	178	178	0	153	129	24	15.69
Smoker	42476	chr9	137645738	G	C	*COL5A1*	MISSENSE	ENSG00000130635:MISSENSE[ENST00000371817(c.1762G>C,Gtg>Ctg):ENSP00000360882(p.Val588Leu)];	NA	NA	NA	NA	NA	NA	NA	NA	BN	NA	MODERATE	U	0.998	36	36	0	30	23	7	23.33
Smoker	42476	chr9	90344632	T	C	*CTSL*	MISSENSE	ENSG00000135047:MISSENSE[ENST00000340342(c.766T>C,Ttc>Ctc):ENSP00000365061(p.Phe256Leu)];ENSG00000135047:MISSENSE[ENST00000343150(c.766T>C,Ttc>Ctc):ENSP00000345344(p.Phe256Leu)];ENSG00000135047:EXONIC-NC[ENST00000375894(n.773T>C)];ENSG00000135047:EXONIC-NC[ENST00000495822(n.466T>C)];	NA	NA	NA	NA	NA	NA	NA	NA	NA	NA	MODIFIER	NA	NA	116	116	0	120	101	19	15.83
Smoker	42476	chr9	98643196	A	G	*ERCC6L2*	MISSENSE	ENSG00000182150:EXONIC-NC[ENST00000466840(n.1009A>G)];ENSG00000182150:MISSENSE[ENST00000288985(c.125A>G,aAt>aGt):ENSP00000288985(p.Asn42Ser)];	NA	NA	NA	NA	NA	NA	NA	T_lc	BN	N	MODERATE	N	0.529	105	105	0	86	70	16	18.6
Smoker	42476	chr9	79117604	T	A	*GCNT1*	MISSENSE	ENSG00000187210:MISSENSE[ENST00000536223(c.307T>A,Ttc>Atc):ENSP00000440883(p.Phe103Ile)];ENSG00000187210:MISSENSE[ENST00000376730(c.307T>A,Ttc>Atc):ENSP00000365920(p.Phe103Ile)];ENSG00000187210:MISSENSE[ENST00000442371(c.307T>A,Ttc>Atc):ENSP00000415454(p.Phe103Ile)];ENSG00000187210:MISSENSE[ENST00000444201(c.307T>A,Ttc>Atc):ENSP00000390703(p.Phe103Ile)];	NA	NA	NA	NA	NA	NA	NA	D	PrD	D	MODERATE	D	0.866	158	157	0	135	27	108	80
Smoker	42476	chr9	99721170	C	G	*HIATL2*	MISSENSE	ENSG00000196312:MISSENSE[ENST00000602917(c.214G>C,Gaa>Caa):ENSP00000473444(p.Glu72Gln)];ENSG00000196312:MISSENSE[ENST00000375223(c.214G>C,Gaa>Caa):ENSP00000364371(p.Glu72Gln)];	NA	NA	NA	NA	NA	NA	NA	T	BN	N	MODERATE	U	1	104	103	0	85	75	10	11.76
Smoker	42476	chr9	27029130	G	C	*IFT74*	INTRONIC	ENSG00000096872:[ENST00000429045(c.1054+28G>C)];ENSG00000096872:[ENST00000380062(c.1054+28G>C)];ENSG00000096872:[ENST00000443698(c.1054+28G>C)];ENSG00000096872:[ENST00000482986(n.297+28G>C)];ENSG00000096872:[ENST00000433700(c.1054+28G>C)];	NA	NA	NA	NA	NA	NA	NA	NA	NA	NA	MODIFIER	NA	NA	68	68	0	38	26	12	31.58
Smoker	42476	chr9	112899775	G	A	*PALM2-AKAP2*	MISSENSE	ENSG00000157654:MISSENSE[ENST00000374530(c.1951G>A,Gag>Aag):ENSP00000363654(p.Glu651Lys)];ENSG00000157654:MISSENSE[ENST00000302798(c.1951G>A,Gag>Aag):ENSP00000305861(p.Glu651Lys)];	NA	NA	NA	NA	NA	NA	NA	T	PrD	N	MODERATE	NA	0.95	65	65	0	67	6	61	91.04
Smoker	42476	chr9	72090884	A	G	*RP11-470P21.2*	INTRONIC	ENSG00000229312:[ENST00000429567(n.215+1616A>G)];	NA	NA	NA	NA	NA	NA	NA	NA	NA	NA	MODIFIER	NA	NA	62	62	0	48	43	5	10.42
Smoker	42476	chr9	131360792	C	T	*SPTAN1*	INTRONIC-SS-DNR	ENSG00000197694:[ENST00000372739(c.3519+9C>T)];ENSG00000197694:[ENST00000358161(c.3519+9C>T)];ENSG00000197694:[ENST00000475367(n.1495+9C>T)];ENSG00000197694:[ENST00000372731(c.3519+9C>T)];	NA	NA	NA	NA	NA	NA	NA	NA	NA	NA	MODIFIER	NA	NA	31	31	0	38	31	7	18.42
Smoker	42476	chr9	125674031	G	A	*ZBTB6*	SILENT	ENSG00000186130:SILENT[ENST00000373659(c.321C>T,gcC>gcT):ENSP00000362763(p.Ala107(=))];	NA	NA	NA	NA	NA	NA	NA	NA	NA	NA	LOW	NA	NA	170	170	0	187	150	37	19.79
Smoker	42476	chrX	83129222	G	C	*CYLC1*	MISSENSE	ENSG00000183035:MISSENSE[ENST00000329312(c.1506G>C,aaG>aaC):ENSP00000331556(p.Lys502Asn)];	chrX*83129	chrX*83129	chrX*83129222*83129222*G*T*rs768170993*NA*NA*NA	NA	NA	NA	NA	T	BN	N	MODERATE	NA	NA	43	43	0	59	39	20	33.9
Smoker	42476	chrX	147062971	C	A	*FMR1NB*	MISSENSE	ENSG00000176988:MISSENSE[ENST00000370467(c.49C>A,Cgt>Agt):ENSP00000359498(p.Arg17Ser)];	NA	NA	chrX*147062971*147062971*C*T*rs782607537*NA*NA*NA	NA	NA	NA	NA	D	BN	N	MODERATE	NA	NA	34	34	0	34	19	15	44.12
Smoker	42476	chrX	44401921	G	C	*FUNDC1*	INTRONIC	ENSG00000069509:[ENST00000378045(c.28+130C>G)];	NA	NA	NA	NA	NA	NA	NA	NA	NA	NA	MODIFIER	NA	NA	16	16	0	25	20	5	20
Smoker	42476	chrX	132091119	C	G	*HS6ST2*	MISSENSE	ENSG00000171004:MISSENSE[ENST00000521489(c.664G>C,Gac>Cac):ENSP00000429473(p.Asp222His)];ENSG00000171004:MISSENSE[ENST00000370836(c.664G>C,Gac>Cac):ENSP00000359873(p.Asp222His)];ENSG00000171004:MISSENSE[ENST00000370833(c.226G>C,Gac>Cac):ENSP00000359870(p.Asp76His)];	NA	NA	NA	NA	NA	NA	NA	T	PrD	D	MODERATE	D	1	88	88	0	73	66	7	9.59

Mangalaparthi *et al.* , 2020. Mutational landscape of esophageal squamous cell carcinoma in an Indian cohort
Supplementary Table 4. List of somatic single nucleotide variants identified using whole exome sequencing of ESCC patients

Cohort	Sample	Chromosome	Genomic position	Reference allele	Alternate allele	Gene symbol	Variant type	Variant annotation	OncoMD	COSMIC	dbSNP	ExAC	ExAC_SAS	1000G	1000G_SAS	SIFT	Polyphen2	CONDEL	VEP	LRT	PhastCon7 Way	Total read (Adjacent normal)	Reference allel read support (Adjacent normal)	Alternative read support (Adjacent normal)	Total read (Tumor)	Reference allel read support (Tumor)	Alternative read support (Tumor)	Alternate allele frequency
Smoker	42476	chrX	69623864	G	C	*KIF4A*	MISSENSE	ENSG00000090889:MISSENSE[ENST00000374403(c.2770G>C,Gag>Cag):ENSP00000363524(p.Glu924Gln)];ENSG00000090889:MISSENSE[ENST00000374388(c.2770G>C,Gag>Cag):ENSP00000363509(p.Glu924Gln)];	NA	NA	NA	NA	NA	NA	NA	T	PoD	N	MODERATE	D	0.823	64	64	0	65	46	19	29.23
Smoker	42476	chrX	21674295	A	C	*KLHL34*	MISSENSE	ENSG00000185915:MISSENSE[ENST00000379499(c.1612T>G,Ttc>Gtc):ENSP00000368813(p.Phe538Val)];	NA	NA	chrX*21674278*21674305*GCGCTCGATCTCAGAGAAGGGCTCGTAT*G*rs748193988*NA*NA*NA	NA	NA	NA	NA	D	BN	D	MODERATE	D	1	56	56	0	58	45	12	20.69
Smoker	42476	chrX	131351195	G	A	*RAP2C*	SILENT	ENSG00000123728:SILENT[ENST00000370874(c.102C>T,ccC>ccT):ENSP00000359911(p.Pro34(=))];ENSG00000123728:SILENT[ENST00000342983(c.102C>T,ccC>ccT):ENSP00000340274(p.Pro34(=))];ENSG00000123728:INTRONIC[ENST00000460462(n.192-11C>T)];	NA	NA	NA	NA	NA	NA	NA	NA	NA	NA	MODIFIER	NA	NA	34	34	0	46	4	42	91.3
Smoker	42476	chrX	131351195	G	A	*RAP2C-AS1*	EXONIC-NC	ENSG00000232160:[ENST00000421483(n.21G>A)];	NA	NA	NA	NA	NA	NA	NA	NA	NA	NA	MODIFIER	NA	NA	34	34	0	46	4	42	91.3
Smoker	42477	chr1	94485254	G	A	*ABCA4*	MISSENSE	ENSG00000198691:MISSENSE[ENST00000370225(c.5080C>T,Cca>Tca):ENSP00000359245(p.Pro1694Ser)];ENSG00000198691:EXONIC-NC[ENST00000460514(n.574C>T)];ENSG00000198691:EXONIC-NC[ENST00000470771(n.190C>T)];	NA	NA	NA	NA	NA	NA	NA	NA	NA	NA	MODIFIER	NA	NA	66	64	1	50	40	10	20
Smoker	42477	chr1	145556632	G	A	*ANKRD35*	MISSENSE	ENSG00000198483:MISSENSE[ENST00000355594(c.202G>A,Gaa>Aaa):ENSP00000347802(p.Glu68Lys)];ENSG00000198483:MISSENSE[ENST00000544626(c.202G>A,Gaa>Aaa):ENSP00000442671(p.Glu68Lys)];	NA	NA	NA	NA	NA	NA	NA	D	PrD	D	MODERATE	NA	NA	46	46	0	71	50	21	29.58
Smoker	42477	chr1	43652353	G	A	*EBNA1BP2*	INTRONIC	ENSG00000117395:[ENST00000461557(n.234-13819C>T)];ENSG00000117395:[ENST00000466927(n.198-4898C>T)];	NA	NA	NA	NA	NA	NA	NA	NA	NA	NA	MODIFIER	NA	NA	82	82	0	129	117	12	9.3
Smoker	42477	chr1	79472166	A	C	*ELTD1*	INTRONIC	ENSG00000162618:[ENST00000370742(c.22+152T>G)];	NA	NA	NA	NA	NA	NA	NA	NA	NA	NA	MODIFIER	NA	NA	67	67	0	83	74	9	10.84
Smoker	42477	chr1	187610744	T	C	*ERVMER61-1*	INTRONIC-SS-ACR	ENSG00000230426:[ENST00000429725(n.151-6T>C)];	NA	NA	NA	5.77E-05	0	NA	NA	NA	NA	NA	LOW	NA	NA	111	111	0	113	91	21	18.58
Smoker	42477	chr1	101339779	C	G	*EXTL2*	MISSENSE	ENSG00000162694:MISSENSE[ENST00000370113(c.712G>C,Gat>Cat):ENSP00000359131(p.Asp238His)];ENSG00000162694:MISSENSE[ENST00000370114(c.712G>C,Gat>Cat):ENSP00000359132(p.Asp238His)];ENSG00000162694:MISSENSE[ENST00000450240(c.736G>C,Gat>Cat):ENSP00000403363(p.Asp246His)];ENSG00000162694:MISSENSE[ENST00000535414(c.673G>C,Gat>Cat):ENSP00000444385(p.Asp225His)];	NA	NA	chr1*101339776*101339780*CATCT*C*rs747720871*NA*NA*NA	NA	NA	NA	NA	NA	PrD	NA	MODERATE	D	0.98	51	51	0	76	60	16	21.05
Smoker	42477	chr1	240370270	G	C	*FMN2*	MISSENSE	ENSG00000155816:MISSENSE[ENST00000447095(c.469G>C,Gat>Cat):ENSP00000409308(p.Asp157His)];ENSG00000155816:MISSENSE[ENST00000319653(c.2158G>C,Gat>Cat):ENSP00000318884(p.Asp720His)];	NA	NA	chr1*240370270*240370270*G*A*rs757718908*NA*NA*NA	NA	NA	NA	NA	NA	UN	NA	MODERATE	NA	NA	82	81	0	136	122	14	10.29
Smoker	42477	chr1	42693619	C	G	*FOXJ3*	MISSENSE	ENSG00000198815:MISSENSE[ENST00000372572(c.463G>C,Gac>Cac):ENSP00000361653(p.Asp155His)];ENSG00000198815:MISSENSE[ENST00000361776(c.463G>C,Gac>Cac):ENSP00000354449(p.Asp155His)];ENSG00000198815:MISSENSE[ENST00000372573(c.463G>C,Gac>Cac):ENSP00000361654(p.Asp155His)];ENSG00000198815:MISSENSE[ENST00000545068(c.463G>C,Gac>Cac):ENSP00000439044(p.Asp155His)];ENSG00000198815:MISSENSE[ENST00000361346(c.463G>C,Gac>Cac):ENSP00000354620(p.Asp155His)];ENSG00000198815:MISSENSE[ENST00000445886(c.463G>C,Gac>Cac):ENSP00000393408(p.Asp155His)];	chr1*42693	chr1*42693	NA	NA	NA	NA	NA	D	PrD	D	MODERATE	NA	NA	138	138	0	165	134	31	18.79
Smoker	42477	chr1	74665467	C	A	*FPGT*	MISSENSE	ENSG00000254685:MISSENSE[ENST00000529485(c.202C>A,Ctt>Att):ENSP00000431733(p.Leu68Ile)];ENSG00000254685:MISSENSE[ENST00000370898(c.241C>A,Ctt>Att):ENSP00000359935(p.Leu81Ile)];ENSG00000254685:MISSENSE[ENST00000609362(c.202C>A,Ctt>Att):ENSP00000476680(p.Leu68Ile)];ENSG00000254685:MISSENSE[ENST00000370894(c.202C>A,Ctt>Att):ENSP00000359931(p.Leu68Ile)];ENSG00000254685:MISSENSE[ENST00000482102(c.268C>A,Ctt>Att):ENSP00000433044(p.Leu90Ile)];ENSG00000254685:MISSENSE[ENST00000467578(c.241C>A,Ctt>Att):ENSP00000434934(p.Leu81Ile)];ENSG00000254685:MISSENSE[ENST00000534056(c.202C>A,Ctt>Att):ENSP00000432819(p.Leu68Ile)];ENSG00000254685:MISSENSE[ENST00000472069(c.196C>A,Ctt>Att):ENSP00000433499(p.Leu66Ile)];ENSG00000254685:EXONIC-NC[ENST00000524915(n.241C>A)];	chr1*74665	chr1*74665	chr1*74665467*74665467*C*T*rs754999716*NA*NA*NA	NA	NA	NA	NA	D_lc	BN	N	MODERATE	NA	0.985	178	178	0	204	183	20	9.8
Smoker	42477	chr1	74665467	C	A	*FPGT-TNNI3K*	MISSENSE	ENSG00000259030:MISSENSE[ENST00000370893(c.202C>A,Ctt>Att):ENSP00000359930(p.Leu68Ile)];ENSG00000259030:MISSENSE[ENST00000370899(c.202C>A,Ctt>Att):ENSP00000359936(p.Leu68Ile)];ENSG00000259030:MISSENSE[ENST00000370895(c.202C>A,Ctt>Att):ENSP00000359932(p.Leu68Ile)];ENSG00000259030:MISSENSE[ENST00000534632(c.202C>A,Ctt>Att):ENSP00000433587(p.Leu68Ile)];ENSG00000259030:MISSENSE[ENST00000557284(c.241C>A,Ctt>Att):ENSP00000450895(p.Leu81Ile)];ENSG00000259030:EXONIC-NC[ENST00000533006(n.228C>A)];	chr1*74665	chr1*74665	chr1*74665467*74665467*C*T*rs754999716*NA*NA*NA	NA	NA	NA	NA	D_lc	BN	N	MODERATE	NA	0.985	178	178	0	204	183	20	9.8
Smoker	42477	chr1	18704861	C	G	*IGSF21*	3UTR	ENSG00000117154:[ENST00000251296(c.*41C>G)];ENSG00000117154:[ENST00000497331(n.1769C>G)];	NA	NA	chr1*18704860*18704862*CCA*CTG*rs386629125*NA*NA*NA|chr1*18704861*18704861*C*T*rs11806254*1*0.9846*0.01538|chr1*18704861*18704863*CAC*CGT*rs386629126*NA*NA*NA	NA	NA	NA	NA	NA	NA	NA	MODIFIER	NA	NA	86	86	0	111	96	15	13.51
Smoker	42477	chr1	6674134	C	G	*KLHL21*	INTRONIC	ENSG00000162413:[ENST00000463043(c.-81+376G>C)];ENSG00000162413:[ENST00000467612(c.-81+376G>C)];	NA	NA	NA	NA	NA	NA	NA	NA	NA	NA	MODIFIER	NA	NA	48	48	0	22	17	5	22.73
Smoker	42477	chr1	235901886	C	T	*LDHAP2*	EXONIC-NC	ENSG00000235674:[ENST00000437325(n.582C>T)];	NA	NA	NA	NA	NA	NA	NA	NA	NA	NA	MODIFIER	NA	NA	144	144	0	186	150	36	19.35
Smoker	42477	chr1	54432113	C	T	*LRRC42*	INTRONIC	ENSG00000116212:[ENST00000477905(n.542+60C>T)];ENSG00000116212:[ENST00000319223(c.1012+60C>T)];ENSG00000116212:[ENST00000371370(c.1012+60C>T)];	NA	NA	0.0001997	NA	NA	0.0001997	0	NA	NA	NA	MODIFIER	NA	NA	59	59	0	99	80	19	19.19
Smoker	42477	chr1	235901886	C	T	*LYST*	EXONIC-NC	ENSG00000143669:[ENST00000461526(n.270G>A)];ENSG00000143669:[ENST00000389793(c.8358+2836G>A)];ENSG00000143669:[ENST00000389794(c.8358+2836G>A)];	NA	NA	NA	NA	NA	NA	NA	NA	NA	NA	MODIFIER	NA	NA	144	144	0	186	150	36	19.35
Smoker	42477	chr1	236144918	C	A	*NID1*	MISSENSE	ENSG00000116962:MISSENSE[ENST00000264187(c.3220G>T,Gtg>Ttg):ENSP00000264187(p.Val1074Leu)];ENSG00000116962:MISSENSE[ENST00000366595(c.2821G>T,Gtg>Ttg):ENSP00000355554(p.Val941Leu)];	NA	NA	NA	NA	NA	NA	NA	T	BN	N	MODERATE	N	0.816	94	94	0	111	93	18	16.22
Smoker	42477	chr1	161989834	C	G	*OLFML2B*	MISSENSE	ENSG00000162745:MISSENSE[ENST00000367940(c.313G>C,Gtg>Ctg):ENSP00000356917(p.Val105Leu)];ENSG00000162745:MISSENSE[ENST00000294794(c.313G>C,Gtg>Ctg):ENSP00000294794(p.Val105Leu)];	NA	NA	.	4.94E-05	0	NA	NA	D	PrD	D	MODERATE	NA	NA	113	112	0	131	109	22	16.79

Supplementary Table 4. List of somatic single nucleotide variants identified using whole exome sequencing of ESCC patients

Cohort	Sample	Chromosome	Genomic position	Reference allele	Alternate allele	Gene symbol	Variant type	Variant annotation	OncoMD	COSMIC	dbSNP	ExAC	ExAC_SAS	1000G	1000G_SAS	SIFT	Polyphen2	CONDEL	VEP	LRT	PhastCon7 Way	Total read (Adjacent normal)	Reference allel read support (Adjacent normal)	Alternative read support (Adjacent normal)	Total read (Tumor)	Reference allel read support (Tumor)	Alternative read support (Tumor)	Alternate allele frequency
Smoker	42477	chr1	36913483	C	G	*OSCP1*	MISSENSE	ENSG00000116885:INTRONIC[ENST00000315643(c.112+2376G>C)];ENSG00000116885:INTRONIC[ENST00000356637(c.112+2376G>C)];ENSG00000116885:INTRONIC[ENST00000235532(c.112+2376G>C)];ENSG00000116885:INTRONIC[ENST00000354267(c.112+2376G>C)];ENSG00000116885:INTRONIC[ENST00000445843(c.22+2376G>C)];ENSG00000116885:MISSENSE[ENST00000524789(c.36G>C,Gac>Cac):ENSP00000434268(p.Asp13His)];	NA	NA	NA	NA	NA	NA	NA	NA	UN	NA	MODERATE	NA	NA	58	58	0	81	66	15	18.52
Smoker	42477	chr1	6674134	C	G	*PHF13*	MISSENSE	ENSG00000116273:MISSENSE[ENST00000377648(c.8C>G,tCt>tGt):ENSP00000366876(p.Ser3Cys)];ENSG00000116273:EXONIC-NC[ENST00000495385(n.313C>G)];	NA	NA	NA	NA	NA	NA	NA	NA	NA	NA	MODIFIER	NA	NA	48	48	0	22	17	5	22.73
Smoker	42477	chr1	3329297	C	T	*PRDM16*	MISSENSE	ENSG00000142611:MISSENSE[ENST00000441472(c.2536C>T,Cgg>Tgg):ENSP00000407968(p.Arg846Trp)];ENSG00000142611:MISSENSE[ENST00000514189(c.2539C>T,Cgg>Tgg):ENSP00000421400(p.Arg847Trp)];ENSG00000142611:MISSENSE[ENST00000509860(c.1963C>T,Cgg>Tgg):ENSP00000425796(p.Arg655Trp)];ENSG00000142611:MISSENSE[ENST00000270722(c.2536C>T,Cgg>Tgg):ENSP00000270722(p.Arg846Trp)];ENSG00000142611:MISSENSE[ENST00000442529(c.2536C>T,Cgg>Tgg):ENSP00000405253(p.Arg846Trp)];ENSG00000142611:MISSENSE[ENST00000511072(c.2539C>T,Cgg>Tgg):ENSP00000426975(p.Arg847Trp)];ENSG00000142611:MISSENSE[ENST00000378391(c.2536C>T,Cgg>Tgg):ENSP00000367643(p.Arg846Trp)];ENSG00000142611:MISSENSE[ENST00000378398(c.2539C>T,Cgg>Tgg):ENSP00000367651(p.Arg847Trp)];ENSG00000142611:EXONIC-NC[ENST00000512462(n.2314C>T)];	NA	NA	NA	0	0	NA	NA	NA	NA	NA	MODIFIER	NA	NA	60	60	0	61	40	21	34.43
Smoker	42477	chr1	45293468	C	G	*PTCH2*	INTRONIC	ENSG00000117425:[ENST00000447098(c.2058+47G>C)];ENSG00000117425:[ENST00000372192(c.2058+47G>C)];	NA	NA	chr1*45293468*45293468*C*T*rs373001101*NA*NA*NA	NA	NA	NA	NA	NA	NA	NA	MODIFIER	NA	NA	49	47	1	57	43	14	24.56
Smoker	42477	chr1	204128547	G	A	*REN*	SILENT	ENSG00000143839:SILENT[ENST00000272190(c.669C>T,gtC>gtT):ENSP00000272190(p.Val223(=))];ENSG00000143839:SILENT[ENST00000367195(c.669C>T,gtC>gtT):ENSP00000356163(p.Val223(=))];	NA	NA	NA	NA	NA	NA	NA	NA	NA	NA	LOW	NA	NA	79	79	0	85	68	17	20
Smoker	42477	chr1	156783987	C	T	*SH2D2A*	INTRONIC-SS-DNR	ENSG00000027869:[ENST00000368199(c.398+10G>A)];ENSG00000027869:[ENST00000368198(c.344+10G>A)];ENSG00000027869:[ENST00000486350(n.465+10G>A)];ENSG00000027869:[ENST00000392306(c.428+10G>A)];	NA	NA	NA	NA	NA	NA	NA	NA	NA	NA	MODIFIER	NA	NA	65	65	0	72	54	18	25
Smoker	42477	chr1	74665467	C	A	*TNNI3K*	MISSENSE	ENSG00000116783:MISSENSE[ENST00000370891(c.202C>A,Ctt>Att):ENSP00000359928(p.Leu68Ile)];	chr1*74665[illegible]	chr1*74665[illegible]	chr1*74665467*74665467*C*T*rs754999716*NA*NA*NA	NA	NA	NA	NA	D_lc	BN	N	MODERATE	NA	0.985	178	178	0	204	183	20	9.8
Smoker	42477	chr1	43652353	G	A	*WDR65*	INTRONIC	ENSG00000243710:[ENST00000533339(c.*869-25G>A)];ENSG00000243710:[ENST00000534615(n.480-25G>A)];ENSG00000243710:[ENST00000528956(c.970-25G>A)];ENSG00000243710:[ENST00000372492(c.970-25G>A)];	NA	NA	NA	NA	NA	NA	NA	NA	NA	NA	MODIFIER	NA	NA	82	82	0	129	117	12	9.3
Smoker	42477	chr1	67299380	C	T	*WDR78*	MISSENSE	ENSG00000152763:MISSENSE[ENST00000431318(c.1139G>A,cGa>cAa):ENSP00000393182(p.Arg380Gln)];ENSG00000152763:MISSENSE[ENST00000371026(c.1901G>A,cGa>cAa):ENSP00000360065(p.Arg634Gln)];ENSG00000152763:MISSENSE[ENST00000464352(c.1199G>A,cGa>cAa):ENSP00000433682(p.Arg400Gln)];ENSG00000152763:3UTR[ENST00000491297(c.*1843G>A)];	NA	NA	NA	NA	NA	NA	NA	NA	NA	NA	MODIFIER	NA	NA	112	112	0	147	109	38	25.85
Smoker	42477	chr10	96354575	G	A	*HELLS*	MISSENSE	ENSG00000119969:MISSENSE[ENST00000348459(c.2212G>A,Gct>Act):ENSP00000239027(p.Ala738Thr)];ENSG00000119969:MISSENSE[ENST00000371332(c.2350G>A,Gct>Act):ENSP00000360383(p.Ala784Thr)];ENSG00000119969:MISSENSE[ENST00000394045(c.1918G>A,Gct>Act):ENSP00000377609(p.Ala640Thr)];ENSG00000119969:MISSENSE[ENST00000371327(c.523G>A,Gct>Act):ENSP00000360378(p.Ala175Thr)];ENSG00000119969:3UTR[ENST00000394036(c.*1757G>A)];ENSG00000119969:3UTR[ENST00000239026(c.*1962G>A)];	NA	NA	chr10*96354575*96354575*G*T*rs111693730*NA*NA*NA	NA	NA	NA	NA	NA	NA	NA	MODIFIER	NA	NA	90	90	0	134	105	28	20.9
Smoker	42477	chr10	102822668	G	C	*KAZALD1*	MISSENSE	ENSG00000107821:MISSENSE[ENST00000370200(c.319G>C,Gag>Cag):ENSP00000359219(p.Glu107Gln)];ENSG00000107821:INTRONIC[ENST00000477979(n.112+796G>C)];ENSG00000107821:INTRONIC[ENST00000470106(n.44-75G>C)];	NA	NA	NA	NA	NA	NA	NA	NA	NA	NA	MODIFIER	NA	NA	91	91	0	83	71	12	14.46
Smoker	42477	chr10	24669797	G	T	*KIAA1217*	INTRONIC-SS-ACR	ENSG00000120549:INTRONIC-SS-ACR[ENST00000430453(c.118-1G>T)];ENSG00000120549:INTRONIC-SS-ACR[ENST00000376454(c.355-1G>T)];ENSG00000120549:INTRONIC-SS-ACR[ENST00000376456(c.355-1G>T)];ENSG00000120549:INTRONIC-SS-ACR[ENST00000376452(c.355-1G>T)];ENSG00000120549:INTRONIC-SS-ACR[ENST00000458595(c.355-1G>T)];ENSG00000120549:INTRONIC-SS-ACR[ENST00000438429(c.-96-1G>T)];ENSG00000120549:INTRONIC-SS-ACR[ENST00000376462(c.115-1G>T)];	NA	NA	NA	NA	NA	NA	NA	NA	NA	NA	HIGH	NA	NA	69	69	0	97	76	21	21.65
Smoker	42477	chr10	6531441	G	A	*PRKCQ*	INTRONIC	ENSG00000065675:[ENST00000397176(c.790+2204C>T)];ENSG00000065675:[ENST00000263125(c.790+2204C>T)];ENSG00000065675:[ENST00000539722(c.415+2204C>T)];	NA	NA	chr10*6531441*6531441*G*C*rs746242666*NA*NA*NA	NA	NA	NA	NA	NA	NA	NA	MODIFIER	NA	NA	53	53	0	71	58	13	18.31
Smoker	42477	chr10	96354575	G	A	*RP11-119K6.6*	INTRONIC	ENSG00000244332:[ENST00000432120(n.208-15983G>A)];	NA	NA	chr10*96354575*96354575*G*T*rs111693730*NA*NA*NA	NA	NA	NA	NA	NA	NA	NA	MODIFIER	NA	NA	90	90	0	134	105	28	20.9
Smoker	42477	chr10	99126302	G	A	*RRP12*	MISSENSE	ENSG00000052749:MISSENSE[ENST00000315563(c.2992C>T,Cgg>Tgg):ENSP00000324315(p.Arg998Trp)];ENSG00000052749:MISSENSE[ENST00000370992(c.3292C>T,Cgg>Tgg):ENSP00000360031(p.Arg1098Trp)];ENSG00000052749:MISSENSE[ENST00000536831(c.2446C>T,Cgg>Tgg):ENSP00000446184(p.Arg816Trp)];ENSG00000052749:MISSENSE[ENST00000414986(c.3109C>T,Cgg>Tgg):ENSP00000414863(p.Arg1037Trp)];ENSG00000052749:EXONIC-NC[ENST00000479481(n.2276C>T)];ENSG00000052749:EXONIC-NC[ENST00000491313(n.581C>T)];ENSG00000052749:EXONIC-NC[ENST00000487612(n.16C>T)];	NA	NA	0.0001997	3.30E-05	0.000182349	0.0001997	0.001	NA	NA	NA	MODIFIER	NA	NA	154	153	0	114	91	23	20.18

Mangalaparthi *et al.* , 2020. Mutational landscape of esophageal squamous cell carcinoma in an Indian cohort
Supplementary Table 4. List of somatic single nucleotide variants identified using whole exome sequencing of ESCC patients

Cohort	Sample	Chromosome	Genomic position	Reference allele	Alternate allele	Gene symbol	Variant type	Variant annotation	OncoMD	COSMIC	dbSNP	ExAC	ExAC_SAS	1000G	1000G_SAS	SIFT	Polyphen2	CONDEL	VEP	LRT	PhastCon7 Way	Total read (Adjacent normal)	Reference allel read support (Adjacent normal)	Alternative read support (Adjacent normal)	Total read (Tumor)	Reference allel read support (Tumor)	Alternative read support (Tumor)	Alternate allele frequency
Smoker	42477	chr10	97131008	A	G	*SORBS1*	INTRONIC	ENSG00000095637:[ENST00000371239(c.1236+75T>C)];ENSG00000095637:[ENST00000607232(c.1206+75T>C)];ENSG00000095637:[ENST00000353505(c.1458+733T>C)];ENSG00000095637:[ENST00000371227(c.1767+75T>C)];ENSG00000095637:[ENST00000371241(c.1024-3552T>C)];ENSG00000095637:[ENST00000371245(c.1458+733T>C)];ENSG00000095637:[ENST00000306402(c.1147-3552T>C)];ENSG00000095637:[ENST00000371247(c.1905+75T>C)];ENSG00000095637:[ENST00000347291(c.1342-3552T>C)];ENSG00000095637:[ENST00000361941(c.1905+75T>C)];ENSG00000095637:[ENST00000277982(c.1971+75T>C)];ENSG00000095637:[ENST00000393949(c.1815+75T>C)];ENSG00000095637:[ENST00000354106(c.1815+75T>C)];ENSG00000095637:[ENST00000371249(c.1420-3552T>C)];ENSG00000095637:[ENST00000474353(n.2295-3552T>C)];ENSG00000095637:[ENST00000371246(c.1971+75T>C)];	NA	NA	NA	NA	NA	NA	NA	NA	NA	NA	MODIFIER	NA	NA	93	93	0	120	101	19	15.83
Smoker	42477	chr10	108448071	A	C	*SORCS1*	MISSENSE	ENSG00000108018:MISSENSE[ENST00000263054(c.1439T>G,tTg>tGg):ENSP00000263054(p.Leu480Trp)];ENSG00000108018:MISSENSE[ENST00000369698(c.44T>G,tTg>tGg):ENSP00000358712(p.Leu15Trp)];ENSG00000108018:MISSENSE[ENST00000344440(c.1439T>G,tTg>tGg):ENSP00000345964(p.Leu480Trp)];	chr10*1084	NA	NA	NA	NA	NA	NA	D	PrD	D	MODERATE	D	1	65	65	0	115	87	28	24.35
Smoker	42477	chr11	64039082	C	T	*BAD*	INTRONIC-SS-DNR	ENSG00000002330:[ENST00000493798(c.123+3G>A)];ENSG00000002330:[ENST00000309032(c.378+3G>A)];ENSG00000002330:[ENST00000492141(c.123+3G>A)];ENSG00000002330:[ENST00000394531(c.*26+3G>A)];ENSG00000002330:[ENST00000394532(c.378+3G>A)];ENSG00000002330:[ENST00000544785(c.188-1273G>A)];	NA	NA	NA	NA	NA	NA	NA	NA	NA	NA	MODIFIER	NA	NA	56	56	0	52	32	20	38.46
Smoker	42477	chr11	76255871	C	T	*C11orf30*	EXONIC-NC	ENSG00000158636:[ENST00000531641(n.1398C>T)];ENSG00000158636:[ENST00000533248(c.3000+5C>T)];ENSG00000158636:[ENST00000524767(c.3318+5C>T)];ENSG00000158636:[ENST00000532719(c.693+5C>T)];ENSG00000158636:[ENST00000524490(c.2979+5C>T)];ENSG00000158636:[ENST00000334736(c.3273+5C>T)];ENSG00000158636:[ENST00000525919(c.3276+5C>T)];ENSG00000158636:[ENST00000525038(c.3276+5C>T)];ENSG00000158636:[ENST00000343878(c.3273+5C>T)];ENSG00000158636:[ENST00000529032(c.3273+5C>T)];	NA	NA	chr11*76255871*76255871*C*G,T*rs747333794*NA*NA*NA	3.37E-05	0.000123503	NA	NA	NA	NA	NA	LOW	NA	NA	70	70	0	69	59	10	14.49
Smoker	42477	chr11	64039082	C	T	*GPR137*	INTRONIC	ENSG00000173264:[ENST00000546139(c.3+949C>T)];	NA	NA	NA	NA	NA	NA	NA	NA	NA	NA	MODIFIER	NA	NA	56	56	0	52	32	20	38.46
Smoker	42477	chr11	47317565	G	C	*MADD*	MISSENSE	ENSG00000110514:MISSENSE[ENST00000395336(c.3718G>C,Gat>Cat):ENSP00000378745(p.Asp1240His)];ENSG00000110514:MISSENSE[ENST00000349238(c.3664G>C,Gat>Cat):ENSP00000304505(p.Asp1222His)];ENSG00000110514:MISSENSE[ENST00000402192(c.3601G>C,Gat>Cat):ENSP00000384287(p.Asp1201His)];ENSG00000110514:MISSENSE[ENST00000311027(c.3718G>C,Gat>Cat):ENSP00000310933(p.Asp1240His)];ENSG00000110514:MISSENSE[ENST00000402799(c.3475G>C,Gat>Cat):ENSP00000385585(p.Asp1159His)];ENSG00000110514:MISSENSE[ENST00000406482(c.3475G>C,Gat>Cat):ENSP00000384435(p.Asp1159His)];ENSG00000110514:MISSENSE[ENST00000395344(c.3463G>C,Gat>Cat):ENSP00000378753(p.Asp1155His)];ENSG00000110514:MISSENSE[ENST00000342922(c.3604G>C,Gat>Cat):ENSP00000343902(p.Asp1202His)];ENSG00000110514:MISSENSE[ENST00000407859(c.3535G>C,Gat>Cat):ENSP00000384204(p.Asp1179His)];ENSG00000110514:MISSENSE[ENST00000405573(c.148G>C,Gat>Cat):ENSP00000384483(p.Asp50His)];	NA	NA	NA	NA	NA	NA	NA	D_lc	PrD	D	MODERATE	D	0.998	110	110	0	97	84	13	13.4
Smoker	42477	chr11	1016809	G	T	*MUC6*	MISSENSE	ENSG00000184956:MISSENSE[ENST00000421673(c.5992C>A,Cca>Aca):ENSP00000406861(p.Pro1998Thr)];	NA	NA	NA	0.0074291	0.012899709	NA	NA	NA	PrD	NA	MODERATE	NA	NA	863	759	14	765	686	20	2.61
Smoker	42477	chr11	1017363	C	G	*MUC6*	MISSENSE	ENSG00000184956:MISSENSE[ENST00000421673(c.5438G>C,gGt>gCt):ENSP00000406861(p.Gly1813Ala)];	NA	NA	NA	0.0001913	6.07E-05	NA	NA	NA	PrD	NA	MODERATE	NA	NA	531	523	5	466	451	13	2.79
Smoker	42477	chr11	57983122	C	G	*OR1S1*	MISSENSE	ENSG00000172774:MISSENSE[ENST00000309433(c.906C>G,atC>atG):ENSP00000311688(p.Ile302Met)];	NA	NA	NA	NA	NA	NA	NA	D	PrD	D	MODERATE	NA	0.999	215	215	0	282	251	30	10.64
Smoker	42477	chr11	801515	G	A	*PIDD*	MISSENSE	ENSG00000177595:MISSENSE[ENST00000347755(c.1412C>T,tCg>tTg):ENSP00000337797(p.Ser471Leu)];ENSG00000177595:MISSENSE[ENST00000531286(c.562C>T,tCg>tTg):ENSP00000435008(p.Ser188Leu)];ENSG00000177595:MISSENSE[ENST00000411829(c.1412C>T,tCg>tTg):ENSP00000416801(p.Ser471Leu)];ENSG00000177595:EXONIC-NC[ENST00000534525(n.2363C>T)];ENSG00000177595:EXONIC-NC[ENST00000527812(n.232C>T)];ENSG00000177595:EXONIC-NC[ENST00000527357(n.2667C>T)];ENSG00000177595:3UTR[ENST00000525028(c.*1145C>T)];ENSG00000177595:3UTR[ENST00000524486(c.*1427C>T)];	NA	NA	NA	0.000127	0	NA	NA	NA	NA	NA	MODIFIER	NA	NA	58	58	0	41	30	11	26.83
Smoker	42477	chr11	32956384	G	A	*QSER1*	MISSENSE	ENSG00000060749:MISSENSE[ENST00000527788(c.2476G>A,Gat>Aat):ENSP00000432766(p.Asp826Asn)];ENSG00000060749:MISSENSE[ENST00000399302(c.3193G>A,Gat>Aat):ENSP00000382241(p.Asp1065Asn)];ENSG00000060749:MISSENSE[ENST00000524678(c.255G>A,Gat>Aat):ENSP00000432136(p.Asp86Asn)];	NA	NA	NA	NA	NA	NA	NA	D	PrD	D	MODERATE	NA	NA	154	154	0	215	173	41	19.07
Smoker	42477	chr11	70348384	C	T	*SHANK2*	SILENT	ENSG00000162105:SILENT[ENST00000409530(c.438G>A,ccG>ccA):ENSP00000387324(p.Pro146(=))];ENSG00000162105:SILENT[ENST00000409161(c.438G>A,ccG>ccA):ENSP00000386491(p.Pro146(=))];ENSG00000162105:SILENT[ENST00000412252(c.436G>A,ccG>ccA):ENSP00000414876(p.Pro146(=))];ENSG00000162105:SILENT[ENST00000424924(c.72G>A,ccG>ccA):ENSP00000402944(p.Pro24(=))];ENSG00000162105:SILENT[ENST00000423696(c.1068G>A,ccG>ccA):ENSP00000394536(p.Pro356(=))];ENSG00000162105:SILENT[ENST00000357171(c.441G>A,ccG>ccA):ENSP00000349694(p.Pro147(=))];ENSG00000162105:SILENT[ENST00000294018(c.1098G>A,ccG>ccA):ENSP00000294018(p.Pro366(=))];ENSG00000162105:SILENT[ENST00000449116(c.441G>A,ccG>ccA):ENSP00000394939(p.Pro147(=))];ENSG00000162105:SILENT[ENST00000338508(c.2208G>A,ccG>ccA):ENSP00000345193(p.Pro736(=))];ENSG00000162105:SILENT[ENST00000449833(c.441G>A,ccG>ccA):ENSP00000399423(p.Pro147(=))];ENSG00000162105:INTRONIC[ENST00000426687(c.426+517G>A)];	NA	NA	NA	NA	NA	NA	NA	NA	NA	NA	LOW	NA	NA	23	23	0	39	24	15	38.46

Mangalaparthi *et al.* , 2020. Mutational landscape of esophageal squamous cell carcinoma in an Indian cohort
Supplementary Table 4. List of somatic single nucleotide variants identified using whole exome sequencing of ESCC patients

Cohort	Sample	Chromosome	Genomic position	Reference allele	Alternate allele	Gene symbol	Variant type	Variant annotation	OncoMD	COSMIC	dbSNP	ExAC	ExAC_SAS	1000G	1000G_SAS	SIFT	Polyphen2	CONDEL	VEP	LRT	PhastCon7 Way	Total read (Adjacent normal)	Reference allel read support (Adjacent normal)	Alternative read support (Adjacent normal)	Total read (Tumor)	Reference allel read support (Tumor)	Alternative read support (Tumor)	Alternate allele frequency
Smoker	42477	chr11	126277184	G	T	*ST3GAL4*	MISSENSE	ENSG00000110080:MISSENSE[ENST00000534083(c.220G>T,Gat>Tat):ENSP00000433318(p.Asp74Tyr)];ENSG00000110080:MISSENSE[ENST00000356132(c.238G>T,Gat>Tat):ENSP00000348451(p.Asp80Tyr)];ENSG00000110080:MISSENSE[ENST00000444328(c.220G>T,Gat>Tat):ENSP00000394354(p.Asp74Tyr)];ENSG00000110080:MISSENSE[ENST00000534457(c.205G>T,Gat>Tat):ENSP00000434668(p.Asp69Tyr)];ENSG00000110080:MISSENSE[ENST00000392669(c.220G>T,Gat>Tat):ENSP00000376437(p.Asp74Tyr)];ENSG00000110080:MISSENSE[ENST00000526727(c.220G>T,Gat>Tat):ENSP00000436047(p.Asp74Tyr)];ENSG00000110080:MISSENSE[ENST00000449406(c.187G>T,Gat>Tat):ENSP00000399444(p.Asp63Tyr)];ENSG00000110080:MISSENSE[ENST00000532243(c.217G>T,Gat>Tat):ENSP00000434349(p.Asp73Tyr)];ENSG00000110080:MISSENSE[ENST00000530591(c.208G>T,Gat>Tat):ENSP00000433989(p.Asp70Tyr)];ENSG00000110080:MISSENSE[ENST00000227495(c.208G>T,Gat>Tat):ENSP00000227495(p.Asp70Tyr)];ENSG00000110080:MISSENSE[ENST00000526311(c.220G>T,Gat>Tat):ENSP00000432647(p.Asp74Tyr)];ENSG00000110080:MISSENSE[ENST00000534452(c.220G>T,Gat>Tat):ENSP00000434756(p.Asp74Tyr)];ENSG00000110080:MISSENSE[ENST00000528858(c.220G>T,Gat>Tat):ENSP00000432424(p.Asp74Tyr)];ENSG00000110080:MISSENSE[ENST00000531217(c.220G>T,Gat>Tat):ENSP00000432929(p.Asp74Tyr)];ENSG00000110080:5UTR[ENST00000524860(c.-273G>T)];ENSG00000110080:EXONIC-NC[ENST00000524834(n.174G>T)];ENSG00000110080:EXONIC-NC[ENST00000526756(n.393G>T)];ENSG00000110080:EXONIC-NC[ENST00000528605(n.385G>T)];	NA	NA	NA	NA	NA	NA	NA	NA	NA	NA	MODIFIER	NA	NA	91	90	0	66	51	15	22.73
Smoker	42477	chr12	53917083	T	A	*ATF7*	MISSENSE	ENSG00000170653:MISSENSE[ENST00000420353(c.1211A>T,cAg>cTg):ENSP00000399465(p.Gln404Leu)];ENSG00000170653:MISSENSE[ENST00000456903(c.1211A>T,cAg>cTg):ENSP00000387406(p.Gln404Leu)];ENSG00000170653:MISSENSE[ENST00000328463(c.1244A>T,cAg>cTg):ENSP00000329212(p.Gln415Leu)];ENSG00000170653:MISSENSE[ENST00000548446(c.1244A>T,cAg>cTg):ENSP00000449938(p.Gln415Leu)];ENSG00000170653:MISSENSE[ENST00000415113(c.1148A>T,cAg>cTg):ENSP00000404880(p.Gln383Leu)];ENSG00000170653:EXONIC-NC[ENST00000546661(n.396A>T)];	NA	NA	NA	NA	NA	NA	NA	D	PoD	D	MODERATE	NA	1	47	47	0	62	57	5	8.06
Smoker	42477	chr12	103695884	G	A	*C12orf42*	INTRONIC	ENSG00000179088:[ENST00000546526(n.338-24896C>T)];ENSG00000179088:[ENST00000550650(n.306-24896C>T)];ENSG00000179088:[ENST00000552977(n.310-24896C>T)];ENSG00000179088:[ENST00000547347(c.260-24896C>T)];ENSG00000179088:[ENST00000315192(c.260-24896C>T)];ENSG00000179088:[ENST00000378113(c.*2C>T)];ENSG00000179088:[ENST00000551134(c.*907C>T)];ENSG00000179088:[ENST00000548048(c.*2C>T)];ENSG00000179088:[ENST00000548883(c.*2C>T)];	NA	NA	0.0001997	8.73E-06	0	0.0001997	0	NA	NA	NA	MODIFIER	NA	NA	92	92	0	118	93	25	21.19
Smoker	42477	chr12	123741365	T	A	*C12orf65*	MISSENSE	ENSG00000130921:MISSENSE[ENST00000543139(c.288T>A,caT>caA):ENSP00000444843(p.His96Gln)];ENSG00000130921:MISSENSE[ENST00000366329(c.288T>A,caT>caA):ENSP00000390647(p.His96Gln)];ENSG00000130921:MISSENSE[ENST00000253233(c.288T>A,caT>caA):ENSP00000253233(p.His96Gln)];ENSG00000130921:MISSENSE[ENST00000429587(c.288T>A,caT>caA):ENSP00000391513(p.His96Gln)];ENSG00000130921:EXONIC-NC[ENST00000425637(n.1488T>A)];ENSG00000130921:EXONIC-NC[ENST00000538888(n.654T>A)];	NA	NA	NA	NA	NA	NA	NA	NA	NA	NA	MODIFIER	NA	NA	113	113	0	106	86	20	18.87
Smoker	42477	chr12	75685548	T	A	*CAPS2*	MISSENSE	ENSG00000180881:MISSENSE[ENST00000409445(c.1273A>T,Agg>Tgg):ENSP00000386959(p.Arg425Trp)];ENSG00000180881:MISSENSE[ENST00000409799(c.1027A>T,Agg>Tgg):ENSP00000386977(p.Arg343Trp)];ENSG00000180881:MISSENSE[ENST00000393284(c.577A>T,Agg>Tgg):ENSP00000376963(p.Arg193Trp)];ENSG00000180881:MISSENSE[ENST00000442339(c.43A>T,Agg>Tgg):ENSP00000389633(p.Arg15Trp)];ENSG00000180881:EXONIC-NC[ENST00000409004(n.746A>T)];ENSG00000180881:3UTR[ENST00000336815(c.*292A>T)];ENSG00000180881:3UTR[ENST00000328705(c.*1420A>T)];	NA	NA	NA	NA	NA	NA	NA	NA	NA	NA	MODIFIER	NA	NA	49	49	0	69	56	13	18.84
Smoker	42477	chr12	120204884	T	C	*CIT*	INTRONIC-SS-DNR	ENSG00000122966:[ENST00000392520(c.1061+7A>G)];ENSG00000122966:[ENST00000261833(c.2178+7A>G)];ENSG00000122966:[ENST00000545913(n.2220+7A>G)];ENSG00000122966:[ENST00000392521(c.2304+7A>G)];ENSG00000122966:[ENST00000537607(n.1076+7A>G)];ENSG00000122966:[ENST00000536325(c.268-46494A>G)];	NA	NA	NA	NA	NA	NA	NA	NA	NA	NA	LOW	NA	NA	30	30	0	59	48	11	18.64
Smoker	42477	chr12	32754209	C	G	*FGD4*	INTRONIC-SS-ACR	ENSG00000139132:[ENST00000381025(c.-54-3C>G)];ENSG00000139132:[ENST00000494977(c.*83-3C>G)];ENSG00000139132:[ENST00000546442(c.412-3C>G)];ENSG00000139132:[ENST00000531134(c.946-3C>G)];ENSG00000139132:[ENST00000395740(c.*83-3C>G)];ENSG00000139132:[ENST00000493087(c.*83-3C>G)];ENSG00000139132:[ENST00000427716(c.691-3C>G)];ENSG00000139132:[ENST00000525053(c.1027-3C>G)];ENSG00000139132:[ENST00000266482(c.-54-3C>G)];ENSG00000139132:[ENST00000534526(c.1102-3C>G)];ENSG00000139132:[ENST00000551984(c.*60-3C>G)];	NA	NA	NA	NA	NA	NA	NA	NA	NA	NA	LOW	NA	NA	76	76	0	99	77	22	22.22
Smoker	42477	chr12	95603349	G	A	*FGD6*	MISSENSE	ENSG00000180263:MISSENSE[ENST00000549499(c.1711C>T,Cat>Tat):ENSP00000449005(p.His571Tyr)];ENSG00000180263:MISSENSE[ENST00000546711(c.1711C>T,Cat>Tat):ENSP00000450342(p.His571Tyr)];ENSG00000180263:MISSENSE[ENST00000343958(c.1711C>T,Cat>Tat):ENSP00000344446(p.His571Tyr)];ENSG00000180263:INTRONIC[ENST00000451107(c.16+7652C>T)];	NA	NA	NA	8.27E-06	0	NA	NA	NA	NA	NA	MODIFIER	NA	NA	191	189	1	217	175	41	18.89
Smoker	42477	chr12	59307988	T	C	*LRIG3*	INTRONIC	ENSG00000139263:[ENST00000379141(c.128+58A>G)];ENSG00000139263:[ENST00000320743(c.308+58A>G)];ENSG00000139263:[ENST00000552267(c.29+58A>G)];ENSG00000139263:[ENST00000433272(c.308+58A>G)];ENSG00000139263:[ENST00000548968(n.229+58A>G)];	NA	NA	0.01158	NA	NA	0.0115815	0	NA	NA	NA	MODIFIER	NA	NA	36	36	0	71	59	12	16.9
Smoker	42477	chr12	15668462	C	G	*PTPRO*	MISSENSE	ENSG00000151490:MISSENSE[ENST00000348962(c.1495C>G,Ctg>Gtg):ENSP00000343434(p.Leu499Val)];ENSG00000151490:MISSENSE[ENST00000543886(c.1495C>G,Ctg>Gtg):ENSP00000444173(p.Leu499Val)];ENSG00000151490:MISSENSE[ENST00000281171(c.1495C>G,Ctg>Gtg):ENSP00000281171(p.Leu499Val)];	chr12*1566	chr12*1566	NA	NA	NA	NA	NA	D	PrD	D	MODERATE	D	1	83	83	0	103	82	21	20.39

Supplementary Table 4. List of somatic single nucleotide variants identified using whole exome sequencing of ESCC patients

Cohort	Sample	Chromosome	Genomic position	Reference allele	Alternate allele	Gene symbol	Variant type	Variant annotation	OncoMD	COSMIC	dbSNP	ExAC	ExAC_SAS	1000G	1000G_SAS	SIFT	Polyphen2	CONDEL	VEP	LRT	PhastCon7 Way	Total read (Adjacent normal)	Reference allel read support (Adjacent normal)	Alternative read support (Adjacent normal)	Total read (Tumor)	Reference allel read support (Tumor)	Alternative read support (Tumor)	Alternate allele frequency
Smoker	42477	chr12	123741365	T	A	*RP11-282O18.3*	INTRONIC	ENSG00000235423:[ENST00000541002(n.809+2225A>T)];ENSG00000235423:[ENST00000543217(n.280+2225A>T)];	NA	NA	NA	NA	NA	NA	NA	NA	NA	NA	MODIFIER	NA	NA	113	113	0	106	86	20	18.87
Smoker	42477	chr12	75685548	T	A	*RP11-560G2.1*	INTRONIC	ENSG00000254451:[ENST00000549953(n.399-3275T>A)];	NA	NA	NA	NA	NA	NA	NA	NA	NA	NA	MODIFIER	NA	NA	49	49	0	69	56	13	18.84
Smoker	42477	chr12	53917083	T	A	*RP11-793H13.10*	MISSENSE	ENSG00000267281:MISSENSE[ENST00000591834(c.1211A>T,cAg>cTg):ENSP00000466174(p.Gln404Leu)];	NA	NA	NA	NA	NA	NA	NA	D	PoD	D	MODERATE	NA	1	47	47	0	62	57	5	8.06
Smoker	42477	chr12	129189681	C	T	*TMEM132C*	MISSENSE	ENSG00000181234:MISSENSE[ENST00000537538(c.323C>T,aCg>aTg):ENSP00000438477(p.Thr108Met)];ENSG00000181234:MISSENSE[ENST00000315208(c.1016C>T,aCg>aTg):ENSP00000324458(p.Thr339Met)];ENSG00000181234:MISSENSE[ENST00000435159(c.2168C>T,aCg>aTg):ENSP00000410852(p.Thr723Met)];	NA	NA	NA	0.0002286	0	NA	NA	T	BN	N	MODERATE	D	0.952	42	42	0	52	42	10	19.23
Smoker	42477	chr12	133634953	C	T	*ZNF84*	MISSENSE	ENSG00000198040:MISSENSE[ENST00000327668(c.1652C>T,gCc>gTc):ENSP00000331465(p.Ala551Val)];ENSG00000198040:MISSENSE[ENST00000543758(c.1649C>T,gCc>gTc):ENSP00000437949(p.Ala550Val)];ENSG00000198040:MISSENSE[ENST00000539354(c.1652C>T,gCc>gTc):ENSP00000445549(p.Ala551Val)];ENSG00000198040:MISSENSE[ENST00000392319(c.1652C>T,gCc>gTc):ENSP00000376133(p.Ala551Val)];ENSG00000198040:INTRONIC[ENST00000535439(c.239-4712C>T)];	NA	NA	NA	NA	NA	NA	NA	NA	NA	NA	MODIFIER	NA	NA	122	122	0	127	98	29	22.83
Smoker	42477	chr13	110802757	G	A	*COL4A1*	SILENT	ENSG00000187498:SILENT[ENST00000375820(c.4963C>T,Ctg>Ttg):ENSP00000364979(p.Leu1655(=))];	NA	NA	NA	NA	NA	NA	NA	NA	NA	NA	LOW	NA	0.998	150	150	0	245	196	49	20
Smoker	42477	chr13	103391791	G	T	*NA*	INTERGENIC		NA	NA	chr13*103391791*103391792*GC*G*rs771007952*NA*NA*NA	NA	NA	NA	NA	NA	NA	NA	MODIFIER	NA	NA	129	129	0	164	143	21	12.8
Smoker	42477	chr13	58208507	T	A	*PCDH17*	SILENT	ENSG00000118946:SILENT[ENST00000484979(c.1827T>A,acT>acA):ENSP00000432899(p.Thr609(=))];ENSG00000118946:SILENT[ENST00000377918(c.1827T>A,acT>acA):ENSP00000367151(p.Thr609(=))];	NA	NA	NA	8.57E-06	0	NA	NA	NA	NA	NA	LOW	NA	NA	124	124	0	166	133	33	19.88
Smoker	42477	chr13	21714785	A	T	*SAP18*	NONSENSE	ENSG00000150459:NONSENSE[ENST00000607003(c.37A>T,Aag>Tag):ENSP00000475925(p.Lys13Ter)];ENSG00000150459:NONSENSE[ENST00000492245(c.32A>T,Aag>Tag):ENSP00000475435(p.Lys12Ter)];ENSG00000150459:NONSENSE[ENST00000382533(c.94A>T,Aag>Tag):ENSP00000371973(p.Lys32Ter)];ENSG00000150459:EXONIC-NC[ENST00000485646(n.35A>T)];	NA	NA	NA	NA	NA	NA	NA	NA	NA	NA	MODIFIER	NA	NA	73	73	0	87	71	16	18.39
Smoker	42477	chr13	29286901	G	A	*SLC46A3*	MISSENSE	ENSG00000139508:MISSENSE[ENST00000266943(c.976C>T,Cat>Tat):ENSP00000266943(p.His326Tyr)];ENSG00000139508:MISSENSE[ENST00000380814(c.976C>T,Cat>Tat):ENSP00000370192(p.His326Tyr)];	NA	NA	chr13*29286900*29286903*TGAA*T*rs762739498*NA*NA*NA	NA	NA	NA	NA	T	BN	N	MODERATE	N	0.991	161	161	0	166	134	32	19.28
Smoker	42477	chr13	29287405	G	A	*SLC46A3*	NONSENSE	ENSG00000139508:NONSENSE[ENST00000266943(c.472C>T,Cag>Tag):ENSP00000266943(p.Gln158Ter)];ENSG00000139508:NONSENSE[ENST00000380814(c.472C>T,Cag>Tag):ENSP00000370192(p.Gln158Ter)];	NA	NA	NA	NA	NA	NA	NA	NA	NA	NA	HIGH	NA	0.98	88	88	0	95	77	18	18.95
Smoker	42477	chr13	52234823	G	A	*WDFY2*	INTRONIC	ENSG00000139668:[ENST00000298125(c.205+24G>A)];	NA	NA	NA	NA	NA	NA	NA	NA	NA	NA	MODIFIER	NA	NA	167	167	0	205	165	40	19.51
Smoker	42477	chr13	52234832	G	T	*WDFY2*	INTRONIC	ENSG00000139668:[ENST00000298125(c.205+33G>T)];	NA	NA	NA	NA	NA	NA	NA	NA	NA	NA	MODIFIER	NA	NA	162	162	0	190	152	38	20
Smoker	42477	chr13	32886046	C	T	*ZAR1L*	MISSENSE	ENSG00000189167:MISSENSE[ENST00000345108(c.17G>A,cGt>cAt):ENSP00000344616(p.Arg6His)];ENSG00000189167:MISSENSE[ENST00000533490(c.17G>A,cGt>cAt):ENSP00000437289(p.Arg6His)];	NA	NA	NA	NA	NA	NA	NA	T	BN	N	MODERATE	U	0.62	36	36	0	41	36	5	12.2
Smoker	42477	chr14	59827651	G	A	*DAAM1*	INTRONIC	ENSG00000100592:[ENST00000553472(n.221+71G>A)];ENSG00000100592:[ENST00000351081(c.2724+71G>A)];ENSG00000100592:[ENST00000553966(n.457+1429G>A)];ENSG00000100592:[ENST00000557029(n.199+71G>A)];ENSG00000100592:[ENST00000395125(c.2724+71G>A)];ENSG00000100592:[ENST00000553307(n.342+71G>A)];ENSG00000100592:[ENST00000557628(n.311+71G>A)];ENSG00000100592:[ENST00000360909(c.2694+71G>A)];	NA	NA	NA	NA	NA	NA	NA	NA	NA	NA	MODIFIER	NA	NA	66	66	0	124	90	34	27.42
Smoker	42477	chr14	26949168	C	G	*NOVA1*	INTRONIC	ENSG00000139910:[ENST00000549146(c.81+15G>C)];ENSG00000139910:[ENST00000347476(c.81+15G>C)];ENSG00000139910:[ENST00000465357(c.447+15G>C)];ENSG00000139910:[ENST00000547619(c.447+15G>C)];ENSG00000139910:[ENST00000549571(c.336+15G>C)];ENSG00000139910:[ENST00000483536(c.*183+15G>C)];ENSG00000139910:[ENST00000344429(c.447+15G>C)];ENSG00000139910:[ENST00000539517(c.447+15G>C)];ENSG00000139910:[ENST00000267422(c.81+15G>C)];ENSG00000139910:[ENST00000546546(c.*183+15G>C)];ENSG00000139910:[ENST00000449198(c.324+15G>C)];ENSG00000139910:[ENST00000574031(c.*3G>C)];	NA	NA	NA	NA	NA	NA	NA	NA	NA	NA	MODIFIER	NA	NA	104	103	0	199	125	74	37.19
Smoker	42477	chr14	23996046	C	T	*RP11-66N24.4*	INTRONIC	ENSG00000157306:[ENST00000553985(n.238+12421C>T)];ENSG00000157306:[ENST00000556354(n.465+12421C>T)];ENSG00000157306:[ENST00000554403(n.1068+12421C>T)];	NA	NA	NA	NA	NA	NA	NA	NA	NA	NA	MODIFIER	NA	NA	80	80	0	80	71	9	11.25
Smoker	42477	chr14	23996046	C	T	*ZFHX2*	INTRONIC-SS-DNR	ENSG00000136367:[ENST00000419474(c.3262+10G>A)];	NA	NA	NA	NA	NA	NA	NA	NA	NA	NA	MODIFIER	NA	NA	80	80	0	80	71	9	11.25
Smoker	42477	chr15	69238474	C	T	*NOX5*	INTRONIC	ENSG00000255346:[ENST00000455873(c.29+15418C>T)];ENSG00000255346:[ENST00000448182(c.-109+15418C>T)];ENSG00000255346:[ENST00000260364(c.-109+15418C>T)];	NA	NA	NA	NA	NA	NA	NA	NA	NA	NA	HIGH	NA	0.041	177	177	0	213	175	38	17.84
Smoker	42477	chr15	28230264	G	T	*OCA2*	MISSENSE	ENSG00000104044:MISSENSE[ENST00000354638(c.1310C>A,tCt>tAt):ENSP00000346659(p.Ser437Tyr)];ENSG00000104044:MISSENSE[ENST00000353809(c.1238C>A,tCt>tAt):ENSP00000261276(p.Ser413Tyr)];ENSG00000104044:MISSENSE[ENST00000382996(c.1310C>A,tCt>tAt):ENSP00000372457(p.Ser437Tyr)];	NA	NA	NA	NA	NA	NA	NA	D	PrD	D	MODERATE	D	0.992	100	100	0	118	99	19	16.1
Smoker	42477	chr15	69238474	C	T	*RP11-809H16.2*	INTRONIC	ENSG00000137808:[ENST00000557966(n.214+15418C>T)];	NA	NA	NA	NA	NA	NA	NA	NA	NA	NA	HIGH	NA	0.041	177	177	0	213	175	38	17.84
Smoker	42477	chr15	69238474	C	T	*SPESP1*	NONSENSE	ENSG00000258484:NONSENSE[ENST00000310673(c.601C>T,Cag>Tag):ENSP00000312284(p.Gln201Ter)];	NA	NA	NA	NA	NA	NA	NA	NA	NA	NA	HIGH	NA	0.041	177	177	0	213	175	38	17.84
Smoker	42477	chr15	50784955	C	A	*USP8*	MISSENSE	ENSG00000138592:MISSENSE[ENST00000307179(c.2292C>A,aaC>aaA):ENSP00000302239(p.Asn764Lys)];ENSG00000138592:MISSENSE[ENST00000396444(c.2292C>A,aaC>aaA):ENSP00000379721(p.Asn764Lys)];ENSG00000138592:MISSENSE[ENST00000433963(c.2292C>A,aaC>aaA):ENSP00000405537(p.Asn764Lys)];ENSG00000138592:MISSENSE[ENST00000425032(c.1974C>A,aaC>aaA):ENSP00000412682(p.Asn658Lys)];	chr15*5078-	chr15*5078-	NA	0.3443056	0.382427148	NA	NA	D	BN	N	MODERATE	N	0.999	213	211	2	263	251	11	4.18

Mangalaparthi *et al.* , 2020. Mutational landscape of esophageal squamous cell carcinoma in an Indian cohort
Supplementary Table 4. List of somatic single nucleotide variants identified using whole exome sequencing of ESCC patients

Cohort	Sample	Chromosome	Genomic position	Reference allele	Alternate allele	Gene symbol	Variant type	Variant annotation	OncoMD	COSMIC	dbSNP	ExAC	ExAC_SAS	1000G	1000G_SAS	SIFT	Polyphen2	CONDEL	VEP	LRT	PhastCon7 Way	Total read (Adjacent normal)	Reference allel read support (Adjacent normal)	Alternative read support (Adjacent normal)	Total read (Tumor)	Reference allel read support (Tumor)	Alternative read support (Tumor)	Alternate allele frequency
Smoker	42477	chr16	1394265	G	A	*BAIAP3*	MISSENSE	ENSG00000007516:MISSENSE[ENST00000421665(c.1441G>A,Gag>Aag):ENSP00000409533(p.Glu481Lys)];ENSG00000007516:MISSENSE[ENST00000568887(c.1465G>A,Gag>Aag):ENSP00000457644(p.Glu489Lys)];ENSG00000007516:MISSENSE[ENST00000397488(c.1600G>A,Gag>Aag):ENSP00000380625(p.Glu534Lys)];ENSG00000007516:MISSENSE[ENST00000426824(c.1549G>A,Gag>Aag):ENSP00000407242(p.Glu517Lys)];ENSG00000007516:MISSENSE[ENST00000562208(c.1480G>A,Gag>Aag):ENSP00000458134(p.Glu494Lys)];ENSG00000007516:MISSENSE[ENST00000324385(c.1654G>A,Gag>Aag):ENSP00000324510(p.Glu552Lys)];ENSG00000007516:MISSENSE[ENST00000397489(c.1600G>A,Gag>Aag):ENSP00000380626(p.Glu534Lys)];ENSG00000007516:EXONIC-NC[ENST00000568198(n.439G>A)];	NA	NA	NA	NA	NA	NA	NA	NA	NA	NA	MODIFIER	NA	NA	114	114	0	104	94	10	9.62
Smoker	42477	chr16	1394482	G	A	*BAIAP3*	MISSENSE	ENSG00000007516:MISSENSE[ENST00000421665(c.1507G>A,Gag>Aag):ENSP00000409533(p.Glu503Lys)];ENSG00000007516:MISSENSE[ENST00000568887(c.1531G>A,Gag>Aag):ENSP00000457644(p.Glu511Lys)];ENSG00000007516:MISSENSE[ENST00000397488(c.1666G>A,Gag>Aag):ENSP00000380625(p.Glu556Lys)];ENSG00000007516:MISSENSE[ENST00000562208(c.1546G>A,Gag>Aag):ENSP00000458134(p.Glu516Lys)];ENSG00000007516:MISSENSE[ENST00000397489(c.1666G>A,Gag>Aag):ENSP00000380626(p.Glu556Lys)];ENSG00000007516:MISSENSE[ENST00000426824(c.1615G>A,Gag>Aag):ENSP00000407242(p.Glu539Lys)];ENSG00000007516:MISSENSE[ENST00000324385(c.1720G>A,Gag>Aag):ENSP00000324510(p.Glu574Lys)];ENSG00000007516:EXONIC-NC[ENST00000568198(n.505G>A)];	NA	NA	NA	NA	NA	NA	NA	NA	NA	NA	MODIFIER	NA	NA	62	62	0	51	38	13	25.49
Smoker	42477	chr16	1394776	G	A	*BAIAP3*	INTRONIC	ENSG00000007516:[ENST00000421665(c.1650-23G>A)];ENSG00000007516:[ENST00000568887(c.1674-23G>A)];ENSG00000007516:[ENST00000397488(c.1809-23G>A)];ENSG00000007516:[ENST00000426824(c.1758-23G>A)];ENSG00000007516:[ENST00000562208(c.1689-23G>A)];ENSG00000007516:[ENST00000566162(c.32-23G>A)];ENSG00000007516:[ENST00000324385(c.1863-23G>A)];ENSG00000007516:[ENST00000397489(c.1809-23G>A)];ENSG00000007516:[ENST00000568198(n.648-23G>A)];	NA	NA	NA	8.28E-06	0	NA	NA	NA	NA	NA	MODIFIER	NA	NA	87	87	0	92	68	24	26.09
Smoker	42477	chr16	89713646	C	T	*CHMP1A*	MISSENSE	ENSG00000131165:MISSENSE[ENST00000397901(c.346G>A,Gag>Aag):ENSP00000380998(p.Glu116Lys)];ENSG00000131165:MISSENSE[ENST00000550102(c.319G>A,Gag>Aag):ENSP00000449243(p.Glu107Lys)];ENSG00000131165:MISSENSE[ENST00000549328(c.346G>A,Gag>Aag):ENSP00000447899(p.Glu116Lys)];ENSG00000131165:MISSENSE[ENST00000535997(c.154G>A,Gag>Aag):ENSP00000442120(p.Glu52Lys)];ENSG00000131165:MISSENSE[ENST00000253475(c.326G>A,cGa>cAa):ENSP00000253475(p.Arg109Gln)];ENSG00000131165:EXONIC-NC[ENST00000548650(n.283G>A)];ENSG00000131165:EXONIC-NC[ENST00000547614(n.459G>A)];ENSG00000131165:EXONIC-NC[ENST00000549139(n.337G>A)];ENSG00000131165:EXONIC-NC[ENST00000551981(n.363G>A)];	NA	NA	NA	NA	NA	NA	NA	NA	NA	NA	MODIFIER	NA	NA	49	49	0	34	25	9	26.47
Smoker	42477	chr16	71570803	G	A	*CHST4*	MISSENSE	ENSG00000140835:MISSENSE[ENST00000539698(c.223G>A,Gcc>Acc):ENSP00000441204(p.Ala75Thr)];ENSG00000140835:MISSENSE[ENST00000572450(c.223G>A,Gcc>Acc):ENSP00000461839(p.Ala75Thr)];ENSG00000140835:MISSENSE[ENST00000338482(c.223G>A,Gcc>Acc):ENSP00000341206(p.Ala75Thr)];ENSG00000140835:EXONIC-NC[ENST00000572693(n.315G>A)];	chr16*7157	chr16*7157	NA	3.52E-05	9.19E-05	NA	NA	NA	NA	NA	MODIFIER	NA	NA	112	112	0	135	106	29	21.48
Smoker	42477	chr16	57492290	G	A	*COQ9*	INTRONIC	ENSG00000088682:[ENST00000567933(c.379-1187G>A)];ENSG00000088682:[ENST00000262507(c.711+28G>A)];ENSG00000088682:[ENST00000569980(n.226+28G>A)];ENSG00000088682:[ENST00000565964(c.483+28G>A)];ENSG00000088682:[ENST00000564655(c.723+28G>A)];ENSG00000088682:[ENST00000567072(c.606+28G>A)];ENSG00000088682:[ENST00000563166(c.304-2794G>A)];ENSG00000088682:[ENST00000564115(c.*59+28G>A)];	NA	NA	NA	NA	NA	NA	NA	NA	NA	NA	MODIFIER	NA	NA	48	47	0	36	30	6	16.67
Smoker	42477	chr16	80581704	G	A	*DYNLRB2*	SILENT	ENSG00000168589:SILENT[ENST00000338542(c.129G>A,caG>caA):ENSP00000342009(p.Gln43(=))];ENSG00000168589:INTRONIC[ENST00000568035(c.80-2708G>A)];ENSG00000168589:INTRONIC[ENST00000561880(c.80-1136G>A)];ENSG00000168589:INTRONIC[ENST00000570222(n.100-1677G>A)];ENSG00000168589:INTRONIC[ENST00000562982(c.167-1677G>A)];ENSG00000168589:INTRONIC[ENST00000305904(c.80-1677G>A)];	NA	NA	NA	NA	NA	NA	NA	NA	NA	NA	MODIFIER	NA	NA	139	139	0	213	169	44	20.66
Smoker	42477	chr16	30676739	G	A	*FBRS*	INTRONIC	ENSG00000156860:[ENST00000395073(c.-121+17G>A)];ENSG00000156860:[ENST00000543786(c.587-1501G>A)];ENSG00000156860:[ENST00000356166(c.1704+17G>A)];ENSG00000156860:[ENST00000568722(c.-21-1501G>A)];ENSG00000156860:[ENST00000287468(c.144+17G>A)];	NA	NA	NA	NA	NA	NA	NA	NA	NA	NA	MODIFIER	NA	NA	76	76	0	70	52	17	24.29
Smoker	42477	chr16	30676971	G	A	*FBRS*	MISSENSE	ENSG00000156860:MISSENSE[ENST00000287468(c.157G>A,Gag>Aag):ENSP00000287468(p.Glu53Lys)];ENSG00000156860:MISSENSE[ENST00000356166(c.1717G>A,Gag>Aag):ENSP00000348489(p.Glu573Lys)];ENSG00000156860:5UTR[ENST00000395073(c.-108G>A)];ENSG00000156860:INTRONIC[ENST00000543786(c.587-1269G>A)];ENSG00000156860:INTRONIC[ENST00000568722(c.-21-1269G>A)];	NA	NA	NA	NA	NA	NA	NA	NA	NA	NA	MODIFIER	NA	NA	38	38	0	43	32	11	25.58
Smoker	42477	chr16	30677061	G	A	*FBRS*	INTRONIC	ENSG00000156860:[ENST00000395073(c.-52+34G>A)];ENSG00000156860:[ENST00000543786(c.587-1179G>A)];ENSG00000156860:[ENST00000356166(c.1773+34G>A)];ENSG00000156860:[ENST00000568722(c.-21-1179G>A)];ENSG00000156860:[ENST00000287468(c.213+34G>A)];	NA	NA	NA	NA	NA	NA	NA	NA	NA	NA	MODIFIER	NA	NA	43	42	0	47	34	13	27.66
Smoker	42477	chr16	30678151	G	A	*FBRS*	INTRONIC	ENSG00000156860:[ENST00000570170(n.568-89G>A)];ENSG00000156860:[ENST00000395073(c.-21-89G>A)];ENSG00000156860:[ENST00000543786(c.587-89G>A)];ENSG00000156860:[ENST00000356166(c.1804-89G>A)];ENSG00000156860:[ENST00000568722(c.-21-89G>A)];ENSG00000156860:[ENST00000287468(c.244-89G>A)];	NA	NA	NA	NA	NA	NA	NA	NA	NA	NA	MODIFIER	NA	NA	60	60	0	58	45	13	22.41

Supplementary Table 4. List of somatic single nucleotide variants identified using whole exome sequencing of ESCC patients

Cohort	Sample	Chromosome	Genomic position	Reference allele	Alternate allele	Gene symbol	Variant type	Variant annotation	OncoMD	COSMIC	dbSNP	ExAC	ExAC_SAS	1000G	1000G_SAS	SIFT	Polyphen2	CONDEL	VEP	LRT	PhastCon7 Way	Total read (Adjacent normal)	Reference allel read support (Adjacent normal)	Alternative read support (Adjacent normal)	Total read (Tumor)	Reference allel read support (Tumor)	Alternative read support (Tumor)	Alternate allele frequency
Smoker	42477	chr16	30680218	G	A	*FBRS*	MISSENSE	ENSG00000156860:MISSENSE[ENST00000287468(c.724G>A,Gag>Aag):ENSP00000287468(p.Glu242Lys)];ENSG00000156860:MISSENSE[ENST00000568722(c.460G>A,Gag>Aag):ENSP00000454920(p.Glu154Lys)];ENSG00000156860:MISSENSE[ENST00000395073(c.460G>A,Gag>Aag):ENSP00000378511(p.Glu154Lys)];ENSG00000156860:MISSENSE[ENST00000356166(c.2284G>A,Gag>Aag):ENSP00000348489(p.Glu762Lys)];ENSG00000156860:EXONIC-NC[ENST00000570170(n.1048G>A)];ENSG00000156860:EXONIC-NC[ENST00000494101(n.959G>A)];ENSG00000156860:3UTR[ENST00000543786(c.*435G>A)];	NA	NA	NA	NA	NA	NA	NA	NA	NA	NA	MODIFIER	NA	NA	44	44	0	31	25	6	19.35
Smoker	42477	chr16	33965582	C	T	*NA*	INTERGENIC		NA	NA	NA	0.2372648	0.168472975	NA	NA	NA	NA	NA	MODIFIER	NA	NA	1780	1664	19	1363	1184	38	2.79
Smoker	42477	chr16	33965595	C	A	*NA*	INTERGENIC		NA	NA	0.0001997	0.0048794	0.003693145	0.0001997	0	NA	NA	NA	MODIFIER	NA	NA	1827	1741	11	1404	1275	29	2.07
Smoker	42477	chr16	2835981	C	T	*PRSS33*	INTRONIC	ENSG00000103355:[ENST00000571674(c.47-19G>A)];ENSG00000103355:[ENST00000576886(c.80-19G>A)];ENSG00000103355:[ENST00000293851(c.80-19G>A)];ENSG00000103355:[ENST00000570702(c.80-19G>A)];	NA	NA	NA	8.94E-06	0	NA	NA	NA	NA	NA	MODIFIER	NA	NA	79	77	1	82	64	17	20.73
Smoker	42477	chr16	1538797	G	A	*PTX4*	SILENT	ENSG00000251692:SILENT[ENST00000447419(c.114C>T,ttC>ttT):ENSP00000445277(p.Phe38(=))];ENSG00000251692:SILENT[ENST00000440447(c.114C>T,ttC>ttT):ENSP00000456816(p.Phe38(=))];	NA	NA	NA	NA	NA	NA	NA	NA	NA	NA	LOW	NA	NA	100	100	0	88	75	13	14.77
Smoker	42477	chr16	80581704	G	A	*RP11-525K10.3*	INTRONIC	ENSG00000259867:[ENST00000568776(n.107+15222C>T)];	NA	NA	NA	NA	NA	NA	NA	NA	NA	NA	MODIFIER	NA	NA	139	139	0	213	169	44	20.66
Smoker	42477	chr16	31499872	C	T	*SLC5A2*	INTRONIC	ENSG00000140675:[ENST00000568891(n.281+61C>T)];ENSG00000140675:[ENST00000330498(c.1129+61C>T)];ENSG00000140675:[ENST00000419665(c.1129+61C>T)];ENSG00000140675:[ENST00000568188(n.323C>T)];	NA	NA	NA	NA	NA	NA	NA	NA	NA	NA	MODIFIER	NA	NA	27	27	0	25	20	5	20
Smoker	42477	chr16	18848624	G	A	*SMG1*	SILENT	ENSG00000157106:SILENT[ENST00000389467(c.7555C>T,Cta>Tta):ENSP00000374118(p.Leu2519(=))];ENSG00000157106:SILENT[ENST00000565324(c.7225C>T,Cta>Tta):ENSP00000456259(p.Leu2409(=))];ENSG00000157106:SILENT[ENST00000446231(c.7555C>T,Cta>Tta):ENSP00000402515(p.Leu2519(=))];	NA	NA	chr16*18848624*18848624*G*C*rs201277380*0*0.9998*0.0001997	NA	NA	NA	NA	NA	NA	NA	LOW	NA	1	155	155	0	203	162	40	19.7
Smoker	42477	chr16	30371025	C	T	*TBC1D10B*	INTRONIC	ENSG00000169221:[ENST00000409939(c.1386+23G>A)];ENSG00000169221:[ENST00000566671(n.317+23G>A)];ENSG00000169221:[ENST00000490703(c.330+23G>A)];ENSG00000169221:[ENST00000478158(n.2736+23G>A)];ENSG00000169221:[ENST00000475872(n.450+23G>A)];	NA	NA	NA	4.05E-05	0	NA	NA	NA	NA	NA	MODIFIER	NA	NA	42	42	0	44	29	15	34.09
Smoker	42477	chr16	71570803	G	A	*ZNF19*	INTRONIC	ENSG00000157429:[ENST00000564225(c.-255+9377C>T)];ENSG00000157429:[ENST00000568446(n.105+9377C>T)];ENSG00000157429:[ENST00000566202(c.-190+9302C>T)];	chr16*7157	chr16*7157	NA	3.52E-05	9.19E-05	NA	NA	NA	NA	NA	MODIFIER	NA	NA	112	112	0	135	106	29	21.48
Smoker	42477	chr17	48753835	C	G	*ABCC3*	SILENT	ENSG00000108846:INTRONIC[ENST00000513745(c.688+21C>G)];ENSG00000108846:3UTR[ENST00000502426(c.*1786C>G)];ENSG00000108846:SILENT[ENST00000505699(c.3264C>G,ctC>ctG):ENSP00000427521(p.Leu1088(=))];ENSG00000108846:SILENT[ENST00000285238(c.3264C>G,ctC>ctG):ENSP00000285238(p.Leu1088(=))];	NA	NA	NA	NA	NA	NA	NA	NA	NA	NA	LOW	NA	NA	174	174	0	205	166	39	19.02
Smoker	42477	chr17	962202	C	G	*ABR*	INTRONIC	ENSG00000159842:[ENST00000576964(n.524+8205G>C)];ENSG00000159842:[ENST00000291107(c.1072-95G>C)];ENSG00000159842:[ENST00000574544(c.182-95G>C)];ENSG00000159842:[ENST00000536794(c.529-95G>C)];ENSG00000159842:[ENST00000544583(c.1045-95G>C)];ENSG00000159842:[ENST00000302538(c.1183-95G>C)];ENSG00000159842:[ENST00000574437(c.1045-95G>C)];	NA	NA	NA	NA	NA	NA	NA	NA	NA	NA	MODIFIER	NA	NA	71	71	0	71	59	12	16.9
Smoker	42477	chr17	27936315	C	T	*ANKRD13B*	INTRONIC	ENSG00000198720:[ENST00000487527(n.718+22C>T)];ENSG00000198720:[ENST00000493506(n.704+22C>T)];ENSG00000198720:[ENST00000488766(c.755+22C>T)];ENSG00000198720:[ENST00000394859(c.755+22C>T)];ENSG00000198720:[ENST00000578943(n.555C>T)];	NA	NA	NA	NA	NA	NA	NA	NA	NA	NA	MODIFIER	NA	NA	60	60	0	69	50	19	27.54
Smoker	42477	chr17	9676174	C	T	*DHRS7C*	MISSENSE	ENSG00000184544:MISSENSE[ENST00000330255(c.640G>A,Gat>Aat):ENSP00000327975(p.Asp214Asn)];ENSG00000184544:MISSENSE[ENST00000571134(c.637G>A,Gat>Aat):ENSP00000459579(p.Asp213Asn)];ENSG00000184544:INTRONIC[ENST00000571771(c.191-1161G>A)];	chr17*9676	chr17*9676	0.0001997	8.29E-05	0	0.0001997	0	NA	NA	NA	MODIFIER	NA	NA	86	86	0	100	90	10	10
Smoker	42477	chr17	40705190	C	T	*HSD17B1*	MISSENSE	ENSG00000108786:MISSENSE[ENST00000225929(c.146C>T,gCg>gTg):ENSP00000225929(p.Ala49Val)];ENSG00000108786:MISSENSE[ENST00000585807(c.146C>T,gCg>gTg):ENSP00000466799(p.Ala49Val)];ENSG00000108786:MISSENSE[ENST00000590299(c.146C>T,gCg>gTg):ENSP00000465128(p.Ala49Val)];ENSG00000108786:EXONIC-NC[ENST00000593215(n.158C>T)];ENSG00000108786:EXONIC-NC[ENST00000587280(n.157C>T)];	NA	NA	NA	3.38E-05	0	NA	NA	NA	NA	NA	MODIFIER	NA	NA	129	128	1	137	131	6	4.38
Smoker	42477	chr17	4634777	G	C	*MED11*	5UTR	ENSG00000161920:[ENST00000575284(c.-8G>C)];ENSG00000161920:[ENST00000573708(c.-8G>C)];ENSG00000161920:[ENST00000293777(c.-8G>C)];	NA	NA	chr17*4634777*4634777*G*A*rs774265519*NA*NA*NA	NA	NA	NA	NA	NA	NA	NA	MODIFIER	NA	NA	109	109	0	123	96	27	21.95
Smoker	42477	chr17	72733451	G	A	*RAB37*	MISSENSE	ENSG00000172794:INTRONIC[ENST00000528438(c.12+581G>A)];ENSG00000172794:INTRONIC[ENST00000392615(c.108+195G>A)];ENSG00000172794:INTRONIC[ENST00000392617(n.591-4942G>A)];ENSG00000172794:INTRONIC[ENST00000340415(c.184-4942G>A)];ENSG00000172794:INTRONIC[ENST00000392614(c.108+195G>A)];ENSG00000172794:INTRONIC[ENST00000402449(c.184-4942G>A)];ENSG00000172794:EXONIC-NC[ENST00000527040(n.92G>A)];ENSG00000172794:EXONIC-NC[ENST00000533530(n.94G>A)];ENSG00000172794:EXONIC-NC[ENST00000577548(n.92G>A)];ENSG00000172794:MISSENSE[ENST00000392610(c.40G>A,Gag>Aag):ENSP00000376387(p.Glu14Lys)];ENSG00000172794:MISSENSE[ENST00000392613(c.40G>A,Gag>Aag):ENSP00000376389(p.Glu14Lys)];ENSG00000172794:MISSENSE[ENST00000392612(c.40G>A,Gag>Aag):ENSP00000376388(p.Glu14Lys)];ENSG00000172794:MISSENSE[ENST00000481224(c.40G>A,Gag>Aag):ENSP00000436563(p.Glu14Lys)];	NA	NA	NA	NA	NA	NA	NA	T_lc	BN	N	MODERATE	NA	NA	41	41	0	57	42	15	26.32
Smoker	42477	chr17	40705190	C	T	*RP11-400F19.6*	EXONIC-NC	ENSG00000266962:[ENST00000590513(n.1577G>A)];	NA	NA	NA	3.38E-05	0	NA	NA	NA	NA	NA	MODIFIER	NA	NA	129	128	1	137	131	6	4.38
Smoker	42477	chr17	40705190	C	T	*RP11-400F19.8*	INTRONIC	ENSG00000266929:[ENST00000585572(n.380-8447C>T)];	NA	NA	NA	3.38E-05	0	NA	NA	NA	NA	NA	MODIFIER	NA	NA	129	128	1	137	131	6	4.38
Smoker	42477	chr17	27936315	C	T	*RP11-68I3.2*	INTRONIC	ENSG00000264031:[ENST00000581474(n.153+48598C>T)];	NA	NA	NA	NA	NA	NA	NA	NA	NA	NA	MODIFIER	NA	NA	60	60	0	69	50	19	27.54
Smoker	42477	chr17	28548959	C	G	*SLC6A4*	MISSENSE	ENSG00000108576:MISSENSE[ENST00000261707(c.18G>C,ttG>ttC):ENSP00000261707(p.Leu6Phe)];ENSG00000108576:MISSENSE[ENST00000394821(c.18G>C,ttG>ttC):ENSP00000378298(p.Leu6Phe)];ENSG00000108576:MISSENSE[ENST00000401766(c.18G>C,ttG>ttC):ENSP00000385822(p.Leu6Phe)];	NA	NA	NA	NA	NA	NA	NA	D_lc	PoD	D	MODERATE	N	0.746	46	46	0	93	68	25	26.88

Supplementary Table 4. List of somatic single nucleotide variants identified using whole exome sequencing of ESCC patients

Cohort	Sample	Chromosome	Genomic position	Reference allele	Alternate allele	Gene symbol	Variant type	Variant annotation	OncoMD	COSMIC	dbSNP	ExAC	ExAC_SAS	1000G	1000G_SAS	SIFT	Polyphen2	CONDEL	VEP	LRT	PhastCon7 Way	Total read (Adjacent normal)	Reference allel read support (Adjacent normal)	Alternative read support (Adjacent normal)	Total read (Tumor)	Reference allel read support (Tumor)	Alternative read support (Tumor)	Alternate allele frequency
Smoker	42477	chr17	7577539	G	A	*TP53*	MISSENSE	ENSG00000141510:MISSENSE[ENST00000413465(c.742C>T,Cgg>Tgg):ENSP00000410739(p.Arg248Trp)];ENSG00000141510:MISSENSE[ENST00000420246(c.742C>T,Cgg>Tgg):ENSP00000391127(p.Arg248Trp)];ENSG00000141510:MISSENSE[ENST00000269305(c.742C>T,Cgg>Tgg):ENSP00000269305(p.Arg248Trp)];ENSG00000141510:MISSENSE[ENST00000359597(c.742C>T,Cgg>Tgg):ENSP00000352610(p.Arg248Trp)];ENSG00000141510:MISSENSE[ENST00000445888(c.742C>T,Cgg>Tgg):ENSP00000391478(p.Arg248Trp)];ENSG00000141510:MISSENSE[ENST00000455263(c.742C>T,Cgg>Tgg):ENSP00000398846(p.Arg248Trp)];ENSG00000141510:MISSENSE[ENST00000509690(c.346C>T,Cgg>Tgg):ENSP00000425104(p.Arg116Trp)];ENSG00000141510:MISSENSE[ENST00000514944(c.463C>T,Cgg>Tgg):ENSP00000423862(p.Arg155Trp)];ENSG00000141510:EXONIC-NC[ENST00000504937(n.624C>T)];ENSG00000141510:EXONIC-NC[ENST00000504290(n.624C>T)];ENSG00000141510:EXONIC-NC[ENST00000510385(n.624C>T)];	chr17*7577	chr17*7577	NA	8.24E-06	0	NA	NA	NA	NA	NA	MODIFIER	NA	NA	35	35	0	59	40	19	32.2
Smoker	42477	chr17	9632267	C	G	*USP43*	MISSENSE	ENSG00000154914:MISSENSE[ENST00000574408(c.2691C>G,tCt>tGt):ENSP00000459328(p.Ser898Cys)];ENSG00000154914:MISSENSE[ENST00000570475(c.3317C>G,tCt>tGt):ENSP00000458963(p.Ser1106Cys)];ENSG00000154914:MISSENSE[ENST00000285199(c.3332C>G,tCt>tGt):ENSP00000285199(p.Ser1111Cys)];ENSG00000154914:MISSENSE[ENST00000573955(c.1907C>G,tCt>tGt):ENSP00000477318(p.Ser636Cys)];ENSG00000154914:EXONIC-NC[ENST00000570827(n.3473C>G)];	NA	NA	NA	NA	NA	NA	NA	NA	NA	NA	MODIFIER	NA	NA	102	102	0	157	125	32	20.38
Smoker	42477	chr17	7369526	C	T	*ZBTB4*	MISSENSE	ENSG00000174282:MISSENSE[ENST00000311403(c.595G>A,Gaa>Aaa):ENSP00000307858(p.Glu199Lys)];ENSG00000174282:MISSENSE[ENST00000380599(c.595G>A,Gaa>Aaa):ENSP00000369973(p.Glu199Lys)];	NA	NA	NA	NA	NA	NA	NA	NA	PoD	NA	MODERATE	N	0.032	40	40	0	36	25	11	30.56
Smoker	42477	chr18	72114037	G	A	*FAM69C*	MISSENSE	ENSG00000187773:MISSENSE[ENST00000343998(c.680C>T,gCg>gTg):ENSP00000344331(p.Ala227Val)];ENSG00000187773:INTRONIC[ENST00000400291(c.-21-4686C>T)];	chr18*7211	chr18*7211	0.0003994	0.0001048	0	0.0003994	0	NA	NA	NA	MODIFIER	NA	NA	52	52	0	43	33	10	23.26
Smoker	42477	chr18	11689839	G	A	*GNAL*	MISSENSE	ENSG00000141404:EXONIC-NC[ENST00000585590(n.151G>A)];ENSG00000141404:MISSENSE[ENST00000334049(c.277G>A,Gag>Aag):ENSP00000334051(p.Glu93Lys)];	NA	NA	NA	0	0	NA	NA	T	BN	N	MODERATE	U	0.316	87	87	0	79	66	13	16.46
Smoker	42477	chr19	47774624	G	A	*CCDC9*	MISSENSE	ENSG00000105321:MISSENSE[ENST00000221922(c.1285G>A,Gag>Aag):ENSP00000221922(p.Glu429Lys)];	NA	NA	NA	NA	NA	NA	NA	T	UN	NA	MODERATE	N	0.316	60	60	0	31	21	10	32.26
Smoker	42477	chr19	39422960	C	T	*CTC-360G5.8*	INTRONIC	ENSG00000269547:[ENST00000599996(c.476-6020G>A)];	NA	NA	NA	4.64E-05	0.000222074	NA	NA	NA	NA	NA	MODIFIER	NA	NA	100	100	0	79	57	22	27.85
Smoker	42477	chr19	10335096	G	A	*DNMT1*	INTRONIC	ENSG00000130816:[ENST00000588952(c.-401-5551C>T)];ENSG00000130816:[ENST00000592342(c.-284+6784C>T)];	chr19*1033	chr19*1033	NA	2.50E-05	6.06E-05	NA	NA	NA	NA	NA	LOW	NA	NA	79	79	0	73	58	14	19.18
Smoker	42477	chr19	10795122	A	G	*ILF3*	MISSENSE	ENSG00000129351:INTRONIC[ENST00000590261(c.2059+476A>G)];ENSG00000129351:INTRONIC[ENST00000318511(c.2059+476A>G)];ENSG00000129351:INTRONIC[ENST00000593061(c.233+476A>G)];ENSG00000129351:INTRONIC[ENST00000590869(c.88+476A>G)];ENSG00000129351:INTRONIC[ENST00000250241(c.2060-413A>G)];ENSG00000129351:INTRONIC[ENST00000588657(c.2071+476A>G)];ENSG00000129351:INTRONIC[ENST00000420083(c.2060-413A>G)];ENSG00000129351:INTRONIC[ENST00000449870(c.2071+476A>G)];ENSG00000129351:EXONIC-NC[ENST00000587928(n.3039A>G)];ENSG00000129351:MISSENSE[ENST00000589998(c.2090A>G,cAt>cGt):ENSP00000465219(p.His697Arg)];ENSG00000129351:MISSENSE[ENST00000407004(c.2102A>G,cAt>cGt):ENSP00000384660(p.His701Arg)];ENSG00000129351:3UTR[ENST00000592763(c.*450A>G)];	NA	NA	NA	NA	NA	NA	NA	NA	NA	NA	MODIFIER	NA	NA	210	210	0	197	157	39	19.8
Smoker	42477	chr19	18391466	G	A	*JUND*	MISSENSE	ENSG00000130522:MISSENSE[ENST00000252818(c.829C>T,Cgc>Tgc):ENSP00000252818(p.Arg277Cys)];ENSG00000130522:MISSENSE[ENST00000600972(c.274C>T,Cgc>Tgc):ENSP00000475153(p.Arg92Cys)];	NA	NA	chr19*18391461*18391473*GTTGCGCAGCCGC*G*rs772178889*NA*NA*NA	NA	NA	NA	NA	D	PrD	D	MODERATE	NA	NA	119	116	1	116	88	28	24.14
Smoker	42477	chr19	6420040	C	T	*KHSRP*	INTRONIC	ENSG00000088247:[ENST00000599395(c.303+393G>A)];ENSG00000088247:[ENST00000595548(c.115+44G>A)];ENSG00000088247:[ENST00000595112(n.211+44G>A)];ENSG00000088247:[ENST00000595258(c.115+44G>A)];ENSG00000088247:[ENST00000398148(c.547+44G>A)];	NA	NA	NA	NA	NA	NA	NA	NA	NA	NA	MODIFIER	NA	NA	73	72	0	70	60	10	14.29
Smoker	42477	chr19	39422960	C	T	*MRPS12*	INTRONIC	ENSG00000128626:[ENST00000407800(c.50-13C>T)];ENSG00000128626:[ENST00000308018(c.50-13C>T)];ENSG00000128626:[ENST00000402029(c.50-13C>T)];	NA	NA	NA	4.64E-05	0.000222074	NA	NA	NA	NA	NA	MODIFIER	NA	NA	100	100	0	79	57	22	27.85
Smoker	42477	chr19	14076324	C	G	*RFX1*	MISSENSE	ENSG00000132005:MISSENSE[ENST00000254325(c.2148G>C,aaG>aaC):ENSP00000254325(p.Lys716Asn)];	NA	NA	NA	NA	NA	NA	NA	D	PoD	D	MODERATE	D	1	59	59	0	37	30	7	18.92
Smoker	42477	chr19	10335096	G	A	*S1PR2*	SILENT	ENSG00000267534:SILENT[ENST00000590320(c.486C>T,ctC>ctT):ENSP00000466933(p.Leu162(=))];	chr19*1033	chr19*1033	NA	2.50E-05	6.06E-05	NA	NA	NA	NA	NA	LOW	NA	NA	79	79	0	73	58	14	19.18
Smoker	42477	chr19	39422960	C	T	*SARS2*	INTRONIC	ENSG00000104835:[ENST00000448145(c.268-6020G>A)];	NA	NA	NA	4.64E-05	0.000222074	NA	NA	NA	NA	NA	MODIFIER	NA	NA	100	100	0	79	57	22	27.85
Smoker	42477	chr19	55944913	C	T	*SHISA7*	SILENT	ENSG00000187902:SILENT[ENST00000376325(c.1227G>A,tcG>tcA):ENSP00000365503(p.Ser409(=))];ENSG00000187902:3UTR[ENST00000416792(c.*290G>A)];	NA	NA	NA	NA	NA	NA	NA	NA	NA	NA	MODIFIER	NA	NA	68	68	0	55	48	7	12.73
Smoker	42477	chr19	33702284	G	T	*SLC7A10*	EXONIC-NC	ENSG00000130876:[ENST00000590490(n.312C>A)];ENSG00000130876:[ENST00000253188(c.912+36C>A)];ENSG00000130876:[ENST00000590036(c.912+36C>A)];	NA	NA	chr19*33702273*33702303*GGCAGGGGCCTGGGCAGGAGCCAGGGACCGA*G*rs771603397*NA*NA*NA	NA	NA	NA	NA	NA	NA	NA	MODIFIER	NA	NA	105	105	0	66	41	25	37.88
Smoker	42477	chr2	202626292	G	A	*ALS2*	MISSENSE	ENSG00000003393:MISSENSE[ENST00000467448(c.425C>T,tCt>tTt):ENSP00000429223(p.Ser142Phe)];ENSG00000003393:MISSENSE[ENST00000264276(c.425C>T,tCt>tTt):ENSP00000264276(p.Ser142Phe)];ENSG00000003393:EXONIC-NC[ENST00000482891(n.565C>T)];ENSG00000003393:EXONIC-NC[ENST00000482789(n.565C>T)];ENSG00000003393:EXONIC-NC[ENST00000496244(n.602C>T)];	NA	NA	NA	NA	NA	NA	NA	NA	NA	NA	MODIFIER	NA	NA	109	109	0	131	104	27	20.61

Mangalaparthi *et al.* , 2020. Mutational landscape of esophageal squamous cell carcinoma in an Indian cohort

Supplementary Table 4. List of somatic single nucleotide variants identified using whole exome sequencing of ESCC patients

Cohort	Sample	Chromosome	Genomic position	Reference allele	Alternate allele	Gene symbol	Variant type	Variant annotation	OncoMD	COSMIC	dbSNP	ExAC	ExAC_SAS	1000G	1000G_SAS	SIFT	Polyphen2	CONDEL	VEP	LRT	PhastCon7 Way	Total read (Adjacent normal)	Reference allel read support (Adjacent normal)	Alternative read support (Adjacent normal)	Total read (Tumor)	Reference allel read support (Tumor)	Alternative read support (Tumor)	Alternate allele frequency
Smoker	42477	chr2	238243326	C	A	*COL6A3*	MISSENSE	ENSG00000163359:MISSENSE[ENST00000472056(c.7351G>T,Gtg>Ttg):ENSP00000418285(p.Val2451Leu)];ENSG00000163359:MISSENSE[ENST00000409809(c.8554G>T,Gtg>Ttg):ENSP00000386844(p.Val2852Leu)];ENSG00000163359:MISSENSE[ENST00000295550(c.9172G>T,Gtg>Ttg):ENSP00000295550(p.Val3058Leu)];ENSG00000163359:MISSENSE[ENST00000347401(c.8569G>T,Gtg>Ttg):ENSP00000315609(p.Val2857Leu)];ENSG00000163359:MISSENSE[ENST00000346358(c.8572G>T,Gtg>Ttg):ENSP00000295546(p.Val2858Leu)];ENSG00000163359:MISSENSE[ENST00000353578(c.8554G>T,Gtg>Ttg):ENSP00000315873(p.Val2852Leu)];ENSG00000163359:EXONIC-NC[ENST00000493608(n.104G>T)];ENSG00000163359:EXONIC-NC[ENST00000491769(n.5614G>T)];	NA	NA	NA	NA	NA	NA	NA	NA	NA	NA	MODIFIER	NA	NA	25	25	0	25	18	7	28
Smoker	42477	chr2	166611459	G	A	*GALNT3*	MISSENSE	ENSG00000115339:MISSENSE[ENST00000392701(c.1507C>T,Cct>Tct):ENSP00000376465(p.Pro503Ser)];ENSG00000115339:MISSENSE[ENST00000409882(c.721C>T,Cct>Tct):ENSP00000386955(p.Pro241Ser)];	NA	NA	NA	NA	NA	NA	NA	T	PrD	D	MODERATE	D	1	67	67	0	85	80	5	5.88
Smoker	42477	chr2	138722149	C	A	*HNMT*	MISSENSE	ENSG00000150540:MISSENSE[ENST00000280096(c.88C>A,Cag>Aag):ENSP00000280096(p.Gln30Lys)];ENSG00000150540:MISSENSE[ENST00000329366(c.88C>A,Cag>Aag):ENSP00000333259(p.Gln30Lys)];ENSG00000150540:MISSENSE[ENST00000280097(c.88C>A,Cag>Aag):ENSP00000280097(p.Gln30Lys)];ENSG00000150540:MISSENSE[ENST00000475675(c.88C>A,Cag>Aag):ENSP00000419415(p.Gln30Lys)];ENSG00000150540:MISSENSE[ENST00000410115(c.88C>A,Cag>Aag):ENSP00000386940(p.Gln30Lys)];ENSG00000150540:EXONIC-NC[ENST00000485653(n.73C>A)];ENSG00000150540:EXONIC-NC[ENST00000467390(n.100C>A)];ENSG00000150540:EXONIC-NC[ENST00000480534(n.66C>A)];	NA	NA	NA	NA	NA	NA	NA	NA	NA	NA	MODIFIER	NA	NA	94	94	0	141	134	7	4.96
Smoker	42477	chr2	155555545	C	A	*KCNJ3*	SILENT	ENSG00000162989:SILENT[ENST00000295101(c.258C>A,atC>atA):ENSP00000295101(p.Ile86(=))];ENSG00000162989:SILENT[ENST00000544049(c.258C>A,atC>atA):ENSP00000438410(p.Ile86(=))];	NA	NA	NA	NA	NA	NA	NA	NA	NA	NA	LOW	NA	1	58	57	0	103	84	19	18.45
Smoker	42477	chr2	149227011	C	A	*MBD5*	MISSENSE	ENSG00000204406:MISSENSE[ENST00000407073(c.1499C>A,cCa>cAa):ENSP00000386049(p.Pro500Gln)];ENSG00000204406:MISSENSE[ENST00000404807(c.1499C>A,cCa>cAa):ENSP00000384672(p.Pro500Gln)];ENSG00000204406:MISSENSE[ENST00000416015(c.718C>A,cCa>cAa):ENSP00000393168(p.Pro240Gln)];	NA	NA	NA	NA	NA	NA	NA	D	PrD	D	MODERATE	NA	NA	103	103	0	141	130	11	7.8
Smoker	42477	chr2	101604715	G	C	*NPAS2*	MISSENSE	ENSG00000170485:MISSENSE[ENST00000433408(c.299G>C,Gaa>Caa):ENSP00000393396(p.Glu101Gln)];ENSG00000170485:MISSENSE[ENST00000335681(c.1804G>C,Gaa>Caa):ENSP00000338283(p.Glu602Gln)];ENSG00000170485:MISSENSE[ENST00000542504(c.1999G>C,Gaa>Caa):ENSP00000438428(p.Glu667Gln)];ENSG00000170485:EXONIC-NC[ENST00000474550(n.6839G>C)];ENSG00000170485:INTRONIC[ENST00000450763(c.427-5094G>C)];	NA	NA	NA	NA	NA	NA	NA	NA	NA	NA	MODIFIER	NA	NA	61	61	0	77	56	21	27.27
Smoker	42477	chr2	136374230	C	G	*R3HDM1*	INTRONIC	ENSG00000048991:[ENST00000488035(n.108-4834C>G)];ENSG00000048991:[ENST00000436436(c.171+11644C>G)];ENSG00000048991:[ENST00000409478(c.172-4834C>G)];ENSG00000048991:[ENST00000410054(c.136-4834C>G)];ENSG00000048991:[ENST00000329971(c.172-4834C>G)];ENSG00000048991:[ENST00000264160(c.214-8C>G)];ENSG00000048991:[ENST00000456040(c.162-8C>G)];ENSG00000048991:[ENST00000409606(c.214-8C>G)];	NA	NA	NA	NA	NA	NA	NA	NA	NA	NA	MODIFIER	NA	NA	74	74	0	115	92	22	19.13
Smoker	42477	chr2	1168662	C	A	*SNTG2*	INTRONIC	ENSG00000172554:[ENST00000308624(c.500-116C>A)];ENSG00000172554:[ENST00000467759(n.132-116C>A)];ENSG00000172554:[ENST00000463442(n.378-116C>A)];ENSG00000172554:[ENST00000475201(n.132-116C>A)];ENSG00000172554:[ENST00000407292(c.211-36127C>A)];ENSG00000172554:[ENST00000494178(n.252-116C>A)];	NA	NA	NA	NA	NA	NA	NA	NA	NA	NA	MODIFIER	NA	NA	26	26	0	40	31	9	22.5
Smoker	42477	chr2	210694092	C	T	*UNC80*	MISSENSE	ENSG00000144406:MISSENSE[ENST00000439458(c.2615C>T,cCg>cTg):ENSP00000391088(p.Pro872Leu)];ENSG00000144406:MISSENSE[ENST00000272845(c.2600C>T,cCg>cTg):ENSP00000272845(p.Pro867Leu)];ENSG00000144406:EXONIC-NC[ENST00000489023(n.140C>T)];	NA	NA	NA	4.63E-05	0	NA	NA	NA	NA	NA	MODIFIER	NA	NA	85	84	0	150	119	31	20.67
Smoker	42477	chr2	168104414	C	A	*XIRP2*	MISSENSE	ENSG00000163092:INTRONIC[ENST00000409605(c.510+5994C>A)];ENSG00000163092:INTRONIC[ENST00000420519(c.1275+5994C>A)];ENSG00000163092:INTRONIC[ENST00000409756(c.1176+5994C>A)];ENSG00000163092:INTRONIC[ENST00000409043(c.1176+5994C>A)];ENSG00000163092:INTRONIC[ENST00000409728(c.1275+5994C>A)];ENSG00000163092:MISSENSE[ENST00000409273(c.5846C>A,cCa>cAa):ENSP00000387255(p.Pro1949Gln)];ENSG00000163092:MISSENSE[ENST00000409195(c.6512C>A,cCa>cAa):ENSP00000386840(p.Pro2171Gln)];ENSG00000163092:MISSENSE[ENST00000295237(c.6512C>A,cCa>cAa):ENSP00000295237(p.Pro2171Gln)];	chr2*16810	NA	NA	NA	NA	NA	NA	NA	BN	NA	MODERATE	N	0.019	231	231	0	222	175	46	20.72
Smoker	42477	chr20	61907452	G	C	*ARFGAP1*	MISSENSE	ENSG00000101199:MISSENSE[ENST00000370275(c.70G>C,Gag>Cag):ENSP00000359298(p.Glu24Gln)];ENSG00000101199:MISSENSE[ENST00000370283(c.70G>C,Gag>Cag):ENSP00000359306(p.Glu24Gln)];ENSG00000101199:MISSENSE[ENST00000353546(c.70G>C,Gag>Cag):ENSP00000314615(p.Glu24Gln)];ENSG00000101199:MISSENSE[ENST00000550188(c.70G>C,Gag>Cag):ENSP00000449515(p.Glu24Gln)];ENSG00000101199:MISSENSE[ENST00000520485(c.70G>C,Gag>Cag):ENSP00000427717(p.Glu24Gln)];ENSG00000101199:MISSENSE[ENST00000523114(c.70G>C,Gag>Cag):ENSP00000428355(p.Glu24Gln)];ENSG00000101199:MISSENSE[ENST00000522403(c.70G>C,Gag>Cag):ENSP00000430929(p.Glu24Gln)];ENSG00000101199:EXONIC-NC[ENST00000522959(n.193G>C)];ENSG00000101199:EXONIC-NC[ENST00000519531(n.166G>C)];ENSG00000101199:EXONIC-NC[ENST00000524192(n.170G>C)];ENSG00000101199:INTRONIC[ENST00000523460(c.42+478G>C)];ENSG00000101199:INTRONIC[ENST00000518601(c.-52-380G>C)];ENSG00000101199:INTRONIC[ENST00000519604(c.12-380G>C)];ENSG00000101199:INTRONIC[ENST00000547204(c.-52-380G>C)];ENSG00000101199:INTRONIC[ENST00000549047(c.-52-380G>C)];ENSG00000101199:5UTR[ENST00000519273(c.-169G>C)];	NA	NA	NA	NA	NA	NA	NA	NA	NA	NA	MODIFIER	NA	NA	190	190	0	166	129	37	22.29
Smoker	42477	chr20	33857655	C	T	*EDEM2*	INTRONIC	ENSG00000088298:[ENST00000540582(c.-502+2153G>A)];	NA	NA	0.001997	0.0018781	0.003656999	0.0019968	0.0051	NA	NA	NA	MODIFIER	NA	NA	78	78	0	73	65	8	10.96
Smoker	42477	chr20	47990088	C	T	*KCNB1*	MISSENSE	ENSG00000158445:MISSENSE[ENST00000371741(c.2009G>A,cGa>cAa):ENSP00000360806(p.Arg670Gln)];	NA	NA	NA	1.65E-05	0	NA	NA	D_lc	PoD	D	MODERATE	N	0.991	96	96	0	114	86	28	24.56

Mangalaparthi *et al.* , 2020. Mutational landscape of esophageal squamous cell carcinoma in an Indian cohort
Supplementary Table 4. List of somatic single nucleotide variants identified using whole exome sequencing of ESCC patients

Cohort	Sample	Chromosome	Genomic position	Reference allele	Alternate allele	Gene symbol	Variant type	Variant annotation	OncoMD	COSMIC	dbSNP	ExAC	ExAC_SAS	1000G	1000G_SAS	SIFT	Polyphen2	CONDEL	VEP	LRT	PhastCon7 Way	Total read (Adjacent normal)	Reference allel read support (Adjacent normal)	Alternative read support (Adjacent normal)	Total read (Tumor)	Reference allel read support (Tumor)	Alternative read support (Tumor)	Alternate allele frequency
Smoker	42477	chr20	33857655	C	T	*MMP24*	SILENT	ENSG00000125966:SILENT[ENST00000246186(c.1287C>T,atC>atT):ENSP00000246186(p.Ile429(=))];	NA	NA	0.001997	0.0018781	0.003656999	0.0019968	0.0051	NA	NA	NA	MODIFIER	NA	NA	78	78	0	73	65	8	10.96
Smoker	42477	chr20	33857655	C	T	*MMP24-AS1*	INTRONIC	ENSG00000126005:[ENST00000453892(n.52+2153G>A)];ENSG00000126005:[ENST00000456350(n.236+2153G>A)];ENSG00000126005:[ENST00000438751(n.237+2153G>A)];ENSG00000126005:[ENST00000566203(n.255+2153G>A)];ENSG00000126005:[ENST00000454184(n.227+2153G>A)];ENSG00000126005:[ENST00000433764(n.221+2153G>A)];	NA	NA	0.001997	0.0018781	0.003656999	0.0019968	0.0051	NA	NA	NA	MODIFIER	NA	NA	78	78	0	73	65	8	10.96
Smoker	42477	chr20	33857655	C	T	*RP4-614O4.11*	INTRONIC	ENSG00000261582:[ENST00000444717(n.426-1864G>A)];	NA	NA	0.001997	0.0018781	0.003656999	0.0019968	0.0051	NA	NA	NA	MODIFIER	NA	NA	78	78	0	73	65	8	10.96
Smoker	42477	chr20	44986452	A	C	*SLC35C2*	INTRONIC	ENSG00000080189:[ENST00000543605(c.208-40T>G)];ENSG00000080189:[ENST00000424568(c.121-40T>G)];ENSG00000080189:[ENST00000372229(c.9+829T>G)];ENSG00000080189:[ENST00000372230(c.121-40T>G)];ENSG00000080189:[ENST00000484318(n.398-111T>G)];ENSG00000080189:[ENST00000317734(c.121-40T>G)];ENSG00000080189:[ENST00000243896(c.121-40T>G)];ENSG00000080189:[ENST00000372227(c.121-40T>G)];ENSG00000080189:[ENST00000420518(c.121-40T>G)];ENSG00000080189:[ENST00000481809(n.389-111T>G)];	NA	NA	NA	NA	NA	NA	NA	NA	NA	NA	MODIFIER	NA	NA	40	40	0	57	42	15	26.32
Smoker	42477	chr20	62421310	C	T	*ZBTB46*	SILENT	ENSG00000130584:SILENT[ENST00000302995(c.801G>A,acG>acA):ENSP00000303102(p.Thr267(=))];ENSG00000130584:SILENT[ENST00000395104(c.801G>A,acG>acA):ENSP00000378536(p.Thr267(=))];ENSG00000130584:SILENT[ENST00000245663(c.801G>A,acG>acA):ENSP00000245663(p.Thr267(=))];	NA	NA	0.001597	0.0045116	0.000666182	0.0015974	0	NA	NA	NA	LOW	NA	NA	106	104	0	123	96	27	21.95
Smoker	42477	chr21	16015455	C	G	*AF165138.7*	INTRONIC-SS-ACR	ENSG00000243440:INTRONIC-SS-ACR[ENST00000400562(c.25-1G>C)];	NA	NA	NA	NA	NA	NA	NA	NA	NA	NA	HIGH	NA	NA	69	69	0	85	65	20	23.53
Smoker	42477	chr21	32202031	G	C	*AP000244.1*	EXONIC-NC	ENSG00000256386:[ENST00000539876(n.21C>G)];	NA	NA	NA	NA	NA	NA	NA	NA	NA	NA	MODIFIER	NA	NA	47	47	0	79	62	17	21.52
Smoker	42477	chr21	32202031	G	C	*KRTAP7-1*	EXONIC-NC	ENSG00000184586:[ENST00000452750(n.48C>G)];	NA	NA	NA	NA	NA	NA	NA	NA	NA	NA	MODIFIER	NA	NA	47	47	0	79	62	17	21.52
Smoker	42477	chr21	45089796	C	A	*RRP1B*	SILENT	ENSG00000160208:SILENT[ENST00000340648(c.162C>A,atC>atA):ENSP00000339145(p.Ile54(=))];	NA	NA	NA	NA	NA	NA	NA	NA	NA	NA	LOW	NA	0.999	47	47	0	66	60	6	9.09
Smoker	42477	chr21	45478939	G	C	*TRAPPC10*	INTRONIC	ENSG00000160218:[ENST00000291574(c.679-45G>C)];ENSG00000160218:[ENST00000422875(c.679-45G>C)];ENSG00000160218:[ENST00000380221(c.679-45G>C)];	NA	NA	NA	NA	NA	NA	NA	NA	NA	NA	MODIFIER	NA	NA	16	16	0	19	14	5	26.32
Smoker	42477	chr22	41545804	A	T	*EP300*	MISSENSE	ENSG00000100393:MISSENSE[ENST00000263253(c.2419A>T,Ata>Tta):ENSP00000263253(p.Ile807Leu)];	chr22*4154	NA	chr22*41545804*41545804*A*G*rs201054979*0*0.9998*0.0001997	NA	NA	NA	NA	NA	UN	NA	MODERATE	N	1	126	126	0	158	87	71	44.94
Smoker	42477	chr3	194177790	G	A	*ATP13A3*	INTRONIC	ENSG00000133657:[ENST00000256031(c.560+33C>T)];ENSG00000133657:[ENST00000439040(c.560+33C>T)];	NA	NA	NA	NA	NA	NA	NA	NA	NA	NA	MODIFIER	NA	NA	55	55	0	116	101	15	12.93
Smoker	42477	chr3	107770753	C	A	*CD47*	INTRONIC	ENSG00000196776:[ENST00000398258(c.197+33G>T)];ENSG00000196776:[ENST00000471694(n.2801+33G>T)];ENSG00000196776:[ENST00000517766(c.299+33G>T)];ENSG00000196776:[ENST00000361309(c.909+33G>T)];ENSG00000196776:[ENST00000355354(c.909+33G>T)];	NA	NA	NA	NA	NA	NA	NA	NA	NA	NA	MODIFIER	NA	NA	41	41	0	53	39	14	26.42
Smoker	42477	chr3	100328699	C	T	*GPR128*	5UTR	ENSG00000144820:[ENST00000273352(c.-2C>T)];	NA	NA	NA	NA	NA	NA	NA	NA	NA	NA	MODIFIER	NA	NA	61	60	0	81	67	14	17.28
Smoker	42477	chr3	126751293	G	A	*PLXNA1*	SILENT	ENSG00000114554:EXONIC-NC[ENST00000503234(n.2488G>A)];ENSG00000114554:EXONIC-NC[ENST00000505278(n.171G>A)];ENSG00000114554:SILENT[ENST00000393409(c.5295G>A,aaG>aaA):ENSP00000377061(p.Lys1765(=))];ENSG00000114554:SILENT[ENST00000251772(c.5226G>A,aaG>aaA):ENSP00000251772(p.Lys1742(=))];	NA	NA	NA	NA	NA	NA	NA	NA	NA	NA	MODIFIER	NA	NA	196	196	0	234	190	43	18.38
Smoker	42477	chr3	155571039	G	C	*SLC33A1*	MISSENSE	ENSG00000169359:MISSENSE[ENST00000392845(c.748C>G,Caa>Gaa):ENSP00000376587(p.Gln250Glu)];ENSG00000169359:MISSENSE[ENST00000359479(c.748C>G,Caa>Gaa):ENSP00000352456(p.Gln250Glu)];ENSG00000169359:MISSENSE[ENST00000468581(c.68C>G,Caa>Gaa):ENSP00000418847(p.Gln24Glu)];ENSG00000169359:EXONIC-NC[ENST00000460729(n.113C>G)];ENSG00000169359:INTRONIC[ENST00000475842(c.121+121C>G)];	NA	NA	NA	NA	NA	NA	NA	NA	NA	NA	MODIFIER	NA	NA	65	65	0	85	69	16	18.82
Smoker	42477	chr3	161089993	G	T	*SPTSSB*	5UTR	ENSG00000196542:[ENST00000359175(c.-896C>A)];	NA	NA	NA	NA	NA	NA	NA	NA	NA	NA	MODIFIER	NA	NA	56	55	0	99	78	21	21.21
Smoker	42477	chr3	133474337	C	T	*TF*	SILENT-SS-PRX	ENSG00000091513:[ENST00000482271(c.252C>T,ttC>ttT):ENSP00000419338(p.Phe84(=))];ENSG00000091513:[ENST00000402696(c.633C>T,ttC>ttT):ENSP00000385834(p.Phe211(=))];ENSG00000091513:[ENST00000264998(c.252C>T,ttC>ttT):ENSP00000264998(p.Phe84(=))];ENSG00000091513:[ENST00000493011(n.681C>T)];ENSG00000091513:[ENST00000485977(c.158-1439C>T)];	NA	NA	NA	NA	NA	NA	NA	NA	NA	NA	LOW	NA	0.881	61	61	0	82	52	29	35.37
Smoker	42477	chr3	179483558	G	C	*USP13*	MISSENSE	ENSG00000058056:MISSENSE[ENST00000263966(c.2335G>C,Gag>Cag):ENSP00000263966(p.Glu779Gln)];ENSG00000058056:MISSENSE[ENST00000496897(c.2140G>C,Gag>Cag):ENSP00000417146(p.Glu714Gln)];	chr3*17948	chr3*17948	NA	NA	NA	NA	NA	T	PoD	N	MODERATE	N	0.997	185	184	0	448	366	82	18.3
Smoker	42477	chr4	13578553	C	G	*BOD1L1*	MISSENSE	ENSG00000038219:MISSENSE[ENST00000507943(c.273G>C,Gag>Cag):ENSP00000425492(p.Glu92Gln)];ENSG00000038219:MISSENSE[ENST00000040738(c.8947G>C,Gag>Cag):ENSP00000040738(p.Glu2983Gln)];ENSG00000038219:EXONIC-NC[ENST00000505343(n.768G>C)];	NA	NA	chr4*13578553*13578553*C*T*rs757828138*NA*NA*NA	NA	NA	NA	NA	NA	BN	NA	MODERATE	N	0.993	64	64	0	105	80	25	23.81
Smoker	42477	chr4	144619112	C	G	*FREM3*	MISSENSE	ENSG00000183090:MISSENSE[ENST00000329798(c.2717G>C,aGa>aCa):ENSP00000332886(p.Arg906Thr)];	NA	NA	NA	NA	NA	NA	NA	D	PoD	D	MODERATE	D	0.023	103	102	0	173	144	29	16.76
Smoker	42477	chr4	154388431	C	G	*KIAA0922*	MISSENSE-SS-PRX	ENSG00000121210:[ENST00000440693(c.193C>G,Cag>Gag):ENSP00000409663(p.Gln65Glu)];ENSG00000121210:[ENST00000409959(c.193C>G,Cag>Gag):ENSP00000386787(p.Gln65Glu)];ENSG00000121210:[ENST00000409663(c.193C>G,Cag>Gag):ENSP00000386574(p.Gln65Glu)];ENSG00000121210:[ENST00000445960(c.193C>G,Cag>Gag):ENSP00000413054(p.Gln65Glu)];	NA	NA	NA	5.05E-05	0.00012639	NA	NA	D	BN	N	MODERATE	NA	NA	69	69	0	99	72	27	27.27
Smoker	42477	chr4	41634728	C	T	*LIMCH1*	INTRONIC	ENSG00000064042:[ENST00000512632(c.936-6221C>T)];ENSG00000064042:[ENST00000512820(c.936-11789C>T)];ENSG00000064042:[ENST00000503057(c.1602-38C>T)];ENSG00000064042:[ENST00000508501(c.936-6221C>T)];ENSG00000064042:[ENST00000313860(c.936-6221C>T)];ENSG00000064042:[ENST00000513024(c.459-11789C>T)];ENSG00000064042:[ENST00000396595(c.474-6221C>T)];ENSG00000064042:[ENST00000514096(c.495-11789C>T)];ENSG00000064042:[ENST00000509277(c.474-11789C>T)];ENSG00000064042:[ENST00000508466(c.440-6221C>T)];ENSG00000064042:[ENST00000511496(c.459-6221C>T)];ENSG00000064042:[ENST00000381753(c.474-11789C>T)];ENSG00000064042:[ENST00000512946(c.936-6221C>T)];	NA	NA	NA	NA	NA	NA	NA	NA	NA	NA	MODIFIER	NA	NA	108	108	0	87	62	25	28.74

Mangalaparthi *et al.* , 2020. Mutational landscape of esophageal squamous cell carcinoma in an Indian cohort

Supplementary Table 4. List of somatic single nucleotide variants identified using whole exome sequencing of ESCC patients

Cohort	Sample	Chromosome	Genomic position	Reference allele	Alternate allele	Gene symbol	Variant type	Variant annotation	OncoMD	COSMIC	dbSNP	ExAC	ExAC_SAS	1000G	1000G_SAS	SIFT	Polyphen2	CONDEL	VEP	LRT	PhastCon7 Way	Total read (Adjacent normal)	Reference allel read support (Adjacent normal)	Alternative read support (Adjacent normal)	Total read (Tumor)	Reference allel read support (Tumor)	Alternative read support (Tumor)	Alternate allele frequency
Smoker	42477	chr4	90857132	C	T	*MMRN1*	SILENT	ENSG00000138722:SILENT[ENST00000394980(c.2301C>T,tcC>tcT):ENSP00000378431(p.Ser767(=))];ENSG00000138722:SILENT[ENST00000508372(c.1527C>T,tcC>tcT):ENSP00000426461(p.Ser509(=))];ENSG00000138722:SILENT[ENST00000264790(c.2301C>T,tcC>tcT):ENSP00000264790(p.Ser767(=))];ENSG00000138722:INTRONIC[ENST00000394981(c.1027+8013C>T)];	NA	NA	0.0001997	8.31E-05	0.000427403	0.0001997	0.001	NA	NA	NA	MODIFIER	NA	NA	100	99	0	125	97	28	22.4
Smoker	42477	chr4	56482489	C	T	*NMU*	INTRONIC	ENSG00000109255:[ENST00000511469(c.172-7143G>A)];ENSG00000109255:[ENST00000507338(c.219+16G>A)];ENSG00000109255:[ENST00000505262(c.219+16G>A)];ENSG00000109255:[ENST00000264218(c.219+16G>A)];ENSG00000109255:[ENST00000509371(n.109+16G>A)];ENSG00000109255:[ENST00000515325(n.262+16G>A)];	NA	NA	NA	NA	NA	NA	NA	NA	NA	NA	MODIFIER	NA	NA	65	65	0	104	97	7	6.73
Smoker	42477	chr4	2948264	G	A	*NOP14*	MISSENSE	ENSG00000087269:MISSENSE[ENST00000398071(c.1510C>T,Cat>Tat):ENSP00000381146(p.His504Tyr)];ENSG00000087269:MISSENSE[ENST00000416614(c.1510C>T,Cat>Tat):ENSP00000405068(p.His504Tyr)];ENSG00000087269:MISSENSE[ENST00000502735(c.1510C>T,Cat>Tat):ENSP00000427415(p.His504Tyr)];ENSG00000087269:MISSENSE[ENST00000314262(c.1510C>T,Cat>Tat):ENSP00000315674(p.His504Tyr)];	NA	NA	NA	NA	NA	NA	NA	NA	NA	NA	MODIFIER	NA	NA	134	134	0	185	150	34	18.38
Smoker	42477	chr4	2948264	G	A	*NOP14-AS1*	INTRONIC	ENSG00000249673:[ENST00000515194(n.2698-336G>A)];ENSG00000249673:[ENST00000503709(n.580-336G>A)];ENSG00000249673:[ENST00000507702(n.251-336G>A)];ENSG00000249673:[ENST00000505731(n.1199-336G>A)];	NA	NA	NA	NA	NA	NA	NA	NA	NA	NA	MODIFIER	NA	NA	134	134	0	185	150	34	18.38
Smoker	42477	chr4	1087472	A	G	*RNF212*	MISSENSE	ENSG00000178222:INTRONIC[ENST00000508428(c.*26-2846T>C)];ENSG00000178222:INTRONIC[ENST00000433731(c.247-2846T>C)];ENSG00000178222:INTRONIC[ENST00000511620(c.247-2846T>C)];ENSG00000178222:INTRONIC[ENST00000510715(c.*111-2846T>C)];ENSG00000178222:INTRONIC[ENST00000506730(c.*26-2846T>C)];ENSG00000178222:INTRONIC[ENST00000512552(n.221-2846T>C)];ENSG00000178222:INTRONIC[ENST00000382968(c.247-2846T>C)];ENSG00000178222:MISSENSE[ENST00000333673(c.577T>C,Tgc>Cgc):ENSP00000327481(p.Cys193Arg)];	NA	NA	NA	NA	NA	NA	NA	D_lc	UN	NA	MODERATE	N	0.002	71	71	0	88	71	17	19.32
Smoker	42477	chr4	20620585	G	A	*SLIT2*	MISSENSE	ENSG00000145147:MISSENSE[ENST00000273739(c.4582G>A,Gag>Aag):ENSP00000273739(p.Glu1528Lys)];ENSG00000145147:MISSENSE[ENST00000504154(c.4543G>A,Gag>Aag):ENSP00000422591(p.Glu1515Lys)];ENSG00000145147:MISSENSE[ENST00000503823(c.4519G>A,Gag>Aag):ENSP00000427548(p.Glu1507Lys)];ENSG00000145147:MISSENSE[ENST00000503837(c.4531G>A,Gag>Aag):ENSP00000422261(p.Glu1511Lys)];ENSG00000145147:INTRONIC[ENST00000512993(c.237-1442G>A)];ENSG00000145147:EXONIC-NC[ENST00000508541(n.2743G>A)];	NA	NA	NA	8.24E-06	6.06E-05	NA	NA	NA	NA	NA	MODIFIER	NA	NA	85	85	0	76	56	20	26.32
Smoker	42477	chr4	96469785	G	A	*UNC5C*	INTRONIC	ENSG00000182168:[ENST00000506749(c.124+100C>T)];ENSG00000182168:[ENST00000513796(c.124+100C>T)];ENSG00000182168:[ENST00000453304(c.124+100C>T)];ENSG00000182168:[ENST00000504962(c.124+100C>T)];	NA	NA	NA	NA	NA	NA	NA	NA	NA	NA	MODIFIER	NA	NA	32	32	0	40	30	10	25
Smoker	42477	chr4	184207186	G	T	*WWC2*	NONSENSE	ENSG00000151718:NONSENSE[ENST00000504005(c.2161G>T,Gaa>Taa):ENSP00000427569(p.Glu721Ter)];ENSG00000151718:NONSENSE[ENST00000508747(c.499G>T,Gaa>Taa):ENSP00000420835(p.Glu167Ter)];ENSG00000151718:NONSENSE[ENST00000513834(c.2968G>T,Gaa>Taa):ENSP00000425054(p.Glu990Ter)];ENSG00000151718:NONSENSE[ENST00000403733(c.3115G>T,Gaa>Taa):ENSP00000384222(p.Glu1039Ter)];ENSG00000151718:NONSENSE[ENST00000448232(c.3187G>T,Gaa>Taa):ENSP00000398577(p.Glu1063Ter)];ENSG00000151718:3UTR[ENST00000427431(c.*2507G>T)];ENSG00000151718:3UTR[ENST00000438543(c.*911G>T)];	NA	NA	chr4*184207186*184207186*G*A*rs778041497*NA*NA*NA	NA	NA	NA	NA	NA	NA	NA	MODIFIER	NA	NA	60	60	0	79	71	8	10.13
Smoker	42477	chr4	60116	C	T	*ZNF595*	EXONIC-NC	ENSG00000197701:[ENST00000502981(n.456C>T)];ENSG00000197701:[ENST00000526473(c.230+66C>T)];ENSG00000197701:[ENST00000507368(n.206+6731C>T)];ENSG00000197701:[ENST00000339368(n.430+70C>T)];ENSG00000197701:[ENST00000512065(n.324+667C>T)];ENSG00000197701:[ENST00000509152(c.226+70C>T)];	NA	NA	NA	NA	NA	NA	NA	NA	NA	NA	MODIFIER	NA	NA	345	344	0	292	277	14	4.79
Smoker	42477	chr5	140480569	C	G	*AC005754.7*	INTRONIC	ENSG00000272154:[ENST00000607216(n.71-230G>C)];	NA	NA	NA	NA	NA	NA	NA	T_lc	BN	N	MODERATE	NA	NA	177	177	0	260	206	54	20.77
Smoker	42477	chr5	179280396	C	T	*C5orf45*	MISSENSE	ENSG00000161010:MISSENSE[ENST00000403396(c.154G>A,Gaa>Aaa):ENSP00000384599(p.Glu52Lys)];ENSG00000161010:SILENT[ENST00000520698(c.108G>A,gaG>gaA):ENSP00000427849(p.Glu36(=))];ENSG00000161010:SILENT[ENST00000518219(c.108G>A,gaG>gaA):ENSP00000428460(p.Glu36(=))];ENSG00000161010:SILENT[ENST00000292586(c.108G>A,gaG>gaA):ENSP00000292586(p.Glu36(=))];ENSG00000161010:SILENT[ENST00000376931(c.108G>A,gaG>gaA):ENSP00000366130(p.Glu36(=))];ENSG00000161010:SILENT[ENST00000521333(c.108G>A,gaG>gaA):ENSP00000429651(p.Glu36(=))];ENSG00000161010:SILENT[ENST00000523835(c.108G>A,gaG>gaA):ENSP00000429427(p.Glu36(=))];ENSG00000161010:SILENT[ENST00000522663(c.108G>A,gaG>gaA):ENSP00000429835(p.Glu36(=))];ENSG00000161010:SILENT[ENST00000521299(c.108G>A,gaG>gaA):ENSP00000429563(p.Glu36(=))];ENSG00000161010:SILENT[ENST00000520995(c.108G>A,gaG>gaA):ENSP00000429047(p.Glu36(=))];ENSG00000161010:SILENT[ENST00000519213(c.108G>A,gaG>gaA):ENSP00000430187(p.Glu36(=))];ENSG00000161010:SILENT[ENST00000523737(c.108G>A,gaG>gaA):ENSP00000431019(p.Glu36(=))];ENSG00000161010:SILENT[ENST00000524068(c.108G>A,gaG>gaA):ENSP00000430766(p.Glu36(=))];ENSG00000161010:SILENT[ENST00000522157(c.108G>A,gaG>gaA):ENSP00000428081(p.Glu36(=))];ENSG00000161010:SILENT[ENST00000519208(c.108G>A,gaG>gaA):ENSP00000430814(p.Glu36(=))];ENSG00000161010:SILENT[ENST00000518235(c.108G>A,gaG>gaA):ENSP00000430298(p.Glu36(=))];ENSG00000161010:EXONIC-NC[ENST00000519398(n.449G>A)];ENSG00000161010:EXONIC-NC[ENST00000520150(n.158G>A)];ENSG00000161010:EXONIC-NC[ENST00000519318(n.120G>A)];ENSG00000161010:EXONIC-NC[ENST00000517338(n.118G>A)];ENSG00000161010:5UTR[ENST00000523084(c.-206G>A)];	NA	NA	NA	NA	NA	NA	NA	NA	NA	NA	LOW	NA	0.994	83	83	0	92	73	19	20.65
Smoker	42477	chr5	24593449	G	A	*CDH10*	MISSENSE	ENSG00000040731:MISSENSE[ENST00000264463(c.151C>T,Cgt>Tgt):ENSP00000264463(p.Arg51Cys)];ENSG00000040731:MISSENSE[ENST00000510477(c.151C>T,Cgt>Tgt):ENSP00000425653(p.Arg51Cys)];	chr5*24593	chr5*24593	NA	2.47E-05	0	NA	NA	NA	NA	NA	MODIFIER	NA	NA	129	129	0	180	154	26	14.44
Smoker	42477	chr5	89989765	G	T	*GPR98*	MISSENSE	ENSG00000164199:MISSENSE[ENST00000405460(c.7192G>T,Gct>Tct):ENSP00000384582(p.Ala2398Ser)];	NA	NA	NA	9.29E-06	0	NA	NA	NA	BN	NA	MODERATE	D	0.998	45	45	0	52	43	9	17.31

Mangalaparthi *et al.* , 2020. Mutational landscape of esophageal squamous cell carcinoma in an Indian cohort
Supplementary Table 4. List of somatic single nucleotide variants identified using whole exome sequencing of ESCC patients

Cohort	Sample	Chromosome	Genomic position	Reference allele	Alternate allele	Gene symbol	Variant type	Variant annotation	OncoMD	COSMIC	dbSNP	ExAC	ExAC_SAS	1000G	1000G_SAS	SIFT	Polyphen2	CONDEL	VEP	LRT	PhastCon7 Way	Total read (Adjacent normal)	Reference allel read support (Adjacent normal)	Alternative read support (Adjacent normal)	Total read (Tumor)	Reference allel read support (Tumor)	Alternative read support (Tumor)	Alternate allele frequency
Smoker	42477	chr5	141008162	G	A	*HDAC3*	SILENT	ENSG00000171720:3UTR[ENST00000519474(c.*350C>T)];ENSG00000171720:EXONIC-NC[ENST00000495485(n.761C>T)];ENSG00000171720:EXONIC-NC[ENST00000467533(n.334C>T)];ENSG00000171720:EXONIC-NC[ENST00000469550(n.728C>T)];ENSG00000171720:EXONIC-NC[ENST00000492407(n.131C>T)];ENSG00000171720:EXONIC-NC[ENST00000476739(n.97C>T)];ENSG00000171720:SILENT[ENST00000523088(c.430C>T,Ctg>Ttg):ENSP00000429099(p.Leu144(=))];ENSG00000171720:SILENT[ENST00000305264(c.655C>T,Ctg>Ttg):ENSP00000302967(p.Leu219(=))];	NA	NA	NA	NA	NA	NA	NA	NA	NA	NA	LOW	NA	1	69	69	0	64	56	8	12.5
Smoker	42477	chr5	41057201	C	T	*MROH2B*	INTRONIC-SS-DNR	ENSG00000171495:[ENST00000515297(n.307+10G>A)];ENSG00000171495:[ENST00000508575(n.1409+10G>A)];ENSG00000171495:[ENST00000399564(c.919+10G>A)];ENSG00000171495:[ENST00000506092(c.-130+964G>A)];	NA	NA	NA	NA	NA	NA	NA	NA	NA	NA	MODIFIER	NA	NA	57	57	0	75	55	20	26.67
Smoker	42477	chr5	140480569	C	G	*PCDHB3*	MISSENSE	ENSG00000113205:MISSENSE[ENST00000231130(c.336C>G,aaC>aaG):ENSP00000231130(p.Asn112Lys)];	NA	NA	NA	NA	NA	NA	NA	T_lc	BN	N	MODERATE	NA	NA	177	177	0	260	206	54	20.77
Smoker	42477	chr5	24593449	G	A	*RP11-116O11.1*	INTRONIC	ENSG00000251294:[ENST00000510391(n.171-16420G>A)];	chr5*24593	chr5*24593	NA	2.47E-05	0	NA	NA	NA	NA	NA	MODIFIER	NA	NA	129	129	0	180	154	26	14.44
Smoker	42477	chr6	31504418	C	T	*ATP6V1G2-DDX39*	3UTR	ENSG00000254870:[ENST00000376185(c.*689G>A)];	NA	NA	NA	NA	NA	NA	NA	T	BN	N	MODERATE	NA	NA	118	118	0	124	113	11	8.87
Smoker	42477	chr6	70861308	G	A	*COL19A1*	MISSENSE	ENSG00000082293:MISSENSE[ENST00000322773(c.2057G>A,gGa>gAa):ENSP00000316030(p.Gly686Glu)];ENSG00000082293:MISSENSE[ENST00000393344(c.923G>A,gGa>gAa):ENSP00000377013(p.Gly308Glu)];	chr6*70861	NA	NA	NA	NA	NA	NA	D	PrD	D	MODERATE	D	1	41	41	0	41	27	14	34.15
Smoker	42477	chr6	132271649	C	T	*CTGF*	SILENT	ENSG00000118523:SILENT[ENST00000367976(c.324G>A,acG>acA):ENSP00000356954(p.Thr108(=))];	NA	NA	NA	NA	NA	NA	NA	NA	NA	NA	LOW	NA	NA	82	80	0	86	74	12	13.95
Smoker	42477	chr6	31504418	C	T	*DDX39B*	MISSENSE	ENSG00000198563:MISSENSE[ENST00000417556(c.520G>A,Gag>Aag):ENSP00000412582(p.Glu174Lys)];ENSG00000198563:MISSENSE[ENST00000415382(c.241G>A,Gag>Aag):ENSP00000392669(p.Glu81Lys)];ENSG00000198563:MISSENSE[ENST00000458640(c.475G>A,Gag>Aag):ENSP00000416269(p.Glu159Lys)];ENSG00000198563:MISSENSE[ENST00000396172(c.475G>A,Gag>Aag):ENSP00000379475(p.Glu159Lys)];ENSG00000198563:MISSENSE[ENST00000453105(c.334G>A,Gag>Aag):ENSP00000400328(p.Glu112Lys)];ENSG00000198563:MISSENSE[ENST00000376177(c.475G>A,Gag>Aag):ENSP00000365347(p.Glu159Lys)];ENSG00000198563:MISSENSE[ENST00000427214(c.475G>A,Gag>Aag):ENSP00000399371(p.Glu159Lys)];ENSG00000198563:MISSENSE[ENST00000431908(c.241G>A,Gag>Aag):ENSP00000408000(p.Glu81Lys)];ENSG00000198563:MISSENSE[ENST00000456662(c.475G>A,Gag>Aag):ENSP00000416350(p.Glu159Lys)];ENSG00000198563:MISSENSE[ENST00000449757(c.544G>A,Gag>Aag):ENSP00000409426(p.Glu182Lys)];ENSG00000198563:MISSENSE[ENST00000419338(c.475G>A,Gag>Aag):ENSP00000410313(p.Glu159Lys)];ENSG00000198563:MISSENSE[ENST00000428450(c.559G>A,Gag>Aag):ENSP00000405707(p.Glu187Lys)];ENSG00000198563:MISSENSE[ENST00000428098(c.475G>A,Gag>Aag):ENSP00000392672(p.Glu159Lys)];ENSG00000198563:3UTR[ENST00000449074(c.*175G>A)];ENSG00000198563:EXONIC-NC[ENST00000481456(n.1944G>A)];ENSG00000198563:EXONIC-NC[ENST00000482195(n.708G>A)];ENSG00000198563:EXONIC-NC[ENST00000462256(n.545G>A)];	NA	NA	NA	NA	NA	NA	NA	T	BN	N	MODERATE	NA	NA	118	118	0	124	113	11	8.87
Smoker	42477	chr6	56335051	C	T	*DST*	MISSENSE	ENSG00000151914:MISSENSE[ENST00000446842(c.20746G>A,Gca>Aca):ENSP00000393645(p.Ala6916Thr)];ENSG00000151914:MISSENSE[ENST00000370788(c.15133G>A,Gca>Aca):ENSP00000359824(p.Ala5045Thr)];ENSG00000151914:MISSENSE[ENST00000244364(c.14482G>A,Gca>Aca):ENSP00000244364(p.Ala4828Thr)];ENSG00000151914:MISSENSE[ENST00000361203(c.21391G>A,Gca>Aca):ENSP00000354508(p.Ala7131Thr)];ENSG00000151914:MISSENSE[ENST00000370754(c.22258G>A,Gca>Aca):ENSP00000359790(p.Ala7420Thr)];ENSG00000151914:MISSENSE[ENST00000370769(c.21724G>A,Gca>Aca):ENSP00000359805(p.Ala7242Thr)];ENSG00000151914:MISSENSE[ENST00000421834(c.15379G>A,Gca>Aca):ENSP00000400883(p.Ala5127Thr)];ENSG00000151914:3UTR[ENST00000312431(c.*9062G>A)];	chr6*56335	chr6*56335	NA	NA	NA	NA	NA	NA	NA	NA	MODIFIER	NA	NA	95	95	0	143	134	9	6.29
Smoker	42477	chr6	145948605	C	T	*EPM2A*	MISSENSE	ENSG00000112425:MISSENSE[ENST00000367519(c.943G>A,Gaa>Aaa):ENSP00000356489(p.Glu315Lys)];ENSG00000112425:INTRONIC[ENST00000435470(c.683+19G>A)];ENSG00000112425:INTRONIC[ENST00000450221(c.417+7776G>A)];	NA	NA	NA	NA	NA	NA	NA	NA	NA	NA	MODIFIER	NA	NA	65	65	0	126	100	26	20.63
Smoker	42477	chr6	26045730	C	T	*HIST1H3C*	MISSENSE	ENSG00000196532:MISSENSE[ENST00000540144(c.92C>T,cCg>cTg):ENSP00000439493(p.Pro31Leu)];	chr6*26045	chr6*26045	NA	NA	NA	NA	NA	D_lc	BN	N	MODERATE	NA	NA	117	117	0	91	62	29	31.87
Smoker	42477	chr6	150690300	G	C	*IYD*	MISSENSE	ENSG00000009765:MISSENSE[ENST00000422583(c.10G>C,Gat>Cat):ENSP00000397342(p.Asp4His)];ENSG00000009765:MISSENSE[ENST00000500320(c.133G>C,Gat>Cat):ENSP00000441276(p.Asp45His)];ENSG00000009765:MISSENSE[ENST00000344419(c.133G>C,Gat>Cat):ENSP00000343763(p.Asp45His)];ENSG00000009765:MISSENSE[ENST00000392255(c.133G>C,Gat>Cat):ENSP00000376084(p.Asp45His)];ENSG00000009765:MISSENSE[ENST00000229447(c.133G>C,Gat>Cat):ENSP00000229447(p.Asp45His)];ENSG00000009765:MISSENSE[ENST00000367335(c.133G>C,Gat>Cat):ENSP00000356304(p.Asp45His)];ENSG00000009765:MISSENSE[ENST00000392256(c.133G>C,Gat>Cat):ENSP00000376085(p.Asp45His)];ENSG00000009765:EXONIC-NC[ENST00000546121(n.76G>C)];	NA	NA	NA	NA	NA	NA	NA	T	BN	N	MODERATE	N	0.998	34	34	0	36	26	9	25
Smoker	42477	chr6	90447982	C	G	*MDN1*	INTRONIC	ENSG00000112159:[ENST00000369393(c.4723+63G>C)];ENSG00000112159:[ENST00000428876(c.4723+63G>C)];	NA	NA	NA	NA	NA	NA	NA	NA	NA	NA	MODIFIER	NA	NA	88	88	0	148	128	20	13.51
Smoker	42477	chr6	110797137	G	C	*SLC22A16*	INTRONIC	ENSG00000004809:[ENST00000461487(n.120+588C>G)];ENSG00000004809:[ENST00000368919(c.53+588C>G)];ENSG00000004809:[ENST00000434949(c.-96+382C>G)];ENSG00000004809:[ENST00000437378(c.-77+382C>G)];ENSG00000004809:[ENST00000439654(c.53+588C>G)];ENSG00000004809:[ENST00000456137(c.53+588C>G)];ENSG00000004809:[ENST00000424139(c.-77+324C>G)];	NA	NA	NA	NA	NA	NA	NA	NA	NA	NA	MODIFIER	NA	NA	139	139	0	194	161	33	17.01

Supplementary Table 4. List of somatic single nucleotide variants identified using whole exome sequencing of ESCC patients

Cohort	Sample	Chromosome	Genomic position	Reference allele	Alternate allele	Gene symbol	Variant type	Variant annotation	OncoMD	COSMIC	dbSNP	ExAC	ExAC_SAS	1000G	1000G_SAS	SIFT	Polyphen2	CONDEL	VEP	LRT	PhastCon7 Way	Total read (Adjacent normal)	Reference allel read support (Adjacent normal)	Alternative read support (Adjacent normal)	Total read (Tumor)	Reference allel read support (Tumor)	Alternative read support (Tumor)	Alternate allele frequency
Smoker	42477	chr6	152841601	C	T	*SYNE1*	MISSENSE	ENSG00000131018:MISSENSE[ENST00000367248(c.302G>A,gGa>gAa):ENSP00000356217(p.Gly101Glu)];ENSG00000131018:MISSENSE[ENST00000413186(c.302G>A,gGa>gAa):ENSP00000414510(p.Gly101Glu)];ENSG00000131018:MISSENSE[ENST00000367253(c.302G>A,gGa>gAa):ENSP00000356222(p.Gly101Glu)];ENSG00000131018:MISSENSE[ENST00000466159(c.302G>A,gGa>gAa):ENSP00000446021(p.Gly101Glu)];ENSG00000131018:MISSENSE[ENST00000423061(c.302G>A,gGa>gAa):ENSP00000396024(p.Gly101Glu)];ENSG00000131018:MISSENSE[ENST00000448038(c.302G>A,gGa>gAa):ENSP00000390975(p.Gly101Glu)];ENSG00000131018:MISSENSE[ENST00000367255(c.302G>A,gGa>gAa):ENSP00000356224(p.Gly101Glu)];ENSG00000131018:MISSENSE[ENST00000537750(c.302G>A,gGa>gAa):ENSP00000441264(p.Gly101Glu)];ENSG00000131018:MISSENSE[ENST00000341594(c.302G>A,gGa>gAa):ENSP00000341887(p.Gly101Glu)];ENSG00000131018:MISSENSE[ENST00000265368(c.302G>A,gGa>gAa):ENSP00000265368(p.Gly101Glu)];ENSG00000131018:EXONIC-NC[ENST00000461872(n.571G>A)];	NA	NA	NA	NA	NA	NA	NA	NA	NA	NA	MODIFIER	NA	NA	113	113	0	118	95	23	19.49
Smoker	42477	chr6	150291237	C	A	*ULBP1*	SILENT	ENSG00000111981:SILENT[ENST00000229708(c.711C>A,ctC>ctA):ENSP00000229708(p.Leu237(=))];	NA	NA	NA	NA	NA	NA	NA	NA	NA	NA	LOW	NA	NA	128	128	0	127	110	17	13.39
Smoker	42477	chr6	149773952	C	A	*ZC3H12D*	INTRONIC	ENSG00000178199:[ENST00000416573(c.681-94G>T)];ENSG00000178199:[ENST00000542614(c.681-94G>T)];ENSG00000178199:[ENST00000458251(c.205-94G>T)];ENSG00000178199:[ENST00000409806(c.681-94G>T)];ENSG00000178199:[ENST00000389942(c.681-94G>T)];	NA	NA	NA	NA	NA	NA	NA	NA	NA	NA	MODIFIER	NA	NA	68	67	1	83	67	16	19.28
Smoker	42477	chr6	43323549	G	A	*ZNF318*	MISSENSE	ENSG00000171467:MISSENSE[ENST00000318149(c.1523C>T,tCc>tTc):ENSP00000323032(p.Ser508Phe)];ENSG00000171467:MISSENSE[ENST00000605935(c.1523C>T,tCc>tTc):ENSP00000475748(p.Ser508Phe)];ENSG00000171467:MISSENSE[ENST00000361428(c.1523C>T,tCc>tTc):ENSP00000354964(p.Ser508Phe)];	NA	chr6*43323	NA	8.24E-06	0	NA	NA	NA	PrD	NA	MODERATE	D	0.997	115	115	0	98	79	19	19.39
Smoker	42477	chr7	91955289	G	A	*ANKIB1*	3UTR	ENSG00000001629:[ENST00000439883(c.*42G>A)];ENSG00000001629:[ENST00000265742(c.670-1808G>A)];	NA	NA	NA	NA	NA	NA	NA	NA	NA	NA	MODIFIER	NA	NA	66	66	0	109	93	16	14.68
Smoker	42477	chr7	147600689	G	A	*CNTNAP2*	MISSENSE	ENSG00000174469:MISSENSE[ENST00000455301(c.304G>A,Gcc>Acc):ENSP00000392208(p.Ala102Thr)];ENSG00000174469:MISSENSE[ENST00000361727(c.2131G>A,Gcc>Acc):ENSP00000354778(p.Ala711Thr)];	NA	NA	NA	NA	NA	NA	NA	T	BN	N	MODERATE	N	1	55	55	0	74	61	13	17.57
Smoker	42477	chr7	155255164	G	A	*EN2*	MISSENSE	ENSG00000164778:MISSENSE[ENST00000297375(c.784G>A,Gag>Aag):ENSP00000297375(p.Glu262Lys)];	NA	NA	NA	NA	NA	NA	NA	D	PrD	D	MODERATE	D	0.976	160	159	0	197	156	41	20.81
Smoker	42477	chr7	27182742	G	T	*HOXA3*	INTRONIC	ENSG00000105997:[ENST00000518451(n.295-2937C>A)];	chr7*27182	chr7*27182	NA	NA	NA	NA	NA	NA	NA	NA	MODIFIER	NA	NA	73	72	0	65	51	14	21.54
Smoker	42477	chr7	27182742	G	T	*HOXA5*	MISSENSE	ENSG00000106004:MISSENSE[ENST00000222726(c.485C>A,gCg>gAg):ENSP00000222726(p.Ala162Glu)];	chr7*27182	chr7*27182	NA	NA	NA	NA	NA	NA	NA	NA	MODIFIER	NA	NA	73	72	0	65	51	14	21.54
Smoker	42477	chr7	27182742	G	T	*HOXA-AS3*	INTRONIC	ENSG00000254369:[ENST00000521197(n.176+2584G>T)];ENSG00000254369:[ENST00000518848(n.173-8452G>T)];	chr7*27182	chr7*27182	NA	NA	NA	NA	NA	NA	NA	NA	MODIFIER	NA	NA	73	72	0	65	51	14	21.54
Smoker	42477	chr7	100551053	C	G	*MUC3A*	EXONIC-NC	ENSG00000169894:[ENST00000483133(n.724C>G)];	NA	NA	NA	NA	NA	NA	NA	NA	NA	NA	MODIFIER	NA	NA	514	355	6	506	333	12	2.37
Smoker	42477	chr7	124427968	C	T	*NA*	INTERGENIC		NA	NA	0.03415	0.0043282	0.000280348	0.0341454	0	NA	NA	NA	MODIFIER	NA	NA	50	50	0	51	38	13	25.49
Smoker	42477	chr7	158468175	G	A	*NCAPG2*	SILENT	ENSG00000146918:SILENT[ENST00000275830(c.696C>T,gtC>gtT):ENSP00000275830(p.Val232(=))];ENSG00000146918:SILENT[ENST00000409423(c.1320C>T,gtC>gtT):ENSP00000386569(p.Val440(=))];ENSG00000146918:SILENT[ENST00000441982(c.725C>T,gtC>gtT):ENSP00000408080(p.Val242(=))];ENSG00000146918:SILENT[ENST00000449727(c.1320C>T,gtC>gtT):ENSP00000388326(p.Val440(=))];ENSG00000146918:SILENT[ENST00000356309(c.1320C>T,gtC>gtT):ENSP00000348657(p.Val440(=))];ENSG00000146918:SILENT[ENST00000409339(c.1320C>T,gtC>gtT):ENSP00000387007(p.Val440(=))];ENSG00000146918:EXONIC-NC[ENST00000467785(n.1084C>T)];ENSG00000146918:3UTR[ENST00000432615(c.*110C>T)];	NA	NA	NA	2.66E-05	0.00019742	NA	NA	NA	NA	NA	LOW	NA	NA	130	129	0	138	109	29	21.01
Smoker	42477	chr7	144101703	G	T	*NOBOX*	MISSENSE	ENSG00000106410:MISSENSE[ENST00000483238(c.156C>A,ttC>ttA):ENSP00000419565(p.Phe52Leu)];ENSG00000106410:MISSENSE[ENST00000467773(c.156C>A,ttC>ttA):ENSP00000419457(p.Phe52Leu)];	NA	NA	chr7*144101703*144101703*G*GA*rs755912961*NA*NA*NA	NA	NA	NA	NA	T_lc	BN	N	MODERATE	NA	NA	60	60	0	71	54	17	23.94
Smoker	42477	chr7	24902899	C	G	*OSBPL3*	MISSENSE	ENSG00000070882:MISSENSE[ENST00000396429(c.790G>C,Gaa>Caa):ENSP00000379706(p.Glu264Gln)];ENSG00000070882:MISSENSE[ENST00000353930(c.790G>C,Gaa>Caa):ENSP00000315277(p.Glu264Gln)];ENSG00000070882:MISSENSE[ENST00000313367(c.790G>C,Gaa>Caa):ENSP00000315410(p.Glu264Gln)];ENSG00000070882:MISSENSE[ENST00000409452(c.790G>C,Gaa>Caa):ENSP00000386801(p.Glu264Gln)];ENSG00000070882:MISSENSE[ENST00000409759(c.790G>C,Gaa>Caa):ENSP00000386325(p.Glu264Gln)];ENSG00000070882:INTRONIC[ENST00000431825(c.777+216G>C)];ENSG00000070882:INTRONIC[ENST00000409555(c.777+216G>C)];ENSG00000070882:INTRONIC[ENST00000352860(c.777+216G>C)];ENSG00000070882:INTRONIC[ENST00000409069(c.777+216G>C)];ENSG00000070882:INTRONIC[ENST00000409863(c.777+216G>C)];ENSG00000070882:INTRONIC[ENST00000396431(c.777+216G>C)];	NA	NA	NA	NA	NA	NA	NA	NA	NA	NA	MODIFIER	NA	NA	166	165	0	204	166	38	18.63
Smoker	42477	chr7	27182742	G	T	*RP1-170O19.22*	INTRONIC	ENSG00000273433:[ENST00000467897(n.275-2937C>A)];	chr7*27182	chr7*27182	NA	NA	NA	NA	NA	NA	NA	NA	MODIFIER	NA	NA	73	72	0	65	51	14	21.54
Smoker	42477	chr7	141137555	C	T	*TMEM178B*	INTRONIC-SS-DNR	ENSG00000261115:[ENST00000565468(c.634+10C>T)];	NA	NA	NA	8.05E-05	0	NA	NA	NA	NA	NA	MODIFIER	NA	NA	39	39	0	46	37	9	19.57
Smoker	42477	chr7	156931944	C	T	*UBE3C*	5UTR	ENSG00000009335:[ENST00000389103(c.-23C>T)];ENSG00000009335:[ENST00000348165(c.-23C>T)];	NA	NA	NA	NA	NA	NA	NA	NA	NA	NA	MODIFIER	NA	NA	139	139	0	132	113	19	14.39
Smoker	42477	chr7	158663997	G	C	*WDR60*	MISSENSE	ENSG00000126870:MISSENSE[ENST00000407559(c.234G>C,gaG>gaC):ENSP00000384290(p.Glu78Asp)];ENSG00000126870:MISSENSE[ENST00000397143(c.264G>C,gaG>gaC):ENSP00000380330(p.Glu88Asp)];	NA	NA	NA	NA	NA	NA	NA	T	BN	N	MODERATE	NA	NA	62	62	0	58	40	17	29.31
Smoker	42477	chr8	75925128	T	C	*CRISPLD1*	SILENT	ENSG00000121005:SILENT[ENST00000262207(c.381T>C,taT>taC):ENSP00000262207(p.Tyr127(=))];ENSG00000121005:5UTR[ENST00000517786(c.-62T>C)];ENSG00000121005:5UTR[ENST00000523524(c.-184T>C)];	NA	NA	0.0001997	NA	NA	0.0001997	0	NA	NA	NA	MODIFIER	NA	NA	134	134	0	185	149	35	18.92
Smoker	42477	chr8	113988098	C	G	*CSMD3*	MISSENSE	ENSG00000164796:MISSENSE[ENST00000352409(c.1310G>C,aGa>aCa):ENSP00000343124(p.Arg437Thr)];ENSG00000164796:MISSENSE[ENST00000297405(c.1310G>C,aGa>aCa):ENSP00000297405(p.Arg437Thr)];ENSG00000164796:MISSENSE[ENST00000343508(c.1190G>C,aGa>aCa):ENSP00000345799(p.Arg397Thr)];ENSG00000164796:INTRONIC[ENST00000455883(c.1031-21108G>C)];	chr8*11398	chr8*11398	chr8*113988095*113988099*GTTCT*G*rs753710667*NA*NA*NA|chr8*113988098*113988098*C*A,T*rs566426423*0*0.9998*0.0001997,.	NA	NA	NA	NA	NA	NA	NA	MODIFIER	NA	NA	83	83	0	121	94	26	21.49

Mangalaparthi *et al.* , 2020. Mutational landscape of esophageal squamous cell carcinoma in an Indian cohort
Supplementary Table 4. List of somatic single nucleotide variants identified using whole exome sequencing of ESCC patients

Cohort	Sample	Chromosome	Genomic position	Reference allele	Alternate allele	Gene symbol	Variant type	Variant annotation	OncoMD	COSMIC	dbSNP	ExAC	ExAC_SAS	1000G	1000G_SAS	SIFT	Polyphen2	CONDEL	VEP	LRT	PhastCon7 Way	Total read (Adjacent normal)	Reference allel read support (Adjacent normal)	Alternative read support (Adjacent normal)	Total read (Tumor)	Reference allel read support (Tumor)	Alternative read support (Tumor)	Alternate allele frequency
Smoker	42477	chr8	110346619	G	A	*ENY2*	5UTR	ENSG00000120533:[ENST00000517311(c.-79G>A)];ENSG00000120533:[ENST00000521662(c.-22G>A)];ENSG00000120533:[ENST00000522766(c.-22G>A)];ENSG00000120533:[ENST00000339942(c.-17G>A)];ENSG00000120533:[ENST00000520147(c.-26G>A)];ENSG00000120533:[ENST00000521688(c.-79G>A)];ENSG00000120533:[ENST00000523335(c.-79G>A)];ENSG00000120533:[ENST00000517756(n.23G>A)];ENSG00000120533:[ENST00000518584(n.14G>A)];ENSG00000120533:[ENST00000522632(n.42G>A)];ENSG00000120533:[ENST00000517350(n.10G>A)];ENSG00000120533:[ENST00000519754(n.45G>A)];ENSG00000120533:[ENST00000522407(n.44G>A)];	NA	NA	NA	NA	NA	NA	NA	NA	NA	NA	MODIFIER	NA	NA	74	73	0	94	73	21	22.34
Smoker	42477	chr8	136470371	G	A	*KHDRBS3*	5UTR	ENSG00000131773:[ENST00000524199(c.-52G>A)];ENSG00000131773:[ENST00000355849(c.88+174G>A)];	NA	NA	NA	NA	NA	NA	NA	NA	NA	NA	MODIFIER	NA	NA	84	84	0	97	70	27	27.84
Smoker	42477	chr8	125740192	T	C	*MTSS1*	MISSENSE	ENSG00000170873:MISSENSE[ENST00000518547(c.5A>G,gAg>gGg):ENSP00000429064(p.Glu2Gly)];ENSG00000170873:MISSENSE[ENST00000378017(c.5A>G,gAg>gGg):ENSP00000367256(p.Glu2Gly)];ENSG00000170873:MISSENSE[ENST00000325064(c.5A>G,gAg>gGg):ENSP00000322804(p.Glu2Gly)];ENSG00000170873:5UTR[ENST00000354184(c.-596A>G)];ENSG00000170873:EXONIC-NC[ENST00000472084(n.68A>G)];ENSG00000170873:EXONIC-NC[ENST00000524243(n.48A>G)];	NA	NA	NA	NA	NA	NA	NA	NA	NA	NA	MODIFIER	NA	NA	163	163	0	122	97	25	20.49
Smoker	42477	chr8	2148810	C	T	*NA*	INTERGENIC		NA	NA	NA	NA	NA	NA	NA	NA	NA	NA	MODIFIER	NA	NA	33	33	0	44	37	7	15.91
Smoker	42477	chr8	69046296	A	C	*PREX2*	MISSENSE-SS-PRX	ENSG00000046889:[ENST00000288368(c.3769A>C,Agt>Cgt):ENSP00000288368(p.Ser1257Arg)];	NA	NA	chr8*69046296*69046296*A*G*rs565195590*NA*NA*NA	NA	NA	NA	NA	D	BN	N	MODERATE	D	0.98	46	46	0	88	78	10	11.36
Smoker	42477	chr8	33424152	C	T	*RNF122*	INTRONIC	ENSG00000133874:[ENST00000256257(c.25+65G>A)];	NA	NA	NA	NA	NA	NA	NA	NA	NA	NA	MODIFIER	NA	NA	154	154	0	124	82	42	33.87
Smoker	42477	chr9	97767458	C	T	*C9orf3*	MISSENSE	ENSG00000148120:MISSENSE[ENST00000424143(c.1168C>T,Cgc>Tgc):ENSP00000402171(p.Arg390Cys)];ENSG00000148120:MISSENSE[ENST00000297979(c.1699C>T,Cgc>Tgc):ENSP00000297979(p.Arg567Cys)];ENSG00000148120:MISSENSE[ENST00000451893(c.194C>T,Cgc>Tgc):ENSP00000411451(p.Arg66Cys)];ENSG00000148120:MISSENSE[ENST00000428313(c.1342C>T,Cgc>Tgc):ENSP00000401854(p.Arg448Cys)];ENSG00000148120:MISSENSE[ENST00000375315(c.1996C>T,Cgc>Tgc):ENSP00000364464(p.Arg666Cys)];ENSG00000148120:MISSENSE[ENST00000445181(c.89C>T,Cgc>Tgc):ENSP00000413927(p.Arg31Cys)];ENSG00000148120:MISSENSE[ENST00000425634(c.82C>T,Cgc>Tgc):ENSP00000411815(p.Arg28Cys)];ENSG00000148120:MISSENSE[ENST00000478473(c.77C>T,Cgc>Tgc):ENSP00000431923(p.Arg27Cys)];ENSG00000148120:EXONIC-NC[ENST00000460573(n.98C>T)];ENSG00000148120:EXONIC-NC[ENST00000471978(n.105C>T)];ENSG00000148120:EXONIC-NC[ENST00000496567(n.72C>T)];ENSG00000148120:EXONIC-NC[ENST00000479161(n.718C>T)];ENSG00000148120:EXONIC-NC[ENST00000463372(n.80C>T)];ENSG00000148120:EXONIC-NC[ENST00000462125(n.662C>T)];ENSG00000148120:EXONIC-NC[ENST00000478603(n.389C>T)];ENSG00000148120:INTRONIC[ENST00000433691(c.64-366C>T)];	NA	NA	NA	NA	NA	NA	NA	T	BN	N	MODERATE	NA	NA	25	25	0	15	8	7	46.67
Smoker	42477	chr9	136256471	C	T	*C9orf96*	MISSENSE	ENSG00000198870:MISSENSE[ENST00000371957(c.482C>T,tCc>tTc):ENSP00000361025(p.Ser161Phe)];ENSG00000198870:5UTR[ENST00000371955(c.-644C>T)];	NA	NA	NA	NA	NA	NA	NA	NA	NA	NA	MODIFIER	NA	NA	85	85	0	98	62	36	36.73
Smoker	42477	chr9	118139030	G	A	*DEC1*	INTRONIC	ENSG00000173077:[ENST00000374016(c.13+36G>A)];	NA	NA	NA	NA	NA	NA	NA	NA	NA	NA	MODIFIER	NA	NA	142	142	0	183	122	61	33.33
Smoker	42477	chr9	14746495	A	G	*FREM1*	INTRONIC	ENSG00000164946:[ENST00000427623(c.*320-29T>C)];ENSG00000164946:[ENST00000380881(c.6142-29T>C)];ENSG00000164946:[ENST00000380875(c.*705-29T>C)];ENSG00000164946:[ENST00000380880(c.6139-29T>C)];ENSG00000164946:[ENST00000422223(c.6139-29T>C)];ENSG00000164946:[ENST00000380894(c.1747-29T>C)];	NA	NA	0.0001997	NA	NA	0.0001997	0	NA	NA	NA	MODIFIER	NA	NA	28	28	0	31	21	10	32.26
Smoker	42477	chr9	101125014	C	G	*GABBR2*	MISSENSE	ENSG00000136928:MISSENSE[ENST00000259455(c.1876G>C,Gag>Cag):ENSP00000259455(p.Glu626Gln)];	NA	NA	NA	NA	NA	NA	NA	T	PoD	N	MODERATE	D	0.995	92	92	0	105	60	45	42.86
Smoker	42477	chr9	139412584	C	T	*NOTCH1*	INTRONIC-SS-DNR	ENSG00000148400:[ENST00000277541(c.1255+5G>A)];	NA	NA	NA	NA	NA	NA	NA	NA	NA	NA	LOW	NA	NA	41	40	0	31	8	23	74.19
Smoker	42477	chr9	101900262	G	C	*TGFBR1*	MISSENSE	ENSG00000106799:MISSENSE[ENST00000550253(c.489G>C,aaG>aaC):ENSP00000450052(p.Lys163Asn)];ENSG00000106799:MISSENSE[ENST00000552516(c.708G>C,aaG>aaC):ENSP00000447297(p.Lys236Asn)];ENSG00000106799:MISSENSE[ENST00000374990(c.465G>C,aaG>aaC):ENSP00000364129(p.Lys155Asn)];ENSG00000106799:MISSENSE[ENST00000374994(c.696G>C,aaG>aaC):ENSP00000364133(p.Lys232Asn)];ENSG00000106799:MISSENSE[ENST00000549766(c.708G>C,aaG>aaC):ENSP00000446685(p.Lys236Asn)];ENSG00000106799:MISSENSE[ENST00000549021(c.258G>C,aaG>aaC):ENSP00000449028(p.Lys86Asn)];	NA	NA	NA	NA	NA	NA	NA	D_lc	PrD	D	MODERATE	NA	NA	202	202	0	287	172	115	40.07
Smoker	42477	chrX	34962857	G	A	*FAM47B*	MISSENSE	ENSG00000189132:MISSENSE[ENST00000329357(c.1909G>A,Gac>Aac):ENSP00000328307(p.Asp637Asn)];	NA	NA	NA	NA	NA	NA	NA	T	BN	N	MODERATE	NA	NA	78	78	0	107	67	40	37.38
Smoker	42477	chrX	106845394	G	A	*FRMPD3*	SILENT	ENSG00000147234:SILENT[ENST00000276185(c.4224G>A,acG>acA):ENSP00000276185(p.Thr1408(=))];ENSG00000147234:SILENT[ENST00000439554(c.4068G>A,acG>acA):ENSP00000398668(p.Thr1356(=))];	NA	NA	NA	0.0001226	0	NA	NA	NA	NA	NA	LOW	NA	NA	67	67	0	85	54	31	36.47
Smoker	42477	chrX	24741457	C	G	*POLA1*	INTRONIC	ENSG00000101868:[ENST00000379059(c.1182+73C>G)];ENSG00000101868:[ENST00000379068(c.1200+73C>G)];	NA	NA	NA	NA	NA	NA	NA	NA	NA	NA	MODIFIER	NA	NA	70	70	0	100	66	34	34
Smoker	42477	chrX	79278762	C	T	*TBX22*	5UTR	ENSG00000122145:[ENST00000373291(c.-45C>T)];ENSG00000122145:[ENST00000373294(c.356+23C>T)];ENSG00000122145:[ENST00000442340(c.-5+27C>T)];ENSG00000122145:[ENST00000373296(c.356+23C>T)];	NA	NA	NA	NA	NA	NA	NA	NA	NA	NA	MODIFIER	NA	NA	43	43	0	79	44	35	44.3
Smoker	42477	chrX	70464246	G	C	*ZMYM3*	SILENT	ENSG00000147130:EXONIC-NC[ENST00000470832(n.75C>G)];ENSG00000147130:EXONIC-NC[ENST00000489332(n.3527C>G)];ENSG00000147130:SILENT[ENST00000373988(c.3192C>G,ctC>ctG):ENSP00000363100(p.Leu1064(=))];ENSG00000147130:SILENT[ENST00000373984(c.3192C>G,ctC>ctG):ENSP00000363096(p.Leu1064(=))];ENSG00000147130:SILENT[ENST00000314425(c.3186C>G,ctC>ctG):ENSP00000322845(p.Leu1062(=))];ENSG00000147130:SILENT[ENST00000373998(c.3150C>G,ctC>ctG):ENSP00000363110(p.Leu1050(=))];ENSG00000147130:SILENT[ENST00000353904(c.3186C>G,ctC>ctG):ENSP00000343909(p.Leu1062(=))];	NA	NA	chrX*70464246*70464246*G*A*rs760450840*NA*NA*NA	NA	NA	NA	NA	NA	NA	NA	LOW	NA	NA	60	60	0	71	49	22	30.99

Supplementary Table 4. List of somatic single nucleotide variants identified using whole exome sequencing of ESCC patients

Cohort	Sample	Chromosome	Genomic position	Reference allele	Alternate allele	Gene symbol	Variant type	Variant annotation	OncoMD	COSMIC	dbSNP	ExAC	ExAC_SAS	1000G	1000G_SAS	SIFT	Polyphen2	CONDEL	VEP	LRT	PhastCon7 Way	Total read (Adjacent normal)	Reference allel read support (Adjacent normal)	Alternative read support (Adjacent normal)	Total read (Tumor)	Reference allel read support (Tumor)	Alternative read support (Tumor)	Alternate allele frequency
Smoker	42477	chrX	152610188	G	T	*ZNF275*	SILENT	ENSG00000063587:INTRONIC[ENST00000370251(c.32-790G>T)];ENSG00000063587:INTRONIC[ENST00000421401(c.32-790G>T)];ENSG00000063587:SILENT[ENST00000440091(c.69G>T,ctG>ctT):ENSP00000411097(p.Leu23(=))];ENSG00000063587:5UTR[ENST00000370249(c.-918G>T)];	NA	NA	NA	NA	NA	NA	NA	NA	NA	NA	MODIFIER	NA	NA	25	25	0	39	34	5	12.82
Smoker	42478	chr1	92447177	G	A	*BRDT*	MISSENSE-SS-PRX	ENSG00000137948:[ENST00000402388(c.1867G>A,Gat>Aat):ENSP00000384051(p.Asp623Asn)];ENSG00000137948:[ENST00000394530(c.1729G>A,Gat>Aat):ENSP00000378038(p.Asp577Asn)];ENSG00000137948:[ENST00000370389(c.1648G>A,Gat>Aat):ENSP00000359416(p.Asp550Asn)];ENSG00000137948:[ENST00000399546(c.1867G>A,Gat>Aat):ENSP00000387822(p.Asp623Asn)];ENSG00000137948:[ENST00000362005(c.1867G>A,Gat>Aat):ENSP00000354568(p.Asp623Asn)];	NA	NA	NA	NA	NA	NA	NA	T	BN	N	MODERATE	N	0.808	135	134	0	105	93	11	10.48
Smoker	42478	chr1	190067264	G	A	*BRINP3*	MISSENSE	ENSG00000162670:MISSENSE[ENST00000367462(c.2185C>T,Cgt>Tgt):ENSP00000356432(p.Arg729Cys)];ENSG00000162670:MISSENSE[ENST00000534846(c.1879C>T,Cgt>Tgt):ENSP00000438022(p.Arg627Cys)];	NA	NA	NA	NA	NA	NA	NA	NA	PrD	NA	MODERATE	D	1	124	124	0	141	121	20	14.18
Smoker	42478	chr1	57397428	C	T	*C8B*	INTRONIC	ENSG00000021852:[ENST00000535057(c.1435+55G>A)];ENSG00000021852:[ENST00000543257(c.1465+55G>A)];ENSG00000021852:[ENST00000371237(c.1621+55G>A)];ENSG00000021852:[ENST00000465658(n.171G>A)];	NA	NA	chr1*57397428*57397428*C*A*rs575690878*0*0.9998*0.0001997	NA	NA	NA	NA	NA	NA	NA	MODIFIER	NA	NA	73	73	0	80	68	12	15
Smoker	42478	chr1	11896109	A	G	*CLCN6*	MISSENSE	ENSG00000011021:MISSENSE[ENST00000376487(c.1813A>G,Acc>Gcc):ENSP00000365670(p.Thr605Ala)];ENSG00000011021:MISSENSE[ENST00000346436(c.1879A>G,Acc>Gcc):ENSP00000234488(p.Thr627Ala)];ENSG00000011021:MISSENSE[ENST00000376496(c.1879A>G,Acc>Gcc):ENSP00000365679(p.Thr627Ala)];ENSG00000011021:3UTR[ENST00000312413(c.*650A>G)];	NA	NA	NA	NA	NA	NA	NA	NA	NA	NA	MODIFIER	NA	NA	99	98	1	71	62	9	12.68
Smoker	42478	chr1	23191465	G	A	*EPHB2*	MISSENSE	ENSG00000133216:MISSENSE[ENST00000544305(c.1063G>A,Gac>Aac):ENSP00000444174(p.Asp355Asn)];ENSG00000133216:MISSENSE[ENST00000374627(c.1045G>A,Gac>Aac):ENSP00000363758(p.Asp349Asn)];ENSG00000133216:MISSENSE[ENST00000400191(c.1063G>A,Gac>Aac):ENSP00000383053(p.Asp355Asn)];ENSG00000133216:MISSENSE[ENST00000374630(c.1063G>A,Gac>Aac):ENSP00000363761(p.Asp355Asn)];ENSG00000133216:MISSENSE[ENST00000374632(c.1063G>A,Gac>Aac):ENSP00000363763(p.Asp355Asn)];ENSG00000133216:EXONIC-NC[ENST00000465676(n.304G>A)];	NA	NA	NA	NA	NA	NA	NA	NA	NA	NA	MODIFIER	NA	NA	111	111	0	92	77	15	16.3
Smoker	42478	chr1	157514657	G	T	*FCRL5*	MISSENSE	ENSG00000143297:MISSENSE[ENST00000361835(c.523C>A,Cct>Act):ENSP00000354691(p.Pro175Thr)];ENSG00000143297:MISSENSE[ENST00000368190(c.523C>A,Cct>Act):ENSP00000357173(p.Pro175Thr)];ENSG00000143297:MISSENSE[ENST00000368189(c.523C>A,Cct>Act):ENSP00000357172(p.Pro175Thr)];ENSG00000143297:MISSENSE[ENST00000356953(c.523C>A,Cct>Act):ENSP00000349434(p.Pro175Thr)];ENSG00000143297:MISSENSE[ENST00000368191(c.268C>A,Cct>Act):ENSP00000357174(p.Pro90Thr)];ENSG00000143297:EXONIC-NC[ENST00000481082(n.721C>A)];	NA	NA	NA	NA	NA	NA	NA	T	BN	N	MODERATE	NA	NA	168	168	0	170	150	20	11.76
Smoker	42478	chr1	32796283	C	G	*HDAC1*	MISSENSE	ENSG00000116478:MISSENSE[ENST00000373548(c.834C>G,atC>atG):ENSP00000362649(p.Ile278Met)];ENSG00000116478:MISSENSE[ENST00000373541(c.255C>G,atC>atG):ENSP00000362642(p.Ile85Met)];ENSG00000116478:EXONIC-NC[ENST00000476391(n.1203C>G)];ENSG00000116478:EXONIC-NC[ENST00000484305(n.449C>G)];ENSG00000116478:EXONIC-NC[ENST00000490081(n.687C>G)];ENSG00000116478:EXONIC-NC[ENST00000482310(n.525C>G)];	NA	NA	NA	NA	NA	NA	NA	NA	NA	NA	MODIFIER	NA	NA	89	88	1	70	53	17	24.29
Smoker	42478	chr1	222716929	C	G	*HHIPL2*	MISSENSE	ENSG00000143512:MISSENSE[ENST00000343410(c.924G>C,gaG>gaC):ENSP00000342118(p.Glu308Asp)];	NA	NA	NA	NA	NA	NA	NA	D	BN	N	MODERATE	D	0.998	169	169	0	199	178	21	10.55
Smoker	42478	chr1	10380152	C	G	*KIF1B*	MISSENSE	ENSG00000054523:MISSENSE[ENST00000263934(c.2029C>G,Ctg>Gtg):ENSP00000263934(p.Leu677Val)];ENSG00000054523:MISSENSE[ENST00000377081(c.2167C>G,Ctg>Gtg):ENSP00000366284(p.Leu723Val)];ENSG00000054523:MISSENSE[ENST00000377086(c.2167C>G,Ctg>Gtg):ENSP00000366290(p.Leu723Val)];	NA	NA	NA	NA	NA	NA	NA	T	BN	N	MODERATE	D	0.927	144	144	0	117	99	18	15.38
Smoker	42478	chr1	167741736	G	C	*MPZL1*	INTRONIC	ENSG00000197965:[ENST00000359523(c.472+11G>C)];ENSG00000197965:[ENST00000474859(c.472+11G>C)];ENSG00000197965:[ENST00000448405(c.259-737G>C)];ENSG00000197965:[ENST00000465787(n.631+11G>C)];ENSG00000197965:[ENST00000474729(n.906+11G>C)];ENSG00000197965:[ENST00000367853(c.394+11G>C)];ENSG00000197965:[ENST00000392121(c.258+6750G>C)];	NA	NA	NA	NA	NA	NA	NA	NA	NA	NA	MODIFIER	NA	NA	271	271	0	238	219	17	7.14
Smoker	42478	chr1	247588235	C	A	*NLRP3*	MISSENSE	ENSG00000162711:MISSENSE[ENST00000366496(c.1490C>A,gCg>gAg):ENSP00000355452(p.Ala497Glu)];ENSG00000162711:MISSENSE[ENST00000336119(c.1490C>A,gCg>gAg):ENSP00000337383(p.Ala497Glu)];ENSG00000162711:MISSENSE[ENST00000391827(c.1490C>A,gCg>gAg):ENSP00000375703(p.Ala497Glu)];ENSG00000162711:MISSENSE[ENST00000366497(c.1490C>A,gCg>gAg):ENSP00000355453(p.Ala497Glu)];ENSG00000162711:MISSENSE[ENST00000391828(c.1490C>A,gCg>gAg):ENSP00000375704(p.Ala497Glu)];ENSG00000162711:MISSENSE[ENST00000348069(c.1490C>A,gCg>gAg):ENSP00000294752(p.Ala497Glu)];ENSG00000162711:EXONIC-NC[ENST00000474792(n.1665C>A)];	NA	NA	chr1*247588235*247588235*C*T*rs180177474*NA*NA*NA	NA	NA	NA	NA	NA	NA	NA	MODIFIER	NA	NA	109	109	0	113	95	18	15.93
Smoker	42478	chr1	247614997	G	A	*OR2B11*	SILENT	ENSG00000177535:SILENT[ENST00000318749(c.288C>T,atC>atT):ENSP00000325682(p.Ile96(=))];	NA	NA	NA	NA	NA	NA	NA	NA	NA	NA	LOW	NA	0.593	111	111	0	102	92	10	9.8
Smoker	42478	chr1	12024180	C	T	*PLOD1*	INTRONIC	ENSG00000083444:[ENST00000376369(c.1344-52C>T)];ENSG00000083444:[ENST00000196061(c.1203-52C>T)];	NA	NA	NA	NA	NA	NA	NA	NA	NA	NA	MODIFIER	NA	NA	78	78	0	67	54	13	19.4

Mangalaparthi *et al.* , 2020. Mutational landscape of esophageal squamous cell carcinoma in an Indian cohort
Supplementary Table 4. List of somatic single nucleotide variants identified using whole exome sequencing of ESCC patients

Cohort	Sample	Chromosome	Genomic position	Reference allele	Alternate allele	Gene symbol	Variant type	Variant annotation	OncoMD	COSMIC	dbSNP	ExAC	ExAC_SAS	1000G	1000G_SAS	SIFT	Polyphen2	CONDEL	VEP	LRT	PhastCon7 Way	Total read (Adjacent normal)	Reference allel read support (Adjacent normal)	Alternative read support (Adjacent normal)	Total read (Tumor)	Reference allel read support (Tumor)	Alternative read support (Tumor)	Alternate allele frequency
Smoker	42478	chr1	240969516	T	G	*RGS7*	MISSENSE	ENSG00000182901:MISSENSE[ENST00000331110(c.1115A>C,aAc>aCc):ENSP00000331485(p.Asn372Thr)];ENSG00000182901:MISSENSE[ENST00000446183(c.941A>C,aAc>aCc):ENSP00000390138(p.Asn314Thr)];ENSG00000182901:MISSENSE[ENST00000401882(c.1034A>C,aAc>aCc):ENSP00000385508(p.Asn345Thr)];ENSG00000182901:MISSENSE[ENST00000366564(c.1193A>C,aAc>aCc):ENSP00000355522(p.Asn398Thr)];ENSG00000182901:MISSENSE[ENST00000366565(c.1193A>C,aAc>aCc):ENSP00000355523(p.Asn398Thr)];ENSG00000182901:MISSENSE[ENST00000366562(c.1193A>C,aAc>aCc):ENSP00000355520(p.Asn398Thr)];ENSG00000182901:MISSENSE[ENST00000407727(c.1193A>C,aAc>aCc):ENSP00000384428(p.Asn398Thr)];ENSG00000182901:MISSENSE[ENST00000366563(c.1193A>C,aAc>aCc):ENSP00000355521(p.Asn398Thr)];ENSG00000182901:MISSENSE[ENST00000348120(c.1034A>C,aAc>aCc):ENSP00000341242(p.Asn345Thr)];ENSG00000182901:MISSENSE[ENST00000440928(c.686A>C,aAc>aCc):ENSP00000404399(p.Asn229Thr)];	NA	NA	NA	NA	NA	NA	NA	D	PrD	D	MODERATE	NA	NA	75	75	0	85	79	6	7.06
Smoker	42478	chr1	38677514	G	A	*RP11-214L19.1*	INTRONIC	ENSG00000237290:[ENST00000431311(n.406-20C>T)];	NA	NA	NA	NA	NA	NA	NA	NA	NA	NA	MODIFIER	NA	NA	101	99	0	97	91	6	6.19
Smoker	42478	chr1	48865258	C	T	*SPATA6*	MISSENSE	ENSG00000132122:MISSENSE[ENST00000396199(c.329G>A,aGa>aAa):ENSP00000379502(p.Arg110Lys)];ENSG00000132122:MISSENSE[ENST00000371843(c.545G>A,aGa>aAa):ENSP00000360909(p.Arg182Lys)];ENSG00000132122:MISSENSE[ENST00000371847(c.545G>A,aGa>aAa):ENSP00000360913(p.Arg182Lys)];ENSG00000132122:MISSENSE[ENST00000603831(c.86G>A,aGa>aAa):ENSP00000474844(p.Arg29Lys)];ENSG00000132122:MISSENSE[ENST00000371841(c.68G>A,aGa>aAa):ENSP00000360907(p.Arg23Lys)];ENSG00000132122:EXONIC-NC[ENST00000490850(n.174G>A)];ENSG00000132122:EXONIC-NC[ENST00000463938(n.504G>A)];ENSG00000132122:3UTR[ENST00000487543(c.*258G>A)];	NA	chr1*48865	NA	NA	NA	NA	NA	T	PrD	N	MODERATE	NA	NA	79	79	0	64	58	6	9.38
Smoker	42478	chr1	36060088	G	C	*TFAP2E*	MISSENSE	ENSG00000116819:MISSENSE[ENST00000373235(c.1140G>C,caG>caC):ENSP00000362332(p.Gln380His)];	NA	NA	NA	NA	NA	NA	NA	D	PrD	D	MODERATE	D	0.993	102	102	0	74	57	17	22.97
Smoker	42478	chr10	116233759	C	T	*ABLIM1*	INTRONIC	ENSG00000099204:[ENST00000392955(c.862-44G>A)];ENSG00000099204:[ENST00000533213(c.862-44G>A)];ENSG00000099204:[ENST00000277895(c.1042-44G>A)];ENSG00000099204:[ENST00000392952(c.178-44G>A)];ENSG00000099204:[ENST00000369256(c.946-44G>A)];ENSG00000099204:[ENST00000369252(c.862-44G>A)];ENSG00000099204:[ENST00000369253(c.94-868G>A)];ENSG00000099204:[ENST00000369266(c.178-44G>A)];ENSG00000099204:[ENST00000428430(c.94-44G>A)];ENSG00000099204:[ENST00000440467(c.37-44G>A)];	NA	NA	NA	NA	NA	NA	NA	NA	NA	NA	MODIFIER	NA	NA	71	71	0	77	68	9	11.69
Smoker	42478	chr10	28250610	C	A	*ARMC4*	MISSENSE	ENSG00000169126:MISSENSE[ENST00000305242(c.1273G>T,Gat>Tat):ENSP00000306410(p.Asp425Tyr)];ENSG00000169126:MISSENSE[ENST00000239715(c.844G>T,Gat>Tat):ENSP00000239715(p.Asp282Tyr)];ENSG00000169126:MISSENSE[ENST00000434029(c.955G>T,Gat>Tat):ENSP00000398155(p.Asp319Tyr)];ENSG00000169126:MISSENSE[ENST00000537576(c.349G>T,Gat>Tat):ENSP00000443208(p.Asp117Tyr)];ENSG00000169126:5UTR[ENST00000545014(c.-153G>T)];ENSG00000169126:EXONIC-NC[ENST00000480504(n.530G>T)];	chr10*2825	chr10*2825	NA	0.0744483	0.148747304	NA	NA	NA	NA	NA	MODIFIER	NA	NA	82	81	1	84	74	10	11.9
Smoker	42478	chr10	28250635	A	C	*ARMC4*	SILENT	ENSG00000169126:5UTR[ENST00000545014(c.-178T>G)];ENSG00000169126:SILENT[ENST00000305242(c.1248T>G,gcT>gcG):ENSP00000306410(p.Ala416(=))];ENSG00000169126:SILENT[ENST00000239715(c.819T>G,gcT>gcG):ENSP00000239715(p.Ala273(=))];ENSG00000169126:SILENT[ENST00000434029(c.930T>G,gcT>gcG):ENSP00000398155(p.Ala310(=))];ENSG00000169126:SILENT[ENST00000537576(c.324T>G,gcT>gcG):ENSP00000443208(p.Ala108(=))];ENSG00000169126:EXONIC-NC[ENST00000480504(n.505T>G)];	chr10*2825	chr10*2825	NA	0.0799922	0.162695711	NA	NA	NA	NA	NA	LOW	NA	NA	101	99	2	88	79	9	10.23
Smoker	42478	chr10	68138921	A	G	*CTNNA3*	MISSENSE	ENSG00000183230:MISSENSE[ENST00000433211(c.1721T>C,cTt>cCt):ENSP00000389714(p.Leu574Pro)];ENSG00000183230:MISSENSE[ENST00000373744(c.1721T>C,cTt>cCt):ENSP00000362849(p.Leu574Pro)];	NA	NA	NA	8.25E-06	0	NA	NA	D	UN	NA	MODERATE	D	0.999	70	69	0	76	70	6	7.89
Smoker	42478	chr10	30316420	G	T	*KIAA1462*	MISSENSE	ENSG00000165757:MISSENSE[ENST00000375377(c.2657C>A,cCg>cAg):ENSP00000364526(p.Pro886Gln)];	chr10*3031	chr10*3031	NA	NA	NA	NA	NA	T	BN	N	MODERATE	N	0.005	156	156	0	107	97	10	9.35
Smoker	42478	chr10	50193607	C	A	*MIR4294*	MATURE-MIRNA	ENSG00000264800:[ENST00000584548(n.26G>T)];	NA	NA	NA	NA	NA	NA	NA	NA	NA	NA	MODIFIER	NA	NA	63	63	0	48	33	15	31.25
Smoker	42478	chr10	115364676	G	C	*NRAP*	MISSENSE	ENSG00000197893:MISSENSE[ENST00000369360(c.3838C>G,Ctc>Gtc):ENSP00000358367(p.Leu1280Val)];ENSG00000197893:MISSENSE[ENST00000369358(c.3943C>G,Ctc>Gtc):ENSP00000358365(p.Leu1315Val)];ENSG00000197893:MISSENSE[ENST00000360478(c.3814C>G,Ctc>Gtc):ENSP00000353666(p.Leu1272Val)];ENSG00000197893:MISSENSE[ENST00000359988(c.3919C>G,Ctc>Gtc):ENSP00000353078(p.Leu1307Val)];	chr10*1153	chr10*1153	chr10*115364676*115364676*G*A*rs186178037*0*0.9998*0.0001997	NA	NA	NA	NA	D	BN	D	MODERATE	D	0.955	138	138	0	97	89	8	8.25

Mangalaparthi *et al.* , 2020. Mutational landscape of esophageal squamous cell carcinoma in an Indian cohort
Supplementary Table 4. List of somatic single nucleotide variants identified using whole exome sequencing of ESCC patients

Cohort	Sample	Chromosome	Genomic position	Reference allele	Alternate allele	Gene symbol	Variant type	Variant annotation	OncoMD	COSMIC	dbSNP	ExAC	ExAC_SAS	1000G	1000G_SAS	SIFT	Polyphen2	CONDEL	VEP	LRT	PhastCon7 Way	Total read (Adjacent normal)	Reference allel read support (Adjacent normal)	Alternative read support (Adjacent normal)	Total read (Tumor)	Reference allel read support (Tumor)	Alternative read support (Tumor)	Alternate allele frequency
Smoker	42478	chr10	55912864	G	T	*PCDH15*	SILENT	ENSG00000150275:SILENT[ENST00000437009(c.1780C>A,Cga>Aga):ENSP00000412628(p.Arg594(=))];ENSG00000150275:SILENT[ENST00000395433(c.1714C>A,Cga>Aga):ENSP00000378821(p.Arg572(=))];ENSG00000150275:SILENT[ENST00000395445(c.1801C>A,Cga>Aga):ENSP00000378832(p.Arg601(=))];ENSG00000150275:SILENT[ENST00000320301(c.1780C>A,Cga>Aga):ENSP00000322604(p.Arg594(=))];ENSG00000150275:SILENT[ENST00000448885(c.1780C>A,Cga>Aga):ENSP00000412320(p.Arg594(=))];ENSG00000150275:SILENT[ENST00000409834(c.613C>A,Cga>Aga):ENSP00000386693(p.Arg205(=))];ENSG00000150275:SILENT[ENST00000395432(c.1669C>A,Cga>Aga):ENSP00000378820(p.Arg557(=))];ENSG00000150275:SILENT[ENST00000373955(c.1780C>A,Cga>Aga):ENSP00000363066(p.Arg594(=))];ENSG00000150275:SILENT[ENST00000373957(c.1714C>A,Cga>Aga):ENSP00000363068(p.Arg572(=))];ENSG00000150275:SILENT[ENST00000361849(c.1780C>A,Cga>Aga):ENSP00000354950(p.Arg594(=))];ENSG00000150275:SILENT[ENST00000395438(c.1780C>A,Cga>Aga):ENSP00000378826(p.Arg594(=))];ENSG00000150275:SILENT[ENST00000395446(c.1780C>A,Cga>Aga):ENSP00000378833(p.Arg594(=))];ENSG00000150275:SILENT[ENST00000414778(c.1795C>A,Cga>Aga):ENSP00000410304(p.Arg599(=))];ENSG00000150275:SILENT[ENST00000373965(c.1801C>A,Cga>Aga):ENSP00000363076(p.Arg601(=))];ENSG00000150275:SILENT[ENST00000395430(c.1780C>A,Cga>Aga):ENSP00000378818(p.Arg594(=))];ENSG00000150275:INTRONIC[ENST00000373956(c.92-20097C>A)];ENSG00000150275:INTRONIC[ENST00000495484(n.368-295841C>A)];ENSG00000150275:INTRONIC[ENST00000395440(c.1305+42579C>A)];ENSG00000150275:INTRONIC[ENST00000414367(c.876+164167C>A)];ENSG00000150275:INTRONIC[ENST00000395442(c.1098+60832C>A)];	chr10*5591	chr10*5591	NA	NA	NA	NA	NA	NA	NA	NA	LOW	NA	NA	104	104	0	94	74	20	21.28
Smoker	42478	chr10	50193607	C	A	*RP11-523O18.1*	INTRONIC	ENSG00000226576:[ENST00000422966(n.402+597C>A)];	NA	NA	NA	NA	NA	NA	NA	NA	NA	NA	MODIFIER	NA	NA	63	63	0	48	33	15	31.25
Smoker	42478	chr10	50193607	C	A	*RP11-523O18.5*	INTRONIC	ENSG00000233665:[ENST00000428825(n.638-2133G>T)];	NA	NA	NA	NA	NA	NA	NA	NA	NA	NA	MODIFIER	NA	NA	63	63	0	48	33	15	31.25
Smoker	42478	chr10	7214005	C	A	*SFMBT2*	MISSENSE	ENSG00000198879:MISSENSE[ENST00000361972(c.2267G>T,cGg>cTg):ENSP00000355109(p.Arg756Leu)];ENSG00000198879:MISSENSE[ENST00000397167(c.2267G>T,cGg>cTg):ENSP00000380353(p.Arg756Leu)];	NA	NA	chr10*7214005*7214005*C*T*rs778340548*NA*NA*NA	NA	NA	NA	NA	D	PoD	D	MODERATE	D	0.162	53	52	0	36	30	6	16.67
Smoker	42478	chr10	69667761	A	G	*SIRT1*	INTRONIC	ENSG00000096717:[ENST00000432464(c.206-42A>G)];ENSG00000096717:[ENST00000403579(c.182-42A>G)];ENSG00000096717:[ENST00000212015(c.1091-42A>G)];ENSG00000096717:[ENST00000406900(c.182-42A>G)];	NA	NA	NA	NA	NA	NA	NA	NA	NA	NA	MODIFIER	NA	NA	53	53	0	51	43	8	15.69
Smoker	42478	chr11	5475409	C	A	*AC104389.28*	INTRONIC	ENSG00000167355:[ENST00000418729(n.84+51390G>T)];ENSG00000167355:[ENST00000420726(n.302+51172G>T)];ENSG00000167355:[ENST00000415970(n.84+51390G>T)];ENSG00000167355:[ENST00000420465(n.45+51390G>T)];	NA	NA	chr11*5475409*5475409*C*T*rs141274915*1*0.9904*0.009585	NA	NA	NA	NA	T	BN	N	MODERATE	N	0.649	184	184	0	202	165	37	18.32
Smoker	42478	chr11	76731290	C	T	*ACER3*	INTRONIC	ENSG00000078124:[ENST00000278544(c.*591-28C>T)];ENSG00000078124:[ENST00000532485(c.751-28C>T)];ENSG00000078124:[ENST00000533873(c.640-28C>T)];ENSG00000078124:[ENST00000530921(n.241-28C>T)];ENSG00000078124:[ENST00000525325(n.522-28C>T)];ENSG00000078124:[ENST00000531461(c.*473-28C>T)];ENSG00000078124:[ENST00000525861(c.*701-28C>T)];ENSG00000078124:[ENST00000531352(c.*632-28C>T)];ENSG00000078124:[ENST00000538157(c.625-28C>T)];ENSG00000078124:[ENST00000544113(c.352-28C>T)];ENSG00000078124:[ENST00000526597(c.466-28C>T)];	NA	NA	NA	NA	NA	NA	NA	NA	NA	NA	MODIFIER	NA	NA	236	231	3	153	120	33	21.57
Smoker	42478	chr11	63426697	C	G	*ATL3*	MISSENSE	ENSG00000184743:MISSENSE[ENST00000540699(c.230G>C,gGt>gCt):ENSP00000441842(p.Gly77Ala)];ENSG00000184743:MISSENSE[ENST00000538786(c.20G>C,gGt>gCt):ENSP00000437593(p.Gly7Ala)];ENSG00000184743:MISSENSE[ENST00000398868(c.74G>C,gGt>gCt):ENSP00000381844(p.Gly25Ala)];ENSG00000184743:EXONIC-NC[ENST00000535789(n.641G>C)];ENSG00000184743:INTRONIC-SS-DNR-PRX[ENST00000332645(c.224+6G>C)];	NA	NA	NA	NA	NA	NA	NA	NA	NA	NA	LOW	NA	NA	124	124	0	130	118	11	8.46
Smoker	42478	chr11	108150326	G	A	*ATM*	MISSENSE	ENSG00000149311:MISSENSE[ENST00000278616(c.3393G>A,atG>atA):ENSP00000278616(p.Met1131Ile)];ENSG00000149311:MISSENSE[ENST00000527805(c.3393G>A,atG>atA):ENSP00000435747(p.Met1131Ile)];ENSG00000149311:MISSENSE[ENST00000452508(c.3393G>A,atG>atA):ENSP00000388058(p.Met1131Ile)];	NA	NA	NA	NA	NA	NA	NA	T	BN	N	MODERATE	N	0.997	94	94	0	86	77	9	10.47
Smoker	42478	chr11	124856689	G	C	*CCDC15*	MISSENSE	ENSG00000149548:MISSENSE[ENST00000344762(c.805G>C,Gat>Cat):ENSP00000341684(p.Asp269His)];ENSG00000149548:MISSENSE[ENST00000529051(c.805G>C,Gat>Cat):ENSP00000435403(p.Asp269His)];	NA	NA	NA	NA	NA	NA	NA	D	PrD	D	MODERATE	N	0.001	204	203	0	162	146	16	9.88
Smoker	42478	chr11	46341865	G	A	*CREB3L1*	MISSENSE	ENSG00000157613:MISSENSE[ENST00000288400(c.1309G>A,Gat>Aat):ENSP00000288400(p.Asp437Asn)];ENSG00000157613:MISSENSE[ENST00000529193(c.1309G>A,Gat>Aat):ENSP00000434939(p.Asp437Asn)];ENSG00000157613:EXONIC-NC[ENST00000530244(n.903G>A)];	NA	NA	NA	NA	NA	NA	NA	NA	NA	NA	MODIFIER	NA	NA	63	63	0	82	77	5	6.1
Smoker	42478	chr11	84028018	C	A	*DLG2*	SILENT	ENSG00000150672:INTRONIC[ENST00000532653(c.205-65684G>T)];ENSG00000150672:INTRONIC[ENST00000524982(c.205-65684G>T)];ENSG00000150672:INTRONIC[ENST00000529111(n.483-65684G>T)];ENSG00000150672:INTRONIC[ENST00000527466(n.423-65684G>T)];ENSG00000150672:INTRONIC[ENST00000543673(c.520-65684G>T)];ENSG00000150672:INTRONIC[ENST00000398309(c.205-65684G>T)];ENSG00000150672:INTRONIC[ENST00000376104(c.520-65684G>T)];ENSG00000150672:SILENT[ENST00000398301(c.171G>T,ggG>ggT):ENSP00000381346(p.Gly57(=))];ENSG00000150672:SILENT[ENST00000280241(c.171G>T,ggG>ggT):ENSP00000280241(p.Gly57(=))];	NA	NA	NA	NA	NA	NA	NA	NA	NA	NA	MODIFIER	NA	NA	76	76	0	67	42	25	37.31
Smoker	42478	chr11	5475409	C	A	*HBE1*	INTRONIC	ENSG00000213931:[ENST00000396895(c.-267+51390G>T)];ENSG00000213931:[ENST00000380237(c.-310+51390G>T)];	NA	NA	chr11*5475409*5475409*C*T*rs141274915*1*0.9904*0.009585	NA	NA	NA	NA	T	BN	N	MODERATE	N	0.649	184	184	0	202	165	37	18.32
Smoker	42478	chr11	5475409	C	A	*HBG2*	INTRONIC	ENSG00000196565:[ENST00000380252(c.62+51172G>T)];ENSG00000196565:[ENST00000380259(c.-564-108280G>T)];	NA	NA	chr11*5475409*5475409*C*T*rs141274915*1*0.9904*0.009585	NA	NA	NA	NA	T	BN	N	MODERATE	N	0.649	184	184	0	202	165	37	18.32

Mangalaparthi *et al.* , 2020. Mutational landscape of esophageal squamous cell carcinoma in an Indian cohort
Supplementary Table 4. List of somatic single nucleotide variants identified using whole exome sequencing of ESCC patients

Cohort	Sample	Chromosome	Genomic position	Reference allele	Alternate allele	Gene symbol	Variant type	Variant annotation	OncoMD	COSMIC	dbSNP	ExAC	ExAC_SAS	1000G	1000G_SAS	SIFT	Polyphen2	CONDEL	VEP	LRT	PhastCon7 Way	Total read (Adjacent normal)	Reference allel read support (Adjacent normal)	Alternative read support (Adjacent normal)	Total read (Tumor)	Reference allel read support (Tumor)	Alternative read support (Tumor)	Alternate allele frequency
Smoker	42478	chr11	21555892	G	C	*NELL1*	INTRONIC	ENSG00000165973:[ENST00000529218(n.1000-28G>C)];ENSG00000165973:[ENST00000325319(c.1475-28G>C)];ENSG00000165973:[ENST00000357134(c.1646-28G>C)];ENSG00000165973:[ENST00000298925(c.1730-28G>C)];ENSG00000165973:[ENST00000532434(c.1646-25843G>C)];	NA	NA	NA	NA	NA	NA	NA	NA	NA	NA	MODIFIER	NA	NA	171	171	0	139	119	20	14.39
Smoker	42478	chr11	119054092	C	T	*NLRX1*	NONSENSE	ENSG00000160703:NONSENSE[ENST00000292199(c.2872C>T,Cga>Tga):ENSP00000292199(p.Arg958Ter)];ENSG00000160703:NONSENSE[ENST00000409991(c.2872C>T,Cga>Tga):ENSP00000386851(p.Arg958Ter)];ENSG00000160703:NONSENSE[ENST00000409109(c.2872C>T,Cga>Tga):ENSP00000387334(p.Arg958Ter)];ENSG00000160703:INTRONIC[ENST00000409265(c.2607-438C>T)];ENSG00000160703:INTRONIC[ENST00000525863(c.2607-438C>T)];	NA	NA	NA	NA	NA	NA	NA	NA	NA	NA	MODIFIER	NA	NA	133	133	0	96	82	14	14.58
Smoker	42478	chr11	123810958	T	A	*OR4D5*	MISSENSE	ENSG00000171014:MISSENSE[ENST00000307033(c.635T>A,cTg>cAg):ENSP00000305970(p.Leu212Gln)];	chr11*1238	chr11*1238	NA	NA	NA	NA	NA	D	PrD	D	MODERATE	N	1	215	214	0	169	153	16	9.47
Smoker	42478	chr11	5475409	C	A	*OR51I2*	MISSENSE	ENSG00000187918:MISSENSE[ENST00000341449(c.691C>A,Cgt>Agt):ENSP00000341987(p.Arg231Ser)];	NA	NA	chr11*5475409*5475409*C*T*rs141274915*1*0.9904*0.009585	NA	NA	NA	NA	T	BN	N	MODERATE	N	0.649	184	184	0	202	165	37	18.32
Smoker	42478	chr11	5776840	G	T	*OR52N4*	MISSENSE	ENSG00000181074:MISSENSE[ENST00000317254(c.870G>T,atG>atT):ENSP00000323224(p.Met290Ile)];	NA	NA	NA	NA	NA	NA	NA	NA	NA	NA	MODIFIER	NA	NA	180	180	0	153	123	30	19.61
Smoker	42478	chr11	800806	G	A	*PIDD*	MISSENSE	ENSG00000177595:MISSENSE[ENST00000347755(c.1873C>T,Cgg>Tgg):ENSP00000337797(p.Arg625Trp)];ENSG00000177595:MISSENSE[ENST00000411829(c.1873C>T,Cgg>Tgg):ENSP00000416801(p.Arg625Trp)];ENSG00000177595:MISSENSE[ENST00000531286(c.1006C>T,cCg>cTg):ENSP00000435008(p.Pro336Leu)];ENSG00000177595:EXONIC-NC[ENST00000534525(n.2896C>T)];ENSG00000177595:EXONIC-NC[ENST00000527357(n.3200C>T)];ENSG00000177595:INTRONIC[ENST00000527812(n.332+530C>T)];ENSG00000177595:3UTR[ENST00000525028(c.*1573C>T)];ENSG00000177595:3UTR[ENST00000524486(c.*1888C>T)];	NA	NA	NA	0	0	NA	NA	NA	NA	NA	MODIFIER	NA	NA	53	53	0	21	16	5	23.81
Smoker	42478	chr11	125128638	G	C	*PKNOX2*	INTRONIC	ENSG00000165495:[ENST00000532623(c.-130+23627G>C)];ENSG00000165495:[ENST00000561399(n.96+23627G>C)];ENSG00000165495:[ENST00000531116(n.83-73077G>C)];ENSG00000165495:[ENST00000531212(c.-130+23627G>C)];ENSG00000165495:[ENST00000530517(n.161+23627G>C)];ENSG00000165495:[ENST00000558729(n.206+18474G>C)];ENSG00000165495:[ENST00000561115(n.196+18474G>C)];ENSG00000165495:[ENST00000559662(n.122+23627G>C)];ENSG00000165495:[ENST00000298282(c.-130+23627G>C)];ENSG00000165495:[ENST00000527238(c.-203+19014G>C)];ENSG00000165495:[ENST00000542175(c.-182+23627G>C)];ENSG00000165495:[ENST00000557814(n.120+23627G>C)];	NA	NA	NA	NA	NA	NA	NA	NA	NA	NA	MODIFIER	NA	NA	101	100	1	81	69	12	14.81
Smoker	42478	chr11	125128638	G	C	*RP11-687M24.8*	EXONIC-NC	ENSG00000254932:[ENST00000526796(n.529C>G)];	NA	NA	NA	NA	NA	NA	NA	NA	NA	NA	MODIFIER	NA	NA	101	100	1	81	69	12	14.81
Smoker	42478	chr11	49598145	C	A	*RP11-707M1.1*	EXONIC-NC	ENSG00000205035:[ENST00000527477(n.749C>A)];ENSG00000205035:[ENST00000534201(n.718C>A)];ENSG00000205035:[ENST00000530858(n.749C>A)];	NA	NA	NA	NA	NA	NA	NA	NA	NA	NA	MODIFIER	NA	NA	205	202	1	207	178	29	14.01
Smoker	42478	chr11	5776840	G	T	*TRIM5*	INTRONIC	ENSG00000132256:[ENST00000412903(c.-61-75372C>A)];ENSG00000132256:[ENST00000380027(c.-440-70216C>A)];	NA	NA	NA	NA	NA	NA	NA	NA	NA	NA	MODIFIER	NA	NA	180	180	0	153	123	30	19.61
Smoker	42478	chr11	11964590	G	A	*USP47*	MISSENSE	ENSG00000170242:MISSENSE[ENST00000527733(c.3022G>A,Gaa>Aaa):ENSP00000433146(p.Glu1008Lys)];ENSG00000170242:MISSENSE[ENST00000339865(c.2818G>A,Gaa>Aaa):ENSP00000339957(p.Glu940Lys)];ENSG00000170242:MISSENSE[ENST00000399455(c.3082G>A,Gaa>Aaa):ENSP00000382382(p.Glu1028Lys)];ENSG00000170242:EXONIC-NC[ENST00000531513(n.94G>A)];ENSG00000170242:EXONIC-NC[ENST00000525078(n.872G>A)];ENSG00000170242:5UTR[ENST00000539466(c.-573G>A)];	NA	NA	NA	NA	NA	NA	NA	NA	NA	NA	MODIFIER	NA	NA	245	245	0	222	204	18	8.11
Smoker	42478	chr12	9225315	G	A	*A2M*	SILENT	ENSG00000175899:INTRONIC[ENST00000543436(c.452-4907C>T)];ENSG00000175899:EXONIC-NC-SS-PRX[ENST00000545828(n.351C>T)];ENSG00000175899:SILENT[ENST00000318602(c.3909C>T,gtC>gtT):ENSP00000323929(p.Val1303(=))];	NA	NA	NA	NA	NA	NA	NA	NA	NA	NA	LOW	NA	NA	101	99	0	123	112	11	8.94
Smoker	42478	chr12	105446644	G	A	*ALDH1L2*	SILENT	ENSG00000136010:SILENT[ENST00000424857(c.1353C>T,ttC>ttT):ENSP00000389608(p.Phe451(=))];ENSG00000136010:SILENT[ENST00000258494(c.1353C>T,ttC>ttT):ENSP00000258494(p.Phe451(=))];ENSG00000136010:3UTR[ENST00000552270(c.*737C>T)];ENSG00000136010:EXONIC-NC[ENST00000549335(n.695C>T)];	NA	NA	0.0001997	8.25E-06	0	0.0001997	0	NA	NA	NA	MODIFIER	NA	NA	273	272	0	326	317	9	2.76
Smoker	42478	chr12	547665	G	A	*CCDC77*	MISSENSE	ENSG00000120647:MISSENSE[ENST00000540180(c.890G>A,gGa>gAa):ENSP00000440554(p.Gly297Glu)];ENSG00000120647:MISSENSE[ENST00000239830(c.986G>A,gGa>gAa):ENSP00000239830(p.Gly329Glu)];ENSG00000120647:MISSENSE[ENST00000412006(c.890G>A,gGa>gAa):ENSP00000412925(p.Gly297Glu)];ENSG00000120647:MISSENSE[ENST00000543504(c.890G>A,gGa>gAa):ENSP00000445873(p.Gly297Glu)];ENSG00000120647:MISSENSE[ENST00000422000(c.890G>A,gGa>gAa):ENSP00000391870(p.Gly297Glu)];ENSG00000120647:EXONIC-NC[ENST00000540118(n.277G>A)];ENSG00000120647:EXONIC-NC[ENST00000537286(n.39G>A)];	NA	NA	NA	NA	NA	NA	NA	T	BN	N	MODERATE	N	0.044	43	43	0	61	55	6	9.84
Smoker	42478	chr12	7293912	C	G	*CLSTN3*	MISSENSE	ENSG00000139182:MISSENSE[ENST00000537408(c.1434C>G,atC>atG):ENSP00000440679(p.Ile478Met)];ENSG00000139182:MISSENSE[ENST00000266546(c.1398C>G,atC>atG):ENSP00000266546(p.Ile466Met)];ENSG00000139182:EXONIC-NC[ENST00000544584(n.210C>G)];	NA	NA	NA	NA	NA	NA	NA	NA	NA	NA	MODIFIER	NA	NA	192	192	0	221	202	19	8.6
Smoker	42478	chr12	52863103	G	C	*KRT6C*	INTRONIC	ENSG00000170465:[ENST00000252250(c.1460-22C>G)];	NA	NA	NA	NA	NA	NA	NA	NA	NA	NA	MODIFIER	NA	NA	134	134	0	107	102	5	4.67
Smoker	42478	chr12	53165805	G	T	*KRT76*	INTRONIC	ENSG00000185069:[ENST00000332411(c.1138-25C>A)];	NA	NA	chr12*53165805*53165805*G*A*rs199872104*0*0.9998*0.0001997	NA	NA	NA	NA	NA	NA	NA	MODIFIER	NA	NA	184	183	0	163	149	14	8.59
Smoker	42478	chr12	57919758	C	T	*MBD6*	MISSENSE	ENSG00000166987:MISSENSE[ENST00000355673(c.1007C>T,cCc>cTc):ENSP00000347896(p.Pro336Leu)];ENSG00000166987:MISSENSE[ENST00000431731(c.1007C>T,cCc>cTc):ENSP00000390211(p.Pro336Leu)];ENSG00000166987:INTRONIC[ENST00000552659(c.365-336C>T)];	NA	NA	chr12*57919758*57919758*C*A*rs753517788*NA*NA*NA	NA	NA	NA	NA	D_lc	UN	NA	MODERATE	N	0.93	100	99	0	76	65	11	14.47

Mangalaparthi *et al.*, 2020. Mutational landscape of esophageal squamous cell carcinoma in an Indian cohort

Supplementary Table 4. List of somatic single nucleotide variants identified using whole exome sequencing of ESCC patients

Cohort	Sample	Chromosome	Genomic position	Reference allele	Alternate allele	Gene symbol	Variant type	Variant annotation	OncoMD	COSMIC	dbSNP	ExAC	ExAC_SAS	1000G	1000G_SAS	SIFT	Polyphen2	CONDEL	VEP	LRT	PhastCon7 Way	Total read (Adjacent normal)	Reference allel read support (Adjacent normal)	Alternative read support (Adjacent normal)	Total read (Tumor)	Reference allel read support (Tumor)	Alternative read support (Tumor)	Alternate allele frequency
Smoker	42478	chr12	124922569	G	A	*NCOR2*	INTRONIC	ENSG00000196498:[ENST00000356219(c.816-27C>T)];ENSG00000196498:[ENST00000542927(c.584-27C>T)];ENSG00000196498:[ENST00000458234(c.816-27C>T)];ENSG00000196498:[ENST00000420698(c.816-27C>T)];ENSG00000196498:[ENST00000404121(c.-475-27C>T)];ENSG00000196498:[ENST00000448008(c.522-27C>T)];ENSG00000196498:[ENST00000429285(c.816-27C>T)];ENSG00000196498:[ENST00000405201(c.816-27C>T)];ENSG00000196498:[ENST00000404621(c.816-27C>T)];ENSG00000196498:[ENST00000397355(c.816-27C>T)];	NA	NA	NA	NA	NA	NA	NA	NA	NA	NA	MODIFIER	NA	NA	108	107	1	94	66	28	29.79
Smoker	42478	chr12	4768294	C	A	*NDUFA9*	NONSENSE	ENSG00000139180:NONSENSE[ENST00000266544(c.503C>A,tCa>tAa):ENSP00000266544(p.Ser168Ter)];ENSG00000139180:EXONIC-NC[ENST00000539573(n.266C>A)];ENSG00000139180:EXONIC-NC[ENST00000396655(n.513C>A)];ENSG00000139180:EXONIC-NC[ENST00000535726(n.759C>A)];ENSG00000139180:EXONIC-NC[ENST00000544675(n.560C>A)];	NA	NA	NA	NA	NA	NA	NA	NA	NA	NA	MODIFIER	NA	NA	65	65	0	65	54	11	16.92
Smoker	42478	chr12	4768294	C	A	*RP11-234B24.6*	EXONIC-NC	ENSG00000255639:[ENST00000543979(n.299C>A)];	NA	NA	NA	NA	NA	NA	NA	NA	NA	NA	MODIFIER	NA	NA	65	65	0	65	54	11	16.92
Smoker	42478	chr12	4768294	C	A	*RP11-500M8.7*	3UTR	ENSG00000272921:[ENST00000536588(c.*503C>A)];	NA	NA	NA	NA	NA	NA	NA	NA	NA	NA	MODIFIER	NA	NA	65	65	0	65	54	11	16.92
Smoker	42478	chr12	16055865	G	A	*STRAP*	MISSENSE	ENSG00000023734:MISSENSE[ENST00000025399(c.1045G>A,Gag>Aag):ENSP00000025399(p.Glu349Lys)];ENSG00000023734:MISSENSE[ENST00000419869(c.1006G>A,Gag>Aag):ENSP00000392270(p.Glu336Lys)];ENSG00000023734:MISSENSE[ENST00000538352(c.724G>A,Gag>Aag):ENSP00000439761(p.Glu242Lys)];ENSG00000023734:MISSENSE[ENST00000538718(c.239G>A,Gag>Aag):ENSP00000445814(p.Glu81Lys)];ENSG00000023734:EXONIC-NC[ENST00000539887(n.129G>A)];ENSG00000023734:3UTR[ENST00000541731(c.*723G>A)];	NA	NA	NA	NA	NA	NA	NA	T	PoD	N	MODERATE	NA	NA	29	29	0	15	10	5	33.33
Smoker	42478	chr12	29709962	T	C	*TMTC1*	INTRONIC	ENSG00000133687:[ENST00000553189(n.1654-29A>G)];ENSG00000133687:[ENST00000256062(c.1209-29A>G)];ENSG00000133687:[ENST00000381224(c.1395-29A>G)];ENSG00000133687:[ENST00000551659(c.1719-29A>G)];ENSG00000133687:[ENST00000539277(c.1533-29A>G)];ENSG00000133687:[ENST00000319685(n.1274-29A>G)];ENSG00000133687:[ENST00000552618(c.1605-29A>G)];	NA	NA	NA	NA	NA	NA	NA	NA	NA	NA	MODIFIER	NA	NA	46	46	0	55	48	7	12.73
Smoker	42478	chr13	26455034	A	G	*AL138815.1*	INTRONIC	ENSG00000215549:[ENST00000400553(c.56-12A>G)];	NA	NA	NA	NA	NA	NA	NA	NA	NA	NA	MODIFIER	NA	NA	95	95	0	75	68	7	9.33
Smoker	42478	chr13	26455034	A	G	*ATP8A2*	INTRONIC	ENSG00000132932:[ENST00000281620(c.*962+18488A>G)];ENSG00000132932:[ENST00000255283(c.2988+18488A>G)];ENSG00000132932:[ENST00000381655(c.3183+18488A>G)];ENSG00000132932:[ENST00000491840(n.2054+18488A>G)];	NA	NA	NA	NA	NA	NA	NA	NA	NA	NA	MODIFIER	NA	NA	95	95	0	75	68	7	9.33
Smoker	42478	chr13	76432043	C	A	*LMO7*	INTRONIC	ENSG00000136153:[ENST00000357063(c.4921-14C>A)];ENSG00000136153:[ENST00000321797(c.4175-14C>A)];ENSG00000136153:[ENST00000526202(c.3806-14C>A)];ENSG00000136153:[ENST00000465261(c.4066-14C>A)];ENSG00000136153:[ENST00000341547(c.4028-14C>A)];ENSG00000136153:[ENST00000377534(c.4818-14C>A)];ENSG00000136153:[ENST00000605961(n.1271C>A)];	NA	NA	NA	NA	NA	NA	NA	NA	NA	NA	MODIFIER	NA	NA	138	138	0	138	126	12	8.7
Smoker	42478	chr13	58208760	C	T	*PCDH17*	MISSENSE	ENSG00000118946:MISSENSE[ENST00000377918(c.2080C>T,Cgg>Tgg):ENSP00000367151(p.Arg694Trp)];ENSG00000118946:MISSENSE[ENST00000484979(c.2080C>T,Cgg>Tgg):ENSP00000432899(p.Arg694Trp)];	NA	NA	chr13*58208760*58208760*C*G*rs776873573*NA*NA*NA	NA	NA	NA	NA	D	PoD	D	MODERATE	NA	NA	127	127	0	105	92	13	12.38
Smoker	42478	chr13	101266671	T	C	*TMTC4*	MISSENSE	ENSG00000125247:MISSENSE[ENST00000376234(c.1793A>G,aAt>aGt):ENSP00000365408(p.Asn598Ser)];ENSG00000125247:MISSENSE[ENST00000328767(c.1460A>G,aAt>aGt):ENSP00000365409(p.Asn487Ser)];ENSG00000125247:MISSENSE[ENST00000342624(c.1850A>G,aAt>aGt):ENSP00000343871(p.Asn617Ser)];	chr13*1012	chr13*1012	chr13*101266671*101266671*T*A,C*rs767185745*NA*NA*NA	0.0002142	6.06E-05	NA	NA	T	BN	N	MODERATE	D	1	122	121	0	96	84	12	12.5
Smoker	42478	chr14	58818022	G	A	*ARID4A*	MISSENSE	ENSG00000032219:MISSENSE[ENST00000355431(c.1636G>A,Gaa>Aaa):ENSP00000347602(p.Glu546Lys)];ENSG00000032219:MISSENSE[ENST00000395168(c.1636G>A,Gaa>Aaa):ENSP00000378597(p.Glu546Lys)];ENSG00000032219:MISSENSE[ENST00000431317(c.1636G>A,Gaa>Aaa):ENSP00000397368(p.Glu546Lys)];ENSG00000032219:MISSENSE[ENST00000348476(c.1636G>A,Gaa>Aaa):ENSP00000344556(p.Glu546Lys)];ENSG00000032219:MISSENSE[ENST00000417477(c.670G>A,Gaa>Aaa):ENSP00000416053(p.Glu224Lys)];ENSG00000032219:EXONIC-NC[ENST00000553355(n.8G>A)];	NA	NA	NA	NA	NA	NA	NA	NA	NA	NA	MODIFIER	NA	NA	115	115	0	105	90	15	14.29
Smoker	42478	chr14	64694689	G	A	*ESR2*	INTRONIC	ENSG00000140009:[ENST00000344288(c.*296-357C>T)];ENSG00000140009:[ENST00000555278(c.1407-42C>T)];ENSG00000140009:[ENST00000556275(c.*6999C>T)];ENSG00000140009:[ENST00000542956(c.1406+6999C>T)];ENSG00000140009:[ENST00000357782(c.1407-42C>T)];ENSG00000140009:[ENST00000353772(c.1407-357C>T)];ENSG00000140009:[ENST00000554572(c.1407-357C>T)];ENSG00000140009:[ENST00000358599(c.1407-357C>T)];ENSG00000140009:[ENST00000553796(c.*56C>T)];	NA	NA	NA	NA	NA	NA	NA	NA	NA	NA	MODIFIER	NA	NA	97	96	0	97	89	8	8.25
Smoker	42478	chr14	77494033	A	G	*IRF2BPL*	MISSENSE	ENSG00000119669:MISSENSE[ENST00000238647(c.103T>C,Tgc>Cgc):ENSP00000238647(p.Cys35Arg)];	NA	NA	NA	NA	NA	NA	NA	D	PrD	D	MODERATE	U	0.986	188	185	3	97	70	27	27.84
Smoker	42478	chr14	64463930	C	T	*SYNE2*	INTRONIC	ENSG00000054654:[ENST00000554584(c.3152+54C>T)];ENSG00000054654:[ENST00000357395(c.-7763+54C>T)];ENSG00000054654:[ENST00000358025(c.3152+54C>T)];ENSG00000054654:[ENST00000344113(c.3152+54C>T)];	NA	NA	NA	NA	NA	NA	NA	NA	NA	NA	MODIFIER	NA	NA	253	252	0	292	263	29	9.93
Smoker	42478	chr15	29346111	C	T	*APBA2*	SILENT	ENSG00000034053:SILENT[ENST00000558259(c.24C>T,agC>agT):ENSP00000454171(p.Ser8(=))];ENSG00000034053:SILENT[ENST00000561069(c.24C>T,agC>agT):ENSP00000453144(p.Ser8(=))];ENSG00000034053:SILENT[ENST00000558206(c.24C>T,agC>agT):ENSP00000453981(p.Ser8(=))];ENSG00000034053:SILENT[ENST00000560283(c.24C>T,agC>agT):ENSP00000453034(p.Ser8(=))];ENSG00000034053:SILENT[ENST00000558402(c.24C>T,agC>agT):ENSP00000453293(p.Ser8(=))];ENSG00000034053:SILENT[ENST00000558330(c.24C>T,agC>agT):ENSP00000452722(p.Ser8(=))];ENSG00000034053:SILENT[ENST00000558358(c.24C>T,agC>agT):ENSP00000453715(p.Ser8(=))];ENSG00000034053:SILENT[ENST00000558804(c.24C>T,agC>agT):ENSP00000454001(p.Ser8(=))];ENSG00000034053:SILENT[ENST00000411764(c.24C>T,agC>agT):ENSP00000409312(p.Ser8(=))];ENSG00000034053:EXONIC-NC[ENST00000559814(n.347C>T)];	chr15*2934	chr15*2934	NA	2.56E-05	0	NA	NA	NA	NA	NA	LOW	NA	0.293	56	56	0	42	36	6	14.29

Mangalaparthi *et al.* , 2020. Mutational landscape of esophageal squamous cell carcinoma in an Indian cohort
Supplementary Table 4. List of somatic single nucleotide variants identified using whole exome sequencing of ESCC patients

Cohort	Sample	Chromosome	Genomic position	Reference allele	Alternate allele	Gene symbol	Variant type	Variant annotation	OncoMD	COSMIC	dbSNP	ExAC	ExAC_SAS	1000G	1000G_SAS	SIFT	Polyphen2	CONDEL	VEP	LRT	PhastCon7 Way	Total read (Adjacent normal)	Reference allel read support (Adjacent normal)	Alternative read support (Adjacent normal)	Total read (Tumor)	Reference allel read support (Tumor)	Alternative read support (Tumor)	Alternate allele frequency
Smoker	42478	chr15	72875618	G	C	*ARIH1*	MISSENSE	ENSG00000166233:MISSENSE[ENST00000379887(c.1659G>C,gaG>gaC):ENSP00000369217(p.Glu553Asp)];ENSG00000166233:EXONIC-NC[ENST00000563310(n.786G>C)];ENSG00000166233:EXONIC-NC[ENST00000562891(n.339G>C)];ENSG00000166233:EXONIC-NC[ENST00000566063(n.617G>C)];	NA	NA	chr15*72875618*72875618*G*A*rs772654041*NA*NA*NA	NA	NA	NA	NA	NA	NA	NA	MODIFIER	NA	NA	263	263	0	200	187	13	6.5
Smoker	42478	chr15	74922144	G	A	*CLK3*	MISSENSE	ENSG00000179335:MISSENSE[ENST00000352989(c.1324G>A,Gag>Aag):ENSP00000323106(p.Glu442Lys)];ENSG00000179335:MISSENSE[ENST00000395066(c.1837G>A,Gag>Aag):ENSP00000378505(p.Glu613Lys)];ENSG00000179335:MISSENSE[ENST00000345005(c.1393G>A,Gag>Aag):ENSP00000344112(p.Glu465Lys)];ENSG00000179335:EXONIC-NC[ENST00000562078(n.2142G>A)];ENSG00000179335:EXONIC-NC[ENST00000454830(n.3390G>A)];ENSG00000179335:EXONIC-NC[ENST00000569406(n.3199G>A)];ENSG00000179335:EXONIC-NC[ENST00000568232(n.643G>A)];ENSG00000179335:INTRONIC[ENST00000561673(n.240+771G>A)];ENSG00000179335:INTRONIC[ENST00000563418(c.181+771G>A)];ENSG00000179335:3UTR[ENST00000483723(c.*837G>A)];ENSG00000179335:3UTR[ENST00000348245(c.*837G>A)];ENSG00000179335:3UTR[ENST00000564468(c.*592G>A)];	NA	NA	NA	NA	NA	NA	NA	NA	NA	NA	MODIFIER	NA	NA	51	51	0	38	31	7	18.42
Smoker	42478	chr16	20693655	C	T	*ACSM1*	SILENT	ENSG00000166743:SILENT[ENST00000519745(c.534G>A,caG>caA):ENSP00000428650(p.Gln178(=))];ENSG00000166743:SILENT[ENST00000307493(c.534G>A,caG>caA):ENSP00000301956(p.Gln178(=))];ENSG00000166743:SILENT[ENST00000520010(c.534G>A,caG>caA):ENSP00000428047(p.Gln178(=))];ENSG00000166743:5UTR[ENST00000219151(c.-610G>A)];	chr16*2069[illegible]	chr16*2069[illegible]	NA	NA	NA	NA	NA	NA	NA	NA	MODIFIER	NA	NA	37	37	0	33	27	6	18.18
Smoker	42478	chr16	20693655	C	T	*ACSM3*	INTRONIC	ENSG00000005187:[ENST00000561584(c.-190+7511C>T)];ENSG00000005187:[ENST00000568235(c.-189-67577C>T)];ENSG00000005187:[ENST00000501740(n.605+2884C>T)];	chr16*2069[illegible]	chr16*2069[illegible]	NA	NA	NA	NA	NA	NA	NA	NA	MODIFIER	NA	NA	37	37	0	33	27	6	18.18
Smoker	42478	chr16	15677772	G	C	*C16orf45*	INTRONIC	ENSG00000166780:[ENST00000565857(c.272+677G>C)];ENSG00000166780:[ENST00000452191(c.451+677G>C)];ENSG00000166780:[ENST00000565913(n.293+677G>C)];ENSG00000166780:[ENST00000564389(c.396+677G>C)];ENSG00000166780:[ENST00000300006(c.502+677G>C)];ENSG00000166780:[ENST00000566490(c.419+2584G>C)];ENSG00000166780:[ENST00000561692(c.359-92G>C)];	NA	NA	NA	NA	NA	NA	NA	NA	NA	NA	MODIFIER	NA	NA	80	80	0	52	46	6	11.54
Smoker	42478	chr16	88871108	G	A	*CDT1*	INTRONIC	ENSG00000167513:[ENST00000301019(c.351+33G>A)];ENSG00000167513:[ENST00000562747(n.57+33G>A)];	NA	NA	NA	8.80E-06	6.12E-05	NA	NA	NA	NA	NA	MODIFIER	NA	NA	190	189	1	111	94	17	15.32
Smoker	42478	chr16	89865482	G	A	*FANCA*	MISSENSE	ENSG00000187741:INTRONIC[ENST00000389301(c.893+92C>T)];ENSG00000187741:INTRONIC[ENST00000567621(c.370+92C>T)];ENSG00000187741:INTRONIC[ENST00000568369(c.893+92C>T)];ENSG00000187741:MISSENSE[ENST00000534992(c.893C>T,tCa>tTa):ENSP00000443675(p.Ser298Leu)];ENSG00000187741:3UTR[ENST00000543736(c.*5C>T)];ENSG00000187741:3UTR[ENST00000563673(c.*34C>T)];ENSG00000187741:3UTR[ENST00000566409(c.*34C>T)];ENSG00000187741:3UTR[ENST00000389302(c.*5C>T)];ENSG00000187741:3UTR[ENST00000565582(c.*96C>T)];ENSG00000187741:EXONIC-NC[ENST00000566889(n.1481C>T)];	NA	NA	NA	NA	NA	NA	NA	NA	NA	NA	MODIFIER	NA	NA	71	70	0	84	76	8	9.52
Smoker	42478	chr16	33630195	G	A	*NA*	INTERGENIC		NA	NA	0.0007987	NA	NA	0.0007987	0	NA	NA	NA	MODIFIER	NA	NA	146	146	0	111	96	15	13.51
Smoker	42478	chr16	57063964	C	T	*NLRC5*	SILENT	ENSG00000140853:SILENT[ENST00000543030(c.282C>T,gtC>gtT):ENSP00000440153(p.Val94(=))];ENSG00000140853:SILENT[ENST00000308149(c.2385C>T,gtC>gtT):ENSP00000308886(p.Val795(=))];ENSG00000140853:SILENT[ENST00000538805(c.1642C>T,gtC>gtT):ENSP00000442906(p.Val548(=))];ENSG00000140853:SILENT[ENST00000538110(c.906C>T,gtC>gtT):ENSP00000441597(p.Val302(=))];ENSG00000140853:SILENT[ENST00000539144(c.2385C>T,gtC>gtT):ENSP00000441727(p.Val795(=))];ENSG00000140853:SILENT[ENST00000436936(c.2385C>T,gtC>gtT):ENSP00000389739(p.Val795(=))];ENSG00000140853:SILENT[ENST00000545081(c.1567C>T,gtC>gtT):ENSP00000443825(p.Val523(=))];ENSG00000140853:SILENT[ENST00000262510(c.2385C>T,gtC>gtT):ENSP00000262510(p.Val795(=))];ENSG00000140853:3UTR[ENST00000539881(c.*270C>T)];ENSG00000140853:EXONIC-NC[ENST00000536231(n.836C>T)];	NA	NA	NA	NA	NA	NA	NA	NA	NA	NA	LOW	NA	NA	155	152	0	93	81	12	12.9
Smoker	42478	chr16	727043	A	C	*RHBDL1*	MISSENSE	ENSG00000103269:MISSENSE[ENST00000352681(c.499A>C,Aag>Cag):ENSP00000344206(p.Lys167Gln)];ENSG00000103269:MISSENSE[ENST00000561556(c.274A>C,Aag>Cag):ENSP00000455608(p.Lys92Gln)];ENSG00000103269:MISSENSE[ENST00000219551(c.694A>C,Aag>Cag):ENSP00000219551(p.Lys232Gln)];ENSG00000103269:EXONIC-NC[ENST00000450775(n.568A>C)];	NA	NA	NA	NA	NA	NA	NA	T	BN	N	MODERATE	N	1	165	165	0	105	84	21	20
Smoker	42478	chr16	30616152	C	T	*RP11-146F11.5*	INTRONIC	ENSG00000260167:[ENST00000563540(n.189-3469C>T)];	NA	NA	0.002396	0.0009032	0	0.0023962	0	NA	NA	NA	MODIFIER	NA	NA	88	87	0	62	51	11	17.74
Smoker	42478	chr16	71610300	G	A	*RP11-432I5.1*	INTRONIC	ENSG00000260886:[ENST00000561529(n.751-1668G>A)];	NA	NA	NA	NA	NA	NA	NA	NA	NA	NA	MODIFIER	NA	NA	77	77	0	56	49	7	12.5
Smoker	42478	chr16	71610300	G	A	*TAT*	NONSENSE	ENSG00000198650:NONSENSE[ENST00000355962(c.19C>T,Cag>Tag):ENSP00000348234(p.Gln7Ter)];ENSG00000198650:EXONIC-NC[ENST00000566010(n.115C>T)];ENSG00000198650:EXONIC-NC[ENST00000566094(n.115C>T)];	NA	NA	NA	NA	NA	NA	NA	NA	NA	NA	MODIFIER	NA	NA	77	77	0	56	49	7	12.5
Smoker	42478	chr16	30616152	C	T	*ZNF689*	SILENT	ENSG00000156853:EXONIC-NC[ENST00000564262(n.667G>A)];ENSG00000156853:EXONIC-NC[ENST00000565440(n.940G>A)];ENSG00000156853:EXONIC-NC[ENST00000566673(n.1199G>A)];ENSG00000156853:EXONIC-NC[ENST00000563304(n.916G>A)];ENSG00000156853:SILENT[ENST00000287461(c.936G>A,acG>acA):ENSP00000287461(p.Thr312(=))];	NA	NA	0.002396	0.0009032	0	0.0023962	0	NA	NA	NA	MODIFIER	NA	NA	88	87	0	62	51	11	17.74

Supplementary Table 4. List of somatic single nucleotide variants identified using whole exome sequencing of ESCC patients

Cohort	Sample	Chromosome	Genomic position	Reference allele	Alternate allele	Gene symbol	Variant type	Variant annotation	OncoMD	COSMIC	dbSNP	ExAC	ExAC_SAS	1000G	1000G_SAS	SIFT	Polyphen2	CONDEL	VEP	LRT	PhastCon7 Way	Total read (Adjacent normal)	Reference allel read support (Adjacent normal)	Alternative read support (Adjacent normal)	Total read (Tumor)	Reference allel read support (Tumor)	Alternative read support (Tumor)	Alternate allele frequency
Smoker	42478	chr17	7124923	C	G	*ACADVL*	MISSENSE	ENSG00000072778:MISSENSE[ENST00000356839(c.544C>G,Cag>Gag):ENSP00000349297(p.Gln182Glu)];ENSG00000072778:MISSENSE[ENST00000543245(c.613C>G,Cag>Gag):ENSP00000438689(p.Gln205Glu)];ENSG00000072778:MISSENSE[ENST00000350303(c.478C>G,Cag>Gag):ENSP00000344152(p.Gln160Glu)];ENSG00000072778:MISSENSE[ENST00000583312(c.544C>G,Cag>Gag):ENSP00000467920(p.Gln182Glu)];ENSG00000072778:MISSENSE[ENST00000579886(c.382C>G,Cag>Gag):ENSP00000463246(p.Gln128Glu)];ENSG00000072778:3UTR[ENST00000322910(c.*499C>G)];ENSG00000072778:3UTR[ENST00000581378(c.*32C>G)];ENSG00000072778:EXONIC-NC[ENST00000579286(n.725C>G)];ENSG00000072778:EXONIC-NC[ENST00000577191(n.621C>G)];ENSG00000072778:EXONIC-NC[ENST00000583760(n.326C>G)];ENSG00000072778:EXONIC-NC[ENST00000577433(n.752C>G)];ENSG00000072778:EXONIC-NC[ENST00000582166(n.525C>G)];ENSG00000072778:EXONIC-NC[ENST00000577857(n.360C>G)];ENSG00000072778:EXONIC-NC[ENST00000580365(n.275C>G)];ENSG00000072778:INTRONIC[ENST00000581562(n.525-348C>G)];	NA	NA	chr17*7124923*7124923*C*CAG*rs757395724*NA*NA*NA	NA	NA	NA	NA	NA	NA	NA	MODIFIER	NA	NA	54	54	0	42	36	6	14.29
Smoker	42478	chr17	54543842	G	C	*ANKFN1*	MISSENSE	ENSG00000153930:MISSENSE[ENST00000318698(c.1692G>C,tgG>tgC):ENSP00000321627(p.Trp564Cys)];ENSG00000153930:MISSENSE[ENST00000566473(c.1692G>C,tgG>tgC):ENSP00000454224(p.Trp564Cys)];	NA	NA	NA	NA	NA	NA	NA	D	PrD	D	MODERATE	D	1	88	88	0	94	86	8	8.51
Smoker	42478	chr17	64208331	A	G	*APOH*	INTRONIC	ENSG00000091583:[ENST00000205948(c.983-25T>C)];ENSG00000091583:[ENST00000585162(c.258-25T>C)];	NA	NA	NA	3.33E-05	0	NA	NA	NA	NA	NA	MODIFIER	NA	NA	99	99	0	104	89	15	14.42
Smoker	42478	chr17	79514605	C	T	*C17orf70*	SILENT	ENSG00000185504:SILENT[ENST00000425898(c.450G>A,acG>acA):ENSP00000399674(p.Thr150(=))];ENSG00000185504:SILENT[ENST00000537152(c.1050G>A,acG>acA):ENSP00000440151(p.Thr350(=))];ENSG00000185504:SILENT[ENST00000327787(c.1503G>A,acG>acA):ENSP00000333283(p.Thr501(=))];ENSG00000185504:3UTR[ENST00000443656(c.*1405G>A)];	chr17*7951	NA	NA	NA	NA	NA	NA	NA	NA	NA	LOW	NA	NA	122	122	0	89	84	5	5.62
Smoker	42478	chr17	10348092	G	C	*CTC-297N7.11*	INTRONIC	ENSG00000272975:[ENST00000587182(n.155+38537G>C)];	NA	NA	NA	NA	NA	NA	NA	NA	NA	NA	MODIFIER	NA	NA	202	200	0	171	157	14	8.19
Smoker	42478	chr17	9683298	C	T	*DHRS7C*	MISSENSE	ENSG00000184544:MISSENSE[ENST00000330255(c.325G>A,Gat>Aat):ENSP00000327975(p.Asp109Asn)];ENSG00000184544:MISSENSE[ENST00000571134(c.322G>A,Gat>Aat):ENSP00000459579(p.Asp108Asn)];	NA	NA	NA	NA	NA	NA	NA	T	PrD	N	MODERATE	D	0.991	58	58	0	44	36	8	18.18
Smoker	42478	chr17	9830119	C	T	*GAS7*	INTRONIC	ENSG00000007237:[ENST00000396115(c.334-8703G>A)];ENSG00000007237:[ENST00000432992(c.886-33G>A)];ENSG00000007237:[ENST00000583882(c.196-9795G>A)];ENSG00000007237:[ENST00000580811(n.528-33G>A)];ENSG00000007237:[ENST00000581871(n.440-33G>A)];ENSG00000007237:[ENST00000323816(c.706-33G>A)];ENSG00000007237:[ENST00000580865(c.466-33G>A)];ENSG00000007237:[ENST00000579158(c.694-33G>A)];ENSG00000007237:[ENST00000437099(c.694-33G>A)];ENSG00000007237:[ENST00000540214(c.334-8703G>A)];ENSG00000007237:[ENST00000585266(c.706-33G>A)];ENSG00000007237:[ENST00000542249(c.694-33G>A)];ENSG00000007237:[ENST00000578456(n.193G>A)];	NA	NA	NA	NA	NA	NA	NA	NA	NA	NA	MODIFIER	NA	NA	107	106	1	69	61	8	11.59
Smoker	42478	chr17	28811967	C	T	*GOSR1*	INTRONIC	ENSG00000108587:[ENST00000467635(n.359+179C>T)];ENSG00000108587:[ENST00000467337(c.153+179C>T)];ENSG00000108587:[ENST00000451249(c.342+179C>T)];ENSG00000108587:[ENST00000225724(c.348+179C>T)];ENSG00000108587:[ENST00000427274(c.342+179C>T)];ENSG00000108587:[ENST00000581721(c.348+179C>T)];ENSG00000108587:[ENST00000414833(c.362+179C>T)];ENSG00000108587:[ENST00000537788(c.*259+179C>T)];ENSG00000108587:[ENST00000491489(n.538C>T)];	NA	NA	NA	NA	NA	NA	NA	NA	NA	NA	MODIFIER	NA	NA	67	67	0	85	78	7	8.24
Smoker	42478	chr17	14204925	G	A	*HS3ST3B1*	SILENT	ENSG00000125430:SILENT[ENST00000466596(c.90G>A,agG>agA):ENSP00000436078(p.Arg30(=))];ENSG00000125430:SILENT[ENST00000360954(c.90G>A,agG>agA):ENSP00000354213(p.Arg30(=))];	NA	NA	NA	NA	NA	NA	NA	NA	NA	NA	LOW	NA	1	113	113	0	65	58	7	10.77
Smoker	42478	chr17	38975147	G	T	*KRT10*	MISSENSE	ENSG00000186395:MISSENSE[ENST00000269576(c.1640C>A,tCc>tAc):ENSP00000269576(p.Ser547Tyr)];	chr17*3897	chr17*3897	NA	0.0187951	0.023841414	NA	NA	D_lc	UN	NA	MODERATE	N	0	100	97	1	43	35	8	18.6
Smoker	42478	chr17	38975149	G	T	*KRT10*	MISSENSE	ENSG00000186395:MISSENSE[ENST00000269576(c.1638C>A,agC>agA):ENSP00000269576(p.Ser546Arg)];	NA	NA	chr17*38975136*38975163*TGCCGCCGCCGGAGCTGCCGCCCCCGTA*T*rs753460356*NA*NA*NA|chr17*38975138*38975149*CCGCCGCCGGAG*C*rs747376338*NA*NA*NA|chr17*38975143*38975158*GCCGGAGCTGCCGCCC*G*rs755330996*NA*NA*NA|chr17*38975148*38975150*AGC*A*rs781768887*NA*NA*NA|chr17*38975149*38975149*G*A,T*rs758343254*NA*NA*NA	0.0175027	0.021221249	NA	NA	T_lc	UN	NA	MODERATE	N	0.001	99	97	1	41	35	6	14.63
Smoker	42478	chr17	38935765	G	A	*KRT27*	MISSENSE	ENSG00000171446:MISSENSE[ENST00000301656(c.961C>T,Ctc>Ttc):ENSP00000301656(p.Leu321Phe)];	NA	NA	NA	NA	NA	NA	NA	T	PrD	N	MODERATE	N	0.997	89	89	0	120	112	8	6.67
Smoker	42478	chr17	10348092	G	C	*MYH4*	INTRONIC	ENSG00000264424:[ENST00000255381(c.5571+20C>G)];	NA	NA	NA	NA	NA	NA	NA	NA	NA	NA	MODIFIER	NA	NA	202	200	0	171	157	14	8.19
Smoker	42478	chr17	42085843	C	T	*NAGS*	SILENT	ENSG00000161653:SILENT[ENST00000293404(c.1479C>T,ttC>ttT):ENSP00000293404(p.Phe493(=))];ENSG00000161653:SILENT[ENST00000589767(c.1410C>T,ttC>ttT):ENSP00000465408(p.Phe470(=))];ENSG00000161653:EXONIC-NC[ENST00000592915(n.1367C>T)];	chr17*4208	chr17*4208	0.0001997	5.77E-05	0	0.0001997	0	NA	NA	NA	LOW	NA	NA	102	101	1	109	80	29	26.61

Mangalaparthi *et al.* , 2020. Mutational landscape of esophageal squamous cell carcinoma in an Indian cohort
Supplementary Table 4. List of somatic single nucleotide variants identified using whole exome sequencing of ESCC patients

Cohort	Sample	Chromosome	Genomic position	Reference allele	Alternate allele	Gene symbol	Variant type	Variant annotation	OncoMD	COSMIC	dbSNP	ExAC	ExAC_SAS	1000G	1000G_SAS	SIFT	Polyphen2	CONDEL	VEP	LRT	PhastCon7 Way	Total read (Adjacent normal)	Reference allel read support (Adjacent normal)	Alternative read support (Adjacent normal)	Total read (Tumor)	Reference allel read support (Tumor)	Alternative read support (Tumor)	Alternate allele frequency
Smoker	42478	chr17	29587459	C	T	*NF1*	SILENT	ENSG00000196712:SILENT[ENST00000456735(c.3438C>T,gaC>gaT):ENSP00000389907(p.Asp1146(=))];ENSG00000196712:SILENT[ENST00000358273(c.4503C>T,gaC>gaT):ENSP00000351015(p.Asp1501(=))];ENSG00000196712:SILENT[ENST00000579081(c.4542C>T,gaC>gaT):ENSP00000462408(p.Asp1514(=))];ENSG00000196712:SILENT[ENST00000356175(c.4440C>T,gaC>gaT):ENSP00000348498(p.Asp1480(=))];ENSG00000196712:3UTR[ENST00000466819(c.*337C>T)];ENSG00000196712:3UTR[ENST00000479614(c.*337C>T)];ENSG00000196712:EXONIC-NC[ENST00000493220(n.2976C>T)];	NA	NA	NA	4.12E-05	0.000121124	NA	NA	NA	NA	NA	LOW	NA	0.996	290	290	0	325	311	14	4.31
Smoker	42478	chr17	40822654	G	A	*PLEKHH3*	SILENT	ENSG00000068137:SILENT[ENST00000412503(c.1482C>T,caC>caT):ENSP00000411885(p.His494(=))];ENSG00000068137:SILENT[ENST00000591022(c.1491C>T,caC>caT):ENSP00000468678(p.His497(=))];ENSG00000068137:SILENT[ENST00000591196(c.1491C>T,caC>caT):ENSP00000467824(p.His497(=))];ENSG00000068137:SILENT[ENST00000293349(c.1482C>T,caC>caT):ENSP00000293349(p.His494(=))];ENSG00000068137:EXONIC-NC[ENST00000456950(n.1330C>T)];ENSG00000068137:EXONIC-NC[ENST00000591490(n.1816C>T)];	NA	NA	NA	NA	NA	NA	NA	NA	NA	NA	MODIFIER	NA	NA	89	89	0	78	70	8	10.26
Smoker	42478	chr17	19318132	G	A	*RNF112*	MISSENSE	ENSG00000128482:MISSENSE[ENST00000461366(c.1058G>A,cGa>cAa):ENSP00000454919(p.Arg353Gln)];	NA	NA	NA	2.50E-05	0	NA	NA	T	BN	N	MODERATE	NA	NA	90	90	0	93	62	31	33.33
Smoker	42478	chr17	10348092	G	C	*RP11-799N11.1*	INTRONIC	ENSG00000272736:[ENST00000399342(n.206+38498G>C)];ENSG00000272736:[ENST00000581304(n.143+38537G>C)];	NA	NA	NA	NA	NA	NA	NA	NA	NA	NA	MODIFIER	NA	NA	202	200	0	171	157	14	8.19
Smoker	42478	chr17	19458591	C	T	*SLC47A1*	SILENT	ENSG00000142494:SILENT[ENST00000436810(c.541C>T,Ctg>Ttg):ENSP00000407155(p.Leu181(=))];ENSG00000142494:SILENT[ENST00000270570(c.610C>T,Ctg>Ttg):ENSP00000270570(p.Leu204(=))];ENSG00000142494:SILENT[ENST00000457293(c.610C>T,Ctg>Ttg):ENSP00000415586(p.Leu204(=))];ENSG00000142494:SILENT[ENST00000395585(c.610C>T,Ctg>Ttg):ENSP00000378951(p.Leu204(=))];ENSG00000142494:INTRONIC[ENST00000571335(c.99-462C>T)];ENSG00000142494:INTRONIC[ENST00000542886(c.544-315C>T)];ENSG00000142494:INTRONIC[ENST00000575023(c.498+5601C>T)];ENSG00000142494:EXONIC-NC[ENST00000584348(n.572C>T)];ENSG00000142494:EXONIC-NC[ENST00000497548(n.672C>T)];	NA	NA	NA	NA	NA	NA	NA	NA	NA	NA	LOW	NA	0.957	122	122	0	138	122	16	11.59
Smoker	42478	chr17	32956017	C	T	*TMEM132E*	INTRONIC	ENSG00000181291:[ENST00000321639(c.876-14C>T)];	NA	NA	NA	NA	NA	NA	NA	NA	NA	NA	MODIFIER	NA	NA	123	123	0	119	113	6	5.04
Smoker	42478	chr17	7578272	G	A	*TP53*	MISSENSE	ENSG00000141510:MISSENSE[ENST00000413465(c.577C>T,Cat>Tat):ENSP00000410739(p.His193Tyr)];ENSG00000141510:MISSENSE[ENST00000420246(c.577C>T,Cat>Tat):ENSP00000391127(p.His193Tyr)];ENSG00000141510:MISSENSE[ENST00000269305(c.577C>T,Cat>Tat):ENSP00000269305(p.His193Tyr)];ENSG00000141510:MISSENSE[ENST00000359597(c.577C>T,Cat>Tat):ENSP00000352610(p.His193Tyr)];ENSG00000141510:MISSENSE[ENST00000445888(c.577C>T,Cat>Tat):ENSP00000391478(p.His193Tyr)];ENSG00000141510:MISSENSE[ENST00000455263(c.577C>T,Cat>Tat):ENSP00000398846(p.His193Tyr)];ENSG00000141510:MISSENSE[ENST00000509690(c.181C>T,Cat>Tat):ENSP00000425104(p.His61Tyr)];ENSG00000141510:MISSENSE[ENST00000514944(c.298C>T,Cat>Tat):ENSP00000423862(p.His100Tyr)];ENSG00000141510:EXONIC-NC[ENST00000504937(n.459C>T)];ENSG00000141510:EXONIC-NC[ENST00000505014(n.833C>T)];ENSG00000141510:EXONIC-NC[ENST00000504290(n.459C>T)];ENSG00000141510:EXONIC-NC[ENST00000510385(n.459C>T)];ENSG00000141510:INTRONIC[ENST00000574684(n.67+99C>T)];	chr17*7578	chr17*7578	NA	NA	NA	NA	NA	NA	NA	NA	MODIFIER	NA	NA	141	141	0	69	64	5	7.25
Smoker	42478	chr17	40765141	C	T	*TUBG1*	INTRONIC	ENSG00000131462:[ENST00000251413(c.606+50C>T)];ENSG00000131462:[ENST00000589688(c.*118+50C>T)];ENSG00000131462:[ENST00000591509(c.645+50C>T)];	NA	NA	0.0001997	4.15E-05	0.000126088	0.0001997	0	NA	NA	NA	MODIFIER	NA	NA	140	140	0	141	126	15	10.64
Smoker	42478	chr17	6023845	G	A	*WSCD1*	MISSENSE	ENSG00000179314:MISSENSE[ENST00000573634(c.1244G>A,cGc>cAc):ENSP00000460396(p.Arg415His)];ENSG00000179314:MISSENSE[ENST00000574232(c.1592G>A,cGc>cAc):ENSP00000458374(p.Arg531His)];ENSG00000179314:MISSENSE[ENST00000317744(c.1592G>A,cGc>cAc):ENSP00000323087(p.Arg531His)];ENSG00000179314:MISSENSE[ENST00000539421(c.1592G>A,cGc>cAc):ENSP00000446032(p.Arg531His)];ENSG00000179314:MISSENSE[ENST00000574946(c.1592G>A,cGc>cAc):ENSP00000460825(p.Arg531His)];ENSG00000179314:3UTR[ENST00000571494(c.*982G>A)];	chr17*6023	chr17*6023	0.0001997	1.66E-05	6.06E-05	0.0001997	0.001	NA	NA	NA	MODIFIER	NA	NA	153	152	0	101	82	19	18.81
Smoker	42478	chr18	5445102	C	T	*EPB41L3*	INTRONIC	ENSG00000082397:[ENST00000342933(c.486+37G>A)];ENSG00000082397:[ENST00000341928(c.486+37G>A)];ENSG00000082397:[ENST00000545076(n.696+37G>A)];ENSG00000082397:[ENST00000544123(c.486+37G>A)];ENSG00000082397:[ENST00000584670(c.37-20744G>A)];ENSG00000082397:[ENST00000542652(n.169+37G>A)];ENSG00000082397:[ENST00000400111(c.486+37G>A)];ENSG00000082397:[ENST00000540638(c.486+37G>A)];	NA	NA	NA	NA	NA	NA	NA	NA	NA	NA	MODIFIER	NA	NA	255	255	0	266	241	25	9.4
Smoker	42478	chr18	29847856	T	C	*GAREM*	MISSENSE	ENSG00000141441:MISSENSE[ENST00000399218(c.2606A>G,aAt>aGt):ENSP00000382165(p.Asn869Ser)];ENSG00000141441:MISSENSE[ENST00000269209(c.2609A>G,aAt>aGt):ENSP00000269209(p.Asn870Ser)];ENSG00000141441:INTRONIC[ENST00000583696(c.65+19138A>G)];	NA	NA	NA	NA	NA	NA	NA	NA	NA	NA	MODIFIER	NA	NA	61	60	1	69	51	18	26.09
Smoker	42478	chr18	33048602	C	T	*INO80C*	SILENT	ENSG00000153391:SILENT[ENST00000334598(c.552G>A,ctG>ctA):ENSP00000334473(p.Leu184(=))];ENSG00000153391:SILENT[ENST00000441607(c.660G>A,ctG>ctA):ENSP00000391457(p.Leu220(=))];ENSG00000153391:SILENT[ENST00000586489(c.387G>A,ctG>ctA):ENSP00000464948(p.Leu129(=))];ENSG00000153391:SILENT[ENST00000590757(c.261G>A,ctG>ctA):ENSP00000467708(p.Leu87(=))];ENSG00000153391:INTRONIC[ENST00000592173(c.447+9644G>A)];	NA	NA	NA	NA	NA	NA	NA	NA	NA	NA	MODIFIER	NA	NA	91	91	0	44	37	7	15.91
Smoker	42478	chr18	33048602	C	T	*RP11-322E11.6*	INTRONIC	ENSG00000267140:[ENST00000589258(c.157-21683G>A)];	NA	NA	NA	NA	NA	NA	NA	NA	NA	NA	MODIFIER	NA	NA	91	91	0	44	37	7	15.91

Mangalaparthi *et al.* , 2020. Mutational landscape of esophageal squamous cell carcinoma in an Indian cohort
Supplementary Table 4. List of somatic single nucleotide variants identified using whole exome sequencing of ESCC patients

Cohort	Sample	Chromosome	Genomic position	Reference allele	Alternate allele	Gene symbol	Variant type	Variant annotation	OncoMD	COSMIC	dbSNP	ExAC	ExAC_SAS	1000G	1000G_SAS	SIFT	Polyphen2	CONDEL	VEP	LRT	PhastCon7 Way	Total read (Adjacent normal)	Reference allel read support (Adjacent normal)	Alternative read support (Adjacent normal)	Total read (Tumor)	Reference allel read support (Tumor)	Alternative read support (Tumor)	Alternate allele frequency
Smoker	42478	chr19	6177248	G	T	*ACSBG2*	MISSENSE	ENSG00000130377:MISSENSE[ENST00000586696(c.747G>T,tgG>tgT):ENSP00000465589(p.Trp249Cys)];ENSG00000130377:MISSENSE[ENST00000591403(c.747G>T,tgG>tgT):ENSP00000467755(p.Trp249Cys)];ENSG00000130377:MISSENSE[ENST00000588485(c.186G>T,tgG>tgT):ENSP00000466336(p.Trp62Cys)];ENSG00000130377:MISSENSE[ENST00000252669(c.747G>T,tgG>tgT):ENSP00000252669(p.Trp249Cys)];ENSG00000130377:MISSENSE[ENST00000588304(c.597G>T,tgG>tgT):ENSP00000464938(p.Trp199Cys)];ENSG00000130377:3UTR[ENST00000587227(c.*127G>T)];ENSG00000130377:EXONIC-NC[ENST00000585526(n.439G>T)];ENSG00000130377:EXONIC-NC[ENST00000591741(n.266G>T)];	NA	NA	NA	NA	NA	NA	NA	NA	NA	NA	MODIFIER	NA	NA	115	115	0	60	52	8	13.33
Smoker	42478	chr19	49967084	C	T	*ALDH16A1*	INTRONIC	ENSG00000161618:[ENST00000293350(c.1099-27C>T)];ENSG00000161618:[ENST00000540132(c.610-27C>T)];ENSG00000161618:[ENST00000433981(c.604-27C>T)];ENSG00000161618:[ENST00000599652(n.1288-27C>T)];ENSG00000161618:[ENST00000455361(c.946-27C>T)];ENSG00000161618:[ENST00000593417(c.*557-27C>T)];	NA	NA	0.001797	0.0002398	0.000749064	0.0017971	0	NA	NA	NA	MODIFIER	NA	NA	104	104	0	81	70	11	13.58
Smoker	42478	chr19	17514961	C	A	*BST2*	SILENT	ENSG00000130303:SILENT[ENST00000252593(c.390G>T,gcG>gcT):ENSP00000252593(p.Ala130(=))];ENSG00000130303:EXONIC-NC[ENST00000527220(n.278G>T)];ENSG00000130303:EXONIC-NC[ENST00000533098(n.205G>T)];	NA	NA	NA	NA	NA	NA	NA	NA	NA	NA	MODIFIER	NA	NA	60	60	0	44	38	6	13.64
Smoker	42478	chr19	6708007	G	A	*C3*	INTRONIC	ENSG00000125730:[ENST00000245907(c.1846-67C>T)];	NA	NA	NA	NA	NA	NA	NA	NA	NA	NA	MODIFIER	NA	NA	99	99	0	74	68	6	8.11
Smoker	42478	chr19	39090533	G	A	*CTB-186G2.1*	INTRONIC	ENSG00000267291:[ENST00000589557(n.235-1520G>A)];	NA	NA	NA	NA	NA	NA	NA	NA	NA	NA	MODIFIER	NA	NA	109	109	0	102	96	6	5.88
Smoker	42478	chr19	37309637	T	C	*CTD-2162K18.5*	INTRONIC	ENSG00000267254:[ENST00000588906(n.308-4996T>C)];ENSG00000267254:[ENST00000587278(n.187+6129T>C)];	NA	NA	NA	NA	NA	NA	NA	T	BN	N	MODERATE	NA	NA	240	239	1	272	233	39	14.34
Smoker	42478	chr19	52729180	G	A	*CTD-2525I3.3*	INTRONIC	ENSG00000268015:[ENST00000593857(n.376+2528C>T)];	NA	NA	NA	NA	NA	NA	NA	NA	NA	NA	MODIFIER	NA	NA	124	124	0	120	113	7	5.83
Smoker	42478	chr19	52795361	C	T	*CTD-2525I3.5*	INTRONIC	ENSG00000269102:[ENST00000594865(n.150+5663G>A)];	NA	NA	NA	NA	NA	NA	NA	NA	NA	NA	MODIFIER	NA	NA	105	104	0	88	76	11	12.5
Smoker	42478	chr19	49967084	C	T	*CTD-3148I10.9*	INTRONIC	ENSG00000269469:[ENST00000599536(c.5-27C>T)];	NA	NA	0.001797	0.0002398	0.000749064	0.0017971	0	NA	NA	NA	MODIFIER	NA	NA	104	104	0	81	70	11	13.58
Smoker	42478	chr19	47151925	G	A	*DACT3*	SILENT	ENSG00000197380:SILENT[ENST00000300875(c.1029C>T,gaC>gaT):ENSP00000300875(p.Asp343(=))];ENSG00000197380:SILENT[ENST00000391916(c.1704C>T,gaC>gaT):ENSP00000375783(p.Asp568(=))];	chr19*4715	NA	NA	NA	NA	NA	NA	NA	NA	NA	LOW	NA	0.995	129	128	0	76	65	11	14.47
Smoker	42478	chr19	6988693	A	G	*EMR4P*	INTRONIC	ENSG00000268758:[ENST00000597372(n.52+127T>C)];ENSG00000268758:[ENST00000600751(n.133+105T>C)];	NA	NA	NA	NA	NA	NA	NA	NA	NA	NA	MODIFIER	NA	NA	23	23	0	40	26	14	35
Smoker	42478	chr19	54744105	G	T	*LILRA6*	INTRONIC	ENSG00000244482:[ENST00000245621(c.1258+45C>A)];ENSG00000244482:[ENST00000430421(c.*590+45C>A)];ENSG00000244482:[ENST00000270464(c.658+1347C>A)];ENSG00000244482:[ENST00000391735(c.*590+45C>A)];ENSG00000244482:[ENST00000440558(c.955+602C>A)];ENSG00000244482:[ENST00000396365(c.1258+45C>A)];ENSG00000244482:[ENST00000419410(c.1258+45C>A)];	NA	NA	NA	NA	NA	NA	NA	NA	NA	NA	MODIFIER	NA	NA	120	120	0	61	49	12	19.67
Smoker	42478	chr19	54744105	G	T	*LILRB3*	INTRONIC	ENSG00000204577:[ENST00000407860(c.355+1797C>A)];	NA	NA	NA	NA	NA	NA	NA	NA	NA	NA	MODIFIER	NA	NA	120	120	0	61	49	12	19.67
Smoker	42478	chr19	39090533	G	A	*MAP4K1*	INTRONIC	ENSG00000104814:[ENST00000396857(c.1669+32C>T)];ENSG00000104814:[ENST00000589130(c.1657+32C>T)];ENSG00000104814:[ENST00000591517(c.1669+32C>T)];ENSG00000104814:[ENST00000586296(c.1004-4350C>T)];ENSG00000104814:[ENST00000585583(c.*770+184C>T)];ENSG00000104814:[ENST00000423454(c.594+184C>T)];ENSG00000104814:[ENST00000591921(c.780+32C>T)];	NA	NA	NA	NA	NA	NA	NA	NA	NA	NA	MODIFIER	NA	NA	109	109	0	102	96	6	5.88
Smoker	42478	chr19	4847829	G	A	*PLIN3*	SILENT	ENSG00000105355:SILENT-SS-PRX[ENST00000589163(c.407C>T,ttC>ttT):ENSP00000468476(p.Phe136(=))];ENSG00000105355:SILENT[ENST00000585479(c.708C>T,ttC>ttT):ENSP00000465596(p.Phe236(=))];ENSG00000105355:SILENT[ENST00000221957(c.708C>T,ttC>ttT):ENSP00000221957(p.Phe236(=))];ENSG00000105355:SILENT[ENST00000592528(c.672C>T,ttC>ttT):ENSP00000467803(p.Phe224(=))];	NA	NA	NA	NA	NA	NA	NA	NA	NA	NA	LOW	NA	0.233	104	104	0	66	57	9	13.64
Smoker	42478	chr19	52729180	G	A	*PPP2R1A*	3UTR	ENSG00000105568:[ENST00000391791(c.*108G>A)];ENSG00000105568:[ENST00000462990(c.1217-38G>A)];ENSG00000105568:[ENST00000444322(c.1589-38G>A)];ENSG00000105568:[ENST00000322088(c.1754-38G>A)];	NA	NA	NA	NA	NA	NA	NA	NA	NA	NA	MODIFIER	NA	NA	124	124	0	120	113	7	5.83
Smoker	42478	chr19	6177248	G	T	*RFX2*	INTRONIC	ENSG00000087903:[ENST00000585324(n.46-794C>A)];ENSG00000087903:[ENST00000589712(n.89-794C>A)];ENSG00000087903:[ENST00000592883(c.-108+22239C>A)];	NA	NA	NA	NA	NA	NA	NA	NA	NA	NA	MODIFIER	NA	NA	115	115	0	60	52	8	13.33
Smoker	42478	chr19	54744105	G	T	*RPS9*	INTRONIC	ENSG00000170889:[ENST00000448962(c.*183-6846G>T)];	NA	NA	NA	NA	NA	NA	NA	NA	NA	NA	MODIFIER	NA	NA	120	120	0	61	49	12	19.67
Smoker	42478	chr19	1119101	C	A	*SBNO2*	MISSENSE	ENSG00000064932:MISSENSE[ENST00000438103(c.1265G>T,cGc>cTc):ENSP00000400762(p.Arg422Leu)];ENSG00000064932:MISSENSE[ENST00000587024(c.1436G>T,cGc>cTc):ENSP00000468520(p.Arg479Leu)];ENSG00000064932:MISSENSE[ENST00000361757(c.1436G>T,cGc>cTc):ENSP00000354733(p.Arg479Leu)];ENSG00000064932:EXONIC-NC[ENST00000592222(n.1289G>T)];ENSG00000064932:EXONIC-NC[ENST00000590446(n.382G>T)];	chr19*1119	chr19*1119	NA	NA	NA	NA	NA	NA	NA	NA	MODIFIER	NA	NA	121	121	0	80	69	11	13.75
Smoker	42478	chr19	51165451	G	A	*SHANK1*	MISSENSE	ENSG00000161681:MISSENSE[ENST00000391813(c.4418C>T,cCg>cTg):ENSP00000375689(p.Pro1473Leu)];ENSG00000161681:MISSENSE[ENST00000391814(c.6281C>T,cCg>cTg):ENSP00000375690(p.Pro2094Leu)];ENSG00000161681:MISSENSE[ENST00000293441(c.6257C>T,cCg>cTg):ENSP00000293441(p.Pro2086Leu)];ENSG00000161681:MISSENSE[ENST00000359082(c.6230C>T,cCg>cTg):ENSP00000351984(p.Pro2077Leu)];	NA	NA	NA	NA	NA	NA	NA	NA	NA	NA	MODIFIER	NA	NA	173	173	0	110	101	9	8.18
Smoker	42478	chr19	51918777	C	T	*SIGLEC10*	INTRONIC	ENSG00000142512:[ENST00000436984(c.928+45G>A)];ENSG00000142512:[ENST00000525998(c.802+45G>A)];ENSG00000142512:[ENST00000432469(c.823+45G>A)];ENSG00000142512:[ENST00000339313(c.1072+45G>A)];ENSG00000142512:[ENST00000357375(n.960+45G>A)];ENSG00000142512:[ENST00000442846(c.628+45G>A)];ENSG00000142512:[ENST00000353836(c.1072+45G>A)];ENSG00000142512:[ENST00000356298(c.1072+45G>A)];ENSG00000142512:[ENST00000441969(c.898+45G>A)];ENSG00000142512:[ENST00000439889(c.898+45G>A)];	NA	NA	NA	NA	NA	NA	NA	NA	NA	NA	MODIFIER	NA	NA	108	107	1	83	73	10	12.05
Smoker	42478	chr19	51165451	G	A	*SYT3*	INTRONIC	ENSG00000213023:[ENST00000544769(c.-518+6159C>T)];	NA	NA	NA	NA	NA	NA	NA	NA	NA	NA	MODIFIER	NA	NA	173	173	0	110	101	9	8.18

Mangalaparthi *et al.* , 2020. Mutational landscape of esophageal squamous cell carcinoma in an Indian cohort

Supplementary Table 4. List of somatic single nucleotide variants identified using whole exome sequencing of ESCC patients

Cohort	Sample	Chromosome	Genomic position	Reference allele	Alternate allele	Gene symbol	Variant type	Variant annotation	OncoMD	COSMIC	dbSNP	ExAC	ExAC_SAS	1000G	1000G_SAS	SIFT	Polyphen2	CONDEL	VEP	LRT	PhastCon7 Way	Total read (Adjacent normal)	Reference allel read support (Adjacent normal)	Alternative read support (Adjacent normal)	Total read (Tumor)	Reference allel read support (Tumor)	Alternative read support (Tumor)	Alternate allele frequency
Smoker	42478	chr19	17758211	C	T	*UNC13A*	MISSENSE	ENSG00000130477:MISSENSE[ENST00000252773(c.1907G>A,cGc>cAc):ENSP00000252773(p.Arg636His)];ENSG00000130477:MISSENSE[ENST00000519716(c.1907G>A,cGc>cAc):ENSP00000429562(p.Arg636His)];ENSG00000130477:MISSENSE[ENST00000550896(c.1901G>A,cGc>cAc):ENSP00000446831(p.Arg634His)];ENSG00000130477:MISSENSE[ENST00000552293(c.1907G>A,cGc>cAc):ENSP00000447572(p.Arg636His)];ENSG00000130477:MISSENSE[ENST00000551649(c.1907G>A,cGc>cAc):ENSP00000447236(p.Arg636His)];ENSG00000130477:MISSENSE[ENST00000428389(c.2171G>A,cGc>cAc):ENSP00000400409(p.Arg724His)];	NA	NA	chr19*17758211*17758211*C*A*rs191081718*0*0.9998*0.0001997	NA	NA	NA	NA	D	PrD	D	MODERATE	U	0.999	144	144	0	128	102	26	20.31
Smoker	42478	chr19	37118087	C	T	*ZNF382*	MISSENSE	ENSG00000161298:MISSENSE[ENST00000435416(c.1285C>T,Cac>Tac):ENSP00000410113(p.His429Tyr)];ENSG00000161298:MISSENSE[ENST00000292928(c.1288C>T,Cac>Tac):ENSP00000292928(p.His430Tyr)];ENSG00000161298:MISSENSE[ENST00000439428(c.1285C>T,Cac>Tac):ENSP00000407593(p.His429Tyr)];ENSG00000161298:MISSENSE[ENST00000423582(c.1141C>T,Cac>Tac):ENSP00000389722(p.His381Tyr)];	NA	NA	NA	NA	NA	NA	NA	D	PrD	D	MODERATE	D	0.989	109	108	1	109	93	16	14.68
Smoker	42478	chr19	22496911	C	G	*ZNF729*	MISSENSE	ENSG00000196350:MISSENSE[ENST00000357491(c.692C>G,aCt>aGt):ENSP00000350085(p.Thr231Ser)];ENSG00000196350:MISSENSE[ENST00000601693(c.692C>G,aCt>aGt):ENSP00000469582(p.Thr231Ser)];	NA	NA	NA	NA	NA	NA	NA	T	BN	N	MODERATE	NA	NA	68	68	0	72	66	6	8.33
Smoker	42478	chr19	52795361	C	T	*ZNF766*	INTRONIC	ENSG00000196214:[ENST00000359102(c.1379-41C>T)];ENSG00000196214:[ENST00000593612(c.1379-41C>T)];ENSG00000196214:[ENST00000439461(c.*910C>T)];	NA	NA	NA	NA	NA	NA	NA	NA	NA	NA	MODIFIER	NA	NA	105	104	0	88	76	11	12.5
Smoker	42478	chr19	40541313	G	C	*ZNF780B*	MISSENSE	ENSG00000128000:MISSENSE[ENST00000434248(c.1453C>G,Ctg>Gtg):ENSP00000391641(p.Leu485Val)];ENSG00000128000:MISSENSE[ENST00000221355(c.1009C>G,Ctg>Gtg):ENSP00000221355(p.Leu337Val)];	NA	NA	chr19*40541311*40541313*CAG*C*rs754993102*NA*NA*NA	NA	NA	NA	NA	T	BN	N	MODERATE	NA	NA	153	153	0	150	138	12	8
Smoker	42478	chr19	37309637	T	C	*ZNF790*	MISSENSE	ENSG00000197863:MISSENSE[ENST00000356725(c.1609A>G,Aaa>Gaa):ENSP00000349161(p.Lys537Glu)];	NA	NA	NA	NA	NA	NA	NA	T	BN	N	MODERATE	NA	NA	240	239	1	272	233	39	14.34
Smoker	42478	chr19	52888474	G	C	*ZNF880*	SILENT	ENSG00000221923:SILENT[ENST00000422689(c.1641G>C,ccG>ccC):ENSP00000406318(p.Pro547(=))];	NA	NA	chr19*52888474*52888474*G*A*rs546626361*0*0.9998*0.0001997	NA	NA	NA	NA	NA	NA	NA	LOW	NA	NA	53	53	0	77	71	6	7.79
Smoker	42478	chr19	23545454	C	T	*ZNF91*	SILENT	ENSG00000167232:SILENT[ENST00000595533(c.30G>A,ctG>ctA):ENSP00000471709(p.Leu10(=))];ENSG00000167232:SILENT[ENST00000397082(c.231G>A,ctG>ctA):ENSP00000380272(p.Leu77(=))];ENSG00000167232:SILENT[ENST00000300619(c.327G>A,ctG>ctA):ENSP00000300619(p.Leu109(=))];ENSG00000167232:INTRONIC[ENST00000599743(c.253+11090G>A)];ENSG00000167232:INTRONIC[ENST00000596989(n.370+11090G>A)];	NA	NA	NA	NA	NA	NA	NA	NA	NA	NA	MODIFIER	NA	NA	146	146	0	149	138	11	7.38
Smoker	42478	chr2	66667646	C	G	*AC092669.1*	INTRONIC	ENSG00000244522:[ENST00000454595(n.340-825G>C)];	NA	NA	NA	NA	NA	NA	NA	NA	NA	NA	MODIFIER	NA	NA	107	107	0	81	76	5	6.17
Smoker	42478	chr2	228109627	T	G	*AC097662.2*	INTRONIC	ENSG00000236432:[ENST00000437673(n.612-6737A>C)];ENSG00000236432:[ENST00000606119(n.22-6737A>C)];ENSG00000236432:[ENST00000439598(n.1593-6737A>C)];	NA	NA	0.009585	0.072214	0.006683375	0.0095847	0.002	NA	NA	NA	MODIFIER	NA	NA	91	89	1	97	92	5	5.15
Smoker	42478	chr2	223797943	G	C	*ACSL3*	MISSENSE	ENSG00000123983:MISSENSE[ENST00000392066(c.1792G>C,Gag>Cag):ENSP00000375918(p.Glu598Gln)];ENSG00000123983:MISSENSE[ENST00000357430(c.1792G>C,Gag>Cag):ENSP00000350012(p.Glu598Gln)];ENSG00000123983:MISSENSE[ENST00000407441(c.294G>C,Gag>Cag):ENSP00000385157(p.Glu99Gln)];ENSG00000123983:EXONIC-NC[ENST00000495541(n.208G>C)];ENSG00000123983:EXONIC-NC[ENST00000474422(n.262G>C)];	NA	NA	NA	NA	NA	NA	NA	NA	NA	NA	MODIFIER	NA	NA	178	177	0	150	133	16	10.67
Smoker	42478	chr2	216212341	C	G	*ATIC*	INTRONIC	ENSG00000138363:[ENST00000426233(c.508+677C>G)];ENSG00000138363:[ENST00000479093(n.418+677C>G)];ENSG00000138363:[ENST00000540518(c.1326+677C>G)];ENSG00000138363:[ENST00000443953(c.*1600+677C>G)];ENSG00000138363:[ENST00000236959(c.1503+677C>G)];ENSG00000138363:[ENST00000435675(c.1500+677C>G)];ENSG00000138363:[ENST00000446622(c.584-3C>G)];	NA	NA	chr2*216212341*216212341*C*T*rs558240043*0*0.9998*0.0001997	NA	NA	NA	NA	NA	NA	NA	MODIFIER	NA	NA	163	163	0	174	159	14	8.05
Smoker	42478	chr2	28464221	G	A	*BRE*	MISSENSE	ENSG00000158019:MISSENSE[ENST00000361704(c.812G>A,aGa>aAa):ENSP00000354699(p.Arg271Lys)];ENSG00000158019:MISSENSE[ENST00000344773(c.812G>A,aGa>aAa):ENSP00000343412(p.Arg271Lys)];ENSG00000158019:MISSENSE[ENST00000379632(c.812G>A,aGa>aAa):ENSP00000368953(p.Arg271Lys)];ENSG00000158019:MISSENSE[ENST00000342045(c.812G>A,aGa>aAa):ENSP00000339371(p.Arg271Lys)];ENSG00000158019:MISSENSE[ENST00000379624(c.812G>A,aGa>aAa):ENSP00000368945(p.Arg271Lys)];	NA	NA	NA	NA	NA	NA	NA	T	BN	N	MODERATE	D	1	95	95	0	77	70	7	9.09
Smoker	42478	chr2	228109627	T	G	*COL4A3*	INTRONIC	ENSG00000169031:[ENST00000396578(c.280-40T>G)];	NA	NA	0.009585	0.072214	0.006683375	0.0095847	0.002	NA	NA	NA	MODIFIER	NA	NA	91	89	1	97	92	5	5.15
Smoker	42478	chr2	116535335	T	C	*DPP10*	INTRONIC	ENSG00000175497:[ENST00000393147(c.1326-28T>C)];ENSG00000175497:[ENST00000409163(c.1164-28T>C)];ENSG00000175497:[ENST00000310323(c.1293-28T>C)];ENSG00000175497:[ENST00000410059(c.1314-28T>C)];	NA	NA	NA	8.28E-06	0	NA	NA	NA	NA	NA	MODIFIER	NA	NA	93	93	0	122	95	27	22.13
Smoker	42478	chr2	116572527	G	A	*DPP10*	MISSENSE	ENSG00000175497:MISSENSE[ENST00000393147(c.1871G>A,gGt>gAt):ENSP00000376855(p.Gly624Asp)];ENSG00000175497:MISSENSE[ENST00000409163(c.1709G>A,gGt>gAt):ENSP00000387038(p.Gly570Asp)];ENSG00000175497:MISSENSE[ENST00000310323(c.1838G>A,gGt>gAt):ENSP00000309066(p.Gly613Asp)];ENSG00000175497:MISSENSE[ENST00000410059(c.1859G>A,gGt>gAt):ENSP00000386565(p.Gly620Asp)];ENSG00000175497:EXONIC-NC[ENST00000473362(n.289G>A)];	NA	NA	NA	NA	NA	NA	NA	NA	NA	NA	MODIFIER	NA	NA	262	261	0	217	198	19	8.76
Smoker	42478	chr2	97369326	G	A	*FER1L5*	EXONIC-NC	ENSG00000249715:[ENST00000424961(n.5878G>A)];ENSG00000249715:[ENST00000457909(n.5261G>A)];ENSG00000249715:[ENST00000436930(n.5878G>A)];ENSG00000249715:[ENST00000414152(n.5866G>A)];ENSG00000249715:[ENST00000397978(n.2069G>A)];	NA	NA	NA	NA	NA	NA	NA	NA	NA	NA	MODIFIER	NA	NA	97	97	0	95	73	22	23.16
Smoker	42478	chr2	234113062	C	T	*INPP5D*	MISSENSE	ENSG00000168918:MISSENSE[ENST00000455936(c.2522C>T,tCa>tTa):ENSP00000404610(p.Ser841Leu)];ENSG00000168918:MISSENSE[ENST00000450745(c.2522C>T,tCa>tTa):ENSP00000407916(p.Ser841Leu)];ENSG00000168918:MISSENSE[ENST00000359570(c.3230C>T,tCa>tTa):ENSP00000352575(p.Ser1077Leu)];ENSG00000168918:MISSENSE[ENST00000445964(c.2129C>T,tCa>tTa):ENSP00000405338(p.Ser710Leu)];ENSG00000168918:MISSENSE[ENST00000415617(c.2129C>T,tCa>tTa):ENSP00000397421(p.Ser710Leu)];ENSG00000168918:MISSENSE[ENST00000435188(c.2129C>T,tCa>tTa):ENSP00000400151(p.Ser710Leu)];ENSG00000168918:INTRONIC[ENST00000417661(c.342-2290C>T)];	NA	NA	NA	NA	NA	NA	NA	NA	NA	NA	MODIFIER	NA	NA	132	130	1	73	64	9	12.33
Smoker	42478	chr2	141242937	C	A	*LRP1B*	MISSENSE	ENSG00000168702:MISSENSE[ENST00000389484(c.9400G>T,Gac>Tac):ENSP00000374135(p.Asp3134Tyr)];	chr2*14124[illegible]	NA	NA	NA	NA	NA	NA	NA	PoD	NA	MODERATE	U	0.989	186	185	0	186	130	56	30.11

Mangalaparthi *et al.* , 2020. Mutational landscape of esophageal squamous cell carcinoma in an Indian cohort
Supplementary Table 4. List of somatic single nucleotide variants identified using whole exome sequencing of ESCC patients

Cohort	Sample	Chromosome	Genomic position	Reference allele	Alternate allele	Gene symbol	Variant type	Variant annotation	OncoMD	COSMIC	dbSNP	ExAC	ExAC_SAS	1000G	1000G_SAS	SIFT	Polyphen2	CONDEL	VEP	LRT	PhastCon7 Way	Total read (Adjacent normal)	Reference allel read support (Adjacent normal)	Alternative read support (Adjacent normal)	Total read (Tumor)	Reference allel read support (Tumor)	Alternative read support (Tumor)	Alternate allele frequency
Smoker	42478	chr2	150017226	G	C	*LYPD6B*	INTRONIC	ENSG00000150556:[ENST00000437627(c.6-26714G>C)];ENSG00000150556:[ENST00000442722(c.5+29759G>C)];ENSG00000150556:[ENST00000409029(c.5+29759G>C)];ENSG00000150556:[ENST00000409876(c.5+29759G>C)];ENSG00000150556:[ENST00000450639(c.6-52G>C)];ENSG00000150556:[ENST00000473975(n.402-52G>C)];ENSG00000150556:[ENST00000498249(n.279-52G>C)];ENSG00000150556:[ENST00000280115(c.6-52G>C)];ENSG00000150556:[ENST00000409642(c.6-52G>C)];	NA	NA	chr2*150017226*150017226*G*A*rs771702862*NA*NA*NA	NA	NA	NA	NA	NA	NA	NA	MODIFIER	NA	NA	149	145	2	144	120	24	16.67
Smoker	42478	chr2	150071151	G	A	*LYPD6B*	MISSENSE	ENSG00000150556:MISSENSE[ENST00000280115(c.551G>A,aGa>aAa):ENSP00000280115(p.Arg184Lys)];ENSG00000150556:MISSENSE[ENST00000409029(c.479G>A,aGa>aAa):ENSP00000386650(p.Arg160Lys)];ENSG00000150556:MISSENSE[ENST00000409876(c.479G>A,aGa>aAa):ENSP00000386479(p.Arg160Lys)];ENSG00000150556:MISSENSE[ENST00000409642(c.551G>A,aGa>aAa):ENSP00000387077(p.Arg184Lys)];ENSG00000150556:EXONIC-NC[ENST00000498249(n.1094G>A)];	chr2*15007	chr2*15007	NA	NA	NA	NA	NA	NA	NA	NA	MODIFIER	NA	NA	133	133	0	110	99	11	10
Smoker	42478	chr2	136627910	C	T	*MCM6*	SILENT	ENSG00000076003:SILENT[ENST00000264156(c.276G>A,cgG>cgA):ENSP00000264156(p.Arg92(=))];	chr2*13662	chr2*13662	NA	NA	NA	NA	NA	NA	NA	NA	LOW	NA	NA	147	146	0	115	108	7	6.09
Smoker	42478	chr2	63821650	G	T	*MDH1*	MISSENSE	ENSG00000014641:MISSENSE[ENST00000539945(c.86G>T,gGa>gTa):ENSP00000438144(p.Gly29Val)];ENSG00000014641:MISSENSE[ENST00000233114(c.32G>T,gGa>gTa):ENSP00000233114(p.Gly11Val)];ENSG00000014641:MISSENSE[ENST00000394423(c.32G>T,gGa>gTa):ENSP00000377945(p.Gly11Val)];ENSG00000014641:MISSENSE[ENST00000442225(c.35G>T,gGa>gTa):ENSP00000399004(p.Gly12Val)];ENSG00000014641:MISSENSE[ENST00000454035(c.35G>T,gGa>gTa):ENSP00000409027(p.Gly12Val)];ENSG00000014641:MISSENSE[ENST00000432309(c.86G>T,gGa>gTa):ENSP00000410073(p.Gly29Val)];ENSG00000014641:MISSENSE[ENST00000421012(c.86G>T,gGa>gTa):ENSP00000400937(p.Gly29Val)];ENSG00000014641:EXONIC-NC[ENST00000485781(n.467G>T)];ENSG00000014641:EXONIC-NC[ENST00000485155(n.97G>T)];ENSG00000014641:EXONIC-NC[ENST00000472098(n.78G>T)];ENSG00000014641:INTRONIC[ENST00000409476(c.4-4654G>T)];ENSG00000014641:INTRONIC[ENST00000409908(c.3+5470G>T)];ENSG00000014641:5UTR[ENST00000436321(c.-104G>T)];ENSG00000014641:5UTR[ENST00000544381(c.-139G>T)];	NA	NA	NA	NA	NA	NA	NA	NA	NA	NA	MODIFIER	NA	NA	106	106	0	126	116	10	7.94
Smoker	42478	chr2	207613857	G	A	*MDH1B*	MISSENSE	ENSG00000138400:MISSENSE[ENST00000454776(c.1103C>T,aCa>aTa):ENSP00000389916(p.Thr368Ile)];ENSG00000138400:MISSENSE[ENST00000374412(c.1103C>T,aCa>aTa):ENSP00000363533(p.Thr368Ile)];ENSG00000138400:MISSENSE[ENST00000392214(c.464C>T,aCa>aTa):ENSP00000376049(p.Thr155Ile)];ENSG00000138400:MISSENSE[ENST00000449792(c.809C>T,aCa>aTa):ENSP00000416577(p.Thr270Ile)];ENSG00000138400:MISSENSE[ENST00000432911(c.464C>T,aCa>aTa):ENSP00000392464(p.Thr155Ile)];ENSG00000138400:3UTR[ENST00000436472(c.*48C>T)];	NA	NA	NA	NA	NA	NA	NA	NA	NA	NA	MODIFIER	NA	NA	180	180	0	172	164	8	4.65
Smoker	42478	chr2	66667646	C	G	*MEIS1*	INTRONIC	ENSG00000143995:[ENST00000490726(n.397-48C>G)];ENSG00000143995:[ENST00000488550(c.382-48C>G)];ENSG00000143995:[ENST00000560281(c.382-48C>G)];ENSG00000143995:[ENST00000495021(c.187-48C>G)];ENSG00000143995:[ENST00000444274(c.286-48C>G)];ENSG00000143995:[ENST00000409622(c.*234+169C>G)];ENSG00000143995:[ENST00000407092(c.382-48C>G)];ENSG00000143995:[ENST00000398506(c.376-48C>G)];ENSG00000143995:[ENST00000272369(c.382-48C>G)];ENSG00000143995:[ENST00000491706(c.240-48C>G)];	NA	NA	NA	NA	NA	NA	NA	NA	NA	NA	MODIFIER	NA	NA	107	107	0	81	76	5	6.17
Smoker	42478	chr2	172930460	C	T	*METAP1D*	SILENT	ENSG00000172878:INTRONIC[ENST00000488581(n.234-532C>T)];ENSG00000172878:EXONIC-NC[ENST00000392582(n.129C>T)];ENSG00000172878:SILENT[ENST00000315796(c.477C>T,ctC>ctT):ENSP00000315152(p.Leu159(=))];	NA	NA	NA	NA	NA	NA	NA	NA	NA	NA	LOW	NA	NA	209	209	0	208	189	18	8.65
Smoker	42478	chr2	128367125	C	T	*MYO7B*	SILENT	ENSG00000169994:SILENT[ENST00000389524(c.2859C>T,gaC>gaT):ENSP00000374175(p.Asp953(=))];ENSG00000169994:SILENT[ENST00000409816(c.2859C>T,gaC>gaT):ENSP00000386461(p.Asp953(=))];ENSG00000169994:SILENT[ENST00000428314(c.2859C>T,gaC>gaT):ENSP00000415090(p.Asp953(=))];	NA	NA	NA	8.57E-06	0	NA	NA	NA	NA	NA	LOW	NA	0.964	167	166	0	126	98	28	22.22
Smoker	42478	chr2	71304636	G	A	*NAGK*	INTRONIC	ENSG00000124357:[ENST00000529236(c.*5-52G>A)];ENSG00000124357:[ENST00000468601(n.326-52G>A)];ENSG00000124357:[ENST00000524537(c.59-52G>A)];ENSG00000124357:[ENST00000450272(c.*942-52G>A)];ENSG00000124357:[ENST00000490998(n.2083-52G>A)];ENSG00000124357:[ENST00000489309(n.3991-52G>A)];ENSG00000124357:[ENST00000418807(c.613-52G>A)];ENSG00000124357:[ENST00000244204(c.766-52G>A)];ENSG00000124357:[ENST00000455662(c.904-52G>A)];ENSG00000124357:[ENST00000475709(n.2589-52G>A)];ENSG00000124357:[ENST00000524736(c.*171-52G>A)];ENSG00000124357:[ENST00000443938(c.766-64G>A)];ENSG00000124357:[ENST00000472519(n.2476-52G>A)];ENSG00000124357:[ENST00000443872(c.322-52G>A)];	NA	NA	NA	NA	NA	NA	NA	NA	NA	NA	MODIFIER	NA	NA	113	113	0	91	85	6	6.59
Smoker	42478	chr2	27898484	G	C	*SLC4A1AP*	SILENT	ENSG00000163798:SILENT[ENST00000326019(c.1431G>C,ctG>ctC):ENSP00000323837(p.Leu477(=))];ENSG00000163798:3UTR[ENST00000427424(c.*357G>C)];	chr2*27898	chr2*27898	NA	NA	NA	NA	NA	NA	NA	NA	MODIFIER	NA	NA	149	147	0	108	101	7	6.48
Smoker	42478	chr2	220326947	C	G	*SPEG*	INTRONIC	ENSG00000072195:[ENST00000396695(c.241-73C>G)];ENSG00000072195:[ENST00000498378(n.2805-73C>G)];ENSG00000072195:[ENST00000396688(c.70-73C>G)];ENSG00000072195:[ENST00000396686(c.70-73C>G)];ENSG00000072195:[ENST00000496786(n.256-73C>G)];ENSG00000072195:[ENST00000485813(n.1860-73C>G)];ENSG00000072195:[ENST00000464989(n.2615-73C>G)];ENSG00000072195:[ENST00000396689(c.70-73C>G)];ENSG00000072195:[ENST00000462545(n.970-73C>G)];ENSG00000072195:[ENST00000475921(n.819-73C>G)];ENSG00000072195:[ENST00000463218(n.244-73C>G)];ENSG00000072195:[ENST00000396698(c.2305-73C>G)];ENSG00000072195:[ENST00000312358(c.2617-73C>G)];	NA	NA	NA	NA	NA	NA	NA	NA	NA	NA	MODIFIER	NA	NA	51	51	0	40	33	7	17.5

Mangalaparthi *et al.* , 2020. Mutational landscape of esophageal squamous cell carcinoma in an Indian cohort
Supplementary Table 4. List of somatic single nucleotide variants identified using whole exome sequencing of ESCC patients

Cohort	Sample	Chromosome	Genomic position	Reference allele	Alternate allele	Gene symbol	Variant type	Variant annotation	OncoMD	COSMIC	dbSNP	ExAC	ExAC_SAS	1000G	1000G_SAS	SIFT	Polyphen2	CONDEL	VEP	LRT	PhastCon7 Way	Total read (Adjacent normal)	Reference allel read support (Adjacent normal)	Alternative read support (Adjacent normal)	Total read (Tumor)	Reference allel read support (Tumor)	Alternative read support (Tumor)	Alternate allele frequency
Smoker	42478	chr2	37129752	C	A	*STRN*	NONSENSE	ENSG00000115808:NONSENSE[ENST00000263918(c.634G>T,Gaa>Taa):ENSP00000263918(p.Glu212Ter)];ENSG00000115808:NONSENSE[ENST00000379213(c.598G>T,Gaa>Taa):ENSP00000368513(p.Glu200Ter)];ENSG00000115808:EXONIC-NC[ENST00000495595(n.320G>T)];	NA	NA	NA	NA	NA	NA	NA	NA	NA	NA	MODIFIER	NA	NA	250	250	0	222	189	33	14.86
Smoker	42478	chr2	179447057	G	C	*TTN*	SILENT	ENSG00000155657:SILENT[ENST00000359218(c.39306C>G,gtC>gtG):ENSP00000352154(p.Val13102(=))];ENSG00000155657:SILENT[ENST00000591111(c.61203C>G,gtC>gtG):ENSP00000465570(p.Val20401(=))];ENSG00000155657:SILENT[ENST00000589042(c.66126C>G,gtC>gtG):ENSP00000467141(p.Val22042(=))];ENSG00000155657:SILENT[ENST00000342175(c.39507C>G,gtC>gtG):ENSP00000340554(p.Val13169(=))];ENSG00000155657:SILENT[ENST00000342992(c.58422C>G,gtC>gtG):ENSP00000343764(p.Val19474(=))];ENSG00000155657:SILENT[ENST00000460472(c.38931C>G,gtC>gtG):ENSP00000434586(p.Val12977(=))];	NA	NA	NA	NA	NA	NA	NA	NA	NA	NA	MODIFIER	NA	NA	200	200	0	235	214	20	8.51
Smoker	42478	chr2	179447057	G	C	*TTN-AS1*	INTRONIC	ENSG00000237298:[ENST00000592630(n.434-15266G>C)];ENSG00000237298:[ENST00000592689(n.463-15266G>C)];ENSG00000237298:[ENST00000438095(n.345-242G>C)];ENSG00000237298:[ENST00000590807(n.75-1194G>C)];ENSG00000237298:[ENST00000586707(n.346-1383G>C)];ENSG00000237298:[ENST00000586831(n.215-242G>C)];ENSG00000237298:[ENST00000585451(n.198+58694G>C)];ENSG00000237298:[ENST00000592600(n.346-2243G>C)];ENSG00000237298:[ENST00000592750(n.346-15266G>C)];ENSG00000237298:[ENST00000419746(n.2044-242G>C)];ENSG00000237298:[ENST00000456053(n.563+10881G>C)];ENSG00000237298:[ENST00000586452(n.225-8809G>C)];ENSG00000237298:[ENST00000591332(n.443-15266G>C)];ENSG00000237298:[ENST00000590932(n.616-15266G>C)];	NA	NA	NA	NA	NA	NA	NA	NA	NA	NA	MODIFIER	NA	NA	200	200	0	235	214	20	8.51
Smoker	42478	chr2	63821650	G	T	*WDPCP*	INTRONIC	ENSG00000143951:[ENST00000490935(n.583+56143C>A)];ENSG00000143951:[ENST00000467687(n.488+56143C>A)];	NA	NA	NA	NA	NA	NA	NA	NA	NA	NA	MODIFIER	NA	NA	106	106	0	126	116	10	7.94
Smoker	42478	chr2	20174350	G	A	*WDR35*	SILENT	ENSG00000118965:SILENT[ENST00000414212(c.615C>T,atC>atT):ENSP00000390802(p.Ile205(=))];ENSG00000118965:SILENT[ENST00000445063(c.152C>T,atC>atT):ENSP00000390105(p.Ile51(=))];ENSG00000118965:SILENT[ENST00000345530(c.615C>T,atC>atT):ENSP00000314444(p.Ile205(=))];ENSG00000118965:SILENT[ENST00000281405(c.615C>T,atC>atT):ENSP00000281405(p.Ile205(=))];ENSG00000118965:5UTR[ENST00000416055(c.-597C>T)];	NA	NA	NA	NA	NA	NA	NA	NA	NA	NA	LOW	NA	0.982	136	136	0	128	119	9	7.03
Smoker	42478	chr20	3305564	A	G	*C20orf194*	SILENT	ENSG00000088854:SILENT[ENST00000252032(c.1240T>C,Ttg>Ctg):ENSP00000252032(p.Leu414(=))];ENSG00000088854:SILENT[ENST00000453730(c.454T>C,Ttg>Ctg):ENSP00000407229(p.Leu152(=))];	NA	NA	NA	NA	NA	NA	NA	NA	NA	NA	LOW	NA	0.162	102	102	0	126	118	7	5.56
Smoker	42478	chr20	61513227	C	T	*DIDO1*	MISSENSE	ENSG00000101191:MISSENSE[ENST00000266070(c.4081G>A,Gat>Aat):ENSP00000266070(p.Asp1361Asn)];ENSG00000101191:MISSENSE[ENST00000395343(c.4081G>A,Gat>Aat):ENSP00000378752(p.Asp1361Asn)];	NA	NA	NA	NA	NA	NA	NA	NA	PrD	NA	MODERATE	D	0.936	177	176	0	109	102	7	6.42
Smoker	42478	chr20	49247331	G	A	*FAM65C*	SILENT	ENSG00000042062:SILENT[ENST00000327979(c.54C>T,gtC>gtT):ENSP00000332663(p.Val18(=))];ENSG00000042062:SILENT[ENST00000535356(c.66C>T,gtC>gtT):ENSP00000439802(p.Val22(=))];ENSG00000042062:SILENT[ENST00000045083(c.54C>T,gtC>gtT):ENSP00000045083(p.Val18(=))];ENSG00000042062:EXONIC-NC[ENST00000462493(n.372C>T)];	NA	NA	chr20*49247331*49247331*G*T*rs760966539*NA*NA*NA	NA	NA	NA	NA	NA	NA	NA	LOW	NA	NA	146	146	0	93	88	5	5.38
Smoker	42478	chr20	29625885	A	T	*FRG1B*	MISSENSE	ENSG00000149531:MISSENSE[ENST00000278882(c.129A>T,aaA>aaT):ENSP00000278882(p.Lys43Asn)];ENSG00000149531:MISSENSE[ENST00000439954(c.144A>T,aaA>aaT):ENSP00000408863(p.Lys48Asn)];ENSG00000149531:MISSENSE[ENST00000358464(c.129A>T,aaA>aaT):ENSP00000351249(p.Lys43Asn)];ENSG00000149531:MISSENSE[ENST00000479318(c.13A>T,aaA>aaT):ENSP00000436872(p.Lys5Asn)];	NA	chr20*2962	NA	0.0002584	0.000181995	NA	NA	D	PrD	D	MODERATE	NA	NA	2646	2597	37	2071	2020	48	2.32
Smoker	42478	chr20	29630738	G	A	*FRG1B*	INTRONIC	ENSG00000149531:[ENST00000278882(c.334-800G>A)];ENSG00000149531:[ENST00000358464(c.334-800G>A)];ENSG00000149531:[ENST00000479318(c.*31G>A)];	NA	NA	NA	0.0025599	0	NA	NA	NA	NA	NA	MODIFIER	NA	NA	661	646	11	544	522	20	3.68
Smoker	42478	chr20	13773803	C	T	*NDUFAF5*	INTRONIC	ENSG00000101247:[ENST00000481249(n.309-23C>T)];ENSG00000101247:[ENST00000378106(c.328-23C>T)];ENSG00000101247:[ENST00000477732(n.311-23C>T)];ENSG00000101247:[ENST00000469177(n.266-23C>T)];ENSG00000101247:[ENST00000485738(n.327-23C>T)];ENSG00000101247:[ENST00000475968(n.309-23C>T)];ENSG00000101247:[ENST00000378081(c.328-23C>T)];ENSG00000101247:[ENST00000463598(c.328-3C>T)];ENSG00000101247:[ENST00000476536(n.268-3C>T)];	NA	NA	NA	NA	NA	NA	NA	NA	NA	NA	MODIFIER	NA	NA	53	53	0	47	42	5	10.64
Smoker	42478	chr20	3686420	G	A	*SIGLEC1*	MISSENSE	ENSG00000088827:MISSENSE[ENST00000344754(c.677C>T,gCt>gTt):ENSP00000341141(p.Ala226Val)];ENSG00000088827:MISSENSE[ENST00000202578(c.677C>T,gCt>gTt):ENSP00000202578(p.Ala226Val)];	NA	NA	NA	NA	NA	NA	NA	NA	BN	NA	MODERATE	N	0	90	90	0	98	91	7	7.14
Smoker	42478	chr20	31592104	G	A	*SUN5*	MISSENSE	ENSG00000167098:MISSENSE[ENST00000375519(c.43C>T,Ctc>Ttc):ENSP00000364669(p.Leu15Phe)];ENSG00000167098:MISSENSE[ENST00000420875(c.10C>T,Ctc>Ttc):ENSP00000400089(p.Leu4Phe)];ENSG00000167098:MISSENSE[ENST00000356173(c.43C>T,Ctc>Ttc):ENSP00000348496(p.Leu15Phe)];ENSG00000167098:MISSENSE[ENST00000375523(c.43C>T,Ctc>Ttc):ENSP00000364673(p.Leu15Phe)];	NA	NA	NA	NA	NA	NA	NA	D_lc	BN	N	MODERATE	N	0.001	45	45	0	43	38	5	11.63
Smoker	42478	chr20	35506444	C	T	*TLDC2*	MISSENSE	ENSG00000101342:MISSENSE[ENST00000602922(c.176C>T,tCa>tTa):ENSP00000473323(p.Ser59Leu)];ENSG00000101342:MISSENSE[ENST00000217320(c.176C>T,tCa>tTa):ENSP00000217320(p.Ser59Leu)];	NA	NA	NA	NA	NA	NA	NA	T	BN	N	MODERATE	N	0.042	64	64	0	58	52	6	10.34
Smoker	42478	chr21	47542321	G	A	*COL6A2*	INTRONIC	ENSG00000142173:[ENST00000413758(c.196-89G>A)];ENSG00000142173:[ENST00000397763(c.1573-89G>A)];ENSG00000142173:[ENST00000310645(c.1573-89G>A)];ENSG00000142173:[ENST00000300527(c.1573-89G>A)];ENSG00000142173:[ENST00000357838(c.1573-89G>A)];ENSG00000142173:[ENST00000409416(c.1573-89G>A)];	NA	NA	NA	NA	NA	NA	NA	NA	NA	NA	MODIFIER	NA	NA	46	46	0	42	36	6	14.29
Smoker	42478	chr22	50885798	G	A	*SBF1*	MISSENSE	ENSG00000100241:MISSENSE[ENST00000348911(c.5461C>T,Ccc>Tcc):ENSP00000252027(p.Pro1821Ser)];ENSG00000100241:MISSENSE[ENST00000380817(c.5536C>T,Ccc>Tcc):ENSP00000370196(p.Pro1846Ser)];ENSG00000100241:MISSENSE[ENST00000390679(c.5458C>T,Ccc>Tcc):ENSP00000375097(p.Pro1820Ser)];ENSG00000100241:MISSENSE[ENST00000418590(c.1100C>T,Ccc>Tcc):ENSP00000401538(p.Pro368Ser)];ENSG00000100241:EXONIC-NC[ENST00000473724(n.241C>T)];ENSG00000100241:EXONIC-NC[ENST00000470434(n.1677C>T)];	NA	NA	NA	NA	NA	NA	NA	NA	NA	NA	MODIFIER	NA	NA	103	103	0	100	81	19	19

Cohort	Sample	Chromosome	Genomic position	Reference allele	Alternate allele	Gene symbol	Variant type	Variant annotation	OncoMD	COSMIC	dbSNP	ExAC	ExAC_SAS	1000G	1000G_SAS	SIFT	Polyphen2	CONDEL	VEP	LRT	PhastCon7 Way	Total read (Adjacent normal)	Reference allel read support (Adjacent normal)	Alternative read support (Adjacent normal)	Total read (Tumor)	Reference allel read support (Tumor)	Alternative read support (Tumor)	Alternate allele frequency
Smoker	42478	chr22	26884363	G	A	*SRRD*	MISSENSE	ENSG00000100104:EXONIC-NC[ENST00000477945(n.315G>A)];ENSG00000100104:MISSENSE[ENST00000215917(c.518G>A,aGa>aAa):ENSP00000215917(p.Arg173Lys)];	NA	NA	NA	NA	NA	NA	NA	T	BN	N	MODERATE	N	1	226	226	0	258	244	14	5.43
Smoker	42478	chr22	40661466	C	T	*TNRC6B*	MISSENSE	ENSG00000100354:MISSENSE[ENST00000454349(c.1232C>T,tCa>tTa):ENSP00000401946(p.Ser411Leu)];ENSG00000100354:MISSENSE[ENST00000335727(c.1232C>T,tCa>tTa):ENSP00000338371(p.Ser411Leu)];ENSG00000100354:MISSENSE[ENST00000446273(c.460C>T,tCa>tTa):ENSP00000409429(p.Ser154Leu)];ENSG00000100354:INTRONIC[ENST00000402203(c.565+3289C>T)];ENSG00000100354:INTRONIC[ENST00000301923(c.565+3289C>T)];	NA	NA	NA	NA	NA	NA	NA	NA	NA	NA	MODIFIER	NA	NA	130	130	0	142	127	15	10.56
Smoker	42478	chr3	183669278	C	T	*ABCC5*	SILENT	ENSG00000114770:SILENT[ENST00000265586(c.2895G>A,acG>acA):ENSP00000265586(p.Thr965(=))];ENSG00000114770:SILENT[ENST00000334444(c.2895G>A,acG>acA):ENSP00000333926(p.Thr965(=))];ENSG00000114770:3UTR[ENST00000437205(c.*1588G>A)];	NA	NA	NA	2.49E-05	6.06E-05	NA	NA	NA	NA	NA	MODIFIER	NA	NA	117	116	0	139	129	10	7.19
Smoker	42478	chr3	88190804	G	A	*CGGBP1*	INTRONIC	ENSG00000163320:[ENST00000462901(c.-337-576C>T)];ENSG00000163320:[ENST00000467332(c.-132-576C>T)];	NA	NA	NA	NA	NA	NA	NA	NA	NA	NA	MODIFIER	NA	NA	78	78	0	88	82	6	6.82
Smoker	42478	chr3	99643071	T	G	*CMSS1*	INTRONIC	ENSG00000184220:[ENST00000491299(c.64+106184T>G)];ENSG00000184220:[ENST00000463526(c.-114+106184T>G)];ENSG00000184220:[ENST00000496116(n.133-87476T>G)];ENSG00000184220:[ENST00000421999(c.64+106184T>G)];	NA	NA	NA	NA	NA	NA	NA	NA	NA	NA	LOW	NA	NA	172	172	0	182	129	53	29.12
Smoker	42478	chr3	148614349	G	T	*CPA3*	MISSENSE	ENSG00000163751:MISSENSE[ENST00000296046(c.1109G>T,gGc>gTc):ENSP00000296046(p.Gly370Val)];	NA	NA	NA	NA	NA	NA	NA	NA	NA	NA	MODIFIER	NA	NA	169	169	0	166	156	10	6.02
Smoker	42478	chr3	184402377	G	A	*EIF2B5*	3UTR	ENSG00000145191:[ENST00000444495(c.*92G>A)];	NA	NA	NA	NA	NA	NA	NA	NA	NA	NA	MODIFIER	NA	NA	66	65	1	89	84	5	5.62
Smoker	42478	chr3	89498365	A	T	*EPHA3*	INTRONIC-SS-ACR	ENSG00000044524:[ENST00000494014(c.2347-10A>T)];ENSG00000044524:[ENST00000336596(c.2347-10A>T)];	NA	NA	NA	NA	NA	NA	NA	NA	NA	NA	MODIFIER	NA	NA	103	103	0	94	89	5	5.32
Smoker	42478	chr3	99643071	T	G	*FILIP1L*	INTRONIC-SS-DNR	ENSG00000168386:[ENST00000398326(c.605+3A>C)];ENSG00000168386:[ENST00000354552(c.605+3A>C)];ENSG00000168386:[ENST00000331335(c.605+3A>C)];	NA	NA	NA	NA	NA	NA	NA	NA	NA	NA	LOW	NA	NA	172	172	0	182	129	53	29.12
Smoker	42478	chr3	75714660	C	A	*FRG2C*	INTRONIC	ENSG00000172969:[ENST00000464571(c.332-18C>A)];ENSG00000172969:[ENST00000308062(c.335-18C>A)];	NA	NA	NA	NA	NA	NA	NA	NA	NA	NA	MODIFIER	NA	NA	348	348	0	230	212	17	7.39
Smoker	42478	chr3	115382716	C	T	*GAP43*	NONSENSE	ENSG00000172020:NONSENSE[ENST00000393780(c.91C>T,Cga>Tga):ENSP00000377372(p.Arg31Ter)];ENSG00000172020:INTRONIC[ENST00000305124(c.31-12144C>T)];	NA	NA	NA	NA	NA	NA	NA	NA	NA	NA	MODIFIER	NA	NA	87	87	0	72	65	7	9.72
Smoker	42478	chr3	7620936	C	G	*GRM7*	SILENT	ENSG00000196277:SILENT[ENST00000402647(c.2343C>G,ccC>ccG):ENSP00000384585(p.Pro781(=))];ENSG00000196277:SILENT[ENST00000486284(c.2343C>G,ccC>ccG):ENSP00000417536(p.Pro781(=))];ENSG00000196277:SILENT[ENST00000389335(c.2343C>G,ccC>ccG):ENSP00000373986(p.Pro781(=))];ENSG00000196277:SILENT[ENST00000467425(c.2343C>G,ccC>ccG):ENSP00000419835(p.Pro781(=))];ENSG00000196277:SILENT[ENST00000389336(c.2343C>G,ccC>ccG):ENSP00000373987(p.Pro781(=))];ENSG00000196277:SILENT[ENST00000357716(c.2343C>G,ccC>ccG):ENSP00000350348(p.Pro781(=))];ENSG00000196277:SILENT[ENST00000440923(c.2343C>G,ccC>ccG):ENSP00000412329(p.Pro781(=))];ENSG00000196277:SILENT[ENST00000403881(c.2343C>G,ccC>ccG):ENSP00000385664(p.Pro781(=))];ENSG00000196277:EXONIC-NC[ENST00000463676(n.1608C>G)];ENSG00000196277:EXONIC-NC[ENST00000458641(n.1547C>G)];	chr3*76209[illegible]	chr3*76209[illegible]	chr3*7620936*7620936*C*T*rs750316462*NA*NA*NA	NA	NA	NA	NA	NA	NA	NA	LOW	NA	NA	109	109	0	99	94	5	5.05
Smoker	42478	chr3	127642084	C	T	*KBTBD12*	SILENT	ENSG00000187715:INTRONIC[ENST00000476626(n.112+7655C>T)];ENSG00000187715:INTRONIC[ENST00000407609(c.-109-4523C>T)];ENSG00000187715:SILENT[ENST00000405256(c.180C>T,ttC>ttT):ENSP00000385879(p.Phe60(=))];ENSG00000187715:SILENT[ENST00000405109(c.180C>T,ttC>ttT):ENSP00000385957(p.Phe60(=))];ENSG00000187715:EXONIC-NC[ENST00000497045(n.181C>T)];	NA	NA	NA	NA	NA	NA	NA	NA	NA	NA	MODIFIER	NA	NA	149	149	0	164	152	12	7.32
Smoker	42478	chr3	108284228	C	T	*KIAA1524*	MISSENSE	ENSG00000163507:MISSENSE[ENST00000295746(c.1489G>A,Gaa>Aaa):ENSP00000295746(p.Glu497Lys)];ENSG00000163507:MISSENSE[ENST00000491772(c.1012G>A,Gaa>Aaa):ENSP00000419487(p.Glu338Lys)];ENSG00000163507:3UTR[ENST00000481530(c.*1059G>A)];ENSG00000163507:EXONIC-NC[ENST00000487834(n.1758G>A)];	NA	NA	chr3*108284227*108284228*TC*T*rs771156389*NA*NA*NA	NA	NA	NA	NA	NA	NA	NA	MODIFIER	NA	NA	55	55	0	73	68	5	6.85
Smoker	42478	chr3	47299005	C	T	*KIF9*	MISSENSE	ENSG00000088727:MISSENSE[ENST00000265529(c.1027G>A,Gag>Aag):ENSP00000265529(p.Glu343Lys)];ENSG00000088727:MISSENSE[ENST00000444589(c.1027G>A,Gag>Aag):ENSP00000414987(p.Glu343Lys)];ENSG00000088727:MISSENSE[ENST00000352910(c.748G>A,Gag>Aag):ENSP00000292334(p.Glu250Lys)];ENSG00000088727:MISSENSE[ENST00000335044(c.1027G>A,Gag>Aag):ENSP00000333942(p.Glu343Lys)];ENSG00000088727:MISSENSE[ENST00000452770(c.1027G>A,Gag>Aag):ENSP00000391100(p.Glu343Lys)];ENSG00000088727:3UTR[ENST00000443784(c.*92G>A)];ENSG00000088727:EXONIC-NC[ENST00000487440(n.1385G>A)];	NA	NA	NA	NA	NA	NA	NA	NA	NA	NA	MODIFIER	NA	NA	65	65	0	49	40	9	18.37
Smoker	42478	chr3	173322789	G	T	*NLGN1*	MISSENSE	ENSG00000169760:MISSENSE[ENST00000361589(c.401G>T,tGg>tTg):ENSP00000354541(p.Trp134Leu)];ENSG00000169760:MISSENSE[ENST00000401917(c.401G>T,tGg>tTg):ENSP00000385750(p.Trp134Leu)];ENSG00000169760:MISSENSE[ENST00000457714(c.401G>T,tGg>tTg):ENSP00000392500(p.Trp134Leu)];ENSG00000169760:MISSENSE[ENST00000545397(c.401G>T,tGg>tTg):ENSP00000441108(p.Trp134Leu)];ENSG00000169760:MISSENSE[ENST00000415045(c.401G>T,tGg>tTg):ENSP00000410374(p.Trp134Leu)];	NA	NA	chr3*173322789*173322789*G*A*rs77794557*NA*NA*NA	NA	NA	NA	NA	D	PoD	D	MODERATE	NA	NA	167	165	2	202	172	30	14.85
Smoker	42478	chr3	51992204	G	T	*PCBP4*	MISSENSE	ENSG00000090097:MISSENSE[ENST00000484633(c.956C>A,cCc>cAc):ENSP00000417100(p.Pro319His)];ENSG00000090097:MISSENSE[ENST00000395014(c.1148C>A,cCc>cAc):ENSP00000378461(p.Pro383His)];ENSG00000090097:MISSENSE[ENST00000395013(c.605C>A,cCc>cAc):ENSP00000378460(p.Pro202His)];ENSG00000090097:MISSENSE[ENST00000428823(c.956C>A,cCc>cAc):ENSP00000395030(p.Pro319His)];ENSG00000090097:MISSENSE[ENST00000322099(c.1085C>A,cCc>cAc):ENSP00000322341(p.Pro362His)];ENSG00000090097:MISSENSE[ENST00000461554(c.1085C>A,cCc>cAc):ENSP00000417196(p.Pro362His)];ENSG00000090097:MISSENSE[ENST00000355852(c.1085C>A,cCc>cAc):ENSP00000348111(p.Pro362His)];ENSG00000090097:EXONIC-NC[ENST00000498822(n.1819C>A)];ENSG00000090097:EXONIC-NC[ENST00000492809(n.1869C>A)];ENSG00000090097:EXONIC-NC[ENST00000497390(n.1704C>A)];ENSG00000090097:3UTR[ENST00000471308(c.*657C>A)];ENSG00000090097:INTRONIC[ENST00000471622(c.924-36C>A)];	NA	NA	NA	NA	NA	NA	NA	T	PoD	N	MODERATE	N	0.997	66	66	0	33	26	7	21.21

Mangalaparthi *et al.* , 2020. Mutational landscape of esophageal squamous cell carcinoma in an Indian cohort
Supplementary Table 4. List of somatic single nucleotide variants identified using whole exome sequencing of ESCC patients

Cohort	Sample	Chromosome	Genomic position	Reference allele	Alternate allele	Gene symbol	Variant type	Variant annotation	OncoMD	COSMIC	dbSNP	ExAC	ExAC_SAS	1000G	1000G_SAS	SIFT	Polyphen2	CONDEL	VEP	LRT	PhastCon7 Way	Total read (Adjacent normal)	Reference allel read support (Adjacent normal)	Alternative read support (Adjacent normal)	Total read (Tumor)	Reference allel read support (Tumor)	Alternative read support (Tumor)	Alternate allele frequency
Smoker	42478	chr3	178936091	G	A	*PIK3CA*	MISSENSE	ENSG00000121879:MISSENSE[ENST00000263967(c.1633G>A,Gag>Aag):ENSP00000263967(p.Glu545Lys)];	chr3*17893	chr3*17893	NA	8.34E-06	0	NA	NA	D	PrD	D	MODERATE	D	1	145	144	1	129	119	10	7.75
Smoker	42478	chr3	97852494	C	G	*RP11-343D2.11*	INTRONIC	ENSG00000249225:[ENST00000508964(n.94+14391G>C)];	NA	NA	NA	NA	NA	NA	NA	NA	NA	NA	MODIFIER	NA	NA	77	77	0	103	95	8	7.77
Smoker	42478	chr3	148614349	G	T	*RP11-680B3.2*	INTRONIC	ENSG00000240521:[ENST00000488190(n.162+14695C>A)];	NA	NA	NA	NA	NA	NA	NA	NA	NA	NA	MODIFIER	NA	NA	169	169	0	166	156	10	6.02
Smoker	42478	chr3	38798574	C	A	*SCN10A*	MISSENSE	ENSG00000185313:MISSENSE[ENST00000449082(c.1027G>T,Gct>Tct):ENSP00000390600(p.Ala343Ser)];	NA	NA	NA	NA	NA	NA	NA	T	BN	N	MODERATE	N	0.992	75	72	2	47	33	14	29.79
Smoker	42478	chr3	164727132	C	G	*SI*	MISSENSE	ENSG00000090402:MISSENSE[ENST00000264382(c.4114G>C,Gag>Cag):ENSP00000264382(p.Glu1372Gln)];	chr3*16472	chr3*16472	NA	8.25E-06	0	NA	NA	T	BN	N	MODERATE	N	0.945	234	233	0	284	262	22	7.75
Smoker	42478	chr3	133698327	C	T	*SLCO2A1*	MISSENSE-SS-PRX	ENSG00000174640:[ENST00000493729(c.232G>A,Gag>Aag):ENSP00000418893(p.Glu78Lys)];ENSG00000174640:[ENST00000310926(c.232G>A,Gag>Aag):ENSP00000311291(p.Glu78Lys)];ENSG00000174640:[ENST00000481359(c.232G>A,Gag>Aag):ENSP00000420028(p.Glu78Lys)];ENSG00000174640:[ENST00000464676(n.494G>A)];ENSG00000174640:[ENST00000462770(n.355G>A)];ENSG00000174640:[ENST00000478651(n.355G>A)];	NA	NA	NA	NA	NA	NA	NA	NA	NA	NA	LOW	NA	NA	161	161	0	118	110	8	6.78
Smoker	42478	chr3	190999905	G	C	*UTS2B*	MISSENSE	ENSG00000188958:MISSENSE[ENST00000446788(c.74C>G,tCt>tGt):ENSP00000415628(p.Ser25Cys)];ENSG00000188958:MISSENSE[ENST00000425357(c.74C>G,tCt>tGt):ENSP00000404406(p.Ser25Cys)];ENSG00000188958:MISSENSE[ENST00000440476(c.74C>G,tCt>tGt):ENSP00000398540(p.Ser25Cys)];ENSG00000188958:MISSENSE[ENST00000427544(c.74C>G,tCt>tGt):ENSP00000398761(p.Ser25Cys)];ENSG00000188958:MISSENSE[ENST00000432514(c.74C>G,tCt>tGt):ENSP00000401028(p.Ser25Cys)];ENSG00000188958:MISSENSE[ENST00000340524(c.74C>G,tCt>tGt):ENSP00000340526(p.Ser25Cys)];ENSG00000188958:EXONIC-NC[ENST00000463450(n.381C>G)];	NA	NA	chr3*190999905*190999905*G*A*rs746877745*NA*NA*NA	NA	NA	NA	NA	NA	NA	NA	MODIFIER	NA	NA	153	151	1	243	216	27	11.11
Smoker	42478	chr3	46062906	G	T	*XCR1*	SILENT	ENSG00000173578:SILENT[ENST00000542109(c.534C>A,tcC>tcA):ENSP00000438119(p.Ser178(=))];ENSG00000173578:SILENT[ENST00000309285(c.534C>A,tcC>tcA):ENSP00000310405(p.Ser178(=))];	chr3*46062	chr3*46062	chr3*46062906*46062906*G*A*rs148266059*1*0.9984*0.001597	NA	NA	NA	NA	NA	NA	NA	LOW	NA	NA	137	137	0	127	89	38	29.92
Smoker	42478	chr3	141163924	G	A	*ZBTB38*	SILENT	ENSG00000177311:SILENT[ENST00000441582(c.2694G>A,gaG>gaA):ENSP00000406955(p.Glu898(=))];ENSG00000177311:SILENT[ENST00000321464(c.2697G>A,gaG>gaA):ENSP00000372635(p.Glu899(=))];ENSG00000177311:SILENT[ENST00000514251(c.2694G>A,gaG>gaA):ENSP00000426387(p.Glu898(=))];	chr3*14116	NA	0.0001997	NA	NA	0.0001997	0	NA	NA	NA	LOW	NA	0.951	164	164	0	198	184	14	7.07
Smoker	42478	chr3	88190804	G	A	*ZNF654*	3UTR	ENSG00000175105:[ENST00000309495(c.*2G>A)];	NA	NA	NA	NA	NA	NA	NA	NA	NA	NA	MODIFIER	NA	NA	78	78	0	88	82	6	6.82
Smoker	42478	chr4	15480899	C	T	*CC2D2A*	MISSENSE	ENSG00000048342:INTRONIC[ENST00000503292(c.123+469C>T)];ENSG00000048342:INTRONIC[ENST00000507954(c.123+469C>T)];ENSG00000048342:INTRONIC[ENST00000413206(c.123+469C>T)];ENSG00000048342:INTRONIC[ENST00000513811(n.303+469C>T)];ENSG00000048342:INTRONIC[ENST00000514450(n.278+469C>T)];ENSG00000048342:INTRONIC[ENST00000424120(c.123+469C>T)];ENSG00000048342:INTRONIC[ENST00000512702(c.123+469C>T)];ENSG00000048342:INTRONIC[ENST00000515124(c.123+469C>T)];ENSG00000048342:5UTR[ENST00000389652(c.-78C>T)];ENSG00000048342:MISSENSE[ENST00000503658(c.176C>T,gCa>gTa):ENSP00000426846(p.Ala59Val)];ENSG00000048342:MISSENSE[ENST00000511544(c.176C>T,gCa>gTa):ENSP00000426109(p.Ala59Val)];ENSG00000048342:MISSENSE[ENST00000438599(c.176C>T,gCa>gTa):ENSP00000401154(p.Ala59Val)];	NA	NA	NA	NA	NA	NA	NA	NA	NA	NA	MODIFIER	NA	NA	61	61	0	58	37	21	36.21
Smoker	42478	chr4	109780917	C	T	*COL25A1*	INTRONIC	ENSG00000188517:[ENST00000399127(c.1186-28G>A)];ENSG00000188517:[ENST00000494183(c.1027-28G>A)];ENSG00000188517:[ENST00000399132(c.1243-28G>A)];ENSG00000188517:[ENST00000399126(c.1243-28G>A)];	NA	NA	NA	NA	NA	NA	NA	NA	NA	NA	MODIFIER	NA	NA	95	95	0	80	73	7	8.75
Smoker	42478	chr4	169362499	G	T	*DDX60L*	NONSENSE	ENSG00000181381:NONSENSE[ENST00000511577(c.1283C>A,tCg>tAg):ENSP00000422423(p.Ser428Ter)];ENSG00000181381:NONSENSE[ENST00000505890(c.1283C>A,tCg>tAg):ENSP00000422202(p.Ser428Ter)];ENSG00000181381:NONSENSE[ENST00000505863(c.467C>A,tCg>tAg):ENSP00000421026(p.Ser156Ter)];ENSG00000181381:NONSENSE[ENST00000260184(c.1283C>A,tCg>tAg):ENSP00000260184(p.Ser428Ter)];	NA	NA	chr4*169362499*169362499*G*A*rs377140092*NA*NA*NA	NA	NA	NA	NA	NA	NA	NA	HIGH	NA	0.005	93	93	0	89	83	6	6.74
Smoker	42478	chr4	153245513	C	T	*FBXW7*	MISSENSE	ENSG00000109670:MISSENSE[ENST00000603548(c.1678G>A,Gat>Aat):ENSP00000474725(p.Asp560Asn)];ENSG00000109670:MISSENSE[ENST00000263981(c.1438G>A,Gat>Aat):ENSP00000263981(p.Asp480Asn)];ENSG00000109670:MISSENSE[ENST00000296555(c.1324G>A,Gat>Aat):ENSP00000296555(p.Asp442Asn)];ENSG00000109670:MISSENSE[ENST00000393956(c.1150G>A,Gat>Aat):ENSP00000377528(p.Asp384Asn)];ENSG00000109670:MISSENSE[ENST00000281708(c.1678G>A,Gat>Aat):ENSP00000281708(p.Asp560Asn)];ENSG00000109670:MISSENSE[ENST00000603841(c.1678G>A,Gat>Aat):ENSP00000474971(p.Asp560Asn)];ENSG00000109670:EXONIC-NC[ENST00000604316(n.76G>A)];ENSG00000109670:EXONIC-NC[ENST00000603821(n.2737G>A)];	NA	NA	NA	NA	NA	NA	NA	NA	NA	NA	MODIFIER	NA	NA	183	180	3	145	99	46	31.72
Smoker	42478	chr4	22416316	C	T	*GPR125*	INTRONIC	ENSG00000152990:[ENST00000508133(c.1132-87G>A)];ENSG00000152990:[ENST00000334304(c.1810-879G>A)];ENSG00000152990:[ENST00000282943(n.1435-933G>A)];	NA	NA	NA	NA	NA	NA	NA	NA	NA	NA	MODIFIER	NA	NA	95	95	0	85	74	10	11.76
Smoker	42478	chr4	108608225	A	T	*PAPSS1*	MISSENSE	ENSG00000138801:MISSENSE[ENST00000265174(c.520T>A,Tac>Aac):ENSP00000265174(p.Tyr174Asn)];ENSG00000138801:EXONIC-NC[ENST00000502431(n.641T>A)];ENSG00000138801:EXONIC-NC[ENST00000511304(n.212T>A)];	NA	NA	NA	NA	NA	NA	NA	NA	NA	NA	MODIFIER	NA	NA	207	206	1	135	96	39	28.89
Smoker	42478	chr4	20255634	C	T	*SLIT2*	INTRONIC	ENSG00000145147:[ENST00000273739(c.179+17C>T)];ENSG00000145147:[ENST00000504154(c.179+17C>T)];ENSG00000145147:[ENST00000503823(c.179+17C>T)];ENSG00000145147:[ENST00000503837(c.179+17C>T)];	NA	NA	NA	1.70E-05	0	NA	NA	NA	NA	NA	MODIFIER	NA	NA	177	172	1	140	114	26	18.57
Smoker	42478	chr5	35788716	G	A	*CTD-2113L7.1*	INTRONIC	ENSG00000248969:[ENST00000510433(n.173+38232C>T)];	NA	NA	NA	NA	NA	NA	NA	NA	NA	NA	MODIFIER	NA	NA	177	177	0	182	145	37	20.33
Smoker	42478	chr5	11346445	C	T	*CTNND2*	INTRONIC	ENSG00000169862:[ENST00000511377(c.1355+39G>A)];ENSG00000169862:[ENST00000304623(c.1628+39G>A)];ENSG00000169862:[ENST00000513588(c.890+39G>A)];ENSG00000169862:[ENST00000359640(c.1628+39G>A)];ENSG00000169862:[ENST00000503622(c.617+39G>A)];ENSG00000169862:[ENST00000458100(c.329+39G>A)];ENSG00000169862:[ENST00000495388(n.713+39G>A)];ENSG00000169862:[ENST00000504499(c.*367+39G>A)];	NA	NA	NA	NA	NA	NA	NA	NA	NA	NA	MODIFIER	NA	NA	165	163	2	157	145	12	7.64

Supplementary Table 4. List of somatic single nucleotide variants identified using whole exome sequencing of ESCC patients

Cohort	Sample	Chromosome	Genomic position	Reference allele	Alternate allele	Gene symbol	Variant type	Variant annotation	OncoMD	COSMIC	dbSNP	ExAC	ExAC_SAS	1000G	1000G_SAS	SIFT	Polyphen2	CONDEL	VEP	LRT	PhastCon7 Way	Total read (Adjacent normal)	Reference allel read support (Adjacent normal)	Alternative read support (Adjacent normal)	Total read (Tumor)	Reference allel read support (Tumor)	Alternative read support (Tumor)	Alternate allele frequency
Smoker	42478	chr5	169174463	C	T	*DOCK2*	SILENT	ENSG00000134516:5UTR[ENST00000540750(c.-487C>T)];ENSG00000134516:SILENT[ENST00000256935(c.2331C>T,atC>atT):ENSP00000256935(p.Ile777(=))];ENSG00000134516:SILENT[ENST00000524185(c.2331C>T,atC>atT):ENSP00000428850(p.Ile777(=))];ENSG00000134516:SILENT[ENST00000520908(c.807C>T,atC>atT):ENSP00000429283(p.Ile269(=))];ENSG00000134516:EXONIC-NC[ENST00000520181(n.34C>T)];ENSG00000134516:EXONIC-NC[ENST00000520836(n.110C>T)];	NA	NA	NA	NA	NA	NA	NA	NA	NA	NA	MODIFIER	NA	NA	87	87	0	75	65	10	13.33
Smoker	42478	chr5	177613844	G	C	*GMCL1P1*	EXONIC-NC	ENSG00000244234:[ENST00000463439(n.457C>G)];	NA	NA	NA	NA	NA	NA	NA	NA	NA	NA	MODIFIER	NA	NA	148	147	0	127	120	7	5.51
Smoker	42478	chr5	126140499	G	T	*LMNB1*	MISSENSE	ENSG00000113368:MISSENSE[ENST00000261366(c.391G>T,Gcc>Tcc):ENSP00000261366(p.Ala131Ser)];ENSG00000113368:MISSENSE[ENST00000395354(c.391G>T,Gcc>Tcc):ENSP00000378761(p.Ala131Ser)];ENSG00000113368:EXONIC-NC[ENST00000472034(n.124G>T)];ENSG00000113368:EXONIC-NC[ENST00000460265(n.1277G>T)];ENSG00000113368:EXONIC-NC[ENST00000463908(n.230G>T)];ENSG00000113368:EXONIC-NC[ENST00000492190(n.631G>T)];ENSG00000113368:EXONIC-NC[ENST00000504788(n.124G>T)];	chr5*12614(	chr5*12614(	chr5*126140499*126140499*G*A*rs373303194*NA*NA*NA	NA	NA	NA	NA	NA	NA	NA	MODIFIER	NA	NA	159	159	0	97	82	15	15.46
Smoker	42478	chr5	150050176	C	T	*MYOZ3*	SILENT	ENSG00000164591:SILENT[ENST00000297130(c.192C>T,ttC>ttT):ENSP00000297130(p.Phe64(=))];ENSG00000164591:SILENT[ENST00000517768(c.192C>T,ttC>ttT):ENSP00000428815(p.Phe64(=))];ENSG00000164591:EXONIC-NC[ENST00000517861(n.193C>T)];	NA	NA	NA	1.79E-05	0	NA	NA	NA	NA	NA	LOW	NA	0.819	61	61	0	66	47	19	28.79
Smoker	42478	chr5	23526353	G	A	*PRDM9*	MISSENSE	ENSG00000164256:MISSENSE[ENST00000296682(c.1156G>A,Gag>Aag):ENSP00000296682(p.Glu386Lys)];	NA	NA	NA	NA	NA	NA	NA	T	BN	N	MODERATE	N	0.156	273	270	2	231	220	11	4.76
Smoker	42478	chr5	35788716	G	A	*SPEF2*	INTRONIC	ENSG00000152582:[ENST00000513078(c.180-271G>A)];ENSG00000152582:[ENST00000440995(c.4433-3726G>A)];ENSG00000152582:[ENST00000303129(c.-45-271G>A)];ENSG00000152582:[ENST00000356031(c.4448-3726G>A)];ENSG00000152582:[ENST00000506526(c.*458G>A)];	NA	NA	NA	NA	NA	NA	NA	NA	NA	NA	MODIFIER	NA	NA	177	177	0	182	145	37	20.33
Smoker	42478	chr5	14399045	G	T	*TRIO*	MISSENSE	ENSG00000038382:MISSENSE[ENST00000509967(c.4333G>T,Gtg>Ttg):ENSP00000445592(p.Val1445Leu)];ENSG00000038382:MISSENSE[ENST00000512070(c.4303G>T,Gtg>Ttg):ENSP00000421555(p.Val1435Leu)];ENSG00000038382:MISSENSE[ENST00000537187(c.4480G>T,Gtg>Ttg):ENSP00000446348(p.Val1494Leu)];ENSG00000038382:MISSENSE[ENST00000344204(c.4480G>T,Gtg>Ttg):ENSP00000339299(p.Val1494Leu)];ENSG00000038382:MISSENSE[ENST00000513206(c.3679G>T,Gtg>Ttg):ENSP00000426342(p.Val1227Leu)];ENSG00000038382:EXONIC-NC[ENST00000515144(n.3398G>T)];	NA	NA	NA	NA	NA	NA	NA	NA	PrD	NA	MODERATE	NA	NA	157	155	0	139	101	37	26.62
Smoker	42478	chr5	796008	G	T	*ZDHHC11*	3UTR	ENSG00000188818:[ENST00000283441(c.*695C>A)];ENSG00000188818:[ENST00000424784(c.*689+6C>A)];ENSG00000188818:[ENST00000503758(n.3636C>A)];	NA	NA	chr5*796006*796008*TGG*T*rs751228458*NA*NA*NA|chr5*796008*796008*G*A,T*rs1057004*NA*NA*NA|chr5*796008*796029*GGCTCCCATTTCCCAGTACTGT*G*rs199757139*NA*NA*NA	NA	NA	NA	NA	NA	NA	NA	MODIFIER	NA	NA	288	284	1	318	302	13	4.09
Smoker	42478	chr6	70092851	C	A	*BAI3*	INTRONIC	ENSG00000135298:[ENST00000546190(c.1272+24C>A)];ENSG00000135298:[ENST00000238918(c.1998+24C>A)];ENSG00000135298:[ENST00000370598(c.4380+24C>A)];	NA	NA	NA	NA	NA	NA	NA	NA	NA	NA	MODIFIER	NA	NA	161	161	0	151	135	16	10.6
Smoker	42478	chr6	32303690	G	A	*C6orf10*	SILENT-SS-PRX	ENSG00000204296:[ENST00000375007(c.444C>T,atC>atT):ENSP00000364146(p.Ile148(=))];ENSG00000204296:[ENST00000533191(c.402C>T,atC>atT):ENSP00000431199(p.Ile134(=))];ENSG00000204296:[ENST00000375015(c.402C>T,atC>atT):ENSP00000364155(p.Ile134(=))];ENSG00000204296:[ENST00000442822(c.381C>T,atC>atT):ENSP00000411164(p.Ile127(=))];ENSG00000204296:[ENST00000527965(c.381C>T,atC>atT):ENSP00000435103(p.Ile127(=))];ENSG00000204296:[ENST00000447241(c.450C>T,atC>atT):ENSP00000415517(p.Ile150(=))];	NA	NA	NA	NA	NA	NA	NA	NA	NA	NA	LOW	NA	NA	122	120	2	95	86	9	9.47
Smoker	42478	chr6	17541424	G	A	*CAP2*	INTRONIC	ENSG00000112186:[ENST00000465994(c.810+45G>A)];ENSG00000112186:[ENST00000378990(c.924+45G>A)];ENSG00000112186:[ENST00000489374(c.666+45G>A)];ENSG00000112186:[ENST00000229922(c.1002+45G>A)];ENSG00000112186:[ENST00000479291(c.*443+45G>A)];ENSG00000112186:[ENST00000493172(c.223-1644G>A)];	NA	NA	NA	NA	NA	NA	NA	NA	NA	NA	MODIFIER	NA	NA	116	116	0	108	101	7	6.48
Smoker	42478	chr6	24353486	C	A	*DCDC2*	3UTR	ENSG00000146038:[ENST00000436313(c.*47G>T)];ENSG00000146038:[ENST00000378454(c.348+311G>T)];	NA	NA	NA	NA	NA	NA	NA	NA	NA	NA	MODIFIER	NA	NA	89	89	0	108	103	5	4.63
Smoker	42478	chr6	30640650	A	G	*DHX16*	INTRONIC	ENSG00000204560:[ENST00000415603(c.27+83T>C)];ENSG00000204560:[ENST00000376442(c.-32T>C)];	NA	NA	NA	NA	NA	NA	NA	NA	NA	NA	MODIFIER	NA	NA	107	107	0	73	66	7	9.59
Smoker	42478	chr6	127648128	T	A	*ECHDC1*	INTRONIC	ENSG00000093144:[ENST00000454859(c.363+19A>T)];ENSG00000093144:[ENST00000368292(c.363+19A>T)];ENSG00000093144:[ENST00000528402(c.138+19A>T)];ENSG00000093144:[ENST00000531582(c.363+19A>T)];ENSG00000093144:[ENST00000368289(c.363+19A>T)];ENSG00000093144:[ENST00000531967(c.381+19A>T)];ENSG00000093144:[ENST00000368295(c.367+15A>T)];ENSG00000093144:[ENST00000417628(c.136+19A>T)];ENSG00000093144:[ENST00000454591(c.138+19A>T)];ENSG00000093144:[ENST00000525745(c.363+19A>T)];ENSG00000093144:[ENST00000368291(c.363+19A>T)];ENSG00000093144:[ENST00000474240(n.235+19A>T)];ENSG00000093144:[ENST00000309620(c.363+18A>T)];ENSG00000093144:[ENST00000526299(c.*172+19A>T)];ENSG00000093144:[ENST00000460558(c.11+19A>T)];ENSG00000093144:[ENST00000430841(c.363+19A>T)];ENSG00000093144:[ENST00000436638(c.284+19A>T)];ENSG00000093144:[ENST00000479525(c.363+19A>T)];ENSG00000093144:[ENST00000474289(c.363+19A>T)];ENSG00000093144:[ENST00000534442(c.363+19A>T)];ENSG00000093144:[ENST00000368287(n.407A>T)];	NA	NA	NA	NA	NA	NA	NA	NA	NA	NA	MODIFIER	NA	NA	160	159	1	131	115	16	12.21

Mangalaparthi *et al.* , 2020. Mutational landscape of esophageal squamous cell carcinoma in an Indian cohort
Supplementary Table 4. List of somatic single nucleotide variants identified using whole exome sequencing of ESCC patients

Cohort	Sample	Chromosome	Genomic position	Reference allele	Alternate allele	Gene symbol	Variant type	Variant annotation	OncoMD	COSMIC	dbSNP	ExAC	ExAC_SAS	1000G	1000G_SAS	SIFT	Polyphen2	CONDEL	VEP	LRT	PhastCon7 Way	Total read (Adjacent normal)	Reference allel read support (Adjacent normal)	Alternative read support (Adjacent normal)	Total read (Tumor)	Reference allel read support (Tumor)	Alternative read support (Tumor)	Alternate allele frequency
Smoker	42478	chr6	142726849	G	T	*GPR126*	MISSENSE	ENSG00000112414:MISSENSE[ENST00000367609(c.2152G>T,Gat>Tat):ENSP00000356581(p.Asp718Tyr)];ENSG00000112414:MISSENSE[ENST0000296932(c.2068G>T,Gat>Tat):ENSP00000296932(p.Asp690Tyr)];ENSG00000112414:MISSENSE[ENST00000367608(c.2068G>T,Gat>Tat):ENSP00000356580(p.Asp690Tyr)];ENSG00000112414:MISSENSE[ENST00000230173(c.2152G>T,Gat>Tat):ENSP00000230173(p.Asp718Tyr)];ENSG00000112414:EXONIC-NC[ENST00000538281(n.404G>T)];ENSG00000112414:3UTR[ENST00000540208(c.*14G>T)];	NA	NA	NA	NA	NA	NA	NA	NA	NA	NA	MODIFIER	NA	NA	135	134	0	114	98	15	13.16
Smoker	42478	chr6	12123639	G	T	*HIVEP1*	MISSENSE	ENSG00000095951:MISSENSE[ENST00000379388(c.3611G>T,gGa>gTa):ENSP00000368698(p.Gly1204Val)];ENSG00000095951:INTRONIC[ENST00000442081(c.166+3472G>T)];	NA	NA	NA	NA	NA	NA	NA	NA	NA	NA	MODIFIER	NA	NA	115	114	0	96	86	10	10.42
Smoker	42478	chr6	31322866	C	T	*HLA-B*	INTRONIC	ENSG00000234745:[ENST00000412585(c.1012+18G>A)];	NA	NA	NA	0.0011885	0.003056546	NA	NA	NA	NA	NA	MODIFIER	NA	NA	137	133	2	96	90	6	6.25
Smoker	42478	chr6	31322910	G	A	*HLA-B*	MISSENSE	ENSG00000234745:MISSENSE[ENST00000412585(c.986C>T,gCt>gTt):ENSP00000399168(p.Ala329Val)];ENSG00000234745:EXONIC-NC[ENST00000463574(n.577C>T)];	chr6*31322	chr6*31322	NA	0.0017885	0.001774569	NA	NA	NA	NA	NA	MODIFIER	NA	NA	176	173	1	135	127	6	4.44
Smoker	42478	chr6	29032981	C	G	*NA*	INTERGENIC		NA	NA	NA	NA	NA	NA	NA	NA	NA	NA	MODIFIER	NA	NA	144	144	0	129	116	13	10.08
Smoker	42478	chr6	88374473	A	C	*ORC3*	MISSENSE	ENSG00000135336:MISSENSE[ENST00000546266(c.1417A>C,Aaa>Caa):ENSP00000444695(p.Lys473Gln)];ENSG00000135336:MISSENSE[ENST00000392844(c.1846A>C,Aaa>Caa):ENSP00000376586(p.Lys616Gln)];ENSG00000135336:MISSENSE[ENST00000257789(c.1849A>C,Aaa>Caa):ENSP00000257789(p.Lys617Gln)];	NA	NA	NA	NA	NA	NA	NA	T	BN	N	MODERATE	D	1	151	151	0	130	117	13	10
Smoker	42478	chr6	30570166	G	A	*PPP1R10*	MISSENSE	ENSG00000204569:MISSENSE[ENST00000376511(c.2260C>T,Cac>Tac):ENSP00000365694(p.His754Tyr)];	chr6*30570	chr6*30570	chr6*30570119*30570167*GGGACGATGTCCACTGCTGTTGCCCATGCCCCCACCAGGGCCTTCGTGA*G*rs770931808*NA*NA*NA	NA	NA	NA	NA	D_lc	UN	NA	MODERATE	N	0.77	102	102	0	67	55	12	17.91
Smoker	42478	chr6	160679538	G	T	*SLC22A2*	SILENT	ENSG00000112499:EXONIC-NC[ENST00000489644(n.234C>A)];ENSG00000112499:EXONIC-NC[ENST00000491092(n.253C>A)];ENSG00000112499:SILENT[ENST00000366952(c.189C>A,gcC>gcA):ENSP00000355919(p.Ala63(=))];ENSG00000112499:SILENT[ENST00000366953(c.252C>A,gcC>gcA):ENSP00000355920(p.Ala84(=))];	NA	NA	NA	NA	NA	NA	NA	NA	NA	NA	LOW	NA	NA	148	147	1	96	89	7	7.29
Smoker	42478	chr6	144747494	G	T	*UTRN*	MISSENSE	ENSG00000152818:MISSENSE[ENST00000367545(c.477G>T,tgG>tgT):ENSP00000356515(p.Trp159Cys)];	NA	NA	NA	NA	NA	NA	NA	D	PrD	D	MODERATE	D	0.998	85	85	0	67	61	6	8.96
Smoker	42478	chr6	149777956	C	G	*ZC3H12D*	MISSENSE	ENSG00000178199:MISSENSE[ENST00000416573(c.526G>C,Gtc>Ctc):ENSP00000408686(p.Val176Leu)];ENSG00000178199:MISSENSE[ENST00000542614(c.526G>C,Gtc>Ctc):ENSP00000440813(p.Val176Leu)];ENSG00000178199:MISSENSE[ENST00000409806(c.526G>C,Gtc>Ctc):ENSP00000386616(p.Val176Leu)];ENSG00000178199:MISSENSE[ENST00000389942(c.526G>C,Gtc>Ctc):ENSP00000374592(p.Val176Leu)];ENSG00000178199:MISSENSE[ENST00000458251(c.50G>C,Gtc>Ctc):ENSP00000395209(p.Val18Leu)];	NA	NA	NA	NA	NA	NA	NA	D_lc	PrD	D	MODERATE	NA	NA	196	196	0	94	85	9	9.57
Smoker	42478	chr7	116955218	G	C	*AC002465.2*	INTRONIC	ENSG00000238202:[ENST00000436097(n.171-6894G>C)];	NA	NA	chr7*116955218*116955218*G*T*rs142048882*0*0.9998*0.0001997	NA	NA	NA	NA	T	BN	N	MODERATE	N	1	193	193	0	141	135	6	4.26
Smoker	42478	chr7	45699738	C	G	*ADCY1*	MISSENSE	ENSG00000164742:MISSENSE[ENST00000432715(c.730C>G,Cat>Gat):ENSP00000392721(p.His244Asp)];ENSG00000164742:MISSENSE[ENST00000297323(c.1405C>G,Cat>Gat):ENSP00000297323(p.His469Asp)];	NA	NA	NA	NA	NA	NA	NA	D	PrD	D	MODERATE	D	0.993	135	135	0	104	96	8	7.69
Smoker	42478	chr7	135069439	C	T	*CNOT4*	SILENT	ENSG00000080802:SILENT[ENST00000356162(c.1290G>A,ctG>ctA):ENSP00000348485(p.Leu430(=))];ENSG00000080802:SILENT[ENST00000414802(c.1290G>A,ctG>ctA):ENSP00000416532(p.Leu430(=))];ENSG00000080802:INTRONIC[ENST00000541284(c.1627+9231G>A)];ENSG00000080802:INTRONIC[ENST00000451834(c.1618+9231G>A)];ENSG00000080802:INTRONIC[ENST00000473470(n.306+2895G>A)];ENSG00000080802:INTRONIC[ENST00000423368(c.1627+9231G>A)];ENSG00000080802:INTRONIC[ENST00000361528(c.1618+9231G>A)];	NA	NA	NA	NA	NA	NA	NA	NA	NA	NA	MODIFIER	NA	NA	234	233	0	175	155	20	11.43
Smoker	42478	chr7	137080292	C	G	*DGKI*	INTRONIC	ENSG00000157680:[ENST00000494390(n.703+52G>C)];ENSG00000157680:[ENST00000477835(n.283+52G>C)];ENSG00000157680:[ENST00000453654(c.2088+52G>C)];ENSG00000157680:[ENST00000497321(n.450+52G>C)];ENSG00000157680:[ENST00000288490(c.3081+52G>C)];ENSG00000157680:[ENST00000424189(c.3120+52G>C)];ENSG00000157680:[ENST00000486153(n.376+52G>C)];ENSG00000157680:[ENST00000446122(c.3027+52G>C)];	NA	NA	NA	NA	NA	NA	NA	NA	NA	NA	MODIFIER	NA	NA	184	184	0	131	122	9	6.87
Smoker	42478	chr7	35707075	G	A	*HERPUD2*	NONSENSE	ENSG00000122557:NONSENSE[ENST00000311350(c.463C>T,Cag>Tag):ENSP00000310729(p.Gln155Ter)];ENSG00000122557:NONSENSE[ENST00000396081(c.463C>T,Cag>Tag):ENSP00000379390(p.Gln155Ter)];ENSG00000122557:NONSENSE[ENST00000413517(c.385C>T,Cag>Tag):ENSP00000391015(p.Gln129Ter)];ENSG00000122557:NONSENSE[ENST00000438224(c.271C>T,Cag>Tag):ENSP00000415475(p.Gln91Ter)];ENSG00000122557:EXONIC-NC[ENST00000426180(n.161C>T)];	NA	NA	NA	NA	NA	NA	NA	NA	NA	NA	MODIFIER	NA	NA	116	116	0	83	73	10	12.05
Smoker	42478	chr7	100551048	C	G	*MUC3A*	EXONIC-NC	ENSG00000169894:[ENST00000483133(n.719C>G)];	NA	NA	NA	NA	NA	NA	NA	NA	NA	NA	MODIFIER	NA	NA	626	403	9	572	371	13	2.27
Smoker	42478	chr7	141871162	G	A	*RP11-1220K2.2*	SILENT	ENSG00000257743:EXONIC-NC[ENST00000496337(n.193G>A)];ENSG00000257743:SILENT[ENST00000477922(c.3273G>A,ggG>ggA):ENSP00000420449(p.Gly1091(=))];	NA	NA	NA	NA	NA	NA	NA	NA	NA	NA	LOW	NA	NA	130	130	0	122	116	6	4.92
Smoker	42478	chr7	11676040	C	G	*THSD7A*	MISSENSE	ENSG00000005108:MISSENSE[ENST00000423059(c.739G>C,Gag>Cag):ENSP00000406482(p.Glu247Gln)];	chr7*11676	chr7*11676	chr7*11676040*11676040*C*T*rs756049301*NA*NA*NA	NA	NA	NA	NA	T	BN	N	MODERATE	D	0.154	220	220	0	117	99	18	15.38
Smoker	42478	chr7	44621116	G	A	*TMED4*	MISSENSE	ENSG00000158604:MISSENSE[ENST00000289577(c.319C>T,Ccc>Tcc):ENSP00000289577(p.Pro107Ser)];ENSG00000158604:MISSENSE[ENST00000457408(c.319C>T,Ccc>Tcc):ENSP00000404042(p.Pro107Ser)];ENSG00000158604:MISSENSE[ENST00000481238(c.319C>T,Ccc>Tcc):ENSP00000417443(p.Pro107Ser)];ENSG00000158604:EXONIC-NC[ENST00000444131(n.330C>T)];ENSG00000158604:EXONIC-NC[ENST00000477639(n.546C>T)];	NA	NA	NA	1.65E-05	0	NA	NA	NA	NA	NA	MODIFIER	NA	NA	182	182	0	168	155	13	7.74
Smoker	42478	chr7	57069817	G	T	*TNRC18P3*	EXONIC-NC	ENSG00000189166:[ENST00000441896(n.799G>T)];	NA	NA	NA	NA	NA	NA	NA	NA	NA	NA	MODIFIER	NA	NA	51	51	0	42	37	5	11.9

Cohort	Sample	Chromosome	Genomic position	Reference allele	Alternate allele	Gene symbol	Variant type	Variant annotation	OncoMD	COSMIC	dbSNP	ExAC	ExAC_SAS	1000G	1000G_SAS	SIFT	Polyphen2	CONDEL	VEP	LRT	PhastCon7 Way	Total read (Adjacent normal)	Reference allel read support (Adjacent normal)	Alternative read support (Adjacent normal)	Total read (Tumor)	Reference allel read support (Tumor)	Alternative read support (Tumor)	Alternate allele frequency
Smoker	42478	chr7	116955218	G	C	*WNT2*	MISSENSE	ENSG00000105989:INTRONIC[ENST00000491214(c.310+5403C>G)];ENSG00000105989:3UTR[ENST00000449446(c.*98C>G)];ENSG00000105989:MISSENSE[ENST00000265441(c.495C>G,atC>atG):ENSP00000265441(p.Ile165Met)];	NA	NA	chr7*116955218*116955218*G*T*rs142048882*0*0.9998*0.0001997	NA	NA	NA	NA	T	BN	N	MODERATE	N	1	193	193	0	141	135	6	4.26
Smoker	42478	chr7	88962785	G	A	*ZNF804B*	SILENT	ENSG00000182348:SILENT[ENST00000333190(c.489G>A,aaG>aaA):ENSP00000329638(p.Lys163(=))];	NA	NA	NA	NA	NA	NA	NA	NA	NA	NA	LOW	NA	0.986	252	252	0	271	253	17	6.27
Smoker	42478	chr8	39349522	G	A	*ADAM3A*	EXONIC-NC	ENSG00000197475:[ENST00000490268(n.1015C>T)];ENSG00000197475:[ENST00000495171(n.1015C>T)];ENSG00000197475:[ENST00000460383(n.1013C>T)];ENSG00000197475:[ENST00000484602(n.703C>T)];ENSG00000197475:[ENST00000497806(n.1015C>T)];ENSG00000197475:[ENST00000481351(n.1015C>T)];ENSG00000197475:[ENST00000424066(n.584C>T)];ENSG00000197475:[ENST00000461344(n.1060C>T)];ENSG00000197475:[ENST00000474764(n.1075C>T)];	NA	NA	NA	NA	NA	NA	NA	NA	NA	NA	MODIFIER	NA	NA	78	78	0	97	76	21	21.65
Smoker	42478	chr8	26716568	C	A	*ADRA1A*	3UTR	ENSG00000120907:[ENST00000521711(c.*63G>T)];ENSG00000120907:[ENST00000519096(c.*63G>T)];ENSG00000120907:[ENST00000380573(c.883+5036G>T)];ENSG00000120907:[ENST00000276393(c.883+5036G>T)];ENSG00000120907:[ENST00000380586(c.883+5036G>T)];ENSG00000120907:[ENST00000380572(c.883+5036G>T)];ENSG00000120907:[ENST00000354550(c.883+5036G>T)];ENSG00000120907:[ENST00000380587(c.883+5036G>T)];ENSG00000120907:[ENST00000380581(c.883+5036G>T)];ENSG00000120907:[ENST00000380582(c.883+5036G>T)];ENSG00000120907:[ENST00000519229(c.883+5036G>T)];	NA	NA	NA	NA	NA	NA	NA	NA	NA	NA	MODIFIER	NA	NA	168	168	0	167	158	9	5.39
Smoker	42478	chr8	139732957	T	A	*COL22A1*	INTRONIC	ENSG00000169436:[ENST00000303045(c.2355+25A>T)];ENSG00000169436:[ENST00000341807(n.100+25A>T)];ENSG00000169436:[ENST00000435777(c.2355+25A>T)];	NA	NA	NA	NA	NA	NA	NA	NA	NA	NA	MODIFIER	NA	NA	103	103	0	89	78	11	12.36
Smoker	42478	chr8	113651052	C	A	*CSMD3*	SILENT	ENSG00000164796:SILENT[ENST00000352409(c.3399G>T,ctG>ctT):ENSP00000343124(p.Leu1133(=))];ENSG00000164796:SILENT[ENST00000455883(c.3087G>T,ctG>ctT):ENSP00000412263(p.Leu1029(=))];ENSG00000164796:SILENT[ENST00000297405(c.3399G>T,ctG>ctT):ENSP00000297405(p.Leu1133(=))];ENSG00000164796:SILENT[ENST00000339701(c.1419G>T,ctG>ctT):ENSP00000341558(p.Leu473(=))];ENSG00000164796:SILENT[ENST00000343508(c.3279G>T,ctG>ctT):ENSP00000345799(p.Leu1093(=))];	NA	NA	NA	NA	NA	NA	NA	NA	NA	NA	LOW	NA	NA	232	231	1	263	219	43	16.35
Smoker	42478	chr8	133634877	G	A	*LRRC6*	SILENT	ENSG00000129295:EXONIC-NC[ENST00000522597(n.163C>T)];ENSG00000129295:SILENT[ENST00000519595(c.894C>T,gcC>gcT):ENSP00000429791(p.Ala298(=))];ENSG00000129295:SILENT[ENST00000519085(c.58C>T,gcC>gcT):ENSP00000429158(p.Ala20(=))];ENSG00000129295:SILENT[ENST00000518642(c.894C>T,gcC>gcT):ENSP00000428610(p.Ala298(=))];ENSG00000129295:SILENT[ENST00000250173(c.894C>T,gcC>gcT):ENSP00000250173(p.Ala298(=))];ENSG00000129295:SILENT[ENST00000522789(c.174C>T,gcC>gcT):ENSP00000428015(p.Ala58(=))];	NA	NA	NA	NA	NA	NA	NA	NA	NA	NA	LOW	NA	NA	278	275	0	276	244	32	11.59
Smoker	42478	chr8	89128831	T	A	*MMP16*	MISSENSE	ENSG00000156103:MISSENSE[ENST00000286614(c.988A>T,Acc>Tcc):ENSP00000286614(p.Thr330Ser)];ENSG00000156103:EXONIC-NC[ENST00000544227(n.988A>T)];	chr8*89128	chr8*89128	NA	NA	NA	NA	NA	NA	NA	NA	MODIFIER	NA	NA	115	114	1	106	97	9	8.49
Smoker	42478	chr8	120795660	G	A	*TAF2*	SILENT	ENSG00000064313:SILENT[ENST00000378164(c.2073C>T,ttC>ttT):ENSP00000367406(p.Phe691(=))];	NA	NA	NA	NA	NA	NA	NA	NA	NA	NA	LOW	NA	1	268	268	0	230	211	18	7.83
Smoker	42478	chr8	71499176	G	A	*TRAM1*	MISSENSE	ENSG00000067167:MISSENSE[ENST00000536748(c.607C>T,Cac>Tac):ENSP00000439359(p.His203Tyr)];ENSG00000067167:MISSENSE[ENST00000262213(c.700C>T,Cac>Tac):ENSP00000262213(p.His234Tyr)];ENSG00000067167:MISSENSE[ENST00000521425(c.442C>T,Cac>Tac):ENSP00000428052(p.His148Tyr)];ENSG00000067167:EXONIC-NC[ENST00000521049(n.738C>T)];	NA	NA	NA	NA	NA	NA	NA	NA	NA	NA	MODIFIER	NA	NA	99	99	0	89	70	19	21.35
Smoker	42478	chr9	123594133	C	T	*PSMD5*	MISSENSE	ENSG00000095261:MISSENSE[ENST00000210313(c.409G>A,Gag>Aag):ENSP00000210313(p.Glu137Lys)];ENSG00000095261:MISSENSE[ENST00000373904(c.409G>A,Gag>Aag):ENSP00000363011(p.Glu137Lys)];ENSG00000095261:EXONIC-NC[ENST00000471789(n.382G>A)];ENSG00000095261:3UTR[ENST00000476949(c.*45G>A)];	NA	NA	chr9*123594133*123594133*C*A*rs756059765*NA*NA*NA	NA	NA	NA	NA	NA	NA	NA	MODIFIER	NA	NA	139	138	1	144	133	10	6.94
Smoker	42478	chr9	123594133	C	T	*PSMD5-AS1*	INTRONIC	ENSG00000226752:[ENST00000589026(n.143+6885C>T)];	NA	NA	chr9*123594133*123594133*C*A*rs756059765*NA*NA*NA	NA	NA	NA	NA	NA	NA	NA	MODIFIER	NA	NA	139	138	1	144	133	10	6.94
Smoker	42478	chr9	85612044	G	C	*RASEF*	INTRONIC-SS-ACR	ENSG00000165105:[ENST00000376447(c.1806-3C>G)];	NA	NA	NA	NA	NA	NA	NA	NA	NA	NA	LOW	NA	NA	199	199	0	181	150	31	17.13
Smoker	42478	chrX	135772796	G	A	*ARHGEF6*	SILENT	ENSG00000129675:SILENT[ENST00000370620(c.696C>T,ctC>ctT):ENSP00000359654(p.Leu232(=))];ENSG00000129675:SILENT[ENST00000250617(c.1158C>T,ctC>ctT):ENSP00000250617(p.Leu386(=))];ENSG00000129675:SILENT[ENST00000370622(c.696C>T,ctC>ctT):ENSP00000359656(p.Leu232(=))];ENSG00000129675:SILENT[ENST00000535227(c.777C>T,ctC>ctT):ENSP00000439483(p.Leu259(=))];	NA	NA	NA	NA	NA	NA	NA	NA	NA	NA	LOW	NA	NA	134	134	0	127	101	26	20.47
Smoker	42478	chrX	70838346	C	G	*CXCR3*	5UTR	ENSG00000186810:[ENST00000373693(c.-47G>C)];ENSG00000186810:[ENST00000373691(c.-150G>C)];	NA	NA	NA	NA	NA	NA	NA	NA	NA	NA	MODIFIER	NA	NA	26	26	0	30	23	7	23.33
Smoker	42478	chrX	27997833	G	C	*DCAF8L1*	MISSENSE	ENSG00000226372:MISSENSE[ENST00000441525(c.1619C>G,aCg>aGg):ENSP00000405222(p.Thr540Arg)];	NA	NA	chrX*27997833*27997833*G*A*rs138100395*NA*NA*NA	NA	NA	NA	NA	T	BN	N	MODERATE	N	0.322	86	83	1	87	71	16	18.39
Smoker	42478	chrX	152751331	C	A	*HAUS7*	INTRONIC	ENSG00000213397:[ENST00000370210(c.-589+9501G>T)];ENSG00000213397:[ENST00000421080(c.-1021+9501G>T)];	NA	NA	NA	NA	NA	NA	NA	NA	NA	NA	MODIFIER	NA	NA	20	20	0	20	15	5	25
Smoker	42478	chrX	105450332	G	A	*MUM1L1*	MISSENSE	ENSG00000157502:MISSENSE[ENST00000357175(c.907G>A,Gag>Aag):ENSP00000349699(p.Glu303Lys)];ENSG00000157502:MISSENSE[ENST00000337685(c.907G>A,Gag>Aag):ENSP00000338641(p.Glu303Lys)];ENSG00000157502:MISSENSE[ENST00000372552(c.907G>A,Gag>Aag):ENSP00000361632(p.Glu303Lys)];	NA	NA	NA	NA	NA	NA	NA	T	PrD	D	MODERATE	N	0.005	86	86	0	74	58	16	21.62
Smoker	42478	chrX	119293090	C	A	*NKAPP1*	INTRONIC	ENSG00000233382:[ENST00000555168(n.126-21991G>T)];	NA	chrX*11929	chrX*119293090*119293090*C*T*rs782040477*NA*NA*NA	NA	NA	NA	NA	NA	NA	NA	LOW	NA	NA	106	105	1	84	73	11	13.1
Smoker	42478	chrX	119293090	C	A	*RHOXF2*	SILENT	ENSG00000131721:SILENT[ENST00000371388(c.249C>A,gcC>gcA):ENSP00000360441(p.Ala83(=))];	NA	chrX*11929	chrX*119293090*119293090*C*T*rs782040477*NA*NA*NA	NA	NA	NA	NA	NA	NA	NA	LOW	NA	NA	106	105	1	84	73	11	13.1

Supplementary Table 4. List of somatic single nucleotide variants identified using whole exome sequencing of ESCC patients

Cohort	Sample	Chromosome	Genomic position	Reference allele	Alternate allele	Gene symbol	Variant type	Variant annotation	OncoMD	COSMIC	dbSNP	ExAC	ExAC_SAS	1000G	1000G_SAS	SIFT	Polyphen2	CONDEL	VEP	LRT	PhastCon7 Way	Total read (Adjacent normal)	Reference allel read support (Adjacent normal)	Alternative read support (Adjacent normal)	Total read (Tumor)	Reference allel read support (Tumor)	Alternative read support (Tumor)	Alternate allele frequency
Smoker	42478	chrX	77388964	G	A	*TAF9B*	INTRONIC	ENSG00000187325:[ENST00000341864(c.482-19C>T)];ENSG00000187325:[ENST00000480681(n.571-19C>T)];	NA	NA	NA	NA	NA	NA	NA	NA	NA	NA	MODIFIER	NA	NA	79	79	0	58	51	6	10.34
Smoker	42478	chrX	152751331	C	A	*U82695.9*	5UTR	ENSG00000224963:[ENST00000428676(c.-27C>A)];	NA	NA	NA	NA	NA	NA	NA	NA	NA	NA	MODIFIER	NA	NA	20	20	0	20	15	5	25
Smoker	42479	chr1	112042554	G	T	*ADORA3*	INTRONIC	ENSG00000121933:[ENST00000369716(c.350+3073C>A)];ENSG00000121933:[ENST00000443498(c.90-11065C>A)];ENSG00000121933:[ENST00000369717(c.1089170C>A)];ENSG00000121933:[ENST00000495493(n.760C>A)];ENSG00000121933:[ENST00000241356(c.*18C>A)];	NA	NA	NA	NA	NA	NA	NA	NA	NA	NA	MODIFIER	NA	NA	101	101	0	55	18	37	67.27
Smoker	42479	chr1	155307583	C	T	*ASH1L*	INTRONIC	ENSG00000116539:[ENST00000368346(c.8819-41G>A)];ENSG00000116539:[ENST00000392403(c.8804-41G>A)];	NA	NA	NA	NA	NA	NA	NA	NA	NA	NA	MODIFIER	NA	NA	116	116	0	139	104	35	25.18
Smoker	42479	chr1	176915135	C	A	*ASTN1*	MISSENSE	ENSG00000152092:MISSENSE[ENST00000367654(c.2200G>T,Ggg>Tgg):ENSP00000356626(p.Gly734Trp)];ENSG00000152092:MISSENSE[ENST00000424564(c.2176G>T,Ggg>Tgg):ENSP00000395041(p.Gly726Trp)];ENSG00000152092:MISSENSE[ENST00000361833(c.2176G>T,Ggg>Tgg):ENSP00000354536(p.Gly726Trp)];ENSG00000152092:MISSENSE[ENST00000367657(c.2176G>T,Ggg>Tgg):ENSP00000356629(p.Gly726Trp)];ENSG00000152092:EXONIC-NC[ENST00000281881(n.2473G>T)];	chr1*17691	chr1*17691	NA	NA	NA	NA	NA	NA	NA	NA	MODIFIER	NA	NA	122	121	1	112	78	33	29.46
Smoker	42479	chr1	169697397	C	T	*C1orf112*	INTRONIC	ENSG00000000460:[ENST00000498289(n.851+44324C>T)];	NA	NA	NA	NA	NA	NA	NA	NA	NA	NA	MODIFIER	NA	NA	95	95	0	78	62	16	20.51
Smoker	42479	chr1	228290414	C	G	*C1orf35*	INTRONIC	ENSG00000143793:[ENST00000469781(n.460-25G>C)];ENSG00000143793:[ENST00000492757(n.105-25G>C)];ENSG00000143793:[ENST00000465199(n.277-25G>C)];ENSG00000143793:[ENST00000491293(n.408-25G>C)];ENSG00000143793:[ENST00000472617(n.2430-25G>C)];ENSG00000143793:[ENST00000272139(c.246-25G>C)];ENSG00000143793:[ENST00000485896(n.339-25G>C)];	NA	NA	NA	NA	NA	NA	NA	NA	NA	NA	MODIFIER	NA	NA	25	25	0	32	27	5	15.62
Smoker	42479	chr1	181620515	C	T	*CACNA1E*	SILENT	ENSG00000198216:5UTR[ENST00000367567(c.-187C>T)];ENSG00000198216:SILENT[ENST00000360108(c.993C>T,ttC>ttT):ENSP00000353222(p.Phe331(=))];ENSG00000198216:SILENT[ENST00000524607(c.993C>T,ttC>ttT):ENSP00000432038(p.Phe331(=))];ENSG00000198216:SILENT[ENST00000358338(c.846C>T,ttC>ttT):ENSP00000351101(p.Phe282(=))];ENSG00000198216:SILENT[ENST00000357570(c.846C>T,ttC>ttT):ENSP00000350183(p.Phe282(=))];ENSG00000198216:SILENT[ENST00000526775(c.993C>T,ttC>ttT):ENSP00000434814(p.Phe331(=))];ENSG00000198216:SILENT[ENST00000367570(c.993C>T,ttC>ttT):ENSP00000356542(p.Phe331(=))];ENSG00000198216:SILENT[ENST00000367573(c.993C>T,ttC>ttT):ENSP00000356545(p.Phe331(=))];ENSG00000198216:EXONIC-NC[ENST00000533229(n.1427C>T)];	NA	NA	NA	NA	NA	NA	NA	NA	NA	NA	LOW	NA	1	154	154	0	161	113	48	29.81
Smoker	42479	chr1	201020185	C	G	*CACNA1S*	MISSENSE	ENSG00000081248:MISSENSE[ENST00000367338(c.3983G>C,gGg>gCg):ENSP00000356307(p.Gly1328Ala)];ENSG00000081248:MISSENSE[ENST00000362061(c.4040G>C,gGg>gCg):ENSP00000355192(p.Gly1347Ala)];	NA	NA	chr1*201020185*201020185*C*A*rs752954170*NA*NA*NA	NA	NA	NA	NA	D	PrD	D	MODERATE	D	0.561	61	61	0	53	34	19	35.85
Smoker	42479	chr1	227300422	C	G	*CDC42BPA*	MISSENSE	ENSG00000143776:MISSENSE[ENST00000366769(c.1840G>C,Gtt>Ctt):ENSP00000355731(p.Val614Leu)];ENSG00000143776:MISSENSE[ENST00000366765(c.1840G>C,Gtt>Ctt):ENSP00000355727(p.Val614Leu)];ENSG00000143776:MISSENSE[ENST00000366764(c.1840G>C,Gtt>Ctt):ENSP00000355726(p.Val614Leu)];ENSG00000143776:MISSENSE[ENST00000334218(c.1840G>C,Gtt>Ctt):ENSP00000335341(p.Val614Leu)];ENSG00000143776:MISSENSE[ENST00000535525(c.1840G>C,Gtt>Ctt):ENSP00000443275(p.Val614Leu)];ENSG00000143776:MISSENSE[ENST00000366766(c.1840G>C,Gtt>Ctt):ENSP00000355728(p.Val614Leu)];ENSG00000143776:EXONIC-NC[ENST00000466538(n.79G>C)];ENSG00000143776:INTRONIC[ENST00000366767(c.1648-299G>C)];	NA	NA	NA	NA	NA	NA	NA	NA	NA	NA	MODIFIER	NA	NA	255	255	0	309	234	74	23.95
Smoker	42479	chr1	227300432	C	G	*CDC42BPA*	SILENT	ENSG00000143776:SILENT[ENST00000366769(c.1830G>C,gtG>gtC):ENSP00000355731(p.Val610(=))];ENSG00000143776:SILENT[ENST00000366765(c.1830G>C,gtG>gtC):ENSP00000355727(p.Val610(=))];ENSG00000143776:SILENT[ENST00000366764(c.1830G>C,gtG>gtC):ENSP00000355726(p.Val610(=))];ENSG00000143776:SILENT[ENST00000334218(c.1830G>C,gtG>gtC):ENSP00000335341(p.Val610(=))];ENSG00000143776:SILENT[ENST00000535525(c.1830G>C,gtG>gtC):ENSP00000443275(p.Val610(=))];ENSG00000143776:SILENT[ENST00000366766(c.1830G>C,gtG>gtC):ENSP00000355728(p.Val610(=))];ENSG00000143776:EXONIC-NC[ENST00000466538(n.69G>C)];ENSG00000143776:INTRONIC[ENST00000366767(c.1648-309G>C)];	NA	NA	NA	8.24E-06	0	NA	NA	NA	NA	NA	LOW	NA	NA	243	243	0	328	251	76	23.17
Smoker	42479	chr1	151039779	C	A	*CDC42SE1*	5UTR	ENSG00000197622:[ENST00000439374(c.-730G>T)];	NA	NA	NA	NA	NA	NA	NA	T	PrD	D	MODERATE	U	0.999	67	67	0	88	66	22	25
Smoker	42479	chr1	91978638	T	G	*CDC7*	MISSENSE	ENSG00000097046:MISSENSE[ENST00000430031(c.512T>G,tTg>tGg):ENSP00000407477(p.Leu171Trp)];ENSG00000097046:MISSENSE[ENST00000428239(c.596T>G,tTg>tGg):ENSP00000393139(p.Leu199Trp)];ENSG00000097046:MISSENSE[ENST00000234626(c.596T>G,tTg>tGg):ENSP00000234626(p.Leu199Trp)];	NA	NA	NA	NA	NA	NA	NA	D	PrD	D	MODERATE	D	1	75	75	0	94	48	46	48.94
Smoker	42479	chr1	243349644	C	G	*CEP170*	MISSENSE	ENSG00000143702:MISSENSE[ENST00000336415(c.894G>C,Gag>Cag):ENSP00000338161(p.Glu299Gln)];ENSG00000143702:MISSENSE[ENST00000366544(c.1189G>C,Gag>Cag):ENSP00000355502(p.Glu397Gln)];ENSG00000143702:MISSENSE[ENST00000366543(c.1189G>C,Gag>Cag):ENSP00000355501(p.Glu397Gln)];ENSG00000143702:MISSENSE[ENST00000522895(c.68G>C,Gag>Cag):ENSP00000428996(p.Glu24Gln)];ENSG00000143702:MISSENSE[ENST00000366542(c.1189G>C,Gag>Cag):ENSP00000355500(p.Glu397Gln)];ENSG00000143702:EXONIC-NC[ENST00000521911(n.158G>C)];	NA	NA	NA	NA	NA	NA	NA	NA	NA	NA	MODIFIER	NA	NA	176	176	0	201	159	42	20.9
Smoker	42479	chr1	6173545	G	A	*CHD5*	INTRONIC	ENSG00000116254:[ENST00000378021(c.1484-487C>T)];ENSG00000116254:[ENST00000377999(c.*1483-487C>T)];ENSG00000116254:[ENST00000262450(c.4913-487C>T)];ENSG00000116254:[ENST00000496404(c.3631-487C>T)];ENSG00000116254:[ENST00000462991(c.3060-69C>T)];	NA	NA	NA	NA	NA	NA	NA	NA	NA	NA	MODIFIER	NA	NA	19	19	0	32	27	5	15.62

Mangalaparthi *et al.* , 2020. Mutational landscape of esophageal squamous cell carcinoma in an Indian cohort
Supplementary Table 4. List of somatic single nucleotide variants identified using whole exome sequencing of ESCC patients

Cohort	Sample	Chromosome	Genomic position	Reference allele	Alternate allele	Gene symbol	Variant type	Variant annotation	OncoMD	COSMIC	dbSNP	ExAC	ExAC_SAS	1000G	1000G_SAS	SIFT	Polyphen2	CONDEL	VEP	LRT	PhastCon7 Way	Total read (Adjacent normal)	Reference allel read support (Adjacent normal)	Alternative read support (Adjacent normal)	Total read (Tumor)	Reference allel read support (Tumor)	Alternative read support (Tumor)	Alternate allele frequency
Smoker	42479	chr1	111858443	C	G	*CHIA*	MISSENSE	ENSG00000134216:INTRONIC[ENST00000369740(c.480+386C>G)];ENSG00000134216:INTRONIC[ENST00000343320(c.480+386C>G)];ENSG00000134216:INTRONIC[ENST00000489524(c.-4+1225C>G)];ENSG00000134216:INTRONIC[ENST00000430615(c.156+386C>G)];ENSG00000134216:INTRONIC[ENST00000483391(c.-4+1225C>G)];ENSG00000134216:INTRONIC[ENST00000422815(c.312+386C>G)];ENSG00000134216:MISSENSE[ENST00000352594(c.244C>G,Cca>Gca):ENSP00000271312(p.Pro82Ala)];ENSG00000134216:EXONIC-NC[ENST00000477918(n.1344C>G)];ENSG00000134216:5UTR[ENST00000353665(c.-55C>G)];ENSG00000134216:5UTR[ENST00000451398(c.-55C>G)];	NA	NA	NA	NA	NA	NA	NA	NA	NA	NA	MODIFIER	NA	NA	153	151	2	95	46	49	51.58
Smoker	42479	chr1	1249023	C	T	*CPSF3L*	INTRONIC	ENSG00000127054:[ENST00000531377(c.*315-51G>A)];ENSG00000127054:[ENST00000419704(c.655-51G>A)];ENSG00000127054:[ENST00000531292(n.555-51G>A)];ENSG00000127054:[ENST00000450926(c.892-51G>A)];ENSG00000127054:[ENST00000532772(c.*380-51G>A)];ENSG00000127054:[ENST00000323275(n.1346-51G>A)];ENSG00000127054:[ENST00000470030(n.866-51G>A)];ENSG00000127054:[ENST00000528879(c.*56-51G>A)];ENSG00000127054:[ENST00000545578(c.871-51G>A)];ENSG00000127054:[ENST00000478641(n.1440-51G>A)];ENSG00000127054:[ENST00000467408(n.593-51G>A)];ENSG00000127054:[ENST00000435064(c.958-51G>A)];ENSG00000127054:[ENST00000461514(n.50-51G>A)];ENSG00000127054:[ENST00000458452(c.*1242-51G>A)];ENSG00000127054:[ENST00000421495(c.184-51G>A)];ENSG00000127054:[ENST00000485710(n.95-51G>A)];ENSG00000127054:[ENST00000540437(c.976-51G>A)];ENSG00000127054:[ENST00000462432(n.1823-51G>A)];ENSG00000127054:[ENST00000527383(c.*380-51G>A)];ENSG00000127054:[ENST00000411962(c.664-51G>A)];ENSG00000127054:[ENST00000525164(n.35-51G>A)];ENSG00000127054:[ENST00000527098(c.*786-51G>A)];ENSG00000127054:[ENST00000525769(n.20-51G>A)];	NA	NA	NA	1.67E-05	0	NA	NA	NA	NA	NA	MODIFIER	NA	NA	138	138	0	201	148	53	26.37
Smoker	42479	chr1	1256078	G	A	*CPSF3L*	INTRONIC	ENSG00000127054:[ENST00000531019(c.127-16C>T)];ENSG00000127054:[ENST00000419704(c.126+298C>T)];ENSG00000127054:[ENST00000470679(c.122-16C>T)];ENSG00000127054:[ENST00000450926(c.127-169C>T)];ENSG00000127054:[ENST00000430786(c.*5-169C>T)];ENSG00000127054:[ENST00000532772(c.126+298C>T)];ENSG00000127054:[ENST00000490853(n.168-169C>T)];ENSG00000127054:[ENST00000488042(n.148-169C>T)];ENSG00000127054:[ENST00000434694(c.127-169C>T)];ENSG00000127054:[ENST00000528879(c.127-169C>T)];ENSG00000127054:[ENST00000527719(c.145-169C>T)];ENSG00000127054:[ENST00000545578(c.40-169C>T)];ENSG00000127054:[ENST00000530031(c.268-169C>T)];ENSG00000127054:[ENST00000532952(n.161-169C>T)];ENSG00000127054:[ENST00000435064(c.127-169C>T)];ENSG00000127054:[ENST00000526332(c.-172-1174C>T)];ENSG00000127054:[ENST00000525285(c.*334-169C>T)];ENSG00000127054:[ENST00000534345(c.130-169C>T)];ENSG00000127054:[ENST00000526904(n.78+3883C>T)];ENSG00000127054:[ENST00000429572(n.185-169C>T)];ENSG00000127054:[ENST00000458452(c.*411-169C>T)];ENSG00000127054:[ENST00000421495(c.-530+3883C>T)];ENSG00000127054:[ENST00000498476(c.307-169C>T)];ENSG00000127054:[ENST00000526797(c.127-169C>T)];ENSG00000127054:[ENST00000540437(c.145-169C>T)];ENSG00000127054:[ENST00000493534(n.177-169C>T)];ENSG00000127054:[ENST00000526113(n.57+3883C>T)];ENSG00000127054:[ENST00000411962(c.135+2483C>T)];ENSG00000127054:[ENST000005270	NA	NA	NA	0	0	NA	NA	NA	NA	NA	MODIFIER	NA	NA	49	49	0	109	88	21	19.27
Smoker	42479	chr1	17197581	C	A	*CROCC*	INTRONIC	ENSG00000058453:[ENST00000466256(n.126-59200C>A)];	NA	NA	NA	NA	NA	NA	NA	NA	NA	NA	MODIFIER	NA	NA	355	351	0	480	428	44	9.17
Smoker	42479	chr1	173800681	A	G	*DARS2*	SILENT	ENSG00000117593:SILENT[ENST00000361951(c.405A>G,ccA>ccG):ENSP00000355086(p.Pro135(=))];ENSG00000117593:5UTR[ENST00000239457(c.-850A>G)];	NA	NA	NA	NA	NA	NA	NA	NA	NA	NA	MODIFIER	NA	NA	111	111	0	119	38	81	68.07
Smoker	42479	chr1	197616194	A	G	*DENND1B*	SILENT	ENSG00000213047:INTRONIC[ENST00000235453(c.418-1321T>C)];ENSG00000213047:INTRONIC[ENST00000496935(n.133-1321T>C)];ENSG00000213047:INTRONIC[ENST00000422998(c.340-1321T>C)];ENSG00000213047:INTRONIC[ENST00000400967(c.418-1321T>C)];ENSG00000213047:INTRONIC[ENST00000294737(c.448-1321T>C)];ENSG00000213047:SILENT[ENST00000367396(c.498T>C,gcT>gcC):ENSP00000356366(p.Ala166(=))];	NA	NA	NA	NA	NA	NA	NA	NA	NA	NA	MODIFIER	NA	NA	39	39	0	34	15	19	55.88
Smoker	42479	chr1	205589780	C	A	*ELK4*	MISSENSE	ENSG00000158711:EXONIC-NC[ENST00000468523(n.439G>T)];ENSG00000158711:MISSENSE[ENST00000357992(c.394G>T,Ggt>Tgt):ENSP00000350681(p.Gly132Cys)];ENSG00000158711:MISSENSE[ENST00000289703(c.394G>T,Ggt>Tgt):ENSP00000289703(p.Gly132Cys)];	NA	NA	NA	NA	NA	NA	NA	T	BN	N	MODERATE	N	0.947	161	161	0	177	132	45	25.42
Smoker	42479	chr1	109158889	C	T	*FAM102B*	INTRONIC	ENSG00000162636:[ENST00000370035(c.406-115C>T)];ENSG00000162636:[ENST00000405454(c.406-115C>T)];	NA	NA	NA	NA	NA	NA	NA	NA	NA	NA	MODIFIER	NA	NA	20	20	0	19	5	14	73.68
Smoker	42479	chr1	184764161	C	T	*FAM129A*	MISSENSE	ENSG00000135842:INTRONIC[ENST00000417056(c.260+3052G>A)];ENSG00000135842:EXONIC-NC[ENST00000487074(n.2209G>A)];ENSG00000135842:MISSENSE[ENST00000367511(c.2737G>A,Gag>Aag):ENSP00000356481(p.Glu913Lys)];	NA	NA	NA	NA	NA	NA	NA	T_lc	BN	N	MODERATE	N	0.007	196	196	0	207	149	58	28.02
Smoker	42479	chr1	159275976	C	T	*FCER1A*	MISSENSE	ENSG00000179639:MISSENSE[ENST00000368115(c.530C>T,aCg>aTg):ENSP00000357097(p.Thr177Met)];ENSG00000179639:MISSENSE[ENST00000368114(c.431C>T,aCg>aTg):ENSP00000357096(p.Thr144Met)];	NA	NA	0.01098	0.0053976	0.007085756	0.0109824	0.0082	T	BN	N	MODERATE	N	0.023	42	42	0	28	14	14	50
Smoker	42479	chr1	152192623	G	T	*FLG-AS1*	INTRONIC	ENSG00000237975:[ENST00000420707(n.159-28748G>T)];ENSG00000237975:[ENST00000593011(n.296+51727G>T)];	chr1*15219	NA	chr1*152192623*152192623*G*A*rs77355406*0*0.9998*0.0001997	NA	NA	NA	NA	NA	UN	NA	MODERATE	NA	NA	214	214	0	251	176	75	29.88

Supplementary Table 4. List of somatic single nucleotide variants identified using whole exome sequencing of ESCC patients

Cohort	Sample	Chromosome	Genomic position	Reference allele	Alternate allele	Gene symbol	Variant type	Variant annotation	OncoMD	COSMIC	dbSNP	ExAC	ExAC_SAS	1000G	1000G_SAS	SIFT	Polyphen2	CONDEL	VEP	LRT	PhastCon7 Way	Total read (Adjacent normal)	Reference allel read support (Adjacent normal)	Alternative read support (Adjacent normal)	Total read (Tumor)	Reference allel read support (Tumor)	Alternative read support (Tumor)	Alternate allele frequency
Smoker	42479	chr1	240370464	G	C	*FMN2*	MISSENSE	ENSG00000155816:MISSENSE[ENST00000447095(c.663G>C,gaG>gaC):ENSP00000409308(p.Glu221Asp)];ENSG00000155816:MISSENSE[ENST00000319653(c.2352G>C,gaG>gaC):ENSP00000318884(p.Glu784Asp)];	NA	NA	NA	NA	NA	NA	NA	NA	UN	NA	MODERATE	NA	NA	104	104	0	145	130	15	10.34
Smoker	42479	chr1	37316124	C	A	*GRIK3*	INTRONIC	ENSG00000163873:[ENST00000373093(c.1213-99G>T)];ENSG00000163873:[ENST00000479620(n.74-99G>T)];ENSG00000163873:[ENST00000373091(c.1213-99G>T)];ENSG00000163873:[ENST00000462621(n.138-99G>T)];	NA	NA	NA	NA	NA	NA	NA	NA	NA	NA	MODIFIER	NA	NA	72	72	0	89	47	42	47.19
Smoker	42479	chr1	152192623	G	T	*HRNR*	MISSENSE	ENSG00000197915:MISSENSE[ENST00000368801(c.1482C>A,caC>caA):ENSP00000357791(p.His494Gln)];	chr1*15219	NA	chr1*152192623*152192623*G*A*rs77355406*0*0.9998*0.0001997	NA	NA	NA	NA	NA	UN	NA	MODERATE	NA	NA	214	214	0	251	176	75	29.88
Smoker	42479	chr1	120056486	C	A	*HSD3B1*	MISSENSE	ENSG00000203857:MISSENSE[ENST00000528909(c.340C>A,Caa>Aaa):ENSP00000432268(p.Gln114Lys)];ENSG00000203857:MISSENSE[ENST00000235547(c.346C>A,Caa>Aaa):ENSP00000235547(p.Gln116Lys)];ENSG00000203857:MISSENSE[ENST00000369413(c.340C>A,Caa>Aaa):ENSP00000358421(p.Gln114Lys)];	NA	NA	NA	NA	NA	NA	NA	T	BN	N	MODERATE	N	0.018	117	117	0	344	250	94	27.33
Smoker	42479	chr1	22151240	C	T	*HSPG2*	MISSENSE	ENSG00000142798:EXONIC-NC[ENST00000486901(n.1961G>A)];ENSG00000142798:MISSENSE[ENST00000374695(c.12622G>A,Gat>Aat):ENSP00000363827(p.Asp4208Asn)];	NA	NA	NA	9.25E-06	0	NA	NA	NA	NA	NA	MODIFIER	NA	NA	54	54	0	80	64	16	20
Smoker	42479	chr1	22160515	G	A	*HSPG2*	INTRONIC	ENSG00000142798:[ENST00000426143(c.377-97C>T)];ENSG00000142798:[ENST00000374695(c.10721-97C>T)];	NA	NA	NA	NA	NA	NA	NA	NA	NA	NA	MODIFIER	NA	NA	45	45	0	78	69	9	11.54
Smoker	42479	chr1	41249900	C	T	*KCNQ4*	SILENT	ENSG00000117013:SILENT[ENST00000509682(c.135C>T,ctC>ctT):ENSP00000423756(p.Leu45(=))];ENSG00000117013:SILENT[ENST00000347132(c.135C>T,ctC>ctT):ENSP00000262916(p.Leu45(=))];	NA	NA	NA	NA	NA	NA	NA	NA	NA	NA	LOW	NA	NA	41	41	0	46	36	10	21.74
Smoker	42479	chr1	23376988	T	C	*KDM1A*	MISSENSE	ENSG00000004487:MISSENSE[ENST00000356634(c.626T>C,gTt>gCt):ENSP00000349049(p.Val209Ala)];ENSG00000004487:MISSENSE[ENST00000542151(c.686T>C,gTt>gCt):ENSP00000439072(p.Val229Ala)];ENSG00000004487:MISSENSE[ENST00000400181(c.686T>C,gTt>gCt):ENSP00000383042(p.Val229Ala)];ENSG00000004487:EXONIC-NC[ENST00000481879(n.71T>C)];	NA	NA	NA	NA	NA	NA	NA	NA	NA	NA	MODIFIER	NA	NA	199	198	1	257	208	49	19.07
Smoker	42479	chr1	156025240	C	A	*LAMTOR2*	INTRONIC	ENSG00000116586:[ENST00000368302(c.231+24C>A)];ENSG00000116586:[ENST00000368305(c.231+24C>A)];ENSG00000116586:[ENST00000487106(n.341+24C>A)];ENSG00000116586:[ENST00000368304(c.231+24C>A)];ENSG00000116586:[ENST00000489664(n.324C>A)];	NA	NA	NA	NA	NA	NA	NA	NA	NA	NA	MODIFIER	NA	NA	143	143	0	149	111	38	25.5
Smoker	42479	chr1	22151240	C	T	*LDLRAD2*	3UTR	ENSG00000187942:[ENST00000344642(c.*2532C>T)];	NA	NA	NA	9.25E-06	0	NA	NA	NA	NA	NA	MODIFIER	NA	NA	54	54	0	80	64	16	20
Smoker	42479	chr1	70618294	G	C	*LRRC40*	INTRONIC	ENSG00000066557:[ENST00000370952(c.1329-68C>G)];	NA	NA	NA	NA	NA	NA	NA	NA	NA	NA	MODIFIER	NA	NA	42	41	0	67	46	20	29.85
Smoker	42479	chr1	70504712	G	T	*LRRC7*	MISSENSE	ENSG00000033122:MISSENSE[ENST00000415775(c.943G>T,Gac>Tac):ENSP00000394867(p.Asp315Tyr)];ENSG00000033122:MISSENSE[ENST00000035383(c.3091G>T,Gac>Tac):ENSP00000035383(p.Asp1031Tyr)];ENSG00000033122:MISSENSE[ENST00000310961(c.3106G>T,Gac>Tac):ENSP00000309245(p.Asp1036Tyr)];	NA	NA	NA	NA	NA	NA	NA	D	PrD	D	MODERATE	D	1	179	179	0	285	188	97	34.04
Smoker	42479	chr1	39853034	G	A	*MACF1*	SILENT	ENSG00000127603:SILENT[ENST00000372925(c.5671G>A,caG>caA):ENSP00000362016(p.Gln1891(=))];ENSG00000127603:SILENT[ENST00000361689(c.8334G>A,caG>caA):ENSP00000354573(p.Gln2778(=))];ENSG00000127603:SILENT[ENST00000289893(c.9840G>A,caG>caA):ENSP00000289893(p.Gln3280(=))];ENSG00000127603:SILENT[ENST00000564288(c.14520G>A,caG>caA):ENSP00000455274(p.Gln4840(=))];ENSG00000127603:SILENT[ENST00000539005(c.8271G>A,caG>caA):ENSP00000444364(p.Gln2757(=))];ENSG00000127603:SILENT[ENST00000372915(c.14535G>A,caG>caA):ENSP00000362006(p.Gln4845(=))];ENSG00000127603:SILENT[ENST00000317713(c.8334G>A,caG>caA):ENSP00000313438(p.Gln2778(=))];ENSG00000127603:SILENT[ENST00000567887(c.14631G>A,caG>caA):ENSP00000455823(p.Gln4877(=))];ENSG00000127603:SILENT[ENST00000545844(c.8334G>A,caG>caA):ENSP00000439537(p.Gln2778(=))];	NA	NA	NA	NA	NA	NA	NA	NA	NA	NA	LOW	NA	0.998	155	155	0	243	187	56	23.05
Smoker	42479	chr1	3477191	C	A	*MEGF6*	INTRONIC	ENSG00000162591:[ENST00000356575(c.481+19198G>T)];ENSG00000162591:[ENST00000485002(c.377-13504G>T)];	NA	NA	NA	NA	NA	NA	NA	NA	NA	NA	MODIFIER	NA	NA	77	77	0	113	65	48	42.48
Smoker	42479	chr1	151039779	C	A	*MLLT11*	MISSENSE	ENSG00000213190:MISSENSE[ENST00000368921(c.79C>A,Ctg>Atg):ENSP00000357917(p.Leu27Met)];	NA	NA	NA	NA	NA	NA	NA	T	PrD	D	MODERATE	U	0.999	67	67	0	88	66	22	25
Smoker	42479	chr1	1290928	G	A	*MXRA8*	MISSENSE	ENSG00000162576:MISSENSE[ENST00000445648(c.278C>T,tCg>tTg):ENSP00000399229(p.Ser93Leu)];ENSG00000162576:MISSENSE[ENST00000477278(c.251C>T,tCg>tTg):ENSP00000436135(p.Ser84Leu)];ENSG00000162576:MISSENSE[ENST00000309212(c.278C>T,tCg>tTg):ENSP00000307887(p.Ser93Leu)];ENSG00000162576:INTRONIC[ENST00000342753(c.74-203C>T)];	NA	NA	NA	NA	NA	NA	NA	NA	NA	NA	MODIFIER	NA	NA	25	25	0	39	27	12	30.77
Smoker	42479	chr1	121310623	G	T	*NA*	INTERGENIC		NA	NA	NA	NA	NA	NA	NA	NA	NA	NA	MODIFIER	NA	NA	177	176	1	123	25	98	79.67
Smoker	42479	chr1	248263570	G	A	*OR2L13*	MISSENSE	ENSG00000196071:MISSENSE[ENST00000358120(c.893G>A,gGg>gAg):ENSP00000350836(p.Gly298Glu)];ENSG00000196071:MISSENSE[ENST00000366478(c.893G>A,gGg>gAg):ENSP00000355434(p.Gly298Glu)];	NA	NA	NA	NA	NA	NA	NA	T	PoD	D	MODERATE	D	0.052	108	108	0	207	52	155	74.88
Smoker	42479	chr1	248059219	G	C	*OR2W3*	MISSENSE	ENSG00000238243:MISSENSE[ENST00000537741(c.331G>C,Gag>Cag):ENSP00000445853(p.Glu111Gln)];ENSG00000238243:MISSENSE[ENST00000360358(c.331G>C,Gag>Cag):ENSP00000353516(p.Glu111Gln)];	NA	NA	NA	NA	NA	NA	NA	D	PoD	D	MODERATE	D	0.022	133	133	0	204	172	32	15.69
Smoker	42479	chr1	17603048	G	A	*PADI3*	MISSENSE	ENSG00000142619:MISSENSE[ENST00000375460(c.1342G>A,Gac>Aac):ENSP00000364609(p.Asp448Asn)];	NA	NA	NA	NA	NA	NA	NA	T	BN	N	MODERATE	N	0.986	48	48	0	69	41	28	40.58
Smoker	42479	chr1	144911890	G	A	*PDE4DIP*	MISSENSE	ENSG00000178104:MISSENSE[ENST00000369356(c.2219C>T,tCc>tTc):ENSP00000358363(p.Ser740Phe)];ENSG00000178104:MISSENSE[ENST00000479408(c.1580C>T,tCc>tTc):ENSP00000436791(p.Ser527Phe)];ENSG00000178104:MISSENSE[ENST00000313431(c.2708C>T,tCc>tTc):ENSP00000316434(p.Ser903Phe)];ENSG00000178104:MISSENSE[ENST00000369351(c.2219C>T,tCc>tTc):ENSP00000358357(p.Ser740Phe)];ENSG00000178104:MISSENSE[ENST00000369354(c.2219C>T,tCc>tTc):ENSP00000358360(p.Ser740Phe)];ENSG00000178104:MISSENSE[ENST00000369349(c.2219C>T,tCc>tTc):ENSP00000358355(p.Ser740Phe)];ENSG00000178104:MISSENSE[ENST00000369359(c.2630C>T,tCc>tTc):ENSP00000358366(p.Ser877Phe)];ENSG00000178104:MISSENSE[ENST00000530740(c.2630C>T,tCc>tTc):ENSP00000435654(p.Ser877Phe)];ENSG00000178104:MISSENSE[ENST00000313382(c.2417C>T,tCc>tTc):ENSP00000327209(p.Ser806Phe)];ENSG00000178104:MISSENSE[ENST00000529945(c.2708C>T,tCc>tTc):ENSP00000433392(p.Ser903Phe)];ENSG00000178104:INTRONIC[ENST00000533963(n.9+4395C>T)];ENSG00000178104:INTRONIC[ENST00000525886(n.293+5929C>T)];ENSG00000178104:EXONIC-NC[ENST00000467859(n.1094C>T)];	NA	NA	chr1*144911890*144911890*G*C*rs782361257*NA*NA*NA	NA	NA	NA	NA	NA	PrD	NA	MODERATE	NA	NA	430	428	0	386	258	127	32.9

Mangalaparthi *et al.*, 2020. Mutational landscape of esophageal squamous cell carcinoma in an Indian cohort
Supplementary Table 4. List of somatic single nucleotide variants identified using whole exome sequencing of ESCC patients

Cohort	Sample	Chromosome	Genomic position	Reference allele	Alternate allele	Gene symbol	Variant type	Variant annotation	OncoMD	COSMIC	dbSNP	ExAC	ExAC_SAS	1000G	1000G_SAS	SIFT	Polyphen2	CONDEL	VEP	LRT	PhastCon7 Way	Total read (Adjacent normal)	Reference allel read support (Adjacent normal)	Alternative read support (Adjacent normal)	Total read (Tumor)	Reference allel read support (Tumor)	Alternative read support (Tumor)	Alternate allele frequency
Smoker	42479	chr1	7868951	A	T	*PER3*	INTRONIC-SS-ACR	ENSG00000049246:INTRONIC-SS-ACR[ENST00000377532(c.1137-2A>T)];ENSG00000049246:INTRONIC-SS-ACR[ENST00000361923(c.1134-2A>T)];ENSG00000049246:INTRONIC[ENST00000463106(n.261-1002A>T)];	NA	NA	NA	NA	NA	NA	NA	NA	NA	NA	MODIFIER	NA	NA	37	37	0	66	36	30	45.45
Smoker	42479	chr1	2337177	C	G	*PEX10*	3UTR	ENSG00000157911:[ENST00000507596(c.*28G>C)];ENSG00000157911:[ENST00000447513(c.*28G>C)];ENSG00000157911:[ENST00000288774(c.*28G>C)];	NA	NA	chr1*2337177*2337179*CTG*C*rs759013217*NA*NA*NA	NA	NA	NA	NA	NA	NA	NA	MODIFIER	NA	NA	68	68	0	103	83	20	19.42
Smoker	42479	chr1	151274767	G	C	*PI4KB*	MISSENSE	ENSG00000143393:MISSENSE[ENST00000368874(c.1483C>G,Ctt>Gtt):ENSP00000357868(p.Leu495Val)];ENSG00000143393:MISSENSE[ENST00000529142(c.532C>G,Ctt>Gtt):ENSP00000433149(p.Leu178Val)];ENSG00000143393:MISSENSE[ENST00000368873(c.1528C>G,Ctt>Gtt):ENSP00000357867(p.Leu510Val)];ENSG00000143393:MISSENSE[ENST00000368872(c.1483C>G,Ctt>Gtt):ENSP00000357866(p.Leu495Val)];ENSG00000143393:MISSENSE[ENST00000271657(c.1564C>G,Ctt>Gtt):ENSP00000271657(p.Leu522Val)];ENSG00000143393:MISSENSE[ENST00000368875(c.1564C>G,Ctt>Gtt):ENSP00000357869(p.Leu522Val)];ENSG00000143393:MISSENSE[ENST00000430800(c.532C>G,Ctt>Gtt):ENSP00000413599(p.Leu178Val)];	NA	NA	NA	NA	NA	NA	NA	D	PrD	D	MODERATE	NA	NA	54	54	0	39	30	9	23.08
Smoker	42479	chr1	107600511	G	A	*PRMT6*	3UTR	ENSG00000198890:[ENST00000370078(c.*46G>A)];ENSG00000198890:[ENST00000361318(c.*46G>A)];	NA	NA	NA	NA	NA	NA	NA	NA	NA	NA	MODIFIER	NA	NA	55	55	0	26	17	9	34.62
Smoker	42479	chr1	167673932	G	A	*RCSD1*	SILENT-STOPCODO	ENSG00000198771:SILENT-STOPCODON[ENST00000537350(c.1160G>A,tGa>tAa):ENSP00000439409(p.Ter387Ter)];ENSG00000198771:SILENT-STOPCODON[ENST00000367854(c.1250G>A,tGa>tAa):ENSP00000356828(p.Ter417Ter)];	NA	NA	NA	NA	NA	NA	NA	NA	NA	NA	LOW	NA	NA	120	120	0	114	86	28	24.56
Smoker	42479	chr1	241519020	G	T	*RGS7*	SILENT	ENSG00000182901:SILENT[ENST00000401882(c.57C>A,ccC>ccA):ENSP00000385508(p.Pro19(=))];ENSG00000182901:SILENT[ENST00000366564(c.57C>A,ccC>ccA):ENSP00000355522(p.Pro19(=))];ENSG00000182901:SILENT[ENST00000366562(c.57C>A,ccC>ccA):ENSP00000355520(p.Pro19(=))];ENSG00000182901:SILENT[ENST00000366565(c.57C>A,ccC>ccA):ENSP00000355523(p.Pro19(=))];ENSG00000182901:SILENT[ENST00000407727(c.57C>A,ccC>ccA):ENSP00000384428(p.Pro19(=))];ENSG00000182901:SILENT[ENST00000366563(c.57C>A,ccC>ccA):ENSP00000355521(p.Pro19(=))];ENSG00000182901:SILENT[ENST00000348120(c.57C>A,ccC>ccA):ENSP00000341242(p.Pro19(=))];	NA	NA	NA	NA	NA	NA	NA	NA	NA	NA	LOW	NA	NA	109	109	0	131	105	26	19.85
Smoker	42479	chr1	40704965	A	T	*RLF*	NONSENSE	ENSG00000117000:NONSENSE[ENST00000372771(c.4591A>T,Aaa>Taa):ENSP00000361857(p.Lys1531Ter)];	NA	NA	NA	NA	NA	NA	NA	NA	NA	NA	HIGH	NA	0.989	177	176	0	229	185	44	19.21
Smoker	42479	chr1	23376988	T	C	*RP1-184J9.2*	INTRONIC	ENSG00000240553:[ENST00000427154(n.262-29944A>G)];	NA	NA	NA	NA	NA	NA	NA	NA	NA	NA	MODIFIER	NA	NA	199	198	1	257	208	49	19.07
Smoker	42479	chr1	151139044	G	C	*SCNM1*	INTRONIC	ENSG00000163156:[ENST00000602841(c.17+27G>C)];ENSG00000163156:[ENST00000368905(c.122+27G>C)];ENSG00000163156:[ENST00000461862(n.253+27G>C)];ENSG00000163156:[ENST00000368902(c.17+27G>C)];ENSG00000163156:[ENST00000471039(n.236G>C)];	NA	NA	chr1*151139043*151139045*TGA*T*rs781128389*NA*NA*NA	NA	NA	NA	NA	NA	NA	NA	MODIFIER	NA	NA	48	48	0	28	20	8	28.57
Smoker	42479	chr1	169697397	C	T	*SELE*	INTRONIC	ENSG00000007908:[ENST00000367776(c.1091-329G>A)];ENSG00000007908:[ENST00000367779(c.1091-731G>A)];ENSG00000007908:[ENST00000367775(c.905-329G>A)];ENSG00000007908:[ENST00000367781(c.1091-329G>A)];ENSG00000007908:[ENST00000367780(c.905-329G>A)];ENSG00000007908:[ENST00000367774(c.1091-731G>A)];ENSG00000007908:[ENST00000333360(c.1091-10G>A)];ENSG00000007908:[ENST00000367782(c.1091-10G>A)];ENSG00000007908:[ENST00000367777(c.1091-10G>A)];	NA	NA	NA	NA	NA	NA	NA	NA	NA	NA	MODIFIER	NA	NA	95	95	0	78	62	16	20.51
Smoker	42479	chr1	158647473	G	C	*SPTA1*	INTRONIC-SS-DNR	ENSG00000163554:[ENST00000368147(c.957+7C>G)];	NA	NA	chr1*158647473*158647473*G*A*rs369937649*NA*NA*NA	NA	NA	NA	NA	NA	NA	NA	LOW	NA	NA	155	155	0	154	117	37	24.03
Smoker	42479	chr1	77510272	C	A	*ST6GALNAC5*	SILENT	ENSG00000117069:EXONIC-NC[ENST00000488940(n.448C>A)];ENSG00000117069:SILENT[ENST00000477717(c.645C>A,ctC>ctA):ENSP00000417583(p.Leu215(=))];ENSG00000117069:SILENT[ENST00000318803(c.645C>A,ctC>ctA):ENSP00000436263(p.Leu215(=))];	NA	NA	NA	9.28E-06	0	NA	NA	NA	NA	NA	LOW	NA	NA	98	98	0	109	56	53	48.62
Smoker	42479	chr1	45879507	C	A	*TESK2*	STOPLOSS	ENSG00000070759:STOPLOSS[ENST00000451835(c.380G>T,tGa>tTa):ENSP00000397244(p.Ter127LeuextTer2)];ENSG00000070759:INTRONIC[ENST00000493974(n.180+7890G>T)];ENSG00000070759:INTRONIC[ENST00000341771(c.344+7890G>T)];ENSG00000070759:INTRONIC[ENST00000372086(c.344+7890G>T)];ENSG00000070759:INTRONIC[ENST00000486676(n.740+7890G>T)];ENSG00000070759:INTRONIC[ENST00000538496(c.95+7890G>T)];ENSG00000070759:INTRONIC[ENST00000372084(c.344+7890G>T)];	NA	NA	NA	NA	NA	NA	NA	NA	NA	NA	MODIFIER	NA	NA	59	59	0	91	77	14	15.38
Smoker	42479	chr1	43779668	A	G	*TIE1*	INTRONIC	ENSG00000066056:[ENST00000461061(n.429+29A>G)];ENSG00000066056:[ENST00000372476(c.2409+29A>G)];ENSG00000066056:[ENST00000473014(n.677+29A>G)];ENSG00000066056:[ENST00000433781(c.1344+29A>G)];ENSG00000066056:[ENST00000471187(n.472A>G)];	NA	NA	NA	NA	NA	NA	NA	NA	NA	NA	MODIFIER	NA	NA	98	97	0	87	55	31	35.63
Smoker	42479	chr1	62149153	C	G	*TM2D1*	SILENT	ENSG00000162604:SILENT[ENST00000606498(c.579G>C,ctG>ctC):ENSP00000475700(p.Leu193(=))];ENSG00000162604:SILENT[ENST00000294613(c.579G>C,ctG>ctC):ENSP00000294613(p.Leu193(=))];ENSG00000162604:SILENT[ENST00000371180(c.765G>C,ctG>ctC):ENSP00000360222(p.Leu255(=))];ENSG00000162604:3UTR[ENST00000488206(c.*437G>C)];ENSG00000162604:EXONIC-NC[ENST00000472357(n.377G>C)];ENSG00000162604:EXONIC-NC[ENST00000488410(n.447G>C)];ENSG00000162604:EXONIC-NC[ENST00000472989(n.322G>C)];	NA	NA	NA	NA	NA	NA	NA	NA	NA	NA	MODIFIER	NA	NA	40	40	0	39	21	18	46.15
Smoker	42479	chr1	109635610	G	A	*TMEM167B*	MISSENSE	ENSG00000215717:MISSENSE[ENST00000338272(c.109G>A,Gag>Aag):ENSP00000342148(p.Glu37Lys)];ENSG00000215717:EXONIC-NC[ENST00000479160(n.291G>A)];ENSG00000215717:EXONIC-NC[ENST00000473828(n.176G>A)];	NA	NA	NA	NA	NA	NA	NA	NA	NA	NA	MODIFIER	NA	NA	109	109	0	79	15	64	81.01
Smoker	42479	chr1	226055766	G	C	*TMEM63A*	INTRONIC	ENSG00000196187:[ENST00000366835(c.372-36C>G)];	NA	NA	NA	NA	NA	NA	NA	NA	NA	NA	MODIFIER	NA	NA	27	27	0	29	21	8	27.59
Smoker	42479	chr1	175365874	G	C	*TNR*	MISSENSE	ENSG00000116147:MISSENSE[ENST00000422274(c.219C>G,cCg>cGg):ENSP00000403413(p.Pro74Arg)];ENSG00000116147:MISSENSE[ENST00000367674(c.1046C>G,cCg>cGg):ENSP00000356646(p.Pro349Arg)];ENSG00000116147:MISSENSE[ENST00000263525(c.1046C>G,cCg>cGg):ENSP00000263525(p.Pro349Arg)];	NA	NA	NA	NA	NA	NA	NA	T	PrD	D	MODERATE	D	1	70	70	0	67	51	16	23.88
Smoker	42479	chr1	17197581	C	A	*U1*	EXONIC-NC	ENSG00000228549:[ENST00000438002(n.142C>A)];	NA	NA	NA	NA	NA	NA	NA	NA	NA	NA	MODIFIER	NA	NA	355	351	0	480	428	44	9.17

Mangalaparthi *et al.* , 2020. Mutational landscape of esophageal squamous cell carcinoma in an Indian cohort
Supplementary Table 4. List of somatic single nucleotide variants identified using whole exome sequencing of ESCC patients

Cohort	Sample	Chromosome	Genomic position	Reference allele	Alternate allele	Gene symbol	Variant type	Variant annotation	OncoMD	COSMIC	dbSNP	ExAC	ExAC_SAS	1000G	1000G_SAS	SIFT	Polyphen2	CONDEL	VEP	LRT	PhastCon7 Way	Total read (Adjacent normal)	Reference allel read support (Adjacent normal)	Alternative read support (Adjacent normal)	Total read (Tumor)	Reference allel read support (Tumor)	Alternative read support (Tumor)	Alternate allele frequency
Smoker	42479	chr1	154242817	C	G	*UBAP2L*	3UTR	ENSG00000143569:[ENST00000428595(c.*46C>G)];ENSG00000143569:[ENST00000361546(c.*46C>G)];ENSG00000143569:[ENST00000428931(c.*46C>G)];ENSG00000143569:[ENST00000433615(c.1160-540C>G)];ENSG00000143569:[ENST00000271877(c.3199-540C>G)];ENSG00000143569:[ENST00000493867(n.385-540C>G)];	NA	NA	chr1*154242817*154242817*C*T*rs754745944*NA*NA*NA	NA	NA	NA	NA	NA	NA	NA	MODIFIER	NA	NA	106	105	0	110	78	32	29.09
Smoker	42479	chr1	154530735	C	G	*UBE2Q1*	MISSENSE	ENSG00000160714:MISSENSE[ENST00000292211(c.295G>C,Ggg>Cgg):ENSP00000292211(p.Gly99Arg)];ENSG00000160714:EXONIC-NC[ENST00000497453(n.228G>C)];	NA	NA	NA	NA	NA	NA	NA	NA	NA	NA	MODIFIER	NA	NA	30	30	0	52	45	7	13.46
Smoker	42479	chr1	55599760	C	G	*USP24*	MISSENSE	ENSG00000162402:MISSENSE[ENST00000294383(c.3364G>C,Gat>Cat):ENSP00000294383(p.Asp1122His)];ENSG00000162402:MISSENSE[ENST00000407756(c.2884G>C,Gat>Cat):ENSP00000385700(p.Asp962His)];	NA	NA	NA	NA	NA	NA	NA	NA	BN	NA	MODERATE	N	0.999	184	184	0	196	157	39	19.9
Smoker	42479	chr1	101194700	G	T	*VCAM1*	SILENT	ENSG00000162692:INTRONIC[ENST00000347652(c.929-2054G>T)];ENSG00000162692:INTRONIC[ENST00000370115(c.929-3274G>T)];ENSG00000162692:SILENT[ENST00000294728(c.966G>T,cgG>cgT):ENSP00000294728(p.Arg322(=))];ENSG00000162692:SILENT[ENST00000370119(c.780G>T,cgG>cgT):ENSP00000359137(p.Arg260(=))];	NA	NA	NA	NA	NA	NA	NA	NA	NA	NA	LOW	NA	NA	175	174	0	175	96	79	45.14
Smoker	42479	chr10	1405675	C	A	*ADARB2*	MISSENSE	ENSG00000185736:MISSENSE[ENST00000381312(c.625G>T,Ggc>Tgc):ENSP00000370713(p.Gly209Cys)];	NA	NA	NA	NA	NA	NA	NA	T	BN	N	MODERATE	N	0.049	152	151	1	253	129	124	49.01
Smoker	42479	chr10	106121891	G	T	*CCDC147*	SILENT	ENSG00000120051:5UTR[ENST00000312902(c.-733G>T)];ENSG00000120051:SILENT[ENST00000369704(c.402G>T,gtG>gtT):ENSP00000358718(p.Val134(=))];	NA	NA	NA	NA	NA	NA	NA	NA	NA	NA	LOW	NA	NA	84	83	1	69	15	54	78.26
Smoker	42479	chr10	35842090	G	A	*CCNY*	SILENT	ENSG00000108100:EXONIC-NC[ENST00000492478(n.788G>A)];ENSG00000108100:EXONIC-NC[ENST00000465416(n.303G>A)];ENSG00000108100:EXONIC-NC[ENST00000497692(n.192G>A)];ENSG00000108100:SILENT[ENST00000374704(c.723G>A,ctG>ctA):ENSP00000363836(p.Leu241(=))];ENSG00000108100:SILENT[ENST00000265375(c.561G>A,ctG>ctA):ENSP00000265375(p.Leu187(=))];ENSG00000108100:SILENT[ENST00000339497(c.648G>A,ctG>ctA):ENSP00000344275(p.Leu216(=))];ENSG00000108100:SILENT[ENST00000374706(c.561G>A,ctG>ctA):ENSP00000363838(p.Leu187(=))];	NA	NA	NA	NA	NA	NA	NA	NA	NA	NA	LOW	NA	NA	41	41	0	63	44	19	30.16
Smoker	42479	chr10	17164632	T	A	*CUBN*	INTRONIC	ENSG00000107611:[ENST00000433666(c.255-48A>T)];ENSG00000107611:[ENST00000377833(c.593+162A>T)];	NA	NA	chr10*17164632*17164633*TA*T*rs777164925*NA*NA*NA	NA	NA	NA	NA	NA	NA	NA	MODIFIER	NA	NA	90	90	0	121	89	32	26.45
Smoker	42479	chr10	135368939	C	T	*CYP2E1*	EXONIC-NC	ENSG00000130649:[ENST00000368520(n.2133C>T)];	NA	NA	NA	NA	NA	NA	NA	NA	NA	NA	MODIFIER	NA	NA	36	36	0	31	22	9	29.03
Smoker	42479	chr10	127413886	G	C	*EDRF1*	INTRONIC-SS-ACR	ENSG00000107938:INTRONIC-SS-ACR[ENST00000368815(c.511-1G>C)];ENSG00000107938:INTRONIC-SS-ACR[ENST00000337623(c.511-1G>C)];ENSG00000107938:INTRONIC-SS-ACR[ENST00000525091(n.617-1G>C)];ENSG00000107938:INTRONIC-SS-ACR[ENST00000481600(c.511-1G>C)];ENSG00000107938:INTRONIC-SS-ACR[ENST00000419769(c.511-1G>C)];ENSG00000107938:INTRONIC-SS-ACR[ENST00000356792(c.511-1G>C)];	NA	NA	NA	NA	NA	NA	NA	NA	NA	NA	HIGH	NA	NA	107	107	0	61	8	52	85.25
Smoker	42479	chr10	64967797	G	A	*JMJD1C*	MISSENSE	ENSG00000171988:MISSENSE[ENST00000399251(c.2975C>T,cCa>cTa):ENSP00000382195(p.Pro992Leu)];ENSG00000171988:MISSENSE[ENST00000399262(c.3632C>T,cCa>cTa):ENSP00000382204(p.Pro1211Leu)];ENSG00000171988:MISSENSE[ENST00000542921(c.3086C>T,cCa>cTa):ENSP00000444682(p.Pro1029Leu)];ENSG00000171988:MISSENSE[ENST00000402544(c.2975C>T,cCa>cTa):ENSP00000384990(p.Pro992Leu)];	NA	NA	NA	NA	NA	NA	NA	D	PrD	D	MODERATE	D	0.999	158	157	0	1150	418	731	63.57
Smoker	42479	chr10	94409666	G	C	*KIF11*	MISSENSE	ENSG00000138160:MISSENSE[ENST00000260731(c.2845G>C,Gat>Cat):ENSP00000260731(p.Asp949His)];	NA	NA	NA	8.25E-06	0	NA	NA	D	BN	N	MODERATE	N	0.963	211	211	0	149	86	63	42.28
Smoker	42479	chr10	30918485	G	A	*LYZL2*	INTRONIC	ENSG00000151033:[ENST00000375318(c.113+37C>T)];	NA	NA	NA	1.78E-05	6.66E-05	NA	NA	NA	NA	NA	MODIFIER	NA	NA	160	160	0	224	185	38	16.96
Smoker	42479	chr10	26482179	G	A	*MYO3A*	MISSENSE	ENSG00000095777:MISSENSE[ENST00000265944(c.4484G>A,cGa>cAa):ENSP00000265944(p.Arg1495Gln)];ENSG00000095777:INTRONIC[ENST00000543632(c.1777-18593G>A)];	NA	NA	0.000599	4.16E-05	0	0.000599	0	NA	NA	NA	MODIFIER	NA	NA	92	92	0	128	116	12	9.38
Smoker	42479	chr10	55698611	C	T	*PCDH15*	MISSENSE	ENSG00000150275:MISSENSE[ENST00000437009(c.3124G>A,Gaa>Aaa):ENSP00000412628(p.Glu1042Lys)];ENSG00000150275:MISSENSE[ENST00000395433(c.3271G>A,Gaa>Aaa):ENSP00000378821(p.Glu1091Lys)];ENSG00000150275:MISSENSE[ENST00000395445(c.3358G>A,Gaa>Aaa):ENSP00000378832(p.Glu1120Lys)];ENSG00000150275:MISSENSE[ENST00000320301(c.3337G>A,Gaa>Aaa):ENSP00000322604(p.Glu1113Lys)];ENSG00000150275:MISSENSE[ENST00000409834(c.2170G>A,Gaa>Aaa):ENSP00000386693(p.Glu724Lys)];ENSG00000150275:MISSENSE[ENST00000395432(c.3226G>A,Gaa>Aaa):ENSP00000378820(p.Glu1076Lys)];ENSG00000150275:MISSENSE[ENST00000361849(c.3337G>A,Gaa>Aaa):ENSP00000354950(p.Glu1113Lys)];ENSG00000150275:MISSENSE[ENST00000395438(c.3337G>A,Gaa>Aaa):ENSP00000378826(p.Glu1113Lys)];ENSG00000150275:MISSENSE[ENST00000414778(c.3352G>A,Gaa>Aaa):ENSP00000410304(p.Glu1118Lys)];ENSG00000150275:MISSENSE[ENST00000373965(c.3358G>A,Gaa>Aaa):ENSP00000363076(p.Glu1120Lys)];ENSG00000150275:MISSENSE[ENST00000395430(c.3337G>A,Gaa>Aaa):ENSP00000378818(p.Glu1113Lys)];ENSG00000150275:3UTR[ENST00000448885(c.*1292G>A)];ENSG00000150275:3UTR[ENST00000373956(c.*1479G>A)];ENSG00000150275:INTRONIC[ENST00000373957(c.2155-116718G>A)];ENSG00000150275:INTRONIC[ENST00000495484(n.368-81588G>A)];ENSG00000150275:INTRONIC[ENST00000395440(c.1306-129305G>A)];ENSG00000150275:INTRONIC[ENST00000414367(c.877-98355G>A)];ENSG00000150275:INTRONIC[ENST00000395446(c.2092-129305G>A)];ENSG00000150275:INTRONIC[ENST00000395442(c.1099-129305G>A)];	NA	chr10*5569	NA	NA	NA	NA	NA	T	BN	N	MODERATE	NA	NA	200	200	0	139	104	35	25.18
Smoker	42479	chr10	135368939	C	T	*SPRN*	INTRONIC	ENSG00000203772:[ENST00000541506(c.-17+13837G>A)];	NA	NA	NA	NA	NA	NA	NA	NA	NA	NA	MODIFIER	NA	NA	36	36	0	31	22	9	29.03
Smoker	42479	chr10	135368939	C	T	*SYCE1*	SILENT	ENSG00000171772:SILENT[ENST00000303903(c.834G>A,ctG>ctA):ENSP00000303978(p.Leu278(=))];ENSG00000171772:SILENT[ENST00000432597(c.726G>A,ctG>ctA):ENSP00000411779(p.Leu242(=))];ENSG00000171772:SILENT[ENST00000343131(c.834G>A,ctG>ctA):ENSP00000341282(p.Leu278(=))];ENSG00000171772:SILENT[ENST00000368517(c.726G>A,ctG>ctA):ENSP00000357503(p.Leu242(=))];ENSG00000171772:EXONIC-NC[ENST00000479535(n.1967G>A)];	NA	NA	NA	NA	NA	NA	NA	NA	NA	NA	MODIFIER	NA	NA	36	36	0	31	22	9	29.03
Smoker	42479	chr10	134698709	C	G	*TTC40*	MISSENSE	ENSG00000171811:MISSENSE[ENST00000368582(c.3525G>C,gaG>gaC):ENSP00000357571(p.Glu1175Asp)];ENSG00000171811:MISSENSE[ENST00000368586(c.3525G>C,gaG>gaC):ENSP00000357575(p.Glu1175Asp)];	NA	NA	NA	NA	NA	NA	NA	D	PoD	D	MODERATE	NA	NA	27	27	0	52	4	48	92.31

Mangalaparthi *et al.* , 2020. Mutational landscape of esophageal squamous cell carcinoma in an Indian cohort
Supplementary Table 4. List of somatic single nucleotide variants identified using whole exome sequencing of ESCC patients

Cohort	Sample	Chromosome	Genomic position	Reference allele	Alternate allele	Gene symbol	Variant type	Variant annotation	OncoMD	COSMIC	dbSNP	ExAC	ExAC_SAS	1000G	1000G_SAS	SIFT	Polyphen2	CONDEL	VEP	LRT	PhastCon7 Way	Total read (Adjacent normal)	Reference allel read support (Adjacent normal)	Alternative read support (Adjacent normal)	Total read (Tumor)	Reference allel read support (Tumor)	Alternative read support (Tumor)	Alternate allele frequency
Smoker	42479	chr11	134253627	C	T	*B3GAT1*	MISSENSE	ENSG00000109956:MISSENSE[ENST00000524765(c.568G>A,Gtg>Atg):ENSP00000433847(p.Val190Met)];ENSG00000109956:MISSENSE[ENST00000537389(c.607G>A,Gtg>Atg):ENSP00000445983(p.Val203Met)];ENSG00000109956:MISSENSE[ENST00000312527(c.568G>A,Gtg>Atg):ENSP00000307875(p.Val190Met)];ENSG00000109956:MISSENSE[ENST00000392580(c.568G>A,Gtg>Atg):ENSP00000376359(p.Val190Met)];ENSG00000109956:EXONIC-NC[ENST00000531778(n.3465G>A)];	NA	NA	NA	NA	NA	NA	NA	NA	NA	NA	MODIFIER	NA	NA	35	35	0	37	22	15	40.54
Smoker	42479	chr11	64111884	C	G	*CCDC88B*	MISSENSE	ENSG00000168071:EXONIC-NC[ENST00000494566(n.891C>G)];ENSG00000168071:EXONIC-NC[ENST00000463837(n.1915C>G)];ENSG00000168071:EXONIC-NC[ENST00000492980(n.630C>G)];ENSG00000168071:EXONIC-NC[ENST00000494080(n.1353C>G)];ENSG00000168071:5UTR[ENST00000301897(c.-1974C>G)];ENSG00000168071:MISSENSE[ENST00000356786(c.1871C>G,aCa>aGa):ENSP00000349238(p.Thr624Arg)];	NA	NA	chr11*64111884*64111884*C*T*rs753176045*NA*NA*NA	NA	NA	NA	NA	T_lc	BN	N	MODERATE	NA	NA	59	58	0	77	37	39	50.65
Smoker	42479	chr11	103908018	T	C	*DDI1*	SILENT	ENSG00000170967:SILENT[ENST00000302259(c.468T>C,gaT>gaC):ENSP00000302805(p.Asp156(=))];	NA	NA	NA	NA	NA	NA	NA	NA	NA	NA	MODIFIER	NA	NA	165	164	0	119	26	93	78.15
Smoker	42479	chr11	47758281	C	T	*FNBP4*	MISSENSE	ENSG00000109920:MISSENSE[ENST00000263773(c.1468G>A,Gaa>Aaa):ENSP00000263773(p.Glu490Lys)];ENSG00000109920:EXONIC-NC[ENST00000527894(n.203G>A)];ENSG00000109920:EXONIC-NC[ENST00000528388(n.75G>A)];ENSG00000109920:EXONIC-NC[ENST00000534003(n.1466G>A)];	NA	NA	NA	NA	NA	NA	NA	NA	NA	NA	MODIFIER	NA	NA	55	55	0	55	40	15	27.27
Smoker	42479	chr11	49175969	C	A	*FOLH1*	MISSENSE	ENSG00000086205:MISSENSE[ENST00000340334(c.1654G>T,Gat>Tat):ENSP00000344131(p.Asp552Tyr)];ENSG00000086205:MISSENSE[ENST00000356696(c.1699G>T,Gat>Tat):ENSP00000349129(p.Asp567Tyr)];ENSG00000086205:MISSENSE[ENST00000256999(c.1699G>T,Gat>Tat):ENSP00000256999(p.Asp567Tyr)];ENSG00000086205:MISSENSE[ENST00000533034(c.1654G>T,Gat>Tat):ENSP00000431463(p.Asp552Tyr)];ENSG00000086205:MISSENSE[ENST00000343844(c.775G>T,Gat>Tat):ENSP00000344086(p.Asp259Tyr)];ENSG00000086205:EXONIC-NC[ENST00000458311(n.1301G>T)];ENSG00000086205:EXONIC-NC[ENST00000532018(n.113G>T)];ENSG00000086205:3UTR[ENST00000525826(c.*1100G>T)];	NA	NA	NA	NA	NA	NA	NA	NA	NA	NA	MODIFIER	NA	NA	89	87	1	88	69	19	21.59
Smoker	42479	chr11	17758142	G	A	*KCNC1*	INTRONIC	ENSG00000129159:[ENST00000379472(c.570+23G>A)];ENSG00000129159:[ENST00000265969(c.570+23G>A)];	NA	NA	NA	NA	NA	NA	NA	NA	NA	NA	MODIFIER	NA	NA	53	53	0	79	52	27	34.18
Smoker	42479	chr11	30439163	C	G	*MPPED2*	MISSENSE	ENSG00000066382:MISSENSE[ENST00000358117(c.554G>C,gGa>gCa):ENSP00000350833(p.Gly185Ala)];ENSG00000066382:MISSENSE[ENST00000448418(c.554G>C,gGa>gCa):ENSP00000388258(p.Gly185Ala)];ENSG00000066382:EXONIC-NC[ENST00000529220(n.158G>C)];ENSG00000066382:EXONIC-NC[ENST00000524667(n.70G>C)];ENSG00000066382:3UTR[ENST00000526437(c.*298G>C)];	NA	NA	NA	NA	NA	NA	NA	NA	NA	NA	MODIFIER	NA	NA	56	56	0	60	46	14	23.33
Smoker	42479	chr11	1101904	C	G	*MUC2*	INTRONIC-SS-ACR	ENSG00000198788:[ENST00000441003(c.7676-8C>G)];	NA	NA	NA	NA	NA	NA	NA	NA	NA	NA	LOW	NA	NA	51	51	0	26	5	21	80.77
Smoker	42479	chr11	76841623	C	G	*MYO7A*	INTRONIC	ENSG00000137474:[ENST00000409893(c.-46-12C>G)];ENSG00000137474:[ENST00000409619(c.-159-12C>G)];ENSG00000137474:[ENST00000409709(c.-46-12C>G)];ENSG00000137474:[ENST00000458637(c.-46-12C>G)];	NA	NA	NA	NA	NA	NA	NA	NA	NA	NA	MODIFIER	NA	NA	70	70	0	90	76	14	15.56
Smoker	42479	chr11	5142409	G	C	*OR52A4*	EXONIC-NC	ENSG00000205494:[ENST00000498233(n.990C>G)];ENSG00000205494:[ENST00000472867(n.400C>G)];ENSG00000205494:[ENST00000380369(n.439C>G)];ENSG00000205494:[ENST00000481634(n.775C>G)];	NA	NA	NA	NA	NA	NA	NA	NA	NA	NA	MODIFIER	NA	NA	137	137	0	84	11	73	86.9
Smoker	42479	chr11	59368790	C	G	*OSBP*	MISSENSE	ENSG00000110048:MISSENSE[ENST00000263847(c.1090G>C,Gag>Cag):ENSP00000263847(p.Glu364Gln)];	NA	NA	NA	NA	NA	NA	NA	D	PoD	D	MODERATE	D	0.997	235	234	1	290	227	63	21.72
Smoker	42479	chr11	103908018	T	C	*PDGFD*	INTRONIC	ENSG00000170962:[ENST00000302251(c.125-37053A>G)];ENSG00000170962:[ENST00000393158(c.125-37035A>G)];	NA	NA	NA	NA	NA	NA	NA	NA	NA	NA	MODIFIER	NA	NA	165	164	0	119	26	93	78.15
Smoker	42479	chr11	45204361	C	G	*PRDM11*	INTRONIC	ENSG00000019485:[ENST00000530656(c.326-51C>G)];ENSG00000019485:[ENST00000526442(c.224-51C>G)];ENSG00000019485:[ENST00000263765(c.326-51C>G)];ENSG00000019485:[ENST00000534751(c.114-51C>G)];ENSG00000019485:[ENST00000424263(c.224-51C>G)];	NA	NA	NA	NA	NA	NA	NA	NA	NA	NA	MODIFIER	NA	NA	55	55	0	114	57	57	50
Smoker	42479	chr11	36595649	G	C	*RAG1*	MISSENSE	ENSG00000166349:MISSENSE[ENST00000299440(c.795G>C,aaG>aaC):ENSP00000299440(p.Lys265Asn)];ENSG00000166349:MISSENSE[ENST00000534663(c.795G>C,aaG>aaC):ENSP00000434610(p.Lys265Asn)];	NA	NA	NA	NA	NA	NA	NA	T_lc	BN	N	MODERATE	NA	NA	121	121	0	158	127	31	19.62
Smoker	42479	chr11	36596423	G	T	*RAG1*	MISSENSE	ENSG00000166349:MISSENSE[ENST00000299440(c.1569G>T,caG>caT):ENSP00000299440(p.Gln523His)];ENSG00000166349:MISSENSE[ENST00000534663(c.1569G>T,caG>caT):ENSP00000434610(p.Gln523His)];	chr11*3659	chr11*3659	NA	NA	NA	NA	NA	D_lc	BN	N	MODERATE	NA	NA	85	85	0	122	66	56	45.9
Smoker	42479	chr11	66824488	G	C	*RHOD*	MISSENSE	ENSG00000173156:MISSENSE[ENST00000308831(c.115G>C,Gat>Cat):ENSP00000308576(p.Asp39His)];ENSG00000173156:MISSENSE[ENST00000532559(c.115G>C,Gat>Cat):ENSP00000432003(p.Asp39His)];ENSG00000173156:MISSENSE[ENST00000533360(c.115G>C,Gat>Cat):ENSP00000431167(p.Asp39His)];	NA	NA	NA	7.19E-05	0	NA	NA	D	PoD	D	MODERATE	N	0.855	26	26	0	47	23	24	51.06
Smoker	42479	chr11	62744718	A	C	*SLC22A6*	SILENT	ENSG00000197901:SILENT[ENST00000377871(c.1503T>G,acT>acG):ENSP00000367102(p.Thr501(=))];ENSG00000197901:SILENT[ENST00000421062(c.1371T>G,acT>acG):ENSP00000404441(p.Thr457(=))];ENSG00000197901:SILENT[ENST00000458333(c.1371T>G,acT>acG):ENSP00000396401(p.Thr457(=))];ENSG00000197901:SILENT[ENST00000360421(c.1503T>G,acT>acG):ENSP00000353597(p.Thr501(=))];ENSG00000197901:3UTR[ENST00000540654(c.*694T>G)];	NA	NA	NA	NA	NA	NA	NA	NA	NA	NA	LOW	NA	NA	55	55	0	82	40	42	51.22
Smoker	42479	chr11	33076184	C	T	*TCP11L1*	MISSENSE	ENSG00000176148:MISSENSE[ENST00000528107(c.209C>T,gCg>gTg):ENSP00000431200(p.Ala70Val)];ENSG00000176148:MISSENSE[ENST00000531632(c.209C>T,gCg>gTg):ENSP00000433067(p.Ala70Val)];ENSG00000176148:MISSENSE[ENST00000432887(c.209C>T,gCg>gTg):ENSP00000395070(p.Ala70Val)];ENSG00000176148:MISSENSE[ENST00000334274(c.209C>T,gCg>gTg):ENSP00000335595(p.Ala70Val)];ENSG00000176148:MISSENSE[ENST00000527661(c.209C>T,gCg>gTg):ENSP00000435667(p.Ala70Val)];ENSG00000176148:MISSENSE[ENST00000530419(c.209C>T,gCg>gTg):ENSP00000436428(p.Ala70Val)];ENSG00000176148:MISSENSE[ENST00000602733(c.209C>T,gCg>gTg):ENSP00000473628(p.Ala70Val)];ENSG00000176148:INTRONIC[ENST00000530171(n.394-3281C>T)];	NA	NA	NA	NA	NA	NA	NA	T	BN	N	MODERATE	NA	NA	112	112	0	110	53	57	51.82
Smoker	42479	chr11	78602429	G	A	*TENM4*	INTRONIC	ENSG00000149256:[ENST00000278550(c.750-48C>T)];ENSG00000149256:[ENST00000533013(n.334-48C>T)];ENSG00000149256:[ENST00000533074(c.285-48C>T)];	NA	NA	NA	NA	NA	NA	NA	NA	NA	NA	MODIFIER	NA	NA	93	92	0	187	160	27	14.44

Mangalaparthi *et al.* , 2020. Mutational landscape of esophageal squamous cell carcinoma in an Indian cohort
Supplementary Table 4. List of somatic single nucleotide variants identified using whole exome sequencing of ESCC patients

Cohort	Sample	Chromosome	Genomic position	Reference allele	Alternate allele	Gene symbol	Variant type	Variant annotation	OncoMD	COSMIC	dbSNP	ExAC	ExAC_SAS	1000G	1000G_SAS	SIFT	Polyphen2	CONDEL	VEP	LRT	PhastCon7 Way	Total read (Adjacent normal)	Reference allel read support (Adjacent normal)	Alternative read support (Adjacent normal)	Total read (Tumor)	Reference allel read support (Tumor)	Alternative read support (Tumor)	Alternate allele frequency
Smoker	42479	chr11	60701232	A	T	*TMEM132A*	EXONIC-NC	ENSG00000006118:[ENST00000539899(n.177A>T)];ENSG00000006118:[ENST00000544098(n.3124A>T)];ENSG00000006118:[ENST00000534983(n.1630A>T)];ENSG00000006118:[ENST00000536409(c.333+16A>T)];ENSG00000006118:[ENST00000005286(c.1562+16A>T)];ENSG00000006118:[ENST00000453848(c.1559+16A>T)];	NA	NA	NA	NA	NA	NA	NA	NA	NA	NA	MODIFIER	NA	NA	23	23	0	37	24	13	35.14
Smoker	42479	chr11	35685365	C	T	*TRIM44*	INTRONIC	ENSG00000166326:[ENST00000299413(c.669+37C>T)];	NA	NA	NA	NA	NA	NA	NA	NA	NA	NA	MODIFIER	NA	NA	83	83	0	105	70	35	33.33
Smoker	42479	chr11	73686673	G	A	*UCP2*	SILENT	ENSG00000175567:SILENT[ENST00000545212(c.291C>T,ttC>ttT):ENSP00000439706(p.Phe97(=))];ENSG00000175567:SILENT[ENST00000544615(c.597C>T,ttC>ttT):ENSP00000439951(p.Phe199(=))];ENSG00000175567:SILENT[ENST00000310473(c.678C>T,ttC>ttT):ENSP00000312029(p.Phe226(=))];ENSG00000175567:INTRONIC[ENST00000536983(c.635-507C>T)];	NA	NA	NA	NA	NA	NA	NA	NA	NA	NA	LOW	NA	1	55	55	0	104	91	13	12.5
Smoker	42479	chr11	57466759	G	T	*ZDHHC5*	SILENT	ENSG00000156599:EXONIC-NC[ENST00000529480(n.2098G>T)];ENSG00000156599:SILENT[ENST00000287169(c.1851G>T,cgG>cgT):ENSP00000287169(p.Arg617(=))];ENSG00000156599:SILENT[ENST00000527985(c.1692G>T,cgG>cgT):ENSP00000432202(p.Arg564(=))];	NA	NA	NA	NA	NA	NA	NA	NA	NA	NA	LOW	NA	NA	152	149	3	220	125	95	43.18
Smoker	42479	chr12	109644686	G	A	*ACACB*	INTRONIC	ENSG00000076555:[ENST00000377854(c.3060+25G>A)];ENSG00000076555:[ENST00000377848(c.3060+25G>A)];ENSG00000076555:[ENST00000338432(c.3060+25G>A)];	NA	NA	0.0007987	6.60E-05	0.00012133	0.0007987	0	NA	NA	NA	MODIFIER	NA	NA	66	66	0	58	53	5	8.62
Smoker	42479	chr12	43750356	C	T	*ADAMTS20*	MISSENSE	ENSG00000173157:MISSENSE[ENST00000389420(c.5574G>A,atG>atA):ENSP00000374071(p.Met1858Ile)];	NA	NA	NA	NA	NA	NA	NA	D	BN	D	MODERATE	N	0.994	174	173	0	121	91	30	24.79
Smoker	42479	chr12	110437523	C	G	*ANKRD13A*	MISSENSE	ENSG00000076513:MISSENSE[ENST00000261739(c.30C>G,caC>caG):ENSP00000261739(p.His10Gln)];ENSG00000076513:EXONIC-NC[ENST00000550404(n.289C>G)];ENSG00000076513:INTRONIC[ENST00000553025(c.-169+399C>G)];	NA	NA	NA	NA	NA	NA	NA	NA	NA	NA	MODIFIER	NA	NA	57	57	0	62	22	40	64.52
Smoker	42479	chr12	50357908	C	A	*AQP5*	MISSENSE	ENSG00000161798:MISSENSE[ENST00000293599(c.562C>A,Cgc>Agc):ENSP00000293599(p.Arg188Ser)];ENSG00000161798:EXONIC-NC[ENST00000553132(n.551C>A)];	NA	NA	chr12*50357908*50357908*C*T*rs368292687*NA*NA*NA	NA	NA	NA	NA	NA	NA	NA	MODIFIER	NA	NA	91	91	0	75	35	40	53.33
Smoker	42479	chr12	110729735	A	G	*ATP2A2*	INTRONIC	ENSG00000174437:[ENST00000308664(c.220-90A>G)];ENSG00000174437:[ENST00000377685(c.*60-90A>G)];ENSG00000174437:[ENST00000395494(c.220-90A>G)];ENSG00000174437:[ENST00000539276(c.220-90A>G)];ENSG00000174437:[ENST00000552636(c.-82-90A>G)];	NA	NA	NA	NA	NA	NA	NA	NA	NA	NA	MODIFIER	NA	NA	64	64	0	37	26	11	29.73
Smoker	42479	chr12	97020634	A	C	*C12orf55*	SILENT	ENSG00000188596:SILENT[ENST00000524981(c.4020A>C,acA>acC):ENSP00000431759(p.Thr1340(=))];ENSG00000188596:SILENT[ENST00000550977(c.259A>C,acA>acC):ENSP00000449035(p.Thr87(=))];ENSG00000188596:EXONIC-NC[ENST00000554108(n.1994A>C)];	NA	NA	NA	NA	NA	NA	NA	NA	NA	NA	LOW	NA	NA	47	47	0	64	56	8	12.5
Smoker	42479	chr12	94706801	G	C	*CCDC41*	INTRONIC-SS-ACR	ENSG00000173588:[ENST00000397809(c.1708-8C>G)];ENSG00000173588:[ENST00000339839(c.1708-8C>G)];ENSG00000173588:[ENST00000547232(c.*6-8C>G)];ENSG00000173588:[ENST00000552632(c.100-8C>G)];ENSG00000173588:[ENST00000546783(n.154C>G)];	NA	NA	NA	NA	NA	NA	NA	NA	NA	NA	LOW	NA	NA	28	28	0	52	41	11	21.15
Smoker	42479	chr12	133463915	C	G	*CHFR*	INTRONIC	ENSG00000072609:[ENST00000499045(n.412-9675G>C)];ENSG00000072609:[ENST00000541817(c.-251-9675G>C)];ENSG00000072609:[ENST00000540537(c.-251-9675G>C)];ENSG00000072609:[ENST00000542714(c.-251-9675G>C)];ENSG00000072609:[ENST00000536932(c.-251-9675G>C)];ENSG00000072609:[ENST00000450056(c.-1G>C)];ENSG00000072609:[ENST00000432561(c.-1G>C)];ENSG00000072609:[ENST00000315585(c.-1G>C)];ENSG00000072609:[ENST00000443047(c.-1G>C)];ENSG00000072609:[ENST00000540963(c.-1G>C)];ENSG00000072609:[ENST00000266880(c.-1G>C)];ENSG00000072609:[ENST00000536196(n.38G>C)];ENSG00000072609:[ENST00000535181(n.30G>C)];ENSG00000072609:[ENST00000537551(n.194G>C)];	NA	NA	NA	NA	NA	NA	NA	NA	NA	NA	MODIFIER	NA	NA	66	66	0	62	36	26	41.94
Smoker	42479	chr12	122837288	G	C	*CLIP1*	SILENT	ENSG00000130779:SILENT[ENST00000537178(c.1353C>G,acC>acG):ENSP00000445531(p.Thr451(=))];ENSG00000130779:SILENT[ENST00000302528(c.1353C>G,acC>acG):ENSP00000303585(p.Thr451(=))];ENSG00000130779:SILENT[ENST00000361654(c.1353C>G,acC>acG):ENSP00000355314(p.Thr451(=))];ENSG00000130779:SILENT[ENST00000545889(c.456C>G,acC>acG):ENSP00000438743(p.Thr152(=))];ENSG00000130779:SILENT[ENST00000358808(c.1353C>G,acC>acG):ENSP00000351665(p.Thr451(=))];ENSG00000130779:SILENT[ENST00000537004(c.1155C>G,acC>acG):ENSP00000441409(p.Thr385(=))];ENSG00000130779:SILENT[ENST00000540304(c.1155C>G,acC>acG):ENSP00000437786(p.Thr385(=))];ENSG00000130779:SILENT[ENST00000540338(c.1353C>G,acC>acG):ENSP00000439093(p.Thr451(=))];ENSG00000130779:EXONIC-NC[ENST00000541410(n.1754C>G)];	NA	NA	NA	NA	NA	NA	NA	NA	NA	NA	LOW	NA	NA	117	117	0	130	67	63	48.46
Smoker	42479	chr12	56215916	G	C	*DNAJC14*	MISSENSE	ENSG00000135392:MISSENSE[ENST00000317287(c.1954C>G,Caa>Gaa):ENSP00000317500(p.Gln652Glu)];ENSG00000135392:MISSENSE[ENST00000357606(c.1954C>G,Caa>Gaa):ENSP00000350223(p.Gln652Glu)];ENSG00000135392:MISSENSE[ENST00000317269(c.1954C>G,Caa>Gaa):ENSP00000316240(p.Gln652Glu)];	NA	NA	NA	NA	NA	NA	NA	NA	NA	NA	MODIFIER	NA	NA	34	34	0	33	26	7	21.21
Smoker	42479	chr12	95611022	C	A	*FGD6*	5UTR	ENSG00000180263:[ENST00000546711(c.-6G>T)];ENSG00000180263:[ENST00000451107(c.-6G>T)];ENSG00000180263:[ENST00000549499(c.-6G>T)];ENSG00000180263:[ENST00000343958(c.-6G>T)];	NA	NA	NA	NA	NA	NA	NA	NA	NA	NA	MODIFIER	NA	NA	109	109	0	157	51	106	67.52
Smoker	42479	chr12	131561322	C	G	*GPR133*	MISSENSE	ENSG00000111452:MISSENSE[ENST00000543617(c.7C>G,Cgt>Ggt):ENSP00000438021(p.Arg3Gly)];ENSG00000111452:INTRONIC[ENST00000376682(c.532-24C>G)];ENSG00000111452:INTRONIC[ENST00000535015(c.1570-24C>G)];ENSG00000111452:INTRONIC[ENST00000446583(n.1679-24C>G)];ENSG00000111452:INTRONIC[ENST00000261654(c.1474-24C>G)];ENSG00000111452:INTRONIC[ENST00000335486(c.39-24C>G)];	NA	NA	NA	NA	NA	NA	NA	NA	NA	NA	MODIFIER	NA	NA	69	67	2	103	41	62	60.19
Smoker	42479	chr12	6933343	C	G	*GPR162*	MISSENSE	ENSG00000250510:EXONIC-NC[ENST00000541431(n.537C>G)];ENSG00000250510:EXONIC-NC[ENST00000535220(n.987C>G)];ENSG00000250510:INTRONIC[ENST00000428545(c.15+414C>G)];ENSG00000250510:INTRONIC[ENST00000382315(c.-45-1306C>G)];ENSG00000250510:MISSENSE[ENST00000311268(c.279C>G,ttC>ttG):ENSP00000311528(p.Phe93Leu)];	NA	NA	chr12*6933343*6933343*C*T*rs370573119*NA*NA*NA	NA	NA	NA	NA	T	PoD	N	MODERATE	NA	NA	136	136	0	149	109	40	26.85

Mangalaparthi *et al.* , 2020. Mutational landscape of esophageal squamous cell carcinoma in an Indian cohort
Supplementary Table 4. List of somatic single nucleotide variants identified using whole exome sequencing of ESCC patients

Cohort	Sample	Chromosome	Genomic position	Reference allele	Alternate allele	Gene symbol	Variant type	Variant annotation	OncoMD	COSMIC	dbSNP	ExAC	ExAC_SAS	1000G	1000G_SAS	SIFT	Polyphen2	CONDEL	VEP	LRT	PhastCon7 Way	Total read (Adjacent normal)	Reference allel read support (Adjacent normal)	Alternative read support (Adjacent normal)	Total read (Tumor)	Reference allel read support (Tumor)	Alternative read support (Tumor)	Alternate allele frequency
Smoker	42479	chr12	13716564	T	C	*GRIN2B*	MISSENSE	ENSG00000273079:MISSENSE[ENST00000609686(c.3608A>G,aAc>aGc):ENSP00000477455(p.Asn1203Ser)];	NA	NA	NA	NA	NA	NA	NA	T	BN	N	MODERATE	D	0.997	120	119	0	120	56	64	53.33
Smoker	42479	chr12	14923882	C	T	*HIST4H4*	MISSENSE	ENSG00000197837:MISSENSE[ENST00000539745(c.137G>A,cGc>cAc):ENSP00000443017(p.Arg46His)];ENSG00000197837:MISSENSE[ENST00000358064(c.137G>A,cGc>cAc):ENSP00000350767(p.Arg46His)];	NA	NA	NA	NA	NA	NA	NA	T_lc	PoD	D	MODERATE	NA	NA	174	174	0	134	61	73	54.48
Smoker	42479	chr12	39750599	C	T	*KIF21A*	INTRONIC	ENSG00000139116:[ENST00000541463(c.1469+35G>A)];ENSG00000139116:[ENST00000361961(c.1469+35G>A)];ENSG00000139116:[ENST00000361418(c.1469+35G>A)];ENSG00000139116:[ENST00000544797(c.1469+35G>A)];ENSG00000139116:[ENST00000395670(c.1469+35G>A)];ENSG00000139116:[ENST00000550429(n.139G>A)];	NA	NA	chr12*39750599*39750601*CAT*C*rs775196778*NA*NA*NA	NA	NA	NA	NA	NA	NA	NA	MODIFIER	NA	NA	54	54	0	50	31	19	38
Smoker	42479	chr12	40714897	C	T	*LRRK2*	NONSENSE	ENSG00000188906:NONSENSE[ENST00000298910(c.5077C>T,Cga>Tga):ENSP00000298910(p.Arg1693Ter)];ENSG00000188906:EXONIC-NC[ENST00000479187(n.1758C>T)];ENSG00000188906:3UTR[ENST00000430804(c.*1750C>T)];	chr12*4071	chr12*4071	NA	8.24E-06	6.06E-05	NA	NA	NA	NA	NA	MODIFIER	NA	NA	161	161	0	155	108	47	30.32
Smoker	42479	chr12	21205218	C	A	*LST3*	INTRONIC	ENSG00000257046:[ENST00000540229(c.1866-15035C>A)];ENSG00000257046:[ENST00000381541(c.1497+23C>A)];	NA	NA	chr12*21205218*21205218*C*T*rs548502652*0*0.9998*0.0001997	NA	NA	NA	NA	NA	NA	NA	MODIFIER	NA	NA	91	91	0	73	58	15	20.55
Smoker	42479	chr12	21205219	C	T	*LST3*	INTRONIC	ENSG00000257046:[ENST00000540229(c.1866-15034C>T)];ENSG00000257046:[ENST00000381541(c.1497+24C>T)];	NA	NA	NA	NA	NA	NA	NA	NA	NA	NA	MODIFIER	NA	NA	92	92	0	69	54	15	21.74
Smoker	42479	chr12	27916231	T	C	*MANSC4*	MISSENSE	ENSG00000205693:MISSENSE[ENST00000381273(c.463A>G,Atg>Gtg):ENSP00000370673(p.Met155Val)];	NA	NA	NA	NA	NA	NA	NA	T	BN	N	MODERATE	N	0.867	78	78	0	65	42	23	35.38
Smoker	42479	chr12	62960103	C	T	*MON2*	MISSENSE-SS-PRX	ENSG00000061987:[ENST00000546600(c.4196C>T,gCg>gTg):ENSP00000447407(p.Ala1399Val)];ENSG00000061987:[ENST00000393630(c.4199C>T,gCg>gTg):ENSP00000377250(p.Ala1400Val)];ENSG00000061987:[ENST00000280379(c.4199C>T,gCg>gTg):ENSP00000280379(p.Ala1400Val)];ENSG00000061987:[ENST00000393629(c.4178C>T,gCg>gTg):ENSP00000377249(p.Ala1393Val)];ENSG00000061987:[ENST00000393632(c.4196C>T,gCg>gTg):ENSP00000377252(p.Ala1399Val)];ENSG00000061987:[ENST00000552738(c.4109C>T,gCg>gTg):ENSP00000449215(p.Ala1370Val)];ENSG00000061987:[ENST00000547287(n.410C>T)];ENSG00000061987:[ENST00000551307(n.1350C>T)];ENSG00000061987:[ENST00000547095(c.*4142C>T)];ENSG00000061987:[ENST00000549539(n.372C>T)];	NA	NA	chr12*62960103*62960103*C*A,T*rs776062821*NA*NA*NA	8.25E-06	6.07E-05	NA	NA	NA	NA	NA	MODIFIER	NA	NA	132	131	0	98	42	56	57.14
Smoker	42479	chr12	40910363	C	T	*MUC19*	INTRONIC	ENSG00000205592:[ENST00000454784(c.*5782+25233C>T)];	NA	NA	NA	NA	NA	NA	NA	NA	NA	NA	MODIFIER	NA	NA	135	135	0	139	93	46	33.09
Smoker	42479	chr12	102028226	C	A	*MYBPC1*	INTRONIC	ENSG00000196091:[ENST00000536007(c.482-106C>A)];ENSG00000196091:[ENST00000547405(c.404-106C>A)];ENSG00000196091:[ENST00000550270(c.482-106C>A)];ENSG00000196091:[ENST00000361685(c.557-106C>A)];ENSG00000196091:[ENST00000551300(c.185-106C>A)];ENSG00000196091:[ENST00000361466(c.557-106C>A)];ENSG00000196091:[ENST00000550514(c.185-106C>A)];ENSG00000196091:[ENST00000452455(c.482-106C>A)];ENSG00000196091:[ENST00000550501(n.574+19504C>A)];ENSG00000196091:[ENST00000549145(c.521-106C>A)];ENSG00000196091:[ENST00000392934(c.443-106C>A)];ENSG00000196091:[ENST00000552198(n.444-106C>A)];ENSG00000196091:[ENST00000553190(c.482-106C>A)];ENSG00000196091:[ENST00000545503(c.482-106C>A)];ENSG00000196091:[ENST00000441232(c.482-106C>A)];ENSG00000196091:[ENST00000550312(n.438-106C>A)];ENSG00000196091:[ENST00000360610(c.482-106C>A)];ENSG00000196091:[ENST00000547509(c.440-106C>A)];ENSG00000196091:[ENST00000541119(c.446-106C>A)];	NA	NA	NA	NA	NA	NA	NA	NA	NA	NA	MODIFIER	NA	NA	209	209	0	175	129	45	25.71
Smoker	42479	chr12	96180866	G	A	*NTN4*	MISSENSE	ENSG00000074527:MISSENSE[ENST00000344911(c.325C>T,Cgc>Tgc):ENSP00000339436(p.Arg109Cys)];ENSG00000074527:MISSENSE[ENST00000538383(c.325C>T,Cgc>Tgc):ENSP00000444432(p.Arg109Cys)];ENSG00000074527:MISSENSE[ENST00000553059(c.436C>T,Cgc>Tgc):ENSP00000447292(p.Arg146Cys)];ENSG00000074527:MISSENSE[ENST00000343702(c.436C>T,Cgc>Tgc):ENSP00000340998(p.Arg146Cys)];ENSG00000074527:MISSENSE[ENST00000547980(c.325C>T,Cgc>Tgc):ENSP00000447594(p.Arg109Cys)];	NA	NA	NA	NA	NA	NA	NA	D	PrD	D	MODERATE	NA	NA	153	153	0	283	234	49	17.31
Smoker	42479	chr12	55759304	G	A	*OR6C75*	MISSENSE	ENSG00000187857:MISSENSE[ENST00000343399(c.410G>A,aGa>aAa):ENSP00000368987(p.Arg137Lys)];	NA	NA	NA	NA	NA	NA	NA	T_lc	BN	N	MODERATE	N	0.01	126	126	0	132	54	78	59.09
Smoker	42479	chr12	29642561	C	A	*OVCH1*	MISSENSE	ENSG00000187950:MISSENSE[ENST00000318184(c.694G>T,Gcc>Tcc):ENSP00000326708(p.Ala232Ser)];	NA	NA	chr12*29642561*29642561*C*G,T*rs150657987*1*0.9986*0.0003994,0.0009984	NA	NA	NA	NA	D	PoD	D	MODERATE	NA	NA	62	62	0	34	25	9	26.47
Smoker	42479	chr12	20522514	A	C	*PDE3A*	MISSENSE	ENSG00000172572:MISSENSE[ENST00000359062(c.296A>C,gAg>gCg):ENSP00000351957(p.Glu99Ala)];	chr12*2052	chr12*2052	0.003195	0.0023413	0	0.0031949	0.001	NA	NA	NA	MODIFIER	NA	NA	62	62	0	52	38	14	26.92
Smoker	42479	chr12	20766642	G	T	*PDE3A*	INTRONIC-SS-DNR	ENSG00000172572:[ENST00000359062(c.1269+8G>T)];ENSG00000172572:[ENST00000544307(n.566+8G>T)];	NA	NA	NA	NA	NA	NA	NA	NA	NA	NA	LOW	NA	NA	74	74	0	63	46	17	26.98
Smoker	42479	chr12	93162827	T	C	*PLEKHG7*	SILENT	ENSG00000187510:SILENT[ENST00000344636(c.1003T>C,Tta>Cta):ENSP00000344961(p.Leu335(=))];	NA	NA	NA	NA	NA	NA	NA	NA	NA	NA	LOW	NA	0.568	84	84	0	137	109	28	20.44
Smoker	42479	chr12	81660808	G	A	*PPFIA2*	INTRONIC-SS-ACR	ENSG00000139220:[ENST00000333447(c.3451-9C>T)];ENSG00000139220:[ENST00000407050(c.3184-9C>T)];ENSG00000139220:[ENST00000551461(c.*1782-9C>T)];ENSG00000139220:[ENST00000541570(c.2095-9C>T)];ENSG00000139220:[ENST00000443686(c.3172-9C>T)];ENSG00000139220:[ENST00000550359(c.3028-9C>T)];ENSG00000139220:[ENST00000548586(c.3469-9C>T)];ENSG00000139220:[ENST00000549396(c.3487-9C>T)];ENSG00000139220:[ENST00000549325(c.3442-9C>T)];ENSG00000139220:[ENST00000541017(c.1045-9C>T)];ENSG00000139220:[ENST00000550584(c.3487-9C>T)];ENSG00000139220:[ENST00000552948(c.3424-9C>T)];ENSG00000139220:[ENST00000545296(n.1244-7633C>T)];	NA	NA	0.0001997	0.0004219	6.57E-05	0.0001997	0	NA	NA	NA	MODIFIER	NA	NA	152	151	0	223	180	42	18.83

Mangalaparthi *et al.* , 2020. Mutational landscape of esophageal squamous cell carcinoma in an Indian cohort
Supplementary Table 4. List of somatic single nucleotide variants identified using whole exome sequencing of ESCC patients

Cohort	Sample	Chromosome	Genomic position	Reference allele	Alternate allele	Gene symbol	Variant type	Variant annotation	OncoMD	COSMIC	dbSNP	ExAC	ExAC_SAS	1000G	1000G_SAS	SIFT	Polyphen2	CONDEL	VEP	LRT	PhastCon7 Way	Total read (Adjacent normal)	Reference allel read support (Adjacent normal)	Alternative read support (Adjacent normal)	Total read (Tumor)	Reference allel read support (Tumor)	Alternative read support (Tumor)	Alternate allele frequency
Smoker	42479	chr12	81738468	C	G	*PPFIA2*	MISSENSE	ENSG00000139220:MISSENSE[ENST00000333447(c.2183G>C,aGt>aCt):ENSP00000327416(p.Ser728Thr)];ENSG00000139220:MISSENSE[ENST00000550359(c.1778G>C,aGt>aCt):ENSP00000447273(p.Ser593Thr)];ENSG00000139220:MISSENSE[ENST00000549396(c.2237G>C,aGt>aCt):ENSP00000450337(p.Ser746Thr)];ENSG00000139220:MISSENSE[ENST00000407050(c.2015G>C,aGt>aCt):ENSP00000385093(p.Ser672Thr)];ENSG00000139220:MISSENSE[ENST00000541570(c.938G>C,aGt>aCt):ENSP00000438337(p.Ser313Thr)];ENSG00000139220:MISSENSE[ENST00000443686(c.1940G>C,aGt>aCt):ENSP00000388373(p.Ser647Thr)];ENSG00000139220:MISSENSE[ENST00000549325(c.2183G>C,aGt>aCt):ENSP00000450298(p.Ser728Thr)];ENSG00000139220:MISSENSE[ENST00000550584(c.2237G>C,aGt>aCt):ENSP00000449558(p.Ser746Thr)];ENSG00000139220:MISSENSE[ENST00000552948(c.2237G>C,aGt>aCt):ENSP00000447868(p.Ser746Thr)];ENSG00000139220:MISSENSE[ENST00000548586(c.2237G>C,aGt>aCt):ENSP00000449338(p.Ser746Thr)];ENSG00000139220:3UTR[ENST00000551461(c.*625G>C)];ENSG00000139220:EXONIC-NC[ENST00000549917(n.297G>C)];ENSG00000139220:EXONIC-NC[ENST00000549344(n.80G>C)];ENSG00000139220:5UTR[ENST00000541017(c.-113G>C)];ENSG00000139220:INTRONIC[ENST00000545296(n.1243+30060G>C)];	NA	NA	NA	NA	NA	NA	NA	NA	NA	NA	MODIFIER	NA	NA	70	70	0	98	81	17	17.35
Smoker	42479	chr12	122337629	G	A	*PSMD9*	MISSENSE	ENSG00000110801:MISSENSE[ENST00000541212(c.331G>A,Gac>Aac):ENSP00000440485(p.Asp111Asn)];ENSG00000110801:MISSENSE[ENST00000340175(c.331G>A,Gac>Aac):ENSP00000340847(p.Asp111Asn)];ENSG00000110801:MISSENSE[ENST00000537407(c.331G>A,Gac>Aac):ENSP00000445058(p.Asp111Asn)];ENSG00000110801:MISSENSE[ENST00000261817(c.331G>A,Gac>Aac):ENSP00000261817(p.Asp111Asn)];ENSG00000110801:MISSENSE[ENST00000538613(c.331G>A,Gac>Aac):ENSP00000443081(p.Asp111Asn)];ENSG00000110801:MISSENSE[ENST00000543699(c.331G>A,Gac>Aac):ENSP00000440500(p.Asp111Asn)];ENSG00000110801:MISSENSE[ENST00000535293(c.178G>A,Gac>Aac):ENSP00000443968(p.Asp60Asn)];ENSG00000110801:INTRONIC[ENST00000542602(c.139-3283G>A)];ENSG00000110801:INTRONIC[ENST00000540962(c.242-3283G>A)];ENSG00000110801:EXONIC-NC[ENST00000544724(n.2453G>A)];	NA	NA	NA	NA	NA	NA	NA	NA	NA	NA	MODIFIER	NA	NA	47	47	0	76	45	31	40.79
Smoker	42479	chr12	70986164	C	T	*PTPRB*	MISSENSE	ENSG00000127329:MISSENSE[ENST00000551525(c.1675G>A,Gaa>Aaa):ENSP00000448349(p.Glu559Lys)];ENSG00000127329:MISSENSE[ENST00000261266(c.1024G>A,Gaa>Aaa):ENSP00000261266(p.Glu342Lys)];ENSG00000127329:MISSENSE[ENST00000334414(c.1678G>A,Gaa>Aaa):ENSP00000334928(p.Glu560Lys)];ENSG00000127329:MISSENSE[ENST00000550358(c.1678G>A,Gaa>Aaa):ENSP00000448058(p.Glu560Lys)];ENSG00000127329:MISSENSE[ENST00000548122(c.1315G>A,Gaa>Aaa):ENSP00000446982(p.Glu439Lys)];ENSG00000127329:MISSENSE[ENST00000550857(c.1024G>A,Gaa>Aaa):ENSP00000447302(p.Glu342Lys)];ENSG00000127329:MISSENSE[ENST00000538708(c.1024G>A,Gaa>Aaa):ENSP00000438927(p.Glu342Lys)];ENSG00000127329:MISSENSE[ENST00000451516(c.1024G>A,Gaa>Aaa):ENSP00000393028(p.Glu342Lys)];ENSG00000127329:EXONIC-NC[ENST00000552253(n.1723G>A)];ENSG00000127329:EXONIC-NC[ENST00000538174(n.1697G>A)];	NA	NA	NA	NA	NA	NA	NA	NA	NA	NA	MODIFIER	NA	NA	163	163	0	248	159	89	35.89
Smoker	42479	chr12	120651854	G	A	*PXN*	INTRONIC	ENSG00000089159:[ENST00000228307(c.1361-61C>T)];ENSG00000089159:[ENST00000458477(c.860-61C>T)];ENSG00000089159:[ENST00000323871(n.3179-61C>T)];ENSG00000089159:[ENST00000267257(c.1403-61C>T)];ENSG00000089159:[ENST00000424649(c.1259-61C>T)];ENSG00000089159:[ENST00000397506(c.797-61C>T)];ENSG00000089159:[ENST00000536957(c.1355-61C>T)];ENSG00000089159:[ENST00000538144(n.1390-61C>T)];	NA	NA	NA	NA	NA	NA	NA	NA	NA	NA	MODIFIER	NA	NA	65	65	0	64	31	33	51.56
Smoker	42479	chr12	107105339	G	C	*RFX4*	INTRONIC	ENSG00000111783:[ENST00000392842(c.993+50G>C)];ENSG00000111783:[ENST00000229387(c.711+50G>C)];ENSG00000111783:[ENST00000536688(n.1125+50G>C)];ENSG00000111783:[ENST00000536722(c.*245+50G>C)];ENSG00000111783:[ENST00000357881(c.1020+50G>C)];ENSG00000111783:[ENST00000539967(c.291+50G>C)];	NA	NA	chr12*107105339*107105339*G*A*rs778836014*NA*NA*NA	NA	NA	NA	NA	NA	NA	NA	MODIFIER	NA	NA	153	153	0	123	83	40	32.52
Smoker	42479	chr12	21205218	C	A	*RP11-125O5.2*	INTRONIC	ENSG00000257062:[ENST00000543498(c.40-2295C>A)];	NA	NA	chr12*21205218*21205218*C*T*rs548502652*0*0.9998*0.0001997	NA	NA	NA	NA	NA	NA	NA	MODIFIER	NA	NA	91	91	0	73	58	15	20.55
Smoker	42479	chr12	21205219	C	T	*RP11-125O5.2*	INTRONIC	ENSG00000257062:[ENST00000543498(c.40-2294C>T)];	NA	NA	NA	NA	NA	NA	NA	NA	NA	NA	MODIFIER	NA	NA	92	92	0	69	54	15	21.74
Smoker	42479	chr12	21294496	C	G	*RP11-125O5.2*	3UTR	ENSG00000257062:[ENST00000592513(c.*330C>G)];ENSG00000257062:[ENST00000543498(c.*45C>G)];ENSG00000257062:[ENST00000590779(c.*303C>G)];ENSG00000257062:[ENST00000585342(c.*263C>G)];	NA	NA	NA	NA	NA	NA	NA	NA	NA	NA	MODIFIER	NA	NA	55	55	0	37	29	8	21.62
Smoker	42479	chr12	107105339	G	C	*RP11-144F15.1*	INTRONIC	ENSG00000257545:[ENST00000549203(c.142+63129C>G)];ENSG00000257545:[ENST00000551505(c.209+63129C>G)];	NA	NA	chr12*107105339*107105339*G*A*rs778836014*NA*NA*NA	NA	NA	NA	NA	NA	NA	NA	MODIFIER	NA	NA	153	153	0	123	83	40	32.52
Smoker	42479	chr12	20522514	A	C	*RP11-284H19.1*	INTRONIC	ENSG00000256879:[ENST00000535755(n.422+261T>G)];	chr12*2052	chr12*2052	0.003195	0.0023413	0	0.0031949	0.001	NA	NA	NA	MODIFIER	NA	NA	62	62	0	52	38	14	26.92
Smoker	42479	chr12	56215916	G	C	*RP11-762I7.5*	MISSENSE	ENSG00000257390:MISSENSE-SS-PRX[ENST00000550124(c.247C>G,Caa>Gaa):ENSP00000447077(p.Gln83Glu)];ENSG00000257390:MISSENSE-SS-PRX[ENST00000548593(c.277C>G,Caa>Gaa):ENSP00000449809(p.Gln93Glu)];ENSG00000257390:MISSENSE[ENST00000546813(c.264C>G,Caa>Gaa):ENSP00000447649(p.Gln89Glu)];ENSG00000257390:MISSENSE[ENST00000546837(c.842C>G,Caa>Gaa):ENSP00000447000(p.Gln282Glu)];ENSG00000257390:EXONIC-NC[ENST00000552719(n.307C>G)];	NA	NA	NA	NA	NA	NA	NA	NA	NA	NA	MODIFIER	NA	NA	34	34	0	33	26	7	21.21
Smoker	42479	chr12	54520122	C	A	*RP11-834C11.4*	EXONIC-NC	ENSG00000250742:[ENST00000508564(n.241C>A)];	NA	NA	NA	NA	NA	NA	NA	NA	NA	NA	MODIFIER	NA	NA	28	28	0	41	14	27	65.85
Smoker	42479	chr12	122337629	G	A	*RP11-87C12.2*	INTRONIC	ENSG00000256950:[ENST00000546333(c.242-3283G>A)];	NA	NA	NA	NA	NA	NA	NA	NA	NA	NA	MODIFIER	NA	NA	47	47	0	76	45	31	40.79
Smoker	42479	chr12	126942690	C	G	*RP5-944M2.3*	INTRONIC	ENSG00000214043:[ENST00000539969(n.316-197C>G)];ENSG00000214043:[ENST00000545767(n.575+14C>G)];ENSG00000214043:[ENST00000540774(n.512+14C>G)];ENSG00000214043:[ENST00000544759(n.585C>G)];ENSG00000214043:[ENST00000397346(n.634C>G)];	NA	NA	chr12*126942690*126942690*C*T*rs535116425*0*0.9996*0.0003994	NA	NA	NA	NA	NA	NA	NA	MODIFIER	NA	NA	46	46	0	33	21	12	36.36

Mangalaparthi *et al.* , 2020. Mutational landscape of esophageal squamous cell carcinoma in an Indian cohort

Supplementary Table 4. List of somatic single nucleotide variants identified using whole exome sequencing of ESCC patients

Cohort	Sample	Chromosome	Genomic position	Reference allele	Alternate allele	Gene symbol	Variant type	Variant annotation	OncoMD	COSMIC	dbSNP	ExAC	ExAC_SAS	1000G	1000G_SAS	SIFT	Polyphen2	CONDEL	VEP	LRT	PhastCon7 Way	Total read (Adjacent normal)	Reference allel read support (Adjacent normal)	Alternative read support (Adjacent normal)	Total read (Tumor)	Reference allel read support (Tumor)	Alternative read support (Tumor)	Alternate allele frequency
Smoker	42479	chr12	109017295	G	C	*SELPLG*	SILENT	ENSG00000110876:SILENT[ENST00000388962(c.759C>G,tcC>tcG):ENSP00000373614(p.Ser253(=))];ENSG00000110876:SILENT[ENST00000228463(c.837C>G,tcC>tcG):ENSP00000228463(p.Ser279(=))];ENSG00000110876:SILENT[ENST00000550948(c.789C>G,tcC>tcG):ENSP00000447752(p.Ser263(=))];	NA	NA	NA	NA	NA	NA	NA	NA	NA	NA	LOW	NA	NA	94	94	0	106	82	24	22.64
Smoker	42479	chr12	46764428	G	A	*SLC38A2*	INTRONIC	ENSG00000134294:[ENST00000549258(c.198+131C>T)];ENSG00000134294:[ENST00000551405(n.411-18C>T)];ENSG00000134294:[ENST00000256689(c.199-18C>T)];ENSG00000134294:[ENST00000547252(n.339-18C>T)];	NA	NA	NA	NA	NA	NA	NA	NA	NA	NA	MODIFIER	NA	NA	118	118	0	103	80	23	22.33
Smoker	42479	chr12	21294496	C	G	*SLCO1B1*	5UTR	ENSG00000134538:[ENST00000256958(c.-13C>G)];	NA	NA	NA	NA	NA	NA	NA	NA	NA	NA	MODIFIER	NA	NA	55	55	0	37	29	8	21.62
Smoker	42479	chr12	21205218	C	A	*SLCO1B3*	INTRONIC	ENSG00000111700:[ENST00000553473(c.1866-15035C>A)];	NA	NA	chr12*21205218*21205218*C*T*rs548502652*0*0.9998*0.0001997	NA	NA	NA	NA	NA	NA	NA	MODIFIER	NA	NA	91	91	0	73	58	15	20.55
Smoker	42479	chr12	21205219	C	T	*SLCO1B3*	INTRONIC	ENSG00000111700:[ENST00000553473(c.1866-15034C>T)];	NA	NA	NA	NA	NA	NA	NA	NA	NA	NA	MODIFIER	NA	NA	92	92	0	69	54	15	21.74
Smoker	42479	chr12	21205218	C	A	*SLCO1B7*	INTRONIC	ENSG00000205754:[ENST00000554957(c.1497+23C>A)];ENSG00000205754:[ENST00000421593(c.1356+23C>A)];	NA	NA	chr12*21205218*21205218*C*T*rs548502652*0*0.9998*0.0001997	NA	NA	NA	NA	NA	NA	NA	MODIFIER	NA	NA	91	91	0	73	58	15	20.55
Smoker	42479	chr12	21205219	C	T	*SLCO1B7*	INTRONIC	ENSG00000205754:[ENST00000554957(c.1497+24C>T)];ENSG00000205754:[ENST00000421593(c.1356+24C>T)];	NA	NA	NA	NA	NA	NA	NA	NA	NA	NA	MODIFIER	NA	NA	92	92	0	69	54	15	21.74
Smoker	42479	chr12	20859000	A	G	*SLCO1C1*	MISSENSE	ENSG00000139155:MISSENSE[ENST00000381552(c.389A>G,cAg>cGg):ENSP00000370964(p.Gln130Arg)];ENSG00000139155:MISSENSE[ENST00000545604(c.389A>G,cAg>cGg):ENSP00000444149(p.Gln130Arg)];ENSG00000139155:MISSENSE[ENST00000266509(c.389A>G,cAg>cGg):ENSP00000266509(p.Gln130Arg)];ENSG00000139155:MISSENSE[ENST00000545102(c.35A>G,cAg>cGg):ENSP00000444527(p.Gln12Arg)];ENSG00000139155:MISSENSE[ENST00000540354(c.389A>G,cAg>cGg):ENSP00000438665(p.Gln130Arg)];ENSG00000139155:INTRONIC[ENST00000539415(c.130-5320A>G)];	NA	NA	NA	NA	NA	NA	NA	NA	NA	NA	MODIFIER	NA	NA	153	153	0	163	157	6	3.68
Smoker	42479	chr12	56743203	C	T	*STAT2*	MISSENSE	ENSG00000170581:MISSENSE[ENST00000418572(c.1336G>A,Gaa>Aaa):ENSP00000387354(p.Glu446Lys)];ENSG00000170581:INTRONIC-SS-DNR-PRX[ENST00000557235(c.1329+7G>A)];ENSG00000170581:INTRONIC-SS-DNR-PRX[ENST00000314128(c.1341+7G>A)];ENSG00000170581:INTRONIC-SS-DNR-PRX[ENST00000556539(n.271+7G>A)];	NA	NA	NA	NA	NA	NA	NA	NA	NA	NA	LOW	NA	NA	124	124	0	146	112	34	23.29
Smoker	42479	chr12	33559763	C	A	*SYT10*	MISSENSE	ENSG00000110975:MISSENSE[ENST00000228567(c.1038G>T,agG>agT):ENSP00000228567(p.Arg346Ser)];ENSG00000110975:MISSENSE[ENST00000535526(c.495G>T,agG>agT):ENSP00000438691(p.Arg165Ser)];ENSG00000110975:3UTR[ENST00000539102(c.*633G>T)];	NA	NA	NA	NA	NA	NA	NA	NA	NA	NA	MODIFIER	NA	NA	50	50	0	39	27	12	30.77
Smoker	42479	chr12	33559764	C	A	*SYT10*	MISSENSE	ENSG00000110975:MISSENSE[ENST00000228567(c.1037G>T,aGg>aTg):ENSP00000228567(p.Arg346Met)];ENSG00000110975:MISSENSE[ENST00000535526(c.494G>T,aGg>aTg):ENSP00000438691(p.Arg165Met)];ENSG00000110975:3UTR[ENST00000539102(c.*632G>T)];	NA	NA	NA	NA	NA	NA	NA	NA	NA	NA	MODIFIER	NA	NA	51	51	0	40	27	13	32.5
Smoker	42479	chr12	129154049	G	C	*TMEM132C*	MISSENSE	ENSG00000181234:MISSENSE[ENST00000315208(c.241G>C,Gtg>Ctg):ENSP00000324458(p.Val81Leu)];ENSG00000181234:MISSENSE[ENST00000435159(c.1393G>C,Gtg>Ctg):ENSP00000410852(p.Val465Leu)];	chr12*1291	chr12*1291	chr12*129154049*129154049*G*A*rs773936735*NA*NA*NA	NA	NA	NA	NA	D	PoD	D	MODERATE	N	0.994	97	97	0	78	56	22	28.21
Smoker	42479	chr12	129180537	C	T	*TMEM132C*	SILENT	ENSG00000181234:5UTR[ENST00000537538(c.-28C>T)];ENSG00000181234:SILENT[ENST00000315208(c.666C>T,ttC>ttT):ENSP00000324458(p.Phe222(=))];ENSG00000181234:SILENT[ENST00000435159(c.1818C>T,ttC>ttT):ENSP00000410852(p.Phe606(=))];	NA	NA	NA	NA	NA	NA	NA	NA	NA	NA	LOW	NA	0.99	71	71	0	72	52	20	27.78
Smoker	42479	chr13	26445263	C	T	*AL138815.1*	INTRONIC	ENSG00000215549:[ENST00000400553(c.33-67C>T)];	NA	NA	NA	NA	NA	NA	NA	NA	NA	NA	MODIFIER	NA	NA	86	86	0	47	5	42	89.36
Smoker	42479	chr13	26445263	C	T	*ATP8A2*	INTRONIC	ENSG00000132932:[ENST00000281620(c.*962+8717C>T)];ENSG00000132932:[ENST00000255283(c.2988+8717C>T)];ENSG00000132932:[ENST00000381655(c.3183+8717C>T)];ENSG00000132932:[ENST00000491840(n.2054+8717C>T)];	NA	NA	NA	NA	NA	NA	NA	NA	NA	NA	MODIFIER	NA	NA	86	86	0	47	5	42	89.36
Smoker	42479	chr13	60348888	C	T	*DIAPH3*	MISSENSE	ENSG00000139734:MISSENSE[ENST00000400324(c.3233G>A,aGa>aAa):ENSP00000383178(p.Arg1078Lys)];ENSG00000139734:MISSENSE[ENST00000267215(c.3233G>A,aGa>aAa):ENSP00000267215(p.Arg1078Lys)];ENSG00000139734:MISSENSE[ENST00000377908(c.3200G>A,aGa>aAa):ENSP00000367141(p.Arg1067Lys)];ENSG00000139734:MISSENSE[ENST00000400320(c.3095G>A,aGa>aAa):ENSP00000383174(p.Arg1032Lys)];ENSG00000139734:MISSENSE[ENST00000400319(c.3023G>A,aGa>aAa):ENSP00000383173(p.Arg1008Lys)];ENSG00000139734:MISSENSE[ENST00000400330(c.3233G>A,aGa>aAa):ENSP00000383184(p.Arg1078Lys)];ENSG00000139734:EXONIC-NC[ENST00000465066(n.2623G>A)];	NA	NA	NA	NA	NA	NA	NA	NA	NA	NA	MODIFIER	NA	NA	115	115	0	81	53	28	34.57
Smoker	42479	chr13	113744427	G	A	*MCF2L*	MISSENSE	ENSG00000126217:INTRONIC[ENST00000423482(c.2947+385G>A)];ENSG00000126217:INTRONIC[ENST00000261963(c.465+385G>A)];ENSG00000126217:INTRONIC[ENST00000397017(c.1934+385G>A)];ENSG00000126217:INTRONIC[ENST00000375608(c.3043+385G>A)];ENSG00000126217:INTRONIC[ENST00000535094(c.2953+385G>A)];ENSG00000126217:INTRONIC[ENST00000420013(c.93+385G>A)];ENSG00000126217:INTRONIC[ENST00000434480(c.2971+385G>A)];ENSG00000126217:INTRONIC[ENST00000442652(c.3043+385G>A)];ENSG00000126217:INTRONIC[ENST00000397030(c.3052+385G>A)];ENSG00000126217:INTRONIC[ENST00000375601(c.2965+385G>A)];ENSG00000126217:INTRONIC[ENST00000421756(c.2965+385G>A)];ENSG00000126217:INTRONIC[ENST00000441756(c.38+385G>A)];ENSG00000126217:INTRONIC[ENST00000375604(c.3124+385G>A)];ENSG00000126217:MISSENSE[ENST00000413354(c.688G>A,aGa>aAa):ENSP00000407392(p.Arg230Lys)];ENSG00000126217:MISSENSE[ENST00000439475(c.315G>A,aGa>aAa):ENSP00000411315(p.Arg106Lys)];ENSG00000126217:MISSENSE[ENST00000453297(c.462G>A,aGa>aAa):ENSP00000392953(p.Arg155Lys)];ENSG00000126217:3UTR[ENST00000375597(c.*231G>A)];	NA	NA	NA	NA	NA	NA	NA	T	BN	N	MODERATE	NA	NA	62	62	0	50	31	19	38
Smoker	42479	chr13	114178525	A	G	*TMCO3*	INTRONIC	ENSG00000150403:[ENST00000375391(c.1225+13786A>G)];ENSG00000150403:[ENST00000434316(c.1343+3477A>G)];ENSG00000150403:[ENST00000465556(n.188+3477A>G)];ENSG00000150403:[ENST00000474393(n.1601+3477A>G)];	NA	NA	NA	NA	NA	NA	NA	NA	NA	NA	MODIFIER	NA	NA	133	133	0	75	11	64	85.33
Smoker	42479	chr13	28562574	G	A	*URAD*	INTRONIC	ENSG00000183463:[ENST00000332715(c.175+26C>T)];	NA	NA	NA	NA	NA	NA	NA	NA	NA	NA	MODIFIER	NA	NA	70	70	0	60	38	22	36.67

Mangalaparthi *et al.* , 2020. Mutational landscape of esophageal squamous cell carcinoma in an Indian cohort
Supplementary Table 4. List of somatic single nucleotide variants identified using whole exome sequencing of ESCC patients

Cohort	Sample	Chromosome	Genomic position	Reference allele	Alternate allele	Gene symbol	Variant type	Variant annotation	OncoMD	COSMIC	dbSNP	ExAC	ExAC_SAS	1000G	1000G_SAS	SIFT	Polyphen2	CONDEL	VEP	LRT	PhastCon7 Way	Total read (Adjacent normal)	Reference allel read support (Adjacent normal)	Alternative read support (Adjacent normal)	Total read (Tumor)	Reference allel read support (Tumor)	Alternative read support (Tumor)	Alternate allele frequency
Smoker	42479	chr14	103390277	G	T	*AMN*	SILENT	ENSG00000166126:SILENT[ENST00000299155(c.168G>T,gtG>gtT):ENSP00000299155(p.Val56(=))];ENSG00000166126:EXONIC-NC[ENST00000541086(n.914G>T)];	NA	NA	NA	NA	NA	NA	NA	NA	NA	NA	MODIFIER	NA	NA	75	75	0	95	77	18	18.95
Smoker	42479	chr14	103410212	G	T	*CDC42BPB*	INTRONIC	ENSG00000198752:[ENST00000361246(c.4408+16C>A)];ENSG00000198752:[ENST00000559245(n.676C>A)];	NA	NA	NA	NA	NA	NA	NA	NA	NA	NA	MODIFIER	NA	NA	32	32	0	43	20	23	53.49
Smoker	42479	chr14	35008455	G	C	*EAPP*	INTRONIC-SS-DNR	ENSG00000129518:[ENST00000555705(c.*43+7C>G)];ENSG00000129518:[ENST00000555683(n.150+306C>G)];ENSG00000129518:[ENST00000250454(c.74+306C>G)];	NA	NA	NA	NA	NA	NA	NA	NA	NA	NA	MODIFIER	NA	NA	103	103	0	99	76	23	23.23
Smoker	42479	chr14	52433423	G	C	*GNG2*	3UTR	ENSG00000186469:[ENST00000557376(c.*18G>C)];ENSG00000186469:[ENST00000556522(c.*254G>C)];ENSG00000186469:[ENST00000554736(c.*18G>C)];ENSG00000186469:[ENST00000555472(c.*18G>C)];ENSG00000186469:[ENST00000556752(c.*18G>C)];ENSG00000186469:[ENST00000556766(c.*18G>C)];ENSG00000186469:[ENST00000335281(c.*18G>C)];ENSG00000186469:[ENST00000553432(c.*18G>C)];ENSG00000186469:[ENST00000554840(n.99-6G>C)];ENSG00000186469:[ENST00000553560(c.88-12569G>C)];ENSG00000186469:[ENST00000553299(n.1207G>C)];	NA	NA	NA	NA	NA	NA	NA	NA	NA	NA	MODIFIER	NA	NA	118	118	0	127	46	81	63.78
Smoker	42479	chr14	106878020	C	T	*IGHV4-39*	SILENT	ENSG00000211959:SILENT[ENST00000390619(c.57G>A,gcG>gcA):ENSP00000375028(p.Ala19(=))];	NA	NA	NA	0.1952236	0.222625908	NA	NA	NA	NA	NA	LOW	NA	NA	42	41	1	19	8	11	57.89
Smoker	42479	chr14	104129155	G	T	*KLC1*	NONSENSE	ENSG00000126214:NONSENSE[ENST00000246489(c.688G>T,Gag>Tag):ENSP00000246489(p.Glu230Ter)];ENSG00000126214:NONSENSE[ENST00000348520(c.688G>T,Gag>Tag):ENSP00000341154(p.Glu230Ter)];ENSG00000126214:NONSENSE[ENST00000452929(c.688G>T,Gag>Tag):ENSP00000414982(p.Glu230Ter)];ENSG00000126214:NONSENSE[ENST00000555836(c.688G>T,Gag>Tag):ENSP00000452481(p.Glu230Ter)];ENSG00000126214:NONSENSE[ENST00000380038(c.688G>T,Gag>Tag):ENSP00000369377(p.Glu230Ter)];ENSG00000126214:NONSENSE[ENST00000557575(c.688G>T,Gag>Tag):ENSP00000450617(p.Glu230Ter)];ENSG00000126214:NONSENSE[ENST00000347839(c.688G>T,Gag>Tag):ENSP00000334618(p.Glu230Ter)];ENSG00000126214:NONSENSE[ENST00000334553(c.688G>T,Gag>Tag):ENSP00000334523(p.Glu230Ter)];ENSG00000126214:NONSENSE[ENST00000445352(c.688G>T,Gag>Tag):ENSP00000412693(p.Glu230Ter)];ENSG00000126214:NONSENSE[ENST00000553286(c.688G>T,Gag>Tag):ENSP00000452487(p.Glu230Ter)];ENSG00000126214:NONSENSE[ENST00000554228(c.688G>T,Gag>Tag):ENSP00000450616(p.Glu230Ter)];ENSG00000126214:NONSENSE[ENST00000557450(c.688G>T,Gag>Tag):ENSP00000450648(p.Glu230Ter)];ENSG00000126214:NONSENSE[ENST00000389744(c.688G>T,Gag>Tag):ENSP00000374394(p.Glu230Ter)];ENSG00000126214:NONSENSE[ENST00000554280(c.688G>T,Gag>Tag):ENSP00000451242(p.Glu230Ter)];	chr14*10412	NA	NA	NA	NA	NA	NA	NA	NA	NA	HIGH	NA	0.986	80	80	0	78	61	17	21.79
Smoker	42479	chr14	95875830	G	A	*LINC00341*	EXONIC-NC	ENSG00000229645:[ENST00000541579(n.147C>T)];ENSG00000229645:[ENST00000464767(n.598C>T)];	NA	NA	NA	NA	NA	NA	NA	NA	NA	NA	MODIFIER	NA	NA	213	213	0	265	212	52	19.62
Smoker	42479	chr14	60485095	G	A	*LRRC9*	SILENT	ENSG00000131951:SILENT[ENST00000445360(c.3324G>A,gtG>gtA):ENSP00000454748(p.Val1108(=))];ENSG00000131951:3UTR[ENST00000254271(c.*13G>A)];	NA	NA	NA	NA	NA	NA	NA	NA	NA	NA	MODIFIER	NA	NA	65	65	0	78	31	47	60.26
Smoker	42479	chr14	60485096	G	A	*LRRC9*	MISSENSE	ENSG00000131951:MISSENSE[ENST00000445360(c.3325G>A,Gat>Aat):ENSP00000454748(p.Asp1109Asn)];ENSG00000131951:3UTR[ENST00000254271(c.*14G>A)];	NA	NA	NA	NA	NA	NA	NA	NA	NA	NA	MODIFIER	NA	NA	64	64	0	78	31	47	60.26
Smoker	42479	chr14	90769109	T	C	*NRDE2*	MISSENSE	ENSG00000119720:3UTR[ENST00000553409(c.*891A>G)];ENSG00000119720:INTRONIC[ENST00000556189(c.571+1170A>G)];ENSG00000119720:MISSENSE[ENST00000357904(c.673A>G,Atc>Gtc):ENSP00000350579(p.Ile225Val)];ENSG00000119720:MISSENSE[ENST00000354366(c.1366A>G,Atc>Gtc):ENSP00000346335(p.Ile456Val)];ENSG00000119720:MISSENSE[ENST00000554464(c.73A>G,Atc>Gtc):ENSP00000452071(p.Ile25Val)];	NA	NA	NA	8.34E-06	0	NA	NA	T	BN	N	MODERATE	NA	NA	72	72	0	114	91	23	20.18
Smoker	42479	chr14	20296257	C	G	*OR4N2*	MISSENSE	ENSG00000176294:MISSENSE[ENST00000315947(c.650C>G,tCc>tGc):ENSP00000319601(p.Ser217Cys)];ENSG00000176294:3UTR[ENST00000568211(c.*111C>G)];	NA	NA	chr14*20296257*20296257*C*T*rs868482041*NA*NA*NA	NA	NA	NA	NA	NA	NA	NA	MODIFIER	NA	NA	460	460	0	665	606	59	8.87
Smoker	42479	chr14	50141054	G	C	*POLE2*	INTRONIC	ENSG00000100479:[ENST00000539565(c.246-120C>G)];ENSG00000100479:[ENST00000216367(c.323+14C>G)];ENSG00000100479:[ENST00000556584(n.240-120C>G)];ENSG00000100479:[ENST00000554396(c.323+14C>G)];	NA	NA	0.0001997	2.48E-05	0	0.0001997	0	NA	NA	NA	MODIFIER	NA	NA	48	48	0	55	43	12	21.82
Smoker	42479	chr14	94712012	A	G	*PPP4R4*	INTRONIC-SS-DNR	ENSG00000119698:[ENST00000304338(c.1428+5A>G)];	NA	NA	chr14*94712012*94712012*A*T*rs760397065*NA*NA*NA	NA	NA	NA	NA	NA	NA	NA	LOW	NA	NA	40	39	0	62	47	15	24.19
Smoker	42479	chr14	94712801	C	A	*PPP4R4*	NONSENSE	ENSG00000119698:NONSENSE[ENST00000304338(c.1536C>A,tgC>tgA):ENSP00000305924(p.Cys512Ter)];	NA	NA	NA	NA	NA	NA	NA	NA	NA	NA	HIGH	NA	0.988	153	152	0	162	13	149	91.98
Smoker	42479	chr14	60485095	G	A	*RP11-16B13.1*	INTRONIC	ENSG00000258553:[ENST00000555432(n.153+11919C>T)];ENSG00000258553:[ENST00000554123(n.164+11919C>T)];	NA	NA	NA	NA	NA	NA	NA	NA	NA	NA	MODIFIER	NA	NA	65	65	0	78	31	47	60.26
Smoker	42479	chr14	60485096	G	A	*RP11-16B13.1*	INTRONIC	ENSG00000258553:[ENST00000555432(n.153+11918C>T)];ENSG00000258553:[ENST00000554123(n.164+11918C>T)];	NA	NA	NA	NA	NA	NA	NA	NA	NA	NA	MODIFIER	NA	NA	64	64	0	78	31	47	60.26
Smoker	42479	chr14	104129155	G	T	*RP11-73M18.2*	NONSENSE	ENSG00000256500:NONSENSE[ENST00000472726(c.1204G>T,Gag>Tag):ENSP00000439065(p.Glu402Ter)];	chr14*10412	NA	NA	NA	NA	NA	NA	NA	NA	NA	HIGH	NA	0.986	80	80	0	78	61	17	21.79
Smoker	42479	chr14	101350064	G	T	*RTL1*	SILENT	ENSG00000254656:SILENT[ENST00000534062(c.1062C>A,atC>atA):ENSP00000435342(p.Ile354(=))];	NA	NA	NA	NA	NA	NA	NA	NA	NA	NA	LOW	NA	NA	121	121	0	126	103	23	18.25
Smoker	42479	chr14	21834723	C	G	*SUPT16H*	INTRONIC	ENSG00000092201:[ENST00000216297(c.956-35G>C)];	NA	NA	NA	NA	NA	NA	NA	NA	NA	NA	MODIFIER	NA	NA	51	51	0	48	24	24	50
Smoker	42479	chr14	92264790	C	G	*TC2N*	INTRONIC	ENSG00000165929:[ENST00000556590(c.11-47G>C)];ENSG00000165929:[ENST00000556018(c.638-47G>C)];ENSG00000165929:[ENST00000435962(c.638-47G>C)];ENSG00000165929:[ENST00000360594(c.638-47G>C)];ENSG00000165929:[ENST00000340892(c.638-47G>C)];	NA	NA	NA	NA	NA	NA	NA	NA	NA	NA	MODIFIER	NA	NA	77	77	0	72	58	14	19.44
Smoker	42479	chr14	77812870	C	G	*TMED8*	INTRONIC	ENSG00000100580:[ENST00000216468(c.198-49G>C)];	NA	NA	chr14*77812866*77812870*GACTC*G*rs769900872*NA*NA*NA	NA	NA	NA	NA	NA	NA	NA	MODIFIER	NA	NA	67	67	0	89	72	17	19.1
Smoker	42479	chr14	22554687	G	A	*TRAV23DV6*	5UTR	ENSG00000211803:[ENST00000390451(c.-42G>A)];	NA	NA	NA	NA	NA	NA	NA	NA	NA	NA	MODIFIER	NA	NA	136	136	0	178	169	8	4.49
Smoker	42479	chr14	38260080	G	T	*TTC6*	INTRONIC	ENSG00000139865:[ENST00000553443(c.3557+38G>T)];ENSG00000139865:[ENST00000476979(c.-99-4395G>T)];ENSG00000139865:[ENST00000533625(c.*1062+51720G>T)];	NA	NA	NA	NA	NA	NA	NA	NA	NA	NA	MODIFIER	NA	NA	32	32	0	64	4	60	93.75

Mangalaparthi *et al.* , 2020. Mutational landscape of esophageal squamous cell carcinoma in an Indian cohort
Supplementary Table 4. List of somatic single nucleotide variants identified using whole exome sequencing of ESCC patients

Cohort	Sample	Chromosome	Genomic position	Reference allele	Alternate allele	Gene symbol	Variant type	Variant annotation	OncoMD	COSMIC	dbSNP	ExAC	ExAC_SAS	1000G	1000G_SAS	SIFT	Polyphen2	CONDEL	VEP	LRT	PhastCon7 Way	Total read (Adjacent normal)	Reference allel read support (Adjacent normal)	Alternative read support (Adjacent normal)	Total read (Tumor)	Reference allel read support (Tumor)	Alternative read support (Tumor)	Alternate allele frequency
Smoker	42479	chr15	84651022	G	C	*ADAMTSL3*	INTRONIC-SS-ACR	ENSG00000156218:[ENST00000562296(n.335-3G>C)];ENSG00000156218:[ENST00000561483(n.2860-3G>C)];ENSG00000156218:[ENST00000567716(n.543-3G>C)];ENSG00000156218:[ENST00000286744(c.2645-3G>C)];ENSG00000156218:[ENST00000567476(c.2645-3G>C)];	NA	NA	NA	NA	NA	NA	NA	NA	NA	NA	LOW	NA	NA	82	82	0	98	58	39	39.8
Smoker	42479	chr15	58350310	G	T	*ALDH1A2*	INTRONIC	ENSG00000128918:[ENST00000249750(c.117+7422C>A)];ENSG00000128918:[ENST00000560122(n.542-43831C>A)];ENSG00000128918:[ENST00000558073(n.416-43831C>A)];ENSG00000128918:[ENST00000558595(n.178+7422C>A)];ENSG00000128918:[ENST00000558231(c.31-43831C>A)];ENSG00000128918:[ENST00000559297(n.425-43831C>A)];ENSG00000128918:[ENST00000557967(c.-171-43831C>A)];ENSG00000128918:[ENST00000347587(c.117+7422C>A)];ENSG00000128918:[ENST00000558239(c.-171-43831C>A)];ENSG00000128918:[ENST00000430119(c.118-4C>A)];ENSG00000128918:[ENST00000537372(c.-43-4C>A)];	NA	NA	NA	NA	NA	NA	NA	NA	NA	NA	MODIFIER	NA	NA	152	152	0	156	122	34	21.79
Smoker	42479	chr15	44630036	C	T	*CASC4*	MISSENSE	ENSG00000166734:MISSENSE[ENST00000558847(c.75C>T,Cca>Tca):ENSP00000453465(p.Pro26Ser)];ENSG00000166734:MISSENSE[ENST00000559222(c.295C>T,Cca>Tca):ENSP00000453703(p.Pro99Ser)];ENSG00000166734:MISSENSE[ENST00000299957(c.652C>T,Cca>Tca):ENSP00000299957(p.Pro218Ser)];ENSG00000166734:MISSENSE[ENST00000345795(c.652C>T,Cca>Tca):ENSP00000335063(p.Pro218Ser)];ENSG00000166734:3UTR[ENST00000360824(c.*27C>T)];ENSG00000166734:3UTR[ENST00000557945(c.*27C>T)];ENSG00000166734:EXONIC-NC[ENST00000559037(n.255C>T)];	NA	NA	chr15*44630036*44630036*C*A*rs777238187*NA*NA*NA	NA	NA	NA	NA	NA	NA	NA	MODIFIER	NA	NA	65	65	0	76	63	13	17.11
Smoker	42479	chr15	78894455	A	G	*CHRNA3*	MISSENSE	ENSG00000080644:MISSENSE[ENST00000326828(c.529T>C,Ttc>Ctc):ENSP00000315602(p.Phe177Leu)];ENSG00000080644:MISSENSE[ENST00000348639(c.529T>C,Ttc>Ctc):ENSP00000267951(p.Phe177Leu)];ENSG00000080644:MISSENSE[ENST00000559658(c.529T>C,Ttc>Ctc):ENSP00000452896(p.Phe177Leu)];ENSG00000080644:EXONIC-NC[ENST00000558903(n.236T>C)];	NA	NA	NA	NA	NA	NA	NA	NA	NA	NA	MODIFIER	NA	NA	145	145	0	196	162	34	17.35
Smoker	42479	chr15	45559816	G	C	*CTD-2651B20.3*	INTRONIC	ENSG00000259520:[ENST00000560344(n.31+11579C>G)];ENSG00000259520:[ENST00000561404(n.56-2809C>G)];	NA	NA	chr15*45559816*45559816*G*A*rs16941213*1*0.9369*0.0631	NA	NA	NA	NA	NA	NA	NA	MODIFIER	NA	NA	103	103	0	140	94	46	32.86
Smoker	42479	chr15	75043616	G	C	*CYP1A2*	MISSENSE	ENSG00000140505:MISSENSE[ENST00000343932(c.918G>C,aaG>aaC):ENSP00000342007(p.Lys306Asn)];	NA	NA	NA	NA	NA	NA	NA	T	BN	N	MODERATE	N	0.825	138	136	0	152	77	75	49.34
Smoker	42479	chr15	45388290	G	A	*DUOX2*	INTRONIC	ENSG00000140279:[ENST00000389039(c.3848-32C>T)];ENSG00000140279:[ENST00000603300(c.3848-32C>T)];	NA	NA	NA	NA	NA	NA	NA	NA	NA	NA	MODIFIER	NA	NA	82	82	0	109	65	44	40.37
Smoker	42479	chr15	60297132	G	T	*FOXB1*	INTRONIC	ENSG00000171956:[ENST00000560857(n.410+290G>T)];ENSG00000171956:[ENST00000396057(c.-31G>T)];	NA	NA	NA	NA	NA	NA	NA	NA	NA	NA	MODIFIER	NA	NA	61	61	0	82	45	36	43.9
Smoker	42479	chr15	91034570	A	G	*IQGAP1*	SILENT	ENSG00000140575:SILENT[ENST00000560738(c.2538A>G,gcA>gcG):ENSP00000453181(p.Ala846(=))];ENSG00000140575:SILENT[ENST00000268182(c.4254A>G,gcA>gcG):ENSP00000268182(p.Ala1418(=))];ENSG00000140575:EXONIC-NC[ENST00000561132(n.268A>G)];ENSG00000140575:EXONIC-NC[ENST00000559674(n.120A>G)];ENSG00000140575:EXONIC-NC[ENST00000561461(n.441A>G)];ENSG00000140575:EXONIC-NC[ENST00000559031(n.301A>G)];	chr15*91034	NA	NA	NA	NA	NA	NA	NA	NA	NA	MODIFIER	NA	NA	114	114	0	122	64	58	47.54
Smoker	42479	chr15	68603678	T	A	*ITGA11*	MISSENSE	ENSG00000137809:MISSENSE[ENST00000315757(c.3037A>T,Acc>Tcc):ENSP00000327290(p.Thr1013Ser)];ENSG00000137809:MISSENSE[ENST00000423218(c.3037A>T,Acc>Tcc):ENSP00000403392(p.Thr1013Ser)];	NA	NA	NA	NA	NA	NA	NA	T	BN	N	MODERATE	D	0.997	28	28	0	47	29	18	38.3
Smoker	42479	chr15	94945798	T	A	*MCTP2*	3UTR	ENSG00000140563:[ENST00000557742(c.*12T>A)];ENSG00000140563:[ENST00000456504(c.*1623+550T>A)];ENSG00000140563:[ENST00000357742(c.2085+550T>A)];ENSG00000140563:[ENST00000451018(c.2085+550T>A)];	NA	NA	NA	NA	NA	NA	NA	NA	NA	NA	MODIFIER	NA	NA	263	263	0	324	184	140	43.21
Smoker	42479	chr15	66274614	C	G	*MEGF11*	MISSENSE	ENSG00000157890:5UTR[ENST00000395614(c.-1875G>C)];ENSG00000157890:MISSENSE[ENST00000409699(c.607G>C,Gag>Cag):ENSP00000386908(p.Glu203Gln)];ENSG00000157890:MISSENSE[ENST00000360698(c.607G>C,Gag>Cag):ENSP00000353919(p.Glu203Gln)];ENSG00000157890:MISSENSE[ENST00000395625(c.382G>C,Gag>Cag):ENSP00000378987(p.Glu128Gln)];ENSG00000157890:MISSENSE[ENST00000422354(c.607G>C,Gag>Cag):ENSP00000414475(p.Glu203Gln)];ENSG00000157890:MISSENSE[ENST00000288745(c.382G>C,Gag>Cag):ENSP00000288745(p.Glu128Gln)];	NA	NA	chr15*66274614*66274614*C*A*rs766239463*NA*NA*NA	NA	NA	NA	NA	T	BN	N	MODERATE	N	0.989	33	33	0	42	20	22	52.38
Smoker	42479	chr15	66416315	G	A	*MEGF11*	MISSENSE	ENSG00000157890:MISSENSE[ENST00000409699(c.122C>T,tCg>tTg):ENSP00000386908(p.Ser41Leu)];ENSG00000157890:MISSENSE[ENST00000422354(c.122C>T,tCg>tTg):ENSP00000414475(p.Ser41Leu)];ENSG00000157890:MISSENSE[ENST00000360698(c.122C>T,tCg>tTg):ENSP00000353919(p.Ser41Leu)];ENSG00000157890:5UTR[ENST00000395614(c.-2360C>T)];ENSG00000157890:5UTR[ENST00000395625(c.-3C>T)];ENSG00000157890:5UTR[ENST00000288745(c.-3C>T)];	chr15*6641	chr15*6641	NA	0.000173	0	NA	NA	NA	NA	NA	MODIFIER	NA	NA	91	91	0	146	120	26	17.81
Smoker	42479	chr15	42360937	C	T	*PLA2G4D*	SILENT	ENSG00000159337:SILENT[ENST00000290472(c.2433G>A,gcG>gcA):ENSP00000290472(p.Ala811(=))];ENSG00000159337:EXONIC-NC[ENST00000560932(n.1586G>A)];	NA	NA	NA	NA	NA	NA	NA	NA	NA	NA	MODIFIER	NA	NA	77	77	0	120	94	25	20.83

Supplementary Table 4. List of somatic single nucleotide variants identified using whole exome sequencing of ESCC patients

Cohort	Sample	Chromosome	Genomic position	Reference allele	Alternate allele	Gene symbol	Variant type	Variant annotation	OncoMD	COSMIC	dbSNP	ExAC	ExAC_SAS	1000G	1000G_SAS	SIFT	Polyphen2	CONDEL	VEP	LRT	PhastCon7 Way	Total read (Adjacent normal)	Reference allel read support (Adjacent normal)	Alternative read support (Adjacent normal)	Total read (Tumor)	Reference allel read support (Tumor)	Alternative read support (Tumor)	Alternate allele frequency
Smoker	42479	chr15	74315568	G	C	*PML*	MISSENSE	ENSG00000140464:MISSENSE[ENST00000563500(c.1002G>C,caG>caC):ENSP00000457032(p.Gln334His)];ENSG00000140464:MISSENSE[ENST00000395135(c.1002G>C,caG>caC):ENSP00000378567(p.Gln334His)];ENSG00000140464:MISSENSE[ENST00000569477(c.1002G>C,caG>caC):ENSP00000455612(p.Gln334His)];ENSG00000140464:MISSENSE[ENST00000565239(c.316G>C,caG>caC):ENSP00000456989(p.Gln106His)];ENSG00000140464:MISSENSE[ENST00000565898(c.1002G>C,caG>caC):ENSP00000455838(p.Gln334His)];ENSG00000140464:MISSENSE[ENST00000435786(c.1002G>C,caG>caC):ENSP00000395576(p.Gln334His)];ENSG00000140464:MISSENSE[ENST00000436891(c.1002G>C,caG>caC):ENSP00000394642(p.Gln334His)];ENSG00000140464:MISSENSE[ENST00000359928(c.1002G>C,caG>caC):ENSP00000353004(p.Gln334His)];ENSG00000140464:MISSENSE[ENST00000564428(c.1002G>C,caG>caC):ENSP00000457023(p.Gln334His)];ENSG00000140464:MISSENSE[ENST00000567543(c.1002G>C,caG>caC):ENSP00000456277(p.Gln334His)];ENSG00000140464:MISSENSE[ENST00000567606(c.247G>C,caG>caC):ENSP00000457669(p.Gln83His)];ENSG00000140464:MISSENSE[ENST00000569965(c.1002G>C,caG>caC):ENSP00000456486(p.Gln334His)];ENSG00000140464:MISSENSE[ENST00000354026(c.1002G>C,caG>caC):ENSP00000315434(p.Gln334His)];ENSG00000140464:MISSENSE[ENST00000268059(c.1002G>C,caG>caC):ENSP00000268059(p.Gln334His)];ENSG00000140464:MISSENSE[ENST00000268058(c.1002G>C,caG>caC):ENSP00000268058(p.Gln334His)];ENSG00000140464:MISSENSE[ENST00000395132(c.1002G>C,caG>caC):ENSP00000378564(p.Gln334His)];ENSG00000140464:EXONIC-NC[ENST00000564725(n.853G>C)];ENSG00000140464:EXONIC-NC[ENST00000569161(n.323G>C)];	NA	NA	NA	NA	NA	NA	NA	NA	NA	NA	MODIFIER	NA	NA	51	51	0	44	29	15	34.09
Smoker	42479	chr15	74317112	G	A	*PML*	INTRONIC	ENSG00000140464:[ENST00000563500(c.1184-86G>A)];ENSG00000140464:[ENST00000395135(c.1184-86G>A)];ENSG00000140464:[ENST00000569477(c.1184-86G>A)];ENSG00000140464:[ENST00000565239(c.498-86G>A)];ENSG00000140464:[ENST00000565898(c.1184-86G>A)];ENSG00000140464:[ENST00000435786(c.1184-86G>A)];ENSG00000140464:[ENST00000436891(c.1184-86G>A)];ENSG00000140464:[ENST00000564725(n.1035-86G>A)];ENSG00000140464:[ENST00000569161(n.462-86G>A)];ENSG00000140464:[ENST00000359928(c.1184-86G>A)];ENSG00000140464:[ENST00000564428(c.1184-86G>A)];ENSG00000140464:[ENST00000567543(c.1184-86G>A)];ENSG00000140464:[ENST00000566068(c.115-86G>A)];ENSG00000140464:[ENST00000567606(c.429-86G>A)];ENSG00000140464:[ENST00000569965(c.1184-86G>A)];ENSG00000140464:[ENST00000354026(c.1184-86G>A)];ENSG00000140464:[ENST00000268059(c.1184-86G>A)];ENSG00000140464:[ENST00000268058(c.1184-86G>A)];ENSG00000140464:[ENST00000395132(c.1184-86G>A)];	NA	NA	NA	NA	NA	NA	NA	NA	NA	NA	MODIFIER	NA	NA	28	28	0	77	41	36	46.75
Smoker	42479	chr15	22440736	C	G	*RP11-2F9.3*	EXONIC-NC	ENSG00000223877:[ENST00000415697(n.32G>C)];ENSG00000223877:[ENST00000460876(n.113G>C)];	NA	NA	NA	NA	NA	NA	NA	NA	NA	NA	MODIFIER	NA	NA	147	147	0	204	143	61	29.9
Smoker	42479	chr15	34038369	G	A	*RYR3*	INTRONIC	ENSG00000198838:[ENST00000389232(c.7989+11G>A)];ENSG00000198838:[ENST00000415757(c.7989+11G>A)];	NA	NA	NA	NA	NA	NA	NA	NA	NA	NA	MODIFIER	NA	NA	101	101	0	135	67	68	50.37
Smoker	42479	chr15	45559816	G	C	*SLC28A2*	INTRONIC	ENSG00000137860:[ENST00000559924(c.*467+38G>C)];ENSG00000137860:[ENST00000347644(c.1068+38G>C)];	NA	NA	chr15*45559816*45559816*G*A*rs16941213*1*0.9369*0.0631	NA	NA	NA	NA	NA	NA	NA	MODIFIER	NA	NA	103	103	0	140	94	46	32.86
Smoker	42479	chr15	25468523	G	T	*SNHG14*	INTRONIC	ENSG00000224078:[ENST00000453082(n.885+186G>T)];	NA	NA	chr15*25468523*25468523*G*A,C*rs554023312*0*0.9998*.,0.0001997	NA	NA	NA	NA	NA	NA	NA	MODIFIER	NA	NA	182	182	0	216	113	103	47.69
Smoker	42479	chr15	42171466	C	A	*SPTBN5*	SILENT	ENSG00000137877:SILENT[ENST00000320955(c.3174G>T,gtG>gtT):ENSP00000317790(p.Val1058(=))];	NA	NA	NA	NA	NA	NA	NA	NA	NA	NA	LOW	NA	NA	29	29	0	47	27	20	42.55
Smoker	42479	chr15	50888567	C	A	*TRPM7*	MISSENSE	ENSG00000092439:MISSENSE[ENST00000560955(c.3175G>T,Gat>Tat):ENSP00000453277(p.Asp1059Tyr)];ENSG00000092439:MISSENSE[ENST00000313478(c.3175G>T,Gat>Tat):ENSP00000320239(p.Asp1059Tyr)];ENSG00000092439:EXONIC-NC[ENST00000560284(n.556G>T)];	NA	NA	NA	NA	NA	NA	NA	NA	NA	NA	MODIFIER	NA	NA	62	62	0	76	71	5	6.58
Smoker	42479	chr15	71055699	G	A	*UACA*	SILENT	ENSG00000137831:SILENT[ENST00000539319(c.48C>T,ccC>ccT):ENSP00000438667(p.Pro16(=))];ENSG00000137831:SILENT[ENST00000322954(c.48C>T,ccC>ccT):ENSP00000314556(p.Pro16(=))];	NA	NA	NA	NA	NA	NA	NA	NA	NA	NA	LOW	NA	NA	21	21	0	54	45	9	16.67
Smoker	42479	chr15	90617726	G	C	*ZNF710*	MISSENSE	ENSG00000140548:MISSENSE[ENST00000559360(c.471G>C,Gag>Cag):ENSP00000453147(p.Glu158Gln)];ENSG00000140548:MISSENSE[ENST00000558883(c.1153G>C,Gag>Cag):ENSP00000453404(p.Glu385Gln)];ENSG00000140548:INTRONIC[ENST00000268154(c.1825+204G>C)];	NA	NA	NA	NA	NA	NA	NA	NA	BN	NA	MODERATE	NA	NA	51	51	0	63	40	23	36.51
Smoker	42479	chr16	48122555	G	C	*ABCC12*	MISSENSE	ENSG00000140798:3UTR[ENST00000532494(c.*367C>G)];ENSG00000140798:3UTR[ENST00000529504(c.*362C>G)];ENSG00000140798:3UTR[ENST00000416054(c.*1422C>G)];ENSG00000140798:3UTR[ENST00000448542(c.*362C>G)];ENSG00000140798:3UTR[ENST00000497206(c.*367C>G)];ENSG00000140798:3UTR[ENST00000534418(c.*367C>G)];ENSG00000140798:3UTR[ENST00000529084(c.*1422C>G)];ENSG00000140798:EXONIC-NC[ENST00000526251(n.210C>G)];ENSG00000140798:MISSENSE[ENST00000311303(c.3376C>G,Cag>Gag):ENSP00000311030(p.Gln1126Glu)];	NA	NA	NA	NA	NA	NA	NA	NA	NA	NA	MODIFIER	NA	NA	85	85	0	152	41	111	73.03
Smoker	42479	chr16	20448482	G	A	*ACSM5*	MISSENSE	ENSG00000183549:MISSENSE[ENST00000331849(c.1417G>A,Gat>Aat):ENSP00000327916(p.Asp473Asn)];ENSG00000183549:EXONIC-NC[ENST00000577024(n.242G>A)];	NA	NA	NA	8.24E-06	0	NA	NA	NA	NA	NA	MODIFIER	NA	NA	184	184	0	130	83	47	36.15
Smoker	42479	chr16	89349926	C	T	*ANKRD11*	SILENT	ENSG00000167522:3UTR[ENST00000330736(c.*2827G>A)];ENSG00000167522:SILENT[ENST00000378330(c.3024G>A,gaG>gaA):ENSP00000367581(p.Glu1008(=))];ENSG00000167522:SILENT[ENST00000301030(c.3024G>A,gaG>gaA):ENSP00000301030(p.Glu1008(=))];ENSG00000167522:INTRONIC[ENST00000562194(c.151+5010G>A)];	NA	NA	NA	NA	NA	NA	NA	NA	NA	NA	MODIFIER	NA	NA	168	168	0	119	82	37	31.09

Mangalaparthi *et al.* , 2020. Mutational landscape of esophageal squamous cell carcinoma in an Indian cohort
Supplementary Table 4. List of somatic single nucleotide variants identified using whole exome sequencing of ESCC patients

Cohort	Sample	Chromosome	Genomic position	Reference allele	Alternate allele	Gene symbol	Variant type	Variant annotation	OncoMD	COSMIC	dbSNP	ExAC	ExAC_SAS	1000G	1000G_SAS	SIFT	Polyphen2	CONDEL	VEP	LRT	PhastCon7 Way	Total read (Adjacent normal)	Reference allel read support (Adjacent normal)	Alternative read support (Adjacent normal)	Total read (Tumor)	Reference allel read support (Tumor)	Alternative read support (Tumor)	Alternate allele frequency
Smoker	42479	chr16	61851463	T	C	*CDH8*	SILENT	ENSG00000150394:SILENT[ENST00000584337(c.1197A>G,gaA>gaG):ENSP00000463511(p.Glu399(=))];ENSG00000150394:SILENT[ENST00000577390(c.1197A>G,gaA>gaG):ENSP00000462701(p.Glu399(=))];ENSG00000150394:SILENT[ENST00000577730(c.1197A>G,gaA>gaG):ENSP00000462018(p.Glu399(=))];ENSG00000150394:SILENT[ENST00000299345(c.1197A>G,gaA>gaG):ENSP00000299345(p.Glu399(=))];ENSG00000150394:SILENT[ENST00000583382(c.1197A>G,gaA>gaG):ENSP00000462976(p.Glu399(=))];ENSG00000150394:SILENT[ENST00000585315(c.1197A>G,gaA>gaG):ENSP00000463266(p.Glu399(=))];ENSG00000150394:EXONIC-NC[ENST00000582242(n.485A>G)];	NA	NA	NA	NA	NA	NA	NA	NA	NA	NA	LOW	NA	NA	81	81	0	42	23	19	45.24
Smoker	42479	chr16	53355384	C	G	*CHD9*	INTRONIC	ENSG00000177200:[ENST00000564641(c.*23-54C>G)];ENSG00000177200:[ENST00000566029(c.7666-54C>G)];ENSG00000177200:[ENST00000219084(n.5203-54C>G)];ENSG00000177200:[ENST00000447540(c.7669-54C>G)];ENSG00000177200:[ENST00000564845(c.7666-54C>G)];ENSG00000177200:[ENST00000564600(c.*134-54C>G)];ENSG00000177200:[ENST00000398510(c.7714-54C>G)];	NA	NA	NA	NA	NA	NA	NA	NA	NA	NA	MODIFIER	NA	NA	48	47	0	64	47	17	26.56
Smoker	42479	chr16	67573692	C	T	*CTD-2012K14.2*	INTRONIC	ENSG00000261396:[ENST00000567122(n.62+256G>A)];	NA	NA	NA	NA	NA	NA	NA	NA	NA	NA	MODIFIER	NA	NA	50	50	0	40	18	22	55
Smoker	42479	chr16	67573692	C	T	*FAM65A*	INTRONIC	ENSG00000039523:[ENST00000428437(c.402+38C>T)];ENSG00000039523:[ENST00000566522(n.151+38C>T)];ENSG00000039523:[ENST00000566559(c.360+38C>T)];ENSG00000039523:[ENST00000569253(c.249+38C>T)];ENSG00000039523:[ENST00000422602(c.420+38C>T)];ENSG00000039523:[ENST00000561534(n.128+38C>T)];ENSG00000039523:[ENST00000540839(c.420+38C>T)];ENSG00000039523:[ENST00000042381(c.360+38C>T)];ENSG00000039523:[ENST00000566815(c.343+38C>T)];ENSG00000039523:[ENST00000565176(c.360+38C>T)];ENSG00000039523:[ENST00000379312(c.372+38C>T)];	NA	NA	NA	NA	NA	NA	NA	NA	NA	NA	MODIFIER	NA	NA	50	50	0	40	18	22	55
Smoker	42479	chr16	71065741	G	C	*HYDIN*	MISSENSE	ENSG00000157423:MISSENSE[ENST00000448089(c.2609C>G,aCc>aGc):ENSP00000398544(p.Thr870Ser)];ENSG00000157423:MISSENSE[ENST00000541601(c.2660C>G,aCc>aGc):ENSP00000437341(p.Thr887Ser)];ENSG00000157423:MISSENSE[ENST00000538568(c.163C>G,aCc>aGc):ENSP00000462031(p.Thr55Ser)];ENSG00000157423:MISSENSE[ENST00000448691(c.2609C>G,aCc>aGc):ENSP00000394826(p.Thr870Ser)];ENSG00000157423:MISSENSE[ENST00000393552(c.1463C>G,aCc>aGc):ENSP00000463767(p.Thr488Ser)];ENSG00000157423:MISSENSE[ENST00000393567(c.2609C>G,aCc>aGc):ENSP00000377197(p.Thr870Ser)];ENSG00000157423:MISSENSE[ENST00000538248(c.2690C>G,aCc>aGc):ENSP00000444970(p.Thr897Ser)];ENSG00000157423:MISSENSE[ENST00000321489(c.2609C>G,aCc>aGc):ENSP00000314736(p.Thr870Ser)];ENSG00000157423:3UTR[ENST00000545230(c.*582C>G)];ENSG00000157423:3UTR[ENST00000539447(c.*1857C>G)];ENSG00000157423:EXONIC-NC[ENST00000540892(n.1097C>G)];	NA	NA	NA	NA	NA	NA	NA	T	BN	N	MODERATE	U	0.495	368	368	0	279	231	48	17.2
Smoker	42479	chr16	71167190	C	T	*HYDIN*	INTRONIC	ENSG00000157423:[ENST00000288168(c.1095-3464G>A)];ENSG00000157423:[ENST00000448089(c.1044-3464G>A)];ENSG00000157423:[ENST00000393550(c.1044-3464G>A)];ENSG00000157423:[ENST00000545230(c.408-3464G>A)];ENSG00000157423:[ENST00000448691(c.1044-3464G>A)];ENSG00000157423:[ENST00000539447(c.*292-3464G>A)];ENSG00000157423:[ENST00000393567(c.1044-3464G>A)];ENSG00000157423:[ENST00000541601(c.1095-3464G>A)];ENSG00000157423:[ENST00000538248(c.1125-3464G>A)];ENSG00000157423:[ENST00000321489(c.1044-3464G>A)];ENSG00000157423:[ENST00000538382(c.560-4G>A)];ENSG00000157423:[ENST00000536785(n.436-4G>A)];	NA	NA	NA	NA	NA	NA	NA	NA	NA	NA	MODIFIER	NA	NA	268	268	0	227	185	42	18.5
Smoker	42479	chr16	55360358	C	A	*IRX6*	NONSENSE	ENSG00000159387:NONSENSE[ENST00000290552(c.156C>A,taC>taA):ENSP00000290552(p.Tyr52Ter)];ENSG00000159387:EXONIC-NC[ENST00000558315(n.322C>A)];	NA	NA	chr16*55360356*55360367*TACGATAGTCGA*T*rs751117139*NA*NA*NA|chr16*55360358*55360358*C*T*rs779816494*NA*NA*NA	NA	NA	NA	NA	NA	NA	NA	MODIFIER	NA	NA	58	58	0	55	33	22	40
Smoker	42479	chr16	27230305	C	G	*KDM8*	MISSENSE	ENSG00000155666:MISSENSE[ENST00000441782(c.981C>G,atC>atG):ENSP00000398410(p.Ile327Met)];ENSG00000155666:MISSENSE[ENST00000286096(c.867C>G,atC>atG):ENSP00000286096(p.Ile289Met)];ENSG00000155666:MISSENSE[ENST00000567735(c.107C>G,atC>atG):ENSP00000455463(p.Ile36Met)];ENSG00000155666:INTRONIC[ENST00000380948(c.499-1582C>G)];ENSG00000155666:INTRONIC[ENST00000568965(c.499-1582C>G)];ENSG00000155666:EXONIC-NC[ENST00000567785(n.363C>G)];ENSG00000155666:EXONIC-NC[ENST00000563571(n.502C>G)];ENSG00000155666:EXONIC-NC[ENST00000567366(n.355C>G)];	NA	NA	chr16*27230305*27230305*C*A*rs780097184*NA*NA*NA	NA	NA	NA	NA	NA	NA	NA	MODIFIER	NA	NA	64	64	0	54	27	27	50
Smoker	42479	chr16	3441762	C	G	*NAA60*	INTRONIC	ENSG00000262621:[ENST00000575785(c.-12-12664C>G)];	NA	NA	NA	NA	NA	NA	NA	NA	NA	NA	MODIFIER	NA	NA	127	126	0	87	51	36	41.38
Smoker	42479	chr16	5009331	C	G	*PPL*	INTRONIC	ENSG00000118898:[ENST00000592772(c.-92+1234G>C)];	NA	NA	NA	NA	NA	NA	NA	D	BN	D	MODERATE	NA	NA	89	89	0	75	45	30	40
Smoker	42479	chr16	24574561	C	T	*RBBP6*	MISSENSE	ENSG00000122257:MISSENSE[ENST00000348022(c.1331C>T,tCa>tTa):ENSP00000316291(p.Ser444Leu)];ENSG00000122257:MISSENSE[ENST00000319715(c.1331C>T,tCa>tTa):ENSP00000317872(p.Ser444Leu)];ENSG00000122257:MISSENSE[ENST00000564314(c.932C>T,tCa>tTa):ENSP00000456750(p.Ser311Leu)];ENSG00000122257:INTRONIC[ENST00000381039(c.1289+1079C>T)];ENSG00000122257:EXONIC-NC[ENST00000562430(n.2121C>T)];	NA	NA	NA	1.67E-05	0	NA	NA	NA	NA	NA	MODIFIER	NA	NA	135	135	0	73	41	31	42.47
Smoker	42479	chr16	55360358	C	A	*RP11-26L20.3*	INTRONIC	ENSG00000259283:[ENST00000558730(n.88+7055G>T)];	NA	NA	chr16*55360356*55360367*TACGATAGTCGA*T*rs751117139*NA*NA*NA|chr16*55360358*55360358*C*T*rs779816494*NA*NA*NA	NA	NA	NA	NA	NA	NA	NA	MODIFIER	NA	NA	58	58	0	55	33	22	40
Smoker	42479	chr16	5009331	C	G	*SEC14L5*	MISSENSE	ENSG00000103184:MISSENSE[ENST00000251170(c.7C>G,Caa>Gaa):ENSP00000251170(p.Gln3Glu)];ENSG00000103184:MISSENSE[ENST00000587469(c.7C>G,Caa>Gaa):ENSP00000468423(p.Gln3Glu)];	NA	NA	NA	NA	NA	NA	NA	D	BN	D	MODERATE	NA	NA	89	89	0	75	45	30	40

Mangalaparthi *et al.* , 2020. Mutational landscape of esophageal squamous cell carcinoma in an Indian cohort
Supplementary Table 4. List of somatic single nucleotide variants identified using whole exome sequencing of ESCC patients

Cohort	Sample	Chromosome	Genomic position	Reference allele	Alternate allele	Gene symbol	Variant type	Variant annotation	OncoMD	COSMIC	dbSNP	ExAC	ExAC_SAS	1000G	1000G_SAS	SIFT	Polyphen2	CONDEL	VEP	LRT	PhastCon7 Way	Total read (Adjacent normal)	Reference allel read support (Adjacent normal)	Alternative read support (Adjacent normal)	Total read (Tumor)	Reference allel read support (Tumor)	Alternative read support (Tumor)	Alternate allele frequency
Smoker	42479	chr16	30976495	G	A	*SETD1A*	MISSENSE	ENSG00000099381:MISSENSE[ENST00000262519(c.1432G>A,Gag>Aag):ENSP00000262519(p.Glu478Lys)];	NA	NA	NA	NA	NA	NA	NA	NA	PoD	NA	MODERATE	D	0.998	54	54	0	59	30	29	49.15
Smoker	42479	chr16	87866539	C	T	*SLC7A5*	3UTR	ENSG00000103257:[ENST00000261622(c.*37G>A)];ENSG00000103257:[ENST00000565644(c.*37G>A)];ENSG00000103257:[ENST00000563489(n.579G>A)];	NA	NA	NA	NA	NA	NA	NA	NA	NA	NA	MODIFIER	NA	NA	41	41	0	27	15	12	44.44
Smoker	42479	chr16	2820874	C	T	*SRRM2*	3UTR	ENSG00000167978:[ENST00000573692(c.*6C>T)];ENSG00000167978:[ENST00000301740(c.*6C>T)];ENSG00000167978:[ENST00000574866(n.577C>T)];ENSG00000167978:[ENST00000574331(n.1222C>T)];	NA	NA	chr16*2820874*2820874*C*G,T*rs370538116*NA*NA*NA	2.55E-05	0.00019101	NA	NA	NA	NA	NA	MODIFIER	NA	NA	32	32	0	33	16	17	51.52
Smoker	42479	chr16	731517	C	T	*STUB1*	SILENT	ENSG00000103266:EXONIC-NC[ENST00000569248(n.1012C>T)];ENSG00000103266:EXONIC-NC[ENST00000563505(n.534C>T)];ENSG00000103266:EXONIC-NC[ENST00000566181(n.207C>T)];ENSG00000103266:SILENT[ENST00000565677(c.222C>T,cgC>cgT):ENSP00000457228(p.Arg74(=))];ENSG00000103266:SILENT[ENST00000567173(c.381C>T,cgC>cgT):ENSP00000456591(p.Arg127(=))];ENSG00000103266:SILENT[ENST00000219548(c.438C>T,cgC>cgT):ENSP00000219548(p.Arg146(=))];ENSG00000103266:SILENT[ENST00000564316(c.37C>T,cgC>cgT):ENSP00000457090(p.Arg13(=))];ENSG00000103266:SILENT[ENST00000564370(c.222C>T,cgC>cgT):ENSP00000456875(p.Arg74(=))];ENSG00000103266:SILENT[ENST00000566408(c.155C>T,cgC>cgT):ENSP00000457583(p.Arg52(=))];	NA	NA	0.000599	0.0001114	0	0.000599	0	NA	NA	NA	LOW	NA	NA	42	42	0	37	25	12	32.43
Smoker	42479	chr16	717456	G	C	*WDR90*	MISSENSE	ENSG00000161996:MISSENSE[ENST00000549091(c.5120G>C,tGt>tCt):ENSP00000448122(p.Cys1707Ser)];ENSG00000161996:MISSENSE[ENST00000547944(c.911G>C,tGt>tCt):ENSP00000449576(p.Cys304Ser)];ENSG00000161996:MISSENSE[ENST00000315764(c.767G>C,tGt>tCt):ENSP00000322808(p.Cys256Ser)];ENSG00000161996:MISSENSE[ENST00000293879(c.5114G>C,tGt>tCt):ENSP00000293879(p.Cys1705Ser)];ENSG00000161996:3UTR[ENST00000551100(c.*13G>C)];ENSG00000161996:3UTR[ENST00000552728(c.*1331G>C)];ENSG00000161996:EXONIC-NC[ENST00000553080(n.1568G>C)];ENSG00000161996:EXONIC-NC[ENST00000549024(n.1884G>C)];ENSG00000161996:EXONIC-NC[ENST00000547407(n.3474G>C)];ENSG00000161996:INTRONIC[ENST00000546896(c.80-58G>C)];	NA	NA	NA	NA	NA	NA	NA	NA	NA	NA	MODIFIER	NA	NA	33	33	0	26	19	7	26.92
Smoker	42479	chr16	3441762	C	G	*ZSCAN32*	INTRONIC	ENSG00000140987:[ENST00000571906(c.367-1245G>C)];ENSG00000140987:[ENST00000573830(c.-116-6821G>C)];ENSG00000140987:[ENST00000573719(c.532+1887G>C)];ENSG00000140987:[ENST00000574940(c.533-1245G>C)];ENSG00000140987:[ENST00000396846(c.533-1245G>C)];ENSG00000140987:[ENST00000574084(n.200+5430G>C)];ENSG00000140987:[ENST00000439568(c.-116-6821G>C)];ENSG00000140987:[ENST00000575350(c.-154-1245G>C)];ENSG00000140987:[ENST00000573327(c.533-1245G>C)];ENSG00000140987:[ENST00000304926(c.-154-1245G>C)];ENSG00000140987:[ENST00000422427(c.-59-1579G>C)];ENSG00000140987:[ENST00000396852(c.533-1245G>C)];ENSG00000140987:[ENST00000576500(c.366+5430G>C)];	NA	NA	NA	NA	NA	NA	NA	NA	NA	NA	MODIFIER	NA	NA	127	126	0	87	51	36	41.38
Smoker	42479	chr17	67161174	G	C	*ABCA10*	SILENT	ENSG00000154263:3UTR[ENST00000416101(c.*1636C>G)];ENSG00000154263:3UTR[ENST00000522406(c.*2141C>G)];ENSG00000154263:3UTR[ENST00000518929(c.*2259C>G)];ENSG00000154263:EXONIC-NC[ENST00000519732(n.436C>G)];ENSG00000154263:SILENT[ENST00000269081(c.3213C>G,ctC>ctG):ENSP00000269081(p.Leu1071(=))];ENSG00000154263:SILENT[ENST00000523419(c.3213C>G,ctC>ctG):ENSP00000428032(p.Leu1071(=))];	NA	NA	NA	NA	NA	NA	NA	NA	NA	NA	MODIFIER	NA	NA	86	86	0	83	65	18	21.69
Smoker	42479	chr17	66865951	T	C	*ABCA8*	INTRONIC	ENSG00000141338:[ENST00000269080(c.4512-31A>G)];ENSG00000141338:[ENST00000430352(c.4632-31A>G)];ENSG00000141338:[ENST00000586539(c.4632-31A>G)];	NA	NA	NA	8.27E-06	0	NA	NA	NA	NA	NA	MODIFIER	NA	NA	94	93	1	104	44	60	57.69
Smoker	42479	chr17	61566088	G	C	*ACE*	SILENT	ENSG00000159640:3UTR[ENST00000582005(c.*305G>C)];ENSG00000159640:3UTR[ENST00000578839(c.*455G>C)];ENSG00000159640:3UTR[ENST00000579314(c.*114G>C)];ENSG00000159640:3UTR[ENST00000579204(c.*533G>C)];ENSG00000159640:SILENT[ENST00000490216(c.663G>C,ggG>ggC):ENSP00000464539(p.Gly221(=))];ENSG00000159640:SILENT[ENST00000428043(c.2385G>C,ggG>ggC):ENSP00000397593(p.Gly795(=))];ENSG00000159640:SILENT[ENST00000290866(c.2385G>C,ggG>ggC):ENSP00000290866(p.Gly795(=))];ENSG00000159640:SILENT[ENST00000582761(c.153G>C,ggG>ggC):ENSP00000462909(p.Gly51(=))];ENSG00000159640:SILENT[ENST00000290863(c.663G>C,ggG>ggC):ENSP00000290863(p.Gly221(=))];ENSG00000264813:SILENT[ENST00000577647(c.663G>C,ggG>ggC):ENSP00000464149(p.Gly221(=))];ENSG00000159640:SILENT[ENST00000413513(c.663G>C,ggG>ggC):ENSP00000392247(p.Gly221(=))];ENSG00000159640:SILENT[ENST00000421982(c.315G>C,ggG>ggC):ENSP00000387760(p.Gly105(=))];ENSG00000159640:EXONIC-NC[ENST00000584865(n.331G>C)];	NA	NA	NA	NA	NA	NA	NA	NA	NA	NA	LOW	NA	NA	96	96	0	128	92	36	28.12
Smoker	42479	chr17	61574285	G	A	*ACE*	SILENT	ENSG00000159640:SILENT[ENST00000490216(c.1908G>A,acG>acA):ENSP00000464539(p.Thr636(=))];ENSG00000159640:SILENT[ENST00000290866(c.3630G>A,acG>acA):ENSP00000290866(p.Thr1210(=))];ENSG00000159640:SILENT[ENST00000290863(c.1908G>A,acG>acA):ENSP00000290863(p.Thr636(=))];ENSG00000264813:SILENT[ENST00000577647(c.1908G>A,acG>acA):ENSP00000464149(p.Thr636(=))];ENSG00000159640:SILENT[ENST00000413513(c.1785G>A,acG>acA):ENSP00000392247(p.Thr595(=))];ENSG00000159640:SILENT[ENST00000579409(c.317G>A,acG>acA):ENSP00000464428(p.Thr106(=))];ENSG00000159640:SILENT[ENST00000421982(c.1245G>A,acG>acA):ENSP00000387760(p.Thr415(=))];ENSG00000159640:3UTR[ENST00000578839(c.*1385G>A)];ENSG00000159640:3UTR[ENST00000579314(c.*1359G>A)];ENSG00000159640:EXONIC-NC[ENST00000582244(n.504G>A)];ENSG00000159640:EXONIC-NC[ENST00000577418(n.640G>A)];ENSG00000159640:INTRONIC[ENST00000428043(c.3410+501G>A)];	NA	NA	NA	9.58E-05	0	NA	NA	NA	NA	NA	LOW	NA	NA	20	20	0	22	13	9	40.91
Smoker	42479	chr17	63531708	C	T	*AXIN2*	INTRONIC	ENSG00000168646:[ENST00000375702(c.2042+36G>A)];ENSG00000168646:[ENST00000578251(n.459+36G>A)];ENSG00000168646:[ENST00000307078(c.2237+36G>A)];	NA	NA	NA	3.33E-05	0	NA	NA	NA	NA	NA	MODIFIER	NA	NA	100	100	0	82	71	11	13.41

Cohort	Sample	Chromosome	Genomic position	Reference allele	Alternate allele	Gene symbol	Variant type	Variant annotation	OncoMD	COSMIC	dbSNP	ExAC	ExAC_SAS	1000G	1000G_SAS	SIFT	Polyphen2	CONDEL	VEP	LRT	PhastCon7 Way	Total read (Adjacent normal)	Reference allel read support (Adjacent normal)	Alternative read support (Adjacent normal)	Total read (Tumor)	Reference allel read support (Tumor)	Alternative read support (Tumor)	Alternate allele frequency
Smoker	42479	chr17	1326945	C	A	*CRK*	INTRONIC-SS-ACR	ENSG00000167193:INTRONIC-SS-ACR[ENST00000572145(n.577-1G>T)];ENSG00000167193:INTRONIC-SS-ACR[ENST00000398970(c.608-1G>T)];ENSG00000167193:INTRONIC-SS-ACR[ENST00000300574(c.778-1G>T)];ENSG00000167193:INTRONIC[ENST00000574295(c.400-466G>T)];	NA	NA	NA	NA	NA	NA	NA	NA	NA	NA	MODIFIER	NA	NA	86	86	0	90	61	29	32.22
Smoker	42479	chr17	42289636	G	C	*CTB-175E5.7*	INTRONIC	ENSG00000267394:[ENST00000586560(n.54-3823G>C)];	NA	NA	NA	NA	NA	NA	NA	NA	NA	NA	MODIFIER	NA	NA	24	24	0	26	11	15	57.69
Smoker	42479	chr17	63052428	C	T	*GNA13*	INTRONIC-SS-DNR	ENSG00000120063:INTRONIC-SS-DNR[ENST00000439174(c.283+1G>A)];	NA	NA	NA	NA	NA	NA	NA	NA	NA	NA	HIGH	NA	NA	23	22	0	22	17	5	22.73
Smoker	42479	chr17	44145034	C	T	*KANSL1*	INTRONIC-SS-ACR	ENSG00000120071:INTRONIC-SS-ACR[ENST00000393476(c.-396-1G>A)];ENSG00000120071:INTRONIC-SS-ACR[ENST00000432791(c.1534-1G>A)];ENSG00000120071:INTRONIC-SS-ACR[ENST00000577114(n.331-1G>A)];ENSG00000120071:INTRONIC-SS-ACR[ENST00000262419(c.1534-1G>A)];ENSG00000120071:INTRONIC-SS-ACR[ENST00000572904(c.1534-1G>A)];ENSG00000120071:INTRONIC-SS-ACR[ENST00000575318(c.1534-1G>A)];ENSG00000120071:INTRONIC-SS-ACR[ENST00000574590(c.1534-1G>A)];	NA	NA	NA	NA	NA	NA	NA	NA	NA	NA	HIGH	NA	NA	206	205	0	221	154	67	30.32
Smoker	42479	chr17	39553279	G	C	*KRT31*	INTRONIC	ENSG00000094796:[ENST00000251645(c.349-32C>G)];	NA	NA	NA	NA	NA	NA	NA	NA	NA	NA	MODIFIER	NA	NA	37	37	0	27	20	7	25.93
Smoker	42479	chr17	60060470	C	G	*MED13*	MISSENSE	ENSG00000108510:MISSENSE[ENST00000397786(c.2894G>C,gGa>gCa):ENSP00000380888(p.Gly965Ala)];	NA	NA	NA	NA	NA	NA	NA	T	BN	N	MODERATE	D	1	19	19	0	22	9	13	59.09
Smoker	42479	chr17	16041449	T	G	*NCOR1*	INTRONIC	ENSG00000141027:[ENST00000582357(c.1434+23A>C)];ENSG00000141027:[ENST00000395848(c.1080+23A>C)];ENSG00000141027:[ENST00000395851(c.1407+23A>C)];ENSG00000141027:[ENST00000460276(c.*1165+23A>C)];ENSG00000141027:[ENST00000436828(c.1434+23A>C)];ENSG00000141027:[ENST00000411510(c.1407+23A>C)];ENSG00000141027:[ENST00000268712(c.1407+23A>C)];	NA	NA	NA	NA	NA	NA	NA	NA	NA	NA	MODIFIER	NA	NA	64	64	0	60	6	54	90
Smoker	42479	chr17	36918619	C	G	*PSMB3*	INTRONIC	ENSG00000108294:[ENST00000584662(c.*74-45C>G)];ENSG00000108294:[ENST00000225426(c.475-45C>G)];ENSG00000108294:[ENST00000579729(c.297-45C>G)];ENSG00000108294:[ENST00000581388(n.225-45C>G)];ENSG00000108294:[ENST00000583873(n.316C>G)];	NA	NA	NA	NA	NA	NA	NA	NA	NA	NA	MODIFIER	NA	NA	43	42	1	56	24	32	57.14
Smoker	42479	chr17	33680393	G	C	*SLFN11*	SILENT	ENSG00000172716:SILENT[ENST00000394566(c.1884C>G,ctC>ctG):ENSP00000378067(p.Leu628(=))];ENSG00000172716:SILENT[ENST00000308377(c.1884C>G,ctC>ctG):ENSP00000312402(p.Leu628(=))];	NA	NA	NA	NA	NA	NA	NA	NA	NA	NA	LOW	NA	NA	171	171	0	187	133	54	28.88
Smoker	42479	chr17	27005183	G	C	*SUPT6H*	SILENT	ENSG00000109111:SILENT[ENST00000314616(c.1089G>C,ctG>ctC):ENSP00000319104(p.Leu363(=))];ENSG00000109111:SILENT[ENST00000347486(c.1089G>C,ctG>ctC):ENSP00000338143(p.Leu363(=))];	NA	NA	NA	NA	NA	NA	NA	NA	NA	NA	LOW	NA	NA	67	67	0	58	7	51	87.93
Smoker	42479	chr17	35837005	G	C	*TADA2A*	MISSENSE	ENSG00000108264:MISSENSE[ENST00000490992(c.1250G>C,aGa>aCa):ENSP00000432503(p.Arg417Thr)];ENSG00000108264:MISSENSE[ENST00000394395(c.1250G>C,aGa>aCa):ENSP00000377918(p.Arg417Thr)];ENSG00000108264:MISSENSE[ENST00000225396(c.1250G>C,aGa>aCa):ENSP00000225396(p.Arg417Thr)];ENSG00000108264:EXONIC-NC[ENST00000591933(n.728G>C)];	NA	NA	NA	NA	NA	NA	NA	NA	NA	NA	MODIFIER	NA	NA	111	111	0	86	65	21	24.42
Smoker	42479	chr17	80789537	G	T	*TBCD*	INTRONIC	ENSG00000141556:[ENST00000539345(c.1318+16727G>T)];ENSG00000141556:[ENST00000397466(c.160+16727G>T)];ENSG00000141556:[ENST00000355528(c.1318+16727G>T)];	NA	NA	NA	NA	NA	NA	NA	NA	NA	NA	MODIFIER	NA	NA	119	119	0	115	13	102	88.7
Smoker	42479	chr17	42289636	G	C	*UBTF*	INTRONIC	ENSG00000108312:[ENST00000393606(c.661-262C>G)];ENSG00000108312:[ENST00000302904(c.771+76C>G)];ENSG00000108312:[ENST00000529383(c.771+76C>G)];ENSG00000108312:[ENST00000436088(c.771+76C>G)];ENSG00000108312:[ENST00000533177(c.661-262C>G)];ENSG00000108312:[ENST00000526094(c.661-262C>G)];ENSG00000108312:[ENST00000527034(c.661-262C>G)];ENSG00000108312:[ENST00000343638(c.661-262C>G)];ENSG00000108312:[ENST00000529042(n.447C>G)];	NA	NA	NA	NA	NA	NA	NA	NA	NA	NA	MODIFIER	NA	NA	24	24	0	26	11	15	57.69
Smoker	42479	chr17	5076236	C	G	*USP6*	MISSENSE	ENSG00000129204:MISSENSE[ENST00000304328(c.3233C>G,tCt>tGt):ENSP00000305473(p.Ser1078Cys)];ENSG00000129204:MISSENSE[ENST00000250066(c.4184C>G,tCt>tGt):ENSP00000250066(p.Ser1395Cys)];ENSG00000129204:MISSENSE[ENST00000574788(c.4184C>G,tCt>tGt):ENSP00000460380(p.Ser1395Cys)];ENSG00000129204:3UTR[ENST00000572949(c.*1711C>G)];ENSG00000129204:3UTR[ENST00000575709(c.*3264C>G)];ENSG00000129204:3UTR[ENST00000332776(c.*1711C>G)];	NA	NA	NA	NA	NA	NA	NA	NA	NA	NA	MODIFIER	NA	NA	111	111	0	146	98	48	32.88
Smoker	42479	chr17	9536380	C	T	*WDR16*	INTRONIC	ENSG00000166596:[ENST00000576714(n.68+30C>T)];ENSG00000166596:[ENST00000576630(c.*1381+30C>T)];ENSG00000166596:[ENST00000352665(c.1320+30C>T)];ENSG00000166596:[ENST00000396219(c.1116+30C>T)];ENSG00000166596:[ENST00000299764(c.1350+30C>T)];	NA	NA	NA	5.17E-05	0	NA	NA	NA	NA	NA	MODIFIER	NA	NA	100	100	0	80	47	33	41.25
Smoker	42479	chr17	44910671	G	T	*WNT9B*	5UTR	ENSG00000158955:[ENST00000575372(c.-41G>T)];	NA	NA	chr17*44910671*44910671*G*A*rs757575201*NA*NA*NA	NA	NA	NA	NA	NA	NA	NA	MODIFIER	NA	NA	51	51	0	66	37	29	43.94
Smoker	42479	chr17	80789537	G	T	*ZNF750*	NONSENSE	ENSG00000141579:NONSENSE[ENST00000269394(c.794C>A,tCg>tAg):ENSP00000269394(p.Ser265Ter)];ENSG00000141579:INTRONIC[ENST00000572562(c.-229-175C>A)];	NA	NA	NA	NA	NA	NA	NA	NA	NA	NA	MODIFIER	NA	NA	119	119	0	115	13	102	88.7
Smoker	42479	chr18	9204407	G	T	*ANKRD12*	INTRONIC	ENSG00000101745:[ENST00000546007(c.236-4248G>T)];ENSG00000101745:[ENST00000581635(c.*117+3765G>T)];ENSG00000101745:[ENST00000262126(c.236-67G>T)];ENSG00000101745:[ENST00000383440(c.236-4248G>T)];ENSG00000101745:[ENST00000581758(n.139-4248G>T)];ENSG00000101745:[ENST00000400020(c.236-4248G>T)];ENSG00000101745:[ENST00000540578(n.448-67G>T)];	NA	NA	NA	NA	NA	NA	NA	NA	NA	NA	MODIFIER	NA	NA	81	80	0	129	107	22	17.05
Smoker	42479	chr18	13038469	C	T	*CEP192*	MISSENSE	ENSG00000101639:MISSENSE[ENST00000510237(c.500C>T,tCa>tTa):ENSP00000423147(p.Ser167Leu)];ENSG00000101639:MISSENSE[ENST00000506447(c.1700C>T,tCa>tTa):ENSP00000427550(p.Ser567Leu)];ENSG00000101639:INTRONIC[ENST00000325971(c.22-2360C>T)];ENSG00000101639:INTRONIC[ENST00000589596(c.968-45C>T)];ENSG00000101639:INTRONIC[ENST00000430049(c.384+1169C>T)];ENSG00000101639:INTRONIC[ENST00000511820(c.425+1169C>T)];ENSG00000101639:INTRONIC[ENST00000513432(c.1201-2360C>T)];	NA	NA	NA	NA	NA	NA	NA	NA	NA	NA	MODIFIER	NA	NA	164	164	0	199	117	82	41.21
Smoker	42479	chr18	19154767	G	C	*ESCO1*	MISSENSE	ENSG00000141446:MISSENSE[ENST00000269214(c.38C>G,tCc>tGc):ENSP00000269214(p.Ser13Cys)];ENSG00000141446:MISSENSE[ENST00000383276(c.38C>G,tCc>tGc):ENSP00000372763(p.Ser13Cys)];	NA	NA	NA	NA	NA	NA	NA	D	PrD	D	MODERATE	NA	NA	60	60	0	40	28	12	30

Mangalaparthi *et al.* , 2020. Mutational landscape of esophageal squamous cell carcinoma in an Indian cohort
Supplementary Table 4. List of somatic single nucleotide variants identified using whole exome sequencing of ESCC patients

Cohort	Sample	Chromosome	Genomic position	Reference allele	Alternate allele	Gene symbol	Variant type	Variant annotation	OncoMD	COSMIC	dbSNP	ExAC	ExAC_SAS	1000G	1000G_SAS	SIFT	Polyphen2	CONDEL	VEP	LRT	PhastCon7 Way	Total read (Adjacent normal)	Reference allel read support (Adjacent normal)	Alternative read support (Adjacent normal)	Total read (Tumor)	Reference allel read support (Tumor)	Alternative read support (Tumor)	Alternate allele frequency
Smoker	42479	chr18	77659557	C	A	*KCNG2*	MISSENSE	ENSG00000178342:MISSENSE[ENST00000316249(c.1142C>A,cCc>cAc):ENSP00000315654(p.Pro381His)];ENSG00000178342:EXONIC-NC[ENST00000590307(n.911C>A)];	NA	NA	NA	NA	NA	NA	NA	NA	NA	NA	MODIFIER	NA	NA	88	88	0	133	62	71	53.38
Smoker	42479	chr18	6962030	C	A	*LAMA1*	MISSENSE	ENSG00000101680:MISSENSE[ENST00000389658(c.7366G>T,Ggc>Tgc):ENSP00000374309(p.Gly2456Cys)];ENSG00000101680:EXONIC-NC[ENST00000488064(n.840G>T)];ENSG00000101680:EXONIC-NC[ENST00000579014(n.8381G>T)];	NA	NA	NA	NA	NA	NA	NA	NA	NA	NA	MODIFIER	NA	NA	178	176	0	233	118	115	49.36
Smoker	42479	chr18	47581751	G	A	*MYO5B*	INTRONIC-SS-ACR	ENSG00000167306:[ENST00000285039(c.28-3C>T)];ENSG00000167306:[ENST00000586036(n.378-3C>T)];	NA	NA	chr18*47581751*47581751*G*T*rs371360783*NA*NA*NA	NA	NA	NA	NA	NA	NA	NA	LOW	NA	NA	77	75	2	57	12	45	78.95
Smoker	42479	chr18	9204407	G	T	*RP11-21J18.1*	INTRONIC	ENSG00000265257:[ENST00000578850(n.63-49802G>T)];	NA	NA	NA	NA	NA	NA	NA	NA	NA	NA	MODIFIER	NA	NA	81	80	0	129	107	22	17.05
Smoker	42479	chr18	44775273	G	T	*SKOR2*	NONSENSE	ENSG00000215474:NONSENSE[ENST00000425639(c.282C>A,tgC>tgA):ENSP00000414750(p.Cys94Ter)];ENSG00000215474:NONSENSE[ENST00000400404(c.282C>A,tgC>tgA):ENSP00000383255(p.Cys94Ter)];	NA	NA	chr18*44775273*44775273*G*A*rs777794065*NA*NA*NA	NA	NA	NA	NA	NA	NA	NA	HIGH	NA	0.999	96	96	0	81	22	59	72.84
Smoker	42479	chr19	32083614	G	A	*AC011525.2*	EXONIC-NC	ENSG00000261400:[ENST00000562167(n.1008C>T)];	NA	NA	NA	NA	NA	NA	NA	NA	NA	NA	MODIFIER	NA	NA	194	194	0	189	88	101	53.44
Smoker	42479	chr19	32083614	G	A	*AC011525.4*	EXONIC-NC	ENSG00000267465:[ENST00000592788(n.512G>A)];ENSG00000267465:[ENST00000590611(n.584G>A)];ENSG00000267465:[ENST00000592644(n.116G>A)];	NA	NA	NA	NA	NA	NA	NA	NA	NA	NA	MODIFIER	NA	NA	194	194	0	189	88	101	53.44
Smoker	42479	chr19	58469945	C	T	*C19orf18*	3UTR	ENSG00000177025:[ENST00000314391(c.*25G>A)];	NA	NA	NA	NA	NA	NA	NA	NA	NA	NA	MODIFIER	NA	NA	58	58	0	90	62	28	31.11
Smoker	42479	chr19	43865566	C	A	*CD177*	INTRONIC	ENSG00000204936:[ENST00000457794(c.*655-31C>A)];ENSG00000204936:[ENST00000378009(c.*319-31C>A)];ENSG00000204936:[ENST00000607517(c.947-31C>A)];	NA	NA	NA	NA	NA	NA	NA	NA	NA	NA	MODIFIER	NA	NA	113	112	1	141	118	22	15.6
Smoker	42479	chr19	37681128	C	T	*CTC-454I21.3*	INTRONIC	ENSG00000267360:[ENST00000585860(c.73-76G>A)];ENSG00000267360:[ENST00000588873(c.35+16814G>A)];ENSG00000267360:[ENST00000590245(c.*164+5655G>A)];	NA	NA	NA	NA	NA	NA	NA	NA	NA	NA	MODIFIER	NA	NA	116	116	0	88	62	26	29.55
Smoker	42479	chr19	43865566	C	A	*CTC-490G23.4*	INTRONIC	ENSG00000272396:[ENST00000607109(n.301-571G>T)];	NA	NA	NA	NA	NA	NA	NA	NA	NA	NA	MODIFIER	NA	NA	113	112	1	141	118	22	15.6
Smoker	42479	chr19	11485533	C	G	*CTD-2342J14.6*	EXONIC-NC	ENSG00000267277:[ENST00000590399(n.79G>C)];	NA	NA	chr19*11485533*11485533*C*T*rs372274006*NA*NA*NA|chr19*11485533*11485539*CTTCCTG*C*rs779290871*NA*NA*NA	NA	NA	NA	NA	NA	NA	NA	LOW	NA	NA	66	66	0	37	21	16	43.24
Smoker	42479	chr19	53787804	C	A	*FAM90A27P*	EXONIC-NC	ENSG00000189348:[ENST00000593323(n.977C>A)];ENSG00000189348:[ENST00000338885(n.987C>A)];	NA	chr19*5378	NA	NA	NA	NA	NA	NA	NA	NA	MODIFIER	NA	NA	47	46	0	59	37	22	37.29
Smoker	42479	chr19	8191691	C	A	*FBN3*	SILENT	ENSG00000142449:SILENT[ENST00000600128(c.2322G>T,ccG>ccT):ENSP00000470498(p.Pro774(=))];ENSG00000142449:SILENT[ENST00000601739(c.2322G>T,ccG>ccT):ENSP00000472324(p.Pro774(=))];ENSG00000142449:SILENT[ENST00000270509(c.2322G>T,ccG>ccT):ENSP00000270509(p.Pro774(=))];	NA	NA	chr19*8191691*8191691*C*T*rs768212719*NA*NA*NA	NA	NA	NA	NA	NA	NA	NA	LOW	NA	NA	45	45	0	48	8	40	83.33
Smoker	42479	chr19	55333134	G	C	*KIR3DL1*	MISSENSE	ENSG00000167633:MISSENSE[ENST00000541392(c.770G>C,aGg>aCg):ENSP00000442355(p.Arg257Thr)];ENSG00000167633:MISSENSE[ENST00000358178(c.485G>C,aGg>aCg):ENSP00000350901(p.Arg162Thr)];ENSG00000167633:MISSENSE[ENST00000326542(c.770G>C,aGg>aCg):ENSP00000326868(p.Arg257Thr)];ENSG00000167633:MISSENSE[ENST00000402254(c.770G>C,aGg>aCg):ENSP00000384528(p.Arg257Thr)];ENSG00000167633:MISSENSE[ENST00000538269(c.770G>C,aGg>aCg):ENSP00000443350(p.Arg257Thr)];ENSG00000167633:MISSENSE[ENST00000391728(c.770G>C,aGg>aCg):ENSP00000375608(p.Arg257Thr)];	NA	NA	NA	NA	NA	NA	NA	D	BN	N	MODERATE	U	0.032	134	134	0	104	50	54	51.92
Smoker	42479	chr19	49001446	C	T	*LMTK3*	SILENT	ENSG00000142235:SILENT[ENST00000600059(c.2880G>A,gaG>gaA):ENSP00000472020(p.Glu960(=))];ENSG00000142235:SILENT[ENST00000270238(c.2967G>A,gaG>gaA):ENSP00000270238(p.Glu989(=))];	NA	NA	NA	NA	NA	NA	NA	NA	NA	NA	LOW	NA	0.025	253	253	0	271	110	161	59.41
Smoker	42479	chr19	9065977	G	A	*MUC16*	MISSENSE	ENSG00000181143:MISSENSE[ENST00000397910(c.21469C>T,Cac>Tac):ENSP00000381008(p.His7157Tyr)];	NA	NA	NA	NA	NA	NA	NA	NA	UN	NA	MODERATE	NA	NA	154	154	0	96	57	39	40.62
Smoker	42479	chr19	9074063	G	C	*MUC16*	SILENT	ENSG00000181143:SILENT[ENST00000397910(c.13383C>G,gtC>gtG):ENSP00000381008(p.Val4461(=))];	NA	NA	NA	NA	NA	NA	NA	NA	NA	NA	LOW	NA	NA	104	104	0	74	14	60	81.08
Smoker	42479	chr19	55421379	C	G	*NCR1*	SILENT-SS-PRX	ENSG00000189430:[ENST00000594765(c.636C>G,ggC>ggG):ENSP00000471986(p.Gly212(=))];ENSG00000189430:[ENST00000338835(c.636C>G,ggC>ggG):ENSP00000339515(p.Gly212(=))];ENSG00000189430:[ENST00000291890(c.636C>G,ggC>ggG):ENSP00000291890(p.Gly212(=))];ENSG00000189430:[ENST00000447255(c.636C>G,ggC>ggG):ENSP00000404434(p.Gly212(=))];ENSG00000189430:[ENST00000350790(c.351C>G,ggC>ggG):ENSP00000344358(p.Gly117(=))];ENSG00000189430:[ENST00000598576(c.600C>G,ggC>ggG):ENSP00000469750(p.Gly200(=))];ENSG00000189430:[ENST00000357397(c.315C>G,ggC>ggG):ENSP00000349972(p.Gly105(=))];	chr19*5542	chr19*5542	chr19*55421379*55421379*C*A*rs770811879*NA*NA*NA	NA	NA	NA	NA	NA	NA	NA	LOW	NA	NA	117	117	0	107	83	24	22.43
Smoker	42479	chr19	57335884	T	G	*PEG3*	MISSENSE	ENSG00000198300:MISSENSE[ENST00000423103(c.140A>C,cAg>cCg):ENSP00000403051(p.Gln47Pro)];ENSG00000198300:MISSENSE[ENST00000599534(c.140A>C,cAg>cCg):ENSP00000472395(p.Gln47Pro)];ENSG00000198300:MISSENSE[ENST00000326441(c.140A>C,cAg>cCg):ENSP00000326581(p.Gln47Pro)];ENSG00000198300:MISSENSE[ENST00000599577(c.140A>C,cAg>cCg):ENSP00000469486(p.Gln47Pro)];ENSG00000198300:INTRONIC[ENST00000596261(n.421-89A>C)];ENSG00000198300:INTRONIC[ENST00000594706(n.442-89A>C)];ENSG00000198300:INTRONIC[ENST00000598410(c.-150-89A>C)];ENSG00000198300:INTRONIC[ENST00000593695(c.-150-89A>C)];ENSG00000198300:5UTR[ENST00000600833(c.-239A>C)];	NA	NA	chr19*57335884*57335884*T*C*rs767490719*NA*NA*NA	NA	NA	NA	NA	NA	NA	NA	MODIFIER	NA	NA	84	84	0	87	66	21	24.14
Smoker	42479	chr19	39909463	C	G	*PLEKHG2*	SILENT	ENSG00000090924:SILENT[ENST00000205135(c.863C>G,ctC>ctG):ENSP00000205135(p.Leu288(=))];ENSG00000090924:SILENT[ENST00000378550(c.1173C>G,ctC>ctG):ENSP00000367812(p.Leu391(=))];ENSG00000090924:SILENT[ENST00000409797(c.1173C>G,ctC>ctG):ENSP00000386492(p.Leu391(=))];ENSG00000090924:SILENT[ENST00000409794(c.1173C>G,ctC>ctG):ENSP00000386733(p.Leu391(=))];ENSG00000090924:SILENT[ENST00000425673(c.1173C>G,ctC>ctG):ENSP00000392906(p.Leu391(=))];ENSG00000090924:SILENT[ENST00000458508(c.996C>G,ctC>ctG):ENSP00000408857(p.Leu332(=))];ENSG00000090924:EXONIC-NC[ENST00000478523(n.351C>G)];	chr19*3990	chr19*3990	NA	NA	NA	NA	NA	NA	NA	NA	LOW	NA	NA	67	67	0	75	28	47	62.67

Mangalaparthi *et al.* , 2020. Mutational landscape of esophageal squamous cell carcinoma in an Indian cohort
Supplementary Table 4. List of somatic single nucleotide variants identified using whole exome sequencing of ESCC patients

Cohort	Sample	Chromosome	Genomic position	Reference allele	Alternate allele	Gene symbol	Variant type	Variant annotation	OncoMD	COSMIC	dbSNP	ExAC	ExAC_SAS	1000G	1000G_SAS	SIFT	Polyphen2	CONDEL	VEP	LRT	PhastCon7 Way	Total read (Adjacent normal)	Reference allel read support (Adjacent normal)	Alternative read support (Adjacent normal)	Total read (Tumor)	Reference allel read support (Tumor)	Alternative read support (Tumor)	Alternate allele frequency
Smoker	42479	chr19	14224999	A	G	*PRKACA*	INTRONIC	ENSG00000072062:[ENST00000589284(c.-129+3700T>C)];ENSG00000072062:[ENST00000308677(c.46+3315T>C)];ENSG00000072062:[ENST00000590853(c.46+3315T>C)];ENSG00000072062:[ENST00000589994(c.-32T>C)];	NA	NA	NA	NA	NA	NA	NA	NA	NA	NA	MODIFIER	NA	NA	53	53	0	31	6	25	80.65
Smoker	42479	chr19	54410124	G	A	*PRKCG*	MISSENSE	ENSG00000126583:MISSENSE[ENST00000542049(c.1622G>A,aGc>aAc):ENSP00000438090(p.Ser541Asn)];ENSG00000126583:MISSENSE[ENST00000540413(c.2069G>A,aGc>aAc):ENSP00000443493(p.Ser690Asn)];ENSG00000126583:MISSENSE[ENST00000263431(c.2069G>A,aGc>aAc):ENSP00000263431(p.Ser690Asn)];	NA	NA	NA	NA	NA	NA	NA	D_lc	BN	N	MODERATE	NA	NA	47	47	0	40	23	17	42.5
Smoker	42479	chr19	34583502	G	T	*RPS4XP21*	EXONIC-NC	ENSG00000186008:[ENST00000603814(n.714C>A)];ENSG00000186008:[ENST00000469064(n.714C>A)];	NA	NA	NA	NA	NA	NA	NA	NA	NA	NA	MODIFIER	NA	NA	144	144	0	125	51	74	59.2
Smoker	42479	chr19	51914409	C	G	*SIGLEC10*	MISSENSE	ENSG00000142512:MISSENSE[ENST00000436984(c.1609G>C,Gag>Cag):ENSP00000414324(p.Glu537Gln)];ENSG00000142512:MISSENSE[ENST00000353836(c.1753G>C,Gag>Cag):ENSP00000342389(p.Glu585Gln)];ENSG00000142512:MISSENSE[ENST00000525998(c.1483G>C,Gag>Cag):ENSP00000431444(p.Glu495Gln)];ENSG00000142512:MISSENSE[ENST00000441969(c.1579G>C,Gag>Cag):ENSP00000408387(p.Glu527Gln)];ENSG00000142512:MISSENSE[ENST00000439889(c.1864G>C,Gag>Cag):ENSP00000389132(p.Glu622Gln)];ENSG00000142512:MISSENSE[ENST00000442846(c.1309G>C,Gag>Cag):ENSP00000395475(p.Glu437Gln)];ENSG00000142512:MISSENSE[ENST00000356298(c.2038G>C,Gag>Cag):ENSP00000348646(p.Glu680Gln)];ENSG00000142512:MISSENSE[ENST00000432469(c.1504G>C,Gag>Cag):ENSP00000396742(p.Glu502Gln)];ENSG00000142512:MISSENSE[ENST00000339313(c.2038G>C,Gag>Cag):ENSP00000345243(p.Glu680Gln)];ENSG00000142512:EXONIC-NC[ENST00000357375(n.1926G>C)];	NA	NA	chr19*51914409*51914409*C*T*rs756960551*NA*NA*NA	NA	NA	NA	NA	NA	NA	NA	MODIFIER	NA	NA	171	171	0	196	145	51	26.02
Smoker	42479	chr19	11101925	G	A	*SMARCA4*	MISSENSE	ENSG00000127616:MISSENSE[ENST00000450717(c.1345G>A,Gag>Aag):ENSP00000397783(p.Glu449Lys)];ENSG00000127616:MISSENSE[ENST00000358026(c.1345G>A,Gag>Aag):ENSP00000350720(p.Glu449Lys)];ENSG00000127616:MISSENSE[ENST00000413806(c.1345G>A,Gag>Aag):ENSP00000414727(p.Glu449Lys)];ENSG00000127616:MISSENSE[ENST00000590574(c.1345G>A,Gag>Aag):ENSP00000466963(p.Glu449Lys)];ENSG00000127616:MISSENSE[ENST00000444061(c.1345G>A,Gag>Aag):ENSP00000392837(p.Glu449Lys)];ENSG00000127616:MISSENSE[ENST00000344626(c.1345G>A,Gag>Aag):ENSP00000343896(p.Glu449Lys)];ENSG00000127616:MISSENSE[ENST00000541122(c.1345G>A,Gag>Aag):ENSP00000445036(p.Glu449Lys)];ENSG00000127616:MISSENSE[ENST00000429416(c.1345G>A,Gag>Aag):ENSP00000395654(p.Glu449Lys)];ENSG00000127616:MISSENSE[ENST00000589677(c.1345G>A,Gag>Aag):ENSP00000464778(p.Glu449Lys)];ENSG00000127616:EXONIC-NC[ENST00000591545(n.1219G>A)];	NA	NA	NA	NA	NA	NA	NA	NA	NA	NA	MODIFIER	NA	NA	48	48	0	35	10	25	71.43
Smoker	42479	chr19	11485533	C	G	*SWSAP1*	SILENT	ENSG00000173928:SILENT[ENST00000312423(c.114C>G,ctC>ctG):ENSP00000310008(p.Leu38(=))];	NA	NA	chr19*11485533*11485533*C*T*rs372274006*NA*NA*NA|chr19*11485533*11485539*CTTCCTG*C*rs779290871*NA*NA*NA	NA	NA	NA	NA	NA	NA	NA	LOW	NA	NA	66	66	0	37	21	16	43.24
Smoker	42479	chr19	57335884	T	G	*ZIM2*	INTRONIC-SS-ACR	ENSG00000269699:INTRONIC-SS-ACR[ENST00000391708(c.-237-2A>C)];ENSG00000269699:INTRONIC-SS-ACR[ENST00000599935(c.-237-2A>C)];ENSG00000269699:INTRONIC[ENST00000221722(c.-150-89A>C)];ENSG00000269699:INTRONIC[ENST00000601070(c.-150-89A>C)];ENSG00000269699:INTRONIC[ENST00000593711(c.-150-89A>C)];ENSG00000269699:INTRONIC[ENST00000597281(c.-150-89A>C)];ENSG00000269699:INTRONIC[ENST00000595671(c.-150-89A>C)];	NA	NA	chr19*57335884*57335884*T*C*rs767490719*NA*NA*NA	NA	NA	NA	NA	NA	NA	NA	MODIFIER	NA	NA	84	84	0	87	66	21	24.14
Smoker	42479	chr19	52537012	G	A	*ZNF432*	SILENT	ENSG00000256087:SILENT[ENST00000221315(c.1920C>T,ctC>ctT):ENSP00000221315(p.Leu640(=))];ENSG00000256087:SILENT[ENST00000594154(c.1920C>T,ctC>ctT):ENSP00000470488(p.Leu640(=))];	NA	NA	NA	NA	NA	NA	NA	NA	NA	NA	LOW	NA	NA	111	111	0	109	79	30	27.52
Smoker	42479	chr19	12383859	G	A	*ZNF44*	MISSENSE	ENSG00000197857:MISSENSE[ENST00000356109(c.1355C>T,tCt>tTt):ENSP00000348419(p.Ser452Phe)];ENSG00000197857:MISSENSE[ENST00000393337(c.1355C>T,tCt>tTt):ENSP00000377008(p.Ser452Phe)];ENSG00000197857:MISSENSE[ENST00000355684(c.1211C>T,tCt>tTt):ENSP00000347910(p.Ser404Phe)];ENSG00000197857:MISSENSE[ENST00000397742(c.1211C>T,tCt>tTt):ENSP00000380850(p.Ser404Phe)];ENSG00000197857:INTRONIC[ENST00000600003(c.335+1929C>T)];	chr19*1238	NA	NA	NA	NA	NA	NA	T	BN	N	MODERATE	NA	NA	148	148	0	93	62	31	33.33
Smoker	42479	chr19	37681128	C	T	*ZNF585B*	INTRONIC	ENSG00000245680:[ENST00000586320(c.28-76G>A)];ENSG00000245680:[ENST00000591273(c.*29-76G>A)];ENSG00000245680:[ENST00000591492(c.28-76G>A)];ENSG00000245680:[ENST00000392156(c.*18-76G>A)];ENSG00000245680:[ENST00000526705(c.*179-76G>A)];ENSG00000245680:[ENST00000527838(c.73-76G>A)];ENSG00000245680:[ENST00000532828(c.73-76G>A)];ENSG00000245680:[ENST00000531805(c.-169G>A)];	NA	NA	NA	NA	NA	NA	NA	NA	NA	NA	MODIFIER	NA	NA	116	116	0	88	62	26	29.55
Smoker	42479	chr19	11998675	C	T	*ZNF69*	5UTR	ENSG00000198429:[ENST00000340180(c.-64C>T)];ENSG00000198429:[ENST00000429654(c.-64C>T)];ENSG00000198429:[ENST00000445911(c.-64C>T)];	NA	NA	NA	NA	NA	NA	NA	NA	NA	NA	MODIFIER	NA	NA	92	92	0	61	45	16	26.23
Smoker	42479	chr19	23170001	T	C	*ZNF728*	INTRONIC	ENSG00000269067:[ENST00000599851(c.60+109A>G)];ENSG00000269067:[ENST00000594710(c.226+109A>G)];	NA	NA	NA	NA	NA	NA	NA	NA	NA	NA	MODIFIER	NA	NA	227	227	0	111	17	94	84.68
Smoker	42479	chr19	12088265	G	C	*ZNF763*	INTRONIC	ENSG00000197054:[ENST00000538752(c.251+19G>C)];ENSG00000197054:[ENST00000343949(c.200+19G>C)];ENSG00000267179:[ENST00000591441(c.*157+19G>C)];ENSG00000197054:[ENST00000586494(c.-175-666G>C)];ENSG00000197054:[ENST00000545530(c.-175-666G>C)];ENSG00000197054:[ENST00000481745(c.284+19G>C)];ENSG00000267179:[ENST00000590798(c.251+19G>C)];ENSG00000267179:[ENST00000591944(c.337+286G>C)];ENSG00000197054:[ENST00000592625(c.130+286G>C)];ENSG00000197054:[ENST00000358987(c.191+19G>C)];	NA	NA	NA	NA	NA	NA	NA	NA	NA	NA	MODIFIER	NA	NA	105	104	0	78	24	54	69.23
Smoker	42479	chr2	132201846	G	C	*AC073869.20*	EXONIC-NC	ENSG00000217950:[ENST00000434348(n.622C>G)];ENSG00000217950:[ENST00000407594(n.315C>G)];	NA	NA	NA	NA	NA	NA	NA	NA	NA	NA	MODIFIER	NA	NA	140	140	0	216	86	130	60.19
Smoker	42479	chr2	203052859	G	C	*AC079354.1*	INTRONIC	ENSG00000182329:[ENST00000469462(n.3262-16G>C)];ENSG00000182329:[ENST00000498697(c.1126-16G>C)];	NA	NA	NA	NA	NA	NA	NA	NA	NA	NA	MODIFIER	NA	NA	133	132	0	124	19	104	83.87

Mangalaparthi *et al.* , 2020. Mutational landscape of esophageal squamous cell carcinoma in an Indian cohort
Supplementary Table 4. List of somatic single nucleotide variants identified using whole exome sequencing of ESCC patients

Cohort	Sample	Chromosome	Genomic position	Reference allele	Alternate allele	Gene symbol	Variant type	Variant annotation	OncoMD	COSMIC	dbSNP	ExAC	ExAC_SAS	1000G	1000G_SAS	SIFT	Polyphen2	CONDEL	VEP	LRT	PhastCon7 Way	Total read (Adjacent normal)	Reference allel read support (Adjacent normal)	Alternative read support (Adjacent normal)	Total read (Tumor)	Reference allel read support (Tumor)	Alternative read support (Tumor)	Alternate allele frequency
Smoker	42479	chr2	25062869	C	T	*ADCY3*	MISSENSE	ENSG00000138031:MISSENSE[ENST00000260600(c.1228G>A,Gac>Aac):ENSP00000260600(p.Asp410Asn)];ENSG00000138031:MISSENSE[ENST00000405392(c.61G>A,Gac>Aac):ENSP00000384484(p.Asp21Asn)];ENSG00000138031:MISSENSE[ENST00000435135(c.1078G>A,Gac>Aac):ENSP00000389799(p.Asp360Asn)];ENSG00000138031:MISSENSE[ENST00000606682(c.241G>A,Gac>Aac):ENSP00000475652(p.Asp81Asn)];ENSG00000138031:MISSENSE[ENST00000427849(c.496G>A,Gac>Aac):ENSP00000399275(p.Asp166Asn)];ENSG00000138031:EXONIC-NC[ENST00000454027(n.111G>A)];	NA	NA	NA	NA	NA	NA	NA	T	PoD	N	MODERATE	NA	NA	77	77	0	130	19	111	85.38
Smoker	42479	chr2	202154213	A	G	*ALS2CR12*	MISSENSE	ENSG00000155749:MISSENSE[ENST00000405148(c.1178T>C,aTa>aCa):ENSP00000385098(p.Ile393Thr)];ENSG00000155749:MISSENSE[ENST00000286190(c.1178T>C,aTa>aCa):ENSP00000286190(p.Ile393Thr)];ENSG00000155749:MISSENSE[ENST00000392257(c.1109T>C,aTa>aCa):ENSP00000376086(p.Ile370Thr)];ENSG00000155749:MISSENSE[ENST00000439709(c.1109T>C,aTa>aCa):ENSP00000412073(p.Ile370Thr)];ENSG00000155749:INTRONIC[ENST00000415745(c.356+206T>C)];ENSG00000155749:EXONIC-NC[ENST00000494171(n.1685T>C)];ENSG00000155749:EXONIC-NC[ENST00000494223(n.104T>C)];	NA	NA	NA	NA	NA	NA	NA	NA	NA	NA	MODIFIER	NA	NA	184	183	0	109	21	88	80.73
Smoker	42479	chr2	86008275	C	T	*ATOH8*	INTRONIC	ENSG00000168874:[ENST00000306279(c.961-5733C>T)];ENSG00000168874:[ENST00000469442(n.712-5733C>T)];ENSG00000168874:[ENST00000489682(n.119C>T)];ENSG00000168874:[ENST00000473116(n.700C>T)];	NA	NA	NA	NA	NA	NA	NA	NA	NA	NA	MODIFIER	NA	NA	124	124	0	133	104	29	21.8
Smoker	42479	chr2	86849872	C	T	*CHMP3*	INTRONIC	ENSG00000115561:[ENST00000439940(c.-8-2280G>A)];	NA	NA	NA	NA	NA	NA	NA	NA	NA	NA	MODIFIER	NA	NA	125	125	0	152	100	52	34.21
Smoker	42479	chr2	208986408	C	G	*CRYGD*	MISSENSE	ENSG00000118231:MISSENSE[ENST00000264376(c.514G>C,Gat>Cat):ENSP00000264376(p.Asp172His)];	NA	NA	NA	NA	NA	NA	NA	D	BN	N	MODERATE	N	0.992	53	52	0	48	31	17	35.42
Smoker	42479	chr2	204116847	A	T	*CYP20A1*	INTRONIC	ENSG00000119004:[ENST00000431118(c.*130+15A>T)];ENSG00000119004:[ENST00000428265(c.*130+15A>T)];ENSG00000119004:[ENST00000449301(c.432+15A>T)];ENSG00000119004:[ENST00000356079(c.432+15A>T)];ENSG00000119004:[ENST00000489360(n.257+15A>T)];ENSG00000119004:[ENST00000443941(c.432+15A>T)];ENSG00000119004:[ENST00000429815(c.432+15A>T)];ENSG00000119004:[ENST00000463131(n.267+15A>T)];ENSG00000119004:[ENST00000461371(n.296+15A>T)];	NA	NA	NA	NA	NA	NA	NA	NA	NA	NA	MODIFIER	NA	NA	127	127	0	93	85	8	8.6
Smoker	42479	chr2	75720521	G	T	*EVA1A*	MISSENSE	ENSG00000115363:MISSENSE[ENST00000410113(c.300C>A,caC>caA):ENSP00000386435(p.His100Gln)];ENSG00000115363:MISSENSE[ENST00000233712(c.300C>A,caC>caA):ENSP00000233712(p.His100Gln)];ENSG00000115363:MISSENSE[ENST00000452003(c.300C>A,caC>caA):ENSP00000388105(p.His100Gln)];ENSG00000115363:MISSENSE[ENST00000393913(c.300C>A,caC>caA):ENSP00000377490(p.His100Gln)];ENSG00000115363:MISSENSE[ENST00000410071(c.300C>A,caC>caA):ENSP00000386930(p.His100Gln)];ENSG00000115363:MISSENSE[ENST00000410010(c.264C>A,caC>caA):ENSP00000386835(p.His88Gln)];ENSG00000115363:MISSENSE[ENST00000432649(c.300C>A,caC>caA):ENSP00000398249(p.His100Gln)];ENSG00000115363:INTRONIC[ENST00000485891(n.411-24025C>A)];ENSG00000115363:INTRONIC[ENST00000490746(n.521-24025C>A)];	NA	NA	chr2*75720521*75720521*G*A,C*rs146541154*0*0.9998*.,0.0001997	NA	NA	NA	NA	NA	NA	NA	MODIFIER	NA	NA	82	82	0	71	28	43	60.56
Smoker	42479	chr2	186673691	A	T	*FSIP2*	MISSENSE	ENSG00000188738:MISSENSE[ENST00000343098(c.19925A>T,aAa>aTa):ENSP00000344403(p.Lys6642Ile)];ENSG00000188738:MISSENSE[ENST00000424728(c.19658A>T,aAa>aTa):ENSP00000401306(p.Lys6553Ile)];ENSG00000188738:INTRONIC[ENST00000415915(c.2910-4581A>T)];	NA	NA	NA	NA	NA	NA	NA	NA	NA	NA	MODIFIER	NA	NA	107	107	0	95	75	20	21.05
Smoker	42479	chr2	97270137	C	A	*KANSL3*	INTRONIC	ENSG00000114982:[ENST00000597150(c.*1541-20G>T)];ENSG00000114982:[ENST00000441706(c.1558+1034G>T)];ENSG00000114982:[ENST00000354204(c.*1861-20G>T)];ENSG00000114982:[ENST00000599854(c.1758-20G>T)];ENSG00000114982:[ENST00000487070(n.2103-20G>T)];ENSG00000114982:[ENST00000431828(c.2019-20G>T)];ENSG00000114982:[ENST00000447759(c.*1673-20G>T)];ENSG00000114982:[ENST00000476534(n.1595-20G>T)];ENSG00000114982:[ENST00000444759(c.*1463-20G>T)];ENSG00000114982:[ENST00000420155(c.2097-20G>T)];ENSG00000114982:[ENST00000440133(c.1479-20G>T)];	NA	NA	NA	NA	NA	NA	NA	NA	NA	NA	MODIFIER	NA	NA	42	42	0	49	40	9	18.37
Smoker	42479	chr2	30748569	G	T	*LCLAT1*	MISSENSE	ENSG00000172954:MISSENSE[ENST00000359433(c.227G>T,tGg>tTg):ENSP00000352406(p.Trp76Leu)];ENSG00000172954:MISSENSE[ENST00000540623(c.113G>T,tGg>tTg):ENSP00000442857(p.Trp38Leu)];ENSG00000172954:MISSENSE[ENST00000319406(c.227G>T,tGg>tTg):ENSP00000368826(p.Trp76Leu)];ENSG00000172954:MISSENSE[ENST00000379509(c.113G>T,tGg>tTg):ENSP00000368823(p.Trp38Leu)];ENSG00000172954:MISSENSE[ENST00000309052(c.227G>T,tGg>tTg):ENSP00000310551(p.Trp76Leu)];ENSG00000172954:MISSENSE[ENST00000488144(c.113G>T,tGg>tTg):ENSP00000417951(p.Trp38Leu)];ENSG00000172954:MISSENSE[ENST00000476038(c.113G>T,tGg>tTg):ENSP00000419646(p.Trp38Leu)];ENSG00000172954:MISSENSE[ENST00000465538(c.113G>T,tGg>tTg):ENSP00000417565(p.Trp38Leu)];ENSG00000172954:MISSENSE[ENST00000497423(c.227G>T,tGg>tTg):ENSP00000417875(p.Trp76Leu)];ENSG00000172954:MISSENSE[ENST00000476535(c.113G>T,tGg>tTg):ENSP00000419444(p.Trp38Leu)];ENSG00000172954:MISSENSE[ENST00000465200(c.113G>T,tGg>tTg):ENSP00000420481(p.Trp38Leu)];ENSG00000172954:MISSENSE[ENST00000466477(c.113G>T,tGg>tTg):ENSP00000419966(p.Trp38Leu)];ENSG00000172954:INTRONIC[ENST00000491680(n.160-7413G>T)];ENSG00000172954:3UTR[ENST00000478015(c.*260G>T)];	NA	NA	NA	NA	NA	NA	NA	T	BN	N	MODERATE	NA	NA	141	141	0	164	26	137	83.54
Smoker	42479	chr2	71368496	C	A	*MPHOSPH10*	MISSENSE	ENSG00000124383:MISSENSE[ENST00000244230(c.1443C>A,aaC>aaA):ENSP00000244230(p.Asn481Lys)];	NA	NA	chr2*71368493*71368505*CAACCAGGTGAGG*C*rs772948248*NA*NA*NA|chr2*71368496*71368496*C*T*rs200379927*0*0.9998*0.0001997	NA	NA	NA	NA	T	BN	N	MODERATE	N	0.999	79	79	0	54	41	13	24.07
Smoker	42479	chr2	234740844	C	T	*MROH2A*	MISSENSE	ENSG00000185038:MISSENSE[ENST00000389758(c.4756C>T,Ctc>Ttc):ENSP00000374408(p.Leu1586Phe)];ENSG00000185038:EXONIC-NC[ENST00000487979(n.718C>T)];	NA	NA	NA	NA	NA	NA	NA	NA	NA	NA	MODIFIER	NA	NA	164	164	0	121	79	42	34.71

Mangalaparthi *et al.* , 2020. Mutational landscape of esophageal squamous cell carcinoma in an Indian cohort
Supplementary Table 4. List of somatic single nucleotide variants identified using whole exome sequencing of ESCC patients

Cohort	Sample	Chromosome	Genomic position	Reference allele	Alternate allele	Gene symbol	Variant type	Variant annotation	OncoMD	COSMIC	dbSNP	ExAC	ExAC_SAS	1000G	1000G_SAS	SIFT	Polyphen2	CONDEL	VEP	LRT	PhastCon7 Way	Total read (Adjacent normal)	Reference allel read support (Adjacent normal)	Alternative read support (Adjacent normal)	Total read (Tumor)	Reference allel read support (Tumor)	Alternative read support (Tumor)	Alternate allele frequency
Smoker	42479	chr2	73441314	C	A	*NA*	INTERGENIC		NA	NA	NA	NA	NA	NA	NA	NA	NA	NA	MODIFIER	NA	NA	103	103	0	84	62	22	26.19
Smoker	42479	chr2	145121259	C	T	*NA*	INTERGENIC		NA	NA	NA	NA	NA	NA	NA	NA	NA	NA	MODIFIER	NA	NA	114	114	0	111	10	100	90.09
Smoker	42479	chr2	203964406	G	A	*NBEAL1*	MISSENSE	ENSG00000144426:MISSENSE[ENST00000449802(c.1153G>A,Gca>Aca):ENSP00000399903(p.Ala385Thr)];	NA	NA	0.002196	0.0007642	0	0.0021965	0	T	BN	N	MODERATE	NA	NA	246	246	0	174	102	72	41.38
Smoker	42479	chr2	113999169	G	T	*PAX8*	MISSENSE	ENSG00000125618:MISSENSE[ENST00000263335(c.736C>A,Cca>Aca):ENSP00000263335(p.Pro246Thr)];ENSG00000125618:MISSENSE[ENST00000263334(c.736C>A,Cca>Aca):ENSP00000263334(p.Pro246Thr)];ENSG00000125618:MISSENSE[ENST00000429538(c.736C>A,Cca>Aca):ENSP00000395498(p.Pro246Thr)];ENSG00000125618:MISSENSE[ENST00000397647(c.736C>A,Cca>Aca):ENSP00000380768(p.Pro246Thr)];ENSG00000125618:MISSENSE[ENST00000468980(c.92C>A,Cca>Aca):ENSP00000451240(p.Pro32Thr)];ENSG00000125618:MISSENSE[ENST00000348715(c.736C>A,Cca>Aca):ENSP00000314750(p.Pro246Thr)];ENSG00000125618:3UTR[ENST00000497038(c.*181C>A)];ENSG00000125618:3UTR[ENST00000554830(c.*276C>A)];ENSG00000125618:EXONIC-NC[ENST00000467778(n.900C>A)];	NA	NA	NA	NA	NA	NA	NA	NA	NA	NA	MODIFIER	NA	NA	61	61	0	61	25	36	59.02
Smoker	42479	chr2	113999169	G	T	*PAX8-AS1*	INTRONIC	ENSG00000189223:[ENST00000436293(n.354+4970G>T)];ENSG00000189223:[ENST00000556070(n.35+6022G>T)];ENSG00000189223:[ENST00000451179(n.164+3380G>T)];ENSG00000189223:[ENST00000445745(n.377+4979G>T)];ENSG00000189223:[ENST00000422956(n.35+6022G>T)];ENSG00000189223:[ENST00000333145(n.132+4979G>T)];ENSG00000189223:[ENST00000553869(n.154+3380G>T)];	NA	NA	NA	NA	NA	NA	NA	NA	NA	NA	MODIFIER	NA	NA	61	61	0	61	25	36	59.02
Smoker	42479	chr2	153535667	C	G	*PRPF40A*	INTRONIC	ENSG00000196504:[ENST00000410080(c.578-44G>C)];ENSG00000196504:[ENST00000545856(c.659-44G>C)];ENSG00000196504:[ENST00000493468(c.637+199G>C)];ENSG00000196504:[ENST00000354363(c.*591-44G>C)];ENSG00000196504:[ENST00000448428(c.437-44G>C)];ENSG00000196504:[ENST00000467114(n.522G>C)];	NA	NA	NA	NA	NA	NA	NA	NA	NA	NA	MODIFIER	NA	NA	61	60	0	53	16	37	69.81
Smoker	42479	chr2	79254963	T	C	*REG3G*	MISSENSE	ENSG00000143954:MISSENSE[ENST00000393897(c.364T>C,Tgg>Cgg):ENSP00000377475(p.Trp122Arg)];ENSG00000143954:MISSENSE[ENST00000409471(c.226T>C,Tgg>Cgg):ENSP00000387105(p.Trp76Arg)];ENSG00000143954:MISSENSE[ENST00000272324(c.364T>C,Tgg>Cgg):ENSP00000272324(p.Trp122Arg)];	NA	NA	NA	NA	NA	NA	NA	D	PrD	D	MODERATE	D	0.307	69	69	0	84	67	17	20.24
Smoker	42479	chr2	86849872	C	T	*RNF103*	SILENT	ENSG00000239305:EXONIC-NC[ENST00000477307(n.130G>A)];ENSG00000239305:EXONIC-NC[ENST00000472680(n.129G>A)];ENSG00000239305:EXONIC-NC[ENST00000463333(n.80G>A)];ENSG00000239305:EXONIC-NC[ENST00000465629(n.297G>A)];ENSG00000239305:SILENT[ENST00000237455(c.138G>A,aaG>aaA):ENSP00000237455(p.Lys46(=))];	NA	NA	NA	NA	NA	NA	NA	NA	NA	NA	MODIFIER	NA	NA	125	125	0	152	100	52	34.21
Smoker	42479	chr2	86849872	C	T	*RNF103-CHMP3*	SILENT	ENSG00000249884:SILENT[ENST00000440757(c.138G>A,aaG>aaA):ENSP00000392995(p.Lys46(=))];ENSG00000249884:INTRONIC[ENST00000604011(c.-8-2280G>A)];	NA	NA	NA	NA	NA	NA	NA	NA	NA	NA	MODIFIER	NA	NA	125	125	0	152	100	52	34.21
Smoker	42479	chr2	231074650	C	T	*SP110*	SILENT	ENSG00000135899:SILENT[ENST00000338556(c.81G>A,aaG>aaA):ENSP00000344049(p.Lys27(=))];ENSG00000135899:SILENT[ENST00000392048(c.825G>A,aaG>aaA):ENSP00000375902(p.Lys275(=))];ENSG00000135899:SILENT[ENST00000358662(c.825G>A,aaG>aaA):ENSP00000351488(p.Lys275(=))];ENSG00000135899:SILENT[ENST00000258381(c.825G>A,aaG>aaA):ENSP00000258381(p.Lys275(=))];ENSG00000135899:SILENT[ENST00000258382(c.825G>A,aaG>aaA):ENSP00000258382(p.Lys275(=))];ENSG00000135899:SILENT[ENST00000540870(c.843G>A,aaG>aaA):ENSP00000439558(p.Lys281(=))];ENSG00000135899:EXONIC-NC[ENST00000486146(n.387G>A)];ENSG00000135899:EXONIC-NC[ENST00000490880(n.152G>A)];	NA	NA	NA	NA	NA	NA	NA	NA	NA	NA	MODIFIER	NA	NA	110	110	0	79	47	32	40.51
Smoker	42479	chr2	231074650	C	T	*SP140*	INTRONIC	ENSG00000079263:[ENST00000441657(n.71-3719C>T)];ENSG00000079263:[ENST00000456542(c.-322-3719C>T)];	NA	NA	NA	NA	NA	NA	NA	NA	NA	NA	MODIFIER	NA	NA	110	110	0	79	47	32	40.51
Smoker	42479	chr2	220299945	C	T	*SPEG*	SILENT	ENSG00000072195:SILENT[ENST00000312358(c.246C>T,ctC>ctT):ENSP00000311684(p.Leu82(=))];	NA	NA	NA	NA	NA	NA	NA	NA	NA	NA	LOW	NA	NA	20	20	0	19	12	7	36.84
Smoker	42479	chr2	98430739	G	A	*TMEM131*	MISSENSE	ENSG00000075568:MISSENSE[ENST00000186436(c.1405C>T,Cac>Tac):ENSP00000186436(p.His469Tyr)];	NA	NA	NA	NA	NA	NA	NA	T	PrD	D	MODERATE	D	1	171	171	0	194	93	101	52.06
Smoker	42479	chr2	234873287	C	G	*TRPM8*	MISSENSE	ENSG00000144481:MISSENSE[ENST00000324695(c.1765C>G,Ctg>Gtg):ENSP00000323926(p.Leu589Val)];ENSG00000144481:MISSENSE[ENST00000433712(c.829C>G,Ctg>Gtg):ENSP00000404423(p.Leu277Val)];ENSG00000144481:3UTR[ENST00000444298(c.*1044C>G)];	NA	NA	NA	NA	NA	NA	NA	NA	NA	NA	MODIFIER	NA	NA	141	141	0	88	53	35	39.77
Smoker	42479	chr2	179438813	C	T	*TTN*	MISSENSE	ENSG00000155657:MISSENSE[ENST00000359218(c.45226G>A,Gat>Aat):ENSP00000352154(p.Asp15076Asn)];ENSG00000155657:MISSENSE[ENST00000591111(c.67123G>A,Gat>Aat):ENSP00000465570(p.Asp22375Asn)];ENSG00000155657:MISSENSE[ENST00000589042(c.72046G>A,Gat>Aat):ENSP00000467141(p.Asp24016Asn)];ENSG00000155657:MISSENSE[ENST00000342175(c.45427G>A,Gat>Aat):ENSP00000340554(p.Asp15143Asn)];ENSG00000155657:MISSENSE[ENST00000342992(c.64342G>A,Gat>Aat):ENSP00000343764(p.Asp21448Asn)];ENSG00000155657:MISSENSE[ENST00000460472(c.44851G>A,Gat>Aat):ENSP00000434586(p.Asp14951Asn)];	NA	NA	NA	NA	NA	NA	NA	NA	NA	NA	MODIFIER	NA	NA	166	166	0	135	51	84	62.22
Smoker	42479	chr2	179502184	C	T	*TTN*	INTRONIC	ENSG00000155657:[ENST00000359218(c.14057-38G>A)];ENSG00000155657:[ENST00000591111(c.35954-38G>A)];ENSG00000155657:[ENST00000589042(c.40877-38G>A)];ENSG00000155657:[ENST00000414766(c.2840-658G>A)];ENSG00000155657:[ENST00000342992(c.33173-38G>A)];ENSG00000155657:[ENST00000342175(c.14258-38G>A)];ENSG00000155657:[ENST00000460472(c.13682-38G>A)];	NA	NA	NA	NA	NA	NA	NA	NA	NA	NA	MODIFIER	NA	NA	94	94	0	84	64	20	23.81

Mangalaparthi *et al.* , 2020. Mutational landscape of esophageal squamous cell carcinoma in an Indian cohort
Supplementary Table 4. List of somatic single nucleotide variants identified using whole exome sequencing of ESCC patients

Cohort	Sample	Chromosome	Genomic position	Reference allele	Alternate allele	Gene symbol	Variant type	Variant annotation	OncoMD	COSMIC	dbSNP	ExAC	ExAC_SAS	1000G	1000G_SAS	SIFT	Polyphen2	CONDEL	VEP	LRT	PhastCon7 Way	Total read (Adjacent normal)	Reference allel read support (Adjacent normal)	Alternative read support (Adjacent normal)	Total read (Tumor)	Reference allel read support (Tumor)	Alternative read support (Tumor)	Alternate allele frequency
Smoker	42479	chr2	179438813	C	T	*TTN-AS1*	INTRONIC	ENSG00000237298:[ENST00000592630(n.434-23510C>T)];ENSG00000237298:[ENST00000592689(n.463-23510C>T)];ENSG00000237298:[ENST00000438095(n.344+2637C>T)];ENSG00000237298:[ENST00000590807(n.75-9438C>T)];ENSG00000237298:[ENST00000586707(n.346-9627C>T)];ENSG00000237298:[ENST00000586831(n.215-8486C>T)];ENSG00000237298:[ENST00000585451(n.198+50450C>T)];ENSG00000237298:[ENST00000592600(n.346-10487C>T)];ENSG00000237298:[ENST00000592750(n.346-23510C>T)];ENSG00000237298:[ENST00000419746(n.2044-8486C>T)];ENSG00000237298:[ENST00000456053(n.563+2637C>T)];ENSG00000237298:[ENST00000586452(n.224+2637C>T)];ENSG00000237298:[ENST00000591332(n.443-23510C>T)];ENSG00000237298:[ENST00000590932(n.616-23510C>T)];	NA	NA	NA	NA	NA	NA	NA	NA	NA	NA	MODIFIER	NA	NA	166	166	0	135	51	84	62.22
Smoker	42479	chr2	179502184	C	T	*TTN-AS1*	INTRONIC	ENSG00000237298:[ENST00000418062(n.139+759C>T)];ENSG00000237298:[ENST00000592630(n.519+39776C>T)];ENSG00000237298:[ENST00000590807(n.520+759C>T)];ENSG00000237298:[ENST00000585451(n.198+113821C>T)];ENSG00000237298:[ENST00000589830(n.211+759C>T)];ENSG00000237298:[ENST00000589487(n.111-6837C>T)];ENSG00000237298:[ENST00000431752(n.124+759C>T)];	NA	NA	NA	NA	NA	NA	NA	NA	NA	NA	MODIFIER	NA	NA	94	94	0	84	64	20	23.81
Smoker	42479	chr2	29117667	G	A	*WDR43*	MISSENSE	ENSG00000163811:MISSENSE[ENST00000407426(c.103G>A,Gac>Aac):ENSP00000384302(p.Asp35Asn)];	NA	NA	NA	NA	NA	NA	NA	D	PrD	D	MODERATE	D	0.559	118	118	0	173	109	64	36.99
Smoker	42479	chr20	31878935	C	T	*BPIFB1*	INTRONIC	ENSG00000125999:[ENST00000253354(c.515+23C>T)];	NA	NA	NA	8.74E-06	0	NA	NA	NA	NA	NA	MODIFIER	NA	NA	54	54	0	92	55	37	40.22
Smoker	42479	chr20	61936748	G	A	*COL20A1*	INTRONIC	ENSG00000101203:[ENST00000358894(c.194-21G>A)];ENSG00000101203:[ENST00000422202(c.194-21G>A)];ENSG00000101203:[ENST00000326996(c.194-21G>A)];ENSG00000101203:[ENST00000479501(n.256-21G>A)];ENSG00000101203:[ENST00000435874(c.194-21G>A)];	NA	NA	NA	NA	NA	NA	NA	NA	NA	NA	MODIFIER	NA	NA	66	66	0	100	48	52	52
Smoker	42479	chr20	47860326	C	T	*DDX27*	INTRONIC	ENSG00000124228:[ENST00000371764(c.2373-27C>T)];ENSG00000124228:[ENST00000484427(n.4668-27C>T)];ENSG00000124228:[ENST00000471144(n.3351C>T)];	NA	NA	NA	NA	NA	NA	NA	NA	NA	NA	MODIFIER	NA	NA	89	89	0	155	125	30	19.35
Smoker	42479	chr20	13273077	G	C	*ISM1*	MISSENSE	ENSG00000101230:MISSENSE[ENST00000262487(c.844G>C,Gag>Cag):ENSP00000262487(p.Glu282Gln)];	chr20*1327.	chr20*1327.	NA	NA	NA	NA	NA	NA	NA	NA	MODIFIER	NA	NA	170	170	0	120	21	99	82.5
Smoker	42479	chr20	43726544	C	T	*KCNS1*	MISSENSE	ENSG00000124134:MISSENSE[ENST00000537075(c.869G>A,cGc>cAc):ENSP00000445595(p.Arg290His)];ENSG00000124134:MISSENSE[ENST00000306117(c.869G>A,cGc>cAc):ENSP00000307694(p.Arg290His)];	NA	NA	NA	1.41E-05	0	NA	NA	D	PrD	D	MODERATE	D	0.999	155	155	0	395	331	64	16.2
Smoker	42479	chr20	48431493	G	C	*SLC9A8*	INTRONIC	ENSG00000197818:[ENST00000489787(n.56-52G>C)];ENSG00000197818:[ENST00000541138(c.-711-52G>C)];ENSG00000197818:[ENST00000539601(c.-512-52G>C)];ENSG00000197818:[ENST00000417961(c.27-52G>C)];ENSG00000197818:[ENST00000361573(c.27-52G>C)];	NA	NA	NA	NA	NA	NA	NA	NA	NA	NA	MODIFIER	NA	NA	64	64	0	81	49	32	39.51
Smoker	42479	chr20	13273077	G	C	*TASP1*	INTRONIC	ENSG00000089123:[ENST00000539805(c.*284+23540C>G)];	chr20*1327.	chr20*1327.	NA	NA	NA	NA	NA	NA	NA	NA	MODIFIER	NA	NA	170	170	0	120	21	99	82.5
Smoker	42479	chr20	47860326	C	T	*ZNFX1*	INTRONIC	ENSG00000124201:[ENST00000371754(c.3313-1866G>A)];ENSG00000124201:[ENST00000469991(n.168+796G>A)];	NA	NA	NA	NA	NA	NA	NA	NA	NA	NA	MODIFIER	NA	NA	89	89	0	155	125	30	19.35
Smoker	42479	chr21	47532644	C	G	*COL6A2*	INTRONIC	ENSG00000142173:[ENST00000397763(c.715-74C>G)];ENSG00000142173:[ENST00000310645(c.715-74C>G)];ENSG00000142173:[ENST00000300527(c.715-74C>G)];ENSG00000142173:[ENST00000460886(n.161-74C>G)];ENSG00000142173:[ENST00000357838(c.715-74C>G)];ENSG00000142173:[ENST00000409416(c.715-74C>G)];	NA	NA	NA	NA	NA	NA	NA	NA	NA	NA	MODIFIER	NA	NA	116	116	0	118	95	23	19.49
Smoker	42479	chr21	44592203	G	T	*CRYAA*	MISSENSE	ENSG00000160202:MISSENSE[ENST00000398132(c.224G>T,cGt>cTt):ENSP00000381200(p.Arg75Leu)];ENSG00000160202:MISSENSE[ENST00000398133(c.275G>T,cGt>cTt):ENSP00000381201(p.Arg92Leu)];ENSG00000160202:MISSENSE[ENST00000291554(c.335G>T,cGt>cTt):ENSP00000291554(p.Arg112Leu)];ENSG00000160202:EXONIC-NC[ENST00000468016(n.436G>T)];ENSG00000160202:EXONIC-NC[ENST00000482775(n.416G>T)];	NA	NA	chr21*44592203*44592203*G*A*rs200594555*NA*NA*NA	NA	NA	NA	NA	NA	NA	NA	MODIFIER	NA	NA	42	42	0	62	44	18	29.03
Smoker	42479	chr21	47952146	G	A	*DIP2A*	MISSENSE	ENSG00000160305:MISSENSE[ENST00000427143(c.1109G>A,aGa>aAa):ENSP00000400528(p.Arg370Lys)];ENSG00000160305:MISSENSE[ENST00000318711(c.1304G>A,aGa>aAa):ENSP00000323633(p.Arg435Lys)];ENSG00000160305:MISSENSE[ENST00000435722(c.1301G>A,aGa>aAa):ENSP00000415089(p.Arg434Lys)];ENSG00000160305:MISSENSE[ENST00000400274(c.1289G>A,aGa>aAa):ENSP00000383133(p.Arg430Lys)];ENSG00000160305:MISSENSE[ENST00000457905(c.1301G>A,aGa>aAa):ENSP00000393434(p.Arg434Lys)];ENSG00000160305:MISSENSE[ENST00000417564(c.1301G>A,aGa>aAa):ENSP00000392066(p.Arg434Lys)];ENSG00000160305:MISSENSE[ENST00000466639(c.1172G>A,aGa>aAa):ENSP00000430249(p.Arg391Lys)];ENSG00000160305:EXONIC-NC[ENST00000494435(n.627G>A)];ENSG00000160305:EXONIC-NC[ENST00000473752(n.694G>A)];	NA	NA	NA	NA	NA	NA	NA	T	BN	N	MODERATE	N	0.982	102	101	0	111	60	50	45.05
Smoker	42479	chr21	39772502	T	A	*ERG*	MISSENSE	ENSG00000157554:MISSENSE[ENST00000453032(c.463A>T,Agg>Tgg):ENSP00000396268(p.Arg155Trp)];ENSG00000157554:MISSENSE[ENST00000398910(c.760A>T,Agg>Tgg):ENSP00000381881(p.Arg254Trp)];ENSG00000157554:MISSENSE[ENST00000288319(c.739A>T,Agg>Tgg):ENSP00000288319(p.Arg247Trp)];ENSG00000157554:MISSENSE[ENST00000398919(c.760A>T,Agg>Tgg):ENSP00000381891(p.Arg254Trp)];ENSG00000157554:MISSENSE[ENST00000417133(c.760A>T,Agg>Tgg):ENSP00000414150(p.Arg254Trp)];ENSG00000157554:MISSENSE[ENST00000398907(c.739A>T,Agg>Tgg):ENSP00000381879(p.Arg247Trp)];ENSG00000157554:INTRONIC[ENST00000398897(c.397+1977A>T)];ENSG00000157554:INTRONIC[ENST00000481609(n.844+1977A>T)];ENSG00000157554:INTRONIC[ENST00000442448(c.694+1977A>T)];ENSG00000157554:INTRONIC[ENST00000429727(c.389-8136A>T)];ENSG00000157554:INTRONIC[ENST00000398905(c.673+1977A>T)];ENSG00000157554:INTRONIC[ENST00000398911(c.694+1977A>T)];ENSG00000157554:EXONIC-NC[ENST00000492833(n.721A>T)];ENSG00000157554:EXONIC-NC[ENST00000468474(n.946A>T)];	NA	NA	NA	NA	NA	NA	NA	NA	NA	NA	MODIFIER	NA	NA	69	69	0	62	40	22	35.48

Supplementary Table 4. List of somatic single nucleotide variants identified using whole exome sequencing of ESCC patients

Cohort	Sample	Chromosome	Genomic position	Reference allele	Alternate allele	Gene symbol	Variant type	Variant annotation	OncoMD	COSMIC	dbSNP	ExAC	ExAC_SAS	1000G	1000G_SAS	SIFT	Polyphen2	CONDEL	VEP	LRT	PhastCon7 Way	Total read (Adjacent normal)	Reference allel read support (Adjacent normal)	Alternative read support (Adjacent normal)	Total read (Tumor)	Reference allel read support (Tumor)	Alternative read support (Tumor)	Alternate allele frequency
Smoker	42479	chr21	33044119	G	T	*SCAF4*	SILENT	ENSG00000156304:SILENT[ENST00000399804(c.2971C>A,Cgg>Agg):ENSP00000382703(p.Arg991(=))];ENSG00000156304:SILENT[ENST00000286835(c.3037C>A,Cgg>Agg):ENSP00000286835(p.Arg1013(=))];ENSG00000156304:SILENT[ENST00000434667(c.2992C>A,Cgg>Agg):ENSP00000402377(p.Arg998(=))];	NA	NA	chr21*33044119*33044119*G*A*rs373258764*NA*NA*NA	NA	NA	NA	NA	NA	NA	NA	LOW	NA	1	154	152	0	215	121	94	43.72
Smoker	42479	chr21	34003593	C	T	*SYNJ1*	SILENT	ENSG00000159082:3UTR[ENST00000382499(c.*522G>A)];ENSG00000159082:3UTR[ENST00000438952(c.*522G>A)];ENSG00000159082:3UTR[ENST00000357345(c.*522G>A)];ENSG00000159082:SILENT[ENST00000382491(c.4293G>A,ccG>ccA):ENSP00000371931(p.Pro1431(=))];ENSG00000159082:SILENT[ENST00000322229(c.4434G>A,ccG>ccA):ENSP00000322234(p.Pro1478(=))];ENSG00000159082:SILENT[ENST00000433931(c.4551G>A,ccG>ccA):ENSP00000409667(p.Pro1517(=))];	NA	NA	NA	2.47E-05	0	NA	NA	NA	NA	NA	MODIFIER	NA	NA	300	299	0	386	299	86	22.28
Smoker	42479	chr22	41922396	C	G	*ACO2*	MISSENSE	ENSG00000100412:MISSENSE[ENST00000216254(c.1892C>G,tCc>tGc):ENSP00000216254(p.Ser631Cys)];ENSG00000100412:MISSENSE[ENST00000396512(c.1967C>G,tCc>tGc):ENSP00000379769(p.Ser656Cys)];	NA	NA	NA	NA	NA	NA	NA	NA	NA	NA	MODIFIER	NA	NA	34	34	0	72	58	14	19.44
Smoker	42479	chr22	30188457	C	G	*ASCC2*	MISSENSE	ENSG00000100325:MISSENSE[ENST00000307790(c.1987G>C,Gat>Cat):ENSP00000305502(p.Asp663His)];ENSG00000100325:MISSENSE[ENST00000397771(c.1987G>C,Gat>Cat):ENSP00000380877(p.Asp663His)];ENSG00000100325:MISSENSE[ENST00000542393(c.1759G>C,Gat>Cat):ENSP00000437570(p.Asp587His)];ENSG00000100325:3UTR[ENST00000458594(c.*1876G>C)];ENSG00000100325:EXONIC-NC[ENST00000487486(n.907G>C)];ENSG00000100325:INTRONIC[ENST00000472433(n.297-3616G>C)];	NA	NA	NA	NA	NA	NA	NA	NA	NA	NA	MODIFIER	NA	NA	56	56	0	83	43	40	48.19
Smoker	42479	chr22	41545099	C	G	*EP300*	MISSENSE	ENSG00000100393:MISSENSE[ENST00000263253(c.2299C>G,Cag>Gag):ENSP00000263253(p.Gln767Glu)];	chr22*4154	chr22*4154	NA	NA	NA	NA	NA	NA	UN	NA	MODERATE	D	0.973	68	68	0	128	85	43	33.59
Smoker	42479	chr22	40415332	G	C	*FAM83F*	MISSENSE	ENSG00000133477:MISSENSE[ENST00000473717(c.146G>C,cGg>cCg):ENSP00000476600(p.Arg49Pro)];ENSG00000133477:MISSENSE[ENST00000333407(c.650G>C,cGg>cCg):ENSP00000330432(p.Arg217Pro)];	chr22*4041	chr22*4041	NA	NA	NA	NA	NA	D	PrD	D	MODERATE	D	0.983	41	41	0	78	57	21	26.92
Smoker	42479	chr22	31521982	C	T	*INPP5J*	SILENT	ENSG00000185133:INTRONIC[ENST00000420017(c.-116-298C>T)];ENSG00000185133:INTRONIC[ENST00000461241(n.146-380C>T)];ENSG00000185133:INTRONIC[ENST00000404390(c.168-380C>T)];ENSG00000185133:SILENT[ENST00000412277(c.1056C>T,agC>agT):ENSP00000392924(p.Ser352(=))];ENSG00000185133:SILENT[ENST00000405300(c.156C>T,agC>agT):ENSP00000384596(p.Ser52(=))];ENSG00000185133:SILENT[ENST00000331075(c.1257C>T,agC>agT):ENSP00000333262(p.Ser419(=))];ENSG00000185133:SILENT[ENST00000400294(c.156C>T,agC>agT):ENSP00000383150(p.Ser52(=))];	NA	NA	NA	9.03E-05	0.000518135	NA	NA	NA	NA	NA	LOW	NA	0.993	62	62	0	148	62	86	58.11
Smoker	42479	chr22	28202503	G	C	*NA*	INTERGENIC		NA	NA	NA	NA	NA	NA	NA	NA	NA	NA	MODIFIER	NA	NA	57	57	0	103	62	41	39.81
Smoker	42479	chr22	31289244	G	C	*OSBP2*	INTRONIC	ENSG00000184792:[ENST00000433183(c.2+15G>C)];ENSG00000184792:[ENST00000496575(n.26-206G>C)];ENSG00000184792:[ENST00000431368(c.1005+15G>C)];ENSG00000184792:[ENST00000437268(c.1216+15G>C)];ENSG00000184792:[ENST00000452656(c.883+15G>C)];ENSG00000184792:[ENST00000401475(c.889+15G>C)];ENSG00000184792:[ENST00000332585(c.1990+15G>C)];ENSG00000184792:[ENST00000446658(c.1987+15G>C)];ENSG00000184792:[ENST00000407373(c.1471+15G>C)];ENSG00000184792:[ENST00000382310(c.1843+15G>C)];ENSG00000184792:[ENST00000403222(c.1492+15G>C)];ENSG00000184792:[ENST00000535268(c.622+15G>C)];	NA	NA	chr22*31289244*31289244*G*A*rs201315645*0*0.9992*0.0007987	NA	NA	NA	NA	NA	NA	NA	MODIFIER	NA	NA	50	50	0	86	38	48	55.81
Smoker	42479	chr22	41922396	C	G	*POLR3H*	3UTR	ENSG00000100413:[ENST00000396504(c.*2891G>C)];ENSG00000100413:[ENST00000355209(c.*2891G>C)];	NA	NA	NA	NA	NA	NA	NA	NA	NA	NA	MODIFIER	NA	NA	34	34	0	72	58	14	19.44
Smoker	42479	chr22	37603009	G	T	*SSTR3*	SILENT	ENSG00000183473:SILENT[ENST00000328544(c.834C>A,atC>atA):ENSP00000330138(p.Ile278(=))];ENSG00000183473:SILENT[ENST00000402501(c.834C>A,atC>atA):ENSP00000384904(p.Ile278(=))];	NA	NA	chr22*37603009*37603009*G*A*rs568818068*0*0.9998*0.0001997	NA	NA	NA	NA	NA	NA	NA	LOW	NA	0.998	118	118	0	226	152	74	32.74
Smoker	42479	chr22	50989686	C	T	*SYCE3*	SILENT	ENSG00000217442:SILENT[ENST00000402753(c.255G>A,aaG>aaA):ENSP00000385122(p.Lys85(=))];ENSG00000217442:SILENT[ENST00000406915(c.255G>A,aaG>aaA):ENSP00000385480(p.Lys85(=))];	NA	NA	NA	NA	NA	NA	NA	NA	NA	NA	LOW	NA	NA	62	61	1	129	77	52	40.31
Smoker	42479	chr22	37465286	A	G	*TMPRSS6*	MISSENSE	ENSG00000187045:MISSENSE[ENST00000406856(c.1940T>C,cTg>cCg):ENSP00000384964(p.Leu647Pro)];ENSG00000187045:MISSENSE[ENST00000381792(c.1940T>C,cTg>cCg):ENSP00000371211(p.Leu647Pro)];ENSG00000187045:MISSENSE[ENST00000406725(c.1940T>C,cTg>cCg):ENSP00000385453(p.Leu647Pro)];ENSG00000187045:MISSENSE[ENST00000346753(c.1967T>C,cTg>cCg):ENSP00000334962(p.Leu656Pro)];	NA	NA	NA	NA	NA	NA	NA	T	PrD	D	MODERATE	D	0.999	51	51	0	80	71	8	10
Smoker	42479	chr3	100557219	G	T	*ABI3BP*	INTRONIC	ENSG00000154175:[ENST00000495591(c.249+27C>A)];ENSG00000154175:[ENST00000528490(c.148-2715C>A)];ENSG00000154175:[ENST00000466947(c.60-3602C>A)];ENSG00000154175:[ENST00000284322(c.1597+7997C>A)];ENSG00000154175:[ENST00000383691(c.-57+27C>A)];ENSG00000154175:[ENST00000495063(c.2029+27C>A)];ENSG00000154175:[ENST00000528305(c.300+23936C>A)];ENSG00000154175:[ENST00000470336(n.178+7997C>A)];ENSG00000154175:[ENST00000534256(c.213+1194C>A)];ENSG00000154175:[ENST00000534413(c.180+1194C>A)];ENSG00000154175:[ENST00000471714(c.2029+27C>A)];ENSG00000154175:[ENST00000533795(c.202+27C>A)];	NA	NA	NA	NA	NA	NA	NA	NA	NA	NA	MODIFIER	NA	NA	173	173	0	152	109	43	28.29
Smoker	42479	chr3	132337591	C	T	*ACAD11*	NONSENSE	ENSG00000240303:NONSENSE[ENST00000481970(c.1301G>A,tGg>tAg):ENSP00000420907(p.Trp434Ter)];ENSG00000240303:NONSENSE[ENST00000264990(c.1301G>A,tGg>tAg):ENSP00000264990(p.Trp434Ter)];ENSG00000240303:NONSENSE[ENST00000355458(c.1301G>A,tGg>tAg):ENSP00000347636(p.Trp434Ter)];ENSG00000240303:EXONIC-NC[ENST00000507705(n.132G>A)];ENSG00000240303:EXONIC-NC[ENST00000496418(n.1199G>A)];ENSG00000240303:EXONIC-NC[ENST00000487024(n.488G>A)];ENSG00000240303:EXONIC-NC[ENST00000469042(n.1632G>A)];ENSG00000240303:INTRONIC[ENST00000485198(c.1197+7944G>A)];ENSG00000240303:INTRONIC[ENST00000545291(c.-12+7944G>A)];	NA	NA	NA	NA	NA	NA	NA	NA	NA	NA	MODIFIER	NA	NA	225	224	0	193	138	55	28.5
Smoker	42479	chr3	132337591	C	T	*ACKR4*	3UTR	ENSG00000129048:[ENST00000509820(c.*141C>T)];	NA	NA	NA	NA	NA	NA	NA	NA	NA	NA	MODIFIER	NA	NA	225	224	0	193	138	55	28.5
Smoker	42479	chr3	49756772	C	T	*AMIGO3*	MISSENSE	ENSG00000176020:MISSENSE[ENST00000535833(c.127G>A,Gac>Aac):ENSP00000439268(p.Asp43Asn)];ENSG00000176020:MISSENSE[ENST00000320431(c.127G>A,Gac>Aac):ENSP00000323096(p.Asp43Asn)];	NA	NA	NA	8.33E-06	0	NA	NA	NA	NA	NA	MODIFIER	NA	NA	36	36	0	29	15	14	48.28

Mangalaparthi *et al.* , 2020. Mutational landscape of esophageal squamous cell carcinoma in an Indian cohort

Supplementary Table 4. List of somatic single nucleotide variants identified using whole exome sequencing of ESCC patients

Cohort	Sample	Chromosome	Genomic position	Reference allele	Alternate allele	Gene symbol	Variant type	Variant annotation	OncoMD	COSMIC	dbSNP	ExAC	ExAC_SAS	1000G	1000G_SAS	SIFT	Polyphen2	CONDEL	VEP	LRT	PhastCon7 Way	Total read (Adjacent normal)	Reference allel read support (Adjacent normal)	Alternative read support (Adjacent normal)	Total read (Tumor)	Reference allel read support (Tumor)	Alternative read support (Tumor)	Alternate allele frequency
Smoker	42479	chr3	93699237	G	A	*ARL13B*	EXONIC-NC	ENSG00000169379:[ENST00000486562(n.180G>A)];ENSG00000169379:[ENST00000492165(n.106G>A)];ENSG00000169379:[ENST00000335438(c.-31G>A)];ENSG00000169379:[ENST00000394222(c.-31G>A)];ENSG00000169379:[ENST00000303097(c.-31G>A)];ENSG00000169379:[ENST00000478400(c.-146G>A)];ENSG00000169379:[ENST00000460371(c.-31G>A)];ENSG00000169379:[ENST00000475206(c.-31G>A)];ENSG00000169379:[ENST00000535334(c.-269G>A)];ENSG00000169379:[ENST00000471138(c.-31G>A)];ENSG00000169379:[ENST00000539730(c.-623G>A)];	NA	NA	NA	NA	NA	NA	NA	NA	NA	NA	MODIFIER	NA	NA	73	73	0	57	22	35	61.4
Smoker	42479	chr3	130735047	C	G	*ASTE1*	MISSENSE	ENSG00000034533:MISSENSE[ENST00000514044(c.1650G>C,caG>caC):ENSP00000426421(p.Gln550His)];ENSG00000034533:MISSENSE[ENST00000264992(c.1650G>C,caG>caC):ENSP00000264992(p.Gln550His)];ENSG00000034533:3UTR[ENST00000507978(c.*147G>C)];	NA	NA	0.0001997	8.24E-06	0	0.0001997	0	NA	NA	NA	MODIFIER	NA	NA	97	97	0	87	62	24	27.59
Smoker	42479	chr3	130735047	C	G	*ATP2C1*	MISSENSE	ENSG00000017260:MISSENSE[ENST00000533801(c.2791C>G,Ctg>Gtg):ENSP00000432956(p.Leu931Val)];ENSG00000017260:MISSENSE[ENST00000504612(c.2667C>G,Ctg>Gtg):ENSP00000425228(p.Leu890Val)];ENSG00000017260:MISSENSE[ENST00000359644(c.2806C>G,Ctg>Gtg):ENSP00000352665(p.Leu936Val)];ENSG00000017260:MISSENSE[ENST00000507488(c.2728C>G,Ctg>Gtg):ENSP00000421326(p.Leu910Val)];ENSG00000017260:MISSENSE[ENST00000393221(c.2878C>G,Ctg>Gtg):ENSP00000376914(p.Leu960Val)];ENSG00000017260:MISSENSE[ENST00000422190(c.2776C>G,Ctg>Gtg):ENSP00000402677(p.Leu926Val)];ENSG00000017260:MISSENSE[ENST00000513801(c.2728C>G,Ctg>Gtg):ENSP00000422872(p.Leu910Val)];ENSG00000017260:MISSENSE[ENST00000504381(c.2641C>G,Ctg>Gtg):ENSP00000425320(p.Leu881Val)];ENSG00000017260:3UTR[ENST00000328560(c.*14C>G)];	NA	NA	0.0001997	8.24E-06	0	0.0001997	0	NA	NA	NA	MODIFIER	NA	NA	97	97	0	87	62	24	27.59
Smoker	42479	chr3	49832157	G	C	*CDHR4*	INTRONIC	ENSG00000187492:[ENST00000412678(c.1186-23C>G)];	NA	NA	NA	NA	NA	NA	NA	NA	NA	NA	MODIFIER	NA	NA	36	36	0	27	7	20	74.07
Smoker	42479	chr3	130368379	C	G	*COL6A6*	MISSENSE	ENSG00000206384:INTRONIC[ENST00000506143(c.*752+4533C>G)];ENSG00000206384:MISSENSE[ENST00000453409(c.5706C>G,aaC>aaG):ENSP00000399236(p.Asn1902Lys)];ENSG00000206384:MISSENSE[ENST00000358511(c.5706C>G,aaC>aaG):ENSP00000351310(p.Asn1902Lys)];	NA	NA	chr3*130368379*130368379*C*T*rs755436627*NA*NA*NA	NA	NA	NA	NA	T	BN	N	MODERATE	NA	NA	89	88	0	94	68	26	27.66
Smoker	42479	chr3	16264110	C	A	*GALNT15*	INTRONIC	ENSG00000131386:[ENST00000339732(c.1630-12C>A)];ENSG00000131386:[ENST00000437509(c.1630-12C>A)];	NA	NA	chr3*16264110*16264110*C*T*rs768871314*NA*NA*NA	NA	NA	NA	NA	NA	NA	NA	MODIFIER	NA	NA	75	75	0	93	80	13	13.98
Smoker	42479	chr3	49756772	C	T	*GMPPB*	3UTR	ENSG00000173540:[ENST00000480687(c.*2413G>A)];	NA	NA	NA	8.33E-06	0	NA	NA	NA	NA	NA	MODIFIER	NA	NA	36	36	0	29	15	14	48.28
Smoker	42479	chr3	120347344	C	T	*HGD*	SILENT	ENSG00000113924:3UTR[ENST00000492108(c.*203G>A)];ENSG00000113924:SILENT[ENST00000283871(c.1221G>A,gcG>gcA):ENSP00000283871(p.Ala407(=))];	NA	NA	0.01757	0.0054291	0.000121124	0.0175719	0	NA	NA	NA	LOW	NA	NA	42	42	0	34	19	15	44.12
Smoker	42479	chr3	127336196	C	T	*MCM2*	SILENT	ENSG00000073111:3UTR[ENST00000477668(c.*1426C>T)];ENSG00000073111:3UTR[ENST00000474964(c.*1426C>T)];ENSG00000073111:SILENT[ENST00000265056(c.1848C>T,atC>atT):ENSP00000265056(p.Ile616(=))];ENSG00000073111:SILENT[ENST00000491422(c.1643C>T,atC>atT):ENSP00000420528(p.Ile548(=))];ENSG00000073111:EXONIC-NC[ENST00000473785(n.216C>T)];	NA	NA	chr3*127336196*127336196*C*G,T*rs755049339*NA*NA*NA	3.33E-05	0	NA	NA	NA	NA	NA	MODIFIER	NA	NA	62	62	0	81	61	20	24.69
Smoker	42479	chr3	196750735	A	T	*MFI2*	SILENT	ENSG00000163975:SILENT[ENST00000439320(c.534T>A,gcT>gcA):ENSP00000393439(p.Ala178(=))];ENSG00000163975:INTRONIC[ENST00000296350(c.487+439T>A)];ENSG00000163975:INTRONIC[ENST00000296351(c.487+439T>A)];	NA	NA	NA	NA	NA	NA	NA	NA	NA	NA	MODIFIER	NA	NA	37	37	0	48	35	13	27.08
Smoker	42479	chr3	132337591	C	T	*NPHP3*	3UTR	ENSG00000113971:[ENST00000471702(c.*3288G>A)];	NA	NA	NA	NA	NA	NA	NA	NA	NA	NA	MODIFIER	NA	NA	225	224	0	193	138	55	28.5
Smoker	42479	chr3	13381402	G	T	*NUP210*	SILENT	ENSG00000132182:EXONIC-NC[ENST00000485755(n.205C>A)];ENSG00000132182:SILENT[ENST00000254508(c.3423C>A,ctC>ctA):ENSP00000254508(p.Leu1141(=))];	NA	NA	chr3*13381402*13381402*G*A*rs140367875*NA*NA*NA	NA	NA	NA	NA	NA	NA	NA	LOW	NA	NA	71	71	0	106	101	5	4.72
Smoker	42479	chr3	196533512	G	C	*PAK2*	MISSENSE	ENSG00000180370:EXONIC-NC[ENST00000481344(n.63G>C)];ENSG00000180370:MISSENSE[ENST00000327134(c.531G>C,gaG>gaC):ENSP00000314067(p.Glu177Asp)];	NA	NA	NA	NA	NA	NA	NA	T	BN	N	MODERATE	N	0.976	134	134	0	168	108	60	35.71
Smoker	42479	chr3	122880831	C	T	*PDIA5*	EXONIC-NC	ENSG00000065485:[ENST00000467157(n.2113C>T)];ENSG00000065485:[ENST00000316218(c.*24C>T)];ENSG00000065485:[ENST00000489923(c.*639C>T)];ENSG00000065485:[ENST00000469649(c.232+529C>T)];	NA	NA	NA	NA	NA	NA	NA	NA	NA	NA	MODIFIER	NA	NA	128	128	0	104	81	23	22.12
Smoker	42479	chr3	126733054	G	A	*PLXNA1*	MISSENSE	ENSG00000114554:MISSENSE[ENST00000393409(c.2440G>A,Gag>Aag):ENSP00000377061(p.Glu814Lys)];ENSG00000114554:MISSENSE[ENST00000251772(c.2371G>A,Gag>Aag):ENSP00000251772(p.Glu791Lys)];	NA	NA	NA	1.01E-05	0	NA	NA	T	BN	N	MODERATE	N	0.98	58	58	0	85	67	18	21.18
Smoker	42479	chr3	152880844	A	C	*RAP2B*	MISSENSE	ENSG00000181467:MISSENSE[ENST00000323534(c.362A>C,gAg>gCg):ENSP00000319096(p.Glu121Ala)];	NA	NA	NA	NA	NA	NA	NA	T	BN	D	MODERATE	U	1	69	69	0	94	35	59	62.77
Smoker	42479	chr3	49756772	C	T	*RNF123*	INTRONIC	ENSG00000164068:[ENST00000327697(c.3501-1172C>T)];ENSG00000164068:[ENST00000433785(c.837-1172C>T)];ENSG00000164068:[ENST00000486102(n.3896-1172C>T)];ENSG00000164068:[ENST00000487805(n.5216-1172C>T)];ENSG00000164068:[ENST00000497099(n.372+735C>T)];ENSG00000164068:[ENST00000457726(c.*1575-1172C>T)];	NA	NA	NA	8.33E-06	0	NA	NA	NA	NA	NA	MODIFIER	NA	NA	36	36	0	29	15	14	48.28
Smoker	42479	chr3	125179693	G	T	*SNX4*	MISSENSE	ENSG00000114520:MISSENSE[ENST00000536067(c.431C>A,tCt>tAt):ENSP00000440824(p.Ser144Tyr)];ENSG00000114520:MISSENSE[ENST00000251775(c.866C>A,tCt>tAt):ENSP00000251775(p.Ser289Tyr)];ENSG00000114520:3UTR[ENST00000471751(c.*594C>A)];	NA	NA	NA	NA	NA	NA	NA	NA	NA	NA	MODIFIER	NA	NA	20	19	0	17	10	7	41.18
Smoker	42479	chr3	9146540	G	A	*SRGAP3*	INTRONIC	ENSG00000196220:[ENST00000360413(c.261-14C>T)];ENSG00000196220:[ENST00000470951(n.301-14C>T)];ENSG00000196220:[ENST00000433332(n.330-14C>T)];ENSG00000196220:[ENST00000383836(c.261-14C>T)];	NA	NA	NA	NA	NA	NA	NA	NA	NA	NA	MODIFIER	NA	NA	64	64	0	43	6	37	86.05
Smoker	42479	chr3	136323277	C	A	*STAG1*	MISSENSE	ENSG00000118007:MISSENSE[ENST00000487065(c.171G>T,aaG>aaT):ENSP00000418472(p.Lys57Asn)];ENSG00000118007:MISSENSE[ENST00000483235(c.171G>T,aaG>aaT):ENSP00000419093(p.Lys57Asn)];ENSG00000118007:MISSENSE[ENST00000480733(c.171G>T,aaG>aaT):ENSP00000420789(p.Lys57Asn)];ENSG00000118007:MISSENSE[ENST00000383202(c.171G>T,aaG>aaT):ENSP00000372689(p.Lys57Asn)];ENSG00000118007:MISSENSE[ENST00000236698(c.171G>T,aaG>aaT):ENSP00000236698(p.Lys57Asn)];ENSG00000118007:EXONIC-NC[ENST00000462818(n.39G>T)];ENSG00000118007:5UTR[ENST00000434713(c.-906G>T)];	NA	NA	NA	NA	NA	NA	NA	T	BN	N	MODERATE	N	0.999	127	127	0	117	96	21	17.95

Mangalaparthi *et al.* , 2020. Mutational landscape of esophageal squamous cell carcinoma in an Indian cohort
Supplementary Table 4. List of somatic single nucleotide variants identified using whole exome sequencing of ESCC patients

Cohort	Sample	Chromosome	Genomic position	Reference allele	Alternate allele	Gene symbol	Variant type	Variant annotation	OncoMD	COSMIC	dbSNP	ExAC	ExAC_SAS	1000G	1000G_SAS	SIFT	Polyphen2	CONDEL	VEP	LRT	PhastCon7 Way	Total read (Adjacent normal)	Reference allel read support (Adjacent normal)	Alternative read support (Adjacent normal)	Total read (Tumor)	Reference allel read support (Tumor)	Alternative read support (Tumor)	Alternate allele frequency
Smoker	42479	chr3	12779611	T	C	*TMEM40*	INTRONIC	ENSG00000088726:[ENST00000435575(c.196+24A>G)];ENSG00000088726:[ENST00000476331(n.182+24A>G)];ENSG00000088726:[ENST00000431022(c.472+24A>G)];ENSG00000088726:[ENST00000435218(c.334+24A>G)];ENSG00000088726:[ENST00000428020(c.65-1277A>G)];ENSG00000088726:[ENST00000485124(n.690+24A>G)];ENSG00000088726:[ENST00000314124(c.424+24A>G)];ENSG00000088726:[ENST00000264728(c.424+24A>G)];	NA	NA	chr3*12779609*12779612*TCTC*T*rs753694096*NA*NA*NA	NA	NA	NA	NA	NA	NA	NA	MODIFIER	NA	NA	90	90	0	82	47	35	42.68
Smoker	42479	chr3	21478695	T	A	*ZNF385D*	MISSENSE-SS-PRX	ENSG00000151789:[ENST00000281523(c.440A>T,gAc>gTc):ENSP00000281523(p.Asp147Val)];ENSG00000151789:[ENST00000446749(c.*264A>T)];ENSG00000151789:[ENST00000495739(n.184A>T)];ENSG00000151789:[ENST00000494118(n.859A>T)];	NA	NA	NA	NA	NA	NA	NA	NA	NA	NA	LOW	NA	NA	106	105	0	215	163	51	23.72
Smoker	42479	chr4	73164099	C	T	*ADAMTS3*	MISSENSE	ENSG00000156140:MISSENSE[ENST00000286657(c.2485G>A,Gac>Aac):ENSP00000286657(p.Asp829Asn)];ENSG00000156140:EXONIC-NC[ENST00000511274(n.708G>A)];	chr4*73164(	NA	NA	NA	NA	NA	NA	NA	NA	NA	MODIFIER	NA	NA	277	277	0	377	318	59	15.65
Smoker	42479	chr4	74319500	G	A	*AFP*	SILENT	ENSG00000081051:EXONIC-NC[ENST00000506820(n.160G>A)];ENSG00000081051:EXONIC-NC[ENST00000514279(n.244G>A)];ENSG00000081051:EXONIC-NC[ENST00000508838(n.448G>A)];ENSG00000081051:SILENT[ENST00000226359(c.1671G>A,gtG>gtA):ENSP00000226359(p.Val557(=))];ENSG00000081051:SILENT[ENST00000395792(c.1671G>A,gtG>gtA):ENSP00000379138(p.Val557(=))];	NA	NA	NA	NA	NA	NA	NA	NA	NA	NA	LOW	NA	NA	132	132	0	219	93	126	57.53
Smoker	42479	chr4	36126483	C	A	*ARAP2*	INTRONIC-SS-DNR	ENSG00000047365:INTRONIC-SS-DNR[ENST00000303965(c.3746+1G>T)];	NA	NA	NA	NA	NA	NA	NA	NA	NA	NA	HIGH	NA	NA	233	232	0	164	23	141	85.98
Smoker	42479	chr4	123663575	C	G	*BBS12*	SILENT	ENSG00000181004:SILENT[ENST00000542236(c.528C>G,ctC>ctG):ENSP00000438273(p.Leu176(=))];ENSG00000181004:SILENT[ENST00000314218(c.528C>G,ctC>ctG):ENSP00000319062(p.Leu176(=))];ENSG00000181004:SILENT[ENST00000433287(c.528C>G,ctC>ctG):ENSP00000398912(p.Leu176(=))];	NA	NA	NA	NA	NA	NA	NA	NA	NA	NA	LOW	NA	NA	207	207	0	205	157	48	23.41
Smoker	42479	chr4	77305440	C	T	*CCDC158*	MISSENSE	ENSG00000163749:MISSENSE[ENST00000434846(c.527G>A,cGa>cAa):ENSP00000401742(p.Arg176Gln)];ENSG00000163749:MISSENSE[ENST00000388914(c.527G>A,cGa>cAa):ENSP00000373566(p.Arg176Gln)];	NA	NA	NA	0.0001906	0.00018173	NA	NA	T	PrD	D	MODERATE	D	0.99	228	225	2	345	224	120	34.78
Smoker	42479	chr4	78528885	G	C	*CXCL13*	MISSENSE	ENSG00000156234:MISSENSE[ENST00000286758(c.93G>C,ttG>ttC):ENSP00000286758(p.Leu31Phe)];ENSG00000156234:EXONIC-NC[ENST00000506590(n.127G>C)];	NA	NA	chr4*78528885*78528885*G*A*rs143408885*1*0.9978*0.002196	NA	NA	NA	NA	NA	NA	NA	MODIFIER	NA	NA	262	261	0	434	267	167	38.48
Smoker	42479	chr4	126389693	A	C	*FAT4*	MISSENSE	ENSG00000196159:MISSENSE[ENST00000394329(c.11926A>C,Aac>Cac):ENSP00000377862(p.Asn3976His)];ENSG00000196159:MISSENSE[ENST00000335110(c.6715A>C,Aac>Cac):ENSP00000335169(p.Asn2239His)];	NA	NA	chr4*126389693*126389695*AAC*A*rs751648701*NA*NA*NA	NA	NA	NA	NA	T	BN	N	MODERATE	U	0.998	187	186	0	144	109	35	24.31
Smoker	42479	chr4	144620737	G	A	*FREM3*	SILENT	ENSG00000183090:SILENT[ENST00000329798(c.1092C>T,gtC>gtT):ENSP00000332886(p.Val364(=))];	NA	NA	NA	NA	NA	NA	NA	NA	NA	NA	LOW	NA	NA	62	62	0	59	48	11	18.64
Smoker	42479	chr4	190878627	T	C	*FRG1*	SILENT	ENSG00000109536:SILENT[ENST00000531991(c.318T>C,agT>agC):ENSP00000435943(p.Ser106(=))];ENSG00000109536:SILENT[ENST00000226798(c.507T>C,agT>agC):ENSP00000226798(p.Ser169(=))];ENSG00000109536:SILENT[ENST00000524583(c.123T>C,agT>agC):ENSP00000435067(p.Ser41(=))];ENSG00000109536:EXONIC-NC[ENST00000514482(n.493T>C)];	NA	NA	NA	NA	NA	NA	NA	NA	NA	NA	LOW	NA	NA	1243	1220	23	1033	985	45	4.36
Smoker	42479	chr4	76581034	C	T	*G3BP2*	MISSENSE	ENSG00000138757:MISSENSE[ENST00000395719(c.463G>A,Gag>Aag):ENSP00000379069(p.Glu155Lys)];ENSG00000138757:MISSENSE[ENST00000359707(c.463G>A,Gag>Aag):ENSP00000352738(p.Glu155Lys)];ENSG00000138757:MISSENSE[ENST00000357854(c.463G>A,Gag>Aag):ENSP00000350518(p.Glu155Lys)];ENSG00000138757:MISSENSE[ENST00000503660(c.463G>A,Gag>Aag):ENSP00000424522(p.Glu155Lys)];ENSG00000138757:3UTR[ENST00000510902(c.*3G>A)];ENSG00000138757:EXONIC-NC[ENST00000507947(n.250G>A)];ENSG00000138757:EXONIC-NC[ENST00000502654(n.539G>A)];	NA	NA	NA	NA	NA	NA	NA	NA	NA	NA	MODIFIER	NA	NA	115	115	0	198	165	33	16.67
Smoker	42479	chr4	46099229	G	T	*GABRG1*	MISSENSE	ENSG00000163285:MISSENSE[ENST00000295452(c.242C>A,cCa>cAa):ENSP00000295452(p.Pro81Gln)];	NA	NA	NA	NA	NA	NA	NA	D	PrD	D	MODERATE	D	0.998	105	104	0	63	18	45	71.43
Smoker	42479	chr4	877800	C	A	*GAK*	INTRONIC	ENSG00000178950:[ENST00000504947(n.354+25G>T)];ENSG00000178950:[ENST00000511163(c.1018+25G>T)];ENSG00000178950:[ENST00000314167(c.1255+25G>T)];	NA	NA	NA	NA	NA	NA	NA	NA	NA	NA	MODIFIER	NA	NA	57	57	0	57	40	17	29.82
Smoker	42479	chr4	95255832	G	A	*HPGDS*	INTRONIC	ENSG00000163106:[ENST00000514774(n.72-43C>T)];ENSG00000163106:[ENST00000506331(n.83-43C>T)];ENSG00000163106:[ENST00000295256(c.-9-43C>T)];	NA	NA	NA	NA	NA	NA	NA	NA	NA	NA	MODIFIER	NA	NA	45	45	0	44	15	29	65.91
Smoker	42479	chr4	37358063	C	A	*KIAA1239*	MISSENSE	ENSG00000174145:MISSENSE[ENST00000309447(c.316C>A,Ctg>Atg):ENSP00000309501(p.Leu106Met)];	NA	NA	NA	NA	NA	NA	NA	D	PrD	D	MODERATE	N	0.996	70	70	0	49	10	39	79.59
Smoker	42479	chr4	187172398	G	A	*KLKB1*	MISSENSE	ENSG00000164344:EXONIC-NC[ENST00000511406(n.845G>A)];ENSG00000164344:EXONIC-NC[ENST00000467271(n.213G>A)];ENSG00000164344:MISSENSE[ENST00000511608(c.927G>A,Gaa>Aaa):ENSP00000426629(p.Glu310Lys)];ENSG00000164344:MISSENSE[ENST00000264690(c.784G>A,Gaa>Aaa):ENSP00000264690(p.Glu262Lys)];ENSG00000164344:MISSENSE[ENST00000513864(c.784G>A,Gaa>Aaa):ENSP00000424469(p.Glu262Lys)];	NA	NA	NA	NA	NA	NA	NA	T	BN	N	MODERATE	N	0.961	65	65	0	47	8	39	82.98
Smoker	42479	chr4	151186808	G	C	*LRBA*	EXONIC-NC	ENSG00000198589:[ENST00000503716(n.2715C>G)];ENSG00000198589:[ENST00000510157(n.1006C>G)];ENSG00000198589:[ENST00000515096(n.1917C>G)];ENSG00000198589:[ENST00000535741(c.*66C>G)];ENSG00000198589:[ENST00000357115(c.*66C>G)];ENSG00000198589:[ENST00000510413(c.*66C>G)];ENSG00000198589:[ENST00000509835(c.*66C>G)];ENSG00000198589:[ENST00000508606(c.*100C>G)];	NA	NA	NA	NA	NA	NA	NA	NA	NA	NA	MODIFIER	NA	NA	80	79	1	51	22	29	56.86

Mangalaparthi *et al.* , 2020. Mutational landscape of esophageal squamous cell carcinoma in an Indian cohort
Supplementary Table 4. List of somatic single nucleotide variants identified using whole exome sequencing of ESCC patients

Cohort	Sample	Chromosome	Genomic position	Reference allele	Alternate allele	Gene symbol	Variant type	Variant annotation	OncoMD	COSMIC	dbSNP	ExAC	ExAC_SAS	1000G	1000G_SAS	SIFT	Polyphen2	CONDEL	VEP	LRT	PhastCon7 Way	Total read (Adjacent normal)	Reference allel read support (Adjacent normal)	Alternative read support (Adjacent normal)	Total read (Tumor)	Reference allel read support (Tumor)	Alternative read support (Tumor)	Alternate allele frequency
Smoker	42479	chr4	83785777	C	G	*SEC31A*	INTRONIC	ENSG00000138674:[ENST00000448323(c.1198-26G>C)];ENSG00000138674:[ENST00000505984(c.1198-26G>C)];ENSG00000138674:[ENST00000264405(c.514-26G>C)];ENSG00000138674:[ENST00000311785(c.1198-26G>C)];ENSG00000138674:[ENST00000500777(c.1198-26G>C)];ENSG00000138674:[ENST00000395310(c.1198-26G>C)];ENSG00000138674:[ENST00000509142(c.1198-26G>C)];ENSG00000138674:[ENST00000508502(c.1198-26G>C)];ENSG00000138674:[ENST00000505472(c.1198-26G>C)];ENSG00000138674:[ENST00000432794(c.1198-26G>C)];ENSG00000138674:[ENST00000507828(c.125-26G>C)];ENSG00000138674:[ENST00000513858(c.1198-26G>C)];ENSG00000138674:[ENST00000355196(c.1198-26G>C)];ENSG00000138674:[ENST00000443462(c.1183-26G>C)];ENSG00000138674:[ENST00000508479(c.1198-26G>C)];ENSG00000138674:[ENST00000436790(n.1299-26G>C)];ENSG00000138674:[ENST00000326950(c.1198-26G>C)];ENSG00000138674:[ENST00000348405(c.1198-26G>C)];	NA	NA	NA	NA	NA	NA	NA	NA	NA	NA	MODIFIER	NA	NA	62	62	0	48	32	16	33.33
Smoker	42479	chr4	20619091	A	G	*SLIT2*	MISSENSE	ENSG00000145147:MISSENSE[ENST00000273739(c.4205A>G,aAt>aGt):ENSP00000273739(p.Asn1402Ser)];ENSG00000145147:MISSENSE[ENST00000504154(c.4166A>G,aAt>aGt):ENSP00000422591(p.Asn1389Ser)];ENSG00000145147:MISSENSE[ENST00000503823(c.4142A>G,aAt>aGt):ENSP00000427548(p.Asn1381Ser)];ENSG00000145147:MISSENSE[ENST00000503837(c.4154A>G,aAt>aGt):ENSP00000422261(p.Asn1385Ser)];ENSG00000145147:INTRONIC[ENST00000512993(c.236+521A>G)];ENSG00000145147:EXONIC-NC[ENST00000508541(n.2366A>G)];	NA	NA	0.0001997	1.67E-05	0	0.0001997	0	NA	NA	NA	MODIFIER	NA	NA	76	76	0	84	62	22	26.19
Smoker	42479	chr4	107181681	G	C	*TBCK*	INTRONIC	ENSG00000145348:[ENST00000361687(c.267-8517C>G)];ENSG00000145348:[ENST00000273980(c.382-14C>G)];ENSG00000145348:[ENST00000394708(c.382-14C>G)];ENSG00000145348:[ENST00000432496(c.382-14C>G)];ENSG00000145348:[ENST00000509532(c.382-14C>G)];ENSG00000145348:[ENST00000467183(c.382-14C>G)];ENSG00000145348:[ENST00000394706(c.382-14C>G)];ENSG00000145348:[ENST00000506280(n.217-14C>G)];	NA	NA	NA	NA	NA	NA	NA	NA	NA	NA	MODIFIER	NA	NA	25	25	0	12	5	7	58.33
Smoker	42479	chr5	35048004	C	A	*AGXT2*	5UTR	ENSG00000113492:[ENST00000231420(c.-7G>T)];ENSG00000113492:[ENST00000510428(c.-7G>T)];	NA	NA	chr5*35048004*35048004*C*T*rs776561710*NA*NA*NA	NA	NA	NA	NA	NA	NA	NA	MODIFIER	NA	NA	149	149	0	64	11	53	82.81
Smoker	42479	chr5	156533950	C	G	*CTB-120L21.1*	INTRONIC	ENSG00000254246:[ENST00000517708(n.147+2005C>G)];	NA	NA	NA	NA	NA	NA	NA	NA	NA	NA	MODIFIER	NA	NA	73	73	0	41	10	31	75.61
Smoker	42479	chr5	121786747	C	T	*CTC-210G5.1*	INTRONIC	ENSG00000250328:[ENST00000510972(n.464-386G>A)];ENSG00000250328:[ENST00000505546(n.304-6139G>A)];ENSG00000250328:[ENST00000503529(n.496-13715G>A)];ENSG00000250328:[ENST00000506053(n.77-13715G>A)];ENSG00000250328:[ENST00000509993(n.85-386G>A)];	NA	NA	NA	NA	NA	NA	NA	NA	NA	NA	LOW	NA	0.984	95	95	0	78	47	31	39.74
Smoker	42479	chr5	169302227	C	A	*DOCK2*	INTRONIC	ENSG00000134516:[ENST00000523351(n.564+34371C>A)];ENSG00000134516:[ENST00000519628(c.411+34371C>A)];ENSG00000134516:[ENST00000540750(c.-19+34371C>A)];ENSG00000134516:[ENST00000256935(c.2799+34371C>A)];ENSG00000134516:[ENST00000524185(c.2799+34371C>A)];ENSG00000134516:[ENST00000520908(c.1275+34371C>A)];ENSG00000134516:[ENST00000520181(n.503-3C>A)];	NA	NA	NA	NA	NA	NA	NA	NA	NA	NA	MODIFIER	NA	NA	163	163	0	110	32	78	70.91
Smoker	42479	chr5	31515114	C	A	*DROSHA*	MISSENSE	ENSG00000113360:MISSENSE[ENST00000512302(c.572G>T,aGt>aTt):ENSP00000428782(p.Ser191Ile)];ENSG00000113360:MISSENSE[ENST00000512076(c.555G>T,aGt>aTt):ENSP00000422745(p.Ser186Ile)];ENSG00000113360:MISSENSE[ENST00000442743(c.1160G>T,aGt>aTt):ENSP00000409335(p.Ser387Ile)];ENSG00000113360:MISSENSE[ENST00000511367(c.1271G>T,aGt>aTt):ENSP00000425979(p.Ser424Ile)];ENSG00000113360:MISSENSE[ENST00000513349(c.1160G>T,aGt>aTt):ENSP00000424161(p.Ser387Ile)];ENSG00000113360:MISSENSE[ENST00000344624(c.1271G>T,aGt>aTt):ENSP00000339845(p.Ser424Ile)];	NA	NA	NA	NA	NA	NA	NA	T	BN	N	MODERATE	D	0.997	58	58	0	54	13	41	75.93
Smoker	42479	chr5	169302227	C	A	*FAM196B*	INTRONIC	ENSG00000204767:[ENST00000377365(c.1421+6145G>T)];	NA	NA	NA	NA	NA	NA	NA	NA	NA	NA	MODIFIER	NA	NA	163	163	0	110	32	78	70.91
Smoker	42479	chr5	179745874	G	A	*GFPT2*	MISSENSE	ENSG00000131459:MISSENSE[ENST00000518906(c.583C>T,Cac>Tac):ENSP00000431125(p.His195Tyr)];ENSG00000131459:MISSENSE[ENST00000253778(c.877C>T,Cac>Tac):ENSP00000253778(p.His293Tyr)];ENSG00000131459:EXONIC-NC[ENST00000518185(n.578C>T)];ENSG00000131459:EXONIC-NC[ENST00000520165(n.382C>T)];ENSG00000131459:EXONIC-NC[ENST00000510122(n.260C>T)];	NA	NA	NA	NA	NA	NA	NA	NA	NA	NA	MODIFIER	NA	NA	84	83	0	95	58	37	38.95
Smoker	42479	chr5	156533950	C	G	*HAVCR2*	MISSENSE	ENSG00000135077:MISSENSE[ENST00000307851(c.82G>C,Gcg>Ccg):ENSP00000312002(p.Ala28Pro)];ENSG00000135077:MISSENSE[ENST00000522593(c.82G>C,Gcg>Ccg):ENSP00000430873(p.Ala28Pro)];ENSG00000135077:EXONIC-NC[ENST00000522902(n.191G>C)];ENSG00000135077:EXONIC-NC[ENST00000521665(n.345G>C)];ENSG00000135077:EXONIC-NC[ENST00000517358(n.281G>C)];ENSG00000135077:5UTR[ENST00000524219(c.-270G>C)];	NA	NA	NA	NA	NA	NA	NA	NA	NA	NA	MODIFIER	NA	NA	73	73	0	41	10	31	75.61
Smoker	42479	chr5	61847135	A	G	*IPO11*	MISSENSE	ENSG00000086200:MISSENSE[ENST00000511713(c.226A>G,aAt>aGt):ENSP00000421517(p.Asn76Ser)];ENSG00000086200:MISSENSE[ENST00000409296(c.2552A>G,aAt>aGt):ENSP00000386992(p.Asn851Ser)];ENSG00000086200:MISSENSE[ENST00000325324(c.2432A>G,aAt>aGt):ENSP00000316651(p.Asn811Ser)];ENSG00000086200:MISSENSE[ENST00000424533(c.2432A>G,aAt>aGt):ENSP00000395685(p.Asn811Ser)];	NA	NA	NA	NA	NA	NA	NA	T	BN	N	MODERATE	NA	NA	31	31	0	12	0	12	100
Smoker	42479	chr5	93788748	G	C	*KIAA0825*	MISSENSE	ENSG00000185261:MISSENSE[ENST00000427991(c.2273C>G,tCt>tGt):ENSP00000400288(p.Ser758Cys)];ENSG00000185261:MISSENSE[ENST00000312498(c.2273C>G,tCt>tGt):ENSP00000312205(p.Ser758Cys)];ENSG00000185261:MISSENSE[ENST00000513200(c.2273C>G,tCt>tGt):ENSP00000424618(p.Ser758Cys)];ENSG00000185261:EXONIC-NC[ENST00000504117(n.1120C>G)];	NA	NA	NA	NA	NA	NA	NA	NA	NA	NA	MODIFIER	NA	NA	73	73	0	44	13	31	70.45

Supplementary Table 4. List of somatic single nucleotide variants identified using whole exome sequencing of ESCC patients

Cohort	Sample	Chromosome	Genomic position	Reference allele	Alternate allele	Gene symbol	Variant type	Variant annotation	OncoMD	COSMIC	dbSNP	ExAC	ExAC_SAS	1000G	1000G_SAS	SIFT	Polyphen2	CONDEL	VEP	LRT	PhastCon7 Way	Total read (Adjacent normal)	Reference allel read support (Adjacent normal)	Alternative read support (Adjacent normal)	Total read (Tumor)	Reference allel read support (Tumor)	Alternative read support (Tumor)	Alternate allele frequency
Smoker	42479	chr5	6750562	C	G	*PAPD7*	SILENT	ENSG00000112941:SILENT[ENST00000230859(c.1056C>G,ctC>ctG):ENSP00000230859(p.Leu352(=))];ENSG00000112941:EXONIC-NC[ENST00000514410(n.70C>G)];ENSG00000112941:EXONIC-NC[ENST00000514697(n.787C>G)];ENSG00000112941:EXONIC-NC[ENST00000505457(n.222C>G)];	NA	NA	NA	NA	NA	NA	NA	NA	NA	NA	MODIFIER	NA	NA	72	72	0	76	64	12	15.79
Smoker	42479	chr5	140347512	C	A	*PCDHA1*	INTRONIC	ENSG00000204970:[ENST00000504120(c.2395-11022C>A)];ENSG00000204970:[ENST00000394633(c.1603-11022C>A)];	NA	NA	chr5*140347512*140347512*C*T*rs782426020*NA*NA*NA	NA	NA	NA	NA	NA	NA	NA	LOW	NA	NA	176	176	0	139	84	55	39.57
Smoker	42479	chr5	140347512	C	A	*PCDHA10*	INTRONIC	ENSG00000250120:[ENST00000506939(c.1600-11022C>A)];ENSG00000250120:[ENST00000307360(c.2389-11022C>A)];	NA	NA	chr5*140347512*140347512*C*T*rs782426020*NA*NA*NA	NA	NA	NA	NA	NA	NA	NA	LOW	NA	NA	176	176	0	139	84	55	39.57
Smoker	42479	chr5	140347512	C	A	*PCDHA11*	INTRONIC	ENSG00000249158:[ENST00000398640(c.2392-11022C>A)];	NA	NA	chr5*140347512*140347512*C*T*rs782426020*NA*NA*NA	NA	NA	NA	NA	NA	NA	NA	LOW	NA	NA	176	176	0	139	84	55	39.57
Smoker	42479	chr5	140347512	C	A	*PCDHA12*	INTRONIC	ENSG00000251664:[ENST00000398631(c.2368-11022C>A)];	NA	NA	chr5*140347512*140347512*C*T*rs782426020*NA*NA*NA	NA	NA	NA	NA	NA	NA	NA	LOW	NA	NA	176	176	0	139	84	55	39.57
Smoker	42479	chr5	140347512	C	A	*PCDHA13*	INTRONIC	ENSG00000239389:[ENST00000289272(c.2395-11022C>A)];ENSG00000239389:[ENST00000409494(c.2395-11022C>A)];	NA	NA	chr5*140347512*140347512*C*T*rs782426020*NA*NA*NA	NA	NA	NA	NA	NA	NA	NA	LOW	NA	NA	176	176	0	139	84	55	39.57
Smoker	42479	chr5	140347512	C	A	*PCDHA2*	INTRONIC	ENSG00000204969:[ENST00000526136(c.2389-11022C>A)];	NA	NA	chr5*140347512*140347512*C*T*rs782426020*NA*NA*NA	NA	NA	NA	NA	NA	NA	NA	LOW	NA	NA	176	176	0	139	84	55	39.57
Smoker	42479	chr5	140347512	C	A	*PCDHA3*	INTRONIC	ENSG00000255408:[ENST00000522353(c.2395-11022C>A)];	NA	NA	chr5*140347512*140347512*C*T*rs782426020*NA*NA*NA	NA	NA	NA	NA	NA	NA	NA	LOW	NA	NA	176	176	0	139	84	55	39.57
Smoker	42479	chr5	140347512	C	A	*PCDHA4*	INTRONIC	ENSG00000204967:[ENST00000512229(c.2386-11022C>A)];ENSG00000204967:[ENST00000530339(c.2386-11022C>A)];	NA	NA	chr5*140347512*140347512*C*T*rs782426020*NA*NA*NA	NA	NA	NA	NA	NA	NA	NA	LOW	NA	NA	176	176	0	139	84	55	39.57
Smoker	42479	chr5	140347512	C	A	*PCDHA5*	INTRONIC	ENSG00000204965:[ENST00000529619(c.2353-11022C>A)];ENSG00000204965:[ENST00000529859(c.2353-11022C>A)];	NA	NA	chr5*140347512*140347512*C*T*rs782426020*NA*NA*NA	NA	NA	NA	NA	NA	NA	NA	LOW	NA	NA	176	176	0	139	84	55	39.57
Smoker	42479	chr5	140347512	C	A	*PCDHA6*	INTRONIC	ENSG00000081842:[ENST00000529310(c.2395-11022C>A)];ENSG00000081842:[ENST00000527624(c.1603-11022C>A)];	NA	NA	chr5*140347512*140347512*C*T*rs782426020*NA*NA*NA	NA	NA	NA	NA	NA	NA	NA	LOW	NA	NA	176	176	0	139	84	55	39.57
Smoker	42479	chr5	140347512	C	A	*PCDHA7*	INTRONIC	ENSG00000204963:[ENST00000525929(c.2356-11022C>A)];	NA	NA	chr5*140347512*140347512*C*T*rs782426020*NA*NA*NA	NA	NA	NA	NA	NA	NA	NA	LOW	NA	NA	176	176	0	139	84	55	39.57
Smoker	42479	chr5	140347512	C	A	*PCDHA8*	INTRONIC	ENSG00000204962:[ENST00000531613(c.2395-11022C>A)];	NA	NA	chr5*140347512*140347512*C*T*rs782426020*NA*NA*NA	NA	NA	NA	NA	NA	NA	NA	LOW	NA	NA	176	176	0	139	84	55	39.57
Smoker	42479	chr5	140347512	C	A	*PCDHA9*	INTRONIC	ENSG00000204961:[ENST00000532602(c.2395-11022C>A)];	NA	NA	chr5*140347512*140347512*C*T*rs782426020*NA*NA*NA	NA	NA	NA	NA	NA	NA	NA	LOW	NA	NA	176	176	0	139	84	55	39.57
Smoker	42479	chr5	140347512	C	A	*PCDHAC1*	INTRONIC	ENSG00000248383:[ENST00000253807(c.2434-11022C>A)];	NA	NA	chr5*140347512*140347512*C*T*rs782426020*NA*NA*NA	NA	NA	NA	NA	NA	NA	NA	LOW	NA	NA	176	176	0	139	84	55	39.57
Smoker	42479	chr5	140347512	C	A	*PCDHAC2*	SILENT	ENSG00000243232:SILENT[ENST00000289269(c.1161C>A,ctC>ctA):ENSP00000289269(p.Leu387(=))];	NA	NA	chr5*140347512*140347512*C*T*rs782426020*NA*NA*NA	NA	NA	NA	NA	NA	NA	NA	LOW	NA	NA	176	176	0	139	84	55	39.57
Smoker	42479	chr5	140625528	G	A	*PCDHB15*	MISSENSE	ENSG00000113248:MISSENSE[ENST00000231173(c.382G>A,Gat>Aat):ENSP00000231173(p.Asp128Asn)];	NA	NA	NA	NA	NA	NA	NA	D_lc	PoD	D	MODERATE	NA	NA	235	235	0	190	37	153	80.53
Smoker	42479	chr5	140807294	G	C	*PCDHGA1*	INTRONIC	ENSG00000204956:[ENST00000517417(c.2422-67080G>C)];	NA	NA	NA	NA	NA	NA	NA	NA	NA	NA	MODIFIER	NA	NA	191	191	0	125	26	99	79.2
Smoker	42479	chr5	140807294	G	C	*PCDHGA10*	INTRONIC	ENSG00000253846:[ENST00000398610(c.2436+12116G>C)];	NA	NA	NA	NA	NA	NA	NA	NA	NA	NA	MODIFIER	NA	NA	191	191	0	125	26	99	79.2
Smoker	42479	chr5	140807294	G	C	*PCDHGA11*	INTRONIC	ENSG00000253873:[ENST00000398587(c.2433+4067G>C)];ENSG00000253873:[ENST00000518882(c.1878+4622G>C)];	NA	NA	NA	NA	NA	NA	NA	NA	NA	NA	MODIFIER	NA	NA	191	191	0	125	26	99	79.2
Smoker	42479	chr5	140807294	G	C	*PCDHGA2*	INTRONIC	ENSG00000081853:[ENST00000394576(c.2425-67080G>C)];	NA	NA	NA	NA	NA	NA	NA	NA	NA	NA	MODIFIER	NA	NA	191	191	0	125	26	99	79.2
Smoker	42479	chr5	140807294	G	C	*PCDHGA3*	INTRONIC	ENSG00000254245:[ENST00000253812(c.2425-67080G>C)];	NA	NA	NA	NA	NA	NA	NA	NA	NA	NA	MODIFIER	NA	NA	191	191	0	125	26	99	79.2
Smoker	42479	chr5	140807294	G	C	*PCDHGA4*	INTRONIC	ENSG00000262576:[ENST00000571252(c.2422-67080G>C)];	NA	NA	NA	NA	NA	NA	NA	NA	NA	NA	MODIFIER	NA	NA	191	191	0	125	26	99	79.2
Smoker	42479	chr5	140807294	G	C	*PCDHGA5*	INTRONIC	ENSG00000253485:[ENST00000518069(c.2421+60976G>C)];	NA	NA	NA	NA	NA	NA	NA	NA	NA	NA	MODIFIER	NA	NA	191	191	0	125	26	99	79.2
Smoker	42479	chr5	140807294	G	C	*PCDHGA6*	INTRONIC	ENSG00000253731:[ENST00000517434(c.2424+51220G>C)];	NA	NA	NA	NA	NA	NA	NA	NA	NA	NA	MODIFIER	NA	NA	191	191	0	125	26	99	79.2
Smoker	42479	chr5	140807294	G	C	*PCDHGA7*	INTRONIC	ENSG00000253537:[ENST00000518325(c.2424+42404G>C)];	NA	NA	NA	NA	NA	NA	NA	NA	NA	NA	MODIFIER	NA	NA	191	191	0	125	26	99	79.2
Smoker	42479	chr5	140807294	G	C	*PCDHGA8*	INTRONIC	ENSG00000253767:[ENST00000398604(c.2424+32490G>C)];	NA	NA	NA	NA	NA	NA	NA	NA	NA	NA	MODIFIER	NA	NA	191	191	0	125	26	99	79.2
Smoker	42479	chr5	140807294	G	C	*PCDHGA9*	INTRONIC	ENSG00000261934:[ENST00000573521(c.2424+22351G>C)];	NA	NA	NA	NA	NA	NA	NA	NA	NA	NA	MODIFIER	NA	NA	191	191	0	125	26	99	79.2
Smoker	42479	chr5	140807294	G	C	*PCDHGB1*	INTRONIC	ENSG00000254221:[ENST00000523390(c.2410-67080G>C)];	NA	NA	NA	NA	NA	NA	NA	NA	NA	NA	MODIFIER	NA	NA	191	191	0	125	26	99	79.2
Smoker	42479	chr5	140807294	G	C	*PCDHGB2*	INTRONIC	ENSG00000253910:[ENST00000522605(c.2421+65171G>C)];	NA	NA	NA	NA	NA	NA	NA	NA	NA	NA	MODIFIER	NA	NA	191	191	0	125	26	99	79.2
Smoker	42479	chr5	140807294	G	C	*PCDHGB3*	INTRONIC	ENSG00000262209:[ENST00000576222(c.2415+54918G>C)];	NA	NA	NA	NA	NA	NA	NA	NA	NA	NA	MODIFIER	NA	NA	191	191	0	125	26	99	79.2
Smoker	42479	chr5	140807294	G	C	*PCDHGB4*	INTRONIC	ENSG00000253953:[ENST00000519479(c.2397+37446G>C)];	NA	NA	NA	NA	NA	NA	NA	NA	NA	NA	MODIFIER	NA	NA	191	191	0	125	26	99	79.2
Smoker	42479	chr5	140807294	G	C	*PCDHGB6*	INTRONIC	ENSG00000253305:[ENST00000520790(c.2418+17107G>C)];	NA	NA	NA	NA	NA	NA	NA	NA	NA	NA	MODIFIER	NA	NA	191	191	0	125	26	99	79.2
Smoker	42479	chr5	140807294	G	C	*PCDHGB7*	INTRONIC	ENSG00000254122:[ENST00000398594(c.2415+7453G>C)];	NA	NA	NA	NA	NA	NA	NA	NA	NA	NA	MODIFIER	NA	NA	191	191	0	125	26	99	79.2
Smoker	42479	chr5	140807294	G	C	*PCDHGB8P*	EXONIC-NC	ENSG00000248449:[ENST00000507007(n.1442G>C)];ENSG00000248449:[ENST00000502926(n.1442G>C)];	NA	NA	NA	NA	NA	NA	NA	NA	NA	NA	MODIFIER	NA	NA	191	191	0	125	26	99	79.2
Smoker	42479	chr5	149210523	G	A	*PPARGC1B*	INTRONIC	ENSG00000155846:[ENST00000360453(c.466-1696G>A)];ENSG00000155846:[ENST00000403750(c.391-1696G>A)];ENSG00000155846:[ENST00000309241(c.582+77G>A)];ENSG00000155846:[ENST00000394320(c.582+77G>A)];	NA	NA	NA	NA	NA	NA	NA	NA	NA	NA	MODIFIER	NA	NA	72	72	0	53	32	21	39.62

Supplementary Table 4. List of somatic single nucleotide variants identified using whole exome sequencing of ESCC patients

Cohort	Sample	Chromosome	Genomic position	Reference allele	Alternate allele	Gene symbol	Variant type	Variant annotation	OncoMD	COSMIC	dbSNP	ExAC	ExAC_SAS	1000G	1000G_SAS	SIFT	Polyphen2	CONDEL	VEP	LRT	PhastCon7 Way	Total read (Adjacent normal)	Reference allel read support (Adjacent normal)	Alternative read support (Adjacent normal)	Total read (Tumor)	Reference allel read support (Tumor)	Alternative read support (Tumor)	Alternate allele frequency
Smoker	42479	chr5	170345816	C	T	*RANBP17*	MISSENSE	ENSG00000204764:MISSENSE[ENST00000523189(c.1054C>T,Cct>Tct):ENSP00000427975(p.Pro352Ser)];ENSG00000204764:MISSENSE[ENST00000519949(c.1054C>T,Cct>Tct):ENSP00000430444(p.Pro352Ser)];ENSG00000204764:MISSENSE[ENST00000389118(c.1054C>T,Cct>Tct):ENSP00000373770(p.Pro352Ser)];ENSG00000204764:MISSENSE[ENST00000519256(c.1054C>T,Cct>Tct):ENSP00000429298(p.Pro352Ser)];ENSG00000204764:MISSENSE[ENST00000522533(c.1054C>T,Cct>Tct):ENSP00000430599(p.Pro352Ser)];ENSG00000204764:MISSENSE[ENST00000522066(c.1054C>T,Cct>Tct):ENSP00000430958(p.Pro352Ser)];ENSG00000204764:MISSENSE[ENST00000520864(c.987C>T,Cct>Tct):ENSP00000429845(p.Pro330Ser)];ENSG00000204764:INTRONIC[ENST00000522734(c.302-629C>T)];ENSG00000204764:EXONIC-NC[ENST00000520879(n.251C>T)];	NA	NA	NA	NA	NA	NA	NA	T	BN	N	MODERATE	NA	NA	63	62	0	40	12	28	70
Smoker	42479	chr5	177309546	C	G	*RP11-423H2.1*	INTRONIC	ENSG00000170089:[ENST00000512851(n.790+15C>G)];ENSG00000170089:[ENST00000511074(n.221+15C>G)];ENSG00000170089:[ENST00000502601(n.480+15C>G)];ENSG00000170089:[ENST00000515065(n.277+15C>G)];ENSG00000170089:[ENST00000507037(n.598+15C>G)];ENSG00000170089:[ENST00000504756(n.140+15C>G)];ENSG00000170089:[ENST00000303154(n.833+15C>G)];ENSG00000170089:[ENST00000358442(n.88+15C>G)];ENSG00000170089:[ENST00000506672(n.863+15C>G)];	NA	NA	NA	NA	NA	NA	NA	NA	NA	NA	MODIFIER	NA	NA	297	297	0	296	230	66	22.3
Smoker	42479	chr5	101595880	G	C	*SLCO4C1*	INTRONIC	ENSG00000173930:[ENST00000310954(c.1128+37C>G)];	NA	NA	NA	NA	NA	NA	NA	NA	NA	NA	MODIFIER	NA	NA	55	54	0	36	23	13	36.11
Smoker	42479	chr5	121786747	C	T	*SNCAIP*	SILENT	ENSG00000064692:3UTR[ENST00000503116(c.*804C>T)];ENSG00000064692:3UTR[ENST00000508017(c.*952C>T)];ENSG00000064692:3UTR[ENST00000395466(c.*1066C>T)];ENSG00000064692:3UTR[ENST00000512146(c.*952C>T)];ENSG00000064692:3UTR[ENST00000512385(c.*952C>T)];ENSG00000064692:3UTR[ENST00000504884(c.*1066C>T)];ENSG00000064692:3UTR[ENST00000395469(c.*804C>T)];ENSG00000064692:3UTR[ENST00000510658(c.*1007C>T)];ENSG00000064692:3UTR[ENST00000509023(c.*850C>T)];ENSG00000064692:3UTR[ENST00000515215(c.*952C>T)];ENSG00000064692:SILENT[ENST00000379536(c.2025C>T,ttC>ttT):ENSP00000368851(p.Phe675(=))];ENSG00000064692:SILENT[ENST00000509154(c.2025C>T,ttC>ttT):ENSP00000422106(p.Phe675(=))];ENSG00000064692:SILENT[ENST00000261368(c.2205C>T,ttC>ttT):ENSP00000261368(p.Phe735(=))];ENSG00000064692:SILENT[ENST00000379538(c.1107C>T,ttC>ttT):ENSP00000368854(p.Phe369(=))];ENSG00000064692:SILENT[ENST00000542191(c.879C>T,ttC>ttT):ENSP00000441681(p.Phe293(=))];ENSG00000064692:SILENT[ENST00000379533(c.2346C>T,ttC>ttT):ENSP00000368848(p.Phe782(=))];ENSG00000064692:SILENT[ENST00000414317(c.1011C>T,ttC>ttT):ENSP00000394392(p.Phe337(=))];ENSG00000064692:SILENT[ENST00000261367(c.2346C>T,ttC>ttT):ENSP00000261367(p.Phe782(=))];ENSG00000064692:EXONIC-NC[ENST00000513719(n.1190C>T)];	NA	NA	NA	NA	NA	NA	NA	NA	NA	NA	LOW	NA	0.984	95	95	0	78	47	31	39.74
Smoker	42479	chr5	133481429	C	T	*TCF7*	INTRONIC	ENSG00000081059:[ENST00000517799(c.*46+398C>T)];ENSG00000081059:[ENST00000517855(c.441-486C>T)];ENSG00000081059:[ENST00000432532(c.804-486C>T)];ENSG00000081059:[ENST00000524342(n.1354-486C>T)];ENSG00000081059:[ENST00000321603(c.1076-463C>T)];ENSG00000081059:[ENST00000520699(c.249+2098C>T)];ENSG00000081059:[ENST00000342854(c.1076-486C>T)];ENSG00000081059:[ENST00000395023(c.731-486C>T)];ENSG00000081059:[ENST00000519238(n.1162-486C>T)];ENSG00000081059:[ENST00000520958(c.731-486C>T)];ENSG00000081059:[ENST00000378560(c.804-463C>T)];ENSG00000081059:[ENST00000395029(c.1149-486C>T)];ENSG00000081059:[ENST00000522653(n.3095C>T)];ENSG00000081059:[ENST00000321584(c.1076-3C>T)];ENSG00000081059:[ENST00000518915(c.731-3C>T)];	NA	NA	NA	NA	NA	NA	NA	NA	NA	NA	MODIFIER	NA	NA	89	89	0	53	13	40	75.47
Smoker	42479	chr5	1272321	C	A	*TERT*	MISSENSE	ENSG00000164362:MISSENSE[ENST00000310581(c.2361G>T,agG>agT):ENSP00000309572(p.Arg787Ser)];ENSG00000164362:MISSENSE[ENST00000334602(c.2361G>T,agG>agT):ENSP00000334346(p.Arg787Ser)];ENSG00000164362:INTRONIC[ENST00000296820(c.2287-3573G>T)];ENSG00000164362:INTRONIC[ENST00000484238(n.1100-3573G>T)];ENSG00000164362:INTRONIC[ENST00000508104(c.2287-3573G>T)];ENSG00000164362:INTRONIC[ENST00000460137(c.2251-3573G>T)];	NA	NA	NA	NA	NA	NA	NA	NA	NA	NA	MODIFIER	NA	NA	58	58	0	61	22	39	63.93
Smoker	42479	chr5	677840	C	T	*TPPP*	INTRONIC	ENSG00000171368:[ENST00000360578(c.311+25G>A)];	NA	NA	NA	4.71E-05	0.000224517	NA	NA	NA	NA	NA	MODIFIER	NA	NA	87	87	0	99	70	29	29.29
Smoker	42479	chr5	14488195	G	C	*TRIO*	MISSENSE	ENSG00000038382:MISSENSE[ENST00000344204(c.7458G>C,tgG>tgC):ENSP00000339299(p.Trp2486Cys)];ENSG00000038382:MISSENSE[ENST00000513206(c.6657G>C,tgG>tgC):ENSP00000426342(p.Trp2219Cys)];ENSG00000038382:INTRONIC[ENST00000537187(c.7102+356G>C)];ENSG00000038382:INTRONIC[ENST00000512070(c.6925+356G>C)];ENSG00000038382:EXONIC-NC[ENST00000515144(n.6376G>C)];	NA	NA	chr5*14488193*14488205*TGGAGCTCCATCC*T*rs140308852*1*0.9864*0.01358	NA	NA	NA	NA	NA	UN	NA	MODERATE	NA	NA	39	39	0	36	30	6	16.67
Smoker	42479	chr6	70909381	G	C	*COL19A1*	MISSENSE	ENSG00000082293:MISSENSE[ENST00000322773(c.3164G>C,aGa>aCa):ENSP00000316030(p.Arg1055Thr)];ENSG00000082293:MISSENSE[ENST00000393344(c.2030G>C,aGa>aCa):ENSP00000377013(p.Arg677Thr)];	NA	NA	NA	NA	NA	NA	NA	T	PoD	N	MODERATE	N	0.974	38	38	0	48	28	20	41.67
Smoker	42479	chr6	56374498	C	G	*DST*	MISSENSE	ENSG00000151914:EXONIC-NC[ENST00000340834(n.2727G>C)];ENSG00000151914:MISSENSE[ENST00000446842(c.17349G>C,caG>caC):ENSP00000393645(p.Gln5783His)];ENSG00000151914:MISSENSE[ENST00000370788(c.11736G>C,caG>caC):ENSP00000359824(p.Gln3912His)];ENSG00000151914:MISSENSE[ENST00000244364(c.11085G>C,caG>caC):ENSP00000244364(p.Gln3695His)];ENSG00000151914:MISSENSE[ENST00000370754(c.18861G>C,caG>caC):ENSP00000359790(p.Gln6287His)];ENSG00000151914:MISSENSE[ENST00000361203(c.17994G>C,caG>caC):ENSP00000354508(p.Gln5998His)];ENSG00000151914:MISSENSE[ENST00000370769(c.18327G>C,caG>caC):ENSP00000359805(p.Gln6109His)];ENSG00000151914:MISSENSE[ENST00000421834(c.12063G>C,caG>caC):ENSP00000400883(p.Gln4021His)];ENSG00000151914:3UTR[ENST00000312431(c.*5665G>C)];	NA	NA	NA	NA	NA	NA	NA	NA	BN	NA	MODERATE	N	0.986	285	284	0	345	183	162	46.96

Mangalaparthi *et al.* , 2020. Mutational landscape of esophageal squamous cell carcinoma in an Indian cohort
Supplementary Table 4. List of somatic single nucleotide variants identified using whole exome sequencing of ESCC patients

Cohort	Sample	Chromosome	Genomic position	Reference allele	Alternate allele	Gene symbol	Variant type	Variant annotation	OncoMD	COSMIC	dbSNP	ExAC	ExAC_SAS	1000G	1000G_SAS	SIFT	Polyphen2	CONDEL	VEP	LRT	PhastCon7 Way	Total read (Adjacent normal)	Reference allel read support (Adjacent normal)	Alternative read support (Adjacent normal)	Total read (Tumor)	Reference allel read support (Tumor)	Alternative read support (Tumor)	Alternate allele frequency
Smoker	42479	chr6	131222199	C	T	*EPB41L2*	MISSENSE	ENSG00000079819:MISSENSE[ENST00000527659(c.1051G>A,Gaa>Aaa):ENSP00000431647(p.Glu351Lys)];ENSG00000079819:MISSENSE[ENST00000529208(c.1051G>A,Gaa>Aaa):ENSP00000436641(p.Glu351Lys)];ENSG00000079819:MISSENSE[ENST00000368128(c.1051G>A,Gaa>Aaa):ENSP00000357110(p.Glu351Lys)];ENSG00000079819:MISSENSE[ENST00000527411(c.1051G>A,Gaa>Aaa):ENSP00000436348(p.Glu351Lys)];ENSG00000079819:MISSENSE[ENST00000337057(c.1051G>A,Gaa>Aaa):ENSP00000338481(p.Glu351Lys)];ENSG00000079819:MISSENSE[ENST00000530481(c.1051G>A,Gaa>Aaa):ENSP00000434576(p.Glu351Lys)];ENSG00000079819:MISSENSE[ENST00000525193(c.1051G>A,Gaa>Aaa):ENSP00000431988(p.Glu351Lys)];ENSG00000079819:MISSENSE[ENST00000392427(c.1051G>A,Gaa>Aaa):ENSP00000376222(p.Glu351Lys)];ENSG00000079819:MISSENSE[ENST00000445890(c.1051G>A,Gaa>Aaa):ENSP00000402041(p.Glu351Lys)];ENSG00000079819:MISSENSE[ENST00000525271(c.1051G>A,Gaa>Aaa):ENSP00000432803(p.Glu351Lys)];ENSG00000079819:MISSENSE[ENST00000528282(c.1051G>A,Gaa>Aaa):ENSP00000434308(p.Glu351Lys)];ENSG00000079819:EXONIC-NC[ENST00000530148(n.482G>A)];ENSG00000079819:EXONIC-NC[ENST00000526333(n.448G>A)];ENSG00000079819:EXONIC-NC[ENST00000528179(n.437G>A)];	NA	NA	NA	NA	NA	NA	NA	NA	NA	NA	MODIFIER	NA	NA	62	62	0	73	53	20	27.4
Smoker	42479	chr6	133783952	G	A	*EYA4*	INTRONIC	ENSG00000112319:[ENST00000430974(c.562+50G>A)];ENSG00000112319:[ENST00000421413(n.797+50G>A)];ENSG00000112319:[ENST00000431403(c.724+50G>A)];ENSG00000112319:[ENST00000355286(c.655+50G>A)];ENSG00000112319:[ENST00000355167(c.724+50G>A)];ENSG00000112319:[ENST00000531901(c.724+50G>A)];ENSG00000112319:[ENST00000367895(c.724+50G>A)];ENSG00000112319:[ENST00000531861(n.724+50G>A)];ENSG00000112319:[ENST00000532518(n.52+50G>A)];ENSG00000112319:[ENST00000452339(c.562+50G>A)];ENSG00000112319:[ENST00000525849(c.655+50G>A)];	NA	NA	NA	NA	NA	NA	NA	NA	NA	NA	MODIFIER	NA	NA	29	29	0	30	22	8	26.67
Smoker	42479	chr6	24861115	A	T	*FAM65B*	INTRONIC	ENSG00000111913:[ENST00000538035(c.715+86T>A)];ENSG00000111913:[ENST00000378023(c.628+86T>A)];ENSG00000111913:[ENST00000540914(c.628+86T>A)];ENSG00000111913:[ENST00000259698(c.628+86T>A)];ENSG00000111913:[ENST00000510784(c.730+86T>A)];	NA	NA	NA	NA	NA	NA	NA	NA	NA	NA	MODIFIER	NA	NA	82	82	0	67	16	51	76.12
Smoker	42479	chr6	26285585	G	C	*HIST1H4H*	MISSENSE	ENSG00000158406:MISSENSE[ENST00000289352(c.143C>G,tCt>tGt):ENSP00000289352(p.Ser48Cys)];ENSG00000158406:MISSENSE[ENST00000377727(c.143C>G,tCt>tGt):ENSP00000366956(p.Ser48Cys)];	NA	NA	chr6*26285585*26285586*GA*G*rs750395074*NA*NA*NA	NA	NA	NA	NA	D	PrD	D	MODERATE	U	0.978	178	178	0	163	92	71	43.56
Smoker	42479	chr6	111587946	C	T	*KIAA1919*	MISSENSE	ENSG00000173214:MISSENSE[ENST00000368847(c.1181C>T,tCa>tTa):ENSP00000357840(p.Ser394Leu)];	NA	NA	NA	NA	NA	NA	NA	D	BN	N	MODERATE	N	0.003	238	237	0	221	168	52	23.53
Smoker	42479	chr6	142396973	C	T	*NMBR*	MISSENSE	ENSG00000135577:MISSENSE[ENST00000258042(c.985G>A,Gaa>Aaa):ENSP00000258042(p.Glu329Lys)];ENSG00000135577:EXONIC-NC[ENST00000480652(n.87G>A)];	NA	NA	NA	NA	NA	NA	NA	NA	NA	NA	MODIFIER	NA	NA	160	159	0	164	107	57	34.76
Smoker	42479	chr6	86203567	G	C	*NT5E*	MISSENSE	ENSG00000135318:MISSENSE[ENST00000369651(c.1420G>C,Gat>Cat):ENSP00000358665(p.Asp474His)];ENSG00000135318:MISSENSE[ENST00000257770(c.1570G>C,Gat>Cat):ENSP00000257770(p.Asp524His)];	NA	NA	chr6*86203567*86203567*G*T*rs777310796*NA*NA*NA	NA	NA	NA	NA	D	PrD	D	MODERATE	D	1	51	51	0	37	28	9	24.32
Smoker	42479	chr6	17662225	T	C	*NUP153*	INTRONIC	ENSG00000124789:[ENST00000537253(c.1268+24A>G)];ENSG00000124789:[ENST00000262077(c.1268+24A>G)];	NA	NA	NA	NA	NA	NA	NA	NA	NA	NA	MODIFIER	NA	NA	64	64	0	41	12	29	70.73
Smoker	42479	chr6	160222162	C	T	*PNLDC1*	MISSENSE	ENSG00000146453:MISSENSE[ENST00000392167(c.152C>T,tCg>tTg):ENSP00000376007(p.Ser51Leu)];ENSG00000146453:MISSENSE[ENST00000610273(c.119C>T,tCg>tTg):ENSP00000476448(p.Ser40Leu)];ENSG00000146453:EXONIC-NC[ENST00000609334(n.162C>T)];ENSG00000146453:INTRONIC[ENST00000610048(c.*113+301C>T)];ENSG00000146453:3UTR[ENST00000275275(c.*131C>T)];ENSG00000146453:3UTR[ENST00000610041(c.*131C>T)];	NA	NA	NA	NA	NA	NA	NA	NA	NA	NA	MODIFIER	NA	NA	157	157	0	198	149	49	24.75
Smoker	42479	chr6	27279658	C	A	*POM121L2*	NONSENSE	ENSG00000158553:NONSENSE[ENST00000444565(c.292G>T,Gaa>Taa):ENSP00000392726(p.Glu98Ter)];ENSG00000158553:NONSENSE[ENST00000377451(c.292G>T,Gaa>Taa):ENSP00000366671(p.Glu98Ter)];	NA	NA	NA	NA	NA	NA	NA	NA	NA	NA	HIGH	NA	0.993	133	133	0	73	16	57	78.08
Smoker	42479	chr6	27279660	C	A	*POM121L2*	MISSENSE	ENSG00000158553:MISSENSE[ENST00000444565(c.290G>T,tGg>tTg):ENSP00000392726(p.Trp97Leu)];ENSG00000158553:MISSENSE[ENST00000377451(c.290G>T,tGg>tTg):ENSP00000366671(p.Trp97Leu)];	NA	NA	NA	NA	NA	NA	NA	D	PrD	D	MODERATE	N	0.994	131	130	1	74	16	58	78.38
Smoker	42479	chr6	128643022	T	G	*PTPRK*	SILENT	ENSG00000152894:3UTR[ENST00000392449(c.*541A>C)];ENSG00000152894:INTRONIC[ENST00000532331(c.495+162A>C)];ENSG00000152894:INTRONIC[ENST00000524534(n.652+162A>C)];ENSG00000152894:INTRONIC[ENST00000368215(c.495+162A>C)];ENSG00000152894:INTRONIC[ENST00000368227(c.495+162A>C)];ENSG00000152894:INTRONIC[ENST00000524481(n.736+162A>C)];ENSG00000152894:INTRONIC[ENST00000490332(c.143+162A>C)];ENSG00000152894:INTRONIC[ENST00000495748(n.521+162A>C)];ENSG00000152894:INTRONIC[ENST00000429595(n.135+162A>C)];ENSG00000152894:INTRONIC[ENST00000368207(c.495+162A>C)];ENSG00000152894:INTRONIC[ENST00000368226(c.495+162A>C)];ENSG00000152894:INTRONIC[ENST00000368210(c.495+162A>C)];ENSG00000152894:INTRONIC[ENST00000531050(c.52+75689A>C)];ENSG00000152894:INTRONIC[ENST00000368213(c.495+162A>C)];ENSG00000152894:SILENT[ENST00000525459(c.657A>C,gcA>gcC):ENSP00000434116(p.Ala219(=))];	NA	NA	NA	NA	NA	NA	NA	NA	NA	NA	MODIFIER	NA	NA	111	111	0	67	25	42	62.69
Smoker	42479	chr6	56989647	G	C	*RP11-203B9.4*	INTRONIC	ENSG00000226803:[ENST00000416069(n.444-9286C>G)];ENSG00000226803:[ENST00000587815(n.46-8302C>G)];ENSG00000226803:[ENST00000586432(n.115-8302C>G)];ENSG00000226803:[ENST00000586053(n.283-9286C>G)];ENSG00000226803:[ENST00000588811(n.214-7928C>G)];ENSG00000226803:[ENST00000591553(n.199-9286C>G)];ENSG00000226803:[ENST00000585792(n.115-9286C>G)];ENSG00000226803:[ENST00000592038(n.258-7928C>G)];ENSG00000226803:[ENST00000592500(n.46-9591C>G)];ENSG00000226803:[ENST00000586668(n.115-8722C>G)];	NA	NA	chr6*56989647*56989647*G*A*rs764838188*NA*NA*NA	NA	NA	NA	NA	NA	NA	NA	MODIFIER	NA	NA	144	144	0	129	70	59	45.74
Smoker	42479	chr6	10913116	C	A	*RP11-637O19.3*	3UTR	ENSG00000272162:[ENST00000480294(c.*990C>A)];	NA	NA	NA	NA	NA	NA	NA	NA	NA	NA	MODIFIER	NA	NA	41	41	0	34	4	30	88.24

Mangalaparthi *et al.* , 2020. Mutational landscape of esophageal squamous cell carcinoma in an Indian cohort
Supplementary Table 4. List of somatic single nucleotide variants identified using whole exome sequencing of ESCC patients

Cohort	Sample	Chromosome	Genomic position	Reference allele	Alternate allele	Gene symbol	Variant type	Variant annotation	OncoMD	COSMIC	dbSNP	ExAC	ExAC_SAS	1000G	1000G_SAS	SIFT	Polyphen2	CONDEL	VEP	LRT	PhastCon7 Way	Total read (Adjacent normal)	Reference allel read support (Adjacent normal)	Alternative read support (Adjacent normal)	Total read (Tumor)	Reference allel read support (Tumor)	Alternative read support (Tumor)	Alternate allele frequency
Smoker	42479	chr6	10913116	C	A	*SYCP2L*	NONSENSE	ENSG00000153157:NONSENSE[ENST00000543878(c.551C>A,tCa>tAa):ENSP00000440676(p.Ser184Ter)];ENSG00000153157:NONSENSE[ENST00000283141(c.1028C>A,tCa>tAa):ENSP00000283141(p.Ser343Ter)];ENSG00000153157:3UTR[ENST00000341041(c.*106C>A)];ENSG00000153157:3UTR[ENST00000487561(c.*181C>A)];	NA	NA	NA	NA	NA	NA	NA	NA	NA	NA	MODIFIER	NA	NA	41	41	0	34	4	30	88.24
Smoker	42479	chr6	99979533	G	A	*TSTD3*	EXONIC-NC	ENSG00000228439:[ENST00000452647(n.701G>A)];	NA	NA	NA	NA	NA	NA	NA	NA	NA	NA	MODIFIER	NA	NA	68	68	0	29	23	6	20.69
Smoker	42479	chr6	144832268	C	G	*UTRN*	INTRONIC-SS-DNR	ENSG00000152818:[ENST00000367545(c.4944+9C>G)];	NA	NA	NA	NA	NA	NA	NA	NA	NA	NA	MODIFIER	NA	NA	53	53	0	48	35	13	27.08
Smoker	42479	chr6	56989647	G	C	*ZNF451*	MISSENSE	ENSG00000112200:MISSENSE[ENST00000357489(c.302G>C,aGa>aCa):ENSP00000350083(p.Arg101Thr)];ENSG00000112200:MISSENSE[ENST00000370706(c.302G>C,aGa>aCa):ENSP00000359740(p.Arg101Thr)];ENSG00000112200:MISSENSE[ENST00000510483(c.218G>C,aGa>aCa):ENSP00000427558(p.Arg73Thr)];ENSG00000112200:MISSENSE[ENST00000491832(c.302G>C,aGa>aCa):ENSP00000421645(p.Arg101Thr)];ENSG00000112200:EXONIC-NC[ENST00000509071(n.409G>C)];ENSG00000112200:EXONIC-NC[ENST00000509251(n.435G>C)];ENSG00000112200:EXONIC-NC[ENST00000444273(n.443G>C)];ENSG00000112200:3UTR[ENST00000502749(c.*61G>C)];ENSG00000112200:3UTR[ENST00000370711(c.*174G>C)];ENSG00000112200:INTRONIC[ENST00000504603(c.106-17099G>C)];ENSG00000112200:5UTR[ENST00000515290(c.-116G>C)];	NA	NA	chr6*56989647*56989647*G*A*rs764838188*NA*NA*NA	NA	NA	NA	NA	NA	NA	NA	MODIFIER	NA	NA	144	144	0	129	70	59	45.74
Smoker	42479	chr7	134730564	G	C	*AGBL3*	SILENT	ENSG00000146856:SILENT[ENST00000275763(c.1743G>C,ctG>ctC):ENSP00000275763(p.Leu581(=))];ENSG00000146856:SILENT[ENST00000458078(c.1665G>C,ctG>ctC):ENSP00000395969(p.Leu555(=))];ENSG00000146856:SILENT[ENST00000436302(c.1743G>C,ctG>ctC):ENSP00000388275(p.Leu581(=))];ENSG00000146856:SILENT[ENST00000435976(c.1743G>C,ctG>ctC):ENSP00000401220(p.Leu581(=))];	NA	NA	NA	NA	NA	NA	NA	NA	NA	NA	LOW	NA	NA	89	89	0	63	30	33	52.38
Smoker	42479	chr7	91625046	C	T	*AKAP9*	NONSENSE	ENSG00000127914:NONSENSE[ENST00000356239(c.862C>T,Cag>Tag):ENSP00000348573(p.Gln288Ter)];ENSG00000127914:NONSENSE[ENST00000359028(c.898C>T,Cag>Tag):ENSP00000351922(p.Gln300Ter)];ENSG00000127914:NONSENSE[ENST00000394564(c.862C>T,Cag>Tag):ENSP00000378065(p.Gln288Ter)];ENSG00000127914:NONSENSE[ENST00000358100(c.898C>T,Cag>Tag):ENSP00000350813(p.Gln300Ter)];ENSG00000127914:EXONIC-NC[ENST00000493453(n.1082C>T)];	NA	NA	NA	NA	NA	NA	NA	NA	NA	NA	MODIFIER	NA	NA	93	93	0	95	39	56	58.95
Smoker	42479	chr7	102727363	G	T	*ARMC10*	SILENT	ENSG00000170632:INTRONIC[ENST00000434153(c.561+152G>T)];ENSG00000170632:INTRONIC[ENST00000454559(c.423+152G>T)];ENSG00000170632:INTRONIC[ENST00000431642(c.126+152G>T)];ENSG00000170632:INTRONIC[ENST00000306450(c.*503+152G>T)];ENSG00000170632:INTRONIC[ENST00000428183(c.528+152G>T)];ENSG00000170632:INTRONIC[ENST00000541300(c.423+152G>T)];ENSG00000170632:INTRONIC[ENST00000441711(c.423+152G>T)];ENSG00000170632:INTRONIC[ENST00000323716(c.528+152G>T)];ENSG00000170632:INTRONIC[ENST00000425331(c.423+152G>T)];ENSG00000170632:SILENT[ENST00000323735(c.576G>T,ggG>ggT):ENSP00000319798(p.Gly192(=))];	NA	NA	NA	NA	NA	NA	NA	NA	NA	NA	MODIFIER	NA	NA	42	41	0	53	32	21	39.62
Smoker	42479	chr7	123257775	G	A	*ASB15*	SILENT	ENSG00000146809:SILENT[ENST00000275699(c.435G>A,gaG>gaA):ENSP00000275699(p.Glu145(=))];ENSG00000146809:SILENT[ENST00000540573(c.435G>A,gaG>gaA):ENSP00000438643(p.Glu145(=))];ENSG00000146809:SILENT[ENST00000434204(c.435G>A,gaG>gaA):ENSP00000390963(p.Glu145(=))];ENSG00000146809:SILENT[ENST00000451558(c.435G>A,gaG>gaA):ENSP00000397655(p.Glu145(=))];ENSG00000146809:SILENT[ENST00000451215(c.435G>A,gaG>gaA):ENSP00000416433(p.Glu145(=))];ENSG00000146809:SILENT[ENST00000447789(c.435G>A,gaG>gaA):ENSP00000401166(p.Glu145(=))];	NA	NA	NA	NA	NA	NA	NA	NA	NA	NA	MODIFIER	NA	NA	126	126	0	146	82	64	43.84
Smoker	42479	chr7	97498317	G	A	*ASNS*	MISSENSE	ENSG00000070669:MISSENSE[ENST00000394309(c.152C>T,gCg>gTg):ENSP00000377846(p.Ala51Val)];ENSG00000070669:MISSENSE[ENST00000442734(c.152C>T,gCg>gTg):ENSP00000400422(p.Ala51Val)];ENSG00000070669:MISSENSE[ENST00000175506(c.152C>T,gCg>gTg):ENSP00000175506(p.Ala51Val)];ENSG00000070669:MISSENSE[ENST00000444334(c.89C>T,gCg>gTg):ENSP00000406994(p.Ala30Val)];ENSG00000070669:MISSENSE[ENST00000394308(c.152C>T,gCg>gTg):ENSP00000377845(p.Ala51Val)];ENSG00000070669:MISSENSE[ENST00000454046(c.152C>T,gCg>gTg):ENSP00000401651(p.Ala51Val)];ENSG00000070669:MISSENSE[ENST00000422745(c.89C>T,gCg>gTg):ENSP00000414901(p.Ala30Val)];ENSG00000070669:MISSENSE[ENST00000448127(c.152C>T,gCg>gTg):ENSP00000402350(p.Ala51Val)];ENSG00000070669:MISSENSE[ENST00000437657(c.152C>T,gCg>gTg):ENSP00000394242(p.Ala51Val)];ENSG00000070669:MISSENSE[ENST00000453600(c.89C>T,gCg>gTg):ENSP00000408797(p.Ala30Val)];ENSG00000070669:INTRONIC[ENST00000437628(c.-1+3036C>T)];ENSG00000070669:INTRONIC[ENST00000455086(c.-1+3346C>T)];ENSG00000070669:EXONIC-NC[ENST00000495255(n.180C>T)];	NA	NA	NA	1.65E-05	6.06E-05	NA	NA	T	BN	N	MODERATE	NA	NA	193	190	3	224	170	54	24.11
Smoker	42479	chr7	33427631	G	C	*BBS9*	MISSENSE	ENSG00000122507:MISSENSE[ENST00000396127(c.1885G>C,Gaa>Caa):ENSP00000379433(p.Glu629Gln)];ENSG00000122507:MISSENSE[ENST00000242067(c.1990G>C,Gaa>Caa):ENSP00000242067(p.Glu664Gln)];ENSG00000122507:MISSENSE[ENST00000355070(c.1975G>C,Gaa>Caa):ENSP00000347182(p.Glu659Gln)];ENSG00000122507:MISSENSE[ENST00000354265(c.1885G>C,Gaa>Caa):ENSP00000346214(p.Glu629Gln)];ENSG00000122507:MISSENSE[ENST00000350941(c.1870G>C,Gaa>Caa):ENSP00000313122(p.Glu624Gln)];ENSG00000122507:MISSENSE[ENST00000434373(c.689G>C,Gaa>Caa):ENSP00000388114(p.Glu231Gln)];ENSG00000122507:3UTR[ENST00000433714(c.*751G>C)];ENSG00000122507:EXONIC-NC[ENST00000496029(n.113G>C)];	chr7*33427	chr7*33427	NA	NA	NA	NA	NA	NA	NA	NA	MODIFIER	NA	NA	139	138	0	282	196	85	30.14
Smoker	42479	chr7	122526146	C	A	*CADPS2*	MISSENSE	ENSG00000081803:MISSENSE[ENST00000334010(c.246G>T,agG>agT):ENSP00000333940(p.Arg82Ser)];ENSG00000081803:MISSENSE[ENST00000449022(c.246G>T,agG>agT):ENSP00000398481(p.Arg82Ser)];ENSG00000081803:MISSENSE[ENST00000313070(c.246G>T,agG>agT):ENSP00000325581(p.Arg82Ser)];ENSG00000081803:MISSENSE[ENST00000412584(c.246G>T,agG>agT):ENSP00000400401(p.Arg82Ser)];	NA	NA	NA	NA	NA	NA	NA	D	BN	N	MODERATE	N	0.986	55	55	0	121	46	75	61.98
Smoker	42479	chr7	40087413	C	T	*CDK13*	INTRONIC-SS-ACR	ENSG00000065883:[ENST00000484589(n.1096-7C>T)];ENSG00000065883:[ENST00000340829(c.2544-7C>T)];ENSG00000065883:[ENST00000181839(c.2544-7C>T)];	NA	NA	NA	NA	NA	NA	NA	NA	NA	NA	LOW	NA	NA	70	70	0	88	81	7	7.95
Smoker	42479	chr7	150751191	G	A	*CDK5*	INTRONIC-SS-ACR	ENSG00000164885:[ENST00000485972(c.793-9C>T)];ENSG00000164885:[ENST00000297518(c.697-9C>T)];	NA	NA	NA	NA	NA	NA	NA	NA	NA	NA	MODIFIER	NA	NA	54	54	0	32	2	30	93.75

Mangalaparthi *et al.* , 2020. Mutational landscape of esophageal squamous cell carcinoma in an Indian cohort
Supplementary Table 4. List of somatic single nucleotide variants identified using whole exome sequencing of ESCC patients

Cohort	Sample	Chromosome	Genomic position	Reference allele	Alternate allele	Gene symbol	Variant type	Variant annotation	OncoMD	COSMIC	dbSNP	ExAC	ExAC_SAS	1000G	1000G_SAS	SIFT	Polyphen2	CONDEL	VEP	LRT	PhastCon7 Way	Total read (Adjacent normal)	Reference allel read support (Adjacent normal)	Alternative read support (Adjacent normal)	Total read (Tumor)	Reference allel read support (Tumor)	Alternative read support (Tumor)	Alternate allele frequency
Smoker	42479	chr7	94051275	C	A	*COL1A2*	EXONIC-NC	ENSG00000164692:[ENST00000473573(n.751C>A)];ENSG00000164692:[ENST00000297268(c.2403+11C>A)];ENSG00000164692:[ENST00000497316(n.800+11C>A)];	NA	NA	chr7*94051275*94051275*C*G,T*rs754417165*NA*NA*NA	NA	NA	NA	NA	NA	NA	NA	MODIFIER	NA	NA	93	93	0	80	41	39	48.75
Smoker	42479	chr7	94053603	A	C	*COL1A2*	EXONIC-NC	ENSG00000164692:[ENST00000469732(n.304A>C)];ENSG00000164692:[ENST00000481570(n.1821A>C)];ENSG00000164692:[ENST00000297268(c.2566-45A>C)];	NA	NA	NA	NA	NA	NA	NA	NA	NA	NA	MODIFIER	NA	NA	49	49	0	50	37	13	26
Smoker	42479	chr7	101844998	G	C	*CUX1*	SILENT	ENSG00000257923:INTRONIC[ENST00000560541(n.1843+6115G>C)];ENSG00000257923:INTRONIC[ENST00000437600(c.1249+6115G>C)];ENSG00000257923:INTRONIC[ENST00000547394(c.1207+6115G>C)];ENSG00000257923:INTRONIC[ENST00000292538(c.1255+6115G>C)];ENSG00000257923:INTRONIC[ENST00000558836(n.1361+6115G>C)];ENSG00000257923:INTRONIC[ENST00000425244(c.1117+6115G>C)];ENSG00000257923:INTRONIC[ENST00000393824(c.1138+6115G>C)];ENSG00000257923:SILENT[ENST00000546411(c.2115G>C,ctG>ctC):ENSP00000450125(p.Leu705(=))];ENSG00000257923:SILENT[ENST00000556210(c.1947G>C,ctG>ctC):ENSP00000451558(p.Leu649(=))];ENSG00000257923:SILENT[ENST00000549414(c.2355G>C,ctG>ctC):ENSP00000446630(p.Leu785(=))];ENSG00000257923:SILENT[ENST00000360264(c.2454G>C,ctG>ctC):ENSP00000353401(p.Leu818(=))];ENSG00000257923:SILENT[ENST00000292535(c.2421G>C,ctG>ctC):ENSP00000292535(p.Leu807(=))];ENSG00000257923:SILENT[ENST00000550008(c.2253G>C,ctG>ctC):ENSP00000447373(p.Leu751(=))];	NA	NA	NA	NA	NA	NA	NA	NA	NA	NA	LOW	NA	NA	41	41	0	71	59	12	16.9
Smoker	42479	chr7	101845003	A	C	*CUX1*	MISSENSE	ENSG00000257923:INTRONIC[ENST00000560541(n.1843+6120A>C)];ENSG00000257923:INTRONIC[ENST00000437600(c.1249+6120A>C)];ENSG00000257923:INTRONIC[ENST00000547394(c.1207+6120A>C)];ENSG00000257923:INTRONIC[ENST00000292538(c.1255+6120A>C)];ENSG00000257923:INTRONIC[ENST00000558836(n.1361+6120A>C)];ENSG00000257923:INTRONIC[ENST00000425244(c.1117+6120A>C)];ENSG00000257923:INTRONIC[ENST00000393824(c.1138+6120A>C)];ENSG00000257923:MISSENSE[ENST00000546411(c.2120A>C,cAg>cCg):ENSP00000450125(p.Gln707Pro)];ENSG00000257923:MISSENSE[ENST00000556210(c.1952A>C,cAg>cCg):ENSP00000451558(p.Gln651Pro)];ENSG00000257923:MISSENSE[ENST00000549414(c.2360A>C,cAg>cCg):ENSP00000446630(p.Gln787Pro)];ENSG00000257923:MISSENSE[ENST00000360264(c.2459A>C,cAg>cCg):ENSP00000353401(p.Gln820Pro)];ENSG00000257923:MISSENSE[ENST00000292535(c.2426A>C,cAg>cCg):ENSP00000292535(p.Gln809Pro)];ENSG00000257923:MISSENSE[ENST00000550008(c.2258A>C,cAg>cCg):ENSP00000447373(p.Gln753Pro)];	NA	NA	NA	NA	NA	NA	NA	T	UN	NA	MODERATE	N	0.228	38	38	0	71	58	13	18.31
Smoker	42479	chr7	36934625	G	T	*ELMO1*	INTRONIC-SS-ACR	ENSG00000155849:[ENST00000396045(c.-3-3C>A)];ENSG00000155849:[ENST00000442504(c.1438-3C>A)];ENSG00000155849:[ENST00000420636(c.*169-3C>A)];ENSG00000155849:[ENST00000487843(n.228-3C>A)];ENSG00000155849:[ENST00000472359(n.475-3C>A)];ENSG00000155849:[ENST00000310758(c.1438-3C>A)];ENSG00000155849:[ENST00000396040(c.-3-3C>A)];ENSG00000155849:[ENST00000448602(c.1438-3C>A)];ENSG00000155849:[ENST00000464262(n.195-3C>A)];ENSG00000155849:[ENST00000341056(c.544-3C>A)];	NA	NA	NA	NA	NA	NA	NA	NA	NA	NA	LOW	NA	NA	46	46	0	45	38	7	15.56
Smoker	42479	chr7	37283987	C	T	*ELMO1*	SILENT	ENSG00000155849:SILENT[ENST00000442504(c.423G>A,caG>caA):ENSP00000406952(p.Gln141(=))];ENSG00000155849:SILENT[ENST00000310758(c.423G>A,caG>caA):ENSP00000312185(p.Gln141(=))];ENSG00000155849:SILENT[ENST00000455879(c.423G>A,caG>caA):ENSP00000416090(p.Gln141(=))];ENSG00000155849:SILENT[ENST00000448602(c.423G>A,caG>caA):ENSP00000394458(p.Gln141(=))];ENSG00000155849:INTRONIC[ENST00000455119(c.414-11188G>A)];	NA	NA	NA	NA	NA	NA	NA	NA	NA	NA	MODIFIER	NA	NA	141	139	1	261	152	109	41.76
Smoker	42479	chr7	37889882	A	C	*EPDR1*	INTRONIC	ENSG00000086289:[ENST00000476620(c.-109-6994A>C)];	NA	NA	NA	NA	NA	NA	NA	NA	UN	NA	MODERATE	NA	NA	57	57	0	94	76	18	19.15
Smoker	42479	chr7	158581086	C	G	*ESYT2*	MISSENSE	ENSG00000117868:MISSENSE[ENST00000251527(c.751G>C,Gat>Cat):ENSP00000251527(p.Asp251His)];ENSG00000117868:MISSENSE[ENST00000275418(c.661G>C,Gat>Cat):ENSP00000275418(p.Asp221His)];ENSG00000117868:EXONIC-NC[ENST00000483958(n.376G>C)];	NA	NA	chr7*158581086*158581086*C*A*rs764264988*NA*NA*NA	NA	NA	NA	NA	T	BN	N	MODERATE	NA	NA	85	85	0	34	4	30	88.24
Smoker	42479	chr7	102608461	C	T	*FBXL13*	INTRONIC-SS-DNR	ENSG00000161040:[ENST00000379306(c.591+8G>A)];ENSG00000161040:[ENST00000393772(c.591+8G>A)];ENSG00000161040:[ENST00000456695(c.591+8G>A)];ENSG00000161040:[ENST00000313221(c.591+8G>A)];ENSG00000161040:[ENST00000455112(c.591+8G>A)];ENSG00000161040:[ENST00000448002(c.861+8G>A)];ENSG00000161040:[ENST00000379305(c.591+8G>A)];ENSG00000161040:[ENST00000477915(n.166+8G>A)];ENSG00000161040:[ENST00000471074(n.859+8G>A)];ENSG00000161040:[ENST00000436908(c.591+8G>A)];ENSG00000161040:[ENST00000379308(c.591+8G>A)];	NA	NA	NA	NA	NA	NA	NA	NA	NA	NA	LOW	NA	NA	53	53	0	112	92	20	17.86
Smoker	42479	chr7	93538911	G	C	*GNGT1*	3UTR	ENSG00000127928:[ENST00000455502(c.*72G>C)];ENSG00000127928:[ENST00000430875(c.*72G>C)];ENSG00000127928:[ENST00000428834(c.*72G>C)];ENSG00000127928:[ENST00000429473(c.97-1191G>C)];ENSG00000127928:[ENST00000248572(c.97-1191G>C)];	NA	NA	NA	NA	NA	NA	NA	NA	NA	NA	MODIFIER	NA	NA	145	145	0	141	99	42	29.79
Smoker	42479	chr7	43532730	G	A	*HECW1*	MISSENSE	ENSG00000002746:MISSENSE[ENST00000453890(c.3286G>A,Gaa>Aaa):ENSP00000407774(p.Glu1096Lys)];ENSG00000002746:MISSENSE[ENST00000395891(c.3388G>A,Gaa>Aaa):ENSP00000379228(p.Glu1130Lys)];ENSG00000002746:INTRONIC[ENST00000429529(c.92+13387G>A)];	NA	NA	NA	NA	NA	NA	NA	NA	NA	NA	MODIFIER	NA	NA	133	133	0	212	156	56	26.42
Smoker	42479	chr7	138529075	G	T	*KIAA1549*	SILENT	ENSG00000122778:SILENT[ENST00000422774(c.5439C>A,gtC>gtA):ENSP00000416040(p.Val1813(=))];ENSG00000122778:SILENT[ENST00000242365(c.5289C>A,gtC>gtA):ENSP00000242365(p.Val1763(=))];ENSG00000122778:SILENT[ENST00000440172(c.5439C>A,gtC>gtA):ENSP00000406661(p.Val1813(=))];	NA	NA	NA	NA	NA	NA	NA	NA	NA	NA	LOW	NA	NA	105	104	1	59	15	44	74.58
Smoker	42479	chr7	104750886	C	G	*KMT2E*	INTRONIC	ENSG00000005483:[ENST00000473063(c.278+71C>G)];ENSG00000005483:[ENST00000334914(c.1010-38C>G)];ENSG00000005483:[ENST00000478079(c.*453-38C>G)];ENSG00000005483:[ENST00000257745(c.3845-38C>G)];ENSG00000005483:[ENST00000334884(c.*948-38C>G)];ENSG00000005483:[ENST00000311117(c.3845-38C>G)];ENSG00000005483:[ENST00000334877(c.3844+71C>G)];	NA	NA	NA	NA	NA	NA	NA	NA	NA	NA	MODIFIER	NA	NA	288	288	0	406	342	64	15.76

Mangalaparthi *et al.* , 2020. Mutational landscape of esophageal squamous cell carcinoma in an Indian cohort
Supplementary Table 4. List of somatic single nucleotide variants identified using whole exome sequencing of ESCC patients

Cohort	Sample	Chromosome	Genomic position	Reference allele	Alternate allele	Gene symbol	Variant type	Variant annotation	OncoMD	COSMIC	dbSNP	ExAC	ExAC_SAS	1000G	1000G_SAS	SIFT	Polyphen2	CONDEL	VEP	LRT	PhastCon7 Way	Total read (Adjacent normal)	Reference allel read support (Adjacent normal)	Alternative read support (Adjacent normal)	Total read (Tumor)	Reference allel read support (Tumor)	Alternative read support (Tumor)	Alternate allele frequency
Smoker	42479	chr7	100555598	G	A	*MUC3A*	INTRONIC	ENSG00000169894:[ENST00000422757(c.181+2532G>A)];ENSG00000169894:[ENST00000319509(c.1711+2532G>A)];ENSG00000169894:[ENST00000483133(n.2631+2532G>A)];ENSG00000169894:[ENST00000414964(c.181+2532G>A)];	NA	NA	NA	NA	NA	NA	NA	NA	NA	NA	MODIFIER	NA	NA	55	55	0	122	75	46	37.7
Smoker	42479	chr7	37889882	A	C	*NME8*	MISSENSE	ENSG00000086288:MISSENSE[ENST00000440017(c.14A>C,aAa>aCa):ENSP00000397063(p.Lys5Thr)];ENSG00000086288:MISSENSE[ENST00000199447(c.14A>C,aAa>aCa):ENSP00000199447(p.Lys5Thr)];ENSG00000086288:MISSENSE[ENST00000444718(c.14A>C,aAa>aCa):ENSP00000390596(p.Lys5Thr)];ENSG00000086288:MISSENSE[ENST00000455500(c.14A>C,aAa>aCa):ENSP00000390047(p.Lys5Thr)];ENSG00000086288:MISSENSE[ENST00000426106(c.14A>C,aAa>aCa):ENSP00000408841(p.Lys5Thr)];	NA	NA	NA	NA	NA	NA	NA	NA	UN	NA	MODERATE	NA	NA	57	57	0	94	76	18	19.15
Smoker	42479	chr7	135279290	C	G	*NUP205*	INTRONIC	ENSG00000155561:[ENST00000440390(c.722-2858C>G)];ENSG00000155561:[ENST00000285968(c.1831-5C>G)];	NA	NA	NA	NA	NA	NA	NA	NA	NA	NA	LOW	NA	NA	124	124	0	78	10	68	87.18
Smoker	42479	chr7	143771895	G	C	*OR2A25*	MISSENSE	ENSG00000221933:MISSENSE[ENST00000408898(c.583G>C,Gag>Cag):ENSP00000386167(p.Glu195Gln)];	NA	NA	NA	NA	NA	NA	NA	T	PrD	N	MODERATE	NA	NA	196	193	0	133	88	45	33.83
Smoker	42479	chr7	82478173	C	T	*PCLO*	INTRONIC	ENSG00000186472:[ENST00000333891(c.13655-1610G>A)];ENSG00000186472:[ENST00000456006(c.*34-1610G>A)];ENSG00000186472:[ENST00000413807(c.*40+11G>A)];ENSG00000186472:[ENST00000423517(c.13655-1610G>A)];ENSG00000186472:[ENST00000426442(n.185-1610G>A)];	NA	NA	NA	NA	NA	NA	NA	NA	NA	NA	MODIFIER	NA	NA	48	48	0	56	33	23	41.07
Smoker	42479	chr7	82544614	T	C	*PCLO*	MISSENSE	ENSG00000186472:MISSENSE[ENST00000437081(c.2848A>G,Att>Gtt):ENSP00000393760(p.Ile950Val)];ENSG00000186472:MISSENSE[ENST00000333891(c.12688A>G,Att>Gtt):ENSP00000334319(p.Ile4230Val)];ENSG00000186472:MISSENSE[ENST00000423517(c.12688A>G,Att>Gtt):ENSP00000388393(p.Ile4230Val)];	NA	NA	NA	NA	NA	NA	NA	NA	PoD	NA	MODERATE	NA	NA	178	174	3	175	73	102	58.29
Smoker	42479	chr7	32209342	G	A	*PDE1C*	INTRONIC	ENSG00000154678:[ENST00000396193(c.308+55C>T)];	NA	NA	NA	NA	NA	NA	NA	NA	NA	NA	MODIFIER	NA	NA	119	118	0	205	127	78	38.05
Smoker	42479	chr7	150035686	C	A	*RARRES2*	INTRONIC	ENSG00000106538:[ENST00000223271(c.*10+18G>T)];ENSG00000106538:[ENST00000467793(c.376-158G>T)];ENSG00000106538:[ENST00000466675(c.*10+18G>T)];ENSG00000106538:[ENST00000478771(n.1784+18G>T)];	NA	NA	NA	NA	NA	NA	NA	NA	NA	NA	MODIFIER	NA	NA	29	29	0	25	11	14	56
Smoker	42479	chr7	75511601	G	T	*RHBDD2*	INTRONIC	ENSG00000005486:[ENST00000006777(c.586+47G>T)];ENSG00000005486:[ENST00000318622(c.163+47G>T)];ENSG00000005486:[ENST00000428119(c.163+47G>T)];ENSG00000005486:[ENST00000468304(n.211+797G>T)];ENSG00000005486:[ENST00000454791(c.*468+47G>T)];	NA	NA	NA	NA	NA	NA	NA	NA	NA	NA	MODIFIER	NA	NA	85	85	0	101	82	18	17.82
Smoker	42479	chr7	123257775	G	A	*RP11-390E23.3*	INTRONIC	ENSG00000230442:[ENST00000418409(n.506-2192C>T)];ENSG00000230442:[ENST00000440504(n.387-2321C>T)];ENSG00000230442:[ENST00000422401(n.40+6822C>T)];	NA	NA	NA	NA	NA	NA	NA	NA	NA	NA	MODIFIER	NA	NA	126	126	0	146	82	64	43.84
Smoker	42479	chr7	35840836	G	A	*SEPT7*	MISSENSE	ENSG00000122545:MISSENSE[ENST00000469679(c.17G>A,aGa>aAa):ENSP00000444501(p.Arg6Lys)];ENSG00000122545:MISSENSE[ENST00000350320(c.17G>A,aGa>aAa):ENSP00000344868(p.Arg6Lys)];ENSG00000122545:MISSENSE[ENST00000399034(c.17G>A,aGa>aAa):ENSP00000381992(p.Arg6Lys)];ENSG00000122545:MISSENSE[ENST00000399035(c.17G>A,aGa>aAa):ENSP00000381993(p.Arg6Lys)];ENSG00000122545:EXONIC-NC[ENST00000493626(n.8G>A)];ENSG00000122545:EXONIC-NC[ENST00000475109(n.8G>A)];ENSG00000122545:5UTR[ENST00000435235(c.-138G>A)];	NA	NA	chr7*35840836*35840836*G*C*rs758649882*NA*NA*NA	NA	NA	NA	NA	NA	NA	NA	MODIFIER	NA	NA	23	23	0	56	43	12	21.43
Smoker	42479	chr7	26386003	G	T	*SNX10*	INTRONIC	ENSG00000086300:[ENST00000338523(c.-23-37G>T)];ENSG00000086300:[ENST00000416246(c.-66-37G>T)];ENSG00000086300:[ENST00000446848(c.-66-37G>T)];ENSG00000086300:[ENST00000412416(c.-194-37G>T)];ENSG00000086300:[ENST00000396376(c.-23-37G>T)];	NA	NA	NA	NA	NA	NA	NA	NA	NA	NA	MODIFIER	NA	NA	86	86	0	111	92	19	17.12
Smoker	42479	chr7	21469548	A	C	*SP4*	SILENT	ENSG00000105866:INTRONIC[ENST00000448246(c.123+1138A>C)];ENSG00000105866:INTRONIC[ENST00000432066(c.7+1672A>C)];ENSG00000105866:SILENT[ENST00000222584(c.765A>C,acA>acC):ENSP00000222584(p.Thr255(=))];	NA	NA	NA	NA	NA	NA	NA	NA	NA	NA	MODIFIER	NA	NA	148	148	0	196	152	44	22.45
Smoker	42479	chr7	20824927	G	C	*SP8*	MISSENSE	ENSG00000164651:MISSENSE[ENST00000418710(c.509C>G,tCg>tGg):ENSP00000408792(p.Ser170Trp)];ENSG00000164651:MISSENSE[ENST00000361443(c.455C>G,tCg>tGg):ENSP00000354482(p.Ser152Trp)];	NA	NA	NA	NA	NA	NA	NA	D	PoD	D	MODERATE	U	0.675	48	48	0	65	53	12	18.46
Smoker	42479	chr7	105029766	C	T	*SRPK2*	MISSENSE	ENSG00000135250:INTRONIC[ENST00000462282(n.153+9837G>A)];ENSG00000135250:MISSENSE[ENST00000482897(c.52G>A,Gag>Aag):ENSP00000419240(p.Glu18Lys)];ENSG00000135250:EXONIC-NC[ENST00000482862(n.72G>A)];	NA	NA	NA	NA	NA	NA	NA	NA	NA	NA	MODIFIER	NA	NA	216	216	0	377	311	66	17.51
Smoker	42479	chr7	88965869	G	A	*ZNF804B*	SILENT	ENSG00000182348:SILENT[ENST00000333190(c.3573G>A,ccG>ccA):ENSP00000329638(p.Pro1191(=))];	chr7*88965[illegible]	chr7*88965[illegible]	NA	2.47E-05	0	NA	NA	NA	NA	NA	LOW	NA	NA	181	180	0	163	111	52	31.9
Smoker	42479	chr8	39442826	T	A	*ADAM18*	SILENT	ENSG00000168619:SILENT[ENST00000520772(c.87T>A,gtT>gtA):ENSP00000429908(p.Val29(=))];ENSG00000168619:SILENT[ENST00000520001(c.87T>A,gtT>gtA):ENSP00000429335(p.Val29(=))];ENSG00000168619:SILENT[ENST00000265707(c.87T>A,gtT>gtA):ENSP00000265707(p.Val29(=))];ENSG00000168619:SILENT[ENST00000520087(c.87T>A,gtT>gtA):ENSP00000428083(p.Val29(=))];ENSG00000168619:SILENT[ENST00000379866(c.87T>A,gtT>gtA):ENSP00000369195(p.Val29(=))];ENSG00000168619:5UTR[ENST00000541111(c.-1310T>A)];	NA	NA	NA	NA	NA	NA	NA	NA	NA	NA	LOW	NA	NA	139	139	0	125	96	29	23.2
Smoker	42479	chr8	17917022	C	G	*ASAH1*	INTRONIC	ENSG00000104763:[ENST00000520781(c.843-49G>C)];ENSG00000104763:[ENST00000314146(c.900-49G>C)];ENSG00000104763:[ENST00000417108(c.648-49G>C)];ENSG00000104763:[ENST00000262097(c.918-49G>C)];ENSG00000104763:[ENST00000381733(c.966-49G>C)];	NA	NA	NA	NA	NA	NA	NA	NA	NA	NA	MODIFIER	NA	NA	177	177	0	195	97	98	50.26
Smoker	42479	chr8	144789140	C	T	*CCDC166*	SILENT	ENSG00000255181:SILENT[ENST00000542437(c.1044G>A,tcG>tcA):ENSP00000437468(p.Ser348(=))];	NA	NA	chr8*144789140*144789140*C*A*rs781990610*NA*NA*NA	NA	NA	NA	NA	NA	NA	NA	MODIFIER	NA	NA	96	96	0	248	53	195	78.63

Mangalaparthi *et al.* , 2020. Mutational landscape of esophageal squamous cell carcinoma in an Indian cohort
Supplementary Table 4. List of somatic single nucleotide variants identified using whole exome sequencing of ESCC patients

Cohort	Sample	Chromosome	Genomic position	Reference allele	Alternate allele	Gene symbol	Variant type	Variant annotation	OncoMD	COSMIC	dbSNP	ExAC	ExAC_SAS	1000G	1000G_SAS	SIFT	Polyphen2	CONDEL	VEP	LRT	PhastCon7 Way	Total read (Adjacent normal)	Reference allel read support (Adjacent normal)	Alternative read support (Adjacent normal)	Total read (Tumor)	Reference allel read support (Tumor)	Alternative read support (Tumor)	Alternate allele frequency
Smoker	42479	chr8	67974106	C	T	*COPS5*	SILENT	ENSG00000121022:INTRONIC[ENST00000517793(n.309-2426G>A)];ENSG00000121022:INTRONIC[ENST00000517736(c.-49-2426G>A)];ENSG00000121022:INTRONIC[ENST00000518747(c.-50+2230G>A)];ENSG00000121022:EXONIC-NC[ENST00000521509(n.385G>A)];ENSG00000121022:EXONIC-NC[ENST00000519963(n.674G>A)];ENSG00000121022:EXONIC-NC[ENST00000519057(n.470G>A)];ENSG00000121022:SILENT[ENST00000523890(c.126G>A,gcG>gcA):ENSP00000427997(p.Ala42(=))];ENSG00000121022:SILENT[ENST00000357849(c.126G>A,gcG>gcA):ENSP00000350512(p.Ala42(=))];ENSG00000121022:SILENT[ENST00000517406(c.126G>A,gcG>gcA):ENSP00000428124(p.Ala42(=))];ENSG00000121022:SILENT[ENST00000518374(c.126G>A,gcG>gcA):ENSP00000427869(p.Ala42(=))];	chr8*67974	chr8*67974	NA	NA	NA	NA	NA	NA	NA	NA	LOW	NA	NA	116	116	0	242	209	33	13.64
Smoker	42479	chr8	113316964	C	G	*CSMD3*	MISSENSE	ENSG00000164796:MISSENSE[ENST00000352409(c.8042G>C,aGa>aCa):ENSP00000343124(p.Arg2681Thr)];ENSG00000164796:MISSENSE[ENST00000297405(c.8252G>C,aGa>aCa):ENSP00000297405(p.Arg2751Thr)];ENSG00000164796:MISSENSE[ENST00000343508(c.8132G>C,aGa>aCa):ENSP00000345799(p.Arg2711Thr)];ENSG00000164796:MISSENSE[ENST00000339701(c.6062G>C,aGa>aCa):ENSP00000341558(p.Arg2021Thr)];ENSG00000164796:INTRONIC[ENST00000455883(c.7759+1272G>C)];	NA	NA	NA	NA	NA	NA	NA	T	BN	N	MODERATE	NA	NA	158	158	0	265	234	31	11.7
Smoker	42479	chr8	114448945	A	G	*CSMD3*	MISSENSE	ENSG00000164796:MISSENSE[ENST00000455883(c.139T>C,Tgg>Cgg):ENSP00000412263(p.Trp47Arg)];ENSG00000164796:MISSENSE[ENST00000352409(c.139T>C,Tgg>Cgg):ENSP00000343124(p.Trp47Arg)];ENSG00000164796:MISSENSE[ENST00000297405(c.139T>C,Tgg>Cgg):ENSP00000297405(p.Trp47Arg)];ENSG00000164796:EXONIC-NC[ENST00000497026(n.224T>C)];ENSG00000164796:EXONIC-NC[ENST00000493303(n.75T>C)];	chr8*11444	chr8*11444	NA	NA	NA	NA	NA	D_lc	BN	N	MODERATE	N	1	86	85	0	179	53	126	70.39
Smoker	42479	chr8	105367392	G	T	*DCSTAMP*	SILENT	ENSG00000164935:INTRONIC[ENST00000518051(n.146-960G>T)];ENSG00000164935:INTRONIC[ENST00000517991(c.828-960G>T)];ENSG00000164935:EXONIC-NC[ENST00000520080(n.109G>T)];ENSG00000164935:SILENT[ENST00000297581(c.1317G>T,cgG>cgT):ENSP00000297581(p.Arg439(=))];	NA	NA	NA	NA	NA	NA	NA	NA	NA	NA	MODIFIER	NA	NA	117	117	0	197	39	158	80.2
Smoker	42479	chr8	105367392	G	T	*DPYS*	INTRONIC	ENSG00000147647:[ENST00000521601(n.329-23200C>A)];	NA	NA	NA	NA	NA	NA	NA	NA	NA	NA	MODIFIER	NA	NA	117	117	0	197	39	158	80.2
Smoker	42479	chr8	28837610	G	T	*HMBOX1*	NONSENSE	ENSG00000147421:NONSENSE[ENST00000403668(c.523G>T,Gag>Tag):ENSP00000384261(p.Glu175Ter)];ENSG00000147421:NONSENSE[ENST00000521516(c.523G>T,Gag>Tag):ENSP00000430238(p.Glu175Ter)];ENSG00000147421:NONSENSE[ENST00000518080(c.107G>T,Gag>Tag):ENSP00000430222(p.Glu37Ter)];ENSG00000147421:NONSENSE[ENST00000444075(c.523G>T,Gag>Tag):ENSP00000401769(p.Glu175Ter)];ENSG00000147421:NONSENSE[ENST00000524238(c.523G>T,Gag>Tag):ENSP00000430110(p.Glu175Ter)];ENSG00000147421:NONSENSE[ENST00000523613(c.523G>T,Gag>Tag):ENSP00000452827(p.Glu175Ter)];ENSG00000147421:NONSENSE[ENST00000397358(c.523G>T,Gag>Tag):ENSP00000380516(p.Glu175Ter)];ENSG00000147421:NONSENSE[ENST00000519047(c.523G>T,Gag>Tag):ENSP00000430059(p.Glu175Ter)];ENSG00000147421:NONSENSE[ENST00000558662(c.523G>T,Gag>Tag):ENSP00000453211(p.Glu175Ter)];ENSG00000147421:NONSENSE[ENST00000355231(c.523G>T,Gag>Tag):ENSP00000475196(p.Glu175Ter)];ENSG00000147421:NONSENSE[ENST00000560599(c.523G>T,Gag>Tag):ENSP00000453185(p.Glu175Ter)];ENSG00000147421:NONSENSE[ENST00000287701(c.523G>T,Gag>Tag):ENSP00000287701(p.Glu175Ter)];	chr8*28837	NA	NA	NA	NA	NA	NA	NA	NA	NA	HIGH	NA	1	154	154	0	238	188	50	21.01
Smoker	42479	chr8	39777580	C	G	*IDO1*	INTRONIC	ENSG00000131203:[ENST00000519154(c.423-39C>G)];ENSG00000131203:[ENST00000518237(c.423-39C>G)];ENSG00000131203:[ENST00000253513(c.433+1117C>G)];ENSG00000131203:[ENST00000522495(c.423-39C>G)];ENSG00000131203:[ENST00000521636(n.326-39C>G)];	NA	NA	NA	NA	NA	NA	NA	NA	NA	NA	MODIFIER	NA	NA	93	93	0	94	73	21	22.34
Smoker	42479	chr8	140630675	G	C	*KCNK9*	SILENT	ENSG00000169427:SILENT[ENST00000520439(c.951C>G,tcC>tcG):ENSP00000430676(p.Ser317(=))];ENSG00000169427:SILENT[ENST00000522317(c.951C>G,tcC>tcG):ENSP00000429847(p.Ser317(=))];ENSG00000169427:SILENT[ENST00000303015(c.951C>G,tcC>tcG):ENSP00000302166(p.Ser317(=))];	NA	NA	chr8*140630673*140630676*AAGG*A*rs768577329*NA*NA*NA	NA	NA	NA	NA	NA	NA	NA	LOW	NA	NA	77	77	0	119	105	14	11.76
Smoker	42479	chr8	72756576	G	C	*MSC*	5UTR	ENSG00000178860:[ENST00000325509(c.-163C>G)];	chr8*72756	NA	NA	NA	NA	NA	NA	NA	NA	NA	MODIFIER	NA	NA	83	83	0	168	115	53	31.55
Smoker	42479	chr8	37729577	C	A	*RAB11FIP1*	NONSENSE	ENSG00000156675:NONSENSE[ENST00000330843(c.2743G>T,Gga>Tga):ENSP00000331342(p.Gly915Ter)];ENSG00000156675:INTRONIC[ENST00000524118(c.1179-1531G>T)];ENSG00000156675:INTRONIC[ENST00000522727(c.1179-1531G>T)];ENSG00000156675:INTRONIC[ENST00000287263(c.1623-1531G>T)];ENSG00000156675:EXONIC-NC[ENST00000523182(n.1220G>T)];ENSG00000156675:EXONIC-NC[ENST00000522774(n.1220G>T)];	NA	NA	chr8*37729577*37729577*C*T*rs148019875*NA*NA*NA	NA	NA	NA	NA	NA	NA	NA	MODIFIER	NA	NA	130	130	0	187	141	46	24.6
Smoker	42479	chr8	30402741	G	C	*RBPMS*	INTRONIC	ENSG00000157110:[ENST00000320203(c.528+600G>C)];ENSG00000157110:[ENST00000538486(c.398-13582G>C)];ENSG00000157110:[ENST00000339877(c.528+600G>C)];ENSG00000157110:[ENST00000520161(c.216+600G>C)];ENSG00000157110:[ENST00000397323(c.528+600G>C)];ENSG00000157110:[ENST00000519359(c.*242+600G>C)];ENSG00000157110:[ENST00000519647(c.216+600G>C)];ENSG00000157110:[ENST00000287771(c.528+600G>C)];ENSG00000157110:[ENST00000522694(c.*45+4G>C)];ENSG00000157110:[ENST00000522099(n.25G>C)];ENSG00000157110:[ENST00000520191(c.*453G>C)];	NA	NA	NA	NA	NA	NA	NA	NA	NA	NA	MODIFIER	NA	NA	98	98	0	81	61	20	24.69
Smoker	42479	chr8	104898184	T	C	*RIMS2*	MISSENSE	ENSG00000176406:MISSENSE[ENST00000406091(c.1357T>C,Tca>Cca):ENSP00000384892(p.Ser453Pro)];ENSG00000176406:MISSENSE[ENST00000408894(c.781T>C,Tca>Cca):ENSP00000386228(p.Ser261Pro)];ENSG00000176406:MISSENSE[ENST00000504942(c.1357T>C,Tca>Cca):ENSP00000427018(p.Ser453Pro)];ENSG00000176406:MISSENSE[ENST00000507740(c.781T>C,Tca>Cca):ENSP00000423559(p.Ser261Pro)];ENSG00000176406:MISSENSE[ENST00000262231(c.781T>C,Tca>Cca):ENSP00000262231(p.Ser261Pro)];ENSG00000176406:MISSENSE[ENST00000515551(c.781T>C,Tca>Cca):ENSP00000425205(p.Ser261Pro)];ENSG00000176406:MISSENSE[ENST00000436393(c.691T>C,Tca>Cca):ENSP00000390665(p.Ser231Pro)];	NA	NA	NA	NA	NA	NA	NA	T_lc	BN	N	MODERATE	NA	NA	163	163	0	223	205	18	8.07

Mangalaparthi *et al.* , 2020. Mutational landscape of esophageal squamous cell carcinoma in an Indian cohort
Supplementary Table 4. List of somatic single nucleotide variants identified using whole exome sequencing of ESCC patients

Cohort	Sample	Chromosome	Genomic position	Reference allele	Alternate allele	Gene symbol	Variant type	Variant annotation	OncoMD	COSMIC	dbSNP	ExAC	ExAC_SAS	1000G	1000G_SAS	SIFT	Polyphen2	CONDEL	VEP	LRT	PhastCon7 Way	Total read (Adjacent normal)	Reference allel read support (Adjacent normal)	Alternative read support (Adjacent normal)	Total read (Tumor)	Reference allel read support (Tumor)	Alternative read support (Tumor)	Alternate allele frequency
Smoker	42479	chr8	72756576	G	C	*RP11-383H13.1*	MISSENSE	ENSG00000235531:MISSENSE[ENST00000537896(c.416G>C,cGa>cCa):ENSP00000440866(p.Arg139Pro)];ENSG00000235531:EXONIC-NC[ENST00000519751(n.198G>C)];ENSG00000235531:EXONIC-NC[ENST00000457356(n.239G>C)];ENSG00000235531:EXONIC-NC[ENST00000518916(n.184G>C)];ENSG00000235531:SILENT[ENST00000522519(c.345G>C,gcG>gcC):ENSP00000427808(p.Ala115(=))];ENSG00000235531:INTRONIC[ENST00000521467(c.-91+16126G>C)];ENSG00000235531:INTRONIC[ENST00000524152(c.-91+637G>C)];	chr8*72756	NA	NA	NA	NA	NA	NA	NA	NA	NA	MODIFIER	NA	NA	83	83	0	168	115	53	31.55
Smoker	42479	chr8	39777580	C	G	*RP11-44K6.3*	INTRONIC	ENSG00000253939:[ENST00000517623(n.256-16040G>C)];	NA	NA	NA	NA	NA	NA	NA	NA	NA	NA	MODIFIER	NA	NA	93	93	0	94	73	21	22.34
Smoker	42479	chr8	39777580	C	G	*RP11-44K6.4*	INTRONIC	ENSG00000254287:[ENST00000522970(n.256+574G>C)];	NA	NA	NA	NA	NA	NA	NA	NA	NA	NA	MODIFIER	NA	NA	93	93	0	94	73	21	22.34
Smoker	42479	chr8	110655124	G	A	*SYBU*	MISSENSE	ENSG00000147642:MISSENSE[ENST00000533821(c.59C>T,tCt>tTt):ENSP00000431469(p.Ser20Phe)];ENSG00000147642:MISSENSE[ENST00000529190(c.59C>T,tCt>tTt):ENSP00000432835(p.Ser20Phe)];ENSG00000147642:MISSENSE[ENST00000534578(c.62C>T,tCt>tTt):ENSP00000431895(p.Ser21Phe)];ENSG00000147642:MISSENSE[ENST00000527600(c.62C>T,tCt>tTt):ENSP00000434612(p.Ser21Phe)];ENSG00000147642:MISSENSE[ENST00000526302(c.62C>T,tCt>tTt):ENSP00000434061(p.Ser21Phe)];ENSG00000147642:MISSENSE[ENST00000534501(c.62C>T,tCt>tTt):ENSP00000434840(p.Ser21Phe)];ENSG00000147642:MISSENSE[ENST00000419099(c.59C>T,tCt>tTt):ENSP00000402652(p.Ser20Phe)];ENSG00000147642:MISSENSE[ENST00000528647(c.59C>T,tCt>tTt):ENSP00000432501(p.Ser20Phe)];ENSG00000147642:MISSENSE[ENST00000446070(c.59C>T,tCt>tTt):ENSP00000414748(p.Ser20Phe)];ENSG00000147642:MISSENSE[ENST00000534184(c.59C>T,tCt>tTt):ENSP00000432291(p.Ser20Phe)];ENSG00000147642:MISSENSE[ENST00000533895(c.59C>T,tCt>tTt):ENSP00000433446(p.Ser20Phe)];ENSG00000147642:MISSENSE[ENST00000528716(c.62C>T,tCt>tTt):ENSP00000436520(p.Ser21Phe)];ENSG00000147642:MISSENSE[ENST00000422135(c.62C>T,tCt>tTt):ENSP00000407118(p.Ser21Phe)];ENSG00000147642:MISSENSE[ENST00000424158(c.77C>T,tCt>tTt):ENSP00000415654(p.Ser26Phe)];ENSG00000147642:MISSENSE[ENST00000408908(c.62C>T,tCt>tTt):ENSP00000386154(p.Ser21Phe)];ENSG00000147642:MISSENSE[ENST00000524720(c.62C>T,tCt>tTt):ENSP00000435224(p.Ser21Phe)];ENSG00000147642:MISSENSE[ENST00000533171(c.62C>T,tCt>tTt):ENSP00000434679(p.Ser21Phe)];ENSG00000147642:MISSENSE[ENST00000433638(c.62C>T,tCt>tTt):ENSP00000403544(p.Ser21Phe)];ENSG00000147642:MISSENSE[ENST00000440310(c.62C>T,tCt>tTt):ENSP00000404369(p.Ser21Phe)];ENSG00000147642:MISSENSE[ENST00000399066(c.53C>T,tCt>tTt):ENSP00000382019(p.Ser18Phe)];ENSG00000147642:MISSENSE[ENST00000276646(c.62C>T,tCt>tTt):ENSP00000276646(p.Ser21Phe)];ENSG00000147642:INTRONIC[ENST00000532779(c.26-23856C>T)];ENSG00000147642:INTRONIC[ENST00000528569(c.-	chr8*11065	NA	NA	NA	NA	NA	NA	T	PrD	N	MODERATE	N	0.998	102	102	0	194	139	55	28.35
Smoker	42479	chr8	120770317	C	A	*TAF2*	MISSENSE	ENSG00000064313:MISSENSE[ENST00000378164(c.2764G>T,Gta>Tta):ENSP00000367406(p.Val922Leu)];ENSG00000064313:MISSENSE[ENST00000529653(c.136G>T,Gta>Tta):ENSP00000436750(p.Val46Leu)];ENSG00000064313:EXONIC-NC[ENST00000523078(n.700G>T)];ENSG00000064313:EXONIC-NC[ENST00000519355(n.526G>T)];	NA	NA	NA	NA	NA	NA	NA	NA	NA	NA	MODIFIER	NA	NA	102	101	0	133	82	51	38.35
Smoker	42479	chr8	98289424	C	G	*TSPYL5*	MISSENSE	ENSG00000180543:MISSENSE[ENST00000322128(c.649G>C,Gcc>Ccc):ENSP00000322802(p.Ala217Pro)];	NA	NA	NA	NA	NA	NA	NA	D	PrD	D	MODERATE	N	0.719	131	131	0	209	169	40	19.14
Smoker	42479	chr8	100733141	G	A	*VPS13B*	MISSENSE	ENSG00000132549:MISSENSE[ENST00000358544(c.6991G>A,Gaa>Aaa):ENSP00000351346(p.Glu2331Lys)];ENSG00000132549:MISSENSE[ENST00000357162(c.6916G>A,Gaa>Aaa):ENSP00000349685(p.Glu2306Lys)];ENSG00000132549:EXONIC-NC[ENST00000518569(n.46G>A)];ENSG00000132549:3UTR[ENST00000395996(c.*2774G>A)];	NA	NA	NA	NA	NA	NA	NA	NA	NA	NA	MODIFIER	NA	NA	169	169	0	242	131	111	45.87
Smoker	42479	chr8	135522388	G	A	*ZFAT*	MISSENSE	ENSG00000066827:INTRONIC[ENST00000522723(n.388-396C>T)];ENSG00000066827:INTRONIC[ENST00000523924(c.*3344-396C>T)];ENSG00000066827:INTRONIC[ENST00000518408(n.725-396C>T)];ENSG00000066827:INTRONIC[ENST00000523243(c.*531-396C>T)];ENSG00000066827:INTRONIC[ENST00000429442(c.3326-396C>T)];ENSG00000066827:INTRONIC[ENST00000520214(c.3326-396C>T)];ENSG00000066827:INTRONIC[ENST00000523399(c.3176-396C>T)];ENSG00000066827:INTRONIC[ENST00000377838(c.3362-396C>T)];ENSG00000066827:INTRONIC[ENST00000520727(c.3326-396C>T)];ENSG00000066827:INTRONIC[ENST00000520356(c.3198+10738C>T)];ENSG00000066827:MISSENSE[ENST00000521673(c.38C>T,tCt>tTt):ENSP00000429600(p.Ser13Phe)];	NA	NA	0.0003994	0.0003706	0.0004	0.0003994	0.002	NA	NA	NA	MODIFIER	NA	NA	54	54	0	82	43	39	47.56
Smoker	42479	chr8	144789140	C	T	*ZNF707*	INTRONIC	ENSG00000181135:[ENST00000527561(n.1089-2283C>T)];	NA	NA	chr8*144789140*144789140*C*A*rs781990610*NA*NA*NA	NA	NA	NA	NA	NA	NA	NA	MODIFIER	NA	NA	96	96	0	248	53	195	78.63
Smoker	42479	chr9	116930318	C	T	*COL27A1*	SILENT	ENSG00000196739:SILENT[ENST00000451716(c.324C>T,ctC>ctT):ENSP00000391328(p.Leu108(=))];ENSG00000196739:SILENT[ENST00000356083(c.483C>T,ctC>ctT):ENSP00000348385(p.Leu161(=))];	NA	NA	0.0001997	0.0002182	0.001531675	0.0001997	0.001	NA	NA	NA	LOW	NA	NA	60	60	0	65	27	38	58.46
Smoker	42479	chr9	90264869	C	A	*DAPK1*	MISSENSE	ENSG00000196730:MISSENSE[ENST00000358077(c.1462C>A,Cac>Aac):ENSP00000350785(p.His488Asn)];ENSG00000196730:MISSENSE[ENST00000491893(c.1462C>A,Cac>Aac):ENSP00000419026(p.His488Asn)];ENSG00000196730:MISSENSE[ENST00000408954(c.1462C>A,Cac>Aac):ENSP00000386135(p.His488Asn)];ENSG00000196730:MISSENSE[ENST00000472284(c.1462C>A,Cac>Aac):ENSP00000417076(p.His488Asn)];ENSG00000196730:MISSENSE[ENST00000469640(c.1462C>A,Cac>Aac):ENSP00000418885(p.His488Asn)];ENSG00000196730:EXONIC-NC[ENST00000469067(n.1942C>A)];ENSG00000196730:EXONIC-NC[ENST00000495281(n.328C>A)];ENSG00000196730:EXONIC-NC[ENST00000475804(n.339C>A)];ENSG00000196730:3UTR[ENST00000489291(c.*846C>A)];	NA	NA	NA	NA	NA	NA	NA	NA	NA	NA	MODIFIER	NA	NA	249	247	2	157	33	124	78.98
Smoker	42479	chr9	135545404	G	A	*DDX31*	MISSENSE	ENSG00000125485:MISSENSE[ENST00000372159(c.233C>T,tCc>tTc):ENSP00000361232(p.Ser78Phe)];ENSG00000125485:MISSENSE[ENST00000310532(c.233C>T,tCc>tTc):ENSP00000310539(p.Ser78Phe)];ENSG00000125485:MISSENSE[ENST00000372153(c.233C>T,tCc>tTc):ENSP00000361226(p.Ser78Phe)];ENSG00000125485:EXONIC-NC[ENST00000480876(n.385C>T)];ENSG00000125485:EXONIC-NC[ENST00000482620(n.385C>T)];	NA	NA	NA	NA	NA	NA	NA	NA	NA	NA	MODIFIER	NA	NA	41	41	0	37	4	33	89.19

Cohort	Sample	Chromosome	Genomic position	Reference allele	Alternate allele	Gene symbol	Variant type	Variant annotation	OncoMD	COSMIC	dbSNP	ExAC	ExAC_SAS	1000G	1000G_SAS	SIFT	Polyphen2	CONDEL	VEP	LRT	PhastCon7 Way	Total read (Adjacent normal)	Reference allel read support (Adjacent normal)	Alternative read support (Adjacent normal)	Total read (Tumor)	Reference allel read support (Tumor)	Alternative read support (Tumor)	Alternate allele frequency
Smoker	42479	chr9	22451265	G	C	*DMRTA1*	MISSENSE	ENSG00000176399:MISSENSE[ENST00000325870(c.870G>C,gaG>gaC):ENSP00000319651(p.Glu290Asp)];	NA	NA	NA	NA	NA	NA	NA	T	BN	N	MODERATE	N	0.978	124	124	0	90	18	72	80
Smoker	42479	chr9	71395451	G	C	*FAM122A*	MISSENSE	ENSG00000187866:MISSENSE[ENST00000394264(c.371G>C,aGt>aCt):ENSP00000377807(p.Ser124Thr)];	NA	NA	.	4.95E-05	0	NA	NA	NA	NA	NA	MODIFIER	NA	NA	170	168	2	102	20	82	80.39
Smoker	42479	chr9	115965203	C	G	*FKBP15*	INTRONIC	ENSG00000119321:[ENST00000238256(c.399+38G>C)];ENSG00000119321:[ENST00000414250(c.474+38G>C)];ENSG00000119321:[ENST00000446284(c.474+38G>C)];	NA	NA	NA	NA	NA	NA	NA	NA	NA	NA	MODIFIER	NA	NA	106	106	0	123	18	104	84.55
Smoker	42479	chr9	132686322	G	C	*FNBP1*	INTRONIC	ENSG00000187239:[ENST00000355681(c.988-104C>G)];ENSG00000187239:[ENST00000449089(c.873-47C>G)];ENSG00000187239:[ENST00000420781(c.988-47C>G)];ENSG00000187239:[ENST00000478129(n.231+917C>G)];ENSG00000187239:[ENST00000482107(n.110+917C>G)];ENSG00000187239:[ENST00000446176(c.988-17C>G)];	NA	NA	NA	NA	NA	NA	NA	NA	NA	NA	MODIFIER	NA	NA	41	41	0	20	12	8	40
Smoker	42479	chr9	124989124	C	T	*LHX6*	INTRONIC-SS-DNR	ENSG00000106852:[ENST00000373755(c.252+3G>A)];ENSG00000106852:[ENST00000560152(n.276+3G>A)];ENSG00000106852:[ENST00000340587(c.339+3G>A)];ENSG00000106852:[ENST00000394319(c.339+3G>A)];ENSG00000106852:[ENST00000373754(c.252+3G>A)];ENSG00000106852:[ENST00000541397(c.306+3G>A)];	NA	NA	NA	NA	NA	NA	NA	NA	NA	NA	LOW	NA	NA	27	27	0	16	4	12	75
Smoker	42479	chr9	131767794	G	C	*NUP188*	MISSENSE	ENSG00000095319:MISSENSE[ENST00000372577(c.4722G>C,caG>caC):ENSP00000361658(p.Gln1574His)];	NA	NA	NA	NA	NA	NA	NA	D	PrD	D	MODERATE	D	0.996	95	95	0	124	45	79	63.71
Smoker	42479	chr9	119093529	C	T	*PAPPA*	NONSENSE	ENSG00000182752:NONSENSE[ENST00000328252(c.3154C>T,Cga>Tga):ENSP00000330658(p.Arg1052Ter)];ENSG00000182752:NONSENSE[ENST00000534838(c.268C>T,Cga>Tga):ENSP00000441461(p.Arg90Ter)];	NA	NA	NA	NA	NA	NA	NA	NA	NA	NA	HIGH	NA	1	101	101	0	96	74	22	22.92
Smoker	42479	chr9	71395451	G	C	*PIP5K1B*	INTRONIC	ENSG00000107242:[ENST00000478500(c.-86+37878G>C)];ENSG00000107242:[ENST00000437200(c.-86+37878G>C)];ENSG00000107242:[ENST00000472907(n.654-5548G>C)];ENSG00000107242:[ENST00000377284(c.-86+37878G>C)];ENSG00000107242:[ENST00000440050(c.-86+37878G>C)];ENSG00000107242:[ENST00000541509(c.-85-37926G>C)];ENSG00000107242:[ENST00000265382(c.-86+37878G>C)];	NA	NA	.	4.95E-05	0	NA	NA	NA	NA	NA	MODIFIER	NA	NA	170	168	2	102	20	82	80.39
Smoker	42479	chr9	140123265	C	A	*RNF224*	SILENT	ENSG00000233198:SILENT[ENST00000445101(c.198C>A,ccC>ccA):ENSP00000406665(p.Pro66(=))];	NA	NA	chr9*140123264*140123266*CCT*C*rs59816380*1*0.9732*0.02676	NA	NA	NA	NA	NA	NA	NA	LOW	NA	NA	19	19	0	28	3	25	89.29
Smoker	42479	chrX	36091465	C	A	*CHDC2*	MISSENSE	ENSG00000176034:MISSENSE[ENST00000313548(c.400C>A,Cca>Aca):ENSP00000324767(p.Pro134Thr)];ENSG00000176034:MISSENSE[ENST00000378660(c.928C>A,Cca>Aca):ENSP00000367929(p.Pro310Thr)];	chrX*36091	chrX*36091	NA	NA	NA	NA	NA	D	PrD	D	MODERATE	NA	NA	110	109	0	124	14	110	88.71
Smoker	42479	chrX	16711375	C	T	*CTPS2*	INTRONIC	ENSG00000047230:[ENST00000359276(c.556-28G>A)];ENSG00000047230:[ENST00000443824(c.556-28G>A)];ENSG00000047230:[ENST00000380241(c.556-28G>A)];	NA	NA	NA	NA	NA	NA	NA	NA	NA	NA	MODIFIER	NA	NA	33	33	0	47	7	40	85.11
Smoker	42479	chrX	83128439	T	C	*CYLC1*	SILENT	ENSG00000183035:SILENT[ENST00000329312(c.723T>C,aaT>aaC):ENSP00000331556(p.Asn241(=))];	NA	NA	NA	NA	NA	NA	NA	NA	NA	NA	LOW	NA	NA	40	40	0	15	0	15	100
Smoker	42479	chrX	154255194	C	G	*F8*	5UTR	ENSG00000185010:[ENST00000423959(c.-102G>C)];	NA	NA	NA	NA	NA	NA	NA	NA	NA	NA	MODIFIER	NA	NA	28	28	0	50	31	19	38
Smoker	42479	chrX	154255194	C	G	*FUNDC2*	INTRONIC	ENSG00000165775:[ENST00000475165(n.242+239C>G)];ENSG00000165775:[ENST00000369498(c.-20C>G)];	NA	NA	NA	NA	NA	NA	NA	NA	NA	NA	MODIFIER	NA	NA	28	28	0	50	31	19	38
Smoker	42479	chrX	105183991	G	C	*NRK*	MISSENSE	ENSG00000123572:MISSENSE[ENST00000428173(c.3928G>C,Gat>Cat):ENSP00000438378(p.Asp1310His)];ENSG00000123572:MISSENSE[ENST00000243300(c.3925G>C,Gat>Cat):ENSP00000434830(p.Asp1309His)];	NA	NA	NA	NA	NA	NA	NA	D	PrD	D	MODERATE	N	0.197	96	96	0	89	13	76	85.39
Smoker	42479	chrX	128599732	G	T	*SMARCA1*	INTRONIC	ENSG00000102038:[ENST00000371122(c.2818-23C>A)];ENSG00000102038:[ENST00000371121(c.2782-23C>A)];ENSG00000102038:[ENST00000371123(c.2782-23C>A)];	NA	NA	NA	NA	NA	NA	NA	NA	NA	NA	MODIFIER	NA	NA	59	59	0	88	65	23	26.14
Smoker	42480	chr1	154562305	G	A	*ADAR*	MISSENSE	ENSG00000160710:MISSENSE[ENST00000368471(c.1711C>T,Cgc>Tgc):ENSP00000357456(p.Arg571Cys)];ENSG00000160710:MISSENSE[ENST00000368474(c.2596C>T,Cgc>Tgc):ENSP00000357459(p.Arg866Cys)];ENSG00000160710:MISSENSE[ENST00000529168(c.2503C>T,Cgc>Tgc):ENSP00000431794(p.Arg835Cys)];ENSG00000160710:MISSENSE[ENST00000292205(c.2725C>T,Cgc>Tgc):ENSP00000292205(p.Arg909Cys)];	NA	NA	NA	NA	NA	NA	NA	NA	PrD	NA	MODERATE	D	0.997	187	187	0	164	141	22	13.41
Smoker	42480	chr1	94620168	G	A	*ARHGAP29*	NONSENSE	ENSG00000137962:NONSENSE[ENST00000552844(c.3088C>T,Caa>Taa):ENSP00000449764(p.Gln1030Ter)];ENSG00000137962:EXONIC-NC[ENST00000546444(n.218C>T)];	NA	NA	chr1*94620168*94620168*G*T*rs1330862*1*0.9253*0.07468	NA	NA	NA	NA	NA	NA	NA	MODIFIER	NA	NA	73	73	0	92	77	15	16.3
Smoker	42480	chr1	154172976	T	C	*C1orf189*	MISSENSE	ENSG00000163263:MISSENSE[ENST00000368525(c.163A>G,Atg>Gtg):ENSP00000357511(p.Met55Val)];	NA	NA	0.0001997	NA	NA	0.0001997	0.001	T	BN	N	MODERATE	NA	NA	84	84	0	84	66	18	21.43
Smoker	42480	chr1	150252226	A	C	*C1orf54*	INTRONIC	ENSG00000118292:[ENST00000369099(c.*3+74A>C)];ENSG00000118292:[ENST00000369098(c.301-1020A>C)];ENSG00000118292:[ENST00000369102(c.*3+74A>C)];	NA	NA	NA	NA	NA	NA	NA	NA	NA	NA	MODIFIER	NA	NA	64	64	0	64	52	12	18.75
Smoker	42480	chr1	34673845	C	T	*C1orf94*	INTRONIC-SS-DNR	ENSG00000142698:[ENST00000488417(c.1524+10C>T)];ENSG00000142698:[ENST00000373374(c.954+10C>T)];	NA	NA	0.02077	0.00689	0.000283487	0.0207668	0	NA	NA	NA	MODIFIER	NA	NA	76	76	0	43	34	9	20.93
Smoker	42480	chr1	7798155	G	A	*CAMTA1*	SILENT	ENSG00000171735:SILENT[ENST00000303635(c.3795G>A,ctG>ctA):ENSP00000306522(p.Leu1265(=))];ENSG00000171735:SILENT[ENST00000495233(c.665G>A,ctG>ctA):ENSP00000451720(p.Leu222(=))];ENSG00000171735:SILENT[ENST00000439411(c.3795G>A,ctG>ctA):ENSP00000402561(p.Leu1265(=))];	NA	NA	NA	NA	NA	NA	NA	NA	NA	NA	LOW	NA	NA	148	148	0	159	140	19	11.95
Smoker	42480	chr1	206318346	A	T	*CTSE*	MISSENSE	ENSG00000196188:MISSENSE[ENST00000361052(c.104A>T,aAg>aTg):ENSP00000354337(p.Lys35Met)];ENSG00000196188:MISSENSE[ENST00000360218(c.104A>T,aAg>aTg):ENSP00000353350(p.Lys35Met)];ENSG00000196188:MISSENSE[ENST00000358184(c.104A>T,aAg>aTg):ENSP00000350911(p.Lys35Met)];ENSG00000196188:INTRONIC[ENST00000432969(c.-1+702A>T)];	NA	NA	NA	NA	NA	NA	NA	T	BN	N	MODERATE	NA	NA	147	147	0	89	63	25	28.09
Smoker	42480	chr1	44788518	G	A	*ERI3*	MISSENSE	ENSG00000117419:INTRONIC[ENST00000495828(n.626-3102C>T)];ENSG00000117419:INTRONIC[ENST00000452396(c.136-3102C>T)];ENSG00000117419:INTRONIC[ENST00000457571(c.484-3102C>T)];ENSG00000117419:INTRONIC[ENST00000537474(c.-42-3102C>T)];ENSG00000117419:INTRONIC[ENST00000372257(c.490-3102C>T)];ENSG00000117419:MISSENSE[ENST00000372259(c.23C>T,cCc>cTc):ENSP00000361333(p.Pro8Leu)];	NA	NA	NA	NA	NA	NA	NA	NA	NA	NA	MODIFIER	NA	NA	19	19	0	36	13	23	63.89
Smoker	42480	chr1	169497385	C	T	*F5*	INTRONIC	ENSG00000198734:[ENST00000367796(c.5435-53G>A)];ENSG00000198734:[ENST00000367797(c.5420-53G>A)];	NA	NA	0.0003994	NA	NA	0.0003994	0.001	NA	NA	NA	MODIFIER	NA	NA	104	104	0	105	74	31	29.52
Smoker	42480	chr1	75596421	A	C	*LHX8*	INTRONIC-SS-DNR	ENSG00000162624:[ENST00000294638(c.18+4A>C)];	NA	NA	NA	NA	NA	NA	NA	NA	NA	NA	MODIFIER	NA	NA	124	123	0	72	64	8	11.11
Smoker	42480	chr1	232940851	C	T	*MAP10*	MISSENSE	ENSG00000212916:MISSENSE[ENST00000418460(c.82C>T,Ctc>Ttc):ENSP00000403208(p.Leu28Phe)];	NA	NA	NA	NA	NA	NA	NA	NA	PoD	NA	MODERATE	NA	NA	209	209	0	141	119	21	14.89
Smoker	42480	chr1	76355107	G	T	*MSH4*	INTRONIC	ENSG00000057468:[ENST00000263187(c.2226+53G>T)];	NA	NA	NA	NA	NA	NA	NA	NA	NA	NA	MODIFIER	NA	NA	67	67	0	74	60	14	18.92

Mangalaparthi *et al.* , 2020. Mutational landscape of esophageal squamous cell carcinoma in an Indian cohort
Supplementary Table 4. List of somatic single nucleotide variants identified using whole exome sequencing of ESCC patients

Cohort	Sample	Chromosome	Genomic position	Reference allele	Alternate allele	Gene symbol	Variant type	Variant annotation	OncoMD	COSMIC	dbSNP	ExAC	ExAC_SAS	1000G	1000G_SAS	SIFT	Polyphen2	CONDEL	VEP	LRT	PhastCon7 Way	Total read (Adjacent normal)	Reference allel read support (Adjacent normal)	Alternative read support (Adjacent normal)	Total read (Tumor)	Reference allel read support (Tumor)	Alternative read support (Tumor)	Alternate allele frequency
Smoker	42480	chr1	163298017	G	A	*NUF2*	INTRONIC-SS-ACR	ENSG00000143228:INTRONIC-SS-ACR[ENST00000367900(c.199-1G>A)];ENSG00000143228:INTRONIC-SS-ACR[ENST00000524800(c.199-1G>A)];ENSG00000143228:INTRONIC-SS-ACR[ENST00000450453(c.199-1G>A)];ENSG00000143228:INTRONIC-SS-ACR[ENST00000442820(c.199-1G>A)];ENSG00000143228:INTRONIC-SS-ACR[ENST00000497990(c.199-1G>A)];ENSG00000143228:INTRONIC-SS-ACR[ENST00000534289(c.199-1G>A)];ENSG00000143228:INTRONIC-SS-ACR[ENST00000487578(n.446-1G>A)];ENSG00000143228:INTRONIC-SS-ACR[ENST00000271452(c.199-1G>A)];ENSG00000143228:INTRONIC-SS-ACR[ENST00000490881(n.312-1G>A)];	NA	NA	NA	NA	NA	NA	NA	NA	NA	NA	HIGH	NA	NA	85	85	0	129	121	7	5.43
Smoker	42480	chr1	77627087	G	A	*PIGK*	NONSENSE	ENSG00000142892:NONSENSE[ENST00000445065(c.424C>T,Caa>Taa):ENSP00000388854(p.Gln142Ter)];ENSG00000142892:NONSENSE[ENST00000370813(c.478C>T,Caa>Taa):ENSP00000359849(p.Gln160Ter)];ENSG00000142892:NONSENSE[ENST00000370812(c.706C>T,Caa>Taa):ENSP00000359848(p.Gln236Ter)];ENSG00000142892:NONSENSE[ENST00000359130(c.706C>T,Caa>Taa):ENSP00000352041(p.Gln236Ter)];ENSG00000142892:EXONIC-NC[ENST00000478391(n.502C>T)];ENSG00000142892:3UTR[ENST00000487906(c.*195C>T)];	NA	NA	NA	NA	NA	NA	NA	NA	NA	NA	MODIFIER	NA	NA	52	52	0	82	53	28	34.15
Smoker	42480	chr1	202533562	C	G	*PPP1R12B*	INTRONIC-SS-ACR	ENSG00000077157:[ENST00000391959(c.331-9C>G)];ENSG00000077157:[ENST00000367269(n.422-9C>G)];ENSG00000077157:[ENST00000367270(c.331-9C>G)];ENSG00000077157:[ENST00000491336(n.1158-9C>G)];ENSG00000077157:[ENST00000608999(c.2653-9C>G)];ENSG00000077157:[ENST00000336894(c.2653-9C>G)];ENSG00000077157:[ENST00000290419(n.1158-9C>G)];ENSG00000077157:[ENST00000466273(n.311-9C>G)];	NA	NA	NA	NA	NA	NA	NA	NA	NA	NA	MODIFIER	NA	NA	68	67	0	84	73	11	13.1
Smoker	42480	chr1	40544250	C	T	*PPT1*	SILENT	ENSG00000131238:SILENT[ENST00000433473(c.708G>A,gtG>gtA):ENSP00000394863(p.Val236(=))];ENSG00000131238:SILENT[ENST00000372779(c.795G>A,gtG>gtA):ENSP00000361865(p.Val265(=))];ENSG00000131238:SILENT[ENST00000530076(c.51G>A,gtG>gtA):ENSP00000434007(p.Val17(=))];ENSG00000131238:SILENT[ENST00000529905(c.708G>A,gtG>gtA):ENSP00000432053(p.Val236(=))];ENSG00000131238:SILENT[ENST00000449045(c.399G>A,gtG>gtA):ENSP00000392293(p.Val133(=))];ENSG00000131238:SILENT[ENST00000439754(c.393G>A,gtG>gtA):ENSP00000403207(p.Val131(=))];ENSG00000131238:SILENT[ENST00000527311(c.483G>A,gtG>gtA):ENSP00000436695(p.Val161(=))];ENSG00000131238:3UTR[ENST00000530704(c.*331G>A)];ENSG00000131238:EXONIC-NC[ENST00000372775(n.105G>A)];	NA	NA	NA	NA	NA	NA	NA	NA	NA	NA	LOW	NA	NA	286	286	0	254	241	13	5.12
Smoker	42480	chr1	75596421	A	C	*RP11-510C10.2*	INTRONIC	ENSG00000224127:[ENST00000446238(n.246+2077T>G)];	NA	NA	NA	NA	NA	NA	NA	NA	NA	NA	MODIFIER	NA	NA	124	123	0	72	64	8	11.11
Smoker	42480	chr1	75596421	A	C	*RP11-510C10.3*	EXONIC-NC	ENSG00000224149:[ENST00000427892(n.1441T>G)];	NA	NA	NA	NA	NA	NA	NA	NA	NA	NA	MODIFIER	NA	NA	124	123	0	72	64	8	11.11
Smoker	42480	chr1	161326622	C	T	*SDHC*	NONSENSE	ENSG00000143252:NONSENSE[ENST00000432287(c.295C>T,Cga>Tga):ENSP00000390558(p.Arg99Ter)];ENSG00000143252:NONSENSE[ENST00000392169(c.238C>T,Cga>Tga):ENSP00000376009(p.Arg80Ter)];ENSG00000143252:NONSENSE[ENST00000367975(c.397C>T,Cga>Tga):ENSP00000356953(p.Arg133Ter)];ENSG00000143252:EXONIC-NC[ENST00000470743(n.495C>T)];ENSG00000143252:INTRONIC[ENST00000513009(c.140-5497C>T)];ENSG00000143252:INTRONIC[ENST00000342751(c.242-5497C>T)];ENSG00000143252:INTRONIC[ENST00000513700(n.132-6018C>T)];ENSG00000143252:3UTR[ENST00000504963(c.*220C>T)];	NA	NA	NA	2.47E-05	0.000121124	NA	NA	NA	NA	NA	MODIFIER	NA	NA	249	249	0	305	268	37	12.13
Smoker	42480	chr1	108700078	G	C	*SLC25A24*	INTRONIC-SS-DNR	ENSG00000085491:[ENST00000565488(c.669+6C>G)];ENSG00000085491:[ENST00000370041(c.612+6C>G)];ENSG00000085491:[ENST00000264128(c.*248+6C>G)];	NA	NA	0.0001997	0.0001072	0	0.0001997	0	NA	NA	NA	LOW	NA	NA	184	184	0	139	84	55	39.57
Smoker	42480	chr1	153752246	T	G	*SLC27A3*	INTRONIC	ENSG00000143554:[ENST00000458027(c.1131-161T>G)];ENSG00000143554:[ENST00000524676(c.400-56T>G)];ENSG00000143554:[ENST00000483574(n.2098-56T>G)];ENSG00000143554:[ENST00000484014(n.1500-56T>G)];ENSG00000143554:[ENST00000271857(c.2260-56T>G)];ENSG00000143554:[ENST00000368660(n.293-56T>G)];ENSG00000143554:[ENST00000368659(n.274-56T>G)];ENSG00000143554:[ENST00000368661(c.2017-56T>G)];ENSG00000143554:[ENST00000468403(n.4118T>G)];	NA	NA	NA	NA	NA	NA	NA	NA	NA	NA	MODIFIER	NA	NA	83	83	0	46	40	6	13.04
Smoker	42480	chr1	77334185	C	G	*ST6GALNAC5*	MISSENSE	ENSG00000117069:MISSENSE[ENST00000477717(c.19C>G,Cat>Gat):ENSP00000417583(p.His7Asp)];ENSG00000117069:MISSENSE[ENST00000318803(c.19C>G,Cat>Gat):ENSP00000436263(p.His7Asp)];ENSG00000117069:EXONIC-NC[ENST00000480428(n.215C>G)];ENSG00000117069:EXONIC-NC[ENST00000496845(n.213C>G)];	NA	NA	NA	4.32E-05	0	NA	NA	NA	PrD	NA	MODERATE	NA	NA	117	115	2	74	37	37	50
Smoker	42480	chr1	175334223	G	T	*TNR*	MISSENSE	ENSG00000116147:MISSENSE[ENST00000367674(c.2510C>A,gCc>gAc):ENSP00000356646(p.Ala837Asp)];ENSG00000116147:MISSENSE[ENST00000263525(c.2510C>A,gCc>gAc):ENSP00000263525(p.Ala837Asp)];	NA	NA	NA	NA	NA	NA	NA	T	PoD	D	MODERATE	D	1	98	98	0	39	31	8	20.51
Smoker	42480	chr1	114964137	G	A	*TRIM33*	MISSENSE	ENSG00000197323:MISSENSE[ENST00000369543(c.1982C>T,tCt>tTt):ENSP00000358556(p.Ser661Phe)];ENSG00000197323:MISSENSE[ENST00000358465(c.1982C>T,tCt>tTt):ENSP00000351250(p.Ser661Phe)];ENSG00000197323:MISSENSE[ENST00000450349(c.806C>T,tCt>tTt):ENSP00000412077(p.Ser269Phe)];ENSG00000197323:MISSENSE[ENST00000448034(c.1192C>T,tCt>tTt):ENSP00000402333(p.Ser398Phe)];	NA	NA	NA	NA	NA	NA	NA	T	PoD	N	MODERATE	NA	NA	86	86	0	107	90	17	15.89

Supplementary Table 4. List of somatic single nucleotide variants identified using whole exome sequencing of ESCC patients

Cohort	Sample	Chromosome	Genomic position	Reference allele	Alternate allele	Gene symbol	Variant type	Variant annotation	OncoMD	COSMIC	dbSNP	ExAC	ExAC_SAS	1000G	1000G_SAS	SIFT	Polyphen2	CONDEL	VEP	LRT	PhastCon7 Way	Total read (Adjacent normal)	Reference allel read support (Adjacent normal)	Alternative read support (Adjacent normal)	Total read (Tumor)	Reference allel read support (Tumor)	Alternative read support (Tumor)	Alternate allele frequency
Smoker	42480	chr10	111860517	A	G	*ADD3*	MISSENSE	ENSG00000148700:EXONIC-NC[ENST00000487085(n.227A>G)];ENSG00000148700:EXONIC-NC[ENST00000495661(n.296A>G)];ENSG00000148700:EXONIC-NC[ENST00000496517(n.321A>G)];ENSG00000148700:EXONIC-NC[ENST00000468345(n.249A>G)];ENSG00000148700:EXONIC-NC[ENST00000473669(n.307A>G)];ENSG00000148700:EXONIC-NC[ENST00000468251(n.214A>G)];ENSG00000148700:EXONIC-NC[ENST00000497125(n.204A>G)];ENSG00000148700:EXONIC-NC[ENST00000475954(n.183A>G)];ENSG00000148700:MISSENSE[ENST00000360162(c.106A>G,Att>Gtt):ENSP00000353286(p.Ile36Val)];ENSG00000148700:MISSENSE[ENST00000356080(c.106A>G,Att>Gtt):ENSP00000348381(p.Ile36Val)];ENSG00000148700:MISSENSE[ENST00000277900(c.106A>G,Att>Gtt):ENSP00000277900(p.Ile36Val)];ENSG00000148700:INTRONIC[ENST00000486014(n.110+84A>G)];ENSG00000148700:INTRONIC[ENST00000484622(n.93-12018A>G)];	NA	NA	NA	NA	NA	NA	NA	NA	NA	NA	MODIFIER	NA	NA	161	161	0	134	86	48	35.82
Smoker	42480	chr10	98742888	C	T	*C10orf12*	MISSENSE	ENSG00000155640:MISSENSE[ENST00000286067(c.1741C>T,Cgc>Tgc):ENSP00000286067(p.Arg581Cys)];	NA	NA	NA	8.31E-06	0	NA	NA	T	BN	N	MODERATE	NA	NA	86	86	0	78	73	5	6.41
Smoker	42480	chr10	73550938	G	A	*CDH23*	SILENT	ENSG00000107736:SILENT[ENST00000224721(c.6114G>A,tcG>tcA):ENSP00000224721(p.Ser2038(=))];	NA	NA	NA	2.00E-05	7.30E-05	NA	NA	NA	NA	NA	LOW	NA	NA	76	76	0	41	36	5	12.2
Smoker	42480	chr10	410386	C	T	*DIP2C*	MISSENSE	ENSG00000151240:MISSENSE[ENST00000540204(c.368G>A,cGc>cAc):ENSP00000443826(p.Arg123His)];ENSG00000151240:MISSENSE[ENST00000280886(c.2405G>A,cGc>cAc):ENSP00000280886(p.Arg802His)];ENSG00000151240:3UTR[ENST00000381496(c.*320G>A)];	NA	NA	0.0001997	0.0004379	0	0.0001997	0	NA	NA	NA	MODIFIER	NA	NA	137	137	0	76	44	32	42.11
Smoker	42480	chr10	74992861	C	T	*DNAJC9*	INTRONIC	ENSG00000213551:[ENST00000469143(n.147+10740G>A)];	NA	NA	chr10*74992861*74992861*C*G,T*rs756665429*NA*NA*NA	4.61E-05	0.00012639	NA	NA	NA	NA	NA	MODIFIER	NA	NA	145	145	0	92	77	15	16.3
Smoker	42480	chr10	129245741	G	A	*DOCK1*	MISSENSE	ENSG00000150760:MISSENSE[ENST00000280333(c.5434G>A,Ggg>Agg):ENSP00000280333(p.Gly1812Arg)];	NA	NA	NA	8.60E-06	6.41E-05	NA	NA	T	PrD	N	MODERATE	D	0.985	176	176	0	88	70	18	20.45
Smoker	42480	chr10	127420577	G	A	*EDRF1*	INTRONIC	ENSG00000107938:[ENST00000368815(c.1129-1396G>A)];ENSG00000107938:[ENST00000530795(c.*16+83G>A)];ENSG00000107938:[ENST00000481600(c.*113-1396G>A)];ENSG00000107938:[ENST00000419769(c.1129-1396G>A)];ENSG00000107938:[ENST00000356792(c.1129-1396G>A)];ENSG00000107938:[ENST00000337623(c.1027-1396G>A)];ENSG00000107938:[ENST00000525091(n.1133-1396G>A)];	NA	NA	NA	NA	NA	NA	NA	NA	NA	NA	MODIFIER	NA	NA	50	50	0	66	56	10	15.15
Smoker	42480	chr10	74992861	C	T	*FAM149B1*	MISSENSE	ENSG00000138286:MISSENSE[ENST00000242505(c.1292C>T,aCg>aTg):ENSP00000242505(p.Thr431Met)];ENSG00000138286:MISSENSE[ENST00000445951(c.677C>T,aCg>aTg):ENSP00000402293(p.Thr226Met)];ENSG00000138286:MISSENSE[ENST00000475829(c.566C>T,aCg>aTg):ENSP00000476913(p.Thr189Met)];ENSG00000138286:MISSENSE[ENST00000372955(c.1090C>T,aCg>aTg):ENSP00000362046(p.Thr364Met)];ENSG00000138286:EXONIC-NC[ENST00000466261(n.395C>T)];ENSG00000138286:INTRONIC[ENST00000470798(n.79-1714C>T)];	NA	NA	chr10*74992861*74992861*C*G,T*rs756665429*NA*NA*NA	4.61E-05	0.00012639	NA	NA	NA	NA	NA	MODIFIER	NA	NA	145	145	0	92	77	15	16.3
Smoker	42480	chr10	78799315	G	A	*KCNMA1*	SILENT	ENSG00000156113:SILENT[ENST00000404857(c.1830C>T,ttC>ttT):ENSP00000385806(p.Phe610(=))];ENSG00000156113:SILENT[ENST00000406533(c.1830C>T,ttC>ttT):ENSP00000385552(p.Phe610(=))];ENSG00000156113:SILENT[ENST00000372408(c.1641C>T,ttC>ttT):ENSP00000361485(p.Phe547(=))];ENSG00000156113:SILENT[ENST00000372403(c.1682C>T,ttC>ttT):ENSP00000361480(p.Phe561(=))];ENSG00000156113:SILENT[ENST00000372440(c.1830C>T,ttC>ttT):ENSP00000361517(p.Phe610(=))];ENSG00000156113:SILENT[ENST00000286628(c.1830C>T,ttC>ttT):ENSP00000286628(p.Phe610(=))];ENSG00000156113:SILENT[ENST00000604624(c.1413C>T,ttC>ttT):ENSP00000473714(p.Phe471(=))];ENSG00000156113:SILENT[ENST00000372437(c.1635C>T,ttC>ttT):ENSP00000361514(p.Phe545(=))];ENSG00000156113:SILENT[ENST00000450795(c.307C>T,ttC>ttT):ENSP00000388370(p.Phe103(=))];ENSG00000156113:SILENT[ENST00000428546(c.281C>T,ttC>ttT):ENSP00000402254(p.Phe94(=))];ENSG00000156113:SILENT[ENST00000354353(c.1830C>T,ttC>ttT):ENSP00000346321(p.Phe610(=))];ENSG00000156113:SILENT[ENST00000457953(c.1752C>T,ttC>ttT):ENSP00000396608(p.Phe584(=))];ENSG00000156113:SILENT[ENST00000372443(c.1830C>T,ttC>ttT):ENSP00000361520(p.Phe610(=))];ENSG00000156113:SILENT[ENST00000286627(c.1830C>T,ttC>ttT):ENSP00000286627(p.Phe610(=))];ENSG00000156113:SILENT[ENST00000434208(c.865C>T,ttC>ttT):ENSP00000402150(p.Phe289(=))];ENSG00000156113:SILENT[ENST00000404771(c.1830C>T,ttC>ttT):ENSP00000385717(p.Phe610(=))];ENSG00000156113:SILENT[ENST00000372421(c.1750C>T,ttC>ttT):ENSP00000361498(p.Phe584(=))];	chr10*7879	chr10*7879	NA	4.96E-05	0	NA	NA	NA	NA	NA	LOW	NA	NA	222	222	0	154	126	28	18.18
Smoker	42480	chr10	90565792	G	A	*LIPM*	INTRONIC	ENSG00000173239:[ENST00000404743(c.148-2263G>A)];ENSG00000173239:[ENST00000539337(c.27+30G>A)];	NA	NA	NA	NA	NA	NA	NA	NA	NA	NA	MODIFIER	NA	NA	63	63	0	46	41	5	10.87
Smoker	42480	chr11	5443608	C	T	*AC104389.28*	INTRONIC	ENSG00000167355:[ENST00000418729(n.85-75468G>A)];ENSG00000167355:[ENST00000420726(n.303-75468G>A)];ENSG00000167355:[ENST00000415970(n.85-75468G>A)];ENSG00000167355:[ENST00000420465(n.46-75468G>A)];	NA	NA	NA	8.24E-06	0	NA	NA	D	BN	N	MODERATE	D	0.959	317	317	0	216	185	30	13.89
Smoker	42480	chr11	26681995	A	G	*ANO3*	3UTR	ENSG00000134343:[ENST00000531568(c.*4A>G)];ENSG00000134343:[ENST00000525139(c.*4A>G)];ENSG00000134343:[ENST00000256737(c.*4A>G)];ENSG00000134343:[ENST00000537978(c.*4A>G)];	NA	NA	NA	NA	NA	NA	NA	NA	NA	NA	MODIFIER	NA	NA	387	386	0	397	359	38	9.57
Smoker	42480	chr11	115085341	C	T	*CADM1*	MISSENSE	ENSG00000182985:MISSENSE[ENST00000452722(c.981G>A,atG>atA):ENSP00000395359(p.Met327Ile)];ENSG00000182985:MISSENSE[ENST00000331581(c.981G>A,atG>atA):ENSP00000329797(p.Met327Ile)];ENSG00000182985:MISSENSE[ENST00000536727(c.981G>A,atG>atA):ENSP00000440322(p.Met327Ile)];ENSG00000182985:MISSENSE[ENST00000545380(c.976G>A,atG>atA):ENSP00000442387(p.Met326Ile)];ENSG00000182985:MISSENSE[ENST00000542447(c.981G>A,atG>atA):ENSP00000439176(p.Met327Ile)];ENSG00000182985:MISSENSE[ENST00000537058(c.981G>A,atG>atA):ENSP00000439817(p.Met327Ile)];ENSG00000182985:EXONIC-NC[ENST00000541434(n.981G>A)];ENSG00000182985:EXONIC-NC[ENST00000441886(n.74G>A)];ENSG00000182985:EXONIC-NC[ENST00000537140(n.1110G>A)];	NA	NA	NA	NA	NA	NA	NA	T	BN	N	MODERATE	N	1	328	327	1	194	163	31	15.98

Mangalaparthi *et al.* , 2020. Mutational landscape of esophageal squamous cell carcinoma in an Indian cohort
Supplementary Table 4. List of somatic single nucleotide variants identified using whole exome sequencing of ESCC patients

Cohort	Sample	Chromosome	Genomic position	Reference allele	Alternate allele	Gene symbol	Variant type	Variant annotation	OncoMD	COSMIC	dbSNP	ExAC	ExAC_SAS	1000G	1000G_SAS	SIFT	Polyphen2	CONDEL	VEP	LRT	PhastCon7 Way	Total read (Adjacent normal)	Reference allel read support (Adjacent normal)	Alternative read support (Adjacent normal)	Total read (Tumor)	Reference allel read support (Tumor)	Alternative read support (Tumor)	Alternate allele frequency
Smoker	42480	chr11	61089794	C	T	*DDB1*	MISSENSE	ENSG00000167986:MISSENSE[ENST00000301764(c.1096G>A,Gac>Aac):ENSP00000301764(p.Asp366Asn)];ENSG00000167986:MISSENSE[ENST00000540166(c.1096G>A,Gac>Aac):ENSP00000440269(p.Asp366Asn)];ENSG00000167986:MISSENSE[ENST00000541513(c.541G>A,Gac>Aac):ENSP00000442660(p.Asp181Asn)];ENSG00000167986:MISSENSE[ENST00000535174(c.445G>A,Gac>Aac):ENSP00000446044(p.Asp149Asn)];ENSG00000167986:MISSENSE[ENST00000539739(c.253G>A,Gac>Aac):ENSP00000445563(p.Asp85Asn)];ENSG00000167986:MISSENSE[ENST00000535967(c.49G>A,Gac>Aac):ENSP00000437713(p.Asp17Asn)];ENSG00000167986:EXONIC-NC[ENST00000545930(n.738G>A)];ENSG00000167986:EXONIC-NC[ENST00000540784(n.463G>A)];ENSG00000167986:EXONIC-NC[ENST00000539712(n.551G>A)];ENSG00000167986:INTRONIC[ENST00000450997(c.210+9221G>A)];ENSG00000167986:INTRONIC[ENST00000543658(c.210+9221G>A)];	NA	NA	NA	NA	NA	NA	NA	NA	NA	NA	MODIFIER	NA	NA	72	71	0	80	72	8	10
Smoker	42480	chr11	5443608	C	T	*HBE1*	INTRONIC	ENSG00000213931:[ENST00000396895(c.-267+83191G>A)];ENSG00000213931:[ENST00000380237(c.-310+83191G>A)];	NA	NA	NA	8.24E-06	0	NA	NA	D	BN	N	MODERATE	D	0.959	317	317	0	216	185	30	13.89
Smoker	42480	chr11	5443608	C	T	*HBG2*	INTRONIC	ENSG00000196565:[ENST00000380252(c.62+82973G>A)];ENSG00000196565:[ENST00000380259(c.-564-76479G>A)];	NA	NA	NA	8.24E-06	0	NA	NA	D	BN	N	MODERATE	D	0.959	317	317	0	216	185	30	13.89
Smoker	42480	chr11	134054772	C	T	*NCAPD3*	INTRONIC	ENSG00000151503:[ENST00000534548(c.2335+26G>A)];ENSG00000151503:[ENST00000525964(c.2335+26G>A)];ENSG00000151503:[ENST00000534532(c.2335+26G>A)];	NA	NA	NA	NA	NA	NA	NA	NA	NA	NA	MODIFIER	NA	NA	130	130	0	84	62	20	23.81
Smoker	42480	chr11	48510724	C	A	*OR4A47*	MISSENSE	ENSG00000237388:MISSENSE[ENST00000446524(c.380C>A,cCc>cAc):ENSP00000412752(p.Pro127His)];	NA	NA	NA	NA	NA	NA	NA	D_lc	PrD	D	MODERATE	D	0.943	247	245	1	245	209	36	14.69
Smoker	42480	chr11	50003804	C	A	*OR4C12*	MISSENSE	ENSG00000221954:MISSENSE[ENST00000335238(c.234G>T,aaG>aaT):ENSP00000334418(p.Lys78Asn)];	chr11*5000	chr11*5000	chr11*50003804*50003804*C*T*rs372332230*NA*NA*NA	NA	NA	NA	NA	D_lc	PoD	D	MODERATE	U	0.972	223	223	0	235	218	17	7.23
Smoker	42480	chr11	5443608	C	T	*OR51Q1*	MISSENSE	ENSG00000167360:MISSENSE[ENST00000300778(c.178C>T,Cgc>Tgc):ENSP00000300778(p.Arg60Cys)];	NA	NA	NA	8.24E-06	0	NA	NA	D	BN	N	MODERATE	D	0.959	317	317	0	216	185	30	13.89
Smoker	42480	chr11	134054772	C	T	*RP11-700F16.3*	INTRONIC	ENSG00000255348:[ENST00000531710(n.97-1052C>T)];	NA	NA	NA	NA	NA	NA	NA	NA	NA	NA	MODIFIER	NA	NA	130	130	0	84	62	20	23.81
Smoker	42480	chr11	116767099	C	T	*SIK3*	INTRONIC-SS-ACR	ENSG00000160584:[ENST00000434315(c.265-7G>A)];ENSG00000160584:[ENST00000497049(n.469-7G>A)];ENSG00000160584:[ENST00000445177(c.722-7G>A)];ENSG00000160584:[ENST00000292055(c.568-7G>A)];ENSG00000160584:[ENST00000446921(c.742-7G>A)];ENSG00000160584:[ENST00000415541(c.*266-7G>A)];ENSG00000160584:[ENST00000375300(c.742-7G>A)];ENSG00000160584:[ENST00000413553(c.449-7G>A)];ENSG00000160584:[ENST00000542607(c.568-7G>A)];ENSG00000160584:[ENST00000375288(c.-1138-7G>A)];	NA	NA	NA	NA	NA	NA	NA	NA	NA	NA	LOW	NA	NA	49	49	0	38	31	7	18.42
Smoker	42480	chr11	125462190	C	G	*STT3A*	INTRONIC	ENSG00000134910:[ENST00000527606(c.-36+408C>G)];	NA	NA	NA	NA	NA	NA	NA	NA	NA	NA	MODIFIER	NA	NA	148	148	0	87	71	15	17.24
Smoker	42480	chr11	125462190	C	G	*STT3A-AS1*	INTRONIC	ENSG00000254671:[ENST00000532714(n.161+123G>C)];ENSG00000254671:[ENST00000530526(n.151+123G>C)];	NA	NA	NA	NA	NA	NA	NA	NA	NA	NA	MODIFIER	NA	NA	148	148	0	87	71	15	17.24
Smoker	42480	chr11	43436195	G	A	*TTC17*	MISSENSE	ENSG00000052841:MISSENSE[ENST00000039989(c.2120G>A,gGg>gAg):ENSP00000039989(p.Gly707Glu)];ENSG00000052841:MISSENSE[ENST00000299240(c.2120G>A,gGg>gAg):ENSP00000299240(p.Gly707Glu)];ENSG00000052841:EXONIC-NC[ENST00000526774(n.2030G>A)];	chr11*4343	NA	chr11*43436195*43436195*G*C*rs267602876*NA*NA*NA	NA	NA	NA	NA	NA	NA	NA	MODIFIER	NA	NA	169	169	0	198	161	36	18.18
Smoker	42480	chr11	43513659	C	T	*TTC17*	SILENT	ENSG00000052841:EXONIC-NC[ENST00000533072(n.1392C>T)];ENSG00000052841:EXONIC-NC[ENST00000525135(n.993C>T)];ENSG00000052841:EXONIC-NC[ENST00000529140(n.652C>T)];ENSG00000052841:EXONIC-NC[ENST00000525543(n.1933C>T)];ENSG00000052841:SILENT[ENST00000418561(c.332C>T,atC>atT):ENSP00000407314(p.Ile111(=))];ENSG00000052841:SILENT[ENST00000039989(c.3240C>T,atC>atT):ENSP00000039989(p.Ile1080(=))];	chr11*4351	chr11*4351	NA	NA	NA	NA	NA	NA	NA	NA	MODIFIER	NA	NA	180	180	0	134	115	19	14.18
Smoker	42480	chr11	118240117	C	G	*UBE4A*	INTRONIC	ENSG00000110344:[ENST00000252108(c.296-21C>G)];ENSG00000110344:[ENST00000431736(c.296-21C>G)];	NA	NA	NA	NA	NA	NA	NA	NA	NA	NA	MODIFIER	NA	NA	130	130	0	134	79	54	40.3
Smoker	42480	chr12	2602493	G	A	*CACNA1C*	MISSENSE	ENSG00000151067:MISSENSE[ENST00000399644(c.1054G>A,Gcc>Acc):ENSP00000382552(p.Ala352Thr)];ENSG00000151067:MISSENSE[ENST00000399595(c.1054G>A,Gcc>Acc):ENSP00000382504(p.Ala352Thr)];ENSG00000151067:MISSENSE[ENST00000406454(c.1054G>A,Gcc>Acc):ENSP00000385896(p.Ala352Thr)];ENSG00000151067:MISSENSE[ENST00000399617(c.1054G>A,Gcc>Acc):ENSP00000382526(p.Ala352Thr)];ENSG00000151067:MISSENSE[ENST00000347598(c.1054G>A,Gcc>Acc):ENSP00000266376(p.Ala352Thr)];ENSG00000151067:MISSENSE[ENST00000402845(c.1054G>A,Gcc>Acc):ENSP00000385724(p.Ala352Thr)];ENSG00000151067:MISSENSE[ENST00000327702(c.1054G>A,Gcc>Acc):ENSP00000329877(p.Ala352Thr)];ENSG00000151067:MISSENSE[ENST00000399634(c.1054G>A,Gcc>Acc):ENSP00000382542(p.Ala352Thr)];ENSG00000151067:MISSENSE[ENST00000399641(c.1054G>A,Gcc>Acc):ENSP00000382549(p.Ala352Thr)];ENSG00000151067:MISSENSE[ENST00000399603(c.1054G>A,Gcc>Acc):ENSP00000382512(p.Ala352Thr)];ENSG00000151067:MISSENSE[ENST00000399629(c.1054G>A,Gcc>Acc):ENSP00000382537(p.Ala352Thr)];ENSG00000151067:MISSENSE[ENST00000480911(c.1054G>A,Gcc>Acc):ENSP00000437936(p.Ala352Thr)];ENSG00000151067:MISSENSE[ENST00000399601(c.1054G>A,Gcc>Acc):ENSP00000382510(p.Ala352Thr)];ENSG00000151067:MISSENSE[ENST00000399597(c.1054G>A,Gcc>Acc):ENSP00000382506(p.Ala352Thr)];ENSG00000151067:MISSENSE[ENST00000399655(c.1054G>A,Gcc>Acc):ENSP00000382563(p.Ala352Thr)];ENSG00000151067:MISSENSE[ENST00000399606(c.1054G>A,Gcc>Acc):ENSP00000382515(p.Ala352Thr)];ENSG00000151067:MISSENSE[ENST00000399637(c.1054G>A,Gcc>Acc):ENSP00000382546(p.Ala352Thr)];ENSG00000151067:MISSENSE[ENST00000335762(c.1054G>A,Gcc>Acc):ENSP00000336982(p.Ala352Thr)];ENSG00000151067:MISSENSE[ENST00000399621(c.1054G>A,Gcc>Acc):ENSP00000382530(p.Ala352Thr)];ENSG00000151067:MISSENSE[ENST00000399638(c.1054G>A,Gcc>Acc):ENSP00000382547(p.Ala352Thr)];ENSG00000151067:MISSENSE[ENST00000399591(c.1054G>A,Gcc>Acc):ENSP00000382500(p.Ala352Thr)];ENSG00000151067:MISS	chr12*2602	chr12*2602	NA	NA	NA	NA	NA	D	PrD	D	MODERATE	D	1	162	162	0	75	49	26	34.67

Supplementary Table 4. List of somatic single nucleotide variants identified using whole exome sequencing of ESCC patients

Cohort	Sample	Chromosome	Genomic position	Reference allele	Alternate allele	Gene symbol	Variant type	Variant annotation	OncoMD	COSMIC	dbSNP	ExAC	ExAC_SAS	1000G	1000G_SAS	SIFT	Polyphen2	CONDEL	VEP	LRT	PhastCon7 Way	Total read (Adjacent normal)	Reference allel read support (Adjacent normal)	Alternative read support (Adjacent normal)	Total read (Tumor)	Reference allel read support (Tumor)	Alternative read support (Tumor)	Alternate allele frequency
Smoker	42480	chr12	2077018	A	T	*DCP1B*	SILENT	ENSG00000151065:EXONIC-NC[ENST00000537725(n.374T>A)];ENSG00000151065:5UTR[ENST00000540622(c.-1T>A)];ENSG00000151065:SILENT[ENST00000280665(c.378T>A,ctT>ctA):ENSP00000280665(p.Leu126(=))];ENSG00000151065:SILENT[ENST00000397173(c.72T>A,ctT>ctA):ENSP00000380358(p.Leu24(=))];ENSG00000151065:3UTR[ENST00000543381(c.*144T>A)];ENSG00000151065:INTRONIC[ENST00000541700(n.437-14564T>A)];	NA	NA	NA	NA	NA	NA	NA	NA	NA	NA	MODIFIER	NA	NA	58	58	0	77	71	6	7.79
Smoker	42480	chr12	49225068	C	A	*DDX23*	MISSENSE	ENSG00000174243:MISSENSE[ENST00000308025(c.2096G>T,gGc>gTc):ENSP00000310723(p.Gly699Val)];ENSG00000174243:EXONIC-NC[ENST00000551331(n.635G>T)];ENSG00000174243:INTRONIC[ENST00000550834(c.411-770G>T)];	NA	NA	NA	NA	NA	NA	NA	NA	NA	NA	MODIFIER	NA	NA	249	249	0	160	138	22	13.75
Smoker	42480	chr12	30830048	A	G	*IPO8*	MISSENSE	ENSG00000133704:INTRONIC[ENST00000256079(c.640-527T>C)];ENSG00000133704:MISSENSE[ENST00000542464(c.13T>C,Tgt>Cgt):ENSP00000439556(p.Cys5Arg)];ENSG00000133704:5UTR[ENST00000544829(c.-195T>C)];	NA	NA	NA	NA	NA	NA	NA	NA	NA	NA	MODIFIER	NA	NA	226	222	2	204	175	29	14.22
Smoker	42480	chr12	108957970	G	A	*ISCU*	INTRONIC	ENSG00000136003:[ENST00000431221(c.115-85G>A)];ENSG00000136003:[ENST00000338291(c.39+39G>A)];ENSG00000136003:[ENST00000547005(c.115-85G>A)];ENSG00000136003:[ENST00000311893(c.115-85G>A)];ENSG00000136003:[ENST00000539593(c.115-85G>A)];ENSG00000136003:[ENST00000392807(c.39+39G>A)];ENSG00000136003:[ENST00000552072(n.137-85G>A)];ENSG00000136003:[ENST00000539580(c.*69+39G>A)];ENSG00000136003:[ENST00000535729(c.115-85G>A)];ENSG00000136003:[ENST00000535405(n.255G>A)];ENSG00000136003:[ENST00000545932(n.1613G>A)];	NA	NA	NA	NA	NA	NA	NA	NA	NA	NA	MODIFIER	NA	NA	235	235	0	155	132	23	14.84
Smoker	42480	chr12	49446730	G	A	*KMT2D*	SILENT	ENSG00000167548:SILENT[ENST00000301067(c.1080C>T,tcC>tcT):ENSP00000301067(p.Ser360(=))];	NA	NA	NA	6.63E-05	6.06E-05	NA	NA	NA	NA	NA	LOW	NA	NA	131	131	0	53	47	6	11.32
Smoker	42480	chr12	116446791	C	A	*MED13L*	MISSENSE	ENSG00000123066:MISSENSE[ENST00000281928(c.1427G>T,gGa>gTa):ENSP00000281928(p.Gly476Val)];ENSG00000123066:EXONIC-NC[ENST00000549755(n.160G>T)];	NA	NA	NA	NA	NA	NA	NA	NA	NA	NA	MODIFIER	NA	NA	285	284	0	208	177	31	14.9
Smoker	42480	chr12	54963005	C	T	*PDE1B*	MISSENSE	ENSG00000123360:MISSENSE[ENST00000538346(c.142C>T,Cgg>Tgg):ENSP00000442559(p.Arg48Trp)];ENSG00000123360:MISSENSE[ENST00000243052(c.265C>T,Cgg>Tgg):ENSP00000243052(p.Arg89Trp)];ENSG00000123360:MISSENSE[ENST00000550620(c.205C>T,Cgg>Tgg):ENSP00000448519(p.Arg69Trp)];ENSG00000123360:INTRONIC[ENST00000394277(n.309-60C>T)];ENSG00000123360:INTRONIC[ENST00000542335(n.252-1020C>T)];ENSG00000123360:EXONIC-NC[ENST00000548855(n.427C>T)];ENSG00000123360:SILENT[ENST00000550285(c.327C>T,tgC>tgT):ENSP00000447354(p.Cys109(=))];	chr12*5496	chr12*5496	NA	NA	NA	NA	NA	NA	NA	NA	LOW	NA	NA	95	95	0	61	55	6	9.84
Smoker	42480	chr12	113837055	G	A	*SDS*	INTRONIC	ENSG00000135094:[ENST00000546639(n.275-99C>T)];ENSG00000135094:[ENST00000553112(n.275-99C>T)];ENSG00000135094:[ENST00000547342(c.436-99C>T)];ENSG00000135094:[ENST00000552280(c.153+306C>T)];ENSG00000135094:[ENST00000257549(c.154-99C>T)];	NA	NA	NA	NA	NA	NA	NA	NA	NA	NA	MODIFIER	NA	NA	123	123	0	55	49	6	10.91
Smoker	42480	chr12	21445208	G	A	*SLCO1A2*	SILENT	ENSG00000084453:SILENT[ENST00000390670(c.1494C>T,tgC>tgT):ENSP00000375088(p.Cys498(=))];ENSG00000084453:SILENT[ENST00000307378(c.1500C>T,tgC>tgT):ENSP00000305974(p.Cys500(=))];ENSG00000084453:SILENT[ENST00000537524(c.1104C>T,tgC>tgT):ENSP00000439401(p.Cys368(=))];ENSG00000084453:SILENT[ENST00000452078(c.1500C>T,tgC>tgT):ENSP00000393973(p.Cys500(=))];ENSG00000084453:SILENT[ENST00000458504(c.1104C>T,tgC>tgT):ENSP00000394854(p.Cys368(=))];ENSG00000084453:EXONIC-NC[ENST00000480394(n.3014C>T)];ENSG00000084453:EXONIC-NC[ENST00000463718(n.1791C>T)];ENSG00000084453:3UTR[ENST00000544020(c.*1079C>T)];ENSG00000084453:3UTR[ENST00000544290(c.*1079C>T)];	chr12*2144	chr12*2144	0.0001997	1.66E-05	0	0.0001997	0	NA	NA	NA	MODIFIER	NA	NA	138	137	0	157	149	8	5.1
Smoker	42480	chr12	104713352	C	T	*TXNRD1*	SILENT	ENSG00000198431:SILENT[ENST00000354940(c.528C>T,taC>taT):ENSP00000347020(p.Tyr176(=))];ENSG00000198431:SILENT[ENST00000388854(c.684C>T,taC>taT):ENSP00000373506(p.Tyr228(=))];ENSG00000198431:SILENT[ENST00000529546(c.414C>T,taC>taT):ENSP00000434919(p.Tyr138(=))];ENSG00000198431:SILENT[ENST00000526950(c.735C>T,taC>taT):ENSP00000432812(p.Tyr245(=))];ENSG00000198431:SILENT[ENST00000526691(c.684C>T,taC>taT):ENSP00000435929(p.Tyr228(=))];ENSG00000198431:SILENT[ENST00000524698(c.528C>T,taC>taT):ENSP00000433425(p.Tyr176(=))];ENSG00000198431:SILENT[ENST00000526390(c.660C>T,taC>taT):ENSP00000435123(p.Tyr220(=))];ENSG00000198431:SILENT[ENST00000429002(c.978C>T,taC>taT):ENSP00000412045(p.Tyr326(=))];ENSG00000198431:SILENT[ENST00000427956(c.873C>T,taC>taT):ENSP00000393328(p.Tyr291(=))];ENSG00000198431:SILENT[ENST00000397736(c.660C>T,taC>taT):ENSP00000380844(p.Tyr220(=))];ENSG00000198431:SILENT[ENST00000503506(c.528C>T,taC>taT):ENSP00000421934(p.Tyr176(=))];ENSG00000198431:SILENT[ENST00000525566(c.978C>T,taC>taT):ENSP00000434516(p.Tyr326(=))];ENSG00000198431:SILENT[ENST00000527688(c.528C>T,taC>taT):ENSP00000433935(p.Tyr176(=))];ENSG00000198431:SILENT[ENST00000542918(c.678C>T,taC>taT):ENSP00000440978(p.Tyr226(=))];ENSG00000198431:SILENT[ENST00000540716(c.414C>T,taC>taT):ENSP00000442709(p.Tyr138(=))];ENSG00000198431:SILENT[ENST00000378070(c.825C>T,taC>taT):ENSP00000367310(p.Tyr275(=))];ENSG00000198431:INTRONIC[ENST00000529751(c.-96-5763C>T)];	NA	NA	NA	NA	NA	NA	NA	NA	NA	NA	LOW	NA	1	118	118	0	121	103	18	14.88
Smoker	42480	chr13	31891664	A	G	*B3GALTL*	INTRONIC	ENSG00000187676:[ENST00000343307(c.1065-39A>G)];	NA	NA	NA	NA	NA	NA	NA	NA	NA	NA	MODIFIER	NA	NA	169	169	0	106	89	17	16.04
Smoker	42480	chr13	49848402	G	A	*CDADC1*	INTRONIC	ENSG00000102543:[ENST00000444959(c.407-25G>A)];ENSG00000102543:[ENST00000251108(c.1001-25G>A)];	NA	NA	NA	NA	NA	NA	NA	NA	NA	NA	MODIFIER	NA	NA	25	25	0	39	34	5	12.82
Smoker	42480	chr13	110802741	C	T	*COL4A1*	MISSENSE	ENSG00000187498:MISSENSE[ENST00000375820(c.4979G>A,aGc>aAc):ENSP00000364979(p.Ser1660Asn)];	NA	NA	NA	NA	NA	NA	NA	NA	UN	NA	MODERATE	D	0.999	338	337	0	218	183	35	16.06
Smoker	42480	chr13	26620873	C	T	*SHISA2*	SILENT	ENSG00000180730:SILENT[ENST00000319420(c.666G>A,acG>acA):ENSP00000313079(p.Thr222(=))];	NA	NA	chr13*26620873*26620873*C*G,T*rs376149775*NA*NA*NA	NA	NA	NA	NA	NA	NA	NA	LOW	NA	NA	292	292	0	164	131	33	20.12

Mangalaparthi *et al.* , 2020. Mutational landscape of esophageal squamous cell carcinoma in an Indian cohort
Supplementary Table 4. List of somatic single nucleotide variants identified using whole exome sequencing of ESCC patients

Cohort	Sample	Chromosome	Genomic position	Reference allele	Alternate allele	Gene symbol	Variant type	Variant annotation	OncoMD	COSMIC	dbSNP	ExAC	ExAC_SAS	1000G	1000G_SAS	SIFT	Polyphen2	CONDEL	VEP	LRT	PhastCon7 Way	Total read (Adjacent normal)	Reference allel read support (Adjacent normal)	Alternative read support (Adjacent normal)	Total read (Tumor)	Reference allel read support (Tumor)	Alternative read support (Tumor)	Alternate allele frequency
Smoker	42480	chr13	46288276	G	A	*SPERT*	SILENT	ENSG00000174015:SILENT[ENST00000378966(c.1008G>A,gaG>gaA):ENSP00000368249(p.Glu336(=))];ENSG00000174015:SILENT[ENST00000310521(c.1116G>A,gaG>gaA):ENSP00000309189(p.Glu372(=))];	NA	NA	NA	NA	NA	NA	NA	NA	NA	NA	LOW	NA	0.996	82	82	0	56	48	8	14.29
Smoker	42480	chr14	44975261	C	T	*FSCB*	SILENT	ENSG00000189139:SILENT[ENST00000340446(c.930G>A,gcG>gcA):ENSP00000344579(p.Ala310(=))];	NA	NA	chr14*44975261*44975261*C*G,T*rs773036021*NA*NA*NA	8.25E-06	6.06E-05	NA	NA	NA	NA	NA	LOW	NA	NA	112	112	0	104	83	21	20.19
Smoker	42480	chr14	42356919	G	T	*LRFN5*	MISSENSE	ENSG00000165379:MISSENSE[ENST00000298119(c.1091G>T,gGg>gTg):ENSP00000298119(p.Gly364Val)];ENSG00000165379:MISSENSE[ENST00000554120(c.1091G>T,gGg>gTg):ENSP00000451897(p.Gly364Val)];ENSG00000165379:MISSENSE[ENST00000554171(c.1091G>T,gGg>gTg):ENSP00000451067(p.Gly364Val)];	NA	NA	NA	NA	NA	NA	NA	D	PrD	D	MODERATE	D	0.999	212	212	0	236	153	82	34.75
Smoker	42480	chr14	71048114	G	A	*MED6*	3UTR	ENSG00000133997:[ENST00000554963(c.*79C>T)];	NA	NA	NA	NA	NA	NA	NA	NA	NA	NA	MODIFIER	NA	NA	65	65	0	77	68	9	11.69
Smoker	42480	chr14	24476433	C	G	*NA*	INTERGENIC		NA	NA	NA	NA	NA	NA	NA	NA	NA	NA	MODIFIER	NA	NA	43	42	1	18	12	6	33.33
Smoker	42480	chr14	24845335	G	A	*NFATC4*	INTRONIC	ENSG00000100968:[ENST00000554050(c.2056+28G>A)];ENSG00000100968:[ENST00000422617(c.2020+28G>A)];ENSG00000100968:[ENST00000250373(c.2056+28G>A)];ENSG00000100968:[ENST00000554473(c.661+28G>A)];ENSG00000100968:[ENST00000555590(c.2095+28G>A)];ENSG00000100968:[ENST00000556169(c.2020+28G>A)];ENSG00000100968:[ENST00000553469(c.2152+28G>A)];ENSG00000100968:[ENST00000539237(c.2152+28G>A)];ENSG00000100968:[ENST00000554661(c.1846+28G>A)];ENSG00000100968:[ENST00000555167(c.661+28G>A)];ENSG00000100968:[ENST00000555453(c.2020+28G>A)];ENSG00000100968:[ENST00000553708(c.2056+28G>A)];ENSG00000100968:[ENST00000557451(c.1846+28G>A)];ENSG00000100968:[ENST00000413692(c.2245+28G>A)];ENSG00000100968:[ENST00000556279(c.2152+28G>A)];ENSG00000100968:[ENST00000554655(n.2712+28G>A)];ENSG00000100968:[ENST00000553879(c.1846+28G>A)];ENSG00000100968:[ENST00000557767(c.-81+28G>A)];ENSG00000100968:[ENST00000424781(c.2095+28G>A)];ENSG00000100968:[ENST00000554591(c.2245+28G>A)];ENSG00000100968:[ENST00000556759(c.661+28G>A)];ENSG00000100968:[ENST00000555821(n.28+28G>A)];ENSG00000100968:[ENST00000555802(c.-81+28G>A)];ENSG00000100968:[ENST00000555393(c.-81+28G>A)];ENSG00000100968:[ENST00000554966(c.2095+28G>A)];ENSG00000100968:[ENST00000554344(c.1846+28G>A)];	NA	NA	NA	NA	NA	NA	NA	NA	NA	NA	MODIFIER	NA	NA	123	123	0	44	29	15	34.09
Smoker	42480	chr14	27064628	C	A	*NOVA1*	MISSENSE	ENSG00000139910:MISSENSE[ENST00000574031(c.268G>T,Gat>Tat):ENSP00000459545(p.Asp90Tyr)];ENSG00000139910:MISSENSE[ENST00000547619(c.268G>T,Gat>Tat):ENSP00000448157(p.Asp90Tyr)];ENSG00000139910:MISSENSE[ENST00000344429(c.268G>T,Gat>Tat):ENSP00000342387(p.Asp90Tyr)];ENSG00000139910:MISSENSE[ENST00000465357(c.268G>T,Gat>Tat):ENSP00000447391(p.Asp90Tyr)];ENSG00000139910:MISSENSE[ENST00000549571(c.157G>T,Gat>Tat):ENSP00000449185(p.Asp53Tyr)];ENSG00000139910:MISSENSE[ENST00000483536(c.268G>T,Gat>Tat):ENSP00000448956(p.Asp90Tyr)];ENSG00000139910:MISSENSE[ENST00000539517(c.268G>T,Gat>Tat):ENSP00000438875(p.Asp90Tyr)];ENSG00000139910:MISSENSE[ENST00000449198(c.145G>T,Gat>Tat):ENSP00000408914(p.Asp49Tyr)];ENSG00000139910:MISSENSE[ENST00000546546(c.52G>T,Gat>Tat):ENSP00000448144(p.Asp18Tyr)];ENSG00000139910:5UTR[ENST00000549146(c.-99G>T)];ENSG00000139910:5UTR[ENST00000267422(c.-201G>T)];ENSG00000139910:5UTR[ENST00000347476(c.-145G>T)];ENSG00000139910:EXONIC-NC[ENST00000551754(n.40G>T)];ENSG00000139910:EXONIC-NC[ENST00000547415(n.57G>T)];	chr14*2706	chr14*2706	NA	NA	NA	NA	NA	NA	NA	NA	MODIFIER	NA	NA	327	326	1	290	268	19	6.55
Smoker	42480	chr14	77706951	G	A	*TMEM63C*	MISSENSE-SS-PRX	ENSG00000165548:[ENST00000298351(c.1064G>A,cGt>cAt):ENSP00000298351(p.Arg355His)];	NA	NA	NA	NA	NA	NA	NA	T	BN	N	MODERATE	N	0.004	101	101	0	57	47	10	17.54
Smoker	42480	chr15	73044827	T	G	*ADPGK*	MISSENSE	ENSG00000159322:MISSENSE[ENST00000456471(c.524A>C,aAg>aCg):ENSP00000397694(p.Lys175Thr)];ENSG00000159322:MISSENSE[ENST00000311669(c.1346A>C,aAg>aCg):ENSP00000312250(p.Lys449Thr)];ENSG00000159322:3UTR[ENST00000569693(c.*623A>C)];ENSG00000159322:3UTR[ENST00000567941(c.*1322A>C)];ENSG00000159322:3UTR[ENST00000563907(c.*372A>C)];ENSG00000159322:3UTR[ENST00000569534(c.*718A>C)];ENSG00000159322:EXONIC-NC[ENST00000562621(n.3462A>C)];	NA	NA	NA	NA	NA	NA	NA	T	BN	N	MODERATE	N	0.938	165	165	0	159	116	43	27.04
Smoker	42480	chr15	101717920	G	C	*CHSY1*	SILENT	ENSG00000131873:SILENT[ENST00000254190(c.2082C>G,ggC>ggG):ENSP00000254190(p.Gly694(=))];ENSG00000131873:EXONIC-NC[ENST00000543813(n.1335C>G)];	NA	NA	NA	NA	NA	NA	NA	NA	NA	NA	MODIFIER	NA	NA	175	174	0	130	123	7	5.38
Smoker	42480	chr15	30336962	G	A	*CTD-2022H16.3*	EXONIC-NC	ENSG00000260174:[ENST00000569699(n.136G>A)];	NA	NA	NA	NA	NA	NA	NA	NA	NA	NA	MODIFIER	NA	NA	367	366	1	244	220	24	9.84
Smoker	42480	chr15	84759086	T	C	*EFTUD1P1*	INTRONIC	ENSG00000259404:[ENST00000558187(n.412+41T>C)];ENSG00000259404:[ENST00000560401(n.412+41T>C)];ENSG00000259404:[ENST00000558550(n.69+41T>C)];	NA	NA	NA	NA	NA	NA	NA	NA	NA	NA	MODIFIER	NA	NA	254	254	0	243	231	12	4.94
Smoker	42480	chr15	72461360	C	T	*GRAMD2*	EXONIC-NC	ENSG00000175318:[ENST00000567637(n.125G>A)];ENSG00000175318:[ENST00000309731(c.135-23G>A)];ENSG00000175318:[ENST00000564129(c.105-23G>A)];ENSG00000175318:[ENST00000570275(c.99-23G>A)];ENSG00000175318:[ENST00000562288(c.*102-23G>A)];ENSG00000175318:[ENST00000568594(c.114-23G>A)];	NA	NA	NA	NA	NA	NA	NA	NA	NA	NA	MODIFIER	NA	NA	56	56	0	34	25	9	26.47
Smoker	42480	chr15	89758455	G	A	*RLBP1*	MISSENSE	ENSG00000140522:MISSENSE[ENST00000268125(c.361C>T,Cgg>Tgg):ENSP00000268125(p.Arg121Trp)];ENSG00000140522:INTRONIC[ENST00000567787(c.184-29C>T)];	NA	NA	0.0001997	4.12E-05	0	0.0001997	0	NA	NA	NA	MODIFIER	NA	NA	93	93	0	69	53	15	21.74
Smoker	42480	chr15	30336962	G	A	*RP11-143J24.1*	INTRONIC	ENSG00000259647:[ENST00000561392(n.138+952C>T)];	NA	NA	NA	NA	NA	NA	NA	NA	NA	NA	MODIFIER	NA	NA	367	366	1	244	220	24	9.84
Smoker	42480	chr15	85213270	C	T	*SEC11A*	MISSENSE	ENSG00000140612:MISSENSE[ENST00000455959(c.442G>A,Gtg>Atg):ENSP00000413121(p.Val148Met)];ENSG00000140612:MISSENSE[ENST00000268220(c.520G>A,Gtg>Atg):ENSP00000268220(p.Val174Met)];ENSG00000140612:MISSENSE[ENST00000559729(c.370G>A,Gtg>Atg):ENSP00000452932(p.Val124Met)];ENSG00000140612:MISSENSE[ENST00000558217(c.388G>A,Gtg>Atg):ENSP00000453930(p.Val130Met)];ENSG00000140612:EXONIC-NC[ENST00000559376(n.1709G>A)];ENSG00000140612:EXONIC-NC[ENST00000558924(n.3133G>A)];ENSG00000140612:3UTR[ENST00000560266(c.*63G>A)];ENSG00000140612:3UTR[ENST00000560409(c.*90G>A)];ENSG00000140612:SILENT[ENST00000558134(c.462G>A,tcG>tcA):ENSP00000452697(p.Ser154(=))];	NA	NA	chr15*85213270*85213270*C*A*rs780135173*NA*NA*NA	NA	NA	NA	NA	NA	NA	NA	MODIFIER	NA	NA	150	150	0	136	125	11	8.09

Mangalaparthi *et al.* , 2020. Mutational landscape of esophageal squamous cell carcinoma in an Indian cohort
Supplementary Table 4. List of somatic single nucleotide variants identified using whole exome sequencing of ESCC patients

Cohort	Sample	Chromosome	Genomic position	Reference allele	Alternate allele	Gene symbol	Variant type	Variant annotation	OncoMD	COSMIC	dbSNP	ExAC	ExAC_SAS	1000G	1000G_SAS	SIFT	Polyphen2	CONDEL	VEP	LRT	PhastCon7 Way	Total read (Adjacent normal)	Reference allel read support (Adjacent normal)	Alternative read support (Adjacent normal)	Total read (Tumor)	Reference allel read support (Tumor)	Alternative read support (Tumor)	Alternate allele frequency
Smoker	42480	chr15	90772587	C	T	*SEMA4B*	EXONIC-NC	ENSG00000185033:[ENST00000558065(n.3427C>T)];ENSG00000185033:[ENST00000332496(c.*712C>T)];ENSG00000185033:[ENST00000559074(c.*8C>T)];ENSG00000185033:[ENST00000411539(c.*712C>T)];	NA	NA	NA	NA	NA	NA	NA	NA	NA	NA	MODIFIER	NA	NA	312	312	0	213	168	45	21.13
Smoker	42480	chr15	49217144	G	A	*SHC4*	SILENT-SS-PRX	ENSG00000185634:[ENST00000332408(c.588C>T,taC>taT):ENSP00000329668(p.Tyr196(=))];	NA	NA	NA	2.47E-05	0.00018184	NA	NA	NA	NA	NA	LOW	NA	0.992	172	172	0	206	164	41	19.9
Smoker	42480	chr15	25221591	C	G	*SNRPN*	INTRONIC	ENSG00000128739:[ENST00000400100(c.267+28C>G)];ENSG00000128739:[ENST00000400098(c.267+28C>G)];ENSG00000128739:[ENST00000400097(c.267+28C>G)];ENSG00000128739:[ENST00000554227(c.279+28C>G)];ENSG00000128739:[ENST00000390687(c.267+28C>G)];ENSG00000128739:[ENST00000579070(c.267+28C>G)];ENSG00000128739:[ENST00000444203(c.279+28C>G)];ENSG00000128739:[ENST00000577565(c.267+28C>G)];ENSG00000128739:[ENST00000346403(c.267+28C>G)];	NA	NA	NA	NA	NA	NA	NA	NA	NA	NA	MODIFIER	NA	NA	51	50	0	63	51	12	19.05
Smoker	42480	chr15	25221591	C	G	*SNURF*	INTRONIC	ENSG00000214265:[ENST00000551312(c.*46-3092C>G)];ENSG00000273173:[ENST00000580062(c.*309+28C>G)];ENSG00000273173:[ENST00000338094(c.*455+28C>G)];ENSG00000214265:[ENST00000557230(c.111-1334C>G)];	NA	NA	NA	NA	NA	NA	NA	NA	NA	NA	MODIFIER	NA	NA	51	50	0	63	51	12	19.05
Smoker	42480	chr16	4774861	T	C	*ANKS3*	INTRONIC	ENSG00000168096:[ENST00000587005(c.*107-29A>G)];ENSG00000168096:[ENST00000304283(c.492-29A>G)];ENSG00000168096:[ENST00000586605(c.-28-29A>G)];ENSG00000168096:[ENST00000592421(c.171-29A>G)];ENSG00000168096:[ENST00000592068(c.171-29A>G)];ENSG00000168096:[ENST00000591653(n.1223-29A>G)];ENSG00000168096:[ENST00000592077(c.*107-29A>G)];ENSG00000168096:[ENST00000446014(c.105-29A>G)];ENSG00000168096:[ENST00000592711(c.171-29A>G)];ENSG00000168096:[ENST00000450067(c.-46+5120A>G)];ENSG00000168096:[ENST00000586166(c.-46+9296A>G)];ENSG00000168096:[ENST00000585773(c.273-29A>G)];ENSG00000168096:[ENST00000590193(c.370-29A>G)];ENSG00000168096:[ENST00000592698(c.-127-29A>G)];ENSG00000168096:[ENST00000590730(c.170+5120A>G)];ENSG00000168096:[ENST00000593120(n.210-29A>G)];ENSG00000168096:[ENST00000590147(c.492-29A>G)];ENSG00000168096:[ENST00000590803(n.388-29A>G)];ENSG00000168096:[ENST00000586159(c.*107-29A>G)];ENSG00000168096:[ENST00000589065(c.171-29A>G)];ENSG00000168096:[ENST00000591281(c.*107-29A>G)];ENSG00000168096:[ENST00000586632(n.263A>G)];	NA	NA	NA	6.20E-05	6.47E-05	NA	NA	NA	NA	NA	MODIFIER	NA	NA	63	62	0	60	44	16	26.67
Smoker	42480	chr16	28944725	A	T	*CD19*	MISSENSE	ENSG00000177455:MISSENSE[ENST00000538922(c.730A>T,Acg>Tcg):ENSP00000437940(p.Thr244Ser)];ENSG00000177455:MISSENSE[ENST00000324662(c.730A>T,Acg>Tcg):ENSP00000313419(p.Thr244Ser)];ENSG00000177455:MISSENSE[ENST00000567541(c.730A>T,Acg>Tcg):ENSP00000456201(p.Thr244Ser)];ENSG00000177455:EXONIC-NC[ENST00000566890(n.353A>T)];ENSG00000177455:EXONIC-NC[ENST00000565089(n.1064A>T)];	NA	NA	NA	8.24E-06	0	NA	NA	NA	NA	NA	MODIFIER	NA	NA	105	104	0	42	34	8	19.05
Smoker	42480	chr16	57015029	G	T	*CETP*	INTRONIC	ENSG00000087237:[ENST00000566128(c.952-41G>T)];ENSG00000087237:[ENST00000200676(c.1147-41G>T)];ENSG00000087237:[ENST00000379780(c.967-41G>T)];	NA	NA	chr16*57015029*57015029*G*A*rs773311643*NA*NA*NA	NA	NA	NA	NA	NA	NA	NA	MODIFIER	NA	NA	59	57	1	33	23	10	30.3
Smoker	42480	chr16	310737	C	T	*ITFG3*	SILENT	ENSG00000167930:INTRONIC[ENST00000442458(c.577+578C>T)];ENSG00000167930:INTRONIC[ENST00000420046(c.577+578C>T)];ENSG00000167930:INTRONIC[ENST00000450082(c.577+578C>T)];ENSG00000167930:INTRONIC[ENST00000301679(c.577+578C>T)];ENSG00000167930:INTRONIC[ENST00000453430(c.577+578C>T)];ENSG00000167930:INTRONIC[ENST00000301678(c.577+578C>T)];ENSG00000167930:INTRONIC[ENST00000399932(c.577+578C>T)];ENSG00000269881:INTRONIC[ENST00000600536(c.577+578C>T)];ENSG00000167930:INTRONIC[ENST00000449945(c.550+578C>T)];ENSG00000167930:SILENT[ENST00000421000(c.689C>T,taC>taT):ENSP00000412581(p.Tyr230(=))];	NA	NA	NA	NA	NA	NA	NA	NA	NA	NA	MODIFIER	NA	NA	133	133	0	71	58	13	18.31
Smoker	42480	chr16	15835822	G	A	*MYH11*	EXONIC-NC	ENSG00000133392:[ENST00000574119(n.227C>T)];ENSG00000133392:[ENST00000452625(c.2542-74C>T)];ENSG00000133392:[ENST00000576790(c.2521-74C>T)];ENSG00000133392:[ENST00000300036(c.2521-74C>T)];ENSG00000133392:[ENST00000396324(c.2542-74C>T)];	NA	NA	NA	NA	NA	NA	NA	NA	NA	NA	MODIFIER	NA	NA	68	68	0	50	37	13	26
Smoker	42480	chr16	15781329	C	G	*NDE1*	MISSENSE	ENSG00000072864:MISSENSE[ENST00000574109(c.46C>G,Ctc>Gtc):ENSP00000459875(p.Leu16Val)];ENSG00000072864:MISSENSE[ENST00000396353(c.484C>G,Ctc>Gtc):ENSP00000379641(p.Leu162Val)];ENSG00000072864:MISSENSE[ENST00000396355(c.484C>G,Ctc>Gtc):ENSP00000379643(p.Leu162Val)];ENSG00000072864:MISSENSE[ENST00000342673(c.484C>G,Ctc>Gtc):ENSP00000345892(p.Leu162Val)];ENSG00000072864:MISSENSE[ENST00000577101(c.123C>G,Ctc>Gtc):ENSP00000461729(p.Leu42Val)];ENSG00000072864:MISSENSE[ENST00000396354(c.484C>G,Ctc>Gtc):ENSP00000379642(p.Leu162Val)];ENSG00000072864:3UTR[ENST00000572756(c.*105C>G)];ENSG00000072864:3UTR[ENST00000573203(c.*236C>G)];	NA	NA	NA	NA	NA	NA	NA	NA	NA	NA	MODIFIER	NA	NA	75	75	0	74	50	24	32.43
Smoker	42480	chr16	47531462	G	C	*PHKB*	INTRONIC	ENSG00000102893:[ENST00000299167(c.166+63G>C)];ENSG00000102893:[ENST00000563376(c.145+63G>C)];ENSG00000102893:[ENST00000323584(c.166+63G>C)];ENSG00000102893:[ENST00000564873(c.145+63G>C)];ENSG00000102893:[ENST00000566044(c.145+63G>C)];ENSG00000102893:[ENST00000455779(c.145+63G>C)];ENSG00000102893:[ENST00000565424(n.96+36125G>C)];ENSG00000102893:[ENST00000567402(n.181+63G>C)];ENSG00000102893:[ENST00000566037(c.145+63G>C)];	NA	NA	NA	NA	NA	NA	NA	NA	NA	NA	MODIFIER	NA	NA	106	106	0	82	68	14	17.07
Smoker	42480	chr16	28944725	A	T	*RABEP2*	INTRONIC	ENSG00000177548:[ENST00000566762(c.-150+2860T>A)];	NA	NA	NA	8.24E-06	0	NA	NA	NA	NA	NA	MODIFIER	NA	NA	105	104	0	42	34	8	19.05
Smoker	42480	chr16	56901000	C	A	*SLC12A3*	MISSENSE	ENSG00000070915:MISSENSE[ENST00000566786(c.298C>A,Cat>Aat):ENSP00000457552(p.His100Asn)];ENSG00000070915:MISSENSE[ENST00000563236(c.301C>A,Cat>Aat):ENSP00000456149(p.His101Asn)];ENSG00000070915:MISSENSE[ENST00000438926(c.301C>A,Cat>Aat):ENSP00000402152(p.His101Asn)];ENSG00000070915:MISSENSE[ENST00000262502(c.298C>A,Cat>Aat):ENSP00000262502(p.His100Asn)];	NA	NA	0.0001997	8.36E-06	0	0.0001997	0	T	BN	N	MODERATE	D	0.996	77	77	0	41	36	5	12.2

Mangalaparthi *et al.* , 2020. Mutational landscape of esophageal squamous cell carcinoma in an Indian cohort
Supplementary Table 4. List of somatic single nucleotide variants identified using whole exome sequencing of ESCC patients

Cohort	Sample	Chromosome	Genomic position	Reference allele	Alternate allele	Gene symbol	Variant type	Variant annotation	OncoMD	COSMIC	dbSNP	ExAC	ExAC_SAS	1000G	1000G_SAS	SIFT	Polyphen2	CONDEL	VEP	LRT	PhastCon7 Way	Total read (Adjacent normal)	Reference allel read support (Adjacent normal)	Alternative read support (Adjacent normal)	Total read (Tumor)	Reference allel read support (Tumor)	Alternative read support (Tumor)	Alternate allele frequency
Smoker	42480	chr16	18826540	C	T	*SMG1*	SILENT	ENSG00000157106:SILENT[ENST00000389467(c.10581G>A,tcG>tcA):ENSP00000374118(p.Ser3527(=))];ENSG00000157106:SILENT[ENST00000565324(c.10248G>A,tcG>tcA):ENSP00000456259(p.Ser3416(=))];ENSG00000157106:SILENT[ENST00000446231(c.10578G>A,tcG>tcA):ENSP00000402515(p.Ser3526(=))];ENSG00000157106:EXONIC-NC[ENST00000561940(n.804G>A)];	NA	NA	chr16*18826540*18826540*C*A,T*rs749536329*NA*NA*NA	0	0	NA	NA	NA	NA	NA	LOW	NA	NA	129	129	0	218	156	62	28.44
Smoker	42480	chr16	30749885	G	A	*SRCAP*	MISSENSE	ENSG00000080603:MISSENSE[ENST00000262518(c.8524G>A,Gag>Aag):ENSP00000262518(p.Glu2842Lys)];ENSG00000080603:MISSENSE[ENST00000344771(c.8050G>A,Gag>Aag):ENSP00000343042(p.Glu2684Lys)];ENSG00000080603:MISSENSE[ENST00000395059(c.8338G>A,Gag>Aag):ENSP00000378499(p.Glu2780Lys)];ENSG00000080603:MISSENSE[ENST00000380361(c.7993G>A,Gag>Aag):ENSP00000369719(p.Glu2665Lys)];	NA	NA	0.0001997	1.69E-05	0	0.0001997	0	NA	UN	NA	MODERATE	NA	NA	65	65	0	46	40	6	13.04
Smoker	42480	chr16	67434906	C	T	*ZDHHC1*	MISSENSE	ENSG00000159714:MISSENSE[ENST00000348579(c.382G>A,Gca>Aca):ENSP00000340299(p.Ala128Thr)];ENSG00000159714:EXONIC-NC[ENST00000562122(n.159G>A)];	NA	NA	NA	8.24E-06	6.06E-05	NA	NA	NA	NA	NA	MODIFIER	NA	NA	151	151	0	76	66	10	13.16
Smoker	42480	chr16	85010876	G	A	*ZDHHC7*	INTRONIC	ENSG00000153786:[ENST00000564466(c.731-45C>T)];ENSG00000153786:[ENST00000569488(n.503-45C>T)];ENSG00000153786:[ENST00000566909(c.269-45C>T)];ENSG00000153786:[ENST00000569377(n.204-45C>T)];ENSG00000153786:[ENST00000344861(c.731-45C>T)];ENSG00000153786:[ENST00000313732(c.620-45C>T)];ENSG00000153786:[ENST00000564526(n.12C>T)];	NA	NA	.	1.67E-05	0	NA	NA	NA	NA	NA	MODIFIER	NA	NA	75	75	0	64	51	13	20.31
Smoker	42480	chr16	2052365	C	T	*ZNF598*	MISSENSE	ENSG00000167962:MISSENSE[ENST00000431526(c.737G>A,cGc>cAc):ENSP00000411409(p.Arg246His)];ENSG00000167962:MISSENSE[ENST00000562103(c.572G>A,cGc>cAc):ENSP00000455308(p.Arg191His)];ENSG00000167962:MISSENSE[ENST00000563630(c.572G>A,cGc>cAc):ENSP00000455882(p.Arg191His)];ENSG00000167962:EXONIC-NC[ENST00000564824(n.853G>A)];ENSG00000167962:EXONIC-NC[ENST00000565396(n.1540G>A)];ENSG00000167962:EXONIC-NC[ENST00000562988(n.853G>A)];	NA	NA	NA	0.0001004	0.000606208	NA	NA	NA	NA	NA	MODIFIER	NA	NA	142	140	0	50	43	7	14
Smoker	42480	chr17	41003363	G	C	*AOC3*	STARTLOSS	ENSG00000131471:STARTLOSS[ENST00000308423(c.3G>C,atG>atC):ENSP00000312326(p.Met1?)];	NA	NA	NA	NA	NA	NA	NA	D	BN	N	HIGH	D	0.933	113	113	0	53	44	9	16.98
Smoker	42480	chr17	48655878	G	A	*CACNA1G*	MISSENSE	ENSG00000006283:MISSENSE[ENST00000416767(c.2254G>A,Gcc>Acc):ENSP00000392390(p.Ala752Thr)];ENSG00000006283:MISSENSE[ENST00000515165(c.2254G>A,Gcc>Acc):ENSP00000426098(p.Ala752Thr)];ENSG00000006283:MISSENSE[ENST00000360761(c.2254G>A,Gcc>Acc):ENSP00000353990(p.Ala752Thr)];ENSG00000006283:MISSENSE[ENST00000354983(c.2254G>A,Gcc>Acc):ENSP00000347078(p.Ala752Thr)];ENSG00000006283:MISSENSE[ENST00000513689(c.2254G>A,Gcc>Acc):ENSP00000426172(p.Ala752Thr)];ENSG00000006283:MISSENSE[ENST00000507510(c.2254G>A,Gcc>Acc):ENSP00000423112(p.Ala752Thr)];ENSG00000006283:MISSENSE[ENST00000514717(c.2254G>A,Gcc>Acc):ENSP00000422407(p.Ala752Thr)];ENSG00000006283:MISSENSE[ENST00000513964(c.2254G>A,Gcc>Acc):ENSP00000425451(p.Ala752Thr)];ENSG00000006283:MISSENSE[ENST00000359106(c.2254G>A,Gcc>Acc):ENSP00000352011(p.Ala752Thr)];ENSG00000006283:MISSENSE[ENST00000515765(c.2254G>A,Gcc>Acc):ENSP00000426232(p.Ala752Thr)];ENSG00000006283:MISSENSE[ENST00000502264(c.2254G>A,Gcc>Acc):ENSP00000425522(p.Ala752Thr)];ENSG00000006283:MISSENSE[ENST00000514181(c.2254G>A,Gcc>Acc):ENSP00000425698(p.Ala752Thr)];ENSG00000006283:MISSENSE[ENST00000352832(c.2254G>A,Gcc>Acc):ENSP00000339302(p.Ala752Thr)];ENSG00000006283:MISSENSE[ENST00000507336(c.2254G>A,Gcc>Acc):ENSP00000420918(p.Ala752Thr)];ENSG00000006283:MISSENSE[ENST00000510366(c.2254G>A,Gcc>Acc):ENSP00000426814(p.Ala752Thr)];ENSG00000006283:MISSENSE[ENST00000505165(c.2254G>A,Gcc>Acc):ENSP00000422268(p.Ala752Thr)];ENSG00000006283:MISSENSE[ENST00000510115(c.2254G>A,Gcc>Acc):ENSP00000427173(p.Ala752Thr)];ENSG00000006283:MISSENSE[ENST00000429973(c.2254G>A,Gcc>Acc):ENSP00000414388(p.Ala752Thr)];ENSG00000006283:MISSENSE[ENST00000512389(c.2254G>A,Gcc>Acc):ENSP00000426261(p.Ala752Thr)];ENSG00000006283:MISSENSE[ENST00000514079(c.2254G>A,Gcc>Acc):ENSP00000423317(p.Ala752Thr)];ENSG00000006283:MISSENSE[ENST00000507896(c.2254G>A,Gcc>Acc):ENSP00000421518(p.Ala752Thr)];ENSG00000006283:MISS	NA	NA	NA	NA	NA	NA	NA	D	PrD	D	MODERATE	NA	NA	340	339	0	87	72	15	17.24
Smoker	42480	chr17	72694670	C	T	*CD300LF*	INTRONIC	ENSG00000186074:[ENST00000343125(c.456-2314G>A)];ENSG00000186074:[ENST00000581500(c.501-50G>A)];ENSG00000186074:[ENST00000301573(c.492+45G>A)];ENSG00000186074:[ENST00000326165(c.447-50G>A)];ENSG00000186074:[ENST00000583937(c.492-50G>A)];ENSG00000186074:[ENST00000462044(c.456-2672G>A)];ENSG00000186074:[ENST00000361254(c.501-50G>A)];ENSG00000186074:[ENST00000469092(c.456-2314G>A)];ENSG00000186074:[ENST00000464910(c.456-50G>A)];	NA	NA	NA	NA	NA	NA	NA	NA	NA	NA	MODIFIER	NA	NA	29	29	0	24	8	15	62.5
Smoker	42480	chr17	80395117	G	A	*HEXDC*	SILENT	ENSG00000169660:SILENT[ENST00000577944(c.777G>A,acG>acA):ENSP00000463129(p.Thr259(=))];ENSG00000169660:SILENT[ENST00000337014(c.777G>A,acG>acA):ENSP00000337854(p.Thr259(=))];ENSG00000169660:SILENT[ENST00000582429(c.353G>A,acG>acA):ENSP00000464912(p.Thr118(=))];ENSG00000169660:SILENT[ENST00000578130(c.84G>A,acG>acA):ENSP00000462000(p.Thr28(=))];ENSG00000169660:SILENT[ENST00000578616(c.27G>A,acG>acA):ENSP00000463241(p.Thr9(=))];ENSG00000169660:SILENT[ENST00000327949(c.777G>A,acG>acA):ENSP00000332634(p.Thr259(=))];ENSG00000169660:EXONIC-NC[ENST00000585077(n.115G>A)];ENSG00000169660:3UTR[ENST00000582315(c.*621G>A)];ENSG00000169660:3UTR[ENST00000578775(c.*527G>A)];	NA	NA	NA	2.54E-05	6.21E-05	NA	NA	NA	NA	NA	LOW	NA	NA	91	91	0	39	29	10	25.64
Smoker	42480	chr17	74289677	G	A	*QRICH2*	SILENT	ENSG00000129646:SILENT[ENST00000262765(c.633C>T,ccC>ccT):ENSP00000262765(p.Pro211(=))];ENSG00000129646:INTRONIC[ENST00000524722(c.306-3519C>T)];	NA	NA	NA	8.24E-06	0	NA	NA	NA	NA	NA	MODIFIER	NA	NA	252	252	0	461	427	34	7.38
Smoker	42480	chr17	72694670	C	T	*RAB37*	INTRONIC	ENSG00000172794:[ENST00000392617(n.479+26873C>T)];ENSG00000172794:[ENST00000340415(c.72+26873C>T)];ENSG00000172794:[ENST00000402449(c.72+26873C>T)];	NA	NA	NA	NA	NA	NA	NA	NA	NA	NA	MODIFIER	NA	NA	29	29	0	24	8	15	62.5

Mangalaparthi *et al.* , 2020. Mutational landscape of esophageal squamous cell carcinoma in an Indian cohort
Supplementary Table 4. List of somatic single nucleotide variants identified using whole exome sequencing of ESCC patients

Cohort	Sample	Chromosome	Genomic position	Reference allele	Alternate allele	Gene symbol	Variant type	Variant annotation	OncoMD	COSMIC	dbSNP	ExAC	ExAC_SAS	1000G	1000G_SAS	SIFT	Polyphen2	CONDEL	VEP	LRT	PhastCon7 Way	Total read (Adjacent normal)	Reference allel read support (Adjacent normal)	Alternative read support (Adjacent normal)	Total read (Tumor)	Reference allel read support (Tumor)	Alternative read support (Tumor)	Alternate allele frequency
Smoker	42480	chr17	28537613	C	T	*SLC6A4*	MISSENSE	ENSG00000108576:MISSENSE[ENST00000261707(c.1369G>A,Gtc>Atc):ENSP00000261707(p.Val457Ile)];ENSG00000108576:MISSENSE[ENST00000394821(c.1369G>A,Gtc>Atc):ENSP00000378298(p.Val457Ile)];ENSG00000108576:MISSENSE[ENST00000579221(c.110G>A,Gtc>Atc):ENSP00000463172(p.Val38Ile)];ENSG00000108576:MISSENSE[ENST00000401766(c.1369G>A,Gtc>Atc):ENSP00000385822(p.Val457Ile)];	chr17*2853	chr17*2853	0.0001997	6.61E-05	0.000426465	0.0001997	0	T	BN	N	MODERATE	N	0.998	27	27	0	39	26	13	33.33
Smoker	42480	chr17	18220088	G	A	*SMCR8*	MISSENSE	ENSG00000176994:MISSENSE[ENST00000406438(c.985G>A,Gaa>Aaa):ENSP00000385025(p.Glu329Lys)];	NA	NA	NA	NA	NA	NA	NA	T	BN	N	MODERATE	N	1	143	143	0	75	59	16	21.33
Smoker	42480	chr17	27010730	C	T	*SUPT6H*	MISSENSE	ENSG00000109111:MISSENSE[ENST00000314616(c.2125C>T,Cgc>Tgc):ENSP00000319104(p.Arg709Cys)];ENSG00000109111:MISSENSE[ENST00000347486(c.2125C>T,Cgc>Tgc):ENSP00000338143(p.Arg709Cys)];	NA	NA	NA	NA	NA	NA	NA	D	PrD	D	MODERATE	D	1	91	91	0	80	67	13	16.25
Smoker	42480	chr17	6716307	C	T	*TEKT1*	MISSENSE	ENSG00000167858:MISSENSE[ENST00000338694(c.695G>A,cGg>cAg):ENSP00000341346(p.Arg232Gln)];ENSG00000167858:MISSENSE[ENST00000535086(c.257G>A,cGg>cAg):ENSP00000444142(p.Arg86Gln)];ENSG00000167858:MISSENSE[ENST00000572291(c.81G>A,cGg>cAg):ENSP00000458518(p.Arg28Gln)];ENSG00000167858:MISSENSE[ENST00000571744(c.29G>A,cGg>cAg):ENSP00000460197(p.Arg10Gln)];ENSG00000167858:3UTR[ENST00000575592(c.*286G>A)];	NA	NA	0.005391	0.0021586	0.000121168	0.0053914	0	NA	NA	NA	MODIFIER	NA	NA	78	78	0	57	41	16	28.07
Smoker	42480	chr17	15234796	C	T	*TEKT3*	MISSENSE	ENSG00000125409:MISSENSE[ENST00000395931(c.107G>A,cGc>cAc):ENSP00000379264(p.Arg36His)];ENSG00000125409:MISSENSE[ENST00000338696(c.107G>A,cGc>cAc):ENSP00000343995(p.Arg36His)];ENSG00000125409:MISSENSE[ENST00000539316(c.107G>A,cGc>cAc):ENSP00000439713(p.Arg36His)];ENSG00000125409:MISSENSE[ENST00000395930(c.107G>A,cGc>cAc):ENSP00000379263(p.Arg36His)];ENSG00000125409:MISSENSE[ENST00000536146(c.107G>A,cGc>cAc):ENSP00000446111(p.Arg36His)];ENSG00000125409:MISSENSE[ENST00000543896(c.107G>A,cGc>cAc):ENSP00000444180(p.Arg36His)];ENSG00000125409:INTRONIC[ENST00000539245(c.-239+98G>A)];ENSG00000125409:EXONIC-NC[ENST00000470325(n.136G>A)];	chr17*1523	chr17*1523	chr17*15234796*15234796*C*A*rs753700946*NA*NA*NA	NA	NA	NA	NA	T	BN	N	MODERATE	NA	NA	140	140	0	138	97	40	28.99
Smoker	42480	chr18	63548061	T	A	*CDH7*	MISSENSE	ENSG00000081138:MISSENSE[ENST00000397968(c.2289T>A,agT>agA):ENSP00000381058(p.Ser763Arg)];ENSG00000081138:MISSENSE[ENST00000323011(c.2289T>A,agT>agA):ENSP00000319166(p.Ser763Arg)];	NA	NA	NA	NA	NA	NA	NA	T	BN	N	MODERATE	D	1	164	164	0	239	203	36	15.06
Smoker	42480	chr18	28934690	C	T	*DSG1*	MISSENSE	ENSG00000134760:MISSENSE[ENST00000462981(c.608C>T,tCt>tTt):ENSP00000462666(p.Ser203Phe)];ENSG00000134760:MISSENSE[ENST00000257192(c.2531C>T,tCt>tTt):ENSP00000257192(p.Ser844Phe)];	NA	NA	NA	NA	NA	NA	NA	NA	NA	NA	MODIFIER	NA	NA	246	246	0	232	221	11	4.74
Smoker	42480	chr18	5416047	C	T	*EPB41L3*	MISSENSE	ENSG00000082397:MISSENSE[ENST00000342933(c.1837G>A,Gaa>Aaa):ENSP00000341138(p.Glu613Lys)];ENSG00000082397:MISSENSE[ENST00000341928(c.1837G>A,Gaa>Aaa):ENSP00000343158(p.Glu613Lys)];ENSG00000082397:INTRONIC[ENST00000578524(n.103+3663G>A)];ENSG00000082397:INTRONIC[ENST00000578196(n.128+3663G>A)];ENSG00000082397:INTRONIC[ENST00000580316(n.251+3663G>A)];ENSG00000082397:INTRONIC[ENST00000542652(n.1124+3663G>A)];ENSG00000082397:INTRONIC[ENST00000581292(n.90+3663G>A)];ENSG00000082397:INTRONIC[ENST00000427684(c.-82+3663G>A)];ENSG00000082397:INTRONIC[ENST00000400111(c.1560+3663G>A)];ENSG00000082397:INTRONIC[ENST00000544123(c.1560+3663G>A)];ENSG00000082397:INTRONIC[ENST00000545076(n.1770+3663G>A)];ENSG00000082397:INTRONIC[ENST00000581387(n.105+3663G>A)];ENSG00000082397:INTRONIC[ENST00000578395(n.386+3663G>A)];ENSG00000082397:INTRONIC[ENST00000580647(n.84+3663G>A)];ENSG00000082397:INTRONIC[ENST00000542146(c.-82+3663G>A)];ENSG00000082397:INTRONIC[ENST00000579271(n.91+3663G>A)];ENSG00000082397:INTRONIC[ENST00000540638(c.1560+3663G>A)];ENSG00000082397:EXONIC-NC[ENST00000582729(n.104G>A)];	NA	NA	NA	NA	NA	NA	NA	NA	NA	NA	MODIFIER	NA	NA	93	93	0	84	70	14	16.67
Smoker	42480	chr18	5416104	C	T	*EPB41L3*	MISSENSE	ENSG00000082397:MISSENSE[ENST00000342933(c.1780G>A,Gag>Aag):ENSP00000341138(p.Glu594Lys)];ENSG00000082397:MISSENSE[ENST00000341928(c.1780G>A,Gag>Aag):ENSP00000343158(p.Glu594Lys)];ENSG00000082397:INTRONIC[ENST00000578524(n.103+3606G>A)];ENSG00000082397:INTRONIC[ENST00000578196(n.128+3606G>A)];ENSG00000082397:INTRONIC[ENST00000580316(n.251+3606G>A)];ENSG00000082397:INTRONIC[ENST00000542652(n.1124+3606G>A)];ENSG00000082397:INTRONIC[ENST00000581292(n.90+3606G>A)];ENSG00000082397:INTRONIC[ENST00000427684(c.-82+3606G>A)];ENSG00000082397:INTRONIC[ENST00000400111(c.1560+3606G>A)];ENSG00000082397:INTRONIC[ENST00000544123(c.1560+3606G>A)];ENSG00000082397:INTRONIC[ENST00000545076(n.1770+3606G>A)];ENSG00000082397:INTRONIC[ENST00000581387(n.105+3606G>A)];ENSG00000082397:INTRONIC[ENST00000578395(n.386+3606G>A)];ENSG00000082397:INTRONIC[ENST00000580647(n.84+3606G>A)];ENSG00000082397:INTRONIC[ENST00000542146(c.-82+3606G>A)];ENSG00000082397:INTRONIC[ENST00000579271(n.91+3606G>A)];ENSG00000082397:INTRONIC[ENST00000540638(c.1560+3606G>A)];ENSG00000082397:EXONIC-NC[ENST00000582729(n.47G>A)];	NA	NA	NA	NA	NA	NA	NA	NA	NA	NA	MODIFIER	NA	NA	111	111	0	103	86	17	16.5
Smoker	42480	chr18	21390470	A	G	*LAMA3*	INTRONIC-SS-DNR	ENSG00000053747:[ENST00000313654(c.1741+3A>G)];ENSG00000053747:[ENST00000399516(c.1741+3A>G)];	NA	NA	NA	NA	NA	NA	NA	NA	NA	NA	LOW	NA	NA	90	90	0	54	41	13	24.07
Smoker	42480	chr18	14866502	C	T	*NA*	INTERGENIC		NA	NA	NA	NA	NA	NA	NA	NA	NA	NA	MODIFIER	NA	NA	70	70	0	35	29	6	17.14
Smoker	42480	chr18	70531971	C	G	*NETO1*	INTRONIC	ENSG00000166342:[ENST00000580049(n.319+72G>C)];ENSG00000166342:[ENST00000327305(c.220+72G>C)];ENSG00000166342:[ENST00000579730(n.302+72G>C)];ENSG00000166342:[ENST00000397929(c.217+72G>C)];ENSG00000166342:[ENST00000299430(c.217+72G>C)];ENSG00000166342:[ENST00000579169(c.208+72G>C)];ENSG00000166342:[ENST00000583169(c.220+72G>C)];	NA	NA	NA	NA	NA	NA	NA	NA	NA	NA	MODIFIER	NA	NA	167	167	0	107	95	11	10.28
Smoker	42480	chr18	23738289	A	C	*PSMA8*	INTRONIC	ENSG00000154611:[ENST00000538664(c.*350+81A>C)];ENSG00000154611:[ENST00000308268(c.495+81A>C)];ENSG00000154611:[ENST00000415576(c.477+81A>C)];ENSG00000154611:[ENST00000343848(c.363+81A>C)];	NA	NA	NA	NA	NA	NA	NA	NA	NA	NA	MODIFIER	NA	NA	18	18	0	42	35	7	16.67
Smoker	42480	chr18	28934690	C	T	*RP11-534N16.1*	INTRONIC	ENSG00000266729:[ENST00000581856(n.96-274G>A)];ENSG00000266729:[ENST00000578477(n.294-274G>A)];ENSG00000266729:[ENST00000581452(n.227-274G>A)];	NA	NA	NA	NA	NA	NA	NA	NA	NA	NA	MODIFIER	NA	NA	246	246	0	232	221	11	4.74
Smoker	42480	chr18	43246187	G	C	*SLC14A2*	INTRONIC-SS-DNR	ENSG00000132874:[ENST00000589658(c.-13+3G>C)];ENSG00000132874:[ENST00000255226(c.1557+3G>C)];ENSG00000132874:[ENST00000586448(c.1557+3G>C)];	NA	NA	NA	NA	NA	NA	NA	NA	NA	NA	LOW	NA	NA	126	126	0	70	57	13	18.57

Cohort	Sample	Chromosome	Genomic position	Reference allele	Alternate allele	Gene symbol	Variant type	Variant annotation	OncoMD	COSMIC	dbSNP	ExAC	ExAC_SAS	1000G	1000G_SAS	SIFT	Polyphen2	CONDEL	VEP	LRT	PhastCon7 Way	Total read (Adjacent normal)	Reference allel read support (Adjacent normal)	Alternative read support (Adjacent normal)	Total read (Tumor)	Reference allel read support (Tumor)	Alternative read support (Tumor)	Alternate allele frequency
Smoker	42480	chr19	49300246	A	C	*BCAT2*	MISSENSE	ENSG00000105552:MISSENSE[ENST00000402551(c.752T>G,gTt>gGt):ENSP00000385161(p.Val251Gly)];ENSG00000105552:MISSENSE[ENST00000597011(c.752T>G,gTt>gGt):ENSP00000469651(p.Val251Gly)];ENSG00000105552:MISSENSE[ENST00000599246(c.596T>G,gTt>gGt):ENSP00000470680(p.Val199Gly)];ENSG00000105552:MISSENSE[ENST00000316273(c.872T>G,gTt>gGt):ENSP00000322991(p.Val291Gly)];ENSG00000105552:MISSENSE[ENST00000598162(c.872T>G,gTt>gGt):ENSP00000470216(p.Val291Gly)];ENSG00000105552:MISSENSE[ENST00000593515(c.848T>G,gTt>gGt):ENSP00000469139(p.Val283Gly)];ENSG00000105552:MISSENSE[ENST00000545387(c.596T>G,gTt>gGt):ENSP00000440973(p.Val199Gly)];ENSG00000105552:EXONIC-NC[ENST00000599510(n.387T>G)];	NA	NA	NA	NA	NA	NA	NA	NA	NA	NA	MODIFIER	NA	NA	94	94	0	41	23	18	43.9
Smoker	42480	chr19	13325143	C	T	*CACNA1A*	SILENT	ENSG00000141837:SILENT[ENST00000573710(c.5847G>A,acG>acA):ENSP00000460092(p.Thr1949(=))];ENSG00000141837:SILENT[ENST00000360228(c.5844G>A,acG>acA):ENSP00000353362(p.Thr1948(=))];ENSG00000141837:SILENT[ENST00000585802(c.1902G>A,acG>acA):ENSP00000465598(p.Thr634(=))];ENSG00000141837:SILENT[ENST00000587525(c.1305G>A,acG>acA):ENSP00000467729(p.Thr435(=))];	NA	NA	0.0001997	2.59E-05	6.30E-05	0.0001997	0.001	NA	NA	NA	LOW	NA	NA	124	124	0	71	56	15	21.13
Smoker	42480	chr19	4171483	G	A	*CREB3L3*	INTRONIC-SS-DNR	ENSG00000060566:[ENST00000602147(c.965+7G>A)];ENSG00000060566:[ENST00000252587(c.738+7G>A)];ENSG00000060566:[ENST00000598894(n.556+7G>A)];ENSG00000060566:[ENST00000078445(c.1072+7G>A)];ENSG00000060566:[ENST00000602257(c.1066+7G>A)];ENSG00000060566:[ENST00000595923(c.1069+7G>A)];	NA	NA	chr19*4171483*4171483*G*T*rs752856335*NA*NA*NA	NA	NA	NA	NA	NA	NA	NA	LOW	NA	NA	62	62	0	41	28	13	31.71
Smoker	42480	chr19	37676331	G	A	*CTC-454I21.3*	INTRONIC	ENSG00000267360:[ENST00000585860(c.292+4232C>T)];ENSG00000267360:[ENST00000588873(c.35+21611C>T)];ENSG00000267360:[ENST00000590245(c.*164+10452C>T)];	NA	NA	NA	NA	NA	NA	NA	NA	NA	NA	MODIFIER	NA	NA	100	100	0	201	179	22	10.95
Smoker	42480	chr19	12154816	C	T	*CTD-2006C1.10*	INTRONIC	ENSG00000257355:[ENST00000547473(c.191+2367G>A)];	chr19*1215	NA	0.0003994	8.28E-05	0	0.0003994	0	T	BN	N	MODERATE	NA	NA	141	140	1	98	68	30	30.61
Smoker	42480	chr19	12154816	C	T	*CTD-2006C1.2*	INTRONIC	ENSG00000219665:[ENST00000591898(n.163-2081C>T)];ENSG00000219665:[ENST00000476474(n.414-2081C>T)];ENSG00000219665:[ENST00000591838(n.237-2081C>T)];	chr19*1215	NA	0.0003994	8.28E-05	0	0.0003994	0	T	BN	N	MODERATE	NA	NA	141	140	1	98	68	30	30.61
Smoker	42480	chr19	38189897	G	A	*CTD-2528L19.4*	INTRONIC	ENSG00000267552:[ENST00000586606(c.346+789C>T)];ENSG00000267552:[ENST00000585547(c.148+789C>T)];ENSG00000267552:[ENST00000591114(c.73+789C>T)];	NA	NA	0.0001997	0.0002805	0.00018173	0.0001997	0	NA	NA	NA	HIGH	NA	NA	128	127	0	481	468	13	2.7
Smoker	42480	chr19	10254447	G	C	*DNMT1*	MISSENSE	ENSG00000130816:MISSENSE[ENST00000340748(c.3063C>G,ttC>ttG):ENSP00000345739(p.Phe1021Leu)];ENSG00000130816:MISSENSE[ENST00000540357(c.3063C>G,ttC>ttG):ENSP00000440457(p.Phe1021Leu)];ENSG00000130816:MISSENSE[ENST00000359526(c.3111C>G,ttC>ttG):ENSP00000352516(p.Phe1037Leu)];ENSG00000130816:EXONIC-NC[ENST00000589538(n.131C>G)];ENSG00000130816:EXONIC-NC[ENST00000591239(n.21C>G)];ENSG00000130816:EXONIC-NC[ENST00000586588(n.984C>G)];ENSG00000130816:3UTR[ENST00000592705(c.*2801C>G)];	NA	NA	NA	NA	NA	NA	NA	NA	NA	NA	MODIFIER	NA	NA	71	71	0	49	44	5	10.2
Smoker	42480	chr19	40377258	G	C	*FCGBP*	INTRONIC	ENSG00000090920:[ENST00000221347(c.11197-33C>G)];ENSG00000090920:[ENST00000595713(n.175-33C>G)];	NA	NA	NA	NA	NA	NA	NA	NA	NA	NA	MODIFIER	NA	NA	50	50	0	113	101	12	10.62
Smoker	42480	chr19	35657133	C	T	*FXYD5*	INTRONIC	ENSG00000089327:[ENST00000592290(c.*157-21C>T)];ENSG00000089327:[ENST00000392217(c.200-21C>T)];ENSG00000089327:[ENST00000586925(n.40-21C>T)];ENSG00000089327:[ENST00000590686(c.413-21C>T)];ENSG00000089327:[ENST00000423817(c.413-21C>T)];ENSG00000089327:[ENST00000496493(c.*340-21C>T)];ENSG00000089327:[ENST00000588699(c.413-21C>T)];ENSG00000089327:[ENST00000392219(c.413-21C>T)];ENSG00000089327:[ENST00000342879(c.413-21C>T)];ENSG00000089327:[ENST00000541435(c.413-21C>T)];ENSG00000089327:[ENST00000543307(c.413-21C>T)];ENSG00000089327:[ENST00000591716(n.272C>T)];	NA	NA	NA	NA	NA	NA	NA	NA	NA	NA	MODIFIER	NA	NA	75	75	0	90	82	8	8.89
Smoker	42480	chr19	10800130	C	T	*ILF3*	3UTR	ENSG00000129351:[ENST00000590261(c.*142C>T)];ENSG00000129351:[ENST00000318511(c.*142C>T)];ENSG00000129351:[ENST00000590869(c.*142C>T)];ENSG00000129351:[ENST00000449870(c.*142C>T)];ENSG00000129351:[ENST00000591649(n.2186C>T)];ENSG00000129351:[ENST00000587928(n.3837C>T)];ENSG00000129351:[ENST00000586544(n.915C>T)];	NA	NA	.	8.82E-06	0	NA	NA	NA	NA	NA	MODIFIER	NA	NA	276	276	0	124	105	18	14.52
Smoker	42480	chr19	55325214	C	T	*KIR2DL4*	INTRONIC	ENSG00000189013:[ENST00000396284(c.924+17C>T)];ENSG00000189013:[ENST00000357494(c.708+17C>T)];ENSG00000189013:[ENST00000396289(c.925+17C>T)];ENSG00000189013:[ENST00000345540(c.759+17C>T)];ENSG00000189013:[ENST00000463062(n.824+17C>T)];ENSG00000189013:[ENST00000486965(n.667-83C>T)];ENSG00000189013:[ENST00000346587(c.474+17C>T)];ENSG00000189013:[ENST00000396293(c.423+17C>T)];ENSG00000189013:[ENST00000359085(c.*41+17C>T)];	NA	NA	NA	NA	NA	NA	NA	NA	NA	NA	MODIFIER	NA	NA	231	222	2	137	70	63	45.99
Smoker	42480	chr19	55325214	C	T	*KIR3DL1*	INTRONIC	ENSG00000167633:[ENST00000541392(c.35-3775C>T)];ENSG00000167633:[ENST00000538269(c.35-3775C>T)];ENSG00000167633:[ENST00000402254(c.35-3775C>T)];	NA	NA	NA	NA	NA	NA	NA	NA	NA	NA	MODIFIER	NA	NA	231	222	2	137	70	63	45.99
Smoker	42480	chr19	55365343	G	A	*KIR3DL1*	INTRONIC	ENSG00000167633:[ENST00000402254(c.1001-11917G>A)];	NA	NA	NA	0.0004545	0	NA	NA	T	BN	N	MODERATE	NA	NA	263	262	1	152	120	32	21.05
Smoker	42480	chr19	55365343	G	A	*KIR3DL2*	MISSENSE	ENSG00000240403:MISSENSE[ENST00000270442(c.497G>A,cGc>cAc):ENSP00000270442(p.Arg166His)];ENSG00000240403:MISSENSE[ENST00000326321(c.497G>A,cGc>cAc):ENSP00000325525(p.Arg166His)];	NA	NA	NA	0.0004545	0	NA	NA	T	BN	N	MODERATE	NA	NA	263	262	1	152	120	32	21.05
Smoker	42480	chr19	39798899	C	G	*LRFN1*	MISSENSE	ENSG00000128011:MISSENSE[ENST00000248668(c.1690G>C,Gac>Cac):ENSP00000248668(p.Asp564His)];	NA	NA	NA	NA	NA	NA	NA	T	BN	N	MODERATE	N	0.261	121	118	3	259	166	93	35.91
Smoker	42480	chr19	9204691	C	T	*OR1M1*	SILENT	ENSG00000170929:SILENT[ENST00000429566(c.771C>T,ggC>ggT):ENSP00000401966(p.Gly257(=))];	NA	NA	0.000599	0.00014	0	0.000599	0	NA	NA	NA	LOW	NA	NA	74	73	1	67	51	16	23.88
Smoker	42480	chr19	39914364	C	T	*PLEKHG2*	INTRONIC	ENSG00000090924:[ENST00000596443(c.291+1436C>T)];ENSG00000090924:[ENST00000378550(c.1677+1436C>T)];ENSG00000090924:[ENST00000409797(c.1677+1436C>T)];ENSG00000090924:[ENST00000205135(c.2203-9C>T)];ENSG00000090924:[ENST00000425673(c.2513-9C>T)];ENSG00000090924:[ENST00000458508(c.2423-9C>T)];ENSG00000090924:[ENST00000600210(c.227-9C>T)];ENSG00000090924:[ENST00000409794(c.2600-9C>T)];	NA	NA	NA	NA	NA	NA	NA	NA	NA	NA	MODIFIER	NA	NA	149	148	1	220	209	11	5
Smoker	42480	chr19	897461	G	A	*R3HDM4*	SILENT	ENSG00000198858:3UTR[ENST00000589428(c.*364C>T)];ENSG00000198858:SILENT[ENST00000587975(c.720C>T,tcC>tcT):ENSP00000464744(p.Ser240(=))];ENSG00000198858:SILENT[ENST00000361574(c.783C>T,tcC>tcT):ENSP00000355385(p.Ser261(=))];	NA	NA	NA	7.25E-05	7.17E-05	NA	NA	NA	NA	NA	LOW	NA	NA	38	38	0	23	15	8	34.78

Mangalaparthi *et al.* , 2020. Mutational landscape of esophageal squamous cell carcinoma in an Indian cohort
Supplementary Table 4. List of somatic single nucleotide variants identified using whole exome sequencing of ESCC patients

Cohort	Sample	Chromosome	Genomic position	Reference allele	Alternate allele	Gene symbol	Variant type	Variant annotation	OncoMD	COSMIC	dbSNP	ExAC	ExAC_SAS	1000G	1000G_SAS	SIFT	Polyphen2	CONDEL	VEP	LRT	PhastCon7 Way	Total read (Adjacent normal)	Reference allel read support (Adjacent normal)	Alternative read support (Adjacent normal)	Total read (Tumor)	Reference allel read support (Tumor)	Alternative read support (Tumor)	Alternate allele frequency
Smoker	42480	chr19	51647962	C	T	*SIGLEC7*	INTRONIC	ENSG00000168995:[ENST00000601682(c.524+21C>T)];ENSG00000168995:[ENST00000600577(c.433+1903C>T)];ENSG00000168995:[ENST00000536156(c.433+1903C>T)];ENSG00000168995:[ENST00000317643(c.712+21C>T)];ENSG00000168995:[ENST00000305628(c.434-207C>T)];	NA	NA	NA	NA	NA	NA	NA	NA	NA	NA	MODIFIER	NA	NA	52	52	0	29	14	15	51.72
Smoker	42480	chr19	44116597	C	T	*SRRM5*	SILENT	ENSG00000226763:SILENT[ENST00000526798(c.369C>T,gaC>gaT):ENSP00000432514(p.Asp123(=))];ENSG00000226763:SILENT[ENST00000417606(c.324C>T,gaC>gaT):ENSP00000414512(p.Asp108(=))];ENSG00000226763:SILENT[ENST00000607544(c.324C>T,gaC>gaT):ENSP00000476253(p.Asp108(=))];	NA	NA	NA	NA	NA	NA	NA	NA	NA	NA	MODIFIER	NA	NA	110	110	0	56	43	13	23.21
Smoker	42480	chr19	56009494	C	T	*SSC5D*	SILENT	ENSG00000179954:SILENT[ENST00000389623(c.1635C>T,tcC>tcT):ENSP00000374274(p.Ser545(=))];ENSG00000179954:SILENT[ENST00000587166(c.1635C>T,tcC>tcT):ENSP00000467252(p.Ser545(=))];ENSG00000179954:INTRONIC[ENST00000589020(c.197-1689C>T)];	NA	NA	NA	NA	NA	NA	NA	NA	NA	NA	MODIFIER	NA	NA	68	68	0	46	40	6	13.04
Smoker	42480	chr19	58131654	G	T	*ZNF134*	MISSENSE	ENSG00000213762:EXONIC-NC[ENST00000597975(n.398G>T)];ENSG00000213762:MISSENSE[ENST00000396161(c.167G>T,gGc>gTc):ENSP00000379464(p.Gly56Val)];ENSG00000213762:MISSENSE[ENST00000600883(c.167G>T,gGc>gTc):ENSP00000469065(p.Gly56Val)];	NA	NA	NA	NA	NA	NA	NA	T	BN	N	MODERATE	NA	NA	119	119	0	68	57	11	16.18
Smoker	42480	chr19	58131655	C	T	*ZNF134*	SILENT	ENSG00000213762:EXONIC-NC[ENST00000597975(n.399C>T)];ENSG00000213762:SILENT[ENST00000396161(c.168C>T,ggC>ggT):ENSP00000379464(p.Gly56(=))];ENSG00000213762:SILENT[ENST00000600883(c.168C>T,ggC>ggT):ENSP00000469065(p.Gly56(=))];	NA	NA	NA	NA	NA	NA	NA	NA	NA	NA	LOW	NA	NA	122	122	0	69	58	11	15.94
Smoker	42480	chr19	9528630	G	C	*ZNF266*	INTRONIC	ENSG00000174652:[ENST00000590306(c.35-46C>G)];ENSG00000174652:[ENST00000361151(c.35-46C>G)];ENSG00000174652:[ENST00000361451(c.35-46C>G)];ENSG00000174652:[ENST00000588933(c.35-46C>G)];ENSG00000174652:[ENST00000592904(c.35-46C>G)];ENSG00000174652:[ENST00000591213(c.35-46C>G)];ENSG00000174652:[ENST00000588221(c.35-46C>G)];ENSG00000174652:[ENST00000592292(c.35-46C>G)];	NA	NA	NA	NA	NA	NA	NA	NA	NA	NA	MODIFIER	NA	NA	130	127	3	123	81	42	34.15
Smoker	42480	chr19	44116597	C	T	*ZNF428*	INTRONIC	ENSG00000131116:[ENST00000598676(c.76+1784G>A)];ENSG00000131116:[ENST00000300811(c.76+1784G>A)];	NA	NA	NA	NA	NA	NA	NA	NA	NA	NA	MODIFIER	NA	NA	110	110	0	56	43	13	23.21
Smoker	42480	chr19	52544913	G	A	*ZNF432*	INTRONIC	ENSG00000256087:[ENST00000221315(c.16-54C>T)];ENSG00000256087:[ENST00000598745(c.16-54C>T)];ENSG00000256087:[ENST00000594154(c.16-54C>T)];ENSG00000256087:[ENST00000601310(c.16-54C>T)];ENSG00000256087:[ENST00000597273(c.16-54C>T)];ENSG00000256087:[ENST00000600368(c.16-54C>T)];	NA	NA	NA	NA	NA	NA	NA	NA	NA	NA	MODIFIER	NA	NA	28	27	1	28	15	13	46.43
Smoker	42480	chr19	2841470	T	C	*ZNF555*	5UTR	ENSG00000186300:[ENST00000585966(c.-105T>C)];ENSG00000186300:[ENST00000591539(c.-101T>C)];ENSG00000186300:[ENST00000334241(c.-101T>C)];	NA	NA	NA	NA	NA	NA	NA	NA	NA	NA	MODIFIER	NA	NA	57	57	0	32	26	6	18.75
Smoker	42480	chr19	37676331	G	A	*ZNF585B*	MISSENSE	ENSG00000245680:MISSENSE[ENST00000312908(c.872C>T,tCa>tTa):ENSP00000442139(p.Ser291Leu)];ENSG00000245680:MISSENSE[ENST00000531805(c.1943C>T,tCa>tTa):ENSP00000436774(p.Ser648Leu)];ENSG00000245680:MISSENSE[ENST00000532828(c.2108C>T,tCa>tTa):ENSP00000433773(p.Ser703Leu)];ENSG00000245680:3UTR[ENST00000527838(c.*1464C>T)];	NA	NA	NA	NA	NA	NA	NA	NA	NA	NA	MODIFIER	NA	NA	100	100	0	201	179	22	10.95
Smoker	42480	chr19	38189897	G	A	*ZNF607*	NONSENSE	ENSG00000198182:NONSENSE[ENST00000395835(c.1132C>T,Cga>Tga):ENSP00000438015(p.Arg378Ter)];ENSG00000198182:NONSENSE[ENST00000355202(c.1135C>T,Cga>Tga):ENSP00000347338(p.Arg379Ter)];	NA	NA	0.0001997	0.0002805	0.00018173	0.0001997	0	NA	NA	NA	HIGH	NA	NA	128	127	0	481	468	13	2.7
Smoker	42480	chr19	40540432	C	T	*ZNF780B*	SILENT	ENSG00000128000:SILENT[ENST00000434248(c.2334G>A,gaG>gaA):ENSP00000391641(p.Glu778(=))];ENSG00000128000:SILENT[ENST00000221355(c.1890G>A,gaG>gaA):ENSP00000221355(p.Glu630(=))];	NA	NA	NA	NA	NA	NA	NA	NA	NA	NA	LOW	NA	NA	209	209	0	633	588	44	6.95
Smoker	42480	chr19	57175104	G	A	*ZNF835*	MISSENSE	ENSG00000127903:MISSENSE[ENST00000537055(c.1463C>T,gCg>gTg):ENSP00000444747(p.Ala488Val)];	chr19*5717	chr19*5717	NA	NA	NA	NA	NA	T	BN	N	MODERATE	NA	NA	187	187	0	87	74	13	14.94
Smoker	42480	chr19	12154816	C	T	*ZNF878*	MISSENSE	ENSG00000257446:MISSENSE[ENST00000602107(c.1541G>A,cGc>cAc):ENSP00000472036(p.Arg514His)];ENSG00000257446:MISSENSE[ENST00000547628(c.1400G>A,cGc>cAc):ENSP00000447931(p.Arg467His)];	chr19*1215	NA	0.0003994	8.28E-05	0	0.0003994	0	T	BN	N	MODERATE	NA	NA	141	140	1	98	68	30	30.61
Smoker	42480	chr2	80831341	G	C	*AC008067.2*	INTRONIC	ENSG00000237031:[ENST00000599412(n.34-45C>G)];ENSG00000237031:[ENST00000609950(n.193+1386C>G)];ENSG00000237031:[ENST00000595478(n.34-45C>G)];ENSG00000237031:[ENST00000596783(n.181-45C>G)];ENSG00000237031:[ENST00000430876(n.186+1386C>G)];ENSG00000237031:[ENST00000596887(n.32+1386C>G)];	NA	NA	NA	NA	NA	NA	NA	NA	NA	NA	MODIFIER	NA	NA	222	222	0	166	148	18	10.84
Smoker	42480	chr2	96605666	G	A	*ANKRD36C*	INTRONIC	ENSG00000174501:[ENST00000528268(c.1532-903C>T)];ENSG00000174501:[ENST00000534304(c.117-903C>T)];ENSG00000174501:[ENST00000456556(c.1532-903C>T)];	NA	NA	NA	NA	NA	NA	NA	NA	NA	NA	MODIFIER	NA	NA	80	80	0	106	96	7	6.6
Smoker	42480	chr2	96605667	C	T	*ANKRD36C*	INTRONIC	ENSG00000174501:[ENST00000528268(c.1532-904G>A)];ENSG00000174501:[ENST00000534304(c.117-904G>A)];ENSG00000174501:[ENST00000456556(c.1532-904G>A)];	NA	NA	NA	NA	NA	NA	NA	NA	NA	NA	MODIFIER	NA	NA	81	80	0	106	96	7	6.6
Smoker	42480	chr2	96605668	A	G	*ANKRD36C*	INTRONIC	ENSG00000174501:[ENST00000528268(c.1532-905T>C)];ENSG00000174501:[ENST00000534304(c.117-905T>C)];ENSG00000174501:[ENST00000456556(c.1532-905T>C)];	NA	NA	NA	NA	NA	NA	NA	NA	NA	NA	MODIFIER	NA	NA	85	84	1	110	101	7	6.36
Smoker	42480	chr2	80801430	G	A	*CTNNA2*	SILENT	ENSG00000066032:SILENT[ENST00000496558(c.1884G>A,ctG>ctA):ENSP00000419295(p.Leu628(=))];ENSG00000066032:SILENT[ENST00000361291(c.1986G>A,ctG>ctA):ENSP00000355398(p.Leu662(=))];ENSG00000066032:SILENT[ENST00000541047(c.1884G>A,ctG>ctA):ENSP00000444675(p.Leu628(=))];ENSG00000066032:SILENT[ENST00000343114(c.921G>A,ctG>ctA):ENSP00000341500(p.Leu307(=))];ENSG00000066032:SILENT[ENST00000540488(c.1884G>A,ctG>ctA):ENSP00000441705(p.Leu628(=))];ENSG00000066032:SILENT[ENST00000466387(c.1884G>A,ctG>ctA):ENSP00000418191(p.Leu628(=))];ENSG00000066032:SILENT[ENST00000402739(c.1884G>A,ctG>ctA):ENSP00000384638(p.Leu628(=))];	NA	NA	NA	NA	NA	NA	NA	NA	NA	NA	LOW	NA	NA	75	75	0	50	31	19	38
Smoker	42480	chr2	80831341	G	C	*CTNNA2*	INTRONIC	ENSG00000066032:[ENST00000496558(c.2295+37G>C)];ENSG00000066032:[ENST00000361291(c.2397+37G>C)];ENSG00000066032:[ENST00000467892(n.253+37G>C)];ENSG00000066032:[ENST00000541047(c.2295+37G>C)];ENSG00000066032:[ENST00000343114(c.1332+37G>C)];ENSG00000066032:[ENST00000540488(c.2295+37G>C)];ENSG00000066032:[ENST00000466387(c.2295+37G>C)];ENSG00000066032:[ENST00000402739(c.2295+37G>C)];	NA	NA	NA	NA	NA	NA	NA	NA	NA	NA	MODIFIER	NA	NA	222	222	0	166	148	18	10.84

Supplementary Table 4. List of somatic single nucleotide variants identified using whole exome sequencing of ESCC patients

Cohort	Sample	Chromosome	Genomic position	Reference allele	Alternate allele	Gene symbol	Variant type	Variant annotation	OncoMD	COSMIC	dbSNP	ExAC	ExAC_SAS	1000G	1000G_SAS	SIFT	Polyphen2	CONDEL	VEP	LRT	PhastCon7 Way	Total read (Adjacent normal)	Reference allel read support (Adjacent normal)	Alternative read support (Adjacent normal)	Total read (Tumor)	Reference allel read support (Tumor)	Alternative read support (Tumor)	Alternate allele frequency
Smoker	42480	chr2	26653710	G	T	*DRC1*	MISSENSE	ENSG00000157856:MISSENSE[ENST00000288710(c.735G>T,gaG>gaT):ENSP00000288710(p.Glu245Asp)];ENSG00000157856:MISSENSE[ENST00000442810(c.222G>T,gaG>gaT):ENSP00000413942(p.Glu74Asp)];ENSG00000157856:3UTR[ENST00000421869(c.*188G>T)];ENSG00000157856:EXONIC-NC[ENST00000483675(n.336G>T)];ENSG00000157856:EXONIC-NC[ENST00000487307(n.442G>T)];	NA	NA	NA	NA	NA	NA	NA	NA	NA	NA	MODIFIER	NA	NA	115	115	0	122	87	34	27.87
Smoker	42480	chr2	241396805	C	G	*GPC1*	INTRONIC	ENSG00000063660:[ENST00000469694(n.181-1642C>G)];ENSG00000063660:[ENST00000264039(c.167-1642C>G)];ENSG00000063660:[ENST00000426280(c.17-1642C>G)];ENSG00000063660:[ENST00000420138(c.285-1642C>G)];	NA	NA	chr2*241396805*241396805*C*T*rs781677876*NA*NA*NA	NA	NA	NA	NA	NA	NA	NA	MODIFIER	NA	NA	75	75	0	32	23	9	28.12
Smoker	42480	chr2	11767141	C	T	*GREB1*	MISSENSE	ENSG00000196208:MISSENSE[ENST00000234142(c.4360C>T,Cgg>Tgg):ENSP00000234142(p.Arg1454Trp)];ENSG00000196208:MISSENSE[ENST00000396123(c.1354C>T,Cgg>Tgg):ENSP00000379429(p.Arg452Trp)];ENSG00000196208:MISSENSE[ENST00000381486(c.4360C>T,Cgg>Tgg):ENSP00000370896(p.Arg1454Trp)];	chr2*11767	NA	NA	2.49E-05	6.08E-05	NA	NA	D	PrD	D	MODERATE	D	0.982	151	151	0	97	89	7	7.22
Smoker	42480	chr2	99513855	G	A	*KIAA1211L*	MISSENSE	ENSG00000196872:MISSENSE[ENST00000420294(c.17C>T,tCc>tTc):ENSP00000403428(p.Ser6Phe)];ENSG00000196872:MISSENSE[ENST00000415261(c.17C>T,tCc>tTc):ENSP00000390799(p.Ser6Phe)];ENSG00000196872:MISSENSE[ENST00000428096(c.17C>T,tCc>tTc):ENSP00000387902(p.Ser6Phe)];ENSG00000196872:INTRONIC[ENST00000397899(c.-11+38546C>T)];ENSG00000196872:INTRONIC[ENST00000462314(n.237+38546C>T)];	NA	NA	NA	NA	NA	NA	NA	D_lc	UN	NA	MODERATE	NA	NA	186	186	0	133	114	19	14.29
Smoker	42480	chr2	128331513	C	T	*MYO7B*	MISSENSE	ENSG00000169994:MISSENSE[ENST00000389524(c.611C>T,aCa>aTa):ENSP00000374175(p.Thr204Ile)];ENSG00000169994:MISSENSE[ENST00000409816(c.611C>T,aCa>aTa):ENSP00000386461(p.Thr204Ile)];ENSG00000169994:MISSENSE[ENST00000428314(c.611C>T,aCa>aTa):ENSP00000415090(p.Thr204Ile)];	NA	NA	NA	NA	NA	NA	NA	D	PrD	D	MODERATE	D	0.992	193	191	2	135	116	19	14.07
Smoker	42480	chr2	1895801	G	A	*MYT1L*	INTRONIC-SS-DNR	ENSG00000186487:[ENST00000399161(c.2283+8C>T)];ENSG00000186487:[ENST00000490585(n.299+8C>T)];ENSG00000186487:[ENST00000602387(c.*173+8C>T)];ENSG00000186487:[ENST00000428368(c.2277+8C>T)];	NA	NA	NA	NA	NA	NA	NA	NA	NA	NA	LOW	NA	NA	53	53	0	17	9	8	47.06
Smoker	42480	chr2	240984458	C	T	*NA*	INTERGENIC		NA	NA	0.0001997	8.41E-05	0.000439367	0.0001997	0.001	NA	NA	NA	MODIFIER	NA	NA	124	124	0	74	62	12	16.22
Smoker	42480	chr2	11809917	G	A	*NTSR2*	SILENT	ENSG00000169006:SILENT[ENST00000306928(c.339C>T,ttC>ttT):ENSP00000303686(p.Phe113(=))];	NA	NA	NA	NA	NA	NA	NA	NA	NA	NA	LOW	NA	0.991	111	111	0	69	48	21	30.43
Smoker	42480	chr2	182850851	G	A	*PPP1R1C*	MISSENSE	ENSG00000150722:MISSENSE[ENST00000409137(c.14G>A,aGt>aAt):ENSP00000386359(p.Ser5Asn)];ENSG00000150722:MISSENSE[ENST00000452904(c.14G>A,aGt>aAt):ENSP00000399602(p.Ser5Asn)];ENSG00000150722:MISSENSE[ENST00000409702(c.14G>A,aGt>aAt):ENSP00000386778(p.Ser5Asn)];ENSG00000150722:MISSENSE[ENST00000280295(c.14G>A,aGt>aAt):ENSP00000280295(p.Ser5Asn)];ENSG00000150722:INTRONIC[ENST00000486067(n.253-1715G>A)];ENSG00000150722:INTRONIC[ENST00000475249(n.185+10860G>A)];ENSG00000150722:INTRONIC[ENST00000464264(n.158-1715G>A)];ENSG00000150722:INTRONIC[ENST00000495820(n.440+10860G>A)];ENSG00000150722:INTRONIC[ENST00000490645(n.213+10860G>A)];ENSG00000150722:INTRONIC[ENST00000465612(n.122+10860G>A)];	NA	NA	NA	NA	NA	NA	NA	NA	NA	NA	MODIFIER	NA	NA	235	234	0	217	205	12	5.53
Smoker	42480	chr2	201171067	C	A	*SPATS2L*	5UTR	ENSG00000196141:[ENST00000444012(c.-473C>A)];ENSG00000196141:[ENST00000409988(c.-330C>A)];ENSG00000196141:[ENST00000439395(c.-147C>A)];ENSG00000196141:[ENST00000358677(c.-73+184C>A)];ENSG00000196141:[ENST00000409718(c.-73+52C>A)];ENSG00000196141:[ENST00000409385(c.-192+52C>A)];ENSG00000196141:[ENST00000439084(c.-23+184C>A)];	NA	NA	NA	NA	NA	NA	NA	NA	NA	NA	MODIFIER	NA	NA	241	241	0	129	116	12	9.3
Smoker	42480	chr2	99757621	T	C	*TSGA10*	INTRONIC	ENSG00000135951:[ENST00000494483(n.78+186A>G)];ENSG00000135951:[ENST00000355053(c.-485+270A>G)];ENSG00000135951:[ENST00000471174(n.270+13535A>G)];ENSG00000135951:[ENST00000476849(n.272+13535A>G)];ENSG00000135951:[ENST00000539964(c.-313+186A>G)];ENSG00000135951:[ENST00000393483(c.-621+13535A>G)];ENSG00000135951:[ENST00000393482(c.-313+186A>G)];ENSG00000135951:[ENST00000462782(n.78+186A>G)];ENSG00000135951:[ENST00000497915(n.23A>G)];	NA	NA	NA	NA	NA	NA	NA	NA	NA	NA	MODIFIER	NA	NA	60	60	0	53	34	19	35.85
Smoker	42480	chr2	179458334	G	A	*TTN*	MISSENSE	ENSG00000155657:MISSENSE[ENST00000359218(c.31873C>T,Cct>Tct):ENSP00000352154(p.Pro10625Ser)];ENSG00000155657:MISSENSE[ENST00000591111(c.53770C>T,Cct>Tct):ENSP00000465570(p.Pro17924Ser)];ENSG00000155657:MISSENSE[ENST00000589042(c.58693C>T,Cct>Tct):ENSP00000467141(p.Pro19565Ser)];ENSG00000155657:MISSENSE[ENST00000342175(c.32074C>T,Cct>Tct):ENSP00000340554(p.Pro10692Ser)];ENSG00000155657:MISSENSE[ENST00000342992(c.50989C>T,Cct>Tct):ENSP00000343764(p.Pro16997Ser)];ENSG00000155657:MISSENSE[ENST00000460472(c.31498C>T,Cct>Tct):ENSP00000434586(p.Pro10500Ser)];	NA	NA	NA	NA	NA	NA	NA	NA	NA	NA	MODIFIER	NA	NA	171	169	2	136	90	46	33.82
Smoker	42480	chr2	179472820	G	A	*TTN*	INTRONIC	ENSG00000155657:[ENST00000359218(c.25886-12C>T)];ENSG00000155657:[ENST00000591111(c.47783-12C>T)];ENSG00000155657:[ENST00000589042(c.52706-12C>T)];ENSG00000155657:[ENST00000342992(c.45002-12C>T)];ENSG00000155657:[ENST00000342175(c.26087-12C>T)];ENSG00000155657:[ENST00000460472(c.25511-12C>T)];	NA	NA	NA	NA	NA	NA	NA	NA	NA	NA	MODIFIER	NA	NA	185	185	0	178	153	25	14.04
Smoker	42480	chr2	179458334	G	A	*TTN-AS1*	INTRONIC	ENSG00000237298:[ENST00000592630(n.434-3989G>A)];ENSG00000237298:[ENST00000589234(n.157+2293G>A)];ENSG00000237298:[ENST00000592689(n.463-3989G>A)];ENSG00000237298:[ENST00000590807(n.200-3989G>A)];ENSG00000237298:[ENST00000585451(n.198+69971G>A)];ENSG00000237298:[ENST00000592750(n.346-3989G>A)];ENSG00000237298:[ENST00000419746(n.3364+2293G>A)];ENSG00000237298:[ENST00000456053(n.564-3989G>A)];ENSG00000237298:[ENST00000586452(n.400+2293G>A)];ENSG00000237298:[ENST00000591332(n.443-3989G>A)];ENSG00000237298:[ENST00000590932(n.616-3989G>A)];ENSG00000237298:[ENST00000589907(n.421-3989G>A)];	NA	NA	NA	NA	NA	NA	NA	NA	NA	NA	MODIFIER	NA	NA	171	169	2	136	90	46	33.82

Supplementary Table 4. List of somatic single nucleotide variants identified using whole exome sequencing of ESCC patients

Cohort	Sample	Chromosome	Genomic position	Reference allele	Alternate allele	Gene symbol	Variant type	Variant annotation	OncoMD	COSMIC	dbSNP	ExAC	ExAC_SAS	1000G	1000G_SAS	SIFT	Polyphen2	CONDEL	VEP	LRT	PhastCon7 Way	Total read (Adjacent normal)	Reference allel read support (Adjacent normal)	Alternative read support (Adjacent normal)	Total read (Tumor)	Reference allel read support (Tumor)	Alternative read support (Tumor)	Alternate allele frequency
Smoker	42480	chr2	179472820	G	A	*TTN-AS1*	INTRONIC	ENSG00000237298:[ENST00000592630(n.519+10412G>A)];ENSG00000237298:[ENST00000589234(n.511-5942G>A)];ENSG00000237298:[ENST00000590807(n.395+7426G>A)];ENSG00000237298:[ENST00000585451(n.198+84457G>A)];ENSG00000237298:[ENST00000592750(n.432-7230G>A)];ENSG00000237298:[ENST00000589830(n.86+10412G>A)];ENSG00000237298:[ENST00000589487(n.110+7426G>A)];ENSG00000237298:[ENST00000456053(n.650-74G>A)];ENSG00000237298:[ENST00000589907(n.506+10412G>A)];	NA	NA	NA	NA	NA	NA	NA	NA	NA	NA	MODIFIER	NA	NA	185	185	0	178	153	25	14.04
Smoker	42480	chr20	31598901	G	A	*BPIFB2*	MISSENSE	ENSG00000078898:MISSENSE[ENST00000170150(c.181G>A,Gga>Aga):ENSP00000170150(p.Gly61Arg)];	NA	NA	NA	NA	NA	NA	NA	T	PoD	D	MODERATE	N	0.037	65	65	0	42	29	13	30.95
Smoker	42480	chr20	5170706	C	T	*CDS2*	INTRONIC	ENSG00000101290:[ENST00000535100(c.516-42C>T)];ENSG00000101290:[ENST00000460006(c.1206-42C>T)];ENSG00000101290:[ENST00000379062(c.846-42C>T)];ENSG00000101290:[ENST00000379070(n.1461-42C>T)];	NA	NA	NA	NA	NA	NA	NA	NA	NA	NA	MODIFIER	NA	NA	88	88	0	94	86	8	8.51
Smoker	42480	chr20	46307535	C	T	*CTD-2653D5.1*	INTRONIC	ENSG00000255438:[ENST00000526566(n.481+55C>T)];	NA	NA	0.0001997	0.000661	0	0.0001997	0	T	PrD	D	MODERATE	D	1	72	72	0	34	21	13	38.24
Smoker	42480	chr20	44519478	G	A	*CTSA*	5UTR	ENSG00000064601:[ENST00000606788(c.-487G>A)];	NA	NA	NA	NA	NA	NA	NA	NA	NA	NA	LOW	NA	0.997	91	91	0	72	65	7	9.72
Smoker	42480	chr20	25196239	A	G	*ENTPD6*	INTRONIC	ENSG00000197586:[ENST00000354989(c.623-73A>G)];ENSG00000197586:[ENST00000417467(c.476-73A>G)];ENSG00000197586:[ENST00000427553(c.247-73A>G)];ENSG00000197586:[ENST00000376666(c.144-73A>G)];ENSG00000197586:[ENST00000376652(c.674-73A>G)];ENSG00000197586:[ENST00000433259(c.674-73A>G)];ENSG00000197586:[ENST00000447877(c.352-73A>G)];ENSG00000197586:[ENST00000425813(c.530-73A>G)];ENSG00000197586:[ENST00000439162(c.620-73A>G)];ENSG00000197586:[ENST00000360031(c.671-73A>G)];ENSG00000197586:[ENST00000418890(c.479-73A>G)];ENSG00000197586:[ENST00000433417(c.436-73A>G)];	NA	NA	0.0001997	NA	NA	0.0001997	0	NA	NA	NA	MODIFIER	NA	NA	268	268	0	173	155	18	10.4
Smoker	42480	chr20	6064757	C	T	*FERMT1*	MISSENSE	ENSG00000101311:MISSENSE[ENST00000536936(c.877G>A,Gaa>Aaa):ENSP00000441063(p.Glu293Lys)];ENSG00000101311:MISSENSE[ENST00000217289(c.1648G>A,Gaa>Aaa):ENSP00000217289(p.Glu550Lys)];ENSG00000101311:EXONIC-NC[ENST00000478194(n.608G>A)];	NA	NA	NA	6.14E-05	6.45E-05	NA	NA	NA	NA	NA	MODIFIER	NA	NA	73	73	0	75	53	22	29.33
Smoker	42480	chr20	29633992	A	G	*FRG1B*	3UTR	ENSG00000149531:[ENST00000278882(c.*82A>G)];ENSG00000149531:[ENST00000358464(c.*82A>G)];	NA	NA	NA	NA	NA	NA	NA	NA	NA	NA	MODIFIER	NA	NA	499	487	9	539	522	15	2.78
Smoker	42480	chr20	31427607	G	A	*MAPRE1*	MISSENSE	ENSG00000101367:MISSENSE[ENST00000375571(c.542G>A,cGa>cAa):ENSP00000364721(p.Arg181Gln)];	NA	NA	0.0001997	NA	NA	0.0001997	0	T	BN	N	MODERATE	N	0.997	82	82	0	80	61	19	23.75
Smoker	42480	chr20	33567505	G	A	*MYH7B*	SILENT	ENSG00000078814:SILENT[ENST00000262873(c.366G>A,ccG>ccA):ENSP00000262873(p.Pro122(=))];ENSG00000078814:EXONIC-NC[ENST00000470929(n.326G>A)];	chr20*3356	chr20*3356	NA	1.66E-05	0	NA	NA	NA	NA	NA	MODIFIER	NA	NA	196	195	0	140	116	24	17.14
Smoker	42480	chr20	44519478	G	A	*NEURL2*	SILENT	ENSG00000124257:SILENT[ENST00000372518(c.153C>T,caC>caT):ENSP00000361596(p.His51(=))];	NA	NA	NA	NA	NA	NA	NA	NA	NA	NA	LOW	NA	0.997	91	91	0	72	65	7	9.72
Smoker	42480	chr20	41077028	G	A	*PTPRT*	INTRONIC	ENSG00000196090:[ENST00000373201(c.1451-59C>T)];ENSG00000196090:[ENST00000356100(c.1451-59C>T)];ENSG00000196090:[ENST00000373198(c.1451-59C>T)];ENSG00000196090:[ENST00000373193(c.1451-59C>T)];ENSG00000196090:[ENST00000373184(c.1451-59C>T)];ENSG00000196090:[ENST00000373190(c.1451-59C>T)];ENSG00000196090:[ENST00000373187(c.1451-59C>T)];	NA	NA	0.004393	NA	NA	0.004393	0.0031	NA	NA	NA	MODIFIER	NA	NA	80	80	0	80	75	5	6.25
Smoker	42480	chr20	1212190	G	C	*RAD21L1*	MISSENSE	ENSG00000244588:MISSENSE[ENST00000409241(c.295G>C,Gag>Cag):ENSP00000386414(p.Glu99Gln)];ENSG00000244588:MISSENSE[ENST00000402452(c.295G>C,Gag>Cag):ENSP00000385925(p.Glu99Gln)];ENSG00000244588:MISSENSE[ENST00000381882(c.295G>C,Gag>Cag):ENSP00000371306(p.Glu99Gln)];ENSG00000244588:EXONIC-NC[ENST00000477283(n.457G>C)];	NA	NA	NA	NA	NA	NA	NA	NA	NA	NA	MODIFIER	NA	NA	67	67	0	90	72	18	20
Smoker	42480	chr20	42204932	C	T	*SGK2*	SILENT	ENSG00000101049:SILENT[ENST00000496343(c.762C>T,ccC>ccT):ENSP00000436841(p.Pro254(=))];ENSG00000101049:SILENT[ENST00000373100(c.762C>T,ccC>ccT):ENSP00000362192(p.Pro254(=))];ENSG00000101049:SILENT[ENST00000423407(c.762C>T,ccC>ccT):ENSP00000392795(p.Pro254(=))];ENSG00000101049:SILENT[ENST00000373077(c.759C>T,ccC>ccT):ENSP00000362168(p.Pro253(=))];ENSG00000101049:SILENT[ENST00000341458(c.942C>T,ccC>ccT):ENSP00000340608(p.Pro314(=))];ENSG00000101049:SILENT[ENST00000373092(c.762C>T,ccC>ccT):ENSP00000362184(p.Pro254(=))];ENSG00000101049:SILENT[ENST00000426287(c.840C>T,ccC>ccT):ENSP00000412214(p.Pro280(=))];	NA	NA	NA	4.12E-05	0	NA	NA	NA	NA	NA	LOW	NA	NA	129	129	0	111	88	23	20.72
Smoker	42480	chr20	46307535	C	T	*SULF2*	MISSENSE	ENSG00000196562:MISSENSE[ENST00000359930(c.1078G>A,Gtc>Atc):ENSP00000353007(p.Val360Ile)];ENSG00000196562:MISSENSE[ENST00000361612(c.1078G>A,Gtc>Atc):ENSP00000354662(p.Val360Ile)];ENSG00000196562:MISSENSE[ENST00000467815(c.1078G>A,Gtc>Atc):ENSP00000418442(p.Val360Ile)];ENSG00000196562:MISSENSE[ENST00000484875(c.1078G>A,Gtc>Atc):ENSP00000418290(p.Val360Ile)];	NA	NA	0.0001997	0.000661	0	0.0001997	0	T	PrD	D	MODERATE	D	1	72	72	0	34	21	13	38.24
Smoker	42480	chr20	45130682	G	T	*ZNF334*	SILENT	ENSG00000198185:SILENT[ENST00000593880(c.1365C>A,ccC>ccA):ENSP00000473194(p.Pro455(=))];ENSG00000198185:SILENT[ENST00000347606(c.1296C>A,ccC>ccA):ENSP00000255129(p.Pro432(=))];ENSG00000198185:SILENT[ENST00000457685(c.1182C>A,ccC>ccA):ENSP00000402582(p.Pro394(=))];	NA	NA	NA	NA	NA	NA	NA	NA	NA	NA	LOW	NA	NA	197	196	0	181	149	32	17.68
Smoker	42480	chr20	32336833	C	T	*ZNF341*	SILENT	ENSG00000131061:3UTR[ENST00000497876(c.*28C>T)];ENSG00000131061:SILENT[ENST00000375200(c.444C>T,ttC>ttT):ENSP00000364346(p.Phe148(=))];ENSG00000131061:SILENT[ENST00000483118(c.444C>T,ttC>ttT):ENSP00000432933(p.Phe148(=))];ENSG00000131061:SILENT[ENST00000342427(c.444C>T,ttC>ttT):ENSP00000344308(p.Phe148(=))];	NA	NA	NA	NA	NA	NA	NA	NA	NA	NA	LOW	NA	0.998	52	52	0	38	32	6	15.79
Smoker	42480	chr21	11038748	C	T	*BAGE2*	EXONIC-NC	ENSG00000187172:[ENST00000496773(n.1150G>A)];ENSG00000187172:[ENST00000470054(n.1456G>A)];	NA	NA	NA	NA	NA	NA	NA	NA	NA	NA	MODIFIER	NA	NA	473	467	5	517	495	21	4.06
Smoker	42480	chr21	43134277	G	A	*LINC00479*	EXONIC-NC	ENSG00000236384:[ENST00000589300(n.273C>T)];ENSG00000236384:[ENST00000412102(n.333C>T)];ENSG00000236384:[ENST00000586552(n.176+1003C>T)];	NA	NA	chr21*43134277*43134277*G*T*rs116463308*1*0.9988*0.001198	NA	NA	NA	NA	NA	NA	NA	MODIFIER	NA	NA	90	89	1	55	43	12	21.82
Smoker	42480	chr21	32589935	C	A	*TIAM1*	MISSENSE	ENSG00000156299:MISSENSE[ENST00000286827(c.2076G>T,agG>agT):ENSP00000286827(p.Arg692Ser)];ENSG00000156299:MISSENSE[ENST00000541036(c.2076G>T,agG>agT):ENSP00000441570(p.Arg692Ser)];ENSG00000156299:EXONIC-NC[ENST00000469412(n.1405G>T)];	NA	NA	NA	NA	NA	NA	NA	NA	NA	NA	MODIFIER	NA	NA	175	175	0	94	76	18	19.15

Mangalaparthi *et al.* , 2020. Mutational landscape of esophageal squamous cell carcinoma in an Indian cohort
Supplementary Table 4. List of somatic single nucleotide variants identified using whole exome sequencing of ESCC patients

Cohort	Sample	Chromosome	Genomic position	Reference allele	Alternate allele	Gene symbol	Variant type	Variant annotation	OncoMD	COSMIC	dbSNP	ExAC	ExAC_SAS	1000G	1000G_SAS	SIFT	Polyphen2	CONDEL	VEP	LRT	PhastCon7 Way	Total read (Adjacent normal)	Reference allel read support (Adjacent normal)	Alternative read support (Adjacent normal)	Total read (Tumor)	Reference allel read support (Tumor)	Alternative read support (Tumor)	Alternate allele frequency
Smoker	42480	chr22	49042503	G	A	*FAM19A5*	SILENT	ENSG00000219438:SILENT[ENST00000358295(c.186G>A,gcG>gcA):ENSP00000351043(p.Ala62(=))];ENSG00000219438:SILENT[ENST00000402357(c.207G>A,gcG>gcA):ENSP00000383933(p.Ala69(=))];ENSG00000219438:SILENT[ENST00000336769(c.207G>A,gcG>gcA):ENSP00000336812(p.Ala69(=))];ENSG00000219438:INTRONIC[ENST00000473898(n.120-61026G>A)];	NA	NA	NA	6.36E-05	0	NA	NA	NA	NA	NA	LOW	NA	NA	81	81	0	35	29	6	17.14
Smoker	42480	chr3	151474874	G	A	*AADACL2*	MISSENSE	ENSG00000197953:3UTR[ENST00000445270(c.*313G>A)];ENSG00000197953:MISSENSE[ENST00000356517(c.698G>A,cGa>cAa):ENSP00000348911(p.Arg233Gln)];	NA	NA	NA	2.47E-05	0	NA	NA	NA	NA	NA	MODIFIER	NA	NA	240	237	2	369	330	39	10.57
Smoker	42480	chr3	180327579	C	G	*CCDC39*	3UTR	ENSG00000145075:[ENST00000489868(c.*42G>C)];ENSG00000145075:[ENST00000473854(c.*45+4023G>C)];	NA	NA	NA	NA	NA	NA	NA	NA	NA	NA	MODIFIER	NA	NA	169	169	0	235	193	42	17.87
Smoker	42480	chr3	180327611	A	G	*CCDC39*	INTRONIC	ENSG00000145075:[ENST00000489868(c.*33-23T>C)];ENSG00000145075:[ENST00000473854(c.*45+3991T>C)];	NA	NA	NA	NA	NA	NA	NA	NA	NA	NA	MODIFIER	NA	NA	183	183	0	310	254	56	18.06
Smoker	42480	chr3	1425012	A	T	*CNTN6*	MISSENSE	ENSG00000134115:MISSENSE[ENST00000539053(c.2221A>T,Agt>Tgt):ENSP00000442791(p.Ser741Cys)];ENSG00000134115:MISSENSE[ENST00000446702(c.2437A>T,Agt>Tgt):ENSP00000407822(p.Ser813Cys)];ENSG00000134115:MISSENSE[ENST00000350110(c.2437A>T,Agt>Tgt):ENSP00000341882(p.Ser813Cys)];ENSG00000134115:3UTR[ENST00000397479(c.*2575A>T)];	NA	NA	NA	NA	NA	NA	NA	NA	NA	NA	MODIFIER	NA	NA	287	286	0	243	211	31	12.76
Smoker	42480	chr3	196812524	C	A	*DLG1*	NONSENSE	ENSG00000075711:NONSENSE[ENST00000452595(c.1516G>T,Gaa>Taa):ENSP00000398939(p.Glu506Ter)];ENSG00000075711:NONSENSE[ENST00000392381(c.1864G>T,Gaa>Taa):ENSP00000376186(p.Glu622Ter)];ENSG00000075711:NONSENSE[ENST00000448528(c.1864G>T,Gaa>Taa):ENSP00000391732(p.Glu622Ter)];ENSG00000075711:NONSENSE[ENST00000422288(c.1711G>T,Gaa>Taa):ENSP00000413238(p.Glu571Ter)];ENSG00000075711:NONSENSE[ENST00000450955(c.1765G>T,Gaa>Taa):ENSP00000411278(p.Glu589Ter)];ENSG00000075711:NONSENSE[ENST00000346964(c.1864G>T,Gaa>Taa):ENSP00000345731(p.Glu622Ter)];ENSG00000075711:NONSENSE[ENST00000443183(c.1516G>T,Gaa>Taa):ENSP00000396658(p.Glu506Ter)];ENSG00000075711:NONSENSE[ENST00000314062(c.1711G>T,Gaa>Taa):ENSP00000321087(p.Glu571Ter)];ENSG00000075711:NONSENSE[ENST00000419354(c.1864G>T,Gaa>Taa):ENSP00000407531(p.Glu622Ter)];ENSG00000075711:NONSENSE[ENST00000392382(c.1765G>T,Gaa>Taa):ENSP00000376187(p.Glu589Ter)];ENSG00000075711:NONSENSE[ENST00000357674(c.1765G>T,Gaa>Taa):ENSP00000350303(p.Glu589Ter)];ENSG00000075711:EXONIC-NC[ENST00000471733(n.1892G>T)];ENSG00000075711:EXONIC-NC[ENST00000493937(n.6G>T)];ENSG00000075711:3UTR[ENST00000419227(c.*217G>T)];	NA	NA	NA	NA	NA	NA	NA	NA	NA	NA	MODIFIER	NA	NA	183	182	0	280	267	13	4.64
Smoker	42480	chr3	97356813	C	T	*EPHA6*	NONSENSE	ENSG00000080224:NONSENSE[ENST00000477384(c.643C>T,Cga>Tga):ENSP00000419470(p.Arg215Ter)];ENSG00000080224:NONSENSE[ENST00000389672(c.2671C>T,Cga>Tga):ENSP00000374323(p.Arg891Ter)];ENSG00000080224:NONSENSE[ENST00000502694(c.847C>T,Cga>Tga):ENSP00000423950(p.Arg283Ter)];ENSG00000080224:NONSENSE[ENST00000514100(c.847C>T,Cga>Tga):ENSP00000421711(p.Arg283Ter)];ENSG00000080224:3UTR[ENST00000442602(c.*141C>T)];ENSG00000080224:3UTR[ENST00000508345(c.*141C>T)];	NA	NA	NA	NA	NA	NA	NA	NA	NA	NA	MODIFIER	NA	NA	128	128	0	166	148	18	10.84
Smoker	42480	chr3	58120433	T	C	*FLNB*	SILENT	ENSG00000136068:SILENT[ENST00000429972(c.4605T>C,agT>agC):ENSP00000415599(p.Ser1535(=))];ENSG00000136068:SILENT[ENST00000295956(c.4605T>C,agT>agC):ENSP00000295956(p.Ser1535(=))];ENSG00000136068:SILENT[ENST00000358537(c.4605T>C,agT>agC):ENSP00000351339(p.Ser1535(=))];ENSG00000136068:SILENT[ENST00000490882(c.4698T>C,agT>agC):ENSP00000420213(p.Ser1566(=))];ENSG00000136068:SILENT[ENST00000348383(c.4605T>C,agT>agC):ENSP00000232447(p.Ser1535(=))];ENSG00000136068:SILENT[ENST00000493452(c.4098T>C,agT>agC):ENSP00000418510(p.Ser1366(=))];ENSG00000136068:SILENT[ENST00000419752(c.4098T>C,agT>agC):ENSP00000414532(p.Ser1366(=))];ENSG00000136068:SILENT[ENST00000357272(c.4605T>C,agT>agC):ENSP00000349819(p.Ser1535(=))];ENSG00000136068:EXONIC-NC[ENST00000481470(n.945T>C)];	NA	NA	NA	NA	NA	NA	NA	NA	NA	NA	LOW	NA	0.95	133	133	0	85	61	24	28.24
Smoker	42480	chr3	58132650	C	T	*FLNB*	SILENT	ENSG00000136068:SILENT[ENST00000429972(c.5625C>T,ggC>ggT):ENSP00000415599(p.Gly1875(=))];ENSG00000136068:SILENT[ENST00000295956(c.5658C>T,ggC>ggT):ENSP00000295956(p.Gly1886(=))];ENSG00000136068:SILENT[ENST00000358537(c.5586C>T,ggC>ggT):ENSP00000351339(p.Gly1862(=))];ENSG00000136068:SILENT[ENST00000490882(c.5751C>T,ggC>ggT):ENSP00000420213(p.Gly1917(=))];ENSG00000136068:SILENT[ENST00000348383(c.5658C>T,ggC>ggT):ENSP00000232447(p.Gly1886(=))];ENSG00000136068:SILENT[ENST00000493452(c.5079C>T,ggC>ggT):ENSP00000418510(p.Gly1693(=))];ENSG00000136068:SILENT[ENST00000419752(c.5118C>T,ggC>ggT):ENSP00000414532(p.Gly1706(=))];ENSG00000136068:SILENT[ENST00000357272(c.5658C>T,ggC>ggT):ENSP00000349819(p.Gly1886(=))];ENSG00000136068:EXONIC-NC[ENST00000481470(n.1926C>T)];ENSG00000136068:EXONIC-NC[ENST00000491408(n.246C>T)];	NA	NA	NA	8.24E-06	6.06E-05	NA	NA	NA	NA	NA	LOW	NA	NA	78	78	0	47	41	6	12.77
Smoker	42480	chr3	141889109	G	A	*GK5*	MISSENSE	ENSG00000175066:INTRONIC[ENST00000480757(c.*1618+58C>T)];ENSG00000175066:INTRONIC[ENST00000392993(c.1441+58C>T)];ENSG00000175066:INTRONIC[ENST00000492097(c.*1647+58C>T)];ENSG00000175066:INTRONIC[ENST00000463349(c.*396+58C>T)];ENSG00000175066:INTRONIC[ENST00000460515(n.642+58C>T)];ENSG00000175066:MISSENSE[ENST00000486459(c.461C>T,gCa>gTa):ENSP00000420593(p.Ala154Val)];	NA	NA	NA	NA	NA	NA	NA	T_lc	UN	NA	MODERATE	NA	NA	143	143	0	275	242	33	12
Smoker	42480	chr3	7721771	G	C	*GRM7*	MISSENSE	ENSG00000196277:MISSENSE[ENST00000402647(c.2487G>C,atG>atC):ENSP00000384585(p.Met829Ile)];ENSG00000196277:MISSENSE[ENST00000486284(c.2487G>C,atG>atC):ENSP00000417536(p.Met829Ile)];ENSG00000196277:MISSENSE[ENST00000389336(c.2487G>C,atG>atC):ENSP00000373987(p.Met829Ile)];ENSG00000196277:MISSENSE[ENST00000357716(c.2487G>C,atG>atC):ENSP00000350348(p.Met829Ile)];ENSG00000196277:MISSENSE[ENST00000403881(c.2487G>C,atG>atC):ENSP00000385664(p.Met829Ile)];ENSG00000196277:MISSENSE[ENST00000389335(c.2487G>C,atG>atC):ENSP00000373986(p.Met829Ile)];ENSG00000196277:MISSENSE[ENST00000467425(c.2487G>C,atG>atC):ENSP00000419835(p.Met829Ile)];ENSG00000196277:MISSENSE[ENST00000440923(c.2487G>C,atG>atC):ENSP00000412329(p.Met829Ile)];ENSG00000196277:EXONIC-NC[ENST00000463676(n.1752G>C)];	NA	NA	NA	NA	NA	NA	NA	T	BN	N	MODERATE	NA	NA	113	113	0	82	58	24	29.27

Mangalaparthi *et al.* , 2020. Mutational landscape of esophageal squamous cell carcinoma in an Indian cohort
Supplementary Table 4. List of somatic single nucleotide variants identified using whole exome sequencing of ESCC patients

Cohort	Sample	Chromosome	Genomic position	Reference allele	Alternate allele	Gene symbol	Variant type	Variant annotation	OncoMD	COSMIC	dbSNP	ExAC	ExAC_SAS	1000G	1000G_SAS	SIFT	Polyphen2	CONDEL	VEP	LRT	PhastCon7 Way	Total read (Adjacent normal)	Reference allel read support (Adjacent normal)	Alternative read support (Adjacent normal)	Total read (Tumor)	Reference allel read support (Tumor)	Alternative read support (Tumor)	Alternate allele frequency
Smoker	42480	chr3	121518217	C	T	*IQCB1*	MISSENSE	ENSG00000173226:INTRONIC[ENST00000349820(c.587+7974G>A)];ENSG00000173226:MISSENSE[ENST00000310864(c.592G>A,Gat>Aat):ENSP00000311505(p.Asp198Asn)];ENSG00000173226:MISSENSE[ENST00000498104(c.40G>A,Gat>Aat):ENSP00000417832(p.Asp14Asn)];ENSG00000173226:MISSENSE[ENST00000460108(c.40G>A,Gat>Aat):ENSP00000419168(p.Asp14Asn)];ENSG00000173226:MISSENSE[ENST00000393650(c.592G>A,Gat>Aat):ENSP00000377261(p.Asp198Asn)];	NA	NA	NA	NA	NA	NA	NA	T	BN	N	MODERATE	NA	NA	30	30	0	56	45	11	19.64
Smoker	42480	chr3	178936091	G	A	*PIK3CA*	MISSENSE	ENSG00000121879:MISSENSE[ENST00000263967(c.1633G>A,Gag>Aag):ENSP00000263967(p.Glu545Lys)];	chr3*17893	chr3*17893	NA	8.34E-06	0	NA	NA	D	PrD	D	MODERATE	D	1	116	116	0	189	154	35	18.52
Smoker	42480	chr3	151474874	G	A	*RP11-454C18.2*	INTRONIC	ENSG00000242908:[ENST00000475855(n.380-1955C>T)];ENSG00000242908:[ENST00000483843(n.500-1955C>T)];	NA	NA	NA	2.47E-05	0	NA	NA	NA	NA	NA	MODIFIER	NA	NA	240	237	2	369	330	39	10.57
Smoker	42480	chr3	38760343	G	A	*SCN10A*	INTRONIC	ENSG00000185313:[ENST00000449082(c.3508-26C>T)];	NA	NA	NA	NA	NA	NA	NA	NA	NA	NA	MODIFIER	NA	NA	57	57	0	38	19	19	50
Smoker	42480	chr3	38888356	T	A	*SCN11A*	MISSENSE	ENSG00000168356:MISSENSE[ENST00000450244(c.5205A>T,agA>agT):ENSP00000400945(p.Arg1735Ser)];ENSG00000168356:MISSENSE[ENST00000302328(c.5205A>T,agA>agT):ENSP00000307599(p.Arg1735Ser)];ENSG00000168356:MISSENSE[ENST00000456224(c.5091A>T,agA>agT):ENSP00000416757(p.Arg1697Ser)];	NA	NA	NA	NA	NA	NA	NA	D	BN	N	MODERATE	N	0.012	193	193	0	109	69	40	36.7
Smoker	42480	chr3	180327579	C	G	*TTC14*	MISSENSE	ENSG00000163728:MISSENSE[ENST00000296015(c.1562C>G,tCt>tGt):ENSP00000296015(p.Ser521Cys)];ENSG00000163728:MISSENSE[ENST00000382584(c.1562C>G,tCt>tGt):ENSP00000372027(p.Ser521Cys)];ENSG00000163728:3UTR[ENST00000412756(c.*1996C>G)];ENSG00000163728:3UTR[ENST00000465065(c.*5274C>G)];ENSG00000163728:3UTR[ENST00000470669(c.*1567C>G)];ENSG00000163728:EXONIC-NC[ENST00000465625(n.381C>G)];	NA	NA	NA	NA	NA	NA	NA	NA	NA	NA	MODIFIER	NA	NA	169	169	0	235	193	42	17.87
Smoker	42480	chr3	180327611	A	G	*TTC14*	MISSENSE	ENSG00000163728:MISSENSE[ENST00000382584(c.1594A>G,Agg>Ggg):ENSP00000372027(p.Arg532Gly)];ENSG00000163728:MISSENSE[ENST00000296015(c.1594A>G,Agg>Ggg):ENSP00000296015(p.Arg532Gly)];ENSG00000163728:3UTR[ENST00000412756(c.*2028A>G)];ENSG00000163728:3UTR[ENST00000465065(c.*5306A>G)];ENSG00000163728:3UTR[ENST00000470669(c.*1599A>G)];ENSG00000163728:EXONIC-NC[ENST00000465625(n.413A>G)];	NA	NA	NA	NA	NA	NA	NA	NA	NA	NA	MODIFIER	NA	NA	183	183	0	310	254	56	18.06
Smoker	42480	chr3	39230321	G	A	*XIRP1*	MISSENSE	ENSG00000168334:MISSENSE[ENST00000396251(c.616C>T,Cgc>Tgc):ENSP00000379550(p.Arg206Cys)];ENSG00000168334:MISSENSE[ENST00000340369(c.616C>T,Cgc>Tgc):ENSP00000343140(p.Arg206Cys)];ENSG00000168334:INTRONIC[ENST00000421646(c.-167-3169C>T)];	NA	NA	0.0001997	0.000117	0	0.0001997	0	T	BN	N	MODERATE	N	0.99	164	164	0	58	37	21	36.21
Smoker	42480	chr4	91246798	A	G	*CCSER1*	MISSENSE	ENSG00000184305:INTRONIC[ENST00000509176(c.1509+12600A>G)];ENSG00000184305:INTRONIC[ENST00000505073(c.1509+12600A>G)];ENSG00000184305:INTRONIC[ENST00000514352(n.88+12600A>G)];ENSG00000184305:INTRONIC[ENST00000508086(n.35+12600A>G)];ENSG00000184305:INTRONIC[ENST00000333691(c.1509+12600A>G)];ENSG00000184305:INTRONIC[ENST00000432775(c.1509+12600A>G)];ENSG00000184305:MISSENSE[ENST00000508550(c.354A>G,Aca>Gca):ENSP00000426310(p.Thr119Ala)];	NA	NA	NA	NA	NA	NA	NA	NA	NA	NA	MODIFIER	NA	NA	87	87	0	100	77	23	23
Smoker	42480	chr4	10509655	G	A	*CLNK*	SILENT	ENSG00000109684:SILENT[ENST00000226951(c.912C>T,gtC>gtT):ENSP00000226951(p.Val304(=))];ENSG00000109684:SILENT[ENST00000515667(c.126C>T,gtC>gtT):ENSP00000427256(p.Val42(=))];	NA	NA	NA	NA	NA	NA	NA	NA	NA	NA	LOW	NA	NA	178	178	0	119	106	13	10.92
Smoker	42480	chr4	66189901	G	A	*EPHA5*	SILENT	ENSG00000145242:SILENT[ENST00000273854(c.3045C>T,caC>caT):ENSP00000273854(p.His1015(=))];ENSG00000145242:SILENT[ENST00000432638(c.2556C>T,caC>caT):ENSP00000389208(p.His852(=))];ENSG00000145242:SILENT[ENST00000354839(c.2979C>T,caC>caT):ENSP00000346899(p.His993(=))];	NA	NA	NA	NA	NA	NA	NA	NA	NA	NA	LOW	NA	1	282	282	0	311	227	84	27.01
Smoker	42480	chr4	44699464	A	G	*GNPDA2*	INTRONIC	ENSG00000163281:[ENST00000608855(c.127+10305T>C)];ENSG00000163281:[ENST00000609092(c.246+10305T>C)];	NA	NA	NA	8.28E-06	0	NA	NA	NA	NA	NA	LOW	NA	NA	103	102	0	64	51	13	20.31
Smoker	42480	chr4	44699464	A	G	*GUF1*	INTRONIC-SS-DNR	ENSG00000151806:[ENST00000511493(n.142+3A>G)];ENSG00000151806:[ENST00000281543(c.1872+3A>G)];ENSG00000151806:[ENST00000506793(n.1754+3A>G)];	NA	NA	NA	8.28E-06	0	NA	NA	NA	NA	NA	LOW	NA	NA	103	102	0	64	51	13	20.31
Smoker	42480	chr4	88732687	C	A	*IBSP*	MISSENSE	ENSG00000029559:MISSENSE[ENST00000226284(c.579C>A,agC>agA):ENSP00000226284(p.Ser193Arg)];	NA	NA	NA	NA	NA	NA	NA	D	PrD	D	MODERATE	N	0.164	108	108	0	105	99	6	5.71
Smoker	42480	chr4	6083345	G	A	*JAKMIP1*	SILENT	ENSG00000152969:SILENT[ENST00000473053(c.1092C>T,aaC>aaT):ENSP00000422004(p.Asn364(=))];ENSG00000152969:SILENT[ENST00000409021(c.1092C>T,aaC>aaT):ENSP00000386711(p.Asn364(=))];ENSG00000152969:SILENT[ENST00000409831(c.1092C>T,aaC>aaT):ENSP00000386925(p.Asn364(=))];ENSG00000152969:SILENT[ENST00000282924(c.1092C>T,aaC>aaT):ENSP00000282924(p.Asn364(=))];ENSG00000152969:SILENT[ENST00000409371(c.597C>T,aaC>aaT):ENSP00000387042(p.Asn199(=))];ENSG00000152969:SILENT[ENST00000410077(c.597C>T,aaC>aaT):ENSP00000386745(p.Asn199(=))];ENSG00000152969:EXONIC-NC[ENST00000457227(n.652C>T)];	NA	NA	0.01538	0.0050025	6.06E-05	0.0153754	0	NA	NA	NA	LOW	NA	0.994	123	123	0	68	59	9	13.24
Smoker	42480	chr4	41749475	C	T	*PHOX2B*	MISSENSE	ENSG00000109132:MISSENSE[ENST00000226382(c.320G>A,aGt>aAt):ENSP00000226382(p.Ser107Asn)];ENSG00000109132:MISSENSE[ENST00000510424(c.139G>A,aGt>aAt):ENSP00000426733(p.Ser47Asn)];	NA	NA	NA	NA	NA	NA	NA	NA	NA	NA	MODERATE	NA	NA	195	195	0	87	69	18	20.69
Smoker	42480	chr4	103228735	G	A	*SLC39A8*	MISSENSE	ENSG00000138821:MISSENSE[ENST00000356736(c.410C>T,aCg>aTg):ENSP00000349174(p.Thr137Met)];ENSG00000138821:MISSENSE[ENST00000394833(c.410C>T,aCg>aTg):ENSP00000378310(p.Thr137Met)];ENSG00000138821:MISSENSE[ENST00000424970(c.410C>T,aCg>aTg):ENSP00000394548(p.Thr137Met)];ENSG00000138821:EXONIC-NC[ENST00000512657(n.329C>T)];ENSG00000138821:EXONIC-NC[ENST00000502903(n.549C>T)];ENSG00000138821:EXONIC-NC[ENST00000510255(n.338C>T)];ENSG00000138821:INTRONIC[ENST00000514000(n.257-2467C>T)];	NA	NA	NA	NA	NA	NA	NA	NA	NA	NA	MODIFIER	NA	NA	155	155	0	425	148	277	65.18
Smoker	42480	chr4	20552481	C	A	*SLIT2*	MISSENSE	ENSG00000145147:MISSENSE[ENST00000504154(c.2521C>A,Cct>Act):ENSP00000422591(p.Pro841Thr)];ENSG00000145147:MISSENSE[ENST00000503837(c.2509C>A,Cct>Act):ENSP00000422261(p.Pro837Thr)];ENSG00000145147:MISSENSE[ENST00000273739(c.2533C>A,Cct>Act):ENSP00000273739(p.Pro845Thr)];ENSG00000145147:MISSENSE[ENST00000503823(c.2497C>A,Cct>Act):ENSP00000427548(p.Pro833Thr)];ENSG00000145147:MISSENSE[ENST00000511508(c.124C>A,Cct>Act):ENSP00000421975(p.Pro42Thr)];ENSG00000145147:EXONIC-NC[ENST00000509394(n.378C>A)];	NA	NA	NA	NA	NA	NA	NA	NA	NA	NA	MODIFIER	NA	NA	152	152	0	105	81	24	22.86

Mangalaparthi *et al.* , 2020. Mutational landscape of esophageal squamous cell carcinoma in an Indian cohort
Supplementary Table 4. List of somatic single nucleotide variants identified using whole exome sequencing of ESCC patients

Cohort	Sample	Chromosome	Genomic position	Reference allele	Alternate allele	Gene symbol	Variant type	Variant annotation	OncoMD	COSMIC	dbSNP	ExAC	ExAC_SAS	1000G	1000G_SAS	SIFT	Polyphen2	CONDEL	VEP	LRT	PhastCon7 Way	Total read (Adjacent normal)	Reference allel read support (Adjacent normal)	Alternative read support (Adjacent normal)	Total read (Tumor)	Reference allel read support (Tumor)	Alternative read support (Tumor)	Alternate allele frequency
Smoker	42480	chr4	186551763	A	C	*SORBS2*	INTRONIC	ENSG00000154556:[ENST00000319454(c.1039-11T>G)];ENSG00000154556:[ENST00000487836(n.505-11T>G)];ENSG00000154556:[ENST00000418609(c.397-11T>G)];ENSG00000154556:[ENST00000448662(c.949-11T>G)];ENSG00000154556:[ENST00000449407(c.898-11T>G)];ENSG00000154556:[ENST00000495932(n.411-11T>G)];ENSG00000154556:[ENST00000498125(n.944-11T>G)];ENSG00000154556:[ENST00000437304(c.1438-11T>G)];ENSG00000154556:[ENST00000451974(c.313-11T>G)];ENSG00000154556:[ENST00000438278(c.515-11T>G)];ENSG00000154556:[ENST00000431808(c.685-11T>G)];ENSG00000154556:[ENST00000284776(c.685-11T>G)];ENSG00000154556:[ENST00000355634(c.985-11T>G)];ENSG00000154556:[ENST00000393528(c.964-11T>G)];ENSG00000154556:[ENST00000445625(c.537-26T>G)];ENSG00000154556:[ENST00000319471(c.1159-11T>G)];ENSG00000154556:[ENST00000490562(n.544-11T>G)];	NA	NA	NA	NA	NA	NA	NA	NA	NA	NA	MODIFIER	NA	NA	45	45	0	51	43	8	15.69
Smoker	42480	chr4	167656203	C	T	*SPOCK3*	MISSENSE	ENSG00000196104:MISSENSE[ENST00000357154(c.1180G>A,Gat>Aat):ENSP00000349677(p.Asp394Asn)];ENSG00000196104:MISSENSE[ENST00000511531(c.1180G>A,Gat>Aat):ENSP00000423421(p.Asp394Asn)];ENSG00000196104:MISSENSE[ENST00000357545(c.1171G>A,Gat>Aat):ENSP00000350153(p.Asp391Asn)];ENSG00000196104:MISSENSE[ENST00000506886(c.1180G>A,Gat>Aat):ENSP00000420920(p.Asp394Asn)];ENSG00000196104:MISSENSE[ENST00000511269(c.1171G>A,Gat>Aat):ENSP00000425502(p.Asp391Asn)];ENSG00000196104:MISSENSE[ENST00000421836(c.1027G>A,Gat>Aat):ENSP00000411344(p.Asp343Asn)];ENSG00000196104:MISSENSE[ENST00000510741(c.1051G>A,Gat>Aat):ENSP00000426716(p.Asp351Asn)];ENSG00000196104:MISSENSE[ENST00000535728(c.784G>A,Gat>Aat):ENSP00000441396(p.Asp262Asn)];ENSG00000196104:MISSENSE[ENST00000541637(c.886G>A,Gat>Aat):ENSP00000445430(p.Asp296Asn)];ENSG00000196104:MISSENSE[ENST00000541354(c.820G>A,Gat>Aat):ENSP00000444789(p.Asp274Asn)];ENSG00000196104:MISSENSE[ENST00000512681(c.886G>A,Gat>Aat):ENSP00000426318(p.Asp296Asn)];ENSG00000196104:MISSENSE[ENST00000534949(c.892G>A,Gat>Aat):ENSP00000438142(p.Asp298Asn)];ENSG00000196104:MISSENSE[ENST00000502330(c.1180G>A,Gat>Aat):ENSP00000423606(p.Asp394Asn)];ENSG00000196104:MISSENSE[ENST00000504953(c.1171G>A,Gat>Aat):ENSP00000425570(p.Asp391Asn)];ENSG00000196104:3UTR[ENST00000502821(c.*1060G>A)];ENSG00000196104:3UTR[ENST00000511905(c.*816G>A)];ENSG00000196104:3UTR[ENST00000505187(c.*930G>A)];ENSG00000196104:3UTR[ENST00000507370(c.*794G>A)];ENSG00000196104:EXONIC-NC[ENST00000507137(n.1102G>A)];	chr4*16765	chr4*16765	NA	8.28E-06	0	NA	NA	NA	NA	NA	MODIFIER	NA	NA	88	88	0	78	63	15	19.23
Smoker	42480	chr4	184605174	C	T	*TRAPPC11*	SILENT	ENSG00000168538:SILENT[ENST00000512476(c.72C>T,gcC>gcT):ENSP00000421004(p.Ala24(=))];ENSG00000168538:SILENT[ENST00000334690(c.1254C>T,gcC>gcT):ENSP00000335371(p.Ala418(=))];ENSG00000168538:SILENT[ENST00000357207(c.1254C>T,gcC>gcT):ENSP00000349738(p.Ala418(=))];ENSG00000168538:INTRONIC[ENST00000505676(c.348+15C>T)];	NA	NA	NA	NA	NA	NA	NA	NA	NA	NA	LOW	NA	NA	129	129	0	131	113	18	13.74
Smoker	42480	chr4	10090093	C	G	*WDR1*	INTRONIC	ENSG00000071127:[ENST00000510848(n.309-96G>C)];ENSG00000071127:[ENST00000505543(n.372-96G>C)];ENSG00000071127:[ENST00000382452(c.637-96G>C)];ENSG00000071127:[ENST00000515743(n.1576-96G>C)];ENSG00000071127:[ENST00000502962(n.192-96G>C)];ENSG00000071127:[ENST00000499869(c.637-96G>C)];ENSG00000071127:[ENST00000504739(n.134-96G>C)];ENSG00000071127:[ENST00000506246(n.151-96G>C)];ENSG00000071127:[ENST00000508949(n.237-96G>C)];ENSG00000071127:[ENST00000509695(n.293-96G>C)];ENSG00000071127:[ENST00000508079(c.649-96G>C)];ENSG00000071127:[ENST00000502702(c.217-96G>C)];ENSG00000071127:[ENST00000382451(c.217-96G>C)];	NA	NA	NA	NA	NA	NA	NA	NA	NA	NA	MODIFIER	NA	NA	117	117	0	66	54	12	18.18
Smoker	42480	chr5	80640015	C	T	*ACOT12*	MISSENSE	ENSG00000172497:MISSENSE[ENST00000307624(c.944G>A,cGg>cAg):ENSP00000303246(p.Arg315Gln)];ENSG00000172497:EXONIC-NC[ENST00000508234(n.8G>A)];	chr5*80640	chr5*80640	NA	6.60E-05	0.000364343	NA	NA	NA	NA	NA	MODIFIER	NA	NA	114	114	0	95	73	22	23.16
Smoker	42480	chr5	150632834	C	T	*GM2A*	SILENT	ENSG00000196743:INTRONIC[ENST00000523466(c.127-6482C>T)];ENSG00000196743:SILENT[ENST00000357164(c.57C>T,gcC>gcT):ENSP00000349687(p.Ala19(=))];	NA	NA	chr5*150632834*150632834*C*G*rs780278367*NA*NA*NA	NA	NA	NA	NA	NA	NA	NA	LOW	NA	NA	132	132	0	60	49	11	18.33
Smoker	42480	chr5	89914967	A	G	*GPR98*	MISSENSE	ENSG00000164199:MISSENSE[ENST00000405460(c.422A>G,gAc>gGc):ENSP00000384582(p.Asp141Gly)];ENSG00000164199:INTRONIC[ENST00000508842(c.369+1197A>G)];	NA	NA	NA	NA	NA	NA	NA	NA	NA	NA	MODIFIER	NA	NA	64	64	0	40	35	5	12.5
Smoker	42480	chr5	169702774	G	A	*LCP2*	EXONIC-NC	ENSG00000043462:[ENST00000522823(n.483C>T)];ENSG00000043462:[ENST00000519594(n.370+25C>T)];ENSG00000043462:[ENST00000522760(n.372+25C>T)];ENSG00000043462:[ENST00000046794(c.254+25C>T)];	NA	NA	NA	3.74E-05	0	NA	NA	NA	NA	NA	MODIFIER	NA	NA	114	114	0	49	39	10	20.41
Smoker	42480	chr5	1037624	G	A	*NKD2*	INTRONIC	ENSG00000145506:[ENST00000523688(n.362-296G>A)];ENSG00000145506:[ENST00000537972(c.788-11G>A)];ENSG00000145506:[ENST00000513296(n.581-296G>A)];ENSG00000145506:[ENST00000274150(c.788-11G>A)];ENSG00000145506:[ENST00000296849(c.788-296G>A)];ENSG00000145506:[ENST00000382730(c.124+606G>A)];	NA	NA	0.0003994	0.0004625	0.000530363	0.0003994	0.002	NA	NA	NA	MODIFIER	NA	NA	107	107	0	52	43	9	17.31
Smoker	42480	chr5	65088497	T	C	*NLN*	INTRONIC	ENSG00000123213:[ENST00000511299(c.657+69T>C)];ENSG00000123213:[ENST00000506799(n.1087+15T>C)];ENSG00000123213:[ENST00000509935(c.324+15T>C)];ENSG00000123213:[ENST00000502464(c.1215+15T>C)];ENSG00000123213:[ENST00000380985(c.1527+15T>C)];ENSG00000123213:[ENST00000506539(n.1658T>C)];	NA	NA	0.004992	NA	NA	0.004992	0.0031	NA	NA	NA	MODIFIER	NA	NA	300	299	0	200	147	53	26.5
Smoker	42480	chr5	140772461	G	A	*PCDHGA1*	INTRONIC	ENSG00000204956:[ENST00000517417(c.2421+59789G>A)];	chr5*14077	chr5*14077	NA	NA	NA	NA	NA	NA	NA	NA	MODIFIER	NA	NA	122	122	0	95	85	10	10.53
Smoker	42480	chr5	140772461	G	A	*PCDHGA2*	INTRONIC	ENSG00000081853:[ENST00000394576(c.2424+51499G>A)];	chr5*14077	chr5*14077	NA	NA	NA	NA	NA	NA	NA	NA	MODIFIER	NA	NA	122	122	0	95	85	10	10.53
Smoker	42480	chr5	140772461	G	A	*PCDHGA3*	INTRONIC	ENSG00000254245:[ENST00000253812(c.2424+46437G>A)];	chr5*14077	chr5*14077	NA	NA	NA	NA	NA	NA	NA	NA	MODIFIER	NA	NA	122	122	0	95	85	10	10.53
Smoker	42480	chr5	140772461	G	A	*PCDHGA4*	INTRONIC	ENSG00000262576:[ENST00000571252(c.2421+35273G>A)];	chr5*14077	chr5*14077	NA	NA	NA	NA	NA	NA	NA	NA	MODIFIER	NA	NA	122	122	0	95	85	10	10.53
Smoker	42480	chr5	140772461	G	A	*PCDHGA5*	INTRONIC	ENSG00000253485:[ENST00000518069(c.2421+26143G>A)];	chr5*14077	chr5*14077	NA	NA	NA	NA	NA	NA	NA	NA	MODIFIER	NA	NA	122	122	0	95	85	10	10.53
Smoker	42480	chr5	140772461	G	A	*PCDHGA6*	INTRONIC	ENSG00000253731:[ENST00000517434(c.2424+16387G>A)];	chr5*14077	chr5*14077	NA	NA	NA	NA	NA	NA	NA	NA	MODIFIER	NA	NA	122	122	0	95	85	10	10.53
Smoker	42480	chr5	140772461	G	A	*PCDHGA7*	INTRONIC	ENSG00000253537:[ENST00000518325(c.2424+7571G>A)];	chr5*14077	chr5*14077	NA	NA	NA	NA	NA	NA	NA	NA	MODIFIER	NA	NA	122	122	0	95	85	10	10.53

Mangalaparthi *et al.* , 2020. Mutational landscape of esophageal squamous cell carcinoma in an Indian cohort
Supplementary Table 4. List of somatic single nucleotide variants identified using whole exome sequencing of ESCC patients

Cohort	Sample	Chromosome	Genomic position	Reference allele	Alternate allele	Gene symbol	Variant type	Variant annotation	OncoMD	COSMIC	dbSNP	ExAC	ExAC_SAS	1000G	1000G_SAS	SIFT	Polyphen2	CONDEL	VEP	LRT	PhastCon7 Way	Total read (Adjacent normal)	Reference allel read support (Adjacent normal)	Alternative read support (Adjacent normal)	Total read (Tumor)	Reference allel read support (Tumor)	Alternative read support (Tumor)	Alternate allele frequency
Smoker	42480	chr5	140772461	G	A	*PCDHGA8*	SILENT	ENSG00000253767:SILENT[ENST00000398604(c.81G>A,ggG>ggA):ENSP00000381605(p.Gly27(=))];	chr5*140772	chr5*140772	NA	NA	NA	NA	NA	NA	NA	NA	MODIFIER	NA	NA	122	122	0	95	85	10	10.53
Smoker	42480	chr5	140772461	G	A	*PCDHGB1*	INTRONIC	ENSG00000254221:[ENST00000523390(c.2409+40225G>A)];	chr5*140772	chr5*140772	NA	NA	NA	NA	NA	NA	NA	NA	MODIFIER	NA	NA	122	122	0	95	85	10	10.53
Smoker	42480	chr5	140772461	G	A	*PCDHGB2*	INTRONIC	ENSG00000253910:[ENST00000522605(c.2421+30338G>A)];	chr5*140772	chr5*140772	NA	NA	NA	NA	NA	NA	NA	NA	MODIFIER	NA	NA	122	122	0	95	85	10	10.53
Smoker	42480	chr5	140772461	G	A	*PCDHGB3*	INTRONIC	ENSG00000262209:[ENST00000576222(c.2415+20085G>A)];	chr5*140772	chr5*140772	NA	NA	NA	NA	NA	NA	NA	NA	MODIFIER	NA	NA	122	122	0	95	85	10	10.53
Smoker	42480	chr5	140772461	G	A	*PCDHGB4*	INTRONIC	ENSG00000253953:[ENST00000519479(c.2397+2613G>A)];	chr5*140772	chr5*140772	NA	NA	NA	NA	NA	NA	NA	NA	MODIFIER	NA	NA	122	122	0	95	85	10	10.53
Smoker	42480	chr5	150838430	C	T	*SLC36A1*	MISSENSE	ENSG00000123643:MISSENSE[ENST00000520111(c.77C>T,tCg>tTg):ENSP00000429796(p.Ser26Leu)];ENSG00000123643:MISSENSE[ENST00000519829(c.77C>T,tCg>tTg):ENSP00000428471(p.Ser26Leu)];ENSG00000123643:MISSENSE[ENST00000429484(c.77C>T,tCg>tTg):ENSP00000395640(p.Ser26Leu)];ENSG00000123643:MISSENSE[ENST00000521925(c.77C>T,tCg>tTg):ENSP00000430305(p.Ser26Leu)];ENSG00000123643:MISSENSE[ENST00000520701(c.77C>T,tCg>tTg):ENSP00000428140(p.Ser26Leu)];ENSG00000123643:MISSENSE[ENST00000243389(c.77C>T,tCg>tTg):ENSP00000243389(p.Ser26Leu)];ENSG00000123643:MISSENSE[ENST00000517945(c.77C>T,tCg>tTg):ENSP00000427804(p.Ser26Leu)];ENSG00000123643:EXONIC-NC[ENST00000521351(n.161C>T)];	NA	NA	NA	3.32E-05	0	NA	NA	T	BN	N	MODERATE	NA	NA	146	146	0	102	84	18	17.65
Smoker	42480	chr5	150853173	A	T	*SLC36A1*	INTRONIC	ENSG00000123643:[ENST00000521925(c.724-61A>T)];ENSG00000123643:[ENST00000243389(c.724-61A>T)];ENSG00000123643:[ENST00000520701(c.724-61A>T)];	NA	NA	NA	NA	NA	NA	NA	NA	NA	NA	MODIFIER	NA	NA	26	26	0	38	29	9	23.68
Smoker	42480	chr6	57048676	A	G	*BAG2*	SILENT	ENSG00000112208:SILENT[ENST00000370693(c.324A>G,gaA>gaG):ENSP00000359727(p.Glu108(=))];ENSG00000112208:SILENT[ENST00000545080(c.225A>G,gaA>gaG):ENSP00000441795(p.Glu75(=))];	NA	NA	NA	NA	NA	NA	NA	NA	NA	NA	LOW	NA	0.999	423	421	0	506	492	14	2.77
Smoker	42480	chr6	70048804	G	T	*BAI3*	INTRONIC-SS-ACR	ENSG00000135298:[ENST00000546190(c.81-4G>T)];ENSG00000135298:[ENST00000238918(c.807-4G>T)];ENSG00000135298:[ENST00000370598(c.3189-4G>T)];	NA	NA	NA	NA	NA	NA	NA	NA	NA	NA	LOW	NA	NA	170	170	0	163	138	25	15.34
Smoker	42480	chr6	56484560	C	T	*DST*	SILENT	ENSG00000151914:INTRONIC[ENST00000522360(c.312-2344G>A)];ENSG00000151914:INTRONIC[ENST00000439203(c.3318+4768G>A)];ENSG00000151914:INTRONIC[ENST00000446842(c.3318+4768G>A)];ENSG00000151914:INTRONIC[ENST00000370788(c.4296+4768G>A)];ENSG00000151914:INTRONIC[ENST00000244364(c.3318+4768G>A)];ENSG00000151914:INTRONIC[ENST00000520645(c.4416+4768G>A)];ENSG00000151914:INTRONIC[ENST00000370754(c.4830+4768G>A)];ENSG00000151914:INTRONIC[ENST00000361203(c.4296+4768G>A)];ENSG00000151914:INTRONIC[ENST00000370769(c.4296+4768G>A)];ENSG00000151914:INTRONIC[ENST00000312431(c.4296+4768G>A)];ENSG00000151914:INTRONIC[ENST00000421834(c.4296+4768G>A)];ENSG00000151914:SILENT[ENST00000370765(c.4272G>A,ctG>ctA):ENSP00000359801(p.Leu1424(=))];	NA	NA	NA	NA	NA	NA	NA	NA	NA	NA	MODIFIER	NA	NA	397	397	0	497	433	62	12.47
Smoker	42480	chr6	66204918	C	T	*EYS*	MISSENSE	ENSG00000188107:EXONIC-NC[ENST00000489873(n.913G>A)];ENSG00000188107:MISSENSE[ENST00000342421(c.386G>A,aGa>aAa):ENSP00000341818(p.Arg129Lys)];ENSG00000188107:MISSENSE[ENST00000393380(c.386G>A,aGa>aAa):ENSP00000377042(p.Arg129Lys)];ENSG00000188107:MISSENSE[ENST00000370616(c.386G>A,aGa>aAa):ENSP00000359650(p.Arg129Lys)];ENSG00000188107:MISSENSE[ENST00000503581(c.386G>A,aGa>aAa):ENSP00000424243(p.Arg129Lys)];ENSG00000188107:MISSENSE[ENST00000471279(c.77G>A,aGa>aAa):ENSP00000420530(p.Arg26Lys)];ENSG00000188107:MISSENSE[ENST00000370621(c.386G>A,aGa>aAa):ENSP00000359655(p.Arg129Lys)];ENSG00000188107:MISSENSE[ENST00000370618(c.386G>A,aGa>aAa):ENSP00000359652(p.Arg129Lys)];	NA	NA	NA	NA	NA	NA	NA	T	BN	N	MODERATE	NA	NA	247	247	0	247	213	33	13.36
Smoker	42480	chr6	159191841	G	A	*EZR*	SILENT	ENSG00000092820:SILENT[ENST00000337147(c.1045C>T,Ctg>Ttg):ENSP00000338934(p.Leu349(=))];ENSG00000092820:SILENT[ENST00000392177(c.949C>T,Ctg>Ttg):ENSP00000376016(p.Leu317(=))];ENSG00000092820:SILENT[ENST00000367075(c.1045C>T,Ctg>Ttg):ENSP00000356042(p.Leu349(=))];	NA	NA	NA	NA	NA	NA	NA	NA	NA	NA	LOW	NA	0.986	98	98	0	65	51	14	21.54
Smoker	42480	chr6	159653920	C	T	*FNDC1*	SILENT	ENSG00000164694:SILENT[ENST00000340366(c.2187C>T,ggC>ggT):ENSP00000342460(p.Gly729(=))];ENSG00000164694:SILENT[ENST00000297267(c.2376C>T,ggC>ggT):ENSP00000297267(p.Gly792(=))];ENSG00000164694:SILENT[ENST00000329629(c.2062C>T,ggC>ggT):ENSP00000333297(p.Gly688(=))];	NA	NA	NA	NA	NA	NA	NA	NA	NA	NA	LOW	NA	NA	282	282	0	168	125	43	25.6
Smoker	42480	chr6	26156843	G	A	*HIST1H1E*	SILENT	ENSG00000168298:SILENT[ENST00000304218(c.225G>A,aaG>aaA):ENSP00000307705(p.Lys75(=))];	chr6*261568	chr6*261568	0.004193	NA	NA	0.0041933	0.0082	NA	NA	NA	LOW	NA	1	108	108	0	66	60	6	9.09
Smoker	42480	chr6	29911155	G	T	*HLA-A*	NONSENSE	ENSG00000206503:NONSENSE[ENST00000396634(c.454G>T,Gag>Tag):ENSP00000379873(p.Glu152Ter)];ENSG00000206503:NONSENSE[ENST00000376806(c.454G>T,Gag>Tag):ENSP00000366002(p.Glu152Ter)];ENSG00000206503:NONSENSE[ENST00000376809(c.454G>T,Gag>Tag):ENSP00000366005(p.Glu152Ter)];ENSG00000206503:NONSENSE[ENST00000376802(c.454G>T,Gag>Tag):ENSP00000365998(p.Glu152Ter)];ENSG00000206503:EXONIC-NC[ENST00000479320(n.695G>T)];ENSG00000206503:EXONIC-NC[ENST00000495183(n.697G>T)];ENSG00000206503:EXONIC-NC[ENST00000496081(n.271G>T)];ENSG00000206503:EXONIC-NC[ENST00000461903(n.695G>T)];	NA	NA	chr6*29911155*29911155*G*A,C,T*rs45482492*NA*NA*NA|chr6*29911155*29911156*GA*G*rs199474523*NA*NA*NA	NA	NA	NA	NA	NA	NA	NA	MODIFIER	NA	NA	62	62	0	30	16	14	46.67
Smoker	42480	chr6	53784277	A	T	*LRRC1*	INTRONIC	ENSG00000137269:[ENST00000370888(c.1107-19A>T)];ENSG00000137269:[ENST00000490222(n.240A>T)];	NA	NA	NA	0.0341948	0.020484171	NA	NA	NA	NA	NA	MODIFIER	NA	NA	36	36	0	79	71	5	6.33
Smoker	42480	chr6	30996600	T	C	*MUC22*	MISSENSE	ENSG00000261272:MISSENSE[ENST00000561890(c.3392T>C,aTa>aCa):ENSP00000455906(p.Ile1131Thr)];	NA	NA	0.0009984	NA	NA	0.0009984	0	NA	UN	NA	MODERATE	NA	NA	595	581	9	283	263	16	5.65
Smoker	42480	chr6	138892931	G	A	*NHSL1*	MISSENSE	ENSG00000135540:MISSENSE[ENST00000427025(c.118C>T,Cgc>Tgc):ENSP00000394546(p.Arg40Cys)];ENSG00000135540:EXONIC-NC[ENST00000468095(n.364C>T)];ENSG00000135540:EXONIC-NC[ENST00000534376(n.345C>T)];ENSG00000135540:INTRONIC[ENST00000491526(c.97-75423C>T)];	chr6*13889	chr6*13889	NA	4.36E-05	0.000126358	NA	NA	NA	NA	NA	MODIFIER	NA	NA	116	116	0	99	75	24	24.24
Smoker	42480	chr6	53784277	A	T	*RP3-523E19.2*	INTRONIC	ENSG00000271218:[ENST00000474641(n.244-293T>A)];	NA	NA	NA	0.0341948	0.020484171	NA	NA	NA	NA	NA	MODIFIER	NA	NA	36	36	0	79	71	5	6.33
Smoker	42480	chr6	108227936	T	G	*SEC63*	MISSENSE	ENSG00000025796:MISSENSE[ENST00000369002(c.779A>C,tAt>tCt):ENSP00000357998(p.Tyr260Ser)];ENSG00000025796:EXONIC-NC[ENST00000489455(n.279A>C)];	NA	NA	chr6*108227936*108227936*T*C*rs762097086*NA*NA*NA	NA	NA	NA	NA	NA	NA	NA	MODIFIER	NA	NA	119	119	0	155	112	43	27.74
Smoker	42480	chr6	168944332	G	A	*SMOC2*	MISSENSE	ENSG00000112562:MISSENSE[ENST00000356284(c.491G>A,cGc>cAc):ENSP00000348630(p.Arg164His)];ENSG00000112562:MISSENSE[ENST00000354536(c.491G>A,cGc>cAc):ENSP00000346537(p.Arg164His)];	NA	NA	0.001198	0.0003288	0.002298993	0.0011981	0.0061	T	BN	N	MODERATE	N	0.958	117	115	1	51	32	19	37.25

Mangalaparthi *et al.* , 2020. Mutational landscape of esophageal squamous cell carcinoma in an Indian cohort
Supplementary Table 4. List of somatic single nucleotide variants identified using whole exome sequencing of ESCC patients

Cohort	Sample	Chromosome	Genomic position	Reference allele	Alternate allele	Gene symbol	Variant type	Variant annotation	OncoMD	COSMIC	dbSNP	ExAC	ExAC_SAS	1000G	1000G_SAS	SIFT	Polyphen2	CONDEL	VEP	LRT	PhastCon7 Way	Total read (Adjacent normal)	Reference allel read support (Adjacent normal)	Alternative read support (Adjacent normal)	Total read (Tumor)	Reference allel read support (Tumor)	Alternative read support (Tumor)	Alternate allele frequency
Smoker	42480	chr6	155561702	C	T	*TIAM2*	SILENT	ENSG00000146426:SILENT[ENST00000461783(c.3207C>T,aaC>aaT):ENSP00000437188(p.Asn1069(=))];ENSG00000146426:SILENT[ENST00000318981(c.3207C>T,aaC>aaT):ENSP00000327315(p.Asn1069(=))];ENSG00000146426:SILENT[ENST00000528391(c.1215C>T,aaC>aaT):ENSP00000435335(p.Asn405(=))];ENSG00000146426:SILENT[ENST00000528535(c.3207C>T,aaC>aaT):ENSP00000434901(p.Asn1069(=))];ENSG00000146426:SILENT[ENST00000456144(c.3207C>T,aaC>aaT):ENSP00000407746(p.Asn1069(=))];ENSG00000146426:SILENT[ENST00000367174(c.1335C>T,aaC>aaT):ENSP00000356142(p.Asn445(=))];ENSG00000146426:SILENT[ENST00000462408(c.21C>T,aaC>aaT):ENSP00000431269(p.Asn7(=))];ENSG00000146426:SILENT[ENST00000529824(c.3207C>T,aaC>aaT):ENSP00000433348(p.Asn1069(=))];ENSG00000146426:SILENT[ENST00000360366(c.3279C>T,aaC>aaT):ENSP00000353528(p.Asn1093(=))];ENSG00000146426:SILENT[ENST00000456877(c.1143C>T,aaC>aaT):ENSP00000407183(p.Asn381(=))];ENSG00000146426:3UTR[ENST00000543712(c.*31C>T)];ENSG00000146426:EXONIC-NC[ENST00000546145(n.164C>T)];ENSG00000146426:5UTR[ENST00000275246(c.-19C>T)];	NA	NA	NA	1.66E-05	6.10E-05	NA	NA	NA	NA	NA	LOW	NA	0.092	96	96	0	73	64	9	12.33
Smoker	42480	chr6	80747734	C	T	*TTK*	SILENT	ENSG00000112742:SILENT[ENST00000369798(c.2100C>T,tcC>tcT):ENSP00000358813(p.Ser700(=))];ENSG00000112742:SILENT[ENST00000509894(c.2097C>T,tcC>tcT):ENSP00000422936(p.Ser699(=))];ENSG00000112742:SILENT[ENST00000230510(c.2097C>T,tcC>tcT):ENSP00000230510(p.Ser699(=))];ENSG00000112742:EXONIC-NC[ENST00000504590(n.465C>T)];	NA	NA	NA	NA	NA	NA	NA	NA	NA	NA	LOW	NA	NA	63	63	0	46	36	10	21.74
Smoker	42480	chr7	34698099	T	C	*AC005493.1*	INTRONIC	ENSG00000214841:[ENST00000399077(c.31+1643A>G)];	NA	NA	NA	8.24E-06	0	NA	NA	NA	NA	NA	MODIFIER	NA	NA	86	86	0	72	61	11	15.28
Smoker	42480	chr7	102462609	A	C	*FBXL13*	MISSENSE	ENSG00000161040:MISSENSE[ENST00000393772(c.1812T>G,caT>caG):ENSP00000377367(p.His604Gln)];ENSG00000161040:MISSENSE[ENST00000379305(c.1812T>G,caT>caG):ENSP00000368607(p.His604Gln)];ENSG00000161040:MISSENSE[ENST00000455112(c.1761T>G,caT>caG):ENSP00000391550(p.His587Gln)];ENSG00000161040:MISSENSE[ENST00000379308(c.1761T>G,caT>caG):ENSP00000368610(p.His587Gln)];ENSG00000161040:MISSENSE[ENST00000313221(c.1896T>G,caT>caG):ENSP00000321927(p.His632Gln)];ENSG00000161040:MISSENSE[ENST00000436908(c.1896T>G,caT>caG):ENSP00000388608(p.His632Gln)];ENSG00000161040:MISSENSE[ENST00000379306(c.1050T>G,caT>caG):ENSP00000368608(p.His350Gln)];ENSG00000161040:MISSENSE[ENST00000456695(c.1050T>G,caT>caG):ENSP00000409716(p.His350Gln)];ENSG00000161040:3UTR[ENST00000448002(c.*34T>G)];	NA	NA	NA	NA	NA	NA	NA	T	BN	N	MODERATE	N	0.999	164	164	0	226	175	51	22.57
Smoker	42480	chr7	107696119	G	A	*LAMB4*	MISSENSE	ENSG00000091128:MISSENSE[ENST00000388781(c.3713C>T,tCt>tTt):ENSP00000373433(p.Ser1238Phe)];ENSG00000091128:MISSENSE[ENST00000422975(c.791C>T,tCt>tTt):ENSP00000416562(p.Ser264Phe)];ENSG00000091128:MISSENSE[ENST00000388780(c.3713C>T,tCt>tTt):ENSP00000373432(p.Ser1238Phe)];ENSG00000091128:MISSENSE[ENST00000205386(c.3713C>T,tCt>tTt):ENSP00000205386(p.Ser1238Phe)];	NA	NA	NA	NA	NA	NA	NA	T	BN	N	MODERATE	N	0.997	73	73	0	94	82	12	12.77
Smoker	42480	chr7	107704411	G	A	*LAMB4*	SILENT	ENSG00000091128:SILENT[ENST00000388781(c.2856C>T,tgC>tgT):ENSP00000373433(p.Cys952(=))];ENSG00000091128:SILENT[ENST00000388780(c.2856C>T,tgC>tgT):ENSP00000373432(p.Cys952(=))];ENSG00000091128:SILENT[ENST00000205386(c.2856C>T,tgC>tgT):ENSP00000205386(p.Cys952(=))];ENSG00000091128:EXONIC-NC[ENST00000471677(n.117C>T)];ENSG00000091128:EXONIC-NC[ENST00000475469(n.2940C>T)];	chr7*10770	chr7*10770	NA	NA	NA	NA	NA	NA	NA	NA	LOW	NA	0.398	128	128	0	154	83	71	46.1
Smoker	42480	chr7	140706403	C	T	*MRPS33*	INTRONIC	ENSG00000090263:[ENST00000496958(c.216-68G>A)];ENSG00000090263:[ENST00000324787(c.216-68G>A)];ENSG00000090263:[ENST00000472343(n.421-68G>A)];ENSG00000090263:[ENST00000467334(c.186-68G>A)];ENSG00000090263:[ENST00000484502(n.99-68G>A)];ENSG00000090263:[ENST00000469351(c.216-68G>A)];ENSG00000090263:[ENST00000393008(c.216-68G>A)];	NA	NA	0.0001997	NA	NA	0.0001997	0	NA	NA	NA	MODIFIER	NA	NA	108	108	0	58	50	7	12.07
Smoker	42480	chr7	100551203	C	G	*MUC3A*	EXONIC-NC	ENSG00000169894:[ENST00000483133(n.874C>G)];	NA	NA	NA	NA	NA	NA	NA	NA	NA	NA	MODIFIER	NA	NA	447	333	3	480	329	20	4.17
Smoker	42480	chr7	100817982	A	T	*NAT16*	MISSENSE	ENSG00000167011:MISSENSE[ENST00000444446(c.107T>A,gTg>gAg):ENSP00000391769(p.Val36Glu)];ENSG00000167011:MISSENSE[ENST00000455377(c.107T>A,gTg>gAg):ENSP00000395125(p.Val36Glu)];ENSG00000167011:MISSENSE[ENST00000443096(c.107T>A,gTg>gAg):ENSP00000394435(p.Val36Glu)];ENSG00000167011:MISSENSE[ENST00000300303(c.107T>A,gTg>gAg):ENSP00000300303(p.Val36Glu)];	NA	NA	NA	NA	NA	NA	NA	T	BN	N	MODERATE	NA	NA	110	110	0	113	72	41	36.28
Smoker	42480	chr7	34698099	T	C	*NPSR1*	SILENT	ENSG00000187258:SILENT[ENST00000465305(c.75T>C,gcT>gcC):ENSP00000434955(p.Ala25(=))];ENSG00000187258:SILENT[ENST00000359791(c.75T>C,gcT>gcC):ENSP00000352839(p.Ala25(=))];ENSG00000187258:SILENT[ENST00000381544(c.75T>C,gcT>gcC):ENSP00000370955(p.Ala25(=))];ENSG00000187258:SILENT[ENST00000531252(c.75T>C,gcT>gcC):ENSP00000433258(p.Ala25(=))];ENSG00000187258:SILENT[ENST00000381539(c.75T>C,gcT>gcC):ENSP00000370950(p.Ala25(=))];ENSG00000187258:SILENT[ENST00000381542(c.75T>C,gcT>gcC):ENSP00000370953(p.Ala25(=))];ENSG00000187258:SILENT[ENST00000396095(c.75T>C,gcT>gcC):ENSP00000379402(p.Ala25(=))];ENSG00000187258:SILENT[ENST00000381553(c.75T>C,gcT>gcC):ENSP00000370965(p.Ala25(=))];ENSG00000187258:SILENT[ENST00000360581(c.75T>C,gcT>gcC):ENSP00000353788(p.Ala25(=))];	NA	NA	NA	8.24E-06	0	NA	NA	NA	NA	NA	MODIFIER	NA	NA	86	86	0	72	61	11	15.28
Smoker	42480	chr7	34698099	T	C	*NPSR1-AS1*	INTRONIC	ENSG00000197085:[ENST00000419766(n.160+70250A>G)];ENSG00000197085:[ENST00000544556(n.280-41484A>G)];ENSG00000197085:[ENST00000537560(n.279+70250A>G)];ENSG00000197085:[ENST00000535640(n.245-41484A>G)];ENSG00000197085:[ENST00000431669(n.245-15136A>G)];ENSG00000197085:[ENST00000439852(n.399+45593A>G)];ENSG00000197085:[ENST00000358772(n.279+70250A>G)];ENSG00000197085:[ENST00000539747(n.164+70250A>G)];	NA	NA	NA	8.24E-06	0	NA	NA	NA	NA	NA	MODIFIER	NA	NA	86	86	0	72	61	11	15.28
Smoker	42480	chr7	100859441	C	T	*PLOD3*	INTRONIC	ENSG00000106397:[ENST00000424135(c.267+37G>A)];ENSG00000106397:[ENST00000223127(c.502+3G>A)];ENSG00000106397:[ENST00000489927(n.449+3G>A)];ENSG00000106397:[ENST00000478082(n.539+3G>A)];	NA	NA	NA	NA	NA	NA	NA	NA	NA	NA	LOW	NA	NA	133	132	0	98	87	11	11.22

Supplementary Table 4. List of somatic single nucleotide variants identified using whole exome sequencing of ESCC patients

Cohort	Sample	Chromosome	Genomic position	Reference allele	Alternate allele	Gene symbol	Variant type	Variant annotation	OncoMD	COSMIC	dbSNP	ExAC	ExAC_SAS	1000G	1000G_SAS	SIFT	Polyphen2	CONDEL	VEP	LRT	PhastCon7 Way	Total read (Adjacent normal)	Reference allel read support (Adjacent normal)	Alternative read support (Adjacent normal)	Total read (Tumor)	Reference allel read support (Tumor)	Alternative read support (Tumor)	Alternate allele frequency
Smoker	42480	chr7	132192481	C	G	*PLXNA4*	MISSENSE	ENSG00000221866:MISSENSE[ENST00000321063(c.972G>C,agG>agC):ENSP00000323194(p.Arg324Ser)];ENSG00000221866:MISSENSE[ENST00000423507(c.972G>C,agG>agC):ENSP00000392772(p.Arg324Ser)];ENSG00000221866:MISSENSE[ENST00000359827(c.972G>C,agG>agC):ENSP00000352882(p.Arg324Ser)];ENSG00000221866:MISSENSE[ENST00000378539(c.972G>C,agG>agC):ENSP00000367800(p.Arg324Ser)];	NA	NA	NA	NA	NA	NA	NA	T	BN	N	MODERATE	U	0.292	133	131	1	69	55	14	20.29
Smoker	42480	chr7	53104142	G	A	*POM121L12*	MISSENSE	ENSG00000221900:MISSENSE[ENST00000408890(c.778G>A,Gcc>Acc):ENSP00000386133(p.Ala260Thr)];	chr7*53104	chr7*53104	.	8.30E-06	0	NA	NA	T_lc	PrD	N	MODERATE	NA	NA	135	135	0	82	68	14	17.07
Smoker	42480	chr7	39491081	T	C	*POU6F2*	INTRONIC	ENSG00000106536:[ENST00000403058(c.1234-51T>C)];ENSG00000106536:[ENST00000559001(c.1069-51T>C)];ENSG00000106536:[ENST00000524147(c.1069-51T>C)];ENSG00000106536:[ENST00000416452(c.331-9065T>C)];ENSG00000106536:[ENST00000518318(c.1234-51T>C)];	NA	NA	0.0007987	NA	NA	0.0007987	0.001	NA	NA	NA	MODIFIER	NA	NA	35	35	0	41	24	17	41.46
Smoker	42480	chr7	103205778	C	T	*RELN*	SILENT	ENSG00000189056:SILENT[ENST00000424685(c.5157G>A,tcG>tcA):ENSP00000388446(p.Ser1719(=))];ENSG00000189056:SILENT[ENST00000428762(c.5157G>A,tcG>tcA):ENSP00000392423(p.Ser1719(=))];ENSG00000189056:SILENT[ENST00000343529(c.5157G>A,tcG>tcA):ENSP00000345694(p.Ser1719(=))];	NA	chr7*10320:	NA	4.12E-05	0	NA	NA	NA	NA	NA	LOW	NA	NA	287	286	0	369	307	61	16.53
Smoker	42480	chr7	84697586	T	G	*SEMA3D*	MISSENSE	ENSG00000153993:MISSENSE[ENST00000444867(c.510A>C,aaA>aaC):ENSP00000401366(p.Lys170Asn)];ENSG00000153993:MISSENSE[ENST00000284136(c.510A>C,aaA>aaC):ENSP00000284136(p.Lys170Asn)];	NA	NA	NA	NA	NA	NA	NA	T	BN	N	MODERATE	D	0.997	151	151	0	175	125	50	28.57
Smoker	42480	chr7	142206888	G	A	*TRBV10-2*	INTRONIC	ENSG00000229769:[ENST00000426318(c.49+24C>T)];	NA	NA	NA	NA	NA	NA	NA	NA	NA	NA	MODIFIER	NA	NA	73	73	0	52	45	6	11.54
Smoker	42480	chr7	142206893	T	A	*TRBV10-2*	INTRONIC	ENSG00000229769:[ENST00000426318(c.49+19A>T)];	NA	NA	NA	NA	NA	NA	NA	NA	NA	NA	MODIFIER	NA	NA	73	73	0	55	48	6	10.91
Smoker	42480	chr8	141569558	A	G	*AGO2*	SILENT	ENSG00000123908:SILENT[ENST00000519980(c.726T>C,agT>agC):ENSP00000430176(p.Ser242(=))];ENSG00000123908:SILENT[ENST00000220592(c.726T>C,agT>agC):ENSP00000220592(p.Ser242(=))];ENSG00000123908:3UTR[ENST00000523609(c.*311T>C)];	NA	NA	NA	NA	NA	NA	NA	NA	NA	NA	MODIFIER	NA	NA	97	97	0	55	49	6	10.91
Smoker	42480	chr8	4851981	C	A	*CSMD1*	5UTR	ENSG00000183117:[ENST00000400186(c.-43G>T)];ENSG00000183117:[ENST00000602723(c.-43G>T)];ENSG00000183117:[ENST00000602557(c.-43G>T)];ENSG00000183117:[ENST00000520002(c.-43G>T)];	NA	NA	chr8*4851981*4851981*C*T*rs778784296*NA*NA*NA	NA	NA	NA	NA	NA	NA	NA	MODIFIER	NA	NA	105	105	0	63	50	13	20.63
Smoker	42480	chr8	113599298	A	T	*CSMD3*	SILENT	ENSG00000164796:SILENT[ENST00000352409(c.3882T>A,ctT>ctA):ENSP00000343124(p.Leu1294(=))];ENSG00000164796:SILENT[ENST00000455883(c.3570T>A,ctT>ctA):ENSP00000412263(p.Leu1190(=))];ENSG00000164796:SILENT[ENST00000297405(c.3882T>A,ctT>ctA):ENSP00000297405(p.Leu1294(=))];ENSG00000164796:SILENT[ENST00000339701(c.1902T>A,ctT>ctA):ENSP00000341558(p.Leu634(=))];ENSG00000164796:SILENT[ENST00000343508(c.3762T>A,ctT>ctA):ENSP00000345799(p.Leu1254(=))];	NA	NA	NA	NA	NA	NA	NA	NA	NA	NA	LOW	NA	NA	59	58	1	109	92	17	15.6
Smoker	42480	chr8	113697714	A	G	*CSMD3*	SILENT	ENSG00000164796:SILENT[ENST00000352409(c.2403T>C,acT>acC):ENSP00000343124(p.Thr801(=))];ENSG00000164796:SILENT[ENST00000455883(c.2091T>C,acT>acC):ENSP00000412263(p.Thr697(=))];ENSG00000164796:SILENT[ENST00000297405(c.2403T>C,acT>acC):ENSP00000297405(p.Thr801(=))];ENSG00000164796:SILENT[ENST00000339701(c.423T>C,acT>acC):ENSP00000341558(p.Thr141(=))];ENSG00000164796:SILENT[ENST00000343508(c.2283T>C,acT>acC):ENSP00000345799(p.Thr761(=))];	NA	NA	NA	1.65E-05	0	NA	NA	NA	NA	NA	LOW	NA	NA	152	152	0	191	152	39	20.42
Smoker	42480	chr8	89339309	C	T	*MMP16*	MISSENSE	ENSG00000156103:MISSENSE[ENST00000286614(c.127G>A,Gtg>Atg):ENSP00000286614(p.Val43Met)];ENSG00000156103:MISSENSE[ENST00000522726(c.178G>A,Gtg>Atg):ENSP00000429147(p.Val60Met)];ENSG00000156103:EXONIC-NC[ENST00000544227(n.127G>A)];ENSG00000156103:EXONIC-NC[ENST00000520568(n.177G>A)];	NA	NA	NA	NA	NA	NA	NA	NA	NA	NA	MODIFIER	NA	NA	217	217	0	146	131	15	10.27
Smoker	42480	chr8	48710965	A	T	*PRKDC*	INTRONIC-SS-ACR	ENSG00000253729:[ENST00000338368(c.10295-7T>A)];ENSG00000253729:[ENST00000314191(c.10295-7T>A)];ENSG00000253729:[ENST00000518216(n.10349-7T>A)];ENSG00000253729:[ENST00000523565(n.10354-7T>A)];	NA	NA	NA	NA	NA	NA	NA	NA	NA	NA	LOW	NA	NA	58	58	0	53	47	6	11.32
Smoker	42480	chr8	89339309	C	T	*RP11-586K2.1*	EXONIC-NC	ENSG00000253553:[ENST00000523254(n.245C>T)];	NA	NA	NA	NA	NA	NA	NA	NA	NA	NA	MODIFIER	NA	NA	217	217	0	146	131	15	10.27
Smoker	42480	chr8	41166267	C	G	*SFRP1*	MISSENSE	ENSG00000104332:MISSENSE[ENST00000220772(c.412G>C,Gac>Cac):ENSP00000220772(p.Asp138His)];	NA	NA	NA	NA	NA	NA	NA	D	PrD	D	MODERATE	D	0.999	206	205	0	87	71	15	17.24
Smoker	42480	chr8	51664677	C	T	*SNTG1*	INTRONIC	ENSG00000147481:[ENST00000517473(c.1285-40554C>T)];ENSG00000147481:[ENST00000276467(c.1285-40554C>T)];ENSG00000147481:[ENST00000520825(c.*374+6C>T)];ENSG00000147481:[ENST00000518864(c.1395+6C>T)];ENSG00000147481:[ENST00000522124(c.1395+6C>T)];	NA	NA	NA	NA	NA	NA	NA	NA	NA	NA	LOW	NA	NA	72	72	0	88	63	25	28.41
Smoker	42480	chr8	103372900	G	T	*UBR5*	INTRONIC-SS-ACR	ENSG00000104517:[ENST00000520539(c.190-7C>A)];ENSG00000104517:[ENST00000522327(n.86-7C>A)];ENSG00000104517:[ENST00000220959(c.190-7C>A)];ENSG00000104517:[ENST00000521922(c.190-7C>A)];	NA	NA	NA	NA	NA	NA	NA	NA	NA	NA	LOW	NA	NA	41	41	0	92	70	22	23.91
Smoker	42480	chr8	56015167	C	T	*XKR4*	MISSENSE	ENSG00000206579:MISSENSE[ENST00000327381(c.119C>T,tCg>tTg):ENSP00000328326(p.Ser40Leu)];	NA	NA	NA	NA	NA	NA	NA	T_lc	BN	N	MODERATE	N	1	46	46	0	27	19	8	29.63
Smoker	42480	chr9	112705443	A	G	*AKAP2*	INTRONIC	ENSG00000241978:[ENST00000510514(c.496+11135A>G)];ENSG00000241978:[ENST00000555236(c.496+11135A>G)];	NA	NA	NA	NA	NA	NA	NA	NA	NA	NA	MODIFIER	NA	NA	115	115	0	147	131	16	10.88
Smoker	42480	chr9	99961638	C	G	*ANKRD18CP*	INTRONIC	ENSG00000159712:[ENST00000354752(n.602+1103G>C)];	NA	NA	NA	0.0034397	0.002275161	NA	NA	NA	NA	NA	MODIFIER	NA	NA	263	262	1	116	110	6	5.17
Smoker	42480	chr9	100109789	G	A	*CCDC180*	INTRONIC	ENSG00000197816:[ENST00000357054(c.3047+3944G>A)];ENSG00000197816:[ENST00000375202(c.2806+18G>A)];ENSG00000197816:[ENST00000530011(n.236-1604G>A)];ENSG00000197816:[ENST00000528678(n.595-2902G>A)];ENSG00000197816:[ENST00000529487(c.2806+18G>A)];ENSG00000197816:[ENST00000395220(c.*913+3944G>A)];	NA	NA	NA	NA	NA	NA	NA	NA	NA	NA	MODIFIER	NA	NA	45	45	0	31	22	9	29.03
Smoker	42480	chr9	135940444	G	A	*CEL*	MISSENSE	ENSG00000170835:MISSENSE[ENST00000372080(c.367G>A,Gtt>Att):ENSP00000361151(p.Val123Ile)];ENSG00000170835:MISSENSE[ENST00000351304(c.358G>A,Gtt>Att):ENSP00000342217(p.Val120Ile)];	chr9*13594(	chr9*13594(	0.0001997	0.0002592	0	0.0001997	0	D	BN	N	MODERATE	N	0.863	140	140	0	68	37	28	41.18
Smoker	42480	chr9	131196802	C	T	*CERCAM*	MISSENSE	ENSG00000167123:MISSENSE[ENST00000372842(c.1211C>T,gCg>gTg):ENSP00000361933(p.Ala404Val)];ENSG00000167123:MISSENSE[ENST00000372838(c.1445C>T,gCg>gTg):ENSP00000361929(p.Ala482Val)];ENSG00000167123:EXONIC-NC[ENST00000463535(n.1121C>T)];ENSG00000167123:INTRONIC[ENST00000487001(n.451-1130C>T)];	chr9*13119(	chr9*13119(	NA	9.20E-05	0	NA	NA	NA	NA	NA	MODIFIER	NA	NA	44	43	0	49	33	16	32.65
Smoker	42480	chr9	124329533	A	G	*DAB2IP*	INTRONIC	ENSG00000136848:[ENST00000259371(c.40+26A>G)];ENSG00000136848:[ENST00000436835(c.-115+26A>G)];ENSG00000136848:[ENST00000394340(c.40+26A>G)];	NA	NA	NA	NA	NA	NA	NA	NA	NA	NA	MODIFIER	NA	NA	63	63	0	56	42	14	25
Smoker	42480	chr9	111909424	G	C	*FRRS1L*	SILENT	ENSG00000260230:SILENT[ENST00000561981(c.522C>G,ctC>ctG):ENSP00000477141(p.Leu174(=))];	NA	NA	NA	NA	NA	NA	NA	NA	NA	NA	LOW	NA	NA	164	164	0	172	131	41	23.84

Mangalaparthi *et al.*, 2020. Mutational landscape of esophageal squamous cell carcinoma in an Indian cohort
Supplementary Table 4. List of somatic single nucleotide variants identified using whole exome sequencing of ESCC patients

Cohort	Sample	Chromosome	Genomic position	Reference allele	Alternate allele	Gene symbol	Variant type	Variant annotation	OncoMD	COSMIC	dbSNP	ExAC	ExAC_SAS	1000G	1000G_SAS	SIFT	Polyphen2	CONDEL	VEP	LRT	PhastCon7 Way	Total read (Adjacent normal)	Reference allel read support (Adjacent normal)	Alternative read support (Adjacent normal)	Total read (Tumor)	Reference allel read support (Tumor)	Alternative read support (Tumor)	Alternate allele frequency
Smoker	42480	chr9	128104518	A	G	*GAPVD1*	MISSENSE	ENSG00000165219:MISSENSE[ENST00000394105(c.3067A>G,Aaa>Gaa):ENSP00000377665(p.Lys1023Glu)];ENSG00000165219:MISSENSE[ENST00000297933(c.2986A>G,Aaa>Gaa):ENSP00000297933(p.Lys996Glu)];ENSG00000165219:MISSENSE[ENST00000312123(c.2923A>G,Aaa>Gaa):ENSP00000309582(p.Lys975Glu)];ENSG00000165219:MISSENSE[ENST00000394104(c.2986A>G,Aaa>Gaa):ENSP00000377664(p.Lys996Glu)];ENSG00000165219:MISSENSE[ENST00000265956(c.2908A>G,Aaa>Gaa):ENSP00000265956(p.Lys970Glu)];ENSG00000165219:MISSENSE[ENST00000495955(c.2986A>G,Aaa>Gaa):ENSP00000419063(p.Lys996Glu)];ENSG00000165219:MISSENSE[ENST00000431329(c.2496A>G,Aaa>Gaa):ENSP00000413082(p.Lys833Glu)];ENSG00000165219:INTRONIC[ENST00000470056(c.2965+975A>G)];ENSG00000165219:INTRONIC[ENST00000394083(c.2902+975A>G)];	NA	NA	NA	NA	NA	NA	NA	NA	NA	NA	MODIFIER	NA	NA	141	140	1	187	144	43	22.99
Smoker	42480	chr9	68372327	C	A	*NA*	INTERGENIC		NA	NA	NA	NA	NA	NA	NA	NA	NA	NA	MODIFIER	NA	NA	160	158	2	153	143	10	6.54
Smoker	42480	chr9	134072630	G	A	*NUP214*	MISSENSE	ENSG00000126883:EXONIC-NC[ENST00000525384(n.197G>A)];ENSG00000126883:EXONIC-NC[ENST00000465486(n.468G>A)];ENSG00000126883:EXONIC-NC[ENST00000470765(n.176G>A)];ENSG00000126883:EXONIC-NC[ENST00000531929(n.506G>A)];ENSG00000126883:EXONIC-NC[ENST00000528114(n.205G>A)];ENSG00000126883:MISSENSE[ENST00000451030(c.3752G>A,aGc>aAc):ENSP00000405014(p.Ser1251Asn)];ENSG00000126883:MISSENSE[ENST00000359428(c.3749G>A,aGc>aAc):ENSP00000352400(p.Ser1250Asn)];ENSG00000126883:MISSENSE[ENST00000483497(c.227G>A,aGc>aAc):ENSP00000436793(p.Ser76Asn)];ENSG00000126883:MISSENSE[ENST00000531600(c.80G>A,aGc>aAc):ENSP00000435364(p.Ser27Asn)];ENSG00000126883:MISSENSE[ENST00000453861(c.307G>A,aGc>aAc):ENSP00000416934(p.Ser103Asn)];ENSG00000126883:MISSENSE[ENST00000411637(c.3719G>A,aGc>aAc):ENSP00000396576(p.Ser1240Asn)];	NA	NA	NA	NA	NA	NA	NA	NA	BN	NA	MODERATE	N	0.434	135	135	0	141	86	55	39.01
Smoker	42480	chr9	117092701	C	A	*ORM2*	MISSENSE	ENSG00000228278:MISSENSE[ENST00000412657(c.517C>A,Ccc>Acc):ENSP00000407099(p.Pro173Thr)];ENSG00000228278:INTRONIC[ENST00000431067(c.115-13C>A)];	chr9*11709	NA	NA	0.0045257	0.005819661	NA	NA	NA	NA	NA	MODIFIER	NA	NA	268	265	3	51	42	9	17.65
Smoker	42480	chr9	112705443	A	G	*PALM2*	MISSENSE	ENSG00000243444:MISSENSE[ENST00000448454(c.980A>G,gAg>gGg):ENSP00000400206(p.Glu327Gly)];ENSG00000243444:MISSENSE[ENST00000374531(c.878A>G,gAg>gGg):ENSP00000363656(p.Glu293Gly)];ENSG00000243444:MISSENSE[ENST00000314527(c.974A>G,gAg>gGg):ENSP00000323805(p.Glu325Gly)];ENSG00000243444:MISSENSE[ENST00000483909(c.872A>G,gAg>gGg):ENSP00000417525(p.Glu291Gly)];	NA	NA	NA	NA	NA	NA	NA	NA	NA	NA	MODIFIER	NA	NA	115	115	0	147	131	16	10.88
Smoker	42480	chr9	112705443	A	G	*PALM2-AKAP2*	MISSENSE	ENSG00000157654:MISSENSE[ENST00000413420(c.974A>G,gAg>gGg):ENSP00000397839(p.Glu325Gly)];ENSG00000157654:INTRONIC[ENST00000374530(c.496+11135A>G)];ENSG00000157654:INTRONIC[ENST00000302798(c.496+11135A>G)];	NA	NA	NA	NA	NA	NA	NA	NA	NA	NA	MODIFIER	NA	NA	115	115	0	147	131	16	10.88
Smoker	42480	chr9	78600962	T	C	*PCSK5*	INTRONIC	ENSG00000099139:[ENST00000376767(c.298-86T>C)];ENSG00000099139:[ENST00000376752(c.298-86T>C)];ENSG00000099139:[ENST00000545128(c.298-86T>C)];	NA	NA	NA	NA	NA	NA	NA	NA	NA	NA	MODIFIER	NA	NA	134	134	0	168	83	85	50.6
Smoker	42480	chr9	140356686	C	T	*PNPLA7*	MISSENSE	ENSG00000130653:MISSENSE[ENST00000277531(c.3515G>A,cGc>cAc):ENSP00000277531(p.Arg1172His)];ENSG00000130653:MISSENSE[ENST00000406427(c.3590G>A,cGc>cAc):ENSP00000384610(p.Arg1197His)];ENSG00000130653:MISSENSE[ENST00000371457(c.2333G>A,cGc>cAc):ENSP00000360512(p.Arg778His)];ENSG00000130653:MISSENSE[ENST00000434090(c.3488G>A,cGc>cAc):ENSP00000400582(p.Arg1163His)];ENSG00000130653:EXONIC-NC[ENST00000469998(n.2473G>A)];ENSG00000130653:EXONIC-NC[ENST00000492278(n.3184G>A)];	NA	NA	NA	3.50E-05	0	NA	NA	NA	NA	NA	MODIFIER	NA	NA	121	121	0	86	75	10	11.63
Smoker	42480	chr9	96847711	G	A	*PTPDC1*	INTRONIC-SS-DNR	ENSG00000158079:[ENST00000288976(c.410+7G>A)];ENSG00000158079:[ENST00000375360(c.254+7G>A)];	NA	NA	NA	NA	NA	NA	NA	NA	NA	NA	LOW	NA	NA	72	72	0	53	35	18	33.96
Smoker	42480	chr9	100109789	G	A	*RP11-23J9.4*	INTRONIC	ENSG00000255036:[ENST00000532526(n.3343+3944G>A)];ENSG00000255036:[ENST00000375206(n.3982+3944G>A)];ENSG00000255036:[ENST00000534123(n.3959+18G>A)];ENSG00000255036:[ENST00000529787(n.3183G>A)];	NA	NA	NA	NA	NA	NA	NA	NA	NA	NA	MODIFIER	NA	NA	45	45	0	31	22	9	29.03
Smoker	42480	chr9	66457563	G	A	*RP11-262H14.1*	INTRONIC	ENSG00000238113:[ENST00000424345(n.67+208G>A)];ENSG00000238113:[ENST00000427509(n.71+208G>A)];	NA	NA	NA	NA	NA	NA	NA	NA	NA	NA	MODIFIER	NA	NA	318	298	3	223	189	10	4.48
Smoker	42480	chr9	4576642	G	A	*SLC1A1*	MISSENSE	ENSG00000106688:MISSENSE[ENST00000422398(c.359G>A,Gtg>Atg):ENSP00000414620(p.Val121Met)];ENSG00000106688:MISSENSE[ENST00000262352(c.1072G>A,Gtg>Atg):ENSP00000262352(p.Val358Met)];	chr9*45766	NA	NA	3.29E-05	0	NA	NA	NA	NA	NA	MODIFIER	NA	NA	117	116	0	94	85	9	9.57
Smoker	42480	chr9	106858510	G	A	*SMC2*	MISSENSE	ENSG00000136824:MISSENSE[ENST00000303219(c.250G>A,Gat>Aat):ENSP00000306152(p.Asp84Asn)];ENSG00000136824:MISSENSE[ENST00000374793(c.250G>A,Gat>Aat):ENSP00000363925(p.Asp84Asn)];ENSG00000136824:MISSENSE[ENST00000286398(c.250G>A,Gat>Aat):ENSP00000286398(p.Asp84Asn)];ENSG00000136824:MISSENSE[ENST00000374787(c.250G>A,Gat>Aat):ENSP00000363919(p.Asp84Asn)];ENSG00000136824:INTRONIC[ENST00000440179(c.-118+1781G>A)];	NA	NA	NA	NA	NA	NA	NA	NA	NA	NA	MODIFIER	NA	NA	111	111	0	127	60	67	52.76
Smoker	42480	chr9	4576642	G	A	*SPATA6L*	INTRONIC	ENSG00000106686:[ENST00000485616(c.*782-22254C>T)];	chr9*45766	NA	NA	3.29E-05	0	NA	NA	NA	NA	NA	MODIFIER	NA	NA	117	116	0	94	85	9	9.57
Smoker	42480	chr9	114688695	T	C	*UGCG*	SILENT	ENSG00000148154:EXONIC-NC[ENST00000495085(n.524T>C)];ENSG00000148154:EXONIC-NC[ENST00000490110(n.570T>C)];ENSG00000148154:SILENT[ENST00000374279(c.477T>C,aaT>aaC):ENSP00000363397(p.Asn159(=))];	NA	NA	NA	NA	NA	NA	NA	NA	NA	NA	MODIFIER	NA	NA	119	119	0	134	99	35	26.12
Smoker	42480	chr9	99961638	C	G	*ZNF322P1*	EXONIC-NC	ENSG00000188801:[ENST00000375210(n.273G>C)];ENSG00000188801:[ENST00000568013(n.156G>C)];	NA	NA	NA	0.0034397	0.002275161	NA	NA	NA	NA	NA	MODIFIER	NA	NA	263	262	1	116	110	6	5.17
Smoker	42480	chrX	55028739	G	A	*APEX2*	SILENT	ENSG00000169188:INTRONIC[ENST00000471758(n.272-656G>A)];ENSG00000169188:SILENT[ENST00000374987(c.297G>A,ctG>ctA):ENSP00000364126(p.Leu99(=))];	chrX*55028	NA	NA	NA	NA	NA	NA	NA	NA	NA	LOW	NA	NA	48	48	0	36	22	14	38.89

Mangalaparthi *et al.* , 2020. Mutational landscape of esophageal squamous cell carcinoma in an Indian cohort
Supplementary Table 4. List of somatic single nucleotide variants identified using whole exome sequencing of ESCC patients

Cohort	Sample	Chromosome	Genomic position	Reference allele	Alternate allele	Gene symbol	Variant type	Variant annotation	OncoMD	COSMIC	dbSNP	ExAC	ExAC_SAS	1000G	1000G_SAS	SIFT	Polyphen2	CONDEL	VEP	LRT	PhastCon7 Way	Total read (Adjacent normal)	Reference allel read support (Adjacent normal)	Alternative read support (Adjacent normal)	Total read (Tumor)	Reference allel read support (Tumor)	Alternative read support (Tumor)	Alternate allele frequency
Smoker	42480	chrX	101857107	A	T	*ARMCX5*	MISSENSE	ENSG00000125962:MISSENSE[ENST00000246174(c.38A>T,aAg>aTg):ENSP00000246174(p.Lys13Met)];ENSG00000125962:MISSENSE[ENST00000604957(c.38A>T,aAg>aTg):ENSP00000474720(p.Lys13Met)];ENSG00000125962:MISSENSE[ENST00000541409(c.38A>T,aAg>aTg):ENSP00000446385(p.Lys13Met)];ENSG00000125962:MISSENSE[ENST00000537008(c.38A>T,aAg>aTg):ENSP00000439001(p.Lys13Met)];ENSG00000125962:MISSENSE[ENST00000372742(c.38A>T,aAg>aTg):ENSP00000361827(p.Lys13Met)];ENSG00000125962:MISSENSE[ENST00000479502(c.38A>T,aAg>aTg):ENSP00000474470(p.Lys13Met)];ENSG00000125962:MISSENSE[ENST00000477663(c.38A>T,aAg>aTg):ENSP00000474484(p.Lys13Met)];ENSG00000125962:MISSENSE[ENST00000536530(c.38A>T,aAg>aTg):ENSP00000445851(p.Lys13Met)];	NA	NA	NA	NA	NA	NA	NA	NA	NA	NA	MODIFIER	NA	NA	39	39	0	26	20	6	23.08
Smoker	42480	chrX	110644486	C	A	*DCX*	MISSENSE	ENSG00000077279:MISSENSE[ENST00000358070(c.655G>T,tGg>tTg):ENSP00000350776(p.Trp219Leu)];ENSG00000077279:MISSENSE[ENST00000371993(c.437G>T,tGg>tTg):ENSP00000361061(p.Trp146Leu)];ENSG00000077279:MISSENSE[ENST00000356220(c.437G>T,tGg>tTg):ENSP00000348553(p.Trp146Leu)];ENSG00000077279:MISSENSE[ENST00000338081(c.680G>T,tGg>tTg):ENSP00000337697(p.Trp227Leu)];ENSG00000077279:MISSENSE[ENST00000488120(c.437G>T,tGg>tTg):ENSP00000419861(p.Trp146Leu)];ENSG00000077279:MISSENSE[ENST00000356915(c.437G>T,tGg>tTg):ENSP00000349385(p.Trp146Leu)];ENSG00000077279:EXONIC-NC[ENST00000496551(n.677G>T)];	NA	NA	NA	NA	NA	NA	NA	NA	NA	NA	MODIFIER	NA	NA	126	126	0	108	99	9	8.33
Smoker	42480	chrX	50122907	T	G	*DGKK*	INTRONIC	ENSG00000204466:[ENST00000376025(n.2814+72A>C)];ENSG00000204466:[ENST00000546288(n.2814+72A>C)];	NA	NA	NA	NA	NA	NA	NA	NA	NA	NA	MODIFIER	NA	NA	74	74	0	79	73	6	7.59
Smoker	42480	chrX	33229479	C	A	*DMD*	EXONIC-NC	ENSG00000198947:[ENST00000488902(n.193G>T)];ENSG00000198947:[ENST00000463609(n.71G>T)];ENSG00000198947:[ENST00000357033(c.-50G>T)];ENSG00000198947:[ENST00000288447(c.7+127897G>T)];	NA	NA	NA	NA	NA	NA	NA	NA	NA	NA	MODIFIER	NA	NA	48	48	0	83	68	15	18.07
Smoker	42480	chrX	92964528	C	G	*FAM133A*	MISSENSE	ENSG00000179083:MISSENSE[ENST00000355813(c.110C>G,cCa>cGa):ENSP00000348067(p.Pro37Arg)];ENSG00000179083:MISSENSE[ENST00000538690(c.110C>G,cCa>cGa):ENSP00000441389(p.Pro37Arg)];ENSG00000179083:MISSENSE[ENST00000332647(c.110C>G,cCa>cGa):ENSP00000362169(p.Pro37Arg)];ENSG00000179083:MISSENSE[ENST00000322139(c.110C>G,cCa>cGa):ENSP00000318974(p.Pro37Arg)];	NA	NA	NA	NA	NA	NA	NA	D_lc	PrD	D	MODERATE	D	0.995	60	58	1	70	43	27	38.57
Smoker	42480	chrX	9001194	A	T	*FAM9B*	INTRONIC	ENSG00000177138:[ENST00000461107(n.373-78T>A)];ENSG00000177138:[ENST00000327220(c.-89-78T>A)];ENSG00000177138:[ENST00000472522(c.88-78T>A)];ENSG00000177138:[ENST00000362066(c.-32T>A)];	NA	NA	NA	NA	NA	NA	NA	NA	NA	NA	MODIFIER	NA	NA	68	68	0	62	46	15	24.19
Smoker	42480	chrX	73963371	C	T	*KIAA2022*	MISSENSE	ENSG00000050030:MISSENSE[ENST00000055682(c.1021G>A,Gtc>Atc):ENSP00000055682(p.Val341Ile)];	NA	NA	chrX*73963371*73963371*C*A*rs745417882*NA*NA*NA	NA	NA	NA	NA	D	PrD	D	MODERATE	D	0.999	106	104	2	193	95	98	50.78
Smoker	42480	chrX	27840312	G	A	*MAGEB10*	MISSENSE	ENSG00000177689:MISSENSE[ENST00000356790(c.889G>A,Gat>Aat):ENSP00000368304(p.Asp297Asn)];	NA	NA	NA	NA	NA	NA	NA	T	BN	N	MODERATE	N	0.046	83	83	0	51	38	12	23.53
Smoker	42480	chrX	26235820	G	C	*MAGEB5*	MISSENSE	ENSG00000188408:MISSENSE[ENST00000379029(c.402G>C,atG>atC):ENSP00000368315(p.Met134Ile)];ENSG00000188408:MISSENSE[ENST00000602297(c.402G>C,atG>atC):ENSP00000473493(p.Met134Ile)];	NA	chrX*26235	NA	NA	NA	NA	NA	T	PoD	D	MODERATE	U	0.31	115	115	0	136	106	30	22.06
Smoker	42480	chrX	141291183	C	T	*MAGEC2*	SILENT	ENSG00000046774:SILENT[ENST00000247452(c.591G>A,ctG>ctA):ENSP00000354660(p.Leu197(=))];	chrX*14129	NA	NA	NA	NA	NA	NA	NA	NA	NA	LOW	NA	NA	52	52	0	37	25	12	32.43
Smoker	42480	chrX	55478885	G	T	*MAGEH1*	MISSENSE	ENSG00000187601:MISSENSE[ENST00000342972(c.78G>T,caG>caT):ENSP00000343706(p.Gln26His)];	NA	NA	NA	NA	NA	NA	NA	D_lc	BN	N	MODERATE	N	0.987	40	40	0	60	51	9	15
Smoker	42480	chrX	78156793	T	C	*NA*	INTERGENIC		NA	NA	NA	NA	NA	NA	NA	NA	NA	NA	MODIFIER	NA	NA	89	89	0	164	152	12	7.32
Smoker	42480	chrX	91066297	G	T	*PCDH11X*	INTRONIC-SS-DNR	ENSG00000102290:[ENST00000395337(c.-45+3G>T)];	NA	NA	NA	NA	NA	NA	NA	NA	NA	NA	LOW	NA	NA	124	124	0	222	194	28	12.61
Smoker	42480	chrX	91132326	C	T	*PCDH11X*	MISSENSE	ENSG00000102290:MISSENSE[ENST00000373088(c.1087C>T,Cct>Tct):ENSP00000362180(p.Pro363Ser)];ENSG00000102290:MISSENSE[ENST00000361724(c.1087C>T,Cct>Tct):ENSP00000355040(p.Pro363Ser)];ENSG00000102290:MISSENSE[ENST00000395337(c.1087C>T,Cct>Tct):ENSP00000378746(p.Pro363Ser)];ENSG00000102290:MISSENSE[ENST00000298274(c.1087C>T,Cct>Tct):ENSP00000298274(p.Pro363Ser)];ENSG00000102290:MISSENSE[ENST00000504220(c.1087C>T,Cct>Tct):ENSP00000423762(p.Pro363Ser)];ENSG00000102290:MISSENSE[ENST00000406881(c.1087C>T,Cct>Tct):ENSP00000384758(p.Pro363Ser)];ENSG00000102290:MISSENSE[ENST00000373094(c.1087C>T,Cct>Tct):ENSP00000362186(p.Pro363Ser)];ENSG00000102290:MISSENSE[ENST00000373097(c.1087C>T,Cct>Tct):ENSP00000362189(p.Pro363Ser)];ENSG00000102290:MISSENSE[ENST00000361655(c.1087C>T,Cct>Tct):ENSP00000355105(p.Pro363Ser)];	NA	NA	NA	NA	NA	NA	NA	T	PrD	D	MODERATE	D	0.901	81	81	0	156	78	77	49.36
Smoker	42480	chrX	101857107	A	T	*RP4-769N13.6*	INTRONIC	ENSG00000271147:[ENST00000475738(n.368+670A>T)];ENSG00000271147:[ENST00000602366(n.322+2332A>T)];ENSG00000271147:[ENST00000466616(n.466+202A>T)];ENSG00000271147:[ENST00000486740(n.236+670A>T)];ENSG00000271147:[ENST00000602463(n.197+2332A>T)];ENSG00000271147:[ENST00000476910(n.568+202A>T)];ENSG00000271147:[ENST00000460793(n.308+2332A>T)];ENSG00000271147:[ENST00000460026(n.334+202A>T)];ENSG00000271147:[ENST00000465548(n.191+77A>T)];	NA	NA	NA	NA	NA	NA	NA	NA	NA	NA	MODIFIER	NA	NA	39	39	0	26	20	6	23.08
Smoker	42480	chrX	101857107	A	T	*RP4-769N13.7*	INTRONIC	ENSG00000270050:[ENST00000602441(n.129-2264T>A)];	NA	NA	NA	NA	NA	NA	NA	NA	NA	NA	MODIFIER	NA	NA	39	39	0	26	20	6	23.08
Smoker	42480	chrX	79278628	A	G	*TBX22*	MISSENSE	ENSG00000122145:5UTR[ENST00000373291(c.-179A>G)];ENSG00000122145:5UTR[ENST00000442340(c.-112A>G)];ENSG00000122145:MISSENSE[ENST00000373294(c.245A>G,aAc>aGc):ENSP00000362390(p.Asn82Ser)];ENSG00000122145:MISSENSE[ENST00000373296(c.245A>G,aAc>aGc):ENSP00000362393(p.Asn82Ser)];	NA	NA	NA	NA	NA	NA	NA	T	BN	N	MODERATE	N	0.069	52	51	0	69	62	7	10.14
Smoker	42481	chr1	156212408	C	T	*BGLAP*	INTRONIC	ENSG00000242252:[ENST00000471413(n.403+26C>T)];ENSG00000242252:[ENST00000368272(c.103+26C>T)];	NA	NA	NA	NA	NA	NA	NA	NA	NA	NA	MODIFIER	NA	NA	57	56	0	49	44	5	10.2

Mangalaparthi *et al.* , 2020. Mutational landscape of esophageal squamous cell carcinoma in an Indian cohort
Supplementary Table 4. List of somatic single nucleotide variants identified using whole exome sequencing of ESCC patients

Cohort	Sample	Chromosome	Genomic position	Reference allele	Alternate allele	Gene symbol	Variant type	Variant annotation	OncoMD	COSMIC	dbSNP	ExAC	ExAC_SAS	1000G	1000G_SAS	SIFT	Polyphen2	CONDEL	VEP	LRT	PhastCon7 Way	Total read (Adjacent normal)	Reference allel read support (Adjacent normal)	Alternative read support (Adjacent normal)	Total read (Tumor)	Reference allel read support (Tumor)	Alternative read support (Tumor)	Alternate allele frequency
Smoker	42481	chr1	245246919	C	A	*EFCAB2*	INTRONIC-SS-ACR	ENSG00000203666:[ENST00000551317(c.282-5C>A)];ENSG00000203666:[ENST00000366523(c.307-5C>A)];ENSG00000203666:[ENST00000549220(n.348-5C>A)];ENSG00000203666:[ENST00000473686(n.290-5C>A)];ENSG00000203666:[ENST00000391837(n.376-5C>A)];ENSG00000203666:[ENST00000495271(n.341-5C>A)];ENSG00000203666:[ENST00000366521(c.482-5C>A)];ENSG00000203666:[ENST00000487845(n.251-5C>A)];ENSG00000203666:[ENST00000447569(c.307-5C>A)];ENSG00000203666:[ENST00000427529(c.30-5C>A)];ENSG00000203666:[ENST00000497591(n.355-5C>A)];ENSG00000203666:[ENST00000425550(c.282-5C>A)];ENSG00000203666:[ENST00000366522(c.715-5C>A)];	NA	NA	NA	NA	NA	NA	NA	NA	NA	NA	LOW	NA	NA	61	61	0	57	44	13	22.81
Smoker	42481	chr1	155206267	A	C	*GBA*	INTRONIC-SS-ACR	ENSG00000177628:[ENST00000491081(n.605-7T>G)];ENSG00000177628:[ENST00000497670(n.623-7T>G)];ENSG00000177628:[ENST00000536770(c.661-7T>G)];ENSG00000177628:[ENST00000427500(c.853-7T>G)];ENSG00000177628:[ENST00000327247(c.1000-7T>G)];ENSG00000177628:[ENST00000428024(c.739-7T>G)];ENSG00000177628:[ENST00000368373(c.1000-7T>G)];ENSG00000177628:[ENST00000484489(n.340-188T>G)];	NA	NA	NA	NA	NA	NA	NA	NA	NA	NA	LOW	NA	NA	116	116	0	101	84	17	16.83
Smoker	42481	chr1	149908010	A	C	*MTMR11*	INTRONIC	ENSG00000014914:[ENST00000406732(c.58+37T>G)];ENSG00000014914:[ENST00000482343(n.329+37T>G)];ENSG00000014914:[ENST00000492824(n.329+37T>G)];ENSG00000014914:[ENST00000369140(c.11+37T>G)];ENSG00000014914:[ENST00000361405(c.142+37T>G)];ENSG00000014914:[ENST00000439741(c.142+37T>G)];ENSG00000014914:[ENST00000482025(n.379+37T>G)];ENSG00000014914:[ENST00000466496(n.191+37T>G)];	NA	NA	NA	NA	NA	NA	NA	NA	NA	NA	MODIFIER	NA	NA	122	122	0	87	60	26	29.89
Smoker	42481	chr1	145578079	C	T	*PIAS3*	SILENT	ENSG00000131788:SILENT[ENST00000393045(c.42C>T,ttC>ttT):ENSP00000376765(p.Phe14(=))];ENSG00000131788:SILENT[ENST00000369298(c.42C>T,ttC>ttT):ENSP00000358304(p.Phe14(=))];ENSG00000131788:SILENT[ENST00000463514(c.48C>T,ttC>ttT):ENSP00000476245(p.Phe16(=))];ENSG00000131788:SILENT[ENST00000369299(c.15C>T,ttC>ttT):ENSP00000358305(p.Phe5(=))];ENSG00000131788:SILENT[ENST00000393046(c.15C>T,ttC>ttT):ENSP00000376766(p.Phe5(=))];ENSG00000131788:EXONIC-NC[ENST00000484423(n.87C>T)];	NA	NA	NA	8.25E-06	0	NA	NA	NA	NA	NA	LOW	NA	NA	173	172	0	113	96	17	15.04
Smoker	42481	chr1	156212408	C	T	*PMF1*	INTRONIC	ENSG00000160783:[ENST00000567140(c.542+26C>T)];ENSG00000160783:[ENST00000565805(c.407+26C>T)];	NA	NA	NA	NA	NA	NA	NA	NA	NA	NA	MODIFIER	NA	NA	57	56	0	49	44	5	10.2
Smoker	42481	chr1	156212408	C	T	*PMF1-BGLAP*	INTRONIC	ENSG00000260238:[ENST00000490491(c.603+26C>T)];ENSG00000260238:[ENST00000368276(c.542+26C>T)];ENSG00000260238:[ENST00000320139(c.407+26C>T)];	NA	NA	NA	NA	NA	NA	NA	NA	NA	NA	MODIFIER	NA	NA	57	56	0	49	44	5	10.2
Smoker	42481	chr1	52442808	G	C	*RAB3B*	INTRONIC	ENSG00000169213:[ENST00000371655(c.1-19C>G)];	NA	NA	chr1*52442807*52442831*AGTCACTCAGGAACAGACCTTCGGT*A*rs775083359*NA*NA*NA	NA	NA	NA	NA	NA	NA	NA	MODIFIER	NA	NA	133	133	0	122	81	41	33.61
Smoker	42481	chr1	19180859	C	T	*RP13-279N23.2*	3UTR	ENSG00000255275:[ENST00000494072(c.*3050G>A)];	chr1*19180	chr1*19180	NA	2.54E-05	6.19E-05	NA	NA	T	BN	N	MODERATE	N	0.003	141	140	1	123	116	7	5.69
Smoker	42481	chr1	19180859	C	T	*TAS1R2*	MISSENSE	ENSG00000179002:MISSENSE[ENST00000375371(c.1105G>A,Gcc>Acc):ENSP00000364520(p.Ala369Thr)];	chr1*19180	chr1*19180	NA	2.54E-05	6.19E-05	NA	NA	T	BN	N	MODERATE	N	0.003	141	140	1	123	116	7	5.69
Smoker	42481	chr1	162467116	G	A	*UHMK1*	INTRONIC	ENSG00000152332:[ENST00000545294(c.46+30G>A)];	NA	NA	NA	NA	NA	NA	NA	NA	NA	NA	MODIFIER	NA	NA	131	129	1	113	76	37	32.74
Smoker	42481	chr1	43316470	G	C	*ZNF691*	INTRONIC	ENSG00000164011:[ENST00000372507(c.-9-151G>C)];ENSG00000164011:[ENST00000372503(c.85-151G>C)];ENSG00000164011:[ENST00000372504(c.57+56G>C)];ENSG00000164011:[ENST00000372508(c.-9-151G>C)];ENSG00000164011:[ENST00000372502(c.57+56G>C)];ENSG00000164011:[ENST00000397034(c.57+56G>C)];ENSG00000164011:[ENST00000372506(c.-9-151G>C)];ENSG00000164011:[ENST00000397044(c.85-151G>C)];	NA	NA	NA	NA	NA	NA	NA	NA	NA	NA	MODIFIER	NA	NA	122	122	0	99	74	25	25.25
Smoker	42481	chr10	33559729	T	C	*NRP1*	MISSENSE	ENSG00000099250:MISSENSE[ENST00000395995(c.304A>G,Aag>Gag):ENSP00000379317(p.Lys102Glu)];ENSG00000099250:MISSENSE[ENST00000374867(c.304A>G,Aag>Gag):ENSP00000364001(p.Lys102Glu)];ENSG00000099250:MISSENSE[ENST00000265371(c.304A>G,Aag>Gag):ENSP00000265371(p.Lys102Glu)];ENSG00000099250:MISSENSE[ENST00000374816(c.304A>G,Aag>Gag):ENSP00000363949(p.Lys102Glu)];ENSG00000099250:MISSENSE[ENST00000374822(c.304A>G,Aag>Gag):ENSP00000363955(p.Lys102Glu)];ENSG00000099250:MISSENSE[ENST00000432372(c.304A>G,Aag>Gag):ENSP00000408911(p.Lys102Glu)];ENSG00000099250:MISSENSE[ENST00000374823(c.304A>G,Aag>Gag):ENSP00000363956(p.Lys102Glu)];ENSG00000099250:MISSENSE[ENST00000374821(c.304A>G,Aag>Gag):ENSP00000363954(p.Lys102Glu)];ENSG00000099250:INTRONIC[ENST00000374875(c.-113-6928A>G)];	NA	NA	NA	NA	NA	NA	NA	NA	NA	NA	MODIFIER	NA	NA	130	130	0	131	101	30	22.9
Smoker	42481	chr10	98469454	C	T	*PIK3AP1*	SILENT	ENSG00000155629:SILENT[ENST00000339364(c.300G>A,agG>agA):ENSP00000339826(p.Arg100(=))];	NA	NA	NA	NA	NA	NA	NA	NA	NA	NA	LOW	NA	0.06	118	117	0	82	74	8	9.76
Smoker	42481	chr10	75523713	A	G	*SEC24C*	MISSENSE	ENSG00000176986:MISSENSE[ENST00000345254(c.1199A>G,aAa>aGa):ENSP00000321845(p.Lys400Arg)];ENSG00000176986:MISSENSE[ENST00000411652(c.842A>G,aAa>aGa):ENSP00000402913(p.Lys281Arg)];ENSG00000176986:MISSENSE[ENST00000339365(c.1199A>G,aAa>aGa):ENSP00000343405(p.Lys400Arg)];ENSG00000176986:MISSENSE[ENST00000465076(c.1199A>G,aAa>aGa):ENSP00000437000(p.Lys400Arg)];ENSG00000176986:INTRONIC[ENST00000546025(c.562-1496A>G)];ENSG00000176986:INTRONIC[ENST00000540668(c.-75-4855A>G)];ENSG00000176986:INTRONIC[ENST00000535742(c.-75-4855A>G)];	NA	NA	NA	NA	NA	NA	NA	NA	NA	NA	MODIFIER	NA	NA	55	55	0	43	24	19	44.19
Smoker	42481	chr11	130289214	G	A	*ADAMTS8*	INTRONIC	ENSG00000134917:[ENST00000257359(c.721-27C>T)];	NA	NA	NA	0.0001344	6.20E-05	NA	NA	NA	NA	NA	MODIFIER	NA	NA	39	39	0	37	31	6	16.22
Smoker	42481	chr11	64674125	C	T	*ATG2A*	INTRONIC	ENSG00000110046:[ENST00000418259(c.2387+12G>A)];ENSG00000110046:[ENST00000377264(c.2983+12G>A)];ENSG00000110046:[ENST00000421419(c.2983+12G>A)];	NA	NA	NA	NA	NA	NA	NA	NA	NA	NA	MODIFIER	NA	NA	58	58	0	33	18	14	42.42

Mangalaparthi *et al.* , 2020. Mutational landscape of esophageal squamous cell carcinoma in an Indian cohort
Supplementary Table 4. List of somatic single nucleotide variants identified using whole exome sequencing of ESCC patients

Cohort	Sample	Chromosome	Genomic position	Reference allele	Alternate allele	Gene symbol	Variant type	Variant annotation	OncoMD	COSMIC	dbSNP	ExAC	ExAC_SAS	1000G	1000G_SAS	SIFT	Polyphen2	CONDEL	VEP	LRT	PhastCon7 Way	Total read (Adjacent normal)	Reference allel read support (Adjacent normal)	Alternative read support (Adjacent normal)	Total read (Tumor)	Reference allel read support (Tumor)	Alternative read support (Tumor)	Alternate allele frequency
Smoker	42481	chr11	61106845	G	A	*DAK*	MISSENSE	ENSG00000149476:MISSENSE[ENST00000394900(c.424G>A,Gcc>Acc):ENSP00000378360(p.Ala142Thr)];ENSG00000149476:MISSENSE[ENST00000529479(c.421G>A,Gcc>Acc):ENSP00000432539(p.Ala141Thr)];ENSG00000149476:MISSENSE[ENST00000532173(c.424G>A,Gcc>Acc):ENSP00000431844(p.Ala142Thr)];ENSG00000149476:EXONIC-NC[ENST00000529092(n.179G>A)];	NA	NA	NA	2.52E-05	0	NA	NA	NA	NA	NA	MODIFIER	NA	NA	82	82	0	80	39	41	51.25
Smoker	42481	chr11	61106845	G	A	*DDB1*	INTRONIC	ENSG00000167986:[ENST00000540166(c.-166+2995C>T)];	NA	NA	NA	2.52E-05	0	NA	NA	NA	NA	NA	MODIFIER	NA	NA	82	82	0	80	39	41	51.25
Smoker	42481	chr11	58919483	C	T	*FAM111A*	SILENT	ENSG00000166801:SILENT[ENST00000361723(c.342C>T,gtC>gtT):ENSP00000355264(p.Val114(=))];ENSG00000166801:SILENT[ENST00000420244(c.342C>T,gtC>gtT):ENSP00000406683(p.Val114(=))];ENSG00000166801:SILENT[ENST00000528737(c.342C>T,gtC>gtT):ENSP00000434435(p.Val114(=))];ENSG00000166801:SILENT[ENST00000531147(c.342C>T,gtC>gtT):ENSP00000431631(p.Val114(=))];ENSG00000166801:SILENT[ENST00000533703(c.342C>T,gtC>gtT):ENSP00000433154(p.Val114(=))];ENSG00000166801:SILENT[ENST00000527629(c.342C>T,gtC>gtT):ENSP00000436128(p.Val114(=))];	NA	NA	NA	NA	NA	NA	NA	NA	NA	NA	LOW	NA	NA	291	290	0	198	184	13	6.57
Smoker	42481	chr11	102713494	T	C	*MMP3*	MISSENSE	ENSG00000149968:3UTR[ENST00000524478(c.*101A>G)];ENSG00000149968:MISSENSE[ENST00000299855(c.259A>G,Atg>Gtg):ENSP00000299855(p.Met87Val)];	NA	NA	NA	NA	NA	NA	NA	T	BN	N	MODERATE	N	0.741	166	165	0	91	72	19	20.88
Smoker	42481	chr11	47587244	C	A	*NDUFS3*	EXONIC-NC	ENSG00000213619:[ENST00000533507(n.357C>A)];	NA	NA	NA	NA	NA	NA	NA	T	PoD	D	MODERATE	U	0.986	73	73	0	30	23	7	23.33
Smoker	42481	chr11	57958283	T	A	*OR9Q2*	MISSENSE	ENSG00000186513:MISSENSE[ENST00000311591(c.321T>A,ttT>ttA):ENSP00000308714(p.Phe107Leu)];	NA	NA	NA	NA	NA	NA	NA	T	BN	N	MODERATE	N	0.991	267	267	0	129	96	33	25.58
Smoker	42481	chr11	47587244	C	A	*PTPMT1*	MISSENSE	ENSG00000110536:MISSENSE[ENST00000534775(c.70C>A,Ctg>Atg):ENSP00000436160(p.Leu24Met)];ENSG00000110536:MISSENSE[ENST00000426530(c.70C>A,Ctg>Atg):ENSP00000410272(p.Leu24Met)];ENSG00000110536:MISSENSE[ENST00000326674(c.70C>A,Ctg>Atg):ENSP00000325958(p.Leu24Met)];ENSG00000110536:MISSENSE[ENST00000326656(c.70C>A,Ctg>Atg):ENSP00000325882(p.Leu24Met)];	NA	NA	NA	NA	NA	NA	NA	T	PoD	D	MODERATE	U	0.986	73	73	0	30	23	7	23.33
Smoker	42481	chr11	123513328	G	A	*SCN3B*	MISSENSE	ENSG00000166257:MISSENSE[ENST00000299333(c.271C>T,Cgc>Tgc):ENSP00000299333(p.Arg91Cys)];ENSG00000166257:MISSENSE[ENST00000530277(c.271C>T,Cgc>Tgc):ENSP00000432785(p.Arg91Cys)];ENSG00000166257:MISSENSE[ENST00000392770(c.271C>T,Cgc>Tgc):ENSP00000376523(p.Arg91Cys)];ENSG00000166257:MISSENSE[ENST00000528267(c.271C>T,Cgc>Tgc):ENSP00000434363(p.Arg91Cys)];ENSG00000166257:MISSENSE[ENST00000527836(c.271C>T,Cgc>Tgc):ENSP00000435554(p.Arg91Cys)];	NA	NA	NA	8.26E-06	0	NA	NA	D	PrD	D	MODERATE	NA	NA	129	129	0	81	65	16	19.75
Smoker	42481	chr11	117058086	C	A	*SIDT2*	INTRONIC-SS-ACR	ENSG00000149577:[ENST00000324225(c.1016-8C>A)];ENSG00000149577:[ENST00000278951(c.1016-8C>A)];ENSG00000149577:[ENST00000529441(n.327-8C>A)];ENSG00000149577:[ENST00000431081(c.1028-8C>A)];ENSG00000149577:[ENST00000525339(n.210-4150C>A)];	NA	NA	chr11*117058086*117058086*C*T*rs773037253*NA*NA*NA	NA	NA	NA	NA	NA	NA	NA	LOW	NA	NA	135	135	0	87	76	11	12.64
Smoker	42481	chr11	107677516	G	T	*SLC35F2*	SILENT	ENSG00000110660:SILENT[ENST00000532513(c.501C>A,gcC>gcA):ENSP00000433783(p.Ala167(=))];ENSG00000110660:SILENT[ENST00000525815(c.501C>A,gcC>gcA):ENSP00000436785(p.Ala167(=))];ENSG00000110660:SILENT[ENST00000429869(c.501C>A,gcC>gcA):ENSP00000393571(p.Ala167(=))];ENSG00000110660:SILENT[ENST00000375682(c.360C>A,gcC>gcA):ENSP00000364834(p.Ala120(=))];ENSG00000110660:SILENT[ENST00000525071(c.501C>A,gcC>gcA):ENSP00000434307(p.Ala167(=))];ENSG00000110660:SILENT[ENST00000265836(c.57C>A,gcC>gcA):ENSP00000265836(p.Ala19(=))];ENSG00000110660:3UTR[ENST00000533664(c.*208C>A)];	NA	NA	chr11*107677516*107677516*G*A*rs554201280*0*0.9998*0.0001997	NA	NA	NA	NA	NA	NA	NA	LOW	NA	NA	300	299	0	201	119	81	40.3
Smoker	42481	chr12	50370796	G	T	*AQP6*	EXONIC-NC	ENSG00000086159:[ENST00000489786(n.5003G>T)];	NA	NA	NA	0.0014864	0	NA	NA	NA	NA	NA	LOW	NA	NA	142	142	0	164	111	53	32.32
Smoker	42481	chr12	52641938	C	T	*KRT7*	EXONIC-NC	ENSG00000135480:[ENST00000549638(n.237C>T)];ENSG00000135480:[ENST00000550153(n.134-23C>T)];ENSG00000135480:[ENST00000331817(c.1206-23C>T)];ENSG00000135480:[ENST00000552322(n.517-23C>T)];ENSG00000135480:[ENST00000548657(n.195-23C>T)];ENSG00000135480:[ENST00000549127(n.197-23C>T)];	NA	NA	NA	NA	NA	NA	NA	NA	NA	NA	MODIFIER	NA	NA	59	59	0	48	32	16	33.33
Smoker	42481	chr12	124941730	G	A	*NCOR2*	INTRONIC	ENSG00000196498:[ENST00000356219(c.706-22C>T)];ENSG00000196498:[ENST00000542927(c.474-22C>T)];ENSG00000196498:[ENST00000458234(c.706-22C>T)];ENSG00000196498:[ENST00000420698(c.706-22C>T)];ENSG00000196498:[ENST00000404121(c.-585-22C>T)];ENSG00000196498:[ENST00000448008(c.469-7317C>T)];ENSG00000196498:[ENST00000429285(c.706-22C>T)];ENSG00000196498:[ENST00000405201(c.706-22C>T)];ENSG00000196498:[ENST00000404621(c.706-22C>T)];ENSG00000196498:[ENST00000397355(c.706-22C>T)];	NA	NA	NA	NA	NA	NA	NA	NA	NA	NA	MODIFIER	NA	NA	16	16	0	24	17	7	29.17
Smoker	42481	chr12	50370796	G	T	*RACGAP1*	SILENT	ENSG00000161800:SILENT[ENST00000548961(c.291C>A,gcC>gcA):ENSP00000446889(p.Ala97(=))];	NA	NA	NA	0.0014864	0	NA	NA	NA	NA	NA	LOW	NA	NA	142	142	0	164	111	53	32.32
Smoker	42481	chr12	98984974	G	T	*RP11-481K9.4*	EXONIC-NC	ENSG00000225185:[ENST00000420972(n.10C>A)];ENSG00000225185:[ENST00000557282(n.19C>A)];	NA	NA	NA	NA	NA	NA	NA	NA	NA	NA	MODIFIER	NA	NA	94	94	0	91	80	11	12.09
Smoker	42481	chr13	34392216	C	G	*RFC3*	5UTR	ENSG00000133119:[ENST00000434425(c.-100C>G)];ENSG00000133119:[ENST00000380071(c.-100C>G)];	NA	NA	NA	NA	NA	NA	NA	NA	NA	NA	MODIFIER	NA	NA	164	161	0	95	79	16	16.84
Smoker	42481	chr13	100623262	G	A	*ZIC5*	MISSENSE	ENSG00000139800:MISSENSE[ENST00000267294(c.668C>T,tCc>tTc):ENSP00000267294(p.Ser223Phe)];	NA	NA	NA	NA	NA	NA	NA	D	UN	NA	MODERATE	NA	NA	43	43	0	29	16	13	44.83
Smoker	42481	chr14	92189455	C	G	*CATSPERB*	MISSENSE	ENSG00000133962:MISSENSE[ENST00000256343(c.247G>C,Gct>Cct):ENSP00000256343(p.Ala83Pro)];ENSG00000133962:MISSENSE[ENST00000554560(c.247G>C,Gct>Cct):ENSP00000452224(p.Ala83Pro)];ENSG00000133962:INTRONIC[ENST00000553329(c.168+1969G>C)];	NA	NA	NA	NA	NA	NA	NA	NA	NA	NA	MODIFIER	NA	NA	71	71	0	55	40	15	27.27
Smoker	42481	chr14	74156083	T	C	*DNAL1*	MISSENSE	ENSG00000119661:MISSENSE[ENST00000553645(c.397T>C,Ttt>Ctt):ENSP00000452037(p.Phe133Leu)];ENSG00000119661:MISSENSE[ENST00000540526(c.280T>C,Ttt>Ctt):ENSP00000439695(p.Phe94Leu)];ENSG00000119661:MISSENSE[ENST00000554339(c.136T>C,Ttt>Ctt):ENSP00000450744(p.Phe46Leu)];ENSG00000119661:MISSENSE[ENST00000554871(c.280T>C,Ttt>Ctt):ENSP00000451834(p.Phe94Leu)];ENSG00000119661:MISSENSE[ENST00000311089(c.58T>C,Ttt>Ctt):ENSP00000310360(p.Phe20Leu)];ENSG00000119661:MISSENSE[ENST00000554113(c.58T>C,Ttt>Ctt):ENSP00000452368(p.Phe20Leu)];ENSG00000119661:MISSENSE[ENST00000559993(c.58T>C,Ttt>Ctt):ENSP00000453439(p.Phe20Leu)];ENSG00000119661:MISSENSE[ENST00000555631(c.280T>C,Ttt>Ctt):ENSP00000451547(p.Phe94Leu)];ENSG00000119661:INTRONIC[ENST00000554159(c.*74+1995T>C)];	NA	NA	NA	NA	NA	NA	NA	T	BN	N	MODERATE	NA	NA	136	134	0	175	137	37	21.14

Cohort	Sample	Chromosome	Genomic position	Reference allele	Alternate allele	Gene symbol	Variant type	Variant annotation	OncoMD	COSMIC	dbSNP	ExAC	ExAC_SAS	1000G	1000G_SAS	SIFT	Polyphen2	CONDEL	VEP	LRT	PhastCon7 Way	Total read (Adjacent normal)	Reference allel read support (Adjacent normal)	Alternative read support (Adjacent normal)	Total read (Tumor)	Reference allel read support (Tumor)	Alternative read support (Tumor)	Alternate allele frequency
Smoker	42481	chr14	76129497	C	G	*FLVCR2*	3UTR	ENSG00000119686:[ENST00000553587(c.*66C>G)];ENSG00000119686:[ENST00000555385(n.158C>G)];ENSG00000119686:[ENST00000556241(n.508C>G)];	NA	NA	NA	NA	NA	NA	NA	NA	NA	NA	MODIFIER	NA	NA	120	120	0	108	50	58	53.7
Smoker	42481	chr14	73730961	G	A	*PAPLN*	SILENT	ENSG00000100767:SILENT[ENST00000559759(c.509G>A,ctG>ctA):ENSP00000453664(p.Leu170(=))];ENSG00000100767:SILENT[ENST00000555445(c.2856G>A,ctG>ctA):ENSP00000451729(p.Leu952(=))];ENSG00000100767:SILENT[ENST00000381166(c.2904G>A,ctG>ctA):ENSP00000370558(p.Leu968(=))];ENSG00000100767:SILENT[ENST00000427855(c.2904G>A,ctG>ctA):ENSP00000403403(p.Leu968(=))];ENSG00000100767:SILENT[ENST00000554301(c.2904G>A,ctG>ctA):ENSP00000451803(p.Leu968(=))];ENSG00000100767:SILENT[ENST00000340738(c.2823G>A,ctG>ctA):ENSP00000345395(p.Leu941(=))];ENSG00000100767:EXONIC-NC[ENST00000555700(n.1776G>A)];ENSG00000100767:INTRONIC-SS-ACR-PRX[ENST00000554314(n.608-4G>A)];ENSG00000100767:3UTR[ENST00000557061(c.*175G>A)];ENSG00000100767:3UTR[ENST00000216658(c.*102G>A)];ENSG00000100767:3UTR[ENST00000555123(c.*68G>A)];	NA	NA	NA	NA	NA	NA	NA	NA	NA	NA	LOW	NA	NA	63	63	0	85	63	22	25.88
Smoker	42481	chr14	94844876	C	A	*SERPINA1*	MISSENSE	ENSG00000197249:MISSENSE[ENST00000448921(c.1167G>T,aaG>aaT):ENSP00000416066(p.Lys389Asn)];ENSG00000197249:MISSENSE[ENST00000449399(c.1167G>T,aaG>aaT):ENSP00000416354(p.Lys389Asn)];ENSG00000197249:MISSENSE[ENST00000355814(c.1167G>T,aaG>aaT):ENSP00000348068(p.Lys389Asn)];ENSG00000197249:MISSENSE[ENST00000404814(c.1167G>T,aaG>aaT):ENSP00000385960(p.Lys389Asn)];ENSG00000197249:MISSENSE[ENST00000393088(c.1167G>T,aaG>aaT):ENSP00000376803(p.Lys389Asn)];ENSG00000197249:MISSENSE[ENST00000393087(c.1167G>T,aaG>aaT):ENSP00000376802(p.Lys389Asn)];ENSG00000197249:MISSENSE[ENST00000437397(c.1167G>T,aaG>aaT):ENSP00000408474(p.Lys389Asn)];ENSG00000197249:MISSENSE[ENST00000440909(c.1167G>T,aaG>aaT):ENSP00000390299(p.Lys389Asn)];ENSG00000197249:3UTR[ENST00000489769(c.*466G>T)];	NA	NA	NA	NA	NA	NA	NA	T	BN	N	MODERATE	N	0.01	166	165	0	131	94	37	28.24
Smoker	42481	chr14	76129497	C	G	*TTLL5*	MISSENSE	ENSG00000119685:MISSENSE[ENST00000557636(c.5C>G,cCa>cGa):ENSP00000450713(p.Pro2Arg)];ENSG00000119685:MISSENSE[ENST00000298832(c.5C>G,cCa>cGa):ENSP00000298832(p.Pro2Arg)];ENSG00000119685:MISSENSE[ENST00000286650(c.5C>G,cCa>cGa):ENSP00000286650(p.Pro2Arg)];ENSG00000119685:MISSENSE[ENST00000556977(c.5C>G,cCa>cGa):ENSP00000451917(p.Pro2Arg)];ENSG00000119685:EXONIC-NC[ENST00000554132(n.172C>G)];ENSG00000119685:EXONIC-NC[ENST00000556685(n.505C>G)];ENSG00000119685:EXONIC-NC[ENST00000556265(n.276C>G)];ENSG00000119685:EXONIC-NC[ENST00000555290(n.135C>G)];	NA	NA	NA	NA	NA	NA	NA	NA	NA	NA	MODIFIER	NA	NA	120	120	0	108	50	58	53.7
Smoker	42481	chr15	86809590	A	C	*AGBL1*	INTRONIC	ENSG00000166748:[ENST00000389298(c.723-15A>C)];ENSG00000166748:[ENST00000567715(n.742-15A>C)];ENSG00000166748:[ENST00000441037(c.1530-15A>C)];ENSG00000166748:[ENST00000421325(c.1530-15A>C)];ENSG00000166748:[ENST00000568785(n.852-15A>C)];	NA	NA	NA	NA	NA	NA	NA	NA	NA	NA	MODIFIER	NA	NA	130	130	0	75	49	26	34.67
Smoker	42481	chr15	75798315	C	T	*PTPN9*	SILENT	ENSG00000169410:SILENT[ENST00000306726(c.669G>A,acG>acA):ENSP00000303554(p.Thr223(=))];	NA	NA	chr15*75798315*75798315*C*G*rs760119034*NA*NA*NA	NA	NA	NA	NA	NA	NA	NA	LOW	NA	NA	75	75	0	68	58	10	14.71
Smoker	42481	chr15	45965871	G	A	*RP11-96O20.4*	MISSENSE	ENSG00000260170:MISSENSE[ENST00000564080(c.526G>A,Gag>Aag):ENSP00000455047(p.Glu176Lys)];	NA	NA	chr15*45965871*45965871*G*C*rs762187581*NA*NA*NA	NA	NA	NA	NA	T	BN	N	MODERATE	NA	NA	138	138	0	146	133	13	8.9
Smoker	42481	chr15	45965871	G	A	*SQRDL*	MISSENSE	ENSG00000137767:MISSENSE[ENST00000260324(c.526G>A,Gag>Aag):ENSP00000260324(p.Glu176Lys)];ENSG00000137767:MISSENSE[ENST00000568606(c.526G>A,Gag>Aag):ENSP00000456019(p.Glu176Lys)];ENSG00000137767:MISSENSE[ENST00000566934(c.369G>A,Gag>Aag):ENSP00000454520(p.Glu124Lys)];	NA	NA	chr15*45965871*45965871*G*C*rs762187581*NA*NA*NA	NA	NA	NA	NA	T	BN	N	MODERATE	NA	NA	138	138	0	146	133	13	8.9
Smoker	42481	chr15	99673304	C	T	*SYNM*	3UTR	ENSG00000182253:[ENST00000328642(c.*41C>T)];ENSG00000182253:[ENST00000560674(c.*41C>T)];ENSG00000182253:[ENST00000336292(c.*41C>T)];ENSG00000182253:[ENST00000558420(n.3922C>T)];ENSG00000182253:[ENST00000561306(n.1475C>T)];ENSG00000182253:[ENST00000561323(n.4903C>T)];	NA	NA	NA	9.68E-06	0	NA	NA	NA	NA	NA	MODIFIER	NA	NA	89	89	0	54	34	20	37.04
Smoker	42481	chr16	30961148	C	G	*AC135048.13*	INTRONIC	ENSG00000261487:[ENST00000562642(n.38+1259C>G)];ENSG00000261487:[ENST00000566056(n.183+1259C>G)];	NA	NA	NA	NA	NA	NA	NA	NA	NA	NA	MODIFIER	NA	NA	50	49	1	41	29	12	29.27
Smoker	42481	chr16	88780212	C	G	*CTU2*	INTRONIC	ENSG00000174177:[ENST00000312060(c.1005+26C>G)];ENSG00000174177:[ENST00000378384(c.744+26C>G)];ENSG00000174177:[ENST00000564105(c.*716+26C>G)];ENSG00000174177:[ENST00000564584(n.299+26C>G)];ENSG00000174177:[ENST00000566637(n.841+26C>G)];ENSG00000174177:[ENST00000567949(c.1218+26C>G)];ENSG00000174177:[ENST00000453996(c.1005+26C>G)];	NA	NA	chr16*88780185*88780230*AGGTGGGCCTTGTGGGCTGGGCAACCTCTCTCACCATTGACACCGG*A*rs761470399*NA*NA*NA|chr16*88780208*88780253*ACCTCTCTCACCATTGACACCGGGGTGGGCCATGTGGGCTGGGCAG*A*rs769728314*NA*NA*NA	NA	NA	NA	NA	NA	NA	NA	MODIFIER	NA	NA	46	46	0	77	66	11	14.29
Smoker	42481	chr16	30961148	C	G	*ORAI3*	INTRONIC	ENSG00000175938:[ENST00000318663(c.228+310C>G)];ENSG00000175938:[ENST00000566237(c.228+310C>G)];ENSG00000175938:[ENST00000562699(c.228+310C>G)];ENSG00000175938:[ENST00000563161(c.*62C>G)];	NA	NA	NA	NA	NA	NA	NA	NA	NA	NA	MODIFIER	NA	NA	50	49	1	41	29	12	29.27
Smoker	42481	chr16	552944	G	A	*RAB11FIP3*	INTRONIC	ENSG00000090565:[ENST00000434585(c.1065-60G>A)];ENSG00000090565:[ENST00000450428(c.414-60G>A)];ENSG00000090565:[ENST00000457159(c.1437-60G>A)];ENSG00000090565:[ENST00000452814(c.372-60G>A)];ENSG00000090565:[ENST00000449879(c.414-60G>A)];ENSG00000090565:[ENST00000262305(c.1302-60G>A)];ENSG00000090565:[ENST00000487899(n.195G>A)];	NA	NA	NA	NA	NA	NA	NA	NA	NA	NA	MODIFIER	NA	NA	73	73	0	63	51	12	19.05

Mangalaparthi *et al.* , 2020. Mutational landscape of esophageal squamous cell carcinoma in an Indian cohort
Supplementary Table 4. List of somatic single nucleotide variants identified using whole exome sequencing of ESCC patients

Cohort	Sample	Chromosome	Genomic position	Reference allele	Alternate allele	Gene symbol	Variant type	Variant annotation	OncoMD	COSMIC	dbSNP	ExAC	ExAC_SAS	1000G	1000G_SAS	SIFT	Polyphen2	CONDEL	VEP	LRT	PhastCon7 Way	Total read (Adjacent normal)	Reference allel read support (Adjacent normal)	Alternative read support (Adjacent normal)	Total read (Tumor)	Reference allel read support (Tumor)	Alternative read support (Tumor)	Alternate allele frequency
Smoker	42481	chr16	68344363	A	G	*SLC7A6OS*	MISSENSE	ENSG00000103061:EXONIC-NC[ENST00000561933(n.487T>C)];ENSG00000103061:EXONIC-NC[ENST00000568538(n.242T>C)];ENSG00000103061:MISSENSE[ENST00000263997(c.346T>C,Tcg>Ccg):ENSP00000263997(p.Ser116Pro)];	NA	NA	chr16*68344363*68344363*A*C*rs8063446*1*0.8882*0.1118	NA	NA	NA	NA	NA	NA	NA	MODIFIER	NA	NA	114	114	0	94	85	9	9.57
Smoker	42481	chr17	46926628	G	T	*CALCOCO2*	MISSENSE	ENSG00000136436:MISSENSE[ENST00000448105(c.504G>T,gaG>gaT):ENSP00000398523(p.Glu168Asp)];ENSG00000136436:MISSENSE[ENST00000258947(c.432G>T,gaG>gaT):ENSP00000258947(p.Glu144Asp)];ENSG00000136436:MISSENSE[ENST00000509507(c.495G>T,gaG>gaT):ENSP00000424352(p.Glu165Asp)];ENSG00000136436:MISSENSE[ENST00000508679(c.216G>T,gaG>gaT):ENSP00000423437(p.Glu72Asp)];ENSG00000136436:MISSENSE[ENST00000509415(c.348G>T,gaG>gaT):ENSP00000425692(p.Glu116Asp)];ENSG00000136436:MISSENSE[ENST00000513119(c.216G>T,gaG>gaT):ENSP00000425090(p.Glu72Asp)];ENSG00000136436:MISSENSE[ENST00000502761(c.432G>T,gaG>gaT):ENSP00000424889(p.Glu144Asp)];ENSG00000136436:MISSENSE[ENST00000505071(c.459G>T,gaG>gaT):ENSP00000422697(p.Glu153Asp)];ENSG00000136436:INTRONIC[ENST00000416445(c.417+811G>T)];ENSG00000136436:EXONIC-NC[ENST00000514962(n.262G>T)];	NA	NA	NA	NA	NA	NA	NA	NA	NA	NA	MODIFIER	NA	NA	57	57	0	22	9	13	59.09
Smoker	42481	chr17	10366366	C	T	*CTC-297N7.11*	INTRONIC	ENSG00000272975:[ENST00000587182(n.155+56811C>T)];	NA	NA	NA	NA	NA	NA	NA	NA	NA	NA	MODIFIER	NA	NA	295	292	1	141	110	31	21.99
Smoker	42481	chr17	39525650	C	T	*KRT33B*	INTRONIC-SS-DNR	ENSG00000131738:[ENST00000251646(c.348+5G>A)];	NA	NA	NA	NA	NA	NA	NA	NA	NA	NA	LOW	NA	NA	134	134	0	87	68	19	21.84
Smoker	42481	chr17	39150329	T	G	*KRTAP3-3*	SILENT	ENSG00000212899:SILENT[ENST00000391586(c.21A>C,cgA>cgC):ENSP00000375428(p.Arg7(=))];	chr17*3915(	chr17*3915(	NA	0.0041267	0.009823427	NA	NA	NA	NA	NA	LOW	NA	NA	225	222	2	116	107	9	7.76
Smoker	42481	chr17	39324333	T	A	*KRTAP4-3*	MISSENSE	ENSG00000196156:MISSENSE[ENST00000391356(c.92A>T,cAg>cTg):ENSP00000375151(p.Gln31Leu)];	chr17*3932	chr17*3932	NA	NA	NA	NA	NA	D	UN	NA	MODERATE	NA	NA	64	63	0	58	51	7	12.07
Smoker	42481	chr17	10366366	C	T	*MYH4*	INTRONIC	ENSG00000264424:[ENST00000255381(c.904+41G>A)];	NA	NA	NA	NA	NA	NA	NA	NA	NA	NA	MODIFIER	NA	NA	295	292	1	141	110	31	21.99
Smoker	42481	chr17	43181217	G	A	*NMT1*	MISSENSE	ENSG00000136448:MISSENSE[ENST00000592782(c.1305G>A,atG>atA):ENSP00000468424(p.Met435Ile)];ENSG00000136448:MISSENSE[ENST00000258960(c.1305G>A,atG>atA):ENSP00000258960(p.Met435Ile)];ENSG00000136448:EXONIC-NC[ENST00000587014(n.300G>A)];	NA	NA	chr17*43181217*43181217*G*T*rs750807200*NA*NA*NA	NA	NA	NA	NA	NA	NA	NA	MODIFIER	NA	NA	138	136	0	101	86	15	14.85
Smoker	42481	chr17	7324026	G	A	*RP11-104H15.7*	INTRONIC	ENSG00000262880:[ENST00000575310(n.118+20G>A)];	NA	NA	NA	NA	NA	NA	NA	NA	NA	NA	MODIFIER	NA	NA	87	87	0	56	48	8	14.29
Smoker	42481	chr17	10366366	C	T	*RP11-799N11.1*	INTRONIC	ENSG00000272736:[ENST00000399342(n.206+56772C>T)];ENSG00000272736:[ENST00000581304(n.143+56811C>T)];	NA	NA	NA	NA	NA	NA	NA	NA	NA	NA	MODIFIER	NA	NA	295	292	1	141	110	31	21.99
Smoker	42481	chr17	7324026	G	A	*SPEM1*	INTRONIC	ENSG00000181323:[ENST00000323675(c.205+20G>A)];	NA	NA	NA	NA	NA	NA	NA	NA	NA	NA	MODIFIER	NA	NA	87	87	0	56	48	8	14.29
Smoker	42481	chr17	7578406	C	T	*TP53*	MISSENSE	ENSG00000141510:MISSENSE[ENST00000413465(c.524G>A,cGc>cAc):ENSP00000410739(p.Arg175His)];ENSG00000141510:MISSENSE[ENST00000420246(c.524G>A,cGc>cAc):ENSP00000391127(p.Arg175His)];ENSG00000141510:MISSENSE[ENST00000269305(c.524G>A,cGc>cAc):ENSP00000269305(p.Arg175His)];ENSG00000141510:MISSENSE[ENST00000359597(c.524G>A,cGc>cAc):ENSP00000352610(p.Arg175His)];ENSG00000141510:MISSENSE[ENST00000514944(c.245G>A,cGc>cAc):ENSP00000423862(p.Arg82His)];ENSG00000141510:MISSENSE[ENST00000445888(c.524G>A,cGc>cAc):ENSP00000391478(p.Arg175His)];ENSG00000141510:MISSENSE[ENST00000455263(c.524G>A,cGc>cAc):ENSP00000398846(p.Arg175His)];ENSG00000141510:MISSENSE[ENST00000509690(c.128G>A,cGc>cAc):ENSP00000425104(p.Arg43His)];ENSG00000141510:EXONIC-NC[ENST00000504937(n.406G>A)];ENSG00000141510:EXONIC-NC[ENST00000505014(n.780G>A)];ENSG00000141510:EXONIC-NC[ENST00000504290(n.406G>A)];ENSG00000141510:EXONIC-NC[ENST00000574684(n.32G>A)];ENSG00000141510:EXONIC-NC[ENST00000510385(n.406G>A)];	chr17*7578	chr17*7578	chr17*7578406*7578406*C*A,T*rs28934578*NA*NA*NA	8.24E-06	0	NA	NA	NA	NA	NA	MODIFIER	NA	NA	72	72	0	53	17	36	67.92
Smoker	42481	chr18	6894794	G	T	*ARHGAP28*	INTRONIC	ENSG00000088756:[ENST00000418986(c.1372-40G>T)];ENSG00000088756:[ENST00000262227(c.1693-40G>T)];ENSG00000088756:[ENST00000419673(c.1372-40G>T)];ENSG00000088756:[ENST00000383472(c.1849-40G>T)];ENSG00000088756:[ENST00000400091(c.1849-40G>T)];ENSG00000088756:[ENST00000531294(c.1357-40G>T)];ENSG00000088756:[ENST00000532996(c.1318-40G>T)];ENSG00000088756:[ENST00000314319(c.1372-40G>T)];	NA	NA	NA	NA	NA	NA	NA	NA	NA	NA	MODIFIER	NA	NA	249	247	1	130	61	69	53.08
Smoker	42481	chr19	47425174	G	A	*ARHGAP35*	MISSENSE	ENSG00000160007:MISSENSE[ENST00000404338(c.3242G>A,gGg>gAg):ENSP00000385720(p.Gly1081Glu)];	NA	NA	NA	NA	NA	NA	NA	D	PoD	D	MODERATE	D	0.999	172	171	0	178	114	64	35.96
Smoker	42481	chr19	16006318	G	A	*CYP4F2*	MISSENSE-SS-PRX	ENSG00000186115:[ENST00000221700(c.341C>T,tCa>tTa):ENSP00000221700(p.Ser114Leu)];ENSG00000186115:[ENST00000586927(c.341C>T,tCa>tTa):ENSP00000465514(p.Ser114Leu)];ENSG00000186115:[ENST00000608168(n.394C>T)];ENSG00000186115:[ENST00000392846(n.192C>T)];ENSG00000186115:[ENST00000011989(c.-105+1906C>T)];ENSG00000186115:[ENST00000587671(c.198+1906C>T)];	chr19*1600(	NA	NA	NA	NA	NA	NA	NA	NA	NA	MODIFIER	NA	NA	188	188	0	157	150	7	4.46
Smoker	42481	chr19	18368564	C	T	*KIAA1683*	MISSENSE	ENSG00000130518:MISSENSE[ENST00000600359(c.2831G>A,cGg>cAg):ENSP00000472912(p.Arg944Gln)];ENSG00000130518:MISSENSE[ENST00000600328(c.2969G>A,cGg>cAg):ENSP00000470780(p.Arg990Gln)];ENSG00000130518:MISSENSE[ENST00000392413(c.3530G>A,cGg>cAg):ENSP00000376213(p.Arg1177Gln)];ENSG00000130518:EXONIC-NC[ENST00000599638(n.4865G>A)];	NA	NA	0.001198	0.0005404	0	0.0011981	0	T	BN	N	MODERATE	N	0.115	53	53	0	56	48	8	14.29
Smoker	42481	chr19	56481908	A	G	*NLRP8*	INTRONIC-SS-ACR	ENSG00000179709:INTRONIC-SS-ACR[ENST00000590542(c.2382-2A>G)];ENSG00000179709:INTRONIC-SS-ACR[ENST00000291971(c.2382-2A>G)];	NA	NA	NA	NA	NA	NA	NA	NA	NA	NA	HIGH	NA	NA	242	242	0	191	170	21	10.99
Smoker	42481	chr19	19243499	C	A	*TMEM161A*	NONSENSE	ENSG00000064545:NONSENSE[ENST00000162044(c.253G>T,Gag>Tag):ENSP00000162044(p.Glu85Ter)];ENSG00000064545:NONSENSE[ENST00000587583(c.253G>T,Gag>Tag):ENSP00000467300(p.Glu85Ter)];ENSG00000064545:NONSENSE[ENST00000450333(c.253G>T,Gag>Tag):ENSP00000404208(p.Glu85Ter)];ENSG00000064545:NONSENSE[ENST00000587915(c.202G>T,Gag>Tag):ENSP00000467793(p.Glu68Ter)];ENSG00000064545:NONSENSE[ENST00000587985(c.253G>T,Gag>Tag):ENSP00000476205(p.Glu85Ter)];ENSG00000064545:NONSENSE[ENST00000587096(c.202G>T,Gag>Tag):ENSP00000467649(p.Glu68Ter)];ENSG00000064545:EXONIC-NC[ENST00000587406(n.318G>T)];ENSG00000064545:EXONIC-NC[ENST00000592147(n.315G>T)];ENSG00000064545:EXONIC-NC[ENST00000589448(n.183G>T)];ENSG00000064545:5UTR[ENST00000592369(c.-123G>T)];ENSG00000064545:3UTR[ENST00000590216(c.*226G>T)];	chr19*1924	chr19*1924	NA	NA	NA	NA	NA	NA	NA	NA	MODIFIER	NA	NA	60	60	0	56	29	27	48.21

Supplementary Table 4. List of somatic single nucleotide variants identified using whole exome sequencing of ESCC patients

Cohort	Sample	Chromosome	Genomic position	Reference allele	Alternate allele	Gene symbol	Variant type	Variant annotation	OncoMD	COSMIC	dbSNP	ExAC	ExAC_SAS	1000G	1000G_SAS	SIFT	Polyphen2	CONDEL	VEP	LRT	PhastCon7 Way	Total read (Adjacent normal)	Reference allel read support (Adjacent normal)	Alternative read support (Adjacent normal)	Total read (Tumor)	Reference allel read support (Tumor)	Alternative read support (Tumor)	Alternate allele frequency
Smoker	42481	chr19	55657815	C	A	*TNNT1*	SILENT	ENSG00000105048:SILENT-SS-PRX[ENST00000536926(c.93G>T,ccG>ccT):ENSP00000439640(p.Pro31(=))];ENSG00000105048:EXONIC-NC[ENST00000586282(n.148G>T)];ENSG00000105048:EXONIC-NC[ENST00000592920(n.171G>T)];ENSG00000105048:INTRONIC[ENST00000589226(c.73+234G>T)];ENSG00000105048:INTRONIC[ENST00000585321(c.-105+234G>T)];ENSG00000105048:INTRONIC[ENST00000588426(c.48+234G>T)];ENSG00000105048:INTRONIC[ENST00000356783(c.73+234G>T)];ENSG00000105048:INTRONIC[ENST00000588147(c.94+213G>T)];ENSG00000105048:INTRONIC[ENST00000587758(c.73+234G>T)];ENSG00000105048:SILENT[ENST00000588981(c.93G>T,ccG>ccT):ENSP00000467176(p.Pro31(=))];ENSG00000105048:SILENT[ENST00000587089(c.8G>T,ccG>ccT):ENSP00000465544(p.Pro3(=))];ENSG00000105048:SILENT[ENST00000593046(c.93G>T,ccG>ccT):ENSP00000470777(p.Pro31(=))];ENSG00000105048:SILENT[ENST00000291901(c.93G>T,ccG>ccT):ENSP00000291901(p.Pro31(=))];ENSG00000105048:5UTR[ENST00000587465(c.-118G>T)];	NA	NA	NA	NA	NA	NA	NA	NA	NA	NA	MODIFIER	NA	NA	26	26	0	17	7	10	58.82
Smoker	42481	chr2	228159737	G	A	*AC097662.2*	INTRONIC	ENSG00000236432:[ENST00000433324(n.35-13232C>T)];ENSG00000236432:[ENST00000396588(n.397+6772C>T)];ENSG00000236432:[ENST00000439598(n.243+10439C>T)];	chr2*22815	chr2*22815	0.0003994	0.0003318	6.06E-05	0.0003994	0	NA	UN	NA	MODERATE	N	0.86	166	166	0	117	101	16	13.68
Smoker	42481	chr2	201721629	C	T	*CLK1*	SILENT	ENSG00000013441:EXONIC-NC[ENST00000473565(n.2440G>A)];ENSG00000013441:EXONIC-NC[ENST00000461981(n.2400G>A)];ENSG00000013441:SILENT[ENST00000321356(c.912G>A,gcG>gcA):ENSP00000326830(p.Ala304(=))];ENSG00000013441:SILENT[ENST00000409403(c.912G>A,gcG>gcA):ENSP00000386875(p.Ala304(=))];ENSG00000013441:SILENT[ENST00000434813(c.1038G>A,gcG>gcA):ENSP00000394734(p.Ala346(=))];ENSG00000013441:SILENT[ENST00000409769(c.381G>A,gcG>gcA):ENSP00000386358(p.Ala127(=))];ENSG00000013441:3UTR[ENST00000432425(c.*410G>A)];	NA	NA	chr2*201721629*201721629*C*G,T*rs144181237*NA*NA*NA	0.0003628	0.00036368	NA	NA	NA	NA	NA	LOW	NA	NA	84	84	0	76	44	32	42.11
Smoker	42481	chr2	228159737	G	A	*COL4A3*	MISSENSE	ENSG00000169031:MISSENSE[ENST00000396578(c.3476G>A,cGt>cAt):ENSP00000379823(p.Arg1159His)];	chr2*22815	chr2*22815	0.0003994	0.0003318	6.06E-05	0.0003994	0	NA	UN	NA	MODERATE	N	0.86	166	166	0	117	101	16	13.68
Smoker	42481	chr2	48035273	G	T	*FBXO11*	MISSENSE	ENSG00000138081:MISSENSE[ENST00000316377(c.2516C>A,aCa>aAa):ENSP00000323822(p.Thr839Lys)];ENSG00000138081:MISSENSE[ENST00000403359(c.2768C>A,aCa>aAa):ENSP00000384823(p.Thr923Lys)];ENSG00000138081:MISSENSE[ENST00000434234(c.230C>A,aCa>aAa):ENSP00000402692(p.Thr77Lys)];ENSG00000138081:MISSENSE[ENST00000402508(c.2516C>A,aCa>aAa):ENSP00000385398(p.Thr839Lys)];ENSG00000138081:MISSENSE[ENST00000434523(c.1040C>A,aCa>aAa):ENSP00000397359(p.Thr347Lys)];ENSG00000138081:INTRONIC[ENST00000405808(c.169+61C>A)];ENSG00000138081:EXONIC-NC[ENST00000465204(n.1776C>A)];	NA	NA	NA	NA	NA	NA	NA	NA	NA	NA	MODIFIER	NA	NA	105	104	0	118	79	39	33.05
Smoker	42481	chr2	176957676	G	A	*HOXD13*	MISSENSE	ENSG00000128714:MISSENSE[ENST00000392539(c.58G>A,Ggt>Agt):ENSP00000376322(p.Gly20Ser)];	NA	NA	NA	NA	NA	NA	NA	T_lc	BN	N	MODERATE	U	0.158	33	33	0	37	29	8	21.62
Smoker	42481	chr2	86669315	C	G	*KDM3A*	MISSENSE	ENSG00000115548:MISSENSE[ENST00000409064(c.145C>G,Cga>Gga):ENSP00000386516(p.Arg49Gly)];ENSG00000115548:MISSENSE[ENST00000542128(c.145C>G,Cga>Gga):ENSP00000438324(p.Arg49Gly)];ENSG00000115548:MISSENSE[ENST00000409556(c.145C>G,Cga>Gga):ENSP00000386660(p.Arg49Gly)];ENSG00000115548:MISSENSE[ENST00000312912(c.145C>G,Cga>Gga):ENSP00000323659(p.Arg49Gly)];ENSG00000115548:MISSENSE[ENST00000452034(c.145C>G,Cga>Gga):ENSP00000396561(p.Arg49Gly)];ENSG00000115548:MISSENSE[ENST00000427678(c.145C>G,Cga>Gga):ENSP00000397660(p.Arg49Gly)];ENSG00000115548:MISSENSE[ENST00000441719(c.145C>G,Cga>Gga):ENSP00000394691(p.Arg49Gly)];	NA	NA	NA	NA	NA	NA	NA	D	PrD	D	MODERATE	NA	NA	159	159	0	134	123	11	8.21
Smoker	42481	chr2	128396954	C	T	*LIMS2*	MISSENSE	ENSG00000072163:MISSENSE[ENST00000324938(c.1000G>A,Gag>Aag):ENSP00000326888(p.Glu334Lys)];ENSG00000072163:MISSENSE[ENST00000545738(c.994G>A,Gag>Aag):ENSP00000443794(p.Glu332Lys)];ENSG00000072163:MISSENSE[ENST00000409254(c.472G>A,Gag>Aag):ENSP00000386907(p.Glu158Lys)];ENSG00000072163:MISSENSE[ENST00000410038(c.472G>A,Gag>Aag):ENSP00000386570(p.Glu158Lys)];ENSG00000072163:MISSENSE[ENST00000409455(c.913G>A,Gag>Aag):ENSP00000386383(p.Glu305Lys)];ENSG00000072163:MISSENSE[ENST00000355119(c.928G>A,Gag>Aag):ENSP00000347240(p.Glu310Lys)];ENSG00000072163:MISSENSE[ENST00000409286(c.472G>A,Gag>Aag):ENSP00000386252(p.Glu158Lys)];ENSG00000072163:MISSENSE[ENST00000410011(c.913G>A,Gag>Aag):ENSP00000387002(p.Glu305Lys)];ENSG00000072163:MISSENSE[ENST00000409754(c.472G>A,Gag>Aag):ENSP00000386345(p.Glu158Lys)];ENSG00000072163:MISSENSE[ENST00000409808(c.913G>A,Gag>Aag):ENSP00000386637(p.Glu305Lys)];ENSG00000072163:MISSENSE[ENST00000426981(c.411G>A,Gag>Aag):ENSP00000397253(p.Glu138Lys)];ENSG00000072163:EXONIC-NC[ENST00000476932(n.1316G>A)];ENSG00000072163:EXONIC-NC[ENST00000494613(n.184G>A)];ENSG00000072163:EXONIC-NC[ENST00000466410(n.1641G>A)];ENSG00000072163:EXONIC-NC[ENST00000469300(n.3853G>A)];ENSG00000072163:EXONIC-NC[ENST00000484252(n.1148G>A)];ENSG00000072163:3UTR[ENST00000413578(c.*138G>A)];	chr2*12839	chr2*12839	NA	2.47E-05	0	NA	NA	NA	NA	NA	MODIFIER	NA	NA	104	104	0	72	65	7	9.72
Smoker	42481	chr2	119729182	G	T	*MARCO*	INTRONIC	ENSG00000019169:[ENST00000327097(c.460+72G>T)];ENSG00000019169:[ENST00000541757(c.226+72G>T)];	NA	NA	NA	NA	NA	NA	NA	NA	NA	NA	MODIFIER	NA	NA	202	201	0	159	136	23	14.47
Smoker	42481	chr2	48035273	G	T	*MSH6*	3UTR	ENSG00000116062:[ENST00000234420(c.*1274G>T)];	NA	NA	NA	NA	NA	NA	NA	NA	NA	NA	MODIFIER	NA	NA	105	104	0	118	79	39	33.05
Smoker	42481	chr2	152552093	T	A	*NEB*	MISSENSE-SS-PRX	ENSG00000183091:[ENST00000603639(c.1673A>T,gAt>gTt):ENSP00000473894(p.Asp558Val)];ENSG00000183091:[ENST00000172853(c.1673A>T,gAt>gTt):ENSP00000172853(p.Asp558Val)];ENSG00000183091:[ENST00000427231(c.1673A>T,gAt>gTt):ENSP00000416578(p.Asp558Val)];ENSG00000183091:[ENST00000397345(c.1673A>T,gAt>gTt):ENSP00000380505(p.Asp558Val)];ENSG00000183091:[ENST00000604864(c.1673A>T,gAt>gTt):ENSP00000474498(p.Asp558Val)];ENSG00000183091:[ENST00000409198(c.1673A>T,gAt>gTt):ENSP00000386259(p.Asp558Val)];ENSG00000183091:[ENST00000489048(n.572A>T)];	NA	NA	chr2*152552093*152552093*T*C*rs752094861*NA*NA*NA	NA	NA	NA	NA	NA	NA	NA	LOW	NA	NA	502	500	1	351	320	31	8.83
Smoker	42481	chr2	120640125	C	G	*PTPN4*	SILENT	ENSG00000088179:SILENT[ENST00000433888(c.267C>G,ctC>ctG):ENSP00000411364(p.Leu89(=))];ENSG00000088179:SILENT[ENST00000263708(c.513C>G,ctC>ctG):ENSP00000263708(p.Leu171(=))];ENSG00000088179:SILENT[ENST00000430976(c.47C>G,ctC>ctG):ENSP00000395603(p.Leu16(=))];ENSG00000088179:EXONIC-NC[ENST00000485247(n.560C>G)];	NA	NA	NA	2.15E-05	0	NA	NA	NA	NA	NA	MODIFIER	NA	NA	93	93	0	106	88	18	16.98

Supplementary Table 4. List of somatic single nucleotide variants identified using whole exome sequencing of ESCC patients

Cohort	Sample	Chromosome	Genomic position	Reference allele	Alternate allele	Gene symbol	Variant type	Variant annotation	OncoMD	COSMIC	dbSNP	ExAC	ExAC_SAS	1000G	1000G_SAS	SIFT	Polyphen2	CONDEL	VEP	LRT	PhastCon7 Way	Total read (Adjacent normal)	Reference allel read support (Adjacent normal)	Alternative read support (Adjacent normal)	Total read (Tumor)	Reference allel read support (Tumor)	Alternative read support (Tumor)	Alternate allele frequency
Smoker	42481	chr2	179641735	G	C	*RP11-88L24.4*	EXONIC-NC	ENSG00000266413:[ENST00000582038(n.83G>C)];	NA	NA	NA	NA	NA	NA	NA	NA	NA	NA	MODERATE	NA	NA	200	200	0	147	125	21	14.29
Smoker	42481	chr2	138000065	G	A	*THSD7B*	MISSENSE	ENSG00000144229:MISSENSE[ENST00000409968(c.2189G>A,tGt>tAt):ENSP00000387145(p.Cys730Tyr)];ENSG00000144229:MISSENSE[ENST00000413152(c.2096G>A,tGt>tAt):ENSP00000413841(p.Cys699Tyr)];ENSG00000144229:MISSENSE[ENST00000272643(c.2189G>A,tGt>tAt):ENSP00000272643(p.Cys730Tyr)];ENSG00000144229:INTRONIC[ENST00000543459(c.813+127335G>A)];	NA	NA	NA	NA	NA	NA	NA	NA	NA	NA	MODIFIER	NA	NA	183	183	0	133	118	15	11.28
Smoker	42481	chr2	216964875	G	A	*TMEM169*	SILENT	ENSG00000163449:SILENT[ENST00000406027(c.504G>A,ctG>ctA):ENSP00000384100(p.Leu168(=))];ENSG00000163449:SILENT[ENST00000295658(c.504G>A,ctG>ctA):ENSP00000295658(p.Leu168(=))];ENSG00000163449:SILENT[ENST00000437356(c.504G>A,ctG>ctA):ENSP00000401305(p.Leu168(=))];ENSG00000163449:SILENT[ENST00000454545(c.504G>A,ctG>ctA):ENSP00000412524(p.Leu168(=))];	NA	NA	NA	NA	NA	NA	NA	NA	NA	NA	LOW	NA	NA	149	149	0	108	58	50	46.3
Smoker	42481	chr2	179641735	G	C	*TTN*	MISSENSE	ENSG00000155657:MISSENSE[ENST00000360870(c.4856C>G,tCc>tGc):ENSP00000354117(p.Ser1619Cys)];ENSG00000155657:MISSENSE[ENST00000359218(c.4718C>G,tCc>tGc):ENSP00000352154(p.Ser1573Cys)];ENSG00000155657:MISSENSE[ENST00000342992(c.4856C>G,tCc>tGc):ENSP00000343764(p.Ser1619Cys)];ENSG00000155657:MISSENSE[ENST00000342175(c.4718C>G,tCc>tGc):ENSP00000340554(p.Ser1573Cys)];ENSG00000155657:MISSENSE[ENST00000589042(c.4856C>G,tCc>tGc):ENSP00000467141(p.Ser1619Cys)];ENSG00000155657:MISSENSE[ENST00000591111(c.4856C>G,tCc>tGc):ENSP00000465570(p.Ser1619Cys)];ENSG00000155657:MISSENSE[ENST00000460472(c.4718C>G,tCc>tGc):ENSP00000434586(p.Ser1573Cys)];	NA	NA	NA	NA	NA	NA	NA	NA	NA	NA	MODERATE	NA	NA	200	200	0	147	125	21	14.29
Smoker	42481	chr2	220115402	C	T	*TUBA4A*	MISSENSE	ENSG00000127824:MISSENSE[ENST00000392088(c.974G>A,aGc>aAc):ENSP00000375938(p.Ser325Asn)];ENSG00000127824:MISSENSE[ENST00000248437(c.1019G>A,aGc>aAc):ENSP00000248437(p.Ser340Asn)];	NA	NA	NA	NA	NA	NA	NA	T_lc	BN	N	MODERATE	D	0.998	126	126	0	116	110	6	5.17
Smoker	42481	chr20	35422937	C	T	*SOGA1*	MISSENSE	ENSG00000149639:MISSENSE[ENST00000279034(c.2834G>A,gGc>gAc):ENSP00000279034(p.Gly945Asp)];ENSG00000149639:MISSENSE[ENST00000237536(c.3548G>A,gGc>gAc):ENSP00000237536(p.Gly1183Asp)];ENSG00000149639:MISSENSE[ENST00000465671(c.2388G>A,gGc>gAc):ENSP00000433939(p.Gly797Asp)];ENSG00000149639:MISSENSE[ENST00000357779(c.2834G>A,gGc>gAc):ENSP00000350424(p.Gly945Asp)];ENSG00000149639:MISSENSE[ENST00000456801(c.2357G>A,gGc>gAc):ENSP00000413886(p.Gly786Asp)];	NA	NA	NA	1.66E-05	0	NA	NA	T	BN	N	MODERATE	N	0.819	121	121	0	148	91	57	38.51
Smoker	42481	chr21	38884785	C	A	*DYRK1A*	MISSENSE	ENSG00000157540:MISSENSE[ENST00000339659(c.2216C>A,tCc>tAc):ENSP00000340373(p.Ser739Tyr)];ENSG00000157540:MISSENSE[ENST00000455387(c.1559C>A,tCc>tAc):ENSP00000407854(p.Ser520Tyr)];ENSG00000157540:MISSENSE[ENST00000398960(c.2243C>A,tCc>tAc):ENSP00000381932(p.Ser748Tyr)];ENSG00000157540:3UTR[ENST00000338785(c.*619C>A)];	NA	NA	NA	NA	NA	NA	NA	NA	NA	NA	MODIFIER	NA	NA	110	110	0	40	21	19	47.5
Smoker	42481	chr22	43089191	C	T	*A4GALT*	MISSENSE	ENSG00000128274:MISSENSE[ENST00000381278(c.767G>A,cGg>cAg):ENSP00000370678(p.Arg256Gln)];ENSG00000128274:MISSENSE[ENST00000249005(c.767G>A,cGg>cAg):ENSP00000249005(p.Arg256Gln)];ENSG00000128274:MISSENSE[ENST00000401850(c.767G>A,cGg>cAg):ENSP00000384794(p.Arg256Gln)];	NA	NA	NA	NA	NA	NA	NA	D	PrD	D	MODERATE	D	0.986	86	86	0	83	56	27	32.53
Smoker	42481	chr3	165503990	G	T	*BCHE*	MISSENSE	ENSG00000114200:MISSENSE[ENST00000540653(c.13C>A,Cgt>Agt):ENSP00000443583(p.Arg5Ser)];ENSG00000114200:MISSENSE[ENST00000264381(c.1627C>A,Cgt>Agt):ENSP00000264381(p.Arg543Ser)];ENSG00000114200:MISSENSE[ENST00000479451(c.217C>A,Cgt>Agt):ENSP00000418325(p.Arg73Ser)];ENSG00000114200:MISSENSE[ENST00000497011(c.1627C>A,Cgt>Agt):ENSP00000419505(p.Arg543Ser)];ENSG00000114200:MISSENSE[ENST00000488954(c.217C>A,Cgt>Agt):ENSP00000418504(p.Arg73Ser)];ENSG00000114200:3UTR[ENST00000482958(c.*133C>A)];	chr3*16550	NA	chr3*165503990*165503990*G*A*rs199660374*NA*NA*NA	NA	NA	NA	NA	NA	NA	NA	MODIFIER	NA	NA	116	116	0	145	137	8	5.52
Smoker	42481	chr3	74411194	G	A	*CNTN3*	INTRONIC-SS-ACR	ENSG00000113805:[ENST00000263665(c.1214-3C>T)];	NA	NA	NA	NA	NA	NA	NA	NA	NA	NA	LOW	NA	NA	49	48	0	21	13	8	38.1
Smoker	42481	chr3	130107829	G	C	*COL6A5*	SILENT	ENSG00000172752:SILENT[ENST00000432398(c.2268G>C,gtG>gtC):ENSP00000390895(p.Val756(=))];ENSG00000172752:SILENT[ENST00000265379(c.2268G>C,gtG>gtC):ENSP00000265379(p.Val756(=))];ENSG00000172752:SILENT[ENST00000312481(c.2268G>C,gtG>gtC):ENSP00000309762(p.Val756(=))];	NA	NA	NA	NA	NA	NA	NA	NA	NA	NA	LOW	NA	NA	381	379	2	414	397	16	3.86
Smoker	42481	chr3	99513460	C	T	*COL8A1*	MISSENSE	ENSG00000144810:MISSENSE[ENST00000261037(c.715C>T,Cct>Tct):ENSP00000261037(p.Pro239Ser)];ENSG00000144810:MISSENSE[ENST00000273342(c.715C>T,Cct>Tct):ENSP00000273342(p.Pro239Ser)];	NA	NA	NA	NA	NA	NA	NA	T	UN	NA	MODERATE	D	0.996	142	142	0	188	173	15	7.98
Smoker	42481	chr3	195452960	G	A	*LINC00969*	INTRONIC	ENSG00000242086:[ENST00000597662(n.378-3254G>A)];ENSG00000242086:[ENST00000595086(n.211-3559G>A)];ENSG00000242086:[ENST00000600197(n.689-7061G>A)];ENSG00000242086:[ENST00000594446(n.211-3254G>A)];	chr3*19545	NA	NA	0.0163489	0.015312423	NA	NA	T_lc	BN	N	MODERATE	N	0.026	200	197	2	143	134	9	6.29
Smoker	42481	chr3	195452960	G	A	*MUC20*	MISSENSE	ENSG00000176945:MISSENSE[ENST00000320736(c.973G>A,Gtt>Att):ENSP00000325431(p.Val325Ile)];ENSG00000176945:MISSENSE[ENST00000445522(c.1381G>A,Gtt>Att):ENSP00000405629(p.Val461Ile)];ENSG00000176945:MISSENSE[ENST00000447234(c.1486G>A,Gtt>Att):ENSP00000414350(p.Val496Ile)];ENSG00000176945:MISSENSE[ENST00000436408(c.1486G>A,Gtt>Att):ENSP00000396774(p.Val496Ile)];	chr3*19545	NA	NA	0.0163489	0.015312423	NA	NA	T_lc	BN	N	MODERATE	N	0.026	200	197	2	143	134	9	6.29
Smoker	42481	chr3	123452835	C	G	*MYLK*	SILENT	ENSG00000065534:SILENT[ENST00000360304(c.1008G>C,ccG>ccC):ENSP00000353452(p.Pro336(=))];ENSG00000065534:SILENT[ENST00000360772(c.1008G>C,ccG>ccC):ENSP00000354004(p.Pro336(=))];ENSG00000065534:SILENT[ENST00000346322(c.1008G>C,ccG>ccC):ENSP00000320622(p.Pro336(=))];ENSG00000065534:SILENT[ENST00000359169(c.1008G>C,ccG>ccC):ENSP00000352088(p.Pro336(=))];ENSG00000065534:SILENT[ENST00000475616(c.1008G>C,ccG>ccC):ENSP00000418335(p.Pro336(=))];ENSG00000065534:3UTR[ENST00000464489(c.*587G>C)];ENSG00000065534:EXONIC-NC[ENST00000514623(n.1783G>C)];	NA	NA	chr3*123452835*123452835*C*T*rs143682943*NA*NA*NA	NA	NA	NA	NA	NA	NA	NA	LOW	NA	NA	83	81	0	105	98	7	6.67
Smoker	42481	chr3	98188778	T	G	*OR5K1*	MISSENSE	ENSG00000232382:MISSENSE[ENST00000332650(c.358T>G,Tat>Gat):ENSP00000373193(p.Tyr120Asp)];	NA	NA	NA	NA	NA	NA	NA	D	PrD	D	MODERATE	N	1	401	400	0	515	411	104	20.19
Smoker	42481	chr3	178922292	T	C	*PIK3CA*	MISSENSE-SS-PRX	ENSG00000121879:[ENST00000263967(c.1061T>C,aTc>aCc):ENSP00000263967(p.Ile354Thr)];	NA	NA	NA	NA	NA	NA	NA	D	PrD	D	MODERATE	D	0.997	93	93	0	124	97	27	21.77

Mangalaparthi *et al.* , 2020. Mutational landscape of esophageal squamous cell carcinoma in an Indian cohort
Supplementary Table 4. List of somatic single nucleotide variants identified using whole exome sequencing of ESCC patients

Cohort	Sample	Chromosome	Genomic position	Reference allele	Alternate allele	Gene symbol	Variant type	Variant annotation	OncoMD	COSMIC	dbSNP	ExAC	ExAC_SAS	1000G	1000G_SAS	SIFT	Polyphen2	CONDEL	VEP	LRT	PhastCon7 Way	Total read (Adjacent normal)	Reference allel read support (Adjacent normal)	Alternative read support (Adjacent normal)	Total read (Tumor)	Reference allel read support (Tumor)	Alternative read support (Tumor)	Alternate allele frequency
Smoker	42481	chr3	58383309	C	T	*PXK*	INTRONIC	ENSG00000168297:[ENST00000536660(c.574-23C>T)];ENSG00000168297:[ENST00000484288(c.985-23C>T)];ENSG00000168297:[ENST00000463280(c.886-23C>T)];ENSG00000168297:[ENST00000302779(c.934-23C>T)];ENSG00000168297:[ENST00000479241(c.934-23C>T)];ENSG00000168297:[ENST00000356151(c.985-23C>T)];ENSG00000168297:[ENST00000383716(c.886-23C>T)];ENSG00000168297:[ENST00000468776(c.*528-23C>T)];ENSG00000168297:[ENST00000479134(c.249-23C>T)];ENSG00000168297:[ENST00000477308(c.*332-23C>T)];ENSG00000168297:[ENST00000459676(c.64-23C>T)];ENSG00000168297:[ENST00000383715(c.934-23C>T)];	NA	NA	NA	1.65E-05	0	NA	NA	NA	NA	NA	MODIFIER	NA	NA	100	100	0	42	36	6	14.29
Smoker	42481	chr3	39450267	A	T	*RPSA*	INTRONIC	ENSG00000168028:[ENST00000490490(n.521+52A>T)];ENSG00000168028:[ENST00000478027(n.188+52A>T)];ENSG00000168028:[ENST00000443003(c.267+37A>T)];ENSG00000168028:[ENST00000444512(c.*267+52A>T)];ENSG00000168028:[ENST00000458478(c.252+52A>T)];ENSG00000168028:[ENST00000301821(c.252+52A>T)];ENSG00000168028:[ENST00000477325(n.386A>T)];	NA	NA	chr3*39450267*39450267*A*G*rs766594253*NA*NA*NA	NA	NA	NA	NA	NA	NA	NA	MODIFIER	NA	NA	50	49	0	29	13	16	55.17
Smoker	42481	chr3	136170895	G	C	*STAG1*	MISSENSE	ENSG00000118007:MISSENSE[ENST00000536929(c.160C>G,Ctt>Gtt):ENSP00000445787(p.Leu54Val)];ENSG00000118007:MISSENSE[ENST00000434713(c.730C>G,Ctt>Gtt):ENSP00000404396(p.Leu244Val)];ENSG00000118007:MISSENSE[ENST00000383202(c.1408C>G,Ctt>Gtt):ENSP00000372689(p.Leu470Val)];ENSG00000118007:MISSENSE[ENST00000236698(c.1408C>G,Ctt>Gtt):ENSP00000236698(p.Leu470Val)];ENSG00000118007:MISSENSE[ENST00000492318(c.240C>G,Ctt>Gtt):ENSP00000419317(p.Leu81Val)];ENSG00000118007:3UTR[ENST00000483235(c.*1500C>G)];ENSG00000118007:3UTR[ENST00000487065(c.*1047C>G)];	NA	NA	NA	NA	NA	NA	NA	NA	NA	NA	MODIFIER	NA	NA	141	140	0	283	269	13	4.59
Smoker	42481	chr3	170895156	G	C	*TNIK*	MISSENSE	ENSG00000154310:MISSENSE[ENST00000470834(c.653C>G,tCt>tGt):ENSP00000419990(p.Ser218Cys)];ENSG00000154310:MISSENSE[ENST00000341852(c.653C>G,tCt>tGt):ENSP00000345352(p.Ser218Cys)];ENSG00000154310:MISSENSE[ENST00000475336(c.653C>G,tCt>tGt):ENSP00000418156(p.Ser218Cys)];ENSG00000154310:MISSENSE[ENST00000369326(c.653C>G,tCt>tGt):ENSP00000358332(p.Ser218Cys)];ENSG00000154310:MISSENSE[ENST00000357327(c.653C>G,tCt>tGt):ENSP00000349880(p.Ser218Cys)];ENSG00000154310:MISSENSE[ENST00000436636(c.653C>G,tCt>tGt):ENSP00000399511(p.Ser218Cys)];ENSG00000154310:MISSENSE[ENST00000538048(c.653C>G,tCt>tGt):ENSP00000443278(p.Ser218Cys)];ENSG00000154310:MISSENSE[ENST00000488470(c.653C>G,tCt>tGt):ENSP00000418378(p.Ser218Cys)];ENSG00000154310:MISSENSE[ENST00000284483(c.653C>G,tCt>tGt):ENSP00000284483(p.Ser218Cys)];ENSG00000154310:MISSENSE[ENST00000460047(c.653C>G,tCt>tGt):ENSP00000418916(p.Ser218Cys)];ENSG00000154310:MISSENSE[ENST00000468757(c.575C>G,tCt>tGt):ENSP00000417338(p.Ser192Cys)];	NA	NA	NA	NA	NA	NA	NA	D_lc	PrD	D	MODERATE	NA	NA	185	185	0	181	144	37	20.44
Smoker	42481	chr3	170928892	C	T	*TNIK*	INTRONIC	ENSG00000154310:[ENST00000470834(c.306+13G>A)];ENSG00000154310:[ENST00000341852(c.306+13G>A)];ENSG00000154310:[ENST00000436636(c.306+13G>A)];ENSG00000154310:[ENST00000475336(c.306+13G>A)];ENSG00000154310:[ENST00000468757(c.228+13G>A)];ENSG00000154310:[ENST00000538048(c.306+13G>A)];ENSG00000154310:[ENST00000488470(c.306+13G>A)];ENSG00000154310:[ENST00000369326(c.306+13G>A)];ENSG00000154310:[ENST00000284483(c.306+13G>A)];ENSG00000154310:[ENST00000460047(c.306+13G>A)];ENSG00000154310:[ENST00000357327(c.306+13G>A)];	NA	NA	chr3*170928892*170928892*C*G,T*rs369434719*NA*NA*NA	0.0001585	0.000182682	NA	NA	NA	NA	NA	MODIFIER	NA	NA	84	83	0	62	56	6	9.68
Smoker	42481	chr4	70807824	C	T	*CSN1S1*	INTRONIC	ENSG00000126545:[ENST00000505782(c.312+43C>T)];ENSG00000126545:[ENST00000510936(c.34-430C>T)];ENSG00000126545:[ENST00000507763(c.333+43C>T)];ENSG00000126545:[ENST00000246891(c.360+43C>T)];ENSG00000126545:[ENST00000444405(c.333+43C>T)];ENSG00000126545:[ENST00000507772(c.336+43C>T)];	NA	NA	NA	NA	NA	NA	NA	NA	NA	NA	MODIFIER	NA	NA	22	22	0	27	17	10	37.04
Smoker	42481	chr4	109668062	C	G	*ETNPPL*	INTRONIC	ENSG00000164089:[ENST00000510706(c.963-55G>C)];ENSG00000164089:[ENST00000411864(c.1065-55G>C)];ENSG00000164089:[ENST00000296486(c.1083-55G>C)];ENSG00000164089:[ENST00000512646(c.909-55G>C)];	NA	NA	NA	NA	NA	NA	NA	NA	NA	NA	MODIFIER	NA	NA	76	76	0	43	27	16	37.21
Smoker	42481	chr4	42965035	G	T	*GRXCR1*	NONSENSE	ENSG00000215203:NONSENSE[ENST00000399770(c.511G>T,Gaa>Taa):ENSP00000382670(p.Glu171Ter)];	NA	NA	NA	NA	NA	NA	NA	NA	NA	NA	HIGH	NA	1	361	360	0	186	144	42	22.58
Smoker	42481	chr4	83406777	C	T	*TMEM150C*	MISSENSE	ENSG00000249242:MISSENSE[ENST00000449862(c.637G>A,Gtg>Atg):ENSP00000403438(p.Val213Met)];ENSG00000249242:MISSENSE[ENST00000515780(c.637G>A,Gtg>Atg):ENSP00000420919(p.Val213Met)];	NA	NA	0.000599	0.0002048	0.000762195	0.000599	0.001	D	PoD	D	MODERATE	NA	NA	68	67	0	26	18	8	30.77
Smoker	42481	chr5	115298759	C	A	*AQPEP*	MISSENSE	ENSG00000172901:MISSENSE[ENST00000504467(c.445C>A,Ctc>Atc):ENSP00000423604(p.Leu149Ile)];ENSG00000172901:MISSENSE[ENST00000357872(c.445C>A,Ctc>Atc):ENSP00000350541(p.Leu149Ile)];ENSG00000172901:5UTR[ENST00000395528(c.-1005C>A)];	NA	NA	chr5*115298759*115298759*C*T*rs747900646*NA*NA*NA	NA	NA	NA	NA	NA	NA	NA	MODIFIER	NA	NA	124	124	0	71	59	12	16.9
Smoker	42481	chr5	39341697	C	T	*C9*	MISSENSE	ENSG00000113600:EXONIC-NC[ENST00000509186(n.260G>A)];ENSG00000113600:EXONIC-NC[ENST00000467285(n.320G>A)];ENSG00000113600:EXONIC-NC[ENST00000483232(n.331G>A)];ENSG00000113600:MISSENSE[ENST00000263408(c.289G>A,Gct>Act):ENSP00000263408(p.Ala97Thr)];	NA	NA	NA	NA	NA	NA	NA	NA	NA	NA	MODIFIER	NA	NA	225	225	0	157	127	30	19.11
Smoker	42481	chr5	140517421	C	T	*PCDHB5*	3UTR	ENSG00000113209:[ENST00000231134(c.*17C>T)];	NA	NA	NA	NA	NA	NA	NA	NA	NA	NA	MODIFIER	NA	NA	117	116	1	67	55	12	17.91
Smoker	42481	chr6	32373117	G	A	*BTNL2*	INTRONIC	ENSG00000204290:[ENST00000544175(c.-229-54C>T)];ENSG00000204290:[ENST00000465865(c.80-54C>T)];ENSG00000204290:[ENST00000414363(c.79+1705C>T)];ENSG00000204290:[ENST00000429232(c.80-54C>T)];ENSG00000204290:[ENST00000454136(c.80-54C>T)];ENSG00000204290:[ENST00000446536(c.80-57C>T)];ENSG00000204290:[ENST00000374995(c.80-54C>T)];ENSG00000204290:[ENST00000374993(c.80-54C>T)];ENSG00000204290:[ENST00000540315(c.98+6C>T)];	NA	NA	NA	NA	NA	NA	NA	NA	NA	NA	MODIFIER	NA	NA	151	151	0	81	49	32	39.51

Mangalaparthi *et al.* , 2020. Mutational landscape of esophageal squamous cell carcinoma in an Indian cohort
Supplementary Table 4. List of somatic single nucleotide variants identified using whole exome sequencing of ESCC patients

Cohort	Sample	Chromosome	Genomic position	Reference allele	Alternate allele	Gene symbol	Variant type	Variant annotation	OncoMD	COSMIC	dbSNP	ExAC	ExAC_SAS	1000G	1000G_SAS	SIFT	Polyphen2	CONDEL	VEP	LRT	PhastCon7 Way	Total read (Adjacent normal)	Reference allel read support (Adjacent normal)	Alternative read support (Adjacent normal)	Total read (Tumor)	Reference allel read support (Tumor)	Alternative read support (Tumor)	Alternate allele frequency
Smoker	42481	chr6	38939391	C	T	*DNAH8*	NONSENSE	ENSG00000124721:NONSENSE[ENST00000359357(c.11824C>T,Caa>Taa):ENSP00000352312(p.Gln3942Ter)];ENSG00000124721:NONSENSE[ENST00000327475(c.12439C>T,Caa>Taa):ENSP00000333363(p.Gln4147Ter)];ENSG00000124721:NONSENSE[ENST00000441566(c.11716C>T,Caa>Taa):ENSP00000402294(p.Gln3906Ter)];ENSG00000124721:NONSENSE[ENST00000449981(c.12475C>T,Caa>Taa):ENSP00000415331(p.Gln4159Ter)];	NA	NA	NA	NA	NA	NA	NA	NA	NA	NA	HIGH	NA	0.998	209	209	0	126	115	11	8.73
Smoker	42481	chr6	64454915	T	A	*EYS*	INTRONIC-SS-ACR	ENSG00000188107:INTRONIC-SS-ACR[ENST00000398580(c.1386-2A>T)];ENSG00000188107:INTRONIC[ENST00000370616(c.8072-3047A>T)];ENSG00000188107:INTRONIC[ENST00000370621(c.8072-3047A>T)];ENSG00000188107:INTRONIC[ENST00000503581(c.8071+17439A>T)];	NA	NA	NA	NA	NA	NA	NA	NA	NA	NA	MODIFIER	NA	NA	59	59	0	40	28	12	30
Smoker	42481	chr6	90422866	C	T	*MDN1*	INTRONIC-SS-DNR	ENSG00000112159:INTRONIC-SS-DNR[ENST00000369393(c.7218+1G>A)];ENSG00000112159:INTRONIC-SS-DNR[ENST00000428876(c.7218+1G>A)];	NA	NA	NA	NA	NA	NA	NA	NA	NA	NA	HIGH	NA	NA	94	94	0	71	60	9	12.68
Smoker	42481	chr6	29012396	T	G	*OR2W1*	MISSENSE	ENSG00000204704:MISSENSE[ENST00000377175(c.557A>C,aAg>aCg):ENSP00000366380(p.Lys186Thr)];	NA	NA	NA	NA	NA	NA	NA	D	PoD	D	MODERATE	N	0.28	267	265	0	158	149	9	5.7
Smoker	42481	chr6	64454915	T	A	*PHF3*	INTRONIC	ENSG00000118482:[ENST00000505138(c.364-33044T>A)];	NA	NA	NA	NA	NA	NA	NA	NA	NA	NA	MODIFIER	NA	NA	59	59	0	40	28	12	30
Smoker	42481	chr6	152804240	G	A	*SYNE1*	MISSENSE	ENSG00000131018:MISSENSE[ENST00000466159(c.1330C>T,Cgg>Tgg):ENSP00000446021(p.Arg444Trp)];ENSG00000131018:MISSENSE[ENST00000367248(c.1300C>T,Cgg>Tgg):ENSP00000356217(p.Arg434Trp)];ENSG00000131018:MISSENSE[ENST00000537750(c.1279C>T,Cgg>Tgg):ENSP00000441264(p.Arg427Trp)];ENSG00000131018:MISSENSE[ENST00000413186(c.1330C>T,Cgg>Tgg):ENSP00000414510(p.Arg444Trp)];ENSG00000131018:MISSENSE[ENST00000367253(c.1330C>T,Cgg>Tgg):ENSP00000356222(p.Arg444Trp)];ENSG00000131018:MISSENSE[ENST00000423061(c.1351C>T,Cgg>Tgg):ENSP00000396024(p.Arg451Trp)];ENSG00000131018:MISSENSE[ENST00000448038(c.1351C>T,Cgg>Tgg):ENSP00000390975(p.Arg451Trp)];ENSG00000131018:MISSENSE[ENST00000367255(c.1330C>T,Cgg>Tgg):ENSP00000356224(p.Arg444Trp)];ENSG00000131018:MISSENSE[ENST00000341594(c.1330C>T,Cgg>Tgg):ENSP00000341887(p.Arg444Trp)];ENSG00000131018:MISSENSE[ENST00000265368(c.1330C>T,Cgg>Tgg):ENSP00000265368(p.Arg444Trp)];ENSG00000131018:EXONIC-NC[ENST00000535896(n.133C>T)];ENSG00000131018:EXONIC-NC[ENST00000461872(n.1548C>T)];	NA	NA	0.0001997	9.88E-05	0	0.0001997	0	NA	PoD	NA	MODERATE	N	0.996	183	183	0	98	89	9	9.18
Smoker	42481	chr6	28967286	A	G	*ZNF311*	SILENT	ENSG00000197935:EXONIC-NC[ENST00000483450(n.1098T>C)];ENSG00000197935:SILENT[ENST00000377179(c.288T>C,aaT>aaC):ENSP00000366384(p.Asn96(=))];	NA	NA	NA	NA	NA	NA	NA	NA	NA	NA	LOW	NA	NA	124	122	0	82	60	21	25.61
Smoker	42481	chr7	34698171	G	C	*AC005493.1*	INTRONIC	ENSG00000214841:[ENST00000399077(c.31+1571C>G)];	NA	NA	NA	NA	NA	NA	NA	NA	NA	NA	MODIFIER	NA	NA	155	155	0	121	111	10	8.26
Smoker	42481	chr7	100159946	C	T	*AGFG2*	SILENT	ENSG00000106351:EXONIC-NC[ENST00000474713(n.597C>T)];ENSG00000106351:SILENT[ENST00000429987(c.167C>T,ctC>ctT):ENSP00000388594(p.Leu56(=))];ENSG00000106351:SILENT[ENST00000300176(c.942C>T,ctC>ctT):ENSP00000300176(p.Leu314(=))];ENSG00000106351:3UTR[ENST00000430857(c.*154C>T)];ENSG00000106351:3UTR[ENST00000262935(c.*154C>T)];	NA	NA	NA	8.32E-06	0	NA	NA	NA	NA	NA	LOW	NA	NA	45	45	0	40	27	12	30
Smoker	42481	chr7	31683330	C	A	*CCDC129*	SILENT	ENSG00000180347:SILENT[ENST00000451887(c.2424C>A,tcC>tcA):ENSP00000395835(p.Ser808(=))];ENSG00000180347:SILENT[ENST00000409210(c.2070C>A,tcC>tcA):ENSP00000387214(p.Ser690(=))];ENSG00000180347:SILENT[ENST00000319386(c.1902C>A,tcC>tcA):ENSP00000313062(p.Ser634(=))];ENSG00000180347:SILENT[ENST00000407970(c.2346C>A,tcC>tcA):ENSP00000384416(p.Ser782(=))];	NA	NA	chr7*31683330*31683330*C*T*rs529502816*0*0.9998*0.0001997	NA	NA	NA	NA	NA	NA	NA	LOW	NA	NA	242	242	0	185	163	22	11.89
Smoker	42481	chr7	150775865	G	A	*FASTK*	INTRONIC	ENSG00000164896:[ENST00000489884(n.686-77C>T)];ENSG00000164896:[ENST00000460980(n.362+64C>T)];ENSG00000164896:[ENST00000540185(c.404-686C>T)];ENSG00000164896:[ENST00000461979(n.262-79C>T)];ENSG00000164896:[ENST00000466855(n.458-77C>T)];ENSG00000164896:[ENST00000483953(n.70+64C>T)];ENSG00000164896:[ENST00000297532(c.685+64C>T)];ENSG00000164896:[ENST00000478477(n.433-686C>T)];ENSG00000164896:[ENST00000353841(c.262+64C>T)];ENSG00000164896:[ENST00000482806(n.709+64C>T)];ENSG00000164896:[ENST00000469237(n.487+64C>T)];ENSG00000164896:[ENST00000482571(c.605-77C>T)];ENSG00000164896:[ENST00000467237(n.1655+64C>T)];ENSG00000164896:[ENST00000478883(n.272C>T)];	NA	NA	NA	NA	NA	NA	NA	NA	NA	NA	MODIFIER	NA	NA	49	49	0	66	50	16	24.24
Smoker	42481	chr7	15666458	T	C	*MEOX2*	SILENT	ENSG00000106511:SILENT[ENST00000262041(c.603A>G,gaA>gaG):ENSP00000262041(p.Glu201(=))];	NA	NA	NA	NA	NA	NA	NA	NA	NA	NA	LOW	NA	0.998	264	263	0	170	97	73	42.94
Smoker	42481	chr7	34698171	G	C	*NPSR1*	MISSENSE-SS-PRX	ENSG00000187258:[ENST00000465305(c.147G>C,aaG>aaC):ENSP00000434955(p.Lys49Asn)];ENSG00000187258:[ENST00000359791(c.147G>C,aaG>aaC):ENSP00000352839(p.Lys49Asn)];ENSG00000187258:[ENST00000531252(c.147G>C,aaG>aaC):ENSP00000433258(p.Lys49Asn)];ENSG00000187258:[ENST00000381539(c.147G>C,aaG>aaC):ENSP00000370950(p.Lys49Asn)];ENSG00000187258:[ENST00000381542(c.147G>C,aaG>aaC):ENSP00000370953(p.Lys49Asn)];ENSG00000187258:[ENST00000396095(c.147G>C,aaG>aaC):ENSP00000379402(p.Lys49Asn)];ENSG00000187258:[ENST00000381553(c.147G>C,aaG>aaC):ENSP00000370965(p.Lys49Asn)];ENSG00000187258:[ENST00000360581(c.147G>C,aaG>aaC):ENSP00000353788(p.Lys49Asn)];ENSG00000187258:[ENST00000381544(c.147G>C,aaG>aaC):ENSP00000370955(p.Lys49Asn)];	NA	NA	NA	NA	NA	NA	NA	NA	NA	NA	MODIFIER	NA	NA	155	155	0	121	111	10	8.26
Smoker	42481	chr7	34698171	G	C	*NPSR1-AS1*	INTRONIC	ENSG00000197085:[ENST00000419766(n.160+70178C>G)];ENSG00000197085:[ENST00000544556(n.280-41556C>G)];ENSG00000197085:[ENST00000537560(n.279+70178C>G)];ENSG00000197085:[ENST00000535640(n.245-41556C>G)];ENSG00000197085:[ENST00000431669(n.245-15208C>G)];ENSG00000197085:[ENST00000439852(n.399+45521C>G)];ENSG00000197085:[ENST00000358772(n.279+70178C>G)];ENSG00000197085:[ENST00000539747(n.164+70178C>G)];	NA	NA	NA	NA	NA	NA	NA	NA	NA	NA	MODIFIER	NA	NA	155	155	0	121	111	10	8.26

Mangalaparthi *et al.* , 2020. Mutational landscape of esophageal squamous cell carcinoma in an Indian cohort
Supplementary Table 4. List of somatic single nucleotide variants identified using whole exome sequencing of ESCC patients

Cohort	Sample	Chromosome	Genomic position	Reference allele	Alternate allele	Gene symbol	Variant type	Variant annotation	OncoMD	COSMIC	dbSNP	ExAC	ExAC_SAS	1000G	1000G_SAS	SIFT	Polyphen2	CONDEL	VEP	LRT	PhastCon7 Way	Total read (Adjacent normal)	Reference allel read support (Adjacent normal)	Alternative read support (Adjacent normal)	Total read (Tumor)	Reference allel read support (Tumor)	Alternative read support (Tumor)	Alternate allele frequency
Smoker	42481	chr7	151048643	T	G	*NUB1*	INTRONIC	ENSG00000013374:[ENST00000470316(c.344+75T>G)];ENSG00000013374:[ENST00000493588(n.363+75T>G)];ENSG00000013374:[ENST00000468404(c.344+75T>G)];ENSG00000013374:[ENST00000355851(c.344+75T>G)];ENSG00000013374:[ENST00000566856(c.344+75T>G)];ENSG00000013374:[ENST00000480907(n.155+75T>G)];ENSG00000013374:[ENST00000568733(c.416+75T>G)];ENSG00000013374:[ENST00000470229(c.344+75T>G)];ENSG00000013374:[ENST00000483358(c.344+75T>G)];ENSG00000013374:[ENST00000413040(c.416+75T>G)];	NA	NA	NA	NA	NA	NA	NA	NA	NA	NA	MODIFIER	NA	NA	85	85	0	50	32	18	36
Smoker	42481	chr7	151048644	C	A	*NUB1*	INTRONIC	ENSG00000013374:[ENST00000470316(c.344+76C>A)];ENSG00000013374:[ENST00000493588(n.363+76C>A)];ENSG00000013374:[ENST00000468404(c.344+76C>A)];ENSG00000013374:[ENST00000355851(c.344+76C>A)];ENSG00000013374:[ENST00000566856(c.344+76C>A)];ENSG00000013374:[ENST00000480907(n.155+76C>A)];ENSG00000013374:[ENST00000568733(c.416+76C>A)];ENSG00000013374:[ENST00000470229(c.344+76C>A)];ENSG00000013374:[ENST00000483358(c.344+76C>A)];ENSG00000013374:[ENST00000413040(c.416+76C>A)];	NA	NA	NA	NA	NA	NA	NA	NA	NA	NA	MODIFIER	NA	NA	85	85	0	50	32	18	36
Smoker	42481	chr7	77256804	C	T	*PTPN12*	MISSENSE	ENSG00000127947:MISSENSE[ENST00000415482(c.1451C>T,tCt>tTt):ENSP00000392429(p.Ser484Phe)];ENSG00000127947:MISSENSE[ENST00000435495(c.1418C>T,tCt>tTt):ENSP00000397991(p.Ser473Phe)];ENSG00000127947:MISSENSE[ENST00000248594(c.1808C>T,tCt>tTt):ENSP00000248594(p.Ser603Phe)];ENSG00000127947:MISSENSE[ENST00000407343(c.332C>T,tCt>tTt):ENSP00000385079(p.Ser111Phe)];	NA	NA	NA	NA	NA	NA	NA	D	PrD	D	MODERATE	NA	NA	414	414	0	380	231	149	39.21
Smoker	42481	chr7	123672507	C	A	*RP5-921G16.1*	INTRONIC	ENSG00000242593:[ENST00000484322(n.75+174C>A)];	NA	NA	NA	NA	NA	NA	NA	T_lc	BN	N	MODERATE	NA	NA	73	73	0	76	58	18	23.68
Smoker	42481	chr7	123672507	C	A	*TMEM229A*	MISSENSE	ENSG00000234224:MISSENSE[ENST00000455783(c.551G>T,cGa>cTa):ENSP00000395244(p.Arg184Leu)];	NA	NA	NA	NA	NA	NA	NA	T_lc	BN	N	MODERATE	NA	NA	73	73	0	76	58	18	23.68
Smoker	42481	chr7	66548448	C	T	*TYW1*	MISSENSE	ENSG00000198874:MISSENSE[ENST00000359626(c.1306C>T,Cgg>Tgg):ENSP00000352645(p.Arg436Trp)];ENSG00000198874:EXONIC-NC[ENST00000495971(n.298C>T)];ENSG00000198874:3UTR[ENST00000361660(c.*98C>T)];	NA	NA	0.03914	0.0103217	0.000181708	0.0391374	0	NA	NA	NA	MODIFIER	NA	NA	227	227	0	210	198	12	5.71
Smoker	42481	chr8	24193188	C	T	*ADAM28*	MISSENSE	ENSG00000042980:MISSENSE[ENST00000437154(c.1601C>T,gCg>gTg):ENSP00000393699(p.Ala534Val)];ENSG00000042980:MISSENSE[ENST00000540823(c.902C>T,gCg>gTg):ENSP00000443743(p.Ala301Val)];ENSG00000042980:INTRONIC[ENST00000521629(c.464+34C>T)];ENSG00000042980:INTRONIC[ENST00000397649(c.808+34C>T)];ENSG00000042980:INTRONIC[ENST00000265769(c.1567+34C>T)];ENSG00000042980:INTRONIC[ENST00000520448(c.*975+34C>T)];	NA	NA	0.01558	0.0086781	0.000954198	0.0155751	0	NA	NA	NA	MODIFIER	NA	NA	191	190	0	189	170	19	10.05
Smoker	42481	chr8	61654888	C	T	*CHD7*	SILENT	ENSG00000171316:INTRONIC[ENST00000527825(c.31+444C>T)];ENSG00000171316:SILENT[ENST00000423902(c.897C>T,gtC>gtT):ENSP00000392028(p.Val299(=))];ENSG00000171316:SILENT[ENST00000524602(c.897C>T,gtC>gtT):ENSP00000437061(p.Val299(=))];ENSG00000171316:SILENT[ENST00000525508(c.897C>T,gtC>gtT):ENSP00000436027(p.Val299(=))];	NA	NA	NA	NA	NA	NA	NA	NA	NA	NA	LOW	NA	NA	202	202	0	201	86	115	57.21
Smoker	42481	chr8	113304870	T	A	*CSMD3*	MISSENSE	ENSG00000164796:MISSENSE[ENST00000297405(c.8684A>T,gAt>gTt):ENSP00000297405(p.Asp2895Val)];ENSG00000164796:MISSENSE[ENST00000343508(c.8564A>T,gAt>gTt):ENSP00000345799(p.Asp2855Val)];ENSG00000164796:MISSENSE[ENST00000455883(c.8177A>T,gAt>gTt):ENSP00000412263(p.Asp2726Val)];ENSG00000164796:MISSENSE[ENST00000339701(c.6494A>T,gAt>gTt):ENSP00000341558(p.Asp2165Val)];ENSG00000164796:MISSENSE[ENST00000352409(c.8474A>T,gAt>gTt):ENSP00000343124(p.Asp2825Val)];	NA	NA	NA	NA	NA	NA	NA	NA	PrD	NA	MODERATE	D	1	197	196	0	154	129	25	16.23
Smoker	42481	chr8	113304871	C	A	*CSMD3*	MISSENSE	ENSG00000164796:MISSENSE[ENST00000297405(c.8683G>T,Gat>Tat):ENSP00000297405(p.Asp2895Tyr)];ENSG00000164796:MISSENSE[ENST00000343508(c.8563G>T,Gat>Tat):ENSP00000345799(p.Asp2855Tyr)];ENSG00000164796:MISSENSE[ENST00000455883(c.8176G>T,Gat>Tat):ENSP00000412263(p.Asp2726Tyr)];ENSG00000164796:MISSENSE[ENST00000339701(c.6493G>T,Gat>Tat):ENSP00000341558(p.Asp2165Tyr)];ENSG00000164796:MISSENSE[ENST00000352409(c.8473G>T,Gat>Tat):ENSP00000343124(p.Asp2825Tyr)];	NA	NA	NA	NA	NA	NA	NA	NA	PrD	NA	MODERATE	D	1	197	196	0	155	131	24	15.48
Smoker	42481	chr8	11705882	C	G	*CTSB*	INTRONIC	ENSG00000164733:[ENST00000453527(c.447-221G>C)];ENSG00000164733:[ENST00000530640(c.447-221G>C)];ENSG00000164733:[ENST00000530290(n.216+39G>C)];ENSG00000164733:[ENST00000530296(c.447-221G>C)];ENSG00000164733:[ENST00000530624(n.98+39G>C)];ENSG00000164733:[ENST00000533455(c.447-221G>C)];ENSG00000164733:[ENST00000532409(n.534-221G>C)];ENSG00000164733:[ENST00000345125(c.447-221G>C)];ENSG00000164733:[ENST00000533110(n.170-221G>C)];ENSG00000164733:[ENST00000353047(c.447-221G>C)];ENSG00000164733:[ENST00000415599(c.328-221G>C)];ENSG00000164733:[ENST00000534636(c.447-221G>C)];ENSG00000164733:[ENST00000531089(c.447-221G>C)];ENSG00000164733:[ENST00000434271(c.447-221G>C)];ENSG00000164733:[ENST00000531551(c.328-221G>C)];ENSG00000164733:[ENST00000533572(c.447-221G>C)];ENSG00000164733:[ENST00000534510(c.447-221G>C)];ENSG00000164733:[ENST00000420692(n.331G>C)];	NA	NA	NA	NA	NA	NA	NA	NA	NA	NA	MODIFIER	NA	NA	54	54	0	55	27	28	50.91
Smoker	42481	chr8	33451178	G	A	*DUSP26*	SILENT	ENSG00000133878:SILENT[ENST00000523956(c.309C>T,ccC>ccT):ENSP00000429176(p.Pro103(=))];ENSG00000133878:SILENT[ENST00000256261(c.309C>T,ccC>ccT):ENSP00000256261(p.Pro103(=))];ENSG00000133878:SILENT[ENST00000522982(c.309C>T,ccC>ccT):ENSP00000430922(p.Pro103(=))];	chr8*33451	chr8*33451	chr8*33451178*33451178*G*T*rs754649348*NA*NA*NA	NA	NA	NA	NA	NA	NA	NA	LOW	NA	NA	93	93	0	82	65	17	20.73
Smoker	42481	chr8	101724897	T	C	*PABPC1*	MISSENSE	ENSG00000070756:MISSENSE[ENST00000519100(c.465A>G,Agg>Ggg):ENSP00000427914(p.Arg156Gly)];ENSG00000070756:MISSENSE[ENST00000519004(c.724A>G,Agg>Ggg):ENSP00000429594(p.Arg242Gly)];ENSG00000070756:MISSENSE[ENST00000522387(c.763A>G,Agg>Ggg):ENSP00000429395(p.Arg255Gly)];ENSG00000070756:MISSENSE[ENST00000318607(c.859A>G,Agg>Ggg):ENSP00000313007(p.Arg287Gly)];ENSG00000070756:MISSENSE[ENST00000519622(c.251A>G,Agg>Ggg):ENSP00000427805(p.Arg85Gly)];ENSG00000070756:MISSENSE[ENST00000517403(c.93A>G,Agg>Ggg):ENSP00000428190(p.Arg32Gly)];ENSG00000070756:EXONIC-NC[ENST00000519596(n.356A>G)];	chr8*10172	NA	NA	NA	NA	NA	NA	NA	NA	NA	MODIFIER	NA	NA	247	242	0	261	246	9	3.45

Mangalaparthi *et al.* , 2020. Mutational landscape of esophageal squamous cell carcinoma in an Indian cohort
Supplementary Table 4. List of somatic single nucleotide variants identified using whole exome sequencing of ESCC patients

Cohort	Sample	Chromosome	Genomic position	Reference allele	Alternate allele	Gene symbol	Variant type	Variant annotation	OncoMD	COSMIC	dbSNP	ExAC	ExAC_SAS	1000G	1000G_SAS	SIFT	Polyphen2	CONDEL	VEP	LRT	PhastCon7 Way	Total read (Adjacent normal)	Reference allel read support (Adjacent normal)	Alternative read support (Adjacent normal)	Total read (Tumor)	Reference allel read support (Tumor)	Alternative read support (Tumor)	Alternate allele frequency
Smoker	42481	chr8	48690330	C	T	*PRKDC*	MISSENSE	ENSG00000253729:MISSENSE[ENST00000314191(c.11956G>A,Gca>Aca):ENSP00000313420(p.Ala3986Thr)];ENSG00000253729:MISSENSE[ENST00000338368(c.11863G>A,Gca>Aca):ENSP00000345182(p.Ala3955Thr)];ENSG00000253729:MISSENSE[ENST00000536429(c.185G>A,Gca>Aca):ENSP00000442177(p.Ala63Thr)];ENSG00000253729:EXONIC-NC[ENST00000523565(n.12015G>A)];ENSG00000253729:EXONIC-NC[ENST00000536483(n.367G>A)];ENSG00000253729:EXONIC-NC[ENST00000518216(n.11917G>A)];	NA	NA	NA	8.28E-06	0	NA	NA	NA	NA	NA	MODIFIER	NA	NA	110	110	0	100	92	8	8
Smoker	42481	chr8	24193188	C	T	*RP11-624C23.1*	INTRONIC	ENSG00000253535:[ENST00000518988(n.356-34746G>A)];ENSG00000253535:[ENST00000519689(n.607-34746G>A)];ENSG00000253535:[ENST00000523578(n.502-34746G>A)];ENSG00000253535:[ENST00000523700(n.165-34746G>A)];	NA	NA	0.01558	0.0086781	0.000954198	0.0155751	0	NA	NA	NA	MODIFIER	NA	NA	191	190	0	189	170	19	10.05
Smoker	42481	chr8	103266649	G	A	*UBR5*	MISSENSE	ENSG00000104517:MISSENSE[ENST00000520539(c.8281C>T,Cat>Tat):ENSP00000429084(p.His2761Tyr)];ENSG00000104517:MISSENSE[ENST00000521922(c.8260C>T,Cat>Tat):ENSP00000427819(p.His2754Tyr)];ENSG00000104517:MISSENSE[ENST00000518205(c.1465C>T,Cat>Tat):ENSP00000428693(p.His489Tyr)];ENSG00000104517:MISSENSE[ENST00000220959(c.8278C>T,Cat>Tat):ENSP00000220959(p.His2760Tyr)];ENSG00000104517:EXONIC-NC[ENST00000517465(n.620C>T)];	NA	NA	NA	NA	NA	NA	NA	NA	NA	NA	MODIFIER	NA	NA	218	218	0	192	170	22	11.46
Smoker	42481	chr8	82627224	C	G	*ZFAND1*	EXONIC-NC-SS-PR	ENSG00000104231:[ENST00000517353(n.360G>C)];ENSG00000104231:[ENST00000521885(n.344G>C)];ENSG00000104231:[ENST00000524339(n.238G>C)];ENSG00000104231:[ENST00000519820(n.283G>C)];ENSG00000104231:[ENST00000524305(n.51G>C)];ENSG00000104231:[ENST00000519523(c.264G>C,ctG>ctC):ENSP00000429167(p.Leu88(=))];ENSG00000104231:[ENST00000523096(c.264G>C,ctG>ctC):ENSP00000430736(p.Leu88(=))];ENSG00000104231:[ENST00000220669(c.264G>C,ctG>ctC):ENSP00000220669(p.Leu88(=))];ENSG00000104231:[ENST00000519464(c.264G>C,ctG>ctC):ENSP00000428375(p.Leu88(=))];ENSG00000104231:[ENST00000517450(c.-149G>C)];ENSG00000104231:[ENST00000520635(c.-149G>C)];ENSG00000104231:[ENST00000522520(c.-58G>C)];ENSG00000104231:[ENST00000521895(c.-58G>C)];ENSG00000104231:[ENST00000523431(c.-58G>C)];ENSG00000104231:[ENST00000517588(c.-58G>C)];ENSG00000104231:[ENST00000520604(c.-58G>C)];ENSG00000104231:[ENST00000520076(c.-58G>C)];ENSG00000104231:[ENST00000523361(c.-58G>C)];ENSG00000104231:[ENST00000518419(c.-58G>C)];ENSG00000104231:[ENST00000521287(c.-58G>C)];ENSG00000104231:[ENST00000521742(c.-58G>C)];ENSG00000104231:[ENST00000520941(n.590G>C)];	NA	NA	NA	NA	NA	NA	NA	NA	NA	NA	LOW	NA	NA	386	386	0	354	308	45	12.71
Smoker	42481	chr9	139369833	C	T	*SEC16A*	SILENT	ENSG00000148396:SILENT[ENST00000290037(c.1701G>A,ccG>ccA):ENSP00000290037(p.Pro567(=))];ENSG00000148396:SILENT[ENST00000313050(c.2235G>A,ccG>ccA):ENSP00000325827(p.Pro745(=))];ENSG00000148396:SILENT[ENST00000431893(c.1701G>A,ccG>ccA):ENSP00000387583(p.Pro567(=))];ENSG00000148396:SILENT[ENST00000371706(c.1701G>A,ccG>ccA):ENSP00000360771(p.Pro567(=))];	chr9*13936	chr9*13936	0.001398	0.0016438	0.009654472	0.0013978	0.0061	NA	NA	NA	LOW	NA	NA	158	157	0	166	158	7	4.22
Smoker	42481	chr9	106896781	T	C	*SMC2*	MISSENSE	ENSG00000136824:MISSENSE[ENST00000303219(c.3194T>C,tTg>tCg):ENSP00000306152(p.Leu1065Ser)];ENSG00000136824:MISSENSE[ENST00000374793(c.3194T>C,tTg>tCg):ENSP00000363925(p.Leu1065Ser)];ENSG00000136824:MISSENSE[ENST00000286398(c.3194T>C,tTg>tCg):ENSP00000286398(p.Leu1065Ser)];ENSG00000136824:MISSENSE[ENST00000374787(c.3194T>C,tTg>tCg):ENSP00000363919(p.Leu1065Ser)];ENSG00000136824:MISSENSE[ENST00000493955(c.395T>C,tTg>tCg):ENSP00000431534(p.Leu132Ser)];	NA	NA	NA	NA	NA	NA	NA	T	PrD	D	MODERATE	NA	NA	253	251	0	209	198	11	5.26
Smoker	42481	chr9	113220473	C	G	*SVEP1*	INTRONIC	ENSG00000165124:[ENST00000401783(c.3576+278G>C)];ENSG00000165124:[ENST00000374469(c.3507+278G>C)];ENSG00000165124:[ENST00000302728(c.3572-74G>C)];ENSG00000165124:[ENST00000467821(n.2995+278G>C)];	NA	NA	NA	NA	NA	NA	NA	NA	NA	NA	MODIFIER	NA	NA	263	260	1	213	201	11	5.16
Smoker	42481	chrX	40990709	G	T	*USP9X*	INTRONIC-SS-ACR	ENSG00000124486:INTRONIC-SS-ACR[ENST00000378308(c.243-1G>T)];ENSG00000124486:INTRONIC-SS-ACR[ENST00000324545(c.243-1G>T)];	NA	NA	NA	NA	NA	NA	NA	NA	NA	NA	HIGH	NA	NA	37	37	0	23	6	17	73.91
Chewer	42482	chr1	180283929	G	A	*ACBD6*	INTRONIC	ENSG00000135847:[ENST00000367595(c.664-72C>T)];ENSG00000135847:[ENST00000475338(n.266-72C>T)];ENSG00000135847:[ENST00000496993(n.280-72C>T)];	NA	NA	NA	NA	NA	NA	NA	NA	NA_NA	NA	MODIFIER	NA	NA	39	39	0	53	48	5	9.43
Chewer	42482	chr1	247014085	C	T	*AHCTF1*	SILENT	ENSG00000153207:SILENT[ENST00000391829(c.5223G>A,acG>acA):ENSP00000375705(p.Thr1741(=))];ENSG00000153207:SILENT[ENST00000366508(c.5328G>A,acG>acA):ENSP00000355464(p.Thr1776(=))];ENSG00000153207:SILENT[ENST00000326225(c.5250G>A,acG>acA):ENSP00000355465(p.Thr1750(=))];ENSG00000153207:EXONIC-NC[ENST00000470300(n.3838G>A)];ENSG00000153207:EXONIC-NC[ENST00000483900(n.2539G>A)];	chr1*24701	chr1*24701	NA	0.0001916	0.000547445	NA	NA	NA	NA_NA	NA	LOW	NA	NA	184	182	1	250	235	14	5.6
Chewer	42482	chr1	17949566	G	A	*ARHGEF10L*	MISSENSE	ENSG00000074964:MISSENSE[ENST00000375415(c.979G>A,Gtg>Atg):ENSP00000364564(p.Val327Met)];ENSG00000074964:MISSENSE[ENST00000361221(c.1096G>A,Gtg>Atg):ENSP00000355060(p.Val366Met)];ENSG00000074964:MISSENSE[ENST00000434513(c.1096G>A,Gtg>Atg):ENSP00000394621(p.Val366Met)];ENSG00000074964:MISSENSE[ENST00000375408(c.430G>A,Gtg>Atg):ENSP00000364557(p.Val144Met)];ENSG00000074964:MISSENSE[ENST00000452522(c.979G>A,Gtg>Atg):ENSP00000399401(p.Val327Met)];ENSG00000074964:MISSENSE[ENST00000375420(c.370G>A,Gtg>Atg):ENSP00000364569(p.Val124Met)];ENSG00000074964:MISSENSE[ENST00000167825(c.430G>A,Gtg>Atg):ENSP00000167825(p.Val144Met)];ENSG00000074964:EXONIC-NC[ENST00000469726(n.1376G>A)];	NA	NA	NA	NA	NA	NA	NA	NA	NA_NA	NA	MODIFIER	NA	NA	76	76	0	97	74	23	23.71
Chewer	42482	chr1	22965493	G	A	*C1QA*	MISSENSE	ENSG00000173372:MISSENSE[ENST00000402322(c.331G>A,Gac>Aac):ENSP00000385564(p.Asp111Asn)];ENSG00000173372:MISSENSE[ENST00000374642(c.331G>A,Gac>Aac):ENSP00000363773(p.Asp111Asn)];ENSG00000173372:MISSENSE[ENST00000438241(c.331G>A,Gac>Aac):ENSP00000416841(p.Asp111Asn)];	NA	NA	NA	NA	NA	NA	NA	T	BN_BN	N	MODERATE	N	NA	105	105	0	132	108	24	18.18

Mangalaparthi *et al.* , 2020. Mutational landscape of esophageal squamous cell carcinoma in an Indian cohort
Supplementary Table 4. List of somatic single nucleotide variants identified using whole exome sequencing of ESCC patients

Cohort	Sample	Chromosome	Genomic position	Reference allele	Alternate allele	Gene symbol	Variant type	Variant annotation	OncoMD	COSMIC	dbSNP	ExAC	ExAC_SAS	1000G	1000G_SAS	SIFT	Polyphen2	CONDEL	VEP	LRT	PhastCon7 Way	Total read (Adjacent normal)	Reference allel read support (Adjacent normal)	Alternative read support (Adjacent normal)	Total read (Tumor)	Reference allel read support (Tumor)	Alternative read support (Tumor)	Alternate allele frequency
Chewer	42482	chr1	36216983	C	T	*CLSPN*	SILENT	ENSG00000092853:SILENT[ENST00000318121(c.1896G>A,gaG>gaA):ENSP00000312995(p.Glu632(=))];ENSG00000092853:SILENT[ENST00000520551(c.1896G>A,gaG>gaA):ENSP00000428848(p.Glu632(=))];ENSG00000092853:SILENT[ENST00000373220(c.1704G>A,gaG>gaA):ENSP00000362317(p.Glu568(=))];ENSG00000092853:SILENT[ENST00000251195(c.1896G>A,gaG>gaA):ENSP00000251195(p.Glu632(=))];	NA	NA	NA	8.30E-06	0	NA	NA	NA	NA_NA	NA	LOW	NA	0.999	112	112	0	175	152	22	12.57
Chewer	42482	chr1	1255981	G	C	*CPSF3L*	3UTR	ENSG00000127054:[ENST00000531019(c.*37C>G)];ENSG00000127054:[ENST00000470679(c.*37C>G)];ENSG00000127054:[ENST00000419704(c.126+395C>G)];ENSG00000127054:[ENST00000450926(c.127-72C>G)];ENSG00000127054:[ENST00000430786(c.*5-72C>G)];ENSG00000127054:[ENST00000532772(c.126+395C>G)];ENSG00000127054:[ENST00000490853(n.168-72C>G)];ENSG00000127054:[ENST00000488042(n.148-72C>G)];ENSG00000127054:[ENST00000434694(c.127-72C>G)];ENSG00000127054:[ENST00000528879(c.127-72C>G)];ENSG00000127054:[ENST00000527719(c.145-72C>G)];ENSG00000127054:[ENST00000545578(c.40-72C>G)];ENSG00000127054:[ENST00000530031(c.268-72C>G)];ENSG00000127054:[ENST00000532952(n.161-72C>G)];ENSG00000127054:[ENST00000435064(c.127-72C>G)];ENSG00000127054:[ENST00000526332(c.-172-1077C>G)];ENSG00000127054:[ENST00000525285(c.*334-72C>G)];ENSG00000127054:[ENST00000534345(c.130-72C>G)];ENSG00000127054:[ENST00000526904(n.78+3980C>G)];ENSG00000127054:[ENST00000429572(n.185-72C>G)];ENSG00000127054:[ENST00000458452(c.*411-72C>G)];ENSG00000127054:[ENST00000421495(c.-530+3980C>G)];ENSG00000127054:[ENST00000498476(c.307-72C>G)];ENSG00000127054:[ENST00000526797(c.127-72C>G)];ENSG00000127054:[ENST00000540437(c.145-72C>G)];ENSG00000127054:[ENST00000493534(n.177-72C>G)];ENSG00000127054:[ENST00000526113(n.57+3980C>G)];ENSG00000127054:[ENST00000411962(c.135+2580C>G)];ENSG00000127054:[ENST00000527098(c.234-	NA	NA	chr1*1255981*1255981*G*A*rs748549781*NA*NA*NA	NA	NA	NA	NA	NA	NA_NA	NA	MODIFIER	NA	NA	62	62	0	80	73	7	8.75
Chewer	42482	chr1	34033353	C	A	*CSMD2*	SILENT	ENSG00000121904:SILENT[ENST00000373381(c.8220G>T,ggG>ggT):ENSP00000362479(p.Gly2740(=))];ENSG00000121904:SILENT[ENST00000241312(c.8151G>T,ggG>ggT):ENSP00000241312(p.Gly2717(=))];ENSG00000121904:EXONIC-NC[ENST00000465819(n.468G>T)];	NA	NA	NA	NA	NA	NA	NA	NA	NA_NA	NA	MODIFIER	NA	NA	175	173	1	250	226	24	9.6
Chewer	42482	chr1	6705917	T	A	*DNAJC11*	MISSENSE	ENSG00000007923:MISSENSE[ENST00000377573(c.556A>T,Atc>Ttc):ENSP00000366796(p.Ile186Phe)];ENSG00000007923:MISSENSE[ENST00000294401(c.826A>T,Atc>Ttc):ENSP00000294401(p.Ile276Phe)];ENSG00000007923:MISSENSE[ENST00000377577(c.826A>T,Atc>Ttc):ENSP00000366800(p.Ile276Phe)];ENSG00000007923:MISSENSE[ENST00000542246(c.712A>T,Atc>Ttc):ENSP00000444020(p.Ile238Phe)];ENSG00000007923:MISSENSE[ENST00000426784(c.916A>T,Atc>Ttc):ENSP00000410194(p.Ile306Phe)];ENSG00000007923:INTRONIC[ENST00000349363(c.698+14A>T)];ENSG00000007923:INTRONIC[ENST00000451196(c.740+14A>T)];	NA	NA	NA	NA	NA	NA	NA	NA	NA_NA	NA	MODIFIER	NA	NA	160	160	0	182	156	26	14.29
Chewer	42482	chr1	167095746	C	A	*DUSP27*	MISSENSE	ENSG00000198842:INTRONIC[ENST00000485151(n.418+173C>A)];ENSG00000198842:MISSENSE[ENST00000361200(c.1378C>A,Ccg>Acg):ENSP00000354483(p.Pro460Thr)];ENSG00000198842:MISSENSE[ENST00000443333(c.1378C>A,Ccg>Acg):ENSP00000404874(p.Pro460Thr)];ENSG00000198842:MISSENSE[ENST00000271385(c.1378C>A,Ccg>Acg):ENSP00000271385(p.Pro460Thr)];	NA	NA	NA	NA	NA	NA	NA	T	BN_BN	N	MODERATE	N	0.008	59	58	1	88	66	22	25
Chewer	42482	chr1	167096519	C	G	*DUSP27*	SILENT	ENSG00000198842:INTRONIC[ENST00000485151(n.418+946C>G)];ENSG00000198842:SILENT[ENST00000361200(c.2151C>G,acC>acG):ENSP00000354483(p.Thr717(=))];ENSG00000198842:SILENT[ENST00000443333(c.2151C>G,acC>acG):ENSP00000404874(p.Thr717(=))];ENSG00000198842:SILENT[ENST00000271385(c.2151C>G,acC>acG):ENSP00000271385(p.Thr717(=))];	NA	NA	chr1*167096519*167096519*C*A*rs752808434*NA*NA*NA	NA	NA	NA	NA	NA	NA_NA	NA	LOW	NA	NA	88	88	0	113	104	9	7.96
Chewer	42482	chr1	157095211	G	C	*ETV3*	MISSENSE	ENSG00000117036:MISSENSE[ENST00000368192(c.961C>G,Cgt>Ggt):ENSP00000357175(p.Arg321Gly)];	NA	NA	chr1*157095211*157095211*G*A*rs779086898*NA*NA*NA	NA	NA	NA	NA	D	PrD_PrD	D	MODERATE	D	1	112	111	1	150	138	12	8
Chewer	42482	chr1	157771290	C	T	*FCRL1*	MISSENSE	ENSG00000163534:MISSENSE[ENST00000368176(c.964G>A,Gtg>Atg):ENSP00000357158(p.Val322Met)];ENSG00000163534:MISSENSE[ENST00000491942(c.964G>A,Gtg>Atg):ENSP00000418130(p.Val322Met)];ENSG00000163534:INTRONIC[ENST00000489998(n.1107+415G>A)];ENSG00000163534:INTRONIC[ENST00000358292(c.886+415G>A)];ENSG00000163534:EXONIC-NC[ENST00000368175(n.744G>A)];ENSG00000163534:EXONIC-NC[ENST00000463001(n.131G>A)];	NA	NA	0.0003994	5.77E-05	6.06E-05	0.0003994	0	NA	NA_NA	NA	MODIFIER	NA	NA	48	48	0	65	51	14	21.54
Chewer	42482	chr1	63788764	T	A	*FOXD3*	MISSENSE	ENSG00000187140:MISSENSE[ENST00000371116(c.35T>A,aTg>aAg):ENSP00000360157(p.Met12Lys)];	NA	NA	NA	NA	NA	NA	NA	NA	NA_NA	NA	MODIFIER	NA	NA	27	27	0	96	78	18	18.75
Chewer	42482	chr1	35260778	C	T	*GJA4*	MISSENSE	ENSG00000187513:MISSENSE[ENST00000342280(c.964C>T,Cgt>Tgt):ENSP00000343676(p.Arg322Cys)];	chr1*35260	chr1*35260	NA	1.70E-05	0	NA	NA	NA	NA_NA	NA	MODIFIER	NA	NA	76	75	0	109	96	13	11.93
Chewer	42482	chr1	91779049	C	A	*HFM1*	MISSENSE	ENSG00000162669:MISSENSE[ENST00000370424(c.2285G>T,gGg>gTg):ENSP00000359453(p.Gly762Val)];ENSG00000162669:MISSENSE[ENST00000370425(c.3248G>T,gGg>gTg):ENSP00000359454(p.Gly1083Val)];ENSG00000162669:MISSENSE[ENST00000294696(c.944G>T,gGg>gTg):ENSP00000294696(p.Gly315Val)];ENSG00000162669:MISSENSE[ENST00000430465(c.883G>T,gGg>gTg):ENSP00000387661(p.Gly295Val)];ENSG00000162669:EXONIC-NC[ENST00000462405(n.1174G>T)];	NA	NA	NA	NA	NA	NA	NA	NA	NA_NA	NA	MODIFIER	NA	NA	36	36	0	45	39	6	13.33

Mangalaparthi *et al.* , 2020. Mutational landscape of esophageal squamous cell carcinoma in an Indian cohort
Supplementary Table 4. List of somatic single nucleotide variants identified using whole exome sequencing of ESCC patients

Cohort	Sample	Chromosome	Genomic position	Reference allele	Alternate allele	Gene symbol	Variant type	Variant annotation	OncoMD	COSMIC	dbSNP	ExAC	ExAC_SAS	1000G	1000G_SAS	SIFT	Polyphen2	CONDEL	VEP	LRT	PhastCon7 Way	Total read (Adjacent normal)	Reference allel read support (Adjacent normal)	Alternative read support (Adjacent normal)	Total read (Tumor)	Reference allel read support (Tumor)	Alternative read support (Tumor)	Alternate allele frequency
Chewer	42482	chr1	109704542	G	T	*KIAA1324*	SILENT	ENSG00000116299:SILENT[ENST00000529753(c.180G>T,gcG>gcT):ENSP00000434595(p.Ala60(=))];ENSG00000116299:SILENT[ENST00000533147(c.180G>T,gcG>gcT):ENSP00000431134(p.Ala60(=))];ENSG00000116299:SILENT[ENST00000534476(c.180G>T,gcG>gcT):ENSP00000432164(p.Ala60(=))];ENSG00000116299:SILENT[ENST00000369939(c.180G>T,gcG>gcT):ENSP00000358955(p.Ala60(=))];ENSG00000116299:SILENT[ENST00000531664(c.180G>T,gcG>gcT):ENSP00000431349(p.Ala60(=))];ENSG00000116299:SILENT[ENST00000526264(c.180G>T,gcG>gcT):ENSP00000435066(p.Ala60(=))];ENSG00000116299:SILENT[ENST00000457623(c.180G>T,gcG>gcT):ENSP00000393964(p.Ala60(=))];ENSG00000116299:EXONIC-NC[ENST00000533374(n.315G>T)];ENSG00000116299:INTRONIC[ENST00000490758(n.165-2579G>T)];ENSG00000116299:INTRONIC[ENST00000527996(c.154-2579G>T)];	NA	NA	chr1*109704542*109704542*G*A*rs77326388*1*0.998*0.001997	NA	NA	NA	NA	NA	NA_NA	NA	LOW	NA	NA	99	99	0	115	107	8	6.96
Chewer	42482	chr1	44595304	C	A	*KLF17*	MISSENSE	ENSG00000171872:MISSENSE[ENST00000372299(c.361C>A,Cag>Aag):ENSP00000361373(p.Gln121Lys)];ENSG00000171872:INTRONIC[ENST00000476802(n.327+72C>A)];	NA	NA	NA	NA	NA	NA	NA	NA	NA_NA	NA	MODIFIER	NA	NA	142	141	1	152	141	11	7.24
Chewer	42482	chr1	70397092	C	T	*LRRC7*	INTRONIC	ENSG00000033122:[ENST00000415775(c.-872-98C>T)];ENSG00000033122:[ENST00000035383(c.534-98C>T)];ENSG00000033122:[ENST00000310961(c.549-98C>T)];	NA	NA	0.0003994	NA	NA	0.0003994	0.001	NA	NA_NA	NA	MODIFIER	NA	NA	175	173	1	202	182	20	9.9
Chewer	42482	chr1	203144523	C	A	*MYBPH*	MISSENSE	ENSG00000133055:MISSENSE[ENST00000255416(c.272G>T,tGg>tTg):ENSP00000255416(p.Trp91Leu)];	NA	NA	NA	NA	NA	NA	NA	D	PrD_PrD	D	MODERATE	D	0.997	88	88	0	93	68	25	26.88
Chewer	42482	chr1	145281568	C	A	*NOTCH2NL*	NONSENSE	ENSG00000213240:NONSENSE[ENST00000369340(c.498C>A,taC>taA):ENSP00000358346(p.Tyr166Ter)];ENSG00000213240:NONSENSE[ENST00000362074(c.498C>A,taC>taA):ENSP00000354929(p.Tyr166Ter)];ENSG00000213240:NONSENSE[ENST00000344859(c.498C>A,taC>taA):ENSP00000344557(p.Tyr166Ter)];	NA	NA	chr1*145281567*145281568*AC*A*rs782402169*NA*NA*NA	NA	NA	NA	NA	NA	NA_NA	NA	HIGH	NA	NA	508	506	0	644	590	54	8.39
Chewer	42482	chr1	176769268	G	C	*PAPPA2*	MISSENSE-SS-PRX	ENSG00000116183:[ENST00000367662(c.5202G>C,gaG>gaC):ENSP00000356634(p.Glu1734Asp)];ENSG00000116183:[ENST00000479836(n.220G>C)];	NA	NA	NA	NA	NA	NA	NA	NA	NA_NA	NA	LOW	NA	NA	85	85	0	113	101	12	10.62
Chewer	42482	chr1	28167668	G	A	*PPP1R8*	MISSENSE	ENSG00000117751:MISSENSE[ENST00000311772(c.415G>A,Gga>Aga):ENSP00000311677(p.Gly139Arg)];ENSG00000117751:INTRONIC[ENST00000236412(c.-180-2029G>A)];ENSG00000117751:5UTR[ENST00000373931(c.-12G>A)];ENSG00000117751:5UTR[ENST00000431586(c.-12G>A)];	NA	NA	NA	NA	NA	NA	NA	NA	NA_NA	NA	MODIFIER	NA	NA	73	73	0	102	96	6	5.88
Chewer	42482	chr1	145281568	C	A	*RP11-458D21.5*	NONSENSE	ENSG00000255168:NONSENSE[ENST00000468030(c.498C>A,taC>taA):ENSP00000457682(p.Tyr166Ter)];	NA	NA	chr1*145281567*145281568*AC*A*rs782402169*NA*NA*NA	NA	NA	NA	NA	NA	NA_NA	NA	HIGH	NA	NA	508	506	0	644	590	54	8.39
Chewer	42482	chr1	63788764	T	A	*RP4-792G4.2*	INTRONIC	ENSG00000230798:[ENST00000431294(n.92+398A>T)];ENSG00000230798:[ENST00000427268(n.87+1262A>T)];	NA	NA	NA	NA	NA	NA	NA	NA	NA_NA	NA	MODIFIER	NA	NA	27	27	0	96	78	18	18.75
Chewer	42482	chr1	169562802	T	C	*SELP*	INTRONIC	ENSG00000174175:[ENST00000263686(c.2407+41A>G)];ENSG00000174175:[ENST00000367793(c.2221+41A>G)];ENSG00000174175:[ENST00000458599(c.1855+41A>G)];ENSG00000174175:[ENST00000367794(c.2221+41A>G)];ENSG00000174175:[ENST00000426706(c.2284+1128A>G)];ENSG00000174175:[ENST00000367786(c.2221+41A>G)];ENSG00000174175:[ENST00000367792(c.1855+41A>G)];ENSG00000174175:[ENST00000367788(c.2221+41A>G)];ENSG00000174175:[ENST00000367791(c.1849+41A>G)];	NA	NA	NA	NA	NA	NA	NA	NA	NA_NA	NA	MODIFIER	NA	NA	52	52	0	93	79	14	15.05
Chewer	42482	chr1	31901899	C	G	*SERINC2*	SILENT	ENSG00000168528:SILENT[ENST00000536384(c.867C>G,gcC>gcG):ENSP00000439048(p.Ala289(=))];ENSG00000168528:SILENT[ENST00000373710(c.882C>G,gcC>gcG):ENSP00000362814(p.Ala294(=))];ENSG00000168528:SILENT[ENST00000536859(c.867C>G,gcC>gcG):ENSP00000444307(p.Ala289(=))];ENSG00000168528:SILENT[ENST00000373709(c.855C>G,gcC>gcG):ENSP00000362813(p.Ala285(=))];ENSG00000168528:EXONIC-NC[ENST00000491976(n.1816C>G)];	chr1*319018	chr1*319018	NA	NA	NA	NA	NA	NA	NA_NA	NA	LOW	NA	NA	96	96	0	121	112	9	7.44
Chewer	42482	chr1	108697718	C	T	*SLC25A24*	MISSENSE	ENSG00000085491:MISSENSE[ENST00000565488(c.709G>A,Ggc>Agc):ENSP00000457733(p.Gly237Ser)];ENSG00000085491:MISSENSE[ENST00000370041(c.652G>A,Ggc>Agc):ENSP00000359058(p.Gly218Ser)];ENSG00000085491:3UTR[ENST00000264128(c.*288G>A)];	NA	NA	NA	NA	NA	NA	NA	NA	NA_NA	NA	MODIFIER	NA	NA	183	183	0	166	155	11	6.63
Chewer	42482	chr1	35260778	C	T	*SMIM12*	INTRONIC	ENSG00000163866:[ENST00000426886(c.207+60594G>A)];	chr1*35260	chr1*35260	NA	1.70E-05	0	NA	NA	NA	NA_NA	NA	MODIFIER	NA	NA	76	75	0	109	96	13	11.93
Chewer	42482	chr1	179561819	A	G	*TDRD5*	SILENT	ENSG00000162782:SILENT[ENST00000367614(c.69A>G,aaA>aaG):ENSP00000356586(p.Lys23(=))];ENSG00000162782:SILENT[ENST00000444136(c.69A>G,aaA>aaG):ENSP00000406052(p.Lys23(=))];ENSG00000162782:SILENT[ENST00000294848(c.69A>G,aaA>aaG):ENSP00000294848(p.Lys23(=))];	NA	NA	NA	NA	NA	NA	NA	NA	NA_NA	NA	LOW	NA	0.995	81	81	0	162	149	13	8.02
Chewer	42482	chr1	54513031	G	A	*TMEM59*	SILENT	ENSG00000116209:SILENT[ENST00000371337(c.204C>T,taC>taT):ENSP00000360388(p.Tyr68(=))];ENSG00000116209:SILENT[ENST00000234831(c.204C>T,taC>taT):ENSP00000234831(p.Tyr68(=))];ENSG00000116209:SILENT[ENST00000452421(c.237C>T,taC>taT):ENSP00000397772(p.Tyr79(=))];ENSG00000116209:INTRONIC[ENST00000440019(c.-98-1572C>T)];ENSG00000116209:5UTR[ENST00000371348(c.-190C>T)];ENSG00000116209:5UTR[ENST00000420738(c.-190C>T)];ENSG00000116209:5UTR[ENST00000371341(c.-190C>T)];	chr1*54513	NA	0.0001997	8.24E-06	0	0.0001997	0	NA	NA_NA	NA	MODIFIER	NA	NA	163	163	0	198	154	43	21.72
Chewer	42482	chr1	156255108	C	T	*TMEM79*	MISSENSE	ENSG00000163472:MISSENSE[ENST00000405535(c.91C>T,Cgg>Tgg):ENSP00000384748(p.Arg31Trp)];ENSG00000163472:MISSENSE[ENST00000295694(c.91C>T,Cgg>Tgg):ENSP00000295694(p.Arg31Trp)];ENSG00000163472:INTRONIC[ENST00000463670(n.215+50C>T)];ENSG00000163472:INTRONIC[ENST00000456810(c.41+50C>T)];ENSG00000163472:INTRONIC[ENST00000357501(c.41+50C>T)];ENSG00000163472:EXONIC-NC[ENST00000495881(n.227C>T)];	NA	NA	0.0007987	0.0002007	0.00190878	0.0007987	0.0041	NA	NA_NA	NA	MODIFIER	NA	NA	97	97	0	167	161	6	3.59
Chewer	42482	chr1	101198059	G	T	*VCAM1*	MISSENSE	ENSG00000162692:MISSENSE[ENST00000347652(c.1335G>T,caG>caT):ENSP00000304611(p.Gln445His)];ENSG00000162692:MISSENSE[ENST00000370115(c.1014G>T,caG>caT):ENSP00000359133(p.Gln338His)];ENSG00000162692:MISSENSE[ENST00000294728(c.1611G>T,caG>caT):ENSP00000294728(p.Gln537His)];ENSG00000162692:MISSENSE[ENST00000370119(c.1425G>T,caG>caT):ENSP00000359137(p.Gln475His)];	NA	NA	NA	NA	NA	NA	NA	T	BN_BN	N	MODERATE	N	0.002	107	107	0	139	114	24	17.27
Chewer	42482	chr1	6640855	T	G	*ZBTB48*	MISSENSE	ENSG00000204859:MISSENSE[ENST00000435905(c.186T>G,agT>agG):ENSP00000416054(p.Ser62Arg)];ENSG00000204859:MISSENSE[ENST00000319084(c.186T>G,agT>agG):ENSP00000313416(p.Ser62Arg)];ENSG00000204859:MISSENSE[ENST00000377674(c.186T>G,agT>agG):ENSP00000366902(p.Ser62Arg)];ENSG00000204859:INTRONIC[ENST00000488936(c.-46+659T>G)];	NA	NA	NA	NA	NA	NA	NA	T	PoD_PoD	N	MODERATE	N	0.947	115	114	0	230	219	11	4.78

Supplementary Table 4. List of somatic single nucleotide variants identified using whole exome sequencing of ESCC patients

Cohort	Sample	Chromosome	Genomic position	Reference allele	Alternate allele	Gene symbol	Variant type	Variant annotation	OncoMD	COSMIC	dbSNP	ExAC	ExAC_SAS	1000G	1000G_SAS	SIFT	Polyphen2	CONDEL	VEP	LRT	PhastCon7 Way	Total read (Adjacent normal)	Reference allel read support (Adjacent normal)	Alternative read support (Adjacent normal)	Total read (Tumor)	Reference allel read support (Tumor)	Alternative read support (Tumor)	Alternate allele frequency
Chewer	42482	chr1	37948356	G	C	*ZC3H12A*	MISSENSE	ENSG00000163874:MISSENSE[ENST00000373087(c.944G>C,gGg>gCg):ENSP00000362179(p.Gly315Ala)];	NA	NA	NA	NA	NA	NA	NA	D	PrD_PrD	D	MODERATE	D	0.925	175	175	0	268	241	27	10.07
Chewer	42482	chr10	92675585	G	C	*ANKRD1*	MISSENSE	ENSG00000148677:MISSENSE[ENST00000371697(c.704C>G,gCg>gGg):ENSP00000360762(p.Ala235Gly)];	chr10*9267	chr10*9267	NA	NA	NA	NA	NA	T	BN_BN	N	MODERATE	D	0.987	37	37	0	33	28	5	15.15
Chewer	42482	chr10	23493204	G	C	*C10orf115*	INTRONIC	ENSG00000204566:[ENST00000376501(c.203-20C>G)];	NA	NA	chr10*23493204*23493204*G*T*rs760003764*NA*NA*NA	NA	NA	NA	NA	NA	NA_NA	NA	MODIFIER	NA	NA	141	141	0	176	143	33	18.75
Chewer	42482	chr10	122357869	C	A	*C10orf85*	MISSENSE	ENSG00000177234:MISSENSE[ENST00000369071(c.47C>A,cCc>cAc):ENSP00000358067(p.Pro16His)];	NA	NA	NA	NA	NA	NA	NA	D_lc	PrD_PrD	D	MODERATE	NA	NA	81	81	0	77	70	7	9.09
Chewer	42482	chr10	32832232	C	G	*CCDC7*	MISSENSE	ENSG00000216937:EXONIC-NC[ENST00000493685(n.338C>G)];ENSG00000216937:MISSENSE[ENST00000277657(c.998C>G,aCt>aGt):ENSP00000277657(p.Thr333Ser)];ENSG00000216937:MISSENSE[ENST00000435402(c.5C>G,aCt>aGt):ENSP00000401923(p.Thr2Ser)];ENSG00000216937:MISSENSE[ENST00000362006(c.998C>G,aCt>aGt):ENSP00000355078(p.Thr333Ser)];ENSG00000216937:3UTR[ENST00000476558(c.*118C>G)];	NA	NA	NA	NA	NA	NA	NA	T	BN_BN	N	MODERATE	NA	NA	44	44	0	64	59	5	7.81
Chewer	42482	chr10	32344800	C	G	*KIF5B*	MISSENSE	ENSG00000170759:MISSENSE[ENST00000302418(c.102G>C,caG>caC):ENSP00000307078(p.Gln34His)];	NA	NA	NA	NA	NA	NA	NA	T	BN_PoD	N	MODERATE	D	0.998	55	55	0	140	119	21	15
Chewer	42482	chr10	27964120	G	T	*MKX*	3UTR	ENSG00000150051:[ENST00000375790(c.*38C>A)];ENSG00000150051:[ENST00000419761(c.*38C>A)];	NA	NA	chr10*27964119*27964120*CG*C*rs776903893*NA*NA*NA|chr10*27964120*27964120*G*A*rs750712873*NA*NA*NA	NA	NA	NA	NA	NA	NA_NA	NA	MODIFIER	NA	NA	104	104	0	160	135	25	15.62
Chewer	42482	chr10	21957927	G	T	*MLLT10*	INTRONIC	ENSG00000078403:[ENST00000446906(c.796-1451G>T)];ENSG00000078403:[ENST00000438473(c.16-1451G>T)];ENSG00000078403:[ENST00000377059(c.796-1451G>T)];ENSG00000078403:[ENST00000307729(c.796-1451G>T)];ENSG00000078403:[ENST00000377072(c.796-1451G>T)];ENSG00000078403:[ENST00000468309(n.27G>T)];	NA	NA	NA	NA	NA	NA	NA	NA	NA_NA	NA	MODIFIER	NA	NA	67	66	0	106	100	6	5.66
Chewer	42482	chr10	9281455	C	T	*NA*	INTERGENIC		NA	NA	NA	NA	NA	NA	NA	NA	NA_NA	NA	MODIFIER	NA	NA	86	86	0	129	111	18	13.95
Chewer	42482	chr10	11985129	C	T	*UPF2*	SILENT	ENSG00000151461:SILENT[ENST00000357604(c.3213G>A,gaG>gaA):ENSP00000350221(p.Glu1071(=))];ENSG00000151461:SILENT[ENST00000356352(c.3213G>A,gaG>gaA):ENSP00000348708(p.Glu1071(=))];ENSG00000151461:SILENT[ENST00000397053(c.3213G>A,gaG>gaA):ENSP00000380244(p.Glu1071(=))];	NA	NA	NA	8.26E-06	6.06E-05	NA	NA	NA	NA_NA	NA	LOW	NA	0.993	183	182	0	243	221	20	8.23
Chewer	42482	chr11	67431149	C	A	*ALDH3B2*	SILENT	ENSG00000132746:SILENT[ENST00000349015(c.957G>T,gtG>gtT):ENSP00000255084(p.Val319(=))];ENSG00000132746:SILENT[ENST00000530069(c.957G>T,gtG>gtT):ENSP00000431595(p.Val319(=))];ENSG00000132746:INTRONIC[ENST00000531248(c.160-279G>T)];	NA	NA	NA	NA	NA	NA	NA	NA	NA_NA	NA	MODIFIER	NA	NA	67	67	0	88	76	11	12.5
Chewer	42482	chr11	62678186	C	A	*AP000438.2*	EXONIC-NC	ENSG00000257002:[ENST00000543624(n.182C>A)];	NA	NA	NA	NA	NA	NA	NA	NA	NA_NA	NA	LOW	NA	NA	91	91	0	145	138	6	4.14
Chewer	42482	chr11	67132750	G	A	*AP003419.11*	INTRONIC	ENSG00000256514:[ENST00000543494(n.61+8245C>T)];	NA	NA	NA	8.33E-06	6.06E-05	NA	NA	T	PrD_PrD	D	MODERATE	D	0.878	69	69	0	91	85	6	6.59
Chewer	42482	chr11	62678186	C	A	*CHRM1*	SILENT	ENSG00000168539:SILENT[ENST00000536524(c.387G>T,cgG>cgT):ENSP00000444482(p.Arg129(=))];ENSG00000168539:SILENT[ENST00000543973(c.387G>T,cgG>cgT):ENSP00000441188(p.Arg129(=))];ENSG00000168539:SILENT[ENST00000306960(c.387G>T,cgG>cgT):ENSP00000306490(p.Arg129(=))];	NA	NA	NA	NA	NA	NA	NA	NA	NA_NA	NA	LOW	NA	NA	91	91	0	145	138	6	4.14
Chewer	42482	chr11	67132750	G	A	*CLCF1*	MISSENSE	ENSG00000175505:MISSENSE[ENST00000312438(c.535C>T,Ctc>Ttc):ENSP00000309338(p.Leu179Phe)];ENSG00000175505:MISSENSE[ENST00000528474(c.505C>T,Ctc>Ttc):ENSP00000432553(p.Leu169Phe)];ENSG00000175505:MISSENSE[ENST00000533438(c.505C>T,Ctc>Ttc):ENSP00000434122(p.Leu169Phe)];	NA	NA	NA	8.33E-06	6.06E-05	NA	NA	T	PrD_PrD	D	MODERATE	D	0.878	69	69	0	91	85	6	6.59
Chewer	42482	chr11	30926685	T	C	*DCDC1*	SILENT	ENSG00000170959:SILENT[ENST00000303697(c.193A>G,gaA>gaG):ENSP00000306898(p.Glu65(=))];ENSG00000170959:SILENT[ENST00000597505(c.4131A>G,gaA>gaG):ENSP00000472625(p.Glu1377(=))];ENSG00000170959:SILENT[ENST00000444572(c.1234A>G,gaA>gaG):ENSP00000404672(p.Glu412(=))];ENSG00000170959:SILENT[ENST00000339794(c.1368A>G,gaA>gaG):ENSP00000341700(p.Glu456(=))];ENSG00000170959:SILENT[ENST00000406071(c.336A>G,gaA>gaG):ENSP00000385936(p.Glu112(=))];ENSG00000170959:EXONIC-NC[ENST00000483396(n.1766A>G)];	NA	NA	NA	NA	NA	NA	NA	NA	NA_NA	NA	LOW	NA	0.574	109	109	0	136	119	17	12.5
Chewer	42482	chr11	58940188	G	T	*DTX4*	SILENT	ENSG00000110042:SILENT[ENST00000227451(c.120G>T,gcG>gcT):ENSP00000227451(p.Ala40(=))];ENSG00000110042:INTRONIC[ENST00000532982(c.-108+1227G>T)];	NA	NA	NA	2.34E-05	7.68E-05	NA	NA	NA	NA_NA	NA	MODIFIER	NA	NA	37	37	0	70	59	11	15.71
Chewer	42482	chr11	103228995	G	T	*DYNC2H1*	MISSENSE	ENSG00000187240:MISSENSE[ENST00000533197(c.25G>T,Ggc>Tgc):ENSP00000436736(p.Gly9Cys)];ENSG00000187240:MISSENSE[ENST00000398093(c.12085G>T,Ggc>Tgc):ENSP00000381167(p.Gly4029Cys)];ENSG00000187240:MISSENSE[ENST00000375735(c.12064G>T,Ggc>Tgc):ENSP00000364887(p.Gly4022Cys)];ENSG00000187240:3UTR[ENST00000528670(c.*110G>T)];ENSG00000187240:INTRONIC[ENST00000334267(c.2206-77676G>T)];	NA	NA	NA	NA	NA	NA	NA	NA	NA_NA	NA	MODIFIER	NA	NA	128	128	0	147	119	28	19.05
Chewer	42482	chr11	65639535	C	A	*EFEMP2*	INTRONIC	ENSG00000172638:[ENST00000307998(c.112-46G>T)];ENSG00000172638:[ENST00000530850(c.149+142G>T)];ENSG00000172638:[ENST00000531972(c.112-46G>T)];ENSG00000172638:[ENST00000529870(n.209-46G>T)];ENSG00000172638:[ENST00000526624(c.112-46G>T)];ENSG00000172638:[ENST00000533347(c.112-46G>T)];ENSG00000172638:[ENST00000528176(c.112-46G>T)];ENSG00000172638:[ENST00000527378(c.112-46G>T)];	NA	NA	NA	NA	NA	NA	NA	NA	NA_NA	NA	MODIFIER	NA	NA	56	56	0	106	81	25	23.58
Chewer	42482	chr11	128396949	C	A	*ETS1*	INTRONIC	ENSG00000134954:[ENST00000392668(c.214+29237G>T)];	NA	NA	NA	NA	NA	NA	NA	NA	NA_NA	NA	MODIFIER	NA	NA	87	87	0	181	168	13	7.18
Chewer	42482	chr11	134177163	T	A	*GLB1L3*	INTRONIC	ENSG00000166105:[ENST00000486034(n.536+61T>A)];ENSG00000166105:[ENST00000455971(c.17+61T>A)];ENSG00000166105:[ENST00000498012(n.1360-5080T>A)];ENSG00000166105:[ENST00000431683(c.961+61T>A)];	NA	NA	NA	NA	NA	NA	NA	NA	NA_NA	NA	MODIFIER	NA	NA	122	122	0	103	94	9	8.74
Chewer	42482	chr11	58478058	C	G	*GLYAT*	3UTR	ENSG00000149124:[ENST00000278400(c.*1G>C)];ENSG00000149124:[ENST00000529732(c.488+5G>C)];ENSG00000149124:[ENST00000344743(c.488+5G>C)];ENSG00000149124:[ENST00000586098(c.90+2175G>C)];	NA	NA	NA	NA	NA	NA	NA	NA	NA_NA	NA	LOW	NA	NA	92	92	0	150	138	12	8

Supplementary Table 4. List of somatic single nucleotide variants identified using whole exome sequencing of ESCC patients

Cohort	Sample	Chromosome	Genomic position	Reference allele	Alternate allele	Gene symbol	Variant type	Variant annotation	OncoMD	COSMIC	dbSNP	ExAC	ExAC_SAS	1000G	1000G_SAS	SIFT	Polyphen2	CONDEL	VEP	LRT	PhastCon7 Way	Total read (Adjacent normal)	Reference allel read support (Adjacent normal)	Alternative read support (Adjacent normal)	Total read (Tumor)	Reference allel read support (Tumor)	Alternative read support (Tumor)	Alternate allele frequency
Chewer	42482	chr11	105733011	A	T	*GRIA4*	INTRONIC	ENSG00000152578:[ENST00000525187(c.672+77A>T)];ENSG00000152578:[ENST00000530497(c.672+77A>T)];ENSG00000152578:[ENST00000282499(c.672+77A>T)];ENSG00000152578:[ENST00000428631(c.672+77A>T)];ENSG00000152578:[ENST00000393127(c.672+77A>T)];ENSG00000152578:[ENST00000393125(c.672+77A>T)];ENSG00000152578:[ENST00000527687(n.315+77A>T)];ENSG00000152578:[ENST00000525032(n.1075A>T)];	NA	NA	NA	NA	NA	NA	NA	NA	NA_NA	NA	MODIFIER	NA	NA	82	82	0	108	82	26	24.07
Chewer	42482	chr11	120742551	G	T	*GRIK4*	INTRONIC	ENSG00000149403:[ENST00000438375(c.907-2224G>T)];ENSG00000149403:[ENST00000533291(n.1305-2224G>T)];ENSG00000149403:[ENST00000527524(c.907-2224G>T)];	NA	NA	NA	NA	NA	NA	NA	NA	NA_NA	NA	MODIFIER	NA	NA	139	139	0	219	203	16	7.31
Chewer	42482	chr11	101832870	A	G	*KIAA1377*	SILENT	ENSG00000110318:SILENT[ENST00000537689(c.507A>G,gtA>gtG):ENSP00000443184(p.Val169(=))];ENSG00000110318:SILENT[ENST00000263468(c.1104A>G,gtA>gtG):ENSP00000263468(p.Val368(=))];ENSG00000110318:3UTR[ENST00000532529(c.*600A>G)];	NA	NA	NA	8.31E-06	0	NA	NA	NA	NA_NA	NA	LOW	NA	NA	170	170	0	175	147	27	15.43
Chewer	42482	chr11	18472520	G	T	*LDHC*	MISSENSE	ENSG00000166796:MISSENSE[ENST00000280704(c.845G>T,gGa>gTa):ENSP00000280704(p.Gly282Val)];ENSG00000166796:MISSENSE[ENST00000536880(c.803G>T,gGa>gTa):ENSP00000439555(p.Gly268Val)];ENSG00000166796:MISSENSE[ENST00000541669(c.845G>T,gGa>gTa):ENSP00000437783(p.Gly282Val)];ENSG00000166796:MISSENSE[ENST00000535809(c.603G>T,tgG>tgT):ENSP00000443997(p.Trp201Cys)];ENSG00000166796:MISSENSE[ENST00000537486(c.429G>T,tgG>tgT):ENSP00000441478(p.Trp143Cys)];ENSG00000166796:NONSENSE[ENST00000546146(c.547G>T,Gaa>Taa):ENSP00000443414(p.Glu183Ter)];ENSG00000166796:NONSENSE[ENST00000544105(c.721G>T,Gaa>Taa):ENSP00000439060(p.Glu241Ter)];ENSG00000166796:3UTR[ENST00000545848(c.*329G>T)];ENSG00000166796:3UTR[ENST00000396215(c.*571G>T)];ENSG00000166796:3UTR[ENST00000539369(c.*415G>T)];	NA	NA	NA	NA	NA	NA	NA	NA	NA_NA	NA	MODIFIER	NA	NA	68	68	0	88	79	9	10.23
Chewer	42482	chr11	102576469	C	A	*MMP27*	5UTR	ENSG00000137675:[ENST00000260229(c.-24G>T)];	NA	NA	chr11*102576469*102576469*C*T*rs374493970*NA*NA*NA	NA	NA	NA	NA	NA	NA_NA	NA	MODIFIER	NA	NA	129	128	0	174	151	23	13.22
Chewer	42482	chr11	92714702	G	C	*MTNR1B*	MISSENSE	ENSG00000134640:MISSENSE[ENST00000257068(c.313G>C,Ggc>Cgc):ENSP00000257068(p.Gly105Arg)];ENSG00000134640:INTRONIC[ENST00000528076(c.166-3271G>C)];ENSG00000134640:3UTR[ENST00000532482(c.*204G>C)];	chr11*9271	chr11*9271	chr11*92714702*92714702*G*A*rs762043101*NA*NA*NA	NA	NA	NA	NA	NA	NA_NA	NA	MODIFIER	NA	NA	95	95	0	123	102	21	17.07
Chewer	42482	chr11	61547004	G	A	*MYRF*	MISSENSE	ENSG00000124920:MISSENSE[ENST00000278836(c.2209G>A,Gtc>Atc):ENSP00000278836(p.Val737Ile)];ENSG00000124920:MISSENSE[ENST00000389602(c.382G>A,Gtc>Atc):ENSP00000374253(p.Val128Ile)];ENSG00000124920:MISSENSE[ENST00000265460(c.2182G>A,Gtc>Atc):ENSP00000265460(p.Val728Ile)];ENSG00000124920:MISSENSE[ENST00000327797(c.1141G>A,Gtc>Atc):ENSP00000333261(p.Val381Ile)];ENSG00000124920:EXONIC-NC[ENST00000536352(n.704G>A)];ENSG00000124920:EXONIC-NC[ENST00000537318(n.630G>A)];	NA	NA	NA	0.0001739	0.000866551	NA	NA	NA	NA_NA	NA	MODIFIER	NA	NA	22	22	0	34	26	8	23.53
Chewer	42482	chr11	47282923	C	A	*NR1H3*	MISSENSE	ENSG00000025434:MISSENSE[ENST00000407404(c.631C>A,Ctg>Atg):ENSP00000385801(p.Leu211Met)];ENSG00000025434:MISSENSE[ENST00000531660(c.229C>A,Ctg>Atg):ENSP00000434650(p.Leu77Met)];ENSG00000025434:MISSENSE[ENST00000481889(c.496C>A,Ctg>Atg):ENSP00000433271(p.Leu166Met)];ENSG00000025434:MISSENSE[ENST00000405853(c.631C>A,Ctg>Atg):ENSP00000384745(p.Leu211Met)];ENSG00000025434:MISSENSE[ENST00000436029(c.631C>A,Ctg>Atg):ENSP00000403696(p.Leu211Met)];ENSG00000025434:MISSENSE[ENST00000395397(c.496C>A,Ctg>Atg):ENSP00000378793(p.Leu166Met)];ENSG00000025434:MISSENSE[ENST00000441012(c.631C>A,Ctg>Atg):ENSP00000387946(p.Leu211Met)];ENSG00000025434:MISSENSE[ENST00000467728(c.631C>A,Ctg>Atg):ENSP00000420656(p.Leu211Met)];ENSG00000025434:MISSENSE[ENST00000527949(c.358C>A,Ctg>Atg):ENSP00000432073(p.Leu120Met)];ENSG00000025434:MISSENSE[ENST00000405576(c.496C>A,Ctg>Atg):ENSP00000385073(p.Leu166Met)];ENSG00000025434:EXONIC-NC[ENST00000481020(n.664C>A)];ENSG00000025434:EXONIC-NC[ENST00000525441(n.561C>A)];ENSG00000025434:EXONIC-NC[ENST00000527464(n.870C>A)];ENSG00000025434:EXONIC-NC[ENST00000487913(n.205C>A)];ENSG00000025434:EXONIC-NC[ENST00000529540(n.818C>A)];ENSG00000025434:INTRONIC[ENST00000532630(n.74-6541C>A)];	NA	NA	NA	NA	NA	NA	NA	NA	NA_NA	NA	MODIFIER	NA	NA	112	112	0	167	152	15	8.98
Chewer	42482	chr11	57970826	G	A	*OR1S2*	SILENT	ENSG00000197887:SILENT[ENST00000302592(c.828C>T,tcC>tcT):ENSP00000305469(p.Ser276(=))];	chr11*5797	chr11*5797	NA	NA	NA	NA	NA	NA	NA_NA	NA	LOW	NA	NA	292	292	0	397	375	22	5.54
Chewer	42482	chr11	55322196	C	A	*OR4C15*	SILENT	ENSG00000181939:SILENT[ENST00000314644(c.414C>A,tcC>tcA):ENSP00000324958(p.Ser138(=))];	NA	NA	NA	NA	NA	NA	NA	NA	NA_NA	NA	LOW	NA	NA	124	123	0	164	142	21	12.8
Chewer	42482	chr11	56019784	A	T	*OR5T3*	MISSENSE	ENSG00000172489:MISSENSE[ENST00000303059(c.109A>T,Act>Tct):ENSP00000305403(p.Thr37Ser)];	NA	NA	NA	NA	NA	NA	NA	T	BN_PoD	N	MODERATE	D	0.026	80	80	0	92	78	14	15.22
Chewer	42482	chr11	56019926	G	T	*OR5T3*	MISSENSE	ENSG00000172489:MISSENSE[ENST00000303059(c.251G>T,tGg>tTg):ENSP00000305403(p.Trp84Leu)];	NA	NA	chr11*56019926*56019926*G*A*rs557388691*NA*NA*NA	NA	NA	NA	NA	D	BN_BN	N	MODERATE	U	0.029	101	101	0	118	107	11	9.32
Chewer	42482	chr11	124096062	C	A	*OR8G2P*	EXONIC-NC	ENSG00000181214:[ENST00000412796(n.626C>A)];ENSG00000181214:[ENST00000366149(n.626C>A)];	chr11*1240	NA	chr11*124096062*124096062*C*T*rs186627433*1*0.9978*0.002196	NA	NA	NA	NA	NA	NA_NA	NA	MODIFIER	NA	NA	183	183	0	245	221	24	9.8
Chewer	42482	chr11	56085981	G	C	*OR8K3*	MISSENSE	ENSG00000181689:MISSENSE[ENST00000312711(c.199G>C,Gct>Cct):ENSP00000323555(p.Ala67Pro)];	chr11*5608	chr11*5608	NA	NA	NA	NA	NA	D_lc	PrD_PrD	D	MODERATE	D	0.73	249	248	0	245	208	37	15.1
Chewer	42482	chr11	72299851	G	A	*PDE2A*	SILENT	ENSG00000186642:SILENT[ENST00000540345(c.1020C>T,gcC>gcT):ENSP00000446399(p.Ala340(=))];ENSG00000186642:SILENT[ENST00000334456(c.1047C>T,gcC>gcT):ENSP00000334910(p.Ala349(=))];ENSG00000186642:SILENT[ENST00000418754(c.702C>T,gcC>gcT):ENSP00000410310(p.Ala234(=))];ENSG00000186642:SILENT[ENST00000475807(c.519C>T,gcC>gcT):ENSP00000439077(p.Ala173(=))];ENSG00000186642:SILENT[ENST00000544570(c.1026C>T,gcC>gcT):ENSP00000442256(p.Ala342(=))];ENSG00000186642:SILENT[ENST00000444035(c.1020C>T,gcC>gcT):ENSP00000411657(p.Ala340(=))];ENSG00000186642:SILENT[ENST00000538299(c.331C>T,gcC>gcT):ENSP00000441322(p.Ala111(=))];ENSG00000186642:3UTR[ENST00000539367(c.*890C>T)];ENSG00000186642:INTRONIC[ENST00000376450(c.303-2626C>T)];	NA	NA	NA	NA	NA	NA	NA	NA	NA_NA	NA	MODIFIER	NA	NA	70	70	0	106	95	11	10.38

Mangalaparthi *et al.* , 2020. Mutational landscape of esophageal squamous cell carcinoma in an Indian cohort
Supplementary Table 4. List of somatic single nucleotide variants identified using whole exome sequencing of ESCC patients

Cohort	Sample	Chromosome	Genomic position	Reference allele	Alternate allele	Gene symbol	Variant type	Variant annotation	OncoMD	COSMIC	dbSNP	ExAC	ExAC_SAS	1000G	1000G_SAS	SIFT	Polyphen2	CONDEL	VEP	LRT	PhastCon7 Way	Total read (Adjacent normal)	Reference allel read support (Adjacent normal)	Alternative read support (Adjacent normal)	Total read (Tumor)	Reference allel read support (Tumor)	Alternative read support (Tumor)	Alternate allele frequency
Chewer	42482	chr11	107799312	C	T	*RAB39A*	SILENT	ENSG00000179331:SILENT[ENST00000320578(c.18C>T,atC>atT):ENSP00000322594(p.Ile6(=))];	NA	NA	NA	NA	NA	NA	NA	NA	NA_NA	NA	LOW	NA	1	100	100	0	159	132	27	16.98
Chewer	42482	chr11	128396949	C	A	*RP11-1007G5.2*	EXONIC-NC	ENSG00000254588:[ENST00000525706(n.696C>A)];	NA	NA	NA	NA	NA	NA	NA	NA	NA_NA	NA	MODIFIER	NA	NA	87	87	0	181	168	13	7.18
Chewer	42482	chr11	120742551	G	T	*RP11-640N11.2*	EXONIC-NC	ENSG00000250493:[ENST00000505153(n.2405C>A)];	NA	NA	NA	NA	NA	NA	NA	NA	NA_NA	NA	MODIFIER	NA	NA	139	139	0	219	203	16	7.31
Chewer	42482	chr11	62886534	G	T	*SLC22A24*	MISSENSE	ENSG00000197658:MISSENSE[ENST00000326192(c.680C>A,cCc>cAc):ENSP00000321549(p.Pro227His)];ENSG00000197658:MISSENSE[ENST00000417740(c.680C>A,cCc>cAc):ENSP00000396586(p.Pro227His)];	NA	NA	NA	NA	NA	NA	NA	T	BN_BN	N	MODERATE	U	0.003	76	76	0	95	81	14	14.74
Chewer	42482	chr11	61547004	G	A	*TMEM258*	INTRONIC	ENSG00000134825:[ENST00000535042(n.249-449C>T)];	NA	NA	NA	0.0001739	0.000866551	NA	NA	NA	NA_NA	NA	MODIFIER	NA	NA	22	22	0	34	26	8	23.53
Chewer	42482	chr11	101344424	T	C	*TRPC6*	MISSENSE	ENSG00000137672:MISSENSE[ENST00000532133(c.1591A>G,Agg>Ggg):ENSP00000435574(p.Arg531Gly)];ENSG00000137672:MISSENSE[ENST00000348423(c.1477A>G,Agg>Ggg):ENSP00000343672(p.Arg493Gly)];ENSG00000137672:MISSENSE[ENST00000344327(c.1825A>G,Agg>Ggg):ENSP00000340913(p.Arg609Gly)];ENSG00000137672:MISSENSE[ENST00000360497(c.1660A>G,Agg>Ggg):ENSP00000353687(p.Arg554Gly)];	NA	NA	NA	NA	NA	NA	NA	D	PrD_PrD	D	MODERATE	N	0.988	44	44	0	79	69	10	12.66
Chewer	42482	chr12	22061102	C	A	*ABCC9*	MISSENSE	ENSG00000069431:MISSENSE[ENST00000345162(c.1364G>T,aGt>aTt):ENSP00000261202(p.Ser455Ile)];ENSG00000069431:MISSENSE[ENST00000261201(,aGt>aTt):ENSP00000261201(p.Ser455Ile)];ENSG00000069431:MISSENSE[ENST00000544039(c.353G>T,aGt>aTt):ENSP00000440521(p.Ser118Ile)];ENSG00000069431:MISSENSE[ENST00000261200(c.1364G>T,aGt>aTt):ENSP00000261200(p.Ser455Ile)];	NA	NA	chr12*22061102*22061102*C*G*rs775938318*NA*NA*NA	NA	NA	NA	NA	D	PoD_PoD	D	MODERATE	D	0.973	213	213	0	273	221	52	19.05
Chewer	42482	chr12	22061137	C	A	*ABCC9*	MISSENSE	ENSG00000069431:MISSENSE[ENST00000261201(c.1329G>T,atG>atT):ENSP00000261201(p.Met443Ile)];ENSG00000069431:MISSENSE[ENST00000261200(c.1329G>T,atG>atT):ENSP00000261200(p.Met443Ile)];ENSG00000069431:MISSENSE[ENST00000345162(c.1329G>T,atG>atT):ENSP00000261202(p.Met443Ile)];ENSG00000069431:MISSENSE[ENST00000544039(c.318G>T,atG>atT):ENSP00000440521(p.Met106Ile)];	chr12*2206	chr12*2206	chr12*22061137*22061137*C*T*rs151197166*0*0.9996*0.0003994	NA	NA	NA	NA	T	BN_BN	N	MODERATE	D	NA	166	166	0	224	181	43	19.2
Chewer	42482	chr12	109577798	G	T	*ACACB*	MISSENSE	ENSG00000076555:MISSENSE[ENST00000377854(c.588G>T,ttG>ttT):ENSP00000367085(p.Leu196Phe)];ENSG00000076555:MISSENSE[ENST00000377848(c.588G>T,ttG>ttT):ENSP00000367079(p.Leu196Phe)];ENSG00000076555:MISSENSE[ENST00000338432(c.588G>T,ttG>ttT):ENSP00000341044(p.Leu196Phe)];ENSG00000076555:EXONIC-NC[ENST00000546328(n.15G>T)];	NA	NA	NA	NA	NA	NA	NA	NA	NA_NA	NA	MODIFIER	NA	NA	84	84	0	106	96	10	9.43
Chewer	42482	chr12	122460114	C	G	*BCL7A*	INTRONIC	ENSG00000110987:[ENST00000261822(c.92+25C>G)];ENSG00000110987:[ENST00000432926(n.206+25C>G)];ENSG00000110987:[ENST00000538010(c.92+25C>G)];	NA	NA	NA	7.10E-05	0	NA	NA	NA	NA_NA	NA	MODIFIER	NA	NA	80	80	0	143	130	13	9.09
Chewer	42482	chr12	2162670	G	C	*CACNA1C*	INTRONIC	ENSG00000151067:[ENST00000543114(c.140-61720G>C)];ENSG00000151067:[ENST00000399655(c.-59G>C)];ENSG00000151067:[ENST00000335762(c.-59G>C)];	NA	NA	NA	NA	NA	NA	NA	NA	NA_NA	NA	MODIFIER	NA	NA	32	32	0	68	49	19	27.94
Chewer	42482	chr12	1995238	G	A	*CACNA2D4*	INTRONIC	ENSG00000151062:[ENST00000280663(n.1187-33C>T)];ENSG00000151062:[ENST00000585732(c.782-73C>T)];ENSG00000151062:[ENST00000587995(c.994-33C>T)];ENSG00000151062:[ENST00000586184(c.994-33C>T)];ENSG00000151062:[ENST00000382722(c.994-33C>T)];ENSG00000151062:[ENST00000444595(c.994-33C>T)];ENSG00000151062:[ENST00000588077(c.802-33C>T)];ENSG00000151062:[ENST00000585708(c.802-33C>T)];	NA	NA	NA	NA	NA	NA	NA	NA	NA_NA	NA	MODIFIER	NA	NA	68	68	0	94	80	14	14.89
Chewer	42482	chr12	111321838	C	G	*CCDC63*	SILENT	ENSG00000173093:SILENT[ENST00000308208(c.858C>G,ctC>ctG):ENSP00000312399(p.Leu286(=))];ENSG00000173093:SILENT[ENST00000552694(c.621C>G,ctC>ctG):ENSP00000450217(p.Leu207(=))];ENSG00000173093:SILENT[ENST00000545036(c.738C>G,ctC>ctG):ENSP00000445881(p.Leu246(=))];	NA	NA	NA	NA	NA	NA	NA	NA	NA_NA	NA	LOW	NA	NA	27	27	0	38	32	6	15.79
Chewer	42482	chr12	58145052	G	C	*CDK4*	MISSENSE	ENSG00000135446:MISSENSE[ENST00000552254(c.292C>G,Cag>Gag):ENSP00000449179(p.Gln98Glu)];ENSG00000135446:MISSENSE[ENST00000547281(c.70C>G,Cag>Gag):ENSP00000447274(p.Gln24Glu)];ENSG00000135446:MISSENSE[ENST00000552862(c.292C>G,Cag>Gag):ENSP00000446763(p.Gln98Glu)];ENSG00000135446:MISSENSE[ENST00000551800(c.70C>G,Cag>Gag):ENSP00000449391(p.Gln24Glu)];ENSG00000135446:MISSENSE[ENST00000546489(c.70C>G,Cag>Gag):ENSP00000447779(p.Gln24Glu)];ENSG00000135446:MISSENSE[ENST00000257904(c.292C>G,Cag>Gag):ENSP00000257904(p.Gln98Glu)];ENSG00000135446:MISSENSE[ENST00000552388(c.292C>G,Cag>Gag):ENSP00000448963(p.Gln98Glu)];ENSG00000135446:MISSENSE[ENST00000550419(c.292C>G,Cag>Gag):ENSP00000448098(p.Gln98Glu)];ENSG00000135446:INTRONIC[ENST00000540325(c.-6-179C>G)];ENSG00000135446:INTRONIC[ENST00000553237(c.219-179C>G)];ENSG00000135446:INTRONIC[ENST00000549606(c.-158+906C>G)];ENSG00000135446:INTRONIC[ENST00000312990(c.264+28C>G)];ENSG00000135446:INTRONIC[ENST00000551888(n.442+28C>G)];ENSG00000135446:EXONIC-NC[ENST00000551706(n.658C>G)];	NA	NA	NA	NA	NA	NA	NA	NA	NA_NA	NA	MODIFIER	NA	NA	78	78	0	119	110	9	7.56
Chewer	42482	chr12	48371128	C	G	*COL2A1*	MISSENSE	ENSG00000139219:MISSENSE[ENST00000380518(c.3248G>C,gGc>gCc):ENSP00000369889(p.Gly1083Ala)];ENSG00000139219:MISSENSE[ENST00000337299(c.3041G>C,gGc>gCc):ENSP00000338213(p.Gly1014Ala)];ENSG00000139219:EXONIC-NC[ENST00000493991(n.2334G>C)];ENSG00000139219:EXONIC-NC[ENST00000546974(n.101G>C)];	NA	NA	NA	NA	NA	NA	NA	NA	NA_NA	NA	MODIFIER	NA	NA	80	80	0	120	96	24	20
Chewer	42482	chr12	124256254	G	T	*DNAH10*	INTRONIC-SS-DNR	ENSG00000197653:[ENST00000409039(c.213+9G>T)];	NA	NA	chr12*124256254*124256254*G*A*rs766533805*NA*NA*NA	NA	NA	NA	NA	NA	NA_NA	NA	MODIFIER	NA	NA	79	79	0	96	85	11	11.46
Chewer	42482	chr12	124366278	G	C	*DNAH10*	MISSENSE	ENSG00000197653:3UTR[ENST00000497783(c.*1437G>C)];ENSG00000197653:MISSENSE[ENST00000409039(c.8387G>C,cGc>cCc):ENSP00000386770(p.Arg2796Pro)];	chr12*1243	chr12*1243	chr12*124366278*124366278*G*A*rs763152052*NA*NA*NA	NA	NA	NA	NA	NA	PoD_PoD	NA	MODERATE	U	0.962	143	143	0	176	157	19	10.8
Chewer	42482	chr12	3747503	C	T	*EFCAB4B*	SILENT	ENSG00000130038:3UTR[ENST00000333750(c.*386G>A)];ENSG00000130038:SILENT[ENST00000440314(c.1389G>A,ccG>ccA):ENSP00000409382(p.Pro463(=))];	NA	NA	NA	5.33E-05	0.000126486	NA	NA	NA	NA_NA	NA	LOW	NA	NA	80	78	2	115	84	30	26.09

Supplementary Table 4. List of somatic single nucleotide variants identified using whole exome sequencing of ESCC patients

Cohort	Sample	Chromosome	Genomic position	Reference allele	Alternate allele	Gene symbol	Variant type	Variant annotation	OncoMD	COSMIC	dbSNP	ExAC	ExAC_SAS	1000G	1000G_SAS	SIFT	Polyphen2	CONDEL	VEP	LRT	PhastCon7 Way	Total read (Adjacent normal)	Reference allel read support (Adjacent normal)	Alternative read support (Adjacent normal)	Total read (Tumor)	Reference allel read support (Tumor)	Alternative read support (Tumor)	Alternate allele frequency
Chewer	42482	chr12	52913992	G	T	*KRT5*	MISSENSE	ENSG00000186081:MISSENSE[ENST00000546577(c.89C>A,aCc>aAc):ENSP00000449651(p.Thr30Asn)];ENSG00000186081:MISSENSE[ENST00000551275(c.89C>A,aCc>aAc):ENSP00000448041(p.Thr30Asn)];ENSG00000186081:MISSENSE[ENST00000252242(c.89C>A,aCc>aAc):ENSP00000252242(p.Thr30Asn)];ENSG00000186081:EXONIC-NC[ENST00000552629(n.187C>A)];ENSG00000186081:INTRONIC[ENST00000549420(c.43+46C>A)];	NA	NA	NA	NA	NA	NA	NA	NA	NA_NA	NA	MODIFIER	NA	NA	44	44	0	55	46	9	16.36
Chewer	42482	chr12	40834954	G	T	*MUC19*	MISSENSE	ENSG00000205592:MISSENSE[ENST00000454784(c.1961G>T,tGc>tTc):ENSP00000476404(p.Cys654Phe)];ENSG00000205592:3UTR[ENST00000425730(c.*2023G>T)];	NA	NA	NA	NA	NA	NA	NA	NA	NA_NA	NA	MODIFIER	NA	NA	202	199	2	281	257	24	8.54
Chewer	42482	chr12	81101770	C	A	*MYF6*	MISSENSE	ENSG00000111046:MISSENSE[ENST00000228641(c.272C>A,cCc>cAc):ENSP00000228641(p.Pro91His)];	NA	NA	chr12*81101770*81101770*C*T*rs372392737*NA*NA*NA	NA	NA	NA	NA	D	PrD_PrD	D	MODERATE	D	0.984	127	127	0	166	158	8	4.82
Chewer	42482	chr12	133250005	C	T	*POLE*	INTRONIC	ENSG00000177084:[ENST00000537064(c.*407-142G>A)];ENSG00000177084:[ENST00000320574(c.1360-142G>A)];ENSG00000177084:[ENST00000535934(c.214-142G>A)];ENSG00000177084:[ENST00000539215(n.68-142G>A)];ENSG00000177084:[ENST00000535270(c.1279-142G>A)];ENSG00000177084:[ENST00000539006(c.700-142G>A)];	NA	NA	chr12*133250005*133250005*C*A*rs531921107*0*0.9998*0.0001997	NA	NA	NA	NA	NA	NA_NA	NA	MODIFIER	NA	NA	48	48	0	82	76	6	7.32
Chewer	42482	chr12	11286689	G	C	*PRR4*	INTRONIC	ENSG00000111215:[ENST00000536668(n.177+37332C>G)];ENSG00000111215:[ENST00000546265(n.188-12910C>G)];ENSG00000111215:[ENST00000541175(n.40-12910C>G)];ENSG00000111215:[ENST00000541977(n.123+37332C>G)];ENSG00000111215:[ENST00000535024(n.192+37332C>G)];	NA	NA	chr12*11286689*11286689*G*A*rs374458254*NA*NA*NA	NA	NA	NA	NA	D	PrD_PrD	D	MODERATE	NA	NA	202	201	0	249	235	13	5.22
Chewer	42482	chr12	15654959	C	T	*PTPRO*	MISSENSE	ENSG00000151490:MISSENSE[ENST00000543886(c.1067C>T,aCt>aTt):ENSP00000444173(p.Thr356Ile)];ENSG00000151490:MISSENSE[ENST00000348962(c.1067C>T,aCt>aTt):ENSP00000343434(p.Thr356Ile)];ENSG00000151490:MISSENSE[ENST00000281171(c.1067C>T,aCt>aTt):ENSP00000281171(p.Thr356Ile)];	NA	NA	NA	NA	NA	NA	NA	D_lc	PoD_PrD	D	MODERATE	D	0.989	207	206	0	248	230	17	6.85
Chewer	42482	chr12	80848868	G	T	*PTPRQ*	INTRONIC	ENSG00000139304:[ENST00000266688(c.402-6759G>T)];	NA	NA	NA	NA	NA	NA	NA	NA	NA_NA	NA	MODIFIER	NA	NA	47	47	0	84	79	5	5.95
Chewer	42482	chr12	40834954	G	T	*RP11-115F18.1*	INTRONIC	ENSG00000258167:[ENST00000552757(n.25+2671C>A)];	NA	NA	NA	NA	NA	NA	NA	NA	NA_NA	NA	MODIFIER	NA	NA	202	199	2	281	257	24	8.54
Chewer	42482	chr12	335496	G	T	*SLC6A13*	INTRONIC	ENSG00000010379:[ENST00000543722(n.290+60C>A)];ENSG00000010379:[ENST00000542379(n.220-3096C>A)];ENSG00000010379:[ENST00000445055(c.784+60C>A)];ENSG00000010379:[ENST00000542947(n.444+60C>A)];ENSG00000010379:[ENST00000343164(c.1060+60C>A)];	NA	NA	chr12*335496*335496*G*A*rs866403465*NA*NA*NA	NA	NA	NA	NA	NA	NA_NA	NA	MODIFIER	NA	NA	43	43	0	65	51	14	21.54
Chewer	42482	chr12	98849000	A	T	*SLC9A7P1*	EXONIC-NC	ENSG00000227825:[ENST00000556476(n.1894T>A)];ENSG00000227825:[ENST00000554295(n.1924T>A)];ENSG00000227825:[ENST00000370696(n.1924T>A)];	NA	NA	NA	NA	NA	NA	NA	NA	NA_NA	NA	MODIFIER	NA	NA	34	33	0	49	44	5	10.2
Chewer	42482	chr12	21392010	G	T	*SLCO1B1*	MISSENSE	ENSG00000134538:MISSENSE[ENST00000256958(c.1963G>T,Gat>Tat):ENSP00000256958(p.Asp655Tyr)];	NA	NA	NA	NA	NA	NA	NA	D	PrD_PrD	D	MODERATE	N	0.063	76	76	0	114	89	24	21.05
Chewer	42482	chr12	11286689	G	C	*TAS2R14*	INTRONIC	ENSG00000212127:[ENST00000381852(n.152+37332C>G)];	NA	NA	chr12*11286689*11286689*G*A*rs374458254*NA*NA*NA	NA	NA	NA	NA	D	PrD_PrD	D	MODERATE	NA	NA	202	201	0	249	235	13	5.22
Chewer	42482	chr12	11286689	G	C	*TAS2R30*	MISSENSE	ENSG00000256188:MISSENSE[ENST00000539585(c.155C>G,gCg>gGg):ENSP00000444736(p.Ala52Gly)];	NA	NA	chr12*11286689*11286689*G*A*rs374458254*NA*NA*NA	NA	NA	NA	NA	D	PrD_PrD	D	MODERATE	NA	NA	202	201	0	249	235	13	5.22
Chewer	42482	chr12	128899948	C	A	*TMEM132C*	MISSENSE	ENSG00000181234:MISSENSE[ENST00000435159(c.757C>A,Cgt>Agt):ENSP00000410852(p.Arg253Ser)];	chr12*1288	NA	NA	NA	NA	NA	NA	D	PoD_PoD	D	MODERATE	NA	NA	108	107	1	128	102	26	20.31
Chewer	42482	chr13	74420369	C	A	*KLF12*	MISSENSE	ENSG00000118922:MISSENSE[ENST00000377666(c.265G>T,Gcc>Tcc):ENSP00000366894(p.Ala89Ser)];ENSG00000118922:MISSENSE[ENST00000377669(c.265G>T,Gcc>Tcc):ENSP00000366897(p.Ala89Ser)];ENSG00000118922:EXONIC-NC[ENST00000472022(n.299G>T)];	NA	NA	NA	NA	NA	NA	NA	NA	NA_NA	NA	MODIFIER	NA	NA	65	65	0	89	47	42	47.19
Chewer	42482	chr13	70275859	C	A	*KLHL1*	MISSENSE	ENSG00000150361:MISSENSE[ENST00000545028(c.1643G>T,tGt>tTt):ENSP00000439602(p.Cys548Phe)];ENSG00000150361:MISSENSE[ENST00000377844(c.2222G>T,tGt>tTt):ENSP00000367075(p.Cys741Phe)];	NA	NA	NA	NA	NA	NA	NA	D	PrD_PoD	D	MODERATE	D	1	60	60	0	49	43	6	12.24
Chewer	42482	chr13	50121278	G	A	*RCBTB1*	INTRONIC	ENSG00000136144:[ENST00000258646(c.1046-2279C>T)];ENSG00000136144:[ENST00000378302(c.1046-2279C>T)];ENSG00000136144:[ENST00000546015(c.*7C>T)];	NA	NA	NA	NA	NA	NA	NA	NA	NA_NA	NA	MODIFIER	NA	NA	46	46	0	66	52	14	21.21
Chewer	42482	chr13	25436812	G	A	*RNF17*	INTRONIC	ENSG00000132972:[ENST00000255324(c.3895-39G>A)];ENSG00000132972:[ENST00000381921(c.3895-39G>A)];ENSG00000132972:[ENST00000339524(c.1051-39G>A)];ENSG00000132972:[ENST00000418120(c.1867-39G>A)];	NA	NA	NA	NA	NA	NA	NA	NA	NA_NA	NA	MODIFIER	NA	NA	58	58	0	67	52	15	22.39
Chewer	42482	chr13	26788155	T	A	*RNF6*	MISSENSE	ENSG00000127870:MISSENSE[ENST00000346166(c.1864A>T,Aac>Tac):ENSP00000342121(p.Asn622Tyr)];ENSG00000127870:MISSENSE[ENST00000381570(c.1864A>T,Aac>Tac):ENSP00000370982(p.Asn622Tyr)];ENSG00000127870:MISSENSE[ENST00000381588(c.1864A>T,Aac>Tac):ENSP00000371000(p.Asn622Tyr)];ENSG00000127870:MISSENSE[ENST00000399762(c.796A>T,Aac>Tac):ENSP00000382665(p.Asn266Tyr)];ENSG00000127870:INTRONIC[ENST00000468480(n.768+1456A>T)];	NA	NA	NA	NA	NA	NA	NA	T	BN_PoD	N	MODERATE	N	0.039	113	113	0	153	115	38	24.84
Chewer	42482	chr14	105350413	G	C	*CEP170B*	MISSENSE	ENSG00000099814:MISSENSE[ENST00000414716(c.1297G>C,Gac>Cac):ENSP00000404151(p.Asp433His)];ENSG00000099814:MISSENSE[ENST00000556508(c.1087G>C,Gac>Cac):ENSP00000451249(p.Asp363His)];ENSG00000099814:MISSENSE[ENST00000418279(c.1087G>C,Gac>Cac):ENSP00000415006(p.Asp363His)];ENSG00000099814:MISSENSE[ENST00000453495(c.1300G>C,Gac>Cac):ENSP00000407238(p.Asp434His)];	NA	NA	chr14*105350413*105350413*G*A*rs377006406*NA*NA*NA	NA	NA	NA	NA	D	PrD_PrD	D	MODERATE	N	0.972	78	78	0	91	80	11	12.09
Chewer	42482	chr14	103568472	G	T	*EXOC3L4*	MISSENSE	ENSG00000205436:MISSENSE[ENST00000380069(c.412G>T,Gac>Tac):ENSP00000369409(p.Asp138Tyr)];ENSG00000205436:INTRONIC[ENST00000559116(c.286+1522G>T)];	NA	NA	NA	0	0	NA	NA	NA	NA_NA	NA	MODIFIER	NA	NA	40	40	0	84	69	15	17.86
Chewer	42482	chr14	107034849	G	T	*IGHV5-51*	SILENT	ENSG00000211966:SILENT[ENST00000390626(c.231C>A,acC>acA):ENSP00000375035(p.Thr77(=))];	NA	NA	chr14*107034849*107034849*G*A*rs782596821*NA*NA*NA	NA	NA	NA	NA	NA	NA_NA	NA	LOW	NA	NA	201	125	0	282	165	23	8.16

Mangalaparthi *et al.* , 2020. Mutational landscape of esophageal squamous cell carcinoma in an Indian cohort
Supplementary Table 4. List of somatic single nucleotide variants identified using whole exome sequencing of ESCC patients

Cohort	Sample	Chromosome	Genomic position	Reference allele	Alternate allele	Gene symbol	Variant type	Variant annotation	OncoMD	COSMIC	dbSNP	ExAC	ExAC_SAS	1000G	1000G_SAS	SIFT	Polyphen2	CONDEL	VEP	LRT	PhastCon7 Way	Total read (Adjacent normal)	Reference allel read support (Adjacent normal)	Alternative read support (Adjacent normal)	Total read (Tumor)	Reference allel read support (Tumor)	Alternative read support (Tumor)	Alternate allele frequency
Chewer	42482	chr14	104121186	G	A	*KLC1*	INTRONIC	ENSG00000126214:[ENST00000557172(c.261+24G>A)];ENSG00000126214:[ENST00000246489(c.261+24G>A)];ENSG00000126214:[ENST00000348520(c.261+24G>A)];ENSG00000126214:[ENST00000452929(c.261+24G>A)];ENSG00000126214:[ENST00000380038(c.261+24G>A)];ENSG00000126214:[ENST00000557575(c.261+24G>A)];ENSG00000126214:[ENST00000445352(c.261+24G>A)];ENSG00000126214:[ENST00000553286(c.261+24G>A)];ENSG00000126214:[ENST00000554228(c.261+24G>A)];ENSG00000126214:[ENST00000557450(c.261+24G>A)];ENSG00000126214:[ENST00000554280(c.261+24G>A)];ENSG00000126214:[ENST00000555836(c.261+24G>A)];ENSG00000126214:[ENST00000347839(c.261+24G>A)];ENSG00000126214:[ENST00000334553(c.261+24G>A)];ENSG00000126214:[ENST00000389744(c.261+24G>A)];	NA	NA	0.003594	0.0016802	0.012794882	0.0035943	0.0184	NA	NA_NA	NA	MODIFIER	NA	NA	112	112	0	130	101	29	22.31
Chewer	42482	chr14	20470860	T	G	*OR4Q2*	MISSENSE	ENSG00000196383:MISSENSE[ENST00000541415(c.539T>G,tTg>tGg):ENSP00000438013(p.Leu180Trp)];	NA	NA	NA	NA	NA	NA	NA	D_lc	PoD_PrD	D	MODERATE	NA	NA	242	242	0	316	308	8	2.53
Chewer	42482	chr14	93677002	G	T	*RP11-371E8.4*	MISSENSE	ENSG00000259066:MISSENSE[ENST00000557574(c.350G>T,cGt>cTt):ENSP00000451369(p.Arg117Leu)];ENSG00000259066:EXONIC-NC[ENST00000557048(n.202G>T)];	NA	NA	chr14*93677002*93677002*G*A*rs751474426*NA*NA*NA	NA	NA	NA	NA	NA	NA_NA	NA	MODIFIER	NA	NA	35	35	0	67	56	11	16.42
Chewer	42482	chr14	104121186	G	A	*RP11-73M18.2*	INTRONIC	ENSG00000256500:[ENST00000472726(c.777+24G>A)];	NA	NA	0.003594	0.0016802	0.012794882	0.0035943	0.0184	NA	NA_NA	NA	MODIFIER	NA	NA	112	112	0	130	101	29	22.31
Chewer	42482	chr14	58862781	G	A	*TOMM20L*	MISSENSE	ENSG00000196860:MISSENSE[ENST00000360945(c.106G>A,Gac>Aac):ENSP00000354204(p.Asp36Asn)];ENSG00000196860:MISSENSE[ENST00000557754(c.106G>A,Gac>Aac):ENSP00000451683(p.Asp36Asn)];	NA	NA	NA	NA	NA	NA	NA	D	PoD_PrD	D	MODERATE	NA	NA	110	109	0	293	252	41	13.99
Chewer	42482	chr14	22573671	G	C	*TRAV24*	MISSENSE	ENSG00000211805:MISSENSE[ENST00000390453(c.52G>C,Gac>Cac):ENSP00000451837(p.Asp18His)];	NA	NA	NA	NA	NA	NA	NA	D	BN_BN	N	MODERATE	NA	NA	127	127	0	203	165	37	18.23
Chewer	42482	chr14	38296516	C	T	*TTC6*	MISSENSE	ENSG00000139865:MISSENSE[ENST00000267368(c.1145C>T,tCg>tTg):ENSP00000267368(p.Ser382Leu)];ENSG00000139865:MISSENSE[ENST00000553443(c.5243C>T,tCg>tTg):ENSP00000451131(p.Ser1748Leu)];ENSG00000139865:MISSENSE[ENST00000382320(c.1385C>T,tCg>tTg):ENSP00000371757(p.Ser462Leu)];ENSG00000139865:MISSENSE[ENST00000476979(c.1145C>T,tCg>tTg):ENSP00000417788(p.Ser382Leu)];ENSG00000139865:MISSENSE[ENST00000478811(c.1436C>T,tCg>tTg):ENSP00000419204(p.Ser479Leu)];ENSG00000139865:MISSENSE[ENST00000555921(c.11C>T,tCg>tTg):ENSP00000450568(p.Ser4Leu)];ENSG00000139865:INTRONIC[ENST00000533625(c.*1063-47149C>T)];	NA	NA	NA	NA	NA	NA	NA	NA	NA_NA	NA	MODIFIER	NA	NA	265	265	0	321	312	9	2.8
Chewer	42482	chr14	93677002	G	T	*UBR7*	MISSENSE	ENSG00000012963:MISSENSE[ENST00000553857(c.70G>T,cGt>cTt):ENSP00000451785(p.Arg24Leu)];ENSG00000012963:MISSENSE[ENST00000554232(c.293G>T,cGt>cTt):ENSP00000450645(p.Arg98Leu)];ENSG00000012963:MISSENSE[ENST00000013070(c.293G>T,cGt>cTt):ENSP00000013070(p.Arg98Leu)];ENSG00000012963:MISSENSE[ENST00000416753(c.65G>T,cGt>cTt):ENSP00000391706(p.Arg22Leu)];ENSG00000012963:INTRONIC[ENST00000555113(c.283-19G>T)];ENSG00000012963:INTRONIC[ENST00000556871(c.150+3216G>T)];ENSG00000012963:SILENT[ENST00000553674(c.159G>T,tcG>tcT):ENSP00000450470(p.Ser53(=))];	NA	NA	chr14*93677002*93677002*G*A*rs751474426*NA*NA*NA	NA	NA	NA	NA	NA	NA_NA	NA	MODIFIER	NA	NA	35	35	0	67	56	11	16.42
Chewer	42482	chr15	90347186	C	G	*ANPEP*	SILENT	ENSG00000166825:SILENT[ENST00000300060(c.1227G>C,ctG>ctC):ENSP00000300060(p.Leu409(=))];ENSG00000166825:SILENT[ENST00000560030(c.15G>C,ctG>ctC):ENSP00000453545(p.Leu5(=))];	NA	NA	NA	NA	NA	NA	NA	NA	NA_NA	NA	LOW	NA	NA	55	55	0	98	92	6	6.12
Chewer	42482	chr15	41523602	T	G	*CHP1*	MISSENSE	ENSG00000187446:MISSENSE[ENST00000334660(c.22T>G,Tta>Gta):ENSP00000335632(p.Leu8Val)];ENSG00000187446:MISSENSE[ENST00000560397(c.22T>G,Tta>Gta):ENSP00000454007(p.Leu8Val)];ENSG00000187446:MISSENSE[ENST00000392151(c.22T>G,Tta>Gta):ENSP00000440490(p.Leu8Val)];ENSG00000187446:MISSENSE[ENST00000560784(c.22T>G,Tta>Gta):ENSP00000452772(p.Leu8Val)];ENSG00000187446:MISSENSE[ENST00000560411(c.22T>G,Tta>Gta):ENSP00000453375(p.Leu8Val)];ENSG00000187446:EXONIC-NC[ENST00000561280(n.166T>G)];ENSG00000187446:INTRONIC[ENST00000558351(n.128+438T>G)];	NA	NA	chr15*41523602*41523602*T*A*rs371891740*NA*NA*NA	NA	NA	NA	NA	D_lc	UN_UN	NA	MODERATE	NA	NA	86	86	0	147	110	37	25.17
Chewer	42482	chr15	65499239	G	C	*CILP*	MISSENSE	ENSG00000138615:MISSENSE[ENST00000261883(c.305C>G,gCg>gGg):ENSP00000261883(p.Ala102Gly)];	NA	NA	chr15*65499239*65499239*G*A*rs567307208*0*0.9998*0.0001997	NA	NA	NA	NA	T	PoD_PoD	N	MODERATE	D	0.39	46	46	0	46	37	9	19.57
Chewer	42482	chr15	57999631	G	A	*GCOM1*	INTRONIC	ENSG00000137878:[ENST00000484300(n.367-1752G>A)];ENSG00000137878:[ENST00000463717(c.*33-4551G>A)];ENSG00000137878:[ENST00000460962(c.*777+1061G>A)];ENSG00000137878:[ENST00000380568(c.1305-7103G>A)];ENSG00000137878:[ENST00000587652(c.1305-1281G>A)];ENSG00000137878:[ENST00000496627(c.*911-4551G>A)];ENSG00000137878:[ENST00000477282(c.1120-4551G>A)];ENSG00000137878:[ENST00000380569(c.1305-4551G>A)];ENSG00000137878:[ENST00000482814(c.1204-4551G>A)];	NA	NA	NA	NA	NA	NA	NA	NA	NA_NA	NA	MODIFIER	NA	NA	136	136	0	231	219	11	4.76
Chewer	42482	chr15	59500155	G	A	*LDHAL6B*	MISSENSE	ENSG00000171989:MISSENSE[ENST00000307144(c.1016G>A,aGt>aAt):ENSP00000302393(p.Ser339Asn)];	NA	NA	NA	NA	NA	NA	NA	NA	NA_NA	NA	MODIFIER	NA	NA	183	181	1	253	238	8	3.16
Chewer	42482	chr15	59500155	G	A	*MYO1E*	INTRONIC	ENSG00000157483:[ENST00000559269(c.597+9935C>T)];ENSG00000157483:[ENST00000560642(n.320+725C>T)];ENSG00000157483:[ENST00000560749(c.101+725C>T)];ENSG00000157483:[ENST00000288235(c.1530+725C>T)];	NA	NA	NA	NA	NA	NA	NA	NA	NA_NA	NA	MODIFIER	NA	NA	183	181	1	253	238	8	3.16
Chewer	42482	chr15	23932111	G	T	*NDN*	MISSENSE	ENSG00000182636:MISSENSE[ENST00000331837(c.254C>A,gCg>gAg):ENSP00000332643(p.Ala85Glu)];	NA	NA	chr15*23932111*23932111*G*A*rs763474594*NA*NA*NA	NA	NA	NA	NA	T	BN_BN	N	MODERATE	N	0.023	66	66	0	110	85	25	22.73
Chewer	42482	chr15	57999631	G	A	*POLR2M*	INTRONIC	ENSG00000255529:[ENST00000494490(c.*186+288G>A)];ENSG00000255529:[ENST00000299638(c.113+478G>A)];ENSG00000255529:[ENST00000482852(c.113+478G>A)];ENSG00000255529:[ENST00000380557(c.113+478G>A)];ENSG00000255529:[ENST00000464308(n.123+223G>A)];ENSG00000255529:[ENST00000567643(n.243-34G>A)];ENSG00000255529:[ENST00000380563(c.113+478G>A)];ENSG00000255529:[ENST00000464277(c.*182+292G>A)];	NA	NA	NA	NA	NA	NA	NA	NA	NA_NA	NA	MODIFIER	NA	NA	136	136	0	231	219	11	4.76
Chewer	42482	chr15	78587359	A	T	*RP11-762H8.1*	INTRONIC	ENSG00000259322:[ENST00000560057(n.68-651A>T)];	NA	NA	NA	NA	NA	NA	NA	NA	NA_NA	NA	MODIFIER	NA	NA	156	155	0	179	142	37	20.67

Mangalaparthi *et al.* , 2020. Mutational landscape of esophageal squamous cell carcinoma in an Indian cohort
Supplementary Table 4. List of somatic single nucleotide variants identified using whole exome sequencing of ESCC patients

Cohort	Sample	Chromosome	Genomic position	Reference allele	Alternate allele	Gene symbol	Variant type	Variant annotation	OncoMD	COSMIC	dbSNP	ExAC	ExAC_SAS	1000G	1000G_SAS	SIFT	Polyphen2	CONDEL	VEP	LRT	PhastCon7 Way	Total read (Adjacent normal)	Reference allel read support (Adjacent normal)	Alternative read support (Adjacent normal)	Total read (Tumor)	Reference allel read support (Tumor)	Alternative read support (Tumor)	Alternate allele frequency
Chewer	42482	chr15	49293215	G	A	*SECISBP2L*	MISSENSE	ENSG00000138593:MISSENSE[ENST00000261847(c.1972C>T,Cgc>Tgc):ENSP00000261847(p.Arg658Cys)];ENSG00000138593:MISSENSE[ENST00000559471(c.2107C>T,Cgc>Tgc):ENSP00000453854(p.Arg703Cys)];ENSG00000138593:MISSENSE[ENST00000380927(c.1393C>T,Cgc>Tgc):ENSP00000370314(p.Arg465Cys)];ENSG00000138593:EXONIC-NC[ENST00000559122(n.331C>T)];	NA	NA	NA	NA	NA	NA	NA	NA	NA_NA	NA	MODIFIER	NA	NA	234	234	0	327	247	79	24.16
Chewer	42482	chr15	44944132	C	A	*SPG11*	MISSENSE	ENSG00000104133:MISSENSE[ENST00000535302(c.1013G>T,tGg>tTg):ENSP00000445278(p.Trp338Leu)];ENSG00000104133:MISSENSE[ENST00000559193(c.1013G>T,tGg>tTg):ENSP00000453848(p.Trp338Leu)];ENSG00000104133:MISSENSE[ENST00000261866(c.1013G>T,tGg>tTg):ENSP00000261866(p.Trp338Leu)];ENSG00000104133:MISSENSE[ENST00000558319(c.1013G>T,tGg>tTg):ENSP00000453599(p.Trp338Leu)];ENSG00000104133:MISSENSE[ENST00000427534(c.1013G>T,tGg>tTg):ENSP00000396110(p.Trp338Leu)];	NA	NA	NA	NA	NA	NA	NA	D	PrD_PrD	D	MODERATE	D	0.996	61	60	0	91	84	7	7.69
Chewer	42482	chr15	42982495	G	T	*STARD9*	MISSENSE	ENSG00000159433:MISSENSE[ENST00000290607(c.8719G>T,Gca>Tca):ENSP00000290607(p.Ala2907Ser)];ENSG00000159433:MISSENSE[ENST00000562619(c.703G>T,Gca>Tca):ENSP00000454648(p.Ala235Ser)];	chr15*4298[illegible]	chr15*4298[illegible]	chr15*42982495*42982495*G*A*rs534412465*0*0.9998*0.0001997	NA	NA	NA	NA	T	BN_BN	N	MODERATE	NA	NA	71	71	0	78	61	17	21.79
Chewer	42482	chr15	102242452	T	A	*TARSL2*	MISSENSE	ENSG00000185418:MISSENSE[ENST00000539112(c.1211A>T,aAg>aTg):ENSP00000439899(p.Lys404Met)];ENSG00000185418:MISSENSE[ENST00000335968(c.1211A>T,aAg>aTg):ENSP00000338093(p.Lys404Met)];ENSG00000185418:MISSENSE[ENST00000558533(c.317A>T,aAg>aTg):ENSP00000452740(p.Lys106Met)];	NA	NA	NA	NA	NA	NA	NA	D	PoD_PrD	D	MODERATE	NA	NA	199	197	0	262	235	27	10.31
Chewer	42482	chr15	78587359	A	T	*WDR61*	INTRONIC	ENSG00000140395:[ENST00000559848(c.13-44T>A)];ENSG00000140395:[ENST00000267973(c.13-44T>A)];ENSG00000140395:[ENST00000558311(c.13-44T>A)];ENSG00000140395:[ENST00000560063(n.98-44T>A)];ENSG00000140395:[ENST00000558840(n.194+658T>A)];ENSG00000140395:[ENST00000560569(c.13-44T>A)];ENSG00000140395:[ENST00000558459(c.13-44T>A)];ENSG00000140395:[ENST00000559700(n.100-44T>A)];ENSG00000140395:[ENST00000560946(n.105-44T>A)];ENSG00000140395:[ENST00000560610(c.13-44T>A)];ENSG00000140395:[ENST00000561347(n.851T>A)];	NA	NA	NA	NA	NA	NA	NA	NA	NA_NA	NA	MODIFIER	NA	NA	156	155	0	179	142	37	20.67
Chewer	42482	chr15	66824653	A	T	*ZWILCH*	SILENT	ENSG00000174442:SILENT[ENST00000307897(c.1224A>T,acA>acT):ENSP00000311429(p.Thr408(=))];ENSG00000174442:SILENT[ENST00000535141(c.882A>T,acA>acT):ENSP00000437749(p.Thr294(=))];ENSG00000174442:SILENT[ENST00000565627(c.882A>T,acA>acT):ENSP00000454737(p.Thr294(=))];ENSG00000174442:SILENT[ENST00000446801(c.882A>T,acA>acT):ENSP00000402217(p.Thr294(=))];	NA	NA	chr15*66824653*66824653*A*G*rs775407199*NA*NA*NA	NA	NA	NA	NA	NA	NA_NA	NA	LOW	NA	NA	103	103	0	135	129	6	4.44
Chewer	42482	chr16	31473512	G	A	*ARMC5*	MISSENSE	ENSG00000140691:MISSENSE[ENST00000268314(c.644G>A,aGc>aAc):ENSP00000268314(p.Ser215Asn)];ENSG00000140691:MISSENSE[ENST00000563544(c.644G>A,aGc>aAc):ENSP00000456877(p.Ser215Asn)];ENSG00000140691:MISSENSE[ENST00000457010(c.644G>A,aGc>aAc):ENSP00000399561(p.Ser215Asn)];ENSG00000140691:MISSENSE[ENST00000408912(c.929G>A,aGc>aAc):ENSP00000386125(p.Ser310Asn)];ENSG00000140691:MISSENSE[ENST00000538189(c.740G>A,aGc>aAc):ENSP00000443995(p.Ser247Asn)];ENSG00000140691:MISSENSE[ENST00000412665(c.152G>A,aGc>aAc):ENSP00000400183(p.Ser51Asn)];ENSG00000140691:MISSENSE[ENST00000564900(c.208G>A,aGc>aAc):ENSP00000456571(p.Ser70Asn)];ENSG00000140691:3UTR[ENST00000564514(c.*422G>A)];	NA	NA	NA	NA	NA	NA	NA	NA	NA_NA	NA	MODIFIER	NA	NA	69	69	0	84	77	7	8.33
Chewer	42482	chr16	67227385	G	T	*E2F4*	MISSENSE	ENSG00000205250:MISSENSE[ENST00000379378(c.418G>T,Gtc>Ttc):ENSP00000368686(p.Val140Phe)];ENSG00000205250:EXONIC-NC[ENST00000561904(n.569G>T)];ENSG00000205250:EXONIC-NC[ENST00000563238(n.734G>T)];ENSG00000205250:EXONIC-NC[ENST00000567007(n.997G>T)];ENSG00000205250:EXONIC-NC[ENST00000564718(n.453G>T)];ENSG00000205250:SILENT[ENST00000568839(c.306G>T,acG>acT):ENSP00000458082(p.Thr102(=))];ENSG00000205250:3UTR[ENST00000565849(c.*126G>T)];ENSG00000205250:INTRONIC[ENST00000569573(c.245+612G>T)];	NA	NA	chr16*67227385*67227385*G*A,T*rs771157075*NA*NA*NA	8.24E-06	0	NA	NA	NA	NA_NA	NA	MODIFIER	NA	NA	57	57	0	77	66	11	14.29
Chewer	42482	chr16	70841431	C	A	*HYDIN*	3UTR	ENSG00000157423:[ENST00000378856(c.*4298G>T)];ENSG00000157423:[ENST00000393567(c.*52G>T)];	NA	NA	NA	NA	NA	NA	NA	NA	NA_NA	NA	MODIFIER	NA	NA	37	37	0	50	42	8	16
Chewer	42482	chr16	70841498	C	A	*HYDIN*	MISSENSE	ENSG00000157423:3UTR[ENST00000378856(c.*4231G>T)];ENSG00000157423:MISSENSE[ENST00000393567(c.15351G>T,aaG>aaT):ENSP00000377197(p.Lys5117Asn)];	NA	NA	NA	NA	NA	NA	NA	NA	PoD_PrD	NA	MODERATE	U	0.998	63	62	1	75	59	16	21.33
Chewer	42482	chr16	27374285	G	C	*IL4R*	MISSENSE	ENSG00000077238:MISSENSE[ENST00000395762(c.1612G>C,Gag>Cag):ENSP00000379111(p.Glu538Gln)];ENSG00000077238:MISSENSE[ENST00000380922(c.1567G>C,Gag>Cag):ENSP00000370309(p.Glu523Gln)];ENSG00000077238:MISSENSE[ENST00000543915(c.1612G>C,Gag>Cag):ENSP00000441667(p.Glu538Gln)];ENSG00000077238:MISSENSE[ENST00000170630(c.1612G>C,Gag>Cag):ENSP00000170630(p.Glu538Gln)];ENSG00000077238:3UTR[ENST00000568746(c.*1655G>C)];ENSG00000077238:INTRONIC[ENST00000565352(c.230-1139G>C)];	NA	NA	NA	NA	NA	NA	NA	T	PoD_PrD	N	MODERATE	N	0.013	116	115	1	172	158	14	8.14
Chewer	42482	chr16	31368722	C	A	*ITGAX*	INTRONIC	ENSG00000140678:[ENST00000562522(c.430+37C>A)];ENSG00000140678:[ENST00000268296(c.430+37C>A)];ENSG00000140678:[ENST00000562918(n.467C>A)];ENSG00000140678:[ENST00000567409(n.685C>A)];	NA	NA	NA	3.74E-05	0	NA	NA	NA	NA_NA	NA	MODIFIER	NA	NA	28	28	0	54	48	6	11.11

Mangalaparthi *et al.* , 2020. Mutational landscape of esophageal squamous cell carcinoma in an Indian cohort
Supplementary Table 4. List of somatic single nucleotide variants identified using whole exome sequencing of ESCC patients

Cohort	Sample	Chromosome	Genomic position	Reference allele	Alternate allele	Gene symbol	Variant type	Variant annotation	OncoMD	COSMIC	dbSNP	ExAC	ExAC_SAS	1000G	1000G_SAS	SIFT	Polyphen2	CONDEL	VEP	LRT	PhastCon7 Way	Total read (Adjacent normal)	Reference allel read support (Adjacent normal)	Alternative read support (Adjacent normal)	Total read (Tumor)	Reference allel read support (Tumor)	Alternative read support (Tumor)	Alternate allele frequency
Chewer	42482	chr16	3294249	C	A	*MEFV*	MISSENSE	ENSG00000103313:INTRONIC-SS-DNR-PRX[ENST00000538326(c.*384+5G>T)];ENSG00000103313:INTRONIC-SS-DNR-PRX[ENST00000576315(c.*276+5G>T)];ENSG00000103313:INTRONIC-SS-DNR-PRX[ENST00000219596(c.1759+5G>T)];ENSG00000103313:INTRONIC-SS-DNR-PRX[ENST00000536379(c.1126+5G>T)];ENSG00000103313:INTRONIC-SS-DNR-PRX[ENST00000339854(c.1219+5G>T)];ENSG00000103313:INTRONIC-SS-DNR-PRX[ENST00000541159(c.1126+5G>T)];ENSG00000103313:INTRONIC-SS-DNR-PRX[ENST00000572244(c.*143+5G>T)];ENSG00000103313:INTRONIC-SS-DNR-PRX[ENST00000539145(c.*392+5G>T)];ENSG00000103313:MISSENSE[ENST00000536980(c.1131G>T,gaG>gaT):ENSP00000444178(p.Glu377Asp)];ENSG00000103313:MISSENSE[ENST00000542898(c.1857G>T,gaG>gaT):ENSP00000444615(p.Glu619Asp)];ENSG00000103313:MISSENSE[ENST00000537682(c.1764G>T,gaG>gaT):ENSP00000438611(p.Glu588Asp)];ENSG00000103313:INTRONIC[ENST00000570511(c.*244-357G>T)];ENSG00000103313:INTRONIC[ENST00000574583(c.*244-357G>T)];	NA	NA	NA	NA	NA	NA	NA	NA	NA_NA	NA	LOW	NA	NA	68	68	0	76	65	11	14.47
Chewer	42482	chr16	18549867	C	A	*NOMO2*	MISSENSE	ENSG00000185164:3UTR[ENST00000564991(c.*677G>T)];ENSG00000185164:MISSENSE[ENST00000330537(c.1201G>T,Gct>Tct):ENSP00000331851(p.Ala401Ser)];ENSG00000185164:MISSENSE[ENST00000543392(c.700G>T,Gct>Tct):ENSP00000439970(p.Ala234Ser)];ENSG00000185164:MISSENSE[ENST00000381474(c.1201G>T,Gct>Tct):ENSP00000370883(p.Ala401Ser)];	NA	NA	NA	NA	NA	NA	NA	T	BN_BN	N	MODERATE	N	1	409	406	2	600	574	26	4.33
Chewer	42482	chr16	66919470	T	A	*PDP2*	MISSENSE	ENSG00000172840:INTRONIC[ENST00000568720(n.59+4927T>A)];ENSG00000172840:INTRONIC[ENST00000566805(c.87-980T>A)];ENSG00000172840:MISSENSE[ENST00000311765(c.1283T>A,gTg>gAg):ENSP00000309548(p.Val428Glu)];	NA	NA	chr16*66919470*66919471*TG*T*rs753693284*NA*NA*NA	NA	NA	NA	NA	T	BN_BN	N	MODERATE	N	0.958	95	95	0	126	114	12	9.52
Chewer	42482	chr16	72166843	C	A	*PMFBP1*	SILENT	ENSG00000118557:SILENT[ENST00000355636(c.816G>T,ctG>ctT):ENSP00000347854(p.Leu272(=))];ENSG00000118557:SILENT[ENST00000237353(c.1251G>T,ctG>ctT):ENSP00000237353(p.Leu417(=))];ENSG00000118557:SILENT[ENST00000537465(c.1251G>T,ctG>ctT):ENSP00000443817(p.Leu417(=))];ENSG00000118557:SILENT[ENST00000537392(c.660G>T,ctG>ctT):ENSP00000439564(p.Leu220(=))];ENSG00000118557:3UTR[ENST00000379073(c.*1061G>T)];	NA	NA	NA	NA	NA	NA	NA	NA	NA_NA	NA	LOW	NA	NA	102	102	0	122	99	23	18.85
Chewer	42482	chr16	68379656	G	T	*PRMT7*	MISSENSE	ENSG00000132600:MISSENSE[ENST00000441236(c.856G>T,Gta>Tta):ENSP00000409324(p.Val286Leu)];ENSG00000132600:MISSENSE[ENST00000339507(c.1006G>T,Gta>Tta):ENSP00000343103(p.Val336Leu)];ENSG00000132600:MISSENSE[ENST00000348497(c.784G>T,Gta>Tta):ENSP00000345775(p.Val262Leu)];ENSG00000132600:MISSENSE[ENST00000449359(c.856G>T,Gta>Tta):ENSP00000414716(p.Val286Leu)];ENSG00000132600:MISSENSE[ENST00000568975(c.1006G>T,Gta>Tta):ENSP00000454776(p.Val336Leu)];ENSG00000132600:EXONIC-NC[ENST00000565356(n.1125G>T)];ENSG00000132600:EXONIC-NC[ENST00000567542(n.1352G>T)];ENSG00000132600:EXONIC-NC[ENST00000564050(n.661G>T)];	NA	NA	NA	8.26E-06	0	NA	NA	NA	NA_NA	NA	MODIFIER	NA	NA	63	63	0	49	39	10	20.41
Chewer	42482	chr16	716830	G	A	*WDR90*	INTRONIC	ENSG00000161996:[ENST00000547944(c.801+38G>A)];ENSG00000161996:[ENST00000551100(c.223+38G>A)];ENSG00000161996:[ENST00000553080(n.1458+38G>A)];ENSG00000161996:[ENST00000552728(c.*1221+38G>A)];ENSG00000161996:[ENST00000315764(c.657+38G>A)];ENSG00000161996:[ENST00000549024(n.1774+38G>A)];ENSG00000161996:[ENST00000549091(c.5010+38G>A)];ENSG00000161996:[ENST00000547407(n.3364+38G>A)];ENSG00000161996:[ENST00000293879(c.5004+38G>A)];	NA	NA	NA	NA	NA	NA	NA	NA	NA_NA	NA	MODIFIER	NA	NA	55	55	0	64	55	9	14.06
Chewer	42482	chr16	3139246	C	T	*ZSCAN10*	MISSENSE	ENSG00000130182:MISSENSE[ENST00000252463(c.2024G>A,gGg>gAg):ENSP00000252463(p.Gly675Glu)];ENSG00000130182:MISSENSE[ENST00000538082(c.1778G>A,gGg>gAg):ENSP00000440047(p.Gly593Glu)];ENSG00000130182:MISSENSE[ENST00000575108(c.1007G>A,gGg>gAg):ENSP00000459520(p.Gly336Glu)];	NA	NA	chr16*3139246*3139246*C*G,T*rs773482636*NA*NA*NA	1.40E-05	0	NA	NA	D	PrD_PrD	D	MODERATE	D	0.941	55	55	0	74	68	6	8.11
Chewer	42482	chr17	12693241	C	A	*ARHGAP44*	INTRONIC	ENSG00000006740:[ENST00000544416(c.53+33C>A)];ENSG00000006740:[ENST00000340825(c.53+33C>A)];ENSG00000006740:[ENST00000580768(c.53+33C>A)];ENSG00000006740:[ENST00000379672(c.53+33C>A)];ENSG00000006740:[ENST00000262444(c.53+33C>A)];	NA	NA	chr17*12693241*12693241*C*T*rs763769208*NA*NA*NA	NA	NA	NA	NA	NA	NA_NA	NA	MODIFIER	NA	NA	77	77	0	84	75	9	10.71
Chewer	42482	chr17	40967992	C	G	*BECN1*	MISSENSE	ENSG00000126581:MISSENSE[ENST00000590099(c.764G>C,cGt>cCt):ENSP00000465364(p.Arg255Pro)];ENSG00000126581:MISSENSE[ENST00000361523(c.764G>C,cGt>cCt):ENSP00000355231(p.Arg255Pro)];ENSG00000126581:MISSENSE[ENST00000438274(c.536G>C,cGt>cCt):ENSP00000416173(p.Arg179Pro)];ENSG00000126581:MISSENSE[ENST00000586589(c.385G>C,cGt>cCt):ENSP00000466281(p.Arg129Pro)];ENSG00000126581:MISSENSE[ENST00000586754(c.22G>C,cGt>cCt):ENSP00000468700(p.Arg8Pro)];ENSG00000126581:INTRONIC[ENST00000593205(c.456-1301G>C)];ENSG00000126581:INTRONIC[ENST00000589663(c.230+2247G>C)];ENSG00000126581:EXONIC-NC[ENST00000589493(n.415G>C)];ENSG00000126581:EXONIC-NC[ENST00000543382(n.1569G>C)];	NA	NA	chr17*40967992*40967992*C*T*rs371551932*NA*NA*NA	NA	NA	NA	NA	NA	NA_NA	NA	MODIFIER	NA	NA	108	108	0	150	121	29	19.33
Chewer	42482	chr17	57725037	T	C	*CLTC*	INTRONIC-SS-DNR	ENSG00000141367:[ENST00000269122(c.519+10T>C)];ENSG00000141367:[ENST00000585198(n.21+10T>C)];ENSG00000141367:[ENST00000393043(c.519+10T>C)];ENSG00000141367:[ENST00000584313(c.251-564T>C)];ENSG00000141367:[ENST00000579456(c.300+229T>C)];	NA	NA	NA	NA	NA	NA	NA	NA	NA_NA	NA	MODIFIER	NA	NA	86	86	0	97	92	5	5.15
Chewer	42482	chr17	18009790	C	T	*DRG2*	MISSENSE	ENSG00000108591:MISSENSE[ENST00000225729(c.998C>T,gCc>gTc):ENSP00000225729(p.Ala333Val)];ENSG00000108591:EXONIC-NC[ENST00000582528(n.4515C>T)];ENSG00000108591:EXONIC-NC[ENST00000497744(n.166C>T)];ENSG00000108591:EXONIC-NC[ENST00000482564(n.1030C>T)];ENSG00000108591:3UTR[ENST00000467099(c.*1375C>T)];ENSG00000108591:3UTR[ENST00000580055(c.*261C>T)];ENSG00000108591:INTRONIC-SS-DNR-PRX[ENST00000395726(c.992+6C>T)];ENSG00000108591:SILENT[ENST00000583355(c.417C>T,cgC>cgT):ENSP00000463256(p.Arg139(=))];	NA	NA	NA	NA	NA	NA	NA	NA	NA_NA	NA	LOW	NA	0.984	60	60	0	51	36	15	29.41

Mangalaparthi *et al.* , 2020. Mutational landscape of esophageal squamous cell carcinoma in an Indian cohort
Supplementary Table 4. List of somatic single nucleotide variants identified using whole exome sequencing of ESCC patients

Cohort	Sample	Chromosome	Genomic position	Reference allele	Alternate allele	Gene symbol	Variant type	Variant annotation	OncoMD	COSMIC	dbSNP	ExAC	ExAC_SAS	1000G	1000G_SAS	SIFT	Polyphen2	CONDEL	VEP	LRT	PhastCon7 Way	Total read (Adjacent normal)	Reference allel read support (Adjacent normal)	Alternative read support (Adjacent normal)	Total read (Tumor)	Reference allel read support (Tumor)	Alternative read support (Tumor)	Alternate allele frequency
Chewer	42482	chr17	7612716	G	T	*EFNB3*	MISSENSE	ENSG00000108947:MISSENSE[ENST00000226091(c.845G>T,gGg>gTg):ENSP00000226091(p.Gly282Val)];	NA	NA	NA	NA	NA	NA	NA	D	PrD_PrD	D	MODERATE	N	0.72	40	40	0	46	36	10	21.74
Chewer	42482	chr17	61902359	C	T	*FTSJ3*	INTRONIC	ENSG00000108592:[ENST00000427159(c.711+49G>A)];	NA	NA	NA	NA	NA	NA	NA	NA	NA_NA	NA	MODIFIER	NA	NA	104	103	0	100	87	13	13
Chewer	42482	chr17	679112	C	A	*GLOD4*	SILENT	ENSG00000167699:3UTR[ENST00000574581(c.*205G>T)];ENSG00000167699:3UTR[ENST00000575851(c.*264G>T)];ENSG00000167699:3UTR[ENST00000572220(c.*188G>T)];ENSG00000167699:3UTR[ENST00000574554(c.*332G>T)];ENSG00000167699:3UTR[ENST00000575790(c.*202G>T)];ENSG00000167699:SILENT[ENST00000301329(c.309G>T,ctG>ctT):ENSP00000301329(p.Leu103(=))];ENSG00000167699:SILENT[ENST00000301328(c.354G>T,ctG>ctT):ENSP00000301328(p.Leu118(=))];ENSG00000167699:SILENT[ENST00000576419(c.309G>T,ctG>ctT):ENSP00000460634(p.Leu103(=))];ENSG00000167699:SILENT[ENST00000536578(c.282G>T,ctG>ctT):ENSP00000444315(p.Leu94(=))];ENSG00000167699:EXONIC-NC[ENST00000571073(n.873G>T)];ENSG00000167699:EXONIC-NC[ENST00000576239(n.348G>T)];	NA	NA	NA	NA	NA	NA	NA	NA	NA_NA	NA	MODIFIER	NA	NA	45	45	0	67	53	12	17.91
Chewer	42482	chr17	9793341	A	T	*GLP2R*	3UTR	ENSG00000065325:[ENST00000574745(c.*319A>T)];ENSG00000065325:[ENST00000262441(c.*319A>T)];	NA	NA	NA	NA	NA	NA	NA	NA	NA_NA	NA	MODIFIER	NA	NA	94	94	0	132	116	16	12.12
Chewer	42482	chr17	65105793	C	A	*HELZ*	NONSENSE	ENSG00000198265:NONSENSE[ENST00000580168(c.3931G>T,Gaa>Taa):ENSP00000464512(p.Glu1311Ter)];ENSG00000198265:NONSENSE[ENST00000358691(c.3928G>T,Gaa>Taa):ENSP00000351524(p.Glu1310Ter)];ENSG00000198265:3UTR[ENST00000579953(c.*595G>T)];	chr17*6510	NA	NA	NA	NA	NA	NA	NA	NA_NA	NA	MODIFIER	NA	NA	57	57	0	96	76	20	20.83
Chewer	42482	chr17	17061831	G	C	*MPRIP*	MISSENSE	ENSG00000133030:MISSENSE[ENST00000395811(c.1561G>C,Gag>Cag):ENSP00000379156(p.Glu521Gln)];ENSG00000133030:MISSENSE[ENST00000395804(c.1561G>C,Gag>Cag):ENSP00000379149(p.Glu521Gln)];ENSG00000133030:MISSENSE[ENST00000584067(c.979G>C,Gag>Cag):ENSP00000462688(p.Glu327Gln)];ENSG00000133030:MISSENSE[ENST00000341712(c.1561G>C,Gag>Cag):ENSP00000342379(p.Glu521Gln)];ENSG00000133030:MISSENSE[ENST00000444976(c.1447G>C,Gag>Cag):ENSP00000400189(p.Glu483Gln)];ENSG00000133030:MISSENSE[ENST00000313485(c.21G>C,Gag>Cag):ENSP00000317786(p.Glu8Gln)];ENSG00000133030:INTRONIC[ENST00000579832(c.64+4102G>C)];ENSG00000133030:EXONIC-NC[ENST00000581955(n.670G>C)];	NA	NA	NA	NA	NA	NA	NA	NA	NA_NA	NA	MODIFIER	NA	NA	24	24	0	28	20	8	28.57
Chewer	42482	chr17	17070245	G	A	*MPRIP*	SILENT	ENSG00000133030:INTRONIC[ENST00000579832(c.64+12516G>A)];ENSG00000133030:INTRONIC[ENST00000395811(c.2164-4787G>A)];ENSG00000133030:INTRONIC[ENST00000341712(c.2164-4787G>A)];ENSG00000133030:INTRONIC[ENST00000444976(c.2050-4787G>A)];ENSG00000133030:INTRONIC[ENST00000395804(c.2164-4787G>A)];ENSG00000133030:INTRONIC[ENST00000584067(c.1582-4787G>A)];ENSG00000133030:SILENT[ENST00000313485(c.3446G>A,gaG>gaA):ENSP00000317786(p.Glu1149(=))];ENSG00000133030:SILENT[ENST00000414263(c.1376G>A,gaG>gaA):ENSP00000412147(p.Glu459(=))];	NA	NA	NA	NA	NA	NA	NA	NA	NA_NA	NA	MODIFIER	NA	NA	70	70	0	86	78	8	9.3
Chewer	42482	chr17	56569211	C	A	*MTMR4*	INTRONIC-SS-ACR	ENSG00000108389:[ENST00000323456(c.3411-10G>T)];ENSG00000108389:[ENST00000578259(n.229-10G>T)];ENSG00000108389:[ENST00000579925(c.3240-10G>T)];	NA	NA	NA	NA	NA	NA	NA	NA	NA_NA	NA	MODIFIER	NA	NA	54	54	0	70	56	14	20
Chewer	42482	chr17	56588840	G	A	*MTMR4*	SILENT	ENSG00000108389:SILENT[ENST00000583243(c.21C>T,agC>agT):ENSP00000464588(p.Ser7(=))];ENSG00000108389:SILENT[ENST00000583656(c.285C>T,agC>agT):ENSP00000463479(p.Ser95(=))];ENSG00000108389:SILENT[ENST00000582663(c.243C>T,agC>agT):ENSP00000464247(p.Ser81(=))];ENSG00000108389:SILENT[ENST00000323456(c.243C>T,agC>agT):ENSP00000325285(p.Ser81(=))];ENSG00000108389:SILENT[ENST00000582390(c.285C>T,agC>agT):ENSP00000462869(p.Ser95(=))];ENSG00000108389:SILENT[ENST00000579925(c.243C>T,agC>agT):ENSP00000464067(p.Ser81(=))];ENSG00000108389:SILENT[ENST00000579921(c.255C>T,agC>agT):ENSP00000463259(p.Ser85(=))];ENSG00000108389:EXONIC-NC[ENST00000580983(n.33C>T)];	NA	NA	NA	NA	NA	NA	NA	NA	NA_NA	NA	LOW	NA	NA	43	43	0	78	71	7	8.97
Chewer	42482	chr17	56588841	C	A	*MTMR4*	MISSENSE	ENSG00000108389:MISSENSE[ENST00000323456(c.242G>T,aGc>aTc):ENSP00000325285(p.Ser81Ile)];ENSG00000108389:MISSENSE[ENST00000583243(c.20G>T,aGc>aTc):ENSP00000464588(p.Ser7Ile)];ENSG00000108389:MISSENSE[ENST00000583656(c.284G>T,aGc>aTc):ENSP00000463479(p.Ser95Ile)];ENSG00000108389:MISSENSE[ENST00000582663(c.242G>T,aGc>aTc):ENSP00000464247(p.Ser81Ile)];ENSG00000108389:MISSENSE[ENST00000579925(c.242G>T,aGc>aTc):ENSP00000464067(p.Ser81Ile)];ENSG00000108389:MISSENSE[ENST00000579921(c.254G>T,aGc>aTc):ENSP00000463259(p.Ser85Ile)];ENSG00000108389:MISSENSE[ENST00000582390(c.284G>T,aGc>aTc):ENSP00000462869(p.Ser95Ile)];ENSG00000108389:EXONIC-NC[ENST00000580983(n.32G>T)];	NA	NA	NA	NA	NA	NA	NA	NA	NA_NA	NA	MODIFIER	NA	NA	42	42	0	78	71	7	8.97
Chewer	42482	chr17	18063124	G	T	*MYO15A*	INTRONIC	ENSG00000091536:[ENST00000557655(c.92-125G>T)];ENSG00000091536:[ENST00000445289(n.317-1507G>T)];ENSG00000091536:[ENST00000451725(c.-21-125G>T)];ENSG00000091536:[ENST00000418233(c.1096-125G>T)];ENSG00000091536:[ENST00000557190(n.132-125G>T)];ENSG00000091536:[ENST00000205890(c.9304-125G>T)];ENSG00000091536:[ENST00000578472(c.166-125G>T)];ENSG00000091536:[ENST00000579848(c.19-125G>T)];ENSG00000091536:[ENST00000556535(c.166-125G>T)];ENSG00000091536:[ENST00000433411(n.629G>T)];	NA	NA	NA	NA	NA	NA	NA	NA	NA_NA	NA	MODIFIER	NA	NA	59	59	0	123	102	21	17.07
Chewer	42482	chr17	79804365	C	T	*P4HB*	SILENT	ENSG00000185624:EXONIC-NC[ENST00000478034(n.682G>A)];ENSG00000185624:EXONIC-NC[ENST00000476482(n.183G>A)];ENSG00000185624:EXONIC-NC[ENST00000477607(n.1068G>A)];ENSG00000185624:EXONIC-NC[ENST00000474712(n.19G>A)];ENSG00000185624:3UTR[ENST00000575069(c.*811G>A)];ENSG00000185624:SILENT[ENST00000576380(c.543G>A,gaG>gaA):ENSP00000460285(p.Glu181(=))];ENSG00000185624:SILENT[ENST00000415593(c.292G>A,gaG>gaA):ENSP00000388117(p.Glu98(=))];ENSG00000185624:SILENT[ENST00000331483(c.996G>A,gaG>gaA):ENSP00000327801(p.Glu332(=))];ENSG00000185624:SILENT[ENST00000439918(c.864G>A,gaG>gaA):ENSP00000388374(p.Glu288(=))];ENSG00000185624:INTRONIC[ENST00000576390(c.234-2889G>A)];ENSG00000185624:INTRONIC[ENST00000571617(c.244-842G>A)];	NA	NA	chr17*79804365*79804365*C*A*rs779399776*NA*NA*NA	NA	NA	NA	NA	NA	NA_NA	NA	LOW	NA	0.1	64	64	0	97	92	5	5.15

Supplementary Table 4. List of somatic single nucleotide variants identified using whole exome sequencing of ESCC patients

Cohort	Sample	Chromosome	Genomic position	Reference allele	Alternate allele	Gene symbol	Variant type	Variant annotation	OncoMD	COSMIC	dbSNP	ExAC	ExAC_SAS	1000G	1000G_SAS	SIFT	Polyphen2	CONDEL	VEP	LRT	PhastCon7 Way	Total read (Adjacent normal)	Reference allel read support (Adjacent normal)	Alternative read support (Adjacent normal)	Total read (Tumor)	Reference allel read support (Tumor)	Alternative read support (Tumor)	Alternate allele frequency
Chewer	42482	chr17	79865034	C	A	*PCYT2*	INTRONIC	ENSG00000185813:[ENST00000571581(n.745-260G>T)];ENSG00000185813:[ENST00000572995(c.68-260G>T)];ENSG00000185813:[ENST00000331285(c.304-260G>T)];ENSG00000185813:[ENST00000538936(c.538-260G>T)];ENSG00000185813:[ENST00000576343(c.376-260G>T)];ENSG00000185813:[ENST00000571105(c.538-260G>T)];ENSG00000185813:[ENST00000573401(c.*571+46G>T)];ENSG00000185813:[ENST00000538721(c.591+46G>T)];ENSG00000185813:[ENST00000573636(c.493-260G>T)];ENSG00000185813:[ENST00000570391(c.442-260G>T)];ENSG00000185813:[ENST00000570388(c.304-260G>T)];	NA	NA	NA	NA	NA	NA	NA	NA	NA_NA	NA	MODIFIER	NA	NA	32	32	0	41	30	11	26.83
Chewer	42482	chr17	12693241	C	A	*RP11-1090M7.1*	INTRONIC	ENSG00000265489:[ENST00000584772(n.100+219G>T)];	NA	NA	chr17*12693241*12693241*C*T*rs763769208*NA*NA*NA	NA	NA	NA	NA	NA	NA_NA	NA	MODIFIER	NA	NA	77	77	0	84	75	9	10.71
Chewer	42482	chr17	74044133	C	A	*SRP68*	EXONIC-NC	ENSG00000167881:[ENST00000588736(n.1596G>T)];ENSG00000167881:[ENST00000542536(n.308-82G>T)];ENSG00000167881:[ENST00000592704(c.*809-82G>T)];ENSG00000167881:[ENST00000307877(c.1078-82G>T)];ENSG00000167881:[ENST00000602720(c.61-82G>T)];ENSG00000167881:[ENST00000355113(c.775-82G>T)];ENSG00000167881:[ENST00000539137(c.964-82G>T)];	NA	NA	NA	NA	NA	NA	NA	NA	NA_NA	NA	MODIFIER	NA	NA	98	98	0	137	102	35	25.55
Chewer	42482	chr17	7578469	C	A	*TP53*	MISSENSE	ENSG00000141510:MISSENSE[ENST00000508793(c.461G>T,gGc>gTc):ENSP00000424104(p.Gly154Val)];ENSG00000141510:MISSENSE[ENST00000413465(c.461G>T,gGc>gTc):ENSP00000410739(p.Gly154Val)];ENSG00000141510:MISSENSE[ENST00000420246(c.461G>T,gGc>gTc):ENSP00000391127(p.Gly154Val)];ENSG00000141510:MISSENSE[ENST00000269305(c.461G>T,gGc>gTc):ENSP00000269305(p.Gly154Val)];ENSG00000141510:MISSENSE[ENST00000509690(c.65G>T,gGc>gTc):ENSP00000425104(p.Gly22Val)];ENSG00000141510:MISSENSE[ENST00000359597(c.461G>T,gGc>gTc):ENSP00000352610(p.Gly154Val)];ENSG00000141510:MISSENSE[ENST00000514944(c.182G>T,gGc>gTc):ENSP00000423862(p.Gly61Val)];ENSG00000141510:MISSENSE[ENST00000445888(c.461G>T,gGc>gTc):ENSP00000391478(p.Gly154Val)];ENSG00000141510:MISSENSE[ENST00000455263(c.461G>T,gGc>gTc):ENSP00000398846(p.Gly154Val)];ENSG00000141510:EXONIC-NC[ENST00000504937(n.343G>T)];ENSG00000141510:EXONIC-NC[ENST00000505014(n.717G>T)];ENSG00000141510:EXONIC-NC[ENST00000504290(n.343G>T)];ENSG00000141510:EXONIC-NC[ENST00000510385(n.343G>T)];	chr17*7578[illegible]	chr17*7578[illegible]	chr17*7578469*7578469*C*T*rs762846821*NA*NA*NA	NA	NA	NA	NA	NA	NA_NA	NA	MODIFIER	NA	NA	53	53	0	60	39	21	35
Chewer	42482	chr17	3419776	C	A	*TRPV3*	NONSENSE	ENSG00000167723:NONSENSE[ENST00000301365(c.2173G>T,Gag>Tag):ENSP00000301365(p.Glu725Ter)];ENSG00000167723:NONSENSE[ENST00000572519(c.2173G>T,Gag>Tag):ENSP00000460215(p.Glu725Ter)];ENSG00000167723:NONSENSE[ENST00000576742(c.2173G>T,Gag>Tag):ENSP00000461518(p.Glu725Ter)];ENSG00000167723:3UTR[ENST00000577016(c.*251G>T)];ENSG00000167723:3UTR[ENST00000381913(c.*416G>T)];ENSG00000167723:3UTR[ENST00000573539(c.*2183G>T)];ENSG00000167723:3UTR[ENST00000571139(c.*2165G>T)];	NA	NA	chr17*3419776*3419776*C*T*rs375812083*NA*NA*NA	NA	NA	NA	NA	NA	NA_NA	NA	MODIFIER	NA	NA	127	126	1	153	146	7	4.58
Chewer	42482	chr18	21209888	C	G	*ANKRD29*	MISSENSE	ENSG00000154065:MISSENSE[ENST00000284207(c.457G>C,Gcc>Ccc):ENSP00000284207(p.Ala153Pro)];ENSG00000154065:MISSENSE[ENST00000592179(c.457G>C,Gcc>Ccc):ENSP00000468354(p.Ala153Pro)];ENSG00000154065:MISSENSE[ENST00000586087(c.358G>C,Gcc>Ccc):ENSP00000465936(p.Ala120Pro)];ENSG00000154065:MISSENSE[ENST00000322980(c.457G>C,Gcc>Ccc):ENSP00000323387(p.Ala153Pro)];ENSG00000154065:EXONIC-NC[ENST00000591280(n.1936G>C)];	NA	NA	chr18*21209888*21209888*C*A,T*rs770121349*NA*NA*NA	NA	NA	NA	NA	NA	NA_NA	NA	MODIFIER	NA	NA	65	64	0	98	88	10	10.2
Chewer	42482	chr18	55398727	G	C	*ATP8B1*	INTRONIC	ENSG00000081923:[ENST00000283684(c.181+132C>G)];ENSG00000081923:[ENST00000591728(c.181+132C>G)];ENSG00000081923:[ENST00000536015(c.181+132C>G)];	NA	NA	chr18*55398727*55398727*G*A*rs565694859*0*0.9998*0.0001997	NA	NA	NA	NA	NA	NA_NA	NA	MODIFIER	NA	NA	53	53	0	78	70	8	10.26
Chewer	42482	chr18	54814710	G	T	*BOD1L2*	MISSENSE	ENSG00000228075:MISSENSE[ENST00000585477(c.167G>T,cGc>cTc):ENSP00000467843(p.Arg56Leu)];	NA	NA	chr18*54814710*54814710*G*A*rs751292958*NA*NA*NA	NA	NA	NA	NA	D	PoD_PoD	D	MODERATE	NA	NA	109	106	1	130	104	26	20
Chewer	42482	chr18	64172233	G	A	*CDH19*	MISSENSE	ENSG00000071991:MISSENSE[ENST00000262150(c.2135C>T,gCc>gTc):ENSP00000262150(p.Ala712Val)];ENSG00000071991:3UTR[ENST00000579658(c.*481C>T)];	NA	NA	NA	NA	NA	NA	NA	NA	NA_NA	NA	MODIFIER	NA	NA	97	97	0	113	100	13	11.5
Chewer	42482	chr18	44089678	C	A	*LOXHD1*	NONSENSE	ENSG00000167210:NONSENSE[ENST00000582408(c.1981G>T,Gag>Tag):ENSP00000461964(p.Glu661Ter)];ENSG00000167210:NONSENSE[ENST00000300591(c.2167G>T,Gag>Tag):ENSP00000300591(p.Glu723Ter)];ENSG00000167210:NONSENSE[ENST00000441551(c.4882G>T,Gag>Tag):ENSP00000387621(p.Glu1628Ter)];ENSG00000167210:NONSENSE[ENST00000398705(c.217G>T,Gag>Tag):ENSP00000381692(p.Glu73Ter)];ENSG00000167210:NONSENSE[ENST00000414184(c.217G>T,Gag>Tag):ENSP00000392440(p.Glu73Ter)];ENSG00000167210:NONSENSE[ENST00000579038(c.1879G>T,Gag>Tag):ENSP00000463285(p.Glu627Ter)];ENSG00000167210:NONSENSE[ENST00000536736(c.5314G>T,Gag>Tag):ENSP00000444586(p.Glu1772Ter)];ENSG00000167210:NONSENSE[ENST00000398686(c.217G>T,Gag>Tag):ENSP00000381676(p.Glu73Ter)];ENSG00000167210:NONSENSE[ENST00000441893(c.2113G>T,Gag>Tag):ENSP00000409062(p.Glu705Ter)];ENSG00000167210:NONSENSE[ENST00000398722(c.4666G>T,Gag>Tag):ENSP00000381707(p.Glu1556Ter)];ENSG00000167210:EXONIC-NC[ENST00000335730(n.4627G>T)];	NA	NA	NA	NA	NA	NA	NA	NA	NA_NA	NA	MODIFIER	NA	NA	32	32	0	40	33	5	12.5
Chewer	42482	chr18	44100317	C	A	*LOXHD1*	INTRONIC	ENSG00000167210:[ENST00000582408(c.1752+1747G>T)];ENSG00000167210:[ENST00000300591(c.1938+743G>T)];ENSG00000167210:[ENST00000441551(c.4653+743G>T)];ENSG00000167210:[ENST00000441893(c.1884+1747G>T)];ENSG00000167210:[ENST00000414184(c.-13+223G>T)];ENSG00000167210:[ENST00000335730(n.4398+1747G>T)];ENSG00000167210:[ENST00000579038(c.1650+743G>T)];ENSG00000167210:[ENST00000536736(c.5085+1747G>T)];ENSG00000167210:[ENST00000398722(c.4437+743G>T)];ENSG00000167210:[ENST00000452425(c.-18G>T)];	NA	NA	NA	NA	NA	NA	NA	NA	NA_NA	NA	MODIFIER	NA	NA	57	57	0	72	63	9	12.5

Mangalaparthi *et al.* , 2020. Mutational landscape of esophageal squamous cell carcinoma in an Indian cohort
Supplementary Table 4. List of somatic single nucleotide variants identified using whole exome sequencing of ESCC patients

Cohort	Sample	Chromosome	Genomic position	Reference allele	Alternate allele	Gene symbol	Variant type	Variant annotation	OncoMD	COSMIC	dbSNP	ExAC	ExAC_SAS	1000G	1000G_SAS	SIFT	Polyphen2	CONDEL	VEP	LRT	PhastCon7 Way	Total read (Adjacent normal)	Reference allel read support (Adjacent normal)	Alternative read support (Adjacent normal)	Total read (Tumor)	Reference allel read support (Tumor)	Alternative read support (Tumor)	Alternate allele frequency
Chewer	42482	chr18	7231636	A	T	*LRRC30*	MISSENSE	ENSG00000206422:MISSENSE[ENST00000383467(c.500A>T,aAg>aTg):ENSP00000372959(p.Lys167Met)];	NA	NA	chr18*7231636*7231636*A*C*rs200482387*1*0.9994*0.000599	NA	NA	NA	NA	D	PrD_PrD	D	MODERATE	D	0.744	154	153	1	186	163	23	12.37
Chewer	42482	chr18	48255939	C	T	*MAPK4*	SILENT	ENSG00000141639:SILENT[ENST00000540640(c.846C>T,atC>atT):ENSP00000439231(p.Ile282(=))];ENSG00000141639:SILENT[ENST00000400384(c.1479C>T,atC>atT):ENSP00000383234(p.Ile493(=))];ENSG00000141639:3UTR[ENST00000592595(c.*401C>T)];	NA	NA	NA	NA	NA	NA	NA	NA	NA_NA	NA	LOW	NA	0.99	52	52	0	76	55	21	27.63
Chewer	42482	chr18	48333122	G	C	*MRO*	MISSENSE	ENSG00000134042:3UTR[ENST00000585524(c.*10C>G)];ENSG00000134042:MISSENSE[ENST00000431965(c.240C>G,caC>caG):ENSP00000392614(p.His80Gln)];ENSG00000134042:MISSENSE[ENST00000428869(c.198C>G,caC>caG):ENSP00000409509(p.His66Gln)];ENSG00000134042:MISSENSE[ENST00000590988(c.134C>G,aCa>aGa):ENSP00000466218(p.Thr45Arg)];ENSG00000134042:MISSENSE[ENST00000436348(c.240C>G,caC>caG):ENSP00000397900(p.His80Gln)];ENSG00000134042:MISSENSE[ENST00000588444(c.198C>G,caC>caG):ENSP00000465653(p.His66Gln)];ENSG00000134042:MISSENSE[ENST00000398439(c.198C>G,caC>caG):ENSP00000381465(p.His66Gln)];ENSG00000134042:MISSENSE[ENST00000592966(c.198C>G,caC>caG):ENSP00000467427(p.His66Gln)];ENSG00000134042:MISSENSE[ENST00000256425(c.198C>G,caC>caG):ENSP00000256425(p.His66Gln)];ENSG00000134042:INTRONIC[ENST00000587291(n.47-1416C>G)];	NA	NA	chr18*48333122*48333122*G*A*rs200310054*0*0.9998*0.0001997	NA	NA	NA	NA	NA	NA_NA	NA	MODIFIER	NA	NA	96	93	0	151	125	26	17.22
Chewer	42482	chr18	55398727	G	C	*RP11-35G9.3*	EXONIC-NC	ENSG00000267040:[ENST00000599199(n.652G>C)];	NA	NA	chr18*55398727*55398727*G*A*rs565694859*0*0.9998*0.0001997	NA	NA	NA	NA	NA	NA_NA	NA	MODIFIER	NA	NA	53	53	0	78	70	8	10.26
Chewer	42482	chr18	55398727	G	C	*RP11-35G9.5*	EXONIC-NC	ENSG00000267787:[ENST00000588925(n.652G>C)];	NA	NA	chr18*55398727*55398727*G*A*rs565694859*0*0.9998*0.0001997	NA	NA	NA	NA	NA	NA_NA	NA	MODIFIER	NA	NA	53	53	0	78	70	8	10.26
Chewer	42482	chr18	67871376	G	A	*RTTN*	MISSENSE	ENSG00000176225:MISSENSE[ENST00000255674(c.343C>T,Ctt>Ttt):ENSP00000255674(p.Leu115Phe)];ENSG00000176225:MISSENSE[ENST00000454359(c.343C>T,Ctt>Ttt):ENSP00000402352(p.Leu115Phe)];ENSG00000176225:MISSENSE[ENST00000437017(c.343C>T,Ctt>Ttt):ENSP00000399520(p.Leu115Phe)];ENSG00000176225:MISSENSE[ENST00000581161(c.343C>T,Ctt>Ttt):ENSP00000462926(p.Leu115Phe)];ENSG00000176225:EXONIC-NC[ENST00000581583(n.411C>T)];	NA	NA	NA	NA	NA	NA	NA	NA	NA_NA	NA	MODIFIER	NA	NA	212	212	0	338	255	82	24.26
Chewer	42482	chr18	67871377	A	T	*RTTN*	SILENT	ENSG00000176225:EXONIC-NC[ENST00000581583(n.410T>A)];ENSG00000176225:SILENT[ENST00000581161(c.342T>A,ctT>ctA):ENSP00000462926(p.Leu114(=))];ENSG00000176225:SILENT[ENST00000255674(c.342T>A,ctT>ctA):ENSP00000255674(p.Leu114(=))];ENSG00000176225:SILENT[ENST00000454359(c.342T>A,ctT>ctA):ENSP00000402352(p.Leu114(=))];ENSG00000176225:SILENT[ENST00000437017(c.342T>A,ctT>ctA):ENSP00000399520(p.Leu114(=))];	NA	NA	NA	NA	NA	NA	NA	NA	NA_NA	NA	LOW	NA	NA	212	212	0	330	249	80	24.24
Chewer	42482	chr18	61387254	C	A	*SERPINB11*	SILENT	ENSG00000206072:EXONIC-NC[ENST00000489748(n.1182C>A)];ENSG00000206072:SILENT[ENST00000382749(c.483C>A,gtC>gtA):ENSP00000421854(p.Val161(=))];ENSG00000206072:SILENT[ENST00000544088(c.483C>A,gtC>gtA):ENSP00000441497(p.Val161(=))];ENSG00000206072:5UTR[ENST00000536691(c.-43C>A)];ENSG00000206072:INTRONIC[ENST00000538847(c.169-2975C>A)];	NA	NA	chr18*61387254*61387254*C*T*rs778932489*NA*NA*NA	NA	NA	NA	NA	NA	NA_NA	NA	LOW	NA	NA	70	70	0	70	62	8	11.43
Chewer	42482	chr18	20878001	C	G	*TMEM241*	MISSENSE	ENSG00000134490:MISSENSE[ENST00000383233(c.861G>C,ttG>ttC):ENSP00000372720(p.Leu287Phe)];ENSG00000134490:3UTR[ENST00000473688(c.*332G>C)];ENSG00000134490:3UTR[ENST00000460322(c.*499G>C)];ENSG00000134490:3UTR[ENST00000477053(c.*360G>C)];ENSG00000134490:3UTR[ENST00000542162(c.*308G>C)];ENSG00000134490:INTRONIC[ENST00000578790(n.251+11643G>C)];ENSG00000134490:INTRONIC[ENST00000581444(c.*632+11643G>C)];ENSG00000134490:INTRONIC[ENST00000450466(c.467+11643G>C)];ENSG00000134490:EXONIC-NC[ENST00000577448(n.416G>C)];ENSG00000134490:EXONIC-NC[ENST00000583048(n.397G>C)];ENSG00000134490:EXONIC-NC[ENST00000475185(n.326G>C)];	NA	NA	NA	NA	NA	NA	NA	NA	NA_NA	NA	MODIFIER	NA	NA	77	77	0	115	88	27	23.48
Chewer	42482	chr18	5293249	C	G	*ZBTB14*	5UTR	ENSG00000198081:[ENST00000357006(c.-4G>C)];ENSG00000198081:[ENST00000582135(c.-4G>C)];ENSG00000198081:[ENST00000582388(c.-4G>C)];ENSG00000198081:[ENST00000400143(c.-4G>C)];ENSG00000198081:[ENST00000578327(c.-4G>C)];ENSG00000198081:[ENST00000585253(n.398G>C)];	NA	NA	NA	NA	NA	NA	NA	NA	NA_NA	NA	MODIFIER	NA	NA	68	68	0	69	53	16	23.19
Chewer	42482	chr18	14105563	C	A	*ZNF519*	MISSENSE	ENSG00000175322:MISSENSE[ENST00000590202(c.976G>T,Ggc>Tgc):ENSP00000464872(p.Gly326Cys)];ENSG00000175322:INTRONIC[ENST00000587419(c.130+18786G>T)];ENSG00000175322:INTRONIC[ENST00000591393(n.155-20488G>T)];ENSG00000175322:INTRONIC[ENST00000592345(n.217-20488G>T)];ENSG00000175322:INTRONIC[ENST00000592411(n.282+18786G>T)];ENSG00000175322:INTRONIC[ENST00000589203(n.180-20488G>T)];ENSG00000175322:INTRONIC[ENST00000592049(n.161-20488G>T)];ENSG00000175322:INTRONIC[ENST00000589498(c.131-3791G>T)];	NA	NA	NA	NA	NA	NA	NA	NA	NA_NA	NA	MODIFIER	NA	NA	134	134	0	165	132	33	20
Chewer	42482	chr19	2853056	G	T	*AC006130.3*	INTRONIC	ENSG00000267063:[ENST00000589365(n.397+5262C>A)];	NA	NA	NA	NA	NA	NA	NA	T	BN_BN	N	MODERATE	NA	NA	99	99	0	77	63	14	18.18
Chewer	42482	chr19	55805637	G	A	*BRSK1*	INTRONIC	ENSG00000160469:[ENST00000585418(c.609+22G>A)];ENSG00000160469:[ENST00000309383(c.609+22G>A)];ENSG00000160469:[ENST00000590333(c.657+22G>A)];	NA	NA	0.0001997	1.65E-05	0.000121581	0.0001997	0	NA	NA_NA	NA	MODIFIER	NA	NA	51	51	0	91	81	10	10.99
Chewer	42482	chr19	46498703	G	T	*CCDC61*	MISSENSE	ENSG00000104983:MISSENSE[ENST00000263284(c.101G>T,tGg>tTg):ENSP00000263284(p.Trp34Leu)];	NA	NA	NA	NA	NA	NA	NA	D_lc	PoD_PoD	D	MODERATE	NA	NA	83	83	0	163	137	26	15.95
Chewer	42482	chr19	19655335	G	C	*CILP2*	MISSENSE	ENSG00000160161:MISSENSE[ENST00000291495(c.1981G>C,Gtg>Ctg):ENSP00000291495(p.Val661Leu)];ENSG00000160161:MISSENSE[ENST00000586018(c.1999G>C,Gtg>Ctg):ENSP00000467413(p.Val667Leu)];	NA	NA	NA	NA	NA	NA	NA	D	BN_BN	N	MODERATE	D	0.118	66	66	0	114	101	13	11.4

Cohort	Sample	Chromosome	Genomic position	Reference allele	Alternate allele	Gene symbol	Variant type	Variant annotation	OncoMD	COSMIC	dbSNP	ExAC	ExAC_SAS	1000G	1000G_SAS	SIFT	Polyphen2	CONDEL	VEP	LRT	PhastCon7 Way	Total read (Adjacent normal)	Reference allel read support (Adjacent normal)	Alternative read support (Adjacent normal)	Total read (Tumor)	Reference allel read support (Tumor)	Alternative read support (Tumor)	Alternate allele frequency
Chewer	42482	chr19	45556423	G	T	*CLASRP*	MISSENSE	ENSG00000104859:MISSENSE[ENST00000391953(c.181G>T,Gac>Tac):ENSP00000375815(p.Asp61Tyr)];ENSG00000104859:MISSENSE[ENST00000221455(c.367G>T,Gac>Tac):ENSP00000221455(p.Asp123Tyr)];ENSG00000104859:MISSENSE[ENST00000391952(c.367G>T,Gac>Tac):ENSP00000375814(p.Asp123Tyr)];ENSG00000104859:MISSENSE[ENST00000544944(c.367G>T,Gac>Tac):ENSP00000438702(p.Asp123Tyr)];ENSG00000104859:MISSENSE[ENST00000587112(c.310G>T,Gac>Tac):ENSP00000466371(p.Asp104Tyr)];ENSG00000104859:EXONIC-NC[ENST00000588016(n.366G>T)];ENSG00000104859:INTRONIC[ENST00000591410(c.100-3285G>T)];	NA	NA	chr19*45556423*45556423*G*A*rs763462753*NA*NA*NA	NA	NA	NA	NA	NA	PrD_PrD	NA	MODERATE	NA	NA	105	105	0	178	127	51	28.65
Chewer	42482	chr19	48219895	G	T	*CTD-2571L23.8*	INTRONIC	ENSG00000268746:[ENST00000599924(n.87-15427G>T)];	NA	NA	NA	NA	NA	NA	NA	NA	NA_NA	NA	MODIFIER	NA	NA	29	29	0	70	55	15	21.43
Chewer	42482	chr19	55687198	A	T	*CTD-2587H24.5*	INTRONIC	ENSG00000267577:[ENST00000591665(n.1187-1254A>T)];	NA	NA	NA	NA	NA	NA	NA	NA	NA_NA	NA	MODIFIER	NA	NA	58	58	0	105	95	10	9.52
Chewer	42482	chr19	48219895	G	T	*EHD2*	MISSENSE	ENSG00000024422:MISSENSE[ENST00000263277(c.26G>T,gGg>gTg):ENSP00000263277(p.Gly9Val)];ENSG00000024422:INTRONIC[ENST00000538399(c.-4-1871G>T)];	NA	NA	NA	NA	NA	NA	NA	NA	NA_NA	NA	MODIFIER	NA	NA	29	29	0	70	55	15	21.43
Chewer	42482	chr19	56204265	G	T	*EPN1*	INTRONIC	ENSG00000063245:[ENST00000270460(c.1178-52G>T)];ENSG00000063245:[ENST00000085079(c.1103-55G>T)];ENSG00000063245:[ENST00000589704(c.28-52G>T)];ENSG00000063245:[ENST00000411543(c.1436-52G>T)];ENSG00000063245:[ENST00000586194(n.150-55G>T)];	NA	NA	NA	NA	NA	NA	NA	NA	NA_NA	NA	MODIFIER	NA	NA	102	100	2	144	103	41	28.47
Chewer	42482	chr19	35842536	C	A	*FFAR1*	SILENT	ENSG00000126266:SILENT[ENST00000246553(c.82C>A,Cga>Aga):ENSP00000246553(p.Arg28(=))];	chr19*3584[illegible]	chr19*3584[illegible]	NA	NA	NA	NA	NA	NA	NA_NA	NA	LOW	NA	0.002	75	75	0	184	152	31	16.85
Chewer	42482	chr19	39734580	G	T	*IFNL3*	INTRONIC	ENSG00000197110:[ENST00000413851(c.409-33C>A)];	NA	NA	chr19*39734580*39734580*G*A*rs202181938*NA*NA*NA	NA	NA	NA	NA	NA	NA_NA	NA	MODIFIER	NA	NA	42	41	1	60	41	19	31.67
Chewer	42482	chr19	17943314	G	A	*JAK3*	INTRONIC	ENSG00000105639:[ENST00000527031(n.2278+4222C>T)];ENSG00000105639:[ENST00000458235(c.2680+14C>T)];ENSG00000105639:[ENST00000534444(c.2680+14C>T)];ENSG00000105639:[ENST00000527670(c.2680+14C>T)];	NA	NA	NA	8.26E-06	0	NA	NA	NA	NA_NA	NA	MODIFIER	NA	NA	69	69	0	98	77	21	21.43
Chewer	42482	chr19	51883847	G	T	*LIM2*	SILENT	ENSG00000105370:SILENT[ENST00000221973(c.498C>A,gtC>gtA):ENSP00000221973(p.Val166(=))];ENSG00000105370:SILENT[ENST00000596399(c.372C>A,gtC>gtA):ENSP00000472090(p.Val124(=))];	NA	NA	NA	NA	NA	NA	NA	NA	NA_NA	NA	LOW	NA	NA	54	54	0	122	90	32	26.23
Chewer	42482	chr19	16145526	G	T	*LINC00905*	EXONIC-NC	ENSG00000167459:[ENST00000588117(n.412G>T)];ENSG00000167459:[ENST00000602744(n.335G>T)];ENSG00000167459:[ENST00000602372(n.473G>T)];ENSG00000167459:[ENST00000397365(n.38-25G>T)];ENSG00000167459:[ENST00000588838(n.88-25G>T)];ENSG00000167459:[ENST00000602814(n.130-138G>T)];	NA	NA	NA	NA	NA	NA	NA	NA	NA_NA	NA	MODIFIER	NA	NA	170	169	0	223	175	48	21.52
Chewer	42482	chr19	39096962	A	G	*MAP4K1*	INTRONIC	ENSG00000104814:[ENST00000396857(c.1158-107T>C)];ENSG00000104814:[ENST00000589130(c.1146-107T>C)];ENSG00000104814:[ENST00000591517(c.1158-107T>C)];ENSG00000104814:[ENST00000586296(c.1003+3277T>C)];ENSG00000104814:[ENST00000585583(c.*320-107T>C)];ENSG00000104814:[ENST00000423454(c.144-107T>C)];ENSG00000104814:[ENST00000591921(c.269-107T>C)];ENSG00000104814:[ENST00000589002(n.528-107T>C)];	NA	NA	NA	NA	NA	NA	NA	NA	NA_NA	NA	MODIFIER	NA	NA	33	33	0	48	43	5	10.42
Chewer	42482	chr19	39096963	G	T	*MAP4K1*	INTRONIC	ENSG00000104814:[ENST00000396857(c.1158-108C>A)];ENSG00000104814:[ENST00000589130(c.1146-108C>A)];ENSG00000104814:[ENST00000591517(c.1158-108C>A)];ENSG00000104814:[ENST00000586296(c.1003+3276C>A)];ENSG00000104814:[ENST00000585583(c.*320-108C>A)];ENSG00000104814:[ENST00000423454(c.144-108C>A)];ENSG00000104814:[ENST00000591921(c.269-108C>A)];ENSG00000104814:[ENST00000589002(n.528-108C>A)];	NA	NA	NA	NA	NA	NA	NA	NA	NA_NA	NA	MODIFIER	NA	NA	33	33	0	47	42	5	10.64
Chewer	42482	chr19	9027646	C	T	*MUC16*	INTRONIC	ENSG00000181143:[ENST00000397910(c.36569-71G>A)];	NA	NA	NA	NA	NA	NA	NA	NA	NA_NA	NA	MODIFIER	NA	NA	51	51	0	78	67	11	14.1
Chewer	42482	chr19	9027653	G	T	*MUC16*	INTRONIC	ENSG00000181143:[ENST00000397910(c.36569-78C>A)];	NA	NA	NA	NA	NA	NA	NA	NA	NA_NA	NA	MODIFIER	NA	NA	51	51	0	81	66	10	12.35
Chewer	42482	chr19	9073240	G	T	*MUC16*	MISSENSE	ENSG00000181143:MISSENSE[ENST00000397910(c.14206C>A,Cct>Act):ENSP00000381008(p.Pro4736Thr)];	NA	NA	NA	0.0001161	0	NA	NA	NA	UN_UN	NA	MODERATE	NA	NA	71	71	0	78	69	9	11.54
Chewer	42482	chr19	9074705	T	G	*MUC16*	SILENT	ENSG00000181143:SILENT[ENST00000397910(c.12741A>C,acA>acC):ENSP00000381008(p.Thr4247(=))];	NA	NA	chr19*9074705*9074705*T*A*rs754124701*NA*NA*NA	NA	NA	NA	NA	NA	NA_NA	NA	LOW	NA	NA	69	69	0	66	58	8	12.12
Chewer	42482	chr19	9083531	T	A	*MUC16*	MISSENSE	ENSG00000181143:MISSENSE[ENST00000397910(c.8284A>T,Agt>Tgt):ENSP00000381008(p.Ser2762Cys)];	NA	NA	NA	NA	NA	NA	NA	NA	UN_UN	NA	MODERATE	NA	NA	86	85	0	79	66	13	16.46
Chewer	42482	chr19	1369276	G	C	*MUM1*	MISSENSE	ENSG00000160953:3UTR[ENST00000587460(c.*1311G>C)];ENSG00000160953:3UTR[ENST00000591433(c.*1311G>C)];ENSG00000160953:EXONIC-NC[ENST00000585399(n.174G>C)];ENSG00000160953:MISSENSE[ENST00000415183(c.1438G>C,Gag>Cag):ENSP00000394925(p.Glu480Gln)];ENSG00000160953:MISSENSE[ENST00000591806(c.1438G>C,Gag>Cag):ENSP00000467083(p.Glu480Gln)];ENSG00000160953:MISSENSE[ENST00000344663(c.1438G>C,Gag>Cag):ENSP00000345789(p.Glu480Gln)];ENSG00000160953:MISSENSE[ENST00000311401(c.1231G>C,Gag>Cag):ENSP00000309135(p.Glu411Gln)];	NA	NA	NA	NA	NA	NA	NA	T	BN_BN	N	MODERATE	N	0.658	42	42	0	72	57	15	20.83
Chewer	42482	chr19	55602833	C	T	*PPP1R12C*	3UTR	ENSG00000125503:[ENST00000435544(c.*7G>A)];ENSG00000125503:[ENST00000263433(c.*7G>A)];ENSG00000125503:[ENST00000591938(c.*7G>A)];ENSG00000125503:[ENST00000592993(c.*7G>A)];ENSG00000125503:[ENST00000376393(c.*7G>A)];ENSG00000125503:[ENST00000590268(n.573G>A)];	NA	NA	NA	7.12E-05	6.49E-05	NA	NA	NA	NA_NA	NA	MODIFIER	NA	NA	75	75	0	123	113	10	8.13
Chewer	42482	chr19	52725357	G	T	*PPP2R1A*	SILENT	ENSG00000105568:SILENT[ENST00000391791(c.157G>T,ctG>ctT):ENSP00000375668(p.Leu53(=))];ENSG00000105568:SILENT[ENST00000462990(c.987G>T,ctG>ctT):ENSP00000470504(p.Leu329(=))];ENSG00000105568:SILENT[ENST00000444322(c.1359G>T,ctG>ctT):ENSP00000415067(p.Leu453(=))];ENSG00000105568:SILENT[ENST00000322088(c.1524G>T,ctG>ctT):ENSP00000324804(p.Leu508(=))];	NA	NA	chr19*52725357*52725357*G*A*rs80165164*NA*NA*NA	NA	NA	NA	NA	NA	NA_NA	NA	LOW	NA	NA	24	24	0	35	28	7	20
Chewer	42482	chr19	38976648	G	C	*RYR1*	MISSENSE	ENSG00000196218:MISSENSE[ENST00000355481(c.5353G>C,Gct>Cct):ENSP00000347667(p.Ala1785Pro)];ENSG00000196218:MISSENSE[ENST00000360985(c.5353G>C,Gct>Cct):ENSP00000354254(p.Ala1785Pro)];ENSG00000196218:MISSENSE[ENST00000359596(c.5353G>C,Gct>Cct):ENSP00000352608(p.Ala1785Pro)];	NA	NA	NA	NA	NA	NA	NA	NA	PoD_PrD	NA	MODERATE	NA	NA	105	104	1	186	139	47	25.27

Mangalaparthi *et al.* , 2020. Mutational landscape of esophageal squamous cell carcinoma in an Indian cohort

Supplementary Table 4. List of somatic single nucleotide variants identified using whole exome sequencing of ESCC patients

Cohort	Sample	Chromosome	Genomic position	Reference allele	Alternate allele	Gene symbol	Variant type	Variant annotation	OncoMD	COSMIC	dbSNP	ExAC	ExAC_SAS	1000G	1000G_SAS	SIFT	Polyphen2	CONDEL	VEP	LRT	PhastCon7 Way	Total read (Adjacent normal)	Reference allel read support (Adjacent normal)	Alternative read support (Adjacent normal)	Total read (Tumor)	Reference allel read support (Tumor)	Alternative read support (Tumor)	Alternate allele frequency
Chewer	42482	chr19	41097155	G	A	*SHKBP1*	3UTR	ENSG00000160410:[ENST00000600718(c.*42G>A)];ENSG00000160410:[ENST00000600733(c.*42G>A)];ENSG00000160410:[ENST00000596163(c.*349G>A)];ENSG00000160410:[ENST00000593764(c.*42G>A)];ENSG00000160410:[ENST00000600320(c.*42G>A)];ENSG00000160410:[ENST00000594862(c.*42G>A)];ENSG00000160410:[ENST00000594298(c.*42G>A)];ENSG00000160410:[ENST00000291842(c.*42G>A)];ENSG00000160410:[ENST00000595726(c.*42G>A)];ENSG00000160410:[ENST00000597396(c.*42G>A)];ENSG00000160410:[ENST00000598201(n.1923G>A)];ENSG00000160410:[ENST00000594973(n.2193G>A)];	NA	NA	NA	NA	NA	NA	NA	NA	NA_NA	NA	MODIFIER	NA	NA	61	61	0	109	104	5	4.59
Chewer	42482	chr19	55687198	A	T	*SYT5*	MISSENSE	ENSG00000129990:MISSENSE[ENST00000589172(c.419T>A,cTt>cAt):ENSP00000467685(p.Leu140His)];ENSG00000129990:MISSENSE[ENST00000592470(c.419T>A,cTt>cAt):ENSP00000465110(p.Leu140His)];ENSG00000129990:MISSENSE[ENST00000537500(c.419T>A,cTt>cAt):ENSP00000442896(p.Leu140His)];ENSG00000129990:MISSENSE[ENST00000354308(c.419T>A,cTt>cAt):ENSP00000346265(p.Leu140His)];ENSG00000129990:MISSENSE[ENST00000590851(c.410T>A,cTt>cAt):ENSP00000465576(p.Leu137His)];ENSG00000129990:EXONIC-NC[ENST00000588305(n.300T>A)];ENSG00000129990:3UTR[ENST00000585461(c.*96T>A)];	NA	NA	NA	NA	NA	NA	NA	NA	NA_NA	NA	MODIFIER	NA	NA	58	58	0	105	95	10	9.52
Chewer	42482	chr19	41848126	C	A	*TGFB1*	MISSENSE	ENSG00000105329:MISSENSE[ENST00000221930(c.661G>T,Gcc>Tcc):ENSP00000221930(p.Ala221Ser)];ENSG00000105329:EXONIC-NC[ENST00000597453(n.192G>T)];ENSG00000105329:EXONIC-NC[ENST00000600196(n.121G>T)];	NA	NA	NA	1.44E-05	0	NA	NA	NA	NA_NA	NA	MODIFIER	NA	NA	93	93	0	138	120	18	13.04
Chewer	42482	chr19	59060756	C	G	*TRIM28*	MISSENSE	ENSG00000130726:MISSENSE[ENST00000341753(c.1475C>G,aCc>aGc):ENSP00000342232(p.Thr492Ser)];ENSG00000130726:MISSENSE[ENST00000253024(c.1721C>G,aCc>aGc):ENSP00000253024(p.Thr574Ser)];ENSG00000130726:MISSENSE[ENST00000597136(c.596C>G,aCc>aGc):ENSP00000471303(p.Thr199Ser)];ENSG00000130726:EXONIC-NC[ENST00000595028(n.431C>G)];	NA	NA	NA	NA	NA	NA	NA	NA	NA_NA	NA	MODIFIER	NA	NA	86	85	0	106	97	8	7.55
Chewer	42482	chr19	44739561	C	T	*ZNF227*	SILENT	ENSG00000131115:SILENT[ENST00000589005(c.825C>T,tgC>tgT):ENSP00000467577(p.Cys275(=))];ENSG00000131115:SILENT[ENST00000313040(c.978C>T,tgC>tgT):ENSP00000321049(p.Cys326(=))];ENSG00000131115:SILENT[ENST00000391961(c.825C>T,tgC>tgT):ENSP00000375823(p.Cys275(=))];	chr19*44739	chr19*44739	NA	4.13E-05	0	NA	NA	NA	NA_NA	NA	MODIFIER	NA	NA	219	219	0	253	231	22	8.7
Chewer	42482	chr19	44739561	C	T	*ZNF235*	INTRONIC	ENSG00000159917:[ENST00000592844(c.239-539G>A)];	chr19*44739	chr19*44739	NA	4.13E-05	0	NA	NA	NA	NA_NA	NA	MODIFIER	NA	NA	219	219	0	253	231	22	8.7
Chewer	42482	chr19	11943081	G	T	*ZNF440*	NONSENSE	ENSG00000171295:NONSENSE[ENST00000304060(c.1090G>T,Gga>Tga):ENSP00000305373(p.Gly364Ter)];	NA	NA	NA	NA	NA	NA	NA	NA	NA_NA	NA	HIGH	NA	NA	177	177	0	181	167	14	7.73
Chewer	42482	chr19	30935426	G	T	*ZNF536*	SILENT	ENSG00000198597:SILENT[ENST00000585628(c.957G>T,gtG>gtT):ENSP00000465771(p.Val319(=))];ENSG00000198597:SILENT[ENST00000355537(c.957G>T,gtG>gtT):ENSP00000347730(p.Val319(=))];	NA	NA	0.008586	0.0027811	0.001527184	0.0085863	0.002	NA	NA_NA	NA	LOW	NA	NA	141	141	0	229	186	43	18.78
Chewer	42482	chr19	2853056	G	T	*ZNF555*	MISSENSE	ENSG00000186300:MISSENSE[ENST00000591539(c.990G>T,atG>atT):ENSP00000467893(p.Met330Ile)];ENSG00000186300:MISSENSE[ENST00000334241(c.993G>T,atG>atT):ENSP00000334853(p.Met331Ile)];	NA	NA	NA	NA	NA	NA	NA	T	BN_BN	N	MODERATE	NA	NA	99	99	0	77	63	14	18.18
Chewer	42482	chr19	12203283	A	G	*ZNF788*	STARTLOSS-SS-PR	ENSG00000214189:[ENST00000430298(c.1A>G,Atg>Gtg):ENSP00000391703(p.Met1?)];ENSG00000214189:[ENST00000596883(c.1A>G,Atg>Gtg):ENSP00000473243(p.Met1?)];ENSG00000214189:[ENST00000397755(n.133A>G)];ENSG00000214189:[ENST00000596096(n.206A>G)];ENSG00000214189:[ENST00000601686(n.162A>G)];ENSG00000214189:[ENST00000595703(n.162A>G)];ENSG00000188474:[ENST00000339302(c.-432A>G)];	NA	NA	NA	NA	NA	NA	NA	NA	NA_NA	NA	LOW	NA	NA	140	140	0	184	170	14	7.61
Chewer	42482	chr2	120932522	T	C	*AC012363.4*	EXONIC-NC	ENSG00000224789:[ENST00000455707(n.514A>G)];	NA	NA	NA	NA	NA	NA	NA	NA	NA_NA	NA	MODIFIER	NA	NA	80	80	0	160	131	28	17.5
Chewer	42482	chr2	98128234	T	C	*ANKRD36B*	EXONIC-NC	ENSG00000196912:[ENST00000377003(n.3367A>G)];ENSG00000196912:[ENST00000258459(n.3367A>G)];ENSG00000196912:[ENST00000359901(n.3123A>G)];ENSG00000196912:[ENST00000438709(n.3346A>G)];ENSG00000196912:[ENST00000447597(n.1996A>G)];	NA	NA	NA	NA	NA	NA	NA	NA	NA_NA	NA	MODIFIER	NA	NA	166	163	3	164	155	9	5.49
Chewer	42482	chr2	219099664	A	G	*ARPC2*	EXONIC-NC	ENSG00000163466:[ENST00000484961(n.187A>G)];ENSG00000163466:[ENST00000480062(n.345A>G)];ENSG00000163466:[ENST00000489598(n.928A>G)];ENSG00000163466:[ENST00000472753(n.150-3723A>G)];ENSG00000163466:[ENST00000315717(c.268+544A>G)];ENSG00000163466:[ENST00000295685(c.268+544A>G)];ENSG00000163466:[ENST00000470146(n.1398+544A>G)];ENSG00000163466:[ENST00000465395(n.284+544A>G)];ENSG00000163466:[ENST00000414983(c.*253+544A>G)];ENSG00000163466:[ENST00000478612(n.210-3723A>G)];ENSG00000163466:[ENST00000491780(n.383-255A>G)];ENSG00000163466:[ENST00000420201(c.*85A>G)];	NA	NA	NA	NA	NA	NA	NA	NA	NA_NA	NA	MODIFIER	NA	NA	137	137	0	182	169	13	7.14
Chewer	42482	chr2	27800896	C	T	*C2orf16*	MISSENSE	ENSG00000221843:MISSENSE[ENST00000408964(c.1457C>T,aCa>aTa):ENSP00000386190(p.Thr486Ile)];	NA	NA	NA	NA	NA	NA	NA	D	BN_BN	N	MODERATE	NA	NA	142	142	0	200	172	28	14
Chewer	42482	chr2	202139592	T	C	*CASP8*	INTRONIC	ENSG00000064012:[ENST00000450491(c.242-20T>C)];ENSG00000064012:[ENST00000392266(c.551-1958T>C)];ENSG00000064012:[ENST00000323492(c.551-20T>C)];ENSG00000064012:[ENST00000264275(c.647-20T>C)];ENSG00000064012:[ENST00000358485(c.773-20T>C)];ENSG00000064012:[ENST00000447616(c.551-1958T>C)];ENSG00000064012:[ENST00000444430(c.139+2093T>C)];ENSG00000064012:[ENST00000392259(c.595+1927T>C)];ENSG00000064012:[ENST00000392258(c.595+1927T>C)];ENSG00000064012:[ENST00000339403(n.681-20T>C)];ENSG00000064012:[ENST00000392263(c.551-20T>C)];ENSG00000064012:[ENST00000432109(c.596-20T>C)];ENSG00000064012:[ENST00000264274(c.550+2093T>C)];ENSG00000064012:[ENST00000424461(c.140-1958T>C)];	NA	NA	NA	NA	NA	NA	NA	NA	NA_NA	NA	MODIFIER	NA	NA	72	71	0	90	69	20	22.22
Chewer	42482	chr2	225368457	C	T	*CUL3*	MISSENSE	ENSG00000036257:MISSENSE[ENST00000344951(c.1091G>A,cGt>cAt):ENSP00000343601(p.Arg364His)];ENSG00000036257:MISSENSE[ENST00000264414(c.1289G>A,cGt>cAt):ENSP00000264414(p.Arg430His)];ENSG00000036257:MISSENSE[ENST00000409777(c.1217G>A,cGt>cAt):ENSP00000386525(p.Arg406His)];ENSG00000036257:MISSENSE[ENST00000409096(c.1217G>A,cGt>cAt):ENSP00000387200(p.Arg406His)];ENSG00000036257:EXONIC-NC[ENST00000481135(n.585G>A)];	NA	NA	NA	NA	NA	NA	NA	NA	NA_NA	NA	MODIFIER	NA	NA	74	73	1	91	76	14	15.38
Chewer	42482	chr2	180858058	C	A	*CWC22*	MISSENSE	ENSG00000163510:MISSENSE[ENST00000404136(c.11G>T,aGt>aTt):ENSP00000384159(p.Ser4Ile)];ENSG00000163510:MISSENSE[ENST00000410053(c.11G>T,aGt>aTt):ENSP00000387006(p.Ser4Ile)];ENSG00000163510:MISSENSE[ENST00000295749(c.11G>T,aGt>aTt):ENSP00000295749(p.Ser4Ile)];	NA	NA	NA	NA	NA	NA	NA	NA	PoD_PoD	NA	MODERATE	D	0.999	85	85	0	116	103	13	11.21

Mangalaparthi *et al.* , 2020. Mutational landscape of esophageal squamous cell carcinoma in an Indian cohort
Supplementary Table 4. List of somatic single nucleotide variants identified using whole exome sequencing of ESCC patients

Cohort	Sample	Chromosome	Genomic position	Reference allele	Alternate allele	Gene symbol	Variant type	Variant annotation	OncoMD	COSMIC	dbSNP	ExAC	ExAC_SAS	1000G	1000G_SAS	SIFT	Polyphen2	CONDEL	VEP	LRT	PhastCon7 Way	Total read (Adjacent normal)	Reference allel read support (Adjacent normal)	Alternative read support (Adjacent normal)	Total read (Tumor)	Reference allel read support (Tumor)	Alternative read support (Tumor)	Alternate allele frequency
Chewer	42482	chr2	71909625	G	A	*DYSF*	INTRONIC	ENSG00000135636:[ENST00000479049(n.2942-35G>A)];ENSG00000135636:[ENST00000409582(c.6171-35G>A)];ENSG00000135636:[ENST00000410020(c.6174-35G>A)];ENSG00000135636:[ENST00000409744(c.6081-35G>A)];ENSG00000135636:[ENST00000429174(c.6120-35G>A)];ENSG00000135636:[ENST00000409762(c.6108-35G>A)];ENSG00000135636:[ENST00000410041(c.6111-35G>A)];ENSG00000135636:[ENST00000409651(c.6153-35G>A)];ENSG00000135636:[ENST00000394120(c.6060-35G>A)];ENSG00000135636:[ENST00000409366(c.6123-35G>A)];ENSG00000135636:[ENST00000413539(c.6150-35G>A)];ENSG00000135636:[ENST00000258104(c.6057-35G>A)];	NA	NA	chr2*71909625*71909625*G*T*rs202056990*0*0.9998*0.0001997	NA	NA	NA	NA	NA	NA_NA	NA	MODIFIER	NA	NA	103	102	0	132	122	10	7.58
Chewer	42482	chr2	88879142	C	G	*EIF2AK3*	MISSENSE	ENSG00000172071:MISSENSE[ENST00000419748(c.1327G>C,Gag>Cag):ENSP00000408325(p.Glu443Gln)];ENSG00000172071:MISSENSE[ENST00000303236(c.1780G>C,Gag>Cag):ENSP00000307235(p.Glu594Gln)];ENSG00000172071:MISSENSE[ENST00000415570(c.1417G>C,Gag>Cag):ENSP00000412076(p.Glu473Gln)];ENSG00000172071:EXONIC-NC[ENST00000478003(n.346G>C)];	NA	NA	NA	NA	NA	NA	NA	NA	NA_NA	NA	MODIFIER	NA	NA	131	131	0	169	150	19	11.24
Chewer	42482	chr2	120932522	T	C	*EPB41L5*	3UTR	ENSG00000115109:[ENST00000443902(c.*46T>C)];ENSG00000115109:[ENST00000263713(c.*39T>C)];ENSG00000115109:[ENST00000452780(c.*39T>C)];	NA	NA	NA	NA	NA	NA	NA	NA	NA_NA	NA	MODIFIER	NA	NA	80	80	0	160	131	28	17.5
Chewer	42482	chr2	187618817	A	G	*FAM171B*	INTRONIC	ENSG00000144369:[ENST00000304698(c.1012+41A>G)];	NA	NA	NA	NA	NA	NA	NA	NA	NA_NA	NA	MODIFIER	NA	NA	132	130	0	168	143	25	14.88
Chewer	42482	chr2	164466896	T	G	*FIGN*	SILENT	ENSG00000182263:SILENT[ENST00000333129(c.1446A>C,ccA>ccC):ENSP00000333836(p.Pro482(=))];ENSG00000182263:INTRONIC[ENST00000409634(c.26-16590A>C)];	NA	NA	NA	NA	NA	NA	NA	NA	NA_NA	NA	MODIFIER	NA	NA	124	124	0	157	134	23	14.65
Chewer	42482	chr2	88747976	C	T	*FOXI3*	EXONIC-NC	ENSG00000214336:[ENST00000398142(n.1090G>A)];ENSG00000214336:[ENST00000428390(n.1010G>A)];	NA	NA	NA	NA	NA	NA	NA	NA	NA_NA	NA	MODIFIER	NA	NA	88	87	0	135	106	29	21.48
Chewer	42482	chr2	186620978	G	T	*FSIP2*	MISSENSE	ENSG00000188738:INTRONIC[ENST00000546113(c.391+2403G>T)];ENSG00000188738:INTRONIC[ENST00000429412(c.409+2403G>T)];ENSG00000188738:MISSENSE[ENST00000343098(c.1318G>T,Gac>Tac):ENSP00000344403(p.Asp440Tyr)];ENSG00000188738:MISSENSE[ENST00000424728(c.1051G>T,Gac>Tac):ENSP00000401306(p.Asp351Tyr)];	NA	NA	NA	NA	NA	NA	NA	NA	NA_NA	NA	MODIFIER	NA	NA	75	75	0	130	89	41	31.54
Chewer	42482	chr2	200713597	G	A	*FTCDNL1*	MISSENSE	ENSG00000226124:MISSENSE[ENST00000420128(c.89C>T,gCa>gTa):ENSP00000457780(p.Ala30Val)];ENSG00000226124:MISSENSE[ENST00000416668(c.89C>T,gCa>gTa):ENSP00000454447(p.Ala30Val)];ENSG00000226124:MISSENSE[ENST00000420922(c.89C>T,gCa>gTa):ENSP00000456442(p.Ala30Val)];	NA	NA	NA	NA	NA	NA	NA	T	BN_BN	N	MODERATE	NA	NA	151	151	0	412	167	245	59.47
Chewer	42482	chr2	37295977	G	A	*HEATR5B*	MISSENSE	ENSG00000008869:MISSENSE[ENST00000233099(c.1024C>T,Cgg>Tgg):ENSP00000233099(p.Arg342Trp)];ENSG00000008869:MISSENSE[ENST00000354531(c.1024C>T,Cgg>Tgg):ENSP00000346531(p.Arg342Trp)];	NA	NA	NA	8.25E-06	0	NA	NA	D	PrD_PrD	D	MODERATE	D	1	93	93	0	133	122	11	8.27
Chewer	42482	chr2	9621431	G	T	*IAH1*	MISSENSE	ENSG00000134330:MISSENSE[ENST00000481688(c.225G>T,caG>caT):ENSP00000420532(p.Gln75His)];ENSG00000134330:MISSENSE[ENST00000497473(c.300G>T,caG>caT):ENSP00000417580(p.Gln100His)];ENSG00000134330:MISSENSE[ENST00000481367(c.239G>T,caG>caT):ENSP00000419807(p.Gln80His)];ENSG00000134330:EXONIC-NC[ENST00000495494(n.532G>T)];ENSG00000134330:EXONIC-NC[ENST00000487850(n.50G>T)];ENSG00000134330:EXONIC-NC[ENST00000484826(n.19G>T)];ENSG00000134330:EXONIC-NC[ENST00000489468(n.345G>T)];ENSG00000134330:EXONIC-NC[ENST00000495050(n.250G>T)];ENSG00000134330:5UTR[ENST00000496603(c.-40G>T)];ENSG00000134330:5UTR[ENST00000545602(c.-40G>T)];ENSG00000134330:5UTR[ENST00000470914(c.-40G>T)];ENSG00000134330:5UTR[ENST00000482918(c.-40G>T)];ENSG00000134330:5UTR[ENST00000495797(c.-40G>T)];ENSG00000134330:3UTR[ENST00000351760(c.*31G>T)];ENSG00000134330:3UTR[ENST00000492223(c.*92G>T)];	NA	NA	NA	3.31E-05	0.000242365	NA	NA	NA	NA_NA	NA	MODIFIER	NA	NA	58	58	0	71	64	7	9.86
Chewer	42482	chr2	77745779	G	T	*LRRTM4*	MISSENSE	ENSG00000176204:MISSENSE[ENST00000409088(c.1216C>A,Cca>Aca):ENSP00000386236(p.Pro406Thr)];ENSG00000176204:MISSENSE[ENST00000409093(c.1216C>A,Cca>Aca):ENSP00000386357(p.Pro406Thr)];ENSG00000176204:MISSENSE[ENST00000409282(c.1219C>A,Cca>Aca):ENSP00000386286(p.Pro407Thr)];ENSG00000176204:MISSENSE[ENST00000409884(c.1216C>A,Cca>Aca):ENSP00000387297(p.Pro406Thr)];ENSG00000176204:MISSENSE[ENST00000409911(c.1219C>A,Cca>Aca):ENSP00000387228(p.Pro407Thr)];	chr2*77745	chr2*77745	NA	NA	NA	NA	NA	T	BN_BN	N	MODERATE	N	0.995	93	93	0	125	119	6	4.8
Chewer	42482	chr2	33447194	C	T	*LTBP1*	MISSENSE	ENSG00000049323:MISSENSE[ENST00000407925(c.874C>T,Cgg>Tgg):ENSP00000384091(p.Arg292Trp)];ENSG00000049323:MISSENSE[ENST00000402934(c.874C>T,Cgg>Tgg):ENSP00000384373(p.Arg292Trp)];ENSG00000049323:MISSENSE[ENST00000404816(c.1852C>T,Cgg>Tgg):ENSP00000386043(p.Arg618Trp)];ENSG00000049323:MISSENSE[ENST00000354476(c.1852C>T,Cgg>Tgg):ENSP00000346467(p.Arg618Trp)];ENSG00000049323:MISSENSE[ENST00000418533(c.874C>T,Cgg>Tgg):ENSP00000393057(p.Arg292Trp)];ENSG00000049323:MISSENSE[ENST00000404525(c.874C>T,Cgg>Tgg):ENSP00000385359(p.Arg292Trp)];ENSG00000049323:MISSENSE[ENST00000390003(c.874C>T,Cgg>Tgg):ENSP00000374653(p.Arg292Trp)];	NA	NA	NA	4.12E-05	0.000181818	NA	NA	D	PoD_PrD	D	MODERATE	NA	NA	178	177	1	226	193	33	14.6
Chewer	42482	chr2	169713251	C	G	*NOSTRIN*	MISSENSE	ENSG00000163072:3UTR[ENST00000427068(c.*354C>G)];ENSG00000163072:MISSENSE[ENST00000444448(c.1187C>G,gCa>gGa):ENSP00000394051(p.Ala396Gly)];ENSG00000163072:MISSENSE[ENST00000458381(c.1187C>G,gCa>gGa):ENSP00000402140(p.Ala396Gly)];ENSG00000163072:MISSENSE[ENST00000397209(c.932C>G,gCa>gGa):ENSP00000380392(p.Ala311Gly)];ENSG00000163072:MISSENSE[ENST00000445023(c.782C>G,gCa>gGa):ENSP00000404413(p.Ala261Gly)];ENSG00000163072:MISSENSE[ENST00000397206(c.782C>G,gCa>gGa):ENSP00000380390(p.Ala261Gly)];ENSG00000163072:MISSENSE[ENST00000317647(c.1016C>G,gCa>gGa):ENSP00000318921(p.Ala339Gly)];ENSG00000163072:MISSENSE[ENST00000421711(c.932C>G,gCa>gGa):ENSP00000401316(p.Ala311Gly)];ENSG00000163072:EXONIC-NC[ENST00000472260(n.1056C>G)];	NA	NA	NA	NA	NA	NA	NA	NA	NA_NA	NA	MODIFIER	NA	NA	178	177	1	208	197	11	5.29
Chewer	42482	chr2	178990909	G	A	*RBM45*	3UTR-SS-PRX	ENSG00000155636:[ENST00000286070(c.*6G>A)];ENSG00000155636:[ENST00000424099(c.*6G>A)];ENSG00000155636:[ENST00000455903(c.*6G>A)];ENSG00000155636:[ENST00000424000(n.1730G>A)];ENSG00000155636:[ENST00000464647(n.44G>A)];	NA	NA	NA	8.41E-06	0	NA	NA	NA	NA_NA	NA	LOW	NA	NA	162	162	0	234	145	89	38.03

Mangalaparthi *et al.* , 2020. Mutational landscape of esophageal squamous cell carcinoma in an Indian cohort
Supplementary Table 4. List of somatic single nucleotide variants identified using whole exome sequencing of ESCC patients

Cohort	Sample	Chromosome	Genomic position	Reference allele	Alternate allele	Gene symbol	Variant type	Variant annotation	OncoMD	COSMIC	dbSNP	ExAC	ExAC_SAS	1000G	1000G_SAS	SIFT	Polyphen2	CONDEL	VEP	LRT	PhastCon7 Way	Total read (Adjacent normal)	Reference allel read support (Adjacent normal)	Alternative read support (Adjacent normal)	Total read (Tumor)	Reference allel read support (Tumor)	Alternative read support (Tumor)	Alternate allele frequency
Chewer	42482	chr2	79314614	C	T	*REG1B*	INTRONIC	ENSG00000172023:[ENST00000476554(n.169+61G>A)];ENSG00000172023:[ENST00000479258(n.171+61G>A)];ENSG00000172023:[ENST00000305089(c.64+61G>A)];ENSG00000172023:[ENST00000469052(n.206G>A)];	NA	NA	NA	NA	NA	NA	NA	NA	NA_NA	NA	MODIFIER	NA	NA	118	118	0	146	121	25	17.12
Chewer	42482	chr2	192700912	C	T	*SDPR*	MISSENSE	ENSG00000168497:MISSENSE[ENST00000304141(c.1015G>A,Gaa>Aaa):ENSP00000305675(p.Glu339Lys)];	NA	chr2*19270(	0.0007987	0.0004049	0.000484907	0.0007987	0.0031	D	BN_PrD	D	MODERATE	N	0.005	201	200	1	419	366	53	12.65
Chewer	42482	chr2	172693715	C	G	*SLC25A12*	MISSENSE	ENSG00000115840:STOPLOSS[ENST00000426896(c.632G>C,tGa>tCa):ENSP00000413968(p.Ter211SerextTer26)];ENSG00000115840:MISSENSE[ENST00000422440(c.528G>C,atG>atC):ENSP00000388658(p.Met176Ile)];ENSG00000115840:MISSENSE[ENST00000392592(c.207G>C,atG>atC):ENSP00000376371(p.Met69Ile)];ENSG00000115840:3UTR[ENST00000263812(c.*148G>C)];ENSG00000115840:3UTR[ENST00000475360(c.*245G>C)];	NA	NA	NA	NA	NA	NA	NA	T	BN_BN	N	MODERATE	N	0.932	211	211	0	277	231	46	16.61
Chewer	42482	chr2	190443104	T	C	*SLC40A1*	EXONIC-NC	ENSG00000138449:[ENST00000418714(n.529A>G)];ENSG00000138449:[ENST00000479598(n.392+1435A>G)];ENSG00000138449:[ENST00000261024(c.111+1435A>G)];ENSG00000138449:[ENST00000455320(c.111+1435A>G)];ENSG00000138449:[ENST00000427241(c.111+1435A>G)];	NA	NA	NA	NA	NA	NA	NA	NA	NA_NA	NA	MODIFIER	NA	NA	179	179	0	517	499	17	3.29
Chewer	42482	chr2	169713251	C	G	*SPC25*	INTRONIC	ENSG00000152253:[ENST00000479309(n.419+16844G>C)];	NA	NA	NA	NA	NA	NA	NA	NA	NA_NA	NA	MODIFIER	NA	NA	178	177	1	208	197	11	5.29
Chewer	42482	chr2	182785387	C	A	*SSFA2*	MISSENSE-SS-PRX	ENSG00000138434:[ENST00000409136(c.1735C>A,Cca>Aca):ENSP00000386916(p.Pro579Thr)];ENSG00000138434:[ENST00000431877(c.3208C>A,Cca>Aca):ENSP00000388731(p.Pro1070Thr)];ENSG00000138434:[ENST00000320370(c.3208C>A,Cca>Aca):ENSP00000314669(p.Pro1070Thr)];ENSG00000138434:[ENST00000409001(c.3144+1214C>A)];ENSG00000138434:[ENST00000428267(c.2685+1214C>A)];ENSG00000138434:[ENST00000454579(c.*2681+1214C>A)];ENSG00000138434:[ENST00000416081(c.*2745C>A)];ENSG00000138434:[ENST00000440623(c.*3323C>A)];ENSG00000138434:[ENST00000491720(n.4710C>A)];ENSG00000138434:[ENST00000467172(n.237C>A)];ENSG00000138434:[ENST00000451836(c.45+10C>A)];	NA	NA	NA	NA	NA	NA	NA	NA	NA_NA	NA	LOW	NA	NA	136	136	0	192	179	13	6.77
Chewer	42482	chr2	1497590	G	A	*TPO*	SILENT	ENSG00000115705:SILENT[ENST00000329066(c.1785G>A,agG>agA):ENSP00000329869(p.Arg595(=))];ENSG00000115705:SILENT[ENST00000337415(c.1785G>A,agG>agA):ENSP00000337263(p.Arg595(=))];ENSG00000115705:SILENT[ENST00000382198(c.1266G>A,agG>agA):ENSP00000371633(p.Arg422(=))];ENSG00000115705:SILENT[ENST00000382201(c.1614G>A,agG>agA):ENSP00000371636(p.Arg538(=))];ENSG00000115705:SILENT[ENST00000446278(c.209G>A,agG>agA):ENSP00000400033(p.Arg70(=))];ENSG00000115705:SILENT[ENST00000349624(c.1266G>A,agG>agA):ENSP00000332044(p.Arg422(=))];ENSG00000115705:SILENT[ENST00000346956(c.1785G>A,agG>agA):ENSP00000263886(p.Arg595(=))];ENSG00000115705:SILENT[ENST00000469607(c.207G>A,agG>agA):ENSP00000419461(p.Arg69(=))];ENSG00000115705:SILENT[ENST00000422464(c.1572G>A,agG>agA):ENSP00000405788(p.Arg524(=))];ENSG00000115705:SILENT[ENST00000345913(c.1785G>A,agG>agA):ENSP00000318820(p.Arg595(=))];ENSG00000115705:EXONIC-NC[ENST00000462973(n.203G>A)];ENSG00000115705:EXONIC-NC[ENST00000497517(n.456G>A)];	NA	NA	NA	NA	NA	NA	NA	NA	NA_NA	NA	LOW	NA	0.97	93	93	0	146	130	16	10.96
Chewer	42482	chr2	166781137	C	A	*TTC21B*	MISSENSE	ENSG00000123607:MISSENSE[ENST00000243344(c.1438G>T,Gtc>Ttc):ENSP00000243344(p.Val480Phe)];	NA	NA	chr2*166781137*166781137*C*T*rs763244073*NA*NA*NA	NA	NA	NA	NA	D	BN_PoD	D	MODERATE	N	0.995	131	131	0	142	125	17	11.97
Chewer	42482	chr2	179465764	G	T	*TTN*	MISSENSE	ENSG00000155657:MISSENSE[ENST00000359218(c.29047C>A,Cct>Act):ENSP00000352154(p.Pro9683Thr)];ENSG00000155657:MISSENSE[ENST00000591111(c.50944C>A,Cct>Act):ENSP00000465570(p.Pro16982Thr)];ENSG00000155657:MISSENSE[ENST00000589042(c.55867C>A,Cct>Act):ENSP00000467141(p.Pro18623Thr)];ENSG00000155657:MISSENSE[ENST00000342175(c.29248C>A,Cct>Act):ENSP00000340554(p.Pro9750Thr)];ENSG00000155657:MISSENSE[ENST00000342992(c.48163C>A,Cct>Act):ENSP00000343764(p.Pro16055Thr)];ENSG00000155657:MISSENSE[ENST00000460472(c.28672C>A,Cct>Act):ENSP00000434586(p.Pro9558Thr)];	NA	NA	chr2*179465764*179465764*G*A*rs754308357*NA*NA*NA	NA	NA	NA	NA	NA	NA_NA	NA	MODIFIER	NA	NA	90	90	0	191	178	13	6.81
Chewer	42482	chr2	179480201	G	C	*TTN*	MISSENSE	ENSG00000155657:MISSENSE[ENST00000359218(c.21651C>G,gaC>gaG):ENSP00000352154(p.Asp7217Glu)];ENSG00000155657:MISSENSE[ENST00000591111(c.43548C>G,gaC>gaG):ENSP00000465570(p.Asp14516Glu)];ENSG00000155657:MISSENSE[ENST00000589042(c.48471C>G,gaC>gaG):ENSP00000467141(p.Asp16157Glu)];ENSG00000155657:MISSENSE[ENST00000342992(c.40767C>G,gaC>gaG):ENSP00000343764(p.Asp13589Glu)];ENSG00000155657:MISSENSE[ENST00000342175(c.21852C>G,gaC>gaG):ENSP00000340554(p.Asp7284Glu)];ENSG00000155657:MISSENSE[ENST00000460472(c.21276C>G,gaC>gaG):ENSP00000434586(p.Asp7092Glu)];	chr2*17948(	chr2*17948(	NA	NA	NA	NA	NA	NA	NA_NA	NA	MODIFIER	NA	NA	95	95	0	161	97	64	39.75
Chewer	42482	chr2	179539821	C	T	*TTN*	SILENT	ENSG00000155657:INTRONIC[ENST00000414766(c.2267-685G>A)];ENSG00000155657:INTRONIC[ENST00000359218(c.13658-32777G>A)];ENSG00000155657:INTRONIC[ENST00000342175(c.13859-32777G>A)];ENSG00000155657:INTRONIC[ENST00000460472(c.13283-32777G>A)];ENSG00000155657:SILENT[ENST00000342992(c.30654G>A,aaG>aaA):ENSP00000343764(p.Lys10218(=))];ENSG00000155657:SILENT[ENST00000589042(c.34557G>A,aaG>aaA):ENSP00000467141(p.Lys11519(=))];ENSG00000155657:SILENT[ENST00000591111(c.33435G>A,aaG>aaA):ENSP00000465570(p.Lys11145(=))];	chr2*17953(	chr2*17953(	NA	NA	NA	NA	NA	NA	NA_NA	NA	MODIFIER	NA	NA	63	63	0	120	108	12	10
Chewer	42482	chr2	179544081	G	T	*TTN*	MISSENSE	ENSG00000155657:INTRONIC[ENST00000414766(c.2015-524C>A)];ENSG00000155657:INTRONIC[ENST00000359218(c.13658-37037C>A)];ENSG00000155657:INTRONIC[ENST00000342175(c.13859-37037C>A)];ENSG00000155657:INTRONIC[ENST00000460472(c.13283-37037C>A)];ENSG00000155657:MISSENSE[ENST00000342992(c.29995C>A,Cct>Act):ENSP00000343764(p.Pro9999Thr)];ENSG00000155657:MISSENSE[ENST00000589042(c.33727C>A,Cct>Act):ENSP00000467141(p.Pro11243Thr)];ENSG00000155657:MISSENSE[ENST00000591111(c.32776C>A,Cct>Act):ENSP00000465570(p.Pro10926Thr)];	NA	NA	NA	NA	NA	NA	NA	NA	NA_NA	NA	MODIFIER	NA	NA	260	260	0	393	377	16	4.07
Chewer	42482	chr2	179594422	C	A	*TTN*	SILENT	ENSG00000155657:SILENT[ENST00000342992(c.14826G>T,acG>acT):ENSP00000343764(p.Thr4942(=))];ENSG00000155657:SILENT[ENST00000589042(c.18558G>T,acG>acT):ENSP00000467141(p.Thr6186(=))];ENSG00000155657:SILENT[ENST00000591111(c.17607G>T,acG>acT):ENSP00000465570(p.Thr5869(=))];ENSG00000155657:INTRONIC[ENST00000342175(c.13858+8387G>T)];ENSG00000155657:INTRONIC[ENST00000359218(c.13657+8387G>T)];ENSG00000155657:INTRONIC[ENST00000460472(c.13282+8387G>T)];	chr2*17959(	chr2*17959(	chr2*179594422*179594422*C*T*rs778145097*NA*NA*NA	NA	NA	NA	NA	NA	NA_NA	NA	MODIFIER	NA	NA	161	160	0	242	218	24	9.92

Mangalaparthi *et al.* , 2020. Mutational landscape of esophageal squamous cell carcinoma in an Indian cohort
Supplementary Table 4. List of somatic single nucleotide variants identified using whole exome sequencing of ESCC patients

Cohort	Sample	Chromosome	Genomic position	Reference allele	Alternate allele	Gene symbol	Variant type	Variant annotation	OncoMD	COSMIC	dbSNP	ExAC	ExAC_SAS	1000G	1000G_SAS	SIFT	Polyphen2	CONDEL	VEP	LRT	PhastCon7 Way	Total read (Adjacent normal)	Reference allel read support (Adjacent normal)	Alternative read support (Adjacent normal)	Total read (Tumor)	Reference allel read support (Tumor)	Alternative read support (Tumor)	Alternate allele frequency
Chewer	42482	chr2	179465764	G	T	*TTN-AS1*	INTRONIC	ENSG00000237298:[ENST00000592630(n.519+3356G>T)];ENSG00000237298:[ENST00000589234(n.510+370G>T)];ENSG00000237298:[ENST00000590807(n.395+370G>T)];ENSG00000237298:[ENST00000585451(n.198+77401G>T)];ENSG00000237298:[ENST00000592750(n.431+3356G>T)];ENSG00000237298:[ENST00000589830(n.86+3356G>T)];ENSG00000237298:[ENST00000589487(n.110+370G>T)];ENSG00000237298:[ENST00000419746(n.3917+370G>T)];ENSG00000237298:[ENST00000456053(n.649+3356G>T)];ENSG00000237298:[ENST00000586452(n.753+370G>T)];ENSG00000237298:[ENST00000589907(n.506+3356G>T)];	NA	NA	chr2*179465764*179465764*G*A*rs754308357*NA*NA*NA	NA	NA	NA	NA	NA	NA_NA	NA	MODIFIER	NA	NA	90	90	0	191	178	13	6.81
Chewer	42482	chr2	179480201	G	C	*TTN-AS1*	INTRONIC	ENSG00000237298:[ENST00000592630(n.519+17793G>C)];ENSG00000237298:[ENST00000590807(n.395+14807G>C)];ENSG00000237298:[ENST00000585451(n.198+91838G>C)];ENSG00000237298:[ENST00000592750(n.483+100G>C)];ENSG00000237298:[ENST00000589830(n.86+17793G>C)];ENSG00000237298:[ENST00000589487(n.110+14807G>C)];ENSG00000237298:[ENST00000456053(n.1571+100G>C)];ENSG00000237298:[ENST00000589907(n.507-16901G>C)];	chr2*17948	chr2*17948	NA	NA	NA	NA	NA	NA	NA_NA	NA	MODIFIER	NA	NA	95	95	0	161	97	64	39.75
Chewer	42482	chr2	179539821	C	T	*TTN-AS1*	INTRONIC	ENSG00000237298:[ENST00000592630(n.520-38660C>T)];ENSG00000237298:[ENST00000585451(n.199-85846C>T)];ENSG00000237298:[ENST00000431752(n.124+38396C>T)];ENSG00000237298:[ENST00000589830(n.211+38396C>T)];ENSG00000237298:[ENST00000589487(n.319-8C>T)];	chr2*17953	chr2*17953	NA	NA	NA	NA	NA	NA	NA_NA	NA	MODIFIER	NA	NA	63	63	0	120	108	12	10
Chewer	42482	chr2	179544081	G	T	*TTN-AS1*	INTRONIC	ENSG00000237298:[ENST00000592630(n.520-34400G>T)];ENSG00000237298:[ENST00000589487(n.939+775G>T)];ENSG00000237298:[ENST00000585451(n.199-81586G>T)];ENSG00000237298:[ENST00000431752(n.125-34400G>T)];ENSG00000237298:[ENST00000589830(n.212-34400G>T)];	NA	NA	NA	NA	NA	NA	NA	NA	NA_NA	NA	MODIFIER	NA	NA	260	260	0	393	377	16	4.07
Chewer	42482	chr2	179594422	C	A	*TTN-AS1*	INTRONIC	ENSG00000237298:[ENST00000585451(n.199-31245C>A)];	chr2*17959	chr2*17959	chr2*179594422*179594422*C*T*rs778145097*NA*NA*NA	NA	NA	NA	NA	NA	NA_NA	NA	MODIFIER	NA	NA	161	160	0	242	218	24	9.92
Chewer	42482	chr2	234581202	C	T	*UGT1A10*	INTRONIC	ENSG00000242515:[ENST00000373445(c.855+35179C>T)];ENSG00000242515:[ENST00000344644(c.855+35179C>T)];	NA	NA	0.0007987	0.0001236	0.000121124	0.0007987	0	T	BN_BN	N	MODERATE	NA	NA	455	454	1	506	400	106	20.95
Chewer	42482	chr2	234581202	C	T	*UGT1A8*	MISSENSE	ENSG00000241635:INTRONIC[ENST00000373450(c.855+53994C>T)];ENSG00000241635:MISSENSE[ENST00000609637(c.622C>T,Cgg>Tgg):ENSP00000476286(p.Arg208Trp)];	NA	NA	0.0007987	0.0001236	0.000121124	0.0007987	0	T	BN_BN	N	MODERATE	NA	NA	455	454	1	506	400	106	20.95
Chewer	42482	chr2	234581202	C	T	*UGT1A9*	MISSENSE	ENSG00000241119:MISSENSE[ENST00000354728(c.622C>T,Cgg>Tgg):ENSP00000346768(p.Arg208Trp)];	NA	NA	0.0007987	0.0001236	0.000121124	0.0007987	0	T	BN_BN	N	MODERATE	NA	NA	455	454	1	506	400	106	20.95
Chewer	42482	chr2	219296767	A	C	*VIL1*	INTRONIC-SS-ACR	ENSG00000127831:INTRONIC-SS-ACR[ENST00000392114(c.271-2A>C)];ENSG00000127831:INTRONIC-SS-ACR[ENST00000248444(c.1204-2A>C)];	NA	NA	NA	NA	NA	NA	NA	NA	NA_NA	NA	HIGH	NA	NA	51	51	0	64	58	6	9.38
Chewer	42482	chr2	63436661	G	T	*WDPCP*	INTRONIC	ENSG00000143951:[ENST00000409199(c.1340-34694C>A)];ENSG00000143951:[ENST00000409120(c.1340-34694C>A)];ENSG00000143951:[ENST00000398544(c.1439-34694C>A)];ENSG00000143951:[ENST00000272321(c.1916-34694C>A)];ENSG00000143951:[ENST00000409354(c.1174-34694C>A)];	NA	NA	NA	NA	NA	NA	NA	NA	NA_NA	NA	MODIFIER	NA	NA	72	72	0	106	91	15	14.15
Chewer	42482	chr20	31601803	G	T	*BPIFB2*	INTRONIC	ENSG00000078898:[ENST00000170150(c.455+41G>T)];	NA	NA	chr20*31601803*31601803*G*A*rs865839112*NA*NA*NA	NA	NA	NA	NA	NA	NA_NA	NA	MODIFIER	NA	NA	59	59	0	67	60	7	10.45
Chewer	42482	chr20	2777831	T	A	*CPXM1*	INTRONIC-SS-DNR	ENSG00000088882:[ENST00000380605(c.832+7A>T)];	NA	NA	NA	NA	NA	NA	NA	NA	NA_NA	NA	LOW	NA	NA	47	47	0	60	53	7	11.67
Chewer	42482	chr20	4913180	G	C	*SLC23A2*	MISSENSE	ENSG00000089057:MISSENSE[ENST00000424750(c.29C>G,tCc>tGc):ENSP00000406601(p.Ser10Cys)];ENSG00000089057:MISSENSE[ENST00000379333(c.29C>G,tCc>tGc):ENSP00000368637(p.Ser10Cys)];ENSG00000089057:MISSENSE[ENST00000338244(c.29C>G,tCc>tGc):ENSP00000344322(p.Ser10Cys)];ENSG00000089057:EXONIC-NC[ENST00000468355(n.395C>G)];	NA	NA	NA	NA	NA	NA	NA	NA	NA_NA	NA	MODIFIER	NA	NA	106	105	0	122	113	9	7.38
Chewer	42482	chr21	15974304	G	A	*AF165138.7*	INTRONIC	ENSG00000243440:[ENST00000400562(c.399+40C>T)];ENSG00000243440:[ENST00000435732(c.375+40C>T)];	NA	NA	NA	NA	NA	NA	NA	NA	NA_NA	NA	MODIFIER	NA	NA	26	26	0	78	67	11	14.1
Chewer	42482	chr21	47978412	C	T	*DIP2A*	INTRONIC	ENSG00000160305:[ENST00000400274(c.3957+106C>T)];ENSG00000160305:[ENST00000417564(c.3969+106C>T)];ENSG00000160305:[ENST00000318711(c.3972+106C>T)];ENSG00000160305:[ENST00000481883(n.2512C>T)];ENSG00000160305:[ENST00000472364(n.2303C>T)];	NA	NA	NA	NA	NA	NA	NA	NA	NA_NA	NA	MODIFIER	NA	NA	117	116	0	172	143	28	16.28
Chewer	42482	chr21	41648028	A	G	*DSCAM*	SILENT	ENSG00000171587:SILENT[ENST00000404019(c.1608T>C,gtT>gtC):ENSP00000385342(p.Val536(=))];ENSG00000171587:SILENT[ENST00000400454(c.2352T>C,gtT>gtC):ENSP00000383303(p.Val784(=))];	NA	NA	NA	NA	NA	NA	NA	NA	NA_NA	NA	LOW	NA	NA	65	65	0	100	81	19	19
Chewer	42482	chr21	9825769	C	A	*NA*	INTERGENIC		NA	NA	NA	NA	NA	NA	NA	NA	NA_NA	NA	MODIFIER	NA	NA	550	540	10	757	734	23	3.04
Chewer	42482	chr21	43239978	G	A	*PRDM15*	SILENT	ENSG00000141956:SILENT[ENST00000433067(c.3318C>T,caC>caT):ENSP00000415471(p.His1106(=))];ENSG00000141956:SILENT[ENST00000269844(c.3318C>T,caC>caT):ENSP00000269844(p.His1106(=))];ENSG00000141956:SILENT[ENST00000422911(c.2391C>T,caC>caT):ENSP00000408592(p.His797(=))];ENSG00000141956:SILENT[ENST00000447016(c.2220C>T,caC>caT):ENSP00000431410(p.His740(=))];ENSG00000141956:SILENT[ENST00000398548(c.2331C>T,caC>caT):ENSP00000381556(p.His777(=))];ENSG00000141956:SILENT[ENST00000447207(c.2220C>T,caC>caT):ENSP00000390245(p.His740(=))];ENSG00000141956:SILENT[ENST00000538201(c.2280C>T,caC>caT):ENSP00000444044(p.His760(=))];ENSG00000141956:3UTR[ENST00000449395(c.*671C>T)];ENSG00000141956:3UTR[ENST00000441787(c.*671C>T)];ENSG00000141956:EXONIC-NC[ENST00000495217(n.162C>T)];	NA	NA	0.0001997	5.56E-05	0.000340832	0.0001997	0.001	NA	NA_NA	NA	LOW	NA	NA	110	109	1	151	119	32	21.19
Chewer	42482	chr21	32491038	G	C	*TIAM1*	3UTR	ENSG00000156299:[ENST00000286827(c.*1648C>G)];ENSG00000156299:[ENST00000423206(c.271-50C>G)];	NA	NA	chr21*32491037*32491038*AG*A*rs773969647*NA*NA*NA	NA	NA	NA	NA	NA	NA_NA	NA	MODIFIER	NA	NA	108	108	0	149	134	15	10.07
Chewer	42482	chr21	10942717	C	A	*TPTE*	MISSENSE	ENSG00000166157:MISSENSE[ENST00000361285(c.724G>T,Gtt>Ttt):ENSP00000355208(p.Val242Phe)];ENSG00000166157:MISSENSE[ENST00000342420(c.610G>T,Gtt>Ttt):ENSP00000344441(p.Val204Phe)];ENSG00000166157:MISSENSE[ENST00000298232(c.670G>T,Gtt>Ttt):ENSP00000298232(p.Val224Phe)];ENSG00000166157:EXONIC-NC[ENST00000415664(n.2346G>T)];ENSG00000166157:EXONIC-NC[ENST00000447568(n.778G>T)];	chr21*1094	chr21*1094	NA	8.24E-06	0	NA	NA	NA	NA_NA	NA	MODIFIER	NA	NA	761	761	0	1032	930	101	9.79
Chewer	42482	chr22	29819080	C	A	*AC000041.8*	INTRONIC	ENSG00000242156:[ENST00000439655(n.69+34G>T)];	NA	NA	NA	NA	NA	NA	NA	NA	NA_NA	NA	MODIFIER	NA	NA	49	49	0	35	29	6	17.14

Mangalaparthi *et al.* , 2020. Mutational landscape of esophageal squamous cell carcinoma in an Indian cohort
Supplementary Table 4. List of somatic single nucleotide variants identified using whole exome sequencing of ESCC patients

Cohort	Sample	Chromosome	Genomic position	Reference allele	Alternate allele	Gene symbol	Variant type	Variant annotation	OncoMD	COSMIC	dbSNP	ExAC	ExAC_SAS	1000G	1000G_SAS	SIFT	Polyphen2	CONDEL	VEP	LRT	PhastCon7 Way	Total read (Adjacent normal)	Reference allel read support (Adjacent normal)	Alternative read support (Adjacent normal)	Total read (Tumor)	Reference allel read support (Tumor)	Alternative read support (Tumor)	Alternate allele frequency
Chewer	42482	chr22	29763162	C	A	*AP1B1*	INTRONIC	ENSG00000100280:[ENST00000356015(c.37+34G>T)];ENSG00000100280:[ENST00000402502(c.37+34G>T)];ENSG00000100280:[ENST00000421126(c.37+34G>T)];ENSG00000100280:[ENST00000473606(n.167+34G>T)];ENSG00000100280:[ENST00000357586(c.37+34G>T)];ENSG00000100280:[ENST00000405198(c.37+34G>T)];ENSG00000100280:[ENST00000432560(c.37+34G>T)];ENSG00000100280:[ENST00000415447(c.144-7214G>T)];ENSG00000100280:[ENST00000317368(c.37+34G>T)];	NA	NA	NA	NA	NA	NA	NA	NA	NA_NA	NA	MODIFIER	NA	NA	190	190	0	275	263	12	4.36
Chewer	42482	chr22	29819080	C	A	*AP1B1*	INTRONIC	ENSG00000100280:[ENST00000415447(c.37+34G>T)];	NA	NA	NA	NA	NA	NA	NA	NA	NA_NA	NA	MODIFIER	NA	NA	49	49	0	35	29	6	17.14
Chewer	42482	chr22	40075096	C	T	*CACNA1I*	INTRONIC	ENSG00000100346:[ENST00000401624(c.5056-16C>T)];ENSG00000100346:[ENST00000407673(c.4951-16C>T)];ENSG00000100346:[ENST00000402142(c.5056-16C>T)];ENSG00000100346:[ENST00000400164(c.4951-16C>T)];ENSG00000100346:[ENST00000336649(c.5074-16C>T)];ENSG00000100346:[ENST00000404898(c.4951-16C>T)];	NA	NA	NA	NA	NA	NA	NA	NA	NA_NA	NA	MODIFIER	NA	NA	53	53	0	65	46	19	29.23
Chewer	42482	chr22	19226784	C	G	*CLTCL1*	INTRONIC	ENSG00000070371:[ENST00000413132(n.815+14G>C)];ENSG00000070371:[ENST00000427926(c.795+14G>C)];ENSG00000070371:[ENST00000505027(c.795+14G>C)];ENSG00000070371:[ENST00000263200(c.795+14G>C)];ENSG00000070371:[ENST00000353891(c.795+14G>C)];	NA	NA	NA	NA	NA	NA	NA	NA	NA_NA	NA	MODIFIER	NA	NA	103	103	0	157	145	12	7.64
Chewer	42482	chr22	37707557	C	T	*CYTH4*	MISSENSE	ENSG00000100055:MISSENSE[ENST00000248901(c.946C>T,Ccc>Tcc):ENSP00000248901(p.Pro316Ser)];ENSG00000100055:MISSENSE[ENST00000446506(c.203C>T,Ccc>Tcc):ENSP00000409579(p.Pro69Ser)];ENSG00000100055:EXONIC-NC[ENST00000462927(n.1106C>T)];	NA	NA	NA	NA	NA	NA	NA	NA	NA_NA	NA	MODIFIER	NA	NA	104	102	0	188	179	9	4.79
Chewer	42482	chr22	25434812	C	T	*KIAA1671*	MISSENSE	ENSG00000197077:MISSENSE[ENST00000406486(c.1715C>T,aCg>aTg):ENSP00000385152(p.Thr572Met)];ENSG00000197077:MISSENSE[ENST00000358431(c.1715C>T,aCg>aTg):ENSP00000351207(p.Thr572Met)];	NA	NA	NA	NA	NA	NA	NA	D	PoD_PrD	D	MODERATE	N	0.282	95	94	0	93	85	8	8.6
Chewer	42482	chr22	38421515	C	T	*POLR2F*	INTRONIC	ENSG00000100142:[ENST00000407936(c.453-15560C>T)];ENSG00000100142:[ENST00000333418(c.121-361C>T)];ENSG00000100142:[ENST00000427034(c.115-361C>T)];ENSG00000100142:[ENST00000405557(c.294-71C>T)];	NA	NA	NA	NA	NA	NA	NA	NA	NA_NA	NA	MODIFIER	NA	NA	101	101	0	104	88	16	15.38
Chewer	42482	chr22	19164702	C	A	*SLC25A1*	MISSENSE	ENSG00000100075:MISSENSE[ENST00000215882(c.457G>T,Gac>Tac):ENSP00000215882(p.Asp153Tyr)];ENSG00000100075:MISSENSE[ENST00000451283(c.148G>T,Gac>Tac):ENSP00000401480(p.Asp50Tyr)];ENSG00000100075:EXONIC-NC[ENST00000461267(n.603G>T)];ENSG00000100075:EXONIC-NC[ENST00000470922(n.599G>T)];	chr22*1916	chr22*1916	NA	NA	NA	NA	NA	NA	NA_NA	NA	MODIFIER	NA	NA	80	80	0	140	116	24	17.14
Chewer	42482	chr3	183689645	C	T	*ABCC5*	SILENT	ENSG00000114770:SILENT[ENST00000265586(c.1467G>A,aaG>aaA):ENSP00000265586(p.Lys489(=))];ENSG00000114770:SILENT[ENST00000334444(c.1467G>A,aaG>aaA):ENSP00000333926(p.Lys489(=))];ENSG00000114770:3UTR[ENST00000437205(c.*160G>A)];	NA	NA	NA	NA	NA	NA	NA	NA	NA_NA	NA	MODIFIER	NA	NA	97	96	0	135	77	58	42.96
Chewer	42482	chr3	169485960	C	G	*ACTRT3*	MISSENSE	ENSG00000184378:MISSENSE[ENST00000330368(c.379G>C,Ggt>Cgt):ENSP00000333037(p.Gly127Arg)];	NA	NA	NA	NA	NA	NA	NA	T	BN_BN	N	MODERATE	N	0.502	114	114	0	210	203	7	3.33
Chewer	42482	chr3	119305386	A	T	*ADPRH*	MISSENSE	ENSG00000144843:MISSENSE[ENST00000478399(c.553A>T,Agc>Tgc):ENSP00000420200(p.Ser185Cys)];ENSG00000144843:MISSENSE[ENST00000465513(c.553A>T,Agc>Tgc):ENSP00000417430(p.Ser185Cys)];ENSG00000144843:MISSENSE[ENST00000357003(c.553A>T,Agc>Tgc):ENSP00000349496(p.Ser185Cys)];ENSG00000144843:MISSENSE[ENST00000478927(c.553A>T,Agc>Tgc):ENSP00000417528(p.Ser185Cys)];ENSG00000144843:EXONIC-NC[ENST00000471850(n.643A>T)];ENSG00000144843:EXONIC-NC[ENST00000485496(n.579A>T)];	NA	NA	NA	NA	NA	NA	NA	NA	NA_NA	NA	MODIFIER	NA	NA	73	72	0	98	79	19	19.39
Chewer	42482	chr3	119248793	G	T	*CD80*	INTRONIC	ENSG00000121594:[ENST00000383668(c.419-2105C>A)];ENSG00000121594:[ENST00000383669(c.701-2105C>A)];ENSG00000121594:[ENST00000264246(c.701-9C>A)];ENSG00000121594:[ENST00000478182(c.701-9C>A)];	NA	NA	NA	NA	NA	NA	NA	NA	NA_NA	NA	MODIFIER	NA	NA	110	109	1	154	122	32	20.78
Chewer	42482	chr3	98518584	G	A	*DCBLD2*	MISSENSE	ENSG00000057019:MISSENSE[ENST00000326840(c.1960C>T,Cct>Tct):ENSP00000321573(p.Pro654Ser)];ENSG00000057019:MISSENSE[ENST00000326857(c.2002C>T,Cct>Tct):ENSP00000321646(p.Pro668Ser)];	NA	NA	NA	NA	NA	NA	NA	NA	NA_NA	NA	MODIFIER	NA	NA	122	122	0	161	152	9	5.59
Chewer	42482	chr3	38103753	G	C	*DLEC1*	MISSENSE	ENSG00000008226:EXONIC-NC[ENST00000440294(n.788G>C)];ENSG00000008226:MISSENSE[ENST00000308059(c.767G>C,tGc>tCc):ENSP00000308597(p.Cys256Ser)];ENSG00000008226:MISSENSE[ENST00000346219(c.767G>C,tGc>tCc):ENSP00000315914(p.Cys256Ser)];ENSG00000008226:MISSENSE[ENST00000452631(c.767G>C,tGc>tCc):ENSP00000410427(p.Cys256Ser)];	NA	NA	NA	NA	NA	NA	NA	T	BN_BN	N	MODERATE	N	0.004	178	177	0	174	133	41	23.56
Chewer	42482	chr3	109047934	A	G	*DPPA4*	EXONIC-NC-SS-PR	ENSG00000121570:[ENST00000487299(n.662T>C)];ENSG00000121570:[ENST00000495679(c.*401T>C)];ENSG00000121570:[ENST00000463966(n.396-6T>C)];ENSG00000121570:[ENST00000475135(n.1674T>C)];ENSG00000121570:[ENST00000335658(c.681T>C,ggT>ggC):ENSP00000335306(p.Gly227(=))];	NA	NA	NA	NA	NA	NA	NA	NA	NA_NA	NA	LOW	NA	NA	125	124	0	158	149	8	5.06
Chewer	42482	chr3	183888172	G	T	*DVL3*	MISSENSE	ENSG00000161202:MISSENSE[ENST00000313143(c.1780G>T,Ggg>Tgg):ENSP00000316054(p.Gly594Trp)];ENSG00000161202:MISSENSE[ENST00000431765(c.1729G>T,Ggg>Tgg):ENSP00000405885(p.Gly577Trp)];ENSG00000161202:EXONIC-NC[ENST00000478247(n.1780G>T)];	NA	NA	chr3*183888172*183888172*G*A*rs374856983*NA*NA*NA	NA	NA	NA	NA	NA	NA_NA	NA	MODIFIER	NA	NA	107	104	3	167	104	62	37.13
Chewer	42482	chr3	183888172	G	T	*EIF2B5*	INTRONIC	ENSG00000145191:[ENST00000444495(c.2106+25677G>T)];	NA	NA	chr3*183888172*183888172*G*A*rs374856983*NA*NA*NA	NA	NA	NA	NA	NA	NA_NA	NA	MODIFIER	NA	NA	107	104	3	167	104	62	37.13
Chewer	42482	chr3	183956551	A	G	*EIF2B5*	INTRONIC	ENSG00000145191:[ENST00000444495(c.2106+94056A>G)];	NA	NA	NA	NA	NA	NA	NA	T	BN_BN	N	MODERATE	N	0.03	74	74	0	126	85	41	32.54
Chewer	42482	chr3	43095198	A	G	*FAM198A*	INTRONIC	ENSG00000144649:[ENST00000430121(c.1413+63A>G)];ENSG00000144649:[ENST00000488863(c.126+63A>G)];ENSG00000144649:[ENST00000273146(c.1413+63A>G)];ENSG00000144649:[ENST00000492161(n.505A>G)];ENSG00000144649:[ENST00000434206(n.1525A>G)];	NA	NA	NA	NA	NA	NA	NA	NA	NA_NA	NA	MODIFIER	NA	NA	171	171	0	169	162	7	4.14
Chewer	42482	chr3	159997130	T	A	*IFT80*	MISSENSE	ENSG00000068885:MISSENSE[ENST00000483465(c.1276A>T,Att>Ttt):ENSP00000418196(p.Ile426Phe)];ENSG00000068885:MISSENSE[ENST00000496589(c.1276A>T,Att>Ttt):ENSP00000420646(p.Ile426Phe)];ENSG00000068885:MISSENSE[ENST00000326448(c.1687A>T,Att>Ttt):ENSP00000312778(p.Ile563Phe)];ENSG00000068885:EXONIC-NC[ENST00000487943(n.2906A>T)];	NA	NA	NA	NA	NA	NA	NA	D	PrD_PrD	D	MODERATE	NA	NA	79	79	0	97	77	19	19.59
Chewer	42482	chr3	43095198	A	G	*KRBOX1*	INTRONIC	ENSG00000273291:[ENST00000443313(c.*79+63A>G)];	NA	NA	NA	NA	NA	NA	NA	NA	NA_NA	NA	MODIFIER	NA	NA	171	171	0	169	162	7	4.14

Mangalaparthi *et al.* , 2020. Mutational landscape of esophageal squamous cell carcinoma in an Indian cohort
Supplementary Table 4. List of somatic single nucleotide variants identified using whole exome sequencing of ESCC patients

Cohort	Sample	Chromosome	Genomic position	Reference allele	Alternate allele	Gene symbol	Variant type	Variant annotation	OncoMD	COSMIC	dbSNP	ExAC	ExAC_SAS	1000G	1000G_SAS	SIFT	Polyphen2	CONDEL	VEP	LRT	PhastCon7 Way	Total read (Adjacent normal)	Reference allel read support (Adjacent normal)	Alternative read support (Adjacent normal)	Total read (Tumor)	Reference allel read support (Tumor)	Alternative read support (Tumor)	Alternate allele frequency
Chewer	42482	chr3	195391062	C	G	*LINC00969*	EXONIC-NC	ENSG00000242086:[ENST00000425425(n.428C>G)];ENSG00000242086:[ENST00000539717(n.368C>G)];ENSG00000242086:[ENST00000453324(n.448C>G)];ENSG00000242086:[ENST00000445430(n.588C>G)];ENSG00000242086:[ENST00000429897(n.456C>G)];ENSG00000242086:[ENST00000457233(n.603C>G)];ENSG00000242086:[ENST00000539252(n.208C>G)];ENSG00000242086:[ENST00000414625(n.588C>G)];	NA	NA	NA	0.0023465	0.001543011	NA	NA	NA	NA_NA	NA	MODIFIER	NA	NA	62	62	0	128	117	11	8.59
Chewer	42482	chr3	172428856	G	C	*NCEH1*	SILENT	ENSG00000144959:5UTR[ENST00000543711(c.-252C>G)];ENSG00000144959:5UTR[ENST00000475381(c.-82C>G)];ENSG00000144959:SILENT[ENST00000538775(c.15C>G,cgC>cgG):ENSP00000442464(p.Arg5(=))];ENSG00000144959:SILENT[ENST00000421723(c.15C>G,cgC>cgG):ENSP00000402196(p.Arg5(=))];ENSG00000144959:SILENT[ENST00000273512(c.15C>G,cgC>cgG):ENSP00000273512(p.Arg5(=))];	NA	NA	NA	8.84E-06	0	NA	NA	NA	NA_NA	NA	LOW	NA	NA	69	69	0	169	105	64	37.87
Chewer	42482	chr3	64084785	C	A	*PRICKLE2*	MISSENSE	ENSG00000163637:MISSENSE[ENST00000564377(c.2645G>T,gGc>gTc):ENSP00000455004(p.Gly882Val)];ENSG00000163637:MISSENSE[ENST00000295902(c.2477G>T,gGc>gTc):ENSP00000295902(p.Gly826Val)];	NA	NA	NA	NA	NA	NA	NA	NA	NA_NA	NA	MODIFIER	NA	NA	92	91	0	101	93	8	7.92
Chewer	42482	chr3	64084786	C	A	*PRICKLE2*	MISSENSE	ENSG00000163637:MISSENSE[ENST00000295902(c.2476G>T,Ggc>Tgc):ENSP00000295902(p.Gly826Cys)];ENSG00000163637:MISSENSE[ENST00000564377(c.2644G>T,Ggc>Tgc):ENSP00000455004(p.Gly882Cys)];	NA	NA	NA	NA	NA	NA	NA	NA	NA_NA	NA	MODIFIER	NA	NA	92	92	0	102	94	8	7.84
Chewer	42482	chr3	141259377	T	C	*RASA2*	SILENT-SS-PRX	ENSG00000155903:[ENST00000286364(c.453T>C,ggT>ggC):ENSP00000286364(p.Gly151(=))];ENSG00000155903:[ENST00000452898(c.453T>C,ggT>ggC):ENSP00000391677(p.Gly151(=))];ENSG00000155903:[ENST00000515549(c.*275T>C)];	NA	NA	NA	NA	NA	NA	NA	NA	NA_NA	NA	LOW	NA	NA	53	53	0	116	104	12	10.34
Chewer	42482	chr3	64084785	C	A	*RP11-129B22.1*	EXONIC-NC	ENSG00000241111:[ENST00000482609(n.2483C>A)];	NA	NA	NA	NA	NA	NA	NA	NA	NA_NA	NA	MODIFIER	NA	NA	92	91	0	101	93	8	7.92
Chewer	42482	chr3	64084786	C	A	*RP11-129B22.1*	EXONIC-NC	ENSG00000241111:[ENST00000482609(n.2484C>A)];	NA	NA	NA	NA	NA	NA	NA	NA	NA_NA	NA	MODIFIER	NA	NA	92	92	0	102	94	8	7.84
Chewer	42482	chr3	159997130	T	A	*RP11-432B6.3*	MISSENSE	ENSG0000248710:MISSENSE[ENST00000483754(c.2200A>T,Att>Ttt):ENSP00000456272(p.Ile734Phe)];	NA	NA	NA	NA	NA	NA	NA	D	PrD_PrD	D	MODERATE	NA	NA	79	79	0	97	77	19	19.59
Chewer	42482	chr3	18457527	C	A	*SATB1*	MISSENSE	ENSG00000182568:MISSENSE[ENST00000440737(c.487G>T,Gtg>Ttg):ENSP00000402982(p.Val163Leu)];ENSG00000182568:MISSENSE[ENST00000454909(c.487G>T,Gtg>Ttg):ENSP00000399708(p.Val163Leu)];ENSG00000182568:MISSENSE[ENST00000415069(c.487G>T,Gtg>Ttg):ENSP00000390529(p.Val163Leu)];ENSG00000182568:MISSENSE[ENST00000338745(c.487G>T,Gtg>Ttg):ENSP00000341024(p.Val163Leu)];ENSG00000182568:MISSENSE[ENST00000417717(c.487G>T,Gtg>Ttg):ENSP00000399518(p.Val163Leu)];ENSG00000182568:MISSENSE[ENST00000452260(c.487G>T,Gtg>Ttg):ENSP00000406727(p.Val163Leu)];ENSG00000182568:EXONIC-NC[ENST00000491519(n.591G>T)];ENSG00000182568:EXONIC-NC[ENST00000475083(n.329G>T)];ENSG00000182568:EXONIC-NC[ENST00000482788(n.92G>T)];	NA	NA	NA	NA	NA	NA	NA	NA	NA_NA	NA	MODIFIER	NA	NA	141	140	1	160	130	29	18.12
Chewer	42482	chr3	195391062	C	G	*SDHAP2*	EXONIC-NC	ENSG00000215837:[ENST00000455183(n.569C>G)];	NA	NA	NA	0.0023465	0.001543011	NA	NA	NA	NA_NA	NA	MODIFIER	NA	NA	62	62	0	128	117	11	8.59
Chewer	42482	chr3	196236482	C	A	*SMCO1*	MISSENSE	ENSG00000214097:MISSENSE[ENST00000397537(c.109G>T,Gat>Tat):ENSP00000380671(p.Asp37Tyr)];ENSG00000214097:3UTR[ENST00000452776(c.*54G>T)];	NA	NA	NA	NA	NA	NA	NA	NA	NA_NA	NA	MODIFIER	NA	NA	150	150	0	262	252	10	3.82
Chewer	42482	chr3	43344818	G	A	*SNRK*	SILENT	ENSG00000163788:SILENT[ENST00000296088(c.123G>A,aaG>aaA):ENSP00000296088(p.Lys41(=))];ENSG00000163788:SILENT[ENST00000454177(c.123G>A,aaG>aaA):ENSP00000401246(p.Lys41(=))];ENSG00000163788:SILENT[ENST00000429705(c.123G>A,aaG>aaA):ENSP00000411375(p.Lys41(=))];ENSG00000163788:INTRONIC[ENST00000437827(c.-30+16651G>A)];ENSG00000163788:EXONIC-NC[ENST00000462810(n.215G>A)];	NA	NA	NA	NA	NA	NA	NA	NA	NA_NA	NA	LOW	NA	0.997	209	209	0	261	238	23	8.81
Chewer	42482	chr3	43344819	G	T	*SNRK*	MISSENSE	ENSG00000163788:MISSENSE[ENST00000296088(c.124G>T,Gtg>Ttg):ENSP00000296088(p.Val42Leu)];ENSG00000163788:MISSENSE[ENST00000454177(c.124G>T,Gtg>Ttg):ENSP00000401246(p.Val42Leu)];ENSG00000163788:MISSENSE[ENST00000429705(c.124G>T,Gtg>Ttg):ENSP00000411375(p.Val42Leu)];ENSG00000163788:INTRONIC[ENST00000437827(c.-30+16652G>T)];ENSG00000163788:EXONIC-NC[ENST00000462810(n.216G>T)];	NA	NA	NA	NA	NA	NA	NA	NA	NA_NA	NA	MODIFIER	NA	NA	211	211	0	261	238	23	8.81
Chewer	42482	chr3	98518584	G	A	*ST3GAL6*	INTRONIC	ENSG00000064225:[ENST00000491912(n.254-1682G>A)];	NA	NA	NA	NA	NA	NA	NA	NA	NA_NA	NA	MODIFIER	NA	NA	122	122	0	161	152	9	5.59
Chewer	42482	chr3	111732274	G	A	*TAGLN3*	MISSENSE	ENSG00000144834:MISSENSE[ENST00000478951(c.476G>A,cGg>cAg):ENSP00000419105(p.Arg159Gln)];ENSG00000144834:MISSENSE[ENST00000469385(c.193G>A,Gga>Aga):ENSP00000420346(p.Gly65Arg)];ENSG00000144834:MISSENSE[ENST00000486460(c.224G>A,cGg>cAg):ENSP00000417904(p.Arg75Gln)];ENSG00000144834:MISSENSE[ENST00000455401(c.476G>A,cGg>cAg):ENSP00000391160(p.Arg159Gln)];ENSG00000144834:MISSENSE[ENST00000273368(c.476G>A,cGg>cAg):ENSP00000273368(p.Arg159Gln)];ENSG00000144834:MISSENSE[ENST00000393917(c.476G>A,cGg>cAg):ENSP00000377494(p.Arg159Gln)];ENSG00000144834:SILENT[ENST00000494932(c.198G>A,tcG>tcA):ENSP00000420675(p.Ser66(=))];	NA	NA	NA	NA	NA	NA	NA	NA	NA_NA	NA	LOW	NA	NA	59	59	0	99	83	16	16.16
Chewer	42482	chr3	18457527	C	A	*TBC1D5*	INTRONIC	ENSG00000131374:[ENST00000414318(n.211+28572G>T)];	NA	NA	NA	NA	NA	NA	NA	NA	NA_NA	NA	MODIFIER	NA	NA	141	140	1	160	130	29	18.12
Chewer	42482	chr3	127292386	C	G	*TPRA1*	MISSENSE	ENSG00000163870:MISSENSE[ENST00000450633(c.1100G>C,cGc>cCc):ENSP00000413428(p.Arg367Pro)];ENSG00000163870:MISSENSE[ENST00000355552(c.1100G>C,cGc>cCc):ENSP00000347748(p.Arg367Pro)];ENSG00000163870:MISSENSE[ENST00000489960(c.1100G>C,cGc>cCc):ENSP00000418298(p.Arg367Pro)];ENSG00000163870:3UTR[ENST00000483868(c.*319G>C)];ENSG00000163870:3UTR[ENST00000296210(c.*54G>C)];ENSG00000163870:3UTR[ENST00000393400(c.*319G>C)];	NA	NA	chr3*127292386*127292386*C*T*rs576865290*0*0.9998*0.0001997	NA	NA	NA	NA	NA	NA_NA	NA	MODIFIER	NA	NA	37	37	0	47	39	8	17.02
Chewer	42482	chr3	49848231	A	G	*UBA7*	MISSENSE	ENSG00000182179:MISSENSE[ENST00000333486(c.1265T>C,tTt>tCt):ENSP00000333266(p.Phe422Ser)];ENSG00000182179:EXONIC-NC[ENST00000460703(n.844T>C)];ENSG00000182179:EXONIC-NC[ENST00000460516(n.326T>C)];	NA	NA	NA	NA	NA	NA	NA	NA	NA_NA	NA	MODIFIER	NA	NA	94	94	0	94	85	9	9.57
Chewer	42482	chr3	183956551	A	G	*VWA5B2*	MISSENSE	ENSG00000145198:MISSENSE[ENST00000273794(c.1438A>G,Agt>Ggt):ENSP00000273794(p.Ser480Gly)];ENSG00000145198:MISSENSE[ENST00000426955(c.2092A>G,Agt>Ggt):ENSP00000398688(p.Ser698Gly)];ENSG00000145198:EXONIC-NC[ENST00000461141(n.1383A>G)];	NA	NA	NA	NA	NA	NA	NA	T	BN_BN	N	MODERATE	N	0.03	74	74	0	126	85	41	32.54
Chewer	42482	chr3	102195930	G	T	*ZPLD1*	INTRONIC	ENSG00000170044:[ENST00000466937(c.1072+45G>T)];ENSG00000170044:[ENST00000306176(c.1120+45G>T)];ENSG00000170044:[ENST00000491959(c.1072+45G>T)];	NA	NA	NA	NA	NA	NA	NA	NA	NA_NA	NA	MODIFIER	NA	NA	54	54	0	85	61	24	28.24
Chewer	42482	chr4	125593640	C	T	*ANKRD50*	SILENT	ENSG00000151458:SILENT[ENST00000504087(c.792G>A,aaG>aaA):ENSP00000425658(p.Lys264(=))];ENSG00000151458:SILENT[ENST00000515641(c.255G>A,aaG>aaA):ENSP00000425355(p.Lys85(=))];	NA	NA	NA	NA	NA	NA	NA	NA	NA_NA	NA	LOW	NA	0.999	157	157	0	130	117	13	10

Mangalaparthi *et al.* , 2020. Mutational landscape of esophageal squamous cell carcinoma in an Indian cohort
Supplementary Table 4. List of somatic single nucleotide variants identified using whole exome sequencing of ESCC patients

Cohort	Sample	Chromosome	Genomic position	Reference allele	Alternate allele	Gene symbol	Variant type	Variant annotation	OncoMD	COSMIC	dbSNP	ExAC	ExAC_SAS	1000G	1000G_SAS	SIFT	Polyphen2	CONDEL	VEP	LRT	PhastCon7 Way	Total read (Adjacent normal)	Reference allel read support (Adjacent normal)	Alternative read support (Adjacent normal)	Total read (Tumor)	Reference allel read support (Tumor)	Alternative read support (Tumor)	Alternate allele frequency
Chewer	42482	chr4	148796273	C	T	*ARHGAP10*	SILENT	ENSG00000071205:SILENT[ENST00000336498(c.804C>T,gcC>gcT):ENSP00000336923(p.Ala268(=))];ENSG00000071205:EXONIC-NC[ENST00000506054(n.5936C>T)];	chr4*14879	chr4*14879	0.001597	0.0014274	0.010855518	0.0015974	0.0082	NA	NA_NA	NA	MODIFIER	NA	NA	117	117	0	138	104	34	24.64
Chewer	42482	chr4	15480781	C	A	*CC2D2A*	INTRONIC	ENSG00000048342:[ENST00000503292(c.123+351C>A)];ENSG00000048342:[ENST00000507954(c.123+351C>A)];ENSG00000048342:[ENST00000503658(c.124-66C>A)];ENSG00000048342:[ENST00000413206(c.123+351C>A)];ENSG00000048342:[ENST00000511544(c.124-66C>A)];ENSG00000048342:[ENST00000513811(n.303+351C>A)];ENSG00000048342:[ENST00000514450(n.278+351C>A)];ENSG00000048342:[ENST00000438599(c.124-66C>A)];ENSG00000048342:[ENST00000424120(c.123+351C>A)];ENSG00000048342:[ENST00000512702(c.123+351C>A)];ENSG00000048342:[ENST00000515124(c.123+351C>A)];	NA	NA	NA	NA	NA	NA	NA	NA	NA_NA	NA	MODIFIER	NA	NA	59	58	0	75	54	21	28
Chewer	42482	chr4	187539491	G	A	*FAT1*	MISSENSE	ENSG00000083857:MISSENSE[ENST00000441802(c.8249C>T,tCc>tTc):ENSP00000406229(p.Ser2750Phe)];	NA	NA	NA	NA	NA	NA	NA	NA	BN_BN	NA	MODERATE	N	0.992	180	180	0	165	151	14	8.48
Chewer	42482	chr4	126355546	T	A	*FAT4*	MISSENSE	ENSG00000196159:MISSENSE[ENST00000394329(c.7165T>A,Tca>Aca):ENSP00000377862(p.Ser2389Thr)];ENSG00000196159:MISSENSE[ENST00000335110(c.2059T>A,Tca>Aca):ENSP00000335169(p.Ser687Thr)];	NA	NA	NA	NA	NA	NA	NA	T	PoD_PrD	N	MODERATE	U	1	178	178	0	203	181	22	10.84
Chewer	42482	chr4	3219592	G	T	*HTT*	SILENT	ENSG00000197386:EXONIC-NC[ENST00000510626(n.8283G>T)];ENSG00000197386:SILENT[ENST00000355072(c.7155G>T,ccG>ccT):ENSP00000347184(p.Pro2385(=))];	NA	NA	chr4*3219592*3219592*G*A,T*rs375913560*NA*NA*NA	2.49E-05	0	NA	NA	NA	NA_NA	NA	LOW	NA	NA	85	85	0	87	62	25	28.74
Chewer	42482	chr4	62679488	C	T	*LPHN3*	INTRONIC	ENSG00000150471:[ENST00000506720(c.1400-39C>T)];ENSG00000150471:[ENST00000545650(c.1196-39C>T)];ENSG00000150471:[ENST00000507625(c.1400-39C>T)];ENSG00000150471:[ENST00000504896(c.1196-39C>T)];ENSG00000150471:[ENST00000508946(c.1196-39C>T)];ENSG00000150471:[ENST00000506700(c.1196-39C>T)];ENSG00000150471:[ENST00000514591(c.1196-39C>T)];ENSG00000150471:[ENST00000514996(c.1196-39C>T)];ENSG00000150471:[ENST00000506746(c.1400-39C>T)];ENSG00000150471:[ENST00000508693(c.1400-39C>T)];ENSG00000150471:[ENST00000514157(c.1196-39C>T)];ENSG00000150471:[ENST00000512091(c.1196-39C>T)];ENSG00000150471:[ENST00000509896(c.1400-39C>T)];ENSG00000150471:[ENST00000511324(c.1400-39C>T)];ENSG00000150471:[ENST00000507164(c.1400-39C>T)];	NA	NA	NA	3.45E-05	6.36E-05	NA	NA	NA	NA_NA	NA	MODIFIER	NA	NA	109	108	0	115	100	15	13.04
Chewer	42482	chr4	146085354	T	A	*OTUD4*	MISSENSE	ENSG00000164164:MISSENSE[ENST00000447906(c.366A>T,gaA>gaT):ENSP00000395487(p.Glu122Asp)];ENSG00000164164:MISSENSE[ENST00000454497(c.171A>T,gaA>gaT):ENSP00000409279(p.Glu57Asp)];ENSG00000164164:MISSENSE[ENST00000514973(c.171A>T,gaA>gaT):ENSP00000425972(p.Glu57Asp)];ENSG00000164164:MISSENSE[ENST00000504501(c.171A>T,gaA>gaT):ENSP00000423453(p.Glu57Asp)];ENSG00000164164:MISSENSE[ENST00000296579(c.171A>T,gaA>gaT):ENSP00000296579(p.Glu57Asp)];ENSG00000164164:MISSENSE[ENST00000509620(c.171A>T,gaA>gaT):ENSP00000424192(p.Glu57Asp)];ENSG00000164164:EXONIC-NC[ENST00000505976(n.321A>T)];ENSG00000164164:EXONIC-NC[ENST00000455611(n.270A>T)];ENSG00000164164:EXONIC-NC[ENST00000509985(n.221A>T)];	NA	NA	NA	NA	NA	NA	NA	NA	NA_NA	NA	MODIFIER	NA	NA	25	25	0	32	27	5	15.62
Chewer	42482	chr5	35048042	C	A	*AGXT2*	5UTR	ENSG00000113492:[ENST00000231420(c.-45G>T)];	NA	NA	0.0001997	NA	NA	0.0001997	0	NA	NA_NA	NA	MODIFIER	NA	NA	81	81	0	119	104	15	12.61
Chewer	42482	chr5	7851027	C	A	*C5orf49*	SILENT	ENSG00000215217:SILENT[ENST00000509627(c.108G>T,ctG>ctT):ENSP00000426019(p.Leu36(=))];ENSG00000215217:SILENT[ENST00000399810(c.108G>T,ctG>ctT):ENSP00000382708(p.Leu36(=))];	NA	NA	NA	NA	NA	NA	NA	NA	NA_NA	NA	LOW	NA	NA	28	28	0	56	47	9	16.07
Chewer	42482	chr5	178046852	C	G	*CLK4*	3UTR	ENSG00000113240:[ENST00000522556(c.*714G>C)];ENSG00000113240:[ENST00000521621(c.*889G>C)];ENSG00000113240:[ENST00000520878(n.350G>C)];ENSG00000113240:[ENST00000522749(n.269G>C)];ENSG00000113240:[ENST00000519583(n.303G>C)];ENSG00000113240:[ENST00000520126(n.269-1073G>C)];ENSG00000113240:[ENST00000520909(n.224-1073G>C)];ENSG00000113240:[ENST00000522136(c.162-1073G>C)];ENSG00000113240:[ENST00000316308(c.162-1073G>C)];ENSG00000113240:[ENST00000520199(n.271-1073G>C)];	NA	NA	NA	NA	NA	NA	NA	NA	NA_NA	NA	MODIFIER	NA	NA	78	77	1	120	98	22	18.33
Chewer	42482	chr5	31529080	C	G	*DROSHA*	INTRONIC	ENSG00000113360:[ENST00000442743(c.20+67G>C)];ENSG00000113360:[ENST00000511367(c.20+67G>C)];ENSG00000113360:[ENST00000513349(c.20+67G>C)];ENSG00000113360:[ENST00000344624(c.20+67G>C)];ENSG00000113360:[ENST00000504361(n.354+67G>C)];ENSG00000113360:[ENST00000507438(c.20+67G>C)];	NA	NA	NA	NA	NA	NA	NA	NA	NA_NA	NA	MODIFIER	NA	NA	139	139	0	143	129	13	9.09
Chewer	42482	chr5	108203468	G	T	*FER*	MISSENSE-SS-PRX	ENSG00000151422:[ENST00000281092(c.482G>T,gGg>gTg):ENSP00000281092(p.Gly161Val)];ENSG00000151422:[ENST00000536402(c.482G>T,gGg>gTg):ENSP00000442627(p.Gly161Val)];ENSG00000151422:[ENST00000504143(c.208G>T,Gga>Tga):ENSP00000421951(p.Gly70Ter)];ENSG00000151422:[ENST00000438717(c.-44G>T)];	chr5*10820	chr5*10820	NA	NA	NA	NA	NA	NA	NA_NA	NA	LOW	NA	NA	149	148	0	163	131	32	19.63
Chewer	42482	chr5	55246013	G	C	*IL6ST*	INTRONIC	ENSG00000134352:[ENST00000522633(c.*767+1279C>G)];ENSG00000134352:[ENST00000583149(n.174+1279C>G)];ENSG00000134352:[ENST00000381287(c.*767+1279C>G)];ENSG00000134352:[ENST00000381293(c.476-7434C>G)];ENSG00000134352:[ENST00000336909(c.1840+1279C>G)];ENSG00000134352:[ENST00000503773(c.*884+1279C>G)];ENSG00000134352:[ENST00000423954(n.44-1203C>G)];ENSG00000134352:[ENST00000381298(c.1840+1279C>G)];ENSG00000134352:[ENST00000536319(c.*584+1279C>G)];ENSG00000134352:[ENST00000381294(c.1657+1279C>G)];ENSG00000134352:[ENST00000502326(c.1840+1279C>G)];ENSG00000134352:[ENST00000381286(c.65-7434C>G)];	NA	NA	NA	NA	NA	NA	NA	NA	NA_NA	NA	MODIFIER	NA	NA	47	47	0	43	38	5	11.63
Chewer	42482	chr5	75871658	A	C	*IQGAP2*	INTRONIC	ENSG00000145703:[ENST00000514350(c.377+64A>C)];ENSG00000145703:[ENST00000505766(c.308+64A>C)];ENSG00000145703:[ENST00000274364(c.458+64A>C)];ENSG00000145703:[ENST00000379730(c.-866+64A>C)];	NA	NA	NA	NA	NA	NA	NA	NA	NA_NA	NA	MODIFIER	NA	NA	46	46	0	44	35	9	20.45

Supplementary Table 4. List of somatic single nucleotide variants identified using whole exome sequencing of ESCC patients

Cohort	Sample	Chromosome	Genomic position	Reference allele	Alternate allele	Gene symbol	Variant type	Variant annotation	OncoMD	COSMIC	dbSNP	ExAC	ExAC_SAS	1000G	1000G_SAS	SIFT	Polyphen2	CONDEL	VEP	LRT	PhastCon7 Way	Total read (Adjacent normal)	Reference allel read support (Adjacent normal)	Alternative read support (Adjacent normal)	Total read (Tumor)	Reference allel read support (Tumor)	Alternative read support (Tumor)	Alternate allele frequency
Chewer	42482	chr5	71537485	C	T	*MRPS27*	SILENT	ENSG00000113048:EXONIC-NC[ENST00000522404(n.300G>A)];ENSG00000113048:INTRONIC[ENST00000457646(c.114-3530G>A)];ENSG00000113048:INTRONIC[ENST00000508863(c.114-3530G>A)];ENSG00000113048:INTRONIC[ENST00000519143(n.195-3530G>A)];ENSG00000113048:INTRONIC[ENST00000522042(n.59-3530G>A)];ENSG00000113048:INTRONIC[ENST00000515404(c.114-3530G>A)];ENSG00000113048:INTRONIC[ENST00000261413(c.282-3530G>A)];ENSG00000113048:SILENT[ENST00000513900(c.312G>A,caG>caA):ENSP00000426941(p.Gln104(=))];	NA	NA	NA	NA	NA	NA	NA	NA	NA_NA	NA	MODIFIER	NA	NA	88	88	0	115	93	21	18.26
Chewer	42482	chr5	32780749	A	C	*NPR3*	INTRONIC	ENSG00000113389:[ENST00000265074(c.1196-79A>C)];ENSG00000113389:[ENST00000434067(c.548-79A>C)];ENSG00000113389:[ENST00000415167(c.1196-79A>C)];ENSG00000113389:[ENST00000506712(n.557-79A>C)];ENSG00000113389:[ENST00000509104(c.527-79A>C)];ENSG00000113389:[ENST00000415685(c.548-79A>C)];	NA	NA	NA	NA	NA	NA	NA	NA	NA_NA	NA	MODIFIER	NA	NA	85	85	0	160	152	8	5
Chewer	42482	chr5	140433306	C	A	*PCDHB1*	SILENT	ENSG00000171815:SILENT[ENST00000306549(c.2251C>A,Cgg>Agg):ENSP00000307234(p.Arg751(=))];	NA	NA	chr5*140433306*140433306*C*T*rs143394864*1*0.9996*0.0003994	NA	NA	NA	NA	NA	NA_NA	NA	LOW	NA	0.072	87	87	0	80	65	15	18.75
Chewer	42482	chr5	155551	C	G	*PLEKHG4B*	MISSENSE	ENSG00000153404:MISSENSE[ENST00000502646(c.875C>G,aCa>aGa):ENSP00000422493(p.Thr292Arg)];ENSG00000153404:MISSENSE[ENST00000283426(c.1133C>G,aCa>aGa):ENSP00000283426(p.Thr378Arg)];	NA	NA	NA	NA	NA	NA	NA	T	PrD_PrD	D	MODERATE	NA	NA	99	99	0	138	108	30	21.74
Chewer	42482	chr5	178046852	C	G	*RN7SKP70*	EXONIC-NC	ENSG00000252464:[ENST00000516655(n.148G>C)];	NA	NA	NA	NA	NA	NA	NA	NA	NA_NA	NA	MODIFIER	NA	NA	78	77	1	120	98	22	18.33
Chewer	42482	chr5	1406365	C	G	*SLC6A3*	MISSENSE	ENSG00000142319:MISSENSE[ENST00000270349(c.1537G>C,Ggg>Cgg):ENSP00000270349(p.Gly513Arg)];ENSG00000142319:MISSENSE[ENST00000453492(c.1537G>C,Ggg>Cgg):ENSP00000399806(p.Gly513Arg)];	NA	NA	chr5*1406365*1406365*C*T*rs777812094*NA*NA*NA	NA	NA	NA	NA	D	PrD_PrD	D	MODERATE	D	0.981	105	105	0	178	158	19	10.67
Chewer	42482	chr5	122189479	G	A	*SNX24*	INTRONIC	ENSG00000064652:[ENST00000506996(c.60+8091G>A)];ENSG00000064652:[ENST00000511211(n.304+9249G>A)];ENSG00000064652:[ENST00000395451(c.60+8091G>A)];ENSG00000064652:[ENST00000261369(c.60+8091G>A)];ENSG00000064652:[ENST00000513881(c.60+8091G>A)];ENSG00000064652:[ENST00000511545(c.*58+17G>A)];ENSG00000064652:[ENST00000513613(n.177+10169G>A)];	NA	NA	NA	NA	NA	NA	NA	NA	NA_NA	NA	MODIFIER	NA	NA	81	81	0	91	67	24	26.37
Chewer	42482	chr5	79616928	G	A	*SPZ1*	SILENT	ENSG00000164299:SILENT[ENST00000296739(c.894G>A,tcG>tcA):ENSP00000369611(p.Ser298(=))];	NA	NA	NA	2.51E-05	6.09E-05	NA	NA	NA	NA_NA	NA	LOW	NA	NA	174	174	0	168	128	40	23.81
Chewer	42482	chr5	82841375	G	T	*VCAN*	MISSENSE	ENSG00000038427:MISSENSE[ENST00000342785(c.4023G>T,atG>atT):ENSP00000342768(p.Met1341Ile)];ENSG00000038427:MISSENSE[ENST00000265077(c.9285G>T,atG>atT):ENSP00000265077(p.Met3095Ile)];ENSG00000038427:MISSENSE[ENST00000502527(c.1062G>T,atG>atT):ENSP00000421362(p.Met354Ile)];ENSG00000038427:MISSENSE[ENST00000512590(c.3879G>T,atG>atT):ENSP00000425959(p.Met1293Ile)];ENSG00000038427:MISSENSE[ENST00000343200(c.6324G>T,atG>atT):ENSP00000340062(p.Met2108Ile)];ENSG00000038427:EXONIC-NC[ENST00000513016(n.6675G>T)];ENSG00000038427:EXONIC-NC[ENST00000507162(n.447G>T)];	NA	NA	NA	NA	NA	NA	NA	NA	NA_NA	NA	MODIFIER	NA	NA	74	73	1	110	91	17	15.45
Chewer	42482	chr5	82841375	G	T	*VCAN-AS1*	INTRONIC	ENSG00000249835:[ENST00000513899(n.229-3855C>A)];ENSG00000249835:[ENST00000512090(n.285-11383C>A)];	NA	NA	NA	NA	NA	NA	NA	NA	NA_NA	NA	MODIFIER	NA	NA	74	73	1	110	91	17	15.45
Chewer	42482	chr6	165715240	C	A	*C6orf118*	MISSENSE	ENSG00000112539:MISSENSE[ENST00000543069(c.259G>T,Ggg>Tgg):ENSP00000439288(p.Gly87Trp)];ENSG00000112539:MISSENSE[ENST00000230301(c.571G>T,Ggg>Tgg):ENSP00000230301(p.Gly191Trp)];	NA	NA	chr6*165715240*165715240*C*T*rs750250177*NA*NA*NA	NA	NA	NA	NA	D	PrD_PrD	D	MODERATE	N	NA	98	98	0	95	80	15	15.79
Chewer	42482	chr6	168708482	C	A	*DACT2*	MISSENSE	ENSG00000164488:MISSENSE[ENST00000366795(c.1955G>T,cGc>cTc):ENSP00000355760(p.Arg652Leu)];ENSG00000164488:MISSENSE[ENST00000610183(c.1445G>T,cGc>cTc):ENSP00000476573(p.Arg482Leu)];ENSG00000164488:MISSENSE[ENST00000607983(c.731G>T,cGc>cTc):ENSP00000476434(p.Arg244Leu)];ENSG00000164488:INTRONIC[ENST00000366796(c.658+2366G>T)];	NA	NA	chr6*168708481*168708502*GCGGACCAGTGAGGGACGGCCC*G*rs750987985*NA*NA*NA|chr6*168708482*168708482*C*T*rs754586349*NA*NA*NA	NA	NA	NA	NA	NA	NA_NA	NA	MODIFIER	NA	NA	67	67	0	69	61	8	11.59
Chewer	42482	chr6	38917200	G	T	*DNAH8*	INTRONIC	ENSG00000124721:[ENST00000359357(c.11479-28G>T)];ENSG00000124721:[ENST00000327475(c.12094-28G>T)];ENSG00000124721:[ENST00000441566(c.11371-28G>T)];ENSG00000124721:[ENST00000449981(c.12130-28G>T)];	NA	NA	NA	NA	NA	NA	NA	NA	NA_NA	NA	MODIFIER	NA	NA	85	85	0	116	99	17	14.66
Chewer	42482	chr6	83829554	T	C	*DOPEY1*	MISSENSE	ENSG00000083097:MISSENSE[ENST00000237163(c.941T>C,tTt>tCt):ENSP00000237163(p.Phe314Ser)];ENSG00000083097:MISSENSE[ENST00000369739(c.941T>C,tTt>tCt):ENSP00000358754(p.Phe314Ser)];ENSG00000083097:MISSENSE[ENST00000349129(c.968T>C,tTt>tCt):ENSP00000195654(p.Phe323Ser)];ENSG00000083097:3UTR[ENST00000536812(c.*274T>C)];	NA	NA	NA	NA	NA	NA	NA	NA	NA_NA	NA	MODIFIER	NA	NA	75	74	0	69	61	8	11.59
Chewer	42482	chr6	1742655	C	T	*GMDS*	INTRONIC	ENSG00000112699:[ENST00000380805(n.1016+47G>A)];ENSG00000112699:[ENST00000531690(n.369+47G>A)];ENSG00000112699:[ENST00000380815(c.890+47G>A)];ENSG00000112699:[ENST00000530927(c.800+47G>A)];	NA	NA	chr6*1742655*1742655*C*A*rs773215942*NA*NA*NA	NA	NA	NA	NA	NA	NA_NA	NA	MODIFIER	NA	NA	114	114	0	123	98	25	20.33
Chewer	42482	chr6	46826763	G	C	*GPR116*	MISSENSE	ENSG00000069122:MISSENSE[ENST00000545669(c.1164C>G,ttC>ttG):ENSP00000441581(p.Phe388Leu)];ENSG00000069122:MISSENSE[ENST00000265417(c.2877C>G,ttC>ttG):ENSP00000265417(p.Phe959Leu)];ENSG00000069122:MISSENSE[ENST00000456426(c.2451C>G,ttC>ttG):ENSP00000412866(p.Phe817Leu)];ENSG00000069122:MISSENSE[ENST00000283296(c.2877C>G,ttC>ttG):ENSP00000283296(p.Phe959Leu)];ENSG00000069122:MISSENSE[ENST00000362015(c.2877C>G,ttC>ttG):ENSP00000354563(p.Phe959Leu)];	NA	NA	NA	NA	NA	NA	NA	NA	PrD_PrD	NA	MODERATE	D	0.603	298	298	0	331	300	31	9.37
Chewer	42482	chr6	27776071	A	T	*HIST1H2AI*	SILENT	ENSG00000196747:SILENT[ENST00000358739(c.84A>T,gtA>gtT):ENSP00000351589(p.Val28(=))];	NA	NA	chr6*27776071*27776071*A*C*rs777256938*NA*NA*NA	NA	NA	NA	NA	NA	NA_NA	NA	LOW	NA	NA	102	102	0	127	110	17	13.39
Chewer	42482	chr6	29858013	C	T	*HLA-H*	INTRONIC	ENSG00000206341:[ENST00000383620(n.1020-28C>T)];	NA	NA	NA	0.2294574	0.232313725	NA	NA	NA	NA_NA	NA	MODIFIER	NA	NA	119	118	1	161	147	13	8.07
Chewer	42482	chr6	76744572	A	T	*IMPG1*	INTRONIC	ENSG00000112706:[ENST00000369950(c.302-68T>A)];ENSG00000112706:[ENST00000369963(c.68-68T>A)];	NA	NA	NA	NA	NA	NA	NA	NA	NA_NA	NA	MODIFIER	NA	NA	77	77	0	58	49	9	15.52

Mangalaparthi *et al.* , 2020. Mutational landscape of esophageal squamous cell carcinoma in an Indian cohort
Supplementary Table 4. List of somatic single nucleotide variants identified using whole exome sequencing of ESCC patients

Cohort	Sample	Chromosome	Genomic position	Reference allele	Alternate allele	Gene symbol	Variant type	Variant annotation	OncoMD	COSMIC	dbSNP	ExAC	ExAC_SAS	1000G	1000G_SAS	SIFT	Polyphen2	CONDEL	VEP	LRT	PhastCon7 Way	Total read (Adjacent normal)	Reference allel read support (Adjacent normal)	Alternative read support (Adjacent normal)	Total read (Tumor)	Reference allel read support (Tumor)	Alternative read support (Tumor)	Alternate allele frequency
Chewer	42482	chr6	73879397	G	T	*KCNQ5*	INTRONIC	ENSG00000185760:[ENST00000342056(c.1526-72G>T)];ENSG00000185760:[ENST00000402622(c.1499-72G>T)];ENSG00000185760:[ENST00000355194(c.1469-72G>T)];ENSG00000185760:[ENST00000370398(c.1469-72G>T)];ENSG00000185760:[ENST00000414165(c.1248-20899G>T)];ENSG00000185760:[ENST00000355635(c.1472-72G>T)];ENSG00000185760:[ENST00000403813(c.1442-72G>T)];	NA	NA	chr6*73879397*73879397*G*A*rs571673349*0*0.9998*0.0001997	NA	NA	NA	NA	NA	NA_NA	NA	MODIFIER	NA	NA	157	157	0	217	186	31	14.29
Chewer	42482	chr6	17800177	T	A	*KIF13A*	INTRONIC-SS-DNR	ENSG00000137177:[ENST00000378814(c.2616+6A>T)];ENSG00000137177:[ENST00000259711(c.2616+6A>T)];ENSG00000137177:[ENST00000378826(c.2616+6A>T)];ENSG00000137177:[ENST00000358380(c.797+6A>T)];ENSG00000137177:[ENST00000378843(c.2616+6A>T)];ENSG00000137177:[ENST00000378816(c.2616+6A>T)];	NA	NA	NA	NA	NA	NA	NA	NA	NA_NA	NA	LOW	NA	NA	163	162	0	206	176	29	14.08
Chewer	42482	chr6	129465089	C	A	*LAMA2*	MISSENSE	ENSG00000196569:MISSENSE[ENST00000421865(c.683C>A,cCt>cAt):ENSP00000400365(p.Pro228His)];	NA	NA	NA	NA	NA	NA	NA	D	PrD_PrD	D	MODERATE	D	0.993	156	156	0	162	137	25	15.43
Chewer	42482	chr6	129799872	C	T	*LAMA2*	MISSENSE	ENSG00000196569:MISSENSE[ENST00000421865(c.7486C>T,Ctc>Ttc):ENSP00000400365(p.Leu2496Phe)];	NA	NA	NA	NA	NA	NA	NA	D	PrD_PrD	D	MODERATE	D	0.997	169	168	0	290	240	49	16.9
Chewer	42482	chr6	90408688	T	C	*MDN1*	MISSENSE	ENSG00000112159:MISSENSE[ENST00000369393(c.9064A>G,Agc>Ggc):ENSP00000358400(p.Ser3022Gly)];ENSG00000112159:MISSENSE[ENST00000428876(c.9064A>G,Agc>Ggc):ENSP00000413970(p.Ser3022Gly)];	NA	NA	NA	NA	NA	NA	NA	NA	BN_PoD	NA	MODERATE	D	0.992	56	56	0	53	44	8	15.09
Chewer	42482	chr6	99572636	G	A	*NA*	INTERGENIC		NA	NA	NA	NA	NA	NA	NA	NA	NA_NA	NA	MODIFIER	NA	NA	119	118	0	170	153	17	10
Chewer	42482	chr6	160240223	C	A	*PNLDC1*	INTRONIC	ENSG00000146453:[ENST00000610273(c.1401-63C>A)];ENSG00000146453:[ENST00000275275(c.*1413-63C>A)];ENSG00000146453:[ENST00000392167(c.1434-63C>A)];	NA	NA	NA	NA	NA	NA	NA	NA	NA_NA	NA	MODIFIER	NA	NA	85	85	0	154	140	14	9.09
Chewer	42482	chr6	160240224	C	G	*PNLDC1*	INTRONIC	ENSG00000146453:[ENST00000610273(c.1401-62C>G)];ENSG00000146453:[ENST00000275275(c.*1413-62C>G)];ENSG00000146453:[ENST00000392167(c.1434-62C>G)];	NA	NA	NA	NA	NA	NA	NA	NA	NA_NA	NA	MODIFIER	NA	NA	86	86	0	154	141	13	8.44
Chewer	42482	chr6	57512541	G	T	*PRIM2*	EXONIC-NC	ENSG00000146143:[ENST00000389488(n.1456G>T)];ENSG00000146143:[ENST00000607273(c.*328G>T)];	NA	NA	chr6*57512541*57512541*G*A,T*rs753221894*NA*NA*NA	8.28E-06	0	NA	NA	NA	NA_NA	NA	MODIFIER	NA	NA	544	543	0	626	608	18	2.88
Chewer	42482	chr6	170845467	C	T	*PSMB1*	INTRONIC	ENSG00000008018:[ENST00000462957(n.1755+855G>A)];ENSG00000008018:[ENST00000262193(c.540+855G>A)];	NA	NA	0.01558	NA	NA	0.0155751	0	NA	NA_NA	NA	MODIFIER	NA	NA	82	82	0	120	103	17	14.17
Chewer	42482	chr6	38917200	G	T	*RP1-207H1.3*	INTRONIC	ENSG00000231150:[ENST00000416948(n.52+3624C>A)];	NA	NA	NA	NA	NA	NA	NA	NA	NA_NA	NA	MODIFIER	NA	NA	85	85	0	116	99	17	14.66
Chewer	42482	chr6	155129206	C	T	*SCAF8*	SILENT-SS-PRX	ENSG00000213079:[ENST00000367186(c.1182C>T,gcC>gcT):ENSP00000356154(p.Ala394(=))];ENSG00000213079:[ENST00000367178(c.984C>T,gcC>gcT):ENSP00000356146(p.Ala328(=))];ENSG00000213079:[ENST00000417268(c.984C>T,gcC>gcT):ENSP00000413098(p.Ala328(=))];	NA	NA	NA	NA	NA	NA	NA	NA	NA_NA	NA	LOW	NA	NA	154	154	0	156	143	13	8.33
Chewer	42482	chr6	122779741	T	C	*SERINC1*	MISSENSE	ENSG00000111897:MISSENSE[ENST00000339697(c.125A>G,tAt>tGt):ENSP00000342962(p.Tyr42Cys)];	NA	NA	NA	NA	NA	NA	NA	D	PrD_PrD	D	MODERATE	D	0.999	220	220	0	238	203	34	14.29
Chewer	42482	chr6	160543014	G	T	*SLC22A1*	MISSENSE	ENSG00000175003:MISSENSE[ENST00000324965(c.47G>T,tGg>tTg):ENSP00000318103(p.Trp16Leu)];ENSG00000175003:MISSENSE[ENST00000366963(c.47G>T,tGg>tTg):ENSP00000355930(p.Trp16Leu)];ENSG00000175003:MISSENSE[ENST00000457470(c.47G>T,tGg>tTg):ENSP00000409557(p.Trp16Leu)];ENSG00000175003:MISSENSE[ENST00000539263(c.47G>T,tGg>tTg):ENSP00000443245(p.Trp16Leu)];ENSG00000175003:MISSENSE[ENST00000460902(c.47G>T,tGg>tTg):ENSP00000439274(p.Trp16Leu)];	NA	NA	NA	NA	NA	NA	NA	T	BN_BN	N	MODERATE	NA	NA	90	90	0	104	89	14	13.46
Chewer	42482	chr6	152563535	C	A	*SYNE1*	MISSENSE	ENSG00000131018:MISSENSE[ENST00000356820(c.3305G>T,aGg>aTg):ENSP00000349276(p.Arg1102Met)];ENSG00000131018:MISSENSE[ENST00000448038(c.19520G>T,aGg>aTg):ENSP00000390975(p.Arg6507Met)];ENSG00000131018:MISSENSE[ENST00000367255(c.19733G>T,aGg>aTg):ENSP00000356224(p.Arg6578Met)];ENSG00000131018:MISSENSE[ENST00000423061(c.19520G>T,aGg>aTg):ENSP00000396024(p.Arg6507Met)];ENSG00000131018:MISSENSE[ENST00000341594(c.18569G>T,aGg>aTg):ENSP00000341887(p.Arg6190Met)];ENSG00000131018:MISSENSE[ENST00000265368(c.19733G>T,aGg>aTg):ENSP00000265368(p.Arg6578Met)];ENSG00000131018:EXONIC-NC[ENST00000367256(n.3425G>T)];ENSG00000131018:EXONIC-NC[ENST00000409694(n.3317G>T)];	NA	NA	NA	NA	NA	NA	NA	NA	NA_NA	NA	MODIFIER	NA	NA	60	60	0	80	70	10	12.5
Chewer	42482	chr6	32803528	C	T	*TAP2*	MISSENSE	ENSG00000204267:MISSENSE[ENST00000374899(c.631G>A,Gga>Aga):ENSP00000364034(p.Gly211Arg)];ENSG00000204267:MISSENSE[ENST00000374897(c.631G>A,Gga>Aga):ENSP00000364032(p.Gly211Arg)];ENSG00000250264:MISSENSE[ENST00000452392(c.631G>A,Gga>Aga):ENSP00000391806(p.Gly211Arg)];	NA	NA	NA	NA	NA	NA	NA	D	PrD_PrD	D	MODERATE	NA	NA	69	69	0	91	81	10	10.99
Chewer	42482	chr6	155571713	C	T	*TIAM2*	SILENT	ENSG00000146426:INTRONIC[ENST00000461783(c.3952-334C>T)];ENSG00000146426:INTRONIC[ENST00000318981(c.3952-334C>T)];ENSG00000146426:INTRONIC[ENST00000528391(c.1960-334C>T)];ENSG00000146426:INTRONIC[ENST00000275246(c.727-334C>T)];ENSG00000146426:INTRONIC[ENST00000528535(c.3952-334C>T)];ENSG00000146426:INTRONIC[ENST00000367174(c.2080-334C>T)];ENSG00000146426:INTRONIC[ENST00000360366(c.4024-334C>T)];ENSG00000146426:INTRONIC[ENST00000456877(c.1888-334C>T)];ENSG00000146426:SILENT[ENST00000456144(c.3993C>T,ccC>ccT):ENSP00000407746(p.Pro1331(=))];ENSG00000146426:SILENT[ENST00000529824(c.3993C>T,ccC>ccT):ENSP00000433348(p.Pro1331(=))];	NA	NA	NA	NA	NA	NA	NA	NA	NA_NA	NA	MODIFIER	NA	NA	67	67	0	94	80	14	14.89
Chewer	42482	chr6	47251890	G	A	*TNFRSF21*	MISSENSE	ENSG00000146072:MISSENSE[ENST00000296861(c.1027C>T,Cat>Tat):ENSP00000296861(p.His343Tyr)];	NA	NA	NA	NA	NA	NA	NA	D	PrD_PrD	D	MODERATE	D	0.998	110	110	0	122	96	26	21.31
Chewer	42482	chr6	80736264	G	A	*TTK*	INTRONIC	ENSG00000112742:[ENST00000369798(c.1394+33G>A)];ENSG00000112742:[ENST00000509894(c.1391+33G>A)];ENSG00000112742:[ENST00000515751(n.518+33G>A)];ENSG00000112742:[ENST00000230510(c.1391+33G>A)];	NA	NA	NA	NA	NA	NA	NA	NA	NA_NA	NA	MODIFIER	NA	NA	78	78	0	62	54	8	12.9
Chewer	42482	chr6	28056534	G	A	*ZNF165*	SILENT	ENSG00000197279:SILENT[ENST00000377325(c.744G>A,tcG>tcA):ENSP00000366542(p.Ser248(=))];	chr6*28056	NA	NA	4.13E-05	0.000121197	NA	NA	NA	NA_NA	NA	LOW	NA	NA	114	112	1	173	145	28	16.18
Chewer	42482	chr6	28402597	G	C	*ZSCAN23*	MISSENSE	ENSG00000187987:MISSENSE[ENST00000289788(c.815C>G,aCa>aGa):ENSP00000289788(p.Thr272Arg)];ENSG00000187987:3UTR[ENST00000481983(c.*226C>G)];	NA	NA	NA	NA	NA	NA	NA	NA	NA_NA	NA	MODIFIER	NA	NA	71	70	0	106	95	11	10.38
Chewer	42482	chr7	87258313	C	A	*ABCB1*	INTRONIC	ENSG00000085563:[ENST00000265724(c.-330-27919G>T)];ENSG00000085563:[ENST00000476862(n.136-25857G>T)];ENSG00000085563:[ENST00000416177(c.-183-27919G>T)];	NA	NA	chr7*87258313*87258313*C*T*rs747828595*NA*NA*NA	NA	NA	NA	NA	NA	NA_NA	NA	MODIFIER	NA	NA	38	38	0	55	41	14	25.45
Chewer	42482	chr7	142983874	C	A	*AC073342.12*	INTRONIC	ENSG00000231840:[ENST00000446192(n.338+262G>T)];	NA	NA	NA	NA	NA	NA	NA	T	BN_BN	N	MODERATE	N	0.616	84	84	0	107	98	9	8.41

Mangalaparthi *et al.* , 2020. Mutational landscape of esophageal squamous cell carcinoma in an Indian cohort
Supplementary Table 4. List of somatic single nucleotide variants identified using whole exome sequencing of ESCC patients

Cohort	Sample	Chromosome	Genomic position	Reference allele	Alternate allele	Gene symbol	Variant type	Variant annotation	OncoMD	COSMIC	dbSNP	ExAC	ExAC_SAS	1000G	1000G_SAS	SIFT	Polyphen2	CONDEL	VEP	LRT	PhastCon7 Way	Total read (Adjacent normal)	Reference allel read support (Adjacent normal)	Alternative read support (Adjacent normal)	Total read (Tumor)	Reference allel read support (Tumor)	Alternative read support (Tumor)	Alternate allele frequency
Chewer	42482	chr7	44147247	G	A	*AEBP1*	SILENT	ENSG00000106624:SILENT[ENST00000449162(c.435G>A,gaG>gaA):ENSP00000401758(p.Glu145(=))];ENSG00000106624:SILENT[ENST00000223357(c.687G>A,gaG>gaA):ENSP00000223357(p.Glu229(=))];ENSG00000106624:INTRONIC[ENST00000455443(c.539+138G>A)];	NA	NA	NA	NA	NA	NA	NA	NA	NA_NA	NA	LOW	NA	0.91	144	142	1	214	176	38	17.76
Chewer	42482	chr7	36571844	G	A	*AOAH*	INTRONIC	ENSG00000136250:[ENST00000491444(n.507-32C>T)];ENSG00000136250:[ENST00000535891(c.1270-32C>T)];ENSG00000136250:[ENST00000538464(c.532-32C>T)];ENSG00000136250:[ENST00000431169(c.1366-32C>T)];ENSG00000136250:[ENST00000258749(c.1366-32C>T)];	NA	NA	NA	NA	NA	NA	NA	NA	NA_NA	NA	MODIFIER	NA	NA	53	53	0	63	54	9	14.29
Chewer	42482	chr7	94045796	C	A	*COL1A2*	MISSENSE	ENSG00000164692:MISSENSE[ENST00000297268(c.1844C>A,cCa>cAa):ENSP00000297268(p.Pro615Gln)];ENSG00000164692:EXONIC-NC[ENST00000488298(n.268C>A)];ENSG00000164692:EXONIC-NC[ENST00000473573(n.181C>A)];	NA	NA	chr7*94045796*94045796*C*T*rs780264582*NA*NA*NA	NA	NA	NA	NA	NA	NA_NA	NA	MODIFIER	NA	NA	46	46	0	41	34	7	17.07
Chewer	42482	chr7	100319728	G	T	*EPO*	INTRONIC	ENSG00000130427:[ENST00000252723(c.246+57G>T)];	NA	NA	NA	NA	NA	NA	NA	NA	NA_NA	NA	MODIFIER	NA	NA	47	47	0	97	79	18	18.56
Chewer	42482	chr7	38726313	G	T	*FAM183B*	5UTR	ENSG00000164556:[ENST00000409072(c.-611C>A)];	NA	NA	NA	NA	NA	NA	NA	NA	NA_NA	NA	MODIFIER	NA	NA	65	65	0	77	69	8	10.39
Chewer	42482	chr7	114296632	A	G	*FOXP2*	3UTR	ENSG00000128573:[ENST00000441290(c.*1249A>G)];ENSG00000128573:[ENST00000393500(c.*30A>G)];ENSG00000128573:[ENST00000393489(c.991-1489A>G)];ENSG00000128573:[ENST00000408937(c.1342-1489A>G)];ENSG00000128573:[ENST00000412402(c.*1186+2568A>G)];ENSG00000128573:[ENST00000393498(c.1204-1489A>G)];ENSG00000128573:[ENST00000393491(c.990+2568A>G)];ENSG00000128573:[ENST00000403559(c.1318-1489A>G)];ENSG00000128573:[ENST00000393494(c.1267-1489A>G)];ENSG00000128573:[ENST00000350908(c.1267-1489A>G)];	NA	NA	NA	NA	NA	NA	NA	NA	NA_NA	NA	MODIFIER	NA	NA	168	168	0	299	276	23	7.69
Chewer	42482	chr7	6537658	C	G	*GRID2IP*	INTRONIC	ENSG00000215045:[ENST00000457091(c.3564+37G>C)];ENSG00000215045:[ENST00000452113(c.2991+37G>C)];ENSG00000215045:[ENST00000435185(c.3012+37G>C)];	NA	NA	NA	NA	NA	NA	NA	NA	NA_NA	NA	MODIFIER	NA	NA	72	72	0	113	89	24	21.24
Chewer	42482	chr7	50467876	G	A	*IKZF1*	MISSENSE	ENSG00000185811:MISSENSE[ENST00000331340(c.1111G>A,Gag>Aag):ENSP00000331614(p.Glu371Lys)];ENSG00000185811:MISSENSE[ENST00000349824(c.682G>A,Gag>Aag):ENSP00000342485(p.Glu228Lys)];ENSG00000185811:MISSENSE[ENST00000343574(c.850G>A,Gag>Aag):ENSP00000342750(p.Glu284Lys)];ENSG00000185811:MISSENSE[ENST00000346667(c.421G>A,Gag>Aag):ENSP00000340080(p.Glu141Lys)];ENSG00000185811:MISSENSE[ENST00000357364(c.850G>A,Gag>Aag):ENSP00000349928(p.Glu284Lys)];ENSG00000185811:MISSENSE[ENST00000438033(c.850G>A,Gag>Aag):ENSP00000396554(p.Glu284Lys)];ENSG00000185811:MISSENSE[ENST00000359197(c.985G>A,Gag>Aag):ENSP00000352123(p.Glu329Lys)];ENSG00000185811:MISSENSE[ENST00000439701(c.985G>A,Gag>Aag):ENSP00000413025(p.Glu329Lys)];ENSG00000185811:3UTR[ENST00000440768(c.*302G>A)];ENSG00000185811:EXONIC-NC[ENST00000471793(n.1330G>A)];	NA	NA	NA	NA	NA	NA	NA	NA	NA_NA	NA	MODIFIER	NA	NA	122	121	1	182	143	39	21.43
Chewer	42482	chr7	155099935	T	C	*INSIG1*	INTRONIC	ENSG00000186480:[ENST00000344756(c.420-16T>C)];ENSG00000186480:[ENST00000340368(c.805-16T>C)];ENSG00000186480:[ENST00000342407(c.*18-16T>C)];ENSG00000186480:[ENST00000476756(c.601-16T>C)];	NA	NA	NA	NA	NA	NA	NA	NA	NA_NA	NA	MODIFIER	NA	NA	61	61	0	73	52	21	28.77
Chewer	42482	chr7	119915387	C	T	*KCND2*	MISSENSE	ENSG00000184408:MISSENSE[ENST00000331113(c.701C>T,aCg>aTg):ENSP00000333496(p.Thr234Met)];	chr7*11991	chr7*11991	NA	NA	NA	NA	NA	D	PrD_PrD	D	MODERATE	D	0.998	138	138	0	129	121	8	6.2
Chewer	42482	chr7	128543771	G	T	*KCP*	INTRONIC-SS-DNR	ENSG00000135253:[ENST00000476647(n.792+9C>A)];ENSG00000135253:[ENST00000487494(n.792+9C>A)];ENSG00000135253:[ENST00000441244(n.617+575C>A)];ENSG00000135253:[ENST00000297801(n.618+575C>A)];	NA	NA	NA	NA	NA	NA	NA	NA	NA_NA	NA	MODIFIER	NA	NA	76	75	0	99	86	13	13.13
Chewer	42482	chr7	151891211	G	A	*KMT2C*	MISSENSE-SS-PRX	ENSG00000055609:[ENST00000355193(c.4543C>T,Ctt>Ttt):ENSP00000347325(p.Leu1515Phe)];ENSG00000055609:[ENST00000262189(c.4543C>T,Ctt>Ttt):ENSP00000262189(p.Leu1515Phe)];ENSG00000055609:[ENST00000473186(n.2254C>T)];ENSG00000055609:[ENST00000558084(c.*2063C>T)];	NA	NA	NA	NA	NA	NA	NA	NA	NA_NA	NA	LOW	NA	NA	84	84	0	92	86	6	6.52
Chewer	42482	chr7	77807348	C	T	*MAGI2*	MISSENSE	ENSG00000187391:MISSENSE[ENST00000354212(c.2383G>A,Gga>Aga):ENSP00000346151(p.Gly795Arg)];ENSG00000187391:MISSENSE[ENST00000419488(c.2341G>A,Gga>Aga):ENSP00000405766(p.Gly781Arg)];ENSG00000187391:MISSENSE[ENST00000522391(c.2383G>A,Gga>Aga):ENSP00000428389(p.Gly795Arg)];ENSG00000187391:EXONIC-NC[ENST00000519748(n.1429G>A)];ENSG00000187391:EXONIC-NC[ENST00000522342(n.37G>A)];	NA	NA	NA	8.25E-06	0	NA	NA	NA	NA_NA	NA	MODIFIER	NA	NA	159	158	0	215	198	16	7.44
Chewer	42482	chr7	34819157	C	A	*NPSR1*	INTRONIC	ENSG00000187258:[ENST00000359791(c.384+980C>A)];ENSG00000187258:[ENST00000381544(c.281-32225C>A)];ENSG00000187258:[ENST00000531252(c.351+980C>A)];ENSG00000187258:[ENST00000381539(c.384+980C>A)];ENSG00000187258:[ENST00000381542(c.281-47856C>A)];ENSG00000187258:[ENST00000396095(c.384+980C>A)];ENSG00000187258:[ENST00000360581(c.384+980C>A)];ENSG00000187258:[ENST00000381553(c.387C>A,gtC>gtA):ENSP00000370965(p.Val129(=))];	NA	NA	NA	NA	NA	NA	NA	NA	NA_NA	NA	MODIFIER	NA	NA	41	41	0	54	44	10	18.52
Chewer	42482	chr7	34819158	A	T	*NPSR1*	MISSENSE	ENSG00000187258:INTRONIC[ENST00000359791(c.384+981A>T)];ENSG00000187258:INTRONIC[ENST00000381544(c.281-32224A>T)];ENSG00000187258:INTRONIC[ENST00000531252(c.351+981A>T)];ENSG00000187258:INTRONIC[ENST00000381539(c.384+981A>T)];ENSG00000187258:INTRONIC[ENST00000381542(c.281-47855A>T)];ENSG00000187258:INTRONIC[ENST00000396095(c.384+981A>T)];ENSG00000187258:INTRONIC[ENST00000360581(c.384+981A>T)];ENSG00000187258:MISSENSE[ENST00000381553(c.388A>T,Atg>Ttg):ENSP00000370965(p.Met130Leu)];	NA	NA	NA	NA	NA	NA	NA	NA	NA_NA	NA	MODIFIER	NA	NA	41	41	0	55	45	10	18.18
Chewer	42482	chr7	34819157	C	A	*NPSR1-AS1*	INTRONIC	ENSG00000197085:[ENST00000436945(n.169-11105G>T)];ENSG00000197085:[ENST00000535640(n.85-18354G>T)];ENSG00000197085:[ENST00000431669(n.85-18354G>T)];ENSG00000197085:[ENST00000442669(n.194-18354G>T)];ENSG00000197085:[ENST00000539747(n.85-50729G>T)];	NA	NA	NA	NA	NA	NA	NA	NA	NA_NA	NA	MODIFIER	NA	NA	41	41	0	54	44	10	18.52
Chewer	42482	chr7	34819158	A	T	*NPSR1-AS1*	INTRONIC	ENSG00000197085:[ENST00000436945(n.169-11106T>A)];ENSG00000197085:[ENST00000535640(n.85-18355T>A)];ENSG00000197085:[ENST00000431669(n.85-18355T>A)];ENSG00000197085:[ENST00000442669(n.194-18355T>A)];ENSG00000197085:[ENST00000539747(n.85-50730T>A)];	NA	NA	NA	NA	NA	NA	NA	NA	NA_NA	NA	MODIFIER	NA	NA	41	41	0	55	45	10	18.18
Chewer	42482	chr7	135262775	A	G	*NUP205*	INTRONIC-SS-DNR	ENSG00000155561:[ENST00000440390(c.259+3A>G)];ENSG00000155561:[ENST00000285968(c.877+3A>G)];	NA	NA	NA	NA	NA	NA	NA	NA	NA_NA	NA	LOW	NA	NA	108	108	0	164	148	16	9.76

Mangalaparthi *et al.*, 2020. Mutational landscape of esophageal squamous cell carcinoma in an Indian cohort
Supplementary Table 4. List of somatic single nucleotide variants identified using whole exome sequencing of ESCC patients

Cohort	Sample	Chromosome	Genomic position	Reference allele	Alternate allele	Gene symbol	Variant type	Variant annotation	OncoMD	COSMIC	dbSNP	ExAC	ExAC_SAS	1000G	1000G_SAS	SIFT	Polyphen2	CONDEL	VEP	LRT	PhastCon7 Way	Total read (Adjacent normal)	Reference allel read support (Adjacent normal)	Alternative read support (Adjacent normal)	Total read (Tumor)	Reference allel read support (Tumor)	Alternative read support (Tumor)	Alternate allele frequency
Chewer	42482	chr7	94293265	G	T	*PEG10*	MISSENSE	ENSG00000242265:MISSENSE[ENST00000482108(c.397G>T,Gcc>Tcc):ENSP00000417587(p.Ala133Ser)];ENSG00000242265:MISSENSE[ENST00000488574(c.397G>T,Gcc>Tcc):ENSP00000418944(p.Ala133Ser)];ENSG00000242265:INTRONIC[ENST00000493935(n.307+524G>T)];	NA	NA	NA	NA	NA	NA	NA	NA	NA_NA	NA	MODIFIER	NA	NA	204	203	0	261	222	38	14.56
Chewer	42482	chr7	141866010	C	A	*RP11-1220K2.2*	MISSENSE	ENSG00000257743:MISSENSE[ENST00000477922(c.2765C>A,aCa>aAa):ENSP00000420449(p.Thr922Lys)];	NA	NA	NA	NA	NA	NA	NA	NA	BN_BN	NA	MODERATE	NA	NA	66	66	0	76	56	20	26.32
Chewer	42482	chr7	87258313	C	A	*RUNDC3B*	INTRONIC	ENSG00000105784:[ENST00000338056(c.122+52C>A)];ENSG00000105784:[ENST00000394654(c.122+52C>A)];ENSG00000105784:[ENST00000493037(c.122+52C>A)];ENSG00000105784:[ENST00000497788(n.185+1141C>A)];ENSG00000105784:[ENST00000466676(n.66+1384C>A)];ENSG00000105784:[ENST00000476114(n.496+52C>A)];	NA	NA	chr7*87258313*87258313*C*T*rs747828595*NA*NA*NA	NA	NA	NA	NA	NA	NA_NA	NA	MODIFIER	NA	NA	38	38	0	55	41	14	25.45
Chewer	42482	chr7	94228233	C	T	*SGCE*	SILENT	ENSG00000127990:SILENT[ENST00000447873(c.1080G>A,aaG>aaA):ENSP00000388734(p.Lys360(=))];ENSG00000127990:SILENT[ENST00000415788(c.1215G>A,aaG>aaA):ENSP00000405313(p.Lys405(=))];ENSG00000127990:SILENT[ENST00000445866(c.1107G>A,aaG>aaA):ENSP00000398930(p.Lys369(=))];ENSG00000127990:SILENT[ENST00000437425(c.984G>A,aaG>aaA):ENSP00000394061(p.Lys328(=))];ENSG00000127990:SILENT[ENST00000265735(c.1107G>A,aaG>aaA):ENSP00000265735(p.Lys369(=))];ENSG00000127990:SILENT[ENST00000428696(c.1080G>A,aaG>aaA):ENSP00000397536(p.Lys360(=))];ENSG00000127990:EXONIC-NC[ENST00000472326(n.217G>A)];ENSG00000127990:INTRONIC[ENST00000522045(c.122+776G>A)];	chr7*94228[illegible]	chr7*94228[illegible]	NA	NA	NA	NA	NA	NA	NA_NA	NA	LOW	NA	0.998	99	99	0	110	105	5	4.55
Chewer	42482	chr7	100456481	C	T	*SLC12A9*	MISSENSE	ENSG00000146828:MISSENSE[ENST00000354161(c.782C>T,aCg>aTg):ENSP00000275730(p.Thr261Met)];ENSG00000146828:MISSENSE[ENST00000416675(c.206C>T,aCg>aTg):ENSP00000410692(p.Thr69Met)];ENSG00000146828:MISSENSE[ENST00000275729(c.515C>T,aCg>aTg):ENSP00000275729(p.Thr172Met)];ENSG00000146828:MISSENSE[ENST00000415287(c.515C>T,aCg>aTg):ENSP00000413796(p.Thr172Met)];ENSG00000146828:MISSENSE[ENST00000428758(c.782C>T,aCg>aTg):ENSP00000408301(p.Thr261Met)];ENSG00000146828:MISSENSE[ENST00000540482(c.782C>T,aCg>aTg):ENSP00000443702(p.Thr261Met)];ENSG00000146828:INTRONIC[ENST00000461016(n.229-1026C>T)];ENSG00000146828:EXONIC-NC[ENST00000487651(n.124C>T)];ENSG00000146828:EXONIC-NC[ENST00000467972(n.2066C>T)];ENSG00000146828:EXONIC-NC[ENST00000475687(n.1761C>T)];ENSG00000146828:EXONIC-NC[ENST00000497958(n.172C>T)];ENSG00000146828:5UTR[ENST00000418037(c.-11C>T)];ENSG00000146828:5UTR[ENST00000448342(c.-11C>T)];	chr7*10045[illegible]	chr7*10045[illegible]	NA	NA	NA	NA	NA	NA	NA_NA	NA	MODIFIER	NA	NA	54	54	0	81	56	25	30.86
Chewer	42482	chr7	87465597	G	T	*SLC25A40*	MISSENSE	ENSG00000075303:MISSENSE[ENST00000341119(c.984C>A,ttC>ttA):ENSP00000344831(p.Phe328Leu)];ENSG00000075303:EXONIC-NC[ENST00000470328(n.715C>A)];ENSG00000075303:EXONIC-NC[ENST00000496348(n.270C>A)];ENSG00000075303:3UTR[ENST00000446236(c.*347C>A)];ENSG00000075303:3UTR[ENST00000429674(c.*801C>A)];	NA	NA	NA	NA	NA	NA	NA	NA	NA_NA	NA	MODIFIER	NA	NA	74	74	0	95	85	10	10.53
Chewer	42482	chr7	142983874	C	A	*TMEM139*	MISSENSE	ENSG00000178826:MISSENSE[ENST00000359333(c.603C>A,caC>caA):ENSP00000352284(p.His201Gln)];ENSG00000178826:MISSENSE[ENST00000409244(c.603C>A,caC>caA):ENSP00000386335(p.His201Gln)];ENSG00000178826:MISSENSE[ENST00000410004(c.603C>A,caC>caA):ENSP00000386564(p.His201Gln)];ENSG00000178826:MISSENSE[ENST00000409102(c.603C>A,caC>caA):ENSP00000386879(p.His201Gln)];ENSG00000178826:MISSENSE[ENST00000409541(c.603C>A,caC>caA):ENSP00000387044(p.His201Gln)];ENSG00000178826:EXONIC-NC[ENST00000487419(n.386C>A)];ENSG00000178826:EXONIC-NC[ENST00000480421(n.483C>A)];ENSG00000178826:EXONIC-NC[ENST00000471161(n.457C>A)];	NA	NA	NA	NA	NA	NA	NA	T	BN_BN	N	MODERATE	N	0.616	84	84	0	107	98	9	8.41
Chewer	42482	chr7	142161977	C	T	*TRBV6-6*	MISSENSE	ENSG00000211724:MISSENSE[ENST00000390371(c.298G>A,Gct>Act):ENSP00000374894(p.Ala100Thr)];	NA	NA	NA	NA	NA	NA	NA	T	PoD_BN	N	MODERATE	NA	NA	181	181	0	262	237	25	9.54
Chewer	42482	chr8	39646174	A	G	*ADAM2*	INTRONIC	ENSG00000104755:[ENST00000521880(c.642+14T>C)];ENSG00000104755:[ENST00000265708(c.642+14T>C)];ENSG00000104755:[ENST00000347580(c.585+14T>C)];ENSG00000104755:[ENST00000379853(c.514-11494T>C)];	NA	NA	NA	NA	NA	NA	NA	NA	NA_NA	NA	MODIFIER	NA	NA	53	53	0	98	91	7	7.14
Chewer	42482	chr8	86376391	C	T	*CA2*	INTRONIC	ENSG00000104267:[ENST00000520996(n.113+47C>T)];ENSG00000104267:[ENST00000522742(c.34+47C>T)];ENSG00000104267:[ENST00000518231(n.105+47C>T)];ENSG00000104267:[ENST00000520127(c.34+47C>T)];ENSG00000104267:[ENST00000285379(c.34+47C>T)];	NA	NA	chr8*86376375*86376396*GCCCCGATCCCCGATCCCCGAT*G*rs765677557*NA*NA*NA	NA	NA	NA	NA	NA	NA_NA	NA	MODIFIER	NA	NA	68	67	0	138	128	10	7.25
Chewer	42482	chr8	88394860	A	G	*CNBD1*	INTRONIC	ENSG00000176571:[ENST00000521593(c.213+28846A>G)];ENSG00000176571:[ENST00000523299(c.378+28846A>G)];ENSG00000176571:[ENST00000518476(c.*5A>G)];	NA	NA	NA	NA	NA	NA	NA	NA	NA_NA	NA	MODIFIER	NA	NA	48	48	0	66	59	7	10.61
Chewer	42482	chr8	145738192	C	A	*CTD-2517M22.17*	EXONIC-NC	ENSG00000265393:[ENST00000580385(n.243C>A)];	NA	NA	chr8*145738192*145738192*C*G*rs750985011*NA*NA*NA	NA	NA	NA	NA	NA	NA_NA	NA	MODIFIER	NA	NA	107	107	0	136	130	6	4.41
Chewer	42482	chr8	145151359	A	C	*CYC1*	SILENT	ENSG00000179091:EXONIC-NC[ENST00000525122(n.112A>C)];ENSG00000179091:EXONIC-NC[ENST00000533444(n.1238A>C)];ENSG00000179091:EXONIC-NC[ENST00000528618(n.772A>C)];ENSG00000179091:SILENT[ENST00000318911(c.573A>C,ggA>ggC):ENSP00000317159(p.Gly191(=))];	NA	NA	NA	NA	NA	NA	NA	NA	NA_NA	NA	MODIFIER	NA	NA	186	186	0	282	264	18	6.38
Chewer	42482	chr8	53454992	T	A	*FAM150A*	MISSENSE	ENSG00000196711:MISSENSE[ENST00000358543(c.204A>T,agA>agT):ENSP00000351345(p.Arg68Ser)];ENSG00000196711:MISSENSE[ENST00000523939(c.204A>T,agA>agT):ENSP00000430953(p.Arg68Ser)];	NA	NA	chr8*53454991*53454992*CT*C*rs763103255*NA*NA*NA	NA	NA	NA	NA	D	BN_PoD	D	MODERATE	D	0.981	64	64	0	110	104	6	5.45
Chewer	42482	chr8	76463629	A	C	*HNF4G*	MISSENSE	ENSG00000164749:MISSENSE[ENST00000396423(c.359A>C,cAa>cCa):ENSP00000379701(p.Gln120Pro)];ENSG00000164749:MISSENSE[ENST00000354370(c.248A>C,cAa>cCa):ENSP00000346339(p.Gln83Pro)];ENSG00000164749:EXONIC-NC[ENST00000396419(n.245A>C)];	NA	NA	NA	NA	NA	NA	NA	NA	NA_NA	NA	MODIFIER	NA	NA	75	74	0	84	67	17	20.24

Supplementary Table 4. List of somatic single nucleotide variants identified using whole exome sequencing of ESCC patients

Cohort	Sample	Chromosome	Genomic position	Reference allele	Alternate allele	Gene symbol	Variant type	Variant annotation	OncoMD	COSMIC	dbSNP	ExAC	ExAC_SAS	1000G	1000G_SAS	SIFT	Polyphen2	CONDEL	VEP	LRT	PhastCon7 Way	Total read (Adjacent normal)	Reference allel read support (Adjacent normal)	Alternative read support (Adjacent normal)	Total read (Tumor)	Reference allel read support (Tumor)	Alternative read support (Tumor)	Alternate allele frequency
Chewer	42482	chr8	29102945	G	T	*KIF13B*	MISSENSE	ENSG00000197892:MISSENSE[ENST00000521515(c.67C>A,Cat>Aat):ENSP00000429201(p.His23Asn)];ENSG00000197892:MISSENSE[ENST00000524189(c.67C>A,Cat>Aat):ENSP00000427900(p.His23Asn)];ENSG00000197892:MISSENSE[ENST00000522355(c.67C>A,Cat>Aat):ENSP00000429027(p.His23Asn)];ENSG00000197892:EXONIC-NC[ENST00000522846(n.126C>A)];ENSG00000197892:3UTR[ENST00000523968(c.*5C>A)];	NA	NA	NA	NA	NA	NA	NA	NA	NA_NA	NA	MODIFIER	NA	NA	170	170	0	215	203	12	5.58
Chewer	42482	chr8	133051039	C	A	*OC90*	MISSENSE-SS-PRX	ENSG00000258417:[ENST00000262283(c.1214G>T,aGa>aTa):ENSP00000262283(p.Arg405Ile)];ENSG00000258417:[ENST00000603859(c.626G>T,aGa>aTa):ENSP00000473802(p.Arg209Ile)];ENSG00000253117:[ENST00000254627(c.626G>T,aGa>aTa):ENSP00000254627(p.Arg209Ile)];ENSG00000258417:[ENST00000443356(c.626G>T,aGa>aTa):ENSP00000390050(p.Arg209Ile)];	chr8*13305	NA	NA	NA	NA	NA	NA	T	BN_PoD	N	MODERATE	NA	0.006	32	32	0	48	43	5	10.42
Chewer	42482	chr8	110442231	T	G	*PKHD1L1*	MISSENSE	ENSG00000205038:MISSENSE[ENST00000378402(c.3194T>G,aTt>aGt):ENSP00000367655(p.Ile1065Ser)];	NA	NA	NA	NA	NA	NA	NA	T	BN_BN	N	MODERATE	N	0.198	134	134	0	206	193	13	6.31
Chewer	42482	chr8	145738192	C	A	*RECQL4*	INTRONIC	ENSG00000160957:[ENST00000532237(n.2882+38G>T)];ENSG00000160957:[ENST00000534626(c.926+38G>T)];ENSG00000160957:[ENST00000428558(c.2755+38G>T)];ENSG00000160957:[ENST00000532269(n.2798+38G>T)];	NA	NA	chr8*145738192*145738192*C*G*rs750985011*NA*NA*NA	NA	NA	NA	NA	NA	NA_NA	NA	MODIFIER	NA	NA	107	107	0	136	130	6	4.41
Chewer	42482	chr8	86376391	C	T	*RP11-317J10.2*	INTRONIC	ENSG00000253549:[ENST00000521761(n.334+420G>A)];ENSG00000253549:[ENST00000517697(n.106+66G>A)];	NA	NA	chr8*86376375*86376396*GCCCCGATCCCCGATCCCCGAT*G*rs765677557*NA*NA*NA	NA	NA	NA	NA	NA	NA_NA	NA	MODIFIER	NA	NA	68	67	0	138	128	10	7.25
Chewer	42482	chr8	92982963	G	T	*RUNX1T1*	MISSENSE	ENSG00000079102:MISSENSE[ENST00000436581(c.1495C>A,Cgc>Agc):ENSP00000402257(p.Arg499Ser)];ENSG00000079102:MISSENSE[ENST00000265814(c.1462C>A,Cgc>Agc):ENSP00000265814(p.Arg488Ser)];ENSG00000079102:MISSENSE[ENST00000518844(c.1381C>A,Cgc>Agc):ENSP00000430728(p.Arg461Ser)];ENSG00000079102:MISSENSE[ENST00000523629(c.1462C>A,Cgc>Agc):ENSP00000428543(p.Arg488Ser)];ENSG00000079102:MISSENSE[ENST00000360348(c.1351C>A,Cgc>Agc):ENSP00000353504(p.Arg451Ser)];ENSG00000079102:MISSENSE[ENST00000520724(c.1351C>A,Cgc>Agc):ENSP00000428742(p.Arg451Ser)];ENSG00000079102:MISSENSE[ENST00000396218(c.1381C>A,Cgc>Agc):ENSP00000379520(p.Arg461Ser)];ENSG00000079102:MISSENSE[ENST00000422361(c.1351C>A,Cgc>Agc):ENSP00000390137(p.Arg451Ser)];ENSG00000079102:EXONIC-NC[ENST00000521078(n.426C>A)];ENSG00000079102:3UTR[ENST00000521751(c.*333C>A)];	chr8*92982	chr8*92982	chr8*92982963*92982963*G*A*rs770002524*NA*NA*NA	NA	NA	NA	NA	NA	NA_NA	NA	MODIFIER	NA	NA	66	66	0	98	76	21	21.43
Chewer	42482	chr8	11188922	T	C	*SLC35G5*	MISSENSE	ENSG00000177710:MISSENSE[ENST00000382435(c.307T>C,Tgg>Cgg):ENSP00000371872(p.Trp103Arg)];	chr8*11188	chr8*11188	NA	0.0233675	0.010824536	NA	NA	T	BN_BN	N	MODERATE	N	0.104	176	176	0	179	171	8	4.47
Chewer	42482	chr8	51415402	C	A	*SNTG1*	MISSENSE	ENSG00000147481:MISSENSE[ENST00000517473(c.428C>A,cCt>cAt):ENSP00000431123(p.Pro143His)];ENSG00000147481:MISSENSE[ENST00000518864(c.428C>A,cCt>cAt):ENSP00000429276(p.Pro143His)];ENSG00000147481:MISSENSE[ENST00000276467(c.428C>A,cCt>cAt):ENSP00000276467(p.Pro143His)];ENSG00000147481:MISSENSE[ENST00000522124(c.428C>A,cCt>cAt):ENSP00000429842(p.Pro143His)];ENSG00000147481:MISSENSE[ENST00000520825(c.428C>A,cCt>cAt):ENSP00000428558(p.Pro143His)];	chr8*51415	NA	NA	NA	NA	NA	NA	D	PrD_PrD	D	MODERATE	NA	NA	45	44	0	96	50	46	47.92
Chewer	42482	chr8	51465613	G	A	*SNTG1*	SILENT	ENSG00000147481:SILENT[ENST00000517473(c.684G>A,caG>caA):ENSP00000431123(p.Gln228(=))];ENSG00000147481:SILENT[ENST00000520825(c.684G>A,caG>caA):ENSP00000428558(p.Gln228(=))];ENSG00000147481:SILENT[ENST00000524004(c.4G>A,caG>caA):ENSP00000430748(p.Gln2(=))];ENSG00000147481:SILENT[ENST00000518864(c.684G>A,caG>caA):ENSP00000429276(p.Gln228(=))];ENSG00000147481:SILENT[ENST00000276467(c.684G>A,caG>caA):ENSP00000276467(p.Gln228(=))];ENSG00000147481:SILENT[ENST00000522124(c.684G>A,caG>caA):ENSP00000429842(p.Gln228(=))];	NA	NA	NA	NA	NA	NA	NA	NA	NA_NA	NA	LOW	NA	0.998	138	138	0	219	208	11	5.02
Chewer	42482	chr8	53079465	G	T	*ST18*	MISSENSE	ENSG00000147488:MISSENSE[ENST00000517580(c.1151C>A,cCg>cAg):ENSP00000428521(p.Pro384Gln)];ENSG00000147488:MISSENSE[ENST00000276480(c.1151C>A,cCg>cAg):ENSP00000276480(p.Pro384Gln)];ENSG00000147488:3UTR[ENST00000521824(c.*862C>A)];ENSG00000147488:3UTR[ENST00000522251(c.*1113C>A)];ENSG00000147488:3UTR[ENST00000521582(c.*111C>A)];	NA	NA	NA	NA	NA	NA	NA	NA	NA_NA	NA	MODIFIER	NA	NA	51	51	0	101	64	37	36.63
Chewer	42482	chr8	27097502	G	C	*STMN4*	MISSENSE	ENSG00000015592:MISSENSE[ENST00000519614(c.496C>G,Cgg>Ggg):ENSP00000430699(p.Arg166Gly)];ENSG00000015592:MISSENSE[ENST00000523048(c.577C>G,Cgg>Ggg):ENSP00000428428(p.Arg193Gly)];ENSG00000015592:MISSENSE[ENST00000350889(c.577C>G,Cgg>Ggg):ENSP00000342538(p.Arg193Gly)];ENSG00000015592:MISSENSE[ENST00000265770(c.496C>G,Cgg>Ggg):ENSP00000265770(p.Arg166Gly)];ENSG00000015592:MISSENSE[ENST00000519997(c.469C>G,Cgg>Ggg):ENSP00000428474(p.Arg157Gly)];ENSG00000015592:MISSENSE[ENST00000522908(c.577C>G,Cgg>Ggg):ENSP00000430389(p.Arg193Gly)];	NA	NA	chr8*27097502*27097502*G*A,T*rs764508091*NA*NA*NA	NA	NA	NA	NA	D	PrD_PrD	D	MODERATE	D	0.999	117	117	0	146	107	39	26.71
Chewer	42482	chr8	133973190	G	A	*TG*	INTRONIC	ENSG00000042832:[ENST00000220616(c.5402-63G>A)];ENSG00000042832:[ENST00000523756(c.*1615-63G>A)];ENSG00000042832:[ENST00000377869(c.5231-63G>A)];ENSG00000042832:[ENST00000542445(c.512-63G>A)];ENSG00000042832:[ENST00000519178(c.768-63G>A)];	NA	NA	NA	NA	NA	NA	NA	NA	NA_NA	NA	MODIFIER	NA	NA	142	142	0	175	156	19	10.86
Chewer	42482	chr8	134145670	G	A	*TG*	INTRONIC	ENSG00000042832:[ENST00000220616(c.7998-44G>A)];ENSG00000042832:[ENST00000523756(c.*4211-44G>A)];ENSG00000042832:[ENST00000542445(c.3108-44G>A)];ENSG00000042832:[ENST00000521107(c.210-44G>A)];ENSG00000042832:[ENST00000377869(c.7827-44G>A)];ENSG00000042832:[ENST00000519543(c.2397-44G>A)];ENSG00000042832:[ENST00000519178(c.3364-44G>A)];ENSG00000042832:[ENST00000522691(n.40G>A)];	NA	NA	NA	NA	NA	NA	NA	NA	NA_NA	NA	MODIFIER	NA	NA	120	119	0	214	193	20	9.35
Chewer	42482	chr9	75531932	T	A	*ALDH1A1*	MISSENSE	ENSG00000165092:MISSENSE[ENST00000297785(c.939A>T,gaA>gaT):ENSP00000297785(p.Glu313Asp)];ENSG00000165092:3UTR[ENST00000376939(c.*26A>T)];	NA	NA	NA	NA	NA	NA	NA	NA	NA_NA	NA	MODIFIER	NA	NA	130	129	0	203	165	38	18.72

Supplementary Table 4. List of somatic single nucleotide variants identified using whole exome sequencing of ESCC patients

Cohort	Sample	Chromosome	Genomic position	Reference allele	Alternate allele	Gene symbol	Variant type	Variant annotation	OncoMD	COSMIC	dbSNP	ExAC	ExAC_SAS	1000G	1000G_SAS	SIFT	Polyphen2	CONDEL	VEP	LRT	PhastCon7 Way	Total read (Adjacent normal)	Reference allel read support (Adjacent normal)	Alternative read support (Adjacent normal)	Total read (Tumor)	Reference allel read support (Tumor)	Alternative read support (Tumor)	Alternate allele frequency
Chewer	42482	chr9	16435032	C	A	*BNC2*	INTRONIC	ENSG00000173068:[ENST00000545497(c.2354+521G>T)];ENSG00000173068:[ENST00000380667(c.2438+521G>T)];ENSG00000173068:[ENST00000380672(c.2639+521G>T)];ENSG00000173068:[ENST00000380666(c.2639+521G>T)];ENSG00000173068:[ENST00000484726(c.2639+521G>T)];ENSG00000173068:[ENST00000411752(c.880+4G>T)];ENSG00000173068:[ENST00000418777(c.2572+4G>T)];	NA	NA	NA	NA	NA	NA	NA	NA	NA_NA	NA	LOW	NA	NA	200	199	0	158	144	14	8.86
Chewer	42482	chr9	16435033	T	C	*BNC2*	INTRONIC	ENSG00000173068:[ENST00000545497(c.2354+520A>G)];ENSG00000173068:[ENST00000380667(c.2438+520A>G)];ENSG00000173068:[ENST00000380672(c.2639+520A>G)];ENSG00000173068:[ENST00000380666(c.2639+520A>G)];ENSG00000173068:[ENST00000484726(c.2639+520A>G)];ENSG00000173068:[ENST00000411752(c.880+3A>G)];ENSG00000173068:[ENST00000418777(c.2572+3A>G)];	NA	NA	NA	NA	NA	NA	NA	NA	NA_NA	NA	LOW	NA	NA	198	197	0	156	142	14	8.97
Chewer	42482	chr9	74674210	C	T	*C9orf57*	MISSENSE	ENSG00000204669:MISSENSE[ENST00000377024(c.104G>A,cGc>cAc):ENSP00000366223(p.Arg35His)];ENSG00000204669:INTRONIC[ENST00000424431(c.62-2451G>A)];	NA	NA	0.0001997	NA	NA	0.0001997	0	NA	NA_NA	NA	MODIFIER	NA	NA	195	193	2	279	199	80	28.67
Chewer	42482	chr9	137642442	G	A	*COL5A1*	MISSENSE	ENSG00000130635:MISSENSE[ENST00000371817(c.1549G>A,Gga>Aga):ENSP00000360882(p.Gly517Arg)];	NA	NA	NA	NA	NA	NA	NA	NA	PrD_PrD	NA	MODERATE	U	0.892	78	77	0	138	131	7	5.07
Chewer	42482	chr9	97912291	C	G	*FANCC*	SILENT	ENSG00000158169:EXONIC-NC[ENST00000493098(n.25G>C)];ENSG00000158169:SILENT[ENST00000375305(c.600G>C,gtG>gtC):ENSP00000364454(p.Val200(=))];ENSG00000158169:SILENT[ENST00000289081(c.600G>C,gtG>gtC):ENSP00000289081(p.Val200(=))];	NA	NA	NA	NA	NA	NA	NA	NA	NA_NA	NA	LOW	NA	NA	55	55	0	61	34	27	44.26
Chewer	42482	chr9	37746593	G	A	*FRMPD1*	MISSENSE	ENSG00000070601:MISSENSE[ENST00000539465(c.4564G>A,Ggg>Agg):ENSP00000444411(p.Gly1522Arg)];ENSG00000070601:MISSENSE[ENST00000377765(c.4564G>A,Ggg>Agg):ENSP00000366995(p.Gly1522Arg)];	NA	NA	NA	1.66E-05	0	NA	NA	NA	NA_NA	NA	MODIFIER	NA	NA	112	112	0	188	175	13	6.91
Chewer	42482	chr9	68415317	C	T	*MIR4477A*	EXONIC-NC	ENSG00000266017:[ENST00000581659(n.10C>T)];	NA	NA	NA	0.0055498	0.008928571	NA	NA	NA	NA_NA	NA	MODIFIER	NA	NA	290	285	4	478	464	14	2.93
Chewer	42482	chr9	133769077	T	G	*QRFP*	MISSENSE	ENSG00000188710:MISSENSE[ENST00000343079(c.149A>C,cAc>cCc):ENSP00000345487(p.His50Pro)];	NA	NA	NA	NA	NA	NA	NA	T	BN_BN	N	MODERATE	N	NA	57	56	0	140	128	12	8.57
Chewer	42482	chr9	133769078	G	T	*QRFP*	MISSENSE	ENSG00000188710:MISSENSE[ENST00000343079(c.148C>A,Cac>Aac):ENSP00000345487(p.His50Asn)];	NA	NA	NA	NA	NA	NA	NA	T	BN_BN	N	MODERATE	N	NA	56	55	0	138	126	12	8.7
Chewer	42482	chr9	37746593	G	A	*RP11-613M10.9*	INTRONIC	ENSG00000255872:[ENST00000540557(c.*911-18568C>T)];	NA	NA	NA	1.66E-05	0	NA	NA	NA	NA_NA	NA	MODIFIER	NA	NA	112	112	0	188	175	13	6.91
Chewer	42482	chr9	111853216	G	A	*TMEM245*	MISSENSE	ENSG00000106771:MISSENSE[ENST00000491854(c.386C>T,cCa>cTa):ENSP00000417842(p.Pro129Leu)];ENSG00000106771:MISSENSE[ENST00000374586(c.1136C>T,cCa>cTa):ENSP00000363714(p.Pro379Leu)];	NA	NA	NA	NA	NA	NA	NA	T	PoD_PoD	N	MODERATE	D	1	284	283	0	345	318	27	7.83
Chewer	42482	chr9	35846297	G	T	*TMEM8B*	MISSENSE	ENSG00000137103:MISSENSE[ENST00000377988(c.416G>T,aGg>aTg):ENSP00000367227(p.Arg139Met)];ENSG00000137103:MISSENSE[ENST00000377991(c.416G>T,aGg>aTg):ENSP00000367230(p.Arg139Met)];ENSG00000137103:MISSENSE[ENST00000377996(c.416G>T,aGg>aTg):ENSP00000367235(p.Arg139Met)];ENSG00000137103:MISSENSE[ENST00000439587(c.416G>T,aGg>aTg):ENSP00000395810(p.Arg139Met)];ENSG00000137103:EXONIC-NC[ENST00000473947(n.919G>T)];	NA	NA	NA	NA	NA	NA	NA	NA	NA_NA	NA	MODIFIER	NA	NA	71	71	0	167	123	44	26.35
Chewer	42482	chr9	140173384	C	G	*TOR4A*	MISSENSE	ENSG00000198113:MISSENSE[ENST00000357503(c.243C>G,agC>agG):ENSP00000350102(p.Ser81Arg)];	NA	NA	NA	NA	NA	NA	NA	T	BN_PoD	N	MODERATE	U	0.094	132	131	0	185	175	10	5.41
Chewer	42482	chrX	110952232	G	T	*ALG13*	MISSENSE	ENSG00000101901:MISSENSE[ENST00000251943(c.478G>T,Gct>Tct):ENSP00000251943(p.Ala160Ser)];ENSG00000101901:MISSENSE[ENST00000495283(c.478G>T,Gct>Tct):ENSP00000427093(p.Ala160Ser)];ENSG00000101901:MISSENSE[ENST00000394780(c.790G>T,Gct>Tct):ENSP00000378260(p.Ala264Ser)];ENSG00000101901:EXONIC-NC[ENST00000470704(n.511G>T)];ENSG00000101901:INTRONIC[ENST00000487141(n.571-2671G>T)];	NA	NA	NA	NA	NA	NA	NA	NA	NA_NA	NA	MODIFIER	NA	NA	170	170	0	163	156	7	4.29
Chewer	42482	chrX	110952232	G	T	*ALG13-AS1*	INTRONIC	ENSG00000229487:[ENST00000430794(n.106+1992C>A)];	NA	NA	NA	NA	NA	NA	NA	NA	NA_NA	NA	MODIFIER	NA	NA	170	170	0	163	156	7	4.29
Chewer	42482	chrX	119509251	A	T	*ATP1B4*	MISSENSE	ENSG00000101892:MISSENSE[ENST00000539306(c.458A>T,gAa>gTa):ENSP00000443334(p.Glu153Val)];ENSG00000101892:MISSENSE[ENST00000218008(c.587A>T,gAa>gTa):ENSP00000218008(p.Glu196Val)];ENSG00000101892:MISSENSE[ENST00000361319(c.575A>T,gAa>gTa):ENSP00000355346(p.Glu192Val)];	NA	NA	NA	NA	NA	NA	NA	D	PrD_PrD	D	MODERATE	D	0.999	83	83	0	118	88	30	25.42
Chewer	42482	chrX	101409054	C	T	*BEX5*	MISSENSE	ENSG00000184515:MISSENSE[ENST00000543160(c.184G>A,Gat>Aat):ENSP00000446054(p.Asp62Asn)];ENSG00000184515:MISSENSE[ENST00000333643(c.184G>A,Gat>Aat):ENSP00000328030(p.Asp62Asn)];	NA	NA	NA	NA	NA	NA	NA	T	PrD_PrD	D	MODERATE	N	0.779	120	120	0	162	154	8	4.94
Chewer	42482	chrX	31893386	A	T	*DMD*	MISSENSE	ENSG00000198947:MISSENSE[ENST00000357033(c.7017T>A,caT>caA):ENSP00000354923(p.His2339Gln)];ENSG00000198947:MISSENSE[ENST00000358062(c.105T>A,caT>caA):ENSP00000350765(p.His35Gln)];ENSG00000198947:MISSENSE[ENST00000378677(c.7005T>A,caT>caA):ENSP00000367948(p.His2335Gln)];ENSG00000198947:5UTR[ENST00000343523(c.-364T>A)];ENSG00000198947:5UTR[ENST00000378707(c.-364T>A)];ENSG00000198947:5UTR[ENST00000474231(c.-364T>A)];ENSG00000198947:5UTR[ENST00000359836(c.-364T>A)];ENSG00000198947:5UTR[ENST00000541735(c.-364T>A)];	NA	NA	NA	NA	NA	NA	NA	NA	NA_NA	NA	MODIFIER	NA	NA	143	141	0	163	139	22	13.5
Chewer	42482	chrX	32360270	G	C	*DMD*	MISSENSE	ENSG00000198947:INTRONIC[ENST00000488902(n.336-125090C>G)];ENSG00000198947:MISSENSE[ENST00000357033(c.5869C>G,Cgg>Ggg):ENSP00000354923(p.Arg1957Gly)];ENSG00000198947:MISSENSE[ENST00000378677(c.5857C>G,Cgg>Ggg):ENSP00000367948(p.Arg1953Gly)];	NA	NA	chrX*32360270*32360270*G*A*rs755477994*NA*NA*NA	NA	NA	NA	NA	NA	PoD_PoD	NA	MODERATE	U	1	98	98	0	104	82	22	21.15
Chewer	42482	chrX	154157866	G	A	*F8*	MISSENSE	ENSG00000185010:MISSENSE[ENST00000360256(c.4199C>T,cCt>cTt):ENSP00000353393(p.Pro1400Leu)];	NA	NA	NA	NA	NA	NA	NA	T	BN_BN	N	MODERATE	N	0.001	202	201	1	179	166	11	6.15
Chewer	42482	chrX	140995005	C	A	*MAGEC1*	SILENT	ENSG00000155495:INTRONIC[ENST00000406005(c.-114-871C>A)];ENSG00000155495:SILENT[ENST00000285879(c.1815C>A,tcC>tcA):ENSP00000285879(p.Ser605(=))];	NA	NA	chrX*140995005*140995005*C*T*rs766020160*NA*NA*NA	NA	NA	NA	NA	NA	NA_NA	NA	LOW	NA	NA	75	74	0	56	50	6	10.71
Chewer	42482	chrX	40540049	T	C	*MED14*	SILENT	ENSG00000180182:SILENT[ENST00000324817(c.2571A>G,gaA>gaG):ENSP00000323720(p.Glu857(=))];ENSG00000180182:EXONIC-NC[ENST00000496531(n.435A>G)];	NA	NA	NA	NA	NA	NA	NA	NA	NA_NA	NA	MODIFIER	NA	NA	126	126	0	106	86	18	16.98

Mangalaparthi *et al.* , 2020. Mutational landscape of esophageal squamous cell carcinoma in an Indian cohort
Supplementary Table 4. List of somatic single nucleotide variants identified using whole exome sequencing of ESCC patients

Cohort	Sample	Chromosome	Genomic position	Reference allele	Alternate allele	Gene symbol	Variant type	Variant annotation	OncoMD	COSMIC	dbSNP	ExAC	ExAC_SAS	1000G	1000G_SAS	SIFT	Polyphen2	CONDEL	VEP	LRT	PhastCon7 Way	Total read (Adjacent normal)	Reference allel read support (Adjacent normal)	Alternative read support (Adjacent normal)	Total read (Tumor)	Reference allel read support (Tumor)	Alternative read support (Tumor)	Alternate allele frequency
Chewer	42482	chrX	154018136	G	T	*MPP1*	INTRONIC	ENSG00000130830:[ENST00000439370(c.*404+93C>A)];ENSG00000130830:[ENST00000393531(c.480+93C>A)];ENSG00000130830:[ENST00000413259(c.390+93C>A)];ENSG00000130830:[ENST00000417435(c.*359+93C>A)];ENSG00000130830:[ENST00000488754(n.282+93C>A)];ENSG00000130830:[ENST00000393529(c.342+93C>A)];ENSG00000130830:[ENST00000494170(n.171-6354C>A)];ENSG00000130830:[ENST00000462825(n.72-3461C>A)];ENSG00000130830:[ENST00000488694(n.515+93C>A)];ENSG00000130830:[ENST00000475943(n.207-3461C>A)];ENSG00000130830:[ENST00000428488(c.171+93C>A)];ENSG00000130830:[ENST00000453245(c.103-3461C>A)];ENSG00000130830:[ENST00000369534(c.480+93C>A)];	NA	NA	NA	NA	NA	NA	NA	NA	NA_NA	NA	MODIFIER	NA	NA	53	53	0	56	46	10	17.86
Chewer	42482	chrX	154018137	A	T	*MPP1*	INTRONIC	ENSG00000130830:[ENST00000439370(c.*404+92T>A)];ENSG00000130830:[ENST00000393531(c.480+92T>A)];ENSG00000130830:[ENST00000413259(c.390+92T>A)];ENSG00000130830:[ENST00000417435(c.*359+92T>A)];ENSG00000130830:[ENST00000488754(n.282+92T>A)];ENSG00000130830:[ENST00000393529(c.342+92T>A)];ENSG00000130830:[ENST00000494170(n.171-6355T>A)];ENSG00000130830:[ENST00000462825(n.72-3462T>A)];ENSG00000130830:[ENST00000488694(n.515+92T>A)];ENSG00000130830:[ENST00000475943(n.207-3462T>A)];ENSG00000130830:[ENST00000428488(c.171+92T>A)];ENSG00000130830:[ENST00000453245(c.103-3462T>A)];ENSG00000130830:[ENST00000369534(c.480+92T>A)];	NA	NA	NA	NA	NA	NA	NA	NA	NA_NA	NA	MODIFIER	NA	NA	54	54	0	57	47	10	17.54
Chewer	42482	chrX	111624369	A	C	*NA*	INTERGENIC		NA	NA	NA	NA	NA	NA	NA	NA	NA_NA	NA	MODIFIER	NA	NA	127	127	0	135	116	19	14.07
Chewer	42482	chrX	101615548	C	G	*NXF2B*	MISSENSE	ENSG00000185945:MISSENSE[ENST00000372749(c.1855G>C,Gcg>Ccg):ENSP00000361835(p.Ala619Pro)];ENSG00000185945:MISSENSE[ENST00000457521(c.1855G>C,Gcg>Ccg):ENSP00000396447(p.Ala619Pro)];ENSG00000185945:MISSENSE[ENST00000372750(c.1855G>C,Gcg>Ccg):ENSP00000361836(p.Ala619Pro)];ENSG00000185945:MISSENSE[ENST00000412230(c.1855G>C,Gcg>Ccg):ENSP00000413087(p.Ala619Pro)];ENSG00000185945:3UTR[ENST00000372752(c.*62G>C)];	NA	NA	chrX*101615548*101615548*C*T*rs370144475*NA*NA*NA	NA	NA	NA	NA	NA	NA_NA	NA	MODIFIER	NA	NA	239	239	0	283	249	34	12.01
Chewer	42482	chrX	152936382	C	G	*PNCK*	MISSENSE	ENSG00000130822:MISSENSE[ENST00000447676(c.1046G>C,cGg>cCg):ENSP00000405950(p.Arg349Pro)];ENSG00000130822:MISSENSE[ENST00000370150(c.797G>C,cGg>cCg):ENSP00000359169(p.Arg266Pro)];ENSG00000130822:MISSENSE[ENST00000393831(c.866G>C,cGg>cCg):ENSP00000377417(p.Arg289Pro)];ENSG00000130822:MISSENSE[ENST00000370145(c.848G>C,cGg>cCg):ENSP00000359164(p.Arg283Pro)];ENSG00000130822:MISSENSE[ENST00000370142(c.866G>C,cGg>cCg):ENSP00000359161(p.Arg289Pro)];ENSG00000130822:MISSENSE[ENST00000340888(c.797G>C,cGg>cCg):ENSP00000340586(p.Arg266Pro)];ENSG00000130822:EXONIC-NC[ENST00000466074(n.924G>C)];ENSG00000130822:EXONIC-NC[ENST00000488994(n.1275G>C)];ENSG00000130822:EXONIC-NC[ENST00000472324(n.1505G>C)];	NA	NA	chrX*152936382*152936382*C*T*rs782668687*NA*NA*NA	NA	NA	NA	NA	NA	NA_NA	NA	MODIFIER	NA	NA	70	70	0	109	91	18	16.51
Chewer	42482	chrX	84363524	G	T	*SATL1*	MISSENSE	ENSG00000184788:MISSENSE[ENST00000509231(c.451C>A,Cca>Aca):ENSP00000425421(p.Pro151Thr)];ENSG00000184788:5UTR[ENST00000332921(c.-111C>A)];ENSG00000184788:5UTR[ENST00000395409(c.-111C>A)];	NA	NA	NA	NA	NA	NA	NA	NA	NA_NA	NA	MODIFIER	NA	NA	117	117	0	135	92	42	31.11
Chewer	42482	chrX	19817899	T	C	*SH3KBP1*	INTRONIC	ENSG00000147010:[ENST00000397821(c.162+36344A>G)];ENSG00000147010:[ENST00000379697(c.162+36344A>G)];	NA	NA	NA	NA	NA	NA	NA	NA	NA_NA	NA	MODIFIER	NA	NA	80	79	0	91	85	6	6.59
Chewer	42482	chrX	47062493	G	T	*UBA1*	INTRONIC	ENSG00000130985:[ENST00000335972(c.1339-40G>T)];ENSG00000130985:[ENST00000377351(c.1339-40G>T)];ENSG00000130985:[ENST00000490869(n.228-40G>T)];	NA	NA	NA	NA	NA	NA	NA	NA	NA_NA	NA	MODIFIER	NA	NA	71	70	0	76	56	20	26.32
Chewer	42482	chrX	73063792	G	A	*XIST*	EXONIC-NC	ENSG00000229807:[ENST00000429829(n.8797C>T)];	NA	NA	NA	0.0001065	0.000777605	NA	NA	NA	NA_NA	NA	MODIFIER	NA	NA	146	145	0	154	145	9	5.84
Chewer	42482	chrX	100169295	C	T	*XKRX*	3UTR	ENSG00000182489:[ENST00000328526(c.*32G>A)];ENSG00000182489:[ENST00000372956(c.*32G>A)];ENSG00000182489:[ENST00000468904(c.*693G>A)];	NA	NA	NA	NA	NA	NA	NA	NA	NA_NA	NA	MODIFIER	NA	NA	130	130	0	134	125	9	6.72
Chewer	42483	chr1	150526447	G	A	*ADAMTSL4*	MISSENSE	ENSG00000143382:MISSENSE[ENST00000271643(c.980G>A,cGc>cAc):ENSP00000271643(p.Arg327His)];ENSG00000143382:MISSENSE[ENST00000369038(c.980G>A,cGc>cAc):ENSP00000358034(p.Arg327His)];ENSG00000143382:MISSENSE[ENST00000369039(c.980G>A,cGc>cAc):ENSP00000358035(p.Arg327His)];ENSG00000143382:MISSENSE[ENST00000369041(c.980G>A,cGc>cAc):ENSP00000358037(p.Arg327His)];	NA	NA	NA	6.03E-05	0	NA	NA	NA	NA_NA	NA	MODIFIER	NA	NA	63	63	0	76	25	51	67.11
Chewer	42483	chr1	49224820	C	G	*AGBL4*	INTRONIC	ENSG00000186094:[ENST00000371839(c.635-95907G>C)];ENSG00000186094:[ENST00000416121(c.172-95907G>C)];ENSG00000186094:[ENST00000371838(c.635-95907G>C)];	NA	NA	chr1*49224820*49224820*C*T*rs367903944*NA*NA*NA	NA	NA	NA	NA	NA	NA_NA	NA	MODIFIER	NA	NA	151	148	0	130	84	46	35.38
Chewer	42483	chr1	247063603	C	A	*AHCTF1*	INTRONIC	ENSG00000153207:[ENST00000391829(c.1250+36G>T)];ENSG00000153207:[ENST00000366508(c.1355+36G>T)];ENSG00000153207:[ENST00000326225(c.1277+36G>T)];	NA	NA	NA	NA	NA	NA	NA	NA	NA_NA	NA	MODIFIER	NA	NA	37	37	0	48	10	37	77.08
Chewer	42483	chr1	49224820	C	G	*BEND5*	MISSENSE	ENSG00000162373:MISSENSE[ENST00000371833(c.497G>C,gGc>gCc):ENSP00000360899(p.Gly166Ala)];ENSG00000162373:EXONIC-NC[ENST00000463562(n.680G>C)];ENSG00000162373:EXONIC-NC[ENST00000476096(n.108G>C)];	NA	NA	chr1*49224820*49224820*C*T*rs367903944*NA*NA*NA	NA	NA	NA	NA	NA	NA_NA	NA	MODIFIER	NA	NA	151	148	0	130	84	46	35.38
Chewer	42483	chr1	11821984	G	C	*C1orf167*	SILENT	ENSG00000215910:SILENT[ENST00000433342(c.141G>C,gcG>gcC):ENSP00000414909(p.Ala47(=))];	NA	NA	NA	NA	NA	NA	NA	NA	NA_NA	NA	LOW	NA	NA	36	36	0	41	28	13	31.71
Chewer	42483	chr1	75039005	G	T	*C1orf173*	MISSENSE	ENSG00000178965:MISSENSE[ENST00000326665(c.2389C>A,Ctg>Atg):ENSP00000322609(p.Leu797Met)];ENSG00000178965:EXONIC-NC[ENST00000433746(n.531C>A)];	NA	NA	NA	NA	NA	NA	NA	NA	NA_NA	NA	MODIFIER	NA	NA	121	121	0	80	53	27	33.75
Chewer	42483	chr1	75072439	G	C	*C1orf173*	MISSENSE	ENSG00000178965:MISSENSE[ENST00000420661(c.744C>G,atC>atG):ENSP00000398581(p.Ile248Met)];ENSG00000178965:MISSENSE[ENST00000326665(c.1335C>G,atC>atG):ENSP00000322609(p.Ile445Met)];	NA	NA	NA	NA	NA	NA	NA	NA	NA_NA	NA	MODIFIER	NA	NA	324	323	0	263	176	86	32.7
Chewer	42483	chr1	158300625	G	C	*CD1B*	MISSENSE	ENSG00000158485:MISSENSE[ENST00000368168(c.289C>G,Cga>Gga):ENSP00000357150(p.Arg97Gly)];ENSG00000158485:MISSENSE[ENST00000451207(c.191C>G,Cga>Gga):ENSP00000395161(p.Arg65Gly)];	chr1*15830[illegible]	chr1*15830[illegible]	chr1*158300625*158300625*G*A,T*rs142860948*NA*NA*NA	NA	NA	NA	NA	T	BN_BN	N	MODERATE	NA	NA	165	164	1	152	52	100	65.79
Chewer	42483	chr1	86894249	G	T	*CLCA2*	MISSENSE-SS-PRX	ENSG00000137975:[ENST00000370565(c.473G>T,cGa>cTa):ENSP00000359596(p.Arg158Leu)];	NA	NA	chr1*86894249*86894249*G*A,C*rs143721621*0*0.9998*.,0.0001997	NA	NA	NA	NA	D	PrD_PrD	D	MODERATE	D	0.884	125	125	0	87	67	20	22.99
Chewer	42483	chr1	167511431	G	A	*CREG1*	3UTR	ENSG00000143162:[ENST00000370509(c.*104C>T)];ENSG00000143162:[ENST00000466652(n.80+3907C>T)];	NA	NA	NA	NA	NA	NA	NA	NA	NA_NA	NA	MODIFIER	NA	NA	76	75	1	93	80	13	13.98

Supplementary Table 4. List of somatic single nucleotide variants identified using whole exome sequencing of ESCC patients

Cohort	Sample	Chromosome	Genomic position	Reference allele	Alternate allele	Gene symbol	Variant type	Variant annotation	OncoMD	COSMIC	dbSNP	ExAC	ExAC_SAS	1000G	1000G_SAS	SIFT	Polyphen2	CONDEL	VEP	LRT	PhastCon7 Way	Total read (Adjacent normal)	Reference allel read support (Adjacent normal)	Alternative read support (Adjacent normal)	Total read (Tumor)	Reference allel read support (Tumor)	Alternative read support (Tumor)	Alternate allele frequency
Chewer	42483	chr1	9932223	C	A	*CTNNBIP1*	INTRONIC	ENSG00000178585:[ENST00000377263(c.-24-77G>T)];ENSG00000178585:[ENST00000377258(c.-24-77G>T)];ENSG00000178585:[ENST00000400904(c.-24-77G>T)];ENSG00000178585:[ENST00000377256(c.-24-77G>T)];	NA	NA	NA	NA	NA	NA	NA	NA	NA_NA	NA	MODIFIER	NA	NA	80	80	0	67	60	7	10.45
Chewer	42483	chr1	220154076	T	C	*EPRS*	INTRONIC	ENSG00000136628:[ENST00000366923(c.3555+22A>G)];	NA	NA	NA	NA	NA	NA	NA	NA	NA_NA	NA	MODIFIER	NA	NA	97	96	1	100	80	20	20
Chewer	42483	chr1	8075358	G	A	*ERRFI1*	MISSENSE	ENSG00000116285:INTRONIC[ENST00000469499(c.125+197C>T)];ENSG00000116285:INTRONIC[ENST00000474874(c.125+197C>T)];ENSG00000116285:INTRONIC-SS-DNR-PRX[ENST00000377482(c.202+10C>T)];ENSG00000116285:INTRONIC-SS-DNR-PRX[ENST00000487559(c.*59+10C>T)];ENSG00000116285:MISSENSE[ENST00000467067(c.212C>T,tCt>tTt):ENSP00000465100(p.Ser71Phe)];	NA	NA	NA	NA	NA	NA	NA	NA	NA_NA	NA	MODIFIER	NA	NA	90	90	0	67	47	20	29.85
Chewer	42483	chr1	157514074	C	T	*FCRL5*	SILENT	ENSG00000143297:SILENT[ENST00000361835(c.822G>A,ccG>ccA):ENSP00000354691(p.Pro274(=))];ENSG00000143297:SILENT[ENST00000368190(c.822G>A,ccG>ccA):ENSP00000357173(p.Pro274(=))];ENSG00000143297:SILENT[ENST00000368189(c.822G>A,ccG>ccA):ENSP00000357172(p.Pro274(=))];ENSG00000143297:SILENT[ENST00000356953(c.822G>A,ccG>ccA):ENSP00000349434(p.Pro274(=))];ENSG00000143297:SILENT[ENST00000368191(c.567G>A,ccG>ccA):ENSP00000357174(p.Pro189(=))];ENSG00000143297:EXONIC-NC[ENST00000481082(n.1020G>A)];	NA	NA	NA	8.24E-06	0	NA	NA	NA	NA_NA	NA	LOW	NA	NA	69	67	2	66	23	43	65.15
Chewer	42483	chr1	101005075	G	C	*GPR88*	MISSENSE	ENSG00000181656:MISSENSE[ENST00000315033(c.553G>C,Gcc>Ccc):ENSP00000314223(p.Ala185Pro)];	NA	NA	NA	NA	NA	NA	NA	T	BN_PoD	N	MODERATE	U	0.022	64	64	0	73	54	19	26.03
Chewer	42483	chr1	114472027	T	C	*HIPK1*	5UTR	ENSG00000163349:[ENST00000369554(c.-130T>C)];ENSG00000163349:[ENST00000426820(c.-130T>C)];ENSG00000163349:[ENST00000369559(c.-130T>C)];	NA	NA	chr1*114472027*114472027*T*G*rs577160926*0*0.9998*0.0001997	NA	NA	NA	NA	NA	NA_NA	NA	MODIFIER	NA	NA	76	76	0	29	11	18	62.07
Chewer	42483	chr1	226924541	C	T	*ITPKB*	MISSENSE	ENSG00000143772:MISSENSE[ENST00000366784(c.619G>A,Gag>Aag):ENSP00000355748(p.Glu207Lys)];ENSG00000143772:MISSENSE[ENST00000272117(c.619G>A,Gag>Aag):ENSP00000272117(p.Glu207Lys)];ENSG00000143772:MISSENSE[ENST00000429204(c.619G>A,Gag>Aag):ENSP00000411152(p.Glu207Lys)];	chr1*22692	chr1*22692	NA	NA	NA	NA	NA	D_lc	BN_PoD	N	MODERATE	N	0.98	126	126	0	127	43	84	66.14
Chewer	42483	chr1	21014381	A	C	*KIF17*	MISSENSE	ENSG00000117245:MISSENSE[ENST00000247986(c.1438T>G,Ttt>Gtt):ENSP00000247986(p.Phe480Val)];ENSG00000117245:MISSENSE[ENST00000400463(c.1438T>G,Ttt>Gtt):ENSP00000383311(p.Phe480Val)];ENSG00000117245:MISSENSE[ENST00000375044(c.1138T>G,Ttt>Gtt):ENSP00000364184(p.Phe380Val)];ENSG00000117245:INTRONIC[ENST00000490034(n.210-1762T>G)];	NA	NA	NA	NA	NA	NA	NA	NA	NA_NA	NA	MODIFIER	NA	NA	79	79	0	54	32	22	40.74
Chewer	42483	chr1	226126988	A	T	*LEFTY2*	INTRONIC	ENSG00000143768:[ENST00000366820(c.737+73T>A)];ENSG00000143768:[ENST00000420304(c.635+73T>A)];	NA	NA	NA	NA	NA	NA	NA	NA	NA_NA	NA	MODIFIER	NA	NA	35	35	0	32	10	21	65.62
Chewer	42483	chr1	156106831	G	T	*LMNA*	INTRONIC	ENSG00000160789:[ENST00000498722(n.720+12G>T)];ENSG00000160789:[ENST00000392353(c.1245+12G>T)];ENSG00000160789:[ENST00000368299(c.1488+12G>T)];ENSG00000160789:[ENST00000347559(c.1488+12G>T)];ENSG00000160789:[ENST00000368301(c.1488+12G>T)];ENSG00000160789:[ENST00000473598(c.1191+12G>T)];ENSG00000160789:[ENST00000368297(c.1245+12G>T)];ENSG00000160789:[ENST00000459904(n.736+12G>T)];ENSG00000160789:[ENST00000508500(c.366+12G>T)];ENSG00000160789:[ENST00000448611(c.1152+12G>T)];ENSG00000160789:[ENST00000361308(c.1488+12G>T)];ENSG00000160789:[ENST00000368300(c.1488+12G>T)];ENSG00000160789:[ENST00000496738(n.885G>T)];ENSG00000160789:[ENST00000368298(n.1248G>T)];	NA	NA	NA	NA	NA	NA	NA	NA	NA_NA	NA	MODIFIER	NA	NA	95	94	0	87	24	63	72.41
Chewer	42483	chr1	39797100	G	T	*MACF1*	NONSENSE	ENSG00000127603:NONSENSE[ENST00000289893(c.160G>T,Gag>Tag):ENSP00000289893(p.Glu54Ter)];ENSG00000127603:NONSENSE[ENST00000564288(c.4840G>T,Gag>Tag):ENSP00000455274(p.Glu1614Ter)];ENSG00000127603:NONSENSE[ENST00000372915(c.4855G>T,Gag>Tag):ENSP00000362006(p.Glu1619Ter)];ENSG00000127603:NONSENSE[ENST00000567887(c.4951G>T,Gag>Tag):ENSP00000455823(p.Glu1651Ter)];ENSG00000127603:INTRONIC[ENST00000372925(c.2029+4075G>T)];ENSG00000127603:INTRONIC[ENST00000361689(c.4629+4075G>T)];ENSG00000127603:INTRONIC[ENST00000530262(c.5076+4075G>T)];ENSG00000127603:INTRONIC[ENST00000539005(c.4629+4075G>T)];ENSG00000127603:INTRONIC[ENST00000317713(c.4629+4075G>T)];ENSG00000127603:INTRONIC[ENST00000545844(c.4629+4075G>T)];ENSG00000127603:INTRONIC[ENST00000476350(n.2029+4075G>T)];	NA	NA	NA	NA	NA	NA	NA	NA	NA_NA	NA	MODIFIER	NA	NA	96	96	0	129	63	66	51.16
Chewer	42483	chr1	3432007	C	A	*MEGF6*	MISSENSE	ENSG00000162591:MISSENSE[ENST00000485002(c.710G>T,cGg>cTg):ENSP00000419033(p.Arg237Leu)];ENSG00000162591:MISSENSE[ENST00000356575(c.689G>T,cGg>cTg):ENSP00000348982(p.Arg230Leu)];ENSG00000162591:MISSENSE[ENST00000294599(c.374G>T,cGg>cTg):ENSP00000294599(p.Arg125Leu)];	NA	NA	chr1*3432007*3432007*C*T*rs772320790*NA*NA*NA	NA	NA	NA	NA	D	BN_BN	N	MODERATE	N	0.997	36	36	0	37	23	14	37.84
Chewer	42483	chr1	38306273	C	A	*MTF1*	INTRONIC	ENSG00000188786:[ENST00000373036(c.409-443G>T)];	NA	NA	NA	NA	NA	NA	NA	NA	NA_NA	NA	MODIFIER	NA	NA	53	53	0	67	39	28	41.79
Chewer	42483	chr1	161183290	G	A	*NDUFS2*	INTRONIC	ENSG00000158864:[ENST00000468828(n.319+25G>A)];ENSG00000158864:[ENST00000392179(c.1212+25G>A)];ENSG00000158864:[ENST00000492153(n.226+25G>A)];ENSG00000158864:[ENST00000483804(n.836+25G>A)];ENSG00000158864:[ENST00000367993(c.1212+25G>A)];ENSG00000158864:[ENST00000493849(n.262+25G>A)];ENSG00000158864:[ENST00000465923(n.577+25G>A)];	NA	NA	NA	NA	NA	NA	NA	NA	NA_NA	NA	MODIFIER	NA	NA	41	41	0	37	30	7	18.92
Chewer	42483	chr1	247597444	C	T	*NLRP3*	SILENT	ENSG00000162711:SILENT[ENST00000366496(c.2367C>T,gaC>gaT):ENSP00000355452(p.Asp789(=))];ENSG00000162711:SILENT[ENST00000336119(c.2367C>T,gaC>gaT):ENSP00000337383(p.Asp789(=))];ENSG00000162711:SILENT[ENST00000391827(c.2196C>T,gaC>gaT):ENSP00000375703(p.Asp732(=))];ENSG00000162711:SILENT[ENST00000366497(c.2367C>T,gaC>gaT):ENSP00000355453(p.Asp789(=))];ENSG00000162711:SILENT[ENST00000391828(c.2367C>T,gaC>gaT):ENSP00000375704(p.Asp789(=))];ENSG00000162711:SILENT[ENST00000348069(c.2196C>T,gaC>gaT):ENSP00000294752(p.Asp732(=))];	NA	NA	NA	NA	NA	NA	NA	NA	NA_NA	NA	LOW	NA	0.939	153	153	0	148	104	44	29.73
Chewer	42483	chr1	120510742	T	A	*NOTCH2*	NONSENSE	ENSG00000134250:NONSENSE[ENST00000256646(c.1222A>T,Aaa>Taa):ENSP00000256646(p.Lys408Ter)];ENSG00000134250:EXONIC-NC[ENST00000479412(n.1360A>T)];	NA	NA	NA	NA	NA	NA	NA	NA	NA_NA	NA	MODIFIER	NA	NA	157	156	0	144	53	91	63.19
Chewer	42483	chr1	102462401	T	A	*OLFM3*	EXONIC-NC	ENSG00000118733:[ENST00000462354(n.61A>T)];ENSG00000118733:[ENST00000370103(c.-29A>T)];	NA	NA	NA	NA	NA	NA	NA	NA	NA_NA	NA	MODIFIER	NA	NA	120	119	0	91	66	25	27.47
Chewer	42483	chr1	102462402	C	A	*OLFM3*	EXONIC-NC	ENSG00000118733:[ENST00000462354(n.60G>T)];ENSG00000118733:[ENST00000370103(c.-30G>T)];	NA	NA	NA	NA	NA	NA	NA	NA	NA_NA	NA	MODIFIER	NA	NA	120	119	0	91	65	26	28.57

Mangalaparthi *et al.* , 2020. Mutational landscape of esophageal squamous cell carcinoma in an Indian cohort
Supplementary Table 4. List of somatic single nucleotide variants identified using whole exome sequencing of ESCC patients

Cohort	Sample	Chromosome	Genomic position	Reference allele	Alternate allele	Gene symbol	Variant type	Variant annotation	OncoMD	COSMIC	dbSNP	ExAC	ExAC_SAS	1000G	1000G_SAS	SIFT	Polyphen2	CONDEL	VEP	LRT	PhastCon7 Way	Total read (Adjacent normal)	Reference allel read support (Adjacent normal)	Alternative read support (Adjacent normal)	Total read (Tumor)	Reference allel read support (Tumor)	Alternative read support (Tumor)	Alternate allele frequency
Chewer	42483	chr1	17681116	C	A	*PADI4*	MISSENSE	ENSG00000159339:MISSENSE[ENST00000375448(c.1244C>A,cCa>cAa):ENSP00000364597(p.Pro415Gln)];ENSG00000159339:EXONIC-NC[ENST00000467001(n.145C>A)];ENSG00000159339:EXONIC-NC[ENST00000487048(n.211C>A)];	NA	NA	NA	2.48E-05	0	NA	NA	NA	NA_NA	NA	MODIFIER	NA	NA	85	85	0	78	51	27	34.62
Chewer	42483	chr1	176668780	T	G	*PAPPA2*	INTRONIC	ENSG00000116183:[ENST00000367662(c.3236+55T>G)];	NA	NA	NA	NA	NA	NA	NA	NA	NA_NA	NA	MODIFIER	NA	NA	30	30	0	42	24	18	42.86
Chewer	42483	chr1	156877796	C	G	*PEAR1*	MISSENSE	ENSG00000187800:MISSENSE[ENST00000338302(c.855C>G,gaC>gaG):ENSP00000344465(p.Asp285Glu)];ENSG00000187800:MISSENSE[ENST00000292357(c.855C>G,gaC>gaG):ENSP00000292357(p.Asp285Glu)];ENSG00000187800:EXONIC-NC[ENST00000469390(n.507C>G)];	NA	NA	NA	NA	NA	NA	NA	NA	NA_NA	NA	MODIFIER	NA	NA	114	114	0	130	38	91	70
Chewer	42483	chr1	7870031	T	A	*PER3*	MISSENSE	ENSG00000049246:MISSENSE[ENST00000377532(c.1321T>A,Tcc>Acc):ENSP00000366755(p.Ser441Thr)];ENSG00000049246:MISSENSE[ENST00000361923(c.1318T>A,Tcc>Acc):ENSP00000355031(p.Ser440Thr)];ENSG00000049246:EXONIC-NC[ENST00000463106(n.339T>A)];	NA	NA	NA	NA	NA	NA	NA	NA	NA_NA	NA	MODIFIER	NA	NA	34	34	0	32	22	10	31.25
Chewer	42483	chr1	167659292	C	A	*RCSD1*	MISSENSE	ENSG00000198771:MISSENSE[ENST00000537350(c.115C>A,Cca>Aca):ENSP00000439409(p.Pro39Thr)];ENSG00000198771:MISSENSE[ENST00000367854(c.205C>A,Cca>Aca):ENSP00000356828(p.Pro69Thr)];ENSG00000198771:INTRONIC[ENST00000361496(c.199-4044C>A)];	NA	NA	NA	NA	NA	NA	NA	NA	NA_NA	NA	MODIFIER	NA	NA	67	67	0	58	22	36	62.07
Chewer	42483	chr1	175951549	A	G	*RFWD2*	INTRONIC	ENSG00000143207:[ENST00000367667(c.*1309+4530T>C)];ENSG00000143207:[ENST00000308769(c.2061+4530T>C)];ENSG00000143207:[ENST00000367666(c.1638+4530T>C)];ENSG00000143207:[ENST00000367669(c.2133+4530T>C)];ENSG00000143207:[ENST00000461830(n.1746+4530T>C)];	NA	NA	NA	NA	NA	NA	NA	NA	NA_NA	NA	MODIFIER	NA	NA	196	196	0	164	120	44	26.83
Chewer	42483	chr1	150526447	G	A	*RP11-54A4.2*	INTRONIC	ENSG00000237781:[ENST00000442435(n.476+564C>T)];	NA	NA	NA	6.03E-05	0	NA	NA	NA	NA_NA	NA	MODIFIER	NA	NA	63	63	0	76	25	51	67.11
Chewer	42483	chr1	75072439	G	C	*RP4-612J11.1*	INTRONIC	ENSG00000234497:[ENST00000416017(n.174-8451G>C)];	NA	NA	NA	NA	NA	NA	NA	NA	NA_NA	NA	MODIFIER	NA	NA	324	323	0	263	176	86	32.7
Chewer	42483	chr1	114472027	T	C	*RP5-1073O3.7*	EXONIC-NC	ENSG00000235527:[ENST00000426237(n.88A>G)];	NA	NA	chr1*114472027*114472027*T*G*rs577160926*0*0.9998*0.0001997	NA	NA	NA	NA	NA	NA_NA	NA	MODIFIER	NA	NA	76	76	0	29	11	18	62.07
Chewer	42483	chr1	38433807	C	G	*SF3A3*	INTRONIC-SS-ACR	ENSG00000183431:INTRONIC-SS-ACR[ENST00000373019(c.1282-1G>C)];ENSG00000183431:INTRONIC-SS-ACR[ENST00000448721(c.1123-1G>C)];	NA	NA	NA	NA	NA	NA	NA	NA	NA_NA	NA	HIGH	NA	NA	206	204	0	250	192	57	22.8
Chewer	42483	chr1	77509991	C	T	*ST6GALNAC5*	MISSENSE	ENSG00000117069:MISSENSE[ENST00000477717(c.364C>T,Cgc>Tgc):ENSP00000417583(p.Arg122Cys)];ENSG00000117069:MISSENSE[ENST00000318803(c.364C>T,Cgc>Tgc):ENSP00000436263(p.Arg122Cys)];ENSG00000117069:EXONIC-NC[ENST00000488940(n.167C>T)];	chr1*77509	chr1*77509	NA	4.97E-05	0	NA	NA	NA	PrD_PrD	NA	MODERATE	NA	NA	215	215	0	150	87	63	42
Chewer	42483	chr1	27676514	C	G	*SYTL1*	MISSENSE	ENSG00000142765:MISSENSE[ENST00000318074(c.763C>G,Cgc>Ggc):ENSP00000316464(p.Arg255Gly)];ENSG00000142765:MISSENSE[ENST00000543823(c.799C>G,Cgc>Ggc):ENSP00000440704(p.Arg267Gly)];ENSG00000142765:EXONIC-NC[ENST00000485269(n.71C>G)];ENSG00000142765:EXONIC-NC[ENST00000490170(n.844C>G)];ENSG00000142765:EXONIC-NC[ENST00000483926(n.1301C>G)];ENSG00000142765:EXONIC-NC[ENST00000496001(n.342C>G)];	NA	NA	NA	NA	NA	NA	NA	NA	NA_NA	NA	MODIFIER	NA	NA	40	40	0	55	40	15	27.27
Chewer	42483	chr1	43892427	G	C	*SZT2*	MISSENSE	ENSG00000198198:MISSENSE[ENST00000372442(c.559G>C,Gag>Cag):ENSP00000361519(p.Glu187Gln)];ENSG00000198198:MISSENSE[ENST00000562955(c.3085G>C,Gag>Cag):ENSP00000457168(p.Glu1029Gln)];ENSG00000198198:EXONIC-NC[ENST00000470139(n.1917G>C)];	NA	NA	NA	NA	NA	NA	NA	NA	NA_NA	NA	MODIFIER	NA	NA	79	79	0	84	51	33	39.29
Chewer	42483	chr1	226041437	C	T	*TMEM63A*	MISSENSE	ENSG00000196187:MISSENSE[ENST00000366835(c.1690G>A,Gcc>Acc):ENSP00000355800(p.Ala564Thr)];	chr1*22604	chr1*22604	NA	NA	NA	NA	NA	D	PrD_PrD	D	MODERATE	D	0.998	90	90	0	113	85	28	24.78
Chewer	42483	chr1	100605759	G	A	*TRMT13*	MISSENSE	ENSG00000122435:MISSENSE[ENST00000370141(c.346G>A,Gaa>Aaa):ENSP00000359160(p.Glu116Lys)];ENSG00000122435:MISSENSE[ENST00000370139(c.253G>A,Gaa>Aaa):ENSP00000359158(p.Glu85Lys)];ENSG00000122435:MISSENSE[ENST00000482437(c.346G>A,Gaa>Aaa):ENSP00000432616(p.Glu116Lys)];ENSG00000122435:INTRONIC[ENST00000370143(c.325-205G>A)];	chr1*10060	NA	NA	NA	NA	NA	NA	T	BN_BN	N	MODERATE	NA	NA	37	37	0	47	30	17	36.17
Chewer	42483	chr1	215848894	C	T	*USH2A*	MISSENSE	ENSG00000042781:MISSENSE[ENST00000366943(c.12359G>A,cGc>cAc):ENSP00000355910(p.Arg4120His)];ENSG00000042781:MISSENSE[ENST00000307340(c.12359G>A,cGc>cAc):ENSP00000305941(p.Arg4120His)];	chr1*21584	chr1*21584	0.000599	4.14E-05	6.07E-05	0.000599	0	NA	PrD_PrD	NA	MODERATE	N	NA	78	77	0	83	19	64	77.11
Chewer	42483	chr1	216420388	T	C	*USH2A*	MISSENSE	ENSG00000042781:MISSENSE[ENST00000366942(c.2348A>G,aAc>aGc):ENSP00000355909(p.Asn783Ser)];ENSG00000042781:MISSENSE[ENST00000366943(c.2348A>G,aAc>aGc):ENSP00000355910(p.Asn783Ser)];ENSG00000042781:MISSENSE[ENST00000307340(c.2348A>G,aAc>aGc):ENSP00000305941(p.Asn783Ser)];	NA	NA	NA	NA	NA	NA	NA	NA	BN_BN	NA	MODERATE	N	NA	163	162	0	106	79	27	25.47
Chewer	42483	chr1	219783913	G	C	*ZC3H11B*	EXONIC-NC	ENSG00000215817:[ENST00000431766(n.2183C>G)];ENSG00000215817:[ENST00000367211(n.1492C>G)];	NA	NA	NA	NA	NA	NA	NA	NA	NA_NA	NA	MODIFIER	NA	NA	120	120	0	104	84	20	19.23
Chewer	42483	chr10	27368096	G	A	*ANKRD26*	INTRONIC-SS-ACR	ENSG00000107890:[ENST00000376087(c.741-6C>T)];ENSG00000107890:[ENST00000466890(n.73-6C>T)];ENSG00000107890:[ENST00000436985(c.888-6C>T)];	NA	NA	NA	NA	NA	NA	NA	NA	NA_NA	NA	LOW	NA	NA	152	152	0	74	37	37	50
Chewer	42483	chr10	61612293	G	C	*CCDC6*	INTRONIC	ENSG00000108091:[ENST00000263102(c.453+18C>G)];	NA	NA	chr10*61612293*61612293*G*A*rs372369142*NA*NA*NA	NA	NA	NA	NA	NA	NA_NA	NA	MODIFIER	NA	NA	30	30	0	11	3	8	72.73
Chewer	42483	chr10	87373271	C	G	*GRID1*	MISSENSE	ENSG00000182771:MISSENSE[ENST00000536331(c.1207G>C,Gcc>Ccc):ENSP00000444455(p.Ala403Pro)];ENSG00000182771:MISSENSE[ENST00000327946(c.2494G>C,Gcc>Ccc):ENSP00000330148(p.Ala832Pro)];ENSG00000182771:3UTR[ENST00000464741(c.*59G>C)];	NA	NA	chr10*87373271*87373271*C*T*rs150878754*NA*NA*NA	NA	NA	NA	NA	NA	NA_NA	NA	MODIFIER	NA	NA	50	50	0	35	14	21	60
Chewer	42483	chr10	7642651	C	G	*ITIH5*	INTRONIC	ENSG00000123243:[ENST00000397146(c.940-14619G>C)];ENSG00000123243:[ENST00000446830(c.286-14619G>C)];ENSG00000123243:[ENST00000434980(n.565-14619G>C)];ENSG00000123243:[ENST00000298441(c.298-14619G>C)];ENSG00000123243:[ENST00000256861(c.940-14619G>C)];ENSG00000123243:[ENST00000397145(c.940-14619G>C)];ENSG00000123243:[ENST00000461751(c.347-123G>C)];ENSG00000123243:[ENST00000476417(n.5483-14619G>C)];	NA	NA	NA	NA	NA	NA	NA	NA	NA_NA	NA	MODIFIER	NA	NA	85	85	0	49	26	23	46.94
Chewer	42483	chr10	90365351	G	A	*LIPJ*	INTRONIC	ENSG00000204022:[ENST00000371939(c.796-39G>A)];ENSG00000204022:[ENST00000531458(c.241-39G>A)];	NA	NA	NA	NA	NA	NA	NA	NA	NA_NA	NA	MODIFIER	NA	NA	119	119	0	96	68	28	29.17
Chewer	42483	chr10	22856765	C	A	*PIP4K2A*	INTRONIC	ENSG00000150867:[ENST00000422321(n.534+15G>T)];ENSG00000150867:[ENST00000376573(c.678+15G>T)];ENSG00000150867:[ENST00000604912(c.216+15G>T)];ENSG00000150867:[ENST00000545335(c.501+15G>T)];ENSG00000150867:[ENST00000323883(c.258+15G>T)];	NA	NA	chr10*22856765*22856765*C*T*rs780538754*NA*NA*NA	NA	NA	NA	NA	NA	NA_NA	NA	MODIFIER	NA	NA	143	143	0	66	38	28	42.42

Supplementary Table 4. List of somatic single nucleotide variants identified using whole exome sequencing of ESCC patients

Cohort	Sample	Chromosome	Genomic position	Reference allele	Alternate allele	Gene symbol	Variant type	Variant annotation	OncoMD	COSMIC	dbSNP	ExAC	ExAC_SAS	1000G	1000G_SAS	SIFT	Polyphen2	CONDEL	VEP	LRT	PhastCon7 Way	Total read (Adjacent normal)	Reference allel read support (Adjacent normal)	Alternative read support (Adjacent normal)	Total read (Tumor)	Reference allel read support (Tumor)	Alternative read support (Tumor)	Alternate allele frequency
Chewer	42483	chr10	95995871	G	A	*PLCE1*	MISSENSE	ENSG00000138193:MISSENSE[ENST00000371380(c.2414G>A,cGa>cAa):ENSP00000360431(p.Arg805Gln)];ENSG00000138193:MISSENSE[ENST00000260766(c.2414G>A,cGa>cAa):ENSP00000260766(p.Arg805Gln)];ENSG00000138193:MISSENSE[ENST00000371375(c.1490G>A,cGa>cAa):ENSP00000360426(p.Arg497Gln)];ENSG00000138193:MISSENSE[ENST00000371385(c.1490G>A,cGa>cAa):ENSP00000360438(p.Arg497Gln)];	NA	NA	NA	6.11E-05	0	NA	NA	T	BN_BN	N	MODERATE	N	0.977	143	142	0	93	40	53	56.99
Chewer	42483	chr10	81838841	G	A	*TMEM254*	SILENT	ENSG00000133678:INTRONIC[ENST00000372277(c.87+299G>A)];ENSG00000133678:INTRONIC[ENST00000472622(n.141+299G>A)];ENSG00000133678:INTRONIC[ENST00000372274(c.87+299G>A)];ENSG00000133678:INTRONIC[ENST00000372275(c.87+299G>A)];ENSG00000133678:INTRONIC[ENST00000372281(c.87+299G>A)];ENSG00000133678:SILENT[ENST00000372273(c.50G>A,agG>agA):ENSP00000361347(p.Arg17(=))];	NA	NA	NA	NA	NA	NA	NA	NA	NA_NA	NA	MODIFIER	NA	NA	119	119	0	85	32	53	62.35
Chewer	42483	chr10	134672660	G	T	*TTC40*	MISSENSE	ENSG00000171811:MISSENSE[ENST00000368586(c.5290C>A,Ctg>Atg):ENSP00000357575(p.Leu1764Met)];	NA	NA	chr10*134672660*134672660*G*A*rs535223921*0*0.9998*0.0001997	NA	NA	NA	NA	D	PrD_PrD	D	MODERATE	NA	NA	187	187	0	162	69	93	57.41
Chewer	42483	chr10	81203857	C	A	*ZCCHC24*	INTRONIC-SS-DNR	ENSG00000165424:INTRONIC-SS-DNR[ENST00000372333(c.67+1G>T)];ENSG00000165424:INTRONIC[ENST00000372336(c.246+1094G>T)];	NA	NA	NA	NA	NA	NA	NA	NA	NA_NA	NA	MODIFIER	NA	NA	111	111	0	115	43	70	60.87
Chewer	42483	chr11	67786739	C	A	*ALDH3B1*	INTRONIC	ENSG00000006534:[ENST00000342456(c.369+15C>A)];ENSG00000006534:[ENST00000530046(n.396+15C>A)];ENSG00000006534:[ENST00000539229(c.477+15C>A)];ENSG00000006534:[ENST00000526609(n.367+15C>A)];ENSG00000006534:[ENST00000531459(n.530+15C>A)];ENSG00000006534:[ENST00000007633(c.477+15C>A)];ENSG00000006534:[ENST00000433100(n.595+15C>A)];ENSG00000006534:[ENST00000434449(n.521+15C>A)];ENSG00000006534:[ENST00000529232(n.3613+15C>A)];ENSG00000006534:[ENST00000316367(c.477+15C>A)];ENSG00000006534:[ENST00000527179(n.483+15C>A)];ENSG00000006534:[ENST00000525518(n.588+15C>A)];	NA	NA	NA	NA	NA	NA	NA	NA	NA_NA	NA	MODIFIER	NA	NA	56	56	0	113	62	51	45.13
Chewer	42483	chr11	70172392	A	T	*AP000487.6*	INTRONIC	ENSG00000254604:[ENST00000528607(n.493+36590T>A)];	NA	NA	NA	NA	NA	NA	NA	T	BN_BN	N	MODERATE	U	0.991	105	105	0	384	33	350	91.15
Chewer	42483	chr11	100827261	A	T	*ARHGAP42*	INTRONIC	ENSG00000165895:[ENST00000524892(c.1061-3353A>T)];ENSG00000165895:[ENST00000298815(c.1163-3353A>T)];	NA	NA	NA	NA	NA	NA	NA	NA	NA_NA	NA	MODIFIER	NA	NA	114	114	0	76	48	28	36.84
Chewer	42483	chr11	117253568	A	G	*CEP164*	MISSENSE	ENSG00000110274:EXONIC-NC[ENST00000529153(n.121A>G)];ENSG00000110274:EXONIC-NC[ENST00000533706(n.958A>G)];ENSG00000110274:INTRONIC[ENST00000533675(n.1832+984A>G)];ENSG00000110274:MISSENSE[ENST00000278935(c.1634A>G,cAg>cGg):ENSP00000278935(p.Gln545Arg)];	NA	NA	NA	NA	NA	NA	NA	T	BN_BN	N	MODERATE	N	0.007	43	43	0	46	37	9	19.57
Chewer	42483	chr11	46341946	C	A	*CREB3L1*	SILENT	ENSG00000157613:EXONIC-NC[ENST00000530244(n.984C>A)];ENSG00000157613:SILENT[ENST00000288400(c.1390C>A,Cgg>Agg):ENSP00000288400(p.Arg464(=))];ENSG00000157613:SILENT[ENST00000529193(c.1390C>A,Cgg>Agg):ENSP00000434939(p.Arg464(=))];	NA	NA	chr11*46341946*46341946*C*T*rs35636632*1*0.9994*0.000599	NA	NA	NA	NA	NA	NA_NA	NA	LOW	NA	0.98	44	44	0	53	33	20	37.74
Chewer	42483	chr11	70172392	A	T	*CTA-797E19.2*	INTRONIC	ENSG00000255539:[ENST00000526017(n.110+814T>A)];	NA	NA	NA	NA	NA	NA	NA	T	BN_BN	N	MODERATE	U	0.991	105	105	0	384	33	350	91.15
Chewer	42483	chr11	6661762	G	T	*DCHS1*	SILENT	ENSG00000166341:SILENT[ENST00000299441(c.1083C>A,tcC>tcA):ENSP00000299441(p.Ser361(=))];	NA	chr11*6661	chr11*6661762*6661762*G*A*rs749233301*NA*NA*NA	NA	NA	NA	NA	NA	NA_NA	NA	LOW	NA	NA	194	194	0	159	113	46	28.93
Chewer	42483	chr11	58920341	G	A	*FAM111A*	NONSENSE	ENSG00000166801:NONSENSE[ENST00000361723(c.1200G>A,tgG>tgA):ENSP00000355264(p.Trp400Ter)];ENSG00000166801:NONSENSE[ENST00000420244(c.1200G>A,tgG>tgA):ENSP00000406683(p.Trp400Ter)];ENSG00000166801:NONSENSE[ENST00000528737(c.1200G>A,tgG>tgA):ENSP00000434435(p.Trp400Ter)];ENSG00000166801:NONSENSE[ENST00000531147(c.1200G>A,tgG>tgA):ENSP00000431631(p.Trp400Ter)];ENSG00000166801:NONSENSE[ENST00000533703(c.1200G>A,tgG>tgA):ENSP00000433154(p.Trp400Ter)];	NA	NA	NA	NA	NA	NA	NA	NA	NA_NA	NA	HIGH	N	0.016	346	345	0	244	175	69	28.28
Chewer	42483	chr11	119003676	C	T	*HINFP*	SILENT	ENSG00000172273:SILENT[ENST00000350777(c.978C>T,ctC>ctT):ENSP00000318085(p.Leu326(=))];ENSG00000172273:SILENT[ENST00000527410(c.978C>T,ctC>ctT):ENSP00000436815(p.Leu326(=))];ENSG00000172273:EXONIC-NC[ENST00000532860(n.128C>T)];ENSG00000172273:EXONIC-NC[ENST00000527755(n.1359C>T)];ENSG00000172273:EXONIC-NC[ENST00000531022(n.1662C>T)];ENSG00000172273:EXONIC-NC[ENST00000529808(n.592C>T)];	NA	NA	NA	NA	NA	NA	NA	NA	NA_NA	NA	MODIFIER	NA	NA	77	77	0	58	42	16	27.59
Chewer	42483	chr11	6630604	G	A	*ILK*	SILENT	ENSG00000166333:SILENT[ENST00000528995(c.510G>A,aaG>aaA):ENSP00000435323(p.Lys170(=))];ENSG00000166333:SILENT[ENST00000420936(c.693G>A,aaG>aaA):ENSP00000403487(p.Lys231(=))];ENSG00000166333:SILENT[ENST00000396751(c.693G>A,aaG>aaA):ENSP00000379975(p.Lys231(=))];ENSG00000166333:SILENT[ENST00000537806(c.291G>A,aaG>aaA):ENSP00000439606(p.Lys97(=))];ENSG00000166333:SILENT[ENST00000299421(c.693G>A,aaG>aaA):ENSP00000299421(p.Lys231(=))];ENSG00000166333:EXONIC-NC[ENST00000534706(n.535G>A)];ENSG00000166333:EXONIC-NC[ENST00000526711(n.433G>A)];ENSG00000166333:EXONIC-NC[ENST00000528784(n.566G>A)];ENSG00000166333:EXONIC-NC[ENST00000530016(n.353G>A)];ENSG00000166333:3UTR[ENST00000527394(c.*260G>A)];ENSG00000166333:3UTR[ENST00000527327(c.*127G>A)];ENSG00000166333:3UTR[ENST00000532063(c.*404G>A)];ENSG00000166333:3UTR[ENST00000527121(c.*673G>A)];ENSG00000166333:INTRONIC[ENST00000526318(n.327+885G>A)];	chr11*6630	chr11*6630	NA	NA	NA	NA	NA	NA	NA_NA	NA	MODIFIER	NA	NA	113	113	0	109	69	40	36.7
Chewer	42483	chr11	62343408	G	C	*MIR3654*	INTRONIC	ENSG00000255508:[ENST00000534745(n.196+721C>G)];ENSG00000255508:[ENST00000496634(c.1474+721C>G)];ENSG00000255508:[ENST00000526409(n.344+721C>G)];	NA	NA	NA	NA	NA	NA	NA	NA	NA_NA	NA	MODIFIER	NA	NA	73	73	0	58	43	15	25.86
Chewer	42483	chr11	1219498	G	T	*MUC5AC*	EXONIC-NC	ENSG00000215182:[ENST00000358378(n.2751G>T)];	NA	NA	NA	NA	NA	NA	NA	NA	NA_NA	NA	MODIFIER	NA	NA	71	71	0	37	18	19	51.35
Chewer	42483	chr11	89885638	G	T	*NAALAD2*	MISSENSE	ENSG00000077616:MISSENSE[ENST00000375944(c.782G>T,gGc>gTc):ENSP00000365111(p.Gly261Val)];ENSG00000077616:MISSENSE[ENST00000534061(c.782G>T,gGc>gTc):ENSP00000432481(p.Gly261Val)];ENSG00000077616:MISSENSE[ENST00000321955(c.782G>T,gGc>gTc):ENSP00000320083(p.Gly261Val)];ENSG00000077616:MISSENSE[ENST00000527493(c.782G>T,gGc>gTc):ENSP00000433710(p.Gly261Val)];ENSG00000077616:MISSENSE[ENST00000525171(c.782G>T,gGc>gTc):ENSP00000435249(p.Gly261Val)];ENSG00000077616:EXONIC-NC[ENST00000524501(n.842G>T)];ENSG00000077616:EXONIC-NC[ENST00000529090(n.877G>T)];	NA	NA	NA	NA	NA	NA	NA	NA	NA_NA	NA	MODIFIER	NA	NA	107	107	0	69	36	33	47.83

Supplementary Table 4. List of somatic single nucleotide variants identified using whole exome sequencing of ESCC patients

Cohort	Sample	Chromosome	Genomic position	Reference allele	Alternate allele	Gene symbol	Variant type	Variant annotation	OncoMD	COSMIC	dbSNP	ExAC	ExAC_SAS	1000G	1000G_SAS	SIFT	Polyphen2	CONDEL	VEP	LRT	PhastCon7 Way	Total read (Adjacent normal)	Reference allel read support (Adjacent normal)	Alternative read support (Adjacent normal)	Total read (Tumor)	Reference allel read support (Tumor)	Alternative read support (Tumor)	Alternate allele frequency
Chewer	42483	chr11	89911221	A	G	*NAALAD2*	SILENT	ENSG00000077616:INTRONIC[ENST00000375944(c.797-13505A>G)];ENSG00000077616:SILENT[ENST00000534061(c.1794A>G,gcA>gcG):ENSP00000432481(p.Ala598(=))];ENSG00000077616:SILENT[ENST00000321955(c.1695A>G,gcA>gcG):ENSP00000320083(p.Ala565(=))];	NA	NA	NA	NA	NA	NA	NA	NA	NA_NA	NA	LOW	NA	NA	47	47	0	37	21	16	43.24
Chewer	42483	chr11	50003185	A	G	*OR4C12*	MISSENSE	ENSG00000221954:MISSENSE[ENST00000335238(c.853T>C,Tac>Cac):ENSP00000334418(p.Tyr285His)];	NA	NA	0.0001997	8.26E-06	0	0.0001997	0	D_lc	PrD_PrD	D	MODERATE	U	0.995	148	148	0	150	108	42	28
Chewer	42483	chr11	48373769	G	A	*OR4C4P*	EXONIC-NC	ENSG00000197161:[ENST00000415304(n.231C>T)];ENSG00000197161:[ENST00000551663(n.231C>T)];	NA	NA	NA	NA	NA	NA	NA	NA	NA_NA	NA	MODIFIER	NA	NA	166	166	0	110	83	26	23.64
Chewer	42483	chr11	48373958	G	A	*OR4C4P*	EXONIC-NC	ENSG00000197161:[ENST00000415304(n.42C>T)];ENSG00000197161:[ENST00000551663(n.42C>T)];	NA	NA	NA	NA	NA	NA	NA	NA	NA_NA	NA	MODIFIER	NA	NA	163	162	0	137	112	25	18.25
Chewer	42483	chr11	55433228	T	C	*OR4C6*	SILENT	ENSG00000181903:SILENT[ENST00000314259(c.586T>C,Tta>Cta):ENSP00000324769(p.Leu196(=))];	chr11*55433	NA	NA	NA	NA	NA	NA	NA	NA_NA	NA	LOW	NA	0.257	112	112	0	88	72	16	18.18
Chewer	42483	chr11	5842502	G	A	*OR52N2*	MISSENSE	ENSG00000180988:MISSENSE[ENST00000317037(c.937G>A,Gac>Aac):ENSP00000322801(p.Asp313Asn)];	NA	NA	NA	8.34E-06	0	NA	NA	NA	NA_NA	NA	MODIFIER	NA	NA	142	142	0	127	79	48	37.8
Chewer	42483	chr11	56431171	G	T	*OR5AR1*	NONSENSE	ENSG00000172459:NONSENSE[ENST00000302969(c.10G>T,Gaa>Taa):ENSP00000302639(p.Glu4Ter)];	NA	chr11*5643	NA	5.28E-05	0.00052283	NA	NA	NA	NA_NA	NA	HIGH	N	0.989	73	73	0	49	35	14	28.57
Chewer	42483	chr11	56185143	C	A	*OR5R1*	MISSENSE	ENSG00000174942:MISSENSE[ENST00000312253(c.566G>T,tGc>tTc):ENSP00000308595(p.Cys189Phe)];	NA	NA	NA	NA	NA	NA	NA	D	PrD_PrD	D	MODERATE	NA	NA	173	171	0	150	107	43	28.67
Chewer	42483	chr11	70172392	A	T	*PPFIA1*	MISSENSE	ENSG00000131626:MISSENSE[ENST00000532504(c.631A>T,Aat>Tat):ENSP00000433619(p.Asn211Tyr)];ENSG00000131626:MISSENSE[ENST00000253925(c.631A>T,Aat>Tat):ENSP00000253925(p.Asn211Tyr)];ENSG00000131626:MISSENSE[ENST00000389547(c.631A>T,Aat>Tat):ENSP00000374198(p.Asn211Tyr)];	NA	NA	NA	NA	NA	NA	NA	T	BN_BN	N	MODERATE	U	0.991	105	105	0	384	33	350	91.15
Chewer	42483	chr11	64088107	G	A	*PRDX5*	INTRONIC	ENSG00000126432:[ENST00000265462(c.307-26G>A)];ENSG00000126432:[ENST00000352435(c.307-230G>A)];ENSG00000126432:[ENST00000347941(c.172-230G>A)];	NA	NA	NA	NA	NA	NA	NA	NA	NA_NA	NA	MODIFIER	NA	NA	91	91	0	108	67	41	37.96
Chewer	42483	chr11	64521443	C	A	*PYGM*	NONSENSE	ENSG00000068976:NONSENSE[ENST00000164139(c.1147G>T,Gag>Tag):ENSP00000164139(p.Glu383Ter)];ENSG00000068976:NONSENSE[ENST00000377432(c.883G>T,Gag>Tag):ENSP00000366650(p.Glu295Ter)];ENSG00000068976:EXONIC-NC[ENST00000460413(n.224G>T)];	NA	NA	chr11*64521443*64521443*C*T*rs757681143*NA*NA*NA	NA	NA	NA	NA	NA	NA_NA	NA	MODIFIER	NA	NA	34	34	0	51	33	18	35.29
Chewer	42483	chr11	61675598	C	G	*RAB3IL1*	SILENT	ENSG00000167994:SILENT[ENST00000531922(c.333G>C,ctG>ctC):ENSP00000435444(p.Leu111(=))];ENSG00000167994:SILENT[ENST00000301773(c.333G>C,ctG>ctC):ENSP00000301773(p.Leu111(=))];ENSG00000167994:SILENT[ENST00000394836(c.192G>C,ctG>ctC):ENSP00000378313(p.Leu64(=))];	NA	NA	NA	NA	NA	NA	NA	NA	NA_NA	NA	LOW	NA	NA	61	61	0	76	50	25	32.89
Chewer	42483	chr11	64503003	C	G	*RASGRP2*	INTRONIC	ENSG00000068831:[ENST00000464324(n.1645+11G>C)];ENSG00000068831:[ENST00000354024(c.1296+11G>C)];ENSG00000068831:[ENST00000394432(c.1296+11G>C)];ENSG00000068831:[ENST00000497441(n.703+11G>C)];ENSG00000068831:[ENST00000494001(n.721+11G>C)];ENSG00000068831:[ENST00000377494(c.1296+11G>C)];ENSG00000068831:[ENST00000421556(c.*1024+11G>C)];ENSG00000068831:[ENST00000377497(c.1296+11G>C)];ENSG00000068831:[ENST00000465852(n.704G>C)];	NA	NA	NA	NA	NA	NA	NA	NA	NA_NA	NA	MODIFIER	NA	NA	92	92	0	111	61	50	45.05
Chewer	42483	chr11	6630604	G	A	*RP11-732A19.2*	INTRONIC	ENSG00000254641:[ENST00000527398(n.229-395C>T)];	chr11*6630(	chr11*6630(	NA	NA	NA	NA	NA	NA	NA_NA	NA	MODIFIER	NA	NA	113	113	0	109	69	40	36.7
Chewer	42483	chr11	16362587	C	A	*SOX6*	MISSENSE	ENSG00000110693:MISSENSE[ENST00000533411(c.208G>T,Gac>Tac):ENSP00000436170(p.Asp70Tyr)];ENSG00000110693:MISSENSE[ENST00000526673(c.208G>T,Gac>Tac):ENSP00000434892(p.Asp70Tyr)];ENSG00000110693:MISSENSE[ENST00000527619(c.217G>T,Gac>Tac):ENSP00000434455(p.Asp73Tyr)];ENSG00000110693:MISSENSE[ENST00000528429(c.208G>T,Gac>Tac):ENSP00000433233(p.Asp70Tyr)];ENSG00000110693:MISSENSE[ENST00000352083(c.208G>T,Gac>Tac):ENSP00000339876(p.Asp70Tyr)];ENSG00000110693:MISSENSE[ENST00000530378(c.208G>T,Gac>Tac):ENSP00000432577(p.Asp70Tyr)];ENSG00000110693:MISSENSE[ENST00000396356(c.208G>T,Gac>Tac):ENSP00000379644(p.Asp70Tyr)];ENSG00000110693:MISSENSE[ENST00000528252(c.208G>T,Gac>Tac):ENSP00000432134(p.Asp70Tyr)];ENSG00000110693:MISSENSE[ENST00000529469(c.208G>T,Gac>Tac):ENSP00000432596(p.Asp70Tyr)];ENSG00000110693:MISSENSE[ENST00000316399(c.208G>T,Gac>Tac):ENSP00000324948(p.Asp70Tyr)];ENSG00000110693:EXONIC-NC[ENST00000533870(n.229G>T)];ENSG00000110693:EXONIC-NC[ENST00000533658(n.545G>T)];	NA	NA	NA	NA	NA	NA	NA	NA	NA_NA	NA	MODIFIER	NA	NA	106	106	0	84	55	29	34.52
Chewer	42483	chr11	6630604	G	A	*TAF10*	EXONIC-NC	ENSG00000166337:[ENST00000526743(n.2315C>T)];	chr11*6630(	chr11*6630(	NA	NA	NA	NA	NA	NA	NA_NA	NA	MODIFIER	NA	NA	113	113	0	109	69	40	36.7
Chewer	42483	chr11	64856032	C	T	*TMEM262*	EXONIC-NC	ENSG00000187066:[ENST00000528029(n.548G>A)];ENSG00000187066:[ENST00000334821(n.404G>A)];ENSG00000187066:[ENST00000525544(n.235-29G>A)];ENSG00000187066:[ENST00000524603(c.275-29G>A)];ENSG00000187066:[ENST00000530719(c.275-29G>A)];ENSG00000187066:[ENST00000524632(c.237-29G>A)];	NA	NA	NA	NA	NA	NA	NA	NA	NA_NA	NA	MODIFIER	NA	NA	112	112	0	147	113	34	23.13
Chewer	42483	chr11	5842502	G	A	*TRIM5*	INTRONIC	ENSG00000132256:[ENST00000412903(c.-62+116129C>T)];ENSG00000132256:[ENST00000380027(c.-441+34480C>T)];	NA	NA	NA	8.34E-06	0	NA	NA	NA	NA_NA	NA	MODIFIER	NA	NA	142	142	0	127	79	48	37.8
Chewer	42483	chr11	48997160	C	A	*TRIM51GP*	EXONIC-NC	ENSG00000220948:[ENST00000534741(n.1243G>T)];	NA	NA	NA	NA	NA	NA	NA	NA	NA_NA	NA	MODIFIER	NA	NA	142	142	0	119	82	37	31.09
Chewer	42483	chr11	8644533	C	A	*TRIM66*	INTRONIC	ENSG00000166436:[ENST00000402157(c.2579-110G>T)];ENSG00000166436:[ENST00000299550(c.2492-110G>T)];ENSG00000166436:[ENST00000481014(n.2941-110G>T)];	NA	NA	NA	NA	NA	NA	NA	NA	NA_NA	NA	MODIFIER	NA	NA	126	126	0	129	89	39	30.23
Chewer	42483	chr11	62343408	G	C	*TUT1*	MISSENSE	ENSG00000149016:MISSENSE[ENST00000476907(c.1783C>G,Ctg>Gtg):ENSP00000419607(p.Leu595Val)];ENSG00000149016:MISSENSE[ENST00000308436(c.1897C>G,Ctg>Gtg):ENSP00000308000(p.Leu633Val)];ENSG00000149016:EXONIC-NC[ENST00000469480(n.1158C>G)];	NA	NA	NA	NA	NA	NA	NA	NA	NA_NA	NA	MODIFIER	NA	NA	73	73	0	58	43	15	25.86
Chewer	42483	chr11	64875774	C	T	*VPS51*	SILENT	ENSG00000149823:SILENT[ENST00000279281(c.831C>T,cgC>cgT):ENSP00000279281(p.Arg277(=))];ENSG00000149823:SILENT[ENST00000530773(c.684C>T,cgC>cgT):ENSP00000434138(p.Arg228(=))];ENSG00000149823:SILENT[ENST00000529180(c.906C>T,cgC>cgT):ENSP00000435245(p.Arg302(=))];ENSG00000149823:SILENT[ENST00000534557(c.573C>T,cgC>cgT):ENSP00000435691(p.Arg191(=))];ENSG00000149823:3UTR[ENST00000534124(c.*525C>T)];ENSG00000149823:EXONIC-NC[ENST00000527646(n.506C>T)];	NA	NA	NA	NA	NA	NA	NA	NA	NA_NA	NA	LOW	NA	NA	50	50	0	50	22	28	56
Chewer	42483	chr11	60643084	G	C	*ZP1*	SILENT	ENSG00000149506:SILENT[ENST00000278853(c.1872G>C,ctG>ctC):ENSP00000278853(p.Leu624(=))];ENSG00000149506:EXONIC-NC[ENST00000537203(n.1491G>C)];	NA	NA	NA	NA	NA	NA	NA	NA	NA_NA	NA	MODIFIER	NA	NA	67	67	0	75	42	31	41.33
Chewer	42483	chr12	9241861	A	C	*A2M*	INTRONIC	ENSG00000175899:[ENST00000543436(c.451+647T>G)];ENSG00000175899:[ENST00000545828(n.348+12285T>G)];ENSG00000175899:[ENST00000462568(n.298-14T>G)];ENSG00000175899:[ENST00000318602(c.2719-14T>G)];	NA	NA	NA	NA	NA	NA	NA	NA	NA_NA	NA	MODIFIER	NA	NA	108	108	0	96	73	23	23.96

Mangalaparthi *et al.* , 2020. Mutational landscape of esophageal squamous cell carcinoma in an Indian cohort
Supplementary Table 4. List of somatic single nucleotide variants identified using whole exome sequencing of ESCC patients

Cohort	Sample	Chromosome	Genomic position	Reference allele	Alternate allele	Gene symbol	Variant type	Variant annotation	OncoMD	COSMIC	dbSNP	ExAC	ExAC_SAS	1000G	1000G_SAS	SIFT	Polyphen2	CONDEL	VEP	LRT	PhastCon7 Way	Total read (Adjacent normal)	Reference allel read support (Adjacent normal)	Alternative read support (Adjacent normal)	Total read (Tumor)	Reference allel read support (Tumor)	Alternative read support (Tumor)	Alternate allele frequency
Chewer	42483	chr12	43822513	G	T	*ADAMTS20*	SILENT	ENSG00000173157:SILENT[ENST00000553158(c.3579C>A,gcC>gcA):ENSP00000448341(p.Ala1193(=))];ENSG00000173157:SILENT[ENST00000389420(c.3579C>A,gcC>gcA):ENSP00000374071(p.Ala1193(=))];ENSG00000173157:SILENT[ENST00000549670(c.969C>A,gcC>gcA):ENSP00000447427(p.Ala323(=))];ENSG00000173157:SILENT[ENST00000395541(c.933C>A,gcC>gcA):ENSP00000378911(p.Ala311(=))];	NA	NA	chr12*43822501*43822519*TCGGGGTAAGTGGGCACAA*T*rs773735378*NA*NA*NA|chr12*43822513*43822513*G*A*rs756802583*NA*NA*NA	NA	NA	NA	NA	NA	NA_NA	NA	LOW	NA	NA	50	50	0	31	17	14	45.16
Chewer	42483	chr12	50467484	G	A	*ASIC1*	SILENT	ENSG00000110881:INTRONIC[ENST00000551199(n.154+2808G>A)];ENSG00000110881:INTRONIC[ENST00000550558(c.559-3267G>A)];ENSG00000110881:INTRONIC[ENST00000228468(c.559-3512G>A)];ENSG00000110881:INTRONIC[ENST00000453327(c.69-3267G>A)];ENSG00000110881:INTRONIC[ENST00000447966(c.559-3512G>A)];ENSG00000110881:SILENT[ENST00000552438(c.117G>A,tcG>tcA):ENSP00000450247(p.Ser39(=))];	NA	NA	NA	7.91E-05	0.000131234	NA	NA	NA	NA_NA	NA	MODIFIER	NA	NA	59	59	0	76	48	28	36.84
Chewer	42483	chr12	15035910	C	G	*C12orf60*	INTRONIC	ENSG00000182993:[ENST00000527783(n.76-16193C>G)];ENSG00000182993:[ENST00000533472(n.87-21031C>G)];	NA	NA	NA	NA	NA	NA	NA	NA	NA_NA	NA	MODIFIER	NA	NA	346	345	0	332	175	157	47.29
Chewer	42483	chr12	51102163	C	T	*DIP2B*	INTRONIC	ENSG00000066084:[ENST00000546732(c.*869-68C>T)];ENSG00000066084:[ENST00000301180(c.2535-68C>T)];	NA	NA	NA	NA	NA	NA	NA	NA	NA_NA	NA	MODIFIER	NA	NA	119	119	0	94	40	54	57.45
Chewer	42483	chr12	272750	C	G	*IQSEC3*	MISSENSE	ENSG00000120645:MISSENSE[ENST00000538872(c.2674C>G,Cag>Gag):ENSP00000437554(p.Gln892Glu)];ENSG00000120645:MISSENSE[ENST00000382841(c.1765C>G,Cag>Gag):ENSP00000372292(p.Gln589Glu)];ENSG00000120645:MISSENSE[ENST00000326261(c.2674C>G,Cag>Gag):ENSP00000315662(p.Gln892Glu)];	NA	NA	NA	NA	NA	NA	NA	D	BN_PoD	D	MODERATE	D	0.593	86	85	0	100	71	28	28
Chewer	42483	chr12	32137029	C	A	*KIAA1551*	MISSENSE	ENSG00000174718:MISSENSE[ENST00000312561(c.3140C>A,cCt>cAt):ENSP00000310338(p.Pro1047His)];ENSG00000174718:INTRONIC[ENST00000535596(n.318-3144C>A)];ENSG00000174718:INTRONIC[ENST00000541981(n.218-8283C>A)];ENSG00000174718:INTRONIC[ENST00000397578(n.139-3144C>A)];	NA	NA	NA	NA	NA	NA	NA	NA	NA_NA	NA	MODIFIER	NA	NA	175	175	0	192	153	39	20.31
Chewer	42483	chr12	10588032	A	G	*KLRC2*	INTRONIC	ENSG00000205809:[ENST00000381902(c.188-23T>C)];ENSG00000205809:[ENST00000535069(c.107-23T>C)];ENSG00000205809:[ENST00000536833(c.11-23T>C)];ENSG00000205809:[ENST00000381901(c.188-23T>C)];	NA	NA	NA	NA	NA	NA	NA	NA	NA_NA	NA	MODIFIER	NA	NA	125	125	0	117	103	14	11.97
Chewer	42483	chr12	49444861	C	A	*KMT2D*	NONSENSE	ENSG00000167548:NONSENSE[ENST00000301067(c.2605G>T,Gag>Tag):ENSP00000301067(p.Glu869Ter)];	NA	NA	NA	NA	NA	NA	NA	NA	NA_NA	NA	HIGH	NA	NA	86	86	0	97	36	61	62.89
Chewer	42483	chr12	52799731	G	A	*KRT82*	MISSENSE	ENSG00000161850:MISSENSE[ENST00000257974(c.331C>T,Ccg>Tcg):ENSP00000257974(p.Pro111Ser)];	NA	NA	chr12*52799731*52799731*G*T*rs762330238*NA*NA*NA	NA	NA	NA	NA	D	PrD_PrD	D	MODERATE	N	0.534	188	187	0	127	38	89	70.08
Chewer	42483	chr12	81239513	C	A	*LIN7A*	MISSENSE	ENSG00000111052:MISSENSE[ENST00000552864(c.479G>T,gGa>gTa):ENSP00000447488(p.Gly160Val)];ENSG00000111052:MISSENSE[ENST00000549417(c.461G>T,gGa>gTa):ENSP00000448975(p.Gly154Val)];ENSG00000111052:MISSENSE[ENST00000552093(c.376G>T,gGa>gTa):ENSP00000448891(p.Gly126Val)];ENSG00000111052:3UTR[ENST00000261203(c.*250G>T)];	NA	chr12*8123	NA	NA	NA	NA	NA	NA	NA_NA	NA	MODIFIER	NA	NA	50	48	2	29	12	17	58.62
Chewer	42483	chr12	21176122	G	T	*LST3*	INTRONIC-SS-ACR	ENSG00000257046:INTRONIC-SS-ACR[ENST00000381541(c.629-1G>T)];ENSG00000257046:INTRONIC[ENST00000540229(c.1866-44131G>T)];	NA	NA	NA	NA	NA	NA	NA	NA	NA_NA	NA	HIGH	NA	NA	210	210	0	192	102	90	46.88
Chewer	42483	chr12	15035910	C	G	*MGP*	MISSENSE	ENSG00000111341:MISSENSE[ENST00000539261(c.166G>C,Gag>Cag):ENSP00000445907(p.Glu56Gln)];ENSG00000111341:MISSENSE[ENST00000228938(c.241G>C,Gag>Cag):ENSP00000228938(p.Glu81Gln)];ENSG00000111341:INTRONIC[ENST00000545199(c.34-696G>C)];	NA	NA	NA	NA	NA	NA	NA	NA	NA_NA	NA	MODIFIER	NA	NA	346	345	0	332	175	157	47.29
Chewer	42483	chr12	10588032	A	G	*NKG2-E*	INTRONIC	ENSG00000255641:[ENST00000539033(c.188-23T>C)];	NA	NA	NA	NA	NA	NA	NA	NA	NA_NA	NA	MODIFIER	NA	NA	125	125	0	117	103	14	11.97
Chewer	42483	chr12	86276084	G	C	*NTS*	SILENT	ENSG00000133636:EXONIC-NC[ENST00000550879(n.625G>C)];ENSG00000133636:SILENT[ENST00000256010(c.444G>C,ctG>ctC):ENSP00000256010(p.Leu148(=))];ENSG00000133636:SILENT[ENST00000551529(c.219G>C,ctG>ctC):ENSP00000448328(p.Leu73(=))];	NA	NA	NA	NA	NA	NA	NA	NA	NA_NA	NA	LOW	NA	NA	99	99	0	79	38	41	51.9
Chewer	42483	chr12	70149363	G	A	*RAB3IP*	MISSENSE	ENSG00000127328:MISSENSE[ENST00000552199(c.223G>A,Gat>Aat):ENSP00000448944(p.Asp75Asn)];ENSG00000127328:MISSENSE[ENST00000549760(c.175G>A,Gat>Aat):ENSP00000450355(p.Asp59Asn)];ENSG00000127328:MISSENSE[ENST00000378809(c.223G>A,Gat>Aat):ENSP00000368086(p.Asp75Asn)];ENSG00000127328:MISSENSE[ENST00000483530(c.175G>A,Gat>Aat):ENSP00000419216(p.Asp59Asn)];ENSG00000127328:MISSENSE[ENST00000362025(c.223G>A,Gat>Aat):ENSP00000355381(p.Asp75Asn)];ENSG00000127328:MISSENSE[ENST00000417413(c.175G>A,Gat>Aat):ENSP00000436304(p.Asp59Asn)];ENSG00000127328:MISSENSE[ENST00000247833(c.175G>A,Gat>Aat):ENSP00000247833(p.Asp59Asn)];ENSG00000127328:MISSENSE[ENST00000550536(c.223G>A,Gat>Aat):ENSP00000447300(p.Asp75Asn)];ENSG00000127328:MISSENSE[ENST00000378815(c.175G>A,Gat>Aat):ENSP00000368092(p.Asp59Asn)];ENSG00000127328:5UTR[ENST00000325555(c.-444G>A)];ENSG00000127328:3UTR[ENST00000547591(c.*46G>A)];	NA	NA	NA	NA	NA	NA	NA	NA	NA_NA	NA	MODIFIER	NA	NA	263	263	0	205	69	136	66.34
Chewer	42483	chr12	27849619	G	C	*REP15*	MISSENSE	ENSG00000174236:MISSENSE[ENST00000310791(c.124G>C,Gaa>Caa):ENSP00000310335(p.Glu42Gln)];	NA	NA	NA	NA	NA	NA	NA	NA	NA_NA	NA	MODIFIER	NA	NA	95	95	0	128	93	35	27.34
Chewer	42483	chr12	27849619	G	C	*RP11-1060J15.4*	EXONIC-NC	ENSG00000256377:[ENST00000536922(n.542C>G)];ENSG00000256377:[ENST00000536317(n.812C>G)];	NA	NA	NA	NA	NA	NA	NA	NA	NA_NA	NA	MODIFIER	NA	NA	95	95	0	128	93	35	27.34
Chewer	42483	chr12	113745587	C	A	*SLC8B1*	SILENT	ENSG00000089060:SILENT[ENST00000550672(c.929G>T,gtG>gtT):ENSP00000448758(p.Val310(=))];ENSG00000089060:SILENT[ENST00000552014(c.1320G>T,gtG>gtT):ENSP00000447091(p.Val440(=))];ENSG00000089060:SILENT[ENST00000202831(c.1320G>T,gtG>gtT):ENSP00000202831(p.Val440(=))];ENSG00000089060:SILENT[ENST00000546737(c.1152G>T,gtG>gtT):ENSP00000450081(p.Val384(=))];ENSG00000089060:EXONIC-NC[ENST00000552565(n.848G>T)];ENSG00000089060:5UTR[ENST00000550047(c.-136G>T)];	NA	NA	chr12*113745587*113745587*C*T*rs750688602*NA*NA*NA	NA	NA	NA	NA	NA	NA_NA	NA	LOW	NA	NA	44	44	0	50	23	27	54
Chewer	42483	chr12	21176122	G	T	*SLCO1B3*	INTRONIC	ENSG00000111700:[ENST00000553473(c.1866-44131G>T)];	NA	NA	NA	NA	NA	NA	NA	NA	NA_NA	NA	HIGH	NA	NA	210	210	0	192	102	90	46.88
Chewer	42483	chr12	21176122	G	T	*SLCO1B7*	INTRONIC-SS-ACR	ENSG00000205754:INTRONIC-SS-ACR[ENST00000554957(c.629-1G>T)];ENSG00000205754:INTRONIC-SS-ACR[ENST00000421593(c.488-1G>T)];	NA	NA	NA	NA	NA	NA	NA	NA	NA_NA	NA	HIGH	NA	NA	210	210	0	192	102	90	46.88

Mangalaparthi *et al.*, 2020. Mutational landscape of esophageal squamous cell carcinoma in an Indian cohort

Supplementary Table 4. List of somatic single nucleotide variants identified using whole exome sequencing of ESCC patients

Cohort	Sample	Chromosome	Genomic position	Reference allele	Alternate allele	Gene symbol	Variant type	Variant annotation	OncoMD	COSMIC	dbSNP	ExAC	ExAC_SAS	1000G	1000G_SAS	SIFT	Polyphen2	CONDEL	VEP	LRT	PhastCon7 Way	Total read (Adjacent normal)	Reference allel read support (Adjacent normal)	Alternative read support (Adjacent normal)	Total read (Tumor)	Reference allel read support (Tumor)	Alternative read support (Tumor)	Alternate allele frequency
Chewer	42483	chr12	88588779	G	A	*TMTC3*	MISSENSE	ENSG00000139324:MISSENSE[ENST00000266712(c.2098G>A,Gac>Aac):ENSP00000266712(p.Asp700Asn)];	NA	NA	NA	NA	NA	NA	NA	T	BN_BN	N	MODERATE	D	0.975	293	292	0	220	71	149	67.73
Chewer	42483	chr13	99478270	C	T	*DOCK9*	INTRONIC	ENSG00000088387:[ENST00000376460(c.4955-76G>A)];ENSG00000088387:[ENST00000419908(c.208-76G>A)];ENSG00000088387:[ENST00000340449(c.886+814G>A)];ENSG00000088387:[ENST00000481051(n.249-41G>A)];ENSG00000088387:[ENST00000448493(c.*846-76G>A)];ENSG00000088387:[ENST00000400228(c.716+814G>A)];ENSG00000088387:[ENST00000451563(c.118+814G>A)];ENSG00000088387:[ENST00000339416(c.4958-76G>A)];	NA	NA	NA	NA	NA	NA	NA	NA	NA_NA	NA	MODIFIER	NA	NA	88	87	1	61	33	28	45.9
Chewer	42483	chr13	21553899	C	G	*LATS2*	SILENT	ENSG00000150457:SILENT[ENST00000542899(c.2703G>C,gtG>gtC):ENSP00000441817(p.Val901(=))];ENSG00000150457:SILENT[ENST00000382592(c.2703G>C,gtG>gtC):ENSP00000372035(p.Val901(=))];	NA	NA	NA	NA	NA	NA	NA	NA	NA_NA	NA	LOW	NA	NA	113	112	1	96	41	55	57.29
Chewer	42483	chr13	48651328	C	A	*MED4*	MISSENSE	ENSG00000136146:MISSENSE[ENST00000258648(c.760G>T,Gat>Tat):ENSP00000258648(p.Asp254Tyr)];ENSG00000136146:MISSENSE[ENST00000417167(c.694G>T,Gat>Tat):ENSP00000413595(p.Asp232Tyr)];ENSG00000136146:MISSENSE[ENST00000378586(c.622G>T,Gat>Tat):ENSP00000367849(p.Asp208Tyr)];ENSG00000136146:EXONIC-NC[ENST00000495013(n.252G>T)];	NA	NA	NA	NA	NA	NA	NA	NA	NA_NA	NA	MODIFIER	NA	NA	74	74	0	41	20	21	51.22
Chewer	42483	chr13	48651328	C	A	*MED4-AS1*	EXONIC-NC	ENSG00000229111:[ENST00000422483(n.56C>A)];	NA	NA	NA	NA	NA	NA	NA	NA	NA_NA	NA	MODIFIER	NA	NA	74	74	0	41	20	21	51.22
Chewer	42483	chr13	20000598	C	G	*TPTE2*	SILENT	ENSG00000132958:SILENT[ENST00000382977(c.1362G>C,ctG>ctC):ENSP00000372437(p.Leu454(=))];ENSG00000132958:SILENT[ENST00000255310(c.1131G>C,ctG>ctC):ENSP00000255310(p.Leu377(=))];ENSG00000132958:SILENT[ENST00000457266(c.1029G>C,ctG>ctC):ENSP00000442218(p.Leu343(=))];ENSG00000132958:SILENT[ENST00000382975(c.1242G>C,ctG>ctC):ENSP00000372435(p.Leu414(=))];ENSG00000132958:SILENT[ENST00000382978(c.1242G>C,ctG>ctC):ENSP00000372438(p.Leu414(=))];ENSG00000132958:SILENT[ENST00000390680(c.1131G>C,ctG>ctC):ENSP00000375098(p.Leu377(=))];ENSG00000132958:SILENT[ENST00000400103(c.1029G>C,ctG>ctC):ENSP00000382974(p.Leu343(=))];ENSG00000132958:SILENT[ENST00000400230(c.1362G>C,ctG>ctC):ENSP00000383089(p.Leu454(=))];ENSG00000132958:INTRONIC[ENST00000462409(c.910-625G>C)];	NA	NA	chr13*20000598*20000598*C*A,G*rs781154706*NA*NA*NA	0.0008262	0.000789985	NA	NA	NA	NA_NA	NA	LOW	NA	NA	294	290	4	239	226	13	5.44
Chewer	42483	chr14	93730271	G	A	*BTBD7*	MISSENSE	ENSG00000011114:MISSENSE[ENST00000334746(c.1231C>T,Cat>Tat):ENSP00000335615(p.His411Tyr)];ENSG00000011114:MISSENSE[ENST00000554565(c.178C>T,Cat>Tat):ENSP00000451010(p.His60Tyr)];ENSG00000011114:INTRONIC[ENST00000393170(c.170-6570C>T)];ENSG00000011114:INTRONIC[ENST00000553975(c.293-6570C>T)];ENSG00000011114:INTRONIC[ENST00000355125(c.72-2248C>T)];ENSG00000011114:EXONIC-NC[ENST00000554644(n.829C>T)];	NA	NA	0.0001997	NA	NA	0.0001997	0	NA	NA_NA	NA	MODIFIER	NA	NA	188	188	0	169	133	36	21.3
Chewer	42483	chr14	29242071	A	T	*C14orf23*	INTRONIC-SS-DNR	ENSG00000186960:[ENST00000399387(c.48+10A>T)];ENSG00000186960:[ENST00000548213(c.48+10A>T)];ENSG00000186960:[ENST00000552957(c.48+10A>T)];	NA	NA	NA	NA	NA	NA	NA	NA	NA_NA	NA	MODIFIER	NA	NA	196	196	0	241	199	42	17.43
Chewer	42483	chr14	60007434	G	C	*CCDC175*	MISSENSE	ENSG00000151838:MISSENSE[ENST00000537690(c.1314C>G,atC>atG):ENSP00000453940(p.Ile438Met)];ENSG00000151838:MISSENSE[ENST00000281581(c.1314C>G,atC>atG):ENSP00000452964(p.Ile438Met)];ENSG00000151838:EXONIC-NC[ENST00000556936(n.540C>G)];	NA	NA	NA	5.16E-05	0.000127129	NA	NA	NA	NA_NA	NA	MODIFIER	NA	NA	49	47	0	92	78	14	15.22
Chewer	42483	chr14	103406333	C	A	*CDC42BPB*	INTRONIC	ENSG00000198752:[ENST00000361246(c.4592-49G>T)];ENSG00000198752:[ENST00000559790(n.682-49G>T)];ENSG00000198752:[ENST00000560492(n.607-49G>T)];	NA	NA	chr14*103406333*103406333*C*T*rs200373977*0*0.9996*0.0003994	NA	NA	NA	NA	NA	NA_NA	NA	MODIFIER	NA	NA	92	91	0	110	33	76	69.09
Chewer	42483	chr14	73181150	G	C	*DPF3*	MISSENSE	ENSG00000205683:MISSENSE[ENST00000366353(c.750C>G,gaC>gaG):ENSP00000381791(p.Asp250Glu)];ENSG00000205683:MISSENSE[ENST00000546183(c.615C>G,gaC>gaG):ENSP00000444662(p.Asp205Glu)];ENSG00000205683:MISSENSE[ENST00000541685(c.585C>G,gaC>gaG):ENSP00000441640(p.Asp195Glu)];ENSG00000205683:MISSENSE[ENST00000556509(c.585C>G,gaC>gaG):ENSP00000450518(p.Asp195Glu)];ENSG00000205683:MISSENSE[ENST00000381216(c.585C>G,gaC>gaG):ENSP00000370614(p.Asp195Glu)];ENSG00000205683:EXONIC-NC[ENST00000557704(n.297C>G)];ENSG00000205683:EXONIC-NC[ENST00000554594(n.63C>G)];ENSG00000205683:EXONIC-NC[ENST00000556238(n.199C>G)];	NA	NA	NA	NA	NA	NA	NA	T	BN_BN	N	MODERATE	NA	NA	71	71	0	81	24	57	70.37
Chewer	42483	chr14	31926509	C	A	*DTD2*	MISSENSE	ENSG00000129480:MISSENSE[ENST00000356180(c.91G>T,Gtc>Ttc):ENSP00000348503(p.Val31Phe)];ENSG00000129480:MISSENSE[ENST00000310850(c.91G>T,Gtc>Ttc):ENSP00000312224(p.Val31Phe)];ENSG00000129480:MISSENSE[ENST00000549850(c.91G>T,Gtc>Ttc):ENSP00000449624(p.Val31Phe)];	NA	NA	chr14*31926509*31926509*C*CG*rs764921013*NA*NA*NA	NA	NA	NA	NA	T_lc	UN_UN	NA	MODERATE	NA	NA	46	46	0	57	8	49	85.96
Chewer	42483	chr14	106967221	G	T	*IGHV1-46*	MISSENSE	ENSG00000211962:MISSENSE[ENST00000390622(c.179C>A,cCt>cAt):ENSP00000375031(p.Pro60His)];	NA	NA	NA	NA	NA	NA	NA	NA	NA_NA	NA	MODIFIER	NA	NA	262	262	0	294	91	203	69.05
Chewer	42483	chr14	63268754	A	T	*KCNH5*	INTRONIC	ENSG00000140015:[ENST00000394968(c.1648+293T>A)];ENSG00000140015:[ENST00000322893(c.1822+293T>A)];ENSG00000140015:[ENST00000420622(c.1822+293T>A)];	NA	NA	NA	NA	NA	NA	NA	NA	NA_NA	NA	MODIFIER	NA	NA	78	78	0	70	22	48	68.57
Chewer	42483	chr14	104643124	G	T	*KIF26A*	SILENT	ENSG00000066735:SILENT[ENST00000315264(c.3582G>T,tcG>tcT):ENSP00000325452(p.Ser1194(=))];ENSG00000066735:SILENT[ENST00000423312(c.3999G>T,tcG>tcT):ENSP00000388241(p.Ser1333(=))];	NA	NA	chr14*104643124*104643124*G*A*rs769663594*NA*NA*NA	NA	NA	NA	NA	NA	NA_NA	NA	LOW	NA	NA	53	53	0	66	19	47	71.21
Chewer	42483	chr14	20897374	G	T	*KLHL33*	SILENT	ENSG00000185271:SILENT[ENST00000344581(c.1236C>A,gtC>gtA):ENSP00000341549(p.Val412(=))];	NA	NA	NA	NA	NA	NA	NA	NA	NA_NA	NA	LOW	NA	NA	73	70	3	134	38	96	71.64
Chewer	42483	chr14	106967221	G	T	*LINC00221*	INTRONIC	ENSG00000187156:[ENST00000433371(n.1037+4841G>T)];	NA	NA	NA	NA	NA	NA	NA	NA	NA_NA	NA	MODIFIER	NA	NA	262	262	0	294	91	203	69.05

Mangalaparthi *et al.* , 2020. Mutational landscape of esophageal squamous cell carcinoma in an Indian cohort
Supplementary Table 4. List of somatic single nucleotide variants identified using whole exome sequencing of ESCC patients

Cohort	Sample	Chromosome	Genomic position	Reference allele	Alternate allele	Gene symbol	Variant type	Variant annotation	OncoMD	COSMIC	dbSNP	ExAC	ExAC_SAS	1000G	1000G_SAS	SIFT	Polyphen2	CONDEL	VEP	LRT	PhastCon7 Way	Total read (Adjacent normal)	Reference allel read support (Adjacent normal)	Alternative read support (Adjacent normal)	Total read (Tumor)	Reference allel read support (Tumor)	Alternative read support (Tumor)	Alternate allele frequency
Chewer	42483	chr14	24838979	G	A	*NFATC4*	SILENT	ENSG00000100968:SILENT[ENST00000554050(c.375G>A,ccG>ccA):ENSP00000451151(p.Pro125(=))];ENSG00000100968:SILENT[ENST00000422617(c.339G>A,ccG>ccA):ENSP00000396788(p.Pro113(=))];ENSG00000100968:SILENT[ENST00000250373(c.375G>A,ccG>ccA):ENSP00000250373(p.Pro125(=))];ENSG00000100968:SILENT[ENST00000555590(c.414G>A,ccG>ccA):ENSP00000451224(p.Pro138(=))];ENSG00000100968:SILENT[ENST00000556169(c.339G>A,ccG>ccA):ENSP00000451454(p.Pro113(=))];ENSG00000100968:SILENT[ENST00000553469(c.471G>A,ccG>ccA):ENSP00000451502(p.Pro157(=))];ENSG00000100968:SILENT[ENST00000539237(c.471G>A,ccG>ccA):ENSP00000439350(p.Pro157(=))];ENSG00000100968:SILENT[ENST00000554661(c.165G>A,ccG>ccA):ENSP00000450733(p.Pro55(=))];ENSG00000100968:SILENT[ENST00000555453(c.339G>A,ccG>ccA):ENSP00000450686(p.Pro113(=))];ENSG00000100968:SILENT[ENST00000553708(c.375G>A,ccG>ccA):ENSP00000450590(p.Pro125(=))];ENSG00000100968:SILENT[ENST00000557451(c.165G>A,ccG>ccA):ENSP00000451284(p.Pro55(=))];ENSG00000100968:SILENT[ENST00000413692(c.564G>A,ccG>ccA):ENSP00000388910(p.Pro188(=))];ENSG00000100968:SILENT[ENST00000556279(c.471G>A,ccG>ccA):ENSP00000452270(p.Pro157(=))];ENSG00000100968:SILENT[ENST00000553879(c.165G>A,ccG>ccA):ENSP00000452349(p.Pro55(=))];ENSG00000100968:SILENT[ENST00000424781(c.414G>A,ccG>ccA):ENSP00000388668(p.Pro138(=))];ENSG00000100968:SILENT[ENST00000554591(c.564G>A,ccG>ccA):ENSP00000452039(p.Pro188(=))];ENSG00000100968:SILENT[ENST00000557674(c.306G>A,ccG>ccA):ENSP00000452352(p.Pro102(=))];ENSG00000100968:SILENT[ENST00000554966(c.414G>A,ccG>ccA):ENSP00000450644(p.Pro138(=))];ENSG00000100968:SILENT[ENST00000554903(c.375G>A,ccG>ccA):ENSP00000451853(p.Pro125(=))];ENSG00000100968:SILENT[ENST00000554344(c.165G>A,ccG>ccA):ENSP00000450469(p.Pro55(=))];ENSG00000100968:INTRONIC[ENST00000554779(c.272+103G>A)];ENSG00000100968:INTRONIC[ENST00000557028(n.427+103G>A)];ENSG00000100968:EXONIC-NC[ENST00000440487(n.575G>A)];ENSG00000100968:EXONIC-	NA	NA	NA	NA	NA	NA	NA	NA	NA_NA	NA	LOW	NA	NA	40	40	0	77	45	32	41.56
Chewer	42483	chr14	31926509	C	A	*RP11-176H8.1*	MISSENSE	ENSG00000203546:MISSENSE[ENST00000547378(c.91G>T,Gtc>Ttc):ENSP00000447056(p.Val31Phe)];ENSG00000203546:MISSENSE[ENST00000549185(c.91G>T,Gtc>Ttc):ENSP00000446654(p.Val31Phe)];ENSG00000203546:MISSENSE[ENST00000547760(c.74G>T,Gtc>Ttc):ENSP00000449799(p.Val26Phe)];	NA	NA	chr14*31926509*31926509*C*CG*rs764921013*NA*NA*NA	NA	NA	NA	NA	T_lc	UN_UN	NA	MODERATE	NA	NA	46	46	0	57	8	49	85.96
Chewer	42483	chr14	57052572	C	T	*TMEM260*	MISSENSE	ENSG00000070269:MISSENSE[ENST00000261556(c.286C>T,Ctt>Ttt):ENSP00000261556(p.Leu96Phe)];ENSG00000070269:MISSENSE[ENST00000538838(c.286C>T,Ctt>Ttt):ENSP00000441934(p.Leu96Phe)];ENSG00000070269:MISSENSE[ENST00000555497(c.286C>T,Ctt>Ttt):ENSP00000452065(p.Leu96Phe)];ENSG00000070269:MISSENSE[ENST00000539559(c.286C>T,Ctt>Ttt):ENSP00000442602(p.Leu96Phe)];ENSG00000070269:MISSENSE[ENST00000556810(c.175C>T,Ctt>Ttt):ENSP00000451677(p.Leu59Phe)];ENSG00000070269:5UTR[ENST00000556422(c.-183C>T)];ENSG00000070269:5UTR[ENST00000536419(c.-763C>T)];ENSG00000070269:EXONIC-NC[ENST00000554981(n.161C>T)];ENSG00000070269:EXONIC-NC[ENST00000557626(n.370C>T)];ENSG00000070269:EXONIC-NC[ENST00000557657(n.376C>T)];ENSG00000070269:EXONIC-NC[ENST00000556975(n.375C>T)];ENSG00000070269:EXONIC-NC[ENST00000556079(n.162C>T)];	NA	NA	NA	8.24E-06	6.06E-05	NA	NA	NA	NA_NA	NA	MODIFIER	NA	NA	130	130	0	177	45	132	74.58
Chewer	42483	chr14	22280027	C	A	*TRAV9-1*	SILENT	ENSG00000211783:SILENT[ENST00000390431(c.216C>A,gcC>gcA):ENSP00000438446(p.Ala72(=))];	NA	NA	NA	NA	NA	NA	NA	NA	NA_NA	NA	LOW	NA	NA	95	95	0	148	49	99	66.89
Chewer	42483	chr15	35182528	A	C	*AQR*	INTRONIC-SS-ACR	ENSG00000021776:[ENST00000543879(c.*657-3T>G)];ENSG00000021776:[ENST00000156471(c.2572-3T>G)];	NA	NA	NA	NA	NA	NA	NA	NA	NA_NA	NA	LOW	NA	NA	133	133	0	111	83	28	25.23
Chewer	42483	chr15	45895301	G	C	*BLOC1S6*	MISSENSE	ENSG00000104164:MISSENSE[ENST00000565323(c.243G>C,caG>caC):ENSP00000456851(p.Gln81His)];ENSG00000104164:MISSENSE[ENST00000565216(c.105G>C,caG>caC):ENSP00000456067(p.Gln35His)];ENSG00000104164:MISSENSE[ENST00000569076(c.78G>C,caG>caC):ENSP00000454803(p.Gln26His)];ENSG00000104164:MISSENSE[ENST00000220531(c.228G>C,caG>caC):ENSP00000220531(p.Gln76His)];ENSG00000104164:INTRONIC-SS-ACR[ENST00000567523(c.225-1G>C)];ENSG00000104164:INTRONIC-SS-ACR[ENST00000568963(c.83-1G>C)];ENSG00000104164:INTRONIC-SS-ACR[ENST00000564310(c.154-1G>C)];ENSG00000104164:INTRONIC[ENST00000566184(n.148-2325G>C)];ENSG00000104164:INTRONIC[ENST00000565727(n.106-2325G>C)];ENSG00000104164:INTRONIC[ENST00000567740(n.313-2325G>C)];ENSG00000104164:INTRONIC[ENST00000563000(n.238-2325G>C)];ENSG00000104164:INTRONIC[ENST00000563160(n.194-2325G>C)];ENSG00000104164:INTRONIC[ENST00000568597(c.225-2325G>C)];ENSG00000104164:5UTR[ENST00000566801(c.-64G>C)];ENSG00000104164:5UTR[ENST00000562384(c.-64G>C)];ENSG00000104164:5UTR[ENST00000564765(c.-64G>C)];ENSG00000104164:5UTR[ENST00000568816(c.-64G>C)];ENSG00000104164:5UTR[ENST00000567461(c.-64G>C)];ENSG00000104164:5UTR[ENST00000565409(c.-64G>C)];	NA	NA	NA	NA	NA	NA	NA	NA	NA_NA	NA	MODIFIER	NA	NA	87	87	0	59	37	22	37.29
Chewer	42483	chr15	40632963	C	T	*C15orf52*	INTRONIC	ENSG00000188549:[ENST00000557973(n.113+46G>A)];ENSG00000188549:[ENST00000559313(c.144+46G>A)];	NA	NA	NA	NA	NA	NA	NA	NA	NA_NA	NA	MODIFIER	NA	NA	26	26	0	26	19	7	26.92
Chewer	42483	chr15	78858023	G	C	*CHRNA5*	5UTR	ENSG00000169684:[ENST00000559554(c.-39G>C)];ENSG00000169684:[ENST00000299565(c.-39G>C)];	NA	NA	NA	NA	NA	NA	NA	NA	NA_NA	NA	MODIFIER	NA	NA	110	110	0	110	82	28	25.45
Chewer	42483	chr15	27193326	G	C	*GABRA5*	MISSENSE	ENSG00000186297:MISSENSE[ENST00000355395(c.1335G>C,tgG>tgC):ENSP00000347557(p.Trp445Cys)];ENSG00000186297:MISSENSE[ENST00000400081(c.1335G>C,tgG>tgC):ENSP00000382953(p.Trp445Cys)];ENSG00000186297:MISSENSE[ENST00000335625(c.1335G>C,tgG>tgC):ENSP00000335592(p.Trp445Cys)];	NA	NA	NA	8.69E-06	0	NA	NA	D	PrD_PrD	D	MODERATE	D	1	74	74	0	77	49	27	35.06
Chewer	42483	chr15	68650971	G	T	*ITGA11*	INTRONIC	ENSG00000137809:[ENST00000562826(n.247-48C>A)];ENSG00000137809:[ENST00000568677(n.369-48C>A)];ENSG00000137809:[ENST00000315757(c.473-48C>A)];ENSG00000137809:[ENST00000565868(n.53-48C>A)];ENSG00000137809:[ENST00000423218(c.473-48C>A)];	NA	NA	chr15*68650971*68650971*G*A*rs199902389*0*0.9998*0.0001997	NA	NA	NA	NA	NA	NA_NA	NA	MODIFIER	NA	NA	43	43	0	27	17	10	37.04

Cohort	Sample	Chromosome	Genomic position	Reference allele	Alternate allele	Gene symbol	Variant type	Variant annotation	OncoMD	COSMIC	dbSNP	ExAC	ExAC_SAS	1000G	1000G_SAS	SIFT	Polyphen2	CONDEL	VEP	LRT	PhastCon7 Way	Total read (Adjacent normal)	Reference allel read support (Adjacent normal)	Alternative read support (Adjacent normal)	Total read (Tumor)	Reference allel read support (Tumor)	Alternative read support (Tumor)	Alternate allele frequency
Chewer	42483	chr15	23812406	G	T	*MKRN3*	MISSENSE	ENSG00000179455:INTRONIC[ENST00000570112(c.*141+275G>T)];ENSG00000179455:INTRONIC[ENST00000564592(c.482+275G>T)];ENSG00000179455:INTRONIC[ENST00000568945(n.430+275G>T)];ENSG00000179455:INTRONIC[ENST00000568252(c.305+1172G>T)];ENSG00000179455:MISSENSE[ENST00000314520(c.1477G>T,Gac>Tac):ENSP00000313881(p.Asp493Tyr)];	NA	NA	NA	NA	NA	NA	NA	NA	NA_NA	NA	MODIFIER	NA	NA	202	201	1	153	108	45	29.41
Chewer	42483	chr15	23891969	C	G	*NA*	INTERGENIC		NA	NA	chr15*23891969*23891969*C*T*rs550046693*0*0.9998*0.0001997	NA	NA	NA	NA	NA	NA_NA	NA	MODIFIER	NA	NA	47	47	0	56	44	12	21.43
Chewer	42483	chr15	32620175	C	G	*NA*	INTERGENIC		NA	NA	NA	NA	NA	NA	NA	NA	NA_NA	NA	MODIFIER	NA	NA	127	127	0	112	92	20	17.86
Chewer	42483	chr15	24921090	G	T	*NPAP1*	MISSENSE	ENSG00000185823:MISSENSE[ENST00000329468(c.76G>T,Gct>Tct):ENSP00000333735(p.Ala26Ser)];	NA	NA	chr15*24921090*24921090*G*C*rs759520625*NA*NA*NA	NA	NA	NA	NA	T_lc	BN_BN	N	MODERATE	NA	NA	59	59	0	35	20	15	42.86
Chewer	42483	chr15	88678391	T	C	*NTRK3*	MISSENSE	ENSG00000140538:MISSENSE[ENST00000317501(c.1145A>G,aAc>aGc):ENSP00000318328(p.Asn382Ser)];ENSG00000140538:MISSENSE[ENST00000558676(c.1145A>G,aAc>aGc):ENSP00000453511(p.Asn382Ser)];ENSG00000140538:MISSENSE[ENST00000557856(c.1145A>G,aAc>aGc):ENSP00000453959(p.Asn382Ser)];ENSG00000140538:MISSENSE[ENST00000540489(c.1145A>G,aAc>aGc):ENSP00000444673(p.Asn382Ser)];ENSG00000140538:MISSENSE[ENST00000357724(c.1145A>G,aAc>aGc):ENSP00000350356(p.Asn382Ser)];ENSG00000140538:MISSENSE[ENST00000394480(c.1145A>G,aAc>aGc):ENSP00000377990(p.Asn382Ser)];ENSG00000140538:MISSENSE[ENST00000542733(c.851A>G,aAc>aGc):ENSP00000437773(p.Asn284Ser)];ENSG00000140538:MISSENSE[ENST00000355254(c.1145A>G,aAc>aGc):ENSP00000347397(p.Asn382Ser)];ENSG00000140538:MISSENSE[ENST00000360948(c.1145A>G,aAc>aGc):ENSP00000354207(p.Asn382Ser)];ENSG00000140538:EXONIC-NC[ENST00000559764(n.392A>G)];ENSG00000140538:INTRONIC[ENST00000560017(c.40+739A>G)];	chr15*8867[illegible]	chr15*8867[illegible]	NA	NA	NA	NA	NA	NA	NA_NA	NA	MODIFIER	NA	NA	85	85	0	63	49	14	22.22
Chewer	42483	chr15	45895301	G	C	*RP11-96O20.4*	INTRONIC	ENSG00000260170:[ENST00000564080(c.-18+15578G>C)];	NA	NA	NA	NA	NA	NA	NA	NA	NA_NA	NA	MODIFIER	NA	NA	87	87	0	59	37	22	37.29
Chewer	42483	chr15	51974128	G	C	*SCG3*	INTRONIC	ENSG00000104112:[ENST00000542355(c.-562+94G>C)];ENSG00000104112:[ENST00000220478(c.82+94G>C)];ENSG00000104112:[ENST00000558709(c.-419+94G>C)];	NA	NA	NA	NA	NA	NA	NA	NA	NA_NA	NA	MODIFIER	NA	NA	163	163	0	138	89	49	35.51
Chewer	42483	chr15	42149587	C	T	*SPTBN5*	MISSENSE	ENSG00000137877:MISSENSE[ENST00000320955(c.8470G>A,Gag>Aag):ENSP00000317790(p.Glu2824Lys)];	NA	NA	chr15*42149587*42149587*C*A,T*rs763325678*NA*NA*NA	8.01E-05	0	NA	NA	NA	BN_BN	NA	MODERATE	N	0.144	56	55	1	55	39	16	29.09
Chewer	42483	chr16	48244930	C	T	*ABCC11*	MISSENSE	ENSG00000121270:MISSENSE[ENST00000394747(c.1537G>A,Gcc>Acc):ENSP00000378230(p.Ala513Thr)];ENSG00000121270:MISSENSE[ENST00000394748(c.1537G>A,Gcc>Acc):ENSP00000378231(p.Ala513Thr)];ENSG00000121270:MISSENSE[ENST00000537808(c.1537G>A,Gcc>Acc):ENSP00000438530(p.Ala513Thr)];ENSG00000121270:MISSENSE[ENST00000353782(c.1537G>A,Gcc>Acc):ENSP00000311326(p.Ala513Thr)];ENSG00000121270:MISSENSE[ENST00000356608(c.1537G>A,Gcc>Acc):ENSP00000349017(p.Ala513Thr)];ENSG00000121270:EXONIC-NC[ENST00000567385(n.1935G>A)];	NA	NA	NA	NA	NA	NA	NA	T	BN_BN	N	MODERATE	N	0.01	86	86	0	109	82	27	24.77
Chewer	42483	chr16	25026606	G	A	*ARHGAP17*	5UTR	ENSG00000140750:[ENST00000455311(c.-121C>T)];ENSG00000140750:[ENST00000573625(c.-24C>T)];ENSG00000140750:[ENST00000441763(c.-24C>T)];ENSG00000140750:[ENST00000289968(c.-24C>T)];ENSG00000140750:[ENST00000303665(c.-24C>T)];ENSG00000140750:[ENST00000571480(c.-24C>T)];ENSG00000140750:[ENST00000575447(n.53C>T)];ENSG00000140750:[ENST00000573449(c.-179+216C>T)];	NA	NA	NA	NA	NA	NA	NA	NA	NA_NA	NA	MODIFIER	NA	NA	30	30	0	21	8	13	61.9
Chewer	42483	chr16	31122582	C	T	*BCKDK*	INTRONIC	ENSG00000103507:[ENST00000287507(c.845+42C>T)];ENSG00000103507:[ENST00000566568(n.1864-39C>T)];ENSG00000103507:[ENST00000394951(c.846-39C>T)];ENSG00000103507:[ENST00000567530(c.*118-39C>T)];ENSG00000103507:[ENST00000394950(c.846-39C>T)];ENSG00000103507:[ENST00000219794(c.846-39C>T)];	NA	NA	0.0001997	5.77E-05	6.06E-05	0.0001997	0.001	NA	NA_NA	NA	MODIFIER	NA	NA	78	78	0	112	90	22	19.64
Chewer	42483	chr16	71417354	G	T	*CALB2*	INTRONIC-SS-DNR	ENSG00000172137:[ENST00000562305(c.476+7G>T)];ENSG00000172137:[ENST00000302628(c.477+7G>T)];ENSG00000172137:[ENST00000490520(c.268+7G>T)];ENSG00000172137:[ENST00000349553(c.477+7G>T)];	NA	NA	NA	NA	NA	NA	NA	NA	NA_NA	NA	LOW	NA	NA	52	52	0	71	56	15	21.13
Chewer	42483	chr16	69177235	G	C	*CIRH1A*	SILENT	ENSG00000141076:SILENT[ENST00000314423(c.681G>C,acG>acC):ENSP00000327179(p.Thr227(=))];ENSG00000141076:SILENT[ENST00000566227(c.537G>C,acG>acC):ENSP00000462497(p.Thr179(=))];ENSG00000141076:SILENT[ENST00000563094(c.681G>C,acG>acC):ENSP00000456622(p.Thr227(=))];ENSG00000141076:SILENT[ENST00000352319(c.681G>C,acG>acC):ENSP00000339164(p.Thr227(=))];ENSG00000141076:SILENT[ENST00000562237(c.723G>C,acG>acC):ENSP00000456709(p.Thr241(=))];ENSG00000141076:EXONIC-NC[ENST00000569615(n.339G>C)];ENSG00000141076:EXONIC-NC[ENST00000569014(n.345G>C)];ENSG00000141076:EXONIC-NC[ENST00000564408(n.398G>C)];	NA	NA	chr16*69177235*69177235*G*T*rs777258895*NA*NA*NA	NA	NA	NA	NA	NA	NA_NA	NA	LOW	NA	NA	113	113	0	96	71	25	26.04
Chewer	42483	chr16	11219946	G	A	*CLEC16A*	MISSENSE	ENSG00000038532:MISSENSE[ENST00000436973(c.163G>A,Ggc>Agc):ENSP00000389963(p.Gly55Ser)];ENSG00000038532:MISSENSE[ENST00000409790(c.2584G>A,Ggc>Agc):ENSP00000387122(p.Gly862Ser)];ENSG00000038532:MISSENSE[ENST00000261657(c.158G>A,Ggc>Agc):ENSP00000261657(p.Gly54Ser)];ENSG00000038532:MISSENSE[ENST00000409552(c.2530G>A,Ggc>Agc):ENSP00000386495(p.Gly844Ser)];ENSG00000038532:MISSENSE[ENST00000428742(c.315G>A,Ggc>Agc):ENSP00000389743(p.Gly106Ser)];ENSG00000038532:5UTR[ENST00000381822(c.-280G>A)];ENSG00000038532:EXONIC-NC[ENST00000459723(n.80G>A)];	NA	NA	NA	NA	NA	NA	NA	T	PoD_PoD	N	MODERATE	NA	NA	64	64	0	78	65	13	16.67

Mangalaparthi *et al.* , 2020. Mutational landscape of esophageal squamous cell carcinoma in an Indian cohort
Supplementary Table 4. List of somatic single nucleotide variants identified using whole exome sequencing of ESCC patients

Cohort	Sample	Chromosome	Genomic position	Reference allele	Alternate allele	Gene symbol	Variant type	Variant annotation	OncoMD	COSMIC	dbSNP	ExAC	ExAC_SAS	1000G	1000G_SAS	SIFT	Polyphen2	CONDEL	VEP	LRT	PhastCon7 Way	Total read (Adjacent normal)	Reference allel read support (Adjacent normal)	Alternative read support (Adjacent normal)	Total read (Tumor)	Reference allel read support (Tumor)	Alternative read support (Tumor)	Alternate allele frequency
Chewer	42483	chr16	11219990	G	T	*CLEC16A*	SILENT	ENSG00000038532:SILENT[ENST00000436973(c.207G>T,gtG>gtT):ENSP00000389963(p.Val69(=))];ENSG00000038532:SILENT[ENST00000409790(c.2628G>T,gtG>gtT):ENSP00000387122(p.Val876(=))];ENSG00000038532:SILENT[ENST00000261657(c.202G>T,gtG>gtT):ENSP00000261657(p.Val68(=))];ENSG00000038532:SILENT[ENST00000428742(c.359G>T,gtG>gtT):ENSP00000389743(p.Val120(=))];ENSG00000038532:SILENT[ENST00000409552(c.2574G>T,gtG>gtT):ENSP00000386495(p.Val858(=))];ENSG00000038532:5UTR[ENST00000381822(c.-236G>T)];ENSG00000038532:EXONIC-NC[ENST00000459723(n.124G>T)];	NA	NA	chr16*11219990*11219990*G*C*rs201261636*NA*NA*NA	NA	NA	NA	NA	NA	NA_NA	NA	LOW	NA	NA	70	70	0	83	59	24	28.92
Chewer	42483	chr16	58562521	T	C	*CNOT1*	MISSENSE	ENSG00000125107:MISSENSE[ENST00000317147(c.6311A>G,cAt>cGt):ENSP00000320949(p.His2104Arg)];ENSG00000125107:MISSENSE[ENST00000569240(c.6296A>G,cAt>cGt):ENSP00000455635(p.His2099Arg)];ENSG00000125107:MISSENSE[ENST00000245138(c.2864A>G,cAt>cGt):ENSP00000245138(p.His955Arg)];ENSG00000125107:MISSENSE[ENST00000567188(c.6296A>G,cAt>cGt):ENSP00000456649(p.His2099Arg)];ENSG00000125107:EXONIC-NC[ENST00000570139(n.455A>G)];ENSG00000125107:EXONIC-NC[ENST00000563130(n.1186A>G)];	NA	NA	NA	NA	NA	NA	NA	NA	NA_NA	NA	MODIFIER	NA	NA	148	148	0	142	83	59	41.55
Chewer	42483	chr16	90021710	G	A	*DEF8*	INTRONIC	ENSG00000140995:[ENST00000561959(c.222+36G>A)];ENSG00000140995:[ENST00000567884(c.222+36G>A)];ENSG00000140995:[ENST00000568096(n.349+36G>A)];ENSG00000140995:[ENST00000567874(c.42+36G>A)];ENSG00000140995:[ENST00000418391(c.222+36G>A)];ENSG00000140995:[ENST00000561741(c.324+36G>A)];ENSG00000140995:[ENST00000563594(c.222+36G>A)];ENSG00000140995:[ENST00000569061(c.222+36G>A)];ENSG00000140995:[ENST00000569803(n.349+36G>A)];ENSG00000140995:[ENST00000569453(c.222+36G>A)];ENSG00000140995:[ENST00000562986(c.42+36G>A)];ENSG00000140995:[ENST00000268676(c.405+36G>A)];ENSG00000140995:[ENST00000567243(n.349+36G>A)];ENSG00000140995:[ENST00000566820(c.222+36G>A)];ENSG00000140995:[ENST00000562578(c.204+36G>A)];ENSG00000140995:[ENST00000570182(c.222+36G>A)];ENSG00000140995:[ENST00000567999(c.222+36G>A)];ENSG00000140995:[ENST00000561784(c.222+36G>A)];ENSG00000140995:[ENST00000566079(c.234+36G>A)];ENSG00000140995:[ENST00000563795(c.222+36G>A)];	NA	NA	NA	NA	NA	NA	NA	NA	NA_NA	NA	MODIFIER	NA	NA	90	90	0	120	60	60	50
Chewer	42483	chr16	85687976	C	T	*GSE1*	SILENT	ENSG00000131149:SILENT[ENST00000253458(c.519C>T,ccC>ccT):ENSP00000253458(p.Pro173(=))];ENSG00000131149:SILENT[ENST00000411612(c.207C>T,ccC>ccT):ENSP00000409006(p.Pro69(=))];ENSG00000131149:SILENT[ENST00000405402(c.207C>T,ccC>ccT):ENSP00000384839(p.Pro69(=))];ENSG00000131149:SILENT[ENST00000393243(c.300C>T,ccC>ccT):ENSP00000376934(p.Pro100(=))];	NA	NA	chr16*85687976*85687976*C*G,T*rs372447672*NA*NA*NA	7.63E-05	0	NA	NA	NA	NA_NA	NA	LOW	NA	NA	33	33	0	38	19	19	50
Chewer	42483	chr16	70937635	G	C	*HYDIN*	MISSENSE	ENSG00000157423:EXONIC-NC[ENST00000309900(n.3195C>G)];ENSG00000157423:MISSENSE[ENST00000393567(c.8742C>G,atC>atG):ENSP00000377197(p.Ile2914Met)];	NA	NA	NA	NA	NA	NA	NA	NA	PoD_PrD	NA	MODERATE	U	1	231	230	0	261	225	36	13.79
Chewer	42483	chr16	70989305	G	A	*HYDIN*	NONSENSE	ENSG00000157423:NONSENSE[ENST00000393567(c.6289C>T,Cag>Tag):ENSP00000377197(p.Gln2097Ter)];ENSG00000157423:EXONIC-NC[ENST00000543521(n.1416C>T)];ENSG00000157423:EXONIC-NC[ENST00000309900(n.1162C>T)];	NA	NA	NA	8.44E-06	0	NA	NA	NA	NA_NA	NA	MODIFIER	NA	NA	94	93	0	88	62	26	29.55
Chewer	42483	chr16	31425810	G	T	*ITGAD*	MISSENSE	ENSG00000156886:MISSENSE[ENST00000389202(c.2035G>T,Gac>Tac):ENSP00000373854(p.Asp679Tyr)];	NA	NA	NA	NA	NA	NA	NA	D	PrD_PrD	D	MODERATE	NA	NA	174	174	0	189	90	99	52.38
Chewer	42483	chr16	70164447	G	C	*PDPR*	MISSENSE-SS-PRX	ENSG00000090857:[ENST00000288050(c.729G>C,caG>caC):ENSP00000288050(p.Gln243His)];ENSG00000090857:[ENST00000398122(c.429G>C,caG>caC):ENSP00000381190(p.Gln143His)];ENSG00000090857:[ENST00000568530(c.729G>C,caG>caC):ENSP00000457916(p.Gln243His)];ENSG00000090857:[ENST00000565186(c.729G>C,caG>caC):ENSP00000455764(p.Gln243His)];ENSG00000090857:[ENST00000561920(n.951G>C)];	NA	NA	NA	NA	NA	NA	NA	NA	NA_NA	NA	LOW	NA	NA	442	442	0	565	446	119	21.06
Chewer	42483	chr16	72001837	C	A	*PKD1L3*	EXONIC-NC	ENSG00000187008:[ENST00000534738(n.2254G>T)];ENSG00000187008:[ENST00000335106(n.2254G>T)];	NA	NA	chr16*72001837*72001837*C*T*rs369308650*NA*NA*NA	NA	NA	NA	NA	NA	NA_NA	NA	MODIFIER	NA	NA	113	113	0	119	97	22	18.49
Chewer	42483	chr16	30663079	G	T	*PRR14*	INTRONIC	ENSG00000156858:[ENST00000569864(c.24-38G>T)];ENSG00000156858:[ENST00000563399(n.164-38G>T)];ENSG00000156858:[ENST00000287463(n.217-38G>T)];ENSG00000156858:[ENST00000563211(n.103-38G>T)];ENSG00000156858:[ENST00000565410(n.266-38G>T)];ENSG00000156858:[ENST00000542965(c.24-38G>T)];ENSG00000156858:[ENST00000568754(c.24-38G>T)];ENSG00000156858:[ENST00000300835(c.24-38G>T)];ENSG00000156858:[ENST00000564946(n.190G>T)];	NA	NA	NA	NA	NA	NA	NA	NA	NA_NA	NA	MODIFIER	NA	NA	77	77	0	92	72	20	21.74
Chewer	42483	chr16	29675991	C	A	*QPRT*	INTRONIC	ENSG00000103485:[ENST00000449759(c.-79+1354C>A)];	chr16*2967	chr16*2967	NA	0	0	NA	NA	NA	NA_NA	NA	LOW	NA	NA	85	85	0	91	67	24	26.37
Chewer	42483	chr16	67995562	G	A	*SLC12A4*	SILENT	ENSG00000124067:SILENT[ENST00000537830(c.240C>T,ctC>ctT):ENSP00000445962(p.Leu80(=))];ENSG00000124067:SILENT[ENST00000541864(c.165C>T,ctC>ctT):ENSP00000438334(p.Leu55(=))];ENSG00000124067:SILENT[ENST00000576616(c.258C>T,ctC>ctT):ENSP00000458902(p.Leu86(=))];ENSG00000124067:SILENT[ENST00000338335(c.258C>T,ctC>ctT):ENSP00000343374(p.Leu86(=))];ENSG00000124067:SILENT[ENST00000572037(c.114C>T,ctC>ctT):ENSP00000461403(p.Leu38(=))];ENSG00000124067:SILENT[ENST00000316341(c.258C>T,ctC>ctT):ENSP00000318557(p.Leu86(=))];ENSG00000124067:SILENT[ENST00000422611(c.264C>T,ctC>ctT):ENSP00000395983(p.Leu88(=))];ENSG00000124067:EXONIC-NC[ENST00000572010(n.299C>T)];ENSG00000124067:EXONIC-NC[ENST00000573023(n.1580C>T)];ENSG00000124067:EXONIC-NC[ENST00000576462(n.174C>T)];ENSG00000124067:EXONIC-NC[ENST00000570802(n.258C>T)];ENSG00000124067:5UTR[ENST00000571299(c.-94C>T)];ENSG00000124067:INTRONIC[ENST00000576377(c.115+6780C>T)];	NA	NA	NA	8.25E-06	0	NA	NA	NA	NA_NA	NA	MODIFIER	NA	NA	124	124	0	124	95	29	23.39
Chewer	42483	chr16	29675991	C	A	*SPN*	SILENT	ENSG00000197471:SILENT[ENST00000360121(c.942C>A,gcC>gcA):ENSP00000353238(p.Ala314(=))];ENSG00000197471:SILENT[ENST00000436527(c.942C>A,gcC>gcA):ENSP00000412907(p.Ala314(=))];ENSG00000197471:SILENT[ENST00000563039(c.942C>A,gcC>gcA):ENSP00000455266(p.Ala314(=))];ENSG00000197471:SILENT[ENST00000395389(c.942C>A,gcC>gcA):ENSP00000378787(p.Ala314(=))];	chr16*2967	chr16*2967	NA	0	0	NA	NA	NA	NA_NA	NA	LOW	NA	NA	85	85	0	91	67	24	26.37

Supplementary Table 4. List of somatic single nucleotide variants identified using whole exome sequencing of ESCC patients

Cohort	Sample	Chromosome	Genomic position	Reference allele	Alternate allele	Gene symbol	Variant type	Variant annotation	OncoMD	COSMIC	dbSNP	ExAC	ExAC_SAS	1000G	1000G_SAS	SIFT	Polyphen2	CONDEL	VEP	LRT	PhastCon7 Way	Total read (Adjacent normal)	Reference allel read support (Adjacent normal)	Alternative read support (Adjacent normal)	Total read (Tumor)	Reference allel read support (Tumor)	Alternative read support (Tumor)	Alternate allele frequency
Chewer	42483	chr16	4257439	G	C	*SRL*	MISSENSE	ENSG00000185739:MISSENSE[ENST00000572111(c.728C>G,cCt>cGt):ENSP00000461179(p.Pro243Arg)];ENSG00000185739:INTRONIC[ENST00000399609(c.62-2804C>G)];ENSG00000185739:INTRONIC[ENST00000537996(c.-65-2804C>G)];	NA	NA	NA	NA	NA	NA	NA	NA	NA_NA	NA	MODIFIER	NA	NA	90	90	0	154	73	80	51.95
Chewer	42483	chr16	2817546	G	T	*SRRM2*	SILENT	ENSG00000167978:EXONIC-NC[ENST00000574593(n.39G>T)];ENSG00000167978:SILENT[ENST00000301740(c.7017G>T,gtG>gtT):ENSP00000301740(p.Val2339(=))];	NA	NA	chr16*2817546*2817546*G*A*rs760734597*NA*NA*NA	NA	NA	NA	NA	NA	NA_NA	NA	LOW	NA	NA	132	132	0	233	190	43	18.45
Chewer	42483	chr16	1551729	G	A	*TELO2*	MISSENSE	ENSG00000100726:INTRONIC[ENST00000497339(c.1227+1083G>A)];ENSG00000100726:EXONIC-NC[ENST00000564507(n.22G>A)];ENSG00000100726:MISSENSE[ENST00000262319(c.1427G>A,gGc>gAc):ENSP00000262319(p.Gly476Asp)];	NA	NA	chr16*1551729*1551729*G*C*rs756519824*NA*NA*NA	NA	NA	NA	NA	T	BN_BN	N	MODERATE	N	0	84	84	0	133	110	23	17.29
Chewer	42483	chr16	31235842	G	C	*TRIM72*	SILENT	ENSG00000177238:SILENT[ENST00000322122(c.1200G>C,ccG>ccC):ENSP00000312675(p.Pro400(=))];	NA	NA	NA	NA	NA	NA	NA	NA	NA_NA	NA	LOW	NA	NA	59	58	0	126	96	30	23.81
Chewer	42483	chr16	88698031	G	C	*ZC3H18*	INTRONIC	ENSG00000158545:[ENST00000564341(c.212-49G>C)];ENSG00000158545:[ENST00000566496(n.1638G>C)];ENSG00000158545:[ENST00000301011(c.*324G>C)];ENSG00000158545:[ENST00000565583(c.*170G>C)];	NA	NA	NA	NA	NA	NA	NA	NA	NA_NA	NA	MODIFIER	NA	NA	40	40	0	45	25	20	44.44
Chewer	42483	chr16	88505525	G	T	*ZNF469*	MISSENSE	ENSG00000225614:MISSENSE[ENST00000565624(c.11647G>T,Ggc>Tgc):ENSP00000456500(p.Gly3883Cys)];ENSG00000225614:MISSENSE[ENST00000437464(c.11563G>T,Ggc>Tgc):ENSP00000402343(p.Gly3855Cys)];	NA	NA	NA	NA	NA	NA	NA	NA	PrD_PrD	NA	MODERATE	NA	NA	72	72	0	116	63	52	44.83
Chewer	42483	chr17	28952089	G	T	*AC005562.1*	INTRONIC	ENSG00000214719:[ENST00000431308(n.203-6684G>T)];ENSG00000214719:[ENST00000398849(n.616-6684G>T)];ENSG00000214719:[ENST00000440026(n.196-8027G>T)];ENSG00000214719:[ENST00000578265(n.287+14129G>T)];	NA	NA	NA	NA	NA	NA	NA	NA	NA_NA	NA	MODIFIER	NA	NA	35	35	0	39	34	5	12.82
Chewer	42483	chr17	42852922	G	A	*ADAM11*	INTRONIC	ENSG00000073670:[ENST00000535346(c.793-30G>A)];ENSG00000073670:[ENST00000587773(c.1075-30G>A)];ENSG00000073670:[ENST00000355638(c.*663-30G>A)];ENSG00000073670:[ENST00000200557(c.1393-30G>A)];	NA	NA	NA	NA	NA	NA	NA	NA	NA_NA	NA	MODIFIER	NA	NA	36	36	0	41	34	7	17.07
Chewer	42483	chr17	42852923	C	G	*ADAM11*	INTRONIC	ENSG00000073670:[ENST00000535346(c.793-29C>G)];ENSG00000073670:[ENST00000587773(c.1075-29C>G)];ENSG00000073670:[ENST00000355638(c.*663-29C>G)];ENSG00000073670:[ENST00000200557(c.1393-29C>G)];	NA	NA	NA	NA	NA	NA	NA	NA	NA_NA	NA	MODIFIER	NA	NA	36	36	0	41	34	7	17.07
Chewer	42483	chr17	56389528	G	T	*BZRAP1*	MISSENSE	ENSG00000005379:MISSENSE[ENST00000343736(c.2654C>A,cCc>cAc):ENSP00000345824(p.Pro885His)];ENSG00000005379:MISSENSE[ENST00000268893(c.2474C>A,cCc>cAc):ENSP00000268893(p.Pro825His)];ENSG00000005379:MISSENSE[ENST00000355701(c.2654C>A,cCc>cAc):ENSP00000347929(p.Pro885His)];	NA	NA	NA	NA	NA	NA	NA	NA	PrD_PrD	NA	MODERATE	D	0.975	109	106	1	114	56	58	50.88
Chewer	42483	chr17	58503265	G	T	*C17orf64*	INTRONIC	ENSG00000141371:[ENST00000464714(n.211+20G>T)];ENSG00000141371:[ENST00000269127(c.153+20G>T)];ENSG00000141371:[ENST00000474834(c.42+20G>T)];	NA	NA	NA	NA	NA	NA	NA	NA	NA_NA	NA	MODIFIER	NA	NA	145	144	1	127	83	44	34.65
Chewer	42483	chr17	45214564	A	T	*CDC27*	MISSENSE	ENSG00000004897:MISSENSE[ENST00000066544(c.1867T>A,Tgt>Agt):ENSP00000066544(p.Cys623Ser)];ENSG00000004897:MISSENSE[ENST00000531206(c.1885T>A,Tgt>Agt):ENSP00000434614(p.Cys629Ser)];ENSG00000004897:MISSENSE[ENST00000527547(c.1864T>A,Tgt>Agt):ENSP00000437339(p.Cys622Ser)];ENSG00000004897:MISSENSE[ENST00000446365(c.1684T>A,Tgt>Agt):ENSP00000392802(p.Cys562Ser)];ENSG00000004897:MISSENSE[ENST00000571643(c.47T>A,Tgt>Agt):ENSP00000460279(p.Cys17Ser)];ENSG00000004897:3UTR[ENST00000573502(c.*501T>A)];ENSG00000004897:3UTR[ENST00000533415(c.*1434T>A)];ENSG00000004897:INTRONIC[ENST00000526866(c.958-4824T>A)];	chr17*4521	chr17*4521	NA	NA	NA	NA	NA	NA	NA_NA	NA	MODIFIER	NA	NA	1683	1662	21	1090	1057	32	2.94
Chewer	42483	chr17	10405087	T	A	*CTC-297N7.11*	INTRONIC	ENSG00000272975:[ENST00000587182(n.156-65767T>A)];	NA	NA	NA	NA	NA	NA	NA	NA	NA_NA	NA	MODIFIER	NA	NA	152	152	0	108	82	26	24.07
Chewer	42483	chr17	78406913	C	G	*ENDOV*	INTRONIC	ENSG00000173818:[ENST00000520284(c.133-3020C>G)];ENSG00000173818:[ENST00000517795(c.257-3020C>G)];ENSG00000173818:[ENST00000518907(c.133-3020C>G)];ENSG00000173818:[ENST00000518901(c.257-3020C>G)];ENSG00000173818:[ENST00000520367(c.704-3020C>G)];ENSG00000173818:[ENST00000518137(c.839-3020C>G)];	NA	NA	NA	NA	NA	NA	NA	NA	NA_NA	NA	MODIFIER	NA	NA	65	65	0	79	67	12	15.19
Chewer	42483	chr17	40876323	C	T	*EZH1*	MISSENSE-SS-PRX	ENSG00000108799:[ENST00000592743(c.366G>A,atG>atA):ENSP00000466924(p.Met122Ile)];ENSG00000108799:[ENST00000593214(c.366G>A,atG>atA):ENSP00000467320(p.Met122Ile)];ENSG00000108799:[ENST00000428826(c.366G>A,atG>atA):ENSP00000404658(p.Met122Ile)];ENSG00000108799:[ENST00000590078(c.156G>A,atG>atA):ENSP00000465220(p.Met52Ile)];ENSG00000108799:[ENST00000586867(c.366G>A,atG>atA):ENSP00000465327(p.Met122Ile)];ENSG00000108799:[ENST00000415827(c.339G>A,atG>atA):ENSP00000407869(p.Met113Ile)];ENSG00000108799:[ENST00000586382(c.220-1390G>A)];ENSG00000108799:[ENST00000588897(c.246+3330G>A)];ENSG00000108799:[ENST00000586089(c.247-1390G>A)];ENSG00000108799:[ENST00000585893(c.247-1390G>A)];ENSG00000108799:[ENST00000435174(c.70+3330G>A)];ENSG00000108799:[ENST00000585912(n.247-1390G>A)];ENSG00000108799:[ENST00000586103(n.473G>A)];ENSG00000108799:[ENST00000591330(n.495G>A)];	chr17*4087	chr17*4087	NA	NA	NA	NA	NA	NA	NA_NA	NA	MODIFIER	NA	NA	123	123	0	153	120	33	21.57
Chewer	42483	chr17	73752723	A	T	*GALK1*	INTRONIC	ENSG00000108479:[ENST00000589643(n.254+1392T>A)];ENSG00000108479:[ENST00000225614(c.*22+1392T>A)];	NA	NA	NA	NA	NA	NA	NA	NA	NA_NA	NA	MODIFIER	NA	NA	37	37	0	47	26	21	44.68
Chewer	42483	chr17	72856624	G	T	*GRIN2C*	EXONIC-NC	ENSG00000161509:[ENST00000584496(n.341C>A)];ENSG00000161509:[ENST00000347612(c.-68C>A)];ENSG00000161509:[ENST00000578159(n.77+927C>A)];	NA	NA	NA	NA	NA	NA	NA	NA	NA_NA	NA	MODIFIER	NA	NA	71	71	0	98	71	26	26.53
Chewer	42483	chr17	43246889	G	A	*HEXIM2*	MISSENSE	ENSG00000168517:MISSENSE[ENST00000591576(c.574G>A,Gac>Aac):ENSP00000465727(p.Asp192Asn)];ENSG00000168517:MISSENSE[ENST00000307275(c.574G>A,Gac>Aac):ENSP00000302276(p.Asp192Asn)];ENSG00000168517:MISSENSE[ENST00000592695(c.574G>A,Gac>Aac):ENSP00000467517(p.Asp192Asn)];	NA	NA	chr17*43246889*43246889*G*T*rs556732963*NA*NA*NA	NA	NA	NA	NA	NA	NA_NA	NA	MODIFIER	NA	NA	100	100	0	120	101	19	15.83
Chewer	42483	chr17	73752723	A	T	*ITGB4*	INTRONIC	ENSG00000132470:[ENST00000579662(c.4687+25A>T)];ENSG00000132470:[ENST00000450894(c.4687+25A>T)];ENSG00000132470:[ENST00000339591(c.4846+25A>T)];ENSG00000132470:[ENST00000449880(c.4846+25A>T)];ENSG00000132470:[ENST00000582629(c.266-984A>T)];ENSG00000132470:[ENST00000584939(c.387+25A>T)];ENSG00000132470:[ENST00000200181(c.4897+25A>T)];	NA	NA	NA	NA	NA	NA	NA	NA	NA_NA	NA	MODIFIER	NA	NA	37	37	0	47	26	21	44.68

Mangalaparthi *et al.* , 2020. Mutational landscape of esophageal squamous cell carcinoma in an Indian cohort
Supplementary Table 4. List of somatic single nucleotide variants identified using whole exome sequencing of ESCC patients

Cohort	Sample	Chromosome	Genomic position	Reference allele	Alternate allele	Gene symbol	Variant type	Variant annotation	OncoMD	COSMIC	dbSNP	ExAC	ExAC_SAS	1000G	1000G_SAS	SIFT	Polyphen2	CONDEL	VEP	LRT	PhastCon7 Way	Total read (Adjacent normal)	Reference allel read support (Adjacent normal)	Alternative read support (Adjacent normal)	Total read (Tumor)	Reference allel read support (Tumor)	Alternative read support (Tumor)	Alternate allele frequency
Chewer	42483	chr17	39925695	G	T	*JUP*	MISSENSE	ENSG00000173801:MISSENSE[ENST00000437187(c.443C>A,aCc>aAc):ENSP00000394146(p.Thr148Asn)];ENSG00000173801:MISSENSE[ENST00000393931(c.443C>A,aCc>aAc):ENSP00000377508(p.Thr148Asn)];ENSG00000173801:MISSENSE[ENST00000424457(c.443C>A,aCc>aAc):ENSP00000401034(p.Thr148Asn)];ENSG00000173801:MISSENSE[ENST00000420370(c.443C>A,aCc>aAc):ENSP00000411449(p.Thr148Asn)];ENSG00000173801:MISSENSE[ENST00000310706(c.443C>A,aCc>aAc):ENSP00000311113(p.Thr148Asn)];ENSG00000173801:MISSENSE[ENST00000449889(c.443C>A,aCc>aAc):ENSP00000389886(p.Thr148Asn)];ENSG00000173801:MISSENSE[ENST00000393930(c.443C>A,aCc>aAc):ENSP00000377507(p.Thr148Asn)];ENSG00000173801:MISSENSE[ENST00000540235(c.443C>A,aCc>aAc):ENSP00000441751(p.Thr148Asn)];	chr17*3992:	NA	NA	NA	NA	NA	NA	D_lc	PrD_PrD	D	MODERATE	D	1	76	76	0	119	98	21	17.65
Chewer	42483	chr17	21318864	C	T	*KCNJ12*	SILENT	ENSG00000184185:SILENT[ENST00000331718(c.210C>T,gaC>gaT):ENSP00000328150(p.Asp70(=))];ENSG00000184185:SILENT[ENST00000583088(c.210C>T,gaC>gaT):ENSP00000463778(p.Asp70(=))];	NA	NA	NA	NA	NA	NA	NA	NA	NA_NA	NA	LOW	NA	1	355	353	0	284	259	24	8.45
Chewer	42483	chr17	39258385	G	T	*KRTAP4-16P*	MISSENSE	ENSG00000241241:MISSENSE[ENST00000440582(c.77C>A,cCc>cAc):ENSP00000411198(p.Pro26His)];	NA	NA	chr17*39258385*39258385*G*A*rs776563550*NA*NA*NA	NA	NA	NA	NA	NA	UN_UN	NA	MODERATE	NA	NA	85	85	0	68	39	29	42.65
Chewer	42483	chr17	30368120	G	T	*LRRC37B*	INTRONIC	ENSG00000185158:[ENST00000327564(c.2205-4599G>T)];ENSG00000185158:[ENST00000578674(c.2159+5462G>T)];ENSG00000185158:[ENST00000584368(c.2007-4599G>T)];ENSG00000185158:[ENST00000583758(n.352-4599G>T)];ENSG00000185158:[ENST00000394713(c.1971-4599G>T)];ENSG00000185158:[ENST00000543378(c.1878-4599G>T)];ENSG00000185158:[ENST00000341671(c.2124-4599G>T)];ENSG00000185158:[ENST00000582815(n.195-4599G>T)];	NA	NA	NA	NA	NA	NA	NA	NA	NA_NA	NA	MODIFIER	NA	NA	94	94	0	97	81	16	16.49
Chewer	42483	chr17	81052364	G	T	*METRNL*	3UTR	ENSG00000176845:[ENST00000320095(c.*44G>T)];ENSG00000176845:[ENST00000570778(c.*44G>T)];ENSG00000176845:[ENST00000574053(c.*44G>T)];ENSG00000176845:[ENST00000571814(c.*44G>T)];	NA	NA	NA	NA	NA	NA	NA	NA	NA_NA	NA	MODIFIER	NA	NA	69	69	0	93	51	42	45.16
Chewer	42483	chr17	10405087	T	A	*MYH1*	NONSENSE	ENSG00000109061:NONSENSE[ENST00000226207(c.3253A>T,Aaa>Taa):ENSP00000226207(p.Lys1085Ter)];	NA	NA	NA	NA	NA	NA	NA	NA	NA_NA	NA	MODIFIER	NA	NA	152	152	0	108	82	26	24.07
Chewer	42483	chr17	3143962	T	C	*NA*	INTERGENIC		NA	NA	NA	NA	NA	NA	NA	NA	NA_NA	NA	MODIFIER	NA	NA	242	241	0	178	109	69	38.76
Chewer	42483	chr17	29664889	T	C	*NF1*	MISSENSE	ENSG00000196712:MISSENSE[ENST00000358273(c.6695T>C,cTa>cCa):ENSP00000351015(p.Leu2232Pro)];ENSG00000196712:MISSENSE[ENST00000456735(c.5630T>C,cTa>cCa):ENSP00000389907(p.Leu1877Pro)];ENSG00000196712:MISSENSE[ENST00000356175(c.6632T>C,cTa>cCa):ENSP00000348498(p.Leu2211Pro)];ENSG00000196712:SILENT[ENST00000417592(c.51T>C,acT>acC):ENSP00000398991(p.Thr17(=))];ENSG00000196712:SILENT[ENST00000444181(c.51T>C,acT>acC):ENSP00000396481(p.Thr17(=))];ENSG00000196712:SILENT[ENST00000581790(c.55T>C,acT>acC):ENSP00000462700(p.Thr19(=))];ENSG00000196712:SILENT[ENST00000471572(c.55T>C,acT>acC):ENSP00000464678(p.Thr19(=))];ENSG00000196712:EXONIC-NC[ENST00000584328(n.109T>C)];ENSG00000196712:3UTR[ENST00000579081(c.*1860T>C)];	NA	NA	NA	NA	NA	NA	NA	NA	NA_NA	NA	LOW	NA	NA	134	134	0	124	94	30	24.19
Chewer	42483	chr17	8926412	G	T	*NTN1*	MISSENSE	ENSG00000065320:MISSENSE[ENST00000546090(c.722G>T,tGg>tTg):ENSP00000441611(p.Trp241Leu)];ENSG00000065320:MISSENSE[ENST00000538852(c.722G>T,tGg>tTg):ENSP00000443259(p.Trp241Leu)];ENSG00000065320:MISSENSE[ENST00000173229(c.722G>T,tGg>tTg):ENSP00000173229(p.Trp241Leu)];	NA	NA	NA	NA	NA	NA	NA	D	BN_BN	N	MODERATE	D	1	90	90	0	113	51	62	54.87
Chewer	42483	chr17	72939795	G	T	*OTOP3*	MISSENSE	ENSG00000182938:MISSENSE[ENST00000328801(c.781G>T,Ggc>Tgc):ENSP00000328090(p.Gly261Cys)];ENSG00000182938:3UTR[ENST00000580749(c.*736G>T)];	NA	NA	NA	NA	NA	NA	NA	NA	NA_NA	NA	MODIFIER	NA	NA	61	61	0	93	77	16	17.2
Chewer	42483	chr17	79864655	A	G	*PCYT2*	SILENT	ENSG00000185813:EXONIC-NC[ENST00000571581(n.864T>C)];ENSG00000185813:EXONIC-NC[ENST00000574155(n.51T>C)];ENSG00000185813:SILENT[ENST00000572995(c.187T>C,gcT>gcC):ENSP00000460767(p.Ala63(=))];ENSG00000185813:SILENT[ENST00000331285(c.423T>C,gcT>gcC):ENSP00000331719(p.Ala141(=))];ENSG00000185813:SILENT[ENST00000538936(c.657T>C,gcT>gcC):ENSP00000439245(p.Ala219(=))];ENSG00000185813:SILENT[ENST00000576343(c.495T>C,gcT>gcC):ENSP00000458931(p.Ala165(=))];ENSG00000185813:SILENT[ENST00000571105(c.657T>C,gcT>gcC):ENSP00000459044(p.Ala219(=))];ENSG00000185813:SILENT[ENST00000538721(c.711T>C,gcT>gcC):ENSP00000442050(p.Ala237(=))];ENSG00000185813:SILENT[ENST00000573636(c.612T>C,gcT>gcC):ENSP00000459836(p.Ala204(=))];ENSG00000185813:SILENT[ENST00000570391(c.561T>C,gcT>gcC):ENSP00000461190(p.Ala187(=))];ENSG00000185813:SILENT[ENST00000570388(c.423T>C,gcT>gcC):ENSP00000458330(p.Ala141(=))];ENSG00000185813:3UTR[ENST00000573401(c.*691T>C)];	NA	NA	chr17*79864655*79864655*A*C,T*rs768421542*NA*NA*NA	NA	NA	NA	NA	NA	NA_NA	NA	LOW	NA	NA	86	86	0	157	61	95	60.51
Chewer	42483	chr17	36934601	T	G	*PIP4K2B*	MISSENSE	ENSG00000141720:MISSENSE[ENST00000269554(c.679A>C,Agc>Cgc):ENSP00000269554(p.Ser227Arg)];ENSG00000141720:EXONIC-NC[ENST00000583278(n.251A>C)];ENSG00000141720:EXONIC-NC[ENST00000311500(n.777A>C)];	NA	NA	NA	NA	NA	NA	NA	NA	NA_NA	NA	MODIFIER	NA	NA	79	79	0	109	56	53	48.62
Chewer	42483	chr17	57774870	G	C	*PTRH2*	MISSENSE	ENSG00000141378:MISSENSE[ENST00000470557(c.470C>G,aCt>aGt):ENSP00000464327(p.Thr157Ser)];ENSG00000141378:MISSENSE[ENST00000537860(c.470C>G,aCt>aGt):ENSP00000437690(p.Thr157Ser)];ENSG00000141378:MISSENSE[ENST00000409433(c.473C>G,aCt>aGt):ENSP00000387180(p.Thr158Ser)];ENSG00000141378:MISSENSE[ENST00000393038(c.470C>G,aCt>aGt):ENSP00000376758(p.Thr157Ser)];ENSG00000141378:INTRONIC[ENST00000587935(n.45+9862C>G)];	NA	NA	NA	NA	NA	NA	NA	NA	NA_NA	NA	MODIFIER	NA	NA	101	101	0	98	81	17	17.35
Chewer	42483	chr17	59482832	G	A	*RP11-332H18.4*	INTRONIC	ENSG00000267280:[ENST00000592009(n.40+6045C>T)];	NA	NA	NA	NA	NA	NA	NA	NA	NA_NA	NA	MODIFIER	NA	NA	37	37	0	51	38	13	25.49
Chewer	42483	chr17	10405087	T	A	*RP11-799N11.1*	INTRONIC	ENSG00000272736:[ENST00000399342(n.207-31554T>A)];ENSG00000272736:[ENST00000581304(n.144-31554T>A)];	NA	NA	NA	NA	NA	NA	NA	NA	NA_NA	NA	MODIFIER	NA	NA	152	152	0	108	82	26	24.07
Chewer	42483	chr17	43246889	G	A	*RP13-890H12.2*	EXONIC-NC	ENSG00000267288:[ENST00000589796(n.1331C>T)];	NA	NA	chr17*43246889*43246889*G*T*rs556732963*NA*NA*NA	NA	NA	NA	NA	NA	NA_NA	NA	MODIFIER	NA	NA	100	100	0	120	101	19	15.83

Mangalaparthi *et al.* , 2020. Mutational landscape of esophageal squamous cell carcinoma in an Indian cohort
Supplementary Table 4. List of somatic single nucleotide variants identified using whole exome sequencing of ESCC patients

Cohort	Sample	Chromosome	Genomic position	Reference allele	Alternate allele	Gene symbol	Variant type	Variant annotation	OncoMD	COSMIC	dbSNP	ExAC	ExAC_SAS	1000G	1000G_SAS	SIFT	Polyphen2	CONDEL	VEP	LRT	PhastCon7 Way	Total read (Adjacent normal)	Reference allel read support (Adjacent normal)	Alternative read support (Adjacent normal)	Total read (Tumor)	Reference allel read support (Tumor)	Alternative read support (Tumor)	Alternate allele frequency
Chewer	42483	chr17	48243600	C	A	*SGCA*	INTRONIC	ENSG00000108823:[ENST00000514934(c.96+103C>A)];ENSG00000108823:[ENST00000451235(c.-115+162C>A)];ENSG00000108823:[ENST00000513821(c.37+162C>A)];ENSG00000108823:[ENST00000262018(c.37+162C>A)];ENSG00000108823:[ENST00000511303(c.33+162C>A)];ENSG00000108823:[ENST00000543315(c.37+162C>A)];ENSG00000108823:[ENST00000344627(c.37+162C>A)];ENSG00000108823:[ENST00000502555(c.37+162C>A)];ENSG00000108823:[ENST00000513942(n.104-1708C>A)];	NA	NA	NA	NA	NA	NA	NA	NA	NA_NA	NA	MODIFIER	NA	NA	21	21	0	31	24	7	22.58
Chewer	42483	chr17	30368120	G	T	*SH3GL1P1*	EXONIC-NC	ENSG00000266777:[ENST00000583601(n.665G>T)];ENSG00000266777:[ENST00000579186(n.218G>T)];	NA	NA	NA	NA	NA	NA	NA	NA	NA_NA	NA	MODIFIER	NA	NA	94	94	0	97	81	16	16.49
Chewer	42483	chr17	28952089	G	T	*SH3GL1P2*	EXONIC-NC	ENSG00000264943:[ENST00000582245(n.659G>T)];	NA	NA	NA	NA	NA	NA	NA	NA	NA_NA	NA	MODIFIER	NA	NA	35	35	0	39	34	5	12.82
Chewer	42483	chr17	4864150	T	A	*SPAG7*	INTRONIC-SS-ACR	ENSG00000091640:INTRONIC-SS-ACR[ENST00000206020(c.86-2A>T)];ENSG00000091640:INTRONIC-SS-ACR[ENST00000575142(c.53-2A>T)];ENSG00000091640:INTRONIC-SS-ACR[ENST00000573366(c.-68-2A>T)];ENSG00000091640:INTRONIC-SS-ACR[ENST00000573805(n.67-2A>T)];ENSG00000091640:INTRONIC-SS-ACR[ENST00000575784(c.53-2A>T)];	NA	NA	NA	NA	NA	NA	NA	NA	NA_NA	NA	HIGH	NA	NA	38	38	0	60	19	41	68.33
Chewer	42483	chr17	59482832	G	A	*TBX2*	MISSENSE	ENSG00000121068:EXONIC-NC[ENST00000477081(n.1565G>A)];ENSG00000121068:MISSENSE[ENST00000240328(c.1321G>A,Gag>Aag):ENSP00000240328(p.Glu441Lys)];ENSG00000121068:3UTR[ENST00000419047(c.*858G>A)];	NA	NA	NA	NA	NA	NA	NA	NA	NA_NA	NA	MODIFIER	NA	NA	37	37	0	51	38	13	25.49
Chewer	42483	chr17	72791089	A	G	*TMEM104*	INTRONIC	ENSG00000109066:[ENST00000582330(c.428-62A>G)];ENSG00000109066:[ENST00000582773(c.428-62A>G)];ENSG00000109066:[ENST00000335464(c.428-62A>G)];ENSG00000109066:[ENST00000578764(c.224-62A>G)];ENSG00000109066:[ENST00000417024(c.467-62A>G)];	NA	NA	0.01937	NA	NA	0.019369	0.0123	NA	NA_NA	NA	MODIFIER	NA	NA	74	73	1	104	98	6	5.77
Chewer	42483	chr17	7578394	T	C	*TP53*	MISSENSE	ENSG00000141510:MISSENSE[ENST00000413465(c.536A>G,cAt>cGt):ENSP00000410739(p.His179Arg)];ENSG00000141510:MISSENSE[ENST00000420246(c.536A>G,cAt>cGt):ENSP00000391127(p.His179Arg)];ENSG00000141510:MISSENSE[ENST00000269305(c.536A>G,cAt>cGt):ENSP00000269305(p.His179Arg)];ENSG00000141510:MISSENSE[ENST00000509690(c.140A>G,cAt>cGt):ENSP00000425104(p.His47Arg)];ENSG00000141510:MISSENSE[ENST00000359597(c.536A>G,cAt>cGt):ENSP00000352610(p.His179Arg)];ENSG00000141510:MISSENSE[ENST00000514944(c.257A>G,cAt>cGt):ENSP00000423862(p.His86Arg)];ENSG00000141510:MISSENSE[ENST00000445888(c.536A>G,cAt>cGt):ENSP00000391478(p.His179Arg)];ENSG00000141510:MISSENSE[ENST00000455263(c.536A>G,cAt>cGt):ENSP00000398846(p.His179Arg)];ENSG00000141510:EXONIC-NC[ENST00000504937(n.418A>G)];ENSG00000141510:EXONIC-NC[ENST00000505014(n.792A>G)];ENSG00000141510:EXONIC-NC[ENST00000504290(n.418A>G)];ENSG00000141510:EXONIC-NC[ENST00000574684(n.44A>G)];ENSG00000141510:EXONIC-NC[ENST00000510385(n.418A>G)];	chr17*7578	chr17*7578	NA	NA	NA	NA	NA	NA	NA_NA	NA	MODIFIER	NA	NA	58	58	0	67	29	38	56.72
Chewer	42483	chr18	34339028	C	T	*FHOD3*	INTRONIC	ENSG00000134775:[ENST00000445677(c.3787-1543C>T)];ENSG00000134775:[ENST00000592930(c.3182-1543C>T)];ENSG00000134775:[ENST00000257209(c.3901-1543C>T)];ENSG00000134775:[ENST00000590592(c.4426-53C>T)];ENSG00000134775:[ENST00000591635(c.1489-1543C>T)];ENSG00000134775:[ENST00000585579(c.950+3754C>T)];ENSG00000134775:[ENST00000592128(c.838-1543C>T)];ENSG00000134775:[ENST00000359247(c.3850-1543C>T)];	NA	NA	NA	NA	NA	NA	NA	NA	NA_NA	NA	MODIFIER	NA	NA	169	169	0	142	111	31	21.83
Chewer	42483	chr18	29790491	G	T	*GAREM*	INTRONIC	ENSG00000141441:[ENST00000583696(c.66-74068C>A)];	NA	NA	NA	NA	NA	NA	NA	D	BN_BN	D	MODERATE	D	0.938	96	96	0	89	61	28	31.46
Chewer	42483	chr18	44181498	C	A	*LOXHD1*	EXONIC-NC	ENSG00000167210:[ENST00000335730(n.129G>T)];ENSG00000167210:[ENST00000441551(c.884-68G>T)];ENSG00000167210:[ENST00000536736(c.884-68G>T)];	NA	NA	NA	NA	NA	NA	NA	NA	NA_NA	NA	MODIFIER	NA	NA	36	36	0	30	21	9	30
Chewer	42483	chr18	29790491	G	T	*MEP1B*	MISSENSE	ENSG00000141434:MISSENSE[ENST00000269202(c.947G>T,aGc>aTc):ENSP00000269202(p.Ser316Ile)];ENSG00000141434:MISSENSE[ENST00000581447(c.947G>T,aGc>aTc):ENSP00000463280(p.Ser316Ile)];	NA	NA	NA	NA	NA	NA	NA	D	BN_BN	D	MODERATE	D	0.938	96	96	0	89	61	28	31.46
Chewer	42483	chr18	47489488	C	T	*MYO5B*	INTRONIC	ENSG00000167306:[ENST00000285039(c.1323-88G>A)];	NA	NA	NA	NA	NA	NA	NA	NA	NA_NA	NA	MODIFIER	NA	NA	63	63	0	63	38	25	39.68
Chewer	42483	chr18	18628997	A	T	*ROCK1*	INTRONIC	ENSG00000067900:[ENST00000399799(c.414+56T>A)];ENSG00000067900:[ENST00000582445(n.120+56T>A)];	NA	NA	NA	NA	NA	NA	NA	NA	NA_NA	NA	MODIFIER	NA	NA	125	125	0	89	66	23	25.84
Chewer	42483	chr18	20948634	C	A	*TMEM241*	INTRONIC	ENSG00000134490:[ENST00000482411(c.*209+45G>T)];ENSG00000134490:[ENST00000578520(c.484+2752G>T)];ENSG00000134490:[ENST00000473688(c.*37+45G>T)];ENSG00000134490:[ENST00000497608(c.*253+45G>T)];ENSG00000134490:[ENST00000383233(c.566+45G>T)];ENSG00000134490:[ENST00000581444(c.*368+45G>T)];ENSG00000134490:[ENST00000477053(c.*100+5042G>T)];ENSG00000134490:[ENST00000460322(c.*204+45G>T)];ENSG00000134490:[ENST00000577531(c.*212+45G>T)];ENSG00000134490:[ENST00000450466(c.203+45G>T)];ENSG00000134490:[ENST00000583048(n.102+45G>T)];ENSG00000134490:[ENST00000582336(c.530+1546G>T)];ENSG00000134490:[ENST00000542162(c.*13+45G>T)];	NA	NA	NA	NA	NA	NA	NA	NA	NA_NA	NA	MODIFIER	NA	NA	25	25	0	30	23	7	23.33
Chewer	42483	chr19	46640574	C	G	*AC007193.6*	INTRONIC	ENSG00000267922:[ENST00000597989(n.16+36179C>G)];	NA	NA	NA	NA	NA	NA	NA	NA	NA_NA	NA	MODIFIER	NA	NA	291	288	0	250	90	159	63.6
Chewer	42483	chr19	54627327	G	T	*AC012314.8*	EXONIC-NC	ENSG00000237017:[ENST00000452097(n.1396C>A)];	NA	NA	chr19*54627327*54627327*G*A*rs201265344*0*0.9998*0.0001997	NA	NA	NA	NA	NA	NA_NA	NA	MODIFIER	NA	NA	67	67	0	56	26	30	53.57
Chewer	42483	chr19	4222636	G	T	*ANKRD24*	INTRONIC	ENSG00000089847:[ENST00000262970(c.3442-31G>T)];ENSG00000089847:[ENST00000600132(c.3172-31G>T)];ENSG00000089847:[ENST00000318934(c.3172-31G>T)];	NA	NA	NA	NA	NA	NA	NA	NA	NA_NA	NA	MODIFIER	NA	NA	79	78	1	82	38	44	53.66
Chewer	42483	chr19	7505291	G	T	*ARHGEF18*	MISSENSE	ENSG00000104880:MISSENSE[ENST00000359920(c.465G>T,agG>agT):ENSP00000352995(p.Arg155Ser)];ENSG00000104880:5UTR[ENST00000319670(c.-10G>T)];ENSG00000104880:5UTR[ENST00000599752(c.-10G>T)];	NA	NA	NA	NA	NA	NA	NA	T	BN_BN	N	MODERATE	NA	NA	80	80	0	70	31	39	55.71
Chewer	42483	chr19	6682132	C	T	*C3*	INTRONIC	ENSG00000125730:[ENST00000601008(c.242-4163G>A)];ENSG00000125730:[ENST00000596548(c.381+21G>A)];ENSG00000125730:[ENST00000245907(c.4260+21G>A)];ENSG00000125730:[ENST00000599899(n.1219+21G>A)];	NA	NA	NA	NA	NA	NA	NA	NA	NA_NA	NA	MODIFIER	NA	NA	78	77	1	62	22	40	64.52

Supplementary Table 4. List of somatic single nucleotide variants identified using whole exome sequencing of ESCC patients

Cohort	Sample	Chromosome	Genomic position	Reference allele	Alternate allele	Gene symbol	Variant type	Variant annotation	OncoMD	COSMIC	dbSNP	ExAC	ExAC_SAS	1000G	1000G_SAS	SIFT	Polyphen2	CONDEL	VEP	LRT	PhastCon7 Way	Total read (Adjacent normal)	Reference allel read support (Adjacent normal)	Alternative read support (Adjacent normal)	Total read (Tumor)	Reference allel read support (Tumor)	Alternative read support (Tumor)	Alternate allele frequency
Chewer	42483	chr19	33444538	C	G	*CEP89*	MISSENSE	ENSG00000121289:MISSENSE[ENST00000590597(c.475G>C,Gtg>Ctg):ENSP00000466442(p.Val159Leu)];ENSG00000121289:MISSENSE[ENST00000593276(c.389G>C,Gtg>Ctg):ENSP00000467839(p.Val131Leu)];ENSG00000121289:MISSENSE[ENST00000591698(c.223G>C,Gtg>Ctg):ENSP00000467544(p.Val75Leu)];ENSG00000121289:MISSENSE[ENST00000586984(c.475G>C,Gtg>Ctg):ENSP00000465141(p.Val159Leu)];ENSG00000121289:MISSENSE[ENST00000305768(c.475G>C,Gtg>Ctg):ENSP00000306105(p.Val159Leu)];	NA	NA	chr19*33444538*33444538*C*T*rs751090550*NA*NA*NA	NA	NA	NA	NA	T	BN_BN	N	MODERATE	N	0.198	489	489	0	298	285	13	4.36
Chewer	42483	chr19	54652143	G	T	*CNOT3*	SILENT	ENSG00000088038:3UTR[ENST00000447684(c.*647G>T)];ENSG00000088038:EXONIC-NC[ENST00000482247(n.1668G>T)];ENSG00000088038:SILENT[ENST00000358389(c.612G>T,acG>acT):ENSP00000351159(p.Thr204(=))];ENSG00000088038:SILENT[ENST00000440571(c.920G>T,acG>acT):ENSP00000398463(p.Thr307(=))];ENSG00000088038:SILENT[ENST00000406403(c.1155G>T,acG>acT):ENSP00000383954(p.Thr385(=))];ENSG00000088038:SILENT[ENST00000221232(c.1155G>T,acG>acT):ENSP00000221232(p.Thr385(=))];	NA	NA	chr19*54652143*54652143*G*A*rs368619014*NA*NA*NA	NA	NA	NA	NA	NA	NA_NA	NA	LOW	NA	NA	29	29	0	34	6	27	79.41
Chewer	42483	chr19	50549040	A	G	*CTD-2126E3.3*	INTRONIC	ENSG00000269091:[ENST00000599410(n.296-996T>C)];ENSG00000269091:[ENST00000599914(n.182-996T>C)];	NA	NA	0.0001997	8.25E-06	0	0.0001997	0	NA	NA_NA	NA	MODIFIER	NA	NA	125	125	0	119	77	42	35.29
Chewer	42483	chr19	7505291	G	T	*CTD-2207O23.3*	MISSENSE	ENSG00000268861:MISSENSE[ENST00000593531(c.338G>T,agG>agT):ENSP00000471635(p.Arg113Ser)];	NA	NA	NA	NA	NA	NA	NA	T	BN_BN	N	MODERATE	NA	NA	80	80	0	70	31	39	55.71
Chewer	42483	chr19	46295647	C	G	*DMWD*	MISSENSE	ENSG00000185800:MISSENSE[ENST00000377735(c.368G>C,gGa>gCa):ENSP00000366964(p.Gly123Ala)];ENSG00000185800:MISSENSE[ENST00000270223(c.368G>C,gGa>gCa):ENSP00000270223(p.Gly123Ala)];ENSG00000185800:MISSENSE[ENST00000598237(c.49G>C,gGa>gCa):ENSP00000473415(p.Gly17Ala)];ENSG00000185800:EXONIC-NC[ENST00000601370(n.385G>C)];	NA	NA	NA	NA	NA	NA	NA	D_lc	BN_PoD	N	MODERATE	NA	NA	115	115	0	124	47	77	62.1
Chewer	42483	chr19	54140014	G	A	*DPRX*	SILENT	ENSG00000204595:SILENT[ENST00000376650(c.348G>A,ccG>ccA):ENSP00000365838(p.Pro116(=))];	NA	NA	NA	1.65E-05	0	NA	NA	NA	NA_NA	NA	LOW	NA	NA	130	130	0	131	56	75	57.25
Chewer	42483	chr19	1068427	G	A	*HMHA1*	SILENT	ENSG00000180448:INTRONIC[ENST00000587602(c.90+933G>A)];ENSG00000180448:INTRONIC[ENST00000536472(c.-60+933G>A)];ENSG00000180448:INTRONIC[ENST00000591293(n.177+933G>A)];ENSG00000180448:SILENT[ENST00000313093(c.105G>A,aaG>aaA):ENSP00000316772(p.Lys35(=))];ENSG00000180448:SILENT[ENST00000590214(c.186G>A,aaG>aaA):ENSP00000466401(p.Lys62(=))];ENSG00000180448:SILENT[ENST00000539243(c.153G>A,aaG>aaA):ENSP00000439601(p.Lys51(=))];ENSG00000180448:SILENT[ENST00000587186(c.120G>A,aaG>aaA):ENSP00000467741(p.Lys40(=))];ENSG00000180448:SILENT[ENST00000586866(c.117G>A,aaG>aaA):ENSP00000468615(p.Lys39(=))];	NA	NA	NA	NA	NA	NA	NA	NA	NA_NA	NA	MODIFIER	NA	NA	20	20	0	29	20	9	31.03
Chewer	42483	chr19	54868495	A	G	*LAIR1*	INTRONIC	ENSG00000167613:[ENST00000391741(c.360+42T>C)];ENSG00000167613:[ENST00000474878(c.400+42T>C)];ENSG00000167613:[ENST00000391742(c.454+42T>C)];ENSG00000167613:[ENST00000460312(n.702+42T>C)];ENSG00000167613:[ENST00000420483(c.*259+42T>C)];ENSG00000167613:[ENST00000348231(c.403+42T>C)];ENSG00000167613:[ENST00000467269(n.457+42T>C)];ENSG00000167613:[ENST00000440716(c.*635+42T>C)];ENSG00000167613:[ENST00000434277(c.451+42T>C)];ENSG00000167613:[ENST00000498511(n.274+42T>C)];ENSG00000167613:[ENST00000463489(n.387+42T>C)];ENSG00000167613:[ENST00000436513(c.*353+42T>C)];ENSG00000167613:[ENST00000391743(c.400+42T>C)];ENSG00000167613:[ENST00000468656(n.487+42T>C)];ENSG00000167613:[ENST00000313038(c.433+42T>C)];ENSG00000167613:[ENST00000475389(n.462T>C)];ENSG00000167613:[ENST00000418556(c.*34T>C)];	NA	NA	NA	NA	NA	NA	NA	NA	NA_NA	NA	MODIFIER	NA	NA	100	100	0	112	78	34	30.36
Chewer	42483	chr19	54782351	C	A	*LILRB2*	MISSENSE	ENSG00000131042:MISSENSE[ENST00000434421(c.673G>T,Gtg>Ttg):ENSP00000410117(p.Val225Leu)];ENSG00000131042:MISSENSE[ENST00000455108(c.48G>T,Gtg>Ttg):ENSP00000394130(p.Val17Leu)];ENSG00000131042:MISSENSE[ENST00000314446(c.1021G>T,Gtg>Ttg):ENSP00000319960(p.Val341Leu)];ENSG00000131042:MISSENSE[ENST00000391748(c.1021G>T,Gtg>Ttg):ENSP00000375628(p.Val341Leu)];ENSG00000131042:MISSENSE[ENST00000391749(c.1021G>T,Gtg>Ttg):ENSP00000375629(p.Val341Leu)];ENSG00000131042:MISSENSE[ENST00000391746(c.1021G>T,Gtg>Ttg):ENSP00000375626(p.Val341Leu)];ENSG00000131042:EXONIC-NC[ENST00000493242(n.1206G>T)];	NA	NA	chr19*54782351*54782351*C*G,T*rs773342765*NA*NA*NA	NA	NA	NA	NA	NA	NA_NA	NA	MODIFIER	NA	NA	153	153	0	140	73	67	47.86
Chewer	42483	chr19	54254668	G	T	*NA*	INTERGENIC		NA	NA	NA	NA	NA	NA	NA	NA	NA_NA	NA	MODIFIER	NA	NA	100	100	0	61	36	25	40.98
Chewer	42483	chr19	54627327	G	T	*PRPF31*	INTRONIC	ENSG00000105618:[ENST00000498612(n.480+30G>T)];ENSG00000105618:[ENST00000391755(c.697+30G>T)];ENSG00000105618:[ENST00000445124(c.697+30G>T)];ENSG00000105618:[ENST00000419967(c.697+30G>T)];ENSG00000105618:[ENST00000321030(c.697+30G>T)];ENSG00000105618:[ENST00000447810(c.697+30G>T)];ENSG00000105618:[ENST00000445811(c.697+30G>T)];ENSG00000105618:[ENST00000466404(n.567+30G>T)];	NA	NA	chr19*54627327*54627327*G*A*rs201265344*0*0.9998*0.0001997	NA	NA	NA	NA	NA	NA_NA	NA	MODIFIER	NA	NA	67	67	0	56	26	30	53.57
Chewer	42483	chr19	6459632	C	A	*SLC25A23*	MISSENSE	ENSG00000125648:MISSENSE[ENST00000301454(c.8G>T,gGg>gTg):ENSP00000301454(p.Gly3Val)];ENSG00000125648:MISSENSE[ENST00000334510(c.8G>T,gGg>gTg):ENSP00000334537(p.Gly3Val)];ENSG00000125648:MISSENSE[ENST00000264088(c.8G>T,gGg>gTg):ENSP00000264088(p.Gly3Val)];ENSG00000125648:INTRONIC[ENST00000597307(c.-97+1124G>T)];ENSG00000125648:EXONIC-NC[ENST00000595267(n.150G>T)];	NA	NA	NA	NA	NA	NA	NA	NA	NA_NA	NA	MODIFIER	NA	NA	75	75	0	91	35	56	61.54
Chewer	42483	chr19	38885292	G	T	*SPRED3*	MISSENSE	ENSG00000188766:MISSENSE[ENST00000587947(c.433G>T,Gac>Tac):ENSP00000467532(p.Asp145Tyr)];ENSG00000188766:MISSENSE[ENST00000587013(c.565G>T,Gac>Tac):ENSP00000467540(p.Asp189Tyr)];ENSG00000188766:MISSENSE[ENST00000338502(c.433G>T,Gac>Tac):ENSP00000345405(p.Asp145Tyr)];ENSG00000188766:MISSENSE[ENST00000586301(c.433G>T,Gac>Tac):ENSP00000466568(p.Asp145Tyr)];ENSG00000188766:3UTR[ENST00000586958(c.*90G>T)];	NA	NA	NA	NA	NA	NA	NA	T	BN_PoD	N	MODERATE	N	0.999	57	57	0	61	19	42	68.85
Chewer	42483	chr19	46640574	C	G	*TGIF1P1*	EXONIC-NC	ENSG00000259339:[ENST00000558095(n.231G>C)];	NA	NA	NA	NA	NA	NA	NA	NA	NA_NA	NA	MODIFIER	NA	NA	291	288	0	250	90	159	63.6
Chewer	42483	chr19	57802704	G	A	*ZNF460*	SILENT	ENSG00000197714:SILENT[ENST00000537645(c.672G>A,tcG>tcA):ENSP00000446167(p.Ser224(=))];ENSG00000197714:SILENT[ENST00000360338(c.795G>A,tcG>tcA):ENSP00000353491(p.Ser265(=))];	chr19*5780	chr19*5780	NA	5.77E-05	0	NA	NA	NA	NA_NA	NA	LOW	NA	NA	139	139	0	127	47	80	62.99

Mangalaparthi *et al.* , 2020. Mutational landscape of esophageal squamous cell carcinoma in an Indian cohort
Supplementary Table 4. List of somatic single nucleotide variants identified using whole exome sequencing of ESCC patients

Cohort	Sample	Chromosome	Genomic position	Reference allele	Alternate allele	Gene symbol	Variant type	Variant annotation	OncoMD	COSMIC	dbSNP	ExAC	ExAC_SAS	1000G	1000G_SAS	SIFT	Polyphen2	CONDEL	VEP	LRT	PhastCon7 Way	Total read (Adjacent normal)	Reference allel read support (Adjacent normal)	Alternative read support (Adjacent normal)	Total read (Tumor)	Reference allel read support (Tumor)	Alternative read support (Tumor)	Alternate allele frequency
Chewer	42483	chr19	50549040	A	G	*ZNF473*	MISSENSE	ENSG00000142528:MISSENSE[ENST00000270617(c.1340A>G,aAt>aGt):ENSP00000270617(p.Asn447Ser)];ENSG00000142528:MISSENSE[ENST00000595661(c.1340A>G,aAt>aGt):ENSP00000472808(p.Asn447Ser)];ENSG00000142528:MISSENSE[ENST00000391821(c.1340A>G,aAt>aGt):ENSP00000375697(p.Asn447Ser)];ENSG00000142528:MISSENSE[ENST00000445728(c.1304A>G,aAt>aGt):ENSP00000388961(p.Asn435Ser)];ENSG00000142528:INTRONIC[ENST00000601364(c.226+3964A>G)];	NA	NA	0.0001997	8.25E-06	0	0.0001997	0	NA	NA_NA	NA	MODIFIER	NA	NA	125	125	0	119	77	42	35.29
Chewer	42483	chr19	11916849	C	G	*ZNF491*	MISSENSE	ENSG00000177599:MISSENSE[ENST00000450087(c.81C>G,aaC>aaG):ENSP00000392176(p.Asn27Lys)];ENSG00000177599:MISSENSE[ENST00000323169(c.81C>G,aaC>aaG):ENSP00000313443(p.Asn27Lys)];ENSG00000177599:INTRONIC[ENST00000492230(n.266+1367C>G)];	NA	NA	NA	NA	NA	NA	NA	NA	NA_NA	NA	MODIFIER	NA	NA	129	129	0	87	45	42	48.28
Chewer	42483	chr19	53888271	A	G	*ZNF525*	INTRONIC	ENSG00000203326:[ENST00000593918(c.143-6850A>G)];ENSG00000203326:[ENST00000467003(c.*2999A>G)];ENSG00000203326:[ENST00000475179(c.*38A>G)];ENSG00000203326:[ENST00000601790(n.183A>G)];	NA	NA	NA	NA	NA	NA	NA	NA	NA_NA	NA	MODIFIER	NA	NA	149	149	0	131	91	39	29.77
Chewer	42483	chr2	159519854	C	T	*AC005042.4*	INTRONIC	ENSG00000204380:[ENST00000342892(n.654-1592G>A)];	NA	NA	NA	8.25E-06	0	NA	NA	NA	NA_NA	NA	MODIFIER	NA	NA	40	40	0	46	38	8	17.39
Chewer	42483	chr2	91766576	C	G	*AC018696.4*	EXONIC-NC	ENSG00000230964:[ENST00000454518(n.40C>G)];	NA	NA	NA	NA	NA	NA	NA	NA	NA_NA	NA	MODIFIER	NA	NA	462	462	0	318	288	30	9.43
Chewer	42483	chr2	130988992	A	T	*AC018804.7*	EXONIC-NC	ENSG00000213225:[ENST00000452600(n.2041A>T)];ENSG00000213225:[ENST00000450578(n.65A>T)];	NA	NA	NA	NA	NA	NA	NA	NA	NA_NA	NA	MODIFIER	NA	NA	71	71	0	80	49	31	38.75
Chewer	42483	chr2	114709303	G	T	*ACTR3*	MISSENSE	ENSG00000115091:MISSENSE[ENST00000536059(c.772G>T,Gtc>Ttc):ENSP00000445257(p.Val258Phe)];ENSG00000115091:MISSENSE[ENST00000535589(c.805G>T,Gtc>Ttc):ENSP00000444987(p.Val269Phe)];ENSG00000115091:MISSENSE[ENST00000263238(c.958G>T,Gtc>Ttc):ENSP00000263238(p.Val320Phe)];ENSG00000115091:EXONIC-NC[ENST00000478928(n.755G>T)];	NA	NA	NA	NA	NA	NA	NA	NA	NA_NA	NA	MODIFIER	NA	NA	180	179	0	156	136	19	12.18
Chewer	42483	chr2	241814476	C	A	*AGXT*	INTRONIC	ENSG00000172482:[ENST00000476698(n.333-50C>A)];ENSG00000172482:[ENST00000307503(c.681-50C>A)];	NA	NA	NA	NA	NA	NA	NA	NA	NA_NA	NA	MODIFIER	NA	NA	69	69	0	50	38	12	24
Chewer	42483	chr2	198051857	G	C	*ANKRD44*	INTRONIC	ENSG00000065413:[ENST00000443014(c.-48-27C>G)];ENSG00000065413:[ENST00000409153(c.28-27C>G)];ENSG00000065413:[ENST00000539527(c.-110-27C>G)];ENSG00000065413:[ENST00000463879(n.204-27C>G)];ENSG00000065413:[ENST00000447713(c.28-27C>G)];ENSG00000065413:[ENST00000409919(c.28-27C>G)];ENSG00000065413:[ENST00000328737(c.-48-27C>G)];	NA	NA	NA	NA	NA	NA	NA	NA	NA_NA	NA	MODIFIER	NA	NA	103	103	0	86	66	20	23.26
Chewer	42483	chr2	131672610	A	G	*ARHGEF4*	MISSENSE	ENSG00000136002:MISSENSE[ENST00000409359(c.101A>G,aAg>aGg):ENSP00000386794(p.Lys34Arg)];	NA	NA	NA	NA	NA	NA	NA	T	PoD_PoD	N	MODERATE	NA	NA	71	71	0	100	60	40	40
Chewer	42483	chr2	127827499	C	A	*BIN1*	INTRONIC	ENSG00000136717:[ENST00000351659(c.411+72G>T)];ENSG00000136717:[ENST00000316724(c.411+72G>T)];ENSG00000136717:[ENST00000393041(c.411+72G>T)];ENSG00000136717:[ENST00000393040(c.411+72G>T)];ENSG00000136717:[ENST00000348750(c.411+72G>T)];ENSG00000136717:[ENST00000357970(c.411+72G>T)];ENSG00000136717:[ENST00000346226(c.411+72G>T)];ENSG00000136717:[ENST00000409400(c.411+72G>T)];ENSG00000136717:[ENST00000259238(c.411+72G>T)];ENSG00000136717:[ENST00000352848(c.411+72G>T)];ENSG00000136717:[ENST00000376113(c.411+72G>T)];	NA	NA	chr2*127827499*127827499*C*T*rs578076705*0*0.9998*0.0001997	NA	NA	NA	NA	NA	NA_NA	NA	MODIFIER	NA	NA	41	41	0	66	53	13	19.7
Chewer	42483	chr2	241530352	G	C	*CAPN10*	MISSENSE	ENSG00000142330:MISSENSE[ENST00000270361(c.394G>C,Gca>Cca):ENSP00000270361(p.Ala132Pro)];ENSG00000142330:MISSENSE[ENST00000391984(c.394G>C,Gca>Cca):ENSP00000375844(p.Ala132Pro)];ENSG00000142330:MISSENSE[ENST00000416591(c.394G>C,Gca>Cca):ENSP00000400144(p.Ala132Pro)];ENSG00000142330:MISSENSE[ENST00000391982(c.394G>C,Gca>Cca):ENSP00000375842(p.Ala132Pro)];ENSG00000142330:MISSENSE[ENST00000391983(c.394G>C,Gca>Cca):ENSP00000375843(p.Ala132Pro)];ENSG00000142330:MISSENSE[ENST00000354082(c.394G>C,Gca>Cca):ENSP00000270362(p.Ala132Pro)];ENSG00000142330:MISSENSE[ENST00000404753(c.394G>C,Gca>Cca):ENSP00000384422(p.Ala132Pro)];ENSG00000142330:MISSENSE[ENST00000357048(c.394G>C,Gca>Cca):ENSP00000349556(p.Ala132Pro)];ENSG00000142330:INTRONIC[ENST00000270364(c.273+1461G>C)];ENSG00000142330:INTRONIC[ENST00000352879(c.141+3883G>C)];ENSG00000142330:EXONIC-NC[ENST00000494738(n.1052G>C)];	NA	NA	chr2*241530352*241530352*G*T*rs748016007*NA*NA*NA	NA	NA	NA	NA	NA	NA_NA	NA	MODIFIER	NA	NA	56	56	0	64	42	22	34.38
Chewer	42483	chr2	132290304	G	A	*CCDC74A*	MISSENSE	ENSG00000163040:MISSENSE[ENST00000295171(c.826G>A,Gaa>Aaa):ENSP00000295171(p.Glu276Lys)];ENSG00000163040:MISSENSE[ENST00000409856(c.628G>A,Gaa>Aaa):ENSP00000387009(p.Glu210Lys)];ENSG00000163040:SILENT[ENST00000434330(c.492G>A,gcG>gcA):ENSP00000406839(p.Ala164(=))];ENSG00000163040:3UTR[ENST00000467992(c.*925G>A)];	chr2*13229	chr2*13229	NA	1.66E-05	0	NA	NA	NA	NA_NA	NA	MODIFIER	NA	NA	43	43	0	54	45	9	16.67
Chewer	42483	chr2	56599548	G	C	*CCDC85A*	MISSENSE	ENSG00000055813:MISSENSE[ENST00000407595(c.1387G>C,Gcc>Ccc):ENSP00000384040(p.Ala463Pro)];	NA	NA	NA	NA	NA	NA	NA	NA	NA_NA	NA	MODIFIER	NA	NA	44	44	0	39	27	12	30.77
Chewer	42483	chr2	80874768	T	C	*CTNNA2*	MISSENSE	ENSG00000066032:MISSENSE[ENST00000496558(c.2489T>C,cTc>cCc):ENSP00000419295(p.Leu830Pro)];ENSG00000066032:MISSENSE[ENST00000540488(c.2354T>C,cTc>cCc):ENSP00000441705(p.Leu785Pro)];ENSG00000066032:MISSENSE[ENST00000361291(c.2591T>C,cTc>cCc):ENSP00000355398(p.Leu864Pro)];ENSG00000066032:MISSENSE[ENST00000466387(c.2489T>C,cTc>cCc):ENSP00000418191(p.Leu830Pro)];ENSG00000066032:MISSENSE[ENST00000541047(c.2489T>C,cTc>cCc):ENSP00000444675(p.Leu830Pro)];ENSG00000066032:MISSENSE[ENST00000343114(c.1526T>C,cTc>cCc):ENSP00000341500(p.Leu509Pro)];ENSG00000066032:MISSENSE[ENST00000402739(c.2633T>C,cTc>cCc):ENSP00000384638(p.Leu878Pro)];	NA	NA	NA	NA	NA	NA	NA	T	PrD_PrD	D	MODERATE	N	1	74	74	0	51	37	14	27.45

Supplementary Table 4. List of somatic single nucleotide variants identified using whole exome sequencing of ESCC patients

Cohort	Sample	Chromosome	Genomic position	Reference allele	Alternate allele	Gene symbol	Variant type	Variant annotation	OncoMD	COSMIC	dbSNP	ExAC	ExAC_SAS	1000G	1000G_SAS	SIFT	Polyphen2	CONDEL	VEP	LRT	PhastCon7 Way	Total read (Adjacent normal)	Reference allel read support (Adjacent normal)	Alternative read support (Adjacent normal)	Total read (Tumor)	Reference allel read support (Tumor)	Alternative read support (Tumor)	Alternate allele frequency
Chewer	42483	chr2	74593071	G	A	*DCTN1*	SILENT	ENSG00000204843:SILENT[ENST00000407639(c.2433C>T,ctC>ctT):ENSP00000384844(p.Leu811(=))];ENSG00000204843:SILENT[ENST00000409240(c.2724C>T,ctC>ctT):ENSP00000386406(p.Leu908(=))];ENSG00000204843:SILENT[ENST00000394003(c.2814C>T,ctC>ctT):ENSP00000377571(p.Leu938(=))];ENSG00000204843:SILENT[ENST00000361874(c.2835C>T,ctC>ctT):ENSP00000354791(p.Leu945(=))];ENSG00000204843:SILENT[ENST00000409567(c.2775C>T,ctC>ctT):ENSP00000386843(p.Leu925(=))];ENSG00000204843:SILENT[ENST00000409868(c.2784C>T,ctC>ctT):ENSP00000387327(p.Leu928(=))];ENSG00000204843:SILENT[ENST00000409438(c.2433C>T,ctC>ctT):ENSP00000387270(p.Leu811(=))];ENSG00000204843:3UTR[ENST00000434055(c.*134C>T)];ENSG00000204843:EXONIC-NC[ENST00000495895(n.251C>T)];ENSG00000204843:EXONIC-NC[ENST00000466110(n.3814C>T)];ENSG00000204843:INTRONIC[ENST00000497666(n.96+3356C>T)];	NA	NA	.	1.65E-05	0	NA	NA	NA	NA_NA	NA	MODIFIER	NA	NA	77	77	0	83	52	31	37.35
Chewer	42483	chr2	196749356	G	T	*DNAH7*	MISSENSE	ENSG00000118997:MISSENSE[ENST00000312428(c.5716C>A,Cca>Aca):ENSP00000311273(p.Pro1906Thr)];	NA	NA	NA	NA	NA	NA	NA	T	BN_BN	N	MODERATE	N	0.685	112	112	0	87	70	17	19.54
Chewer	42483	chr2	225760985	C	A	*DOCK10*	INTRONIC	ENSG00000135905:[ENST00000474102(n.352+27G>T)];ENSG00000135905:[ENST00000258390(c.416+27G>T)];ENSG00000135905:[ENST00000435582(c.416+27G>T)];ENSG00000135905:[ENST00000492369(n.484+27G>T)];ENSG00000135905:[ENST00000471810(n.390+27G>T)];ENSG00000135905:[ENST00000409592(c.398+27G>T)];	NA	NA	NA	NA	NA	NA	NA	NA	NA_NA	NA	MODIFIER	NA	NA	204	203	0	190	125	65	34.21
Chewer	42483	chr2	162891796	G	A	*DPP4*	MISSENSE	ENSG00000197635:MISSENSE[ENST00000360534(c.652C>T,Cca>Tca):ENSP00000353731(p.Pro218Ser)];ENSG00000197635:3UTR[ENST00000434918(c.*140C>T)];ENSG00000197635:EXONIC-NC[ENST00000490286(n.646C>T)];	NA	NA	NA	NA	NA	NA	NA	NA	NA_NA	NA	MODIFIER	NA	NA	135	134	0	109	64	45	41.28
Chewer	42483	chr2	242626206	C	G	*DTYMK*	5UTR	ENSG00000168393:[ENST00000400770(c.-8G>C)];ENSG00000168393:[ENST00000305784(c.-8G>C)];	NA	NA	NA	NA	NA	NA	NA	NA	NA_NA	NA	MODIFIER	NA	NA	43	43	0	31	19	12	38.71
Chewer	42483	chr2	141272325	C	T	*LRP1B*	NONSENSE	ENSG00000168702:NONSENSE[ENST00000389484(c.8166G>A,tgG>tgA):ENSP00000374135(p.Trp2722Ter)];	chr2*14127	chr2*14127	NA	NA	NA	NA	NA	NA	NA_NA	NA	HIGH	U	1	167	167	0	94	70	23	24.47
Chewer	42483	chr2	44173249	C	A	*LRPPRC*	INTRONIC-SS-DNR	ENSG00000138095:[ENST00000260665(c.2210+3G>T)];	NA	NA	NA	NA	NA	NA	NA	NA	NA_NA	NA	LOW	NA	NA	191	190	0	143	101	42	29.37
Chewer	42483	chr2	149241092	G	T	*MBD5*	MISSENSE	ENSG00000204406:MISSENSE[ENST00000404807(c.2932G>T,Ggg>Tgg):ENSP00000384672(p.Gly978Trp)];ENSG00000204406:MISSENSE[ENST00000416015(c.2151G>T,Ggg>Tgg):ENSP00000393168(p.Gly718Trp)];ENSG00000204406:INTRONIC[ENST00000407073(c.2845+87G>T)];	NA	NA	NA	NA	NA	NA	NA	NA	NA_NA	NA	MODIFIER	NA	NA	158	155	2	102	78	24	23.53
Chewer	42483	chr2	128364971	G	T	*MYO7B*	MISSENSE	ENSG00000169994:MISSENSE[ENST00000389524(c.2615G>T,cGg>cTg):ENSP00000374175(p.Arg872Leu)];ENSG00000169994:MISSENSE[ENST00000409816(c.2615G>T,cGg>cTg):ENSP00000386461(p.Arg872Leu)];ENSG00000169994:MISSENSE[ENST00000428314(c.2615G>T,cGg>cTg):ENSP00000415090(p.Arg872Leu)];	NA	NA	chr2*128364971*128364971*G*A*rs772262646*NA*NA*NA	NA	NA	NA	NA	D	PrD_PrD	D	MODERATE	D	0.196	41	41	0	68	43	25	36.76
Chewer	42483	chr2	132233753	C	A	*MZT2A*	INTRONIC	ENSG00000173272:[ENST00000445782(n.331-3998G>T)];ENSG00000173272:[ENST00000427024(c.279-3998G>T)];	NA	NA	chr2*132233753*132233753*C*T*rs769925507*NA*NA*NA	NA	NA	NA	NA	NA	NA_NA	NA	MODIFIER	NA	NA	30	30	0	53	36	17	32.08
Chewer	42483	chr2	203914565	C	G	*NBEAL1*	MISSENSE	ENSG00000144426:MISSENSE[ENST00000449802(c.172C>G,Ctg>Gtg):ENSP00000399903(p.Leu58Val)];ENSG00000144426:EXONIC-NC[ENST00000460416(n.192C>G)];ENSG00000144426:EXONIC-NC[ENST00000478884(n.459C>G)];ENSG00000144426:EXONIC-NC[ENST00000497505(n.505C>G)];	NA	NA	NA	NA	NA	NA	NA	NA	NA_NA	NA	MODIFIER	NA	NA	171	171	0	126	93	33	26.19
Chewer	42483	chr2	203977856	G	T	*NBEAL1*	MISSENSE	ENSG00000144426:MISSENSE[ENST00000449802(c.2234G>T,gGa>gTa):ENSP00000399903(p.Gly745Val)];	NA	NA	NA	NA	NA	NA	NA	D	PoD_PoD	D	MODERATE	NA	NA	149	149	0	126	99	26	20.63
Chewer	42483	chr2	50780009	G	C	*NRXN1*	MISSENSE	ENSG00000179915:MISSENSE[ENST00000405472(c.1451C>G,tCt>tGt):ENSP00000434015(p.Ser484Cys)];ENSG00000179915:MISSENSE[ENST00000406316(c.1475C>G,tCt>tGt):ENSP00000384311(p.Ser492Cys)];ENSG00000179915:MISSENSE[ENST00000401669(c.1475C>G,tCt>tGt):ENSP00000385017(p.Ser492Cys)];ENSG00000179915:MISSENSE[ENST00000406859(c.1475C>G,tCt>tGt):ENSP00000385681(p.Ser492Cys)];ENSG00000179915:MISSENSE[ENST00000404971(c.1595C>G,tCt>tGt):ENSP00000385142(p.Ser532Cys)];ENSG00000179915:MISSENSE[ENST00000402717(c.1451C>G,tCt>tGt):ENSP00000385434(p.Ser484Cys)];ENSG00000179915:EXONIC-NC[ENST00000495871(n.186C>G)];ENSG00000179915:EXONIC-NC[ENST00000331040(n.682C>G)];	NA	NA	NA	NA	NA	NA	NA	NA	NA_NA	NA	MODIFIER	NA	NA	203	203	0	156	97	59	37.82
Chewer	42483	chr2	159519854	C	T	*PKP4*	MISSENSE	ENSG00000144283:MISSENSE[ENST00000389759(c.2474C>T,tCg>tTg):ENSP00000374409(p.Ser825Leu)];ENSG00000144283:MISSENSE[ENST00000389757(c.2474C>T,tCg>tTg):ENSP00000374407(p.Ser825Leu)];ENSG00000144283:3UTR[ENST00000426248(c.*1743C>T)];ENSG00000144283:EXONIC-NC[ENST00000483881(n.102C>T)];ENSG00000144283:EXONIC-NC[ENST00000495123(n.170C>T)];ENSG00000144283:EXONIC-NC[ENST00000480171(n.918C>T)];	NA	NA	NA	8.25E-06	0	NA	NA	NA	NA_NA	NA	MODIFIER	NA	NA	40	40	0	46	38	8	17.39
Chewer	42483	chr2	220162056	C	G	*PTPRN*	MISSENSE	ENSG00000054356:MISSENSE[ENST00000409251(c.1900G>C,Gac>Cac):ENSP00000386638(p.Asp634His)];ENSG00000054356:MISSENSE[ENST00000423636(c.1717G>C,Gac>Cac):ENSP00000392598(p.Asp573His)];ENSG00000054356:MISSENSE[ENST00000295718(c.1987G>C,Gac>Cac):ENSP00000295718(p.Asp663His)];ENSG00000054356:EXONIC-NC[ENST00000462351(n.2461G>C)];	NA	NA	chr2*220162056*220162056*C*T*rs752312263*NA*NA*NA	NA	NA	NA	NA	NA	NA_NA	NA	MODIFIER	NA	NA	67	67	0	69	59	10	14.49
Chewer	42483	chr2	56599548	G	C	*RP11-482H16.1*	INTRONIC	ENSG00000271894:[ENST00000607540(n.384-11853G>C)];	NA	NA	NA	NA	NA	NA	NA	NA	NA_NA	NA	MODIFIER	NA	NA	44	44	0	39	27	12	30.77
Chewer	42483	chr2	242283249	G	T	*SEPT2*	MISSENSE	ENSG00000168385:MISSENSE[ENST00000391973(c.779G>T,tGg>tTg):ENSP00000375834(p.Trp260Leu)];ENSG00000168385:MISSENSE[ENST00000360051(c.779G>T,tGg>tTg):ENSP00000353157(p.Trp260Leu)];ENSG00000168385:MISSENSE[ENST00000407971(c.659G>T,tGg>tTg):ENSP00000384525(p.Trp220Leu)];ENSG00000168385:MISSENSE[ENST00000391971(c.779G>T,tGg>tTg):ENSP00000375832(p.Trp260Leu)];ENSG00000168385:MISSENSE[ENST00000401990(c.809G>T,tGg>tTg):ENSP00000385109(p.Trp270Leu)];ENSG00000168385:MISSENSE[ENST00000402092(c.779G>T,tGg>tTg):ENSP00000385172(p.Trp260Leu)];ENSG00000168385:MISSENSE[ENST00000421717(c.344G>T,tGg>tTg):ENSP00000408296(p.Trp115Leu)];ENSG00000168385:EXONIC-NC[ENST00000481500(n.256G>T)];ENSG00000168385:EXONIC-NC[ENST00000484167(n.665G>T)];	NA	NA	NA	NA	NA	NA	NA	NA	NA_NA	NA	MODIFIER	NA	NA	121	121	0	108	87	21	19.44

Mangalaparthi *et al.* , 2020. Mutational landscape of esophageal squamous cell carcinoma in an Indian cohort
Supplementary Table 4. List of somatic single nucleotide variants identified using whole exome sequencing of ESCC patients

Cohort	Sample	Chromosome	Genomic position	Reference allele	Alternate allele	Gene symbol	Variant type	Variant annotation	OncoMD	COSMIC	dbSNP	ExAC	ExAC_SAS	1000G	1000G_SAS	SIFT	Polyphen2	CONDEL	VEP	LRT	PhastCon7 Way	Total read (Adjacent normal)	Reference allele read support (Adjacent normal)	Alternative read support (Adjacent normal)	Total read (Tumor)	Reference allele read support (Tumor)	Alternative read support (Tumor)	Alternate allele frequency
Chewer	42483	chr2	201397791	G	C	*SGOL2*	MISSENSE	ENSG00000163535:MISSENSE[ENST00000409203(c.93G>C,ttG>ttC):ENSP00000386249(p.Leu31Phe)];ENSG00000163535:MISSENSE[ENST00000357799(c.93G>C,ttG>ttC):ENSP00000350447(p.Leu31Phe)];ENSG00000163535:MISSENSE[ENST00000418045(c.93G>C,ttG>ttC):ENSP00000393325(p.Leu31Phe)];ENSG00000163535:EXONIC-NC[ENST00000460534(n.222G>C)];	NA	NA	NA	NA	NA	NA	NA	NA	NA_NA	NA	MODIFIER	NA	NA	36	36	0	21	13	8	38.1
Chewer	42483	chr2	174829901	G	C	*SP3*	INTRONIC	ENSG00000172845:[ENST00000462904(n.123+15C>G)];ENSG00000172845:[ENST00000455789(c.-38C>G)];ENSG00000172845:[ENST00000310015(c.-2C>G)];	NA	NA	NA	NA	NA	NA	NA	NA	NA_NA	NA	MODIFIER	NA	NA	31	31	0	18	12	6	33.33
Chewer	42483	chr2	160019989	G	T	*TANC1*	MISSENSE	ENSG00000115183:MISSENSE[ENST00000263635(c.878G>T,gGa>gTa):ENSP00000263635(p.Gly293Val)];ENSG00000115183:MISSENSE[ENST00000454300(c.560G>T,gGa>gTa):ENSP00000396339(p.Gly187Val)];	NA	NA	NA	NA	NA	NA	NA	T	PoD_PoD	D	MODERATE	D	0.998	37	37	0	30	18	12	40
Chewer	42483	chr2	39996918	G	C	*THUMPD2*	MISSENSE	ENSG00000138050:MISSENSE[ENST00000454352(c.514C>G,Cag>Gag):ENSP00000392122(p.Gln172Glu)];ENSG00000138050:MISSENSE[ENST00000510781(c.517C>G,Cag>Gag):ENSP00000426202(p.Gln173Glu)];ENSG00000138050:MISSENSE[ENST00000505747(c.604C>G,Cag>Gag):ENSP00000423933(p.Gln202Glu)];ENSG00000138050:MISSENSE[ENST00000378727(c.604C>G,Cag>Gag):ENSP00000368001(p.Gln202Glu)];ENSG00000138050:MISSENSE[ENST00000260619(c.514C>G,Cag>Gag):ENSP00000260619(p.Gln172Glu)];ENSG00000138050:EXONIC-NC[ENST00000403537(n.604C>G)];ENSG00000138050:EXONIC-NC[ENST00000460072(n.236C>G)];ENSG00000138050:3UTR[ENST00000530522(c.*309C>G)];	NA	NA	NA	NA	NA	NA	NA	T	BN_BN	N	MODERATE	N	0.141	101	101	0	41	28	13	31.71
Chewer	42483	chr2	193049159	C	G	*TMEFF2*	MISSENSE	ENSG00000144339:MISSENSE[ENST00000409056(c.333G>C,caG>caC):ENSP00000386871(p.Gln111His)];ENSG00000144339:MISSENSE[ENST00000272771(c.333G>C,caG>caC):ENSP00000272771(p.Gln111His)];ENSG00000144339:MISSENSE[ENST00000392314(c.333G>C,caG>caC):ENSP00000376128(p.Gln111His)];	NA	NA	NA	NA	NA	NA	NA	D	BN_PoD	N	MODERATE	D	1	57	57	0	58	48	10	17.24
Chewer	42483	chr2	179431867	C	T	*TTN*	MISSENSE	ENSG00000155657:MISSENSE[ENST00000359218(c.52172G>A,cGa>cAa):ENSP00000352154(p.Arg17391Gln)];ENSG00000155657:MISSENSE[ENST00000591111(c.74069G>A,cGa>cAa):ENSP00000465570(p.Arg24690Gln)];ENSG00000155657:MISSENSE[ENST00000589042(c.78992G>A,cGa>cAa):ENSP00000467141(p.Arg26331Gln)];ENSG00000155657:MISSENSE[ENST00000342175(c.52373G>A,cGa>cAa):ENSP00000340554(p.Arg17458Gln)];ENSG00000155657:MISSENSE[ENST00000342992(c.71288G>A,cGa>cAa):ENSP00000343764(p.Arg23763Gln)];ENSG00000155657:MISSENSE[ENST00000460472(c.51797G>A,cGa>cAa):ENSP00000434586(p.Arg17266Gln)];	NA	NA	NA	4.15E-05	6.06E-05	NA	NA	NA	NA_NA	NA	MODIFIER	NA	NA	237	237	0	166	102	64	38.55
Chewer	42483	chr2	179439987	G	A	*TTN*	SILENT	ENSG00000155657:SILENT[ENST00000359218(c.44052C>T,gtC>gtT):ENSP00000352154(p.Val14684(=))];ENSG00000155657:SILENT[ENST00000591111(c.65949C>T,gtC>gtT):ENSP00000465570(p.Val21983(=))];ENSG00000155657:SILENT[ENST00000589042(c.70872C>T,gtC>gtT):ENSP00000467141(p.Val23624(=))];ENSG00000155657:SILENT[ENST00000342175(c.44253C>T,gtC>gtT):ENSP00000340554(p.Val14751(=))];ENSG00000155657:SILENT[ENST00000342992(c.63168C>T,gtC>gtT):ENSP00000343764(p.Val21056(=))];ENSG00000155657:SILENT[ENST00000460472(c.43677C>T,gtC>gtT):ENSP00000434586(p.Val14559(=))];	NA	NA	NA	NA	NA	NA	NA	NA	NA_NA	NA	MODIFIER	NA	NA	127	127	0	127	96	31	24.41
Chewer	42483	chr2	179596487	G	T	*TTN*	SILENT	ENSG00000155657:SILENT[ENST00000342992(c.13383C>A,ggC>ggA):ENSP00000343764(p.Gly4461(=))];ENSG00000155657:SILENT[ENST00000589042(c.17115C>A,ggC>ggA):ENSP00000467141(p.Gly5705(=))];ENSG00000155657:SILENT[ENST00000591111(c.16164C>A,ggC>ggA):ENSP00000465570(p.Gly5388(=))];ENSG00000155657:INTRONIC[ENST00000342175(c.13858+6322C>A)];ENSG00000155657:INTRONIC[ENST00000359218(c.13657+6322C>A)];ENSG00000155657:INTRONIC[ENST00000460472(c.13282+6322C>A)];	NA	NA	chr2*179596487*179596487*G*A,C*rs370036981*1*0.9992*0.0007987,.	NA	NA	NA	NA	NA	NA_NA	NA	MODIFIER	NA	NA	171	171	0	132	102	30	22.73
Chewer	42483	chr2	179669353	G	T	*TTN*	MISSENSE	ENSG00000155657:MISSENSE[ENST00000412264(c.17C>A,cCg>cAg):ENSP00000394672(p.Pro6Gln)];ENSG00000155657:MISSENSE[ENST00000359218(c.17C>A,cCg>cAg):ENSP00000352154(p.Pro6Gln)];ENSG00000155657:MISSENSE[ENST00000342175(c.17C>A,cCg>cAg):ENSP00000340554(p.Pro6Gln)];ENSG00000155657:MISSENSE[ENST00000342992(c.17C>A,cCg>cAg):ENSP00000343764(p.Pro6Gln)];ENSG00000155657:MISSENSE[ENST00000591111(c.17C>A,cCg>cAg):ENSP00000465570(p.Pro6Gln)];ENSG00000155657:MISSENSE[ENST00000589042(c.17C>A,cCg>cAg):ENSP00000467141(p.Pro6Gln)];ENSG00000155657:MISSENSE[ENST00000360870(c.17C>A,cCg>cAg):ENSP00000354117(p.Pro6Gln)];ENSG00000155657:MISSENSE[ENST00000460472(c.17C>A,cCg>cAg):ENSP00000434586(p.Pro6Gln)];ENSG00000155657:EXONIC-NC[ENST00000470257(n.227C>A)];	NA	NA	chr2*179669353*179669353*G*A*rs201490999*NA*NA*NA	NA	NA	NA	NA	NA	NA_NA	NA	MODERATE	NA	NA	59	59	0	48	35	13	27.08
Chewer	42483	chr2	179431867	C	T	*TTN-AS1*	INTRONIC	ENSG00000237298:[ENST00000592630(n.433+29449C>T)];ENSG00000237298:[ENST00000592689(n.462+24779C>T)];ENSG00000237298:[ENST00000438095(n.195-4160C>T)];ENSG00000237298:[ENST00000590807(n.75-16384C>T)];ENSG00000237298:[ENST00000586707(n.346-16573C>T)];ENSG00000237298:[ENST00000586831(n.215-15432C>T)];ENSG00000237298:[ENST00000585451(n.198+43504C>T)];ENSG00000237298:[ENST00000592600(n.346-17433C>T)];ENSG00000237298:[ENST00000592750(n.346-30456C>T)];ENSG00000237298:[ENST00000419746(n.2044-15432C>T)];ENSG00000237298:[ENST00000456053(n.414-4160C>T)];ENSG00000237298:[ENST00000586452(n.75-4160C>T)];ENSG00000237298:[ENST00000591332(n.443-30456C>T)];ENSG00000237298:[ENST00000590932(n.615+24779C>T)];	NA	NA	NA	4.15E-05	6.06E-05	NA	NA	NA	NA_NA	NA	MODIFIER	NA	NA	237	237	0	166	102	64	38.55

Mangalaparthi *et al.* , 2020. Mutational landscape of esophageal squamous cell carcinoma in an Indian cohort

Supplementary Table 4. List of somatic single nucleotide variants identified using whole exome sequencing of ESCC patients

Cohort	Sample	Chromosome	Genomic position	Reference allele	Alternate allele	Gene symbol	Variant type	Variant annotation	OncoMD	COSMIC	dbSNP	ExAC	ExAC_SAS	1000G	1000G_SAS	SIFT	Polyphen2	CONDEL	VEP	LRT	PhastCon7 Way	Total read (Adjacent normal)	Reference allel read support (Adjacent normal)	Alternative read support (Adjacent normal)	Total read (Tumor)	Reference allel read support (Tumor)	Alternative read support (Tumor)	Alternate allele frequency
Chewer	42483	chr2	179439987	G	A	*TTN-AS1*	INTRONIC	ENSG00000237298:[ENST00000592630(n.434-22336G>A)];ENSG00000237298:[ENST00000592689(n.463-22336G>A)];ENSG00000237298:[ENST00000438095(n.344+3811G>A)];ENSG00000237298:[ENST00000590807(n.75-8264G>A)];ENSG00000237298:[ENST00000586707(n.346-8453G>A)];ENSG00000237298:[ENST00000586831(n.215-7312G>A)];ENSG00000237298:[ENST00000585451(n.198+51624G>A)];ENSG00000237298:[ENST00000592600(n.346-9313G>A)];ENSG00000237298:[ENST00000592750(n.346-22336G>A)];ENSG00000237298:[ENST00000419746(n.2044-7312G>A)];ENSG00000237298:[ENST00000456053(n.563+3811G>A)];ENSG00000237298:[ENST00000586452(n.224+3811G>A)];ENSG00000237298:[ENST00000591332(n.443-22336G>A)];ENSG00000237298:[ENST00000590932(n.616-22336G>A)];	NA	NA	NA	NA	NA	NA	NA	NA	NA_NA	NA	MODIFIER	NA	NA	127	127	0	127	96	31	24.41
Chewer	42483	chr2	179596487	G	T	*TTN-AS1*	INTRONIC	ENSG00000237298:[ENST00000585451(n.199-29180G>T)];	NA	NA	chr2*179596487*179596487*G*A,C*rs370036981*1*0.9992*0.0007987,.	NA	NA	NA	NA	NA	NA_NA	NA	MODIFIER	NA	NA	171	171	0	132	102	30	22.73
Chewer	42483	chr2	132233753	C	A	*TUBA3D*	5UTR	ENSG00000075886:[ENST00000321253(c.-20C>A)];	NA	NA	chr2*132233753*132233753*C*T*rs769925507*NA*NA*NA	NA	NA	NA	NA	NA	NA_NA	NA	MODIFIER	NA	NA	30	30	0	53	36	17	32.08
Chewer	42483	chr2	234621763	C	T	*UGT1A10*	INTRONIC	ENSG00000242515:[ENST00000373445(c.856-53917C>T)];ENSG00000242515:[ENST00000344644(c.856-53917C>T)];	NA	NA	NA	NA	NA	NA	NA	NA	NA_NA	NA	MODIFIER	NA	NA	45	45	0	54	43	11	20.37
Chewer	42483	chr2	234621763	C	T	*UGT1A5*	SILENT	ENSG00000240224:SILENT[ENST00000373414(c.126C>T,ctC>ctT):ENSP00000362513(p.Leu42(=))];	NA	NA	NA	NA	NA	NA	NA	NA	NA_NA	NA	MODIFIER	NA	NA	45	45	0	54	43	11	20.37
Chewer	42483	chr2	234621763	C	T	*UGT1A6*	INTRONIC	ENSG00000167165:[ENST00000305139(c.861+19252C>T)];ENSG00000167165:[ENST00000446481(c.60+19252C>T)];ENSG00000167165:[ENST00000373424(c.60+19252C>T)];ENSG00000167165:[ENST00000480628(n.186+19252C>T)];ENSG00000167165:[ENST00000478062(n.182-16387C>T)];ENSG00000167165:[ENST00000406651(c.60+19252C>T)];ENSG00000167165:[ENST00000484784(c.202-4676C>T)];	NA	NA	NA	NA	NA	NA	NA	NA	NA_NA	NA	MODIFIER	NA	NA	45	45	0	54	43	11	20.37
Chewer	42483	chr2	234621763	C	T	*UGT1A7*	INTRONIC	ENSG00000244122:[ENST00000373426(c.855+30325C>T)];	NA	NA	NA	NA	NA	NA	NA	NA	NA_NA	NA	MODIFIER	NA	NA	45	45	0	54	43	11	20.37
Chewer	42483	chr2	234621763	C	T	*UGT1A8*	SILENT	ENSG00000241635:SILENT[ENST00000608381(c.126C>T,ctC>ctT):ENSP00000477394(p.Leu42(=))];ENSG00000241635:INTRONIC[ENST00000609637(c.855+40328C>T)];ENSG00000241635:INTRONIC[ENST00000373450(c.856-53917C>T)];	NA	NA	NA	NA	NA	NA	NA	NA	NA_NA	NA	MODIFIER	NA	NA	45	45	0	54	43	11	20.37
Chewer	42483	chr2	234621763	C	T	*UGT1A9*	INTRONIC	ENSG00000241119:[ENST00000354728(c.855+40328C>T)];	NA	NA	NA	NA	NA	NA	NA	NA	NA_NA	NA	MODIFIER	NA	NA	45	45	0	54	43	11	20.37
Chewer	42483	chr20	3565421	G	C	*ATRN*	MISSENSE	ENSG00000088812:MISSENSE[ENST00000446916(c.3078G>C,aaG>aaC):ENSP00000416587(p.Lys1026Asn)];ENSG00000088812:MISSENSE[ENST00000262919(c.3078G>C,aaG>aaC):ENSP00000262919(p.Lys1026Asn)];	NA	NA	NA	NA	NA	NA	NA	T	BN_BN	N	MODERATE	N	0.998	162	162	0	155	116	39	25.16
Chewer	42483	chr20	31609001	G	T	*BPIFB2*	INTRONIC	ENSG00000078898:[ENST00000170150(c.1195-86G>T)];	NA	NA	NA	NA	NA	NA	NA	NA	NA_NA	NA	MODIFIER	NA	NA	63	63	0	62	46	16	25.81
Chewer	42483	chr20	36470813	G	A	*CTNNBL1*	MISSENSE	ENSG00000132792:MISSENSE[ENST00000405275(c.1303G>A,Gaa>Aaa):ENSP00000384355(p.Glu435Lys)];ENSG00000132792:MISSENSE[ENST00000373469(c.628G>A,Gaa>Aaa):ENSP00000362568(p.Glu210Lys)];ENSG00000132792:MISSENSE[ENST00000361383(c.1384G>A,Gaa>Aaa):ENSP00000355050(p.Glu462Lys)];ENSG00000132792:MISSENSE[ENST00000373473(c.823G>A,Gaa>Aaa):ENSP00000362572(p.Glu275Lys)];ENSG00000132792:EXONIC-NC[ENST00000472237(n.158G>A)];ENSG00000132792:EXONIC-NC[ENST00000473857(n.2566G>A)];	NA	NA	NA	NA	NA	NA	NA	NA	NA_NA	NA	MODIFIER	NA	NA	56	56	0	51	23	28	54.9
Chewer	42483	chr20	5540758	C	A	*GPCPD1*	INTRONIC	ENSG00000125772:[ENST00000481038(n.2804-36G>T)];ENSG00000125772:[ENST00000418646(c.307-1293G>T)];ENSG00000125772:[ENST00000379019(c.1396-36G>T)];	NA	NA	NA	NA	NA	NA	NA	NA	NA_NA	NA	MODIFIER	NA	NA	81	81	0	100	78	22	22
Chewer	42483	chr20	746185	C	A	*SLC52A3*	SILENT	ENSG00000101276:EXONIC-NC[ENST00000473664(n.285G>T)];ENSG00000101276:SILENT[ENST00000381944(c.234G>T,ctG>ctT):ENSP00000371370(p.Leu78(=))];ENSG00000101276:SILENT[ENST00000217254(c.234G>T,ctG>ctT):ENSP00000217254(p.Leu78(=))];	NA	NA	NA	NA	NA	NA	NA	NA	NA_NA	NA	LOW	NA	NA	68	67	1	95	71	24	25.26
Chewer	42483	chr20	36784358	G	T	*TGM2*	SILENT	ENSG00000198959:SILENT[ENST00000373403(c.324C>A,gcC>gcA):ENSP00000362502(p.Ala108(=))];ENSG00000198959:SILENT[ENST00000453095(c.324C>A,gcC>gcA):ENSP00000387642(p.Ala108(=))];ENSG00000198959:SILENT[ENST00000361475(c.324C>A,gcC>gcA):ENSP00000355330(p.Ala108(=))];ENSG00000198959:SILENT[ENST00000536724(c.144C>A,gcC>gcA):ENSP00000437479(p.Ala48(=))];ENSG00000198959:EXONIC-NC[ENST00000468262(n.408C>A)];ENSG00000198959:EXONIC-NC[ENST00000474777(n.385C>A)];ENSG00000198959:INTRONIC[ENST00000536701(c.191-4899C>A)];	NA	NA	NA	NA	NA	NA	NA	NA	NA_NA	NA	MODIFIER	NA	NA	38	37	0	47	22	24	51.06
Chewer	42483	chr20	57766308	G	A	*ZNF831*	SILENT	ENSG00000124203:SILENT[ENST00000371030(c.234G>A,gtG>gtA):ENSP00000360069(p.Val78(=))];	NA	NA	NA	NA	NA	NA	NA	NA	NA_NA	NA	LOW	NA	NA	70	70	0	80	44	36	45
Chewer	42483	chr21	46074368	C	A	*KRTAP12-4*	MISSENSE	ENSG00000212933:MISSENSE[ENST00000391618(c.164G>T,tGc>tTc):ENSP00000375476(p.Cys55Phe)];	NA	NA	NA	NA	NA	NA	NA	NA	NA_NA	NA	MODIFIER	NA	NA	28	28	0	33	13	19	57.58
Chewer	42483	chr21	46074368	C	A	*TSPEAR*	INTRONIC	ENSG00000175894:[ENST00000323084(c.82+56980G>T)];	NA	NA	NA	NA	NA	NA	NA	NA	NA_NA	NA	MODIFIER	NA	NA	28	28	0	33	13	19	57.58
Chewer	42483	chr22	19965585	C	T	*ARVCF*	MISSENSE	ENSG00000099889:MISSENSE[ENST00000263207(c.1594G>A,Gat>Aat):ENSP00000263207(p.Asp532Asn)];ENSG00000099889:MISSENSE[ENST00000406522(c.1405G>A,Gat>Aat):ENSP00000384732(p.Asp469Asn)];ENSG00000099889:MISSENSE[ENST00000344269(c.1405G>A,Gat>Aat):ENSP00000342042(p.Asp469Asn)];ENSG00000099889:MISSENSE[ENST00000406259(c.1594G>A,Gat>Aat):ENSP00000385444(p.Asp532Asn)];ENSG00000099889:MISSENSE[ENST00000401994(c.1405G>A,Gat>Aat):ENSP00000384341(p.Asp469Asn)];ENSG00000099889:EXONIC-NC[ENST00000495096(n.534G>A)];	NA	NA	NA	5.16E-05	0.000203087	NA	NA	NA	NA_NA	NA	MODIFIER	NA	NA	86	86	0	73	50	23	31.51
Chewer	42483	chr22	18003242	G	A	*CECR2*	SILENT	ENSG00000099954:SILENT[ENST00000400573(c.987G>A,caG>caA):ENSP00000383417(p.Gln329(=))];ENSG00000099954:SILENT[ENST00000400585(c.564G>A,caG>caA):ENSP00000383428(p.Gln188(=))];ENSG00000099954:SILENT[ENST00000342247(c.843G>A,caG>caA):ENSP00000341219(p.Gln281(=))];ENSG00000099954:SILENT[ENST00000262608(c.930G>A,caG>caA):ENSP00000262608(p.Gln310(=))];	NA	NA	NA	NA	NA	NA	NA	NA	NA_NA	NA	LOW	NA	1	30	30	0	34	24	10	29.41
Chewer	42483	chr22	37705249	G	A	*CYTH4*	INTRONIC-SS-ACR	ENSG00000100055:[ENST00000462927(n.857-4G>A)];ENSG00000100055:[ENST00000248901(c.697-4G>A)];	NA	NA	NA	4.14E-05	0.000306598	NA	NA	NA	NA_NA	NA	LOW	NA	NA	65	64	1	43	31	12	27.91

Mangalaparthi *et al.* , 2020. Mutational landscape of esophageal squamous cell carcinoma in an Indian cohort
Supplementary Table 4. List of somatic single nucleotide variants identified using whole exome sequencing of ESCC patients

Cohort	Sample	Chromosome	Genomic position	Reference allele	Alternate allele	Gene symbol	Variant type	Variant annotation	OncoMD	COSMIC	dbSNP	ExAC	ExAC_SAS	1000G	1000G_SAS	SIFT	Polyphen2	CONDEL	VEP	LRT	PhastCon7 Way	Total read (Adjacent normal)	Reference allel read support (Adjacent normal)	Alternative read support (Adjacent normal)	Total read (Tumor)	Reference allel read support (Tumor)	Alternative read support (Tumor)	Alternate allele frequency
Chewer	42483	chr22	44011810	G	T	*EFCAB6*	INTRONIC-SS-ACR	ENSG00000186976:[ENST00000262726(c.2466-8C>A)];ENSG00000186976:[ENST00000396231(c.2010-8C>A)];ENSG00000186976:[ENST00000468552(n.1012-7330C>A)];	NA	NA	chr22*44011810*44011810*G*GA*rs760951612*NA*NA*NA|chr22*44011810*44011811*GA*G*rs766374347*NA*NA*NA	NA	NA	NA	NA	NA	NA_NA	NA	LOW	NA	NA	25	25	0	26	18	8	30.77
Chewer	42483	chr22	35480314	G	T	*ISX*	INTRONIC	ENSG00000175329:[ENST00000308700(c.382-62G>T)];ENSG00000175329:[ENST00000404699(c.382-62G>T)];	NA	NA	NA	NA	NA	NA	NA	NA	NA_NA	NA	MODIFIER	NA	NA	49	49	0	43	29	12	27.91
Chewer	42483	chr22	25436420	G	T	*KIAA1671*	MISSENSE	ENSG00000197077:MISSENSE[ENST00000406486(c.3323G>T,cGt>cTt):ENSP00000385152(p.Arg1108Leu)];ENSG00000197077:MISSENSE[ENST00000358431(c.3323G>T,cGt>cTt):ENSP00000351207(p.Arg1108Leu)];	NA	NA	chr22*25436420*25436420*G*A*rs796087654*NA*NA*NA	NA	NA	NA	NA	T	BN_BN	N	MODERATE	N	0.008	176	176	0	133	100	33	24.81
Chewer	42483	chr22	24123203	C	T	*MMP11*	SILENT	ENSG00000099953:EXONIC-NC[ENST00000493132(n.76C>T)];ENSG00000099953:EXONIC-NC[ENST00000465385(n.2358C>T)];ENSG00000099953:EXONIC-NC[ENST00000492464(n.60C>T)];ENSG00000099953:EXONIC-NC[ENST00000460352(n.834C>T)];ENSG00000099953:SILENT[ENST00000215743(c.774C>T,ggC>ggT):ENSP00000215743(p.Gly258(=))];ENSG00000099953:SILENT[ENST00000437086(c.726C>T,ggC>ggT):ENSP00000408070(p.Gly242(=))];ENSG00000099953:SILENT[ENST00000434318(c.137C>T,ggC>ggT):ENSP00000412107(p.Gly46(=))];	NA	NA	NA	NA	NA	NA	NA	NA	NA_NA	NA	MODIFIER	NA	NA	146	146	0	149	110	39	26.17
Chewer	42483	chr22	25275533	C	A	*SGSM1*	INTRONIC	ENSG00000167037:[ENST00000480523(n.1588+40C>A)];ENSG00000167037:[ENST00000473458(n.2869+40C>A)];ENSG00000167037:[ENST00000400359(c.1660+40C>A)];ENSG00000167037:[ENST00000400358(c.1495+40C>A)];	NA	NA	chr22*25275527*25275539*GTGGAGCAGCTAT*G*rs778759541*NA*NA*NA	NA	NA	NA	NA	NA	NA_NA	NA	MODIFIER	NA	NA	46	45	0	45	31	14	31.11
Chewer	42483	chr22	51159753	G	T	*SHANK3*	SILENT	ENSG00000251322:SILENT[ENST00000262795(c.3540G>T,cgG>cgT):ENSP00000442518(p.Arg1180(=))];ENSG00000251322:SILENT[ENST00000445220(c.3498G>T,cgG>cgT):ENSP00000446078(p.Arg1166(=))];ENSG00000251322:SILENT[ENST00000414786(c.3450G>T,cgG>cgT):ENSP00000464552(p.Arg1150(=))];	NA	NA	chr22*51159753*51159753*G*C*rs750499367*NA*NA*NA	NA	NA	NA	NA	NA	NA_NA	NA	LOW	NA	NA	58	58	0	67	46	21	31.34
Chewer	42483	chr3	43753297	G	T	*ABHD5*	MISSENSE	ENSG00000011198:MISSENSE[ENST00000413300(c.99G>T,ttG>ttT):ENSP00000392159(p.Leu33Phe)];ENSG00000011198:MISSENSE[ENST00000458276(c.603G>T,ttG>ttT):ENSP00000390849(p.Leu201Phe)];ENSG00000011198:3UTR[ENST00000013894(c.*597G>T)];	NA	NA	NA	NA	NA	NA	NA	NA	NA_NA	NA	MODIFIER	NA	NA	158	157	0	129	56	73	56.59
Chewer	42483	chr3	48698417	C	T	*CELSR3*	MISSENSE	ENSG00000008300:MISSENSE[ENST00000164024(c.1651G>A,Gtg>Atg):ENSP00000164024(p.Val551Met)];ENSG00000008300:MISSENSE[ENST00000544264(c.1651G>A,Gtg>Atg):ENSP00000445694(p.Val551Met)];	NA	NA	NA	NA	NA	NA	NA	NA	PoD_PrD	NA	MODERATE	NA	NA	99	99	0	84	56	28	33.33
Chewer	42483	chr3	170244539	C	T	*CLDN11*	INTRONIC	ENSG00000013297:[ENST00000486975(c.391+103424C>T)];ENSG00000013297:[ENST00000480067(n.218+49878C>T)];ENSG00000013297:[ENST00000451576(c.391+103424C>T)];	chr3*17024	chr3*17024	NA	8.24E-06	6.06E-05	NA	NA	T	PrD_PrD	D	MODERATE	D	0.997	98	98	0	140	121	19	13.57
Chewer	42483	chr3	1418726	A	G	*CNTN6*	SILENT	ENSG00000134115:SILENT[ENST00000539053(c.1917A>G,ggA>ggG):ENSP00000442791(p.Gly639(=))];ENSG00000134115:SILENT[ENST00000446702(c.2133A>G,ggA>ggG):ENSP00000407822(p.Gly711(=))];ENSG00000134115:SILENT[ENST00000350110(c.2133A>G,ggA>ggG):ENSP00000341882(p.Gly711(=))];ENSG00000134115:3UTR[ENST00000397479(c.*2271A>G)];	NA	NA	NA	NA	NA	NA	NA	NA	NA_NA	NA	MODIFIER	NA	NA	242	242	0	153	69	84	54.9
Chewer	42483	chr3	130113889	G	T	*COL6A5*	MISSENSE	ENSG00000172752:MISSENSE[ENST00000432398(c.3149G>T,gGa>gTa):ENSP00000390895(p.Gly1050Val)];ENSG00000172752:MISSENSE[ENST00000265379(c.3149G>T,gGa>gTa):ENSP00000265379(p.Gly1050Val)];ENSG00000172752:MISSENSE[ENST00000312481(c.3149G>T,gGa>gTa):ENSP00000309762(p.Gly1050Val)];	NA	NA	NA	NA	NA	NA	NA	D	PrD_PrD	D	MODERATE	NA	NA	87	87	0	96	77	19	19.79
Chewer	42483	chr3	130312028	C	T	*COL6A6*	INTRONIC	ENSG00000206384:[ENST00000506143(c.749-15C>T)];ENSG00000206384:[ENST00000453409(c.4417-15C>T)];ENSG00000206384:[ENST00000358511(c.4417-15C>T)];ENSG00000206384:[ENST00000511332(c.753-15C>T)];	NA	NA	chr3*130312027*130312030*CCTT*C*rs756723208*NA*NA*NA|chr3*130312028*130312028*C*G*rs780269587*NA*NA*NA	NA	NA	NA	NA	NA	NA_NA	NA	MODIFIER	NA	NA	107	106	0	135	92	43	31.85
Chewer	42483	chr3	130394264	C	G	*COL6A6*	3UTR	ENSG00000206384:[ENST00000506143(c.*1834C>G)];ENSG00000206384:[ENST00000453409(c.*23C>G)];ENSG00000206384:[ENST00000358511(c.*23C>G)];	NA	NA	NA	NA	NA	NA	NA	NA	NA_NA	NA	MODIFIER	NA	NA	57	57	0	87	62	25	28.74
Chewer	42483	chr3	56696530	G	T	*FAM208A*	INTRONIC	ENSG00000163946:[ENST00000355628(c.1055-12C>A)];ENSG00000163946:[ENST00000478052(n.250+1039C>A)];ENSG00000163946:[ENST00000493960(c.1055-12C>A)];ENSG00000163946:[ENST00000431842(c.-134-12C>A)];	NA	NA	NA	NA	NA	NA	NA	NA	NA_NA	NA	MODIFIER	NA	NA	41	41	0	27	16	11	40.74
Chewer	42483	chr3	120499920	G	T	*GTF2E1*	MISSENSE	ENSG00000153767:MISSENSE[ENST00000283875(c.923G>T,cGt>cTt):ENSP00000283875(p.Arg308Leu)];	NA	NA	chr3*120499920*120499920*G*A*rs200375471*NA*NA*NA	NA	NA	NA	NA	T	BN_BN	N	MODERATE	N	0.805	47	47	0	59	32	27	45.76
Chewer	42483	chr3	178560576	C	A	*KCNMB2*	MISSENSE	ENSG00000197584:MISSENSE[ENST00000452583(c.559C>A,Cta>Ata):ENSP00000397483(p.Leu187Ile)];ENSG00000197584:MISSENSE[ENST00000358316(c.559C>A,Cta>Ata):ENSP00000351068(p.Leu187Ile)];ENSG00000197584:MISSENSE[ENST00000432997(c.559C>A,Cta>Ata):ENSP00000407592(p.Leu187Ile)];ENSG00000197584:MISSENSE[ENST00000420517(c.559C>A,Cta>Ata):ENSP00000408252(p.Leu187Ile)];	NA	NA	NA	NA	NA	NA	NA	NA	NA_NA	NA	MODIFIER	NA	NA	131	131	0	132	88	44	33.33
Chewer	42483	chr3	120053830	G	A	*LRRC58*	SILENT	ENSG00000163428:SILENT[ENST00000295628(c.786C>T,ctC>ctT):ENSP00000295628(p.Leu262(=))];	NA	NA	NA	NA	NA	NA	NA	NA	NA_NA	NA	LOW	NA	NA	161	161	0	188	147	41	21.81

Supplementary Table 4. List of somatic single nucleotide variants identified using whole exome sequencing of ESCC patients

Cohort	Sample	Chromosome	Genomic position	Reference allele	Alternate allele	Gene symbol	Variant type	Variant annotation	OncoMD	COSMIC	dbSNP	ExAC	ExAC_SAS	1000G	1000G_SAS	SIFT	Polyphen2	CONDEL	VEP	LRT	PhastCon7 Way	Total read (Adjacent normal)	Reference allel read support (Adjacent normal)	Alternative read support (Adjacent normal)	Total read (Tumor)	Reference allel read support (Tumor)	Alternative read support (Tumor)	Alternate allele frequency
Chewer	42483	chr3	168833319	T	G	*MECOM*	MISSENSE	ENSG00000085276:MISSENSE[ENST00000472280(c.1780A>C,Agt>Cgt):ENSP00000420048(p.Ser594Arg)];ENSG00000085276:MISSENSE[ENST00000460814(c.1777A>C,Agt>Cgt):ENSP00000420466(p.Ser593Arg)];ENSG00000085276:MISSENSE[ENST00000464456(c.1777A>C,Agt>Cgt):ENSP00000419770(p.Ser593Arg)];ENSG00000085276:MISSENSE[ENST00000494292(c.2341A>C,Agt>Cgt):ENSP00000417899(p.Ser781Arg)];ENSG00000085276:MISSENSE[ENST00000468789(c.1777A>C,Agt>Cgt):ENSP00000419995(p.Ser593Arg)];ENSG00000085276:MISSENSE[ENST00000433243(c.1780A>C,Agt>Cgt):ENSP00000394302(p.Ser594Arg)];ENSG00000085276:MISSENSE[ENST00000392736(c.1777A>C,Agt>Cgt):ENSP00000376493(p.Ser593Arg)];ENSG00000085276:MISSENSE[ENST00000492586(c.730A>C,Agt>Cgt):ENSP00000417506(p.Ser244Arg)];ENSG00000085276:MISSENSE[ENST00000264674(c.1972A>C,Agt>Cgt):ENSP00000264674(p.Ser658Arg)];	NA	NA	chr3*168833317*168833320*ACTC*A*rs762849606*NA*NA*NA	NA	NA	NA	NA	T	PoD_PoD	N	MODERATE	N	0.996	146	146	0	194	155	39	20.1
Chewer	42483	chr3	168851802	G	A	*MECOM*	INTRONIC	ENSG00000085276:[ENST00000481315(c.-54-2483C>T)];ENSG00000085276:[ENST00000472280(c.-54-2483C>T)];ENSG00000085276:[ENST00000460890(c.-54-2483C>T)];ENSG00000085276:[ENST00000487503(c.-54-2483C>T)];ENSG00000085276:[ENST00000484519(c.-54-2483C>T)];ENSG00000085276:[ENST00000464456(c.-54-2483C>T)];ENSG00000085276:[ENST00000392736(c.-54-2483C>T)];ENSG00000085276:[ENST00000494292(c.511-2483C>T)];ENSG00000085276:[ENST00000468789(c.-54-2483C>T)];ENSG00000085276:[ENST00000433243(c.-54-2483C>T)];ENSG00000085276:[ENST00000475754(c.-179-540C>T)];ENSG00000085276:[ENST00000264674(c.139-2483C>T)];ENSG00000085276:[ENST00000494597(c.-54-2483C>T)];	NA	NA	NA	NA	NA	NA	NA	NA	NA_NA	NA	MODIFIER	NA	NA	78	78	0	63	53	10	15.87
Chewer	42483	chr3	151085881	G	A	*MED12L*	INTRONIC	ENSG00000144893:[ENST00000474524(c.3344-57G>A)];ENSG00000144893:[ENST00000273432(c.2924-57G>A)];	NA	NA	NA	NA	NA	NA	NA	NA	NA_NA	NA	MODIFIER	NA	NA	98	98	0	130	111	19	14.62
Chewer	42483	chr3	124627120	C	G	*MUC13*	SILENT	ENSG00000173702:SILENT[ENST00000311075(c.1410G>C,tcG>tcC):ENSP00000312235(p.Ser470(=))];	NA	NA	chr3*124627120*124627120*C*A,T*rs151214305*0*0.9994*.,0.000599	NA	NA	NA	NA	NA	NA_NA	NA	LOW	NA	NA	140	140	0	137	71	66	48.18
Chewer	42483	chr3	151085881	G	A	*P2RY12*	INTRONIC	ENSG00000169313:[ENST00000302632(c.-180+16599C>T)];	NA	NA	NA	NA	NA	NA	NA	NA	NA_NA	NA	MODIFIER	NA	NA	98	98	0	130	111	19	14.62
Chewer	42483	chr3	142671724	G	C	*PAQR9*	INTRONIC	ENSG00000188582:[ENST00000498470(c.45-32C>G)];ENSG00000188582:[ENST00000492509(c.352-32C>G)];	NA	NA	NA	NA	NA	NA	NA	NA	NA_NA	NA	MODIFIER	NA	NA	64	64	0	78	65	13	16.67
Chewer	42483	chr3	129251287	C	G	*RHO*	INTRONIC	ENSG00000163914:[ENST00000296271(c.696+28C>G)];	NA	NA	chr3*129251287*129251287*C*T*rs750763646*NA*NA*NA	NA	NA	NA	NA	NA	NA_NA	NA	MODIFIER	NA	NA	82	81	0	132	92	40	30.3
Chewer	42483	chr3	178560576	C	A	*RP11-385J1.2*	INTRONIC	ENSG00000237978:[ENST00000451742(n.192-1172G>T)];ENSG00000237978:[ENST00000437488(n.194+16701G>T)];ENSG00000237978:[ENST00000425330(n.113-1172G>T)];ENSG00000237978:[ENST00000432385(n.63-1172G>T)];	NA	NA	NA	NA	NA	NA	NA	NA	NA_NA	NA	MODIFIER	NA	NA	131	131	0	132	88	44	33.33
Chewer	42483	chr3	38608019	A	T	*SCN5A*	MISSENSE	ENSG00000183873:MISSENSE[ENST00000449557(c.3559T>A,Tat>Aat):ENSP00000413996(p.Tyr1187Asn)];ENSG00000183873:MISSENSE[ENST00000413689(c.3721T>A,Tat>Aat):ENSP00000410257(p.Tyr1241Asn)];ENSG00000183873:MISSENSE[ENST00000423572(c.3718T>A,Tat>Aat):ENSP00000398266(p.Tyr1240Asn)];ENSG00000183873:MISSENSE[ENST00000425664(c.3721T>A,Tat>Aat):ENSP00000416634(p.Tyr1241Asn)];ENSG00000183873:MISSENSE[ENST00000414099(c.3721T>A,Tat>Aat):ENSP00000398962(p.Tyr1241Asn)];ENSG00000183873:MISSENSE[ENST00000451551(c.3559T>A,Tat>Aat):ENSP00000388797(p.Tyr1187Asn)];ENSG00000183873:MISSENSE[ENST00000450102(c.3559T>A,Tat>Aat):ENSP00000403355(p.Tyr1187Asn)];ENSG00000183873:MISSENSE[ENST00000333535(c.3721T>A,Tat>Aat):ENSP00000328968(p.Tyr1241Asn)];ENSG00000183873:MISSENSE[ENST00000455624(c.3718T>A,Tat>Aat):ENSP00000399524(p.Tyr1240Asn)];ENSG00000183873:MISSENSE[ENST00000443581(c.3718T>A,Tat>Aat):ENSP00000397915(p.Tyr1240Asn)];	chr3*38608(	chr3*38608(	NA	NA	NA	NA	NA	D	PrD_PrD	D	MODERATE	N	0.997	81	81	0	79	62	17	21.52
Chewer	42483	chr3	122640818	C	T	*SEMA5B*	SILENT	ENSG00000082684:SILENT[ENST00000357599(c.1632G>A,ctG>ctA):ENSP00000350215(p.Leu544(=))];ENSG00000082684:SILENT[ENST00000475244(c.1632G>A,ctG>ctA):ENSP00000417570(p.Leu544(=))];ENSG00000082684:SILENT[ENST00000195173(c.1632G>A,ctG>ctA):ENSP00000195173(p.Leu544(=))];ENSG00000082684:SILENT[ENST00000451055(c.1794G>A,ctG>ctA):ENSP00000389588(p.Leu598(=))];ENSG00000082684:SILENT[ENST00000393583(c.1632G>A,ctG>ctA):ENSP00000377208(p.Leu544(=))];	NA	NA	NA	NA	NA	NA	NA	NA	NA_NA	NA	LOW	NA	NA	31	31	0	35	17	18	51.43
Chewer	42483	chr3	125734346	C	G	*SLC41A3*	MISSENSE	ENSG00000114544:MISSENSE[ENST00000315891(c.961G>C,Gtc>Ctc):ENSP00000326070(p.Val321Leu)];ENSG00000114544:MISSENSE[ENST00000360370(c.961G>C,Gtc>Ctc):ENSP00000353533(p.Val321Leu)];ENSG00000114544:MISSENSE[ENST00000346785(c.853G>C,Gtc>Ctc):ENSP00000264471(p.Val285Leu)];ENSG00000114544:MISSENSE[ENST00000508835(c.610G>C,Gtc>Ctc):ENSP00000427409(p.Val204Leu)];ENSG00000114544:MISSENSE[ENST00000383598(c.883G>C,Gtc>Ctc):ENSP00000373092(p.Val295Leu)];ENSG00000114544:EXONIC-NC[ENST00000512557(n.3221G>C)];ENSG00000114544:EXONIC-NC[ENST00000506102(n.1195G>C)];	NA	NA	chr3*125734346*125734346*C*T*rs138712564*NA*NA*NA	NA	NA	NA	NA	NA	NA_NA	NA	MODIFIER	NA	NA	114	114	0	138	67	71	51.45
Chewer	42483	chr3	170244539	C	T	*SLC7A14*	MISSENSE	ENSG00000013293:MISSENSE[ENST00000231706(c.187G>A,Gtt>Att):ENSP00000231706(p.Val63Ile)];	chr3*17024	chr3*17024	NA	8.24E-06	6.06E-05	NA	NA	T	PrD_PrD	D	MODERATE	D	0.997	98	98	0	140	121	19	13.57
Chewer	42483	chr3	196241972	T	C	*SMCO1*	INTRONIC	ENSG00000214097:[ENST00000397537(c.50+59A>G)];ENSG00000214097:[ENST00000452776(c.106+3A>G)];	NA	NA	NA	NA	NA	NA	NA	NA	NA_NA	NA	LOW	NA	NA	202	202	0	241	204	37	15.35
Chewer	42483	chr3	9055124	G	T	*SRGAP3*	MISSENSE	ENSG00000196220:MISSENSE[ENST00000360413(c.1943C>A,cCt>cAt):ENSP00000353587(p.Pro648His)];ENSG00000196220:MISSENSE[ENST00000383836(c.2015C>A,cCt>cAt):ENSP00000373347(p.Pro672His)];ENSG00000196220:EXONIC-NC[ENST00000475560(n.683C>A)];ENSG00000196220:EXONIC-NC[ENST00000518265(n.202C>A)];	NA	NA	NA	NA	NA	NA	NA	NA	NA_NA	NA	MODIFIER	NA	NA	90	90	0	82	77	5	6.1

Mangalaparthi *et al.* , 2020. Mutational landscape of esophageal squamous cell carcinoma in an Indian cohort
Supplementary Table 4. List of somatic single nucleotide variants identified using whole exome sequencing of ESCC patients

Cohort	Sample	Chromosome	Genomic position	Reference allele	Alternate allele	Gene symbol	Variant type	Variant annotation	OncoMD	COSMIC	dbSNP	ExAC	ExAC_SAS	1000G	1000G_SAS	SIFT	Polyphen2	CONDEL	VEP	LRT	PhastCon7 Way	Total read (Adjacent normal)	Reference allel read support (Adjacent normal)	Alternative read support (Adjacent normal)	Total read (Tumor)	Reference allel read support (Tumor)	Alternative read support (Tumor)	Alternate allele frequency
Chewer	42483	chr3	156261065	G	C	*SSR3*	INTRONIC-SS-ACR	ENSG00000114850:[ENST00000464138(n.326-7C>G)];ENSG00000114850:[ENST00000265044(c.492-7C>G)];ENSG00000114850:[ENST00000498205(n.328-7C>G)];ENSG00000114850:[ENST00000463503(c.336-7C>G)];ENSG00000114850:[ENST00000478842(n.351-7C>G)];ENSG00000114850:[ENST00000496050(c.336-7C>G)];ENSG00000114850:[ENST00000467789(c.531-7C>G)];ENSG00000114850:[ENST00000476217(c.506-7C>G)];	NA	NA	chr3*156261062*156261065*TAAG*T*rs750060306*NA*NA*NA|chr3*156261065*156261065*G*A*rs758353838*NA*NA*NA	NA	NA	NA	NA	NA	NA_NA	NA	LOW	NA	NA	84	84	0	93	73	20	21.51
Chewer	42483	chr3	121100146	G	A	*STXBP5L*	MISSENSE	ENSG00000145087:MISSENSE[ENST00000497029(c.2348G>A,cGa>cAa):ENSP00000420287(p.Arg783Gln)];ENSG00000145087:MISSENSE[ENST00000471262(c.2255G>A,cGa>cAa):ENSP00000420167(p.Arg752Gln)];ENSG00000145087:MISSENSE[ENST00000472879(c.2354G>A,cGa>cAa):ENSP00000419627(p.Arg785Gln)];ENSG00000145087:MISSENSE[ENST00000273666(c.2426G>A,cGa>cAa):ENSP00000273666(p.Arg809Gln)];ENSG00000145087:MISSENSE[ENST00000471454(c.2354G>A,cGa>cAa):ENSP00000420019(p.Arg785Gln)];ENSG00000145087:MISSENSE[ENST00000492541(c.2426G>A,cGa>cAa):ENSP00000420666(p.Arg809Gln)];	NA	NA	NA	NA	NA	NA	NA	T	BN_PoD	N	MODERATE	D	0.998	91	91	0	75	53	22	29.33
Chewer	42483	chr3	176743289	T	C	*TBL1XR1*	SILENT	ENSG00000177565:EXONIC-NC[ENST00000474363(n.1482A>G)];ENSG00000177565:SILENT[ENST00000457928(c.1542A>G,aaA>aaG):ENSP00000413251(p.Lys514(=))];ENSG00000177565:SILENT[ENST00000430069(c.1542A>G,aaA>aaG):ENSP00000405574(p.Lys514(=))];	NA	NA	NA	NA	NA	NA	NA	NA	NA_NA	NA	LOW	NA	NA	60	60	0	50	32	18	36
Chewer	42483	chr3	100277340	C	T	*TMEM45A*	SILENT	ENSG00000181458:SILENT[ENST00000449609(c.543C>T,ctC>ctT):ENSP00000405597(p.Leu181(=))];ENSG00000181458:SILENT[ENST00000403410(c.543C>T,ctC>ctT):ENSP00000385089(p.Leu181(=))];ENSG00000181458:SILENT[ENST00000323523(c.495C>T,ctC>ctT):ENSP00000319009(p.Leu165(=))];	chr3*10027	chr3*10027	NA	4.12E-05	0	NA	NA	NA	NA_NA	NA	LOW	NA	NA	105	105	0	67	53	14	20.9
Chewer	42483	chr3	194877260	T	A	*XXYLT1*	MISSENSE	ENSG00000173950:MISSENSE[ENST00000355729(c.94A>T,Atc>Ttc):ENSP00000347967(p.Ile32Phe)];ENSG00000173950:MISSENSE[ENST00000310380(c.703A>T,Atc>Ttc):ENSP00000309640(p.Ile235Phe)];ENSG00000173950:MISSENSE[ENST00000437101(c.94A>T,Atc>Ttc):ENSP00000409865(p.Ile32Phe)];ENSG00000173950:MISSENSE[ENST00000429994(c.265A>T,Atc>Ttc):ENSP00000399422(p.Ile89Phe)];ENSG00000173950:3UTR[ENST00000418940(c.*84A>T)];ENSG00000173950:EXONIC-NC[ENST00000491138(n.581A>T)];	NA	NA	NA	NA	NA	NA	NA	NA	NA_NA	NA	MODIFIER	NA	NA	108	107	0	130	106	24	18.46
Chewer	42483	chr4	165118710	C	T	*ANP32C*	EXONIC-NC	ENSG00000248546:[ENST00000512835(n.154G>A)];	NA	NA	chr4*165118710*165118710*C*A,T*rs754003794*NA*NA*NA	8.24E-06	0	NA	NA	NA	NA_NA	NA	MODIFIER	NA	NA	215	215	0	228	161	67	29.39
Chewer	42483	chr4	169215041	C	A	*DDX60*	MISSENSE	ENSG00000137628:MISSENSE[ENST00000393743(c.779G>T,cGg>cTg):ENSP00000377344(p.Arg260Leu)];	chr4*16921	chr4*16921	chr4*169215041*169215041*C*T*rs768314712*NA*NA*NA	NA	NA	NA	NA	D	PoD_PrD	D	MODERATE	D	0.995	225	224	0	199	147	50	25.13
Chewer	42483	chr4	126384740	C	G	*FAT4*	SILENT	ENSG00000196159:SILENT[ENST00000394329(c.11817C>G,gtC>gtG):ENSP00000377862(p.Val3939(=))];ENSG00000196159:INTRONIC[ENST00000335110(c.6689-4927C>G)];	NA	NA	NA	NA	NA	NA	NA	NA	NA_NA	NA	MODIFIER	NA	NA	56	56	0	42	29	13	30.95
Chewer	42483	chr4	79202662	G	A	*FRAS1*	INTRONIC	ENSG00000138759:[ENST00000502446(c.893+75G>A)];ENSG00000138759:[ENST00000325942(c.1107+75G>A)];ENSG00000138759:[ENST00000264899(c.1107+75G>A)];ENSG00000138759:[ENST00000264895(c.1107+75G>A)];ENSG00000138759:[ENST00000508900(c.634+75G>A)];	NA	NA	NA	NA	NA	NA	NA	NA	NA_NA	NA	MODIFIER	NA	NA	66	66	0	54	44	10	18.52
Chewer	42483	chr4	79400611	G	T	*FRAS1*	MISSENSE	ENSG00000138759:MISSENSE[ENST00000512123(c.2867G>T,Ggc>Tgc):ENSP00000422834(p.Gly957Cys)];ENSG00000138759:MISSENSE[ENST00000264895(c.8182G>T,Ggc>Tgc):ENSP00000264895(p.Gly2728Cys)];	NA	NA	NA	NA	NA	NA	NA	NA	PrD_PrD	NA	MODERATE	D	0.987	121	121	0	143	93	49	34.27
Chewer	42483	chr4	70918829	C	T	*HTN1*	SILENT	ENSG00000126550:EXONIC-NC[ENST00000506754(n.231C>T)];ENSG00000126550:EXONIC-NC[ENST00000503645(n.107C>T)];ENSG00000126550:SILENT[ENST00000511674(c.36C>T,ctC>ctT):ENSP00000424501(p.Leu12(=))];ENSG00000126550:SILENT[ENST00000246896(c.36C>T,ctC>ctT):ENSP00000246896(p.Leu12(=))];	NA	NA	NA	NA	NA	NA	NA	NA	NA_NA	NA	MODIFIER	NA	NA	51	51	0	65	45	20	30.77
Chewer	42483	chr4	57976116	G	T	*IGFBP7*	SILENT	ENSG00000163453:SILENT[ENST00000537922(c.402C>A,cgC>cgA):ENSP00000444146(p.Arg134(=))];ENSG00000163453:SILENT[ENST00000295666(c.402C>A,cgC>cgA):ENSP00000295666(p.Arg134(=))];	NA	NA	NA	NA	NA	NA	NA	NA	NA_NA	NA	MODIFIER	NA	NA	57	57	0	60	37	23	38.33
Chewer	42483	chr4	57976116	G	T	*IGFBP7-AS1*	EXONIC-NC	ENSG00000245067:[ENST00000508328(n.98G>T)];ENSG00000245067:[ENST00000499667(n.189G>T)];	NA	NA	NA	NA	NA	NA	NA	NA	NA_NA	NA	MODIFIER	NA	NA	57	57	0	60	37	23	38.33
Chewer	42483	chr4	165118710	C	T	*MARCH1*	INTRONIC	ENSG00000145416:[ENST00000503008(c.-113-85896G>A)];ENSG00000145416:[ENST00000515471(n.381-85896G>A)];ENSG00000145416:[ENST00000507270(c.-113-85896G>A)];ENSG00000145416:[ENST00000505391(n.312-85896G>A)];ENSG00000145416:[ENST00000510075(n.332-67495G>A)];ENSG00000145416:[ENST00000514618(c.-322-85896G>A)];ENSG00000145416:[ENST00000508725(n.415+37275G>A)];	NA	NA	chr4*165118710*165118710*C*A,T*rs754003794*NA*NA*NA	8.24E-06	0	NA	NA	NA	NA_NA	NA	MODIFIER	NA	NA	215	215	0	228	161	67	29.39
Chewer	42483	chr4	183090475	G	A	*MIR1305*	EXONIC-NC	ENSG00000221227:[ENST00000408300(n.30G>A)];	NA	NA	NA	NA	NA	NA	NA	NA	NA_NA	NA	MODIFIER	NA	NA	74	74	0	83	44	39	46.99
Chewer	42483	chr4	140270644	G	T	*NAA15*	MISSENSE	ENSG00000164134:MISSENSE[ENST00000296543(c.720G>T,ttG>ttT):ENSP00000296543(p.Leu240Phe)];ENSG00000164134:MISSENSE[ENST00000398947(c.720G>T,ttG>ttT):ENSP00000381920(p.Leu240Phe)];ENSG00000164134:EXONIC-NC[ENST00000480277(n.556G>T)];	NA	NA	NA	NA	NA	NA	NA	NA	NA_NA	NA	MODIFIER	NA	NA	132	132	0	109	81	28	25.69
Chewer	42483	chr4	56458131	C	A	*PDCL2*	INTRONIC	ENSG00000163440:[ENST00000295645(c.6+140G>T)];	NA	NA	NA	NA	NA	NA	NA	NA	NA_NA	NA	MODIFIER	NA	NA	62	62	0	52	27	25	48.08
Chewer	42483	chr4	76797520	C	A	*PPEF2*	NONSENSE	ENSG00000156194:NONSENSE[ENST00000286719(c.1240G>T,Gag>Tag):ENSP00000286719(p.Glu414Ter)];ENSG00000156194:3UTR[ENST00000511880(c.*1478G>T)];	NA	NA	NA	NA	NA	NA	NA	NA	NA_NA	NA	MODIFIER	NA	NA	47	47	0	39	25	14	35.9
Chewer	42483	chr4	183090475	G	A	*RP11-402C9.1*	INTRONIC	ENSG00000248266:[ENST00000505389(n.121-20895G>A)];	NA	NA	NA	NA	NA	NA	NA	NA	NA_NA	NA	MODIFIER	NA	NA	74	74	0	83	44	39	46.99
Chewer	42483	chr4	54218833	C	A	*SCFD2*	SILENT	ENSG00000184178:SILENT[ENST00000388940(c.939G>T,gcG>gcT):ENSP00000373592(p.Ala313(=))];ENSG00000184178:SILENT[ENST00000503450(c.18G>T,gcG>gcT):ENSP00000427202(p.Ala6(=))];ENSG00000184178:SILENT[ENST00000401642(c.939G>T,gcG>gcT):ENSP00000384182(p.Ala313(=))];	NA	NA	NA	8.24E-06	0	NA	NA	NA	NA_NA	NA	LOW	NA	NA	95	95	0	82	66	16	19.51
Chewer	42483	chr4	146468099	T	C	*SMAD1*	INTRONIC	ENSG00000170365:[ENST00000511125(n.2747+23T>C)];ENSG00000170365:[ENST00000302085(c.997+23T>C)];ENSG00000170365:[ENST00000511255(n.405+23T>C)];ENSG00000170365:[ENST00000510948(n.317+23T>C)];ENSG00000170365:[ENST00000515385(c.997+23T>C)];ENSG00000170365:[ENST00000394092(c.997+23T>C)];	NA	NA	NA	NA	NA	NA	NA	NA	NA_NA	NA	MODIFIER	NA	NA	69	69	0	69	53	16	23.19

Supplementary Table 4. List of somatic single nucleotide variants identified using whole exome sequencing of ESCC patients

Cohort	Sample	Chromosome	Genomic position	Reference allele	Alternate allele	Gene symbol	Variant type	Variant annotation	OncoMD	COSMIC	dbSNP	ExAC	ExAC_SAS	1000G	1000G_SAS	SIFT	Polyphen2	CONDEL	VEP	LRT	PhastCon7 Way	Total read (Adjacent normal)	Reference allel read support (Adjacent normal)	Alternative read support (Adjacent normal)	Total read (Tumor)	Reference allel read support (Tumor)	Alternative read support (Tumor)	Alternate allele frequency
Chewer	42483	chr4	48158719	G	T	*TEC*	MISSENSE	ENSG00000135605:MISSENSE[ENST00000381501(c.770C>A,gCa>gAa):ENSP00000370912(p.Ala257Glu)];ENSG00000135605:3UTR[ENST00000505452(c.*360C>A)];	NA	NA	NA	NA	NA	NA	NA	NA	NA_NA	NA	MODIFIER	NA	NA	163	162	0	228	155	73	32.02
Chewer	42483	chr4	183090475	G	A	*TENM3*	INTRONIC	ENSG00000218336:[ENST00000512480(c.-76+24568G>A)];ENSG00000218336:[ENST00000513201(n.175+25161G>A)];	NA	NA	NA	NA	NA	NA	NA	NA	NA_NA	NA	MODIFIER	NA	NA	74	74	0	83	44	39	46.99
Chewer	42483	chr4	949204	G	C	*TMEM175*	SILENT	ENSG00000127419:EXONIC-NC[ENST00000506669(n.217G>C)];ENSG00000127419:EXONIC-NC[ENST00000515876(n.750G>C)];ENSG00000127419:EXONIC-NC[ENST00000438836(n.1200G>C)];ENSG00000127419:EXONIC-NC[ENST00000452360(n.1212G>C)];ENSG00000127419:SILENT[ENST00000510493(c.393G>C,ctG>ctC):ENSP00000424208(p.Leu131(=))];ENSG00000127419:SILENT[ENST00000264771(c.639G>C,ctG>ctC):ENSP00000264771(p.Leu213(=))];ENSG00000127419:SILENT[ENST00000515492(c.393G>C,ctG>ctC):ENSP00000425867(p.Leu131(=))];ENSG00000127419:SILENT[ENST00000515740(c.291G>C,ctG>ctC):ENSP00000427039(p.Leu97(=))];ENSG00000127419:SILENT[ENST00000509508(c.357G>C,ctG>ctC):ENSP00000421750(p.Leu119(=))];ENSG00000127419:SILENT[ENST00000508204(c.393G>C,ctG>ctC):ENSP00000423669(p.Leu131(=))];ENSG00000127419:3UTR[ENST00000513952(c.*661G>C)];	NA	NA	NA	NA	NA	NA	NA	NA	NA_NA	NA	MODIFIER	NA	NA	57	57	0	50	25	25	50
Chewer	42483	chr4	69343299	C	A	*TMPRSS11E*	MISSENSE	ENSG00000087128:MISSENSE[ENST00000305363(c.920C>A,cCa>cAa):ENSP00000307519(p.Pro307Gln)];ENSG00000087128:3UTR[ENST00000510647(c.*369C>A)];	NA	NA	NA	NA	NA	NA	NA	NA	NA_NA	NA	MODIFIER	NA	NA	302	299	1	270	160	110	40.74
Chewer	42483	chr5	74912694	C	T	*ANKDD1B*	SILENT	ENSG00000189045:SILENT[ENST00000601380(c.259C>T,Ctg>Ttg):ENSP00000471417(p.Leu87(=))];ENSG00000189045:INTRONIC[ENST00000506596(n.352+5042C>T)];	NA	NA	chr5*74912694*74912694*C*G*rs763172727*NA*NA*NA	NA	NA	NA	NA	NA	NA_NA	NA	MODIFIER	NA	NA	130	130	0	105	46	59	56.19
Chewer	42483	chr5	172574007	A	G	*BNIP1*	INTRONIC	ENSG00000113734:[ENST00000352523(c.177+46A>G)];ENSG00000113734:[ENST00000393770(c.177+46A>G)];ENSG00000113734:[ENST00000351486(c.177+46A>G)];ENSG00000113734:[ENST00000231668(c.177+46A>G)];	NA	NA	NA	NA	NA	NA	NA	NA	NA_NA	NA	MODIFIER	NA	NA	71	71	0	59	50	9	15.25
Chewer	42483	chr5	205466	G	A	*CCDC127*	SILENT	ENSG00000164366:SILENT[ENST00000296824(c.729C>T,ctC>ctT):ENSP00000296824(p.Leu243(=))];	NA	NA	NA	NA	NA	NA	NA	NA	NA_NA	NA	LOW	NA	NA	154	154	0	142	97	44	30.99
Chewer	42483	chr5	205811	C	A	*CCDC127*	MISSENSE	ENSG00000164366:MISSENSE[ENST00000296824(c.384G>T,atG>atT):ENSP00000296824(p.Met128Ile)];	NA	NA	NA	NA	NA	NA	NA	T	BN_BN	N	MODERATE	N	0.845	105	104	1	114	66	48	42.11
Chewer	42483	chr5	175995896	C	T	*CDHR2*	INTRONIC	ENSG00000074276:[ENST00000261944(c.265-65C>T)];ENSG00000074276:[ENST00000506348(c.265-65C>T)];ENSG00000074276:[ENST00000510636(c.265-65C>T)];	NA	NA	NA	NA	NA	NA	NA	NA	NA_NA	NA	MODIFIER	NA	NA	105	105	0	106	64	42	39.62
Chewer	42483	chr5	1341991	C	T	*CLPTM1L*	INTRONIC	ENSG00000049656:[ENST00000320927(c.264-16G>A)];ENSG00000049656:[ENST00000320895(c.264-16G>A)];	NA	NA	NA	NA	NA	NA	NA	NA	NA_NA	NA	MODIFIER	NA	NA	35	35	0	45	27	18	40
Chewer	42483	chr5	13754327	T	C	*DNAH5*	MISSENSE	ENSG00000039139:MISSENSE[ENST00000265104(c.10540A>G,Act>Gct):ENSP00000265104(p.Thr3514Ala)];	NA	NA	NA	NA	NA	NA	NA	NA	PoD_PoD	NA	MODERATE	D	0.977	141	141	0	160	127	33	20.62
Chewer	42483	chr5	15936643	G	C	*FBXL7*	MISSENSE	ENSG00000183580:MISSENSE[ENST00000329673(c.788G>C,cGc>cCc):ENSP00000329632(p.Arg263Pro)];ENSG00000183580:MISSENSE[ENST00000510662(c.683G>C,cGc>cCc):ENSP00000425184(p.Arg228Pro)];ENSG00000183580:MISSENSE[ENST00000504595(c.824G>C,cGc>cCc):ENSP00000423630(p.Arg275Pro)];	chr5*15936	chr5*15936	NA	NA	NA	NA	NA	D	BN_PoD	D	MODERATE	D	0.999	119	119	0	135	83	52	38.52
Chewer	42483	chr5	147019229	G	T	*JAKMIP2*	MISSENSE	ENSG00000176049:MISSENSE[ENST00000265272(c.1496C>A,aCg>aAg):ENSP00000265272(p.Thr499Lys)];ENSG00000176049:MISSENSE[ENST00000333010(c.1370C>A,aCg>aAg):ENSP00000328989(p.Thr457Lys)];ENSG00000176049:MISSENSE[ENST00000507386(c.1496C>A,aCg>aAg):ENSP00000421398(p.Thr499Lys)];ENSG00000176049:EXONIC-NC[ENST00000504845(n.294C>A)];	chr5*14701	NA	NA	NA	NA	NA	NA	NA	NA_NA	NA	MODIFIER	NA	NA	184	182	2	133	55	78	58.65
Chewer	42483	chr5	16701187	C	G	*MYO10*	MISSENSE	ENSG00000145555:MISSENSE[ENST00000274203(c.1388G>C,aGc>aCc):ENSP00000274203(p.Ser463Thr)];ENSG00000145555:MISSENSE[ENST00000427430(c.1388G>C,aGc>aCc):ENSP00000391106(p.Ser463Thr)];ENSG00000145555:MISSENSE[ENST00000515803(c.1334G>C,aGc>aCc):ENSP00000425051(p.Ser445Thr)];ENSG00000145555:MISSENSE[ENST00000505695(c.1334G>C,aGc>aCc):ENSP00000421170(p.Ser445Thr)];ENSG00000145555:MISSENSE[ENST00000513610(c.3317G>C,aGc>aCc):ENSP00000421280(p.Ser1106Thr)];	NA	NA	NA	NA	NA	NA	NA	T_lc	PrD_PrD	D	MODERATE	NA	NA	159	156	0	179	107	72	40.22
Chewer	42483	chr5	177482586	C	T	*NA*	INTERGENIC		NA	NA	NA	8.83E-06	0	NA	NA	NA	NA_NA	NA	MODIFIER	NA	NA	34	34	0	43	33	7	16.28
Chewer	42483	chr5	37302883	C	T	*NUP155*	MISSENSE-SS-PRX	ENSG00000113569:[ENST00000513532(c.3253G>A,Gag>Aag):ENSP00000422019(p.Glu1085Lys)];ENSG00000113569:[ENST00000381843(c.3268G>A,Gag>Aag):ENSP00000371265(p.Glu1090Lys)];ENSG00000113569:[ENST00000231498(c.3445G>A,Gag>Aag):ENSP00000231498(p.Glu1149Lys)];ENSG00000113569:[ENST00000502533(n.1103G>A)];ENSG00000113569:[ENST00000515054(n.285G>A)];	NA	NA	NA	NA	NA	NA	NA	NA	NA_NA	NA	LOW	NA	NA	235	233	0	306	255	51	16.67
Chewer	42483	chr5	140797989	G	C	*PCDHGA1*	INTRONIC	ENSG00000204956:[ENST00000517417(c.2422-76385G>C)];	NA	NA	NA	NA	NA	NA	NA	D	PrD_PrD	D	MODERATE	U	0.997	165	165	0	171	74	97	56.73
Chewer	42483	chr5	140797989	G	C	*PCDHGA10*	INTRONIC	ENSG00000253846:[ENST00000398610(c.2436+2811G>C)];	NA	NA	NA	NA	NA	NA	NA	D	PrD_PrD	D	MODERATE	U	0.997	165	165	0	171	74	97	56.73
Chewer	42483	chr5	140797989	G	C	*PCDHGA2*	INTRONIC	ENSG00000081853:[ENST00000394576(c.2425-76385G>C)];	NA	NA	NA	NA	NA	NA	NA	D	PrD_PrD	D	MODERATE	U	0.997	165	165	0	171	74	97	56.73
Chewer	42483	chr5	140797989	G	C	*PCDHGA3*	INTRONIC	ENSG00000254245:[ENST00000253812(c.2424+71965G>C)];	NA	NA	NA	NA	NA	NA	NA	D	PrD_PrD	D	MODERATE	U	0.997	165	165	0	171	74	97	56.73
Chewer	42483	chr5	140797989	G	C	*PCDHGA4*	INTRONIC	ENSG00000262576:[ENST00000571252(c.2421+60801G>C)];	NA	NA	NA	NA	NA	NA	NA	D	PrD_PrD	D	MODERATE	U	0.997	165	165	0	171	74	97	56.73
Chewer	42483	chr5	140797989	G	C	*PCDHGA5*	INTRONIC	ENSG00000253485:[ENST00000518069(c.2421+51671G>C)];	NA	NA	NA	NA	NA	NA	NA	D	PrD_PrD	D	MODERATE	U	0.997	165	165	0	171	74	97	56.73
Chewer	42483	chr5	140797989	G	C	*PCDHGA6*	INTRONIC	ENSG00000253731:[ENST00000517434(c.2424+41915G>C)];	NA	NA	NA	NA	NA	NA	NA	D	PrD_PrD	D	MODERATE	U	0.997	165	165	0	171	74	97	56.73
Chewer	42483	chr5	140797989	G	C	*PCDHGA7*	INTRONIC	ENSG00000253537:[ENST00000518325(c.2424+33099G>C)];	NA	NA	NA	NA	NA	NA	NA	D	PrD_PrD	D	MODERATE	U	0.997	165	165	0	171	74	97	56.73
Chewer	42483	chr5	140797989	G	C	*PCDHGA8*	INTRONIC	ENSG00000253767:[ENST00000398604(c.2424+23185G>C)];	NA	NA	NA	NA	NA	NA	NA	D	PrD_PrD	D	MODERATE	U	0.997	165	165	0	171	74	97	56.73
Chewer	42483	chr5	140797989	G	C	*PCDHGA9*	INTRONIC	ENSG00000261934:[ENST00000573521(c.2424+13046G>C)];	NA	NA	NA	NA	NA	NA	NA	D	PrD_PrD	D	MODERATE	U	0.997	165	165	0	171	74	97	56.73
Chewer	42483	chr5	140797989	G	C	*PCDHGB1*	INTRONIC	ENSG00000254221:[ENST00000523390(c.2409+65753G>C)];	NA	NA	NA	NA	NA	NA	NA	D	PrD_PrD	D	MODERATE	U	0.997	165	165	0	171	74	97	56.73
Chewer	42483	chr5	140797989	G	C	*PCDHGB2*	INTRONIC	ENSG00000253910:[ENST00000522605(c.2421+55866G>C)];	NA	NA	NA	NA	NA	NA	NA	D	PrD_PrD	D	MODERATE	U	0.997	165	165	0	171	74	97	56.73
Chewer	42483	chr5	140797989	G	C	*PCDHGB3*	INTRONIC	ENSG00000262209:[ENST00000576222(c.2415+45613G>C)];	NA	NA	NA	NA	NA	NA	NA	D	PrD_PrD	D	MODERATE	U	0.997	165	165	0	171	74	97	56.73
Chewer	42483	chr5	140797989	G	C	*PCDHGB4*	INTRONIC	ENSG00000253953:[ENST00000519479(c.2397+28141G>C)];	NA	NA	NA	NA	NA	NA	NA	D	PrD_PrD	D	MODERATE	U	0.997	165	165	0	171	74	97	56.73
Chewer	42483	chr5	140797989	G	C	*PCDHGB6*	INTRONIC	ENSG00000253305:[ENST00000520790(c.2418+7802G>C)];	NA	NA	NA	NA	NA	NA	NA	D	PrD_PrD	D	MODERATE	U	0.997	165	165	0	171	74	97	56.73
Chewer	42483	chr5	140797989	G	C	*PCDHGB7*	MISSENSE	ENSG00000254122:MISSENSE[ENST00000398594(c.563G>C,gGt>gCt):ENSP00000381594(p.Gly188Ala)];	NA	NA	NA	NA	NA	NA	NA	D	PrD_PrD	D	MODERATE	U	0.997	165	165	0	171	74	97	56.73

Supplementary Table 4. List of somatic single nucleotide variants identified using whole exome sequencing of ESCC patients

Cohort	Sample	Chromosome	Genomic position	Reference allele	Alternate allele	Gene symbol	Variant type	Variant annotation	OncoMD	COSMIC	dbSNP	ExAC	ExAC_SAS	1000G	1000G_SAS	SIFT	Polyphen2	CONDEL	VEP	LRT	PhastCon7 Way	Total read (Adjacent normal)	Reference allel read support (Adjacent normal)	Alternative read support (Adjacent normal)	Total read (Tumor)	Reference allel read support (Tumor)	Alternative read support (Tumor)	Alternate allele frequency
Chewer	42483	chr5	170319382	T	C	*RANBP17*	INTRONIC-SS-ACR	ENSG00000204764:[ENST00000519949(c.257-9T>C)];ENSG00000204764:[ENST00000389118(c.257-9T>C)];ENSG00000204764:[ENST00000523727(n.59-9T>C)];ENSG00000204764:[ENST00000519256(c.257-9T>C)];ENSG00000204764:[ENST00000519944(c.11-9T>C)];ENSG00000204764:[ENST00000443155(n.421-9T>C)];ENSG00000204764:[ENST00000522533(c.257-9T>C)];ENSG00000204764:[ENST00000522066(c.257-9T>C)];ENSG00000204764:[ENST00000523189(c.257-9T>C)];ENSG00000204764:[ENST00000520864(c.256-9T>C)];ENSG00000204764:[ENST00000519130(n.268-9T>C)];	NA	NA	NA	NA	NA	NA	NA	NA	NA_NA	NA	MODIFIER	NA	NA	122	122	0	85	69	15	17.65
Chewer	42483	chr5	236653	C	A	*SDHA*	SILENT	ENSG00000073578:EXONIC-NC[ENST00000514027(n.1326C>A)];ENSG00000073578:EXONIC-NC[ENST00000511810(n.2118C>A)];ENSG00000073578:EXONIC-NC[ENST00000505555(n.1411C>A)];ENSG00000073578:EXONIC-NC[ENST00000515752(n.957C>A)];ENSG00000073578:SILENT[ENST000005158 15(c.26C>A,ctC>ctA):ENSP00000422404(p.Leu9(=))];ENSG00000073578:SILENT[ENST00000264932(c.1371C>A,ctC>ctA):ENSP00000264932(p.Leu457(=))];ENSG00000073578:SILENT[ENST00000504309(c.1371C>A,ctC>ctA):ENSP00000426514(p.Leu457(=))];ENSG00000073578:SILENT[ENST00000510361(c.1227C>A,ctC>ctA):ENSP00000427703(p.Leu409(=))];	chr5*23665	NA	NA	0.0208867	0.034893828	NA	NA	NA	NA_NA	NA	MODIFIER	NA	NA	128	128	0	150	143	7	4.67
Chewer	42483	chr5	236676	G	A	*SDHA*	MISSENSE	ENSG00000073578:MISSENSE[ENST00000515815(c.49G>A,cGg>cAg):ENSP00000422404(p.Arg17Gln)];ENSG00000073578:MISSENSE[ENST00000504309(c.1394G>A,cGg>cAg):ENSP00000426514(p.Arg465Gln)];ENSG00000073578:MISSENSE[ENST00000510361(c.1250G>A,cGg>cAg):ENSP00000427703(p.Arg417Gln)];ENSG00000073578:MISSENSE[ENST00000264932(c.1394G>A,cGg>cAg):ENSP00000264932(p.Arg465Gln)];ENSG00000073578:EXONIC-NC[ENST00000514027(n.1349G>A)];ENSG00000073578:EXONIC-NC[ENST00000511810(n.2141G>A)];ENSG00000073578:EXONIC-NC[ENST00000505555(n.1434G>A)];ENSG00000073578:EXONIC-NC[ENST00000515752(n.980G>A)];	NA	NA	NA	0.016016	0.008037019	NA	NA	NA	NA_NA	NA	MODIFIER	NA	NA	125	125	0	158	148	10	6.33
Chewer	42483	chr5	236678	G	A	*SDHA*	MISSENSE	ENSG00000073578:MISSENSE[ENST00000515815(c.51G>A,Gca>Aca):ENSP00000422404(p.Ala18Thr)];ENSG00000073578:MISSENSE[ENST00000510361(c.1252G>A,Gca>Aca):ENSP00000427703(p.Ala418Thr)];ENSG00000073578:MISSENSE[ENST00000264932(c.1396G>A,Gca>Aca):ENSP00000264932(p.Ala466Thr)];ENSG00000073578:MISSENSE[ENST00000504309(c.1396G>A,Gca>Aca):ENSP00000426514(p.Ala466Thr)];ENSG00000073578:EXONIC-NC[ENST00000514027(n.1351G>A)];ENSG00000073578:EXONIC-NC[ENST00000511810(n.2143G>A)];ENSG00000073578:EXONIC-NC[ENST00000505555(n.1436G>A)];ENSG00000073578:EXONIC-NC[ENST00000515752(n.982G>A)];	NA	NA	NA	0.0382381	0.054865814	NA	NA	NA	NA_NA	NA	MODIFIER	NA	NA	127	127	0	160	149	10	6.25
Chewer	42483	chr5	1088548	G	C	*SLC12A7*	INTRONIC	ENSG00000113504:[ENST00000264930(c.490-73C>G)];	NA	NA	NA	NA	NA	NA	NA	NA	NA_NA	NA	MODIFIER	NA	NA	36	36	0	61	32	29	47.54
Chewer	42483	chr5	140698865	C	G	*TAF7*	SILENT	ENSG00000178913:SILENT[ENST00000313368(c.747G>C,acG>acC):ENSP00000312709(p.Thr249(=))];	NA	NA	NA	NA	NA	NA	NA	NA	NA_NA	NA	LOW	NA	NA	169	168	0	124	45	79	63.71
Chewer	42483	chr6	26598341	G	A	*ABT1*	INTRONIC-SS-DNR	ENSG00000146109:[ENST00000274849(c.438+3G>A)];	NA	NA	NA	NA	NA	NA	NA	NA	NA_NA	NA	LOW	NA	NA	45	45	0	25	20	5	20
Chewer	42483	chr6	109894805	A	T	*AK9*	INTRONIC-SS-ACR	ENSG00000155085:[ENST00000341338(c.-572-8T>A)];ENSG00000155085:[ENST00000424296(c.2192-8T>A)];ENSG00000155085:[ENST00000466992(n.903-8T>A)];	NA	NA	NA	NA	NA	NA	NA	NA	NA_NA	NA	LOW	NA	NA	85	85	0	56	40	15	26.79
Chewer	42483	chr6	7879348	G	A	*BMP6*	MISSENSE	ENSG00000153162:MISSENSE[ENST00000283147(c.1246G>A,Gag>Aag):ENSP00000283147(p.Glu416Lys)];	NA	NA	NA	NA	NA	NA	NA	D	BN_PoD	N	MODERATE	D	0.999	58	58	0	53	34	19	35.85
Chewer	42483	chr6	38690612	C	A	*DNAH8*	SILENT	ENSG00000124721:SILENT[ENST00000373278(c.27C>A,gcC>gcA):ENSP00000362375(p.Ala9(=))];ENSG00000124721:SILENT[ENST00000449981(c.27C>A,gcC>gcA):ENSP00000415331(p.Ala9(=))];	NA	NA	NA	NA	NA	NA	NA	NA	NA_NA	NA	LOW	NA	NA	49	49	0	35	22	13	37.14
Chewer	42483	chr6	38905946	C	G	*DNAH8*	MISSENSE	ENSG00000124721:MISSENSE[ENST00000449981(c.11760C>G,atC>atG):ENSP00000415331(p.Ile3920Met)];ENSG00000124721:MISSENSE[ENST00000359357(c.11109C>G,atC>atG):ENSP00000352312(p.Ile3703Met)];ENSG00000124721:MISSENSE[ENST00000441566(c.11001C>G,atC>atG):ENSP00000402294(p.Ile3667Met)];ENSG00000124721:MISSENSE[ENST00000327475(c.11724C>G,atC>atG):ENSP00000333363(p.Ile3908Met)];	NA	NA	NA	NA	NA	NA	NA	NA	NA_NA	NA	MODIFIER	NA	NA	190	190	0	123	79	44	35.77
Chewer	42483	chr6	28478537	G	A	*GPX6*	NONSENSE	ENSG00000198704:NONSENSE[ENST00000361902(c.232C>T,Cag>Tag):ENSP00000354581(p.Gln78Ter)];ENSG00000198704:NONSENSE[ENST00000474923(c.232C>T,Cag>Tag):ENSP00000417364(p.Gln78Ter)];ENSG00000198704:EXONIC-NC[ENST00000483058(n.451C>T)];	NA	NA	NA	NA	NA	NA	NA	NA	NA_NA	NA	MODIFIER	NA	NA	116	116	0	67	50	17	25.37
Chewer	42483	chr6	70407499	G	C	*LMBRD1*	NONSENSE	ENSG00000168216:NONSENSE[ENST00000370570(c.1154C>G,tCa>tGa):ENSP00000359602(p.Ser385Ter)];ENSG00000168216:NONSENSE[ENST00000370577(c.1373C>G,tCa>tGa):ENSP00000359609(p.Ser458Ter)];ENSG00000168216:3UTR[ENST00000472827(c.*127C>G)];	NA	NA	NA	NA	NA	NA	NA	NA	NA_NA	NA	MODIFIER	NA	NA	84	84	0	39	31	8	20.51
Chewer	42483	chr6	84108185	C	G	*ME1*	MISSENSE	ENSG00000065833:MISSENSE[ENST00000369705(c.263G>C,aGa>aCa):ENSP00000358719(p.Arg88Thr)];ENSG00000065833:MISSENSE[ENST00000543031(c.38G>C,aGa>aCa):ENSP00000446114(p.Arg13Thr)];ENSG00000065833:INTRONIC[ENST00000541327(c.-137+32411G>C)];	NA	NA	NA	NA	NA	NA	NA	NA	NA_NA	NA	MODIFIER	NA	NA	89	88	0	72	28	44	61.11
Chewer	42483	chr6	31726877	G	A	*MSH5*	MISSENSE	ENSG00000204410:MISSENSE[ENST00000395853(c.352G>A,Ggc>Agc):ENSP00000379194(p.Gly118Ser)];ENSG00000204410:MISSENSE[ENST00000375740(c.1381G>A,Ggc>Agc):ENSP00000364892(p.Gly461Ser)];ENSG00000204410:MISSENSE[ENST00000375755(c.1330G>A,Ggc>Agc):ENSP00000364908(p.Gly444Ser)];ENSG00000204410:MISSENSE[ENST00000375750(c.1330G>A,Ggc>Agc):ENSP00000364903(p.Gly444Ser)];ENSG00000204410:MISSENSE[ENST00000534153(c.1381G>A,Ggc>Agc):ENSP00000431693(p.Gly461Ser)];ENSG00000204410:MISSENSE[ENST00000450148(c.856G>A,Ggc>Agc):ENSP00000394971(p.Gly286Ser)];ENSG00000204410:MISSENSE[ENST00000375742(c.1381G>A,Ggc>Agc):ENSP00000364894(p.Gly461Ser)];ENSG00000204410:MISSENSE[ENST00000375703(c.1330G>A,Ggc>Agc):ENSP00000364855(p.Gly444Ser)];ENSG00000204410:MISSENSE[ENST00000431848(c.427G>A,Ggc>Agc):ENSP00000416784(p.Gly143Ser)];ENSG00000204410:MISSENSE[ENST00000423982(c.1330G>A,Ggc>Agc):ENSP00000406352(p.Gly444Ser)];ENSG00000204410:EXONIC-NC[ENST00000467319(n.286G>A)];ENSG00000204410:EXONIC-NC[ENST00000468602(n.603G>A)];ENSG00000204410:EXONIC-NC[ENST00000468136(n.536G>A)];ENSG00000204410:3UTR[ENST00000463144(c.*539G>A)];	NA	NA	NA	NA	NA	NA	NA	D	PrD_PrD	D	MODERATE	NA	NA	51	51	0	29	19	10	34.48

Mangalaparthi *et al.* , 2020. Mutational landscape of esophageal squamous cell carcinoma in an Indian cohort
Supplementary Table 4. List of somatic single nucleotide variants identified using whole exome sequencing of ESCC patients

Cohort	Sample	Chromosome	Genomic position	Reference allele	Alternate allele	Gene symbol	Variant type	Variant annotation	OncoMD	COSMIC	dbSNP	ExAC	ExAC_SAS	1000G	1000G_SAS	SIFT	Polyphen2	CONDEL	VEP	LRT	PhastCon7 Way	Total read (Adjacent normal)	Reference allel read support (Adjacent normal)	Alternative read support (Adjacent normal)	Total read (Tumor)	Reference allel read support (Tumor)	Alternative read support (Tumor)	Alternate allele frequency
Chewer	42483	chr6	31726877	G	A	*MSH5-SAPCD1*	MISSENSE	ENSG00000255152:MISSENSE[ENST00000493662(c.1381G>A,Ggc>Agc):ENSP00000417871(p.Gly461Ser)];	NA	NA	NA	NA	NA	NA	NA	D	PrD_PrD	D	MODERATE	NA	NA	51	51	0	29	19	10	34.48
Chewer	42483	chr6	72596888	C	T	*RIMS1*	SILENT-SS-PRX	ENSG00000079841:[ENST00000518273(c.162C>T,ctC>ctT):ENSP00000430408(p.Leu54(=))];ENSG00000079841:[ENST00000491071(c.162C>T,ctC>ctT):ENSP00000430101(p.Leu54(=))];ENSG00000079841:[ENST00000517960(c.162C>T,ctC>ctT):ENSP00000429959(p.Leu54(=))];ENSG00000079841:[ENST00000522291(c.162C>T,ctC>ctT):ENSP00000430932(p.Leu54(=))];ENSG00000079841:[ENST00000264839(c.162C>T,ctC>ctT):ENSP00000264839(p.Leu54(=))];ENSG00000079841:[ENST00000348717(c.162C>T,ctC>ctT):ENSP00000275037(p.Leu54(=))];ENSG00000079841:[ENST00000520567(c.162C>T,ctC>ctT):ENSP00000430502(p.Leu54(=))];ENSG00000079841:[ENST00000521978(c.162C>T,ctC>ctT):ENSP00000428417(p.Leu54(=))];ENSG00000079841:[ENST00000370419(n.483C>T)];	NA	NA	NA	NA	NA	NA	NA	NA	NA_NA	NA	LOW	NA	NA	49	49	0	38	13	25	65.79
Chewer	42483	chr6	38905946	C	G	*RP1-207H1.3*	INTRONIC	ENSG00000231150:[ENST00000416948(n.53-1778G>C)];	NA	NA	NA	NA	NA	NA	NA	NA	NA_NA	NA	MODIFIER	NA	NA	190	190	0	123	79	44	35.77
Chewer	42483	chr6	7229721	G	T	*RREB1*	SILENT	ENSG00000124782:SILENT[ENST00000349384(c.1389G>T,tcG>tcT):ENSP00000305560(p.Ser463(=))];ENSG00000124782:SILENT[ENST00000334984(c.1389G>T,tcG>tcT):ENSP00000335574(p.Ser463(=))];ENSG00000124782:SILENT[ENST00000379938(c.1389G>T,tcG>tcT):ENSP00000369270(p.Ser463(=))];ENSG00000124782:SILENT[ENST00000483150(c.1389G>T,tcG>tcT):ENSP00000419511(p.Ser463(=))];ENSG00000124782:SILENT[ENST00000379933(c.1389G>T,tcG>tcT):ENSP00000369265(p.Ser463(=))];	NA	NA	chr6*7229721*7229721*G*A,T*rs756737413*NA*NA*NA	8.32E-06	0	NA	NA	NA	NA_NA	NA	LOW	NA	NA	126	126	0	141	92	49	34.75
Chewer	42483	chr6	45513067	G	T	*RUNX2*	INTRONIC	ENSG00000124813:[ENST00000359524(c.1045+48G>T)];ENSG00000124813:[ENST00000352853(c.1291+48G>T)];ENSG00000124813:[ENST00000483377(c.*608+48G>T)];ENSG00000124813:[ENST00000541979(c.1226-1497G>T)];ENSG00000124813:[ENST00000371436(c.1022-1497G>T)];ENSG00000124813:[ENST00000465038(c.1087+48G>T)];ENSG00000124813:[ENST00000576263(c.1021+32923G>T)];ENSG00000124813:[ENST00000371438(c.1087+48G>T)];ENSG00000124813:[ENST00000371432(c.980-1497G>T)];ENSG00000124813:[ENST00000478660(c.*178+31677G>T)];	NA	NA	NA	NA	NA	NA	NA	NA	NA_NA	NA	MODIFIER	NA	NA	34	34	0	32	24	8	25
Chewer	42483	chr6	25862076	T	A	*SLC17A3*	MISSENSE	ENSG00000124564:MISSENSE[ENST00000397060(c.485A>T,gAc>gTc):ENSP00000380250(p.Asp162Val)];ENSG00000124564:INTRONIC[ENST00000360657(c.304-137A>T)];ENSG00000124564:INTRONIC[ENST00000503922(n.409-137A>T)];ENSG00000124564:INTRONIC[ENST00000449356(c.303+385A>T)];ENSG00000124564:INTRONIC[ENST00000361703(c.304-137A>T)];ENSG00000124564:INTRONIC[ENST00000506105(c.303+385A>T)];ENSG00000124564:INTRONIC[ENST00000509714(n.406-137A>T)];ENSG00000124564:EXONIC-NC[ENST00000308453(n.558A>T)];	NA	NA	NA	NA	NA	NA	NA	NA	NA_NA	NA	MODIFIER	NA	NA	218	216	2	128	92	36	28.12
Chewer	42483	chr6	152792828	C	A	*SYNE1*	SILENT	ENSG00000131018:SILENT[ENST00000466159(c.1536G>T,ctG>ctT):ENSP00000446021(p.Leu512(=))];ENSG00000131018:SILENT[ENST00000423061(c.1557G>T,ctG>ctT):ENSP00000396024(p.Leu519(=))];ENSG00000131018:SILENT[ENST00000537750(c.1485G>T,ctG>ctT):ENSP00000441264(p.Leu495(=))];ENSG00000131018:SILENT[ENST00000367255(c.1536G>T,ctG>ctT):ENSP00000356224(p.Leu512(=))];ENSG00000131018:SILENT[ENST00000413186(c.1536G>T,ctG>ctT):ENSP00000414510(p.Leu512(=))];ENSG00000131018:SILENT[ENST00000341594(c.1557G>T,ctG>ctT):ENSP00000341887(p.Leu519(=))];ENSG00000131018:SILENT[ENST00000367248(c.1506G>T,ctG>ctT):ENSP00000356217(p.Leu502(=))];ENSG00000131018:SILENT[ENST00000495090(c.237G>T,ctG>ctT):ENSP00000438508(p.Leu79(=))];ENSG00000131018:SILENT[ENST00000448038(c.1557G>T,ctG>ctT):ENSP00000390975(p.Leu519(=))];ENSG00000131018:SILENT[ENST00000367253(c.1536G>T,ctG>ctT):ENSP00000356222(p.Leu512(=))];ENSG00000131018:SILENT[ENST00000265368(c.1536G>T,ctG>ctT):ENSP00000265368(p.Leu512(=))];ENSG00000131018:EXONIC-NC[ENST00000535896(n.356G>T)];ENSG00000131018:EXONIC-NC[ENST00000468937(n.292G>T)];ENSG00000131018:EXONIC-NC[ENST00000461872(n.1754G>T)];ENSG00000131018:INTRONIC[ENST00000481502(n.170-23G>T)];ENSG00000131018:INTRONIC[ENST00000474655(c.161-23G>T)];	NA	NA	NA	NA	NA	NA	NA	NA	NA_NA	NA	LOW	NA	NA	87	87	0	52	23	29	55.77
Chewer	42483	chr6	83667170	C	G	*UBE3D*	INTRONIC-SS-ACR	ENSG00000118420:INTRONIC-SS-ACR[ENST00000430071(c.*706-1G>C)];ENSG00000118420:INTRONIC-SS-ACR[ENST00000369747(c.1011-1G>C)];ENSG00000118420:INTRONIC-SS-ACR[ENST00000237186(c.*862-1G>C)];ENSG00000118420:INTRONIC-SS-ACR[ENST00000509102(c.*130-1G>C)];	NA	NA	NA	NA	NA	NA	NA	NA	NA_NA	NA	HIGH	NA	NA	104	104	0	54	19	35	64.81
Chewer	42483	chr6	33282918	A	T	*ZBTB22*	SILENT	ENSG00000236104:SILENT[ENST00000431845(c.1776T>A,tcT>tcA):ENSP00000407545(p.Ser592(=))];ENSG00000236104:SILENT[ENST00000418724(c.1776T>A,tcT>tcA):ENSP00000404403(p.Ser592(=))];	NA	NA	NA	NA	NA	NA	NA	NA	NA_NA	NA	LOW	NA	NA	115	115	0	98	74	24	24.49
Chewer	42483	chr6	28964252	G	T	*ZNF311*	MISSENSE	ENSG00000197935:EXONIC-NC[ENST00000483450(n.1337C>A)];ENSG00000197935:MISSENSE[ENST00000377179(c.527C>A,tCc>tAc):ENSP00000366384(p.Ser176Tyr)];	NA	NA	NA	NA	NA	NA	NA	T	PrD_PrD	N	MODERATE	NA	NA	132	132	0	103	73	30	29.13
Chewer	42483	chr7	20795182	C	T	*ABCB5*	MISSENSE	ENSG00000004846:MISSENSE[ENST00000258738(c.2374C>T,Cat>Tat):ENSP00000258738(p.His792Tyr)];ENSG00000004846:MISSENSE[ENST00000404938(c.3709C>T,Cat>Tat):ENSP00000384881(p.His1237Tyr)];ENSG00000004846:INTRONIC[ENST00000441315(c.931-7697C>T)];	NA	NA	NA	NA	NA	NA	NA	NA	NA_NA	NA	MODIFIER	NA	NA	71	71	0	63	46	17	26.98
Chewer	42483	chr7	87758062	G	C	*ADAM22*	INTRONIC	ENSG00000008277:[ENST00000439864(c.753+71G>C)];ENSG00000008277:[ENST00000398209(c.753+71G>C)];ENSG00000008277:[ENST00000478920(n.459+71G>C)];ENSG00000008277:[ENST00000398203(c.654+71G>C)];ENSG00000008277:[ENST00000315984(c.753+71G>C)];ENSG00000008277:[ENST00000265727(c.753+71G>C)];ENSG00000008277:[ENST00000398201(c.753+71G>C)];ENSG00000008277:[ENST00000398204(c.753+71G>C)];	NA	NA	NA	NA	NA	NA	NA	NA	NA_NA	NA	MODIFIER	NA	NA	63	62	0	78	49	29	37.18
Chewer	42483	chr7	45662328	G	A	*ADCY1*	MISSENSE	ENSG00000164742:MISSENSE[ENST00000432715(c.331G>A,Gat>Aat):ENSP00000392721(p.Asp111Asn)];ENSG00000164742:MISSENSE[ENST00000297323(c.1006G>A,Gat>Aat):ENSP00000297323(p.Asp336Asn)];	chr7*45662	chr7*45662	NA	NA	NA	NA	NA	D	PrD_PrD	D	MODERATE	D	0.995	45	45	0	73	48	25	34.25
Chewer	42483	chr7	88423997	C	A	*C7orf62*	MISSENSE	ENSG00000164645:MISSENSE[ENST00000297203(c.260G>T,gGa>gTa):ENSP00000297203(p.Gly87Val)];	NA	chr7*88423	chr7*88423997*88423997*C*G*rs377125725*NA*NA*NA	NA	NA	NA	NA	NA	NA_NA	NA	MODIFIER	NA	NA	162	160	0	190	133	57	30

Supplementary Table 4. List of somatic single nucleotide variants identified using whole exome sequencing of ESCC patients

Cohort	Sample	Chromosome	Genomic position	Reference allele	Alternate allele	Gene symbol	Variant type	Variant annotation	OncoMD	COSMIC	dbSNP	ExAC	ExAC_SAS	1000G	1000G_SAS	SIFT	Polyphen2	CONDEL	VEP	LRT	PhastCon7 Way	Total read (Adjacent normal)	Reference allel read support (Adjacent normal)	Alternative read support (Adjacent normal)	Total read (Tumor)	Reference allel read support (Tumor)	Alternative read support (Tumor)	Alternate allele frequency
Chewer	42483	chr7	89887495	A	G	*C7orf63*	INTRONIC	ENSG00000105792:[ENST00000427396(c.246+18A>G)];ENSG00000105792:[ENST00000463311(n.246+18A>G)];ENSG00000105792:[ENST00000457170(c.66+18A>G)];ENSG00000105792:[ENST00000451029(c.246+18A>G)];ENSG00000105792:[ENST00000389297(c.246+18A>G)];ENSG00000105792:[ENST00000316089(c.246+18A>G)];ENSG00000105792:[ENST00000497910(c.246+18A>G)];ENSG00000105792:[ENST00000485791(n.476+18A>G)];	NA	NA	NA	NA	NA	NA	NA	NA	NA_NA	NA	MODIFIER	NA	NA	38	38	0	47	35	12	25.53
Chewer	42483	chr7	90528711	G	T	*CDK14*	INTRONIC	ENSG00000058091:[ENST00000436577(c.315+43G>T)];ENSG00000058091:[ENST00000487145(n.321+43G>T)];ENSG00000058091:[ENST00000406263(c.564+43G>T)];ENSG00000058091:[ENST00000265741(c.648+43G>T)];ENSG00000058091:[ENST00000380050(c.702+43G>T)];ENSG00000058091:[ENST00000431029(c.*127+43G>T)];	NA	NA	chr7*90528711*90528711*G*A*rs566859740*0*0.9998*0.0001997	NA	NA	NA	NA	NA	NA_NA	NA	MODIFIER	NA	NA	115	115	0	201	134	67	33.33
Chewer	42483	chr7	28449848	G	T	*CREB5*	INTRONIC	ENSG00000146592:[ENST00000424599(c.-63+215G>T)];ENSG00000146592:[ENST00000469531(n.89+806G>T)];ENSG00000146592:[ENST00000396299(c.-24-84676G>T)];ENSG00000146592:[ENST00000482692(n.620G>T)];ENSG00000146592:[ENST00000478078(n.391G>T)];	NA	NA	NA	NA	NA	NA	NA	NA	NA_NA	NA	MODIFIER	NA	NA	26	26	0	11	4	7	63.64
Chewer	42483	chr7	155251145	C	A	*EN2*	MISSENSE	ENSG00000164778:MISSENSE[ENST00000297375(c.73C>A,Ccc>Acc):ENSP00000297375(p.Pro25Thr)];	NA	NA	NA	NA	NA	NA	NA	T_lc	BN_BN	N	MODERATE	NA	NA	39	39	0	58	25	33	56.9
Chewer	42483	chr7	31003741	G	C	*GHRHR*	INTRONIC-SS-DNR	ENSG00000106128:INTRONIC-SS-DNR[ENST00000326139(c.57+1G>C)];ENSG00000106128:INTRONIC[ENST00000466427(n.285-4708G>C)];	NA	NA	chr7*31003741*31003741*G*A*rs2302022*NA*NA*NA	NA	NA	NA	NA	NA	NA_NA	NA	MODIFIER	NA	NA	24	24	0	17	8	9	52.94
Chewer	42483	chr7	100280413	G	C	*GIGYF1*	INTRONIC	ENSG00000146830:[ENST00000275732(c.2413-13C>G)];	NA	NA	NA	NA	NA	NA	NA	NA	NA_NA	NA	MODIFIER	NA	NA	52	52	0	56	38	18	32.14
Chewer	42483	chr7	100285026	C	T	*GIGYF1*	MISSENSE	ENSG00000146830:MISSENSE[ENST00000275732(c.377G>A,cGc>cAc):ENSP00000275732(p.Arg126His)];ENSG00000146830:EXONIC-NC[ENST00000471340(n.377G>A)];	NA	NA	0.0001997	8.34E-06	6.12E-05	0.0001997	0.001	NA	NA_NA	NA	MODIFIER	NA	NA	143	143	0	160	118	42	26.25
Chewer	42483	chr7	92855757	C	A	*HEPACAM2*	STARTLOSS	ENSG00000188175:STARTLOSS[ENST00000453812(c.3G>T,atG>atT):ENSP00000390204(p.Met1?)];ENSG00000188175:STARTLOSS[ENST00000394468(c.3G>T,atG>atT):ENSP00000377980(p.Met1?)];	NA	NA	NA	NA	NA	NA	NA	D_lc	BN_NA	N	HIGH	U	0.966	75	75	0	137	76	61	44.53
Chewer	42483	chr7	110603131	C	T	*IMMP2L*	SILENT	ENSG00000184903:INTRONIC[ENST00000489381(n.246+425G>A)];ENSG00000184903:INTRONIC[ENST00000450877(c.251+425G>A)];ENSG00000184903:INTRONIC[ENST00000415362(c.305+425G>A)];ENSG00000184903:INTRONIC[ENST00000452895(c.305+425G>A)];ENSG00000184903:INTRONIC[ENST00000405709(c.305+425G>A)];ENSG00000184903:INTRONIC[ENST00000331762(c.305+425G>A)];ENSG00000184903:INTRONIC[ENST00000487733(n.66+425G>A)];ENSG00000184903:SILENT[ENST00000437687(c.321G>A,ctG>ctA):ENSP00000394387(p.Leu107(=))];ENSG00000184903:SILENT[ENST00000447215(c.321G>A,ctG>ctA):ENSP00000388327(p.Leu107(=))];	NA	NA	chr7*110603126*110603133*CTTTTCAG*C*rs550790457*1*0.9988*0.001198	NA	NA	NA	NA	NA	NA_NA	NA	MODIFIER	NA	NA	91	91	0	81	48	33	40.74
Chewer	42483	chr7	86567500	G	A	*KIAA1324L*	MISSENSE	ENSG00000164659:MISSENSE[ENST00000444627(c.1031C>T,cCt>cTt):ENSP00000397377(p.Pro344Leu)];ENSG00000164659:MISSENSE[ENST00000416314(c.530C>T,cCt>cTt):ENSP00000402390(p.Pro177Leu)];ENSG00000164659:MISSENSE[ENST00000297222(c.311C>T,cCt>cTt):ENSP00000297222(p.Pro104Leu)];ENSG00000164659:MISSENSE[ENST00000423294(c.912C>T,cCt>cTt):ENSP00000406961(p.Pro305Leu)];ENSG00000164659:MISSENSE[ENST00000450689(c.1031C>T,cCt>cTt):ENSP00000413445(p.Pro344Leu)];ENSG00000164659:3UTR[ENST00000394714(c.*303C>T)];	NA	NA	NA	NA	NA	NA	NA	NA	NA_NA	NA	MODIFIER	NA	NA	178	176	1	393	205	188	47.84
Chewer	42483	chr7	86567501	G	A	*KIAA1324L*	MISSENSE	ENSG00000164659:MISSENSE[ENST00000416314(c.529C>T,Cct>Tct):ENSP00000402390(p.Pro177Ser)];ENSG00000164659:MISSENSE[ENST00000297222(c.310C>T,Cct>Tct):ENSP00000297222(p.Pro104Ser)];ENSG00000164659:MISSENSE[ENST00000444627(c.1030C>T,Cct>Tct):ENSP00000397377(p.Pro344Ser)];ENSG00000164659:MISSENSE[ENST00000450689(c.1030C>T,Cct>Tct):ENSP00000413445(p.Pro344Ser)];ENSG00000164659:MISSENSE[ENST00000423294(c.911C>T,Cct>Tct):ENSP00000406961(p.Pro305Ser)];ENSG00000164659:3UTR[ENST00000394714(c.*302C>T)];	NA	NA	NA	NA	NA	NA	NA	NA	NA_NA	NA	MODIFIER	NA	NA	178	175	1	394	205	189	47.97
Chewer	42483	chr7	152012336	A	T	*KMT2C*	MISSENSE	ENSG00000055609:MISSENSE[ENST00000355193(c.477T>A,ttT>ttA):ENSP00000347325(p.Phe159Leu)];ENSG00000055609:MISSENSE[ENST00000262189(c.477T>A,ttT>ttA):ENSP00000262189(p.Phe159Leu)];ENSG00000055609:MISSENSE[ENST00000558084(c.477T>A,ttT>ttA):ENSP00000453752(p.Phe159Leu)];	NA	NA	NA	NA	NA	NA	NA	T	BN_BN	N	MODERATE	NA	NA	203	203	0	142	59	83	58.45
Chewer	42483	chr7	141767118	G	C	*MGAM*	INTRONIC	ENSG00000257335:[ENST00000475668(c.4920-23G>C)];ENSG00000257335:[ENST00000549489(c.4618+1850G>C)];	NA	NA	chr7*141767118*141767118*G*A*rs754775931*NA*NA*NA	NA	NA	NA	NA	NA	NA_NA	NA	MODIFIER	NA	NA	65	65	0	43	18	25	58.14
Chewer	42483	chr7	141793403	G	T	*MGAM*	INTRONIC	ENSG00000257335:[ENST00000475668(c.7172+53G>T)];ENSG00000257335:[ENST00000549489(c.4619-1017G>T)];	NA	NA	NA	NA	NA	NA	NA	NA	NA_NA	NA	MODIFIER	NA	NA	146	146	0	111	47	64	57.66
Chewer	42483	chr7	100842124	G	C	*MOGAT3*	INTRONIC	ENSG00000106384:[ENST00000223114(c.289-13C>G)];ENSG00000106384:[ENST00000379423(c.289-13C>G)];ENSG00000106384:[ENST00000440203(c.289-13C>G)];	NA	NA	NA	NA	NA	NA	NA	NA	NA_NA	NA	MODIFIER	NA	NA	52	52	0	60	31	29	48.33
Chewer	42483	chr7	142211053	T	C	*NA*	INTERGENIC		NA	NA	NA	NA	NA	NA	NA	NA	NA_NA	NA	MODIFIER	NA	NA	108	108	0	74	28	46	62.16
Chewer	42483	chr7	31378559	G	T	*NEUROD6*	MISSENSE	ENSG00000164600:MISSENSE[ENST00000297142(c.324C>A,caC>caA):ENSP00000297142(p.His108Gln)];	NA	NA	chr7*31378559*31378559*G*A*rs148251003*0*0.9998*0.0001997	NA	NA	NA	NA	D	PrD_PrD	D	MODERATE	D	0.995	220	220	0	218	159	59	27.06
Chewer	42483	chr7	82580315	C	T	*PCLO*	MISSENSE	ENSG00000186472:MISSENSE[ENST00000333891(c.9589G>A,Gat>Aat):ENSP00000334319(p.Asp3197Asn)];ENSG00000186472:MISSENSE[ENST00000423517(c.9589G>A,Gat>Aat):ENSP00000388393(p.Asp3197Asn)];	NA	NA	NA	8.31E-06	0	NA	NA	NA	BN_PoD	NA	MODERATE	NA	NA	318	317	0	264	198	66	25
Chewer	42483	chr7	39379177	G	A	*POU6F2*	INTRONIC	ENSG00000106536:[ENST00000520104(c.488-64G>A)];ENSG00000106536:[ENST00000517348(n.973-64G>A)];ENSG00000106536:[ENST00000403058(c.512-64G>A)];ENSG00000106536:[ENST00000559001(c.488-64G>A)];ENSG00000106536:[ENST00000524147(c.488-64G>A)];ENSG00000106536:[ENST00000518318(c.512-64G>A)];	NA	NA	NA	NA	NA	NA	NA	NA	NA_NA	NA	MODIFIER	NA	NA	81	81	0	75	39	36	48
Chewer	42483	chr7	141539212	C	A	*PRSS37*	MISSENSE	ENSG00000165076:MISSENSE[ENST00000419085(c.102G>T,aaG>aaT):ENSP00000398810(p.Lys34Asn)];ENSG00000165076:MISSENSE[ENST00000438520(c.102G>T,aaG>aaT):ENSP00000414461(p.Lys34Asn)];ENSG00000165076:MISSENSE[ENST00000452758(c.70G>T,Gtc>Ttc):ENSP00000395287(p.Val24Phe)];ENSG00000165076:MISSENSE[ENST00000350549(c.102G>T,aaG>aaT):ENSP00000297767(p.Lys34Asn)];	NA	NA	NA	NA	NA	NA	NA	T	PrD_PrD	N	MODERATE	N	0.216	43	43	0	51	46	5	9.8

Mangalaparthi *et al.* , 2020. Mutational landscape of esophageal squamous cell carcinoma in an Indian cohort
Supplementary Table 4. List of somatic single nucleotide variants identified using whole exome sequencing of ESCC patients

Cohort	Sample	Chromosome	Genomic position	Reference allele	Alternate allele	Gene symbol	Variant type	Variant annotation	OncoMD	COSMIC	dbSNP	ExAC	ExAC_SAS	1000G	1000G_SAS	SIFT	Polyphen2	CONDEL	VEP	LRT	PhastCon7 Way	Total read (Adjacent normal)	Reference allel read support (Adjacent normal)	Alternative read support (Adjacent normal)	Total read (Tumor)	Reference allel read support (Tumor)	Alternative read support (Tumor)	Alternate allele frequency
Chewer	42483	chr7	100459297	G	T	*SLC12A9*	INTRONIC	ENSG00000146828:[ENST00000415287(c.1270-62G>T)];ENSG00000146828:[ENST00000354161(c.1537-62G>T)];ENSG00000146828:[ENST00000487651(n.2850-62G>T)];ENSG00000146828:[ENST00000467972(n.2821-62G>T)];ENSG00000146828:[ENST00000428758(c.1537-62G>T)];ENSG00000146828:[ENST00000482184(n.66-62G>T)];ENSG00000146828:[ENST00000475687(n.2516-62G>T)];ENSG00000146828:[ENST00000540482(c.1537-62G>T)];ENSG00000146828:[ENST00000275729(c.1270-62G>T)];	NA	NA	NA	NA	NA	NA	NA	NA	NA_NA	NA	MODIFIER	NA	NA	51	50	0	58	25	33	56.9
Chewer	42483	chr7	20824458	G	C	*SP8*	MISSENSE	ENSG00000164651:MISSENSE[ENST00000418710(c.978C>G,agC>agG):ENSP00000408792(p.Ser326Arg)];ENSG00000164651:MISSENSE[ENST00000361443(c.924C>G,agC>agG):ENSP00000354482(p.Ser308Arg)];	NA	NA	NA	NA	NA	NA	NA	D	BN_PoD	N	MODERATE	U	0.991	31	31	0	43	33	10	23.26
Chewer	42483	chr7	134931114	G	A	*STRA8*	INTRONIC	ENSG00000146857:[ENST00000275764(c.447-55G>A)];	NA	NA	NA	NA	NA	NA	NA	NA	NA_NA	NA	MODIFIER	NA	NA	73	72	1	52	20	32	61.54
Chewer	42483	chr7	111958321	G	C	*ZNF277*	MISSENSE	ENSG00000198839:MISSENSE[ENST00000450657(c.550G>C,Gga>Cga):ENSP00000402292(p.Gly184Arg)];ENSG00000198839:MISSENSE[ENST00000361822(c.550G>C,Gga>Cga):ENSP00000354501(p.Gly184Arg)];ENSG00000198839:3UTR[ENST00000361946(c.*393G>C)];ENSG00000198839:3UTR[ENST00000457808(c.*137G>C)];ENSG00000198839:INTRONIC[ENST00000425229(c.294-9451G>C)];	NA	NA	NA	1.65E-05	0.000121462	NA	NA	NA	NA_NA	NA	MODIFIER	NA	NA	50	50	0	45	17	27	60
Chewer	42483	chr7	99092120	C	G	*ZNF394*	MISSENSE	ENSG00000160908:MISSENSE[ENST00000337673(c.718G>C,Gag>Cag):ENSP00000337363(p.Glu240Gln)];ENSG00000160908:INTRONIC[ENST00000462024(n.83+4219G>C)];ENSG00000160908:3UTR[ENST00000426306(c.*102G>C)];	NA	NA	NA	NA	NA	NA	NA	NA	NA_NA	NA	MODIFIER	NA	NA	160	160	0	148	108	40	27.03
Chewer	42483	chr7	63721360	G	A	*ZNF679*	INTRONIC	ENSG00000197123:[ENST00000255746(c.262+53G>A)];ENSG00000197123:[ENST00000421025(c.262+53G>A)];	NA	NA	NA	NA	NA	NA	NA	NA	NA_NA	NA	MODIFIER	NA	NA	116	116	0	187	152	34	18.18
Chewer	42483	chr7	99092120	C	G	*ZNF789*	INTRONIC	ENSG00000198556:[ENST00000493485(c.131-9050C>G)];ENSG00000198556:[ENST00000494186(n.115-6226C>G)];	NA	NA	NA	NA	NA	NA	NA	NA	NA_NA	NA	MODIFIER	NA	NA	160	160	0	148	108	40	27.03
Chewer	42483	chr7	88423997	C	A	*ZNF804B*	INTRONIC	ENSG00000182348:[ENST00000333190(c.108+34599C>A)];	NA	chr7*88423	chr7*88423997*88423997*C*G*rs377125725*NA*NA*NA	NA	NA	NA	NA	NA	NA_NA	NA	MODIFIER	NA	NA	162	160	0	190	133	57	30
Chewer	42483	chr7	88956725	A	C	*ZNF804B*	MISSENSE	ENSG00000182348:MISSENSE[ENST00000333190(c.317A>C,aAa>aCa):ENSP00000329638(p.Lys106Thr)];	chr7*88956	NA	chr7*88956725*88956725*A*G*rs778201335*NA*NA*NA	NA	NA	NA	NA	D	PrD_PrD	D	MODERATE	D	0.998	168	168	0	249	206	43	17.27
Chewer	42483	chr8	42614173	T	C	*CHRNA6*	INTRONIC	ENSG00000147434:[ENST00000530869(n.510+39A>G)];ENSG00000147434:[ENST00000276410(c.264+39A>G)];ENSG00000147434:[ENST00000533810(c.27+39A>G)];ENSG00000147434:[ENST00000534622(c.220-1993A>G)];	NA	NA	NA	8.24E-06	0	NA	NA	NA	NA_NA	NA	MODIFIER	NA	NA	211	210	0	374	335	39	10.43
Chewer	42483	chr8	3057246	G	A	*CSMD1*	SILENT	ENSG00000183117:SILENT[ENST00000400186(c.5187C>T,ttC>ttT):ENSP00000383047(p.Phe1729(=))];ENSG00000183117:SILENT[ENST00000542608(c.5184C>T,ttC>ttT):ENSP00000446243(p.Phe1728(=))];ENSG00000183117:SILENT[ENST00000602723(c.5187C>T,ttC>ttT):ENSP00000473617(p.Phe1729(=))];ENSG00000183117:SILENT[ENST00000335551(c.3625C>T,ttC>ttT):ENSP00000334828(p.Phe1209(=))];ENSG00000183117:SILENT[ENST00000537824(c.5184C>T,ttC>ttT):ENSP00000441462(p.Phe1728(=))];ENSG00000183117:SILENT[ENST00000520002(c.5187C>T,ttC>ttT):ENSP00000430733(p.Phe1729(=))];ENSG00000183117:SILENT[ENST00000539096(c.5184C>T,ttC>ttT):ENSP00000441675(p.Phe1728(=))];ENSG00000183117:SILENT[ENST00000602557(c.5187C>T,ttC>ttT):ENSP00000473359(p.Phe1729(=))];ENSG00000183117:EXONIC-NC[ENST00000523387(n.1417C>T)];ENSG00000183117:EXONIC-NC[ENST00000523488(n.2717C>T)];	chr8*30572	chr8*30572	NA	0	0	NA	NA	NA	NA_NA	NA	LOW	NA	0.995	60	59	0	40	22	18	45
Chewer	42483	chr8	113585818	C	A	*CSMD3*	SILENT	ENSG00000164796:SILENT[ENST00000352409(c.3954G>T,ctG>ctT):ENSP00000343124(p.Leu1318(=))];ENSG00000164796:SILENT[ENST00000455883(c.3642G>T,ctG>ctT):ENSP00000412263(p.Leu1214(=))];ENSG00000164796:SILENT[ENST00000297405(c.3954G>T,ctG>ctT):ENSP00000297405(p.Leu1318(=))];ENSG00000164796:SILENT[ENST00000339701(c.1974G>T,ctG>ctT):ENSP00000341558(p.Leu658(=))];ENSG00000164796:SILENT[ENST00000343508(c.3834G>T,ctG>ctT):ENSP00000345799(p.Leu1278(=))];	NA	NA	NA	NA	NA	NA	NA	NA	NA_NA	NA	LOW	NA	NA	228	227	0	257	74	183	71.21
Chewer	42483	chr8	113812485	T	C	*CSMD3*	SILENT	ENSG00000164796:SILENT[ENST00000352409(c.1878A>G,gtA>gtG):ENSP00000343124(p.Val626(=))];ENSG00000164796:SILENT[ENST00000455883(c.1566A>G,gtA>gtG):ENSP00000412263(p.Val522(=))];ENSG00000164796:SILENT[ENST00000297405(c.1878A>G,gtA>gtG):ENSP00000297405(p.Val626(=))];ENSG00000164796:SILENT[ENST00000343508(c.1758A>G,gtA>gtG):ENSP00000345799(p.Val586(=))];	NA	NA	NA	NA	NA	NA	NA	NA	NA_NA	NA	LOW	NA	NA	166	166	0	244	205	39	15.98
Chewer	42483	chr8	109254466	T	C	*EIF3E*	MISSENSE	ENSG00000104408:INTRONIC[ENST00000519030(c.-74-2162A>G)];ENSG00000104408:INTRONIC[ENST00000518442(n.191-324A>G)];ENSG00000104408:INTRONIC[ENST00000518634(n.525-324A>G)];ENSG00000104408:INTRONIC[ENST00000521440(c.87-324A>G)];ENSG00000104408:INTRONIC[ENST00000521297(c.57-324A>G)];ENSG00000104408:INTRONIC[ENST00000518345(c.-57-324A>G)];ENSG00000104408:INTRONIC[ENST00000220849(c.91-324A>G)];ENSG00000104408:INTRONIC[ENST00000519627(c.90+6376A>G)];ENSG00000104408:INTRONIC[ENST00000522445(c.91-324A>G)];ENSG00000104408:3UTR[ENST00000518100(c.*62A>G)];ENSG00000104408:EXONIC-NC[ENST00000521614(n.597A>G)];ENSG00000104408:MISSENSE[ENST00000522887(c.110A>G,tAt>tGt):ENSP00000430649(p.Tyr37Cys)];	NA	NA	NA	NA	NA	NA	NA	NA	UN_UN	NA	MODERATE	NA	NA	206	206	0	254	157	97	38.19

Mangalaparthi *et al.* , 2020. Mutational landscape of esophageal squamous cell carcinoma in an Indian cohort
Supplementary Table 4. List of somatic single nucleotide variants identified using whole exome sequencing of ESCC patients

Cohort	Sample	Chromosome	Genomic position	Reference allele	Alternate allele	Gene symbol	Variant type	Variant annotation	OncoMD	COSMIC	dbSNP	ExAC	ExAC_SAS	1000G	1000G_SAS	SIFT	Polyphen2	CONDEL	VEP	LRT	PhastCon7 Way	Total read (Adjacent normal)	Reference allel read support (Adjacent normal)	Alternative read support (Adjacent normal)	Total read (Tumor)	Reference allel read support (Tumor)	Alternative read support (Tumor)	Alternate allele frequency
Chewer	42483	chr8	144940217	T	C	*EPPK1*	MISSENSE	ENSG00000227184:MISSENSE[ENST00000525985(c.7205A>G,cAg>cGg):ENSP00000436337(p.Gln2402Arg)];	NA	chr8*14494	chr8*144940205*144940217*AGGGTGGCCCTCT*A*rs782603773*NA*NA*NA|chr8*144940211*144940220*GCCCTCTGCA*G*rs782374379*NA*NA*NA|chr8*144940212*144940217*CCCTCT*C*rs145250036*NA*NA*NA|chr8*144940217*144940217*T*A,C*rs201070164*NA*NA*NA	0.0060423	0.026666667	NA	NA	NA	BN_BN	NA	MODERATE	NA	NA	84	84	0	122	115	7	5.74
Chewer	42483	chr8	28567932	G	T	*EXTL3*	INTRONIC	ENSG00000012232:[ENST00000520940(n.486-3042G>T)];ENSG00000012232:[ENST00000522725(n.423-3042G>T)];ENSG00000012232:[ENST00000519288(n.179-3042G>T)];ENSG00000012232:[ENST00000523149(c.-52-3042G>T)];ENSG00000012232:[ENST00000519886(n.56-3042G>T)];ENSG00000012232:[ENST00000220562(c.-569-3042G>T)];ENSG00000012232:[ENST00000454906(n.420-3042G>T)];ENSG00000012232:[ENST00000521532(c.-47G>T)];ENSG00000012232:[ENST00000518223(n.61G>T)];	NA	NA	NA	NA	NA	NA	NA	NA	NA_NA	NA	MODIFIER	NA	NA	180	179	1	166	110	55	33.13
Chewer	42483	chr8	41355247	G	A	*GOLGA7*	INTRONIC	ENSG00000147533:[ENST00000357743(c.264+67G>A)];ENSG00000147533:[ENST00000518270(c.264+67G>A)];ENSG00000147533:[ENST00000405786(c.264+67G>A)];ENSG00000147533:[ENST00000523420(n.327+67G>A)];ENSG00000147533:[ENST00000520817(c.264+67G>A)];	NA	NA	NA	NA	NA	NA	NA	NA	NA_NA	NA	MODIFIER	NA	NA	55	55	0	67	42	25	37.31
Chewer	42483	chr8	89198806	G	T	*MMP16*	NONSENSE	ENSG00000156103:NONSENSE[ENST00000286614(c.303C>A,tgC>tgA):ENSP00000286614(p.Cys101Ter)];ENSG00000156103:NONSENSE[ENST00000522726(c.354C>A,tgC>tgA):ENSP00000429147(p.Cys118Ter)];ENSG00000156103:EXONIC-NC[ENST00000544227(n.303C>A)];ENSG00000156103:EXONIC-NC[ENST00000520568(n.353C>A)];	chr8*89198	chr8*89198	chr8*89198806*89198806*G*A*rs371332118*NA*NA*NA	NA	NA	NA	NA	NA	NA_NA	NA	MODIFIER	NA	NA	123	122	0	175	43	130	74.29
Chewer	42483	chr8	107694778	G	C	*OXR1*	INTRONIC	ENSG00000164830:[ENST00000497705(c.103-649G>C)];ENSG00000164830:[ENST00000442977(c.307-649G>C)];ENSG00000164830:[ENST00000312046(c.283-649G>C)];ENSG00000164830:[ENST00000517686(c.97-649G>C)];ENSG00000164830:[ENST00000445937(c.304-649G>C)];ENSG00000164830:[ENST00000531443(c.304-649G>C)];ENSG00000164830:[ENST00000452423(c.-1227-649G>C)];ENSG00000164830:[ENST00000517566(c.304-649G>C)];ENSG00000164830:[ENST00000435082(c.199+24365G>C)];ENSG00000164830:[ENST00000438229(c.*60-649G>C)];ENSG00000164830:[ENST00000517455(n.53+5G>C)];	NA	NA	NA	NA	NA	NA	NA	NA	NA_NA	NA	MODIFIER	NA	NA	105	105	0	139	44	95	68.35
Chewer	42483	chr8	52325800	G	C	*PXDNL*	MISSENSE	ENSG00000147485:MISSENSE[ENST00000543296(c.1814C>G,gCt>gGt):ENSP00000444865(p.Ala605Gly)];ENSG00000147485:MISSENSE[ENST00000356297(c.1814C>G,gCt>gGt):ENSP00000348645(p.Ala605Gly)];	NA	NA	NA	NA	NA	NA	NA	T	BN_PoD	N	MODERATE	NA	NA	198	197	0	145	96	49	33.79
Chewer	42483	chr8	85618215	G	A	*RALYL*	5UTR	ENSG00000184672:[ENST00000523850(c.-44G>A)];ENSG00000184672:[ENST00000521376(c.-44G>A)];ENSG00000184672:[ENST00000521268(c.257-68599G>A)];ENSG00000184672:[ENST00000522455(c.257-68599G>A)];ENSG00000184672:[ENST00000517638(c.296-68599G>A)];ENSG00000184672:[ENST00000517988(c.*1-68599G>A)];ENSG00000184672:[ENST00000521695(c.257-68599G>A)];ENSG00000184672:[ENST00000518566(c.257-68599G>A)];	NA	NA	chr8*85618215*85618215*G*T*rs761618499*NA*NA*NA	NA	NA	NA	NA	NA	NA_NA	NA	MODIFIER	NA	NA	91	91	0	93	32	61	65.59
Chewer	42483	chr8	41122911	C	A	*SFRP1*	MISSENSE	ENSG00000104332:MISSENSE[ENST00000379845(c.312G>T,aaG>aaT):ENSP00000369174(p.Lys104Asn)];ENSG00000104332:MISSENSE[ENST00000220772(c.720G>T,aaG>aaT):ENSP00000220772(p.Lys240Asn)];	NA	NA	NA	NA	NA	NA	NA	T	PoD_PrD	D	MODERATE	D	1	63	63	0	50	36	13	26
Chewer	42483	chr8	92355774	C	T	*SLC26A7*	INTRONIC	ENSG00000147606:[ENST00000520249(n.268+80C>T)];ENSG00000147606:[ENST00000276609(c.1140+80C>T)];ENSG00000147606:[ENST00000309536(c.1140+80C>T)];ENSG00000147606:[ENST00000523719(c.1140+80C>T)];ENSG00000147606:[ENST00000522181(c.*247+80C>T)];	NA	NA	NA	NA	NA	NA	NA	NA	NA_NA	NA	MODIFIER	NA	NA	30	30	0	35	11	24	68.57

Mangalaparthi *et al.* , 2020. Mutational landscape of esophageal squamous cell carcinoma in an Indian cohort
Supplementary Table 4. List of somatic single nucleotide variants identified using whole exome sequencing of ESCC patients

Cohort	Sample	Chromosome	Genomic position	Reference allele	Alternate allele	Gene symbol	Variant type	Variant annotation	OncoMD	COSMIC	dbSNP	ExAC	ExAC_SAS	1000G	1000G_SAS	SIFT	Polyphen2	CONDEL	VEP	LRT	PhastCon7 Way	Total read (Adjacent normal)	Reference allel read support (Adjacent normal)	Alternative read support (Adjacent normal)	Total read (Tumor)	Reference allel read support (Tumor)	Alternative read support (Tumor)	Alternate allele frequency
Chewer	42483	chr8	110655056	C	T	*SYBU*	MISSENSE	ENSG00000147642:MISSENSE[ENST00000422135(c.130G>A,Gcc>Acc):ENSP00000407118(p.Ala44Thr)];ENSG00000147642:MISSENSE[ENST00000534578(c.130G>A,Gcc>Acc):ENSP00000431895(p.Ala44Thr)];ENSG00000147642:MISSENSE[ENST00000424158(c.145G>A,Gcc>Acc):ENSP00000415654(p.Ala49Thr)];ENSG00000147642:MISSENSE[ENST00000527600(c.130G>A,Gcc>Acc):ENSP00000434612(p.Ala44Thr)];ENSG00000147642:MISSENSE[ENST00000533821(c.127G>A,Gcc>Acc):ENSP00000431469(p.Ala43Thr)];ENSG00000147642:MISSENSE[ENST00000408908(c.130G>A,Gcc>Acc):ENSP00000386154(p.Ala44Thr)];ENSG00000147642:MISSENSE[ENST00000526302(c.130G>A,Gcc>Acc):ENSP00000434061(p.Ala44Thr)];ENSG00000147642:MISSENSE[ENST00000524720(c.130G>A,Gcc>Acc):ENSP00000435224(p.Ala44Thr)];ENSG00000147642:MISSENSE[ENST00000534501(c.130G>A,Gcc>Acc):ENSP00000434840(p.Ala44Thr)];ENSG00000147642:MISSENSE[ENST00000419099(c.127G>A,Gcc>Acc):ENSP00000402652(p.Ala43Thr)];ENSG00000147642:MISSENSE[ENST00000528647(c.127G>A,Gcc>Acc):ENSP00000432501(p.Ala43Thr)];ENSG00000147642:MISSENSE[ENST00000533171(c.130G>A,Gcc>Acc):ENSP00000434679(p.Ala44Thr)];ENSG00000147642:MISSENSE[ENST00000433638(c.130G>A,Gcc>Acc):ENSP00000403544(p.Ala44Thr)];ENSG00000147642:MISSENSE[ENST00000440310(c.130G>A,Gcc>Acc):ENSP00000404369(p.Ala44Thr)];ENSG00000147642:MISSENSE[ENST00000529190(c.127G>A,Gcc>Acc):ENSP00000432835(p.Ala43Thr)];ENSG00000147642:MISSENSE[ENST00000446070(c.127G>A,Gcc>Acc):ENSP00000414748(p.Ala43Thr)];ENSG00000147642:MISSENSE[ENST00000534184(c.127G>A,Gcc>Acc):ENSP00000432291(p.Ala43Thr)];ENSG00000147642:MISSENSE[ENST00000399066(c.121G>A,Gcc>Acc):ENSP00000382019(p.Ala41Thr)];ENSG00000147642:MISSENSE[ENST00000276646(c.130G>A,Gcc>Acc):ENSP00000276646(p.Ala44Thr)];ENSG00000147642:MISSENSE[ENST00000533895(c.127G>A,Gcc>Acc):ENSP00000433446(p.Ala43Thr)];ENSG00000147642:MISSENSE[ENST00000528716(c.130G>A,Gcc>Acc):ENSP00000436520(p.Ala44Thr)];ENSG00000147642:INTRONIC[ENST00000532779(c.26-	NA	NA	chr8*110655056*110655056*C*A*rs752117798*NA*NA*NA	NA	NA	NA	NA	T_lc	BN_BN	N	MODERATE	N	NA	57	57	0	83	66	17	20.48
Chewer	42483	chr8	35453154	A	G	*UNC5D*	SILENT	ENSG00000156687:SILENT[ENST00000453357(c.534A>G,ccA>ccG):ENSP00000394303(p.Pro178(=))];ENSG00000156687:SILENT[ENST00000416672(c.549A>G,ccA>ccG):ENSP00000412652(p.Pro183(=))];ENSG00000156687:SILENT[ENST00000404895(c.549A>G,ccA>ccG):ENSP00000385143(p.Pro183(=))];ENSG00000156687:SILENT[ENST00000287272(c.549A>G,ccA>ccG):ENSP00000287272(p.Pro183(=))];ENSG00000156687:SILENT[ENST00000420357(c.549A>G,ccA>ccG):ENSP00000392739(p.Pro183(=))];	NA	NA	NA	8.31E-06	0	NA	NA	NA	NA_NA	NA	LOW	NA	NA	96	95	1	75	51	24	32
Chewer	42483	chr9	140964124	C	T	*CACNA1B*	INTRONIC	ENSG00000148408:[ENST00000371355(c.4677+1059C>T)];ENSG00000148408:[ENST00000371357(c.4672-3816C>T)];ENSG00000148408:[ENST00000277549(c.2257-3816C>T)];ENSG00000148408:[ENST00000277551(c.4669-86C>T)];ENSG00000148408:[ENST00000371363(c.4669-3816C>T)];ENSG00000148408:[ENST00000371372(c.4669-86C>T)];	NA	NA	NA	NA	NA	NA	NA	NA	NA_NA	NA	MODIFIER	NA	NA	116	116	0	164	131	33	20.12
Chewer	42483	chr9	114393885	G	A	*DNAJC25*	SILENT	ENSG00000059769:SILENT[ENST00000463589(c.198G>A,gcG>gcA):ENSP00000464086(p.Ala66(=))];ENSG00000059769:SILENT[ENST00000556107(c.198G>A,gcG>gcA):ENSP00000451984(p.Ala66(=))];ENSG00000059769:SILENT[ENST00000447096(c.198G>A,gcG>gcA):ENSP00000399325(p.Ala66(=))];ENSG00000059769:SILENT[ENST00000313525(c.198G>A,gcG>gcA):ENSP00000320650(p.Ala66(=))];	NA	NA	NA	NA	NA	NA	NA	NA	NA_NA	NA	LOW	NA	NA	39	37	2	81	9	72	88.89
Chewer	42483	chr9	114393885	G	A	*DNAJC25-GNG10*	SILENT	ENSG00000244115:SILENT[ENST00000374294(c.198G>A,gcG>gcA):ENSP00000363412(p.Ala66(=))];	NA	NA	NA	NA	NA	NA	NA	NA	NA_NA	NA	LOW	NA	NA	39	37	2	81	9	72	88.89
Chewer	42483	chr9	368283	T	C	*DOCK8*	MISSENSE	ENSG00000107099:INTRONIC[ENST00000432829(c.1593+148T>C)];ENSG00000107099:INTRONIC[ENST00000483757(c.*1046+148T>C)];ENSG00000107099:INTRONIC[ENST00000453981(c.1797+148T>C)];ENSG00000107099:INTRONIC[ENST00000382331(c.-159+148T>C)];ENSG00000107099:INTRONIC[ENST00000469391(c.1593+148T>C)];ENSG00000107099:EXONIC-NC[ENST00000495184(n.1806T>C)];ENSG00000107099:MISSENSE[ENST00000382329(c.181T>C,Tat>Cat):ENSP00000371766(p.Tyr61His)];	NA	NA	NA	NA	NA	NA	NA	T	BN_BN	N	MODERATE	NA	NA	99	99	0	60	28	32	53.33
Chewer	42483	chr9	140007566	C	T	*DPP7*	MISSENSE	ENSG00000176978:MISSENSE[ENST00000473532(c.340G>A,cGa>cAa):ENSP00000473430(p.Arg114Gln)];ENSG00000176978:MISSENSE[ENST00000371579(c.709G>A,Gac>Aac):ENSP00000360635(p.Asp237Asn)];ENSG00000176978:EXONIC-NC[ENST00000482088(n.143G>A)];ENSG00000176978:EXONIC-NC[ENST00000460830(n.50G>A)];ENSG00000176978:EXONIC-NC[ENST00000485456(n.648G>A)];ENSG00000176978:EXONIC-NC[ENST00000478597(n.792G>A)];ENSG00000176978:3UTR[ENST00000472306(c.*185G>A)];	NA	NA	0.0003994	8.28E-05	0	0.0003994	0	NA	NA_NA	NA	MODIFIER	NA	NA	82	82	0	169	143	26	15.38
Chewer	42483	chr9	112042089	T	C	*EPB41L4B*	INTRONIC-SS-DNR	ENSG00000095203:[ENST00000374566(c.411+8A>G)];ENSG00000095203:[ENST00000374557(c.411+8A>G)];	NA	NA	NA	NA	NA	NA	NA	NA	NA_NA	NA	LOW	NA	NA	101	101	0	185	124	61	32.97
Chewer	42483	chr9	34833115	C	G	*FAM205B*	EXONIC-NC	ENSG00000257198:[ENST00000455647(n.3279G>C)];ENSG00000257198:[ENST00000399773(n.3186G>C)];	NA	NA	NA	NA	NA	NA	NA	NA	NA_NA	NA	MODIFIER	NA	NA	99	99	0	93	69	24	25.81
Chewer	42483	chr9	69002366	T	A	*NA*	INTERGENIC		NA	NA	NA	NA	NA	NA	NA	NA	NA_NA	NA	MODIFIER	NA	NA	139	137	1	94	88	6	6.38
Chewer	42483	chr9	69002370	G	T	*NA*	INTERGENIC		NA	NA	chr9*69002370*69002370*G*A*rs757159213*NA*NA*NA	NA	NA	NA	NA	NA	NA_NA	NA	MODIFIER	NA	NA	133	131	1	91	85	6	6.59
Chewer	42483	chr9	139393442	G	A	*NOTCH1*	MISSENSE	ENSG00000148400:MISSENSE[ENST00000277541(c.6089C>T,tCc>tTc):ENSP00000277541(p.Ser2030Phe)];	NA	NA	NA	NA	NA	NA	NA	D	PrD_PrD	D	MODERATE	D	0.968	165	165	0	275	60	215	78.18
Chewer	42483	chr9	27157924	C	T	*TEK*	MISSENSE	ENSG00000120156:MISSENSE[ENST00000406359(c.148C>T,Cgc>Tgc):ENSP00000383977(p.Arg50Cys)];ENSG00000120156:MISSENSE[ENST00000380036(c.148C>T,Cgc>Tgc):ENSP00000369375(p.Arg50Cys)];ENSG00000120156:INTRONIC[ENST00000519097(c.53-10569C>T)];ENSG00000120156:INTRONIC[ENST00000519080(c.53-11569C>T)];	NA	NA	NA	5.77E-05	0	NA	NA	T	BN_BN	N	MODERATE	N	0.975	205	205	0	148	103	45	30.41
Chewer	42483	chrX	49957759	A	C	*AKAP4*	SILENT	ENSG00000147081:INTRONIC[ENST00000448865(c.542-86T>G)];ENSG00000147081:INTRONIC[ENST00000376058(c.542-86T>G)];ENSG00000147081:SILENT[ENST00000358526(c.1605T>G,gcT>gcG):ENSP00000351327(p.Ala535(=))];ENSG00000147081:SILENT[ENST00000376056(c.1578T>G,gcT>gcG):ENSP00000365224(p.Ala526(=))];ENSG00000147081:SILENT[ENST00000376064(c.1578T>G,gcT>gcG):ENSP00000365232(p.Ala526(=))];ENSG00000147081:EXONIC-NC[ENST00000481402(n.1717T>G)];	NA	NA	NA	NA	NA	NA	NA	NA	NA_NA	NA	MODIFIER	NA	NA	108	108	0	69	23	46	66.67

Mangalaparthi *et al.* , 2020. Mutational landscape of esophageal squamous cell carcinoma in an Indian cohort
Supplementary Table 4. List of somatic single nucleotide variants identified using whole exome sequencing of ESCC patients

Cohort	Sample	Chromosome	Genomic position	Reference allele	Alternate allele	Gene symbol	Variant type	Variant annotation	OncoMD	COSMIC	dbSNP	ExAC	ExAC_SAS	1000G	1000G_SAS	SIFT	Polyphen2	CONDEL	VEP	LRT	PhastCon7 Way	Total read (Adjacent normal)	Reference allel read support (Adjacent normal)	Alternative read support (Adjacent normal)	Total read (Tumor)	Reference allel read support (Tumor)	Alternative read support (Tumor)	Alternate allele frequency
Chewer	42483	chrX	152988965	G	A	*BCAP31*	INTRONIC	ENSG00000185825:[ENST00000423827(c.-44-222C>T)];ENSG00000185825:[ENST00000429550(c.-44-222C>T)];ENSG00000185825:[ENST00000442093(c.-44-222C>T)];ENSG00000185825:[ENST00000441714(c.-44-222C>T)];ENSG00000185825:[ENST00000416815(c.-44-222C>T)];ENSG00000185825:[ENST00000345046(c.-44-222C>T)];ENSG00000185825:[ENST00000458587(c.155C>T,gCa>gTa):ENSP00000392330(p.Ala52Val)];	NA	NA	NA	NA	NA	NA	NA	NA	NA_NA	NA	MODIFIER	NA	NA	43	43	0	49	43	6	12.24
Chewer	42483	chrX	36371699	G	T	*CXorf30*	SILENT	ENSG00000205081:SILENT[ENST00000378657(c.1092G>T,ctG>ctT):ENSP00000367926(p.Leu364(=))];ENSG00000205081:SILENT[ENST00000378653(c.1947G>T,ctG>ctT):ENSP00000367922(p.Leu649(=))];	NA	NA	NA	NA	NA	NA	NA	NA	NA_NA	NA	LOW	NA	NA	86	86	0	58	26	32	55.17
Chewer	42483	chrX	32305646	C	A	*DMD*	INTRONIC	ENSG00000198947:[ENST00000488902(n.336-70466G>T)];ENSG00000198947:[ENST00000357033(c.6290G>T,gGg>gTg):ENSP00000354923(p.Gly2097Val)];ENSG00000198947:[ENST00000378677(c.6278G>T,gGg>gTg):ENSP00000367948(p.Gly2093Val)];	NA	NA	NA	NA	NA	NA	NA	NA	PrD_PrD	NA	MODERATE	U	0.992	70	70	0	47	19	28	59.57
Chewer	42483	chrX	8767024	G	A	*FAM9A*	MISSENSE	ENSG00000183304:MISSENSE[ENST00000543214(c.203C>T,cCg>cTg):ENSP00000440163(p.Pro68Leu)];ENSG00000183304:MISSENSE[ENST00000381003(c.203C>T,cCg>cTg):ENSP00000370391(p.Pro68Leu)];	NA	NA	0.00106	3.45E-05	0	0.0010596	0	T_lc	PrD_PrD	N	MODERATE	NA	NA	91	89	1	67	27	40	59.7
Chewer	42483	chrX	48826550	C	A	*KCND1*	SILENT	ENSG00000102057:SILENT[ENST00000218176(c.129G>T,gtG>gtT):ENSP00000218176(p.Val43(=))];	NA	NA	NA	NA	NA	NA	NA	NA	NA_NA	NA	LOW	NA	NA	112	111	0	94	88	6	6.38
Chewer	42483	chrX	138708366	G	T	*MCF2*	MISSENSE	ENSG00000101977:MISSENSE[ENST00000519895(c.853C>A,Cag>Aag):ENSP00000430276(p.Gln285Lys)];ENSG00000101977:MISSENSE[ENST00000370573(c.673C>A,Cag>Aag):ENSP00000359605(p.Gln225Lys)];ENSG00000101977:MISSENSE[ENST00000536274(c.556C>A,Cag>Aag):ENSP00000438155(p.Gln186Lys)];ENSG00000101977:MISSENSE[ENST00000520602(c.853C>A,Cag>Aag):ENSP00000427745(p.Gln285Lys)];ENSG00000101977:MISSENSE[ENST00000414978(c.853C>A,Cag>Aag):ENSP00000397055(p.Gln285Lys)];ENSG00000101977:MISSENSE[ENST00000370578(c.1108C>A,Cag>Aag):ENSP00000359610(p.Gln370Lys)];ENSG00000101977:MISSENSE[ENST00000370576(c.673C>A,Cag>Aag):ENSP00000359608(p.Gln225Lys)];ENSG00000101977:MISSENSE[ENST00000338585(c.673C>A,Cag>Aag):ENSP00000342204(p.Gln225Lys)];	NA	NA	NA	NA	NA	NA	NA	T	BN_BN	N	MODERATE	D	0.937	44	43	0	23	10	13	56.52
Chewer	42483	chrX	69478546	C	A	*P2RY4*	MISSENSE	ENSG00000186912:MISSENSE[ENST00000374519(c.929G>T,gGg>gTg):ENSP00000363643(p.Gly310Val)];	NA	NA	NA	NA	NA	NA	NA	D	PoD_PoD	D	MODERATE	U	0.991	111	111	0	131	33	98	74.81
Chewer	42483	chrX	91873298	G	C	*PCDH11X*	MISSENSE	ENSG00000102290:MISSENSE[ENST00000406881(c.3379G>C,Gcc>Ccc):ENSP00000384758(p.Ala1127Pro)];ENSG00000102290:MISSENSE[ENST00000373097(c.3373G>C,Gcc>Ccc):ENSP00000362189(p.Ala1125Pro)];ENSG00000102290:MISSENSE[ENST00000373094(c.3403G>C,Gcc>Ccc):ENSP00000362186(p.Ala1135Pro)];ENSG00000102290:MISSENSE[ENST00000373088(c.3292G>C,Gcc>Ccc):ENSP00000362180(p.Ala1098Pro)];ENSG00000102290:MISSENSE[ENST00000361655(c.3349G>C,Gcc>Ccc):ENSP00000355105(p.Ala1117Pro)];ENSG00000102290:MISSENSE[ENST00000298274(c.3292G>C,Gcc>Ccc):ENSP00000298274(p.Ala1098Pro)];ENSG00000102290:3UTR[ENST00000504220(c.*42G>C)];	NA	NA	NA	NA	NA	NA	NA	NA	NA_NA	NA	MODIFIER	NA	NA	121	121	0	72	34	38	52.78
Chewer	42483	chrX	73744297	C	T	*SLC16A2*	MISSENSE	ENSG00000147100:MISSENSE[ENST00000587091(c.679C>T,Cgc>Tgc):ENSP00000465734(p.Arg227Cys)];ENSG00000147100:MISSENSE[ENST00000276033(c.901C>T,Cgc>Tgc):ENSP00000276033(p.Arg301Cys)];ENSG00000147100:MISSENSE[ENST00000590447(c.119C>T,Cgc>Tgc):ENSP00000466213(p.Arg41Cys)];	NA	NA	NA	NA	NA	NA	NA	D	PrD_PrD	D	MODERATE	NA	NA	42	42	0	71	46	25	35.21
Chewer	42483	chrX	100169483	C	A	*XKRX*	MISSENSE	ENSG00000182489:MISSENSE[ENST00000328526(c.1233G>T,atG>atT):ENSP00000327570(p.Met411Ile)];ENSG00000182489:MISSENSE[ENST00000372956(c.1194G>T,atG>atT):ENSP00000362047(p.Met398Ile)];ENSG00000182489:3UTR[ENST00000468904(c.*505G>T)];	NA	NA	NA	NA	NA	NA	NA	NA	NA_NA	NA	MODIFIER	NA	NA	87	87	0	118	24	94	79.66
Chewer	42484	chr1	167780109	G	A	*ADCY10*	SILENT	ENSG00000143199:3UTR[ENST00000485964(c.*1460C>T)];ENSG00000143199:SILENT[ENST00000545172(c.4065C>T,gtC>gtT):ENSP00000441992(p.Val1355(=))];ENSG00000143199:SILENT[ENST00000367851(c.4524C>T,gtC>gtT):ENSP00000356825(p.Val1508(=))];ENSG00000143199:SILENT[ENST00000367848(c.4248C>T,gtC>gtT):ENSP00000356822(p.Val1416(=))];	NA	NA	chr1*167780107*167780111*AAGAC*A*rs746942962*NA*NA*NA	NA	NA	NA	NA	NA	NA_NA	NA	LOW	NA	NA	108	108	0	104	87	17	16.35
Chewer	42484	chr1	116916893	T	G	*AL136376.1*	INTRONIC	ENSG00000269279:[ENST00000598661(c.86-747A>C)];	NA	NA	NA	NA	NA	NA	NA	NA	NA_NA	NA	MODIFIER	NA	NA	36	36	0	25	20	5	20
Chewer	42484	chr1	3390140	C	T	*ARHGEF16*	INTRONIC	ENSG00000130762:[ENST00000413250(c.417+54C>T)];ENSG00000130762:[ENST00000378371(c.441+54C>T)];ENSG00000130762:[ENST00000378378(c.1305+54C>T)];ENSG00000130762:[ENST00000378373(c.441+54C>T)];ENSG00000130762:[ENST00000485984(n.592+54C>T)];ENSG00000130762:[ENST00000418137(c.417+54C>T)];ENSG00000130762:[ENST00000464620(n.522C>T)];	NA	NA	NA	NA	NA	NA	NA	NA	NA_NA	NA	MODIFIER	NA	NA	65	65	0	41	32	9	21.95
Chewer	42484	chr1	116916893	T	G	*ATP1A1*	INTRONIC	ENSG00000163399:[ENST00000537345(c.12+30T>G)];ENSG00000163399:[ENST00000418797(c.-82+1350T>G)];ENSG00000163399:[ENST00000488733(n.255+748T>G)];ENSG00000163399:[ENST00000295598(c.12+748T>G)];	NA	NA	NA	NA	NA	NA	NA	NA	NA_NA	NA	MODIFIER	NA	NA	36	36	0	25	20	5	20
Chewer	42484	chr1	111436999	C	T	*CD53*	SILENT	ENSG00000143119:SILENT[ENST00000429072(c.303C>T,atC>atT):ENSP00000412250(p.Ile101(=))];ENSG00000143119:SILENT[ENST00000271324(c.303C>T,atC>atT):ENSP00000271324(p.Ile101(=))];ENSG00000143119:EXONIC-NC[ENST00000497404(n.69C>T)];ENSG00000143119:INTRONIC[ENST00000476408(n.365+1844C>T)];	NA	NA	NA	NA	NA	NA	NA	NA	NA_NA	NA	LOW	NA	1	112	112	0	68	54	14	20.59
Chewer	42484	chr1	43826907	C	A	*CDC20*	SILENT	ENSG00000117399:EXONIC-NC[ENST00000482046(n.179C>A)];ENSG00000117399:SILENT[ENST00000310955(c.1194C>A,gcC>gcA):ENSP00000308450(p.Ala398(=))];ENSG00000117399:SILENT[ENST00000372462(c.1194C>A,gcC>gcA):ENSP00000361540(p.Ala398(=))];	NA	NA	chr1*43826907*43826907*C*T*rs747229782*NA*NA*NA	NA	NA	NA	NA	NA	NA_NA	NA	LOW	NA	NA	122	121	0	104	75	29	27.88
Chewer	42484	chr1	3742974	C	A	*CEP104*	MISSENSE	ENSG00000116198:MISSENSE[ENST00000438539(c.105G>T,Gct>Tct):ENSP00000395283(p.Ala36Ser)];ENSG00000116198:MISSENSE[ENST00000461667(c.172G>T,Gct>Tct):ENSP00000463605(p.Ala58Ser)];ENSG00000116198:MISSENSE[ENST00000378230(c.2215G>T,Gct>Tct):ENSP00000367476(p.Ala739Ser)];	NA	NA	NA	NA	NA	NA	NA	T	BN_BN	N	MODERATE	N	0.001	72	71	0	59	53	6	10.17
Chewer	42484	chr1	196762615	G	A	*CFHR3*	MISSENSE	ENSG00000116785:MISSENSE[ENST00000367425(c.965G>A,gGg>gAg):ENSP00000356395(p.Gly322Glu)];ENSG00000116785:MISSENSE[ENST00000391985(c.782G>A,gGg>gAg):ENSP00000375845(p.Gly261Glu)];ENSG00000116785:EXONIC-NC[ENST00000461558(n.637G>A)];ENSG00000116785:3UTR[ENST00000367427(c.*466G>A)];	NA	NA	NA	NA	NA	NA	NA	NA	NA_NA	NA	MODIFIER	NA	NA	187	187	0	160	119	41	25.62

Mangalaparthi *et al.* , 2020. Mutational landscape of esophageal squamous cell carcinoma in an Indian cohort
Supplementary Table 4. List of somatic single nucleotide variants identified using whole exome sequencing of ESCC patients

Cohort	Sample	Chromosome	Genomic position	Reference allele	Alternate allele	Gene symbol	Variant type	Variant annotation	OncoMD	COSMIC	dbSNP	ExAC	ExAC_SAS	1000G	1000G_SAS	SIFT	Polyphen2	CONDEL	VEP	LRT	PhastCon7 Way	Total read (Adjacent normal)	Reference allel read support (Adjacent normal)	Alternative read support (Adjacent normal)	Total read (Tumor)	Reference allel read support (Tumor)	Alternative read support (Tumor)	Alternate allele frequency
Chewer	42484	chr1	9816515	G	A	*CLSTN1*	INTRONIC	ENSG00000171603:[ENST00000377298(c.244+24C>T)];ENSG00000171603:[ENST00000377288(c.244+24C>T)];ENSG00000171603:[ENST00000361311(c.215-1148C>T)];	NA	NA	NA	NA	NA	NA	NA	NA	NA_NA	NA	MODIFIER	NA	NA	63	63	0	51	45	6	11.76
Chewer	42484	chr1	197396746	G	A	*CRB1*	MISSENSE	ENSG00000134376:MISSENSE[ENST00000367399(c.1955G>A,cGt>cAt):ENSP00000356369(p.Arg652His)];ENSG00000134376:MISSENSE[ENST00000484075(c.2291G>A,cGt>cAt):ENSP00000433932(p.Arg764His)];ENSG00000134376:MISSENSE[ENST00000535699(c.2084G>A,cGt>cAt):ENSP00000438786(p.Arg695His)];ENSG00000134376:MISSENSE[ENST00000367397(c.434G>A,cGt>cAt):ENSP00000356367(p.Arg145His)];ENSG00000134376:MISSENSE[ENST00000544212(c.734G>A,cGt>cAt):ENSP00000444556(p.Arg245His)];ENSG00000134376:MISSENSE[ENST00000367400(c.2291G>A,cGt>cAt):ENSP00000356370(p.Arg764His)];ENSG00000134376:EXONIC-NC[ENST00000480086(n.192G>A)];ENSG00000134376:3UTR[ENST00000543483(c.*298G>A)];ENSG00000134376:INTRONIC[ENST00000538660(c.2128+5660G>A)];	NA	NA	NA	1.66E-05	6.07E-05	NA	NA	T	PrD_PrD	N	MODERATE	NA	NA	210	210	0	124	101	22	17.74
Chewer	42484	chr1	34330095	A	G	*CSMD2*	INTRONIC	ENSG00000121904:[ENST00000373381(c.921-17498T>C)];ENSG00000121904:[ENST00000241312(c.801-17498T>C)];	NA	NA	NA	NA	NA	NA	NA	NA	NA_NA	NA	LOW	NA	NA	160	160	0	105	87	18	17.14
Chewer	42484	chr1	44685842	G	T	*DMAP1*	MISSENSE	ENSG00000178028:MISSENSE[ENST00000315913(c.1205G>T,gGt>gTt):ENSP00000312697(p.Gly402Val)];ENSG00000178028:MISSENSE[ENST00000361745(c.1205G>T,gGt>gTt):ENSP00000354697(p.Gly402Val)];ENSG00000178028:MISSENSE[ENST00000372289(c.1205G>T,gGt>gTt):ENSP00000361363(p.Gly402Val)];ENSG00000178028:EXONIC-NC[ENST00000488433(n.4290G>T)];ENSG00000178028:EXONIC-NC[ENST00000494092(n.285G>T)];	NA	NA	chr1*44685842*44685842*G*A,T*rs760275631*NA*NA*NA	9.04E-06	0	NA	NA	NA	NA_NA	NA	MODIFIER	NA	NA	60	60	0	65	53	12	18.46
Chewer	42484	chr1	65851446	C	G	*DNAJC6*	MISSENSE	ENSG00000116675:MISSENSE[ENST00000371069(c.852C>G,caC>caG):ENSP00000360108(p.His284Gln)];ENSG00000116675:MISSENSE[ENST00000395325(c.681C>G,caC>caG):ENSP00000378735(p.His227Gln)];ENSG00000116675:MISSENSE[ENST00000263441(c.642C>G,caC>caG):ENSP00000263441(p.His214Gln)];ENSG00000116675:MISSENSE[ENST00000494710(c.774C>G,caC>caG):ENSP00000473821(p.His258Gln)];ENSG00000116675:EXONIC-NC[ENST00000498720(n.293C>G)];	NA	NA	NA	NA	NA	NA	NA	NA	NA_NA	NA	MODIFIER	NA	NA	174	174	0	145	130	15	10.34
Chewer	42484	chr1	98349278	C	T	*DPYD*	INTRONIC	ENSG00000188641:[ENST00000423006(c.39+37162G>A)];ENSG00000188641:[ENST00000306031(c.40-348G>A)];ENSG00000188641:[ENST00000370192(c.40-348G>A)];ENSG00000188641:[ENST00000460019(n.115-348G>A)];	NA	NA	NA	NA	NA	NA	NA	NA	NA_NA	NA	MODIFIER	NA	NA	34	34	0	17	10	7	41.18
Chewer	42484	chr1	1957048	G	A	*GABRD*	MISSENSE	ENSG00000187730:MISSENSE[ENST00000378585(c.341G>A,cGc>cAc):ENSP00000367848(p.Arg114His)];	NA	NA	NA	1.66E-05	6.06E-05	NA	NA	D	PrD_PrD	D	MODERATE	D	1	213	213	0	215	167	48	22.33
Chewer	42484	chr1	54059991	C	A	*GLIS1*	MISSENSE	ENSG00000174332:MISSENSE[ENST00000312233(c.585G>T,caG>caT):ENSP00000309653(p.Gln195His)];	NA	NA	NA	NA	NA	NA	NA	T	PrD_PrD	N	MODERATE	D	0.888	99	98	0	91	62	29	31.87
Chewer	42484	chr1	34330095	A	G	*HMGB4*	SILENT	ENSG00000176256:EXONIC-NC[ENST00000425537(n.333A>G)];ENSG00000176256:SILENT[ENST00000522796(c.303A>G,ctA>ctG):ENSP00000430919(p.Leu101(=))];ENSG00000176256:SILENT[ENST00000519684(c.303A>G,ctA>ctG):ENSP00000429214(p.Leu101(=))];	NA	NA	NA	NA	NA	NA	NA	NA	NA_NA	NA	LOW	NA	NA	160	160	0	105	87	18	17.14
Chewer	42484	chr1	156811413	C	T	*INSRR*	INTRONIC	ENSG00000027644:[ENST00000368195(c.3527+44G>A)];	NA	NA	NA	NA	NA	NA	NA	NA	NA_NA	NA	MODIFIER	NA	NA	89	89	0	58	46	12	20.69
Chewer	42484	chr1	165183006	C	T	*LMX1A*	MISSENSE	ENSG00000162761:MISSENSE[ENST00000294816(c.541G>A,Gca>Aca):ENSP00000294816(p.Ala181Thr)];ENSG00000162761:MISSENSE[ENST00000342310(c.541G>A,Gca>Aca):ENSP00000340226(p.Ala181Thr)];ENSG00000162761:MISSENSE[ENST00000367893(c.541G>A,Gca>Aca):ENSP00000356868(p.Ala181Thr)];	NA	NA	NA	NA	NA	NA	NA	T	BN_BN	N	MODERATE	N	0.997	66	66	0	44	39	5	11.36
Chewer	42484	chr1	46066077	G	T	*NASP*	MISSENSE	ENSG00000132780:INTRONIC[ENST00000527470(c.108-6077G>T)];ENSG00000132780:INTRONIC[ENST00000350030(c.108-1850G>T)];ENSG00000132780:INTRONIC[ENST00000537798(c.108-6077G>T)];ENSG00000132780:INTRONIC[ENST00000527359(c.107+9135G>T)];ENSG00000132780:INTRONIC[ENST00000351223(c.108-1850G>T)];ENSG00000132780:INTRONIC[ENST00000528238(c.108-6077G>T)];ENSG00000132780:INTRONIC[ENST00000528084(c.*17-1850G>T)];ENSG00000132780:INTRONIC[ENST00000470768(c.108-4531G>T)];ENSG00000132780:INTRONIC[ENST00000372052(c.108-1850G>T)];ENSG00000132780:INTRONIC[ENST00000525515(c.60-1850G>T)];ENSG00000132780:INTRONIC[ENST00000464190(n.214-1850G>T)];ENSG00000132780:INTRONIC[ENST00000437901(c.108-1850G>T)];ENSG00000132780:INTRONIC[ENST00000530840(c.*238-1850G>T)];ENSG00000132780:INTRONIC[ENST00000534101(n.195-1850G>T)];ENSG00000132780:3UTR[ENST00000529333(c.*11G>T)];ENSG00000132780:3UTR[ENST00000437362(c.*11G>T)];ENSG00000132780:3UTR[ENST00000531532(c.*37G>T)];ENSG00000132780:3UTR[ENST00000453748(c.*11G>T)];ENSG00000132780:MISSENSE[ENST00000402363(c.61G>T,Gtc>Ttc):ENSP00000384529(p.Val21Phe)];	NA	NA	NA	NA	NA	NA	NA	NA	NA_NA	NA	MODIFIER	NA	NA	211	210	1	152	107	45	29.61
Chewer	42484	chr1	156811413	C	T	*NTRK1*	INTRONIC	ENSG00000198400:[ENST00000392302(c.10-460C>T)];ENSG00000198400:[ENST00000489021(n.200-460C>T)];ENSG00000198400:[ENST00000497019(c.10-460C>T)];ENSG00000198400:[ENST00000530298(n.158-460C>T)];	NA	NA	NA	NA	NA	NA	NA	NA	NA_NA	NA	MODIFIER	NA	NA	89	89	0	58	46	12	20.69
Chewer	42484	chr1	248617078	G	C	*OR2T2*	3UTR	ENSG00000196240:[ENST00000342927(c.*5G>C)];	NA	NA	chr1*248617051*248617100*CGACTGTGATCAGGAAGGGCTAGCAGGGACTCCCACAGCATCAGAGTGGT*C*rs757757641*NA*NA*NA|chr1*248617078*248617078*G*A*rs764099203*NA*NA*NA	NA	NA	NA	NA	NA	NA_NA	NA	MODIFIER	NA	NA	101	95	0	125	110	13	10.4

Mangalaparthi *et al.* , 2020. Mutational landscape of esophageal squamous cell carcinoma in an Indian cohort
Supplementary Table 4. List of somatic single nucleotide variants identified using whole exome sequencing of ESCC patients

Cohort	Sample	Chromosome	Genomic position	Reference allele	Alternate allele	Gene symbol	Variant type	Variant annotation	OncoMD	COSMIC	dbSNP	ExAC	ExAC_SAS	1000G	1000G_SAS	SIFT	Polyphen2	CONDEL	VEP	LRT	PhastCon7 Way	Total read (Adjacent normal)	Reference allel read support (Adjacent normal)	Alternative read support (Adjacent normal)	Total read (Tumor)	Reference allel read support (Tumor)	Alternative read support (Tumor)	Alternate allele frequency
Chewer	42484	chr1	114254404	C	G	*PHTF1*	SILENT	ENSG00000116793:EXONIC-NC[ENST00000474926(n.2162G>C)];ENSG00000116793:SILENT[ENST00000412670(c.278G>C,ctG>ctC):ENSP00000416810(p.Leu93(=))];ENSG00000116793:SILENT[ENST00000357783(c.1011G>C,ctG>ctC):ENSP00000350428(p.Leu337(=))];ENSG00000116793:SILENT[ENST00000369596(c.852G>C,ctG>ctC):ENSP00000358609(p.Leu284(=))];ENSG00000116793:SILENT[ENST00000369600(c.852G>C,ctG>ctC):ENSP00000358613(p.Leu284(=))];ENSG00000116793:SILENT[ENST00000369604(c.1011G>C,ctG>ctC):ENSP00000358617(p.Leu337(=))];ENSG00000116793:SILENT[ENST00000393357(c.1011G>C,ctG>ctC):ENSP00000377025(p.Leu337(=))];ENSG00000116793:SILENT[ENST00000369598(c.876G>C,ctG>ctC):ENSP00000358611(p.Leu292(=))];ENSG00000116793:3UTR[ENST00000447664(c.*149G>C)];	NA	NA	NA	NA	NA	NA	NA	NA	NA_NA	NA	LOW	NA	NA	123	123	0	73	54	19	26.03
Chewer	42484	chr1	186645715	A	G	*PTGS2*	MISSENSE	ENSG00000073756:MISSENSE[ENST00000367468(c.854T>C,aTg>aCg):ENSP00000356438(p.Met285Thr)];ENSG00000073756:3UTR[ENST00000559627(c.*252T>C)];ENSG00000073756:EXONIC-NC[ENST00000490885(n.987T>C)];	NA	NA	NA	NA	NA	NA	NA	NA	NA_NA	NA	MODIFIER	NA	NA	126	126	0	86	81	5	5.81
Chewer	42484	chr1	186647495	C	G	*PTGS2*	MISSENSE	ENSG00000073756:MISSENSE[ENST00000559627(c.355G>C,Gac>Cac):ENSP00000454130(p.Asp119His)];ENSG00000073756:MISSENSE[ENST00000367468(c.355G>C,Gac>Cac):ENSP00000356438(p.Asp119His)];ENSG00000073756:EXONIC-NC[ENST00000490885(n.488G>C)];ENSG00000073756:EXONIC-NC[ENST00000559800(n.484G>C)];	NA	NA	NA	NA	NA	NA	NA	NA	NA_NA	NA	MODIFIER	NA	NA	69	69	0	62	39	23	37.1
Chewer	42484	chr1	180165717	C	T	*QSOX1*	MISSENSE	ENSG00000116260:MISSENSE[ENST00000367600(c.1789C>T,Ccg>Tcg):ENSP00000356572(p.Pro597Ser)];ENSG00000116260:MISSENSE[ENST00000367602(c.1789C>T,Ccg>Tcg):ENSP00000356574(p.Pro597Ser)];	NA	NA	NA	NA	NA	NA	NA	T	BN_BN	N	MODERATE	N	0.003	116	116	0	101	86	15	14.85
Chewer	42484	chr1	192780093	C	G	*RGS2*	INTRONIC	ENSG00000116741:[ENST00000487236(n.282-18C>G)];ENSG00000116741:[ENST00000464302(n.407-18C>G)];ENSG00000116741:[ENST00000235382(c.275-18C>G)];	NA	NA	chr1*192780091*192780093*CTC*C,CCT*rs34717272*NA*NA*NA|chr1*192780093*192780093*C*T*rs201709865*NA*NA*NA	NA	NA	NA	NA	NA	NA_NA	NA	MODIFIER	NA	NA	283	283	0	191	140	51	26.7
Chewer	42484	chr1	45088450	C	A	*RNF220*	SILENT	ENSG00000187147:SILENT[ENST00000372247(c.795C>A,ggC>ggA):ENSP00000361321(p.Gly265(=))];ENSG00000187147:SILENT[ENST00000361799(c.795C>A,ggC>ggA):ENSP00000354872(p.Gly265(=))];ENSG00000187147:SILENT[ENST00000355387(c.795C>A,ggC>ggA):ENSP00000347548(p.Gly265(=))];ENSG00000187147:SILENT[ENST00000443020(c.78C>A,ggC>ggA):ENSP00000414640(p.Gly26(=))];ENSG00000187147:EXONIC-NC[ENST00000496262(n.70C>A)];	NA	NA	NA	NA	NA	NA	NA	NA	NA_NA	NA	LOW	NA	NA	102	102	0	74	60	14	18.92
Chewer	42484	chr1	154461628	C	A	*SHE*	MISSENSE	ENSG00000169291:MISSENSE[ENST00000555188(c.15G>T,cGg>cTg):ENSP00000451961(p.Arg6Leu)];ENSG00000169291:MISSENSE[ENST00000304760(c.923G>T,cGg>cTg):ENSP00000307369(p.Arg308Leu)];	NA	NA	chr1*154461628*154461628*C*T*rs201328310*0*0.9998*0.0001997	NA	NA	NA	NA	T	BN_BN	N	MODERATE	N	0.189	51	51	0	61	46	15	24.59
Chewer	42484	chr1	205896725	G	C	*SLC26A9*	MISSENSE	ENSG00000174502:MISSENSE[ENST00000367135(c.1110C>G,atC>atG):ENSP00000356103(p.Ile370Met)];ENSG00000174502:MISSENSE[ENST00000340781(c.1110C>G,atC>atG):ENSP00000341682(p.Ile370Met)];ENSG00000174502:MISSENSE[ENST00000367134(c.1110C>G,atC>atG):ENSP00000356102(p.Ile370Met)];ENSG00000174502:EXONIC-NC[ENST00000491127(n.494C>G)];ENSG00000174502:EXONIC-NC[ENST00000469392(n.794C>G)];	NA	NA	chr1*205896725*205896725*G*A*rs781507869*NA*NA*NA	NA	NA	NA	NA	NA	NA_NA	NA	MODIFIER	NA	NA	66	66	0	53	46	7	13.21
Chewer	42484	chr1	9098914	G	A	*SLC2A5*	SILENT	ENSG00000142583:SILENT[ENST00000377424(c.1071C>T,tgC>tgT):ENSP00000366641(p.Cys357(=))];ENSG00000142583:SILENT[ENST00000536305(c.894C>T,tgC>tgT):ENSP00000440688(p.Cys298(=))];ENSG00000142583:SILENT[ENST00000535586(c.726C>T,tgC>tgT):ENSP00000442744(p.Cys242(=))];ENSG00000142583:EXONIC-NC[ENST00000487492(n.419C>T)];	NA	NA	NA	NA	NA	NA	NA	NA	NA_NA	NA	LOW	NA	0.025	49	49	0	43	33	10	23.26
Chewer	42484	chr1	158583983	T	C	*SPTA1*	INTRONIC	ENSG00000163554:[ENST00000368147(c.6842+60A>G)];ENSG00000163554:[ENST00000481212(n.283+60A>G)];ENSG00000163554:[ENST00000498708(n.274+60A>G)];	NA	NA	NA	NA	NA	NA	NA	NA	NA_NA	NA	MODIFIER	NA	NA	45	45	0	23	18	5	21.74
Chewer	42484	chr1	158639291	C	G	*SPTA1*	MISSENSE	ENSG00000163554:MISSENSE[ENST00000368147(c.1740G>C,gaG>gaC):ENSP00000357129(p.Glu580Asp)];	NA	NA	chr1*158639291*158639291*C*T*rs188262107*0*0.9998*0.0001997	NA	NA	NA	NA	T	BN_BN	N	MODERATE	N	0.032	167	167	0	128	120	8	6.25
Chewer	42484	chr1	246720075	C	G	*TFB2M*	INTRONIC	ENSG00000162851:[ENST00000544618(c.403-52G>C)];ENSG00000162851:[ENST00000366514(c.557-52G>C)];	NA	NA	NA	NA	NA	NA	NA	NA	NA_NA	NA	MODIFIER	NA	NA	58	58	0	44	25	19	43.18
Chewer	42484	chr1	55247286	T	G	*TTC22*	MISSENSE	ENSG00000006555:MISSENSE[ENST00000371276(c.1340A>C,gAg>gCg):ENSP00000360323(p.Glu447Ala)];ENSG00000006555:EXONIC-NC[ENST00000488771(n.2333A>C)];	NA	NA	NA	NA	NA	NA	NA	NA	NA_NA	NA	MODIFIER	NA	NA	50	50	0	44	38	6	13.64
Chewer	42484	chr1	150082659	G	T	*VPS45*	MISSENSE	ENSG00000136631:MISSENSE[ENST00000369130(c.1542G>T,gaG>gaT):ENSP00000358126(p.Glu514Asp)];ENSG00000136631:MISSENSE[ENST00000369128(c.1227G>T,gaG>gaT):ENSP00000358124(p.Glu409Asp)];ENSG00000136631:EXONIC-NC[ENST00000484306(n.1371G>T)];ENSG00000136631:EXONIC-NC[ENST00000491789(n.274G>T)];ENSG00000136631:3UTR[ENST00000535106(c.*261G>T)];	NA	NA	NA	NA	NA	NA	NA	NA	NA_NA	NA	MODIFIER	NA	NA	87	87	0	45	30	14	31.11
Chewer	42484	chr1	241953926	C	T	*WDR64*	SILENT	ENSG00000162843:INTRONIC[ENST00000472717(c.1538+2590C>T)];ENSG00000162843:SILENT[ENST00000437684(c.2394C>T,ttC>ttT):ENSP00000402446(p.Phe798(=))];ENSG00000162843:SILENT[ENST00000366552(c.2895C>T,ttC>ttT):ENSP00000355510(p.Phe965(=))];ENSG00000162843:SILENT[ENST00000414635(c.1707C>T,ttC>ttT):ENSP00000406656(p.Phe569(=))];ENSG00000162843:SILENT[ENST00000425826(c.1331C>T,ttC>ttT):ENSP00000406342(p.Phe444(=))];ENSG00000162843:3UTR[ENST00000468967(c.*1150C>T)];	NA	NA	NA	NA	NA	NA	NA	NA	NA_NA	NA	LOW	NA	NA	271	267	2	234	205	27	11.54
Chewer	42484	chr1	53222235	G	C	*ZYG11B*	MISSENSE	ENSG00000162378:MISSENSE[ENST00000294353(c.136G>C,Gaa>Caa):ENSP00000294353(p.Glu46Gln)];ENSG00000162378:MISSENSE[ENST00000443756(c.136G>C,Gaa>Caa):ENSP00000400522(p.Glu46Gln)];ENSG00000162378:MISSENSE[ENST00000545132(c.136G>C,Gaa>Caa):ENSP00000441315(p.Glu46Gln)];	NA	NA	NA	NA	NA	NA	NA	D	BN_BN	N	MODERATE	D	0.998	182	182	0	108	76	32	29.63

Mangalaparthi *et al.* , 2020. Mutational landscape of esophageal squamous cell carcinoma in an Indian cohort
Supplementary Table 4. List of somatic single nucleotide variants identified using whole exome sequencing of ESCC patients

Cohort	Sample	Chromosome	Genomic position	Reference allele	Alternate allele	Gene symbol	Variant type	Variant annotation	OncoMD	COSMIC	dbSNP	ExAC	ExAC_SAS	1000G	1000G_SAS	SIFT	Polyphen2	CONDEL	VEP	LRT	PhastCon7 Way	Total read (Adjacent normal)	Reference allel read support (Adjacent normal)	Alternative read support (Adjacent normal)	Total read (Tumor)	Reference allel read support (Tumor)	Alternative read support (Tumor)	Alternate allele frequency
Chewer	42484	chr10	28250610	C	A	*ARMC4*	MISSENSE	ENSG00000169126:MISSENSE[ENST00000305242(c.1273G>T,Gat>Tat):ENSP00000306410(p.Asp425Tyr)];ENSG00000169126:MISSENSE[ENST00000239715(c.844G>T,Gat>Tat):ENSP00000239715(p.Asp282Tyr)];ENSG00000169126:MISSENSE[ENST00000434029(c.955G>T,Gat>Tat):ENSP00000398155(p.Asp319Tyr)];ENSG00000169126:MISSENSE[ENST00000537576(c.349G>T,Gat>Tat):ENSP00000443208(p.Asp117Tyr)];ENSG00000169126:5UTR[ENST00000545014(c.-153G>T)];ENSG00000169126:EXONIC-NC[ENST00000480504(n.530G>T)];	chr10*2825(	chr10*2825(	NA	0.0744483	0.148747304	NA	NA	NA	NA_NA	NA	MODIFIER	NA	NA	107	107	0	73	61	12	16.44
Chewer	42484	chr10	28250635	A	C	*ARMC4*	SILENT	ENSG00000169126:5UTR[ENST00000545014(c.-178T>G)];ENSG00000169126:SILENT[ENST00000305242(c.1248T>G,gcT>gcG):ENSP00000306410(p.Ala416(=))];ENSG00000169126:SILENT[ENST00000239715(c.819T>G,gcT>gcG):ENSP00000239715(p.Ala273(=))];ENSG00000169126:SILENT[ENST00000434029(c.930T>G,gcT>gcG):ENSP00000398155(p.Ala310(=))];ENSG00000169126:SILENT[ENST00000537576(c.324T>G,gcT>gcG):ENSP00000443208(p.Ala108(=))];ENSG00000169126:EXONIC-NC[ENST00000480504(n.505T>G)];	chr10*2825(	chr10*2825(	NA	0.0799922	0.162695711	NA	NA	NA	NA_NA	NA	LOW	NA	NA	113	113	0	76	65	11	14.47
Chewer	42484	chr10	73337695	G	A	*CDH23*	MISSENSE	ENSG00000107736:MISSENSE[ENST00000398809(c.778G>A,Gcc>Acc):ENSP00000381789(p.Ala260Thr)];ENSG00000107736:MISSENSE[ENST00000398842(c.778G>A,Gcc>Acc):ENSP00000381822(p.Ala260Thr)];ENSG00000107736:MISSENSE[ENST00000224721(c.793G>A,Gcc>Acc):ENSP00000224721(p.Ala265Thr)];ENSG00000107736:MISSENSE[ENST00000461841(c.913G>A,Gcc>Acc):ENSP00000473454(p.Ala305Thr)];ENSG00000107736:MISSENSE[ENST00000299366(c.913G>A,Gcc>Acc):ENSP00000299366(p.Ala305Thr)];ENSG00000107736:MISSENSE[ENST00000466757(c.149G>A,Gcc>Acc):ENSP00000473539(p.Ala51Thr)];	NA	NA	0.0001997	3.53E-05	0.000104319	0.0001997	0	D	PrD_PrD	D	MODERATE	NA	NA	123	123	0	80	43	37	46.25
Chewer	42484	chr10	102709841	C	A	*FAM178A*	SILENT	ENSG00000119906:SILENT[ENST00000370269(c.3129C>A,gtC>gtA):ENSP00000359292(p.Val1043(=))];ENSG00000119906:SILENT[ENST00000238961(c.3129C>A,gtC>gtA):ENSP00000238961(p.Val1043(=))];	NA	NA	NA	NA	NA	NA	NA	NA	NA_NA	NA	LOW	NA	NA	56	56	0	45	39	6	13.33
Chewer	42484	chr10	1070636	C	T	*IDI2*	MISSENSE	ENSG00000148377:MISSENSE[ENST00000277517(c.28G>A,Gac>Aac):ENSP00000277517(p.Asp10Asn)];	NA	NA	NA	NA	NA	NA	NA	NA	NA_NA	NA	MODIFIER	NA	NA	191	191	0	153	93	60	39.22
Chewer	42484	chr10	1070636	C	T	*IDI2-AS1*	INTRONIC	ENSG00000232656:[ENST00000536039(n.118+1913C>T)];ENSG00000232656:[ENST00000420381(n.116+1913C>T)];ENSG00000232656:[ENST00000437374(n.118+1913C>T)];ENSG00000232656:[ENST00000434470(n.110+1913C>T)];ENSG00000232656:[ENST00000428780(n.116+1913C>T)];	NA	NA	NA	NA	NA	NA	NA	NA	NA_NA	NA	MODIFIER	NA	NA	191	191	0	153	93	60	39.22
Chewer	42484	chr10	129913862	G	A	*MKI67*	SILENT	ENSG00000148773:EXONIC-NC[ENST00000478293(n.566C>T)];ENSG00000148773:SILENT[ENST00000368654(c.810C>T,taC>taT):ENSP00000357643(p.Tyr270(=))];ENSG00000148773:INTRONIC[ENST00000368653(c.400+893C>T)];	chr10*1299	chr10*1299	0.0001997	6.60E-05	6.06E-05	0.0001997	0	NA	NA_NA	NA	MODIFIER	NA	NA	214	213	1	152	110	42	27.63
Chewer	42484	chr10	26385468	T	C	*MYO3A*	INTRONIC	ENSG00000095777:[ENST00000265944(c.1662-29T>C)];ENSG00000095777:[ENST00000543632(c.1662-29T>C)];	NA	NA	chr10*26385467*26385468*CT*C*rs751151983*NA*NA*NA	NA	NA	NA	NA	NA	NA_NA	NA	MODIFIER	NA	NA	64	64	0	50	38	12	24
Chewer	42484	chr10	55583115	A	G	*PCDH15*	SILENT	ENSG00000150275:SILENT[ENST00000437009(c.4164T>C,ctT>ctC):ENSP00000412628(p.Leu1388(=))];ENSG00000150275:SILENT[ENST00000395433(c.4302T>C,ctT>ctC):ENSP00000378821(p.Leu1434(=))];ENSG00000150275:SILENT[ENST00000320301(c.4371T>C,ctT>ctC):ENSP00000322604(p.Leu1457(=))];ENSG00000150275:SILENT[ENST00000395432(c.4251T>C,ctT>ctC):ENSP00000378820(p.Leu1417(=))];ENSG00000150275:SILENT[ENST00000361849(c.4377T>C,ctT>ctC):ENSP00000354950(p.Leu1459(=))];ENSG00000150275:SILENT[ENST00000395430(c.4362T>C,ctT>ctC):ENSP00000378818(p.Leu1454(=))];ENSG00000150275:INTRONIC[ENST00000395445(c.4394+1781T>C)];ENSG00000150275:INTRONIC[ENST00000409834(c.3206+1781T>C)];ENSG00000150275:INTRONIC[ENST00000373957(c.2155-1222T>C)];ENSG00000150275:INTRONIC[ENST00000495484(n.853-5342T>C)];ENSG00000150275:INTRONIC[ENST00000395440(c.1306-13809T>C)];ENSG00000150275:INTRONIC[ENST00000395438(c.4373+1781T>C)];ENSG00000150275:INTRONIC[ENST00000414367(c.*447+4038T>C)];ENSG00000150275:INTRONIC[ENST00000395446(c.2092-13809T>C)];ENSG00000150275:INTRONIC[ENST00000395442(c.1099-13809T>C)];ENSG00000150275:INTRONIC[ENST00000414778(c.4373+4038T>C)];ENSG00000150275:INTRONIC[ENST00000373965(c.4394+1781T>C)];ENSG00000150275:3UTR[ENST00000448885(c.*2332T>C)];ENSG00000150275:3UTR[ENST00000373956(c.*2519T>C)];ENSG00000150275:EXONIC-NC[ENST00000463095(n.1390T>C)];	NA	NA	chr10*55583115*55583115*A*AGACTT*rs866568225*NA*NA*NA	NA	NA	NA	NA	NA	NA_NA	NA	MODIFIER	NA	NA	103	103	0	55	35	20	36.36
Chewer	42484	chr10	16553116	G	A	*PTER*	MISSENSE	ENSG00000165983:MISSENSE[ENST00000298942(c.911G>A,cGg>cAg):ENSP00000298942(p.Arg304Gln)];ENSG00000165983:MISSENSE[ENST00000378000(c.911G>A,cGg>cAg):ENSP00000367239(p.Arg304Gln)];ENSG00000165983:MISSENSE[ENST00000535784(c.911G>A,cGg>cAg):ENSP00000439485(p.Arg304Gln)];ENSG00000165983:MISSENSE[ENST00000423462(c.770G>A,cGg>cAg):ENSP00000389535(p.Arg257Gln)];	NA	NA	0.0001997	0.0002152	0	0.0001997	0	D	PoD_PoD	D	MODERATE	D	0.821	342	342	0	227	176	51	22.47
Chewer	42484	chr10	119768718	C	T	*RAB11FIP2*	MISSENSE	ENSG00000107560:EXONIC-NC[ENST00000476207(n.65G>A)];ENSG00000107560:MISSENSE[ENST00000369199(c.1390G>A,Gcc>Acc):ENSP00000358200(p.Ala464Thr)];ENSG00000107560:MISSENSE[ENST00000355624(c.1330G>A,Gcc>Acc):ENSP00000347839(p.Ala444Thr)];	NA	NA	NA	NA	NA	NA	NA	T	BN_BN	N	MODERATE	N	1	83	83	0	57	48	9	15.79
Chewer	42484	chr11	1011467	G	A	*AP2A2*	3UTR	ENSG00000183020:[ENST00000448903(c.*842G>A)];	NA	NA	NA	0.0001562	0	NA	NA	NA	NA_NA	NA	MODIFIER	NA	NA	83	83	0	76	46	30	39.47

Mangalaparthi *et al.* , 2020. Mutational landscape of esophageal squamous cell carcinoma in an Indian cohort
Supplementary Table 4. List of somatic single nucleotide variants identified using whole exome sequencing of ESCC patients

Cohort	Sample	Chromosome	Genomic position	Reference allele	Alternate allele	Gene symbol	Variant type	Variant annotation	OncoMD	COSMIC	dbSNP	ExAC	ExAC_SAS	1000G	1000G_SAS	SIFT	Polyphen2	CONDEL	VEP	LRT	PhastCon7 Way	Total read (Adjacent normal)	Reference allel read support (Adjacent normal)	Alternative read support (Adjacent normal)	Total read (Tumor)	Reference allel read support (Tumor)	Alternative read support (Tumor)	Alternate allele frequency
Chewer	42484	chr11	46689400	G	A	*ATG13*	MISSENSE	ENSG00000175224:MISSENSE[ENST00000525009(c.311G>A,cGa>cAa):ENSP00000436174(p.Arg104Gln)];ENSG00000175224:MISSENSE[ENST00000528494(c.1214G>A,cGa>cAa):ENSP00000432412(p.Arg405Gln)];ENSG00000175224:MISSENSE[ENST00000530500(c.767G>A,cGa>cAa):ENSP00000434390(p.Arg256Gln)];ENSG00000175224:MISSENSE[ENST00000312040(c.1115G>A,cGa>cAa):ENSP00000310321(p.Arg372Gln)];ENSG00000175224:MISSENSE[ENST00000451945(c.1004G>A,cGa>cAa):ENSP00000404900(p.Arg335Gln)];ENSG00000175224:MISSENSE[ENST00000526508(c.1115G>A,cGa>cAa):ENSP00000431974(p.Arg372Gln)];ENSG00000175224:MISSENSE[ENST00000359513(c.1115G>A,cGa>cAa):ENSP00000352500(p.Arg372Gln)];ENSG00000175224:MISSENSE[ENST00000524625(c.1004G>A,cGa>cAa):ENSP00000433543(p.Arg335Gln)];ENSG00000175224:MISSENSE[ENST00000529655(c.1004G>A,cGa>cAa):ENSP00000433756(p.Arg335Gln)];ENSG00000175224:MISSENSE[ENST00000434074(c.1115G>A,cGa>cAa):ENSP00000400642(p.Arg372Gln)];ENSG00000175224:MISSENSE[ENST00000529395(c.89G>A,cGa>cAa):ENSP00000431877(p.Arg30Gln)];ENSG00000175224:EXONIC-NC[ENST00000533316(n.366G>A)];ENSG00000175224:EXONIC-NC[ENST00000527907(n.647G>A)];ENSG00000175224:INTRONIC[ENST00000531933(c.65-4405G>A)];	NA	NA	0.0001997	4.96E-05	0.00024263	0.0001997	0.001	NA	NA_NA	NA	MODIFIER	NA	NA	90	89	1	63	49	14	22.22
Chewer	42484	chr11	62383716	A	C	*B3GAT3*	INTRONIC-SS-ACR	ENSG00000149541:[ENST00000534026(c.910-4T>G)];ENSG00000149541:[ENST00000531383(c.910-8T>G)];ENSG00000149541:[ENST00000265471(c.909+262T>G)];ENSG00000149541:[ENST00000532585(c.*1031+262T>G)];	NA	NA	NA	0.2058824	0.125	NA	NA	NA	NA_NA	NA	MODIFIER	NA	NA	18	18	0	19	14	5	26.32
Chewer	42484	chr11	67208640	C	A	*CORO1B*	MISSENSE	ENSG00000172725:MISSENSE[ENST00000537042(c.712G>T,Ggc>Tgc):ENSP00000445330(p.Gly238Cys)];ENSG00000172725:MISSENSE[ENST00000341356(c.712G>T,Ggc>Tgc):ENSP00000340211(p.Gly238Cys)];ENSG00000172725:MISSENSE[ENST00000453768(c.712G>T,Ggc>Tgc):ENSP00000416006(p.Gly238Cys)];ENSG00000172725:MISSENSE[ENST00000393893(c.712G>T,Ggc>Tgc):ENSP00000377471(p.Gly238Cys)];	NA	NA	NA	NA	NA	NA	NA	D	PrD_PrD	D	MODERATE	N	0.864	134	134	0	111	105	6	5.41
Chewer	42484	chr11	61068428	C	G	*DDB1*	INTRONIC	ENSG00000167986:[ENST00000540166(c.3216-20G>C)];ENSG00000167986:[ENST00000451943(c.177-24G>C)];ENSG00000167986:[ENST00000301764(c.3216-24G>C)];ENSG00000167986:[ENST00000450997(c.1149-24G>C)];ENSG00000167986:[ENST00000538470(c.357-24G>C)];ENSG00000167986:[ENST00000545894(n.1596G>C)];	NA	NA	chr11*61068428*61068428*C*T*rs776234499*NA*NA*NA	NA	NA	NA	NA	NA	NA_NA	NA	MODIFIER	NA	NA	53	53	0	49	38	11	22.45
Chewer	42484	chr11	58920321	G	C	*FAM111A*	MISSENSE	ENSG00000166801:MISSENSE[ENST00000361723(c.1180G>C,Gga>Cga):ENSP00000355264(p.Gly394Arg)];ENSG00000166801:MISSENSE[ENST00000420244(c.1180G>C,Gga>Cga):ENSP00000406683(p.Gly394Arg)];ENSG00000166801:MISSENSE[ENST00000528737(c.1180G>C,Gga>Cga):ENSP00000434435(p.Gly394Arg)];ENSG00000166801:MISSENSE[ENST00000531147(c.1180G>C,Gga>Cga):ENSP00000431631(p.Gly394Arg)];ENSG00000166801:MISSENSE[ENST00000533703(c.1180G>C,Gga>Cga):ENSP00000433154(p.Gly394Arg)];	NA	NA	chr11*58920321*58920321*G*A,C*rs144962024*NA*NA*NA	8.24E-06	0	NA	NA	D	PrD_PrD	D	MODERATE	N	0.055	358	358	0	227	197	30	13.22
Chewer	42484	chr11	105781185	A	T	*GRIA4*	NONSENSE	ENSG00000152578:NONSENSE[ENST00000282499(c.1183A>T,Aag>Tag):ENSP00000282499(p.Lys395Ter)];ENSG00000152578:NONSENSE[ENST00000530497(c.1183A>T,Aag>Tag):ENSP00000435775(p.Lys395Ter)];ENSG00000152578:NONSENSE[ENST00000393127(c.1183A>T,Aag>Tag):ENSP00000376835(p.Lys395Ter)];ENSG00000152578:NONSENSE[ENST00000525187(c.1183A>T,Aag>Tag):ENSP00000432180(p.Lys395Ter)];ENSG00000152578:NONSENSE[ENST00000428631(c.1183A>T,Aag>Tag):ENSP00000415551(p.Lys395Ter)];ENSG00000152578:NONSENSE[ENST00000393125(c.1183A>T,Aag>Tag):ENSP00000376833(p.Lys395Ter)];	NA	NA	NA	NA	NA	NA	NA	NA	NA_NA	NA	HIGH	D	1	223	222	0	159	150	9	5.66
Chewer	42484	chr11	65272059	A	G	*MALAT1*	EXONIC-NC	ENSG00000251562:[ENST00000534336(n.6827A>G)];	NA	NA	NA	NA	NA	NA	NA	NA	NA_NA	NA	MODIFIER	NA	NA	144	144	0	146	113	33	22.6
Chewer	42484	chr11	65272061	T	A	*MALAT1*	EXONIC-NC	ENSG00000251562:[ENST00000534336(n.6829T>A)];	NA	NA	chr11*65272061*65272061*T*C*rs763814739*NA*NA*NA	NA	NA	NA	NA	NA	NA_NA	NA	MODIFIER	NA	NA	141	141	0	145	112	33	22.76
Chewer	42484	chr11	68517736	C	T	*MTL5*	SILENT	ENSG00000132749:EXONIC-NC[ENST00000432435(n.344G>A)];ENSG00000132749:SILENT[ENST00000255087(c.393G>A,gcG>gcA):ENSP00000255087(p.Ala131(=))];ENSG00000132749:SILENT[ENST00000544963(c.393G>A,gcG>gcA):ENSP00000440968(p.Ala131(=))];ENSG00000132749:SILENT[ENST00000443940(c.393G>A,gcG>gcA):ENSP00000403086(p.Ala131(=))];	NA	NA	NA	NA	NA	NA	NA	NA	NA_NA	NA	LOW	NA	NA	111	111	0	129	114	15	11.63
Chewer	42484	chr11	1024067	C	T	*MUC6*	MISSENSE	ENSG00000184956:MISSENSE[ENST00000421673(c.3262G>A,Gac>Aac):ENSP00000406861(p.Asp1088Asn)];	NA	NA	NA	1.72E-05	0	NA	NA	NA	UN_UN	NA	MODERATE	U	0.351	70	70	0	66	55	11	16.67
Chewer	42484	chr11	76901165	C	G	*MYO7A*	MISSENSE	ENSG00000137474:MISSENSE[ENST00000458169(c.1274C>G,cCc>cGc):ENSP00000417017(p.Pro425Arg)];ENSG00000137474:MISSENSE[ENST00000409619(c.3698C>G,cCc>cGc):ENSP00000386635(p.Pro1233Arg)];ENSG00000137474:MISSENSE[ENST00000409709(c.3731C>G,cCc>cGc):ENSP00000386331(p.Pro1244Arg)];ENSG00000137474:MISSENSE[ENST00000458637(c.3731C>G,cCc>cGc):ENSP00000392185(p.Pro1244Arg)];ENSG00000137474:EXONIC-NC[ENST00000481328(n.1274C>G)];ENSG00000137474:EXONIC-NC[ENST00000467137(n.258C>G)];	NA	NA	NA	NA	NA	NA	NA	NA	NA_NA	NA	MODIFIER	NA	NA	43	43	0	36	29	7	19.44
Chewer	42484	chr11	21594780	A	T	*NELL1*	MISSENSE	ENSG00000165973:MISSENSE[ENST00000325319(c.2036A>T,gAg>gTg):ENSP00000317837(p.Glu679Val)];ENSG00000165973:MISSENSE[ENST00000357134(c.2207A>T,gAg>gTg):ENSP00000349654(p.Glu736Val)];ENSG00000165973:MISSENSE[ENST00000298925(c.2291A>T,gAg>gTg):ENSP00000298925(p.Glu764Val)];ENSG00000165973:MISSENSE[ENST00000532434(c.2066A>T,gAg>gTg):ENSP00000437170(p.Glu689Val)];ENSG00000165973:EXONIC-NC[ENST00000529218(n.1367A>T)];	NA	NA	NA	NA	NA	NA	NA	NA	NA_NA	NA	MODIFIER	NA	NA	97	97	0	72	61	10	13.89

Supplementary Table 4. List of somatic single nucleotide variants identified using whole exome sequencing of ESCC patients

Cohort	Sample	Chromosome	Genomic position	Reference allele	Alternate allele	Gene symbol	Variant type	Variant annotation	OncoMD	COSMIC	dbSNP	ExAC	ExAC_SAS	1000G	1000G_SAS	SIFT	Polyphen2	CONDEL	VEP	LRT	PhastCon7 Way	Total read (Adjacent normal)	Reference allel read support (Adjacent normal)	Alternative read support (Adjacent normal)	Total read (Tumor)	Reference allel read support (Tumor)	Alternative read support (Tumor)	Alternate allele frequency
Chewer	42484	chr11	71730010	G	T	*NUMA1*	MISSENSE	ENSG00000137497:MISSENSE[ENST00000542977(c.601C>A,Cca>Aca):ENSP00000444880(p.Pro201Thr)];ENSG00000137497:MISSENSE[ENST00000544238(c.601C>A,Cca>Aca):ENSP00000442761(p.Pro201Thr)];ENSG00000137497:MISSENSE[ENST00000537217(c.601C>A,Cca>Aca):ENSP00000442936(p.Pro201Thr)];ENSG00000137497:MISSENSE[ENST00000543937(c.601C>A,Cca>Aca):ENSP00000439759(p.Pro201Thr)];ENSG00000137497:MISSENSE[ENST00000393695(c.601C>A,Cca>Aca):ENSP00000377298(p.Pro201Thr)];ENSG00000137497:MISSENSE[ENST00000351960(c.601C>A,Cca>Aca):ENSP00000260051(p.Pro201Thr)];ENSG00000137497:MISSENSE[ENST00000358965(c.601C>A,Cca>Aca):ENSP00000351851(p.Pro201Thr)];ENSG00000137497:EXONIC-NC[ENST00000540843(n.740C>A)];ENSG00000137497:EXONIC-NC[ENST00000534987(n.982C>A)];ENSG00000137497:EXONIC-NC[ENST00000536119(n.761C>A)];	NA	NA	NA	NA	NA	NA	NA	NA	NA_NA	NA	MODIFIER	NA	NA	49	49	0	50	34	16	32
Chewer	42484	chr11	51412017	G	T	*OR4A5*	MISSENSE	ENSG00000221840:MISSENSE[ENST00000319760(c.379C>A,Cca>Aca):ENSP00000367664(p.Pro127Thr)];	NA	NA	NA	NA	NA	NA	NA	D_lc	PoD_PrD	D	MODERATE	NA	NA	135	135	0	126	90	36	28.57
Chewer	42484	chr11	5221740	A	G	*OR51V1*	MISSENSE	ENSG00000176742:MISSENSE[ENST00000321255(c.191T>C,cTg>cCg):ENSP00000321729(p.Leu64Pro)];	NA	NA	NA	NA	NA	NA	NA	D	PrD_PrD	D	MODERATE	N	0.959	95	95	0	60	33	27	45
Chewer	42484	chr11	56468332	A	G	*OR9G1*	MISSENSE	ENSG00000174914:MISSENSE[ENST00000312153(c.469A>G,Atc>Gtc):ENSP00000309012(p.Ile157Val)];	NA	NA	NA	6.59E-05	0	NA	NA	T	BN_BN	N	MODERATE	N	0.001	279	279	0	302	284	17	5.63
Chewer	42484	chr11	67205045	C	G	*PTPRCAP*	INTRONIC	ENSG00000213402:[ENST00000326294(c.3+43G>C)];	NA	NA	chr11*67205042*67205080*CGCCTGTGGCCCCCATCCCAAGAACCCGGGGGGCTCCGA*C*rs750093964*NA*NA*NA	NA	NA	NA	NA	NA	NA_NA	NA	MODIFIER	NA	NA	79	79	0	82	66	16	19.51
Chewer	42484	chr11	71730010	G	T	*RP11-849H4.4*	EXONIC-NC	ENSG00000251143:[ENST00000502284(n.3603G>T)];	NA	NA	NA	NA	NA	NA	NA	NA	NA_NA	NA	MODIFIER	NA	NA	49	49	0	50	34	16	32
Chewer	42484	chr11	64900684	C	A	*SYVN1*	MISSENSE	ENSG00000162298:MISSENSE[ENST00000307289(c.172G>T,Ggc>Tgc):ENSP00000302035(p.Gly58Cys)];ENSG00000162298:MISSENSE[ENST00000377190(c.172G>T,Ggc>Tgc):ENSP00000366395(p.Gly58Cys)];ENSG00000162298:MISSENSE[ENST00000526060(c.172G>T,Ggc>Tgc):ENSP00000436984(p.Gly58Cys)];ENSG00000162298:MISSENSE[ENST00000294256(c.172G>T,Ggc>Tgc):ENSP00000294256(p.Gly58Cys)];ENSG00000162298:MISSENSE[ENST00000527142(c.172G>T,Ggc>Tgc):ENSP00000432888(p.Gly58Cys)];ENSG00000162298:MISSENSE[ENST00000532771(c.119G>T,Ggc>Tgc):ENSP00000432166(p.Gly41Cys)];ENSG00000162298:5UTR[ENST00000531018(c.-9G>T)];ENSG00000162298:EXONIC-NC[ENST00000533685(n.108G>T)];ENSG00000162298:EXONIC-NC[ENST00000525874(n.115G>T)];ENSG00000162298:EXONIC-NC[ENST00000449943(n.221G>T)];ENSG00000162298:EXONIC-NC[ENST00000529207(n.112G>T)];ENSG00000162298:INTRONIC-SS-ACR-PRX[ENST00000528487(c.133-6G>T)];	NA	NA	NA	NA	NA	NA	NA	NA	NA_NA	NA	MODIFIER	NA	NA	90	90	0	89	67	22	24.72
Chewer	42484	chr11	2436640	C	T	*TRPM5*	MISSENSE	ENSG00000070985:MISSENSE[ENST00000533060(c.1190G>A,cGc>cAc):ENSP00000434121(p.Arg397His)];ENSG00000070985:MISSENSE[ENST00000528453(c.1190G>A,cGc>cAc):ENSP00000436809(p.Arg397His)];ENSG00000070985:MISSENSE[ENST00000533881(c.1172G>A,cGc>cAc):ENSP00000434383(p.Arg391His)];ENSG00000070985:MISSENSE[ENST00000452833(c.1196G>A,cGc>cAc):ENSP00000387965(p.Arg399His)];ENSG00000070985:MISSENSE[ENST00000155858(c.1190G>A,cGc>cAc):ENSP00000155858(p.Arg397His)];	NA	NA	0.0001997	6.68E-05	0.000368256	0.0001997	0	D	PrD_PrD	D	MODERATE	D	0.077	140	140	0	86	74	12	13.95
Chewer	42484	chr11	113704141	C	T	*USP28*	INTRONIC-SS-DNR	ENSG00000048028:INTRONIC-SS-DNR[ENST00000545540(c.384+1G>A)];ENSG00000048028:INTRONIC-SS-DNR[ENST00000537642(c.456+1G>A)];ENSG00000048028:INTRONIC-SS-DNR[ENST00000535607(c.98+1G>A)];ENSG00000048028:INTRONIC-SS-DNR[ENST00000003302(c.759+1G>A)];ENSG00000048028:INTRONIC-SS-DNR[ENST00000260188(c.759+1G>A)];ENSG00000048028:INTRONIC-SS-DNR[ENST00000537706(c.759+1G>A)];ENSG00000048028:INTRONIC-SS-DNR[ENST00000545608(c.133+1G>A)];ENSG00000048028:INTRONIC-SS-DNR[ENST00000540438(c.*295+1G>A)];ENSG00000048028:INTRONIC-SS-DNR[ENST00000542033(n.791+1G>A)];ENSG00000048028:INTRONIC-SS-DNR[ENST00000538475(c.51+1G>A)];	NA	NA	NA	NA	NA	NA	NA	NA	NA_NA	NA	HIGH	NA	NA	146	146	0	127	121	6	4.72
Chewer	42484	chr12	30784853	C	T	*IPO8*	MISSENSE	ENSG00000133704:MISSENSE[ENST00000256079(c.2992G>A,Gca>Aca):ENSP00000256079(p.Ala998Thr)];ENSG00000133704:MISSENSE[ENST00000544829(c.2377G>A,Gca>Aca):ENSP00000444520(p.Ala793Thr)];ENSG00000133704:MISSENSE[ENST00000535598(c.465G>A,Gca>Aca):ENSP00000446232(p.Ala156Thr)];	NA	NA	NA	NA	NA	NA	NA	D	PoD_PrD	D	MODERATE	NA	NA	37	37	0	47	39	8	17.02
Chewer	42484	chr12	21176061	A	G	*LST3*	INTRONIC	ENSG00000257046:[ENST00000540229(c.1866-44192A>G)];ENSG00000257046:[ENST00000381541(c.629-62A>G)];	NA	NA	NA	NA	NA	NA	NA	NA	NA_NA	NA	MODIFIER	NA	NA	109	109	0	99	84	15	15.15
Chewer	42484	chr12	17826416	G	T	*NA*	INTERGENIC		NA	NA	NA	NA	NA	NA	NA	NA	NA_NA	NA	MODIFIER	NA	NA	35	35	0	31	24	7	22.58
Chewer	42484	chr12	4758315	G	T	*NDUFA9*	MISSENSE	ENSG00000139180:EXONIC-NC[ENST00000539573(n.55G>T)];ENSG00000139180:EXONIC-NC[ENST00000542369(n.35G>T)];ENSG00000139180:EXONIC-NC[ENST00000396655(n.33G>T)];ENSG00000139180:EXONIC-NC[ENST00000544679(n.31G>T)];ENSG00000139180:MISSENSE[ENST00000266544(c.23G>T,cGg>cTg):ENSP00000266544(p.Arg8Leu)];	NA	NA	chr12*4758315*4758315*G*A*rs762977169*NA*NA*NA	NA	NA	NA	NA	NA	NA_NA	NA	MODIFIER	NA	NA	45	45	0	38	32	6	15.79
Chewer	42484	chr12	18496248	C	T	*PIK3C2G*	INTRONIC	ENSG00000139144:[ENST00000546003(c.*693-13C>T)];ENSG00000139144:[ENST00000535651(c.1396-13C>T)];ENSG00000139144:[ENST00000538779(c.1396-13C>T)];ENSG00000139144:[ENST00000266497(c.1396-13C>T)];ENSG00000139144:[ENST00000433979(c.1396-13C>T)];	NA	NA	NA	NA	NA	NA	NA	NA	NA_NA	NA	MODIFIER	NA	NA	63	63	0	43	34	9	20.93
Chewer	42484	chr12	11083369	A	T	*PRH2*	MISSENSE	ENSG00000134551:MISSENSE[ENST00000396400(c.209A>T,cAg>cTg):ENSP00000379682(p.Gln70Leu)];ENSG00000134551:MISSENSE[ENST00000381847(c.209A>T,cAg>cTg):ENSP00000371271(p.Gln70Leu)];	NA	NA	NA	NA	NA	NA	NA	NA	NA_NA	NA	MODIFIER	NA	NA	119	119	0	109	98	11	10.09
Chewer	42484	chr12	11083369	A	T	*PRR4*	INTRONIC	ENSG00000111215:[ENST00000539853(n.238+42885T>A)];ENSG00000111215:[ENST00000541977(n.359+42885T>A)];ENSG00000111215:[ENST00000535024(n.428+42885T>A)];ENSG00000111215:[ENST00000536668(n.517+42885T>A)];ENSG00000111215:[ENST00000536086(n.90-38845T>A)];ENSG00000111215:[ENST00000546317(n.53-35441T>A)];	NA	NA	NA	NA	NA	NA	NA	NA	NA_NA	NA	MODIFIER	NA	NA	119	119	0	109	98	11	10.09

Mangalaparthi *et al.* , 2020. Mutational landscape of esophageal squamous cell carcinoma in an Indian cohort
Supplementary Table 4. List of somatic single nucleotide variants identified using whole exome sequencing of ESCC patients

Cohort	Sample	Chromosome	Genomic position	Reference allele	Alternate allele	Gene symbol	Variant type	Variant annotation	OncoMD	COSMIC	dbSNP	ExAC	ExAC_SAS	1000G	1000G_SAS	SIFT	Polyphen2	CONDEL	VEP	LRT	PhastCon7 Way	Total read (Adjacent normal)	Reference allel read support (Adjacent normal)	Alternative read support (Adjacent normal)	Total read (Tumor)	Reference allel read support (Tumor)	Alternative read support (Tumor)	Alternate allele frequency
Chewer	42484	chr12	71032965	C	T	*PTPRR*	SILENT-STOPCODO	ENSG00000153233:SILENT-STOPCODON[ENST00000342084(c.1637G>A,tGa>tAa):ENSP00000339605(p.Ter546Ter)];ENSG00000153233:SILENT-STOPCODON[ENST00000440835(c.1238G>A,tGa>tAa):ENSP00000391750(p.Ter413Ter)];ENSG00000153233:SILENT-STOPCODON[ENST00000378778(c.1355G>A,tGa>tAa):ENSP00000368054(p.Ter452Ter)];ENSG00000153233:SILENT-STOPCODON[ENST00000283228(c.1973G>A,tGa>tAa):ENSP00000283228(p.Ter658Ter)];ENSG00000153233:SILENT-STOPCODON[ENST00000549308(c.1238G>A,tGa>tAa):ENSP00000446943(p.Ter413Ter)];ENSG00000153233:3UTR[ENST00000551219(c.*755G>A)];ENSG00000153233:EXONIC-NC[ENST00000549107(n.807G>A)];ENSG00000153233:EXONIC-NC[ENST00000537619(n.821G>A)];ENSG00000153233:EXONIC-NC[ENST00000547752(n.626G>A)];	NA	NA	chr12*71032965*71032965*C*G*rs754066021*NA*NA*NA	NA	NA	NA	NA	NA	NA_NA	NA	MODIFIER	NA	NA	64	64	0	34	28	6	17.65
Chewer	42484	chr12	18238628	C	A	*RERGL*	INTRONIC-SS-ACR	ENSG00000111404:INTRONIC-SS-ACR[ENST00000540148(n.119-1G>T)];ENSG00000111404:INTRONIC-SS-ACR[ENST00000229002(c.113-1G>T)];ENSG00000111404:INTRONIC-SS-ACR[ENST00000541632(n.399-1G>T)];ENSG00000111404:INTRONIC-SS-ACR[ENST00000536890(c.110-1G>T)];ENSG00000111404:INTRONIC-SS-ACR[ENST00000538724(c.110-1G>T)];	NA	NA	chr12*18238628*18238628*C*G*rs202151617*NA*NA*NA	NA	NA	NA	NA	NA	NA_NA	NA	HIGH	NA	NA	57	57	0	38	29	9	23.68
Chewer	42484	chr12	4758315	G	T	*RP11-500M8.7*	INTRONIC	ENSG00000272921:[ENST00000536588(c.*50-5143G>T)];	NA	NA	chr12*4758315*4758315*G*A*rs762977169*NA*NA*NA	NA	NA	NA	NA	NA	NA_NA	NA	MODIFIER	NA	NA	45	45	0	38	32	6	15.79
Chewer	42484	chr12	21176061	A	G	*SLCO1B3*	INTRONIC	ENSG00000111700:[ENST00000553473(c.1866-44192A>G)];	NA	NA	NA	NA	NA	NA	NA	NA	NA_NA	NA	MODIFIER	NA	NA	109	109	0	99	84	15	15.15
Chewer	42484	chr12	21176061	A	G	*SLCO1B7*	INTRONIC	ENSG00000205754:[ENST00000554957(c.629-62A>G)];ENSG00000205754:[ENST00000421593(c.488-62A>G)];	NA	NA	NA	NA	NA	NA	NA	NA	NA_NA	NA	MODIFIER	NA	NA	109	109	0	99	84	15	15.15
Chewer	42484	chr12	98931452	G	T	*TMPO*	INTRONIC	ENSG00000120802:[ENST00000393053(c.663+102G>T)];ENSG00000120802:[ENST00000343315(c.663+102G>T)];ENSG00000120802:[ENST00000556029(c.663+102G>T)];ENSG00000120802:[ENST00000556678(c.384+102G>T)];ENSG00000120802:[ENST00000261210(c.*18G>T)];	NA	NA	NA	NA	NA	NA	NA	NA	NA_NA	NA	MODIFIER	NA	NA	126	126	0	96	55	41	42.71
Chewer	42484	chr12	113715179	G	A	*TPCN1*	INTRONIC-SS-DNR	ENSG00000186815:[ENST00000546503(n.112+10G>A)];ENSG00000186815:[ENST00000551127(n.158+10G>A)];ENSG00000186815:[ENST00000392569(c.980+10G>A)];ENSG00000186815:[ENST00000552077(n.1450+10G>A)];ENSG00000186815:[ENST00000541517(c.1400+10G>A)];ENSG00000186815:[ENST00000546781(c.243+10G>A)];ENSG00000186815:[ENST00000428632(n.1313+10G>A)];ENSG00000186815:[ENST00000335509(c.1184+10G>A)];ENSG00000186815:[ENST00000550785(c.1400+10G>A)];	NA	NA	NA	NA	NA	NA	NA	NA	NA_NA	NA	MODIFIER	NA	NA	151	151	0	108	57	50	46.3
Chewer	42484	chr12	108620830	C	G	*WSCD2*	MISSENSE	ENSG00000075035:MISSENSE[ENST00000549903(c.868C>G,Ccg>Gcg):ENSP00000447272(p.Pro290Ala)];ENSG00000075035:MISSENSE[ENST00000332082(c.868C>G,Ccg>Gcg):ENSP00000331933(p.Pro290Ala)];ENSG00000075035:MISSENSE[ENST00000547525(c.868C>G,Ccg>Gcg):ENSP00000448047(p.Pro290Ala)];ENSG00000075035:MISSENSE[ENST00000261400(c.868C>G,Ccg>Gcg):ENSP00000261400(p.Pro290Ala)];ENSG00000075035:EXONIC-NC[ENST00000546629(n.64C>G)];	NA	NA	NA	NA	NA	NA	NA	NA	NA_NA	NA	MODIFIER	NA	NA	126	126	0	87	77	10	11.49
Chewer	42484	chr12	77209736	G	T	*ZDHHC17*	SILENT	ENSG00000186908:SILENT[ENST00000426126(c.708G>T,ggG>ggT):ENSP00000403397(p.Gly236(=))];ENSG00000186908:SILENT[ENST00000550876(c.219G>T,ggG>ggT):ENSP00000449734(p.Gly73(=))];ENSG00000186908:SILENT[ENST00000334822(c.708G>T,ggG>ggT):ENSP00000334868(p.Gly236(=))];ENSG00000186908:SILENT[ENST00000359019(c.558G>T,ggG>ggT):ENSP00000351913(p.Gly186(=))];ENSG00000186908:3UTR[ENST00000549944(c.*200G>T)];ENSG00000186908:3UTR[ENST00000550163(c.*398G>T)];ENSG00000186908:3UTR[ENST00000552453(c.*55G>T)];ENSG00000186908:3UTR[ENST00000546778(c.*496G>T)];	NA	NA	NA	NA	NA	NA	NA	NA	NA_NA	NA	LOW	NA	NA	172	172	0	89	67	22	24.72
Chewer	42484	chr13	41515184	G	A	*ELF1*	MISSENSE	ENSG00000120690:EXONIC-NC[ENST00000498824(n.1435C>T)];ENSG00000120690:MISSENSE[ENST00000239882(c.1129C>T,Cca>Tca):ENSP00000239882(p.Pro377Ser)];ENSG00000120690:MISSENSE[ENST00000442101(c.1057C>T,Cca>Tca):ENSP00000405580(p.Pro353Ser)];	NA	NA	NA	NA	NA	NA	NA	T	BN_BN	N	MODERATE	N	0.955	232	232	0	174	107	67	38.51
Chewer	42484	chr13	32722036	G	T	*FRY*	MISSENSE	ENSG00000073910:MISSENSE[ENST00000380250(c.1344G>T,agG>agT):ENSP00000369600(p.Arg448Ser)];	chr13*32722	chr13*32722	NA	NA	NA	NA	NA	D	PrD_PrD	D	MODERATE	D	0.995	154	154	0	106	63	43	40.57
Chewer	42484	chr13	103339467	C	T	*METTL21C*	INTRONIC	ENSG00000139780:[ENST00000267273(c.283-60G>A)];	NA	NA	NA	NA	NA	NA	NA	NA	NA_NA	NA	MODIFIER	NA	NA	241	241	0	131	106	25	19.08
Chewer	42484	chr13	109318469	C	A	*MYO16*	MISSENSE	ENSG00000041515:MISSENSE[ENST00000356711(c.198C>A,gaC>gaA):ENSP00000349145(p.Asp66Glu)];ENSG00000041515:MISSENSE[ENST00000357550(c.198C>A,gaC>gaA):ENSP00000350160(p.Asp66Glu)];ENSG00000041515:MISSENSE[ENST00000251041(c.198C>A,gaC>gaA):ENSP00000251041(p.Asp66Glu)];	chr13*1093	NA	NA	NA	NA	NA	NA	T	PrD_PrD	D	MODERATE	U	0.982	212	212	0	182	122	60	32.97
Chewer	42484	chr13	73293160	T	A	*MZT1*	MISSENSE	ENSG00000204899:MISSENSE[ENST00000377818(c.155A>T,gAa>gTa):ENSP00000367049(p.Glu52Val)];	NA	NA	NA	NA	NA	NA	NA	D	PrD_PrD	D	MODERATE	D	1	42	42	0	46	40	6	13.04
Chewer	42484	chr13	73293161	C	A	*MZT1*	NONSENSE	ENSG00000204899:NONSENSE[ENST00000377818(c.154G>T,Gaa>Taa):ENSP00000367049(p.Glu52Ter)];	NA	NA	NA	NA	NA	NA	NA	NA	NA_NA	NA	HIGH	D	1	42	42	0	46	40	6	13.04
Chewer	42484	chr13	23907910	C	T	*SACS*	MISSENSE	ENSG00000151835:MISSENSE[ENST00000382292(c.10105G>A,Gtc>Atc):ENSP00000371729(p.Val3369Ile)];ENSG00000151835:MISSENSE[ENST00000402364(c.7855G>A,Gtc>Atc):ENSP00000385844(p.Val2619Ile)];ENSG00000151835:MISSENSE[ENST00000382298(c.10105G>A,Gtc>Atc):ENSP00000371735(p.Val3369Ile)];ENSG00000151835:INTRONIC[ENST00000455470(c.2130-4287G>A)];ENSG00000151835:INTRONIC[ENST00000423156(c.1058-4287G>A)];	NA	NA	NA	NA	NA	NA	NA	NA	NA_NA	NA	MODIFIER	NA	NA	147	147	0	108	73	34	31.48
Chewer	42484	chr13	24200942	G	A	*TNFRSF19*	INTRONIC	ENSG00000127863:[ENST00000382258(c.445+11G>A)];ENSG00000127863:[ENST00000382263(c.445+11G>A)];ENSG00000127863:[ENST00000403372(c.49+11G>A)];ENSG00000127863:[ENST00000248484(c.445+11G>A)];	NA	NA	NA	2.50E-05	0	NA	NA	NA	NA_NA	NA	MODIFIER	NA	NA	98	98	0	61	40	21	34.43

Mangalaparthi *et al.* , 2020. Mutational landscape of esophageal squamous cell carcinoma in an Indian cohort
Supplementary Table 4. List of somatic single nucleotide variants identified using whole exome sequencing of ESCC patients

Cohort	Sample	Chromosome	Genomic position	Reference allele	Alternate allele	Gene symbol	Variant type	Variant annotation	OncoMD	COSMIC	dbSNP	ExAC	ExAC_SAS	1000G	1000G_SAS	SIFT	Polyphen2	CONDEL	VEP	LRT	PhastCon7 Way	Total read (Adjacent normal)	Reference allel read support (Adjacent normal)	Alternative read support (Adjacent normal)	Total read (Tumor)	Reference allel read support (Tumor)	Alternative read support (Tumor)	Alternate allele frequency
Chewer	42484	chr13	38213293	G	C	*TRPC4*	INTRONIC	ENSG00000133107:[ENST00000379673(c.1927-27C>G)];ENSG00000133107:[ENST00000488717(c.*813-27C>G)];ENSG00000133107:[ENST00000338947(c.1603-27C>G)];ENSG00000133107:[ENST00000379681(c.2137-27C>G)];ENSG00000133107:[ENST00000447043(c.2122-27C>G)];ENSG00000133107:[ENST00000379679(c.1603-27C>G)];ENSG00000133107:[ENST00000355779(c.2122-27C>G)];ENSG00000133107:[ENST00000358477(c.2122-27C>G)];ENSG00000133107:[ENST00000379705(c.2122-27C>G)];ENSG00000133107:[ENST00000426868(c.*526C>G)];	NA	NA	NA	NA	NA	NA	NA	NA	NA_NA	NA	MODIFIER	NA	NA	196	195	1	151	96	55	36.42
Chewer	42484	chr14	105177403	C	A	*INF2*	INTRONIC	ENSG00000203485:[ENST00000252527(c.715-13C>A)];ENSG00000203485:[ENST00000330634(c.2311-13C>A)];ENSG00000203485:[ENST00000392634(c.2311-13C>A)];ENSG00000203485:[ENST00000474229(n.804-13C>A)];ENSG00000203485:[ENST00000480763(n.283+59C>A)];	NA	NA	NA	NA	NA	NA	NA	NA	NA_NA	NA	MODIFIER	NA	NA	89	89	0	121	115	6	4.96
Chewer	42484	chr14	60591077	G	C	*PCNXL4*	MISSENSE	ENSG00000126773:MISSENSE[ENST00000556360(c.54G>C,Gtt>Ctt):ENSP00000450994(p.Val19Leu)];ENSG00000126773:MISSENSE[ENST00000406854(c.2188G>C,Gtt>Ctt):ENSP00000384801(p.Val730Leu)];ENSG00000126773:MISSENSE[ENST00000404681(c.2188G>C,Gtt>Ctt):ENSP00000385713(p.Val730Leu)];ENSG00000126773:MISSENSE[ENST00000555740(c.496G>C,Gtt>Ctt):ENSP00000450441(p.Val166Leu)];ENSG00000126773:MISSENSE[ENST00000317623(c.1486G>C,Gtt>Ctt):ENSP00000317396(p.Val496Leu)];ENSG00000126773:MISSENSE[ENST00000406949(c.1486G>C,Gtt>Ctt):ENSP00000385201(p.Val496Leu)];ENSG00000126773:5UTR[ENST00000535349(c.-192G>C)];ENSG00000126773:EXONIC-NC[ENST00000483571(n.901G>C)];ENSG00000126773:INTRONIC[ENST00000555929(c.266+49G>C)];	NA	NA	chr14*60591077*60591077*G*T*rs764152147*NA*NA*NA	NA	NA	NA	NA	NA	NA_NA	NA	MODIFIER	NA	NA	168	168	0	134	74	59	44.03
Chewer	42484	chr14	20942977	C	T	*PNP*	SILENT	ENSG00000198805:EXONIC-NC[ENST00000557229(n.647C>T)];ENSG00000198805:EXONIC-NC[ENST00000554056(n.639C>T)];ENSG00000198805:EXONIC-NC[ENST00000556754(n.1435C>T)];ENSG00000198805:SILENT[ENST00000553591(c.448C>T,Ctg>Ttg):ENSP00000452421(p.Leu150(=))];ENSG00000198805:SILENT[ENST00000554065(c.94C>T,Ctg>Ttg):ENSP00000451108(p.Leu32(=))];ENSG00000198805:SILENT[ENST00000361505(c.331C>T,Ctg>Ttg):ENSP00000354532(p.Leu111(=))];	NA	NA	NA	NA	NA	NA	NA	NA	NA_NA	NA	MODIFIER	NA	NA	100	97	1	65	31	34	52.31
Chewer	42484	chr14	20942977	C	T	*RP11-203M5.8*	EXONIC-NC	ENSG00000258908:[ENST00000554678(n.463G>A)];	NA	NA	NA	NA	NA	NA	NA	NA	NA_NA	NA	MODIFIER	NA	NA	100	97	1	65	31	34	52.31
Chewer	42484	chr14	24026046	G	C	*RP11-66N24.4*	INTRONIC	ENSG00000157306:[ENST00000553985(n.239-1858G>C)];ENSG00000157306:[ENST00000555446(n.356+494G>C)];ENSG00000157306:[ENST00000556354(n.466-1858G>C)];	NA	NA	chr14*24026046*24026046*G*A,T*rs779993651*NA*NA*NA	NA	NA	NA	NA	T	BN_BN	N	MODERATE	N	0.991	120	120	0	103	41	62	60.19
Chewer	42484	chr14	24026046	G	C	*THTPA*	MISSENSE	ENSG00000259431:MISSENSE[ENST00000557630(c.80G>C,gGc>gCc):ENSP00000452281(p.Gly27Ala)];ENSG00000259431:MISSENSE[ENST00000404535(c.80G>C,gGc>gCc):ENSP00000384580(p.Gly27Ala)];ENSG00000259431:MISSENSE[ENST00000554970(c.80G>C,gGc>gCc):ENSP00000452465(p.Gly27Ala)];ENSG00000259431:MISSENSE[ENST00000556015(c.80G>C,gGc>gCc):ENSP00000451835(p.Gly27Ala)];ENSG00000259431:MISSENSE[ENST00000554789(c.80G>C,gGc>gCc):ENSP00000450459(p.Gly27Ala)];ENSG00000259431:MISSENSE[ENST00000288014(c.80G>C,gGc>gCc):ENSP00000288014(p.Gly27Ala)];	NA	NA	chr14*24026046*24026046*G*A,T*rs779993651*NA*NA*NA	NA	NA	NA	NA	T	BN_BN	N	MODERATE	N	0.991	120	120	0	103	41	62	60.19
Chewer	42484	chr15	63915110	A	T	*HERC1*	INTRONIC	ENSG00000103657:[ENST00000558324(c.*317-20T>A)];ENSG00000103657:[ENST00000443617(c.13612-20T>A)];	NA	NA	chr15*63915110*63915111*AT*A*rs780609796*NA*NA*NA	NA	NA	NA	NA	NA	NA_NA	NA	MODIFIER	NA	NA	54	54	0	46	24	22	47.83
Chewer	42484	chr15	74219349	G	A	*LOXL1*	SILENT	ENSG00000129038:SILENT[ENST00000566011(c.225G>A,gtG>gtA):ENSP00000457827(p.Val75(=))];ENSG00000129038:SILENT[ENST00000261921(c.225G>A,gtG>gtA):ENSP00000261921(p.Val75(=))];	NA	NA	NA	NA	NA	NA	NA	NA	NA_NA	NA	MODIFIER	NA	NA	96	96	0	94	68	26	27.66
Chewer	42484	chr15	74219349	G	A	*LOXL1-AS1*	INTRONIC	ENSG00000261801:[ENST00000564963(n.42+646C>T)];ENSG00000261801:[ENST00000565756(n.183+769C>T)];ENSG00000261801:[ENST00000562965(n.184+769C>T)];ENSG00000261801:[ENST00000564194(n.184+1057C>T)];ENSG00000261801:[ENST00000565416(n.164+769C>T)];ENSG00000261801:[ENST00000568087(n.166+769C>T)];ENSG00000261801:[ENST00000566675(n.139+1057C>T)];ENSG00000261801:[ENST00000567257(n.133+646C>T)];ENSG00000261801:[ENST00000562739(n.42+646C>T)];	NA	NA	NA	NA	NA	NA	NA	NA	NA_NA	NA	MODIFIER	NA	NA	96	96	0	94	68	26	27.66
Chewer	42484	chr15	31779416	C	T	*OTUD7A*	MISSENSE	ENSG00000169918:MISSENSE[ENST00000382902(c.1352G>A,cGg>cAg):ENSP00000372358(p.Arg451Gln)];ENSG00000169918:MISSENSE[ENST00000307050(c.1331G>A,cGg>cAg):ENSP00000305926(p.Arg444Gln)];	NA	NA	NA	NA	NA	NA	NA	T	PrD_PrD	N	MODERATE	D	0.998	45	45	0	47	38	9	19.15
Chewer	42484	chr15	42976496	G	T	*STARD9*	MISSENSE	ENSG00000159433:MISSENSE[ENST00000290607(c.2720G>T,aGa>aTa):ENSP00000290607(p.Arg907Ile)];	NA	NA	NA	NA	NA	NA	NA	NA	PoD_PrD	NA	MODERATE	NA	NA	114	114	0	80	69	11	13.75
Chewer	42484	chr15	91801617	C	A	*SV2B*	INTRONIC	ENSG00000185518:[ENST00000330276(c.785-34C>A)];ENSG00000185518:[ENST00000394232(c.785-34C>A)];ENSG00000185518:[ENST00000545111(c.332-34C>A)];ENSG00000185518:[ENST00000557410(c.785-34C>A)];	NA	NA	NA	NA	NA	NA	NA	NA	NA_NA	NA	MODIFIER	NA	NA	61	61	0	44	31	12	27.27
Chewer	42484	chr16	76486508	T	C	*CNTNAP4*	MISSENSE	ENSG00000152910:EXONIC-NC[ENST00000469667(n.1030T>C)];ENSG00000152910:EXONIC-NC[ENST00000469589(n.1559T>C)];ENSG00000152910:EXONIC-NC[ENST00000471618(n.1144T>C)];ENSG00000152910:MISSENSE[ENST00000478060(c.956T>C,gTt>gCt):ENSP00000418741(p.Val319Ala)];ENSG00000152910:MISSENSE[ENST00000377504(c.1028T>C,gTt>gCt):ENSP00000439733(p.Val343Ala)];ENSG00000152910:MISSENSE[ENST00000307431(c.1172T>C,gTt>gCt):ENSP00000306893(p.Val391Ala)];ENSG00000152910:MISSENSE[ENST00000476707(c.1184T>C,gTt>gCt):ENSP00000417628(p.Val395Ala)];	NA	NA	NA	NA	NA	NA	NA	T	BN_BN	N	MODERATE	N	0.025	204	204	0	142	109	33	23.24
Chewer	42484	chr16	68023343	C	G	*DPEP2*	INTRONIC	ENSG00000167261:[ENST00000572888(c.995-42G>C)];ENSG00000167261:[ENST00000393847(c.995-42G>C)];ENSG00000167261:[ENST00000575203(c.*160-42G>C)];ENSG00000167261:[ENST00000412757(c.995-42G>C)];	NA	NA	NA	NA	NA	NA	NA	NA	NA_NA	NA	MODIFIER	NA	NA	102	101	1	76	59	17	22.37
Chewer	42484	chr16	68023343	C	G	*DUS2*	INTRONIC	ENSG00000167264:[ENST00000571081(c.-99+1487C>G)];ENSG00000167264:[ENST00000570709(c.-19+1487C>G)];	NA	NA	NA	NA	NA	NA	NA	NA	NA_NA	NA	MODIFIER	NA	NA	102	101	1	76	59	17	22.37

Cohort	Sample	Chromosome	Genomic position	Reference allele	Alternate allele	Gene symbol	Variant type	Variant annotation	OncoMD	COSMIC	dbSNP	ExAC	ExAC_SAS	1000G	1000G_SAS	SIFT	Polyphen2	CONDEL	VEP	LRT	PhastCon7 Way	Total read (Adjacent normal)	Reference allel read support (Adjacent normal)	Alternative read support (Adjacent normal)	Total read (Tumor)	Reference allel read support (Tumor)	Alternative read support (Tumor)	Alternate allele frequency
Chewer	42484	chr16	54967290	G	A	*IRX5*	SILENT	ENSG00000176842:SILENT[ENST00000558597(c.759G>A,tcG>tcA):ENSP00000453725(p.Ser253(=))];ENSG00000176842:SILENT[ENST00000320990(c.954G>A,tcG>tcA):ENSP00000316250(p.Ser318(=))];ENSG00000176842:SILENT[ENST00000394636(c.957G>A,tcG>tcA):ENSP00000378132(p.Ser319(=))];ENSG00000176842:INTRONIC[ENST00000560154(c.406-109G>A)];	NA	NA	NA	NA	NA	NA	NA	NA	NA_NA	NA	LOW	NA	NA	46	46	0	70	40	30	42.86
Chewer	42484	chr16	87637973	G	A	*JPH3*	INTRONIC	ENSG00000154118:[ENST00000537256(n.96+2437G>A)];ENSG00000154118:[ENST00000284262(c.382+839G>A)];ENSG00000154118:[ENST00000301008(n.770G>A)];	NA	NA	NA	NA	NA	NA	NA	NA	NA_NA	NA	MODIFIER	NA	NA	74	74	0	86	73	12	13.95
Chewer	42484	chr16	87637974	C	T	*JPH3*	INTRONIC	ENSG00000154118:[ENST00000537256(n.96+2438C>T)];ENSG00000154118:[ENST00000284262(c.382+840C>T)];ENSG00000154118:[ENST00000301008(n.771C>T)];	NA	NA	NA	NA	NA	NA	NA	NA	NA_NA	NA	MODIFIER	NA	NA	74	74	0	87	74	12	13.79
Chewer	42484	chr16	33965595	C	A	*NA*	INTERGENIC		NA	NA	0.0001997	0.0048794	0.003693145	0.0001997	0	NA	NA_NA	NA	MODIFIER	NA	NA	2066	1937	25	1245	1167	27	2.17
Chewer	42484	chr16	72003774	C	T	*PKD1L3*	EXONIC-NC-SS-PR	ENSG00000187008:[ENST00000534738(n.2184G>A)];ENSG00000187008:[ENST00000335106(n.2184G>A)];	NA	NA	NA	NA	NA	NA	NA	NA	NA_NA	NA	LOW	NA	NA	138	137	0	109	78	31	28.44
Chewer	42484	chr16	723419	C	A	*RHOT2*	INTRONIC	ENSG00000140983:[ENST00000569197(c.281-61C>A)];ENSG00000140983:[ENST00000568636(n.2453-61C>A)];ENSG00000140983:[ENST00000569675(n.1865-61C>A)];ENSG00000140983:[ENST00000562957(n.340-61C>A)];ENSG00000140983:[ENST00000315082(c.1731-61C>A)];ENSG00000140983:[ENST00000564659(n.362-61C>A)];	NA	NA	chr16*723419*723419*C*T*rs576006171*0*0.9998*0.0001997	NA	NA	NA	NA	NA	NA_NA	NA	MODIFIER	NA	NA	32	32	0	35	28	6	17.14
Chewer	42484	chr16	22152952	C	A	*VWA3A*	SILENT	ENSG00000175267:5UTR[ENST00000389397(c.-334C>A)];ENSG00000175267:SILENT[ENST00000389398(c.2433C>A,acC>acA):ENSP00000374049(p.Thr811(=))];ENSG00000175267:3UTR[ENST00000299840(c.*20C>A)];	NA	NA	NA	NA	NA	NA	NA	NA	NA_NA	NA	MODIFIER	NA	NA	109	108	1	84	73	11	13.1
Chewer	42484	chr17	79096958	G	T	*AATK*	INTRONIC	ENSG00000181409:[ENST00000573469(n.432-4836C>A)];ENSG00000181409:[ENST00000374792(c.1112+36C>A)];ENSG00000181409:[ENST00000326724(c.1112+36C>A)];ENSG00000181409:[ENST00000573441(n.357+36C>A)];ENSG00000181409:[ENST00000417379(c.803+36C>A)];	NA	NA	NA	NA	NA	NA	NA	NA	NA_NA	NA	MODIFIER	NA	NA	27	27	0	31	22	9	29.03
Chewer	42484	chr17	66929352	C	T	*ABCA8*	MISSENSE	ENSG00000141338:MISSENSE[ENST00000269080(c.527G>A,gGt>gAt):ENSP00000269080(p.Gly176Asp)];ENSG00000141338:MISSENSE[ENST00000430352(c.527G>A,gGt>gAt):ENSP00000402814(p.Gly176Asp)];ENSG00000141338:MISSENSE[ENST00000586539(c.527G>A,gGt>gAt):ENSP00000467271(p.Gly176Asp)];ENSG00000141338:EXONIC-NC[ENST00000428549(n.826G>A)];ENSG00000141338:EXONIC-NC[ENST00000541225(n.538G>A)];ENSG00000141338:EXONIC-NC[ENST00000585850(n.706G>A)];	NA	NA	NA	NA	NA	NA	NA	NA	NA_NA	NA	MODIFIER	NA	NA	39	39	0	40	28	12	30
Chewer	42484	chr17	34190496	A	G	*C17orf66*	INTRONIC-SS-DNR	ENSG00000172653:[ENST00000589015(c.523+10T>C)];ENSG00000172653:[ENST00000587573(c.403+10T>C)];ENSG00000172653:[ENST00000462028(n.1408+10T>C)];ENSG00000172653:[ENST00000585840(c.625+10T>C)];ENSG00000172653:[ENST00000311880(c.625+10T>C)];ENSG00000172653:[ENST00000592980(c.505+10T>C)];	NA	NA	NA	NA	NA	NA	NA	NA	NA_NA	NA	MODIFIER	NA	NA	57	57	0	61	47	14	22.95
Chewer	42484	chr17	10351385	A	T	*CTC-297N7.11*	INTRONIC	ENSG00000272975:[ENST00000587182(n.155+41830A>T)];	NA	NA	NA	NA	NA	NA	NA	NA	NA_NA	NA	MODIFIER	NA	NA	141	141	0	83	77	6	7.23
Chewer	42484	chr17	10397971	T	A	*CTC-297N7.11*	INTRONIC	ENSG00000272975:[ENST00000587182(n.156-72883T>A)];	NA	NA	NA	NA	NA	NA	NA	NA	NA_NA	NA	MODIFIER	NA	NA	203	202	1	176	149	27	15.34
Chewer	42484	chr17	10426709	C	T	*CTC-297N7.11*	INTRONIC	ENSG00000272975:[ENST00000587182(n.156-44145C>T)];	NA	NA	NA	8.24E-06	6.06E-05	NA	NA	NA	NA_NA	NA	MODIFIER	NA	NA	134	134	0	106	86	20	18.87
Chewer	42484	chr17	17948411	C	T	*GID4*	INTRONIC	ENSG00000141034:[ENST00000268719(c.439-50C>T)];ENSG00000141034:[ENST00000376345(c.439-50C>T)];	NA	NA	NA	1.68E-05	0	NA	NA	NA	NA_NA	NA	MODIFIER	NA	NA	52	50	1	38	32	6	15.79
Chewer	42484	chr17	36486059	C	A	*GPR179*	MISSENSE	ENSG00000188888:MISSENSE[ENST00000342292(c.3393G>T,agG>agT):ENSP00000345060(p.Arg1131Ser)];	NA	NA	NA	NA	NA	NA	NA	D	PrD_PrD	D	MODERATE	NA	0.473	136	136	0	142	109	33	23.24
Chewer	42484	chr17	7216753	G	C	*GPS2*	MISSENSE	ENSG00000132522:MISSENSE[ENST00000573684(c.190C>G,Cag>Gag):ENSP00000458837(p.Gln64Glu)];ENSG00000132522:MISSENSE[ENST00000380728(c.670C>G,Cag>Gag):ENSP00000370104(p.Gln224Glu)];ENSG00000132522:MISSENSE[ENST00000570780(c.741C>G,Cag>Gag):ENSP00000460836(p.Gln248Glu)];ENSG00000132522:MISSENSE[ENST00000389167(c.670C>G,Cag>Gag):ENSP00000379841(p.Gln224Glu)];ENSG00000132522:MISSENSE[ENST00000577040(c.670C>G,Cag>Gag):ENSP00000461624(p.Gln224Glu)];ENSG00000132522:MISSENSE[ENST00000391950(c.670C>G,Cag>Gag):ENSP00000438697(p.Gln224Glu)];ENSG00000132522:EXONIC-NC[ENST00000572172(n.1123C>G)];ENSG00000132522:EXONIC-NC[ENST00000572363(n.1089C>G)];ENSG00000132522:EXONIC-NC[ENST00000571569(n.1175C>G)];ENSG00000132522:EXONIC-NC[ENST00000571697(n.1516C>G)];ENSG00000132522:EXONIC-NC[ENST00000573807(n.324C>G)];	chr17*7216	chr17*7216	chr17*7216753*7216753*G*A*rs745671551*NA*NA*NA	NA	NA	NA	NA	NA	NA_NA	NA	MODIFIER	NA	NA	133	133	0	114	101	13	11.4
Chewer	42484	chr17	10397971	T	A	*MYH1*	MISSENSE	ENSG00000109061:MISSENSE[ENST00000226207(c.5486A>T,gAa>gTa):ENSP00000226207(p.Glu1829Val)];	NA	NA	NA	NA	NA	NA	NA	NA	NA_NA	NA	MODIFIER	NA	NA	203	202	1	176	149	27	15.34
Chewer	42484	chr17	10426709	C	T	*MYH2*	SILENT	ENSG00000125414:SILENT[ENST00000245503(c.5493G>A,gaG>gaA):ENSP00000245503(p.Glu1831(=))];ENSG00000125414:SILENT[ENST00000397183(c.5493G>A,gaG>gaA):ENSP00000380367(p.Glu1831(=))];ENSG00000125414:INTRONIC[ENST00000532183(c.1975-1960G>A)];	NA	NA	NA	8.24E-06	6.06E-05	NA	NA	NA	NA_NA	NA	MODIFIER	NA	NA	134	134	0	106	86	20	18.87
Chewer	42484	chr17	10351385	A	T	*MYH4*	MISSENSE	ENSG00000264424:MISSENSE[ENST00000255381(c.4715T>A,gTg>gAg):ENSP00000255381(p.Val1572Glu)];	NA	NA	NA	NA	NA	NA	NA	NA	NA_NA	NA	MODIFIER	NA	NA	141	141	0	83	77	6	7.23
Chewer	42484	chr17	29560113	C	T	*NF1*	MISSENSE	ENSG00000196712:MISSENSE[ENST00000358273(c.3590C>T,gCa>gTa):ENSP00000351015(p.Ala1197Val)];ENSG00000196712:MISSENSE[ENST00000456735(c.2588C>T,gCa>gTa):ENSP00000389907(p.Ala863Val)];ENSG00000196712:MISSENSE[ENST00000356175(c.3590C>T,gCa>gTa):ENSP00000348498(p.Ala1197Val)];ENSG00000196712:MISSENSE[ENST00000466819(c.66C>T,gCa>gTa):ENSP00000463819(p.Ala23Val)];ENSG00000196712:MISSENSE[ENST00000579081(c.3692C>T,gCa>gTa):ENSP00000462408(p.Ala1231Val)];ENSG00000196712:MISSENSE[ENST00000479614(c.66C>T,gCa>gTa):ENSP00000462157(p.Ala23Val)];ENSG00000196712:3UTR[ENST00000495910(c.*2991C>T)];ENSG00000196712:EXONIC-NC[ENST00000493220(n.2126C>T)];	NA	NA	chr17*29560113*29560113*C*G,T*rs370820478*NA*NA*NA	NA	NA	NA	NA	NA	NA_NA	NA	MODIFIER	NA	NA	232	231	1	211	184	27	12.8
Chewer	42484	chr17	7216753	G	C	*RP11-542C16.2*	3UTR	ENSG00000261915:[ENST00000315601(c.*945C>G)];ENSG00000261915:[ENST00000575474(c.*945C>G)];	chr17*7216	chr17*7216	chr17*7216753*7216753*G*A*rs745671551*NA*NA*NA	NA	NA	NA	NA	NA	NA_NA	NA	MODIFIER	NA	NA	133	133	0	114	101	13	11.4
Chewer	42484	chr17	10351385	A	T	*RP11-799N11.1*	INTRONIC	ENSG00000272736:[ENST00000399342(n.206+41791A>T)];ENSG00000272736:[ENST00000581304(n.143+41830A>T)];	NA	NA	NA	NA	NA	NA	NA	NA	NA_NA	NA	MODIFIER	NA	NA	141	141	0	83	77	6	7.23
Chewer	42484	chr17	10397971	T	A	*RP11-799N11.1*	INTRONIC	ENSG00000272736:[ENST00000399342(n.207-38670T>A)];ENSG00000272736:[ENST00000581304(n.144-38670T>A)];	NA	NA	NA	NA	NA	NA	NA	NA	NA_NA	NA	MODIFIER	NA	NA	203	202	1	176	149	27	15.34

Mangalaparthi *et al.* , 2020. Mutational landscape of esophageal squamous cell carcinoma in an Indian cohort
Supplementary Table 4. List of somatic single nucleotide variants identified using whole exome sequencing of ESCC patients

Cohort	Sample	Chromosome	Genomic position	Reference allele	Alternate allele	Gene symbol	Variant type	Variant annotation	OncoMD	COSMIC	dbSNP	ExAC	ExAC_SAS	1000G	1000G_SAS	SIFT	Polyphen2	CONDEL	VEP	LRT	PhastCon7 Way	Total read (Adjacent normal)	Reference allel read support (Adjacent normal)	Alternative read support (Adjacent normal)	Total read (Tumor)	Reference allel read support (Tumor)	Alternative read support (Tumor)	Alternate allele frequency
Chewer	42484	chr17	10426709	C	T	*RP11-799N11.1*	INTRONIC	ENSG00000272736:[ENST00000399342(n.207-9932C>T)];ENSG00000272736:[ENST00000581304(n.144-9932C>T)];	NA	NA	NA	8.24E-06	6.06E-05	NA	NA	NA	NA_NA	NA	MODIFIER	NA	NA	134	134	0	106	86	20	18.87
Chewer	42484	chr17	53108630	T	A	*STXBP4*	INTRONIC-SS-DNR	ENSG00000166263:[ENST00000398391(c.538+5T>A)];ENSG00000166263:[ENST00000405898(c.763+5T>A)];ENSG00000166263:[ENST00000376352(c.763+5T>A)];ENSG00000166263:[ENST00000468837(n.458+5T>A)];ENSG00000166263:[ENST00000299341(c.538+5T>A)];ENSG00000166263:[ENST00000434978(c.763+5T>A)];ENSG00000166263:[ENST00000463234(c.200+5T>A)];	NA	NA	NA	NA	NA	NA	NA	NA	NA_NA	NA	LOW	NA	NA	262	262	0	234	177	56	23.93
Chewer	42484	chr17	34190496	A	G	*TAF15*	INTRONIC	ENSG00000172660:[ENST00000586593(c.105-572A>G)];	NA	NA	NA	NA	NA	NA	NA	NA	NA_NA	NA	MODIFIER	NA	NA	57	57	0	61	47	14	22.95
Chewer	42484	chr17	7577058	C	A	*TP53*	NONSENSE	ENSG00000141510:NONSENSE[ENST00000420246(c.880G>T,Gag>Tag):ENSP00000391127(p.Glu294Ter)];ENSG00000141510:NONSENSE[ENST00000269305(c.880G>T,Gag>Tag):ENSP00000269305(p.Glu294Ter)];ENSG00000141510:NONSENSE[ENST00000509690(c.484G>T,Gag>Tag):ENSP00000425104(p.Glu162Ter)];ENSG00000141510:NONSENSE[ENST00000359597(c.880G>T,Gag>Tag):ENSP00000352610(p.Glu294Ter)];ENSG00000141510:NONSENSE[ENST00000445888(c.880G>T,Gag>Tag):ENSP00000391478(p.Glu294Ter)];ENSG00000141510:NONSENSE[ENST00000455263(c.880G>T,Gag>Tag):ENSP00000398846(p.Glu294Ter)];ENSG00000141510:EXONIC-NC[ENST00000504937(n.762G>T)];ENSG00000141510:EXONIC-NC[ENST00000504290(n.762G>T)];ENSG00000141510:EXONIC-NC[ENST00000510385(n.762G>T)];ENSG00000141510:INTRONIC[ENST00000413465(c.782+441G>T)];	chr17*7577	chr17*7577	NA	NA	NA	NA	NA	NA	NA_NA	NA	MODIFIER	NA	NA	107	107	0	75	53	22	29.33
Chewer	42484	chr18	12057669	C	T	*AP002414.1*	EXONIC-NC	ENSG00000212712:[ENST00000391405(n.153G>A)];	NA	NA	chr18*12057669*12057669*C*G*rs770384766*NA*NA*NA	NA	NA	NA	NA	NA	NA_NA	NA	MODIFIER	NA	NA	43	43	0	70	52	18	25.71
Chewer	42484	chr18	65180744	G	A	*CTD-2541J13.2*	EXONIC-NC	ENSG00000263424:[ENST00000583493(n.2766G>A)];	NA	NA	NA	NA	NA	NA	NA	NA	NA_NA	NA	HIGH	D	0.999	165	165	0	111	89	21	18.92
Chewer	42484	chr18	67406333	G	A	*DOK6*	SILENT	ENSG00000206052:EXONIC-NC[ENST00000577609(n.113G>A)];ENSG00000206052:SILENT[ENST00000582992(c.443G>A,aaG>aaA):ENSP00000462984(p.Lys148(=))];ENSG00000206052:SILENT[ENST00000382713(c.732G>A,aaG>aaA):ENSP00000372160(p.Lys244(=))];	NA	NA	NA	NA	NA	NA	NA	NA	NA_NA	NA	LOW	NA	0.999	95	95	0	65	52	13	20
Chewer	42484	chr18	28598191	T	C	*DSC3*	MISSENSE	ENSG00000134762:MISSENSE[ENST00000360428(c.1109A>G,aAt>aGt):ENSP00000353608(p.Asn370Ser)];ENSG00000134762:MISSENSE[ENST00000434452(c.1109A>G,aAt>aGt):ENSP00000392068(p.Asn370Ser)];	chr18*2859	chr18*2859	NA	NA	NA	NA	NA	D	PoD_PoD	D	MODERATE	N	0.422	48	48	0	31	25	6	19.35
Chewer	42484	chr18	65180744	G	A	*DSEL*	NONSENSE	ENSG00000171451:NONSENSE[ENST00000310045(c.1132C>T,Cga>Tga):ENSP00000310565(p.Arg378Ter)];	NA	NA	NA	NA	NA	NA	NA	NA	NA_NA	NA	HIGH	D	0.999	165	165	0	111	89	21	18.92
Chewer	42484	chr18	10773930	C	A	*PIEZO2*	INTRONIC	ENSG00000154864:[ENST00000580640(c.2567+74G>T)];ENSG00000154864:[ENST00000503781(c.2493-303G>T)];ENSG00000154864:[ENST00000583325(c.480-303G>T)];ENSG00000154864:[ENST00000582913(c.2535-303G>T)];ENSG00000154864:[ENST00000302079(c.2493-303G>T)];ENSG00000154864:[ENST00000383408(c.356+74G>T)];	NA	NA	NA	NA	NA	NA	NA	NA	NA_NA	NA	MODIFIER	NA	NA	58	58	0	60	53	6	10
Chewer	42484	chr18	9845646	G	A	*RAB31*	MISSENSE	ENSG00000168461:MISSENSE[ENST00000578921(c.448G>A,Gca>Aca):ENSP00000461945(p.Ala150Thr)];ENSG00000168461:EXONIC-NC[ENST00000577371(n.242G>A)];ENSG00000168461:EXONIC-NC[ENST00000583921(n.396G>A)];ENSG00000168461:EXONIC-NC[ENST00000577796(n.252G>A)];ENSG00000168461:INTRONIC[ENST00000435762(n.154-13579G>A)];ENSG00000168461:3UTR[ENST00000578734(c.*290G>A)];ENSG00000168461:3UTR[ENST00000581109(c.*44G>A)];	NA	NA	NA	NA	NA	NA	NA	NA	NA_NA	NA	MODIFIER	NA	NA	177	176	0	145	115	30	20.69
Chewer	42484	chr18	61582942	T	G	*SERPINB10*	INTRONIC	ENSG00000242550:[ENST00000397996(c.805+30T>G)];ENSG00000242550:[ENST00000238508(c.168+30T>G)];ENSG00000242550:[ENST00000418725(c.724+30T>G)];	NA	NA	NA	NA	NA	NA	NA	NA	NA_NA	NA	MODIFIER	NA	NA	107	107	0	45	34	11	24.44
Chewer	42484	chr18	60028931	C	G	*TNFRSF11A*	MISSENSE	ENSG00000141655:INTRONIC[ENST00000269485(c.616+1649C>G)];ENSG00000141655:MISSENSE[ENST00000586569(c.635C>G,cCc>cGc):ENSP00000465500(p.Pro212Arg)];	NA	NA	NA	NA	NA	NA	NA	T	PoD_PrD	N	MODERATE	N	0.203	243	243	0	125	104	21	16.8
Chewer	42484	chr18	54444132	C	T	*WDR7*	INTRONIC	ENSG00000091157:[ENST00000591524(n.118-2530C>T)];ENSG00000091157:[ENST00000254442(c.2947+21C>T)];ENSG00000091157:[ENST00000589935(c.-1+125325C>T)];ENSG00000091157:[ENST00000357574(c.2849-2530C>T)];	NA	NA	chr18*54444132*54444132*C*A*rs748384341*NA*NA*NA	NA	NA	NA	NA	NA	NA_NA	NA	MODIFIER	NA	NA	94	94	0	45	34	11	24.44
Chewer	42484	chr19	36034236	C	G	*AD000090.2*	INTRONIC	ENSG00000236144:[ENST00000444728(n.186-237G>C)];ENSG00000236144:[ENST00000589137(n.94-61G>C)];ENSG00000236144:[ENST00000588286(n.2159G>C)];ENSG00000236144:[ENST00000590717(n.1707G>C)];	NA	NA	NA	NA	NA	NA	NA	NA	NA_NA	NA	LOW	NA	NA	86	86	0	123	56	67	54.47
Chewer	42484	chr19	54496309	G	A	*CACNG6*	MISSENSE	ENSG00000130433:MISSENSE[ENST00000252729(c.178G>A,Gag>Aag):ENSP00000252729(p.Glu60Lys)];ENSG00000130433:MISSENSE[ENST00000346968(c.178G>A,Gag>Aag):ENSP00000319097(p.Glu60Lys)];ENSG00000130433:MISSENSE[ENST00000352529(c.178G>A,Gag>Aag):ENSP00000319135(p.Glu60Lys)];	NA	NA	NA	NA	NA	NA	NA	D	PoD_PoD	D	MODERATE	U	0.883	83	83	0	82	68	14	17.07
Chewer	42484	chr19	47109250	G	C	*CALM3*	INTRONIC	ENSG00000160014:[ENST00000599839(c.-75+136G>C)];ENSG00000160014:[ENST00000482455(n.144+136G>C)];ENSG00000160014:[ENST00000291295(c.34+136G>C)];ENSG00000160014:[ENST00000477244(n.160+136G>C)];ENSG00000160014:[ENST00000597743(c.34+136G>C)];ENSG00000160014:[ENST00000597868(n.102+136G>C)];ENSG00000160014:[ENST00000391918(c.-75+136G>C)];ENSG00000160014:[ENST00000598871(c.-74-2204G>C)];ENSG00000160014:[ENST00000594523(c.-67+136G>C)];ENSG00000160014:[ENST00000596362(c.34+136G>C)];ENSG00000160014:[ENST00000602169(n.241G>C)];	NA	NA	NA	NA	NA	NA	NA	NA	NA_NA	NA	MODIFIER	NA	NA	75	75	0	152	116	36	23.68

Mangalaparthi *et al.* , 2020. Mutational landscape of esophageal squamous cell carcinoma in an Indian cohort
Supplementary Table 4. List of somatic single nucleotide variants identified using whole exome sequencing of ESCC patients

Cohort	Sample	Chromosome	Genomic position	Reference allele	Alternate allele	Gene symbol	Variant type	Variant annotation	OncoMD	COSMIC	dbSNP	ExAC	ExAC_SAS	1000G	1000G_SAS	SIFT	Polyphen2	CONDEL	VEP	LRT	PhastCon7 Way	Total read (Adjacent normal)	Reference allel read support (Adjacent normal)	Alternative read support (Adjacent normal)	Total read (Tumor)	Reference allel read support (Tumor)	Alternative read support (Tumor)	Alternate allele frequency
Chewer	42484	chr19	47111520	G	A	*CALM3*	MISSENSE	ENSG00000160014:MISSENSE[ENST00000291295(c.101G>A,gGg>gAg):ENSP00000291295(p.Gly34Glu)];ENSG00000160014:MISSENSE[ENST00000597743(c.101G>A,gGg>gAg):ENSP00000470308(p.Gly34Glu)];ENSG00000160014:MISSENSE[ENST00000596362(c.101G>A,gGg>gAg):ENSP00000472141(p.Gly34Glu)];ENSG00000160014:5UTR[ENST00000599839(c.-8G>A)];ENSG00000160014:5UTR[ENST00000391918(c.-8G>A)];ENSG00000160014:5UTR[ENST00000598871(c.-8G>A)];ENSG00000160014:5UTR[ENST00000594523(c.-8G>A)];ENSG00000160014:EXONIC-NC[ENST00000482455(n.211G>A)];ENSG00000160014:EXONIC-NC[ENST00000477244(n.225G>A)];ENSG00000160014:EXONIC-NC[ENST00000595072(n.291G>A)];ENSG00000160014:EXONIC-NC[ENST00000597868(n.169G>A)];ENSG00000160014:EXONIC-NC[ENST00000486500(n.302G>A)];	NA	NA	NA	NA	NA	NA	NA	NA	NA_NA	NA	MODIFIER	NA	NA	72	72	0	149	105	43	28.86
Chewer	42484	chr19	47111530	G	A	*CALM3*	MISSENSE	ENSG00000160014:STARTLOSS[ENST00000599839(c.3G>A,atG>atA):ENSP00000471225(p.Met1?)];ENSG00000160014:STARTLOSS[ENST00000391918(c.3G>A,atG>atA):ENSP00000375785(p.Met1?)];ENSG00000160014:STARTLOSS[ENST00000598871(c.3G>A,atG>atA):ENSP00000470502(p.Met1?)];ENSG00000160014:STARTLOSS[ENST00000594523(c.3G>A,atG>atA):ENSP00000468877(p.Met1?)];ENSG00000160014:MISSENSE[ENST00000597743(c.111G>A,atG>atA):ENSP00000470308(p.Met37Ile)];ENSG00000160014:MISSENSE[ENST00000291295(c.111G>A,atG>atA):ENSP00000291295(p.Met37Ile)];ENSG00000160014:MISSENSE[ENST00000596362(c.111G>A,atG>atA):ENSP00000472141(p.Met37Ile)];ENSG00000160014:EXONIC-NC[ENST00000482455(n.221G>A)];ENSG00000160014:EXONIC-NC[ENST00000477244(n.235G>A)];ENSG00000160014:EXONIC-NC[ENST00000595072(n.301G>A)];ENSG00000160014:EXONIC-NC[ENST00000597868(n.179G>A)];ENSG00000160014:EXONIC-NC[ENST00000486500(n.312G>A)];	NA	NA	NA	NA	NA	NA	NA	NA	NA_NA	NA	MODIFIER	NA	NA	76	76	0	172	122	49	28.49
Chewer	42484	chr19	47111545	G	A	*CALM3*	SILENT	ENSG00000160014:SILENT[ENST00000599839(c.18G>A,caG>caA):ENSP00000471225(p.Gln6(=))];ENSG00000160014:SILENT[ENST00000291295(c.126G>A,caG>caA):ENSP00000291295(p.Gln42(=))];ENSG00000160014:SILENT[ENST00000597743(c.126G>A,caG>caA):ENSP00000470308(p.Gln42(=))];ENSG00000160014:SILENT[ENST00000391918(c.18G>A,caG>caA):ENSP00000375785(p.Gln6(=))];ENSG00000160014:SILENT[ENST00000598871(c.18G>A,caG>caA):ENSP00000470502(p.Gln6(=))];ENSG00000160014:SILENT[ENST00000594523(c.18G>A,caG>caA):ENSP00000468877(p.Gln6(=))];ENSG00000160014:SILENT[ENST00000596362(c.126G>A,caG>caA):ENSP00000472141(p.Gln42(=))];ENSG00000160014:EXONIC-NC[ENST00000482455(n.236G>A)];ENSG00000160014:EXONIC-NC[ENST00000477244(n.250G>A)];ENSG00000160014:EXONIC-NC[ENST00000595072(n.316G>A)];ENSG00000160014:EXONIC-NC[ENST00000597868(n.194G>A)];ENSG00000160014:EXONIC-NC[ENST00000486500(n.327G>A)];	NA	NA	NA	NA	NA	NA	NA	NA	NA_NA	NA	LOW	NA	0.998	80	80	0	184	128	53	28.8
Chewer	42484	chr19	47111672	G	A	*CALM3*	INTRONIC	ENSG00000160014:[ENST00000599839(c.71-67G>A)];ENSG00000160014:[ENST00000482455(n.289-67G>A)];ENSG00000160014:[ENST00000291295(c.179-67G>A)];ENSG00000160014:[ENST00000477244(n.303-67G>A)];ENSG00000160014:[ENST00000595072(n.369-67G>A)];ENSG00000160014:[ENST00000597743(c.165+88G>A)];ENSG00000160014:[ENST00000597868(n.247-67G>A)];ENSG00000160014:[ENST00000486500(n.380-67G>A)];ENSG00000160014:[ENST00000391918(c.71-67G>A)];ENSG00000160014:[ENST00000598871(c.71-67G>A)];ENSG00000160014:[ENST00000594523(c.71-67G>A)];ENSG00000160014:[ENST00000596362(c.179-67G>A)];	NA	NA	NA	NA	NA	NA	NA	NA	NA_NA	NA	MODIFIER	NA	NA	87	87	0	266	171	95	35.71
Chewer	42484	chr19	47111784	G	C	*CALM3*	MISSENSE	ENSG00000160014:MISSENSE[ENST00000599839(c.116G>C,aGa>aCa):ENSP00000471225(p.Arg39Thr)];ENSG00000160014:MISSENSE[ENST00000291295(c.224G>C,aGa>aCa):ENSP00000291295(p.Arg75Thr)];ENSG00000160014:MISSENSE[ENST00000391918(c.116G>C,aGa>aCa):ENSP00000375785(p.Arg39Thr)];ENSG00000160014:MISSENSE[ENST00000598871(c.116G>C,aGa>aCa):ENSP00000470502(p.Arg39Thr)];ENSG00000160014:MISSENSE[ENST00000594523(c.116G>C,aGa>aCa):ENSP00000468877(p.Arg39Thr)];ENSG00000160014:MISSENSE[ENST00000596362(c.224G>C,aGa>aCa):ENSP00000472141(p.Arg75Thr)];ENSG00000160014:EXONIC-NC[ENST00000482455(n.334G>C)];ENSG00000160014:EXONIC-NC[ENST00000477244(n.348G>C)];ENSG00000160014:EXONIC-NC[ENST00000595072(n.414G>C)];ENSG00000160014:EXONIC-NC[ENST00000597868(n.292G>C)];ENSG00000160014:EXONIC-NC[ENST00000486500(n.425G>C)];ENSG00000160014:INTRONIC[ENST00000597743(c.165+200G>C)];	NA	NA	NA	NA	NA	NA	NA	NA	NA_NA	NA	MODIFIER	NA	NA	100	100	0	236	166	70	29.66
Chewer	42484	chr19	47111788	G	A	*CALM3*	SILENT	ENSG00000160014:SILENT[ENST00000599839(c.120G>A,aaG>aaA):ENSP00000471225(p.Lys40(=))];ENSG00000160014:SILENT[ENST00000291295(c.228G>A,aaG>aaA):ENSP00000291295(p.Lys76(=))];ENSG00000160014:SILENT[ENST00000391918(c.120G>A,aaG>aaA):ENSP00000375785(p.Lys40(=))];ENSG00000160014:SILENT[ENST00000598871(c.120G>A,aaG>aaA):ENSP00000470502(p.Lys40(=))];ENSG00000160014:SILENT[ENST00000594523(c.120G>A,aaG>aaA):ENSP00000468877(p.Lys40(=))];ENSG00000160014:SILENT[ENST00000596362(c.228G>A,aaG>aaA):ENSP00000472141(p.Lys76(=))];ENSG00000160014:EXONIC-NC[ENST00000482455(n.338G>A)];ENSG00000160014:EXONIC-NC[ENST00000477244(n.352G>A)];ENSG00000160014:EXONIC-NC[ENST00000595072(n.418G>A)];ENSG00000160014:EXONIC-NC[ENST00000597868(n.296G>A)];ENSG00000160014:EXONIC-NC[ENST00000486500(n.429G>A)];ENSG00000160014:INTRONIC[ENST00000597743(c.165+204G>A)];	NA	NA	NA	NA	NA	NA	NA	NA	NA_NA	NA	LOW	NA	1	97	96	0	237	168	69	29.11

Mangalaparthi *et al.* , 2020. Mutational landscape of esophageal squamous cell carcinoma in an Indian cohort
Supplementary Table 4. List of somatic single nucleotide variants identified using whole exome sequencing of ESCC patients

Cohort	Sample	Chromosome	Genomic position	Reference allele	Alternate allele	Gene symbol	Variant type	Variant annotation	OncoMD	COSMIC	dbSNP	ExAC	ExAC_SAS	1000G	1000G_SAS	SIFT	Polyphen2	CONDEL	VEP	LRT	PhastCon7 Way	Total read (Adjacent normal)	Reference allel read support (Adjacent normal)	Alternative read support (Adjacent normal)	Total read (Tumor)	Reference allel read support (Tumor)	Alternative read support (Tumor)	Alternate allele frequency
Chewer	42484	chr19	47111822	G	C	*CALM3*	MISSENSE	ENSG00000160014:MISSENSE[ENST00000599839(c.154G>C,Gag>Cag):ENSP00000471225(p.Glu52Gln)];ENSG00000160014:MISSENSE[ENST00000291295(c.262G>C,Gag>Cag):ENSP00000291295(p.Glu88Gln)];ENSG00000160014:MISSENSE[ENST00000391918(c.154G>C,Gag>Cag):ENSP00000375785(p.Glu52Gln)];ENSG00000160014:MISSENSE[ENST00000598871(c.154G>C,Gag>Cag):ENSP00000470502(p.Glu52Gln)];ENSG00000160014:MISSENSE[ENST00000594523(c.154G>C,Gag>Cag):ENSP00000468877(p.Glu52Gln)];ENSG00000160014:MISSENSE[ENST00000596362(c.262G>C,Gag>Cag):ENSP00000472141(p.Glu88Gln)];ENSG00000160014:EXONIC-NC[ENST00000482455(n.372G>C)];ENSG00000160014:EXONIC-NC[ENST00000477244(n.386G>C)];ENSG00000160014:EXONIC-NC[ENST00000595072(n.452G>C)];ENSG00000160014:EXONIC-NC[ENST00000597868(n.330G>C)];ENSG00000160014:EXONIC-NC[ENST00000486500(n.463G>C)];ENSG00000160014:INTRONIC[ENST00000597743(c.165+238G>C)];	NA	NA	NA	NA	NA	NA	NA	NA	NA_NA	NA	MODIFIER	NA	NA	80	80	0	170	120	50	29.41
Chewer	42484	chr19	47111840	G	A	*CALM3*	MISSENSE	ENSG00000160014:MISSENSE[ENST00000599839(c.172G>A,Gac>Aac):ENSP00000471225(p.Asp58Asn)];ENSG00000160014:MISSENSE[ENST00000291295(c.280G>A,Gac>Aac):ENSP00000291295(p.Asp94Asn)];ENSG00000160014:MISSENSE[ENST00000391918(c.172G>A,Gac>Aac):ENSP00000375785(p.Asp58Asn)];ENSG00000160014:MISSENSE[ENST00000598871(c.172G>A,Gac>Aac):ENSP00000470502(p.Asp58Asn)];ENSG00000160014:MISSENSE[ENST00000594523(c.172G>A,Gac>Aac):ENSP00000468877(p.Asp58Asn)];ENSG00000160014:MISSENSE[ENST00000596362(c.280G>A,Gac>Aac):ENSP00000472141(p.Asp94Asn)];ENSG00000160014:EXONIC-NC[ENST00000482455(n.390G>A)];ENSG00000160014:EXONIC-NC[ENST00000477244(n.404G>A)];ENSG00000160014:EXONIC-NC[ENST00000595072(n.470G>A)];ENSG00000160014:EXONIC-NC[ENST00000597868(n.348G>A)];ENSG00000160014:EXONIC-NC[ENST00000486500(n.481G>A)];ENSG00000160014:INTRONIC[ENST00000597743(c.165+256G>A)];	NA	NA	NA	NA	NA	NA	NA	NA	NA_NA	NA	MODIFIER	NA	NA	67	67	0	130	92	38	29.23
Chewer	42484	chr19	47112292	G	T	*CALM3*	INTRONIC	ENSG00000160014:[ENST00000599839(c.313+54G>T)];ENSG00000160014:[ENST00000482455(n.531+54G>T)];ENSG00000160014:[ENST00000291295(c.421+54G>T)];ENSG00000160014:[ENST00000477244(n.545+54G>T)];ENSG00000160014:[ENST00000595072(n.611+54G>T)];ENSG00000160014:[ENST00000597743(c.223+54G>T)];ENSG00000160014:[ENST00000597868(n.746+54G>T)];ENSG00000160014:[ENST00000486500(n.879+54G>T)];ENSG00000160014:[ENST00000391918(c.313+54G>T)];ENSG00000160014:[ENST00000598871(c.313+54G>T)];ENSG00000160014:[ENST00000594523(c.313+54G>T)];ENSG00000160014:[ENST00000596362(c.421+54G>T)];	NA	NA	NA	NA	NA	NA	NA	NA	NA_NA	NA	MODIFIER	NA	NA	82	82	0	249	167	82	32.93
Chewer	42484	chr19	19637035	C	A	*CTC-260F20.3*	MISSENSE	ENSG00000258674:EXONIC-NC[ENST00000586674(n.149C>A)];ENSG00000258674:MISSENSE[ENST00000555938(c.139C>A,Cac>Aac):ENSP00000452549(p.His47Asn)];	NA	NA	chr19*19637035*19637035*C*T*rs766346722*NA*NA*NA	NA	NA	NA	NA	D_lc	BN_BN	N	MODERATE	NA	0.144	147	147	0	130	124	6	4.62
Chewer	42484	chr19	41594436	G	C	*CYP2A13*	MISSENSE	ENSG00000197838:MISSENSE[ENST00000330436(c.60G>C,ttG>ttC):ENSP00000332679(p.Leu20Phe)];	NA	NA	chr19*41594436*41594436*G*A*rs866594422*NA*NA*NA	NA	NA	NA	NA	T	UN_UN	NA	MODERATE	U	0.612	91	91	0	118	107	11	9.32
Chewer	42484	chr19	47152526	C	T	*DACT3*	MISSENSE	ENSG00000197380:MISSENSE[ENST00000300875(c.428G>A,cGa>cAa):ENSP00000300875(p.Arg143Gln)];ENSG00000197380:MISSENSE[ENST00000391916(c.1103G>A,cGa>cAa):ENSP00000375783(p.Arg368Gln)];	NA	NA	NA	NA	NA	NA	NA	T	BN_PoD	N	MODERATE	U	0.142	42	42	0	166	158	8	4.82
Chewer	42484	chr19	36001239	G	T	*DMKN*	INTRONIC	ENSG00000161249:[ENST00000486450(n.61+34C>A)];ENSG00000161249:[ENST00000450261(c.57+34C>A)];ENSG00000161249:[ENST00000498211(c.49+42C>A)];ENSG00000161249:[ENST00000419602(c.736-85C>A)];ENSG00000161249:[ENST00000429837(c.736-85C>A)];ENSG00000161249:[ENST00000424570(c.919-85C>A)];ENSG00000161249:[ENST00000462126(n.28-85C>A)];ENSG00000161249:[ENST00000467637(c.57+34C>A)];ENSG00000161249:[ENST00000472065(n.87+34C>A)];ENSG00000161249:[ENST00000462538(c.49+42C>A)];ENSG00000161249:[ENST00000480502(c.57+34C>A)];ENSG00000161249:[ENST00000418261(c.919-85C>A)];ENSG00000161249:[ENST00000440396(c.919-85C>A)];ENSG00000161249:[ENST00000482321(n.100+34C>A)];ENSG00000161249:[ENST00000460051(c.49+42C>A)];ENSG00000161249:[ENST00000392206(c.57+34C>A)];ENSG00000161249:[ENST00000602781(c.57+34C>A)];ENSG00000161249:[ENST00000464709(c.49+42C>A)];ENSG00000161249:[ENST00000471786(n.57+34C>A)];ENSG00000161249:[ENST00000447113(c.919-85C>A)];ENSG00000161249:[ENST00000476246(c.49+42C>A)];ENSG00000161249:[ENST00000470746(c.49+42C>A)];ENSG00000161249:[ENST00000463292(c.49+42C>A)];ENSG00000161249:[ENST00000490622(c.49+42C>A)];ENSG00000161249:[ENST00000493517(c.49+42C>A)];ENSG00000161249:[ENST00000451297(c.919-85C>A)];ENSG00000161249:[ENST00000472252(c.-91+42C>A)];ENSG00000161249:[ENST00000458071(c.57+34C>A)];ENSG00000161249:[ENST00000443640(c.57+34C>A)];ENSG00000161249:[ENST00000474992(n.45+42C>A)];ENSG00000161249:[ENST00000470857(n.48+34C>A)];ENSG00000161249:[ENST00000462721(c.49+42C>A)];ENSG00000161249:[ENST00000480507(c.49+42C>A)];ENSG00000161249:[ENST00000414866(c.57+34C>A)];ENSG00000161249:[ENST00000498593(c.49+42C>A)];ENSG00000161249:[ENST00000402589(c.57+34C>A)];ENSG00000161249:[ENST00000488892(c.57+34C>A)];ENSG	NA	NA	NA	NA	NA	NA	NA	NA	NA_NA	NA	MODIFIER	NA	NA	167	167	0	218	202	16	7.34
Chewer	42484	chr19	36034236	C	G	*GAPDHS*	INTRONIC-SS-ACR	ENSG00000105679:[ENST00000222286(c.742-6C>G)];	NA	NA	NA	NA	NA	NA	NA	NA	NA_NA	NA	LOW	NA	NA	86	86	0	123	56	67	54.47
Chewer	42484	chr19	16254651	G	A	*HSH2D*	INTRONIC	ENSG00000196684:[ENST00000535834(c.-105+67G>A)];ENSG00000196684:[ENST00000593154(c.-28+67G>A)];ENSG00000196684:[ENST00000587963(c.-28+67G>A)];ENSG00000196684:[ENST00000588246(c.-28+67G>A)];ENSG00000196684:[ENST00000593031(c.-28+67G>A)];ENSG00000196684:[ENST00000397372(c.42+67G>A)];ENSG00000196684:[ENST00000253680(c.-28+67G>A)];ENSG00000196684:[ENST00000586872(c.-28+67G>A)];ENSG00000196684:[ENST00000589463(c.-28+67G>A)];	NA	NA	NA	NA	NA	NA	NA	NA	NA_NA	NA	MODIFIER	NA	NA	46	46	0	42	27	14	33.33
Chewer	42484	chr19	39804563	G	A	*LRFN1*	INTRONIC-SS-DNR	ENSG00000128011:[ENST00000248668(c.1406+8C>T)];	NA	NA	NA	1.80E-05	8.67E-05	NA	NA	NA	NA_NA	NA	LOW	NA	NA	94	94	0	146	84	62	42.47

Supplementary Table 4. List of somatic single nucleotide variants identified using whole exome sequencing of ESCC patients

Cohort	Sample	Chromosome	Genomic position	Reference allele	Alternate allele	Gene symbol	Variant type	Variant annotation	OncoMD	COSMIC	dbSNP	ExAC	ExAC_SAS	1000G	1000G_SAS	SIFT	Polyphen2	CONDEL	VEP	LRT	PhastCon7 Way	Total read (Adjacent normal)	Reference allel read support (Adjacent normal)	Alternative read support (Adjacent normal)	Total read (Tumor)	Reference allel read support (Tumor)	Alternative read support (Tumor)	Alternate allele frequency
Chewer	42484	chr19	41115428	C	T	*LTBP4*	INTRONIC-SS-ACR	ENSG00000090006:[ENST00000545697(c.-14-8C>T)];ENSG00000090006:[ENST00000308370(c.1628-8C>T)];ENSG00000090006:[ENST00000600509(n.1628-8C>T)];ENSG00000090006:[ENST00000601209(n.1444-8C>T)];ENSG00000090006:[ENST00000396819(c.1427-8C>T)];ENSG00000090006:[ENST00000602240(n.1517-8C>T)];ENSG00000090006:[ENST00000204005(c.1517-8C>T)];ENSG00000090006:[ENST00000598717(n.429-1852C>T)];	NA	NA	NA	1.72E-05	0	NA	NA	NA	NA_NA	NA	MODIFIER	NA	NA	95	95	0	113	104	9	7.96
Chewer	42484	chr19	19637035	C	A	*NDUFA13*	MISSENSE	ENSG00000186010:MISSENSE[ENST00000428459(c.139C>A,Cac>Aac):ENSP00000465129(p.His47Asn)];ENSG00000186010:MISSENSE[ENST00000606722(c.117C>A,Cac>Aac):ENSP00000476093(p.His40Asn)];ENSG00000186010:MISSENSE[ENST00000512771(c.139C>A,Cac>Aac):ENSP00000465471(p.His47Asn)];ENSG00000186010:MISSENSE[ENST00000507754(c.139C>A,Cac>Aac):ENSP00000423673(p.His47Asn)];ENSG00000186010:MISSENSE[ENST00000252576(c.388C>A,Cac>Aac):ENSP00000252576(p.His130Asn)];ENSG00000186010:MISSENSE[ENST00000503283(c.139C>A,Cac>Aac):ENSP00000468317(p.His47Asn)];ENSG00000186010:MISSENSE[ENST00000502506(c.296C>A,gCa>gAa):ENSP00000467274(p.Ala99Glu)];ENSG00000186010:EXONIC-NC[ENST00000511584(n.148C>A)];ENSG00000186010:EXONIC-NC[ENST00000511180(n.137C>A)];	NA	NA	chr19*19637035*19637035*C*T*rs766346722*NA*NA*NA	NA	NA	NA	NA	D_lc	BN_BN	N	MODERATE	NA	0.144	147	147	0	130	124	6	4.62
Chewer	42484	chr19	56320662	C	A	*NLRP11*	SILENT	ENSG00000179873:SILENT[ENST00000589093(c.1314G>T,ccG>ccT):ENSP00000466285(p.Pro438(=))];ENSG00000179873:SILENT[ENST00000592953(c.1017G>T,ccG>ccT):ENSP00000468196(p.Pro339(=))];ENSG00000179873:SILENT[ENST00000589824(c.1314G>T,ccG>ccT):ENSP00000468082(p.Pro438(=))];ENSG00000179873:SILENT[ENST00000443188(c.1314G>T,ccG>ccT):ENSP00000409898(p.Pro438(=))];ENSG00000179873:SILENT[ENST00000593244(c.1314G>T,ccG>ccT):ENSP00000467988(p.Pro438(=))];ENSG00000179873:SILENT[ENST00000360133(c.1314G>T,ccG>ccT):ENSP00000353251(p.Pro438(=))];ENSG00000179873:SILENT[ENST00000590409(c.1017G>T,ccG>ccT):ENSP00000466582(p.Pro339(=))];	NA	NA	chr19*56320662*56320662*C*T*rs144528374*0*0.9996*0.0003994	NA	NA	NA	NA	NA	NA_NA	NA	LOW	NA	NA	150	150	0	104	90	14	13.46
Chewer	42484	chr19	56372770	C	T	*NLRP4*	SILENT	ENSG00000160505:SILENT[ENST00000589437(c.572C>T,atC>atT):ENSP00000468754(p.Ile191(=))];ENSG00000160505:SILENT[ENST00000301295(c.1875C>T,atC>atT):ENSP00000301295(p.Ile625(=))];ENSG00000160505:SILENT[ENST00000346986(c.1875C>T,atC>atT):ENSP00000344787(p.Ile625(=))];ENSG00000160505:SILENT[ENST00000587891(c.1650C>T,atC>atT):ENSP00000465463(p.Ile550(=))];	NA	NA	NA	8.27E-06	6.14E-05	NA	NA	NA	NA_NA	NA	LOW	NA	NA	77	77	0	82	54	28	34.15
Chewer	42484	chr19	617755	G	A	*POLRMT*	INTRONIC	ENSG00000099821:[ENST00000588649(c.3495+22C>T)];ENSG00000099821:[ENST00000592633(n.212+22C>T)];ENSG00000099821:[ENST00000590336(c.235+22C>T)];ENSG00000099821:[ENST00000589961(n.48C>T)];ENSG00000099821:[ENST00000587057(n.879C>T)];	NA	NA	NA	NA	NA	NA	NA	NA	NA_NA	NA	MODIFIER	NA	NA	92	91	1	87	73	14	16.09
Chewer	42484	chr19	38980895	C	A	*RYR1*	MISSENSE	ENSG00000196218:MISSENSE[ENST00000355481(c.5994C>A,ttC>ttA):ENSP00000347667(p.Phe1998Leu)];ENSG00000196218:MISSENSE[ENST00000360985(c.5994C>A,ttC>ttA):ENSP00000354254(p.Phe1998Leu)];ENSG00000196218:MISSENSE[ENST00000359596(c.5994C>A,ttC>ttA):ENSP00000352608(p.Phe1998Leu)];	NA	NA	NA	NA	NA	NA	NA	NA	BN_PoD	NA	MODERATE	U	0.997	66	66	0	118	88	30	25.42
Chewer	42484	chr19	38980952	C	G	*RYR1*	INTRONIC	ENSG00000196218:[ENST00000355481(c.6015+36C>G)];ENSG00000196218:[ENST00000360985(c.6015+36C>G)];ENSG00000196218:[ENST00000359596(c.6015+36C>G)];	NA	NA	NA	NA	NA	NA	NA	NA	NA_NA	NA	MODIFIER	NA	NA	47	47	0	92	72	20	21.74
Chewer	42484	chr19	38990623	C	T	*RYR1*	SILENT	ENSG00000196218:SILENT[ENST00000355481(c.7290C>T,atC>atT):ENSP00000347667(p.Ile2430(=))];ENSG00000196218:SILENT[ENST00000360985(c.7290C>T,atC>atT):ENSP00000354254(p.Ile2430(=))];ENSG00000196218:SILENT[ENST00000594335(c.742C>T,atC>atT):ENSP00000470927(p.Ile248(=))];ENSG00000196218:SILENT[ENST00000359596(c.7290C>T,atC>atT):ENSP00000352608(p.Ile2430(=))];	NA	NA	chr19*38990623*38990623*C*G,T*rs752588287*NA*NA*NA	4.95E-05	0.000121168	NA	NA	NA	NA_NA	NA	LOW	NA	0.995	61	61	0	101	77	24	23.76
Chewer	42484	chr19	33281860	G	A	*TDRD12*	INTRONIC-SS-ACR	ENSG00000173809:[ENST00000444215(c.1183-10G>A)];	NA	NA	NA	NA	NA	NA	NA	NA	NA_NA	NA	MODIFIER	NA	NA	95	95	0	127	109	17	13.39
Chewer	42484	chr19	49705355	G	A	*TRPM4*	MISSENSE	ENSG00000130529:MISSENSE[ENST00000355712(c.2026G>A,Gtg>Atg):ENSP00000347944(p.Val676Met)];ENSG00000130529:MISSENSE[ENST00000427978(c.2653G>A,Gtg>Atg):ENSP00000407492(p.Val885Met)];ENSG00000130529:MISSENSE[ENST00000252826(c.3088G>A,Gtg>Atg):ENSP00000252826(p.Val1030Met)];ENSG00000130529:3UTR[ENST00000595519(c.*2498G>A)];ENSG00000130529:3UTR[ENST00000598502(c.*2201G>A)];ENSG00000130529:3UTR[ENST00000598697(c.*2043G>A)];ENSG00000130529:EXONIC-NC[ENST00000596338(n.3185G>A)];ENSG00000130529:EXONIC-NC[ENST00000595071(n.2052G>A)];	NA	NA	NA	NA	NA	NA	NA	NA	NA_NA	NA	MODIFIER	NA	NA	128	128	0	74	63	11	14.86
Chewer	42484	chr19	31768403	G	A	*TSHZ3*	MISSENSE	ENSG00000121297:MISSENSE[ENST00000240587(c.2296C>T,Ccg>Tcg):ENSP00000240587(p.Pro766Ser)];	NA	NA	NA	NA	NA	NA	NA	T	BN_PoD	N	MODERATE	D	0.998	90	90	0	282	266	16	5.67
Chewer	42484	chr19	31768550	G	T	*TSHZ3*	MISSENSE	ENSG00000121297:MISSENSE[ENST00000240587(c.2149C>A,Cct>Act):ENSP00000240587(p.Pro717Thr)];	NA	NA	NA	NA	NA	NA	NA	D	PrD_PrD	D	MODERATE	D	0.998	139	139	0	335	311	24	7.16
Chewer	42484	chr19	992028	G	A	*WDR18*	SILENT	ENSG00000065268:SILENT[ENST00000607440(c.890G>A,ccG>ccA):ENSP00000475744(p.Pro297(=))];ENSG00000065268:SILENT[ENST00000251289(c.1005G>A,ccG>ccA):ENSP00000251289(p.Pro335(=))];ENSG00000065268:SILENT[ENST00000587001(c.1005G>A,ccG>ccA):ENSP00000464855(p.Pro335(=))];ENSG00000065268:3UTR[ENST00000591985(c.*188G>A)];ENSG00000065268:3UTR[ENST00000590354(c.*26G>A)];	chr19*9920	chr19*9920	NA	1.30E-05	8.77E-05	NA	NA	NA	NA_NA	NA	LOW	NA	NA	31	31	0	35	30	5	14.29
Chewer	42484	chr19	19637035	C	A	*YJEFN3*	MISSENSE	ENSG00000250067:MISSENSE[ENST00000608404(c.139C>A,Cac>Aac):ENSP00000477181(p.His47Asn)];	NA	NA	chr19*19637035*19637035*C*T*rs766346722*NA*NA*NA	NA	NA	NA	NA	D_lc	BN_BN	N	MODERATE	NA	0.144	147	147	0	130	124	6	4.62
Chewer	42484	chr19	36207004	C	T	*ZBTB32*	INTRONIC	ENSG00000011590:[ENST00000392197(c.1025-31C>T)];ENSG00000011590:[ENST00000262630(c.1025-31C>T)];ENSG00000011590:[ENST00000426659(c.29-31C>T)];ENSG00000011590:[ENST00000481182(c.882-31C>T)];	NA	NA	NA	NA	NA	NA	NA	NA	NA_NA	NA	MODIFIER	NA	NA	87	87	0	144	59	84	58.33
Chewer	42484	chr19	44681065	G	A	*ZNF226*	SILENT	ENSG00000167380:SILENT[ENST00000590089(c.1650G>A,caG>caA):ENSP00000465121(p.Gln550(=))];ENSG00000167380:SILENT[ENST00000337433(c.1650G>A,caG>caA):ENSP00000336719(p.Gln550(=))];ENSG00000167380:SILENT[ENST00000454662(c.1650G>A,caG>caA):ENSP00000393265(p.Gln550(=))];ENSG00000167380:3UTR[ENST00000588883(c.*3925G>A)];	NA	NA	NA	NA	NA	NA	NA	NA	NA_NA	NA	LOW	NA	0.001	79	79	0	81	72	9	11.11
Chewer	42484	chr19	22585578	C	A	*ZNF98*	INTRONIC	ENSG00000197360:[ENST00000357774(c.253+13G>T)];ENSG00000197360:[ENST00000593657(c.130+13G>T)];ENSG00000197360:[ENST00000601553(c.253+13G>T)];	NA	NA	NA	NA	NA	NA	NA	NA	NA_NA	NA	MODIFIER	NA	NA	143	141	0	97	81	15	15.46

Mangalaparthi *et al.* , 2020. Mutational landscape of esophageal squamous cell carcinoma in an Indian cohort
Supplementary Table 4. List of somatic single nucleotide variants identified using whole exome sequencing of ESCC patients

Cohort	Sample	Chromosome	Genomic position	Reference allele	Alternate allele	Gene symbol	Variant type	Variant annotation	OncoMD	COSMIC	dbSNP	ExAC	ExAC_SAS	1000G	1000G_SAS	SIFT	Polyphen2	CONDEL	VEP	LRT	PhastCon7 Way	Total read (Adjacent normal)	Reference allel read support (Adjacent normal)	Alternative read support (Adjacent normal)	Total read (Tumor)	Reference allel read support (Tumor)	Alternative read support (Tumor)	Alternate allele frequency
Chewer	42484	chr19	58564076	G	T	*ZSCAN1*	INTRONIC	ENSG00000152467:[ENST00000282326(c.604+80G>T)];	NA	NA	NA	NA	NA	NA	NA	NA	NA_NA	NA	MODIFIER	NA	NA	64	64	0	63	47	16	25.4
Chewer	42484	chr2	166868748	C	G	*AC010127.3*	INTRONIC	ENSG00000236107:[ENST00000595647(n.195-3375C>G)];ENSG00000236107:[ENST00000597623(n.164-3375C>G)];	chr2*16686	chr2*16686	chr2*166868748*166868748*C*A,T*rs771494149*NA*NA*NA	NA	NA	NA	NA	NA	NA_NA	NA	LOW	NA	NA	97	97	0	74	44	30	40.54
Chewer	42484	chr2	99870628	G	T	*C2orf15*	INTRONIC	ENSG00000241962:[ENST00000424491(c.*292+38051G>T)];	NA	NA	chr2*99870628*99870628*G*A*rs372269079*NA*NA*NA	NA	NA	NA	NA	NA	NA_NA	NA	MODIFIER	NA	NA	197	196	1	193	112	81	41.97
Chewer	42484	chr2	26785547	A	G	*C2orf70*	MISSENSE	ENSG00000173557:MISSENSE[ENST00000329615(c.67A>G,Atg>Gtg):ENSP00000332875(p.Met23Val)];ENSG00000173557:5UTR[ENST00000409392(c.-8A>G)];	NA	NA	NA	NA	NA	NA	NA	NA	NA_NA	NA	MODIFIER	NA	NA	101	101	0	76	66	10	13.16
Chewer	42484	chr2	135694419	G	A	*CCNT2*	SILENT	ENSG00000082258:SILENT[ENST00000264157(c.249G>A,tcG>tcA):ENSP00000264157(p.Ser83(=))];ENSG00000082258:SILENT[ENST00000295238(c.249G>A,tcG>tcA):ENSP00000295238(p.Ser83(=))];ENSG00000082258:SILENT[ENST00000452839(c.249G>A,tcG>tcA):ENSP00000400298(p.Ser83(=))];ENSG00000082258:SILENT[ENST00000417175(c.249G>A,tcG>tcA):ENSP00000402512(p.Ser83(=))];ENSG00000082258:SILENT[ENST00000419781(c.249G>A,tcG>tcA):ENSP00000404653(p.Ser83(=))];ENSG00000082258:EXONIC-NC[ENST00000475094(n.267G>A)];ENSG00000082258:5UTR[ENST00000446247(c.-166G>A)];ENSG00000082258:5UTR[ENST00000537343(c.-364G>A)];	chr2*13569	chr2*13569	0.01058	0.0033992	0	0.0105831	0	NA	NA_NA	NA	LOW	NA	NA	114	114	0	105	89	16	15.24
Chewer	42484	chr2	233128076	G	A	*DIS3L2*	MISSENSE	ENSG00000144535:MISSENSE[ENST00000424049(c.490G>A,Gtc>Atc):ENSP00000415419(p.Val164Ile)];ENSG00000144535:MISSENSE[ENST00000390005(c.1585G>A,Gtc>Atc):ENSP00000374655(p.Val529Ile)];ENSG00000144535:MISSENSE[ENST00000325385(c.1585G>A,Gtc>Atc):ENSP00000315569(p.Val529Ile)];ENSG00000144535:MISSENSE[ENST00000409307(c.1585G>A,Gtc>Atc):ENSP00000386799(p.Val529Ile)];ENSG00000144535:3UTR[ENST00000445090(c.*811G>A)];ENSG00000144535:3UTR[ENST00000433430(c.*1668G>A)];ENSG00000144535:INTRONIC-SS-DNR-PRX[ENST00000273009(c.1581+4G>A)];ENSG00000144535:EXONIC-NC[ENST00000498319(n.167G>A)];	chr2*23312	chr2*23312	NA	2.48E-05	0	NA	NA	T	BN_BN	N	MODERATE	N	0.973	138	138	0	123	76	47	38.21
Chewer	42484	chr2	84934679	C	T	*DNAH6*	MISSENSE	ENSG00000115423:MISSENSE[ENST00000237449(c.8887C>T,Cgt>Tgt):ENSP00000237449(p.Arg2963Cys)];ENSG00000115423:MISSENSE[ENST00000389394(c.8887C>T,Cgt>Tgt):ENSP00000374045(p.Arg2963Cys)];	NA	NA	NA	NA	NA	NA	NA	D	PoD_PrD	D	MODERATE	D	0.91	190	190	0	194	160	34	17.53
Chewer	42484	chr2	73145483	T	G	*EMX1*	MISSENSE	ENSG00000135638:MISSENSE[ENST00000258106(c.502T>G,Ttc>Gtc):ENSP00000258106(p.Phe168Val)];ENSG00000135638:MISSENSE[ENST00000473732(c.136T>G,Ttc>Gtc):ENSP00000446992(p.Phe46Val)];ENSG00000135638:INTRONIC[ENST00000394111(n.801+1294T>G)];	NA	NA	NA	NA	NA	NA	NA	NA	NA_NA	NA	MODIFIER	NA	NA	77	77	0	105	61	44	41.9
Chewer	42484	chr2	203590967	T	G	*FAM117B*	INTRONIC-SS-ACR	ENSG00000138439:[ENST00000392238(c.847-6T>G)];ENSG00000138439:[ENST00000303116(c.115-6T>G)];	NA	NA	chr2*203590967*203590967*T*C*rs763129785*NA*NA*NA	NA	NA	NA	NA	NA	NA_NA	NA	LOW	NA	NA	138	138	0	108	97	11	10.19
Chewer	42484	chr2	239072994	C	G	*FAM132B*	MISSENSE	ENSG00000178752:EXONIC-NC[ENST00000479091(n.326C>G)];ENSG00000178752:EXONIC-NC[ENST00000486834(n.906C>G)];ENSG00000178752:EXONIC-NC[ENST00000481917(n.442C>G)];ENSG00000178752:EXONIC-NC[ENST00000473274(n.276C>G)];ENSG00000178752:MISSENSE[ENST00000344233(c.21C>G,Cgc>Ggc):ENSP00000343261(p.Arg8Gly)];ENSG00000178752:MISSENSE[ENST00000546354(c.880C>G,Cgc>Ggc):ENSP00000442304(p.Arg294Gly)];	NA	NA	chr2*239072994*239072994*C*T*rs114568419*1*0.9964*0.003594	NA	NA	NA	NA	NA	NA_NA	NA	MODIFIER	NA	NA	23	23	0	31	14	17	54.84
Chewer	42484	chr2	48040349	C	G	*FBXO11*	MISSENSE	ENSG00000138081:MISSENSE[ENST00000493962(c.1625G>C,Gtg>Ctg):ENSP00000428118(p.Val543Leu)];ENSG00000138081:INTRONIC[ENST00000470899(n.652+24G>C)];ENSG00000138081:INTRONIC[ENST00000316377(c.1975+24G>C)];ENSG00000138081:INTRONIC[ENST00000403359(c.2227+24G>C)];ENSG00000138081:INTRONIC[ENST00000402508(c.1975+24G>C)];ENSG00000138081:INTRONIC[ENST00000434523(c.499+24G>C)];	NA	NA	NA	NA	NA	NA	NA	NA	NA_NA	NA	MODIFIER	NA	NA	124	124	0	193	173	19	9.84
Chewer	42484	chr2	179334446	C	A	*FKBP7*	MISSENSE	ENSG00000079150:MISSENSE[ENST00000434643(c.437G>T,gGa>gTa):ENSP00000415486(p.Gly146Val)];ENSG00000079150:MISSENSE[ENST00000424785(c.440G>T,gGa>gTa):ENSP00000413152(p.Gly147Val)];ENSG00000079150:3UTR[ENST00000412612(c.*167G>T)];ENSG00000079150:3UTR[ENST00000233092(c.*170G>T)];ENSG00000079150:3UTR[ENST00000419184(c.*27G>T)];ENSG00000079150:3UTR[ENST00000435079(c.*177G>T)];ENSG00000079150:INTRONIC[ENST00000470945(n.323-3788G>T)];ENSG00000079150:INTRONIC[ENST00000464248(n.428-3788G>T)];	NA	NA	NA	NA	NA	NA	NA	NA	NA_NA	NA	MODIFIER	NA	NA	167	167	0	168	143	25	14.88
Chewer	42484	chr2	177054463	A	T	*HOXD1*	INTRONIC	ENSG00000128645:[ENST00000331462(c.653-73A>T)];	NA	NA	NA	NA	NA	NA	NA	NA	NA_NA	NA	MODIFIER	NA	NA	207	207	0	185	137	48	25.95
Chewer	42484	chr2	201371748	C	G	*KCTD18*	EXONIC-NC	ENSG00000155729:[ENST00000478816(n.125G>C)];ENSG00000155729:[ENST00000447556(n.125G>C)];ENSG00000155729:[ENST00000468413(n.154G>C)];ENSG00000155729:[ENST00000359878(c.-9G>C)];ENSG00000155729:[ENST00000409157(c.-9G>C)];	NA	NA	NA	NA	NA	NA	NA	NA	NA_NA	NA	MODIFIER	NA	NA	112	112	0	63	53	9	14.29
Chewer	42484	chr2	100900868	C	A	*LONRF2*	MISSENSE	ENSG00000170500:MISSENSE[ENST00000393437(c.2157G>T,atG>atT):ENSP00000377086(p.Met719Ile)];ENSG00000170500:MISSENSE[ENST00000409647(c.1428G>T,atG>atT):ENSP00000386823(p.Met476Ile)];	NA	NA	NA	NA	NA	NA	NA	D	BN_PoD	D	MODERATE	D	0.892	194	193	0	144	111	33	22.92
Chewer	42484	chr2	141291620	C	T	*LRP1B*	MISSENSE	ENSG00000168702:MISSENSE[ENST00000389484(c.7732G>A,Gga>Aga):ENSP00000374135(p.Gly2578Arg)];	NA	NA	NA	4.13E-05	0.00018173	NA	NA	NA	PrD_PrD	NA	MODERATE	U	0.521	155	155	0	143	121	22	15.38
Chewer	42484	chr2	44177687	G	A	*LRPPRC*	INTRONIC	ENSG00000138095:[ENST00000260665(c.1677+25C>T)];ENSG00000138095:[ENST00000467058(n.406+25C>T)];	NA	NA	NA	NA	NA	NA	NA	NA	NA_NA	NA	MODIFIER	NA	NA	120	120	0	144	130	14	9.72
Chewer	42484	chr2	99870628	G	T	*LYG2*	INTRONIC	ENSG00000185674:[ENST00000409679(c.43+53C>A)];ENSG00000185674:[ENST00000423306(c.43+53C>A)];ENSG00000185674:[ENST00000423800(c.43+53C>A)];ENSG00000185674:[ENST00000409238(c.43+53C>A)];ENSG00000185674:[ENST00000333017(c.43+53C>A)];	NA	NA	chr2*99870628*99870628*G*A*rs372269079*NA*NA*NA	NA	NA	NA	NA	NA	NA_NA	NA	MODIFIER	NA	NA	197	196	1	193	112	81	41.97
Chewer	42484	chr2	120317209	G	C	*PCDP1*	INTRONIC	ENSG00000163075:[ENST00000413369(c.241-56G>C)];ENSG00000163075:[ENST00000602047(c.-763-56G>C)];ENSG00000163075:[ENST00000295220(c.241-56G>C)];ENSG00000163075:[ENST00000598644(c.241-56G>C)];ENSG00000163075:[ENST00000442513(c.241-56G>C)];ENSG00000163075:[ENST00000594033(c.*366-56G>C)];ENSG00000163075:[ENST00000594371(c.210-56G>C)];ENSG00000163075:[ENST00000463985(c.*39-56G>C)];ENSG00000163075:[ENST00000413057(c.140-56G>C)];	NA	NA	NA	NA	NA	NA	NA	NA	NA_NA	NA	MODIFIER	NA	NA	193	193	0	152	105	47	30.92

Mangalaparthi *et al.* , 2020. Mutational landscape of esophageal squamous cell carcinoma in an Indian cohort
Supplementary Table 4. List of somatic single nucleotide variants identified using whole exome sequencing of ESCC patients

Cohort	Sample	Chromosome	Genomic position	Reference allele	Alternate allele	Gene symbol	Variant type	Variant annotation	OncoMD	COSMIC	dbSNP	ExAC	ExAC_SAS	1000G	1000G_SAS	SIFT	Polyphen2	CONDEL	VEP	LRT	PhastCon7 Way	Total read (Adjacent normal)	Reference allel read support (Adjacent normal)	Alternative read support (Adjacent normal)	Total read (Tumor)	Reference allel read support (Tumor)	Alternative read support (Tumor)	Alternate allele frequency
Chewer	42484	chr2	119591628	G	A	*RP11-19E11.1*	EXONIC-NC	ENSG00000258910:[ENST00000557729(n.468C>T)];ENSG00000258910:[ENST00000584273(n.260C>T)];	NA	NA	chr2*119591628*119591628*G*T*rs773712785*NA*NA*NA	NA	NA	NA	NA	NA	NA_NA	NA	MODIFIER	NA	NA	35	35	0	50	41	9	18
Chewer	42484	chr2	166868748	C	G	*SCN1A*	SILENT	ENSG00000144285:SILENT[ENST00000409050(c.3666G>C,acG>acC):ENSP00000386312(p.Thr1222(=))];ENSG00000144285:SILENT[ENST00000423058(c.3750G>C,acG>acC):ENSP00000407030(p.Thr1250(=))];ENSG00000144285:SILENT[ENST00000303395(c.3750G>C,acG>acC):ENSP00000303540(p.Thr1250(=))];ENSG00000144285:SILENT[ENST00000375405(c.3717G>C,acG>acC):ENSP00000364554(p.Thr1239(=))];	chr2*16686	chr2*16686	chr2*166868748*166868748*C*A,T*rs771494149*NA*NA*NA	NA	NA	NA	NA	NA	NA_NA	NA	LOW	NA	NA	97	97	0	74	44	30	40.54
Chewer	42484	chr2	31572580	G	A	*XDH*	MISSENSE	ENSG00000158125:MISSENSE[ENST00000379416(c.2941C>T,Cgg>Tgg):ENSP00000368727(p.Arg981Trp)];	chr2*31572	chr2*31572	NA	1.65E-05	0	NA	NA	D	PrD_PrD	D	MODERATE	D	0.92	103	103	0	86	66	20	23.26
Chewer	42484	chr20	62070044	C	T	*KCNQ2*	SILENT	ENSG00000075043:SILENT[ENST00000359689(c.957G>A,aaG>aaA):ENSP00000352718(p.Lys319(=))];ENSG00000075043:SILENT[ENST00000354587(c.957G>A,aaG>aaA):ENSP00000346601(p.Lys319(=))];ENSG00000075043:SILENT[ENST00000360480(c.957G>A,aaG>aaA):ENSP00000353668(p.Lys319(=))];ENSG00000075043:SILENT[ENST00000359125(c.957G>A,aaG>aaA):ENSP00000352035(p.Lys319(=))];ENSG00000075043:SILENT[ENST00000344425(c.957G>A,aaG>aaA):ENSP00000345523(p.Lys319(=))];ENSG00000075043:SILENT[ENST00000370224(c.957G>A,aaG>aaA):ENSP00000359244(p.Lys319(=))];ENSG00000075043:SILENT[ENST00000357249(c.957G>A,aaG>aaA):ENSP00000349789(p.Lys319(=))];ENSG00000075043:SILENT[ENST00000344462(c.957G>A,aaG>aaA):ENSP00000339611(p.Lys319(=))];ENSG00000075043:EXONIC-NC[ENST00000370221(n.1132G>A)];	NA	NA	NA	NA	NA	NA	NA	NA	NA_NA	NA	LOW	NA	0.995	58	58	0	74	63	11	14.86
Chewer	42484	chr20	36854037	T	C	*KIAA1755*	INTRONIC	ENSG00000149633:[ENST00000435901(c.66+1519A>G)];ENSG00000149633:[ENST00000279024(c.2169+30A>G)];ENSG00000149633:[ENST00000496900(n.2471A>G)];	NA	NA	NA	NA	NA	NA	NA	NA	NA_NA	NA	MODIFIER	NA	NA	64	64	0	50	42	6	12
Chewer	42484	chr20	33575008	C	T	*MYH7B*	SILENT	ENSG00000078814:SILENT[ENST00000262873(c.1191C>T,atC>atT):ENSP00000262873(p.Ile397(=))];	NA	NA	0.0001997	0.0001155	0	0.0001997	0	NA	NA_NA	NA	LOW	NA	0.869	43	43	0	31	21	10	32.26
Chewer	42484	chr20	30414694	G	T	*MYLK2*	MISSENSE	ENSG00000101306:MISSENSE[ENST00000375985(c.1177G>T,Ggg>Tgg):ENSP00000365152(p.Gly393Trp)];ENSG00000101306:MISSENSE[ENST00000375994(c.1177G>T,Ggg>Tgg):ENSP00000365162(p.Gly393Trp)];ENSG00000101306:EXONIC-NC[ENST00000468730(n.115G>T)];	NA	NA	chr20*30414694*30414694*G*A*rs757155062*NA*NA*NA	NA	NA	NA	NA	NA	NA_NA	NA	MODIFIER	NA	NA	91	89	0	64	25	39	60.94
Chewer	42484	chr20	58422200	G	A	*PHACTR3*	3UTR	ENSG00000087495:[ENST00000541461(c.*15G>A)];ENSG00000087495:[ENST00000395636(c.*15G>A)];ENSG00000087495:[ENST00000395639(c.*15G>A)];ENSG00000087495:[ENST00000359926(c.*15G>A)];ENSG00000087495:[ENST00000355648(c.*15G>A)];ENSG00000087495:[ENST00000361300(c.*15G>A)];ENSG00000087495:[ENST00000371015(c.*15G>A)];ENSG00000087495:[ENST00000473657(n.1842G>A)];ENSG00000087495:[ENST00000492611(n.1019G>A)];	NA	NA	NA	NA	NA	NA	NA	NA	NA_NA	NA	MODIFIER	NA	NA	99	99	0	89	76	13	14.61
Chewer	42484	chr20	19698195	G	A	*SLC24A3*	SILENT	ENSG00000185052:SILENT[ENST00000328041(c.1743G>A,ctG>ctA):ENSP00000333519(p.Leu581(=))];	NA	NA	NA	NA	NA	NA	NA	NA	NA_NA	NA	LOW	NA	NA	75	75	0	87	73	14	16.09
Chewer	42484	chr20	32319876	C	G	*ZNF341*	INTRONIC	ENSG00000131061:[ENST00000497876(c.31+18C>G)];ENSG00000131061:[ENST00000375200(c.31+18C>G)];ENSG00000131061:[ENST00000483118(c.31+18C>G)];ENSG00000131061:[ENST00000342427(c.31+18C>G)];	NA	NA	NA	NA	NA	NA	NA	NA	NA_NA	NA	MODIFIER	NA	NA	34	34	0	38	31	7	18.42
Chewer	42484	chr21	34968050	C	T	*AP000304.12*	INTRONIC	ENSG00000249209:[ENST00000429238(c.287-9563G>A)];	NA	NA	NA	NA	NA	NA	NA	NA	NA_NA	NA	MODIFIER	NA	NA	132	132	0	78	62	16	20.51
Chewer	42484	chr21	45837892	C	T	*AP001065.2*	INTRONIC	ENSG00000230061:[ENST00000423310(n.230-3065G>A)];	NA	NA	0.000599	4.99E-05	0	0.000599	0	NA	NA_NA	NA	MODIFIER	NA	NA	184	184	0	142	117	24	16.9
Chewer	42484	chr21	34968050	C	T	*CRYZL1*	SILENT	ENSG00000205758:INTRONIC[ENST00000445393(c.464-5823G>A)];ENSG00000205758:INTRONIC[ENST00000440526(c.629-216G>A)];ENSG00000205758:3UTR[ENST00000420072(c.*331G>A)];ENSG00000205758:3UTR[ENST00000437996(c.*331G>A)];ENSG00000205758:3UTR[ENST00000429827(c.*331G>A)];ENSG00000205758:3UTR[ENST00000452420(c.*331G>A)];ENSG00000205758:SILENT[ENST00000441940(c.217G>A,caG>caA):ENSP00000397318(p.Gln73(=))];ENSG00000205758:SILENT[ENST00000361534(c.963G>A,caG>caA):ENSP00000355075(p.Gln321(=))];ENSG00000205758:SILENT[ENST00000381554(c.891G>A,caG>caA):ENSP00000370966(p.Gln297(=))];ENSG00000205758:SILENT[ENST00000381540(c.891G>A,caG>caA):ENSP00000370951(p.Gln297(=))];ENSG00000205758:SILENT[ENST00000290244(c.846G>A,caG>caA):ENSP00000290244(p.Gln282(=))];ENSG00000205758:EXONIC-NC[ENST00000480893(n.214G>A)];ENSG00000205758:EXONIC-NC[ENST00000479964(n.215G>A)];	NA	NA	NA	NA	NA	NA	NA	NA	NA_NA	NA	MODIFIER	NA	NA	132	132	0	78	62	16	20.51
Chewer	42484	chr21	45837892	C	T	*TRPM2*	MISSENSE	ENSG00000142185:MISSENSE[ENST00000300481(c.3169C>T,Cgc>Tgc):ENSP00000300481(p.Arg1057Cys)];ENSG00000142185:MISSENSE[ENST00000397928(c.3229C>T,Cgc>Tgc):ENSP00000381023(p.Arg1077Cys)];ENSG00000142185:MISSENSE[ENST00000397932(c.3229C>T,Cgc>Tgc):ENSP00000381026(p.Arg1077Cys)];ENSG00000142185:MISSENSE[ENST00000300482(c.3229C>T,Cgc>Tgc):ENSP00000300482(p.Arg1077Cys)];ENSG00000142185:EXONIC-NC[ENST00000498430(n.2681C>T)];	NA	NA	0.000599	4.99E-05	0	0.000599	0	NA	NA_NA	NA	MODIFIER	NA	NA	184	184	0	142	117	24	16.9
Chewer	42484	chr22	45258318	G	C	*ARHGAP8*	MISSENSE	ENSG00000241484:EXONIC-NC[ENST00000469342(n.338G>C)];ENSG00000241484:3UTR[ENST00000336963(c.*123G>C)];ENSG00000241484:3UTR[ENST00000389772(c.*970G>C)];ENSG00000241484:MISSENSE[ENST00000356099(c.1145G>C,aGc>aCc):ENSP00000348407(p.Ser382Thr)];ENSG00000241484:MISSENSE[ENST00000517296(c.1775G>C,aGc>aCc):ENSP00000429240(p.Ser592Thr)];ENSG00000241484:MISSENSE[ENST00000389773(c.1511G>C,aGc>aCc):ENSP00000374423(p.Ser504Thr)];ENSG00000241484:MISSENSE[ENST00000389774(c.1238G>C,aGc>aCc):ENSP00000374424(p.Ser413Thr)];	chr22*4525	chr22*4525	chr22*45258318*45258318*G*T*rs369230909*NA*NA*NA	NA	NA	NA	NA	T	BN_BN	N	MODERATE	NA	0.317	125	124	0	110	82	26	23.64
Chewer	42484	chr22	41488526	C	A	*EP300*	5UTR	ENSG00000100393:[ENST00000263253(c.-483C>A)];	NA	NA	chr22*41488526*41488526*C*T*rs376284350*NA*NA*NA	NA	NA	NA	NA	NA	NA_NA	NA	MODIFIER	NA	NA	38	37	0	35	26	8	22.86
Chewer	42484	chr22	19137334	C	G	*GSC2*	MISSENSE	ENSG00000063515:MISSENSE[ENST00000086933(c.355G>C,Ggc>Cgc):ENSP00000086933(p.Gly119Arg)];	NA	NA	NA	NA	NA	NA	NA	T	PrD_PrD	D	MODERATE	N	0.619	89	89	0	100	66	34	34

Mangalaparthi *et al.* , 2020. Mutational landscape of esophageal squamous cell carcinoma in an Indian cohort
Supplementary Table 4. List of somatic single nucleotide variants identified using whole exome sequencing of ESCC patients

Cohort	Sample	Chromosome	Genomic position	Reference allele	Alternate allele	Gene symbol	Variant type	Variant annotation	OncoMD	COSMIC	dbSNP	ExAC	ExAC_SAS	1000G	1000G_SAS	SIFT	Polyphen2	CONDEL	VEP	LRT	PhastCon7 Way	Total read (Adjacent normal)	Reference allel read support (Adjacent normal)	Alternative read support (Adjacent normal)	Total read (Tumor)	Reference allel read support (Tumor)	Alternative read support (Tumor)	Alternate allele frequency
Chewer	42484	chr22	18389415	G	A	*MICAL3*	MISSENSE	ENSG00000243156:MISSENSE[ENST00000429452(c.164C>T,tCc>tTc):ENSP00000414846(p.Ser55Phe)];ENSG00000243156:MISSENSE[ENST00000585038(c.164C>T,tCc>tTc):ENSP00000462033(p.Ser55Phe)];ENSG00000243156:MISSENSE[ENST00000424046(c.164C>T,tCc>tTc):ENSP00000406193(p.Ser55Phe)];ENSG00000243156:MISSENSE[ENST00000207726(c.164C>T,tCc>tTc):ENSP00000207726(p.Ser55Phe)];ENSG00000243156:MISSENSE[ENST00000444520(c.164C>T,tCc>tTc):ENSP00000410315(p.Ser55Phe)];ENSG00000243156:MISSENSE[ENST00000383094(c.164C>T,tCc>tTc):ENSP00000372574(p.Ser55Phe)];ENSG00000243156:MISSENSE[ENST00000414725(c.164C>T,tCc>tTc):ENSP00000391827(p.Ser55Phe)];ENSG00000243156:MISSENSE[ENST00000400561(c.164C>T,tCc>tTc):ENSP00000383406(p.Ser55Phe)];ENSG00000243156:MISSENSE[ENST00000495076(c.164C>T,tCc>tTc):ENSP00000434678(p.Ser55Phe)];ENSG00000243156:MISSENSE[ENST00000441493(c.164C>T,tCc>tTc):ENSP00000416015(p.Ser55Phe)];	NA	NA	NA	NA	NA	NA	NA	NA	PrD_PrD	NA	MODERATE	D	1	94	94	0	48	41	7	14.58
Chewer	42484	chr22	41488526	C	A	*MIR1281*	EXONIC-NC	ENSG00000221160:[ENST00000408233(n.10C>A)];	NA	NA	chr22*41488526*41488526*C*T*rs376284350*NA*NA*NA	NA	NA	NA	NA	NA	NA_NA	NA	MODIFIER	NA	NA	38	37	0	35	26	8	22.86
Chewer	42484	chr22	45258318	G	C	*PRR5-ARHGAP8*	MISSENSE	ENSG00000248405:MISSENSE[ENST00000515632(c.1357G>C,aGc>aCc):ENSP00000425026(p.Ser453Thr)];ENSG00000248405:MISSENSE[ENST00000352766(c.1775G>C,aGc>aCc):ENSP00000262731(p.Ser592Thr)];ENSG00000248405:MISSENSE[ENST00000361473(c.1538G>C,aGc>aCc):ENSP00000354732(p.Ser513Thr)];	chr22*4525	chr22*4525	chr22*45258318*45258318*G*T*rs369230909*NA*NA*NA	NA	NA	NA	NA	T	BN_BN	N	MODERATE	NA	0.317	125	124	0	110	82	26	23.64
Chewer	42484	chr22	26706625	C	G	*SEZ6L*	INTRONIC	ENSG00000100095:[ENST00000529632(c.1515-11C>G)];ENSG00000100095:[ENST00000248933(c.1515-11C>G)];ENSG00000100095:[ENST00000360929(c.1515-11C>G)];ENSG00000100095:[ENST00000343706(c.1515-11C>G)];ENSG00000100095:[ENST00000402979(c.834-11C>G)];ENSG00000100095:[ENST00000403121(c.834-11C>G)];ENSG00000100095:[ENST00000404234(c.1515-11C>G)];	NA	NA	NA	NA	NA	NA	NA	NA	NA_NA	NA	MODIFIER	NA	NA	187	187	0	193	168	25	12.95
Chewer	42484	chr22	19747184	C	A	*TBX1*	SILENT	ENSG00000184058:SILENT[ENST00000332710(c.18C>A,gtC>gtA):ENSP00000331791(p.Val6(=))];ENSG00000184058:SILENT[ENST00000359500(c.18C>A,gtC>gtA):ENSP00000352483(p.Val6(=))];ENSG00000184058:SILENT[ENST00000329705(c.18C>A,gtC>gtA):ENSP00000331176(p.Val6(=))];	NA	NA	NA	NA	NA	NA	NA	NA	NA_NA	NA	LOW	NA	NA	106	106	0	95	66	29	30.53
Chewer	42484	chr22	29445469	G	T	*ZNRF3*	MISSENSE	ENSG00000183579:MISSENSE[ENST00000406323(c.1000G>T,Ggc>Tgc):ENSP00000384553(p.Gly334Cys)];ENSG00000183579:MISSENSE[ENST00000332811(c.1000G>T,Ggc>Tgc):ENSP00000328614(p.Gly334Cys)];ENSG00000183579:MISSENSE[ENST00000402174(c.1000G>T,Ggc>Tgc):ENSP00000384456(p.Gly334Cys)];ENSG00000183579:MISSENSE[ENST00000544604(c.1300G>T,Ggc>Tgc):ENSP00000443824(p.Gly434Cys)];	chr22*2944	chr22*2944	chr22*29445469*29445469*G*A*rs779844636*NA*NA*NA	NA	NA	NA	NA	D	BN_BN	N	MODERATE	D	0.999	78	78	0	94	88	6	6.38
Chewer	42484	chr3	180337678	G	C	*CCDC39*	NONSENSE	ENSG00000145075:NONSENSE[ENST00000442201(c.2079C>G,taC>taG):ENSP00000405708(p.Tyr693Ter)];ENSG00000145075:INTRONIC[ENST00000476379(c.1999-525C>G)];ENSG00000145075:INTRONIC[ENST00000273654(c.2251-525C>G)];	NA	NA	chr3*180337678*180337678*G*A*rs376782159*NA*NA*NA	NA	NA	NA	NA	NA	NA_NA	NA	MODIFIER	NA	NA	224	224	0	325	302	21	6.46
Chewer	42484	chr3	424268	G	T	*CHL1*	MISSENSE	ENSG00000134121:MISSENSE[ENST00000397491(c.2042G>T,aGa>aTa):ENSP00000380628(p.Arg681Ile)];ENSG00000134121:MISSENSE[ENST00000256509(c.2090G>T,aGa>aTa):ENSP00000256509(p.Arg697Ile)];ENSG00000134121:3UTR[ENST00000453040(c.*2380G>T)];ENSG00000134121:EXONIC-NC[ENST00000470880(n.405G>T)];ENSG00000134121:EXONIC-NC[ENST00000470005(n.207G>T)];	NA	NA	NA	NA	NA	NA	NA	NA	NA_NA	NA	MODIFIER	NA	NA	99	99	0	81	64	17	20.99
Chewer	42484	chr3	424268	G	T	*CHL1-AS1*	EXONIC-NC	ENSG00000234661:[ENST00000417612(n.241C>A)];	NA	NA	NA	NA	NA	NA	NA	NA	NA_NA	NA	MODIFIER	NA	NA	99	99	0	81	64	17	20.99
Chewer	42484	chr3	194408329	C	T	*FAM43A*	SILENT	ENSG00000185112:SILENT[ENST00000329759(c.774C>T,ctC>ctT):ENSP00000371397(p.Leu258(=))];	NA	NA	NA	8.00E-05	0	NA	NA	NA	NA_NA	NA	LOW	NA	NA	58	58	0	78	70	8	10.26
Chewer	42484	chr3	50232328	C	T	*GNAT1*	SILENT	ENSG00000114349:SILENT[ENST00000433068(c.993C>T,gtC>gtT):ENSP00000387555(p.Val331(=))];ENSG00000114349:SILENT[ENST00000232461(c.993C>T,gtC>gtT):ENSP00000232461(p.Val331(=))];	NA	NA	NA	NA	NA	NA	NA	NA	NA_NA	NA	LOW	NA	NA	135	135	0	75	56	19	25.33
Chewer	42484	chr3	48725799	G	C	*IP6K2*	SILENT	ENSG00000068745:SILENT[ENST00000328631(c.1188C>G,acC>acG):ENSP00000331103(p.Thr396(=))];	NA	NA	chr3*48725799*48725799*G*A*rs141135563*NA*NA*NA	NA	NA	NA	NA	NA	NA_NA	NA	LOW	NA	NA	101	101	0	59	48	11	18.64
Chewer	42484	chr3	159606541	T	G	*IQCJ-SCHIP1*	INTRONIC	ENSG00000250588:[ENST00000527095(c.499-68T>G)];ENSG00000250588:[ENST00000476809(c.1342-68T>G)];ENSG00000250588:[ENST00000473061(c.156-3345T>G)];ENSG00000250588:[ENST00000337808(c.1195-68T>G)];ENSG00000250588:[ENST00000485419(c.1423-68T>G)];ENSG00000250588:[ENST00000460298(c.1075-68T>G)];ENSG00000250588:[ENST00000412423(c.1156-68T>G)];	NA	NA	NA	NA	NA	NA	NA	NA	NA_NA	NA	MODIFIER	NA	NA	102	102	0	183	173	10	5.46
Chewer	42484	chr3	197427672	C	A	*KIAA0226*	MISSENSE	ENSG00000145016:MISSENSE[ENST00000296343(c.1073G>T,aGc>aTc):ENSP00000296343(p.Ser358Ile)];ENSG00000145016:MISSENSE[ENST00000273582(c.893G>T,aGc>aTc):ENSP00000273582(p.Ser298Ile)];ENSG00000145016:MISSENSE[ENST00000389665(c.1073G>T,aGc>aTc):ENSP00000374316(p.Ser358Ile)];ENSG00000145016:MISSENSE[ENST00000449205(c.1073G>T,aGc>aTc):ENSP00000390962(p.Ser358Ile)];ENSG00000145016:MISSENSE[ENST00000413360(c.1008G>T,aGc>aTc):ENSP00000405115(p.Ser337Ile)];ENSG00000145016:MISSENSE[ENST00000415452(c.349G>T,aGc>aTc):ENSP00000409618(p.Ser117Ile)];	NA	NA	NA	NA	NA	NA	NA	T_lc	BN_BN	N	MODERATE	NA	NA	133	133	0	141	52	89	63.12
Chewer	42484	chr3	197581344	C	T	*LRCH3*	INTRONIC	ENSG00000186001:[ENST00000466845(n.56+28C>T)];ENSG00000186001:[ENST00000334859(c.1644+28C>T)];ENSG00000186001:[ENST00000452660(c.177+28C>T)];ENSG00000186001:[ENST00000438796(c.1644+28C>T)];ENSG00000186001:[ENST00000433298(c.135+28C>T)];ENSG00000186001:[ENST00000441090(c.1182+28C>T)];ENSG00000186001:[ENST00000481048(n.347+28C>T)];ENSG00000186001:[ENST00000425562(c.1644+28C>T)];ENSG00000186001:[ENST00000461674(n.291+28C>T)];ENSG00000186001:[ENST00000414675(c.1560+28C>T)];ENSG00000186001:[ENST00000536618(c.429+28C>T)];	NA	NA	chr3*197581344*197581344*C*A,T*rs758426521*NA*NA*NA	9.07E-05	0	NA	NA	NA	NA_NA	NA	MODIFIER	NA	NA	177	177	0	258	230	28	10.85

Supplementary Table 4. List of somatic single nucleotide variants identified using whole exome sequencing of ESCC patients

Cohort	Sample	Chromosome	Genomic position	Reference allele	Alternate allele	Gene symbol	Variant type	Variant annotation	OncoMD	COSMIC	dbSNP	ExAC	ExAC_SAS	1000G	1000G_SAS	SIFT	Polyphen2	CONDEL	VEP	LRT	PhastCon7 Way	Total read (Adjacent normal)	Reference allel read support (Adjacent normal)	Alternative read support (Adjacent normal)	Total read (Tumor)	Reference allel read support (Tumor)	Alternative read support (Tumor)	Alternate allele frequency
Chewer	42484	chr3	182751858	G	A	*MCCC1*	MISSENSE	ENSG00000078070:MISSENSE[ENST00000497959(c.1271C>T,tCt>tTt):ENSP00000420648(p.Ser424Phe)];ENSG00000078070:SILENT[ENST00000539926(c.1197C>T,ttC>ttT):ENSP00000441253(p.Phe399(=))];ENSG00000078070:SILENT[ENST00000265594(c.1602C>T,ttC>ttT):ENSP00000265594(p.Phe534(=))];ENSG00000078070:SILENT[ENST00000476176(c.1461C>T,ttC>ttT):ENSP00000420433(p.Phe487(=))];ENSG00000078070:SILENT[ENST00000492597(c.1275C>T,ttC>ttT):ENSP00000419898(p.Phe425(=))];ENSG00000078070:3UTR[ENST00000497830(c.*1199C>T)];ENSG00000078070:3UTR[ENST00000495767(c.*1183C>T)];ENSG00000078070:EXONIC-NC[ENST00000489909(n.146C>T)];	chr3*18275	chr3*18275	NA	NA	NA	NA	NA	NA	NA_NA	NA	LOW	NA	0.997	41	41	0	42	37	5	11.9
Chewer	42484	chr3	16358761	G	T	*OXNAD1*	INTRONIC	ENSG00000154814:[ENST00000606098(c.*30+13662G>T)];ENSG00000154814:[ENST00000435829(c.*30+13662G>T)];ENSG00000154814:[ENST00000544043(c.*30+13662G>T)];ENSG00000154814:[ENST00000605932(c.*30+13662G>T)];	NA	NA	NA	NA	NA	NA	NA	NA	NA_NA	NA	MODIFIER	NA	NA	87	87	0	40	22	18	45
Chewer	42484	chr3	16358761	G	T	*RFTN1*	INTRONIC	ENSG00000131378:[ENST00000432519(c.1225-22C>A)];ENSG00000131378:[ENST00000334133(c.1333-22C>A)];ENSG00000131378:[ENST00000607320(c.178-22C>A)];ENSG00000131378:[ENST00000483671(n.612-22C>A)];	NA	NA	NA	NA	NA	NA	NA	NA	NA_NA	NA	MODIFIER	NA	NA	87	87	0	40	22	18	45
Chewer	42484	chr3	159606541	T	G	*SCHIP1*	EXONIC-NC	ENSG00000151967:[ENST00000482885(n.3131T>G)];ENSG00000151967:[ENST00000495954(c.150-3345T>G)];ENSG00000151967:[ENST00000482804(c.514-68T>G)];ENSG00000151967:[ENST00000445224(c.466-68T>G)];	NA	NA	NA	NA	NA	NA	NA	NA	NA_NA	NA	MODIFIER	NA	NA	102	102	0	183	173	10	5.46
Chewer	42484	chr4	148653616	G	T	*ARHGAP10*	INTRONIC-SS-DNR	ENSG00000071205:[ENST00000510379(n.393+10G>T)];ENSG00000071205:[ENST00000336498(c.154+10G>T)];	NA	NA	NA	NA	NA	NA	NA	NA	NA_NA	NA	MODIFIER	NA	NA	161	161	0	124	100	24	19.35
Chewer	42484	chr4	79780776	A	G	*BMP2K*	MISSENSE	ENSG00000138756:MISSENSE[ENST00000502871(c.922A>G,Ata>Gta):ENSP00000421768(p.Ile308Val)];ENSG00000138756:MISSENSE[ENST00000335016(c.922A>G,Ata>Gta):ENSP00000334836(p.Ile308Val)];ENSG00000138756:MISSENSE[ENST00000389010(c.922A>G,Ata>Gta):ENSP00000373662(p.Ile308Val)];ENSG00000138756:EXONIC-NC[ENST00000515075(n.456A>G)];ENSG00000138756:EXONIC-NC[ENST00000505725(n.204A>G)];	NA	NA	NA	NA	NA	NA	NA	NA	NA_NA	NA	MODIFIER	NA	NA	75	75	0	45	36	9	20
Chewer	42484	chr4	187541193	C	G	*FAT1*	MISSENSE	ENSG00000083857:MISSENSE[ENST00000441802(c.6547G>C,Gaa>Caa):ENSP00000406229(p.Glu2183Gln)];	NA	NA	NA	NA	NA	NA	NA	NA	PrD_PrD	NA	MODERATE	D	1	176	176	0	104	62	41	39.42
Chewer	42484	chr4	187541260	C	T	*FAT1*	SILENT	ENSG00000083857:SILENT[ENST00000441802(c.6480G>A,ggG>ggA):ENSP00000406229(p.Gly2160(=))];	NA	NA	NA	NA	NA	NA	NA	NA	NA_NA	NA	LOW	NA	NA	233	232	1	165	97	68	41.21
Chewer	42484	chr4	187541310	C	G	*FAT1*	MISSENSE	ENSG00000083857:MISSENSE[ENST00000441802(c.6430G>C,Gac>Cac):ENSP00000406229(p.Asp2144His)];	NA	NA	NA	NA	NA	NA	NA	NA	PrD_PrD	NA	MODERATE	D	0.954	250	249	0	154	98	56	36.36
Chewer	42484	chr4	187541329	C	T	*FAT1*	SILENT	ENSG00000083857:SILENT[ENST00000441802(c.6411G>A,ctG>ctA):ENSP00000406229(p.Leu2137(=))];	NA	NA	NA	NA	NA	NA	NA	NA	NA_NA	NA	LOW	NA	NA	236	236	0	151	100	51	33.77
Chewer	42484	chr4	187541417	C	T	*FAT1*	MISSENSE	ENSG00000083857:MISSENSE[ENST00000441802(c.6323G>A,aGa>aAa):ENSP00000406229(p.Arg2108Lys)];	NA	NA	chr4*187541417*187541417*C*G*rs780389120*NA*NA*NA	NA	NA	NA	NA	NA	BN_BN	NA	MODERATE	N	0.959	200	200	0	108	70	38	35.19
Chewer	42484	chr4	187541421	C	G	*FAT1*	MISSENSE	ENSG00000083857:MISSENSE[ENST00000441802(c.6319G>C,Gac>Cac):ENSP00000406229(p.Asp2107His)];	NA	NA	NA	NA	NA	NA	NA	NA	PrD_PrD	NA	MODERATE	D	0.704	198	198	0	108	69	39	36.11
Chewer	42484	chr4	187584589	G	A	*FAT1*	SILENT	ENSG00000083857:SILENT[ENST00000441802(c.3444C>T,atC>atT):ENSP00000406229(p.Ile1148(=))];	NA	NA	NA	NA	NA	NA	NA	NA	NA_NA	NA	LOW	NA	0.985	208	208	0	103	55	48	46.6
Chewer	42484	chr4	144918765	A	T	*GYPB*	SILENT	ENSG00000250361:3UTR[ENST00000506516(c.*196T>A)];ENSG00000250361:3UTR[ENST00000505583(c.*184T>A)];ENSG00000250361:3UTR[ENST00000504951(c.*277T>A)];ENSG00000250361:3UTR[ENST00000506679(c.*152T>A)];ENSG00000250361:INTRONIC[ENST00000511198(c.*162-1308T>A)];ENSG00000250361:INTRONIC[ENST00000429670(c.176-54T>A)];ENSG00000250361:INTRONIC[ENST00000513677(n.114-1308T>A)];ENSG00000250361:INTRONIC[ENST00000507009(n.226-1308T>A)];ENSG00000250361:EXONIC-NC[ENST00000513557(n.97T>A)];ENSG00000250361:EXONIC-NC[ENST00000510196(n.328T>A)];ENSG00000250361:EXONIC-NC[ENST00000503255(n.213T>A)];ENSG00000250361:SILENT[ENST00000283126(c.198T>A,atT>atA):ENSP00000283126(p.Ile66(=))];ENSG00000250361:SILENT[ENST00000502664(c.198T>A,atT>atA):ENSP00000427690(p.Ile66(=))];ENSG00000250361:SILENT[ENST00000513128(c.99T>A,atT>atA):ENSP00000425244(p.Ile33(=))];	NA	NA	NA	NA	NA	NA	NA	NA	NA_NA	NA	MODIFIER	NA	NA	159	158	0	67	59	8	11.94
Chewer	42484	chr4	174448430	A	G	*HAND2*	STOPLOSS	ENSG00000164107:STOPLOSS[ENST00000359562(c.652T>C,Tga>Cga):ENSP00000352565(p.Ter218ArgextTer12)];ENSG00000164107:STOPLOSS[ENST00000503024(c.275T>C,Tga>Cga):ENSP00000427084(p.Ter93ArgextTer12)];	NA	NA	NA	NA	NA	NA	NA	NA	NA_NA	NA	MODIFIER	NA	NA	99	99	0	56	40	16	28.57
Chewer	42484	chr4	174448430	A	G	*HAND2-AS1*	EXONIC-NC	ENSG00000237125:[ENST00000512099(n.10A>G)];	NA	NA	NA	NA	NA	NA	NA	NA	NA_NA	NA	MODIFIER	NA	NA	99	99	0	56	40	16	28.57
Chewer	42484	chr4	3180149	A	C	*HTT*	MISSENSE	ENSG00000197386:MISSENSE[ENST00000355072(c.4588A>C,Agt>Cgt):ENSP00000347184(p.Ser1530Arg)];ENSG00000197386:EXONIC-NC[ENST00000510626(n.4687A>C)];ENSG00000197386:EXONIC-NC[ENST00000509043(n.76A>C)];ENSG00000197386:EXONIC-NC[ENST00000513639(n.76A>C)];ENSG00000197386:EXONIC-NC[ENST00000513326(n.76A>C)];	NA	NA	NA	NA	NA	NA	NA	NA	NA_NA	NA	MODIFIER	NA	NA	73	73	0	48	30	18	37.5
Chewer	42484	chr4	88727360	G	A	*IBSP*	INTRONIC	ENSG00000029559:[ENST00000226284(c.246+24G>A)];	NA	NA	0.0003994	5.90E-05	0	0.0003994	0	NA	NA_NA	NA	MODIFIER	NA	NA	50	50	0	59	41	18	30.51
Chewer	42484	chr4	37446868	G	C	*KIAA1239*	SILENT	ENSG00000174145:SILENT[ENST00000309447(c.3258G>C,ctG>ctC):ENSP00000309501(p.Leu1086(=))];	NA	NA	NA	NA	NA	NA	NA	NA	NA_NA	NA	LOW	NA	NA	160	160	0	89	61	28	31.46
Chewer	42484	chr4	71339800	T	C	*MUC7*	INTRONIC	ENSG00000171195:[ENST00000514512(n.95+1792T>C)];ENSG00000171195:[ENST00000413702(c.54+8T>C)];ENSG00000171195:[ENST00000505411(c.54+8T>C)];ENSG00000171195:[ENST00000504482(n.348+8T>C)];ENSG00000171195:[ENST00000456088(c.54+8T>C)];ENSG00000171195:[ENST00000304887(c.54+8T>C)];	NA	NA	NA	NA	NA	NA	NA	NA	NA_NA	NA	LOW	NA	NA	171	171	0	121	104	17	14.05
Chewer	42484	chr4	119035991	G	T	*NDST3*	MISSENSE	ENSG00000164100:INTRONIC[ENST00000433996(c.982-23218G>T)];ENSG00000164100:MISSENSE[ENST00000296499(c.1100G>T,tGt>tTt):ENSP00000296499(p.Cys367Phe)];	NA	NA	NA	NA	NA	NA	NA	T	BN_BN	N	MODERATE	N	0.718	211	210	0	141	129	10	7.09
Chewer	42484	chr4	144918765	A	T	*RP11-673E1.4*	INTRONIC	ENSG00000248828:[ENST00000506982(n.184+28233A>T)];	NA	NA	NA	NA	NA	NA	NA	NA	NA_NA	NA	MODIFIER	NA	NA	159	158	0	67	59	8	11.94
Chewer	42484	chr4	83619122	G	T	*SCD5*	INTRONIC	ENSG00000145284:[ENST00000282709(c.*27+51C>A)];ENSG00000145284:[ENST00000319540(c.363+7314C>A)];ENSG00000145284:[ENST00000273908(c.363+7314C>A)];	NA	NA	NA	NA	NA	NA	NA	NA	NA_NA	NA	MODIFIER	NA	NA	152	152	0	129	123	6	4.65
Chewer	42484	chr4	77661491	A	C	*SHROOM3*	MISSENSE	ENSG00000138771:INTRONIC[ENST00000469923(n.415-8679A>C)];ENSG00000138771:MISSENSE[ENST00000296043(c.2165A>C,tAc>tCc):ENSP00000296043(p.Tyr722Ser)];ENSG00000138771:EXONIC-NC[ENST00000486758(n.1974A>C)];	NA	NA	NA	NA	NA	NA	NA	NA	NA_NA	NA	MODIFIER	NA	NA	215	215	0	163	74	88	53.99

Mangalaparthi *et al.* , 2020. Mutational landscape of esophageal squamous cell carcinoma in an Indian cohort
Supplementary Table 4. List of somatic single nucleotide variants identified using whole exome sequencing of ESCC patients

Cohort	Sample	Chromosome	Genomic position	Reference allele	Alternate allele	Gene symbol	Variant type	Variant annotation	OncoMD	COSMIC	dbSNP	ExAC	ExAC_SAS	1000G	1000G_SAS	SIFT	Polyphen2	CONDEL	VEP	LRT	PhastCon7 Way	Total read (Adjacent normal)	Reference allel read support (Adjacent normal)	Alternative read support (Adjacent normal)	Total read (Tumor)	Reference allel read support (Tumor)	Alternative read support (Tumor)	Alternate allele frequency
Chewer	42484	chr5	14758612	C	T	*ANKH*	MISSENSE	ENSG00000154122:MISSENSE[ENST00000284268(c.409G>A,Gcc>Acc):ENSP00000284268(p.Ala137Thr)];	NA	chr5*14758	NA	1.65E-05	0	NA	NA	T	PoD_PrD	D	MODERATE	D	1	161	161	0	184	174	9	4.89
Chewer	42484	chr5	14871460	C	A	*ANKH*	MISSENSE	ENSG00000154122:INTRONIC-SS-DNR[ENST00000284268(c.96+1G>T)];ENSG00000154122:INTRONIC-SS-DNR[ENST00000513115(n.121+1G>T)];ENSG00000154122:MISSENSE[ENST00000505140(c.97G>T,Gta>Tta):ENSP00000426332(p.Val33Leu)];	NA	NA	NA	NA	NA	NA	NA	NA	UN_UN	NA	MODERATE	NA	NA	91	90	0	130	110	20	15.38
Chewer	42484	chr5	74403321	T	C	*ANKRD31*	INTRONIC	ENSG00000145700:[ENST00000506364(c.4340+25A>G)];ENSG00000145700:[ENST00000504022(n.1130+25A>G)];ENSG00000145700:[ENST00000274361(c.4169+25A>G)];	NA	NA	NA	NA	NA	NA	NA	NA	NA_NA	NA	MODIFIER	NA	NA	98	98	0	41	29	12	29.27
Chewer	42484	chr5	73048859	C	T	*ARHGEF28*	SILENT	ENSG00000214944:SILENT[ENST00000437974(c.307C>T,Ctg>Ttg):ENSP00000411459(p.Leu103(=))];ENSG00000214944:SILENT[ENST00000513042(c.307C>T,Ctg>Ttg):ENSP00000441436(p.Leu103(=))];ENSG00000214944:SILENT[ENST00000287898(c.307C>T,Ctg>Ttg):ENSP00000287898(p.Leu103(=))];ENSG00000214944:SILENT[ENST00000545377(c.307C>T,Ctg>Ttg):ENSP00000441913(p.Leu103(=))];ENSG00000214944:SILENT[ENST00000426542(c.307C>T,Ctg>Ttg):ENSP00000412175(p.Leu103(=))];ENSG00000214944:SILENT[ENST00000296794(c.307C>T,Ctg>Ttg):ENSP00000296794(p.Leu103(=))];	NA	NA	NA	NA	NA	NA	NA	NA	NA_NA	NA	LOW	NA	NA	81	81	0	52	46	6	11.54
Chewer	42484	chr5	24537714	G	C	*CDH10*	MISSENSE	ENSG00000040731:MISSENSE[ENST00000264463(c.301C>G,Ctt>Gtt):ENSP00000264463(p.Leu101Val)];ENSG00000040731:MISSENSE[ENST00000510477(c.301C>G,Ctt>Gtt):ENSP00000425653(p.Leu101Val)];	chr5*24537	chr5*24537	NA	NA	NA	NA	NA	T	PrD_PoD	N	MODERATE	NA	NA	198	198	0	199	187	12	6.03
Chewer	42484	chr5	156811439	A	G	*CTB-109A12.1*	INTRONIC	ENSG00000251405:[ENST00000519499(n.72-19826T>C)];	NA	NA	NA	NA	NA	NA	NA	NA	NA_NA	NA	MODIFIER	NA	NA	120	120	0	58	40	18	31.03
Chewer	42484	chr5	156811439	A	G	*CTB-47B11.3*	EXONIC-NC	ENSG00000248544:[ENST00000508443(n.509T>C)];ENSG00000248544:[ENST00000520658(n.410+6T>C)];	NA	NA	NA	NA	NA	NA	NA	NA	NA_NA	NA	MODIFIER	NA	NA	120	120	0	58	40	18	31.03
Chewer	42484	chr5	156811439	A	G	*CYFIP2*	INTRONIC	ENSG00000055163:[ENST00000523969(c.*2993+1072A>G)];ENSG00000055163:[ENST00000524058(n.122+1072A>G)];ENSG00000055163:[ENST00000523383(n.128+1072A>G)];ENSG00000055163:[ENST00000442283(c.*407+1072A>G)];ENSG00000055163:[ENST00000519663(n.104+1072A>G)];ENSG00000055163:[ENST00000521420(c.3129+1072A>G)];ENSG00000055163:[ENST00000519153(c.*2918+1072A>G)];ENSG00000055163:[ENST00000523119(c.*2510+1072A>G)];ENSG00000055163:[ENST00000517753(c.*2918+1072A>G)];ENSG00000055163:[ENST00000377576(c.3207+1072A>G)];ENSG00000055163:[ENST00000541131(c.2982+1072A>G)];ENSG00000055163:[ENST00000318218(c.3282+1072A>G)];ENSG00000055163:[ENST00000435847(c.2304+1072A>G)];ENSG00000055163:[ENST00000347377(c.3207+1072A>G)];ENSG00000055163:[ENST00000522463(c.2619+1072A>G)];ENSG00000055163:[ENST00000520759(c.*2918+1072A>G)];ENSG00000055163:[ENST00000522775(n.126A>G)];ENSG00000055163:[ENST00000522884(n.126A>G)];	NA	NA	NA	NA	NA	NA	NA	NA	NA_NA	NA	MODIFIER	NA	NA	120	120	0	58	40	18	31.03
Chewer	42484	chr5	49695104	T	A	*EMB*	3UTR	ENSG00000170571:[ENST00000514111(c.*3A>T)];ENSG00000170571:[ENST00000303221(c.*3A>T)];ENSG00000170571:[ENST00000508934(c.*3A>T)];ENSG00000170571:[ENST00000505896(n.1406A>T)];	NA	NA	NA	NA	NA	NA	NA	NA	NA_NA	NA	MODIFIER	NA	NA	199	197	0	115	93	22	19.13
Chewer	42484	chr5	93727230	G	T	*KIAA0825*	INTRONIC-SS-DNR	ENSG00000185261:[ENST00000427991(c.3456+10C>A)];ENSG00000185261:[ENST00000513200(c.3456+10C>A)];	NA	NA	chr5*93727226*93727245*GGCCGTTTCCCTACCTCATT*G*rs766167375*NA*NA*NA|chr5*93727230*93727230*G*A*rs574293739*0*0.9998*0.0001997	NA	NA	NA	NA	NA	NA_NA	NA	MODIFIER	NA	NA	76	76	0	48	38	10	20.83
Chewer	42484	chr5	7878278	A	C	*MTRR*	MISSENSE	ENSG00000124275:MISSENSE[ENST00000514220(c.408A>C,aAg>aCg):ENSP00000423863(p.Lys137Thr)];ENSG00000124275:MISSENSE[ENST00000440940(c.623A>C,aAg>aCg):ENSP00000402510(p.Lys208Thr)];ENSG00000124275:MISSENSE[ENST00000510525(c.648A>C,aAg>aCg):ENSP00000421991(p.Lys217Thr)];ENSG00000124275:MISSENSE[ENST00000264668(c.704A>C,aAg>aCg):ENSP00000264668(p.Lys235Thr)];ENSG00000124275:3UTR[ENST00000514369(c.*287A>C)];ENSG00000124275:3UTR[ENST00000513439(c.*330A>C)];ENSG00000124275:3UTR[ENST00000341013(c.*292A>C)];ENSG00000124275:3UTR[ENST00000511461(c.*292A>C)];	NA	NA	NA	NA	NA	NA	NA	T	BN_BN	N	MODERATE	N	0.358	202	201	1	233	212	21	9.01
Chewer	42484	chr5	102895795	T	A	*NUDT12*	MISSENSE	ENSG00000112874:MISSENSE[ENST00000230792(c.155A>T,tAt>tTt):ENSP00000230792(p.Tyr52Phe)];ENSG00000112874:INTRONIC-SS-DNR-PRX[ENST00000507423(c.152+3A>T)];ENSG00000112874:EXONIC-NC[ENST00000508889(n.245A>T)];	NA	NA	chr5*102895795*102895795*T*C*rs765324998*NA*NA*NA	NA	NA	NA	NA	NA	NA_NA	NA	MODIFIER	NA	NA	259	259	0	164	114	50	30.49
Chewer	42484	chr5	1201947	T	A	*SLC6A19*	MISSENSE	ENSG00000174358:MISSENSE[ENST00000304460(c.182T>A,cTg>cAg):ENSP00000305302(p.Leu61Gln)];ENSG00000174358:MISSENSE[ENST00000515652(c.182T>A,cTg>cAg):ENSP00000425701(p.Leu61Gln)];	NA	NA	NA	8.54E-06	0	NA	NA	D_lc	PrD_PrD	D	MODERATE	NA	NA	88	88	0	92	85	7	7.61
Chewer	42484	chr6	10683345	C	G	*C6orf52*	INTRONIC	ENSG00000137434:[ENST00000503680(c.49+75G>C)];ENSG00000137434:[ENST00000467832(n.168+75G>C)];ENSG00000137434:[ENST00000426700(c.316+75G>C)];ENSG00000137434:[ENST00000259983(c.316+75G>C)];ENSG00000137434:[ENST00000379586(c.55+75G>C)];ENSG00000137434:[ENST00000460742(c.49+75G>C)];	NA	NA	NA	NA	NA	NA	NA	NA	NA_NA	NA	MODIFIER	NA	NA	161	161	0	159	143	16	10.06
Chewer	42484	chr6	31084691	G	A	*CDSN*	MISSENSE	ENSG00000204539:MISSENSE[ENST00000376288(c.701C>T,tCg>tTg):ENSP00000365465(p.Ser234Leu)];	chr6*31084	NA	0.0001997	8.25E-06	0	0.0001997	0	NA	NA_NA	NA	MODIFIER	NA	NA	82	82	0	70	59	11	15.71
Chewer	42484	chr6	70812184	T	A	*COL19A1*	INTRONIC	ENSG00000082293:[ENST00000322773(c.1278+70T>A)];ENSG00000082293:[ENST00000393344(c.144+70T>A)];	NA	NA	NA	NA	NA	NA	NA	NA	NA_NA	NA	MODIFIER	NA	NA	95	95	0	97	87	10	10.31
Chewer	42484	chr6	70831794	T	G	*COL19A1*	MISSENSE	ENSG00000082293:MISSENSE[ENST00000455415(c.23T>G,aTa>aGa):ENSP00000416556(p.Ile8Arg)];ENSG00000082293:MISSENSE[ENST00000322773(c.1301T>G,aTa>aGa):ENSP00000316030(p.Ile434Arg)];ENSG00000082293:MISSENSE[ENST00000393344(c.167T>G,aTa>aGa):ENSP00000377013(p.Ile56Arg)];	NA	NA	NA	NA	NA	NA	NA	T	UN_UN	NA	MODERATE	N	0.919	40	40	0	25	20	5	20
Chewer	42484	chr6	43006721	C	T	*CUL7*	SILENT	ENSG00000044090:SILENT[ENST00000535468(c.4551G>A,caG>caA):ENSP00000438788(p.Gln1517(=))];ENSG00000044090:SILENT[ENST00000265348(c.4299G>A,caG>caA):ENSP00000265348(p.Gln1433(=))];	NA	NA	NA	NA	NA	NA	NA	NA	NA_NA	NA	LOW	NA	0.671	35	35	0	49	35	14	28.57

Cohort	Sample	Chromosome	Genomic position	Reference allele	Alternate allele	Gene symbol	Variant type	Variant annotation	OncoMD	COSMIC	dbSNP	ExAC	ExAC_SAS	1000G	1000G_SAS	SIFT	Polyphen2	CONDEL	VEP	LRT	PhastCon7 Way	Total read (Adjacent normal)	Reference allel read support (Adjacent normal)	Alternative read support (Adjacent normal)	Total read (Tumor)	Reference allel read support (Tumor)	Alternative read support (Tumor)	Alternate allele frequency
Chewer	42484	chr6	152129288	G	A	*ESR1*	MISSENSE	ENSG00000091831:MISSENSE[ENST00000406599(c.241G>A,Ggc>Agc):ENSP00000384064(p.Gly81Ser)];ENSG00000091831:MISSENSE[ENST00000440973(c.241G>A,Ggc>Agc):ENSP00000405330(p.Gly81Ser)];ENSG00000091831:MISSENSE[ENST00000338799(c.241G>A,Ggc>Agc):ENSP00000342630(p.Gly81Ser)];ENSG00000091831:MISSENSE[ENST00000443427(c.241G>A,Ggc>Agc):ENSP00000387500(p.Gly81Ser)];ENSG00000091831:MISSENSE[ENST00000404742(c.241G>A,Ggc>Agc):ENSP00000385373(p.Gly81Ser)];ENSG00000091831:MISSENSE[ENST00000456483(c.241G>A,Ggc>Agc):ENSP00000415934(p.Gly81Ser)];ENSG00000091831:MISSENSE[ENST00000206249(c.241G>A,Ggc>Agc):ENSP00000206249(p.Gly81Ser)];ENSG00000091831:MISSENSE[ENST00000446550(c.241G>A,Ggc>Agc):ENSP00000411105(p.Gly81Ser)];ENSG00000091831:EXONIC-NC[ENST00000488573(n.49G>A)];	NA	NA	NA	1.76E-05	9.25E-05	NA	NA	T	BN_PoD	N	MODERATE	N	NA	63	63	0	78	31	47	60.26
Chewer	42484	chr6	65301602	A	T	*EYS*	MISSENSE	ENSG00000188107:MISSENSE[ENST00000370616(c.4158T>A,gaT>gaA):ENSP00000359650(p.Asp1386Glu)];ENSG00000188107:MISSENSE[ENST00000370621(c.4158T>A,gaT>gaA):ENSP00000359655(p.Asp1386Glu)];ENSG00000188107:MISSENSE[ENST00000503581(c.4158T>A,gaT>gaA):ENSP00000424243(p.Asp1386Glu)];	NA	NA	NA	NA	NA	NA	NA	T	BN_BN	N	MODERATE	NA	NA	266	266	0	239	206	32	13.39
Chewer	42484	chr6	46988254	T	C	*GPR110*	INTRONIC	ENSG00000153292:[ENST00000449332(n.582+213A>G)];ENSG00000153292:[ENST00000419892(n.664-21A>G)];ENSG00000153292:[ENST00000475745(c.473+213A>G)];ENSG00000153292:[ENST00000487323(c.381+213A>G)];ENSG00000153292:[ENST00000493249(n.415+213A>G)];ENSG00000153292:[ENST00000477771(n.677-21A>G)];ENSG00000153292:[ENST00000371253(c.611+213A>G)];ENSG00000153292:[ENST00000471487(n.644+213A>G)];ENSG00000153292:[ENST00000371243(c.612-21A>G)];ENSG00000153292:[ENST00000491283(n.777A>G)];	NA	NA	chr6*46988254*46988254*T*A*rs548003431*1*0.9792*0.02077	NA	NA	NA	NA	NA	NA_NA	NA	MODIFIER	NA	NA	59	59	0	65	59	5	7.69
Chewer	42484	chr6	84836116	C	T	*KIAA1009*	MISSENSE	ENSG00000135315:MISSENSE[ENST00000403245(c.3986G>A,aGa>aAa):ENSP00000385215(p.Arg1329Lys)];ENSG00000135315:MISSENSE[ENST00000257766(c.3758G>A,aGa>aAa):ENSP00000257766(p.Arg1253Lys)];ENSG00000135315:EXONIC-NC[ENST00000487999(n.1289G>A)];ENSG00000135315:EXONIC-NC[ENST00000461137(n.3110G>A)];	NA	NA	chr6*84836116*84836117*CT*C*rs760221837*NA*NA*NA	NA	NA	NA	NA	NA	NA_NA	NA	MODIFIER	NA	NA	105	105	0	63	30	33	52.38
Chewer	42484	chr6	39512254	G	C	*KIF6*	INTRONIC	ENSG00000164627:[ENST00000287152(c.1426+77C>G)];ENSG00000164627:[ENST00000373213(c.943+77C>G)];ENSG00000164627:[ENST00000373215(c.1426+77C>G)];ENSG00000164627:[ENST00000373216(c.1426+77C>G)];ENSG00000164627:[ENST00000538893(c.1477+26C>G)];ENSG00000164627:[ENST00000458470(c.1101+77C>G)];	NA	NA	NA	NA	NA	NA	NA	NA	NA_NA	NA	MODIFIER	NA	NA	40	40	0	52	34	18	34.62
Chewer	42484	chr6	63990231	G	T	*LGSN*	MISSENSE	ENSG00000146166:MISSENSE[ENST00000370657(c.1225C>A,Cta>Ata):ENSP00000359691(p.Leu409Ile)];ENSG00000146166:INTRONIC[ENST00000485906(c.529+696C>A)];ENSG00000146166:3UTR[ENST00000370658(c.*177C>A)];	NA	NA	NA	NA	NA	NA	NA	NA	NA_NA	NA	MODIFIER	NA	NA	170	170	0	212	181	30	14.15
Chewer	42484	chr6	13814336	G	T	*MCUR1*	MISSENSE	ENSG00000050393:MISSENSE[ENST00000359495(c.326C>A,gCg>gAg):ENSP00000352475(p.Ala109Glu)];ENSG00000050393:MISSENSE[ENST00000488770(c.326C>A,gCg>gAg):ENSP00000476162(p.Ala109Glu)];ENSG00000050393:MISSENSE[ENST00000379170(c.326C>A,gCg>gAg):ENSP00000368468(p.Ala109Glu)];	NA	NA	NA	NA	NA	NA	NA	T	BN_BN	N	MODERATE	U	0.063	27	27	0	55	46	9	16.36
Chewer	42484	chr6	54095503	C	T	*MLIP*	INTRONIC	ENSG00000146147:[ENST00000358276(c.814-26603C>T)];ENSG00000146147:[ENST00000370876(c.646-26603C>T)];ENSG00000146147:[ENST00000509997(c.675+28472C>T)];ENSG00000146147:[ENST00000370877(c.801+28472C>T)];ENSG00000146147:[ENST00000274897(c.1114-9C>T)];ENSG00000146147:[ENST00000502396(c.2719-9C>T)];ENSG00000146147:[ENST00000514433(c.901-9C>T)];	NA	NA	NA	NA	NA	NA	NA	NA	NA_NA	NA	MODIFIER	NA	NA	112	112	0	118	111	7	5.93
Chewer	42484	chr6	84764970	G	A	*MRAP2*	INTRONIC	ENSG00000135324:[ENST00000257776(c.-7-61G>A)];	NA	NA	NA	NA	NA	NA	NA	NA	NA_NA	NA	MODIFIER	NA	NA	82	82	0	55	45	10	18.18
Chewer	42484	chr6	24141185	C	A	*NRSN1*	MISSENSE	ENSG00000152954:MISSENSE[ENST00000378475(c.223C>A,Caa>Aaa):ENSP00000367736(p.Gln75Lys)];ENSG00000152954:MISSENSE[ENST00000378478(c.223C>A,Caa>Aaa):ENSP00000367739(p.Gln75Lys)];ENSG00000152954:INTRONIC[ENST00000378491(c.190-4591C>A)];ENSG00000152954:INTRONIC[ENST00000378477(c.190-4591C>A)];ENSG00000152954:INTRONIC[ENST00000468195(n.256+6441C>A)];	NA	NA	NA	NA	NA	NA	NA	NA	UN_UN	NA	MODERATE	NA	NA	103	103	0	143	111	32	22.38
Chewer	42484	chr6	51890829	G	A	*PKHD1*	MISSENSE	ENSG00000170927:MISSENSE[ENST00000340994(c.3779C>T,gCg>gTg):ENSP00000341097(p.Ala1260Val)];ENSG00000170927:MISSENSE[ENST00000371117(c.3779C>T,gCg>gTg):ENSP00000360158(p.Ala1260Val)];	chr6*51890[illegible]	NA	NA	8.29E-06	6.07E-05	NA	NA	T	BN_BN	N	MODERATE	N	0.001	85	85	0	74	51	23	31.08
Chewer	42484	chr6	151161838	C	T	*PLEKHG1*	MISSENSE	ENSG00000120278:MISSENSE[ENST00000367328(c.3964C>T,Cat>Tat):ENSP00000356297(p.His1322Tyr)];ENSG00000120278:MISSENSE[ENST00000358517(c.3964C>T,Cat>Tat):ENSP00000351318(p.His1322Tyr)];ENSG00000120278:INTRONIC[ENST00000475490(c.1158-333C>T)];	NA	NA	NA	NA	NA	NA	NA	T	BN_BN	N	MODERATE	N	0.035	243	243	0	177	147	30	16.95
Chewer	42484	chr6	30570014	G	A	*PPP1R10*	SILENT	ENSG00000204569:SILENT[ENST00000376511(c.2412C>T,ggC>ggT):ENSP00000365694(p.Gly804(=))];	NA	NA	NA	2.60E-05	6.28E-05	NA	NA	NA	NA_NA	NA	LOW	NA	NA	173	172	0	176	157	18	10.23
Chewer	42484	chr6	42666235	G	T	*PRPH2*	MISSENSE	ENSG00000112619:MISSENSE[ENST00000230381(c.839C>A,aCa>aAa):ENSP00000230381(p.Thr280Lys)];	NA	NA	NA	NA	NA	NA	NA	D	BN_PoD	N	MODERATE	D	0.387	172	172	0	215	183	32	14.88
Chewer	42484	chr6	42689909	G	T	*PRPH2*	MISSENSE	ENSG00000112619:MISSENSE[ENST00000230381(c.164C>A,tCt>tAt):ENSP00000230381(p.Ser55Tyr)];	NA	NA	NA	NA	NA	NA	NA	T	BN_BN	N	MODERATE	D	0.997	215	215	0	203	141	62	30.54
Chewer	42484	chr6	31084691	G	A	*PSORS1C1*	EXONIC-NC	ENSG00000204540:[ENST00000467107(n.1921G>A)];ENSG00000204540:[ENST00000552747(n.53+2023G>A)];ENSG00000204540:[ENST00000550838(n.58+2023G>A)];ENSG00000204540:[ENST00000479581(n.61+2023G>A)];ENSG00000204540:[ENST00000493289(n.68-176G>A)];ENSG00000204540:[ENST00000259881(c.-229+2023G>A)];ENSG00000204540:[ENST00000548049(n.119+2023G>A)];	chr6*31084[illegible]	NA	0.0001997	8.25E-06	0	0.0001997	0	NA	NA_NA	NA	MODIFIER	NA	NA	82	82	0	70	59	11	15.71
Chewer	42484	chr6	76373001	C	T	*SENP6*	MISSENSE	ENSG00000112701:MISSENSE[ENST00000327284(c.740C>T,aCg>aTg):ENSP00000321820(p.Thr247Met)];ENSG00000112701:MISSENSE[ENST00000370010(c.740C>T,aCg>aTg):ENSP00000359027(p.Thr247Met)];ENSG00000112701:MISSENSE[ENST00000370014(c.761C>T,aCg>aTg):ENSP00000359031(p.Thr254Met)];ENSG00000112701:MISSENSE[ENST00000447266(c.761C>T,aCg>aTg):ENSP00000402527(p.Thr254Met)];ENSG00000112701:MISSENSE[ENST00000424947(c.431C>T,aCg>aTg):ENSP00000391426(p.Thr144Met)];ENSG00000112701:MISSENSE[ENST00000483859(c.431C>T,aCg>aTg):ENSP00000426480(p.Thr144Met)];ENSG00000112701:EXONIC-NC[ENST00000485497(n.721C>T)];	NA	NA	NA	1.67E-05	0	NA	NA	T	BN_BN	N	MODERATE	N	NA	99	99	0	97	89	8	8.25

Cohort	Sample	Chromosome	Genomic position	Reference allele	Alternate allele	Gene symbol	Variant type	Variant annotation	OncoMD	COSMIC	dbSNP	ExAC	ExAC_SAS	1000G	1000G_SAS	SIFT	Polyphen2	CONDEL	VEP	LRT	PhastCon7 Way	Total read (Adjacent normal)	Reference allel read support (Adjacent normal)	Alternative read support (Adjacent normal)	Total read (Tumor)	Reference allel read support (Tumor)	Alternative read support (Tumor)	Alternate allele frequency
Chewer	42484	chr6	160557724	C	T	*SLC22A1*	INTRONIC	ENSG00000175003:[ENST00000324965(c.1061+42C>T)];ENSG00000175003:[ENST00000366963(c.1061+42C>T)];ENSG00000175003:[ENST00000457470(c.1061+42C>T)];ENSG00000175003:[ENST00000539263(c.*534+42C>T)];ENSG00000175003:[ENST00000460902(c.1061+42C>T)];	NA	NA	NA	8.25E-06	0	NA	NA	NA	NA_NA	NA	MODIFIER	NA	NA	95	95	0	94	75	19	20.21
Chewer	42484	chr6	159166551	G	C	*SYTL3*	MISSENSE	ENSG00000164674:MISSENSE[ENST00000367081(c.73G>C,Ggc>Cgc):ENSP00000356048(p.Gly25Arg)];ENSG00000164674:MISSENSE[ENST00000297239(c.895G>C,Ggc>Cgc):ENSP00000297239(p.Gly299Arg)];ENSG00000164674:MISSENSE[ENST00000360448(c.691G>C,Ggc>Cgc):ENSP00000353631(p.Gly231Arg)];	NA	NA	chr6*159166551*159166551*G*A*rs780654203*NA*NA*NA	NA	NA	NA	NA	D	PrD_PrD	D	MODERATE	D	1	99	98	0	75	66	9	12
Chewer	42484	chr6	167772800	T	G	*TCP10*	MISSENSE	ENSG00000203690:MISSENSE[ENST00000366827(c.890A>C,cAa>cCa):ENSP00000355792(p.Gln297Pro)];ENSG00000203690:EXONIC-NC[ENST00000508373(n.2820A>C)];	NA	NA	NA	NA	NA	NA	NA	NA	NA_NA	NA	MODIFIER	NA	NA	245	245	0	141	80	61	43.26
Chewer	42484	chr6	32021387	G	T	*TNXB*	MISSENSE	ENSG00000168477:MISSENSE[ENST00000375247(c.8563C>A,Cct>Act):ENSP00000364396(p.Pro2855Thr)];ENSG00000168477:MISSENSE[ENST00000375244(c.8569C>A,Cct>Act):ENSP00000364393(p.Pro2857Thr)];	NA	NA	chr6*32021387*32021387*G*C*rs774545672*NA*NA*NA	NA	NA	NA	NA	NA	PoD_PrD	NA	MODERATE	NA	NA	30	30	0	56	37	19	33.93
Chewer	42484	chr6	18139973	A	T	*TPMT*	INTRONIC	ENSG00000137364:[ENST00000309983(c.367-25T>A)];	chr6*18139	NA	NA	NA	NA	NA	NA	NA	NA_NA	NA	MODIFIER	NA	NA	26	26	0	19	14	5	26.32
Chewer	42484	chr6	167772800	T	G	*TTLL2*	INTRONIC	ENSG00000120440:[ENST00000515138(c.*645-11T>G)];	NA	NA	NA	NA	NA	NA	NA	NA	NA_NA	NA	MODIFIER	NA	NA	245	245	0	141	80	61	43.26
Chewer	42484	chr6	90039412	C	G	*UBE2J1*	MISSENSE	ENSG00000198833:MISSENSE[ENST00000435041(c.943G>C,Gac>Cac):ENSP00000451261(p.Asp315His)];	NA	NA	NA	NA	NA	NA	NA	D_lc	PoD_PrD	D	MODERATE	D	1	71	71	0	50	34	15	30
Chewer	42484	chr6	133035167	G	C	*VNN1*	MISSENSE	ENSG00000112299:MISSENSE[ENST00000367928(c.8C>G,aCt>aGt):ENSP00000356905(p.Thr3Ser)];	NA	NA	NA	NA	NA	NA	NA	T_lc	BN_BN	N	MODERATE	N	0.014	97	96	0	71	63	8	11.27
Chewer	42484	chr6	38120186	G	C	*ZFAND3*	EXONIC-NC	ENSG00000156639:[ENST00000463847(n.384G>C)];ENSG00000156639:[ENST00000440482(n.387G>C)];ENSG00000156639:[ENST00000373391(c.*21G>C)];ENSG00000156639:[ENST00000287218(c.*21G>C)];	NA	NA	chr6*38120186*38120186*G*A*rs760596239*NA*NA*NA	NA	NA	NA	NA	NA	NA_NA	NA	MODIFIER	NA	NA	103	102	1	116	81	35	30.17
Chewer	42484	chr7	87765309	G	C	*ADAM22*	MISSENSE	ENSG00000008277:MISSENSE[ENST00000398209(c.1183G>C,Gag>Cag):ENSP00000381267(p.Glu395Gln)];ENSG00000008277:MISSENSE[ENST00000398203(c.1084G>C,Gag>Cag):ENSP00000381261(p.Glu362Gln)];ENSG00000008277:MISSENSE[ENST00000315984(c.1183G>C,Gag>Cag):ENSP00000315900(p.Glu395Gln)];ENSG00000008277:MISSENSE[ENST00000265727(c.1183G>C,Gag>Cag):ENSP00000265727(p.Glu395Gln)];ENSG00000008277:MISSENSE[ENST00000398201(c.1183G>C,Gag>Cag):ENSP00000381260(p.Glu395Gln)];ENSG00000008277:MISSENSE[ENST00000398204(c.1183G>C,Gag>Cag):ENSP00000381262(p.Glu395Gln)];	chr7*87765	chr7*87765	chr7*87765309*87765309*G*A*rs779354391*NA*NA*NA	NA	NA	NA	NA	T	PoD_PoD	N	MODERATE	D	1	152	152	0	140	89	50	35.71
Chewer	42484	chr7	2963002	G	A	*CARD11*	INTRONIC	ENSG00000198286:[ENST00000396946(c.1941-35C>T)];ENSG00000198286:[ENST00000355508(c.354-35C>T)];	NA	NA	NA	NA	NA	NA	NA	NA	NA_NA	NA	MODIFIER	NA	NA	77	77	0	78	68	10	12.82
Chewer	42484	chr7	29519894	G	T	*CHN2*	MISSENSE	ENSG00000106069:MISSENSE[ENST00000439711(c.168G>T,caG>caT):ENSP00000387425(p.Gln56His)];ENSG00000106069:MISSENSE[ENST00000409041(c.168G>T,caG>caT):ENSP00000386849(p.Gln56His)];ENSG00000106069:MISSENSE[ENST00000421775(c.168G>T,caG>caT):ENSP00000394284(p.Gln56His)];ENSG00000106069:INTRONIC-SS-ACR[ENST00000493906(n.139-1G>T)];ENSG00000106069:INTRONIC-SS-ACR[ENST00000539406(c.802-1G>T)];ENSG00000106069:INTRONIC-SS-ACR[ENST00000495789(c.616-1G>T)];ENSG00000106069:INTRONIC-SS-ACR[ENST00000539389(c.145-1G>T)];ENSG00000106069:INTRONIC-SS-ACR[ENST00000474070(c.*267-1G>T)];ENSG00000106069:INTRONIC-SS-ACR[ENST00000546235(c.532-1G>T)];ENSG00000106069:INTRONIC-SS-ACR[ENST00000222792(c.577-1G>T)];ENSG00000106069:INTRONIC[ENST00000467441(n.276+57G>T)];ENSG00000106069:INTRONIC[ENST00000446446(c.130-15674G>T)];ENSG00000106069:INTRONIC[ENST00000435288(c.302-29023G>T)];ENSG00000106069:INTRONIC[ENST00000424025(c.111+57G>T)];ENSG00000106069:EXONIC-NC[ENST00000412711(n.567G>T)];	chr7*29519	chr7*29519	NA	NA	NA	NA	NA	NA	NA_NA	NA	MODIFIER	NA	NA	118	118	0	85	73	12	14.12
Chewer	42484	chr7	142563755	A	G	*EPHB6*	SILENT	ENSG00000106123:SILENT[ENST00000442129(c.1143A>G,caA>caG):ENSP00000410789(p.Gln381(=))];ENSG00000106123:SILENT[ENST00000411471(c.312A>G,caA>caG):ENSP00000409061(p.Gln104(=))];ENSG00000106123:SILENT[ENST00000392957(c.1143A>G,caA>caG):ENSP00000376684(p.Gln381(=))];ENSG00000106123:EXONIC-NC[ENST00000466783(n.2129A>G)];ENSG00000106123:3UTR[ENST00000425995(c.*925A>G)];ENSG00000106123:3UTR[ENST00000422643(c.*323A>G)];	NA	NA	NA	NA	NA	NA	NA	NA	NA_NA	NA	MODIFIER	NA	NA	59	59	0	77	46	31	40.26
Chewer	42484	chr7	158527113	C	A	*ESYT2*	MISSENSE-SS-PRX	ENSG00000117868:[ENST00000251527(c.2636G>T,tGg>tTg):ENSP00000251527(p.Trp879Leu)];ENSG00000117868:[ENST00000275418(c.2609G>T,tGg>tTg):ENSP00000275418(p.Trp870Leu)];ENSG00000117868:[ENST00000435514(c.941G>T,tGg>tTg):ENSP00000411488(p.Trp314Leu)];	NA	NA	NA	NA	NA	NA	NA	NA	PrD_PrD	NA	MODERATE	D	0.999	63	63	0	82	56	26	31.71
Chewer	42484	chr7	158557603	A	C	*ESYT2*	INTRONIC	ENSG00000117868:[ENST00000251527(c.1069-59T>G)];ENSG00000117868:[ENST00000275418(c.979-59T>G)];	NA	NA	NA	NA	NA	NA	NA	NA	NA_NA	NA	MODIFIER	NA	NA	207	207	0	192	171	21	10.94
Chewer	42484	chr7	128488880	C	T	*FLNC*	MISSENSE	ENSG00000128591:MISSENSE[ENST00000325888(c.4771C>T,Cgg>Tgg):ENSP00000327145(p.Arg1591Trp)];ENSG00000128591:MISSENSE[ENST00000346177(c.4771C>T,Cgg>Tgg):ENSP00000344002(p.Arg1591Trp)];	NA	NA	0.0001997	2.49E-05	0	0.0001997	0	D	PrD_PrD	D	MODERATE	D	0.998	89	89	0	103	60	42	40.78
Chewer	42484	chr7	127235914	G	T	*FSCN3*	MISSENSE	ENSG00000106328:MISSENSE[ENST00000265825(c.698G>T,gGc>gTc):ENSP00000265825(p.Gly233Val)];ENSG00000106328:MISSENSE[ENST00000420086(c.296G>T,gGc>gTc):ENSP00000412243(p.Gly99Val)];ENSG00000106328:MISSENSE[ENST00000478821(c.296G>T,gGc>gTc):ENSP00000473531(p.Gly99Val)];ENSG00000106328:EXONIC-NC[ENST00000469242(n.420G>T)];	NA	NA	NA	NA	NA	NA	NA	NA	NA_NA	NA	MODIFIER	NA	NA	118	118	0	140	116	24	17.14
Chewer	42484	chr7	127235935	C	A	*FSCN3*	MISSENSE	ENSG00000106328:EXONIC-NC[ENST00000469242(n.441C>A)];ENSG00000106328:MISSENSE[ENST00000265825(c.719C>A,aCg>aAg):ENSP00000265825(p.Thr240Lys)];ENSG00000106328:MISSENSE[ENST00000420086(c.317C>A,aCg>aAg):ENSP00000412243(p.Thr106Lys)];ENSG00000106328:MISSENSE[ENST00000478821(c.317C>A,aCg>aAg):ENSP00000473531(p.Thr106Lys)];	chr7*12723	chr7*12723	chr7*127235935*127235935*C*T*rs115869480*1*0.9926*0.007388	NA	NA	NA	NA	T	BN_BN	N	MODERATE	N	NA	87	87	0	116	73	43	37.07
Chewer	42484	chr7	150417160	G	A	*GIMAP1*	MISSENSE	ENSG00000213203:MISSENSE[ENST00000307194(c.68G>A,cGg>cAg):ENSP00000302833(p.Arg23Gln)];	NA	NA	NA	1.81E-05	0.00015361	NA	NA	T	BN_BN	N	MODERATE	NA	0.002	80	80	0	87	51	36	41.38
Chewer	42484	chr7	128506868	G	A	*KCP*	INTRONIC	ENSG00000135253:[ENST00000297801(n.4214-1804C>T)];ENSG00000135253:[ENST00000492679(n.1241-1804C>T)];	NA	NA	0.0003994	NA	NA	0.0003994	0	NA	NA_NA	NA	MODIFIER	NA	NA	76	76	0	103	86	17	16.5

Supplementary Table 4. List of somatic single nucleotide variants identified using whole exome sequencing of ESCC patients

Cohort	Sample	Chromosome	Genomic position	Reference allele	Alternate allele	Gene symbol	Variant type	Variant annotation	OncoMD	COSMIC	dbSNP	ExAC	ExAC_SAS	1000G	1000G_SAS	SIFT	Polyphen2	CONDEL	VEP	LRT	PhastCon7 Way	Total read (Adjacent normal)	Reference allel read support (Adjacent normal)	Alternative read support (Adjacent normal)	Total read (Tumor)	Reference allel read support (Tumor)	Alternative read support (Tumor)	Alternate allele frequency
Chewer	42484	chr7	11142112	G	A	*PHF14*	INTRONIC-SS-ACR	ENSG00000106443:INTRONIC-SS-ACR[ENST00000521747(c.*1738-1G>A)];ENSG00000106443:INTRONIC-SS-ACR[ENST00000403050(c.2655-1G>A)];ENSG00000106443:INTRONIC[ENST00000470665(n.680-8865G>A)];ENSG00000106443:INTRONIC[ENST00000481418(n.478-883G>A)];ENSG00000106443:INTRONIC[ENST00000423760(c.*1738-8865G>A)];ENSG00000106443:INTRONIC[ENST00000445996(c.1800-8865G>A)];	NA	NA	NA	NA	NA	NA	NA	NA	NA_NA	NA	MODIFIER	NA	NA	266	264	0	177	147	30	16.95
Chewer	42484	chr7	53103758	C	T	*POM121L12*	MISSENSE	ENSG00000221900:MISSENSE[ENST00000408890(c.394C>T,Cgg>Tgg):ENSP00000386133(p.Arg132Trp)];	NA	NA	NA	1.72E-05	0	NA	NA	T	PrD_PrD	N	MODERATE	NA	NA	81	81	0	76	70	6	7.89
Chewer	42484	chr7	77268593	C	A	*PTPN12*	MISSENSE	ENSG00000127947:MISSENSE[ENST00000415482(c.1982C>A,aCa>aAa):ENSP00000392429(p.Thr661Lys)];ENSG00000127947:MISSENSE[ENST00000435495(c.1949C>A,aCa>aAa):ENSP00000397991(p.Thr650Lys)];ENSG00000127947:MISSENSE[ENST00000248594(c.2339C>A,aCa>aAa):ENSP00000248594(p.Thr780Lys)];ENSG00000127947:EXONIC-NC[ENST00000520947(n.450C>A)];	NA	NA	NA	NA	NA	NA	NA	NA	NA_NA	NA	MODIFIER	NA	NA	275	275	0	250	231	19	7.6
Chewer	42484	chr7	141872405	A	T	*RP11-1220K2.2*	INTRONIC	ENSG00000257743:[ENST00000496337(n.369-47A>T)];ENSG00000257743:[ENST00000477922(c.3449-47A>T)];	NA	NA	NA	NA	NA	NA	NA	NA	NA_NA	NA	MODIFIER	NA	NA	115	115	0	129	99	30	23.26
Chewer	42484	chr7	128506868	G	A	*RP11-309L24.4*	EXONIC-NC	ENSG00000239815:[ENST00000461420(n.236G>A)];	NA	NA	0.0003994	NA	NA	0.0003994	0	NA	NA_NA	NA	MODIFIER	NA	NA	76	76	0	103	86	17	16.5
Chewer	42484	chr7	92734678	C	G	*SAMD9*	MISSENSE	ENSG00000205413:MISSENSE[ENST00000446617(c.733G>C,Ggg>Cgg):ENSP00000414529(p.Gly245Arg)];ENSG00000205413:MISSENSE[ENST00000379958(c.733G>C,Ggg>Cgg):ENSP00000369292(p.Gly245Arg)];	NA	NA	NA	NA	NA	NA	NA	D	PrD_PrD	D	MODERATE	D	0.938	197	197	0	208	158	50	24.04
Chewer	42484	chr7	101943921	G	A	*SH2B2*	SILENT	ENSG00000160999:SILENT[ENST00000536178(c.216G>A,ctG>ctA):ENSP00000440273(p.Leu72(=))];ENSG00000160999:SILENT[ENST00000306803(c.87G>A,ctG>ctA):ENSP00000304701(p.Leu29(=))];ENSG00000160999:SILENT[ENST00000444095(c.87G>A,ctG>ctA):ENSP00000401883(p.Leu29(=))];	NA	NA	NA	NA	NA	NA	NA	NA	NA_NA	NA	LOW	NA	NA	240	240	0	314	269	45	14.33
Chewer	42484	chr8	62531482	C	T	*ASPH*	INTRONIC	ENSG00000198363:[ENST00000523927(n.470+19024G>A)];ENSG00000198363:[ENST00000523897(n.248+55G>A)];ENSG00000198363:[ENST00000522349(c.236-34894G>A)];ENSG00000198363:[ENST00000541428(c.889+55G>A)];ENSG00000198363:[ENST00000379454(c.976+55G>A)];ENSG00000198363:[ENST00000522919(c.415+55G>A)];ENSG00000198363:[ENST00000524173(n.352+14760G>A)];ENSG00000198363:[ENST00000521499(n.140+55G>A)];	NA	NA	NA	NA	NA	NA	NA	NA	NA_NA	NA	MODIFIER	NA	NA	137	136	1	100	85	15	15
Chewer	42484	chr8	113326107	C	G	*CSMD3*	INTRONIC	ENSG00000164796:[ENST00000352409(c.7486+28G>C)];ENSG00000164796:[ENST00000455883(c.7384+28G>C)];ENSG00000164796:[ENST00000297405(c.7696+28G>C)];ENSG00000164796:[ENST00000492692(n.888+28G>C)];ENSG00000164796:[ENST00000339701(c.5506+28G>C)];ENSG00000164796:[ENST00000343508(c.7576+28G>C)];	NA	NA	chr8*113326107*113326107*C*T*rs762900477*NA*NA*NA	NA	NA	NA	NA	NA	NA_NA	NA	MODIFIER	NA	NA	115	115	0	123	71	52	42.28
Chewer	42484	chr8	105405075	C	T	*DPYS*	SILENT	ENSG00000147647:SILENT[ENST00000351513(c.1380G>A,ggG>ggA):ENSP00000276651(p.Gly460(=))];ENSG00000147647:SILENT[ENST00000533874(c.10G>A,ggG>ggA):ENSP00000436654(p.Gly4(=))];ENSG00000147647:EXONIC-NC[ENST00000520483(n.284G>A)];ENSG00000147647:EXONIC-NC[ENST00000521372(n.212G>A)];ENSG00000147647:INTRONIC[ENST00000521601(n.198-11533G>A)];	NA	NA	NA	NA	NA	NA	NA	NA	NA_NA	NA	MODIFIER	NA	NA	149	149	0	137	120	17	12.41
Chewer	42484	chr8	97157150	C	A	*GDF6*	MISSENSE	ENSG00000156466:MISSENSE[ENST00000287020(c.1009G>T,Gcc>Tcc):ENSP00000287020(p.Ala337Ser)];	NA	NA	NA	NA	NA	NA	NA	D	PoD_PoD	D	MODERATE	N	0.994	74	74	0	80	72	8	10
Chewer	42484	chr8	57890681	T	C	*IMPAD1*	MISSENSE	ENSG00000104331:MISSENSE[ENST00000262644(c.574A>G,Act>Gct):ENSP00000262644(p.Thr192Ala)];ENSG00000104331:MISSENSE[ENST00000520392(c.102A>G,Act>Gct):ENSP00000428617(p.Thr35Ala)];ENSG00000104331:INTRONIC[ENST00000517461(c.201+2037A>G)];	NA	NA	NA	NA	NA	NA	NA	NA	NA_NA	NA	MODIFIER	NA	NA	91	91	0	89	75	14	15.73
Chewer	42484	chr8	134270549	C	G	*NDRG1*	INTRONIC	ENSG00000104419:[ENST00000414097(c.450+60G>C)];ENSG00000104419:[ENST00000517331(n.168+60G>C)];ENSG00000104419:[ENST00000517599(c.*56+60G>C)];ENSG00000104419:[ENST00000520230(c.501+60G>C)];ENSG00000104419:[ENST00000323851(c.450+60G>C)];ENSG00000104419:[ENST00000519228(c.450+60G>C)];ENSG00000104419:[ENST00000518066(c.37-16250G>C)];ENSG00000104419:[ENST00000354944(c.240+60G>C)];ENSG00000104419:[ENST00000518010(n.526+862G>C)];ENSG00000104419:[ENST00000537882(c.207+60G>C)];ENSG00000104419:[ENST00000522476(c.252+60G>C)];ENSG00000104419:[ENST00000522890(c.450+60G>C)];ENSG00000104419:[ENST00000519580(c.450+60G>C)];ENSG00000104419:[ENST00000518176(c.49-11643G>C)];ENSG00000104419:[ENST00000522377(c.450+60G>C)];ENSG00000104419:[ENST00000518480(c.252+60G>C)];	NA	NA	NA	NA	NA	NA	NA	NA	NA_NA	NA	MODIFIER	NA	NA	109	109	0	139	72	66	47.48
Chewer	42484	chr8	70981785	A	T	*PRDM14*	MISSENSE	ENSG00000147596:MISSENSE[ENST00000276594(c.311T>A,aTt>aAt):ENSP00000276594(p.Ile104Asn)];	NA	NA	NA	NA	NA	NA	NA	D_lc	BN_PoD	D	MODERATE	N	0.005	94	94	0	65	51	14	21.54
Chewer	42484	chr8	48734296	A	C	*PRKDC*	MISSENSE	ENSG00000253729:MISSENSE[ENST00000338368(c.8977T>G,Tgg>Ggg):ENSP00000345182(p.Trp2993Gly)];ENSG00000253729:MISSENSE[ENST00000314191(c.8977T>G,Tgg>Ggg):ENSP00000313420(p.Trp2993Gly)];ENSG00000253729:EXONIC-NC[ENST00000518216(n.9031T>G)];ENSG00000253729:EXONIC-NC[ENST00000523565(n.9036T>G)];	NA	NA	NA	NA	NA	NA	NA	NA	NA_NA	NA	MODIFIER	NA	NA	126	126	0	134	113	21	15.67
Chewer	42484	chr8	106258572	T	G	*RP11-127H5.1*	MISSENSE	ENSG00000253350:MISSENSE[ENST00000518180(c.104T>G,aTg>aGg):ENSP00000429987(p.Met35Arg)];	NA	NA	NA	NA	NA	NA	NA	NA	UN_UN	NA	MODERATE	NA	NA	239	239	0	198	179	19	9.6
Chewer	42484	chr8	51705410	C	A	*SNTG1*	3UTR	ENSG00000147481:[ENST00000517473(c.*21C>A)];ENSG00000147481:[ENST00000520825(c.*554C>A)];ENSG00000147481:[ENST00000276467(c.*21C>A)];ENSG00000147481:[ENST00000522124(c.*21C>A)];	NA	NA	NA	NA	NA	NA	NA	NA	NA_NA	NA	MODIFIER	NA	NA	190	189	1	165	146	19	11.52
Chewer	42484	chr8	53071529	G	T	*ST18*	MISSENSE	ENSG00000147488:MISSENSE[ENST00000517580(c.1735C>A,Ctg>Atg):ENSP00000428521(p.Leu579Met)];ENSG00000147488:MISSENSE[ENST00000276480(c.1735C>A,Ctg>Atg):ENSP00000276480(p.Leu579Met)];ENSG00000147488:3UTR[ENST00000521824(c.*1446C>A)];ENSG00000147488:3UTR[ENST00000522251(c.*1697C>A)];ENSG00000147488:3UTR[ENST00000521582(c.*695C>A)];	chr8*53071	chr8*53071	NA	NA	NA	NA	NA	NA	NA_NA	NA	MODIFIER	NA	NA	102	102	0	191	79	112	58.64
Chewer	42484	chr8	59739436	G	A	*TOX*	MISSENSE	ENSG00000198846:MISSENSE[ENST00000361421(c.950C>T,gCg>gTg):ENSP00000354842(p.Ala317Val)];	chr8*59739	chr8*59739	NA	NA	NA	NA	NA	D	PrD_PrD	D	MODERATE	D	0.999	95	95	0	85	63	22	25.88
Chewer	42484	chr8	100454663	A	G	*VPS13B*	MISSENSE	ENSG00000132549:MISSENSE[ENST00000395996(c.3245A>G,aAt>aGt):ENSP00000379318(p.Asn1082Ser)];ENSG00000132549:MISSENSE[ENST00000358544(c.3245A>G,aAt>aGt):ENSP00000351346(p.Asn1082Ser)];ENSG00000132549:MISSENSE[ENST00000357162(c.3245A>G,aAt>aGt):ENSP00000349685(p.Asn1082Ser)];ENSG00000132549:MISSENSE[ENST00000496144(c.3245A>G,aAt>aGt):ENSP00000430900(p.Asn1082Ser)];	NA	NA	NA	NA	NA	NA	NA	T	BN_BN	N	MODERATE	NA	NA	188	188	0	181	130	50	27.62

Mangalaparthi *et al.* , 2020. Mutational landscape of esophageal squamous cell carcinoma in an Indian cohort
Supplementary Table 4. List of somatic single nucleotide variants identified using whole exome sequencing of ESCC patients

Cohort	Sample	Chromosome	Genomic position	Reference allele	Alternate allele	Gene symbol	Variant type	Variant annotation	OncoMD	COSMIC	dbSNP	ExAC	ExAC_SAS	1000G	1000G_SAS	SIFT	Polyphen2	CONDEL	VEP	LRT	PhastCon7 Way	Total read (Adjacent normal)	Reference allel read support (Adjacent normal)	Alternative read support (Adjacent normal)	Total read (Tumor)	Reference allel read support (Tumor)	Alternative read support (Tumor)	Alternate allele frequency
Chewer	42484	chr9	91930204	A	G	*CKS2*	MISSENSE	ENSG00000123975:MISSENSE[ENST00000314355(c.179A>G,cAt>cGt):ENSP00000364976(p.His60Arg)];	NA	NA	NA	NA	NA	NA	NA	D	PoD_PoD	D	MODERATE	D	1	57	57	0	50	34	16	32
Chewer	42484	chr9	95773547	G	A	*FGD3*	MISSENSE	ENSG00000127084:MISSENSE[ENST00000416701(c.1028G>A,cGg>cAg):ENSP00000413833(p.Arg343Gln)];ENSG00000127084:MISSENSE[ENST00000467786(c.1028G>A,cGg>cAg):ENSP00000432310(p.Arg343Gln)];ENSG00000127084:MISSENSE[ENST00000375482(c.1028G>A,cGg>cAg):ENSP00000364631(p.Arg343Gln)];ENSG00000127084:MISSENSE[ENST00000337352(c.1028G>A,cGg>cAg):ENSP00000336914(p.Arg343Gln)];	NA	NA	NA	0.0002568	6.06E-05	NA	NA	D	BN_PoD	N	MODERATE	N	0.993	62	62	0	87	72	15	17.24
Chewer	42484	chr9	71491570	T	A	*PIP5K1B*	INTRONIC	ENSG00000107242:[ENST00000478500(c.321-23T>A)];ENSG00000107242:[ENST00000437200(c.201-23T>A)];ENSG00000107242:[ENST00000265382(c.201-23T>A)];ENSG00000107242:[ENST00000377284(c.201-23T>A)];ENSG00000107242:[ENST00000440050(c.201-23T>A)];ENSG00000107242:[ENST00000541509(c.201-23T>A)];	NA	NA	NA	NA	NA	NA	NA	NA	NA_NA	NA	MODIFIER	NA	NA	202	202	0	133	98	35	26.32
Chewer	42484	chr9	84530910	C	A	*SPATA31D5P*	EXONIC-NC	ENSG00000240632:[ENST00000527857(n.932C>A)];ENSG00000240632:[ENST00000376459(n.903C>A)];	NA	NA	chr9*84530910*84530910*C*T*rs117202352*0*0.9998*0.0001997	NA	NA	NA	NA	NA	NA_NA	NA	MODIFIER	NA	NA	279	279	0	203	182	21	10.34
Chewer	42484	chr9	35724631	G	A	*TLN1*	MISSENSE	ENSG00000137076:MISSENSE[ENST00000314888(c.449C>T,aCa>aTa):ENSP00000316029(p.Thr150Ile)];ENSG00000137076:MISSENSE[ENST00000540444(c.449C>T,aCa>aTa):ENSP00000442981(p.Thr150Ile)];ENSG00000137076:EXONIC-NC[ENST00000378192(n.512C>T)];	NA	NA	NA	NA	NA	NA	NA	NA	NA_NA	NA	MODIFIER	NA	NA	223	222	1	163	134	29	17.79
Chewer	42484	chr9	35841734	A	G	*TMEM8B*	INTRONIC	ENSG00000137103:[ENST00000490199(n.313+470A>G)];ENSG00000137103:[ENST00000473947(n.783-4238A>G)];ENSG00000137103:[ENST00000377996(c.-105A>G)];ENSG00000137103:[ENST00000439587(c.-105A>G)];ENSG00000137103:[ENST00000377988(c.-105A>G)];ENSG00000137103:[ENST00000377991(c.-105A>G)];ENSG00000137103:[ENST00000464519(n.548A>G)];	NA	NA	NA	NA	NA	NA	NA	NA	NA_NA	NA	MODIFIER	NA	NA	152	151	0	114	71	43	37.72
Chewer	42484	chr9	96062416	G	T	*WNK2*	MISSENSE	ENSG00000165238:MISSENSE[ENST00000448251(c.2589G>T,Gta>Tta):ENSP00000390441(p.Val864Leu)];ENSG00000165238:MISSENSE[ENST00000427277(c.5035G>T,Gta>Tta):ENSP00000411181(p.Val1679Leu)];ENSG00000165238:MISSENSE[ENST00000349097(c.5146G>T,Gta>Tta):ENSP00000297876(p.Val1716Leu)];ENSG00000165238:MISSENSE[ENST00000297954(c.6310G>T,Gta>Tta):ENSP00000297954(p.Val2104Leu)];ENSG00000165238:MISSENSE[ENST00000395477(c.6199G>T,Gta>Tta):ENSP00000378860(p.Val2067Leu)];ENSG00000165238:MISSENSE[ENST00000432730(c.6186G>T,Gta>Tta):ENSP00000415038(p.Val2063Leu)];ENSG00000165238:INTRONIC[ENST00000474009(n.300+2067G>T)];ENSG00000165238:INTRONIC[ENST00000411624(c.4733+2067G>T)];ENSG00000165238:INTRONIC[ENST00000453718(c.1680+873G>T)];ENSG00000165238:3UTR[ENST00000395475(c.*3664G>T)];ENSG00000165238:3UTR[ENST00000356055(c.*189G>T)];ENSG00000165238:EXONIC-NC[ENST00000479696(n.340G>T)];	NA	NA	chr9*96062416*96062416*G*A*rs767200622*NA*NA*NA	NA	NA	NA	NA	NA	NA_NA	NA	MODIFIER	NA	NA	109	109	0	63	40	23	36.51
Chewer	42484	chrX	48337428	G	A	*FTSJ1*	INTRONIC	ENSG00000068438:[ENST00000473235(n.180-2111G>A)];ENSG00000068438:[ENST00000492562(n.245-2386G>A)];ENSG00000068438:[ENST00000487353(n.762G>A)];ENSG00000068438:[ENST00000485486(n.517G>A)];ENSG00000068438:[ENST00000490202(n.30G>A)];ENSG00000068438:[ENST00000396894(c.-74G>A)];ENSG00000068438:[ENST00000496365(n.3G>A)];ENSG00000068438:[ENST00000456787(c.285G>A,ctG>ctA):ENSP00000415457(p.Leu95(=))];ENSG00000068438:[ENST00000348411(c.285G>A,ctG>ctA):ENSP00000326948(p.Leu95(=))];ENSG00000068438:[ENST00000019019(c.285G>A,ctG>ctA):ENSP00000019019(p.Leu95(=))];	NA	NA	NA	NA	NA	NA	NA	NA	NA_NA	NA	LOW	NA	NA	99	98	1	59	30	29	49.15
Chewer	42484	chrX	135439992	C	G	*GPR112*	INTRONIC	ENSG00000156920:[ENST00000287534(c.6539-3622C>G)];ENSG00000156920:[ENST00000370652(c.6980+77C>G)];ENSG00000156920:[ENST00000394143(c.6980+77C>G)];ENSG00000156920:[ENST00000394141(c.6365+77C>G)];ENSG00000156920:[ENST00000412101(c.6365+77C>G)];	NA	NA	NA	NA	NA	NA	NA	NA	NA_NA	NA	MODIFIER	NA	NA	133	133	0	95	52	43	45.26
Chewer	42484	chrX	135440037	G	A	*GPR112*	INTRONIC	ENSG00000156920:[ENST00000287534(c.6539-3577G>A)];ENSG00000156920:[ENST00000370652(c.6980+122G>A)];ENSG00000156920:[ENST00000394143(c.6980+122G>A)];ENSG00000156920:[ENST00000394141(c.6365+122G>A)];ENSG00000156920:[ENST00000412101(c.6365+122G>A)];	NA	NA	NA	NA	NA	NA	NA	NA	NA_NA	NA	MODIFIER	NA	NA	104	104	0	67	38	29	43.28
Chewer	42484	chrX	53267524	G	T	*IQSEC2*	INTRONIC	ENSG00000124313:[ENST00000375365(c.2501-36C>A)];ENSG00000124313:[ENST00000375368(c.3086-36C>A)];ENSG00000124313:[ENST00000396435(c.3116-36C>A)];	NA	NA	NA	NA	NA	NA	NA	NA	NA_NA	NA	MODIFIER	NA	NA	35	35	0	16	11	5	31.25
Chewer	42484	chrX	140982491	C	T	*MAGEC3*	MISSENSE	ENSG00000165509:MISSENSE[ENST00000448920(c.70C>T,Ctc>Ttc):ENSP00000395092(p.Leu24Phe)];ENSG00000165509:INTRONIC[ENST00000298296(c.910-564C>T)];ENSG00000165509:INTRONIC[ENST00000443323(c.-118-1726C>T)];ENSG00000165509:INTRONIC[ENST00000536088(c.-68-1880C>T)];ENSG00000165509:5UTR[ENST00000544766(c.-488C>T)];ENSG00000165509:EXONIC-NC[ENST00000483584(n.40C>T)];	NA	NA	NA	NA	NA	NA	NA	NA	NA_NA	NA	MODIFIER	NA	NA	115	115	0	95	84	11	11.58
Chewer	42484	chrX	63615153	C	T	*MTMR8*	INTRONIC	ENSG00000102043:[ENST00000453546(c.24+67G>A)];ENSG00000102043:[ENST00000374852(c.24+67G>A)];	NA	NA	NA	NA	NA	NA	NA	NA	NA_NA	NA	MODIFIER	NA	NA	100	100	0	56	44	12	21.43
Chewer	42484	chrX	545936	C	A	*NA*	INTERGENIC		NA	NA	NA	NA	NA	NA	NA	NA	NA_NA	NA	MODIFIER	NA	NA	85	85	0	41	23	18	43.9
Chewer	42484	chrX	144905848	T	G	*SLITRK2*	MISSENSE	ENSG00000185985:MISSENSE[ENST00000335565(c.1905T>G,ttT>ttG):ENSP00000334374(p.Phe635Leu)];ENSG00000185985:MISSENSE[ENST00000370490(c.1905T>G,ttT>ttG):ENSP00000359521(p.Phe635Leu)];ENSG00000185985:MISSENSE[ENST00000434188(c.1905T>G,ttT>ttG):ENSP00000397015(p.Phe635Leu)];ENSG00000185985:MISSENSE[ENST00000428560(c.1905T>G,ttT>ttG):ENSP00000412010(p.Phe635Leu)];ENSG00000185985:MISSENSE[ENST00000413937(c.1905T>G,ttT>ttG):ENSP00000407347(p.Phe635Leu)];ENSG00000185985:MISSENSE[ENST00000447897(c.1905T>G,ttT>ttG):ENSP00000411681(p.Phe635Leu)];	NA	NA	NA	1.14E-05	0	NA	NA	T	BN_BN	N	MODERATE	D	0.996	224	224	0	182	114	68	37.36
Chewer	42484	chrX	139586093	G	C	*SOX3*	MISSENSE	ENSG00000134595:MISSENSE[ENST00000370536(c.1133C>G,cCc>cGc):ENSP00000359567(p.Pro378Arg)];	NA	NA	NA	NA	NA	NA	NA	D	BN_BN	N	MODERATE	U	0.998	43	43	0	45	38	7	15.56

Supplementary Table 4. List of somatic single nucleotide variants identified using whole exome sequencing of ESCC patients
[truncated: 3,513,929 more chars]
